# Molecular Regulation of Host Defense Responses Mediated by Biological Anti-TMV Agent Ningnanmycin

**DOI:** 10.3390/v11090815

**Published:** 2019-09-03

**Authors:** Mengnan An, Tao Zhou, Yi Guo, Xiuxiang Zhao, Yuanhua Wu

**Affiliations:** College of Plant Protection, Shenyang Agricultural University, Shenyang 110866, Liaoning, China

**Keywords:** Ningnanmycin, antiviral agents, BY-2 protoplasts, transcriptome analysis, resistance genes

## Abstract

Ningnanmycin (NNM) belongs to microbial pesticides that display comprehensive antiviral activity against plant viruses. NNM treatment has been shown to efficiently delay or suppress the disease symptoms caused by tobacco mosaic virus (TMV) infection in local-inoculated or systemic-uninoculated tobacco leaves, respectively. However, the underlying molecular mechanism of NNM-mediated antiviral activity remains to be further elucidated. In this study, 414 differentially expressed genes (DEGs), including 383 which were up-regulated and 31 down-regulated, caused by NNM treatment in TMV-infected BY-2 protoplasts, were discovered by RNA-seq. In addition, KEGG analysis indicated significant enrichment of DEGs in the plant–pathogen interaction and MAPK signaling pathway. The up-regulated expression of crucial DEGs, including defense-responsive genes, such as the receptor-like kinase *FLS2*, *RLK1,* and the mitogen-activated protein kinase kinase kinase *MAPKKK*, calcium signaling genes, such as the calcium-binding protein *CML19*, as well as phytohormone responsive genes, such as the WRKY transcription factors *WRKY40* and *WRKY70*, were confirmed by RT-qPCR. These findings provided valuable insights into the antiviral mechanisms of NNM, which indicated that the agent induces tobacco systemic resistance against TMV via activating multiple plant defense signaling pathways.

## 1. Introduction

Plant viruses infecting crops, vegetables, and ornamental plants significantly affect product quality and yields in agriculture [1]. Virus infection is a complicated process involving the molecular interaction between viruses and the hosts. Plants utilize multiple defense strategies against viruses, such as the inducement of innate pathogen-associated molecular pattern (PAMP)-triggered immunity (PTI) through immune receptor signaling to inhibit virus infection [2]. Furthermore, plants employ resistance proteins containing a nucleotide-binding leucine-rich repeat (NB-LRR) domain, which recognize viral proteins and subsequently activate effector-triggered immunity (ETI) and a hypersensitive response (HR) to restrict viruses in local necrotic spots from systemic infection [3]. In addition, the plant antiviral defense response is often accompanied by significant changes in phytohormone expression [4]. Studies also indicate that the induction of RNA silencing also significantly contribute to resistance in plants [5]. The tobacco mosaic virus (TMV) is one of the most serious plant viruses infecting multiple host species, which cause diseases in a wide variety of crops [6,7]. TMV belongs to the positive-strand RNA virus and encodes a 126 kDa and a 183 kDa protein that function as replicases, a 30 kDa movement protein (MP) that facilitates the movement of viruses between host cells, and a 17.5 kDa coat protein (CP) that plays an important role in virions formation [7].

In modern agriculture, green prevention strategies or agents for virus control are effective, environmentally friendly, but still under development. Plant virus inhibitors from metabolites of microbes have been considered as a potential alternative for chemical pesticides [8,9]. *Streptomyces* are major members of actinomycetes that have been reported to produce more than 30 secondary metabolites [10]. Ningnanmycin (NNM) is a microbial pesticide isolated from fermentation broth of *Streptomyces noursei* var. *xichangensis* and is characterized by resistance enhancement, high efficiency, and low toxicity in host plants. Studies have shown that NNM promotes the expression of pathogenesis-related proteins, PAL, POD, and SOD activity, enhances salicylic acid (SA) biosynthesis, and induces systemic resistance in the host plants [11,12,13]. Furthermore, NNM has been shown to inhibit the polymerization process of TMV-CP in vitro [14]. However, the precise antiviral mode of action, especially the host signaling pathway and defense responsive genes involved in the resistance against TMV induced by NNM, remains to be further elucidated. The high-throughput sequencing techniques (such as Illumina RNA-seq) provide a powerful tool to clarify the transcriptomic variations of the host plants in response to biological antiviral agents [15,16].

In this study, we show that NNM effectively suppresses the systemic infection of TMV in planta and significantly inhibits the viral RNA accumulation in BY-2 protoplasts. The results of transcriptome analysis and RT-qPCR indicate that various defense-responsive, immune signaling, calcium signaling, and phytohormone responsive genes are significantly up- or down-regulated by NNM treatment. This study provides a novel insight into the identification of crucial genes and pathways involved in resistance against virus infection in response to biological agents.

## 2. Materials and Methods

### 2.1. NNM Treatment on N. benthamiana and BY-2 Protoplasts Inoculated with TMV

**Figure 1:**
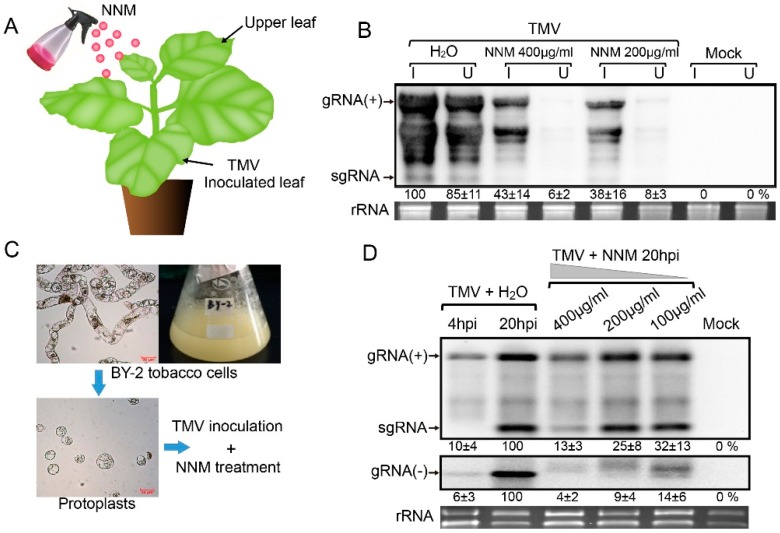
Effects of Ningnanmycin (NNM) treatment on tobacco mosaic virus (TMV)-inoculated *Nicotiana benthamiana* and BY-2 protoplasts. (**A**) Schematic representation of TMV-inoculated leaves (I) and the un-inoculated upper leaves (U) of *N. benthamiana* with the treatment of distilled H_2_O, 100 μg/mL and 50 μg/mL NNM at 7 dpi. (**B**) Northern blot analysis of the accumulation of TMV RNA from the inoculated leaves and upper leaves and relative accumulation of genomic RNA (gRNA) are shown. The ribosomal RNA (rRNA) is shown below the northern blots as a loading control. (**C**) Schematic representation of NNM treatment on TMV-inoculated BY-2 protoplasts. (**D**) RNA accumulations of TMV in BY-2 protoplasts under various concentrations of NNM treatment and relative accumulation of positive- and negative-strand gRNA are shown.

The agent 8% NNM was purchased from Deqiang Biological Co. Ltd. (Haerbin, China). To prepare the virus inoculum, the plant virus of TMV Shenyang isolate was collected from the tobacco planted area in the Shenyang, Liaoning province of northwest China. The complete nucleotide sequence of TMV-SY was determined (no. MG516107) and used for the construction of an infectious clone of pCB-TMV-SY through homologuous recombination techniques [17]. TMV virions were extracted and purified from the pCB-TMV-SY-inoculated *N. benthamiana* leaves following Gooding’s method [18], and adjusted to a concentration of 40 μg/mL with 10 mM PBS buffer. *N. benthamiana* plants at a leaf stage of six to seven, cultivated at the 25 °C climate-control chamber were used for NNM treatment and virus inoculations. The 400 μg/mL and 200 μg/mL concentration of NNM diluted by distilled water was sprayed onto *N. benthamiana* and infiltrated with *Agrobacteriums* (OD_600_ = 0.2) containing pCB-TMV-SY and an empty vector. Moreover, plants with H_2_O treatment were inoculated by TMV, while healthy plants without NNM treatment and TMV inoculation were served as the mock. Three plants were used in each of the treatments (i.e., two concentrations of NNM treatment, H_2_O treatment, and the mock) as biological replicates, and three individual leaves of each plant were sampled and pooled for further study. RNA extracted from the TMV-inoculated (I) leaves and the upper (U) un-infiltrated leaves (Figure 1A) were subjected to a northern blot to detect viral RNA accumulation after 7 days of incubation. The experiments were performed with three biological replicates for statistical analysis (*p* < 0.05). In the BY-2 protoplasts experiment, BY-2 suspension cells were incubated with cell wall hydrolase buffer (cellulose RS 10 mg/mL and Pectolyase Y23 1 mg/mL in 0.4 M Mannitol buffer) at 25 °C for 3 h (Figure 1C). Virus inoculation in the protoplasts was performed using polyethylene glycol (PEG) solution (PEG_6000_ 4g, 0.5 mL H_2_O, 5 mL 0.8M mannitol, 1 mL 1 M Ca(NO_3_)_2_). BY-2 protoplasts inoculated by 0.1 mg of TMV virions were incubated with 400 μg/mL, 200 μg/mL, and 100 μg/mL NNM diluted in W1 buffer (0.5 M Mannitol,4 mM MES, and 20 mM KCl). The BY-2 protoplasts experiment were carried out with three biological replicates for statistical analysis (*p* < 0.05).

### 2.2. Northern Blot Analysis

Total RNA was extracted from NNM and mock treatment *N. benthamiana* leaves or BY-2 protoplasts inoculated by TMV with TRIzon Reagent (CWBIO, Beijing, China) according to the manufacturer’s protocol. The extracted RNA of each treatment group was adjusted to 10 μg and subjected to northern blot analysis as described [19] using a DIG High Prime DNA Labeling and Detection Starter Kit II (Roche, Mannheim, Germany). The digoxigenin-labelled RNA probes for TMV positive- and negative-strand RNA have been previously described [20]. The RNA signals were detected with a chemical luminous imaging system, Tanon 5200 (Tanon, Shanghai, China).

### 2.3. RNA Extraction, cDNA Library Construction, and Illumina Sequencing

To clarify the crucial differentially expressed genes (DEGs) induced by NNM in the TMV-infected plant cells, total RNA was extracted from NNM and mock treatment BY-2 protoplasts at 20 hpi with a TRIzon Reagent (CWBIO, Beijing, China) according to the manufacturer’s protocol. Equal quantities of 10 RNA samples per treatment group were mixed together for the RNA pool. The mRNA was purified by oligo-dT beads (Qiagen, Hilden, Germany) and fragmented into approximately 300 nt pieces. The reverse-transcribed cDNA fragments were purified and washed for end repair and ligated to sequencing adapters. The final cDNA library was constructed by PCR enrichment and sequenced through Hiseq X ten Illumina sequencing platforms by GENEWIZ Technology Co. Ltd. (Suzhou, China). The raw reads were submitted to the SRA database at NCBI with accession numbers SRR8690256 and SRR8568069. The read counts for each matched gene were normalized with RPKM (reads per kilo bases per million reads) to calculate its expression level. Significant differentially expressed genes (DEGs) between the NNM treatment and control treatment groups were shown in a volcano plot based on the significance false discovery rate (FDR) <0.05 and log2 fold change of >1.0 or <−1.0. In addition, all of the DEGs were subjected to the Gene Ontology (GO) analysis and mapped to the Kyoto Encyclopedia of Genes and Genomes (KEGG) pathway to clarify the main biological functions and pathways.

### 2.4. Real-Time Quantitative PCR

To validate the transcriptome results, RNA extracted from NNM and control treatment BY-2 protoplasts at 20 hpi was subjected to reverse transcription to generate cDNA using a FastKing RT Kit (TIANGEN, Beijing, China). RT-qPCR was performed using SYBR Premix Ex Taq II (TaKaRa, Dalian, China) according to the manufacturer’s protocol, and the conditions were as follows: 5 min at 95 °C, followed by 40 cycles at 95 °C for 30 s, 55 °C for 30 s, and 72 °C for 40 s. The relative gene expressions were analyzed by ABI StepOne Plus (Applied biosystems, Foster City, CA, USA) and calculated by the 2^–ΔΔCT^ method using actin as a reference gene with three independent biological replicates. The primers used in RT-qPCR are listed in Table S1.

## 3. Results

### 3.1. NNM Treatment-Inhibited TMV Systemic Infection in Planta and Viral Accumulation in Protoplasts

*N. benthamiana* plants were used to verify the anti-viral effect of NNM on the RNA accumulation and systemic infection of TMV in planta (Figure 1A). The results showed that 400 μg/mL and 200 μg/mL NNM treatment on TMV = inoculated (I) leaves exhibited delays in discoloration or mosaic symptoms. In addition, the upper (U) un-inoculated leaves of the NNM-treated plants did not display any viral disease symptoms at 7 days post-inoculation (dpi) [21]. Furthermore, NNM effectively and reproducibly reduced accumulation of viral RNA in the inoculated and upper leaves of *N. benthamiana* in the biological replicates at 7 dpi (Figure 1B), which indicated that NNM treatment suppressed and delayed the systemic infection of TMV.

Efficient long-distance movement and systemic infection of plant viruses require rapid replication of the viral genome in single cells. Therefore, tobacco BY-2 cells were used to test the effect of NNM on TMV viral accumulation in single plant protoplasts (Figure 1C). The results demonstrated that the accumulation of genome RNA, especially the negative strand RNA of TMV in BY-2 protoplasts at 20 hpi, was progressively inhibited with increased concentration of NNM from 100 to 400 μg/mL (Figure 1D). When the concentration of NNM was lower than 50 μg/mL, the antiviral effect of NNM on TMV in BY-2 protoplasts was extremely low [21]. To exclude the possibility that NNM may affect Agrobacterium infection in the plants, we treated *Nicotiana benthamiana* with 200 μg/mL, 400 μg/mL NNM, and distilled water 1 day after transient expression of green fluorescent protein (GFP) through agro-infiltration using pGD-GFP, and observed the GFP fluorescence 1 to 2 days after the agent treatment. The repeated results indicated that NNM treatment did not affect the GFP expression [21], which suggested that the agent has no obvious impact on Agrobacterium infection or gene expression. Taken together, the results indicate that NNM inhibits the systemic infection of TMV in planta and the viral accumulation in the single cells.

### 3.2. Identification of the Genes in Response to NNM by RNA-Seq

**Figure 2:**
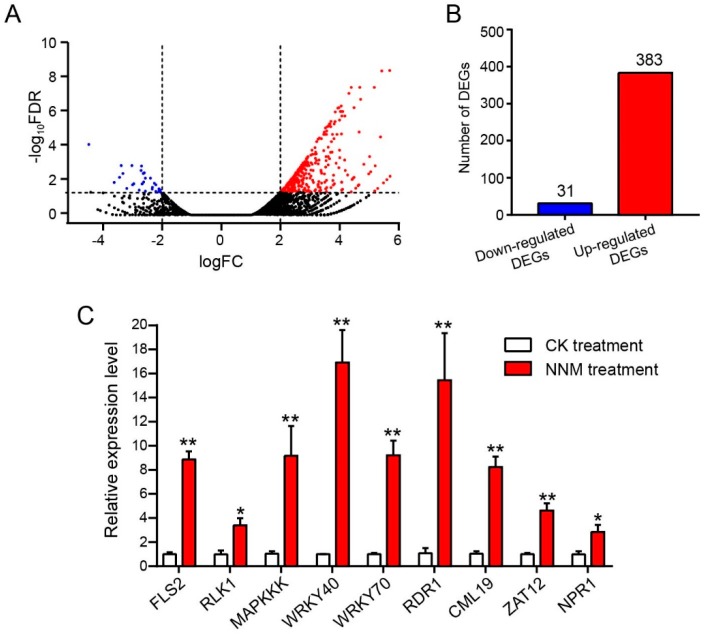
NNM-induced differential gene expression in BY-2 protoplasts. (**A**) Numbers of up-regulated genes (in red) and down-regulated differentially expressed genes (in blue) were shown (false discovery rate (FDR) < 0.05 and ≥ 2-fold change). Black dots indicate non-differentially expressed genes (FDR ≥ 0.05). (**B**) Differentially expressed genes (DEGs)—383 up-regulated, and 31 down-regulated—are listed. (**C**) RT-qPCR verification on regulation of nine DEGs induced by NNM. An equal volume of sterile water treatment was used as a control. * indicate a significant difference (*p* < 0.05) and ** indicate a significant difference (*p* < 0.01).

**Figure 3:**
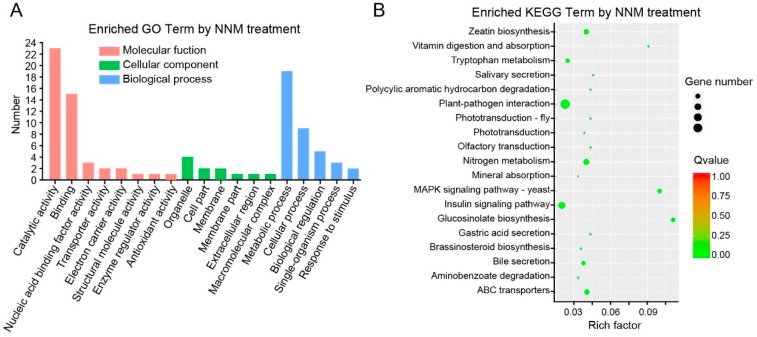
DEGs enriched in Gene Ontology (GO) and Kyoto Encyclopedia of Genes and Genomes (KEGG) analysis induced by NNM. (**A**) GO terms of three categories significantly enriched in DEGs of NNM vs. CK involved in molecular function, cellular component, and biological process. (**B**) KEGG pathways of the significantly enriched DEGs. The rich factor reflects the degree of enriched DEGs in a given pathway. The number of enriched DEGs in the pathway is indicated by the circle area, and the circle color represents the ranges of the corrected *p*-value.

To define the mode of action for NNM leading to inhibition of TMV RNA accumulation, we used transcriptome analysis to clarify crucial genes in TMV-inoculated tobacco BY-2 protoplasts in response to NNM. There were 69,374,590 and 61,272,488 clean reads from NNM treatment and control libraries (Table 1). The total DEGs in response to NNM were shown in Table S2, and annotation of the DEGs in KEGG pathways were shown in Table S3. The volcano plot analysis indicated the significantly up- and down-regulated DEGs between the NNM and control treatment group (Figure 2A). There were 414 genes differentially expressed (FDR < 0.05 and ≥2-fold change) between the NNM treatment and control treatment samples, including 383 up-regulated and 31 down-regulated DEGs (Figure 2B) (Table S4). These crucial DEGs were classified into various functions, including catalytic activity, binding, as well as metabolic and cellular processing by GO analysis (Figure 3A). In addition, a total of 46 DEGs were annotated to 19 different KEGG pathways (Table S5). The results indicated that plant–pathogen interaction is the most enriched category that shows significantly differential expression in KEGG analysis (Figure 3B), especially in the pathway of PAMP-triggered immunity (Figure S1) and MAPK signaling (Figure S2).

### 3.3. Plant Immunity, Metal Ion, and Phytohormone Signaling Response Induced by NNM

In this study, results of RNA-seq indicated that NNM treatment can induce plant immunity through regulating gene expression, such as receptor-like kinases (*RLKs*), *PRRs,* and other genes involved in the defense response and host–pathogen interaction (Table 2). The metal ion, especially Ca^2+^, is also considered to be a critical component of immune-signaling pathways [22]. Here, our results indicated that many lines of genes involved in calcium signaling were significantly up-regulated by NNM, such as the calmodulin-like protein 19 (*CML19*), *CML41*, *CML45,* and various genes involved in the binding, uptake, and transportation of calcium (Table 3). Plant virus infections and host resistance responses were frequently accompanied by phytohormone alterations [3,23]. In particular, phytohormone-responsive genes, such as the WRKY transcription factors *WRKY40* and *WRKY70*, as well as ethylene, auxin, and abscisic acid (ABA)-responsive genes and/or transcription factors were differentially regulated by NNM (Table 4).

### 3.4. Verification of DEGs by Real-Time Quantitative PCR

In order to reveal the regulation of NNM treatment on host gene expression and its antiviral effect, nine crucial DEGs corresponding with resistance, immunity signaling, calcium signaling, and phytohormone response were selected for RT-qPCR verification. The selected genes for comparison included the receptor-like kinase *FLS2* (LOC107827601), S-receptor-like serine/threonine-protein kinase *RLK1* (LOC107816125), mitogen-activated YODA-like protein kinase kinase kinase (*MAPKKK*, LOC107821703), *WRKY40* (LOC107792337), *WRKY70* (LOC107782765), RNA-dependent 1-like RNA polymerase (*RDR1*, LOC107827981), putative calcium-binding protein *CML19* (LOC107808229), putative zinc finger protein *ZAT12* (LOC107806768), and regulatory protein non-expressor pathogenesis-related genes 1 (*NPR1*) (LOC107831756). The results of RT-qPCR showed that the nine DEGs which were selected were significantly up-regulated by NNM, which were consistent with the results of RNA-seq (Figure 2C).

## 4. Discussion

**Figure 4:**
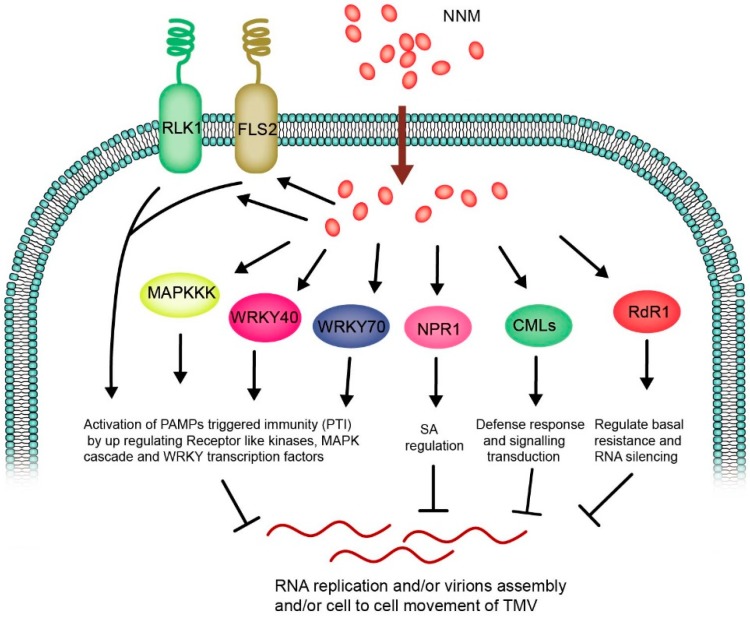
A model for crucial genes regulation involved in anti-TMV mechanisms by treatment of NNM.

Plant viruses are an obligate parasite, which cannot replicate their genomes or move from cell to cell without the assistance of host factors. therefore, virus–host interactions played crucial roles in the multiplication and systemic infection of various plant viruses [65]. The effective plant antiviral agents can inhibit viruses by directly targeting viral nucleic acids or proteins, or indirectly control viruses by regulating the host responses, such as phytohormone expression and various signaling pathways to affect the balance of the virus–host interaction [3]. NNM plays multiple functions and roles by the direct impact on viruses and the induction of the host defense, respectively. For example, a study indicated that NNM can interact with TMV-CP and inhibit the polymerization of TMV virions [14]. On the other hand, a comparative analysis of RNA-seq of tobacco leaves under NNM-treated and non-treated ones was performed, in which the defense-related genes were induced by NNM and identified to contribute the plant response to CMV infection [13]. Therefore, it is possible that NNM has potential roles by regulating host defense-regulated responses and directly affecting the TMV assembly. In the present study, we used the RNA-seq and RT-qPCR to elucidate more crucial genes associated with host-resistance responses by NNM regulation against TMV. A model of the possible induced effects and regulation of the crucial DEGs and signaling pathways involved in TMV resistance are shown in Figure 4.

The tobacco BY-2 suspension cultured cell has been well-applied in the subject of plant physiology and pathology. The BY-2 protoplast is proved to be an effective system for agents screening and clarification of their mode of action. For example, BY-2 cells were treated with microbial-derived metabolite from *Cladosporium herbarum*, which results in significant up-regulation of host defense-related genes [66]. In this study, our results showed that NNM treatment effectively inhibited viral RNA accumulation of TMV and significantly regulated many lines of potential virus resistance genes in the tobacco BY-2 protoplasts.

Application of biological agents is a very effective and environmentally safe strategy to control viruses by inducing host defense responses [67]. PTI is an important defense response induced by perception of viral PAMPs by pattern recognition receptors (PRRs) [2]. FLAGELLIN-SENSING2 (FLS2) and RLK1 are well-conserved PRRs, being critical for the perception of conserved microbe-associated molecular patterns (MAMPs) to activate PTI in different plant species [37,38]. After recognition of invading pathogens by PRRs, mitogen-activated protein kinases (MAPKs) cascades are rapidly activated, and the transduction signal can eventually activate various WRKY transcription factors in plants [2]. Notably, many studies have shown that WRKY transcription factors from various plant species are induced in response to viral infection [68,69]. In this study, our results showed that NMM induced a significant up-regulation of *FLS2*, *RLK1*, *MAPKKK*, *WRKY40,* and *WRKY70*, which indicated that NNM can effectively inhibit TMV, possibly through positively regulating the PTI pathway. Induction of ZAT12 is indicated to be closely related with the oxidative stress response, and serves as an abiotic stress marker [43]. Our previous work has shown that the biological agent of cytosinpeptidemycin significantly induced up-regulation of *ZAT12* [20]. In this work, gene expression of *ZAT12* was significantly up-regulated under NNM treatment. However, the potential functions of ZAT12 involved in the anti-virus mechanism still remains to be clarified. In addition, various receptor-like kinases, as well as defense- and stress-responsive genes (Table 2) were differentially regulated by NNM, and their possible roles involved in virus resistance still need to be further investigated.

Calcium is an essential element needed for the growth and development of plants [70]. Ca^2+^ is a secondary messenger involved in diverse crucial signaling pathways in plants through signals of abiotic and biotic stimuli [71]. The calmodulin-like protein CML19 is possibly involved in drought response [59], and the CML46 and CML47 have been reported to negatively regulate SA [58]. Ca^2+^ signaling is also a critical component of plant immune response [71]. The perception of pathogen attacks induces an influx of Ca^2+^ into the cytosol, which is decoded into downstream responses ultimately leading to defense. For example, the Calmodulin-like protein CML41 localizes at plasmodesmata and induces plasmodesmal closure during bacterial immune responses [72]. In this study, our results showed that a variety of genes involved in calcium signaling were significantly induced by NNM (Table 3). These results indicate that calcium signaling can play vital roles in NNM-mediated resistance to TMV.

Plants can use elaborate strategies by inducing phytohormone-signaling networks to get through abiotic or biotic stresses, as well as inducing resistance response to pathogen infection [3,23]. Our results demonstrated that various phytohormone-responsive genes, including WRKY transcription factors, ethylene, auxin, and ABA-responsive genes or transcription factors were differentially regulated by NNM (Table 4). WRKY40 mainly functions as a central negative regulator of the expression of ABA-responsive genes [29]. In addition, WRKY40 was also reported to associate with a transcription factor BZR1 to mediate plant immune signaling [73]. WRKY70 is an important node of convergence between SA and JA signaling in Arabidopsis [74]. Overexpression of WRKY70 can enhance the expression of SA-responsive PR genes and negatively affects transcription of JA-responsive genes [27,74]. In addition, NNM induced up-regulation of *NPR1* (Figure 2C) that regulates SA and systemic acquired resistance (SAR), which is consistent with the results of a previous study [11]. Such results indicated that NNM induced biosynthesis of SA, that may contribute to virus resistance. Ethylene responsive factors (ERFs) play vital roles in plant pathogen defense responses. A typical transcription factor, ERF109 belongs to the stress responsive genes, and was reported to improve salt tolerance and delay PCD in plants [63]. In the present study, ERF109 was significantly up-regulated by NNM, which suggests the possible involvement of ERF109 in virus defense.

RNA silencing (or RNAi) is an effective defense mechanism against viruses, with remarkable specificity and adaptability [75,76]. Host proteins, such as the DICER-LIKE proteins (DCLs), Argonaute (AGO) proteins, and RNA-dependent RNA polymerase (RDR) proteins, are core components of plant RNA silencing pathways against plant viruses [75,76]. Studies have indicated that the RdR1 induces the basal defense response [31,32], and also regulates RNA silencing-related gene expression to suppress the replication and movement of TMV [32]. In addition, the tobacco calmodulin-like protein was reported to promote RNA silencing by binding to and directing degradation of the virus RNA silencing suppressors [77,78]. Taken together, the remarkable up-regulation of RdR1 and CMLs induced by NNM possibly promoted RNA silencing to suppress virus infection, which provides a new perspective for the development of novel antiviral agents.

In summary, the effect and mode of actions of NNM on TMV through induction of host resistance were explored. We identified various crucial genes involved in host stress responses, signaling transduction, and various phytohormone responses by NNM treatment (Figure 4). We further hypothesized that NNM possibly regulates RNA silencing in controlling the virus. This work provided a novel insight into the antiviral mode of action for the biological agent of NNM, and also provided a theoretical basis for the development of highly specific and effective antiviral agents.

## Figures and Tables

**Table 1:** Read numbers aligned onto the *Nicotiana tabacum* reference genome by Illumina sequencing.

Sample Name	CK	NNM
Raw reads	62,234,835	70,288,896
Clean reads	61,272,488	69,374,590
Total mapped	55,575,990	63,343,398
Total mapped	90.70%	91.31%
Uniquely mapped	51,425,125	58,315,517
Uniquely mapped	83.93%	84.06%
Q30	90.26%	89.19%
GC contents	42.93%	42.79%

**Table 2:** Defense signaling responsive genes induced by NNM.

Gene Symbol	Gene Description	Regulate	logFC	*p*-value	Major Reported Functions	References
LOC107797745	kiwellin-like	Up	4.68729	3.03 × 10^−12^	fungi resistance	[24]
LOC107829465	protein NTM1-like 9	Up	4.00045	8.89 × 10^−10^	activate SA synthesis	[25]
LOC107808010	dehydration-responsive element-binding protein 1D-like	Up	3.84958	1.23 × 10^−9^	cadmium and salt stresses response	[26]
LOC107789568	probable WRKY transcription factor 70	Up	3.66519	1.76 × 10^−9^	regulate immune response	[27]
LOC107802518	probable WRKY transcription factor 50	Up	2.57431	4.13 × 10^−5^	activate PR1	[28]
LOC107792337	probable WRKY transcription factor 40	Up	3.67502	1.92 × 10^−9^	regulate plant immunity, negatively regulate ABA	[29,30]
LOC107765311	RNA-dependent RNA polymerase 1-like	Up	4.06945	1.01 × 10^−8^	basal resistance against TMV	[31,32]
LOC107788900	probable RNA-dependent RNA polymerase 1	Up	2.91618	1.98 × 10^−6^	virus resistance, antiviral RNA silencing	[32,33]
LOC107807916	probable WRKY transcription factor 51	Up	3.51474	1.25 × 10^−8^	defense response	[34]
LOC107790605	aspartic proteinase CDR1-like	Up	5.38706	1.77 × 10^−8^	defense response	[35]
LOC107821703	mitogen-activated protein kinase kinase kinase YODA-like	Up	3.05664	1.59 × 10^−7^	MAPK pathway	[36]
LOC107807021	G-type lectin S-receptor-like serine/threonine-protein kinase RLK1	Up	4.01213	5.03 × 10^−7^	receptor-like protein kinase, PRR in PTI pathway	[37]
LOC107827601	LRR receptor-like serine/threonine-protein kinase FLS2	Up	2.91334	5.76 × 10^−7^	PRR in PTI pathway	[38]
LOC107825406	glucan endo-1,3-beta-glucosidase, acidic	Up	2.87528	6.30 × 10^−7^	defense against pathogen infect, belongs to PR2?	[39,40]
LOC107795723	protein HYPER-SENSITIVITY-RELATED 4-like	Up	3.07585	1.73 × 10^−6^	unknown function	
LOC107798618	basic form of pathogenesis-related protein 1-like	Down	-3.0255	2.25 × 10^−6^	response to pathogen infection?	[41]
LOC107791385	bidirectional sugar transporter SWEET12-like	Up	3.53641	2.93 × 10^−6^	Induced by pathogen	[42]
LOC107771327	receptor-like protein 12	Up	3.11029	5.47 × 10^−6^	Unknown function	
LOC107791128	zinc finger protein ZAT12-like	Up	2.68871	6.34 × 10^−6^	Stress response	[43]
LOC107789836	ribonuclease 3-like protein 3	Up	3.39531	9.08 × 10^−6^	antivital immunity?	[44]
LOC107831090	mitogen-activated protein kinase kinase kinase 2-like	Up	2.62214	1.26 × 10^−5^	Unknown function	
LOC107797667	defensin-like protein 19	Up	2.42846	1.84 × 10^−5^	Unknown function, antibiotics?	[45]
LOC107789688	eukaryotic initiation factor 4A-9-like	Up	2.48755	1.85 × 10^−5^	involved in virus resistance?	[46]
LOC107818786	protein ENHANCED DISEASE RESISTANCE 2-like	Up	2.79094	2.13 × 10^−5^	Unknown function	
LOC107831360	CBL-interacting serine/threonine-protein kinase 25-like	Down	−2.3955	2.24 × 10^−5^	Unknown function	
LOC107765573	disease resistance-like protein CSA1	Up	5.60637	2.86 × 10^−5^	Unknown function, disease resistance	
LOC107828757	receptor protein kinase CLAVATA1-like	Up	2.37093	3.15 × 10^−5^	disease resistance	[47]
LOC107768384	pathogenesis-related leaf protein 4-like	Down	−2.6253	4.41 × 10^−5^	Unknown function	
LOC107763443	leucine-rich repeat receptor protein kinase EMS1-like	Up	3.28444	7.18 × 10^−5^	Cell differentiation	[48]
LOC107772607	thaumatin-like protein 1b	Up	2.20055	0.000118	biotic and abiotic stress response	
LOC107785865	pleiotropic drug resistance protein 1-like	Up	2.15959	0.000125	Resistance to pathogens?	[49]
LOC107765095	E3 ubiquitin-protein ligase ATL6-like	Up	2.10871	0.000158	defense response	[50]
LOC107800503	mitogen-activated protein kinase kinase 5-like	Up	2.10615	0.000159	salinity stress response	[51]
LOC107775435	probable LRR receptor-like serine/threonine-protein kinase At1g67720	Up	2.09775	0.000213	Unknown function	
LOC107779438	G-type lectin S-receptor-like serine/threonine-protein kinase At5g35370	Down	−2.0631	0.000218	Unknown function	
LOC107831947	probable L-type lectin-domain containing receptor kinase S.5	Up	2.91973	0.000249	Unknown function	
LOC107822671	protein NtpR-like	Down	−2.5692	0.000273	Enhance plant resistance?	[52]
LOC107828940	G-type lectin S-receptor-like serine/threonine-protein kinase At4g27290	Up	2.02879	0.00029	Unknown function, RLKs	
LOC107771655	G-type lectin S-receptor-like serine/threonine-protein kinase CES101	Up	2.01366	0.000299	Unknown function, RLKs	
LOC107786338	disease resistance protein TAO1-like	Up	1.98511	0.000372	TIR-NB-LRR protein	[53]

**Table 3:** Metal signaling responsive genes induced by NNM.

Gene Symbol	Gene Description	Regulate	logFC	*p*-value	Major Reported Functions	References
LOC107829449	blue copper protein-like	Up	3.3206	2.13 × 10^−8^	Metal uptake	[54]
LOC107771638	metal tolerance protein 9-like	Up	2.60188	5.76 × 10^−6^	Unknown function	
LOC107789039	heavy metal-associated isoprenylated plant protein 20-like	Up	2.90802	5.67 × 10^−7^	metal homeostasis, plant pathogen interaction	[55]
LOC107760691	metal transporter Nramp5-like	Up	4.71724	2.13 × 10^−11^	regulate cadmium uptake	[56]
LOC107832175	calcium-transporting ATPase 12, plasma membrane-type-like	Up	3.85928	2.50 × 10^−10^	calcium-transporting, involved in plant immunity	[57]
LOC107774700	probable calcium-binding protein CML45	Up	3.17773	2.67 × 10^−7^	Unknown function	
LOC107802864	calmodulin-binding protein 60 A-like	Up	5.02141	4.02 × 10^−7^	calmodulin binding, Regulate plant immunity?	[58]
LOC107782005	putative calcium-transporting ATPase 13, plasma membrane-type	Up	2.73531	7.72 × 10^−6^	calcium-transporting, involved in plant immunity	[57]
LOC107819435	probable calcium-binding protein CML44	Up	2.29199	4.93 × 10^−5^	Unknown function	
LOC107808229	putative calcium-binding protein CML19	Up	2.69525	9.43 × 10^−5^	Drought stress response	[59]
LOC107794908	calcium-binding protein PBP1-like	Up	2.08773	0.000209	Unknown function	
LOC107762012	calcium uniporter protein 2, mitochondrial-like	Up	3.16227	0.000242	Unknown function	
LOC107792571	calmodulin-like	Up	2.38374	0.000126	Regulate plant immunity	[58]
LOC107806239	calmodulin-binding protein 60 D-like	Up	2.03208	0.000273	Unknown function	
LOC107780788	cyclic nucleotide-gated ion channel 1-like	Up	2.46217	1.79 × 10^−5^	ion uptake	
LOC107817341	probable magnesium transporter NIPA8	Up	2.16689	0.00017	Unknown function	

**Table 4:** Phytohormone responsive genes induced by NNM.

Gene Symbol	Gene Description	Regulate	logFC	*p*-value	Major Reported Functions	References
LOC107764158	DELLA protein GAI-like	Up	3.82241	1.85 × 10^−9^	repressors of GA signal pathway, virus defense?	[60]
LOC107802518	probable WRKY transcription factor 50	Up	2.57431	4.13 × 10^−5^	Up-regulate SA	[28]
LOC107792337	probable WRKY transcription factor 40	Up	3.67502	1.92 × 10^−9^	Negatively regulate ABA	[29]
LOC107802866	transcription factor MYB1R1-like	Up	2.91012	2.45 × 10^−6^	enhancement of ripening	[61]
LOC107829401	probable NAD(P)H dehydrogenase (quinone) FQR1-like 1	Up	2.89138	6.41 × 10^−7^	Auxin response	[62]
LOC107820920	ethylene-responsive transcription factor ERF022-like	Up	5.49089	5.49 × 10^−5^	unknown function	
LOC107785865	pleiotropic drug resistance protein 1-like	Up	2.15959	0.000125	Plant hormone transportation	[49]
LOC107806172	ethylene-responsive transcription factor ERF109-like	Up	2.88682	7.04 × 10^−5^	retards PCD and improves salt tolerance	[63]
LOC107801499	transcription factor LHW-like	Up	4.54979	0.000142	regulate Auxin	[64]
LOC107763786	cytokinin hydroxylase-like	Up	2.406	0.000246	unknown function	
LOC107803728	ARF guanine-nucleotide exchange factor GNL2-like	Up	3.04343	0.000321	unknown function	
LOC107797939	ethylene-responsive transcription factor 1B-like	Up	2.02845	0.000338	unknown function	
LOC107766165	ethylene-responsive transcription factor 14-like	Up	2.70111	0.000367	unknown function	

## Supplementary Materials

The following are available online at https://www.mdpi.com/1999-4915/11/9/815/s1, Figure S1: NNM induced KEGG pathway involved in plant-pathogen interaction, Figure S2: NNM induced KEGG pathway involved in MAPK signaling pathway, Table S1: Nucleic acid sequence of oligonucleotide primers used in RT-qPCR, Table S2: Total Genes of NNM-responsive tobacco BY-2 transcriptome, Table S3: Total Annotation of KEGGs of tobacco BY-2 transcriptome, Table S4: Annotation of DEGs of CytPM-responsive tobacco BY-2 transcriptome, Table S5: List of DEGs involved in the KEGG pathways.
