# Supplementary material for: Molecular Regulation of Host Defense Responses Mediated by Biological Anti-TMV Agent Ningnanmycin
Source: Viruses. 2019 Sep 3;11(9):815. doi: 10.3390/v11090815 (PMC6784071; doi:10.3390/v11090815)
Supplement: Supplementary file 1 [file viruses-11-00815-s001.zip › Supplementary file/Supplementary Table S1.pdf.pdf]

Table S1. Nucleic acid sequence of oligonucleotide primers used in RT-qPCR.

Gene name	NCBI Number(Gene symbol)	Primer name and sequence	
Actin	LOC107795948(XM_016618658)	Actin +	CAAGGAAATCACCGCTTTGG
		Actin -	AAGGGATGCGAGGATGGA
FLS2	LOC107827601 (XM_016654767)	FLS2 +	TGGGTGACTCATCTTGTTTC
		FLS2-	ACTGCTGATATTTGCTAGCC
RLK1	LOC107816125 (XM_016641811)	RLK1+	GTATTTGGCACCAGAGTTGC
		RLK1-	CACATTCTCATAGAAGGGC
MAPKKK	LOC107795061 (XM_016617629)	MAPKKK+	AGTTCAAAGTCTACCTCGCC
		MAPKKK-	AGCATAGACATGCCCAATG
WRKY40	LOC107792337(NM_001325540)	WRKY40+	TAGCTACATATGAGGGGGAG
		WRKY40-	AAAATTCCCGGAGATGGCTG
WRKY70	LOC107782765 (XM_016603703)	WRKY70+	TTGATCAAGGATGCGAAGC
		WRKY70-	CAAATCAACAGTTCCCTCC
RDR1	LOC107827981 (XM_016655228)	RDR1+	GACAGAGAACCTGATATGGC
		RDR1-	TGATTTCCTGGCAACATCCC
CML19	LOC107808229(XM_016632733)	CML19+	GCATGGAGACTACTTCTAGC
		CML19-	TTTCCATCTCCGTCCTCGTC
ZAT12	LOC107806768(XM_016630999)	ZAT12+	TATGGAGTTCTCTTTGGGTC
		ZAT12-	AATCGGAGATGAAACGGGTG
NPR1	LOC107831756(NM_001326267)	NPR1+	CAAGTTTCAGAGACACCTAC
		NPR1-	GCCCTATGTATCCTCTTAAC
