# Supplementary material for: Molecular Regulation of Host Defense Responses Mediated by Biological Anti-TMV Agent Ningnanmycin
Source: Viruses. 2019 Sep 3;11(9):815. doi: 10.3390/v11090815 (PMC6784071; doi:10.3390/v11090815)
Supplement: Supplementary file 1 [file viruses-11-00815-s001.zip › Supplementary file/Supplementary Table S2.pdf.pdf]

Table S2. Total Genes of NNM-responsive tobacco BY-2 transcriptome.

Gene ID	baseMeanA	baseMeanB	Fold Change (FC)	log2FC	*P* -value	FDR
gene35747	7.231978776	375.5541995	51.92966008	5.698486877	6.36E-14	3.53E-09
gene27870	9.298258426	400.7202026	43.09626429	5.429490913	1.32E-13	3.68E-09
gene30562	9.298258426	336.8372717	36.22584535	5.178947451	2.55E-12	3.37E-08
gene26522	44.42501248	938.8854988	21.13416399	4.401505139	2.58E-12	3.37E-08
gene6013	18.59651685	479.1219813	25.76407104	4.68728867	3.03E-12	3.37E-08
gene60199	35.12675405	703.6801625	20.03259856	4.324277669	8.24E-12	7.63E-08
gene26669	13.43081773	353.291966	26.30457603	4.717241892	2.13E-11	1.69E-07
gene46157	43.39187265	725.942396	16.72991626	4.064358319	5.91E-11	4.10E-07
gene73356	15.49709738	349.4202733	22.54746581	4.494893388	8.61E-11	5.22E-07
gene65284	50.62385143	801.4404052	15.83128076	3.98470607	9.39E-11	5.22E-07
gene41266	74.38606741	1145.053139	15.39338184	3.944238314	1.11E-10	5.61E-07
gene7862	137.4075967	2246.549735	16.3495308	4.031177328	2.12E-10	8.80E-07
gene9211	27.89477528	471.3785958	16.89845468	4.078819417	2.21E-10	8.80E-07
gene57923	25.82849563	447.1805159	17.31345574	4.113821808	2.22E-10	8.80E-07
gene2435	56.82269038	824.6705618	14.51305027	3.859278863	2.50E-10	9.27E-07
gene25977	23.76221598	402.656049	16.94522301	4.082806719	4.74E-10	1.62E-06
gene15622	19.62965668	357.1636588	18.19510472	4.185478449	4.96E-10	1.62E-06
gene22012	24.7953558	396.8485098	16.00495322	4.000446555	8.89E-10	2.74E-06
gene55266	50.62385143	663.9953115	13.11625435	3.713283879	1.14E-09	3.32E-06
gene6932	32.02733458	461.6993638	14.41579107	3.849578102	1.23E-09	3.42E-06
gene52615	126.0430587	1599.009118	12.68621322	3.665189591	1.76E-09	4.63E-06
gene29775	30.99419475	438.4692072	14.14681719	3.822405601	1.85E-09	4.63E-06
gene55108	135.3413171	1728.710826	12.77297179	3.67502232	1.92E-09	4.63E-06
gene66576	135.3413171	1671.603357	12.35102032	3.626558323	2.66E-09	6.15E-06
gene62238	36.15989388	473.3144422	13.08948648	3.710336595	3.03E-09	6.74E-06
gene34157	129.1424781	1553.516727	12.02947899	3.588502254	3.16E-09	6.74E-06
gene44150	111.5791011	1287.337849	11.53744596	3.528251985	3.95E-09	8.13E-06
gene55356	163.2360924	1935.846389	11.85918115	3.567932493	6.06E-09	1.20E-05
gene38266	7.231978776	183.905407	25.42947272	4.668429643	7.43E-09	1.39E-05
gene11213	141.540156	1588.361962	11.22198821	3.488256397	7.51E-09	1.39E-05
gene30821	336.803583	5654.607303	16.78903548	4.069447445	1.01E-08	1.81E-05
gene47369	163.2360924	1804.208835	11.05275683	3.466334353	1.17E-08	1.93E-05
gene37225	18.59651685	280.6977264	15.09410223	3.915913045	1.17E-08	1.93E-05
gene61808	23.76221598	323.286347	13.60505886	3.766071293	1.24E-08	1.93E-05
gene51723	36.15989388	427.822052	11.83139678	3.564548499	1.25E-08	1.93E-05
gene69152	41.325593	472.346519	11.42987879	3.514738199	1.25E-08	1.93E-05
gene53544	3.099419475	129.7017081	41.84709721	5.387055646	1.77E-08	2.66E-05
gene21997	126.0430587	1259.268076	9.990776879	3.320596866	2.13E-08	3.11E-05
gene62216	333.7041635	4695.395417	14.07053292	3.814605066	2.36E-08	3.35E-05
gene43463	180.7994694	1896.161538	10.48764991	3.390619527	2.41E-08	3.35E-05
gene56099	104.3471223	1012.447662	9.702688861	3.27838461	2.62E-08	3.55E-05
gene32294	106.413402	997.9288137	9.377848984	3.229257047	3.88E-08	5.09E-05
gene25640	347.1349812	4604.410637	13.26403528	3.729447844	3.94E-08	5.09E-05
gene40997	151.8715543	6.775462362	0.04461311	-4.48638847	5.93E-08	7.49E-05

gene69731	21.69593633	267.1468017	12.31321837	3.622135991	8.60E-08	0.000106
gene62237	65.08780898	585.5935328	8.99697719	3.169440365	9.47E-08	0.000114
gene8820	107.4465418	903.0723406	8.404852547	3.071222509	1.36E-07	0.000161
gene53443	11.36453808	181.9695606	16.01205077	4.00108619	1.41E-07	0.000163
gene55625	25.82849563	285.5373424	11.05512867	3.466643911	1.50E-07	0.00017
gene15013	130.175618	1083.106055	8.320345021	3.056643354	1.59E-07	0.000176
gene56617	15.49709738	208.1034868	13.42854612	3.747231211	1.95E-07	0.000209
gene70199	44.42501248	406.5277417	9.150875128	3.19390972	1.96E-07	0.000209
gene36021	16.5302372	214.8789492	12.99914494	3.700344823	2.03E-07	0.000213
gene23519	39.25931335	363.9391212	9.270134653	3.212590295	2.55E-07	0.000262
gene39271	42.35873283	383.2975851	9.048844464	3.177733572	2.67E-07	0.000269
gene65110	233.4896005	1995.857627	8.547950844	3.095578612	2.77E-07	0.000275
gene58626	52.69013108	450.0842855	8.542098421	3.094590521	2.90E-07	0.000283
gene14215	61.9883895	509.1276004	8.213273557	3.03795735	3.27E-07	0.000313
gene6464	3.099419475	100.6640122	32.4783441	5.021406174	4.02E-07	0.000378
gene65625	18.59651685	217.7827188	11.71094138	3.549785146	4.09E-07	0.000378
gene73767	101.2477029	772.4027093	7.628841816	2.931464049	4.24E-07	0.000381
gene12960	16.5302372	203.2638709	12.29648845	3.620174474	4.25E-07	0.000381
gene35571	148.7721348	1125.694675	7.56656935	2.919639337	4.76E-07	0.00042
gene6841	9.298258426	150.0280952	16.1350748	4.01212836	5.03E-07	0.000437
gene66632	162.2029525	1226.358688	7.560643431	2.918509017	5.13E-07	0.000438
gene52148	88.85002496	666.8990811	7.505896385	2.908024375	5.67E-07	0.000477
gene20340	175.6337703	1323.151007	7.533579704	2.913335548	5.76E-07	0.000477
gene29803	372.9634769	3397.410413	9.10923086	3.187329245	5.95E-07	0.000486
gene1828	116.7448002	856.6120273	7.337474779	2.875283639	6.30E-07	0.000507
gene21954	167.3686517	1241.845459	7.419821134	2.891384409	6.41E-07	0.000508
gene70231	14.46395755	182.9374838	12.64781669	3.660816459	6.55E-07	0.000512
gene28148	170.4680711	1255.396383	7.364407745	2.880569507	7.04E-07	0.000543
gene15937	24.7953558	245.8524914	9.915263705	3.309651143	7.90E-07	0.000601
gene20680	721.1315979	13007.91981	18.0382053	4.172983901	8.07E-07	0.000605
gene73687	440.1175655	4111.737731	9.342362253	3.223787387	8.65E-07	0.000641
gene45913	598.1879587	6987.437542	11.68100668	3.546092707	8.98E-07	0.000657
gene46761	96.08200373	680.4500058	7.081971435	2.824151025	1.04E-06	0.000752
gene53708	60.95524968	455.8918247	7.479123243	2.902869157	1.08E-06	0.000769
gene66626	210.7605243	1519.639416	7.210265872	2.850052458	1.12E-06	0.000785
gene55318	0	58.07539168	Inf	Inf	1.19E-06	0.00083
gene27213	50.62385143	388.137201	7.667081624	2.938677539	1.27E-06	0.000874
gene60170	63.02152933	461.6993638	7.326057758	2.873037075	1.29E-06	0.000877
gene4961	38.22617353	314.5750383	8.229310162	3.040771499	1.33E-06	0.000889
gene7209	27.89477528	253.595877	9.091160423	3.184464456	1.39E-06	0.000908
gene34547	137.4075967	937.9175756	6.825805835	2.770999377	1.40E-06	0.000908
gene38225	68.18722846	489.7691365	7.182710716	2.844528412	1.41E-06	0.000908
gene37100	9.298258426	137.4450936	14.78181046	3.885751075	1.47E-06	0.000939
gene19174	22.72907615	220.6864884	9.709435038	3.279387352	1.59E-06	0.001004
gene24185	117.7779401	792.7290964	6.730709469	2.750758584	1.65E-06	0.001031
gene58165	33.0604744	278.7618801	8.431877796	3.075853958	1.73E-06	0.001067
gene144	3.099419475	88.08101071	28.41855109	4.828761096	1.76E-06	0.001076
gene52022	344.0355618	2596.937931	7.548457834	2.916181929	1.98E-06	0.001188

gene39484	732.496136	8993.942325	12.27848433	3.618060578	1.99E-06	0.001188
gene47571	139.4738764	919.5270349	6.592826261	2.720897063	2.03E-06	0.001202
gene30901	63.02152933	443.3088231	7.034244136	2.814395407	2.12E-06	0.001237
gene10267	292.3785705	2090.7141	7.150709085	2.838086311	2.14E-06	0.001237
gene66830	184.9320287	1228.294534	6.64187022	2.731589533	2.16E-06	0.001237
gene64258	39.25931335	307.7995759	7.840167074	2.970884398	2.22E-06	0.001259
gene60778	275.8483333	33.87731181	0.12281137	-3.02548396	2.25E-06	0.001263
gene193	181.8326092	17.4226175	0.095816793	-3.38357766	2.27E-06	0.001263
gene15043	2.06627965	76.46593238	37.00657477	5.209709705	2.36E-06	0.001299
gene64611	44.42501248	333.9335022	7.516790284	2.910116754	2.45E-06	0.001332
gene4787	686.0048439	105.5036282	0.15379429	-2.70092615	2.50E-06	0.001346
gene54251	13.43081773	155.8356343	11.60284039	3.536406117	2.93E-06	0.001552
gene949	71.28664793	478.1540582	6.707484109	2.745771731	2.93E-06	0.001552
gene65354	177.7000499	1135.373907	6.389271741	2.6756515	3.10E-06	0.001626
gene20752	0	52.26785251	Inf	Inf	3.18E-06	0.001636
gene29428	0	52.26785251	Inf	Inf	3.18E-06	0.001636
gene59326	7.231978776	114.214937	15.79304095	3.981217084	3.32E-06	0.001694
gene59115	0	51.29992932	Inf	Inf	3.76E-06	0.001899
gene56330	364.6983583	2577.579467	7.067702415	2.821241297	3.85E-06	0.001924
gene514	350.2344007	2443.038143	6.975437417	2.802283688	3.90E-06	0.001933
gene35340	134.3081773	832.4139474	6.197790517	2.631753993	3.96E-06	0.001949
gene51332	286.1797315	1883.578537	6.581802724	2.718482785	4.23E-06	0.002059
gene31321	60.95524968	405.5598186	6.65340263	2.734092341	4.45E-06	0.002149
gene46729	574.4257427	4708.946342	8.197658969	3.035211974	4.83E-06	0.002311
gene51610	40.29245318	294.2486512	7.302822935	2.868454251	4.87E-06	0.002313
gene70652	788.2856865	8190.566073	10.3903524	3.37717268	5.05E-06	0.002379
gene43970	158.0703932	955.3401931	6.043764259	2.595447388	5.23E-06	0.002439
gene57029	5.165699125	94.85647307	18.36275609	4.198710706	5.37E-06	0.002476
gene22937	387.4274344	59.04331487	0.152398384	-2.71408049	5.39E-06	0.002476
gene36215	23.76221598	205.1997173	8.635546342	3.110287455	5.47E-06	0.002488
gene26270	337.8367228	2237.838426	6.624023604	2.727707814	5.51E-06	0.002488
gene47913	92.98258426	570.1067616	6.131328422	2.616199684	5.63E-06	0.002518
gene28058	126.0430587	756.9159382	6.005217156	2.586216416	5.67E-06	0.002518
gene36497	204.5616854	1241.845459	6.070762746	2.601877792	5.76E-06	0.002538
gene33460	139.4738764	832.4139474	5.96824272	2.577306209	5.92E-06	0.002589
gene54020	323.3727653	2084.906561	6.447378336	2.688712644	6.33E-06	0.002746
gene32217	14.46395755	150.9960184	10.43946775	3.383976253	6.75E-06	0.002906
gene31636	2.06627965	68.72254682	33.25907353	5.055676076	6.82E-06	0.002912
gene39485	244.8541385	1487.69795	6.075853808	2.603087157	6.90E-06	0.002926
gene20310	85.75060548	517.8389091	6.038895075	2.594284607	7.41E-06	0.003117
gene45836	402.9245318	2683.083096	6.659021439	2.735310185	7.72E-06	0.003223
gene51331	289.279151	1778.074909	6.146571235	2.619781851	7.87E-06	0.003261
gene19660	149.8052746	867.2591824	5.789243299	2.533374789	8.14E-06	0.003339
gene15701	145.6727153	843.0611025	5.787364508	2.532906513	8.18E-06	0.003339
gene200	1769.76852	290.3769584	0.164076237	-2.60756179	8.44E-06	0.003423
gene8577	446.3164044	3025.727906	6.779333846	2.761143517	8.55E-06	0.003441
gene55937	161.1698127	924.3666509	5.735358473	2.519883661	9.04E-06	0.003602
gene52857	13.43081773	141.3167864	10.52183041	3.395313797	9.08E-06	0.003602

gene70847	145.6727153	14.51884792	0.099667586	-3.3267318	9.33E-06	0.003674
gene6546	34.09361423	247.7883378	7.267881198	2.861534837	9.51E-06	0.003708
gene62255	373.9966167	60.01123807	0.160459307	-2.63972062	9.55E-06	0.003708
gene11780	2.06627965	65.81877724	31.85376056	4.993391798	1.03E-05	0.003954
gene65671	324.4059051	1974.563317	6.08670584	2.605661645	1.04E-05	0.003954
gene64709	26.86163545	210.0393332	7.819305478	2.967040471	1.04E-05	0.003954
gene64105	11.36453808	127.7658617	11.24250373	3.490891457	1.05E-05	0.003954
gene58998	85.75060548	497.512522	5.801854334	2.536514074	1.16E-05	0.004369
gene18936	131.2087578	734.6537047	5.599120951	2.485200345	1.19E-05	0.004425
gene59778	4.1325593	80.33762515	19.4401627	4.280968388	1.22E-05	0.004516
gene43236	563.0612047	3937.511556	6.993043604	2.805920501	1.25E-05	0.004599
gene23487	57.8558302	356.1957356	6.156609185	2.622135991	1.26E-05	0.004603
gene66871	7.231978776	100.6640122	13.91929033	3.799013753	1.28E-05	0.004638
gene30283	87.81688513	498.4804452	5.676362177	2.504966643	1.44E-05	0.00519
gene26500	21.69593633	178.0978678	8.208812246	3.03717349	1.49E-05	0.005279
gene66186	216.9593633	1200.224761	5.532025644	2.467807844	1.49E-05	0.005279
gene73157	255.1855368	1434.462174	5.621251864	2.490891457	1.49E-05	0.005279
gene39135	1.033139825	54.2036989	52.4650174	5.713283879	1.52E-05	0.005347
gene58347	42.35873283	276.8260337	6.535276558	2.708248289	1.55E-05	0.00538
gene64255	43.39187265	281.6656496	6.49120751	2.698486877	1.55E-05	0.00538
gene50415	257.2518164	1420.91125	5.523425527	2.465563277	1.77E-05	0.006109
gene4477	96.08200373	529.4539875	5.510438656	2.462167168	1.79E-05	0.006124
gene7482	3.099419475	69.69047001	22.48500746	4.490891457	1.83E-05	0.006213
gene59917	126.0430587	678.5141594	5.383193384	2.428462253	1.84E-05	0.006213
gene52727	297.5442696	1668.699588	5.608239707	2.487548014	1.85E-05	0.006213
gene25637	115.7116604	10.64715514	0.092014539	-3.44199435	1.87E-05	0.00623
gene35738	253.1192571	1387.033938	5.479764572	2.454113912	1.87E-05	0.00623
gene55235	288.2460112	1594.169502	5.530586512	2.467432485	2.00E-05	0.006621
gene25330	322.3396254	54.2036989	0.168157107	-2.57211834	2.12E-05	0.006889
gene12379	32.02733458	221.6544116	6.920788586	2.790936434	2.13E-05	0.006889
gene2505	855.4397752	6864.511296	8.024540705	3.00441882	2.13E-05	0.006889
gene71476	87.81688513	481.0578277	5.477965052	2.453640062	2.13E-05	0.006889
gene57045	762.4571909	5644.928071	7.403600016	2.888226955	2.18E-05	0.007002
gene42417	53.7232709	320.3825774	5.963571689	2.576176645	2.20E-05	0.007002
gene9044	126.0430587	666.8990811	5.291041714	2.403551792	2.21E-05	0.007002
gene2356	1008.344469	191.6487925	0.190062819	-2.39545176	2.24E-05	0.007058
gene39458	54.75641073	324.2542702	5.921759039	2.566025787	2.27E-05	0.007126
gene53657	3.099419475	67.75462362	21.86042392	4.450249473	2.38E-05	0.007416
gene21637	97.11514356	519.7747555	5.352149381	2.420118383	2.43E-05	0.007519
gene47036	159.1035331	828.5422546	5.207566662	2.380609402	2.44E-05	0.007519
gene64812	140.5070162	727.8782424	5.180369365	2.373054967	2.63E-05	0.008045
gene33632	203.5285455	1056.972129	5.193237763	2.376634278	2.65E-05	0.008045
gene31807	63.02152933	357.1636588	5.667327699	2.502668625	2.65E-05	0.008045
gene55647	3.099419475	66.78670043	21.54813215	4.429490913	2.71E-05	0.008181
gene234	432.8855867	2487.56261	5.746466703	2.522675167	2.83E-05	0.00851
gene3107	1.033139825	50.33200612	48.71751616	5.606368675	2.86E-05	0.008552
gene15024	30.99419475	211.0072564	6.807960591	2.767222686	2.90E-05	0.008601
gene33951	161.1698127	821.7667922	5.09876371	2.350147481	3.02E-05	0.008894

gene57953	225.2244819	1160.53991	5.152814209	2.365360575	3.03E-05	0.008894
gene52737	30.99419475	210.0393332	6.776731414	2.760589593	3.05E-05	0.008912
gene51729	42.35873283	260.3713394	6.146816063	2.619839315	3.13E-05	0.009092
gene32109	33.0604744	218.750642	6.616681882	2.726107919	3.15E-05	0.00911
gene70396	8.265118601	97.76024266	11.8280508	3.564140439	3.21E-05	0.009205
gene2728	16.5302372	141.3167864	8.54898721	3.095753516	3.21E-05	0.009205
gene59530	221.0919226	1127.630522	5.100279145	2.35057621	3.30E-05	0.009394
gene65437	6.19883895	85.17724113	13.74083789	3.780398075	3.36E-05	0.009496
gene34314	223.1582022	1136.341831	5.092090809	2.348258148	3.37E-05	0.009496
gene57796	378.129176	2055.868865	5.436948524	2.442797169	3.47E-05	0.009724
gene21377	97.11514356	502.352138	5.172747726	2.370930833	3.51E-05	0.009786
gene36437	286.1797315	1482.858334	5.18156309	2.373387373	3.56E-05	0.009881
gene47663	29.96105493	201.3280245	6.719657401	2.74838768	3.80E-05	0.010491
gene49251	118.8110799	597.2086111	5.026539711	2.329565585	3.93E-05	0.010768
gene61004	122.9436392	616.567075	5.015038428	2.326260756	3.94E-05	0.010768
gene15755	11.36453808	112.2790906	9.879776004	3.304478333	4.05E-05	0.011022
gene64304	43.39187265	258.435493	5.95585019	2.574307466	4.13E-05	0.011199
gene71871	338.8698626	1766.45983	5.212797079	2.382057701	4.17E-05	0.011205
gene71714	37.1930337	231.3336435	6.219811091	2.636870763	4.18E-05	0.011205
gene71515	46.49129213	271.0184945	5.829446378	2.543358877	4.27E-05	0.011376
gene14316	309.9419475	1589.329886	5.127830867	2.358348678	4.28E-05	0.011376
gene54986	367.7977777	1932.94262	5.255449426	2.393814143	4.39E-05	0.011596
gene33562	4108.797084	665.9311579	0.162074482	-2.62527114	4.41E-05	0.011596
gene31050	45.4581523	265.2109553	5.834178071	2.544529421	4.52E-05	0.011847
gene40162	51.65699125	291.3448816	5.639989371	2.495692444	4.59E-05	0.011904
gene7315	4.1325593	69.69047001	16.86375559	4.075853958	4.63E-05	0.011904
gene29212	4.1325593	69.69047001	16.86375559	4.075853958	4.63E-05	0.011904
gene29307	4.1325593	69.69047001	16.86375559	4.075853958	4.63E-05	0.011904
gene61837	150.8384145	737.5574743	4.889719087	2.289751585	4.66E-05	0.011935
gene70960	8.265118601	93.88854988	11.35961314	3.505841799	4.74E-05	0.012077
gene58681	221.0919226	1083.106055	4.898894732	2.292456291	4.84E-05	0.01224
gene21401	14.46395755	125.8300153	8.699556457	3.120941848	4.86E-05	0.01224
gene18935	352.3006804	1814.85599	5.151440491	2.364975908	4.87E-05	0.01224
gene8819	36.15989388	223.590258	6.183377051	2.628394981	4.89E-05	0.012246
gene12974	227.2907615	1113.111674	4.897302761	2.291987389	4.93E-05	0.012279
gene47227	133.2750374	649.4764636	4.873204136	2.284870657	5.06E-05	0.012523
gene71106	83.68432583	6.775462362	0.080964533	-3.62656612	5.07E-05	0.012523
gene46645	332.6710237	1688.058051	5.074256341	2.343196403	5.12E-05	0.012592
gene52197	26.86163545	181.9695606	6.77432917	2.76007809	5.22E-05	0.01278
gene67451	14.46395755	124.8620921	8.632636792	3.10980129	5.26E-05	0.012813
gene41660	38.22617353	230.3657203	6.026387134	2.591293354	5.41E-05	0.013114
gene14303	1.033139825	46.46031334	44.97001491	5.490891457	5.49E-05	0.013263
gene2912	37.1930337	225.5261044	6.063665205	2.6001901	5.52E-05	0.013284
gene20284	547.5641073	3057.669372	5.58413039	2.481332629	5.61E-05	0.013438
gene50080	9.298258426	97.76024266	10.51382293	3.394215438	5.65E-05	0.013464
gene22758	59.92210985	319.4146542	5.330497458	2.414270176	5.88E-05	0.013952
gene44547	1179.84568	244.8845682	0.207556439	-2.2684244	5.95E-05	0.01402
gene48355	63.02152933	331.9976558	5.268003796	2.397256385	5.96E-05	0.01402

gene19398	156.0041136	741.4291671	4.752625748	2.248724799	6.16E-05	0.014396
gene38456	200.4291261	953.4043467	4.756815366	2.249996029	6.17E-05	0.014396
gene30945	132.2418976	632.0538461	4.779527952	2.256868138	6.20E-05	0.014402
gene58659	5.165699125	73.56216279	14.24050472	3.831928375	6.32E-05	0.014629
gene55161	921.560724	6238.264989	6.769239213	2.7589937	6.35E-05	0.014646
gene58048	135.3413171	17.4226175	0.128730959	-2.95756904	6.40E-05	0.01469
gene71914	176.6669101	834.3497938	4.722728174	2.239620501	6.49E-05	0.014831
gene31887	185.9651685	29.03769584	0.156145885	-2.67903354	6.74E-05	0.015351
gene67583	19.62965668	145.1884792	7.396384032	2.886820134	7.04E-05	0.015955
gene294	10.33139825	100.6640122	9.743503231	3.28444058	7.18E-05	0.01622
gene31132	97.11514356	468.4748262	4.823911174	2.270203342	7.35E-05	0.016534
gene59697	428.7530274	2169.115879	5.059126678	2.338888364	7.61E-05	0.017047
gene1327	123.976779	15.48677111	0.124916708	-3.00096164	7.69E-05	0.017126
gene66233	2.06627965	52.26785251	25.29563339	4.660816459	7.71E-05	0.017126
gene62254	762.4571909	164.5469431	0.215811386	-2.21215711	7.74E-05	0.017126
gene72888	10.33139825	99.69608905	9.6498157	3.270501389	7.87E-05	0.017348
gene41481	5.165699125	71.6263164	13.8657546	3.793454227	8.01E-05	0.017544
gene32453	45.4581523	251.6600306	5.536081382	2.468865151	8.02E-05	0.017544
gene7104	214.8930836	995.9929673	4.634830263	2.212516505	8.09E-05	0.017616
gene1196	18.59651685	138.4130168	7.442953857	2.895875292	8.20E-05	0.017792
gene72706	78.51862671	385.2334315	4.906268075	2.294626064	8.32E-05	0.017983
gene73601	862.6717539	5324.545494	6.172156987	2.625774757	8.67E-05	0.018664
gene19991	242.7878589	1119.887136	4.612615891	2.205585159	8.96E-05	0.019162
gene63867	2.06627965	51.29992932	24.82719573	4.633849411	8.97E-05	0.019162
gene25365	259.3180961	1199.256838	4.624655418	2.209345875	9.11E-05	0.019351
gene60291	99.18142321	467.506903	4.713653907	2.236845835	9.13E-05	0.019351
gene69436	974.2508551	6309.891306	6.476659757	2.695249956	9.43E-05	0.019851
gene49184	98.14828338	461.6993638	4.704100244	2.233918804	9.44E-05	0.019851
gene48183	51.65699125	271.9864177	5.265239246	2.396499087	9.70E-05	0.020328
gene39912	0	33.87731181	Inf	Inf	9.85E-05	0.020498
gene46125	253.1192571	1158.604064	4.57730509	2.194498455	9.85E-05	0.020498
gene43760	29.96105493	183.905407	6.138148587	2.61780357	0.00010025	0.020777
gene44032	107.4465418	497.512522	4.630326055	2.211113788	0.00010072	0.020797
gene56526	624.0164543	3314.169018	5.311028251	2.408991204	0.00010455	0.021508
gene36363	23.76221598	156.8035575	6.598860884	2.722217003	0.00010842	0.022222
gene42730	9.298258426	90.9847803	9.785142134	3.290592807	0.00011022	0.022427
gene26635	32.02733458	0	0	#NAME?	0.00011023	0.022427
gene17299	15.49709738	120.0224761	7.744835902	2.953234671	0.00011512	0.023337
gene37372	99.18142321	455.8918247	4.596544493	2.200549706	0.0001183	0.023895
gene29250	283.0803121	56.13954529	0.198316672	-2.33412213	0.0001202	0.024105
gene39785	85.75060548	400.7202026	4.673088899	2.224376483	0.0001206	0.024105
gene5336	107.4465418	488.8012133	4.549250307	2.185628816	0.00012066	0.024105
gene7723	115.7116604	522.6785251	4.517077391	2.175389632	0.00012108	0.024105
gene37332	182.865749	808.2158675	4.419722511	2.143955794	0.00012375	0.024513
gene55329	37.1930337	209.07141	5.621251864	2.490891457	0.00012401	0.024513
gene49302	259.3180961	1158.604064	4.467887438	2.15959284	0.00012465	0.024553
gene5565	644.6792509	3364.501025	5.21887593	2.383739105	0.000126	0.024727
gene4346	332.6710237	1518.671492	4.565084977	2.190641716	0.00012683	0.024727

gene37596	6.19883895	73.56216279	11.86708727	3.568893969	0.00012716	0.024727
gene64822	77.48548688	365.8749676	4.721851566	2.23935269	0.00012732	0.024727
gene61234	98.14828338	448.1484391	4.566034409	2.190941732	0.0001282	0.024812
gene44434	8.265118601	84.20931793	10.188519	3.348872452	0.00012917	0.024899
gene31873	5.165699125	67.75462362	13.11625435	3.713283879	0.00012955	0.024899
gene12343	312.0082272	1408.328248	4.513753567	2.174327655	0.0001313	0.025148
gene48354	71.28664793	339.7410413	4.765843972	2.25273172	0.00013448	0.025669
gene13050	516.5699125	2520.471999	4.879246618	2.286658405	0.00013648	0.025962
gene61314	200.4291261	875.002568	4.365645778	2.126195077	0.00014005	0.02655
gene63370	2.06627965	48.39615973	23.42188277	4.549785146	0.0001419	0.026764
gene46514	1264.563146	8972.648014	7.095452721	2.826894737	0.00014215	0.026764
gene2209	63.02152933	304.8958063	4.83796267	2.274399637	0.00014777	0.027729
gene24652	43.39187265	229.3977971	5.286653539	2.402354783	0.00014905	0.027821
gene49421	88.85002496	9.679231946	0.10893899	-3.1984077	0.00014947	0.027821
gene64111	55.78955055	276.8260337	4.961969238	2.310912791	0.00014976	0.027821
gene39498	1.033139825	40.65277417	39.34876305	5.298246379	0.00015139	0.027965
gene63450	186.9983083	809.1837907	4.327225192	2.113442202	0.00015154	0.027965
gene62983	73.35292758	343.6127341	4.684376554	2.227857052	0.00015251	0.02805
gene30629	169.4349313	730.782012	4.313054022	2.108709786	0.00015769	0.028907
gene62478	183.8988889	791.7611732	4.305415754	2.106152559	0.00015906	0.029027
gene48382	6.19883895	71.6263164	11.5547955	3.530419822	0.00015987	0.029027
gene67338	125.0099188	545.9086818	4.366922936	2.126617072	0.00015996	0.029027
gene53681	663.2757677	153.8997879	0.232029867	-2.10761758	0.00016043	0.029027
gene28512	41.325593	219.7185652	5.316767388	2.410549349	0.00016144	0.029114
gene51746	45.4581523	235.2053363	5.17410683	2.371309842	0.00016378	0.02944
gene23618	223.1582022	958.2439627	4.294011841	2.102326169	0.00016743	0.029905
gene43954	98.14828338	436.5333608	4.447692265	2.153056972	0.00016744	0.029905
gene51777	244.8541385	48.39615973	0.197653019	-2.3389581	0.00016814	0.029934
gene63451	88.85002496	399.7522794	4.499180271	2.169662173	0.00016921	0.030027
gene11085	89.88316478	403.6239722	4.490540282	2.166889034	0.00016981	0.030039
gene60908	201.4622659	860.48372	4.271190519	2.094638252	0.00017225	0.030305
gene25606	100.214563	443.3088231	4.423596828	2.145219903	0.00017267	0.030305
gene41474	787.2525467	4172.716892	5.300353628	2.406088616	0.00017295	0.030305
gene50271	123.976779	537.197373	4.333048312	2.115382322	0.00017389	0.030374
gene16798	42.35873283	221.6544116	5.23279137	2.38758074	0.00017727	0.030867
gene73439	104.3471223	457.8276711	4.387544772	2.133413847	0.00017935	0.031131
gene72558	250.0198377	1069.55513	4.277881068	2.096896374	0.00018113	0.031342
gene36869	664.3089075	3313.201095	4.987440418	2.318299606	0.00018249	0.031479
gene51622	138.4407366	592.3689951	4.278863359	2.097227609	0.00018333	0.031526
gene14443	3.099419475	53.23577571	17.17604736	4.102326169	0.00018441	0.031614
gene31743	140.5070162	600.1123807	4.271049211	2.094590521	0.00018516	0.031634
gene1834	938.0909612	5260.662563	5.607838451	2.487444789	0.00018566	0.031634
gene33303	104.3471223	455.8918247	4.368992786	2.127300724	0.00018714	0.03175
gene21810	1139.553227	7046.480857	6.183546928	2.628434616	0.00018749	0.03175
gene43654	34.09361423	188.745023	5.536081382	2.468865151	0.00018967	0.032022
gene51407	16.5302372	118.0866297	7.143674244	2.836666294	0.00019315	0.032511
gene8832	590.9559799	2835.047037	4.797391233	2.262250098	0.00019404	0.032562
gene20704	9.298258426	85.17724113	9.160558594	3.195435574	0.00019828	0.033173

gene64025	142.5732959	603.9840735	4.236305753	2.082806719	0.00019905	0.033203
gene44624	203.5285455	852.7403345	4.189782481	2.066875346	0.0002065	0.034342
gene57434	125.0099188	531.3898339	4.250781368	2.087728058	0.0002089	0.034555
gene7255	234.5227403	984.3778889	4.19736648	2.069484433	0.0002097	0.034555
gene8308	13.43081773	103.5677818	7.711204481	2.946956225	0.00021051	0.034555
gene18070	7.231978776	74.53008599	10.30562842	3.365360575	0.0002109	0.034555
gene51949	119.8442197	17.4226175	0.145377203	-2.78212704	0.00021121	0.034555
gene58956	3.099419475	52.26785251	16.86375559	4.075853958	0.00021276	0.034555
gene69514	3.099419475	52.26785251	16.86375559	4.075853958	0.00021276	0.034555
gene3992	112.6122409	482.0257509	4.280402796	2.097746564	0.00021278	0.034555
gene60752	113.6453808	485.8974437	4.275558236	2.096112797	0.00021338	0.034555
gene41176	17.56337703	120.9903993	6.888789049	2.7842504	0.00021667	0.034985
gene43536	999.0462109	239.0770291	0.239305276	-2.06307589	0.00021776	0.03506
gene38274	0	30.00561903	Inf	Inf	0.00021962	0.035257
gene8547	178.7331897	743.3650135	4.159076524	2.05626323	0.00022149	0.035455
gene57173	91.94944443	400.7202026	4.358049198	2.123682483	0.00022288	0.035574
gene59409	116.7448002	495.5766757	4.24495716	2.085749994	0.00022369	0.035601
gene68123	440.1175655	1950.365237	4.431464204	2.14778346	0.00022565	0.03581
gene6840	13.43081773	102.5998586	7.639137149	2.933409693	0.00022909	0.036168
gene712	126.0430587	530.4219107	4.208259592	2.073223702	0.0002292	0.036168
gene43227	51.65699125	249.7241842	4.834276603	2.273300022	0.00023601	0.037137
gene27852	9.298258426	83.24139474	8.95236408	3.16226871	0.00024186	0.037949
gene29442	36.15989388	191.6487925	5.300037472	2.40600256	0.00024576	0.038392
gene30557	98.14828338	420.0786665	4.280040893	2.097624581	0.00024606	0.038392
gene24255	13.43081773	101.6319354	7.567069817	2.919734756	0.00024936	0.038797
gene50979	73.35292758	327.1580398	4.460054296	2.157061273	0.00025202	0.039018
gene24939	470.0786204	2077.163176	4.41875696	2.143640582	0.00025218	0.039018
gene12644	283.0803121	1175.058758	4.150973092	2.05344958	0.00025348	0.03911
gene35917	1.033139825	37.74900459	36.53813712	5.191331176	0.00025595	0.039301
gene37891	142.5732959	588.4973023	4.127682528	2.045332013	0.00025614	0.039301
gene54930	37.1930337	194.5525621	5.230887152	2.387055646	0.00025794	0.039403
gene34450	48.55757178	236.1732595	4.863778209	2.282077443	0.0002589	0.039403
gene5297	140.5070162	579.7859936	4.126384641	2.044878308	0.00025893	0.039403
gene56401	112.6122409	472.346519	4.194450932	2.068481969	0.00025995	0.039418
gene9990	228.3239013	935.013806	4.095120136	2.033905776	0.00026044	0.039418
gene22678	7.231978776	72.5942396	10.03794976	3.327392725	0.00026293	0.039686
gene45541	248.9866978	1020.191047	4.097371691	2.034698772	0.00026666	0.040123
gene49185	216.9593633	884.6817999	4.077638257	2.027733794	0.00026783	0.040123
gene73206	209.7273845	854.6761809	4.075176844	2.026862667	0.00026799	0.040123
gene5578	0	29.03769584	Inf	Inf	0.00026979	0.040267
gene1868	26.86163545	154.8677111	5.765386527	2.527417333	0.0002704	0.040267
gene13280	146.7058552	601.0803039	4.097180056	2.034631295	0.0002712	0.040278
gene15881	143.6064357	24.19807987	0.168502754	-2.56915593	0.00027304	0.040289
gene67645	149.8052746	612.6953822	4.089945322	2.032081556	0.0002732	0.040289
gene73530	32.02733458	174.226175	5.439921159	2.443585743	0.00027345	0.040289
gene30667	347.1349812	1453.820638	4.188055704	2.066280631	0.00027431	0.040309
gene18757	362.6320786	84.20931793	0.232216957	-2.10645477	0.00027798	0.040719
gene48791	22.72907615	138.4130168	6.08968952	2.606368675	0.00027857	0.040719

gene59636	332.6710237	1384.130168	4.160657436	2.05681151	0.00027933	0.040723
gene53278	95.04886391	402.656049	4.236305753	2.082806719	0.00028322	0.041098
gene71635	427.7198876	1833.246531	4.286091397	2.099662615	0.00028338	0.041098
gene25610	167.3686517	678.5141594	4.054009832	2.019349588	0.00028582	0.041294
gene20701	11.36453808	90.9847803	8.006025383	3.00108619	0.00028622	0.041294
gene54550	3057.060742	657.2198492	0.21498423	-2.21769726	0.00028902	0.041496
gene2148	139.4738764	569.1388384	4.080612464	2.028785704	0.00028953	0.041496
gene50374	141.540156	576.882224	4.075749527	2.027065394	0.00029049	0.041496
gene48551	163.2360924	661.0915419	4.049910362	2.017889977	0.00029061	0.041496
gene34386	13.43081773	99.69608905	7.422935154	2.891989766	0.00029561	0.042102
gene36512	163.2360924	659.1556955	4.038051181	2.013659196	0.0002988	0.042447
gene71229	389.4937141	1634.822276	4.197300795	2.069461856	0.0003016	0.042736
gene36406	1762.536542	417.1748969	0.23669007	-2.07892892	0.00030702	0.043326
gene12748	5.165699125	60.97916126	11.80462892	3.561280785	0.00030733	0.043326
gene10997	2.06627965	43.55654376	21.07969449	4.397782053	0.00031115	0.043754
gene54236	674.6403058	3119.616456	4.624118111	2.209178248	0.00031583	0.044299
gene5263	22.72907615	136.4771704	6.004519037	2.58604869	0.00031798	0.044489
gene40521	454.581523	1937.782236	4.262782664	2.091795502	0.00031893	0.04451
gene65387	10.33139825	85.17724113	8.244502734	3.04343248	0.00032119	0.044713
gene39085	1339.982353	8137.330297	6.072714524	2.60234155	0.00032602	0.045088
gene56714	402.9245318	1682.250512	4.175100744	2.06181101	0.00032645	0.045088
gene37159	361.5989388	1489.633797	4.119574581	2.042495361	0.00032668	0.045088
gene23565	194.2302871	774.3385557	3.98670345	1.995196295	0.00032713	0.045088
gene49690	36.15989388	185.8412534	5.139430276	2.36160844	0.00033266	0.045737
gene36671	41.325593	1.935846389	0.046843766	-4.41599914	0.00033721	0.046237
gene60163	113.6453808	463.6352102	4.079666126	2.028451089	0.00033796	0.046237
gene22180	389.4937141	93.88854988	0.241052799	-2.05257891	0.00034614	0.047239
gene30840	18.59651685	119.0545529	6.40198129	2.678518461	0.00034723	0.047272
gene64147	15.49709738	106.4715514	6.870418945	2.780398075	0.00034849	0.047328
gene45497	304.7762484	1225.390764	4.020624215	2.007419502	0.00035031	0.047459
gene61392	20.6627965	126.7979385	6.136533285	2.617423863	0.0003573	0.048288
gene31582	17.56337703	114.214937	6.503016863	2.701109165	0.00036703	0.049482
gene4990	265.516935	1051.164589	3.958936138	1.985112795	0.00037164	0.049902
gene59211	156.0041136	616.567075	3.952248827	1.98267378	0.00037194	0.049902
gene37207	46.49129213	2.903769584	0.062458354	-4.00096164	0.00037503	0.050195
gene37072	368.8309176	1495.441336	4.054544412	2.019539815	0.00037934	0.050568
gene19527	83.68432583	350.3881965	4.187022994	2.065924841	0.00037964	0.050568
gene37586	229.3570412	900.168571	3.924747923	1.972599996	0.00038297	0.050891
gene3586	119.8442197	480.0899045	4.005949604	2.002144272	0.00038467	0.050995
gene423	5.165699125	59.04331487	11.42987879	3.514738199	0.00039551	0.052306
gene12779	14.46395755	100.6640122	6.959645165	2.799013753	0.00039707	0.05237
gene18383	54.75641073	247.7883378	4.52528452	2.178008502	0.00039788	0.05237
gene58392	6.19883895	63.88293085	10.30562842	3.365360575	0.00040782	0.053425
gene63268	6.19883895	63.88293085	10.30562842	3.365360575	0.00040782	0.053425
gene62397	117.7779401	469.4427494	3.985829173	1.99487988	0.00040972	0.053494
gene18900	126.0430587	499.4483684	3.962521806	1.986418875	0.00041027	0.053494
gene66027	755.2252121	194.5525621	0.25760867	-1.95674695	0.00041358	0.053799
gene9907	77.48548688	325.2221934	4.197201392	2.069427689	0.00041931	0.054417

gene39823	102.2808427	412.3352809	4.031402852	2.011281957	0.00042173	0.054559
gene26551	52.69013108	239.0770291	4.537415721	2.181870846	0.00042325	0.054559
gene54362	4.1325593	53.23577571	12.88203552	3.68728867	0.00042438	0.054559
gene11220	171.501211	665.9311579	3.882953095	1.95715428	0.00042544	0.054559
gene1757	173.5674906	673.6745435	3.881340573	1.95655503	0.0004258	0.054559
gene28947	589.9228401	2536.926693	4.30043816	2.10448366	0.0004263	0.054559
gene73533	845.1083769	218.750642	0.258843301	-1.94984911	0.00043388	0.055402
gene22561	153.9378339	598.1765343	3.885831826	1.958223464	0.00043859	0.055875
gene60557	3.099419475	47.42823654	15.30229674	3.9356763	0.00044055	0.055996
gene8510	9.298258426	77.43385557	8.32778054	3.05793205	0.00044292	0.056169
gene9642	807.9153432	210.0393332	0.259976908	-1.94354461	0.00044971	0.056747
gene66147	188.0314482	724.0065496	3.850454574	1.945028776	0.00045177	0.056747
gene55046	50.62385143	230.3657203	4.550537223	2.186036876	0.00045177	0.056747
gene4502	1100.293914	5629.4413	5.116306861	2.355102795	0.00045235	0.056747
gene41889	1626.162085	405.5598186	0.249396922	-2.00348444	0.00045258	0.056747
gene22439	43.39187265	205.1997173	4.728989664	2.241531988	0.00045477	0.056878
gene61632	179.7663296	692.0650842	3.849803719	1.944784892	0.00045568	0.056878
gene50544	909.1630461	4324.680834	4.756771464	2.249982714	0.0004572	0.056903
gene21213	819.2798813	3773.932536	4.606402064	2.20364034	0.00045793	0.056903
gene19775	7.231978776	67.75462362	9.368753107	3.227857052	0.00046002	0.056908
gene54771	7.231978776	67.75462362	9.368753107	3.227857052	0.00046002	0.056908
gene7127	70.25350811	297.1524208	4.229716476	2.080560961	0.00046193	0.057017
gene50467	54.75641073	243.916645	4.454576949	2.155288426	0.0004641	0.057159
gene60558	19.62965668	119.0545529	6.065034906	2.600515949	0.00047061	0.057832
gene60425	86.78374531	352.3240428	4.059793013	2.021406174	0.00048598	0.059589
gene39555	138.4407366	25.16600306	0.181781777	-2.45972052	0.0004903	0.059986
gene63699	219.0256429	834.3497938	3.809370367	1.929552561	0.00049713	0.060688
gene18636	112.6122409	18.3905407	0.16330854	-2.61432785	0.00050465	0.061468
gene27590	73.35292758	304.8958063	4.156559477	2.055389856	0.00050651	0.061468
gene36198	35.12675405	174.226175	4.959928116	2.310319212	0.00050715	0.061468
gene24356	21.69593633	125.8300153	5.799704304	2.535979347	0.00050794	0.061468
gene35918	3.099419475	46.46031334	14.99000497	3.905928957	0.00051094	0.061562
gene54758	3.099419475	46.46031334	14.99000497	3.905928957	0.00051094	0.061562
gene25321	137.4075967	527.5181411	3.839075521	1.940758941	0.00052166	0.062718
gene18152	286.1797315	1091.817364	3.81514567	1.931738143	0.00053035	0.06361
gene10214	85.75060548	345.5485805	4.029692602	2.010669789	0.00053137	0.06361
gene21084	143.6064357	547.8445282	3.814902344	1.931646127	0.00053924	0.064326
gene33758	92.98258426	369.7466604	3.976515208	1.991504688	0.00053967	0.064326
gene58983	58.88897003	253.595877	4.306339148	2.106461944	0.00054997	0.065414
gene63175	600.2542384	2494.338073	4.155469321	2.055011425	0.00055968	0.066427
gene14834	297.5442696	1127.630522	3.789790753	1.922118195	0.00057245	0.067797
gene35758	394.6594132	1532.222417	3.882391667	1.956945669	0.0005739	0.067824
gene31250	10.33139825	79.36970196	7.682377548	2.941552866	0.00057579	0.067903
gene22118	90.91630461	360.0674284	3.96042745	1.985656149	0.000578	0.068019
gene35968	39.25931335	185.8412534	4.73368578	2.242963944	0.00058338	0.068507
gene65940	54.75641073	238.1091059	4.348515593	2.120523007	0.00058556	0.068618
gene17031	75.41920723	306.8316527	4.068348952	2.024443428	0.00059388	0.069447
gene20872	296.5111298	1117.95129	3.77035186	1.914699166	0.00059696	0.069623

gene51702	693.2368226	185.8412534	0.268077585	-1.8992775	0.00059789	0.069623
gene12505	9.298258426	74.53008599	8.015488769	3.002790496	0.00060244	0.070005
gene31627	42.35873283	195.5204853	4.615824702	2.206588435	0.00060876	0.070592
gene6976	1066.200299	5099.987313	4.783329469	2.258015166	0.00061284	0.070843
gene5793	33.0604744	163.5790199	4.947872735	2.306808393	0.00061347	0.070843
gene39460	19.62965668	115.1828602	5.867797999	2.552819207	0.00062902	0.072487
gene69259	1.033139825	32.90938862	31.85376056	4.993391798	0.00063254	0.072742
gene13370	352.3006804	1335.734009	3.791460202	1.922753579	0.00063395	0.072754
gene60912	47.52443195	211.9751796	4.460341153	2.15715406	0.0006353	0.072759
gene73463	231.4233208	857.5799504	3.705676452	1.889736923	0.00063956	0.072958
gene39916	4.1325593	50.33200612	12.17937904	3.606368675	0.00064088	0.072958
gene9724	209.7273845	776.2744021	3.70134975	1.888051466	0.0006416	0.072958
gene25406	13.43081773	90.9847803	6.77432917	2.76007809	0.0006423	0.072958
gene61250	463.8797815	1810.016374	3.90190831	1.964179877	0.0006482	0.073478
gene41047	294.4448501	1098.592826	3.731064834	1.89958743	0.00065024	0.073559
gene62118	319.2402059	80.33762515	0.251652592	-1.99049464	0.00065645	0.073844
gene11380	318.2070661	1191.513453	3.74445944	1.904757462	0.00065751	0.073844
gene12560	23.76221598	129.7017081	5.458317028	2.448456191	0.00065758	0.073844
gene47113	878.1688513	3896.858782	4.437482354	2.149741383	0.00065807	0.073844
gene539	6.19883895	60.01123807	9.681044877	3.275162766	0.00065973	0.073881
gene38623	66.12094881	7.743385557	0.117109414	-3.09407104	0.00066602	0.074435
gene43110	50.62385143	220.6864884	4.35933818	2.124109127	0.00068134	0.075973
gene70209	1283.159663	6496.700482	5.063049183	2.340006499	0.0006835	0.075973
gene40709	12.3976779	86.14516432	6.948491888	2.796699887	0.00068389	0.075973
gene60003	502.105955	1967.787855	3.919068944	1.970510953	0.00068547	0.075997
gene37249	502.105955	1965.852008	3.91521349	1.969090977	0.00069057	0.076389
gene4751	143.6064357	533.3256802	3.713800692	1.892896392	0.00069175	0.076389
gene8928	57.8558302	243.916645	4.215938898	2.075853958	0.0006989	0.076985
gene6489	64.05466916	264.2430321	4.125273546	2.044489787	0.00069992	0.076985
gene33494	2.06627965	38.71692779	18.73750621	4.227857052	0.00070137	0.076991
gene67153	44.42501248	199.3921781	4.488286372	2.166164729	0.00070392	0.077119
gene19373	312.0082272	1156.668218	3.707172173	1.89031912	0.00070707	0.077311
gene69918	47.52443195	3.871692779	0.081467418	-3.617633	0.00071163	0.077657
gene71822	56.82269038	5.807539168	0.102204579	-3.29046826	0.00071493	0.077855
gene24555	309.9419475	78.40177877	0.252956334	-1.98303973	0.00071664	0.077855
gene23317	36.15989388	171.3224055	4.737912286	2.24425149	0.00071765	0.077855
gene1797	694.2699625	2865.052656	4.126712678	2.044992994	0.00072404	0.078378
gene15191	7.231978776	63.88293085	8.833395787	3.142968154	0.0007258	0.078378
gene63492	11.36453808	81.30554835	7.154320555	2.838814761	0.00072811	0.078378
gene71178	11.36453808	81.30554835	7.154320555	2.838814761	0.00072811	0.078378
gene31298	209.7273845	764.6593238	3.645967958	1.866301883	0.00073367	0.078774
gene17702	97.11514356	371.6825067	3.827235312	1.936302606	0.00073463	0.078774
gene30977	856.472915	3705.209989	4.326126284	2.11307578	0.00073952	0.079146
gene32561	548.5972471	2157.500801	3.932759073	1.975541807	0.00074271	0.079335
gene61386	618.8507552	2485.626764	4.016520531	2.005946251	0.00074438	0.07936
gene31366	206.627965	751.108399	3.635076206	1.861985609	0.00075371	0.080201
gene1080	395.692553	1484.794181	3.752393542	1.907811142	0.0007553	0.080217
gene56520	1576.571373	418.1428201	0.265222893	-1.91472279	0.00075985	0.080373

gene5665	22.72907615	123.8941689	5.450910899	2.446497338	0.00076027	0.080373
gene70432	493.8408364	1903.904924	3.855300703	1.946843391	0.00076112	0.080373
gene72161	26.86163545	138.4130168	5.152814209	2.365360575	0.00076352	0.080474
gene18957	845.1083769	233.2694899	0.276023166	-1.85713874	0.00077419	0.081444
gene26747	37.1930337	173.2582518	4.658352239	2.219819732	0.00077867	0.081761
gene60900	0	24.19807987	Inf	Inf	0.00078154	0.081777
gene11790	608.519357	2419.807987	3.976550555	1.991517512	0.00078177	0.081777
gene22429	208.6942447	754.9800918	3.617637338	1.855047788	0.00078625	0.08209
gene2079	260.3512359	942.7571916	3.621097431	1.856426995	0.00080145	0.083483
gene22688	303.7431086	1107.304135	3.64552842	1.866127949	0.00080259	0.083483
gene45335	1282.126523	6275.046071	4.894248702	2.291087414	0.0008097	0.084065
gene72774	414.2890699	1547.709188	3.735819506	1.901424754	0.00081384	0.084337
gene61411	12.3976779	84.20931793	6.792346003	2.763909952	0.00082077	0.084889
gene62035	9.298258426	71.6263164	7.703196999	2.945457321	0.00082222	0.084889
gene29556	68.18722846	272.9543409	4.003012691	2.00108619	0.00082692	0.085216
gene31557	28.9279151	144.220556	4.985515046	2.317742555	0.00082975	0.085276
gene42962	75.41920723	296.1844976	3.92717596	1.97349224	0.00083057	0.085276
gene4646	4.1325593	48.39615973	11.71094138	3.549785146	0.00084676	0.086777
gene58735	1638.559763	438.4692072	0.267594272	-1.90188086	0.00085141	0.087093
gene1395	139.4738764	506.2238308	3.629524352	1.859780496	0.00086863	0.088691
gene47238	222.1250624	792.7290964	3.5688413	1.835455749	0.00088633	0.090333
gene45274	958.7537577	4152.390505	4.331029184	2.114709894	0.00089379	0.090926
gene2613	539.2989887	150.0280952	0.278190945	-1.84585263	0.00089901	0.091289
gene63781	372.9634769	1358.964165	3.643692344	1.86540115	0.00091241	0.092166
gene56473	13.43081773	87.11308752	6.486059843	2.697342335	0.00091262	0.092166
gene69312	13.43081773	87.11308752	6.486059843	2.697342335	0.00091262	0.092166
gene47865	7.231978776	61.94708446	8.565717127	3.098574035	0.00091428	0.092166
gene42771	60.95524968	246.8204146	4.049206851	2.017639344	0.00091826	0.092181
gene42481	1.033139825	30.97354223	29.98000994	4.905928957	0.00091852	0.092181
gene8561	390.5268539	1427.686712	3.655796517	1.870185772	0.00091975	0.092181
gene34570	14.46395755	90.9847803	6.290448515	2.653162886	0.00092107	0.092181
gene32960	22.72907615	120.9903993	5.323155175	2.412281623	0.00093226	0.093134
gene31056	71.28664793	278.7618801	3.91043608	1.967329501	0.00095105	0.094741
gene38815	6.19883895	57.10746848	9.212607222	3.203609505	0.00095176	0.094741
gene4529	58.88897003	6.775462362	0.115054863	-3.11960614	0.00095706	0.095099
gene42876	266.5500749	67.75462362	0.254190976	-1.97601528	0.0009613	0.095349
gene31405	789.3188264	223.590258	0.283269891	-1.81975083	0.00096676	0.09572
gene45127	11.36453808	78.40177877	6.898809106	2.786347341	0.00096939	0.095796
gene34173	89.88316478	14.51884792	0.161530226	-2.63012394	0.00097365	0.095796
gene10003	4.1325593	47.42823654	11.47672256	3.520638801	0.00097443	0.095796
gene34935	4.1325593	47.42823654	11.47672256	3.520638801	0.00097443	0.095796
gene36916	1055.868901	297.1524208	0.281429276	-1.82915568	0.00097865	0.095892
gene54364	23.76221598	123.8941689	5.213914773	2.382367001	0.00097886	0.095892
gene5384	100.214563	369.7466604	3.689550193	1.883444942	0.00099532	0.097333
gene48259	22.72907615	120.0224761	5.280569933	2.400693648	0.00099807	0.09743
gene30639	14.46395755	90.0168571	6.22352885	2.637732846	0.00100307	0.097595
gene30583	646.7455305	2510.792767	3.882195776	1.956872874	0.00100327	0.097595
gene28956	363.6652184	1305.72839	3.590468165	1.844171971	0.0010091	0.09799

gene22294	100.214563	17.4226175	0.17385315	-2.52405888	0.00101564	0.098453
gene39420	145.6727153	517.8389091	3.554810576	1.829772686	0.00102242	0.098938
gene63485	29.96105493	144.220556	4.813600734	2.267116482	0.00102661	0.09917
gene52646	197.3297066	692.0650842	3.507151032	1.81029956	0.00103838	0.100133
gene33561	49.5907116	4.839615973	0.097591178	-3.35710545	0.00104021	0.100136
gene37347	101.2477029	370.7145835	3.661461674	1.872419694	0.00105532	0.101415
gene65365	35.12675405	160.6752503	4.574155929	2.193505547	0.00106181	0.101862
gene36564	197.3297066	690.1292378	3.49734082	1.806258395	0.00106422	0.101918
gene24352	22.72907615	119.0545529	5.237984692	2.389011843	0.00106863	0.102164
gene63306	151.8715543	535.2615266	3.524435693	1.817392282	0.00108025	0.102749
gene26066	27.89477528	136.4771704	4.892571067	2.290592807	0.00108073	0.102749
gene71079	12.3976779	81.30554835	6.558127175	2.713283879	0.00108146	0.102749
gene1729	8.265118601	64.85085404	7.846330727	2.972018147	0.001084	0.102749
gene67587	8.265118601	64.85085404	7.846330727	2.972018147	0.001084	0.102749
gene45085	57.8558302	6.775462362	0.117109414	-3.09407104	0.00108946	0.103091
gene37839	46.49129213	196.4884085	4.226348624	2.079411778	0.00109962	0.103875
gene67510	47.52443195	199.3921781	4.195572044	2.068867528	0.00111828	0.105458
gene67858	4.1325593	46.46031334	11.24250373	3.490891457	0.00112221	0.105504
gene28296	30.99419475	146.1564024	4.715605731	2.2374431	0.00112256	0.105504
gene9146	542.3984082	2011.344398	3.708241706	1.890735283	0.00112847	0.105879
gene42903	1213.939294	5450.375509	4.489825425	2.166659351	0.00113421	0.106239
gene1924	70.25350811	270.0505713	3.84394429	1.942587427	0.00114282	0.106825
gene24166	22.72907615	118.0866297	5.19539945	2.377234676	0.00114432	0.106825
gene37638	95.04886391	347.4844269	3.655850397	1.870207034	0.00115355	0.10738
gene56829	216.9593633	751.108399	3.461977339	1.791596281	0.00115412	0.10738
gene27530	24.7953558	124.8620921	5.035704795	2.332193711	0.00116661	0.10836
gene33752	69.22036828	9.679231946	0.139832136	-2.83823214	0.00117113	0.10846
gene55860	151.8715543	530.4219107	3.492569186	1.804288695	0.00117159	0.10846
gene47809	16.5302372	95.82439627	5.796915985	2.535285577	0.00117722	0.108628
gene32386	88.85002496	327.1580398	3.682137849	1.880543638	0.00117932	0.108628
gene69653	73.35292758	278.7618801	3.800282951	1.926106839	0.0011796	0.108628
gene49575	339.9030025	1190.545529	3.502603745	1.808427783	0.00118286	0.108628
gene34488	422.5541885	1508.024337	3.568830646	1.835451442	0.00118331	0.108628
gene44465	12.3976779	80.33762515	6.480054232	2.696005887	0.00118629	0.108628
gene7051	14.46395755	88.08101071	6.08968952	2.606368675	0.00119052	0.108628
gene18419	13.43081773	84.20931793	6.269857849	2.648432734	0.001191	0.108628
gene52410	13.43081773	84.20931793	6.269857849	2.648432734	0.001191	0.108628
gene40634	506.2385143	1843.893686	3.642341769	1.8648663	0.00119662	0.108944
gene8021	542.3984082	1994.889704	3.677904791	1.878884134	0.00119839	0.108944
gene20142	1934.037753	528.4860643	0.273255299	-1.87167862	0.00120492	0.109358
gene3921	365.7314981	101.6319354	0.277886745	-1.84743108	0.00121099	0.10973
gene26224	27.89477528	134.5413241	4.823172896	2.269982527	0.00122144	0.110497
gene53658	193.1971473	664.9632347	3.44188951	1.783200786	0.00122807	0.110905
gene49551	265.516935	69.69047001	0.262470904	-1.92977059	0.00122995	0.110905
gene13569	106.413402	380.3938155	3.574679584	1.837813932	0.00124426	0.112013
gene30537	147.738995	512.9992932	3.472335068	1.795906169	0.00125181	0.112511
gene26758	454.581523	1622.239274	3.568643229	1.835375677	0.00125963	0.113023
gene35067	38.22617353	167.4507127	4.380525101	2.131103819	0.00126157	0.113023

gene5324	3.099419475	40.65277417	13.11625435	3.713283879	0.00126779	0.113193
gene26664	3.099419475	40.65277417	13.11625435	3.713283879	0.00126779	0.113193
gene8678	5.165699125	50.33200612	9.743503231	3.28444058	0.00126958	0.113193
gene48149	360.565799	100.6640122	0.279183474	-1.84071455	0.00127695	0.113667
gene58274	540.3321285	156.8035575	0.290198471	-1.78488818	0.00128568	0.114099
gene22519	79.55176653	12.58300153	0.158173754	-2.66041787	0.00128592	0.114099
gene37390	4.1325593	45.49239015	11.0082849	3.460517808	0.00129342	0.114163
gene61292	11.36453808	75.49800918	6.643297658	2.731899557	0.00129427	0.114163
gene36796	2630.373995	694.0009306	0.263841162	-1.92225844	0.00129481	0.114163
gene30498	887.4671097	3546.470585	3.99617129	1.998618424	0.00129485	0.114163
gene3766	19.62965668	105.5036282	5.37470573	2.426185768	0.00131138	0.11529
gene56820	971.1514356	3962.677559	4.080390981	2.028707397	0.00131256	0.11529
gene57666	1064.13402	4455.350465	4.186832092	2.065859062	0.00131386	0.11529
gene25472	2803.941485	733.6857815	0.261662301	-1.93422201	0.00131646	0.115335
gene56437	444.2501248	1570.939345	3.536159603	1.822183392	0.00132416	0.115828
gene34523	24.7953558	122.9262457	4.957631853	2.309651143	0.00132983	0.116141
gene40995	992.8473719	290.3769584	0.29246888	-1.77364497	0.00133601	0.116497
gene39208	73.35292758	274.8901873	3.747501243	1.905928957	0.00134002	0.116664
gene12955	197.3297066	48.39615973	0.245255317	-2.02764368	0.00135055	0.117297
gene19858	48.55757178	198.4242549	4.086371036	2.030820204	0.00135152	0.117297
gene10017	806.8822034	3126.391919	3.874657175	1.954068668	0.00137206	0.118894
gene60956	29.96105493	139.38094	4.652070508	2.217872963	0.00137733	0.119069
gene40113	181.8326092	618.5029214	3.401496157	1.766169459	0.00137992	0.119069
gene27518	1571.405674	7580.77446	4.824199496	2.270289569	0.00138051	0.119069
gene22043	198.3628464	672.7066203	3.391293442	1.761835624	0.00139048	0.119571
gene27652	212.826804	721.10278	3.388214109	1.760525045	0.00139063	0.119571
gene25811	279.9808926	950.5005771	3.394876587	1.76335913	0.00140992	0.120946
gene42708	1067.233439	4414.697691	4.136581115	2.048438873	0.00141699	0.120946
gene51676	1166.414863	340.7089645	0.2920993	-1.7754692	0.0014171	0.120946
gene51761	259.3180961	877.9063375	3.38544186	1.759344143	0.00141748	0.120946
gene44335	199.3959862	674.6424667	3.383430526	1.758486765	0.00141752	0.120946
gene684	39.25931335	168.4186359	4.289902739	2.100944939	0.00142168	0.121116
gene33340	96.08200373	17.4226175	0.181330705	-2.46330485	0.00142493	0.121207
gene61167	201.4622659	680.4500058	3.377555607	1.755979522	0.00143679	0.122028
gene50378	21.69593633	111.3111674	5.130507654	2.359101585	0.00144465	0.122236
gene22512	824.4455804	3182.531464	3.860208047	1.948678604	0.00144936	0.122236
gene18856	5.165699125	49.36408293	9.556128169	3.256426204	0.0014499	0.122236
gene45256	5.165699125	49.36408293	9.556128169	3.256426204	0.0014499	0.122236
gene53441	180.7994694	43.55654376	0.240910794	-2.05342906	0.00145123	0.122236
gene18987	95.04886391	338.7731181	3.564199552	1.833578112	0.00145244	0.122236
gene36093	207.6611048	698.8405465	3.365293405	1.750732292	0.00147706	0.123891
gene35553	403.9576716	1394.777323	3.452780877	1.78775878	0.00147844	0.123891
gene43272	3.099419475	39.68485098	12.80396258	3.678518461	0.00148006	0.123891
gene11099	523.8018913	1862.284226	3.555321692	1.829980103	0.00148102	0.123891
gene67515	1540.411479	7241.033419	4.700713749	2.23287983	0.00148737	0.124235
gene7960	784.1531272	2978.29967	3.798109791	1.92528161	0.00150337	0.125382
gene58256	50.62385143	202.2959477	3.996059999	1.998578245	0.00150885	0.125651
gene52442	34.09361423	150.9960184	4.428865105	2.146937056	0.00151416	0.125904

gene5021	1396.805044	6260.527223	4.482033661	2.164153484	0.0015205	0.125934
gene39469	727.3304369	2716.960407	3.735524144	1.901310687	0.00152113	0.125934
gene11487	19.62965668	103.5677818	5.276087276	2.39946843	0.00152132	0.125934
gene36383	0	21.29431028	Inf	Inf	0.00152664	0.126186
gene61204	56.82269038	220.6864884	3.883774015	1.957459257	0.00153949	0.126907
gene39021	48.55757178	195.5204853	4.026570484	2.009551588	0.00153993	0.126907
gene12730	9.298258426	65.81877724	7.078613459	2.823466796	0.00154736	0.127203
gene73056	88.85002496	317.4788078	3.573198859	1.837216207	0.0015483	0.127203
gene23947	111.5791011	387.1692779	3.469908558	1.794897644	0.00155039	0.127203
gene9319	13.43081773	81.30554835	6.053655854	2.597806661	0.00155799	0.12745
gene42848	13.43081773	81.30554835	6.053655854	2.597806661	0.00155799	0.12745
gene8316	6.19883895	53.23577571	8.588023682	3.102326169	0.00156353	0.127528
gene12302	6.19883895	53.23577571	8.588023682	3.102326169	0.00156353	0.127528
gene8078	1732.575487	8557.408964	4.939126191	2.304255829	0.00156614	0.127553
gene53675	367.7977777	1248.620921	3.394857165	1.763350876	0.0015886	0.129193
gene72632	1188.110799	4974.157297	4.186610628	2.065782748	0.00159802	0.129769
gene37259	41.325593	172.2903286	4.169095133	2.059734293	0.00161873	0.131259
gene69982	118.8110799	407.4956649	3.429778311	1.778115329	0.00162522	0.131593
gene69078	19.62965668	102.5998586	5.226778049	2.385921898	0.00163888	0.132381
gene46567	42.35873283	175.1940982	4.135961738	2.048222839	0.00164081	0.132381
gene42076	7.231978776	57.10746848	7.896520476	2.981217084	0.0016421	0.132381
gene19320	15.49709738	88.08101071	5.683710218	2.506833001	0.00165531	0.133252
gene18627	56.82269038	218.750642	3.849705822	1.944748206	0.00166451	0.13374
gene51112	250.0198377	829.5101778	3.317777443	1.730217113	0.00166913	0.13374
gene25721	23.76221598	116.1507834	4.888045099	2.289257596	0.00167363	0.13374
gene51042	153.9378339	515.9030627	3.351372756	1.744752159	0.00167447	0.13374
gene43086	40.29245318	168.4186359	4.179905232	2.063470234	0.00167647	0.13374
gene63021	83.68432583	299.0882671	3.574005815	1.837541982	0.00167819	0.13374
gene23815	747.9932334	2768.260337	3.700916283	1.887882501	0.00167823	0.13374
gene72194	697.3693819	2546.605925	3.651731767	1.868580798	0.00168368	0.133938
gene53780	53.7232709	209.07141	3.891635906	1.960376741	0.0016887	0.133938
gene63060	8209.32905	1706.448592	0.207866999	-2.26626736	0.00169017	0.133938
gene66753	121.9104994	415.2390505	3.406097528	1.768119745	0.00169134	0.133938
gene10547	8.265118601	60.97916126	7.377893072	2.88320888	0.00169276	0.133938
gene72945	314.0745068	89.04893391	0.283528055	-1.8184366	0.00170519	0.13461
gene48451	522.7687515	1822.599376	3.486435198	1.801752667	0.00170611	0.13461
gene70657	55.78955055	214.8789492	3.8515985	1.945457321	0.00171725	0.135298
gene58328	4.1325593	43.55654376	10.53984725	3.397782053	0.00172222	0.135305
gene68534	4.1325593	43.55654376	10.53984725	3.397782053	0.00172222	0.135305
gene178	3.099419475	38.71692779	12.49167081	3.642894551	0.00172957	0.135499
gene455	3.099419475	38.71692779	12.49167081	3.642894551	0.00172957	0.135499
gene6968	750.059513	2761.484874	3.681687688	1.88036725	0.00174375	0.136417
gene60551	16.5302372	90.9847803	5.50414245	2.460517808	0.00176346	0.137698
gene57738	452.5152434	1543.837495	3.411680641	1.770482606	0.00176552	0.137698
gene34532	139.4738764	467.506903	3.351931667	1.744992738	0.00176755	0.137698
gene52994	77.48548688	278.7618801	3.597601193	1.847035268	0.0017727	0.137905
gene32679	30.99419475	138.4130168	4.465772314	2.158909698	0.00178328	0.138534
gene24458	50.62385143	198.4242549	3.919580382	1.970699212	0.00178779	0.138691

gene42152	52.69013108	204.2317941	3.876091972	1.954602803	0.00181785	0.140758
gene5436	123.976779	418.1428201	3.372751119	1.753925863	0.0018195	0.140758
gene65505	425.6536079	1437.365944	3.376844263	1.755675645	0.00182207	0.14076
gene2834	452.5152434	1537.062033	3.396707748	1.764137094	0.00182514	0.140778
gene48486	39.25931335	163.5790199	4.166629671	2.058880879	0.00182737	0.140778
gene22641	26.86163545	124.8620921	4.648342888	2.216716494	0.00183293	0.141011
gene62895	1807.994694	8784.870915	4.85890304	2.280630644	0.00185644	0.142622
gene61210	175.6337703	43.55654376	0.247996406	-2.01160888	0.00186752	0.143276
gene18954	816.1804618	3014.112828	3.69294901	1.884773343	0.00190588	0.145898
gene20659	145.6727153	33.87731181	0.232557701	-2.10433938	0.00190913	0.145898
gene42630	257.2518164	840.1573329	3.265894657	1.707478257	0.00190959	0.145898
gene59614	620.9170349	2183.634727	3.516789852	1.814259128	0.00191288	0.145949
gene55845	0	20.32638709	Inf	Inf	0.00192129	0.146214
gene40178	39.25931335	162.6110967	4.141975058	2.050318866	0.0019218	0.146214
gene33262	295.47799	84.20931793	0.284993539	-1.81099888	0.00192513	0.146214
gene48543	100.214563	343.6127341	3.428770467	1.777691329	0.00192688	0.146214
gene32388	2.06627965	32.90938862	15.92688028	3.993391798	0.00193534	0.146467
gene9543	418.4216292	1399.616939	3.34499185	1.742002696	0.00193549	0.146467
gene17709	815.147322	250.6921074	0.307542086	-1.70114425	0.00196141	0.148227
gene61874	668.4414668	2368.508057	3.543329035	1.825105441	0.0019696	0.148643
gene20083	1.033139825	27.10184945	26.2325087	4.713283879	0.0019776	0.149044
gene58760	295.47799	964.0515019	3.262684649	1.706059552	0.00198316	0.149075
gene5755	154.9709738	509.1276004	3.285309423	1.716029255	0.00198338	0.149075
gene7787	223.1582022	60.01123807	0.268917913	-1.89476224	0.00199175	0.149503
gene50807	99.18142321	338.7731181	3.415691237	1.772177568	0.00201635	0.151145
gene18043	3.099419475	37.74900459	12.17937904	3.606368675	0.00202316	0.151451
gene60144	65.08780898	238.1091059	3.658275023	1.871163538	0.00203271	0.151961
gene28907	142.5732959	469.4427494	3.292641491	1.719245437	0.00203635	0.152028
gene50924	96.08200373	329.0938862	3.425135545	1.776161082	0.00204234	0.152103
gene41118	13.43081773	78.40177877	5.837453859	2.545339241	0.00204283	0.152103
gene10518	605.4199375	2100.393332	3.469316424	1.79465143	0.00204889	0.152265
gene8460	19.62965668	99.69608905	5.078850369	2.34450197	0.00205048	0.152265
gene26687	98.14828338	19.35846389	0.197236908	-2.34199856	0.00206183	0.152903
gene40077	77.48548688	273.9222641	3.535142839	1.821768509	0.00206919	0.153244
gene36121	7.231978776	55.17162209	7.628841816	2.931464049	0.00208257	0.154029
gene55939	1357.54573	5648.799764	4.16103829	2.056943564	0.00210639	0.155489
gene34476	153.9378339	502.352138	3.263344203	1.706351165	0.00210827	0.155489
gene52648	618.8507552	2144.917799	3.46596943	1.79325893	0.00211069	0.155489
gene27913	18.59651685	95.82439627	5.152814209	2.365360575	0.0021181	0.155669
gene39486	8.265118601	59.04331487	7.143674244	2.836666294	0.00212074	0.155669
gene46185	111.5791011	373.6183531	3.348461759	1.743498492	0.00212155	0.155669
gene66338	59.92210985	221.6544116	3.699042175	1.88715175	0.00213713	0.156606
gene58512	161.1698127	39.68485098	0.24623005	-2.02192126	0.00215197	0.157485
gene37309	63.02152933	230.3657203	3.655349573	1.870009382	0.00216524	0.158248
gene9181	5.165699125	46.46031334	8.994002983	3.168963362	0.00216814	0.158251
gene12174	720.0984581	2545.638002	3.535124917	1.821761195	0.00217911	0.158843
gene12696	158.0703932	512.9992932	3.245385063	1.698389663	0.00218209	0.158852
gene11696	115.7116604	384.2655083	3.320888378	1.731569232	0.00220506	0.160016

gene4468	1358.57887	5605.24322	4.125813631	2.044678654	0.00220527	0.160016
gene18424	208.6942447	669.8028507	3.209493639	1.682345702	0.00220672	0.160016
gene71589	21.69593633	105.5036282	4.862828994	2.281795859	0.00221052	0.160083
gene9861	198.3628464	636.8934621	3.210749763	1.68291023	0.00221756	0.160383
gene64309	125.0099188	28.06977264	0.224540364	-2.15495329	0.00224208	0.161768
gene8694	645.7123907	2232.99881	3.458194147	1.790018866	0.00224254	0.161768
gene34894	94.01572408	319.4146542	3.397459918	1.764456531	0.00225084	0.162157
gene16216	193.1971473	619.4708446	3.206418176	1.680962592	0.00225529	0.162267
gene12965	578.558302	1965.852008	3.397845993	1.764620464	0.00226706	0.162903
gene43347	967.0188763	3602.61013	3.725480669	1.897426577	0.00227399	0.16316
gene6227	236.5890199	65.81877724	0.278198782	-1.84581199	0.00227749	0.16316
gene4941	98.14828338	331.0297326	3.372751119	1.753925863	0.00227945	0.16316
gene39412	12.3976779	73.56216279	5.933543635	2.568893969	0.00228502	0.163348
gene12745	237.6221598	756.9159382	3.185376056	1.671463703	0.00233271	0.166543
gene52879	983.5491135	3661.653445	3.722898425	1.896426254	0.0023394	0.166806
gene73361	114.6785206	378.4579691	3.300164383	1.722537888	0.00234643	0.16692
gene60296	7.231978776	54.2036989	7.495002486	2.905928957	0.00234701	0.16692
gene20233	1045.537503	3943.319095	3.771571162	1.915165647	0.00236485	0.167974
gene17841	768.6560299	241.9807987	0.314810252	-1.66744557	0.00237258	0.168105
gene30139	40.29245318	161.6431735	4.011748125	2.00423103	0.00237275	0.168105
gene55740	152.9046941	491.7049829	3.215761202	1.685160278	0.00239875	0.16973
gene41724	2461.972203	13795.80929	5.603560136	2.486343713	0.00240928	0.170138
gene34452	201.4622659	54.2036989	0.269051371	-1.89404644	0.00241479	0.170138
gene56908	669.4746067	2302.68928	3.439546858	1.78221851	0.00241931	0.170138
gene72050	18.59651685	0	0	#NAME?	0.00242456	0.170138
gene68236	198.3628464	630.1179997	3.17659285	1.667480189	0.0024258	0.170138
gene11435	0	19.35846389	Inf	Inf	0.00242737	0.170138
gene18855	0	19.35846389	Inf	Inf	0.00242737	0.170138
gene21873	65.08780898	233.2694899	3.583919839	1.841538369	0.00242901	0.170138
gene59753	294.4448501	935.9817292	3.178801493	1.668482927	0.0024442	0.17092
gene56427	22.72907615	107.4394746	4.726961795	2.240913204	0.00244634	0.17092
gene48402	18.59651685	93.88854988	5.048716952	2.335916797	0.00247133	0.17245
gene45715	423.5873283	1371.547167	3.237932476	1.6950729	0.00249923	0.174177
gene60614	1631.327784	7031.962009	4.310575765	2.107880583	0.00251962	0.175379
gene62871	1024.874706	3803.938155	3.711612874	1.892046243	0.00252748	0.175543
gene52755	39.25931335	3.871692779	0.098618454	-3.34199856	0.0025283	0.175543
gene14397	147.738995	472.346519	3.197168893	1.676794957	0.00256684	0.177848
gene65452	668.4414668	2278.4912	3.408662259	1.76920566	0.00256903	0.177848
gene41810	45.4581523	175.1940982	3.853964346	1.946343225	0.00257217	0.177848
gene66247	24.7953558	113.2470138	4.56726714	2.191331176	0.0025743	0.177848
gene72988	58.88897003	213.911026	3.63244638	1.860941502	0.00257771	0.177862
gene39100	81.61804618	278.7618801	3.415444171	1.77207321	0.00258901	0.17842
gene1164	33.0604744	138.4130168	4.186661545	2.065800293	0.00259722	0.178764
gene66954	39.25931335	156.8035575	3.994047377	1.997851446	0.00260321	0.178955
gene784	66.12094881	234.2374131	3.542559769	1.824792194	0.00261344	0.179274
gene15640	557.8955055	1848.733302	3.313762673	1.728470283	0.00261674	0.179274
gene67424	88.85002496	299.0882671	3.366214779	1.75112723	0.00261753	0.179274
gene63456	624.0164543	2098.457486	3.362823963	1.749673259	0.00262773	0.17975

gene59200	7.231978776	53.23577571	7.361163156	2.879933748	0.00264632	0.18043
gene62867	7.231978776	53.23577571	7.361163156	2.879933748	0.00264632	0.18043
gene22494	9.298258426	60.97916126	6.558127175	2.713283879	0.00264823	0.18043
gene52882	76.45234706	263.2751089	3.443649791	1.783938432	0.00265098	0.18043
gene60462	54.75641073	201.3280245	3.676793672	1.87844822	0.00265606	0.18043
gene53975	20.6627965	99.69608905	4.82490785	2.270501389	0.00265716	0.18043
gene47450	4.1325593	40.65277417	9.837190763	3.298246379	0.00266208	0.180543
gene62522	18.59651685	92.92062669	4.996668324	2.320966456	0.00266998	0.180858
gene42089	262.4175156	76.46593238	0.291390353	-1.77897498	0.00268297	0.181517
gene14128	108.4796816	354.2598892	3.265679655	1.707383277	0.00270645	0.182883
gene9318	21.69593633	102.5998586	4.728989664	2.241531988	0.0027384	0.184785
gene61354	2.06627965	30.97354223	14.99000497	3.905928957	0.00274284	0.184785
gene11203	238.6552996	745.3008599	3.122917702	1.642894551	0.00274458	0.184785
gene39380	24.7953558	112.2790906	4.528230668	2.178947451	0.00275148	0.184802
gene59299	24.7953558	112.2790906	4.528230668	2.178947451	0.00275148	0.184802
gene54768	85.75060548	288.441112	3.363720995	1.750058046	0.00275949	0.185116
gene69872	443.216985	1420.91125	3.205904327	1.680731372	0.00277454	0.185602
gene8822	3.099419475	35.8131582	11.5547955	3.530419822	0.00277677	0.185602
gene9027	3.099419475	35.8131582	11.5547955	3.530419822	0.00277677	0.185602
gene55536	57.8558302	209.07141	3.613661913	1.853461537	0.00278038	0.18562
gene2092	358.4995193	1130.534291	3.153516896	1.656961663	0.00279167	0.18615
gene3363	293.4117103	916.6232653	3.124017321	1.643402453	0.00280615	0.186892
gene24218	22.72907615	105.5036282	4.641791312	2.214681663	0.00281249	0.18709
gene1015	1103.393333	351.3561197	0.318432339	-1.65094124	0.00284602	0.189094
gene21224	832.710699	2896.994122	3.478992314	1.798669492	0.00287473	0.190773
gene15645	39.25931335	154.8677111	3.94473815	1.979929538	0.00288159	0.191
gene8505	104.3471223	339.7410413	3.255873605	1.703044694	0.0028886	0.191236
gene42509	149.8052746	37.74900459	0.251987153	-1.98857791	0.00290294	0.191957
gene59466	298.5774094	928.2383437	3.108869976	1.636390279	0.0029289	0.193118
gene22989	1.033139825	25.16600306	24.35875808	4.606368675	0.0029344	0.193118
gene53692	1.033139825	25.16600306	24.35875808	4.606368675	0.0029344	0.193118
gene69961	1.033139825	25.16600306	24.35875808	4.606368675	0.0029344	0.193118
gene22727	547.5641073	1780.010755	3.250780559	1.700786172	0.0029503	0.193934
gene29658	105.3802622	341.6768877	3.242323379	1.697027988	0.00296277	0.194385
gene33316	386.3942946	1214.743609	3.143792821	1.652506146	0.00296416	0.194385
gene36578	38.22617353	3.871692779	0.101283817	-3.30352441	0.00297556	0.194903
gene70232	7.231978776	52.26785251	7.227323826	2.853461537	0.00298524	0.195306
gene27279	466.9792009	1488.665873	3.187863336	1.672589782	0.00299895	0.19583
gene24670	1429.865518	5658.478996	3.957350482	1.984534843	0.00300029	0.19583
gene26572	61.9883895	218.750642	3.528897004	1.819217323	0.00301047	0.195851
gene36957	890.5665292	3115.744764	3.498609774	1.806781759	0.00301114	0.195851
gene27398	67.15408863	233.2694899	3.473645383	1.79645048	0.00301119	0.195851
gene63358	262.4175156	810.1517139	3.087262343	1.626328082	0.00302777	0.196699
gene60046	168.4017915	523.6464483	3.109506399	1.636685586	0.0030463	0.197671
gene9452	477.3105992	152.9318648	0.320403245	-1.64203934	0.00305789	0.19808
gene29650	184.9320287	50.33200612	0.272164895	-1.8774471	0.00305972	0.19808
gene25284	20.6627965	97.76024266	4.731220319	2.242212345	0.00307759	0.198426
gene14161	159.1035331	495.5766757	3.114806228	1.639142416	0.00307867	0.198426

gene22415	0	18.3905407	Inf	Inf	0.00307936	0.198426
gene47116	0	18.3905407	Inf	Inf	0.00307936	0.198426
gene39353	4.1325593	39.68485098	9.602971935	3.263480961	0.00308297	0.198428
gene48398	524.8350311	169.3865591	0.322742479	-1.63154462	0.00310382	0.199539
gene65744	67.15408863	232.3015667	3.459231916	1.790451739	0.00311873	0.200266
gene25276	35.12675405	141.3167864	4.023052805	2.008290674	0.00312403	0.200374
gene52546	179.7663296	555.5879137	3.090611657	1.627892387	0.00312908	0.200467
gene32181	143.6064357	449.1163623	3.127411109	1.644968879	0.00314268	0.201106
gene70695	1560.041136	6301.179997	4.039111439	2.01403795	0.00316156	0.202082
gene45776	807.9153432	2750.837719	3.404858866	1.767594999	0.00318817	0.203548
gene34298	117.7779401	373.6183531	3.172226929	1.66549598	0.00321189	0.204528
gene1752	758.3246316	247.7883378	0.326757602	-1.6137073	0.00321703	0.204528
gene63585	10.33139825	62.91500765	6.08968952	2.606368675	0.00321824	0.204528
gene72085	10.33139825	62.91500765	6.08968952	2.606368675	0.00321824	0.204528
gene65462	13.43081773	73.56216279	5.477117201	2.453416752	0.00322527	0.20474
gene6668	50.62385143	184.8733302	3.651901721	1.868647941	0.00325539	0.206354
gene15485	3.099419475	34.84523501	11.24250373	3.490891457	0.00325812	0.206354
gene40544	343.0024219	107.4394746	0.313232408	-1.67469461	0.00327109	0.206649
gene35260	113.6453808	361.0353516	3.176859008	1.667601063	0.00327376	0.206649
gene62905	59.92210985	9.679231946	0.161530226	-2.63012394	0.00327394	0.206649
gene34299	23.76221598	106.4715514	4.480708008	2.163726714	0.00330154	0.208134
gene12061	152.9046941	473.3144422	3.095486669	1.630166246	0.00330688	0.208134
gene53963	809.9816229	2743.094334	3.386613049	1.759843155	0.0033105	0.208134
gene17863	153.9378339	476.2182118	3.093574851	1.629274942	0.00331246	0.208134
gene71271	1097.194494	3952.030404	3.601941519	1.848774758	0.00332595	0.208746
gene44243	8.265118601	55.17162209	6.675236589	2.738818971	0.00334566	0.209414
gene65621	38.22617353	149.060172	3.899426969	1.963262132	0.0033458	0.209414
gene46417	6.19883895	47.42823654	7.651148371	2.9356763	0.00334791	0.209414
gene10171	562.0280648	1799.369219	3.201564711	1.67877717	0.0033616	0.209952
gene12797	1175.713121	4299.514831	3.656942118	1.870637792	0.00336562	0.209952
gene45324	88.85002496	18.3905407	0.20698408	-2.27240828	0.00336786	0.209952
gene40828	43.39187265	163.5790199	3.769807798	1.91449097	0.00338744	0.210937
gene29083	543.431548	1730.646672	3.184663604	1.671140989	0.00339257	0.210976
gene55692	50.62385143	183.905407	3.632781817	1.861074721	0.00339884	0.210976
gene3876	49.5907116	181.0016374	3.649910065	1.867860916	0.00339947	0.210976
gene39870	954.6211984	3319.008634	3.47678078	1.797752104	0.00341551	0.211735
gene65234	601.2873782	197.4563317	0.328389284	-1.60652104	0.00341942	0.211741
gene46117	522.7687515	1655.148663	3.166120121	1.662715992	0.00342829	0.212054
gene20359	22.72907615	102.5998586	4.514035588	2.174417793	0.00346953	0.214128
gene73375	22.72907615	102.5998586	4.514035588	2.174417793	0.00346953	0.214128
gene61305	1742.906885	548.8124514	0.314883403	-1.66711038	0.00347992	0.214531
gene47298	216.9593633	62.91500765	0.289985215	-1.78594875	0.00349409	0.215057
gene35595	269.6494943	817.8950995	3.033178688	1.600830491	0.00349724	0.215057
gene22596	36.15989388	142.2847096	3.934876305	1.976318285	0.00350006	0.215057
gene23663	700.4688014	231.3336435	0.330255456	-1.5983457	0.00350678	0.215231
gene5804	146.7058552	452.0201319	3.081132184	1.623460577	0.00351507	0.215502
gene37848	348.1681211	110.3432442	0.316925179	-1.65778581	0.00351931	0.215524
gene17386	203.5285455	617.5349982	3.034144407	1.601289751	0.00352566	0.215675

gene16336	134.3081773	416.2069737	3.098895259	1.631753993	0.00353872	0.216003
gene38687	618.8507552	1991.018011	3.217282995	1.685842842	0.00353998	0.216003
gene68837	41.325593	156.8035575	3.794345008	1.923850865	0.00354608	0.216003
gene14213	73.35292758	245.8524914	3.351638436	1.744866524	0.00354659	0.216003
gene12308	339.9030025	1034.709895	3.044132849	1.606031321	0.0035894	0.218323
gene73284	206.627965	625.2783837	3.026107254	1.597463122	0.00359253	0.218323
gene43270	909.1630461	3099.290069	3.408948574	1.769326836	0.00363349	0.220571
gene37954	131.2087578	405.5598186	3.090950828	1.628050704	0.00367305	0.222502
gene67949	55.78955055	196.4884085	3.521957187	1.816377372	0.00367332	0.222502
gene32222	1241.83407	4534.720167	3.651631307	1.868541108	0.00367919	0.222615
gene8527	52.69013108	187.7770998	3.563800202	1.833416457	0.00369358	0.223242
gene68439	695.3031023	2257.19689	3.246349516	1.698818335	0.00372074	0.22464
gene40849	170.4680711	516.8709859	3.032069187	1.600302674	0.00373469	0.225237
gene3761	121.9104994	378.4579691	3.104391919	1.634310705	0.00374125	0.225388
gene41169	343.0024219	1038.581588	3.027913278	1.598323886	0.00375186	0.225638
gene26501	8.265118601	54.2036989	6.558127175	2.713283879	0.00375352	0.225638
gene38621	462.8466416	151.9639416	0.328324607	-1.60680522	0.00375929	0.22574
gene21799	23.76221598	104.535705	4.399240589	2.137254503	0.00378625	0.227114
gene35399	85.75060548	277.7939569	3.239556797	1.695796452	0.00379378	0.22732
gene43895	6.19883895	46.46031334	7.495002486	2.905928957	0.00380839	0.227717
gene35304	71.28664793	238.1091059	3.340164151	1.739919005	0.0038086	0.227717
gene15440	44.42501248	5.807539168	0.130726788	-2.9353733	0.0038155	0.227884
gene19850	33.0604744	131.6375545	3.981720071	1.993391798	0.00386078	0.230117
gene33249	419.454769	1280.562387	3.052921271	1.610190386	0.00386118	0.230117
gene21776	1889.61274	7905.028731	4.183412063	2.064680109	0.00386835	0.230297
gene14522	34.09361423	134.5413241	3.946232369	1.98047591	0.00387582	0.230495
gene11503	366.7646379	1109.239981	3.024391848	1.596645071	0.00388396	0.230695
gene23366	47.52443195	172.2903286	3.625300115	1.858100432	0.00389118	0.230695
gene14298	322.3396254	968.8911178	3.005808289	1.587752997	0.00389164	0.230695
gene6264	96.08200373	21.29431028	0.221626418	-2.17379824	0.00390801	0.231219
gene71563	2.06627965	29.03769584	14.05312966	3.812819552	0.00390881	0.231219
gene20377	0	17.4226175	Inf	Inf	0.00392349	0.231841
gene50997	26.86163545	113.2470138	4.215938898	2.075853958	0.00393536	0.232295
gene48491	577.5251622	1812.920144	3.139118885	1.650359668	0.00394893	0.232736
gene34401	644.6792509	2051.997173	3.182973812	1.670375287	0.00395526	0.232736
gene54305	1225.303833	4401.146766	3.591881988	1.844739951	0.0039554	0.232736
gene71917	227.2907615	678.5141594	2.985225422	1.577839877	0.00396053	0.232791
gene56635	224.191342	66.78670043	0.297900444	-1.74709782	0.00396987	0.233094
gene64029	419.454769	1274.754847	3.039075823	1.60363267	0.00399356	0.234237
gene25517	74.38606741	244.8845682	3.292075745	1.71899753	0.00403295	0.236297
gene3749	232.4564606	692.0650842	2.977181543	1.573947197	0.00404061	0.236497
gene59970	46.49129213	168.4186359	3.622584535	1.857019356	0.00408018	0.238056
gene60352	326.4721847	975.6665802	2.988513649	1.579428132	0.00408352	0.238056
gene4139	718.0321784	2309.464742	3.216380563	1.685438117	0.00408527	0.238056
gene59254	33.0604744	130.6696313	3.952442717	1.982744554	0.0040867	0.238056
gene18576	45.4581523	165.5148663	3.641038139	1.864349853	0.00408867	0.238056
gene28441	9.298258426	57.10746848	6.141738148	2.618647005	0.00409802	0.23835
gene56568	4.1325593	37.74900459	9.13453428	3.191331176	0.00414517	0.24084

gene32931	166.3355118	498.4804452	2.996837174	1.583440701	0.00415084	0.240918
gene913	168.4017915	504.2879844	2.994552374	1.582340365	0.00415529	0.240924
gene33560	10306.6029	2281.39497	0.221352757	-2.17558075	0.00420567	0.24345
gene42484	2750.218214	838.2214866	0.304783629	-1.71414268	0.00420762	0.24345
gene24909	2067.31279	8835.202921	4.273762037	2.095506581	0.00423663	0.244874
gene72559	408.0902309	1227.326611	3.007488339	1.588559144	0.00425399	0.245621
gene71883	157.0372534	470.4106726	2.995535533	1.582813947	0.00427621	0.246648
gene32633	29.96105493	120.9903993	4.03825565	2.013732246	0.00428154	0.246699
gene71450	113.6453808	349.4202733	3.074654429	1.62042427	0.00429952	0.247478
gene16436	1116.824151	3863.949393	3.459765255	1.790674154	0.00430853	0.247724
gene16486	42.35873283	155.8356343	3.678949391	1.87929383	0.00431439	0.247724
gene49277	626.082734	211.9751796	0.338573751	-1.56245797	0.00432217	0.247724
gene45503	341.9692821	1016.319354	2.971960955	1.571415162	0.00432233	0.247724
gene71615	33.0604744	129.7017081	3.923165364	1.972018147	0.00432609	0.247724
gene38231	514.5036329	1571.907268	3.055191776	1.611262942	0.00436852	0.249897
gene65841	61.9883895	209.07141	3.372751119	1.753925863	0.00437616	0.24999
gene4047	294.4448501	93.88854988	0.318866334	-1.64897631	0.00438654	0.24999
gene36573	185.9651685	550.7482977	2.961566954	1.566360703	0.00438808	0.24999
gene25876	1.033139825	23.23015667	22.48500746	4.490891457	0.00438937	0.24999
gene35560	503.1390948	169.3865591	0.336659506	-1.57063789	0.00439267	0.24999
gene36133	1102.360193	3784.579691	3.433160698	1.779537388	0.00443015	0.251866
gene48691	216.9593633	638.8293085	2.944465262	1.558005653	0.00444344	0.252363
gene63821	26.86163545	111.3111674	4.143871567	2.050979289	0.00447466	0.253715
gene11611	56.82269038	194.5525621	3.423853408	1.775620934	0.00447638	0.253715
gene58905	3.099419475	32.90938862	10.61792019	3.408429297	0.00449982	0.254783
gene62907	21.69593633	95.82439627	4.416697893	2.142968154	0.00452952	0.256202
gene52696	714.932759	2262.036506	3.163984973	1.661742748	0.00453544	0.256202
gene50594	856.472915	2787.618801	3.254765856	1.702553761	0.0045387	0.256202
gene51137	177.7000499	524.6143715	2.952246619	1.561813243	0.00456872	0.257634
gene24480	252.0861173	738.5253975	2.929655172	1.550730866	0.00458207	0.257929
gene5969	34.09361423	131.6375545	3.861061887	1.948997679	0.00458324	0.257929
gene51226	407.0570911	1209.903993	2.972320148	1.571589517	0.00463531	0.260595
gene44759	23.76221598	101.6319354	4.277039462	2.096612518	0.00465297	0.261062
gene58333	85.75060548	271.0184945	3.160543217	1.660172542	0.00465302	0.261062
gene43838	26.86163545	1.935846389	0.072067332	-3.79451076	0.00471678	0.264373
gene9394	8.265118601	52.26785251	6.323908347	2.660816459	0.00473038	0.264868
gene39731	447.3495443	1334.766085	2.983720678	1.577112483	0.00473594	0.264912
gene35305	1119.92357	3812.649464	3.404383624	1.767393617	0.00474861	0.265353
gene34113	79.55176653	253.595877	3.187809499	1.672565417	0.00478268	0.266989
gene37099	40.29245318	148.0922488	3.675433911	1.87791458	0.00478923	0.267006
gene49862	20.6627965	91.95270349	4.450157726	2.15385647	0.00479404	0.267006
gene45188	27.89477528	113.2470138	4.059793013	2.021406174	0.00479741	0.267006
gene33751	6916.871129	1779.042832	0.257203409	-1.95901833	0.0048027	0.267033
gene51205	4.1325593	36.7810814	8.900315452	3.15385647	0.0048125	0.267043
gene54189	4.1325593	36.7810814	8.900315452	3.15385647	0.0048125	0.267043
gene25758	72.31978776	14.51884792	0.200758995	-2.31646346	0.00484136	0.26824
gene47823	69.22036828	13.55092472	0.19576499	-2.35280531	0.00484382	0.26824
gene64646	33.0604744	127.7658617	3.864610657	1.950323076	0.00484855	0.26824

gene55215	762.4571909	262.3071857	0.344028739	-1.53939901	0.00485375	0.26826
gene65634	914.3287452	313.6071151	0.342991639	-1.54375469	0.00486408	0.268564
gene58176	220.0587827	639.7972317	2.907392396	1.539725798	0.00490472	0.270539
gene7616	5.165699125	40.65277417	7.86975261	2.976318285	0.00493636	0.271745
gene31561	5.165699125	40.65277417	7.86975261	2.976318285	0.00493636	0.271745
gene72237	130.175618	388.137201	2.981642854	1.57610746	0.00496192	0.272881
gene28139	71.28664793	230.3657203	3.231540927	1.692222263	0.00499191	0.27401
gene9778	765.5566104	2411.096678	3.149468825	1.655108531	0.00499231	0.27401
gene59318	266.5500749	85.17724113	0.31955437	-1.64586668	0.00501803	0.27439
gene5851	50.62385143	175.1940982	3.460702678	1.791065	0.00501897	0.27439
gene30883	0	16.45469431	Inf	Inf	0.00502218	0.27439
gene67386	0	16.45469431	Inf	Inf	0.00502218	0.27439
gene57808	1203.607896	409.4315113	0.340170177	-1.55567143	0.00502572	0.27439
gene32772	340.9361423	993.0891977	2.912830512	1.542421758	0.00502888	0.27439
gene13674	403.9576716	136.4771704	0.337850176	-1.56554449	0.0050537	0.275474
gene13716	38.22617353	141.3167864	3.696859334	1.88630015	0.00507431	0.276326
gene53117	264.4837952	764.6593238	2.891138654	1.5316378	0.00508945	0.276879
gene36228	492.8076966	1467.371563	2.977574363	1.574137539	0.00509518	0.27692
gene36699	47.52443195	166.4827895	3.503098988	1.808631755	0.00510563	0.276921
gene51856	27.89477528	112.2790906	4.025093928	2.00902245	0.00510954	0.276921
gene43271	564.0943445	1701.608976	3.016532594	1.592891171	0.00511016	0.276921
gene58514	35.12675405	132.6054777	3.775056399	1.916498198	0.00511691	0.277016
gene57018	160.1366729	468.4748262	2.925468712	1.548667789	0.00513086	0.277501
gene50329	114.6785206	344.5806573	3.004753249	1.587246521	0.00514785	0.277958
gene35623	16.5302372	78.40177877	4.742931261	2.24577896	0.00515396	0.277958
gene62852	1020.742147	3360.629332	3.292339149	1.719112958	0.0051579	0.277958
gene8520	153.9378339	451.0522087	2.930093254	1.550946581	0.00516469	0.277958
gene9885	106.413402	322.3184238	3.028926975	1.598806797	0.00516963	0.277958
gene30659	1262.496866	429.7578984	0.340403141	-1.55468375	0.00517375	0.277958
gene57405	1550.742877	5662.350689	3.651379459	1.868441604	0.00517434	0.277958
gene18251	44.42501248	157.7714807	3.551411062	1.828392356	0.00519122	0.278595
gene24772	90.91630461	280.6977264	3.087430001	1.626406428	0.00524382	0.281146
gene69091	3.099419475	31.94146542	10.30562842	3.365360575	0.00529674	0.28371
gene73859	236.5890199	74.53008599	0.315019209	-1.66648829	0.00532921	0.285174
gene41503	110.5459613	331.9976558	3.003254501	1.586526736	0.00534215	0.285591
gene62048	116.7448002	348.4523501	2.984735503	1.577603091	0.00535125	0.285803
gene63609	240.7215792	691.097161	2.870939793	1.521523076	0.00537338	0.286709
gene42986	1.033139825	22.26223348	21.54813215	4.429490913	0.00538554	0.286897
gene5294	314.0745068	904.0402638	2.878426119	1.525280183	0.00538728	0.286897
gene24211	335.7704432	112.2790906	0.334392419	-1.58038596	0.0053924	0.286897
gene43739	52.69013108	179.065791	3.398469264	1.764885075	0.00539977	0.287015
gene44322	12.3976779	64.85085404	5.230887152	2.387055646	0.00541452	0.28738
gene38406	233.4896005	669.8028507	2.868662456	1.520378222	0.00541699	0.28738
gene10810	15.49709738	0	0	#NAME?	0.00545533	0.289138
gene42995	41.325593	148.0922488	3.583548063	1.841388704	0.00554201	0.293452
gene65208	480.4100187	166.4827895	0.346543126	-1.52889319	0.00558364	0.293972
gene55664	4.1325593	35.8131582	8.666096624	3.115382322	0.00559197	0.293972
gene60398	4.1325593	35.8131582	8.666096624	3.115382322	0.00559197	0.293972

gene25314	116.7448002	346.5165037	2.968153639	1.569565771	0.00559724	0.293972
gene9560	2.06627965	27.10184945	13.11625435	3.713283879	0.00560256	0.293972
gene27041	2.06627965	27.10184945	13.11625435	3.713283879	0.00560256	0.293972
gene44096	2.06627965	27.10184945	13.11625435	3.713283879	0.00560256	0.293972
gene63967	2.06627965	27.10184945	13.11625435	3.713283879	0.00560256	0.293972
gene54567	130.175618	382.3296619	2.937029744	1.554357876	0.00560279	0.293972
gene20160	16.5302372	77.43385557	4.684376554	2.227857052	0.00560475	0.293972
gene3649	643.646111	1944.557698	3.02115971	1.595102452	0.00561811	0.294394
gene16579	6.19883895	43.55654376	7.02656483	2.812819552	0.00562491	0.294473
gene21207	37.1930337	136.4771704	3.6694283	1.875555308	0.00566191	0.296131
gene16325	5.165699125	39.68485098	7.682377548	2.941552866	0.00567526	0.29655
gene23298	879.2019911	2772.132029	3.153009271	1.656729412	0.00569514	0.297309
gene25442	392.5931335	134.5413241	0.342699127	-1.54498558	0.00571996	0.298324
gene44007	164.2692322	473.3144422	2.881333503	1.526736656	0.00572696	0.298409
gene55306	28.9279151	113.2470138	3.914800406	1.968938754	0.00579823	0.301839
gene20529	14.46395755	70.65839321	4.885135549	2.288398593	0.00580633	0.301978
gene50071	153.9378339	444.2767463	2.886078977	1.52911078	0.00582919	0.302884
gene17118	122.9436392	361.0353516	2.93659236	1.554143014	0.00584892	0.303624
gene41482	308.9088077	878.8742607	2.845092917	1.50847577	0.00586501	0.304175
gene24665	301.6768289	857.5799504	2.842710703	1.507267286	0.00587789	0.304559
gene37555	80.58490636	249.7241842	3.098895259	1.631753993	0.00590734	0.3058
gene59941	12.3976779	63.88293085	5.152814209	2.365360575	0.0059671	0.308606
gene6547	479.3768788	1391.873554	2.903505812	1.537795922	0.00599138	0.309573
gene23365	253.1192571	82.27347154	0.325038373	-1.62131805	0.00601258	0.31038
gene36898	120.8773595	354.2598892	2.930738151	1.551264075	0.00602245	0.310601
gene11610	54.75641073	181.9695606	3.323255819	1.732597354	0.00602808	0.310603
gene10961	42.35873283	149.060172	3.51899507	1.815163493	0.00607764	0.312866
gene44083	963.9194568	3053.797679	3.168104614	1.663619976	0.00609488	0.313463
gene54000	813.0810423	2503.049381	3.078474655	1.622215691	0.00612423	0.314681
gene60100	23.76221598	97.76024266	4.114104625	2.040578483	0.00613179	0.314779
gene31249	61.9883895	200.3601013	3.232219822	1.692525319	0.00614379	0.315103
gene17405	343.0024219	117.1187066	0.341451544	-1.55024724	0.00615173	0.31511
gene35173	708.73392	2138.142337	3.016847757	1.593041894	0.00615527	0.31511
gene70044	25.82849563	103.5677818	4.00982633	2.003539753	0.0061699	0.315274
gene51336	46.49129213	159.7073271	3.435209473	1.780398075	0.00617479	0.315274
gene33299	27.89477528	109.375321	3.920996671	1.971220417	0.0061755	0.315274
gene26426	51.65699125	173.2582518	3.354013612	1.745888544	0.00618558	0.315499
gene4066	65.08780898	208.1034868	3.197272886	1.676841883	0.00621357	0.316636
gene31325	500.0396753	176.1620214	0.352296088	-1.50513964	0.00622305	0.316708
gene43346	55.78955055	183.905407	3.29641313	1.720897063	0.0062264	0.316708
gene17666	430.8193071	1234.102073	2.864546813	1.518306915	0.0062361	0.316872
gene23496	112.6122409	331.0297326	2.939553727	1.555597147	0.00624103	0.316872
gene54608	98.14828338	293.280728	2.988139149	1.579247332	0.00626179	0.317548
gene67980	89.88316478	21.29431028	0.236910998	-2.07758292	0.00626578	0.317548
gene34540	276.8814731	779.1781717	2.814121736	1.492684739	0.00628256	0.318108
gene15743	595.0885392	1750.973059	2.942374023	1.556980648	0.00630875	0.319144
gene46629	14.46395755	69.69047001	4.818215884	2.268499036	0.00635421	0.321151
gene55171	9.298258426	53.23577571	5.725349121	2.517363669	0.00637874	0.321962

gene7997	558.9286454	1630.950583	2.917994267	1.544977049	0.00638191	0.321962
gene17894	19.62965668	85.17724113	4.339211965	2.117433062	0.00638765	0.321962
gene25505	86.78374531	20.32638709	0.234218828	-2.09407104	0.00639417	0.321999
gene27500	584.757141	1713.224055	2.929804417	1.550804359	0.00641123	0.322565
gene60721	137.4075967	394.9126634	2.87402351	1.523071863	0.0064267	0.322987
gene72192	131.2087578	378.4579691	2.884395642	1.528269067	0.00643615	0.322987
gene2807	0	15.48677111	Inf	Inf	0.00646032	0.322987
gene9618	0	15.48677111	Inf	Inf	0.00646032	0.322987
gene14572	0	15.48677111	Inf	Inf	0.00646032	0.322987
gene47019	0	15.48677111	Inf	Inf	0.00646032	0.322987
gene50674	0	15.48677111	Inf	Inf	0.00646032	0.322987
gene11586	245.8872784	689.1613146	2.80275303	1.486844624	0.00646667	0.323013
gene43464	4.1325593	34.84523501	8.431877796	3.075853958	0.00650319	0.324291
gene297	253.1192571	83.24139474	0.328862354	-1.60444423	0.00650393	0.324291
gene71835	117.7779401	342.6448109	2.909244386	1.540644493	0.006522	0.324882
gene32930	5.165699125	38.71692779	7.495002486	2.905928957	0.00652919	0.324882
gene63600	448.3826841	1279.594463	2.85379991	1.512884186	0.00653332	0.324882
gene68578	12.3976779	62.91500765	5.074741266	2.343334269	0.00657774	0.326637
gene32057	24.7953558	99.69608905	4.020756542	2.007466983	0.00658596	0.326637
gene806	26.86163545	105.5036282	3.927669572	1.973673563	0.00658873	0.326637
gene597	1.033139825	21.29431028	20.61125684	4.365360575	0.00662252	0.326637
gene12528	1.033139825	21.29431028	20.61125684	4.365360575	0.00662252	0.326637
gene31897	1.033139825	21.29431028	20.61125684	4.365360575	0.00662252	0.326637
gene37892	1.033139825	21.29431028	20.61125684	4.365360575	0.00662252	0.326637
gene68270	1.033139825	21.29431028	20.61125684	4.365360575	0.00662252	0.326637
gene69957	1.033139825	21.29431028	20.61125684	4.365360575	0.00662252	0.326637
gene39519	357.4663795	123.8941689	0.34658971	-1.52869927	0.00662743	0.326637
gene28203	56.82269038	10.64715514	0.187375062	-2.41599914	0.00664533	0.32723
gene9723	142.5732959	406.5277417	2.851359641	1.511650018	0.00669013	0.329144
gene11267	76.45234706	235.2053363	3.076495953	1.621288095	0.00670294	0.329424
gene895	47.52443195	160.6752503	3.38089786	1.757406432	0.00670768	0.329424
gene7096	434.9518664	1232.166227	2.832879502	1.502269237	0.00678453	0.332903
gene32487	40.29245318	5.807539168	0.144134663	-2.79451076	0.00681678	0.333724
gene21067	33.0604744	3.871692779	0.117109414	-3.09407104	0.00682233	0.333724
gene40692	33.0604744	3.871692779	0.117109414	-3.09407104	0.00682233	0.333724
gene59947	18.59651685	81.30554835	4.372084783	2.128321378	0.00682528	0.333724
gene21178	92.98258426	276.8260337	2.977181543	1.573947197	0.00683748	0.334026
gene58830	1035.206105	3258.029473	3.14722784	1.654081625	0.00685292	0.334487
gene70169	39.25931335	138.4130168	3.525609722	1.81787278	0.00688728	0.335693
gene26725	256.2186766	85.17724113	0.332439626	-1.58883574	0.00688974	0.335693
gene68877	123.976779	356.1957356	2.873084286	1.522600317	0.00691434	0.336597
gene47225	25.82849563	101.6319354	3.934876305	1.976318285	0.00704223	0.342452
gene37442	67.15408863	210.0393332	3.127722191	1.645112376	0.00704695	0.342452
gene57961	82.65118601	19.35846389	0.234218828	-2.09407104	0.00708886	0.344188
gene29068	35.12675405	126.7979385	3.609725462	1.851889117	0.00712578	0.345474
gene71012	9.298258426	52.26785251	5.621251864	2.490891457	0.00713121	0.345474
gene13991	811.0147627	2436.262681	3.003968353	1.586869614	0.00713401	0.345474
gene11668	83.68432583	251.6600306	3.007254084	1.588446767	0.00714417	0.345665

gene58790	161.1698127	48.39615973	0.300280548	-1.73561707	0.00715116	0.345702
gene6759	105.3802622	306.8316527	2.911661505	1.541842645	0.00715763	0.345714
gene26711	322.3396254	893.3931087	2.771589461	1.470713575	0.00719461	0.347129
gene70389	536.1995692	1531.254494	2.855754801	1.513872113	0.00719943	0.347129
gene51428	244.8541385	676.5783131	2.763189208	1.466334353	0.0072112	0.347304
gene65662	16.5302372	74.53008599	4.508712433	2.172715497	0.00721557	0.347304
gene54838	12.3976779	61.94708446	4.996668324	2.320966456	0.00725268	0.348788
gene55488	1126.122409	3564.861126	3.165607128	1.662482219	0.0072777	0.349688
gene64836	113.6453808	327.1580398	2.878762318	1.525448679	0.00730545	0.350597
gene72649	531.0338701	191.6487925	0.360897493	-1.47033897	0.00731233	0.350597
gene24200	286.1797315	97.76024266	0.341604355	-1.54960173	0.00731555	0.350597
gene43232	38.22617353	134.5413241	3.519612654	1.815416664	0.00732245	0.350625
gene1170	30.99419475	115.1828602	3.716272066	1.893856124	0.00734103	0.351212
gene42408	621.9501747	1797.433372	2.889995767	1.53106738	0.00735689	0.351431
gene12979	667.408327	1944.557698	2.9135952	1.542800451	0.00735983	0.351431
gene25882	206.627965	571.0746848	2.763782167	1.466643911	0.00736458	0.351431
gene3633	29.96105493	112.2790906	3.747501243	1.905928957	0.00738772	0.352232
gene11325	49.5907116	8.711308752	0.175664121	-2.50910854	0.00740893	0.352941
gene55777	1974.330206	7254.584344	3.674453403	1.877529657	0.00743147	0.353599
gene24741	103.3139825	300.0561903	2.904313463	1.538197172	0.00744299	0.353599
gene62625	1237.70151	442.3408999	0.357388996	-1.48443288	0.00744755	0.353599
gene5493	13.43081773	64.85085404	4.828511217	2.271578429	0.00745167	0.353599
gene6854	214.8930836	592.3689951	2.756575433	1.462877081	0.00745608	0.353599
gene45871	36.15989388	4.839615973	0.13383933	-2.90142597	0.00746095	0.353599
gene21271	106.413402	307.7995759	2.892488823	1.532311385	0.00746944	0.3537
gene29093	10.33139825	55.17162209	5.340189271	2.416890876	0.00747866	0.353769
gene18105	267.5832147	735.6216279	2.749132186	1.458976277	0.0074868	0.353769
gene7980	53.7232709	174.226175	3.243029922	1.697342335	0.00749784	0.353769
gene18821	329.5716042	908.8798798	2.757761495	1.463497691	0.00750016	0.353769
gene30178	964.9525966	349.4202733	0.362111335	-1.46549476	0.00750275	0.353769
gene52371	5.165699125	37.74900459	7.307627424	2.869403081	0.00751672	0.353827
gene59320	5.165699125	37.74900459	7.307627424	2.869403081	0.00751672	0.353827
gene11072	326.4721847	899.2006478	2.754294822	1.461682995	0.0075552	0.3551
gene55395	487.6419974	1371.547167	2.812610837	1.49190995	0.00756469	0.3551
gene64910	3495.112028	1114.079597	0.318753616	-1.64948639	0.00756621	0.3551
gene313	4.1325593	33.87731181	8.197658969	3.035211974	0.00756933	0.3551
gene9402	34.09361423	122.9262457	3.605550438	1.850219524	0.00759926	0.356203
gene28114	79.55176653	239.0770291	3.005301321	1.587509648	0.00764587	0.358086
gene44625	262.4175156	719.1669336	2.740544708	1.45446267	0.00766309	0.358163
gene13492	33.0604744	120.0224761	3.630391829	1.860125267	0.00766578	0.358163
gene51601	456.6478027	1275.722771	2.793668913	1.482161052	0.00766687	0.358163
gene34155	439.0844257	1222.486995	2.784172982	1.477248849	0.00770954	0.359854
gene17059	849.2409362	2535.95877	2.986147584	1.578285469	0.00772451	0.360212
gene53730	281.0140324	769.4989397	2.738293647	1.453277166	0.00773018	0.360212
gene63321	15.49709738	70.65839321	4.559459845	2.18886292	0.00774675	0.360682
gene46173	295.47799	809.1837907	2.738558601	1.453416752	0.00775961	0.360927
gene61398	11.36453808	58.07539168	5.110228968	2.353387934	0.00776501	0.360927
gene45646	42.35873283	144.220556	3.404741983	1.767545472	0.00777165	0.360933

gene38509	46.49129213	154.8677111	3.331112216	1.736003955	0.0077823	0.360993
gene51844	635.3809924	232.3015667	0.365609877	-1.45162305	0.00778594	0.360993
gene13909	452.5152434	1260.235999	2.784958115	1.47765563	0.00780304	0.361485
gene61266	100.214563	290.3769584	2.897552507	1.534834805	0.00781345	0.361665
gene57940	372.9634769	1026.96651	2.753531038	1.461282871	0.00783716	0.362217
gene34521	67.15408863	207.1355637	3.084481792	1.62502813	0.00784543	0.362217
gene18557	113.6453808	30.97354223	0.272545545	-1.87543076	0.00784757	0.362217
gene49392	29.96105493	111.3111674	3.715195198	1.893438012	0.00785146	0.362217
gene2680	96.08200373	279.7298033	2.911365213	1.541695828	0.00786402	0.362496
gene71249	143.6064357	400.7202026	2.790405602	1.480474841	0.00788817	0.363307
gene28071	203.5285455	557.5237601	2.739290249	1.453802139	0.00791218	0.363891
gene40764	396.7256928	142.2847096	0.35864758	-1.4793612	0.00791395	0.363891
gene31722	146.7058552	408.4635881	2.784235078	1.477281026	0.00792914	0.364288
gene14209	154.9709738	429.7578984	2.77315092	1.471526133	0.0079467	0.364296
gene57206	48.55757178	159.7073271	3.289030346	1.717662319	0.00795315	0.364296
gene19781	7.231978776	44.52446695	6.156609185	2.622135991	0.00796511	0.364296
gene28836	7.231978776	44.52446695	6.156609185	2.622135991	0.00796511	0.364296
gene41160	7.231978776	44.52446695	6.156609185	2.622135991	0.00796511	0.364296
gene44311	44.42501248	149.060172	3.35532088	1.746450743	0.00796945	0.364296
gene15514	9.298258426	51.29992932	5.517154608	2.46392441	0.00797523	0.364296
gene11813	117.7779401	333.9335022	2.835280546	1.503491494	0.00798601	0.364489
gene40895	557.8955055	1574.811038	2.822770612	1.497111895	0.00799306	0.364511
gene67253	26.86163545	102.5998586	3.819568574	1.933409693	0.00800188	0.364614
gene26227	97.11514356	281.6656496	2.90032676	1.536215448	0.00800969	0.364671
gene1857	128.1093383	360.0674284	2.810625932	1.490891457	0.00801805	0.364752
gene41848	68.18722846	209.07141	3.066137381	1.616422339	0.00803834	0.365376
gene2806	2.06627965	25.16600306	12.17937904	3.606368675	0.00807866	0.366011
gene7680	2.06627965	25.16600306	12.17937904	3.606368675	0.00807866	0.366011
gene26228	2.06627965	25.16600306	12.17937904	3.606368675	0.00807866	0.366011
gene32286	2.06627965	25.16600306	12.17937904	3.606368675	0.00807866	0.366011
gene9137	177.7000499	487.8332901	2.745262538	1.456944125	0.0081014	0.366295
gene16077	33.0604744	119.0545529	3.601114476	1.848443462	0.00811921	0.366295
gene32480	656.0437889	1878.738921	2.863740123	1.517900578	0.00813444	0.366295
gene50552	518.6361922	189.7129461	0.365791954	-1.45090475	0.00813628	0.366295
gene3349	82.65118601	244.8845682	2.96286817	1.566994436	0.00814276	0.366295
gene48829	101.2477029	291.3448816	2.877545597	1.524838789	0.00816237	0.366295
gene1800	1.033139825	20.32638709	19.67438153	4.298246379	0.00816456	0.366295
gene19212	1.033139825	20.32638709	19.67438153	4.298246379	0.00816456	0.366295
gene28369	1.033139825	20.32638709	19.67438153	4.298246379	0.00816456	0.366295
gene38258	1.033139825	20.32638709	19.67438153	4.298246379	0.00816456	0.366295
gene64537	1.033139825	20.32638709	19.67438153	4.298246379	0.00816456	0.366295
gene55389	184.9320287	506.2238308	2.737350768	1.452780316	0.0081703	0.366295
gene24738	42.35873283	6.775462362	0.159954321	-2.64426813	0.00817067	0.366295
gene68055	56.82269038	180.0337142	3.16834196	1.663728054	0.00818612	0.366692
gene39102	476.2774594	1321.215161	2.774045117	1.471991252	0.0082257	0.368168
gene180	234.5227403	636.8934621	2.71570024	1.441324243	0.00825313	0.368871
gene62688	926.7264231	2774.067876	2.993405396	1.581787676	0.00826103	0.368871
gene60755	284.1134519	98.72816585	0.34749557	-1.52493351	0.00827815	0.368871

gene23136	340.9361423	928.2383437	2.722616433	1.444993747	0.00830243	0.368871
gene58522	129.1424781	361.0353516	2.795635927	1.483176492	0.00831071	0.368871
gene47963	1219.104994	3830.072081	3.141708139	1.651549162	0.00832183	0.368871
gene27583	2878.327553	958.2439627	0.332916927	-1.58676587	0.00834803	0.368871
gene48370	292.3785705	792.7290964	2.711310528	1.438990355	0.00834913	0.368871
gene53352	6.19883895	40.65277417	6.558127175	2.713283879	0.00834988	0.368871
gene67621	6.19883895	40.65277417	6.558127175	2.713283879	0.00834988	0.368871
gene14698	0	14.51884792	Inf	Inf	0.0083543	0.368871
gene14808	0	14.51884792	Inf	Inf	0.0083543	0.368871
gene19653	0	14.51884792	Inf	Inf	0.0083543	0.368871
gene39971	0	14.51884792	Inf	Inf	0.0083543	0.368871
gene58853	0	14.51884792	Inf	Inf	0.0083543	0.368871
gene67859	0	14.51884792	Inf	Inf	0.0083543	0.368871
gene70035	0	14.51884792	Inf	Inf	0.0083543	0.368871
gene48425	794.4845255	2319.143974	2.919054934	1.545501361	0.00839294	0.370283
gene18697	28.9279151	107.4394746	3.71404141	1.892989901	0.00841391	0.370673
gene22586	57.8558302	181.9695606	3.145224257	1.653162886	0.00841512	0.370673
gene46094	291.3454307	788.8574036	2.707636093	1.437033853	0.00843072	0.371065
gene64750	1098.227634	3372.24441	3.070624255	1.618531984	0.00843876	0.371125
gene14995	8.265118601	47.42823654	5.738361278	2.520638801	0.00849492	0.3733
gene59494	42.35873283	142.2847096	3.359040748	1.748049297	0.00857676	0.376598
gene9574	295.47799	797.5687124	2.699249147	1.432558147	0.00863816	0.378994
gene5935	18.59651685	78.40177877	4.215938898	2.075853958	0.00866004	0.379562
gene52836	23.76221598	1.935846389	0.081467418	-3.617633	0.00866476	0.379562
gene30577	3.099419475	29.03769584	9.368753107	3.227857052	0.008696	0.380083
gene59143	3.099419475	29.03769584	9.368753107	3.227857052	0.008696	0.380083
gene43363	22.72907615	90.0168571	3.96042745	1.985656149	0.00869718	0.380083
gene9991	856.472915	2511.76069	2.932679652	1.552219488	0.00870663	0.380197
gene71219	283.0803121	762.7234774	2.694371332	1.429948693	0.00872401	0.380656
gene9714	40.29245318	136.4771704	3.387164585	1.76007809	0.00880289	0.382838
gene36847	4.1325593	32.90938862	7.963440141	2.993391798	0.00881782	0.382838
gene40555	4.1325593	32.90938862	7.963440141	2.993391798	0.00881782	0.382838
gene43864	4.1325593	32.90938862	7.963440141	2.993391798	0.00881782	0.382838
gene72522	4.1325593	32.90938862	7.963440141	2.993391798	0.00881782	0.382838
gene62870	1214.972434	3774.900459	3.106984449	1.63551502	0.00882106	0.382838
gene32380	412.2227902	1121.822983	2.721399712	1.444348871	0.00882642	0.382838
gene25967	69.22036828	209.07141	3.020374136	1.594727268	0.00882915	0.382838
gene63850	96.08200373	25.16600306	0.26192213	-1.93279014	0.00886079	0.38391
gene40655	121.9104994	339.7410413	2.786807068	1.478613128	0.008882	0.384529
gene21664	1226.336972	449.1163623	0.366225901	-1.44919427	0.0089081	0.385359
gene52310	9.298258426	50.33200612	5.413057351	2.436443673	0.00892222	0.385669
gene26602	13.43081773	62.91500765	4.684376554	2.227857052	0.00899848	0.388489
gene41072	34.09361423	120.0224761	3.520379955	1.815731148	0.00900145	0.388489
gene69718	614.7181959	1719.999517	2.798029289	1.484411064	0.0090423	0.389903
gene22570	59.92210985	12.58300153	0.209989294	-2.25161232	0.00904824	0.389903
gene65975	75.41920723	223.590258	2.964632833	1.567853439	0.00908355	0.391121
gene7881	14.46395755	65.81877724	4.550537223	2.186036876	0.00913253	0.392925
gene11140	261.3843757	90.9847803	0.34808806	-1.52247577	0.00913989	0.392937

gene34112	38.22617353	5.807539168	0.151925726	-2.71856191	0.00914934	0.39304
gene49036	15.49709738	68.72254682	4.434543137	2.148785481	0.00923248	0.396305
gene14594	24.7953558	94.85647307	3.825574185	1.9356763	0.00924364	0.396477
gene59468	552.7298064	1525.446955	2.759842037	1.464585695	0.00925518	0.396666
gene73987	68.18722846	15.48677111	0.227121287	-2.13846516	0.00932736	0.399042
gene35438	661.2094881	247.7883378	0.374750124	-1.41599914	0.00933031	0.399042
gene24605	203.5285455	67.75462362	0.332899857	-1.58683985	0.00934173	0.399042
gene38934	220.0587827	588.4973023	2.674273187	1.41914685	0.009347	0.399042
gene3870	120.8773595	334.9014253	2.770588526	1.470192465	0.00935037	0.399042
gene2785	103.3139825	291.3448816	2.819994685	1.495692444	0.00935372	0.399042
gene72906	178.7331897	481.0578277	2.691485719	1.428402771	0.00940324	0.400846
gene15898	64.05466916	194.5525621	3.037289314	1.602784337	0.0094232	0.401389
gene17565	266.5500749	710.4556249	2.665373946	1.414337954	0.00943386	0.401535
gene45283	797.583945	2285.266663	2.86523654	1.518654246	0.00946354	0.402476
gene14859	1115.791011	3367.404794	3.017952969	1.593570323	0.0094805	0.402476
gene65490	57.8558302	179.065791	3.095034509	1.629955495	0.00948744	0.402476
gene3775	47.52443195	8.711308752	0.183301691	-2.447708	0.0095123	0.402476
gene48831	47.52443195	8.711308752	0.183301691	-2.447708	0.0095123	0.402476
gene47565	34.09361423	119.0545529	3.491989794	1.804049343	0.00952487	0.402476
gene29798	11.36453808	56.13954529	4.939888002	2.304478333	0.00953009	0.402476
gene4956	6.19883895	39.68485098	6.40198129	2.678518461	0.00953567	0.402476
gene53641	6.19883895	39.68485098	6.40198129	2.678518461	0.00953567	0.402476
gene54428	6.19883895	39.68485098	6.40198129	2.678518461	0.00953567	0.402476
gene71597	6.19883895	39.68485098	6.40198129	2.678518461	0.00953567	0.402476
gene27592	472.1449001	1280.562387	2.712223274	1.439475948	0.00957611	0.403876
gene54157	27.89477528	102.5998586	3.678103072	1.878961909	0.00962851	0.405778
gene6234	13.43081773	0	0	#NAME?	0.00965874	0.406744
gene53776	282.0471722	749.1725526	2.656195936	1.409361572	0.00969119	0.407801
gene28778	1359.61201	500.4162916	0.368058158	-1.44199435	0.00971612	0.408541
gene54896	2.06627965	24.19807987	11.71094138	3.549785146	0.00972376	0.408552
gene55899	90.91630461	259.4034162	2.853211174	1.512586528	0.00974665	0.40893
gene61784	40.29245318	134.5413241	3.339119697	1.739467811	0.00974748	0.40893
gene30244	316.1407865	114.214937	0.361278715	-1.46881584	0.00976878	0.409515
gene42736	82.65118601	239.0770291	2.892602522	1.532368093	0.00978354	0.409824
gene56660	1066.200299	3172.852232	2.975850066	1.57330184	0.0098144	0.410549
gene61230	204.5616854	68.72254682	0.335950238	-1.57368054	0.00981585	0.410549
gene19795	98.14828338	276.8260337	2.820487778	1.495944685	0.00982303	0.410549
gene60913	2819.438583	11340.18815	4.02214406	2.007964755	0.00983148	0.410594
gene14413	160.1366729	430.7258216	2.689738795	1.427466077	0.00988401	0.412477
gene36464	13.43081773	61.94708446	4.612309222	2.205489239	0.0098916	0.412484
gene70506	722.1647377	2027.799093	2.807945316	1.48951484	0.00993551	0.413823
gene8055	195.2634269	519.7747555	2.661915565	1.41246481	0.00993861	0.413823
gene68843	219.0256429	580.7539168	2.651533898	1.406827193	0.00997256	0.414439
gene21555	9.298258426	49.36408293	5.308960094	2.408429297	0.00998506	0.414439
gene31659	9.298258426	49.36408293	5.308960094	2.408429297	0.00998506	0.414439
gene73799	9.298258426	49.36408293	5.308960094	2.408429297	0.00998506	0.414439
gene44283	429.7861672	1151.828602	2.680003894	1.422235097	0.00999071	0.414439
gene36041	22.72907615	88.08101071	3.875256967	1.954291978	0.01002975	0.415748

gene39325	153.9378339	48.39615973	0.314387688	-1.66938337	0.01004005	0.415865
gene14043	15.49709738	67.75462362	4.372084783	2.128321378	0.01008169	0.416602
gene56383	83.68432583	21.29431028	0.254459961	-1.97448943	0.01008312	0.416602
gene35788	87.81688513	250.6921074	2.854714182	1.513346308	0.01008508	0.416602
gene53540	1773.90108	5888.844716	3.319714263	1.73105907	0.01009435	0.416602
gene54415	1.033139825	19.35846389	18.73750621	4.227857052	0.01009536	0.416602
gene44677	2419.61347	846.9327953	0.350028137	-1.5144572	0.01012006	0.417312
gene52900	365.7314981	970.8269642	2.654480047	1.408429297	0.01014045	0.417676
gene60693	19.62965668	79.36970196	4.043356604	2.015553448	0.01014393	0.417676
gene17111	17.56337703	73.56216279	4.188383742	2.066393629	0.0101534	0.417756
gene45690	497.9733957	1343.477394	2.697889899	1.431831473	0.01019168	0.418896
gene62937	97.11514356	272.9543409	2.810625932	1.490891457	0.01019619	0.418896
gene47914	239.6884394	632.0538461	2.636980939	1.398887143	0.01026393	0.421167
gene9501	3.099419475	28.06977264	9.056461337	3.178947451	0.01028179	0.421167
gene31220	3.099419475	28.06977264	9.056461337	3.178947451	0.01028179	0.421167
gene62364	3.099419475	28.06977264	9.056461337	3.178947451	0.01028179	0.421167
gene67884	10.33139825	52.26785251	5.059126678	2.338888364	0.01031304	0.422135
gene62841	51.65699125	161.6431735	3.129163538	1.645777059	0.01038641	0.424826
gene28445	682.9054244	1891.321922	2.769522477	1.469637247	0.01041114	0.425242
gene57030	1801.795855	5969.182341	3.312907134	1.728097763	0.01041901	0.425242
gene51337	39.25931335	130.6696313	3.328372814	1.73481704	0.01041955	0.425242
gene2376	53.7232709	166.4827895	3.098895259	1.631753993	0.01050046	0.428133
gene62925	35.12675405	120.0224761	3.416839369	1.772662426	0.01050582	0.428133
gene32814	2904.156048	11630.56511	4.004800332	2.001730316	0.01054137	0.429267
gene53605	314.0745068	825.638485	2.628798158	1.394403375	0.01055411	0.429471
gene25774	71.28664793	209.07141	2.93282706	1.552292002	0.01058948	0.430325
gene10218	24.7953558	92.92062669	3.747501243	1.905928957	0.01059058	0.430325
gene50962	221.0919226	76.46593238	0.345855839	-1.53175728	0.01064678	0.432292
gene9745	195.2634269	65.81877724	0.337076831	-1.56885063	0.01066218	0.432436
gene9393	34.09361423	117.1187066	3.435209473	1.780398075	0.0106659	0.432436
gene45063	80.58490636	231.3336435	2.870682042	1.521393546	0.01067434	0.432462
gene29484	164.2692322	436.5333608	2.657426196	1.410029625	0.01070466	0.433375
gene21700	172.5343508	456.8597479	2.64793501	1.404867714	0.01077555	0.435927
gene25076	28.9279151	103.5677818	3.58020208	1.840041021	0.01079525	0.436333
gene40730	285.1465917	746.2687831	2.617140814	1.38799155	0.01080595	0.436333
gene44148	1814.193533	5975.957804	3.294002374	1.719841595	0.01080915	0.436333
gene26340	781.0537078	2183.634727	2.795754895	1.483237885	0.01084781	0.43669
gene27288	1343.081773	502.352138	0.374029451	-1.41877622	0.01086251	0.43669
gene6568	0	13.55092472	Inf	Inf	0.01086516	0.43669
gene20486	0	13.55092472	Inf	Inf	0.01086516	0.43669
gene49587	0	13.55092472	Inf	Inf	0.01086516	0.43669
gene52554	0	13.55092472	Inf	Inf	0.01086516	0.43669
gene32067	238.6552996	624.3104605	2.615950543	1.387335266	0.01089445	0.436873
gene21893	6.19883895	38.71692779	6.245835405	2.642894551	0.01089588	0.436873
gene58684	6.19883895	38.71692779	6.245835405	2.642894551	0.01089588	0.436873
gene4662	69.22036828	203.2638709	2.936474854	1.554085284	0.01090119	0.436873
gene74033	332.6710237	122.9262457	0.369512933	-1.43630323	0.01094486	0.438307
gene2814	100.214563	277.7939569	2.771991899	1.470923041	0.01097376	0.439148

gene55876	98.14828338	27.10184945	0.276131671	-1.85657173	0.01098506	0.439284
gene66009	80.58490636	230.3657203	2.85867082	1.515344501	0.01101397	0.440123
gene6792	17.56337703	72.5942396	4.13327343	2.047284806	0.01102628	0.440159
gene49325	16.5302372	69.69047001	4.215938898	2.075853958	0.01103072	0.440159
gene71395	1287.292222	3880.404087	3.014392553	1.591867306	0.01111809	0.443327
gene64514	52.69013108	162.6110967	3.086177494	1.625821037	0.01113686	0.443757
gene61679	393.6262734	148.0922488	0.376225518	-1.41033039	0.0111565	0.444221
gene56196	57.8558302	12.58300153	0.217488911	-2.20098625	0.01117813	0.444763
gene31696	736.6286953	2033.606632	2.760694289	1.465031137	0.01122931	0.446287
gene53762	563.0612047	215.8468724	0.383345311	-1.38328356	0.01123251	0.446287
gene37751	100.214563	276.8260337	2.76233339	1.465887451	0.01126549	0.447278
gene7250	1092.028795	3183.499387	2.915215607	1.543602588	0.01131654	0.448914
gene53808	326.4721847	120.9903993	0.370599411	-1.43206751	0.01132288	0.448914
gene39259	100.214563	28.06977264	0.280096742	-1.83600289	0.01133599	0.448966
gene24310	1198.442197	3550.342278	2.962464345	1.56679779	0.0113415	0.448966
gene56477	232.4564606	604.9519966	2.602431419	1.379860145	0.01135279	0.448966
gene66594	40.29245318	131.6375545	3.267052366	1.707989579	0.01136011	0.448966
gene28154	80.58490636	229.3977971	2.846659598	1.509269987	0.01136459	0.448966
gene46899	156.0041136	50.33200612	0.322632557	-1.63203606	0.01138521	0.449461
gene45015	58.88897003	177.1299446	3.00786284	1.588738781	0.01139967	0.449563
gene5736	95.04886391	26.13392626	0.274952537	-1.8627455	0.01140398	0.449563
gene31668	294.4448501	764.6593238	2.596952616	1.376819691	0.01145148	0.451052
gene65546	23.76221598	89.04893391	3.747501243	1.905928957	0.01145799	0.451052
gene16317	54.75641073	11.61507834	0.212122712	-2.237029	0.01147856	0.451509
gene18620	10.33139825	51.29992932	4.965439147	2.311921316	0.01148585	0.451509
gene52230	395.692553	1034.709895	2.614933961	1.386774512	0.01152701	0.452806
gene5941	193.1971473	504.2879844	2.610224796	1.384174059	0.01154074	0.453025
gene67559	22.72907615	86.14516432	3.790086484	1.922230769	0.01156958	0.453806
gene46376	162.2029525	426.8541288	2.631605172	1.395943053	0.01158036	0.453806
gene36934	1014.543308	2910.545046	2.868822871	1.520458895	0.01158555	0.453806
gene68216	1126.122409	3286.099246	2.918065761	1.545012396	0.01159688	0.453806
gene16959	351.2675405	913.7194957	2.601206745	1.37918107	0.0116015	0.453806
gene6967	723.1978776	1981.338779	2.739691087	1.454013232	0.01162563	0.454431
gene57892	27.89477528	99.69608905	3.574005815	1.837541982	0.01165606	0.45506
gene61537	32.02733458	110.3432442	3.445283401	1.78462266	0.01165813	0.45506
gene49591	533.1001497	1417.039557	2.658111347	1.41040154	0.01166736	0.455101
gene70914	11.36453808	54.2036989	4.769547036	2.25385226	0.0117086	0.456224
gene44360	108.4796816	295.2165744	2.721399712	1.444348871	0.01171257	0.456224
gene59323	129.1424781	39.68485098	0.307295102	-1.70230332	0.01174585	0.457031
gene52507	130.175618	347.4844269	2.669351084	1.416489067	0.01174977	0.457031
gene25807	82.65118601	233.2694899	2.822336874	1.496890198	0.01176212	0.457192
gene38317	3247.15847	13362.1797	4.115037755	2.040905667	0.01181385	0.458881
gene40361	134.3081773	357.1636588	2.659284536	1.41103815	0.01184279	0.459684
gene588	12.3976779	57.10746848	4.606303611	2.203609505	0.01186231	0.46012
gene3089	18.59651685	74.53008599	4.007744385	2.002790496	0.01191908	0.462
gene1466	40.29245318	130.6696313	3.243029922	1.697342335	0.0119555	0.462765
gene70953	40.29245318	130.6696313	3.243029922	1.697342335	0.0119555	0.462765
gene10471	264.4837952	682.3858522	2.580066774	1.367408404	0.0119755	0.463217

gene52355	2401.016953	8503.205265	3.541501551	1.824361174	0.01199399	0.463426
gene32796	4.1325593	30.97354223	7.495002486	2.905928957	0.01199759	0.463426
gene8497	16.5302372	68.72254682	4.157384191	2.055676076	0.01201373	0.463727
gene7911	15.49709738	65.81877724	4.247168075	2.086501202	0.01202778	0.463946
gene58267	850.274076	2360.764672	2.776474949	1.473254379	0.01210648	0.466534
gene56957	154.9709738	406.5277417	2.62325087	1.391355784	0.01211167	0.466534
gene18907	160.1366729	419.1107433	2.617206513	1.388027766	0.0121325	0.467013
gene37909	3.099419475	27.10184945	8.744169567	3.128321378	0.01217079	0.467838
gene61886	3.099419475	27.10184945	8.744169567	3.128321378	0.01217079	0.467838
gene4213	675.6734456	1823.567299	2.69888851	1.432365381	0.01219132	0.468304
gene34744	64.05466916	187.7770998	2.931513069	1.551645488	0.01223052	0.469485
gene1693	362.6320786	137.4450936	0.379020781	-1.39965114	0.0122917	0.471507
gene33554	36.15989388	5.807539168	0.160607196	-2.63839156	0.0123136	0.472021
gene16883	288.2460112	740.4612439	2.568851658	1.361123582	0.01237009	0.47386
gene56174	89.88316478	248.756261	2.767551205	1.46861001	0.01240095	0.474715
gene71160	172.5343508	448.1484391	2.597444724	1.377093047	0.01243572	0.475718
gene1048	48.55757178	150.0280952	3.089695174	1.62746451	0.01249304	0.477581
gene42035	1.033139825	18.3905407	17.8006309	4.15385647	0.01252133	0.478005
gene46375	1.033139825	18.3905407	17.8006309	4.15385647	0.01252133	0.478005
gene70171	86.78374531	241.0128755	2.7771661	1.473613466	0.01255055	0.478791
gene53703	400.8582521	1035.677818	2.583650986	1.369411196	0.01258304	0.479702
gene56345	110.5459613	297.1524208	2.688044116	1.426556816	0.01263302	0.481276
gene23160	236.5890199	85.17724113	0.360021953	-1.47384321	0.01265863	0.481922
gene28772	115.7116604	34.84523501	0.301138493	-1.73150096	0.01267182	0.482093
gene61884	20.6627965	79.36970196	3.841188774	1.941552866	0.01268646	0.48232
gene58292	17.56337703	0.967923195	0.055110312	-4.18153388	0.01273135	0.483695
gene56445	413.25593	1066.65136	2.581091481	1.367981275	0.0127899	0.485476
gene7144	10.33139825	50.33200612	4.871751616	2.28444058	0.01279569	0.485476
gene21635	329.5716042	844.0290257	2.56098831	1.356700668	0.01280825	0.485621
gene27832	200.4291261	514.9351395	2.569163223	1.36129855	0.01284197	0.486567
gene56863	18.59651685	73.56216279	3.955695756	1.983931469	0.01291416	0.488823
gene8258	171.501211	443.3088231	2.584872845	1.370093313	0.01291912	0.488823
gene23609	407.0570911	156.8035575	0.385212691	-1.37627286	0.01292904	0.488866
gene71735	46.49129213	144.220556	3.102098251	1.633244381	0.01297775	0.490236
gene13337	11.36453808	53.23577571	4.684376554	2.227857052	0.01298293	0.490236
gene12275	703.5682209	1887.45023	2.682682608	1.423676377	0.01301967	0.491289
gene53373	120.8773595	320.3825774	2.650476306	1.406251644	0.01305611	0.49233
gene65191	235.5558801	85.17724113	0.361600997	-1.46752944	0.01307283	0.492382
gene53246	38.22617353	123.8941689	3.241082156	1.696475591	0.01307522	0.492382
gene60209	12.3976779	56.13954529	4.528230668	2.178947451	0.01309787	0.492842
gene45768	32.02733458	108.4073978	3.384839832	1.759087568	0.01311437	0.492842
gene23409	21.69593633	1.935846389	0.08922622	-3.48638847	0.01312391	0.492842
gene63076	67.15408863	16.45469431	0.245028927	-2.02897602	0.01313159	0.492842
gene4319	15.49709738	64.85085404	4.184709721	2.065127551	0.01314069	0.492842
gene49937	15.49709738	64.85085404	4.184709721	2.065127551	0.01314069	0.492842
gene13686	13.43081773	59.04331487	4.396107227	2.136226576	0.01315504	0.493048
gene61482	23.76221598	87.11308752	3.666033825	1.874220097	0.01317481	0.49313
gene67056	761.4240511	299.0882671	0.392801182	-1.34812882	0.01317509	0.49313

gene18633	112.6122409	33.87731181	0.300831522	-1.73297235	0.01318724	0.49313
gene34294	152.9046941	396.8485098	2.59539782	1.375955691	0.01319274	0.49313
gene33809	175.6337703	452.0201319	2.573651589	1.36381676	0.01321798	0.493741
gene57432	996.9799312	2786.650877	2.795092248	1.482895898	0.01328145	0.495778
gene65059	1045.537503	2942.486512	2.814328997	1.49279099	0.01330813	0.496144
gene46665	2572.518164	9130.419495	3.549214782	1.827499882	0.0133091	0.496144
gene52047	487.6419974	1262.171846	2.588316536	1.372014062	0.01335966	0.497695
gene46608	360.565799	919.5270349	2.550233654	1.350629434	0.01340744	0.49914
gene21515	65.08780898	187.7770998	2.884981116	1.528561875	0.01343687	0.4999
gene58109	28.9279151	3.871692779	0.13383933	-2.90142597	0.01350321	0.501319
gene21943	223.1582022	80.33762515	0.360003013	-1.47391911	0.0135058	0.501319
gene17985	227.2907615	577.8501472	2.542338911	1.346156364	0.01350774	0.501319
gene48206	121.9104994	321.3505006	2.635954264	1.398325339	0.0135111	0.501319
gene66908	53.7232709	160.6752503	2.990794261	1.58052867	0.01352963	0.501346
gene30171	633.3147128	1670.635434	2.637922979	1.399402442	0.01352989	0.501346
gene4851	229.3570412	582.6897632	2.540535752	1.345132767	0.01355878	0.501984
gene74018	477.3105992	186.8091766	0.391378647	-1.35336305	0.01357064	0.501984
gene45016	55.78955055	165.5148663	2.966771817	1.568893969	0.01357421	0.501984
gene59261	567.193764	223.590258	0.394204366	-1.34298434	0.0136986	0.506012
gene66048	318.2070661	120.9903993	0.38022537	-1.3950733	0.01370603	0.506012
gene58056	206.627965	525.5822947	2.543616469	1.346881155	0.01371046	0.506012
gene16744	313.041367	792.7290964	2.532346137	1.340474615	0.01378051	0.508259
gene20759	69.22036828	17.4226175	0.251697845	-1.99023523	0.01382322	0.509435
gene70793	1084.796816	3049.925986	2.811518194	1.491349383	0.0138354	0.509435
gene48391	227.2907615	575.9143008	2.533821863	1.341315101	0.01383989	0.509435
gene54497	154.9709738	398.7843562	2.573284187	1.363610793	0.01395642	0.513384
gene59402	205.5948252	521.7106019	2.537566796	1.343445799	0.01396628	0.513406
gene53127	3319.478258	13122.13475	3.953071456	1.982974034	0.01399645	0.513797
gene26233	273.7820536	691.097161	2.524260271	1.335860671	0.01400916	0.513797
gene46361	4.1325593	30.00561903	7.260783658	2.860125267	0.01401286	0.513797
gene30304	9.298258426	46.46031334	4.996668324	2.320966456	0.01402316	0.513797
gene49814	9.298258426	46.46031334	4.996668324	2.320966456	0.01402316	0.513797
gene39893	493.8408364	194.5525621	0.393958028	-1.34388616	0.0140658	0.514818
gene58385	103.3139825	275.8581105	2.670094636	1.416890876	0.01408431	0.514818
gene45145	738.6949749	1966.819932	2.662560324	1.412814212	0.01411166	0.514818
gene54773	23.76221598	86.14516432	3.625300115	1.858100432	0.01412852	0.514818
gene36055	17.56337703	69.69047001	3.967942492	1.988391117	0.01413331	0.514818
gene7325	2.06627965	22.26223348	10.77406607	3.429490913	0.01415618	0.514818
gene51835	2.06627965	22.26223348	10.77406607	3.429490913	0.01415618	0.514818
gene59396	59.92210985	174.226175	2.907544068	1.539801058	0.01418447	0.514818
gene50705	1572.438814	4721.529343	3.002679215	1.586250356	0.01420385	0.514818
gene405	0	12.58300153	Inf	Inf	0.01421785	0.514818
gene1397	0	12.58300153	Inf	Inf	0.01421785	0.514818
gene11488	0	12.58300153	Inf	Inf	0.01421785	0.514818
gene14769	0	12.58300153	Inf	Inf	0.01421785	0.514818
gene17160	0	12.58300153	Inf	Inf	0.01421785	0.514818
gene29681	0	12.58300153	Inf	Inf	0.01421785	0.514818
gene36596	0	12.58300153	Inf	Inf	0.01421785	0.514818

gene45182	0	12.58300153	Inf	Inf	0.01421785	0.514818
gene63149	0	12.58300153	Inf	Inf	0.01421785	0.514818
gene53505	6.19883895	36.7810814	5.933543635	2.568893969	0.01424938	0.515624
gene66869	10.33139825	49.36408293	4.778064085	2.256426204	0.01425886	0.51563
gene2134	120.8773595	316.5108846	2.618446381	1.388711063	0.01427876	0.516014
gene41776	52.69013108	11.61507834	0.22044125	-2.18153388	0.01431826	0.516153
gene22095	781.0537078	2087.810331	2.673068843	1.418496994	0.01432121	0.516153
gene28641	406.0239513	158.7394039	0.390960689	-1.35490454	0.01433356	0.516153
gene9935	883.3345504	2396.57783	2.713103239	1.439943946	0.01434162	0.516153
gene4965	194.2302871	68.72254682	0.353819931	-1.49891278	0.01434701	0.516153
gene21043	15.49709738	63.88293085	4.122251367	2.04343248	0.01435884	0.516153
gene33747	15.49709738	63.88293085	4.122251367	2.04343248	0.01435884	0.516153
gene1156	41.325593	129.7017081	3.138532291	1.650090052	0.01435997	0.516153
gene60117	302.7099687	761.7555542	2.51645348	1.331391928	0.01436622	0.516153
gene6600	3.099419475	26.13392626	8.431877796	3.075853958	0.0144237	0.516954
gene42987	3.099419475	26.13392626	8.431877796	3.075853958	0.0144237	0.516954
gene5098	152.9046941	392.0088938	2.563746627	1.358253689	0.01443371	0.516954
gene39067	334.7373033	843.0611025	2.518575295	1.332607862	0.01443726	0.516954
gene48084	121.9104994	318.446731	2.6121354	1.385229681	0.01444247	0.516954
gene58398	56.82269038	166.4827895	2.929864608	1.550833998	0.01444877	0.516954
gene26000	12.3976779	55.17162209	4.450157726	2.15385647	0.01446524	0.516954
gene35467	493.8408364	1264.107692	2.559747188	1.35600133	0.01446688	0.516954
gene4084	13.43081773	58.07539168	4.324039896	2.112379834	0.0144723	0.516954
gene67646	184.9320287	64.85085404	0.350673999	-1.51179763	0.01448224	0.516977
gene52133	1293.491061	3714.889221	2.871986775	1.522049106	0.01453497	0.518526
gene1278	365.7314981	142.2847096	0.389041443	-1.36200425	0.01460535	0.520492
gene20100	110.5459613	291.3448816	2.635509052	1.398081647	0.01460883	0.520492
gene5317	361.5989388	910.8157262	2.518856193	1.332768758	0.01462131	0.520603
gene47198	396.7256928	1001.800506	2.525171736	1.336381508	0.01468931	0.522298
gene5051	388.4605742	151.9639416	0.391195276	-1.35403915	0.01469536	0.522298
gene27662	189.064588	66.78670043	0.353248068	-1.50124642	0.01469711	0.522298
gene8990	279.9808926	701.7443161	2.506400739	1.3256171	0.01473477	0.523205
gene65936	64.05466916	182.9374838	2.855958608	1.51397507	0.0147415	0.523205
gene51114	20.6627965	77.43385557	3.747501243	1.905928957	0.01476035	0.52354
gene13062	617.8176154	246.8204146	0.399503686	-1.32371928	0.01489361	0.52793
gene40639	7.231978776	39.68485098	5.487412534	2.456126039	0.01493407	0.528716
gene41313	127.0761985	329.0938862	2.589736631	1.372805387	0.01493484	0.528716
gene35700	958.7537577	381.3617387	0.397768181	-1.33000022	0.01495177	0.528978
gene42085	50.62385143	150.9960184	2.982705071	1.576621331	0.01500908	0.530668
gene45140	63.02152933	15.48677111	0.245737786	-2.02480838	0.015024	0.530858
gene303	410.1565106	161.6431735	0.394101201	-1.34336195	0.01507156	0.5322
gene10545	73.35292758	19.35846389	0.263908538	-1.92189007	0.01511623	0.533099
gene14850	73.35292758	19.35846389	0.263908538	-1.92189007	0.01511623	0.533099
gene43943	36.15989388	116.1507834	3.212143922	1.683536535	0.01517467	0.534821
gene53908	199.3959862	500.4162916	2.509660806	1.32749239	0.01524632	0.537005
gene73693	1414.368421	4094.315113	2.894800997	1.533464173	0.01526859	0.537449
gene39587	64.05466916	181.9695606	2.840847716	1.506321498	0.01530305	0.538167
gene52446	33.0604744	108.4073978	3.279063588	1.713283879	0.01531348	0.538167

gene15931	38.22617353	120.9903993	3.165119293	1.662259876	0.01532333	0.538167
gene13410	429.7861672	1082.138132	2.517852398	1.332193711	0.01534072	0.538167
gene31771	5.165699125	32.90938862	6.370752113	2.671463703	0.01535682	0.538167
gene54821	5.165699125	32.90938862	6.370752113	2.671463703	0.01535682	0.538167
gene71799	5.165699125	32.90938862	6.370752113	2.671463703	0.01535682	0.538167
gene3043	8.265118601	42.58862056	5.152814209	2.365360575	0.01540664	0.539232
gene35288	8.265118601	42.58862056	5.152814209	2.365360575	0.01540664	0.539232
gene40994	382.2617353	956.3081163	2.501710289	1.322914728	0.01551297	0.542549
gene62590	16.5302372	65.81877724	3.981720071	1.993391798	0.01553384	0.542549
gene45204	281.0140324	698.8405465	2.486852847	1.314321142	0.01557367	0.542549
gene7871	1.033139825	17.4226175	16.86375559	4.075853958	0.01558059	0.542549
gene12890	1.033139825	17.4226175	16.86375559	4.075853958	0.01558059	0.542549
gene26239	1.033139825	17.4226175	16.86375559	4.075853958	0.01558059	0.542549
gene47143	1.033139825	17.4226175	16.86375559	4.075853958	0.01558059	0.542549
gene65478	1.033139825	17.4226175	16.86375559	4.075853958	0.01558059	0.542549
gene73817	127.0761985	327.1580398	2.574502886	1.364293888	0.0155893	0.542549
gene65865	310.9750873	773.3706325	2.486921506	1.314360973	0.01564009	0.543656
gene70439	76.45234706	210.0393332	2.747323546	1.458026823	0.0156407	0.543656
gene9225	21.69593633	79.36970196	3.658275023	1.871163538	0.01566464	0.543756
gene1618	48.55757178	145.1884792	2.990027587	1.580158795	0.01566923	0.543756
gene49211	1396.805044	4013.009565	2.872991892	1.522553921	0.01568324	0.543756
gene33070	571.3263233	1459.628178	2.554806453	1.353214	0.01569202	0.543756
gene6525	326.4721847	812.0875603	2.487463246	1.314675209	0.01569252	0.543756
gene13302	225.2244819	83.24139474	0.369593012	-1.43599061	0.01570509	0.543852
gene33768	75.41920723	20.32638709	0.269512076	-1.89157818	0.01571935	0.544007
gene50497	39.25931335	122.9262457	3.131135907	1.64668613	0.01579066	0.546135
gene71559	79.55176653	216.8147956	2.725455449	1.446497338	0.01582898	0.546887
gene31859	434.9518664	173.2582518	0.398338909	-1.32793169	0.01583211	0.546887
gene59058	199.3959862	497.512522	2.495097978	1.319096469	0.01589759	0.547808
gene26720	231.4233208	574.9463776	2.484392565	1.312893155	0.01589855	0.547808
gene46155	133.2750374	340.7089645	2.556434956	1.35413332	0.01590195	0.547808
gene5579	2438.209987	8102.485062	3.323128486	1.732542075	0.01590615	0.547808
gene66784	697.3693819	1810.016374	2.595491602	1.376007819	0.01592043	0.547808
gene13509	96.08200373	254.5638002	2.649443083	1.405689135	0.01594105	0.547808
gene19615	257.2518164	637.8613853	2.479521405	1.31006168	0.01594311	0.547808
gene10176	344.0355618	854.6761809	2.484266965	1.312820217	0.01594669	0.547808
gene2123	24.7953558	87.11308752	3.513282415	1.812819552	0.01594752	0.547808
gene16434	11.36453808	51.29992932	4.514035588	2.174417793	0.01597433	0.548051
gene54438	11.36453808	51.29992932	4.514035588	2.174417793	0.01597433	0.548051
gene21788	198.3628464	494.6087525	2.493454603	1.318145937	0.01599512	0.548425
gene7399	55.78955055	161.6431735	2.897373646	1.534745747	0.01600807	0.548531
gene22651	47.52443195	142.2847096	2.993927623	1.582039345	0.01602041	0.5486
gene14633	253.1192571	627.2142301	2.477939597	1.309141021	0.01603362	0.5486
gene993	27.89477528	94.85647307	3.400510387	1.765751299	0.01604059	0.5486
gene39887	27.89477528	3.871692779	0.138796342	-2.84895855	0.01605946	0.5486
gene70748	27.89477528	3.871692779	0.138796342	-2.84895855	0.01605946	0.5486
gene3157	38.22617353	120.0224761	3.139798339	1.650671901	0.01615611	0.550146
gene35937	38.22617353	120.0224761	3.139798339	1.650671901	0.01615611	0.550146

gene58708	38.22617353	120.0224761	3.139798339	1.650671901	0.01615611	0.550146
gene4187	40.29245318	7.743385557	0.192179551	-2.37947326	0.01615877	0.550146
gene48090	417.3884893	1041.485357	2.495242164	1.319179836	0.01615889	0.550146
gene63989	1275.927684	506.2238308	0.396749626	-1.33369923	0.01616417	0.550146
gene26436	33.0604744	107.4394746	3.249786234	1.700344823	0.01622552	0.55173
gene29912	16.5302372	0.967923195	0.058554707	-4.09407104	0.01624464	0.55173
gene73646	16.5302372	0.967923195	0.058554707	-4.09407104	0.01624464	0.55173
gene72735	23.76221598	84.20931793	3.543832697	1.825310496	0.01625042	0.55173
gene12236	854.4066353	2260.100659	2.645228356	1.403392272	0.0162769	0.551825
gene50233	6.19883895	35.8131582	5.777397749	2.530419822	0.01630854	0.551825
gene53887	6.19883895	35.8131582	5.777397749	2.530419822	0.01630854	0.551825
gene20561	546.5309675	220.6864884	0.40379503	-1.30830494	0.01632012	0.551825
gene2233	792.4182458	2076.195252	2.620075022	1.389608122	0.01634793	0.551825
gene6101	574.4257427	232.3015667	0.404406609	-1.30612152	0.01636099	0.551825
gene9063	4.1325593	29.03769584	7.02656483	2.812819552	0.01638097	0.551825
gene9526	4.1325593	29.03769584	7.02656483	2.812819552	0.01638097	0.551825
gene21120	4.1325593	29.03769584	7.02656483	2.812819552	0.01638097	0.551825
gene32499	4.1325593	29.03769584	7.02656483	2.812819552	0.01638097	0.551825
gene36929	4.1325593	29.03769584	7.02656483	2.812819552	0.01638097	0.551825
gene64470	4.1325593	29.03769584	7.02656483	2.812819552	0.01638097	0.551825
gene65032	253.1192571	625.2783837	2.470291636	1.304681372	0.01638805	0.551825
gene67880	322.3396254	796.6007892	2.471308913	1.305275358	0.01639566	0.551825
gene4681	35.12675405	112.2790906	3.196398119	1.676447111	0.0164024	0.551825
gene446	163.2360924	409.4315113	2.508216813	1.326662062	0.01641219	0.551825
gene28766	234.5227403	579.7859936	2.472195203	1.305792662	0.0164336	0.552211
gene45621	2511.562915	8360.920555	3.328971178	1.73507638	0.01650485	0.55427
gene3424	269.6494943	664.9632347	2.466028117	1.302189249	0.01653199	0.554845
gene64840	1034.172965	416.2069737	0.40245393	-1.31310445	0.01657263	0.555874
gene19659	210.7605243	521.7106019	2.475371532	1.307645077	0.01659983	0.55645
gene11038	34.09361423	5.807539168	0.170340966	-2.55350266	0.01661682	0.556684
gene23596	643.646111	261.3392626	0.406029428	-1.3003438	0.01664215	0.557196
gene73490	148.7721348	374.5862763	2.517852398	1.332193711	0.01667465	0.557948
gene2432	17.56337703	67.75462362	3.857721868	1.947749132	0.01668793	0.558056
gene29273	460.780362	1148.924832	2.493432722	1.318133276	0.01676569	0.560319
gene40341	98.14828338	257.4675698	2.62325087	1.391355784	0.01677961	0.560447
gene19559	328.5384644	809.1837907	2.462980377	1.300405133	0.01681829	0.561313
gene5740	415.3222097	166.4827895	0.400852123	-1.31885798	0.01682597	0.561313
gene55630	673.607166	1726.774979	2.563474777	1.358100702	0.01683585	0.561313
gene55207	21.69593633	78.40177877	3.613661913	1.853461537	0.01686484	0.561942
gene66429	178.7331897	63.88293085	0.357420639	-1.48430515	0.01687565	0.561965
gene70739	261.3843757	642.7010012	2.458834808	1.297974813	0.01689753	0.562357
gene30354	91.94944443	242.9487219	2.64219891	1.40173908	0.01696328	0.563391
gene26743	180.7994694	64.85085404	0.358689405	-1.47919296	0.0169763	0.563391
gene46607	30.99419475	101.6319354	3.279063588	1.713283879	0.01698881	0.563391
gene49527	1301.75618	3634.551596	2.792037136	1.481318131	0.01702838	0.563391
gene6930	351.2675405	139.38094	0.396794249	-1.33353698	0.01704385	0.563391
gene59763	24.7953558	86.14516432	3.474245944	1.796699887	0.01707614	0.563391
gene13088	861.6386141	350.3881965	0.406653312	-1.29812873	0.01710648	0.563391

gene18188	3.099419475	25.16600306	8.119586026	3.021406174	0.01711392	0.563391
gene40982	3.099419475	25.16600306	8.119586026	3.021406174	0.01711392	0.563391
gene27275	64.05466916	179.065791	2.79551504	1.483114107	0.01712045	0.563391
gene599	2.06627965	21.29431028	10.30562842	3.365360575	0.0171239	0.563391
gene4478	2.06627965	21.29431028	10.30562842	3.365360575	0.0171239	0.563391
gene4900	2.06627965	21.29431028	10.30562842	3.365360575	0.0171239	0.563391
gene23016	2.06627965	21.29431028	10.30562842	3.365360575	0.0171239	0.563391
gene23276	2.06627965	21.29431028	10.30562842	3.365360575	0.0171239	0.563391
gene24254	2.06627965	21.29431028	10.30562842	3.365360575	0.0171239	0.563391
gene32853	2.06627965	21.29431028	10.30562842	3.365360575	0.0171239	0.563391
gene45508	2.06627965	21.29431028	10.30562842	3.365360575	0.0171239	0.563391
gene62210	2.06627965	21.29431028	10.30562842	3.365360575	0.0171239	0.563391
gene46437	266.5500749	102.5998586	0.384917763	-1.37737784	0.01713147	0.563391
gene52222	284.1134519	696.9047001	2.452909904	1.294494245	0.01715061	0.563687
gene44582	20.6627965	75.49800918	3.653813712	1.869403081	0.01718015	0.564324
gene35186	33.0604744	106.4715514	3.220508881	1.68728867	0.01719223	0.564387
gene6270	254.152397	623.3425373	2.452632927	1.294331329	0.01723235	0.565238
gene50000	90.91630461	240.0449523	2.640284967	1.400693648	0.01724177	0.565238
gene70204	59.92210985	169.3865591	2.826778955	1.499159073	0.01725823	0.565238
gene57530	835.8101185	2183.634727	2.612596664	1.385484417	0.01725888	0.565238
gene27546	119.8442197	305.8637295	2.552177571	1.35172871	0.01730194	0.565768
gene60221	96.08200373	251.6600306	2.619221299	1.389137959	0.01730506	0.565768
gene67680	278.9477528	683.3537754	2.449755442	1.292637733	0.01730559	0.565768
gene59463	14.46395755	59.04331487	4.082099568	2.029311372	0.01735428	0.567025
gene54403	81.61804618	23.23015667	0.284620348	-1.81288929	0.01736796	0.567139
gene52888	29.96105493	98.72816585	3.29521661	1.720373303	0.01738974	0.567516
gene157	23.76221598	83.24139474	3.503098988	1.808631755	0.01742922	0.568471
gene14164	289.279151	707.5518553	2.445913758	1.290373536	0.01749915	0.570417
gene10589	13.43081773	56.13954529	4.179905232	2.063470234	0.01752539	0.570937
gene71040	359.5326591	881.7780303	2.452567265	1.294292705	0.01754333	0.571187
gene6384	1093.061935	2949.261974	2.698165474	1.431978829	0.01758302	0.571822
gene35624	32.02733458	103.5677818	3.233730911	1.693199633	0.01760954	0.571822
gene23155	11.36453808	0	0	#NAME?	0.01761412	0.571822
gene73492	11.36453808	0	0	#NAME?	0.01761412	0.571822
gene34261	9.298258426	44.52446695	4.78847381	2.259565911	0.01761431	0.571822
gene57792	545.4978276	1363.803781	2.500108547	1.321990734	0.01764608	0.572109
gene15308	12.3976779	53.23577571	4.294011841	2.102326169	0.01765405	0.572109
gene71643	12.3976779	53.23577571	4.294011841	2.102326169	0.01765405	0.572109
gene6517	1707.780131	5000.291223	2.927947886	1.549889875	0.01768303	0.572714
gene30618	10.33139825	47.42823654	4.590689023	2.198710706	0.01772056	0.573086
gene8407	11.36453808	50.33200612	4.428865105	2.146937056	0.01772549	0.573086
gene64783	11.36453808	50.33200612	4.428865105	2.146937056	0.01772549	0.573086
gene40264	71.28664793	194.5525621	2.729158514	1.448456191	0.01777141	0.573907
gene54341	34.09361423	108.4073978	3.179698024	1.668889759	0.01777153	0.573907
gene31883	18.59651685	69.69047001	3.747501243	1.905928957	0.01781873	0.574817
gene57342	145.6727153	363.9391212	2.498334162	1.320966456	0.01782042	0.574817
gene33277	489.7082771	1213.775686	2.47856886	1.309507341	0.01783766	0.575039
gene47721	116.7448002	37.74900459	0.323346346	-1.62884779	0.01786366	0.575434

gene63177	391.5599937	960.1798091	2.452190787	1.294071229	0.01787061	0.575434
gene1970	36.15989388	113.2470138	3.131840324	1.647010659	0.01788578	0.575588
gene533	308.9088077	753.0442454	2.437755825	1.285553627	0.0179575	0.577562
gene19583	84.71746566	224.5581812	2.650671611	1.406357947	0.01801371	0.57888
gene29862	42.35873283	127.7658617	3.016281488	1.592771071	0.01801933	0.57888
gene50572	869.9037327	2263.972352	2.60255505	1.37992868	0.01816271	0.583149
gene24054	473.1780399	1166.34745	2.464923034	1.3015426	0.01826129	0.585975
gene21573	195.2634269	478.1540582	2.44876404	1.292053764	0.01827263	0.586001
gene39535	128.1093383	322.3184238	2.515963536	1.331111013	0.018291	0.58615
gene29815	455.6146629	185.8412534	0.407891292	-1.29374339	0.01830457	0.58615
gene36835	484.542578	1195.385145	2.467038398	1.302780171	0.01831712	0.58615
gene37114	78.51862671	210.0393332	2.675025558	1.419552676	0.01831949	0.58615
gene47199	559.9617852	1393.8094	2.489115217	1.315633012	0.01837408	0.587558
gene22873	147.738995	51.29992932	0.347233507	-1.52602193	0.01843336	0.589107
gene48182	16.5302372	63.88293085	3.864610657	1.950323076	0.01844904	0.589107
gene10746	383.2948751	934.0458828	2.436885916	1.285038712	0.01855229	0.589107
gene44147	2717.15774	9040.402638	3.327154145	1.734288707	0.01860761	0.589107
gene33906	736.6286953	303.9278831	0.412593054	-1.27720856	0.01861349	0.589107
gene46523	164.2692322	404.5918954	2.462980377	1.300405133	0.01863694	0.589107
gene43774	4020.980199	16129.47212	4.011328412	2.004080086	0.0186474	0.589107
gene11236	452.5152434	1109.239981	2.451276498	1.293533226	0.0186546	0.589107
gene66566	26.86163545	90.0168571	3.351130919	1.74464805	0.01866878	0.589107
gene675	6.19883895	34.84523501	5.621251864	2.490891457	0.0186752	0.589107
gene14924	6.19883895	34.84523501	5.621251864	2.490891457	0.0186752	0.589107
gene61400	6.19883895	34.84523501	5.621251864	2.490891457	0.0186752	0.589107
gene4825	98.14828338	253.595877	2.583803489	1.36949635	0.01868685	0.589107
gene3304	0	11.61507834	Inf	Inf	0.01873009	0.589107
gene18724	0	11.61507834	Inf	Inf	0.01873009	0.589107
gene18733	0	11.61507834	Inf	Inf	0.01873009	0.589107
gene28171	0	11.61507834	Inf	Inf	0.01873009	0.589107
gene39109	0	11.61507834	Inf	Inf	0.01873009	0.589107
gene40537	0	11.61507834	Inf	Inf	0.01873009	0.589107
gene43762	0	11.61507834	Inf	Inf	0.01873009	0.589107
gene46791	0	11.61507834	Inf	Inf	0.01873009	0.589107
gene55351	0	11.61507834	Inf	Inf	0.01873009	0.589107
gene56272	0	11.61507834	Inf	Inf	0.01873009	0.589107
gene58213	0	11.61507834	Inf	Inf	0.01873009	0.589107
gene62521	0	11.61507834	Inf	Inf	0.01873009	0.589107
gene63852	0	11.61507834	Inf	Inf	0.01873009	0.589107
gene67168	0	11.61507834	Inf	Inf	0.01873009	0.589107
gene67474	0	11.61507834	Inf	Inf	0.01873009	0.589107
gene68372	0	11.61507834	Inf	Inf	0.01873009	0.589107
gene58402	80.58490636	23.23015667	0.288269326	-1.79451076	0.01874739	0.589317
gene11677	1285.225942	520.7426787	0.405175978	-1.30337945	0.0187619	0.58944
gene55530	48.55757178	141.3167864	2.910293518	1.541164664	0.01878663	0.589782
gene69675	85.75060548	225.5261044	2.630023463	1.39507567	0.01879401	0.589782
gene70670	252.0861173	97.76024266	0.387804944	-1.3665969	0.01886229	0.591537
gene52804	553.7629462	1371.547167	2.476776708	1.308463809	0.01887124	0.591537

gene67640	415.3222097	169.3865591	0.40784373	-1.29391162	0.01890026	0.592112
gene23483	1646.824881	656.251926	0.39849527	-1.32736549	0.01891132	0.592125
gene40267	913.2956054	376.5221227	0.412267529	-1.27834726	0.01902432	0.595327
gene37182	25.82849563	87.11308752	3.372751119	1.753925863	0.01911935	0.597964
gene71451	4.1325593	28.06977264	6.792346003	2.763909952	0.01916612	0.59909
gene52248	242.7878589	93.88854988	0.386710235	-1.37067515	0.01919187	0.599557
gene32020	194.2302871	472.346519	2.431889104	1.282077443	0.01921468	0.599647
gene39616	92.98258426	241.0128755	2.592021693	1.374077792	0.01921635	0.599647
gene38543	47.52443195	138.4130168	2.912460205	1.542238337	0.01925836	0.600426
gene35032	7.231978776	37.74900459	5.219733874	2.383976253	0.01926293	0.600426
gene8645	13.43081773	55.17162209	4.107837901	2.038379253	0.01929064	0.600929
gene4086	146.7058552	51.29992932	0.349678813	-1.51589771	0.01931848	0.600929
gene37899	1703.647572	4895.755518	2.87369031	1.522904595	0.01932129	0.600929
gene36407	33.0604744	5.807539168	0.175664121	-2.50910854	0.01932235	0.600929
gene13260	292.3785705	116.1507834	0.397261616	-1.33183869	0.01939092	0.602102
gene52925	669.4746067	1676.442973	2.504117343	1.324302168	0.01940431	0.602102
gene924	41.325593	123.8941689	2.998000994	1.584000862	0.01942948	0.602102
gene14814	37.1930337	114.214937	3.070869074	1.618647005	0.01943669	0.602102
gene9877	1.033139825	16.45469431	15.92688028	3.993391798	0.01945334	0.602102
gene12627	1.033139825	16.45469431	15.92688028	3.993391798	0.01945334	0.602102
gene13210	1.033139825	16.45469431	15.92688028	3.993391798	0.01945334	0.602102
gene17694	1.033139825	16.45469431	15.92688028	3.993391798	0.01945334	0.602102
gene56436	163.2360924	399.7522794	2.448920907	1.29214618	0.01945761	0.602102
gene21897	200.4291261	485.8974437	2.424285598	1.277559668	0.019473	0.602243
gene30044	44.42501248	9.679231946	0.217877979	-2.1984077	0.01950027	0.602751
gene18093	57.8558302	161.6431735	2.793896016	1.482278327	0.019547	0.603378
gene54775	91.94944443	238.1091059	2.589565466	1.372710031	0.01955144	0.603378
gene60843	21.69593633	76.46593238	3.524435693	1.817392282	0.01955316	0.603378
gene73458	8.265118601	40.65277417	4.918595381	2.298246379	0.01959988	0.604484
gene62886	228.3239013	550.7482977	2.412135981	1.27031124	0.01961676	0.604669
gene55213	5905.42724	1987.146319	0.336494929	-1.57134333	0.01964057	0.605067
gene59731	11.36453808	49.36408293	4.343694622	2.11892268	0.01967298	0.605477
gene3152	171.501211	418.1428201	2.438133339	1.285777027	0.01967569	0.605477
gene53273	1859.651685	5430.049122	2.919928052	1.545932821	0.01973235	0.605799
gene16073	559.9617852	1378.322629	2.461458381	1.299513346	0.01973317	0.605799
gene23262	66.12094881	180.0337142	2.722793872	1.445087768	0.01973654	0.605799
gene32088	9.298258426	43.55654376	4.684376554	2.227857052	0.01975023	0.605799
gene33540	9.298258426	43.55654376	4.684376554	2.227857052	0.01975023	0.605799
gene59123	1192.243358	488.8012133	0.40998443	-1.28635897	0.01975635	0.605799
gene399	10.33139825	46.46031334	4.497001491	2.168963362	0.0197625	0.605799
gene23444	458.7140823	1115.04752	2.430811617	1.281438092	0.01979915	0.606373
gene62592	101.2477029	258.435493	2.552507224	1.351915044	0.01980307	0.606373
gene29448	189.064588	457.8276711	2.421541104	1.275925492	0.0199579	0.610778
gene38634	49.5907116	11.61507834	0.234218828	-2.09407104	0.01998573	0.611293
gene69433	19.62965668	1.935846389	0.098618454	-3.34199856	0.02004736	0.61269
gene72167	23.76221598	81.30554835	3.42163157	1.774684423	0.02005349	0.61269
gene57191	633.3147128	264.2430321	0.417238107	-1.26105717	0.02011183	0.613827
gene53820	1884.447041	5497.803746	2.91746259	1.544714157	0.02011281	0.613827

gene40819	1027.974126	2684.051019	2.611010288	1.384608142	0.02015188	0.614579
gene1755	201.4622659	485.8974437	2.411853364	1.270142197	0.02015957	0.614579
gene23666	28.9279151	93.88854988	3.245603755	1.698486877	0.02018117	0.6149
gene2366	431.8524469	1043.421204	2.416152117	1.272711287	0.02022878	0.616013
gene1524	3.099419475	24.19807987	7.807294256	2.964822646	0.02033038	0.617077
gene4854	3.099419475	24.19807987	7.807294256	2.964822646	0.02033038	0.617077
gene42432	3.099419475	24.19807987	7.807294256	2.964822646	0.02033038	0.617077
gene62110	3.099419475	24.19807987	7.807294256	2.964822646	0.02033038	0.617077
gene66089	3.099419475	24.19807987	7.807294256	2.964822646	0.02033038	0.617077
gene69159	3.099419475	24.19807987	7.807294256	2.964822646	0.02033038	0.617077
gene65529	929.8258426	2391.738214	2.572243214	1.363027061	0.02039166	0.618599
gene15710	150.8384145	368.7787372	2.444859544	1.289751585	0.02040396	0.618634
gene3652	199.3959862	480.0899045	2.407721006	1.26766823	0.02045777	0.619522
gene24216	19.62965668	70.65839321	3.599573562	1.847826002	0.02046815	0.619522
gene60869	15.49709738	60.01123807	3.872417951	1.953234671	0.02050027	0.619522
gene68936	35.12675405	108.4073978	3.086177494	1.625821037	0.02050459	0.619522
gene3310	649.84495	271.9864177	0.418540481	-1.25656093	0.02052196	0.619522
gene1208	5.165699125	30.97354223	5.996001989	2.584000862	0.02053361	0.619522
gene17622	5.165699125	30.97354223	5.996001989	2.584000862	0.02053361	0.619522
gene34534	5.165699125	30.97354223	5.996001989	2.584000862	0.02053361	0.619522
gene41583	5.165699125	30.97354223	5.996001989	2.584000862	0.02053361	0.619522
gene32046	22.72907615	78.40177877	3.449404553	1.786347341	0.02054493	0.619527
gene56894	48.55757178	139.38094	2.870426484	1.521265106	0.02057171	0.619998
gene5712	223.1582022	86.14516432	0.386027327	-1.37322511	0.02059834	0.620464
gene18006	209.7273845	503.3200612	2.399877643	1.262960852	0.02066779	0.621853
gene37160	130.175618	321.3505006	2.468592089	1.303688464	0.02070407	0.621853
gene14087	239.6884394	573.0105312	2.390647345	1.257401327	0.02072596	0.621853
gene22861	2.06627965	20.32638709	9.837190763	3.298246379	0.0207502	0.621853
gene29030	2.06627965	20.32638709	9.837190763	3.298246379	0.0207502	0.621853
gene36833	2.06627965	20.32638709	9.837190763	3.298246379	0.0207502	0.621853
gene37365	2.06627965	20.32638709	9.837190763	3.298246379	0.0207502	0.621853
gene40049	2.06627965	20.32638709	9.837190763	3.298246379	0.0207502	0.621853
gene65161	2.06627965	20.32638709	9.837190763	3.298246379	0.0207502	0.621853
gene8529	219.0256429	524.6143715	2.395218955	1.260157543	0.02075643	0.621853
gene58610	534.1332896	1298.952927	2.431889104	1.282077443	0.02088318	0.624993
gene6149	141.540156	346.5165037	2.448185119	1.291712651	0.02088372	0.624993
gene59524	585.7902808	1433.494251	2.447111702	1.291079957	0.02090702	0.625021
gene22066	29.96105493	95.82439627	3.198298475	1.677304582	0.02090718	0.625021
gene4138	1986.727884	5823.025939	2.930963011	1.551374761	0.02092646	0.625117
gene20028	826.5118601	2087.810331	2.526050057	1.336883228	0.02093291	0.625117
gene60143	18.59651685	67.75462362	3.643403986	1.865286972	0.02094416	0.625117
gene45260	215.9262234	83.24139474	0.385508501	-1.37516542	0.02112119	0.630063
gene12240	85.75060548	221.6544116	2.584872845	1.370093313	0.02119488	0.631921
gene48162	220.0587827	85.17724113	0.387065856	-1.36934904	0.0212212	0.632367
gene34641	499.0065355	1205.064377	2.414927043	1.271979605	0.02127056	0.633073
gene5474	26.86163545	88.08101071	3.279063588	1.713283879	0.0212791	0.633073
gene36736	26.86163545	88.08101071	3.279063588	1.713283879	0.0212791	0.633073
gene49730	1304.855599	3494.202733	2.677846296	1.421073155	0.02132816	0.634193

gene21981	126.0430587	310.7033455	2.46505717	1.301621106	0.02135526	0.634493
gene16385	298.5774094	709.4877017	2.376226999	1.248672662	0.02136172	0.634493
gene58929	474.2111797	1141.181446	2.406483641	1.266926616	0.02137253	0.634493
gene45399	6.19883895	33.87731181	5.465105979	2.450249473	0.02139655	0.634867
gene38023	104.3471223	262.3071857	2.513794151	1.329866515	0.02147219	0.636761
gene20722	288.2460112	684.3216986	2.374089049	1.24737405	0.0214833	0.636761
gene59516	562.0280648	1364.771704	2.42829814	1.279945563	0.02154127	0.638054
gene64110	754.1920723	1877.770998	2.489778223	1.31601724	0.02154989	0.638054
gene55475	37.1930337	112.2790906	3.018820446	1.59398495	0.02164879	0.640347
gene10468	87.81688513	225.5261044	2.568140558	1.360724165	0.02165039	0.640347
gene36647	631.2484331	1544.805419	2.447222579	1.291145323	0.02167729	0.640801
gene60566	76.45234706	200.3601013	2.620718775	1.389962549	0.02171885	0.641688
gene7716	122.9436392	302.9599599	2.464218254	1.30113004	0.0217686	0.642475
gene28151	162.2029525	391.0409706	2.410812901	1.269519691	0.02177358	0.642475
gene45507	504.1722346	1213.775686	2.407462376	1.267513252	0.02178019	0.642475
gene56932	248.9866978	98.72816585	0.396519841	-1.33453504	0.02184148	0.643941
gene65976	88.85002496	227.4619507	2.560066256	1.356181149	0.0218753	0.644383
gene59410	722.1647377	1785.818294	2.472868309	1.306185412	0.02188468	0.644383
gene45668	7.231978776	36.7810814	5.085894544	2.346501548	0.02189127	0.644383
gene2791	54.75641073	151.9639416	2.775272147	1.472629251	0.02191914	0.644606
gene13785	16.5302372	61.94708446	3.747501243	1.905928957	0.02192206	0.644606
gene72080	657.0769288	277.7939569	0.422772349	-1.24204707	0.02197179	0.645727
gene21405	217.9925031	517.8389091	2.375489532	1.248224849	0.02199984	0.646119
gene73143	1012.477029	424.9182824	0.419681899	-1.25263185	0.02202373	0.646119
gene68813	525.868171	221.6544116	0.421501859	-1.2463891	0.02203496	0.646119
gene42644	229.3570412	543.9728354	2.37172939	1.245939411	0.02203603	0.646119
gene26913	148.7721348	360.0674284	2.420261219	1.275162766	0.0220433	0.646119
gene6987	319.2402059	130.6696313	0.409314456	-1.28871847	0.022067	0.646132
gene42362	319.2402059	130.6696313	0.409314456	-1.28871847	0.022067	0.646132
gene64071	957.7206178	2438.198527	2.545834852	1.348138835	0.02209621	0.646646
gene49550	9.298258426	42.58862056	4.580279297	2.195435574	0.02215158	0.647584
gene69734	9.298258426	42.58862056	4.580279297	2.195435574	0.02215158	0.647584
gene59514	136.3744569	331.9976558	2.4344563	1.283599603	0.02218078	0.648097
gene15445	534.1332896	1286.369926	2.408331311	1.268033876	0.02221833	0.648852
gene72493	1099.260774	2844.726269	2.587853889	1.371756165	0.02230008	0.650898
gene18635	99.18142321	31.94146542	0.322050888	-1.63463942	0.02233366	0.651535
gene21477	827.5449999	2067.483944	2.498334162	1.320966456	0.02235967	0.651707
gene60647	1376.142247	3684.883602	2.677690922	1.420989444	0.02236301	0.651707
gene59564	73.35292758	192.6167157	2.625889955	1.392806458	0.02241226	0.652373
gene9418	4.1325593	27.10184945	6.558127175	2.713283879	0.02244458	0.652373
gene13821	4.1325593	27.10184945	6.558127175	2.713283879	0.02244458	0.652373
gene24650	4.1325593	27.10184945	6.558127175	2.713283879	0.02244458	0.652373
gene55898	4.1325593	27.10184945	6.558127175	2.713283879	0.02244458	0.652373
gene8900	82.65118601	212.9431028	2.576407104	1.365360575	0.02249127	0.653388
gene69201	48.55757178	137.4450936	2.830559449	1.501087224	0.02252682	0.654078
gene12819	65.08780898	174.226175	2.676786602	1.420502129	0.02255241	0.654479
gene35310	581.6577215	1405.424479	2.4162397	1.272763583	0.02256736	0.654571
gene71572	220.0587827	86.14516432	0.391464332	-1.35304723	0.02267979	0.656907

gene14281	18.59651685	66.78670043	3.591355358	1.844528412	0.02271006	0.656907
gene37341	18.59651685	66.78670043	3.591355358	1.844528412	0.02271006	0.656907
gene44874	18.59651685	66.78670043	3.591355358	1.844528412	0.02271006	0.656907
gene63906	18.59651685	66.78670043	3.591355358	1.844528412	0.02271006	0.656907
gene43434	26.86163545	87.11308752	3.243029922	1.697342335	0.02271883	0.656907
gene223	102.2808427	255.5317234	2.498334162	1.320966456	0.02278743	0.658271
gene20091	25.82849563	3.871692779	0.14990005	-2.73792723	0.02278974	0.658271
gene57608	194.2302871	460.7314406	2.372088553	1.246157868	0.0228517	0.658974
gene43927	54.75641073	150.9960184	2.757595254	1.463410721	0.02285582	0.658974
gene27493	116.7448002	39.68485098	0.33992821	-1.556698	0.02285811	0.658974
gene68106	153.9378339	369.7466604	2.401921938	1.264189264	0.0228615	0.658974
gene29332	635.3809924	1541.901649	2.426735561	1.279016908	0.02290788	0.659839
gene23871	472.1449001	1123.758829	2.380114301	1.251030858	0.02291648	0.659839
gene70472	35.12675405	106.4715514	3.031067182	1.599825829	0.02292715	0.659839
gene31433	33.0604744	101.6319354	3.074122113	1.620174474	0.02296657	0.660384
gene7116	90.91630461	230.3657203	2.533821863	1.341315101	0.02296987	0.660384
gene19778	2830.803121	9045.242254	3.19529189	1.675947727	0.02304627	0.662236
gene48010	3198.600898	1224.422841	0.382799505	-1.38533913	0.02305816	0.662236
gene2565	365.7314981	861.4516432	2.355420979	1.235984932	0.02308503	0.662567
gene34925	52.69013108	146.1564024	2.773885724	1.471908354	0.02309352	0.662567
gene6230	68.18722846	19.35846389	0.283901609	-1.81653707	0.02312434	0.663109
gene58405	73.35292758	191.6487925	2.612694528	1.385538457	0.02318545	0.664518
gene8573	17.56337703	63.88293085	3.637280618	1.862860235	0.02329784	0.667051
gene64924	17.56337703	63.88293085	3.637280618	1.862860235	0.02329784	0.667051
gene21066	172.5343508	64.85085404	0.375872131	-1.41168615	0.02333673	0.66782
gene51862	99.18142321	247.7883378	2.498334162	1.320966456	0.02339359	0.669103
gene69677	523.8018913	1248.620921	2.383765583	1.25324237	0.02353122	0.672692
gene9145	48.55757178	136.4771704	2.810625932	1.490891457	0.02357307	0.673283
gene28639	45.4581523	10.64715514	0.234218828	-2.09407104	0.02357612	0.673283
gene9566	50.62385143	12.58300153	0.248558756	-2.00834117	0.02359817	0.673566
gene63246	1540.411479	4164.005583	2.703177456	1.434656224	0.0236255	0.674
gene48853	29.96105493	93.88854988	3.133686384	1.647860804	0.02364089	0.674093
gene14933	438.0512858	1033.741972	2.359865169	1.238704434	0.02369041	0.675158
gene2188	72.31978776	188.745023	2.609866937	1.383976253	0.02371667	0.67556
gene57133	5.165699125	30.00561903	5.808626926	2.538197172	0.02376744	0.676659
gene38495	12.3976779	50.33200612	4.059793013	2.021406174	0.02383669	0.678283
gene51910	395.692553	929.2062669	2.348303651	1.23161897	0.02388361	0.67927
gene35146	404.9908114	951.4685003	2.349358241	1.232266719	0.02392957	0.680229
gene53196	1347.214332	3544.534739	2.631010267	1.395616878	0.02406404	0.683702
gene28940	266.5500749	108.4073978	0.406705561	-1.29794338	0.02407875	0.68377
gene24408	162.2029525	385.2334315	2.375008749	1.247932828	0.02414488	0.685298
gene53977	100.214563	248.756261	2.482236648	1.311640664	0.02423463	0.687494
gene6379	11.36453808	47.42823654	4.173353657	2.061207182	0.02424864	0.68754
gene31822	119.8442197	291.3448816	2.431029901	1.281567638	0.024334	0.68894
gene7839	1.033139825	15.48677111	14.99000497	3.905928957	0.0243759	0.68894
gene29633	1.033139825	15.48677111	14.99000497	3.905928957	0.0243759	0.68894
gene50061	1.033139825	15.48677111	14.99000497	3.905928957	0.0243759	0.68894
gene4828	174.6006304	412.3352809	2.36159102	1.239759141	0.024389	0.68894

gene14555	104.3471223	257.4675698	2.467414185	1.302999909	0.02441076	0.68894
gene9335	238.6552996	95.82439627	0.40151799	-1.31646346	0.02443464	0.68894
gene61016	98.14828338	243.916645	2.485185035	1.313353272	0.0244723	0.68894
gene19538	1191.210218	3065.412757	2.573360025	1.363653311	0.02447591	0.68894
gene59190	1847.254007	757.8838614	0.410275933	-1.28533357	0.02451373	0.68894
gene18096	15.49709738	58.07539168	3.747501243	1.905928957	0.02451445	0.68894
gene25427	15.49709738	58.07539168	3.747501243	1.905928957	0.02451445	0.68894
gene8685	6.19883895	32.90938862	5.308960094	2.408429297	0.02452715	0.68894
gene55942	6.19883895	32.90938862	5.308960094	2.408429297	0.02452715	0.68894
gene70951	56.82269038	153.8997879	2.708421353	1.437452198	0.02454404	0.68894
gene60332	91.94944443	230.3657203	2.505351955	1.325013289	0.02454845	0.68894
gene13028	142.5732959	340.7089645	2.389710937	1.256836119	0.02459475	0.68894
gene18809	10.33139825	44.52446695	4.309626429	2.107562818	0.02459734	0.68894
gene31362	2342.127983	939.853422	0.401281838	-1.31731223	0.02462513	0.68894
gene62578	18.59651685	65.81877724	3.539306729	1.823466796	0.02462702	0.68894
gene43468	437.018146	1025.030663	2.34551053	1.229901978	0.02462887	0.68894
gene42981	186.9983083	439.4371304	2.349952437	1.232631557	0.02464949	0.68894
gene40045	476.2774594	1120.855059	2.353365748	1.234725554	0.02470741	0.68894
gene36194	246.9204182	574.9463776	2.328468345	1.219381269	0.02473118	0.68894
gene68493	843.0420973	362.0032748	0.429401184	-1.21960193	0.02478764	0.68894
gene2519	649.84495	279.7298033	0.430456224	-1.21606157	0.02481725	0.68894
gene52485	250.0198377	581.72184	2.326702734	1.2182869	0.02482405	0.68894
gene34557	382.2617353	891.4572623	2.332059895	1.221604842	0.0248323	0.68894
gene4666	0	10.64715514	Inf	Inf	0.02485618	0.68894
gene7944	0	10.64715514	Inf	Inf	0.02485618	0.68894
gene21501	0	10.64715514	Inf	Inf	0.02485618	0.68894
gene26446	0	10.64715514	Inf	Inf	0.02485618	0.68894
gene26656	0	10.64715514	Inf	Inf	0.02485618	0.68894
gene33485	0	10.64715514	Inf	Inf	0.02485618	0.68894
gene34689	0	10.64715514	Inf	Inf	0.02485618	0.68894
gene34842	0	10.64715514	Inf	Inf	0.02485618	0.68894
gene35644	0	10.64715514	Inf	Inf	0.02485618	0.68894
gene38076	0	10.64715514	Inf	Inf	0.02485618	0.68894
gene45143	0	10.64715514	Inf	Inf	0.02485618	0.68894
gene50863	0	10.64715514	Inf	Inf	0.02485618	0.68894
gene53029	0	10.64715514	Inf	Inf	0.02485618	0.68894
gene55832	0	10.64715514	Inf	Inf	0.02485618	0.68894
gene60805	0	10.64715514	Inf	Inf	0.02485618	0.68894
gene68908	0	10.64715514	Inf	Inf	0.02485618	0.68894
gene70318	0	10.64715514	Inf	Inf	0.02485618	0.68894
gene66161	18.59651685	1.935846389	0.104097257	-3.26399604	0.02487318	0.689068
gene31812	126.0430587	44.52446695	0.353248068	-1.50124642	0.02490286	0.689546
gene29836	251.0529775	583.6576864	2.324838734	1.217130645	0.02494806	0.690331
gene2015	364.6983583	153.8997879	0.421991995	-1.24471246	0.02495606	0.690331
gene57225	34.09361423	102.5998586	3.009357059	1.589455292	0.02499656	0.691107
gene6894	128.1093383	45.49239015	0.355105965	-1.4936785	0.02502338	0.691504
gene18219	205.5948252	480.0899045	2.335126403	1.223500646	0.02506668	0.692097
gene2263	87.81688513	220.6864884	2.513030245	1.329428035	0.02509483	0.692097

gene16376	184.9320287	433.6295912	2.344805247	1.229468101	0.02510959	0.692097
gene23648	696.3362421	1677.410896	2.408909367	1.268380115	0.02511703	0.692097
gene8825	233.4896005	543.0049122	2.325606413	1.217606955	0.02512686	0.692097
gene40572	14.46395755	55.17162209	3.814420908	1.931464049	0.02513403	0.692097
gene12629	2.06627965	19.35846389	9.368753107	3.227857052	0.02519436	0.692097
gene30957	2.06627965	19.35846389	9.368753107	3.227857052	0.02519436	0.692097
gene32365	2.06627965	19.35846389	9.368753107	3.227857052	0.02519436	0.692097
gene36933	2.06627965	19.35846389	9.368753107	3.227857052	0.02519436	0.692097
gene40748	2.06627965	19.35846389	9.368753107	3.227857052	0.02519436	0.692097
gene60373	2.06627965	19.35846389	9.368753107	3.227857052	0.02519436	0.692097
gene48318	1410.235861	3705.209989	2.627369003	1.393618835	0.02521102	0.692213
gene27281	1139.553227	485.8974437	0.426392934	-1.22974456	0.02537416	0.696348
gene24220	47.52443195	132.6054777	2.790259078	1.480399084	0.025387	0.696356
gene28919	126.0430587	302.9599599	2.403622723	1.265210466	0.02542131	0.696953
gene27990	37.1930337	109.375321	2.940747503	1.556182918	0.02544874	0.697017
gene37968	37.1930337	109.375321	2.940747503	1.556182918	0.02544874	0.697017
gene31848	543.431548	1281.53031	2.358218463	1.237697374	0.02553907	0.699146
gene46681	567.193764	244.8845682	0.431747639	-1.21173981	0.02557066	0.699666
gene61667	1954.700549	5461.022664	2.793789906	1.482223534	0.02560534	0.70027
gene71524	788.2856865	1914.552079	2.428754082	1.280216421	0.02563536	0.700499
gene67471	608.519357	1445.10933	2.374795991	1.247803583	0.02563892	0.700499
gene23752	715.9658988	309.7354223	0.432611976	-1.20885449	0.02567559	0.701156
gene20240	13.43081773	52.26785251	3.891635906	1.960376741	0.02575135	0.702692
gene12726	40.29245318	116.1507834	2.882693264	1.527417333	0.02577275	0.702692
gene53207	1967.098227	5495.867899	2.793896016	1.482278327	0.02577422	0.702692
gene38092	4240.005842	1587.394039	0.374384871	-1.41740596	0.02578247	0.702692
gene6475	220.0587827	88.08101071	0.400261283	-1.32098602	0.02581615	0.703265
gene24647	51.65699125	141.3167864	2.735675907	1.451897326	0.02588311	0.704429
gene14737	60.95524968	161.6431735	2.651833507	1.4069902	0.02588972	0.704429
gene5140	26.86163545	85.17724113	3.17096259	1.664920857	0.02589841	0.704429
gene73205	19.62965668	67.75462362	3.451645882	1.78728446	0.02590961	0.704429
gene70388	28.9279151	90.0168571	3.111764425	1.637732846	0.02593656	0.704692
gene21097	275.8483333	636.8934621	2.308853762	1.207176799	0.02595439	0.704692
gene5123	2438.209987	982.4420426	0.40293578	-1.31137818	0.02596554	0.704692
gene59676	112.6122409	272.9543409	2.423842547	1.277295984	0.02597002	0.704692
gene13623	91.94944443	228.4298739	2.484298577	1.312838575	0.02598724	0.704815
gene63865	16.5302372	60.01123807	3.630391829	1.860125267	0.02606028	0.706213
gene57497	348.1681211	804.3441747	2.310217754	1.208028842	0.02606421	0.706213
gene45808	78.51862671	199.3921781	2.539425184	1.34450197	0.02610693	0.707026
gene73689	85.75060548	214.8789492	2.505859265	1.325305392	0.02616393	0.707616
gene19143	30.99419475	5.807539168	0.187375062	-2.41599914	0.02617727	0.707616
gene18200	259.3180961	598.1765343	2.306728853	1.205848431	0.02621674	0.707616
gene16731	227.2907615	525.5822947	2.312378608	1.209377631	0.02623062	0.707616
gene39018	39.25931335	8.711308752	0.221891521	-2.17207356	0.02625969	0.707616
gene6545	337.8367228	779.1781717	2.306375	1.205627104	0.02627184	0.707616
gene61209	622.9833145	1476.082872	2.369377859	1.244508292	0.02627935	0.707616
gene60772	2969.243857	9323.036211	3.139868821	1.650704287	0.02630457	0.707616
gene3351	4.1325593	26.13392626	6.323908347	2.660816459	0.02630707	0.707616

gene17333	4.1325593	26.13392626	6.323908347	2.660816459	0.02630707	0.707616
gene49600	4.1325593	26.13392626	6.323908347	2.660816459	0.02630707	0.707616
gene62316	4.1325593	26.13392626	6.323908347	2.660816459	0.02630707	0.707616
gene68121	4.1325593	26.13392626	6.323908347	2.660816459	0.02630707	0.707616
gene71604	4.1325593	26.13392626	6.323908347	2.660816459	0.02630707	0.707616
gene30382	127.0761985	45.49239015	0.357993005	-1.4819967	0.02632845	0.707848
gene34531	86.78374531	216.8147956	2.498334162	1.320966456	0.02639434	0.709276
gene46156	3109.750873	9920.244822	3.19004487	1.673576717	0.02645586	0.710585
gene59087	44.42501248	10.64715514	0.239665777	-2.06090418	0.02649684	0.711145
gene42394	180.7994694	421.0465897	2.328804344	1.219589436	0.0265023	0.711145
gene4191	451.4821036	1048.26082	2.321821422	1.215257015	0.02651566	0.71116
gene36524	172.5343508	402.656049	2.333773229	1.222664382	0.02655626	0.711905
gene27287	4756.575755	1756.780598	0.369337248	-1.43698933	0.02658514	0.712044
gene47034	59.92210985	158.7394039	2.649095706	1.405499966	0.02658707	0.712044
gene44958	32.02733458	96.79231946	3.022178422	1.595588836	0.0266227	0.712654
gene6417	333.7041635	141.3167864	0.423479243	-1.23963684	0.02665781	0.713251
gene38184	18.59651685	64.85085404	3.487258101	1.802093146	0.02670796	0.713936
gene48916	21.69593633	2.903769584	0.13383933	-2.90142597	0.02671288	0.713936
gene50540	25.82849563	82.27347154	3.185376056	1.671463703	0.02672202	0.713936
gene51025	67.15408863	174.226175	2.594423937	1.37541424	0.02674836	0.713936
gene37515	321.3064856	738.5253975	2.298507595	1.200697433	0.02677182	0.713936
gene53859	27.89477528	87.11308752	3.122917702	1.642894551	0.0267734	0.713936
gene4432	14.46395755	0.967923195	0.066919665	-3.90142597	0.02679494	0.713936
gene67984	14.46395755	0.967923195	0.066919665	-3.90142597	0.02679494	0.713936
gene39114	42.35873283	120.0224761	2.833476549	1.502573262	0.02680446	0.713936
gene47208	15.49709738	57.10746848	3.685042889	1.88168141	0.02681195	0.713936
gene47372	88.85002496	220.6864884	2.483808963	1.312554216	0.02684787	0.714183
gene53197	632.2815729	1494.473413	2.363620065	1.240998151	0.02685459	0.714183
gene19881	844.0752371	366.8428908	0.434609232	-1.20220927	0.02687706	0.714183
gene71656	1759.437122	4754.438732	2.702249869	1.434161082	0.02688508	0.714183
gene68239	255.1855368	586.561456	2.298568576	1.200735709	0.02688551	0.714183
gene67189	651.9112296	1543.837495	2.368171348	1.24377347	0.02692799	0.714969
gene62535	780.0205679	339.7410413	0.435553952	-1.19907666	0.02701624	0.716969
gene3979	3694.508014	12567.51476	3.401674786	1.76624522	0.02709263	0.718349
gene19170	854.4066353	371.6825067	0.435018282	-1.20085206	0.02709409	0.718349
gene20512	153.9378339	58.07539168	0.377265226	-1.40634897	0.0271759	0.720174
gene72965	850.274076	2059.740558	2.422443088	1.276462772	0.02721479	0.720861
gene72468	61.9883895	162.6110967	2.62325087	1.391355784	0.02723609	0.721081
gene27060	285.1465917	653.3481564	2.29127114	1.196148192	0.02728182	0.721948
gene33148	127.0761985	301.9920367	2.376464203	1.24881667	0.02731914	0.722469
gene23268	377.0960362	866.2912592	2.297269598	1.199920175	0.02732751	0.722469
gene46119	52.69013108	142.2847096	2.700405307	1.43317596	0.02742378	0.723883
gene46415	22.72907615	74.53008599	3.279063588	1.713283879	0.02742635	0.723883
gene73800	22.72907615	74.53008599	3.279063588	1.713283879	0.02742635	0.723883
gene56854	1510.450424	640.7651549	0.424221242	-1.23711123	0.02743314	0.723883
gene4142	51.65699125	13.55092472	0.262325087	-1.93057231	0.02744872	0.72395
gene14516	547.5641073	1276.690694	2.331582141	1.221309256	0.02749074	0.724714
gene19704	14.46395755	54.2036989	3.747501243	1.905928957	0.02758164	0.726571

gene16183	24.7953558	79.36970196	3.200990645	1.678518461	0.02758734	0.726571
gene33908	28.9279151	89.04893391	3.078304592	1.622135991	0.02761621	0.726987
gene24631	100.214563	243.916645	2.433944106	1.283296038	0.02767611	0.728219
gene63654	81.61804618	204.2317941	2.502287222	1.323247397	0.02769787	0.728446
gene35455	1281.093383	3255.125704	2.540896508	1.345337615	0.0277261	0.72845
gene27554	645.7123907	1520.607339	2.354929781	1.235684042	0.02773077	0.72845
gene39966	36.15989388	105.5036282	2.917697396	1.544830264	0.02773738	0.72845
gene24644	544.4646878	1267.011462	2.327077385	1.218519187	0.02776343	0.72879
gene20381	44.42501248	123.8941689	2.788838134	1.479664202	0.02778048	0.728893
gene68745	97.11514356	237.1411827	2.441855863	1.287978044	0.02780447	0.729178
gene6749	9.298258426	40.65277417	4.372084783	2.128321378	0.02788872	0.730698
gene28988	9.298258426	40.65277417	4.372084783	2.128321378	0.02788872	0.730698
gene44030	150.8384145	352.3240428	2.335771323	1.223899038	0.0279258	0.731273
gene51429	82.65118601	206.1676405	2.494430515	1.318710482	0.02793703	0.731273
gene59923	276.8814731	632.0538461	2.282759619	1.190778948	0.02799131	0.732349
gene64171	19.62965668	66.78670043	3.402336655	1.7665259	0.02803192	0.733067
gene68274	57.8558302	152.9318648	2.64332677	1.402354783	0.02808658	0.733906
gene19725	41.325593	9.679231946	0.234218828	-2.09407104	0.02812128	0.733906
gene8859	948.4223594	2311.400589	2.437100481	1.285165734	0.02812849	0.733906
gene24927	6.19883895	31.94146542	5.152814209	2.365360575	0.02813009	0.733906
gene60241	6.19883895	31.94146542	5.152814209	2.365360575	0.02813009	0.733906
gene37125	314.0745068	716.263164	2.280551743	1.189382904	0.02816065	0.734242
gene67979	716.9990386	314.5750383	0.438738438	-1.18856699	0.02819494	0.734242
gene51187	617.8176154	271.0184945	0.438670714	-1.1887897	0.02819557	0.734242
gene47428	8.265118601	37.74900459	4.56726714	2.191331176	0.02819584	0.734242
gene42177	139.4738764	327.1580398	2.345658185	1.229992796	0.02824341	0.734686
gene66735	45.4581523	125.8300153	2.768040691	1.468865151	0.0282487	0.734686
gene39317	2676.865287	7960.200353	2.97370226	1.572260206	0.02825256	0.734686
gene683	7.231978776	34.84523501	4.818215884	2.268499036	0.02830743	0.73508
gene11784	7.231978776	34.84523501	4.818215884	2.268499036	0.02830743	0.73508
gene62059	7.231978776	34.84523501	4.818215884	2.268499036	0.02830743	0.73508
gene32734	297.5442696	677.5462362	2.277127491	1.187215067	0.02841001	0.737095
gene49070	16.5302372	59.04331487	3.571837122	1.836666294	0.02841787	0.737095
gene44723	2877.294413	8749.057756	3.040723854	1.604414803	0.02843766	0.737095
gene18864	525.868171	230.3657203	0.438067434	-1.19077513	0.0284381	0.737095
gene725	165.302372	63.88293085	0.386461066	-1.37160502	0.02846641	0.737484
gene47014	92.98258426	227.4619507	2.446285534	1.290592807	0.02852149	0.738442
gene17202	127.0761985	300.0561903	2.361230458	1.239538857	0.02852995	0.738442
gene59657	67.15408863	172.2903286	2.565597005	1.359294575	0.02875747	0.742915
gene26837	152.9046941	355.2278124	2.323197561	1.216111844	0.02877673	0.742915
gene383	3.099419475	22.26223348	7.182710716	2.844528412	0.0287964	0.742915
gene1185	3.099419475	22.26223348	7.182710716	2.844528412	0.0287964	0.742915
gene11558	3.099419475	22.26223348	7.182710716	2.844528412	0.0287964	0.742915
gene29533	3.099419475	22.26223348	7.182710716	2.844528412	0.0287964	0.742915
gene60868	3.099419475	22.26223348	7.182710716	2.844528412	0.0287964	0.742915
gene18131	102.2808427	246.8204146	2.413163679	1.270925773	0.02882376	0.743276
gene16858	33.0604744	97.76024266	2.957012699	1.564140439	0.0289576	0.74638
gene63740	1305.888739	3299.650171	2.526746783	1.337281093	0.02898049	0.746624

gene2462	348.1681211	790.79325	2.271297118	1.183516444	0.029124	0.749973
gene5713	99.18142321	33.87731181	0.341569124	-1.54975053	0.02924456	0.752609
gene15152	936.0246815	2262.036506	2.416641944	1.273003735	0.02925344	0.752609
gene27680	4033.377877	13935.19023	3.454967687	1.788672218	0.02928482	0.753067
gene10396	15.49709738	56.13954529	3.622584535	1.857019356	0.02932783	0.7534
gene58815	69.22036828	176.1620214	2.544944874	1.347634406	0.02932895	0.7534
gene72821	92.98258426	226.4940275	2.435875808	1.28444058	0.02933843	0.7534
gene66860	607.4862171	268.1147249	0.441351124	-1.18000122	0.02937573	0.754009
gene24916	616.7844756	1433.494251	2.324141265	1.216697761	0.02941665	0.754606
gene66936	551.6966666	1272.819001	2.307099314	1.206080109	0.02942618	0.754606
gene39164	173.5674906	398.7843562	2.297575167	1.200112061	0.02944087	0.754635
gene57170	444.2501248	1013.415585	2.281182443	1.189781834	0.02954259	0.756892
gene1957	34.09361423	99.69608905	2.924186576	1.548035364	0.02964707	0.758876
gene51323	137.4075967	320.3825774	2.331622014	1.221333928	0.02964734	0.758876
gene209	74.38606741	186.8091766	2.511346319	1.328460992	0.0296626	0.758917
gene16570	118.8110799	280.6977264	2.362555131	1.240347996	0.02970278	0.759595
gene16016	319.2402059	722.0707032	2.261841365	1.177497749	0.02974084	0.759887
gene27001	55.78955055	147.1243256	2.637130504	1.398968968	0.02974153	0.759887
gene45999	43.39187265	10.64715514	0.245372105	-2.02695685	0.02978619	0.760678
gene2754	423.5873283	963.0835787	2.273636425	1.185001573	0.02984158	0.761061
gene56414	210.7605243	479.1219813	2.273300386	1.18478833	0.02985488	0.761061
gene29802	182.865749	418.1428201	2.286610928	1.193210909	0.02985911	0.761061
gene64711	499.0065355	1142.14937	2.288846515	1.194620722	0.02987447	0.761061
gene71285	852.3403557	2031.670786	2.383637912	1.253165099	0.02990351	0.761061
gene2449	2501.231517	7184.893874	2.872542516	1.522328246	0.02991248	0.761061
gene42396	37.1930337	106.4715514	2.862674561	1.517363669	0.02991544	0.761061
gene61207	123.976779	291.3448816	2.349995571	1.232658038	0.02992286	0.761061
gene8504	223.1582022	506.2238308	2.26845272	1.181708591	0.02992453	0.761061
gene67711	453.5483832	199.3921781	0.439627139	-1.18564765	0.0300282	0.763348
gene48703	715.9658988	1677.410896	2.342864233	1.228273354	0.03006922	0.764042
gene15553	821.3461609	1948.429391	2.372238994	1.246249363	0.03009634	0.764101
gene63935	125.0099188	293.280728	2.346059662	1.230239703	0.03009909	0.764101
gene43245	2769.847871	8191.533996	2.95739491	1.564326904	0.03013285	0.764591
gene15184	76.45234706	24.19807987	0.316511929	-1.65966822	0.03016285	0.764591
gene17264	219.0256429	90.0168571	0.410987754	-1.28283269	0.03017955	0.764591
gene43534	109.5128215	260.3713394	2.377542062	1.249470865	0.03018165	0.764591
gene4788	84.71746566	208.1034868	2.456441363	1.296569802	0.03018718	0.764591
gene15355	14.46395755	53.23577571	3.680581578	1.879933748	0.0302711	0.766367
gene43635	290.3122908	123.8941689	0.426761707	-1.22849736	0.03033926	0.767742
gene3423	110.5459613	262.3071857	2.372833731	1.246611012	0.0303697	0.768095
gene19605	16371.13367	4530.848474	0.276758383	-1.85330108	0.03041505	0.768095
gene19142	160.1366729	367.810814	2.2968556	1.19966016	0.03043985	0.768095
gene38319	25.82849563	80.33762515	3.110426032	1.637112198	0.03055503	0.768095
gene67003	25.82849563	80.33762515	3.110426032	1.637112198	0.03055503	0.768095
gene13496	644.6792509	1493.505489	2.31666443	1.212049084	0.03058077	0.768095
gene64030	41.325593	115.1828602	2.787204049	1.478818625	0.03059076	0.768095
gene26948	10.33139825	42.58862056	4.122251367	2.04343248	0.03064272	0.768095
gene49823	10.33139825	42.58862056	4.122251367	2.04343248	0.03064272	0.768095

gene570	1.033139825	14.51884792	14.05312966	3.812819552	0.03065995	0.768095
gene15300	1.033139825	14.51884792	14.05312966	3.812819552	0.03065995	0.768095
gene17002	1.033139825	14.51884792	14.05312966	3.812819552	0.03065995	0.768095
gene31057	1.033139825	14.51884792	14.05312966	3.812819552	0.03065995	0.768095
gene34912	1.033139825	14.51884792	14.05312966	3.812819552	0.03065995	0.768095
gene43462	1.033139825	14.51884792	14.05312966	3.812819552	0.03065995	0.768095
gene11200	2.06627965	18.3905407	8.900315452	3.15385647	0.03066034	0.768095
gene20932	2.06627965	18.3905407	8.900315452	3.15385647	0.03066034	0.768095
gene32941	2.06627965	18.3905407	8.900315452	3.15385647	0.03066034	0.768095
gene54087	2.06627965	18.3905407	8.900315452	3.15385647	0.03066034	0.768095
gene56098	2.06627965	18.3905407	8.900315452	3.15385647	0.03066034	0.768095
gene57893	2.06627965	18.3905407	8.900315452	3.15385647	0.03066034	0.768095
gene60441	2.06627965	18.3905407	8.900315452	3.15385647	0.03066034	0.768095
gene9327	33.0604744	96.79231946	2.927735346	1.549785146	0.0306851	0.768095
gene26083	33.0604744	96.79231946	2.927735346	1.549785146	0.0306851	0.768095
gene8707	232.4564606	524.6143715	2.256828526	1.174296807	0.03074062	0.769139
gene66516	242.7878589	101.6319354	0.418603862	-1.25634247	0.03076791	0.769475
gene19324	91.94944443	222.6223348	2.421138443	1.275685577	0.03083213	0.77043
gene20765	4.1325593	25.16600306	6.08968952	2.606368675	0.03086157	0.77043
gene46312	4.1325593	25.16600306	6.08968952	2.606368675	0.03086157	0.77043
gene70187	4.1325593	25.16600306	6.08968952	2.606368675	0.03086157	0.77043
gene60920	5281.410786	20719.3639	3.923073729	1.971984449	0.03090707	0.771211
gene52948	141.540156	327.1580398	2.311415	1.20877631	0.0309222	0.771211
gene4275	17.56337703	1.935846389	0.110220625	-3.18153388	0.03094842	0.771211
gene61885	17.56337703	1.935846389	0.110220625	-3.18153388	0.03094842	0.771211
gene71360	834.7769787	1973.595394	2.364218761	1.241363534	0.03096786	0.771332
gene19235	16.5302372	58.07539168	3.513282415	1.812819552	0.03099145	0.771332
gene73819	384.3280149	168.4186359	0.438215871	-1.19028636	0.03101562	0.771332
gene5015	30.99419475	91.95270349	2.966771817	1.568893969	0.03102271	0.771332
gene20014	30.99419475	91.95270349	2.966771817	1.568893969	0.03102271	0.771332
gene27195	69.22036828	21.29431028	0.307630699	-1.70072862	0.03106895	0.772136
gene10965	842.0089574	1991.018011	2.364604312	1.241598786	0.03108942	0.7723
gene38419	42.35873283	117.1187066	2.764924697	1.467240189	0.03110818	0.77242
gene2172	59.92210985	17.4226175	0.290754407	-1.78212704	0.03115817	0.772639
gene8537	59.92210985	17.4226175	0.290754407	-1.78212704	0.03115817	0.772639
gene70501	278.9477528	119.0545529	0.426798753	-1.22837214	0.03115873	0.772639
gene25835	2317.332628	966.9552714	0.417270814	-1.26094408	0.03125572	0.774698
gene61280	9.298258426	39.68485098	4.267987527	2.09355596	0.03130471	0.77533
gene53849	28.9279151	87.11308752	3.011384927	1.590427131	0.03130911	0.77533
gene68919	107.4465418	254.5638002	2.369213526	1.244408228	0.0313347	0.775618
gene29837	390.5268539	878.8742607	2.250483551	1.17023502	0.03136746	0.776083
gene29557	60.95524968	156.8035575	2.572437294	1.36313591	0.03143558	0.777422
gene10847	226.2576217	509.1276004	2.250211934	1.170060886	0.03148771	0.778364
gene10261	364.6983583	818.8630227	2.2453159	1.166918436	0.03152762	0.778756
gene37368	188.0314482	425.8862056	2.264973279	1.17949403	0.0315316	0.778756
gene20108	323.3727653	140.3488632	0.434015719	-1.2041808	0.03156106	0.778922
gene68561	193.1971473	78.40177877	0.4058123	-1.3011155	0.03156636	0.778922
gene60053	1919.573795	5102.891082	2.658345876	1.410528825	0.03159995	0.77932

gene29622	20.6627965	67.75462362	3.279063588	1.713283879	0.03163527	0.77932
gene54096	40.29245318	112.2790906	2.786603488	1.478507733	0.03163663	0.77932
gene37003	536.1995692	1220.551148	2.276300129	1.186690789	0.03163861	0.77932
gene73154	774.8548688	1811.95222	2.338440776	1.225546891	0.03167448	0.779835
gene4527	109.5128215	258.435493	2.359865169	1.238704434	0.03170944	0.779835
gene71969	3689.342315	11952.88353	3.23984128	1.695923137	0.03172666	0.779835
gene69787	943.2566603	2248.485581	2.383747368	1.253231345	0.03173992	0.779835
gene18653	325.4390449	728.8461656	2.239578124	1.163226993	0.03174236	0.779835
gene30331	61.9883895	158.7394039	2.560792516	1.356590366	0.03175234	0.779835
gene3608	491.7745567	218.750642	0.44481895	-1.16870984	0.03177727	0.779835
gene14777	32.02733458	93.88854988	2.931513069	1.551645488	0.03178412	0.779835
gene10418	108.4796816	38.71692779	0.35690488	-1.48638847	0.03179867	0.779835
gene45177	127.0761985	295.2165744	2.323146096	1.216079884	0.03179993	0.779835
gene44538	288.2460112	123.8941689	0.429820931	-1.21819236	0.03181534	0.779869
gene25832	415.3222097	934.0458828	2.248966853	1.169262398	0.03184914	0.780026
gene71426	8.265118601	36.7810814	4.450157726	2.15385647	0.03184982	0.780026
gene10128	1037.272384	459.7635175	0.443242801	-1.17383089	0.03188842	0.780627
gene35548	5.165699125	28.06977264	5.433876802	2.441981857	0.03190613	0.780716
gene50120	273.7820536	117.1187066	0.427780802	-1.22505636	0.03198847	0.782387
gene31211	58.88897003	151.9639416	2.580516207	1.367659691	0.03202937	0.783042
gene46635	465.9460611	1051.164589	2.25597913	1.173753721	0.03206156	0.783268
gene49014	1468.091691	3703.274143	2.522508754	1.334859276	0.03207757	0.783268
gene53339	531.0338701	237.1411827	0.44656508	-1.16305765	0.03215658	0.783268
gene55415	471.1117602	1062.779668	2.25589713	1.173701281	0.03216192	0.783268
gene25245	38.22617353	107.4394746	2.810625932	1.490891457	0.03216847	0.783268
gene8569	7.231978776	33.87731181	4.684376554	2.227857052	0.03220858	0.783268
gene21400	7.231978776	33.87731181	4.684376554	2.227857052	0.03220858	0.783268
gene25656	7.231978776	33.87731181	4.684376554	2.227857052	0.03220858	0.783268
gene73022	326.4721847	142.2847096	0.435824907	-1.19817945	0.03221404	0.783268
gene16519	12.3976779	47.42823654	3.825574185	1.9356763	0.03223332	0.783268
gene34897	12.3976779	47.42823654	3.825574185	1.9356763	0.03223332	0.783268
gene12970	867.8374531	2045.22171	2.356687538	1.236760491	0.0322343	0.783268
gene6588	6.19883895	30.97354223	4.996668324	2.320966456	0.03227836	0.783268
gene13184	6.19883895	30.97354223	4.996668324	2.320966456	0.03227836	0.783268
gene27245	6.19883895	30.97354223	4.996668324	2.320966456	0.03227836	0.783268
gene33391	6.19883895	30.97354223	4.996668324	2.320966456	0.03227836	0.783268
gene37178	6.19883895	30.97354223	4.996668324	2.320966456	0.03227836	0.783268
gene29169	145.6727153	56.13954529	0.385381333	-1.3756414	0.03232038	0.783928
gene52051	143.6064357	55.17162209	0.384186278	-1.3801221	0.03233567	0.783928
gene59268	141.540156	54.2036989	0.382956331	-1.3847482	0.0323479	0.783928
gene25077	100.214563	238.1091059	2.375993056	1.24853062	0.03246096	0.786325
gene16220	23.76221598	3.871692779	0.162934837	-2.617633	0.03248289	0.786514
gene61526	242.7878589	102.5998586	0.422590566	-1.24266754	0.03250637	0.786739
gene8119	64.05466916	19.35846389	0.302217842	-1.72633926	0.03254386	0.787304
gene24949	47.52443195	12.58300153	0.26476911	-1.91719328	0.03260041	0.788328
gene6055	56.82269038	147.1243256	2.589182677	1.372496757	0.03265258	0.789246
gene45465	254.152397	567.2029921	2.231743626	1.158171306	0.0326935	0.789622
gene54416	36.15989388	102.5998586	2.837393798	1.504566394	0.03269806	0.789622

gene35570	223.1582022	499.4483684	2.23809102	1.16226871	0.03271079	0.789622
gene49360	410.1565106	181.9695606	0.443658837	-1.17247739	0.03276492	0.790585
gene24186	853.3734955	2001.665167	2.345590972	1.229951456	0.03279754	0.791029
gene62929	23.76221598	74.53008599	3.136495605	1.649153541	0.03282442	0.791334
gene27054	21.69593633	69.69047001	3.212143922	1.683536535	0.03288465	0.7917
gene47363	445.2832646	198.4242549	0.445613547	-1.166135	0.03288702	0.7917
gene66226	323.3727653	141.3167864	0.437008931	-1.19426533	0.03291328	0.7917
gene67793	79.55176653	194.5525621	2.445609577	1.290194107	0.03295222	0.7917
gene73613	259.3180961	577.8501472	2.228344863	1.155972524	0.0329546	0.7917
gene10567	533.1001497	239.0770291	0.448465507	-1.15693107	0.0330267	0.7917
gene17978	97.11514356	33.87731181	0.348836552	-1.51937688	0.03305804	0.7917
gene39425	281.0140324	625.2783837	2.225078863	1.15385647	0.03308952	0.7917
gene6535	782.0868476	1815.823913	2.321767613	1.215223579	0.0331713	0.7917
gene1456	34.09361423	97.76024266	2.867406254	1.51974632	0.03321784	0.7917
gene22392	14.46395755	52.26785251	3.613661913	1.853461537	0.03322634	0.7917
gene39745	14.46395755	52.26785251	3.613661913	1.853461537	0.03322634	0.7917
gene22279	11.36453808	44.52446695	3.917842208	1.970059294	0.03323018	0.7917
gene5156	0	9.679231946	Inf	Inf	0.03325494	0.7917
gene7733	0	9.679231946	Inf	Inf	0.03325494	0.7917
gene10673	0	9.679231946	Inf	Inf	0.03325494	0.7917
gene14231	0	9.679231946	Inf	Inf	0.03325494	0.7917
gene27072	0	9.679231946	Inf	Inf	0.03325494	0.7917
gene27705	0	9.679231946	Inf	Inf	0.03325494	0.7917
gene28087	0	9.679231946	Inf	Inf	0.03325494	0.7917
gene45255	0	9.679231946	Inf	Inf	0.03325494	0.7917
gene46557	0	9.679231946	Inf	Inf	0.03325494	0.7917
gene47115	0	9.679231946	Inf	Inf	0.03325494	0.7917
gene55858	0	9.679231946	Inf	Inf	0.03325494	0.7917
gene56767	0	9.679231946	Inf	Inf	0.03325494	0.7917
gene59969	0	9.679231946	Inf	Inf	0.03325494	0.7917
gene64229	0	9.679231946	Inf	Inf	0.03325494	0.7917
gene67714	0	9.679231946	Inf	Inf	0.03325494	0.7917
gene74096	0	9.679231946	Inf	Inf	0.03325494	0.7917
gene32898	9.298258426	0	0	#NAME?	0.03329573	0.7917
gene36604	9.298258426	0	0	#NAME?	0.03329573	0.7917
gene74202	9.298258426	0	0	#NAME?	0.03329573	0.7917
gene50724	288.2460112	124.8620921	0.433178907	-1.2069651	0.03333063	0.792191
gene68772	42.35873283	10.64715514	0.251356791	-1.99219143	0.03349098	0.795661
gene6953	86.78374531	209.07141	2.409107942	1.268499036	0.03350811	0.795728
gene39494	1540.411479	3881.37201	2.519698186	1.333250936	0.03354249	0.796204
gene877	239.6884394	533.3256802	2.225078863	1.15385647	0.03358305	0.796826
gene20093	146.7058552	333.9335022	2.276211142	1.186634389	0.03361278	0.796859
gene24374	32.02733458	6.775462362	0.21155249	-2.24091243	0.03362319	0.796859
gene12621	81.61804618	27.10184945	0.332057072	-1.59049687	0.03362744	0.796859
gene69647	26.86163545	81.30554835	3.026827927	1.597806661	0.03365841	0.797252
gene21077	73.35292758	181.0016374	2.467544832	1.303076297	0.03367311	0.79726
gene53483	87.81688513	211.0072564	2.40280962	1.264722345	0.03372298	0.797797
gene36378	1655.09	4217.241359	2.548043526	1.349389922	0.03372449	0.797797

gene16517	49.5907116	13.55092472	0.273255299	-1.87167862	0.03378406	0.798866
gene16468	961.8531772	2271.715738	2.361811336	1.239893726	0.03380787	0.799089
gene45276	401.891392	179.065791	0.445557667	-1.16631593	0.03387695	0.799554
gene61507	2231.582022	6040.808658	2.706962414	1.436674856	0.03388672	0.799554
gene23912	343.0024219	760.787631	2.218024079	1.149275028	0.03389023	0.799554
gene48041	103.3139825	242.9487219	2.35155703	1.233616321	0.03390664	0.799554
gene38779	38.22617353	106.4715514	2.785304978	1.477835305	0.03391798	0.799554
gene37345	492.8076966	1104.400365	2.241037169	1.164166577	0.03392199	0.799554
gene54992	381.2285955	846.9327953	2.221587796	1.151591157	0.03392832	0.799554
gene53563	79.55176653	193.5846389	2.433442366	1.282998606	0.03402052	0.801387
gene43111	22.72907615	71.6263164	3.151307863	1.655950704	0.03408034	0.802128
gene18740	18.59651685	61.94708446	3.331112216	1.736003955	0.03408086	0.802128
gene64439	224.191342	498.4804452	2.223459839	1.152806346	0.03411735	0.802357
gene48331	141.540156	322.3184238	2.277222471	1.187275241	0.03412367	0.802357
gene37232	109.5128215	255.5317234	2.33334983	1.222402621	0.03414808	0.802357
gene45825	214.8930836	90.0168571	0.418891365	-1.25535195	0.0341678	0.802357
gene3568	1918.540655	826.6064082	0.430851651	-1.21473688	0.03419292	0.802357
gene22401	310.9750873	688.1933914	2.213017761	1.14601503	0.03422772	0.802357
gene20137	174.6006304	392.0088938	2.245174561	1.166827618	0.0342301	0.802357
gene1710	80.58490636	195.5204853	2.42626683	1.278738221	0.03425184	0.802357
gene46179	190.0977278	424.9182824	2.23526229	1.16044413	0.03428575	0.802357
gene17494	276.8814731	612.6953822	2.212843551	1.145901456	0.0343183	0.802357
gene15127	3.099419475	21.29431028	6.870418945	2.780398075	0.03433618	0.802357
gene30835	3.099419475	21.29431028	6.870418945	2.780398075	0.03433618	0.802357
gene36883	3.099419475	21.29431028	6.870418945	2.780398075	0.03433618	0.802357
gene40147	3.099419475	21.29431028	6.870418945	2.780398075	0.03433618	0.802357
gene43065	3.099419475	21.29431028	6.870418945	2.780398075	0.03433618	0.802357
gene45983	3.099419475	21.29431028	6.870418945	2.780398075	0.03433618	0.802357
gene47110	3.099419475	21.29431028	6.870418945	2.780398075	0.03433618	0.802357
gene72660	53.7232709	139.38094	2.594423937	1.37541424	0.03436549	0.802705
gene29067	3798.855137	12154.21156	3.199440652	1.677819705	0.03439305	0.803011
gene37774	13.43081773	49.36408293	3.675433911	1.87791458	0.03441898	0.803053
gene36856	348.1681211	770.4668629	2.212916164	1.145948796	0.03443341	0.803053
gene42978	277.9146129	614.6312286	2.211582983	1.145079376	0.03443824	0.803053
gene61092	111.5791011	259.4034162	2.324838734	1.217130645	0.03450542	0.804223
gene55572	992.8473719	446.2125927	0.449427178	-1.15384072	0.03451737	0.804223
gene72402	4059.206373	13329.27031	3.283713389	1.71532821	0.03460636	0.805958
gene25669	112.6122409	261.3392626	2.320700311	1.214560229	0.03468194	0.80738
gene16340	43.39187265	117.1187066	2.699093157	1.432474771	0.0348528	0.81087
gene49019	671.5408863	1526.414878	2.273003638	1.184599994	0.03486106	0.81087
gene23791	596.1216791	1343.477394	2.253696588	1.172293301	0.03491934	0.811886
gene69272	25.82849563	78.40177877	3.035476007	1.60192277	0.03494205	0.812074
gene61194	44.42501248	11.61507834	0.261453575	-1.9353733	0.03499373	0.812262
gene3235	30.99419475	90.0168571	2.904313463	1.538197172	0.03499402	0.812262
gene67043	30.99419475	90.0168571	2.904313463	1.538197172	0.03499402	0.812262
gene49083	164.2692322	368.7787372	2.244965367	1.166693189	0.03503323	0.812832
gene15189	74.38606741	181.9695606	2.446285534	1.290592807	0.03508334	0.813427
gene53265	188.0314482	77.43385557	0.411813323	-1.27993759	0.0350904	0.813427

gene28016	51.65699125	134.5413241	2.604513364	1.38101384	0.03510279	0.813427
gene571	9.298258426	38.71692779	4.16389027	2.05793205	0.03514763	0.813561
gene51752	9.298258426	38.71692779	4.16389027	2.05793205	0.03514763	0.813561
gene46247	95.04886391	224.5581812	2.362555131	1.240347996	0.03515254	0.813561
gene13917	1164.348583	2791.490493	2.397469739	1.261512606	0.03516883	0.8136
gene34830	145.6727153	57.10746848	0.392025839	-1.35097935	0.03524024	0.814912
gene60795	468.0123408	1039.549511	2.221201068	1.151339995	0.03534471	0.816988
gene19229	121.9104994	279.7298033	2.294550549	1.19821159	0.03540555	0.817904
gene13875	886.4339699	2058.772635	2.32253355	1.215699437	0.03541452	0.817904
gene25195	17.56337703	59.04331487	3.361729056	1.749203453	0.0354285	0.817904
gene52246	66.12094881	164.5469431	2.488575044	1.315319893	0.0354563	0.818125
gene5785	231.4233208	511.0634468	2.208348947	1.142968154	0.03546753	0.818125
gene73896	1099.260774	494.6087525	0.449946695	-1.152174	0.03568214	0.822733
gene28557	123.976779	283.601496	2.287537217	1.193795215	0.03573736	0.823665
gene54999	57.8558302	147.1243256	2.542947272	1.346501548	0.03575984	0.823841
gene53024	53.7232709	15.48677111	0.288269326	-1.79451076	0.03577994	0.823962
gene16633	557.8955055	253.595877	0.454558021	-1.13746364	0.03579553	0.82398
gene443	234.5227403	516.8709859	2.203926942	1.140076401	0.03585413	0.824987
gene8989	63.02152933	157.7714807	2.503453699	1.323919773	0.03592153	0.826195
gene62477	1035.206105	2437.230604	2.354343346	1.235324731	0.0359601	0.826699
gene20080	235.5558801	518.8068323	2.202478801	1.139128133	0.0359823	0.826699
gene62814	8.265118601	35.8131582	4.333048312	2.115382322	0.03598808	0.826699
gene9364	795.5176653	362.0032748	0.455053722	-1.13589122	0.03604705	0.827375
gene22224	120.8773595	276.8260337	2.290139648	1.195435574	0.0360473	0.827375
gene42738	100.214563	234.2374131	2.337359023	1.224879352	0.03610334	0.828319
gene4770	338.8698626	150.9960184	0.445587038	-1.16622083	0.03620851	0.829664
gene57076	564.0943445	1259.268076	2.232371391	1.158577062	0.03623623	0.829664
gene42042	4.1325593	24.19807987	5.855470692	2.549785146	0.03623666	0.829664
gene55384	4.1325593	24.19807987	5.855470692	2.549785146	0.03623666	0.829664
gene57161	4.1325593	24.19807987	5.855470692	2.549785146	0.03623666	0.829664
gene13754	101.2477029	236.1732595	2.332628325	1.22195645	0.03628733	0.830461
gene15526	571.3263233	260.3713394	0.45573139	-1.13374435	0.03630135	0.830461
gene31941	34.09361423	7.743385557	0.227121287	-2.13846516	0.03632358	0.830627
gene24770	85.75060548	204.2317941	2.381695067	1.251988714	0.03643728	0.832571
gene63849	262.4175156	114.214937	0.435241286	-1.20011268	0.03643856	0.832571
gene67934	497.9733957	1103.432442	2.215846171	1.147857729	0.03646222	0.832769
gene37265	36.15989388	100.6640122	2.783858066	1.477085658	0.03648682	0.832988
gene55337	326.4721847	715.2952408	2.190983717	1.131578763	0.03653951	0.833518
gene20264	123.976779	282.6335728	2.279729923	1.18886292	0.03654005	0.833518
gene5425	84.71746566	29.03769584	0.34275926	-1.54473245	0.03658129	0.834117
gene10704	43.39187265	116.1507834	2.676786602	1.420502129	0.03659651	0.834121
gene38441	7.231978776	32.90938862	4.550537223	2.186036876	0.03666115	0.834567
gene49607	7.231978776	32.90938862	4.550537223	2.186036876	0.03666115	0.834567
gene50504	7.231978776	32.90938862	4.550537223	2.186036876	0.03666115	0.834567
gene60318	1325.518396	593.3369183	0.447626318	-1.15963323	0.03668934	0.834867
gene43214	325.4390449	712.3914713	2.189016599	1.130282894	0.03674396	0.835767
gene73871	120.8773595	275.8581105	2.282132167	1.190382346	0.03687524	0.837955
gene18197	11.36453808	43.55654376	3.832671726	1.938350434	0.0369227	0.837955

gene31131	11.36453808	43.55654376	3.832671726	1.938350434	0.0369227	0.837955
gene9361	1464.992272	3590.995052	2.451204092	1.293490611	0.0369664	0.837955
gene44816	18.59651685	60.97916126	3.279063588	1.713283879	0.03697014	0.837955
gene65769	18.59651685	60.97916126	3.279063588	1.713283879	0.03697014	0.837955
gene24873	5.165699125	27.10184945	5.24650174	2.391355784	0.03700345	0.837955
gene25928	5.165699125	27.10184945	5.24650174	2.391355784	0.03700345	0.837955
gene37623	5.165699125	27.10184945	5.24650174	2.391355784	0.03700345	0.837955
gene62597	5.165699125	27.10184945	5.24650174	2.391355784	0.03700345	0.837955
gene17644	172.5343508	70.65839321	0.409532321	-1.28795078	0.03700609	0.837955
gene30654	6.19883895	30.00561903	4.840522439	2.275162766	0.03705636	0.838308
gene65050	6.19883895	30.00561903	4.840522439	2.275162766	0.03705636	0.838308
gene111	1317.253277	3177.691848	2.412362075	1.270446459	0.03706697	0.838308
gene65538	871.9700124	397.816433	0.456227195	-1.13217565	0.03708431	0.838359
gene58404	37.1930337	102.5998586	2.758577304	1.46392441	0.03710913	0.838579
gene58516	259.3180961	567.2029921	2.187286582	1.129142257	0.03717183	0.839654
gene1987	241.7547191	104.535705	0.43240399	-1.20954826	0.0372016	0.839985
gene64466	92.98258426	32.90938862	0.353930673	-1.4984613	0.03725667	0.840377
gene12148	128.1093383	49.36408293	0.385327749	-1.37584201	0.03737243	0.840377
gene6896	2.06627965	17.4226175	8.431877796	3.075853958	0.03740053	0.840377
gene9571	2.06627965	17.4226175	8.431877796	3.075853958	0.03740053	0.840377
gene19264	2.06627965	17.4226175	8.431877796	3.075853958	0.03740053	0.840377
gene30684	2.06627965	17.4226175	8.431877796	3.075853958	0.03740053	0.840377
gene35804	2.06627965	17.4226175	8.431877796	3.075853958	0.03740053	0.840377
gene39139	2.06627965	17.4226175	8.431877796	3.075853958	0.03740053	0.840377
gene45172	2.06627965	17.4226175	8.431877796	3.075853958	0.03740053	0.840377
gene63837	2.06627965	17.4226175	8.431877796	3.075853958	0.03740053	0.840377
gene64097	2.06627965	17.4226175	8.431877796	3.075853958	0.03740053	0.840377
gene70093	2.06627965	17.4226175	8.431877796	3.075853958	0.03740053	0.840377
gene40927	222.1250624	486.8653669	2.191852471	1.132150697	0.03750699	0.842254
gene55590	164.2692322	364.9070444	2.221396177	1.151466715	0.03752296	0.842254
gene16250	121.9104994	46.46031334	0.381101821	-1.39175159	0.03752952	0.842254
gene17394	1872.049363	820.7988691	0.438449373	-1.18951783	0.03761261	0.843463
gene16426	248.9866978	543.9728354	2.184746575	1.127465941	0.0376179	0.843463
gene22816	984.5822533	2286.234586	2.322035135	1.215389802	0.03762895	0.843463
gene62774	385.3611548	842.0931793	2.185205148	1.127768727	0.03769545	0.844612
gene9641	158.0703932	63.88293085	0.404142291	-1.30706477	0.0377186	0.84479
gene8150	23.76221598	72.5942396	3.055028187	1.611185691	0.03780131	0.846171
gene44031	132.2418976	298.1203439	2.254356216	1.172715497	0.03781073	0.846171
gene26456	92.98258426	217.7827188	2.342188277	1.227857052	0.03782999	0.846261
gene14998	65.08780898	160.6752503	2.468592089	1.303688464	0.03787458	0.846918
gene44178	13.43081773	48.39615973	3.60336658	1.849345428	0.03792261	0.847381
gene9825	1409.202721	3416.768877	2.424611325	1.277753496	0.03792582	0.847381
gene57622	1638.559763	4067.213264	2.482187929	1.311612348	0.03794918	0.847562
gene61635	1980.529045	5089.340157	2.569687211	1.361592762	0.03799391	0.84822
gene58486	123.976779	280.6977264	2.264115334	1.178947451	0.03820018	0.851902
gene17508	10.33139825	40.65277417	3.934876305	1.976318285	0.03820483	0.851902
gene20508	10.33139825	40.65277417	3.934876305	1.976318285	0.03820483	0.851902
gene71183	417.3884893	911.7836493	2.184496393	1.127300724	0.0382396	0.852335

gene20476	26.86163545	79.36970196	2.954760595	1.563041243	0.03837135	0.854928
gene40782	61.9883895	153.8997879	2.482719573	1.311921316	0.03845933	0.856202
gene59569	61.9883895	153.8997879	2.482719573	1.311921316	0.03845933	0.856202
gene69990	50.62385143	14.51884792	0.286798565	-1.80189029	0.03851652	0.857131
gene59433	559.9617852	1236.03792	2.207361203	1.142322725	0.03867	0.857176
gene50386	33.0604744	92.92062669	2.810625932	1.490891457	0.0386851	0.857176
gene16233	115.7116604	263.2751089	2.275268612	1.186036876	0.03868524	0.857176
gene2616	1.033139825	13.55092472	13.11625435	3.713283879	0.03871916	0.857176
gene14655	1.033139825	13.55092472	13.11625435	3.713283879	0.03871916	0.857176
gene14846	1.033139825	13.55092472	13.11625435	3.713283879	0.03871916	0.857176
gene24344	1.033139825	13.55092472	13.11625435	3.713283879	0.03871916	0.857176
gene25417	1.033139825	13.55092472	13.11625435	3.713283879	0.03871916	0.857176
gene32900	1.033139825	13.55092472	13.11625435	3.713283879	0.03871916	0.857176
gene48671	1.033139825	13.55092472	13.11625435	3.713283879	0.03871916	0.857176
gene53036	1.033139825	13.55092472	13.11625435	3.713283879	0.03871916	0.857176
gene69940	1.033139825	13.55092472	13.11625435	3.713283879	0.03871916	0.857176
gene72669	1.033139825	13.55092472	13.11625435	3.713283879	0.03871916	0.857176
gene25936	137.4075967	54.2036989	0.394473815	-1.34199856	0.03874504	0.857408
gene17653	648.8118102	299.0882671	0.460978457	-1.11722876	0.03878416	0.857932
gene8671	5238.018913	2036.510402	0.388794015	-1.36292209	0.03880564	0.858065
gene8982	30.99419475	6.775462362	0.218604239	-2.19360672	0.03884052	0.858153
gene72538	30.99419475	6.775462362	0.218604239	-2.19360672	0.03884052	0.858153
gene24122	44.42501248	117.1187066	2.636323549	1.398527439	0.03890081	0.85909
gene24316	317.1739263	142.2847096	0.448601533	-1.15649354	0.03891385	0.85909
gene2149	358.4995193	778.2102485	2.170742795	1.118188795	0.03897975	0.860203
gene33868	2988.873514	8463.520414	2.831675671	1.501656034	0.03901769	0.860698
gene50579	322.3396254	698.8405465	2.168025559	1.116381765	0.03904198	0.860892
gene41668	1363.744569	3271.580398	2.398968599	1.262414274	0.03908092	0.861409
gene45061	474.2111797	217.7827188	0.459252603	-1.1226402	0.03916074	0.862531
gene27654	43.39187265	11.61507834	0.26767866	-1.90142597	0.03916289	0.862531
gene38033	540.3321285	1187.64176	2.197984716	1.136181354	0.0392184	0.862654
gene7782	1470.157971	3562.925279	2.423498256	1.277091045	0.03921926	0.862654
gene42293	215.9262234	470.4106726	2.178571297	1.123382328	0.03922622	0.862654
gene1641	49.5907116	127.7658617	2.576407104	1.365360575	0.03923059	0.862654
gene33437	172.5343508	379.4258923	2.199132466	1.136934508	0.03932475	0.863773
gene2554	142.5732959	317.4788078	2.226776101	1.154956504	0.03932566	0.863773
gene35461	41.325593	110.3432442	2.670094636	1.416890876	0.03935527	0.863773
gene48676	41.325593	110.3432442	2.670094636	1.416890876	0.03935527	0.863773
gene73663	328.5384644	148.0922488	0.450760763	-1.14956616	0.03937023	0.863773
gene55380	59.92210985	149.060172	2.48756548	1.314734502	0.0393748	0.863773
gene51493	34.09361423	94.85647307	2.782235771	1.476244681	0.03939258	0.863822
gene28034	12.3976779	45.49239015	3.6694283	1.875555308	0.0394442	0.863844
gene34618	12.3976779	45.49239015	3.6694283	1.875555308	0.0394442	0.863844
gene48834	675.6734456	1504.152644	2.226153261	1.15455292	0.03947062	0.863844
gene19722	9.298258426	37.74900459	4.059793013	2.021406174	0.03947133	0.863844
gene52263	9.298258426	37.74900459	4.059793013	2.021406174	0.03947133	0.863844
gene70701	19.62965668	2.903769584	0.147927681	-2.75703606	0.03951596	0.86448
gene43164	149.8052746	331.9976558	2.216194701	1.148084633	0.03959852	0.865725

gene48978	109.5128215	249.7241842	2.280319153	1.189235758	0.03960407	0.865725
gene57552	128.1093383	287.4731888	2.243967478	1.166051767	0.03967053	0.866496
gene68124	128.1093383	287.4731888	2.243967478	1.166051767	0.03967053	0.866496
gene66658	55.78955055	140.3488632	2.515683705	1.330950544	0.0397025	0.866853
gene12543	6655.486753	2487.56261	0.373761184	-1.41981134	0.03983045	0.869305
gene8715	170.4680711	70.65839321	0.41449635	-1.2705687	0.039874	0.869652
gene33891	317.1739263	685.2896218	2.160611466	1.111439661	0.039879	0.869652
gene70942	73.35292758	176.1620214	2.401567698	1.263976477	0.0398933	0.869652
gene41150	25.82849563	76.46593238	2.960525982	1.565853515	0.03996278	0.870825
gene1638	51.65699125	131.6375545	2.548300845	1.349535608	0.03999299	0.871142
gene54417	291.3454307	629.1500765	2.159464369	1.110673512	0.04003513	0.871717
gene44855	378.129176	817.8950995	2.163004474	1.11303665	0.04007604	0.872039
gene58173	273.7820536	591.4010719	2.160116282	1.111108977	0.04008128	0.872039
gene64737	74.38606741	178.0978678	2.394236905	1.259565911	0.04012141	0.872464
gene65533	142.5732959	57.10746848	0.40054814	-1.31995245	0.04015147	0.872464
gene20698	148.7721348	329.0938862	2.212066706	1.145394891	0.04020324	0.872464
gene16302	478.343739	220.6864884	0.461355445	-1.11604941	0.04021913	0.872464
gene7756	28.9279151	83.24139474	2.877545597	1.524838789	0.04023964	0.872464
gene22104	28.9279151	83.24139474	2.877545597	1.524838789	0.04023964	0.872464
gene68731	28.9279151	83.24139474	2.877545597	1.524838789	0.04023964	0.872464
gene30968	325.4390449	147.1243256	0.452079515	-1.14535155	0.04023984	0.872464
gene728	32.02733458	90.0168571	2.810625932	1.490891457	0.04024995	0.872464
gene39824	220.0587827	477.186135	2.168448489	1.116663173	0.04025791	0.872464
gene6126	75.41920723	180.0337142	2.387106956	1.255263209	0.04034479	0.874006
gene46062	778.9874281	361.0353516	0.463467495	-1.10945994	0.04042745	0.875455
gene62464	679.8060049	315.5429615	0.464166187	-1.10728666	0.04051494	0.877008
gene57452	70.25350811	169.3865591	2.411076167	1.269677227	0.04057354	0.877934
gene64322	48.55757178	124.8620921	2.571423725	1.36256736	0.04062872	0.878089
gene22750	89.88316478	209.07141	2.326035254	1.217872963	0.04063643	0.878089
gene60161	89.88316478	209.07141	2.326035254	1.217872963	0.04063643	0.878089
gene45379	38.22617353	9.679231946	0.253209543	-1.98159631	0.04064862	0.878089
gene32750	8.265118601	34.84523501	4.215938898	2.075853958	0.04067557	0.878089
gene71325	8.265118601	34.84523501	4.215938898	2.075853958	0.04067557	0.878089
gene36926	461.8135018	1001.800506	2.169275048	1.117212988	0.04072579	0.878832
gene51642	211.7936641	458.7955943	2.166238523	1.115192106	0.04084814	0.8803
gene19730	136.3744569	302.9599599	2.221530093	1.151553684	0.04085306	0.8803
gene67145	169.4349313	370.7145835	2.18794661	1.129577534	0.0408953	0.8803
gene71089	384.3280149	176.1620214	0.458363727	-1.12543521	0.04093562	0.8803
gene15929	580.6245817	1271.851078	2.190487826	1.131252197	0.04096451	0.8803
gene1715	3.099419475	20.32638709	6.558127175	2.713283879	0.04099427	0.8803
gene44628	3.099419475	20.32638709	6.558127175	2.713283879	0.04099427	0.8803
gene49408	3.099419475	20.32638709	6.558127175	2.713283879	0.04099427	0.8803
gene55725	3.099419475	20.32638709	6.558127175	2.713283879	0.04099427	0.8803
gene57978	3.099419475	20.32638709	6.558127175	2.713283879	0.04099427	0.8803
gene72489	3.099419475	20.32638709	6.558127175	2.713283879	0.04099427	0.8803
gene4225	11.36453808	42.58862056	3.747501243	1.905928957	0.04103154	0.8803
gene17559	11.36453808	42.58862056	3.747501243	1.905928957	0.04103154	0.8803
gene42977	11.36453808	42.58862056	3.747501243	1.905928957	0.04103154	0.8803

gene57134	11.36453808	42.58862056	3.747501243	1.905928957	0.04103154	0.8803
gene46349	217.9925031	471.3785958	2.162361499	1.11260773	0.04106482	0.880674
gene60512	29.96105493	85.17724113	2.842931977	1.50737958	0.0411021	0.881133
gene6962	21.69593633	66.78670043	3.078304592	1.622135991	0.04112139	0.881207
gene35783	3070.49156	8640.650359	2.814093506	1.492670267	0.04121398	0.882656
gene12359	244.8541385	527.5181411	2.154417909	1.107298127	0.0412208	0.882656
gene44181	76.45234706	26.13392626	0.341832884	-1.54863691	0.04126631	0.88329
gene22846	1789.398177	799.5045588	0.446800812	-1.16229629	0.04128301	0.883307
gene61868	172.5343508	376.5221227	2.18230237	1.125851009	0.04130933	0.88353
gene73888	360.565799	775.3064789	2.150249639	1.104504163	0.0413752	0.884507
gene18195	41.325593	109.375321	2.646672753	1.404179824	0.04138687	0.884507
gene22550	78.51862671	27.10184945	0.345164588	-1.53464363	0.04148947	0.886359
gene18710	58.88897003	18.3905407	0.31229177	-1.67903354	0.04153595	0.88701
gene53907	19.62965668	61.94708446	3.15579052	1.658001443	0.04158825	0.887786
gene74035	907.0967664	421.0465897	0.464169431	-1.10727658	0.04164393	0.888633
gene55482	80.58490636	28.06977264	0.348325436	-1.52149227	0.04168374	0.888953
gene42234	522.7687515	1135.373907	2.171847311	1.11892268	0.041719	0.888953
gene25229	354.36696	760.787631	2.146892111	1.102249693	0.04171954	0.888953
gene23192	165.302372	361.0353516	2.184090568	1.127032682	0.04173622	0.888953
gene55920	7.231978776	31.94146542	4.416697893	2.142968154	0.04174432	0.888953
gene67221	182.865749	77.43385557	0.423446468	-1.2397485	0.04177019	0.888953
gene10474	13.43081773	47.42823654	3.531299248	1.820199083	0.04178694	0.888953
gene54418	13.43081773	47.42823654	3.531299248	1.820199083	0.04178694	0.888953
gene67004	143.6064357	316.5108846	2.204016019	1.140134709	0.0418499	0.889766
gene52801	97.11514356	222.6223348	2.292354484	1.196830156	0.04185723	0.889766
gene51560	30.99419475	87.11308752	2.810625932	1.490891457	0.04192001	0.890681
gene48658	27.89477528	80.33762515	2.880024103	1.526080886	0.04193232	0.890681
gene42200	83.68432583	195.5204853	2.336405096	1.224290437	0.04198132	0.891381
gene44136	112.6122409	253.595877	2.25193882	1.171167633	0.04206707	0.89286
gene9904	178.7331897	75.49800918	0.422406209	-1.24329705	0.04209708	0.893156
gene33149	97.11514356	35.8131582	0.368770069	-1.43920653	0.04224094	0.895866
gene43857	220.0587827	473.3144422	2.150854587	1.104909991	0.0424021	0.898033
gene61573	533.1001497	248.756261	0.466622006	-1.09967375	0.04242139	0.898033
gene36377	1497.019607	3579.379974	2.391004071	1.257616586	0.04248089	0.898033
gene34934	1455.694014	3466.13296	2.381086223	1.251619864	0.04249572	0.898033
gene31984	176.6669101	383.2975851	2.169605983	1.117433062	0.04252989	0.898033
gene71813	63.02152933	20.32638709	0.322530845	-1.63249096	0.04254769	0.898033
gene31461	6.19883895	29.03769584	4.684376554	2.227857052	0.04256171	0.898033
gene32229	910.1961859	423.9503593	0.465779099	-1.10228219	0.04258482	0.898033
gene719	4.1325593	23.23015667	5.621251864	2.490891457	0.04258562	0.898033
gene6258	4.1325593	23.23015667	5.621251864	2.490891457	0.04258562	0.898033
gene8574	4.1325593	23.23015667	5.621251864	2.490891457	0.04258562	0.898033
gene13409	4.1325593	23.23015667	5.621251864	2.490891457	0.04258562	0.898033
gene31933	4.1325593	23.23015667	5.621251864	2.490891457	0.04258562	0.898033
gene40750	4.1325593	23.23015667	5.621251864	2.490891457	0.04258562	0.898033
gene68034	4.1325593	23.23015667	5.621251864	2.490891457	0.04258562	0.898033
gene33043	10.33139825	39.68485098	3.841188774	1.941552866	0.04267074	0.899486
gene21791	324.4059051	148.0922488	0.456502938	-1.13130395	0.04269379	0.899631

gene42787	173.5674906	376.5221227	2.169312475	1.117237879	0.04282178	0.901758
gene48117	721.1315979	1590.297809	2.205280996	1.140962495	0.04283642	0.901758
gene48024	28.9279151	82.27347154	2.844085765	1.507964971	0.04284345	0.901758
gene54921	624.0164543	292.3128048	0.468437655	-1.09407104	0.04293906	0.903428
gene15374	65.08780898	21.29431028	0.327162807	-1.61191935	0.04296011	0.903529
gene59182	476.2774594	1025.030663	2.152171267	1.10579289	0.04302127	0.904473
gene11001	316.1407865	144.220556	0.456190919	-1.13229037	0.04304103	0.904546
gene29489	89.88316478	207.1355637	2.304497891	1.204452447	0.04306275	0.90466
gene31316	375.0297565	801.4404052	2.137004841	1.095590176	0.0431485	0.906119
gene21483	1141.619507	2625.975627	2.300219654	1.201771635	0.04319352	0.906721
gene36958	564.0943445	1222.486995	2.167167614	1.11581074	0.04322537	0.906765
gene60488	1970.197646	4915.113982	2.494731425	1.318884508	0.04322827	0.906765
gene33334	2572.518164	6802.564212	2.644321158	1.402897406	0.0432601	0.907017
gene50489	228.3239013	100.6640122	0.440882499	-1.18153388	0.04327292	0.907017
gene3295	458.7140823	213.911026	0.466327576	-1.10058435	0.04333424	0.907959
gene48890	711.8333395	333.9335022	0.469117536	-1.09197866	0.04336573	0.908277
gene37495	1689.183614	4097.218883	2.425561584	1.27831881	0.04340583	0.908774
gene68793	867.8374531	1938.750159	2.234001485	1.159630145	0.04345872	0.909327
gene29829	540.3321285	1167.315373	2.160366395	1.111276012	0.04346499	0.909327
gene43295	18.59651685	59.04331487	3.174966331	1.666741293	0.0435098	0.909922
gene38921	108.4796816	243.916645	2.248500746	1.168963362	0.04357153	0.91087
gene57281	921.560724	430.7258216	0.467387347	-1.09730942	0.04360888	0.911264
gene10896	303.7431086	138.4130168	0.455691052	-1.13387205	0.0436232	0.911264
gene42902	144.6395755	316.5108846	2.188273047	1.129792765	0.04364819	0.911443
gene18013	46.49129213	119.0545529	2.560792516	1.356590366	0.04368461	0.911861
gene49220	26.86163545	77.43385557	2.882693264	1.527417333	0.04374619	0.912803
gene5592	42.35873283	11.61507834	0.274207408	-1.86666055	0.04383486	0.91431
gene60164	16.5302372	54.2036989	3.279063588	1.713283879	0.04386796	0.914314
gene60374	16.5302372	54.2036989	3.279063588	1.713283879	0.04386796	0.914314
gene71771	65.08780898	156.8035575	2.409107942	1.268499036	0.04391091	0.914866
gene16241	920.5275841	2063.612251	2.241771226	1.164639058	0.04393698	0.914979
gene24114	72.31978776	171.3224055	2.368956143	1.24425149	0.04394931	0.914979
gene27328	2560.120487	6730.937896	2.629148874	1.394595836	0.04406646	0.916831
gene48405	1313.120718	3060.573141	2.330762968	1.220802294	0.04407127	0.916831
gene48159	24.7953558	4.839615973	0.195182356	-2.35710545	0.04410328	0.91685
gene53088	53.7232709	16.45469431	0.306286159	-1.70704792	0.04417816	0.91685
gene976	2188.19015	5547.167828	2.53504835	1.342013264	0.04418688	0.91685
gene47401	38.22617353	101.6319354	2.658700206	1.410721109	0.04418912	0.91685
gene58403	260.3512359	117.1187066	0.44984886	-1.15248773	0.04425448	0.91685
gene12391	21.69593633	65.81877724	3.033691482	1.601074375	0.0443045	0.91685
gene13503	9.298258426	36.7810814	3.955695756	1.983931469	0.04433636	0.91685
gene24904	9.298258426	36.7810814	3.955695756	1.983931469	0.04433636	0.91685
gene15249	273.7820536	123.8941689	0.452528452	-1.14391959	0.04438292	0.91685
gene54354	67.15408863	160.6752503	2.392635409	1.258600575	0.04438948	0.91685
gene42811	539.2989887	253.595877	0.470232436	-1.08855404	0.04443308	0.91685
gene66812	1768.735381	4300.482754	2.431388438	1.281780396	0.04449669	0.91685
gene37837	30.99419475	86.14516432	2.779396755	1.474771792	0.04451908	0.91685
gene70253	256.2186766	115.1828602	0.44954904	-1.15344959	0.04454124	0.91685

gene22679	827.5449999	389.1051242	0.470192104	-1.08867778	0.04454357	0.91685
gene3751	102.2808427	38.71692779	0.378535479	-1.40149957	0.04458278	0.91685
gene37107	102.2808427	38.71692779	0.378535479	-1.40149957	0.04458278	0.91685
gene28031	460.780362	984.3778889	2.136327783	1.095133021	0.0446519	0.91685
gene15626	100.214563	226.4940275	2.260090956	1.176380834	0.04465828	0.91685
gene38897	518.6361922	243.916645	0.470303941	-1.08833467	0.04469084	0.91685
gene29502	98.14828338	36.7810814	0.374750124	-1.41599914	0.04474782	0.91685
gene12750	35.12675405	94.85647307	2.700405307	1.43317596	0.04479266	0.91685
gene46647	109.5128215	244.8845682	2.236126921	1.161002077	0.04481077	0.91685
gene49646	69.22036828	164.5469431	2.377146311	1.249230702	0.04484249	0.91685
gene2328	0	8.711308752	Inf	Inf	0.04489752	0.91685
gene4909	0	8.711308752	Inf	Inf	0.04489752	0.91685
gene5072	0	8.711308752	Inf	Inf	0.04489752	0.91685
gene8383	0	8.711308752	Inf	Inf	0.04489752	0.91685
gene11287	0	8.711308752	Inf	Inf	0.04489752	0.91685
gene16685	0	8.711308752	Inf	Inf	0.04489752	0.91685
gene18479	0	8.711308752	Inf	Inf	0.04489752	0.91685
gene20235	0	8.711308752	Inf	Inf	0.04489752	0.91685
gene23008	0	8.711308752	Inf	Inf	0.04489752	0.91685
gene25021	0	8.711308752	Inf	Inf	0.04489752	0.91685
gene30359	0	8.711308752	Inf	Inf	0.04489752	0.91685
gene35362	0	8.711308752	Inf	Inf	0.04489752	0.91685
gene39333	0	8.711308752	Inf	Inf	0.04489752	0.91685
gene41262	0	8.711308752	Inf	Inf	0.04489752	0.91685
gene51979	0	8.711308752	Inf	Inf	0.04489752	0.91685
gene52504	0	8.711308752	Inf	Inf	0.04489752	0.91685
gene55603	0	8.711308752	Inf	Inf	0.04489752	0.91685
gene57355	0	8.711308752	Inf	Inf	0.04489752	0.91685
gene62362	0	8.711308752	Inf	Inf	0.04489752	0.91685
gene64189	0	8.711308752	Inf	Inf	0.04489752	0.91685
gene64667	0	8.711308752	Inf	Inf	0.04489752	0.91685
gene65259	0	8.711308752	Inf	Inf	0.04489752	0.91685
gene66596	0	8.711308752	Inf	Inf	0.04489752	0.91685
gene67148	0	8.711308752	Inf	Inf	0.04489752	0.91685
gene71581	0	8.711308752	Inf	Inf	0.04489752	0.91685
gene73179	0	8.711308752	Inf	Inf	0.04489752	0.91685
gene45559	12.3976779	0.967923195	0.078072943	-3.67903354	0.04505955	0.919483
gene61249	12.3976779	0.967923195	0.078072943	-3.67903354	0.04505955	0.919483
gene60797	63.02152933	151.9639416	2.411302029	1.269812368	0.04507675	0.919496
gene46897	44.42501248	12.58300153	0.283241373	-1.81989608	0.04509368	0.919504
gene37285	135.3413171	296.1844976	2.188426298	1.129893798	0.04511734	0.919649
gene5718	247.953558	111.3111674	0.44891942	-1.15547159	0.04515144	0.920006
gene22517	446.3164044	950.5005771	2.129656378	1.090620669	0.04521227	0.920159
gene24816	297.5442696	630.1179997	2.117728567	1.082517688	0.04522108	0.920159
gene72179	157.0372534	339.7410413	2.163442329	1.113328664	0.04522466	0.920159
gene29050	112.6122409	250.6921074	2.226153261	1.15455292	0.04522522	0.920159
gene55478	64.05466916	153.8997879	2.402631845	1.264615602	0.04532362	0.921824
gene25590	488.6751373	1043.421204	2.135204197	1.094374046	0.04538042	0.922641

gene54564	36.15989388	96.79231946	2.676786602	1.420502129	0.04542179	0.922728
gene62777	4273.066317	13135.68567	3.074065484	1.620147898	0.04543207	0.922728
gene1831	2791.543807	7447.20106	2.667771518	1.415635112	0.04544658	0.922728
gene42601	460.780362	981.4741194	2.130025931	1.090870994	0.04545116	0.922728
gene49731	313.041367	662.0594651	2.114926444	1.080607488	0.04556707	0.922743
gene8074	11.36453808	41.62069737	3.66233076	1.872762093	0.04560365	0.922743
gene37354	11.36453808	41.62069737	3.66233076	1.872762093	0.04560365	0.922743
gene46818	28.9279151	81.30554835	2.810625932	1.490891457	0.04561474	0.922743
gene16479	2516.728614	6533.481564	2.596021489	1.376302326	0.04562369	0.922743
gene42130	653.9775093	1416.071634	2.165321611	1.114581321	0.04571199	0.922743
gene0	2.06627965	16.45469431	7.963440141	2.993391798	0.04573432	0.922743
gene510	2.06627965	16.45469431	7.963440141	2.993391798	0.04573432	0.922743
gene3619	2.06627965	16.45469431	7.963440141	2.993391798	0.04573432	0.922743
gene11902	2.06627965	16.45469431	7.963440141	2.993391798	0.04573432	0.922743
gene16433	2.06627965	16.45469431	7.963440141	2.993391798	0.04573432	0.922743
gene19087	2.06627965	16.45469431	7.963440141	2.993391798	0.04573432	0.922743
gene27661	2.06627965	16.45469431	7.963440141	2.993391798	0.04573432	0.922743
gene43667	2.06627965	16.45469431	7.963440141	2.993391798	0.04573432	0.922743
gene60302	2.06627965	16.45469431	7.963440141	2.993391798	0.04573432	0.922743
gene65008	2.06627965	16.45469431	7.963440141	2.993391798	0.04573432	0.922743
gene65570	2.06627965	16.45469431	7.963440141	2.993391798	0.04573432	0.922743
gene71620	41.325593	107.4394746	2.599828987	1.378416728	0.04576683	0.923064
gene63879	250.0198377	529.4539875	2.117647913	1.082462742	0.0458216	0.92353
gene44887	59.92210985	19.35846389	0.323060452	-1.63012394	0.04583982	0.92353
gene62488	59.92210985	19.35846389	0.323060452	-1.63012394	0.04583982	0.92353
gene2042	422.5541885	896.2968782	2.121140679	1.084840307	0.04587648	0.923693
gene72333	454.581523	213.911026	0.470566917	-1.0875282	0.04588116	0.923693
gene25047	109.5128215	243.916645	2.227288475	1.155288426	0.04593129	0.924127
gene2835	15.49709738	51.29992932	3.310292765	1.726958816	0.04598105	0.924127
gene22420	15.49709738	51.29992932	3.310292765	1.726958816	0.04598105	0.924127
gene32283	8.265118601	33.87731181	4.098829484	2.035211974	0.0459859	0.924127
gene70470	8.265118601	33.87731181	4.098829484	2.035211974	0.0459859	0.924127
gene42256	37.1930337	98.72816585	2.654480047	1.408429297	0.0460192	0.924462
gene51097	13.43081773	46.46031334	3.459231916	1.790451739	0.04604894	0.924725
gene41829	4840.260081	15607.76151	3.22457084	1.689107164	0.04608754	0.925164
gene50580	1878.248202	4579.244634	2.438040206	1.285721918	0.04610413	0.925164
gene35514	84.71746566	194.5525621	2.296487042	1.199428643	0.0461897	0.926546
gene28050	589.9228401	1267.011462	2.147757936	1.102831403	0.04621734	0.926766
gene12306	103.3139825	231.3336435	2.239131993	1.162939575	0.04628112	0.927647
gene27414	344.0355618	725.942396	2.110079529	1.077297375	0.04633495	0.927647
gene15686	20.6627965	62.91500765	3.04484476	1.606368675	0.04641356	0.927647
gene59154	179.7663296	384.2655083	2.137583324	1.095980658	0.04645557	0.927647
gene65085	29.96105493	83.24139474	2.778319887	1.474212716	0.04647108	0.927647
gene12584	274.8151935	579.7859936	2.109730493	1.077058714	0.04647543	0.927647
gene70134	353.3338202	745.3008599	2.10933915	1.076791077	0.04650773	0.927647
gene21784	1192.243358	557.5237601	0.467625805	-1.09657355	0.04652442	0.927647
gene6106	8.265118601	0	0	#NAME?	0.04652846	0.927647
gene18970	8.265118601	0	0	#NAME?	0.04652846	0.927647

gene32963	8.265118601	0	0	#NAME?	0.04652846	0.927647
gene40888	8.265118601	0	0	#NAME?	0.04652846	0.927647
gene41044	8.265118601	0	0	#NAME?	0.04652846	0.927647
gene44071	8.265118601	0	0	#NAME?	0.04652846	0.927647
gene49388	8.265118601	0	0	#NAME?	0.04652846	0.927647
gene71499	8.265118601	0	0	#NAME?	0.04652846	0.927647
gene21338	43.39187265	111.3111674	2.565253827	1.359101585	0.04670043	0.930702
gene28711	258.2849563	544.9407586	2.1098432	1.077135784	0.04671524	0.930702
gene33687	71.28664793	167.4507127	2.348977228	1.232032728	0.04686405	0.933332
gene65938	49.5907116	123.8941689	2.498334162	1.320966456	0.04689827	0.933679
gene39674	177.7000499	379.4258923	2.135204197	1.094374046	0.04695489	0.934471
gene31943	144.6395755	60.01123807	0.414901923	-1.26915775	0.04703407	0.935663
gene33801	64.05466916	152.9318648	2.387520953	1.255513394	0.04704844	0.935663
gene2524	381.2285955	803.3762515	2.10733471	1.075419477	0.04708248	0.935895
gene26613	56.82269038	138.4130168	2.435875808	1.28444058	0.0470994	0.935895
gene35495	172.5343508	368.7787372	2.137422116	1.095871852	0.04711068	0.935895
gene58670	50.62385143	125.8300153	2.485587559	1.313586926	0.04724467	0.937878
gene72584	3011.60259	8121.843526	2.696851023	1.431275828	0.04726606	0.937878
gene52240	65.08780898	154.8677111	2.379365868	1.250577128	0.04727686	0.937878
gene25976	30.99419475	85.17724113	2.748167578	1.45846998	0.04727803	0.937878
gene45374	907.0967664	429.7578984	0.473772936	-1.07773231	0.04731807	0.938337
gene32032	1887.54646	4577.308787	2.425004567	1.277987464	0.04743269	0.940274
gene45382	607.4862171	1300.888774	2.141429282	1.098574035	0.04746943	0.940334
gene15599	72.31978776	25.16600306	0.347982258	-1.52291434	0.04749071	0.940334
gene60237	370.8971972	780.1460949	2.103402508	1.072724951	0.04749368	0.940334
gene54822	883.3345504	419.1107433	0.474464339	-1.07562844	0.04754364	0.940334
gene5409	7.231978776	30.97354223	4.282858563	2.098574035	0.04754867	0.940334
gene40229	45.4581523	115.1828602	2.533821863	1.341315101	0.0475509	0.940334
gene9651	245.8872784	111.3111674	0.452691852	-1.14339876	0.04756776	0.940334
gene39894	51.65699125	127.7658617	2.47335082	1.306466886	0.04757704	0.940334
gene2618	734.5624156	1592.233655	2.167594777	1.116095076	0.04758804	0.940334
gene20873	1582.770212	3726.504299	2.354419025	1.235371105	0.04765296	0.941281
gene56129	338.8698626	711.4235481	2.099400468	1.069977392	0.04776531	0.943165
gene11420	88.85002496	32.90938862	0.370392565	-1.43287296	0.04779612	0.943172
gene4953	2597.31352	6715.451124	2.585537353	1.370464148	0.04783277	0.943172
gene19060	24.7953558	71.6263164	2.888698875	1.530419822	0.04783355	0.943172
gene60984	24.7953558	71.6263164	2.888698875	1.530419822	0.04783355	0.943172
gene61590	379.1623158	796.6007892	2.100949266	1.071041324	0.04793171	0.944772
gene30150	1484.621929	689.1613146	0.464199876	-1.10718196	0.04797487	0.945287
gene34419	32.02733458	87.11308752	2.71996058	1.443585743	0.04803859	0.946033
gene9972	303.7431086	636.8934621	2.096816172	1.068200386	0.04806383	0.946033
gene40204	303.7431086	636.8934621	2.096816172	1.068200386	0.04806383	0.946033
gene61672	99.18142321	221.6544116	2.234837981	1.160170244	0.048233	0.949026
gene66149	12.3976779	43.55654376	3.513282415	1.812819552	0.04828985	0.949809
gene41654	15.49709738	1.935846389	0.124916708	-3.00096164	0.04834439	0.949872
gene54611	15.49709738	1.935846389	0.124916708	-3.00096164	0.04834439	0.949872
gene70357	15.49709738	1.935846389	0.124916708	-3.00096164	0.04834439	0.949872
gene60395	26.86163545	5.807539168	0.216201995	-2.20954826	0.04840095	0.950647

gene43956	54.75641073	133.5734009	2.439411186	1.286532959	0.04849683	0.952194
gene23328	149.8052746	321.3505006	2.145121401	1.101059298	0.04867046	0.953503
gene58073	48.55757178	120.9903993	2.491689656	1.31712439	0.04868931	0.953503
gene24066	19.62965668	60.01123807	3.057172067	1.612197754	0.04870845	0.953503
gene3535	984.5822533	467.506903	0.474827676	-1.07452407	0.04870895	0.953503
gene24553	387.4274344	182.9374838	0.472185157	-1.0825754	0.04880088	0.953503
gene59273	302.7099687	633.0217693	2.091182434	1.064318928	0.0488691	0.953503
gene8670	507.2716541	241.9807987	0.477024089	-1.06786597	0.04888704	0.953503
gene24809	6.19883895	28.06977264	4.528230668	2.178947451	0.04890729	0.953503
gene515	22.72907615	66.78670043	2.938381656	1.555021795	0.04897633	0.953503
gene7185	3.099419475	19.35846389	6.245835405	2.642894551	0.04900739	0.953503
gene19319	3.099419475	19.35846389	6.245835405	2.642894551	0.04900739	0.953503
gene26587	3.099419475	19.35846389	6.245835405	2.642894551	0.04900739	0.953503
gene30466	3.099419475	19.35846389	6.245835405	2.642894551	0.04900739	0.953503
gene44169	3.099419475	19.35846389	6.245835405	2.642894551	0.04900739	0.953503
gene45927	3.099419475	19.35846389	6.245835405	2.642894551	0.04900739	0.953503
gene39441	1123.02299	2516.600306	2.240916107	1.16408864	0.04901822	0.953503
gene70282	43.39187265	110.3432442	2.542947272	1.346501548	0.0490303	0.953503
gene51939	518.6361922	1095.689056	2.112635163	1.079043646	0.04903141	0.953503
gene19003	659.1432084	315.5429615	0.478716852	-1.0627555	0.04907684	0.953503
gene61101	84.71746566	192.6167157	2.273636425	1.185001573	0.04907867	0.953503
gene3860	1.033139825	12.58300153	12.17937904	3.606368675	0.04910618	0.953503
gene17286	1.033139825	12.58300153	12.17937904	3.606368675	0.04910618	0.953503
gene18156	1.033139825	12.58300153	12.17937904	3.606368675	0.04910618	0.953503
gene19506	1.033139825	12.58300153	12.17937904	3.606368675	0.04910618	0.953503
gene20637	1.033139825	12.58300153	12.17937904	3.606368675	0.04910618	0.953503
gene23826	1.033139825	12.58300153	12.17937904	3.606368675	0.04910618	0.953503
gene26360	1.033139825	12.58300153	12.17937904	3.606368675	0.04910618	0.953503
gene28785	1.033139825	12.58300153	12.17937904	3.606368675	0.04910618	0.953503
gene45794	1.033139825	12.58300153	12.17937904	3.606368675	0.04910618	0.953503
gene47001	1.033139825	12.58300153	12.17937904	3.606368675	0.04910618	0.953503
gene64950	1.033139825	12.58300153	12.17937904	3.606368675	0.04910618	0.953503
gene49717	38.22617353	99.69608905	2.608058297	1.382976118	0.04911283	0.953503
gene47172	954.6211984	2102.329179	2.202265341	1.138988303	0.0492683	0.956187
gene18616	2653.103071	6843.216986	2.579325719	1.366993969	0.04931013	0.956513
gene66649	204.5616854	90.9847803	0.444779188	-1.16883881	0.04931954	0.956513
gene18212	391.5599937	818.8630227	2.091283675	1.064388772	0.04945683	0.958841
gene17049	56.82269038	18.3905407	0.323647835	-1.62750324	0.04955271	0.960365
gene37129	17.56337703	55.17162209	3.141287807	1.65135613	0.04959539	0.960522
gene64862	17.56337703	55.17162209	3.141287807	1.65135613	0.04959539	0.960522
gene58914	51.65699125	126.7979385	2.454613314	1.295495768	0.0496851	0.961924
gene47681	308.9088077	144.220556	0.466870974	-1.0989042	0.04977861	0.963073
gene3282	9.298258426	35.8131582	3.8515985	1.945457321	0.0498108	0.963073
gene63068	121.9104994	49.36408293	0.404920685	-1.30428875	0.04984217	0.963073
gene47334	45.4581523	114.214937	2.512529242	1.329140387	0.0498499	0.963073
gene41077	5.165699125	25.16600306	4.871751616	2.28444058	0.04986567	0.963073
gene70882	5.165699125	25.16600306	4.871751616	2.28444058	0.04986567	0.963073
gene55261	143.6064357	307.7995759	2.143355027	1.099870839	0.04987861	0.963073

gene18466	100.214563	38.71692779	0.386340334	-1.37205579	0.04988663	0.963073
gene23864	58.88897003	19.35846389	0.328728179	-1.60503296	0.04994162	0.963073
gene69795	569.2600436	1204.096454	2.115195801	1.080791218	0.04994459	0.963073
gene44112	430.8193071	205.1997173	0.476301117	-1.07005416	0.04994909	0.963073
gene19485	52.69013108	128.7337849	2.44322385	1.28878605	0.04998912	0.963073
gene66868	285.1465917	594.3048415	2.084208119	1.059499345	0.04999242	0.963073
gene56948	279.9808926	129.7017081	0.463251999	-1.11013089	0.05005517	0.963073
gene3791	35.12675405	92.92062669	2.645294995	1.403428616	0.05006925	0.963073
gene31871	4.1325593	22.26223348	5.387033037	2.429490913	0.05009123	0.963073
gene44185	4.1325593	22.26223348	5.387033037	2.429490913	0.05009123	0.963073
gene48054	4.1325593	22.26223348	5.387033037	2.429490913	0.05009123	0.963073
gene54646	4.1325593	22.26223348	5.387033037	2.429490913	0.05009123	0.963073
gene62420	4.1325593	22.26223348	5.387033037	2.429490913	0.05009123	0.963073
gene59856	135.3413171	56.13954529	0.414799756	-1.26951305	0.05017234	0.964298
gene4322	46.49129213	116.1507834	2.498334162	1.320966456	0.05023074	0.965087
gene51285	932.925262	2042.317941	2.189154934	1.130374063	0.05029033	0.965311
gene45897	831.6775592	398.7843562	0.479493948	-1.06041549	0.05031097	0.965311
gene37033	15.49709738	50.33200612	3.24783441	1.699478079	0.05031712	0.965311
gene37514	15.49709738	50.33200612	3.24783441	1.699478079	0.05031712	0.965311
gene3558	138.4407366	297.1524208	2.146423286	1.101934612	0.05032931	0.965311
gene52182	202.4954057	424.9182824	2.098409497	1.069296242	0.05042455	0.966804
gene50209	171.501211	74.53008599	0.434574692	-1.20232393	0.0505512	0.968898
gene1708	1053.802622	502.352138	0.476704202	-1.06883375	0.05065096	0.970394
gene33031	830.6444194	398.7843562	0.480090333	-1.05862221	0.05068784	0.970394
gene19820	11.36453808	40.65277417	3.577160277	1.838814761	0.05069116	0.970394
gene58586	109.5128215	240.0449523	2.191934689	1.132204812	0.05069911	0.970394
gene53705	13.43081773	45.49239015	3.387164585	1.76007809	0.05074926	0.970869
gene58211	131.2087578	54.2036989	0.413110373	-1.27540081	0.05075892	0.970869
gene65115	161.1698127	341.6768877	2.119980671	1.084051111	0.05080854	0.971384
gene65958	184.9320287	389.1051242	2.104043994	1.073164871	0.05082489	0.971384
gene189	169.4349313	73.56216279	0.434161729	-1.20369553	0.05084345	0.971384
gene42282	500.0396753	1048.26082	2.096355292	1.067883247	0.05085581	0.971384
gene668	122.9436392	266.1788785	2.165047987	1.114399002	0.05089092	0.971721
gene5631	227.2907615	474.2823654	2.086676828	1.061207182	0.05094652	0.972448
gene41560	2296.669831	5688.484615	2.476840396	1.308500907	0.05096687	0.972502
gene38610	69.22036828	24.19807987	0.34958034	-1.51630404	0.05101628	0.97278
gene4285	201.4622659	422.0145129	2.094757105	1.066782968	0.05103533	0.97278
gene30506	42.35873283	107.4394746	2.536418524	1.342792818	0.05105119	0.97278
gene7564	495.907116	1038.581588	2.094306684	1.066472722	0.05105144	0.97278
gene52029	292.3785705	136.4771704	0.466782399	-1.09917793	0.05107604	0.972914
gene20539	111.5791011	44.52446695	0.399039484	-1.32539659	0.05118901	0.974426
gene32435	75.41920723	27.10184945	0.359349434	-1.47654068	0.05119046	0.974426
gene54262	24.7953558	70.65839321	2.849662403	1.510791015	0.05124319	0.975095
gene19410	660.1763482	1401.552786	2.122997574	1.086102723	0.05132447	0.976307
gene6028	245.8872784	511.0634468	2.078446067	1.055505313	0.05156419	0.980532
gene59555	1653.02372	3852.334315	2.330477335	1.220625482	0.05159282	0.98074
gene43868	80.58490636	182.9374838	2.270120945	1.182769162	0.05161905	0.980903
gene55450	118.8110799	257.4675698	2.167033327	1.115721341	0.05164353	0.981033

gene34159	169.4349313	357.1636588	2.107969449	1.075853958	0.05167651	0.981324
gene57431	185.9651685	82.27347154	0.442413341	-1.1765332	0.0517352	0.982096
gene20834	359.5326591	745.3008599	2.072971233	1.051700096	0.05175252	0.982096
gene23813	44.42501248	111.3111674	2.505596761	1.325154253	0.05187683	0.984118
gene52545	71.28664793	164.5469431	2.308243519	1.206795436	0.05197928	0.984682
gene15407	782.0868476	1676.442973	2.143550909	1.100002681	0.05199057	0.984682
gene27899	8.265118601	32.90938862	3.981720071	1.993391798	0.0520025	0.984682
gene35761	8.265118601	32.90938862	3.981720071	1.993391798	0.0520025	0.984682
gene51901	656.0437889	1388.969784	2.117190663	1.082151197	0.0520088	0.984682
gene29558	83.68432583	188.745023	2.255440563	1.173409268	0.052019	0.984682
gene29989	523.8018913	252.6279538	0.482296758	-1.05200698	0.05203063	0.984682
gene50940	47.52443195	14.51884792	0.305502819	-1.7107424	0.05206685	0.985032
gene62470	23.76221598	4.839615973	0.203668546	-2.2957049	0.05208771	0.985091
gene53118	480.4100187	231.3336435	0.481533762	-1.05429114	0.05217502	0.986328
gene12438	16.5302372	52.26785251	3.161954174	1.660816459	0.05220641	0.986328
gene50090	16.5302372	52.26785251	3.161954174	1.660816459	0.05220641	0.986328
gene3003	318.2070661	658.1877724	2.068426011	1.048533352	0.05222444	0.986333
gene68757	103.3139825	40.65277417	0.393487631	-1.34560981	0.05225405	0.986557
gene60082	34.09361423	90.0168571	2.640284967	1.400693648	0.05231414	0.987287
gene26383	622.9833145	1313.471775	2.108357872	1.07611977	0.05232827	0.987287
gene17383	2228.482603	5450.375509	2.445778801	1.290293931	0.05239554	0.988129
gene25789	746.9600935	1593.201578	2.132913916	1.09282574	0.05242087	0.988129
gene55805	63.02152933	148.0922488	2.349867583	1.232579462	0.05242624	0.988129
gene59643	53.7232709	129.7017081	2.414255608	1.271578429	0.05244654	0.988176
gene52091	101.2477029	39.68485098	0.391958038	-1.35122888	0.05251311	0.989095
gene51125	29.96105493	81.30554835	2.713707797	1.440265384	0.05253537	0.989179
gene64199	666.3751872	1409.296171	2.114868918	1.080568247	0.05262167	0.990468
gene17416	179.7663296	376.5221227	2.094508597	1.066611806	0.05272189	0.992018
gene72057	1056.902041	2319.143974	2.194284696	1.13375072	0.05291178	0.995254
gene45958	177.7000499	78.40177877	0.441202908	-1.1804858	0.05296281	0.995587
gene20812	14.46395755	47.42823654	3.279063588	1.713283879	0.05300117	0.995587
gene28474	14.46395755	47.42823654	3.279063588	1.713283879	0.05300117	0.995587
gene35018	14.46395755	47.42823654	3.279063588	1.713283879	0.05300117	0.995587
gene53077	1108.559032	531.3898339	0.47935186	-1.06084307	0.05303623	0.995809
gene17641	532.0670099	1110.207904	2.086594139	1.061150011	0.05304883	0.995809
gene28343	2660.33505	1197.320992	0.450063984	-1.15179798	0.05307058	0.99588
gene25825	2195.422128	5337.128495	2.4310261	1.281565383	0.05309815	0.996061
gene36809	588.8897003	285.5373424	0.484874064	-1.04431801	0.05319708	0.997471
gene40522	333.7041635	688.1933914	2.062285901	1.044244352	0.05320921	0.997471
gene19088	10.33139825	37.74900459	3.653813712	1.869403081	0.0532541	0.997639
gene54847	10.33139825	37.74900459	3.653813712	1.869403081	0.0532541	0.997639
gene4882	159.1035331	334.9014253	2.104927646	1.073770644	0.0533556	0.999005
gene52928	313.041367	148.0922488	0.473075652	-1.07985718	0.053363	0.999005
gene7165	509.3379338	1058.907975	2.078989026	1.055882143	0.05361373	1
gene11841	468.0123408	969.859041	2.072293734	1.05122851	0.05366263	1
gene60248	398.7919725	191.6487925	0.480573346	-1.05717146	0.05370127	1
gene6099	90.91630461	34.84523501	0.383267173	-1.38357766	0.05371443	1
gene57566	152.9046941	322.3184238	2.107969449	1.075853958	0.05374363	1

gene61881	53.7232709	17.4226175	0.324302992	-1.62458576	0.05376118	1
gene221	23.76221598	67.75462362	2.851359641	1.511650018	0.05381481	1
gene66276	23.76221598	67.75462362	2.851359641	1.511650018	0.05381481	1
gene54878	1322.418976	630.1179997	0.476488928	-1.06948541	0.05385977	1
gene40953	242.7878589	112.2790906	0.4624576	-1.11260699	0.05392591	1
gene864	17.56337703	54.2036989	3.086177494	1.625821037	0.05395032	1
gene45009	156.0041136	328.125963	2.103316095	1.07266568	0.05402767	1
gene20638	60.95524968	143.2526328	2.350127898	1.232739273	0.05406137	1
gene17335	20.6627965	60.97916126	2.951157229	1.561280785	0.05411188	1
gene1601	55.78955055	18.3905407	0.329641313	-1.60103103	0.05412484	1
gene20007	167.3686517	350.3881965	2.093511497	1.065924841	0.05414146	1
gene59744	260.3512359	536.2294498	2.05963858	1.042391199	0.05414484	1
gene34760	27.89477528	76.46593238	2.741227761	1.454822203	0.05417351	1
gene43506	27.89477528	76.46593238	2.741227761	1.454822203	0.05417351	1
gene64168	7.231978776	30.00561903	4.149019233	2.052770345	0.05417775	1
gene48261	210.7605243	95.82439627	0.454660077	-1.13713977	0.0541799	1
gene1352	455.6146629	941.7892684	2.067074098	1.047590106	0.05419319	1
gene19343	61.9883895	145.1884792	2.342188277	1.227857052	0.05425507	1
gene43209	400.8582521	825.638485	2.059676907	1.042418046	0.0543209	1
gene17550	1346.181192	641.733078	0.476706317	-1.06882735	0.05432675	1
gene55917	348.1681211	166.4827895	0.478167814	-1.06441107	0.05440101	1
gene7591	282.0471722	579.7859936	2.055634839	1.039584009	0.0544253	1
gene27350	146.7058552	62.91500765	0.428851375	-1.22145035	0.05447187	1
gene51029	38.22617353	97.76024266	2.557416389	1.354687074	0.05457792	1
gene47507	59.92210985	20.32638709	0.339213475	-1.55973462	0.05462898	1
gene59418	794.4845255	1689.025975	2.125939424	1.08810049	0.05475626	1
gene62372	45.4581523	112.2790906	2.469944001	1.304478333	0.05478164	1
gene8464	315.1076466	646.572694	2.051910517	1.036967817	0.05482136	1
gene47594	104.3471223	41.62069737	0.398867707	-1.32601777	0.05485193	1
gene39463	66.12094881	152.9318648	2.312910923	1.209709705	0.05495563	1
gene20515	72.31978776	26.13392626	0.361366191	-1.46846656	0.05495924	1
gene28196	15.49709738	49.36408293	3.185376056	1.671463703	0.05506386	1
gene49689	15.49709738	49.36408293	3.185376056	1.671463703	0.05506386	1
gene46441	389.4937141	187.7770998	0.482105598	-1.05257891	0.05506937	1
gene22513	1088.929376	2377.219366	2.183079472	1.12636465	0.05518762	1
gene60310	197.3297066	408.4635881	2.069954875	1.049599317	0.05519976	1
gene10747	1223.237553	2703.409483	2.210044546	1.144075449	0.05521604	1
gene49867	142.5732959	60.97916126	0.427703946	-1.22531558	0.05522004	1
gene70994	5700.865555	2337.534515	0.410031511	-1.28619331	0.05522167	1
gene17678	2117.936641	5067.077924	2.392459635	1.258494584	0.05524342	1
gene7885	259.3180961	532.3577571	2.052914027	1.037673211	0.05525679	1
gene17624	1210.839875	2671.468017	2.206293394	1.141624654	0.05537347	1
gene18014	446.3164044	918.5591117	2.058089514	1.041305732	0.05540028	1
gene45528	630.2152933	307.7995759	0.488403851	-1.03385352	0.05544596	1
gene8579	151.8715543	65.81877724	0.433384497	-1.20628055	0.05553464	1
gene65076	18.59651685	56.13954529	3.018820446	1.59398495	0.05556106	1
gene60391	115.7116604	47.42823654	0.409882948	-1.28671612	0.05558423	1
gene65374	86.78374531	192.6167157	2.219502224	1.150236154	0.05560207	1

gene5122	3079.789819	1376.386783	0.446909323	-1.16194595	0.05560698	1
gene8145	319.2402059	653.3481564	2.046572281	1.033209621	0.05568667	1
gene64024	307.8756679	630.1179997	2.04666385	1.033274169	0.0556892	1
gene43873	20.6627965	3.871692779	0.187375062	-2.41599914	0.05573438	1
gene24790	386.3942946	791.7611732	2.049101615	1.03499153	0.05578994	1
gene1248	107.4465418	232.3015667	2.162019948	1.112379834	0.05580185	1
gene25474	29.96105493	80.33762515	2.681401751	1.422987393	0.05585513	1
gene29341	29.96105493	80.33762515	2.681401751	1.422987393	0.05585513	1
gene33600	166.3355118	346.5165037	2.083238269	1.058827856	0.05591489	1
gene45131	13.43081773	44.52446695	3.315097253	1.729051195	0.05593253	1
gene46804	13.43081773	44.52446695	3.315097253	1.729051195	0.05593253	1
gene67541	41.325593	103.5677818	2.506141456	1.325467848	0.05594632	1
gene47640	9.298258426	34.84523501	3.747501243	1.905928957	0.05597114	1
gene33851	142.5732959	300.0561903	2.104574973	1.073528905	0.05599104	1
gene57891	59.92210985	140.3488632	2.342188277	1.227857052	0.05600487	1
gene16221	42.35873283	12.58300153	0.297058025	-1.75118333	0.0560084	1
gene1364	2.06627965	15.48677111	7.495002486	2.905928957	0.05606722	1
gene1798	2.06627965	15.48677111	7.495002486	2.905928957	0.05606722	1
gene8442	2.06627965	15.48677111	7.495002486	2.905928957	0.05606722	1
gene10473	2.06627965	15.48677111	7.495002486	2.905928957	0.05606722	1
gene16350	2.06627965	15.48677111	7.495002486	2.905928957	0.05606722	1
gene28360	2.06627965	15.48677111	7.495002486	2.905928957	0.05606722	1
gene37057	2.06627965	15.48677111	7.495002486	2.905928957	0.05606722	1
gene54911	2.06627965	15.48677111	7.495002486	2.905928957	0.05606722	1
gene63833	2.06627965	15.48677111	7.495002486	2.905928957	0.05606722	1
gene6799	1633.394063	773.3706325	0.473474619	-1.078641	0.0560762	1
gene5420	157.0372534	328.125963	2.089478489	1.063142906	0.05610254	1
gene9439	207.6611048	427.822052	2.060193469	1.042779825	0.05611941	1
gene18210	113.6453808	243.916645	2.146296166	1.101849167	0.05626163	1
gene42391	11.36453808	39.68485098	3.491989794	1.804049343	0.05635184	1
gene66653	832.710699	1767.427753	2.122499153	1.085763978	0.05635816	1
gene35188	61.9883895	144.220556	2.326573688	1.218206882	0.05636395	1
gene50352	148.7721348	311.6712687	2.094957292	1.066920833	0.05647467	1
gene12436	827.5449999	1754.844752	2.120542994	1.084433734	0.05648787	1
gene58100	25.82849563	5.807539168	0.224850075	-2.15296473	0.05652069	1
gene61373	25.82849563	5.807539168	0.224850075	-2.15296473	0.05652069	1
gene70284	25.82849563	5.807539168	0.224850075	-2.15296473	0.05652069	1
gene57162	984.5822533	2118.783873	2.151962282	1.105652792	0.05653427	1
gene24732	22.72907615	64.85085404	2.853211174	1.512586528	0.0566241	1
gene61766	64.05466916	148.0922488	2.311966493	1.209120489	0.05669233	1
gene279	1853.452846	4307.258216	2.32391033	1.216554402	0.05674569	1
gene60691	44.42501248	13.55092472	0.305029171	-1.71298088	0.05687768	1
gene51394	140.5070162	295.2165744	2.10108066	1.071131548	0.05695108	1
gene25816	16.5302372	51.29992932	3.103399467	1.633849411	0.05695428	1
gene65011	154.9709738	323.286347	2.086109025	1.060814559	0.05695822	1
gene21112	33.0604744	8.711308752	0.263496181	-1.92414604	0.05702165	1
gene4687	37.1930337	94.85647307	2.55038279	1.350713799	0.05704138	1
gene11310	19.62965668	58.07539168	2.958553613	1.564892039	0.05704989	1

gene39145	19.62965668	58.07539168	2.958553613	1.564892039	0.05704989	1
gene34035	44.42501248	109.375321	2.462021165	1.299843164	0.05708757	1
gene41659	5389.890467	16882.51636	3.132255927	1.647202095	0.05711493	1
gene24107	616.7844756	1279.594463	2.07462171	1.052848297	0.0571458	1
gene68068	106.413402	229.3977971	2.155722802	1.108171678	0.05714754	1
gene61713	651.9112296	1356.060396	2.080130444	1.056674002	0.05730721	1
gene3966	145.6727153	304.8958063	2.093019311	1.065585623	0.05732625	1
gene54585	45.4581523	111.3111674	2.44865138	1.291987389	0.0574246	1
gene47718	859.5723345	1822.599376	2.120356022	1.084306523	0.05745011	1
gene9670	55.78955055	131.6375545	2.35953782	1.238504296	0.0574911	1
gene40011	55.78955055	131.6375545	2.35953782	1.238504296	0.0574911	1
gene38245	1401.970743	3121.552303	2.226545967	1.154807396	0.05762779	1
gene4265	71.28664793	161.6431735	2.26750981	1.181108792	0.05764398	1
gene32000	262.4175156	123.8941689	0.472126141	-1.08275573	0.05765273	1
gene57996	27.89477528	75.49800918	2.706528675	1.436443673	0.05775439	1
gene65773	72.31978776	163.5790199	2.261884679	1.177525376	0.05775912	1
gene19837	535.1664294	1100.528672	2.056423221	1.040137208	0.05777127	1
gene30649	658.1100686	1367.675474	2.078186521	1.055325145	0.05778831	1
gene44511	94.01572408	205.1997173	2.182610614	1.126054771	0.05782382	1
gene52483	329.5716042	158.7394039	0.481653765	-1.05393165	0.05786962	1
gene38325	5.165699125	24.19807987	4.684376554	2.227857052	0.05794045	1
gene61079	5.165699125	24.19807987	4.684376554	2.227857052	0.05794045	1
gene66824	5.165699125	24.19807987	4.684376554	2.227857052	0.05794045	1
gene54352	245.8872784	501.3842148	2.039081559	1.027919481	0.0579416	1
gene71959	350.2344007	169.3865591	0.483637697	-1.04800139	0.05796192	1
gene14721	39.25931335	98.72816585	2.514770571	1.330426785	0.05798976	1
gene29269	367.7977777	748.2046295	2.034282627	1.02452013	0.05800977	1
gene73166	310.9750873	632.0538461	2.032490292	1.023248461	0.05805339	1
gene38057	943.2566603	461.6993638	0.489473738	-1.03069664	0.05809099	1
gene7077	81.61804618	30.97354223	0.379493797	-1.39785179	0.05809594	1
gene31109	48.55757178	15.48677111	0.318936276	-1.6486599	0.05812802	1
gene26411	741.7943944	364.9070444	0.49192478	-1.02349036	0.05813112	1
gene30080	148.7721348	64.85085404	0.435907263	-1.19790685	0.05817603	1
gene38055	276.8814731	131.6375545	0.475429262	-1.07269739	0.05820494	1
gene11205	1112.691592	2409.160831	2.165164947	1.114476937	0.05821979	1
gene45867	441.1507053	215.8468724	0.489281485	-1.0312634	0.05826044	1
gene22602	126.0430587	266.1788785	2.111809102	1.078479427	0.05839	1
gene71818	126.0430587	266.1788785	2.111809102	1.078479427	0.05839	1
gene6921	747.9932334	1564.163883	2.091147102	1.064294552	0.0584701	1
gene67580	126.0430587	53.23577571	0.42236182	-1.24344867	0.05851457	1
gene13798	596.1216791	1229.262457	2.062099904	1.04411423	0.05853066	1
gene30983	28.9279151	77.43385557	2.676786602	1.420502129	0.05859477	1
gene34394	28.9279151	77.43385557	2.676786602	1.420502129	0.05859477	1
gene16748	447.3495443	911.7836493	2.03819063	1.027288992	0.0586583	1
gene4129	3.099419475	18.3905407	5.933543635	2.568893969	0.05867149	1
gene27891	3.099419475	18.3905407	5.933543635	2.568893969	0.05867149	1
gene58897	3.099419475	18.3905407	5.933543635	2.568893969	0.05867149	1
gene62200	3.099419475	18.3905407	5.933543635	2.568893969	0.05867149	1

gene49461	313.041367	634.9576157	2.028350508	1.020306978	0.05876027	1
gene52581	83.68432583	184.8733302	2.209175115	1.143507782	0.05878393	1
gene2334	24.7953558	68.72254682	2.771589461	1.470713575	0.05880723	1
gene213	8.265118601	31.94146542	3.864610657	1.950323076	0.05881978	1
gene21088	135.3413171	58.07539168	0.429103196	-1.22060345	0.05886055	1
gene64459	35.12675405	9.679231946	0.275551562	-1.85960579	0.0588644	1
gene65455	35.12675405	9.679231946	0.275551562	-1.85960579	0.0588644	1
gene10215	1123.02299	2428.519295	2.162484043	1.112689487	0.05886888	1
gene6118	17.56337703	2.903769584	0.165330937	-2.59657138	0.05887274	1
gene20720	17.56337703	2.903769584	0.165330937	-2.59657138	0.05887274	1
gene72955	73.35292758	27.10184945	0.369471954	-1.43646324	0.0588838	1
gene49711	11.36453808	0.967923195	0.085170483	-3.55350266	0.05890885	1
gene52598	167.3686517	345.5485805	2.064595592	1.045859218	0.05892199	1
gene73402	1917.507515	905.008187	0.471971129	-1.08322948	0.0589684	1
gene47645	4.1325593	21.29431028	5.152814209	2.365360575	0.05897165	1
gene52300	4.1325593	21.29431028	5.152814209	2.365360575	0.05897165	1
gene53504	4.1325593	21.29431028	5.152814209	2.365360575	0.05897165	1
gene62542	4.1325593	21.29431028	5.152814209	2.365360575	0.05897165	1
gene67201	4.1325593	21.29431028	5.152814209	2.365360575	0.05897165	1
gene72812	4.1325593	21.29431028	5.152814209	2.365360575	0.05897165	1
gene14019	86.78374531	190.6808693	2.197195669	1.135663353	0.05900549	1
gene71905	66.12094881	150.9960184	2.28363357	1.191331176	0.05910513	1
gene32144	115.7116604	245.8524914	2.124699365	1.087258721	0.05912979	1
gene26724	90.91630461	198.4242549	2.182493622	1.125977438	0.05927412	1
gene27572	67.15408863	24.19807987	0.360336658	-1.47258267	0.05928202	1
gene26651	604.3867977	1243.781305	2.057922691	1.041188786	0.05943959	1
gene70633	333.7041635	161.6431735	0.484390641	-1.04575711	0.05948194	1
gene37348	10.33139825	36.7810814	3.560126181	1.831928375	0.05950435	1
gene6737	142.5732959	61.94708446	0.434492898	-1.2025955	0.0595105	1
gene65405	189.064588	387.1692779	2.047814887	1.034085308	0.05960091	1
gene19342	71.28664793	160.6752503	2.253931907	1.172443931	0.05966388	1
gene72346	36.15989388	91.95270349	2.542947272	1.346501548	0.05968676	1
gene5766	21.69593633	61.94708446	2.855239042	1.513611534	0.05970216	1
gene49207	99.18142321	213.911026	2.156765038	1.108869015	0.05974379	1
gene18369	119.8442197	50.33200612	0.419978588	-1.25161232	0.05987979	1
gene43364	44.42501248	108.4073978	2.440233367	1.287019124	0.05988269	1
gene69725	1050.703202	2249.453504	2.140902873	1.098219346	0.05993043	1
gene45807	2942.382222	7404.612439	2.516536561	1.331439558	0.05995902	1
gene51303	1706.746991	814.9913299	0.477511508	-1.06639259	0.05999255	1
gene67261	27.89477528	6.775462362	0.242893599	-2.04160362	0.06002483	1
gene14191	75.41920723	168.4186359	2.233100056	1.159047894	0.06002967	1
gene20687	30.99419475	81.30554835	2.62325087	1.391355784	0.06011211	1
gene54160	30.99419475	81.30554835	2.62325087	1.391355784	0.06011211	1
gene28132	1547.643458	744.3329367	0.480946004	-1.05605316	0.06016161	1
gene4040	15.49709738	48.39615973	3.122917702	1.642894551	0.06025955	1
gene47460	15.49709738	48.39615973	3.122917702	1.642894551	0.06025955	1
gene51963	15.49709738	48.39615973	3.122917702	1.642894551	0.06025955	1
gene18224	18.59651685	55.17162209	2.966771817	1.568893969	0.06027942	1

gene38531	18.59651685	55.17162209	2.966771817	1.568893969	0.06027942	1
gene6	37.1930337	10.64715514	0.286267456	-1.80456443	0.06033842	1
gene277	37.1930337	10.64715514	0.286267456	-1.80456443	0.06033842	1
gene44617	138.4407366	288.441112	2.083498825	1.059008287	0.06034124	1
gene60944	1719.144669	3894.922935	2.265616737	1.179903828	0.06044903	1
gene50407	816.1804618	403.6239722	0.494527854	-1.01587631	0.06050078	1
gene16383	629.1821535	1293.145388	2.055279828	1.039334831	0.06059284	1
gene35128	1334.816654	648.5085404	0.485840912	-1.04144411	0.06061996	1
gene35307	38.22617353	95.82439627	2.50677448	1.325832211	0.06064157	1
gene52074	38.22617353	95.82439627	2.50677448	1.325832211	0.06064157	1
gene35324	267.5832147	127.7658617	0.477480853	-1.06648521	0.06067361	1
gene36432	690.1374032	1425.750866	2.065894211	1.046766379	0.06068758	1
gene18454	14.46395755	1.935846389	0.13383933	-2.90142597	0.06070886	1
gene31744	14.46395755	1.935846389	0.13383933	-2.90142597	0.06070886	1
gene38071	14.46395755	1.935846389	0.13383933	-2.90142597	0.06070886	1
gene46268	14.46395755	1.935846389	0.13383933	-2.90142597	0.06070886	1
gene55239	14.46395755	1.935846389	0.13383933	-2.90142597	0.06070886	1
gene43119	127.0761985	54.2036989	0.426544857	-1.22923063	0.06073723	1
gene73891	799.6502246	395.8805866	0.495067186	-1.01430377	0.0607641	1
gene53976	146.7058552	303.9278831	2.071682025	1.050802586	0.06079182	1
gene72223	1943.336011	4480.516468	2.305579911	1.20512967	0.06085192	1
gene8625	282.0471722	569.1388384	2.017885285	1.012844161	0.06085215	1
gene41022	22.72907615	63.88293085	2.810625932	1.490891457	0.06088362	1
gene61389	612.6519163	303.9278831	0.496085746	-1.01133859	0.06090021	1
gene4266	440.1175655	890.4893391	2.023298793	1.016709387	0.06105746	1
gene15977	39.25931335	97.76024266	2.490115957	1.316212926	0.0610705	1
gene56903	433.9187265	213.911026	0.492974866	-1.020414	0.06107947	1
gene5584	92.98258426	201.3280245	2.16522294	1.114515578	0.06109316	1
gene27650	1917.507515	4405.986382	2.297767465	1.200232804	0.06118377	1
gene1369	0	7.743385557	Inf	Inf	0.06124254	1
gene2011	0	7.743385557	Inf	Inf	0.06124254	1
gene2150	0	7.743385557	Inf	Inf	0.06124254	1
gene3512	0	7.743385557	Inf	Inf	0.06124254	1
gene3912	0	7.743385557	Inf	Inf	0.06124254	1
gene9138	0	7.743385557	Inf	Inf	0.06124254	1
gene12146	0	7.743385557	Inf	Inf	0.06124254	1
gene12619	0	7.743385557	Inf	Inf	0.06124254	1
gene14069	0	7.743385557	Inf	Inf	0.06124254	1
gene17672	0	7.743385557	Inf	Inf	0.06124254	1
gene18746	0	7.743385557	Inf	Inf	0.06124254	1
gene19013	0	7.743385557	Inf	Inf	0.06124254	1
gene23057	0	7.743385557	Inf	Inf	0.06124254	1
gene23816	0	7.743385557	Inf	Inf	0.06124254	1
gene28751	0	7.743385557	Inf	Inf	0.06124254	1
gene34129	0	7.743385557	Inf	Inf	0.06124254	1
gene34475	0	7.743385557	Inf	Inf	0.06124254	1
gene38075	0	7.743385557	Inf	Inf	0.06124254	1
gene40409	0	7.743385557	Inf	Inf	0.06124254	1

gene45693	0	7.743385557	Inf	Inf	0.06124254	1
gene45898	0	7.743385557	Inf	Inf	0.06124254	1
gene47744	0	7.743385557	Inf	Inf	0.06124254	1
gene49340	0	7.743385557	Inf	Inf	0.06124254	1
gene49665	0	7.743385557	Inf	Inf	0.06124254	1
gene50458	0	7.743385557	Inf	Inf	0.06124254	1
gene51368	0	7.743385557	Inf	Inf	0.06124254	1
gene51971	0	7.743385557	Inf	Inf	0.06124254	1
gene53528	0	7.743385557	Inf	Inf	0.06124254	1
gene53920	0	7.743385557	Inf	Inf	0.06124254	1
gene56233	0	7.743385557	Inf	Inf	0.06124254	1
gene57028	0	7.743385557	Inf	Inf	0.06124254	1
gene57454	0	7.743385557	Inf	Inf	0.06124254	1
gene59467	0	7.743385557	Inf	Inf	0.06124254	1
gene60748	0	7.743385557	Inf	Inf	0.06124254	1
gene61735	0	7.743385557	Inf	Inf	0.06124254	1
gene68878	0	7.743385557	Inf	Inf	0.06124254	1
gene69326	0	7.743385557	Inf	Inf	0.06124254	1
gene71299	0	7.743385557	Inf	Inf	0.06124254	1
gene52122	521.7356117	1060.843821	2.033297704	1.023821462	0.0612594	1
gene49214	223.1582022	452.0201319	2.025559121	1.018320194	0.06126248	1
gene14929	100.214563	214.8789492	2.144188855	1.100431981	0.06140306	1
gene63717	175.6337703	359.0995052	2.04459259	1.031813397	0.06149446	1
gene710	39.25931335	11.61507834	0.295855361	-1.75703606	0.06150785	1
gene13137	39.25931335	11.61507834	0.295855361	-1.75703606	0.06150785	1
gene16457	39.25931335	11.61507834	0.295855361	-1.75703606	0.06150785	1
gene73117	39.25931335	11.61507834	0.295855361	-1.75703606	0.06150785	1
gene18854	237.6221598	480.0899045	2.020391974	1.014635216	0.06154745	1
gene67311	138.4407366	287.4731888	2.076507219	1.054158887	0.06156949	1
gene13173	69.22036828	155.8356343	2.251297388	1.170756644	0.0615838	1
gene20840	13.43081773	43.55654376	3.243029922	1.697342335	0.06164776	1
gene30393	13.43081773	43.55654376	3.243029922	1.697342335	0.06164776	1
gene17029	471.1117602	953.4043467	2.023732853	1.017018857	0.06166008	1
gene19488	217.9925031	441.3729768	2.024716311	1.017719782	0.0616934	1
gene16853	252.0861173	120.0224761	0.476116961	-1.07061207	0.06172988	1
gene5000	7.231978776	29.03769584	4.015179903	2.00546463	0.06174987	1
gene5210	7.231978776	29.03769584	4.015179903	2.00546463	0.06174987	1
gene38290	7.231978776	29.03769584	4.015179903	2.00546463	0.06174987	1
gene62020	412.2227902	203.2638709	0.493092269	-1.02007046	0.06185817	1
gene32822	52.69013108	123.8941689	2.351373329	1.233503615	0.06188245	1
gene20011	34.09361423	87.11308752	2.555114484	1.353387934	0.0619949	1
gene31679	378.129176	185.8412534	0.491475573	-1.02480838	0.06206524	1
gene48822	290.3122908	583.6576864	2.010447731	1.007516828	0.06209991	1
gene21963	16.5302372	50.33200612	3.04484476	1.606368675	0.06213431	1
gene54866	245.8872784	495.5766757	2.015462853	1.011111193	0.0621454	1
gene28871	152.9046941	314.5750383	2.05732754	1.040771499	0.06227585	1
gene29827	227.2907615	458.7955943	2.018540442	1.013312492	0.06234251	1
gene30197	345.0687016	693.0330074	2.008391385	1.006040441	0.06237081	1

gene72877	1229.436392	2653.077477	2.157962375	1.109669711	0.06238553	1
gene47760	127.0761985	265.2109553	2.08702305	1.061446534	0.06238921	1
gene5217	174.6006304	79.36970196	0.454578553	-1.13739848	0.06241711	1
gene46452	214.8930836	100.6640122	0.468437655	-1.09407104	0.06251126	1
gene5594	1.033139825	11.61507834	11.24250373	3.490891457	0.06256484	1
gene6943	1.033139825	11.61507834	11.24250373	3.490891457	0.06256484	1
gene9112	1.033139825	11.61507834	11.24250373	3.490891457	0.06256484	1
gene15837	1.033139825	11.61507834	11.24250373	3.490891457	0.06256484	1
gene28516	1.033139825	11.61507834	11.24250373	3.490891457	0.06256484	1
gene29187	1.033139825	11.61507834	11.24250373	3.490891457	0.06256484	1
gene36472	1.033139825	11.61507834	11.24250373	3.490891457	0.06256484	1
gene39700	1.033139825	11.61507834	11.24250373	3.490891457	0.06256484	1
gene51923	1.033139825	11.61507834	11.24250373	3.490891457	0.06256484	1
gene62024	1.033139825	11.61507834	11.24250373	3.490891457	0.06256484	1
gene65787	1.033139825	11.61507834	11.24250373	3.490891457	0.06256484	1
gene71009	1.033139825	11.61507834	11.24250373	3.490891457	0.06256484	1
gene8094	255.1855368	121.9583225	0.477920199	-1.06515835	0.06256922	1
gene51817	181.8326092	83.24139474	0.457791345	-1.12723791	0.06264773	1
gene52783	288.2460112	139.38094	0.483548547	-1.04826735	0.06264813	1
gene30996	11.36453808	38.71692779	3.406819312	1.768425433	0.06264976	1
gene25644	162.2029525	331.9976558	2.046804023	1.033372974	0.06276462	1
gene3298	2542.55711	6116.306667	2.405572974	1.266380565	0.06276782	1
gene58795	29.96105493	7.743385557	0.258448362	-1.95205204	0.06278291	1
gene73860	29.96105493	7.743385557	0.258448362	-1.95205204	0.06278291	1
gene46624	44.42501248	107.4394746	2.41844557	1.274080068	0.06281212	1
gene46163	94.01572408	202.2959477	2.151724615	1.105493449	0.062868	1
gene23199	182.865749	371.6825067	2.032543047	1.023285907	0.06293649	1
gene55179	236.5890199	112.2790906	0.474574393	-1.07529384	0.06296632	1
gene2145	247.953558	498.4804452	2.010378271	1.007466983	0.06301924	1
gene47018	20.6627965	59.04331487	2.857469698	1.514738199	0.06308551	1
gene219	61.9883895	141.3167864	2.279729923	1.18886292	0.06318547	1
gene36708	105.3802622	43.55654376	0.413327343	-1.27464329	0.06334044	1
gene20321	3292.616623	8407.380869	2.553404126	1.35242189	0.06335834	1
gene43518	5913.692359	18572.51026	3.140594595	1.651037724	0.06336192	1
gene65805	112.6122409	237.1411827	2.105820653	1.074382571	0.06337536	1
gene56821	70.25350811	26.13392626	0.371994609	-1.42664638	0.06341882	1
gene69431	70.25350811	26.13392626	0.371994609	-1.42664638	0.06341882	1
gene48725	37.1930337	92.92062669	2.498334162	1.320966456	0.0634907	1
gene60909	37.1930337	92.92062669	2.498334162	1.320966456	0.0634907	1
gene40566	574.4257427	1165.379526	2.028773155	1.020607561	0.06349549	1
gene36321	558.9286454	1132.470138	2.026144387	1.018736987	0.06351666	1
gene19010	47.52443195	113.2470138	2.382921986	1.25273172	0.06359479	1
gene63504	121.9104994	254.5638002	2.088120396	1.062204897	0.0636172	1
gene935	177.7000499	361.0353516	2.031712156	1.022696022	0.06363358	1
gene64073	67.15408863	150.9960184	2.248500746	1.168963362	0.06364165	1
gene65255	69.22036828	154.8677111	2.237314175	1.161767861	0.06378672	1
gene53621	66.12094881	24.19807987	0.365966918	-1.45021485	0.06390163	1
gene51673	14.46395755	45.49239015	3.145224257	1.653162886	0.06390796	1

gene59089	14.46395755	45.49239015	3.145224257	1.653162886	0.06390796	1
gene35706	803.7827839	1661.924125	2.067628417	1.047976936	0.06392074	1
gene15704	223.1582022	105.5036282	0.472775041	-1.08077422	0.06399779	1
gene41310	135.3413171	279.7298033	2.06684706	1.047431638	0.06400299	1
gene47892	665.3420474	332.965579	0.500442713	-0.99872317	0.06409971	1
gene14150	264.4837952	529.4539875	2.001839043	1.001325979	0.06415556	1
gene11776	746.9600935	373.6183531	0.500185159	-0.99946584	0.06421465	1
gene39867	294.4448501	588.4973023	1.99866733	0.999038361	0.06425677	1
gene40998	87.81688513	34.84523501	0.396794249	-1.33353698	0.06428549	1
gene45049	213.8599438	100.6640122	0.470700639	-1.08711828	0.06430079	1
gene43920	21.69593633	60.97916126	2.810625932	1.490891457	0.06432254	1
gene59890	21.69593633	60.97916126	2.810625932	1.490891457	0.06432254	1
gene23320	81.61804618	178.0978678	2.182089331	1.125710165	0.0643581	1
gene43771	3051.895043	7604.97254	2.491885347	1.317237691	0.06439764	1
gene26464	59.92210985	21.29431028	0.355366497	-1.49262042	0.06442965	1
gene27564	59.92210985	21.29431028	0.355366497	-1.49262042	0.06442965	1
gene46973	59.92210985	21.29431028	0.355366497	-1.49262042	0.06442965	1
gene11879	223.1582022	448.1484391	2.008209578	1.005909838	0.0644959	1
gene60317	52.69013108	122.9262457	2.333003225	1.222188301	0.06457478	1
gene72683	55.78955055	19.35846389	0.346990856	-1.52703045	0.06458099	1
gene53002	6.19883895	26.13392626	4.215938898	2.075853958	0.06466146	1
gene60390	6.19883895	26.13392626	4.215938898	2.075853958	0.06466146	1
gene62668	6.19883895	26.13392626	4.215938898	2.075853958	0.06466146	1
gene69886	6.19883895	26.13392626	4.215938898	2.075853958	0.06466146	1
gene42030	687.0379837	1402.520709	2.041401993	1.029560306	0.06468768	1
gene49817	99.18142321	211.0072564	2.127487685	1.089150781	0.06472664	1
gene21880	1268.695705	2726.639639	2.149167549	1.10377796	0.06473398	1
gene45186	100.214563	212.9431028	2.124871839	1.087375828	0.06474202	1
gene20880	102.2808427	216.8147956	2.119798683	1.083927259	0.06477203	1
gene20097	26.86163545	71.6263164	2.666491269	1.414942604	0.0648198	1
gene26153	26.86163545	71.6263164	2.666491269	1.414942604	0.0648198	1
gene60288	474.2111797	951.4685003	2.006423596	1.00462622	0.06484466	1
gene39536	5577.921916	16915.42575	3.032567685	1.600539846	0.06490183	1
gene40806	230.390181	461.6993638	2.003988893	1.002874513	0.06493972	1
gene63101	32.02733458	8.711308752	0.271996058	-1.87834235	0.06494146	1
gene73967	32.02733458	8.711308752	0.271996058	-1.87834235	0.06494146	1
gene11723	195.2634269	90.9847803	0.465959149	-1.10172462	0.06498298	1
gene54689	1098.227634	2323.015667	2.115240589	1.080821766	0.06512024	1
gene14186	247.953558	119.0545529	0.480148597	-1.05844713	0.06518948	1
gene65553	83.68432583	32.90938862	0.393256303	-1.3464582	0.06519621	1
gene21628	432.8855867	215.8468724	0.498623375	-1.00397758	0.06526813	1
gene9692	378.129176	754.0121686	1.994059746	0.995708636	0.0653478	1
gene33732	18.59651685	54.2036989	2.914723189	1.543358877	0.06539761	1
gene37997	162.2029525	73.56216279	0.453519259	-1.14076428	0.06543389	1
gene37396	12.3976779	40.65277417	3.279063588	1.713283879	0.06545123	1
gene39784	12.3976779	40.65277417	3.279063588	1.713283879	0.06545123	1
gene44488	12.3976779	40.65277417	3.279063588	1.713283879	0.06545123	1
gene29710	245.8872784	118.0866297	0.480247008	-1.05815147	0.06557036	1

gene62940	533.1001497	1071.490976	2.009924358	1.007141208	0.06571758	1
gene28935	597.1548189	300.0561903	0.502476378	-0.99287232	0.06572272	1
gene9785	837.8763981	1728.710826	2.063205062	1.044887218	0.06573084	1
gene15940	1302.789319	2799.233879	2.148646629	1.103428233	0.06573975	1
gene1531	7.231978776	0	0	#NAME?	0.0658676	1
gene14079	7.231978776	0	0	#NAME?	0.0658676	1
gene22780	7.231978776	0	0	#NAME?	0.0658676	1
gene27829	7.231978776	0	0	#NAME?	0.0658676	1
gene35486	7.231978776	0	0	#NAME?	0.0658676	1
gene39148	7.231978776	0	0	#NAME?	0.0658676	1
gene61303	7.231978776	0	0	#NAME?	0.0658676	1
gene61797	7.231978776	0	0	#NAME?	0.0658676	1
gene66811	7.231978776	0	0	#NAME?	0.0658676	1
gene72993	7.231978776	0	0	#NAME?	0.0658676	1
gene69946	2636.572834	6310.859229	2.393584258	1.259172592	0.06602658	1
gene6173	24.7953558	5.807539168	0.234218828	-2.09407104	0.06603722	1
gene40948	143.6064357	63.88293085	0.44484727	-1.168618	0.06606267	1
gene16235	72.31978776	159.7073271	2.208348947	1.142968154	0.06618537	1
gene7831	553.7629462	1113.111674	2.010086954	1.007257912	0.06626559	1
gene42038	1672.653377	814.0234067	0.486665927	-1.03899632	0.06635983	1
gene52658	672.5740261	1364.771704	2.02917694	1.020894671	0.06638433	1
gene53048	106.413402	223.590258	2.101147542	1.071177471	0.06648489	1
gene34564	10.33139825	35.8131582	3.46643865	1.793454227	0.06649505	1
gene67088	10.33139825	35.8131582	3.46643865	1.793454227	0.06649505	1
gene63088	118.8110799	246.8204146	2.077419167	1.054792343	0.06652543	1
gene45354	120.8773595	250.6921074	2.073937654	1.052372525	0.06653799	1
gene3103	8.265118601	30.97354223	3.747501243	1.905928957	0.0665446	1
gene34645	8.265118601	30.97354223	3.747501243	1.905928957	0.0665446	1
gene26402	122.9436392	254.5638002	2.070573166	1.050030183	0.06655259	1
gene50892	352.3006804	699.8084697	1.986395454	0.990152865	0.06657242	1
gene62796	668.4414668	1355.092472	2.027241785	1.019518166	0.06660118	1
gene39915	34.09361423	9.679231946	0.283901609	-1.81653707	0.06661694	1
gene42868	137.4075967	281.6656496	2.049855003	1.035521864	0.06671918	1
gene58177	19.62965668	56.13954529	2.859935159	1.515982438	0.06681724	1
gene62295	19.62965668	56.13954529	2.859935159	1.515982438	0.06681724	1
gene3585	19.62965668	3.871692779	0.197236908	-2.34199856	0.06687106	1
gene35246	19.62965668	3.871692779	0.197236908	-2.34199856	0.06687106	1
gene34266	268.6163545	130.6696313	0.486454488	-1.03962326	0.06698738	1
gene28173	194.2302871	90.9847803	0.468437655	-1.09407104	0.06700015	1
gene71820	2330.763445	5422.305736	2.326407576	1.218103872	0.06709165	1
gene21200	29.96105493	77.43385557	2.584483616	1.369876056	0.06710894	1
gene19919	90.91630461	36.7810814	0.404559793	-1.30557515	0.06712814	1
gene44108	530.0007303	1060.843821	2.001589358	1.001146024	0.06718388	1
gene67882	328.5384644	651.41231	1.982758126	0.987508696	0.06719111	1
gene20864	543.431548	273.9222641	0.504060291	-0.98833179	0.06731254	1
gene13122	5.165699125	23.23015667	4.497001491	2.168963362	0.06736278	1
gene23699	5.165699125	23.23015667	4.497001491	2.168963362	0.06736278	1
gene24686	5.165699125	23.23015667	4.497001491	2.168963362	0.06736278	1

gene35082	5.165699125	23.23015667	4.497001491	2.168963362	0.06736278	1
gene52277	5.165699125	23.23015667	4.497001491	2.168963362	0.06736278	1
gene57462	5.165699125	23.23015667	4.497001491	2.168963362	0.06736278	1
gene21292	38.22617353	93.88854988	2.456132571	1.296388433	0.06736649	1
gene14860	53.7232709	123.8941689	2.306154611	1.205489239	0.0675069	1
gene41631	1134.387528	2389.802368	2.106689565	1.074977739	0.06751284	1
gene48517	295.47799	585.5935328	1.981851619	0.986848952	0.06752298	1
gene34514	30.99419475	79.36970196	2.560792516	1.356590366	0.06776367	1
gene29937	16.5302372	49.36408293	2.986290053	1.578354299	0.06778499	1
gene2304	605.4199375	305.8637295	0.505209212	-0.98504715	0.06780736	1
gene68391	1368.910268	2937.646896	2.145974768	1.101633113	0.06794099	1
gene35979	13.43081773	42.58862056	3.17096259	1.664920857	0.0679487	1
gene15936	3409.361423	8616.452279	2.527292126	1.337592433	0.06800272	1
gene21907	20.6627965	58.07539168	2.810625932	1.490891457	0.06811359	1
gene70786	69.22036828	26.13392626	0.377546767	-1.40527273	0.0681213	1
gene39379	102.2808427	214.8789492	2.100871909	1.070988203	0.06823207	1
gene21342	86.78374531	34.84523501	0.40151799	-1.31646346	0.06824467	1
gene61284	64.05466916	143.2526328	2.236412032	1.161186012	0.06827211	1
gene21214	297.5442696	588.4973023	1.977847878	0.983931469	0.06830614	1
gene56350	32.02733458	81.30554835	2.538629874	1.344050069	0.06836215	1
gene65098	234.5227403	465.5710566	1.98518513	0.989273553	0.06846552	1
gene51829	476.2774594	946.6288844	1.987557601	0.990996671	0.06847216	1
gene39207	231.4233208	111.3111674	0.480985093	-1.05593591	0.06847387	1
gene19584	483.5094381	243.916645	0.504471321	-0.98715584	0.06850324	1
gene17900	2198.521548	1054.068359	0.479444179	-1.06056524	0.06854346	1
gene34464	195.2634269	91.95270349	0.470916161	-1.08645786	0.06855034	1
gene58526	313.041367	154.8677111	0.494719636	-1.01531693	0.06857019	1
gene7295	914.3287452	460.7314406	0.503901297	-0.98878693	0.06861839	1
gene19442	168.4017915	338.7731181	2.011695452	1.008411914	0.06864697	1
gene19844	4809.265886	13492.84933	2.805594378	1.488306445	0.06868244	1
gene43777	3849.478988	10043.17107	2.608968928	1.383479762	0.06869031	1
gene34623	317.1739263	626.2463069	1.974457088	0.981456013	0.06886759	1
gene26008	38.22617353	11.61507834	0.303851452	-1.71856191	0.06886949	1
gene7620	2.06627965	14.51884792	7.02656483	2.812819552	0.06891584	1
gene10035	2.06627965	14.51884792	7.02656483	2.812819552	0.06891584	1
gene10166	2.06627965	14.51884792	7.02656483	2.812819552	0.06891584	1
gene21747	2.06627965	14.51884792	7.02656483	2.812819552	0.06891584	1
gene22191	2.06627965	14.51884792	7.02656483	2.812819552	0.06891584	1
gene23650	2.06627965	14.51884792	7.02656483	2.812819552	0.06891584	1
gene24307	2.06627965	14.51884792	7.02656483	2.812819552	0.06891584	1
gene42638	2.06627965	14.51884792	7.02656483	2.812819552	0.06891584	1
gene65776	2.06627965	14.51884792	7.02656483	2.812819552	0.06891584	1
gene32194	188.0314482	375.5541995	1.997294618	0.998047159	0.06927566	1
gene34607	21.69593633	60.01123807	2.766012822	1.467807844	0.06929844	1
gene65233	45.4581523	107.4394746	2.363480897	1.240913204	0.06930673	1
gene33345	1239.76779	2621.136011	2.114215285	1.08012229	0.0693234	1
gene1688	82.65118601	32.90938862	0.398172007	-1.3285363	0.06936059	1
gene7151	26.86163545	6.775462362	0.252235661	-1.98715584	0.06945073	1

gene21080	26.86163545	6.775462362	0.252235661	-1.98715584	0.06945073	1
gene42157	26.86163545	6.775462362	0.252235661	-1.98715584	0.06945073	1
gene21688	860.6054743	1760.652291	2.04582976	1.032686099	0.06945774	1
gene57610	985.6153931	2038.446248	2.068196441	1.048373222	0.06947312	1
gene43328	140.5070162	285.5373424	2.03219277	1.02303726	0.06947755	1
gene32700	4.1325593	20.32638709	4.918595381	2.298246379	0.06948736	1
gene43557	4.1325593	20.32638709	4.918595381	2.298246379	0.06948736	1
gene62680	4.1325593	20.32638709	4.918595381	2.298246379	0.06948736	1
gene70230	4.1325593	20.32638709	4.918595381	2.298246379	0.06948736	1
gene73142	40.29245318	12.58300153	0.31229177	-1.67903354	0.06957891	1
gene73984	114.6785206	49.36408293	0.430456224	-1.21606157	0.06963072	1
gene53878	11.36453808	37.74900459	3.321648829	1.731899557	0.06965585	1
gene17670	339.9030025	669.8028507	1.970570562	0.978613411	0.06967005	1
gene51639	117.7779401	242.9487219	2.062769324	1.044582496	0.06967228	1
gene54478	245.8872784	485.8974437	1.976098344	0.982654747	0.0698468	1
gene6254	58.88897003	21.29431028	0.361600997	-1.46752944	0.06986459	1
gene10018	80.58490636	31.94146542	0.396370324	-1.33507914	0.06991414	1
gene42249	27.89477528	72.5942396	2.602431419	1.379860145	0.06997044	1
gene62716	175.6337703	351.3561197	2.00050434	1.000363759	0.06999144	1
gene51412	50.62385143	117.1187066	2.31350842	1.21008235	0.07009158	1
gene25039	14.46395755	44.52446695	3.078304592	1.622135991	0.07017733	1
gene68931	697.3693819	1403.488632	2.012546964	1.00902245	0.07022102	1
gene19557	1844.154588	4083.667958	2.214384838	1.14690597	0.07023186	1
gene46308	167.3686517	77.43385557	0.462654474	-1.11199295	0.07030597	1
gene41874	54.75641073	19.35846389	0.353537853	-1.5000634	0.07035484	1
gene1229	3.099419475	17.4226175	5.621251864	2.490891457	0.07035815	1
gene6959	3.099419475	17.4226175	5.621251864	2.490891457	0.07035815	1
gene14296	3.099419475	17.4226175	5.621251864	2.490891457	0.07035815	1
gene17968	3.099419475	17.4226175	5.621251864	2.490891457	0.07035815	1
gene17995	3.099419475	17.4226175	5.621251864	2.490891457	0.07035815	1
gene24301	3.099419475	17.4226175	5.621251864	2.490891457	0.07035815	1
gene28410	3.099419475	17.4226175	5.621251864	2.490891457	0.07035815	1
gene38380	3.099419475	17.4226175	5.621251864	2.490891457	0.07035815	1
gene41274	3.099419475	17.4226175	5.621251864	2.490891457	0.07035815	1
gene51589	3.099419475	17.4226175	5.621251864	2.490891457	0.07035815	1
gene57626	3.099419475	17.4226175	5.621251864	2.490891457	0.07035815	1
gene69690	3.099419475	17.4226175	5.621251864	2.490891457	0.07035815	1
gene23135	22.72907615	61.94708446	2.725455449	1.446497338	0.07038236	1
gene26374	22.72907615	61.94708446	2.725455449	1.446497338	0.07038236	1
gene31118	22.72907615	61.94708446	2.725455449	1.446497338	0.07038236	1
gene541	7.231978776	28.06977264	3.881340573	1.95655503	0.07040016	1
gene6864	7.231978776	28.06977264	3.881340573	1.95655503	0.07040016	1
gene19782	7.231978776	28.06977264	3.881340573	1.95655503	0.07040016	1
gene65963	7.231978776	28.06977264	3.881340573	1.95655503	0.07040016	1
gene67617	7.231978776	28.06977264	3.881340573	1.95655503	0.07040016	1
gene73097	7.231978776	28.06977264	3.881340573	1.95655503	0.07040016	1
gene70314	86.78374531	184.8733302	2.130276004	1.091040362	0.070492	1
gene62363	57.8558302	130.6696313	2.258538695	1.175389632	0.07066753	1

gene22606	9.298258426	32.90938862	3.539306729	1.823466796	0.07070358	1
gene64561	9.298258426	32.90938862	3.539306729	1.823466796	0.07070358	1
gene63928	456.6478027	901.1364942	1.973373109	0.980663755	0.07083019	1
gene7103	63.02152933	140.3488632	2.226998689	1.155100709	0.07083522	1
gene65001	329.5716042	647.5406172	1.964794931	0.974378744	0.0708426	1
gene48664	71.28664793	155.8356343	2.186042392	1.128321378	0.07085144	1
gene57845	71.28664793	155.8356343	2.186042392	1.128321378	0.07085144	1
gene3314	18.59651685	53.23577571	2.862674561	1.517363669	0.07094861	1
gene30853	110.5459613	47.42823654	0.429036357	-1.22082819	0.07101719	1
gene5666	89.88316478	36.7810814	0.409209906	-1.28908703	0.07111641	1
gene56671	1067.233439	2211.7045	2.072371815	1.051282868	0.0712418	1
gene50432	341.9692821	171.3224055	0.500987704	-0.9971529	0.07125026	1
gene8280	365.7314981	183.905407	0.502842681	-0.99182099	0.07129076	1
gene74031	556.8623657	283.601496	0.509284724	-0.97345565	0.07132089	1
gene23085	29.96105493	76.46593238	2.552177571	1.35172871	0.07133615	1
gene34811	29.96105493	76.46593238	2.552177571	1.35172871	0.07133615	1
gene21003	1480.489369	3172.852232	2.143110446	1.099706201	0.07141156	1
gene34937	113.6453808	234.2374131	2.061125684	1.04343248	0.07143465	1
gene68384	264.4837952	519.7747555	1.965242351	0.974707235	0.07151392	1
gene31597	197.3297066	93.88854988	0.475795315	-1.07158703	0.07167545	1
gene906	2449.574525	1173.122912	0.478908847	-1.06217701	0.07168246	1
gene63943	105.3802622	218.750642	2.075821767	1.053682577	0.07174327	1
gene72463	2185.09073	4945.119601	2.263118658	1.178312229	0.07177034	1
gene43170	559.9617852	285.5373424	0.509922909	-0.97164894	0.07181127	1
gene9250	125.0099188	55.17162209	0.441337956	-1.18004427	0.07189905	1
gene61900	1350.313751	2859.245117	2.117467229	1.082339642	0.07191892	1
gene61738	30.99419475	78.40177877	2.529563339	1.338888364	0.0719413	1
gene47790	426.6867478	838.2214866	1.96448915	0.9741542	0.07201698	1
gene31572	28.9279151	7.743385557	0.26767866	-1.90142597	0.07203661	1
gene24217	16.5302372	2.903769584	0.175664121	-2.50910854	0.07208244	1
gene48493	16.5302372	2.903769584	0.175664121	-2.50910854	0.07208244	1
gene27286	2378.287877	1143.117293	0.480647151	-1.05694991	0.07208281	1
gene21921	72.31978776	28.06977264	0.388134057	-1.36537307	0.07208374	1
gene65589	1209.806735	2531.119154	2.092168179	1.064998827	0.07209528	1
gene10136	388.4605742	761.7555542	1.960959759	0.97155993	0.07210681	1
gene6931	2259.476797	5136.768394	2.273432681	1.184872285	0.07214001	1
gene37866	163.2360924	326.1901166	1.998272023	0.99875299	0.07216164	1
gene44113	15.49709738	46.46031334	2.998000994	1.584000862	0.07216804	1
gene28451	343.0024219	172.2903286	0.502300618	-0.99337704	0.07222175	1
gene68552	19.62965668	55.17162209	2.810625932	1.490891457	0.07230821	1
gene30490	12.3976779	39.68485098	3.200990645	1.678518461	0.07243968	1
gene24685	465.9460611	237.1411827	0.508945568	-0.97441673	0.07244052	1
gene50274	418.4216292	820.7988691	1.961654972	0.972071313	0.07245098	1
gene48131	130.175618	264.2430321	2.029896507	1.021406174	0.07253011	1
gene58713	270.6826342	530.4219107	1.959571261	0.970538038	0.07256687	1
gene72923	6757.767596	21394.00637	3.16583932	1.662588034	0.07265461	1
gene69191	351.2675405	687.2254682	1.95641609	0.968213235	0.07268564	1
gene40201	21.69593633	4.839615973	0.22306555	-2.16446037	0.07281889	1

gene69215	21.69593633	4.839615973	0.22306555	-2.16446037	0.07281889	1
gene31918	33.0604744	82.27347154	2.488575044	1.315319893	0.07298601	1
gene27597	774.8548688	1558.356343	2.011159	1.008027144	0.073034	1
gene62604	113.6453808	233.2694899	2.052608635	1.037458579	0.07315745	1
gene47945	50.62385143	116.1507834	2.294388516	1.198109708	0.07321809	1
gene13601	246.9204182	483.9615973	1.95999019	0.970846433	0.07322718	1
gene63170	51.65699125	118.0866297	2.285975758	1.192810104	0.07329368	1
gene39871	2393.784975	5477.477358	2.288207761	1.19421805	0.07330331	1
gene44382	52.69013108	120.0224761	2.277892912	1.187699925	0.07335795	1
gene43670	120.8773595	53.23577571	0.440411471	-1.18307605	0.07338096	1
gene10551	53.7232709	121.9583225	2.270120945	1.182769162	0.07341177	1
gene8689	34.09361423	84.20931793	2.469944001	1.304478333	0.07343422	1
gene50121	2102.439544	4702.17088	2.23653084	1.161262652	0.07344081	1
gene22553	896.7653682	1820.663529	2.03025629	1.021661858	0.07346064	1
gene35484	746.9600935	382.3296619	0.511847507	-0.96621404	0.07346432	1
gene17986	56.82269038	127.7658617	2.248500746	1.168963362	0.07351823	1
gene64569	57.8558302	129.7017081	2.241808779	1.164663225	0.0735377	1
gene61572	4594.372802	2061.676405	0.448739467	-1.15605002	0.07357304	1
gene31540	183.8988889	87.11308752	0.473701	-1.07795138	0.07360709	1
gene65372	720.0984581	1438.333867	1.997412786	0.998132511	0.07388015	1
gene25763	26.86163545	69.69047001	2.594423937	1.37541424	0.07388358	1
gene27158	26.86163545	69.69047001	2.594423937	1.37541424	0.07388358	1
gene33866	626.082734	1240.877536	1.981970542	0.98693552	0.07388903	1
gene31085	206.627965	406.5277417	1.967438153	0.976318285	0.07392653	1
gene5199	16.5302372	48.39615973	2.927735346	1.549785146	0.07394799	1
gene61080	16.5302372	48.39615973	2.927735346	1.549785146	0.07394799	1
gene46331	99.18142321	206.1676405	2.078692096	1.055676076	0.07395372	1
gene56912	1192.243358	2477.883378	2.078336911	1.055429543	0.07395376	1
gene1894	36.15989388	88.08101071	2.435875808	1.28444058	0.07420214	1
gene51951	1729.476067	856.6120273	0.495301464	-1.01362121	0.07422792	1
gene69535	64.05466916	24.19807987	0.377772303	-1.40441116	0.07423895	1
gene2054	517.6030524	265.2109553	0.512382904	-0.96470575	0.07428955	1
gene71523	126.0430587	56.13954529	0.445399738	-1.16682739	0.07435336	1
gene10605	6.19883895	25.16600306	4.059793013	2.021406174	0.07439506	1
gene44499	6.19883895	25.16600306	4.059793013	2.021406174	0.07439506	1
gene46440	6.19883895	25.16600306	4.059793013	2.021406174	0.07439506	1
gene52955	6.19883895	25.16600306	4.059793013	2.021406174	0.07439506	1
gene53314	6.19883895	25.16600306	4.059793013	2.021406174	0.07439506	1
gene59374	6.19883895	25.16600306	4.059793013	2.021406174	0.07439506	1
gene42576	121.9104994	247.7883378	2.032543047	1.023285907	0.0744587	1
gene50424	79.55176653	31.94146542	0.40151799	-1.31646346	0.07445981	1
gene29960	3109.750873	7484.950064	2.406929162	1.267193683	0.0744866	1
gene20828	109.5128215	47.42823654	0.43308387	-1.20728165	0.07457235	1
gene21146	27.89477528	71.6263164	2.567732333	1.36049482	0.07458651	1
gene1056	395.692553	201.3280245	0.508799124	-0.97483191	0.07462338	1
gene367	21.69593633	59.04331487	2.721399712	1.444348871	0.07465634	1
gene32478	21.69593633	59.04331487	2.721399712	1.444348871	0.07465634	1
gene55522	184.9320287	364.9070444	1.973195487	0.980533893	0.0747111	1

gene34519	241.7547191	472.346519	1.953825434	0.966301575	0.07479981	1
gene23804	13.43081773	41.62069737	3.098895259	1.631753993	0.07489421	1
gene26359	142.5732959	285.5373424	2.0027407	1.001975644	0.07514762	1
gene18444	8.265118601	30.00561903	3.630391829	1.860125267	0.07529773	1
gene26031	8.265118601	30.00561903	3.630391829	1.860125267	0.07529773	1
gene37444	8.265118601	30.00561903	3.630391829	1.860125267	0.07529773	1
gene40346	8.265118601	30.00561903	3.630391829	1.860125267	0.07529773	1
gene67604	8.265118601	30.00561903	3.630391829	1.860125267	0.07529773	1
gene10805	40.29245318	95.82439627	2.378221943	1.249883358	0.0753148	1
gene20911	814.1141822	418.1428201	0.513616922	-0.96123536	0.07549804	1
gene32840	586.8234206	301.9920367	0.51462165	-0.95841594	0.07551144	1
gene44810	411.1896504	800.472482	1.946723322	0.961047855	0.07552091	1
gene24898	17.56337703	50.33200612	2.865736245	1.518905834	0.07554098	1
gene66590	17.56337703	50.33200612	2.865736245	1.518905834	0.07554098	1
gene56504	411.1896504	210.0393332	0.510808901	-0.96914443	0.07564231	1
gene2739	42.35873283	99.69608905	2.353613585	1.234877479	0.07570211	1
gene57634	769.6891697	395.8805866	0.514338258	-0.95921063	0.07571595	1
gene42818	375.0297565	728.8461656	1.943435562	0.958609273	0.07571676	1
gene16219	150.8384145	69.69047001	0.462020701	-1.1139706	0.07572192	1
gene4958	114.6785206	50.33200612	0.438896542	-1.18804719	0.07574775	1
gene12812	243.8209987	475.2502886	1.949177024	0.962865122	0.07574781	1
gene69401	564.0943445	290.3769584	0.514766654	-0.95800949	0.07577608	1
gene42375	533.1001497	1044.389127	1.959086163	0.970180851	0.07579532	1
gene60911	29.96105493	75.49800918	2.519871525	1.33335018	0.07582652	1
gene61415	1027.974126	2096.52164	2.039469269	1.028193769	0.07594493	1
gene15999	70.25350811	151.9639416	2.163079762	1.113086864	0.07594595	1
gene45502	259.3180961	128.7337849	0.496431938	-1.01033216	0.07595487	1
gene7886	618.8507552	1219.583225	1.97072269	0.978724782	0.07596968	1
gene17075	136.3744569	61.94708446	0.454242575	-1.13846516	0.07597421	1
gene14803	205.5948252	402.656049	1.958493112	0.969744054	0.0759937	1
gene47695	2123.102341	1040.517434	0.490092924	-1.02887278	0.07612612	1
gene54996	455.6146629	887.5855695	1.948105805	0.962072035	0.07612924	1
gene71289	67.15408863	146.1564024	2.176433414	1.121965883	0.07613398	1
gene43456	687.0379837	354.2598892	0.51563363	-0.95558174	0.07632741	1
gene54801	35.12675405	10.64715514	0.303106718	-1.72210227	0.07641765	1
gene40461	389.4937141	755.948015	1.940847792	0.956686982	0.07647532	1
gene3174	13.43081773	1.935846389	0.144134663	-2.79451076	0.07648964	1
gene10987	13.43081773	1.935846389	0.144134663	-2.79451076	0.07648964	1
gene11376	13.43081773	1.935846389	0.144134663	-2.79451076	0.07648964	1
gene19937	13.43081773	1.935846389	0.144134663	-2.79451076	0.07648964	1
gene42920	13.43081773	1.935846389	0.144134663	-2.79451076	0.07648964	1
gene45421	13.43081773	1.935846389	0.144134663	-2.79451076	0.07648964	1
gene61057	13.43081773	1.935846389	0.144134663	-2.79451076	0.07648964	1
gene65247	13.43081773	1.935846389	0.144134663	-2.79451076	0.07648964	1
gene69605	57.8558302	128.7337849	2.225078863	1.15385647	0.07652141	1
gene17139	53.7232709	120.9903993	2.252104112	1.171273523	0.07655027	1
gene21395	1491.853907	750.1404758	0.502824353	-0.99187357	0.07656889	1
gene9730	1049.670062	2141.046107	2.039732468	1.02837994	0.07658033	1

gene15880	23.76221598	62.91500765	2.647691096	1.404734814	0.07658913	1
gene65590	23.76221598	62.91500765	2.647691096	1.404734814	0.07658913	1
gene57575	1966.065087	4309.194063	2.191786066	1.132106988	0.07659399	1
gene51650	53.7232709	19.35846389	0.360336658	-1.47258267	0.07664091	1
gene54280	53.7232709	19.35846389	0.360336658	-1.47258267	0.07664091	1
gene48686	1204.641036	612.6953822	0.508612411	-0.97536143	0.07667305	1
gene69558	141.540156	282.6335728	1.996843728	0.997721433	0.07668069	1
gene36249	32.02733458	79.36970196	2.478186306	1.309284651	0.07686192	1
gene57802	18.59651685	52.26785251	2.810625932	1.490891457	0.07696791	1
gene62791	134.3081773	269.0826481	2.003471818	1.002502216	0.07705056	1
gene68860	37.1930337	11.61507834	0.31229177	-1.67903354	0.07711423	1
gene15288	815.147322	1628.046813	1.997242424	0.998009457	0.07712548	1
gene13590	1143.685786	583.6576864	0.510330454	-0.97049636	0.07715922	1
gene25578	23.76221598	5.807539168	0.244402255	-2.0326705	0.07719419	1
gene68471	266.5500749	516.8709859	1.939114015	0.955397633	0.07722199	1
gene2484	2053.881972	4523.105089	2.202222498	1.138960237	0.07722452	1
gene50324	2739.886816	6351.512003	2.318165833	1.212983775	0.07725308	1
gene71429	293.4117103	568.1709153	1.936428899	0.95339853	0.07727233	1
gene67954	33.0604744	81.30554835	2.459297691	1.298246379	0.07729957	1
gene73980	49.5907116	17.4226175	0.351328242	-1.50910854	0.07735214	1
gene63137	24.7953558	64.85085404	2.615443576	1.387055646	0.07742471	1
gene69749	24.7953558	64.85085404	2.615443576	1.387055646	0.07742471	1
gene38808	11.36453808	36.7810814	3.236478346	1.694424851	0.07744864	1
gene4559	10.33139825	0.967923195	0.093687531	-3.41599914	0.07747792	1
gene21421	10.33139825	0.967923195	0.093687531	-3.41599914	0.07747792	1
gene30732	10.33139825	0.967923195	0.093687531	-3.41599914	0.07747792	1
gene45951	10.33139825	0.967923195	0.093687531	-3.41599914	0.07747792	1
gene67254	10.33139825	0.967923195	0.093687531	-3.41599914	0.07747792	1
gene73996	10.33139825	0.967923195	0.093687531	-3.41599914	0.07747792	1
gene55574	1106.492753	2262.036506	2.04433016	1.031628211	0.07749779	1
gene23456	244.8541385	475.2502886	1.940952648	0.956764922	0.07757508	1
gene10739	47.52443195	16.45469431	0.346236528	-1.53017016	0.07761152	1
gene59406	171.501211	81.30554835	0.474081483	-1.07679305	0.07765152	1
gene37942	585.7902808	302.9599599	0.517181609	-0.95125712	0.07768493	1
gene62090	2668.600168	6143.408516	2.302109019	1.202956156	0.07774683	1
gene70937	41.325593	13.55092472	0.327906359	-1.60864422	0.07778488	1
gene18869	69.22036828	27.10184945	0.391529981	-1.35280531	0.07787912	1
gene52724	206.627965	402.656049	1.948700646	0.962512485	0.07814119	1
gene58553	25.82849563	66.78670043	2.585775858	1.370597224	0.07818424	1
gene46357	117.7779401	238.1091059	2.021678302	1.015553448	0.07821053	1
gene12554	74.38606741	158.7394039	2.133993763	1.09355596	0.07822403	1
gene73167	231.4233208	114.214937	0.49353253	-1.01878292	0.07822825	1
gene3967	19.62965668	54.2036989	2.761316705	1.465356365	0.07824708	1
gene19569	443.216985	858.5478736	1.937082519	0.953885413	0.07829868	1
gene54656	36.15989388	87.11308752	2.409107942	1.268499036	0.07834086	1
gene26278	5.165699125	22.26223348	4.309626429	2.107562818	0.07836243	1
gene68693	5.165699125	22.26223348	4.309626429	2.107562818	0.07836243	1
gene36167	72.31978776	154.8677111	2.141429282	1.098574035	0.07842342	1

gene68729	72.31978776	154.8677111	2.141429282	1.098574035	0.07842342	1
gene8382	130.175618	59.04331487	0.453566619	-1.14061363	0.07843675	1
gene27657	313.041367	158.7394039	0.507087627	-0.97969302	0.07844633	1
gene39363	652.9443695	1282.498233	1.964176878	0.973924853	0.07846668	1
gene30163	170.4680711	334.9014253	1.964599136	0.97423497	0.07848444	1
gene28254	80.58490636	32.90938862	0.408381546	-1.29201042	0.07848727	1
gene32213	385.3611548	744.3329367	1.931520413	0.949736924	0.07852842	1
gene24581	168.4017915	331.0297326	1.965713842	0.975053317	0.07853563	1
gene33162	629.1821535	1233.13415	1.959900075	0.970780101	0.07860111	1
gene42032	70.25350811	150.9960184	2.149302183	1.103868334	0.07861981	1
gene52716	466.9792009	905.008187	1.938005344	0.954572549	0.07864064	1
gene36541	334.7373033	645.6047708	1.92869084	0.947621905	0.07880021	1
gene64696	15.49709738	45.49239015	2.93554264	1.553627213	0.07897535	1
gene52099	2949.614201	6902.260301	2.340055286	1.226542615	0.07909935	1
gene60517	611.6187764	1195.385145	1.954461163	0.966770917	0.07920949	1
gene33348	78.51862671	31.94146542	0.406801122	-1.29760444	0.07929487	1
gene36297	41.325593	96.79231946	2.342188277	1.227857052	0.07937826	1
gene46098	20.6627965	56.13954529	2.716938401	1.441981857	0.07939456	1
gene65862	9.298258426	31.94146542	3.435209473	1.780398075	0.07947999	1
gene13568	493.8408364	956.3081163	1.936470308	0.95342938	0.07967499	1
gene1163	44.42501248	102.5998586	2.30950658	1.207584657	0.07969786	1
gene33914	44.42501248	102.5998586	2.30950658	1.207584657	0.07969786	1
gene34601	140.5070162	64.85085404	0.461548866	-1.11544469	0.07972072	1
gene50146	1463.959132	742.3970903	0.507115994	-0.97961232	0.07979801	1
gene29046	319.2402059	162.6110967	0.509369101	-0.97321665	0.07981362	1
gene46030	509.3379338	264.2430321	0.518797079	-0.94675774	0.07984273	1
gene64957	51.65699125	116.1507834	2.248500746	1.168963362	0.07987212	1
gene6820	401.891392	774.3385557	1.926735858	0.946158801	0.07987595	1
gene43697	50.62385143	114.214937	2.256148707	1.173862162	0.07988446	1
gene54915	165.302372	78.40177877	0.474293126	-1.07614914	0.07991343	1
gene71734	714.932759	1406.392402	1.967167379	0.976119717	0.07992254	1
gene35972	28.9279151	72.5942396	2.509487439	1.327392725	0.08007523	1
gene1149	1.033139825	10.64715514	10.30562842	3.365360575	0.08010451	1
gene1598	1.033139825	10.64715514	10.30562842	3.365360575	0.08010451	1
gene5132	1.033139825	10.64715514	10.30562842	3.365360575	0.08010451	1
gene5797	1.033139825	10.64715514	10.30562842	3.365360575	0.08010451	1
gene13586	1.033139825	10.64715514	10.30562842	3.365360575	0.08010451	1
gene15441	1.033139825	10.64715514	10.30562842	3.365360575	0.08010451	1
gene19016	1.033139825	10.64715514	10.30562842	3.365360575	0.08010451	1
gene19303	1.033139825	10.64715514	10.30562842	3.365360575	0.08010451	1
gene21754	1.033139825	10.64715514	10.30562842	3.365360575	0.08010451	1
gene23845	1.033139825	10.64715514	10.30562842	3.365360575	0.08010451	1
gene30705	1.033139825	10.64715514	10.30562842	3.365360575	0.08010451	1
gene32299	1.033139825	10.64715514	10.30562842	3.365360575	0.08010451	1
gene37814	1.033139825	10.64715514	10.30562842	3.365360575	0.08010451	1
gene39798	1.033139825	10.64715514	10.30562842	3.365360575	0.08010451	1
gene43483	1.033139825	10.64715514	10.30562842	3.365360575	0.08010451	1
gene45851	1.033139825	10.64715514	10.30562842	3.365360575	0.08010451	1

gene46255	1.033139825	10.64715514	10.30562842	3.365360575	0.08010451	1
gene53677	1.033139825	10.64715514	10.30562842	3.365360575	0.08010451	1
gene56096	1.033139825	10.64715514	10.30562842	3.365360575	0.08010451	1
gene65753	1.033139825	10.64715514	10.30562842	3.365360575	0.08010451	1
gene66022	1.033139825	10.64715514	10.30562842	3.365360575	0.08010451	1
gene72516	1.033139825	10.64715514	10.30562842	3.365360575	0.08010451	1
gene37172	76.45234706	30.97354223	0.405135269	-1.30352441	0.08010746	1
gene28508	12.3976779	38.71692779	3.122917702	1.642894551	0.08017523	1
gene16629	223.1582022	431.6937448	1.934474021	0.951941354	0.08026077	1
gene60803	229.3570412	443.3088231	1.932832848	0.950716878	0.08026741	1
gene63279	229.3570412	443.3088231	1.932832848	0.950716878	0.08026741	1
gene11959	7.231978776	27.10184945	3.747501243	1.905928957	0.0802828	1
gene13808	7.231978776	27.10184945	3.747501243	1.905928957	0.0802828	1
gene28237	7.231978776	27.10184945	3.747501243	1.905928957	0.0802828	1
gene42499	7.231978776	27.10184945	3.747501243	1.905928957	0.0802828	1
gene51342	7.231978776	27.10184945	3.747501243	1.905928957	0.0802828	1
gene54839	7.231978776	27.10184945	3.747501243	1.905928957	0.0802828	1
gene56046	7.231978776	27.10184945	3.747501243	1.905928957	0.0802828	1
gene26384	18.59651685	3.871692779	0.208194513	-2.26399604	0.08032122	1
gene14193	133.2750374	265.2109553	1.98995221	0.992733784	0.08038167	1
gene59501	356.4332397	182.9374838	0.513244735	-0.96228117	0.08039711	1
gene56755	1377.175387	701.7443161	0.509553339	-0.97269492	0.0804195	1
gene16345	113.6453808	229.3977971	2.018540442	1.013312492	0.0804656	1
gene39805	341.9692821	175.1940982	0.51230946	-0.96491256	0.08054787	1
gene62710	628.1490136	1225.390764	1.950796288	0.964063133	0.08055612	1
gene44798	16.5302372	47.42823654	2.869180639	1.520638801	0.08066844	1
gene67590	16.5302372	47.42823654	2.869180639	1.520638801	0.08066844	1
gene10435	60.95524968	23.23015667	0.381101821	-1.39175159	0.08072516	1
gene65737	75.41920723	159.7073271	2.11759488	1.082426612	0.08072988	1
gene51816	276.8814731	532.3577571	1.922691869	0.943127575	0.08080345	1
gene10401	385.3611548	740.4612439	1.921473492	0.942213074	0.08086701	1
gene20912	339.9030025	174.226175	0.512576158	-0.96416172	0.08092137	1
gene6203	74.38606741	30.00561903	0.40337687	-1.30979973	0.0809299	1
gene1841	1229.436392	2515.632383	2.04616717	1.032924017	0.08094868	1
gene59012	73.35292758	155.8356343	2.124463733	1.087098715	0.08099194	1
gene39488	292.3785705	561.3954529	1.92009781	0.941179804	0.08112076	1
gene41986	644.6792509	1257.33223	1.950322161	0.963712453	0.08121118	1
gene51900	239.6884394	461.6993638	1.926247945	0.945793418	0.08128462	1
gene48249	241.7547191	465.5710566	1.92579925	0.945457321	0.08129141	1
gene13501	2715.09146	6200.515985	2.283722694	1.191387479	0.08129383	1
gene39345	22.72907615	60.01123807	2.640284967	1.400693648	0.08134933	1
gene14470	694.2699625	362.0032748	0.521415724	-0.93949401	0.08136685	1
gene55276	860.6054743	1707.416515	1.983971246	0.988391117	0.08141635	1
gene38139	434.9518664	836.2856402	1.922708476	0.943140036	0.08143434	1
gene14068	243.8209987	121.9583225	0.50019614	-0.99943417	0.08149333	1
gene30698	1194.309638	2433.358911	2.037460667	1.026772209	0.08153908	1
gene8978	551.6966666	1067.619284	1.935156307	0.952450101	0.08163739	1
gene26991	72.31978776	29.03769584	0.40151799	-1.31646346	0.08177071	1

gene61074	186.9983083	362.971198	1.941040008	0.956829855	0.08188208	1
gene62462	929.8258426	482.9936741	0.519445311	-0.94495623	0.08191934	1
gene48533	733.5292758	1438.333867	1.960840439	0.971472143	0.08193546	1
gene23266	4.1325593	19.35846389	4.684376554	2.227857052	0.08194957	1
gene25702	4.1325593	19.35846389	4.684376554	2.227857052	0.08194957	1
gene41674	4.1325593	19.35846389	4.684376554	2.227857052	0.08194957	1
gene63153	4.1325593	19.35846389	4.684376554	2.227857052	0.08194957	1
gene37308	348.1681211	666.8990811	1.915451303	0.937684348	0.08196974	1
gene40251	153.9378339	72.5942396	0.471581532	-1.08442087	0.0819907	1
gene51477	968.0520161	502.352138	0.518930935	-0.94638555	0.08199596	1
gene74215	4838.193801	2203.961114	0.455533863	-1.13436979	0.08202638	1
gene20426	65.08780898	140.3488632	2.156300318	1.108558123	0.0820284	1
gene23626	506.2385143	975.6665802	1.927286353	0.946570941	0.08212612	1
gene67973	526.9013108	274.8901873	0.521710957	-0.93867736	0.08213938	1
gene38595	23.76221598	61.94708446	2.606957386	1.382367001	0.08218003	1
gene51126	23.76221598	61.94708446	2.606957386	1.382367001	0.08218003	1
gene59768	34.09361423	82.27347154	2.413163679	1.270925773	0.08218532	1
gene52707	107.4465418	47.42823654	0.441412406	-1.17980092	0.08221048	1
gene55304	307.8756679	589.4652255	1.914621021	0.937058854	0.0822521	1
gene65361	2388.619276	5310.994569	2.223457971	1.152805135	0.08226939	1
gene8768	392.5931335	752.0763222	1.915663464	0.937844137	0.08235096	1
gene62936	194.2302871	94.85647307	0.488371173	-1.03395005	0.08241626	1
gene51382	176.6669101	85.17724113	0.482134663	-1.05249194	0.08244734	1
gene34124	35.12675405	84.20931793	2.397298589	1.261409611	0.08246483	1
gene45505	35.12675405	84.20931793	2.397298589	1.261409611	0.08246483	1
gene57147	35.12675405	84.20931793	2.397298589	1.261409611	0.08246483	1
gene24917	1360.64515	696.9047001	0.512186958	-0.96525758	0.08246849	1
gene5290	13.43081773	40.65277417	3.026827927	1.597806661	0.08254876	1
gene10006	13.43081773	40.65277417	3.026827927	1.597806661	0.08254876	1
gene26196	13.43081773	40.65277417	3.026827927	1.597806661	0.08254876	1
gene46544	13.43081773	40.65277417	3.026827927	1.597806661	0.08254876	1
gene67740	4039.576716	10108.98984	2.502487403	1.323362807	0.08255333	1
gene17040	4279.265155	1984.242549	0.463687684	-1.10877469	0.08259983	1
gene56916	322.3396254	616.567075	1.912787093	0.9356763	0.08260491	1
gene73426	199.3959862	97.76024266	0.490281898	-1.0283166	0.08263425	1
gene35881	1251.132328	2553.381387	2.040856375	1.029174656	0.08263703	1
gene53853	204.5616854	394.9126634	1.930530943	0.948997679	0.08263814	1
gene38983	27.89477528	7.743385557	0.277592685	-1.84895855	0.08267169	1
gene20444	181.8326092	88.08101071	0.484407121	-1.04570802	0.08270349	1
gene64634	79.55176653	166.4827895	2.092760434	1.065407171	0.08277886	1
gene2967	126.0430587	250.6921074	1.988940209	0.991999907	0.08281517	1
gene43014	204.5616854	100.6640122	0.492096123	-1.02298795	0.08284029	1
gene63283	527.9344506	275.8581105	0.522523412	-0.93643242	0.08284595	1
gene70865	56.82269038	124.8620921	2.197398456	1.135796499	0.08294906	1
gene28770	377.0960362	721.10278	1.912252347	0.935272918	0.08297497	1
gene9488	1131.288108	2285.266663	2.020057177	1.014396128	0.08298746	1
gene71282	119.8442197	54.2036989	0.452284633	-1.14469712	0.08301312	1
gene32809	308.9088077	590.4331487	1.911350968	0.934592715	0.08304256	1

gene54001	10.33139825	33.87731181	3.279063588	1.713283879	0.08305519	1
gene51799	38.22617353	90.0168571	2.354848754	1.235634402	0.0830809	1
gene25093	184.9320287	358.131582	1.936557905	0.95349464	0.08321567	1
gene30590	248.9866978	477.186135	1.916512565	0.938483457	0.08322874	1
gene54301	345.0687016	659.1556955	1.910215828	0.933735652	0.08323076	1
gene57469	40.29245318	93.88854988	2.330177055	1.22043958	0.08333764	1
gene19496	4147.023258	10424.53281	2.513738689	1.329834685	0.08335667	1
gene26347	227.2907615	436.5333608	1.920594387	0.941552866	0.08336367	1
gene56250	41.325593	95.82439627	2.318766394	1.213357482	0.08342779	1
gene56743	694.2699625	1352.188703	1.947641085	0.961727839	0.08350363	1
gene5716	618.8507552	1198.288915	1.936313247	0.953312363	0.08351838	1
gene6094	48.55757178	109.375321	2.252487449	1.171519067	0.08353052	1
gene53333	47.52443195	107.4394746	2.260720858	1.176782867	0.08356115	1
gene63416	47.52443195	107.4394746	2.260720858	1.176782867	0.08356115	1
gene8773	44.42501248	101.6319354	2.287718782	1.19390972	0.08357125	1
gene22408	213.8599438	411.3673577	1.923536266	0.94376103	0.0835803	1
gene24691	45.4581523	103.5677818	2.278310415	1.187964324	0.08358285	1
gene14366	247.953558	124.8620921	0.50357048	-0.98973438	0.08364455	1
gene50609	12324.32497	4723.46519	0.383263603	-1.3835911	0.08384682	1
gene14774	1237.70151	638.8293085	0.516141657	-0.95416102	0.08386255	1
gene2077	322.3396254	614.6312286	1.906781482	0.931139519	0.08408388	1
gene48944	130.175618	257.4675698	1.977847878	0.983931469	0.08413785	1
gene38240	5266.946828	2383.026905	0.452449395	-1.14417165	0.0841391	1
gene18106	631.2484331	1221.519072	1.935084521	0.952396582	0.0842167	1
gene67699	29.96105493	8.711308752	0.290754407	-1.78212704	0.08427218	1
gene9165	723.1978776	1409.296171	1.948700646	0.962512485	0.08427419	1
gene45632	66.12094881	26.13392626	0.395244272	-1.33918354	0.08439824	1
gene16729	77.48548688	31.94146542	0.412225137	-1.27849561	0.08443677	1
gene18428	77.48548688	31.94146542	0.412225137	-1.27849561	0.08443677	1
gene56960	77.48548688	31.94146542	0.412225137	-1.27849561	0.08443677	1
gene55981	84.71746566	175.1940982	2.067980869	1.048222839	0.08450861	1
gene36693	3.099419475	16.45469431	5.308960094	2.408429297	0.08451511	1
gene44598	3.099419475	16.45469431	5.308960094	2.408429297	0.08451511	1
gene50051	3.099419475	16.45469431	5.308960094	2.408429297	0.08451511	1
gene55911	3.099419475	16.45469431	5.308960094	2.408429297	0.08451511	1
gene66093	3.099419475	16.45469431	5.308960094	2.408429297	0.08451511	1
gene2487	0	6.775462362	Inf	Inf	0.0845319	1
gene5084	0	6.775462362	Inf	Inf	0.0845319	1
gene5198	0	6.775462362	Inf	Inf	0.0845319	1
gene9320	0	6.775462362	Inf	Inf	0.0845319	1
gene13664	0	6.775462362	Inf	Inf	0.0845319	1
gene14858	0	6.775462362	Inf	Inf	0.0845319	1
gene15362	0	6.775462362	Inf	Inf	0.0845319	1
gene15641	0	6.775462362	Inf	Inf	0.0845319	1
gene20709	0	6.775462362	Inf	Inf	0.0845319	1
gene21953	0	6.775462362	Inf	Inf	0.0845319	1
gene23481	0	6.775462362	Inf	Inf	0.0845319	1
gene24694	0	6.775462362	Inf	Inf	0.0845319	1

gene24968	0	6.775462362	Inf	Inf	0.0845319	1
gene31215	0	6.775462362	Inf	Inf	0.0845319	1
gene32997	0	6.775462362	Inf	Inf	0.0845319	1
gene34853	0	6.775462362	Inf	Inf	0.0845319	1
gene35117	0	6.775462362	Inf	Inf	0.0845319	1
gene35948	0	6.775462362	Inf	Inf	0.0845319	1
gene36062	0	6.775462362	Inf	Inf	0.0845319	1
gene37181	0	6.775462362	Inf	Inf	0.0845319	1
gene40773	0	6.775462362	Inf	Inf	0.0845319	1
gene42197	0	6.775462362	Inf	Inf	0.0845319	1
gene42808	0	6.775462362	Inf	Inf	0.0845319	1
gene43072	0	6.775462362	Inf	Inf	0.0845319	1
gene45773	0	6.775462362	Inf	Inf	0.0845319	1
gene47056	0	6.775462362	Inf	Inf	0.0845319	1
gene50280	0	6.775462362	Inf	Inf	0.0845319	1
gene51017	0	6.775462362	Inf	Inf	0.0845319	1
gene53797	0	6.775462362	Inf	Inf	0.0845319	1
gene54290	0	6.775462362	Inf	Inf	0.0845319	1
gene54654	0	6.775462362	Inf	Inf	0.0845319	1
gene57660	0	6.775462362	Inf	Inf	0.0845319	1
gene58216	0	6.775462362	Inf	Inf	0.0845319	1
gene58435	0	6.775462362	Inf	Inf	0.0845319	1
gene59174	0	6.775462362	Inf	Inf	0.0845319	1
gene61593	0	6.775462362	Inf	Inf	0.0845319	1
gene66633	0	6.775462362	Inf	Inf	0.0845319	1
gene68874	0	6.775462362	Inf	Inf	0.0845319	1
gene71950	0	6.775462362	Inf	Inf	0.0845319	1
gene2178	367.7977777	700.7763929	1.905330688	0.930041413	0.08457898	1
gene39388	14.46395755	42.58862056	2.944465262	1.558005653	0.08461724	1
gene45147	14.46395755	42.58862056	2.944465262	1.558005653	0.08461724	1
gene72038	577.5251622	1112.143751	1.92570614	0.945387566	0.08464296	1
gene38673	140.5070162	275.8581105	1.963304879	0.973284224	0.08472513	1
gene23304	27.89477528	69.69047001	2.498334162	1.320966456	0.08473974	1
gene59522	27.89477528	69.69047001	2.498334162	1.320966456	0.08473974	1
gene53244	82.65118601	171.3224055	2.072836625	1.051606412	0.08486908	1
gene2140	2.06627965	13.55092472	6.558127175	2.713283879	0.0849407	1
gene3088	2.06627965	13.55092472	6.558127175	2.713283879	0.0849407	1
gene6683	2.06627965	13.55092472	6.558127175	2.713283879	0.0849407	1
gene10476	2.06627965	13.55092472	6.558127175	2.713283879	0.0849407	1
gene13197	2.06627965	13.55092472	6.558127175	2.713283879	0.0849407	1
gene15357	2.06627965	13.55092472	6.558127175	2.713283879	0.0849407	1
gene15895	2.06627965	13.55092472	6.558127175	2.713283879	0.0849407	1
gene15975	2.06627965	13.55092472	6.558127175	2.713283879	0.0849407	1
gene19859	2.06627965	13.55092472	6.558127175	2.713283879	0.0849407	1
gene28733	2.06627965	13.55092472	6.558127175	2.713283879	0.0849407	1
gene36613	2.06627965	13.55092472	6.558127175	2.713283879	0.0849407	1
gene44784	2.06627965	13.55092472	6.558127175	2.713283879	0.0849407	1
gene44913	2.06627965	13.55092472	6.558127175	2.713283879	0.0849407	1

gene46817	2.06627965	13.55092472	6.558127175	2.713283879	0.0849407	1
gene50089	2.06627965	13.55092472	6.558127175	2.713283879	0.0849407	1
gene50521	2.06627965	13.55092472	6.558127175	2.713283879	0.0849407	1
gene54108	2.06627965	13.55092472	6.558127175	2.713283879	0.0849407	1
gene56632	2.06627965	13.55092472	6.558127175	2.713283879	0.0849407	1
gene58564	2.06627965	13.55092472	6.558127175	2.713283879	0.0849407	1
gene56026	1888.5796	4017.849181	2.127444975	1.089121818	0.08501774	1
gene6219	115.7116604	52.26785251	0.451707739	-1.14653846	0.0850294	1
gene70018	593.0222596	311.6712687	0.525564199	-0.92806109	0.08503577	1
gene68950	202.4954057	389.1051242	1.921550382	0.942270804	0.08505351	1
gene73114	136.3744569	268.1147249	1.966018644	0.975277003	0.08517893	1
gene67477	8.265118601	29.03769584	3.513282415	1.812819552	0.08521557	1
gene8798	614.7181959	323.286347	0.525909838	-0.92711261	0.08528382	1
gene50404	128.1093383	59.04331487	0.460882209	-1.11753002	0.08529332	1
gene21629	64.05466916	25.16600306	0.392883195	-1.34782764	0.08530591	1
gene62008	303.7431086	577.8501472	1.902430478	0.927843733	0.08531967	1
gene216	551.6966666	1058.907975	1.919366273	0.940630048	0.08533865	1
gene34138	106.413402	213.911026	2.010188773	1.007330989	0.08548352	1
gene957	287.2128714	147.1243256	0.512248371	-0.9650846	0.08556005	1
gene33169	6.19883895	24.19807987	3.903647128	1.964822646	0.08562551	1
gene37346	6.19883895	24.19807987	3.903647128	1.964822646	0.08562551	1
gene43942	6.19883895	24.19807987	3.903647128	1.964822646	0.08562551	1
gene58136	6.19883895	24.19807987	3.903647128	1.964822646	0.08562551	1
gene59147	6.19883895	24.19807987	3.903647128	1.964822646	0.08562551	1
gene10316	20.6627965	55.17162209	2.670094636	1.416890876	0.08571048	1
gene38714	20.6627965	55.17162209	2.670094636	1.416890876	0.08571048	1
gene61134	196.2965668	96.79231946	0.493092269	-1.02007046	0.08581228	1
gene22597	663.2757677	1281.53031	1.932122915	0.950186876	0.08600022	1
gene26415	34.09361423	10.64715514	0.31229177	-1.67903354	0.086004	1
gene40996	34.09361423	10.64715514	0.31229177	-1.67903354	0.086004	1
gene34187	30.99419475	75.49800918	2.435875808	1.28444058	0.08605059	1
gene6867	428.7530274	815.9592531	1.903098523	0.928350251	0.08605751	1
gene38748	115.7116604	230.3657203	1.990860035	0.993391798	0.08608557	1
gene44809	11.36453808	35.8131582	3.151307863	1.655950704	0.08611486	1
gene72150	11.36453808	35.8131582	3.151307863	1.655950704	0.08611486	1
gene38479	744.8938139	1448.013099	1.943918814	0.958967967	0.08613126	1
gene1124	239.6884394	120.9903993	0.504781956	-0.98626775	0.08613567	1
gene8698	178.7331897	344.5806573	1.927905264	0.94703416	0.08616569	1
gene8307	102.2808427	206.1676405	2.015701426	1.011281957	0.08617939	1
gene50881	20.6627965	4.839615973	0.234218828	-2.09407104	0.08619217	1
gene4538	40.29245318	13.55092472	0.336314214	-1.57211834	0.08633633	1
gene47631	156.0041136	302.9599599	1.941999816	0.957543064	0.08641986	1
gene8947	15.49709738	44.52446695	2.873084286	1.522600317	0.08642118	1
gene12049	15.49709738	44.52446695	2.873084286	1.522600317	0.08642118	1
gene15179	233.4896005	445.2446695	1.906914349	0.931240045	0.08643219	1
gene24795	1288.325362	2611.456779	2.02701651	1.01935784	0.08653018	1
gene67241	112.6122409	224.5581812	1.99408323	0.995725627	0.08657848	1
gene23172	1607.565568	3336.431252	2.075455782	1.053428195	0.08661062	1

gene27979	21.69593633	57.10746848	2.632173492	1.396254583	0.08663309	1
gene33230	118.8110799	54.2036989	0.456217543	-1.13220617	0.08678408	1
gene59015	2140.665718	4615.057792	2.155898398	1.108289189	0.08680275	1
gene3973	304.7762484	577.8501472	1.895981561	0.922944934	0.08695278	1
gene22253	209.7273845	104.535705	0.498436126	-1.00451946	0.08695493	1
gene57050	9542.079424	3909.441783	0.409705433	-1.28734107	0.08698998	1
gene52537	71.28664793	150.0280952	2.104574973	1.073528905	0.08699859	1
gene67358	97.11514356	196.4884085	2.023252001	1.016676022	0.08712166	1
gene29550	164.2692322	317.4788078	1.932673597	0.950598006	0.08720668	1
gene41721	89.88316478	38.71692779	0.430747269	-1.21508644	0.08723981	1
gene63498	108.4796816	216.8147956	1.99866733	0.999038361	0.08728105	1
gene26410	69.22036828	146.1564024	2.111465253	1.078244506	0.08740712	1
gene36618	47.52443195	106.4715514	2.240354004	1.163726714	0.08743553	1
gene47402	47.52443195	106.4715514	2.240354004	1.163726714	0.08743553	1
gene9036	22.72907615	59.04331487	2.597699725	1.377234676	0.08745022	1
gene33492	22.72907615	59.04331487	2.597699725	1.377234676	0.08745022	1
gene30568	95.04886391	192.6167157	2.026502031	1.018991621	0.08752189	1
gene52269	39.25931335	90.9847803	2.317533663	1.212590295	0.08760953	1
gene43264	273.7820536	140.3488632	0.512629887	-0.9640105	0.08761146	1
gene73842	144.6395755	281.6656496	1.947362253	0.961521283	0.08762936	1
gene68641	43.39187265	98.72816585	2.275268612	1.186036876	0.08766229	1
gene20312	41.325593	94.85647307	2.295344511	1.198710706	0.08767822	1
gene25082	41.325593	94.85647307	2.295344511	1.198710706	0.08767822	1
gene63486	80.58490636	33.87731181	0.420392768	-1.25019025	0.08768782	1
gene17491	939.124101	1847.765379	1.967541219	0.97639386	0.08769532	1
gene64626	94.01572408	190.6808693	2.028180618	1.020186136	0.08772711	1
gene45402	1140.586367	2279.459123	1.998497606	0.998915844	0.08773923	1
gene48170	294.4448501	557.5237601	1.893474312	0.921035849	0.08774838	1
gene25511	520.7024718	992.1212745	1.905351574	0.930057228	0.08782727	1
gene70122	217.9925031	109.375321	0.50173891	-0.99499127	0.08789496	1
gene8560	193.1971473	369.7466604	1.913830849	0.936463325	0.0879799	1
gene12510	16.5302372	46.46031334	2.810625932	1.490891457	0.08799512	1
gene26098	253.1192571	480.0899045	1.896694507	0.923487328	0.08809172	1
gene3922	511.4042134	270.0505713	0.528056993	-0.92123445	0.08809714	1
gene20058	962.886317	506.2238308	0.525735824	-0.92759005	0.08814075	1
gene19041	508.3047939	966.9552714	1.902313893	0.927755319	0.08822739	1
gene54042	78.51862671	162.6110967	2.070987529	1.050318866	0.08837943	1
gene20111	772.7885892	408.4635881	0.528557996	-0.91986631	0.0884035	1
gene14975	15.49709738	2.903769584	0.187375062	-2.41599914	0.08844237	1
gene19866	15.49709738	2.903769584	0.187375062	-2.41599914	0.08844237	1
gene59492	15.49709738	2.903769584	0.187375062	-2.41599914	0.08844237	1
gene66323	15.49709738	2.903769584	0.187375062	-2.41599914	0.08844237	1
gene64941	363.6652184	687.2254682	1.889720087	0.918172552	0.08850881	1
gene71622	125.0099188	245.8524914	1.966663875	0.975750406	0.08856818	1
gene52068	183.8988889	352.3240428	1.915857377	0.937990166	0.08857014	1
gene56981	638.4804119	1224.422841	1.917714026	0.939387598	0.08858891	1
gene68742	368.8309176	696.9047001	1.889496425	0.918001789	0.08861048	1
gene43074	275.8483333	521.7106019	1.891295103	0.919374488	0.08872392	1

gene43592	12.3976779	37.74900459	3.04484476	1.606368675	0.0887359	1
gene53604	12.3976779	37.74900459	3.04484476	1.606368675	0.0887359	1
gene49657	657.0769288	1261.203923	1.919415927	0.94066737	0.08881941	1
gene51067	3598.426011	1728.710826	0.480407495	-1.05766944	0.08889468	1
gene57443	380.1954556	199.3921781	0.524446505	-0.93113247	0.08895593	1
gene22607	198.3628464	378.4579691	1.907907534	0.931991253	0.08896811	1
gene30766	241.7547191	122.9262457	0.508475062	-0.97575108	0.0889958	1
gene22776	1893.745299	3986.875639	2.105286091	1.074016297	0.08910693	1
gene46484	133.2750374	260.3713394	1.953639214	0.966164064	0.08916902	1
gene5343	74.38606741	154.8677111	2.081945135	1.05793205	0.08929975	1
gene42592	9.298258426	30.97354223	3.331112216	1.736003955	0.08935341	1
gene43118	17.56337703	48.39615973	2.75551562	1.462322305	0.08936862	1
gene69681	17.56337703	48.39615973	2.75551562	1.462322305	0.08936862	1
gene2940	25.82849563	64.85085404	2.510825833	1.328161957	0.0893783	1
gene33186	535.1664294	1017.287278	1.900880215	0.926667623	0.08938116	1
gene14071	375.0297565	707.5518553	1.88665524	0.915830815	0.08941266	1
gene9055	85.75060548	175.1940982	2.043065437	1.030735412	0.08949468	1
gene55247	328.5384644	619.4708446	1.885535217	0.914974096	0.08950857	1
gene21610	486.6088576	257.4675698	0.529105802	-0.91837186	0.08952311	1
gene4052	725.2641572	1396.71317	1.92579925	0.945457321	0.08969459	1
gene46910	100.214563	44.52446695	0.444291384	-1.17042193	0.08971211	1
gene37594	184.9320287	353.291966	1.910388203	0.933865833	0.08987102	1
gene22610	26.86163545	66.78670043	2.48632294	1.314013695	0.08987669	1
gene12756	628.1490136	1200.224761	1.910732542	0.934125848	0.08994998	1
gene18028	53.7232709	20.32638709	0.378353491	-1.40219334	0.08998961	1
gene20869	265.516935	136.4771704	0.514005521	-0.96014424	0.09001671	1
gene16864	125.0099188	58.07539168	0.46456627	-1.10604369	0.0900458	1
gene25015	196.2965668	97.76024266	0.498023191	-1.00571517	0.090065	1
gene60354	866.8043132	458.7955943	0.529295468	-0.91785479	0.09014536	1
gene39348	755.2252121	400.7202026	0.530596961	-0.91431168	0.09016735	1
gene12886	70.25350811	147.1243256	2.094191871	1.066393629	0.09026006	1
gene31681	22.72907615	5.807539168	0.255511448	-1.96854016	0.09027751	1
gene57308	2609.711198	5758.175085	2.206441498	1.141721496	0.0902928	1
gene432	27.89477528	68.72254682	2.463635076	1.300788574	0.09031541	1
gene4048	556.8623657	1057.940052	1.899823218	0.92586518	0.09032328	1
gene57021	1643.725462	3387.731181	2.061007912	1.043350043	0.09043481	1
gene8130	1822.458651	3803.938155	2.087256219	1.061607708	0.09048211	1
gene6567	837.8763981	1625.143044	1.939597591	0.955757367	0.09049656	1
gene62690	18.59651685	50.33200612	2.706528675	1.436443673	0.09056698	1
gene9324	163.2360924	79.36970196	0.486226427	-1.04029979	0.09057842	1
gene17398	163.2360924	79.36970196	0.486226427	-1.04029979	0.09057842	1
gene70867	505.2053745	268.1147249	0.530704419	-0.91401953	0.09066717	1
gene9071	521.7356117	988.2495817	1.894157806	0.92155653	0.09069075	1
gene60774	83.68432583	35.8131582	0.427955389	-1.22446768	0.0906988	1
gene73387	233.4896005	441.3729768	1.890332485	0.918640008	0.09079115	1
gene36234	1916.474376	4023.65672	2.099509793	1.070052518	0.09079661	1
gene35686	112.6122409	222.6223348	1.976892857	0.983234683	0.09081857	1
gene51684	173.5674906	331.9976558	1.912787093	0.9356763	0.09083491	1

gene6202	134.3081773	261.3392626	1.945817953	0.960376741	0.09086535	1
gene59734	703.5682209	1349.284933	1.917774131	0.939432815	0.09086789	1
gene68580	1147.818346	2278.4912	1.98506254	0.98918446	0.09088378	1
gene61119	194.2302871	96.79231946	0.498337931	-1.00480371	0.09092043	1
gene48395	51.65699125	19.35846389	0.374750124	-1.41599914	0.09092824	1
gene74075	51.65699125	19.35846389	0.374750124	-1.41599914	0.09092824	1
gene63207	13.43081773	39.68485098	2.954760595	1.563041243	0.09098279	1
gene4823	9423.268345	32287.014	3.426307394	1.77665459	0.09102827	1
gene40468	29.96105493	72.5942396	2.42295339	1.276766652	0.09103793	1
gene179	610.5856366	1162.475757	1.903870132	0.928935072	0.09106752	1
gene64948	1160.216024	2303.657203	1.98554162	0.989532602	0.09118756	1
gene6663	122.9436392	57.10746848	0.464501204	-1.10624576	0.09118864	1
gene48017	122.9436392	57.10746848	0.464501204	-1.10624576	0.09118864	1
gene1302	5.165699125	21.29431028	4.122251367	2.04343248	0.09120861	1
gene4337	5.165699125	21.29431028	4.122251367	2.04343248	0.09120861	1
gene4341	5.165699125	21.29431028	4.122251367	2.04343248	0.09120861	1
gene43457	5.165699125	21.29431028	4.122251367	2.04343248	0.09120861	1
gene49697	5.165699125	21.29431028	4.122251367	2.04343248	0.09120861	1
gene67198	5.165699125	21.29431028	4.122251367	2.04343248	0.09120861	1
gene73550	5.165699125	21.29431028	4.122251367	2.04343248	0.09120861	1
gene25906	156.0041136	300.0561903	1.923386399	0.943648623	0.09122079	1
gene62206	761.4240511	1465.435717	1.924598671	0.944557638	0.0912964	1
gene23358	48.55757178	107.4394746	2.212620415	1.145755971	0.09133234	1
gene22907	150.8384145	72.5942396	0.481271564	-1.05507691	0.09135475	1
gene62730	11005.00542	42291.46814	3.842930243	1.942206789	0.09139528	1
gene15358	275.8483333	518.8068323	1.880768414	0.911322215	0.09154026	1
gene21410	7.231978776	26.13392626	3.613661913	1.853461537	0.09157352	1
gene25002	7.231978776	26.13392626	3.613661913	1.853461537	0.09157352	1
gene9565	19.62965668	52.26785251	2.662698252	1.412888945	0.09161196	1
gene67054	19.62965668	52.26785251	2.662698252	1.412888945	0.09161196	1
gene35729	271.715774	511.0634468	1.880875149	0.911404087	0.09163313	1
gene72326	64.05466916	135.5092472	2.115524895	1.081015663	0.09176018	1
gene598	33.0604744	78.40177877	2.37146563	1.24577896	0.09179209	1
gene21048	33.0604744	78.40177877	2.37146563	1.24577896	0.09179209	1
gene68133	49.5907116	18.3905407	0.370846477	-1.43110603	0.09185302	1
gene56304	44.42501248	99.69608905	2.244143186	1.166164729	0.09187474	1
gene15349	341.9692821	641.733078	1.87658106	0.90810661	0.0918947	1
gene60568	315.1076466	591.4010719	1.876822344	0.908292094	0.09192008	1
gene62089	476.2774594	897.2648014	1.883911959	0.913731545	0.09208886	1
gene19171	2530.159432	1267.011462	0.500763488	-0.99779872	0.09213486	1
gene45904	507.2716541	270.0505713	0.532358883	-0.90952895	0.09219666	1
gene18061	37.1930337	86.14516432	2.316163963	1.211737386	0.09220648	1
gene61073	37.1930337	86.14516432	2.316163963	1.211737386	0.09220648	1
gene33240	173.5674906	331.0297326	1.907210454	0.931464049	0.09232932	1
gene18045	225.2244819	424.9182824	1.886643401	0.915821761	0.09233871	1
gene51380	428.7530274	805.3120979	1.878265683	0.909401149	0.09248122	1
gene41758	20.6627965	54.2036989	2.62325087	1.391355784	0.09252232	1
gene66635	108.4796816	49.36408293	0.455053722	-1.13589122	0.09255061	1

gene1075	114.6785206	225.5261044	1.966594121	0.975699235	0.09255893	1
gene67366	159.1035331	77.43385557	0.486688473	-1.03892949	0.09259774	1
gene2385	262.4175156	135.5092472	0.516387967	-0.95347271	0.09265024	1
gene34339	47.52443195	17.4226175	0.366603382	-1.447708	0.09275426	1
gene39590	462.8466416	870.162952	1.88002434	0.91075134	0.09278066	1
gene26026	1510.450424	785.953634	0.52034388	-0.94246272	0.09282677	1
gene66676	10.33139825	32.90938862	3.185376056	1.671463703	0.09282871	1
gene18857	216.9593633	409.4315113	1.887134554	0.916197292	0.09283205	1
gene23828	92.98258426	186.8091766	2.009077055	1.006532898	0.09297653	1
gene44829	92.98258426	186.8091766	2.009077055	1.006532898	0.09297653	1
gene28358	197.3297066	373.6183531	1.893371047	0.920957166	0.09297843	1
gene59999	58.88897003	125.8300153	2.136733165	1.095406756	0.09303971	1
gene43813	196.2965668	371.6825067	1.893474312	0.921035849	0.09306411	1
gene27454	538.2658489	1015.351431	1.886338198	0.915588357	0.09322824	1
gene60587	91.94944443	184.8733302	2.010597577	1.007624354	0.09324599	1
gene67615	21.69593633	56.13954529	2.587560382	1.371592529	0.09331427	1
gene6284	250.0198377	469.4427494	1.877622007	0.908906656	0.09336817	1
gene2106	344.0355618	643.6689244	1.870937182	0.90376112	0.09343956	1
gene34522	511.4042134	272.9543409	0.533735026	-0.90580441	0.09344292	1
gene19193	330.604744	618.5029214	1.870822886	0.903672983	0.09347781	1
gene38398	198.3628464	99.69608905	0.502594568	-0.99253302	0.0935482	1
gene52566	206.627965	390.0730474	1.887803751	0.916708795	0.09355894	1
gene6476	80.58490636	164.5469431	2.041907728	1.029917674	0.09358018	1
gene18984	548.5972471	1034.709895	1.886101143	0.915407044	0.09361401	1
gene72261	45.4581523	16.45469431	0.361974552	-1.46603982	0.09361967	1
gene10582	523.8018913	986.3137353	1.882990023	0.913025356	0.09367263	1
gene72323	268.6163545	503.3200612	1.873750621	0.905928957	0.093693	1
gene4870	22.72907615	58.07539168	2.555114484	1.353387934	0.09400184	1
gene53363	22.72907615	58.07539168	2.555114484	1.353387934	0.09400184	1
gene61169	22.72907615	58.07539168	2.555114484	1.353387934	0.09400184	1
gene27093	171.501211	326.1901166	1.901969757	0.927494306	0.09409093	1
gene47800	1224.270693	645.6047708	0.527338255	-0.92319944	0.09414897	1
gene52913	455.6146629	242.9487219	0.533232887	-0.90716233	0.09418552	1
gene9736	67.15408863	140.3488632	2.089952616	1.063470234	0.09429721	1
gene64693	168.4017915	320.3825774	1.902489128	0.927888209	0.09447585	1
gene55096	2018.755218	4228.856437	2.094784152	1.066801595	0.09449853	1
gene24303	663.2757677	1258.300153	1.897099539	0.923795377	0.09463997	1
gene30984	86.78374531	175.1940982	2.018743229	1.013457421	0.09466496	1
gene3266	6.19883895	0	0	#NAME?	0.09467956	1
gene3990	6.19883895	0	0	#NAME?	0.09467956	1
gene19941	6.19883895	0	0	#NAME?	0.09467956	1
gene42390	6.19883895	0	0	#NAME?	0.09467956	1
gene46334	6.19883895	0	0	#NAME?	0.09467956	1
gene62187	6.19883895	0	0	#NAME?	0.09467956	1
gene64686	6.19883895	0	0	#NAME?	0.09467956	1
gene71115	6.19883895	0	0	#NAME?	0.09467956	1
gene55646	68.18722846	28.06977264	0.411657333	-1.28048417	0.09469074	1
gene35055	565.1274843	1064.715514	1.884027133	0.913819742	0.09469402	1

gene43776	223.1582022	419.1107433	1.878088007	0.909264669	0.09486229	1
gene25717	133.2750374	257.4675698	1.931851416	0.949984137	0.09488576	1
gene72341	2056.981392	1053.100436	0.511964007	-0.96588571	0.09488944	1
gene2687	85.75060548	173.2582518	2.020490128	1.014705303	0.09496346	1
gene43275	95.04886391	189.7129461	1.995951749	0.997076845	0.09501622	1
gene72366	334.7373033	624.3104605	1.86507585	0.899234304	0.0950684	1
gene6134	24.7953558	61.94708446	2.498334162	1.320966456	0.09511116	1
gene7448	1651.99058	3368.372717	2.038978162	1.027846324	0.09511808	1
gene25481	203.5285455	383.2975851	1.883262046	0.913233757	0.09512978	1
gene27744	1032.106685	2011.344398	1.948775671	0.962568028	0.09526029	1
gene70981	174.6006304	331.0297326	1.895925185	0.922902035	0.09526321	1
gene20021	94.01572408	187.7770998	1.997294618	0.998047159	0.09530593	1
gene25927	63.02152933	132.6054777	2.104129796	1.073223702	0.09550282	1
gene27989	25.82849563	63.88293085	2.47335082	1.306466886	0.09555285	1
gene42802	316.1407865	166.4827895	0.526609652	-0.92519413	0.09555849	1
gene10482	3576.730074	8299.941394	2.320538934	1.214459903	0.09556766	1
gene69751	92.98258426	185.8412534	1.99866733	0.999038361	0.09560085	1
gene38313	109.5128215	50.33200612	0.459599209	-1.12155178	0.09568377	1
gene1566	368.8309176	687.2254682	1.863253419	0.897823907	0.09570614	1
gene52123	47.52443195	104.535705	2.199620295	1.137254503	0.09571393	1
gene31741	148.7721348	284.5694192	1.912787093	0.9356763	0.09574496	1
gene14825	11.36453808	34.84523501	3.066137381	1.616422339	0.09575028	1
gene24175	11.36453808	34.84523501	3.066137381	1.616422339	0.09575028	1
gene28234	11.36453808	34.84523501	3.066137381	1.616422339	0.09575028	1
gene3667	404.9908114	215.8468724	0.532967332	-0.90788099	0.09575896	1
gene52664	61.9883895	130.6696313	2.107969449	1.075853958	0.0958099	1
gene71634	217.9925031	111.3111674	0.510619245	-0.96968018	0.09584918	1
gene59291	2315.266348	1176.994605	0.508362507	-0.97607046	0.0958814	1
gene29559	457.6809425	244.8845682	0.5350552	-0.90224036	0.09588629	1
gene18933	16.5302372	45.49239015	2.752071225	1.460517808	0.09598072	1
gene17890	283.0803121	148.0922488	0.523145703	-0.93471528	0.09599389	1
gene65209	877.1357115	469.4427494	0.535199677	-0.90185085	0.09599574	1
gene23943	28.9279151	8.711308752	0.301138493	-1.73150096	0.09601369	1
gene70148	28.9279151	8.711308752	0.301138493	-1.73150096	0.09601369	1
gene12539	89.88316478	39.68485098	0.441515951	-1.17946253	0.0960825	1
gene37333	89.88316478	39.68485098	0.441515951	-1.17946253	0.0960825	1
gene24911	207.6611048	390.0730474	1.878411693	0.909513294	0.09609101	1
gene67630	450.4489637	241.0128755	0.535050349	-0.90225344	0.09609216	1
gene23647	45.4581523	100.6640122	2.214432553	1.146937056	0.09612898	1
gene55788	82.65118601	35.8131582	0.433304831	-1.20654577	0.09614146	1
gene28462	1271.795125	2517.568229	1.979539141	0.985164594	0.09619729	1
gene59277	246.9204182	127.7658617	0.51743741	-0.95054373	0.09621225	1
gene6620	27.89477528	67.75462362	2.428935991	1.280324471	0.09625123	1
gene27002	27.89477528	67.75462362	2.428935991	1.280324471	0.09625123	1
gene2531	310.9750873	578.8180704	1.861300451	0.896310954	0.0962829	1
gene44687	469.0454806	875.9704911	1.867559815	0.901154451	0.09632703	1
gene28759	37.1930337	12.58300153	0.338316084	-1.56355633	0.09633725	1
gene47578	37.1930337	12.58300153	0.338316084	-1.56355633	0.09633725	1

gene39411	71.28664793	147.1243256	2.063841264	1.045332013	0.09635935	1
gene69563	276.8814731	515.9030627	1.863263211	0.897831489	0.09639197	1
gene11506	8.265118601	28.06977264	3.396173001	1.763909952	0.09645199	1
gene13863	8.265118601	28.06977264	3.396173001	1.763909952	0.09645199	1
gene65401	54.75641073	21.29431028	0.388891638	-1.36255988	0.09652743	1
gene45005	300.6436891	559.4596065	1.86087261	0.895979296	0.09654498	1
gene4182	251.0529775	468.4748262	1.866039714	0.89997969	0.09658407	1
gene21569	1107.525892	2164.276263	1.9541541	0.966544239	0.09659489	1
gene48832	281.0140324	147.1243256	0.523547968	-0.93360637	0.09664638	1
gene10423	215.9262234	404.5918954	1.873750621	0.905928957	0.09667063	1
gene8721	70.25350811	145.1884792	2.066636715	1.047284806	0.09668994	1
gene72439	1693.316173	3449.678266	2.037232219	1.026610439	0.09670603	1
gene17377	12.3976779	1.935846389	0.156145885	-2.67903354	0.09670745	1
gene47138	12.3976779	1.935846389	0.156145885	-2.67903354	0.09670745	1
gene985	4.1325593	18.3905407	4.450157726	2.15385647	0.09673026	1
gene7554	4.1325593	18.3905407	4.450157726	2.15385647	0.09673026	1
gene10266	4.1325593	18.3905407	4.450157726	2.15385647	0.09673026	1
gene15194	4.1325593	18.3905407	4.450157726	2.15385647	0.09673026	1
gene36504	4.1325593	18.3905407	4.450157726	2.15385647	0.09673026	1
gene46438	4.1325593	18.3905407	4.450157726	2.15385647	0.09673026	1
gene51489	4.1325593	18.3905407	4.450157726	2.15385647	0.09673026	1
gene53852	4.1325593	18.3905407	4.450157726	2.15385647	0.09673026	1
gene64837	4.1325593	18.3905407	4.450157726	2.15385647	0.09673026	1
gene2665	33.0604744	10.64715514	0.322050888	-1.63463942	0.09679248	1
gene26660	33.0604744	10.64715514	0.322050888	-1.63463942	0.09679248	1
gene64144	41.325593	92.92062669	2.248500746	1.168963362	0.09681944	1
gene36836	514.5036329	962.1156555	1.86998807	0.903029066	0.09691507	1
gene67533	135.3413171	64.85085404	0.479165235	-1.06140485	0.09693962	1
gene44682	114.6785206	223.590258	1.949713484	0.963262132	0.09701451	1
gene16881	97.11514356	192.6167157	1.983384966	0.987964726	0.09702597	1
gene22150	32.02733458	75.49800918	2.357299169	1.237134865	0.09708065	1
gene60545	762.4571909	1449.948946	1.901679154	0.927273859	0.09709191	1
gene31015	1154.017185	613.6633054	0.53176271	-0.91114548	0.09719692	1
gene78	37.1930337	85.17724113	2.290139648	1.195435574	0.09722785	1
gene8056	34.09361423	79.36970196	2.327993196	1.219086842	0.09725201	1
gene64357	299.6105493	157.7714807	0.526588537	-0.92525198	0.09725713	1
gene42826	113.6453808	221.6544116	1.950404056	0.963773031	0.09728464	1
gene49099	64.05466916	26.13392626	0.407994087	-1.29337985	0.09732017	1
gene25695	257.2518164	479.1219813	1.862462967	0.89721174	0.09732973	1
gene33376	417.3884893	776.2744021	1.859836632	0.8951759	0.0973407	1
gene67061	104.3471223	205.1997173	1.966510553	0.975637928	0.09745686	1
gene3986	87.81688513	38.71692779	0.440882499	-1.18153388	0.09747282	1
gene7194	87.81688513	38.71692779	0.440882499	-1.18153388	0.09747282	1
gene63476	253.1192571	131.6375545	0.520061397	-0.94324614	0.09758348	1
gene72702	801.7165043	1527.382801	1.905140774	0.929897604	0.09766812	1
gene23223	67.15408863	139.38094	2.07553915	1.053486145	0.09770684	1
gene1168	147.738995	281.6656496	1.906508499	0.930932963	0.09775733	1
gene48472	634.3478526	1193.449299	1.881379899	0.911791196	0.09776672	1

gene21139	52.69013108	20.32638709	0.385772187	-1.37417896	0.09777077	1
gene15693	111.5791011	217.7827188	1.951823564	0.964822646	0.09784051	1
gene36586	934.9915417	501.3842148	0.536244653	-0.89903674	0.09788101	1
gene64707	102.2808427	201.3280245	1.968384491	0.977012055	0.09805937	1
gene61066	505.2053745	271.9864177	0.538368021	-0.89333538	0.09805972	1
gene39361	430.8193071	800.472482	1.858023698	0.893768902	0.09811832	1
gene17524	148.7721348	72.5942396	0.487955891	-1.03517735	0.09816372	1
gene52195	12.3976779	36.7810814	2.966771817	1.568893969	0.09820733	1
gene21286	138.4407366	66.78670043	0.482420869	-1.05163578	0.09822051	1
gene29660	326.4721847	604.9519966	1.852997054	0.889860588	0.09854962	1
gene5942	6.19883895	23.23015667	3.747501243	1.905928957	0.09858476	1
gene26145	6.19883895	23.23015667	3.747501243	1.905928957	0.09858476	1
gene56388	6.19883895	23.23015667	3.747501243	1.905928957	0.09858476	1
gene35193	52.69013108	113.2470138	2.149302183	1.103868334	0.0986183	1
gene55264	61.9883895	25.16600306	0.405979301	-1.30052192	0.09869716	1
gene72658	172.5343508	325.2221934	1.884970685	0.914542087	0.09870641	1
gene22853	182.865749	343.6127341	1.879043702	0.909998621	0.0988254	1
gene54754	167.3686517	83.24139474	0.49735356	-1.00765629	0.09889218	1
gene6677	50.62385143	19.35846389	0.382398086	-1.38685279	0.09902746	1
gene17913	50.62385143	19.35846389	0.382398086	-1.38685279	0.09902746	1
gene17306	1386.473645	2749.869796	1.983355259	0.987943117	0.09904253	1
gene22650	2638.639113	5696.228	2.158774943	1.110212847	0.09904814	1
gene37137	2350.393102	4978.02899	2.117955922	1.082672565	0.09905015	1
gene45040	63.02152933	131.6375545	2.088771185	1.06265446	0.09911811	1
gene58621	5828.974893	2691.794404	0.461795505	-1.11467396	0.09919262	1
gene67106	334.7373033	619.4708446	1.850617898	0.888007049	0.09921384	1
gene67035	131.2087578	62.91500765	0.479503112	-1.06038792	0.09948514	1
gene41609	1126.122409	602.0482271	0.53462059	-0.90341269	0.0995435	1
gene29184	204.5616854	104.535705	0.511022897	-0.96854016	0.09959277	1
gene55062	113.6453808	220.6864884	1.941887008	0.957459257	0.09961364	1
gene15002	228.3239013	118.0866297	0.517189086	-0.95123627	0.09966059	1
gene20791	317.1739263	586.561456	1.849336933	0.887008095	0.09968889	1
gene18742	271.715774	503.3200612	1.85237704	0.88937778	0.09972234	1
gene59243	715.9658988	387.1692779	0.54076497	-0.8869264	0.09980622	1
gene1590	972.1845754	1868.091766	1.921540223	0.942263176	0.09981475	1
gene17994	821.3461609	1560.29219	1.899676731	0.925753935	0.09983379	1
gene35502	1749.105724	3550.342278	2.029804276	1.021340622	0.09993155	1
gene30998	96.08200373	189.7129461	1.974489902	0.98147999	0.10000761	1
gene41364	706.6676404	382.3296619	0.541031795	-0.88621472	0.10001387	1
gene34004	437.018146	809.1837907	1.851602269	0.888774236	0.10009453	1
gene27807	59.92210985	125.8300153	2.099892938	1.070315775	0.10021342	1
gene24840	108.4796816	50.33200612	0.463976344	-1.10787684	0.10024029	1
gene52481	48.55757178	18.3905407	0.378736828	-1.40073238	0.10029149	1
gene63493	182.865749	342.6448109	1.873750621	0.905928957	0.10037109	1
gene13646	83.68432583	36.7810814	0.439521751	-1.18599353	0.10038047	1
gene42729	46.49129213	101.6319354	2.186042392	1.128321378	0.10042134	1
gene16905	294.4448501	155.8356343	0.529252369	-0.91797227	0.10042715	1
gene26302	9.298258426	30.00561903	3.227014959	1.690200266	0.10045894	1

gene45666	9.298258426	30.00561903	3.227014959	1.690200266	0.10045894	1
gene55412	9.298258426	30.00561903	3.227014959	1.690200266	0.10045894	1
gene46646	110.5459613	214.8789492	1.943797374	0.958877837	0.10052961	1
gene43488	202.4954057	103.5677818	0.51145744	-0.9673139	0.10056336	1
gene40979	192.1640075	359.0995052	1.868713657	0.902045522	0.10060848	1
gene42009	1695.382453	3422.576416	2.018763619	1.013471993	0.10071615	1
gene4607	139.4738764	67.75462362	0.485787198	-1.04160362	0.10072275	1
gene67877	123.976779	59.04331487	0.47624495	-1.0702243	0.10079026	1
gene19178	134.3081773	64.85085404	0.482851122	-1.05034967	0.10079082	1
gene46824	129.1424781	61.94708446	0.479680159	-1.05985533	0.10081703	1
gene19073	22.72907615	57.10746848	2.512529242	1.329140387	0.10103582	1
gene51453	22.72907615	57.10746848	2.512529242	1.329140387	0.10103582	1
gene64106	92.98258426	183.905407	1.977847878	0.983931469	0.1010662	1
gene72389	418.4216292	225.5261044	0.538992463	-0.891663	0.10110499	1
gene22979	1104.426473	2137.174414	1.935098864	0.952407276	0.10112378	1
gene50289	221.0919226	410.3994345	1.856238933	0.892382425	0.10136932	1
gene13659	23.76221598	59.04331487	2.484756259	1.313104338	0.10148052	1
gene4746	305.8093882	162.6110967	0.531740041	-0.91120699	0.10156879	1
gene45348	152.9046941	75.49800918	0.49375861	-1.01812219	0.10157356	1
gene3232	3.099419475	15.48677111	4.996668324	2.320966456	0.10169381	1
gene7495	3.099419475	15.48677111	4.996668324	2.320966456	0.10169381	1
gene9886	3.099419475	15.48677111	4.996668324	2.320966456	0.10169381	1
gene16176	3.099419475	15.48677111	4.996668324	2.320966456	0.10169381	1
gene16503	3.099419475	15.48677111	4.996668324	2.320966456	0.10169381	1
gene24367	3.099419475	15.48677111	4.996668324	2.320966456	0.10169381	1
gene37655	3.099419475	15.48677111	4.996668324	2.320966456	0.10169381	1
gene51405	3.099419475	15.48677111	4.996668324	2.320966456	0.10169381	1
gene52429	3.099419475	15.48677111	4.996668324	2.320966456	0.10169381	1
gene70172	3.099419475	15.48677111	4.996668324	2.320966456	0.10169381	1
gene38136	106.413402	49.36408293	0.463889717	-1.10814623	0.10170358	1
gene11377	312.0082272	574.9463776	1.84272826	0.881843338	0.1017125	1
gene55687	142.5732959	69.69047001	0.48880451	-1.0326705	0.10190605	1
gene45689	587.8565605	1093.75321	1.860578385	0.895751172	0.10191324	1
gene64215	190.0977278	96.79231946	0.509171365	-0.97377681	0.10203568	1
gene30868	19.62965668	4.839615973	0.246546134	-2.02007046	0.10209097	1
gene45923	19.62965668	4.839615973	0.246546134	-2.02007046	0.10209097	1
gene49465	54.75641073	116.1507834	2.121227119	1.084899098	0.10209155	1
gene24851	515.5367727	279.7298033	0.542599128	-0.88204137	0.10212824	1
gene30134	25.82849563	62.91500765	2.435875808	1.28444058	0.10214566	1
gene46633	166.3355118	83.24139474	0.500442713	-0.99872317	0.10214589	1
gene10141	89.88316478	178.0978678	1.981437439	0.986547417	0.10218522	1
gene49125	94.01572408	42.58862056	0.452994656	-1.14243406	0.10225602	1
gene51591	94.01572408	42.58862056	0.452994656	-1.14243406	0.10225602	1
gene45684	128.1093383	244.8845682	1.911527852	0.934726221	0.10232774	1
gene33873	53.7232709	114.214937	2.125986282	1.088132288	0.10247214	1
gene35008	673.607166	1259.268076	1.869439845	0.902606049	0.10247694	1
gene43470	37.1930337	84.20931793	2.264115334	1.178947451	0.10251419	1
gene63422	179.7663296	90.9847803	0.506128041	-0.98242569	0.10254458	1

gene39677	9.298258426	0.967923195	0.104097257	-3.26399604	0.1025683	1
gene41809	9.298258426	0.967923195	0.104097257	-3.26399604	0.1025683	1
gene48237	9.298258426	0.967923195	0.104097257	-3.26399604	0.1025683	1
gene69740	9.298258426	0.967923195	0.104097257	-3.26399604	0.1025683	1
gene73144	9.298258426	0.967923195	0.104097257	-3.26399604	0.1025683	1
gene58543	27.89477528	66.78670043	2.394236905	1.259565911	0.10256915	1
gene36888	127.0761985	242.9487219	1.911834984	0.934958005	0.10261734	1
gene72550	127.0761985	242.9487219	1.911834984	0.934958005	0.10261734	1
gene2227	3882.539463	1904.872847	0.490625495	-1.02730589	0.10268208	1
gene30444	245.8872784	453.9559783	1.846195465	0.884555306	0.10271779	1
gene54187	312.0082272	166.4827895	0.533584614	-0.90621103	0.10273941	1
gene32009	44.42501248	16.45469431	0.370392565	-1.43287296	0.10280736	1
gene15027	174.6006304	88.08101071	0.504471321	-0.98715584	0.1028077	1
gene42814	1292.457921	691.097161	0.534715405	-0.90315685	0.1028635	1
gene13333	33.0604744	76.46593238	2.312910923	1.209709705	0.102892	1
gene58356	33.0604744	76.46593238	2.312910923	1.209709705	0.102892	1
gene24328	32.02733458	74.53008599	2.327077385	1.218519187	0.1028961	1
gene70831	618.8507552	336.8372717	0.544294838	-0.87753974	0.10307387	1
gene21591	285.1465917	524.6143715	1.839805864	0.879553541	0.10310194	1
gene322	1.033139825	9.679231946	9.368753107	3.227857052	0.10310744	1
gene4114	1.033139825	9.679231946	9.368753107	3.227857052	0.10310744	1
gene4708	1.033139825	9.679231946	9.368753107	3.227857052	0.10310744	1
gene4917	1.033139825	9.679231946	9.368753107	3.227857052	0.10310744	1
gene10496	1.033139825	9.679231946	9.368753107	3.227857052	0.10310744	1
gene13045	1.033139825	9.679231946	9.368753107	3.227857052	0.10310744	1
gene13059	1.033139825	9.679231946	9.368753107	3.227857052	0.10310744	1
gene13157	1.033139825	9.679231946	9.368753107	3.227857052	0.10310744	1
gene17283	1.033139825	9.679231946	9.368753107	3.227857052	0.10310744	1
gene19483	1.033139825	9.679231946	9.368753107	3.227857052	0.10310744	1
gene20033	1.033139825	9.679231946	9.368753107	3.227857052	0.10310744	1
gene21903	1.033139825	9.679231946	9.368753107	3.227857052	0.10310744	1
gene23805	1.033139825	9.679231946	9.368753107	3.227857052	0.10310744	1
gene29180	1.033139825	9.679231946	9.368753107	3.227857052	0.10310744	1
gene34627	1.033139825	9.679231946	9.368753107	3.227857052	0.10310744	1
gene42166	1.033139825	9.679231946	9.368753107	3.227857052	0.10310744	1
gene42934	1.033139825	9.679231946	9.368753107	3.227857052	0.10310744	1
gene43221	1.033139825	9.679231946	9.368753107	3.227857052	0.10310744	1
gene44722	1.033139825	9.679231946	9.368753107	3.227857052	0.10310744	1
gene49278	1.033139825	9.679231946	9.368753107	3.227857052	0.10310744	1
gene52940	1.033139825	9.679231946	9.368753107	3.227857052	0.10310744	1
gene53941	1.033139825	9.679231946	9.368753107	3.227857052	0.10310744	1
gene55185	1.033139825	9.679231946	9.368753107	3.227857052	0.10310744	1
gene56163	1.033139825	9.679231946	9.368753107	3.227857052	0.10310744	1
gene56359	1.033139825	9.679231946	9.368753107	3.227857052	0.10310744	1
gene58786	1.033139825	9.679231946	9.368753107	3.227857052	0.10310744	1
gene58889	1.033139825	9.679231946	9.368753107	3.227857052	0.10310744	1
gene60994	1.033139825	9.679231946	9.368753107	3.227857052	0.10310744	1
gene65746	1.033139825	9.679231946	9.368753107	3.227857052	0.10310744	1

gene70653	1.033139825	9.679231946	9.368753107	3.227857052	0.10310744	1
gene70936	1.033139825	9.679231946	9.368753107	3.227857052	0.10310744	1
gene71904	1.033139825	9.679231946	9.368753107	3.227857052	0.10310744	1
gene50270	2594.214101	5522.001825	2.128583691	1.089893815	0.10343119	1
gene39315	72.31978776	30.97354223	0.428285856	-1.22335406	0.10344328	1
gene44994	666.3751872	362.971198	0.544694948	-0.87647961	0.10355142	1
gene6416	159.1035331	79.36970196	0.498855685	-1.00330558	0.10360969	1
gene23587	60.95524968	126.7979385	2.080180775	1.056708909	0.1037106	1
gene55134	10.33139825	31.94146542	3.091688525	1.628394981	0.10375283	1
gene37431	328.5384644	176.1620214	0.536199077	-0.89915936	0.10378086	1
gene26946	349.2012609	640.7651549	1.834945135	0.875736927	0.1039059	1
gene10222	284.1134519	150.9960184	0.531463813	-0.91195663	0.10390743	1
gene44135	145.6727153	274.8901873	1.887039633	0.916124724	0.10395782	1
gene12091	100.214563	195.5204853	1.951018688	0.964227597	0.10398848	1
gene43666	69.22036828	141.3167864	2.041549185	1.029664325	0.10399596	1
gene72929	42.35873283	15.48677111	0.365609877	-1.45162305	0.10403513	1
gene62406	232.4564606	428.7899752	1.84460339	0.883310654	0.10404655	1
gene54993	757.2914918	1419.943327	1.87502876	0.906912724	0.10406813	1
gene69	204.5616854	105.5036282	0.51575459	-0.95524334	0.10409103	1
gene63488	767.62289	1440.269714	1.876272493	0.907869367	0.10411711	1
gene35670	242.7878589	447.1805159	1.841856994	0.881161051	0.10422722	1
gene6485	1853.452846	3751.670302	2.024152009	1.017317637	0.10424691	1
gene1186	336.803583	181.0016374	0.537410071	-0.89590474	0.10430354	1
gene13011	457.6809425	248.756261	0.543514571	-0.87960938	0.10432546	1
gene32647	143.6064357	70.65839321	0.492028041	-1.02318756	0.10436882	1
gene23142	783.1199874	1470.275333	1.877458571	0.908781073	0.10438216	1
gene62204	1093.061935	2099.425409	1.920682939	0.941619382	0.10438347	1
gene21867	7.231978776	25.16600306	3.479822583	1.799013753	0.10447246	1
gene30814	7.231978776	25.16600306	3.479822583	1.799013753	0.10447246	1
gene38907	7.231978776	25.16600306	3.479822583	1.799013753	0.10447246	1
gene49908	7.231978776	25.16600306	3.479822583	1.799013753	0.10447246	1
gene50059	7.231978776	25.16600306	3.479822583	1.799013753	0.10447246	1
gene66405	7.231978776	25.16600306	3.479822583	1.799013753	0.10447246	1
gene33327	206.627965	382.3296619	1.850328739	0.88778161	0.10450522	1
gene72314	180.7994694	91.95270349	0.508589454	-0.97542655	0.10452418	1
gene59913	128.1093383	243.916645	1.903972406	0.92901257	0.10453738	1
gene59829	263.4506554	139.38094	0.529058999	-0.91849948	0.10456097	1
gene57874	244.8541385	128.7337849	0.525757031	-0.92753186	0.10456919	1
gene72262	84.71746566	37.74900459	0.445587038	-1.16622083	0.10462261	1
gene16545	20334.25804	7255.552267	0.356814212	-1.48675501	0.10467226	1
gene3087	16.5302372	44.52446695	2.693516518	1.429490913	0.10468204	1
gene71247	391.5599937	211.9751796	0.541360668	-0.88533802	0.10473156	1
gene43320	407.0570911	220.6864884	0.542151195	-0.88323285	0.10478679	1
gene15351	489.7082771	901.1364942	1.840149608	0.879823065	0.10478951	1
gene45539	812.0479025	442.3408999	0.544722668	-0.87640619	0.10480371	1
gene20475	1774.934219	3568.732819	2.010628213	1.007646337	0.10491446	1
gene4985	2.06627965	12.58300153	6.08968952	2.606368675	0.10498937	1
gene13841	2.06627965	12.58300153	6.08968952	2.606368675	0.10498937	1

gene22186	2.06627965	12.58300153	6.08968952	2.606368675	0.10498937	1
gene22565	2.06627965	12.58300153	6.08968952	2.606368675	0.10498937	1
gene28391	2.06627965	12.58300153	6.08968952	2.606368675	0.10498937	1
gene32827	2.06627965	12.58300153	6.08968952	2.606368675	0.10498937	1
gene40160	2.06627965	12.58300153	6.08968952	2.606368675	0.10498937	1
gene44630	2.06627965	12.58300153	6.08968952	2.606368675	0.10498937	1
gene46535	2.06627965	12.58300153	6.08968952	2.606368675	0.10498937	1
gene49180	2.06627965	12.58300153	6.08968952	2.606368675	0.10498937	1
gene49695	2.06627965	12.58300153	6.08968952	2.606368675	0.10498937	1
gene51739	2.06627965	12.58300153	6.08968952	2.606368675	0.10498937	1
gene51925	2.06627965	12.58300153	6.08968952	2.606368675	0.10498937	1
gene58984	2.06627965	12.58300153	6.08968952	2.606368675	0.10498937	1
gene64635	2.06627965	12.58300153	6.08968952	2.606368675	0.10498937	1
gene65097	2.06627965	12.58300153	6.08968952	2.606368675	0.10498937	1
gene70805	2.06627965	12.58300153	6.08968952	2.606368675	0.10498937	1
gene52386	107.4465418	50.33200612	0.468437655	-1.09407104	0.10500573	1
gene8627	212.826804	392.976817	1.846462991	0.884764347	0.10508307	1
gene70393	639.5135517	349.4202733	0.54638447	-0.87201162	0.10523713	1
gene29280	462.8466416	849.8365649	1.83610831	0.876651164	0.10529654	1
gene15297	463.8797815	851.7724113	1.836192146	0.876717035	0.10529703	1
gene1180	417.3884893	764.6593238	1.832008652	0.873426317	0.10555324	1
gene27837	2161.328514	4450.510849	2.0591552	1.042052571	0.10558033	1
gene64738	961.8531772	1825.503145	1.89790208	0.92440556	0.10561691	1
gene49401	21.69593633	5.807539168	0.26767866	-1.90142597	0.10562239	1
gene54834	1126.122409	2162.340417	1.920164628	0.941230007	0.1056926	1
gene55669	17.56337703	46.46031334	2.645294995	1.403428616	0.10569588	1
gene8140	162.2029525	302.9599599	1.867783263	0.901327055	0.10578294	1
gene42622	162.2029525	302.9599599	1.867783263	0.901327055	0.10578294	1
gene55299	192.1640075	98.72816585	0.513770332	-0.96080451	0.10579194	1
gene35534	44.42501248	96.79231946	2.178779792	1.123520392	0.10584738	1
gene45306	80.58490636	160.6752503	1.993862841	0.995566169	0.10593861	1
gene61981	80.58490636	160.6752503	1.993862841	0.995566169	0.10593861	1
gene13577	232.4564606	121.9583225	0.524650174	-0.93057231	0.10598675	1
gene44307	622.9833145	1152.796525	1.850445265	0.887872463	0.10608664	1
gene23359	358.4995193	655.2840028	1.827851831	0.870149128	0.10612992	1
gene4394	5.165699125	20.32638709	3.934876305	1.976318285	0.10621662	1
gene23215	5.165699125	20.32638709	3.934876305	1.976318285	0.10621662	1
gene24830	5.165699125	20.32638709	3.934876305	1.976318285	0.10621662	1
gene54551	5.165699125	20.32638709	3.934876305	1.976318285	0.10621662	1
gene73448	5.165699125	20.32638709	3.934876305	1.976318285	0.10621662	1
gene28425	204.5616854	377.4900459	1.845360461	0.88390265	0.10626919	1
gene11785	64.05466916	131.6375545	2.055081327	1.039195488	0.10627282	1
gene57047	320.2733458	172.2903286	0.537947759	-0.89446202	0.10630329	1
gene71445	38.22617353	13.55092472	0.354493361	-1.49616949	0.10633938	1
gene37042	126.0430587	60.97916126	0.483796267	-1.04752846	0.10642772	1
gene73807	11.36453808	33.87731181	2.980966898	1.575780355	0.1064604	1
gene15423	18.59651685	48.39615973	2.602431419	1.379860145	0.10653524	1
gene9720	42.35873283	92.92062669	2.193659264	1.133339453	0.1065462	1

gene71701	547.5641073	1007.608046	1.840164525	0.87983476	0.10654676	1
gene70709	3876.340624	8877.791541	2.290250626	1.195505484	0.10663513	1
gene24525	115.7116604	55.17162209	0.476802613	-1.06853595	0.10667128	1
gene46216	71.28664793	144.220556	2.023107555	1.01657302	0.10668906	1
gene57829	182.865749	338.7731181	1.852578298	0.889534518	0.10679468	1
gene63350	53.7232709	113.2470138	2.107969449	1.075853958	0.1068072	1
gene60877	627.1158738	343.6127341	0.547925429	-0.86794854	0.10684027	1
gene70536	253.1192571	463.6352102	1.831686832	0.873172863	0.10684039	1
gene35646	176.6669101	90.0168571	0.509528678	-0.97276475	0.10688229	1
gene19470	85.75060548	169.3865591	1.975339511	0.982100637	0.10688631	1
gene57126	275.8483333	147.1243256	0.533352237	-0.90683946	0.10694799	1
gene53686	1670.587097	887.5855695	0.531301583	-0.91239708	0.10706346	1
gene12495	141.540156	266.1788785	1.880589127	0.911184682	0.10710043	1
gene60091	172.5343508	320.3825774	1.856920526	0.892912071	0.10713052	1
gene65235	353.3338202	644.6368476	1.824441395	0.867454809	0.10718115	1
gene2273	474.2111797	868.2271056	1.830887045	0.872542788	0.10718704	1
gene35071	217.9925031	400.7202026	1.838229283	0.878316725	0.10718736	1
gene12498	238.6552996	437.501284	1.833193249	0.874358878	0.10719859	1
gene3542	40.29245318	89.04893391	2.210064836	1.144088694	0.10720388	1
gene47550	19.62965668	50.33200612	2.564079798	1.358441161	0.10722466	1
gene48866	19.62965668	50.33200612	2.564079798	1.358441161	0.10722466	1
gene19355	52.69013108	111.3111674	2.112561975	1.078993666	0.10726349	1
gene30313	36.15989388	12.58300153	0.347982258	-1.52291434	0.1073607	1
gene51474	36.15989388	12.58300153	0.347982258	-1.52291434	0.1073607	1
gene9443	247.953558	453.9559783	1.830810503	0.872482474	0.10739481	1
gene59790	365.7314981	198.4242549	0.542540787	-0.88219649	0.10743821	1
gene43601	355.4000998	192.6167157	0.541971473	-0.88371118	0.107548	1
gene2337	235.5558801	431.6937448	1.832659599	0.873938842	0.10757527	1
gene17199	484.542578	265.2109553	0.547342932	-0.86948307	0.10760335	1
gene58232	857.5060548	1608.688349	1.876008152	0.907666097	0.10767277	1
gene38646	92.98258426	42.58862056	0.45802793	-1.12649252	0.10770308	1
gene41319	425.6536079	232.3015667	0.545752608	-0.87368097	0.10771818	1
gene4525	60.95524968	125.8300153	2.064301532	1.04565372	0.1077199	1
gene43767	51.65699125	109.375321	2.117338202	1.082251729	0.10772212	1
gene38111	5380.592209	13420.25509	2.494196656	1.318575219	0.10775861	1
gene48306	204.5616854	376.5221227	1.840628767	0.880198682	0.10776168	1
gene7102	856.472915	468.4748262	0.546981484	-0.8704361	0.10776812	1
gene32134	38.22617353	85.17724113	2.228243982	1.15590721	0.107805	1
gene61581	49.5907116	19.35846389	0.390364713	-1.35710545	0.1078352	1
gene68805	49.5907116	19.35846389	0.390364713	-1.35710545	0.1078352	1
gene67152	104.3471223	201.3280245	1.92940658	0.948157192	0.10784887	1
gene13777	147.738995	73.56216279	0.497919746	-1.00601487	0.10788434	1
gene31746	147.738995	73.56216279	0.497919746	-1.00601487	0.10788434	1
gene57753	123.976779	60.01123807	0.484052244	-1.04676533	0.10795097	1
gene33201	184.9320287	341.6768877	1.84758092	0.885637553	0.10806023	1
gene72061	1318.286417	2552.413464	1.936160027	0.953198199	0.10822378	1
gene7524	21.69593633	54.2036989	2.498334162	1.320966456	0.10823358	1
gene42927	21.69593633	54.2036989	2.498334162	1.320966456	0.10823358	1

gene12277	34.09361423	11.61507834	0.340681931	-1.55350266	0.10824258	1
gene38508	34.09361423	11.61507834	0.340681931	-1.55350266	0.10824258	1
gene72065	998.013071	1889.386076	1.893147626	0.920786915	0.10825941	1
gene31690	347.1349812	632.0538461	1.820772553	0.864550715	0.10833444	1
gene66561	546.5309675	300.0561903	0.549019558	-0.86507055	0.10837505	1
gene49194	591.9891198	325.2221934	0.54937191	-0.86414495	0.10843031	1
gene69884	66.12094881	28.06977264	0.424521625	-1.23609005	0.10847255	1
gene54717	1826.591211	3655.845906	2.001458172	1.001051466	0.10856306	1
gene72917	22.72907615	56.13954529	2.469944001	1.304478333	0.10858578	1
gene16719	687.0379837	377.4900459	0.549445671	-0.86395126	0.10861915	1
gene12440	49.5907116	105.5036282	2.127487685	1.089150781	0.10864425	1
gene71297	49.5907116	105.5036282	2.127487685	1.089150781	0.10864425	1
gene51085	158.0703932	294.2486512	1.861503885	0.896468627	0.10865191	1
gene22831	67.15408863	136.4771704	2.032298751	1.023112496	0.10866389	1
gene28368	67.15408863	136.4771704	2.032298751	1.023112496	0.10866389	1
gene1909	12.3976779	35.8131582	2.888698875	1.530419822	0.10868338	1
gene3277	12.3976779	35.8131582	2.888698875	1.530419822	0.10868338	1
gene53377	12.3976779	35.8131582	2.888698875	1.530419822	0.10868338	1
gene10983	115.7116604	220.6864884	1.907210454	0.931464049	0.10869174	1
gene12967	14.46395755	2.903769584	0.200758995	-2.31646346	0.10874699	1
gene29706	14.46395755	2.903769584	0.200758995	-2.31646346	0.10874699	1
gene62214	14.46395755	2.903769584	0.200758995	-2.31646346	0.10874699	1
gene32390	74.38606741	149.060172	2.003872192	1.002790496	0.10880743	1
gene73391	191.1308676	98.72816585	0.516547469	-0.95302716	0.10882643	1
gene64417	85.75060548	38.71692779	0.451506174	-1.14718238	0.10886454	1
gene37879	33.0604744	75.49800918	2.28363357	1.191331176	0.10892057	1
gene338	25.82849563	7.743385557	0.299800099	-1.73792723	0.10893553	1
gene66288	25.82849563	7.743385557	0.299800099	-1.73792723	0.10893553	1
gene14410	499.0065355	273.9222641	0.548935223	-0.86529218	0.10897745	1
gene62589	32.02733458	73.56216279	2.2968556	1.19966016	0.10905133	1
gene66851	48.55757178	103.5677818	2.132886346	1.092807091	0.10910653	1
gene33302	8.265118601	27.10184945	3.279063588	1.713283879	0.10918029	1
gene60014	8.265118601	27.10184945	3.279063588	1.713283879	0.10918029	1
gene61989	8.265118601	27.10184945	3.279063588	1.713283879	0.10918029	1
gene67660	8.265118601	27.10184945	3.279063588	1.713283879	0.10918029	1
gene20309	25.82849563	61.94708446	2.398400795	1.262072767	0.10918343	1
gene61671	25.82849563	61.94708446	2.398400795	1.262072767	0.10918343	1
gene51887	57.8558302	120.0224761	2.074509617	1.052770345	0.10922576	1
gene56580	545.4978276	998.8967369	1.83116538	0.872762093	0.10922674	1
gene73364	221.0919226	116.1507834	0.525350642	-0.92864743	0.10926601	1
gene23056	178.7331897	330.0618094	1.8466733	0.884928658	0.10930694	1
gene57900	29.96105493	9.679231946	0.323060452	-1.63012394	0.10934825	1
gene49709	263.4506554	480.0899045	1.82231433	0.86577183	0.10935777	1
gene7822	269.6494943	144.220556	0.534844526	-0.90280852	0.10939343	1
gene12209	27.89477528	8.711308752	0.31229177	-1.67903354	0.10939662	1
gene34313	27.89477528	8.711308752	0.31229177	-1.67903354	0.10939662	1
gene43046	27.89477528	8.711308752	0.31229177	-1.67903354	0.10939662	1
gene72531	229.3570412	120.9903993	0.527519882	-0.92270263	0.10952497	1

gene17084	56.82269038	23.23015667	0.408818317	-1.29046826	0.10964026	1
gene45471	133.2750374	250.6921074	1.881013221	0.911509989	0.10964895	1
gene58845	338.8698626	615.5991518	1.816624078	0.861259907	0.10967436	1
gene2041	184.9320287	340.7089645	1.84234698	0.881544798	0.10972971	1
gene35520	96.08200373	44.52446695	0.463400691	-1.1096679	0.10978938	1
gene5236	387.4274344	703.6801625	1.816288936	0.860993725	0.11004159	1
gene60409	2528.093152	1311.535929	0.518784653	-0.94679229	0.11017982	1
gene55665	336.803583	182.9374838	0.543157772	-0.88055677	0.11019849	1
gene50771	216.9593633	396.8485098	1.829137511	0.871163538	0.11020119	1
gene42050	71.28664793	30.97354223	0.434492898	-1.2025955	0.11024629	1
gene40653	55.78955055	116.1507834	2.081945135	1.05793205	0.11026065	1
gene43131	1133.354388	2157.500801	1.903641812	0.928762048	0.11034536	1
gene66029	955.6543382	523.6464483	0.547945452	-0.86789581	0.11036456	1
gene15400	45.4581523	97.76024266	2.150554691	1.104708821	0.11048901	1
gene36348	13.43081773	37.74900459	2.810625932	1.490891457	0.11050383	1
gene68952	13.43081773	37.74900459	2.810625932	1.490891457	0.11050383	1
gene18804	900.8979275	494.6087525	0.549017527	-0.86507589	0.11061395	1
gene58276	150.8384145	280.6977264	1.860916713	0.896013488	0.11062815	1
gene40279	170.4680711	87.11308752	0.511022897	-0.96854016	0.11066521	1
gene61164	130.175618	244.8845682	1.88118614	0.911642608	0.11070766	1
gene37659	4977.667677	12021.60608	2.415108211	1.272087832	0.11073595	1
gene72063	44.42501248	95.82439627	2.156991994	1.109020822	0.11094525	1
gene21378	239.6884394	436.5333608	1.821253298	0.864931585	0.11098945	1
gene28966	620.9170349	342.6448109	0.551836705	-0.85768667	0.11099799	1
gene27182	268.6163545	487.8332901	1.816096756	0.860841067	0.11117508	1
gene23346	208.6942447	109.375321	0.524093614	-0.93210356	0.11117659	1
gene33777	138.4407366	68.72254682	0.496404083	-1.01041311	0.11121208	1
gene28117	135.3413171	253.595877	1.873750621	0.905928957	0.11121497	1
gene8965	89.88316478	175.1940982	1.949131394	0.962831348	0.11129237	1
gene17032	836.8432583	460.7314406	0.550558825	-0.86103138	0.11132531	1
gene48455	114.6785206	55.17162209	0.481098133	-1.0555969	0.11141441	1
gene47521	94.01572408	43.55654376	0.463289989	-1.11001259	0.11157758	1
gene40352	108.4796816	207.1355637	1.909441109	0.933150425	0.111594	1
gene15923	82.65118601	162.6110967	1.967438153	0.976318285	0.11161945	1
gene9927	168.4017915	310.7033455	1.845012115	0.88363029	0.11170686	1
gene6282	233.4896005	123.8941689	0.530619645	-0.91425001	0.11175503	1
gene18663	1743.940025	932.1100364	0.534485145	-0.90377824	0.11196255	1
gene2113	14.46395755	39.68485098	2.743706267	1.456126039	0.11198859	1
gene3743	14.46395755	39.68485098	2.743706267	1.456126039	0.11198859	1
gene10664	14.46395755	39.68485098	2.743706267	1.456126039	0.11198859	1
gene19467	14.46395755	39.68485098	2.743706267	1.456126039	0.11198859	1
gene36294	14.46395755	39.68485098	2.743706267	1.456126039	0.11198859	1
gene47289	14.46395755	39.68485098	2.743706267	1.456126039	0.11198859	1
gene51662	14.46395755	39.68485098	2.743706267	1.456126039	0.11198859	1
gene57085	14.46395755	39.68485098	2.743706267	1.456126039	0.11198859	1
gene12986	1196.375917	652.3802332	0.545297029	-0.8748858	0.11210756	1
gene73432	801.7165043	442.3408999	0.55174229	-0.85793353	0.11214648	1
gene63544	159.1035331	294.2486512	1.849416198	0.887069929	0.11227825	1

gene37157	41.325593	90.0168571	2.178235097	1.123159673	0.11228345	1
gene41053	248.9866978	452.0201319	1.81543888	0.86031836	0.11234143	1
gene63768	74.38606741	148.0922488	1.990860035	0.993391798	0.11243436	1
gene65693	100.214563	192.6167157	1.922043163	0.942640735	0.11246303	1
gene8796	172.5343508	317.4788078	1.840090431	0.879776669	0.11251899	1
gene1733	193.1971473	100.6640122	0.521042954	-0.94052579	0.11264774	1
gene10845	301.6768289	163.5790199	0.542232628	-0.88301617	0.11269966	1
gene15957	452.5152434	819.8309459	1.811720064	0.857360056	0.11271934	1
gene29293	352.3006804	192.6167157	0.546739551	-0.87107435	0.11275829	1
gene58980	421.5210486	762.7234774	1.809455257	0.855555434	0.11279544	1
gene1569	3351.505593	1703.544823	0.5082924	-0.97626943	0.11290594	1
gene10202	9.298258426	29.03769584	3.122917702	1.642894551	0.11294744	1
gene31457	9.298258426	29.03769584	3.122917702	1.642894551	0.11294744	1
gene20440	209.7273845	110.3432442	0.526127022	-0.92651695	0.11295292	1
gene63105	149.8052746	277.7939569	1.854366994	0.890926793	0.11301412	1
gene1608	177.7000499	326.1901166	1.835621975	0.876268983	0.11306831	1
gene40245	392.5931335	709.4877017	1.807183165	0.853742736	0.1130796	1
gene33266	217.9925031	115.1828602	0.528379915	-0.92035247	0.1130915	1
gene9208	86.78374531	39.68485098	0.457284378	-1.12883646	0.11310313	1
gene29689	951.5217789	1779.042832	1.869681673	0.902792662	0.11312831	1
gene51750	960.8200373	1797.433372	1.870728443	0.903600151	0.11318172	1
gene35722	15.49709738	41.62069737	2.685709224	1.425303116	0.11319254	1
gene36909	15.49709738	41.62069737	2.685709224	1.425303116	0.11319254	1
gene71722	15.49709738	41.62069737	2.685709224	1.425303116	0.11319254	1
gene8668	2187.15701	4434.056155	2.027314973	1.01957025	0.11324804	1
gene31387	2835.96882	5983.701189	2.109931938	1.077196462	0.11332132	1
gene51645	116.7448002	220.6864884	1.890332485	0.918640008	0.11341764	1
gene73712	122.9436392	231.3336435	1.881623523	0.911978001	0.11342407	1
gene621	279.9808926	506.2238308	1.808065637	0.854447052	0.11343554	1
gene46235	368.8309176	202.2959477	0.548478823	-0.86649218	0.11347334	1
gene56690	242.7878589	129.7017081	0.534218262	-0.9044988	0.11350854	1
gene13682	102.2808427	48.39615973	0.473169349	-1.07957147	0.11352427	1
gene16716	6.19883895	22.26223348	3.591355358	1.844528412	0.11354007	1
gene24436	6.19883895	22.26223348	3.591355358	1.844528412	0.11354007	1
gene41351	6.19883895	22.26223348	3.591355358	1.844528412	0.11354007	1
gene67147	6.19883895	22.26223348	3.591355358	1.844528412	0.11354007	1
gene67938	6.19883895	22.26223348	3.591355358	1.844528412	0.11354007	1
gene48837	38.22617353	84.20931793	2.202923028	1.139419087	0.113542	1
gene8517	72.31978776	144.220556	1.994206019	0.99581446	0.11357299	1
gene58770	147.738995	273.9222641	1.854095895	0.890715863	0.11367619	1
gene25659	309.9419475	559.4596065	1.805046432	0.852035949	0.11374236	1
gene24554	139.4738764	69.69047001	0.499666832	-1.00096164	0.11382139	1
gene34205	773.821729	1426.718789	1.843730585	0.882627857	0.1138488	1
gene42400	191.1308676	99.69608905	0.521611659	-0.93895198	0.11388211	1
gene31642	698.4025218	387.1692779	0.554364089	-0.85109429	0.11399602	1
gene43682	531.0338701	294.2486512	0.554105242	-0.85176808	0.11400761	1
gene71435	67.15408863	29.03769584	0.43240399	-1.20954826	0.11405155	1
gene16820	71.28664793	142.2847096	1.995951749	0.997076845	0.11415862	1

gene58808	16.5302372	43.55654376	2.634961811	1.397782053	0.11416027	1
gene4379	56.82269038	117.1187066	2.061125684	1.04343248	0.11419071	1
gene20308	1285.225942	2453.685298	1.909147036	0.932928218	0.11422748	1
gene10170	4.1325593	17.4226175	4.215938898	2.075853958	0.11428242	1
gene17820	4.1325593	17.4226175	4.215938898	2.075853958	0.11428242	1
gene25973	4.1325593	17.4226175	4.215938898	2.075853958	0.11428242	1
gene31048	4.1325593	17.4226175	4.215938898	2.075853958	0.11428242	1
gene37079	4.1325593	17.4226175	4.215938898	2.075853958	0.11428242	1
gene46858	4.1325593	17.4226175	4.215938898	2.075853958	0.11428242	1
gene58308	4.1325593	17.4226175	4.215938898	2.075853958	0.11428242	1
gene72816	4.1325593	17.4226175	4.215938898	2.075853958	0.11428242	1
gene46983	36.15989388	80.33762515	2.22173288	1.151685371	0.1143086	1
gene61978	165.302372	303.9278831	1.838617797	0.878621611	0.11444607	1
gene66314	1183.97824	648.5085404	0.547736875	-0.86844509	0.11451956	1
gene58032	4871.254275	11562.81048	2.373682389	1.247126908	0.11452038	1
gene60576	504.1722346	912.7515725	1.810396348	0.85630558	0.11454445	1
gene12909	41.325593	15.48677111	0.374750124	-1.41599914	0.11459815	1
gene63810	456.6478027	252.6279538	0.553222751	-0.85406761	0.11459818	1
gene67059	1861.717965	3686.819448	1.980331886	0.985742233	0.11465032	1
gene18673	107.4465418	204.2317941	1.900775871	0.926588427	0.1148378	1
gene62083	340.9361423	186.8091766	0.547930106	-0.86793622	0.11490588	1
gene62956	101.2477029	193.5846389	1.91199043	0.935075302	0.11491923	1
gene1630	17.56337703	45.49239015	2.590184683	1.373054967	0.11492854	1
gene35771	34.09361423	76.46593238	2.242822714	1.165315585	0.11499063	1
gene60075	149.8052746	276.8260337	1.847905785	0.885891203	0.11513434	1
gene2403	449.4158239	248.756261	0.553510241	-0.85331808	0.11513511	1
gene25370	118.8110799	223.590258	1.881897363	0.912187947	0.11514665	1
gene40758	991.8142321	1852.604995	1.867895151	0.901413476	0.11519592	1
gene267	418.4216292	754.0121686	1.802039178	0.849630377	0.11519916	1
gene67407	33.0604744	74.53008599	2.254356216	1.172715497	0.11529087	1
gene25850	69.22036828	138.4130168	1.999599544	0.999711103	0.11536352	1
gene33413	54.75641073	113.2470138	2.068196441	1.048373222	0.11539712	1
gene44134	148.7721348	274.8901873	1.847726307	0.885751075	0.11548156	1
gene50972	302.7099687	544.9407586	1.800207508	0.848163214	0.11551041	1
gene61240	18.59651685	47.42823654	2.55038279	1.350713799	0.11552787	1
gene57046	775.8880086	430.7258216	0.555139165	-0.84907862	0.11556129	1
gene11525	117.7779401	221.6544116	1.881968826	0.912242731	0.11559834	1
gene13628	733.5292758	1344.445317	1.832844798	0.874084626	0.11562217	1
gene29033	167.3686517	306.8316527	1.833268355	0.874417984	0.11580056	1
gene38145	30.99419475	70.65839321	2.279729923	1.18886292	0.115821	1
gene42267	2967.177578	6273.110224	2.11416744	1.080089642	0.11585623	1
gene37189	1403.003882	2690.826481	1.917903803	0.93953036	0.11590831	1
gene20706	10.33139825	30.97354223	2.998000994	1.584000862	0.1159595	1
gene56097	10.33139825	30.97354223	2.998000994	1.584000862	0.1159595	1
gene62572	10.33139825	30.97354223	2.998000994	1.584000862	0.1159595	1
gene72441	10.33139825	30.97354223	2.998000994	1.584000862	0.1159595	1
gene53754	19.62965668	49.36408293	2.514770571	1.330426785	0.11598371	1
gene15683	836.8432583	1543.837495	1.844834717	0.883491568	0.11602158	1

gene39095	541.3652683	979.538273	1.809385142	0.85549953	0.11604201	1
gene38837	29.96105493	68.72254682	2.293729209	1.197695081	0.11605568	1
gene48800	29.96105493	68.72254682	2.293729209	1.197695081	0.11605568	1
gene45907	65.08780898	28.06977264	0.431260064	-1.21336997	0.11607923	1
gene10700	166.3355118	304.8958063	1.833016912	0.874220097	0.11610162	1
gene55766	1791.464457	3521.304582	1.965601142	0.974970601	0.11612502	1
gene8996	2735.754257	5698.163847	2.082849303	1.058558462	0.11625221	1
gene28977	44.42501248	94.85647307	2.135204197	1.094374046	0.11627909	1
gene63041	44.42501248	94.85647307	2.135204197	1.094374046	0.11627909	1
gene4362	39.25931335	14.51884792	0.369819202	-1.43510796	0.11630968	1
gene65860	39.25931335	14.51884792	0.369819202	-1.43510796	0.11630968	1
gene56138	929.8258426	1725.807056	1.856054088	0.892238753	0.11631394	1
gene24338	16.5302372	3.871692779	0.234218828	-2.09407104	0.11631779	1
gene72185	16.5302372	3.871692779	0.234218828	-2.09407104	0.11631779	1
gene65549	257.2518164	463.6352102	1.802262144	0.84980887	0.11635711	1
gene68181	363.6652184	653.3481564	1.796564871	0.84524103	0.1163855	1
gene36698	284.1134519	511.0634468	1.798800597	0.847035268	0.11645975	1
gene44622	171.501211	313.6071151	1.828600004	0.870739528	0.11654789	1
gene36801	547.5641073	990.1854281	1.808346119	0.854670837	0.11658936	1
gene6992	26.86163545	62.91500765	2.342188277	1.227857052	0.11659006	1
gene18940	91.94944443	177.1299446	1.926384066	0.945895364	0.11663926	1
gene68868	255.1855368	459.7635175	1.80168329	0.849345428	0.11666503	1
gene11869	22.72907615	55.17162209	2.42735876	1.279387352	0.11668732	1
gene12980	25.82849563	60.97916126	2.360925783	1.23935269	0.11669431	1
gene22365	438.0512858	787.8894804	1.798623828	0.846893486	0.11674268	1
gene1099	24.7953558	59.04331487	2.381224748	1.251703793	0.11675086	1
gene14734	459.7472222	827.5743314	1.800063799	0.848048041	0.1167606	1
gene69672	85.75060548	166.4827895	1.941476548	0.95715428	0.11676104	1
gene54385	339.9030025	186.8091766	0.549595547	-0.86355778	0.1167837	1
gene6656	547.5641073	304.8958063	0.556822119	-0.84471157	0.11679676	1
gene6851	43.39187265	92.92062669	2.141429282	1.098574035	0.11683795	1
gene23331	483.5094381	871.1308752	1.80168329	0.849345428	0.11684055	1
gene51637	103.3139825	196.4884085	1.901856881	0.927408684	0.11685573	1
gene281	135.3413171	67.75462362	0.500620395	-0.99821103	0.11707464	1
gene1296	103.3139825	49.36408293	0.477806408	-1.06550189	0.11708688	1
gene62985	235.5558801	424.9182824	1.803895883	0.851116072	0.11713603	1
gene38514	98.14828338	46.46031334	0.473368578	-1.07896415	0.11731509	1
gene71038	234.5227403	422.9824361	1.803588153	0.850869939	0.11731932	1
gene22947	87.81688513	40.65277417	0.462926624	-1.11114456	0.11733556	1
gene11628	84.71746566	164.5469431	1.942302473	0.957767888	0.11735584	1
gene14967	376.0628963	674.6424667	1.79396179	0.843149162	0.11736366	1
gene68011	574.4257427	320.3825774	0.557744115	-0.84232471	0.11760234	1
gene62414	217.9925031	116.1507834	0.532820082	-0.90827964	0.11768231	1
gene57239	232.4564606	419.1107433	1.802964487	0.85037098	0.11769325	1
gene72701	209.7273845	111.3111674	0.530742171	-0.91391691	0.1177051	1
gene4509	1705.713851	3323.84825	1.94865525	0.962478876	0.11773977	1
gene3443	168.4017915	87.11308752	0.517293116	-0.9509461	0.11776989	1
gene8479	3630.453345	1846.797455	0.508696099	-0.97512406	0.11777048	1

gene54240	372.9634769	206.1676405	0.552782386	-0.85521645	0.11786311	1
gene69342	2264.642497	1201.192685	0.530411615	-0.91481573	0.11787797	1
gene16411	37.1930337	13.55092472	0.364340399	-1.45664112	0.11799685	1
gene42489	37.1930337	13.55092472	0.364340399	-1.45664112	0.11799685	1
gene53891	37.1930337	13.55092472	0.364340399	-1.45664112	0.11799685	1
gene73293	179.7663296	327.1580398	1.819907213	0.863864897	0.11801576	1
gene60132	71.28664793	141.3167864	1.982373846	0.987229059	0.11807896	1
gene6807	765.5566104	426.8541288	0.557573565	-0.84276593	0.11815568	1
gene7316	222.1250624	400.7202026	1.804029668	0.851223065	0.11818946	1
gene12501	63.02152933	27.10184945	0.430041126	-1.21745346	0.11819694	1
gene29370	439.0844257	787.8894804	1.794391772	0.84349491	0.11820747	1
gene73023	1060.001461	1978.43501	1.866445551	0.900293422	0.11821547	1
gene35503	5110.942715	2500.145612	0.489175041	-1.0315773	0.11822915	1
gene52215	850.274076	473.3144422	0.556661029	-0.84512901	0.11824448	1
gene68020	262.4175156	142.2847096	0.542207365	-0.88308339	0.11826071	1
gene2182	0	5.807539168	Inf	Inf	0.11832652	1
gene2875	0	5.807539168	Inf	Inf	0.11832652	1
gene3564	0	5.807539168	Inf	Inf	0.11832652	1
gene4000	0	5.807539168	Inf	Inf	0.11832652	1
gene4575	0	5.807539168	Inf	Inf	0.11832652	1
gene4585	0	5.807539168	Inf	Inf	0.11832652	1
gene5658	0	5.807539168	Inf	Inf	0.11832652	1
gene5794	0	5.807539168	Inf	Inf	0.11832652	1
gene6916	0	5.807539168	Inf	Inf	0.11832652	1
gene7192	0	5.807539168	Inf	Inf	0.11832652	1
gene7311	0	5.807539168	Inf	Inf	0.11832652	1
gene7771	0	5.807539168	Inf	Inf	0.11832652	1
gene8005	0	5.807539168	Inf	Inf	0.11832652	1
gene12008	0	5.807539168	Inf	Inf	0.11832652	1
gene14199	0	5.807539168	Inf	Inf	0.11832652	1
gene16059	0	5.807539168	Inf	Inf	0.11832652	1
gene16394	0	5.807539168	Inf	Inf	0.11832652	1
gene18026	0	5.807539168	Inf	Inf	0.11832652	1
gene18476	0	5.807539168	Inf	Inf	0.11832652	1
gene18718	0	5.807539168	Inf	Inf	0.11832652	1
gene19203	0	5.807539168	Inf	Inf	0.11832652	1
gene19814	0	5.807539168	Inf	Inf	0.11832652	1
gene20209	0	5.807539168	Inf	Inf	0.11832652	1
gene20400	0	5.807539168	Inf	Inf	0.11832652	1
gene20914	0	5.807539168	Inf	Inf	0.11832652	1
gene22137	0	5.807539168	Inf	Inf	0.11832652	1
gene22374	0	5.807539168	Inf	Inf	0.11832652	1
gene24888	0	5.807539168	Inf	Inf	0.11832652	1
gene25939	0	5.807539168	Inf	Inf	0.11832652	1
gene26046	0	5.807539168	Inf	Inf	0.11832652	1
gene27815	0	5.807539168	Inf	Inf	0.11832652	1
gene28181	0	5.807539168	Inf	Inf	0.11832652	1
gene32939	0	5.807539168	Inf	Inf	0.11832652	1

gene33634	0	5.807539168	Inf	Inf	0.11832652	1
gene34030	0	5.807539168	Inf	Inf	0.11832652	1
gene36622	0	5.807539168	Inf	Inf	0.11832652	1
gene37447	0	5.807539168	Inf	Inf	0.11832652	1
gene37749	0	5.807539168	Inf	Inf	0.11832652	1
gene39730	0	5.807539168	Inf	Inf	0.11832652	1
gene40735	0	5.807539168	Inf	Inf	0.11832652	1
gene41466	0	5.807539168	Inf	Inf	0.11832652	1
gene41632	0	5.807539168	Inf	Inf	0.11832652	1
gene41729	0	5.807539168	Inf	Inf	0.11832652	1
gene41778	0	5.807539168	Inf	Inf	0.11832652	1
gene43986	0	5.807539168	Inf	Inf	0.11832652	1
gene45001	0	5.807539168	Inf	Inf	0.11832652	1
gene45304	0	5.807539168	Inf	Inf	0.11832652	1
gene45818	0	5.807539168	Inf	Inf	0.11832652	1
gene47747	0	5.807539168	Inf	Inf	0.11832652	1
gene49126	0	5.807539168	Inf	Inf	0.11832652	1
gene50073	0	5.807539168	Inf	Inf	0.11832652	1
gene52117	0	5.807539168	Inf	Inf	0.11832652	1
gene52209	0	5.807539168	Inf	Inf	0.11832652	1
gene53011	0	5.807539168	Inf	Inf	0.11832652	1
gene54607	0	5.807539168	Inf	Inf	0.11832652	1
gene54791	0	5.807539168	Inf	Inf	0.11832652	1
gene55322	0	5.807539168	Inf	Inf	0.11832652	1
gene55401	0	5.807539168	Inf	Inf	0.11832652	1
gene55515	0	5.807539168	Inf	Inf	0.11832652	1
gene55896	0	5.807539168	Inf	Inf	0.11832652	1
gene56402	0	5.807539168	Inf	Inf	0.11832652	1
gene59616	0	5.807539168	Inf	Inf	0.11832652	1
gene60216	0	5.807539168	Inf	Inf	0.11832652	1
gene60276	0	5.807539168	Inf	Inf	0.11832652	1
gene61552	0	5.807539168	Inf	Inf	0.11832652	1
gene62149	0	5.807539168	Inf	Inf	0.11832652	1
gene63766	0	5.807539168	Inf	Inf	0.11832652	1
gene65950	0	5.807539168	Inf	Inf	0.11832652	1
gene66771	0	5.807539168	Inf	Inf	0.11832652	1
gene67073	0	5.807539168	Inf	Inf	0.11832652	1
gene68686	0	5.807539168	Inf	Inf	0.11832652	1
gene68904	0	5.807539168	Inf	Inf	0.11832652	1
gene69202	0	5.807539168	Inf	Inf	0.11832652	1
gene69582	0	5.807539168	Inf	Inf	0.11832652	1
gene70958	0	5.807539168	Inf	Inf	0.11832652	1
gene71708	0	5.807539168	Inf	Inf	0.11832652	1
gene72435	0	5.807539168	Inf	Inf	0.11832652	1
gene73852	0	5.807539168	Inf	Inf	0.11832652	1
gene33086	55.78955055	23.23015667	0.416389027	-1.26399604	0.11832673	1
gene74029	125.0099188	61.94708446	0.495537354	-1.01293428	0.11835737	1
gene30334	11.36453808	32.90938862	2.895796415	1.533960179	0.11836131	1

gene60823	11.36453808	32.90938862	2.895796415	1.533960179	0.11836131	1
gene9023	337.8367228	604.9519966	1.79066382	0.840494511	0.11840722	1
gene43862	154.9709738	79.36970196	0.512158503	-0.96533773	0.11843574	1
gene67592	538.2658489	969.859041	1.801821615	0.849456188	0.11845746	1
gene64845	100.214563	190.6808693	1.902726147	0.928067934	0.11847776	1
gene24168	179.7663296	93.88854988	0.522281064	-0.9371017	0.11848128	1
gene23194	111.5791011	210.0393332	1.882425393	0.912592687	0.11850006	1
gene43348	94.01572408	180.0337142	1.914931954	0.937293128	0.11873893	1
gene15612	333.7041635	183.905407	0.551103124	-0.85960579	0.11894901	1
gene17819	892.6328089	1644.501508	1.842304575	0.881511592	0.119042	1
gene25722	7.231978776	24.19807987	3.345983253	1.742430224	0.11920714	1
gene34512	7.231978776	24.19807987	3.345983253	1.742430224	0.11920714	1
gene42386	7.231978776	24.19807987	3.345983253	1.742430224	0.11920714	1
gene46872	7.231978776	24.19807987	3.345983253	1.742430224	0.11920714	1
gene51785	7.231978776	24.19807987	3.345983253	1.742430224	0.11920714	1
gene53427	7.231978776	24.19807987	3.345983253	1.742430224	0.11920714	1
gene57212	7.231978776	24.19807987	3.345983253	1.742430224	0.11920714	1
gene66386	281.0140324	503.3200612	1.791085153	0.840833928	0.11925367	1
gene17206	1067.233439	1989.082165	1.863774214	0.898227096	0.11928574	1
gene13537	90.91630461	42.58862056	0.468437655	-1.09407104	0.11944759	1
gene29266	953.5880585	530.4219107	0.556237996	-0.8462258	0.11947828	1
gene13439	38.22617353	83.24139474	2.177602074	1.122740346	0.11957291	1
gene30764	38.22617353	83.24139474	2.177602074	1.122740346	0.11957291	1
gene8164	35.12675405	12.58300153	0.358217031	-1.48109417	0.11963163	1
gene28323	35.12675405	12.58300153	0.358217031	-1.48109417	0.11963163	1
gene46261	646.7455305	362.0032748	0.559730617	-0.83719543	0.11971778	1
gene17406	355.4000998	634.9576157	1.78659943	0.837216207	0.11981729	1
gene41593	47.52443195	99.69608905	2.097786022	1.068867528	0.11985726	1
gene73499	91.94944443	176.1620214	1.915857377	0.937990166	0.11993293	1
gene59568	108.4796816	204.2317941	1.882673243	0.912782628	0.12008367	1
gene4026	136.3744569	251.6600306	1.845360461	0.88390265	0.12018509	1
gene60116	319.2402059	570.1067616	1.785823812	0.836589753	0.12026207	1
gene16113	12.3976779	34.84523501	2.810625932	1.490891457	0.1202667	1
gene24491	12.3976779	34.84523501	2.810625932	1.490891457	0.1202667	1
gene35653	12.3976779	34.84523501	2.810625932	1.490891457	0.1202667	1
gene37138	12.3976779	34.84523501	2.810625932	1.490891457	0.1202667	1
gene41733	12.3976779	34.84523501	2.810625932	1.490891457	0.1202667	1
gene5125	5043.788626	2481.755071	0.492041847	-1.02314708	0.1204183	1
gene70015	364.6983583	202.2959477	0.554693881	-0.85023628	0.12050771	1
gene33869	36.15989388	79.36970196	2.194965014	1.134197944	0.1206049	1
gene64219	139.4738764	70.65839321	0.506606649	-0.98106208	0.12074036	1
gene8173	53.7232709	110.3432442	2.05391895	1.038379253	0.12089578	1
gene45796	53.7232709	110.3432442	2.05391895	1.038379253	0.12089578	1
gene911	18.59651685	4.839615973	0.260243142	-1.94206795	0.12100034	1
gene20564	18.59651685	4.839615973	0.260243142	-1.94206795	0.12100034	1
gene45995	1042.438084	579.7859936	0.556182667	-0.84636931	0.12109865	1
gene52120	760.3909113	1383.162245	1.8190147	0.863157202	0.12111332	1
gene28818	220.0587827	118.0866297	0.536614028	-0.89804333	0.12116471	1

gene41320	45.4581523	95.82439627	2.107969449	1.075853958	0.12118742	1
gene69333	264.4837952	144.220556	0.545290708	-0.87490252	0.12122878	1
gene36106	781.0537078	437.501284	0.560142382	-0.83613451	0.12123916	1
gene69178	78.51862671	35.8131582	0.456110349	-1.13254519	0.12125195	1
gene31376	256.2186766	139.38094	0.543992116	-0.87834235	0.12127541	1
gene4363	605.4199375	1089.881517	1.800207508	0.848163214	0.12134333	1
gene71468	1209.806735	2267.844045	1.874550686	0.906544835	0.12137145	1
gene68739	1108.559032	615.5991518	0.555314723	-0.84862245	0.12144876	1
gene14696	585.7902808	1053.100436	1.797743101	0.846186873	0.12147062	1
gene64814	88.85002496	41.62069737	0.468437655	-1.09407104	0.12155922	1
gene17144	568.2269038	1020.191047	1.795393777	0.8443003	0.12165439	1
gene3596	1427.799238	2713.088715	1.900189216	0.926143085	0.12166753	1
gene62638	771.7554493	432.661668	0.560620166	-0.83490445	0.12167297	1
gene762	44.42501248	17.4226175	0.392180363	-1.3504108	0.1216917	1
gene42598	462.8466416	826.6064082	1.785918561	0.836666294	0.12170861	1
gene37116	149.8052746	273.9222641	1.828522158	0.870678109	0.12172894	1
gene9484	13.43081773	36.7810814	2.738558601	1.453416752	0.12176583	1
gene21386	13.43081773	36.7810814	2.738558601	1.453416752	0.12176583	1
gene23550	13.43081773	36.7810814	2.738558601	1.453416752	0.12176583	1
gene58007	13.43081773	36.7810814	2.738558601	1.453416752	0.12176583	1
gene55927	660.1763482	370.7145835	0.561538723	-0.83254258	0.12181042	1
gene27970	66.12094881	29.03769584	0.439160302	-1.18718045	0.12186129	1
gene756	822.3793008	460.7314406	0.56024202	-0.8358779	0.12189669	1
gene53037	375.0297565	667.8670043	1.780837367	0.83255577	0.12196278	1
gene3229	94.01572408	179.065791	1.904636621	0.929515777	0.12203104	1
gene2770	927.7595629	1704.512746	1.837235437	0.877536516	0.12203564	1
gene64297	422.5541885	753.0442454	1.782124674	0.833598269	0.12215478	1
gene27132	198.3628464	105.5036282	0.531871921	-0.91084922	0.12222296	1
gene62741	65.08780898	129.7017081	1.992718915	0.994738224	0.12223706	1
gene64493	197.3297066	355.2278124	1.800174026	0.848136381	0.12230648	1
gene68784	245.8872784	439.4371304	1.787148702	0.837659681	0.12235388	1
gene45664	183.8988889	331.9976558	1.805327144	0.852260292	0.12244567	1
gene613	3.099419475	14.51884792	4.684376554	2.227857052	0.12257448	1
gene4093	3.099419475	14.51884792	4.684376554	2.227857052	0.12257448	1
gene26412	3.099419475	14.51884792	4.684376554	2.227857052	0.12257448	1
gene28065	3.099419475	14.51884792	4.684376554	2.227857052	0.12257448	1
gene29410	3.099419475	14.51884792	4.684376554	2.227857052	0.12257448	1
gene30964	3.099419475	14.51884792	4.684376554	2.227857052	0.12257448	1
gene33506	3.099419475	14.51884792	4.684376554	2.227857052	0.12257448	1
gene36457	3.099419475	14.51884792	4.684376554	2.227857052	0.12257448	1
gene38176	3.099419475	14.51884792	4.684376554	2.227857052	0.12257448	1
gene56039	3.099419475	14.51884792	4.684376554	2.227857052	0.12257448	1
gene63244	3.099419475	14.51884792	4.684376554	2.227857052	0.12257448	1
gene65827	3.099419475	14.51884792	4.684376554	2.227857052	0.12257448	1
gene70286	3.099419475	14.51884792	4.684376554	2.227857052	0.12257448	1
gene72476	3.099419475	14.51884792	4.684376554	2.227857052	0.12257448	1
gene2285	962.886317	538.1652962	0.558908447	-0.83931612	0.12268984	1
gene244	11.36453808	1.935846389	0.170340966	-2.55350266	0.12271289	1

gene10554	11.36453808	1.935846389	0.170340966	-2.55350266	0.12271289	1
gene12764	11.36453808	1.935846389	0.170340966	-2.55350266	0.12271289	1
gene16262	11.36453808	1.935846389	0.170340966	-2.55350266	0.12271289	1
gene17710	11.36453808	1.935846389	0.170340966	-2.55350266	0.12271289	1
gene35943	11.36453808	1.935846389	0.170340966	-2.55350266	0.12271289	1
gene38818	11.36453808	1.935846389	0.170340966	-2.55350266	0.12271289	1
gene46147	11.36453808	1.935846389	0.170340966	-2.55350266	0.12271289	1
gene60547	11.36453808	1.935846389	0.170340966	-2.55350266	0.12271289	1
gene67817	11.36453808	1.935846389	0.170340966	-2.55350266	0.12271289	1
gene8753	58.88897003	25.16600306	0.427346633	-1.22652134	0.12272454	1
gene62634	58.88897003	25.16600306	0.427346633	-1.22652134	0.12272454	1
gene68696	173.5674906	90.9847803	0.524204043	-0.93179961	0.12284292	1
gene20149	30.99419475	69.69047001	2.248500746	1.168963362	0.12288059	1
gene36789	30.99419475	69.69047001	2.248500746	1.168963362	0.12288059	1
gene13579	297.5442696	529.4539875	1.779412483	0.831400978	0.12295952	1
gene55550	873.0031522	1595.137425	1.827184038	0.869621953	0.12303663	1
gene4435	555.8292259	312.6391919	0.562473467	-0.83014305	0.12306997	1
gene35606	50.62385143	104.535705	2.064949664	1.046106615	0.12307361	1
gene35187	42.35873283	90.0168571	2.125107412	1.087535763	0.12321014	1
gene9000	487.6419974	273.9222641	0.561728205	-0.83205585	0.12324884	1
gene4090	123.976779	229.3977971	1.850328739	0.88778161	0.12334529	1
gene3291	703.5682209	395.8805866	0.56267548	-0.829625	0.12336252	1
gene361	5042.755486	11778.65736	2.335758176	1.223890918	0.1234774	1
gene66389	296.5111298	163.5790199	0.55167919	-0.85809853	0.12351981	1
gene9179	509.3379338	908.8798798	1.784433908	0.835466468	0.12353512	1
gene49057	219.0256429	392.0088938	1.789785381	0.8397866	0.12356093	1
gene42461	150.8384145	274.8901873	1.822414988	0.865851517	0.12358503	1
gene37321	69.22036828	136.4771704	1.971633117	0.979391119	0.12359346	1
gene16871	8.265118601	26.13392626	3.161954174	1.660816459	0.12359537	1
gene17547	8.265118601	26.13392626	3.161954174	1.660816459	0.12359537	1
gene35594	8.265118601	26.13392626	3.161954174	1.660816459	0.12359537	1
gene36101	8.265118601	26.13392626	3.161954174	1.660816459	0.12359537	1
gene63540	56.82269038	115.1828602	2.02705749	1.019387006	0.12361615	1
gene67401	276.8814731	492.6729061	1.77936393	0.831361612	0.12361689	1
gene46845	1734.641766	945.6609612	0.545162108	-0.87524281	0.12362927	1
gene43490	28.9279151	65.81877724	2.275268612	1.186036876	0.12368257	1
gene5372	5.165699125	19.35846389	3.747501243	1.905928957	0.12375548	1
gene5720	5.165699125	19.35846389	3.747501243	1.905928957	0.12375548	1
gene23149	5.165699125	19.35846389	3.747501243	1.905928957	0.12375548	1
gene36508	5.165699125	19.35846389	3.747501243	1.905928957	0.12375548	1
gene37385	5.165699125	19.35846389	3.747501243	1.905928957	0.12375548	1
gene38238	5.165699125	19.35846389	3.747501243	1.905928957	0.12375548	1
gene67	28.9279151	9.679231946	0.334598325	-1.57949787	0.12376915	1
gene17106	28.9279151	9.679231946	0.334598325	-1.57949787	0.12376915	1
gene69756	28.9279151	9.679231946	0.334598325	-1.57949787	0.12376915	1
gene68023	1913.374956	3732.311839	1.950643196	0.96394991	0.12376943	1
gene46256	217.9925031	390.0730474	1.789387442	0.839465796	0.12380846	1
gene66490	15.49709738	40.65277417	2.62325087	1.391355784	0.12382026	1

gene69544	231.4233208	413.3032041	1.785918561	0.836666294	0.12385784	1
gene16861	1014.543308	567.2029921	0.559072232	-0.8388934	0.12386295	1
gene19315	307.8756679	546.876605	1.776290438	0.828867493	0.12387726	1
gene56349	41.325593	88.08101071	2.131391332	1.091795502	0.12388774	1
gene65129	41.325593	88.08101071	2.131391332	1.091795502	0.12388774	1
gene17404	389.4937141	217.7827188	0.55914309	-0.83871057	0.12395981	1
gene36960	1722.244088	939.853422	0.545714413	-0.87378195	0.12399126	1
gene67507	178.7331897	322.3184238	1.803349587	0.850679096	0.12403901	1
gene27940	1208.773595	671.7386971	0.555719201	-0.84757201	0.12404407	1
gene5870	127.0761985	63.88293085	0.502713581	-0.99219143	0.12423751	1
gene43357	528.9675904	943.7251148	1.784088726	0.835187365	0.12427381	1
gene26189	16.5302372	42.58862056	2.576407104	1.365360575	0.1244812	1
gene61883	16.5302372	42.58862056	2.576407104	1.365360575	0.1244812	1
gene62772	40.29245318	86.14516432	2.137997504	1.096260169	0.12456492	1
gene45003	335.7704432	186.8091766	0.5563598	-0.84590991	0.12459857	1
gene22435	387.4274344	687.2254682	1.773817255	0.826857386	0.12461648	1
gene4905	25.82849563	60.01123807	2.323450771	1.216269077	0.12470795	1
gene34415	364.6983583	646.572694	1.772897189	0.826108876	0.12477478	1
gene23343	113.6453808	56.13954529	0.4939888	-1.01744976	0.12480669	1
gene47016	1380.274806	2599.841701	1.883568177	0.913468254	0.12481652	1
gene29592	1226.336972	2287.202509	1.865068542	0.899228651	0.12481956	1
gene16715	22.72907615	6.775462362	0.29809669	-1.74614774	0.12482504	1
gene42101	22.72907615	6.775462362	0.29809669	-1.74614774	0.12482504	1
gene64392	22.72907615	6.775462362	0.29809669	-1.74614774	0.12482504	1
gene53484	105.3802622	197.4563317	1.873750621	0.905928957	0.12484132	1
gene54937	17.56337703	44.52446695	2.53507437	1.342028071	0.12495186	1
gene56808	17.56337703	44.52446695	2.53507437	1.342028071	0.12495186	1
gene48737	1408.169582	2655.981246	1.886123149	0.915423876	0.12504438	1
gene7605	78.51862671	151.9639416	1.935387155	0.952622192	0.12505062	1
gene26372	24.7953558	7.743385557	0.31229177	-1.67903354	0.1250658	1
gene46674	24.7953558	7.743385557	0.31229177	-1.67903354	0.1250658	1
gene21551	146.7058552	75.49800918	0.51462165	-0.95841594	0.12507076	1
gene49167	67.15408863	132.6054777	1.974644886	0.981593227	0.12512051	1
gene52080	67.15408863	132.6054777	1.974644886	0.981593227	0.12512051	1
gene3448	412.2227902	231.3336435	0.561185963	-0.83344917	0.12512779	1
gene51888	39.25931335	84.20931793	2.144951369	1.100944939	0.12524011	1
gene48769	696.3362421	392.976817	0.564349223	-0.82533991	0.12524231	1
gene30929	18.59651685	46.46031334	2.498334162	1.320966456	0.12526362	1
gene39742	18.59651685	46.46031334	2.498334162	1.320966456	0.12526362	1
gene59953	18.59651685	46.46031334	2.498334162	1.320966456	0.12526362	1
gene8177	49.5907116	20.32638709	0.409882948	-1.28671612	0.12529279	1
gene70348	166.3355118	87.11308752	0.523719118	-0.93313483	0.12531001	1
gene5786	464.9129213	825.638485	1.7758992	0.828549697	0.1253339	1
gene13951	95.04886391	45.49239015	0.478621083	-1.06304415	0.12535404	1
gene65674	22.72907615	54.2036989	2.384773518	1.25385226	0.12537817	1
gene63822	19.62965668	48.39615973	2.465461344	1.301857633	0.12544237	1
gene65516	19.62965668	48.39615973	2.465461344	1.301857633	0.12544237	1
gene5039	21.69593633	52.26785251	2.409107942	1.268499036	0.12548319	1

gene71125	21.69593633	52.26785251	2.409107942	1.268499036	0.12548319	1
gene57517	114.6785206	212.9431028	1.856869985	0.892872804	0.12548334	1
gene37763	20.6627965	50.33200612	2.435875808	1.28444058	0.12550959	1
gene7171	158.0703932	82.27347154	0.520486284	-0.94206795	0.12559393	1
gene25092	162.2029525	293.280728	1.808109676	0.854482191	0.12575509	1
gene53038	145.6727153	265.2109553	1.820594575	0.864409687	0.12577481	1
gene69665	74.38606741	33.87731181	0.455425498	-1.13471303	0.12579545	1
gene34583	259.3180961	142.2847096	0.548687931	-0.86594225	0.12585702	1
gene71442	189.064588	100.6640122	0.532431871	-0.90933116	0.12586306	1
gene27854	687.0379837	388.137201	0.564942857	-0.82382315	0.12586896	1
gene25389	197.3297066	353.291966	1.790363814	0.840252782	0.12587586	1
gene38103	38.22617353	82.27347154	2.152281119	1.105866527	0.12591154	1
gene15706	87.81688513	167.4507127	1.906816809	0.931166248	0.12605037	1
gene73130	79.55176653	36.7810814	0.462354049	-1.11293007	0.12606074	1
gene23597	59.92210985	120.0224761	2.002974802	1.002144272	0.12609824	1
gene62636	134.3081773	245.8524914	1.830510222	0.87224583	0.12611658	1
gene59583	46.49129213	96.79231946	2.081945135	1.05793205	0.12611844	1
gene5607	40.29245318	15.48677111	0.384359102	-1.37947326	0.12621395	1
gene20348	40.29245318	15.48677111	0.384359102	-1.37947326	0.12621395	1
gene56745	2366.923339	4719.593497	1.993978182	0.995649624	0.12631696	1
gene31581	177.7000499	319.4146542	1.797493329	0.845986416	0.1263629	1
gene649	420.4879088	744.3329367	1.770164899	0.82388376	0.12644352	1
gene10600	420.4879088	744.3329367	1.770164899	0.82388376	0.12644352	1
gene19160	394.6594132	221.6544116	0.561634676	-0.83229608	0.12651387	1
gene47096	273.7820536	150.9960184	0.551519051	-0.85851737	0.12656624	1
gene71232	274.8151935	486.8653669	1.77161008	0.825061111	0.12656919	1
gene8612	152.9046941	79.36970196	0.519079564	-0.9459724	0.1265903	1
gene45975	232.4564606	413.3032041	1.777981145	0.830240025	0.12670933	1
gene2223	3000.238052	6199.548062	2.066352054	1.047086074	0.12682128	1
gene52096	3981.720886	8701.62952	2.185394147	1.127893501	0.12688001	1
gene48374	111.5791011	55.17162209	0.49446197	-1.01606853	0.12688725	1
gene43089	319.2402059	564.2992225	1.767632059	0.821818002	0.12690199	1
gene20623	793.4513857	1432.526328	1.805436797	0.852347916	0.12698066	1
gene43662	9.298258426	28.06977264	3.018820446	1.59398495	0.12698696	1
gene59188	574.4257427	325.2221934	0.566169253	-0.82069469	0.12719905	1
gene37056	116.7448002	215.8468724	1.848877826	0.886649894	0.12722727	1
gene56216	36.15989388	78.40177877	2.168197148	1.116495943	0.12723464	1
gene60417	409.1233707	230.3657203	0.563071525	-0.8286099	0.1274349	1
gene22106	67.15408863	30.00561903	0.446817456	-1.16224255	0.1276264	1
gene74068	54.75641073	23.23015667	0.424245424	-1.237029	0.12768253	1
gene69745	151.8715543	274.8901873	1.810017607	0.856003731	0.12776341	1
gene24957	4248.270961	9401.43799	2.213003379	1.146005654	0.12778403	1
gene23025	47.52443195	19.35846389	0.407337092	-1.2957049	0.12781451	1
gene73542	47.52443195	19.35846389	0.407337092	-1.2957049	0.12781451	1
gene46940	69.22036828	135.5092472	1.957649903	0.969122783	0.12791587	1
gene32890	412.2227902	727.8782424	1.765739934	0.820272872	0.12793944	1
gene16948	178.7331897	320.3825774	1.792518658	0.841988136	0.12801435	1
gene62799	178.7331897	320.3825774	1.792518658	0.841988136	0.12801435	1

gene52186	114.6785206	57.10746848	0.497978769	-1.00584386	0.12809304	1
gene26165	1329.650955	2481.755071	1.866471093	0.900313165	0.12814596	1
gene11483	170.4680711	90.0168571	0.528056993	-0.92123445	0.12819198	1
gene31465	949.4554992	1728.710826	1.820739178	0.86452427	0.12824807	1
gene72125	150.8384145	78.40177877	0.519773289	-0.9440456	0.12835827	1
gene72369	150.8384145	78.40177877	0.519773289	-0.9440456	0.12835827	1
gene33650	401.891392	226.4940275	0.563570238	-0.82733267	0.12836551	1
gene72755	82.65118601	38.71692779	0.468437655	-1.09407104	0.12839158	1
gene59550	77.48548688	35.8131582	0.46219182	-1.11343637	0.128456	1
gene73571	77.48548688	35.8131582	0.46219182	-1.11343637	0.128456	1
gene11537	50.62385143	103.5677818	2.04582976	1.032686099	0.12845971	1
gene70791	293.4117103	517.8389091	1.764888349	0.819576918	0.12846863	1
gene6954	43.39187265	90.9847803	2.096816172	1.068200386	0.12849474	1
gene27028	43.39187265	90.9847803	2.096816172	1.068200386	0.12849474	1
gene9380	34.09361423	74.53008599	2.186042392	1.128321378	0.12851397	1
gene46120	38.22617353	14.51884792	0.379814315	-1.39663381	0.12854753	1
gene72654	56.82269038	114.214937	2.010023394	1.007212292	0.12860341	1
gene8944	299.6105493	528.4860643	1.763910068	0.818777008	0.12867731	1
gene48163	149.8052746	271.0184945	1.809138531	0.855302884	0.12868818	1
gene70567	68.18722846	133.5734009	1.958921104	0.970059294	0.12874519	1
gene56686	217.9925031	387.1692779	1.77606694	0.828685958	0.12876301	1
gene42079	3825.716772	1973.595394	0.515875981	-0.95490382	0.12888834	1
gene62274	528.9675904	936.9496524	1.771279884	0.824792194	0.12889054	1
gene70504	118.8110799	218.750642	1.841163654	0.880617868	0.12894447	1
gene43646	336.803583	593.3369183	1.761670446	0.816944066	0.12898118	1
gene39288	109.5128215	54.2036989	0.494952994	-1.01463658	0.12904302	1
gene15416	33.0604744	72.5942396	2.195801509	1.134747647	0.12913171	1
gene60888	33.0604744	72.5942396	2.195801509	1.134747647	0.12913171	1
gene37105	143.6064357	260.3713394	1.81308963	0.858450246	0.12916687	1
gene20908	253.1192571	139.38094	0.550653244	-0.86078398	0.12920383	1
gene21347	61.9883895	122.9262457	1.983052741	0.987723048	0.12922627	1
gene52155	61.9883895	122.9262457	1.983052741	0.987723048	0.12922627	1
gene60734	59.92210985	26.13392626	0.43613161	-1.19716454	0.12923183	1
gene53135	334.7373033	589.4652255	1.760978593	0.816377372	0.12925804	1
gene65077	310.9750873	547.8445282	1.761699089	0.816967523	0.12927793	1
gene59244	459.7472222	260.3713394	0.566335862	-0.82027021	0.1292891	1
gene36049	254.152397	449.1163623	1.767114407	0.821395446	0.12929465	1
gene5108	49.5907116	101.6319354	2.049414742	1.035211974	0.12931557	1
gene52908	23683.69735	8632.906973	0.364508415	-1.45597598	0.12940203	1
gene64806	340.9361423	600.1123807	1.760189978	0.815731148	0.12952321	1
gene1571	331.6378839	185.8412534	0.560374018	-0.83553803	0.12953552	1
gene28614	1554.875437	2935.711049	1.888068317	0.916910968	0.12954289	1
gene68657	10.33139825	30.00561903	2.904313463	1.538197172	0.12959465	1
gene69280	10.33139825	30.00561903	2.904313463	1.538197172	0.12959465	1
gene69717	10.33139825	30.00561903	2.904313463	1.538197172	0.12959465	1
gene49062	413.25593	727.8782424	1.761325584	0.816661619	0.12961387	1
gene19272	693.2368226	1237.973766	1.785787664	0.836560549	0.12973735	1
gene10407	32.02733458	70.65839321	2.206190248	1.141557205	0.12974602	1

gene38452	278.9477528	154.8677111	0.555185369	-0.84895855	0.12989834	1
gene72637	127.0761985	232.3015667	1.828049387	0.870305047	0.12990096	1
gene6674	334.7373033	187.7770998	0.56096855	-0.8340082	0.12994065	1
gene7540	90.91630461	43.55654376	0.479083966	-1.06164957	0.12997089	1
gene27278	207.6611048	368.7787372	1.775868126	0.828524453	0.12999027	1
gene37786	41.325593	87.11308752	2.107969449	1.075853958	0.13011342	1
gene9967	2.06627965	11.61507834	5.621251864	2.490891457	0.13015358	1
gene23529	2.06627965	11.61507834	5.621251864	2.490891457	0.13015358	1
gene23931	2.06627965	11.61507834	5.621251864	2.490891457	0.13015358	1
gene26978	2.06627965	11.61507834	5.621251864	2.490891457	0.13015358	1
gene27007	2.06627965	11.61507834	5.621251864	2.490891457	0.13015358	1
gene29655	2.06627965	11.61507834	5.621251864	2.490891457	0.13015358	1
gene37872	2.06627965	11.61507834	5.621251864	2.490891457	0.13015358	1
gene39204	2.06627965	11.61507834	5.621251864	2.490891457	0.13015358	1
gene41943	2.06627965	11.61507834	5.621251864	2.490891457	0.13015358	1
gene42243	2.06627965	11.61507834	5.621251864	2.490891457	0.13015358	1
gene42344	2.06627965	11.61507834	5.621251864	2.490891457	0.13015358	1
gene47147	2.06627965	11.61507834	5.621251864	2.490891457	0.13015358	1
gene52050	2.06627965	11.61507834	5.621251864	2.490891457	0.13015358	1
gene56418	2.06627965	11.61507834	5.621251864	2.490891457	0.13015358	1
gene57888	2.06627965	11.61507834	5.621251864	2.490891457	0.13015358	1
gene62026	2.06627965	11.61507834	5.621251864	2.490891457	0.13015358	1
gene63555	2.06627965	11.61507834	5.621251864	2.490891457	0.13015358	1
gene66919	2.06627965	11.61507834	5.621251864	2.490891457	0.13015358	1
gene70050	2.06627965	11.61507834	5.621251864	2.490891457	0.13015358	1
gene41392	2246.04598	4412.761844	1.964680102	0.974294425	0.13022449	1
gene22497	112.6122409	56.13954529	0.498520808	-1.00427437	0.13025042	1
gene35105	1080.664257	608.8236894	0.56337913	-0.82782197	0.13025945	1
gene18223	297.5442696	523.6464483	1.759894247	0.815488739	0.13026676	1
gene44210	52.69013108	22.26223348	0.422512395	-1.24293443	0.1303398	1
gene35000	85.75060548	40.65277417	0.474081483	-1.07679305	0.13050177	1
gene21071	364.6983583	640.7651549	1.756972962	0.81309199	0.13075066	1
gene57347	595.0885392	338.7731181	0.569281873	-0.81278493	0.13077009	1
gene11578	6.19883895	21.29431028	3.435209473	1.780398075	0.13079899	1
gene27556	6.19883895	21.29431028	3.435209473	1.780398075	0.13079899	1
gene33939	6.19883895	21.29431028	3.435209473	1.780398075	0.13079899	1
gene64278	6.19883895	21.29431028	3.435209473	1.780398075	0.13079899	1
gene66138	6.19883895	21.29431028	3.435209473	1.780398075	0.13079899	1
gene17165	40.29245318	85.17724113	2.11397506	1.079958356	0.13093022	1
gene44247	40.29245318	85.17724113	2.11397506	1.079958356	0.13093022	1
gene69068	40.29245318	85.17724113	2.11397506	1.079958356	0.13093022	1
gene34817	75.41920723	34.84523501	0.462020701	-1.1139706	0.13094616	1
gene18150	276.8814731	153.8997879	0.55583274	-0.84727728	0.13095355	1
gene2789	29.96105493	66.78670043	2.229117119	1.156472418	0.13096223	1
gene35552	29.96105493	66.78670043	2.229117119	1.156472418	0.13096223	1
gene13399	368.8309176	208.1034868	0.564224627	-0.82565846	0.13106461	1
gene2745	1285.225942	720.1348568	0.56031771	-0.835683	0.13114472	1
gene73319	107.4465418	53.23577571	0.495462905	-1.01315105	0.13127783	1

gene33832	490.7414169	864.3554128	1.761325584	0.816661619	0.1314219	1
gene63420	80.58490636	153.8997879	1.909784287	0.933409693	0.13143185	1
gene64846	129.1424781	235.2053363	1.821285604	0.864957176	0.13150718	1
gene61670	28.9279151	64.85085404	2.241808779	1.164663225	0.13155858	1
gene8931	202.4954057	359.0995052	1.773371124	0.826494489	0.1315971	1
gene62476	1258.364307	2323.98359	1.846828917	0.885050227	0.13168381	1
gene21557	94.01572408	45.49239015	0.483880655	-1.04727683	0.13168552	1
gene912	214.8930836	117.1187066	0.545009195	-0.87564752	0.13170252	1
gene61605	4535.483832	10093.50307	2.225452333	1.1540986	0.13174407	1
gene31734	322.3396254	181.0016374	0.561524625	-0.8325788	0.13175641	1
gene45263	289.279151	508.1596772	1.756641208	0.812819552	0.13176567	1
gene21428	138.4407366	250.6921074	1.81082616	0.856648054	0.13178338	1
gene63258	294.4448501	164.5469431	0.558837905	-0.83949822	0.13188082	1
gene31088	133.2750374	241.9807987	1.815649827	0.860485986	0.13192577	1
gene64673	675.6734456	385.2334315	0.57014736	-0.81059325	0.13193927	1
gene51631	46.49129213	95.82439627	2.061125684	1.04343248	0.13196525	1
gene48733	1472.224251	820.7988691	0.557522992	-0.84289679	0.13213365	1
gene45426	27.89477528	62.91500765	2.255440563	1.173409268	0.13214251	1
gene51157	183.8988889	327.1580398	1.779010421	0.831074962	0.13221454	1
gene41954	64.05466916	125.8300153	1.964415974	0.974100459	0.13224666	1
gene56664	64.05466916	125.8300153	1.964415974	0.974100459	0.13224666	1
gene37679	449.4158239	255.5317234	0.568586395	-0.81454851	0.13226332	1
gene21184	102.2808427	50.33200612	0.492096123	-1.02298795	0.13228482	1
gene58105	122.9436392	224.5581812	1.826513211	0.869092188	0.13230151	1
gene64265	118.8110799	60.01123807	0.505097994	-0.98536478	0.13241706	1
gene63527	278.9477528	489.7691365	1.75577373	0.812106934	0.13247083	1
gene53178	289.279151	161.6431735	0.558779203	-0.83964977	0.13251319	1
gene55864	38.22617353	81.30554835	2.126960165	1.088793014	0.1325722	1
gene27865	271.715774	477.186135	1.756195925	0.812453804	0.13260965	1
gene60881	88.85002496	167.4507127	1.88464452	0.91429243	0.13269364	1
gene24915	26.86163545	60.97916126	2.270120945	1.182769162	0.13270983	1
gene55226	12.3976779	33.87731181	2.73255299	1.450249473	0.13306936	1
gene37006	275.8483333	483.9615973	1.754448147	0.81101731	0.13311698	1
gene33408	50.62385143	21.29431028	0.420637895	-1.24934927	0.13312576	1
gene12801	43.39187265	17.4226175	0.40151799	-1.31646346	0.13317822	1
gene45044	43.39187265	17.4226175	0.40151799	-1.31646346	0.13317822	1
gene42566	175.6337703	93.88854988	0.53457003	-0.90354914	0.13317822	1
gene25501	515.5367727	294.2486512	0.570761712	-0.80903954	0.13325324	1
gene18515	34.09361423	12.58300153	0.369072092	-1.43802544	0.1332848	1
gene21053	34.09361423	12.58300153	0.369072092	-1.43802544	0.1332848	1
gene47228	34.09361423	12.58300153	0.369072092	-1.43802544	0.1332848	1
gene73531	68.18722846	30.97354223	0.454242575	-1.13846516	0.13336905	1
gene19625	37.1930337	79.36970196	2.133993763	1.09355596	0.1333941	1
gene1325	1.033139825	8.711308752	8.431877796	3.075853958	0.13348658	1
gene5697	1.033139825	8.711308752	8.431877796	3.075853958	0.13348658	1
gene6062	1.033139825	8.711308752	8.431877796	3.075853958	0.13348658	1
gene6218	1.033139825	8.711308752	8.431877796	3.075853958	0.13348658	1
gene7832	1.033139825	8.711308752	8.431877796	3.075853958	0.13348658	1

gene8257	1.033139825	8.711308752	8.431877796	3.075853958	0.13348658	1
gene8844	1.033139825	8.711308752	8.431877796	3.075853958	0.13348658	1
gene12241	1.033139825	8.711308752	8.431877796	3.075853958	0.13348658	1
gene13298	1.033139825	8.711308752	8.431877796	3.075853958	0.13348658	1
gene13603	1.033139825	8.711308752	8.431877796	3.075853958	0.13348658	1
gene18788	1.033139825	8.711308752	8.431877796	3.075853958	0.13348658	1
gene18798	1.033139825	8.711308752	8.431877796	3.075853958	0.13348658	1
gene19388	1.033139825	8.711308752	8.431877796	3.075853958	0.13348658	1
gene19650	1.033139825	8.711308752	8.431877796	3.075853958	0.13348658	1
gene21285	1.033139825	8.711308752	8.431877796	3.075853958	0.13348658	1
gene22035	1.033139825	8.711308752	8.431877796	3.075853958	0.13348658	1
gene24099	1.033139825	8.711308752	8.431877796	3.075853958	0.13348658	1
gene24658	1.033139825	8.711308752	8.431877796	3.075853958	0.13348658	1
gene25357	1.033139825	8.711308752	8.431877796	3.075853958	0.13348658	1
gene27563	1.033139825	8.711308752	8.431877796	3.075853958	0.13348658	1
gene28217	1.033139825	8.711308752	8.431877796	3.075853958	0.13348658	1
gene30330	1.033139825	8.711308752	8.431877796	3.075853958	0.13348658	1
gene36552	1.033139825	8.711308752	8.431877796	3.075853958	0.13348658	1
gene36826	1.033139825	8.711308752	8.431877796	3.075853958	0.13348658	1
gene37588	1.033139825	8.711308752	8.431877796	3.075853958	0.13348658	1
gene37632	1.033139825	8.711308752	8.431877796	3.075853958	0.13348658	1
gene39233	1.033139825	8.711308752	8.431877796	3.075853958	0.13348658	1
gene41264	1.033139825	8.711308752	8.431877796	3.075853958	0.13348658	1
gene42935	1.033139825	8.711308752	8.431877796	3.075853958	0.13348658	1
gene43277	1.033139825	8.711308752	8.431877796	3.075853958	0.13348658	1
gene51778	1.033139825	8.711308752	8.431877796	3.075853958	0.13348658	1
gene55017	1.033139825	8.711308752	8.431877796	3.075853958	0.13348658	1
gene59380	1.033139825	8.711308752	8.431877796	3.075853958	0.13348658	1
gene59981	1.033139825	8.711308752	8.431877796	3.075853958	0.13348658	1
gene61390	1.033139825	8.711308752	8.431877796	3.075853958	0.13348658	1
gene65729	1.033139825	8.711308752	8.431877796	3.075853958	0.13348658	1
gene66664	1.033139825	8.711308752	8.431877796	3.075853958	0.13348658	1
gene68005	1.033139825	8.711308752	8.431877796	3.075853958	0.13348658	1
gene68491	1.033139825	8.711308752	8.431877796	3.075853958	0.13348658	1
gene68566	1.033139825	8.711308752	8.431877796	3.075853958	0.13348658	1
gene70770	1.033139825	8.711308752	8.431877796	3.075853958	0.13348658	1
gene73003	1.033139825	8.711308752	8.431877796	3.075853958	0.13348658	1
gene73781	1.033139825	8.711308752	8.431877796	3.075853958	0.13348658	1
gene4438	264.4837952	147.1243256	0.556269716	-0.84614353	0.13350203	1
gene31147	496.9402559	283.601496	0.570695356	-0.80920727	0.13351311	1
gene25022	207.6611048	366.8428908	1.766545984	0.820931304	0.1335969	1
gene71254	2731.621698	1465.435717	0.536470961	-0.89842801	0.13370786	1
gene30661	765.5566104	1364.771704	1.782718203	0.834078672	0.13379005	1
gene32547	133.2750374	68.72254682	0.515644551	-0.95555118	0.13383837	1
gene72875	133.2750374	68.72254682	0.515644551	-0.95555118	0.13383837	1
gene5725	13.43081773	2.903769584	0.216201995	-2.20954826	0.1340005	1
gene23717	13.43081773	2.903769584	0.216201995	-2.20954826	0.1340005	1
gene27031	13.43081773	2.903769584	0.216201995	-2.20954826	0.1340005	1

gene48892	13.43081773	2.903769584	0.216201995	-2.20954826	0.1340005	1
gene50212	13.43081773	2.903769584	0.216201995	-2.20954826	0.1340005	1
gene56030	13.43081773	2.903769584	0.216201995	-2.20954826	0.1340005	1
gene66170	13.43081773	2.903769584	0.216201995	-2.20954826	0.1340005	1
gene3810	77.48548688	148.0922488	1.911225634	0.934498109	0.13400089	1
gene22151	50.62385143	102.5998586	2.026709856	1.019139567	0.13407043	1
gene56621	50.62385143	102.5998586	2.026709856	1.019139567	0.13407043	1
gene56682	50.62385143	102.5998586	2.026709856	1.019139567	0.13407043	1
gene47973	829.6112795	473.3144422	0.570525563	-0.80963657	0.13410501	1
gene25054	13.43081773	35.8131582	2.666491269	1.414942604	0.1341583	1
gene27732	13.43081773	35.8131582	2.666491269	1.414942604	0.1341583	1
gene38073	13.43081773	35.8131582	2.666491269	1.414942604	0.1341583	1
gene34277	72.31978776	139.38094	1.927286353	0.946570941	0.13416106	1
gene45740	119.8442197	218.750642	1.825291554	0.868126924	0.13422826	1
gene29108	23.76221598	55.17162209	2.321821422	1.215257015	0.13425743	1
gene31337	23.76221598	55.17162209	2.321821422	1.215257015	0.13425743	1
gene56698	1050.703202	1906.808693	1.814792883	0.859804907	0.13429418	1
gene38844	1212.906155	2223.319578	1.833051609	0.874247405	0.13445406	1
gene49972	540.3321285	949.5326539	1.757312963	0.813371147	0.13449787	1
gene9115	1130.254969	639.7972317	0.566064516	-0.8209616	0.1345564	1
gene29794	262.4175156	146.1564024	0.556961307	-0.84435099	0.13466582	1
gene63017	43.39187265	90.0168571	2.074509617	1.052770345	0.13473301	1
gene12842	55.78955055	111.3111674	1.995197421	0.996531505	0.13475181	1
gene52460	55.78955055	111.3111674	1.995197421	0.996531505	0.13475181	1
gene36543	108.4796816	54.2036989	0.499666832	-1.00096164	0.13480415	1
gene25761	35.12675405	75.49800918	2.149302183	1.103868334	0.13502938	1
gene27163	21.69593633	51.29992932	2.364494832	1.241531988	0.1350861	1
gene414	4.1325593	16.45469431	3.981720071	1.993391798	0.13516239	1
gene13421	4.1325593	16.45469431	3.981720071	1.993391798	0.13516239	1
gene13883	4.1325593	16.45469431	3.981720071	1.993391798	0.13516239	1
gene28072	4.1325593	16.45469431	3.981720071	1.993391798	0.13516239	1
gene43067	4.1325593	16.45469431	3.981720071	1.993391798	0.13516239	1
gene60630	4.1325593	16.45469431	3.981720071	1.993391798	0.13516239	1
gene65918	4.1325593	16.45469431	3.981720071	1.993391798	0.13516239	1
gene66257	4.1325593	16.45469431	3.981720071	1.993391798	0.13516239	1
gene72621	4.1325593	16.45469431	3.981720071	1.993391798	0.13516239	1
gene4946	66.12094881	128.7337849	1.946944005	0.961211392	0.13516463	1
gene29880	104.3471223	192.6167157	1.845922642	0.884342094	0.13527309	1
gene42497	242.7878589	134.5413241	0.55415178	-0.85164692	0.13528294	1
gene7576	20.6627965	49.36408293	2.389032042	1.256426204	0.13540913	1
gene58226	20.6627965	49.36408293	2.389032042	1.256426204	0.13540913	1
gene72951	20.6627965	49.36408293	2.389032042	1.256426204	0.13540913	1
gene61017	15.49709738	39.68485098	2.560792516	1.356590366	0.13542646	1
gene20537	325.4390449	183.905407	0.565099394	-0.82342345	0.13563377	1
gene44398	32.02733458	11.61507834	0.362661411	-1.46330485	0.1356363	1
gene61895	32.02733458	11.61507834	0.362661411	-1.46330485	0.1356363	1
gene44639	836.8432583	1494.473413	1.785846271	0.836607896	0.13564144	1
gene41279	99.18142321	183.905407	1.854232386	0.890822064	0.13567413	1

gene50353	344.0355618	600.1123807	1.74433241	0.802674995	0.13570143	1
gene49871	16.5302372	41.62069737	2.517852398	1.332193711	0.13571543	1
gene73836	95.04886391	46.46031334	0.48880451	-1.0326705	0.13573763	1
gene10878	18.59651685	45.49239015	2.446285534	1.290592807	0.13580004	1
gene5418	17.56337703	43.55654376	2.479964058	1.310319212	0.13582945	1
gene30248	17.56337703	43.55654376	2.479964058	1.310319212	0.13582945	1
gene35213	17.56337703	43.55654376	2.479964058	1.310319212	0.13582945	1
gene44073	17.56337703	43.55654376	2.479964058	1.310319212	0.13582945	1
gene20297	142.5732959	74.53008599	0.522749268	-0.93580896	0.13599702	1
gene31363	41.325593	16.45469431	0.398172007	-1.3285363	0.13602463	1
gene32581	41.325593	16.45469431	0.398172007	-1.3285363	0.13602463	1
gene3826	70.25350811	135.5092472	1.928860934	0.947749132	0.13602986	1
gene164	7.231978776	23.23015667	3.212143922	1.683536535	0.13603584	1
gene45759	7.231978776	23.23015667	3.212143922	1.683536535	0.13603584	1
gene49628	7.231978776	23.23015667	3.212143922	1.683536535	0.13603584	1
gene60059	7.231978776	23.23015667	3.212143922	1.683536535	0.13603584	1
gene60750	7.231978776	23.23015667	3.212143922	1.683536535	0.13603584	1
gene3261	48.55757178	20.32638709	0.418603862	-1.25634247	0.13604833	1
gene22640	48.55757178	20.32638709	0.418603862	-1.25634247	0.13604833	1
gene66047	76.45234706	35.8131582	0.468437655	-1.09407104	0.13607002	1
gene42305	2468.171042	4855.102744	1.967085207	0.976059451	0.13614693	1
gene4860	3829.849332	8121.843526	2.120669202	1.084519596	0.13615051	1
gene32965	590.9559799	1038.581588	1.757460155	0.813491981	0.13622955	1
gene30363	1798.696435	3404.185876	1.89258499	0.920358089	0.13638161	1
gene35140	440.1175655	251.6600306	0.571801833	-0.80641285	0.13642889	1
gene55505	2360.7245	1286.369926	0.544904721	-0.8759241	0.13646962	1
gene47041	122.9436392	62.91500765	0.511738615	-0.96652099	0.13650841	1
gene22777	134.3081773	69.69047001	0.518884787	-0.94651385	0.13669605	1
gene50619	366.7646379	638.8293085	1.741796352	0.800575957	0.13671691	1
gene35436	185.9651685	100.6640122	0.541305735	-0.88548442	0.13673405	1
gene959	8.265118601	0.967923195	0.117109414	-3.09407104	0.1367637	1
gene6673	8.265118601	0.967923195	0.117109414	-3.09407104	0.1367637	1
gene8617	8.265118601	0.967923195	0.117109414	-3.09407104	0.1367637	1
gene9579	8.265118601	0.967923195	0.117109414	-3.09407104	0.1367637	1
gene11546	8.265118601	0.967923195	0.117109414	-3.09407104	0.1367637	1
gene17020	8.265118601	0.967923195	0.117109414	-3.09407104	0.1367637	1
gene17882	8.265118601	0.967923195	0.117109414	-3.09407104	0.1367637	1
gene18000	8.265118601	0.967923195	0.117109414	-3.09407104	0.1367637	1
gene28877	8.265118601	0.967923195	0.117109414	-3.09407104	0.1367637	1
gene30797	8.265118601	0.967923195	0.117109414	-3.09407104	0.1367637	1
gene38992	8.265118601	0.967923195	0.117109414	-3.09407104	0.1367637	1
gene47895	8.265118601	0.967923195	0.117109414	-3.09407104	0.1367637	1
gene50425	8.265118601	0.967923195	0.117109414	-3.09407104	0.1367637	1
gene53763	8.265118601	0.967923195	0.117109414	-3.09407104	0.1367637	1
gene56885	8.265118601	0.967923195	0.117109414	-3.09407104	0.1367637	1
gene64533	8.265118601	0.967923195	0.117109414	-3.09407104	0.1367637	1
gene66340	8.265118601	0.967923195	0.117109414	-3.09407104	0.1367637	1
gene66720	8.265118601	0.967923195	0.117109414	-3.09407104	0.1367637	1

gene18825	373.9966167	212.9431028	0.569371736	-0.81255722	0.13680526	1
gene51253	584.757141	335.8693485	0.574374086	-0.79993743	0.13693298	1
gene31967	393.6262734	224.5581812	0.570485754	-0.80973724	0.13695883	1
gene28651	147.738995	264.2430321	1.788580139	0.838814761	0.13713527	1
gene62591	246.9204182	431.6937448	1.748311249	0.805962049	0.13715912	1
gene29223	98.14828338	48.39615973	0.493092269	-1.02007046	0.13718084	1
gene65249	2624.175156	5195.811709	1.979979003	0.985485131	0.13726896	1
gene52304	805.8490636	1432.526328	1.777660846	0.829980103	0.13731572	1
gene47073	126.0430587	64.85085404	0.51451349	-0.95871919	0.13734648	1
gene5914	32.02733458	69.69047001	2.175968464	1.121657648	0.13745471	1
gene31031	287.2128714	161.6431735	0.562799197	-0.82930782	0.13749563	1
gene26802	192.1640075	104.535705	0.543992116	-0.87834235	0.1375689	1
gene41808	192.1640075	104.535705	0.543992116	-0.87834235	0.1375689	1
gene13127	2205.753527	4262.733749	1.932552163	0.950507356	0.13772074	1
gene19662	53.7232709	23.23015667	0.43240399	-1.20954826	0.13775543	1
gene21711	53.7232709	23.23015667	0.43240399	-1.20954826	0.13775543	1
gene61494	52.69013108	105.5036282	2.00234135	1.001687939	0.13778478	1
gene18734	345.0687016	600.1123807	1.739109858	0.798349069	0.13779898	1
gene1162	224.191342	123.8941689	0.552626911	-0.85562228	0.13789445	1
gene40801	29.96105493	10.64715514	0.355366497	-1.49262042	0.13792216	1
gene13281	140.5070162	73.56216279	0.523547968	-0.93360637	0.13806079	1
gene32094	129.1424781	66.78670043	0.517155172	-0.95133087	0.13812922	1
gene2392	109.5128215	200.3601013	1.82955839	0.87149546	0.138184	1
gene404	30.99419475	67.75462362	2.186042392	1.128321378	0.13827295	1
gene19522	30.99419475	67.75462362	2.186042392	1.128321378	0.13827295	1
gene26443	30.99419475	67.75462362	2.186042392	1.128321378	0.13827295	1
gene50007	675.6734456	1189.577606	1.760580668	0.816051332	0.13832085	1
gene12843	100.214563	184.8733302	1.844775096	0.883444942	0.1384261	1
gene31235	100.214563	184.8733302	1.844775096	0.883444942	0.1384261	1
gene45891	586.8234206	337.8051949	0.575650499	-0.79673494	0.1384862	1
gene37626	354.36696	615.5991518	1.737179876	0.796747146	0.13856197	1
gene38086	39.25931335	82.27347154	2.095642142	1.067392379	0.138584	1
gene1623	5.165699125	0	0	#NAME?	0.13865732	1
gene1829	5.165699125	0	0	#NAME?	0.13865732	1
gene6831	5.165699125	0	0	#NAME?	0.13865732	1
gene11277	5.165699125	0	0	#NAME?	0.13865732	1
gene13584	5.165699125	0	0	#NAME?	0.13865732	1
gene19405	5.165699125	0	0	#NAME?	0.13865732	1
gene19515	5.165699125	0	0	#NAME?	0.13865732	1
gene21186	5.165699125	0	0	#NAME?	0.13865732	1
gene22838	5.165699125	0	0	#NAME?	0.13865732	1
gene23482	5.165699125	0	0	#NAME?	0.13865732	1
gene24538	5.165699125	0	0	#NAME?	0.13865732	1
gene26528	5.165699125	0	0	#NAME?	0.13865732	1
gene27133	5.165699125	0	0	#NAME?	0.13865732	1
gene30886	5.165699125	0	0	#NAME?	0.13865732	1
gene32077	5.165699125	0	0	#NAME?	0.13865732	1
gene33022	5.165699125	0	0	#NAME?	0.13865732	1

gene33486	5.165699125	0	0	#NAME?	0.13865732	1
gene34307	5.165699125	0	0	#NAME?	0.13865732	1
gene35363	5.165699125	0	0	#NAME?	0.13865732	1
gene41484	5.165699125	0	0	#NAME?	0.13865732	1
gene43087	5.165699125	0	0	#NAME?	0.13865732	1
gene49308	5.165699125	0	0	#NAME?	0.13865732	1
gene50260	5.165699125	0	0	#NAME?	0.13865732	1
gene50561	5.165699125	0	0	#NAME?	0.13865732	1
gene54117	5.165699125	0	0	#NAME?	0.13865732	1
gene56831	5.165699125	0	0	#NAME?	0.13865732	1
gene58257	5.165699125	0	0	#NAME?	0.13865732	1
gene61013	5.165699125	0	0	#NAME?	0.13865732	1
gene65716	5.165699125	0	0	#NAME?	0.13865732	1
gene67830	5.165699125	0	0	#NAME?	0.13865732	1
gene68480	5.165699125	0	0	#NAME?	0.13865732	1
gene71351	5.165699125	0	0	#NAME?	0.13865732	1
gene42631	339.9030025	590.4331487	1.737063646	0.796650615	0.13865798	1
gene50351	58.88897003	26.13392626	0.443783042	-1.17207356	0.13867862	1
gene73010	58.88897003	26.13392626	0.443783042	-1.17207356	0.13867862	1
gene73098	58.88897003	26.13392626	0.443783042	-1.17207356	0.13867862	1
gene63384	741.7943944	1310.568006	1.76675372	0.821100946	0.13869152	1
gene19558	39.25931335	15.48677111	0.394473815	-1.34199856	0.13898136	1
gene49138	39.25931335	15.48677111	0.394473815	-1.34199856	0.13898136	1
gene54824	345.0687016	196.4884085	0.569418228	-0.81243942	0.13900606	1
gene11528	245.8872784	428.7899752	1.743847742	0.802274082	0.1390511	1
gene26405	64.05466916	29.03769584	0.453326763	-1.14137676	0.13906605	1
gene2139	29.96105493	65.81877724	2.196811073	1.135410803	0.13909527	1
gene52738	298.5774094	518.8068323	1.737595732	0.797092465	0.13910948	1
gene7843	251.0529775	437.501284	1.742665187	0.801295415	0.13917962	1
gene21243	1234.602091	2248.485581	1.821222884	0.864907493	0.13923928	1
gene37678	76.45234706	145.1884792	1.899071576	0.925294282	0.13925753	1
gene67359	285.1465917	495.5766757	1.737971591	0.7974045	0.13938511	1
gene1100	631.2484331	363.9391212	0.576538653	-0.79451076	0.13953697	1
gene18805	742.8275342	427.822052	0.575937256	-0.79601644	0.13956426	1
gene13779	38.22617353	80.33762515	2.101639211	1.071515022	0.13956975	1
gene29975	38.22617353	80.33762515	2.101639211	1.071515022	0.13956975	1
gene48394	196.2965668	344.5806573	1.755408477	0.811806779	0.13958831	1
gene40701	547.5641073	955.3401931	1.744709305	0.802986682	0.13964913	1
gene29589	327.5053246	568.1709153	1.734844818	0.79480662	0.13967958	1
gene61428	283.0803121	159.7073271	0.564176738	-0.82578091	0.13973285	1
gene8857	138.4407366	247.7883378	1.78985134	0.839839766	0.13984715	1
gene33613	1513.549844	2800.201802	1.850088924	0.887594615	0.13987357	1
gene4629	8.265118601	25.16600306	3.04484476	1.606368675	0.13991599	1
gene38735	8.265118601	25.16600306	3.04484476	1.606368675	0.13991599	1
gene40253	8.265118601	25.16600306	3.04484476	1.606368675	0.13991599	1
gene73373	8.265118601	25.16600306	3.04484476	1.606368675	0.13991599	1
gene2585	28.9279151	63.88293085	2.208348947	1.142968154	0.13991929	1
gene7253	642.6129712	370.7145835	0.576886244	-0.79364123	0.1399936	1

gene11281	27.89477528	9.679231946	0.346990856	-1.52703045	0.14007867	1
gene26354	27.89477528	9.679231946	0.346990856	-1.52703045	0.14007867	1
gene30974	27.89477528	9.679231946	0.346990856	-1.52703045	0.14007867	1
gene68823	27.89477528	9.679231946	0.346990856	-1.52703045	0.14007867	1
gene3680	231.4233208	403.6239722	1.74409377	0.802477608	0.14011629	1
gene60942	401.891392	696.9047001	1.734062272	0.794155708	0.14016422	1
gene10327	236.5890199	412.3352809	1.742833547	0.801434789	0.14018905	1
gene41328	15.49709738	3.871692779	0.249833416	-2.00096164	0.14025748	1
gene49179	15.49709738	3.871692779	0.249833416	-2.00096164	0.14025748	1
gene52320	15.49709738	3.871692779	0.249833416	-2.00096164	0.14025748	1
gene36591	357.4663795	204.2317941	0.571331476	-0.80760008	0.14033068	1
gene14500	82.65118601	39.68485098	0.480148597	-1.05844713	0.1403595	1
gene2730	174.6006304	307.7995759	1.762877804	0.817932476	0.14037349	1
gene40607	2829.769981	5631.377146	1.990047666	0.992802987	0.14046989	1
gene55618	1005.24505	1801.305065	1.791906427	0.841495302	0.14047837	1
gene57073	595.0885392	1039.549511	1.74688209	0.804782233	0.14049505	1
gene50742	337.8367228	192.6167157	0.57014736	-0.81059325	0.14051036	1
gene23588	37.1930337	78.40177877	2.107969449	1.075853958	0.14056218	1
gene47940	79.55176653	150.0280952	1.885917833	0.915266821	0.14070102	1
gene73268	870.9368725	1546.741265	1.775951063	0.828591829	0.14070171	1
gene64460	329.5716042	187.7770998	0.569761161	-0.81157081	0.14080621	1
gene31985	217.9925031	380.3938155	1.744985768	0.80321527	0.14108611	1
gene62168	223.1582022	123.8941689	0.555185369	-0.84895855	0.14113647	1
gene4902	77.48548688	36.7810814	0.474683491	-1.07496222	0.14116387	1
gene19835	255.1855368	143.2526328	0.561366583	-0.83298491	0.14117413	1
gene12437	1384.407366	2535.95877	1.831800981	0.873262768	0.14125315	1
gene21805	43.39187265	89.04893391	2.052203062	1.03717349	0.14125988	1
gene43112	519.669332	300.0561903	0.577398303	-0.79236123	0.14126191	1
gene38607	899.8647876	517.8389091	0.575463021	-0.79720487	0.14136376	1
gene58113	705.6345005	1238.941689	1.755783891	0.812115283	0.1413852	1
gene5007	127.0761985	228.4298739	1.797581897	0.846057501	0.14145435	1
gene30094	1031.073545	1847.765379	1.792079126	0.841634339	0.14146746	1
gene4094	36.15989388	76.46593238	2.114661416	1.080426688	0.1415596	1
gene21322	36.15989388	76.46593238	2.114661416	1.080426688	0.1415596	1
gene16638	26.86163545	60.01123807	2.234087279	1.159685549	0.14156042	1
gene21575	1971.230786	3733.279762	1.893882638	0.921346931	0.14161691	1
gene35537	72.31978776	33.87731181	0.468437655	-1.09407104	0.14173468	1
gene38249	72.31978776	33.87731181	0.468437655	-1.09407104	0.14173468	1
gene2703	148.7721348	264.2430321	1.776159443	0.828761096	0.14174619	1
gene38267	133.2750374	69.69047001	0.52290715	-0.9353733	0.14176785	1
gene73015	135.3413171	241.9807987	1.787929982	0.83829024	0.14180181	1
gene58822	179.7663296	97.76024266	0.543818427	-0.87880306	0.14194756	1
gene8138	25.82849563	8.711308752	0.337275112	-1.56800223	0.14201292	1
gene11989	25.82849563	8.711308752	0.337275112	-1.56800223	0.14201292	1
gene65760	25.82849563	8.711308752	0.337275112	-1.56800223	0.14201292	1
gene44847	214.8930836	119.0545529	0.554017612	-0.85199626	0.14201418	1
gene68802	37.1930337	14.51884792	0.390364713	-1.35710545	0.14204605	1
gene37762	82.65118601	154.8677111	1.873750621	0.905928957	0.14204743	1

gene72579	278.9477528	157.7714807	0.565595095	-0.82215849	0.14205986	1
gene16442	938.0909612	1669.667511	1.77985673	0.831761116	0.14209645	1
gene38198	190.0977278	332.965579	1.751549494	0.808631755	0.14214347	1
gene69054	1368.910268	777.2423253	0.567781792	-0.81659151	0.1422116	1
gene10618	386.3942946	667.8670043	1.728459798	0.789487048	0.14229881	1
gene28078	42.35873283	87.11308752	2.05655556	1.040230048	0.14235854	1
gene39547	25.82849563	58.07539168	2.248500746	1.168963362	0.14236989	1
gene8526	58.88897003	115.1828602	1.955932666	0.967856706	0.14243832	1
gene44773	53.7232709	106.4715514	1.981851619	0.986848952	0.14246495	1
gene42553	174.6006304	306.8316527	1.757334163	0.813388551	0.14264627	1
gene69562	695.3031023	1217.647379	1.751246866	0.808382469	0.14272866	1
gene71649	183.8988889	322.3184238	1.752693699	0.809573893	0.14273811	1
gene48745	9.298258426	27.10184945	2.914723189	1.543358877	0.14276439	1
gene59441	9.298258426	27.10184945	2.914723189	1.543358877	0.14276439	1
gene70273	9.298258426	27.10184945	2.914723189	1.543358877	0.14276439	1
gene69269	628.1490136	1095.689056	1.744313901	0.802659686	0.14277492	1
gene2260	706.6676404	1237.973766	1.751847255	0.808876991	0.14296127	1
gene23239	949.4554992	546.876605	0.575989718	-0.79588504	0.14297289	1
gene35391	832.710699	481.0578277	0.577701029	-0.79160503	0.14300547	1
gene28622	171.501211	92.92062669	0.541807409	-0.88414797	0.14303676	1
gene4333	464.9129213	804.3441747	1.730096407	0.790852432	0.14305154	1
gene4959	103.3139825	188.745023	1.826906856	0.869403081	0.14307259	1
gene52899	490.7414169	849.8365649	1.731740048	0.792222383	0.14307695	1
gene46275	202.4954057	353.291966	1.744691267	0.802971766	0.14312589	1
gene3017	150.8384145	267.1468017	1.771079355	0.824628855	0.14315875	1
gene49993	24.7953558	56.13954529	2.264115334	1.178947451	0.14316551	1
gene41854	182.865749	320.3825774	1.752009762	0.809010813	0.14321186	1
gene26389	799.6502246	1408.328248	1.761180332	0.816542638	0.14324673	1
gene32176	1133.354388	649.4764636	0.573056822	-0.8032499	0.14325534	1
gene20213	47.52443195	95.82439627	2.016318604	1.011723621	0.14327676	1
gene62490	63.02152933	121.9583225	1.935185068	0.952471543	0.1432865	1
gene3833	684.971704	396.8485098	0.579364823	-0.787456	0.14330157	1
gene15158	216.9593633	377.4900459	1.739911291	0.799013753	0.14331955	1
gene30325	386.3942946	666.8990811	1.725954784	0.787394669	0.14334544	1
gene31261	262.4175156	148.0922488	0.564338278	-0.82536789	0.14335311	1
gene7834	17.56337703	4.839615973	0.275551562	-1.85960579	0.14349811	1
gene23140	17.56337703	4.839615973	0.275551562	-1.85960579	0.14349811	1
gene45043	17.56337703	4.839615973	0.275551562	-1.85960579	0.14349811	1
gene64213	17.56337703	4.839615973	0.275551562	-1.85960579	0.14349811	1
gene22647	89.88316478	166.4827895	1.852213258	0.889250216	0.1435184	1
gene28195	34.09361423	72.5942396	2.12926207	1.090353528	0.14356127	1
gene40313	34.09361423	72.5942396	2.12926207	1.090353528	0.14356127	1
gene67879	34.09361423	72.5942396	2.12926207	1.090353528	0.14356127	1
gene30692	23.76221598	7.743385557	0.325869673	-1.617633	0.14358805	1
gene51054	23.76221598	7.743385557	0.325869673	-1.617633	0.14358805	1
gene70904	23.76221598	7.743385557	0.325869673	-1.617633	0.14358805	1
gene20263	226.2576217	392.976817	1.736855599	0.796477814	0.1436244	1
gene62248	967.0188763	1719.999517	1.778661781	0.830792203	0.14374812	1

gene70142	128.1093383	229.3977971	1.790640715	0.840475895	0.14375518	1
gene40326	210.7605243	366.8428908	1.740567366	0.799557653	0.14377565	1
gene14054	88.85002496	43.55654376	0.490225453	-1.0284827	0.14380749	1
gene12419	67.15408863	128.7337849	1.91699102	0.938843579	0.14393865	1
gene31464	67.15408863	128.7337849	1.91699102	0.938843579	0.14393865	1
gene56559	23.76221598	54.2036989	2.281087713	1.189721923	0.14394119	1
gene58714	23.76221598	54.2036989	2.281087713	1.189721923	0.14394119	1
gene44910	102.2808427	186.8091766	1.826433687	0.869029374	0.14394896	1
gene28561	180.7994694	316.5108846	1.750618438	0.80786467	0.14418318	1
gene61391	180.7994694	316.5108846	1.750618438	0.80786467	0.14418318	1
gene17155	3368.03583	6848.056602	2.033249332	1.02378714	0.1442176	1
gene2075	5.165699125	18.3905407	3.560126181	1.831928375	0.14425672	1
gene4637	5.165699125	18.3905407	3.560126181	1.831928375	0.14425672	1
gene9906	5.165699125	18.3905407	3.560126181	1.831928375	0.14425672	1
gene16197	5.165699125	18.3905407	3.560126181	1.831928375	0.14425672	1
gene19707	5.165699125	18.3905407	3.560126181	1.831928375	0.14425672	1
gene26830	5.165699125	18.3905407	3.560126181	1.831928375	0.14425672	1
gene26862	5.165699125	18.3905407	3.560126181	1.831928375	0.14425672	1
gene41210	5.165699125	18.3905407	3.560126181	1.831928375	0.14425672	1
gene48849	5.165699125	18.3905407	3.560126181	1.831928375	0.14425672	1
gene51389	5.165699125	18.3905407	3.560126181	1.831928375	0.14425672	1
gene9286	994.9136516	1771.299446	1.780354952	0.832164902	0.14427653	1
gene12786	122.9436392	220.6864884	1.795021604	0.844001208	0.14440362	1
gene13921	199.3959862	347.4844269	1.742685163	0.801311953	0.14440892	1
gene38624	1820.392372	1021.15897	0.560955422	-0.83404197	0.14445128	1
gene6364	359.5326591	619.4708446	1.722989077	0.784913556	0.14445964	1
gene663	33.0604744	70.65839321	2.137246803	1.095753516	0.14457404	1
gene8684	988.7148126	1758.716445	1.778790428	0.830896546	0.14460157	1
gene60387	22.72907615	52.26785251	2.299603035	1.20138484	0.14468954	1
gene31262	517.6030524	895.328955	1.729759805	0.790571719	0.14470882	1
gene69592	225.2244819	125.8300153	0.558687112	-0.83988756	0.14473093	1
gene28086	19.62965668	5.807539168	0.295855361	-1.75703606	0.14473291	1
gene51196	19.62965668	5.807539168	0.295855361	-1.75703606	0.14473291	1
gene33113	827.5449999	1456.724408	1.760296308	0.815818296	0.14477677	1
gene24248	2869.029294	1559.324267	0.543502386	-0.87964173	0.14480997	1
gene43822	10.33139825	29.03769584	2.810625932	1.490891457	0.14481922	1
gene56999	10.33139825	29.03769584	2.810625932	1.490891457	0.14481922	1
gene13946	100.214563	50.33200612	0.502242435	-0.99354417	0.14498975	1
gene10030	109.5128215	198.4242549	1.811881497	0.857488602	0.14499068	1
gene13591	730.4298563	1277.658617	1.749187285	0.806684766	0.14499617	1
gene28470	431.8524469	744.3329367	1.723581612	0.785409613	0.14501645	1
gene70890	974.2508551	562.3633761	0.577226464	-0.79279065	0.14503504	1
gene7317	210.7605243	117.1187066	0.55569565	-0.84763315	0.14518327	1
gene66749	111.5791011	57.10746848	0.511811512	-0.9663155	0.14531396	1
gene57179	613.6850561	1065.683437	1.73653151	0.796208589	0.14534675	1
gene52747	231.4233208	400.7202026	1.731546333	0.792060992	0.14538629	1
gene39909	429.7861672	248.756261	0.578790757	-0.78888621	0.14540798	1
gene6166	3326.710237	6732.873742	2.023883435	1.017126201	0.14542677	1

gene55615	215.9262234	374.5862763	1.734788255	0.794759581	0.14560872	1
gene13445	261.3843757	451.0522087	1.725628043	0.787121527	0.14569362	1
gene34021	42.35873283	17.4226175	0.411311112	-1.28169805	0.14571984	1
gene73649	288.2460112	496.5445988	1.7226417	0.78462266	0.14572248	1
gene13908	104.3471223	189.7129461	1.818094662	0.862427318	0.14581104	1
gene48387	275.8483333	156.8035575	0.5684412	-0.81491697	0.14599422	1
gene9790	20.6627965	48.39615973	2.342188277	1.227857052	0.14606646	1
gene28983	20.6627965	48.39615973	2.342188277	1.227857052	0.14606646	1
gene53434	20.6627965	48.39615973	2.342188277	1.227857052	0.14606646	1
gene57118	159.1035331	279.7298033	1.758162109	0.814068098	0.14607536	1
gene47516	874.036292	1539.965803	1.761901441	0.817133224	0.14615089	1
gene46132	114.6785206	59.04331487	0.514859405	-0.95774957	0.14615488	1
gene963	11.36453808	30.97354223	2.725455449	1.446497338	0.1462577	1
gene6122	11.36453808	30.97354223	2.725455449	1.446497338	0.1462577	1
gene45544	11.36453808	30.97354223	2.725455449	1.446497338	0.1462577	1
gene50861	11.36453808	30.97354223	2.725455449	1.446497338	0.1462577	1
gene1428	439.0844257	254.5638002	0.579760486	-0.78647109	0.14625872	1
gene62043	367.7977777	632.0538461	1.71848196	0.781134707	0.14639832	1
gene15070	233.4896005	403.6239722	1.728659312	0.789653568	0.14643654	1
gene66709	164.2692322	89.04893391	0.542091375	-0.88339204	0.14659486	1
gene46861	365.7314981	211.0072564	0.576945813	-0.79349227	0.14662509	1
gene39764	30.99419475	66.78670043	2.154813215	1.107562818	0.1466519	1
gene44699	30.99419475	66.78670043	2.154813215	1.107562818	0.1466519	1
gene65337	2720.257159	5318.737955	1.95523351	0.967340916	0.14665929	1
gene17135	19.62965668	46.46031334	2.36684289	1.242963944	0.14667084	1
gene519	99.18142321	181.0016374	1.824955032	0.867860916	0.14669358	1
gene31469	123.976779	221.6544116	1.787870385	0.838242149	0.14679269	1
gene68897	1006.27819	581.72184	0.578092466	-0.79062782	0.1468082	1
gene29132	378.129176	649.4764636	1.717604736	0.780398075	0.14682553	1
gene64028	136.3744569	241.9807987	1.774385058	0.827319122	0.14689916	1
gene59838	2988.873514	5924.657874	1.982237738	0.987130002	0.14693999	1
gene9511	106.413402	54.2036989	0.509369101	-0.97321665	0.14706777	1
gene7784	253.1192571	143.2526328	0.565949167	-0.82125562	0.14708067	1
gene35721	946.3560798	1672.57128	1.767380499	0.821612671	0.14713524	1
gene41658	73.35292758	34.84523501	0.475035369	-1.07389316	0.14719492	1
gene45728	18.59651685	44.52446695	2.394236905	1.259565911	0.14719845	1
gene54736	1061.0346	1888.418153	1.779789417	0.831706553	0.14719855	1
gene58571	235.5558801	132.6054777	0.562947007	-0.82892897	0.1472795	1
gene19808	174.6006304	304.8958063	1.746246881	0.804257539	0.14729866	1
gene47421	795.5176653	462.667287	0.581592725	-0.78191887	0.14736576	1
gene61751	64.05466916	122.9262457	1.919083298	0.940417333	0.14740229	1
gene23977	808.948483	470.4106726	0.581508814	-0.78212704	0.14746009	1
gene4395	178.7331897	311.6712687	1.74377948	0.802217607	0.14748428	1
gene7301	85.75060548	158.7394039	1.851175313	0.88844153	0.14753838	1
gene16361	17.56337703	42.58862056	2.424853745	1.277897734	0.14762929	1
gene42600	17.56337703	42.58862056	2.424853745	1.277897734	0.14762929	1
gene63848	400.8582521	232.3015667	0.579510501	-0.78709329	0.14766199	1
gene3713	169.4349313	296.1844976	1.748072226	0.805764795	0.14769169	1

gene18068	319.2402059	547.8445282	1.716088757	0.779124172	0.14774029	1
gene12753	279.9808926	159.7073271	0.570422237	-0.80989787	0.14775697	1
gene5489	13.43081773	34.84523501	2.594423937	1.37541424	0.14778865	1
gene30971	16.5302372	40.65277417	2.459297691	1.298246379	0.14793863	1
gene33398	68.18722846	31.94146542	0.468437655	-1.09407104	0.14795859	1
gene61857	426.6867478	247.7883378	0.580726585	-0.78406901	0.14796853	1
gene5061	1645.791741	3027.663753	1.839639656	0.879423202	0.14797636	1
gene18161	3.099419475	13.55092472	4.372084783	2.128321378	0.14799688	1
gene21042	3.099419475	13.55092472	4.372084783	2.128321378	0.14799688	1
gene22760	3.099419475	13.55092472	4.372084783	2.128321378	0.14799688	1
gene25128	3.099419475	13.55092472	4.372084783	2.128321378	0.14799688	1
gene44233	3.099419475	13.55092472	4.372084783	2.128321378	0.14799688	1
gene48655	3.099419475	13.55092472	4.372084783	2.128321378	0.14799688	1
gene55253	3.099419475	13.55092472	4.372084783	2.128321378	0.14799688	1
gene56142	3.099419475	13.55092472	4.372084783	2.128321378	0.14799688	1
gene59371	3.099419475	13.55092472	4.372084783	2.128321378	0.14799688	1
gene60445	3.099419475	13.55092472	4.372084783	2.128321378	0.14799688	1
gene60659	3.099419475	13.55092472	4.372084783	2.128321378	0.14799688	1
gene61707	3.099419475	13.55092472	4.372084783	2.128321378	0.14799688	1
gene62307	3.099419475	13.55092472	4.372084783	2.128321378	0.14799688	1
gene64571	3.099419475	13.55092472	4.372084783	2.128321378	0.14799688	1
gene65060	3.099419475	13.55092472	4.372084783	2.128321378	0.14799688	1
gene65732	3.099419475	13.55092472	4.372084783	2.128321378	0.14799688	1
gene67751	3.099419475	13.55092472	4.372084783	2.128321378	0.14799688	1
gene68622	3.099419475	13.55092472	4.372084783	2.128321378	0.14799688	1
gene69296	3.099419475	13.55092472	4.372084783	2.128321378	0.14799688	1
gene72523	3.099419475	13.55092472	4.372084783	2.128321378	0.14799688	1
gene72578	3.099419475	13.55092472	4.372084783	2.128321378	0.14799688	1
gene69993	306.842528	526.5502179	1.716027505	0.779072677	0.14802649	1
gene31315	14.46395755	36.7810814	2.542947272	1.346501548	0.1480621	1
gene56433	14.46395755	36.7810814	2.542947272	1.346501548	0.1480621	1
gene70482	14.46395755	36.7810814	2.542947272	1.346501548	0.1480621	1
gene7664	15.49709738	38.71692779	2.498334162	1.320966456	0.14809572	1
gene22172	15.49709738	38.71692779	2.498334162	1.320966456	0.14809572	1
gene26955	15.49709738	38.71692779	2.498334162	1.320966456	0.14809572	1
gene69245	15.49709738	38.71692779	2.498334162	1.320966456	0.14809572	1
gene46267	81.61804618	39.68485098	0.486226427	-1.04029979	0.14809691	1
gene59943	247.953558	140.3488632	0.566028834	-0.82105255	0.14824214	1
gene66261	168.4017915	294.2486512	1.747301193	0.805128316	0.14826756	1
gene50936	347.1349812	200.3601013	0.577182111	-0.79290151	0.14836104	1
gene62226	121.9104994	217.7827188	1.786414787	0.837067099	0.14840342	1
gene32359	33.0604744	12.58300153	0.380605595	-1.39363133	0.14846776	1
gene55105	33.0604744	12.58300153	0.380605595	-1.39363133	0.14846776	1
gene69042	33.0604744	12.58300153	0.380605595	-1.39363133	0.14846776	1
gene73316	33.0604744	12.58300153	0.380605595	-1.39363133	0.14846776	1
gene67719	48.55757178	96.79231946	1.993351725	0.995196295	0.14849111	1
gene17012	150.8384145	265.2109553	1.758245446	0.814136481	0.14849874	1
gene12922	63.02152933	120.9903993	1.919826456	0.940975904	0.1486137	1

gene23946	455.6146629	265.2109553	0.582094864	-0.78067381	0.14863103	1
gene62066	484.542578	832.4139474	1.71793767	0.780677694	0.14868567	1
gene49017	4439.401828	9447.898303	2.12819174	1.089628137	0.14887486	1
gene72062	548.5972471	944.693038	1.722015637	0.784098243	0.14897655	1
gene46708	67.15408863	127.7658617	1.902577554	0.927955263	0.14904646	1
gene46335	137.4075967	242.9487219	1.768087992	0.822190075	0.14911553	1
gene22562	1550.742877	2832.143268	1.826313897	0.868934749	0.14914875	1
gene34135	4891.917072	2537.894616	0.518793467	-0.94676778	0.1491495	1
gene6977	698.4025218	1211.83984	1.735159599	0.795068367	0.14930804	1
gene54137	42.35873283	86.14516432	2.033704943	1.024110383	0.14934965	1
gene6578	76.45234706	36.7810814	0.481098133	-1.0555969	0.14934972	1
gene60427	3608.757409	7350.40874	2.036825396	1.026322313	0.14961552	1
gene21821	79.55176653	148.0922488	1.86158341	0.896530259	0.14964059	1
gene52307	52.69013108	103.5677818	1.965601142	0.974970601	0.14968285	1
gene50801	47.52443195	94.85647307	1.995951749	0.997076845	0.14977923	1
gene54140	104.3471223	53.23577571	0.510179625	-0.97092281	0.14984252	1
gene56415	27.89477528	60.97916126	2.186042392	1.128321378	0.14988115	1
gene37131	238.6552996	410.3994345	1.719632605	0.78210037	0.14991787	1
gene38605	214.8930836	370.7145835	1.72511175	0.78668982	0.14992063	1
gene4566	8828.179805	22829.43647	2.58597321	1.370707329	0.149929	1
gene40416	252.0861173	143.2526328	0.568268631	-0.81535502	0.1501217	1
gene59553	541.3652683	316.5108846	0.584653104	-0.77434722	0.15012341	1
gene22052	695.3031023	406.5277417	0.584677014	-0.77428822	0.15014702	1
gene66523	177.7000499	97.76024266	0.550141898	-0.86212432	0.15015719	1
gene42240	71.28664793	33.87731181	0.475226607	-1.07331248	0.15041599	1
gene20500	302.7099687	517.8389091	1.710676762	0.774567184	0.15048785	1
gene58352	358.4995193	612.6953822	1.709054962	0.773198793	0.15048927	1
gene17858	35.12675405	73.56216279	2.094191871	1.066393629	0.15049164	1
gene11574	241.7547191	415.2390505	1.717604736	0.780398075	0.15055613	1
gene60664	555.8292259	955.3401931	1.718765672	0.781372869	0.15060952	1
gene45027	41.325593	84.20931793	2.037703801	1.026944358	0.15063602	1
gene19530	6.19883895	20.32638709	3.279063588	1.713283879	0.15071489	1
gene29036	6.19883895	20.32638709	3.279063588	1.713283879	0.15071489	1
gene34740	6.19883895	20.32638709	3.279063588	1.713283879	0.15071489	1
gene57238	6.19883895	20.32638709	3.279063588	1.713283879	0.15071489	1
gene45918	160.1366729	87.11308752	0.543992116	-0.87834235	0.15078642	1
gene63836	3483.74749	7031.962009	2.018505081	1.013287218	0.15091207	1
gene6887	320.2733458	184.8733302	0.577236079	-0.79276662	0.15096271	1
gene34012	1057.935181	1871.963458	1.76945005	0.823301036	0.15098879	1
gene56740	361.5989388	617.5349982	1.707789852	0.772130459	0.15104881	1
gene50852	853.3734955	1490.60172	1.746716681	0.804645621	0.15123466	1
gene19021	87.81688513	43.55654376	0.495992812	-1.01160888	0.1512408	1
gene30806	146.7058552	257.4675698	1.754991779	0.811464273	0.15125427	1
gene22997	66.12094881	30.97354223	0.468437655	-1.09407104	0.15131873	1
gene50208	66.12094881	30.97354223	0.468437655	-1.09407104	0.15131873	1
gene63161	66.12094881	30.97354223	0.468437655	-1.09407104	0.15131873	1
gene64135	1250.099188	2235.90258	1.788580139	0.838814761	0.15143926	1
gene44973	1866.883664	1056.004205	0.565650783	-0.82201645	0.15146943	1

gene6176	193.1971473	333.9335022	1.728459798	0.789487048	0.15150144	1
gene45017	98.14828338	178.0978678	1.814579549	0.859635304	0.15156319	1
gene23419	169.4349313	92.92062669	0.548414816	-0.86666055	0.15165102	1
gene38162	784.1531272	458.7955943	0.585084186	-0.77328387	0.1517024	1
gene64931	117.7779401	210.0393332	1.783350372	0.834590175	0.15182466	1
gene69126	149.8052746	262.3071857	1.75098765	0.808168908	0.15193864	1
gene6167	60.95524968	28.06977264	0.460498034	-1.1187331	0.15201883	1
gene58685	323.3727653	551.7162209	1.706130758	0.770728219	0.15206553	1
gene50345	25.82849563	57.10746848	2.211025733	1.144715816	0.15208535	1
gene72029	55.78955055	25.16600306	0.451088113	-1.14851883	0.15239857	1
gene62785	332.6710237	567.2029921	1.704996683	0.769768933	0.15244495	1
gene55701	90.91630461	45.49239015	0.500376586	-0.99891381	0.15255974	1
gene65917	74.38606741	35.8131582	0.481449812	-1.05454268	0.15261345	1
gene57949	102.2808427	52.26785251	0.511022897	-0.96854016	0.15272633	1
gene38565	116.7448002	60.97916126	0.522328713	-0.93697008	0.15291498	1
gene36744	33.0604744	69.69047001	2.107969449	1.075853958	0.15294775	1
gene23337	203.5285455	350.3881965	1.72156783	0.783723024	0.15297789	1
gene30701	4001.350543	8259.28862	2.064125233	1.045530503	0.15299117	1
gene72884	38.22617353	15.48677111	0.405135269	-1.30352441	0.15300706	1
gene5152	76.45234706	142.2847096	1.861090144	0.896147936	0.15315813	1
gene39648	676.7065854	1166.34745	1.723564503	0.785395291	0.15324093	1
gene45220	1876.181922	3465.165037	1.846923795	0.885124342	0.1534342	1
gene42770	915.361885	535.2615266	0.584754003	-0.77409826	0.1535426	1
gene42124	854.4066353	500.4162916	0.585688677	-0.77179409	0.15362325	1
gene10143	140.5070162	75.49800918	0.537325546	-0.89613167	0.15369616	1
gene37061	140.5070162	75.49800918	0.537325546	-0.89613167	0.15369616	1
gene12897	210.7605243	362.0032748	1.717604736	0.780398075	0.15377066	1
gene26054	91.94944443	167.4507127	1.821117177	0.864823753	0.1538456	1
gene20747	87.81688513	160.6752503	1.829662372	0.871577452	0.15402477	1
gene1434	63.02152933	120.0224761	1.904467845	0.929387929	0.15412777	1
gene45492	32.02733458	67.75462362	2.115524895	1.081015663	0.15421845	1
gene29331	1114.757871	1969.723701	1.766952046	0.821262886	0.15427337	1
gene59131	23.76221598	53.23577571	2.240354004	1.163726714	0.15429907	1
gene33420	67.15408863	126.7979385	1.888164088	0.916984145	0.15432523	1
gene49038	138.4407366	242.9487219	1.754893306	0.81138332	0.15434509	1
gene16925	146.7058552	79.36970196	0.541012503	-0.88626616	0.15437922	1
gene33172	85.75060548	42.58862056	0.496656791	-1.00967886	0.15441532	1
gene72515	99.18142321	179.065791	1.805436797	0.852347916	0.15450556	1
gene63526	212.826804	120.0224761	0.563944362	-0.82637526	0.15451749	1
gene35050	1546.610318	2800.201802	1.810541265	0.856421059	0.15454571	1
gene42726	299.6105493	173.2582518	0.578278209	-0.79016436	0.15473446	1
gene68127	299.6105493	173.2582518	0.578278209	-0.79016436	0.15473446	1
gene34164	95.04886391	172.2903286	1.812650058	0.858100432	0.15473588	1
gene15289	208.6942447	358.131582	1.716058737	0.779098934	0.1547392	1
gene33382	2469.204182	1377.354706	0.557813208	-0.842146	0.1547458	1
gene12387	64.05466916	30.00561903	0.468437655	-1.09407104	0.15486173	1
gene20423	64.05466916	30.00561903	0.468437655	-1.09407104	0.15486173	1
gene23063	64.05466916	30.00561903	0.468437655	-1.09407104	0.15486173	1

gene12272	145.6727153	254.5638002	1.747505012	0.805296594	0.154865	1
gene70328	152.9046941	83.24139474	0.544400518	-0.87725965	0.15496908	1
gene43698	468.0123408	274.8901873	0.587356707	-0.76769116	0.15502048	1
gene14457	121.9104994	215.8468724	1.770535545	0.824185807	0.15505982	1
gene24059	111.5791011	58.07539168	0.520486284	-0.94206795	0.15506778	1
gene9635	891.599669	1552.548804	1.741307067	0.800170634	0.15508712	1
gene32408	48.55757178	95.82439627	1.973418208	0.980696725	0.15511835	1
gene37371	57.8558302	111.3111674	1.92394037	0.944064086	0.15513997	1
gene1146	28.9279151	10.64715514	0.368058158	-1.44199435	0.15515872	1
gene16357	28.9279151	10.64715514	0.368058158	-1.44199435	0.15515872	1
gene72107	28.9279151	10.64715514	0.368058158	-1.44199435	0.15515872	1
gene26274	86.78374531	158.7394039	1.829137511	0.871163538	0.15517749	1
gene2619	7.231978776	22.26223348	3.078304592	1.622135991	0.15525116	1
gene2966	7.231978776	22.26223348	3.078304592	1.622135991	0.15525116	1
gene12743	7.231978776	22.26223348	3.078304592	1.622135991	0.15525116	1
gene37656	7.231978776	22.26223348	3.078304592	1.622135991	0.15525116	1
gene56413	7.231978776	22.26223348	3.078304592	1.622135991	0.15525116	1
gene71725	7.231978776	22.26223348	3.078304592	1.622135991	0.15525116	1
gene7513	228.3239013	129.7017081	0.568060143	-0.81588441	0.15531863	1
gene2842	403.9576716	686.257545	1.698835282	0.764545977	0.15536147	1
gene44145	10511.16458	28727.96042	2.733090154	1.450533049	0.15537747	1
gene12888	22.72907615	51.29992932	2.257017794	1.174417793	0.15539655	1
gene26144	22.72907615	51.29992932	2.257017794	1.174417793	0.15539655	1
gene8915	868.8705929	1509.960184	1.737842431	0.79729728	0.1555159	1
gene56175	30.99419475	65.81877724	2.123584038	1.086501202	0.15551845	1
gene8259	996.9799312	1746.133443	1.751422861	0.808527448	0.15552305	1
gene44003	113.6453808	202.2959477	1.78006309	0.831928375	0.15553604	1
gene61180	113.6453808	202.2959477	1.78006309	0.831928375	0.15553604	1
gene6514	198.3628464	111.3111674	0.561149275	-0.83354349	0.15568558	1
gene38087	1588.969051	2878.603581	1.811617148	0.857278101	0.15568898	1
gene51771	109.5128215	195.5204853	1.785366158	0.836219985	0.15580019	1
gene56927	109.5128215	195.5204853	1.785366158	0.836219985	0.15580019	1
gene23388	419.454769	712.3914713	1.698374948	0.764154996	0.15580082	1
gene1474	260.3512359	443.3088231	1.7027337	0.767852821	0.15592493	1
gene23855	120.8773595	213.911026	1.769653365	0.823466796	0.15597329	1
gene29508	204.5616854	115.1828602	0.563071525	-0.8286099	0.15599486	1
gene7092	72.31978776	34.84523501	0.481821588	-1.05342906	0.15602514	1
gene33464	880.235131	516.8709859	0.587196498	-0.76808473	0.15607864	1
gene10388	293.4117103	498.4804452	1.698911215	0.764610459	0.15609074	1
gene46177	48.55757178	21.29431028	0.438537379	-1.18922828	0.15617865	1
gene38642	276.8814731	159.7073271	0.576807561	-0.79383802	0.1562213	1
gene54748	80.58490636	39.68485098	0.492460099	-1.02192126	0.15623903	1
gene14708	466.9792009	793.6970196	1.69964105	0.765230094	0.15624491	1
gene22449	10.33139825	1.935846389	0.187375062	-2.41599914	0.15630164	1
gene23232	10.33139825	1.935846389	0.187375062	-2.41599914	0.15630164	1
gene26176	10.33139825	1.935846389	0.187375062	-2.41599914	0.15630164	1
gene34412	10.33139825	1.935846389	0.187375062	-2.41599914	0.15630164	1
gene35689	10.33139825	1.935846389	0.187375062	-2.41599914	0.15630164	1

gene46209	10.33139825	1.935846389	0.187375062	-2.41599914	0.15630164	1
gene58156	10.33139825	1.935846389	0.187375062	-2.41599914	0.15630164	1
gene73452	10.33139825	1.935846389	0.187375062	-2.41599914	0.15630164	1
gene74066	10.33139825	1.935846389	0.187375062	-2.41599914	0.15630164	1
gene64860	1197.409057	2119.751796	1.77028208	0.82397926	0.15630357	1
gene15549	609.5524968	1042.453281	1.710194423	0.774160347	0.15630563	1
gene48724	398.7919725	676.5783131	1.696569539	0.762620565	0.15632723	1
gene8126	21.69593633	49.36408293	2.275268612	1.186036876	0.15647829	1
gene8288	306.842528	178.0978678	0.580421068	-0.78482821	0.1565433	1
gene59703	56.82269038	109.375321	1.924852911	0.944748206	0.15654646	1
gene25527	216.9593633	122.9262457	0.566586497	-0.81963187	0.15657459	1
gene16174	123.976779	218.750642	1.764448502	0.819217323	0.15663541	1
gene36716	177.7000499	98.72816585	0.555588847	-0.84791046	0.15669324	1
gene45658	677.7397253	399.7522794	0.58983156	-0.76162508	0.15674669	1
gene3383	91.94944443	46.46031334	0.505281066	-0.98484197	0.15689571	1
gene55768	415.3222097	243.916645	0.587294971	-0.76784281	0.15690757	1
gene25070	36.15989388	14.51884792	0.40151799	-1.31646346	0.15692669	1
gene7441	234.5227403	399.7522794	1.704535257	0.769378441	0.15709391	1
gene7557	354.36696	207.1355637	0.584522789	-0.77466882	0.1571124	1
gene19512	354.36696	207.1355637	0.584522789	-0.77466882	0.1571124	1
gene51225	88.85002496	161.6431735	1.819281127	0.863368494	0.15727426	1
gene55350	67.15408863	31.94146542	0.475644389	-1.07204474	0.15737536	1
gene36687	20.6627965	47.42823654	2.295344511	1.198710706	0.15753498	1
gene71488	20.6627965	47.42823654	2.295344511	1.198710706	0.15753498	1
gene50986	196.2965668	110.3432442	0.562125186	-0.83103664	0.15759596	1
gene57866	274.8151935	158.7394039	0.577622372	-0.79180147	0.15759911	1
gene36066	149.8052746	260.3713394	1.738065232	0.797482229	0.15762567	1
gene27096	80.58490636	148.0922488	1.837716956	0.87791458	0.15783706	1
gene47081	373.9966167	633.0217693	1.692586887	0.759229895	0.15795687	1
gene39346	430.8193071	253.595877	0.588636286	-0.76455162	0.15797752	1
gene9588	75.41920723	36.7810814	0.487688518	-1.03596809	0.1579866	1
gene36706	55.78955055	107.4394746	1.92579925	0.945457321	0.15798936	1
gene46888	55.78955055	107.4394746	1.92579925	0.945457321	0.15798936	1
gene51633	407.0570911	689.1613146	1.693033556	0.759610568	0.15805142	1
gene54166	400.8582521	678.5141594	1.69265359	0.759286748	0.15814688	1
gene66227	163.2360924	90.0168571	0.551451923	-0.85869298	0.15817036	1
gene35452	72.31978776	134.5413241	1.860366688	0.895587012	0.1582653	1
gene19793	533.1001497	905.9761102	1.699448238	0.765066421	0.15830166	1
gene66259	355.4000998	208.1034868	0.585547069	-0.77214295	0.15835936	1
gene2239	8.265118601	24.19807987	2.927735346	1.549785146	0.15838715	1
gene2270	8.265118601	24.19807987	2.927735346	1.549785146	0.15838715	1
gene47141	8.265118601	24.19807987	2.927735346	1.549785146	0.15838715	1
gene62348	8.265118601	24.19807987	2.927735346	1.549785146	0.15838715	1
gene9755	26.86163545	9.679231946	0.360336658	-1.47258267	0.15851403	1
gene55873	26.86163545	9.679231946	0.360336658	-1.47258267	0.15851403	1
gene73827	26.86163545	9.679231946	0.360336658	-1.47258267	0.15851403	1
gene36549	1320.352696	2346.245824	1.776984157	0.829430819	0.15853672	1
gene25638	19.62965668	45.49239015	2.317533663	1.212590295	0.15855504	1

gene37766	19.62965668	45.49239015	2.317533663	1.212590295	0.15855504	1
gene62253	61.9883895	29.03769584	0.468437655	-1.09407104	0.1586024	1
gene48143	9013.111834	22882.67224	2.538820406	1.344158343	0.15866641	1
gene3769	411.1896504	241.9807987	0.588489517	-0.76491138	0.15867012	1
gene43494	83.68432583	152.9318648	1.827485174	0.869859702	0.15880274	1
gene42641	98.14828338	50.33200612	0.51281596	-0.96348693	0.15884597	1
gene48640	50.62385143	98.72816585	1.950230239	0.963644455	0.15893099	1
gene53480	95.04886391	171.3224055	1.80246663	0.849972551	0.15893301	1
gene47436	86.78374531	43.55654376	0.501897488	-0.99453537	0.15903754	1
gene20711	1040.371804	1817.75976	1.747221285	0.805062336	0.1590451	1
gene18132	1947.46857	1110.207904	0.570077444	-0.81077018	0.15909831	1
gene16932	113.6453808	201.3280245	1.771546042	0.825008961	0.1591867	1
gene34486	41.325593	17.4226175	0.42159389	-1.24607414	0.15940623	1
gene13904	8008.899924	19462.03167	2.430050551	1.280986326	0.15943645	1
gene52258	452.5152434	267.1468017	0.590359785	-0.76033365	0.15943919	1
gene30380	199.3959862	340.7089645	1.70870523	0.772903538	0.15944737	1
gene68026	423.5873283	249.7241842	0.589545927	-0.76232389	0.15948163	1
gene4127	18.59651685	43.55654376	2.342188277	1.227857052	0.15952401	1
gene12859	154.9709738	268.1147249	1.730096407	0.790852432	0.1595512	1
gene4122	27.89477528	60.01123807	2.151343306	1.105237765	0.15959019	1
gene26938	27.89477528	60.01123807	2.151343306	1.105237765	0.15959019	1
gene26114	248.9866978	143.2526328	0.575342514	-0.79750701	0.15960813	1
gene29222	56.82269038	26.13392626	0.459920607	-1.12054325	0.15961159	1
gene21773	40.29245318	81.30554835	2.017885285	1.012844161	0.15963887	1
gene55107	101.2477029	52.26785251	0.516237416	-0.95389339	0.1596598	1
gene57222	78.51862671	38.71692779	0.493092269	-1.02007046	0.1596965	1
gene50815	63.02152933	119.0545529	1.889109233	0.917706124	0.15983446	1
gene1422	151.8715543	83.24139474	0.548103923	-0.86747863	0.15996046	1
gene73579	105.3802622	187.7770998	1.781900101	0.833416457	0.15997512	1
gene24585	4.1325593	15.48677111	3.747501243	1.905928957	0.16002125	1
gene26633	4.1325593	15.48677111	3.747501243	1.905928957	0.16002125	1
gene27121	4.1325593	15.48677111	3.747501243	1.905928957	0.16002125	1
gene27895	4.1325593	15.48677111	3.747501243	1.905928957	0.16002125	1
gene30342	4.1325593	15.48677111	3.747501243	1.905928957	0.16002125	1
gene42397	4.1325593	15.48677111	3.747501243	1.905928957	0.16002125	1
gene43887	4.1325593	15.48677111	3.747501243	1.905928957	0.16002125	1
gene45128	4.1325593	15.48677111	3.747501243	1.905928957	0.16002125	1
gene51141	4.1325593	15.48677111	3.747501243	1.905928957	0.16002125	1
gene52164	4.1325593	15.48677111	3.747501243	1.905928957	0.16002125	1
gene65735	4.1325593	15.48677111	3.747501243	1.905928957	0.16002125	1
gene70635	4.1325593	15.48677111	3.747501243	1.905928957	0.16002125	1
gene36078	903.9973469	1564.163883	1.73027486	0.791001233	0.16020563	1
gene48373	51.65699125	23.23015667	0.449700149	-1.15296473	0.16025742	1
gene65770	386.3942946	227.4619507	0.588678337	-0.76444856	0.16028409	1
gene47597	607.4862171	360.0674284	0.592717033	-0.75458458	0.16033069	1
gene55882	248.9866978	422.0145129	1.694927948	0.761223945	0.16034443	1
gene44176	169.4349313	291.3448816	1.719508954	0.781996629	0.16035811	1
gene35704	3115.949712	6065.006738	1.946439223	0.960837298	0.16035828	1

gene71484	1634.427203	942.7571916	0.57681198	-0.79382697	0.16042177	1
gene17272	1003.17877	590.4331487	0.588562245	-0.7647331	0.16044269	1
gene53122	876.1025717	1512.863953	1.726811451	0.788110565	0.16044317	1
gene51436	161.1698127	89.04893391	0.552516209	-0.85591131	0.16047362	1
gene5172	9.298258426	26.13392626	2.810625932	1.490891457	0.16048705	1
gene55126	9.298258426	26.13392626	2.810625932	1.490891457	0.16048705	1
gene6321	2841.134519	1580.618577	0.556333594	-0.84597787	0.1605125	1
gene71920	915.361885	540.1011426	0.59004111	-0.76111262	0.16055415	1
gene7429	201.4622659	343.6127341	1.705593514	0.770273857	0.16057965	1
gene15205	293.4117103	495.5766757	1.689014645	0.756181837	0.16070501	1
gene62282	1185.011379	2082.970715	1.757764314	0.813741643	0.16073291	1
gene28359	74.38606741	137.4450936	1.847726307	0.885751075	0.16074568	1
gene2470	115.7116604	204.2317941	1.765006166	0.819673223	0.16083034	1
gene38721	357.4663795	210.0393332	0.587577868	-0.76714804	0.16085043	1
gene73330	170.4680711	94.85647307	0.556447154	-0.84568341	0.1608786	1
gene4979	488.6751373	289.4090352	0.592231962	-0.75576574	0.16091259	1
gene10480	431.8524469	728.8461656	1.687720356	0.755075879	0.16092176	1
gene40741	387.4274344	653.3481564	1.686375559	0.753925863	0.16093318	1
gene56550	540.3321285	320.3825774	0.592936382	-0.75405077	0.16099041	1
gene27635	26.86163545	58.07539168	2.162019948	1.112379834	0.16100176	1
gene9331	70.25350811	130.6696313	1.859973043	0.895281712	0.1610162	1
gene6940	1094.095075	642.7010012	0.587427013	-0.76751849	0.161019	1
gene27432	34.09361423	13.55092472	0.397462253	-1.33111024	0.16103747	1
gene47698	2793.610087	1557.38842	0.557482387	-0.84300187	0.16104487	1
gene55446	3654.215561	7296.205041	1.996654253	0.997584533	0.16104604	1
gene22797	141.540156	245.8524914	1.736980503	0.79658156	0.1610902	1
gene64429	44.42501248	88.08101071	1.982689611	0.987458842	0.1611041	1
gene70593	1322.418976	2341.406208	1.77054795	0.824195916	0.16110888	1
gene33355	81.61804618	40.65277417	0.498085608	-1.00553437	0.16118809	1
gene33723	81.61804618	40.65277417	0.498085608	-1.00553437	0.16118809	1
gene21795	320.2733458	540.1011426	1.686375559	0.753925863	0.16121914	1
gene31161	16.5302372	39.68485098	2.400742984	1.263480961	0.16123165	1
gene29045	182.865749	102.5998586	0.56106657	-0.83375614	0.16130446	1
gene15116	366.7646379	215.8468724	0.58851604	-0.76484636	0.16138212	1
gene68684	557.8955055	331.0297326	0.593354363	-0.75303413	0.16138251	1
gene39432	300.6436891	507.191754	1.68701946	0.754476615	0.16140699	1
gene49961	256.2186766	148.0922488	0.577991623	-0.79087951	0.16149023	1
gene55143	1834.856329	1053.100436	0.573941632	-0.80102407	0.16153165	1
gene67060	192.1640075	108.4073978	0.564139972	-0.82587493	0.16156815	1
gene26078	3232.694513	1781.946601	0.551226413	-0.85928308	0.16158474	1
gene23353	110.5459613	58.07539168	0.525350642	-0.92864743	0.16159356	1
gene41816	144.6395755	250.6921074	1.733219325	0.793454227	0.16163387	1
gene30012	24.7953558	8.711308752	0.351328242	-1.50910854	0.16178327	1
gene52941	24.7953558	8.711308752	0.351328242	-1.50910854	0.16178327	1
gene62129	24.7953558	8.711308752	0.351328242	-1.50910854	0.16178327	1
gene68343	24.7953558	8.711308752	0.351328242	-1.50910854	0.16178327	1
gene3498	10.33139825	28.06977264	2.716938401	1.441981857	0.16181039	1
gene20510	10.33139825	28.06977264	2.716938401	1.441981857	0.16181039	1

gene20549	10.33139825	28.06977264	2.716938401	1.441981857	0.16181039	1
gene20900	10.33139825	28.06977264	2.716938401	1.441981857	0.16181039	1
gene21431	10.33139825	28.06977264	2.716938401	1.441981857	0.16181039	1
gene30511	10.33139825	28.06977264	2.716938401	1.441981857	0.16181039	1
gene40513	10.33139825	28.06977264	2.716938401	1.441981857	0.16181039	1
gene1021	2.06627965	10.64715514	5.152814209	2.365360575	0.161845	1
gene1074	2.06627965	10.64715514	5.152814209	2.365360575	0.161845	1
gene3723	2.06627965	10.64715514	5.152814209	2.365360575	0.161845	1
gene3878	2.06627965	10.64715514	5.152814209	2.365360575	0.161845	1
gene4409	2.06627965	10.64715514	5.152814209	2.365360575	0.161845	1
gene4768	2.06627965	10.64715514	5.152814209	2.365360575	0.161845	1
gene8218	2.06627965	10.64715514	5.152814209	2.365360575	0.161845	1
gene9592	2.06627965	10.64715514	5.152814209	2.365360575	0.161845	1
gene14415	2.06627965	10.64715514	5.152814209	2.365360575	0.161845	1
gene16423	2.06627965	10.64715514	5.152814209	2.365360575	0.161845	1
gene16551	2.06627965	10.64715514	5.152814209	2.365360575	0.161845	1
gene27340	2.06627965	10.64715514	5.152814209	2.365360575	0.161845	1
gene30706	2.06627965	10.64715514	5.152814209	2.365360575	0.161845	1
gene32385	2.06627965	10.64715514	5.152814209	2.365360575	0.161845	1
gene38797	2.06627965	10.64715514	5.152814209	2.365360575	0.161845	1
gene39560	2.06627965	10.64715514	5.152814209	2.365360575	0.161845	1
gene40091	2.06627965	10.64715514	5.152814209	2.365360575	0.161845	1
gene41515	2.06627965	10.64715514	5.152814209	2.365360575	0.161845	1
gene41598	2.06627965	10.64715514	5.152814209	2.365360575	0.161845	1
gene41661	2.06627965	10.64715514	5.152814209	2.365360575	0.161845	1
gene42512	2.06627965	10.64715514	5.152814209	2.365360575	0.161845	1
gene51381	2.06627965	10.64715514	5.152814209	2.365360575	0.161845	1
gene56044	2.06627965	10.64715514	5.152814209	2.365360575	0.161845	1
gene61677	2.06627965	10.64715514	5.152814209	2.365360575	0.161845	1
gene61715	2.06627965	10.64715514	5.152814209	2.365360575	0.161845	1
gene62883	2.06627965	10.64715514	5.152814209	2.365360575	0.161845	1
gene63408	2.06627965	10.64715514	5.152814209	2.365360575	0.161845	1
gene66750	2.06627965	10.64715514	5.152814209	2.365360575	0.161845	1
gene25984	114.6785206	202.2959477	1.764026486	0.818872222	0.16186334	1
gene18899	140.5070162	76.46593238	0.544214335	-0.87775314	0.16187587	1
gene18646	232.4564606	393.9447402	1.69470334	0.76103275	0.16189055	1
gene51641	15.49709738	37.74900459	2.435875808	1.28444058	0.1619188	1
gene61452	15.49709738	37.74900459	2.435875808	1.28444058	0.1619188	1
gene69221	48.55757178	94.85647307	1.95348469	0.966049949	0.16202474	1
gene15623	143.6064357	78.40177877	0.545948922	-0.87316211	0.16208197	1
gene45832	96.08200373	49.36408293	0.513770332	-0.96080451	0.16209539	1
gene52752	113.6453808	60.01123807	0.528056993	-0.92123445	0.16209927	1
gene59801	113.6453808	60.01123807	0.528056993	-0.92123445	0.16209927	1
gene5552	361.5989388	212.9431028	0.588893052	-0.76392244	0.16226316	1
gene13947	314.0745068	183.905407	0.585547069	-0.77214295	0.1623151	1
gene34526	25.82849563	56.13954529	2.173550721	1.120053762	0.16243795	1
gene60372	69.22036828	128.7337849	1.859767408	0.895122202	0.16244501	1
gene7572	14.46395755	35.8131582	2.476027607	1.3080274	0.1624485	1

gene32602	14.46395755	35.8131582	2.476027607	1.3080274	0.1624485	1
gene41178	14.46395755	35.8131582	2.476027607	1.3080274	0.1624485	1
gene25584	11.36453808	30.00561903	2.640284967	1.400693648	0.16254559	1
gene34769	11.36453808	30.00561903	2.640284967	1.400693648	0.16254559	1
gene37770	11.36453808	30.00561903	2.640284967	1.400693648	0.16254559	1
gene67011	11.36453808	30.00561903	2.640284967	1.400693648	0.16254559	1
gene68785	11.36453808	30.00561903	2.640284967	1.400693648	0.16254559	1
gene5651	59.92210985	28.06977264	0.468437655	-1.09407104	0.16255722	1
gene54062	38.22617353	77.43385557	2.025676347	1.018403686	0.16274027	1
gene48221	13.43081773	33.87731181	2.522356606	1.334772255	0.16277306	1
gene54476	244.8541385	141.3167864	0.577146816	-0.79298973	0.16281701	1
gene975	12.3976779	31.94146542	2.576407104	1.365360575	0.16283176	1
gene5610	12.3976779	31.94146542	2.576407104	1.365360575	0.16283176	1
gene35764	12.3976779	31.94146542	2.576407104	1.365360575	0.16283176	1
gene44501	12.3976779	31.94146542	2.576407104	1.365360575	0.16283176	1
gene49354	12.3976779	31.94146542	2.576407104	1.365360575	0.16283176	1
gene53126	12.3976779	31.94146542	2.576407104	1.365360575	0.16283176	1
gene32305	238.6552996	403.6239722	1.691242444	0.758083489	0.16295378	1
gene28081	162.2029525	90.0168571	0.554964356	-0.84953298	0.16295851	1
gene11168	291.3454307	490.7370597	1.684382208	0.752219541	0.16298732	1
gene29600	403.9576716	679.4820826	1.682062578	0.75023138	0.16314134	1
gene71456	122.9436392	65.81877724	0.53535732	-0.90142597	0.16329922	1
gene70973	76.45234706	37.74900459	0.49375861	-1.01812219	0.16331215	1
gene60978	174.6006304	97.76024266	0.55990773	-0.836739	0.16331712	1
gene29220	102.2808427	181.9695606	1.779116752	0.831161188	0.16335291	1
gene73728	102.2808427	181.9695606	1.779116752	0.831161188	0.16335291	1
gene5483	177.7000499	99.69608905	0.561035797	-0.83383527	0.1633894	1
gene72636	180.7994694	101.6319354	0.562125186	-0.83103664	0.16345648	1
gene6432	126.0430587	67.75462362	0.537551408	-0.89552536	0.16361197	1
gene33830	87.81688513	44.52446695	0.507014874	-0.97990002	0.16362573	1
gene11222	928.7927027	1601.912887	1.724725961	0.786367153	0.16365074	1
gene39119	315.1076466	184.8733302	0.586698965	-0.76930765	0.16370796	1
gene51184	157.0372534	270.0505713	1.719659287	0.782122755	0.16372176	1
gene20505	196.2965668	111.3111674	0.567056109	-0.8184366	0.16373725	1
gene22549	54.75641073	25.16600306	0.459599209	-1.12155178	0.16374625	1
gene70528	54.75641073	25.16600306	0.459599209	-1.12155178	0.16374625	1
gene38128	39.25931335	16.45469431	0.419128428	-1.25453572	0.16377439	1
gene67675	606.4530773	361.0353516	0.595322812	-0.74825592	0.16383762	1
gene69205	24.7953558	54.2036989	2.186042392	1.128321378	0.16389634	1
gene69859	208.6942447	119.0545529	0.57047358	-0.80976802	0.16392763	1
gene41294	1459.826573	850.8044881	0.58281203	-0.77889744	0.16400006	1
gene26950	514.5036329	867.2591824	1.685623049	0.753281947	0.16405045	1
gene61238	1414.368421	825.638485	0.58375065	-0.77657584	0.16409595	1
gene10208	135.3413171	73.56216279	0.543530715	-0.87956653	0.16435014	1
gene41453	922.5938638	546.876605	0.592759855	-0.75448035	0.16437718	1
gene65152	357.4663795	600.1123807	1.678793909	0.747425134	0.16444994	1
gene32189	49.5907116	22.26223348	0.44891942	-1.15547159	0.16454694	1
gene62781	475.2443195	282.6335728	0.594712154	-0.74973654	0.16456772	1

gene57187	44.42501248	19.35846389	0.435755958	-1.1984077	0.16469916	1
gene61675	44.42501248	19.35846389	0.435755958	-1.1984077	0.16469916	1
gene10639	1528.013801	2721.800023	1.781266649	0.832903499	0.16474226	1
gene13709	79.55176653	39.68485098	0.498855685	-1.00330558	0.16480464	1
gene36679	22.72907615	7.743385557	0.340681931	-1.55350266	0.16484721	1
gene53778	22.72907615	7.743385557	0.340681931	-1.55350266	0.16484721	1
gene58492	22.72907615	7.743385557	0.340681931	-1.55350266	0.16484721	1
gene37771	1342.048633	2365.604288	1.762681493	0.817771811	0.16497462	1
gene10439	416.3553495	698.8405465	1.6784714	0.747147955	0.16502894	1
gene50654	197.3297066	334.9014253	1.697166793	0.763128356	0.16522026	1
gene19958	506.2385143	851.7724113	1.682551578	0.750650731	0.16523896	1
gene16375	823.4124406	1408.328248	1.710355806	0.77429648	0.16525056	1
gene4227	177.7000499	302.9599599	1.704895188	0.769683049	0.16526106	1
gene46865	46.49129213	90.9847803	1.957028427	0.968664712	0.16528203	1
gene69944	46.49129213	90.9847803	1.957028427	0.968664712	0.16528203	1
gene18334	2327.664026	4318.873294	1.8554539	0.891772157	0.16529215	1
gene63116	193.1971473	328.125963	1.698399627	0.76417596	0.16532386	1
gene5508	32.02733458	12.58300153	0.392883195	-1.34782764	0.16534157	1
gene11133	32.02733458	12.58300153	0.392883195	-1.34782764	0.16534157	1
gene61371	71.28664793	34.84523501	0.48880451	-1.0326705	0.16535957	1
gene51895	237.6221598	400.7202026	1.686375559	0.753925863	0.16539061	1
gene45879	1078.597977	1871.963458	1.735552539	0.79539504	0.16547211	1
gene1572	12.3976779	2.903769584	0.234218828	-2.09407104	0.16547329	1
gene18403	12.3976779	2.903769584	0.234218828	-2.09407104	0.16547329	1
gene36394	12.3976779	2.903769584	0.234218828	-2.09407104	0.16547329	1
gene38177	12.3976779	2.903769584	0.234218828	-2.09407104	0.16547329	1
gene70610	12.3976779	2.903769584	0.234218828	-2.09407104	0.16547329	1
gene72845	12.3976779	2.903769584	0.234218828	-2.09407104	0.16547329	1
gene56194	169.4349313	94.85647307	0.559840125	-0.8369132	0.16554706	1
gene52101	114.6785206	60.97916126	0.531740041	-0.91120699	0.16558819	1
gene41052	292.3785705	171.3224055	0.585960883	-0.77112374	0.16566247	1
gene20491	2576.650724	4837.680127	1.877507138	0.908818393	0.16571386	1
gene64487	63.02152933	118.0866297	1.873750621	0.905928957	0.16573962	1
gene39242	117.7779401	62.91500765	0.534183291	-0.90459324	0.16594625	1
gene16813	58.88897003	111.3111674	1.89018703	0.918528993	0.16594625	1
gene7021	219.0256429	125.8300153	0.574499011	-0.79962368	0.1659666	1
gene54076	618.8507552	1045.35705	1.689190877	0.756332361	0.16614592	1
gene25282	291.3454307	488.8012133	1.677737702	0.746517182	0.16620298	1
gene43476	97.11514356	50.33200612	0.518271448	-0.94822018	0.1662341	1
gene43861	97.11514356	50.33200612	0.518271448	-0.94822018	0.1662341	1
gene33271	725.2641572	432.661668	0.596557356	-0.74526724	0.16624385	1
gene670	103.3139825	182.9374838	1.770694337	0.824315191	0.16630704	1
gene52399	96.08200373	171.3224055	1.783085269	0.834375696	0.16637275	1
gene30277	29.96105493	62.91500765	2.099892938	1.070315775	0.16652054	1
gene72260	5096.478757	2701.473636	0.530066692	-0.91575421	0.16654056	1
gene71287	494.8739762	295.2165744	0.596548997	-0.74528746	0.1665858	1
gene71961	485.5757178	814.9913299	1.678402152	0.747088433	0.16660563	1
gene59754	564.0943445	949.5326539	1.683286959	0.751281142	0.16683423	1

gene7074	100.214563	52.26785251	0.521559451	-0.93909638	0.16688916	1
gene29608	77.48548688	141.3167864	1.823783938	0.866934825	0.16689101	1
gene56975	558.9286454	333.9335022	0.597452832	-0.74310328	0.16691863	1
gene48785	66.12094881	122.9262457	1.859111945	0.894613643	0.16696034	1
gene9461	131.2087578	227.4619507	1.733588173	0.793761216	0.16701125	1
gene31548	133.2750374	72.5942396	0.544694948	-0.87647961	0.16714553	1
gene7193	85.75060548	43.55654376	0.507944446	-0.97725738	0.16721337	1
gene8700	885.4008301	1515.767723	1.711956519	0.77564606	0.1672876	1
gene39975	5769.052783	3015.080751	0.522630121	-0.93613782	0.16729986	1
gene40547	299.6105493	176.1620214	0.587970023	-0.76618549	0.1673126	1
gene25140	66.12094881	31.94146542	0.483076332	-1.04967692	0.16736439	1
gene39635	1805.928414	1045.35705	0.578847446	-0.78874492	0.16741113	1
gene26628	20.6627965	6.775462362	0.327906359	-1.60864422	0.16751772	1
gene52229	20.6627965	6.775462362	0.327906359	-1.60864422	0.16751772	1
gene11742	707.7007802	422.9824361	0.597685417	-0.74254175	0.16760003	1
gene29098	35.12675405	71.6263164	2.039081559	1.027919481	0.16763573	1
gene40990	95.04886391	169.3865591	1.782099776	0.833578112	0.16765087	1
gene65502	53.7232709	102.5998586	1.909784287	0.933409693	0.16765379	1
gene7905	312.0082272	183.905407	0.589424864	-0.76262017	0.16767943	1
gene5577	440.1175655	736.5895511	1.67361998	0.74297198	0.1677986	1
gene33215	154.9709738	86.14516432	0.555879351	-0.8471563	0.16781146	1
gene61293	112.6122409	197.4563317	1.753418013	0.810169974	0.16786199	1
gene42915	210.7605243	120.9903993	0.574065754	-0.8007121	0.16787135	1
gene29617	204.5616854	117.1187066	0.572534912	-0.80456443	0.16788403	1
gene36710	192.1640075	109.375321	0.569176936	-0.81305089	0.16791713	1
gene15015	185.9651685	105.5036282	0.567330049	-0.81773981	0.16793203	1
gene36327	185.9651685	105.5036282	0.567330049	-0.81773981	0.16793203	1
gene38182	1877.215062	1085.041901	0.578006177	-0.79084318	0.16801224	1
gene5242	69.22036828	127.7658617	1.845784194	0.884233886	0.16804255	1
gene16607	357.4663795	211.9751796	0.592993333	-0.75391221	0.16805154	1
gene8247	184.9320287	313.6071151	1.695796652	0.761963182	0.16811311	1
gene69882	52.69013108	24.19807987	0.459252603	-1.1226402	0.16813331	1
gene1154	1241.83407	732.7178583	0.590028794	-0.76114273	0.16821	1
gene11346	5.165699125	17.4226175	3.372751119	1.753925863	0.16822433	1
gene20905	5.165699125	17.4226175	3.372751119	1.753925863	0.16822433	1
gene23402	5.165699125	17.4226175	3.372751119	1.753925863	0.16822433	1
gene47163	5.165699125	17.4226175	3.372751119	1.753925863	0.16822433	1
gene50099	5.165699125	17.4226175	3.372751119	1.753925863	0.16822433	1
gene52272	5.165699125	17.4226175	3.372751119	1.753925863	0.16822433	1
gene61064	5.165699125	17.4226175	3.372751119	1.753925863	0.16822433	1
gene64844	5.165699125	17.4226175	3.372751119	1.753925863	0.16822433	1
gene66737	5.165699125	17.4226175	3.372751119	1.753925863	0.16822433	1
gene54673	140.5070162	241.9807987	1.722197262	0.7842504	0.16834542	1
gene6733	21.69593633	48.39615973	2.230655502	1.157467724	0.1683674	1
gene12590	21.69593633	48.39615973	2.230655502	1.157467724	0.1683674	1
gene30792	21.69593633	48.39615973	2.230655502	1.157467724	0.1683674	1
gene12669	37.1930337	15.48677111	0.416389027	-1.26399604	0.1684059	1
gene24975	37.1930337	15.48677111	0.416389027	-1.26399604	0.1684059	1

gene56324	37.1930337	15.48677111	0.416389027	-1.26399604	0.1684059	1
gene69790	37.1930337	15.48677111	0.416389027	-1.26399604	0.1684059	1
gene1132	0	4.839615973	Inf	Inf	0.16850917	1
gene2756	0	4.839615973	Inf	Inf	0.16850917	1
gene3560	0	4.839615973	Inf	Inf	0.16850917	1
gene4200	0	4.839615973	Inf	Inf	0.16850917	1
gene4582	0	4.839615973	Inf	Inf	0.16850917	1
gene5749	0	4.839615973	Inf	Inf	0.16850917	1
gene5987	0	4.839615973	Inf	Inf	0.16850917	1
gene6964	0	4.839615973	Inf	Inf	0.16850917	1
gene7734	0	4.839615973	Inf	Inf	0.16850917	1
gene8477	0	4.839615973	Inf	Inf	0.16850917	1
gene8580	0	4.839615973	Inf	Inf	0.16850917	1
gene9078	0	4.839615973	Inf	Inf	0.16850917	1
gene9330	0	4.839615973	Inf	Inf	0.16850917	1
gene9776	0	4.839615973	Inf	Inf	0.16850917	1
gene9916	0	4.839615973	Inf	Inf	0.16850917	1
gene12244	0	4.839615973	Inf	Inf	0.16850917	1
gene12417	0	4.839615973	Inf	Inf	0.16850917	1
gene12832	0	4.839615973	Inf	Inf	0.16850917	1
gene12854	0	4.839615973	Inf	Inf	0.16850917	1
gene13890	0	4.839615973	Inf	Inf	0.16850917	1
gene14088	0	4.839615973	Inf	Inf	0.16850917	1
gene14570	0	4.839615973	Inf	Inf	0.16850917	1
gene15712	0	4.839615973	Inf	Inf	0.16850917	1
gene19071	0	4.839615973	Inf	Inf	0.16850917	1
gene19328	0	4.839615973	Inf	Inf	0.16850917	1
gene22325	0	4.839615973	Inf	Inf	0.16850917	1
gene22357	0	4.839615973	Inf	Inf	0.16850917	1
gene23195	0	4.839615973	Inf	Inf	0.16850917	1
gene23497	0	4.839615973	Inf	Inf	0.16850917	1
gene24389	0	4.839615973	Inf	Inf	0.16850917	1
gene24750	0	4.839615973	Inf	Inf	0.16850917	1
gene27669	0	4.839615973	Inf	Inf	0.16850917	1
gene28006	0	4.839615973	Inf	Inf	0.16850917	1
gene28160	0	4.839615973	Inf	Inf	0.16850917	1
gene28161	0	4.839615973	Inf	Inf	0.16850917	1
gene28304	0	4.839615973	Inf	Inf	0.16850917	1
gene29940	0	4.839615973	Inf	Inf	0.16850917	1
gene30195	0	4.839615973	Inf	Inf	0.16850917	1
gene30469	0	4.839615973	Inf	Inf	0.16850917	1
gene30637	0	4.839615973	Inf	Inf	0.16850917	1
gene31197	0	4.839615973	Inf	Inf	0.16850917	1
gene32463	0	4.839615973	Inf	Inf	0.16850917	1
gene33246	0	4.839615973	Inf	Inf	0.16850917	1
gene33738	0	4.839615973	Inf	Inf	0.16850917	1
gene33835	0	4.839615973	Inf	Inf	0.16850917	1
gene33988	0	4.839615973	Inf	Inf	0.16850917	1

gene36119	0	4.839615973	Inf	Inf	0.16850917	1
gene36290	0	4.839615973	Inf	Inf	0.16850917	1
gene37300	0	4.839615973	Inf	Inf	0.16850917	1
gene37608	0	4.839615973	Inf	Inf	0.16850917	1
gene37731	0	4.839615973	Inf	Inf	0.16850917	1
gene38493	0	4.839615973	Inf	Inf	0.16850917	1
gene38609	0	4.839615973	Inf	Inf	0.16850917	1
gene38663	0	4.839615973	Inf	Inf	0.16850917	1
gene38805	0	4.839615973	Inf	Inf	0.16850917	1
gene39308	0	4.839615973	Inf	Inf	0.16850917	1
gene40835	0	4.839615973	Inf	Inf	0.16850917	1
gene41292	0	4.839615973	Inf	Inf	0.16850917	1
gene41442	0	4.839615973	Inf	Inf	0.16850917	1
gene41582	0	4.839615973	Inf	Inf	0.16850917	1
gene43605	0	4.839615973	Inf	Inf	0.16850917	1
gene43791	0	4.839615973	Inf	Inf	0.16850917	1
gene44715	0	4.839615973	Inf	Inf	0.16850917	1
gene45030	0	4.839615973	Inf	Inf	0.16850917	1
gene45221	0	4.839615973	Inf	Inf	0.16850917	1
gene45726	0	4.839615973	Inf	Inf	0.16850917	1
gene45792	0	4.839615973	Inf	Inf	0.16850917	1
gene45888	0	4.839615973	Inf	Inf	0.16850917	1
gene46534	0	4.839615973	Inf	Inf	0.16850917	1
gene46950	0	4.839615973	Inf	Inf	0.16850917	1
gene49692	0	4.839615973	Inf	Inf	0.16850917	1
gene49905	0	4.839615973	Inf	Inf	0.16850917	1
gene50520	0	4.839615973	Inf	Inf	0.16850917	1
gene54502	0	4.839615973	Inf	Inf	0.16850917	1
gene54623	0	4.839615973	Inf	Inf	0.16850917	1
gene55846	0	4.839615973	Inf	Inf	0.16850917	1
gene56792	0	4.839615973	Inf	Inf	0.16850917	1
gene57800	0	4.839615973	Inf	Inf	0.16850917	1
gene57972	0	4.839615973	Inf	Inf	0.16850917	1
gene59008	0	4.839615973	Inf	Inf	0.16850917	1
gene60932	0	4.839615973	Inf	Inf	0.16850917	1
gene61093	0	4.839615973	Inf	Inf	0.16850917	1
gene61726	0	4.839615973	Inf	Inf	0.16850917	1
gene62108	0	4.839615973	Inf	Inf	0.16850917	1
gene63387	0	4.839615973	Inf	Inf	0.16850917	1
gene63463	0	4.839615973	Inf	Inf	0.16850917	1
gene63753	0	4.839615973	Inf	Inf	0.16850917	1
gene63780	0	4.839615973	Inf	Inf	0.16850917	1
gene64381	0	4.839615973	Inf	Inf	0.16850917	1
gene64529	0	4.839615973	Inf	Inf	0.16850917	1
gene65105	0	4.839615973	Inf	Inf	0.16850917	1
gene66167	0	4.839615973	Inf	Inf	0.16850917	1
gene66319	0	4.839615973	Inf	Inf	0.16850917	1
gene66477	0	4.839615973	Inf	Inf	0.16850917	1

gene66900	0	4.839615973	Inf	Inf	0.16850917	1
gene67985	0	4.839615973	Inf	Inf	0.16850917	1
gene68286	0	4.839615973	Inf	Inf	0.16850917	1
gene69111	0	4.839615973	Inf	Inf	0.16850917	1
gene69766	0	4.839615973	Inf	Inf	0.16850917	1
gene71514	0	4.839615973	Inf	Inf	0.16850917	1
gene71570	0	4.839615973	Inf	Inf	0.16850917	1
gene72418	0	4.839615973	Inf	Inf	0.16850917	1
gene72609	0	4.839615973	Inf	Inf	0.16850917	1
gene73633	0	4.839615973	Inf	Inf	0.16850917	1
gene73901	0	4.839615973	Inf	Inf	0.16850917	1
gene73938	0	4.839615973	Inf	Inf	0.16850917	1
gene68839	978.3834144	1681.282589	1.71842916	0.78109038	0.16853912	1
gene48756	223.1582022	375.5541995	1.682905651	0.750954297	0.16858572	1
gene37140	77.48548688	38.71692779	0.499666832	-1.00096164	0.1685882	1
gene52550	1124.05613	1946.493544	1.731669347	0.792163482	0.16901108	1
gene11850	86.78374531	155.8356343	1.795677679	0.844528412	0.16910839	1
gene63877	161.1698127	274.8901873	1.705593514	0.770273857	0.16916152	1
gene38369	104.3471223	183.905407	1.762438703	0.817573082	0.16926287	1
gene4937	14.46395755	3.871692779	0.26767866	-1.90142597	0.16933974	1
gene11828	14.46395755	3.871692779	0.26767866	-1.90142597	0.16933974	1
gene13832	14.46395755	3.871692779	0.26767866	-1.90142597	0.16933974	1
gene45309	14.46395755	3.871692779	0.26767866	-1.90142597	0.16933974	1
gene50164	14.46395755	3.871692779	0.26767866	-1.90142597	0.16933974	1
gene54598	14.46395755	3.871692779	0.26767866	-1.90142597	0.16933974	1
gene65728	14.46395755	3.871692779	0.26767866	-1.90142597	0.16933974	1
gene66098	14.46395755	3.871692779	0.26767866	-1.90142597	0.16933974	1
gene15808	42.35873283	18.3905407	0.434161729	-1.20369553	0.1693644	1
gene54638	42.35873283	18.3905407	0.434161729	-1.20369553	0.1693644	1
gene19988	18.59651685	5.807539168	0.31229177	-1.67903354	0.16949237	1
gene54576	18.59651685	5.807539168	0.31229177	-1.67903354	0.16949237	1
gene72355	18.59651685	5.807539168	0.31229177	-1.67903354	0.16949237	1
gene41994	601.2873782	1010.511815	1.680580454	0.74895961	0.16950857	1
gene20478	233.4896005	135.5092472	0.580365237	-0.78496699	0.16968446	1
gene68814	286.1797315	478.1540582	1.670817341	0.740554022	0.16983264	1
gene8343	29.96105493	11.61507834	0.387672542	-1.36708954	0.16983622	1
gene37199	29.96105493	11.61507834	0.387672542	-1.36708954	0.16983622	1
gene38011	29.96105493	11.61507834	0.387672542	-1.36708954	0.16983622	1
gene15104	539.2989887	323.286347	0.599456616	-0.73827275	0.16985218	1
gene3279	82.65118601	149.060172	1.803484973	0.850787402	0.16986872	1
gene44802	27.89477528	59.04331487	2.116644221	1.081778792	0.16990129	1
gene11148	185.9651685	314.5750383	1.691580422	0.758371768	0.16996675	1
gene43891	450.4489637	752.0763222	1.669614946	0.73951542	0.16997546	1
gene44019	186.9983083	106.4715514	0.569371736	-0.81255722	0.17013004	1
gene62417	134.3081773	73.56216279	0.54771172	-0.86851134	0.17014421	1
gene48380	247.953558	415.2390505	1.674664618	0.743872199	0.17025201	1
gene341	16.5302372	4.839615973	0.292773535	-1.77214295	0.17028734	1
gene4875	16.5302372	4.839615973	0.292773535	-1.77214295	0.17028734	1

gene8163	16.5302372	4.839615973	0.292773535	-1.77214295	0.17028734	1
gene13250	16.5302372	4.839615973	0.292773535	-1.77214295	0.17028734	1
gene35672	16.5302372	4.839615973	0.292773535	-1.77214295	0.17028734	1
gene44721	16.5302372	4.839615973	0.292773535	-1.77214295	0.17028734	1
gene68403	16.5302372	4.839615973	0.292773535	-1.77214295	0.17028734	1
gene69521	16.5302372	4.839615973	0.292773535	-1.77214295	0.17028734	1
gene27138	437.018146	261.3392626	0.598005517	-0.7417693	0.17029126	1
gene11709	577.5251622	346.5165037	0.600002435	-0.73695974	0.1703093	1
gene61446	143.6064357	79.36970196	0.552689032	-0.85546011	0.170344	1
gene9731	98.14828338	51.29992932	0.522677805	-0.9360062	0.17034436	1
gene36102	1275.927684	754.0121686	0.590952119	-0.75888685	0.17035853	1
gene16103	146.7058552	81.30554835	0.55420793	-0.85150074	0.17037827	1
gene34771	149.8052746	83.24139474	0.555663977	-0.84771538	0.1703997	1
gene2881	189.064588	319.4146542	1.689447282	0.756551333	0.17041846	1
gene52146	323.3727653	539.1332194	1.667219003	0.737443627	0.17048694	1
gene70368	43.39187265	85.17724113	1.962976842	0.973043153	0.17049807	1
gene54789	85.75060548	153.8997879	1.794737041	0.843772481	0.17052181	1
gene22423	71.28664793	130.6696313	1.833016912	0.874220097	0.17056877	1
gene16769	473.1780399	789.8253268	1.669192693	0.739150511	0.17073472	1
gene65339	391.5599937	652.3802332	1.666105434	0.7364797	0.17077033	1
gene31562	166.3355118	282.6335728	1.699177582	0.764836637	0.17082066	1
gene28096	47.52443195	91.95270349	1.934851185	0.952222609	0.17100428	1
gene34755	47.52443195	91.95270349	1.934851185	0.952222609	0.17100428	1
gene28295	287.2128714	479.1219813	1.668177262	0.738272599	0.17110955	1
gene1756	33.0604744	67.75462362	2.049414742	1.035211974	0.17111124	1
gene37433	33.0604744	67.75462362	2.049414742	1.035211974	0.17111124	1
gene55885	33.0604744	67.75462362	2.049414742	1.035211974	0.17111124	1
gene45830	51.65699125	98.72816585	1.911225634	0.934498109	0.1711278	1
gene33323	380.1954556	633.0217693	1.664990362	0.735513826	0.17125041	1
gene22711	19.62965668	44.52446695	2.268224436	1.181563399	0.17136782	1
gene40611	243.8209987	142.2847096	0.583562164	-0.77704175	0.17140473	1
gene18715	240.7215792	140.3488632	0.583033992	-0.7783481	0.17142815	1
gene21593	806.8822034	1367.675474	1.695012566	0.761295969	0.17144696	1
gene31352	1052.769482	627.2142301	0.595775467	-0.74715938	0.17144729	1
gene63256	234.5227403	136.4771704	0.581935766	-0.78106818	0.17148607	1
gene25535	231.4233208	134.5413241	0.58136459	-0.78248489	0.17152048	1
gene2976	64.05466916	30.97354223	0.483548547	-1.04826735	0.17157463	1
gene24365	3084.955518	5880.133407	1.906067486	0.9305992	0.17158424	1
gene34434	26.86163545	57.10746848	2.125986282	1.088132288	0.17166041	1
gene54372	91.94944443	163.5790199	1.779010421	0.831074962	0.17166871	1
gene69484	215.9262234	124.8620921	0.578262752	-0.79020292	0.17174424	1
gene63764	206.627965	119.0545529	0.576178316	-0.79541273	0.17191606	1
gene64636	281.0140324	468.4748262	1.66708695	0.737329353	0.17194251	1
gene70813	862.6717539	516.8709859	0.599151396	-0.7390075	0.17198876	1
gene23133	301.6768289	502.352138	1.66519961	0.735695126	0.1720617	1
gene20050	686.0048439	412.3352809	0.601067594	-0.73440085	0.17207774	1
gene44329	161.1698127	273.9222641	1.699587903	0.765184981	0.17208515	1
gene16427	742.8275342	1253.460537	1.68741798	0.754817379	0.17216005	1

gene8739	191.1308676	109.375321	0.572253568	-0.80527354	0.17225041	1
gene44927	330.604744	196.4884085	0.594330275	-0.75066322	0.17225876	1
gene50492	58.88897003	110.3432442	1.873750621	0.905928957	0.17236974	1
gene2751	2189.223289	3988.811485	1.82202131	0.865539833	0.17251477	1
gene29401	75.41920723	37.74900459	0.500522426	-0.99849338	0.1725501	1
gene9650	172.5343508	97.76024266	0.566613212	-0.81956385	0.17268504	1
gene44604	119.8442197	64.85085404	0.541126257	-0.88596285	0.17268949	1
gene61535	54.75641073	103.5677818	1.891427514	0.919475488	0.17275363	1
gene12626	50.62385143	23.23015667	0.458877703	-1.12381839	0.1727961	1
gene13840	50.62385143	23.23015667	0.458877703	-1.12381839	0.1727961	1
gene58970	80.58490636	145.1884792	1.80168329	0.849345428	0.17284462	1
gene26605	424.6204681	254.5638002	0.599509019	-0.73814664	0.1728759	1
gene44065	360.565799	599.1444575	1.66167856	0.73264133	0.17288813	1
gene6782	383.2948751	636.8934621	1.66162791	0.732597354	0.17295493	1
gene35812	157.0372534	88.08101071	0.560892456	-0.83420392	0.17300062	1
gene58005	153.9378339	86.14516432	0.559610085	-0.83750613	0.17304913	1
gene53138	37.1930337	74.53008599	2.003872192	1.002790496	0.17308163	1
gene66496	37.1930337	74.53008599	2.003872192	1.002790496	0.17308163	1
gene1753	400.8582521	240.0449523	0.598827518	-0.73978758	0.17311104	1
gene28964	1245.966629	739.4933207	0.593509732	-0.75265641	0.17313452	1
gene32489	144.6395755	80.33762515	0.55543322	-0.84831463	0.17314428	1
gene69038	144.6395755	80.33762515	0.55543322	-0.84831463	0.17314428	1
gene14837	141.540156	78.40177877	0.553918979	-0.85225312	0.17315405	1
gene30873	1702.614432	995.9929673	0.584978577	-0.7735443	0.17322104	1
gene53971	177.7000499	300.0561903	1.688554339	0.755788607	0.17322398	1
gene34483	522.7687515	314.5750383	0.601747976	-0.73276871	0.17323898	1
gene36382	5583.087615	11879.32137	2.127733288	1.08931732	0.17331102	1
gene40700	163.2360924	276.8260337	1.695862904	0.762019545	0.17331784	1
gene3624	35.12675405	14.51884792	0.413327343	-1.27464329	0.17332063	1
gene27675	35.12675405	14.51884792	0.413327343	-1.27464329	0.17332063	1
gene37377	35.12675405	14.51884792	0.413327343	-1.27464329	0.17332063	1
gene61775	764.5234706	459.7635175	0.601372666	-0.7336688	0.17342545	1
gene742	25.82849563	55.17162209	2.136075708	1.094962781	0.17346535	1
gene13179	25.82849563	55.17162209	2.136075708	1.094962781	0.17346535	1
gene11014	199.3959862	334.9014253	1.679579573	0.748100147	0.17352329	1
gene1750	67.15408863	32.90938862	0.490057855	-1.02897602	0.17352433	1
gene34041	67.15408863	32.90938862	0.490057855	-1.02897602	0.17352433	1
gene72913	223.1582022	129.7017081	0.581209684	-0.78286936	0.17352986	1
gene20484	773.821729	1305.72839	1.687376227	0.754781681	0.17353819	1
gene30232	380.1954556	227.4619507	0.598276353	-0.74111605	0.17359636	1
gene51420	216.9593633	125.8300153	0.57997043	-0.78594875	0.1736767	1
gene69387	148.7721348	253.595877	1.704592579	0.769426957	0.17368093	1
gene52	6.19883895	19.35846389	3.122917702	1.642894551	0.17369292	1
gene12845	6.19883895	19.35846389	3.122917702	1.642894551	0.17369292	1
gene15922	6.19883895	19.35846389	3.122917702	1.642894551	0.17369292	1
gene21331	6.19883895	19.35846389	3.122917702	1.642894551	0.17369292	1
gene22799	6.19883895	19.35846389	3.122917702	1.642894551	0.17369292	1
gene37812	6.19883895	19.35846389	3.122917702	1.642894551	0.17369292	1

gene44330	6.19883895	19.35846389	3.122917702	1.642894551	0.17369292	1
gene45821	6.19883895	19.35846389	3.122917702	1.642894551	0.17369292	1
gene46096	6.19883895	19.35846389	3.122917702	1.642894551	0.17369292	1
gene47817	6.19883895	19.35846389	3.122917702	1.642894551	0.17369292	1
gene53972	6.19883895	19.35846389	3.122917702	1.642894551	0.17369292	1
gene56812	6.19883895	19.35846389	3.122917702	1.642894551	0.17369292	1
gene59024	6.19883895	19.35846389	3.122917702	1.642894551	0.17369292	1
gene61106	6.19883895	19.35846389	3.122917702	1.642894551	0.17369292	1
gene70997	6.19883895	19.35846389	3.122917702	1.642894551	0.17369292	1
gene72510	6.19883895	19.35846389	3.122917702	1.642894551	0.17369292	1
gene11825	272.7489138	453.9559783	1.664373185	0.73497895	0.1737511	1
gene30627	58.88897003	28.06977264	0.47665586	-1.06898006	0.17379303	1
gene3625	78.51862671	39.68485098	0.505419576	-0.98444655	0.17381316	1
gene1980	1.033139825	7.743385557	7.495002486	2.905928957	0.17392182	1
gene2059	1.033139825	7.743385557	7.495002486	2.905928957	0.17392182	1
gene2546	1.033139825	7.743385557	7.495002486	2.905928957	0.17392182	1
gene4324	1.033139825	7.743385557	7.495002486	2.905928957	0.17392182	1
gene5852	1.033139825	7.743385557	7.495002486	2.905928957	0.17392182	1
gene6443	1.033139825	7.743385557	7.495002486	2.905928957	0.17392182	1
gene8194	1.033139825	7.743385557	7.495002486	2.905928957	0.17392182	1
gene8195	1.033139825	7.743385557	7.495002486	2.905928957	0.17392182	1
gene8283	1.033139825	7.743385557	7.495002486	2.905928957	0.17392182	1
gene9856	1.033139825	7.743385557	7.495002486	2.905928957	0.17392182	1
gene9925	1.033139825	7.743385557	7.495002486	2.905928957	0.17392182	1
gene10874	1.033139825	7.743385557	7.495002486	2.905928957	0.17392182	1
gene12104	1.033139825	7.743385557	7.495002486	2.905928957	0.17392182	1
gene12910	1.033139825	7.743385557	7.495002486	2.905928957	0.17392182	1
gene13859	1.033139825	7.743385557	7.495002486	2.905928957	0.17392182	1
gene14257	1.033139825	7.743385557	7.495002486	2.905928957	0.17392182	1
gene18021	1.033139825	7.743385557	7.495002486	2.905928957	0.17392182	1
gene19112	1.033139825	7.743385557	7.495002486	2.905928957	0.17392182	1
gene20214	1.033139825	7.743385557	7.495002486	2.905928957	0.17392182	1
gene20767	1.033139825	7.743385557	7.495002486	2.905928957	0.17392182	1
gene21491	1.033139825	7.743385557	7.495002486	2.905928957	0.17392182	1
gene24893	1.033139825	7.743385557	7.495002486	2.905928957	0.17392182	1
gene28541	1.033139825	7.743385557	7.495002486	2.905928957	0.17392182	1
gene30257	1.033139825	7.743385557	7.495002486	2.905928957	0.17392182	1
gene32014	1.033139825	7.743385557	7.495002486	2.905928957	0.17392182	1
gene38641	1.033139825	7.743385557	7.495002486	2.905928957	0.17392182	1
gene40152	1.033139825	7.743385557	7.495002486	2.905928957	0.17392182	1
gene40864	1.033139825	7.743385557	7.495002486	2.905928957	0.17392182	1
gene41817	1.033139825	7.743385557	7.495002486	2.905928957	0.17392182	1
gene42933	1.033139825	7.743385557	7.495002486	2.905928957	0.17392182	1
gene43631	1.033139825	7.743385557	7.495002486	2.905928957	0.17392182	1
gene48878	1.033139825	7.743385557	7.495002486	2.905928957	0.17392182	1
gene49335	1.033139825	7.743385557	7.495002486	2.905928957	0.17392182	1
gene50734	1.033139825	7.743385557	7.495002486	2.905928957	0.17392182	1
gene50878	1.033139825	7.743385557	7.495002486	2.905928957	0.17392182	1

gene54040	1.033139825	7.743385557	7.495002486	2.905928957	0.17392182	1
gene56306	1.033139825	7.743385557	7.495002486	2.905928957	0.17392182	1
gene56456	1.033139825	7.743385557	7.495002486	2.905928957	0.17392182	1
gene58133	1.033139825	7.743385557	7.495002486	2.905928957	0.17392182	1
gene61704	1.033139825	7.743385557	7.495002486	2.905928957	0.17392182	1
gene62104	1.033139825	7.743385557	7.495002486	2.905928957	0.17392182	1
gene62755	1.033139825	7.743385557	7.495002486	2.905928957	0.17392182	1
gene63696	1.033139825	7.743385557	7.495002486	2.905928957	0.17392182	1
gene63994	1.033139825	7.743385557	7.495002486	2.905928957	0.17392182	1
gene66359	1.033139825	7.743385557	7.495002486	2.905928957	0.17392182	1
gene71070	1.033139825	7.743385557	7.495002486	2.905928957	0.17392182	1
gene72746	1.033139825	7.743385557	7.495002486	2.905928957	0.17392182	1
gene42870	45.4581523	20.32638709	0.447145035	-1.16118524	0.17396002	1
gene28578	573.3926029	345.5485805	0.602638713	-0.73063474	0.17399471	1
gene58460	204.5616854	118.0866297	0.577266606	-0.79269033	0.17401493	1
gene71020	151.8715543	258.435493	1.701671483	0.766952543	0.17402751	1
gene57148	653.9775093	1095.689056	1.675423146	0.74452551	0.17408977	1
gene27185	2432.011148	1393.8094	0.573109791	-0.80311655	0.1742286	1
gene66544	594.0553994	358.131582	0.602858896	-0.73010773	0.17423196	1
gene44494	763.4903307	1286.369926	1.684854246	0.752623792	0.1742719	1
gene48368	17.56337703	40.65277417	2.314633121	1.210783538	0.17429001	1
gene46843	985.6153931	590.4331487	0.599050251	-0.73925107	0.17445293	1
gene42206	368.8309176	220.6864884	0.598340535	-0.74096129	0.17447058	1
gene6477	27.89477528	10.64715514	0.381689941	-1.38952693	0.17451022	1
gene18304	27.89477528	10.64715514	0.381689941	-1.38952693	0.17451022	1
gene38358	27.89477528	10.64715514	0.381689941	-1.38952693	0.17451022	1
gene57914	27.89477528	10.64715514	0.381689941	-1.38952693	0.17451022	1
gene60022	27.89477528	10.64715514	0.381689941	-1.38952693	0.17451022	1
gene7094	371.930337	222.6223348	0.598559226	-0.74043409	0.1745498	1
gene19847	235.5558801	392.976817	1.668295509	0.73837486	0.17458203	1
gene57805	972.1845754	582.6897632	0.599361251	-0.73850228	0.17458472	1
gene52571	65.08780898	120.0224761	1.844008548	0.882845344	0.17460877	1
gene21859	72.31978776	131.6375545	1.820214889	0.864108781	0.17462914	1
gene55783	45.4581523	88.08101071	1.937628484	0.954291978	0.17472263	1
gene70520	45.4581523	88.08101071	1.937628484	0.954291978	0.17472263	1
gene57249	231.4233208	386.2013547	1.668809147	0.738818971	0.17474207	1
gene14764	102.2808427	54.2036989	0.529949671	-0.91607274	0.17482622	1
gene42116	524.8350311	316.5108846	0.603067375	-0.7296089	0.17505116	1
gene18020	570.2931834	950.5005771	1.6666876	0.736983714	0.1750787	1
gene39999	105.3802622	56.13954529	0.53273302	-0.90851539	0.17515715	1
gene10221	119.8442197	207.1355637	1.728373418	0.789414948	0.17527581	1
gene10188	24.7953558	53.23577571	2.14700592	1.102326169	0.17531558	1
gene63423	24.7953558	53.23577571	2.14700592	1.102326169	0.17531558	1
gene30255	749.0263732	1259.268076	1.681206592	0.749497018	0.17536985	1
gene50381	629.1821535	1051.164589	1.670684052	0.740438927	0.17538451	1
gene10839	133.2750374	228.4298739	1.713973437	0.777344751	0.1753983	1
gene59014	695.3031023	1165.379526	1.676074107	0.745085939	0.17550984	1
gene24339	16.5302372	38.71692779	2.342188277	1.227857052	0.17568076	1

gene56271	16.5302372	38.71692779	2.342188277	1.227857052	0.17568076	1
gene73484	16.5302372	38.71692779	2.342188277	1.227857052	0.17568076	1
gene42108	233.4896005	389.1051242	1.666477323	0.736801685	0.17572105	1
gene15414	114.6785206	61.94708446	0.540180359	-0.88848691	0.17581261	1
gene73160	114.6785206	61.94708446	0.540180359	-0.88848691	0.17581261	1
gene23516	53.7232709	25.16600306	0.468437655	-1.09407104	0.17590164	1
gene71690	53.7232709	25.16600306	0.468437655	-1.09407104	0.17590164	1
gene8846	628.1490136	379.4258923	0.604038029	-0.72728871	0.17590572	1
gene40345	4648.096073	9452.737919	2.033679548	1.024092368	0.17595155	1
gene45109	56.82269038	106.4715514	1.873750621	0.905928957	0.17596915	1
gene39613	142.5732959	79.36970196	0.556694025	-0.8450435	0.17600077	1
gene32471	61.9883895	30.00561903	0.484052244	-1.04676533	0.17603048	1
gene36942	3243.025911	1822.599376	0.562005801	-0.83134307	0.17611769	1
gene72365	127.0761985	69.69047001	0.548414816	-0.86666055	0.17612906	1
gene45173	648.8118102	392.0088938	0.604195065	-0.72691369	0.17620854	1
gene33354	5136.77121	10663.60984	2.075936303	1.053762177	0.1762103	1
gene58547	914.3287452	1549.645035	1.694844489	0.761152904	0.17632509	1
gene58046	671.5408863	1122.790906	1.671962093	0.741542139	0.17650166	1
gene29725	193.1971473	111.3111674	0.576153266	-0.79547545	0.17655279	1
gene50783	407.0570911	244.8845682	0.601597598	-0.73312929	0.17661727	1
gene24545	191.1308676	320.3825774	1.676247178	0.745234903	0.17665112	1
gene5068	48.55757178	92.92062669	1.913617656	0.936302606	0.17671644	1
gene10008	224.191342	373.6183531	1.66651553	0.736834761	0.17672247	1
gene11532	29.96105493	61.94708446	2.067586893	1.047947962	0.17676267	1
gene52418	1365.810849	811.1196371	0.593874062	-0.75177107	0.17679133	1
gene8106	449.4158239	271.0184945	0.603046177	-0.72965962	0.17681337	1
gene14993	108.4796816	188.745023	1.739911291	0.799013753	0.17681565	1
gene69408	159.1035331	269.0826481	1.691242444	0.758083489	0.17685766	1
gene33013	35.12675405	70.65839321	2.011526402	1.008290674	0.17689066	1
gene40153	148.7721348	252.6279538	1.698086501	0.763909952	0.17690042	1
gene35802	74.38606741	134.5413241	1.808689836	0.854945028	0.17699272	1
gene55432	15.49709738	36.7810814	2.373417454	1.246965874	0.17699278	1
gene63987	15.49709738	36.7810814	2.373417454	1.246965874	0.17699278	1
gene42471	687.0379837	1148.924832	1.672287209	0.741822646	0.17699695	1
gene41518	84.71746566	150.9960184	1.782348152	0.833779171	0.17703983	1
gene35983	609.5524968	1015.351431	1.665732544	0.736156775	0.17704417	1
gene45556	1784.232478	3164.140923	1.773390498	0.826510251	0.17713687	1
gene34246	90.91630461	47.42823654	0.521669207	-0.93879282	0.17717456	1
gene20739	7.231978776	21.29431028	2.944465262	1.558005653	0.17718372	1
gene25436	7.231978776	21.29431028	2.944465262	1.558005653	0.17718372	1
gene26325	7.231978776	21.29431028	2.944465262	1.558005653	0.17718372	1
gene28440	7.231978776	21.29431028	2.944465262	1.558005653	0.17718372	1
gene32037	7.231978776	21.29431028	2.944465262	1.558005653	0.17718372	1
gene41737	7.231978776	21.29431028	2.944465262	1.558005653	0.17718372	1
gene52042	7.231978776	21.29431028	2.944465262	1.558005653	0.17718372	1
gene54929	7.231978776	21.29431028	2.944465262	1.558005653	0.17718372	1
gene72462	7.231978776	21.29431028	2.944465262	1.558005653	0.17718372	1
gene13030	23.76221598	51.29992932	2.158886586	1.110287455	0.17720998	1

gene42194	23.76221598	51.29992932	2.158886586	1.110287455	0.17720998	1
gene25289	67.15408863	122.9262457	1.830510222	0.87224583	0.17725126	1
gene31019	141.540156	241.0128755	1.702787973	0.767898806	0.17733792	1
gene18181	223.1582022	371.6825067	1.665556108	0.736003955	0.17733987	1
gene29380	341.9692821	565.2671457	1.652976379	0.725066109	0.17746784	1
gene40728	5862.035368	12507.50352	2.133645182	1.093320281	0.17749418	1
gene56254	1206.707316	2075.227329	1.719743721	0.782193588	0.1775964	1
gene39053	633.3147128	383.2975851	0.605224507	-0.72445769	0.1775987	1
gene31626	500.0396753	828.5422546	1.656953029	0.728532706	0.17760249	1
gene2241	474.2111797	286.5052656	0.604172314	-0.72696802	0.17762773	1
gene42560	94.01572408	49.36408293	0.525061987	-0.92944034	0.17769865	1
gene41596	48.55757178	22.26223348	0.458470897	-1.12509794	0.17776092	1
gene13666	531.0338701	321.3505006	0.605141251	-0.72465616	0.17788479	1
gene48200	263.4506554	155.8356343	0.591517353	-0.7575076	0.17792998	1
gene45609	65.08780898	31.94146542	0.49074421	-1.02695685	0.17795659	1
gene4056	1318.286417	2279.459123	1.729107646	0.790027687	0.17800816	1
gene68575	703.5682209	1176.026681	1.671517625	0.741158567	0.17807889	1
gene24902	39.25931335	77.43385557	1.972369075	0.979929538	0.17812937	1
gene50857	489.7082771	296.1844976	0.604818239	-0.72542645	0.17815333	1
gene58927	306.842528	507.191754	1.652938259	0.725032837	0.17819692	1
gene68778	203.5285455	118.0866297	0.580196893	-0.78538553	0.17822081	1
gene35334	251.0529775	148.0922488	0.589884455	-0.7614957	0.17824875	1
gene4835	564.0943445	341.6768877	0.605708763	-0.72330381	0.17837145	1
gene36168	51.65699125	97.76024266	1.892488128	0.92028425	0.17838275	1
gene53919	206.627965	344.5806573	1.667638053	0.737806198	0.17848374	1
gene22752	33.0604744	13.55092472	0.409882948	-1.28671612	0.17853876	1
gene29413	33.0604744	13.55092472	0.409882948	-1.28671612	0.17853876	1
gene10152	83.68432583	149.060172	1.781219727	0.832865494	0.17861362	1
gene61501	704.6013607	1176.994605	1.670440437	0.740228541	0.17865118	1
gene15168	43.39187265	84.20931793	1.940670286	0.95655503	0.17865472	1
gene21817	73.35292758	132.6054777	1.807773487	0.85421392	0.178687	1
gene36050	116.7448002	201.3280245	1.724513846	0.786189712	0.1789388	1
gene5382	3.099419475	12.58300153	4.059793013	2.021406174	0.17899822	1
gene10336	3.099419475	12.58300153	4.059793013	2.021406174	0.17899822	1
gene12911	3.099419475	12.58300153	4.059793013	2.021406174	0.17899822	1
gene16088	3.099419475	12.58300153	4.059793013	2.021406174	0.17899822	1
gene17074	3.099419475	12.58300153	4.059793013	2.021406174	0.17899822	1
gene22921	3.099419475	12.58300153	4.059793013	2.021406174	0.17899822	1
gene28870	3.099419475	12.58300153	4.059793013	2.021406174	0.17899822	1
gene29936	3.099419475	12.58300153	4.059793013	2.021406174	0.17899822	1
gene37039	3.099419475	12.58300153	4.059793013	2.021406174	0.17899822	1
gene37226	3.099419475	12.58300153	4.059793013	2.021406174	0.17899822	1
gene39006	3.099419475	12.58300153	4.059793013	2.021406174	0.17899822	1
gene49129	3.099419475	12.58300153	4.059793013	2.021406174	0.17899822	1
gene52652	3.099419475	12.58300153	4.059793013	2.021406174	0.17899822	1
gene62281	3.099419475	12.58300153	4.059793013	2.021406174	0.17899822	1
gene64118	3.099419475	12.58300153	4.059793013	2.021406174	0.17899822	1
gene67572	3.099419475	12.58300153	4.059793013	2.021406174	0.17899822	1

gene72238	3.099419475	12.58300153	4.059793013	2.021406174	0.17899822	1
gene2166	415.3222097	250.6921074	0.60360872	-0.72831445	0.1790165	1
gene31407	66.12094881	120.9903993	1.829834591	0.871713241	0.17903794	1
gene59023	1445.362615	858.5478736	0.594001716	-0.751461	0.17909193	1
gene8410	43.39187265	19.35846389	0.4461311	-1.16446037	0.17913812	1
gene24500	43.39187265	19.35846389	0.4461311	-1.16446037	0.17913812	1
gene26353	43.39187265	19.35846389	0.4461311	-1.16446037	0.17913812	1
gene54624	22.72907615	49.36408293	2.171847311	1.11892268	0.17914668	1
gene17384	13.43081773	32.90938862	2.450289274	1.29295208	0.17923682	1
gene57653	13.43081773	32.90938862	2.450289274	1.29295208	0.17923682	1
gene61376	13.43081773	32.90938862	2.450289274	1.29295208	0.17923682	1
gene72210	13.43081773	32.90938862	2.450289274	1.29295208	0.17923682	1
gene9581	112.6122409	60.97916126	0.541496739	-0.88497544	0.17924237	1
gene62465	112.6122409	60.97916126	0.541496739	-0.88497544	0.17924237	1
gene8041	8.265118601	23.23015667	2.810625932	1.490891457	0.17928247	1
gene27977	8.265118601	23.23015667	2.810625932	1.490891457	0.17928247	1
gene48679	8.265118601	23.23015667	2.810625932	1.490891457	0.17928247	1
gene52392	8.265118601	23.23015667	2.810625932	1.490891457	0.17928247	1
gene58968	8.265118601	23.23015667	2.810625932	1.490891457	0.17928247	1
gene51035	2493.999538	4564.725786	1.830283333	0.872066999	0.17931173	1
gene53	25.82849563	9.679231946	0.374750124	-1.41599914	0.17933873	1
gene23897	118.8110799	64.85085404	0.545831703	-0.8734719	0.17935253	1
gene52475	971.1514356	585.5935328	0.602988897	-0.72979666	0.17970342	1
gene25523	2006.35754	3584.21959	1.786431141	0.837080305	0.17975741	1
gene1747	755.2252121	1263.139769	1.672533899	0.742035452	0.17983151	1
gene15270	372.9634769	614.6312286	1.647966267	0.720686711	0.17994675	1
gene14117	252.0861173	149.060172	0.591306549	-0.75802184	0.17995683	1
gene41744	544.4646878	901.1364942	1.655087124	0.726907163	0.17998714	1
gene42436	252.0861173	417.1748969	1.654890405	0.726735678	0.18005896	1
gene36	12.3976779	30.97354223	2.498334162	1.320966456	0.18006807	1
gene6057	12.3976779	30.97354223	2.498334162	1.320966456	0.18006807	1
gene6888	12.3976779	30.97354223	2.498334162	1.320966456	0.18006807	1
gene42863	12.3976779	30.97354223	2.498334162	1.320966456	0.18006807	1
gene44455	12.3976779	30.97354223	2.498334162	1.320966456	0.18006807	1
gene44222	259.3180961	428.7899752	1.653528935	0.725548291	0.18023028	1
gene40977	818.2467415	1372.51509	1.677385342	0.746214154	0.1802761	1
gene19308	1098.227634	1870.995535	1.703650024	0.768628997	0.18030104	1
gene8582	339.9030025	204.2317941	0.600853163	-0.73491563	0.18034537	1
gene46446	9.298258426	25.16600306	2.706528675	1.436443673	0.18038428	1
gene61627	9.298258426	25.16600306	2.706528675	1.436443673	0.18038428	1
gene17838	1762.536542	3108.001378	1.763368478	0.818333975	0.18039526	1
gene64400	1134.387528	681.417929	0.600692367	-0.73530176	0.1804016	1
gene38528	5595.485293	11739.94043	2.098109425	1.069089922	0.180427	1
gene30128	852.3403557	515.9030627	0.605278231	-0.72432963	0.18045521	1
gene61599	325.4390449	536.2294498	1.647710864	0.720463104	0.18047805	1
gene38566	525.868171	869.1950288	1.652876285	0.724978745	0.18049385	1
gene49552	367.7977777	221.6544116	0.602652939	-0.73060069	0.18054283	1
gene999	85.75060548	44.52446695	0.5192321	-0.94554852	0.18056223	1

gene48749	85.75060548	44.52446695	0.5192321	-0.94554852	0.18056223	1
gene69656	85.75060548	44.52446695	0.5192321	-0.94554852	0.18056223	1
gene9749	11.36453808	29.03769584	2.555114484	1.353387934	0.18061068	1
gene22271	11.36453808	29.03769584	2.555114484	1.353387934	0.18061068	1
gene47083	11.36453808	29.03769584	2.555114484	1.353387934	0.18061068	1
gene56786	11.36453808	29.03769584	2.555114484	1.353387934	0.18061068	1
gene73368	11.36453808	29.03769584	2.555114484	1.353387934	0.18061068	1
gene73887	11.36453808	29.03769584	2.555114484	1.353387934	0.18061068	1
gene46518	102.2808427	178.0978678	1.741263204	0.800134293	0.18062234	1
gene57414	85.75060548	151.9639416	1.772161732	0.825510274	0.1806236	1
gene49403	380.1954556	229.3977971	0.603368067	-0.72888975	0.18068028	1
gene15529	10.33139825	27.10184945	2.62325087	1.391355784	0.18076255	1
gene56967	10.33139825	27.10184945	2.62325087	1.391355784	0.18076255	1
gene45234	737.6618351	1231.198304	1.669055175	0.739031648	0.18076794	1
gene16848	195.2634269	113.2470138	0.57997043	-0.78594875	0.18083118	1
gene27783	195.2634269	113.2470138	0.57997043	-0.78594875	0.18083118	1
gene53358	277.9146129	165.5148663	0.595560142	-0.74768089	0.1808506	1
gene53280	764.5234706	1277.658617	1.671182987	0.74086971	0.18090391	1
gene1700	144.6395755	244.8845682	1.693067526	0.759639514	0.18098715	1
gene24775	171.501211	287.4731888	1.67621667	0.745208646	0.18100462	1
gene53881	2624.175156	4820.257509	1.836865767	0.877246202	0.18100875	1
gene35756	108.4796816	187.7770998	1.730988669	0.791596281	0.18109392	1
gene36103	458.7140823	755.948015	1.647972112	0.720691828	0.18112198	1
gene857	21.69593633	47.42823654	2.186042392	1.128321378	0.18112279	1
gene59674	21.69593633	47.42823654	2.186042392	1.128321378	0.18112279	1
gene28840	150.8384145	85.17724113	0.564691968	-0.82446398	0.18128749	1
gene35803	150.8384145	85.17724113	0.564691968	-0.82446398	0.18128749	1
gene13058	221.0919226	129.7017081	0.58664155	-0.76944884	0.18136288	1
gene42945	221.0919226	129.7017081	0.58664155	-0.76944884	0.18136288	1
gene46698	620.9170349	377.4900459	0.607955692	-0.71796191	0.18145866	1
gene36445	220.0587827	364.9070444	1.658225315	0.72964005	0.18155126	1
gene11064	68.18722846	123.8941689	1.8169703	0.861534837	0.18159758	1
gene22655	205.5948252	341.6768877	1.661894395	0.732828709	0.18164052	1
gene54926	167.3686517	280.6977264	1.67712247	0.745988044	0.18164348	1
gene51937	53.7232709	100.6640122	1.873750621	0.905928957	0.18176739	1
gene31204	306.842528	183.905407	0.599347842	-0.73853456	0.18183987	1
gene51131	358.4995193	589.4652255	1.644256669	0.717435522	0.18192412	1
gene30235	60.95524968	112.2790906	1.841992136	0.881266902	0.18193465	1
gene56139	1926.805774	3417.7368	1.773783765	0.826830147	0.18200549	1
gene65083	101.2477029	176.1620214	1.739911291	0.799013753	0.18206242	1
gene49398	598.1879587	363.9391212	0.60840262	-0.71690173	0.18208809	1
gene44280	208.6942447	121.9583225	0.58438757	-0.7750026	0.18210883	1
gene27967	259.3180961	427.822052	1.649796364	0.722287962	0.18220994	1
gene38220	74.38606741	37.74900459	0.507474127	-0.97859383	0.18228018	1
gene57338	308.9088077	508.1596772	1.645015178	0.718100895	0.18231939	1
gene34333	304.7762484	501.3842148	1.645089529	0.7181661	0.18240943	1
gene65410	49.5907116	93.88854988	1.893268857	0.920879298	0.18241563	1
gene36634	130.175618	221.6544116	1.7027337	0.767852821	0.18244125	1

gene29642	368.8309176	605.9199198	1.642812169	0.71616754	0.18266965	1
gene55289	465.9460611	766.5951702	1.645244448	0.718301953	0.1827243	1
gene71029	107.4465418	58.07539168	0.540504987	-0.88762017	0.18275227	1
gene2469	41.325593	80.33762515	1.94401627	0.959040293	0.18281663	1
gene71621	41.325593	80.33762515	1.94401627	0.959040293	0.18281663	1
gene36022	1956.766829	3471.940499	1.7743251	0.827270371	0.18283062	1
gene17403	3380.433508	1909.712463	0.564931231	-0.82385284	0.18285171	1
gene32168	805.8490636	1346.381164	1.670760971	0.740505347	0.18295258	1
gene35389	196.2965668	114.214937	0.581848877	-0.7812836	0.18296108	1
gene16621	26.86163545	56.13954529	2.089952616	1.063470234	0.18299209	1
gene20353	26.86163545	56.13954529	2.089952616	1.063470234	0.18299209	1
gene42565	26.86163545	56.13954529	2.089952616	1.063470234	0.18299209	1
gene52172	164.2692322	93.88854988	0.571552863	-0.80704116	0.18302459	1
gene31016	32.02733458	64.85085404	2.024859543	1.017821837	0.18308687	1
gene20940	20.6627965	45.49239015	2.20165698	1.138589713	0.18313416	1
gene28377	20.6627965	45.49239015	2.20165698	1.138589713	0.18313416	1
gene50463	20.6627965	45.49239015	2.20165698	1.138589713	0.18313416	1
gene56966	77.48548688	39.68485098	0.512158503	-0.96533773	0.18328459	1
gene55549	410.1565106	248.756261	0.60649107	-0.72144169	0.18335052	1
gene6507	189.064588	314.5750383	1.663849596	0.734525026	0.18348609	1
gene27636	158.0703932	90.0168571	0.569473228	-0.81230007	0.18350365	1
gene15846	54.75641073	26.13392626	0.477276102	-1.067104	0.18355801	1
gene41455	54.75641073	26.13392626	0.477276102	-1.067104	0.18355801	1
gene33129	260.3512359	428.7899752	1.646967312	0.719811922	0.18364986	1
gene69286	151.8715543	255.5317234	1.682551578	0.750650731	0.18369307	1
gene59971	353.3338202	579.7859936	1.640901495	0.714488635	0.18374348	1
gene17645	382.2617353	627.2142301	1.640797841	0.714397499	0.18377334	1
gene21579	241.7547191	398.7843562	1.649541145	0.722064764	0.18379842	1
gene2088	7.231978776	0.967923195	0.13383933	-2.90142597	0.18382571	1
gene7965	7.231978776	0.967923195	0.13383933	-2.90142597	0.18382571	1
gene16329	7.231978776	0.967923195	0.13383933	-2.90142597	0.18382571	1
gene21813	7.231978776	0.967923195	0.13383933	-2.90142597	0.18382571	1
gene21851	7.231978776	0.967923195	0.13383933	-2.90142597	0.18382571	1
gene24108	7.231978776	0.967923195	0.13383933	-2.90142597	0.18382571	1
gene33956	7.231978776	0.967923195	0.13383933	-2.90142597	0.18382571	1
gene38440	7.231978776	0.967923195	0.13383933	-2.90142597	0.18382571	1
gene41311	7.231978776	0.967923195	0.13383933	-2.90142597	0.18382571	1
gene44838	7.231978776	0.967923195	0.13383933	-2.90142597	0.18382571	1
gene53059	7.231978776	0.967923195	0.13383933	-2.90142597	0.18382571	1
gene54593	7.231978776	0.967923195	0.13383933	-2.90142597	0.18382571	1
gene57946	7.231978776	0.967923195	0.13383933	-2.90142597	0.18382571	1
gene59590	7.231978776	0.967923195	0.13383933	-2.90142597	0.18382571	1
gene61105	7.231978776	0.967923195	0.13383933	-2.90142597	0.18382571	1
gene62583	7.231978776	0.967923195	0.13383933	-2.90142597	0.18382571	1
gene69123	7.231978776	0.967923195	0.13383933	-2.90142597	0.18382571	1
gene72688	7.231978776	0.967923195	0.13383933	-2.90142597	0.18382571	1
gene54049	151.8715543	86.14516432	0.567223828	-0.81800996	0.183986	1
gene45754	4608.83676	9216.564659	1.999759406	0.999826437	0.18406868	1

gene47576	289.279151	475.2502886	1.642877777	0.716225154	0.18419377	1
gene3857	23.76221598	8.711308752	0.366603382	-1.447708	0.18427454	1
gene29564	23.76221598	8.711308752	0.366603382	-1.447708	0.18427454	1
gene30166	23.76221598	8.711308752	0.366603382	-1.447708	0.18427454	1
gene69701	23.76221598	8.711308752	0.366603382	-1.447708	0.18427454	1
gene57068	206.627965	120.9903993	0.585547069	-0.77214295	0.1843492	1
gene42607	2580.783283	4707.010496	1.82386895	0.867002071	0.18435792	1
gene20207	636.4141322	1052.132513	1.653219907	0.725278641	0.18443604	1
gene61178	48.55757178	91.95270349	1.893684139	0.921195713	0.18452312	1
gene69641	121.9104994	208.1034868	1.707018575	0.771478757	0.18461195	1
gene3989	1127.155549	1912.616233	1.696852075	0.762860802	0.1846705	1
gene53100	150.8384145	253.595877	1.681241996	0.749527399	0.18474233	1
gene9290	96.08200373	167.4507127	1.742789556	0.801398373	0.18487166	1
gene57048	6214.336048	3299.650171	0.530973888	-0.91328718	0.18497813	1
gene9616	1194.309638	2032.638709	1.701936118	0.767176887	0.1851287	1
gene7546	19.62965668	43.55654376	2.21891521	1.14985454	0.18517494	1
gene17487	19.62965668	43.55654376	2.21891521	1.14985454	0.18517494	1
gene25831	19.62965668	43.55654376	2.21891521	1.14985454	0.18517494	1
gene45369	19.62965668	43.55654376	2.21891521	1.14985454	0.18517494	1
gene47424	19.62965668	43.55654376	2.21891521	1.14985454	0.18517494	1
gene25614	516.5699125	848.8686417	1.643279295	0.716577704	0.18519409	1
gene13616	25.82849563	54.2036989	2.098600696	1.069427689	0.18520685	1
gene63615	25.82849563	54.2036989	2.098600696	1.069427689	0.18520685	1
gene44290	424.6204681	695.9367769	1.638961918	0.712782333	0.18520695	1
gene10701	86.78374531	45.49239015	0.524204043	-0.93179961	0.18529472	1
gene29978	133.2750374	74.53008599	0.559220147	-0.83851176	0.18537113	1
gene60923	220.0587827	129.7017081	0.589395735	-0.76269147	0.18540262	1
gene46400	905.0304868	1515.767723	1.674825042	0.744010394	0.18543606	1
gene5950	271.715774	446.2125927	1.642203491	0.715632908	0.18547503	1
gene69170	350.2344007	211.9751796	0.605238033	-0.72442545	0.18559576	1
gene70108	350.2344007	211.9751796	0.605238033	-0.72442545	0.18559576	1
gene45032	114.6785206	196.4884085	1.713384577	0.776849007	0.18570253	1
gene596	89.88316478	47.42823654	0.527665405	-0.9223047	0.18570635	1
gene45365	57.8558302	28.06977264	0.485167572	-1.04344497	0.18576723	1
gene41662	242.7878589	144.220556	0.594018814	-0.75141947	0.18577172	1
gene45892	1988.794163	1171.187066	0.588893052	-0.76392244	0.18589983	1
gene66348	51.65699125	96.79231946	1.873750621	0.905928957	0.18592514	1
gene60054	1137.486947	1928.103004	1.695055058	0.761332135	0.18594862	1
gene64254	239.6884394	142.2847096	0.59362358	-0.75237969	0.18596898	1
gene56515	185.9651685	308.7674991	1.660351245	0.731488474	0.18597618	1
gene41171	92.98258426	49.36408293	0.530896009	-0.9134988	0.1860184	1
gene47038	123.976779	211.0072564	1.701990148	0.767222686	0.18602021	1
gene54957	199.3959862	330.0618094	1.655308191	0.727099848	0.18615104	1
gene69158	49.5907116	23.23015667	0.468437655	-1.09407104	0.18627106	1
gene11330	91.94944443	160.6752503	1.747430355	0.805234957	0.18628697	1
gene31273	91.94944443	160.6752503	1.747430355	0.805234957	0.18628697	1
gene51688	898.8316478	1503.184721	1.672376273	0.74189948	0.18635585	1
gene53631	72.31978776	129.7017081	1.793447023	0.84273513	0.18640389	1

gene47660	262.4175156	156.8035575	0.597534647	-0.74290573	0.18641174	1
gene56367	95.04886391	165.5148663	1.741366067	0.800219515	0.18644815	1
gene27377	428.7530274	701.7443161	1.63670988	0.710798615	0.18648692	1
gene49746	394.6594132	645.6047708	1.635852964	0.71004308	0.18651406	1
gene19734	35.12675405	69.69047001	1.983971246	0.988391117	0.18663014	1
gene50182	481.4431585	788.8574036	1.638526563	0.712399061	0.18668855	1
gene21889	47.52443195	90.0168571	1.894117476	0.921525812	0.18669473	1
gene70051	942.2235205	1578.68273	1.675486438	0.744580009	0.18679607	1
gene51503	72.31978776	36.7810814	0.508589454	-0.97542655	0.18682855	1
gene47025	431.8524469	263.2751089	0.609641351	-0.71396733	0.18685913	1
gene66112	441.1507053	269.0826481	0.609956291	-0.71322223	0.18694167	1
gene14994	236.5890199	389.1051242	1.644645742	0.71777686	0.18695226	1
gene41708	1118.890431	1892.289846	1.691219975	0.758064321	0.18696591	1
gene10825	110.5459613	189.7129461	1.716145429	0.779171814	0.18700805	1
gene55055	110.5459613	189.7129461	1.716145429	0.779171814	0.18700805	1
gene9434	745.9269537	455.8918247	0.611174891	-0.71034282	0.18719317	1
gene56648	39.25931335	76.46593238	1.947714462	0.961782191	0.18722574	1
gene15427	18.59651685	41.62069737	2.23809102	1.16226871	0.18723708	1
gene22604	122.9436392	209.07141	1.700546782	0.765998696	0.187325	1
gene66198	221.0919226	130.6696313	0.591019472	-0.75872243	0.18732681	1
gene34826	359.5326591	587.5293791	1.634147453	0.708538167	0.18736704	1
gene36161	24.7953558	52.26785251	2.107969449	1.075853958	0.18749387	1
gene69325	180.7994694	300.0561903	1.659607693	0.73084225	0.18750548	1
gene53529	272.7489138	163.5790199	0.59974215	-0.73758573	0.18756169	1
gene15649	217.9925031	128.7337849	0.590542257	-0.7598878	0.18758325	1
gene23938	84.71746566	149.060172	1.759497535	0.815163493	0.18760052	1
gene23773	29.96105493	60.97916126	2.035280847	1.025227885	0.1876035	1
gene47962	29.96105493	60.97916126	2.035280847	1.025227885	0.1876035	1
gene5451	60.95524968	30.00561903	0.492256519	-1.02251778	0.18762266	1
gene11093	60.95524968	30.00561903	0.492256519	-1.02251778	0.18762266	1
gene36537	60.95524968	30.00561903	0.492256519	-1.02251778	0.18762266	1
gene5320	293.4117103	480.0899045	1.636232937	0.710378148	0.18763118	1
gene13400	1026.940986	1726.774979	1.681474401	0.749726815	0.18765218	1
gene27740	1156.083464	699.8084697	0.605326943	-0.72421353	0.18765403	1
gene63082	978.3834144	595.2727647	0.608424832	-0.71684906	0.18772972	1
gene70717	578.558302	354.2598892	0.612314935	-0.70765422	0.1877979	1
gene24199	304.7762484	183.905407	0.603411217	-0.72878658	0.18781617	1
gene11733	68.18722846	122.9262457	1.802775219	0.850219524	0.18787876	1
gene65274	1352.380031	814.0234067	0.601919126	-0.73235844	0.18794729	1
gene15868	370.8971972	225.5261044	0.608055564	-0.71772493	0.18798716	1
gene39035	700.4688014	428.7899752	0.612147143	-0.70804962	0.18805412	1
gene30954	50.62385143	94.85647307	1.873750621	0.905928957	0.1880992	1
gene59927	839.9426778	512.9992932	0.610755123	-0.71133404	0.1881547	1
gene24656	153.9378339	257.4675698	1.672542501	0.742042872	0.18822319	1
gene36627	299.6105493	489.7691365	1.634685887	0.709013441	0.18823528	1
gene37240	100.214563	173.2582518	1.728872996	0.789831892	0.18825811	1
gene16808	3380.433508	6356.351619	1.880336236	0.910990664	0.18826409	1
gene67492	71.28664793	127.7658617	1.792283203	0.841798619	0.18831123	1

gene40208	103.3139825	178.0978678	1.723850572	0.785634723	0.18833426	1
gene23073	306.842528	501.3842148	1.634011485	0.708418124	0.18835361	1
gene35540	78.51862671	40.65277417	0.517746882	-0.94968113	0.18856335	1
gene70893	254.152397	151.9639416	0.597924487	-0.7419648	0.18864853	1
gene41588	220.0587827	362.0032748	1.645029888	0.718113796	0.18865029	1
gene57503	121.9104994	207.1355637	1.699078953	0.764752894	0.18865722	1
gene14232	74.38606741	132.6054777	1.782665522	0.834036038	0.18866351	1
gene37820	74.38606741	132.6054777	1.782665522	0.834036038	0.18866351	1
gene47707	247.953558	406.5277417	1.639531794	0.713283879	0.18867649	1
gene19597	473.1780399	773.3706325	1.634417846	0.708776861	0.18868466	1
gene19156	44.42501248	20.32638709	0.457543756	-1.12801838	0.18872024	1
gene23807	44.42501248	20.32638709	0.457543756	-1.12801838	0.18872024	1
gene53586	1726.376648	3004.433596	1.740311768	0.799345781	0.1887338	1
gene1450	52.69013108	25.16600306	0.477622707	-1.06605667	0.18891593	1
gene72571	34.09361423	67.75462362	1.987311265	0.990817854	0.1889308	1
gene44449	77.48548688	137.4450936	1.773817255	0.826857386	0.18894914	1
gene55316	128.1093383	71.6263164	0.559103008	-0.83881399	0.18896118	1
gene36337	2710.958901	4939.312062	1.821979692	0.865506878	0.18908551	1
gene9379	21.69593633	7.743385557	0.35690488	-1.48638847	0.18923339	1
gene29883	21.69593633	7.743385557	0.35690488	-1.48638847	0.18923339	1
gene69585	21.69593633	7.743385557	0.35690488	-1.48638847	0.18923339	1
gene1433	53.7232709	99.69608905	1.855733789	0.891989766	0.18923414	1
gene43175	53.7232709	99.69608905	1.855733789	0.891989766	0.18923414	1
gene42460	17.56337703	39.68485098	2.259522808	1.17601812	0.18930961	1
gene65197	17.56337703	39.68485098	2.259522808	1.17601812	0.18930961	1
gene67538	17.56337703	39.68485098	2.259522808	1.17601812	0.18930961	1
gene72833	701.5019412	1156.668218	1.648845355	0.721456095	0.18943696	1
gene24805	1000.079351	1675.47505	1.67534211	0.744455729	0.18945299	1
gene29411	250.0198377	409.4315113	1.637596101	0.711579572	0.18956631	1
gene34494	649.84495	1068.587207	1.644372564	0.717537207	0.18959025	1
gene37833	341.9692821	557.5237601	1.630332867	0.705166551	0.18961282	1
gene2407	4.1325593	14.51884792	3.513282415	1.812819552	0.18963675	1
gene23918	4.1325593	14.51884792	3.513282415	1.812819552	0.18963675	1
gene27731	4.1325593	14.51884792	3.513282415	1.812819552	0.18963675	1
gene33804	4.1325593	14.51884792	3.513282415	1.812819552	0.18963675	1
gene37700	4.1325593	14.51884792	3.513282415	1.812819552	0.18963675	1
gene38533	4.1325593	14.51884792	3.513282415	1.812819552	0.18963675	1
gene46578	4.1325593	14.51884792	3.513282415	1.812819552	0.18963675	1
gene50471	4.1325593	14.51884792	3.513282415	1.812819552	0.18963675	1
gene55201	4.1325593	14.51884792	3.513282415	1.812819552	0.18963675	1
gene56748	4.1325593	14.51884792	3.513282415	1.812819552	0.18963675	1
gene65956	4.1325593	14.51884792	3.513282415	1.812819552	0.18963675	1
gene72020	4.1325593	14.51884792	3.513282415	1.812819552	0.18963675	1
gene8781	667.408327	409.4315113	0.613464793	-0.70494754	0.18968593	1
gene21417	648.8118102	1066.65136	1.644007313	0.717216716	0.18973994	1
gene15103	551.6966666	903.0723406	1.636900122	0.710966296	0.18976804	1
gene9389	687.0379837	1131.502215	1.646928178	0.719777641	0.1898158	1
gene60707	23.76221598	50.33200612	2.118152876	1.082806719	0.18985462	1

gene41971	744.8938139	1230.23038	1.651551345	0.723821822	0.18993062	1
gene50845	28.9279151	11.61507834	0.40151799	-1.31646346	0.18996931	1
gene63056	28.9279151	11.61507834	0.40151799	-1.31646346	0.18996931	1
gene15907	56.82269038	104.535705	1.839682428	0.879456745	0.19014852	1
gene9623	136.3744569	229.3977971	1.682117035	0.750278086	0.19015066	1
gene33557	450.4489637	275.8581105	0.612407027	-0.70743726	0.19015701	1
gene46834	190.0977278	111.3111674	0.585547069	-0.77214295	0.19030302	1
gene41323	106.413402	58.07539168	0.545752608	-0.87368097	0.19036221	1
gene10163	94.01572408	50.33200612	0.53535732	-0.90142597	0.19038169	1
gene28801	180.7994694	299.0882671	1.65425412	0.726180873	0.19040628	1
gene5737	210.7605243	346.5165037	1.64412432	0.717319392	0.1904359	1
gene34275	103.3139825	56.13954529	0.54338768	-0.87994624	0.19044515	1
gene21696	100.214563	54.2036989	0.540876468	-0.88662896	0.19048161	1
gene72083	269.6494943	440.4050536	1.633250063	0.707745695	0.19052513	1
gene36565	73.35292758	130.6696313	1.781382633	0.832997434	0.19058742	1
gene45462	39.25931335	17.4226175	0.443783042	-1.17207356	0.19060276	1
gene16271	186.9983083	109.375321	0.584900056	-0.77373797	0.19065231	1
gene35381	509.3379338	831.4460242	1.63240546	0.706999441	0.19079894	1
gene20786	226.2576217	134.5413241	0.594637754	-0.74991703	0.1908658	1
gene17957	59.92210985	109.375321	1.825291554	0.868126924	0.19088182	1
gene23633	1286.259082	2183.634727	1.697663214	0.763550282	0.19090516	1
gene34042	183.8988889	107.4394746	0.584231233	-0.77538861	0.19101115	1
gene9111	79.55176653	140.3488632	1.764245715	0.819051506	0.19102724	1
gene52947	55.78955055	27.10184945	0.485787198	-1.04160362	0.1910991	1
gene35899	82.65118601	145.1884792	1.756641208	0.812819552	0.1911732	1
gene43369	33.0604744	65.81877724	1.990860035	0.993391798	0.19132041	1
gene49663	33.0604744	65.81877724	1.990860035	0.993391798	0.19132041	1
gene27464	34.09361423	14.51884792	0.425852414	-1.23157457	0.19136913	1
gene32844	34.09361423	14.51884792	0.425852414	-1.23157457	0.19136913	1
gene1698	16.5302372	37.74900459	2.28363357	1.191331176	0.19137768	1
gene16370	16.5302372	37.74900459	2.28363357	1.191331176	0.19137768	1
gene28175	16.5302372	37.74900459	2.28363357	1.191331176	0.19137768	1
gene32498	16.5302372	37.74900459	2.28363357	1.191331176	0.19137768	1
gene62094	16.5302372	37.74900459	2.28363357	1.191331176	0.19137768	1
gene66990	16.5302372	37.74900459	2.28363357	1.191331176	0.19137768	1
gene27192	122.9436392	208.1034868	1.692673881	0.759304043	0.19139686	1
gene33905	91.94944443	159.7073271	1.736903666	0.79651774	0.19141214	1
gene24679	497.9733957	305.8637295	0.614217009	-0.70317963	0.19150811	1
gene1492	150.8384145	251.6600306	1.668408088	0.738472211	0.19154178	1
gene26319	262.4175156	157.7714807	0.601223132	-0.73402758	0.19159104	1
gene37880	37.1930337	72.5942396	1.951823564	0.964822646	0.19190077	1
gene19275	242.7878589	396.8485098	1.634548414	0.70889211	0.19191375	1
gene8406	47.52443195	22.26223348	0.468437655	-1.09407104	0.19198684	1
gene41642	194.2302871	114.214937	0.588038759	-0.76601685	0.19212801	1
gene24437	129.1424781	72.5942396	0.562125186	-0.83103664	0.19213128	1
gene11740	245.8872784	401.6881258	1.633627117	0.70807872	0.19214012	1
gene6302	22.72907615	48.39615973	2.12926207	1.090353528	0.19229017	1
gene15800	22.72907615	48.39615973	2.12926207	1.090353528	0.19229017	1

gene22267	22.72907615	48.39615973	2.12926207	1.090353528	0.19229017	1
gene44969	22.72907615	48.39615973	2.12926207	1.090353528	0.19229017	1
gene19670	55.78955055	102.5998586	1.839051536	0.878961909	0.1923586	1
gene29422	27.89477528	57.10746848	2.047246049	1.033684504	0.19246442	1
gene30122	27.89477528	57.10746848	2.047246049	1.033684504	0.19246442	1
gene72190	27.89477528	57.10746848	2.047246049	1.033684504	0.19246442	1
gene8568	471.1117602	766.5951702	1.627204487	0.702395563	0.19258088	1
gene16877	48.55757178	90.9847803	1.873750621	0.905928957	0.19265169	1
gene57551	143.6064357	240.0449523	1.671547317	0.741184194	0.19265905	1
gene47846	149.8052746	249.7241842	1.666991932	0.737247122	0.19268935	1
gene15312	279.9808926	455.8918247	1.628296204	0.703363165	0.19269021	1
gene40282	975.2839949	1625.143044	1.666328016	0.736672422	0.19281839	1
gene22880	246.9204182	148.0922488	0.599756998	-0.73755001	0.19282215	1
gene55035	162.2029525	269.0826481	1.658925709	0.73024928	0.19285067	1
gene4258	378.129176	231.3336435	0.611784697	-0.70890407	0.19287408	1
gene59909	109.5128215	186.8091766	1.705820141	0.770465539	0.1929643	1
gene59473	103.3139825	177.1299446	1.714481819	0.777772605	0.19305446	1
gene50951	58.88897003	107.4394746	1.824441395	0.867454809	0.19306422	1
gene48894	368.8309176	225.5261044	0.611462038	-0.70966516	0.19306488	1
gene15914	87.81688513	152.9318648	1.741485872	0.800318769	0.19313579	1
gene28713	734.5624156	452.0201319	0.615359733	-0.70049805	0.19320463	1
gene16904	15.49709738	35.8131582	2.3109591	1.208491727	0.19342126	1
gene31590	15.49709738	35.8131582	2.3109591	1.208491727	0.19342126	1
gene32447	15.49709738	35.8131582	2.3109591	1.208491727	0.19342126	1
gene26709	500.0396753	307.7995759	0.615550307	-0.70005133	0.19343009	1
gene14378	1528.013801	920.4949581	0.602412725	-0.73117585	0.19348202	1
gene41348	44.42501248	84.20931793	1.895538419	0.922607698	0.19362259	1
gene24900	1691.249894	2919.256355	1.726094036	0.787511063	0.19365578	1
gene12704	921.560724	565.2671457	0.613380248	-0.70514638	0.19373436	1
gene24247	79.55176653	41.62069737	0.523190109	-0.93459283	0.193782	1
gene63533	142.5732959	238.1091059	1.670082076	0.739919005	0.19387775	1
gene33572	139.4738764	233.2694899	1.672495925	0.742002696	0.19390061	1
gene33462	139.4738764	79.36970196	0.569065004	-0.81333464	0.19403974	1
gene5197	19.62965668	6.775462362	0.345164588	-1.53464363	0.19406819	1
gene28992	19.62965668	6.775462362	0.345164588	-1.53464363	0.19406819	1
gene37419	19.62965668	6.775462362	0.345164588	-1.53464363	0.19406819	1
gene53885	741.7943944	456.8597479	0.615884605	-0.69926803	0.19408512	1
gene3785	123.976779	209.07141	1.686375559	0.753925863	0.19413523	1
gene19493	82.65118601	43.55654376	0.526992362	-0.92414604	0.19417888	1
gene46513	82.65118601	43.55654376	0.526992362	-0.92414604	0.19417888	1
gene61222	120.8773595	204.2317941	1.689578552	0.756663426	0.1942029	1
gene39866	40.29245318	77.43385557	1.921795509	0.942454833	0.19424999	1
gene30293	231.4233208	138.4130168	0.598094506	-0.74155463	0.19430372	1
gene49258	1132.321248	1899.065308	1.677143576	0.7460062	0.19432655	1
gene6823	114.6785206	194.5525621	1.696503941	0.762564781	0.19435314	1
gene28285	61.9883895	30.97354223	0.499666832	-1.00096164	0.19439875	1
gene7838	85.75060548	45.49239015	0.530519754	-0.91452162	0.19445236	1
gene10095	85.75060548	45.49239015	0.530519754	-0.91452162	0.19445236	1

gene41428	1538.3452	2632.751089	1.711417626	0.775191854	0.19450595	1
gene21381	248.9866978	405.5598186	1.628841308	0.703846054	0.19455906	1
gene45008	208.6942447	341.6768877	1.637212795	0.711241847	0.19459755	1
gene8364	502.105955	815.9592531	1.625073841	0.700505274	0.19461356	1
gene53549	88.85002496	47.42823654	0.533801049	-0.90562595	0.19462111	1
gene62102	98.14828338	53.23577571	0.542401496	-0.88256694	0.19464552	1
gene24448	74.38606741	131.6375545	1.769653365	0.823466796	0.19476379	1
gene72320	42.35873283	19.35846389	0.457012347	-1.12969495	0.19478767	1
gene32122	21.69593633	46.46031334	2.141429282	1.098574035	0.19480097	1
gene44990	21.69593633	46.46031334	2.141429282	1.098574035	0.19480097	1
gene70201	21.69593633	46.46031334	2.141429282	1.098574035	0.19480097	1
gene9347	496.9402559	807.2479443	1.624436609	0.699939446	0.19481661	1
gene25778	133.2750374	75.49800918	0.566482746	-0.81989608	0.19486896	1
gene22315	26.86163545	55.17162209	2.05391895	1.038379253	0.19503444	1
gene24450	26.86163545	55.17162209	2.05391895	1.038379253	0.19503444	1
gene23634	47.52443195	89.04893391	1.873750621	0.905928957	0.19503623	1
gene61836	47.52443195	89.04893391	1.873750621	0.905928957	0.19503623	1
gene69556	47.52443195	89.04893391	1.873750621	0.905928957	0.19503623	1
gene42061	286.1797315	464.6031334	1.623466242	0.699077386	0.19510458	1
gene21413	14.46395755	33.87731181	2.342188277	1.227857052	0.19541258	1
gene34659	14.46395755	33.87731181	2.342188277	1.227857052	0.19541258	1
gene62825	14.46395755	33.87731181	2.342188277	1.227857052	0.19541258	1
gene24421	440.1175655	713.3593944	1.620838272	0.696740146	0.19544594	1
gene24488	388.4605742	629.1500765	1.619598277	0.695636013	0.19549019	1
gene58422	1026.940986	1710.320285	1.665451382	0.73591324	0.19556065	1
gene25003	300.6436891	182.9374838	0.608486027	-0.71670396	0.1956394	1
gene50421	300.6436891	182.9374838	0.608486027	-0.71670396	0.1956394	1
gene33681	243.8209987	396.8485098	1.627622362	0.702766007	0.19572506	1
gene3809	1435.031217	869.1950288	0.605697645	-0.72333029	0.19576864	1
gene31146	1135.420668	694.0009306	0.611228023	-0.71021741	0.19581838	1
gene69382	297.5442696	181.0016374	0.608318344	-0.71710158	0.19582847	1
gene55020	383.2948751	620.4387678	1.618698313	0.694834127	0.19597872	1
gene1447	544.4646878	884.6817999	1.62486534	0.70032016	0.19609269	1
gene57242	123.976779	69.69047001	0.562125186	-0.83103664	0.19609791	1
gene41074	50.62385143	93.88854988	1.854630717	0.891131955	0.19613725	1
gene17101	263.4506554	427.822052	1.623917205	0.699478079	0.19621287	1
gene3324	26.86163545	10.64715514	0.396370324	-1.33507914	0.19621979	1
gene50652	26.86163545	10.64715514	0.396370324	-1.33507914	0.19621979	1
gene65342	26.86163545	10.64715514	0.396370324	-1.33507914	0.19621979	1
gene485	5.165699125	16.45469431	3.185376056	1.671463703	0.19625453	1
gene8018	5.165699125	16.45469431	3.185376056	1.671463703	0.19625453	1
gene13228	5.165699125	16.45469431	3.185376056	1.671463703	0.19625453	1
gene13978	5.165699125	16.45469431	3.185376056	1.671463703	0.19625453	1
gene15969	5.165699125	16.45469431	3.185376056	1.671463703	0.19625453	1
gene17382	5.165699125	16.45469431	3.185376056	1.671463703	0.19625453	1
gene21759	5.165699125	16.45469431	3.185376056	1.671463703	0.19625453	1
gene22190	5.165699125	16.45469431	3.185376056	1.671463703	0.19625453	1
gene23156	5.165699125	16.45469431	3.185376056	1.671463703	0.19625453	1

gene37330	5.165699125	16.45469431	3.185376056	1.671463703	0.19625453	1
gene52679	5.165699125	16.45469431	3.185376056	1.671463703	0.19625453	1
gene58284	5.165699125	16.45469431	3.185376056	1.671463703	0.19625453	1
gene58319	5.165699125	16.45469431	3.185376056	1.671463703	0.19625453	1
gene72275	5.165699125	16.45469431	3.185376056	1.671463703	0.19625453	1
gene73880	5.165699125	16.45469431	3.185376056	1.671463703	0.19625453	1
gene37758	146.7058552	243.916645	1.662623791	0.733461761	0.19626315	1
gene19809	143.6064357	239.0770291	1.664807207	0.735355116	0.19631954	1
gene2851	30.99419475	61.94708446	1.99866733	0.999038361	0.19638647	1
gene15556	30.99419475	61.94708446	1.99866733	0.999038361	0.19638647	1
gene32418	143.6064357	82.27347154	0.572909363	-0.80362118	0.19649513	1
gene2439	67.15408863	120.0224761	1.787269824	0.837757454	0.19649527	1
gene1774	120.8773595	67.75462362	0.56052369	-0.83515275	0.19649583	1
gene50468	120.8773595	67.75462362	0.56052369	-0.83515275	0.19649583	1
gene13919	811.0147627	1333.798162	1.644604049	0.717740286	0.1965276	1
gene24534	613.6850561	379.4258923	0.618274616	-0.69368032	0.19656806	1
gene37277	245.8872784	399.7522794	1.625754216	0.701109165	0.19659785	1
gene55819	565.1274843	349.4202733	0.61830345	-0.69361304	0.19670899	1
gene54729	53.7232709	26.13392626	0.486454488	-1.03962326	0.19677938	1
gene17080	82.65118601	144.220556	1.744930266	0.803169382	0.19687485	1
gene5410	35.12675405	68.72254682	1.95641609	0.968213235	0.19687636	1
gene50506	79.55176653	139.38094	1.752078503	0.809067417	0.19689341	1
gene49687	368.8309176	226.4940275	0.614086338	-0.70348659	0.19690768	1
gene71937	304.7762484	185.8412534	0.609762914	-0.71367969	0.19693677	1
gene32248	37.1930337	16.45469431	0.442413341	-1.1765332	0.19697007	1
gene65774	37.1930337	16.45469431	0.442413341	-1.1765332	0.19697007	1
gene62615	53.7232709	98.72816585	1.837716956	0.87791458	0.19698616	1
gene33952	114.6785206	63.88293085	0.557060996	-0.84409279	0.19725854	1
gene62534	114.6785206	63.88293085	0.557060996	-0.84409279	0.19725854	1
gene10276	13.43081773	31.94146542	2.378221943	1.249883358	0.19731469	1
gene35940	13.43081773	31.94146542	2.378221943	1.249883358	0.19731469	1
gene49326	13.43081773	31.94146542	2.378221943	1.249883358	0.19731469	1
gene65864	13.43081773	31.94146542	2.378221943	1.249883358	0.19731469	1
gene69764	13.43081773	31.94146542	2.378221943	1.249883358	0.19731469	1
gene70356	179.7663296	295.2165744	1.642223964	0.715650893	0.19732536	1
gene63809	211.7936641	345.5485805	1.631534078	0.706229121	0.19734534	1
gene7580	20.6627965	44.52446695	2.154813215	1.107562818	0.19738665	1
gene51554	20.6627965	44.52446695	2.154813215	1.107562818	0.19738665	1
gene51701	20.6627965	44.52446695	2.154813215	1.107562818	0.19738665	1
gene10631	461.8135018	747.2367063	1.618048635	0.694254973	0.19748502	1
gene43213	145.6727153	241.9807987	1.661126437	0.732161889	0.19749989	1
gene8600	321.3064856	196.4884085	0.611529544	-0.7095059	0.19751039	1
gene19312	142.5732959	237.1411827	1.663293124	0.734042439	0.19758308	1
gene6172	111.5791011	61.94708446	0.555185369	-0.84895855	0.19761672	1
gene66870	111.5791011	61.94708446	0.555185369	-0.84895855	0.19761672	1
gene63186	753.1589325	465.5710566	0.618157784	-0.69395296	0.19765331	1
gene10155	511.4042134	828.5422546	1.62013185	0.696111228	0.19765995	1
gene55156	153.9378339	89.04893391	0.578473346	-0.78967761	0.19779552	1

gene1795	1259.397447	2116.848027	1.680841923	0.749184051	0.19785994	1
gene2408	32.02733458	13.55092472	0.423104979	-1.24091243	0.19787912	1
gene17372	32.02733458	13.55092472	0.423104979	-1.24091243	0.19787912	1
gene58662	32.02733458	13.55092472	0.423104979	-1.24091243	0.19787912	1
gene8405	426.6867478	263.2751089	0.617021996	-0.69660617	0.19788691	1
gene63897	532.0670099	862.4195664	1.620885246	0.696781956	0.19789746	1
gene39202	316.1407865	511.0634468	1.616569164	0.692935233	0.1979142	1
gene53569	316.1407865	511.0634468	1.616569164	0.692935233	0.1979142	1
gene23513	281.0140324	454.9239015	1.618865427	0.694983062	0.1980143	1
gene3506	326.4721847	527.5181411	1.615813431	0.692260628	0.19809258	1
gene52198	45.4581523	21.29431028	0.468437655	-1.09407104	0.19810614	1
gene48698	266.5500749	161.6431735	0.606427042	-0.72159401	0.19822252	1
gene53495	266.5500749	161.6431735	0.606427042	-0.72159401	0.19822252	1
gene49040	286.1797315	174.226175	0.608799841	-0.71596011	0.19822993	1
gene7892	530.0007303	328.125963	0.619104737	-0.6917446	0.19823249	1
gene50530	150.8384145	87.11308752	0.577525876	-0.79204251	0.19830404	1
gene722	123.976779	208.1034868	1.678568265	0.747231211	0.1983154	1
gene3695	167.3686517	97.76024266	0.584101274	-0.77570956	0.19834767	1
gene71024	131.2087578	74.53008599	0.568026763	-0.81596919	0.19841416	1
gene67994	331.6378839	203.2638709	0.612909082	-0.70625501	0.19842974	1
gene20452	120.8773595	203.2638709	1.681571071	0.749809755	0.19847391	1
gene2792	17.56337703	5.807539168	0.330661874	-1.59657138	0.19852206	1
gene8300	17.56337703	5.807539168	0.330661874	-1.59657138	0.19852206	1
gene9812	17.56337703	5.807539168	0.330661874	-1.59657138	0.19852206	1
gene20596	17.56337703	5.807539168	0.330661874	-1.59657138	0.19852206	1
gene64345	17.56337703	5.807539168	0.330661874	-1.59657138	0.19852206	1
gene4119	809.9816229	1328.958546	1.640726788	0.714335023	0.19854009	1
gene7809	102.2808427	56.13954529	0.548876445	-0.86544667	0.19854694	1
gene54284	150.8384145	249.7241842	1.655574179	0.727331653	0.19857538	1
gene11669	77.48548688	40.65277417	0.524650174	-0.93057231	0.19861996	1
gene60673	1761.503402	1059.875898	0.601688249	-0.73291191	0.19871731	1
gene9881	84.71746566	147.1243256	1.736646917	0.796304465	0.1987448	1
gene471	260.3512359	157.7714807	0.605994745	-0.72262281	0.19882208	1
gene12717	3123.181691	5711.714772	1.828812838	0.870907436	0.19894226	1
gene43306	29.96105493	60.01123807	2.002974802	1.002144272	0.19907377	1
gene7672	12.3976779	30.00561903	2.420261219	1.275162766	0.19907784	1
gene13334	12.3976779	30.00561903	2.420261219	1.275162766	0.19907784	1
gene18605	12.3976779	30.00561903	2.420261219	1.275162766	0.19907784	1
gene19804	12.3976779	30.00561903	2.420261219	1.275162766	0.19907784	1
gene26714	12.3976779	30.00561903	2.420261219	1.275162766	0.19907784	1
gene37356	12.3976779	30.00561903	2.420261219	1.275162766	0.19907784	1
gene71658	12.3976779	30.00561903	2.420261219	1.275162766	0.19907784	1
gene71674	12.3976779	30.00561903	2.420261219	1.275162766	0.19907784	1
gene71819	345.0687016	211.9751796	0.614298482	-0.70298828	0.19917865	1
gene21663	3357.704432	1936.814312	0.576826922	-0.79378959	0.1992004	1
gene71802	341.9692821	210.0393332	0.614205264	-0.70320722	0.19932744	1
gene4948	144.6395755	83.24139474	0.575509119	-0.79708931	0.19934813	1
gene10994	144.6395755	83.24139474	0.575509119	-0.79708931	0.19934813	1

gene61566	171.501211	281.6656496	1.642353707	0.715764868	0.19937026	1
gene37991	125.0099188	70.65839321	0.565222295	-0.82310972	0.19938276	1
gene45295	196.2965668	320.3825774	1.63213541	0.706760755	0.19957739	1
gene17544	102.2808427	174.226175	1.703409656	0.768425433	0.19958571	1
gene30180	1146.785206	703.6801625	0.613611127	-0.70460345	0.19969507	1
gene62158	611.6187764	379.4258923	0.620363381	-0.68881456	0.19970503	1
gene23094	277.9146129	449.1163623	1.616022841	0.692447589	0.19983886	1
gene24829	234.5227403	141.3167864	0.602571786	-0.73079497	0.19984945	1
gene6411	9.298258426	1.935846389	0.208194513	-2.26399604	0.19987271	1
gene11836	9.298258426	1.935846389	0.208194513	-2.26399604	0.19987271	1
gene11943	9.298258426	1.935846389	0.208194513	-2.26399604	0.19987271	1
gene16554	9.298258426	1.935846389	0.208194513	-2.26399604	0.19987271	1
gene31421	9.298258426	1.935846389	0.208194513	-2.26399604	0.19987271	1
gene36563	9.298258426	1.935846389	0.208194513	-2.26399604	0.19987271	1
gene37386	9.298258426	1.935846389	0.208194513	-2.26399604	0.19987271	1
gene39245	9.298258426	1.935846389	0.208194513	-2.26399604	0.19987271	1
gene42796	9.298258426	1.935846389	0.208194513	-2.26399604	0.19987271	1
gene44304	9.298258426	1.935846389	0.208194513	-2.26399604	0.19987271	1
gene48873	9.298258426	1.935846389	0.208194513	-2.26399604	0.19987271	1
gene54258	9.298258426	1.935846389	0.208194513	-2.26399604	0.19987271	1
gene44246	188.0314482	111.3111674	0.591981652	-0.75637563	0.20001714	1
gene59162	391.5599937	631.0859229	1.61172217	0.688603073	0.2000282	1
gene6194	45.4581523	85.17724113	1.873750621	0.905928957	0.20003861	1
gene28424	19.62965668	42.58862056	2.169605983	1.117433062	0.20004567	1
gene47500	247.953558	150.0280952	0.605065305	-0.72483723	0.20015709	1
gene6511	140.5070162	233.2694899	1.660198161	0.731355452	0.20018428	1
gene8332	6.19883895	18.3905407	2.966771817	1.568893969	0.2001965	1
gene19163	6.19883895	18.3905407	2.966771817	1.568893969	0.2001965	1
gene28811	6.19883895	18.3905407	2.966771817	1.568893969	0.2001965	1
gene32351	6.19883895	18.3905407	2.966771817	1.568893969	0.2001965	1
gene36185	6.19883895	18.3905407	2.966771817	1.568893969	0.2001965	1
gene37674	6.19883895	18.3905407	2.966771817	1.568893969	0.2001965	1
gene42445	6.19883895	18.3905407	2.966771817	1.568893969	0.2001965	1
gene51421	6.19883895	18.3905407	2.966771817	1.568893969	0.2001965	1
gene62518	6.19883895	18.3905407	2.966771817	1.568893969	0.2001965	1
gene63262	6.19883895	18.3905407	2.966771817	1.568893969	0.2001965	1
gene63621	6.19883895	18.3905407	2.966771817	1.568893969	0.2001965	1
gene68505	1552.809157	941.7892684	0.606506771	-0.72140434	0.20024669	1
gene39261	1326.551535	810.1517139	0.610720121	-0.71141672	0.20026144	1
gene2776	485.5757178	301.0241135	0.619932386	-0.68981722	0.20027509	1
gene67318	138.4407366	79.36970196	0.573311757	-0.80260823	0.20042444	1
gene36089	263.4506554	425.8862056	1.616569164	0.692935233	0.20046203	1
gene66876	24.7953558	51.29992932	2.068932978	1.04888691	0.20047586	1
gene11246	457.6809425	283.601496	0.619648908	-0.69047708	0.20063002	1
gene5456	11.36453808	28.06977264	2.469944001	1.304478333	0.20063386	1
gene7283	11.36453808	28.06977264	2.469944001	1.304478333	0.20063386	1
gene37063	11.36453808	28.06977264	2.469944001	1.304478333	0.20063386	1
gene46206	11.36453808	28.06977264	2.469944001	1.304478333	0.20063386	1

gene53676	11.36453808	28.06977264	2.469944001	1.304478333	0.20063386	1
gene21485	83.68432583	145.1884792	1.734954279	0.794897644	0.20071311	1
gene57395	48.55757178	23.23015667	0.478404414	-1.06369739	0.20074426	1
gene60567	1492.887047	2529.183308	1.694155839	0.760566589	0.20081533	1
gene71422	256.2186766	414.2711273	1.616865456	0.693199633	0.20084436	1
gene13392	10197.09007	5141.60801	0.504223065	-0.98786598	0.20084697	1
gene55774	65.08780898	116.1507834	1.784524401	0.835539629	0.20095242	1
gene55769	135.3413171	77.43385557	0.572137594	-0.80556595	0.20097277	1
gene40397	960.8200373	1584.49027	1.649102025	0.721680657	0.20100369	1
gene11891	68.18722846	120.9903993	1.774385058	0.827319122	0.20105159	1
gene9582	63.02152933	31.94146542	0.506834184	-0.98041426	0.20107994	1
gene73985	63.02152933	31.94146542	0.506834184	-0.98041426	0.20107994	1
gene66853	245.8872784	397.816433	1.617881314	0.694105777	0.2011537	1
gene29503	337.8367228	543.9728354	1.610164907	0.687208451	0.201164	1
gene68512	2009.45696	3486.459347	1.73502564	0.794956983	0.2011685	1
gene65311	41.325593	78.40177877	1.897172504	0.923850865	0.20123671	1
gene20999	278.9477528	450.0842855	1.61350748	0.690200266	0.20125068	1
gene30378	40.29245318	18.3905407	0.456426433	-1.13154575	0.20130472	1
gene74212	40.29245318	18.3905407	0.456426433	-1.13154575	0.20130472	1
gene64176	142.5732959	236.1732595	1.656504173	0.728141837	0.20135444	1
gene37965	132.2418976	75.49800918	0.570908392	-0.80866882	0.2015266	1
gene72461	203.5285455	331.0297326	1.626453585	0.701729652	0.20157999	1
gene42372	178.7331897	105.5036282	0.5902856	-0.76051495	0.20158545	1
gene46663	414.2890699	256.4996466	0.619132063	-0.69168092	0.20161825	1
gene20601	109.5128215	60.97916126	0.556822119	-0.84471157	0.20170506	1
gene72687	2315.266348	4070.117033	1.757947649	0.813892108	0.20171765	1
gene20204	301.6768289	184.8733302	0.612819124	-0.70646677	0.20182506	1
gene29029	682.9054244	1109.239981	1.624295168	0.699813824	0.20185821	1
gene45738	28.9279151	58.07539168	2.007589952	1.00546463	0.20187183	1
gene60711	28.9279151	58.07539168	2.007589952	1.00546463	0.20187183	1
gene25159	10.33139825	26.13392626	2.529563339	1.338888364	0.20188839	1
gene48441	10.33139825	26.13392626	2.529563339	1.338888364	0.20188839	1
gene49535	10.33139825	26.13392626	2.529563339	1.338888364	0.20188839	1
gene62182	10.33139825	26.13392626	2.529563339	1.338888364	0.20188839	1
gene63307	10.33139825	26.13392626	2.529563339	1.338888364	0.20188839	1
gene64642	10.33139825	26.13392626	2.529563339	1.338888364	0.20188839	1
gene66845	10.33139825	26.13392626	2.529563339	1.338888364	0.20188839	1
gene340	2.06627965	9.679231946	4.684376554	2.227857052	0.20189606	1
gene2525	2.06627965	9.679231946	4.684376554	2.227857052	0.20189606	1
gene2727	2.06627965	9.679231946	4.684376554	2.227857052	0.20189606	1
gene4284	2.06627965	9.679231946	4.684376554	2.227857052	0.20189606	1
gene5595	2.06627965	9.679231946	4.684376554	2.227857052	0.20189606	1
gene8327	2.06627965	9.679231946	4.684376554	2.227857052	0.20189606	1
gene14560	2.06627965	9.679231946	4.684376554	2.227857052	0.20189606	1
gene19147	2.06627965	9.679231946	4.684376554	2.227857052	0.20189606	1
gene30050	2.06627965	9.679231946	4.684376554	2.227857052	0.20189606	1
gene32379	2.06627965	9.679231946	4.684376554	2.227857052	0.20189606	1
gene33134	2.06627965	9.679231946	4.684376554	2.227857052	0.20189606	1

gene37450	2.06627965	9.679231946	4.684376554	2.227857052	0.20189606	1
gene39599	2.06627965	9.679231946	4.684376554	2.227857052	0.20189606	1
gene41612	2.06627965	9.679231946	4.684376554	2.227857052	0.20189606	1
gene44249	2.06627965	9.679231946	4.684376554	2.227857052	0.20189606	1
gene49529	2.06627965	9.679231946	4.684376554	2.227857052	0.20189606	1
gene52210	2.06627965	9.679231946	4.684376554	2.227857052	0.20189606	1
gene52465	2.06627965	9.679231946	4.684376554	2.227857052	0.20189606	1
gene52995	2.06627965	9.679231946	4.684376554	2.227857052	0.20189606	1
gene54245	2.06627965	9.679231946	4.684376554	2.227857052	0.20189606	1
gene55616	2.06627965	9.679231946	4.684376554	2.227857052	0.20189606	1
gene55851	2.06627965	9.679231946	4.684376554	2.227857052	0.20189606	1
gene56844	2.06627965	9.679231946	4.684376554	2.227857052	0.20189606	1
gene58278	2.06627965	9.679231946	4.684376554	2.227857052	0.20189606	1
gene60631	2.06627965	9.679231946	4.684376554	2.227857052	0.20189606	1
gene60973	2.06627965	9.679231946	4.684376554	2.227857052	0.20189606	1
gene64687	2.06627965	9.679231946	4.684376554	2.227857052	0.20189606	1
gene65849	2.06627965	9.679231946	4.684376554	2.227857052	0.20189606	1
gene66822	2.06627965	9.679231946	4.684376554	2.227857052	0.20189606	1
gene67066	2.06627965	9.679231946	4.684376554	2.227857052	0.20189606	1
gene67336	2.06627965	9.679231946	4.684376554	2.227857052	0.20189606	1
gene69034	2.06627965	9.679231946	4.684376554	2.227857052	0.20189606	1
gene53481	282.0471722	172.2903286	0.61085643	-0.71109475	0.20189903	1
gene59497	247.953558	400.7202026	1.616109911	0.692525319	0.20200021	1
gene62249	129.1424781	73.56216279	0.569620189	-0.81192781	0.20208472	1
gene33818	953.5880585	1569.971422	1.646383265	0.719300223	0.20210584	1
gene62851	1257.331167	2102.329179	1.67205684	0.741623892	0.20211976	1
gene60382	1401.970743	2360.764672	1.683890113	0.751797994	0.20217341	1
gene4493	7.231978776	20.32638709	2.810625932	1.490891457	0.20220607	1
gene13717	7.231978776	20.32638709	2.810625932	1.490891457	0.20220607	1
gene45667	7.231978776	20.32638709	2.810625932	1.490891457	0.20220607	1
gene53116	7.231978776	20.32638709	2.810625932	1.490891457	0.20220607	1
gene58745	7.231978776	20.32638709	2.810625932	1.490891457	0.20220607	1
gene61041	7.231978776	20.32638709	2.810625932	1.490891457	0.20220607	1
gene61462	7.231978776	20.32638709	2.810625932	1.490891457	0.20220607	1
gene10956	15.49709738	4.839615973	0.31229177	-1.67903354	0.20221529	1
gene26413	15.49709738	4.839615973	0.31229177	-1.67903354	0.20221529	1
gene61683	15.49709738	4.839615973	0.31229177	-1.67903354	0.20221529	1
gene69892	15.49709738	4.839615973	0.31229177	-1.67903354	0.20221529	1
gene15218	33.0604744	64.85085404	1.961582682	0.972018147	0.20225425	1
gene29298	193.1971473	314.5750383	1.62825923	0.703330405	0.20240444	1
gene18295	787.2525467	1284.434079	1.631540075	0.706234425	0.202475	1
gene34175	103.3139825	57.10746848	0.552756433	-0.85528418	0.20252763	1
gene38726	103.3139825	57.10746848	0.552756433	-0.85528418	0.20252763	1
gene52375	103.3139825	57.10746848	0.552756433	-0.85528418	0.20252763	1
gene23227	292.3785705	179.065791	0.612444991	-0.70734783	0.20260369	1
gene5027	9.298258426	24.19807987	2.602431419	1.379860145	0.20270897	1
gene23902	9.298258426	24.19807987	2.602431419	1.379860145	0.20270897	1
gene26774	9.298258426	24.19807987	2.602431419	1.379860145	0.20270897	1

gene29555	9.298258426	24.19807987	2.602431419	1.379860145	0.20270897	1
gene29984	69.22036828	35.8131582	0.517378903	-0.95070687	0.20273684	1
gene17638	18.59651685	40.65277417	2.186042392	1.128321378	0.20277489	1
gene25198	18.59651685	40.65277417	2.186042392	1.128321378	0.20277489	1
gene38212	18.59651685	40.65277417	2.186042392	1.128321378	0.20277489	1
gene50581	18.59651685	40.65277417	2.186042392	1.128321378	0.20277489	1
gene64583	221.0919226	358.131582	1.619831145	0.695843431	0.20280641	1
gene1389	51.65699125	25.16600306	0.487175162	-1.03748751	0.20284259	1
gene33260	51.65699125	25.16600306	0.487175162	-1.03748751	0.20284259	1
gene61781	51.65699125	25.16600306	0.487175162	-1.03748751	0.20284259	1
gene40023	24.7953558	9.679231946	0.390364713	-1.35710545	0.2028443	1
gene48688	24.7953558	9.679231946	0.390364713	-1.35710545	0.2028443	1
gene62040	24.7953558	9.679231946	0.390364713	-1.35710545	0.2028443	1
gene74174	24.7953558	9.679231946	0.390364713	-1.35710545	0.2028443	1
gene10580	522.7687515	325.2221934	0.622114831	-0.6847472	0.20286939	1
gene38129	269.6494943	434.5975144	1.611712699	0.688594595	0.2028839	1
gene25853	100.214563	55.17162209	0.550534976	-0.86109387	0.20289773	1
gene12479	8.265118601	22.26223348	2.693516518	1.429490913	0.20290661	1
gene15081	8.265118601	22.26223348	2.693516518	1.429490913	0.20290661	1
gene16164	8.265118601	22.26223348	2.693516518	1.429490913	0.20290661	1
gene67155	8.265118601	22.26223348	2.693516518	1.429490913	0.20290661	1
gene11298	159.1035331	261.3392626	1.642573597	0.715958013	0.2029995	1
gene31990	117.7779401	197.4563317	1.676513714	0.745464284	0.20309835	1
gene46032	156.0041136	90.9847803	0.583220392	-0.77788693	0.20313106	1
gene64955	169.4349313	99.69608905	0.588403396	-0.76512252	0.2032889	1
gene38415	72.31978776	37.74900459	0.521973387	-0.93795184	0.20330878	1
gene51808	510.3710736	821.7667922	1.610135909	0.687182469	0.20333863	1
gene8926	182.865749	108.4073978	0.592825055	-0.75432167	0.20334745	1
gene11540	23.76221598	49.36408293	2.077419167	1.054792343	0.20335653	1
gene9557	195.2634269	317.4788078	1.62590001	0.701238537	0.20338	1
gene45986	364.6983583	225.5261044	0.618390786	-0.69340927	0.20361289	1
gene56103	364.6983583	225.5261044	0.618390786	-0.69340927	0.20361289	1
gene4994	910.1961859	1492.537566	1.639797649	0.713517798	0.20362222	1
gene63603	250.0198377	151.9639416	0.607807536	-0.71831353	0.20365983	1
gene6427	252.0861173	406.5277417	1.612654223	0.689437137	0.20366998	1
gene9280	75.41920723	39.68485098	0.526190243	-0.9263436	0.20369934	1
gene31142	90.91630461	49.36408293	0.542961828	-0.88107732	0.20375966	1
gene36747	361.5989388	223.590258	0.618337705	-0.69353311	0.20377619	1
gene12138	35.12675405	15.48677111	0.440882499	-1.18153388	0.20382478	1
gene16404	35.12675405	15.48677111	0.440882499	-1.18153388	0.20382478	1
gene52549	35.12675405	15.48677111	0.440882499	-1.18153388	0.20382478	1
gene59544	35.12675405	15.48677111	0.440882499	-1.18153388	0.20382478	1
gene41490	1144.718926	705.6160089	0.616409839	-0.69803821	0.20388141	1
gene60841	193.1971473	115.1828602	0.59619338	-0.74614774	0.20391009	1
gene64580	87.81688513	47.42823654	0.540081061	-0.88875214	0.20393345	1
gene22235	78.51862671	41.62069737	0.530074189	-0.9157338	0.20393347	1
gene10569	1004.21191	1654.18074	1.647242702	0.720053135	0.20394308	1
gene5204	40.29245318	76.46593238	1.897773065	0.924307486	0.20394392	1

gene23564	40.29245318	76.46593238	1.897773065	0.924307486	0.20394392	1
gene56965	136.3744569	78.40177877	0.574900759	-0.79861516	0.2039971	1
gene68240	84.71746566	45.49239015	0.536989507	-0.8970342	0.20403086	1
gene54571	246.9204182	150.0280952	0.607596959	-0.71881345	0.20406151	1
gene63811	817.2136016	1332.830239	1.630944757	0.705707916	0.20416163	1
gene61456	1165.381723	1934.878466	1.660295874	0.731440361	0.20420457	1
gene33251	105.3802622	178.0978678	1.69004958	0.757065571	0.20428477	1
gene45704	5792.814999	3184.46731	0.549727086	-0.86321253	0.20434769	1
gene8718	149.8052746	87.11308752	0.581508814	-0.78212704	0.20436821	1
gene38235	50.62385143	92.92062669	1.835510813	0.876181613	0.20449423	1
gene62442	219.0256429	354.2598892	1.617435678	0.69370834	0.20449752	1
gene39729	54.75641073	27.10184945	0.494952994	-1.01463658	0.20450883	1
gene72316	54.75641073	27.10184945	0.494952994	-1.01463658	0.20450883	1
gene73137	538.2658489	866.2912592	1.609411522	0.686533267	0.20465594	1
gene16212	43.39187265	20.32638709	0.468437655	-1.09407104	0.20467293	1
gene59582	43.39187265	20.32638709	0.468437655	-1.09407104	0.20467293	1
gene2707	13.43081773	3.871692779	0.288269326	-1.79451076	0.20469702	1
gene10727	13.43081773	3.871692779	0.288269326	-1.79451076	0.20469702	1
gene15513	13.43081773	3.871692779	0.288269326	-1.79451076	0.20469702	1
gene23695	13.43081773	3.871692779	0.288269326	-1.79451076	0.20469702	1
gene45802	13.43081773	3.871692779	0.288269326	-1.79451076	0.20469702	1
gene51977	13.43081773	3.871692779	0.288269326	-1.79451076	0.20469702	1
gene59117	13.43081773	3.871692779	0.288269326	-1.79451076	0.20469702	1
gene60162	287.2128714	176.1620214	0.613350024	-0.70521748	0.20473273	1
gene15876	11.36453808	2.903769584	0.255511448	-1.96854016	0.20477315	1
gene26439	11.36453808	2.903769584	0.255511448	-1.96854016	0.20477315	1
gene33447	11.36453808	2.903769584	0.255511448	-1.96854016	0.20477315	1
gene50541	11.36453808	2.903769584	0.255511448	-1.96854016	0.20477315	1
gene57982	11.36453808	2.903769584	0.255511448	-1.96854016	0.20477315	1
gene66882	11.36453808	2.903769584	0.255511448	-1.96854016	0.20477315	1
gene60327	36.15989388	69.69047001	1.927286353	0.946570941	0.20477852	1
gene11428	27.89477528	56.13954529	2.012546964	1.00902245	0.20478709	1
gene52421	27.89477528	56.13954529	2.012546964	1.00902245	0.20478709	1
gene5413	29.96105493	12.58300153	0.419978588	-1.25161232	0.20487758	1
gene19211	29.96105493	12.58300153	0.419978588	-1.25161232	0.20487758	1
gene22258	29.96105493	12.58300153	0.419978588	-1.25161232	0.20487758	1
gene25340	29.96105493	12.58300153	0.419978588	-1.25161232	0.20487758	1
gene37988	29.96105493	12.58300153	0.419978588	-1.25161232	0.20487758	1
gene31349	186.9983083	111.3111674	0.595252269	-0.74842688	0.20504485	1
gene7978	339.9030025	210.0393332	0.617939035	-0.69446358	0.20507346	1
gene34504	32.02733458	62.91500765	1.964415974	0.974100459	0.20511286	1
gene43436	463.8797815	744.3329367	1.604581546	0.68219711	0.20532135	1
gene15872	110.5459613	185.8412534	1.681122053	0.749424471	0.20535932	1
gene34503	190.0977278	308.7674991	1.624256653	0.699779614	0.20536959	1
gene24869	43.39187265	81.30554835	1.873750621	0.905928957	0.20537689	1
gene66139	43.39187265	81.30554835	1.873750621	0.905928957	0.20537689	1
gene66010	17.56337703	38.71692779	2.204412496	1.14039421	0.20556902	1
gene57873	376.0628963	233.2694899	0.620293818	-0.68897635	0.20563615	1

gene67416	183.8988889	109.375321	0.594757922	-0.74962551	0.20563756	1
gene29930	121.9104994	203.2638709	1.667320468	0.737531425	0.20567893	1
gene43385	542.3984082	872.0987984	1.607856486	0.68513864	0.20570004	1
gene26349	171.501211	279.7298033	1.631066053	0.705815208	0.20587201	1
gene12101	738.6949749	460.7314406	0.623709997	-0.68105271	0.20588548	1
gene14540	127.0761985	72.5942396	0.571265433	-0.80776686	0.20588668	1
gene73095	868.8705929	1418.00748	1.632012283	0.706651916	0.20594246	1
gene23562	107.4465418	60.01123807	0.55852182	-0.84031445	0.20595777	1
gene72067	3998.251123	7508.180221	1.877866094	0.909094192	0.20599738	1
gene32223	1188.110799	1970.691624	1.658676637	0.730032657	0.20600789	1
gene26512	617.8176154	995.9929673	1.612114874	0.688954549	0.20604848	1
gene17145	165.302372	270.0505713	1.633676323	0.708122174	0.20615907	1
gene47218	165.302372	270.0505713	1.633676323	0.708122174	0.20615907	1
gene38396	180.7994694	107.4394746	0.594246626	-0.75086629	0.20624761	1
gene61385	192.1640075	311.6712687	1.62190242	0.697687024	0.20633522	1
gene10463	46.49129213	86.14516432	1.85293117	0.889809291	0.20642239	1
gene28544	46.49129213	86.14516432	1.85293117	0.889809291	0.20642239	1
gene13994	153.9378339	90.0168571	0.5847611	-0.77408075	0.20644081	1
gene73641	422.5541885	676.5783131	1.601163428	0.679120569	0.20655829	1
gene27273	396.7256928	634.9576157	1.600495322	0.678518461	0.20667768	1
gene19024	791.385106	1285.402002	1.624243359	0.699767806	0.2066931	1
gene31406	255.1855368	155.8356343	0.61067581	-0.7115214	0.20671909	1
gene74069	3146.943907	1840.989916	0.585008812	-0.77346974	0.20673359	1
gene62982	700.4688014	437.501284	0.62458354	-0.67903354	0.20673694	1
gene4116	39.25931335	74.53008599	1.898405235	0.924787984	0.20674243	1
gene30231	39.25931335	74.53008599	1.898405235	0.924787984	0.20674243	1
gene17181	268.6163545	164.5469431	0.612572319	-0.70704792	0.2068027	1
gene53422	1004.21191	623.3425373	0.620728087	-0.68796667	0.20682146	1
gene19336	177.7000499	105.5036282	0.593717493	-0.75215147	0.20687536	1
gene10248	164.2692322	96.79231946	0.589229755	-0.76309781	0.20704239	1
gene32800	150.8384145	88.08101071	0.583942831	-0.77610096	0.20711901	1
gene66446	228.3239013	367.810814	1.610916824	0.687882006	0.20717081	1
gene28633	77.48548688	134.5413241	1.736342243	0.796051339	0.20724075	1
gene43427	406.0239513	649.4764636	1.599601357	0.677712411	0.20729165	1
gene70773	3695.541154	2135.238567	0.577787793	-0.79138837	0.20740922	1
gene64358	146.7058552	241.0128755	1.64283065	0.716183769	0.20742136	1
gene26184	11606.29279	28378.54014	2.445099451	1.289893146	0.20744761	1
gene63394	250.0198377	401.6881258	1.606625016	0.684033246	0.20745578	1
gene71861	132.2418976	218.750642	1.65417047	0.726107919	0.20746461	1
gene29156	1333.783514	820.7988691	0.615391374	-0.70042387	0.20755682	1
gene30018	35.12675405	67.75462362	1.928860934	0.947749132	0.20765212	1
gene56286	35.12675405	67.75462362	1.928860934	0.947749132	0.20765212	1
gene69030	143.6064357	236.1732595	1.644586876	0.717725221	0.2076943	1
gene18278	471.1117602	294.2486512	0.62458354	-0.67903354	0.20770629	1
gene64367	71.28664793	124.8620921	1.751549494	0.808631755	0.20775794	1
gene27025	433.9187265	694.0009306	1.599379995	0.677512748	0.20780229	1
gene10021	117.7779401	66.78670043	0.567056109	-0.8184366	0.20782316	1
gene5612	26.86163545	54.2036989	2.017885285	1.012844161	0.2078265	1

gene51734	26.86163545	54.2036989	2.017885285	1.012844161	0.2078265	1
gene25628	932.925262	580.7539168	0.622508512	-0.68383453	0.20783284	1
gene17764	95.04886391	52.26785251	0.549905074	-0.8627455	0.20786718	1
gene20954	175.6337703	285.5373424	1.625754216	0.701109165	0.20787709	1
gene7010	154.9709738	253.595877	1.636408876	0.710533268	0.20799538	1
gene1703	4.1325593	0	0	#NAME?	0.20799962	1
gene2920	4.1325593	0	0	#NAME?	0.20799962	1
gene5017	4.1325593	0	0	#NAME?	0.20799962	1
gene5162	4.1325593	0	0	#NAME?	0.20799962	1
gene5550	4.1325593	0	0	#NAME?	0.20799962	1
gene7606	4.1325593	0	0	#NAME?	0.20799962	1
gene9252	4.1325593	0	0	#NAME?	0.20799962	1
gene11803	4.1325593	0	0	#NAME?	0.20799962	1
gene13979	4.1325593	0	0	#NAME?	0.20799962	1
gene14196	4.1325593	0	0	#NAME?	0.20799962	1
gene16732	4.1325593	0	0	#NAME?	0.20799962	1
gene19217	4.1325593	0	0	#NAME?	0.20799962	1
gene20496	4.1325593	0	0	#NAME?	0.20799962	1
gene22463	4.1325593	0	0	#NAME?	0.20799962	1
gene22695	4.1325593	0	0	#NAME?	0.20799962	1
gene23458	4.1325593	0	0	#NAME?	0.20799962	1
gene24852	4.1325593	0	0	#NAME?	0.20799962	1
gene25588	4.1325593	0	0	#NAME?	0.20799962	1
gene28611	4.1325593	0	0	#NAME?	0.20799962	1
gene33196	4.1325593	0	0	#NAME?	0.20799962	1
gene33760	4.1325593	0	0	#NAME?	0.20799962	1
gene33959	4.1325593	0	0	#NAME?	0.20799962	1
gene34048	4.1325593	0	0	#NAME?	0.20799962	1
gene34660	4.1325593	0	0	#NAME?	0.20799962	1
gene37164	4.1325593	0	0	#NAME?	0.20799962	1
gene38203	4.1325593	0	0	#NAME?	0.20799962	1
gene40754	4.1325593	0	0	#NAME?	0.20799962	1
gene44349	4.1325593	0	0	#NAME?	0.20799962	1
gene45056	4.1325593	0	0	#NAME?	0.20799962	1
gene47934	4.1325593	0	0	#NAME?	0.20799962	1
gene49074	4.1325593	0	0	#NAME?	0.20799962	1
gene51338	4.1325593	0	0	#NAME?	0.20799962	1
gene51390	4.1325593	0	0	#NAME?	0.20799962	1
gene53137	4.1325593	0	0	#NAME?	0.20799962	1
gene54651	4.1325593	0	0	#NAME?	0.20799962	1
gene54855	4.1325593	0	0	#NAME?	0.20799962	1
gene55576	4.1325593	0	0	#NAME?	0.20799962	1
gene55677	4.1325593	0	0	#NAME?	0.20799962	1
gene55938	4.1325593	0	0	#NAME?	0.20799962	1
gene55982	4.1325593	0	0	#NAME?	0.20799962	1
gene56600	4.1325593	0	0	#NAME?	0.20799962	1
gene57032	4.1325593	0	0	#NAME?	0.20799962	1
gene58321	4.1325593	0	0	#NAME?	0.20799962	1

gene59067	4.1325593	0	0	#NAME?	0.20799962	1
gene60857	4.1325593	0	0	#NAME?	0.20799962	1
gene62950	4.1325593	0	0	#NAME?	0.20799962	1
gene64993	4.1325593	0	0	#NAME?	0.20799962	1
gene66441	4.1325593	0	0	#NAME?	0.20799962	1
gene66702	4.1325593	0	0	#NAME?	0.20799962	1
gene66953	4.1325593	0	0	#NAME?	0.20799962	1
gene68000	4.1325593	0	0	#NAME?	0.20799962	1
gene70839	4.1325593	0	0	#NAME?	0.20799962	1
gene72145	4.1325593	0	0	#NAME?	0.20799962	1
gene73797	4.1325593	0	0	#NAME?	0.20799962	1
gene74209	4.1325593	0	0	#NAME?	0.20799962	1
gene34077	224.191342	361.0353516	1.610389359	0.687409544	0.20800846	1
gene37112	553.7629462	346.5165037	0.625748808	-0.67634446	0.2080846	1
gene8389	65.08780898	115.1828602	1.769653365	0.823466796	0.20809172	1
gene13606	30.99419475	60.97916126	1.967438153	0.976318285	0.20809393	1
gene42899	195.2634269	117.1187066	0.599798479	-0.73745023	0.20826343	1
gene1600	2191.289569	1316.375545	0.600730987	-0.73520901	0.20827238	1
gene70672	38.22617353	17.4226175	0.455777178	-1.13359941	0.2083233	1
gene14517	299.6105493	184.8733302	0.617045463	-0.69655131	0.20838605	1
gene27699	16.5302372	36.7810814	2.225078863	1.15385647	0.20841972	1
gene42151	16.5302372	36.7810814	2.225078863	1.15385647	0.20841972	1
gene44132	16.5302372	36.7810814	2.225078863	1.15385647	0.20841972	1
gene60937	16.5302372	36.7810814	2.225078863	1.15385647	0.20841972	1
gene18141	205.5948252	123.8941689	0.602613265	-0.73069566	0.20857889	1
gene36417	472.1449001	295.2165744	0.625266892	-0.67745597	0.20872509	1
gene12378	70.25350811	36.7810814	0.523547968	-0.93360637	0.20873784	1
gene72694	70.25350811	36.7810814	0.523547968	-0.93360637	0.20873784	1
gene73084	70.25350811	36.7810814	0.523547968	-0.93360637	0.20873784	1
gene58206	119.8442197	199.3921781	1.663761328	0.734448489	0.20891363	1
gene14124	128.1093383	73.56216279	0.5742139	-0.80033984	0.2091055	1
gene35303	128.1093383	73.56216279	0.5742139	-0.80033984	0.2091055	1
gene50928	793.4513857	1285.402002	1.620013558	0.696005887	0.20916027	1
gene30921	509.3379338	814.9913299	1.600099415	0.678161543	0.20920266	1
gene58567	263.4506554	422.0145129	1.60187308	0.679759845	0.20922395	1
gene19999	312.0082272	498.4804452	1.597651606	0.675952839	0.20926595	1
gene67368	153.9378339	251.6600306	1.634815978	0.709128249	0.20930245	1
gene60257	49.5907116	24.19807987	0.487955891	-1.03517735	0.20932797	1
gene55375	2923.785705	5222.913558	1.786353066	0.837017252	0.2093318	1
gene59019	21.69593633	45.49239015	2.096816172	1.068200386	0.20946111	1
gene3600	38.22617353	72.5942396	1.899071576	0.925294282	0.20963664	1
gene49087	508.3047939	318.446731	0.626487759	-0.67464177	0.20979014	1
gene36864	73.35292758	127.7658617	1.741796352	0.800575957	0.20982402	1
gene18087	125.0099188	71.6263164	0.572965066	-0.80348091	0.20982647	1
gene9938	22.72907615	8.711308752	0.383267173	-1.38357766	0.20984151	1
gene12421	22.72907615	8.711308752	0.383267173	-1.38357766	0.20984151	1
gene43200	22.72907615	8.711308752	0.383267173	-1.38357766	0.20984151	1
gene46103	22.72907615	8.711308752	0.383267173	-1.38357766	0.20984151	1

gene70724	22.72907615	8.711308752	0.383267173	-1.38357766	0.20984151	1
gene5901	1690.216754	2858.277194	1.691071389	0.757937565	0.20986795	1
gene67685	175.6337703	104.535705	0.595191374	-0.74857448	0.20991264	1
gene69232	48.55757178	89.04893391	1.833883587	0.874902061	0.20991993	1
gene37383	70.25350811	122.9262457	1.749752419	0.807150802	0.21012597	1
gene28607	110.5459613	184.8733302	1.672366209	0.741890798	0.21020104	1
gene43192	556.8623657	891.4572623	1.600857442	0.67884484	0.21031962	1
gene55633	428.7530274	268.1147249	0.625336051	-0.6772964	0.21036039	1
gene28465	51.65699125	93.88854988	1.817538103	0.861985609	0.21037521	1
gene41575	67.15408863	118.0866297	1.758442891	0.814298481	0.21038321	1
gene14104	105.3802622	59.04331487	0.560288176	-0.83575905	0.21038405	1
gene14098	1273.861404	2111.040488	1.657197934	0.728745927	0.21038488	1
gene51510	84.71746566	145.1884792	1.7137963	0.777195643	0.21049942	1
gene37961	121.9104994	69.69047001	0.571652732	-0.80678909	0.2105556	1
gene55660	227.2907615	364.9070444	1.605463601	0.682989956	0.21059428	1
gene25713	148.7721348	87.11308752	0.585547069	-0.77214295	0.21060318	1
gene2074	254.152397	155.8356343	0.613158232	-0.70566867	0.21060953	1
gene13355	34.09361423	65.81877724	1.930530943	0.948997679	0.21064853	1
gene13553	34.09361423	65.81877724	1.930530943	0.948997679	0.21064853	1
gene16983	34.09361423	65.81877724	1.930530943	0.948997679	0.21064853	1
gene40923	34.09361423	65.81877724	1.930530943	0.948997679	0.21064853	1
gene35763	415.3222097	662.0594651	1.59408635	0.67272978	0.21072445	1
gene5525	1116.824151	694.0009306	0.621405733	-0.68639254	0.21080938	1
gene66470	684.971704	429.7578984	0.62740971	-0.67252024	0.21094516	1
gene26293	416.3553495	260.3713394	0.625358458	-0.67724471	0.2109672	1
gene43930	25.82849563	52.26785251	2.023650671	1.016960269	0.21099749	1
gene47789	25.82849563	52.26785251	2.023650671	1.016960269	0.21099749	1
gene61076	25.82849563	52.26785251	2.023650671	1.016960269	0.21099749	1
gene63734	354.36696	220.6864884	0.622762597	-0.6832458	0.2110563	1
gene27541	193.1971473	116.1507834	0.601203408	-0.73407491	0.21108177	1
gene72479	370.8971972	231.3336435	0.623713647	-0.68104427	0.2112	1
gene36067	29.96105493	59.04331487	1.970668757	0.978685299	0.21120523	1
gene36033	33.0604744	14.51884792	0.439160302	-1.18718045	0.21122371	1
gene47972	33.0604744	14.51884792	0.439160302	-1.18718045	0.21122371	1
gene49390	33.0604744	14.51884792	0.439160302	-1.18718045	0.21122371	1
gene1081	15.49709738	34.84523501	2.248500746	1.168963362	0.21131434	1
gene5582	15.49709738	34.84523501	2.248500746	1.168963362	0.21131434	1
gene11810	15.49709738	34.84523501	2.248500746	1.168963362	0.21131434	1
gene6478	169.4349313	100.6640122	0.594116051	-0.75118333	0.2113408	1
gene47685	1718.111529	2903.769584	1.690093766	0.757103289	0.21138974	1
gene64200	235.5558801	377.4900459	1.602549874	0.680369256	0.21139255	1
gene62677	544.4646878	870.162952	1.598199059	0.676447111	0.21147208	1
gene56625	364.6983583	580.7539168	1.592422624	0.671223274	0.2115285	1
gene46952	99.18142321	55.17162209	0.556269716	-0.84614353	0.21159626	1
gene22964	656.0437889	1053.100436	1.605228879	0.682779016	0.21161453	1
gene22963	224.191342	136.4771704	0.608753082	-0.71607092	0.2116509	1
gene45316	101.2477029	170.3544823	1.682551578	0.750650731	0.21165328	1
gene53248	695.3031023	436.5333608	0.627831746	-0.67155011	0.21171203	1

gene8219	271.715774	167.4507127	0.616271592	-0.69836181	0.21172578	1
gene21145	41.325593	19.35846389	0.468437655	-1.09407104	0.21173802	1
gene23017	1303.822459	2160.40457	1.65697757	0.728554073	0.21174405	1
gene57915	381.2285955	238.1091059	0.62458354	-0.67903354	0.21180451	1
gene31969	1005.24505	627.2142301	0.623941625	-0.68051704	0.21182186	1
gene13725	554.7960861	886.6176463	1.598096433	0.676354467	0.21189229	1
gene44199	55.78955055	28.06977264	0.503136741	-0.99097755	0.21210309	1
gene73256	96.08200373	53.23577571	0.554066044	-0.85187014	0.21216816	1
gene55238	246.9204182	394.9126634	1.599351995	0.677487491	0.21217403	1
gene61952	246.9204182	394.9126634	1.599351995	0.677487491	0.21217403	1
gene16734	231.4233208	370.7145835	1.601889482	0.679774617	0.21227502	1
gene22482	174.6006304	282.6335728	1.61874314	0.694874079	0.21233853	1
gene72372	27.89477528	11.61507834	0.416389027	-1.26399604	0.21241648	1
gene39126	656.0437889	412.3352809	0.628517925	-0.6699742	0.21242015	1
gene29747	699.4356616	439.4371304	0.628273842	-0.67053458	0.21244853	1
gene56888	186.9983083	112.2790906	0.600428376	-0.73593594	0.21245073	1
gene23200	372.9634769	593.3369183	1.590871372	0.669817193	0.21245458	1
gene56523	106.413402	178.0978678	1.673641332	0.742990386	0.21251987	1
gene70102	290.3122908	462.667287	1.593688251	0.672369444	0.21258625	1
gene13390	335.7704432	209.07141	0.622661745	-0.68347945	0.21261537	1
gene25736	37.1930337	70.65839321	1.899774936	0.925828514	0.21263125	1
gene64834	37.1930337	70.65839321	1.899774936	0.925828514	0.21263125	1
gene46364	83.68432583	143.2526328	1.711821555	0.775532319	0.21268882	1
gene37579	20.6627965	43.55654376	2.107969449	1.075853958	0.21269401	1
gene63535	20.6627965	43.55654376	2.107969449	1.075853958	0.21269401	1
gene65093	20.6627965	43.55654376	2.107969449	1.075853958	0.21269401	1
gene5799	92.98258426	51.29992932	0.551715461	-0.85800369	0.21270827	1
gene5341	450.4489637	282.6335728	0.627448603	-0.67243081	0.2127678	1
gene25366	463.8797815	738.5253975	1.592062054	0.670896569	0.21282769	1
gene3049	2751.251354	4855.102744	1.764688907	0.819413876	0.21291106	1
gene42632	58.88897003	30.00561903	0.509528678	-0.97276475	0.21301028	1
gene26813	122.9436392	203.2638709	1.653309372	0.725356711	0.21302074	1
gene63458	200.4291261	322.3184238	1.608141642	0.685394482	0.21303057	1
gene8162	173.5674906	103.5677818	0.596700347	-0.74492148	0.2130364	1
gene8220	262.4175156	161.6431735	0.615977074	-0.69905144	0.21307889	1
gene64413	312.0082272	496.5445988	1.591447134	0.670339233	0.21307947	1
gene5556	126.0430587	72.5942396	0.575947937	-0.79598969	0.2131003	1
gene6692	792.4182458	497.512522	0.627840821	-0.67152926	0.21316568	1
gene57124	50.62385143	91.95270349	1.816390909	0.861074721	0.21318077	1
gene57278	50.62385143	91.95270349	1.816390909	0.861074721	0.21318077	1
gene73847	50.62385143	91.95270349	1.816390909	0.861074721	0.21318077	1
gene41871	379.1623158	237.1411827	0.625434472	-0.67706936	0.21325145	1
gene70182	53.7232709	96.79231946	1.80168329	0.849345428	0.21338293	1
gene24349	487.6419974	776.2744021	1.591894066	0.670744334	0.21352553	1
gene4845	61.9883895	31.94146542	0.515281421	-0.95656752	0.21367864	1
gene16469	61.9883895	31.94146542	0.515281421	-0.95656752	0.21367864	1
gene2465	33.0604744	63.88293085	1.932305328	0.950323076	0.21377562	1
gene48939	33.0604744	63.88293085	1.932305328	0.950323076	0.21377562	1

gene31901	136.3744569	79.36970196	0.581998299	-0.78091316	0.21377993	1
gene6107	88.85002496	150.9960184	1.699448238	0.765066421	0.21380443	1
gene44752	1719.144669	2896.994122	1.685136902	0.752865802	0.21384995	1
gene6262	156.0041136	253.595877	1.625571731	0.700947219	0.2140403	1
gene55240	167.3686517	271.0184945	1.619290661	0.695361971	0.21407642	1
gene25829	40.29245318	75.49800918	1.873750621	0.905928957	0.2140882	1
gene42492	40.29245318	75.49800918	1.873750621	0.905928957	0.2140882	1
gene48827	144.6395755	236.1732595	1.632839827	0.707383277	0.21413869	1
gene73981	65.08780898	33.87731181	0.520486284	-0.94206795	0.21415341	1
gene15329	14.46395755	32.90938862	2.275268612	1.186036876	0.21423367	1
gene38427	14.46395755	32.90938862	2.275268612	1.186036876	0.21423367	1
gene57055	573.3926029	914.6874189	1.595220124	0.673755515	0.21429006	1
gene8539	24.7953558	50.33200612	2.029896507	1.021406174	0.21430802	1
gene16007	24.7953558	50.33200612	2.029896507	1.021406174	0.21430802	1
gene68510	44.42501248	21.29431028	0.479331554	-1.06090418	0.21432747	1
gene10264	836.8432583	525.5822947	0.628053449	-0.67104075	0.21435138	1
gene30584	80.58490636	43.55654376	0.540504987	-0.88762017	0.21435901	1
gene43731	80.58490636	43.55654376	0.540504987	-0.88762017	0.21435901	1
gene3350	28.9279151	57.10746848	1.974130119	0.981217084	0.21445521	1
gene33337	175.6337703	283.601496	1.614732153	0.691294875	0.21449122	1
gene22445	281.0140324	447.1805159	1.59131027	0.670215157	0.21456479	1
gene9150	77.48548688	41.62069737	0.537141845	-0.89662498	0.2145788	1
gene45612	330.604744	206.1676405	0.623607629	-0.68128952	0.21458782	1
gene51201	702.5350811	442.3408999	0.629635319	-0.66741162	0.21461678	1
gene32412	533.1001497	848.8686417	1.592324898	0.671134734	0.21463354	1
gene27188	74.38606741	39.68485098	0.533498441	-0.90644404	0.21468565	1
gene56140	299.6105493	476.2182118	1.589457424	0.668534372	0.21478749	1
gene12614	364.6983583	578.8180704	1.587114549	0.666406258	0.21481123	1
gene72598	302.7099687	481.0578277	1.589170749	0.668274144	0.21483813	1
gene20885	1623.062665	997.9288137	0.614843059	-0.70170989	0.21490294	1
gene59653	183.8988889	296.1844976	1.610583399	0.687583368	0.2149503	1
gene45224	754.1920723	1211.83984	1.606805327	0.68419515	0.21497085	1
gene40703	103.3139825	58.07539168	0.562125186	-0.83103664	0.2149938	1
gene67343	102.2808427	171.3224055	1.675019495	0.744177887	0.21500223	1
gene70064	43.39187265	80.33762515	1.851444066	0.888650965	0.21508324	1
gene66322	800.6833644	1289.273695	1.610216663	0.687254824	0.21509189	1
gene58387	407.0570911	255.5317234	0.627754015	-0.67172874	0.21513992	1
gene65313	270.6826342	430.7258216	1.591257684	0.670167481	0.21525247	1
gene18767	260.3512359	160.6752503	0.617148022	-0.69631154	0.21525809	1
gene19619	153.9378339	90.9847803	0.591048854	-0.75865071	0.21528065	1
gene64677	465.9460611	293.280728	0.629430641	-0.66788068	0.21534637	1
gene16770	65.08780898	114.214937	1.754782328	0.811292083	0.21546462	1
gene37358	135.3413171	221.6544116	1.637743864	0.711709743	0.21546926	1
gene35693	79.55176653	136.4771704	1.715576868	0.778693768	0.21550694	1
gene56199	99.18142321	166.4827895	1.678568265	0.747231211	0.21560967	1
gene33470	876.1025717	1415.103711	1.615226066	0.691736098	0.21567402	1
gene22818	174.6006304	281.6656496	1.6131995	0.689924863	0.2157053	1
gene19309	100.214563	56.13954529	0.560193485	-0.83600289	0.21571734	1

gene58997	36.15989388	68.72254682	1.900518487	0.926393059	0.21573829	1
gene62358	317.1739263	503.3200612	1.586889777	0.666201924	0.21575686	1
gene8999	203.5285455	326.1901166	1.602675024	0.680481918	0.21584143	1
gene3296	36.15989388	16.45469431	0.455053722	-1.13589122	0.21590388	1
gene45424	87.81688513	149.060172	1.697397622	0.763324561	0.21598728	1
gene58844	282.0471722	448.1484391	1.588913073	0.668040199	0.21600498	1
gene71801	282.0471722	448.1484391	1.588913073	0.668040199	0.21600498	1
gene7134	58.88897003	104.535705	1.775132168	0.827926445	0.21605011	1
gene37174	58.88897003	104.535705	1.775132168	0.827926445	0.21605011	1
gene47887	19.62965668	41.62069737	2.120296756	1.084266198	0.21605271	1
gene47525	49.5907116	90.0168571	1.815195915	0.860125267	0.21608174	1
gene60186	49.5907116	90.0168571	1.815195915	0.860125267	0.21608174	1
gene58245	55.78955055	99.69608905	1.787002907	0.837541982	0.21620353	1
gene69134	161.1698127	95.82439627	0.594555486	-0.75011664	0.21623197	1
gene52749	47.52443195	23.23015667	0.48880451	-1.0326705	0.21628044	1
gene70573	47.52443195	23.23015667	0.48880451	-1.0326705	0.21628044	1
gene19588	127.0761985	73.56216279	0.578882306	-0.78865804	0.21635042	1
gene20725	179.7663296	289.4090352	1.609917919	0.686987135	0.21636526	1
gene44750	113.6453808	64.85085404	0.570642235	-0.80934157	0.21640429	1
gene1516	97.11514356	54.2036989	0.558138483	-0.84130497	0.21642511	1
gene68601	97.11514356	54.2036989	0.558138483	-0.84130497	0.21642511	1
gene50151	84.71746566	144.220556	1.702370991	0.767545472	0.21661413	1
gene48365	328.5384644	520.7426787	1.585028041	0.664508364	0.21662124	1
gene15997	73.35292758	126.7979385	1.728600925	0.789604839	0.21662454	1
gene25363	579.5914419	922.4308045	1.591519022	0.6704044	0.2167625	1
gene73398	373.9966167	592.3689951	1.583888647	0.663470912	0.21679591	1
gene2636	3.099419475	11.61507834	3.747501243	1.905928957	0.2168594	1
gene11307	3.099419475	11.61507834	3.747501243	1.905928957	0.2168594	1
gene26094	3.099419475	11.61507834	3.747501243	1.905928957	0.2168594	1
gene28316	3.099419475	11.61507834	3.747501243	1.905928957	0.2168594	1
gene29955	3.099419475	11.61507834	3.747501243	1.905928957	0.2168594	1
gene37884	3.099419475	11.61507834	3.747501243	1.905928957	0.2168594	1
gene39023	3.099419475	11.61507834	3.747501243	1.905928957	0.2168594	1
gene39110	3.099419475	11.61507834	3.747501243	1.905928957	0.2168594	1
gene39314	3.099419475	11.61507834	3.747501243	1.905928957	0.2168594	1
gene39511	3.099419475	11.61507834	3.747501243	1.905928957	0.2168594	1
gene40032	3.099419475	11.61507834	3.747501243	1.905928957	0.2168594	1
gene43983	3.099419475	11.61507834	3.747501243	1.905928957	0.2168594	1
gene45411	3.099419475	11.61507834	3.747501243	1.905928957	0.2168594	1
gene52376	3.099419475	11.61507834	3.747501243	1.905928957	0.2168594	1
gene54761	3.099419475	11.61507834	3.747501243	1.905928957	0.2168594	1
gene55301	3.099419475	11.61507834	3.747501243	1.905928957	0.2168594	1
gene59870	3.099419475	11.61507834	3.747501243	1.905928957	0.2168594	1
gene60685	3.099419475	11.61507834	3.747501243	1.905928957	0.2168594	1
gene62373	3.099419475	11.61507834	3.747501243	1.905928957	0.2168594	1
gene62674	3.099419475	11.61507834	3.747501243	1.905928957	0.2168594	1
gene72151	3.099419475	11.61507834	3.747501243	1.905928957	0.2168594	1
gene46772	500.0396753	315.5429615	0.63103585	-0.66420613	0.21702216	1

gene61219	109.5128215	181.9695606	1.66162791	0.732597354	0.2170296	1
gene16547	1299.6899	2140.078183	1.646606766	0.71949606	0.21704201	1
gene44393	158.0703932	93.88854988	0.5939667	-0.75154604	0.21708917	1
gene9474	94.01572408	52.26785251	0.555947987	-0.84697818	0.21711108	1
gene46659	94.01572408	52.26785251	0.555947987	-0.84697818	0.21711108	1
gene19046	13.43081773	30.97354223	2.306154611	1.205489239	0.21715106	1
gene62178	13.43081773	30.97354223	2.306154611	1.205489239	0.21715106	1
gene11401	20.6627965	7.743385557	0.374750124	-1.41599914	0.21718592	1
gene61869	20.6627965	7.743385557	0.374750124	-1.41599914	0.21718592	1
gene4910	39.25931335	73.56216279	1.873750621	0.905928957	0.21719901	1
gene38489	110.5459613	62.91500765	0.569129862	-0.81317022	0.21725025	1
gene48859	110.5459613	62.91500765	0.569129862	-0.81317022	0.21725025	1
gene38463	213.8599438	341.6768877	1.597666593	0.675966372	0.21726674	1
gene35306	1240.80093	2036.510402	1.641286972	0.71482751	0.2174316	1
gene15843	1816.259812	3060.573141	1.685096549	0.752831254	0.21748203	1
gene55458	258.2849563	159.7073271	0.618337705	-0.69353311	0.21748279	1
gene20783	89.88316478	151.9639416	1.690683032	0.75760621	0.21754836	1
gene51383	178.7331897	287.4731888	1.608392874	0.68561985	0.21756824	1
gene15764	67.15408863	117.1187066	1.744029425	0.802424381	0.21766192	1
gene21891	67.15408863	117.1187066	1.744029425	0.802424381	0.21766192	1
gene33369	67.15408863	117.1187066	1.744029425	0.802424381	0.21766192	1
gene47345	147.738995	240.0449523	1.624790749	0.70025393	0.21767711	1
gene49973	50.62385143	25.16600306	0.497117512	-1.00834117	0.21773728	1
gene68844	23.76221598	48.39615973	2.036685458	1.02622319	0.21776661	1
gene72054	1638.559763	2738.254718	1.671135091	0.740828362	0.2177862	1
gene62916	1617.896966	997.9288137	0.616806159	-0.69711092	0.21780912	1
gene22696	27.89477528	55.17162209	1.977847878	0.983931469	0.21785304	1
gene31996	27.89477528	55.17162209	1.977847878	0.983931469	0.21785304	1
gene29629	1864.817384	3147.686229	1.687932693	0.755257377	0.21786979	1
gene41745	245.8872784	391.0409706	1.590326158	0.669322676	0.21789868	1
gene66751	165.302372	98.72816585	0.597258011	-0.7435738	0.21793229	1
gene73916	219.0256429	349.4202733	1.595339562	0.673863529	0.21796082	1
gene17598	521.7356117	827.5743314	1.586194833	0.665569989	0.2180272	1
gene64167	339.9030025	212.9431028	0.626481971	-0.6746551	0.21807801	1
gene5247	107.4465418	60.97916126	0.567530236	-0.81723084	0.21809985	1
gene37704	64.05466916	112.2790906	1.752863485	0.809713641	0.21812351	1
gene60456	64.05466916	112.2790906	1.752863485	0.809713641	0.21812351	1
gene27909	236.5890199	376.5221227	1.591460681	0.670351514	0.21816654	1
gene29701	299.6105493	186.8091766	0.623506672	-0.6815231	0.2181778	1
gene44984	666.3751872	1062.779668	1.594866808	0.673435945	0.21821275	1
gene53953	86.78374531	147.1243256	1.695298181	0.761539047	0.21822801	1
gene62499	578.558302	365.8749676	0.632390835	-0.66111164	0.21831238	1
gene36154	95.04886391	159.7073271	1.680265503	0.748689215	0.21834753	1
gene24960	161.1698127	260.3713394	1.61550935	0.6919891	0.21835589	1
gene31400	336.803583	211.0072564	0.626499441	-0.67461487	0.21840831	1
gene13519	252.0861173	155.8356343	0.618184119	-0.6938915	0.2185955	1
gene32649	508.3047939	321.3505006	0.632200413	-0.66154612	0.21870058	1
gene15266	45.4581523	83.24139474	1.83116538	0.872762093	0.21877454	1

gene70688	866.8043132	1394.777323	1.609102888	0.686256576	0.21877824	1
gene15468	177.7000499	285.5373424	1.606850097	0.684235346	0.21879163	1
gene30043	162.2029525	96.79231946	0.596735867	-0.7448356	0.21881078	1
gene70485	116.7448002	192.6167157	1.649895459	0.722374615	0.21884601	1
gene43981	241.7547191	384.2655083	1.589485036	0.668559434	0.21885223	1
gene9997	83.68432583	142.2847096	1.700255194	0.765751299	0.21892043	1
gene46743	108.4796816	180.0337142	1.659607693	0.73084225	0.21895502	1
gene22038	35.12675405	66.78670043	1.901305778	0.926990572	0.21898087	1
gene61118	479.3768788	302.9599599	0.631987009	-0.66203319	0.21898262	1
gene61005	1117.857291	1820.663529	1.628708373	0.703728306	0.21903737	1
gene53297	54.75641073	97.76024266	1.785366158	0.836219985	0.21907669	1
gene10066	48.55757178	88.08101071	1.81395007	0.859134745	0.21908289	1
gene27238	680.8391447	430.7258216	0.632639626	-0.66054417	0.2191213	1
gene40437	262.4175156	416.2069737	1.586048754	0.665437119	0.21914395	1
gene54566	51.65699125	92.92062669	1.798800597	0.847035268	0.21916657	1
gene56540	190.0977278	115.1828602	0.605913924	-0.72281524	0.219177	1
gene56739	138.4407366	225.5261044	1.629044384	0.704025911	0.21918485	1
gene31075	2973.376417	5250.983331	1.766000195	0.820485502	0.21920607	1
gene31701	30.99419475	13.55092472	0.437208478	-1.19360672	0.21923402	1
gene33015	30.99419475	13.55092472	0.437208478	-1.19360672	0.21923402	1
gene53640	30.99419475	13.55092472	0.437208478	-1.19360672	0.21923402	1
gene61362	30.99419475	13.55092472	0.437208478	-1.19360672	0.21923402	1
gene42199	163.2360924	263.2751089	1.612848636	0.68961105	0.21931049	1
gene23960	39.25931335	18.3905407	0.468437655	-1.09407104	0.21936046	1
gene36124	39.25931335	18.3905407	0.468437655	-1.09407104	0.21936046	1
gene59350	39.25931335	18.3905407	0.468437655	-1.09407104	0.21936046	1
gene59925	113.6453808	187.7770998	1.652307366	0.724482085	0.21950898	1
gene30798	18.59651685	39.68485098	2.133993763	1.09355596	0.21954046	1
gene35622	18.59651685	39.68485098	2.133993763	1.09355596	0.21954046	1
gene52945	367.7977777	231.3336435	0.628969661	-0.66893767	0.2195464	1
gene38527	169.4349313	101.6319354	0.599828705	-0.73737753	0.21955651	1
gene15994	159.1035331	94.85647307	0.59619338	-0.74614774	0.21971496	1
gene22426	1120.95671	1824.535222	1.62765895	0.702798437	0.21975794	1
gene35750	97.11514356	162.6110967	1.674415449	0.743657528	0.21976382	1
gene65241	197.3297066	120.0224761	0.608233186	-0.71730356	0.21977413	1
gene62888	138.4407366	81.30554835	0.587294971	-0.76784281	0.21978752	1
gene10540	148.7721348	88.08101071	0.592053148	-0.7562014	0.21980894	1
gene49003	2605.578639	4527.944705	1.737788542	0.797252543	0.2199115	1
gene48110	1846.220867	3106.065532	1.682391087	0.750513112	0.21992194	1
gene3910	887.4671097	1427.686712	1.608720702	0.685913875	0.21995703	1
gene15101	580.6245817	367.810814	0.633474409	-0.65864176	0.22002203	1
gene9021	12.3976779	29.03769584	2.342188277	1.227857052	0.22002841	1
gene17907	12.3976779	29.03769584	2.342188277	1.227857052	0.22002841	1
gene51742	12.3976779	29.03769584	2.342188277	1.227857052	0.22002841	1
gene62941	12.3976779	29.03769584	2.342188277	1.227857052	0.22002841	1
gene23375	255.1855368	404.5918954	1.585481295	0.664920857	0.22010524	1
gene56468	213.8599438	340.7089645	1.593140625	0.671873618	0.22011278	1
gene53479	176.6669101	106.4715514	0.602668329	-0.73056384	0.22022682	1

gene60922	176.6669101	106.4715514	0.602668329	-0.73056384	0.22022682	1
gene45229	228.3239013	362.971198	1.58972055	0.668773183	0.22025687	1
gene22355	77.48548688	132.6054777	1.711358901	0.775142349	0.22032917	1
gene52170	132.2418976	215.8468724	1.632212455	0.706828857	0.22033279	1
gene22400	102.2808427	170.3544823	1.665556108	0.736003955	0.22038023	1
gene48825	242.7878589	150.0280952	0.617939035	-0.69446358	0.22039875	1
gene40169	30.99419475	60.01123807	1.936208975	0.953234671	0.22045688	1
gene46500	30.99419475	60.01123807	1.936208975	0.953234671	0.22045688	1
gene64070	30.99419475	60.01123807	1.936208975	0.953234671	0.22045688	1
gene40805	207.6611048	331.0297326	1.59408635	0.67272978	0.22050967	1
gene53016	25.82849563	10.64715514	0.412225137	-1.27849561	0.22055355	1
gene17262	392.5931335	619.4708446	1.57789526	0.658001443	0.22061458	1
gene48568	667.408327	422.9824361	0.633768593	-0.65797193	0.22078981	1
gene41375	63.02152933	110.3432442	1.750881728	0.808081633	0.22086589	1
gene59414	798.6170848	505.2559076	0.632663535	-0.66048965	0.22089765	1
gene39078	178.7331897	286.5052656	1.60297741	0.680754095	0.22093913	1
gene13939	645.7123907	1025.998586	1.588940527	0.668065127	0.2209507	1
gene37485	129.1424781	211.0072564	1.633910542	0.708328997	0.22095227	1
gene50971	343.0024219	541.0690658	1.577449695	0.657593998	0.22115091	1
gene14431	411.1896504	648.5085404	1.577151905	0.657321621	0.22122732	1
gene56419	142.5732959	231.3336435	1.622559415	0.698271308	0.22123627	1
gene71054	448.3826841	707.5518553	1.578008876	0.65810532	0.22124333	1
gene35828	1723.277228	2878.603581	1.670423965	0.740214315	0.2213667	1
gene1845	163.2360924	97.76024266	0.598888648	-0.73964031	0.22137567	1
gene22292	41.325593	76.46593238	1.850328739	0.88778161	0.22137912	1
gene18903	134.3081773	218.750642	1.628721694	0.703740106	0.22138968	1
gene34057	26.86163545	53.23577571	1.981851619	0.986848952	0.22140874	1
gene34593	26.86163545	53.23577571	1.981851619	0.986848952	0.22140874	1
gene27624	267.5832147	166.4827895	0.622172021	-0.68461458	0.22143998	1
gene63827	2307.001229	1401.552786	0.607521473	-0.71899269	0.22149948	1
gene47399	740.7612546	469.4427494	0.633730161	-0.65805941	0.22161707	1
gene27087	295.47799	466.5389798	1.578929719	0.658946956	0.22164825	1
gene15273	172.5343508	276.8260337	1.604469095	0.682096001	0.22168756	1
gene50174	56.82269038	100.6640122	1.771546042	0.825008961	0.22174833	1
gene60480	286.1797315	452.0201319	1.579497365	0.65946553	0.22175754	1
gene73438	132.2418976	77.43385557	0.585547069	-0.77214295	0.22175772	1
gene36056	432.8855867	273.9222641	0.632782131	-0.66021923	0.22178216	1
gene53128	1811.094113	3035.407138	1.676007401	0.74502852	0.22181873	1
gene55012	226.2576217	139.38094	0.616027602	-0.6989331	0.22188741	1
gene40792	44.42501248	81.30554835	1.830175026	0.871981625	0.22193689	1
gene3131	429.7861672	271.9864177	0.632841256	-0.66008444	0.2220073	1
gene2232	1720.177809	2870.860195	1.668932235	0.738925377	0.22201092	1
gene14582	170.4680711	102.5998586	0.601871412	-0.7324728	0.22202147	1
gene44144	1298.65676	812.0875603	0.625328867	-0.67731298	0.22205225	1
gene19717	87.81688513	148.0922488	1.686375559	0.753925863	0.22207689	1
gene7146	47.52443195	86.14516432	1.812650058	0.858100432	0.22218936	1
gene20463	47.52443195	86.14516432	1.812650058	0.858100432	0.22218936	1
gene15122	50.62385143	90.9847803	1.797271004	0.845807964	0.22220769	1

gene27884	319.2402059	503.3200612	1.576618646	0.656833742	0.22222838	1
gene10273	1002.14563	1617.399658	1.613936747	0.690584038	0.22223033	1
gene51367	91.94944443	51.29992932	0.557914511	-0.84188402	0.22227625	1
gene7139	160.1366729	95.82439627	0.598391327	-0.74083883	0.22232613	1
gene8782	965.9857365	609.7916126	0.631263578	-0.66368558	0.22238295	1
gene22890	205.5948252	125.8300153	0.612029097	-0.70832785	0.22242074	1
gene56154	255.1855368	403.6239722	1.581688278	0.661465299	0.22253648	1
gene63044	282.0471722	445.2446695	1.57861774	0.658661867	0.22256035	1
gene31852	220.0587827	349.4202733	1.587849705	0.667074363	0.22256197	1
gene59360	149.8052746	89.04893391	0.594431232	-0.75041818	0.22258799	1
gene7795	194.2302871	309.7354223	1.59468138	0.6732682	0.22260535	1
gene25301	223.1582022	137.4450936	0.615908769	-0.69921143	0.22260854	1
gene49561	205.5948252	327.1580398	1.591275653	0.670183772	0.22264658	1
gene31257	252.0861173	398.7843562	1.581937	0.661692146	0.22265539	1
gene31052	1292.457921	2113.944257	1.635600063	0.709820023	0.22270309	1
gene11665	11.36453808	27.10184945	2.384773518	1.25385226	0.22281097	1
gene27481	11.36453808	27.10184945	2.384773518	1.25385226	0.22281097	1
gene42405	11.36453808	27.10184945	2.384773518	1.25385226	0.22281097	1
gene67234	11.36453808	27.10184945	2.384773518	1.25385226	0.22281097	1
gene55872	76.45234706	130.6696313	1.709164418	0.773291188	0.22284389	1
gene49636	105.3802622	60.01123807	0.569473228	-0.81230007	0.22286867	1
gene45968	600.2542384	381.3617387	0.635333687	-0.65441358	0.2229613	1
gene39014	556.8623657	879.8421839	1.579999364	0.659923978	0.22306661	1
gene11855	3169.672983	5612.986606	1.770840915	0.824434612	0.22307501	1
gene58533	230.390181	142.2847096	0.617581483	-0.6952986	0.22307578	1
gene1471	17.56337703	37.74900459	2.149302183	1.103868334	0.22315933	1
gene14650	17.56337703	37.74900459	2.149302183	1.103868334	0.22315933	1
gene14907	17.56337703	37.74900459	2.149302183	1.103868334	0.22315933	1
gene32079	17.56337703	37.74900459	2.149302183	1.103868334	0.22315933	1
gene32740	17.56337703	37.74900459	2.149302183	1.103868334	0.22315933	1
gene30351	333.7041635	210.0393332	0.629417778	-0.66791017	0.22325999	1
gene16038	585.7902808	926.3024973	1.5812869	0.661099146	0.22331426	1
gene12823	1405.070162	2308.496819	1.642976188	0.716311571	0.22332585	1
gene39451	138.4407366	224.5581812	1.622052777	0.697820761	0.22344407	1
gene48787	89.88316478	150.9960184	1.67991435	0.74838768	0.22356857	1
gene21673	73.35292758	125.8300153	1.715405498	0.77854965	0.22362718	1
gene41998	73.35292758	125.8300153	1.715405498	0.77854965	0.22362718	1
gene64486	73.35292758	125.8300153	1.715405498	0.77854965	0.22362718	1
gene65889	73.35292758	125.8300153	1.715405498	0.77854965	0.22362718	1
gene37147	265.516935	165.5148663	0.623368397	-0.68184308	0.22367891	1
gene51162	61.9883895	108.4073978	1.748833913	0.806393283	0.22369551	1
gene47811	454.581523	288.441112	0.634520097	-0.65626224	0.22371945	1
gene31068	471.1117602	299.0882671	0.634856296	-0.65549803	0.22374608	1
gene61645	45.4581523	22.26223348	0.489730276	-1.02994071	0.22375162	1
gene64772	126.0430587	73.56216279	0.583627243	-0.77688087	0.22382533	1
gene36748	102.2808427	58.07539168	0.567803219	-0.81653707	0.22383717	1
gene67875	445.2832646	700.7763929	1.573776624	0.654230785	0.2238865	1
gene3501	955.6543382	1536.09411	1.607374182	0.684705814	0.2239067	1

gene4334	1931.971473	1188.609683	0.615231487	-0.70079875	0.22391618	1
gene14399	29.96105493	58.07539168	1.938362712	0.954838557	0.22403065	1
gene23917	181.8326092	110.3432442	0.60683969	-0.72061265	0.22403071	1
gene966	566.1606241	360.0674284	0.63598105	-0.65294432	0.22407248	1
gene51047	34.09361423	15.48677111	0.454242575	-1.13846516	0.22411712	1
gene68427	34.09361423	15.48677111	0.454242575	-1.13846516	0.22411712	1
gene38537	337.8367228	212.9431028	0.630313665	-0.66585816	0.22424861	1
gene29861	273.7820536	431.6937448	1.576778825	0.656980307	0.22427398	1
gene22430	3264.721847	1942.621852	0.595034414	-0.74895499	0.22430739	1
gene64500	314.0745068	197.4563317	0.628692643	-0.66957321	0.22431111	1
gene15285	747.9932334	475.2502886	0.635367096	-0.65433772	0.22435019	1
gene40281	2386.552996	4086.571728	1.712332278	0.775962684	0.22435832	1
gene63212	4561.312328	2636.622782	0.578040396	-0.79075778	0.22438394	1
gene20384	186.9983083	298.1203439	1.59424086	0.67286961	0.22445098	1
gene16316	171.501211	103.5677818	0.603889508	-0.72764349	0.2244737	1
gene6193	1143.685786	1853.572918	1.620701192	0.696618127	0.22467039	1
gene10082	40.29245318	74.53008599	1.849728178	0.887313278	0.2247005	1
gene53477	40.29245318	74.53008599	1.849728178	0.887313278	0.2247005	1
gene12933	55.78955055	98.72816585	1.769653365	0.823466796	0.22474022	1
gene54500	145.6727153	235.2053363	1.614614897	0.691190108	0.22478816	1
gene5052	18.59651685	6.775462362	0.364340399	-1.45664112	0.22480626	1
gene10542	18.59651685	6.775462362	0.364340399	-1.45664112	0.22480626	1
gene40300	18.59651685	6.775462362	0.364340399	-1.45664112	0.22480626	1
gene45989	18.59651685	6.775462362	0.364340399	-1.45664112	0.22480626	1
gene616	505.2053745	321.3505006	0.636078943	-0.65272227	0.22491477	1
gene49721	1034.172965	653.3481564	0.631759076	-0.66255361	0.22493173	1
gene70696	937.0578213	1503.184721	1.604153647	0.681812331	0.22493651	1
gene462	4.1325593	13.55092472	3.279063588	1.713283879	0.22493669	1
gene4590	4.1325593	13.55092472	3.279063588	1.713283879	0.22493669	1
gene15413	4.1325593	13.55092472	3.279063588	1.713283879	0.22493669	1
gene46668	4.1325593	13.55092472	3.279063588	1.713283879	0.22493669	1
gene53019	4.1325593	13.55092472	3.279063588	1.713283879	0.22493669	1
gene54383	4.1325593	13.55092472	3.279063588	1.713283879	0.22493669	1
gene56038	4.1325593	13.55092472	3.279063588	1.713283879	0.22493669	1
gene58484	4.1325593	13.55092472	3.279063588	1.713283879	0.22493669	1
gene59965	4.1325593	13.55092472	3.279063588	1.713283879	0.22493669	1
gene66008	4.1325593	13.55092472	3.279063588	1.713283879	0.22493669	1
gene67122	4.1325593	13.55092472	3.279063588	1.713283879	0.22493669	1
gene68303	4.1325593	13.55092472	3.279063588	1.713283879	0.22493669	1
gene70260	4.1325593	13.55092472	3.279063588	1.713283879	0.22493669	1
gene74099	4.1325593	13.55092472	3.279063588	1.713283879	0.22493669	1
gene29716	178.7331897	108.4073978	0.606531993	-0.72134435	0.22496245	1
gene52728	667.408327	1056.972129	1.583696346	0.663295743	0.22509762	1
gene26947	299.6105493	471.3785958	1.573304401	0.653797829	0.22510586	1
gene1495	52.69013108	93.88854988	1.781900101	0.833416457	0.22511792	1
gene3890	25.82849563	51.29992932	1.986175659	0.989993221	0.22513325	1
gene31867	25.82849563	51.29992932	1.986175659	0.989993221	0.22513325	1
gene71732	25.82849563	51.29992932	1.986175659	0.989993221	0.22513325	1

gene37896	21.69593633	44.52446695	2.052203062	1.03717349	0.22516494	1
gene61801	21.69593633	44.52446695	2.052203062	1.03717349	0.22516494	1
gene46969	26422.55103	11371.16169	0.430358207	-1.21639011	0.22518197	1
gene60036	123.976779	202.2959477	1.6317245	0.706397493	0.22520064	1
gene16736	43.39187265	79.36970196	1.829137511	0.871163538	0.22521401	1
gene283	110.5459613	181.9695606	1.646098677	0.719050822	0.22536505	1
gene48619	110.5459613	181.9695606	1.646098677	0.719050822	0.22536505	1
gene17948	185.9651685	113.2470138	0.608968952	-0.71555942	0.22540604	1
gene64488	185.9651685	113.2470138	0.608968952	-0.71555942	0.22540604	1
gene64002	46.49129213	84.20931793	1.811292267	0.857019356	0.22540663	1
gene9788	10.33139825	25.16600306	2.435875808	1.28444058	0.22541964	1
gene70756	10.33139825	25.16600306	2.435875808	1.28444058	0.22541964	1
gene51459	129.1424781	210.0393332	1.626415539	0.701695904	0.22550496	1
gene53452	1041.404944	658.1877724	0.632019059	-0.66196003	0.22554108	1
gene1398	424.6204681	666.8990811	1.570576859	0.651294546	0.22561734	1
gene32975	109.5128215	62.91500765	0.574499011	-0.79962368	0.2256748	1
gene13419	185.9651685	296.1844976	1.592688028	0.671463703	0.22568428	1
gene37461	140.5070162	83.24139474	0.592435858	-0.75526913	0.22571516	1
gene2670	743.8606741	473.3144422	0.636294482	-0.65223348	0.225778	1
gene26296	1980.529045	3327.719943	1.680217693	0.748648164	0.22587692	1
gene41875	33.0604744	62.91500765	1.903027975	0.92829677	0.22591148	1
gene46616	33.0604744	62.91500765	1.903027975	0.92829677	0.22591148	1
gene15004	64.05466916	111.3111674	1.737752592	0.797222697	0.22591304	1
gene63719	353.3338202	554.6199905	1.569677056	0.650467771	0.22604411	1
gene68317	440.1175655	691.097161	1.570255802	0.6509996	0.22606616	1
gene36492	247.953558	391.0409706	1.57707344	0.657249844	0.22620555	1
gene71066	73.35292758	39.68485098	0.541012503	-0.88626616	0.22622085	1
gene5205	72.31978776	123.8941689	1.713143425	0.77664594	0.22629532	1
gene50290	72.31978776	123.8941689	1.713143425	0.77664594	0.22629532	1
gene46619	147.738995	88.08101071	0.59619338	-0.74614774	0.22641804	1
gene43322	332.6710237	210.0393332	0.631372492	-0.66343669	0.22643408	1
gene24135	419.454769	658.1877724	1.569150767	0.649983976	0.22647797	1
gene11441	207.6611048	127.7658617	0.615261398	-0.70072862	0.22656312	1
gene7861	1825.558071	1129.566368	0.618751266	-0.69256852	0.22660208	1
gene46392	60.95524968	106.4715514	1.746716681	0.804645621	0.22661641	1
gene62408	60.95524968	106.4715514	1.746716681	0.804645621	0.22661641	1
gene52644	196.2965668	311.6712687	1.587757106	0.666990226	0.2266271	1
gene21368	614.7181959	969.859041	1.577729515	0.657849892	0.22667201	1
gene58376	190.0977278	116.1507834	0.611005637	-0.7107424	0.22673059	1
gene65132	190.0977278	116.1507834	0.611005637	-0.7107424	0.22673059	1
gene59344	319.2402059	201.3280245	0.630647458	-0.66509435	0.22676233	1
gene18372	137.4075967	81.30554835	0.591710723	-0.75703606	0.22681694	1
gene74213	67.15408863	35.8131582	0.533298254	-0.90698549	0.22683843	1
gene46654	57.8558302	30.00561903	0.518627404	-0.94722966	0.22688475	1
gene53574	57.8558302	30.00561903	0.518627404	-0.94722966	0.22688475	1
gene56289	57.8558302	30.00561903	0.518627404	-0.94722966	0.22688475	1
gene11204	16.5302372	35.8131582	2.166524156	1.115382322	0.22690969	1
gene41611	16.5302372	35.8131582	2.166524156	1.115382322	0.22690969	1

gene54268	16.5302372	35.8131582	2.166524156	1.115382322	0.22690969	1
gene1290	563.0612047	359.0995052	0.637762826	-0.64890809	0.22695004	1
gene4369	1854.485986	1146.988986	0.618494286	-0.69316783	0.22698	1
gene35935	308.9088077	484.9295205	1.569814484	0.650594076	0.22701216	1
gene1509	116.7448002	67.75462362	0.580365237	-0.78496699	0.22710708	1
gene2581	116.7448002	67.75462362	0.580365237	-0.78496699	0.22710708	1
gene50675	973.2177152	1560.29219	1.603230362	0.680981736	0.2271581	1
gene50043	36.15989388	67.75462362	1.873750621	0.905928957	0.22724614	1
gene52952	36.15989388	67.75462362	1.873750621	0.905928957	0.22724614	1
gene38729	181.8326092	289.4090352	1.591623397	0.670499012	0.22736949	1
gene42299	336.803583	212.9431028	0.632247142	-0.66143948	0.22739475	1
gene11008	1488.754488	2444.006066	1.641644802	0.715142009	0.22741715	1
gene32141	596.1216791	380.3938155	0.63811438	-0.64811305	0.22742703	1
gene43735	144.6395755	86.14516432	0.595585019	-0.74762063	0.22751743	1
gene52066	2747.118795	1660.956202	0.604617538	-0.72590527	0.22752583	1
gene1777	979.4165542	1569.971422	1.602965985	0.680743812	0.22760033	1
gene9612	37.1930337	17.4226175	0.468437655	-1.09407104	0.22760937	1
gene24228	37.1930337	17.4226175	0.468437655	-1.09407104	0.22760937	1
gene57991	567.193764	362.0032748	0.63823564	-0.64783892	0.22769092	1
gene2918	9.298258426	23.23015667	2.498334162	1.320966456	0.22773888	1
gene12172	9.298258426	23.23015667	2.498334162	1.320966456	0.22773888	1
gene21062	9.298258426	23.23015667	2.498334162	1.320966456	0.22773888	1
gene24497	9.298258426	23.23015667	2.498334162	1.320966456	0.22773888	1
gene40497	9.298258426	23.23015667	2.498334162	1.320966456	0.22773888	1
gene47941	9.298258426	23.23015667	2.498334162	1.320966456	0.22773888	1
gene54147	9.298258426	23.23015667	2.498334162	1.320966456	0.22773888	1
gene64293	9.298258426	23.23015667	2.498334162	1.320966456	0.22773888	1
gene3589	1060.001461	1705.480669	1.60894181	0.68611215	0.22774495	1
gene13871	28.9279151	56.13954529	1.940670286	0.95655503	0.22777447	1
gene17439	28.9279151	56.13954529	1.940670286	0.95655503	0.22777447	1
gene69338	28.9279151	56.13954529	1.940670286	0.95655503	0.22777447	1
gene10893	635.3809924	405.5598186	0.638293911	-0.64770721	0.2278251	1
gene9683	28.9279151	12.58300153	0.434977823	-1.20098625	0.22793487	1
gene44764	28.9279151	12.58300153	0.434977823	-1.20098625	0.22793487	1
gene59207	28.9279151	12.58300153	0.434977823	-1.20098625	0.22793487	1
gene68396	28.9279151	12.58300153	0.434977823	-1.20098625	0.22793487	1
gene33014	134.3081773	79.36970196	0.590952119	-0.75888685	0.2279494	1
gene54870	334.7373033	524.6143715	1.567242032	0.648227995	0.22795104	1
gene14093	1522.848102	2502.081458	1.643027597	0.716356713	0.22802159	1
gene22864	472.1449001	301.0241135	0.637567225	-0.64935063	0.22805299	1
gene42464	39.25931335	72.5942396	1.849096008	0.886820134	0.22814599	1
gene72352	39.25931335	72.5942396	1.849096008	0.886820134	0.22814599	1
gene1064	1.033139825	6.775462362	6.558127175	2.713283879	0.22822111	1
gene2204	1.033139825	6.775462362	6.558127175	2.713283879	0.22822111	1
gene2677	1.033139825	6.775462362	6.558127175	2.713283879	0.22822111	1
gene2947	1.033139825	6.775462362	6.558127175	2.713283879	0.22822111	1
gene5854	1.033139825	6.775462362	6.558127175	2.713283879	0.22822111	1
gene8149	1.033139825	6.775462362	6.558127175	2.713283879	0.22822111	1

gene8433	1.033139825	6.775462362	6.558127175	2.713283879	0.22822111	1
gene8885	1.033139825	6.775462362	6.558127175	2.713283879	0.22822111	1
gene9392	1.033139825	6.775462362	6.558127175	2.713283879	0.22822111	1
gene9682	1.033139825	6.775462362	6.558127175	2.713283879	0.22822111	1
gene11423	1.033139825	6.775462362	6.558127175	2.713283879	0.22822111	1
gene11694	1.033139825	6.775462362	6.558127175	2.713283879	0.22822111	1
gene11762	1.033139825	6.775462362	6.558127175	2.713283879	0.22822111	1
gene14670	1.033139825	6.775462362	6.558127175	2.713283879	0.22822111	1
gene16742	1.033139825	6.775462362	6.558127175	2.713283879	0.22822111	1
gene17287	1.033139825	6.775462362	6.558127175	2.713283879	0.22822111	1
gene17371	1.033139825	6.775462362	6.558127175	2.713283879	0.22822111	1
gene17768	1.033139825	6.775462362	6.558127175	2.713283879	0.22822111	1
gene18823	1.033139825	6.775462362	6.558127175	2.713283879	0.22822111	1
gene19710	1.033139825	6.775462362	6.558127175	2.713283879	0.22822111	1
gene19959	1.033139825	6.775462362	6.558127175	2.713283879	0.22822111	1
gene20498	1.033139825	6.775462362	6.558127175	2.713283879	0.22822111	1
gene21582	1.033139825	6.775462362	6.558127175	2.713283879	0.22822111	1
gene22773	1.033139825	6.775462362	6.558127175	2.713283879	0.22822111	1
gene27161	1.033139825	6.775462362	6.558127175	2.713283879	0.22822111	1
gene27461	1.033139825	6.775462362	6.558127175	2.713283879	0.22822111	1
gene27804	1.033139825	6.775462362	6.558127175	2.713283879	0.22822111	1
gene28635	1.033139825	6.775462362	6.558127175	2.713283879	0.22822111	1
gene28722	1.033139825	6.775462362	6.558127175	2.713283879	0.22822111	1
gene29065	1.033139825	6.775462362	6.558127175	2.713283879	0.22822111	1
gene32637	1.033139825	6.775462362	6.558127175	2.713283879	0.22822111	1
gene34683	1.033139825	6.775462362	6.558127175	2.713283879	0.22822111	1
gene38030	1.033139825	6.775462362	6.558127175	2.713283879	0.22822111	1
gene38461	1.033139825	6.775462362	6.558127175	2.713283879	0.22822111	1
gene39761	1.033139825	6.775462362	6.558127175	2.713283879	0.22822111	1
gene41814	1.033139825	6.775462362	6.558127175	2.713283879	0.22822111	1
gene42956	1.033139825	6.775462362	6.558127175	2.713283879	0.22822111	1
gene45113	1.033139825	6.775462362	6.558127175	2.713283879	0.22822111	1
gene46017	1.033139825	6.775462362	6.558127175	2.713283879	0.22822111	1
gene47620	1.033139825	6.775462362	6.558127175	2.713283879	0.22822111	1
gene48923	1.033139825	6.775462362	6.558127175	2.713283879	0.22822111	1
gene50189	1.033139825	6.775462362	6.558127175	2.713283879	0.22822111	1
gene51129	1.033139825	6.775462362	6.558127175	2.713283879	0.22822111	1
gene52949	1.033139825	6.775462362	6.558127175	2.713283879	0.22822111	1
gene53392	1.033139825	6.775462362	6.558127175	2.713283879	0.22822111	1
gene55941	1.033139825	6.775462362	6.558127175	2.713283879	0.22822111	1
gene57322	1.033139825	6.775462362	6.558127175	2.713283879	0.22822111	1
gene58530	1.033139825	6.775462362	6.558127175	2.713283879	0.22822111	1
gene58730	1.033139825	6.775462362	6.558127175	2.713283879	0.22822111	1
gene59184	1.033139825	6.775462362	6.558127175	2.713283879	0.22822111	1
gene59415	1.033139825	6.775462362	6.558127175	2.713283879	0.22822111	1
gene61002	1.033139825	6.775462362	6.558127175	2.713283879	0.22822111	1
gene62830	1.033139825	6.775462362	6.558127175	2.713283879	0.22822111	1
gene63128	1.033139825	6.775462362	6.558127175	2.713283879	0.22822111	1

gene66757	1.033139825	6.775462362	6.558127175	2.713283879	0.22822111	1
gene66858	1.033139825	6.775462362	6.558127175	2.713283879	0.22822111	1
gene67706	1.033139825	6.775462362	6.558127175	2.713283879	0.22822111	1
gene68340	1.033139825	6.775462362	6.558127175	2.713283879	0.22822111	1
gene68389	1.033139825	6.775462362	6.558127175	2.713283879	0.22822111	1
gene69273	1.033139825	6.775462362	6.558127175	2.713283879	0.22822111	1
gene69515	1.033139825	6.775462362	6.558127175	2.713283879	0.22822111	1
gene69837	1.033139825	6.775462362	6.558127175	2.713283879	0.22822111	1
gene69933	1.033139825	6.775462362	6.558127175	2.713283879	0.22822111	1
gene71428	1.033139825	6.775462362	6.558127175	2.713283879	0.22822111	1
gene71980	1.033139825	6.775462362	6.558127175	2.713283879	0.22822111	1
gene72258	1.033139825	6.775462362	6.558127175	2.713283879	0.22822111	1
gene72481	1.033139825	6.775462362	6.558127175	2.713283879	0.22822111	1
gene5523	123.976779	72.5942396	0.585547069	-0.77214295	0.22822924	1
gene15149	123.976779	72.5942396	0.585547069	-0.77214295	0.22822924	1
gene41509	1411.269001	2306.560973	1.634387896	0.708750425	0.22827183	1
gene70494	208.6942447	330.0618094	1.581556836	0.661345403	0.22839506	1
gene62124	135.3413171	218.750642	1.616288704	0.692684918	0.22852905	1
gene35751	593.0222596	933.0779596	1.573428222	0.653911366	0.22856285	1
gene56984	789.3188264	1252.492614	1.586801901	0.666122031	0.22857333	1
gene58497	42.35873283	77.43385557	1.828049387	0.870305047	0.22861214	1
gene64355	626.082734	986.3137353	1.575372841	0.655693309	0.22862083	1
gene61186	208.6942447	128.7337849	0.616853546	-0.69700009	0.22862159	1
gene69625	45.4581523	82.27347154	1.809872759	0.855888274	0.22874062	1
gene36428	116.7448002	190.6808693	1.633313595	0.707801814	0.22876569	1
gene46941	215.9262234	133.5734009	0.618606664	-0.69290572	0.22891308	1
gene2375	100.214563	57.10746848	0.569851993	-0.81134084	0.22893206	1
gene28796	258.2849563	161.6431735	0.625832708	-0.67615104	0.22896613	1
gene39299	1189.143939	1922.295465	1.616537243	0.692906746	0.22902515	1
gene73811	24.7953558	49.36408293	1.990860035	0.993391798	0.22903853	1
gene1865	71.28664793	121.9583225	1.710815785	0.774684423	0.22904348	1
gene10279	5.165699125	15.48677111	2.998000994	1.584000862	0.22906005	1
gene15678	5.165699125	15.48677111	2.998000994	1.584000862	0.22906005	1
gene16555	5.165699125	15.48677111	2.998000994	1.584000862	0.22906005	1
gene19304	5.165699125	15.48677111	2.998000994	1.584000862	0.22906005	1
gene23554	5.165699125	15.48677111	2.998000994	1.584000862	0.22906005	1
gene30196	5.165699125	15.48677111	2.998000994	1.584000862	0.22906005	1
gene31103	5.165699125	15.48677111	2.998000994	1.584000862	0.22906005	1
gene39639	5.165699125	15.48677111	2.998000994	1.584000862	0.22906005	1
gene40870	5.165699125	15.48677111	2.998000994	1.584000862	0.22906005	1
gene44695	5.165699125	15.48677111	2.998000994	1.584000862	0.22906005	1
gene48009	5.165699125	15.48677111	2.998000994	1.584000862	0.22906005	1
gene48037	5.165699125	15.48677111	2.998000994	1.584000862	0.22906005	1
gene51883	5.165699125	15.48677111	2.998000994	1.584000862	0.22906005	1
gene59198	5.165699125	15.48677111	2.998000994	1.584000862	0.22906005	1
gene60522	5.165699125	15.48677111	2.998000994	1.584000862	0.22906005	1
gene63978	5.165699125	15.48677111	2.998000994	1.584000862	0.22906005	1
gene65564	5.165699125	15.48677111	2.998000994	1.584000862	0.22906005	1

gene67158	5.165699125	15.48677111	2.998000994	1.584000862	0.22906005	1
gene67330	5.165699125	15.48677111	2.998000994	1.584000862	0.22906005	1
gene67535	5.165699125	15.48677111	2.998000994	1.584000862	0.22906005	1
gene70769	5.165699125	15.48677111	2.998000994	1.584000862	0.22906005	1
gene38567	164.2692322	262.3071857	1.596812637	0.675195043	0.22907506	1
gene21847	20.6627965	42.58862056	2.061125684	1.04343248	0.22912462	1
gene31916	20.6627965	42.58862056	2.061125684	1.04343248	0.22912462	1
gene18075	482.4762983	755.948015	1.566808603	0.647828955	0.22922816	1
gene5710	267.5832147	420.0786665	1.569899169	0.650671901	0.22923361	1
gene18593	103.3139825	170.3544823	1.648900547	0.721504386	0.2292893	1
gene16280	552.7298064	353.291966	0.639176614	-0.64571347	0.22930018	1
gene73718	120.8773595	70.65839321	0.584546134	-0.7746112	0.22940722	1
gene8441	283.0803121	178.0978678	0.629142544	-0.66854117	0.22945102	1
gene63275	283.0803121	178.0978678	0.629142544	-0.66854117	0.22945102	1
gene36212	83.68432583	46.46031334	0.555185369	-0.84895855	0.22949445	1
gene33193	692.2036828	442.3408999	0.639032861	-0.64603797	0.22950856	1
gene46689	692.2036828	442.3408999	0.639032861	-0.64603797	0.22950856	1
gene60726	3399.030025	6021.450194	1.771520154	0.824987879	0.22951347	1
gene3409	8.265118601	21.29431028	2.576407104	1.365360575	0.22959747	1
gene7205	8.265118601	21.29431028	2.576407104	1.365360575	0.22959747	1
gene20992	8.265118601	21.29431028	2.576407104	1.365360575	0.22959747	1
gene63018	8.265118601	21.29431028	2.576407104	1.365360575	0.22959747	1
gene8273	32.02733458	60.97916126	1.903972406	0.92901257	0.22962016	1
gene42961	376.0628963	239.0770291	0.635736818	-0.65349845	0.22962029	1
gene69257	161.1698127	257.4675698	1.597492517	0.675809173	0.2296768	1
gene27507	390.5268539	610.7595358	1.563937357	0.645182727	0.22967769	1
gene69352	451.4821036	706.5839321	1.565031984	0.646192141	0.22968891	1
gene26195	347.1349812	543.0049122	1.564247171	0.645468494	0.22970927	1
gene35544	596.1216791	936.9496524	1.571742289	0.652364685	0.22976242	1
gene66854	477.3105992	304.8958063	0.638778621	-0.64661207	0.22986565	1
gene34264	2501.231517	1525.446955	0.609878352	-0.71340659	0.22991685	1
gene23198	740.7612546	473.3144422	0.638956802	-0.6462097	0.23004119	1
gene27066	95.04886391	157.7714807	1.659898648	0.731095155	0.23005122	1
gene15483	244.8541385	152.9318648	0.62458354	-0.67903354	0.23006914	1
gene28224	163.2360924	98.72816585	0.604818239	-0.72542645	0.23007166	1
gene50661	520.7024718	332.965579	0.639454577	-0.64508621	0.2300953	1
gene48166	520.7024718	815.9592531	1.56703549	0.648037854	0.23016648	1
gene57605	349.2012609	221.6544116	0.634746882	-0.65574669	0.23018805	1
gene12165	195.2634269	120.0224761	0.614669516	-0.70211716	0.23019121	1
gene66816	598.1879587	939.853422	1.571167404	0.651836904	0.23021155	1
gene53979	873.0031522	556.5558369	0.637518703	-0.64946043	0.23025313	1
gene19518	134.3081773	216.8147956	1.614308228	0.690916066	0.23029384	1
gene29167	128.1093383	75.49800918	0.589324792	-0.76286513	0.23030851	1
gene52166	252.0861173	157.7714807	0.625863425	-0.67608023	0.23036864	1
gene52316	56.82269038	99.69608905	1.754511946	0.81106977	0.23037204	1
gene67710	217.9925031	343.6127341	1.576259409	0.656504982	0.23040422	1
gene25710	289.279151	452.9880551	1.565920162	0.647010659	0.23048946	1
gene6550	107.4465418	61.94708446	0.576538653	-0.79451076	0.23059239	1

gene44857	107.4465418	61.94708446	0.576538653	-0.79451076	0.23059239	1
gene67771	259.3180961	162.6110967	0.627071921	-0.67329717	0.23067589	1
gene38977	257.2518164	403.6239722	1.568983954	0.649830598	0.23068117	1
gene47469	177.7000499	108.4073978	0.610058342	-0.71298088	0.2306864	1
gene1440	214.8930836	338.7731181	1.576472879	0.65670035	0.23073546	1
gene11412	7.231978776	19.35846389	2.676786602	1.420502129	0.23073646	1
gene13212	7.231978776	19.35846389	2.676786602	1.420502129	0.23073646	1
gene32525	7.231978776	19.35846389	2.676786602	1.420502129	0.23073646	1
gene35941	7.231978776	19.35846389	2.676786602	1.420502129	0.23073646	1
gene55728	7.231978776	19.35846389	2.676786602	1.420502129	0.23073646	1
gene63201	7.231978776	19.35846389	2.676786602	1.420502129	0.23073646	1
gene64515	7.231978776	19.35846389	2.676786602	1.420502129	0.23073646	1
gene71657	7.231978776	19.35846389	2.676786602	1.420502129	0.23073646	1
gene73843	7.231978776	19.35846389	2.676786602	1.420502129	0.23073646	1
gene6972	6.19883895	17.4226175	2.810625932	1.490891457	0.23075397	1
gene9038	6.19883895	17.4226175	2.810625932	1.490891457	0.23075397	1
gene38164	6.19883895	17.4226175	2.810625932	1.490891457	0.23075397	1
gene73603	6.19883895	17.4226175	2.810625932	1.490891457	0.23075397	1
gene4214	115.7116604	188.745023	1.631166836	0.705904348	0.23077336	1
gene26403	15.49709738	33.87731181	2.186042392	1.128321378	0.23078879	1
gene51469	15.49709738	33.87731181	2.186042392	1.128321378	0.23078879	1
gene60950	15.49709738	33.87731181	2.186042392	1.128321378	0.23078879	1
gene70412	1369.943408	862.4195664	0.629529338	-0.66765448	0.23084081	1
gene17109	478.343739	748.2046295	1.564156836	0.645385177	0.2308636	1
gene1767	216.9593633	134.5413241	0.620122229	-0.68937549	0.23090344	1
gene66783	2159.262234	1329.926469	0.61591707	-0.69919198	0.23092389	1
gene3931	65.08780898	112.2790906	1.725040255	0.786630028	0.23093636	1
gene26070	65.08780898	112.2790906	1.725040255	0.786630028	0.23093636	1
gene61580	184.9320287	113.2470138	0.612371013	-0.7075221	0.23093948	1
gene48705	126.0430587	204.2317941	1.620333529	0.696290808	0.23101197	1
gene18229	284.1134519	179.065791	0.630261573	-0.66597739	0.23103625	1
gene56120	1609.631847	1006.640122	0.625385317	-0.67718275	0.23115128	1
gene60665	991.8142321	631.0859229	0.636294482	-0.65223348	0.23115159	1
gene9040	160.1366729	255.5317234	1.595710207	0.674198671	0.23118392	1
gene17339	608.519357	955.3401931	1.569942159	0.650711407	0.23140277	1
gene13600	584.757141	374.5862763	0.640584356	-0.64253953	0.23144115	1
gene40771	125.0099188	73.56216279	0.588450608	-0.76500677	0.2315361	1
gene13143	450.4489637	703.6801625	1.562175117	0.643556186	0.23155511	1
gene5524	43.39187265	21.29431028	0.49074421	-1.02695685	0.23180118	1
gene14369	43.39187265	21.29431028	0.49074421	-1.02695685	0.23180118	1
gene40643	43.39187265	21.29431028	0.49074421	-1.02695685	0.23180118	1
gene49247	43.39187265	21.29431028	0.49074421	-1.02695685	0.23180118	1
gene27905	558.9286454	358.131582	0.640746516	-0.64217437	0.23180619	1
gene25530	539.2989887	844.0290257	1.565048412	0.646207285	0.23206319	1
gene2342	41.325593	75.49800918	1.826906856	0.869403081	0.23213799	1
gene64045	41.325593	75.49800918	1.826906856	0.869403081	0.23213799	1
gene50813	449.4158239	287.4731888	0.639659695	-0.64462351	0.23214239	1
gene44856	132.2418976	78.40177877	0.592866408	-0.75422104	0.23224429	1

gene15067	1199.475337	759.8197078	0.633460051	-0.65867446	0.23225831	1
gene18164	605.4199375	388.137201	0.641104095	-0.64136947	0.23228653	1
gene21863	157.0372534	94.85647307	0.604038029	-0.72728871	0.23234414	1
gene11301	235.5558801	147.1243256	0.62458354	-0.67903354	0.2324155	1
gene66582	686.0048439	1079.234362	1.573216824	0.65371752	0.2325011	1
gene39399	71.28664793	38.71692779	0.543116122	-0.88066741	0.23250691	1
gene72334	1409.202721	887.5855695	0.629849457	-0.66692105	0.23250803	1
gene27759	16.5302372	5.807539168	0.351328242	-1.50910854	0.23254345	1
gene32460	16.5302372	5.807539168	0.351328242	-1.50910854	0.23254345	1
gene64819	75.41920723	127.7658617	1.694075904	0.760498517	0.23254439	1
gene33202	153.9378339	245.8524914	1.597089456	0.675445123	0.23256284	1
gene35142	889.5333894	568.1709153	0.638729161	-0.64672378	0.23260377	1
gene9409	1348.247472	850.8044881	0.63104475	-0.66418578	0.23265713	1
gene32363	661.2094881	423.9503593	0.641174041	-0.64121208	0.23268816	1
gene58635	302.7099687	191.6487925	0.633110278	-0.65947128	0.232733	1
gene66947	58.88897003	102.5998586	1.74225935	0.800959397	0.23274938	1
gene62019	121.9104994	71.6263164	0.587531975	-0.76726073	0.23279624	1
gene34986	480.4100187	307.7995759	0.640701825	-0.64227499	0.23290171	1
gene10268	46.49129213	23.23015667	0.499666832	-1.00096164	0.23294708	1
gene55160	46.49129213	23.23015667	0.499666832	-1.00096164	0.23294708	1
gene48580	80.58490636	135.5092472	1.681571071	0.749809755	0.23301469	1
gene4595	68.18722846	36.7810814	0.539413058	-0.89053765	0.23303526	1
gene14521	553.7629462	355.2278124	0.641479924	-0.64052398	0.23303618	1
gene15328	32.02733458	14.51884792	0.453326763	-1.14137676	0.23304618	1
gene64407	32.02733458	14.51884792	0.453326763	-1.14137676	0.23304618	1
gene33892	23.76221598	47.42823654	1.995951749	0.997076845	0.2331377	1
gene61723	348.1681211	221.6544116	0.636630404	-0.65147204	0.2332915	1
gene65172	348.1681211	221.6544116	0.636630404	-0.65147204	0.2332915	1
gene64324	299.6105493	189.7129461	0.633198486	-0.65927029	0.23330458	1
gene49921	337.8367228	214.8789492	0.636043789	-0.652802	0.23333	1
gene17578	87.81688513	49.36408293	0.562125186	-0.83103664	0.23333114	1
gene22336	691.170543	443.3088231	0.641388479	-0.64072966	0.23333937	1
gene19098	193.1971473	119.0545529	0.616233493	-0.698451	0.233374	1
gene47058	547.5641073	351.3561197	0.641671203	-0.64009385	0.23339666	1
gene26527	65.08780898	34.84523501	0.53535732	-0.90142597	0.23348254	1
gene69836	30.99419475	59.04331487	1.904979798	0.929775699	0.23350656	1
gene67461	90.91630461	150.9960184	1.660824414	0.731899557	0.23355551	1
gene4267	49.5907116	25.16600306	0.507474127	-0.97859383	0.23365782	1
gene28011	49.5907116	25.16600306	0.507474127	-0.97859383	0.23365782	1
gene47961	49.5907116	25.16600306	0.507474127	-0.97859383	0.23365782	1
gene16975	96.08200373	158.7394039	1.652124204	0.72432215	0.23369579	1
gene11100	678.7728651	435.5654376	0.641695418	-0.64003941	0.23370704	1
gene225	101.2477029	166.4827895	1.64431177	0.717483867	0.23378242	1
gene2471	1421.600399	896.2968782	0.630484402	-0.66546741	0.23390078	1
gene25158	52.69013108	27.10184945	0.514362916	-0.95914146	0.23403454	1
gene46316	52.69013108	27.10184945	0.514362916	-0.95914146	0.23403454	1
gene71761	52.69013108	27.10184945	0.514362916	-0.95914146	0.23403454	1
gene19005	190.0977278	300.0561903	1.57843123	0.658491406	0.23404965	1

gene7113	58.88897003	30.97354223	0.525965087	-0.92696106	0.23407287	1
gene53987	118.8110799	69.69047001	0.586565412	-0.76963609	0.23408919	1
gene33941	551.6966666	354.2598892	0.642128022	-0.63906714	0.23411024	1
gene37108	55.78955055	29.03769584	0.520486284	-0.94206795	0.2341535	1
gene47371	55.78955055	29.03769584	0.520486284	-0.94206795	0.2341535	1
gene71262	152.9046941	243.916645	1.595220124	0.673755515	0.23417585	1
gene19700	286.1797315	181.0016374	0.63247539	-0.66091875	0.23419335	1
gene72195	98.14828338	56.13954529	0.571987032	-0.80594566	0.23424717	1
gene12409	264.4837952	413.3032041	1.562678741	0.644021216	0.23431204	1
gene13538	243.8209987	152.9318648	0.627230081	-0.67293334	0.23443681	1
gene45517	3172.772403	5534.584827	1.744400204	0.802731064	0.23452475	1
gene57507	448.3826841	287.4731888	0.641133565	-0.64130315	0.23457139	1
gene3531	200.4291261	315.5429615	1.574336862	0.654744269	0.23464766	1
gene67708	87.81688513	146.1564024	1.664331434	0.73494276	0.23473066	1
gene4154	14.46395755	31.94146542	2.208348947	1.142968154	0.23478963	1
gene62192	134.3081773	215.8468724	1.607101495	0.684461044	0.23486832	1
gene23576	98.14828338	161.6431735	1.646928178	0.719777641	0.23492452	1
gene63443	98.14828338	161.6431735	1.646928178	0.719777641	0.23492452	1
gene9502	60.95524968	105.5036282	1.730837438	0.791470232	0.23497961	1
gene27787	158.0703932	95.82439627	0.606213436	-0.72210227	0.23498891	1
gene6795	49.5907116	88.08101071	1.776159443	0.828761096	0.234994	1
gene14363	49.5907116	88.08101071	1.776159443	0.828761096	0.234994	1
gene33994	522.7687515	335.8693485	0.642481685	-0.63827277	0.23499413	1
gene71434	387.4274344	247.7883378	0.639573545	-0.64481783	0.23502304	1
gene23222	356.4332397	227.4619507	0.638161444	-0.64800665	0.23503057	1
gene39598	397.7588327	254.5638002	0.639995342	-0.64386669	0.23507343	1
gene71554	300.6436891	468.4748262	1.558239348	0.639916851	0.23510245	1
gene1919	994.9136516	1583.522346	1.59161787	0.670494002	0.23513961	1
gene29074	165.302372	100.6640122	0.608968952	-0.71555942	0.23516186	1
gene1863	353.3338202	549.7803746	1.555980048	0.637823561	0.23518048	1
gene65176	133.2750374	79.36970196	0.595533143	-0.74774629	0.23535175	1
gene6454	271.715774	423.9503593	1.56027143	0.641797027	0.23539742	1
gene4326	37.1930337	68.72254682	1.847726307	0.885751075	0.23543992	1
gene18612	37.1930337	68.72254682	1.847726307	0.885751075	0.23543992	1
gene25766	46.49129213	83.24139474	1.790472816	0.840340615	0.23549218	1
gene43319	46.49129213	83.24139474	1.790472816	0.840340615	0.23549218	1
gene1707	648.8118102	417.1748969	0.642982896	-0.63714773	0.23555752	1
gene71380	194.2302871	120.0224761	0.617939035	-0.69446358	0.23556777	1
gene64065	201.4622659	124.8620921	0.619779052	-0.6901741	0.2356428	1
gene5453	486.6088576	312.6391919	0.642485616	-0.63826394	0.23566085	1
gene43845	105.3802622	60.97916126	0.57865828	-0.78921646	0.23571228	1
gene53119	431.8524469	671.7386971	1.555481975	0.637361677	0.23571712	1
gene24341	304.7762484	193.5846389	0.635169702	-0.654786	0.23573244	1
gene6639	257.2518164	401.6881258	1.561458851	0.642894551	0.2357486	1
gene55059	43.39187265	78.40177877	1.806830956	0.853461537	0.2357846	1
gene51153	26.86163545	52.26785251	1.945817953	0.960376741	0.23582294	1
gene72907	26.86163545	52.26785251	1.945817953	0.960376741	0.23582294	1
gene54278	510.3710736	328.125963	0.642916458	-0.63729681	0.23589456	1

gene30081	120.8773595	195.5204853	1.61751122	0.69377572	0.23590551	1
gene46732	269.6494943	170.3544823	0.631762662	-0.66254542	0.23597454	1
gene50851	550.6635268	354.2598892	0.643332765	-0.63636293	0.23609272	1
gene63087	122.9436392	72.5942396	0.590467633	-0.76007012	0.2361567	1
gene63841	378.129176	587.5293791	1.553779545	0.635781825	0.23649934	1
gene25657	35.12675405	16.45469431	0.468437655	-1.09407104	0.23656626	1
gene34122	35.12675405	16.45469431	0.468437655	-1.09407104	0.23656626	1
gene34831	35.12675405	16.45469431	0.468437655	-1.09407104	0.23656626	1
gene70806	71.28664793	120.9903993	1.697237882	0.763188785	0.23657127	1
gene41276	348.1681211	541.0690658	1.554045397	0.636028648	0.23657181	1
gene42137	570.2931834	889.5214159	1.559761613	0.641325551	0.23662725	1
gene53214	148.7721348	237.1411827	1.593989244	0.672641894	0.23664782	1
gene59559	138.4407366	221.6544116	1.601077956	0.679043554	0.23668345	1
gene50097	416.3553495	267.1468017	0.641631726	-0.64018262	0.23673482	1
gene63435	728.3635767	1143.117293	1.569432258	0.650242759	0.23678871	1
gene7623	54.75641073	95.82439627	1.750012373	0.807365122	0.23691082	1
gene8878	910.1961859	1440.269714	1.582372829	0.662089559	0.23691296	1
gene7956	344.0355618	219.7185652	0.638650737	-0.64690092	0.23692399	1
gene49634	298.5774094	189.7129461	0.635389484	-0.65428688	0.23694328	1
gene29674	2635.539694	4488.259854	1.702975624	0.768057784	0.23695404	1
gene11515	76.45234706	128.7337849	1.683843464	0.751758027	0.23697566	1
gene49609	117.7779401	190.6808693	1.618986283	0.695090762	0.23697682	1
gene3439	102.2808427	59.04331487	0.577266606	-0.79269033	0.23705135	1
gene5891	220.0587827	344.5806573	1.565857327	0.646952767	0.23706777	1
gene71892	179.7663296	283.601496	1.577611874	0.657742315	0.23710955	1
gene11804	551.6966666	859.5157968	1.557949955	0.639648891	0.23717798	1
gene28613	81.61804618	136.4771704	1.672144542	0.741699561	0.23721184	1
gene46606	4309.22621	7817.915643	1.814227256	0.859355184	0.23737056	1
gene4999	26.86163545	11.61507834	0.43240399	-1.20954826	0.23742001	1
gene36928	26.86163545	11.61507834	0.43240399	-1.20954826	0.23742001	1
gene15345	75.41920723	41.62069737	0.55185806	-0.85763085	0.23743222	1
gene15178	22.72907615	45.49239015	2.001506346	1.00108619	0.23744514	1
gene46547	22.72907615	45.49239015	2.001506346	1.00108619	0.23744514	1
gene65489	22.72907615	45.49239015	2.001506346	1.00108619	0.23744514	1
gene28083	145.6727153	232.3015667	1.59468138	0.6732682	0.23751803	1
gene38898	119.8442197	70.65839321	0.589585325	-0.76222748	0.23753454	1
gene71951	29.96105493	57.10746848	1.906056667	0.930591011	0.23758373	1
gene10744	18.59651685	38.71692779	2.081945135	1.05793205	0.2376189	1
gene30437	18.59651685	38.71692779	2.081945135	1.05793205	0.2376189	1
gene41039	18.59651685	38.71692779	2.081945135	1.05793205	0.2376189	1
gene65646	18.59651685	38.71692779	2.081945135	1.05793205	0.2376189	1
gene37930	230.390181	360.0674284	1.562859263	0.644187868	0.23768522	1
gene54292	283.0803121	440.4050536	1.555760096	0.637619609	0.23772733	1
gene30610	202.4954057	125.8300153	0.62139689	-0.68641307	0.2377572	1
gene19994	51.65699125	90.9847803	1.761325584	0.816661619	0.23777383	1
gene44188	317.1739263	492.6729061	1.553320955	0.635355957	0.23781606	1
gene59779	88.85002496	50.33200612	0.566482746	-0.81989608	0.23800067	1
gene55277	127.0761985	75.49800918	0.594116051	-0.75118333	0.23806942	1

gene14290	227.2907615	355.2278124	1.562878359	0.644205496	0.23807942	1
gene64818	1041.404944	1656.116586	1.590271485	0.669273078	0.23808594	1
gene26	795.5176653	1250.556767	1.572003768	0.652604676	0.23816631	1
gene23299	383.2948751	245.8524914	0.641418676	-0.64066173	0.23828957	1
gene53772	4816.497865	8887.470773	1.845214308	0.883788384	0.23834448	1
gene27516	372.9634769	239.0770291	0.641019949	-0.64155884	0.23836124	1
gene33972	1276.960824	2051.997173	1.606938235	0.684314478	0.2383731	1
gene9214	334.7373033	213.911026	0.639041493	-0.64601849	0.2384654	1
gene27423	274.8151935	174.226175	0.633975774	-0.65750038	0.23852836	1
gene12985	83.68432583	139.38094	1.665556108	0.736003955	0.23863325	1
gene3580	254.152397	395.8805866	1.557650415	0.639371484	0.23863583	1
gene67679	1730.509207	2835.047037	1.638273305	0.712176054	0.23866815	1
gene29025	33.0604744	61.94708446	1.873750621	0.905928957	0.23868947	1
gene55127	442.1838451	284.5694192	0.643554536	-0.63586568	0.23875653	1
gene34487	629.1821535	981.4741194	1.559920468	0.641472476	0.23878342	1
gene5083	341.9692821	218.750642	0.639679215	-0.64457949	0.2387839	1
gene7418	21.69593633	8.711308752	0.40151799	-1.31646346	0.23887797	1
gene16944	21.69593633	8.711308752	0.40151799	-1.31646346	0.23887797	1
gene17295	21.69593633	8.711308752	0.40151799	-1.31646346	0.23887797	1
gene56295	21.69593633	8.711308752	0.40151799	-1.31646346	0.23887797	1
gene38854	666.3751872	429.7578984	0.644918819	-0.63281053	0.23889785	1
gene33511	13.43081773	30.00561903	2.234087279	1.159685549	0.23890006	1
gene38377	13.43081773	30.00561903	2.234087279	1.159685549	0.23890006	1
gene72739	13.43081773	30.00561903	2.234087279	1.159685549	0.23890006	1
gene10539	177.7000499	109.375321	0.615505291	-0.70015684	0.23891876	1
gene15775	38.22617353	18.3905407	0.481098133	-1.0555969	0.23894707	1
gene19233	335.7704432	520.7426787	1.550888976	0.633095411	0.23897393	1
gene61629	5783.516741	11013.03011	1.904209947	0.929192551	0.23927685	1
gene10804	56.82269038	98.72816585	1.737477849	0.796994585	0.23930238	1
gene60801	264.4837952	411.3673577	1.555359403	0.637247988	0.23931598	1
gene664	1634.427203	2664.692555	1.630352548	0.705183967	0.2393799	1
gene25518	356.4332397	228.4298739	0.640877024	-0.64188055	0.2394279	1
gene17301	123.976779	73.56216279	0.593354363	-0.75303413	0.2394887	1
gene61571	8347.769787	4571.501248	0.547631447	-0.8687228	0.23951236	1
gene18901	106.413402	61.94708446	0.582136115	-0.78057157	0.23959002	1
gene66741	106.413402	61.94708446	0.582136115	-0.78057157	0.23959002	1
gene58702	39.25931335	71.6263164	1.824441395	0.867454809	0.23960255	1
gene64128	261.3843757	406.5277417	1.555287077	0.6371809	0.23962221	1
gene68276	101.2477029	165.5148663	1.634751818	0.709071627	0.23963602	1
gene64843	96.08200373	55.17162209	0.5742139	-0.80033984	0.23979582	1
gene7262	432.8855867	278.7618801	0.643962027	-0.63495248	0.23979731	1
gene38468	1764.602821	1108.272058	0.628057512	-0.67103142	0.2398529	1
gene38511	75.41920723	126.7979385	1.681241996	0.749527399	0.23986181	1
gene32851	123.976779	199.3921781	1.608302617	0.685538888	0.23986674	1
gene40184	123.976779	199.3921781	1.608302617	0.685538888	0.23986674	1
gene70037	196.2965668	121.9583225	0.621296259	-0.68664673	0.23992418	1
gene22878	157.0372534	248.756261	1.584058914	0.663625992	0.23995663	1
gene53147	80.58490636	134.5413241	1.669559849	0.739467811	0.23998703	1

gene11619	25.82849563	50.33200612	1.948700646	0.962512485	0.24015623	1
gene17069	25.82849563	50.33200612	1.948700646	0.962512485	0.24015623	1
gene20693	25.82849563	50.33200612	1.948700646	0.962512485	0.24015623	1
gene45707	167.3686517	102.5998586	0.613017179	-0.70600059	0.24019324	1
gene36405	160.1366729	97.76024266	0.610480041	-0.71198397	0.24022984	1
gene67139	141.540156	225.5261044	1.593371879	0.672083018	0.24024473	1
gene65378	441.1507053	683.3537754	1.549025689	0.63136107	0.2402651	1
gene12420	14.46395755	4.839615973	0.334598325	-1.57949787	0.24026581	1
gene16140	14.46395755	4.839615973	0.334598325	-1.57949787	0.24026581	1
gene22985	14.46395755	4.839615973	0.334598325	-1.57949787	0.24026581	1
gene30075	14.46395755	4.839615973	0.334598325	-1.57949787	0.24026581	1
gene45264	14.46395755	4.839615973	0.334598325	-1.57949787	0.24026581	1
gene49830	14.46395755	4.839615973	0.334598325	-1.57949787	0.24026581	1
gene53173	14.46395755	4.839615973	0.334598325	-1.57949787	0.24026581	1
gene54277	14.46395755	4.839615973	0.334598325	-1.57949787	0.24026581	1
gene66505	14.46395755	4.839615973	0.334598325	-1.57949787	0.24026581	1
gene66518	14.46395755	4.839615973	0.334598325	-1.57949787	0.24026581	1
gene39595	61.9883895	106.4715514	1.717604736	0.780398075	0.24029613	1
gene69062	53.7232709	93.88854988	1.747632791	0.805402081	0.24035257	1
gene64137	113.6453808	66.78670043	0.587676331	-0.7669063	0.2404041	1
gene50926	41.325593	20.32638709	0.491859538	-1.02368172	0.24049853	1
gene12364	108.4796816	176.1620214	1.623917205	0.699478079	0.24050782	1
gene34508	63.02152933	33.87731181	0.537551408	-0.89552536	0.24054008	1
gene38739	63.02152933	33.87731181	0.537551408	-0.89552536	0.24054008	1
gene69866	63.02152933	33.87731181	0.537551408	-0.89552536	0.24054008	1
gene35386	339.9030025	217.7827188	0.640720197	-0.64223363	0.24067374	1
gene25626	159.1035331	251.6600306	1.581737538	0.661510229	0.24075299	1
gene50983	153.9378339	243.916645	1.584513948	0.66404036	0.24083201	1
gene36567	347.1349812	222.6223348	0.641313457	-0.64089842	0.24095628	1
gene53034	200.4291261	124.8620921	0.622973789	-0.68275663	0.24095729	1
gene37999	6934.434506	13659.33212	1.969783132	0.978036802	0.24096425	1
gene2311	59.92210985	31.94146542	0.533049746	-0.90765792	0.24112705	1
gene66628	185.9651685	115.1828602	0.619378678	-0.69110638	0.2411686	1
gene66713	92.98258426	53.23577571	0.572534912	-0.80456443	0.24119607	1
gene13812	254.152397	394.9126634	1.553841979	0.635839793	0.2412687	1
gene36192	128.1093383	76.46593238	0.596880238	-0.74448661	0.24129602	1
gene36737	87.81688513	145.1884792	1.653309372	0.725356711	0.24130133	1
gene33799	461.8135018	298.1203439	0.64554272	-0.63141552	0.24135431	1
gene53968	77.48548688	129.7017081	1.673883888	0.743199457	0.24138783	1
gene66448	886.4339699	1394.777323	1.573470073	0.653949739	0.24143211	1
gene67052	133.2750374	212.9431028	1.597771848	0.676061415	0.24145513	1
gene26105	849.2409362	1333.798162	1.570576859	0.651294546	0.24156003	1
gene65983	157.0372534	95.82439627	0.610201683	-0.71264194	0.24156403	1
gene2704	56.82269038	30.00561903	0.528056993	-0.92123445	0.24160109	1
gene42573	149.8052746	90.9847803	0.60735365	-0.71939128	0.24160307	1
gene62347	79.55176653	44.52446695	0.559691744	-0.83729563	0.24171627	1
gene54803	28.9279151	55.17162209	1.907210454	0.931464049	0.24186611	1
gene57145	28.9279151	55.17162209	1.907210454	0.931464049	0.24186611	1

gene31201	145.6727153	231.3336435	1.588036874	0.667244412	0.24190639	1
gene39286	47.52443195	24.19807987	0.509171365	-0.97377681	0.24191668	1
gene53469	53.7232709	28.06977264	0.522488154	-0.93652977	0.24192527	1
gene73339	221.0919226	344.5806573	1.558540237	0.640195401	0.24193323	1
gene73465	954.6211984	1506.088491	1.5776818	0.65780626	0.24193403	1
gene20351	343.0024219	530.4219107	1.546408645	0.628921608	0.24193712	1
gene33947	50.62385143	26.13392626	0.516237416	-0.95389339	0.24205152	1
gene40919	50.62385143	26.13392626	0.516237416	-0.95389339	0.24205152	1
gene18626	1502.185306	952.4364235	0.63403391	-0.65736809	0.24207968	1
gene24894	17.56337703	36.7810814	2.094191871	1.066393629	0.24217627	1
gene28892	17.56337703	36.7810814	2.094191871	1.066393629	0.24217627	1
gene22086	47.52443195	84.20931793	1.771916349	0.825310496	0.24218961	1
gene24634	261.3843757	405.5598186	1.551584013	0.633741816	0.24219398	1
gene16882	183.8988889	288.441112	1.568476644	0.649364046	0.24223239	1
gene51700	492.8076966	762.7234774	1.547710157	0.63013532	0.24230672	1
gene55043	182.865749	113.2470138	0.61929046	-0.69131187	0.24238064	1
gene60530	117.7779401	69.69047001	0.591710723	-0.75703606	0.24245288	1
gene69283	2625.208296	4438.895771	1.690873741	0.757768936	0.24250935	1
gene22733	1210.839875	774.3385557	0.639505332	-0.64497171	0.24251264	1
gene38608	112.6122409	181.9695606	1.615895031	0.692333484	0.24251738	1
gene9441	100.214563	58.07539168	0.579510501	-0.78709329	0.24253275	1
gene1091	258.2849563	400.7202026	1.551465515	0.633631629	0.24254653	1
gene54856	355.4000998	228.4298739	0.642740039	-0.63769275	0.242556	1
gene43663	2199.554688	3656.813829	1.662524624	0.733375709	0.24265908	1
gene848	69.22036828	117.1187066	1.691968845	0.758703003	0.24266357	1
gene11082	671.5408863	434.5975144	0.647164638	-0.62779532	0.2426827	1
gene50050	84.71746566	140.3488632	1.656669757	0.728286042	0.24273694	1
gene11414	29.96105493	13.55092472	0.452284633	-1.14469712	0.24279132	1
gene13097	29.96105493	13.55092472	0.452284633	-1.14469712	0.24279132	1
gene17152	29.96105493	13.55092472	0.452284633	-1.14469712	0.24279132	1
gene19698	29.96105493	13.55092472	0.452284633	-1.14469712	0.24279132	1
gene36912	29.96105493	13.55092472	0.452284633	-1.14469712	0.24279132	1
gene50222	721.1315979	1124.726752	1.559669214	0.641240084	0.24285801	1
gene18264	44.42501248	79.36970196	1.78659943	0.837216207	0.24289425	1
gene49511	44.42501248	79.36970196	1.78659943	0.837216207	0.24289425	1
gene38951	76.45234706	42.58862056	0.557060996	-0.84409279	0.2429069	1
gene21737	233.4896005	362.971198	1.554549741	0.63649678	0.24302428	1
gene53879	1373.042828	2204.929037	1.605870548	0.683355599	0.24302445	1
gene14579	139.4738764	84.20931793	0.603764089	-0.72794314	0.24303684	1
gene50547	139.4738764	84.20931793	0.603764089	-0.72794314	0.24303684	1
gene39172	680.8391447	1059.875898	1.556719978	0.638509456	0.24306707	1
gene23219	8619.485561	4719.593497	0.547549325	-0.86893916	0.24306898	1
gene59860	201.4622659	125.8300153	0.62458354	-0.67903354	0.24308059	1
gene6750	12.3976779	28.06977264	2.264115334	1.178947451	0.24309922	1
gene13824	12.3976779	28.06977264	2.264115334	1.178947451	0.24309922	1
gene20535	12.3976779	28.06977264	2.264115334	1.178947451	0.24309922	1
gene69848	12.3976779	28.06977264	2.264115334	1.178947451	0.24309922	1
gene72740	12.3976779	28.06977264	2.264115334	1.178947451	0.24309922	1

gene14789	344.0355618	531.3898339	1.544578215	0.627212929	0.24319953	1
gene8974	4596.439082	8355.113016	1.817736049	0.862142723	0.243339	1
gene26193	270.6826342	172.2903286	0.636503074	-0.65176061	0.24334058	1
gene30507	35.12675405	64.85085404	1.846195465	0.884555306	0.24339407	1
gene16683	4144.956978	7411.387901	1.788049415	0.838386608	0.24343238	1
gene13770	114.6785206	184.8733302	1.61210076	0.688941918	0.24348112	1
gene48068	114.6785206	184.8733302	1.61210076	0.688941918	0.24348112	1
gene48751	38.22617353	69.69047001	1.823108713	0.866400592	0.24355762	1
gene58506	2166.494213	1351.22078	0.623690002	-0.68109896	0.24357215	1
gene62198	60.95524968	104.535705	1.714958196	0.778173409	0.24362417	1
gene56100	222.1250624	345.5485805	1.555648772	0.637516371	0.24379138	1
gene38865	917.4281647	1442.20556	1.572009249	0.652609706	0.24381305	1
gene13528	371.930337	573.9784544	1.543241831	0.625964155	0.24381531	1
gene34581	172.5343508	106.4715514	0.617103498	-0.69641562	0.24382976	1
gene41590	172.5343508	106.4715514	0.617103498	-0.69641562	0.24382976	1
gene65619	172.5343508	106.4715514	0.617103498	-0.69641562	0.24382976	1
gene60807	356.4332397	229.3977971	0.643592605	-0.63578035	0.24386608	1
gene48459	52.69013108	91.95270349	1.745159893	0.803359223	0.24391717	1
gene26057	203.5285455	317.4788078	1.559873614	0.641429142	0.24391747	1
gene54635	1001.112491	644.6368476	0.643920492	-0.63504553	0.24397225	1
gene60818	114.6785206	67.75462362	0.590822268	-0.75920389	0.24399954	1
gene16275	86.78374531	49.36408293	0.568817153	-0.81396312	0.24403404	1
gene43082	73.35292758	40.65277417	0.55420793	-0.85150074	0.24405239	1
gene32236	121.9104994	195.5204853	1.603803498	0.68149739	0.24405909	1
gene71629	267.5832147	414.2711273	1.548195494	0.630587655	0.24406201	1
gene37508	259.3180961	401.6881258	1.549016948	0.631352929	0.24417123	1
gene53065	673.607166	436.5333608	0.648053321	-0.62581557	0.24418957	1
gene63626	317.1739263	489.7691365	1.544165822	0.626827686	0.24419171	1
gene67943	151.8715543	240.0449523	1.580578755	0.660452922	0.2442958	1
gene30574	237.6221598	368.7787372	1.551954319	0.634086093	0.24433611	1
gene4620	136.3744569	82.27347154	0.60329092	-0.72907423	0.24453228	1
gene13899	129.1424781	206.1676405	1.596435529	0.674854292	0.24459037	1
gene4601	462.8466416	714.3273176	1.543334775	0.626051041	0.24461839	1
gene56384	183.8988889	114.214937	0.621074644	-0.68716142	0.24468272	1
gene15003	234.5227403	363.9391212	1.551828709	0.633969321	0.24479956	1
gene65334	5259.714849	3067.348604	0.583177737	-0.77799245	0.24484996	1
gene53198	1545.577178	2495.305996	1.614481652	0.691071045	0.2449547	1
gene35929	615.7513357	954.3722699	1.549931303	0.632204273	0.24496315	1
gene51638	209.7273845	131.6375545	0.627660307	-0.67194412	0.24497589	1
gene17537	221.0919226	343.6127341	1.554162315	0.636137185	0.24498176	1
gene66637	49.5907116	87.11308752	1.756641208	0.812819552	0.24501018	1
gene37486	153.9378339	242.9487219	1.578226195	0.65830399	0.2450671	1
gene3681	547.5641073	355.2278124	0.64874196	-0.62428334	0.24508423	1
gene71693	3189.30264	5491.996206	1.722005349	0.784089624	0.2451534	1
gene54143	4056.106953	2425.615526	0.598015672	-0.7417448	0.24532118	1
gene47229	880.235131	569.1388384	0.646575919	-0.62910832	0.24538682	1
gene44	0	3.871692779	Inf	Inf	0.24543428	1
gene161	0	3.871692779	Inf	Inf	0.24543428	1

gene702	0	3.871692779	Inf	Inf	0.24543428	1
gene1117	0	3.871692779	Inf	Inf	0.24543428	1
gene1293	0	3.871692779	Inf	Inf	0.24543428	1
gene1460	0	3.871692779	Inf	Inf	0.24543428	1
gene2035	0	3.871692779	Inf	Inf	0.24543428	1
gene2604	0	3.871692779	Inf	Inf	0.24543428	1
gene2950	0	3.871692779	Inf	Inf	0.24543428	1
gene3256	0	3.871692779	Inf	Inf	0.24543428	1
gene3368	0	3.871692779	Inf	Inf	0.24543428	1
gene3799	0	3.871692779	Inf	Inf	0.24543428	1
gene4885	0	3.871692779	Inf	Inf	0.24543428	1
gene5069	0	3.871692779	Inf	Inf	0.24543428	1
gene5170	0	3.871692779	Inf	Inf	0.24543428	1
gene5742	0	3.871692779	Inf	Inf	0.24543428	1
gene6330	0	3.871692779	Inf	Inf	0.24543428	1
gene6684	0	3.871692779	Inf	Inf	0.24543428	1
gene7019	0	3.871692779	Inf	Inf	0.24543428	1
gene7403	0	3.871692779	Inf	Inf	0.24543428	1
gene7836	0	3.871692779	Inf	Inf	0.24543428	1
gene8060	0	3.871692779	Inf	Inf	0.24543428	1
gene8708	0	3.871692779	Inf	Inf	0.24543428	1
gene8941	0	3.871692779	Inf	Inf	0.24543428	1
gene8980	0	3.871692779	Inf	Inf	0.24543428	1
gene9293	0	3.871692779	Inf	Inf	0.24543428	1
gene9360	0	3.871692779	Inf	Inf	0.24543428	1
gene9415	0	3.871692779	Inf	Inf	0.24543428	1
gene9905	0	3.871692779	Inf	Inf	0.24543428	1
gene10272	0	3.871692779	Inf	Inf	0.24543428	1
gene11240	0	3.871692779	Inf	Inf	0.24543428	1
gene11604	0	3.871692779	Inf	Inf	0.24543428	1
gene11662	0	3.871692779	Inf	Inf	0.24543428	1
gene11716	0	3.871692779	Inf	Inf	0.24543428	1
gene11846	0	3.871692779	Inf	Inf	0.24543428	1
gene11851	0	3.871692779	Inf	Inf	0.24543428	1
gene12586	0	3.871692779	Inf	Inf	0.24543428	1
gene13156	0	3.871692779	Inf	Inf	0.24543428	1
gene13178	0	3.871692779	Inf	Inf	0.24543428	1
gene13793	0	3.871692779	Inf	Inf	0.24543428	1
gene14278	0	3.871692779	Inf	Inf	0.24543428	1
gene14552	0	3.871692779	Inf	Inf	0.24543428	1
gene14829	0	3.871692779	Inf	Inf	0.24543428	1
gene15505	0	3.871692779	Inf	Inf	0.24543428	1
gene15765	0	3.871692779	Inf	Inf	0.24543428	1
gene15966	0	3.871692779	Inf	Inf	0.24543428	1
gene16117	0	3.871692779	Inf	Inf	0.24543428	1
gene16169	0	3.871692779	Inf	Inf	0.24543428	1
gene16704	0	3.871692779	Inf	Inf	0.24543428	1
gene17345	0	3.871692779	Inf	Inf	0.24543428	1

gene17864	0	3.871692779	Inf	Inf	0.24543428	1
gene18037	0	3.871692779	Inf	Inf	0.24543428	1
gene18401	0	3.871692779	Inf	Inf	0.24543428	1
gene19027	0	3.871692779	Inf	Inf	0.24543428	1
gene19813	0	3.871692779	Inf	Inf	0.24543428	1
gene20096	0	3.871692779	Inf	Inf	0.24543428	1
gene20824	0	3.871692779	Inf	Inf	0.24543428	1
gene21037	0	3.871692779	Inf	Inf	0.24543428	1
gene21082	0	3.871692779	Inf	Inf	0.24543428	1
gene21606	0	3.871692779	Inf	Inf	0.24543428	1
gene21756	0	3.871692779	Inf	Inf	0.24543428	1
gene22603	0	3.871692779	Inf	Inf	0.24543428	1
gene22869	0	3.871692779	Inf	Inf	0.24543428	1
gene23367	0	3.871692779	Inf	Inf	0.24543428	1
gene23916	0	3.871692779	Inf	Inf	0.24543428	1
gene24990	0	3.871692779	Inf	Inf	0.24543428	1
gene25121	0	3.871692779	Inf	Inf	0.24543428	1
gene25536	0	3.871692779	Inf	Inf	0.24543428	1
gene25806	0	3.871692779	Inf	Inf	0.24543428	1
gene26597	0	3.871692779	Inf	Inf	0.24543428	1
gene26706	0	3.871692779	Inf	Inf	0.24543428	1
gene26874	0	3.871692779	Inf	Inf	0.24543428	1
gene26931	0	3.871692779	Inf	Inf	0.24543428	1
gene27124	0	3.871692779	Inf	Inf	0.24543428	1
gene27295	0	3.871692779	Inf	Inf	0.24543428	1
gene27703	0	3.871692779	Inf	Inf	0.24543428	1
gene28566	0	3.871692779	Inf	Inf	0.24543428	1
gene28620	0	3.871692779	Inf	Inf	0.24543428	1
gene28664	0	3.871692779	Inf	Inf	0.24543428	1
gene29035	0	3.871692779	Inf	Inf	0.24543428	1
gene29612	0	3.871692779	Inf	Inf	0.24543428	1
gene29727	0	3.871692779	Inf	Inf	0.24543428	1
gene30488	0	3.871692779	Inf	Inf	0.24543428	1
gene30549	0	3.871692779	Inf	Inf	0.24543428	1
gene30808	0	3.871692779	Inf	Inf	0.24543428	1
gene30834	0	3.871692779	Inf	Inf	0.24543428	1
gene31416	0	3.871692779	Inf	Inf	0.24543428	1
gene31579	0	3.871692779	Inf	Inf	0.24543428	1
gene31813	0	3.871692779	Inf	Inf	0.24543428	1
gene31847	0	3.871692779	Inf	Inf	0.24543428	1
gene32727	0	3.871692779	Inf	Inf	0.24543428	1
gene33611	0	3.871692779	Inf	Inf	0.24543428	1
gene33787	0	3.871692779	Inf	Inf	0.24543428	1
gene34241	0	3.871692779	Inf	Inf	0.24543428	1
gene34285	0	3.871692779	Inf	Inf	0.24543428	1
gene34363	0	3.871692779	Inf	Inf	0.24543428	1
gene35936	0	3.871692779	Inf	Inf	0.24543428	1
gene36193	0	3.871692779	Inf	Inf	0.24543428	1

gene36381	0	3.871692779	Inf	Inf	0.24543428	1
gene36763	0	3.871692779	Inf	Inf	0.24543428	1
gene37171	0	3.871692779	Inf	Inf	0.24543428	1
gene37434	0	3.871692779	Inf	Inf	0.24543428	1
gene37660	0	3.871692779	Inf	Inf	0.24543428	1
gene37708	0	3.871692779	Inf	Inf	0.24543428	1
gene38064	0	3.871692779	Inf	Inf	0.24543428	1
gene38133	0	3.871692779	Inf	Inf	0.24543428	1
gene38151	0	3.871692779	Inf	Inf	0.24543428	1
gene38351	0	3.871692779	Inf	Inf	0.24543428	1
gene39474	0	3.871692779	Inf	Inf	0.24543428	1
gene40406	0	3.871692779	Inf	Inf	0.24543428	1
gene40543	0	3.871692779	Inf	Inf	0.24543428	1
gene40613	0	3.871692779	Inf	Inf	0.24543428	1
gene40783	0	3.871692779	Inf	Inf	0.24543428	1
gene41456	0	3.871692779	Inf	Inf	0.24543428	1
gene41791	0	3.871692779	Inf	Inf	0.24543428	1
gene42175	0	3.871692779	Inf	Inf	0.24543428	1
gene42412	0	3.871692779	Inf	Inf	0.24543428	1
gene42742	0	3.871692779	Inf	Inf	0.24543428	1
gene44105	0	3.871692779	Inf	Inf	0.24543428	1
gene44225	0	3.871692779	Inf	Inf	0.24543428	1
gene46077	0	3.871692779	Inf	Inf	0.24543428	1
gene46315	0	3.871692779	Inf	Inf	0.24543428	1
gene46653	0	3.871692779	Inf	Inf	0.24543428	1
gene47535	0	3.871692779	Inf	Inf	0.24543428	1
gene47987	0	3.871692779	Inf	Inf	0.24543428	1
gene48356	0	3.871692779	Inf	Inf	0.24543428	1
gene48487	0	3.871692779	Inf	Inf	0.24543428	1
gene48620	0	3.871692779	Inf	Inf	0.24543428	1
gene48707	0	3.871692779	Inf	Inf	0.24543428	1
gene49020	0	3.871692779	Inf	Inf	0.24543428	1
gene49151	0	3.871692779	Inf	Inf	0.24543428	1
gene49188	0	3.871692779	Inf	Inf	0.24543428	1
gene49215	0	3.871692779	Inf	Inf	0.24543428	1
gene49580	0	3.871692779	Inf	Inf	0.24543428	1
gene49801	0	3.871692779	Inf	Inf	0.24543428	1
gene49815	0	3.871692779	Inf	Inf	0.24543428	1
gene50666	0	3.871692779	Inf	Inf	0.24543428	1
gene50998	0	3.871692779	Inf	Inf	0.24543428	1
gene51192	0	3.871692779	Inf	Inf	0.24543428	1
gene51203	0	3.871692779	Inf	Inf	0.24543428	1
gene52261	0	3.871692779	Inf	Inf	0.24543428	1
gene52790	0	3.871692779	Inf	Inf	0.24543428	1
gene52880	0	3.871692779	Inf	Inf	0.24543428	1
gene53070	0	3.871692779	Inf	Inf	0.24543428	1
gene53263	0	3.871692779	Inf	Inf	0.24543428	1
gene53519	0	3.871692779	Inf	Inf	0.24543428	1

gene54165	0	3.871692779	Inf	Inf	0.24543428	1
gene54181	0	3.871692779	Inf	Inf	0.24543428	1
gene54461	0	3.871692779	Inf	Inf	0.24543428	1
gene54631	0	3.871692779	Inf	Inf	0.24543428	1
gene54854	0	3.871692779	Inf	Inf	0.24543428	1
gene55691	0	3.871692779	Inf	Inf	0.24543428	1
gene56525	0	3.871692779	Inf	Inf	0.24543428	1
gene56747	0	3.871692779	Inf	Inf	0.24543428	1
gene56813	0	3.871692779	Inf	Inf	0.24543428	1
gene57963	0	3.871692779	Inf	Inf	0.24543428	1
gene58455	0	3.871692779	Inf	Inf	0.24543428	1
gene58469	0	3.871692779	Inf	Inf	0.24543428	1
gene58755	0	3.871692779	Inf	Inf	0.24543428	1
gene59630	0	3.871692779	Inf	Inf	0.24543428	1
gene60154	0	3.871692779	Inf	Inf	0.24543428	1
gene60158	0	3.871692779	Inf	Inf	0.24543428	1
gene60904	0	3.871692779	Inf	Inf	0.24543428	1
gene61287	0	3.871692779	Inf	Inf	0.24543428	1
gene62258	0	3.871692779	Inf	Inf	0.24543428	1
gene62350	0	3.871692779	Inf	Inf	0.24543428	1
gene62390	0	3.871692779	Inf	Inf	0.24543428	1
gene62720	0	3.871692779	Inf	Inf	0.24543428	1
gene62739	0	3.871692779	Inf	Inf	0.24543428	1
gene62933	0	3.871692779	Inf	Inf	0.24543428	1
gene63046	0	3.871692779	Inf	Inf	0.24543428	1
gene63542	0	3.871692779	Inf	Inf	0.24543428	1
gene63563	0	3.871692779	Inf	Inf	0.24543428	1
gene63861	0	3.871692779	Inf	Inf	0.24543428	1
gene64797	0	3.871692779	Inf	Inf	0.24543428	1
gene64900	0	3.871692779	Inf	Inf	0.24543428	1
gene65299	0	3.871692779	Inf	Inf	0.24543428	1
gene65766	0	3.871692779	Inf	Inf	0.24543428	1
gene66452	0	3.871692779	Inf	Inf	0.24543428	1
gene66983	0	3.871692779	Inf	Inf	0.24543428	1
gene67900	0	3.871692779	Inf	Inf	0.24543428	1
gene68015	0	3.871692779	Inf	Inf	0.24543428	1
gene68726	0	3.871692779	Inf	Inf	0.24543428	1
gene69454	0	3.871692779	Inf	Inf	0.24543428	1
gene69835	0	3.871692779	Inf	Inf	0.24543428	1
gene70096	0	3.871692779	Inf	Inf	0.24543428	1
gene70151	0	3.871692779	Inf	Inf	0.24543428	1
gene70603	0	3.871692779	Inf	Inf	0.24543428	1
gene71207	0	3.871692779	Inf	Inf	0.24543428	1
gene71469	0	3.871692779	Inf	Inf	0.24543428	1
gene71913	0	3.871692779	Inf	Inf	0.24543428	1
gene72136	0	3.871692779	Inf	Inf	0.24543428	1
gene73031	0	3.871692779	Inf	Inf	0.24543428	1
gene73303	0	3.871692779	Inf	Inf	0.24543428	1

gene73684	0	3.871692779	Inf	Inf	0.24543428	1
gene73743	0	3.871692779	Inf	Inf	0.24543428	1
gene74164	0	3.871692779	Inf	Inf	0.24543428	1
gene74188	0	3.871692779	Inf	Inf	0.24543428	1
gene62836	131.2087578	209.07141	1.593425725	0.672131772	0.24543569	1
gene52729	787.2525467	1228.294534	1.560229356	0.641758123	0.24546174	1
gene30772	113.6453808	182.9374838	1.609722125	0.686811667	0.24574994	1
gene51424	189.064588	295.2165744	1.561458851	0.642894551	0.24582592	1
gene44337	73.35292758	122.9262457	1.675819218	0.744866524	0.24589674	1
gene50028	612.6519163	948.5647307	1.548293094	0.630678601	0.24596016	1
gene21263	140.5070162	85.17724113	0.606213436	-0.72210227	0.24598752	1
gene49714	140.5070162	85.17724113	0.606213436	-0.72210227	0.24598752	1
gene54024	46.49129213	82.27347154	1.769653365	0.823466796	0.24599127	1
gene49033	304.7762484	195.5204853	0.641521399	-0.6404307	0.24599316	1
gene13961	96.08200373	156.8035575	1.631976348	0.706620148	0.24604023	1
gene21957	126.0430587	75.49800918	0.598985854	-0.73940616	0.24606772	1
gene33188	67.15408863	36.7810814	0.54771172	-0.86851134	0.24614862	1
gene26181	319.2402059	205.1997173	0.642775294	-0.63761362	0.24623634	1
gene33553	54.75641073	94.85647307	1.73233548	0.792718346	0.24631818	1
gene7275	33.0604744	15.48677111	0.468437655	-1.09407104	0.24632734	1
gene22004	33.0604744	15.48677111	0.468437655	-1.09407104	0.24632734	1
gene39645	163.2360924	256.4996466	1.571341502	0.651996758	0.24635518	1
gene48603	27.89477528	53.23577571	1.908449707	0.932401168	0.24636967	1
gene22713	1356.51259	867.2591824	0.639329991	-0.64536732	0.2465185	1
gene49446	487.6419974	316.5108846	0.649064039	-0.62356727	0.24651941	1
gene67671	603.3536578	392.0088938	0.649716611	-0.6221175	0.2465499	1
gene64770	508.3047939	330.0618094	0.649338376	-0.62295762	0.24656955	1
gene64908	158.0703932	248.756261	1.573705587	0.654165663	0.24657506	1
gene40796	305.8093882	471.3785958	1.541413096	0.624253553	0.24658264	1
gene61153	294.4448501	188.745023	0.641019949	-0.64155884	0.2466415	1
gene5536	20.6627965	41.62069737	2.014281918	1.010265616	0.24674974	1
gene10045	20.6627965	41.62069737	2.014281918	1.010265616	0.24674974	1
gene29163	20.6627965	41.62069737	2.014281918	1.010265616	0.24674974	1
gene38210	20.6627965	41.62069737	2.014281918	1.010265616	0.24674974	1
gene603	217.9925031	137.4450936	0.630503764	-0.66542311	0.2467632	1
gene44574	43.39187265	77.43385557	1.784524401	0.835539629	0.2468107	1
gene61128	43.39187265	77.43385557	1.784524401	0.835539629	0.2468107	1
gene34559	159.1035331	97.76024266	0.614444197	-0.7026461	0.24683258	1
gene67537	310.9750873	479.1219813	1.540708567	0.623593995	0.24687112	1
gene32252	245.8872784	380.3938155	1.547025198	0.629496696	0.24688198	1
gene44836	564.0943445	871.1308752	1.544299963	0.626953007	0.24689512	1
gene6327	16.5302372	34.84523501	2.107969449	1.075853958	0.24695584	1
gene9515	16.5302372	34.84523501	2.107969449	1.075853958	0.24695584	1
gene34700	16.5302372	34.84523501	2.107969449	1.075853958	0.24695584	1
gene45294	16.5302372	34.84523501	2.107969449	1.075853958	0.24695584	1
gene62804	16.5302372	34.84523501	2.107969449	1.075853958	0.24695584	1
gene60800	184.9320287	115.1828602	0.622838894	-0.68306906	0.24697256	1
gene3344	1026.940986	1617.399658	1.574968455	0.655322933	0.24699824	1

gene14642	210.7605243	132.6054777	0.629176067	-0.6684643	0.24701609	1
gene45292	59.92210985	102.5998586	1.712220395	0.775868416	0.24706465	1
gene2628	80.58490636	133.5734009	1.657548627	0.729051195	0.24714877	1
gene11951	90.91630461	52.26785251	0.574900759	-0.79861516	0.24716073	1
gene20188	90.91630461	52.26785251	0.574900759	-0.79861516	0.24716073	1
gene30784	90.91630461	52.26785251	0.574900759	-0.79861516	0.24716073	1
gene26835	30.99419475	58.07539168	1.873750621	0.905928957	0.247275	1
gene27943	30.99419475	58.07539168	1.873750621	0.905928957	0.247275	1
gene63702	30.99419475	58.07539168	1.873750621	0.905928957	0.247275	1
gene72960	30.99419475	58.07539168	1.873750621	0.905928957	0.247275	1
gene7015	707.7007802	459.7635175	0.649658062	-0.62224752	0.24728899	1
gene11439	11.36453808	26.13392626	2.299603035	1.20138484	0.24735313	1
gene33396	11.36453808	26.13392626	2.299603035	1.20138484	0.24735313	1
gene35898	11.36453808	26.13392626	2.299603035	1.20138484	0.24735313	1
gene40222	11.36453808	26.13392626	2.299603035	1.20138484	0.24735313	1
gene41970	11.36453808	26.13392626	2.299603035	1.20138484	0.24735313	1
gene46133	11.36453808	26.13392626	2.299603035	1.20138484	0.24735313	1
gene53591	11.36453808	26.13392626	2.299603035	1.20138484	0.24735313	1
gene54815	11.36453808	26.13392626	2.299603035	1.20138484	0.24735313	1
gene17611	40.29245318	72.5942396	1.80168329	0.849345428	0.24740164	1
gene72039	40.29245318	72.5942396	1.80168329	0.849345428	0.24740164	1
gene53517	704.6013607	457.8276711	0.649768361	-0.6220026	0.24743378	1
gene14249	575.4588826	888.5534927	1.54407816	0.626745783	0.24745587	1
gene51371	360.565799	554.6199905	1.538193562	0.621237059	0.24747733	1
gene65934	142.5732959	225.5261044	1.581825706	0.661590645	0.24748226	1
gene63915	631.2484331	976.6345034	1.547147608	0.629610846	0.24748531	1
gene45325	70.25350811	118.0866297	1.680864528	0.749203453	0.24749849	1
gene71035	770.7223095	1199.256838	1.556016769	0.637857609	0.24751358	1
gene65785	115.7116604	68.72254682	0.593912027	-0.75167885	0.24756058	1
gene6428	51.65699125	90.0168571	1.742588078	0.801231578	0.24761113	1
gene68916	34.09361423	62.91500765	1.845360461	0.88390265	0.24766014	1
gene71126	34.09361423	62.91500765	1.845360461	0.88390265	0.24766014	1
gene20672	130.175618	78.40177877	0.602276985	-0.73150096	0.24766961	1
gene3262	37.1930337	67.75462362	1.821701993	0.865286972	0.24768012	1
gene21911	37.1930337	67.75462362	1.821701993	0.865286972	0.24768012	1
gene40723	37.1930337	67.75462362	1.821701993	0.865286972	0.24768012	1
gene7294	12.3976779	3.871692779	0.31229177	-1.67903354	0.24770661	1
gene11738	12.3976779	3.871692779	0.31229177	-1.67903354	0.24770661	1
gene20158	12.3976779	3.871692779	0.31229177	-1.67903354	0.24770661	1
gene33612	12.3976779	3.871692779	0.31229177	-1.67903354	0.24770661	1
gene35827	12.3976779	3.871692779	0.31229177	-1.67903354	0.24770661	1
gene39678	12.3976779	3.871692779	0.31229177	-1.67903354	0.24770661	1
gene40028	12.3976779	3.871692779	0.31229177	-1.67903354	0.24770661	1
gene43851	12.3976779	3.871692779	0.31229177	-1.67903354	0.24770661	1
gene45244	12.3976779	3.871692779	0.31229177	-1.67903354	0.24770661	1
gene48891	12.3976779	3.871692779	0.31229177	-1.67903354	0.24770661	1
gene51321	12.3976779	3.871692779	0.31229177	-1.67903354	0.24770661	1
gene72417	12.3976779	3.871692779	0.31229177	-1.67903354	0.24770661	1

gene38492	409.1233707	629.1500765	1.537800384	0.620868244	0.24771407	1
gene2959	301.6768289	464.6031334	1.540069004	0.622994993	0.24772646	1
gene23872	24.7953558	10.64715514	0.429401184	-1.21960193	0.24780128	1
gene25480	24.7953558	10.64715514	0.429401184	-1.21960193	0.24780128	1
gene25554	24.7953558	10.64715514	0.429401184	-1.21960193	0.24780128	1
gene28259	24.7953558	10.64715514	0.429401184	-1.21960193	0.24780128	1
gene63234	24.7953558	10.64715514	0.429401184	-1.21960193	0.24780128	1
gene66807	24.7953558	10.64715514	0.429401184	-1.21960193	0.24780128	1
gene65807	495.907116	322.3184238	0.649957247	-0.62158327	0.24783827	1
gene53279	1058.968321	683.3537754	0.645301434	-0.63195486	0.24785934	1
gene63264	542.3984082	836.2856402	1.541829083	0.624642846	0.2478711	1
gene34738	366.7646379	237.1411827	0.646575919	-0.62910832	0.24795305	1
gene34059	163.2360924	100.6640122	0.61667742	-0.69741207	0.24797002	1
gene12312	87.81688513	144.220556	1.642287309	0.715706541	0.24803889	1
gene34770	87.81688513	144.220556	1.642287309	0.715706541	0.24803889	1
gene59618	60.95524968	32.90938862	0.539894247	-0.88925125	0.24804865	1
gene50814	609.5524968	941.7892684	1.545050301	0.627653807	0.24806857	1
gene47798	503.1390948	327.1580398	0.650233789	-0.62096957	0.24816279	1
gene17411	288.2460112	184.8733302	0.641373421	-0.64076353	0.24817539	1
gene6317	513.4704931	333.9335022	0.650346041	-0.62072053	0.24817565	1
gene33177	98.14828338	57.10746848	0.581848877	-0.7812836	0.24825186	1
gene5895	1707.780131	2765.356567	1.619269669	0.695343268	0.24850305	1
gene36547	169.4349313	265.2109553	1.565267287	0.646409035	0.24851653	1
gene31114	765.5566104	497.512522	0.649870323	-0.62177623	0.2485543	1
gene17440	36.15989388	17.4226175	0.481821588	-1.05342906	0.24858165	1
gene57713	95.04886391	154.8677111	1.629348366	0.704295095	0.24868414	1
gene40350	252.0861173	389.1051242	1.543540471	0.62624331	0.2487272	1
gene56737	77.48548688	128.7337849	1.661392218	0.732392702	0.24880503	1
gene43885	1231.502671	791.7611732	0.642922822	-0.63728253	0.2488199	1
gene24778	353.3338202	228.4298739	0.646498752	-0.62928051	0.24892632	1
gene41630	114.6785206	183.905407	1.603660442	0.681368699	0.24897324	1
gene24687	146.7058552	231.3336435	1.576853516	0.657048645	0.24900375	1
gene55157	61.9883895	105.5036282	1.701990148	0.767222686	0.24901157	1
gene69187	61.9883895	105.5036282	1.701990148	0.767222686	0.24901157	1
gene10258	176.6669101	275.8581105	1.561458851	0.642894551	0.24901481	1
gene250	4287.530274	7647.561161	1.78367514	0.834852882	0.24901751	1
gene25460	314.0745068	202.2959477	0.644101776	-0.63463942	0.24903219	1
gene33121	1860.684825	1176.026681	0.632039702	-0.66191291	0.24905265	1
gene25567	19.62965668	7.743385557	0.394473815	-1.34199856	0.24919725	1
gene38244	19.62965668	7.743385557	0.394473815	-1.34199856	0.24919725	1
gene60277	112.6122409	66.78670043	0.593067857	-0.75373091	0.24924721	1
gene5700	6.19883895	0.967923195	0.156145885	-2.67903354	0.24932596	1
gene8880	6.19883895	0.967923195	0.156145885	-2.67903354	0.24932596	1
gene12425	6.19883895	0.967923195	0.156145885	-2.67903354	0.24932596	1
gene12465	6.19883895	0.967923195	0.156145885	-2.67903354	0.24932596	1
gene13680	6.19883895	0.967923195	0.156145885	-2.67903354	0.24932596	1
gene15617	6.19883895	0.967923195	0.156145885	-2.67903354	0.24932596	1
gene19187	6.19883895	0.967923195	0.156145885	-2.67903354	0.24932596	1

gene21694	6.19883895	0.967923195	0.156145885	-2.67903354	0.24932596	1
gene23042	6.19883895	0.967923195	0.156145885	-2.67903354	0.24932596	1
gene23147	6.19883895	0.967923195	0.156145885	-2.67903354	0.24932596	1
gene24371	6.19883895	0.967923195	0.156145885	-2.67903354	0.24932596	1
gene27612	6.19883895	0.967923195	0.156145885	-2.67903354	0.24932596	1
gene29462	6.19883895	0.967923195	0.156145885	-2.67903354	0.24932596	1
gene30800	6.19883895	0.967923195	0.156145885	-2.67903354	0.24932596	1
gene31278	6.19883895	0.967923195	0.156145885	-2.67903354	0.24932596	1
gene34889	6.19883895	0.967923195	0.156145885	-2.67903354	0.24932596	1
gene37394	6.19883895	0.967923195	0.156145885	-2.67903354	0.24932596	1
gene39184	6.19883895	0.967923195	0.156145885	-2.67903354	0.24932596	1
gene42122	6.19883895	0.967923195	0.156145885	-2.67903354	0.24932596	1
gene48275	6.19883895	0.967923195	0.156145885	-2.67903354	0.24932596	1
gene51986	6.19883895	0.967923195	0.156145885	-2.67903354	0.24932596	1
gene55002	6.19883895	0.967923195	0.156145885	-2.67903354	0.24932596	1
gene56892	6.19883895	0.967923195	0.156145885	-2.67903354	0.24932596	1
gene58725	6.19883895	0.967923195	0.156145885	-2.67903354	0.24932596	1
gene58863	6.19883895	0.967923195	0.156145885	-2.67903354	0.24932596	1
gene62117	6.19883895	0.967923195	0.156145885	-2.67903354	0.24932596	1
gene62533	6.19883895	0.967923195	0.156145885	-2.67903354	0.24932596	1
gene64901	6.19883895	0.967923195	0.156145885	-2.67903354	0.24932596	1
gene67295	6.19883895	0.967923195	0.156145885	-2.67903354	0.24932596	1
gene67455	6.19883895	0.967923195	0.156145885	-2.67903354	0.24932596	1
gene68753	6.19883895	0.967923195	0.156145885	-2.67903354	0.24932596	1
gene33933	204.5616854	128.7337849	0.629315234	-0.66814523	0.24939059	1
gene13739	235.5558801	363.9391212	1.545022442	0.627627794	0.24949332	1
gene42889	23.76221598	46.46031334	1.95521804	0.967329501	0.24952448	1
gene62120	23.76221598	46.46031334	1.95521804	0.967329501	0.24952448	1
gene27643	380.1954556	583.6576864	1.535151664	0.618381192	0.24952527	1
gene40362	129.1424781	205.1997173	1.588940527	0.668065127	0.24958695	1
gene10857	793.4513857	515.9030627	0.650201225	-0.62104182	0.24963323	1
gene67244	74.38606741	41.62069737	0.559522755	-0.83773129	0.24968192	1
gene34624	2314.233208	3833.943774	1.656679958	0.728294926	0.24968409	1
gene47863	289.279151	185.8412534	0.642428784	-0.63839156	0.2497374	1
gene29272	373.9966167	573.9784544	1.534715633	0.617971364	0.24986175	1
gene2433	136.3744569	215.8468724	1.582751472	0.662434737	0.24987469	1
gene15903	211.7936641	328.125963	1.549271855	0.63159032	0.24990328	1
gene19114	39.25931335	19.35846389	0.493092269	-1.02007046	0.24992495	1
gene30535	39.25931335	19.35846389	0.493092269	-1.02007046	0.24992495	1
gene47236	39.25931335	19.35846389	0.493092269	-1.02007046	0.24992495	1
gene59728	39.25931335	19.35846389	0.493092269	-1.02007046	0.24992495	1
gene41884	171.501211	106.4715514	0.620820989	-0.68775076	0.25007996	1
gene11771	1123.02299	724.9744728	0.645556217	-0.63138536	0.25009515	1
gene61882	145.6727153	89.04893391	0.611294529	-0.71006044	0.25019827	1
gene33443	208.6942447	131.6375545	0.630767536	-0.66481968	0.25024199	1
gene73757	51.65699125	27.10184945	0.524650174	-0.93057231	0.25025211	1
gene66498	708.73392	461.6993638	0.651442454	-0.61829035	0.25031974	1
gene11175	111.5791011	179.065791	1.604832708	0.682422915	0.25044819	1

gene1053	315.1076466	203.2638709	0.645061689	-0.63249096	0.25048927	1
gene67002	74.38606741	123.8941689	1.665556108	0.736003955	0.25052467	1
gene13193	245.8872784	156.8035575	0.637705043	-0.6490388	0.25059036	1
gene58312	42.35873283	21.29431028	0.502713581	-0.99219143	0.25061014	1
gene39405	58.88897003	100.6640122	1.709386532	0.773478661	0.25062312	1
gene46627	58.88897003	100.6640122	1.709386532	0.773478661	0.25062312	1
gene8602	201.4622659	126.7979385	0.629388029	-0.66797836	0.25063357	1
gene12239	48.55757178	25.16600306	0.518271448	-0.94822018	0.25066405	1
gene74142	48.55757178	25.16600306	0.518271448	-0.94822018	0.25066405	1
gene58837	220.0587827	139.38094	0.633380492	-0.65885566	0.25070021	1
gene6693	239.6884394	369.7466604	1.542613658	0.62537679	0.25072341	1
gene31930	45.4581523	23.23015667	0.511022897	-0.96854016	0.25081422	1
gene69818	64.05466916	108.4073978	1.692419916	0.759087568	0.25083466	1
gene41040	1270.761985	2015.216091	1.585832843	0.66524071	0.25090158	1
gene59376	123.976779	74.53008599	0.601161658	-0.7341751	0.25101398	1
gene30071	71.28664793	39.68485098	0.556694025	-0.8450435	0.25102975	1
gene28414	438.0512858	671.7386971	1.533470438	0.616800355	0.25114108	1
gene25324	422.5541885	274.8901873	0.650544226	-0.62028096	0.25118834	1
gene29230	152.9046941	240.0449523	1.569899169	0.650671901	0.2511929	1
gene48523	200.4291261	310.7033455	1.550190591	0.632445602	0.25131881	1
gene73011	429.7861672	279.7298033	0.650858088	-0.61958508	0.25139273	1
gene43198	81.61804618	134.5413241	1.64842618	0.721089281	0.25143593	1
gene15185	1313.120718	2084.906561	1.587749346	0.666983176	0.25150531	1
gene38824	186.9983083	117.1187066	0.626308909	-0.67505369	0.25151557	1
gene28124	172.5343508	269.0826481	1.559588841	0.641165737	0.25153566	1
gene41065	835.8101185	543.9728354	0.650833034	-0.61964062	0.25157677	1
gene16190	39.25931335	70.65839321	1.799786781	0.847826002	0.25158827	1
gene6272	10.33139825	24.19807987	2.342188277	1.227857052	0.25160772	1
gene10953	10.33139825	24.19807987	2.342188277	1.227857052	0.25160772	1
gene20547	10.33139825	24.19807987	2.342188277	1.227857052	0.25160772	1
gene21218	10.33139825	24.19807987	2.342188277	1.227857052	0.25160772	1
gene48339	10.33139825	24.19807987	2.342188277	1.227857052	0.25160772	1
gene69526	10.33139825	24.19807987	2.342188277	1.227857052	0.25160772	1
gene23766	147.738995	232.3015667	1.572378144	0.652948215	0.25163294	1
gene69862	536.1995692	823.7026386	1.536186685	0.61935355	0.25163707	1
gene4632	743.8606741	484.9295205	0.65190907	-0.61725735	0.2516397	1
gene50382	91.94944443	53.23577571	0.578967889	-0.78844476	0.25165234	1
gene26531	128.1093383	203.2638709	1.586643671	0.665978164	0.25172962	1
gene9080	19.62965668	39.68485098	2.021678302	1.015553448	0.25178351	1
gene19839	19.62965668	39.68485098	2.021678302	1.015553448	0.25178351	1
gene24765	667.408327	435.5654376	0.65262212	-0.61568021	0.25183783	1
gene35325	216.9593633	137.4450936	0.633506162	-0.65856944	0.25187276	1
gene41570	313.041367	480.0899045	1.533630872	0.616951284	0.25188015	1
gene33502	1241.83407	1964.884085	1.582243661	0.661971788	0.25190778	1
gene29831	710.8001997	1098.592826	1.545571915	0.628140784	0.25192897	1
gene61372	323.3727653	209.07141	0.646533761	-0.62920239	0.2519595	1
gene4504	15.49709738	32.90938862	2.123584038	1.086501202	0.25196792	1
gene9971	15.49709738	32.90938862	2.123584038	1.086501202	0.25196792	1

gene10769	15.49709738	32.90938862	2.123584038	1.086501202	0.25196792	1
gene13296	15.49709738	32.90938862	2.123584038	1.086501202	0.25196792	1
gene27944	15.49709738	32.90938862	2.123584038	1.086501202	0.25196792	1
gene55578	108.4796816	174.226175	1.606071961	0.683536535	0.25199489	1
gene42329	36.15989388	65.81877724	1.820214889	0.864108781	0.25199863	1
gene65695	36.15989388	65.81877724	1.820214889	0.864108781	0.25199863	1
gene48123	88.85002496	145.1884792	1.634084844	0.708482892	0.25203632	1
gene19956	33.0604744	60.97916126	1.844473268	0.88320888	0.25213791	1
gene23910	33.0604744	60.97916126	1.844473268	0.88320888	0.25213791	1
gene32102	135.3413171	82.27347154	0.607896194	-0.71810311	0.25220837	1
gene39324	209.7273845	132.6054777	0.632275456	-0.66137488	0.25228918	1
gene19798	130.175618	206.1676405	1.583765406	0.663358653	0.25250602	1
gene35252	128.1093383	77.43385557	0.604435684	-0.72633926	0.25253432	1
gene52075	128.1093383	77.43385557	0.604435684	-0.72633926	0.25253432	1
gene40108	221.0919226	140.3488632	0.634798692	-0.65562894	0.25265582	1
gene126	2.06627965	8.711308752	4.215938898	2.075853958	0.25269445	1
gene3847	2.06627965	8.711308752	4.215938898	2.075853958	0.25269445	1
gene7396	2.06627965	8.711308752	4.215938898	2.075853958	0.25269445	1
gene7731	2.06627965	8.711308752	4.215938898	2.075853958	0.25269445	1
gene8762	2.06627965	8.711308752	4.215938898	2.075853958	0.25269445	1
gene9126	2.06627965	8.711308752	4.215938898	2.075853958	0.25269445	1
gene9672	2.06627965	8.711308752	4.215938898	2.075853958	0.25269445	1
gene11283	2.06627965	8.711308752	4.215938898	2.075853958	0.25269445	1
gene11359	2.06627965	8.711308752	4.215938898	2.075853958	0.25269445	1
gene14106	2.06627965	8.711308752	4.215938898	2.075853958	0.25269445	1
gene14169	2.06627965	8.711308752	4.215938898	2.075853958	0.25269445	1
gene15124	2.06627965	8.711308752	4.215938898	2.075853958	0.25269445	1
gene15861	2.06627965	8.711308752	4.215938898	2.075853958	0.25269445	1
gene16639	2.06627965	8.711308752	4.215938898	2.075853958	0.25269445	1
gene16992	2.06627965	8.711308752	4.215938898	2.075853958	0.25269445	1
gene17788	2.06627965	8.711308752	4.215938898	2.075853958	0.25269445	1
gene18592	2.06627965	8.711308752	4.215938898	2.075853958	0.25269445	1
gene21330	2.06627965	8.711308752	4.215938898	2.075853958	0.25269445	1
gene23187	2.06627965	8.711308752	4.215938898	2.075853958	0.25269445	1
gene26162	2.06627965	8.711308752	4.215938898	2.075853958	0.25269445	1
gene28055	2.06627965	8.711308752	4.215938898	2.075853958	0.25269445	1
gene30969	2.06627965	8.711308752	4.215938898	2.075853958	0.25269445	1
gene31369	2.06627965	8.711308752	4.215938898	2.075853958	0.25269445	1
gene32587	2.06627965	8.711308752	4.215938898	2.075853958	0.25269445	1
gene35407	2.06627965	8.711308752	4.215938898	2.075853958	0.25269445	1
gene36881	2.06627965	8.711308752	4.215938898	2.075853958	0.25269445	1
gene39449	2.06627965	8.711308752	4.215938898	2.075853958	0.25269445	1
gene40956	2.06627965	8.711308752	4.215938898	2.075853958	0.25269445	1
gene44368	2.06627965	8.711308752	4.215938898	2.075853958	0.25269445	1
gene44641	2.06627965	8.711308752	4.215938898	2.075853958	0.25269445	1
gene47047	2.06627965	8.711308752	4.215938898	2.075853958	0.25269445	1
gene47315	2.06627965	8.711308752	4.215938898	2.075853958	0.25269445	1
gene50435	2.06627965	8.711308752	4.215938898	2.075853958	0.25269445	1

gene53069	2.06627965	8.711308752	4.215938898	2.075853958	0.25269445	1
gene53285	2.06627965	8.711308752	4.215938898	2.075853958	0.25269445	1
gene54641	2.06627965	8.711308752	4.215938898	2.075853958	0.25269445	1
gene57650	2.06627965	8.711308752	4.215938898	2.075853958	0.25269445	1
gene59591	2.06627965	8.711308752	4.215938898	2.075853958	0.25269445	1
gene60238	2.06627965	8.711308752	4.215938898	2.075853958	0.25269445	1
gene60349	2.06627965	8.711308752	4.215938898	2.075853958	0.25269445	1
gene62689	2.06627965	8.711308752	4.215938898	2.075853958	0.25269445	1
gene66358	2.06627965	8.711308752	4.215938898	2.075853958	0.25269445	1
gene66424	2.06627965	8.711308752	4.215938898	2.075853958	0.25269445	1
gene71081	2.06627965	8.711308752	4.215938898	2.075853958	0.25269445	1
gene73840	2.06627965	8.711308752	4.215938898	2.075853958	0.25269445	1
gene74206	2.06627965	8.711308752	4.215938898	2.075853958	0.25269445	1
gene14446	120.8773595	72.5942396	0.600561097	-0.73561707	0.25276843	1
gene29279	106.413402	62.91500765	0.591231992	-0.75820376	0.25277325	1
gene67823	2278.073314	3755.541995	1.648560637	0.721206953	0.25279775	1
gene52420	662.2426279	432.661668	0.653328025	-0.61412057	0.25299025	1
gene61661	165.302372	102.5998586	0.620679893	-0.68807868	0.25304969	1
gene48973	171.501211	267.1468017	1.5576963	0.639413982	0.25318733	1
gene64718	78.51862671	129.7017081	1.6518591	0.724090634	0.2532445	1
gene33917	269.6494943	173.2582518	0.642531343	-0.63816126	0.25332629	1
gene29892	608.519357	397.816433	0.653744911	-0.61320028	0.25337684	1
gene40993	331.6378839	214.8789492	0.647932458	-0.62608466	0.2533851	1
gene16690	1025.907846	665.9311579	0.649114012	-0.6234562	0.25339489	1
gene9648	88.85002496	51.29992932	0.577376645	-0.79241534	0.25340316	1
gene58410	88.85002496	51.29992932	0.577376645	-0.79241534	0.25340316	1
gene2242	27.89477528	12.58300153	0.451088113	-1.14851883	0.25347362	1
gene3919	27.89477528	12.58300153	0.451088113	-1.14851883	0.25347362	1
gene6669	27.89477528	12.58300153	0.451088113	-1.14851883	0.25347362	1
gene17698	27.89477528	12.58300153	0.451088113	-1.14851883	0.25347362	1
gene30367	27.89477528	12.58300153	0.451088113	-1.14851883	0.25347362	1
gene21681	139.4738764	85.17724113	0.610703906	-0.71145502	0.25350166	1
gene36325	449.4158239	293.280728	0.652582113	-0.61576865	0.2535331	1
gene61377	178.7331897	277.7939569	1.554238232	0.636207656	0.25353322	1
gene31040	78.51862671	44.52446695	0.567056109	-0.8184366	0.25359914	1
gene56924	78.51862671	44.52446695	0.567056109	-0.8184366	0.25359914	1
gene11302	105.3802622	169.3865591	1.607384112	0.684714727	0.25361692	1
gene18477	324.4059051	496.5445988	1.530627498	0.614123223	0.25367189	1
gene73470	401.891392	614.6312286	1.529346587	0.612915393	0.25374595	1
gene21367	188.0314482	118.0866297	0.628015318	-0.67112835	0.25376878	1
gene72272	85.75060548	140.3488632	1.63670988	0.710798615	0.2538289	1
gene64964	52.69013108	90.9847803	1.726789788	0.788092466	0.25384574	1
gene13309	10.33139825	2.903769584	0.281062593	-1.83103664	0.25393341	1
gene25384	10.33139825	2.903769584	0.281062593	-1.83103664	0.25393341	1
gene26121	10.33139825	2.903769584	0.281062593	-1.83103664	0.25393341	1
gene30465	10.33139825	2.903769584	0.281062593	-1.83103664	0.25393341	1
gene34303	10.33139825	2.903769584	0.281062593	-1.83103664	0.25393341	1
gene38510	10.33139825	2.903769584	0.281062593	-1.83103664	0.25393341	1

gene52826	10.33139825	2.903769584	0.281062593	-1.83103664	0.25393341	1
gene53013	10.33139825	2.903769584	0.281062593	-1.83103664	0.25393341	1
gene60469	10.33139825	2.903769584	0.281062593	-1.83103664	0.25393341	1
gene69182	10.33139825	2.903769584	0.281062593	-1.83103664	0.25393341	1
gene72772	10.33139825	2.903769584	0.281062593	-1.83103664	0.25393341	1
gene33213	132.2418976	80.33762515	0.607505084	-0.71903161	0.25393784	1
gene14632	161.1698127	251.6600306	1.561458851	0.642894551	0.25395991	1
gene14506	547.5641073	358.131582	0.654045028	-0.61253813	0.25403436	1
gene55285	44.42501248	78.40177877	1.764811632	0.819514205	0.25404171	1
gene11042	1969.164507	3202.857851	1.626505983	0.701776129	0.25411072	1
gene73802	2366.923339	3909.441783	1.651697678	0.723949644	0.25413447	1
gene36346	92.98258426	150.9960184	1.623917205	0.699478079	0.25418288	1
gene58142	817.2136016	533.3256802	0.652614787	-0.61569642	0.25421679	1
gene25084	266.5500749	171.3224055	0.642740039	-0.63769275	0.25427211	1
gene48216	266.5500749	171.3224055	0.642740039	-0.63769275	0.25427211	1
gene23448	299.6105493	193.5846389	0.646120904	-0.63012394	0.25427337	1
gene36125	226.2576217	348.4523501	1.540069004	0.622994993	0.25428646	1
gene49114	371.930337	241.9807987	0.650607855	-0.62013986	0.25429169	1
gene759	180.7994694	113.2470138	0.626368065	-0.67491744	0.25433556	1
gene15674	653.9775093	427.822052	0.654184656	-0.61223017	0.25437896	1
gene611	100.214563	161.6431735	1.612970896	0.689720407	0.25439002	1
gene53770	156.0041136	243.916645	1.563527009	0.644804141	0.25442434	1
gene36105	267.5832147	410.3994345	1.533726377	0.617041124	0.25444011	1
gene55635	240.7215792	153.8997879	0.639326929	-0.64537423	0.25449782	1
gene48103	22.72907615	44.52446695	1.958921104	0.970059294	0.25459866	1
gene13181	336.803583	514.9351395	1.528888544	0.612483238	0.25460547	1
gene17174	162.2029525	100.6640122	0.620605301	-0.68825207	0.2546087	1
gene30769	103.3139825	60.97916126	0.590231446	-0.76064731	0.25461469	1
gene48956	103.3139825	60.97916126	0.590231446	-0.76064731	0.25461469	1
gene71335	103.3139825	60.97916126	0.590231446	-0.76064731	0.25461469	1
gene11153	661.2094881	432.661668	0.65434885	-0.61186812	0.25472278	1
gene1451	2067.31279	1306.696313	0.632074797	-0.6618328	0.25472401	1
gene73544	143.6064357	225.5261044	1.570445665	0.651174029	0.25482635	1
gene885	61.9883895	33.87731181	0.546510598	-0.87167862	0.25483927	1
gene20087	61.9883895	33.87731181	0.546510598	-0.87167862	0.25483927	1
gene62901	61.9883895	33.87731181	0.546510598	-0.87167862	0.25483927	1
gene70258	919.4944443	599.1444575	0.651602042	-0.61793697	0.25484273	1
gene22584	254.152397	390.0730474	1.534799798	0.61805048	0.25484371	1
gene49045	173.5674906	108.4073978	0.62458354	-0.67903354	0.2549357	1
gene43575	568.2269038	871.1308752	1.53306869	0.616422339	0.25494027	1
gene18916	722.1647377	472.346519	0.654070317	-0.61248235	0.25498667	1
gene13545	41.325593	73.56216279	1.78006309	0.831928375	0.25511761	1
gene44098	41.325593	73.56216279	1.78006309	0.831928375	0.25511761	1
gene55144	85.75060548	49.36408293	0.575670372	-0.79668513	0.25518402	1
gene65416	85.75060548	49.36408293	0.575670372	-0.79668513	0.25518402	1
gene67690	397.7588327	259.4034162	0.652162554	-0.61669649	0.25520752	1
gene136	75.41920723	42.58862056	0.564691968	-0.82446398	0.25521768	1
gene7919	75.41920723	42.58862056	0.564691968	-0.82446398	0.25521768	1

gene74178	75.41920723	42.58862056	0.564691968	-0.82446398	0.25521768	1
gene20345	165.302372	257.4675698	1.557555204	0.639283297	0.25528644	1
gene53622	165.302372	257.4675698	1.557555204	0.639283297	0.25528644	1
gene66613	165.302372	257.4675698	1.557555204	0.639283297	0.25528644	1
gene59338	49.5907116	86.14516432	1.737122972	0.796699887	0.25541553	1
gene67406	1150.917765	746.2687831	0.648411907	-0.62501751	0.25574016	1
gene1298	9.298258426	22.26223348	2.394236905	1.259565911	0.2557777	1
gene1802	9.298258426	22.26223348	2.394236905	1.259565911	0.2557777	1
gene5386	9.298258426	22.26223348	2.394236905	1.259565911	0.2557777	1
gene11915	9.298258426	22.26223348	2.394236905	1.259565911	0.2557777	1
gene18819	9.298258426	22.26223348	2.394236905	1.259565911	0.2557777	1
gene51443	9.298258426	22.26223348	2.394236905	1.259565911	0.2557777	1
gene56299	9.298258426	22.26223348	2.394236905	1.259565911	0.2557777	1
gene57594	9.298258426	22.26223348	2.394236905	1.259565911	0.2557777	1
gene14539	177.7000499	111.3111674	0.62639919	-0.67484575	0.25581449	1
gene42265	433.9187265	283.601496	0.653582062	-0.61355971	0.25581895	1
gene15471	1450.528314	933.0779596	0.643267663	-0.63650893	0.25594444	1
gene8585	38.22617353	68.72254682	1.797787758	0.846222711	0.25594845	1
gene34132	38.22617353	68.72254682	1.797787758	0.846222711	0.25594845	1
gene27548	189.064588	119.0545529	0.629703078	-0.66725638	0.25600983	1
gene41652	189.064588	119.0545529	0.629703078	-0.66725638	0.25600983	1
gene22062	25.82849563	49.36408293	1.911225634	0.934498109	0.25611339	1
gene71672	25.82849563	49.36408293	1.911225634	0.934498109	0.25611339	1
gene35703	355.4000998	231.3336435	0.650910463	-0.61946899	0.25611453	1
gene4574	140.5070162	220.6864884	1.570643903	0.65135613	0.25615635	1
gene63067	278.9477528	426.8541288	1.530229674	0.613748205	0.25616198	1
gene48101	97.11514356	156.8035575	1.614614897	0.691190108	0.25616567	1
gene42834	118.8110799	188.745023	1.588614657	0.667769219	0.25618302	1
gene15967	154.9709738	241.9807987	1.561458851	0.642894551	0.25628677	1
gene25087	241.7547191	154.8677111	0.640598503	-0.64250767	0.2563086	1
gene53984	624.0164543	957.2760395	1.534055765	0.617350928	0.25634202	1
gene1790	1127.155549	731.7499351	0.64920049	-0.62326401	0.25639573	1
gene56405	1144.718926	1795.497526	1.568505146	0.649390262	0.2564486	1
gene50822	114.6785206	68.72254682	0.599262586	-0.73873979	0.25645164	1
gene54613	114.6785206	68.72254682	0.599262586	-0.73873979	0.25645164	1
gene31077	170.4680711	106.4715514	0.62458354	-0.67903354	0.25647506	1
gene59968	170.4680711	106.4715514	0.62458354	-0.67903354	0.25647506	1
gene26442	255.1855368	391.0409706	1.532379051	0.615773208	0.25651945	1
gene3066	35.12675405	63.88293085	1.818640309	0.862860235	0.25652826	1
gene34548	35.12675405	63.88293085	1.818640309	0.862860235	0.25652826	1
gene37382	35.12675405	63.88293085	1.818640309	0.862860235	0.25652826	1
gene39972	1781.133058	2867.956426	1.610186511	0.687227809	0.2565836	1
gene965	8.265118601	1.935846389	0.234218828	-2.09407104	0.25664654	1
gene1169	8.265118601	1.935846389	0.234218828	-2.09407104	0.25664654	1
gene5894	8.265118601	1.935846389	0.234218828	-2.09407104	0.25664654	1
gene17078	8.265118601	1.935846389	0.234218828	-2.09407104	0.25664654	1
gene18918	8.265118601	1.935846389	0.234218828	-2.09407104	0.25664654	1
gene20246	8.265118601	1.935846389	0.234218828	-2.09407104	0.25664654	1

gene21984	8.265118601	1.935846389	0.234218828	-2.09407104	0.25664654	1
gene26191	8.265118601	1.935846389	0.234218828	-2.09407104	0.25664654	1
gene34722	8.265118601	1.935846389	0.234218828	-2.09407104	0.25664654	1
gene36157	8.265118601	1.935846389	0.234218828	-2.09407104	0.25664654	1
gene39149	8.265118601	1.935846389	0.234218828	-2.09407104	0.25664654	1
gene39946	8.265118601	1.935846389	0.234218828	-2.09407104	0.25664654	1
gene42746	8.265118601	1.935846389	0.234218828	-2.09407104	0.25664654	1
gene42773	8.265118601	1.935846389	0.234218828	-2.09407104	0.25664654	1
gene46028	8.265118601	1.935846389	0.234218828	-2.09407104	0.25664654	1
gene46884	8.265118601	1.935846389	0.234218828	-2.09407104	0.25664654	1
gene52629	8.265118601	1.935846389	0.234218828	-2.09407104	0.25664654	1
gene54554	8.265118601	1.935846389	0.234218828	-2.09407104	0.25664654	1
gene57109	8.265118601	1.935846389	0.234218828	-2.09407104	0.25664654	1
gene63624	8.265118601	1.935846389	0.234218828	-2.09407104	0.25664654	1
gene64043	8.265118601	1.935846389	0.234218828	-2.09407104	0.25664654	1
gene69011	8.265118601	1.935846389	0.234218828	-2.09407104	0.25664654	1
gene70638	8.265118601	1.935846389	0.234218828	-2.09407104	0.25664654	1
gene70845	8.265118601	1.935846389	0.234218828	-2.09407104	0.25664654	1
gene72208	8.265118601	1.935846389	0.234218828	-2.09407104	0.25664654	1
gene73395	8.265118601	1.935846389	0.234218828	-2.09407104	0.25664654	1
gene26624	206.627965	318.446731	1.541159886	0.624016541	0.25664877	1
gene15620	181.8326092	114.214937	0.628132311	-0.67085961	0.25665569	1
gene9363	1399.904463	2218.479962	1.584736688	0.664243149	0.25699539	1
gene6369	30.99419475	14.51884792	0.468437655	-1.09407104	0.2570085	1
gene25176	30.99419475	14.51884792	0.468437655	-1.09407104	0.2570085	1
gene63428	30.99419475	14.51884792	0.468437655	-1.09407104	0.2570085	1
gene65742	30.99419475	14.51884792	0.468437655	-1.09407104	0.2570085	1
gene73536	30.99419475	14.51884792	0.468437655	-1.09407104	0.2570085	1
gene1529	113.6453808	181.0016374	1.592688028	0.671463703	0.25702421	1
gene13381	183.8988889	284.5694192	1.547423266	0.629867871	0.25704406	1
gene55125	72.31978776	120.0224761	1.659607693	0.73084225	0.2570893	1
gene771	18.59651685	37.74900459	2.029896507	1.021406174	0.25709914	1
gene11906	18.59651685	37.74900459	2.029896507	1.021406174	0.25709914	1
gene21716	18.59651685	37.74900459	2.029896507	1.021406174	0.25709914	1
gene42172	18.59651685	37.74900459	2.029896507	1.021406174	0.25709914	1
gene70835	1946.43543	1237.005843	0.635523698	-0.65398217	0.25720252	1
gene14188	14.46395755	30.97354223	2.141429282	1.098574035	0.25722195	1
gene36519	14.46395755	30.97354223	2.141429282	1.098574035	0.25722195	1
gene55421	14.46395755	30.97354223	2.141429282	1.098574035	0.25722195	1
gene59032	14.46395755	30.97354223	2.141429282	1.098574035	0.25722195	1
gene63561	14.46395755	30.97354223	2.141429282	1.098574035	0.25722195	1
gene64760	14.46395755	30.97354223	2.141429282	1.098574035	0.25722195	1
gene17648	275.8483333	178.0978678	0.645636918	-0.63120502	0.25728921	1
gene65054	301.6768289	195.5204853	0.648112372	-0.62568412	0.25729392	1
gene54408	226.2576217	347.4844269	1.535791034	0.618981931	0.25740947	1
gene59061	1758.403982	2826.335728	1.607330145	0.684666289	0.2574114	1
gene55444	665.3420474	1021.15897	1.53478797	0.618039362	0.25751196	1
gene20052	144.6395755	226.4940275	1.565920162	0.647010659	0.25752209	1

gene63065	334.7373033	217.7827188	0.650607855	-0.62013986	0.25754036	1
gene64889	67.15408863	112.2790906	1.671962093	0.741542139	0.25763948	1
gene67281	537.232709	820.7988691	1.52782743	0.611481598	0.2576618	1
gene41432	287.2128714	185.8412534	0.647050574	-0.62804962	0.25769407	1
gene28421	865.7711734	566.2350689	0.654023934	-0.61258466	0.25769997	1
gene13463	541.3652683	355.2278124	0.656170303	-0.60785779	0.25771742	1
gene64449	1047.603783	682.3858522	0.651377805	-0.61843353	0.25785832	1
gene64958	367.7977777	560.4275297	1.523738216	0.607615064	0.25791254	1
gene55798	89.88316478	52.26785251	0.581508814	-0.78212704	0.25799254	1
gene60122	61.9883895	104.535705	1.686375559	0.753925863	0.25801346	1
gene57609	118.8110799	71.6263164	0.602858896	-0.73010773	0.25803947	1
gene46762	367.7977777	240.0449523	0.652654711	-0.61560816	0.25805215	1
gene66038	324.4059051	494.6087525	1.524660139	0.608487689	0.25806712	1
gene35344	566.1606241	371.6825067	0.656496568	-0.60714063	0.25811298	1
gene3225	146.7058552	229.3977971	1.563658089	0.644925086	0.25818731	1
gene16015	6927.202527	3975.26056	0.573862327	-0.80122343	0.25823039	1
gene60087	43.39187265	76.46593238	1.762217846	0.817392282	0.25830822	1
gene28366	69.22036828	38.71692779	0.559328544	-0.83823214	0.25839256	1
gene28659	69.22036828	38.71692779	0.559328544	-0.83823214	0.25839256	1
gene51537	69.22036828	38.71692779	0.559328544	-0.83823214	0.25839256	1
gene37109	297.5442696	453.9559783	1.525675419	0.609448068	0.25842018	1
gene9964	97.11514356	57.10746848	0.588038759	-0.76601685	0.25845845	1
gene22817	97.11514356	57.10746848	0.588038759	-0.76601685	0.25845845	1
gene1356	207.6611048	319.4146542	1.538153495	0.62119948	0.258658	1
gene20798	263.4506554	402.656049	1.528392664	0.612015238	0.25871357	1
gene32378	79.55176653	45.49239015	0.571858956	-0.80626873	0.25881892	1
gene17902	697.3693819	457.8276711	0.656506699	-0.60711836	0.25882567	1
gene30229	549.6303869	361.0353516	0.656869344	-0.60632166	0.25884881	1
gene14102	2045.616854	3319.976558	1.622970867	0.698637103	0.25888547	1
gene42872	1648.891161	2634.686936	1.597853757	0.676135372	0.25888685	1
gene50350	486.6088576	319.4146542	0.656409453	-0.60733208	0.258979	1
gene26885	237.6221598	363.9391212	1.531587464	0.615027757	0.25901206	1
gene2761	134.3081773	211.0072564	1.571067829	0.651745468	0.25901254	1
gene34295	1005.24505	1563.195959	1.555039699	0.636951411	0.25904127	1
gene31573	216.9593633	138.4130168	0.637967473	-0.64844522	0.25906322	1
gene9423	34.09361423	16.45469431	0.482632736	-1.05100232	0.25909442	1
gene57904	34.09361423	16.45469431	0.482632736	-1.05100232	0.25909442	1
gene18471	228.3239013	146.1564024	0.640127475	-0.64356886	0.25914315	1
gene64391	704.6013607	462.667287	0.656636948	-0.60683216	0.25914546	1
gene46706	49.5907116	26.13392626	0.526992362	-0.92414604	0.25919349	1
gene1314	22.72907615	9.679231946	0.425852414	-1.23157457	0.25921312	1
gene1689	22.72907615	9.679231946	0.425852414	-1.23157457	0.25921312	1
gene7440	22.72907615	9.679231946	0.425852414	-1.23157457	0.25921312	1
gene18344	22.72907615	9.679231946	0.425852414	-1.23157457	0.25921312	1
gene25500	22.72907615	9.679231946	0.425852414	-1.23157457	0.25921312	1
gene43083	22.72907615	9.679231946	0.425852414	-1.23157457	0.25921312	1
gene45415	22.72907615	9.679231946	0.425852414	-1.23157457	0.25921312	1
gene28667	2310.100649	1457.692331	0.631008148	-0.66426946	0.25930647	1

gene42699	48.55757178	84.20931793	1.734216001	0.794283601	0.25954158	1
gene45884	698.4025218	458.7955943	0.656921446	-0.60620723	0.25955404	1
gene4456	1989.827303	1266.043539	0.636257999	-0.65231621	0.25961647	1
gene50764	1203.607896	782.0819413	0.649781331	-0.6219738	0.25962609	1
gene1230	64.05466916	107.4394746	1.677309024	0.746148513	0.25968198	1
gene71691	64.05466916	107.4394746	1.677309024	0.746148513	0.25968198	1
gene748	209.7273845	133.5734009	0.636890605	-0.6508825	0.25970803	1
gene39479	8.265118601	20.32638709	2.459297691	1.298246379	0.25972808	1
gene46110	8.265118601	20.32638709	2.459297691	1.298246379	0.25972808	1
gene58634	8.265118601	20.32638709	2.459297691	1.298246379	0.25972808	1
gene67228	8.265118601	20.32638709	2.459297691	1.298246379	0.25972808	1
gene68691	8.265118601	20.32638709	2.459297691	1.298246379	0.25972808	1
gene66508	298.5774094	454.9239015	1.523638049	0.607520221	0.25988551	1
gene51694	46.49129213	24.19807987	0.520486284	-0.94206795	0.25993915	1
gene12650	86.78374531	50.33200612	0.57997043	-0.78594875	0.25994161	1
gene47702	86.78374531	50.33200612	0.57997043	-0.78594875	0.25994161	1
gene58539	86.78374531	50.33200612	0.57997043	-0.78594875	0.25994161	1
gene8017	83.68432583	136.4771704	1.630857022	0.705630306	0.25994983	1
gene20913	83.68432583	136.4771704	1.630857022	0.705630306	0.25994983	1
gene2459	21.69593633	42.58862056	1.962976842	0.973043153	0.25996292	1
gene38591	21.69593633	42.58862056	1.962976842	0.973043153	0.25996292	1
gene53673	21.69593633	42.58862056	1.962976842	0.973043153	0.25996292	1
gene11818	115.7116604	69.69047001	0.602276985	-0.73150096	0.2599997	1
gene24331	246.9204182	377.4900459	1.528792348	0.612392462	0.26001632	1
gene20012	462.8466416	303.9278831	0.656649213	-0.60680522	0.26001796	1
gene13814	678.7728651	446.2125927	0.657381306	-0.60519766	0.26011091	1
gene2345	37.1930337	18.3905407	0.49446197	-1.01606853	0.2601763	1
gene21019	37.1930337	18.3905407	0.49446197	-1.01606853	0.2601763	1
gene30177	37.1930337	18.3905407	0.49446197	-1.01606853	0.2601763	1
gene41833	37.1930337	18.3905407	0.49446197	-1.01606853	0.2601763	1
gene64684	37.1930337	18.3905407	0.49446197	-1.01606853	0.2601763	1
gene44544	565.1274843	371.6825067	0.657696745	-0.60450557	0.26018164	1
gene22504	4556.146629	2739.222641	0.601214769	-0.73404765	0.26018535	1
gene19453	285.1465917	434.5975144	1.524119618	0.607976135	0.26028935	1
gene5071	114.6785206	181.9695606	1.586779806	0.666101942	0.26029623	1
gene5488	17.56337703	6.775462362	0.385772187	-1.37417896	0.26036265	1
gene8516	17.56337703	6.775462362	0.385772187	-1.37417896	0.26036265	1
gene32894	17.56337703	6.775462362	0.385772187	-1.37417896	0.26036265	1
gene50620	17.56337703	6.775462362	0.385772187	-1.37417896	0.26036265	1
gene56969	17.56337703	6.775462362	0.385772187	-1.37417896	0.26036265	1
gene67683	17.56337703	6.775462362	0.385772187	-1.37417896	0.26036265	1
gene8975	108.4796816	64.85085404	0.597815674	-0.74222737	0.26038082	1
gene26707	108.4796816	64.85085404	0.597815674	-0.74222737	0.26038082	1
gene18879	189.064588	291.3448816	1.540980702	0.623848795	0.26045183	1
gene2193	71.28664793	118.0866297	1.656504173	0.728141837	0.26052615	1
gene3222	452.5152434	688.1933914	1.520818141	0.604847647	0.26061226	1
gene62701	950.4886391	1472.211179	1.548899291	0.631243343	0.2606472	1
gene5986	76.45234706	43.55654376	0.569721473	-0.81167131	0.26066104	1

gene20825	176.6669101	272.9543409	1.545022442	0.627627794	0.26076033	1
gene64004	176.6669101	272.9543409	1.545022442	0.627627794	0.26076033	1
gene6929	928.7927027	1436.398021	1.546521648	0.629027028	0.26125943	1
gene12866	24.7953558	47.42823654	1.912787093	0.9356763	0.2613955	1
gene37254	149.8052746	233.2694899	1.557151379	0.638909203	0.26147407	1
gene68207	63.02152933	34.84523501	0.552910019	-0.85488338	0.26150075	1
gene54851	4713.183882	8387.054482	1.779488068	0.831462259	0.26161316	1
gene27721	60.95524968	102.5998586	1.683199711	0.751206362	0.26178758	1
gene41468	60.95524968	102.5998586	1.683199711	0.751206362	0.26178758	1
gene45280	30.99419475	57.10746848	1.842521444	0.88168141	0.261795	1
gene48263	1548.676598	998.8967369	0.645000214	-0.63262846	0.26183594	1
gene25405	95.04886391	152.9318648	1.608981512	0.686147749	0.2618563	1
gene44966	1038.305524	1612.560042	1.553068923	0.635121856	0.26188501	1
gene17590	4157.354656	7256.52019	1.745465757	0.803612054	0.26189286	1
gene40109	27.89477528	52.26785251	1.873750621	0.905928957	0.26190058	1
gene69115	27.89477528	52.26785251	1.873750621	0.905928957	0.26190058	1
gene67753	722.1647377	475.2502886	0.658091241	-0.60364047	0.26190986	1
gene7425	83.68432583	48.39615973	0.578318093	-0.79006486	0.26193058	1
gene55229	322.3396254	489.7691365	1.519419574	0.603520312	0.26205354	1
gene55093	80.58490636	131.6375545	1.633526183	0.707989579	0.26205662	1
gene19548	151.8715543	236.1732595	1.55508555	0.636993949	0.26209047	1
gene32307	111.5791011	177.1299446	1.587483165	0.666741293	0.262092	1
gene64630	176.6669101	111.3111674	0.630062343	-0.66643351	0.26211064	1
gene47312	378.129176	573.9784544	1.517942785	0.602117413	0.26216988	1
gene10420	142.5732959	88.08101071	0.617794589	-0.69480086	0.26226269	1
gene12734	739.7281148	486.8653669	0.658167991	-0.60347223	0.26231918	1
gene6834	389.4937141	255.5317234	0.656061226	-0.60809764	0.26233228	1
gene23508	763.4903307	1171.187066	1.533990698	0.617289735	0.26236784	1
gene64614	720.0984581	1102.464519	1.530991361	0.614466142	0.26257162	1
gene49855	98.14828338	58.07539168	0.591710723	-0.75703606	0.26264135	1
gene35990	954.6211984	1476.082872	1.546249836	0.628773443	0.26265243	1
gene64542	192.1640075	121.9583225	0.634657469	-0.65594993	0.26266024	1
gene4731	1900.977278	1215.711532	0.639519234	-0.64494035	0.26266717	1
gene16314	17.56337703	35.8131582	2.039081559	1.027919481	0.26271997	1
gene31830	17.56337703	35.8131582	2.039081559	1.027919481	0.26271997	1
gene37798	17.56337703	35.8131582	2.039081559	1.027919481	0.26271997	1
gene59534	17.56337703	35.8131582	2.039081559	1.027919481	0.26271997	1
gene64047	17.56337703	35.8131582	2.039081559	1.027919481	0.26271997	1
gene33440	13.43081773	29.03769584	2.162019948	1.112379834	0.26272546	1
gene35816	13.43081773	29.03769584	2.162019948	1.112379834	0.26272546	1
gene50559	13.43081773	29.03769584	2.162019948	1.112379834	0.26272546	1
gene57244	13.43081773	29.03769584	2.162019948	1.112379834	0.26272546	1
gene64104	13.43081773	29.03769584	2.162019948	1.112379834	0.26272546	1
gene68630	13.43081773	29.03769584	2.162019948	1.112379834	0.26272546	1
gene63618	1773.90108	1138.277677	0.641680469	-0.64007302	0.26275629	1
gene63790	320.2733458	209.07141	0.652790539	-0.61530795	0.26279807	1
gene53736	781.0537078	513.9672164	0.658043373	-0.60374542	0.2628391	1
gene61970	230.390181	148.0922488	0.64278889	-0.6375831	0.26291753	1

gene35369	557.8955055	367.810814	0.659282626	-0.60103103	0.26295725	1
gene63231	313.041367	204.2317941	0.65241152	-0.61614584	0.26302346	1
gene6498	241.7547191	368.7787372	1.525425185	0.609211425	0.26311489	1
gene30816	82.65118601	134.5413241	1.627820852	0.702941934	0.26314261	1
gene58966	82.65118601	134.5413241	1.627820852	0.702941934	0.26314261	1
gene2450	135.3413171	83.24139474	0.615047914	-0.70122929	0.26314513	1
gene27892	163.2360924	252.6279538	1.54762314	0.630054205	0.26315321	1
gene1661	3.099419475	10.64715514	3.435209473	1.780398075	0.2631607	1
gene1982	3.099419475	10.64715514	3.435209473	1.780398075	0.2631607	1
gene12099	3.099419475	10.64715514	3.435209473	1.780398075	0.2631607	1
gene15546	3.099419475	10.64715514	3.435209473	1.780398075	0.2631607	1
gene17253	3.099419475	10.64715514	3.435209473	1.780398075	0.2631607	1
gene19155	3.099419475	10.64715514	3.435209473	1.780398075	0.2631607	1
gene25563	3.099419475	10.64715514	3.435209473	1.780398075	0.2631607	1
gene27249	3.099419475	10.64715514	3.435209473	1.780398075	0.2631607	1
gene35424	3.099419475	10.64715514	3.435209473	1.780398075	0.2631607	1
gene36000	3.099419475	10.64715514	3.435209473	1.780398075	0.2631607	1
gene36740	3.099419475	10.64715514	3.435209473	1.780398075	0.2631607	1
gene39142	3.099419475	10.64715514	3.435209473	1.780398075	0.2631607	1
gene39272	3.099419475	10.64715514	3.435209473	1.780398075	0.2631607	1
gene40148	3.099419475	10.64715514	3.435209473	1.780398075	0.2631607	1
gene40947	3.099419475	10.64715514	3.435209473	1.780398075	0.2631607	1
gene41013	3.099419475	10.64715514	3.435209473	1.780398075	0.2631607	1
gene42045	3.099419475	10.64715514	3.435209473	1.780398075	0.2631607	1
gene42952	3.099419475	10.64715514	3.435209473	1.780398075	0.2631607	1
gene46279	3.099419475	10.64715514	3.435209473	1.780398075	0.2631607	1
gene47324	3.099419475	10.64715514	3.435209473	1.780398075	0.2631607	1
gene48372	3.099419475	10.64715514	3.435209473	1.780398075	0.2631607	1
gene50220	3.099419475	10.64715514	3.435209473	1.780398075	0.2631607	1
gene51618	3.099419475	10.64715514	3.435209473	1.780398075	0.2631607	1
gene52673	3.099419475	10.64715514	3.435209473	1.780398075	0.2631607	1
gene52992	3.099419475	10.64715514	3.435209473	1.780398075	0.2631607	1
gene53419	3.099419475	10.64715514	3.435209473	1.780398075	0.2631607	1
gene56015	3.099419475	10.64715514	3.435209473	1.780398075	0.2631607	1
gene58743	3.099419475	10.64715514	3.435209473	1.780398075	0.2631607	1
gene59137	3.099419475	10.64715514	3.435209473	1.780398075	0.2631607	1
gene61182	3.099419475	10.64715514	3.435209473	1.780398075	0.2631607	1
gene61684	3.099419475	10.64715514	3.435209473	1.780398075	0.2631607	1
gene61916	3.099419475	10.64715514	3.435209473	1.780398075	0.2631607	1
gene64590	3.099419475	10.64715514	3.435209473	1.780398075	0.2631607	1
gene65069	3.099419475	10.64715514	3.435209473	1.780398075	0.2631607	1
gene67593	3.099419475	10.64715514	3.435209473	1.780398075	0.2631607	1
gene68872	3.099419475	10.64715514	3.435209473	1.780398075	0.2631607	1
gene70939	3.099419475	10.64715514	3.435209473	1.780398075	0.2631607	1
gene71127	3.099419475	10.64715514	3.435209473	1.780398075	0.2631607	1
gene73995	3.099419475	10.64715514	3.435209473	1.780398075	0.2631607	1
gene45120	158.0703932	98.72816585	0.62458354	-0.67903354	0.26316417	1
gene12424	106.413402	169.3865591	1.591778441	0.670639541	0.26321345	1

gene16972	7.231978776	18.3905407	2.542947272	1.346501548	0.26324235	1
gene22332	7.231978776	18.3905407	2.542947272	1.346501548	0.26324235	1
gene23741	7.231978776	18.3905407	2.542947272	1.346501548	0.26324235	1
gene25191	7.231978776	18.3905407	2.542947272	1.346501548	0.26324235	1
gene40087	7.231978776	18.3905407	2.542947272	1.346501548	0.26324235	1
gene41559	7.231978776	18.3905407	2.542947272	1.346501548	0.26324235	1
gene43311	7.231978776	18.3905407	2.542947272	1.346501548	0.26324235	1
gene12	419.454769	275.8581105	0.657658777	-0.60458885	0.26342126	1
gene64543	808.948483	532.3577571	0.658086106	-0.60365173	0.26346948	1
gene17915	116.7448002	70.65839321	0.605238033	-0.72442545	0.26351204	1
gene63969	1049.670062	687.2254682	0.654706172	-0.61108052	0.26364879	1
gene38503	1735.674906	2769.22826	1.595476348	0.673987221	0.26367574	1
gene68899	718.0321784	473.3144422	0.659182773	-0.60124956	0.26374267	1
gene14896	1363.744569	885.6497231	0.649424931	-0.62276532	0.26382125	1
gene23936	47.52443195	82.27347154	1.731182639	0.791757937	0.26382826	1
gene47877	57.8558302	97.76024266	1.689721543	0.756785517	0.26395035	1
gene12989	80.58490636	46.46031334	0.576538653	-0.79451076	0.26395608	1
gene66989	80.58490636	46.46031334	0.576538653	-0.79451076	0.26395608	1
gene20117	162.2029525	101.6319354	0.62657266	-0.67444627	0.26397545	1
gene66143	273.7820536	416.2069737	1.520212768	0.604273257	0.26401364	1
gene62484	128.1093383	78.40177877	0.61199113	-0.70841735	0.26401812	1
gene4042	250.0198377	161.6431735	0.646521392	-0.62922999	0.26406459	1
gene59639	378.129176	573.0105312	1.515383016	0.599682484	0.26410041	1
gene4611	39.25931335	69.69047001	1.775132168	0.827926445	0.26412306	1
gene63379	39.25931335	69.69047001	1.775132168	0.827926445	0.26412306	1
gene27055	245.8872784	374.5862763	1.523406493	0.607300949	0.26416597	1
gene40227	302.7099687	197.4563317	0.652295438	-0.61640256	0.26417334	1
gene35680	196.2965668	301.0241135	1.533516956	0.616844118	0.26432522	1
gene72040	459.7472222	696.9047001	1.515843199	0.600120527	0.26446157	1
gene50500	295.47799	192.6167157	0.651881772	-0.61731776	0.2645077	1
gene56492	102.2808427	60.97916126	0.59619338	-0.74614774	0.26457265	1
gene32655	407.0570911	616.567075	1.514694348	0.599026699	0.26464156	1
gene1627	276.8814731	180.0337142	0.650219432	-0.62100142	0.2647439	1
gene44757	565.1274843	858.5478736	1.51921097	0.603322228	0.26491142	1
gene69845	154.9709738	96.79231946	0.62458354	-0.67903354	0.2649808	1
gene39930	5490.10503	3258.029473	0.593436638	-0.75283409	0.26498633	1
gene26736	619.883895	409.4315113	0.660497094	-0.59837588	0.2650447	1
gene10947	767.62289	506.2238308	0.659469431	-0.60062231	0.26506691	1
gene39617	318.2070661	208.1034868	0.653987636	-0.61266473	0.26506956	1
gene12285	129.1424781	202.2959477	1.56645552	0.647503804	0.26513848	1
gene72712	86.78374531	140.3488632	1.617225239	0.693520624	0.26515987	1
gene60190	336.803583	220.6864884	0.655237947	-0.60990918	0.26516748	1
gene23882	1823.491791	1171.187066	0.642277125	-0.63873218	0.26517502	1
gene1303	25.82849563	11.61507834	0.449700149	-1.15296473	0.26524101	1
gene28536	25.82849563	11.61507834	0.449700149	-1.15296473	0.26524101	1
gene41720	36.15989388	64.85085404	1.793447023	0.84273513	0.26529069	1
gene47869	36.15989388	64.85085404	1.793447023	0.84273513	0.26529069	1
gene39254	395.692553	260.3713394	0.658014252	-0.60380926	0.26530073	1

gene15913	511.4042134	775.3064789	1.516034594	0.600302674	0.26556794	1
gene4408	703.5682209	464.6031334	0.660352642	-0.59869144	0.2655744	1
gene56447	4727.64784	8371.56771	1.770768042	0.824375242	0.26563895	1
gene19788	20.6627965	40.65277417	1.967438153	0.976318285	0.26564329	1
gene22024	20.6627965	40.65277417	1.967438153	0.976318285	0.26564329	1
gene41615	44.42501248	77.43385557	1.743023834	0.801592297	0.26565531	1
gene55686	59.92210985	100.6640122	1.67991435	0.74838768	0.26569303	1
gene60867	264.4837952	401.6881258	1.518762711	0.602896483	0.26586433	1
gene10475	6.19883895	16.45469431	2.654480047	1.408429297	0.26596539	1
gene10973	6.19883895	16.45469431	2.654480047	1.408429297	0.26596539	1
gene12003	6.19883895	16.45469431	2.654480047	1.408429297	0.26596539	1
gene12611	6.19883895	16.45469431	2.654480047	1.408429297	0.26596539	1
gene16001	6.19883895	16.45469431	2.654480047	1.408429297	0.26596539	1
gene16177	6.19883895	16.45469431	2.654480047	1.408429297	0.26596539	1
gene19549	6.19883895	16.45469431	2.654480047	1.408429297	0.26596539	1
gene31219	6.19883895	16.45469431	2.654480047	1.408429297	0.26596539	1
gene35380	6.19883895	16.45469431	2.654480047	1.408429297	0.26596539	1
gene50505	6.19883895	16.45469431	2.654480047	1.408429297	0.26596539	1
gene55960	6.19883895	16.45469431	2.654480047	1.408429297	0.26596539	1
gene53548	3658.348121	6244.072529	1.706801081	0.77129493	0.26598108	1
gene14264	202.4954057	309.7354223	1.529592344	0.613147207	0.26598576	1
gene45791	77.48548688	44.52446695	0.574616857	-0.79932778	0.26601346	1
gene4141	296.5111298	193.5846389	0.652874781	-0.61512178	0.26603357	1
gene6653	2581.816423	1629.014737	0.630956842	-0.66438677	0.26605823	1
gene15676	508.3047939	335.8693485	0.660763685	-0.5977937	0.26606564	1
gene23984	1556.941716	2460.460761	1.580316549	0.66021357	0.26612884	1
gene18847	241.7547191	367.810814	1.521421445	0.605419845	0.26613998	1
gene69451	136.3744569	84.20931793	0.617486	-0.69552167	0.26616285	1
gene26744	67.15408863	37.74900459	0.562125186	-0.83103664	0.2661793	1
gene2346	49.5907116	85.17724113	1.717604736	0.780398075	0.26622206	1
gene32658	49.5907116	85.17724113	1.717604736	0.780398075	0.26622206	1
gene37068	49.5907116	85.17724113	1.717604736	0.780398075	0.26622206	1
gene52119	123.976779	194.5525621	1.569266145	0.650090052	0.26625762	1
gene15543	216.9593633	331.0297326	1.525768363	0.609535954	0.26628487	1
gene25041	33.0604744	60.01123807	1.815195915	0.860125267	0.26628568	1
gene38802	33.0604744	60.01123807	1.815195915	0.860125267	0.26628568	1
gene55651	33.0604744	60.01123807	1.815195915	0.860125267	0.26628568	1
gene22619	67.15408863	111.3111674	1.657548627	0.729051195	0.26639411	1
gene54051	159.1035331	245.8524914	1.545235902	0.627827103	0.26640642	1
gene20253	190.0977278	291.3448816	1.532605807	0.615986677	0.26641224	1
gene34491	182.865749	280.6977264	1.534993447	0.618232497	0.26644699	1
gene32788	278.9477528	422.9824361	1.51635004	0.600602829	0.2665763	1
gene73416	1516.649263	2391.738214	1.576988347	0.657171999	0.26663213	1
gene8291	546.5309675	828.5422546	1.516002393	0.600272031	0.26667058	1
gene40143	99.18142321	59.04331487	0.595306187	-0.74829621	0.26677231	1
gene9295	330.604744	216.8147956	0.655812718	-0.60864422	0.26678861	1
gene30357	84.71746566	49.36408293	0.582690742	-0.77919771	0.2667959	1
gene34283	151.8715543	94.85647307	0.62458354	-0.67903354	0.26686022	1

gene62380	151.8715543	94.85647307	0.62458354	-0.67903354	0.26686022	1
gene51448	349.2012609	229.3977971	0.656921446	-0.60620723	0.26686324	1
gene26715	613.6850561	932.1100364	1.51887361	0.603001824	0.26695376	1
gene34880	23.76221598	45.49239015	1.914484331	0.936955852	0.26698337	1
gene13462	91.94944443	54.2036989	0.589494578	-0.76244955	0.26698474	1
gene44217	541.3652683	358.131582	0.661534093	-0.59611258	0.26698844	1
gene6712	29.96105493	55.17162209	1.841444576	0.880837976	0.26701214	1
gene788	4.1325593	12.58300153	3.04484476	1.606368675	0.26702426	1
gene3659	4.1325593	12.58300153	3.04484476	1.606368675	0.26702426	1
gene3928	4.1325593	12.58300153	3.04484476	1.606368675	0.26702426	1
gene8326	4.1325593	12.58300153	3.04484476	1.606368675	0.26702426	1
gene9254	4.1325593	12.58300153	3.04484476	1.606368675	0.26702426	1
gene10876	4.1325593	12.58300153	3.04484476	1.606368675	0.26702426	1
gene14982	4.1325593	12.58300153	3.04484476	1.606368675	0.26702426	1
gene19428	4.1325593	12.58300153	3.04484476	1.606368675	0.26702426	1
gene22855	4.1325593	12.58300153	3.04484476	1.606368675	0.26702426	1
gene23130	4.1325593	12.58300153	3.04484476	1.606368675	0.26702426	1
gene28574	4.1325593	12.58300153	3.04484476	1.606368675	0.26702426	1
gene34672	4.1325593	12.58300153	3.04484476	1.606368675	0.26702426	1
gene36925	4.1325593	12.58300153	3.04484476	1.606368675	0.26702426	1
gene57465	4.1325593	12.58300153	3.04484476	1.606368675	0.26702426	1
gene58519	4.1325593	12.58300153	3.04484476	1.606368675	0.26702426	1
gene61743	4.1325593	12.58300153	3.04484476	1.606368675	0.26702426	1
gene63763	4.1325593	12.58300153	3.04484476	1.606368675	0.26702426	1
gene67302	4.1325593	12.58300153	3.04484476	1.606368675	0.26702426	1
gene73488	4.1325593	12.58300153	3.04484476	1.606368675	0.26702426	1
gene10800	1015.576448	1568.035575	1.543985761	0.626659448	0.26704224	1
gene27206	449.4158239	679.4820826	1.511922915	0.596384586	0.26723525	1
gene30703	379.1623158	573.0105312	1.511253907	0.595746069	0.26723767	1
gene24728	100.214563	159.7073271	1.593653879	0.672338329	0.26727691	1
gene9851	26.86163545	50.33200612	1.873750621	0.905928957	0.26732062	1
gene12435	26.86163545	50.33200612	1.873750621	0.905928957	0.26732062	1
gene14101	26.86163545	50.33200612	1.873750621	0.905928957	0.26732062	1
gene21608	26.86163545	50.33200612	1.873750621	0.905928957	0.26732062	1
gene52803	26.86163545	50.33200612	1.873750621	0.905928957	0.26732062	1
gene61378	26.86163545	50.33200612	1.873750621	0.905928957	0.26732062	1
gene18249	109.5128215	173.2582518	1.582081893	0.661824279	0.26740537	1
gene15379	274.8151935	179.065791	0.651586212	-0.61797202	0.26741704	1
gene64717	83.68432583	135.5092472	1.619290661	0.695361971	0.267429	1
gene997	5.165699125	14.51884792	2.810625932	1.490891457	0.26744678	1
gene5144	5.165699125	14.51884792	2.810625932	1.490891457	0.26744678	1
gene5589	5.165699125	14.51884792	2.810625932	1.490891457	0.26744678	1
gene10774	5.165699125	14.51884792	2.810625932	1.490891457	0.26744678	1
gene15022	5.165699125	14.51884792	2.810625932	1.490891457	0.26744678	1
gene16412	5.165699125	14.51884792	2.810625932	1.490891457	0.26744678	1
gene16844	5.165699125	14.51884792	2.810625932	1.490891457	0.26744678	1
gene22359	5.165699125	14.51884792	2.810625932	1.490891457	0.26744678	1
gene26229	5.165699125	14.51884792	2.810625932	1.490891457	0.26744678	1

gene30693	5.165699125	14.51884792	2.810625932	1.490891457	0.26744678	1
gene35457	5.165699125	14.51884792	2.810625932	1.490891457	0.26744678	1
gene35772	5.165699125	14.51884792	2.810625932	1.490891457	0.26744678	1
gene37605	5.165699125	14.51884792	2.810625932	1.490891457	0.26744678	1
gene37738	5.165699125	14.51884792	2.810625932	1.490891457	0.26744678	1
gene43356	5.165699125	14.51884792	2.810625932	1.490891457	0.26744678	1
gene48555	5.165699125	14.51884792	2.810625932	1.490891457	0.26744678	1
gene50963	5.165699125	14.51884792	2.810625932	1.490891457	0.26744678	1
gene51625	5.165699125	14.51884792	2.810625932	1.490891457	0.26744678	1
gene62220	5.165699125	14.51884792	2.810625932	1.490891457	0.26744678	1
gene67803	5.165699125	14.51884792	2.810625932	1.490891457	0.26744678	1
gene43502	182.865749	116.1507834	0.635169702	-0.654786	0.26745328	1
gene18348	565.1274843	856.6120273	1.515785466	0.600065579	0.26746138	1
gene62211	194.2302871	297.1524208	1.529897449	0.61343495	0.2674914	1
gene3528	570.2931834	377.4900459	0.661922774	-0.59526519	0.26749296	1
gene71886	50.62385143	27.10184945	0.53535732	-0.90142597	0.26750973	1
gene17810	186.9983083	286.5052656	1.53212758	0.615536435	0.26752755	1
gene19834	137.4075967	213.911026	1.556762734	0.638549081	0.26757366	1
gene40515	305.8093882	462.667287	1.512927022	0.597342399	0.26764869	1
gene819	240.7215792	155.8356343	0.647368777	-0.62734031	0.26773705	1
gene7718	1035.206105	680.4500058	0.657308726	-0.60535696	0.26776581	1
gene8137	3361.836991	2087.810331	0.621032589	-0.68725912	0.26777821	1
gene59210	1645.791741	2605.64924	1.583219295	0.6628611	0.26779559	1
gene63338	272.7489138	413.3032041	1.51532484	0.599627097	0.26781763	1
gene56303	223.1582022	339.7410413	1.52242238	0.606368675	0.2678541	1
gene4110	959.7868975	632.0538461	0.658535606	-0.60266665	0.26785447	1
gene51252	2459.905924	1559.324267	0.633895895	-0.65768217	0.26794626	1
gene69032	64.05466916	35.8131582	0.559103008	-0.83881399	0.26803511	1
gene55155	237.6221598	361.0353516	1.519367352	0.603470726	0.26820438	1
gene38453	1722.244088	1112.143751	0.645752689	-0.63094635	0.26821879	1
gene43421	244.8541385	158.7394039	0.648301903	-0.62526229	0.26826307	1
gene70649	762.4571909	504.2879844	0.661398424	-0.59640849	0.26834333	1
gene35273	1052.769482	692.0650842	0.657375709	-0.60520995	0.26837632	1
gene72430	158.0703932	243.916645	1.543088747	0.625821037	0.26838241	1
gene2536	12.3976779	27.10184945	2.186042392	1.128321378	0.26848173	1
gene34814	12.3976779	27.10184945	2.186042392	1.128321378	0.26848173	1
gene58060	12.3976779	27.10184945	2.186042392	1.128321378	0.26848173	1
gene5203	324.4059051	212.9431028	0.656409453	-0.60733208	0.26848464	1
gene6182	271.715774	177.1299446	0.651894228	-0.61729019	0.26851137	1
gene64517	5969.481909	10942.37172	1.833052161	0.87424784	0.26860471	1
gene12536	16.5302372	33.87731181	2.049414742	1.035211974	0.26867158	1
gene18440	16.5302372	33.87731181	2.049414742	1.035211974	0.26867158	1
gene27075	16.5302372	33.87731181	2.049414742	1.035211974	0.26867158	1
gene35692	16.5302372	33.87731181	2.049414742	1.035211974	0.26867158	1
gene50959	678.7728651	1031.806125	1.520105146	0.604171119	0.26869224	1
gene8554	141.540156	219.7185652	1.552340843	0.634445361	0.26872115	1
gene2830	1297.62362	847.9007185	0.653425774	-0.61390473	0.26872913	1
gene49164	790.3519662	522.6785251	0.661323749	-0.59657138	0.26874267	1

gene138	28.9279151	13.55092472	0.468437655	-1.09407104	0.26875109	1
gene2589	28.9279151	13.55092472	0.468437655	-1.09407104	0.26875109	1
gene15888	28.9279151	13.55092472	0.468437655	-1.09407104	0.26875109	1
gene20978	28.9279151	13.55092472	0.468437655	-1.09407104	0.26875109	1
gene27547	28.9279151	13.55092472	0.468437655	-1.09407104	0.26875109	1
gene33805	28.9279151	13.55092472	0.468437655	-1.09407104	0.26875109	1
gene34537	28.9279151	13.55092472	0.468437655	-1.09407104	0.26875109	1
gene68326	28.9279151	13.55092472	0.468437655	-1.09407104	0.26875109	1
gene72930	28.9279151	13.55092472	0.468437655	-1.09407104	0.26875109	1
gene43231	113.6453808	179.065791	1.575653932	0.655950704	0.26876467	1
gene60906	148.7721348	92.92062669	0.62458354	-0.67903354	0.26880522	1
gene10541	47.52443195	25.16600306	0.529538219	-0.91719328	0.26881765	1
gene35559	47.52443195	25.16600306	0.529538219	-0.91719328	0.26881765	1
gene11248	317.1739263	208.1034868	0.656117889	-0.60797304	0.2688222	1
gene11491	294.4448501	445.2446695	1.512149624	0.596600899	0.26882617	1
gene44666	38.22617353	67.75462362	1.772466804	0.825758608	0.26891802	1
gene47390	38.22617353	67.75462362	1.772466804	0.825758608	0.26891802	1
gene62938	424.6204681	280.6977264	0.661055572	-0.59715654	0.26904908	1
gene30696	1535.24578	2416.904217	1.574278365	0.654690662	0.26910913	1
gene40195	2281.172734	1453.820638	0.637312825	-0.6499264	0.26913693	1
gene45921	417.3884893	275.8581105	0.660914514	-0.59746442	0.26914262	1
gene6435	137.4075967	85.17724113	0.619887424	-0.68992186	0.26915517	1
gene54111	955.6543382	630.1179997	0.659357651	-0.60086687	0.26918326	1
gene44772	564.0943445	853.7082577	1.513413963	0.597806661	0.26920466	1
gene44921	88.85002496	52.26785251	0.588270544	-0.7654483	0.26925369	1
gene23568	210.7605243	135.5092472	0.642953645	-0.63721337	0.26925948	1
gene46397	87.81688513	141.3167864	1.609221122	0.686362579	0.26928617	1
gene54965	747.9932334	1139.2456	1.523069393	0.606981675	0.26953408	1
gene34396	241.7547191	156.8035575	0.648605984	-0.62458576	0.26954086	1
gene49659	399.8251123	603.0161503	1.50819979	0.592827554	0.26956696	1
gene27155	1218.071854	1892.289846	1.553512496	0.635533846	0.26958062	1
gene16203	199.3959862	127.7658617	0.640764461	-0.64213396	0.26974479	1
gene12674	740.7612546	490.7370597	0.662476684	-0.59405842	0.26985742	1
gene63548	870.9368725	1332.830239	1.53034081	0.61385298	0.26989877	1
gene22140	60.95524968	33.87731181	0.555773489	-0.84743108	0.26992036	1
gene43741	60.95524968	33.87731181	0.555773489	-0.84743108	0.26992036	1
gene67896	60.95524968	33.87731181	0.555773489	-0.84743108	0.26992036	1
gene33439	699.4356616	463.6352102	0.662870419	-0.59320122	0.26992633	1
gene30264	44.42501248	23.23015667	0.52290715	-0.9353733	0.26995429	1
gene34810	44.42501248	23.23015667	0.52290715	-0.9353733	0.26995429	1
gene39775	440.1175655	291.3448816	0.661970583	-0.59516099	0.26999244	1
gene7119	2261.543077	1443.173483	0.638136632	-0.64806274	0.27002099	1
gene21684	117.7779401	184.8733302	1.569677056	0.650467771	0.27004574	1
gene58204	211.7936641	322.3184238	1.521851114	0.605827224	0.27013158	1
gene22320	73.35292758	120.0224761	1.636232937	0.710378148	0.27016293	1
gene46121	43.39187265	75.49800918	1.739911291	0.799013753	0.27029332	1
gene55753	261.3843757	170.3544823	0.651739347	-0.617633	0.27029771	1
gene64861	502.105955	332.965579	0.66313808	-0.59261879	0.27031385	1

gene1589	35.12675405	62.91500765	1.791085153	0.840833928	0.27031481	1
gene18035	35.12675405	62.91500765	1.791085153	0.840833928	0.27031481	1
gene63652	35.12675405	62.91500765	1.791085153	0.840833928	0.27031481	1
gene37566	380.1954556	573.0105312	1.507147239	0.591820366	0.27039237	1
gene67172	138.4407366	214.8789492	1.552136709	0.634255633	0.27041109	1
gene12761	182.865749	279.7298033	1.529700366	0.613249089	0.27042617	1
gene50679	806.8822034	534.2936034	0.662170514	-0.59472532	0.2705656	1
gene34311	119.8442197	187.7770998	1.566843192	0.647860804	0.27065996	1
gene6598	48.55757178	83.24139474	1.714282483	0.77760486	0.27067001	1
gene16658	48.55757178	83.24139474	1.714282483	0.77760486	0.27067001	1
gene69575	48.55757178	83.24139474	1.714282483	0.77760486	0.27067001	1
gene18602	429.7861672	284.5694192	0.662118609	-0.59483842	0.27067071	1
gene38300	192.1640075	122.9262457	0.639694433	-0.64454517	0.27083903	1
gene30540	41.325593	21.29431028	0.515281421	-0.95656752	0.27084035	1
gene16215	242.7878589	367.810814	1.514947311	0.599267618	0.27097797	1
gene13051	215.9262234	328.125963	1.519620719	0.603711288	0.27113718	1
gene37939	201.4622659	306.8316527	1.523022941	0.606937673	0.27116974	1
gene29606	1515.616123	2379.155212	1.569761086	0.650545001	0.27123624	1
gene50752	78.51862671	45.49239015	0.579383416	-0.78740971	0.27127644	1
gene10479	60.95524968	101.6319354	1.667320468	0.737531425	0.27132168	1
gene25826	60.95524968	101.6319354	1.667320468	0.737531425	0.27132168	1
gene231	38.22617353	19.35846389	0.506419087	-0.98159631	0.27136431	1
gene26063	38.22617353	19.35846389	0.506419087	-0.98159631	0.27136431	1
gene31409	38.22617353	19.35846389	0.506419087	-0.98159631	0.27136431	1
gene15465	35.12675405	17.4226175	0.495992812	-1.01160888	0.27136659	1
gene42354	35.12675405	17.4226175	0.495992812	-1.01160888	0.27136659	1
gene43966	35.12675405	17.4226175	0.495992812	-1.01160888	0.27136659	1
gene41834	92.98258426	55.17162209	0.593354363	-0.75303413	0.27138917	1
gene48277	92.98258426	55.17162209	0.593354363	-0.75303413	0.27138917	1
gene33725	312.0082272	470.4106726	1.507686758	0.592336721	0.27142643	1
gene9471	142.5732959	220.6864884	1.547880948	0.630294514	0.27149445	1
gene4203	68.18722846	112.2790906	1.646629334	0.719515832	0.27151172	1
gene52154	32.02733458	58.07539168	1.813307053	0.858623242	0.27155026	1
gene3841	85.75060548	50.33200612	0.586958026	-0.76867076	0.27158805	1
gene3068	637.4472721	965.0194251	1.513881175	0.598251972	0.27161254	1
gene5076	19.62965668	38.71692779	1.972369075	0.979929538	0.2716691	1
gene63847	19.62965668	38.71692779	1.972369075	0.979929538	0.2716691	1
gene13101	596.1216791	901.1364942	1.511665363	0.596138806	0.27167606	1
gene6871	231.4233208	150.0280952	0.648284255	-0.62530156	0.271741	1
gene54739	196.2965668	299.0882671	1.523655111	0.607536376	0.27177092	1
gene2339	20.6627965	8.711308752	0.42159389	-1.24607414	0.27181843	1
gene12362	20.6627965	8.711308752	0.42159389	-1.24607414	0.27181843	1
gene31571	20.6627965	8.711308752	0.42159389	-1.24607414	0.27181843	1
gene37587	20.6627965	8.711308752	0.42159389	-1.24607414	0.27181843	1
gene56408	20.6627965	8.711308752	0.42159389	-1.24607414	0.27181843	1
gene59352	20.6627965	8.711308752	0.42159389	-1.24607414	0.27181843	1
gene7297	57.8558302	31.94146542	0.552087237	-0.85703185	0.27182396	1
gene17828	57.8558302	31.94146542	0.552087237	-0.85703185	0.27182396	1

gene24529	57.8558302	31.94146542	0.552087237	-0.85703185	0.27182396	1
gene28405	528.9675904	351.3561197	0.664229957	-0.59024531	0.27183214	1
gene67561	528.9675904	351.3561197	0.664229957	-0.59024531	0.27183214	1
gene29089	550.6635268	365.8749676	0.664425642	-0.58982034	0.27197793	1
gene6512	144.6395755	223.590258	1.545844263	0.628394981	0.27202201	1
gene41944	144.6395755	223.590258	1.545844263	0.628394981	0.27202201	1
gene54012	745.9269537	1133.438061	1.519502755	0.60359929	0.27205888	1
gene36890	449.4158239	298.1203439	0.663350795	-0.59215609	0.27205999	1
gene23824	138.4407366	86.14516432	0.622253005	-0.6844268	0.27212226	1
gene23798	690.1374032	1046.324973	1.516111094	0.600375472	0.27213412	1
gene30281	40.29245318	70.65839321	1.753638402	0.810351297	0.27219572	1
gene54145	40.29245318	70.65839321	1.753638402	0.810351297	0.27219572	1
gene37020	7024.317671	13201.50445	1.879400259	0.910272353	0.27220827	1
gene24164	55.78955055	93.88854988	1.682905651	0.750954297	0.27224056	1
gene69281	1650.95744	2602.74547	1.576506702	0.656731304	0.27224592	1
gene41709	153.9378339	96.79231946	0.628775376	-0.66938337	0.27225161	1
gene6626	638.4804119	965.9873482	1.512947508	0.597361934	0.27235508	1
gene44896	737.6618351	489.7691365	0.663948049	-0.59085773	0.2723969	1
gene1914	15.49709738	5.807539168	0.374750124	-1.41599914	0.27240922	1
gene9486	15.49709738	5.807539168	0.374750124	-1.41599914	0.27240922	1
gene14335	15.49709738	5.807539168	0.374750124	-1.41599914	0.27240922	1
gene19462	15.49709738	5.807539168	0.374750124	-1.41599914	0.27240922	1
gene20927	15.49709738	5.807539168	0.374750124	-1.41599914	0.27240922	1
gene22518	15.49709738	5.807539168	0.374750124	-1.41599914	0.27240922	1
gene26807	15.49709738	5.807539168	0.374750124	-1.41599914	0.27240922	1
gene32620	15.49709738	5.807539168	0.374750124	-1.41599914	0.27240922	1
gene46602	15.49709738	5.807539168	0.374750124	-1.41599914	0.27240922	1
gene59150	15.49709738	5.807539168	0.374750124	-1.41599914	0.27240922	1
gene68604	15.49709738	5.807539168	0.374750124	-1.41599914	0.27240922	1
gene72682	15.49709738	5.807539168	0.374750124	-1.41599914	0.27240922	1
gene3001	8703.169886	4927.696984	0.566195656	-0.82062741	0.2724459	1
gene31039	266.5500749	174.226175	0.653633938	-0.6134452	0.27245313	1
gene57049	21426.28683	10426.46865	0.486620418	-1.03913124	0.2724565	1
gene37052	460.780362	305.8637295	0.663795063	-0.5911902	0.27246457	1
gene503	28.9279151	53.23577571	1.840290789	0.879933748	0.27251719	1
gene7646	643.646111	427.822052	0.664685212	-0.58925684	0.27252848	1
gene46298	534.1332896	805.3120979	1.507698759	0.592348204	0.27255512	1
gene55534	443.216985	666.8990811	1.504678529	0.589455292	0.2726942	1
gene73643	115.7116604	70.65839321	0.610641944	-0.71160141	0.27272525	1
gene9600	208.6942447	134.5413241	0.644681526	-0.63334145	0.27282397	1
gene59645	50.62385143	86.14516432	1.701671483	0.766952543	0.27283804	1
gene31758	1314.153858	861.4516432	0.655518103	-0.60929247	0.27287485	1
gene73385	158.0703932	99.69608905	0.630706909	-0.66495836	0.2728975	1
gene11272	22.72907615	43.55654376	1.916335863	0.938350434	0.27290507	1
gene35263	45.4581523	78.40177877	1.724702277	0.786347341	0.27292208	1
gene49586	45.4581523	78.40177877	1.724702277	0.786347341	0.27292208	1
gene65796	45.4581523	78.40177877	1.724702277	0.786347341	0.27292208	1
gene52542	544.4646878	820.7988691	1.507533707	0.59219026	0.27300928	1

gene14968	1095.128215	722.0707032	0.659348096	-0.60088777	0.27304298	1
gene29414	4347.452384	7529.474531	1.731928004	0.792378959	0.2730438	1
gene5237	25.82849563	48.39615973	1.873750621	0.905928957	0.27305298	1
gene72495	25.82849563	48.39615973	1.873750621	0.905928957	0.27305298	1
gene52509	2334.896005	1491.569643	0.638816307	-0.64652695	0.27307816	1
gene64401	1295.557341	2012.312322	1.553240647	0.635281367	0.27308676	1
gene35853	231.4233208	350.3881965	1.514057422	0.598419922	0.27310096	1
gene10576	54.75641073	30.00561903	0.547983672	-0.86779519	0.27373036	1
gene2046	216.9593633	140.3488632	0.646890095	-0.62840747	0.27373725	1
gene66621	181.8326092	116.1507834	0.638778621	-0.64661207	0.27378235	1
gene13433	608.519357	918.5591117	1.509498591	0.59406941	0.27378522	1
gene59069	89.88316478	53.23577571	0.592277495	-0.75565483	0.27381009	1
gene23264	37.1930337	65.81877724	1.769653365	0.823466796	0.27394486	1
gene27906	37.1930337	65.81877724	1.769653365	0.823466796	0.27394486	1
gene869	269.6494943	406.5277417	1.507615443	0.592268478	0.27404009	1
gene63891	1480.489369	2314.304358	1.56320228	0.644504477	0.27410705	1
gene46109	90.91630461	145.1884792	1.596946552	0.675316029	0.27418108	1
gene31371	150.8384145	94.85647307	0.62886151	-0.66918576	0.27429496	1
gene63519	150.8384145	94.85647307	0.62886151	-0.66918576	0.27429496	1
gene10019	135.3413171	84.20931793	0.622199634	-0.68455055	0.27430116	1
gene4339	536.1995692	807.2479443	1.505499054	0.590241802	0.27430731	1
gene3148	65.08780898	36.7810814	0.565099394	-0.82342345	0.27444449	1
gene55604	65.08780898	36.7810814	0.565099394	-0.82342345	0.27444449	1
gene9681	11.36453808	25.16600306	2.214432553	1.146937056	0.27448671	1
gene9872	11.36453808	25.16600306	2.214432553	1.146937056	0.27448671	1
gene11854	11.36453808	25.16600306	2.214432553	1.146937056	0.27448671	1
gene23352	11.36453808	25.16600306	2.214432553	1.146937056	0.27448671	1
gene61651	11.36453808	25.16600306	2.214432553	1.146937056	0.27448671	1
gene50548	332.6710237	219.7185652	0.660467999	-0.59843943	0.27457775	1
gene2511	901.9310673	598.1765343	0.663217574	-0.59244586	0.27459826	1
gene886	9615.432352	5384.556732	0.55999112	-0.83652415	0.27478277	1
gene12858	52.69013108	89.04893391	1.69004958	0.757065571	0.27481324	1
gene32998	52.69013108	89.04893391	1.69004958	0.757065571	0.27481324	1
gene42073	175.6337703	268.1147249	1.526555653	0.610280187	0.27486627	1
gene14343	101.2477029	60.97916126	0.602276985	-0.73150096	0.27488089	1
gene62959	101.2477029	60.97916126	0.602276985	-0.73150096	0.27488089	1
gene20675	2397.917534	1532.222417	0.638980447	-0.64615631	0.27497642	1
gene8185	15.49709738	31.94146542	2.061125684	1.04343248	0.27498126	1
gene21617	15.49709738	31.94146542	2.061125684	1.04343248	0.27498126	1
gene32747	15.49709738	31.94146542	2.061125684	1.04343248	0.27498126	1
gene41244	15.49709738	31.94146542	2.061125684	1.04343248	0.27498126	1
gene43156	15.49709738	31.94146542	2.061125684	1.04343248	0.27498126	1
gene13788	305.8093882	459.7635175	1.503431664	0.588259294	0.27503456	1
gene2221	2476.436161	1579.650654	0.637872552	-0.6486599	0.27508645	1
gene12430	112.6122409	68.72254682	0.61025823	-0.71250825	0.27510417	1
gene46176	5637.844025	3372.24441	0.598144325	-0.74143446	0.2751093	1
gene34836	291.3454307	191.6487925	0.657806069	-0.60426578	0.27522026	1
gene73587	2725.422859	4455.350465	1.634737322	0.709058835	0.2752333	1

gene65888	2297.702971	3699.40245	1.610043812	0.687099947	0.27524452	1
gene30296	147.738995	227.4619507	1.539620266	0.622574566	0.27524562	1
gene69419	234.5227403	354.2598892	1.510556668	0.595080308	0.2754155	1
gene45858	67.15408863	110.3432442	1.64313516	0.716451158	0.2754161	1
gene58361	59.92210985	99.69608905	1.663761328	0.734448489	0.27547576	1
gene9420	425.6536079	638.8293085	1.500819673	0.585750644	0.27547605	1
gene15165	340.9361423	225.5261044	0.66149075	-0.59620711	0.27552052	1
gene24719	1951.60113	1261.203923	0.646240619	-0.62985666	0.27554332	1
gene30390	34.09361423	60.97916126	1.788580139	0.838814761	0.27559845	1
gene52444	34.09361423	60.97916126	1.788580139	0.838814761	0.27559845	1
gene7339	178.7331897	114.214937	0.639024778	-0.64605622	0.27560996	1
gene19241	51.65699125	28.06977264	0.54338768	-0.87994624	0.27561786	1
gene30553	6647.221635	3907.505937	0.587840477	-0.76650339	0.27566336	1
gene43780	295.47799	194.5525621	0.658433348	-0.60289069	0.27567337	1
gene28187	315.1076466	473.3144422	1.502072219	0.586954179	0.27569977	1
gene12860	430.8193071	286.5052656	0.665024201	-0.58852125	0.27571099	1
gene7364	149.8052746	230.3657203	1.537767751	0.62083763	0.27572705	1
gene18950	149.8052746	230.3657203	1.537767751	0.62083763	0.27572705	1
gene63324	564.0943445	848.8686417	1.504834519	0.589604848	0.27573564	1
gene44717	533.1001497	801.4404052	1.503358057	0.588188659	0.27586318	1
gene53553	1662.321979	2612.424702	1.571551562	0.652189607	0.27586866	1
gene19643	85.75060548	137.4450936	1.602846917	0.680636645	0.275934	1
gene16982	72.31978776	41.62069737	0.575509119	-0.79708931	0.2759341	1
gene26007	72.31978776	41.62069737	0.575509119	-0.79708931	0.2759341	1
gene71575	72.31978776	41.62069737	0.575509119	-0.79708931	0.2759341	1
gene66107	414.2890699	621.406691	1.499935036	0.584900017	0.27607102	1
gene2752	526.9013108	791.7611732	1.502674518	0.587532553	0.27620397	1
gene53508	116.7448002	71.6263164	0.613528965	-0.70479664	0.27621687	1
gene14532	8317.808732	4758.310425	0.572062977	-0.80575412	0.27623037	1
gene23690	894.6990885	594.3048415	0.664251086	-0.59019941	0.27624934	1
gene45084	379.1623158	251.6600306	0.663726378	-0.59133948	0.27627041	1
gene35058	172.5343508	263.2751089	1.52592865	0.609687505	0.2762965	1
gene15095	337.8367228	223.590258	0.661829348	-0.59546883	0.27641371	1
gene67348	79.55176653	46.46031334	0.584026168	-0.77589508	0.27645152	1
gene16231	206.627965	133.5734009	0.646443964	-0.62940278	0.27647562	1
gene46998	186.9983083	120.0224761	0.641837229	-0.63972062	0.27651905	1
gene38296	142.5732959	219.7185652	1.541091997	0.623952987	0.27654292	1
gene16689	450.4489637	675.6103899	1.499860016	0.584827858	0.2765664	1
gene45985	276.8814731	181.9695606	0.657211039	-0.60557138	0.27660734	1
gene14912	61.9883895	34.84523501	0.562125186	-0.83103664	0.27664435	1
gene31233	153.9378339	236.1732595	1.534211918	0.617497774	0.27666904	1
gene24730	245.8872784	160.6752503	0.653450847	-0.61384938	0.27667955	1
gene29392	437.018146	655.2840028	1.499443464	0.584427127	0.27669174	1
gene34788	240.7215792	362.971198	1.50784653	0.592489597	0.27676092	1
gene68472	768.6560299	512.03137	0.666138494	-0.58610594	0.27682423	1
gene30389	78.51862671	126.7979385	1.61487718	0.691424445	0.27688219	1
gene40243	4122.227902	2542.734232	0.616834948	-0.69704359	0.27696664	1
gene2068	265.516935	174.226175	0.65617726	-0.6078425	0.2769899	1

gene16604	652.9443695	984.3778889	1.507598403	0.592252172	0.27699054	1
gene65676	281.0140324	184.8733302	0.657879354	-0.60410506	0.27703002	1
gene37246	110.5459613	173.2582518	1.567296081	0.648277748	0.27705031	1
gene38598	642.6129712	428.7899752	0.667260069	-0.58367892	0.2770807	1
gene15480	30.99419475	56.13954529	1.811292267	0.857019356	0.27710004	1
gene15601	30.99419475	56.13954529	1.811292267	0.857019356	0.27710004	1
gene20979	30.99419475	56.13954529	1.811292267	0.857019356	0.27710004	1
gene59490	328.5384644	492.6729061	1.499589727	0.584567847	0.27718418	1
gene26122	1577.604513	1030.838202	0.653419912	-0.61391768	0.27722948	1
gene2272	593.0222596	395.8805866	0.667564464	-0.58302094	0.27740514	1
gene25211	49.5907116	84.20931793	1.698086501	0.763909952	0.27744191	1
gene40582	49.5907116	84.20931793	1.698086501	0.763909952	0.27744191	1
gene52895	49.5907116	84.20931793	1.698086501	0.763909952	0.27744191	1
gene58608	49.5907116	84.20931793	1.698086501	0.763909952	0.27744191	1
gene10151	48.55757178	26.13392626	0.538204966	-0.89377239	0.27745625	1
gene66068	48.55757178	26.13392626	0.538204966	-0.89377239	0.27745625	1
gene70744	380.1954556	252.6279538	0.664468631	-0.589727	0.27749924	1
gene38389	123.976779	192.6167157	1.553651557	0.635662982	0.27752981	1
gene55570	273.7820536	411.3673577	1.502535876	0.587399438	0.27771558	1
gene45279	273.7820536	180.0337142	0.657580407	-0.60476078	0.27780405	1
gene48473	977.3502745	1491.569643	1.526136209	0.60988373	0.27787996	1
gene22126	4215.210486	2597.905854	0.616316994	-0.69825552	0.27799391	1
gene16228	18.59651685	36.7810814	1.977847878	0.983931469	0.27807363	1
gene17136	18.59651685	36.7810814	1.977847878	0.983931469	0.27807363	1
gene36691	18.59651685	36.7810814	1.977847878	0.983931469	0.27807363	1
gene49362	18.59651685	36.7810814	1.977847878	0.983931469	0.27807363	1
gene55875	160.1366729	101.6319354	0.634657469	-0.65594993	0.27808073	1
gene19262	125.0099188	77.43385557	0.619421693	-0.69100619	0.27810121	1
gene19228	239.6884394	361.0353516	1.506269357	0.590979782	0.27813142	1
gene69253	275.8483333	414.2711273	1.501807614	0.586700011	0.2781357	1
gene1839	1517.682403	2366.572211	1.559332971	0.640929026	0.27816766	1
gene45944	91.94944443	146.1564024	1.589530022	0.668600265	0.27819202	1
gene72377	64.05466916	105.5036282	1.64708724	0.719916971	0.27822774	1
gene389	56.82269038	94.85647307	1.669341463	0.739279087	0.27826637	1
gene22057	23.76221598	10.64715514	0.448070801	-1.15820138	0.27827663	1
gene29654	69.22036828	39.68485098	0.573311757	-0.80260823	0.27831415	1
gene37870	69.22036828	39.68485098	0.573311757	-0.80260823	0.27831415	1
gene67718	27.89477528	51.29992932	1.839051536	0.878961909	0.27833502	1
gene48840	437.018146	291.3448816	0.66666541	-0.58496522	0.2783484	1
gene30102	431.8524469	646.572694	1.497207434	0.582274117	0.27838982	1
gene51776	375.0297565	561.3954529	1.496935758	0.582012309	0.27843182	1
gene65323	552.7298064	829.5101778	1.500751666	0.585685269	0.2785141	1
gene24120	329.5716042	493.6408293	1.497825732	0.58286978	0.27855485	1
gene32899	164.2692322	104.535705	0.636368136	-0.6520665	0.2785652	1
gene20756	1497.019607	981.4741194	0.655618747	-0.60907099	0.2786398	1
gene55119	605.4199375	404.5918954	0.668283071	-0.58146877	0.27870571	1
gene33621	73.35292758	119.0545529	1.62303751	0.698696342	0.27871881	1
gene60301	188.0314482	120.9903993	0.643458318	-0.6360814	0.27875188	1

gene15893	210.7605243	318.446731	1.510941065	0.595447388	0.27877349	1
gene22109	231.4233208	150.9960184	0.652466734	-0.61602375	0.27877502	1
gene63364	141.540156	217.7827188	1.538663831	0.621678065	0.27887264	1
gene70190	1124.05613	744.3329367	0.662184847	-0.5946941	0.278895	1
gene24030	58.88897003	32.90938862	0.558837905	-0.83949822	0.27889764	1
gene49790	129.1424781	80.33762515	0.622085206	-0.6848159	0.27890201	1
gene71934	1308.988158	2022.959477	1.545437569	0.628015374	0.27897413	1
gene59571	76.45234706	44.52446695	0.58238195	-0.77996245	0.27897801	1
gene900	1137.486947	753.0442454	0.662024516	-0.59504345	0.27900706	1
gene66049	2943.415362	4828.000895	1.640271692	0.7139348	0.2790344	1
gene16553	24.7953558	46.46031334	1.873750621	0.905928957	0.27912614	1
gene17733	24.7953558	46.46031334	1.873750621	0.905928957	0.27912614	1
gene39477	24.7953558	46.46031334	1.873750621	0.905928957	0.27912614	1
gene59537	21.69593633	41.62069737	1.918363731	0.939876289	0.27919241	1
gene60934	21.69593633	41.62069737	1.918363731	0.939876289	0.27919241	1
gene33285	45.4581523	24.19807987	0.532315517	-0.90964647	0.27920331	1
gene44174	45.4581523	24.19807987	0.532315517	-0.90964647	0.27920331	1
gene67862	45.4581523	24.19807987	0.532315517	-0.90964647	0.27920331	1
gene71899	45.4581523	24.19807987	0.532315517	-0.90964647	0.27920331	1
gene67956	556.8623657	835.317717	1.500043401	0.585004243	0.27920346	1
gene32604	489.7082771	327.1580398	0.668067205	-0.58193486	0.279248	1
gene36453	652.9443695	982.4420426	1.504633608	0.589412221	0.279259	1
gene28007	317.1739263	210.0393332	0.662221311	-0.59461466	0.27935176	1
gene18478	51.65699125	87.11308752	1.686375559	0.753925863	0.27938872	1
gene735	196.2965668	297.1524208	1.513793265	0.598168194	0.27940788	1
gene13762	917.4281647	610.7595358	0.665730091	-0.58699071	0.27944955	1
gene62844	66.12094881	108.4073978	1.639531794	0.713283879	0.27945132	1
gene23710	744.8938139	497.512522	0.66789724	-0.58230194	0.27952975	1
gene43404	501.0728152	334.9014253	0.668368778	-0.58128375	0.27955843	1
gene36060	214.8930836	324.2542702	1.508909755	0.593506524	0.27965224	1
gene19166	734.5624156	490.7370597	0.668067205	-0.58193486	0.2796591	1
gene39815	117.7779401	72.5942396	0.616365336	-0.69814237	0.27967181	1
gene64559	152.9046941	96.79231946	0.633023859	-0.65966822	0.27970091	1
gene71168	152.9046941	96.79231946	0.633023859	-0.65966822	0.27970091	1
gene46656	278.9477528	183.905407	0.659282626	-0.60103103	0.27980382	1
gene30916	787.2525467	525.5822947	0.667615871	-0.58290984	0.27981691	1
gene55476	200.4291261	129.7017081	0.64712006	-0.6278947	0.27986757	1
gene65373	200.4291261	129.7017081	0.64712006	-0.6278947	0.27986757	1
gene34156	574.4257427	384.2655083	0.668955932	-0.58001692	0.27989524	1
gene64326	95.04886391	57.10746848	0.60082221	-0.73498995	0.28001883	1
gene49819	1436.064357	2228.159194	1.551573356	0.633731907	0.28006967	1
gene9083	46.49129213	79.36970196	1.707195011	0.771627865	0.28010758	1
gene46819	531.0338701	355.2278124	0.668936263	-0.58005934	0.28013585	1
gene7363	157.0372534	99.69608905	0.634856296	-0.65549803	0.28018952	1
gene27667	325.4390449	215.8468724	0.663248236	-0.59237916	0.2801906	1
gene73790	325.4390449	215.8468724	0.663248236	-0.59237916	0.2801906	1
gene1291	247.953558	162.6110967	0.655812718	-0.60864422	0.28020318	1
gene64865	226.2576217	340.7089645	1.505845248	0.590573516	0.28027832	1

gene72099	2826.670561	1797.433372	0.635883572	-0.65316546	0.28032196	1
gene4580	718.0321784	480.0899045	0.668618927	-0.5807439	0.28038305	1
gene22029	235.5558801	354.2598892	1.50393142	0.588738781	0.28051032	1
gene42295	1473.257391	2288.170432	1.553136911	0.63518501	0.28052738	1
gene18867	252.0861173	165.5148663	0.656580648	-0.60695587	0.28055336	1
gene15318	77.48548688	124.8620921	1.611425534	0.688337522	0.28057972	1
gene70908	540.3321285	809.1837907	1.497567418	0.582620952	0.28060435	1
gene1363	184.9320287	119.0545529	0.643774655	-0.63537232	0.28061604	1
gene37069	161.1698127	102.5998586	0.636594762	-0.65155281	0.28064433	1
gene1769	10.33139825	23.23015667	2.248500746	1.168963362	0.2807239	1
gene20972	10.33139825	23.23015667	2.248500746	1.168963362	0.2807239	1
gene37284	10.33139825	23.23015667	2.248500746	1.168963362	0.2807239	1
gene38065	10.33139825	23.23015667	2.248500746	1.168963362	0.2807239	1
gene48367	10.33139825	23.23015667	2.248500746	1.168963362	0.2807239	1
gene70065	10.33139825	23.23015667	2.248500746	1.168963362	0.2807239	1
gene71495	10.33139825	23.23015667	2.248500746	1.168963362	0.2807239	1
gene46658	42.35873283	22.26223348	0.525564199	-0.92806109	0.2807996	1
gene62444	42.35873283	22.26223348	0.525564199	-0.92806109	0.2807996	1
gene58920	2676.865287	4342.103451	1.622085158	0.697849562	0.28084178	1
gene54580	88.85002496	141.3167864	1.590509248	0.669488761	0.28086353	1
gene33848	212.826804	138.4130168	0.650355191	-0.62070023	0.28089124	1
gene18786	679.8060049	454.9239015	0.669196651	-0.57949787	0.28089843	1
gene11182	2655.16935	4303.386523	1.620757833	0.696668546	0.2809043	1
gene22349	329.5716042	492.6729061	1.494888819	0.580038189	0.28090493	1
gene35576	506.2385143	338.7731181	0.669196651	-0.57949787	0.28094121	1
gene36479	87.81688513	52.26785251	0.595191374	-0.74857448	0.28095738	1
gene67383	261.3843757	392.0088938	1.49974111	0.58471348	0.28099714	1
gene71028	1159.182884	1777.106985	1.53306869	0.616422339	0.28115326	1
gene47608	33.0604744	59.04331487	1.785918561	0.836666294	0.28116219	1
gene65074	3243.025911	2045.22171	0.630652288	-0.6650833	0.28116628	1
gene15156	60.95524968	100.6640122	1.651441226	0.723725625	0.28116761	1
gene32454	60.95524968	100.6640122	1.651441226	0.723725625	0.28116761	1
gene26560	110.5459613	67.75462362	0.612909082	-0.70625501	0.2812467	1
gene17804	3816.418514	6428.945859	1.684549489	0.752362813	0.28134866	1
gene31588	310.9750873	206.1676405	0.662971565	-0.5929811	0.28141922	1
gene55561	169.4349313	108.4073978	0.639817285	-0.64426813	0.28146877	1
gene18183	99.18142321	60.01123807	0.605065305	-0.72483723	0.28150309	1
gene22507	326.4721847	487.8332901	1.494256825	0.579428132	0.28151294	1
gene49995	221.0919226	144.220556	0.65231038	-0.61636951	0.28153922	1
gene73849	409.1233707	610.7595358	1.492849296	0.578068532	0.28166576	1
gene3731	14.46395755	30.00561903	2.074509617	1.052770345	0.28167971	1
gene30206	14.46395755	30.00561903	2.074509617	1.052770345	0.28167971	1
gene30986	14.46395755	30.00561903	2.074509617	1.052770345	0.28167971	1
gene45688	14.46395755	30.00561903	2.074509617	1.052770345	0.28167971	1
gene55976	14.46395755	30.00561903	2.074509617	1.052770345	0.28167971	1
gene6584	26.86163545	12.58300153	0.468437655	-1.09407104	0.28172895	1
gene14916	26.86163545	12.58300153	0.468437655	-1.09407104	0.28172895	1
gene40848	225.2244819	338.7731181	1.504157609	0.588955744	0.28177431	1

gene62594	663.2757677	444.2767463	0.669822068	-0.57815019	0.28183729	1
gene45546	149.8052746	94.85647307	0.633198486	-0.65927029	0.28191481	1
gene37516	199.3959862	301.0241135	1.509679905	0.59424269	0.28197753	1
gene71698	208.6942447	314.5750383	1.507348891	0.592013382	0.28198862	1
gene5357	190.0977278	287.4731888	1.512238953	0.596686121	0.28207268	1
gene64587	400.8582521	598.1765343	1.492239541	0.577479142	0.28212497	1
gene14072	39.25931335	20.32638709	0.517746882	-0.94968113	0.28216063	1
gene31779	39.25931335	20.32638709	0.517746882	-0.94968113	0.28216063	1
gene28771	229.3570412	150.0280952	0.654124654	-0.6123625	0.28216851	1
gene54031	870.9368725	581.72184	0.667926526	-0.58223868	0.28222223	1
gene28746	303.7431086	453.9559783	1.494539186	0.579700724	0.28222335	1
gene25828	205.5948252	133.5734009	0.649692427	-0.62217121	0.28227778	1
gene15707	466.9792009	312.6391919	0.669492755	-0.57885965	0.2823489	1
gene72896	334.7373033	222.6223348	0.665065807	-0.588431	0.28238124	1
gene49337	106.413402	166.4827895	1.56449081	0.645693184	0.28240357	1
gene9893	38.22617353	66.78670043	1.74714585	0.805000048	0.28248878	1
gene16435	38.22617353	66.78670043	1.74714585	0.805000048	0.28248878	1
gene49257	38.22617353	66.78670043	1.74714585	0.805000048	0.28248878	1
gene60189	350.2344007	233.2694899	0.6660382	-0.58632317	0.28260781	1
gene18943	994.9136516	663.0273883	0.666417017	-0.58550285	0.28273717	1
gene42737	238.6552996	358.131582	1.500622792	0.585561376	0.28275773	1
gene62422	43.39187265	74.53008599	1.717604736	0.780398075	0.28278232	1
gene31666	237.6221598	155.8356343	0.655812718	-0.60864422	0.28278511	1
gene30489	95.04886391	150.0280952	1.57843123	0.658491406	0.28279526	1
gene68983	158.0703932	100.6640122	0.636830277	-0.65101917	0.28279622	1
gene42081	4973.535118	8677.43144	1.744721055	0.802996398	0.28293997	1
gene24127	29.96105493	54.2036989	1.809138531	0.855302884	0.28295901	1
gene48509	29.96105493	54.2036989	1.809138531	0.855302884	0.28295901	1
gene69394	567.193764	380.3938155	0.670659375	-0.57634788	0.28296128	1
gene9187	501.0728152	335.8693485	0.67030048	-0.57712013	0.28300171	1
gene3108	445.2832646	298.1203439	0.669507183	-0.57882856	0.28307511	1
gene17452	36.15989388	18.3905407	0.508589454	-0.97542655	0.2831646	1
gene46992	36.15989388	18.3905407	0.508589454	-0.97542655	0.2831646	1
gene57306	36.15989388	18.3905407	0.508589454	-0.97542655	0.2831646	1
gene12618	2986.807234	4884.14044	1.635237917	0.709500554	0.2831717	1
gene17917	63.02152933	35.8131582	0.568268631	-0.81535502	0.2832314	1
gene54066	63.02152933	35.8131582	0.568268631	-0.81535502	0.2832314	1
gene5832	29.96105493	14.51884792	0.484590678	-1.04516144	0.28329236	1
gene41604	29.96105493	14.51884792	0.484590678	-1.04516144	0.28329236	1
gene42937	29.96105493	14.51884792	0.484590678	-1.04516144	0.28329236	1
gene52526	29.96105493	14.51884792	0.484590678	-1.04516144	0.28329236	1
gene72041	29.96105493	14.51884792	0.484590678	-1.04516144	0.28329236	1
gene56513	1176.746261	781.1140181	0.663791375	-0.59119821	0.28331784	1
gene7612	1732.575487	2710.184945	1.564252159	0.645473095	0.28333024	1
gene21126	52.69013108	29.03769584	0.551103124	-0.85960579	0.28352317	1
gene70210	52.69013108	29.03769584	0.551103124	-0.85960579	0.28352317	1
gene35558	1239.76779	821.7667922	0.662839282	-0.59326899	0.28352548	1
gene44392	74.38606741	120.0224761	1.61350748	0.690200266	0.28353677	1

gene50712	65.08780898	106.4715514	1.635814035	0.710008747	0.2836237	1
gene54861	65.08780898	106.4715514	1.635814035	0.710008747	0.2836237	1
gene64863	142.5732959	90.0168571	0.631372492	-0.66343669	0.28374225	1
gene41605	126.0430587	194.5525621	1.543540471	0.62624331	0.28375666	1
gene19083	1331.717235	880.8101071	0.661409257	-0.59638486	0.28379223	1
gene4152	166.3355118	252.6279538	1.518785442	0.602918075	0.28387971	1
gene40731	112.6122409	175.1940982	1.555728727	0.637590519	0.28388531	1
gene13500	244.8541385	366.8428908	1.498209885	0.583239746	0.28394903	1
gene55498	230.390181	150.9960184	0.655392594	-0.60956872	0.28403528	1
gene55217	209.7273845	315.5429615	1.504538676	0.589321194	0.28403793	1
gene40283	705.6345005	473.3144422	0.670764315	-0.57612216	0.2840481	1
gene2018	439.0844257	654.3160796	1.490182847	0.575489362	0.28408936	1
gene55900	771.7554493	1161.507834	1.505020579	0.589783214	0.28409197	1
gene14197	50.62385143	85.17724113	1.682551578	0.750650731	0.28414324	1
gene23325	922.5938638	616.567075	0.668297394	-0.58143785	0.28417442	1
gene38679	70.25350811	40.65277417	0.57865828	-0.78921646	0.28420535	1
gene57669	822.3793008	1239.909612	1.507710142	0.592359096	0.284339	1
gene46517	87.81688513	139.38094	1.587176997	0.666463022	0.28434654	1
gene4904	297.5442696	197.4563317	0.663620012	-0.5915707	0.28436468	1
gene34544	469.0454806	314.5750383	0.670670652	-0.57632362	0.28439346	1
gene5124	3707.938832	2323.015667	0.626497839	-0.67461856	0.28440705	1
gene13478	344.0355618	229.3977971	0.666785131	-0.58470616	0.28449317	1
gene226	26.86163545	49.36408293	1.837716956	0.87791458	0.28449356	1
gene11820	26.86163545	49.36408293	1.837716956	0.87791458	0.28449356	1
gene43971	26.86163545	49.36408293	1.837716956	0.87791458	0.28449356	1
gene45739	26.86163545	49.36408293	1.837716956	0.87791458	0.28449356	1
gene16834	202.4954057	304.8958063	1.505692464	0.590427131	0.28450619	1
gene44197	317.1739263	211.0072564	0.665273022	-0.58798156	0.28468743	1
gene37581	35.12675405	61.94708446	1.763529997	0.818466115	0.28477971	1
gene65984	116.7448002	181.0016374	1.550404275	0.632644454	0.28479453	1
gene56796	1424.699819	940.8213452	0.660364614	-0.59866528	0.28479599	1
gene72703	386.3942946	258.435493	0.668838791	-0.58026957	0.28479821	1
gene33757	667.408327	448.1484391	0.671475648	-0.57459301	0.2848506	1
gene43034	111.5791011	68.72254682	0.615908769	-0.69921143	0.28487995	1
gene22097	17.56337703	34.84523501	1.983971246	0.988391117	0.28489473	1
gene24314	458.7140823	307.7995759	0.67100529	-0.57560395	0.2852966	1
gene51918	223.1582022	146.1564024	0.65494524	-0.61055381	0.28538243	1
gene6148	159.1035331	101.6319354	0.638778621	-0.64661207	0.2853835	1
gene68460	309.9419475	206.1676405	0.665181471	-0.58818011	0.28541726	1
gene65300	40.29245318	69.69047001	1.729615958	0.790451739	0.28542525	1
gene69673	391.5599937	582.6897632	1.488123844	0.573494595	0.2854467	1
gene36090	596.1216791	890.4893391	1.493804655	0.578991499	0.28548952	1
gene21346	2093.141286	3310.297326	1.581497316	0.661291108	0.28551787	1
gene56704	100.214563	60.97916126	0.608486027	-0.71670396	0.28554878	1
gene654	23.76221598	44.52446695	1.873750621	0.905928957	0.28557233	1
gene4775	23.76221598	44.52446695	1.873750621	0.905928957	0.28557233	1
gene22932	23.76221598	44.52446695	1.873750621	0.905928957	0.28557233	1
gene27542	23.76221598	44.52446695	1.873750621	0.905928957	0.28557233	1

gene32026	23.76221598	44.52446695	1.873750621	0.905928957	0.28557233	1
gene60009	23.76221598	44.52446695	1.873750621	0.905928957	0.28557233	1
gene31732	59.92210985	98.72816585	1.647608305	0.720373303	0.28558151	1
gene65477	293.4117103	437.501284	1.491083241	0.5763608	0.28558853	1
gene1444	13.43081773	4.839615973	0.360336658	-1.47258267	0.28559669	1
gene31391	13.43081773	4.839615973	0.360336658	-1.47258267	0.28559669	1
gene33841	13.43081773	4.839615973	0.360336658	-1.47258267	0.28559669	1
gene34780	13.43081773	4.839615973	0.360336658	-1.47258267	0.28559669	1
gene36625	13.43081773	4.839615973	0.360336658	-1.47258267	0.28559669	1
gene43989	13.43081773	4.839615973	0.360336658	-1.47258267	0.28559669	1
gene44534	13.43081773	4.839615973	0.360336658	-1.47258267	0.28559669	1
gene47182	13.43081773	4.839615973	0.360336658	-1.47258267	0.28559669	1
gene50688	13.43081773	4.839615973	0.360336658	-1.47258267	0.28559669	1
gene51869	13.43081773	4.839615973	0.360336658	-1.47258267	0.28559669	1
gene69311	13.43081773	4.839615973	0.360336658	-1.47258267	0.28559669	1
gene74089	13.43081773	4.839615973	0.360336658	-1.47258267	0.28559669	1
gene61435	10297.30464	20605.14897	2.001023539	1.000738139	0.28577152	1
gene6761	18.59651685	7.743385557	0.416389027	-1.26399604	0.28581587	1
gene22779	18.59651685	7.743385557	0.416389027	-1.26399604	0.28581587	1
gene51490	18.59651685	7.743385557	0.416389027	-1.26399604	0.28581587	1
gene70917	18.59651685	7.743385557	0.416389027	-1.26399604	0.28581587	1
gene72686	18.59651685	7.743385557	0.416389027	-1.26399604	0.28581587	1
gene30066	49.5907116	27.10184945	0.546510598	-0.87167862	0.28586175	1
gene67162	49.5907116	27.10184945	0.546510598	-0.87167862	0.28586175	1
gene3665	20.6627965	39.68485098	1.920594387	0.941552866	0.28588164	1
gene9427	20.6627965	39.68485098	1.920594387	0.941552866	0.28588164	1
gene35813	20.6627965	39.68485098	1.920594387	0.941552866	0.28588164	1
gene35953	20.6627965	39.68485098	1.920594387	0.941552866	0.28588164	1
gene51526	20.6627965	39.68485098	1.920594387	0.941552866	0.28588164	1
gene55986	20.6627965	39.68485098	1.920594387	0.941552866	0.28588164	1
gene68797	20.6627965	39.68485098	1.920594387	0.941552866	0.28588164	1
gene26886	302.7099687	451.0522087	1.490047423	0.575358247	0.28592051	1
gene14419	295.47799	440.4050536	1.490483449	0.575780355	0.28595725	1
gene4255	165.302372	250.6921074	1.516566909	0.600809149	0.28598375	1
gene61181	167.3686517	107.4394746	0.641933083	-0.63950518	0.28602555	1
gene71175	441.1507053	656.251926	1.487591243	0.57297816	0.28621231	1
gene18722	937.0578213	627.2142301	0.669344213	-0.57917978	0.28643324	1
gene6265	618.8507552	924.3666509	1.493682674	0.578873687	0.28644319	1
gene45139	360.565799	536.2294498	1.487188889	0.572587896	0.28644622	1
gene8301	337.8367228	225.5261044	0.667559472	-0.58303172	0.28646136	1
gene40122	895.7322283	600.1123807	0.669968504	-0.57783482	0.28649898	1
gene27292	267.5832147	177.1299446	0.661962092	-0.59517949	0.28655047	1
gene43194	555.8292259	828.5422546	1.490641758	0.57593358	0.28659456	1
gene48215	71.28664793	115.1828602	1.615770463	0.692222263	0.28665082	1
gene53811	71.28664793	115.1828602	1.615770463	0.692222263	0.28665082	1
gene55193	2154.096535	3409.993415	1.583027204	0.662686048	0.28671125	1
gene52144	156.0041136	237.1411827	1.520095703	0.604162156	0.28671515	1
gene53420	292.3785705	435.5654376	1.489731059	0.575051905	0.28675452	1

gene36069	2560.120487	1647.405277	0.643487401	-0.63601619	0.28681033	1
gene56090	357.4663795	239.0770291	0.668809832	-0.58033204	0.28688156	1
gene49900	67.15408863	38.71692779	0.576538653	-0.79451076	0.28689748	1
gene67160	2071.445349	1348.31701	0.650906388	-0.61947802	0.28693448	1
gene35471	32.02733458	57.10746848	1.783085269	0.834375696	0.28702891	1
gene45765	32.02733458	57.10746848	1.783085269	0.834375696	0.28702891	1
gene13353	295.47799	196.4884085	0.664984923	-0.58860646	0.28705608	1
gene3653	9.298258426	21.29431028	2.290139648	1.195435574	0.28715564	1
gene8073	9.298258426	21.29431028	2.290139648	1.195435574	0.28715564	1
gene64033	9.298258426	21.29431028	2.290139648	1.195435574	0.28715564	1
gene65726	9.298258426	21.29431028	2.290139648	1.195435574	0.28715564	1
gene45819	1517.682403	2346.245824	1.545939927	0.62848426	0.28722108	1
gene41711	384.3280149	571.0746848	1.485904391	0.57134129	0.28727687	1
gene26192	377.0960362	252.6279538	0.669930017	-0.5779177	0.28736533	1
gene17965	54.75641073	90.9847803	1.66162791	0.732597354	0.28744061	1
gene42685	54.75641073	90.9847803	1.66162791	0.732597354	0.28744061	1
gene50418	54.75641073	90.9847803	1.66162791	0.732597354	0.28744061	1
gene4428	156.0041136	99.69608905	0.639060642	-0.64597526	0.28765527	1
gene5770	453.5483832	304.8958063	0.672245382	-0.57294015	0.28768564	1
gene20174	100.214563	156.8035575	1.564678354	0.645866117	0.28770949	1
gene9450	323.3727653	215.8468724	0.667486244	-0.58318999	0.2878667	1
gene2968	642.6129712	432.661668	0.67328499	-0.57071079	0.28788594	1
gene6401	86.78374531	137.4450936	1.583765406	0.663358653	0.28792901	1
gene15562	64.05466916	104.535705	1.631976348	0.706620148	0.28793994	1
gene34795	160.1366729	102.5998586	0.640701825	-0.64227499	0.28795152	1
gene3545	37.1930337	64.85085404	1.743629051	0.802093146	0.28801528	1
gene13267	37.1930337	64.85085404	1.743629051	0.802093146	0.28801528	1
gene38761	37.1930337	64.85085404	1.743629051	0.802093146	0.28801528	1
gene72404	446.3164044	300.0561903	0.672294783	-0.57283414	0.28802326	1
gene31791	385.3611548	258.435493	0.670631925	-0.57640693	0.28805757	1
gene48184	693.2368226	1036.645741	1.49537028	0.580502765	0.28813678	1
gene5306	412.2227902	276.8260337	0.671544709	-0.57444464	0.28814118	1
gene28535	46.49129213	25.16600306	0.541305735	-0.88548442	0.28818191	1
gene30566	46.49129213	25.16600306	0.541305735	-0.88548442	0.28818191	1
gene70513	46.49129213	25.16600306	0.541305735	-0.88548442	0.28818191	1
gene59335	1012.477029	1533.19034	1.514296421	0.598647638	0.28819273	1
gene60021	2249.145399	1459.628178	0.648970128	-0.62377602	0.28832136	1
gene66272	135.3413171	207.1355637	1.530468065	0.613972942	0.28833138	1
gene21670	85.75060548	51.29992932	0.59824568	-0.74119002	0.28844789	1
gene4927	119.8442197	184.8733302	1.542613658	0.62537679	0.2884831	1
gene53752	119.8442197	184.8733302	1.542613658	0.62537679	0.2884831	1
gene49745	88.85002496	140.3488632	1.579615349	0.659573292	0.28851745	1
gene52505	88.85002496	140.3488632	1.579615349	0.659573292	0.28851745	1
gene28767	596.1216791	401.6881258	0.673835795	-0.56953103	0.2886566	1
gene39811	1540.411479	2380.123136	1.545121656	0.627720434	0.28869299	1
gene34292	172.5343508	111.3111674	0.645153657	-0.63228528	0.28871343	1
gene56979	333.7041635	495.5766757	1.485077892	0.570538602	0.28876187	1
gene4738	13.43081773	28.06977264	2.089952616	1.063470234	0.28880039	1

gene9934	13.43081773	28.06977264	2.089952616	1.063470234	0.28880039	1
gene27241	13.43081773	28.06977264	2.089952616	1.063470234	0.28880039	1
gene31447	13.43081773	28.06977264	2.089952616	1.063470234	0.28880039	1
gene48350	13.43081773	28.06977264	2.089952616	1.063470234	0.28880039	1
gene60585	13.43081773	28.06977264	2.089952616	1.063470234	0.28880039	1
gene64918	13.43081773	28.06977264	2.089952616	1.063470234	0.28880039	1
gene66345	13.43081773	28.06977264	2.089952616	1.063470234	0.28880039	1
gene74238	13.43081773	28.06977264	2.089952616	1.063470234	0.28880039	1
gene10653	969.0851559	649.4764636	0.670195451	-0.5773462	0.28882381	1
gene42841	121.9104994	187.7770998	1.540286528	0.623198749	0.28886092	1
gene22874	106.413402	165.5148663	1.555394933	0.637280944	0.28907338	1
gene21399	28.9279151	52.26785251	1.806830956	0.853461537	0.28915407	1
gene30501	28.9279151	52.26785251	1.806830956	0.853461537	0.28915407	1
gene73682	116.7448002	72.5942396	0.621819896	-0.68543132	0.28919437	1
gene38130	123.976779	190.6808693	1.538036968	0.62109018	0.28922877	1
gene48235	534.1332896	360.0674284	0.674115311	-0.5689327	0.28940077	1
gene60716	197.3297066	296.1844976	1.50096254	0.585887971	0.28940592	1
gene35395	208.6942447	312.6391919	1.498072898	0.583107829	0.28944312	1
gene6371	1610.664987	2493.370149	1.548037717	0.630440622	0.28947303	1
gene2302	172.5343508	260.3713394	1.509098554	0.593687027	0.2894897	1
gene39124	233.4896005	153.8997879	0.65912909	-0.60136705	0.2895823	1
gene42975	233.4896005	153.8997879	0.65912909	-0.60136705	0.2895823	1
gene73539	456.6478027	677.5462362	1.48373918	0.569237509	0.28959162	1
gene63831	2133.433739	1389.937708	0.651502637	-0.61815708	0.28965829	1
gene49310	64.05466916	36.7810814	0.5742139	-0.80033984	0.28968435	1
gene36058	148.7721348	94.85647307	0.637595698	-0.6492862	0.28972319	1
gene45233	313.041367	209.07141	0.667871509	-0.58235752	0.28976915	1
gene28899	231.4233208	345.5485805	1.493145026	0.578354299	0.28978057	1
gene43016	231.4233208	345.5485805	1.493145026	0.578354299	0.28978057	1
gene35069	120.8773595	75.49800918	0.62458354	-0.67903354	0.28982054	1
gene45966	277.9146129	413.3032041	1.48715895	0.572558854	0.28984438	1
gene62703	68.18722846	110.3432442	1.618239173	0.694424851	0.28993142	1
gene25524	994.9136516	1503.184721	1.51086953	0.595379083	0.28994445	1
gene39737	421.5210486	283.601496	0.672805064	-0.57173953	0.28995229	1
gene23043	71.28664793	41.62069737	0.583849831	-0.77633075	0.28998022	1
gene64661	71.28664793	41.62069737	0.583849831	-0.77633075	0.28998022	1
gene19980	176.6669101	266.1788785	1.506670821	0.59136425	0.29015725	1
gene43587	1175.713121	784.9857109	0.66766773	-0.58279778	0.29023453	1
gene32627	205.5948252	134.5413241	0.654400343	-0.61175459	0.29024039	1
gene53027	44.42501248	75.49800918	1.699448238	0.765066421	0.29034287	1
gene44123	4234.840143	2640.494475	0.623516918	-0.68149939	0.29036381	1
gene17752	686.0048439	462.667287	0.674437347	-0.56824367	0.29042388	1
gene31183	81.61804618	129.7017081	1.589130274	0.668237399	0.29042678	1
gene7508	43.39187265	23.23015667	0.53535732	-0.90142597	0.29044179	1
gene14455	43.39187265	23.23015667	0.53535732	-0.90142597	0.29044179	1
gene35789	43.39187265	23.23015667	0.53535732	-0.90142597	0.29044179	1
gene54909	43.39187265	23.23015667	0.53535732	-0.90142597	0.29044179	1
gene23134	203.5285455	304.8958063	1.498049355	0.583085156	0.29046275	1

gene72076	387.4274344	260.3713394	0.67205189	-0.57335547	0.29046385	1
gene9128	2206.786666	1436.398021	0.650900263	-0.6194916	0.29056956	1
gene48113	213.8599438	140.3488632	0.656265314	-0.60764891	0.29057592	1
gene1039	254.152397	168.4186359	0.662667903	-0.59364205	0.29059303	1
gene15460	1126.122409	753.0442454	0.668705497	-0.58055712	0.29062192	1
gene66136	34.09361423	60.01123807	1.760189978	0.815731148	0.29062893	1
gene15795	2204.720387	3482.587654	1.57960514	0.659563967	0.29064163	1
gene21516	149.8052746	227.4619507	1.518384124	0.602536813	0.29067783	1
gene59241	256.2186766	381.3617387	1.488422873	0.573784466	0.29070142	1
gene1478	165.302372	106.4715514	0.644101776	-0.63463942	0.29071474	1
gene32135	129.1424781	81.30554835	0.629580209	-0.66753791	0.29083705	1
gene48951	169.4349313	109.375321	0.64552994	-0.63144409	0.29091218	1
gene64402	7089.40548	4203.690434	0.592953873	-0.75400822	0.29092796	1
gene64250	167.3686517	252.6279538	1.509410223	0.59398495	0.29095196	1
gene40697	762.4571909	513.9672164	0.674093211	-0.56898	0.29101058	1
gene35231	25.82849563	47.42823654	1.836275609	0.876782611	0.29102436	1
gene53428	1595.16789	2464.332454	1.544873407	0.627488623	0.29103415	1
gene20494	563.0612047	836.2856402	1.485248199	0.570704039	0.29117869	1
gene5272	898.8316478	1351.22078	1.50330797	0.588140592	0.29122168	1
gene43574	53.7232709	30.00561903	0.55852182	-0.84031445	0.29123112	1
gene49748	759.3577714	512.03137	0.674295292	-0.56854757	0.29132041	1
gene44287	60.95524968	99.69608905	1.635561983	0.709786434	0.2913331	1
gene28841	796.5508051	1192.481376	1.497056268	0.582128447	0.29136562	1
gene71112	489.7082771	725.942396	1.482397644	0.567932493	0.29137063	1
gene9100	82.65118601	49.36408293	0.597258011	-0.7435738	0.29146527	1
gene59886	82.65118601	49.36408293	0.597258011	-0.7435738	0.29146527	1
gene47299	558.9286454	377.4900459	0.675381462	-0.56622551	0.29151451	1
gene5404	787.2525467	1177.962528	1.496295608	0.581395222	0.29154032	1
gene1552	328.5384644	486.8653669	1.481912834	0.567460591	0.29154284	1
gene29116	288.2460112	427.822052	1.484225403	0.569710204	0.29155144	1
gene10464	1144.718926	1737.422134	1.517771826	0.601954919	0.29158337	1
gene58867	2620.042596	1690.961821	0.645394782	-0.63174618	0.29160814	1
gene69564	137.4075967	87.11308752	0.633975774	-0.65750038	0.2916093	1
gene8169	1361.678289	905.9761102	0.665337853	-0.58784098	0.29173389	1
gene16920	922.5938638	620.4387678	0.672493924	-0.57240686	0.29175311	1
gene26821	322.3396254	215.8468724	0.669625623	-0.57857336	0.29177599	1
gene23687	1485.655068	985.3458121	0.663239963	-0.59239716	0.29179977	1
gene12562	341.9692821	229.3977971	0.670814044	-0.5760152	0.29183809	1
gene51261	302.7099687	202.2959477	0.668283071	-0.58146877	0.29183855	1
gene54976	211.7936641	316.5108846	1.494430374	0.579595683	0.29184916	1
gene52256	194.2302871	126.7979385	0.65282269	-0.61523689	0.29187145	1
gene67666	140.5070162	213.911026	1.52242238	0.606368675	0.29189287	1
gene20868	158.0703932	239.0770291	1.512471907	0.596908346	0.29192579	1
gene28232	361.5989388	242.9487219	0.671873437	-0.5737386	0.29200289	1
gene41302	408.0902309	274.8901873	0.673601489	-0.57003277	0.29204744	1
gene66659	122.9436392	188.745023	1.535215845	0.618441507	0.29205794	1
gene48497	283.0803121	188.745023	0.666754327	-0.58477281	0.29212036	1
gene58302	450.4489637	666.8990811	1.480520847	0.566104805	0.29213581	1

gene39243	863.7048938	581.72184	0.673519213	-0.57020899	0.29214587	1
gene7279	16.5302372	32.90938862	1.990860035	0.993391798	0.29217528	1
gene20915	16.5302372	32.90938862	1.990860035	0.993391798	0.29217528	1
gene43846	16.5302372	32.90938862	1.990860035	0.993391798	0.29217528	1
gene69953	225.2244819	335.8693485	1.491264829	0.576536484	0.29222859	1
gene53381	1383.374226	2117.81595	1.530906034	0.614385734	0.29226808	1
gene47394	53.7232709	89.04893391	1.657548627	0.729051195	0.29229175	1
gene34753	125.0099188	191.6487925	1.53306869	0.616422339	0.29238316	1
gene4180	22.72907615	42.58862056	1.873750621	0.905928957	0.29242824	1
gene14604	22.72907615	42.58862056	1.873750621	0.905928957	0.29242824	1
gene55953	488.6751373	330.0618094	0.675421736	-0.56613949	0.29246735	1
gene2530	98.14828338	60.01123807	0.611434413	-0.70973034	0.29252837	1
gene52393	227.2907615	338.7731181	1.490483449	0.575780355	0.29256369	1
gene21092	40.29245318	21.29431028	0.528493765	-0.92004164	0.29258061	1
gene46878	40.29245318	21.29431028	0.528493765	-0.92004164	0.29258061	1
gene65764	60.95524968	34.84523501	0.571652732	-0.80678909	0.29258743	1
gene5014	327.5053246	484.9295205	1.480676753	0.56625672	0.29260672	1
gene32832	89.88316478	141.3167864	1.572227533	0.65281002	0.29266446	1
gene28321	193.1971473	289.4090352	1.497998491	0.583036171	0.29276338	1
gene45941	934.9915417	629.1500765	0.67289387	-0.57154912	0.29280406	1
gene5426	21.69593633	9.679231946	0.4461311	-1.16446037	0.29281086	1
gene19550	21.69593633	9.679231946	0.4461311	-1.16446037	0.29281086	1
gene30904	21.69593633	9.679231946	0.4461311	-1.16446037	0.29281086	1
gene32486	21.69593633	9.679231946	0.4461311	-1.16446037	0.29281086	1
gene68009	21.69593633	9.679231946	0.4461311	-1.16446037	0.29281086	1
gene74082	21.69593633	9.679231946	0.4461311	-1.16446037	0.29281086	1
gene47197	2260.509937	3569.700742	1.579157288	0.659154874	0.29285939	1
gene1446	296.5111298	439.4371304	1.482025753	0.567570517	0.29287746	1
gene58466	420.4879088	283.601496	0.674458148	-0.56819917	0.29297795	1
gene17895	19.62965668	37.74900459	1.923059848	0.943403662	0.29301425	1
gene72540	19.62965668	37.74900459	1.923059848	0.943403662	0.29301425	1
gene24682	162.2029525	104.535705	0.644474736	-0.63380429	0.29303029	1
gene4727	998.013071	670.7707739	0.672106201	-0.57323888	0.29308134	1
gene16593	181.8326092	272.9543409	1.501129759	0.58604869	0.29314025	1
gene73607	2038.384875	3193.178619	1.566523898	0.647566778	0.29316798	1
gene48630	1488.754488	2286.234586	1.535669316	0.618867586	0.29320161	1
gene60514	30.99419475	55.17162209	1.78006309	0.831928375	0.29322414	1
gene45848	649.84495	439.4371304	0.676218428	-0.56443876	0.2932754	1
gene28965	111.5791011	172.2903286	1.544109308	0.626774885	0.29336388	1
gene32746	243.8209987	161.6431735	0.662958377	-0.5930098	0.29352958	1
gene46333	3301.914881	5394.235964	1.633668995	0.708115702	0.29356421	1
gene18054	126.0430587	79.36970196	0.629703078	-0.66725638	0.29357005	1
gene29195	1373.042828	914.6874189	0.666175447	-0.58602591	0.29359155	1
gene37977	152.9046941	231.3336435	1.512927022	0.597342399	0.29367196	1
gene16677	182.865749	119.0545529	0.651048945	-0.61916209	0.29369769	1
gene20816	8.265118601	19.35846389	2.342188277	1.227857052	0.293708	1
gene21461	8.265118601	19.35846389	2.342188277	1.227857052	0.293708	1
gene43100	8.265118601	19.35846389	2.342188277	1.227857052	0.293708	1

gene60166	8.265118601	19.35846389	2.342188277	1.227857052	0.293708	1
gene66433	8.265118601	19.35846389	2.342188277	1.227857052	0.293708	1
gene71960	8.265118601	19.35846389	2.342188277	1.227857052	0.293708	1
gene10777	78.51862671	124.8620921	1.590222567	0.669228699	0.29372154	1
gene20974	78.51862671	124.8620921	1.590222567	0.669228699	0.29372154	1
gene66885	1126.122409	754.9800918	0.670424534	-0.57685315	0.29373213	1
gene52017	1300.72304	868.2271056	0.667495754	-0.58316944	0.29373944	1
gene16451	350.2344007	517.8389091	1.478549532	0.564182575	0.29377136	1
gene18824	1423.666679	2179.763034	1.531090856	0.614559896	0.29379714	1
gene6966	6522.211716	3916.217246	0.600443134	-0.73590047	0.29388415	1
gene56527	866.8043132	584.6256096	0.674460891	-0.5681933	0.29393567	1
gene12635	130.175618	82.27347154	0.632019059	-0.66196003	0.29394405	1
gene3042	154.9709738	234.2374131	1.511492168	0.595973503	0.29395687	1
gene32255	195.2634269	127.7658617	0.654325614	-0.61191935	0.29401244	1
gene12846	96.08200373	150.0280952	1.561458851	0.642894551	0.29402847	1
gene65602	188.0314482	281.6656496	1.497970964	0.583009659	0.29405793	1
gene20887	1154.017185	1748.06929	1.514768855	0.599097664	0.29421685	1
gene39846	48.55757178	81.30554835	1.674415449	0.743657528	0.29423218	1
gene58999	452.5152434	305.8637295	0.675919174	-0.56507735	0.29435078	1
gene12384	1055.868901	709.4877017	0.671946774	-0.57358114	0.29443423	1
gene62834	98.14828338	152.9318648	1.558171569	0.639854096	0.29443635	1
gene56489	1144.718926	767.5630933	0.67052538	-0.57663615	0.29447031	1
gene17553	90.91630461	55.17162209	0.60683969	-0.72061265	0.29449145	1
gene12193	215.9262234	142.2847096	0.658950577	-0.60175783	0.29450883	1
gene61112	100.214563	155.8356343	1.555019846	0.636932993	0.29482412	1
gene57479	57.8558302	94.85647307	1.639531794	0.713283879	0.29489858	1
gene59533	57.8558302	94.85647307	1.639531794	0.713283879	0.29489858	1
gene21998	677.7397253	458.7955943	0.676949539	-0.5628798	0.29490181	1
gene47919	1067.233439	717.2310872	0.672047053	-0.57336585	0.29494597	1
gene17573	414.2890699	611.727459	1.476571562	0.562251278	0.29502489	1
gene26203	236.5890199	156.8035575	0.662767687	-0.59342483	0.29505006	1
gene44274	970.1182958	1457.692331	1.502592351	0.587453664	0.29507759	1
gene48650	656.0437889	444.2767463	0.677205933	-0.56233348	0.2951367	1
gene73164	401.891392	593.3369183	1.476361351	0.562045875	0.2951557	1
gene71019	356.4332397	240.0449523	0.673463991	-0.57032728	0.2952825	1
gene57332	154.9709738	99.69608905	0.643321047	-0.63638921	0.29529778	1
gene61974	336.803583	226.4940275	0.672481051	-0.57243448	0.29532591	1
gene40628	163.2360924	105.5036282	0.646325373	-0.62966747	0.29554133	1
gene7140	57.8558302	32.90938862	0.568817153	-0.81396312	0.2956084	1
gene18758	167.3686517	108.4073978	0.647716264	-0.62656612	0.29564134	1
gene5551	72.31978776	42.58862056	0.588893052	-0.76392244	0.29564144	1
gene73562	72.31978776	42.58862056	0.588893052	-0.76392244	0.29564144	1
gene671	27.89477528	50.33200612	1.80435245	0.851481173	0.2957154	1
gene31533	171.501211	111.3111674	0.649040125	-0.62362042	0.29573035	1
gene10846	1965.031947	1292.177465	0.657585983	-0.60474855	0.29576495	1
gene31663	225.2244819	334.9014253	1.486967236	0.57237286	0.29579094	1
gene32632	43.39187265	73.56216279	1.695298181	0.761539047	0.29579172	1
gene57293	564.0943445	382.3296619	0.677776095	-0.56111934	0.2958561	1

gene39923	71.28664793	114.214937	1.60219256	0.680047549	0.29586642	1
gene54871	183.8988889	120.0224761	0.652654711	-0.61560816	0.29595235	1
gene5391	65.08780898	37.74900459	0.57997043	-0.78594875	0.29600616	1
gene55481	196.2965668	128.7337849	0.655812718	-0.60864422	0.29614058	1
gene19818	24.7953558	11.61507834	0.468437655	-1.09407104	0.29615902	1
gene63413	24.7953558	11.61507834	0.468437655	-1.09407104	0.29615902	1
gene44173	200.4291261	131.6375545	0.656778568	-0.60652104	0.29620157	1
gene64196	200.4291261	131.6375545	0.656778568	-0.60652104	0.29620157	1
gene59627	215.9262234	321.3505006	1.48824212	0.573609256	0.29623518	1
gene1129	12.3976779	26.13392626	2.107969449	1.075853958	0.2963791	1
gene3366	12.3976779	26.13392626	2.107969449	1.075853958	0.2963791	1
gene16454	12.3976779	26.13392626	2.107969449	1.075853958	0.2963791	1
gene34245	12.3976779	26.13392626	2.107969449	1.075853958	0.2963791	1
gene39872	12.3976779	26.13392626	2.107969449	1.075853958	0.2963791	1
gene53234	12.3976779	26.13392626	2.107969449	1.075853958	0.2963791	1
gene60992	12.3976779	26.13392626	2.107969449	1.075853958	0.2963791	1
gene36018	122.9436392	77.43385557	0.629832142	-0.66696071	0.29641033	1
gene54971	733.5292758	1090.84944	1.487124613	0.572525542	0.29642089	1
gene28037	689.1042633	1023.094817	1.484673468	0.570145667	0.29648988	1
gene56773	760.3909113	514.9351395	0.677197915	-0.56235056	0.29664068	1
gene6001	38.22617353	65.81877724	1.721824895	0.783938432	0.29668266	1
gene41289	38.22617353	65.81877724	1.721824895	0.783938432	0.29668266	1
gene54196	38.22617353	65.81877724	1.721824895	0.783938432	0.29668266	1
gene66077	38.22617353	65.81877724	1.721824895	0.783938432	0.29668266	1
gene43489	229.3570412	151.9639416	0.662564972	-0.59386616	0.29668429	1
gene48736	924.6601434	624.3104605	0.675178297	-0.56665957	0.29670065	1
gene36473	33.0604744	58.07539168	1.756641208	0.812819552	0.29679728	1
gene62021	290.3122908	194.5525621	0.670149244	-0.57744567	0.2969608	1
gene27366	61.9883895	100.6640122	1.623917205	0.699478079	0.29703313	1
gene30289	61.9883895	100.6640122	1.623917205	0.699478079	0.29703313	1
gene33565	61.9883895	100.6640122	1.623917205	0.699478079	0.29703313	1
gene47577	30.99419475	15.48677111	0.499666832	-1.00096164	0.29713841	1
gene57756	30.99419475	15.48677111	0.499666832	-1.00096164	0.29713841	1
gene68072	30.99419475	15.48677111	0.499666832	-1.00096164	0.29713841	1
gene3744	27.89477528	13.55092472	0.485787198	-1.04160362	0.29732502	1
gene23768	27.89477528	13.55092472	0.485787198	-1.04160362	0.29732502	1
gene34528	27.89477528	13.55092472	0.485787198	-1.04160362	0.29732502	1
gene37229	27.89477528	13.55092472	0.485787198	-1.04160362	0.29732502	1
gene48836	27.89477528	13.55092472	0.485787198	-1.04160362	0.29732502	1
gene67832	27.89477528	13.55092472	0.485787198	-1.04160362	0.29732502	1
gene52275	52.69013108	87.11308752	1.653309372	0.725356711	0.29733223	1
gene4491	351.2675405	517.8389091	1.474200857	0.559933102	0.29740989	1
gene2163	290.3122908	428.7899752	1.476995597	0.562665525	0.29747892	1
gene38989	587.8565605	869.1950288	1.478583531	0.564215749	0.29748734	1
gene66103	832.710699	1241.845459	1.491328813	0.576598383	0.29751201	1
gene73044	327.5053246	482.9936741	1.474765868	0.560485932	0.29757556	1
gene47933	579.5914419	856.6120273	1.477958378	0.563605641	0.29771122	1
gene13758	87.81688513	53.23577571	0.606213436	-0.72210227	0.29771265	1

gene46811	144.6395755	218.750642	1.51238443	0.596824902	0.29783247	1
gene43042	76.45234706	45.49239015	0.595042427	-0.74893556	0.29783382	1
gene72795	369.8640574	544.9407586	1.47335419	0.559104291	0.2978348	1
gene24563	251.0529775	371.6825067	1.480494318	0.566078954	0.29786149	1
gene59279	156.0041136	100.6640122	0.645265115	-0.63203606	0.29791112	1
gene64546	156.0041136	100.6640122	0.645265115	-0.63203606	0.29791112	1
gene67472	1054.835761	1586.426116	1.503955567	0.588761945	0.29792884	1
gene12553	448.3826841	303.9278831	0.677831446	-0.56100153	0.29796956	1
gene47100	164.2692322	106.4715514	0.648152731	-0.62559429	0.29803367	1
gene26530	176.6669101	115.1828602	0.651977555	-0.61710579	0.29814406	1
gene33190	296.5111298	437.501284	1.475497005	0.561200992	0.29837121	1
gene69724	544.4646878	369.7466604	0.679101269	-0.55830137	0.2983921	1
gene28492	521.7356117	354.2598892	0.6790027	-0.55851078	0.29843812	1
gene18434	150.8384145	227.4619507	1.507984233	0.592621344	0.29853914	1
gene19030	410.1565106	277.7939569	0.677287693	-0.56215931	0.29861176	1
gene42789	351.2675405	237.1411827	0.675101327	-0.56682404	0.29867837	1
gene53670	327.5053246	220.6864884	0.673840917	-0.56952006	0.29873883	1
gene20343	54.75641073	30.97354223	0.565660565	-0.8219915	0.29874723	1
gene52991	460.780362	312.6391919	0.678499384	-0.55958059	0.29878646	1
gene13467	2535.325131	4016.881258	1.584365338	0.663905044	0.2988404	1
gene35934	174.6006304	261.3392626	1.496783041	0.581865117	0.29884439	1
gene8235	81.61804618	128.7337849	1.577271093	0.657430644	0.29884475	1
gene34047	642.6129712	436.5333608	0.679309912	-0.55785819	0.2988767	1
gene12377	279.9808926	413.3032041	1.476183608	0.561872175	0.29887832	1
gene37524	720.0984581	488.8012133	0.67879775	-0.55894631	0.29888227	1
gene51109	303.7431086	204.2317941	0.672383301	-0.5726442	0.29899823	1
gene20514	154.9709738	233.2694899	1.505246333	0.589999602	0.29900038	1
gene32781	921.560724	1377.354706	1.494589201	0.579749003	0.29903286	1
gene47021	564.0943445	832.4139474	1.475664409	0.561364665	0.29908265	1
gene51076	359.5326591	242.9487219	0.675734779	-0.56547099	0.29912036	1
gene7737	455.6146629	670.7707739	1.472232631	0.558005653	0.29913743	1
gene27280	646.7455305	439.4371304	0.679459091	-0.5575414	0.29918587	1
gene9725	2960.978739	4755.406655	1.606025262	0.683494586	0.29920443	1
gene24715	61.9883895	35.8131582	0.577739775	-0.79150827	0.29921068	1
gene27767	61.9883895	35.8131582	0.577739775	-0.79150827	0.29921068	1
gene67525	61.9883895	35.8131582	0.577739775	-0.79150827	0.29921068	1
gene68544	61.9883895	35.8131582	0.577739775	-0.79150827	0.29921068	1
gene61985	267.5832147	179.065791	0.669196651	-0.57949787	0.29924774	1
gene13338	1117.857291	753.0442454	0.673649715	-0.56992948	0.29935929	1
gene62865	372.9634769	548.8124514	1.471491139	0.557278855	0.29938288	1
gene68440	159.1035331	239.0770291	1.502650661	0.587509648	0.29945594	1
gene28934	21.69593633	40.65277417	1.873750621	0.905928957	0.29973582	1
gene30449	21.69593633	40.65277417	1.873750621	0.905928957	0.29973582	1
gene64888	21.69593633	40.65277417	1.873750621	0.905928957	0.29973582	1
gene53182	109.5128215	168.4186359	1.537889661	0.620951998	0.29974744	1
gene43145	29.96105493	53.23577571	1.776832486	0.829307675	0.29977645	1
gene60712	29.96105493	53.23577571	1.776832486	0.829307675	0.29977645	1
gene64437	44.42501248	24.19807987	0.544694948	-0.87647961	0.29977856	1

gene41265	137.4075967	208.1034868	1.514497683	0.598839371	0.29978768	1
gene5930	1600.333589	2452.717375	1.532628817	0.616008337	0.29979263	1
gene10238	96.08200373	59.04331487	0.614509612	-0.70249252	0.29981586	1
gene16389	96.08200373	59.04331487	0.614509612	-0.70249252	0.29981586	1
gene53085	56.82269038	92.92062669	1.63527327	0.709531744	0.29981938	1
gene27453	565.1274843	384.2655083	0.67996252	-0.55647287	0.29986199	1
gene36603	128.1093383	81.30554835	0.634657469	-0.65594993	0.2998853	1
gene17370	35.12675405	60.97916126	1.73597484	0.795746039	0.29994926	1
gene3855	15.49709738	30.97354223	1.99866733	0.999038361	0.29996422	1
gene43155	15.49709738	30.97354223	1.99866733	0.999038361	0.29996422	1
gene51737	15.49709738	30.97354223	1.99866733	0.999038361	0.29996422	1
gene63185	15.49709738	30.97354223	1.99866733	0.999038361	0.29996422	1
gene63803	15.49709738	30.97354223	1.99866733	0.999038361	0.29996422	1
gene24599	667.408327	987.2816585	1.479276806	0.564892039	0.29998452	1
gene49144	139.4738764	211.0072564	1.512880131	0.597297684	0.30000322	1
gene14388	11.36453808	3.871692779	0.340681931	-1.55350266	0.30003579	1
gene16878	11.36453808	3.871692779	0.340681931	-1.55350266	0.30003579	1
gene17718	11.36453808	3.871692779	0.340681931	-1.55350266	0.30003579	1
gene24537	11.36453808	3.871692779	0.340681931	-1.55350266	0.30003579	1
gene26843	11.36453808	3.871692779	0.340681931	-1.55350266	0.30003579	1
gene44885	11.36453808	3.871692779	0.340681931	-1.55350266	0.30003579	1
gene46420	11.36453808	3.871692779	0.340681931	-1.55350266	0.30003579	1
gene48176	11.36453808	3.871692779	0.340681931	-1.55350266	0.30003579	1
gene49100	11.36453808	3.871692779	0.340681931	-1.55350266	0.30003579	1
gene52506	11.36453808	3.871692779	0.340681931	-1.55350266	0.30003579	1
gene56077	11.36453808	3.871692779	0.340681931	-1.55350266	0.30003579	1
gene59001	11.36453808	3.871692779	0.340681931	-1.55350266	0.30003579	1
gene63958	11.36453808	3.871692779	0.340681931	-1.55350266	0.30003579	1
gene71633	11.36453808	3.871692779	0.340681931	-1.55350266	0.30003579	1
gene18702	956.687478	1430.590482	1.495358217	0.580491128	0.30005732	1
gene26851	132.2418976	84.20931793	0.636782438	-0.65112755	0.30006794	1
gene22101	184.9320287	275.8581105	1.491672981	0.576931288	0.30007221	1
gene13431	465.9460611	316.5108846	0.679286533	-0.55790784	0.30007516	1
gene5515	7.231978776	17.4226175	2.409107942	1.268499036	0.30024339	1
gene8457	7.231978776	17.4226175	2.409107942	1.268499036	0.30024339	1
gene27166	7.231978776	17.4226175	2.409107942	1.268499036	0.30024339	1
gene28700	7.231978776	17.4226175	2.409107942	1.268499036	0.30024339	1
gene30538	7.231978776	17.4226175	2.409107942	1.268499036	0.30024339	1
gene39431	7.231978776	17.4226175	2.409107942	1.268499036	0.30024339	1
gene42513	7.231978776	17.4226175	2.409107942	1.268499036	0.30024339	1
gene48587	7.231978776	17.4226175	2.409107942	1.268499036	0.30024339	1
gene49998	7.231978776	17.4226175	2.409107942	1.268499036	0.30024339	1
gene60217	7.231978776	17.4226175	2.409107942	1.268499036	0.30024339	1
gene63587	7.231978776	17.4226175	2.409107942	1.268499036	0.30024339	1
gene64564	7.231978776	17.4226175	2.409107942	1.268499036	0.30024339	1
gene67047	7.231978776	17.4226175	2.409107942	1.268499036	0.30024339	1
gene69106	7.231978776	17.4226175	2.409107942	1.268499036	0.30024339	1
gene57196	70.25350811	112.2790906	1.598199059	0.676447111	0.30024821	1

gene21140	488.6751373	719.1669336	1.471666714	0.557450984	0.30027002	1
gene39222	214.8930836	142.2847096	0.662118609	-0.59483842	0.30032592	1
gene59785	902.9642071	611.727459	0.6774659	-0.56177976	0.30033824	1
gene66918	485.5757178	330.0618094	0.679732938	-0.55696006	0.30039845	1
gene40165	148.7721348	95.82439627	0.644101776	-0.63463942	0.30044934	1
gene73666	152.9046941	98.72816585	0.645684336	-0.63109907	0.30048445	1
gene672	169.4349313	110.3432442	0.651242594	-0.61873303	0.30049693	1
gene6861	831.6775592	564.2992225	0.678507213	-0.55956394	0.30053143	1
gene11337	89.88316478	140.3488632	1.561458851	0.642894551	0.3005327	1
gene20953	89.88316478	140.3488632	1.561458851	0.642894551	0.3005327	1
gene73775	89.88316478	140.3488632	1.561458851	0.642894551	0.3005327	1
gene44289	189.064588	281.6656496	1.48978533	0.575104461	0.30056349	1
gene72497	100.214563	61.94708446	0.618144535	-0.69398389	0.30058835	1
gene5877	308.9088077	208.1034868	0.673672882	-0.56987987	0.30063296	1
gene4296	18.59651685	35.8131582	1.92579925	0.945457321	0.30063808	1
gene4588	18.59651685	35.8131582	1.92579925	0.945457321	0.30063808	1
gene15962	18.59651685	35.8131582	1.92579925	0.945457321	0.30063808	1
gene13125	965.9857365	1444.141406	1.494992474	0.580138222	0.30077393	1
gene48329	946.3560798	640.7651549	0.677086742	-0.56258742	0.30077591	1
gene13836	248.9866978	367.810814	1.47723078	0.562895229	0.30085597	1
gene60112	6676.14955	11932.55714	1.787341199	0.837815068	0.30085805	1
gene36807	58.88897003	95.82439627	1.627204487	0.702395563	0.3008758	1
gene43343	58.88897003	95.82439627	1.627204487	0.702395563	0.3008758	1
gene2853	91.94944443	143.2526328	1.557949955	0.639648891	0.30088603	1
gene33379	91.94944443	143.2526328	1.557949955	0.639648891	0.30088603	1
gene125	119.8442197	182.9374838	1.526460636	0.610190386	0.30094086	1
gene31576	3745.131866	2381.091059	0.635782969	-0.65339372	0.30096383	1
gene41331	879.2019911	596.2406879	0.678161212	-0.56029982	0.3010221	1
gene24560	84.71746566	51.29992932	0.605541359	-0.72370259	0.30106428	1
gene58528	84.71746566	51.29992932	0.605541359	-0.72370259	0.30106428	1
gene13412	664.3089075	452.0201319	0.680436656	-0.55546723	0.30115741	1
gene15818	73.35292758	43.55654376	0.593794211	-0.75196507	0.30119171	1
gene63855	73.35292758	43.55654376	0.593794211	-0.75196507	0.30119171	1
gene1028	16.5302372	6.775462362	0.409882948	-1.28671612	0.30144865	1
gene14472	16.5302372	6.775462362	0.409882948	-1.28671612	0.30144865	1
gene56900	16.5302372	6.775462362	0.409882948	-1.28671612	0.30144865	1
gene62759	16.5302372	6.775462362	0.409882948	-1.28671612	0.30144865	1
gene66217	16.5302372	6.775462362	0.409882948	-1.28671612	0.30144865	1
gene72319	16.5302372	6.775462362	0.409882948	-1.28671612	0.30144865	1
gene73952	16.5302372	6.775462362	0.409882948	-1.28671612	0.30144865	1
gene15869	42.35873283	71.6263164	1.690945683	0.757830318	0.30148082	1
gene52657	42.35873283	71.6263164	1.690945683	0.757830318	0.30148082	1
gene14438	404.9908114	274.8901873	0.678756603	-0.55903377	0.30156105	1
gene34621	544.4646878	370.7145835	0.680879021	-0.55452961	0.30166062	1
gene52265	333.7041635	225.5261044	0.675826463	-0.56527525	0.30166403	1
gene9536	382.2617353	561.3954529	1.468615352	0.554456586	0.30176151	1
gene44650	600.2542384	884.6817999	1.473845153	0.559584958	0.30186436	1
gene22205	3853.611548	2446.909836	0.634965358	-0.65525021	0.3018758	1

gene1923	1.033139825	5.807539168	5.621251864	2.490891457	0.30189074	1
gene4159	1.033139825	5.807539168	5.621251864	2.490891457	0.30189074	1
gene5449	1.033139825	5.807539168	5.621251864	2.490891457	0.30189074	1
gene5570	1.033139825	5.807539168	5.621251864	2.490891457	0.30189074	1
gene6063	1.033139825	5.807539168	5.621251864	2.490891457	0.30189074	1
gene6147	1.033139825	5.807539168	5.621251864	2.490891457	0.30189074	1
gene7398	1.033139825	5.807539168	5.621251864	2.490891457	0.30189074	1
gene7473	1.033139825	5.807539168	5.621251864	2.490891457	0.30189074	1
gene7926	1.033139825	5.807539168	5.621251864	2.490891457	0.30189074	1
gene8716	1.033139825	5.807539168	5.621251864	2.490891457	0.30189074	1
gene8886	1.033139825	5.807539168	5.621251864	2.490891457	0.30189074	1
gene9178	1.033139825	5.807539168	5.621251864	2.490891457	0.30189074	1
gene9827	1.033139825	5.807539168	5.621251864	2.490891457	0.30189074	1
gene11296	1.033139825	5.807539168	5.621251864	2.490891457	0.30189074	1
gene11365	1.033139825	5.807539168	5.621251864	2.490891457	0.30189074	1
gene12147	1.033139825	5.807539168	5.621251864	2.490891457	0.30189074	1
gene12171	1.033139825	5.807539168	5.621251864	2.490891457	0.30189074	1
gene12455	1.033139825	5.807539168	5.621251864	2.490891457	0.30189074	1
gene14678	1.033139825	5.807539168	5.621251864	2.490891457	0.30189074	1
gene15222	1.033139825	5.807539168	5.621251864	2.490891457	0.30189074	1
gene16502	1.033139825	5.807539168	5.621251864	2.490891457	0.30189074	1
gene17018	1.033139825	5.807539168	5.621251864	2.490891457	0.30189074	1
gene17347	1.033139825	5.807539168	5.621251864	2.490891457	0.30189074	1
gene17401	1.033139825	5.807539168	5.621251864	2.490891457	0.30189074	1
gene20771	1.033139825	5.807539168	5.621251864	2.490891457	0.30189074	1
gene21236	1.033139825	5.807539168	5.621251864	2.490891457	0.30189074	1
gene23600	1.033139825	5.807539168	5.621251864	2.490891457	0.30189074	1
gene23750	1.033139825	5.807539168	5.621251864	2.490891457	0.30189074	1
gene23911	1.033139825	5.807539168	5.621251864	2.490891457	0.30189074	1
gene25772	1.033139825	5.807539168	5.621251864	2.490891457	0.30189074	1
gene27985	1.033139825	5.807539168	5.621251864	2.490891457	0.30189074	1
gene28032	1.033139825	5.807539168	5.621251864	2.490891457	0.30189074	1
gene30770	1.033139825	5.807539168	5.621251864	2.490891457	0.30189074	1
gene31792	1.033139825	5.807539168	5.621251864	2.490891457	0.30189074	1
gene31954	1.033139825	5.807539168	5.621251864	2.490891457	0.30189074	1
gene32432	1.033139825	5.807539168	5.621251864	2.490891457	0.30189074	1
gene34104	1.033139825	5.807539168	5.621251864	2.490891457	0.30189074	1
gene34380	1.033139825	5.807539168	5.621251864	2.490891457	0.30189074	1
gene34626	1.033139825	5.807539168	5.621251864	2.490891457	0.30189074	1
gene34713	1.033139825	5.807539168	5.621251864	2.490891457	0.30189074	1
gene35588	1.033139825	5.807539168	5.621251864	2.490891457	0.30189074	1
gene36421	1.033139825	5.807539168	5.621251864	2.490891457	0.30189074	1
gene37929	1.033139825	5.807539168	5.621251864	2.490891457	0.30189074	1
gene38597	1.033139825	5.807539168	5.621251864	2.490891457	0.30189074	1
gene38851	1.033139825	5.807539168	5.621251864	2.490891457	0.30189074	1
gene38964	1.033139825	5.807539168	5.621251864	2.490891457	0.30189074	1
gene39232	1.033139825	5.807539168	5.621251864	2.490891457	0.30189074	1
gene39367	1.033139825	5.807539168	5.621251864	2.490891457	0.30189074	1

gene40355	1.033139825	5.807539168	5.621251864	2.490891457	0.30189074	1
gene40699	1.033139825	5.807539168	5.621251864	2.490891457	0.30189074	1
gene42686	1.033139825	5.807539168	5.621251864	2.490891457	0.30189074	1
gene42878	1.033139825	5.807539168	5.621251864	2.490891457	0.30189074	1
gene43020	1.033139825	5.807539168	5.621251864	2.490891457	0.30189074	1
gene44202	1.033139825	5.807539168	5.621251864	2.490891457	0.30189074	1
gene45677	1.033139825	5.807539168	5.621251864	2.490891457	0.30189074	1
gene45953	1.033139825	5.807539168	5.621251864	2.490891457	0.30189074	1
gene46704	1.033139825	5.807539168	5.621251864	2.490891457	0.30189074	1
gene47549	1.033139825	5.807539168	5.621251864	2.490891457	0.30189074	1
gene48872	1.033139825	5.807539168	5.621251864	2.490891457	0.30189074	1
gene49241	1.033139825	5.807539168	5.621251864	2.490891457	0.30189074	1
gene50005	1.033139825	5.807539168	5.621251864	2.490891457	0.30189074	1
gene50018	1.033139825	5.807539168	5.621251864	2.490891457	0.30189074	1
gene50064	1.033139825	5.807539168	5.621251864	2.490891457	0.30189074	1
gene50646	1.033139825	5.807539168	5.621251864	2.490891457	0.30189074	1
gene50890	1.033139825	5.807539168	5.621251864	2.490891457	0.30189074	1
gene50961	1.033139825	5.807539168	5.621251864	2.490891457	0.30189074	1
gene51144	1.033139825	5.807539168	5.621251864	2.490891457	0.30189074	1
gene54547	1.033139825	5.807539168	5.621251864	2.490891457	0.30189074	1
gene55897	1.033139825	5.807539168	5.621251864	2.490891457	0.30189074	1
gene56560	1.033139825	5.807539168	5.621251864	2.490891457	0.30189074	1
gene56647	1.033139825	5.807539168	5.621251864	2.490891457	0.30189074	1
gene56729	1.033139825	5.807539168	5.621251864	2.490891457	0.30189074	1
gene57262	1.033139825	5.807539168	5.621251864	2.490891457	0.30189074	1
gene60340	1.033139825	5.807539168	5.621251864	2.490891457	0.30189074	1
gene60972	1.033139825	5.807539168	5.621251864	2.490891457	0.30189074	1
gene62077	1.033139825	5.807539168	5.621251864	2.490891457	0.30189074	1
gene63929	1.033139825	5.807539168	5.621251864	2.490891457	0.30189074	1
gene64495	1.033139825	5.807539168	5.621251864	2.490891457	0.30189074	1
gene65894	1.033139825	5.807539168	5.621251864	2.490891457	0.30189074	1
gene66293	1.033139825	5.807539168	5.621251864	2.490891457	0.30189074	1
gene66631	1.033139825	5.807539168	5.621251864	2.490891457	0.30189074	1
gene68122	1.033139825	5.807539168	5.621251864	2.490891457	0.30189074	1
gene68560	1.033139825	5.807539168	5.621251864	2.490891457	0.30189074	1
gene68894	1.033139825	5.807539168	5.621251864	2.490891457	0.30189074	1
gene71972	1.033139825	5.807539168	5.621251864	2.490891457	0.30189074	1
gene73276	1.033139825	5.807539168	5.621251864	2.490891457	0.30189074	1
gene73388	1.033139825	5.807539168	5.621251864	2.490891457	0.30189074	1
gene73939	1.033139825	5.807539168	5.621251864	2.490891457	0.30189074	1
gene74152	1.033139825	5.807539168	5.621251864	2.490891457	0.30189074	1
gene40395	51.65699125	29.03769584	0.562125186	-0.83103664	0.3020012	1
gene67649	51.65699125	29.03769584	0.562125186	-0.83103664	0.3020012	1
gene54489	369.8640574	250.6921074	0.677795267	-0.56107853	0.30205068	1
gene13761	408.0902309	599.1444575	1.468166626	0.554015713	0.30210875	1
gene27080	1717.078389	1142.14937	0.665170197	-0.58820457	0.30223449	1
gene61242	215.9262234	143.2526328	0.663433234	-0.59197681	0.30227695	1
gene41507	397.7588327	270.0505713	0.67893042	-0.55866437	0.3022822	1

gene60306	102.2808427	157.7714807	1.542532077	0.625300491	0.30235491	1
gene35351	350.2344007	237.1411827	0.677092776	-0.56257457	0.3023756	1
gene7231	78.51862671	123.8941689	1.57789526	0.658001443	0.3024785	1
gene45135	865.7711734	1287.337849	1.486926209	0.572333053	0.30252887	1
gene16942	58.88897003	33.87731181	0.575274314	-0.79767804	0.30256574	1
gene57482	186.9983083	122.9262457	0.65736555	-0.60523224	0.3026291	1
gene62725	41.325593	22.26223348	0.538703304	-0.89243718	0.30263965	1
gene66665	41.325593	22.26223348	0.538703304	-0.89243718	0.30263965	1
gene66840	41.325593	22.26223348	0.538703304	-0.89243718	0.30263965	1
gene67007	277.9146129	186.8091766	0.672181914	-0.57307637	0.3026403	1
gene54565	382.2617353	259.4034162	0.678601576	-0.55936331	0.30264461	1
gene26159	26.86163545	48.39615973	1.80168329	0.849345428	0.30267709	1
gene4653	63.02152933	101.6319354	1.612654223	0.689437137	0.30268135	1
gene47481	63.02152933	101.6319354	1.612654223	0.689437137	0.30268135	1
gene33195	138.4407366	209.07141	1.510187068	0.594727268	0.30271381	1
gene9257	37.1930337	63.88293085	1.717604736	0.780398075	0.3027421	1
gene52779	3059.127022	4910.274366	1.605122746	0.682683627	0.30275274	1
gene60424	207.6611048	307.7995759	1.482220641	0.567760221	0.3027947	1
gene12372	170.4680711	111.3111674	0.652973701	-0.61490321	0.30289843	1
gene35916	170.4680711	111.3111674	0.652973701	-0.61490321	0.30289843	1
gene8899	290.3122908	195.5204853	0.673483319	-0.57028588	0.30291719	1
gene18622	166.3355118	108.4073978	0.651739347	-0.617633	0.30296283	1
gene60680	1733.608626	2661.788785	1.535403519	0.61861786	0.30298889	1
gene59815	869.9037327	1293.145388	1.486538498	0.571956826	0.30302882	1
gene3839	77.48548688	46.46031334	0.599600199	-0.73792723	0.30305777	1
gene49286	77.48548688	46.46031334	0.599600199	-0.73792723	0.30305777	1
gene67093	77.48548688	46.46031334	0.599600199	-0.73792723	0.30305777	1
gene45886	1232.535811	830.478101	0.673796326	-0.56961553	0.30310095	1
gene35125	137.4075967	88.08101071	0.641019949	-0.64155884	0.30314053	1
gene43323	149.8052746	96.79231946	0.646120904	-0.63012394	0.30315221	1
gene33441	110.5459613	169.3865591	1.532272704	0.615673082	0.30327117	1
gene59066	32.02733458	56.13954529	1.752863485	0.809713641	0.30331157	1
gene73723	32.02733458	56.13954529	1.752863485	0.809713641	0.30331157	1
gene8731	488.6751373	332.965579	0.681363862	-0.55350266	0.30333021	1
gene49985	998.013071	1490.60172	1.493569336	0.578764213	0.30337052	1
gene37352	667.408327	454.9239015	0.681627548	-0.55294445	0.30339278	1
gene15669	44.42501248	74.53008599	1.67766044	0.746450743	0.30344814	1
gene19194	174.6006304	260.3713394	1.4912394	0.576511883	0.30344825	1
gene57847	1456.727153	2213.640346	1.519598465	0.60369016	0.30344846	1
gene25975	581.6577215	855.6441041	1.471044005	0.556840404	0.30354569	1
gene29762	379.1623158	257.4675698	0.679043141	-0.55842486	0.30366016	1
gene55814	150.8384145	226.4940275	1.501567279	0.586469117	0.30377483	1
gene1220	53.7232709	88.08101071	1.639531794	0.713283879	0.30381927	1
gene44620	53.7232709	88.08101071	1.639531794	0.713283879	0.30381927	1
gene29602	1631.327784	1088.913594	0.667501409	-0.58315721	0.30390455	1
gene49597	97.11514356	60.01123807	0.617939035	-0.69446358	0.30393754	1
gene66848	97.11514356	60.01123807	0.617939035	-0.69446358	0.30393754	1
gene30573	430.8193071	293.280728	0.680751125	-0.55480063	0.303984	1

gene64508	118.8110799	181.0016374	1.523440723	0.607333366	0.30404046	1
gene18591	229.3570412	152.9318648	0.666785131	-0.58470616	0.30406616	1
gene3985	154.9709738	232.3015667	1.499000497	0.584000862	0.30412112	1
gene59765	470.0786204	320.3825774	0.68155105	-0.55310637	0.30412136	1
gene11584	67.15408863	107.4394746	1.599894761	0.67797701	0.30414819	1
gene11630	216.9593633	144.220556	0.66473534	-0.58914804	0.30421733	1
gene7017	895.7322283	608.8236894	0.679693853	-0.55704302	0.30426579	1
gene42318	619.883895	422.9824361	0.682357518	-0.55140026	0.3043496	1
gene65660	474.2111797	323.286347	0.681734976	-0.55271709	0.30435196	1
gene57602	208.6942447	138.4130168	0.663233512	-0.59241119	0.30435734	1
gene52740	88.85002496	138.4130168	1.557827552	0.639535539	0.30438464	1
gene27209	11.36453808	24.19807987	2.12926207	1.090353528	0.30445307	1
gene34677	11.36453808	24.19807987	2.12926207	1.090353528	0.30445307	1
gene50035	11.36453808	24.19807987	2.12926207	1.090353528	0.30445307	1
gene61846	11.36453808	24.19807987	2.12926207	1.090353528	0.30445307	1
gene69252	11.36453808	24.19807987	2.12926207	1.090353528	0.30445307	1
gene6440	81.61804618	49.36408293	0.604818239	-0.72542645	0.304553	1
gene19757	70.25350811	41.62069737	0.592435858	-0.75526913	0.30466914	1
gene46615	70.25350811	41.62069737	0.592435858	-0.75526913	0.30466914	1
gene13782	224.191342	331.0297326	1.476550029	0.562230239	0.30475024	1
gene41134	69.22036828	110.3432442	1.59408635	0.67272978	0.3047766	1
gene64318	427.7198876	291.3448816	0.681158137	-0.55393832	0.30486344	1
gene56068	380.1954556	258.435493	0.679743772	-0.55693707	0.30487045	1
gene38444	55.78955055	90.9847803	1.630857022	0.705630306	0.30492784	1
gene54744	183.8988889	120.9903993	0.657918055	-0.60402019	0.30493576	1
gene27065	355.4000998	520.7426787	1.46522941	0.551126565	0.30500971	1
gene21541	179.7663296	118.0866297	0.656889586	-0.6062772	0.30504919	1
gene62143	1230.469532	830.478101	0.674927806	-0.5671949	0.3051139	1
gene42485	356.4332397	241.9807987	0.678895153	-0.55873931	0.30511965	1
gene32260	167.3686517	249.7241842	1.49206068	0.577306209	0.30515966	1
gene19490	46.49129213	77.43385557	1.665556108	0.736003955	0.30517463	1
gene3203	39.25931335	66.78670043	1.701168327	0.7665259	0.30522691	1
gene70045	435.9850062	297.1524208	0.681565688	-0.55307539	0.3052725	1
gene73234	435.9850062	297.1524208	0.681565688	-0.55307539	0.3052725	1
gene28793	135.3413171	204.2317941	1.509012905	0.593605144	0.30533283	1
gene69406	135.3413171	204.2317941	1.509012905	0.593605144	0.30533283	1
gene32171	23.76221598	43.55654376	1.833016912	0.874220097	0.30535066	1
gene36640	23.76221598	43.55654376	1.833016912	0.874220097	0.30535066	1
gene46480	48.55757178	27.10184945	0.558138483	-0.84130497	0.30536356	1
gene12475	392.5931335	267.1468017	0.680467331	-0.55540219	0.30540061	1
gene4467	38.22617353	20.32638709	0.531740041	-0.91120699	0.3054217	1
gene7927	38.22617353	20.32638709	0.531740041	-0.91120699	0.3054217	1
gene14198	38.22617353	20.32638709	0.531740041	-0.91120699	0.3054217	1
gene60081	38.22617353	20.32638709	0.531740041	-0.91120699	0.3054217	1
gene60281	163.2360924	106.4715514	0.652254963	-0.61649208	0.30551565	1
gene62821	2439.243127	1599.977041	0.655931761	-0.60838236	0.30552895	1
gene53121	263.4506554	177.1299446	0.672345811	-0.57272464	0.3056771	1
gene475	387.4274344	567.2029921	1.464023819	0.549939026	0.30568832	1

gene37040	63.02152933	36.7810814	0.583627243	-0.77688087	0.30569099	1
gene55112	63.02152933	36.7810814	0.583627243	-0.77688087	0.30569099	1
gene36306	844.0752371	574.9463776	0.681155367	-0.55394419	0.30571443	1
gene61000	246.9204182	165.5148663	0.670316645	-0.57708534	0.30571811	1
gene10762	85.75060548	52.26785251	0.609533335	-0.71422297	0.30574557	1
gene44826	2277.040174	3552.278124	1.560041919	0.641584796	0.3058041	1
gene51941	150.8384145	97.76024266	0.648112372	-0.62568412	0.30583233	1
gene61812	73.35292758	116.1507834	1.583451229	0.663072433	0.30585889	1
gene63377	73.35292758	116.1507834	1.583451229	0.663072433	0.30585889	1
gene6847	130.175618	83.24139474	0.639454577	-0.64508621	0.30607435	1
gene12638	55.78955055	31.94146542	0.572534912	-0.80456443	0.30607704	1
gene26170	55.78955055	31.94146542	0.572534912	-0.80456443	0.30607704	1
gene67411	55.78955055	31.94146542	0.572534912	-0.80456443	0.30607704	1
gene4834	217.9925031	145.1884792	0.666025102	-0.58635154	0.3061471	1
gene58354	981.4828338	666.8990811	0.679481146	-0.55749457	0.30615375	1
gene57390	502.105955	735.6216279	1.465072502	0.550972061	0.30619939	1
gene37553	1791.464457	1193.449299	0.666186423	-0.58600214	0.30624158	1
gene24207	303.7431086	445.2446695	1.46585933	0.551746663	0.30624808	1
gene64536	105.3802622	161.6431735	1.533903695	0.617207907	0.3062764	1
gene20392	365.7314981	535.2615266	1.463536855	0.549459077	0.30630873	1
gene5593	6.19883895	15.48677111	2.498334162	1.320966456	0.30651645	1
gene6856	6.19883895	15.48677111	2.498334162	1.320966456	0.30651645	1
gene9594	6.19883895	15.48677111	2.498334162	1.320966456	0.30651645	1
gene9830	6.19883895	15.48677111	2.498334162	1.320966456	0.30651645	1
gene25471	6.19883895	15.48677111	2.498334162	1.320966456	0.30651645	1
gene25478	6.19883895	15.48677111	2.498334162	1.320966456	0.30651645	1
gene31742	6.19883895	15.48677111	2.498334162	1.320966456	0.30651645	1
gene33853	6.19883895	15.48677111	2.498334162	1.320966456	0.30651645	1
gene40043	6.19883895	15.48677111	2.498334162	1.320966456	0.30651645	1
gene41447	6.19883895	15.48677111	2.498334162	1.320966456	0.30651645	1
gene43029	6.19883895	15.48677111	2.498334162	1.320966456	0.30651645	1
gene64706	6.19883895	15.48677111	2.498334162	1.320966456	0.30651645	1
gene69495	6.19883895	15.48677111	2.498334162	1.320966456	0.30651645	1
gene69507	6.19883895	15.48677111	2.498334162	1.320966456	0.30651645	1
gene27883	2172.693052	3375.14818	1.553439947	0.635466471	0.30659375	1
gene63892	1636.493483	1094.721133	0.668943167	-0.58004445	0.30667711	1
gene18644	89.88316478	55.17162209	0.613814859	-0.70412453	0.30668992	1
gene299	48.55757178	80.33762515	1.654481932	0.726379536	0.30669167	1
gene36478	28.9279151	51.29992932	1.773371124	0.826494489	0.30671793	1
gene56720	28.9279151	51.29992932	1.773371124	0.826494489	0.30671793	1
gene38088	77.48548688	121.9583225	1.573950522	0.65439019	0.30674693	1
gene32720	319.2402059	467.506903	1.464436165	0.550345308	0.30676056	1
gene13465	59.92210985	96.79231946	1.61530226	0.691804151	0.30679421	1
gene26923	2642.771673	1727.742902	0.6537617	-0.61316323	0.30683839	1
gene5190	466.9792009	683.3537754	1.46334949	0.549274368	0.30691576	1
gene67025	158.0703932	236.1732595	1.494101803	0.579278452	0.30692729	1
gene53061	580.6245817	851.7724113	1.466993369	0.55286235	0.30693398	1
gene24235	189.064588	124.8620921	0.660420301	-0.59854363	0.30700853	1

gene26881	604.3867977	413.3032041	0.683838902	-0.5482716	0.30703335	1
gene56954	115.7116604	176.1620214	1.52242238	0.606368675	0.30707377	1
gene42115	381.2285955	557.5237601	1.462439509	0.548376952	0.30710048	1
gene26287	570.2931834	390.0730474	0.683986866	-0.54795947	0.30728979	1
gene25227	260.3512359	175.1940982	0.67291441	-0.57150508	0.30733455	1
gene49283	119.8442197	181.9695606	1.518384124	0.602536813	0.30734724	1
gene64552	676.7065854	462.667287	0.683704425	-0.54855533	0.3073572	1
gene57177	285.1465917	192.6167157	0.675500677	-0.56597088	0.30736996	1
gene5063	289.279151	195.5204853	0.675888617	-0.56514258	0.30740821	1
gene17905	41.325593	69.69047001	1.686375559	0.753925863	0.30742646	1
gene48118	41.325593	69.69047001	1.686375559	0.753925863	0.30742646	1
gene56179	41.325593	69.69047001	1.686375559	0.753925863	0.30742646	1
gene10656	94.01572408	58.07539168	0.617719985	-0.69497509	0.30742992	1
gene36684	1099.260774	1642.565661	1.494245679	0.579417371	0.30743604	1
gene2410	6319.71631	11094.33566	1.755511658	0.811891577	0.30743936	1
gene10360	8069.855174	14795.67395	1.83344975	0.874560726	0.3074548	1
gene58126	933.9584019	635.9255389	0.680892787	-0.55450045	0.30748118	1
gene53525	248.9866978	365.8749676	1.469455882	0.555282045	0.30753694	1
gene46146	20.6627965	38.71692779	1.873750621	0.905928957	0.30754334	1
gene62212	481.4431585	329.0938862	0.683557094	-0.54886625	0.30754404	1
gene36449	1104.426473	749.1725526	0.678336287	-0.55992742	0.30758042	1
gene53663	305.8093882	207.1355637	0.677335529	-0.56205742	0.30764846	1
gene68882	168.4017915	250.6921074	1.488654635	0.57400909	0.30766164	1
gene21598	446.3164044	304.8958063	0.683138247	-0.54975053	0.30778848	1
gene70040	314.0745068	212.9431028	0.67800187	-0.56063884	0.30781163	1
gene32419	170.4680711	253.595877	1.487644433	0.573029744	0.30781165	1
gene69915	1778.033639	2721.800023	1.530792199	0.614278454	0.3079343	1
gene44417	172.5343508	256.4996466	1.486658427	0.572073214	0.30796293	1
gene46319	98.14828338	60.97916126	0.621296259	-0.68664673	0.30800118	1
gene33638	446.3164044	652.3802332	1.46169898	0.547646236	0.30801219	1
gene47954	50.62385143	83.24139474	1.64431177	0.717483867	0.30802596	1
gene62672	50.62385143	83.24139474	1.64431177	0.717483867	0.30802596	1
gene16795	35.12675405	18.3905407	0.523547968	-0.93360637	0.3080326	1
gene19973	35.12675405	18.3905407	0.523547968	-0.93360637	0.3080326	1
gene54300	35.12675405	18.3905407	0.523547968	-0.93360637	0.3080326	1
gene72972	35.12675405	18.3905407	0.523547968	-0.93360637	0.3080326	1
gene35113	370.8971972	542.036989	1.461421097	0.54737194	0.30808582	1
gene26279	78.51862671	47.42823654	0.604038029	-0.72728871	0.30818581	1
gene73411	67.15408863	39.68485098	0.590952119	-0.75888685	0.30826818	1
gene32725	1445.362615	972.7628106	0.673023365	-0.5712715	0.30828158	1
gene39178	136.3744569	205.1997173	1.504678529	0.589455292	0.30830808	1
gene25509	14.46395755	29.03769584	2.007589952	1.00546463	0.30831904	1
gene25855	14.46395755	29.03769584	2.007589952	1.00546463	0.30831904	1
gene28920	14.46395755	29.03769584	2.007589952	1.00546463	0.30831904	1
gene29431	14.46395755	29.03769584	2.007589952	1.00546463	0.30831904	1
gene33133	14.46395755	29.03769584	2.007589952	1.00546463	0.30831904	1
gene34881	14.46395755	29.03769584	2.007589952	1.00546463	0.30831904	1
gene49795	14.46395755	29.03769584	2.007589952	1.00546463	0.30831904	1

gene58836	14.46395755	29.03769584	2.007589952	1.00546463	0.30831904	1
gene46018	723.1978776	494.6087525	0.683918977	-0.54810267	0.30841928	1
gene18948	102.2808427	63.88293085	0.62458354	-0.67903354	0.30843283	1
gene7351	410.1565106	599.1444575	1.460770321	0.546729359	0.30850495	1
gene52090	202.4954057	134.5413241	0.664416674	-0.58983981	0.30864389	1
gene34796	147.738995	95.82439627	0.648605984	-0.62458576	0.30864536	1
gene47839	17.56337703	33.87731181	1.928860934	0.947749132	0.30880856	1
gene51359	17.56337703	33.87731181	1.928860934	0.947749132	0.30880856	1
gene64712	17.56337703	33.87731181	1.928860934	0.947749132	0.30880856	1
gene73113	17.56337703	33.87731181	1.928860934	0.947749132	0.30880856	1
gene73348	17.56337703	33.87731181	1.928860934	0.947749132	0.30880856	1
gene19541	45.4581523	25.16600306	0.553608138	-0.85306294	0.3088215	1
gene40648	45.4581523	25.16600306	0.553608138	-0.85306294	0.3088215	1
gene42640	45.4581523	25.16600306	0.553608138	-0.85306294	0.3088215	1
gene72457	273.7820536	184.8733302	0.675257299	-0.56649076	0.30890188	1
gene56935	94.01572408	145.1884792	1.544299963	0.626953007	0.30900986	1
gene30658	363.6652184	247.7883378	0.681363862	-0.55350266	0.30905026	1
gene50344	114.6785206	72.5942396	0.633023859	-0.65966822	0.30910963	1
gene16913	36.15989388	61.94708446	1.713143425	0.77664594	0.3091226	1
gene32273	36.15989388	61.94708446	1.713143425	0.77664594	0.3091226	1
gene59379	36.15989388	61.94708446	1.713143425	0.77664594	0.3091226	1
gene70162	36.15989388	61.94708446	1.713143425	0.77664594	0.3091226	1
gene363	19.62965668	8.711308752	0.443783042	-1.17207356	0.30913899	1
gene13441	19.62965668	8.711308752	0.443783042	-1.17207356	0.30913899	1
gene25750	19.62965668	8.711308752	0.443783042	-1.17207356	0.30913899	1
gene45537	19.62965668	8.711308752	0.443783042	-1.17207356	0.30913899	1
gene66015	19.62965668	8.711308752	0.443783042	-1.17207356	0.30913899	1
gene59948	310.9750873	211.0072564	0.678534278	-0.5595064	0.30916371	1
gene14567	248.9866978	167.4507127	0.67252875	-0.57233215	0.30916854	1
gene36207	3190.33578	5107.730698	1.601000976	0.678974188	0.30917528	1
gene46396	127.0761985	81.30554835	0.639817285	-0.64426813	0.30918217	1
gene66099	278.9477528	408.4635881	1.464301412	0.550212548	0.3092016	1
gene30072	43.39187265	72.5942396	1.672991626	0.742430224	0.30933811	1
gene10298	323.3727653	219.7185652	0.679459091	-0.5575414	0.30937622	1
gene13846	82.65118601	50.33200612	0.608968952	-0.71555942	0.30940394	1
gene14383	82.65118601	50.33200612	0.608968952	-0.71555942	0.30940394	1
gene1506	68.18722846	108.4073978	1.589849012	0.668889759	0.30945859	1
gene16837	3492.012609	5641.056378	1.615416956	0.691906588	0.30951168	1
gene65003	657.0769288	450.0842855	0.68497959	-0.54586709	0.30951789	1
gene26271	331.6378839	225.5261044	0.680037219	-0.55631439	0.30953592	1
gene55359	296.5111298	433.6295912	1.462439509	0.548376952	0.30964205	1
gene19338	228.3239013	152.9318648	0.669802258	-0.57819285	0.30968833	1
gene22826	339.9030025	231.3336435	0.680587232	-0.55514801	0.30970897	1
gene2496	309.9419475	452.9880551	1.461525485	0.547474986	0.30972178	1
gene40666	52.69013108	30.00561903	0.569473228	-0.81230007	0.30974912	1
gene3166	104.3471223	159.7073271	1.530538874	0.614039688	0.3098055	1
gene58294	219.0256429	322.3184238	1.471601314	0.557386869	0.30985257	1
gene52400	173.5674906	114.214937	0.658043373	-0.60374542	0.30999937	1

gene48709	396.7256928	271.0184945	0.683138247	-0.54975053	0.31007127	1
gene429	25.82849563	46.46031334	1.798800597	0.847035268	0.3100778	1
gene23879	25.82849563	46.46031334	1.798800597	0.847035268	0.3100778	1
gene9096	110.5459613	168.4186359	1.52351686	0.607405466	0.31016155	1
gene40100	30.99419475	54.2036989	1.748833913	0.806393283	0.31020181	1
gene66517	54.75641073	89.04893391	1.626274124	0.701570458	0.31023448	1
gene42123	584.757141	855.6441041	1.463246952	0.549173273	0.31027669	1
gene32173	32.02733458	16.45469431	0.513770332	-0.96080451	0.31032983	1
gene34098	32.02733458	16.45469431	0.513770332	-0.96080451	0.31032983	1
gene52162	71.28664793	42.58862056	0.597427734	-0.74316388	0.31034959	1
gene499	86.78374531	53.23577571	0.613430263	-0.70502875	0.31035018	1
gene50175	72.31978776	114.214937	1.579304095	0.659288989	0.31039981	1
gene70790	116.7448002	177.1299446	1.517240547	0.601449833	0.31046075	1
gene12397	1580.703932	2397.545753	1.516758264	0.600991172	0.3104893	1
gene39444	118.8110799	180.0337142	1.515293981	0.599597717	0.31055147	1
gene63269	1877.215062	1252.492614	0.667207844	-0.58379185	0.31055771	1
gene48093	865.7711734	1278.62654	1.476864302	0.562537274	0.31068283	1
gene18029	384.3280149	560.4275297	1.458201088	0.544189684	0.31078867	1
gene54091	320.2733458	467.506903	1.459712178	0.54568393	0.31085801	1
gene31100	1069.299719	727.8782424	0.68070554	-0.55489724	0.31093795	1
gene2985	209.7273845	308.7674991	1.472232631	0.558005653	0.310963	1
gene25762	308.9088077	451.0522087	1.460146805	0.546113427	0.31098563	1
gene60234	45.4581523	75.49800918	1.660824414	0.731899557	0.311	1
gene17183	404.9908114	590.4331487	1.457892703	0.543884545	0.31101238	1
gene70454	693.2368226	475.2502886	0.685552575	-0.54466079	0.31102147	1
gene54888	131.2087578	197.4563317	1.504902074	0.589669612	0.31104164	1
gene52381	783.1199874	536.2294498	0.684734726	-0.54638292	0.31104661	1
gene46248	764.5234706	523.6464483	0.684931815	-0.54596772	0.31104685	1
gene19132	183.8988889	271.9864177	1.478999788	0.564621846	0.31107498	1
gene54970	237.6221598	159.7073271	0.672106201	-0.57323888	0.31123375	1
gene61525	284.1134519	415.2390505	1.461525485	0.547474986	0.31125722	1
gene46151	613.6850561	421.0465897	0.686095556	-0.54351857	0.31128053	1
gene15244	148.7721348	96.79231946	0.650607855	-0.62013986	0.31134793	1
gene54546	78.51862671	122.9262457	1.565567953	0.64668613	0.31146611	1
gene58020	78.51862671	122.9262457	1.565567953	0.64668613	0.31146611	1
gene25784	38.22617353	64.85085404	1.696503941	0.762564781	0.31152176	1
gene37748	38.22617353	64.85085404	1.696503941	0.762564781	0.31152176	1
gene55497	38.22617353	64.85085404	1.696503941	0.762564781	0.31152176	1
gene67516	38.22617353	64.85085404	1.696503941	0.762564781	0.31152176	1
gene67197	186.9983083	123.8941689	0.662541656	-0.59391693	0.31156784	1
gene10627	1900.977278	2912.480893	1.532096636	0.615507297	0.3115843	1
gene86	95.04886391	59.04331487	0.621189065	-0.68689566	0.31161588	1
gene46106	192.1640075	283.601496	1.475830463	0.561527	0.31164499	1
gene9559	2956.846179	4687.652032	1.585355391	0.664806288	0.31164543	1
gene43573	149.8052746	223.590258	1.492539288	0.577768908	0.31174098	1
gene26598	80.58490636	125.8300153	1.561458851	0.642894551	0.31174819	1
gene35590	470.0786204	322.3184238	0.685669183	-0.54441541	0.31175227	1
gene43972	470.0786204	322.3184238	0.685669183	-0.54441541	0.31175227	1

gene14562	493.8408364	338.7731181	0.685996566	-0.54372674	0.31181585	1
gene2409	153.9378339	229.3977971	1.490197642	0.575503685	0.31190653	1
gene60608	153.9378339	229.3977971	1.490197642	0.575503685	0.31190653	1
gene65954	450.4489637	308.7674991	0.68546611	-0.54484275	0.31196499	1
gene23972	64.05466916	37.74900459	0.589324792	-0.76286513	0.31203198	1
gene29164	222.1250624	326.1901166	1.46849758	0.554340888	0.31204441	1
gene64184	178.7331897	118.0866297	0.660686635	-0.59796193	0.3120689	1
gene4360	28.9279151	14.51884792	0.501897488	-0.99453537	0.31208843	1
gene18985	28.9279151	14.51884792	0.501897488	-0.99453537	0.31208843	1
gene39044	28.9279151	14.51884792	0.501897488	-0.99453537	0.31208843	1
gene48180	28.9279151	14.51884792	0.501897488	-0.99453537	0.31208843	1
gene68688	28.9279151	14.51884792	0.501897488	-0.99453537	0.31208843	1
gene55681	132.2418976	85.17724113	0.644101776	-0.63463942	0.31214016	1
gene16373	541.3652683	371.6825067	0.686565113	-0.54253154	0.31224262	1
gene70535	321.3064856	468.4748262	1.45803103	0.544021424	0.3123015	1
gene7852	22.72907615	10.64715514	0.468437655	-1.09407104	0.31231663	1
gene27522	22.72907615	10.64715514	0.468437655	-1.09407104	0.31231663	1
gene33766	22.72907615	10.64715514	0.468437655	-1.09407104	0.31231663	1
gene45493	22.72907615	10.64715514	0.468437655	-1.09407104	0.31231663	1
gene46418	22.72907615	10.64715514	0.468437655	-1.09407104	0.31231663	1
gene66087	22.72907615	10.64715514	0.468437655	-1.09407104	0.31231663	1
gene550	5.165699125	13.55092472	2.62325087	1.391355784	0.31234142	1
gene1165	5.165699125	13.55092472	2.62325087	1.391355784	0.31234142	1
gene9685	5.165699125	13.55092472	2.62325087	1.391355784	0.31234142	1
gene10309	5.165699125	13.55092472	2.62325087	1.391355784	0.31234142	1
gene11686	5.165699125	13.55092472	2.62325087	1.391355784	0.31234142	1
gene12305	5.165699125	13.55092472	2.62325087	1.391355784	0.31234142	1
gene15831	5.165699125	13.55092472	2.62325087	1.391355784	0.31234142	1
gene16900	5.165699125	13.55092472	2.62325087	1.391355784	0.31234142	1
gene21917	5.165699125	13.55092472	2.62325087	1.391355784	0.31234142	1
gene26327	5.165699125	13.55092472	2.62325087	1.391355784	0.31234142	1
gene33164	5.165699125	13.55092472	2.62325087	1.391355784	0.31234142	1
gene34421	5.165699125	13.55092472	2.62325087	1.391355784	0.31234142	1
gene37885	5.165699125	13.55092472	2.62325087	1.391355784	0.31234142	1
gene39290	5.165699125	13.55092472	2.62325087	1.391355784	0.31234142	1
gene40304	5.165699125	13.55092472	2.62325087	1.391355784	0.31234142	1
gene44240	5.165699125	13.55092472	2.62325087	1.391355784	0.31234142	1
gene46797	5.165699125	13.55092472	2.62325087	1.391355784	0.31234142	1
gene48810	5.165699125	13.55092472	2.62325087	1.391355784	0.31234142	1
gene49381	5.165699125	13.55092472	2.62325087	1.391355784	0.31234142	1
gene66712	5.165699125	13.55092472	2.62325087	1.391355784	0.31234142	1
gene71154	5.165699125	13.55092472	2.62325087	1.391355784	0.31234142	1
gene4452	42.35873283	23.23015667	0.548414816	-0.86666055	0.31235302	1
gene8384	42.35873283	23.23015667	0.548414816	-0.86666055	0.31235302	1
gene31661	42.35873283	23.23015667	0.548414816	-0.86666055	0.31235302	1
gene55506	42.35873283	23.23015667	0.548414816	-0.86666055	0.31235302	1
gene55195	576.4920224	395.8805866	0.686706097	-0.54223532	0.31235368	1
gene19621	123.976779	79.36970196	0.640198129	-0.64340963	0.31241565	1

gene66252	622.9833145	427.822052	0.686731156	-0.54218268	0.31252055	1
gene46432	115.7116604	73.56216279	0.635736818	-0.65349845	0.31254266	1
gene25184	563.0612047	821.7667922	1.45946264	0.545437281	0.31280825	1
gene7844	402.9245318	586.561456	1.455760098	0.541772626	0.31289042	1
gene23414	804.8159237	1183.770067	1.470858158	0.556658127	0.31289341	1
gene25586	735.5955555	1079.234362	1.467157263	0.55302352	0.31294865	1
gene12997	25.82849563	12.58300153	0.487175162	-1.03748751	0.31295004	1
gene29321	557.8955055	383.2975851	0.687041895	-0.54153002	0.31303403	1
gene66791	95.04886391	146.1564024	1.537697521	0.62077174	0.31303479	1
gene619	10.33139825	22.26223348	2.154813215	1.107562818	0.31305906	1
gene4419	10.33139825	22.26223348	2.154813215	1.107562818	0.31305906	1
gene6298	10.33139825	22.26223348	2.154813215	1.107562818	0.31305906	1
gene10355	10.33139825	22.26223348	2.154813215	1.107562818	0.31305906	1
gene12529	10.33139825	22.26223348	2.154813215	1.107562818	0.31305906	1
gene30084	10.33139825	22.26223348	2.154813215	1.107562818	0.31305906	1
gene36288	10.33139825	22.26223348	2.154813215	1.107562818	0.31305906	1
gene43510	10.33139825	22.26223348	2.154813215	1.107562818	0.31305906	1
gene30888	684.971704	470.4106726	0.686759278	-0.5421236	0.31317303	1
gene31126	196.2965668	130.6696313	0.665674563	-0.58711105	0.31320658	1
gene2952	33.0604744	57.10746848	1.727363854	0.788572006	0.3132211	1
gene14263	33.0604744	57.10746848	1.727363854	0.788572006	0.3132211	1
gene46755	33.0604744	57.10746848	1.727363854	0.788572006	0.3132211	1
gene64995	33.0604744	57.10746848	1.727363854	0.788572006	0.3132211	1
gene28706	56.82269038	32.90938862	0.579159283	-0.78796792	0.31322607	1
gene57327	22.72907615	41.62069737	1.83116538	0.872762093	0.31323352	1
gene10696	8601.922184	15837.15931	1.841118644	0.880582599	0.31325053	1
gene20847	236.5890199	346.5165037	1.464634765	0.550540946	0.31329216	1
gene60048	256.2186766	374.5862763	1.461978811	0.547922402	0.31329332	1
gene17600	230.390181	154.8677111	0.672197532	-0.57304285	0.31335976	1
gene58816	192.1640075	127.7658617	0.664879253	-0.58883574	0.31347198	1
gene29118	105.3802622	160.6752503	1.524718643	0.608543046	0.31350043	1
gene13446	186.9983083	275.8581105	1.475190406	0.560901179	0.31352075	1
gene41877	49.5907116	28.06977264	0.566028834	-0.82105255	0.31358442	1
gene50319	570.2931834	392.0088938	0.687381342	-0.5408174	0.31362943	1
gene25053	109.5128215	166.4827895	1.520212768	0.604273257	0.31362957	1
gene73408	111.5791011	169.3865591	1.518084994	0.602252566	0.31368592	1
gene21099	49.5907116	81.30554835	1.639531794	0.713283879	0.31370387	1
gene41477	49.5907116	81.30554835	1.639531794	0.713283879	0.31370387	1
gene48901	272.7489138	184.8733302	0.677815092	-0.56103633	0.31371456	1
gene31364	153.9378339	100.6640122	0.653926391	-0.61279985	0.31373837	1
gene13043	335.7704432	488.8012133	1.455760098	0.541772626	0.31380463	1
gene2923	65.08780898	103.5677818	1.591200925	0.67011602	0.31382167	1
gene46326	517.6030524	754.0121686	1.456738258	0.542741682	0.31382206	1
gene3540	574.4257427	394.9126634	0.687491235	-0.54058677	0.31382819	1
gene67786	914.3287452	1349.284933	1.475710941	0.561410158	0.31385494	1
gene15963	119.8442197	181.0016374	1.510307613	0.594842421	0.31387409	1
gene29047	121.9104994	183.905407	1.508528043	0.593141516	0.31391492	1
gene61527	543.431548	373.6183531	0.687516863	-0.540533	0.31400956	1

gene13799	725.2641572	1062.779668	1.465369076	0.551264075	0.3140181	1
gene58946	422.5541885	614.6312286	1.454561913	0.540584705	0.31402431	1
gene63745	996.9799312	681.417929	0.683482092	-0.54902456	0.31403608	1
gene16703	27.89477528	49.36408293	1.769653365	0.823466796	0.31408478	1
gene73213	27.89477528	49.36408293	1.769653365	0.823466796	0.31408478	1
gene5680	3869.108645	6290.532842	1.625835152	0.701180986	0.31414326	1
gene23221	776.9211485	533.3256802	0.6864605	-0.54275139	0.31415721	1
gene64247	68.18722846	40.65277417	0.59619338	-0.74614774	0.31421048	1
gene39047	138.4407366	207.1355637	1.496203854	0.581306753	0.31422256	1
gene43122	138.4407366	207.1355637	1.496203854	0.581306753	0.31422256	1
gene16702	424.6204681	617.5349982	1.454322258	0.540346987	0.31425122	1
gene6944	895.7322283	613.6633054	0.685096825	-0.5456202	0.31433405	1
gene8240	488.6751373	335.8693485	0.687305989	-0.54097556	0.31436709	1
gene59602	146.7058552	218.750642	1.491083241	0.5763608	0.31439524	1
gene41113	270.6826342	394.9126634	1.458950866	0.544931297	0.31459559	1
gene230	590.9559799	406.5277417	0.687915438	-0.53969686	0.31462442	1
gene18298	157.0372534	233.2694899	1.48544046	0.570890779	0.3146539	1
gene54994	288.2460112	420.0786665	1.457361594	0.543358877	0.31465726	1
gene60255	51.65699125	84.20931793	1.630163041	0.705016263	0.31479858	1
gene71641	302.7099687	206.1676405	0.681073178	-0.55411828	0.31487568	1
gene38620	87.81688513	54.2036989	0.617235499	-0.69610706	0.31487969	1
gene58895	702.5350811	1027.934433	1.463178794	0.549106071	0.31491887	1
gene59717	294.4448501	200.3601013	0.680467331	-0.55540219	0.3149391	1
gene11898	413.25593	283.601496	0.686261165	-0.54317038	0.31497694	1
gene55503	849.2409362	582.6897632	0.686130094	-0.54344595	0.31512836	1
gene28170	1250.099188	1866.155919	1.49280628	0.578026961	0.31514702	1
gene28027	360.565799	246.8204146	0.684536402	-0.54680083	0.31514804	1
gene25801	277.9146129	188.745023	0.67914753	-0.55820309	0.31520289	1
gene50075	277.9146129	188.745023	0.67914753	-0.55820309	0.31520289	1
gene48328	1818.326092	1219.583225	0.670717552	-0.57622274	0.31526145	1
gene73555	912.2624655	1344.445317	1.473748365	0.559490213	0.31537775	1
gene24810	727.3304369	1064.715514	1.463867673	0.549785146	0.31538328	1
gene48388	91.94944443	57.10746848	0.621074644	-0.68716142	0.31539062	1
gene52412	269.6494943	182.9374838	0.678426949	-0.55973462	0.31540734	1
gene15713	73.35292758	115.1828602	1.570255802	0.6509996	0.3154133	1
gene19796	73.35292758	115.1828602	1.570255802	0.6509996	0.3154133	1
gene40056	73.35292758	115.1828602	1.570255802	0.6509996	0.3154133	1
gene53974	296.5111298	431.6937448	1.455910762	0.54192193	0.31542155	1
gene72997	2778.11299	1824.535222	0.656753425	-0.60657628	0.31549621	1
gene13073	9.298258426	2.903769584	0.31229177	-1.67903354	0.31551999	1
gene16027	9.298258426	2.903769584	0.31229177	-1.67903354	0.31551999	1
gene27851	9.298258426	2.903769584	0.31229177	-1.67903354	0.31551999	1
gene28619	9.298258426	2.903769584	0.31229177	-1.67903354	0.31551999	1
gene35481	9.298258426	2.903769584	0.31229177	-1.67903354	0.31551999	1
gene40744	9.298258426	2.903769584	0.31229177	-1.67903354	0.31551999	1
gene40992	9.298258426	2.903769584	0.31229177	-1.67903354	0.31551999	1
gene48047	9.298258426	2.903769584	0.31229177	-1.67903354	0.31551999	1
gene50835	9.298258426	2.903769584	0.31229177	-1.67903354	0.31551999	1

gene51571	9.298258426	2.903769584	0.31229177	-1.67903354	0.31551999	1
gene53821	9.298258426	2.903769584	0.31229177	-1.67903354	0.31551999	1
gene57750	9.298258426	2.903769584	0.31229177	-1.67903354	0.31551999	1
gene60569	9.298258426	2.903769584	0.31229177	-1.67903354	0.31551999	1
gene67940	9.298258426	2.903769584	0.31229177	-1.67903354	0.31551999	1
gene69374	9.298258426	2.903769584	0.31229177	-1.67903354	0.31551999	1
gene71105	9.298258426	2.903769584	0.31229177	-1.67903354	0.31551999	1
gene71401	9.298258426	2.903769584	0.31229177	-1.67903354	0.31551999	1
gene33817	1570.372534	1059.875898	0.674920043	-0.5672115	0.31562632	1
gene27299	163.2360924	107.4394746	0.658184554	-0.60343593	0.31563562	1
gene66430	698.4025218	1021.15897	1.462135285	0.548076804	0.31563837	1
gene9308	261.3843757	177.1299446	0.677660798	-0.56136478	0.315663	1
gene39509	96.08200373	60.01123807	0.62458354	-0.67903354	0.31574037	1
gene12361	478.343739	329.0938862	0.687986189	-0.53954849	0.31587279	1
gene8401	19.62965668	36.7810814	1.873750621	0.905928957	0.31590666	1
gene27784	19.62965668	36.7810814	1.873750621	0.905928957	0.31590666	1
gene38288	19.62965668	36.7810814	1.873750621	0.905928957	0.31590666	1
gene65360	19.62965668	36.7810814	1.873750621	0.905928957	0.31590666	1
gene44308	646.7455305	445.2446695	0.688438727	-0.53859984	0.31590832	1
gene73437	72.31978776	43.55654376	0.602276985	-0.73150096	0.3159132	1
gene46728	39.25931335	21.29431028	0.542401496	-0.88256694	0.31592205	1
gene55745	39.25931335	21.29431028	0.542401496	-0.88256694	0.31592205	1
gene64984	39.25931335	21.29431028	0.542401496	-0.88256694	0.31592205	1
gene46263	116.7448002	74.53008599	0.63840176	-0.64746347	0.31593563	1
gene16976	60.95524968	35.8131582	0.587531975	-0.76726073	0.31600403	1
gene56484	104.3471223	65.81877724	0.630767536	-0.66481968	0.31606885	1
gene58476	1308.988158	889.5214159	0.679548864	-0.5573508	0.31608765	1
gene35892	108.4796816	68.72254682	0.633506162	-0.65856944	0.31609145	1
gene6157	269.6494943	392.976817	1.457361594	0.543358877	0.31610109	1
gene60531	2357.625081	1563.195959	0.663038399	-0.59283567	0.31612703	1
gene48341	248.9866978	168.4186359	0.676416199	-0.56401688	0.31614731	1
gene21924	196.2965668	288.441112	1.469414961	0.555241869	0.31624104	1
gene40059	307.8756679	210.0393332	0.682221283	-0.55168833	0.31630014	1
gene21096	394.6594132	573.0105312	1.451911476	0.537953494	0.31631971	1
gene20134	154.9709738	101.6319354	0.655812718	-0.60864422	0.31632967	1
gene32631	154.9709738	101.6319354	0.655812718	-0.60864422	0.31632967	1
gene22354	81.61804618	126.7979385	1.55355273	0.63557121	0.3163322	1
gene51473	81.61804618	126.7979385	1.55355273	0.63557121	0.3163322	1
gene66785	295.47799	201.3280245	0.681363862	-0.55350266	0.31642742	1
gene51335	291.3454307	423.9503593	1.455146759	0.541164664	0.31642816	1
gene26005	83.68432583	129.7017081	1.549892489	0.632168144	0.31648906	1
gene61286	83.68432583	129.7017081	1.549892489	0.632168144	0.31648906	1
gene40480	727.3304369	1063.747591	1.462536884	0.548473009	0.31651892	1
gene24734	232.4564606	339.7410413	1.461525485	0.547474986	0.31661477	1
gene20878	85.75060548	132.6054777	1.546408645	0.628921608	0.31662316	1
gene63243	712.8664793	490.7370597	0.688399685	-0.53868166	0.31665917	1
gene61396	283.0803121	192.6167157	0.680431339	-0.55547851	0.31665947	1
gene20356	585.7902808	403.6239722	0.689024699	-0.53737239	0.31670693	1

gene55648	1248.032909	849.8365649	0.68094083	-0.55439865	0.3167891	1
gene41152	91.94944443	141.3167864	1.536896577	0.620020085	0.31691371	1
gene49435	91.94944443	141.3167864	1.536896577	0.620020085	0.31691371	1
gene24219	4759.675174	7917.611732	1.663477326	0.734202202	0.31700255	1
gene58106	146.7058552	95.82439627	0.653173632	-0.61446154	0.31703808	1
gene62854	238.6552996	348.4523501	1.460065419	0.546033012	0.31706166	1
gene38469	98.14828338	150.0280952	1.528586033	0.612197754	0.3170789	1
gene60893	98.14828338	150.0280952	1.528586033	0.612197754	0.3170789	1
gene47357	44.42501248	73.56216279	1.655872642	0.727591715	0.31708208	1
gene72202	100.214563	152.9318648	1.526044321	0.609796863	0.31711371	1
gene59483	141.540156	211.0072564	1.49079429	0.576081198	0.31716731	1
gene69772	120.8773595	181.9695606	1.505406482	0.590153089	0.31718076	1
gene69879	110.5459613	167.4507127	1.514761016	0.599090198	0.31718986	1
gene36245	76.45234706	46.46031334	0.607702904	-0.71856191	0.31720331	1
gene6825	4.1325593	11.61507834	2.810625932	1.490891457	0.31720501	1
gene11521	4.1325593	11.61507834	2.810625932	1.490891457	0.31720501	1
gene12270	4.1325593	11.61507834	2.810625932	1.490891457	0.31720501	1
gene17696	4.1325593	11.61507834	2.810625932	1.490891457	0.31720501	1
gene22724	4.1325593	11.61507834	2.810625932	1.490891457	0.31720501	1
gene23344	4.1325593	11.61507834	2.810625932	1.490891457	0.31720501	1
gene24806	4.1325593	11.61507834	2.810625932	1.490891457	0.31720501	1
gene26322	4.1325593	11.61507834	2.810625932	1.490891457	0.31720501	1
gene27189	4.1325593	11.61507834	2.810625932	1.490891457	0.31720501	1
gene36525	4.1325593	11.61507834	2.810625932	1.490891457	0.31720501	1
gene39068	4.1325593	11.61507834	2.810625932	1.490891457	0.31720501	1
gene40663	4.1325593	11.61507834	2.810625932	1.490891457	0.31720501	1
gene46530	4.1325593	11.61507834	2.810625932	1.490891457	0.31720501	1
gene46908	4.1325593	11.61507834	2.810625932	1.490891457	0.31720501	1
gene48945	4.1325593	11.61507834	2.810625932	1.490891457	0.31720501	1
gene49115	4.1325593	11.61507834	2.810625932	1.490891457	0.31720501	1
gene50555	4.1325593	11.61507834	2.810625932	1.490891457	0.31720501	1
gene53302	4.1325593	11.61507834	2.810625932	1.490891457	0.31720501	1
gene54195	4.1325593	11.61507834	2.810625932	1.490891457	0.31720501	1
gene55342	4.1325593	11.61507834	2.810625932	1.490891457	0.31720501	1
gene58316	4.1325593	11.61507834	2.810625932	1.490891457	0.31720501	1
gene59437	4.1325593	11.61507834	2.810625932	1.490891457	0.31720501	1
gene60096	4.1325593	11.61507834	2.810625932	1.490891457	0.31720501	1
gene65052	4.1325593	11.61507834	2.810625932	1.490891457	0.31720501	1
gene71094	4.1325593	11.61507834	2.810625932	1.490891457	0.31720501	1
gene71455	4.1325593	11.61507834	2.810625932	1.490891457	0.31720501	1
gene72931	4.1325593	11.61507834	2.810625932	1.490891457	0.31720501	1
gene74131	4.1325593	11.61507834	2.810625932	1.490891457	0.31720501	1
gene19521	228.3239013	153.8997879	0.674041513	-0.56909065	0.31724175	1
gene15353	830.6444194	571.0746848	0.687508002	-0.54055159	0.31728812	1
gene10102	57.8558302	92.92062669	1.606071961	0.683536535	0.31729564	1
gene68446	57.8558302	92.92062669	1.606071961	0.683536535	0.31729564	1
gene60247	1178.81254	1751.940982	1.486191334	0.571619862	0.31730365	1
gene27221	13.43081773	27.10184945	2.017885285	1.012844161	0.31730665	1

gene28069	13.43081773	27.10184945	2.017885285	1.012844161	0.31730665	1
gene42757	13.43081773	27.10184945	2.017885285	1.012844161	0.31730665	1
gene70375	13.43081773	27.10184945	2.017885285	1.012844161	0.31730665	1
gene4263	2.06627965	7.743385557	3.747501243	1.905928957	0.31736226	1
gene7088	2.06627965	7.743385557	3.747501243	1.905928957	0.31736226	1
gene8110	2.06627965	7.743385557	3.747501243	1.905928957	0.31736226	1
gene11031	2.06627965	7.743385557	3.747501243	1.905928957	0.31736226	1
gene12790	2.06627965	7.743385557	3.747501243	1.905928957	0.31736226	1
gene12810	2.06627965	7.743385557	3.747501243	1.905928957	0.31736226	1
gene13721	2.06627965	7.743385557	3.747501243	1.905928957	0.31736226	1
gene15243	2.06627965	7.743385557	3.747501243	1.905928957	0.31736226	1
gene16439	2.06627965	7.743385557	3.747501243	1.905928957	0.31736226	1
gene17054	2.06627965	7.743385557	3.747501243	1.905928957	0.31736226	1
gene17390	2.06627965	7.743385557	3.747501243	1.905928957	0.31736226	1
gene22653	2.06627965	7.743385557	3.747501243	1.905928957	0.31736226	1
gene24714	2.06627965	7.743385557	3.747501243	1.905928957	0.31736226	1
gene26788	2.06627965	7.743385557	3.747501243	1.905928957	0.31736226	1
gene30726	2.06627965	7.743385557	3.747501243	1.905928957	0.31736226	1
gene31082	2.06627965	7.743385557	3.747501243	1.905928957	0.31736226	1
gene35414	2.06627965	7.743385557	3.747501243	1.905928957	0.31736226	1
gene37976	2.06627965	7.743385557	3.747501243	1.905928957	0.31736226	1
gene38424	2.06627965	7.743385557	3.747501243	1.905928957	0.31736226	1
gene40464	2.06627965	7.743385557	3.747501243	1.905928957	0.31736226	1
gene42019	2.06627965	7.743385557	3.747501243	1.905928957	0.31736226	1
gene42338	2.06627965	7.743385557	3.747501243	1.905928957	0.31736226	1
gene42908	2.06627965	7.743385557	3.747501243	1.905928957	0.31736226	1
gene43137	2.06627965	7.743385557	3.747501243	1.905928957	0.31736226	1
gene44305	2.06627965	7.743385557	3.747501243	1.905928957	0.31736226	1
gene45301	2.06627965	7.743385557	3.747501243	1.905928957	0.31736226	1
gene47346	2.06627965	7.743385557	3.747501243	1.905928957	0.31736226	1
gene49650	2.06627965	7.743385557	3.747501243	1.905928957	0.31736226	1
gene50104	2.06627965	7.743385557	3.747501243	1.905928957	0.31736226	1
gene50556	2.06627965	7.743385557	3.747501243	1.905928957	0.31736226	1
gene51036	2.06627965	7.743385557	3.747501243	1.905928957	0.31736226	1
gene51599	2.06627965	7.743385557	3.747501243	1.905928957	0.31736226	1
gene51903	2.06627965	7.743385557	3.747501243	1.905928957	0.31736226	1
gene55670	2.06627965	7.743385557	3.747501243	1.905928957	0.31736226	1
gene56529	2.06627965	7.743385557	3.747501243	1.905928957	0.31736226	1
gene56964	2.06627965	7.743385557	3.747501243	1.905928957	0.31736226	1
gene62050	2.06627965	7.743385557	3.747501243	1.905928957	0.31736226	1
gene62968	2.06627965	7.743385557	3.747501243	1.905928957	0.31736226	1
gene63992	2.06627965	7.743385557	3.747501243	1.905928957	0.31736226	1
gene64317	2.06627965	7.743385557	3.747501243	1.905928957	0.31736226	1
gene65893	2.06627965	7.743385557	3.747501243	1.905928957	0.31736226	1
gene67511	2.06627965	7.743385557	3.747501243	1.905928957	0.31736226	1
gene69040	2.06627965	7.743385557	3.747501243	1.905928957	0.31736226	1
gene70176	2.06627965	7.743385557	3.747501243	1.905928957	0.31736226	1
gene70277	2.06627965	7.743385557	3.747501243	1.905928957	0.31736226	1

gene70645	2.06627965	7.743385557	3.747501243	1.905928957	0.31736226	1
gene71740	2.06627965	7.743385557	3.747501243	1.905928957	0.31736226	1
gene72562	2.06627965	7.743385557	3.747501243	1.905928957	0.31736226	1
gene73623	2.06627965	7.743385557	3.747501243	1.905928957	0.31736226	1
gene12135	512.4373532	744.3329367	1.452534504	0.538572434	0.3173864	1
gene44442	427.7198876	294.2486512	0.687947088	-0.53963049	0.31745982	1
gene18101	29.96105493	52.26785251	1.744526441	0.802835464	0.31750163	1
gene66003	1609.631847	1086.977748	0.675295875	-0.56640835	0.31751047	1
gene21862	170.4680711	251.6600306	1.476288368	0.561974555	0.31753906	1
gene54207	16.5302372	31.94146542	1.932305328	0.950323076	0.31758972	1
gene56004	16.5302372	31.94146542	1.932305328	0.950323076	0.31758972	1
gene59422	16.5302372	31.94146542	1.932305328	0.950323076	0.31758972	1
gene16043	361.5989388	524.6143715	1.450818338	0.536866886	0.31761226	1
gene13533	1025.907846	702.7122393	0.684966239	-0.54589521	0.31764269	1
gene15291	246.9204182	360.0674284	1.458232701	0.54422096	0.3176601	1
gene41934	313.041367	213.911026	0.683331497	-0.54934247	0.31769341	1
gene16282	168.4017915	111.3111674	0.660985649	-0.59730915	0.31769731	1
gene49024	168.4017915	111.3111674	0.660985649	-0.59730915	0.31769731	1
gene6763	221.0919226	323.286347	1.462225952	0.548166263	0.31773868	1
gene8969	138.4407366	90.0168571	0.650219432	-0.62100142	0.31774258	1
gene23688	304.7762484	208.1034868	0.68280743	-0.55044934	0.31778205	1
gene72767	7131.764213	12604.29584	1.767346124	0.821584611	0.31778574	1
gene16459	290.3122908	422.0145129	1.453657066	0.539686961	0.31783192	1
gene7236	24.7953558	44.52446695	1.795677679	0.844528412	0.31796155	1
gene63292	24.7953558	44.52446695	1.795677679	0.844528412	0.31796155	1
gene71556	737.6618351	508.1596772	0.688878905	-0.53767769	0.31797443	1
gene9900	292.3785705	199.3921781	0.681965774	-0.55222876	0.3179918	1
gene59271	164.2692322	108.4073978	0.659937326	-0.59959908	0.31808858	1
gene29339	703.5682209	484.9295205	0.68924307	-0.53691524	0.31811028	1
gene69808	1314.153858	894.3610318	0.680560367	-0.55520496	0.31813246	1
gene53163	80.58490636	49.36408293	0.612572319	-0.70704792	0.31816299	1
gene1388	191.1308676	280.6977264	1.468615352	0.554456586	0.31819377	1
gene5107	65.08780898	38.71692779	0.594841467	-0.74942287	0.31823723	1
gene23248	279.9808926	190.6808693	0.68104958	-0.55416827	0.31831347	1
gene59866	733.5292758	1071.490976	1.460733759	0.546693249	0.31832949	1
gene53722	211.7936641	142.2847096	0.67180815	-0.5738788	0.31839507	1
gene1760	46.49129213	76.46593238	1.644736657	0.717856608	0.31844759	1
gene33693	46.49129213	76.46593238	1.644736657	0.717856608	0.31844759	1
gene49293	46.49129213	76.46593238	1.644736657	0.717856608	0.31844759	1
gene51592	46.49129213	76.46593238	1.644736657	0.717856608	0.31844759	1
gene56019	6407.533195	3950.094557	0.616476644	-0.69788186	0.31845303	1
gene50469	61.9883895	98.72816585	1.592688028	0.671463703	0.31845428	1
gene60860	994.9136516	682.3858522	0.685874449	-0.54398358	0.31846804	1
gene43806	642.6129712	443.3088231	0.689853525	-0.53563803	0.31854209	1
gene71135	126.0430587	81.30554835	0.645061689	-0.63249096	0.31873252	1
gene49488	629.1821535	915.6553421	1.455310417	0.541326912	0.31877203	1
gene66941	84.71746566	52.26785251	0.616966668	-0.69673555	0.31885674	1
gene9593	6227.766866	10748.78708	1.725945641	0.787387027	0.31889444	1

gene3705	363.6652184	249.7241842	0.686687018	-0.54227541	0.31889456	1
gene8775	64.05466916	101.6319354	1.586643671	0.665978164	0.3189168	1
gene60492	371.930337	255.5317234	0.687041895	-0.54153002	0.31893133	1
gene57962	584.757141	403.6239722	0.690242058	-0.53482571	0.31900384	1
gene7669	14.46395755	5.807539168	0.40151799	-1.31646346	0.31910333	1
gene8871	14.46395755	5.807539168	0.40151799	-1.31646346	0.31910333	1
gene22649	14.46395755	5.807539168	0.40151799	-1.31646346	0.31910333	1
gene24452	14.46395755	5.807539168	0.40151799	-1.31646346	0.31910333	1
gene25917	14.46395755	5.807539168	0.40151799	-1.31646346	0.31910333	1
gene27553	14.46395755	5.807539168	0.40151799	-1.31646346	0.31910333	1
gene42084	14.46395755	5.807539168	0.40151799	-1.31646346	0.31910333	1
gene44883	14.46395755	5.807539168	0.40151799	-1.31646346	0.31910333	1
gene61594	14.46395755	5.807539168	0.40151799	-1.31646346	0.31910333	1
gene63453	14.46395755	5.807539168	0.40151799	-1.31646346	0.31910333	1
gene70654	569.2600436	392.976817	0.690329176	-0.53464363	0.31918206	1
gene9068	525.868171	362.971198	0.690232302	-0.5348461	0.31927974	1
gene32579	301.6768289	206.1676405	0.68340562	-0.54918598	0.31930047	1
gene58847	151.8715543	99.69608905	0.656450048	-0.60724286	0.31931612	1
gene16909	88.85002496	55.17162209	0.620952241	-0.68744578	0.31933568	1
gene11808	36.15989388	19.35846389	0.53535732	-0.90142597	0.31947063	1
gene14920	36.15989388	19.35846389	0.53535732	-0.90142597	0.31947063	1
gene18380	36.15989388	19.35846389	0.53535732	-0.90142597	0.31947063	1
gene25483	36.15989388	19.35846389	0.53535732	-0.90142597	0.31947063	1
gene8910	446.3164044	646.572694	1.448686823	0.534745747	0.31949837	1
gene58029	113.6453808	72.5942396	0.638778621	-0.64661207	0.31951497	1
gene17512	1666.454538	2514.66446	1.508990736	0.593583949	0.31959679	1
gene31264	48.55757178	79.36970196	1.634548414	0.70889211	0.31962065	1
gene60287	48.55757178	79.36970196	1.634548414	0.70889211	0.31962065	1
gene72803	92.98258426	58.07539168	0.62458354	-0.67903354	0.31964064	1
gene20734	173.5674906	115.1828602	0.663620012	-0.5915707	0.3196475	1
gene63235	173.5674906	115.1828602	0.663620012	-0.5915707	0.3196475	1
gene3067	502.105955	727.8782424	1.449650687	0.535705305	0.31969071	1
gene49844	147.738995	96.79231946	0.65515756	-0.61008619	0.31973834	1
gene66692	1131.288108	774.3385557	0.684475113	-0.54693001	0.31983255	1
gene4233	3.099419475	9.679231946	3.122917702	1.642894551	0.31984726	1
gene4540	3.099419475	9.679231946	3.122917702	1.642894551	0.31984726	1
gene11131	3.099419475	9.679231946	3.122917702	1.642894551	0.31984726	1
gene12602	3.099419475	9.679231946	3.122917702	1.642894551	0.31984726	1
gene13453	3.099419475	9.679231946	3.122917702	1.642894551	0.31984726	1
gene14606	3.099419475	9.679231946	3.122917702	1.642894551	0.31984726	1
gene20276	3.099419475	9.679231946	3.122917702	1.642894551	0.31984726	1
gene23674	3.099419475	9.679231946	3.122917702	1.642894551	0.31984726	1
gene24116	3.099419475	9.679231946	3.122917702	1.642894551	0.31984726	1
gene24612	3.099419475	9.679231946	3.122917702	1.642894551	0.31984726	1
gene27502	3.099419475	9.679231946	3.122917702	1.642894551	0.31984726	1
gene30615	3.099419475	9.679231946	3.122917702	1.642894551	0.31984726	1
gene31058	3.099419475	9.679231946	3.122917702	1.642894551	0.31984726	1
gene33684	3.099419475	9.679231946	3.122917702	1.642894551	0.31984726	1

gene33728	3.099419475	9.679231946	3.122917702	1.642894551	0.31984726	1
gene34105	3.099419475	9.679231946	3.122917702	1.642894551	0.31984726	1
gene35222	3.099419475	9.679231946	3.122917702	1.642894551	0.31984726	1
gene35694	3.099419475	9.679231946	3.122917702	1.642894551	0.31984726	1
gene36752	3.099419475	9.679231946	3.122917702	1.642894551	0.31984726	1
gene37128	3.099419475	9.679231946	3.122917702	1.642894551	0.31984726	1
gene38587	3.099419475	9.679231946	3.122917702	1.642894551	0.31984726	1
gene38616	3.099419475	9.679231946	3.122917702	1.642894551	0.31984726	1
gene40400	3.099419475	9.679231946	3.122917702	1.642894551	0.31984726	1
gene41382	3.099419475	9.679231946	3.122917702	1.642894551	0.31984726	1
gene42545	3.099419475	9.679231946	3.122917702	1.642894551	0.31984726	1
gene43018	3.099419475	9.679231946	3.122917702	1.642894551	0.31984726	1
gene45599	3.099419475	9.679231946	3.122917702	1.642894551	0.31984726	1
gene54229	3.099419475	9.679231946	3.122917702	1.642894551	0.31984726	1
gene57074	3.099419475	9.679231946	3.122917702	1.642894551	0.31984726	1
gene58565	3.099419475	9.679231946	3.122917702	1.642894551	0.31984726	1
gene64641	3.099419475	9.679231946	3.122917702	1.642894551	0.31984726	1
gene65537	3.099419475	9.679231946	3.122917702	1.642894551	0.31984726	1
gene66602	3.099419475	9.679231946	3.122917702	1.642894551	0.31984726	1
gene66738	3.099419475	9.679231946	3.122917702	1.642894551	0.31984726	1
gene69499	3.099419475	9.679231946	3.122917702	1.642894551	0.31984726	1
gene70877	3.099419475	9.679231946	3.122917702	1.642894551	0.31984726	1
gene72412	3.099419475	9.679231946	3.122917702	1.642894551	0.31984726	1
gene72946	3.099419475	9.679231946	3.122917702	1.642894551	0.31984726	1
gene59615	281.0140324	191.6487925	0.681990116	-0.55217726	0.31985887	1
gene32585	487.6419974	706.5839321	1.448980883	0.535038561	0.31995746	1
gene19616	165.302372	243.916645	1.475578614	0.561280785	0.31996983	1
gene44793	150.8384145	223.590258	1.482316416	0.567853439	0.31997566	1
gene42904	546.5309675	377.4900459	0.690702025	-0.53386464	0.31998565	1
gene22908	169.4349313	249.7241842	1.473864818	0.559604207	0.32000263	1
gene47636	906.0636266	623.3425373	0.687967731	-0.5395872	0.32000562	1
gene15954	69.22036828	41.62069737	0.601278184	-0.73389548	0.32002314	1
gene64163	3450.687016	5505.547131	1.595493044	0.674002319	0.32013503	1
gene25486	360.565799	247.7883378	0.687220858	-0.54115427	0.32013544	1
gene3861	39.25931335	65.81877724	1.676513714	0.745464284	0.32016681	1
gene47045	39.25931335	65.81877724	1.676513714	0.745464284	0.32016681	1
gene68886	72.31978776	113.2470138	1.565920162	0.647010659	0.32018259	1
gene69501	134.3081773	200.3601013	1.491793764	0.577048101	0.32019455	1
gene28251	350.2344007	507.191754	1.448149448	0.534210495	0.32024645	1
gene72998	1897.877859	2884.41112	1.519808615	0.603889661	0.32029396	1
gene35017	598.1879587	413.3032041	0.690925316	-0.53339832	0.32031704	1
gene41093	913.2956054	1340.573625	1.467841974	0.553696658	0.32034195	1
gene21123	212.826804	143.2526328	0.673094883	-0.5711182	0.32034249	1
gene21262	190.0977278	278.7618801	1.46641353	0.552292002	0.32039901	1
gene22471	32.02733458	55.17162209	1.7226417	0.78462266	0.32043062	1
gene53740	32.02733458	55.17162209	1.7226417	0.78462266	0.32043062	1
gene13688	194.2302871	284.5694192	1.465113518	0.55101245	0.32052402	1
gene20570	1146.785206	1697.737283	1.480431797	0.566018028	0.32053008	1

gene39833	76.45234706	119.0545529	1.557238692	0.638990096	0.32055246	1
gene60763	633.3147128	920.4949581	1.453455825	0.539487224	0.3205542	1
gene21792	310.9750873	212.9431028	0.684759363	-0.54633101	0.32058527	1
gene14800	50.62385143	82.27347154	1.625191866	0.700610049	0.320627	1
gene878	78.51862671	121.9583225	1.553240647	0.635281367	0.32068868	1
gene65591	78.51862671	121.9583225	1.553240647	0.635281367	0.32068868	1
gene48735	543.431548	375.5541995	0.691079127	-0.53307719	0.32072024	1
gene29676	109.5128215	165.5148663	1.511374322	0.595861017	0.32078112	1
gene62988	202.4954057	296.1844976	1.462672679	0.548606955	0.32081825	1
gene25745	309.9419475	449.1163623	1.449033814	0.535091261	0.32087436	1
gene61131	396.7256928	573.9784544	1.446789217	0.53285475	0.32092168	1
gene29149	161.1698127	106.4715514	0.660617206	-0.59811355	0.32097904	1
gene63469	161.1698127	106.4715514	0.660617206	-0.59811355	0.32097904	1
gene33568	959.7868975	660.1236187	0.687781444	-0.5399779	0.32108809	1
gene27890	444.2501248	642.7010012	1.446709782	0.532775538	0.32125192	1
gene16078	508.3047939	351.3561197	0.691231174	-0.53275981	0.32139832	1
gene19631	803.7827839	1174.090835	1.460706622	0.546666447	0.3214322	1
gene67531	131.2087578	85.17724113	0.649173444	-0.62332411	0.32143236	1
gene27988	216.9593633	316.5108846	1.458848698	0.544830264	0.32145312	1
gene4295	200.4291261	134.5413241	0.671266331	-0.57504281	0.32148083	1
gene386	52.69013108	85.17724113	1.616569164	0.692935233	0.32148855	1
gene54211	52.69013108	85.17724113	1.616569164	0.692935233	0.32148855	1
gene35573	21.69593633	39.68485098	1.829137511	0.871163538	0.32166544	1
gene54075	427.7198876	295.2165744	0.690210072	-0.53489257	0.32170747	1
gene27127	43.39187265	24.19807987	0.557663875	-0.84253228	0.32173566	1
gene35970	43.39187265	24.19807987	0.557663875	-0.84253228	0.32173566	1
gene64962	520.7024718	360.0674284	0.691503206	-0.53219216	0.32174244	1
gene69527	324.4059051	222.6223348	0.686246247	-0.54320174	0.32175769	1
gene51289	243.8209987	165.5148663	0.678837619	-0.55886158	0.32176798	1
gene73982	4589.207103	2926.999741	0.637800752	-0.6488223	0.32177448	1
gene61072	217.9925031	147.1243256	0.674905437	-0.56724272	0.32190401	1
gene13524	316.1407865	216.8147956	0.685817221	-0.54410396	0.3219131	1
gene18754	26.86163545	47.42823654	1.765649624	0.820199083	0.32191802	1
gene4331	312.0082272	213.911026	0.685594184	-0.54457322	0.32200208	1
gene50456	312.0082272	213.911026	0.685594184	-0.54457322	0.32200208	1
gene25057	306.842528	444.2767463	1.447898207	0.533960179	0.3220639	1
gene3326	287.2128714	416.2069737	1.449123682	0.535180734	0.32214052	1
gene21244	287.2128714	416.2069737	1.449123682	0.535180734	0.32214052	1
gene73374	3127.314251	2051.029249	0.655843668	-0.60857613	0.32221724	1
gene12806	54.75641073	88.08101071	1.608597232	0.685803142	0.32222404	1
gene7674	9.298258426	20.32638709	2.186042392	1.128321378	0.32222937	1
gene22595	9.298258426	20.32638709	2.186042392	1.128321378	0.32222937	1
gene23420	9.298258426	20.32638709	2.186042392	1.128321378	0.32222937	1
gene25746	9.298258426	20.32638709	2.186042392	1.128321378	0.32222937	1
gene28019	9.298258426	20.32638709	2.186042392	1.128321378	0.32222937	1
gene37144	9.298258426	20.32638709	2.186042392	1.128321378	0.32222937	1
gene37312	9.298258426	20.32638709	2.186042392	1.128321378	0.32222937	1
gene40559	9.298258426	20.32638709	2.186042392	1.128321378	0.32222937	1

gene46727	9.298258426	20.32638709	2.186042392	1.128321378	0.32222937	1
gene52224	9.298258426	20.32638709	2.186042392	1.128321378	0.32222937	1
gene52998	9.298258426	20.32638709	2.186042392	1.128321378	0.32222937	1
gene66471	9.298258426	20.32638709	2.186042392	1.128321378	0.32222937	1
gene69554	9.298258426	20.32638709	2.186042392	1.128321378	0.32222937	1
gene52523	265.516935	385.2334315	1.450880831	0.536929028	0.32230983	1
gene56141	519.669332	752.0763222	1.447220907	0.533285155	0.32231252	1
gene36637	509.3379338	352.3240428	0.691729438	-0.53172024	0.32232564	1
gene14077	192.1640075	128.7337849	0.669916217	-0.57794742	0.32233135	1
gene65658	77.48548688	47.42823654	0.61209187	-0.70817989	0.32233612	1
gene17412	1968.131367	1323.151007	0.672287953	-0.5728488	0.32240613	1
gene68701	388.4605742	561.3954529	1.445180001	0.531249195	0.32241404	1
gene2007	61.9883895	36.7810814	0.593354363	-0.75303413	0.32249381	1
gene35406	61.9883895	36.7810814	0.593354363	-0.75303413	0.32249381	1
gene58239	61.9883895	36.7810814	0.593354363	-0.75303413	0.32249381	1
gene8695	172.5343508	253.595877	1.469828332	0.555647666	0.32250227	1
gene31694	180.7994694	265.2109553	1.466879058	0.552749928	0.32253717	1
gene11345	118.8110799	76.46593238	0.643592605	-0.63578035	0.32260368	1
gene59029	408.0902309	281.6656496	0.690204343	-0.53490454	0.32261993	1
gene8107	415.3222097	600.1123807	1.444932071	0.531001671	0.32262296	1
gene6540	257.2518164	175.1940982	0.681021812	-0.55422709	0.32264778	1
gene32675	193.1971473	282.6335728	1.462928293	0.548859056	0.32271872	1
gene57858	525.868171	363.9391212	0.692072921	-0.53100404	0.32276187	1
gene18815	149.8052746	221.6544116	1.47961687	0.565223655	0.32277296	1
gene1018	3.099419475	0	0	#NAME?	0.32283652	1
gene1321	3.099419475	0	0	#NAME?	0.32283652	1
gene1612	3.099419475	0	0	#NAME?	0.32283652	1
gene2772	3.099419475	0	0	#NAME?	0.32283652	1
gene2895	3.099419475	0	0	#NAME?	0.32283652	1
gene3666	3.099419475	0	0	#NAME?	0.32283652	1
gene4381	3.099419475	0	0	#NAME?	0.32283652	1
gene5163	3.099419475	0	0	#NAME?	0.32283652	1
gene6590	3.099419475	0	0	#NAME?	0.32283652	1
gene7008	3.099419475	0	0	#NAME?	0.32283652	1
gene7115	3.099419475	0	0	#NAME?	0.32283652	1
gene7662	3.099419475	0	0	#NAME?	0.32283652	1
gene7903	3.099419475	0	0	#NAME?	0.32283652	1
gene7942	3.099419475	0	0	#NAME?	0.32283652	1
gene8048	3.099419475	0	0	#NAME?	0.32283652	1
gene8183	3.099419475	0	0	#NAME?	0.32283652	1
gene9424	3.099419475	0	0	#NAME?	0.32283652	1
gene9563	3.099419475	0	0	#NAME?	0.32283652	1
gene9813	3.099419475	0	0	#NAME?	0.32283652	1
gene10506	3.099419475	0	0	#NAME?	0.32283652	1
gene10626	3.099419475	0	0	#NAME?	0.32283652	1
gene12158	3.099419475	0	0	#NAME?	0.32283652	1
gene12369	3.099419475	0	0	#NAME?	0.32283652	1
gene12588	3.099419475	0	0	#NAME?	0.32283652	1

gene13009	3.099419475	0	0	#NAME?	0.32283652	1
gene13223	3.099419475	0	0	#NAME?	0.32283652	1
gene13835	3.099419475	0	0	#NAME?	0.32283652	1
gene14791	3.099419475	0	0	#NAME?	0.32283652	1
gene14939	3.099419475	0	0	#NAME?	0.32283652	1
gene14976	3.099419475	0	0	#NAME?	0.32283652	1
gene15264	3.099419475	0	0	#NAME?	0.32283652	1
gene15287	3.099419475	0	0	#NAME?	0.32283652	1
gene16028	3.099419475	0	0	#NAME?	0.32283652	1
gene16552	3.099419475	0	0	#NAME?	0.32283652	1
gene17359	3.099419475	0	0	#NAME?	0.32283652	1
gene18024	3.099419475	0	0	#NAME?	0.32283652	1
gene18277	3.099419475	0	0	#NAME?	0.32283652	1
gene18794	3.099419475	0	0	#NAME?	0.32283652	1
gene19848	3.099419475	0	0	#NAME?	0.32283652	1
gene21446	3.099419475	0	0	#NAME?	0.32283652	1
gene21522	3.099419475	0	0	#NAME?	0.32283652	1
gene21872	3.099419475	0	0	#NAME?	0.32283652	1
gene22078	3.099419475	0	0	#NAME?	0.32283652	1
gene22573	3.099419475	0	0	#NAME?	0.32283652	1
gene23842	3.099419475	0	0	#NAME?	0.32283652	1
gene23863	3.099419475	0	0	#NAME?	0.32283652	1
gene23974	3.099419475	0	0	#NAME?	0.32283652	1
gene24564	3.099419475	0	0	#NAME?	0.32283652	1
gene24683	3.099419475	0	0	#NAME?	0.32283652	1
gene24749	3.099419475	0	0	#NAME?	0.32283652	1
gene25149	3.099419475	0	0	#NAME?	0.32283652	1
gene25583	3.099419475	0	0	#NAME?	0.32283652	1
gene25652	3.099419475	0	0	#NAME?	0.32283652	1
gene25793	3.099419475	0	0	#NAME?	0.32283652	1
gene25965	3.099419475	0	0	#NAME?	0.32283652	1
gene26850	3.099419475	0	0	#NAME?	0.32283652	1
gene26899	3.099419475	0	0	#NAME?	0.32283652	1
gene28284	3.099419475	0	0	#NAME?	0.32283652	1
gene28312	3.099419475	0	0	#NAME?	0.32283652	1
gene28335	3.099419475	0	0	#NAME?	0.32283652	1
gene29136	3.099419475	0	0	#NAME?	0.32283652	1
gene29864	3.099419475	0	0	#NAME?	0.32283652	1
gene30017	3.099419475	0	0	#NAME?	0.32283652	1
gene30793	3.099419475	0	0	#NAME?	0.32283652	1
gene32071	3.099419475	0	0	#NAME?	0.32283652	1
gene33108	3.099419475	0	0	#NAME?	0.32283652	1
gene34664	3.099419475	0	0	#NAME?	0.32283652	1
gene35036	3.099419475	0	0	#NAME?	0.32283652	1
gene35247	3.099419475	0	0	#NAME?	0.32283652	1
gene35932	3.099419475	0	0	#NAME?	0.32283652	1
gene36365	3.099419475	0	0	#NAME?	0.32283652	1
gene36908	3.099419475	0	0	#NAME?	0.32283652	1

gene38464	3.099419475	0	0	#NAME?	0.32283652	1
gene38482	3.099419475	0	0	#NAME?	0.32283652	1
gene38556	3.099419475	0	0	#NAME?	0.32283652	1
gene38581	3.099419475	0	0	#NAME?	0.32283652	1
gene39351	3.099419475	0	0	#NAME?	0.32283652	1
gene39550	3.099419475	0	0	#NAME?	0.32283652	1
gene39660	3.099419475	0	0	#NAME?	0.32283652	1
gene40040	3.099419475	0	0	#NAME?	0.32283652	1
gene40797	3.099419475	0	0	#NAME?	0.32283652	1
gene41353	3.099419475	0	0	#NAME?	0.32283652	1
gene41510	3.099419475	0	0	#NAME?	0.32283652	1
gene42015	3.099419475	0	0	#NAME?	0.32283652	1
gene43116	3.099419475	0	0	#NAME?	0.32283652	1
gene44072	3.099419475	0	0	#NAME?	0.32283652	1
gene44388	3.099419475	0	0	#NAME?	0.32283652	1
gene44540	3.099419475	0	0	#NAME?	0.32283652	1
gene44705	3.099419475	0	0	#NAME?	0.32283652	1
gene44959	3.099419475	0	0	#NAME?	0.32283652	1
gene45151	3.099419475	0	0	#NAME?	0.32283652	1
gene46921	3.099419475	0	0	#NAME?	0.32283652	1
gene47111	3.099419475	0	0	#NAME?	0.32283652	1
gene47150	3.099419475	0	0	#NAME?	0.32283652	1
gene47410	3.099419475	0	0	#NAME?	0.32283652	1
gene48211	3.099419475	0	0	#NAME?	0.32283652	1
gene48310	3.099419475	0	0	#NAME?	0.32283652	1
gene48323	3.099419475	0	0	#NAME?	0.32283652	1
gene49766	3.099419475	0	0	#NAME?	0.32283652	1
gene50063	3.099419475	0	0	#NAME?	0.32283652	1
gene50318	3.099419475	0	0	#NAME?	0.32283652	1
gene51040	3.099419475	0	0	#NAME?	0.32283652	1
gene52610	3.099419475	0	0	#NAME?	0.32283652	1
gene52984	3.099419475	0	0	#NAME?	0.32283652	1
gene54018	3.099419475	0	0	#NAME?	0.32283652	1
gene54468	3.099419475	0	0	#NAME?	0.32283652	1
gene54494	3.099419475	0	0	#NAME?	0.32283652	1
gene54713	3.099419475	0	0	#NAME?	0.32283652	1
gene54934	3.099419475	0	0	#NAME?	0.32283652	1
gene57094	3.099419475	0	0	#NAME?	0.32283652	1
gene57971	3.099419475	0	0	#NAME?	0.32283652	1
gene58000	3.099419475	0	0	#NAME?	0.32283652	1
gene58763	3.099419475	0	0	#NAME?	0.32283652	1
gene59002	3.099419475	0	0	#NAME?	0.32283652	1
gene59003	3.099419475	0	0	#NAME?	0.32283652	1
gene59587	3.099419475	0	0	#NAME?	0.32283652	1
gene60744	3.099419475	0	0	#NAME?	0.32283652	1
gene61279	3.099419475	0	0	#NAME?	0.32283652	1
gene61283	3.099419475	0	0	#NAME?	0.32283652	1
gene61295	3.099419475	0	0	#NAME?	0.32283652	1

gene61407	3.099419475	0	0	#NAME?	0.32283652	1
gene62280	3.099419475	0	0	#NAME?	0.32283652	1
gene63346	3.099419475	0	0	#NAME?	0.32283652	1
gene63426	3.099419475	0	0	#NAME?	0.32283652	1
gene64746	3.099419475	0	0	#NAME?	0.32283652	1
gene67067	3.099419475	0	0	#NAME?	0.32283652	1
gene67082	3.099419475	0	0	#NAME?	0.32283652	1
gene67462	3.099419475	0	0	#NAME?	0.32283652	1
gene67736	3.099419475	0	0	#NAME?	0.32283652	1
gene68449	3.099419475	0	0	#NAME?	0.32283652	1
gene69003	3.099419475	0	0	#NAME?	0.32283652	1
gene69359	3.099419475	0	0	#NAME?	0.32283652	1
gene69570	3.099419475	0	0	#NAME?	0.32283652	1
gene69777	3.099419475	0	0	#NAME?	0.32283652	1
gene70531	3.099419475	0	0	#NAME?	0.32283652	1
gene73903	3.099419475	0	0	#NAME?	0.32283652	1
gene73983	3.099419475	0	0	#NAME?	0.32283652	1
gene74001	3.099419475	0	0	#NAME?	0.32283652	1
gene31713	145.6727153	215.8468724	1.481724782	0.567277504	0.32287961	1
gene32940	337.8367228	232.3015667	0.687614907	-0.54032727	0.32289899	1
gene34975	33.0604744	17.4226175	0.526992362	-0.92414604	0.32290596	1
gene37360	33.0604744	17.4226175	0.526992362	-0.92414604	0.32290596	1
gene39790	33.0604744	17.4226175	0.526992362	-0.92414604	0.32290596	1
gene46886	33.0604744	17.4226175	0.526992362	-0.92414604	0.32290596	1
gene57621	33.0604744	17.4226175	0.526992362	-0.92414604	0.32290596	1
gene73188	33.0604744	17.4226175	0.526992362	-0.92414604	0.32290596	1
gene21051	648.8118102	449.1163623	0.692213605	-0.5307108	0.32307288	1
gene3795	321.3064856	220.6864884	0.686841064	-0.5419518	0.32321166	1
gene18583	110.5459613	70.65839321	0.639176614	-0.64571347	0.32324891	1
gene50685	274.8151935	187.7770998	0.683285001	-0.54944064	0.32332191	1
gene18268	58.88897003	93.88854988	1.594331669	0.672951785	0.32337897	1
gene4805	162.2029525	107.4394746	0.662376812	-0.59427593	0.32344835	1
gene13907	85.75060548	53.23577571	0.620820989	-0.68775076	0.32346227	1
gene667	217.9925031	317.4788078	1.456374891	0.542381773	0.32349757	1
gene27833	270.6826342	184.8733302	0.682989253	-0.55006522	0.32354115	1
gene64038	304.7762484	209.07141	0.685983278	-0.54375469	0.32364769	1
gene16946	125.0099188	186.8091766	1.494354834	0.579522757	0.32365051	1
gene18262	689.1042633	1000.832583	1.452367423	0.538406476	0.32372687	1
gene34701	531.0338701	367.810814	0.692631553	-0.52983998	0.32376821	1
gene31516	363.6652184	250.6921074	0.689348595	-0.53669437	0.32386655	1
gene11799	433.9187265	300.0561903	0.691503206	-0.53219216	0.32389047	1
gene661	158.0703932	104.535705	0.661323749	-0.59657138	0.32397446	1
gene36080	234.5227403	340.7089645	1.452775812	0.538812088	0.32417604	1
gene69545	214.8930836	145.1884792	0.675631234	-0.56569207	0.32420329	1
gene11111	3165.540424	2078.131099	0.656485409	-0.60716515	0.32425102	1
gene19561	112.6122409	169.3865591	1.504157609	0.588955744	0.32425333	1
gene69800	66.12094881	39.68485098	0.600185746	-0.73651904	0.32431032	1
gene70989	66.12094881	39.68485098	0.600185746	-0.73651904	0.32431032	1

gene19478	422.5541885	609.7916126	1.443108669	0.529179942	0.32431906	1
gene13229	330.604744	227.4619507	0.688017806	-0.53948219	0.32438597	1
gene31033	330.604744	227.4619507	0.688017806	-0.53948219	0.32438597	1
gene16897	3045.696204	4779.604735	1.569297925	0.650119269	0.32451522	1
gene65297	300.6436891	434.5975144	1.445556751	0.531625248	0.32454229	1
gene40237	911.2293257	1332.830239	1.462672679	0.548606955	0.32461121	1
gene18912	246.9204182	358.131582	1.45039274	0.536443609	0.32473318	1
gene51348	359.5326591	518.8068323	1.443003352	0.529074651	0.32474285	1
gene25796	67.15408863	105.5036282	1.571067829	0.651745468	0.32475342	1
gene71711	2489.866978	1658.052432	0.665920086	-0.58657904	0.32476547	1
gene9577	102.2808427	154.8677111	1.514141916	0.598500431	0.32476952	1
gene45137	18.59651685	34.84523501	1.873750621	0.905928957	0.32489093	1
gene68993	18.59651685	34.84523501	1.873750621	0.905928957	0.32489093	1
gene5400	179.7663296	263.2751089	1.464540716	0.550448302	0.32492098	1
gene45652	851.3072159	588.4973023	0.691286637	-0.53264406	0.32493251	1
gene43331	192.1640075	280.6977264	1.46071957	0.546679236	0.32494991	1
gene21171	69.22036828	108.4073978	1.566119922	0.647194688	0.32495411	1
gene54693	69.22036828	108.4073978	1.566119922	0.647194688	0.32495411	1
gene68518	513.4704931	741.4291671	1.443956716	0.530027497	0.32509145	1
gene21136	36.15989388	60.97916126	1.686375559	0.753925863	0.32513465	1
gene23924	161.1698127	237.1411827	1.471374687	0.557164677	0.32520665	1
gene59962	161.1698127	237.1411827	1.471374687	0.557164677	0.32520665	1
gene71966	73.35292758	114.214937	1.557060376	0.638824887	0.32523	1
gene2266	28.9279151	50.33200612	1.739911291	0.799013753	0.32524896	1
gene54753	28.9279151	50.33200612	1.739911291	0.799013753	0.32524896	1
gene56588	87.81688513	134.5413241	1.532066685	0.615479093	0.32532711	1
gene48952	472.1449001	327.1580398	0.69291872	-0.52924196	0.32532961	1
gene21326	1642.692322	2464.332454	1.500178957	0.58513461	0.32535392	1
gene62899	154.9709738	228.4298739	1.474017156	0.559753316	0.32539331	1
gene63091	123.976779	80.33762515	0.648005423	-0.62592221	0.32540455	1
gene72343	2156.162815	1447.045176	0.671120551	-0.57535616	0.32545374	1
gene22432	150.8384145	222.6223348	1.475899462	0.561594449	0.32554354	1
gene16290	1364.777709	933.0779596	0.683684935	-0.54859646	0.32556195	1
gene41886	2507.430355	3866.853163	1.542157753	0.624950351	0.3255915	1
gene70211	741.7943944	513.9672164	0.692870181	-0.52934303	0.32562558	1
gene32928	145.6727153	95.82439627	0.657806069	-0.60426578	0.32563078	1
gene10816	265.516935	384.2655083	1.447235402	0.533299605	0.32563413	1
gene2921	47.52443195	77.43385557	1.629348366	0.704295095	0.32579139	1
gene33471	910.1961859	1329.926469	1.461142653	0.547097037	0.32586662	1
gene65885	163.2360924	108.4073978	0.664114144	-0.59049687	0.32589775	1
gene54561	915.361885	1337.669855	1.461356297	0.547307968	0.32591993	1
gene61315	285.1465917	195.5204853	0.685684104	-0.54438402	0.32597516	1
gene70185	40.29245318	22.26223348	0.552516209	-0.85591131	0.32603029	1
gene39591	10396.48606	19542.3693	1.879709085	0.910509399	0.32603947	1
gene2222	29.96105493	15.48677111	0.516896723	-0.95205204	0.32607616	1
gene54064	215.9262234	146.1564024	0.676881205	-0.56302544	0.3261168	1
gene68024	141.540156	92.92062669	0.656496568	-0.60714063	0.32620408	1
gene66960	641.5798314	445.2446695	0.693981712	-0.52703045	0.32637757	1

gene33770	978.3834144	1432.526328	1.464176832	0.550089802	0.3264056	1
gene39298	276.8814731	189.7129461	0.685177466	-0.54545039	0.32643926	1
gene20460	621.9501747	431.6937448	0.694096991	-0.52679082	0.32645304	1
gene64182	159.1035331	105.5036282	0.663113045	-0.59267326	0.32648042	1
gene48762	8498.608201	15320.28832	1.802682035	0.85014495	0.32651172	1
gene51804	9621.631191	5703.003463	0.592727298	-0.75455959	0.32659019	1
gene547	74.38606741	45.49239015	0.611571383	-0.70940719	0.32670222	1
gene20031	74.38606741	45.49239015	0.611571383	-0.70940719	0.32670222	1
gene52427	111.5791011	71.6263164	0.641933083	-0.63950518	0.32676007	1
gene38070	826.5118601	1203.128531	1.455670014	0.541683348	0.32678782	1
gene35432	246.9204182	168.4186359	0.682076586	-0.55199436	0.32691707	1
gene62986	7930.381297	4814.44997	0.607089343	-0.72001925	0.32695645	1
gene14625	58.88897003	34.84523501	0.591710723	-0.75703606	0.32700355	1
gene5812	12.3976779	25.16600306	2.029896507	1.021406174	0.32700539	1
gene7518	12.3976779	25.16600306	2.029896507	1.021406174	0.32700539	1
gene10384	12.3976779	25.16600306	2.029896507	1.021406174	0.32700539	1
gene18589	12.3976779	25.16600306	2.029896507	1.021406174	0.32700539	1
gene28987	12.3976779	25.16600306	2.029896507	1.021406174	0.32700539	1
gene31276	12.3976779	25.16600306	2.029896507	1.021406174	0.32700539	1
gene53839	12.3976779	25.16600306	2.029896507	1.021406174	0.32700539	1
gene56238	12.3976779	25.16600306	2.029896507	1.021406174	0.32700539	1
gene59535	12.3976779	25.16600306	2.029896507	1.021406174	0.32700539	1
gene70918	12.3976779	25.16600306	2.029896507	1.021406174	0.32700539	1
gene72077	12.3976779	25.16600306	2.029896507	1.021406174	0.32700539	1
gene73793	12.3976779	25.16600306	2.029896507	1.021406174	0.32700539	1
gene63676	38.22617353	63.88293085	1.671182987	0.74086971	0.32702827	1
gene45540	424.6204681	294.2486512	0.6929686	-0.52913811	0.32705115	1
gene88	15.49709738	30.00561903	1.936208975	0.953234671	0.32705742	1
gene6606	15.49709738	30.00561903	1.936208975	0.953234671	0.32705742	1
gene15020	15.49709738	30.00561903	1.936208975	0.953234671	0.32705742	1
gene21496	15.49709738	30.00561903	1.936208975	0.953234671	0.32705742	1
gene26826	15.49709738	30.00561903	1.936208975	0.953234671	0.32705742	1
gene40539	15.49709738	30.00561903	1.936208975	0.953234671	0.32705742	1
gene47560	15.49709738	30.00561903	1.936208975	0.953234671	0.32705742	1
gene54059	15.49709738	30.00561903	1.936208975	0.953234671	0.32705742	1
gene39069	172.5343508	115.1828602	0.667593784	-0.58295757	0.32711291	1
gene65292	8259.952902	14798.57772	1.791605582	0.841253066	0.32712053	1
gene69056	328.5384644	226.4940275	0.689398814	-0.53658928	0.3271854	1
gene28306	699.4356616	1013.415585	1.448904653	0.53496266	0.32720802	1
gene31723	387.4274344	268.1147249	0.692038563	-0.53107566	0.32725729	1
gene71331	296.5111298	427.822052	1.442853266	0.528924589	0.32727473	1
gene33909	182.865749	267.1468017	1.460890315	0.546847863	0.32728299	1
gene55016	78.51862671	48.39615973	0.616365336	-0.69814237	0.327371	1
gene48157	51.65699125	83.24139474	1.611425534	0.688337522	0.32746141	1
gene36820	270.6826342	391.0409706	1.444647426	0.530717438	0.32754048	1
gene19718	221.0919226	150.0280952	0.678577912	-0.55941362	0.32755476	1
gene6448	17.56337703	7.743385557	0.440882499	-1.18153388	0.3276481	1
gene20151	17.56337703	7.743385557	0.440882499	-1.18153388	0.3276481	1

gene20459	17.56337703	7.743385557	0.440882499	-1.18153388	0.3276481	1
gene20526	17.56337703	7.743385557	0.440882499	-1.18153388	0.3276481	1
gene24373	17.56337703	7.743385557	0.440882499	-1.18153388	0.3276481	1
gene29675	17.56337703	7.743385557	0.440882499	-1.18153388	0.3276481	1
gene45261	17.56337703	7.743385557	0.440882499	-1.18153388	0.3276481	1
gene51741	17.56337703	7.743385557	0.440882499	-1.18153388	0.3276481	1
gene55462	17.56337703	7.743385557	0.440882499	-1.18153388	0.3276481	1
gene64403	591.9891198	854.6761809	1.443736299	0.529807256	0.32769425	1
gene3272	82.65118601	51.29992932	0.620679893	-0.68807868	0.32778269	1
gene69825	354.36696	244.8845682	0.69104797	-0.53314223	0.32781539	1
gene10579	162.2029525	238.1091059	1.467970232	0.553822713	0.32782056	1
gene34896	1665.421398	1132.470138	0.679990145	-0.55641426	0.3278993	1
gene71259	95.04886391	60.01123807	0.631372492	-0.66343669	0.32794661	1
gene45933	2767.781591	4292.739368	1.550967526	0.63316848	0.32797477	1
gene12187	1767.702241	1199.256838	0.678426949	-0.55973462	0.32801015	1
gene3104	30.99419475	53.23577571	1.717604736	0.780398075	0.32806784	1
gene33580	234.5227403	159.7073271	0.680988662	-0.55429732	0.32807164	1
gene19729	199.3959862	134.5413241	0.674744395	-0.56758701	0.32807406	1
gene36182	199.3959862	134.5413241	0.674744395	-0.56758701	0.32807406	1
gene63376	849.2409362	588.4973023	0.6929686	-0.52913811	0.32809126	1
gene47920	53.7232709	86.14516432	1.603498128	0.681222669	0.32809616	1
gene29426	1143.685786	1682.250512	1.470902701	0.556701817	0.32819416	1
gene16	181.8326092	121.9583225	0.670717552	-0.57622274	0.32820005	1
gene41796	400.8582521	277.7939569	0.692997975	-0.52907696	0.32827941	1
gene59528	105.3802622	158.7394039	1.506348539	0.591055619	0.3284001	1
gene72902	495.907116	344.5806573	0.694849189	-0.52522821	0.32848009	1
gene65548	360.565799	518.8068323	1.438868672	0.524934921	0.32853514	1
gene9056	147.738995	217.7827188	1.47410451	0.559838811	0.32853741	1
gene53903	147.738995	217.7827188	1.47410451	0.559838811	0.32853741	1
gene64945	1330.684095	1969.723701	1.480233895	0.565825158	0.32853787	1
gene28786	380.1954556	263.2751089	0.692473056	-0.53017016	0.32854765	1
gene3405	293.4117103	422.9824361	1.44160039	0.527671307	0.32862866	1
gene6360	40.29245318	66.78670043	1.657548627	0.729051195	0.32867754	1
gene11570	40.29245318	66.78670043	1.657548627	0.729051195	0.32867754	1
gene28211	40.29245318	66.78670043	1.657548627	0.729051195	0.32867754	1
gene70787	40.29245318	66.78670043	1.657548627	0.729051195	0.32867754	1
gene37690	101.2477029	152.9318648	1.51047244	0.594999861	0.32872496	1
gene9514	26.86163545	13.55092472	0.504471321	-0.98715584	0.32873538	1
gene17789	26.86163545	13.55092472	0.504471321	-0.98715584	0.32873538	1
gene29160	26.86163545	13.55092472	0.504471321	-0.98715584	0.32873538	1
gene30744	26.86163545	13.55092472	0.504471321	-0.98715584	0.32873538	1
gene1704	520.7024718	362.0032748	0.695220965	-0.52445651	0.32881338	1
gene44377	1000.079351	1462.531947	1.462415903	0.548353665	0.32888751	1
gene60273	142.5732959	93.88854988	0.658528298	-0.60268266	0.32897108	1
gene7872	243.8209987	166.4827895	0.68280743	-0.55044934	0.32900414	1
gene13272	1376.142247	2039.414171	1.481979189	0.567525188	0.3290112	1
gene43608	2569.418745	1714.191978	0.667151659	-0.58391334	0.32903109	1
gene55886	57.8558302	91.95270349	1.589342045	0.668429643	0.32904973	1

gene41868	351.2675405	242.9487219	0.691634421	-0.53191843	0.32918602	1
gene45038	540.3321285	778.2102485	1.440244264	0.526313512	0.32920319	1
gene41688	5935.388295	10040.2673	1.691594012	0.758383358	0.32929438	1
gene21493	617.8176154	429.7578984	0.695606418	-0.52365685	0.32931641	1
gene15036	410.1565106	284.5694192	0.693806905	-0.5273939	0.32932155	1
gene70033	51.65699125	30.00561903	0.580862693	-0.78373092	0.32932982	1
gene13665	488.6751373	339.7410413	0.695228825	-0.5244402	0.32934539	1
gene65071	333.7041635	480.0899045	1.438669208	0.524734912	0.32941039	1
gene52573	759.3577714	527.5181411	0.694689856	-0.52555906	0.3294318	1
gene50722	355.4000998	511.0634468	1.437994663	0.524058321	0.32944812	1
gene36611	196.2965668	285.5373424	1.454622193	0.540644493	0.32945483	1
gene3052	278.9477528	191.6487925	0.687041895	-0.54153002	0.32952928	1
gene45978	562.0280648	391.0409706	0.695767694	-0.5233224	0.32954863	1
gene25457	221.0919226	320.3825774	1.449092186	0.535149377	0.32959574	1
gene72744	138.4407366	90.9847803	0.657211039	-0.60557138	0.32962738	1
gene29885	61.9883895	97.76024266	1.57707344	0.657249844	0.32966871	1
gene66684	131.2087578	194.5525621	1.482771161	0.568295961	0.32971168	1
gene18581	917.4281647	1336.701932	1.457009915	0.543010695	0.32973887	1
gene35621	86.78374531	132.6054777	1.527999019	0.611643617	0.32976155	1
gene45232	86.78374531	132.6054777	1.527999019	0.611643617	0.32976155	1
gene59567	783.1199874	543.9728354	0.694622592	-0.52569876	0.32977695	1
gene13958	1029.007266	711.4235481	0.691368829	-0.53247254	0.32985471	1
gene54512	758.3246316	1098.592826	1.44871046	0.534769286	0.3298767	1
gene7037	64.05466916	100.6640122	1.571532779	0.652172364	0.32987883	1
gene34160	64.05466916	100.6640122	1.571532779	0.652172364	0.32987883	1
gene51917	64.05466916	100.6640122	1.571532779	0.652172364	0.32987883	1
gene34960	84.71746566	129.7017081	1.530991361	0.614466142	0.32988001	1
gene61557	318.2070661	457.8276711	1.438772799	0.524838789	0.32991503	1
gene40598	82.65118601	126.7979385	1.534133321	0.617423863	0.32998589	1
gene39284	296.5111298	204.2317941	0.688782894	-0.53787878	0.32999417	1
gene60549	520.7024718	749.1725526	1.438772799	0.524838789	0.33000227	1
gene11772	66.12094881	103.5677818	1.56633841	0.647395943	0.33003395	1
gene38098	42.35873283	69.69047001	1.645244448	0.718301953	0.33008703	1
gene41601	245.8872784	355.2278124	1.444677475	0.530747446	0.33010361	1
gene1788	7574.981197	13308.94393	1.756960655	0.813081884	0.33015595	1
gene17689	76.45234706	118.0866297	1.544578215	0.627212929	0.33020378	1
gene68820	649.84495	937.9175756	1.443294398	0.529365605	0.33022733	1
gene13980	72.31978776	112.2790906	1.552536229	0.634626935	0.33023578	1
gene20245	72.31978776	112.2790906	1.552536229	0.634626935	0.33023578	1
gene71368	67.15408863	40.65277417	0.605365585	-0.72412143	0.33025417	1
gene13107	25.82849563	45.49239015	1.761325584	0.816661619	0.33026432	1
gene42474	25.82849563	45.49239015	1.761325584	0.816661619	0.33026432	1
gene45453	25.82849563	45.49239015	1.761325584	0.816661619	0.33026432	1
gene19911	134.3081773	88.08101071	0.655812718	-0.60864422	0.33029339	1
gene5491	254.152397	366.8428908	1.443397328	0.529468489	0.33032921	1
gene9430	369.8640574	531.3898339	1.436716608	0.522775518	0.33038339	1
gene60358	350.2344007	503.3200612	1.437094872	0.523155307	0.33040165	1
gene32045	163.2360924	239.0770291	1.464608872	0.55051544	0.33042468	1

gene1326	37.1930337	20.32638709	0.546510598	-0.87167862	0.33043704	1
gene10195	37.1930337	20.32638709	0.546510598	-0.87167862	0.33043704	1
gene59391	37.1930337	20.32638709	0.546510598	-0.87167862	0.33043704	1
gene63572	37.1930337	20.32638709	0.546510598	-0.87167862	0.33043704	1
gene1579	33.0604744	56.13954529	1.698086501	0.763909952	0.33046403	1
gene3852	33.0604744	56.13954529	1.698086501	0.763909952	0.33046403	1
gene32550	33.0604744	56.13954529	1.698086501	0.763909952	0.33046403	1
gene43645	33.0604744	56.13954529	1.698086501	0.763909952	0.33046403	1
gene59111	23.76221598	11.61507834	0.48880451	-1.0326705	0.33047095	1
gene66695	23.76221598	11.61507834	0.48880451	-1.0326705	0.33047095	1
gene58950	407.0570911	282.6335728	0.694333987	-0.5262983	0.33048127	1
gene26404	1694.349313	1153.764448	0.680948397	-0.55438262	0.33055101	1
gene1065	20.6627965	9.679231946	0.468437655	-1.09407104	0.33055821	1
gene6233	20.6627965	9.679231946	0.468437655	-1.09407104	0.33055821	1
gene37706	20.6627965	9.679231946	0.468437655	-1.09407104	0.33055821	1
gene39842	20.6627965	9.679231946	0.468437655	-1.09407104	0.33055821	1
gene53913	20.6627965	9.679231946	0.468437655	-1.09407104	0.33055821	1
gene59112	20.6627965	9.679231946	0.468437655	-1.09407104	0.33055821	1
gene63881	20.6627965	9.679231946	0.468437655	-1.09407104	0.33055821	1
gene67509	20.6627965	9.679231946	0.468437655	-1.09407104	0.33055821	1
gene34143	1620.996386	2418.840063	1.492193373	0.577434506	0.33068565	1
gene16398	20.6627965	37.74900459	1.826906856	0.869403081	0.3307087	1
gene24774	20.6627965	37.74900459	1.826906856	0.869403081	0.3307087	1
gene30135	20.6627965	37.74900459	1.826906856	0.869403081	0.3307087	1
gene40002	20.6627965	37.74900459	1.826906856	0.869403081	0.3307087	1
gene63400	108.4796816	69.69047001	0.642428784	-0.63839156	0.3307432	1
gene40460	227.2907615	154.8677111	0.681363862	-0.55350266	0.33077024	1
gene18802	1498.052746	1025.030663	0.684242037	-0.54742135	0.33077375	1
gene14129	44.42501248	25.16600306	0.566482746	-0.81989608	0.33080209	1
gene52794	44.42501248	25.16600306	0.566482746	-0.81989608	0.33080209	1
gene59099	44.42501248	25.16600306	0.566482746	-0.81989608	0.33080209	1
gene1227	7.231978776	1.935846389	0.26767866	-1.90142597	0.33096547	1
gene2986	7.231978776	1.935846389	0.26767866	-1.90142597	0.33096547	1
gene3730	7.231978776	1.935846389	0.26767866	-1.90142597	0.33096547	1
gene5030	7.231978776	1.935846389	0.26767866	-1.90142597	0.33096547	1
gene8047	7.231978776	1.935846389	0.26767866	-1.90142597	0.33096547	1
gene9453	7.231978776	1.935846389	0.26767866	-1.90142597	0.33096547	1
gene10702	7.231978776	1.935846389	0.26767866	-1.90142597	0.33096547	1
gene12033	7.231978776	1.935846389	0.26767866	-1.90142597	0.33096547	1
gene12564	7.231978776	1.935846389	0.26767866	-1.90142597	0.33096547	1
gene12760	7.231978776	1.935846389	0.26767866	-1.90142597	0.33096547	1
gene16957	7.231978776	1.935846389	0.26767866	-1.90142597	0.33096547	1
gene17001	7.231978776	1.935846389	0.26767866	-1.90142597	0.33096547	1
gene18880	7.231978776	1.935846389	0.26767866	-1.90142597	0.33096547	1
gene22677	7.231978776	1.935846389	0.26767866	-1.90142597	0.33096547	1
gene27749	7.231978776	1.935846389	0.26767866	-1.90142597	0.33096547	1
gene29473	7.231978776	1.935846389	0.26767866	-1.90142597	0.33096547	1
gene32484	7.231978776	1.935846389	0.26767866	-1.90142597	0.33096547	1

gene36418	7.231978776	1.935846389	0.26767866	-1.90142597	0.33096547	1
gene38012	7.231978776	1.935846389	0.26767866	-1.90142597	0.33096547	1
gene39919	7.231978776	1.935846389	0.26767866	-1.90142597	0.33096547	1
gene40486	7.231978776	1.935846389	0.26767866	-1.90142597	0.33096547	1
gene43296	7.231978776	1.935846389	0.26767866	-1.90142597	0.33096547	1
gene43705	7.231978776	1.935846389	0.26767866	-1.90142597	0.33096547	1
gene47129	7.231978776	1.935846389	0.26767866	-1.90142597	0.33096547	1
gene47542	7.231978776	1.935846389	0.26767866	-1.90142597	0.33096547	1
gene50693	7.231978776	1.935846389	0.26767866	-1.90142597	0.33096547	1
gene50996	7.231978776	1.935846389	0.26767866	-1.90142597	0.33096547	1
gene51876	7.231978776	1.935846389	0.26767866	-1.90142597	0.33096547	1
gene52014	7.231978776	1.935846389	0.26767866	-1.90142597	0.33096547	1
gene52593	7.231978776	1.935846389	0.26767866	-1.90142597	0.33096547	1
gene52859	7.231978776	1.935846389	0.26767866	-1.90142597	0.33096547	1
gene59022	7.231978776	1.935846389	0.26767866	-1.90142597	0.33096547	1
gene68557	7.231978776	1.935846389	0.26767866	-1.90142597	0.33096547	1
gene73997	7.231978776	1.935846389	0.26767866	-1.90142597	0.33096547	1
gene47156	310.9750873	447.1805159	1.437994663	0.524058321	0.33097981	1
gene49299	378.129176	262.3071857	0.693697293	-0.52762184	0.33100438	1
gene5004	827.5449999	574.9463776	0.694761466	-0.52541035	0.33101533	1
gene4386	279.9808926	192.6167157	0.687963789	-0.53959546	0.33106411	1
gene54017	104.3471223	66.78670043	0.640043529	-0.64375807	0.33122048	1
gene39940	444.2501248	637.8613853	1.435815883	0.521870762	0.33122232	1
gene30315	44.42501248	72.5942396	1.634084844	0.708482892	0.3312605	1
gene32703	71.28664793	43.55654376	0.611005637	-0.7107424	0.33127383	1
gene25847	428.7530274	298.1203439	0.695319508	-0.52425203	0.33134236	1
gene34511	146.7058552	215.8468724	1.471290101	0.557081737	0.33150755	1
gene21089	323.3727653	223.590258	0.691431939	-0.53234085	0.33156357	1
gene28595	211.7936641	306.8316527	1.448729139	0.534787887	0.3315779	1
gene55100	110.5459613	165.5148663	1.497249328	0.582314485	0.33166797	1
gene3607	228.3239013	330.0618094	1.445585887	0.531654326	0.33166984	1
gene10449	143.6064357	94.85647307	0.660530795	-0.59830227	0.33171186	1
gene2553	55.78955055	32.90938862	0.589884455	-0.7614957	0.33178807	1
gene56095	55.78955055	32.90938862	0.589884455	-0.7614957	0.33178807	1
gene17506	219.0256429	149.060172	0.680560367	-0.55520496	0.33179071	1
gene30565	392.5931335	563.3312993	1.434898502	0.520948691	0.33186906	1
gene52792	108.4796816	162.6110967	1.499000497	0.584000862	0.33188699	1
gene7545	315.1076466	217.7827188	0.691137524	-0.53295528	0.33196671	1
gene1497	8.265118601	18.3905407	2.225078863	1.15385647	0.33198379	1
gene2213	8.265118601	18.3905407	2.225078863	1.15385647	0.33198379	1
gene5177	8.265118601	18.3905407	2.225078863	1.15385647	0.33198379	1
gene22042	8.265118601	18.3905407	2.225078863	1.15385647	0.33198379	1
gene27725	8.265118601	18.3905407	2.225078863	1.15385647	0.33198379	1
gene46984	8.265118601	18.3905407	2.225078863	1.15385647	0.33198379	1
gene48662	8.265118601	18.3905407	2.225078863	1.15385647	0.33198379	1
gene48879	8.265118601	18.3905407	2.225078863	1.15385647	0.33198379	1
gene50117	8.265118601	18.3905407	2.225078863	1.15385647	0.33198379	1
gene54661	8.265118601	18.3905407	2.225078863	1.15385647	0.33198379	1

gene62299	8.265118601	18.3905407	2.225078863	1.15385647	0.33198379	1
gene71109	8.265118601	18.3905407	2.225078863	1.15385647	0.33198379	1
gene25193	96.08200373	60.97916126	0.634657469	-0.65594993	0.33200514	1
gene55892	357.4663795	512.9992932	1.435098019	0.521149278	0.33210656	1
gene55004	106.413402	159.7073271	1.500819673	0.585750644	0.33210884	1
gene42110	609.5524968	424.9182824	0.697098748	-0.52056506	0.33213941	1
gene10491	1530.080081	1047.292897	0.684469336	-0.54694218	0.33219178	1
gene54129	939.124101	1366.707551	1.455300263	0.541316846	0.33220185	1
gene10173	1357.54573	933.0779596	0.687327092	-0.54093127	0.33220786	1
gene11208	46.49129213	75.49800918	1.623917205	0.699478079	0.33223252	1
gene14371	442.1838451	307.7995759	0.6960896	-0.52265507	0.3322887	1
gene28054	79.55176653	49.36408293	0.620527803	-0.68843224	0.33231041	1
gene59218	263.4506554	379.4258923	1.440216164	0.526285364	0.33232122	1
gene16279	1225.303833	844.9969489	0.689622383	-0.53612149	0.33244368	1
gene22722	721.1315979	502.352138	0.696616456	-0.52156354	0.33244812	1
gene12120	83.68432583	52.26785251	0.62458354	-0.67903354	0.33246375	1
gene67656	83.68432583	52.26785251	0.62458354	-0.67903354	0.33246375	1
gene68589	83.68432583	52.26785251	0.62458354	-0.67903354	0.33246375	1
gene67916	421.5210486	293.280728	0.695767694	-0.5233224	0.33251058	1
gene21882	1666.454538	1137.309754	0.682472715	-0.55115673	0.33257502	1
gene40980	228.3239013	155.8356343	0.682520023	-0.55105672	0.33258399	1
gene2362	134.3081773	198.4242549	1.477380298	0.563041243	0.33260449	1
gene55733	1201.541617	1764.523984	1.468550036	0.554392421	0.33265499	1
gene29599	631.2484331	908.8798798	1.439813284	0.525881734	0.33266773	1
gene4893	640.5466916	922.4308045	1.440068018	0.526136955	0.33278789	1
gene62016	453.5483832	650.4443868	1.434123482	0.52016925	0.33291377	1
gene43400	1189.143939	1745.16552	1.467581395	0.55344052	0.33292856	1
gene61109	826.5118601	1197.320992	1.448643449	0.534702552	0.332934	1
gene38749	98.14828338	148.0922488	1.508862343	0.593461191	0.33301318	1
gene59600	224.191342	152.9318648	0.682148844	-0.55184153	0.33310953	1
gene67179	224.191342	152.9318648	0.682148844	-0.55184153	0.33310953	1
gene50375	492.8076966	343.6127341	0.69725521	-0.52024128	0.33314142	1
gene3969	525.868171	366.8428908	0.697594779	-0.51953885	0.33330308	1
gene21470	896.7653682	623.3425373	0.695101037	-0.5247054	0.33336066	1
gene64360	1530.080081	1048.26082	0.685101932	-0.54560944	0.33338424	1
gene38741	663.2757677	462.667287	0.697548907	-0.51963372	0.33343573	1
gene20136	27.89477528	48.39615973	1.734954279	0.794897644	0.3334867	1
gene24382	27.89477528	48.39615973	1.734954279	0.794897644	0.3334867	1
gene30258	206.627965	140.3488632	0.6792346	-0.55801814	0.33350809	1
gene37813	206.627965	140.3488632	0.6792346	-0.55801814	0.33350809	1
gene57878	206.627965	140.3488632	0.6792346	-0.55801814	0.33350809	1
gene61680	2483.668139	3799.098539	1.529632111	0.613184715	0.33353664	1
gene41114	804.8159237	1164.411603	1.446804877	0.532870366	0.33359845	1
gene10394	225.2244819	325.2221934	1.443991305	0.530062055	0.33363097	1
gene84	229.3570412	331.0297326	1.443294398	0.529365605	0.33363755	1
gene48921	59.92210985	35.8131582	0.597661836	-0.74259867	0.33364266	1
gene73958	59.92210985	35.8131582	0.597661836	-0.74259867	0.33364266	1
gene32051	113.6453808	73.56216279	0.647295669	-0.62750324	0.33365092	1

gene45801	113.6453808	73.56216279	0.647295669	-0.62750324	0.33365092	1
gene11520	233.4896005	336.8372717	1.44262216	0.52869349	0.33365426	1
gene4208	239.6884394	345.5485805	1.441657267	0.527728226	0.3336973	1
gene53060	123.976779	183.905407	1.483385909	0.568893969	0.33370637	1
gene28374	1286.259082	886.6176463	0.689299425	-0.53679728	0.33372377	1
gene14638	762.4571909	531.3898339	0.696943829	-0.52088571	0.33382362	1
gene55556	162.2029525	108.4073978	0.668344171	-0.58133687	0.33387438	1
gene73845	131.2087578	86.14516432	0.656550415	-0.6070223	0.33394424	1
gene65393	264.4837952	181.9695606	0.688017806	-0.53948219	0.33403007	1
gene6769	493.8408364	344.5806573	0.697756508	-0.51920442	0.33408408	1
gene45270	202.4954057	137.4450936	0.678756603	-0.55903377	0.33413314	1
gene43633	1394.738764	959.2118859	0.687735876	-0.54007349	0.3341474	1
gene54210	149.8052746	219.7185652	1.466694452	0.552568354	0.3341483	1
gene55741	664.3089075	463.6352102	0.69792111	-0.51886412	0.33416485	1
gene14279	393.6262734	273.9222641	0.69589426	-0.52305999	0.33419529	1
gene57401	393.6262734	273.9222641	0.69589426	-0.52305999	0.33419529	1
gene44862	52.69013108	84.20931793	1.598199059	0.676447111	0.33420757	1
gene57935	215.9262234	147.1243256	0.681363862	-0.55350266	0.33423629	1
gene39336	906.0636266	630.1179997	0.695445641	-0.52399034	0.33426422	1
gene43725	325.4390449	225.5261044	0.692990309	-0.52909292	0.33427127	1
gene47128	539.2989887	376.5221227	0.698169532	-0.51835069	0.33428465	1
gene61490	2455.773364	1649.341124	0.671617808	-0.57428761	0.33429116	1
gene17274	1186.044519	819.8309459	0.691231174	-0.53275981	0.334299	1
gene7108	48.55757178	28.06977264	0.578072	-0.7906789	0.33430033	1
gene24121	48.55757178	28.06977264	0.578072	-0.7906789	0.33430033	1
gene43753	48.55757178	28.06977264	0.578072	-0.7906789	0.33430033	1
gene57103	48.55757178	28.06977264	0.578072	-0.7906789	0.33430033	1
gene68416	48.55757178	28.06977264	0.578072	-0.7906789	0.33430033	1
gene37628	85.75060548	130.6696313	1.523833337	0.607705122	0.33432777	1
gene73927	445.2832646	637.8613853	1.432484524	0.518519553	0.3343393	1
gene65650	351.2675405	243.916645	0.694389936	-0.52618206	0.33439384	1
gene49827	260.3512359	179.065791	0.687785446	-0.53996951	0.33444084	1
gene41275	325.4390449	466.5389798	1.433567936	0.519610274	0.33448607	1
gene31494	17.56337703	32.90938862	1.873750621	0.905928957	0.33457257	1
gene68405	17.56337703	32.90938862	1.873750621	0.905928957	0.33457257	1
gene44163	833.7438388	580.7539168	0.696561569	-0.52167722	0.33462036	1
gene22628	81.61804618	124.8620921	1.529834368	0.613375464	0.33471464	1
gene46314	852.3403557	1234.102073	1.447898207	0.533960179	0.33474623	1
gene19177	2778.11299	4282.092213	1.54136719	0.624210587	0.33481168	1
gene36889	79.55176653	121.9583225	1.53306869	0.616422339	0.33489022	1
gene1187	34.09361423	18.3905407	0.539413058	-0.89053765	0.33490441	1
gene17456	113.6453808	169.3865591	1.490483449	0.575780355	0.33496949	1
gene70876	313.041367	216.8147956	0.69260749	-0.5298901	0.3349825	1
gene40712	597.1548189	417.1748969	0.698604254	-0.51745267	0.33499424	1
gene26215	64.05466916	38.71692779	0.604435684	-0.72633926	0.33503619	1
gene39117	64.05466916	38.71692779	0.604435684	-0.72633926	0.33503619	1
gene68522	77.48548688	119.0545529	1.53647551	0.619624771	0.33505061	1
gene44649	562.0280648	806.2800211	1.43459032	0.520638801	0.33505439	1

gene9902	139.4738764	205.1997173	1.471241229	0.557033814	0.33518179	1
gene18314	140.5070162	92.92062669	0.661323749	-0.59657138	0.33522937	1
gene38664	140.5070162	92.92062669	0.661323749	-0.59657138	0.33522937	1
gene73286	140.5070162	92.92062669	0.661323749	-0.59657138	0.33522937	1
gene7360	22.72907615	40.65277417	1.788580139	0.838814761	0.33538737	1
gene9298	22.72907615	40.65277417	1.788580139	0.838814761	0.33538737	1
gene69395	15919.65156	32962.62439	2.070561925	1.050022351	0.33540856	1
gene44991	71.28664793	110.3432442	1.547880948	0.630294514	0.33541004	1
gene19533	153.9378339	102.5998586	0.666501899	-0.58531911	0.33544394	1
gene57080	153.9378339	102.5998586	0.666501899	-0.58531911	0.33544394	1
gene43350	63.02152933	98.72816585	1.566578388	0.647616961	0.33545069	1
gene1766	69.22036828	107.4394746	1.552136709	0.634255633	0.33547689	1
gene14312	101.2477029	64.85085404	0.640516794	-0.6426917	0.33548038	1
gene19109	122.9436392	80.33762515	0.653450847	-0.61384938	0.33548437	1
gene19226	67.15408863	104.535705	1.556654362	0.638448646	0.33550973	1
gene65287	67.15408863	104.535705	1.556654362	0.638448646	0.33550973	1
gene54845	1713.97897	1171.187066	0.683314723	-0.54937788	0.33556909	1
gene22635	238.6552996	343.6127341	1.439786733	0.52585513	0.33558344	1
gene65875	230.390181	331.9976558	1.44102346	0.527093823	0.33559958	1
gene52932	283.0803121	195.5204853	0.690689098	-0.53389164	0.3356283	1
gene33614	234.5227403	160.6752503	0.685115866	-0.5455801	0.33562849	1
gene1930	1139.553227	1665.795818	1.461797289	0.547743263	0.3356392	1
gene51604	1104.426473	765.627247	0.693235146	-0.5285833	0.33565036	1
gene44378	39.25931335	64.85085404	1.6518591	0.724090634	0.33576164	1
gene71938	41.325593	23.23015667	0.562125186	-0.83103664	0.33576559	1
gene73829	41.325593	23.23015667	0.562125186	-0.83103664	0.33576559	1
gene20461	207.6611048	300.0561903	1.444932071	0.531001671	0.33588939	1
gene49952	563.0612047	807.2479443	1.433677081	0.51972011	0.33593228	1
gene38097	1281.093383	884.6817999	0.690567769	-0.53414509	0.33597853	1
gene55909	583.7240012	837.2535634	1.434331228	0.520378222	0.33599937	1
gene30751	1431.931798	985.3458121	0.688123424	-0.53926074	0.33604762	1
gene14617	344.0355618	239.0770291	0.694919525	-0.52508218	0.33604785	1
gene22728	68.18722846	41.62069737	0.61038846	-0.71220041	0.33606453	1
gene44895	68.18722846	41.62069737	0.61038846	-0.71220041	0.33606453	1
gene47501	29.96105493	51.29992932	1.712220395	0.775868416	0.33617205	1
gene23697	190.0977278	128.7337849	0.677197915	-0.56235056	0.33617634	1
gene25151	163.2360924	109.375321	0.670043735	-0.57767283	0.33629845	1
gene72450	365.7314981	254.5638002	0.696040132	-0.5227576	0.33635107	1
gene71582	92.98258426	59.04331487	0.634993266	-0.6551868	0.33645488	1
gene59698	1132.321248	784.9857109	0.693253537	-0.52854502	0.33652751	1
gene39567	438.0512858	305.8637295	0.69823726	-0.51821075	0.33655446	1
gene37571	414.2890699	592.3689951	1.429844614	0.515858373	0.33659502	1
gene51113	127.0761985	187.7770998	1.477673254	0.563327293	0.33668234	1
gene6082	954.6211984	1385.098092	1.45094001	0.536987872	0.33669646	1
gene73630	959.7868975	1392.841477	1.451198678	0.537245047	0.3367051	1
gene2829	357.4663795	248.756261	0.695887153	-0.52307472	0.33671417	1
gene44352	72.31978776	44.52446695	0.615660918	-0.6997921	0.336719	1
gene56649	72.31978776	44.52446695	0.615660918	-0.6997921	0.336719	1

gene39814	152.9046941	223.590258	1.462285113	0.548224632	0.33672122	1
gene66511	425.6536079	297.1524208	0.698108545	-0.51847673	0.33680709	1
gene28903	513.4704931	359.0995052	0.699357626	-0.51589771	0.33684814	1
gene15099	534.1332896	373.6183531	0.699485242	-0.51563448	0.3368638	1
gene18362	132.2418976	87.11308752	0.658740453	-0.60221795	0.33689066	1
gene50770	353.3338202	245.8524914	0.695807979	-0.52323887	0.33690937	1
gene46844	783.1199874	546.876605	0.698330542	-0.51801802	0.33691631	1
gene8757	438.0512858	626.2463069	1.429618693	0.515630404	0.33695554	1
gene2076	41.325593	67.75462362	1.639531794	0.713283879	0.33704951	1
gene26922	41.325593	67.75462362	1.639531794	0.713283879	0.33704951	1
gene72122	76.45234706	47.42823654	0.620363381	-0.68881456	0.33706027	1
gene20952	84.71746566	53.23577571	0.628391977	-0.67026333	0.33706169	1
gene44830	84.71746566	53.23577571	0.628391977	-0.67026333	0.33706169	1
gene11306	159.1035331	106.4715514	0.669196651	-0.57949787	0.3371254	1
gene49767	80.58490636	50.33200612	0.62458354	-0.67903354	0.33715678	1
gene15793	148.7721348	217.7827188	1.463867673	0.549785146	0.33715933	1
gene30096	626.082734	898.2327246	1.434686944	0.520735968	0.33716819	1
gene33498	960.8200373	1393.8094	1.450645642	0.536695147	0.33723518	1
gene68473	97.11514356	146.1564024	1.504980552	0.589744844	0.33725345	1
gene18629	14.46395755	28.06977264	1.940670286	0.95655503	0.33730241	1
gene42183	14.46395755	28.06977264	1.940670286	0.95655503	0.33730241	1
gene46013	14.46395755	28.06977264	1.940670286	0.95655503	0.33730241	1
gene56795	14.46395755	28.06977264	1.940670286	0.95655503	0.33730241	1
gene58778	345.0687016	240.0449523	0.695643943	-0.52357903	0.33732863	1
gene29778	361.5989388	516.8709859	1.429404045	0.515413777	0.33739315	1
gene26585	208.6942447	142.2847096	0.681785498	-0.55261018	0.3374177	1
gene65240	208.6942447	142.2847096	0.681785498	-0.55261018	0.3374177	1
gene28026	256.2186766	367.810814	1.43553475	0.521588255	0.33745907	1
gene16803	400.8582521	279.7298033	0.697827229	-0.5190582	0.33747713	1
gene12376	11.36453808	23.23015667	2.044091587	1.031459839	0.33750759	1
gene23032	11.36453808	23.23015667	2.044091587	1.031459839	0.33750759	1
gene31224	11.36453808	23.23015667	2.044091587	1.031459839	0.33750759	1
gene36229	11.36453808	23.23015667	2.044091587	1.031459839	0.33750759	1
gene37904	11.36453808	23.23015667	2.044091587	1.031459839	0.33750759	1
gene44016	11.36453808	23.23015667	2.044091587	1.031459839	0.33750759	1
gene55874	11.36453808	23.23015667	2.044091587	1.031459839	0.33750759	1
gene57930	11.36453808	23.23015667	2.044091587	1.031459839	0.33750759	1
gene63217	11.36453808	23.23015667	2.044091587	1.031459839	0.33750759	1
gene64747	11.36453808	23.23015667	2.044091587	1.031459839	0.33750759	1
gene4350	195.2634269	132.6054777	0.679110675	-0.55828138	0.33753057	1
gene53102	3725.502209	5884.973023	1.57964556	0.659600884	0.33763585	1
gene69534	172.5343508	250.6921074	1.452998236	0.539032952	0.33764753	1
gene35061	275.8483333	190.6808693	0.69125257	-0.53271516	0.33792217	1
gene45607	358.4995193	249.7241842	0.696581643	-0.52163564	0.33795269	1
gene22477	154.9709738	103.5677818	0.668304388	-0.58142275	0.33798131	1
gene68463	1719.144669	2557.25308	1.487514766	0.57290399	0.33810367	1
gene7950	43.39187265	70.65839321	1.628378516	0.703436093	0.33810845	1
gene41263	43.39187265	70.65839321	1.628378516	0.703436093	0.33810845	1

gene26663	164.2692322	239.0770291	1.455397495	0.541413233	0.33837111	1
gene41337	32.02733458	54.2036989	1.692419916	0.759087568	0.3384186	1
gene72072	32.02733458	54.2036989	1.692419916	0.759087568	0.3384186	1
gene59080	448.3826841	313.6071151	0.699418435	-0.51577227	0.33845746	1
gene22179	106.413402	68.72254682	0.645807253	-0.63082445	0.33854077	1
gene56325	1609.631847	2384.962752	1.481682135	0.567235979	0.33854907	1
gene68779	813.0810423	1172.154989	1.441621349	0.527692281	0.33859167	1
gene42094	123.976779	81.30554835	0.655812718	-0.60864422	0.33861295	1
gene35497	515.5367727	361.0353516	0.700309601	-0.51393523	0.33865913	1
gene3041	511.4042134	358.131582	0.700290636	-0.5139743	0.33867858	1
gene54802	136.3744569	200.3601013	1.469190828	0.555021795	0.33868136	1
gene70332	164.2692322	110.3432442	0.671721921	-0.57406398	0.33870257	1
gene937	495.907116	708.5197785	1.428734849	0.514738199	0.33872852	1
gene39370	326.4721847	466.5389798	1.429031328	0.515037545	0.33874451	1
gene3758	56.82269038	33.87731181	0.59619338	-0.74614774	0.33875496	1
gene33122	56.82269038	33.87731181	0.59619338	-0.74614774	0.33875496	1
gene72487	364.6983583	520.7426787	1.427872287	0.513866946	0.3387943	1
gene24206	341.9692821	238.1091059	0.696287995	-0.52224394	0.3388422	1
gene3394	150.8384145	100.6640122	0.667363235	-0.58345588	0.33886623	1
gene69823	763.4903307	1098.592826	1.43890863	0.524974985	0.33887626	1
gene66929	137.4075967	90.9847803	0.662152475	-0.59476463	0.33888649	1
gene64568	427.7198876	299.0882671	0.699262007	-0.51609497	0.33893529	1
gene42088	45.4581523	73.56216279	1.618239173	0.694424851	0.33896192	1
gene54505	45.4581523	73.56216279	1.618239173	0.694424851	0.33896192	1
gene27146	84.71746566	128.7337849	1.519566053	0.603659388	0.33903118	1
gene21771	24.7953558	43.55654376	1.756641208	0.812819552	0.33917705	1
gene27510	24.7953558	43.55654376	1.756641208	0.812819552	0.33917705	1
gene66280	24.7953558	43.55654376	1.756641208	0.812819552	0.33917705	1
gene70838	24.7953558	43.55654376	1.756641208	0.812819552	0.33917705	1
gene73283	24.7953558	43.55654376	1.756641208	0.812819552	0.33917705	1
gene32598	853.3734955	596.2406879	0.698686672	-0.51728247	0.33919266	1
gene61567	223.1582022	152.9318648	0.68530694	-0.5451778	0.33920321	1
gene34435	340.9361423	486.8653669	1.428025095	0.514021332	0.33920471	1
gene66892	102.2808427	65.81877724	0.643510314	-0.63596482	0.33925965	1
gene29430	969.0851559	675.6103899	0.697163078	-0.52043193	0.33927637	1
gene40377	4469.362883	7194.573106	1.609753626	0.686839899	0.33928531	1
gene56887	1871.016223	1277.658617	0.682868807	-0.55031966	0.33930275	1
gene36561	30.99419475	16.45469431	0.530896009	-0.9134988	0.33934209	1
gene39934	30.99419475	16.45469431	0.530896009	-0.9134988	0.33934209	1
gene45992	30.99419475	16.45469431	0.530896009	-0.9134988	0.33934209	1
gene70017	30.99419475	16.45469431	0.530896009	-0.9134988	0.33934209	1
gene45584	209.7273845	143.2526328	0.683042098	-0.54995359	0.33935427	1
gene13094	461.8135018	659.1556955	1.427320104	0.513308923	0.33942522	1
gene52572	108.4796816	161.6431735	1.490077875	0.575387731	0.33945944	1
gene70631	232.4564606	333.9335022	1.436542143	0.522600317	0.33950653	1
gene546	12.3976779	4.839615973	0.390364713	-1.35710545	0.33955998	1
gene4872	12.3976779	4.839615973	0.390364713	-1.35710545	0.33955998	1
gene6389	12.3976779	4.839615973	0.390364713	-1.35710545	0.33955998	1

gene9595	12.3976779	4.839615973	0.390364713	-1.35710545	0.33955998	1
gene18053	12.3976779	4.839615973	0.390364713	-1.35710545	0.33955998	1
gene21354	12.3976779	4.839615973	0.390364713	-1.35710545	0.33955998	1
gene29491	12.3976779	4.839615973	0.390364713	-1.35710545	0.33955998	1
gene36556	12.3976779	4.839615973	0.390364713	-1.35710545	0.33955998	1
gene44441	12.3976779	4.839615973	0.390364713	-1.35710545	0.33955998	1
gene53307	12.3976779	4.839615973	0.390364713	-1.35710545	0.33955998	1
gene54816	12.3976779	4.839615973	0.390364713	-1.35710545	0.33955998	1
gene73313	12.3976779	4.839615973	0.390364713	-1.35710545	0.33955998	1
gene73832	12.3976779	4.839615973	0.390364713	-1.35710545	0.33955998	1
gene1633	351.2675405	501.3842148	1.427357091	0.513346308	0.33956281	1
gene12467	45.4581523	26.13392626	0.574900759	-0.79861516	0.33956631	1
gene54781	45.4581523	26.13392626	0.574900759	-0.79861516	0.33956631	1
gene63715	45.4581523	26.13392626	0.574900759	-0.79861516	0.33956631	1
gene65058	45.4581523	26.13392626	0.574900759	-0.79861516	0.33956631	1
gene46623	196.2965668	133.5734009	0.680467331	-0.55540219	0.33958076	1
gene40592	569.2600436	814.0234067	1.42996758	0.515982438	0.33958248	1
gene20342	80.58490636	122.9262457	1.525425185	0.609211425	0.33959044	1
gene49968	692.2036828	484.9295205	0.700559001	-0.51342154	0.33962471	1
gene73642	1858.618545	2773.099953	1.492022104	0.577268909	0.33976355	1
gene29097	177.7000499	257.4675698	1.448888562	0.534946637	0.33977448	1
gene20062	78.51862671	120.0224761	1.528586033	0.612197754	0.33985587	1
gene70602	78.51862671	120.0224761	1.528586033	0.612197754	0.33985587	1
gene71321	182.865749	123.8941689	0.677514349	-0.56167659	0.33986726	1
gene51516	98.14828338	62.91500765	0.641019949	-0.64155884	0.33993965	1
gene381	475.2443195	332.965579	0.700619798	-0.51329634	0.34000157	1
gene11935	788.2856865	551.7162209	0.699893745	-0.51479218	0.3400527	1
gene47037	76.45234706	117.1187066	1.531917738	0.615338828	0.34010863	1
gene69767	60.95524968	36.7810814	0.603411217	-0.72878658	0.34012227	1
gene56784	1027.974126	716.263164	0.696771588	-0.5212423	0.34019508	1
gene22672	255.1855368	176.1620214	0.690329176	-0.53464363	0.34027488	1
gene42714	34.09361423	57.10746848	1.675019495	0.744177887	0.34030374	1
gene59898	34.09361423	57.10746848	1.675019495	0.744177887	0.34030374	1
gene47300	74.38606741	114.214937	1.535434537	0.618647005	0.34034593	1
gene8338	19.62965668	35.8131582	1.824441395	0.867454809	0.34043599	1
gene43092	19.62965668	35.8131582	1.824441395	0.867454809	0.34043599	1
gene43542	19.62965668	35.8131582	1.824441395	0.867454809	0.34043599	1
gene46966	19.62965668	35.8131582	1.824441395	0.867454809	0.34043599	1
gene59324	19.62965668	35.8131582	1.824441395	0.867454809	0.34043599	1
gene50946	2735.754257	1836.1503	0.671167849	-0.57525449	0.34053119	1
gene51782	72.31978776	111.3111674	1.539152296	0.622135991	0.34056454	1
gene31714	94.01572408	60.01123807	0.638310651	-0.64766937	0.34056592	1
gene25073	673.607166	472.346519	0.701219558	-0.51206186	0.34066118	1
gene22363	380.1954556	542.036989	1.425679821	0.511650018	0.34067537	1
gene28258	129.1424781	85.17724113	0.659560219	-0.60042371	0.34074668	1
gene61955	348.1681211	496.5445988	1.426163307	0.512139191	0.34078253	1
gene53455	283.0803121	404.5918954	1.429247737	0.515256006	0.34098307	1
gene11976	260.3512359	372.6504299	1.43133728	0.517363669	0.34100813	1

gene29539	55.78955055	88.08101071	1.578808394	0.658836095	0.34106596	1
gene49120	55.78955055	88.08101071	1.578808394	0.658836095	0.34106596	1
gene73652	55.78955055	88.08101071	1.578808394	0.658836095	0.34106596	1
gene645	295.47799	422.0145129	1.428243481	0.514241945	0.34107754	1
gene69552	65.08780898	39.68485098	0.609712504	-0.71379896	0.34109719	1
gene59983	2877.294413	4421.473153	1.53667735	0.61981428	0.34112135	1
gene58373	188.0314482	127.7658617	0.679491984	-0.55747156	0.34118161	1
gene41021	557.8955055	796.6007892	1.427867372	0.51386198	0.34119687	1
gene62990	543.431548	381.3617387	0.701765917	-0.51093821	0.3412072	1
gene32043	59.92210985	93.88854988	1.566843192	0.647860804	0.34123678	1
gene71755	535.1664294	375.5541995	0.701752163	-0.51096649	0.34124318	1
gene53356	309.9419475	442.3408999	1.42717339	0.513160621	0.34126905	1
gene48071	633.3147128	905.9761102	1.430530654	0.516550413	0.34128632	1
gene15560	1045.537503	728.8461656	0.697101886	-0.52055856	0.34134509	1
gene46669	614.7181959	878.8742607	1.429718962	0.515731586	0.34136235	1
gene39689	514.5036329	361.0353516	0.701715845	-0.51104116	0.3413961	1
gene65522	344.0355618	240.0449523	0.697732964	-0.5192531	0.34140492	1
gene6436	233.4896005	160.6752503	0.688147352	-0.53921057	0.34150292	1
gene16478	954.6211984	666.8990811	0.698600746	-0.51745991	0.34165814	1
gene18239	1233.568951	1800.337142	1.459454002	0.545428741	0.34167063	1
gene11403	137.4075967	201.3280245	1.465188456	0.551086239	0.34169816	1
gene46740	125.0099188	82.27347154	0.658135549	-0.60354334	0.341706	1
gene50364	125.0099188	82.27347154	0.658135549	-0.60354334	0.341706	1
gene411	5.165699125	0.967923195	0.187375062	-2.41599914	0.34172872	1
gene3783	5.165699125	0.967923195	0.187375062	-2.41599914	0.34172872	1
gene4370	5.165699125	0.967923195	0.187375062	-2.41599914	0.34172872	1
gene5826	5.165699125	0.967923195	0.187375062	-2.41599914	0.34172872	1
gene5979	5.165699125	0.967923195	0.187375062	-2.41599914	0.34172872	1
gene6185	5.165699125	0.967923195	0.187375062	-2.41599914	0.34172872	1
gene6456	5.165699125	0.967923195	0.187375062	-2.41599914	0.34172872	1
gene9275	5.165699125	0.967923195	0.187375062	-2.41599914	0.34172872	1
gene10230	5.165699125	0.967923195	0.187375062	-2.41599914	0.34172872	1
gene12988	5.165699125	0.967923195	0.187375062	-2.41599914	0.34172872	1
gene13464	5.165699125	0.967923195	0.187375062	-2.41599914	0.34172872	1
gene15443	5.165699125	0.967923195	0.187375062	-2.41599914	0.34172872	1
gene16111	5.165699125	0.967923195	0.187375062	-2.41599914	0.34172872	1
gene17840	5.165699125	0.967923195	0.187375062	-2.41599914	0.34172872	1
gene20721	5.165699125	0.967923195	0.187375062	-2.41599914	0.34172872	1
gene22540	5.165699125	0.967923195	0.187375062	-2.41599914	0.34172872	1
gene22547	5.165699125	0.967923195	0.187375062	-2.41599914	0.34172872	1
gene24060	5.165699125	0.967923195	0.187375062	-2.41599914	0.34172872	1
gene27656	5.165699125	0.967923195	0.187375062	-2.41599914	0.34172872	1
gene30379	5.165699125	0.967923195	0.187375062	-2.41599914	0.34172872	1
gene32205	5.165699125	0.967923195	0.187375062	-2.41599914	0.34172872	1
gene33352	5.165699125	0.967923195	0.187375062	-2.41599914	0.34172872	1
gene33845	5.165699125	0.967923195	0.187375062	-2.41599914	0.34172872	1
gene37677	5.165699125	0.967923195	0.187375062	-2.41599914	0.34172872	1
gene39627	5.165699125	0.967923195	0.187375062	-2.41599914	0.34172872	1

gene39906	5.165699125	0.967923195	0.187375062	-2.41599914	0.34172872	1
gene40579	5.165699125	0.967923195	0.187375062	-2.41599914	0.34172872	1
gene42184	5.165699125	0.967923195	0.187375062	-2.41599914	0.34172872	1
gene42650	5.165699125	0.967923195	0.187375062	-2.41599914	0.34172872	1
gene43874	5.165699125	0.967923195	0.187375062	-2.41599914	0.34172872	1
gene44642	5.165699125	0.967923195	0.187375062	-2.41599914	0.34172872	1
gene44844	5.165699125	0.967923195	0.187375062	-2.41599914	0.34172872	1
gene45486	5.165699125	0.967923195	0.187375062	-2.41599914	0.34172872	1
gene46097	5.165699125	0.967923195	0.187375062	-2.41599914	0.34172872	1
gene47124	5.165699125	0.967923195	0.187375062	-2.41599914	0.34172872	1
gene50302	5.165699125	0.967923195	0.187375062	-2.41599914	0.34172872	1
gene51552	5.165699125	0.967923195	0.187375062	-2.41599914	0.34172872	1
gene53156	5.165699125	0.967923195	0.187375062	-2.41599914	0.34172872	1
gene53361	5.165699125	0.967923195	0.187375062	-2.41599914	0.34172872	1
gene53538	5.165699125	0.967923195	0.187375062	-2.41599914	0.34172872	1
gene57932	5.165699125	0.967923195	0.187375062	-2.41599914	0.34172872	1
gene58013	5.165699125	0.967923195	0.187375062	-2.41599914	0.34172872	1
gene58434	5.165699125	0.967923195	0.187375062	-2.41599914	0.34172872	1
gene59606	5.165699125	0.967923195	0.187375062	-2.41599914	0.34172872	1
gene60176	5.165699125	0.967923195	0.187375062	-2.41599914	0.34172872	1
gene61045	5.165699125	0.967923195	0.187375062	-2.41599914	0.34172872	1
gene62737	5.165699125	0.967923195	0.187375062	-2.41599914	0.34172872	1
gene63398	5.165699125	0.967923195	0.187375062	-2.41599914	0.34172872	1
gene64406	5.165699125	0.967923195	0.187375062	-2.41599914	0.34172872	1
gene66342	5.165699125	0.967923195	0.187375062	-2.41599914	0.34172872	1
gene66509	5.165699125	0.967923195	0.187375062	-2.41599914	0.34172872	1
gene69950	5.165699125	0.967923195	0.187375062	-2.41599914	0.34172872	1
gene70010	5.165699125	0.967923195	0.187375062	-2.41599914	0.34172872	1
gene70560	5.165699125	0.967923195	0.187375062	-2.41599914	0.34172872	1
gene72230	5.165699125	0.967923195	0.187375062	-2.41599914	0.34172872	1
gene72556	5.165699125	0.967923195	0.187375062	-2.41599914	0.34172872	1
gene73064	5.165699125	0.967923195	0.187375062	-2.41599914	0.34172872	1
gene74032	5.165699125	0.967923195	0.187375062	-2.41599914	0.34172872	1
gene74123	5.165699125	0.967923195	0.187375062	-2.41599914	0.34172872	1
gene74138	5.165699125	0.967923195	0.187375062	-2.41599914	0.34172872	1
gene74219	5.165699125	0.967923195	0.187375062	-2.41599914	0.34172872	1
gene10666	69.22036828	42.58862056	0.615261398	-0.70072862	0.34174302	1
gene42849	667.408327	955.3401931	1.431417851	0.517444876	0.34176794	1
gene35087	5187.395062	8486.750571	1.636033205	0.71020203	0.3418532	1
gene58097	470.0786204	669.8028507	1.424874099	0.510834449	0.34188196	1
gene49434	300.6436891	209.07141	0.695412602	-0.52405888	0.34188246	1
gene11392	36.15989388	60.01123807	1.659607693	0.73084225	0.34188914	1
gene59404	81.61804618	51.29992932	0.628536601	-0.66993134	0.34191248	1
gene20265	560.994925	393.9447402	0.702225141	-0.50999445	0.3420125	1
gene65134	135.3413171	198.4242549	1.466102585	0.551986055	0.34202138	1
gene65523	135.3413171	198.4242549	1.466102585	0.551986055	0.34202138	1
gene24299	77.48548688	48.39615973	0.62458354	-0.67903354	0.34208491	1
gene36530	214.8930836	308.7674991	1.436842424	0.522901852	0.34209666	1

gene57156	229.3570412	157.7714807	0.687885926	-0.53975876	0.34215928	1
gene51674	415.3222097	591.4010719	1.423957251	0.509905835	0.3421992	1
gene71955	595.0885392	849.8365649	1.428084241	0.514081085	0.34220322	1
gene15393	26.86163545	46.46031334	1.729615958	0.790451739	0.34226359	1
gene16346	26.86163545	46.46031334	1.729615958	0.790451739	0.34226359	1
gene43691	26.86163545	46.46031334	1.729615958	0.790451739	0.34226359	1
gene56242	26.86163545	46.46031334	1.729615958	0.790451739	0.34226359	1
gene57583	26.86163545	46.46031334	1.729615958	0.790451739	0.34226359	1
gene64442	26.86163545	46.46031334	1.729615958	0.790451739	0.34226359	1
gene40126	1377.175387	953.4043467	0.692289708	-0.53055219	0.34227574	1
gene23091	49.5907116	29.03769584	0.585547069	-0.77214295	0.34227947	1
gene46660	49.5907116	29.03769584	0.585547069	-0.77214295	0.34227947	1
gene65513	49.5907116	29.03769584	0.585547069	-0.77214295	0.34227947	1
gene67678	49.5907116	29.03769584	0.585547069	-0.77214295	0.34227947	1
gene6100	7.231978776	16.45469431	2.275268612	1.186036876	0.34231318	1
gene29800	7.231978776	16.45469431	2.275268612	1.186036876	0.34231318	1
gene33726	7.231978776	16.45469431	2.275268612	1.186036876	0.34231318	1
gene37948	7.231978776	16.45469431	2.275268612	1.186036876	0.34231318	1
gene38472	7.231978776	16.45469431	2.275268612	1.186036876	0.34231318	1
gene46741	7.231978776	16.45469431	2.275268612	1.186036876	0.34231318	1
gene48651	7.231978776	16.45469431	2.275268612	1.186036876	0.34231318	1
gene52069	7.231978776	16.45469431	2.275268612	1.186036876	0.34231318	1
gene54241	7.231978776	16.45469431	2.275268612	1.186036876	0.34231318	1
gene55659	7.231978776	16.45469431	2.275268612	1.186036876	0.34231318	1
gene58300	7.231978776	16.45469431	2.275268612	1.186036876	0.34231318	1
gene59292	7.231978776	16.45469431	2.275268612	1.186036876	0.34231318	1
gene60045	7.231978776	16.45469431	2.275268612	1.186036876	0.34231318	1
gene65061	7.231978776	16.45469431	2.275268612	1.186036876	0.34231318	1
gene66499	7.231978776	16.45469431	2.275268612	1.186036876	0.34231318	1
gene31232	900.8979275	1298.952927	1.441842508	0.527913588	0.34231336	1
gene40149	133.2750374	195.5204853	1.46704506	0.552913184	0.34235483	1
gene8092	147.738995	98.72816585	0.668260711	-0.58151704	0.34241914	1
gene29014	7990.303407	13941.9657	1.744860612	0.803111792	0.34253519	1
gene4604	238.6552996	164.5469431	0.689475337	-0.53642915	0.34260532	1
gene38843	120.8773595	79.36970196	0.656613466	-0.60688376	0.34268136	1
gene67757	120.8773595	79.36970196	0.656613466	-0.60688376	0.34268136	1
gene29277	111.5791011	165.5148663	1.483385909	0.568893969	0.34270665	1
gene16701	680.8391447	478.1540582	0.702301068	-0.50983847	0.34285809	1
gene64879	317.1739263	452.0201319	1.425149088	0.511112851	0.34286112	1
gene12787	557.8955055	392.0088938	0.702656483	-0.50910854	0.34286238	1
gene27882	537.232709	377.4900459	0.702656483	-0.50910854	0.34298324	1
gene8324	103.3139825	66.78670043	0.646443964	-0.62940278	0.34298518	1
gene45724	157.0372534	105.5036282	0.671838216	-0.57381423	0.34298959	1
gene46283	198.3628464	285.5373424	1.439469878	0.5255376	0.34301039	1
gene62443	4415.639612	2878.603581	0.651910897	-0.6172533	0.34303064	1
gene40403	246.9204182	353.291966	1.430792839	0.516814802	0.34303704	1
gene22422	455.6146629	648.5085404	1.423370653	0.509311396	0.34309736	1
gene7387	109.5128215	162.6110967	1.484858983	0.570325925	0.34310126	1

gene53779	793.4513857	556.5558369	0.701436593	-0.5116154	0.34316204	1
gene7057	38.22617353	62.91500765	1.645862032	0.718843404	0.34322443	1
gene26693	38.22617353	62.91500765	1.645862032	0.718843404	0.34322443	1
gene30688	38.22617353	62.91500765	1.645862032	0.718843404	0.34322443	1
gene53242	319.2402059	222.6223348	0.697350555	-0.52004402	0.34323763	1
gene30559	238.6552996	341.6768877	1.431675258	0.517704289	0.34325868	1
gene57546	234.5227403	335.8693485	1.432139792	0.518172322	0.34339058	1
gene17133	85.75060548	129.7017081	1.512545682	0.596978716	0.34350025	1
gene22542	85.75060548	129.7017081	1.512545682	0.596978716	0.34350025	1
gene29750	315.1076466	219.7185652	0.697280969	-0.52018799	0.34358933	1
gene9841	27.89477528	14.51884792	0.520486284	-0.94206795	0.3435952	1
gene46667	27.89477528	14.51884792	0.520486284	-0.94206795	0.3435952	1
gene62802	855.4397752	1230.23038	1.438126232	0.524190314	0.34362251	1
gene47023	393.6262734	275.8581105	0.70081224	-0.51290012	0.34362439	1
gene5439	449.4158239	315.5429615	0.702118049	-0.51021448	0.34363834	1
gene28889	116.7448002	76.46593238	0.654983624	-0.61046926	0.34366926	1
gene26373	130.175618	86.14516432	0.661761132	-0.59561754	0.34371524	1
gene1966	125.0099188	183.905407	1.471126521	0.556921328	0.34379286	1
gene67892	99.18142321	63.88293085	0.644101776	-0.63463942	0.34381809	1
gene31470	83.68432583	126.7979385	1.515193404	0.599501955	0.3438775	1
gene47814	2123.102341	1447.045176	0.68157109	-0.55306395	0.34391938	1
gene22952	230.390181	158.7394039	0.689002471	-0.53741894	0.34393863	1
gene51057	152.9046941	102.5998586	0.67100529	-0.57560395	0.34399366	1
gene61508	437.018146	306.8316527	0.702102774	-0.51024587	0.34404806	1
gene57044	122.9436392	181.0016374	1.472232631	0.558005653	0.34417885	1
gene27586	2405.149513	1629.98266	0.677705336	-0.56126996	0.34422465	1
gene64991	1172.613701	816.9271763	0.696672037	-0.52144844	0.34433648	1
gene26646	306.842528	213.911026	0.697136174	-0.5204876	0.34433684	1
gene53191	882.3014106	618.5029214	0.701010918	-0.51249118	0.34442412	1
gene20453	424.6204681	298.1203439	0.702086608	-0.51027909	0.34451859	1
gene20653	79.55176653	120.9903993	1.520901478	0.604926701	0.34461964	1
gene62387	254.152397	362.971198	1.428163583	0.514161237	0.34471405	1
gene26916	205.5948252	295.2165744	1.435914421	0.521969769	0.34478646	1
gene42320	28.9279151	49.36408293	1.706451459	0.770999377	0.34478763	1
gene67684	395.692553	562.3633761	1.421212939	0.507122728	0.3448578	1
gene27059	156.0041136	226.4940275	1.451846508	0.537888937	0.34492153	1
gene7806	692.2036828	989.2175049	1.429084429	0.515091152	0.34492463	1
gene43081	442.1838451	310.7033455	0.702656483	-0.50910854	0.34493113	1
gene3903	552.7298064	786.9215572	1.423700239	0.509645417	0.34498265	1
gene8763	118.8110799	175.1940982	1.474560272	0.560284793	0.34498304	1
gene40862	539.2989887	767.5630933	1.423260769	0.509200016	0.34501122	1
gene17842	16.5302372	30.97354223	1.873750621	0.905928957	0.34504138	1
gene39093	16.5302372	30.97354223	1.873750621	0.905928957	0.34504138	1
gene40280	16.5302372	30.97354223	1.873750621	0.905928957	0.34504138	1
gene53125	16.5302372	30.97354223	1.873750621	0.905928957	0.34504138	1
gene58068	16.5302372	30.97354223	1.873750621	0.905928957	0.34504138	1
gene69874	16.5302372	30.97354223	1.873750621	0.905928957	0.34504138	1
gene61147	271.715774	188.745023	0.69464139	-0.52565972	0.34504172	1

gene41771	514.5036329	362.0032748	0.703597121	-0.50717852	0.34505318	1
gene11367	21.69593633	38.71692779	1.784524401	0.835539629	0.34505474	1
gene14183	21.69593633	38.71692779	1.784524401	0.835539629	0.34505474	1
gene39020	21.69593633	38.71692779	1.784524401	0.835539629	0.34505474	1
gene52863	21.69593633	38.71692779	1.784524401	0.835539629	0.34505474	1
gene29179	136.3744569	199.3921781	1.462093288	0.548035364	0.34506163	1
gene49833	42.35873283	24.19807987	0.571265433	-0.80776686	0.34514631	1
gene42843	99.18142321	148.0922488	1.493145026	0.578354299	0.34519872	1
gene11495	329.5716042	230.3657203	0.698985342	-0.51666589	0.34522488	1
gene15370	153.9378339	223.590258	1.452471119	0.538509478	0.34522824	1
gene28828	1658.189419	2446.909836	1.47565158	0.561352123	0.34526797	1
gene20543	90.91630461	58.07539168	0.638778621	-0.64661207	0.34535179	1
gene67918	1322.418976	1930.03885	1.45947607	0.545450556	0.34536868	1
gene54943	1576.571373	1088.913594	0.690684616	-0.533901	0.34540203	1
gene7033	197.3297066	283.601496	1.437196157	0.523256983	0.34540336	1
gene11193	57.8558302	34.84523501	0.602276985	-0.73150096	0.34554281	1
gene14845	57.8558302	34.84523501	0.602276985	-0.73150096	0.34554281	1
gene15667	57.8558302	34.84523501	0.602276985	-0.73150096	0.34554281	1
gene19939	325.4390449	227.4619507	0.698938724	-0.51676212	0.34557711	1
gene32052	908.1299062	636.8934621	0.70132418	-0.51184662	0.34570102	1
gene1907	307.8756679	214.8789492	0.697940668	-0.5188237	0.34573779	1
gene2455	315.1076466	448.1484391	1.422207439	0.508131907	0.34580297	1
gene28727	782.0868476	549.7803746	0.702965887	-0.50847341	0.34590543	1
gene69910	705.6345005	496.5445988	0.703685263	-0.5069978	0.34591424	1
gene2269	71.28664793	109.375321	1.534303045	0.617583462	0.34599311	1
gene58473	86.78374531	55.17162209	0.635736818	-0.65349845	0.34601615	1
gene54557	341.9692821	485.8974437	1.420880381	0.506785104	0.34605336	1
gene34765	758.3246316	533.3256802	0.703294681	-0.50779879	0.34607071	1
gene28678	95.04886391	142.2847096	1.496963812	0.582039345	0.34608928	1
gene60267	303.7431086	211.9751796	0.697876507	-0.51895633	0.34615047	1
gene5791	620.9170349	884.6817999	1.424798725	0.510758131	0.34620798	1
gene25316	559.9617852	796.6007892	1.422598488	0.508528536	0.34625034	1
gene8228	112.6122409	166.4827895	1.47837205	0.564009387	0.3462702	1
gene42569	69.22036828	106.4715514	1.538153495	0.62119948	0.34629662	1
gene17942	622.9833145	887.5855695	1.424734096	0.510692688	0.34634392	1
gene2427	35.12675405	19.35846389	0.551103124	-0.85960579	0.34636113	1
gene42414	35.12675405	19.35846389	0.551103124	-0.85960579	0.34636113	1
gene44419	35.12675405	19.35846389	0.551103124	-0.85960579	0.34636113	1
gene51121	35.12675405	19.35846389	0.551103124	-0.85960579	0.34636113	1
gene59348	35.12675405	19.35846389	0.551103124	-0.85960579	0.34636113	1
gene56008	250.0198377	173.2582518	0.692978019	-0.5291185	0.34638593	1
gene71756	227.2907615	156.8035575	0.689880911	-0.53558075	0.34644555	1
gene5574	365.7314981	256.4996466	0.701333213	-0.51182804	0.34649505	1
gene41082	153.9378339	103.5677818	0.672789653	-0.57177258	0.34652276	1
gene68639	92.98258426	139.38094	1.499000497	0.584000862	0.34654361	1
gene51402	82.65118601	52.26785251	0.632390835	-0.66111164	0.34657984	1
gene73823	82.65118601	52.26785251	0.632390835	-0.66111164	0.34657984	1
gene58108	131.2087578	87.11308752	0.663927386	-0.59090263	0.34665164	1

gene21986	466.9792009	663.0273883	1.419822097	0.505710172	0.34667649	1
gene56326	110.5459613	163.5790199	1.47973764	0.565341407	0.34672076	1
gene68118	344.0355618	241.0128755	0.700546404	-0.51344748	0.34679666	1
gene61264	447.3495443	634.9576157	1.419376914	0.505257747	0.34683846	1
gene60903	30.99419475	52.26785251	1.686375559	0.753925863	0.34685721	1
gene66897	30.99419475	52.26785251	1.686375559	0.753925863	0.34685721	1
gene578	117.7779401	77.43385557	0.657456358	-0.60503296	0.34692673	1
gene22280	213.8599438	147.1243256	0.687947088	-0.53963049	0.3469811	1
gene71119	1856.552266	2752.773566	1.482734215	0.568260013	0.34698362	1
gene45866	78.51862671	49.36408293	0.628692643	-0.66957321	0.34701036	1
gene68885	78.51862671	49.36408293	0.628692643	-0.66957321	0.34701036	1
gene42220	282.0471722	196.4884085	0.696650872	-0.52149227	0.34703555	1
gene55103	126.0430587	184.8733302	1.466747413	0.552620447	0.34704006	1
gene1279	180.7994694	260.3713394	1.440111192	0.526180207	0.34704364	1
gene16151	63.02152933	97.76024266	1.551219777	0.633403102	0.34704651	1
gene63724	1405.070162	975.6665802	0.694389936	-0.52618206	0.34718121	1
gene18338	140.5070162	93.88854988	0.668212538	-0.58162104	0.34722217	1
gene43627	60.95524968	94.85647307	1.55616577	0.637995751	0.34722444	1
gene61932	626.082734	891.4572623	1.423864952	0.509812319	0.34727905	1
gene2004	202.4954057	290.3769584	1.433992823	0.520037803	0.34727977	1
gene21930	202.4954057	290.3769584	1.433992823	0.520037803	0.34727977	1
gene64563	70.25350811	43.55654376	0.619991014	-0.68968079	0.34729372	1
gene5	58.88897003	91.95270349	1.561458851	0.642894551	0.34735487	1
gene48490	58.88897003	91.95270349	1.561458851	0.642894551	0.34735487	1
gene58039	58.88897003	91.95270349	1.561458851	0.642894551	0.34735487	1
gene4003	3738.933027	2475.947532	0.662206976	-0.59464589	0.34735985	1
gene27042	52.69013108	83.24139474	1.579828955	0.659768369	0.34736809	1
gene2393	24.7953558	12.58300153	0.507474127	-0.97859383	0.34739874	1
gene2755	24.7953558	12.58300153	0.507474127	-0.97859383	0.34739874	1
gene31122	24.7953558	12.58300153	0.507474127	-0.97859383	0.34739874	1
gene38171	24.7953558	12.58300153	0.507474127	-0.97859383	0.34739874	1
gene41467	24.7953558	12.58300153	0.507474127	-0.97859383	0.34739874	1
gene70082	24.7953558	12.58300153	0.507474127	-0.97859383	0.34739874	1
gene47106	88.85002496	133.5734009	1.503358057	0.588188659	0.34746672	1
gene933	149.8052746	100.6640122	0.67196574	-0.57354042	0.34762364	1
gene50265	675.6734456	476.2182118	0.70480528	-0.50470336	0.34768488	1
gene41154	349.2012609	244.8845682	0.701270573	-0.5119569	0.34772524	1
gene31116	3167.606704	4877.364978	1.539763435	0.622708716	0.34774618	1
gene55456	423.5873283	298.1203439	0.703799014	-0.5067646	0.34788512	1
gene1390	268.6163545	382.3296619	1.423329799	0.509269987	0.34791165	1
gene26741	793.4513857	558.4916833	0.703876373	-0.50660604	0.34792659	1
gene64240	362.6320786	254.5638002	0.701989193	-0.51047927	0.34799879	1
gene42859	113.6453808	74.53008599	0.655812718	-0.60864422	0.34803433	1
gene27048	46.49129213	27.10184945	0.582944638	-0.77856922	0.34804181	1
gene73753	46.49129213	27.10184945	0.582944638	-0.77856922	0.34804181	1
gene21464	2701.660643	4103.994345	1.519063601	0.603182275	0.34808318	1
gene30203	273.7820536	190.6808693	0.696469571	-0.52186777	0.34812172	1
gene34667	104.3471223	154.8677111	1.484158908	0.569645569	0.34813586	1

gene14534	228.3239013	157.7714807	0.690998532	-0.53324545	0.3482301	1
gene15938	228.3239013	157.7714807	0.690998532	-0.53324545	0.3482301	1
gene54026	260.3512359	181.0016374	0.695220965	-0.52445651	0.34835607	1
gene23673	13.43081773	26.13392626	1.945817953	0.960376741	0.34843381	1
gene29684	13.43081773	26.13392626	1.945817953	0.960376741	0.34843381	1
gene39623	13.43081773	26.13392626	1.945817953	0.960376741	0.34843381	1
gene48096	13.43081773	26.13392626	1.945817953	0.960376741	0.34843381	1
gene57022	13.43081773	26.13392626	1.945817953	0.960376741	0.34843381	1
gene47625	135.3413171	197.4563317	1.458950866	0.544931297	0.34849794	1
gene37779	33.0604744	55.17162209	1.668809147	0.738818971	0.34855652	1
gene58909	33.0604744	55.17162209	1.668809147	0.738818971	0.34855652	1
gene69952	33.0604744	55.17162209	1.668809147	0.738818971	0.34855652	1
gene7653	196.2965668	134.5413241	0.685398254	-0.54498558	0.34856997	1
gene69162	96.08200373	61.94708446	0.644731397	-0.63322985	0.34859553	1
gene36709	23.76221598	41.62069737	1.751549494	0.808631755	0.3487176	1
gene37695	23.76221598	41.62069737	1.751549494	0.808631755	0.3487176	1
gene61499	428.7530274	301.9920367	0.704349631	-0.50563635	0.34873739	1
gene14315	346.1018414	490.7370597	1.417897858	0.503753608	0.34884203	1
gene41712	346.1018414	490.7370597	1.417897858	0.503753608	0.34884203	1
gene25157	65891.59176	26355.58067	0.399983973	-1.3219859	0.34884777	1
gene1006	214.8930836	148.0922488	0.689143858	-0.53712292	0.3488584	1
gene532	15.49709738	6.775462362	0.437208478	-1.19360672	0.34886	1
gene5787	15.49709738	6.775462362	0.437208478	-1.19360672	0.34886	1
gene9959	15.49709738	6.775462362	0.437208478	-1.19360672	0.34886	1
gene13273	15.49709738	6.775462362	0.437208478	-1.19360672	0.34886	1
gene15248	15.49709738	6.775462362	0.437208478	-1.19360672	0.34886	1
gene17891	15.49709738	6.775462362	0.437208478	-1.19360672	0.34886	1
gene20195	15.49709738	6.775462362	0.437208478	-1.19360672	0.34886	1
gene20435	15.49709738	6.775462362	0.437208478	-1.19360672	0.34886	1
gene35127	15.49709738	6.775462362	0.437208478	-1.19360672	0.34886	1
gene36919	15.49709738	6.775462362	0.437208478	-1.19360672	0.34886	1
gene37406	15.49709738	6.775462362	0.437208478	-1.19360672	0.34886	1
gene38873	15.49709738	6.775462362	0.437208478	-1.19360672	0.34886	1
gene40311	15.49709738	6.775462362	0.437208478	-1.19360672	0.34886	1
gene48066	15.49709738	6.775462362	0.437208478	-1.19360672	0.34886	1
gene50623	15.49709738	6.775462362	0.437208478	-1.19360672	0.34886	1
gene57079	15.49709738	6.775462362	0.437208478	-1.19360672	0.34886	1
gene66242	15.49709738	6.775462362	0.437208478	-1.19360672	0.34886	1
gene69385	15.49709738	6.775462362	0.437208478	-1.19360672	0.34886	1
gene73929	15.49709738	6.775462362	0.437208478	-1.19360672	0.34886	1
gene48727	662.2426279	942.7571916	1.423582765	0.509526372	0.34890635	1
gene5652	10.33139825	21.29431028	2.061125684	1.04343248	0.34892266	1
gene29037	10.33139825	21.29431028	2.061125684	1.04343248	0.34892266	1
gene31902	10.33139825	21.29431028	2.061125684	1.04343248	0.34892266	1
gene32347	10.33139825	21.29431028	2.061125684	1.04343248	0.34892266	1
gene38841	10.33139825	21.29431028	2.061125684	1.04343248	0.34892266	1
gene50968	10.33139825	21.29431028	2.061125684	1.04343248	0.34892266	1
gene68594	10.33139825	21.29431028	2.061125684	1.04343248	0.34892266	1

gene70468	10.33139825	21.29431028	2.061125684	1.04343248	0.34892266	1
gene67607	166.3355118	240.0449523	1.443137125	0.529208389	0.3489828	1
gene47432	479.3768788	679.4820826	1.417427733	0.503275182	0.34920636	1
gene65417	233.4896005	161.6431735	0.692292818	-0.53054571	0.34921669	1
gene26960	831.6775592	585.5935328	0.704111258	-0.50612468	0.34924488	1
gene58695	398.7919725	280.6977264	0.703870052	-0.50661899	0.34924663	1
gene6729	265.516935	184.8733302	0.696276982	-0.52226676	0.34931195	1
gene10116	131.2087578	191.6487925	1.460640248	0.54660089	0.34935788	1
gene26951	849.2409362	1215.711532	1.431527239	0.517555122	0.34936979	1
gene68910	1270.761985	886.6176463	0.697705516	-0.51930986	0.34937544	1
gene22039	242.7878589	168.4186359	0.6936864	-0.52764449	0.34943249	1
gene6187	315.1076466	220.6864884	0.700352691	-0.51384646	0.34946206	1
gene36511	231.4233208	330.0618094	1.426225361	0.512201964	0.34953532	1
gene39850	132.2418976	88.08101071	0.666059791	-0.5862764	0.34955634	1
gene54708	743.8606741	524.6143715	0.705258914	-0.5037751	0.34964555	1
gene5746	113.6453808	167.4507127	1.473449352	0.559197471	0.34981224	1
gene71565	113.6453808	167.4507127	1.473449352	0.559197471	0.34981224	1
gene36722	927.7595629	652.3802332	0.703178129	-0.5080379	0.34984289	1
gene45460	297.5442696	208.1034868	0.699403444	-0.5158032	0.34990903	1
gene12400	35.12675405	58.07539168	1.653309372	0.725356711	0.34995193	1
gene24640	50.62385143	30.00561903	0.592717033	-0.75458458	0.35001893	1
gene56050	50.62385143	30.00561903	0.592717033	-0.75458458	0.35001893	1
gene56111	50.62385143	30.00561903	0.592717033	-0.75458458	0.35001893	1
gene61123	50.62385143	30.00561903	0.592717033	-0.75458458	0.35001893	1
gene9376	386.3942946	271.9864177	0.70390899	-0.50653918	0.35005326	1
gene46205	368.8309176	259.4034162	0.703312558	-0.50776212	0.35008875	1
gene53784	238.6552996	165.5148663	0.693531074	-0.52796757	0.35017802	1
gene391	1866.883664	2761.484874	1.479194943	0.564812197	0.35018114	1
gene12975	502.105955	711.4235481	1.416879328	0.502716893	0.35020255	1
gene21124	528.9675904	373.6183531	0.706316152	-0.50161401	0.35020288	1
gene29935	21.69593633	10.64715514	0.49074421	-1.02695685	0.35028015	1
gene41883	21.69593633	10.64715514	0.49074421	-1.02695685	0.35028015	1
gene49726	21.69593633	10.64715514	0.49074421	-1.02695685	0.35028015	1
gene50025	21.69593633	10.64715514	0.49074421	-1.02695685	0.35028015	1
gene57001	21.69593633	10.64715514	0.49074421	-1.02695685	0.35028015	1
gene31072	1139.553227	1646.437354	1.444809523	0.530879307	0.35032735	1
gene39217	1993.959862	1369.61132	0.686880085	-0.54186984	0.35034489	1
gene30898	794.4845255	1134.405984	1.427851579	0.513846023	0.35037175	1
gene29695	367.7977777	520.7426787	1.415839655	0.501657888	0.35038075	1
gene49275	371.930337	526.5502179	1.415722692	0.501538702	0.35043076	1
gene50811	188.0314482	128.7337849	0.68463965	-0.54658325	0.35053814	1
gene2733	696.3362421	491.7049829	0.70613154	-0.50199114	0.35057052	1
gene58228	1407.136442	979.538273	0.696121743	-0.52258846	0.35058307	1
gene16352	156.0041136	225.5261044	1.445642036	0.531710362	0.35069688	1
gene62875	546.5309675	386.2013547	0.706641302	-0.50095002	0.35070532	1
gene21482	215.9262234	149.060172	0.690329176	-0.53464363	0.35072412	1
gene44537	74.38606741	113.2470138	1.52242238	0.606368675	0.35072742	1
gene13379	329.5716042	231.3336435	0.701922255	-0.51061685	0.3508654	1

gene6631	18.59651685	33.87731181	1.821701993	0.865286972	0.35093285	1
gene26450	18.59651685	33.87731181	1.821701993	0.865286972	0.35093285	1
gene31375	18.59651685	33.87731181	1.821701993	0.865286972	0.35093285	1
gene40364	18.59651685	33.87731181	1.821701993	0.865286972	0.35093285	1
gene54901	18.59651685	33.87731181	1.821701993	0.865286972	0.35093285	1
gene59864	18.59651685	33.87731181	1.821701993	0.865286972	0.35093285	1
gene66821	18.59651685	33.87731181	1.821701993	0.865286972	0.35093285	1
gene517	39.25931335	22.26223348	0.567056109	-0.8184366	0.35105686	1
gene26964	39.25931335	22.26223348	0.567056109	-0.8184366	0.35105686	1
gene30900	39.25931335	22.26223348	0.567056109	-0.8184366	0.35105686	1
gene42048	39.25931335	22.26223348	0.567056109	-0.8184366	0.35105686	1
gene63332	39.25931335	22.26223348	0.567056109	-0.8184366	0.35105686	1
gene74171	39.25931335	22.26223348	0.567056109	-0.8184366	0.35105686	1
gene20778	276.8814731	392.976817	1.41929618	0.505175683	0.35112124	1
gene72425	276.8814731	392.976817	1.41929618	0.505175683	0.35112124	1
gene59392	83.68432583	53.23577571	0.636149902	-0.65256133	0.35116112	1
gene65434	54.75641073	32.90938862	0.60101435	-0.73452866	0.35133187	1
gene68704	54.75641073	32.90938862	0.60101435	-0.73452866	0.35133187	1
gene10749	18.59651685	8.711308752	0.468437655	-1.09407104	0.35135654	1
gene11641	18.59651685	8.711308752	0.468437655	-1.09407104	0.35135654	1
gene18523	18.59651685	8.711308752	0.468437655	-1.09407104	0.35135654	1
gene22858	18.59651685	8.711308752	0.468437655	-1.09407104	0.35135654	1
gene31009	18.59651685	8.711308752	0.468437655	-1.09407104	0.35135654	1
gene33311	18.59651685	8.711308752	0.468437655	-1.09407104	0.35135654	1
gene47808	18.59651685	8.711308752	0.468437655	-1.09407104	0.35135654	1
gene55546	18.59651685	8.711308752	0.468437655	-1.09407104	0.35135654	1
gene62880	18.59651685	8.711308752	0.468437655	-1.09407104	0.35135654	1
gene70640	18.59651685	8.711308752	0.468437655	-1.09407104	0.35135654	1
gene71277	18.59651685	8.711308752	0.468437655	-1.09407104	0.35135654	1
gene17532	101.2477029	65.81877724	0.650076746	-0.62131805	0.35140333	1
gene71223	485.5757178	687.2254682	1.415279725	0.501087224	0.35141307	1
gene71496	156.0041136	105.5036282	0.676287476	-0.56429146	0.35151311	1
gene64341	418.4216292	295.2165744	0.705548074	-0.50318371	0.35152111	1
gene37687	25.82849563	44.52446695	1.723850572	0.785634723	0.35163532	1
gene37778	25.82849563	44.52446695	1.723850572	0.785634723	0.35163532	1
gene41624	25.82849563	44.52446695	1.723850572	0.785634723	0.35163532	1
gene73638	431.8524469	610.7595358	1.41427828	0.50006602	0.35166524	1
gene49808	612.6519163	869.1950288	1.418742039	0.504612297	0.35168247	1
gene36032	1933.004613	1330.894393	0.688510717	-0.53844898	0.35172162	1
gene16386	414.2890699	292.3128048	0.705576917	-0.50312473	0.35177019	1
gene41226	310.9750873	440.4050536	1.416206865	0.502032015	0.35187884	1
gene65144	475.2443195	335.8693485	0.706729854	-0.50076924	0.35189356	1
gene70950	32.02733458	17.4226175	0.543992116	-0.87834235	0.35193663	1
gene72792	226.2576217	322.3184238	1.424563829	0.510520264	0.35194777	1
gene7012	361.5989388	254.5638002	0.703994876	-0.50636317	0.35196027	1
gene34634	134.3081773	195.5204853	1.455760098	0.541772626	0.35200915	1
gene53541	39.25931335	63.88293085	1.627204487	0.702395563	0.35203232	1
gene58240	39.25931335	63.88293085	1.627204487	0.702395563	0.35203232	1

gene64638	39.25931335	63.88293085	1.627204487	0.702395563	0.35203232	1
gene50183	281.0140324	196.4884085	0.699212089	-0.51619797	0.35208059	1
gene72911	281.0140324	196.4884085	0.699212089	-0.51619797	0.35208059	1
gene3407	619.883895	438.4692072	0.70734086	-0.49952249	0.35212722	1
gene65887	1255.264887	877.9063375	0.699379347	-0.5158529	0.35213951	1
gene6024	58.88897003	35.8131582	0.608147132	-0.71750769	0.35215777	1
gene10705	58.88897003	35.8131582	0.608147132	-0.71750769	0.35215777	1
gene34843	1941.269731	2872.796042	1.479854136	0.565454982	0.35233753	1
gene73485	75.41920723	47.42823654	0.62886151	-0.66918576	0.35237477	1
gene1113	905.0304868	637.8613853	0.704795468	-0.50472345	0.35241078	1
gene65797	514.5036329	363.9391212	0.707359672	-0.49948412	0.352414	1
gene18170	66.12094881	101.6319354	1.537061057	0.620174474	0.35242491	1
gene52331	178.7331897	256.4996466	1.435098019	0.521149278	0.35244004	1
gene29235	132.2418976	192.6167157	1.456548335	0.542553577	0.35248585	1
gene68720	8356.034905	14504.32907	1.735790867	0.795593138	0.35256132	1
gene64805	196.2965668	280.6977264	1.42996758	0.515982438	0.35257002	1
gene43479	110.5459613	72.5942396	0.656688302	-0.60671934	0.35259698	1
gene56709	226.2576217	156.8035575	0.693031052	-0.5290081	0.3526158	1
gene1399	63.02152933	38.71692779	0.614344466	-0.70288029	0.35262295	1
gene35460	63.02152933	38.71692779	0.614344466	-0.70288029	0.35262295	1
gene36837	299.6105493	210.0393332	0.701041181	-0.5124289	0.35275938	1
gene40232	116.7448002	171.3224055	1.467494956	0.553355544	0.35278718	1
gene11885	244.8541385	170.3544823	0.695738627	-0.52338267	0.35279116	1
gene17874	41.325593	66.78670043	1.616109911	0.692525319	0.35279317	1
gene56113	41.325593	66.78670043	1.616109911	0.692525319	0.35279317	1
gene63952	3565.365536	2379.155212	0.667296295	-0.5836006	0.35279342	1
gene30148	64.05466916	98.72816585	1.541310995	0.624157988	0.3527986	1
gene3906	67.15408863	41.62069737	0.619779052	-0.6901741	0.35281559	1
gene27797	67.15408863	41.62069737	0.619779052	-0.6901741	0.35281559	1
gene62392	254.152397	177.1299446	0.696943829	-0.52088571	0.35293198	1
gene26171	1458.793433	2125.559335	1.457066701	0.543066922	0.35293833	1
gene50276	286.1797315	200.3601013	0.700119817	-0.51432625	0.35297468	1
gene57367	761.4240511	538.1652962	0.706787887	-0.50065078	0.35298634	1
gene62251	472.1449001	333.9335022	0.707269108	-0.49966885	0.3530265	1
gene57797	450.4489637	318.446731	0.706954076	-0.50031159	0.35302773	1
gene24426	384.3280149	543.0049122	1.412868412	0.498627106	0.35309231	1
gene19794	996.9799312	701.7443161	0.703870052	-0.50661899	0.35311667	1
gene70796	308.9088077	216.8147956	0.701873142	-0.5107178	0.35314067	1
gene65156	61.9883895	95.82439627	1.545844263	0.628394981	0.35314213	1
gene2925	6.19883895	14.51884792	2.342188277	1.227857052	0.35319793	1
gene3843	6.19883895	14.51884792	2.342188277	1.227857052	0.35319793	1
gene11829	6.19883895	14.51884792	2.342188277	1.227857052	0.35319793	1
gene19180	6.19883895	14.51884792	2.342188277	1.227857052	0.35319793	1
gene19856	6.19883895	14.51884792	2.342188277	1.227857052	0.35319793	1
gene30234	6.19883895	14.51884792	2.342188277	1.227857052	0.35319793	1
gene43239	6.19883895	14.51884792	2.342188277	1.227857052	0.35319793	1
gene47611	6.19883895	14.51884792	2.342188277	1.227857052	0.35319793	1
gene49617	6.19883895	14.51884792	2.342188277	1.227857052	0.35319793	1

gene54844	6.19883895	14.51884792	2.342188277	1.227857052	0.35319793	1
gene55414	6.19883895	14.51884792	2.342188277	1.227857052	0.35319793	1
gene63320	6.19883895	14.51884792	2.342188277	1.227857052	0.35319793	1
gene65038	6.19883895	14.51884792	2.342188277	1.227857052	0.35319793	1
gene69008	6.19883895	14.51884792	2.342188277	1.227857052	0.35319793	1
gene70085	6.19883895	14.51884792	2.342188277	1.227857052	0.35319793	1
gene70140	6.19883895	14.51884792	2.342188277	1.227857052	0.35319793	1
gene42907	2045.616854	3034.439215	1.483385909	0.568893969	0.35322964	1
gene17906	267.5832147	379.4258923	1.417973443	0.503830513	0.35325232	1
gene46771	1997.059282	2957.973283	1.481164485	0.566731862	0.35327923	1
gene47841	43.39187265	69.69047001	1.606071961	0.683536535	0.35336568	1
gene45384	83.68432583	125.8300153	1.503627042	0.588446767	0.35345504	1
gene60695	1232.535811	863.3874896	0.700496879	-0.51354947	0.35360684	1
gene772	250.0198377	174.226175	0.696849405	-0.52108118	0.35367822	1
gene8741	57.8558302	90.0168571	1.555882212	0.637732846	0.35371224	1
gene38226	57.8558302	90.0168571	1.555882212	0.637732846	0.35371224	1
gene40657	57.8558302	90.0168571	1.555882212	0.637732846	0.35371224	1
gene20172	129.1424781	86.14516432	0.667055221	-0.5841219	0.35373359	1
gene1213	170.4680711	244.8845682	1.436542143	0.522600317	0.35376431	1
gene70711	558.9286454	395.8805866	0.708284662	-0.49759879	0.35388034	1
gene12330	499.0065355	353.291966	0.707990659	-0.49819777	0.3538854	1
gene33483	499.0065355	353.291966	0.707990659	-0.49819777	0.3538854	1
gene26040	166.3355118	113.2470138	0.680834853	-0.5546232	0.35390332	1
gene51891	55.78955055	87.11308752	1.561458851	0.642894551	0.35392274	1
gene60432	138.4407366	92.92062669	0.671194252	-0.57519773	0.35393413	1
gene20735	27.89477528	47.42823654	1.700255194	0.765751299	0.3539649	1
gene29910	27.89477528	47.42823654	1.700255194	0.765751299	0.3539649	1
gene45287	27.89477528	47.42823654	1.700255194	0.765751299	0.3539649	1
gene8852	4221.409325	6647.696501	1.57475762	0.655129792	0.35398457	1
gene2826	147.738995	99.69608905	0.674812287	-0.56744185	0.3539974	1
gene10906	47.52443195	75.49800918	1.588614657	0.667769219	0.35400701	1
gene56212	81.61804618	122.9262457	1.506116006	0.590832895	0.35402323	1
gene47993	139.4738764	202.2959477	1.450421777	0.536472492	0.35409282	1
gene54923	1498.052746	1043.421204	0.696518334	-0.52176677	0.35418977	1
gene8450	43.39187265	25.16600306	0.57997043	-0.78594875	0.35418994	1
gene15744	43.39187265	25.16600306	0.57997043	-0.78594875	0.35418994	1
gene52217	43.39187265	25.16600306	0.57997043	-0.78594875	0.35418994	1
gene63765	43.39187265	25.16600306	0.57997043	-0.78594875	0.35418994	1
gene66623	43.39187265	25.16600306	0.57997043	-0.78594875	0.35418994	1
gene9483	448.3826841	633.0217693	1.411789063	0.49752455	0.3542633	1
gene32784	542.3984082	384.2655083	0.708456187	-0.49724946	0.35430055	1
gene50660	464.9129213	329.0938862	0.707861346	-0.4984613	0.3543772	1
gene12443	1133.354388	1631.918506	1.439901344	0.525969967	0.3544171	1
gene17543	2093.141286	1439.30179	0.687627634	-0.54030057	0.35450761	1
gene971	199.3959862	137.4450936	0.689307223	-0.53678096	0.3545779	1
gene39513	79.55176653	120.0224761	1.508734267	0.593338726	0.35459394	1
gene62355	79.55176653	120.0224761	1.508734267	0.593338726	0.35459394	1
gene57579	115.7116604	76.46593238	0.660831692	-0.59764522	0.35464406	1

gene63999	115.7116604	76.46593238	0.660831692	-0.59764522	0.35464406	1
gene9103	88.85002496	57.10746848	0.642740039	-0.63769275	0.35466152	1
gene6457	272.7489138	386.2013547	1.415959276	0.501779774	0.35476703	1
gene58879	756.258352	535.2615266	0.70777602	-0.49863521	0.35483441	1
gene44878	2778.11299	1884.54646	0.678354864	-0.55988791	0.35484845	1
gene70349	2227.449463	3317.072788	1.489179819	0.57451797	0.35495761	1
gene14377	195.2634269	278.7618801	1.427619521	0.513611534	0.35509527	1
gene8619	102.2808427	66.78670043	0.652973701	-0.61490321	0.35511245	1
gene65999	232.4564606	161.6431735	0.695369675	-0.52414794	0.35526636	1
gene52627	918.4613045	648.5085404	0.706081505	-0.50209337	0.35529401	1
gene73701	292.3785705	205.1997173	0.701828855	-0.51080883	0.3553021	1
gene33926	223.1582022	154.8677111	0.693981712	-0.52703045	0.35530436	1
gene66487	223.1582022	154.8677111	0.693981712	-0.52703045	0.35530436	1
gene67720	1533.1795	2234.934656	1.457712326	0.543706038	0.35531738	1
gene8132	20.6627965	36.7810814	1.78006309	0.831928375	0.35545929	1
gene13971	20.6627965	36.7810814	1.78006309	0.831928375	0.35545929	1
gene18041	20.6627965	36.7810814	1.78006309	0.831928375	0.35545929	1
gene25108	20.6627965	36.7810814	1.78006309	0.831928375	0.35545929	1
gene32756	20.6627965	36.7810814	1.78006309	0.831928375	0.35545929	1
gene68771	20.6627965	36.7810814	1.78006309	0.831928375	0.35545929	1
gene70313	20.6627965	36.7810814	1.78006309	0.831928375	0.35545929	1
gene49279	670.5077465	475.2502886	0.708791645	-0.4965665	0.35546554	1
gene8006	84.71746566	54.2036989	0.639817285	-0.64426813	0.35565851	1
gene60475	633.3147128	896.2968782	1.415247207	0.501054076	0.35579309	1
gene36068	29.96105493	50.33200612	1.67991435	0.74838768	0.35582532	1
gene48539	29.96105493	50.33200612	1.67991435	0.74838768	0.35582532	1
gene70469	29.96105493	50.33200612	1.67991435	0.74838768	0.35582532	1
gene1049	2471.270462	3703.274143	1.498530493	0.583548441	0.35587659	1
gene73634	899.8647876	635.9255389	0.706690102	-0.50085039	0.35600365	1
gene33030	246.9204182	172.2903286	0.697756508	-0.51920442	0.35611385	1
gene27681	237.6221598	165.5148663	0.696546427	-0.52170858	0.35611674	1
gene47335	514.5036329	364.9070444	0.709240948	-0.49565226	0.35611743	1
gene65256	131.2087578	190.6808693	1.453263277	0.539296089	0.35612932	1
gene42839	766.5897502	543.0049122	0.708338341	-0.49748946	0.35614803	1
gene18312	536.1995692	756.9159382	1.411631008	0.497363026	0.35617286	1
gene46230	167.3686517	114.214937	0.68241535	-0.551278	0.35622052	1
gene10395	987.6816728	696.9047001	0.705596468	-0.50308476	0.35624333	1
gene31357	488.6751373	346.5165037	0.709093787	-0.49595164	0.35624622	1
gene13345	824.4455804	1173.122912	1.422923404	0.508858004	0.35628677	1
gene7643	73.35292758	111.3111674	1.517474095	0.601671888	0.35630375	1
gene41890	98.14828338	63.88293085	0.650881795	-0.61953253	0.35637763	1
gene60639	98.14828338	63.88293085	0.650881795	-0.61953253	0.35637763	1
gene9456	15.49709738	29.03769584	1.873750621	0.905928957	0.3564058	1
gene51840	15.49709738	29.03769584	1.873750621	0.905928957	0.3564058	1
gene60286	15.49709738	29.03769584	1.873750621	0.905928957	0.3564058	1
gene61480	15.49709738	29.03769584	1.873750621	0.905928957	0.3564058	1
gene71388	15.49709738	29.03769584	1.873750621	0.905928957	0.3564058	1
gene73699	15.49709738	29.03769584	1.873750621	0.905928957	0.3564058	1

gene23735	803.7827839	569.1388384	0.708075428	-0.49802504	0.35641164	1
gene3945	638.4804119	452.9880551	0.709478391	-0.49516935	0.35648592	1
gene34073	200.4291261	138.4130168	0.690583348	-0.53411255	0.35655337	1
gene17279	80.58490636	51.29992932	0.636594762	-0.65155281	0.35657439	1
gene23289	80.58490636	51.29992932	0.636594762	-0.65155281	0.35657439	1
gene20968	271.715774	384.2655083	1.414218625	0.500005164	0.3565865	1
gene43910	271.715774	384.2655083	1.414218625	0.500005164	0.3565865	1
gene12257	139.4738764	93.88854988	0.67316226	-0.5709738	0.35667277	1
gene61998	501.0728152	706.5839321	1.41014222	0.495840673	0.35677412	1
gene8512	934.9915417	1334.766085	1.427570225	0.513561716	0.35684041	1
gene22735	71.28664793	108.4073978	1.520725142	0.604759422	0.35686563	1
gene27314	1671.620237	2444.006066	1.462058195	0.548000737	0.35696753	1
gene36762	181.8326092	124.8620921	0.686687018	-0.54227541	0.35699055	1
gene2021	567.193764	402.656049	0.709909161	-0.49429366	0.35706616	1
gene24483	32.02733458	53.23577571	1.662198132	0.73309236	0.35730812	1
gene51914	32.02733458	53.23577571	1.662198132	0.73309236	0.35730812	1
gene3752	36.15989388	20.32638709	0.562125186	-0.83103664	0.35730969	1
gene11567	36.15989388	20.32638709	0.562125186	-0.83103664	0.35730969	1
gene38148	36.15989388	20.32638709	0.562125186	-0.83103664	0.35730969	1
gene49066	36.15989388	20.32638709	0.562125186	-0.83103664	0.35730969	1
gene51171	36.15989388	20.32638709	0.562125186	-0.83103664	0.35730969	1
gene51216	36.15989388	20.32638709	0.562125186	-0.83103664	0.35730969	1
gene59071	36.15989388	20.32638709	0.562125186	-0.83103664	0.35730969	1
gene69864	36.15989388	20.32638709	0.562125186	-0.83103664	0.35730969	1
gene31610	86.78374531	129.7017081	1.494539186	0.579700724	0.35734155	1
gene56819	107.4465418	70.65839321	0.657614401	-0.6046862	0.35737048	1
gene21699	76.45234706	48.39615973	0.633023859	-0.65966822	0.35737764	1
gene60324	113.6453808	166.4827895	1.464932304	0.550833998	0.35744538	1
gene69667	51.65699125	30.97354223	0.599600199	-0.73792723	0.35752847	1
gene3139	28.9279151	15.48677111	0.53535732	-0.90142597	0.35760347	1
gene3823	28.9279151	15.48677111	0.53535732	-0.90142597	0.35760347	1
gene11634	28.9279151	15.48677111	0.53535732	-0.90142597	0.35760347	1
gene20565	28.9279151	15.48677111	0.53535732	-0.90142597	0.35760347	1
gene26872	28.9279151	15.48677111	0.53535732	-0.90142597	0.35760347	1
gene69632	669.4746067	475.2502886	0.709885459	-0.49434183	0.35761728	1
gene27937	551.6966666	778.2102485	1.410576311	0.496284716	0.35764441	1
gene66040	196.2965668	135.5092472	0.690329176	-0.53464363	0.35765424	1
gene6798	153.9378339	104.535705	0.679077406	-0.55835206	0.35774647	1
gene43130	257.2518164	180.0337142	0.699834569	-0.51491416	0.3577535	1
gene32328	247.953558	173.2582518	0.698752836	-0.51714586	0.35776185	1
gene20764	116.7448002	77.43385557	0.663274556	-0.59232191	0.35788624	1
gene53667	516.5699125	366.8428908	0.710151486	-0.49380129	0.35788933	1
gene62219	313.041367	441.3729768	1.409950963	0.495644987	0.35798741	1
gene65845	3716.203951	2483.690917	0.668340853	-0.58134403	0.35799183	1
gene71467	632.2815729	449.1163623	0.710310693	-0.49347789	0.35808217	1
gene19111	126.0430587	84.20931793	0.668099607	-0.58186489	0.35808769	1
gene62229	1266.629426	889.5214159	0.702274397	-0.50989326	0.35813585	1
gene8902	98.14828338	145.1884792	1.479276806	0.564892039	0.35822399	1

gene20189	55.78955055	33.87731181	0.607233998	-0.71967553	0.35826643	1
gene40622	55.78955055	33.87731181	0.607233998	-0.71967553	0.35826643	1
gene64558	55.78955055	33.87731181	0.607233998	-0.71967553	0.35826643	1
gene54807	1368.910268	959.2118859	0.700712025	-0.51310644	0.35828665	1
gene13475	326.4721847	230.3657203	0.705621278	-0.50303403	0.3583488	1
gene41430	362.6320786	256.4996466	0.707327514	-0.49954971	0.35836595	1
gene62505	355.4000998	500.4162916	1.408036441	0.493684672	0.35837072	1
gene65617	615.7513357	437.501284	0.710516175	-0.4930606	0.35837487	1
gene24022	2917.586866	1979.402933	0.678438389	-0.55971029	0.35838792	1
gene72697	1758.403982	1221.519072	0.694674878	-0.52559017	0.35847549	1
gene10454	34.09361423	56.13954529	1.646629334	0.719515832	0.35848463	1
gene26343	65.08780898	99.69608905	1.531716778	0.61514956	0.35848562	1
gene57470	65.08780898	99.69608905	1.531716778	0.61514956	0.35848562	1
gene60233	1649.924301	1148.924832	0.696350027	-0.52211542	0.35849083	1
gene41850	649.84495	461.6993638	0.71047619	-0.49314179	0.35856924	1
gene26841	298.5774094	421.0465897	1.410175641	0.495874865	0.35858168	1
gene55241	1481.522509	1035.677818	0.699063168	-0.51650527	0.35860441	1
gene23953	59.92210985	36.7810814	0.613814859	-0.70412453	0.35860577	1
gene68599	64.05466916	39.68485098	0.619546576	-0.69071535	0.35865367	1
gene644	82.65118601	123.8941689	1.499000497	0.584000862	0.35866336	1
gene20325	281.0140324	197.4563317	0.702656483	-0.50910854	0.35867673	1
gene31402	134.3081773	194.5525621	1.448553365	0.534612835	0.35869264	1
gene37338	689.1042633	974.698657	1.414442935	0.500233973	0.35869982	1
gene25726	103.3139825	67.75462362	0.655812718	-0.60864422	0.35876744	1
gene64364	103.3139825	67.75462362	0.655812718	-0.60864422	0.35876744	1
gene15432	264.4837952	373.6183531	1.412632304	0.498385994	0.35885285	1
gene28468	225.2244819	156.8035575	0.696210093	-0.52240537	0.35888155	1
gene63663	225.2244819	156.8035575	0.696210093	-0.52240537	0.35888155	1
gene8070	96.08200373	142.2847096	1.480867427	0.56644249	0.35890172	1
gene12018	22.72907615	39.68485098	1.745994897	0.804049343	0.358957	1
gene36218	22.72907615	39.68485098	1.745994897	0.804049343	0.358957	1
gene46262	22.72907615	39.68485098	1.745994897	0.804049343	0.358957	1
gene68328	22.72907615	39.68485098	1.745994897	0.804049343	0.358957	1
gene20575	63.02152933	96.79231946	1.535861165	0.619047809	0.3589904	1
gene62560	182.865749	125.8300153	0.688100511	-0.53930878	0.35912424	1
gene12341	149.8052746	101.6319354	0.678426949	-0.55973462	0.35913112	1
gene46285	421.5210486	299.0882671	0.709545272	-0.49503336	0.35921434	1
gene10916	206.627965	143.2526328	0.69328773	-0.52847387	0.35934538	1
gene25646	36.15989388	59.04331487	1.632839827	0.707383277	0.35941112	1
gene52590	143.6064357	207.1355637	1.442383572	0.52845487	0.35949487	1
gene57256	495.907116	697.8726233	1.40726479	0.49289381	0.3595154	1
gene14306	131.2087578	88.08101071	0.671304356	-0.57496109	0.3595479	1
gene68	579.5914419	816.9271763	1.40948799	0.495171185	0.35956655	1
gene55999	360.565799	507.191754	1.406655194	0.492268732	0.3596427	1
gene67755	1032.106685	1475.114949	1.429227201	0.515235276	0.35965032	1
gene48226	3212.031716	4896.723442	1.524494113	0.608330579	0.35965097	1
gene13153	756.258352	537.197373	0.710335789	-0.49342692	0.35990118	1
gene39827	58.88897003	90.9847803	1.545022442	0.627627794	0.3599157	1

gene13828	105.3802622	154.8677111	1.469608331	0.55543171	0.36006553	1
gene701	38.22617353	61.94708446	1.620541078	0.696475591	0.36013239	1
gene4722	38.22617353	61.94708446	1.620541078	0.696475591	0.36013239	1
gene14411	254.152397	178.0978678	0.700752265	-0.51302359	0.36017036	1
gene47613	99.18142321	64.85085404	0.653860894	-0.61294435	0.36017918	1
gene56148	368.8309176	261.3392626	0.708561159	-0.49703571	0.36031342	1
gene4033	56.82269038	88.08101071	1.550102787	0.632363883	0.36032251	1
gene26225	56.82269038	88.08101071	1.550102787	0.632363883	0.36032251	1
gene10980	178.7331897	122.9262457	0.687763956	-0.54001458	0.36038214	1
gene62391	1416.4347	993.0891977	0.701118942	-0.51226888	0.36056908	1
gene476	12.3976779	24.19807987	1.951823564	0.964822646	0.36058443	1
gene9377	12.3976779	24.19807987	1.951823564	0.964822646	0.36058443	1
gene11513	12.3976779	24.19807987	1.951823564	0.964822646	0.36058443	1
gene37267	12.3976779	24.19807987	1.951823564	0.964822646	0.36058443	1
gene72629	12.3976779	24.19807987	1.951823564	0.964822646	0.36058443	1
gene25692	139.4738764	201.3280245	1.44348196	0.529553078	0.36059944	1
gene40913	206.627965	293.280728	1.419366096	0.50524675	0.36061722	1
gene38000	537.232709	755.948015	1.407114649	0.492739882	0.36065868	1
gene27695	462.8466416	329.0938862	0.711021441	-0.49203503	0.36066114	1
gene8448	54.75641073	85.17724113	1.555566554	0.637440121	0.36068092	1
gene72799	54.75641073	85.17724113	1.555566554	0.637440121	0.36068092	1
gene38717	1276.960824	898.2327246	0.703414473	-0.50755308	0.36068897	1
gene30813	40.29245318	23.23015667	0.576538653	-0.79451076	0.36075792	1
gene49445	40.29245318	23.23015667	0.576538653	-0.79451076	0.36075792	1
gene51975	108.4796816	71.6263164	0.660274029	-0.5988632	0.36084257	1
gene7775	277.9146129	195.5204853	0.703527185	-0.50732192	0.36087516	1
gene2844	400.8582521	284.5694192	0.709900364	-0.49431154	0.36092241	1
gene7990	52.69013108	82.27347154	1.561458851	0.642894551	0.36098069	1
gene17571	52.69013108	82.27347154	1.561458851	0.642894551	0.36098069	1
gene13532	239.6884394	338.7731181	1.413389477	0.499159073	0.36099344	1
gene32228	441.1507053	313.6071151	0.710884311	-0.4923133	0.361075	1
gene69591	42.35873283	67.75462362	1.599543213	0.677659969	0.36108496	1
gene62553	313.041367	440.4050536	1.406858965	0.492477708	0.36109462	1
gene42429	572.3594631	407.4956649	0.711957592	-0.49013679	0.36112004	1
gene70804	628.1490136	447.1805159	0.711901963	-0.49024952	0.36120893	1
gene39630	50.62385143	79.36970196	1.567832153	0.648771117	0.36120969	1
gene47376	81.61804618	52.26785251	0.640395782	-0.64296429	0.36121816	1
gene62103	4291.662833	2847.630039	0.663526039	-0.59177501	0.36130929	1
gene41435	44.42501248	70.65839321	1.590509248	0.669488761	0.36131378	1
gene38791	974.2508551	690.1292378	0.708369137	-0.49742674	0.36133574	1
gene29686	4791.702509	7600.132924	1.586102833	0.665486309	0.36134503	1
gene18934	530.0007303	745.3008599	1.4062261	0.491828577	0.36135001	1
gene4508	1145.752066	1641.597738	1.432768735	0.518805761	0.36137683	1
gene19432	9.298258426	19.35846389	2.081945135	1.05793205	0.36138093	1
gene24197	9.298258426	19.35846389	2.081945135	1.05793205	0.36138093	1
gene27588	9.298258426	19.35846389	2.081945135	1.05793205	0.36138093	1
gene38693	9.298258426	19.35846389	2.081945135	1.05793205	0.36138093	1
gene55943	9.298258426	19.35846389	2.081945135	1.05793205	0.36138093	1

gene57189	9.298258426	19.35846389	2.081945135	1.05793205	0.36138093	1
gene58740	9.298258426	19.35846389	2.081945135	1.05793205	0.36138093	1
gene59359	9.298258426	19.35846389	2.081945135	1.05793205	0.36138093	1
gene60142	9.298258426	19.35846389	2.081945135	1.05793205	0.36138093	1
gene68344	9.298258426	19.35846389	2.081945135	1.05793205	0.36138093	1
gene71204	9.298258426	19.35846389	2.081945135	1.05793205	0.36138093	1
gene72353	9.298258426	19.35846389	2.081945135	1.05793205	0.36138093	1
gene60837	231.4233208	161.6431735	0.698474004	-0.51772167	0.36140706	1
gene33943	95.04886391	61.94708446	0.651739347	-0.617633	0.36159769	1
gene36707	273.7820536	192.6167157	0.703540328	-0.50729497	0.36162446	1
gene2238	233.4896005	330.0618094	1.413603898	0.499377923	0.36165576	1
gene35198	1378.208527	967.9231946	0.70230533	-0.50982971	0.36168699	1
gene19359	174.6006304	120.0224761	0.687411471	-0.54075417	0.36169251	1
gene52338	123.976779	180.0337142	1.452156732	0.538197172	0.3617271	1
gene65186	171.501211	244.8845682	1.427888275	0.5138831	0.36175887	1
gene24129	675.6734456	481.0578277	0.711967935	-0.49011583	0.36182961	1
gene63278	146.7058552	211.0072564	1.438301533	0.524366162	0.36184792	1
gene36195	198.3628464	281.6656496	1.419951643	0.505841799	0.36186808	1
gene52671	738.6949749	525.5822947	0.71150111	-0.49106209	0.36188718	1
gene67928	1133.354388	800.472482	0.706286128	-0.50167533	0.36189794	1
gene60905	365.7314981	259.4034162	0.709272834	-0.4955874	0.36193795	1
gene33631	508.3047939	362.0032748	0.712177574	-0.48969109	0.36201221	1
gene50376	1911.308676	2803.105572	1.466589676	0.552465288	0.36201268	1
gene1968	189.064588	130.6696313	0.691137524	-0.53295528	0.36206112	1
gene16363	141.540156	95.82439627	0.677012086	-0.56274651	0.36206633	1
gene38724	268.6163545	378.4579691	1.408916333	0.494585941	0.36218035	1
gene5212	169.4349313	241.9807987	1.428163583	0.514161237	0.36220951	1
gene42000	329.5716042	233.2694899	0.707796081	-0.49859432	0.3622558	1
gene39277	77.48548688	49.36408293	0.637075211	-0.65046439	0.36227783	1
gene54734	77.48548688	49.36408293	0.637075211	-0.65046439	0.36227783	1
gene1241	17.56337703	31.94146542	1.818640309	0.862860235	0.3623004	1
gene24050	17.56337703	31.94146542	1.818640309	0.862860235	0.3623004	1
gene24051	17.56337703	31.94146542	1.818640309	0.862860235	0.3623004	1
gene40217	17.56337703	31.94146542	1.818640309	0.862860235	0.3623004	1
gene44597	17.56337703	31.94146542	1.818640309	0.862860235	0.3623004	1
gene50682	17.56337703	31.94146542	1.818640309	0.862860235	0.3623004	1
gene67518	17.56337703	31.94146542	1.818640309	0.862860235	0.3623004	1
gene19000	490.7414169	689.1613146	1.404326782	0.489878684	0.36234456	1
gene6534	104.3471223	68.72254682	0.658595515	-0.60253541	0.36236938	1
gene1759	227.2907615	321.3505006	1.413830014	0.499608674	0.3623767	1
gene39214	203.5285455	141.3167864	0.694333987	-0.5262983	0.36239996	1
gene64342	325.4390449	230.3657203	0.707861346	-0.4984613	0.36282014	1
gene60423	548.5972471	391.0409706	0.712801555	-0.48842761	0.3628676	1
gene18787	407.0570911	571.0746848	1.40293511	0.488448281	0.36289818	1
gene36197	407.0570911	571.0746848	1.40293511	0.488448281	0.36289818	1
gene4663	44.42501248	26.13392626	0.588270544	-0.7654483	0.36291313	1
gene70331	44.42501248	26.13392626	0.588270544	-0.7654483	0.36291313	1
gene42163	90.91630461	59.04331487	0.649424931	-0.62276532	0.36301254	1

gene43696	90.91630461	59.04331487	0.649424931	-0.62276532	0.36301254	1
gene57899	90.91630461	59.04331487	0.649424931	-0.62276532	0.36301254	1
gene63920	1294.524201	911.7836493	0.704338821	-0.50565849	0.3630497	1
gene64174	165.302372	236.1732595	1.428734849	0.514738199	0.36315427	1
gene10533	25.82849563	13.55092472	0.524650174	-0.93057231	0.36321347	1
gene37319	25.82849563	13.55092472	0.524650174	-0.93057231	0.36321347	1
gene38923	25.82849563	13.55092472	0.524650174	-0.93057231	0.36321347	1
gene49442	25.82849563	13.55092472	0.524650174	-0.93057231	0.36321347	1
gene63710	25.82849563	13.55092472	0.524650174	-0.93057231	0.36321347	1
gene63718	25.82849563	13.55092472	0.524650174	-0.93057231	0.36321347	1
gene41719	73.35292758	46.46031334	0.633380492	-0.65885566	0.36321807	1
gene49782	73.35292758	46.46031334	0.633380492	-0.65885566	0.36321807	1
gene24433	256.2186766	180.0337142	0.702656483	-0.50910854	0.36336569	1
gene61429	371.930337	521.7106019	1.402710535	0.488217323	0.363419	1
gene53393	1031.073545	1469.307409	1.425026775	0.510989027	0.36348596	1
gene8551	246.9204182	173.2582518	0.701676488	-0.51112207	0.36357106	1
gene14000	140.5070162	202.2959477	1.439756911	0.525825247	0.36358631	1
gene27252	140.5070162	202.2959477	1.439756911	0.525825247	0.36358631	1
gene14520	199.3959862	138.4130168	0.6941615	-0.52665674	0.36359748	1
gene39856	363.6652184	510.0955236	1.402651388	0.48815649	0.36362509	1
gene5138	1203.607896	849.8365649	0.706074269	-0.50210815	0.36363482	1
gene59733	163.2360924	233.2694899	1.429031328	0.515037545	0.3636491	1
gene27355	129.1424781	186.8091766	1.44653548	0.532601709	0.36366304	1
gene37921	295.47799	415.2390505	1.405312966	0.490891457	0.36368297	1
gene5390	10.33139825	3.871692779	0.374750124	-1.41599914	0.36368837	1
gene10975	10.33139825	3.871692779	0.374750124	-1.41599914	0.36368837	1
gene11055	10.33139825	3.871692779	0.374750124	-1.41599914	0.36368837	1
gene13358	10.33139825	3.871692779	0.374750124	-1.41599914	0.36368837	1
gene22238	10.33139825	3.871692779	0.374750124	-1.41599914	0.36368837	1
gene22710	10.33139825	3.871692779	0.374750124	-1.41599914	0.36368837	1
gene25770	10.33139825	3.871692779	0.374750124	-1.41599914	0.36368837	1
gene30917	10.33139825	3.871692779	0.374750124	-1.41599914	0.36368837	1
gene31717	10.33139825	3.871692779	0.374750124	-1.41599914	0.36368837	1
gene41544	10.33139825	3.871692779	0.374750124	-1.41599914	0.36368837	1
gene44454	10.33139825	3.871692779	0.374750124	-1.41599914	0.36368837	1
gene50252	10.33139825	3.871692779	0.374750124	-1.41599914	0.36368837	1
gene50995	10.33139825	3.871692779	0.374750124	-1.41599914	0.36368837	1
gene60184	10.33139825	3.871692779	0.374750124	-1.41599914	0.36368837	1
gene60725	10.33139825	3.871692779	0.374750124	-1.41599914	0.36368837	1
gene68666	10.33139825	3.871692779	0.374750124	-1.41599914	0.36368837	1
gene69432	10.33139825	3.871692779	0.374750124	-1.41599914	0.36368837	1
gene72769	10.33139825	3.871692779	0.374750124	-1.41599914	0.36368837	1
gene73152	10.33139825	3.871692779	0.374750124	-1.41599914	0.36368837	1
gene55036	216.9593633	306.8316527	1.414235588	0.500022469	0.36372113	1
gene70588	1102.360193	780.1460949	0.707705249	-0.49877948	0.36376106	1
gene3163	26.86163545	45.49239015	1.693582292	0.76007809	0.36376124	1
gene25561	26.86163545	45.49239015	1.693582292	0.76007809	0.36376124	1
gene16602	520.7024718	730.782012	1.403454086	0.488981867	0.3638842	1

gene7814	33.0604744	18.3905407	0.556269716	-0.84614353	0.36390661	1
gene8987	33.0604744	18.3905407	0.556269716	-0.84614353	0.36390661	1
gene17619	33.0604744	18.3905407	0.556269716	-0.84614353	0.36390661	1
gene35566	2298.736111	1585.458193	0.689708656	-0.53594102	0.36399151	1
gene33800	69.22036828	43.55654376	0.629244612	-0.66830714	0.36399283	1
gene47567	69.22036828	43.55654376	0.629244612	-0.66830714	0.36399283	1
gene14823	420.4879088	589.4652255	1.401860109	0.48734239	0.3639936	1
gene65905	128.1093383	86.14516432	0.672434699	-0.57253392	0.36400335	1
gene18016	81.61804618	121.9583225	1.494256825	0.579428132	0.36403441	1
gene30303	81.61804618	121.9583225	1.494256825	0.579428132	0.36403441	1
gene46358	66.12094881	100.6640122	1.52242238	0.606368675	0.36410807	1
gene71521	403.9576716	566.2350689	1.401718815	0.487196974	0.36413728	1
gene11659	1730.509207	1207.968147	0.698042023	-0.5186142	0.36415118	1
gene30931	228.3239013	159.7073271	0.699477042	-0.51565139	0.3641677	1
gene50184	151.8715543	103.5677818	0.681943253	-0.5522764	0.36416802	1
gene72158	403.9576716	287.4731888	0.71164186	-0.49077672	0.36419138	1
gene16348	118.8110799	79.36970196	0.66803283	-0.58200909	0.36424892	1
gene66736	172.5343508	245.8524914	1.424948077	0.510909351	0.36428243	1
gene48899	86.78374531	56.13954529	0.646890095	-0.62840747	0.3644102	1
gene21630	92.98258426	137.4450936	1.478181046	0.56382298	0.36451111	1
gene39127	730.4298563	520.7426787	0.712926333	-0.48817509	0.36457866	1
gene54109	275.8483333	194.5525621	0.705288155	-0.50371528	0.36462865	1
gene28059	2941.349082	2004.568936	0.681513442	-0.55318598	0.3646866	1
gene57748	363.6652184	258.435493	0.710641216	-0.49280673	0.36478242	1
gene14749	637.4472721	454.9239015	0.713665148	-0.48668078	0.36479702	1
gene3963	5.165699125	12.58300153	2.435875808	1.28444058	0.3648027	1
gene19427	5.165699125	12.58300153	2.435875808	1.28444058	0.3648027	1
gene19719	5.165699125	12.58300153	2.435875808	1.28444058	0.3648027	1
gene19784	5.165699125	12.58300153	2.435875808	1.28444058	0.3648027	1
gene20895	5.165699125	12.58300153	2.435875808	1.28444058	0.3648027	1
gene26776	5.165699125	12.58300153	2.435875808	1.28444058	0.3648027	1
gene26825	5.165699125	12.58300153	2.435875808	1.28444058	0.3648027	1
gene32901	5.165699125	12.58300153	2.435875808	1.28444058	0.3648027	1
gene34263	5.165699125	12.58300153	2.435875808	1.28444058	0.3648027	1
gene39421	5.165699125	12.58300153	2.435875808	1.28444058	0.3648027	1
gene44120	5.165699125	12.58300153	2.435875808	1.28444058	0.3648027	1
gene45094	5.165699125	12.58300153	2.435875808	1.28444058	0.3648027	1
gene45620	5.165699125	12.58300153	2.435875808	1.28444058	0.3648027	1
gene50325	5.165699125	12.58300153	2.435875808	1.28444058	0.3648027	1
gene54390	5.165699125	12.58300153	2.435875808	1.28444058	0.3648027	1
gene60192	5.165699125	12.58300153	2.435875808	1.28444058	0.3648027	1
gene73709	5.165699125	12.58300153	2.435875808	1.28444058	0.3648027	1
gene18771	52.69013108	31.94146542	0.606213436	-0.72210227	0.36481744	1
gene3724	136.3744569	196.4884085	1.440800667	0.526870754	0.36483394	1
gene35774	195.2634269	135.5092472	0.693981712	-0.52703045	0.36484515	1
gene917	60.95524968	37.74900459	0.61929046	-0.69131187	0.36489254	1
gene69435	60.95524968	37.74900459	0.61929046	-0.69131187	0.36489254	1
gene24203	56.82269038	34.84523501	0.613227476	-0.70550576	0.36501361	1

gene41627	56.82269038	34.84523501	0.613227476	-0.70550576	0.36501361	1
gene38626	472.1449001	336.8372717	0.713419274	-0.4871779	0.36517245	1
gene21295	157.0372534	224.5581812	1.42996758	0.515982438	0.36522783	1
gene28515	133.2750374	90.0168571	0.675421736	-0.56613949	0.36523461	1
gene29521	133.2750374	90.0168571	0.675421736	-0.56613949	0.36523461	1
gene32250	727.3304369	518.8068323	0.713302793	-0.48741347	0.36527583	1
gene47209	1270.761985	1822.599376	1.434257081	0.520303641	0.36529474	1
gene2055	171.501211	118.0866297	0.688546915	-0.53837314	0.36529898	1
gene50030	171.501211	118.0866297	0.688546915	-0.53837314	0.36529898	1
gene25473	102.2808427	150.0280952	1.466824981	0.552696742	0.36536648	1
gene26612	102.2808427	150.0280952	1.466824981	0.552696742	0.36536648	1
gene13118	28.9279151	48.39615973	1.672991626	0.742430224	0.36537445	1
gene13491	28.9279151	48.39615973	1.672991626	0.742430224	0.36537445	1
gene18567	28.9279151	48.39615973	1.672991626	0.742430224	0.36537445	1
gene57751	320.2733458	449.1163623	1.402290788	0.487785547	0.36537764	1
gene22223	497.9733957	697.8726233	1.401425517	0.48689507	0.36540891	1
gene7042	294.4448501	413.3032041	1.403669325	0.489203107	0.36542228	1
gene13621	185.9651685	128.7337849	0.692246757	-0.5306417	0.36542928	1
gene14949	569.2600436	406.5277417	0.714133631	-0.48573403	0.36545609	1
gene23168	134.3081773	193.5846389	1.441346632	0.527417333	0.36548567	1
gene64554	134.3081773	193.5846389	1.441346632	0.527417333	0.36548567	1
gene17613	96.08200373	62.91500765	0.654805325	-0.61086204	0.36549647	1
gene30782	96.08200373	62.91500765	0.654805325	-0.61086204	0.36549647	1
gene66133	96.08200373	62.91500765	0.654805325	-0.61086204	0.36549647	1
gene70986	638.4804119	455.8918247	0.714026329	-0.48595082	0.36552728	1
gene10452	235.5558801	331.9976558	1.409422068	0.495103709	0.36555689	1
gene40506	346.1018414	245.8524914	0.710347251	-0.49340364	0.36565633	1
gene17457	214.8930836	150.0280952	0.698152275	-0.51838636	0.36570333	1
gene39147	214.8930836	150.0280952	0.698152275	-0.51838636	0.36570333	1
gene58477	525.868171	375.5541995	0.714160355	-0.48568005	0.36574451	1
gene42974	463.8797815	331.0297326	0.713611038	-0.48679016	0.3657671	1
gene57037	82.65118601	53.23577571	0.644101776	-0.63463942	0.36577309	1
gene29185	802.7496441	1133.438061	1.411944645	0.497683529	0.36582642	1
gene7688	268.6163545	377.4900459	1.405312966	0.490891457	0.36582747	1
gene3623	253.1192571	178.0978678	0.703612478	-0.50714703	0.36587083	1
gene22413	114.6785206	76.46593238	0.666785131	-0.58470616	0.36592072	1
gene583	162.2029525	111.3111674	0.686246247	-0.54320174	0.36596048	1
gene11819	162.2029525	111.3111674	0.686246247	-0.54320174	0.36596048	1
gene67439	950.4886391	1348.31701	1.418551422	0.504418448	0.36597465	1
gene72390	533.1001497	747.2367063	1.401681666	0.487158738	0.36597918	1
gene38645	111.5791011	162.6110967	1.457361594	0.543358877	0.36598058	1
gene54275	111.5791011	162.6110967	1.457361594	0.543358877	0.36598058	1
gene32785	878.1688513	625.2783837	0.712025236	-0.48999972	0.36602172	1
gene1605	898.8316478	1272.819001	1.416081648	0.50190445	0.36603612	1
gene28665	59.92210985	91.95270349	1.534537147	0.61780357	0.36604038	1
gene10838	787.2525467	561.3954529	0.713107192	-0.48780914	0.36604809	1
gene71167	432.8855867	308.7674991	0.713277385	-0.48746486	0.36612027	1
gene4531	453.5483832	634.9576157	1.399977685	0.485403832	0.36614493	1

gene58288	191.1308676	132.6054777	0.693794149	-0.52742042	0.36614508	1
gene11626	711.8333395	508.1596772	0.713874511	-0.4862576	0.36615809	1
gene38863	243.8209987	171.3224055	0.702656483	-0.50910854	0.36618125	1
gene44600	570.2931834	407.4956649	0.714537148	-0.48491908	0.36626052	1
gene67259	175.6337703	249.7241842	1.42184606	0.507765276	0.3663094	1
gene32278	1029.007266	730.782012	0.710181586	-0.49374014	0.36649421	1
gene26543	30.99419475	51.29992932	1.655146382	0.726958816	0.36660483	1
gene37797	57.8558302	89.04893391	1.539152296	0.622135991	0.36663771	1
gene43450	152.9046941	104.535705	0.683665767	-0.54863691	0.36665094	1
gene20389	260.3512359	365.8749676	1.405312966	0.490891457	0.36665501	1
gene23212	291.3454307	206.1676405	0.707639862	-0.49891278	0.36668704	1
gene5664	19.62965668	34.84523501	1.775132168	0.827926445	0.36669324	1
gene6250	19.62965668	34.84523501	1.775132168	0.827926445	0.36669324	1
gene37134	19.62965668	34.84523501	1.775132168	0.827926445	0.36669324	1
gene33991	397.7588327	556.5558369	1.39922936	0.484632467	0.36669675	1
gene49294	353.3338202	494.6087525	1.399834163	0.485255923	0.36672817	1
gene52184	173.5674906	246.8204146	1.422042882	0.507964971	0.36679542	1
gene45929	181.8326092	125.8300153	0.692010173	-0.53113485	0.3668033	1
gene23925	522.7687515	373.6183531	0.714691443	-0.48460758	0.36683589	1
gene21959	2722.323439	1866.155919	0.685501176	-0.54476895	0.36684472	1
gene12130	130.175618	187.7770998	1.442490558	0.528561875	0.3668473	1
gene33889	129.1424781	87.11308752	0.674550224	-0.56800223	0.36691052	1
gene18877	630.2152933	884.6817999	1.403777105	0.489313879	0.36694068	1
gene7857	98.14828338	144.220556	1.469414961	0.555241869	0.36698665	1
gene4991	12309.86102	7368.799281	0.598609462	-0.74031301	0.36705809	1
gene59764	397.7588327	283.601496	0.712998613	-0.48802882	0.36720804	1
gene18176	73.35292758	110.3432442	1.504278668	0.589071851	0.3672193	1
gene9968	238.6552996	335.8693485	1.407340835	0.492971768	0.36722619	1
gene63670	487.6419974	682.3858522	1.39935825	0.484765355	0.36729681	1
gene24739	65723.18997	27161.86069	0.413276664	-1.27482019	0.36730883	1
gene24510	438.0512858	612.6953822	1.398684131	0.484070192	0.36731286	1
gene51998	305.8093882	216.8147956	0.708986722	-0.49616949	0.36732327	1
gene14070	182.865749	259.4034162	1.418545668	0.504412597	0.36734688	1
gene41623	143.6064357	97.76024266	0.680751125	-0.55480063	0.36735085	1
gene2504	1263.530006	893.3931087	0.707061252	-0.50009289	0.36743983	1
gene72007	560.994925	785.953634	1.400999544	0.486456486	0.36744427	1
gene42659	158.0703932	108.4073978	0.685817221	-0.54410396	0.36750335	1
gene48366	597.1548189	837.2535634	1.402071183	0.487559597	0.36750402	1
gene14140	194.2302871	274.8901873	1.415279725	0.501087224	0.36751902	1
gene23117	310.9750873	435.5654376	1.400644152	0.486090471	0.36752073	1
gene49450	33.0604744	54.2036989	1.639531794	0.713283879	0.36752893	1
gene62232	33.0604744	54.2036989	1.639531794	0.713283879	0.36752893	1
gene47149	128.1093383	184.8733302	1.443090196	0.529161474	0.36755817	1
gene48648	148.7721348	212.9431028	1.43133728	0.517363669	0.36756763	1
gene70799	101.2477029	66.78670043	0.659636698	-0.60025643	0.36760968	1
gene65640	705.6345005	504.2879844	0.714658912	-0.48467325	0.36762838	1
gene43461	110.5459613	73.56216279	0.665444146	-0.58761052	0.36764432	1
gene24559	53.7232709	83.24139474	1.549447629	0.631753993	0.36771959	1

gene60146	267.5832147	375.5541995	1.403504326	0.489033511	0.36776911	1
gene25918	1852.419706	1293.145388	0.698084448	-0.51852652	0.36777201	1
gene28858	37.1930337	21.29431028	0.572534912	-0.80456443	0.36778134	1
gene47971	37.1930337	21.29431028	0.572534912	-0.80456443	0.36778134	1
gene51218	37.1930337	21.29431028	0.572534912	-0.80456443	0.36778134	1
gene63253	37.1930337	21.29431028	0.572534912	-0.80456443	0.36778134	1
gene6858	268.6163545	189.7129461	0.70625985	-0.50172901	0.36781742	1
gene64954	244.8541385	172.2903286	0.703644748	-0.50708086	0.36782621	1
gene66119	554.7960861	396.8485098	0.715305172	-0.48336922	0.3678333	1
gene52787	3724.469069	5710.746848	1.533304947	0.616644652	0.36792616	1
gene14421	259.3180961	182.9374838	0.705455911	-0.50337217	0.36809649	1
gene14475	206.627965	144.220556	0.697972106	-0.51875871	0.36812925	1
gene56781	1191.210218	844.0290257	0.708547503	-0.49706352	0.36813522	1
gene10884	35.12675405	57.10746848	1.625754216	0.701109165	0.36820637	1
gene12005	35.12675405	57.10746848	1.625754216	0.701109165	0.36820637	1
gene24024	35.12675405	57.10746848	1.625754216	0.701109165	0.36820637	1
gene52732	35.12675405	57.10746848	1.625754216	0.701109165	0.36820637	1
gene72359	35.12675405	57.10746848	1.625754216	0.701109165	0.36820637	1
gene13232	902.9642071	643.6689244	0.71283991	-0.48834998	0.36828545	1
gene21144	74.38606741	47.42823654	0.637595698	-0.6492862	0.368296	1
gene58870	3693.474875	5656.543149	1.531496312	0.614941893	0.36832564	1
gene33007	382.2617353	534.2936034	1.39771668	0.483071953	0.36837471	1
gene24043	22.72907615	11.61507834	0.511022897	-0.96854016	0.36848907	1
gene38722	22.72907615	11.61507834	0.511022897	-0.96854016	0.36848907	1
gene47701	22.72907615	11.61507834	0.511022897	-0.96854016	0.36848907	1
gene72961	22.72907615	11.61507834	0.511022897	-0.96854016	0.36848907	1
gene72044	135.3413171	194.5525621	1.437495706	0.523557646	0.3685707	1
gene34527	49.5907116	77.43385557	1.561458851	0.642894551	0.36858479	1
gene3243	243.8209987	342.6448109	1.405312966	0.490891457	0.36859501	1
gene608	125.0099188	84.20931793	0.673621091	-0.56999078	0.36864983	1
gene45641	87.81688513	57.10746848	0.650301686	-0.62081893	0.36866862	1
gene61412	1060.001461	1506.088491	1.420836241	0.506740286	0.36867104	1
gene28317	37.1930337	60.01123807	1.61350748	0.690200266	0.36868415	1
gene56870	82.65118601	122.9262457	1.487289556	0.572685549	0.36869036	1
gene49228	114.6785206	166.4827895	1.451734716	0.537777845	0.36876079	1
gene33077	226.2576217	158.7394039	0.701586991	-0.5113061	0.36876401	1
gene4014	14.46395755	27.10184945	1.873750621	0.905928957	0.36879743	1
gene5520	14.46395755	27.10184945	1.873750621	0.905928957	0.36879743	1
gene13969	14.46395755	27.10184945	1.873750621	0.905928957	0.36879743	1
gene30247	14.46395755	27.10184945	1.873750621	0.905928957	0.36879743	1
gene64343	14.46395755	27.10184945	1.873750621	0.905928957	0.36879743	1
gene3073	197.3297066	137.4450936	0.6965251	-0.52175275	0.36880326	1
gene33596	197.3297066	137.4450936	0.6965251	-0.52175275	0.36880326	1
gene31291	188.0314482	266.1788785	1.415608299	0.501422125	0.36881303	1
gene40323	69.22036828	104.535705	1.510187068	0.594727268	0.36884941	1
gene42596	3374.234669	5122.249546	1.518047809	0.602217227	0.36887866	1
gene22605	47.52443195	74.53008599	1.568247803	0.649153541	0.36890384	1
gene20150	1918.540655	1338.637778	0.697737509	-0.5192437	0.36898402	1

gene12814	39.25931335	62.91500765	1.602549874	0.680369256	0.36899966	1
gene56260	39.25931335	62.91500765	1.602549874	0.680369256	0.36899966	1
gene26440	278.9477528	197.4563317	0.707861346	-0.4984613	0.36908091	1
gene32325	153.9378339	105.5036282	0.68536516	-0.54505524	0.3691106	1
gene63858	153.9378339	219.7185652	1.427320104	0.513308923	0.36913776	1
gene62203	115.7116604	77.43385557	0.669196651	-0.57949787	0.36914346	1
gene95	0	2.903769584	Inf	Inf	0.36916133	1
gene143	0	2.903769584	Inf	Inf	0.36916133	1
gene439	0	2.903769584	Inf	Inf	0.36916133	1
gene730	0	2.903769584	Inf	Inf	0.36916133	1
gene793	0	2.903769584	Inf	Inf	0.36916133	1
gene1379	0	2.903769584	Inf	Inf	0.36916133	1
gene1454	0	2.903769584	Inf	Inf	0.36916133	1
gene1512	0	2.903769584	Inf	Inf	0.36916133	1
gene1657	0	2.903769584	Inf	Inf	0.36916133	1
gene1687	0	2.903769584	Inf	Inf	0.36916133	1
gene1691	0	2.903769584	Inf	Inf	0.36916133	1
gene1709	0	2.903769584	Inf	Inf	0.36916133	1
gene1794	0	2.903769584	Inf	Inf	0.36916133	1
gene1989	0	2.903769584	Inf	Inf	0.36916133	1
gene2333	0	2.903769584	Inf	Inf	0.36916133	1
gene2379	0	2.903769584	Inf	Inf	0.36916133	1
gene2380	0	2.903769584	Inf	Inf	0.36916133	1
gene2654	0	2.903769584	Inf	Inf	0.36916133	1
gene2737	0	2.903769584	Inf	Inf	0.36916133	1
gene3097	0	2.903769584	Inf	Inf	0.36916133	1
gene3543	0	2.903769584	Inf	Inf	0.36916133	1
gene3829	0	2.903769584	Inf	Inf	0.36916133	1
gene3972	0	2.903769584	Inf	Inf	0.36916133	1
gene3993	0	2.903769584	Inf	Inf	0.36916133	1
gene4196	0	2.903769584	Inf	Inf	0.36916133	1
gene4253	0	2.903769584	Inf	Inf	0.36916133	1
gene4277	0	2.903769584	Inf	Inf	0.36916133	1
gene4288	0	2.903769584	Inf	Inf	0.36916133	1
gene4300	0	2.903769584	Inf	Inf	0.36916133	1
gene4490	0	2.903769584	Inf	Inf	0.36916133	1
gene4565	0	2.903769584	Inf	Inf	0.36916133	1
gene4659	0	2.903769584	Inf	Inf	0.36916133	1
gene4942	0	2.903769584	Inf	Inf	0.36916133	1
gene5464	0	2.903769584	Inf	Inf	0.36916133	1
gene5599	0	2.903769584	Inf	Inf	0.36916133	1
gene5708	0	2.903769584	Inf	Inf	0.36916133	1
gene5751	0	2.903769584	Inf	Inf	0.36916133	1
gene6156	0	2.903769584	Inf	Inf	0.36916133	1
gene6573	0	2.903769584	Inf	Inf	0.36916133	1
gene6721	0	2.903769584	Inf	Inf	0.36916133	1
gene6904	0	2.903769584	Inf	Inf	0.36916133	1
gene7228	0	2.903769584	Inf	Inf	0.36916133	1

gene7298	0	2.903769584	Inf	Inf	0.36916133	1
gene7658	0	2.903769584	Inf	Inf	0.36916133	1
gene7710	0	2.903769584	Inf	Inf	0.36916133	1
gene7876	0	2.903769584	Inf	Inf	0.36916133	1
gene7923	0	2.903769584	Inf	Inf	0.36916133	1
gene8096	0	2.903769584	Inf	Inf	0.36916133	1
gene8111	0	2.903769584	Inf	Inf	0.36916133	1
gene8190	0	2.903769584	Inf	Inf	0.36916133	1
gene8237	0	2.903769584	Inf	Inf	0.36916133	1
gene8613	0	2.903769584	Inf	Inf	0.36916133	1
gene9156	0	2.903769584	Inf	Inf	0.36916133	1
gene9307	0	2.903769584	Inf	Inf	0.36916133	1
gene9336	0	2.903769584	Inf	Inf	0.36916133	1
gene9466	0	2.903769584	Inf	Inf	0.36916133	1
gene9561	0	2.903769584	Inf	Inf	0.36916133	1
gene9584	0	2.903769584	Inf	Inf	0.36916133	1
gene9601	0	2.903769584	Inf	Inf	0.36916133	1
gene10113	0	2.903769584	Inf	Inf	0.36916133	1
gene10156	0	2.903769584	Inf	Inf	0.36916133	1
gene10368	0	2.903769584	Inf	Inf	0.36916133	1
gene10647	0	2.903769584	Inf	Inf	0.36916133	1
gene10771	0	2.903769584	Inf	Inf	0.36916133	1
gene10773	0	2.903769584	Inf	Inf	0.36916133	1
gene11223	0	2.903769584	Inf	Inf	0.36916133	1
gene11458	0	2.903769584	Inf	Inf	0.36916133	1
gene11753	0	2.903769584	Inf	Inf	0.36916133	1
gene11759	0	2.903769584	Inf	Inf	0.36916133	1
gene11977	0	2.903769584	Inf	Inf	0.36916133	1
gene12036	0	2.903769584	Inf	Inf	0.36916133	1
gene12064	0	2.903769584	Inf	Inf	0.36916133	1
gene12245	0	2.903769584	Inf	Inf	0.36916133	1
gene12322	0	2.903769584	Inf	Inf	0.36916133	1
gene12503	0	2.903769584	Inf	Inf	0.36916133	1
gene12959	0	2.903769584	Inf	Inf	0.36916133	1
gene12963	0	2.903769584	Inf	Inf	0.36916133	1
gene13004	0	2.903769584	Inf	Inf	0.36916133	1
gene13147	0	2.903769584	Inf	Inf	0.36916133	1
gene13458	0	2.903769584	Inf	Inf	0.36916133	1
gene13617	0	2.903769584	Inf	Inf	0.36916133	1
gene13742	0	2.903769584	Inf	Inf	0.36916133	1
gene13784	0	2.903769584	Inf	Inf	0.36916133	1
gene13831	0	2.903769584	Inf	Inf	0.36916133	1
gene13834	0	2.903769584	Inf	Inf	0.36916133	1
gene14184	0	2.903769584	Inf	Inf	0.36916133	1
gene14246	0	2.903769584	Inf	Inf	0.36916133	1
gene14456	0	2.903769584	Inf	Inf	0.36916133	1
gene14565	0	2.903769584	Inf	Inf	0.36916133	1
gene14719	0	2.903769584	Inf	Inf	0.36916133	1

gene14795	0	2.903769584	Inf	Inf	0.36916133	1
gene15102	0	2.903769584	Inf	Inf	0.36916133	1
gene15434	0	2.903769584	Inf	Inf	0.36916133	1
gene15458	0	2.903769584	Inf	Inf	0.36916133	1
gene15730	0	2.903769584	Inf	Inf	0.36916133	1
gene16292	0	2.903769584	Inf	Inf	0.36916133	1
gene16335	0	2.903769584	Inf	Inf	0.36916133	1
gene16542	0	2.903769584	Inf	Inf	0.36916133	1
gene16617	0	2.903769584	Inf	Inf	0.36916133	1
gene16792	0	2.903769584	Inf	Inf	0.36916133	1
gene16974	0	2.903769584	Inf	Inf	0.36916133	1
gene17003	0	2.903769584	Inf	Inf	0.36916133	1
gene17205	0	2.903769584	Inf	Inf	0.36916133	1
gene17466	0	2.903769584	Inf	Inf	0.36916133	1
gene17639	0	2.903769584	Inf	Inf	0.36916133	1
gene17717	0	2.903769584	Inf	Inf	0.36916133	1
gene17797	0	2.903769584	Inf	Inf	0.36916133	1
gene18009	0	2.903769584	Inf	Inf	0.36916133	1
gene18513	0	2.903769584	Inf	Inf	0.36916133	1
gene19290	0	2.903769584	Inf	Inf	0.36916133	1
gene19384	0	2.903769584	Inf	Inf	0.36916133	1
gene19742	0	2.903769584	Inf	Inf	0.36916133	1
gene19934	0	2.903769584	Inf	Inf	0.36916133	1
gene20047	0	2.903769584	Inf	Inf	0.36916133	1
gene20065	0	2.903769584	Inf	Inf	0.36916133	1
gene20577	0	2.903769584	Inf	Inf	0.36916133	1
gene20620	0	2.903769584	Inf	Inf	0.36916133	1
gene21044	0	2.903769584	Inf	Inf	0.36916133	1
gene21255	0	2.903769584	Inf	Inf	0.36916133	1
gene21269	0	2.903769584	Inf	Inf	0.36916133	1
gene21403	0	2.903769584	Inf	Inf	0.36916133	1
gene21404	0	2.903769584	Inf	Inf	0.36916133	1
gene21449	0	2.903769584	Inf	Inf	0.36916133	1
gene21736	0	2.903769584	Inf	Inf	0.36916133	1
gene21755	0	2.903769584	Inf	Inf	0.36916133	1
gene21945	0	2.903769584	Inf	Inf	0.36916133	1
gene22000	0	2.903769584	Inf	Inf	0.36916133	1
gene22254	0	2.903769584	Inf	Inf	0.36916133	1
gene22902	0	2.903769584	Inf	Inf	0.36916133	1
gene22911	0	2.903769584	Inf	Inf	0.36916133	1
gene23090	0	2.903769584	Inf	Inf	0.36916133	1
gene23285	0	2.903769584	Inf	Inf	0.36916133	1
gene23371	0	2.903769584	Inf	Inf	0.36916133	1
gene23396	0	2.903769584	Inf	Inf	0.36916133	1
gene23423	0	2.903769584	Inf	Inf	0.36916133	1
gene23859	0	2.903769584	Inf	Inf	0.36916133	1
gene24690	0	2.903769584	Inf	Inf	0.36916133	1
gene24729	0	2.903769584	Inf	Inf	0.36916133	1

gene25040	0	2.903769584	Inf	Inf	0.36916133	1
gene25058	0	2.903769584	Inf	Inf	0.36916133	1
gene25222	0	2.903769584	Inf	Inf	0.36916133	1
gene25288	0	2.903769584	Inf	Inf	0.36916133	1
gene25645	0	2.903769584	Inf	Inf	0.36916133	1
gene25650	0	2.903769584	Inf	Inf	0.36916133	1
gene25957	0	2.903769584	Inf	Inf	0.36916133	1
gene25960	0	2.903769584	Inf	Inf	0.36916133	1
gene26061	0	2.903769584	Inf	Inf	0.36916133	1
gene26219	0	2.903769584	Inf	Inf	0.36916133	1
gene26433	0	2.903769584	Inf	Inf	0.36916133	1
gene26562	0	2.903769584	Inf	Inf	0.36916133	1
gene26691	0	2.903769584	Inf	Inf	0.36916133	1
gene26783	0	2.903769584	Inf	Inf	0.36916133	1
gene26794	0	2.903769584	Inf	Inf	0.36916133	1
gene26817	0	2.903769584	Inf	Inf	0.36916133	1
gene26975	0	2.903769584	Inf	Inf	0.36916133	1
gene27008	0	2.903769584	Inf	Inf	0.36916133	1
gene27353	0	2.903769584	Inf	Inf	0.36916133	1
gene27363	0	2.903769584	Inf	Inf	0.36916133	1
gene27472	0	2.903769584	Inf	Inf	0.36916133	1
gene27499	0	2.903769584	Inf	Inf	0.36916133	1
gene27604	0	2.903769584	Inf	Inf	0.36916133	1
gene27934	0	2.903769584	Inf	Inf	0.36916133	1
gene28487	0	2.903769584	Inf	Inf	0.36916133	1
gene28626	0	2.903769584	Inf	Inf	0.36916133	1
gene28696	0	2.903769584	Inf	Inf	0.36916133	1
gene29258	0	2.903769584	Inf	Inf	0.36916133	1
gene29286	0	2.903769584	Inf	Inf	0.36916133	1
gene29309	0	2.903769584	Inf	Inf	0.36916133	1
gene29582	0	2.903769584	Inf	Inf	0.36916133	1
gene29897	0	2.903769584	Inf	Inf	0.36916133	1
gene30253	0	2.903769584	Inf	Inf	0.36916133	1
gene30461	0	2.903769584	Inf	Inf	0.36916133	1
gene30514	0	2.903769584	Inf	Inf	0.36916133	1
gene30997	0	2.903769584	Inf	Inf	0.36916133	1
gene31214	0	2.903769584	Inf	Inf	0.36916133	1
gene31379	0	2.903769584	Inf	Inf	0.36916133	1
gene31524	0	2.903769584	Inf	Inf	0.36916133	1
gene31546	0	2.903769584	Inf	Inf	0.36916133	1
gene31784	0	2.903769584	Inf	Inf	0.36916133	1
gene31808	0	2.903769584	Inf	Inf	0.36916133	1
gene31821	0	2.903769584	Inf	Inf	0.36916133	1
gene31894	0	2.903769584	Inf	Inf	0.36916133	1
gene31951	0	2.903769584	Inf	Inf	0.36916133	1
gene32155	0	2.903769584	Inf	Inf	0.36916133	1
gene32224	0	2.903769584	Inf	Inf	0.36916133	1
gene32288	0	2.903769584	Inf	Inf	0.36916133	1

gene32878	0	2.903769584	Inf	Inf	0.36916133	1
gene32902	0	2.903769584	Inf	Inf	0.36916133	1
gene33000	0	2.903769584	Inf	Inf	0.36916133	1
gene33035	0	2.903769584	Inf	Inf	0.36916133	1
gene33142	0	2.903769584	Inf	Inf	0.36916133	1
gene33336	0	2.903769584	Inf	Inf	0.36916133	1
gene33642	0	2.903769584	Inf	Inf	0.36916133	1
gene33690	0	2.903769584	Inf	Inf	0.36916133	1
gene33796	0	2.903769584	Inf	Inf	0.36916133	1
gene33807	0	2.903769584	Inf	Inf	0.36916133	1
gene33898	0	2.903769584	Inf	Inf	0.36916133	1
gene34262	0	2.903769584	Inf	Inf	0.36916133	1
gene34400	0	2.903769584	Inf	Inf	0.36916133	1
gene34467	0	2.903769584	Inf	Inf	0.36916133	1
gene34552	0	2.903769584	Inf	Inf	0.36916133	1
gene34610	0	2.903769584	Inf	Inf	0.36916133	1
gene34733	0	2.903769584	Inf	Inf	0.36916133	1
gene34928	0	2.903769584	Inf	Inf	0.36916133	1
gene35081	0	2.903769584	Inf	Inf	0.36916133	1
gene35092	0	2.903769584	Inf	Inf	0.36916133	1
gene35203	0	2.903769584	Inf	Inf	0.36916133	1
gene35284	0	2.903769584	Inf	Inf	0.36916133	1
gene35408	0	2.903769584	Inf	Inf	0.36916133	1
gene35474	0	2.903769584	Inf	Inf	0.36916133	1
gene35515	0	2.903769584	Inf	Inf	0.36916133	1
gene35639	0	2.903769584	Inf	Inf	0.36916133	1
gene35905	0	2.903769584	Inf	Inf	0.36916133	1
gene35909	0	2.903769584	Inf	Inf	0.36916133	1
gene36034	0	2.903769584	Inf	Inf	0.36916133	1
gene36140	0	2.903769584	Inf	Inf	0.36916133	1
gene36184	0	2.903769584	Inf	Inf	0.36916133	1
gene36318	0	2.903769584	Inf	Inf	0.36916133	1
gene36608	0	2.903769584	Inf	Inf	0.36916133	1
gene37043	0	2.903769584	Inf	Inf	0.36916133	1
gene37306	0	2.903769584	Inf	Inf	0.36916133	1
gene37363	0	2.903769584	Inf	Inf	0.36916133	1
gene37405	0	2.903769584	Inf	Inf	0.36916133	1
gene37691	0	2.903769584	Inf	Inf	0.36916133	1
gene37693	0	2.903769584	Inf	Inf	0.36916133	1
gene37945	0	2.903769584	Inf	Inf	0.36916133	1
gene38124	0	2.903769584	Inf	Inf	0.36916133	1
gene38168	0	2.903769584	Inf	Inf	0.36916133	1
gene38392	0	2.903769584	Inf	Inf	0.36916133	1
gene38594	0	2.903769584	Inf	Inf	0.36916133	1
gene38746	0	2.903769584	Inf	Inf	0.36916133	1
gene38858	0	2.903769584	Inf	Inf	0.36916133	1
gene38891	0	2.903769584	Inf	Inf	0.36916133	1
gene39396	0	2.903769584	Inf	Inf	0.36916133	1

gene39442	0	2.903769584	Inf	Inf	0.36916133	1
gene39564	0	2.903769584	Inf	Inf	0.36916133	1
gene39647	0	2.903769584	Inf	Inf	0.36916133	1
gene39859	0	2.903769584	Inf	Inf	0.36916133	1
gene39981	0	2.903769584	Inf	Inf	0.36916133	1
gene40092	0	2.903769584	Inf	Inf	0.36916133	1
gene40235	0	2.903769584	Inf	Inf	0.36916133	1
gene40307	0	2.903769584	Inf	Inf	0.36916133	1
gene40759	0	2.903769584	Inf	Inf	0.36916133	1
gene41079	0	2.903769584	Inf	Inf	0.36916133	1
gene41321	0	2.903769584	Inf	Inf	0.36916133	1
gene41551	0	2.903769584	Inf	Inf	0.36916133	1
gene41764	0	2.903769584	Inf	Inf	0.36916133	1
gene41909	0	2.903769584	Inf	Inf	0.36916133	1
gene42107	0	2.903769584	Inf	Inf	0.36916133	1
gene42119	0	2.903769584	Inf	Inf	0.36916133	1
gene42226	0	2.903769584	Inf	Inf	0.36916133	1
gene42250	0	2.903769584	Inf	Inf	0.36916133	1
gene42428	0	2.903769584	Inf	Inf	0.36916133	1
gene42967	0	2.903769584	Inf	Inf	0.36916133	1
gene43001	0	2.903769584	Inf	Inf	0.36916133	1
gene43254	0	2.903769584	Inf	Inf	0.36916133	1
gene43259	0	2.903769584	Inf	Inf	0.36916133	1
gene43366	0	2.903769584	Inf	Inf	0.36916133	1
gene44201	0	2.903769584	Inf	Inf	0.36916133	1
gene44669	0	2.903769584	Inf	Inf	0.36916133	1
gene44776	0	2.903769584	Inf	Inf	0.36916133	1
gene44944	0	2.903769584	Inf	Inf	0.36916133	1
gene45236	0	2.903769584	Inf	Inf	0.36916133	1
gene45649	0	2.903769584	Inf	Inf	0.36916133	1
gene45670	0	2.903769584	Inf	Inf	0.36916133	1
gene46049	0	2.903769584	Inf	Inf	0.36916133	1
gene46426	0	2.903769584	Inf	Inf	0.36916133	1
gene46592	0	2.903769584	Inf	Inf	0.36916133	1
gene46775	0	2.903769584	Inf	Inf	0.36916133	1
gene46854	0	2.903769584	Inf	Inf	0.36916133	1
gene46955	0	2.903769584	Inf	Inf	0.36916133	1
gene46974	0	2.903769584	Inf	Inf	0.36916133	1
gene47195	0	2.903769584	Inf	Inf	0.36916133	1
gene47217	0	2.903769584	Inf	Inf	0.36916133	1
gene47319	0	2.903769584	Inf	Inf	0.36916133	1
gene47783	0	2.903769584	Inf	Inf	0.36916133	1
gene47793	0	2.903769584	Inf	Inf	0.36916133	1
gene47826	0	2.903769584	Inf	Inf	0.36916133	1
gene48212	0	2.903769584	Inf	Inf	0.36916133	1
gene48498	0	2.903769584	Inf	Inf	0.36916133	1
gene48752	0	2.903769584	Inf	Inf	0.36916133	1
gene49054	0	2.903769584	Inf	Inf	0.36916133	1

gene49230	0	2.903769584	Inf	Inf	0.36916133	1
gene49493	0	2.903769584	Inf	Inf	0.36916133	1
gene49703	0	2.903769584	Inf	Inf	0.36916133	1
gene49770	0	2.903769584	Inf	Inf	0.36916133	1
gene49851	0	2.903769584	Inf	Inf	0.36916133	1
gene49979	0	2.903769584	Inf	Inf	0.36916133	1
gene50204	0	2.903769584	Inf	Inf	0.36916133	1
gene50362	0	2.903769584	Inf	Inf	0.36916133	1
gene50585	0	2.903769584	Inf	Inf	0.36916133	1
gene50591	0	2.903769584	Inf	Inf	0.36916133	1
gene51063	0	2.903769584	Inf	Inf	0.36916133	1
gene51087	0	2.903769584	Inf	Inf	0.36916133	1
gene51139	0	2.903769584	Inf	Inf	0.36916133	1
gene51191	0	2.903769584	Inf	Inf	0.36916133	1
gene51577	0	2.903769584	Inf	Inf	0.36916133	1
gene51870	0	2.903769584	Inf	Inf	0.36916133	1
gene51908	0	2.903769584	Inf	Inf	0.36916133	1
gene52193	0	2.903769584	Inf	Inf	0.36916133	1
gene52582	0	2.903769584	Inf	Inf	0.36916133	1
gene52614	0	2.903769584	Inf	Inf	0.36916133	1
gene52889	0	2.903769584	Inf	Inf	0.36916133	1
gene53067	0	2.903769584	Inf	Inf	0.36916133	1
gene53161	0	2.903769584	Inf	Inf	0.36916133	1
gene53183	0	2.903769584	Inf	Inf	0.36916133	1
gene53417	0	2.903769584	Inf	Inf	0.36916133	1
gene53895	0	2.903769584	Inf	Inf	0.36916133	1
gene54303	0	2.903769584	Inf	Inf	0.36916133	1
gene54503	0	2.903769584	Inf	Inf	0.36916133	1
gene54606	0	2.903769584	Inf	Inf	0.36916133	1
gene54640	0	2.903769584	Inf	Inf	0.36916133	1
gene54762	0	2.903769584	Inf	Inf	0.36916133	1
gene54784	0	2.903769584	Inf	Inf	0.36916133	1
gene54948	0	2.903769584	Inf	Inf	0.36916133	1
gene55099	0	2.903769584	Inf	Inf	0.36916133	1
gene55188	0	2.903769584	Inf	Inf	0.36916133	1
gene55273	0	2.903769584	Inf	Inf	0.36916133	1
gene55331	0	2.903769584	Inf	Inf	0.36916133	1
gene55426	0	2.903769584	Inf	Inf	0.36916133	1
gene55809	0	2.903769584	Inf	Inf	0.36916133	1
gene55912	0	2.903769584	Inf	Inf	0.36916133	1
gene56082	0	2.903769584	Inf	Inf	0.36916133	1
gene56375	0	2.903769584	Inf	Inf	0.36916133	1
gene56582	0	2.903769584	Inf	Inf	0.36916133	1
gene56945	0	2.903769584	Inf	Inf	0.36916133	1
gene56973	0	2.903769584	Inf	Inf	0.36916133	1
gene57019	0	2.903769584	Inf	Inf	0.36916133	1
gene57210	0	2.903769584	Inf	Inf	0.36916133	1
gene57510	0	2.903769584	Inf	Inf	0.36916133	1

gene57673	0	2.903769584	Inf	Inf	0.36916133	1
gene57719	0	2.903769584	Inf	Inf	0.36916133	1
gene58018	0	2.903769584	Inf	Inf	0.36916133	1
gene58130	0	2.903769584	Inf	Inf	0.36916133	1
gene58327	0	2.903769584	Inf	Inf	0.36916133	1
gene58447	0	2.903769584	Inf	Inf	0.36916133	1
gene58613	0	2.903769584	Inf	Inf	0.36916133	1
gene58614	0	2.903769584	Inf	Inf	0.36916133	1
gene58750	0	2.903769584	Inf	Inf	0.36916133	1
gene59095	0	2.903769584	Inf	Inf	0.36916133	1
gene59102	0	2.903769584	Inf	Inf	0.36916133	1
gene59139	0	2.903769584	Inf	Inf	0.36916133	1
gene59296	0	2.903769584	Inf	Inf	0.36916133	1
gene59891	0	2.903769584	Inf	Inf	0.36916133	1
gene60070	0	2.903769584	Inf	Inf	0.36916133	1
gene60157	0	2.903769584	Inf	Inf	0.36916133	1
gene60308	0	2.903769584	Inf	Inf	0.36916133	1
gene61533	0	2.903769584	Inf	Inf	0.36916133	1
gene61678	0	2.903769584	Inf	Inf	0.36916133	1
gene61804	0	2.903769584	Inf	Inf	0.36916133	1
gene62012	0	2.903769584	Inf	Inf	0.36916133	1
gene62113	0	2.903769584	Inf	Inf	0.36916133	1
gene62218	0	2.903769584	Inf	Inf	0.36916133	1
gene62786	0	2.903769584	Inf	Inf	0.36916133	1
gene63144	0	2.903769584	Inf	Inf	0.36916133	1
gene63218	0	2.903769584	Inf	Inf	0.36916133	1
gene63461	0	2.903769584	Inf	Inf	0.36916133	1
gene63656	0	2.903769584	Inf	Inf	0.36916133	1
gene63816	0	2.903769584	Inf	Inf	0.36916133	1
gene64362	0	2.903769584	Inf	Inf	0.36916133	1
gene65456	0	2.903769584	Inf	Inf	0.36916133	1
gene65655	0	2.903769584	Inf	Inf	0.36916133	1
gene65738	0	2.903769584	Inf	Inf	0.36916133	1
gene65772	0	2.903769584	Inf	Inf	0.36916133	1
gene65833	0	2.903769584	Inf	Inf	0.36916133	1
gene66063	0	2.903769584	Inf	Inf	0.36916133	1
gene66129	0	2.903769584	Inf	Inf	0.36916133	1
gene66349	0	2.903769584	Inf	Inf	0.36916133	1
gene66714	0	2.903769584	Inf	Inf	0.36916133	1
gene66827	0	2.903769584	Inf	Inf	0.36916133	1
gene66855	0	2.903769584	Inf	Inf	0.36916133	1
gene67135	0	2.903769584	Inf	Inf	0.36916133	1
gene67249	0	2.903769584	Inf	Inf	0.36916133	1
gene67323	0	2.903769584	Inf	Inf	0.36916133	1
gene67391	0	2.903769584	Inf	Inf	0.36916133	1
gene67415	0	2.903769584	Inf	Inf	0.36916133	1
gene68927	0	2.903769584	Inf	Inf	0.36916133	1
gene69204	0	2.903769584	Inf	Inf	0.36916133	1

gene69218	0	2.903769584	Inf	Inf	0.36916133	1
gene69440	0	2.903769584	Inf	Inf	0.36916133	1
gene69566	0	2.903769584	Inf	Inf	0.36916133	1
gene69691	0	2.903769584	Inf	Inf	0.36916133	1
gene69969	0	2.903769584	Inf	Inf	0.36916133	1
gene70009	0	2.903769584	Inf	Inf	0.36916133	1
gene70180	0	2.903769584	Inf	Inf	0.36916133	1
gene70819	0	2.903769584	Inf	Inf	0.36916133	1
gene71281	0	2.903769584	Inf	Inf	0.36916133	1
gene71528	0	2.903769584	Inf	Inf	0.36916133	1
gene71857	0	2.903769584	Inf	Inf	0.36916133	1
gene72087	0	2.903769584	Inf	Inf	0.36916133	1
gene72308	0	2.903769584	Inf	Inf	0.36916133	1
gene72424	0	2.903769584	Inf	Inf	0.36916133	1
gene72546	0	2.903769584	Inf	Inf	0.36916133	1
gene72646	0	2.903769584	Inf	Inf	0.36916133	1
gene73191	0	2.903769584	Inf	Inf	0.36916133	1
gene73295	0	2.903769584	Inf	Inf	0.36916133	1
gene29876	41.325593	65.81877724	1.592688028	0.671463703	0.36918291	1
gene66057	41.325593	65.81877724	1.592688028	0.671463703	0.36918291	1
gene2219	43.39187265	68.72254682	1.583765406	0.663358653	0.36922632	1
gene4993	103.3139825	150.9960184	1.461525485	0.547474986	0.36922713	1
gene67968	485.5757178	347.4844269	0.715613269	-0.48274796	0.36924449	1
gene23218	3676.944637	2481.755071	0.67495035	-0.56714671	0.36925197	1
gene47406	70.25350811	44.52446695	0.633768593	-0.65797193	0.36938675	1
gene51938	358.4995193	255.5317234	0.712781216	-0.48846878	0.36940371	1
gene32891	202.4954057	141.3167864	0.697876507	-0.51895633	0.36941846	1
gene40908	202.4954057	141.3167864	0.697876507	-0.51895633	0.36941846	1
gene569	106.413402	70.65839321	0.663999007	-0.59074701	0.36941847	1
gene1840	106.413402	70.65839321	0.663999007	-0.59074701	0.36941847	1
gene36755	367.7977777	262.3071857	0.713183172	-0.48765543	0.36948804	1
gene50690	938.0909612	668.8349275	0.712974493	-0.48807763	0.36949164	1
gene69295	91.94944443	135.5092472	1.473736444	0.559478543	0.36956992	1
gene21766	260.3512359	183.905407	0.706374242	-0.50149536	0.36965706	1
gene61312	67.15408863	101.6319354	1.513413963	0.597806661	0.36966648	1
gene2130	535.1664294	748.2046295	1.398078408	0.483445273	0.36967602	1
gene60304	589.9228401	825.638485	1.399570298	0.484983953	0.36978103	1
gene11482	130.175618	88.08101071	0.676632169	-0.56355633	0.36978464	1
gene46158	130.175618	88.08101071	0.676632169	-0.56355633	0.36978464	1
gene57532	3059.127022	4601.506867	1.50418954	0.588986369	0.36982732	1
gene69498	159.1035331	109.375321	0.687447468	-0.54067862	0.36988751	1
gene22954	326.4721847	232.3015667	0.711550869	-0.4909612	0.36993632	1
gene34911	427.7198876	305.8637295	0.715102894	-0.48377725	0.36995504	1
gene21977	21.69593633	37.74900459	1.739911291	0.799013753	0.36997793	1
gene24624	21.69593633	37.74900459	1.739911291	0.799013753	0.36997793	1
gene31645	21.69593633	37.74900459	1.739911291	0.799013753	0.36997793	1
gene65847	3980.687746	2674.371787	0.671836617	-0.57381767	0.37000936	1
gene44732	697.3693819	978.5703498	1.403231021	0.488752547	0.37001703	1

gene24988	251.0529775	177.1299446	0.705548074	-0.50318371	0.37005133	1
gene26303	307.8756679	218.750642	0.710516175	-0.4930606	0.37005334	1
gene7237	41.325593	24.19807987	0.585547069	-0.77214295	0.3700826	1
gene37041	41.325593	24.19807987	0.585547069	-0.77214295	0.3700826	1
gene1017	372.9634769	266.1788785	0.713686179	-0.48663826	0.37011461	1
gene3302	298.5774094	211.9751796	0.709950495	-0.49420967	0.37016195	1
gene28660	262.4175156	367.810814	1.401624481	0.487099878	0.37020871	1
gene61100	382.2617353	272.9543409	0.714050912	-0.48590115	0.37021628	1
gene43317	181.8326092	257.4675698	1.415959276	0.501779774	0.37023345	1
gene67090	83.68432583	54.2036989	0.647716264	-0.62656612	0.37024161	1
gene74143	83.68432583	54.2036989	0.647716264	-0.62656612	0.37024161	1
gene69200	1526.980661	2199.121498	1.440176391	0.526245522	0.37024797	1
gene63042	265.516935	187.7770998	0.70721327	-0.49978275	0.37025351	1
gene11332	217.9925031	306.8316527	1.40753305	0.493168798	0.37029149	1
gene623	66.12094881	41.62069737	0.629463099	-0.66780629	0.37029844	1
gene19697	66.12094881	41.62069737	0.629463099	-0.66780629	0.37029844	1
gene40966	66.12094881	41.62069737	0.629463099	-0.66780629	0.37029844	1
gene41666	66.12094881	41.62069737	0.629463099	-0.66780629	0.37029844	1
gene62328	66.12094881	41.62069737	0.629463099	-0.66780629	0.37029844	1
gene68193	66.12094881	41.62069737	0.629463099	-0.66780629	0.37029844	1
gene33270	1534.21264	2209.768653	1.440327498	0.526396885	0.37039378	1
gene7143	120.8773595	81.30554835	0.672628428	-0.57211834	0.37045404	1
gene29514	437.018146	609.7916126	1.395346207	0.480623122	0.3707245	1
gene56265	135.3413171	91.95270349	0.679413393	-0.55763844	0.3707976	1
gene56126	29.96105493	16.45469431	0.549202768	-0.8645892	0.37082796	1
gene64841	29.96105493	16.45469431	0.549202768	-0.8645892	0.37082796	1
gene70922	7126.598513	11793.1762	1.654811364	0.72666677	0.37085993	1
gene48039	684.971704	490.7370597	0.716434061	-0.48109417	0.37088251	1
gene43441	605.4199375	846.9327953	1.398917913	0.484311309	0.37095095	1
gene52492	382.2617353	533.3256802	1.395184584	0.480456005	0.37098199	1
gene53678	328.5384644	458.7955943	1.39647452	0.48178925	0.37099227	1
gene12111	352.3006804	491.7049829	1.395696944	0.480985714	0.37101009	1
gene42189	61.9883895	38.71692779	0.62458354	-0.67903354	0.37102363	1
gene52531	61.9883895	38.71692779	0.62458354	-0.67903354	0.37102363	1
gene71246	61.9883895	38.71692779	0.62458354	-0.67903354	0.37102363	1
gene40681	92.98258426	60.97916126	0.655812718	-0.60864422	0.37108323	1
gene49874	5659.539962	3704.242066	0.654512927	-0.61150641	0.37111923	1
gene68335	102.2808427	67.75462362	0.662437088	-0.59414465	0.37124109	1
gene47331	1190.177078	1694.833514	1.424017942	0.509967324	0.37129332	1
gene67976	45.4581523	27.10184945	0.59619338	-0.74614774	0.37133166	1
gene14656	625.0495942	448.1484391	0.71698061	-0.47999399	0.37133735	1
gene1695	238.6552996	334.9014253	1.403285097	0.488808143	0.37136089	1
gene10404	76.45234706	114.214937	1.493936306	0.57911864	0.37138916	1
gene48319	117.7779401	170.3544823	1.446403988	0.532470561	0.37143354	1
gene50258	1092.028795	777.2423253	0.711741603	-0.49057453	0.3714591	1
gene62528	189.064588	131.6375545	0.696257062	-0.52230804	0.37159365	1
gene20800	126.0430587	85.17724113	0.675778913	-0.56537676	0.37160659	1
gene72015	126.0430587	85.17724113	0.675778913	-0.56537676	0.37160659	1

gene43581	252.0861173	178.0978678	0.706496136	-0.50124642	0.37164955	1
gene44460	492.8076966	353.291966	0.716896202	-0.48016385	0.37167229	1
gene22648	533.1001497	382.3296619	0.717181682	-0.47958946	0.37172239	1
gene2774	1231.502671	874.0346448	0.709730206	-0.49465739	0.37172397	1
gene24169	79.55176653	51.29992932	0.644862227	-0.63293713	0.37178201	1
gene30702	79.55176653	51.29992932	0.644862227	-0.63293713	0.37178201	1
gene46042	79.55176653	51.29992932	0.644862227	-0.63293713	0.37178201	1
gene70871	79.55176653	51.29992932	0.644862227	-0.63293713	0.37178201	1
gene16057	465.9460611	333.9335022	0.716678453	-0.48060211	0.37182978	1
gene53476	767.62289	549.7803746	0.716211543	-0.48154232	0.37185855	1
gene10256	49.5907116	30.00561903	0.605065305	-0.72483723	0.37186207	1
gene50763	566.1606241	790.79325	1.396764834	0.482089142	0.37188216	1
gene3500	53.7232709	32.90938862	0.612572319	-0.70704792	0.37189482	1
gene29468	53.7232709	32.90938862	0.612572319	-0.70704792	0.37189482	1
gene38914	53.7232709	32.90938862	0.612572319	-0.70704792	0.37189482	1
gene23191	97.11514356	142.2847096	1.465113518	0.55101245	0.3718959	1
gene72266	97.11514356	142.2847096	1.465113518	0.55101245	0.3718959	1
gene51833	234.5227403	329.0938862	1.403249364	0.488771405	0.3719861	1
gene72332	196.2965668	276.8260337	1.410243889	0.495944685	0.37207751	1
gene44565	2505.364076	1731.614595	0.691162858	-0.5329024	0.37211823	1
gene63187	194.2302871	135.5092472	0.697673104	-0.51937688	0.37216039	1
gene63280	194.2302871	135.5092472	0.697673104	-0.51937688	0.37216039	1
gene40861	242.7878589	171.3224055	0.705646511	-0.50298244	0.37216864	1
gene2503	665.3420474	477.186135	0.717204236	-0.47954409	0.37217703	1
gene18563	604.3867977	433.6295912	0.717470323	-0.47900894	0.37219983	1
gene53043	511.4042134	366.8428908	0.717324733	-0.47930172	0.3722407	1
gene70145	276.8814731	387.1692779	1.398321359	0.483695956	0.37224773	1
gene2095	116.7448002	78.40177877	0.671565488	-0.5744	0.3723245	1
gene46145	116.7448002	78.40177877	0.671565488	-0.5744	0.3723245	1
gene3760	85.75060548	126.7979385	1.478682719	0.564312527	0.37233936	1
gene51983	494.8739762	690.1292378	1.394555525	0.479805377	0.37233988	1
gene5809	295.47799	210.0393332	0.710845953	-0.49239115	0.37234509	1
gene8038	23.76221598	40.65277417	1.710815785	0.774684423	0.37242873	1
gene50040	23.76221598	40.65277417	1.710815785	0.774684423	0.37242873	1
gene65801	23.76221598	40.65277417	1.710815785	0.774684423	0.37242873	1
gene65822	23.76221598	40.65277417	1.710815785	0.774684423	0.37242873	1
gene67458	23.76221598	40.65277417	1.710815785	0.774684423	0.37242873	1
gene36420	179.7663296	124.8620921	0.694579972	-0.52578728	0.37251065	1
gene34969	859.5723345	1209.903993	1.40756507	0.493201618	0.37253539	1
gene8453	596.1216791	427.822052	0.717675715	-0.47859599	0.37258088	1
gene53348	578.558302	415.2390505	0.717713408	-0.47852022	0.37262351	1
gene69568	288.2460112	402.656049	1.396918026	0.482247363	0.3727941	1
gene26077	88.85002496	58.07539168	0.653633938	-0.6134452	0.37285141	1
gene60092	88.85002496	58.07539168	0.653633938	-0.6134452	0.37285141	1
gene1465	58.88897003	90.0168571	1.528586033	0.612197754	0.37286928	1
gene10403	58.88897003	90.0168571	1.528586033	0.612197754	0.37286928	1
gene41815	58.88897003	90.0168571	1.528586033	0.612197754	0.37286928	1
gene24264	2128.26804	1482.858334	0.696744163	-0.52129908	0.37288105	1

gene21375	952.5549187	1344.445317	1.411409769	0.4971369	0.37288368	1
gene1263	19.62965668	9.679231946	0.493092269	-1.02007046	0.3728966	1
gene19477	19.62965668	9.679231946	0.493092269	-1.02007046	0.3728966	1
gene19604	19.62965668	9.679231946	0.493092269	-1.02007046	0.3728966	1
gene46036	19.62965668	9.679231946	0.493092269	-1.02007046	0.3728966	1
gene52065	19.62965668	9.679231946	0.493092269	-1.02007046	0.3728966	1
gene53685	19.62965668	9.679231946	0.493092269	-1.02007046	0.3728966	1
gene56365	19.62965668	9.679231946	0.493092269	-1.02007046	0.3728966	1
gene55666	165.302372	114.214937	0.690945542	-0.53335609	0.37290238	1
gene3782	323.3727653	451.0522087	1.394836725	0.480096254	0.37290816	1
gene3276	4346.419244	6737.713358	1.550175669	0.632431713	0.37292496	1
gene39103	98.14828338	64.85085404	0.66074364	-0.59783746	0.37310901	1
gene22499	945.3229399	675.6103899	0.714687395	-0.48461575	0.37311514	1
gene19162	398.7919725	285.5373424	0.716005743	-0.48195694	0.37312519	1
gene31926	539.2989887	387.1692779	0.717912115	-0.47812085	0.37314266	1
gene47383	132.2418976	189.7129461	1.43459032	0.520638801	0.37316192	1
gene7068	552.7298064	396.8485098	0.71797921	-0.47798603	0.37320392	1
gene9743	204.5616854	143.2526328	0.700290636	-0.5139743	0.37323285	1
gene32108	75.41920723	48.39615973	0.641695418	-0.64003941	0.37326473	1
gene51284	493.8408364	688.1933914	1.393553025	0.478767898	0.37335031	1
gene73517	2905.189188	1994.889704	0.686664301	-0.54232313	0.37348326	1
gene60525	121.9104994	82.27347154	0.674867809	-0.56732316	0.37349925	1
gene35030	458.7140823	329.0938862	0.71742704	-0.47909597	0.37352727	1
gene18084	315.1076466	439.4371304	1.394561938	0.479812011	0.37358464	1
gene45804	237.6221598	332.965579	1.401239595	0.48670366	0.37360618	1
gene25322	4263.768058	6591.556955	1.545946418	0.628490317	0.3736079	1
gene48385	190.0977278	132.6054777	0.697564769	-0.51960092	0.37361791	1
gene51344	56.82269038	87.11308752	1.53306869	0.616422339	0.37362984	1
gene1246	13.43081773	5.807539168	0.43240399	-1.20954826	0.37372051	1
gene10481	13.43081773	5.807539168	0.43240399	-1.20954826	0.37372051	1
gene10826	13.43081773	5.807539168	0.43240399	-1.20954826	0.37372051	1
gene12916	13.43081773	5.807539168	0.43240399	-1.20954826	0.37372051	1
gene14781	13.43081773	5.807539168	0.43240399	-1.20954826	0.37372051	1
gene16998	13.43081773	5.807539168	0.43240399	-1.20954826	0.37372051	1
gene26905	13.43081773	5.807539168	0.43240399	-1.20954826	0.37372051	1
gene28044	13.43081773	5.807539168	0.43240399	-1.20954826	0.37372051	1
gene38434	13.43081773	5.807539168	0.43240399	-1.20954826	0.37372051	1
gene38860	13.43081773	5.807539168	0.43240399	-1.20954826	0.37372051	1
gene40718	13.43081773	5.807539168	0.43240399	-1.20954826	0.37372051	1
gene46914	13.43081773	5.807539168	0.43240399	-1.20954826	0.37372051	1
gene47179	13.43081773	5.807539168	0.43240399	-1.20954826	0.37372051	1
gene57890	13.43081773	5.807539168	0.43240399	-1.20954826	0.37372051	1
gene60307	13.43081773	5.807539168	0.43240399	-1.20954826	0.37372051	1
gene65715	13.43081773	5.807539168	0.43240399	-1.20954826	0.37372051	1
gene70544	13.43081773	5.807539168	0.43240399	-1.20954826	0.37372051	1
gene74255	13.43081773	5.807539168	0.43240399	-1.20954826	0.37372051	1
gene73679	209.7273845	147.1243256	0.701502696	-0.51147945	0.37374436	1
gene67542	987.6816728	705.6160089	0.714416424	-0.48516285	0.3737526	1

gene29072	329.5716042	235.2053363	0.713669908	-0.48667115	0.3737881	1
gene40785	92.98258426	136.4771704	1.46777132	0.553627213	0.37379194	1
gene9858	272.7489138	193.5846389	0.709754023	-0.49460897	0.37384165	1
gene61617	352.3006804	490.7370597	1.392949509	0.478142965	0.37386031	1
gene7117	11.36453808	22.26223348	1.958921104	0.970059294	0.3739182	1
gene40241	11.36453808	22.26223348	1.958921104	0.970059294	0.3739182	1
gene53534	11.36453808	22.26223348	1.958921104	0.970059294	0.3739182	1
gene64499	11.36453808	22.26223348	1.958921104	0.970059294	0.3739182	1
gene32185	235.5558801	330.0618094	1.401203864	0.486666872	0.37393764	1
gene1670	156.0041136	107.4394746	0.68869642	-0.53805992	0.3739613	1
gene50538	102.2808427	149.060172	1.457361594	0.543358877	0.373992	1
gene28768	188.0314482	265.2109553	1.410460633	0.496166399	0.37399646	1
gene67829	120.8773595	174.226175	1.441346632	0.527417333	0.37400601	1
gene57056	175.6337703	121.9583225	0.694389936	-0.52618206	0.37408298	1
gene41827	70.25350811	105.5036282	1.501756013	0.58665044	0.37420419	1
gene63471	2598.34666	3845.558852	1.48000223	0.56559935	0.37422944	1
gene26510	25.82849563	43.55654376	1.686375559	0.753925863	0.37424234	1
gene57291	25.82849563	43.55654376	1.686375559	0.753925863	0.37424234	1
gene70700	25.82849563	43.55654376	1.686375559	0.753925863	0.37424234	1
gene48400	344.0355618	245.8524914	0.7146136	-0.48476472	0.37425737	1
gene52028	112.6122409	75.49800918	0.670424534	-0.57685315	0.37426228	1
gene32033	992.8473719	709.4877017	0.714598962	-0.48479428	0.37426478	1
gene29199	248.9866978	176.1620214	0.707515795	-0.49916574	0.37431572	1
gene68912	248.9866978	176.1620214	0.707515795	-0.49916574	0.37431572	1
gene40705	141.540156	96.79231946	0.683850592	-0.54824694	0.37434689	1
gene27582	334.7373033	239.0770291	0.714222845	-0.48555381	0.3743611	1
gene61604	656.0437889	916.6232653	1.397198298	0.48253679	0.37448236	1
gene34178	5752.522546	3771.028766	0.6555435	-0.60923658	0.37459461	1
gene68680	638.4804119	458.7955943	0.718574267	-0.47679082	0.37465393	1
gene49377	71.28664793	45.49239015	0.638161444	-0.64800665	0.37465657	1
gene27817	16.5302372	30.00561903	1.815195915	0.860125267	0.37465949	1
gene38236	16.5302372	30.00561903	1.815195915	0.860125267	0.37465949	1
gene43789	16.5302372	30.00561903	1.815195915	0.860125267	0.37465949	1
gene29104	137.4075967	196.4884085	1.42996758	0.515982438	0.37469023	1
gene8483	386.3942946	276.8260337	0.716434061	-0.48109417	0.3746957	1
gene7578	395.692553	283.601496	0.716721843	-0.48051477	0.3747244	1
gene63911	1147.818346	817.8950995	0.71256493	-0.48890661	0.37478688	1
gene56293	6112.055205	3985.907716	0.652138697	-0.61674927	0.37481639	1
gene21288	103.3139825	68.72254682	0.665181471	-0.58818011	0.37481821	1
gene49715	217.9925031	305.8637295	1.403092882	0.488610516	0.37482706	1
gene24049	118.8110799	171.3224055	1.441973304	0.528044456	0.37487527	1
gene9552	1173.646841	1665.795818	1.419333107	0.505213219	0.37496274	1
gene40455	738.6949749	530.4219107	0.718052686	-0.47783839	0.37500733	1
gene1334	94.01572408	61.94708446	0.658901317	-0.60186568	0.37501764	1
gene61554	94.01572408	61.94708446	0.658901317	-0.60186568	0.37501764	1
gene8696	8.265118601	17.4226175	2.107969449	1.075853958	0.37503806	1
gene20733	8.265118601	17.4226175	2.107969449	1.075853958	0.37503806	1
gene23964	8.265118601	17.4226175	2.107969449	1.075853958	0.37503806	1

gene31381	8.265118601	17.4226175	2.107969449	1.075853958	0.37503806	1
gene48477	8.265118601	17.4226175	2.107969449	1.075853958	0.37503806	1
gene48701	8.265118601	17.4226175	2.107969449	1.075853958	0.37503806	1
gene49556	8.265118601	17.4226175	2.107969449	1.075853958	0.37503806	1
gene54083	8.265118601	17.4226175	2.107969449	1.075853958	0.37503806	1
gene58772	8.265118601	17.4226175	2.107969449	1.075853958	0.37503806	1
gene71588	8.265118601	17.4226175	2.107969449	1.075853958	0.37503806	1
gene51617	52.69013108	81.30554835	1.543088747	0.625821037	0.37505585	1
gene43636	487.6419974	350.3881965	0.718535726	-0.47686821	0.37505925	1
gene4356	595.0885392	427.822052	0.718921679	-0.47609349	0.37508647	1
gene60502	1108.559032	1569.971422	1.416227171	0.5020527	0.37511824	1
gene4454	68.18722846	102.5998586	1.504678529	0.589455292	0.37516144	1
gene44418	68.18722846	102.5998586	1.504678529	0.589455292	0.37516144	1
gene70525	1963.998807	1375.41886	0.700315527	-0.51392302	0.37525282	1
gene35208	34.09361423	19.35846389	0.567803219	-0.81653707	0.37529549	1
gene15396	363.6652184	260.3713394	0.715964371	-0.4820403	0.37531189	1
gene6479	16.5302372	7.743385557	0.468437655	-1.09407104	0.37535755	1
gene9166	16.5302372	7.743385557	0.468437655	-1.09407104	0.37535755	1
gene14535	16.5302372	7.743385557	0.468437655	-1.09407104	0.37535755	1
gene31925	16.5302372	7.743385557	0.468437655	-1.09407104	0.37535755	1
gene39804	16.5302372	7.743385557	0.468437655	-1.09407104	0.37535755	1
gene54932	16.5302372	7.743385557	0.468437655	-1.09407104	0.37535755	1
gene61166	16.5302372	7.743385557	0.468437655	-1.09407104	0.37535755	1
gene14384	144.6395755	206.1676405	1.425388866	0.51135556	0.37538008	1
gene32049	519.669332	373.6183531	0.718954016	-0.4760286	0.37541434	1
gene17259	487.6419974	678.5141594	1.391418629	0.476556541	0.37543665	1
gene12183	117.7779401	79.36970196	0.673892767	-0.56940905	0.37546462	1
gene40331	252.0861173	352.3240428	1.39763366	0.482986259	0.37546686	1
gene36352	1847.254007	1297.017081	0.702132504	-0.51018478	0.3754792	1
gene47844	667.408327	932.1100364	1.396611338	0.48193059	0.37550027	1
gene22105	349.2012609	485.8974437	1.391453864	0.476593074	0.37550471	1
gene17261	27.89477528	46.46031334	1.665556108	0.736003955	0.37556313	1
gene24312	27.89477528	46.46031334	1.665556108	0.736003955	0.37556313	1
gene32738	27.89477528	46.46031334	1.665556108	0.736003955	0.37556313	1
gene14954	171.501211	119.0545529	0.694190742	-0.52659597	0.3757219	1
gene36233	151.8715543	104.535705	0.688316555	-0.53885589	0.37574509	1
gene63438	791.385106	568.1709153	0.717944918	-0.47805493	0.37584219	1
gene21941	107.4465418	155.8356343	1.450355048	0.536406117	0.37589532	1
gene39681	107.4465418	155.8356343	1.450355048	0.536406117	0.37589532	1
gene56667	67.15408863	42.58862056	0.634192518	-0.65700724	0.3759133	1
gene58174	67.15408863	42.58862056	0.634192518	-0.65700724	0.3759133	1
gene24828	98.14828338	143.2526328	1.459553116	0.545526714	0.37593289	1
gene16507	6581.100686	10704.26261	1.626515551	0.701784616	0.3760033	1
gene60496	350.2344007	250.6921074	0.715783792	-0.48240422	0.37603357	1
gene61570	211.7936641	297.1524208	1.403027904	0.488543703	0.37605075	1
gene47704	1719.144669	1210.871916	0.704345561	-0.50564469	0.37608375	1
gene53272	66.12094881	99.69608905	1.507783703	0.592429484	0.37612456	1
gene35665	1518.715543	1074.394746	0.707436459	-0.49932752	0.3761358	1

gene65933	2096.240705	3054.765602	1.457258985	0.543257297	0.37622123	1
gene23839	1176.746261	1668.699588	1.418062367	0.503920984	0.37628615	1
gene70016	288.2460112	401.6881258	1.39356005	0.478775171	0.37628724	1
gene14733	48.55757178	75.49800918	1.554814345	0.636742324	0.37628904	1
gene29246	48.55757178	75.49800918	1.554814345	0.636742324	0.37628904	1
gene47734	77.48548688	115.1828602	1.486508826	0.571928029	0.37631748	1
gene68437	157.0372534	108.4073978	0.690329176	-0.53464363	0.37635294	1
gene61464	255.1855368	181.0016374	0.709294264	-0.49554382	0.37639271	1
gene5114	435.9850062	605.9199198	1.38977238	0.474848615	0.3764912	1
gene21801	29.96105493	49.36408293	1.647608305	0.720373303	0.37649893	1
gene41543	29.96105493	49.36408293	1.647608305	0.720373303	0.37649893	1
gene43104	29.96105493	49.36408293	1.647608305	0.720373303	0.37649893	1
gene44379	3042.596785	4547.303168	1.494546761	0.579708036	0.37653673	1
gene43257	1784.232478	1255.396383	0.703605836	-0.50716065	0.37653713	1
gene41399	1865.850524	2700.505713	1.447332291	0.533396186	0.37659319	1
gene44697	240.7215792	170.3544823	0.707682638	-0.49882557	0.37660055	1
gene48843	232.4564606	325.2221934	1.399067131	0.484465188	0.376601	1
gene6123	2071.445349	1448.981022	0.699502414	-0.51559906	0.37665419	1
gene62134	652.9443695	910.8157262	1.394936183	0.480199121	0.37666135	1
gene45682	159.1035331	225.5261044	1.417480178	0.503328561	0.37668623	1
gene20078	610.5856366	439.4371304	0.719697785	-0.47453688	0.37671911	1
gene73115	99.18142321	65.81877724	0.663620012	-0.5915707	0.37682557	1
gene59471	489.7082771	352.3240428	0.71945699	-0.47501965	0.3768729	1
gene43297	289.279151	206.1676405	0.712694433	-0.48864444	0.37687412	1
gene27562	142.5732959	97.76024266	0.685684104	-0.54438402	0.37695912	1
gene31829	142.5732959	97.76024266	0.685684104	-0.54438402	0.37695912	1
gene22266	1397.838183	992.1212745	0.709754023	-0.49460897	0.37696049	1
gene30475	89.88316478	59.04331487	0.656889586	-0.6062772	0.3769605	1
gene137	63.02152933	39.68485098	0.629703078	-0.66725638	0.37700438	1
gene57663	63.02152933	39.68485098	0.629703078	-0.66725638	0.37700438	1
gene3725	4.1325593	10.64715514	2.576407104	1.365360575	0.377013	1
gene4638	4.1325593	10.64715514	2.576407104	1.365360575	0.377013	1
gene5904	4.1325593	10.64715514	2.576407104	1.365360575	0.377013	1
gene8424	4.1325593	10.64715514	2.576407104	1.365360575	0.377013	1
gene9253	4.1325593	10.64715514	2.576407104	1.365360575	0.377013	1
gene16185	4.1325593	10.64715514	2.576407104	1.365360575	0.377013	1
gene19129	4.1325593	10.64715514	2.576407104	1.365360575	0.377013	1
gene21279	4.1325593	10.64715514	2.576407104	1.365360575	0.377013	1
gene23654	4.1325593	10.64715514	2.576407104	1.365360575	0.377013	1
gene24006	4.1325593	10.64715514	2.576407104	1.365360575	0.377013	1
gene24429	4.1325593	10.64715514	2.576407104	1.365360575	0.377013	1
gene28813	4.1325593	10.64715514	2.576407104	1.365360575	0.377013	1
gene30765	4.1325593	10.64715514	2.576407104	1.365360575	0.377013	1
gene37858	4.1325593	10.64715514	2.576407104	1.365360575	0.377013	1
gene38771	4.1325593	10.64715514	2.576407104	1.365360575	0.377013	1
gene43460	4.1325593	10.64715514	2.576407104	1.365360575	0.377013	1
gene47244	4.1325593	10.64715514	2.576407104	1.365360575	0.377013	1
gene48330	4.1325593	10.64715514	2.576407104	1.365360575	0.377013	1

gene49735	4.1325593	10.64715514	2.576407104	1.365360575	0.377013	1
gene50328	4.1325593	10.64715514	2.576407104	1.365360575	0.377013	1
gene57140	4.1325593	10.64715514	2.576407104	1.365360575	0.377013	1
gene60399	4.1325593	10.64715514	2.576407104	1.365360575	0.377013	1
gene60692	4.1325593	10.64715514	2.576407104	1.365360575	0.377013	1
gene62337	4.1325593	10.64715514	2.576407104	1.365360575	0.377013	1
gene63677	4.1325593	10.64715514	2.576407104	1.365360575	0.377013	1
gene64897	4.1325593	10.64715514	2.576407104	1.365360575	0.377013	1
gene66756	4.1325593	10.64715514	2.576407104	1.365360575	0.377013	1
gene67337	4.1325593	10.64715514	2.576407104	1.365360575	0.377013	1
gene68268	4.1325593	10.64715514	2.576407104	1.365360575	0.377013	1
gene7968	557.8955055	776.2744021	1.391433332	0.476571786	0.37709959	1
gene18400	2753.317634	4081.732112	1.482477743	0.568010445	0.3771027	1
gene21781	328.5384644	456.8597479	1.390582222	0.475689051	0.37712803	1
gene45385	32.02733458	52.26785251	1.631976348	0.706620148	0.37713161	1
gene71833	32.02733458	52.26785251	1.631976348	0.706620148	0.37713161	1
gene65838	1339.982353	952.4364235	0.710782811	-0.4925193	0.37719285	1
gene29343	44.42501248	69.69047001	1.568721451	0.649589203	0.37721989	1
gene32884	44.42501248	69.69047001	1.568721451	0.649589203	0.37721989	1
gene44771	44.42501248	69.69047001	1.568721451	0.649589203	0.37721989	1
gene64553	44.42501248	69.69047001	1.568721451	0.649589203	0.37721989	1
gene65916	44.42501248	69.69047001	1.568721451	0.649589203	0.37721989	1
gene27878	279.9808926	390.0730474	1.3932131	0.478415944	0.37729783	1
gene26177	75.41920723	112.2790906	1.48873337	0.574085393	0.37736058	1
gene58462	75.41920723	112.2790906	1.48873337	0.574085393	0.37736058	1
gene20582	121.9104994	175.1940982	1.437071451	0.523131794	0.37737691	1
gene33979	430.8193071	309.7354223	0.718945083	-0.47604652	0.37751363	1
gene12040	658.1100686	917.5911885	1.394282252	0.479522643	0.3775171	1
gene59226	34.09361423	55.17162209	1.618239173	0.694424851	0.3775241	1
gene6010	42.35873283	66.78670043	1.576692596	0.656901409	0.37752449	1
gene33244	540.3321285	389.1051242	0.720122132	-0.47368649	0.37758752	1
gene53316	1724.310368	1215.711532	0.705042175	-0.50421853	0.37759952	1
gene38047	147.738995	101.6319354	0.687915438	-0.53969686	0.37760494	1
gene22998	306.842528	426.8541288	1.391117886	0.476244681	0.37762673	1
gene6852	40.29245318	63.88293085	1.585481295	0.664920857	0.37770074	1
gene42021	36.15989388	58.07539168	1.606071961	0.683536535	0.37772542	1
gene2501	38.22617353	60.97916126	1.595220124	0.673755515	0.3777741	1
gene42215	38.22617353	60.97916126	1.595220124	0.673755515	0.3777741	1
gene57744	38.22617353	60.97916126	1.595220124	0.673755515	0.3777741	1
gene65109	38.22617353	60.97916126	1.595220124	0.673755515	0.3777741	1
gene66199	38.22617353	60.97916126	1.595220124	0.673755515	0.3777741	1
gene69540	38.22617353	60.97916126	1.595220124	0.673755515	0.3777741	1
gene33229	285.1465917	203.2638709	0.71283991	-0.48834998	0.37779515	1
gene47159	262.4175156	365.8749676	1.39424751	0.479486694	0.37784881	1
gene49449	337.8367228	469.4427494	1.389555124	0.474623068	0.37785305	1
gene57321	84.71746566	124.8620921	1.473864818	0.559604207	0.37787492	1
gene10428	468.0123408	336.8372717	0.719718782	-0.47449479	0.37787848	1
gene18946	2381.387297	1657.084509	0.695848387	-0.52315509	0.37788115	1

gene67756	172.5343508	120.0224761	0.695643943	-0.52357903	0.37791414	1
gene46362	256.2186766	181.9695606	0.710211929	-0.4936785	0.37795681	1
gene32027	18358.89469	36783.98517	2.003605652	1.002598586	0.37795853	1
gene17254	58.88897003	36.7810814	0.62458354	-0.67903354	0.37797347	1
gene36830	58.88897003	36.7810814	0.62458354	-0.67903354	0.37797347	1
gene35241	94.01572408	137.4450936	1.461937298	0.547881436	0.37797927	1
gene39706	522.7687515	376.5221227	0.720246039	-0.47343827	0.37798549	1
gene59487	164.2692322	232.3015667	1.414151412	0.499936597	0.37798831	1
gene8805	26.86163545	14.51884792	0.540504987	-0.88762017	0.37801729	1
gene19738	26.86163545	14.51884792	0.540504987	-0.88762017	0.37801729	1
gene42369	26.86163545	14.51884792	0.540504987	-0.88762017	0.37801729	1
gene47964	26.86163545	14.51884792	0.540504987	-0.88762017	0.37801729	1
gene14398	1676.785936	1183.770067	0.705975665	-0.50230964	0.37802906	1
gene45101	61.9883895	93.88854988	1.514615086	0.598951203	0.37805664	1
gene12076	76.45234706	49.36408293	0.645684336	-0.63109907	0.37812763	1
gene38562	76.45234706	49.36408293	0.645684336	-0.63109907	0.37812763	1
gene46838	76.45234706	49.36408293	0.645684336	-0.63109907	0.37812763	1
gene48138	4143.923838	6359.255389	1.534597555	0.617860361	0.37813882	1
gene38409	602.320518	838.2214866	1.391653549	0.476800098	0.37824672	1
gene62843	1817.292952	2623.071857	1.443395163	0.529466325	0.37825636	1
gene44949	5620.280648	8931.027317	1.589071414	0.668183962	0.37828041	1
gene25768	696.3362421	501.3842148	0.720031767	-0.47386754	0.37829812	1
gene2207	177.7000499	123.8941689	0.697209534	-0.5203358	0.37836864	1
gene55405	1419.53412	2024.895323	1.426450619	0.512429804	0.37839038	1
gene17026	73.35292758	109.375321	1.491083241	0.5763608	0.37842171	1
gene45496	202.4954057	142.2847096	0.702656483	-0.50910854	0.37845752	1
gene24986	314.0745068	436.5333608	1.389903833	0.474985066	0.37850859	1
gene16841	792.4182458	1108.272058	1.398594825	0.483978072	0.37852577	1
gene52719	388.4605742	539.1332194	1.387871138	0.472873622	0.37853731	1
gene14488	343.0024219	245.8524914	0.716766051	-0.48042579	0.37862396	1
gene34362	162.2029525	229.3977971	1.414264004	0.500051457	0.37867348	1
gene65338	4695.620505	3139.942843	0.668696041	-0.58057752	0.37868134	1
gene15479	376.0628963	270.0505713	0.718099483	-0.47774437	0.37868353	1
gene1974	54.75641073	33.87731181	0.618691243	-0.69270848	0.37876916	1
gene14122	54.75641073	33.87731181	0.618691243	-0.69270848	0.37876916	1
gene29811	54.75641073	33.87731181	0.618691243	-0.69270848	0.37876916	1
gene54798	18.59651685	32.90938862	1.769653365	0.823466796	0.37886588	1
gene64142	18.59651685	32.90938862	1.769653365	0.823466796	0.37886588	1
gene16956	127.0761985	181.9695606	1.43197202	0.518003303	0.37887588	1
gene17265	127.0761985	181.9695606	1.43197202	0.518003303	0.37887588	1
gene30934	95.04886391	62.91500765	0.661922774	-0.59526519	0.37888689	1
gene61839	85.75060548	56.13954529	0.654683952	-0.61112948	0.37892868	1
gene73656	85.75060548	56.13954529	0.654683952	-0.61112948	0.37892868	1
gene33708	231.4233208	323.286347	1.396948008	0.482278327	0.37895745	1
gene54676	1813.160393	2615.328472	1.442414296	0.528485601	0.37896123	1
gene26138	59.92210985	90.9847803	1.518384124	0.602536813	0.37901818	1
gene27332	42.35873283	25.16600306	0.594116051	-0.75118333	0.37905143	1
gene29443	42.35873283	25.16600306	0.594116051	-0.75118333	0.37905143	1

gene30142	42.35873283	25.16600306	0.594116051	-0.75118333	0.37905143	1
gene32073	42.35873283	25.16600306	0.594116051	-0.75118333	0.37905143	1
gene12574	232.4564606	164.5469431	0.707861346	-0.4984613	0.37907281	1
gene46542	232.4564606	164.5469431	0.707861346	-0.4984613	0.37907281	1
gene42750	784.1531272	564.2992225	0.719628862	-0.47467505	0.37908794	1
gene14672	178.7331897	251.6600306	1.408020698	0.493668542	0.37920184	1
gene67454	1126.122409	805.3120979	0.715119503	-0.48374374	0.37923528	1
gene59092	169.4349313	239.0770291	1.41102562	0.496744184	0.37924661	1
gene10519	278.9477528	388.137201	1.391433332	0.476571786	0.37924996	1
gene71021	229.3570412	320.3825774	1.396872648	0.482200497	0.37935488	1
gene450	46.49129213	28.06977264	0.603764089	-0.72794314	0.37946045	1
gene7761	46.49129213	28.06977264	0.603764089	-0.72794314	0.37946045	1
gene31028	46.49129213	28.06977264	0.603764089	-0.72794314	0.37946045	1
gene71050	46.49129213	28.06977264	0.603764089	-0.72794314	0.37946045	1
gene8269	123.976779	84.20931793	0.6792346	-0.55801814	0.37947827	1
gene23392	143.6064357	98.72816585	0.687491235	-0.54058677	0.37954442	1
gene21631	109.5128215	73.56216279	0.671721921	-0.57406398	0.37962065	1
gene65346	334.7373033	464.6031334	1.387963423	0.472969549	0.37964096	1
gene66535	168.4017915	117.1187066	0.695471856	-0.52393596	0.37966536	1
gene53840	291.3454307	208.1034868	0.714284368	-0.48542955	0.37969503	1
gene17704	377.0960362	271.0184945	0.718698868	-0.47654068	0.37981312	1
gene15395	185.9651685	261.3392626	1.405312966	0.490891457	0.37981928	1
gene25043	479.3768788	345.5485805	0.720828633	-0.47227177	0.37984783	1
gene36401	439.0844257	608.8236894	1.38657546	0.471526133	0.37985941	1
gene25425	328.5384644	455.8918247	1.387636073	0.472629251	0.38022943	1
gene46957	743.8606741	1037.613665	1.39490324	0.480165051	0.38023152	1
gene72090	1066.200299	1502.216798	1.408944266	0.494614543	0.38024856	1
gene15120	247.953558	176.1620214	0.710463777	-0.493167	0.38026774	1
gene57127	213.8599438	299.0882671	1.398524015	0.483905027	0.38030683	1
gene17627	382.2617353	274.8901873	0.719115103	-0.47570538	0.38031088	1
gene37163	344.0355618	477.186135	1.38702561	0.471994426	0.38031263	1
gene45208	677.7397253	488.8012133	0.72122261	-0.47148347	0.38044731	1
gene67931	1543.510899	1094.721133	0.709240948	-0.49565226	0.38045674	1
gene11068	100.214563	66.78670043	0.666437077	-0.58545943	0.38048412	1
gene62001	100.214563	66.78670043	0.666437077	-0.58545943	0.38048412	1
gene25572	443.216985	319.4146542	0.720673316	-0.47258267	0.38053589	1
gene37282	69.22036828	103.5677818	1.496203854	0.581306753	0.38059352	1
gene7099	406.0239513	292.3128048	0.719939806	-0.47405181	0.38061791	1
gene17129	114.6785206	77.43385557	0.675225449	-0.56655881	0.38070349	1
gene26704	139.4738764	198.4242549	1.422662509	0.508593459	0.38074258	1
gene45976	139.4738764	198.4242549	1.422662509	0.508593459	0.38074258	1
gene60285	687.0379837	495.5766757	0.721323548	-0.47128157	0.38077931	1
gene44804	765.5566104	551.7162209	0.720673316	-0.47258267	0.38081898	1
gene8673	372.9634769	516.8709859	1.385848798	0.470769861	0.38087182	1
gene8879	191.1308676	268.1147249	1.402780871	0.488289662	0.38101251	1
gene65866	181.8326092	255.5317234	1.405312966	0.490891457	0.38102306	1
gene36782	213.8599438	150.9960184	0.706050959	-0.50215578	0.38105882	1
gene68373	512.4373532	710.4556249	1.386424351	0.471368899	0.38113951	1

gene56452	433.9187265	601.0803039	1.385237067	0.470132897	0.3812366	1
gene14871	11177.53977	6871.286759	0.614740533	-0.70195048	0.38126274	1
gene40432	386.3942946	535.2615266	1.385272852	0.470170167	0.38129911	1
gene51498	2315.266348	3379.987796	1.459869962	0.545839867	0.38137705	1
gene65873	68.18722846	43.55654376	0.638778621	-0.64661207	0.38139094	1
gene72056	68.18722846	43.55654376	0.638778621	-0.64661207	0.38139094	1
gene14339	1767.702241	1248.620921	0.706352514	-0.50153974	0.38143594	1
gene10912	243.8209987	173.2582518	0.710596104	-0.49289832	0.38149241	1
gene25135	1192.243358	852.7403345	0.715240164	-0.48350034	0.38149603	1
gene15383	789.3188264	1101.496595	1.395502753	0.480784971	0.3815016	1
gene59340	2593.180961	3813.617387	1.470632958	0.556437222	0.38150621	1
gene39786	830.6444194	1160.53991	1.397156091	0.482493209	0.38158491	1
gene4983	119.8442197	81.30554835	0.678426949	-0.55973462	0.38162508	1
gene73988	119.8442197	81.30554835	0.678426949	-0.55973462	0.38162508	1
gene55440	910.1961859	1274.754847	1.400527564	0.485970378	0.38165565	1
gene68558	67.15408863	100.6640122	1.499000497	0.584000862	0.38170123	1
gene45837	722.1647377	1005.672199	1.39258004	0.47776025	0.38171779	1
gene14099	20.6627965	35.8131582	1.733219325	0.793454227	0.3818774	1
gene21052	20.6627965	35.8131582	1.733219325	0.793454227	0.3818774	1
gene23601	20.6627965	35.8131582	1.733219325	0.793454227	0.3818774	1
gene26797	20.6627965	35.8131582	1.733219325	0.793454227	0.3818774	1
gene62613	20.6627965	35.8131582	1.733219325	0.793454227	0.3818774	1
gene47294	169.4349313	118.0866297	0.696943829	-0.52088571	0.38188104	1
gene53767	823.4124406	593.3369183	0.720582893	-0.47276369	0.38188998	1
gene17258	161.1698127	227.4619507	1.411318577	0.497043684	0.38205836	1
gene32350	288.2460112	206.1676405	0.715248893	-0.48348273	0.38205943	1
gene15341	128.1093383	182.9374838	1.427979304	0.51397507	0.38211415	1
gene23860	2217.118065	3226.088008	1.455081738	0.541100198	0.38212184	1
gene37944	527.9344506	381.3617387	0.722365699	-0.4691987	0.38222295	1
gene50965	527.9344506	381.3617387	0.722365699	-0.4691987	0.38222295	1
gene62923	273.7820536	195.5204853	0.714146463	-0.48570811	0.38225453	1
gene29620	6456.090767	10401.30265	1.611083708	0.688031455	0.38225498	1
gene12423	283.0803121	392.976817	1.388216701	0.473232791	0.38230378	1
gene5350	13.43081773	25.16600306	1.873750621	0.905928957	0.38237774	1
gene8929	13.43081773	25.16600306	1.873750621	0.905928957	0.38237774	1
gene35773	13.43081773	25.16600306	1.873750621	0.905928957	0.38237774	1
gene41314	13.43081773	25.16600306	1.873750621	0.905928957	0.38237774	1
gene62840	13.43081773	25.16600306	1.873750621	0.905928957	0.38237774	1
gene18395	559.9617852	404.5918954	0.722534834	-0.46886095	0.38238198	1
gene52317	622.9833145	450.0842855	0.722466036	-0.46899833	0.38240379	1
gene57722	85.75060548	125.8300153	1.467395065	0.553257338	0.38240407	1
gene56023	234.5227403	166.4827895	0.70987909	-0.49435478	0.38242727	1
gene59793	626.082734	869.1950288	1.388306979	0.473326608	0.3825024	1
gene18572	294.4448501	408.4635881	1.387232916	0.472210036	0.38250355	1
gene18774	1805.928414	1275.722771	0.706408272	-0.50142586	0.38265104	1
gene21193	118.8110799	170.3544823	1.433826562	0.519870524	0.38268715	1
gene62911	118.8110799	170.3544823	1.433826562	0.519870524	0.38268715	1
gene68297	96.08200373	63.88293085	0.664879253	-0.58883574	0.38269255	1

gene53564	375.0297565	270.0505713	0.720077718	-0.47377547	0.38271475	1
gene64165	2821.504862	4169.813123	1.477868487	0.563517892	0.38278199	1
gene44906	65.08780898	97.76024266	1.501974704	0.586860516	0.38282087	1
gene60510	510.3710736	368.7787372	0.722569825	-0.46879109	0.38283195	1
gene46339	212.826804	297.1524208	1.396217089	0.481523275	0.38290698	1
gene55169	298.5774094	213.911026	0.716434061	-0.48109417	0.38292235	1
gene53757	110.5459613	74.53008599	0.67419999	-0.56875149	0.38292631	1
gene11620	3630.453345	5475.541512	1.50822528	0.592851936	0.38303907	1
gene10086	284.1134519	203.2638709	0.715432055	-0.48311333	0.3830605	1
gene2790	130.175618	89.04893391	0.684067687	-0.54778901	0.3830913	1
gene37938	1785.265618	1262.171846	0.706993869	-0.50023039	0.38320447	1
gene35119	845.1083769	1179.898374	1.396150371	0.481454334	0.38321115	1
gene8784	190.0977278	133.5734009	0.702656483	-0.50910854	0.38321884	1
gene44182	589.9228401	817.8950995	1.386444199	0.471389552	0.38323695	1
gene59126	92.98258426	135.5092472	1.457361594	0.543358877	0.38327445	1
gene60010	92.98258426	135.5092472	1.457361594	0.543358877	0.38327445	1
gene12708	30.99419475	17.4226175	0.562125186	-0.83103664	0.38333042	1
gene23321	30.99419475	17.4226175	0.562125186	-0.83103664	0.38333042	1
gene59256	30.99419475	17.4226175	0.562125186	-0.83103664	0.38333042	1
gene61787	30.99419475	17.4226175	0.562125186	-0.83103664	0.38333042	1
gene34340	606.4530773	438.4692072	0.723005989	-0.4679205	0.38340074	1
gene44578	49.5907116	76.46593238	1.541940616	0.624747204	0.38355266	1
gene42072	83.68432583	122.9262457	1.468927956	0.554763641	0.38358196	1
gene2107	687.0379837	496.5445988	0.722732383	-0.46846656	0.38364009	1
gene28634	3113.883433	2146.853646	0.689445733	-0.53649109	0.38365576	1
gene23641	116.7448002	167.4507127	1.434331228	0.520378222	0.38371062	1
gene57765	1369.943408	977.6024266	0.713607891	-0.48679653	0.38375253	1
gene62847	230.390181	163.5790199	0.710008644	-0.49409151	0.3837824	1
gene15618	200.4291261	141.3167864	0.70507111	-0.50415933	0.38380684	1
gene22546	100.214563	145.1884792	1.448776254	0.534834805	0.38391225	1
gene65649	100.214563	145.1884792	1.448776254	0.534834805	0.38391225	1
gene62746	63.02152933	94.85647307	1.505143942	0.589901463	0.38394995	1
gene5130	22.72907615	38.71692779	1.703409656	0.768425433	0.38400984	1
gene17045	22.72907615	38.71692779	1.703409656	0.768425433	0.38400984	1
gene70244	22.72907615	38.71692779	1.703409656	0.768425433	0.38400984	1
gene24205	584.757141	422.9824361	0.723347192	-0.46723982	0.38402196	1
gene36568	123.976779	177.1299446	1.428734849	0.514738199	0.38405522	1
gene60827	123.976779	177.1299446	1.428734849	0.514738199	0.38405522	1
gene36369	101.2477029	67.75462362	0.669196651	-0.57949787	0.38408596	1
gene496	205.5948252	145.1884792	0.70618742	-0.50187697	0.38409001	1
gene32597	205.5948252	145.1884792	0.70618742	-0.50187697	0.38409001	1
gene7635	795.5176653	1108.272058	1.393145754	0.478346204	0.38414389	1
gene59652	572.3594631	792.7290964	1.385019638	0.469906432	0.38418483	1
gene61750	140.5070162	96.79231946	0.688878905	-0.53767769	0.38418819	1
gene66904	1728.442927	1224.422841	0.708396454	-0.4973711	0.38431144	1
gene47493	47.52443195	73.56216279	1.547880948	0.630294514	0.38434435	1
gene72274	47.52443195	73.56216279	1.547880948	0.630294514	0.38434435	1
gene72976	357.4663795	257.4675698	0.720256742	-0.47341684	0.38436392	1

gene71963	1195.342778	856.6120273	0.716624589	-0.48071055	0.38436904	1
gene13736	2230.548882	3240.606856	1.452829338	0.538865241	0.38445965	1
gene30202	285.1465917	204.2317941	0.716234386	-0.48149631	0.38448268	1
gene7699	347.1349812	480.0899045	1.383006411	0.467807844	0.38448576	1
gene34222	120.8773595	82.27347154	0.680635909	-0.55504483	0.38464688	1
gene17374	679.8060049	491.7049829	0.723301912	-0.46733013	0.38469877	1
gene51809	245.8872784	175.1940982	0.71249761	-0.48904292	0.38470737	1
gene4311	162.2029525	228.4298739	1.408296645	0.493951257	0.38472508	1
gene19191	72.31978776	107.4394746	1.485616564	0.571061806	0.38473677	1
gene67627	72.31978776	107.4394746	1.485616564	0.571061806	0.38473677	1
gene6768	81.61804618	120.0224761	1.470538462	0.556344519	0.38478924	1
gene52565	81.61804618	120.0224761	1.470538462	0.556344519	0.38478924	1
gene43641	73.35292758	47.42823654	0.646575919	-0.62910832	0.3848401	1
gene70698	73.35292758	47.42823654	0.646575919	-0.62910832	0.3848401	1
gene51913	290.3122908	208.1034868	0.716826305	-0.48030451	0.38486867	1
gene40642	377.0960362	271.9864177	0.72126565	-0.47139738	0.38496263	1
gene36857	549.6303869	397.816433	0.723789009	-0.4663589	0.38496639	1
gene52335	338.8698626	243.916645	0.719794446	-0.47434312	0.3850475	1
gene7180	45.4581523	70.65839321	1.554361311	0.636321897	0.3850684	1
gene21996	45.4581523	70.65839321	1.554361311	0.636321897	0.3850684	1
gene60677	658.1100686	912.7515725	1.386928443	0.471893355	0.38507206	1
gene32292	169.4349313	238.1091059	1.405312966	0.490891457	0.38507733	1
gene25458	1258.364307	1777.106985	1.41223569	0.497980882	0.38517841	1
gene65298	275.8483333	197.4563317	0.715814844	-0.48234163	0.38517983	1
gene11044	191.1308676	134.5413241	0.703922531	-0.50651143	0.38520142	1
gene2568	833.7438388	602.0482271	0.72210216	-0.46972514	0.38521437	1
gene24562	404.9908114	559.4596065	1.381413086	0.466144795	0.38526626	1
gene39203	232.4564606	323.286347	1.39073935	0.475852058	0.38531116	1
gene9042	126.0430587	86.14516432	0.683458218	-0.54907495	0.38531308	1
gene57268	504.1722346	696.9047001	1.382275049	0.467044715	0.38532885	1
gene59760	82.65118601	54.2036989	0.655812718	-0.60864422	0.3853396	1
gene72092	82.65118601	54.2036989	0.655812718	-0.60864422	0.3853396	1
gene905	23.76221598	12.58300153	0.529538219	-0.91719328	0.38534733	1
gene1025	23.76221598	12.58300153	0.529538219	-0.91719328	0.38534733	1
gene30892	23.76221598	12.58300153	0.529538219	-0.91719328	0.38534733	1
gene36713	23.76221598	12.58300153	0.529538219	-0.91719328	0.38534733	1
gene62548	23.76221598	12.58300153	0.529538219	-0.91719328	0.38534733	1
gene64408	23.76221598	12.58300153	0.529538219	-0.91719328	0.38534733	1
gene74180	23.76221598	12.58300153	0.529538219	-0.91719328	0.38534733	1
gene20647	1262.496866	1782.914525	1.41221303	0.497957733	0.38536867	1
gene19887	156.0041136	108.4073978	0.694900893	-0.52512086	0.38538406	1
gene13977	825.4787202	596.2406879	0.72229686	-0.4693362	0.38541641	1
gene65355	406.0239513	293.280728	0.722323713	-0.46928256	0.38541861	1
gene37671	24.7953558	41.62069737	1.678568265	0.747231211	0.38548385	1
gene62645	24.7953558	41.62069737	1.678568265	0.747231211	0.38548385	1
gene54465	3386.632347	5065.142078	1.495627975	0.580751361	0.38558299	1
gene55218	88.85002496	129.7017081	1.459782461	0.545753392	0.3856535	1
gene57167	43.39187265	67.75462362	1.561458851	0.642894551	0.38570669	1

gene67051	634.3478526	878.8742607	1.385476844	0.470382599	0.38572902	1
gene19838	967.0188763	696.9047001	0.720673316	-0.47258267	0.38577591	1
gene51855	143.6064357	203.2638709	1.415423131	0.501233402	0.3858146	1
gene34805	286.1797315	205.1997173	0.717030924	-0.47989275	0.38589801	1
gene31431	70.25350811	104.535705	1.487978435	0.573353618	0.38596336	1
gene40001	434.9518664	314.5750383	0.723241036	-0.46745156	0.3860168	1
gene15615	35.12675405	20.32638709	0.57865828	-0.78921646	0.38614354	1
gene60588	35.12675405	20.32638709	0.57865828	-0.78921646	0.38614354	1
gene59770	111.5791011	75.49800918	0.676632169	-0.56355633	0.38618603	1
gene11437	58.88897003	89.04893391	1.512149624	0.596600899	0.38622356	1
gene56828	191.1308676	267.1468017	1.39771668	0.483071953	0.38626517	1
gene2769	475.2443195	656.251926	1.380872741	0.465580369	0.38629635	1
gene55904	97.11514356	64.85085404	0.667772828	-0.5825707	0.38643613	1
gene17163	425.6536079	587.5293791	1.380299305	0.464981136	0.38644515	1
gene11421	26.86163545	44.52446695	1.657548627	0.729051195	0.38645826	1
gene13864	26.86163545	44.52446695	1.657548627	0.729051195	0.38645826	1
gene57178	26.86163545	44.52446695	1.657548627	0.729051195	0.38645826	1
gene52786	181.8326092	254.5638002	1.399989811	0.485416327	0.38651902	1
gene3271	51.65699125	31.94146542	0.618337705	-0.69353311	0.3865198	1
gene8723	51.65699125	31.94146542	0.618337705	-0.69353311	0.3865198	1
gene73680	1052.769482	757.8838614	0.719895357	-0.47414088	0.38666037	1
gene34608	103.3139825	149.060172	1.442787979	0.528859308	0.38666718	1
gene14776	39.25931335	61.94708446	1.57789526	0.658001443	0.38668424	1
gene29898	39.25931335	61.94708446	1.57789526	0.658001443	0.38668424	1
gene47566	39.25931335	61.94708446	1.57789526	0.658001443	0.38668424	1
gene11761	560.994925	406.5277417	0.724654936	-0.46463391	0.38669037	1
gene11778	519.669332	376.5221227	0.724541741	-0.46485929	0.38672539	1
gene34358	69.22036828	44.52446695	0.643227825	-0.63659828	0.38673619	1
gene54272	69.22036828	44.52446695	0.643227825	-0.63659828	0.38673619	1
gene66229	69.22036828	44.52446695	0.643227825	-0.63659828	0.38673619	1
gene48462	235.5558801	327.1580398	1.388876557	0.473918379	0.38682361	1
gene66677	86.78374531	126.7979385	1.461079354	0.547034535	0.38689153	1
gene47652	2437.176847	3553.246048	1.457935255	0.543926653	0.38692088	1
gene59316	186.9983083	131.6375545	0.70395051	-0.50645409	0.38695631	1
gene56946	37.1930337	59.04331487	1.587483165	0.666741293	0.38704569	1
gene40588	588.8897003	426.8541288	0.724845635	-0.46425431	0.38708157	1
gene31998	146.7058552	101.6319354	0.692759913	-0.52957265	0.38714262	1
gene775	156.0041136	219.7185652	1.408415202	0.494072705	0.3871438	1
gene8289	192.1640075	135.5092472	0.705174965	-0.50394684	0.38716912	1
gene51857	179.7663296	251.6600306	1.399928625	0.485353274	0.38721215	1
gene3488	87.81688513	58.07539168	0.661323749	-0.59657138	0.38729722	1
gene63054	35.12675405	56.13954529	1.598199059	0.676447111	0.38729835	1
gene23967	47.52443195	29.03769584	0.611005637	-0.7107424	0.3873136	1
gene61510	378.129176	521.7106019	1.37971528	0.464370581	0.38732726	1
gene4557	30.99419475	50.33200612	1.623917205	0.699478079	0.38734541	1
gene50058	56.82269038	86.14516432	1.516034594	0.600302674	0.38735987	1
gene65518	56.82269038	86.14516432	1.516034594	0.600302674	0.38735987	1
gene18246	39.25931335	23.23015667	0.591710723	-0.75703606	0.38740853	1

gene54495	39.25931335	23.23015667	0.591710723	-0.75703606	0.38740853	1
gene59484	39.25931335	23.23015667	0.591710723	-0.75703606	0.38740853	1
gene66931	39.25931335	23.23015667	0.591710723	-0.75703606	0.38740853	1
gene69541	39.25931335	23.23015667	0.591710723	-0.75703606	0.38740853	1
gene6214	242.7878589	336.8372717	1.387372801	0.472355506	0.38741395	1
gene13117	3899.0697	5895.620179	1.512058166	0.596513638	0.38745625	1
gene39152	408.0902309	295.2165744	0.72341005	-0.46711445	0.38751122	1
gene66365	391.5599937	540.1011426	1.379357318	0.46399623	0.38755299	1
gene34597	520.7024718	377.4900459	0.724963038	-0.46402065	0.38757209	1
gene67327	520.7024718	377.4900459	0.724963038	-0.46402065	0.38757209	1
gene54522	326.4721847	235.2053363	0.720445255	-0.47303929	0.38758078	1
gene70433	783.1199874	1087.945671	1.389245184	0.474301239	0.38760267	1
gene4852	102.2808427	68.72254682	0.671900475	-0.57368054	0.38763238	1
gene23342	102.2808427	68.72254682	0.671900475	-0.57368054	0.38763238	1
gene37459	102.2808427	68.72254682	0.671900475	-0.57368054	0.38763238	1
gene16145	292.3785705	210.0393332	0.718381422	-0.47717805	0.3876514	1
gene66881	576.4920224	796.6007892	1.381807134	0.466556265	0.38768357	1
gene23363	43.39187265	26.13392626	0.602276985	-0.73150096	0.38768364	1
gene24459	43.39187265	26.13392626	0.602276985	-0.73150096	0.38768364	1
gene51437	43.39187265	26.13392626	0.602276985	-0.73150096	0.38768364	1
gene1947	626.082734	453.9559783	0.725073467	-0.46380091	0.38773558	1
gene49320	272.7489138	195.5204853	0.716851564	-0.48025368	0.38776795	1
gene72837	101.2477029	146.1564024	1.443552775	0.529623852	0.38785494	1
gene55936	331.6378839	239.0770291	0.720897825	-0.4721333	0.38795789	1
gene73658	497.9733957	361.0353516	0.725009317	-0.46392856	0.38797019	1
gene13348	799.6502246	578.8180704	0.723839064	-0.46625913	0.38797117	1
gene61686	212.826804	150.9960184	0.709478391	-0.49516935	0.38798113	1
gene50479	162.2029525	113.2470138	0.698180964	-0.51832707	0.3879856	1
gene4694	250.0198377	346.5165037	1.385956038	0.470881497	0.38800146	1
gene44915	108.4796816	155.8356343	1.436542143	0.522600317	0.3881132	1
gene10174	15.49709738	28.06977264	1.811292267	0.857019356	0.38815751	1
gene14372	15.49709738	28.06977264	1.811292267	0.857019356	0.38815751	1
gene37271	15.49709738	28.06977264	1.811292267	0.857019356	0.38815751	1
gene38042	15.49709738	28.06977264	1.811292267	0.857019356	0.38815751	1
gene38158	15.49709738	28.06977264	1.811292267	0.857019356	0.38815751	1
gene55812	15.49709738	28.06977264	1.811292267	0.857019356	0.38815751	1
gene71107	15.49709738	28.06977264	1.811292267	0.857019356	0.38815751	1
gene79	340.9361423	470.4106726	1.379761821	0.464419246	0.38816058	1
gene15548	217.9925031	154.8677111	0.710426776	-0.49324214	0.3881827	1
gene12610	161.1698127	226.4940275	1.405312966	0.490891457	0.38819059	1
gene51343	161.1698127	226.4940275	1.405312966	0.490891457	0.38819059	1
gene5500	903.9973469	653.3481564	0.722732383	-0.46846656	0.3882505	1
gene56867	2007.39068	1418.00748	0.706393376	-0.50145628	0.38830583	1
gene30855	1988.794163	1405.424479	0.706671663	-0.50088804	0.38833603	1
gene4573	505.2053745	696.9047001	1.37944831	0.464091398	0.38835115	1
gene29663	201.4622659	280.6977264	1.393301744	0.478507733	0.38835339	1
gene11010	640.5466916	464.6031334	0.725322821	-0.46330485	0.38836775	1
gene56650	627.1158738	454.9239015	0.725422399	-0.4631068	0.38845598	1

gene21566	690.1374032	500.4162916	0.72509661	-0.46375487	0.38850252	1
gene7917	300.6436891	415.2390505	1.381166695	0.465887451	0.38857233	1
gene17886	985.6153931	711.4235481	0.72180645	-0.47031606	0.38859571	1
gene67176	177.7000499	124.8620921	0.702656483	-0.50910854	0.3886059	1
gene23053	107.4465418	72.5942396	0.675631234	-0.56569207	0.38860657	1
gene29229	107.4465418	72.5942396	0.675631234	-0.56569207	0.38860657	1
gene13632	10.33139825	20.32638709	1.967438153	0.976318285	0.38864077	1
gene15311	10.33139825	20.32638709	1.967438153	0.976318285	0.38864077	1
gene15637	10.33139825	20.32638709	1.967438153	0.976318285	0.38864077	1
gene17877	10.33139825	20.32638709	1.967438153	0.976318285	0.38864077	1
gene32107	10.33139825	20.32638709	1.967438153	0.976318285	0.38864077	1
gene39954	10.33139825	20.32638709	1.967438153	0.976318285	0.38864077	1
gene48846	10.33139825	20.32638709	1.967438153	0.976318285	0.38864077	1
gene52827	10.33139825	20.32638709	1.967438153	0.976318285	0.38864077	1
gene54003	10.33139825	20.32638709	1.967438153	0.976318285	0.38864077	1
gene54837	10.33139825	20.32638709	1.967438153	0.976318285	0.38864077	1
gene25410	144.6395755	204.2317941	1.412004933	0.497745128	0.38874695	1
gene70836	144.6395755	204.2317941	1.412004933	0.497745128	0.38874695	1
gene65143	734.5624156	1018.255201	1.386206508	0.471142196	0.38875502	1
gene11890	399.8251123	289.4090352	0.723839064	-0.46625913	0.3888014	1
gene72075	1312.087578	940.8213452	0.717041576	-0.47987132	0.38882312	1
gene62827	1542.477759	1100.528672	0.713481064	-0.48705295	0.38883888	1
gene35152	3782.3249	2588.226622	0.68429516	-0.54730935	0.38884434	1
gene19833	1062.06774	765.627247	0.720883629	-0.47216171	0.38893466	1
gene1736	708.73392	513.9672164	0.725190656	-0.46356776	0.38897879	1
gene53478	255.1855368	353.291966	1.38445137	0.469314379	0.38899919	1
gene56276	238.6552996	170.3544823	0.713809761	-0.48638847	0.38901739	1
gene34708	137.4075967	94.85647307	0.690329176	-0.53464363	0.3890214	1
gene69828	137.4075967	94.85647307	0.690329176	-0.53464363	0.3890214	1
gene17380	293.4117103	211.0072564	0.719150767	-0.47563384	0.38903293	1
gene49409	1037.272384	748.2046295	0.721319338	-0.47128999	0.38909058	1
gene669	795.5176653	1104.400365	1.38827887	0.473297397	0.38910734	1
gene39597	784.1531272	568.1709153	0.724566281	-0.46481043	0.38912003	1
gene41339	193.1971473	136.4771704	0.706414004	-0.50141415	0.3891221	1
gene8304	573.3926029	416.2069737	0.725867358	-0.46222215	0.38914891	1
gene7883	940.1572408	679.4820826	0.722732383	-0.46846656	0.38921634	1
gene59845	910.1961859	658.1877724	0.723127368	-0.46767832	0.38921718	1
gene26016	225.2244819	312.6391919	1.388122593	0.473134987	0.3892276	1
gene4476	3.099419475	8.711308752	2.810625932	1.490891457	0.3893028	1
gene5927	3.099419475	8.711308752	2.810625932	1.490891457	0.3893028	1
gene6195	3.099419475	8.711308752	2.810625932	1.490891457	0.3893028	1
gene8172	3.099419475	8.711308752	2.810625932	1.490891457	0.3893028	1
gene8809	3.099419475	8.711308752	2.810625932	1.490891457	0.3893028	1
gene9358	3.099419475	8.711308752	2.810625932	1.490891457	0.3893028	1
gene13297	3.099419475	8.711308752	2.810625932	1.490891457	0.3893028	1
gene18905	3.099419475	8.711308752	2.810625932	1.490891457	0.3893028	1
gene19396	3.099419475	8.711308752	2.810625932	1.490891457	0.3893028	1
gene20220	3.099419475	8.711308752	2.810625932	1.490891457	0.3893028	1

gene23006	3.099419475	8.711308752	2.810625932	1.490891457	0.3893028	1
gene26256	3.099419475	8.711308752	2.810625932	1.490891457	0.3893028	1
gene26268	3.099419475	8.711308752	2.810625932	1.490891457	0.3893028	1
gene29572	3.099419475	8.711308752	2.810625932	1.490891457	0.3893028	1
gene31255	3.099419475	8.711308752	2.810625932	1.490891457	0.3893028	1
gene36046	3.099419475	8.711308752	2.810625932	1.490891457	0.3893028	1
gene36606	3.099419475	8.711308752	2.810625932	1.490891457	0.3893028	1
gene39779	3.099419475	8.711308752	2.810625932	1.490891457	0.3893028	1
gene39873	3.099419475	8.711308752	2.810625932	1.490891457	0.3893028	1
gene40989	3.099419475	8.711308752	2.810625932	1.490891457	0.3893028	1
gene42821	3.099419475	8.711308752	2.810625932	1.490891457	0.3893028	1
gene49372	3.099419475	8.711308752	2.810625932	1.490891457	0.3893028	1
gene50157	3.099419475	8.711308752	2.810625932	1.490891457	0.3893028	1
gene57638	3.099419475	8.711308752	2.810625932	1.490891457	0.3893028	1
gene58271	3.099419475	8.711308752	2.810625932	1.490891457	0.3893028	1
gene60889	3.099419475	8.711308752	2.810625932	1.490891457	0.3893028	1
gene64832	3.099419475	8.711308752	2.810625932	1.490891457	0.3893028	1
gene65344	3.099419475	8.711308752	2.810625932	1.490891457	0.3893028	1
gene66568	3.099419475	8.711308752	2.810625932	1.490891457	0.3893028	1
gene66704	3.099419475	8.711308752	2.810625932	1.490891457	0.3893028	1
gene67841	3.099419475	8.711308752	2.810625932	1.490891457	0.3893028	1
gene70681	3.099419475	8.711308752	2.810625932	1.490891457	0.3893028	1
gene71311	3.099419475	8.711308752	2.810625932	1.490891457	0.3893028	1
gene45572	386.3942946	532.3577571	1.37775781	0.462322305	0.38932608	1
gene17325	437.018146	602.0482271	1.377627525	0.462185874	0.38935703	1
gene47419	298.5774094	214.8789492	0.719675844	-0.47458086	0.38936334	1
gene22587	366.7646379	265.2109553	0.723109395	-0.46771417	0.38939354	1
gene51043	1570.372534	2231.062964	1.420722099	0.506624384	0.3894647	1
gene68367	871.9700124	631.0859229	0.723747278	-0.46644208	0.38949481	1
gene42516	120.8773595	172.2903286	1.425331669	0.511297668	0.38951404	1
gene61897	368.8309176	508.1596772	1.37775781	0.462322305	0.3895905	1
gene26721	52.69013108	80.33762515	1.524718643	0.608543046	0.38960385	1
gene42255	52.69013108	80.33762515	1.524718643	0.608543046	0.38960385	1
gene67568	52.69013108	80.33762515	1.524718643	0.608543046	0.38960385	1
gene73838	147.738995	102.5998586	0.694467014	-0.52602193	0.38962406	1
gene38789	83.68432583	55.17162209	0.659282626	-0.60103103	0.38968302	1
gene9205	223.1582022	309.7354223	1.387963423	0.472969549	0.38974276	1
gene18958	74.38606741	48.39615973	0.650607855	-0.62013986	0.38976152	1
gene40321	64.05466916	95.82439627	1.495978319	0.581089266	0.38976792	1
gene44853	400.8582521	290.3769584	0.724388127	-0.4651652	0.38985852	1
gene17184	73.35292758	108.4073978	1.477887814	0.563536759	0.38991568	1
gene14437	715.9658988	991.1533513	1.384358323	0.469217415	0.38993679	1
gene17378	745.9269537	541.0690658	0.72536468	-0.4632216	0.38998495	1
gene39059	308.9088077	222.6223348	0.720673316	-0.47258267	0.39002854	1
gene8530	7.231978776	15.48677111	2.141429282	1.098574035	0.39007931	1
gene10655	7.231978776	15.48677111	2.141429282	1.098574035	0.39007931	1
gene13558	7.231978776	15.48677111	2.141429282	1.098574035	0.39007931	1
gene21992	7.231978776	15.48677111	2.141429282	1.098574035	0.39007931	1

gene22092	7.231978776	15.48677111	2.141429282	1.098574035	0.39007931	1
gene22762	7.231978776	15.48677111	2.141429282	1.098574035	0.39007931	1
gene28299	7.231978776	15.48677111	2.141429282	1.098574035	0.39007931	1
gene29357	7.231978776	15.48677111	2.141429282	1.098574035	0.39007931	1
gene29872	7.231978776	15.48677111	2.141429282	1.098574035	0.39007931	1
gene30755	7.231978776	15.48677111	2.141429282	1.098574035	0.39007931	1
gene40302	7.231978776	15.48677111	2.141429282	1.098574035	0.39007931	1
gene40481	7.231978776	15.48677111	2.141429282	1.098574035	0.39007931	1
gene43982	7.231978776	15.48677111	2.141429282	1.098574035	0.39007931	1
gene45680	7.231978776	15.48677111	2.141429282	1.098574035	0.39007931	1
gene55120	7.231978776	15.48677111	2.141429282	1.098574035	0.39007931	1
gene56246	7.231978776	15.48677111	2.141429282	1.098574035	0.39007931	1
gene57906	7.231978776	15.48677111	2.141429282	1.098574035	0.39007931	1
gene64778	7.231978776	15.48677111	2.141429282	1.098574035	0.39007931	1
gene66352	7.231978776	15.48677111	2.141429282	1.098574035	0.39007931	1
gene68593	7.231978776	15.48677111	2.141429282	1.098574035	0.39007931	1
gene71516	7.231978776	15.48677111	2.141429282	1.098574035	0.39007931	1
gene34920	505.2053745	366.8428908	0.726126263	-0.46170766	0.39013631	1
gene63220	647.7786703	470.4106726	0.726190432	-0.46158017	0.39021219	1
gene8690	3376.300948	2328.823206	0.689755813	-0.53584238	0.39022299	1
gene47693	349.2012609	481.0578277	1.377594762	0.462151562	0.39023744	1
gene11074	60.95524968	38.71692779	0.635169702	-0.654786	0.39026402	1
gene48683	60.95524968	38.71692779	0.635169702	-0.654786	0.39026402	1
gene54852	335.7704432	462.667287	1.37792738	0.462499857	0.39032307	1
gene30788	896.7653682	1247.652998	1.391281423	0.476414273	0.3903387	1
gene25552	538.2658489	391.0409706	0.726482966	-0.46099912	0.39052192	1
gene32976	367.7977777	266.1788785	0.723709861	-0.46651667	0.39053074	1
gene2664	501.0728152	363.9391212	0.726319829	-0.46132313	0.39059267	1
gene56756	1082.730537	781.1140181	0.721429748	-0.47106918	0.39065376	1
gene60239	50.62385143	77.43385557	1.529592344	0.613147207	0.39069677	1
gene38978	639.5135517	464.6031334	0.726494587	-0.46097605	0.39075397	1
gene13075	556.8623657	767.5630933	1.378371283	0.46296455	0.3907573	1
gene732	2193.355849	1546.741265	0.705193946	-0.50390801	0.39101251	1
gene61274	1211.873015	1701.608976	1.404114916	0.489661014	0.3910714	1
gene28752	61.9883895	92.92062669	1.499000497	0.584000862	0.39107203	1
gene55335	354.36696	487.8332901	1.37663311	0.461144114	0.39113446	1
gene66973	200.4291261	278.7618801	1.390825204	0.475941116	0.39120676	1
gene28715	255.1855368	182.9374838	0.716880298	-0.48019585	0.39123313	1
gene6021	71.28664793	105.5036282	1.479991433	0.565588825	0.39127158	1
gene29697	71.28664793	105.5036282	1.479991433	0.565588825	0.39127158	1
gene6515	653.9775093	475.2502886	0.72670739	-0.46055352	0.39133591	1
gene64100	133.2750374	91.95270349	0.689946934	-0.53544269	0.39136113	1
gene42673	209.7273845	149.060172	0.710732994	-0.49262042	0.39142027	1
gene32187	1044.504363	1458.660254	1.396509489	0.481825376	0.39148666	1
gene13795	1583.803352	2247.517658	1.419063582	0.504939232	0.39150207	1
gene53040	585.7902808	425.8862056	0.72702846	-0.45991625	0.39153514	1
gene39016	2635.539694	3847.494699	1.459850788	0.545820918	0.39156726	1
gene64489	95.04886391	137.4450936	1.446046675	0.53211412	0.39162931	1

gene45272	977.3502745	1361.867935	1.393428713	0.478639197	0.39165428	1
gene6198	265.516935	190.6808693	0.718149557	-0.47764377	0.39166361	1
gene12757	123.976779	176.1620214	1.420927555	0.506833001	0.39175518	1
gene41768	48.55757178	74.53008599	1.534880828	0.618126646	0.39175773	1
gene60128	48.55757178	74.53008599	1.534880828	0.618126646	0.39175773	1
gene60981	48.55757178	74.53008599	1.534880828	0.618126646	0.39175773	1
gene11400	143.6064357	99.69608905	0.694231345	-0.52651159	0.39188582	1
gene2369	27.89477528	15.48677111	0.555185369	-0.84895855	0.3919017	1
gene13418	27.89477528	15.48677111	0.555185369	-0.84895855	0.3919017	1
gene31977	27.89477528	15.48677111	0.555185369	-0.84895855	0.3919017	1
gene52110	27.89477528	15.48677111	0.555185369	-0.84895855	0.3919017	1
gene71027	27.89477528	15.48677111	0.555185369	-0.84895855	0.3919017	1
gene73012	27.89477528	15.48677111	0.555185369	-0.84895855	0.3919017	1
gene44338	1328.617815	954.3722699	0.718319639	-0.47730213	0.39190701	1
gene16243	56.82269038	35.8131582	0.630261573	-0.66597739	0.39194117	1
gene828	230.390181	164.5469431	0.714209878	-0.48558001	0.39195161	1
gene43173	70.25350811	45.49239015	0.647546171	-0.62694503	0.39195368	1
gene66761	70.25350811	45.49239015	0.647546171	-0.62694503	0.39195368	1
gene57185	2637.605973	3849.430545	1.459441093	0.545415981	0.39196708	1
gene18456	432.8855867	595.2727647	1.375127246	0.459565123	0.39203152	1
gene21078	181.8326092	253.595877	1.394666656	0.47992034	0.39208311	1
gene56490	859.5723345	623.3425373	0.725177524	-0.46359388	0.39210498	1
gene13000	17.56337703	30.97354223	1.763529997	0.818466115	0.39210721	1
gene31765	17.56337703	30.97354223	1.763529997	0.818466115	0.39210721	1
gene35089	17.56337703	30.97354223	1.763529997	0.818466115	0.39210721	1
gene36533	17.56337703	30.97354223	1.763529997	0.818466115	0.39210721	1
gene48531	17.56337703	30.97354223	1.763529997	0.818466115	0.39210721	1
gene20633	79.55176653	52.26785251	0.657029439	-0.60597008	0.39211312	1
gene72580	79.55176653	52.26785251	0.657029439	-0.60597008	0.39211312	1
gene64233	430.8193071	592.3689951	1.37498247	0.459413226	0.39217953	1
gene9003	1119.92357	808.2158675	0.721670558	-0.4705877	0.39225598	1
gene12936	379.1623158	274.8901873	0.724993428	-0.46396018	0.39235198	1
gene17087	59.92210985	90.0168571	1.502231102	0.587106773	0.39239013	1
gene16879	444.2501248	610.7595358	1.374810049	0.459232302	0.39244318	1
gene60106	481.4431585	662.0594651	1.375156035	0.459595327	0.39251485	1
gene3663	20.6627965	10.64715514	0.515281421	-0.95656752	0.39254509	1
gene7637	20.6627965	10.64715514	0.515281421	-0.95656752	0.39254509	1
gene24019	20.6627965	10.64715514	0.515281421	-0.95656752	0.39254509	1
gene33397	20.6627965	10.64715514	0.515281421	-0.95656752	0.39254509	1
gene33465	20.6627965	10.64715514	0.515281421	-0.95656752	0.39254509	1
gene39879	20.6627965	10.64715514	0.515281421	-0.95656752	0.39254509	1
gene45678	20.6627965	10.64715514	0.515281421	-0.95656752	0.39254509	1
gene58017	20.6627965	10.64715514	0.515281421	-0.95656752	0.39254509	1
gene34748	1232.535811	887.5855695	0.72012964	-0.47367145	0.39257708	1
gene29900	165.302372	231.3336435	1.399457495	0.48486767	0.39264811	1
gene39664	310.9750873	224.5581812	0.722109874	-0.46970972	0.39265364	1
gene30863	266.5500749	367.810814	1.379893868	0.46455731	0.39268816	1
gene39828	172.5343508	241.0128755	1.396897918	0.482226596	0.39272485	1

gene1235	8.265118601	2.903769584	0.351328242	-1.50910854	0.39275564	1
gene1238	8.265118601	2.903769584	0.351328242	-1.50910854	0.39275564	1
gene3020	8.265118601	2.903769584	0.351328242	-1.50910854	0.39275564	1
gene12025	8.265118601	2.903769584	0.351328242	-1.50910854	0.39275564	1
gene18051	8.265118601	2.903769584	0.351328242	-1.50910854	0.39275564	1
gene18098	8.265118601	2.903769584	0.351328242	-1.50910854	0.39275564	1
gene20005	8.265118601	2.903769584	0.351328242	-1.50910854	0.39275564	1
gene23443	8.265118601	2.903769584	0.351328242	-1.50910854	0.39275564	1
gene23583	8.265118601	2.903769584	0.351328242	-1.50910854	0.39275564	1
gene28142	8.265118601	2.903769584	0.351328242	-1.50910854	0.39275564	1
gene29367	8.265118601	2.903769584	0.351328242	-1.50910854	0.39275564	1
gene32281	8.265118601	2.903769584	0.351328242	-1.50910854	0.39275564	1
gene34200	8.265118601	2.903769584	0.351328242	-1.50910854	0.39275564	1
gene34373	8.265118601	2.903769584	0.351328242	-1.50910854	0.39275564	1
gene36660	8.265118601	2.903769584	0.351328242	-1.50910854	0.39275564	1
gene37510	8.265118601	2.903769584	0.351328242	-1.50910854	0.39275564	1
gene44708	8.265118601	2.903769584	0.351328242	-1.50910854	0.39275564	1
gene47658	8.265118601	2.903769584	0.351328242	-1.50910854	0.39275564	1
gene49162	8.265118601	2.903769584	0.351328242	-1.50910854	0.39275564	1
gene50667	8.265118601	2.903769584	0.351328242	-1.50910854	0.39275564	1
gene50975	8.265118601	2.903769584	0.351328242	-1.50910854	0.39275564	1
gene52818	8.265118601	2.903769584	0.351328242	-1.50910854	0.39275564	1
gene54219	8.265118601	2.903769584	0.351328242	-1.50910854	0.39275564	1
gene55320	8.265118601	2.903769584	0.351328242	-1.50910854	0.39275564	1
gene57715	8.265118601	2.903769584	0.351328242	-1.50910854	0.39275564	1
gene59298	8.265118601	2.903769584	0.351328242	-1.50910854	0.39275564	1
gene59853	8.265118601	2.903769584	0.351328242	-1.50910854	0.39275564	1
gene63252	8.265118601	2.903769584	0.351328242	-1.50910854	0.39275564	1
gene65095	8.265118601	2.903769584	0.351328242	-1.50910854	0.39275564	1
gene65503	8.265118601	2.903769584	0.351328242	-1.50910854	0.39275564	1
gene68064	8.265118601	2.903769584	0.351328242	-1.50910854	0.39275564	1
gene73106	8.265118601	2.903769584	0.351328242	-1.50910854	0.39275564	1
gene74122	8.265118601	2.903769584	0.351328242	-1.50910854	0.39275564	1
gene74172	8.265118601	2.903769584	0.351328242	-1.50910854	0.39275564	1
gene13934	179.7663296	126.7979385	0.705348653	-0.50359154	0.3927607	1
gene23201	46.49129213	71.6263164	1.5406394	0.623529226	0.39277467	1
gene10362	422.5541885	580.7539168	1.374389209	0.458790614	0.39280038	1
gene44673	121.9104994	173.2582518	1.421192209	0.507101685	0.39289371	1
gene3311	190.0977278	134.5413241	0.707748197	-0.49869193	0.39291071	1
gene68210	351.2675405	482.9936741	1.375002294	0.459434026	0.39298964	1
gene12920	418.4216292	303.9278831	0.726367525	-0.46122839	0.39303221	1
gene46379	100.214563	144.220556	1.439117745	0.525184635	0.3930569	1
gene42491	118.8110799	81.30554835	0.684326314	-0.54724367	0.39308229	1
gene63052	118.8110799	81.30554835	0.684326314	-0.54724367	0.39308229	1
gene41783	205.5948252	146.1564024	0.710895336	-0.49229092	0.39313858	1
gene26609	826.5118601	600.1123807	0.726078366	-0.46180283	0.39315409	1
gene25348	210.7605243	150.0280952	0.711841535	-0.49037198	0.39322157	1
gene45703	303.7431086	418.1428201	1.37663311	0.461144114	0.39328157	1

gene53823	123.976779	85.17724113	0.687041895	-0.54153002	0.39348697	1
gene54716	52.69013108	32.90938862	0.62458354	-0.67903354	0.39351384	1
gene67431	52.69013108	32.90938862	0.62458354	-0.67903354	0.39351384	1
gene25838	1572.438814	2227.191271	1.416392963	0.502221581	0.39355558	1
gene64787	611.6187764	445.2446695	0.727977437	-0.45803436	0.39358152	1
gene35976	76.45234706	112.2790906	1.468615352	0.554456586	0.39359149	1
gene40981	76.45234706	112.2790906	1.468615352	0.554456586	0.39359149	1
gene26888	231.4233208	165.5148663	0.71520392	-0.48357345	0.39361554	1
gene36344	1501.152166	2121.687643	1.413372802	0.499142052	0.39368471	1
gene38505	57.8558302	87.11308752	1.505692464	0.590427131	0.3937189	1
gene65800	241.7547191	173.2582518	0.716669575	-0.48061999	0.39385853	1
gene70181	299.6105493	412.3352809	1.376237525	0.460729487	0.39397173	1
gene73217	1835.889469	1306.696313	0.71175108	-0.49055532	0.39403885	1
gene19731	134.3081773	92.92062669	0.691846383	-0.53147636	0.39405514	1
gene2031	119.8442197	170.3544823	1.421465989	0.50737958	0.39406599	1
gene62603	83.68432583	121.9583225	1.457361594	0.543358877	0.39410061	1
gene167	797.583945	579.7859936	0.726927864	-0.46011589	0.3942402	1
gene72229	139.4738764	96.79231946	0.693981712	-0.52703045	0.39424886	1
gene14050	329.5716042	452.9880551	1.374475377	0.458881062	0.39431636	1
gene61899	105.3802622	150.9960184	1.432868122	0.518905834	0.3943296	1
gene70684	105.3802622	150.9960184	1.432868122	0.518905834	0.3943296	1
gene59204	90.91630461	131.6375545	1.447898207	0.533960179	0.39433198	1
gene9035	560.994925	771.4347861	1.37511901	0.459556483	0.39434879	1
gene3764	175.6337703	244.8845682	1.394290904	0.479531595	0.39442542	1
gene29062	267.5832147	368.7787372	1.378183372	0.462767856	0.39443933	1
gene32944	5378.525929	3605.5139	0.670353541	-0.57700593	0.39447133	1
gene34237	149.8052746	104.535705	0.697810576	-0.51909263	0.39451222	1
gene4259	75.41920723	49.36408293	0.654529327	-0.61147026	0.39457428	1
gene19654	75.41920723	49.36408293	0.654529327	-0.61147026	0.39457428	1
gene42341	75.41920723	49.36408293	0.654529327	-0.61147026	0.39457428	1
gene18138	154.9709738	108.4073978	0.699533565	-0.51553481	0.39459891	1
gene18651	154.9709738	108.4073978	0.699533565	-0.51553481	0.39459891	1
gene30924	42.35873283	65.81877724	1.553841979	0.635839793	0.39461397	1
gene41825	160.1366729	112.2790906	0.701145394	-0.51221445	0.39466462	1
gene44334	468.0123408	340.7089645	0.727991411	-0.45800666	0.39466745	1
gene10100	19.62965668	33.87731181	1.725822941	0.78728446	0.39477004	1
gene16616	19.62965668	33.87731181	1.725822941	0.78728446	0.39477004	1
gene19247	19.62965668	33.87731181	1.725822941	0.78728446	0.39477004	1
gene73528	19.62965668	33.87731181	1.725822941	0.78728446	0.39477004	1
gene44271	327.5053246	237.1411827	0.724083442	-0.46577213	0.3947786	1
gene27106	175.6337703	123.8941689	0.705411999	-0.50346198	0.39478535	1
gene1342	48.55757178	30.00561903	0.617939035	-0.69446358	0.39490444	1
gene35959	48.55757178	30.00561903	0.617939035	-0.69446358	0.39490444	1
gene71595	48.55757178	30.00561903	0.617939035	-0.69446358	0.39490444	1
gene18764	9325.120061	5931.433337	0.636070452	-0.65274152	0.39492057	1
gene36871	201.4622659	143.2526328	0.711064338	-0.49194799	0.39492733	1
gene68173	201.4622659	143.2526328	0.711064338	-0.49194799	0.39492733	1
gene4894	518.6361922	712.3914713	1.373586113	0.457947359	0.39492743	1

gene66715	204.5616854	283.601496	1.386386192	0.471329191	0.39500034	1
gene5881	211.7936641	150.9960184	0.712939261	-0.48814892	0.39501058	1
gene6140	32.02733458	18.3905407	0.5742139	-0.80033984	0.39516701	1
gene22950	32.02733458	18.3905407	0.5742139	-0.80033984	0.39516701	1
gene31078	32.02733458	18.3905407	0.5742139	-0.80033984	0.39516701	1
gene37378	32.02733458	18.3905407	0.5742139	-0.80033984	0.39516701	1
gene53370	32.02733458	18.3905407	0.5742139	-0.80033984	0.39516701	1
gene56255	32.02733458	18.3905407	0.5742139	-0.80033984	0.39516701	1
gene16619	487.6419974	355.2278124	0.728460252	-0.45707784	0.39519913	1
gene2750	232.4564606	166.4827895	0.716189126	-0.48158748	0.39526925	1
gene30341	232.4564606	166.4827895	0.716189126	-0.48158748	0.39526925	1
gene2468	357.4663795	259.4034162	0.725672206	-0.46261008	0.39527724	1
gene51669	40.29245318	62.91500765	1.561458851	0.642894551	0.39541796	1
gene40881	1477.38995	2083.938638	1.410554227	0.496262129	0.39543618	1
gene18163	420.4879088	576.882224	1.371935344	0.456212493	0.39546734	1
gene44632	195.2634269	271.0184945	1.387963423	0.472969549	0.39549367	1
gene31472	65.08780898	96.79231946	1.487103668	0.572505223	0.39551147	1
gene35046	65.08780898	96.79231946	1.487103668	0.572505223	0.39551147	1
gene63686	621.9501747	855.6441041	1.375743812	0.460211839	0.39556819	1
gene2447	857.5060548	623.3425373	0.72692494	-0.46012169	0.39563041	1
gene45004	202.4954057	280.6977264	1.386193062	0.471128203	0.39566656	1
gene34669	1077.564838	780.1460949	0.723989933	-0.46595846	0.39570316	1
gene27996	2770.881011	4044.95103	1.459806832	0.545777478	0.3957261	1
gene54067	1174.679981	848.8686417	0.722638212	-0.46865455	0.3958812	1
gene24013	263.4506554	189.7129461	0.720108082	-0.47371464	0.39594145	1
gene13288	171.501211	239.0770291	1.394025312	0.479256757	0.39609828	1
gene56195	796.5508051	579.7859936	0.727870702	-0.4582459	0.39615206	1
gene62718	353.3338202	256.4996466	0.725941396	-0.46207501	0.39615398	1
gene55671	233.4896005	322.3184238	1.38044017	0.465128361	0.39616908	1
gene9230	38.22617353	60.01123807	1.569899169	0.650671901	0.39617125	1
gene60594	38.22617353	60.01123807	1.569899169	0.650671901	0.39617125	1
gene61574	38.22617353	60.01123807	1.569899169	0.650671901	0.39617125	1
gene2804	61.9883895	39.68485098	0.640198129	-0.64340963	0.39617358	1
gene3717	61.9883895	39.68485098	0.640198129	-0.64340963	0.39617358	1
gene32082	61.9883895	39.68485098	0.640198129	-0.64340963	0.39617358	1
gene26463	1279.027103	922.4308045	0.72119723	-0.47153424	0.39617375	1
gene13654	536.1995692	391.0409706	0.729282515	-0.45545029	0.39627197	1
gene69190	1474.29053	1058.907975	0.718249187	-0.47744364	0.39629012	1
gene40382	1171.580562	1636.758122	1.397051279	0.482384976	0.39632458	1
gene50094	586.8234206	806.2800211	1.373973827	0.458354523	0.39633523	1
gene22629	125.0099188	86.14516432	0.689106634	-0.53720085	0.39636083	1
gene41398	1401.970743	1008.575969	0.719398728	-0.47513649	0.39636164	1
gene19412	53.7232709	81.30554835	1.513413963	0.597806661	0.39639119	1
gene54291	53.7232709	81.30554835	1.513413963	0.597806661	0.39639119	1
gene69778	358.4995193	260.3713394	0.72628086	-0.46140053	0.39642986	1
gene6845	493.8408364	360.0674284	0.729116351	-0.45577904	0.39643244	1
gene53952	378.129176	274.8901873	0.726974285	-0.46002376	0.39643887	1
gene71977	95.04886391	63.88293085	0.672106201	-0.57323888	0.39646385	1

gene40055	36.15989388	21.29431028	0.588893052	-0.76392244	0.39648749	1
gene40595	36.15989388	21.29431028	0.588893052	-0.76392244	0.39648749	1
gene73525	36.15989388	21.29431028	0.588893052	-0.76392244	0.39648749	1
gene37355	21.69593633	36.7810814	1.695298181	0.761539047	0.39650932	1
gene57008	21.69593633	36.7810814	1.695298181	0.761539047	0.39650932	1
gene62086	21.69593633	36.7810814	1.695298181	0.761539047	0.39650932	1
gene67321	21.69593633	36.7810814	1.695298181	0.761539047	0.39650932	1
gene24471	437.018146	599.1444575	1.37098302	0.455210703	0.39655656	1
gene72827	80.58490636	53.23577571	0.660617206	-0.59811355	0.39658394	1
gene12785	634.3478526	462.667287	0.729358955	-0.45529908	0.39658576	1
gene3962	284.1134519	205.1997173	0.722245694	-0.4694384	0.39659288	1
gene2509	40.29245318	24.19807987	0.600561097	-0.73561707	0.39661645	1
gene11835	40.29245318	24.19807987	0.600561097	-0.73561707	0.39661645	1
gene51454	40.29245318	24.19807987	0.600561097	-0.73561707	0.39661645	1
gene54499	40.29245318	24.19807987	0.600561097	-0.73561707	0.39661645	1
gene60806	40.29245318	24.19807987	0.600561097	-0.73561707	0.39661645	1
gene73560	40.29245318	24.19807987	0.600561097	-0.73561707	0.39661645	1
gene28744	142.5732959	200.3601013	1.405312966	0.490891457	0.39662911	1
gene26765	266.5500749	366.8428908	1.376262569	0.460755739	0.39663217	1
gene65102	135.3413171	93.88854988	0.693716833	-0.5275812	0.39671871	1
gene49885	417.3884893	303.9278831	0.728165464	-0.45766178	0.39674286	1
gene68497	811.0147627	590.4331487	0.728017757	-0.45795445	0.39677526	1
gene50165	140.5070162	97.76024266	0.695767694	-0.5233224	0.39682013	1
gene27486	186.9983083	132.6054777	0.709126616	-0.49588485	0.39682483	1
gene36664	223.1582022	159.7073271	0.71566864	-0.48263633	0.39682988	1
gene29860	475.2443195	346.5165037	0.729133394	-0.45574532	0.39683488	1
gene8659	694.2699625	506.2238308	0.729145517	-0.45572133	0.39686385	1
gene23937	432.8855867	593.3369183	1.370655288	0.454865787	0.39689511	1
gene57211	171.501211	120.9903993	0.705478397	-0.50332619	0.39689723	1
gene62155	171.501211	120.9903993	0.705478397	-0.50332619	0.39689723	1
gene71856	379.1623158	519.7747555	1.37085025	0.455070982	0.39693172	1
gene73677	489.7082771	357.1636588	0.729339641	-0.45533729	0.39696365	1
gene12958	169.4349313	236.1732595	1.393887657	0.47911429	0.39696998	1
gene16504	169.4349313	236.1732595	1.393887657	0.47911429	0.39696998	1
gene30069	169.4349313	236.1732595	1.393887657	0.47911429	0.39696998	1
gene7567	283.0803121	389.1051242	1.374539689	0.458948565	0.39702467	1
gene56316	3386.632347	5013.842148	1.480480204	0.5660652	0.39707077	1
gene22322	1173.646841	1638.693969	1.39624111	0.481548095	0.39721146	1
gene4936	100.214563	67.75462362	0.676095585	-0.56470087	0.39730889	1
gene22592	456.6478027	332.965579	0.729151826	-0.45570885	0.39734616	1
gene7336	12.3976779	23.23015667	1.873750621	0.905928957	0.39734788	1
gene15071	12.3976779	23.23015667	1.873750621	0.905928957	0.39734788	1
gene17108	12.3976779	23.23015667	1.873750621	0.905928957	0.39734788	1
gene24910	12.3976779	23.23015667	1.873750621	0.905928957	0.39734788	1
gene43556	12.3976779	23.23015667	1.873750621	0.905928957	0.39734788	1
gene50846	12.3976779	23.23015667	1.873750621	0.905928957	0.39734788	1
gene59385	12.3976779	23.23015667	1.873750621	0.905928957	0.39734788	1
gene64036	12.3976779	23.23015667	1.873750621	0.905928957	0.39734788	1

gene22843	245.8872784	338.7731181	1.37775781	0.462322305	0.39735533	1
gene64141	212.826804	294.2486512	1.382573274	0.467355943	0.39738868	1
gene4935	34.09361423	54.2036989	1.589849012	0.668889759	0.39745009	1
gene14311	34.09361423	54.2036989	1.589849012	0.668889759	0.39745009	1
gene21850	34.09361423	54.2036989	1.589849012	0.668889759	0.39745009	1
gene33497	34.09361423	54.2036989	1.589849012	0.668889759	0.39745009	1
gene58829	34.09361423	54.2036989	1.589849012	0.668889759	0.39745009	1
gene29625	269.6494943	194.5525621	0.721501676	-0.47092535	0.39754869	1
gene10581	1903.043558	1356.060396	0.712574544	-0.48888715	0.3975692	1
gene2626	23.76221598	39.68485098	1.670082076	0.739919005	0.39757337	1
gene4676	23.76221598	39.68485098	1.670082076	0.739919005	0.39757337	1
gene19243	23.76221598	39.68485098	1.670082076	0.739919005	0.39757337	1
gene22015	23.76221598	39.68485098	1.670082076	0.739919005	0.39757337	1
gene29359	23.76221598	39.68485098	1.670082076	0.739919005	0.39757337	1
gene11554	359.5326591	261.3392626	0.726886017	-0.46019894	0.39757768	1
gene71957	523.8018913	382.3296619	0.729912717	-0.45420414	0.39767004	1
gene48446	274.8151935	198.4242549	0.722027965	-0.46987338	0.39768412	1
gene19258	601.2873782	825.638485	1.373117938	0.457455545	0.3977064	1
gene1468	113.6453808	161.6431735	1.422347063	0.508273536	0.39779665	1
gene42667	3972.422627	5959.503109	1.500218801	0.585172927	0.39790835	1
gene20916	105.3802622	71.6263164	0.679693853	-0.55704302	0.39795126	1
gene47874	105.3802622	71.6263164	0.679693853	-0.55704302	0.39795126	1
gene55464	105.3802622	71.6263164	0.679693853	-0.55704302	0.39795126	1
gene21261	260.3512359	358.131582	1.375570893	0.460030494	0.39803138	1
gene31788	537.232709	736.5895511	1.371080984	0.455313787	0.39807832	1
gene20254	25.82849563	42.58862056	1.648900547	0.721504386	0.39813663	1
gene20898	25.82849563	42.58862056	1.648900547	0.721504386	0.39813663	1
gene70414	25.82849563	42.58862056	1.648900547	0.721504386	0.39813663	1
gene44716	2365.890199	1671.603357	0.706543084	-0.50115056	0.39817594	1
gene44954	835.8101185	608.8236894	0.728423449	-0.45715073	0.39818253	1
gene2340	29.96105493	48.39615973	1.61530226	0.691804151	0.39823002	1
gene56779	57.8558302	36.7810814	0.635736818	-0.65349845	0.3982541	1
gene56962	443.216985	323.286347	0.72940875	-0.45520059	0.39828801	1
gene11917	2582.849563	1817.75976	0.703780733	-0.50680208	0.39831176	1
gene4802	27.89477528	45.49239015	1.630857022	0.705630306	0.39832477	1
gene30504	27.89477528	45.49239015	1.630857022	0.705630306	0.39832477	1
gene47647	27.89477528	45.49239015	1.630857022	0.705630306	0.39832477	1
gene54479	126.0430587	178.0978678	1.412992272	0.498753575	0.39835519	1
gene64375	2070.412209	2961.844976	1.430558109	0.516578101	0.39836404	1
gene52156	414.2890699	301.9920367	0.728940391	-0.45612725	0.39846523	1
gene65874	330.604744	240.0449523	0.726078366	-0.46180283	0.39849084	1
gene57618	77.48548688	113.2470138	1.461525485	0.547474986	0.39853986	1
gene31159	179.7663296	249.7241842	1.389159943	0.474212716	0.39854276	1
gene39709	179.7663296	249.7241842	1.389159943	0.474212716	0.39854276	1
gene45822	234.5227403	168.4186359	0.718133498	-0.47767603	0.39854632	1
gene7459	208.6942447	149.060172	0.714251475	-0.48549599	0.39858197	1
gene7184	91.94944443	132.6054777	1.442156377	0.528227609	0.39861279	1
gene29974	335.7704432	243.916645	0.726438702	-0.46108703	0.39871483	1

gene6441	305.8093882	221.6544116	0.724812318	-0.46432062	0.3987155	1
gene50333	581.6577215	424.9182824	0.730529772	-0.45298503	0.39871992	1
gene18521	115.7116604	79.36970196	0.685926567	-0.54387396	0.39878373	1
gene38965	115.7116604	79.36970196	0.685926567	-0.54387396	0.39878373	1
gene5889	165.302372	230.3657203	1.393602025	0.478818625	0.39878732	1
gene46774	1646.824881	2327.855283	1.413541482	0.499314221	0.39897925	1
gene6522	17.56337703	8.711308752	0.495992812	-1.01160888	0.39902598	1
gene17728	17.56337703	8.711308752	0.495992812	-1.01160888	0.39902598	1
gene22402	17.56337703	8.711308752	0.495992812	-1.01160888	0.39902598	1
gene24262	17.56337703	8.711308752	0.495992812	-1.01160888	0.39902598	1
gene44200	17.56337703	8.711308752	0.495992812	-1.01160888	0.39902598	1
gene46370	17.56337703	8.711308752	0.495992812	-1.01160888	0.39902598	1
gene49055	17.56337703	8.711308752	0.495992812	-1.01160888	0.39902598	1
gene3601	2735.754257	3979.132253	1.454491844	0.540515206	0.39903808	1
gene10678	49.5907116	75.49800918	1.52242238	0.606368675	0.39904338	1
gene14179	815.147322	594.3048415	0.729076604	-0.45585769	0.39904577	1
gene70170	964.9525966	701.7443161	0.727231906	-0.4595126	0.39908554	1
gene14665	557.8955055	764.6593238	1.37061388	0.454822203	0.39912451	1
gene12162	511.4042134	373.6183531	0.730573475	-0.45289872	0.39916642	1
gene42198	162.2029525	114.214937	0.704148323	-0.50604874	0.39917644	1
gene63032	157.0372534	110.3432442	0.702656483	-0.50910854	0.39924531	1
gene30424	150.8384145	211.0072564	1.398896012	0.484288723	0.39926035	1
gene59900	90.91630461	60.97916126	0.670717552	-0.57622274	0.3992893	1
gene70422	90.91630461	60.97916126	0.670717552	-0.57622274	0.3992893	1
gene15157	177.7000499	246.8204146	1.388972118	0.474017639	0.39939253	1
gene20217	177.7000499	246.8204146	1.388972118	0.474017639	0.39939253	1
gene42195	530.0007303	725.942396	1.369700747	0.453860726	0.39940352	1
gene39377	1905.109837	2711.152868	1.423095307	0.509032284	0.3994045	1
gene49075	1239.76779	1731.614595	1.396724942	0.482047938	0.39944161	1
gene71394	631.2484331	866.2912592	1.372345995	0.456644259	0.39952423	1
gene7038	2152.030256	1528.350724	0.710190166	-0.49372271	0.39969359	1
gene32030	163.2360924	227.4619507	1.393453785	0.478665155	0.39973438	1
gene64086	440.1175655	321.3505006	0.730146956	-0.45374123	0.39990659	1
gene31846	287.2128714	393.9447402	1.371612415	0.455872868	0.39991236	1
gene32661	136.3744569	191.6487925	1.405312966	0.490891457	0.39995797	1
gene27735	569.2600436	416.2069737	0.731136812	-0.4517867	0.39996621	1
gene39327	58.88897003	88.08101071	1.495713215	0.580833583	0.39998635	1
gene390	2.06627965	6.775462362	3.279063588	1.713283879	0.39999304	1
gene2217	2.06627965	6.775462362	3.279063588	1.713283879	0.39999304	1
gene2550	2.06627965	6.775462362	3.279063588	1.713283879	0.39999304	1
gene3064	2.06627965	6.775462362	3.279063588	1.713283879	0.39999304	1
gene5167	2.06627965	6.775462362	3.279063588	1.713283879	0.39999304	1
gene5580	2.06627965	6.775462362	3.279063588	1.713283879	0.39999304	1
gene5625	2.06627965	6.775462362	3.279063588	1.713283879	0.39999304	1
gene8634	2.06627965	6.775462362	3.279063588	1.713283879	0.39999304	1
gene9414	2.06627965	6.775462362	3.279063588	1.713283879	0.39999304	1
gene10314	2.06627965	6.775462362	3.279063588	1.713283879	0.39999304	1
gene11000	2.06627965	6.775462362	3.279063588	1.713283879	0.39999304	1

gene12243	2.06627965	6.775462362	3.279063588	1.713283879	0.39999304	1
gene13470	2.06627965	6.775462362	3.279063588	1.713283879	0.39999304	1
gene15732	2.06627965	6.775462362	3.279063588	1.713283879	0.39999304	1
gene16347	2.06627965	6.775462362	3.279063588	1.713283879	0.39999304	1
gene16538	2.06627965	6.775462362	3.279063588	1.713283879	0.39999304	1
gene18467	2.06627965	6.775462362	3.279063588	1.713283879	0.39999304	1
gene18511	2.06627965	6.775462362	3.279063588	1.713283879	0.39999304	1
gene18556	2.06627965	6.775462362	3.279063588	1.713283879	0.39999304	1
gene20595	2.06627965	6.775462362	3.279063588	1.713283879	0.39999304	1
gene23915	2.06627965	6.775462362	3.279063588	1.713283879	0.39999304	1
gene27325	2.06627965	6.775462362	3.279063588	1.713283879	0.39999304	1
gene28809	2.06627965	6.775462362	3.279063588	1.713283879	0.39999304	1
gene30011	2.06627965	6.775462362	3.279063588	1.713283879	0.39999304	1
gene32145	2.06627965	6.775462362	3.279063588	1.713283879	0.39999304	1
gene32652	2.06627965	6.775462362	3.279063588	1.713283879	0.39999304	1
gene34319	2.06627965	6.775462362	3.279063588	1.713283879	0.39999304	1
gene35361	2.06627965	6.775462362	3.279063588	1.713283879	0.39999304	1
gene36980	2.06627965	6.775462362	3.279063588	1.713283879	0.39999304	1
gene37162	2.06627965	6.775462362	3.279063588	1.713283879	0.39999304	1
gene38668	2.06627965	6.775462362	3.279063588	1.713283879	0.39999304	1
gene40112	2.06627965	6.775462362	3.279063588	1.713283879	0.39999304	1
gene42068	2.06627965	6.775462362	3.279063588	1.713283879	0.39999304	1
gene42990	2.06627965	6.775462362	3.279063588	1.713283879	0.39999304	1
gene43832	2.06627965	6.775462362	3.279063588	1.713283879	0.39999304	1
gene44005	2.06627965	6.775462362	3.279063588	1.713283879	0.39999304	1
gene45610	2.06627965	6.775462362	3.279063588	1.713283879	0.39999304	1
gene45805	2.06627965	6.775462362	3.279063588	1.713283879	0.39999304	1
gene46224	2.06627965	6.775462362	3.279063588	1.713283879	0.39999304	1
gene46805	2.06627965	6.775462362	3.279063588	1.713283879	0.39999304	1
gene46839	2.06627965	6.775462362	3.279063588	1.713283879	0.39999304	1
gene49221	2.06627965	6.775462362	3.279063588	1.713283879	0.39999304	1
gene49845	2.06627965	6.775462362	3.279063588	1.713283879	0.39999304	1
gene50188	2.06627965	6.775462362	3.279063588	1.713283879	0.39999304	1
gene53847	2.06627965	6.775462362	3.279063588	1.713283879	0.39999304	1
gene54329	2.06627965	6.775462362	3.279063588	1.713283879	0.39999304	1
gene54767	2.06627965	6.775462362	3.279063588	1.713283879	0.39999304	1
gene55145	2.06627965	6.775462362	3.279063588	1.713283879	0.39999304	1
gene55287	2.06627965	6.775462362	3.279063588	1.713283879	0.39999304	1
gene56757	2.06627965	6.775462362	3.279063588	1.713283879	0.39999304	1
gene56769	2.06627965	6.775462362	3.279063588	1.713283879	0.39999304	1
gene56789	2.06627965	6.775462362	3.279063588	1.713283879	0.39999304	1
gene56857	2.06627965	6.775462362	3.279063588	1.713283879	0.39999304	1
gene57215	2.06627965	6.775462362	3.279063588	1.713283879	0.39999304	1
gene63733	2.06627965	6.775462362	3.279063588	1.713283879	0.39999304	1
gene63754	2.06627965	6.775462362	3.279063588	1.713283879	0.39999304	1
gene65775	2.06627965	6.775462362	3.279063588	1.713283879	0.39999304	1
gene68100	2.06627965	6.775462362	3.279063588	1.713283879	0.39999304	1
gene68811	2.06627965	6.775462362	3.279063588	1.713283879	0.39999304	1

gene71192	2.06627965	6.775462362	3.279063588	1.713283879	0.39999304	1
gene72285	2.06627965	6.775462362	3.279063588	1.713283879	0.39999304	1
gene72813	2.06627965	6.775462362	3.279063588	1.713283879	0.39999304	1
gene73150	2.06627965	6.775462362	3.279063588	1.713283879	0.39999304	1
gene73615	2.06627965	6.775462362	3.279063588	1.713283879	0.39999304	1
gene73736	2.06627965	6.775462362	3.279063588	1.713283879	0.39999304	1
gene44998	156.0041136	217.7827188	1.396006258	0.481305409	0.39999492	1
gene68160	377.0960362	515.9030627	1.368094632	0.452168026	0.3999964	1
gene55417	197.3297066	272.9543409	1.383239988	0.468051481	0.40005051	1
gene38015	1493.920187	1075.362669	0.719826051	-0.47427978	0.40006392	1
gene18230	235.5558801	324.2542702	1.37654925	0.461056228	0.40009365	1
gene35155	235.5558801	324.2542702	1.37654925	0.461056228	0.40009365	1
gene66423	526.9013108	385.2334315	0.731130144	-0.45179986	0.40014704	1
gene44710	488.6751373	357.1636588	0.730881585	-0.45229041	0.4001509	1
gene5473	96.08200373	64.85085404	0.674953181	-0.56714066	0.40016809	1
gene39262	96.08200373	64.85085404	0.674953181	-0.56714066	0.40016809	1
gene59538	96.08200373	64.85085404	0.674953181	-0.56714066	0.40016809	1
gene36398	211.7936641	292.3128048	1.380177287	0.464853596	0.40018279	1
gene42126	386.3942946	528.4860643	1.367737753	0.451791638	0.40026095	1
gene34883	53.7232709	33.87731181	0.630589151	-0.66522774	0.4002976	1
gene68679	53.7232709	33.87731181	0.630589151	-0.66522774	0.4002976	1
gene1742	214.8930836	153.8997879	0.716169108	-0.48162781	0.40030517	1
gene8144	209.7273845	150.0280952	0.715348144	-0.48328256	0.40037125	1
gene58962	1442.263196	1039.549511	0.720776564	-0.47237599	0.40039042	1
gene48911	3502.344007	2429.487219	0.69367464	-0.52766895	0.40042203	1
gene26499	762.4571909	1049.228743	1.376114955	0.460600992	0.40043088	1
gene54459	199.3959862	142.2847096	0.713578605	-0.48685574	0.40053936	1
gene5667	340.9361423	466.5389798	1.368405757	0.452496079	0.40055106	1
gene58069	322.3396254	234.2374131	0.726678927	-0.46061002	0.40056398	1
gene66587	681.8722846	498.4804452	0.731046644	-0.45196464	0.40060443	1
gene21829	194.2302871	138.4130168	0.712623242	-0.48878856	0.40064008	1
gene29421	189.064588	134.5413241	0.711615673	-0.49082981	0.40075071	1
gene1357	287.2128714	208.1034868	0.724561841	-0.46481927	0.40075728	1
gene51075	455.6146629	332.965579	0.730805231	-0.45244113	0.40076851	1
gene37602	202.4954057	279.7298033	1.381413086	0.466144795	0.40081226	1
gene44366	977.3502745	711.4235481	0.727910522	-0.45816698	0.40082284	1
gene70451	178.7331897	126.7979385	0.709425813	-0.49527627	0.40099683	1
gene12634	24.7953558	13.55092472	0.546510598	-0.87167862	0.4009972	1
gene17169	24.7953558	13.55092472	0.546510598	-0.87167862	0.4009972	1
gene13807	153.9378339	214.8789492	1.395881335	0.481176303	0.40102306	1
gene9894	387.4274344	282.6335728	0.729513575	-0.45499327	0.40103789	1
gene61133	2885.559531	2023.9274	0.701398595	-0.51169356	0.40108579	1
gene62489	173.5674906	122.9262457	0.708233122	-0.49770378	0.4011291	1
gene70842	134.3081773	188.745023	1.405312966	0.490891457	0.40113297	1
gene71033	1603.433009	2260.100659	1.409538563	0.495222949	0.40114334	1
gene51732	4943.574063	3353.853869	0.678426949	-0.55973462	0.40117441	1
gene4758	66.12094881	97.76024266	1.47850635	0.564140439	0.40118157	1
gene56917	168.4017915	119.0545529	0.706967259	-0.50028469	0.40126478	1

gene46913	888.5002496	1226.358688	1.380256998	0.464936916	0.40131043	1
gene6793	163.2360924	115.1828602	0.705621278	-0.50303403	0.40140123	1
gene11402	146.7058552	205.1997173	1.398715253	0.484102292	0.40140124	1
gene49168	4556.146629	3109.937224	0.682580584	-0.55092872	0.40141707	1
gene3661	778.9874281	569.1388384	0.730613638	-0.45281941	0.40142291	1
gene3953	56.82269038	85.17724113	1.499000497	0.584000862	0.40152107	1
gene13959	56.82269038	85.17724113	1.499000497	0.584000862	0.40152107	1
gene32568	56.82269038	85.17724113	1.499000497	0.584000862	0.40152107	1
gene48360	56.82269038	85.17724113	1.499000497	0.584000862	0.40152107	1
gene28740	376.0628963	513.9672164	1.366705467	0.450702367	0.40154996	1
gene635	4969.402559	7602.068771	1.529775196	0.613319661	0.40155204	1
gene14771	45.4581523	69.69047001	1.53306869	0.616422339	0.40160617	1
gene36978	45.4581523	69.69047001	1.53306869	0.616422339	0.40160617	1
gene57143	111.5791011	76.46593238	0.68530694	-0.5451778	0.40161436	1
gene64112	111.5791011	76.46593238	0.68530694	-0.5451778	0.40161436	1
gene39822	87.81688513	126.7979385	1.443890185	0.529961022	0.40164552	1
gene43428	87.81688513	126.7979385	1.443890185	0.529961022	0.40164552	1
gene55524	318.2070661	231.3336435	0.726990907	-0.45999078	0.40164823	1
gene54182	1248.032909	904.0402638	0.724372136	-0.46519704	0.40165752	1
gene17616	915.361885	1264.107692	1.380992275	0.46570525	0.40168764	1
gene8095	185.9651685	257.4675698	1.384493515	0.469358296	0.40169625	1
gene50760	585.7902808	428.7899752	0.731985472	-0.45011308	0.40173073	1
gene9095	257.2518164	185.8412534	0.722409878	-0.46911047	0.40175446	1
gene61727	80.58490636	117.1187066	1.453357854	0.539389975	0.40180721	1
gene55088	139.4738764	195.5204853	1.401843058	0.487324842	0.40184179	1
gene54391	107.4465418	152.9318648	1.423329799	0.509269987	0.40187408	1
gene70483	107.4465418	152.9318648	1.423329799	0.509269987	0.40187408	1
gene10608	121.9104994	84.20931793	0.690747051	-0.5337706	0.40195045	1
gene40690	137.4075967	95.82439627	0.697373352	-0.51999686	0.40195653	1
gene41343	221.0919226	158.7394039	0.71797921	-0.47798603	0.40195869	1
gene49804	328.5384644	239.0770291	0.727698748	-0.45858677	0.40198146	1
gene8692	127.0761985	88.08101071	0.693135392	-0.52879091	0.40200321	1
gene53932	72.31978776	47.42823654	0.655812718	-0.60864422	0.40202291	1
gene12879	283.0803121	205.1997173	0.724881627	-0.46418267	0.40203539	1
gene59339	283.0803121	205.1997173	0.724881627	-0.46418267	0.40203539	1
gene73716	447.3495443	327.1580398	0.7313253	-0.45141482	0.40208738	1
gene6850	119.8442197	169.3865591	1.413389477	0.499159073	0.40215869	1
gene8864	86.78374531	58.07539168	0.669196651	-0.57949787	0.40222016	1
gene11366	49.5907116	30.97354223	0.62458354	-0.67903354	0.40224552	1
gene47644	49.5907116	30.97354223	0.62458354	-0.67903354	0.40224552	1
gene49481	49.5907116	30.97354223	0.62458354	-0.67903354	0.40224552	1
gene56511	865.7711734	632.0538461	0.730047229	-0.4539383	0.40225223	1
gene11960	205.5948252	147.1243256	0.715603252	-0.48276815	0.40226405	1
gene26076	212.826804	293.280728	1.378025336	0.462602413	0.40231991	1
gene67961	415.3222097	567.2029921	1.365693861	0.44963412	0.40233248	1
gene15905	1427.799238	1030.838202	0.721976994	-0.46997523	0.40236746	1
gene55382	100.214563	143.2526328	1.429459237	0.51546948	0.40238565	1
gene14334	1294.524201	1807.112604	1.395966644	0.48126447	0.40239056	1

gene19018	884.3676903	1219.583225	1.379045434	0.463669989	0.40239876	1
gene13047	2985.774094	2092.649947	0.700873502	-0.51277401	0.40244409	1
gene8507	1898.910998	1357.996242	0.715144756	-0.4836928	0.40255186	1
gene817	157.0372534	218.750642	1.392985659	0.478180406	0.40273839	1
gene42669	291.3454307	398.7843562	1.368768184	0.452878131	0.40274613	1
gene2072	43.39187265	66.78670043	1.539152296	0.622135991	0.40282284	1
gene48207	92.98258426	133.5734009	1.436542143	0.522600317	0.40285503	1
gene29209	265.516935	363.9391212	1.370681388	0.454893259	0.40286523	1
gene14365	489.7082771	668.8349275	1.365782362	0.449727608	0.40287194	1
gene10610	5344.432315	3609.385593	0.675354346	-0.56628344	0.4029393	1
gene51874	1384.407366	1000.832583	0.722932143	-0.46806786	0.40295593	1
gene3716	14.46395755	26.13392626	1.806830956	0.853461537	0.40297542	1
gene6604	14.46395755	26.13392626	1.806830956	0.853461537	0.40297542	1
gene26539	14.46395755	26.13392626	1.806830956	0.853461537	0.40297542	1
gene49096	14.46395755	26.13392626	1.806830956	0.853461537	0.40297542	1
gene52216	14.46395755	26.13392626	1.806830956	0.853461537	0.40297542	1
gene68280	14.46395755	26.13392626	1.806830956	0.853461537	0.40297542	1
gene4497	448.3826841	328.125963	0.731798918	-0.45048081	0.40302917	1
gene63083	54.75641073	82.27347154	1.502535876	0.587399438	0.40307204	1
gene132	1.033139825	4.839615973	4.684376554	2.227857052	0.40309621	1
gene452	1.033139825	4.839615973	4.684376554	2.227857052	0.40309621	1
gene1387	1.033139825	4.839615973	4.684376554	2.227857052	0.40309621	1
gene3939	1.033139825	4.839615973	4.684376554	2.227857052	0.40309621	1
gene4222	1.033139825	4.839615973	4.684376554	2.227857052	0.40309621	1
gene4368	1.033139825	4.839615973	4.684376554	2.227857052	0.40309621	1
gene5135	1.033139825	4.839615973	4.684376554	2.227857052	0.40309621	1
gene5430	1.033139825	4.839615973	4.684376554	2.227857052	0.40309621	1
gene6275	1.033139825	4.839615973	4.684376554	2.227857052	0.40309621	1
gene7894	1.033139825	4.839615973	4.684376554	2.227857052	0.40309621	1
gene9688	1.033139825	4.839615973	4.684376554	2.227857052	0.40309621	1
gene10399	1.033139825	4.839615973	4.684376554	2.227857052	0.40309621	1
gene10685	1.033139825	4.839615973	4.684376554	2.227857052	0.40309621	1
gene10709	1.033139825	4.839615973	4.684376554	2.227857052	0.40309621	1
gene12048	1.033139825	4.839615973	4.684376554	2.227857052	0.40309621	1
gene12675	1.033139825	4.839615973	4.684376554	2.227857052	0.40309621	1
gene12861	1.033139825	4.839615973	4.684376554	2.227857052	0.40309621	1
gene13063	1.033139825	4.839615973	4.684376554	2.227857052	0.40309621	1
gene15454	1.033139825	4.839615973	4.684376554	2.227857052	0.40309621	1
gene15998	1.033139825	4.839615973	4.684376554	2.227857052	0.40309621	1
gene17068	1.033139825	4.839615973	4.684376554	2.227857052	0.40309621	1
gene17731	1.033139825	4.839615973	4.684376554	2.227857052	0.40309621	1
gene18047	1.033139825	4.839615973	4.684376554	2.227857052	0.40309621	1
gene18741	1.033139825	4.839615973	4.684376554	2.227857052	0.40309621	1
gene20120	1.033139825	4.839615973	4.684376554	2.227857052	0.40309621	1
gene21057	1.033139825	4.839615973	4.684376554	2.227857052	0.40309621	1
gene21227	1.033139825	4.839615973	4.684376554	2.227857052	0.40309621	1
gene21455	1.033139825	4.839615973	4.684376554	2.227857052	0.40309621	1
gene21879	1.033139825	4.839615973	4.684376554	2.227857052	0.40309621	1

gene21964	1.033139825	4.839615973	4.684376554	2.227857052	0.40309621	1
gene22230	1.033139825	4.839615973	4.684376554	2.227857052	0.40309621	1
gene23175	1.033139825	4.839615973	4.684376554	2.227857052	0.40309621	1
gene23351	1.033139825	4.839615973	4.684376554	2.227857052	0.40309621	1
gene23511	1.033139825	4.839615973	4.684376554	2.227857052	0.40309621	1
gene24546	1.033139825	4.839615973	4.684376554	2.227857052	0.40309621	1
gene24551	1.033139825	4.839615973	4.684376554	2.227857052	0.40309621	1
gene25353	1.033139825	4.839615973	4.684376554	2.227857052	0.40309621	1
gene26407	1.033139825	4.839615973	4.684376554	2.227857052	0.40309621	1
gene27614	1.033139825	4.839615973	4.684376554	2.227857052	0.40309621	1
gene28274	1.033139825	4.839615973	4.684376554	2.227857052	0.40309621	1
gene28624	1.033139825	4.839615973	4.684376554	2.227857052	0.40309621	1
gene29143	1.033139825	4.839615973	4.684376554	2.227857052	0.40309621	1
gene29256	1.033139825	4.839615973	4.684376554	2.227857052	0.40309621	1
gene30286	1.033139825	4.839615973	4.684376554	2.227857052	0.40309621	1
gene30953	1.033139825	4.839615973	4.684376554	2.227857052	0.40309621	1
gene32285	1.033139825	4.839615973	4.684376554	2.227857052	0.40309621	1
gene32405	1.033139825	4.839615973	4.684376554	2.227857052	0.40309621	1
gene32567	1.033139825	4.839615973	4.684376554	2.227857052	0.40309621	1
gene32831	1.033139825	4.839615973	4.684376554	2.227857052	0.40309621	1
gene33569	1.033139825	4.839615973	4.684376554	2.227857052	0.40309621	1
gene35248	1.033139825	4.839615973	4.684376554	2.227857052	0.40309621	1
gene36253	1.033139825	4.839615973	4.684376554	2.227857052	0.40309621	1
gene36714	1.033139825	4.839615973	4.684376554	2.227857052	0.40309621	1
gene37133	1.033139825	4.839615973	4.684376554	2.227857052	0.40309621	1
gene37776	1.033139825	4.839615973	4.684376554	2.227857052	0.40309621	1
gene37871	1.033139825	4.839615973	4.684376554	2.227857052	0.40309621	1
gene38051	1.033139825	4.839615973	4.684376554	2.227857052	0.40309621	1
gene39670	1.033139825	4.839615973	4.684376554	2.227857052	0.40309621	1
gene40784	1.033139825	4.839615973	4.684376554	2.227857052	0.40309621	1
gene40807	1.033139825	4.839615973	4.684376554	2.227857052	0.40309621	1
gene42010	1.033139825	4.839615973	4.684376554	2.227857052	0.40309621	1
gene42047	1.033139825	4.839615973	4.684376554	2.227857052	0.40309621	1
gene42423	1.033139825	4.839615973	4.684376554	2.227857052	0.40309621	1
gene42557	1.033139825	4.839615973	4.684376554	2.227857052	0.40309621	1
gene42869	1.033139825	4.839615973	4.684376554	2.227857052	0.40309621	1
gene43053	1.033139825	4.839615973	4.684376554	2.227857052	0.40309621	1
gene44139	1.033139825	4.839615973	4.684376554	2.227857052	0.40309621	1
gene44184	1.033139825	4.839615973	4.684376554	2.227857052	0.40309621	1
gene45020	1.033139825	4.839615973	4.684376554	2.227857052	0.40309621	1
gene47404	1.033139825	4.839615973	4.684376554	2.227857052	0.40309621	1
gene49700	1.033139825	4.839615973	4.684376554	2.227857052	0.40309621	1
gene49848	1.033139825	4.839615973	4.684376554	2.227857052	0.40309621	1
gene50862	1.033139825	4.839615973	4.684376554	2.227857052	0.40309621	1
gene51565	1.033139825	4.839615973	4.684376554	2.227857052	0.40309621	1
gene52578	1.033139825	4.839615973	4.684376554	2.227857052	0.40309621	1
gene53413	1.033139825	4.839615973	4.684376554	2.227857052	0.40309621	1
gene53739	1.033139825	4.839615973	4.684376554	2.227857052	0.40309621	1

gene53898	1.033139825	4.839615973	4.684376554	2.227857052	0.40309621	1
gene53995	1.033139825	4.839615973	4.684376554	2.227857052	0.40309621	1
gene54286	1.033139825	4.839615973	4.684376554	2.227857052	0.40309621	1
gene54336	1.033139825	4.839615973	4.684376554	2.227857052	0.40309621	1
gene55472	1.033139825	4.839615973	4.684376554	2.227857052	0.40309621	1
gene55734	1.033139825	4.839615973	4.684376554	2.227857052	0.40309621	1
gene55861	1.033139825	4.839615973	4.684376554	2.227857052	0.40309621	1
gene57556	1.033139825	4.839615973	4.684376554	2.227857052	0.40309621	1
gene58885	1.033139825	4.839615973	4.684376554	2.227857052	0.40309621	1
gene59125	1.033139825	4.839615973	4.684376554	2.227857052	0.40309621	1
gene59704	1.033139825	4.839615973	4.684376554	2.227857052	0.40309621	1
gene62101	1.033139825	4.839615973	4.684376554	2.227857052	0.40309621	1
gene63204	1.033139825	4.839615973	4.684376554	2.227857052	0.40309621	1
gene63209	1.033139825	4.839615973	4.684376554	2.227857052	0.40309621	1
gene64207	1.033139825	4.839615973	4.684376554	2.227857052	0.40309621	1
gene65045	1.033139825	4.839615973	4.684376554	2.227857052	0.40309621	1
gene66775	1.033139825	4.839615973	4.684376554	2.227857052	0.40309621	1
gene67526	1.033139825	4.839615973	4.684376554	2.227857052	0.40309621	1
gene68553	1.033139825	4.839615973	4.684376554	2.227857052	0.40309621	1
gene68845	1.033139825	4.839615973	4.684376554	2.227857052	0.40309621	1
gene69133	1.033139825	4.839615973	4.684376554	2.227857052	0.40309621	1
gene69184	1.033139825	4.839615973	4.684376554	2.227857052	0.40309621	1
gene70004	1.033139825	4.839615973	4.684376554	2.227857052	0.40309621	1
gene70291	1.033139825	4.839615973	4.684376554	2.227857052	0.40309621	1
gene70540	1.033139825	4.839615973	4.684376554	2.227857052	0.40309621	1
gene70719	1.033139825	4.839615973	4.684376554	2.227857052	0.40309621	1
gene71460	1.033139825	4.839615973	4.684376554	2.227857052	0.40309621	1
gene71825	1.033139825	4.839615973	4.684376554	2.227857052	0.40309621	1
gene73016	1.033139825	4.839615973	4.684376554	2.227857052	0.40309621	1
gene60844	91.94944443	61.94708446	0.673708089	-0.56980447	0.40314494	1
gene28431	587.8565605	430.7258216	0.732705647	-0.44869436	0.40322432	1
gene10118	258.2849563	186.8091766	0.72326774	-0.46739829	0.40325277	1
gene23467	258.2849563	186.8091766	0.72326774	-0.46739829	0.40325277	1
gene10985	268.6163545	194.5525621	0.724276683	-0.46538717	0.40327722	1
gene29786	1093.061935	1515.767723	1.386717142	0.471673542	0.40339803	1
gene10755	181.8326092	251.6600306	1.384020345	0.468865151	0.40341764	1
gene9451	78.51862671	114.214937	1.454622193	0.540644493	0.40343501	1
gene18255	14.46395755	6.775462362	0.468437655	-1.09407104	0.40350009	1
gene21970	14.46395755	6.775462362	0.468437655	-1.09407104	0.40350009	1
gene22143	14.46395755	6.775462362	0.468437655	-1.09407104	0.40350009	1
gene26559	14.46395755	6.775462362	0.468437655	-1.09407104	0.40350009	1
gene42304	14.46395755	6.775462362	0.468437655	-1.09407104	0.40350009	1
gene44020	14.46395755	6.775462362	0.468437655	-1.09407104	0.40350009	1
gene44740	14.46395755	6.775462362	0.468437655	-1.09407104	0.40350009	1
gene62374	14.46395755	6.775462362	0.468437655	-1.09407104	0.40350009	1
gene63974	14.46395755	6.775462362	0.468437655	-1.09407104	0.40350009	1
gene68569	14.46395755	6.775462362	0.468437655	-1.09407104	0.40350009	1
gene70445	14.46395755	6.775462362	0.468437655	-1.09407104	0.40350009	1

gene21027	823.4124406	602.0482271	0.731162413	-0.45173619	0.40353531	1
gene4552	294.4448501	213.911026	0.726489276	-0.46098659	0.40358963	1
gene68302	164.2692322	116.1507834	0.707075706	-0.5000634	0.40360565	1
gene43996	375.0297565	273.9222641	0.730401413	-0.45323854	0.40363109	1
gene8013	11.36453808	4.839615973	0.425852414	-1.23157457	0.40371728	1
gene8635	11.36453808	4.839615973	0.425852414	-1.23157457	0.40371728	1
gene17699	11.36453808	4.839615973	0.425852414	-1.23157457	0.40371728	1
gene18175	11.36453808	4.839615973	0.425852414	-1.23157457	0.40371728	1
gene19841	11.36453808	4.839615973	0.425852414	-1.23157457	0.40371728	1
gene28427	11.36453808	4.839615973	0.425852414	-1.23157457	0.40371728	1
gene39942	11.36453808	4.839615973	0.425852414	-1.23157457	0.40371728	1
gene40425	11.36453808	4.839615973	0.425852414	-1.23157457	0.40371728	1
gene47033	11.36453808	4.839615973	0.425852414	-1.23157457	0.40371728	1
gene47134	11.36453808	4.839615973	0.425852414	-1.23157457	0.40371728	1
gene49771	11.36453808	4.839615973	0.425852414	-1.23157457	0.40371728	1
gene59477	11.36453808	4.839615973	0.425852414	-1.23157457	0.40371728	1
gene61304	11.36453808	4.839615973	0.425852414	-1.23157457	0.40371728	1
gene63956	11.36453808	4.839615973	0.425852414	-1.23157457	0.40371728	1
gene64263	11.36453808	4.839615973	0.425852414	-1.23157457	0.40371728	1
gene70390	11.36453808	4.839615973	0.425852414	-1.23157457	0.40371728	1
gene71796	11.36453808	4.839615973	0.425852414	-1.23157457	0.40371728	1
gene74228	11.36453808	4.839615973	0.425852414	-1.23157457	0.40371728	1
gene56337	154.9709738	215.8468724	1.392821295	0.478010166	0.40379674	1
gene40454	981.4828338	1356.060396	1.381644537	0.466386494	0.40383655	1
gene42239	77.48548688	51.29992932	0.662058553	-0.59496928	0.40388771	1
gene47803	77.48548688	51.29992932	0.662058553	-0.59496928	0.40388771	1
gene61422	77.48548688	51.29992932	0.662058553	-0.59496928	0.40388771	1
gene60540	651.9112296	892.4251855	1.368936666	0.453055702	0.40392062	1
gene27644	526.9013108	386.2013547	0.732967155	-0.44817954	0.40393857	1
gene39455	41.325593	63.88293085	1.545844263	0.628394981	0.40397876	1
gene3502	45.4581523	28.06977264	0.617486	-0.69552167	0.40400922	1
gene6532	45.4581523	28.06977264	0.617486	-0.69552167	0.40400922	1
gene45141	45.4581523	28.06977264	0.617486	-0.69552167	0.40400922	1
gene47392	45.4581523	28.06977264	0.617486	-0.69552167	0.40400922	1
gene58329	45.4581523	28.06977264	0.617486	-0.69552167	0.40400922	1
gene60536	45.4581523	28.06977264	0.617486	-0.69552167	0.40400922	1
gene20695	110.5459613	156.8035575	1.418446732	0.504311973	0.40414412	1
gene73301	102.2808427	69.69047001	0.681363862	-0.55350266	0.40427289	1
gene48632	58.88897003	37.74900459	0.641019949	-0.64155884	0.40439459	1
gene56466	58.88897003	37.74900459	0.641019949	-0.64155884	0.40439459	1
gene43916	1825.558071	2582.419083	1.414591584	0.500385583	0.40442525	1
gene9367	138.4407366	96.79231946	0.69916068	-0.51630404	0.40453174	1
gene62395	370.8971972	271.0184945	0.730710549	-0.45262806	0.40454317	1
gene48931	697.3693819	511.0634468	0.732844687	-0.44842062	0.40454722	1
gene62837	480.4100187	655.2840028	1.364009861	0.447854074	0.40468223	1
gene41002	425.6536079	311.6712687	0.732218083	-0.44965469	0.40470124	1
gene865	376.0628963	274.8901873	0.730968649	-0.45211856	0.40472364	1
gene22714	499.0065355	365.8749676	0.733206765	-0.447708	0.40477057	1

gene11348	128.1093383	89.04893391	0.695101037	-0.5247054	0.40477299	1
gene14530	254.152397	183.905407	0.723602882	-0.46672994	0.40477763	1
gene61887	648.8118102	887.5855695	1.368016974	0.452086131	0.40480975	1
gene45299	185.9651685	132.6054777	0.713066209	-0.48789206	0.40482777	1
gene50858	248.9866978	180.0337142	0.723065593	-0.46780157	0.40482885	1
gene52493	122.9436392	85.17724113	0.692815356	-0.52945719	0.40483124	1
gene6935	406.0239513	297.1524208	0.731859339	-0.4503617	0.40483869	1
gene52706	484.542578	355.2278124	0.733119913	-0.4478789	0.40484375	1
gene23196	580.6245817	425.8862056	0.733496685	-0.44713765	0.404848	1
gene20881	556.8623657	408.4635881	0.733509056	-0.44711332	0.40488916	1
gene30930	243.8209987	176.1620214	0.722505536	-0.46891945	0.40489671	1
gene2958	28.9279151	16.45469431	0.568817153	-0.81396312	0.40494846	1
gene16692	28.9279151	16.45469431	0.568817153	-0.81396312	0.40494846	1
gene33041	28.9279151	16.45469431	0.568817153	-0.81396312	0.40494846	1
gene36144	28.9279151	16.45469431	0.568817153	-0.81396312	0.40494846	1
gene67821	28.9279151	16.45469431	0.568817153	-0.81396312	0.40494846	1
gene32910	3334.975355	2327.855283	0.698012739	-0.51867473	0.40496132	1
gene16147	470.0786204	344.5806573	0.733027716	-0.44806035	0.40496612	1
gene3079	9.298258426	18.3905407	1.977847878	0.983931469	0.40501534	1
gene7869	9.298258426	18.3905407	1.977847878	0.983931469	0.40501534	1
gene14504	9.298258426	18.3905407	1.977847878	0.983931469	0.40501534	1
gene32013	9.298258426	18.3905407	1.977847878	0.983931469	0.40501534	1
gene34437	9.298258426	18.3905407	1.977847878	0.983931469	0.40501534	1
gene37311	9.298258426	18.3905407	1.977847878	0.983931469	0.40501534	1
gene53871	9.298258426	18.3905407	1.977847878	0.983931469	0.40501534	1
gene66607	9.298258426	18.3905407	1.977847878	0.983931469	0.40501534	1
gene70278	9.298258426	18.3905407	1.977847878	0.983931469	0.40501534	1
gene70830	9.298258426	18.3905407	1.977847878	0.983931469	0.40501534	1
gene45699	69.22036828	101.6319354	1.468237427	0.554085284	0.40504404	1
gene41626	39.25931335	60.97916126	1.553240647	0.635281367	0.40510628	1
gene41750	39.25931335	60.97916126	1.553240647	0.635281367	0.40510628	1
gene11889	305.8093882	222.6223348	0.727977437	-0.45803436	0.40510648	1
gene32875	76.45234706	111.3111674	1.455954875	0.541965642	0.40510994	1
gene62032	503.1390948	686.257545	1.363951941	0.447792812	0.40513693	1
gene43572	219.0256429	301.0241135	1.374378404	0.458779272	0.40515421	1
gene39260	82.65118601	55.17162209	0.667523659	-0.58310912	0.4052561	1
gene20153	600.2542384	440.4050536	0.733697532	-0.44674266	0.40532052	1
gene38614	223.1582022	160.6752503	0.720006026	-0.47391911	0.40533924	1
gene35612	441.1507053	323.286347	0.732825184	-0.44845901	0.40537849	1
gene56898	1724.310368	1239.909612	0.719075658	-0.47578452	0.40541783	1
gene32664	41.325593	25.16600306	0.608968952	-0.71555942	0.40545243	1
gene17871	1732.575487	1245.717151	0.718997331	-0.47594168	0.40550043	1
gene4151	580.6245817	792.7290964	1.365304056	0.449222278	0.40556514	1
gene60309	120.8773595	170.3544823	1.409316707	0.494995856	0.40556568	1
gene42677	190.0977278	262.3071857	1.379854398	0.464516042	0.40556959	1
gene66779	921.560724	673.6745435	0.731014816	-0.45202745	0.40568597	1
gene50927	3118.015992	4552.142784	1.459948504	0.545917483	0.40575792	1
gene47674	377.0960362	275.8581105	0.731532777	-0.45100559	0.40581179	1

gene30181	556.8623657	759.8197078	1.364465898	0.448336338	0.40581828	1
gene18158	337.8367228	460.7314406	1.363769565	0.447599894	0.40581992	1
gene62973	594.0553994	811.1196371	1.365393931	0.449317245	0.40586998	1
gene26647	505.2053745	370.7145835	0.733789865	-0.44656112	0.40588532	1
gene59883	703.5682209	963.0835787	1.368855997	0.452970684	0.4058952	1
gene52232	490.7414169	360.0674284	0.733721296	-0.44669594	0.40597151	1
gene2627	276.8814731	378.4579691	1.366859129	0.450864563	0.40603163	1
gene45982	88.85002496	127.7658617	1.437994663	0.524058321	0.40605443	1
gene38764	476.2774594	349.4202733	0.733648562	-0.44683896	0.4061069	1
gene42458	59.92210985	89.04893391	1.486078079	0.571509918	0.40616364	1
gene6387	265.516935	192.6167157	0.725440416	-0.46307097	0.40618261	1
gene70900	126.0430587	177.1299446	1.405312966	0.490891457	0.40618942	1
gene1932	195.2634269	269.0826481	1.378049399	0.462627605	0.40619268	1
gene7765	50.62385143	76.46593238	1.51047244	0.594999861	0.40620375	1
gene36832	37.1930337	58.07539168	1.561458851	0.642894551	0.40620379	1
gene55954	37.1930337	58.07539168	1.561458851	0.642894551	0.40620379	1
gene61528	260.3512359	188.745023	0.724963038	-0.46402065	0.40622459	1
gene48403	255.1855368	184.8733302	0.724466333	-0.46500945	0.40628357	1
gene62523	741.7943944	543.9728354	0.733320229	-0.44748476	0.4062856	1
gene56646	37.1930337	22.26223348	0.598559226	-0.74043409	0.40636109	1
gene69929	37.1930337	22.26223348	0.598559226	-0.74043409	0.40636109	1
gene72912	37.1930337	22.26223348	0.598559226	-0.74043409	0.40636109	1
gene21682	33.0604744	19.35846389	0.585547069	-0.77214295	0.40638872	1
gene40971	33.0604744	19.35846389	0.585547069	-0.77214295	0.40638872	1
gene14360	448.3826841	610.7595358	1.362138989	0.445873919	0.40639307	1
gene14394	244.8541385	177.1299446	0.72341005	-0.46711445	0.40645393	1
gene25520	312.0082272	227.4619507	0.72902549	-0.45595884	0.40648194	1
gene54425	397.7588327	291.3448816	0.732466152	-0.449166	0.40650891	1
gene57054	397.7588327	291.3448816	0.732466152	-0.449166	0.40650891	1
gene69486	192.1640075	137.4450936	0.715248893	-0.48348273	0.40652605	1
gene27282	222.1250624	304.8958063	1.372631269	0.456944125	0.40653249	1
gene13006	236.5890199	324.2542702	1.370538118	0.454742454	0.40655519	1
gene29021	737.6618351	541.0690658	0.733492015	-0.44714683	0.40656557	1
gene47965	16.5302372	29.03769584	1.756641208	0.812819552	0.40657486	1
gene52432	16.5302372	29.03769584	1.756641208	0.812819552	0.40657486	1
gene56707	16.5302372	29.03769584	1.756641208	0.812819552	0.40657486	1
gene67998	131.2087578	183.905407	1.401624481	0.487099878	0.40677046	1
gene708	144.6395755	101.6319354	0.702656483	-0.50910854	0.40683246	1
gene1996	6.19883895	13.55092472	2.186042392	1.128321378	0.40686395	1
gene4542	6.19883895	13.55092472	2.186042392	1.128321378	0.40686395	1
gene6543	6.19883895	13.55092472	2.186042392	1.128321378	0.40686395	1
gene7750	6.19883895	13.55092472	2.186042392	1.128321378	0.40686395	1
gene16224	6.19883895	13.55092472	2.186042392	1.128321378	0.40686395	1
gene19362	6.19883895	13.55092472	2.186042392	1.128321378	0.40686395	1
gene20836	6.19883895	13.55092472	2.186042392	1.128321378	0.40686395	1
gene26142	6.19883895	13.55092472	2.186042392	1.128321378	0.40686395	1
gene29447	6.19883895	13.55092472	2.186042392	1.128321378	0.40686395	1
gene31008	6.19883895	13.55092472	2.186042392	1.128321378	0.40686395	1

gene32172	6.19883895	13.55092472	2.186042392	1.128321378	0.40686395	1
gene32711	6.19883895	13.55092472	2.186042392	1.128321378	0.40686395	1
gene37045	6.19883895	13.55092472	2.186042392	1.128321378	0.40686395	1
gene37549	6.19883895	13.55092472	2.186042392	1.128321378	0.40686395	1
gene43673	6.19883895	13.55092472	2.186042392	1.128321378	0.40686395	1
gene46269	6.19883895	13.55092472	2.186042392	1.128321378	0.40686395	1
gene53513	6.19883895	13.55092472	2.186042392	1.128321378	0.40686395	1
gene53864	6.19883895	13.55092472	2.186042392	1.128321378	0.40686395	1
gene55667	6.19883895	13.55092472	2.186042392	1.128321378	0.40686395	1
gene55726	6.19883895	13.55092472	2.186042392	1.128321378	0.40686395	1
gene60457	6.19883895	13.55092472	2.186042392	1.128321378	0.40686395	1
gene62657	6.19883895	13.55092472	2.186042392	1.128321378	0.40686395	1
gene64149	6.19883895	13.55092472	2.186042392	1.128321378	0.40686395	1
gene64999	6.19883895	13.55092472	2.186042392	1.128321378	0.40686395	1
gene65213	6.19883895	13.55092472	2.186042392	1.128321378	0.40686395	1
gene69424	6.19883895	13.55092472	2.186042392	1.128321378	0.40686395	1
gene47205	54.75641073	34.84523501	0.636368136	-0.6520665	0.40688016	1
gene48863	73.35292758	48.39615973	0.659771346	-0.59996197	0.40688301	1
gene28930	1490.820768	1078.266439	0.723270337	-0.46739311	0.40692333	1
gene65171	553.7629462	754.9800918	1.363363325	0.44717008	0.40695215	1
gene15902	337.8367228	246.8204146	0.730590839	-0.45286443	0.40695995	1
gene27199	181.8326092	129.7017081	0.713302793	-0.48741347	0.40705542	1
gene44107	181.8326092	129.7017081	0.713302793	-0.48741347	0.40705542	1
gene69824	348.1681211	254.5638002	0.731151949	-0.45175684	0.40718763	1
gene5412	279.9808926	382.3296619	1.365556265	0.449488758	0.40721796	1
gene50853	338.8698626	461.6993638	1.362468059	0.446222408	0.40724038	1
gene42105	418.4216292	306.8316527	0.733307342	-0.44751011	0.40724585	1
gene18838	35.12675405	55.17162209	1.570643903	0.65135613	0.4072538	1
gene58152	35.12675405	55.17162209	1.570643903	0.65135613	0.4072538	1
gene41526	136.3744569	190.6808693	1.398215426	0.483586657	0.40731776	1
gene50692	521.7356117	383.2975851	0.734658659	-0.444854	0.4074888	1
gene44712	398.7919725	292.3128048	0.732995709	-0.44812334	0.40754328	1
gene3848	64.05466916	41.62069737	0.649768361	-0.6220026	0.40754959	1
gene42355	64.05466916	41.62069737	0.649768361	-0.6220026	0.40754959	1
gene51385	64.05466916	41.62069737	0.649768361	-0.6220026	0.40754959	1
gene60149	64.05466916	41.62069737	0.649768361	-0.6220026	0.40754959	1
gene56378	271.715774	197.4563317	0.726701762	-0.46056469	0.4075859	1
gene60830	171.501211	121.9583225	0.711122224	-0.49183055	0.40764258	1
gene19094	103.3139825	70.65839321	0.683918977	-0.54810267	0.40767348	1
gene58576	368.8309176	270.0505713	0.732179865	-0.44973	0.40769656	1
gene48204	3677.977777	2558.221003	0.695550968	-0.52377186	0.40778097	1
gene2848	256.2186766	185.8412534	0.725322821	-0.46330485	0.40778104	1
gene43358	256.2186766	185.8412534	0.725322821	-0.46330485	0.40778104	1
gene13866	111.5791011	157.7714807	1.413987737	0.499769609	0.40778621	1
gene28189	111.5791011	157.7714807	1.413987737	0.499769609	0.40778621	1
gene370	118.8110799	82.27347154	0.692473056	-0.53017016	0.40779291	1
gene39238	118.8110799	82.27347154	0.692473056	-0.53017016	0.40779291	1
gene53570	113.6453808	78.40177877	0.689880911	-0.53558075	0.40784561	1

gene48584	845.1083769	619.4708446	0.733007578	-0.44809998	0.40786364	1
gene61603	1423.666679	1031.806125	0.724752599	-0.46443949	0.40786528	1
gene18226	57.8558302	86.14516432	1.488962547	0.574307466	0.40789086	1
gene32534	166.3355118	118.0866297	0.70993036	-0.49425058	0.40795443	1
gene61652	166.3355118	118.0866297	0.70993036	-0.49425058	0.40795443	1
gene74025	560.994925	412.3352809	0.73500715	-0.44416981	0.4079856	1
gene3237	854.4066353	626.2463069	0.732960491	-0.44819266	0.40799133	1
gene28726	245.8872784	178.0978678	0.724306963	-0.46532685	0.40800198	1
gene17777	33.0604744	52.26785251	1.580977087	0.660816459	0.40823353	1
gene29968	33.0604744	52.26785251	1.580977087	0.660816459	0.40823353	1
gene47653	33.0604744	52.26785251	1.580977087	0.660816459	0.40823353	1
gene58801	33.0604744	52.26785251	1.580977087	0.660816459	0.40823353	1
gene576	161.1698127	114.214937	0.708662094	-0.49683021	0.4082759	1
gene8351	161.1698127	114.214937	0.708662094	-0.49683021	0.4082759	1
gene15398	694.2699625	948.5647307	1.366276495	0.450249473	0.4083435	1
gene28799	2381.387297	3407.089645	1.43071631	0.516737635	0.40837809	1
gene18730	116.7448002	164.5469431	1.409458432	0.49514093	0.40839569	1
gene69241	230.390181	166.4827895	0.722612347	-0.46870619	0.40847753	1
gene6706	237.6221598	325.2221934	1.368652628	0.452756328	0.40849946	1
gene67957	359.5326591	263.2751089	0.732270358	-0.4495517	0.40856241	1
gene17862	349.2012609	475.2502886	1.360963839	0.444628735	0.40858609	1
gene15600	259.3180961	354.2598892	1.366120971	0.450085241	0.4085905	1
gene3290	429.7861672	315.5429615	0.734185941	-0.44578261	0.40859569	1
gene21127	156.0041136	110.3432442	0.707309837	-0.49958577	0.40860446	1
gene21408	156.0041136	110.3432442	0.707309837	-0.49958577	0.40860446	1
gene25338	386.3942946	525.5822947	1.36022271	0.443842885	0.40863855	1
gene30910	225.2244819	162.6110967	0.721995652	-0.46993795	0.40867592	1
gene33778	215.9262234	296.1844976	1.371693039	0.455957667	0.40868102	1
gene73818	91.94944443	131.6375545	1.431629688	0.517658367	0.40871428	1
gene15679	18.59651685	31.94146542	1.717604736	0.780398075	0.40879247	1
gene18875	18.59651685	31.94146542	1.717604736	0.780398075	0.40879247	1
gene33294	18.59651685	31.94146542	1.717604736	0.780398075	0.40879247	1
gene65495	220.0587827	158.7394039	0.721350004	-0.47122866	0.40889476	1
gene996	121.9104994	171.3224055	1.405312966	0.490891457	0.40894898	1
gene57171	6323.848869	9875.720355	1.56166293	0.643083095	0.40906997	1
gene30350	30.99419475	49.36408293	1.592688028	0.671463703	0.40911313	1
gene41232	30.99419475	49.36408293	1.592688028	0.671463703	0.40911313	1
gene73833	1429.865518	1993.921781	1.394482038	0.479729351	0.40913755	1
gene69881	250.0198377	341.6768877	1.36659911	0.450590093	0.40913968	1
gene34186	557.8955055	410.3994345	0.735620614	-0.44296619	0.40926674	1
gene14017	145.6727153	102.5998586	0.704317609	-0.50570194	0.40926947	1
gene48439	875.0694318	641.733078	0.73335104	-0.44742414	0.40931259	1
gene16211	890.5665292	1222.486995	1.372707097	0.457023821	0.40932868	1
gene55435	46.49129213	70.65839321	1.519819948	0.603900419	0.40933012	1
gene52468	3440.355618	5045.783614	1.466645944	0.552520639	0.40934611	1
gene40226	50.62385143	31.94146542	0.630956842	-0.66438677	0.40934871	1
gene67435	50.62385143	31.94146542	0.630956842	-0.66438677	0.40934871	1
gene1416	475.2443195	349.4202733	0.73524345	-0.44370607	0.40943068	1

gene851	84.71746566	121.9583225	1.439588892	0.525656876	0.40945391	1
gene256	127.0761985	178.0978678	1.40150453	0.486976407	0.40945812	1
gene44760	83.68432583	56.13954529	0.670848988	-0.57594005	0.40946274	1
gene56067	83.68432583	56.13954529	0.670848988	-0.57594005	0.40946274	1
gene62778	83.68432583	56.13954529	0.670848988	-0.57594005	0.40946274	1
gene37932	514.5036329	378.4579691	0.735578808	-0.44304818	0.40947525	1
gene16723	3321.544538	2326.88736	0.700543778	-0.51345288	0.40949778	1
gene7590	97.11514356	138.4130168	1.425246483	0.511211442	0.40954486	1
gene41773	1084.796816	1497.377182	1.380329624	0.465012826	0.40958808	1
gene50648	733.5292758	539.1332194	0.734985279	-0.44421274	0.409591	1
gene63739	480.4100187	353.291966	0.735396749	-0.4434053	0.40963844	1
gene19661	55.78955055	83.24139474	1.49206068	0.577306209	0.40964826	1
gene33024	255.1855368	348.4523501	1.365486283	0.449414821	0.40976709	1
gene6667	28.9279151	46.46031334	1.606071961	0.683536535	0.40985326	1
gene27726	28.9279151	46.46031334	1.606071961	0.683536535	0.40985326	1
gene13198	378.129176	513.9672164	1.359237131	0.442797169	0.40986713	1
gene33153	114.6785206	161.6431735	1.409533125	0.495217383	0.4098755	1
gene30243	359.5326591	488.8012133	1.359546069	0.443125038	0.40989837	1
gene55186	135.3413171	94.85647307	0.700868553	-0.5127842	0.40991407	1
gene7561	69.22036828	45.49239015	0.657211039	-0.60557138	0.4099361	1
gene24229	199.3959862	143.2526328	0.718432881	-0.47707471	0.40997889	1
gene61911	149.8052746	208.1034868	1.389159943	0.474212716	0.41000802	1
gene68554	1744.973165	2452.717375	1.405590312	0.491176153	0.41002006	1
gene5308	785.1862671	576.882224	0.734707481	-0.44475813	0.41003039	1
gene526	20.6627965	34.84523501	1.686375559	0.753925863	0.41004519	1
gene6188	20.6627965	34.84523501	1.686375559	0.753925863	0.41004519	1
gene12924	20.6627965	34.84523501	1.686375559	0.753925863	0.41004519	1
gene16845	20.6627965	34.84523501	1.686375559	0.753925863	0.41004519	1
gene29949	20.6627965	34.84523501	1.686375559	0.753925863	0.41004519	1
gene64871	20.6627965	34.84523501	1.686375559	0.753925863	0.41004519	1
gene72847	20.6627965	34.84523501	1.686375559	0.753925863	0.41004519	1
gene73770	20.6627965	34.84523501	1.686375559	0.753925863	0.41004519	1
gene55167	77.48548688	112.2790906	1.449033814	0.535091261	0.41006256	1
gene59393	77.48548688	112.2790906	1.449033814	0.535091261	0.41006256	1
gene486	340.9361423	463.6352102	1.359888709	0.443488588	0.41006971	1
gene8238	544.4646878	400.7202026	0.735989333	-0.44224324	0.41008331	1
gene46366	167.3686517	119.0545529	0.711331254	-0.49140654	0.41009933	1
gene6046	130.175618	90.9847803	0.698938724	-0.51676212	0.41021277	1
gene12512	130.175618	90.9847803	0.698938724	-0.51676212	0.41021277	1
gene19516	194.2302871	139.38094	0.717606621	-0.47873489	0.41030255	1
gene23034	320.2733458	234.2374131	0.731367178	-0.45133221	0.4103127	1
gene44967	226.2576217	163.5790199	0.722976838	-0.46797867	0.41032806	1
gene4190	59.92210985	38.71692779	0.646120904	-0.63012394	0.41036941	1
gene54353	59.92210985	38.71692779	0.646120904	-0.63012394	0.41036941	1
gene26632	253.1192571	345.5485805	1.365161167	0.449071282	0.41037365	1
gene14242	137.4075967	191.6487925	1.394746703	0.480003141	0.41038148	1
gene48450	63.02152933	92.92062669	1.474426719	0.56015412	0.41038322	1
gene20183	26.86163545	43.55654376	1.621514961	0.697342335	0.41040143	1

gene53408	26.86163545	43.55654376	1.621514961	0.697342335	0.41040143	1
gene30502	204.5616854	280.6977264	1.372191112	0.456481427	0.41044006	1
gene58377	335.7704432	245.8524914	0.732204089	-0.44968226	0.41044978	1
gene23204	125.0099188	87.11308752	0.696849405	-0.52108118	0.41048411	1
gene64651	125.0099188	87.11308752	0.696849405	-0.52108118	0.41048411	1
gene72648	8647.380336	5623.633761	0.650328023	-0.6207605	0.41050433	1
gene32089	231.4233208	316.5108846	1.367670654	0.45172086	0.41051578	1
gene8536	21.69593633	11.61507834	0.53535732	-0.90142597	0.41053779	1
gene11688	21.69593633	11.61507834	0.53535732	-0.90142597	0.41053779	1
gene12625	21.69593633	11.61507834	0.53535732	-0.90142597	0.41053779	1
gene19035	21.69593633	11.61507834	0.53535732	-0.90142597	0.41053779	1
gene22129	21.69593633	11.61507834	0.53535732	-0.90142597	0.41053779	1
gene35962	21.69593633	11.61507834	0.53535732	-0.90142597	0.41053779	1
gene36997	21.69593633	11.61507834	0.53535732	-0.90142597	0.41053779	1
gene24779	669.4746067	912.7515725	1.363384904	0.447192914	0.41059702	1
gene2891	22.72907615	37.74900459	1.660824414	0.731899557	0.41061302	1
gene47982	22.72907615	37.74900459	1.660824414	0.731899557	0.41061302	1
gene55533	189.064588	135.5092472	0.71673521	-0.48048786	0.4106471	1
gene31083	94.01572408	63.88293085	0.679491984	-0.55747156	0.41065468	1
gene66269	94.01572408	63.88293085	0.679491984	-0.55747156	0.41065468	1
gene44626	24.7953558	40.65277417	1.639531794	0.713283879	0.41068693	1
gene30083	944.2898001	1297.017081	1.373537108	0.457895887	0.41071703	1
gene10431	683.9385642	503.3200612	0.735914141	-0.44239064	0.4107249	1
gene52841	452.5152434	614.6312286	1.358255302	0.441754679	0.41077785	1
gene12254	763.4903307	561.3954529	0.735301326	-0.44359251	0.41081792	1
gene17312	125.0099188	175.1940982	1.40144158	0.486911606	0.41086233	1
gene20135	114.6785206	79.36970196	0.692106085	-0.53093491	0.41089635	1
gene54266	114.6785206	79.36970196	0.692106085	-0.53093491	0.41089635	1
gene65047	99.18142321	67.75462362	0.683138247	-0.54975053	0.41091652	1
gene44968	1157.116604	844.9969489	0.730260845	-0.45351622	0.41092337	1
gene11934	104.3471223	71.6263164	0.686423495	-0.54282916	0.41102148	1
gene21180	272.7489138	371.6825067	1.362727725	0.446497338	0.41103545	1
gene218	377.0960362	276.8260337	0.734099559	-0.44595236	0.41108473	1
gene43149	3114.916573	2192.346036	0.703821751	-0.506718	0.41111452	1
gene41252	388.4605742	527.5181411	1.357970863	0.441452525	0.41115325	1
gene50321	397.7588327	540.1011426	1.35786084	0.441335633	0.41118445	1
gene39713	82.65118601	119.0545529	1.44044579	0.526515367	0.41123315	1
gene41572	1102.360193	1520.607339	1.379410603	0.464051961	0.41127082	1
gene10844	165.302372	228.4298739	1.381891083	0.466643911	0.41131123	1
gene40791	6352.776784	4258.862056	0.670393782	-0.57691933	0.41140049	1
gene41710	736.6286953	542.036989	0.735834746	-0.44254629	0.4114144	1
gene62800	301.6768289	410.3994345	1.360394287	0.444024852	0.41141445	1
gene32956	325.4390449	442.3408999	1.359212752	0.442771293	0.4114584	1
gene5250	237.6221598	172.2903286	0.725060023	-0.46382766	0.41147574	1
gene8553	2401.016953	3428.383955	1.427888275	0.5138831	0.4114917	1
gene57174	1253.198608	913.7194957	0.729109887	-0.45579183	0.41149262	1
gene61366	308.9088077	420.0786665	1.359879214	0.443478515	0.41155626	1
gene53266	295.47799	215.8468724	0.730500679	-0.45304248	0.41156716	1

gene1534	270.6826342	368.7787372	1.362402647	0.446153143	0.41159688	1
gene47214	290.3122908	211.9751796	0.730162609	-0.4537103	0.41160487	1
gene69339	152.9046941	211.9751796	1.38632225	0.471262651	0.41162965	1
gene34008	74.38606741	49.36408293	0.663620012	-0.5915707	0.41163202	1
gene60704	146.7058552	103.5677818	0.70595534	-0.50235118	0.41168087	1
gene64447	341.9692821	250.6921074	0.733083702	-0.44795016	0.41168139	1
gene22083	232.4564606	168.4186359	0.724516907	-0.46490874	0.41171127	1
gene32090	232.4564606	168.4186359	0.724516907	-0.46490874	0.41171127	1
gene29474	279.9808926	204.2317941	0.729449043	-0.4551209	0.41173418	1
gene7813	46.49129213	29.03769584	0.62458354	-0.67903354	0.41173533	1
gene23384	46.49129213	29.03769584	0.62458354	-0.67903354	0.41173533	1
gene60243	46.49129213	29.03769584	0.62458354	-0.67903354	0.41173533	1
gene54007	1266.629426	1754.844752	1.385444485	0.470348903	0.41178921	1
gene1915	695.3031023	512.03137	0.73641462	-0.44140983	0.41193255	1
gene7810	448.3826841	330.0618094	0.736116316	-0.44199435	0.41194866	1
gene2181	638.4804119	470.4106726	0.736766021	-0.44072157	0.41196388	1
gene48760	227.2907615	164.5469431	0.723949104	-0.46603982	0.41196954	1
gene57065	227.2907615	164.5469431	0.723949104	-0.46603982	0.41196954	1
gene29766	585.7902808	431.6937448	0.736942484	-0.44037607	0.41201648	1
gene11571	168.4017915	120.0224761	0.71271496	-0.48860289	0.41222491	1
gene40466	60.95524968	90.0168571	1.476769558	0.562444718	0.41225217	1
gene36153	483.5094381	356.1957356	0.736688279	-0.44087381	0.41225747	1
gene45938	246.9204182	336.8372717	1.364153172	0.448005645	0.41227002	1
gene66126	68.18722846	99.69608905	1.462093288	0.548035364	0.41230526	1
gene51877	1696.415593	1226.358688	0.722911704	-0.46810865	0.41232039	1
gene28876	562.0280648	414.2711273	0.737100428	-0.4400669	0.41233759	1
gene65622	930.8589824	683.3537754	0.734110954	-0.44592997	0.41233992	1
gene11713	1011.443889	741.4291671	0.733040335	-0.44803551	0.41235719	1
gene27019	42.35873283	64.85085404	1.530991361	0.614466142	0.41237022	1
gene61850	42.35873283	64.85085404	1.530991361	0.614466142	0.41237022	1
gene71092	297.5442696	404.5918954	1.359770416	0.443363087	0.41239247	1
gene14882	254.152397	184.8733302	0.727411318	-0.45915672	0.41239673	1
gene69894	254.152397	184.8733302	0.727411318	-0.45915672	0.41239673	1
gene48870	1865.850524	1344.445317	0.720553603	-0.47282234	0.41242061	1
gene16753	163.2360924	225.5261044	1.381594604	0.466334353	0.41242384	1
gene55116	940.1572408	690.1292378	0.734057249	-0.44603551	0.41247898	1
gene70081	775.8880086	571.0746848	0.736027208	-0.442169	0.41258076	1
gene16069	248.9866978	181.0016374	0.726953042	-0.46006592	0.41259186	1
gene46372	163.2360924	116.1507834	0.711550869	-0.4909612	0.41266486	1
gene34442	7163.791547	4755.406655	0.663811422	-0.59115464	0.41268245	1
gene1455	615.7513357	453.9559783	0.737239129	-0.43979545	0.41276888	1
gene24454	168.4017915	232.3015667	1.37944831	0.464091398	0.41281879	1
gene20755	762.4571909	561.3954529	0.73629767	-0.44163896	0.41287144	1
gene43179	217.9925031	298.1203439	1.367571544	0.451616309	0.41287989	1
gene18339	353.3338202	259.4034162	0.7341596	-0.44583437	0.41292628	1
gene53301	734.5624156	541.0690658	0.736586918	-0.44107232	0.4129438	1
gene31230	302.7099687	411.3673577	1.35894883	0.442491133	0.41298546	1
gene37577	65.08780898	42.58862056	0.654325614	-0.61191935	0.41302628	1

gene115	238.6552996	173.2582518	0.725976972	-0.46200431	0.41304936	1
gene17337	238.6552996	173.2582518	0.725976972	-0.46200431	0.41304936	1
gene54872	16451.71857	9927.020284	0.60340324	-0.72880565	0.41312814	1
gene59129	373.9966167	274.8901873	0.73500715	-0.44416981	0.41315776	1
gene67219	206.627965	149.060172	0.721393989	-0.47114069	0.41323924	1
gene60933	51.65699125	77.43385557	1.499000497	0.584000862	0.41324133	1
gene29137	626.082734	461.6993638	0.737441457	-0.43939957	0.41326558	1
gene2566	55.78955055	35.8131582	0.641933083	-0.63950518	0.41327016	1
gene56528	55.78955055	35.8131582	0.641933083	-0.63950518	0.41327016	1
gene60138	55.78955055	35.8131582	0.641933083	-0.63950518	0.41327016	1
gene52904	392.5931335	532.3577571	1.356003739	0.439361157	0.41333699	1
gene41157	1847.254007	2596.937931	1.405836946	0.491429275	0.41338187	1
gene67452	115.7116604	162.6110967	1.405312966	0.490891457	0.41340904	1
gene4776	678.7728651	500.4162916	0.737236736	-0.43980013	0.41341027	1
gene57948	84.71746566	57.10746848	0.674093211	-0.56898	0.41358648	1
gene38994	152.9046941	108.4073978	0.708986722	-0.49616949	0.41358847	1
gene41682	152.9046941	108.4073978	0.708986722	-0.49616949	0.41358847	1
gene20664	120.8773595	84.20931793	0.696650872	-0.52149227	0.41360213	1
gene35174	120.8773595	84.20931793	0.696650872	-0.52149227	0.41360213	1
gene5419	201.4622659	145.1884792	0.720673316	-0.47258267	0.41361787	1
gene8052	621.9501747	458.7955943	0.737672587	-0.43894747	0.41371644	1
gene47708	636.4141322	469.4427494	0.737637217	-0.43901665	0.41376204	1
gene34139	824.4455804	606.887843	0.736116316	-0.44199435	0.41383281	1
gene59278	824.4455804	606.887843	0.736116316	-0.44199435	0.41383281	1
gene33227	698.4025218	514.9351395	0.737304239	-0.43966804	0.41383473	1
gene12907	42.35873283	26.13392626	0.616966668	-0.69673555	0.41393824	1
gene13372	42.35873283	26.13392626	0.616966668	-0.69673555	0.41393824	1
gene66866	262.4175156	357.1636588	1.36105114	0.444721276	0.41395654	1
gene1243	11.36453808	21.29431028	1.873750621	0.905928957	0.41396288	1
gene5448	11.36453808	21.29431028	1.873750621	0.905928957	0.41396288	1
gene20606	11.36453808	21.29431028	1.873750621	0.905928957	0.41396288	1
gene20634	11.36453808	21.29431028	1.873750621	0.905928957	0.41396288	1
gene39938	11.36453808	21.29431028	1.873750621	0.905928957	0.41396288	1
gene40487	11.36453808	21.29431028	1.873750621	0.905928957	0.41396288	1
gene63698	11.36453808	21.29431028	1.873750621	0.905928957	0.41396288	1
gene69316	11.36453808	21.29431028	1.873750621	0.905928957	0.41396288	1
gene69981	11.36453808	21.29431028	1.873750621	0.905928957	0.41396288	1
gene70405	11.36453808	21.29431028	1.873750621	0.905928957	0.41396288	1
gene70832	11.36453808	21.29431028	1.873750621	0.905928957	0.41396288	1
gene70968	11.36453808	21.29431028	1.873750621	0.905928957	0.41396288	1
gene71419	11.36453808	21.29431028	1.873750621	0.905928957	0.41396288	1
gene39847	420.4879088	309.7354223	0.736609581	-0.44102793	0.41398322	1
gene16074	485.5757178	358.131582	0.737540138	-0.43920653	0.41398955	1
gene71055	3492.012609	2447.877759	0.700993391	-0.51252725	0.41412106	1
gene60966	364.6983583	268.1147249	0.735168445	-0.44385325	0.41413518	1
gene29927	110.5459613	76.46593238	0.691711678	-0.53175728	0.41415036	1
gene48507	110.5459613	76.46593238	0.691711678	-0.53175728	0.41415036	1
gene41902	126.0430587	176.1620214	1.39763366	0.482986259	0.41415323	1

gene17464	369.8640574	271.9864177	0.73536861	-0.4434605	0.41418284	1
gene63033	1649.924301	1195.385145	0.724509085	-0.46492431	0.41418917	1
gene59631	66.12094881	96.79231946	1.463867673	0.549785146	0.41423377	1
gene892	105.3802622	72.5942396	0.688878905	-0.53767769	0.41431808	1
gene63266	105.3802622	72.5942396	0.688878905	-0.53767769	0.41431808	1
gene32355	2185.09073	1567.067652	0.717163654	-0.47962572	0.41435425	1
gene44013	100.214563	68.72254682	0.685754093	-0.54423677	0.41438282	1
gene48108	559.9617852	413.3032041	0.738091804	-0.43812783	0.4144088	1
gene65780	617.8176154	839.1894098	1.358312532	0.441815466	0.41444379	1
gene44409	191.1308676	137.4450936	0.719115103	-0.47570538	0.41445063	1
gene11250	1239.76779	905.9761102	0.730762742	-0.45252501	0.41447345	1
gene33127	131.2087578	182.9374838	1.39424751	0.479486694	0.41448168	1
gene47320	2072.478489	1489.633797	0.718769244	-0.47639942	0.41454087	1
gene25268	142.5732959	100.6640122	0.706050959	-0.50215578	0.41455228	1
gene71131	142.5732959	100.6640122	0.706050959	-0.50215578	0.41455228	1
gene2438	400.8582521	295.2165744	0.736461262	-0.44131845	0.41458867	1
gene3176	2823.571142	4063.341571	1.439078871	0.525145663	0.41459889	1
gene14691	668.4414668	908.8798798	1.359700026	0.443288402	0.41462079	1
gene47194	176.6669101	242.9487219	1.37517955	0.459619996	0.41465798	1
gene11897	90.91630461	129.7017081	1.426605587	0.512586528	0.41474647	1
gene40533	2156.162815	1547.709188	0.717807198	-0.47833171	0.41486611	1
gene13268	780.0205679	574.9463776	0.737091304	-0.44008476	0.4148846	1
gene14168	2701.660643	1920.359618	0.710807119	-0.49246996	0.4149155	1
gene40498	945.3229399	694.9688538	0.735165544	-0.44385894	0.41491927	1
gene66558	70.25350811	46.46031334	0.661323749	-0.59657138	0.41495879	1
gene37903	219.0256429	299.0882671	1.365539958	0.44947153	0.41496644	1
gene1108	287.2128714	390.0730474	1.358132195	0.441623912	0.41500245	1
gene8609	164.2692322	226.4940275	1.378797627	0.463410721	0.41506746	1
gene46841	164.2692322	226.4940275	1.378797627	0.463410721	0.41506746	1
gene54563	49.5907116	74.53008599	1.502904144	0.587752997	0.41506842	1
gene49811	141.540156	196.4884085	1.388216701	0.473232791	0.41508888	1
gene58880	456.6478027	618.5029214	1.354441908	0.437698518	0.41512569	1
gene67909	2714.05832	1929.070927	0.710769887	-0.49254553	0.41514365	1
gene35613	595.0885392	439.4371304	0.738439915	-0.43744756	0.41516893	1
gene49671	496.9402559	366.8428908	0.738203207	-0.43791009	0.41517398	1
gene34766	229.3570412	166.4827895	0.725867358	-0.46222215	0.41522088	1
gene35501	991.8142321	1359.932088	1.371156054	0.455392776	0.41524278	1
gene39654	2371.055899	1695.801437	0.715209388	-0.48356242	0.41532187	1
gene51762	159.1035331	113.2470138	0.711781892	-0.49049286	0.41534533	1
gene37764	96.08200373	136.4771704	1.420423858	0.506321498	0.41535346	1
gene33862	738.6949749	544.9407586	0.737707412	-0.43887936	0.41535696	1
gene24246	180.7994694	129.7017081	0.717378809	-0.47919296	0.41538477	1
gene56521	3689.342315	2580.483237	0.699442615	-0.5157224	0.41541419	1
gene47791	391.5599937	288.441112	0.736646023	-0.44095656	0.41546965	1
gene2335	132.2418976	92.92062669	0.702656483	-0.50910854	0.41552343	1
gene18160	25.82849563	14.51884792	0.562125186	-0.83103664	0.41556309	1
gene26011	25.82849563	14.51884792	0.562125186	-0.83103664	0.41556309	1
gene52388	25.82849563	14.51884792	0.562125186	-0.83103664	0.41556309	1

gene52647	25.82849563	14.51884792	0.562125186	-0.83103664	0.41556309	1
gene38460	947.3892196	1297.017081	1.369043529	0.453168318	0.41561321	1
gene11406	378.129176	512.03137	1.354117594	0.43735303	0.41567865	1
gene66670	378.129176	512.03137	1.354117594	0.43735303	0.41567865	1
gene20077	493.8408364	668.8349275	1.354353221	0.437604049	0.41571816	1
gene47307	2585.948982	1842.925763	0.712669034	-0.48869586	0.41572604	1
gene4537	174.6006304	240.0449523	1.374822941	0.459245831	0.41572931	1
gene25290	1054.835761	774.3385557	0.734084475	-0.445982	0.41577098	1
gene25278	38.22617353	23.23015667	0.607702904	-0.71856191	0.41579534	1
gene27941	38.22617353	23.23015667	0.607702904	-0.71856191	0.41579534	1
gene40170	38.22617353	23.23015667	0.607702904	-0.71856191	0.41579534	1
gene49254	38.22617353	23.23015667	0.607702904	-0.71856191	0.41579534	1
gene63741	38.22617353	23.23015667	0.607702904	-0.71856191	0.41579534	1
gene73612	38.22617353	23.23015667	0.607702904	-0.71856191	0.41579534	1
gene960	283.0803121	207.1355637	0.731720133	-0.45063614	0.41584882	1
gene22516	101.2477029	143.2526328	1.414872918	0.500672478	0.41585133	1
gene51982	197.3297066	142.2847096	0.721050632	-0.47182753	0.41585998	1
gene29192	83.68432583	120.0224761	1.434228871	0.520275264	0.41587253	1
gene41996	245.8872784	179.065791	0.728243414	-0.45750735	0.41587511	1
gene41969	153.9378339	109.375321	0.710516175	-0.4930606	0.41587933	1
gene72673	2312.166929	1656.116586	0.716261688	-0.48144132	0.41588775	1
gene16209	696.3362421	946.6288844	1.359442216	0.44301483	0.41591591	1
gene21770	2316.299488	1659.020356	0.716237414	-0.48149021	0.41594646	1
gene16655	277.9146129	203.2638709	0.731389648	-0.45128789	0.41600149	1
gene22905	782.0868476	576.882224	0.737619135	-0.43905201	0.41602951	1
gene7638	56.82269038	84.20931793	1.481966401	0.567512739	0.41612171	1
gene7955	56.82269038	84.20931793	1.481966401	0.567512739	0.41612171	1
gene72371	56.82269038	84.20931793	1.481966401	0.567512739	0.41612171	1
gene25371	567.193764	419.1107433	0.738919872	-0.43651017	0.41612852	1
gene14658	473.1780399	349.4202733	0.73845412	-0.4374198	0.41614937	1
gene56444	60.95524968	39.68485098	0.651048945	-0.61916209	0.41618502	1
gene17595	51.65699125	32.90938862	0.637075211	-0.65046439	0.41622517	1
gene39563	51.65699125	32.90938862	0.637075211	-0.65046439	0.41622517	1
gene46766	51.65699125	32.90938862	0.637075211	-0.65046439	0.41622517	1
gene57919	51.65699125	32.90938862	0.637075211	-0.65046439	0.41622517	1
gene13724	350.2344007	474.2823654	1.354185552	0.437425433	0.41624628	1
gene4797	789.3188264	1075.362669	1.362393286	0.44614323	0.41626198	1
gene70312	615.7513357	835.317717	1.356582874	0.439977185	0.41630847	1
gene5757	213.8599438	154.8677111	0.72415483	-0.46562991	0.4163277	1
gene39312	213.8599438	154.8677111	0.72415483	-0.46562991	0.4163277	1
gene394	377.0960362	277.7939569	0.73666634	-0.44091677	0.41638107	1
gene33252	148.7721348	105.5036282	0.709162562	-0.49581172	0.41642842	1
gene72103	167.3686517	230.3657203	1.376397061	0.460896717	0.41653096	1
gene54849	295.47799	400.7202026	1.356176149	0.439544577	0.41662227	1
gene29554	1228.403252	899.2006478	0.732007707	-0.45006926	0.41668746	1
gene4424	674.6403058	498.4804452	0.738883285	-0.4365816	0.41679527	1
gene4641	36.15989388	56.13954529	1.552536229	0.634626935	0.41682864	1
gene20645	36.15989388	56.13954529	1.552536229	0.634626935	0.41682864	1

gene59446	36.15989388	56.13954529	1.552536229	0.634626935	0.41682864	1
gene54799	893.6659487	1220.551148	1.365780077	0.449725194	0.41686055	1
gene12237	47.52443195	71.6263164	1.507147239	0.591820366	0.41690802	1
gene12875	47.52443195	71.6263164	1.507147239	0.591820366	0.41690802	1
gene24226	116.7448002	163.5790199	1.4011675	0.486629431	0.41691629	1
gene5234	239.6884394	326.1901166	1.360892154	0.444552743	0.41691895	1
gene23767	143.6064357	101.6319354	0.707711566	-0.4987666	0.41698959	1
gene9663	34.09361423	20.32638709	0.59619338	-0.74614774	0.41704218	1
gene12567	34.09361423	20.32638709	0.59619338	-0.74614774	0.41704218	1
gene25010	34.09361423	20.32638709	0.59619338	-0.74614774	0.41704218	1
gene29363	34.09361423	20.32638709	0.59619338	-0.74614774	0.41704218	1
gene62241	34.09361423	20.32638709	0.59619338	-0.74614774	0.41704218	1
gene16013	513.4704931	694.9688538	1.353473789	0.436666949	0.41707984	1
gene29733	694.2699625	512.9992932	0.738904635	-0.43653992	0.41711647	1
gene19107	80.58490636	54.2036989	0.672628428	-0.57211834	0.41712617	1
gene8939	94.01572408	133.5734009	1.420755966	0.506658773	0.4171628	1
gene2152	121.9104994	170.3544823	1.397373345	0.482717526	0.41718182	1
gene8022	121.9104994	170.3544823	1.397373345	0.482717526	0.41718182	1
gene16991	154.9709738	213.911026	1.380329624	0.465012826	0.41719222	1
gene8835	547.5641073	741.4291671	1.354049977	0.437280989	0.41720015	1
gene68049	284.1134519	208.1034868	0.732466152	-0.449166	0.41720638	1
gene29099	3653.182421	5347.77565	1.463867673	0.549785146	0.41722765	1
gene34385	29.96105493	17.4226175	0.581508814	-0.78212704	0.41723057	1
gene48744	29.96105493	17.4226175	0.581508814	-0.78212704	0.41723057	1
gene50336	29.96105493	17.4226175	0.581508814	-0.78212704	0.41723057	1
gene11782	971.1514356	1328.958546	1.368435959	0.45252792	0.41726109	1
gene66435	578.558302	427.822052	0.739462299	-0.4354515	0.41726579	1
gene19322	278.9477528	204.2317941	0.732150706	-0.44978745	0.41737857	1
gene35359	278.9477528	204.2317941	0.732150706	-0.44978745	0.41737857	1
gene39965	1010.410749	1384.130168	1.369868808	0.454037733	0.41739132	1
gene53340	352.3006804	259.4034162	0.736312561	-0.44160978	0.41744313	1
gene14782	336.803583	247.7883378	0.735705765	-0.4427992	0.41753817	1
gene15305	160.1366729	114.214937	0.713234108	-0.4875524	0.41754919	1
gene33903	160.1366729	114.214937	0.713234108	-0.4875524	0.41754919	1
gene70208	160.1366729	114.214937	0.713234108	-0.4875524	0.41754919	1
gene9982	138.4407366	97.76024266	0.706152286	-0.50194875	0.417559	1
gene26108	106.413402	73.56216279	0.691286637	-0.53264406	0.41756446	1
gene28168	106.413402	73.56216279	0.691286637	-0.53264406	0.41756446	1
gene9744	326.4721847	240.0449523	0.735269231	-0.44365548	0.41764803	1
gene73457	1649.924301	1198.288915	0.726269026	-0.46142404	0.41775409	1
gene25725	101.2477029	69.69047001	0.688316555	-0.53885589	0.4177932	1
gene36271	81.61804618	117.1187066	1.434960919	0.521011446	0.41780676	1
gene47089	137.4075967	190.6808693	1.387702528	0.472698341	0.41785479	1
gene51031	90.91630461	61.94708446	0.681363862	-0.55350266	0.41786884	1
gene23613	96.08200373	65.81877724	0.685027109	-0.54576701	0.41790714	1
gene25061	96.08200373	65.81877724	0.685027109	-0.54576701	0.41790714	1
gene9020	757.2914918	559.4596065	0.738763888	-0.43681475	0.41791813	1
gene61331	341.9692821	462.667287	1.352949844	0.436108358	0.41793285	1

gene32569	2395.851254	3403.217952	1.420462955	0.506361207	0.41795134	1
gene60500	3592.227172	2521.439922	0.701915497	-0.51063074	0.41805354	1
gene44459	54.75641073	81.30554835	1.484858983	0.570325925	0.41812013	1
gene25477	133.2750374	93.88854988	0.704472133	-0.50538546	0.41813175	1
gene3489	445.2832646	329.0938862	0.73906637	-0.43622417	0.41817838	1
gene62457	310.9750873	421.0465897	1.353956014	0.437180871	0.41818584	1
gene49061	175.6337703	241.0128755	1.372246779	0.456539953	0.4182309	1
gene54511	295.47799	216.8147956	0.733776467	-0.44658746	0.41823993	1
gene22122	762.4571909	1036.645741	1.359611731	0.443194715	0.41824867	1
gene56562	61.9883895	90.9847803	1.46777132	0.553627213	0.41825337	1
gene58117	61.9883895	90.9847803	1.46777132	0.553627213	0.41825337	1
gene10288	34.09361423	53.23577571	1.561458851	0.642894551	0.41829002	1
gene12608	34.09361423	53.23577571	1.561458851	0.642894551	0.41829002	1
gene7223	109.5128215	153.8997879	1.405312966	0.490891457	0.41829299	1
gene68653	363.6652184	491.7049829	1.352081414	0.435182025	0.41829844	1
gene65707	952.5549187	701.7443161	0.736696963	-0.4408568	0.41830115	1
gene46757	66.12094881	43.55654376	0.658740453	-0.60221795	0.4183615	1
gene49306	66.12094881	43.55654376	0.658740453	-0.60221795	0.4183615	1
gene62188	66.12094881	43.55654376	0.658740453	-0.60221795	0.4183615	1
gene69028	66.12094881	43.55654376	0.658740453	-0.60221795	0.4183615	1
gene32276	455.6146629	336.8372717	0.739302966	-0.43576239	0.41836236	1
gene2330	274.8151935	372.6504299	1.356003739	0.439361157	0.41847626	1
gene19415	171.501211	122.9262457	0.716766051	-0.48042579	0.41848827	1
gene68421	658.1100686	486.8653669	0.73979322	-0.43480602	0.41849578	1
gene40244	86.78374531	123.8941689	1.427619521	0.513611534	0.4184968	1
gene44268	373.9966167	275.8581105	0.737595203	-0.43909882	0.4185052	1
gene5773	180.7994694	247.7883378	1.37051474	0.454717845	0.41851187	1
gene31908	1033.139825	1415.103711	1.369711704	0.453872268	0.41851543	1
gene57983	10117.53831	6517.994793	0.64422734	-0.63435821	0.41856891	1
gene38896	1083.763677	796.6007892	0.735031821	-0.44412139	0.41858258	1
gene855	1656.12314	2307.528896	1.393331716	0.478538767	0.41865713	1
gene69368	506.2385143	374.5862763	0.739940296	-0.43451923	0.41866579	1
gene22742	128.1093383	90.0168571	0.702656483	-0.50910854	0.41870167	1
gene25648	128.1093383	90.0168571	0.702656483	-0.50910854	0.41870167	1
gene5845	188.0314482	135.5092472	0.720673316	-0.47258267	0.41873536	1
gene20611	45.4581523	68.72254682	1.51177607	0.596244458	0.41875213	1
gene32426	45.4581523	68.72254682	1.51177607	0.596244458	0.41875213	1
gene30452	149.8052746	106.4715514	0.710732994	-0.49262042	0.41876534	1
gene43980	368.8309176	498.4804452	1.351514804	0.434577315	0.41883523	1
gene11684	2166.494213	3057.669372	1.411344352	0.497070032	0.41895351	1
gene63712	125.0099188	174.226175	1.393698809	0.478918816	0.41895797	1
gene56266	253.1192571	343.6127341	1.357513205	0.440966232	0.41902276	1
gene38600	168.4017915	231.3336435	1.373700609	0.45806761	0.41912051	1
gene6350	130.175618	181.0016374	1.390441929	0.475543493	0.41913347	1
gene27595	1721.210949	2401.417446	1.395190664	0.480462291	0.41916725	1
gene5639	47.52443195	30.00561903	0.631372492	-0.66343669	0.41919042	1
gene6990	47.52443195	30.00561903	0.631372492	-0.66343669	0.41919042	1
gene15945	47.52443195	30.00561903	0.631372492	-0.66343669	0.41919042	1

gene46087	47.52443195	30.00561903	0.631372492	-0.66343669	0.41919042	1
gene51472	47.52443195	30.00561903	0.631372492	-0.66343669	0.41919042	1
gene61358	496.9402559	367.810814	0.740150973	-0.43410852	0.41924079	1
gene48708	773.821729	572.042608	0.739243403	-0.43587863	0.4192534	1
gene64259	1499.085886	2079.099022	1.386911211	0.471875431	0.41925919	1
gene59978	716.9990386	530.4219107	0.739780505	-0.43483081	0.41931186	1
gene22077	13.43081773	24.19807987	1.80168329	0.849345428	0.41933862	1
gene27658	13.43081773	24.19807987	1.80168329	0.849345428	0.41933862	1
gene28394	13.43081773	24.19807987	1.80168329	0.849345428	0.41933862	1
gene43908	13.43081773	24.19807987	1.80168329	0.849345428	0.41933862	1
gene56426	13.43081773	24.19807987	1.80168329	0.849345428	0.41933862	1
gene56807	13.43081773	24.19807987	1.80168329	0.849345428	0.41933862	1
gene69243	13.43081773	24.19807987	1.80168329	0.849345428	0.41933862	1
gene33366	1134.387528	1557.38842	1.37288923	0.457215228	0.4193757	1
gene1019	635.3809924	470.4106726	0.740360002	-0.43370114	0.41944911	1
gene14477	56.82269038	36.7810814	0.647295669	-0.62750324	0.41947575	1
gene14739	56.82269038	36.7810814	0.647295669	-0.62750324	0.41947575	1
gene36145	56.82269038	36.7810814	0.647295669	-0.62750324	0.41947575	1
gene55547	1047.603783	1434.462174	1.3692793	0.453416752	0.41958207	1
gene59847	698.4025218	516.8709859	0.740076059	-0.43425455	0.41963247	1
gene52937	32.02733458	50.33200612	1.571532779	0.652172364	0.41970821	1
gene55838	32.02733458	50.33200612	1.571532779	0.652172364	0.41970821	1
gene28599	251.0529775	340.7089645	1.357119792	0.440548072	0.41972134	1
gene44546	631.2484331	854.6761809	1.353945825	0.437170014	0.41976826	1
gene14347	67.15408863	97.76024266	1.455760098	0.541772626	0.41983351	1
gene33314	71.28664793	47.42823654	0.66531725	-0.58788566	0.4198599	1
gene35102	71.28664793	47.42823654	0.66531725	-0.58788566	0.4198599	1
gene50023	572.3594631	423.9503593	0.740706473	-0.43302615	0.41986736	1
gene12027	338.8698626	249.7241842	0.736932409	-0.44039579	0.41987532	1
gene51946	798.6170848	590.4331487	0.739319456	-0.43573021	0.41993907	1
gene70302	107.4465418	150.9960184	1.405312966	0.490891457	0.42002367	1
gene20403	139.4738764	98.72816585	0.707861346	-0.4984613	0.42005005	1
gene8720	206.627965	281.6656496	1.363153577	0.44694811	0.42008655	1
gene26236	323.3727653	238.1091059	0.736330116	-0.44157538	0.42015	1
gene57456	52.69013108	78.40177877	1.487978435	0.573353618	0.42015856	1
gene65911	52.69013108	78.40177877	1.487978435	0.573353618	0.42015856	1
gene25823	582.6908613	431.6937448	0.740862391	-0.4327225	0.42020532	1
gene36444	1163.315443	854.6761809	0.734689964	-0.44479253	0.42024909	1
gene46990	1431.931798	1046.324973	0.730708666	-0.45263178	0.42027269	1
gene3259	18.59651685	9.679231946	0.520486284	-0.94206795	0.42029166	1
gene13417	18.59651685	9.679231946	0.520486284	-0.94206795	0.42029166	1
gene14395	18.59651685	9.679231946	0.520486284	-0.94206795	0.42029166	1
gene25907	18.59651685	9.679231946	0.520486284	-0.94206795	0.42029166	1
gene32169	18.59651685	9.679231946	0.520486284	-0.94206795	0.42029166	1
gene39193	18.59651685	9.679231946	0.520486284	-0.94206795	0.42029166	1
gene41422	18.59651685	9.679231946	0.520486284	-0.94206795	0.42029166	1
gene47813	18.59651685	9.679231946	0.520486284	-0.94206795	0.42029166	1
gene61255	18.59651685	9.679231946	0.520486284	-0.94206795	0.42029166	1

gene63205	18.59651685	9.679231946	0.520486284	-0.94206795	0.42029166		1
gene72003	18.59651685	9.679231946	0.520486284	-0.94206795	0.42029166		1
gene10009	1427.799238	1043.421204	0.730789859	-0.45247148	0.4203122		1
gene16487	236.5890199	321.3505006	1.358264643	0.4417646	0.42034729		1
gene7879	827.5449999	611.727459	0.739207486	-0.43594873	0.4203577		1
gene22243	156.0041136	111.3111674	0.713514309	-0.48698573	0.42039295		1
gene54463	1688.150474	1227.326611	0.727024415	-0.45992428	0.42040001		1
gene38060	4622.267577	6893.548992	1.491378177	0.576646136	0.42040279		1
gene6577	248.9866978	181.9695606	0.730840491	-0.45237153	0.42040606		1
gene50677	568.2269038	421.0465897	0.7409832	-0.43248726	0.42044818		1
gene14288	1010.410749	1381.226399	1.366994957	0.451007921	0.42044959		1
gene47220	227.2907615	165.5148663	0.728207628	-0.45757824	0.42045018		1
gene44817	3435.189918	2421.743833	0.704981061	-0.50434359	0.42052212		1
gene44833	172.5343508	123.8941689	0.71808407	-0.47777534	0.42053909		1
gene39407	122.9436392	171.3224055	1.393503613	0.478716743	0.42054076		1
gene50503	483.5094381	358.131582	0.740692019	-0.4330543	0.42060681		1
gene32551	4514.821036	3130.263611	0.693330608	-0.52838464	0.42062191		1
gene22699	302.7099687	222.6223348	0.735431131	-0.44333785	0.42068218		1
gene16198	222.1250624	161.6431735	0.727712451	-0.4585596	0.42091052		1
gene57806	72.31978776	104.535705	1.445464765	0.531533442	0.42100308		1
gene1983	266.5500749	361.0353516	1.354474771	0.437733521	0.42102363		1
gene45917	292.3785705	214.8789492	0.734933989	-0.44431342	0.42103192		1
gene29000	29.96105493	47.42823654	1.582996215	0.662657806	0.42105507		1
gene32907	29.96105493	47.42823654	1.582996215	0.662657806	0.42105507		1
gene58651	29.96105493	47.42823654	1.582996215	0.662657806	0.42105507		1
gene42091	150.8384145	107.4394746	0.712281914	-0.48947974	0.42107819		1
gene72154	102.2808427	70.65839321	0.690827249	-0.5336031	0.42114902		1
gene59178	186.9983083	255.5317234	1.366492166	0.450477189	0.42117882		1
gene48434	569.2600436	422.0145129	0.741338721	-0.43179523	0.42119368		1
gene71888	167.3686517	120.0224761	0.717114435	-0.47972474	0.42120148		1
gene4252	153.9378339	211.9751796	1.377018074	0.461547496	0.42125703		1
gene43377	474.2111797	351.3561197	0.740927533	-0.43259565	0.42129141		1
gene7410	1249.066049	1718.06367	1.375478641	0.459933737	0.42129395		1
gene33637	95.04886391	134.5413241	1.415496393	0.501308073	0.42132832		1
gene53665	81.61804618	55.17162209	0.675973325	-0.56496178	0.42135599		1
gene55991	81.61804618	55.17162209	0.675973325	-0.56496178	0.42135599		1
gene43150	129.1424781	90.9847803	0.704530234	-0.50526648	0.42137362		1
gene15163	159.1035331	218.750642	1.374894937	0.459321378	0.421392		1
gene72442	164.2692322	225.5261044	1.37290533	0.457232146	0.42153806		1
gene16274	1193.276498	876.9384143	0.734899594	-0.44438094	0.42158931		1
gene62067	233.4896005	170.3544823	0.729602012	-0.45481839	0.42159985		1
gene10397	397.7588327	294.2486512	0.739766479	-0.43485817	0.42161024		1
gene32381	413.25593	305.8637295	0.740131495	-0.43414648	0.421618		1
gene7206	339.9030025	458.7955943	1.349783882	0.432728432	0.42165516		1
gene46031	145.6727153	103.5677818	0.710962115	-0.49215541	0.42178582		1
gene54153	308.9088077	227.4619507	0.736340127	-0.44155577	0.42178774		1
gene53935	3568.464956	2512.728613	0.704148323	-0.50604874	0.4218159		1
gene20707	836.8432583	1137.309754	1.359047519	0.4425959	0.42184349		1

gene41985	77.48548688	111.3111674	1.436542143	0.522600317	0.4218648	1
gene658	1180.87882	868.2271056	0.735238105	-0.44371656	0.42191474	1
gene48685	649.84495	878.8742607	1.352436856	0.435561237	0.42208899	1
gene34276	43.39187265	27.10184945	0.62458354	-0.67903354	0.4220941	1
gene48478	43.39187265	27.10184945	0.62458354	-0.67903354	0.4220941	1
gene50765	43.39187265	27.10184945	0.62458354	-0.67903354	0.4220941	1
gene40644	1069.299719	1462.531947	1.367747434	0.451801849	0.42211472	1
gene27750	345.0687016	465.5710566	1.349212648	0.432117748	0.42213475	1
gene50964	475.2443195	352.3240428	0.741353507	-0.43176645	0.42216055	1
gene45924	3395.930605	2398.513676	0.706290545	-0.50166631	0.42218015	1
gene12375	50.62385143	75.49800918	1.491352535	0.576621331	0.4222342	1
gene7191	27.89477528	44.52446695	1.596157937	0.674603411	0.42229253	1
gene38304	27.89477528	44.52446695	1.596157937	0.674603411	0.42229253	1
gene68166	27.89477528	44.52446695	1.596157937	0.674603411	0.42229253	1
gene33532	605.4199375	449.1163623	0.741826185	-0.4308469	0.42231084	1
gene31335	262.4175156	355.2278124	1.353674169	0.436880523	0.42237337	1
gene49394	41.325593	62.91500765	1.52242238	0.606368675	0.42242175	1
gene8231	330.604744	243.916645	0.737789307	-0.43871921	0.42242446	1
gene8639	15.49709738	27.10184945	1.748833913	0.806393283	0.42246223	1
gene18030	15.49709738	27.10184945	1.748833913	0.806393283	0.42246223	1
gene28095	15.49709738	27.10184945	1.748833913	0.806393283	0.42246223	1
gene38846	15.49709738	27.10184945	1.748833913	0.806393283	0.42246223	1
gene40907	15.49709738	27.10184945	1.748833913	0.806393283	0.42246223	1
gene64917	15.49709738	27.10184945	1.748833913	0.806393283	0.42246223	1
gene73183	15.49709738	27.10184945	1.748833913	0.806393283	0.42246223	1
gene47453	57.8558302	85.17724113	1.472232631	0.558005653	0.42249424	1
gene21932	82.65118601	118.0866297	1.428734849	0.514738199	0.42249467	1
gene39763	536.1995692	397.816433	0.741918599	-0.43066719	0.42251865	1
gene52201	536.1995692	397.816433	0.741918599	-0.43066719	0.42251865	1
gene70826	157.0372534	112.2790906	0.71498379	-0.48401756	0.42261638	1
gene42438	118.8110799	83.24139474	0.700619798	-0.51329634	0.42268716	1
gene60220	364.6983583	491.7049829	1.348251155	0.43108927	0.42269439	1
gene27404	587.8565605	793.6970196	1.350154226	0.433124214	0.42271108	1
gene17472	239.6884394	175.1940982	0.730924273	-0.45220615	0.42271525	1
gene15918	378.129176	279.7298033	0.739773128	-0.4348452	0.42275666	1
gene48413	888.5002496	657.2198492	0.73969574	-0.43499613	0.42286831	1
gene41058	52.69013108	33.87731181	0.642953645	-0.63721337	0.42288544	1
gene62273	87.81688513	124.8620921	1.42184606	0.507765276	0.42294976	1
gene18494	256.2186766	187.7770998	0.732878267	-0.44835451	0.4229944	1
gene14299	70.25350811	101.6319354	1.4466457	0.532711633	0.42314684	1
gene6848	606.4530773	818.8630227	1.350249596	0.433226117	0.42315866	1
gene72596	184.9320287	133.5734009	0.722283759	-0.46936236	0.42316284	1
gene50885	135.3413171	95.82439627	0.708020273	-0.49813742	0.42325713	1
gene12957	92.98258426	131.6375545	1.415722692	0.501538702	0.42327337	1
gene2247	757.2914918	561.3954529	0.741320164	-0.43183134	0.42327656	1
gene51695	113.6453808	79.36970196	0.698397959	-0.51787875	0.42331695	1
gene69809	113.6453808	79.36970196	0.698397959	-0.51787875	0.42331695	1
gene72631	113.6453808	79.36970196	0.698397959	-0.51787875	0.42331695	1

gene21020	151.8715543	108.4073978	0.713809761	-0.48638847	0.42336735	1
gene3430	25.82849563	41.62069737	1.611425534	0.688337522	0.42336873	1
gene72284	25.82849563	41.62069737	1.611425534	0.688337522	0.42336873	1
gene560	8.265118601	16.45469431	1.990860035	0.993391798	0.42338674	1
gene15467	8.265118601	16.45469431	1.990860035	0.993391798	0.42338674	1
gene17263	8.265118601	16.45469431	1.990860035	0.993391798	0.42338674	1
gene23651	8.265118601	16.45469431	1.990860035	0.993391798	0.42338674	1
gene26275	8.265118601	16.45469431	1.990860035	0.993391798	0.42338674	1
gene33675	8.265118601	16.45469431	1.990860035	0.993391798	0.42338674	1
gene48695	8.265118601	16.45469431	1.990860035	0.993391798	0.42338674	1
gene51307	8.265118601	16.45469431	1.990860035	0.993391798	0.42338674	1
gene53252	8.265118601	16.45469431	1.990860035	0.993391798	0.42338674	1
gene56585	8.265118601	16.45469431	1.990860035	0.993391798	0.42338674	1
gene57430	8.265118601	16.45469431	1.990860035	0.993391798	0.42338674	1
gene58890	8.265118601	16.45469431	1.990860035	0.993391798	0.42338674	1
gene71896	8.265118601	16.45469431	1.990860035	0.993391798	0.42338674	1
gene72849	8.265118601	16.45469431	1.990860035	0.993391798	0.42338674	1
gene44138	1403.003882	1027.934433	0.732666848	-0.44877076	0.42340401	1
gene3055	3882.539463	2723.73587	0.701534626	-0.51141378	0.42345157	1
gene15708	210.7605243	286.5052656	1.359387706	0.44295698	0.42349296	1
gene69047	67.15408863	44.52446695	0.663019451	-0.5928769	0.42355786	1
gene21000	1211.873015	891.4572623	0.735602865	-0.443001	0.42361012	1
gene34529	399.8251123	296.1844976	0.740785129	-0.43287296	0.42362521	1
gene34397	212.826804	154.8677111	0.727670144	-0.45864348	0.42362729	1
gene5597	446.3164044	331.0297326	0.741692954	-0.43110603	0.42363123	1
gene1426	439.0844257	591.4010719	1.346896035	0.429638495	0.42365968	1
gene4745	439.0844257	591.4010719	1.346896035	0.429638495	0.42365968	1
gene45687	406.0239513	546.876605	1.346907253	0.429650512	0.42366996	1
gene18347	215.9262234	293.280728	1.358245068	0.441743808	0.42378682	1
gene58388	215.9262234	293.280728	1.358245068	0.441743808	0.42378682	1
gene67983	245.8872784	180.0337142	0.732179865	-0.44973	0.42380002	1
gene71498	245.8872784	180.0337142	0.732179865	-0.44973	0.42380002	1
gene73746	373.9966167	276.8260337	0.740183257	-0.43404559	0.4238758	1
gene59358	129.1424781	179.065791	1.38657546	0.471526133	0.42389135	1
gene60141	108.4796816	75.49800918	0.695964517	-0.52291434	0.42391108	1
gene59549	75.41920723	108.4073978	1.437397737	0.52345932	0.42399362	1
gene32911	130.175618	91.95270349	0.706374242	-0.50149536	0.42401297	1
gene40210	163.2360924	117.1187066	0.717480459	-0.47898855	0.42403575	1
gene11770	262.4175156	192.6167157	0.73400861	-0.44613111	0.42406508	1
gene70777	262.4175156	192.6167157	0.73400861	-0.44613111	0.42406508	1
gene43120	160.1366729	219.7185652	1.372069003	0.456353039	0.4240902	1
gene6256	17.56337703	30.00561903	1.708419684	0.772662426	0.42410845	1
gene11802	17.56337703	30.00561903	1.708419684	0.772662426	0.42410845	1
gene54440	17.56337703	30.00561903	1.708419684	0.772662426	0.42410845	1
gene67365	17.56337703	30.00561903	1.708419684	0.772662426	0.42410845	1
gene69708	17.56337703	30.00561903	1.708419684	0.772662426	0.42410845	1
gene24865	224.191342	163.5790199	0.729640219	-0.45474284	0.42417443	1
gene54233	224.191342	163.5790199	0.729640219	-0.45474284	0.42417443	1

gene70969	203.5285455	276.8260337	1.3601337	0.443748474	0.42418736	1
gene35347	1680.918495	1225.390764	0.729000703	-0.45600789	0.42420609	1
gene22134	23.76221598	38.71692779	1.629348366	0.704295095	0.42421212	1
gene32829	23.76221598	38.71692779	1.629348366	0.704295095	0.42421212	1
gene50373	23.76221598	38.71692779	1.629348366	0.704295095	0.42421212	1
gene4933	39.25931335	60.01123807	1.528586033	0.612197754	0.42428553	1
gene66223	39.25931335	60.01123807	1.528586033	0.612197754	0.42428553	1
gene13396	3465.150973	5020.617611	1.448888562	0.534946637	0.42432333	1
gene71386	48.55757178	72.5942396	1.495013794	0.580158795	0.42434319	1
gene31542	839.9426778	622.3746142	0.740972724	-0.43250766	0.42434794	1
gene54139	1580.703932	1154.732371	0.730517808	-0.45300865	0.42436534	1
gene16776	1052.769482	777.2423253	0.738283488	-0.4377532	0.42443663	1
gene64423	278.9477528	205.1997173	0.735620614	-0.44296619	0.42444947	1
gene52070	103.3139825	71.6263164	0.69328773	-0.52847387	0.42445158	1
gene17973	378.129176	509.1276004	1.346438288	0.429148108	0.42453052	1
gene59735	5399.188726	3707.145835	0.686611642	-0.54243378	0.42454649	1
gene1961	411.1896504	304.8958063	0.741496791	-0.43148765	0.42456032	1
gene30467	243.8209987	330.0618094	1.353705428	0.436913836	0.42456197	1
gene33808	72.31978776	48.39615973	0.669196651	-0.57949787	0.42464379	1
gene33973	72.31978776	48.39615973	0.669196651	-0.57949787	0.42464379	1
gene65908	72.31978776	48.39615973	0.669196651	-0.57949787	0.42464379	1
gene39580	564.0943445	419.1107433	0.742979871	-0.42860497	0.42464596	1
gene1613	658.1100686	488.8012133	0.742734744	-0.42908103	0.42467349	1
gene13401	55.78955055	82.27347154	1.474711137	0.560432391	0.42469837	1
gene27212	55.78955055	82.27347154	1.474711137	0.560432391	0.42469837	1
gene25662	21.69593633	35.8131582	1.650685071	0.723064899	0.42472262	1
gene31838	21.69593633	35.8131582	1.650685071	0.723064899	0.42472262	1
gene46692	21.69593633	35.8131582	1.650685071	0.723064899	0.42472262	1
gene50906	21.69593633	35.8131582	1.650685071	0.723064899	0.42472262	1
gene10593	219.0256429	159.7073271	0.729171822	-0.45566928	0.42472762	1
gene5243	19.62965668	32.90938862	1.676513714	0.745464284	0.42475767	1
gene34580	19.62965668	32.90938862	1.676513714	0.745464284	0.42475767	1
gene45036	125.0099188	88.08101071	0.704592176	-0.50513964	0.42477505	1
gene928	39.25931335	24.19807987	0.616365336	-0.69814237	0.42481878	1
gene3668	39.25931335	24.19807987	0.616365336	-0.69814237	0.42481878	1
gene17298	39.25931335	24.19807987	0.616365336	-0.69814237	0.42481878	1
gene49457	252.0861173	184.8733302	0.733373706	-0.44737955	0.42485737	1
gene11683	248.9866978	336.8372717	1.352832399	0.435983116	0.4248991	1
gene23835	807.9153432	599.1444575	0.741593117	-0.43130024	0.42491274	1
gene41029	98.14828338	67.75462362	0.690329176	-0.53464363	0.42491534	1
gene45877	644.6792509	870.162952	1.349761065	0.432704043	0.42494212	1
gene52290	376.0628963	506.2238308	1.346114801	0.428801453	0.42494315	1
gene58169	295.47799	217.7827188	0.737052255	-0.44016119	0.42494922	1
gene10726	946.3560798	1287.337849	1.360310222	0.443935699	0.42497689	1
gene11792	85.75060548	121.9583225	1.422244448	0.508169449	0.42500672	1
gene58391	201.4622659	273.9222641	1.359670323	0.443256886	0.42518498	1
gene49895	972.1845754	719.1669336	0.739743205	-0.43490356	0.4251977	1
gene14374	77.48548688	52.26785251	0.674550224	-0.56800223	0.42524933	1

gene29483	77.48548688	52.26785251	0.674550224	-0.56800223	0.42524933	1
gene4978	68.18722846	98.72816585	1.447898207	0.533960179	0.42535921	1
gene24280	536.1995692	722.0707032	1.346645437	0.429370049	0.42537432	1
gene41450	147.738995	203.2638709	1.375830876	0.460303138	0.42540317	1
gene12926	698.4025218	518.8068323	0.74284788	-0.42886129	0.42545626	1
gene55804	422.5541885	313.6071151	0.742170173	-0.43017807	0.42547885	1
gene47829	87.81688513	60.01123807	0.683367874	-0.54926567	0.42548408	1
gene21543	57.8558302	37.74900459	0.652466734	-0.61602375	0.42550471	1
gene33944	127.0761985	176.1620214	1.386270785	0.471209092	0.42551458	1
gene49802	312.0082272	230.3657203	0.738332199	-0.43765802	0.42552438	1
gene70344	683.9385642	508.1596772	0.742990239	-0.42858484	0.42554341	1
gene6880	121.9104994	169.3865591	1.389433724	0.474497019	0.42555316	1
gene19918	407.0570911	301.9920367	0.741891109	-0.43072064	0.42557671	1
gene195	111.5791011	155.8356343	1.396638195	0.481958333	0.42560332	1
gene32683	152.9046941	109.375321	0.71531696	-0.48334545	0.42563317	1
gene44432	152.9046941	109.375321	0.71531696	-0.48334545	0.42563317	1
gene8821	630.2152933	468.4748262	0.743356804	-0.42787324	0.42570275	1
gene7573	1055.868901	780.1460949	0.738866439	-0.4366145	0.42574241	1
gene21208	180.7994694	130.6696313	0.722732383	-0.46846656	0.42580633	1
gene2948	5.165699125	11.61507834	2.248500746	1.168963362	0.42602694	1
gene3431	5.165699125	11.61507834	2.248500746	1.168963362	0.42602694	1
gene9933	5.165699125	11.61507834	2.248500746	1.168963362	0.42602694	1
gene10895	5.165699125	11.61507834	2.248500746	1.168963362	0.42602694	1
gene14574	5.165699125	11.61507834	2.248500746	1.168963362	0.42602694	1
gene15195	5.165699125	11.61507834	2.248500746	1.168963362	0.42602694	1
gene16106	5.165699125	11.61507834	2.248500746	1.168963362	0.42602694	1
gene18848	5.165699125	11.61507834	2.248500746	1.168963362	0.42602694	1
gene19920	5.165699125	11.61507834	2.248500746	1.168963362	0.42602694	1
gene20060	5.165699125	11.61507834	2.248500746	1.168963362	0.42602694	1
gene20071	5.165699125	11.61507834	2.248500746	1.168963362	0.42602694	1
gene21356	5.165699125	11.61507834	2.248500746	1.168963362	0.42602694	1
gene23039	5.165699125	11.61507834	2.248500746	1.168963362	0.42602694	1
gene27494	5.165699125	11.61507834	2.248500746	1.168963362	0.42602694	1
gene30923	5.165699125	11.61507834	2.248500746	1.168963362	0.42602694	1
gene31482	5.165699125	11.61507834	2.248500746	1.168963362	0.42602694	1
gene31490	5.165699125	11.61507834	2.248500746	1.168963362	0.42602694	1
gene32942	5.165699125	11.61507834	2.248500746	1.168963362	0.42602694	1
gene35009	5.165699125	11.61507834	2.248500746	1.168963362	0.42602694	1
gene37191	5.165699125	11.61507834	2.248500746	1.168963362	0.42602694	1
gene37210	5.165699125	11.61507834	2.248500746	1.168963362	0.42602694	1
gene37753	5.165699125	11.61507834	2.248500746	1.168963362	0.42602694	1
gene38793	5.165699125	11.61507834	2.248500746	1.168963362	0.42602694	1
gene42367	5.165699125	11.61507834	2.248500746	1.168963362	0.42602694	1
gene43052	5.165699125	11.61507834	2.248500746	1.168963362	0.42602694	1
gene47506	5.165699125	11.61507834	2.248500746	1.168963362	0.42602694	1
gene48592	5.165699125	11.61507834	2.248500746	1.168963362	0.42602694	1
gene50281	5.165699125	11.61507834	2.248500746	1.168963362	0.42602694	1
gene52046	5.165699125	11.61507834	2.248500746	1.168963362	0.42602694	1

gene52311	5.165699125	11.61507834	2.248500746	1.168963362	0.42602694	1
gene52379	5.165699125	11.61507834	2.248500746	1.168963362	0.42602694	1
gene56770	5.165699125	11.61507834	2.248500746	1.168963362	0.42602694	1
gene57922	5.165699125	11.61507834	2.248500746	1.168963362	0.42602694	1
gene59245	5.165699125	11.61507834	2.248500746	1.168963362	0.42602694	1
gene65253	5.165699125	11.61507834	2.248500746	1.168963362	0.42602694	1
gene68314	5.165699125	11.61507834	2.248500746	1.168963362	0.42602694	1
gene71211	5.165699125	11.61507834	2.248500746	1.168963362	0.42602694	1
gene72808	5.165699125	11.61507834	2.248500746	1.168963362	0.42602694	1
gene74124	5.165699125	11.61507834	2.248500746	1.168963362	0.42602694	1
gene55790	1445.362615	1991.985935	1.378191129	0.462775976	0.42605985	1
gene32114	652.9443695	880.8101071	1.348981856	0.431870944	0.42610563	1
gene55889	603.3536578	813.0554835	1.347560378	0.430349915	0.42613052	1
gene47479	525.868171	391.0409706	0.743610266	-0.42738141	0.42615189	1
gene59836	37.1930337	57.10746848	1.535434537	0.618647005	0.42618075	1
gene59566	986.648533	1342.509471	1.360676498	0.444324106	0.42625643	1
gene58281	114.6785206	80.33762515	0.700546404	-0.51344748	0.4262862	1
gene71903	114.6785206	80.33762515	0.700546404	-0.51344748	0.4262862	1
gene50192	691.170543	933.0779596	1.349996711	0.432955892	0.42629265	1
gene73651	360.565799	267.1468017	0.740909988	-0.43262981	0.42632601	1
gene37927	48.55757178	30.97354223	0.637872552	-0.6486599	0.42638836	1
gene18491	147.738995	105.5036282	0.71412174	-0.48575806	0.42648033	1
gene54995	147.738995	105.5036282	0.71412174	-0.48575806	0.42648033	1
gene37322	46.49129213	69.69047001	1.499000497	0.584000862	0.42648037	1
gene8933	3232.694513	4650.87095	1.438697944	0.524763729	0.42651763	1
gene124	695.3031023	516.8709859	0.743375061	-0.42783781	0.42651972	1
gene28861	636.4141322	473.3144422	0.743720823	-0.42716693	0.42652479	1
gene35054	367.7977777	494.6087525	1.344784505	0.427375007	0.42665937	1
gene6949	591.9891198	440.4050536	0.743941128	-0.42673964	0.42670391	1
gene17636	392.5931335	291.3448816	0.742103865	-0.43030697	0.42676055	1
gene54312	5662.639381	8576.767428	1.514623632	0.598959344	0.42678221	1
gene55265	156.0041136	213.911026	1.371188369	0.455426777	0.42678556	1
gene17415	971.1514356	719.1669336	0.740530166	-0.43336959	0.42681612	1
gene4241	109.5128215	76.46593238	0.69823726	-0.51821075	0.42701352	1
gene58425	632.2815729	470.4106726	0.743989217	-0.42664638	0.42705363	1
gene13794	22.72907615	12.58300153	0.553608138	-0.85306294	0.42707516	1
gene32514	22.72907615	12.58300153	0.553608138	-0.85306294	0.42707516	1
gene33384	22.72907615	12.58300153	0.553608138	-0.85306294	0.42707516	1
gene36629	22.72907615	12.58300153	0.553608138	-0.85306294	0.42707516	1
gene43563	22.72907615	12.58300153	0.553608138	-0.85306294	0.42707516	1
gene61789	22.72907615	12.58300153	0.553608138	-0.85306294	0.42707516	1
gene62034	570.2931834	767.5630933	1.34590964	0.428581556	0.42707611	1
gene27861	334.7373033	247.7883378	0.740247159	-0.43392105	0.42711241	1
gene50084	334.7373033	247.7883378	0.740247159	-0.43392105	0.42711241	1
gene2657	394.6594132	530.4219107	1.343999137	0.426532212	0.42714929	1
gene3900	35.12675405	21.29431028	0.606213436	-0.72210227	0.4271695	1
gene67211	35.12675405	21.29431028	0.606213436	-0.72210227	0.4271695	1
gene67229	35.12675405	21.29431028	0.606213436	-0.72210227	0.4271695	1

gene70434	35.12675405	21.29431028	0.606213436	-0.72210227	0.4271695	1
gene3375	125.0099188	173.2582518	1.385956038	0.470881497	0.42718682	1
gene45558	125.0099188	173.2582518	1.385956038	0.470881497	0.42718682	1
gene35869	944.2898001	1282.498233	1.358161692	0.441655246	0.42723529	1
gene32527	476.2774594	354.2598892	0.7438099	-0.42699415	0.42729265	1
gene23707	329.5716042	243.916645	0.740102126	-0.43420373	0.42730845	1
gene24962	617.8176154	459.7635175	0.744173533	-0.42628901	0.42732875	1
gene68042	429.7861672	319.4146542	0.743194357	-0.42818855	0.42734227	1
gene45746	486.6088576	362.0032748	0.743930714	-0.42675983	0.42734349	1
gene44782	63.02152933	41.62069737	0.660420301	-0.59854363	0.42736296	1
gene55199	63.02152933	41.62069737	0.660420301	-0.59854363	0.42736296	1
gene21868	114.6785206	159.7073271	1.392652489	0.477835305	0.42737207	1
gene49913	94.01572408	132.6054777	1.410460633	0.496166399	0.42747587	1
gene24305	769.6891697	572.042608	0.743212495	-0.42815334	0.42750416	1
gene71408	99.18142321	139.38094	1.405312966	0.490891457	0.42751618	1
gene54234	409.1233707	303.9278831	0.742875878	-0.42880691	0.42753918	1
gene60334	1033.139825	764.6593238	0.740131495	-0.43414648	0.42772865	1
gene41869	254.152397	186.8091766	0.735028191	-0.44412851	0.42778165	1
gene44038	3125.247971	2226.223348	0.712334947	-0.48937232	0.42783825	1
gene46122	1142.652647	844.0290257	0.738657569	-0.43702239	0.4278679	1
gene3534	2541.52397	1828.406915	0.719413602	-0.47510666	0.42792389	1
gene7319	548.5972471	408.4635881	0.74456004	-0.42553991	0.42801291	1
gene65480	5744.257427	8703.565366	1.515176762	0.599486109	0.42804998	1
gene48814	35.12675405	54.2036989	1.543088747	0.625821037	0.42809805	1
gene49460	35.12675405	54.2036989	1.543088747	0.625821037	0.42809805	1
gene9818	1021.775287	1389.937708	1.360316427	0.44394228	0.42810973	1
gene28730	451.4821036	335.8693485	0.743926162	-0.42676866	0.42815836	1
gene33748	18115.07369	33251.06551	1.835546798	0.876209897	0.42820635	1
gene45449	372.9634769	276.8260337	0.742233626	-0.43005473	0.42821465	1
gene55607	190.0977278	258.435493	1.359487543	0.443062932	0.42823771	1
gene56733	308.9088077	228.4298739	0.739473489	-0.43542967	0.42824181	1
gene24111	165.302372	119.0545529	0.720222895	-0.47348463	0.42825882	1
gene2648	137.4075967	97.76024266	0.711461702	-0.491142	0.42826432	1
gene600	99.18142321	68.72254682	0.692897365	-0.52928642	0.42833168	1
gene25016	99.18142321	68.72254682	0.692897365	-0.52928642	0.42833168	1
gene50912	99.18142321	68.72254682	0.692897365	-0.52928642	0.42833168	1
gene45838	3133.513089	4491.163623	1.433267867	0.519308264	0.42839728	1
gene9517	138.4407366	190.6808693	1.377346539	0.461891586	0.42848391	1
gene26038	282.0471722	208.1034868	0.737832204	-0.43863533	0.42848933	1
gene28393	68.18722846	45.49239015	0.667168782	-0.58387631	0.4286207	1
gene70616	68.18722846	45.49239015	0.667168782	-0.58387631	0.4286207	1
gene35482	1855.519126	1352.188703	0.728738758	-0.45652637	0.42863425	1
gene44956	800.6833644	595.2727647	0.743455892	-0.42768094	0.42867186	1
gene890	6.19883895	1.935846389	0.31229177	-1.67903354	0.42870389	1
gene1574	6.19883895	1.935846389	0.31229177	-1.67903354	0.42870389	1
gene4195	6.19883895	1.935846389	0.31229177	-1.67903354	0.42870389	1
gene4273	6.19883895	1.935846389	0.31229177	-1.67903354	0.42870389	1
gene4858	6.19883895	1.935846389	0.31229177	-1.67903354	0.42870389	1

gene5246	6.19883895	1.935846389	0.31229177	-1.67903354	0.42870389	1
gene5897	6.19883895	1.935846389	0.31229177	-1.67903354	0.42870389	1
gene6009	6.19883895	1.935846389	0.31229177	-1.67903354	0.42870389	1
gene6077	6.19883895	1.935846389	0.31229177	-1.67903354	0.42870389	1
gene7908	6.19883895	1.935846389	0.31229177	-1.67903354	0.42870389	1
gene10055	6.19883895	1.935846389	0.31229177	-1.67903354	0.42870389	1
gene10585	6.19883895	1.935846389	0.31229177	-1.67903354	0.42870389	1
gene15761	6.19883895	1.935846389	0.31229177	-1.67903354	0.42870389	1
gene16163	6.19883895	1.935846389	0.31229177	-1.67903354	0.42870389	1
gene18738	6.19883895	1.935846389	0.31229177	-1.67903354	0.42870389	1
gene26068	6.19883895	1.935846389	0.31229177	-1.67903354	0.42870389	1
gene27747	6.19883895	1.935846389	0.31229177	-1.67903354	0.42870389	1
gene30322	6.19883895	1.935846389	0.31229177	-1.67903354	0.42870389	1
gene31530	6.19883895	1.935846389	0.31229177	-1.67903354	0.42870389	1
gene32265	6.19883895	1.935846389	0.31229177	-1.67903354	0.42870389	1
gene34395	6.19883895	1.935846389	0.31229177	-1.67903354	0.42870389	1
gene34942	6.19883895	1.935846389	0.31229177	-1.67903354	0.42870389	1
gene36985	6.19883895	1.935846389	0.31229177	-1.67903354	0.42870389	1
gene37606	6.19883895	1.935846389	0.31229177	-1.67903354	0.42870389	1
gene40809	6.19883895	1.935846389	0.31229177	-1.67903354	0.42870389	1
gene44543	6.19883895	1.935846389	0.31229177	-1.67903354	0.42870389	1
gene49996	6.19883895	1.935846389	0.31229177	-1.67903354	0.42870389	1
gene51214	6.19883895	1.935846389	0.31229177	-1.67903354	0.42870389	1
gene54778	6.19883895	1.935846389	0.31229177	-1.67903354	0.42870389	1
gene58369	6.19883895	1.935846389	0.31229177	-1.67903354	0.42870389	1
gene66956	6.19883895	1.935846389	0.31229177	-1.67903354	0.42870389	1
gene68933	6.19883895	1.935846389	0.31229177	-1.67903354	0.42870389	1
gene72226	6.19883895	1.935846389	0.31229177	-1.67903354	0.42870389	1
gene73659	6.19883895	1.935846389	0.31229177	-1.67903354	0.42870389	1
gene74199	6.19883895	1.935846389	0.31229177	-1.67903354	0.42870389	1
gene40818	58.88897003	86.14516432	1.462840397	0.548772373	0.42876772	1
gene26873	30.99419475	18.3905407	0.593354363	-0.75303413	0.42881325	1
gene43500	30.99419475	18.3905407	0.593354363	-0.75303413	0.42881325	1
gene53804	30.99419475	18.3905407	0.593354363	-0.75303413	0.42881325	1
gene20110	188.0314482	136.4771704	0.725820982	-0.46231433	0.42883055	1
gene27083	778.9874281	1052.132513	1.350641197	0.433644468	0.42887414	1
gene68149	310.9750873	418.1428201	1.344618386	0.427196782	0.42901689	1
gene15882	117.7779401	163.5790199	1.388876557	0.473918379	0.42906426	1
gene62693	199.3959862	145.1884792	0.728141433	-0.45770939	0.42908586	1
gene12112	540.3321285	725.942396	1.34351144	0.426008606	0.42909121	1
gene8100	26.86163545	15.48677111	0.576538653	-0.79451076	0.4291538	1
gene72213	26.86163545	15.48677111	0.576538653	-0.79451076	0.4291538	1
gene8274	519.669332	387.1692779	0.745030068	-0.42462944	0.42920029	1
gene50346	426.6867478	317.4788078	0.744055937	-0.42651701	0.42926744	1
gene60554	271.715774	200.3601013	0.737388553	-0.43950308	0.42926977	1
gene16476	73.35292758	49.36408293	0.672966772	-0.57139282	0.42931464	1
gene47400	73.35292758	49.36408293	0.672966772	-0.57139282	0.42931464	1
gene28868	177.7000499	241.9807987	1.36173737	0.445448487	0.42933182	1

gene16865	53.7232709	34.84523501	0.648605984	-0.62458576	0.42933945	1
gene49357	53.7232709	34.84523501	0.648605984	-0.62458576	0.42933945	1
gene10566	210.7605243	153.8997879	0.730211639	-0.45361343	0.42934847	1
gene54526	210.7605243	153.8997879	0.730211639	-0.45361343	0.42934847	1
gene47771	1069.299719	791.7611732	0.740448313	-0.43352906	0.42943733	1
gene10523	1124.05613	831.4460242	0.739683724	-0.43501956	0.42945299	1
gene46150	411.1896504	305.8637295	0.743850749	-0.42691492	0.42948663	1
gene62446	83.68432583	57.10746848	0.68241535	-0.551278	0.42955788	1
gene403	91.94944443	129.7017081	1.410576311	0.496284716	0.42956354	1
gene45722	78.51862671	53.23577571	0.67800187	-0.56063884	0.42959003	1
gene47950	78.51862671	53.23577571	0.67800187	-0.56063884	0.42959003	1
gene2673	15.49709738	7.743385557	0.499666832	-1.00096164	0.4296612	1
gene4342	15.49709738	7.743385557	0.499666832	-1.00096164	0.4296612	1
gene4660	15.49709738	7.743385557	0.499666832	-1.00096164	0.4296612	1
gene5643	15.49709738	7.743385557	0.499666832	-1.00096164	0.4296612	1
gene5996	15.49709738	7.743385557	0.499666832	-1.00096164	0.4296612	1
gene7277	15.49709738	7.743385557	0.499666832	-1.00096164	0.4296612	1
gene10776	15.49709738	7.743385557	0.499666832	-1.00096164	0.4296612	1
gene17010	15.49709738	7.743385557	0.499666832	-1.00096164	0.4296612	1
gene18664	15.49709738	7.743385557	0.499666832	-1.00096164	0.4296612	1
gene19912	15.49709738	7.743385557	0.499666832	-1.00096164	0.4296612	1
gene20454	15.49709738	7.743385557	0.499666832	-1.00096164	0.4296612	1
gene34517	15.49709738	7.743385557	0.499666832	-1.00096164	0.4296612	1
gene39032	15.49709738	7.743385557	0.499666832	-1.00096164	0.4296612	1
gene48086	15.49709738	7.743385557	0.499666832	-1.00096164	0.4296612	1
gene54977	15.49709738	7.743385557	0.499666832	-1.00096164	0.4296612	1
gene57271	15.49709738	7.743385557	0.499666832	-1.00096164	0.4296612	1
gene61793	15.49709738	7.743385557	0.499666832	-1.00096164	0.4296612	1
gene66364	15.49709738	7.743385557	0.499666832	-1.00096164	0.4296612	1
gene45155	400.8582521	298.1203439	0.743705144	-0.42719734	0.42967571	1
gene50388	479.3768788	357.1636588	0.745058167	-0.42457503	0.42985578	1
gene30991	213.8599438	289.4090352	1.353264338	0.436443673	0.42989597	1
gene7413	480.4100187	644.6368476	1.341847219	0.424220418	0.42990681	1
gene6047	1620.996386	1187.64176	0.732661572	-0.44878115	0.42993478	1
gene6041	353.3338202	262.3071857	0.742377805	-0.42977452	0.42993594	1
gene3188	44.42501248	28.06977264	0.63184614	-0.6623548	0.42993877	1
gene65015	44.42501248	28.06977264	0.63184614	-0.6623548	0.42993877	1
gene68774	44.42501248	28.06977264	0.63184614	-0.6623548	0.42993877	1
gene19770	233.4896005	171.3224055	0.733747478	-0.44664446	0.42994255	1
gene50428	520.7024718	388.137201	0.745410713	-0.42389254	0.42999333	1
gene21008	33.0604744	51.29992932	1.551699733	0.633849411	0.43002435	1
gene31678	33.0604744	51.29992932	1.551699733	0.633849411	0.43002435	1
gene39253	33.0604744	51.29992932	1.551699733	0.633849411	0.43002435	1
gene56106	33.0604744	51.29992932	1.551699733	0.633849411	0.43002435	1
gene16070	401.891392	539.1332194	1.341489841	0.423836129	0.43002439	1
gene64949	752.1257927	1014.383508	1.348688634	0.431557318	0.43003793	1
gene54247	110.5459613	77.43385557	0.700467522	-0.51360993	0.43007011	1
gene45671	205.5948252	150.0280952	0.729727001	-0.45457126	0.43007944	1

gene31069	2236.747721	1620.303428	0.72440151	-0.46513854	0.43014133	1
gene73070	127.0761985	90.0168571	0.708369137	-0.49742674	0.43015002	1
gene32497	842.0089574	626.2463069	0.743752547	-0.42710539	0.43024242	1
gene40851	992.8473719	736.5895511	0.741896058	-0.43071102	0.43028203	1
gene3062	244.8541385	180.0337142	0.735269231	-0.44365548	0.43028831	1
gene43689	407.0570911	545.9086818	1.341110851	0.42342849	0.43043392	1
gene50563	252.0861173	339.7410413	1.347718172	0.430518839	0.43045191	1
gene10995	369.8640574	274.8901873	0.74321952	-0.4281397	0.43048029	1
gene14454	711.8333395	530.4219107	0.745149013	-0.42439913	0.43052356	1
gene26280	612.6519163	456.8597479	0.74570851	-0.42331629	0.43053621	1
gene58189	337.8367228	250.6921074	0.742051087	-0.43040958	0.43057157	1
gene16999	175.6337703	239.0770291	1.361224716	0.444905252	0.43058284	1
gene70543	120.8773595	167.4507127	1.385294263	0.470192465	0.43068583	1
gene73727	189.064588	137.4450936	0.726974285	-0.46002376	0.4306892	1
gene69744	480.4100187	358.131582	0.745470677	-0.42377649	0.43070451	1
gene27536	138.4407366	98.72816585	0.713143893	-0.48773489	0.43072487	1
gene7560	42.35873283	63.88293085	1.508140744	0.592771071	0.43081078	1
gene55078	200.4291261	146.1564024	0.729217381	-0.45557915	0.4308528	1
gene51365	819.2798813	609.7916126	0.744301949	-0.42604008	0.4308715	1
gene16641	105.3802622	73.56216279	0.698063957	-0.51856887	0.43090193	1
gene62215	2723.356579	3859.109777	1.417041678	0.502882192	0.43092747	1
gene62580	3775.092921	2667.596324	0.706630639	-0.50097179	0.43095144	1
gene73473	216.9593633	293.280728	1.351777234	0.434857422	0.43102664	1
gene51142	1501.152166	2064.580174	1.375330377	0.459778219	0.4310574	1
gene43577	267.5832147	197.4563317	0.737924955	-0.43845399	0.43109564	1
gene16472	56.82269038	83.24139474	1.464932304	0.550833998	0.43116982	1
gene24366	56.82269038	83.24139474	1.464932304	0.550833998	0.43116982	1
gene55362	56.82269038	83.24139474	1.464932304	0.550833998	0.43116982	1
gene28588	1911.308676	1393.8094	0.729243485	-0.4555275	0.43120257	1
gene17034	424.6204681	569.1388384	1.34034716	0.422606718	0.43127628	1
gene48095	161.1698127	116.1507834	0.720673316	-0.47258267	0.43129509	1
gene63840	334.7373033	449.1163623	1.341697976	0.424059949	0.43129846	1
gene47980	74.38606741	106.4715514	1.43133728	0.517363669	0.4313183	1
gene27745	858.5391946	638.8293085	0.744088694	-0.4264535	0.43134888	1
gene235	58.88897003	38.71692779	0.657456358	-0.60503296	0.43136436	1
gene62250	100.214563	140.3488632	1.400483712	0.485925204	0.43149735	1
gene26820	1277.993964	943.7251148	0.738442545	-0.43744242	0.43151436	1
gene12289	408.0902309	303.9278831	0.744756576	-0.42515914	0.43152984	1
gene65931	1197.409057	885.6497231	0.739638403	-0.43510796	0.43155722	1
gene13354	183.8988889	133.5734009	0.726341533	-0.46128002	0.4315576	1
gene12490	278.9477528	206.1676405	0.739090523	-0.43617702	0.43156018	1
gene19326	1115.791011	826.6064082	0.740825477	-0.43279438	0.43159255	1
gene34061	49.5907116	73.56216279	1.483385909	0.568893969	0.43163895	1
gene62160	49.5907116	73.56216279	1.483385909	0.568893969	0.43163895	1
gene40534	195.2634269	142.2847096	0.728680797	-0.45664112	0.43167008	1
gene49909	393.6262734	527.5181411	1.340149723	0.422394189	0.43168059	1
gene6161	468.0123408	627.2142301	1.340166007	0.422411719	0.43172602	1
gene11177	183.8988889	249.7241842	1.357942866	0.441422781	0.43174854	1

gene55438	295.47799	396.8485098	1.343072998	0.425537719	0.43182153	1
gene57721	716.9990386	965.0194251	1.345914531	0.428586798	0.4318704	1
gene9625	30.99419475	48.39615973	1.561458851	0.642894551	0.43194145	1
gene25949	30.99419475	48.39615973	1.561458851	0.642894551	0.43194145	1
gene72807	30.99419475	48.39615973	1.561458851	0.642894551	0.43194145	1
gene64911	788.2856865	1062.779668	1.348216371	0.431052049	0.43196925	1
gene70269	1038.305524	1409.296171	1.357303933	0.440743811	0.43207128	1
gene18577	163.2360924	222.6223348	1.363805832	0.447638259	0.43209524	1
gene41847	144.6395755	103.5677818	0.716040416	-0.48188707	0.43209698	1
gene50841	144.6395755	103.5677818	0.716040416	-0.48188707	0.43209698	1
gene19368	116.7448002	82.27347154	0.704729216	-0.50485907	0.43210072	1
gene65202	257.2518164	189.7129461	0.737460084	-0.43936313	0.43210462	1
gene66832	532.0670099	713.3593944	1.340732241	0.423021144	0.43213842	1
gene38078	220.0587827	297.1524208	1.350332021	0.433314182	0.43213972	1
gene33428	229.3570412	168.4186359	0.734307676	-0.44554341	0.43214707	1
gene3740	424.6204681	316.5108846	0.745397145	-0.4239188	0.43216606	1
gene58114	156.0041136	112.2790906	0.719718782	-0.47449479	0.43229412	1
gene47694	557.8955055	748.2046295	1.341119658	0.423437964	0.43229826	1
gene18736	600.2542384	448.1484391	0.746597709	-0.42159701	0.43234948	1
gene1420	240.7215792	177.1299446	0.735829107	-0.44255735	0.43238153	1
gene45873	178.7331897	129.7017081	0.725672206	-0.46261008	0.43247241	1
gene66097	645.7123907	482.0257509	0.746502248	-0.42178149	0.43249222	1
gene32755	10.33139825	19.35846389	1.873750621	0.905928957	0.43255291	1
gene38701	10.33139825	19.35846389	1.873750621	0.905928957	0.43255291	1
gene42496	10.33139825	19.35846389	1.873750621	0.905928957	0.43255291	1
gene43071	10.33139825	19.35846389	1.873750621	0.905928957	0.43255291	1
gene46796	10.33139825	19.35846389	1.873750621	0.905928957	0.43255291	1
gene55652	10.33139825	19.35846389	1.873750621	0.905928957	0.43255291	1
gene63895	10.33139825	19.35846389	1.873750621	0.905928957	0.43255291	1
gene72570	10.33139825	19.35846389	1.873750621	0.905928957	0.43255291	1
gene57933	128.1093383	90.9847803	0.710211929	-0.4936785	0.43278696	1
gene33289	113.6453808	157.7714807	1.38827887	0.473297397	0.43279739	1
gene49706	916.3950248	1238.941689	1.351973391	0.435066757	0.4329093	1
gene70437	207.6611048	280.6977264	1.351710647	0.434786355	0.43295044	1
gene17821	316.1407865	423.9503593	1.341017602	0.423328174	0.43297119	1
gene26172	212.826804	287.4731888	1.350737705	0.43374755	0.43302201	1
gene61601	40.29245318	60.97916126	1.513413963	0.597806661	0.43303028	1
gene72106	40.29245318	60.97916126	1.513413963	0.597806661	0.43303028	1
gene17168	4274.099456	6255.687607	1.463627057	0.549547991	0.43303405	1
gene65336	618.8507552	830.478101	1.341968308	0.424350601	0.43304319	1
gene842	89.88316478	61.94708446	0.689195631	-0.53701454	0.43304349	1
gene17446	1277.993964	944.693038	0.739199922	-0.43596349	0.43308874	1
gene4840	905.0304868	673.6745435	0.744366685	-0.42591461	0.43310492	1
gene11806	550.6635268	411.3673577	0.747039413	-0.42074374	0.43324428	1
gene56227	383.2948751	285.5373424	0.744954762	-0.42477528	0.43325807	1
gene6216	49.5907116	31.94146542	0.644101776	-0.63463942	0.43334216	1
gene51018	49.5907116	31.94146542	0.644101776	-0.63463942	0.43334216	1
gene7899	278.9477528	374.5862763	1.342854612	0.425303116	0.4333807	1

gene26482	184.9320287	134.5413241	0.727517699	-0.45894575	0.43344279	1
gene73521	184.9320287	134.5413241	0.727517699	-0.45894575	0.43344279	1
gene29455	378.129176	281.6656496	0.744892665	-0.42489554	0.43344305	1
gene51232	40.29245318	25.16600306	0.62458354	-0.67903354	0.43345761	1
gene59205	40.29245318	25.16600306	0.62458354	-0.67903354	0.43345761	1
gene34776	196.2965668	143.2526328	0.729776558	-0.45447329	0.43346034	1
gene49919	3077.723539	4387.595841	1.425597779	0.511566994	0.43346878	1
gene52530	307.8756679	228.4298739	0.741954944	-0.43059651	0.43349833	1
gene8009	84.71746566	58.07539168	0.68551852	-0.54473245	0.43353518	1
gene12394	84.71746566	58.07539168	0.68551852	-0.54473245	0.43353518	1
gene7840	768.6560299	1034.709895	1.346128639	0.428816283	0.43357353	1
gene56811	219.0256429	160.6752503	0.733591045	-0.44695207	0.43358527	1
gene30750	962.886317	716.263164	0.743870955	-0.42687573	0.43360268	1
gene37542	479.3768788	641.733078	1.338681748	0.420813022	0.43360652	1
gene17458	564.0943445	755.948015	1.34010919	0.422350554	0.43363531	1
gene25210	54.75641073	80.33762515	1.46718209	0.553047933	0.43364187	1
gene57683	72.31978776	103.5677818	1.432080832	0.518112926	0.43371425	1
gene19296	873.0031522	650.4443868	0.745065336	-0.42456115	0.43376523	1
gene5504	28.9279151	45.49239015	1.572612129	0.653162886	0.433824	1
gene6973	28.9279151	45.49239015	1.572612129	0.653162886	0.433824	1
gene29963	28.9279151	45.49239015	1.572612129	0.653162886	0.433824	1
gene43559	28.9279151	45.49239015	1.572612129	0.653162886	0.433824	1
gene48111	28.9279151	45.49239015	1.572612129	0.653162886	0.433824	1
gene64505	28.9279151	45.49239015	1.572612129	0.653162886	0.433824	1
gene64652	28.9279151	45.49239015	1.572612129	0.653162886	0.433824	1
gene70137	28.9279151	45.49239015	1.572612129	0.653162886	0.433824	1
gene20827	269.6494943	199.3921781	0.739449479	-0.43547651	0.43385363	1
gene26773	269.6494943	199.3921781	0.739449479	-0.43547651	0.43385363	1
gene47795	1135.420668	842.0931793	0.741657434	-0.43117512	0.43391514	1
gene45007	87.81688513	123.8941689	1.410823997	0.496538021	0.43395165	1
gene45945	87.81688513	123.8941689	1.410823997	0.496538021	0.43395165	1
gene3706	340.9361423	253.595877	0.743822216	-0.42697026	0.43398303	1
gene4002	417.3884893	558.4916833	1.338062016	0.420144983	0.43398537	1
gene39101	200.4291261	271.0184945	1.35219117	0.435299131	0.43400882	1
gene6790	82.65118601	117.1187066	1.417023907	0.502864099	0.43401306	1
gene6420	195.2634269	264.2430321	1.353264338	0.436443673	0.43402509	1
gene53006	47.52443195	70.65839321	1.486780384	0.572191559	0.43405619	1
gene57633	121.9104994	168.4186359	1.381494102	0.466229403	0.43406422	1
gene64853	190.0977278	257.4675698	1.35439583	0.437649436	0.43406925	1
gene24353	389.4937141	290.3769584	0.74552412	-0.42367307	0.43409467	1
gene49025	389.4937141	290.3769584	0.74552412	-0.42367307	0.43409467	1
gene27446	215.9262234	291.3448816	1.349279754	0.432189501	0.43411936	1
gene16557	1108.559032	822.7347154	0.742165903	-0.43018637	0.43418387	1
gene64870	785.1862671	1056.972129	1.346141894	0.428830489	0.43419773	1
gene71187	297.5442696	220.6864884	0.741692954	-0.43110603	0.43424284	1
gene16961	16168.63826	28615.68133	1.769826306	0.823607778	0.43426011	1
gene58181	1039.338664	772.4027093	0.743167493	-0.4282407	0.43426887	1
gene62582	1853.452846	2565.964389	1.384423884	0.469285737	0.43432254	1

gene15377	299.6105493	401.6881258	1.340700876	0.422987393	0.43433863	1
gene4655	213.8599438	156.8035575	0.733206765	-0.447708	0.4343689	1
gene50301	213.8599438	156.8035575	0.733206765	-0.447708	0.4343689	1
gene13941	598.1879587	447.1805159	0.747558538	-0.41974154	0.4343758	1
gene41485	264.4837952	195.5204853	0.739253175	-0.43585956	0.43438184	1
gene19380	1515.616123	1116.015443	0.736344399	-0.4415474	0.4343905	1
gene43660	179.7663296	130.6696313	0.726886017	-0.46019894	0.43440148	1
gene11136	116.7448002	161.6431735	1.384585636	0.469454287	0.43440434	1
gene6902	1286.259082	951.4685003	0.739717615	-0.43495346	0.43441736	1
gene44858	341.9692821	457.8276711	1.338797649	0.420937923	0.43445319	1
gene15589	157.0372534	113.2470138	0.721147443	-0.47163384	0.43447007	1
gene22335	157.0372534	113.2470138	0.721147443	-0.47163384	0.43447007	1
gene33367	157.0372534	113.2470138	0.721147443	-0.47163384	0.43447007	1
gene71369	157.0372534	113.2470138	0.721147443	-0.47163384	0.43447007	1
gene74092	157.0372534	113.2470138	0.721147443	-0.47163384	0.43447007	1
gene3380	1047.603783	1419.943327	1.355420198	0.438740175	0.43451406	1
gene41993	792.4182458	591.4010719	0.7463244	-0.42212524	0.43454964	1
gene48133	292.3785705	216.8147956	0.741555016	-0.43137436	0.43464848	1
gene20761	241.7547191	325.2221934	1.345256856	0.42788166	0.43466706	1
gene51685	547.5641073	409.4315113	0.747732559	-0.41940574	0.43472388	1
gene64062	442.1838451	591.4010719	1.337455175	0.41949054	0.43472484	1
gene37620	164.2692322	223.590258	1.361120734	0.444795043	0.43473395	1
gene37500	694.2699625	518.8068323	0.747269593	-0.42029928	0.43473647	1
gene33547	111.5791011	154.8677111	1.387963423	0.472969549	0.43475283	1
gene48011	111.5791011	154.8677111	1.387963423	0.472969549	0.43475283	1
gene1505	960.8200373	715.2952408	0.744463285	-0.4257274	0.43479347	1
gene47671	1555.908577	2137.174414	1.373586113	0.457947359	0.43484336	1
gene66273	432.8855867	323.286347	0.746817073	-0.42117318	0.43484632	1
gene11868	59.92210985	87.11308752	1.453772034	0.539801058	0.43494399	1
gene4901	259.3180961	191.6487925	0.73904905	-0.43625798	0.43494513	1
gene31153	259.3180961	191.6487925	0.73904905	-0.43625798	0.43494513	1
gene25136	885.4008301	1194.417222	1.349012991	0.431904242	0.4349505	1
gene70608	932.925262	694.9688538	0.744935186	-0.42481319	0.43503612	1
gene69418	130.175618	179.065791	1.375570893	0.460030494	0.43517823	1
gene10850	185.9651685	135.5092472	0.728680797	-0.45664112	0.43531235	1
gene72025	38.22617353	58.07539168	1.519257261	0.603366187	0.43531623	1
gene72541	38.22617353	58.07539168	1.519257261	0.603366187	0.43531623	1
gene18346	4660.493751	3260.933243	0.699696946	-0.5151979	0.43536006	1
gene59436	129.1424781	91.95270349	0.712025236	-0.48999972	0.43539106	1
gene51009	853.3734955	1149.892755	1.347467154	0.430250106	0.43539592	1
gene30059	174.6006304	126.7979385	0.726216957	-0.46152748	0.43541062	1
gene43390	1644.758602	1208.93607	0.735023406	-0.4441379	0.43546823	1
gene47321	553.7629462	414.2711273	0.748101927	-0.41869325	0.43548607	1
gene13343	895.7322283	667.8670043	0.745610109	-0.42350667	0.43548899	1
gene4594	298.5774094	221.6544116	0.742368326	-0.42979294	0.43550718	1
gene29356	54.75641073	35.8131582	0.654045028	-0.61253813	0.43559657	1
gene52092	54.75641073	35.8131582	0.654045028	-0.61253813	0.43559657	1
gene64602	54.75641073	35.8131582	0.654045028	-0.61253813	0.43559657	1

gene28737	26.86163545	42.58862056	1.585481295	0.664920857	0.43563663	1
gene45691	26.86163545	42.58862056	1.585481295	0.664920857	0.43563663	1
gene53403	5968.448769	4102.058499	0.68729056	-0.54100795	0.43563832	1
gene45814	1258.364307	932.1100364	0.740731465	-0.43297747	0.43568138	1
gene28556	2136.533158	1556.420497	0.728479449	-0.45703982	0.43571757	1
gene34730	65.08780898	93.88854988	1.442490558	0.528561875	0.43574732	1
gene34952	96.08200373	134.5413241	1.400276002	0.485711218	0.43576098	1
gene3599	897.798508	1210.871916	1.348712329	0.431582664	0.43579098	1
gene58860	226.2576217	166.4827895	0.735810746	-0.44259335	0.43601631	1
gene21317	112.6122409	79.36970196	0.70480528	-0.50470336	0.4360498	1
gene12540	326.4721847	242.9487219	0.744163617	-0.42630824	0.43605595	1
gene51751	237.6221598	175.1940982	0.737280136	-0.43971521	0.43607041	1
gene54515	2064.213371	1506.088491	0.72961861	-0.45478557	0.43610273	1
gene35564	1187.077659	880.8101071	0.741998723	-0.43051139	0.43617391	1
gene24125	676.7065854	506.2238308	0.748069905	-0.418755	0.43618466	1
gene30016	52.69013108	77.43385557	1.469608331	0.55543171	0.43618467	1
gene61506	52.69013108	77.43385557	1.469608331	0.55543171	0.43618467	1
gene56942	70.25350811	100.6640122	1.432868122	0.518905834	0.43619293	1
gene38522	455.6146629	340.7089645	0.747800702	-0.41927427	0.43620733	1
gene40295	865.7711734	1166.34745	1.347177505	0.429939954	0.43622075	1
gene63251	554.7960861	415.2390505	0.748453461	-0.41801548	0.43622899	1
gene1563	343.0024219	255.5317234	0.744985187	-0.42471636	0.43623126	1
gene28282	85.75060548	120.9903993	1.410956793	0.49667381	0.4362585	1
gene9194	196.2965668	265.2109553	1.351072817	0.434105431	0.43630319	1
gene69321	1214.972434	901.1364942	0.741692954	-0.43110603	0.43637481	1
gene25558	288.2460112	213.911026	0.742112701	-0.4302898	0.4363821	1
gene22404	1021.775287	1382.194322	1.352738062	0.43588251	0.43639701	1
gene26459	365.7314981	488.8012133	1.336502915	0.418462984	0.43647974	1
gene19608	424.6204681	567.2029921	1.335788156	0.417691227	0.43667082	1
gene45057	90.91630461	62.91500765	0.692010173	-0.53113485	0.43671779	1
gene47997	90.91630461	62.91500765	0.692010173	-0.53113485	0.43671779	1
gene66611	146.7058552	105.5036282	0.719150767	-0.47563384	0.43673346	1
gene56804	135.3413171	96.79231946	0.715171993	-0.48363786	0.43674117	1
gene4592	36.15989388	22.26223348	0.615660918	-0.6997921	0.43680878	1
gene52502	36.15989388	22.26223348	0.615660918	-0.6997921	0.43680878	1
gene65993	36.15989388	22.26223348	0.615660918	-0.6997921	0.43680878	1
gene66295	36.15989388	22.26223348	0.615660918	-0.6997921	0.43680878	1
gene37200	221.0919226	162.6110967	0.735490898	-0.44322061	0.43681352	1
gene55650	243.8209987	180.0337142	0.738384779	-0.43755528	0.43685982	1
gene19234	175.6337703	238.1091059	1.355713685	0.439052526	0.43687287	1
gene73219	898.8316478	670.7707739	0.746269644	-0.42223109	0.43696189	1
gene18442	128.1093383	176.1620214	1.375091182	0.459527286	0.43697868	1
gene2418	1175.713121	873.0667216	0.742584824	-0.42937226	0.43705931	1
gene8059	59.92210985	39.68485098	0.662273927	-0.59450003	0.4370617	1
gene38905	59.92210985	39.68485098	0.662273927	-0.59450003	0.4370617	1
gene71887	509.3379338	381.3617387	0.748740106	-0.41746306	0.43716011	1
gene8065	327.5053246	243.916645	0.74477154	-0.42513015	0.4372206	1
gene65593	1538.3452	1134.405984	0.737419654	-0.43944223	0.43728412	1

gene1953	24.7953558	39.68485098	1.600495322	0.678518461	0.43732967	1
gene68529	24.7953558	39.68485098	1.600495322	0.678518461	0.43732967	1
gene20396	6309.384912	4322.744987	0.685129382	-0.54555164	0.43734835	1
gene40084	122.9436392	169.3865591	1.37775781	0.462322305	0.43741739	1
gene1094	85.75060548	59.04331487	0.688546915	-0.53837314	0.43743337	1
gene21611	492.8076966	658.1877724	1.335587445	0.417474437	0.43745286	1
gene32266	822.3793008	614.6312286	0.747381686	-0.42008288	0.43745349	1
gene591	12.3976779	5.807539168	0.468437655	-1.09407104	0.43748359	1
gene7267	12.3976779	5.807539168	0.468437655	-1.09407104	0.43748359	1
gene9955	12.3976779	5.807539168	0.468437655	-1.09407104	0.43748359	1
gene14001	12.3976779	5.807539168	0.468437655	-1.09407104	0.43748359	1
gene15819	12.3976779	5.807539168	0.468437655	-1.09407104	0.43748359	1
gene15889	12.3976779	5.807539168	0.468437655	-1.09407104	0.43748359	1
gene19249	12.3976779	5.807539168	0.468437655	-1.09407104	0.43748359	1
gene19976	12.3976779	5.807539168	0.468437655	-1.09407104	0.43748359	1
gene25074	12.3976779	5.807539168	0.468437655	-1.09407104	0.43748359	1
gene37735	12.3976779	5.807539168	0.468437655	-1.09407104	0.43748359	1
gene38975	12.3976779	5.807539168	0.468437655	-1.09407104	0.43748359	1
gene49563	12.3976779	5.807539168	0.468437655	-1.09407104	0.43748359	1
gene49641	12.3976779	5.807539168	0.468437655	-1.09407104	0.43748359	1
gene50027	12.3976779	5.807539168	0.468437655	-1.09407104	0.43748359	1
gene50297	12.3976779	5.807539168	0.468437655	-1.09407104	0.43748359	1
gene67791	12.3976779	5.807539168	0.468437655	-1.09407104	0.43748359	1
gene73779	12.3976779	5.807539168	0.468437655	-1.09407104	0.43748359	1
gene5252	45.4581523	29.03769584	0.638778621	-0.64661207	0.43748972	1
gene3071	12.3976779	22.26223348	1.795677679	0.844528412	0.4375326	1
gene4678	12.3976779	22.26223348	1.795677679	0.844528412	0.4375326	1
gene32341	12.3976779	22.26223348	1.795677679	0.844528412	0.4375326	1
gene34171	12.3976779	22.26223348	1.795677679	0.844528412	0.4375326	1
gene38536	12.3976779	22.26223348	1.795677679	0.844528412	0.4375326	1
gene41571	12.3976779	22.26223348	1.795677679	0.844528412	0.4375326	1
gene42437	12.3976779	22.26223348	1.795677679	0.844528412	0.4375326	1
gene65422	12.3976779	22.26223348	1.795677679	0.844528412	0.4375326	1
gene12320	227.2907615	167.4507127	0.736724676	-0.44080253	0.43758601	1
gene62853	507.2716541	677.5462362	1.335667449	0.417560854	0.43759869	1
gene26808	215.9262234	158.7394039	0.735155746	-0.44387817	0.43765688	1
gene55332	289.279151	214.8789492	0.742808282	-0.42893819	0.43767392	1
gene60245	773.821729	578.8180704	0.747999247	-0.41889128	0.43770855	1
gene54016	572.3594631	428.7899752	0.749162026	-0.41665032	0.43771538	1
gene47489	4230.707584	2984.107209	0.705344709	-0.50359961	0.43774016	1
gene28115	587.8565605	440.4050536	0.749170943	-0.41663315	0.43776072	1
gene67307	183.8988889	248.756261	1.352679522	0.435820075	0.43779869	1
gene27800	603.3536578	452.0201319	0.749179401	-0.41661686	0.43784114	1
gene42675	462.8466416	346.5165037	0.748663753	-0.41761019	0.43784405	1
gene1458	117.7779401	162.6110967	1.380658353	0.465356365	0.43787936	1
gene5249	117.7779401	162.6110967	1.380658353	0.465356365	0.43787936	1
gene72265	562.0280648	751.108399	1.336425076	0.418378958	0.43790195	1
gene12794	136.3744569	186.8091766	1.369825265	0.453991875	0.4379065	1

gene23555	272.7489138	202.2959477	0.741692954	-0.43110603	0.43793403	1
gene1573	634.3478526	848.8686417	1.338175322	0.420267144	0.43794264	1
gene32612	65.08780898	43.55654376	0.669196651	-0.57949787	0.43797141	1
gene50130	65.08780898	43.55654376	0.669196651	-0.57949787	0.43797141	1
gene21865	317.1739263	236.1732595	0.744617511	-0.42542855	0.43797356	1
gene65517	193.1971473	141.3167864	0.731464146	-0.45114094	0.43797575	1
gene70772	1104.426473	821.7667922	0.744066547	-0.42649644	0.43804913	1
gene43265	1015.576448	756.9159382	0.745306707	-0.42409385	0.43809258	1
gene2096	1328.617815	984.3778889	0.740903726	-0.43264201	0.43817582	1
gene26281	1512.516704	2070.387713	1.368836263	0.452949886	0.43817942	1
gene70195	102.2808427	71.6263164	0.700290636	-0.5139743	0.43824702	1
gene22173	895.7322283	1206.032301	1.346420573	0.429129126	0.43828375	1
gene60636	1063.10088	791.7611732	0.744765796	-0.42514128	0.43831347	1
gene70296	244.8541385	181.0016374	0.739222292	-0.43591983	0.43833422	1
gene41591	63.02152933	90.9847803	1.443709495	0.529780471	0.4383416	1
gene18484	112.6122409	155.8356343	1.383825	0.46866151	0.43836016	1
gene27051	70.25350811	47.42823654	0.675101327	-0.56682404	0.43836603	1
gene28605	312.0082272	232.3015667	0.744536671	-0.42558519	0.43838063	1
gene33849	170.4680711	123.8941689	0.72678812	-0.46039326	0.43847815	1
gene69263	287.2128714	384.2655083	1.337911865	0.419983081	0.438556	1
gene54701	526.9013108	394.9126634	0.749500249	-0.41599914	0.43859756	1
gene65180	254.152397	340.7089645	1.34056955	0.42284607	0.43861785	1
gene1837	83.68432583	118.0866297	1.411096147	0.496816291	0.43864514	1
gene40436	83.68432583	118.0866297	1.411096147	0.496816291	0.43864514	1
gene28240	635.3809924	476.2182118	0.749500249	-0.41599914	0.43875407	1
gene54665	144.6395755	197.4563317	1.365161167	0.449071282	0.43875924	1
gene36254	775.8880086	580.7539168	0.748502246	-0.41792145	0.43881856	1
gene37334	22.72907615	36.7810814	1.618239173	0.694424851	0.4388326	1
gene37746	22.72907615	36.7810814	1.618239173	0.694424851	0.4388326	1
gene57325	22.72907615	36.7810814	1.618239173	0.694424851	0.4388326	1
gene9555	3385.599207	2420.77591	0.715021407	-0.48394166	0.43885047	1
gene5534	186.9983083	252.6279538	1.350963846	0.433989066	0.43889353	1
gene50216	650.8780898	487.8332901	0.749500249	-0.41599914	0.43890949	1
gene67606	290.3122908	215.8468724	0.743498912	-0.42759746	0.43895912	1
gene44192	656.0437889	491.7049829	0.749500249	-0.41599914	0.43896705	1
gene22032	113.6453808	80.33762515	0.706915007	-0.50039133	0.43897486	1
gene19852	946.3560798	706.5839321	0.746636438	-0.42152218	0.43898717	1
gene28459	147.738995	106.4715514	0.720673316	-0.47258267	0.43901432	1
gene19723	43.39187265	64.85085404	1.494539186	0.579700724	0.43902488	1
gene29296	43.39187265	64.85085404	1.494539186	0.579700724	0.43902488	1
gene39506	43.39187265	64.85085404	1.494539186	0.579700724	0.43902488	1
gene58563	1844.154588	2543.702156	1.37933239	0.463970158	0.43908258	1
gene11311	489.7082771	653.3481564	1.334157879	0.4159294	0.43910368	1
gene27886	1179.84568	1599.977041	1.356090095	0.439453031	0.43912466	1
gene3022	136.3744569	97.76024266	0.716851564	-0.48025368	0.43919698	1
gene47939	139.4738764	190.6808693	1.367143972	0.451165179	0.43920175	1
gene41992	125.0099188	89.04893391	0.712334947	-0.48937232	0.43922541	1
gene12827	216.9593633	159.7073271	0.736116316	-0.44199435	0.43928355	1

gene39143	97.11514356	67.75462362	0.697673104	-0.51937688	0.43931156	1
gene41061	97.11514356	67.75462362	0.697673104	-0.51937688	0.43931156	1
gene49309	97.11514356	67.75462362	0.697673104	-0.51937688	0.43931156	1
gene10054	176.6669101	239.0770291	1.353264338	0.436443673	0.43935604	1
gene18402	727.3304369	544.9407586	0.749234091	-0.41651155	0.43944579	1
gene13457	205.5948252	150.9960184	0.734434917	-0.44529345	0.43949003	1
gene2324	19.62965668	10.64715514	0.542401496	-0.88256694	0.43950374	1
gene17071	19.62965668	10.64715514	0.542401496	-0.88256694	0.43950374	1
gene28215	19.62965668	10.64715514	0.542401496	-0.88256694	0.43950374	1
gene41008	19.62965668	10.64715514	0.542401496	-0.88256694	0.43950374	1
gene55493	19.62965668	10.64715514	0.542401496	-0.88256694	0.43950374	1
gene20417	1085.829956	1468.339486	1.352273879	0.435387373	0.43953277	1
gene37702	318.2070661	424.9182824	1.335351498	0.417219545	0.43960558	1
gene20681	3035.364806	2183.634727	0.719397788	-0.47513837	0.43960569	1
gene72980	1368.910268	1014.383508	0.74101534	-0.43242469	0.43963379	1
gene25491	7818.802196	12149.37194	1.553866134	0.63586222	0.43968967	1
gene1701	32.02733458	19.35846389	0.604435684	-0.72633926	0.43975479	1
gene23862	32.02733458	19.35846389	0.604435684	-0.72633926	0.43975479	1
gene64868	257.2518164	190.6808693	0.741222635	-0.43202116	0.43980783	1
gene72883	1062.06774	791.7611732	0.745490276	-0.42373856	0.43980877	1
gene61943	772.7885892	578.8180704	0.748999246	-0.41696383	0.43981076	1
gene4644	554.7960861	740.4612439	1.334654772	0.416466616	0.43981141	1
gene67497	115.7116604	159.7073271	1.380218092	0.464896249	0.43987702	1
gene6288	340.9361423	254.5638002	0.746661232	-0.42147427	0.43991845	1
gene35360	1010.410749	754.0121686	0.746243218	-0.42228218	0.43992183	1
gene67876	324.4059051	241.9807987	0.745919833	-0.42290751	0.43992957	1
gene31900	147.738995	201.3280245	1.362727725	0.446497338	0.43998852	1
gene19131	328.5384644	438.4692072	1.334605395	0.416413241	0.4399987	1
gene660	544.4646878	408.4635881	0.750211349	-0.41463101	0.43999988	1
gene1702	14.46395755	25.16600306	1.739911291	0.799013753	0.44000926	1
gene17470	14.46395755	25.16600306	1.739911291	0.799013753	0.44000926	1
gene19007	14.46395755	25.16600306	1.739911291	0.799013753	0.44000926	1
gene39532	14.46395755	25.16600306	1.739911291	0.799013753	0.44000926	1
gene40104	14.46395755	25.16600306	1.739911291	0.799013753	0.44000926	1
gene52721	14.46395755	25.16600306	1.739911291	0.799013753	0.44000926	1
gene54123	14.46395755	25.16600306	1.739911291	0.799013753	0.44000926	1
gene64811	14.46395755	25.16600306	1.739911291	0.799013753	0.44000926	1
gene4810	20.6627965	33.87731181	1.639531794	0.713283879	0.44004393	1
gene52686	20.6627965	33.87731181	1.639531794	0.713283879	0.44004393	1
gene56496	20.6627965	33.87731181	1.639531794	0.713283879	0.44004393	1
gene66072	20.6627965	33.87731181	1.639531794	0.713283879	0.44004393	1
gene73540	20.6627965	33.87731181	1.639531794	0.713283879	0.44004393	1
gene48270	50.62385143	32.90938862	0.650076746	-0.62131805	0.44006401	1
gene56641	50.62385143	32.90938862	0.650076746	-0.62131805	0.44006401	1
gene37119	34.09361423	52.26785251	1.53306869	0.616422339	0.44007054	1
gene36781	182.865749	133.5734009	0.730445158	-0.45315214	0.44009047	1
gene28838	211.7936641	155.8356343	0.735789878	-0.44263426	0.44020547	1
gene48242	142.5732959	102.5998586	0.719628862	-0.47467505	0.44029144	1

gene60632	471.1117602	353.291966	0.749911159	-0.4152084	0.44029317	1
gene73657	960.8200373	1294.113311	1.346884183	0.429625801	0.44040983	1
gene28822	179.7663296	242.9487219	1.351469557	0.434529015	0.44044891	1
gene72437	200.4291261	147.1243256	0.734046635	-0.44605637	0.44048402	1
gene36396	131.2087578	93.88854988	0.715566182	-0.48284289	0.44050317	1
gene38003	131.2087578	93.88854988	0.715566182	-0.48284289	0.44050317	1
gene43736	119.8442197	85.17724113	0.710732994	-0.49262042	0.44052533	1
gene33654	274.8151935	204.2317941	0.743160491	-0.42825429	0.44061727	1
gene42955	344.0355618	458.7955943	1.333570262	0.415293838	0.44062176	1
gene21928	1276.960824	948.5647307	0.742829939	-0.42889613	0.44062613	1
gene13544	347.1349812	259.4034162	0.747269593	-0.42029928	0.44066626	1
gene13293	229.3570412	169.3865591	0.738527835	-0.4372758	0.44069422	1
gene69090	2146.864557	1569.003499	0.730834879	-0.45238261	0.44074456	1
gene68136	545.4978276	409.4315113	0.75056488	-0.41395131	0.4407476	1
gene23919	86.78374531	121.9583225	1.405312966	0.490891457	0.44074842	1
gene25636	308.9088077	412.3352809	1.334812316	0.416636903	0.44077072	1
gene487	314.0745068	234.2374131	0.745802057	-0.42313532	0.44078506	1
gene32017	18.59651685	30.97354223	1.665556108	0.736003955	0.44081448	1
gene44583	18.59651685	30.97354223	1.665556108	0.736003955	0.44081448	1
gene56054	18.59651685	30.97354223	1.665556108	0.736003955	0.44081448	1
gene69098	18.59651685	30.97354223	1.665556108	0.736003955	0.44081448	1
gene51293	189.064588	138.4130168	0.732093822	-0.44989954	0.44082924	1
gene24345	457.6809425	609.7916126	1.332350893	0.413974086	0.44089524	1
gene38439	217.9925031	160.6752503	0.73706778	-0.4401308	0.44089883	1
gene20002	16.5302372	28.06977264	1.698086501	0.763909952	0.44091829	1
gene21730	16.5302372	28.06977264	1.698086501	0.763909952	0.44091829	1
gene27243	16.5302372	28.06977264	1.698086501	0.763909952	0.44091829	1
gene30201	16.5302372	28.06977264	1.698086501	0.763909952	0.44091829	1
gene35096	16.5302372	28.06977264	1.698086501	0.763909952	0.44091829	1
gene35922	16.5302372	28.06977264	1.698086501	0.763909952	0.44091829	1
gene40329	16.5302372	28.06977264	1.698086501	0.763909952	0.44091829	1
gene41473	16.5302372	28.06977264	1.698086501	0.763909952	0.44091829	1
gene52174	16.5302372	28.06977264	1.698086501	0.763909952	0.44091829	1
gene68250	16.5302372	28.06977264	1.698086501	0.763909952	0.44091829	1
gene38400	193.1971473	260.3713394	1.347697639	0.430496859	0.44102131	1
gene32333	60.95524968	88.08101071	1.445011072	0.531080547	0.4410249	1
gene36498	60.95524968	88.08101071	1.445011072	0.531080547	0.4410249	1
gene6331	9.298258426	3.871692779	0.416389027	-1.26399604	0.44104578	1
gene19525	9.298258426	3.871692779	0.416389027	-1.26399604	0.44104578	1
gene20928	9.298258426	3.871692779	0.416389027	-1.26399604	0.44104578	1
gene22311	9.298258426	3.871692779	0.416389027	-1.26399604	0.44104578	1
gene30468	9.298258426	3.871692779	0.416389027	-1.26399604	0.44104578	1
gene35141	9.298258426	3.871692779	0.416389027	-1.26399604	0.44104578	1
gene37477	9.298258426	3.871692779	0.416389027	-1.26399604	0.44104578	1
gene43523	9.298258426	3.871692779	0.416389027	-1.26399604	0.44104578	1
gene46346	9.298258426	3.871692779	0.416389027	-1.26399604	0.44104578	1
gene47388	9.298258426	3.871692779	0.416389027	-1.26399604	0.44104578	1
gene49222	9.298258426	3.871692779	0.416389027	-1.26399604	0.44104578	1

gene51119	9.298258426	3.871692779	0.416389027	-1.26399604	0.44104578	1
gene51553	9.298258426	3.871692779	0.416389027	-1.26399604	0.44104578	1
gene53812	9.298258426	3.871692779	0.416389027	-1.26399604	0.44104578	1
gene57299	9.298258426	3.871692779	0.416389027	-1.26399604	0.44104578	1
gene62170	9.298258426	3.871692779	0.416389027	-1.26399604	0.44104578	1
gene66236	9.298258426	3.871692779	0.416389027	-1.26399604	0.44104578	1
gene71314	9.298258426	3.871692779	0.416389027	-1.26399604	0.44104578	1
gene74128	9.298258426	3.871692779	0.416389027	-1.26399604	0.44104578	1
gene68501	2227.449463	1626.110967	0.73003271	-0.45396699	0.44121823	1
gene10562	86.78374531	60.01123807	0.691503206	-0.53219216	0.44125481	1
gene9814	577.5251622	433.6295912	0.750841036	-0.41342059	0.44129209	1
gene19232	76.45234706	108.4073978	1.417973443	0.503830513	0.4413825	1
gene48974	76.45234706	108.4073978	1.417973443	0.503830513	0.4413825	1
gene49870	76.45234706	108.4073978	1.417973443	0.503830513	0.4413825	1
gene16549	807.9153432	1083.106055	1.340618252	0.42289848	0.44139032	1
gene7040	66.12094881	94.85647307	1.43459032	0.520638801	0.4414135	1
gene49896	66.12094881	94.85647307	1.43459032	0.520638801	0.4414135	1
gene6370	960.8200373	1293.145388	1.34587679	0.428546343	0.44154146	1
gene62784	433.9187265	577.8501472	1.331701335	0.41327056	0.44155916	1
gene49088	211.7936641	284.5694192	1.343616299	0.426121202	0.44155966	1
gene61829	224.191342	165.5148663	0.738275014	-0.43776976	0.44157364	1
gene12345	55.78955055	36.7810814	0.659282626	-0.60103103	0.44166565	1
gene15372	55.78955055	36.7810814	0.659282626	-0.60103103	0.44166565	1
gene72842	55.78955055	36.7810814	0.659282626	-0.60103103	0.44166565	1
gene27791	41.325593	26.13392626	0.632390835	-0.66111164	0.44173597	1
gene29420	41.325593	26.13392626	0.632390835	-0.66111164	0.44173597	1
gene72941	41.325593	26.13392626	0.632390835	-0.66111164	0.44173597	1
gene5744	451.4821036	338.7731181	0.7503578	-0.4143494	0.44173915	1
gene8468	1266.629426	941.7892684	0.743539704	-0.42751831	0.44179777	1
gene62023	212.826804	156.8035575	0.736766021	-0.44072157	0.44185065	1
gene24165	126.0430587	90.0168571	0.714175442	-0.48564957	0.44185744	1
gene13440	27.89477528	16.45469431	0.589884455	-0.7614957	0.4418646	1
gene65475	27.89477528	16.45469431	0.589884455	-0.7614957	0.4418646	1
gene38716	264.4837952	196.4884085	0.742912844	-0.42873513	0.44190523	1
gene67552	676.7065854	904.0402638	1.335941283	0.4178566	0.44196015	1
gene17827	625.0495942	469.4427494	0.751048803	-0.41302144	0.44196298	1
gene61342	583.7240012	438.4692072	0.751158435	-0.41281086	0.44197979	1
gene44039	345.0687016	459.7635175	1.332382553	0.414008367	0.44201448	1
gene636	287.2128714	213.911026	0.744782171	-0.42510956	0.44206135	1
gene16660	127.0761985	174.226175	1.37103704	0.455267548	0.44209339	1
gene33212	563.0612047	422.9824361	0.751219286	-0.41269399	0.44209545	1
gene13611	154.9709738	112.2790906	0.724516907	-0.46490874	0.44215512	1
gene4761	4326.789587	3057.669372	0.706683168	-0.50086455	0.44216967	1
gene10898	172.5343508	233.2694899	1.352017664	0.435114	0.44217326	1
gene2500	435.9850062	327.1580398	0.750388282	-0.4142908	0.44229003	1
gene5279	435.9850062	327.1580398	0.750388282	-0.4142908	0.44229003	1
gene2151	23.76221598	13.55092472	0.570271928	-0.81027808	0.44232788	1
gene4818	23.76221598	13.55092472	0.570271928	-0.81027808	0.44232788	1

gene5537	23.76221598	13.55092472	0.570271928	-0.81027808	0.44232788	1
gene8897	23.76221598	13.55092472	0.570271928	-0.81027808	0.44232788	1
gene28204	23.76221598	13.55092472	0.570271928	-0.81027808	0.44232788	1
gene39900	23.76221598	13.55092472	0.570271928	-0.81027808	0.44232788	1
gene52363	23.76221598	13.55092472	0.570271928	-0.81027808	0.44232788	1
gene58722	23.76221598	13.55092472	0.570271928	-0.81027808	0.44232788	1
gene2307	153.9378339	209.07141	1.358154813	0.441647938	0.44234195	1
gene55080	802.7496441	1075.362669	1.339599061	0.42180127	0.44236167	1
gene17385	1321.385836	1794.529603	1.358066322	0.441553936	0.44246828	1
gene49052	372.9634769	496.5445988	1.331349126	0.412888945	0.4424996	1
gene21456	1073.432278	801.4404052	0.746614781	-0.42156402	0.44250784	1
gene47981	32.02733458	49.36408293	1.541310995	0.624157988	0.44252347	1
gene71784	32.02733458	49.36408293	1.541310995	0.624157988	0.44252347	1
gene51497	1520.781823	1125.694675	0.740207871	-0.43399762	0.44253018	1
gene39817	484.542578	363.9391212	0.75109833	-0.4129263	0.44254313	1
gene42052	4369.14832	6354.415773	1.454383167	0.540407407	0.44255882	1
gene51209	214.8930836	288.441112	1.342254051	0.424657759	0.44256133	1
gene8816	287.2128714	383.2975851	1.33454181	0.416344504	0.44259052	1
gene37712	143.6064357	103.5677818	0.721191786	-0.47154513	0.4426165	1
gene8907	190.0977278	139.38094	0.733206765	-0.447708	0.44263823	1
gene52588	1530.080081	1132.470138	0.740137821	-0.43413415	0.44266008	1
gene19495	76.45234706	52.26785251	0.683665767	-0.54863691	0.4426879	1
gene72539	579.5914419	435.5654376	0.75150426	-0.41214681	0.44270873	1
gene53539	121.9104994	167.4507127	1.373554481	0.457914135	0.44271621	1
gene27261	564.0943445	423.9503593	0.751559315	-0.41204112	0.44281926	1
gene63549	793.4513857	595.2727647	0.750232182	-0.41459094	0.44286528	1
gene63752	132.2418976	94.85647307	0.71729516	-0.4793612	0.44301237	1
gene39963	71.28664793	48.39615973	0.678895153	-0.55873931	0.443058	1
gene58594	71.28664793	48.39615973	0.678895153	-0.55873931	0.443058	1
gene67245	66.12094881	44.52446695	0.67337913	-0.57050909	0.44306529	1
gene59766	530.0007303	705.6160089	1.331349126	0.412888945	0.44317645	1
gene25968	109.5128215	77.43385557	0.707075706	-0.5000634	0.44321678	1
gene63165	799.6502246	1070.523053	1.338739139	0.420874871	0.44323305	1
gene14237	344.0355618	257.4675698	0.748374873	-0.41816697	0.4432468	1
gene70925	120.8773595	86.14516432	0.712665835	-0.48870233	0.44325755	1
gene7768	1013.510168	757.8838614	0.747781211	-0.41931187	0.44325812	1
gene53598	196.2965668	144.220556	0.734707481	-0.44475813	0.44329006	1
gene70507	288.2460112	214.8789492	0.745470677	-0.42377649	0.44334335	1
gene33961	116.7448002	160.6752503	1.376294704	0.460789426	0.44337448	1
gene57833	1273.861404	1726.774979	1.35554384	0.438871772	0.44337715	1
gene72270	381.2285955	507.191754	1.33041372	0.411874952	0.44347505	1
gene35279	213.8599438	157.7714807	0.737732733	-0.43882985	0.44348403	1
gene56239	149.8052746	108.4073978	0.723655412	-0.46662521	0.44350332	1
gene50133	565.1274843	424.9182824	0.751898101	-0.41139094	0.44354098	1
gene56458	759.3577714	1015.351431	1.337118641	0.41912748	0.44357168	1
gene57882	689.1042633	517.8389091	0.751466703	-0.41221891	0.44358637	1
gene71407	689.1042633	517.8389091	0.751466703	-0.41221891	0.44358637	1
gene7907	7397.281148	11368.25792	1.536815716	0.619944177	0.44359952	1

gene51677	79.55176653	112.2790906	1.411396572	0.497123411	0.44367309	1
gene466	167.3686517	121.9583225	0.728680797	-0.45664112	0.44370141	1
gene70130	356.4332397	474.2823654	1.330634499	0.412114344	0.44376515	1
gene11020	1806.961554	2481.755071	1.373440993	0.457794929	0.44377298	1
gene8165	58.88897003	85.17724113	1.446403988	0.532470561	0.44380084	1
gene30274	58.88897003	85.17724113	1.446403988	0.532470561	0.44380084	1
gene12632	207.6611048	278.7618801	1.342388505	0.424802267	0.44386265	1
gene13083	92.98258426	64.85085404	0.69745162	-0.51983495	0.44386531	1
gene10528	98.14828338	136.4771704	1.390520198	0.475624701	0.44388892	1
gene23944	202.4954057	149.060172	0.736116316	-0.44199435	0.44391564	1
gene42985	612.6519163	460.7314406	0.752028074	-0.41114157	0.44396273	1
gene38190	151.8715543	206.1676405	1.357513205	0.440966232	0.44399279	1
gene55748	151.8715543	206.1676405	1.357513205	0.440966232	0.44399279	1
gene53462	138.4407366	99.69608905	0.7201355	-0.47365971	0.44402301	1
gene23445	262.4175156	350.3881965	1.335231742	0.417090157	0.44405742	1
gene26368	1136.453808	1534.158264	1.349952152	0.432908273	0.44412592	1
gene7534	69.22036828	98.72816585	1.426287786	0.512265108	0.44420228	1
gene74	7.231978776	14.51884792	2.007589952	1.00546463	0.44422628	1
gene836	7.231978776	14.51884792	2.007589952	1.00546463	0.44422628	1
gene15031	7.231978776	14.51884792	2.007589952	1.00546463	0.44422628	1
gene32262	7.231978776	14.51884792	2.007589952	1.00546463	0.44422628	1
gene32618	7.231978776	14.51884792	2.007589952	1.00546463	0.44422628	1
gene37836	7.231978776	14.51884792	2.007589952	1.00546463	0.44422628	1
gene37960	7.231978776	14.51884792	2.007589952	1.00546463	0.44422628	1
gene38038	7.231978776	14.51884792	2.007589952	1.00546463	0.44422628	1
gene43388	7.231978776	14.51884792	2.007589952	1.00546463	0.44422628	1
gene46832	7.231978776	14.51884792	2.007589952	1.00546463	0.44422628	1
gene49651	7.231978776	14.51884792	2.007589952	1.00546463	0.44422628	1
gene55032	7.231978776	14.51884792	2.007589952	1.00546463	0.44422628	1
gene56506	7.231978776	14.51884792	2.007589952	1.00546463	0.44422628	1
gene59185	7.231978776	14.51884792	2.007589952	1.00546463	0.44422628	1
gene59719	7.231978776	14.51884792	2.007589952	1.00546463	0.44422628	1
gene61243	7.231978776	14.51884792	2.007589952	1.00546463	0.44422628	1
gene61289	7.231978776	14.51884792	2.007589952	1.00546463	0.44422628	1
gene61592	7.231978776	14.51884792	2.007589952	1.00546463	0.44422628	1
gene67890	7.231978776	14.51884792	2.007589952	1.00546463	0.44422628	1
gene69278	7.231978776	14.51884792	2.007589952	1.00546463	0.44422628	1
gene70940	7.231978776	14.51884792	2.007589952	1.00546463	0.44422628	1
gene71991	7.231978776	14.51884792	2.007589952	1.00546463	0.44422628	1
gene74103	7.231978776	14.51884792	2.007589952	1.00546463	0.44422628	1
gene12325	361.5989388	271.0184945	0.749500249	-0.41599914	0.44423949	1
gene73254	361.5989388	271.0184945	0.749500249	-0.41599914	0.44423949	1
gene17445	1296.59048	965.0194251	0.744274649	-0.426093	0.44424257	1
gene37302	317.1739263	237.1411827	0.747669222	-0.41952795	0.44434674	1
gene62647	354.36696	471.3785958	1.330199056	0.411642153	0.44435198	1
gene41553	173.5674906	126.7979385	0.730539677	-0.45296546	0.44438823	1
gene56866	173.5674906	126.7979385	0.730539677	-0.45296546	0.44438823	1
gene56022	191.1308676	140.3488632	0.734307676	-0.44554341	0.44443252	1

gene23255	127.0761985	90.9847803	0.71598601	-0.4819967	0.44445578	1
gene58575	1181.91196	881.7780303	0.746060671	-0.42263514	0.44453854	1
gene11299	328.5384644	245.8524914	0.748321789	-0.41826931	0.44454656	1
gene73796	328.5384644	245.8524914	0.748321789	-0.41826931	0.44454656	1
gene37613	277.9146129	207.1355637	0.745320879	-0.42406642	0.44458775	1
gene26924	104.3471223	73.56216279	0.704975481	-0.50435501	0.4445927	1
gene35320	644.6792509	484.9295205	0.752202774	-0.41080647	0.44460398	1
gene11456	133.2750374	181.9695606	1.36536867	0.449290553	0.44463987	1
gene17171	856.472915	642.7010012	0.75040435	-0.4142599	0.44467384	1
gene58655	1233.568951	919.5270349	0.745420055	-0.42387446	0.44474132	1
gene24140	46.49129213	30.00561903	0.645402992	-0.63172783	0.44476315	1
gene25653	46.49129213	30.00561903	0.645402992	-0.63172783	0.44476315	1
gene26540	46.49129213	30.00561903	0.645402992	-0.63172783	0.44476315	1
gene48654	46.49129213	30.00561903	0.645402992	-0.63172783	0.44476315	1
gene52756	46.49129213	30.00561903	0.645402992	-0.63172783	0.44476315	1
gene73444	422.5541885	317.4788078	0.751332768	-0.41247607	0.44478861	1
gene32764	312.0082272	233.2694899	0.747638907	-0.41958645	0.4448549	1
gene54460	1344.114912	999.8646601	0.743883317	-0.42685175	0.44486315	1
gene43909	400.8582521	301.0241135	0.750949025	-0.41321312	0.44493738	1
gene19777	243.8209987	181.0016374	0.742354589	-0.42981963	0.44497623	1
gene4239	87.81688513	60.97916126	0.694389936	-0.52618206	0.44500178	1
gene31586	29.96105493	46.46031334	1.550690169	0.632910462	0.44500961	1
gene46377	29.96105493	46.46031334	1.550690169	0.632910462	0.44500961	1
gene8480	722.1647377	543.0049122	0.751912803	-0.41136273	0.44506276	1
gene4848	339.9030025	452.0201319	1.329850365	0.411263923	0.44526198	1
gene26645	615.7513357	819.8309459	1.331431859	0.412978596	0.44530624	1
gene73464	1746.006304	1289.273695	0.738412967	-0.43750021	0.44532534	1
gene64471	306.842528	229.3977971	0.747607571	-0.41964692	0.44538794	1
gene40567	536.1995692	403.6239722	0.752749527	-0.4097582	0.44545174	1
gene30400	101.2477029	140.3488632	1.386193062	0.471128203	0.44552204	1
gene51352	1118.890431	836.2856402	0.747424071	-0.42000107	0.44554239	1
gene16148	250.0198377	185.8412534	0.743306032	-0.42797178	0.44557217	1
gene52971	185.9651685	136.4771704	0.73388566	-0.44637279	0.44563646	1
gene53486	149.8052746	203.2638709	1.356853898	0.440265384	0.44569017	1
gene6544	150.8384145	109.375321	0.725115823	-0.46371664	0.44571222	1
gene17309	150.8384145	109.375321	0.725115823	-0.46371664	0.44571222	1
gene14743	278.9477528	371.6825067	1.332444886	0.41407586	0.44580605	1
gene65153	213.8599438	286.5052656	1.339686435	0.421895365	0.44580717	1
gene3788	51.65699125	75.49800918	1.461525485	0.547474986	0.4458253	1
gene70840	51.65699125	75.49800918	1.461525485	0.547474986	0.4458253	1
gene35903	238.6552996	177.1299446	0.742199921	-0.43012025	0.44585771	1
gene43790	626.082734	471.3785958	0.752901446	-0.40946706	0.44592595	1
gene2541	12310.89416	20325.41916	1.651010796	0.723349554	0.44596168	1
gene71412	37.1930337	23.23015667	0.62458354	-0.67903354	0.44599467	1
gene71420	37.1930337	23.23015667	0.62458354	-0.67903354	0.44599467	1
gene6145	82.65118601	57.10746848	0.690945542	-0.53335609	0.44604451	1
gene42819	82.65118601	57.10746848	0.690945542	-0.53335609	0.44604451	1
gene43224	82.65118601	57.10746848	0.690945542	-0.53335609	0.44604451	1

gene20686	438.0512858	581.72184	1.327976561	0.409229683	0.44605845	1
gene16407	122.9436392	168.4186359	1.369884908	0.454054689	0.44606429	1
gene73024	804.8159237	604.9519966	0.751665044	-0.41183818	0.4461647	1
gene36006	158.0703932	213.911026	1.353264338	0.436443673	0.44618868	1
gene36641	158.0703932	213.911026	1.353264338	0.436443673	0.44618868	1
gene11216	475.2443195	631.0859229	1.327918919	0.40916706	0.44639411	1
gene26156	574.4257427	763.6914006	1.329486727	0.410869374	0.44645749	1
gene60356	448.3826841	595.2727647	1.327599807	0.408820324	0.44655477	1
gene53075	51.65699125	33.87731181	0.655812718	-0.60864422	0.44656531	1
gene16022	56.82269038	82.27347154	1.447898207	0.533960179	0.44667315	1
gene25587	266.5500749	355.2278124	1.332686973	0.414337954	0.44668928	1
gene53636	215.9262234	159.7073271	0.739638403	-0.43510796	0.44671592	1
gene7597	72.31978776	102.5998586	1.418696899	0.504566394	0.44674386	1
gene63005	72.31978776	102.5998586	1.418696899	0.504566394	0.44674386	1
gene42835	198.3628464	146.1564024	0.736813395	-0.4406288	0.44676373	1
gene39227	330.604744	247.7883378	0.749500249	-0.41599914	0.44682175	1
gene22501	180.7994694	132.6054777	0.733439529	-0.44725007	0.44690468	1
gene43109	180.7994694	132.6054777	0.733439529	-0.44725007	0.44690468	1
gene71457	77.48548688	53.23577571	0.687041895	-0.54153002	0.44694471	1
gene49554	211.7936641	283.601496	1.339046176	0.421205712	0.44697776	1
gene1960	341.9692821	256.4996466	0.750066336	-0.4149099	0.44698169	1
gene16446	37.1930337	56.13954529	1.509410223	0.59398495	0.44699814	1
gene17127	37.1930337	56.13954529	1.509410223	0.59398495	0.44699814	1
gene26689	37.1930337	56.13954529	1.509410223	0.59398495	0.44699814	1
gene40751	37.1930337	56.13954529	1.509410223	0.59398495	0.44699814	1
gene2916	1580.703932	1172.154989	0.74153987	-0.43140383	0.44705019	1
gene1240	44.42501248	65.81877724	1.481570259	0.567127043	0.44706089	1
gene48722	44.42501248	65.81877724	1.481570259	0.567127043	0.44706089	1
gene68978	104.3471223	144.220556	1.382122983	0.466885994	0.44706571	1
gene36466	163.2360924	119.0545529	0.729339641	-0.45533729	0.44708267	1
gene65331	291.3454307	217.7827188	0.747506897	-0.4198412	0.4471497	1
gene10373	161.1698127	217.7827188	1.351262467	0.434307929	0.44724398	1
gene53468	430.8193071	324.2542702	0.752645633	-0.40995733	0.44726597	1
gene33347	325.4390449	243.916645	0.749500249	-0.41599914	0.44732665	1
gene59976	268.6163545	200.3601013	0.745896882	-0.4229519	0.44733247	1
gene17936	230.390181	307.7995759	1.335992596	0.417912012	0.44733515	1
gene52558	835.8101185	1116.983367	1.336408045	0.418360573	0.44733579	1
gene65040	94.01572408	65.81877724	0.70008265	-0.51440284	0.44734224	1
gene35498	672.5740261	895.328955	1.331197638	0.412724779	0.44740417	1
gene6719	336.803583	252.6279538	0.750075019	-0.4148932	0.44745532	1
gene27415	336.803583	252.6279538	0.750075019	-0.4148932	0.44745532	1
gene42317	736.6286953	554.6199905	0.752916624	-0.40943798	0.44746181	1
gene56062	116.7448002	83.24139474	0.713020148	-0.48798525	0.44750202	1
gene65419	116.7448002	83.24139474	0.713020148	-0.48798525	0.44750202	1
gene10553	27.89477528	43.55654376	1.561458851	0.642894551	0.44750801	1
gene29565	27.89477528	43.55654376	1.561458851	0.642894551	0.44750801	1
gene2898	1345.148052	1821.631452	1.354223759	0.437466136	0.44750818	1
gene45197	620.9170349	825.638485	1.32970822	0.411109707	0.44751317	1

gene6273	975.2839949	731.7499351	0.750294211	-0.41447167	0.44752153	1
gene46744	201.4622659	270.0505713	1.340452368	0.422719955	0.44753355	1
gene31973	56.82269038	37.74900459	0.664329766	-0.59002854	0.44755502	1
gene55363	56.82269038	37.74900459	0.664329766	-0.59002854	0.44755502	1
gene33454	264.4837952	352.3240428	1.332119582	0.413723597	0.44758887	1
gene4001	1785.265618	1319.279314	0.738982088	-0.4363887	0.44759587	1
gene16625	72.31978776	49.36408293	0.682580584	-0.55092872	0.44763546	1
gene48427	72.31978776	49.36408293	0.682580584	-0.55092872	0.44763546	1
gene52577	72.31978776	49.36408293	0.682580584	-0.55092872	0.44763546	1
gene30757	105.3802622	74.53008599	0.707249009	-0.49970984	0.44769002	1
gene4654	85.75060548	120.0224761	1.399669139	0.485085836	0.44775645	1
gene27356	210.7605243	155.8356343	0.739396691	-0.43557951	0.44781125	1
gene21783	382.2617353	507.191754	1.326818008	0.407970498	0.44781911	1
gene35122	3416.593402	2457.556991	0.719300397	-0.47533369	0.44785827	1
gene992	2350.393102	1719.999517	0.731792276	-0.45049391	0.44789604	1
gene45812	274.8151935	205.1997173	0.746682578	-0.42143302	0.44791395	1
gene31988	2404.116373	1757.748521	0.731141197	-0.45177805	0.44792861	1
gene21650	134.3081773	96.79231946	0.720673316	-0.47258267	0.44793994	1
gene37615	134.3081773	96.79231946	0.720673316	-0.47258267	0.44793994	1
gene61474	1191.210218	890.4893391	0.747550118	-0.41975779	0.44795153	1
gene36593	387.4274344	513.9672164	1.32661544	0.407750222	0.44798567	1
gene32782	370.8971972	278.7618801	0.751587993	-0.41198608	0.44800792	1
gene5075	67.15408863	45.49239015	0.677432917	-0.56185	0.44802374	1
gene44877	67.15408863	45.49239015	0.677432917	-0.56185	0.44802374	1
gene58988	67.15408863	45.49239015	0.677432917	-0.56185	0.44802374	1
gene23570	846.1415167	1130.534291	1.336105449	0.418033874	0.44809135	1
gene26356	120.8773595	165.5148663	1.3692793	0.453416752	0.44814248	1
gene54294	120.8773595	165.5148663	1.3692793	0.453416752	0.44814248	1
gene73242	863.7048938	649.4764636	0.75196571	-0.41126122	0.44817444	1
gene59931	410.1565106	543.9728354	1.326256737	0.40736008	0.44819197	1
gene618	4.1325593	9.679231946	2.342188277	1.227857052	0.44823204	1
gene2595	4.1325593	9.679231946	2.342188277	1.227857052	0.44823204	1
gene5590	4.1325593	9.679231946	2.342188277	1.227857052	0.44823204	1
gene9043	4.1325593	9.679231946	2.342188277	1.227857052	0.44823204	1
gene10752	4.1325593	9.679231946	2.342188277	1.227857052	0.44823204	1
gene13038	4.1325593	9.679231946	2.342188277	1.227857052	0.44823204	1
gene14141	4.1325593	9.679231946	2.342188277	1.227857052	0.44823204	1
gene18436	4.1325593	9.679231946	2.342188277	1.227857052	0.44823204	1
gene24410	4.1325593	9.679231946	2.342188277	1.227857052	0.44823204	1
gene25037	4.1325593	9.679231946	2.342188277	1.227857052	0.44823204	1
gene25302	4.1325593	9.679231946	2.342188277	1.227857052	0.44823204	1
gene26898	4.1325593	9.679231946	2.342188277	1.227857052	0.44823204	1
gene27220	4.1325593	9.679231946	2.342188277	1.227857052	0.44823204	1
gene28404	4.1325593	9.679231946	2.342188277	1.227857052	0.44823204	1
gene29504	4.1325593	9.679231946	2.342188277	1.227857052	0.44823204	1
gene29784	4.1325593	9.679231946	2.342188277	1.227857052	0.44823204	1
gene30896	4.1325593	9.679231946	2.342188277	1.227857052	0.44823204	1
gene31157	4.1325593	9.679231946	2.342188277	1.227857052	0.44823204	1

gene31704	4.1325593	9.679231946	2.342188277	1.227857052	0.44823204	1
gene34666	4.1325593	9.679231946	2.342188277	1.227857052	0.44823204	1
gene35754	4.1325593	9.679231946	2.342188277	1.227857052	0.44823204	1
gene39382	4.1325593	9.679231946	2.342188277	1.227857052	0.44823204	1
gene43444	4.1325593	9.679231946	2.342188277	1.227857052	0.44823204	1
gene46299	4.1325593	9.679231946	2.342188277	1.227857052	0.44823204	1
gene47259	4.1325593	9.679231946	2.342188277	1.227857052	0.44823204	1
gene49012	4.1325593	9.679231946	2.342188277	1.227857052	0.44823204	1
gene50656	4.1325593	9.679231946	2.342188277	1.227857052	0.44823204	1
gene50767	4.1325593	9.679231946	2.342188277	1.227857052	0.44823204	1
gene51689	4.1325593	9.679231946	2.342188277	1.227857052	0.44823204	1
gene51745	4.1325593	9.679231946	2.342188277	1.227857052	0.44823204	1
gene52776	4.1325593	9.679231946	2.342188277	1.227857052	0.44823204	1
gene53338	4.1325593	9.679231946	2.342188277	1.227857052	0.44823204	1
gene58981	4.1325593	9.679231946	2.342188277	1.227857052	0.44823204	1
gene59331	4.1325593	9.679231946	2.342188277	1.227857052	0.44823204	1
gene61795	4.1325593	9.679231946	2.342188277	1.227857052	0.44823204	1
gene62418	4.1325593	9.679231946	2.342188277	1.227857052	0.44823204	1
gene65680	4.1325593	9.679231946	2.342188277	1.227857052	0.44823204	1
gene67991	4.1325593	9.679231946	2.342188277	1.227857052	0.44823204	1
gene69183	4.1325593	9.679231946	2.342188277	1.227857052	0.44823204	1
gene70229	4.1325593	9.679231946	2.342188277	1.227857052	0.44823204	1
gene71483	4.1325593	9.679231946	2.342188277	1.227857052	0.44823204	1
gene71884	4.1325593	9.679231946	2.342188277	1.227857052	0.44823204	1
gene74260	4.1325593	9.679231946	2.342188277	1.227857052	0.44823204	1
gene43353	175.6337703	128.7337849	0.732967155	-0.44817954	0.44824013	1
gene66260	491.7745567	370.7145835	0.753830345	-0.40768822	0.44824152	1
gene67175	1354.446311	1009.543892	0.745355415	-0.42399957	0.44825536	1
gene27112	293.4117103	390.0730474	1.329439261	0.410817866	0.44828652	1
gene34863	380.1954556	504.2879844	1.326391405	0.407506563	0.44837422	1
gene20962	504.1722346	668.8349275	1.326600081	0.407733519	0.44839676	1
gene43330	977.3502745	733.6857815	0.750688674	-0.41371338	0.44841594	1
gene57854	486.6088576	366.8428908	0.753876312	-0.40760025	0.44842678	1
gene66013	410.1565106	308.7674991	0.752804091	-0.40965363	0.44843578	1
gene30852	540.3321285	407.4956649	0.754157755	-0.40706176	0.44843834	1
gene54645	1482.555649	1102.464519	0.743624376	-0.42735403	0.44849402	1
gene60000	1410.235861	1050.196666	0.74469576	-0.42527695	0.44856129	1
gene50782	535.1664294	403.6239722	0.754202712	-0.40697576	0.4485635	1
gene52903	567.193764	427.822052	0.754278483	-0.40683082	0.44862669	1
gene1988	1781.133058	2436.262681	1.367816216	0.451874399	0.44865754	1
gene53520	88.85002496	61.94708446	0.697209534	-0.5203358	0.44867648	1
gene2246	94.01572408	130.6696313	1.389869966	0.474949914	0.44873899	1
gene4545	94.01572408	130.6696313	1.389869966	0.474949914	0.44873899	1
gene25401	181.8326092	133.5734009	0.734595414	-0.44497821	0.44876258	1
gene42353	49.5907116	72.5942396	1.463867673	0.549785146	0.44876577	1
gene30429	432.8855867	326.1901166	0.753525011	-0.4082727	0.44906438	1
gene29511	729.3967165	549.7803746	0.753746709	-0.4078483	0.44910779	1
gene47409	180.7994694	242.9487219	1.343746874	0.426261399	0.44915874	1

gene39015	145.6727153	197.4563317	1.355479173	0.438802946	0.44923108	1
gene56183	145.6727153	197.4563317	1.355479173	0.438802946	0.44923108	1
gene46780	229.3570412	170.3544823	0.742747994	-0.42905529	0.4492951	1
gene8686	2586.982122	1887.45023	0.72959539	-0.45483148	0.44931128	1
gene55154	231.4233208	308.7674991	1.334210822	0.415986648	0.44931506	1
gene44569	2752.284494	2002.63309	0.727625757	-0.45873148	0.44932204	1
gene66410	146.7058552	106.4715514	0.72574848	-0.46245845	0.44943895	1
gene24023	376.0628963	498.4804452	1.325524135	0.406562939	0.44950906	1
gene2453	600.2542384	452.9880551	0.754660319	-0.40610068	0.4495224	1
gene62896	153.9378339	208.1034868	1.351867059	0.434953286	0.4495625	1
gene7370	70.25350811	99.69608905	1.419090544	0.504966643	0.44957253	1
gene4404	54.75641073	79.36970196	1.449505198	0.535560507	0.44964568	1
gene44561	54.75641073	79.36970196	1.449505198	0.535560507	0.44964568	1
gene30052	170.4680711	124.8620921	0.732466152	-0.449166	0.44964596	1
gene39028	3618.055667	2597.905854	0.718039216	-0.47786546	0.44968343	1
gene33418	132.2418976	180.0337142	1.361396936	0.445087768	0.44971025	1
gene15347	65.08780898	92.92062669	1.427619521	0.513611534	0.4498996	1
gene21199	65.08780898	92.92062669	1.427619521	0.513611534	0.4498996	1
gene32165	65.08780898	92.92062669	1.427619521	0.513611534	0.4498996	1
gene20141	83.68432583	58.07539168	0.693981712	-0.52703045	0.44994535	1
gene67068	83.68432583	58.07539168	0.693981712	-0.52703045	0.44994535	1
gene2767	42.35873283	62.91500765	1.485290127	0.570744765	0.4499529	1
gene12982	42.35873283	62.91500765	1.485290127	0.570744765	0.4499529	1
gene68792	42.35873283	62.91500765	1.485290127	0.570744765	0.4499529	1
gene7660	59.92210985	86.14516432	1.437619011	0.523681392	0.44996457	1
gene4728	25.82849563	40.65277417	1.573950522	0.65439019	0.44998793	1
gene51922	25.82849563	40.65277417	1.573950522	0.65439019	0.44998793	1
gene37480	270.6826342	202.2959477	0.747354733	-0.42013491	0.45000056	1
gene36946	762.4571909	1015.351431	1.331683199	0.413250913	0.45004026	1
gene58926	827.5449999	623.3425373	0.753243071	-0.4088126	0.45004398	1
gene14869	501.0728152	663.9953115	1.325147347	0.406152786	0.45010661	1
gene20397	33.0604744	20.32638709	0.614824423	-0.70175362	0.45010755	1
gene46277	33.0604744	20.32638709	0.614824423	-0.70175362	0.45010755	1
gene47969	33.0604744	20.32638709	0.614824423	-0.70175362	0.45010755	1
gene51319	33.0604744	20.32638709	0.614824423	-0.70175362	0.45010755	1
gene56056	33.0604744	20.32638709	0.614824423	-0.70175362	0.45010755	1
gene58199	33.0604744	20.32638709	0.614824423	-0.70175362	0.45010755	1
gene64599	33.0604744	20.32638709	0.614824423	-0.70175362	0.45010755	1
gene49422	4525.152434	3207.697467	0.708859539	-0.49642831	0.45011923	1
gene66519	1119.92357	1505.120568	1.343949362	0.42647878	0.45014456	1
gene9660	505.2053745	381.3617387	0.75486477	-0.40570988	0.45025934	1
gene52872	170.4680711	229.3977971	1.345693628	0.428349991	0.45025974	1
gene33749	288.2460112	215.8468724	0.748828653	-0.41729246	0.45033948	1
gene41960	97.11514356	134.5413241	1.385379449	0.470281178	0.45034867	1
gene64158	356.4332397	268.1147249	0.752215829	-0.41078143	0.45035438	1
gene66939	135.3413171	97.76024266	0.722323713	-0.46928256	0.45035942	1
gene72775	135.3413171	97.76024266	0.722323713	-0.46928256	0.45035942	1
gene7999	478.343739	361.0353516	0.754761319	-0.40590761	0.45047213	1

gene20063	574.4257427	761.7555542	1.326116672	0.407207709	0.45050467	1
gene37145	521.7356117	393.9447402	0.755065844	-0.40532564	0.45051157	1
gene48771	442.1838451	585.5935328	1.324321409	0.405253302	0.45051452	1
gene46543	1259.397447	941.7892684	0.747809415	-0.41925746	0.45054037	1
gene1344	192.1640075	257.4675698	1.339832434	0.422052581	0.45056691	1
gene2201	423.5873283	319.4146542	0.754070372	-0.40722893	0.4505698	1
gene15725	206.627965	152.9318648	0.740131495	-0.43414648	0.45062445	1
gene12047	307.8756679	408.4635881	1.326716044	0.407859625	0.45064006	1
gene38295	205.5948252	274.8901873	1.337048182	0.419051456	0.45066288	1
gene48500	1338.949213	999.8646601	0.746753238	-0.42129651	0.45071714	1
gene5312	106.413402	75.49800918	0.709478391	-0.49516935	0.45073861	1
gene26147	106.413402	75.49800918	0.709478391	-0.49516935	0.45073861	1
gene54860	106.413402	75.49800918	0.709478391	-0.49516935	0.45073861	1
gene7059	95.04886391	66.78670043	0.702656483	-0.50910854	0.45075696	1
gene50338	95.04886391	66.78670043	0.702656483	-0.50910854	0.45075696	1
gene63868	95.04886391	66.78670043	0.702656483	-0.50910854	0.45075696	1
gene32605	339.9030025	255.5317234	0.751778365	-0.4116207	0.45077074	1
gene59679	3573.630655	2569.836082	0.719110711	-0.4757142	0.4508334	1
gene58734	675.6734456	510.0955236	0.754943867	-0.40555872	0.45083556	1
gene55555	362.6320786	272.9543409	0.752703241	-0.40984691	0.45093372	1
gene26685	4792.735649	6978.726233	1.456104977	0.54211437	0.45095964	1
gene20586	1855.519126	1372.51509	0.739693313	-0.43500086	0.45100042	1
gene29385	3203.766598	2317.208128	0.723276199	-0.46738142	0.45100745	1
gene20330	1012.477029	1356.060396	1.339349296	0.421532258	0.45102299	1
gene5729	189.064588	139.38094	0.737213359	-0.43984588	0.45104481	1
gene36181	189.064588	139.38094	0.737213359	-0.43984588	0.45104481	1
gene50466	189.064588	139.38094	0.737213359	-0.43984588	0.45104481	1
gene38809	78.51862671	54.2036989	0.690329176	-0.53464363	0.45110674	1
gene63567	869.9037327	655.2840028	0.753283356	-0.40873544	0.45113748	1
gene23023	606.4530773	804.3441747	1.326309	0.40741693	0.45115416	1
gene15399	462.8466416	349.4202733	0.754937471	-0.40557094	0.45119103	1
gene35961	78.51862671	110.3432442	1.405312966	0.490891457	0.45120277	1
gene21313	385.3611548	290.3769584	0.753519017	-0.40828417	0.451214	1
gene34133	173.5674906	233.2694899	1.34396994	0.42650087	0.45121989	1
gene73350	173.5674906	233.2694899	1.34396994	0.42650087	0.45121989	1
gene9677	501.0728152	378.4579691	0.755295354	-0.40488718	0.45123555	1
gene1401	775.8880086	1032.774049	1.331086493	0.41260432	0.45124514	1
gene42815	236.5890199	176.1620214	0.744590858	-0.42548019	0.45125601	1
gene29434	710.8001997	944.693038	1.329055673	0.41040154	0.45126567	1
gene12102	1054.835761	1414.135787	1.340621772	0.422902269	0.45128803	1
gene1494	91.94944443	127.7658617	1.389522933	0.474589645	0.45129073	1
gene272	312.0082272	234.2374131	0.750741143	-0.41361255	0.45135894	1
gene55117	736.6286953	979.538273	1.329758506	0.411164265	0.45135922	1
gene1910	856.472915	1142.14937	1.333549899	0.415271809	0.45146434	1
gene28697	424.6204681	320.3825774	0.754515153	-0.40637822	0.45147804	1
gene41798	1254.231748	1689.993898	1.347433519	0.430214094	0.45149449	1
gene1159	160.1366729	215.8468724	1.347891576	0.430704451	0.45157642	1
gene40816	160.1366729	215.8468724	1.347891576	0.430704451	0.45157642	1

gene64968	171.501211	125.8300153	0.733697532	-0.44674266	0.45159094	1
gene52160	1538.3452	1145.053139	0.744340828	-0.42596472	0.45163201	1
gene50929	242.7878589	181.0016374	0.745513545	-0.42369353	0.45170188	1
gene36948	47.52443195	30.97354223	0.651739347	-0.617633	0.45177416	1
gene44554	47.52443195	30.97354223	0.651739347	-0.617633	0.45177416	1
gene59671	47.52443195	30.97354223	0.651739347	-0.617633	0.45177416	1
gene61579	47.52443195	30.97354223	0.651739347	-0.617633	0.45177416	1
gene63027	47.52443195	30.97354223	0.651739347	-0.617633	0.45177416	1
gene70527	47.52443195	30.97354223	0.651739347	-0.617633	0.45177416	1
gene18991	800.6833644	603.9840735	0.754335734	-0.40672132	0.45178	1
gene52404	100.214563	138.4130168	1.381166695	0.465887451	0.45186292	1
gene34044	201.4622659	149.060172	0.739891271	-0.43461482	0.45187367	1
gene60575	201.4622659	149.060172	0.739891271	-0.43461482	0.45187367	1
gene61945	138.4407366	187.7770998	1.356371719	0.439752608	0.45192591	1
gene47827	112.6122409	80.33762515	0.713400466	-0.48721594	0.45197493	1
gene61782	306.842528	230.3657203	0.750762034	-0.4135724	0.45199718	1
gene1655	177.7000499	130.6696313	0.73533818	-0.4435202	0.45202374	1
gene41603	4062.305792	2902.801661	0.714569953	-0.48485284	0.45204787	1
gene9705	430.8193071	325.2221934	0.754892337	-0.40565719	0.45205299	1
gene55964	430.8193071	325.2221934	0.754892337	-0.40565719	0.45205299	1
gene43384	130.175618	93.88854988	0.721245279	-0.47143812	0.45205516	1
gene47605	130.175618	93.88854988	0.721245279	-0.47143812	0.45205516	1
gene49470	375.0297565	282.6335728	0.753629727	-0.40807222	0.45206927	1
gene2458	1557.974856	1159.571987	0.74428158	-0.42607956	0.45208212	1
gene57302	216.9593633	289.4090352	1.33393199	0.415685113	0.45210778	1
gene44777	2987.840374	2170.083802	0.726305134	-0.46135232	0.45214881	1
gene27215	153.9378339	112.2790906	0.729379437	-0.45525857	0.45220109	1
gene14053	1338.949213	1000.832583	0.747476135	-0.41990058	0.45223456	1
gene33573	86.78374531	120.9903993	1.394159689	0.479395819	0.45223698	1
gene20133	108.4796816	149.060172	1.374083789	0.45846998	0.45224034	1
gene8592	254.152397	337.8051949	1.329144242	0.410497678	0.45235334	1
gene27320	425.6536079	321.3505006	0.754957775	-0.40553214	0.45238282	1
gene34783	1413.335281	1055.036282	0.746486907	-0.42181114	0.4524012	1
gene15901	23.76221598	37.74900459	1.588614657	0.667769219	0.45240419	1
gene22888	23.76221598	37.74900459	1.588614657	0.667769219	0.45240419	1
gene57860	23.76221598	37.74900459	1.588614657	0.667769219	0.45240419	1
gene74106	23.76221598	37.74900459	1.588614657	0.667769219	0.45240419	1
gene27651	535.1664294	404.5918954	0.756011351	-0.4035202	0.45243767	1
gene70471	116.7448002	159.7073271	1.368003772	0.452072208	0.45249707	1
gene58378	3302.948021	2387.866521	0.722950076	-0.46803207	0.45254129	1
gene21820	731.4629962	971.7948874	1.328563294	0.409866961	0.45258847	1
gene13737	832.710699	628.1821533	0.754382229	-0.4066324	0.45259871	1
gene67296	381.2285955	287.4731888	0.754070372	-0.40722893	0.45262615	1
gene64710	917.4281647	1224.422841	1.334625302	0.41643476	0.45262809	1
gene62537	1232.535811	923.3987277	0.749186124	-0.41660392	0.45263573	1
gene59079	932.925262	702.7122393	0.7532353	-0.40882748	0.4526422	1
gene3562	213.8599438	158.7394039	0.7422587	-0.430006	0.45265943	1
gene65149	486.6088576	367.810814	0.755865431	-0.40379868	0.452681	1

gene4769	16.5302372	8.711308752	0.526992362	-0.92414604	0.45273407	1
gene6004	16.5302372	8.711308752	0.526992362	-0.92414604	0.45273407	1
gene15583	16.5302372	8.711308752	0.526992362	-0.92414604	0.45273407	1
gene17467	16.5302372	8.711308752	0.526992362	-0.92414604	0.45273407	1
gene24057	16.5302372	8.711308752	0.526992362	-0.92414604	0.45273407	1
gene27044	16.5302372	8.711308752	0.526992362	-0.92414604	0.45273407	1
gene34956	16.5302372	8.711308752	0.526992362	-0.92414604	0.45273407	1
gene39961	16.5302372	8.711308752	0.526992362	-0.92414604	0.45273407	1
gene68448	16.5302372	8.711308752	0.526992362	-0.92414604	0.45273407	1
gene22397	33.0604744	50.33200612	1.52242238	0.606368675	0.45281192	1
gene38826	33.0604744	50.33200612	1.52242238	0.606368675	0.45281192	1
gene67739	33.0604744	50.33200612	1.52242238	0.606368675	0.45281192	1
gene72840	190.0977278	140.3488632	0.738298479	-0.43772391	0.45281789	1
gene40892	68.18722846	46.46031334	0.681363862	-0.55350266	0.45285216	1
gene64902	68.18722846	46.46031334	0.681363862	-0.55350266	0.45285216	1
gene11063	52.69013108	34.84523501	0.661323749	-0.59657138	0.4528568	1
gene52817	52.69013108	34.84523501	0.661323749	-0.59657138	0.4528568	1
gene39091	95.04886391	131.6375545	1.384946111	0.469829842	0.45288696	1
gene40391	95.04886391	131.6375545	1.384946111	0.469829842	0.45288696	1
gene1778	63.02152933	90.0168571	1.428350884	0.51435043	0.45290788	1
gene44400	63.02152933	90.0168571	1.428350884	0.51435043	0.45290788	1
gene31551	2105.538964	1552.548804	0.737364082	-0.43955095	0.45292057	1
gene53110	481.4431585	363.9391212	0.755933727	-0.40366834	0.45292091	1
gene30695	431.8524469	326.1901166	0.755327703	-0.40482539	0.45294568	1
gene4558	353.3338202	266.1788785	0.753335411	-0.40863575	0.45296026	1
gene63178	40.29245318	60.01123807	1.48939152	0.574723048	0.45296148	1
gene28548	220.0587827	293.280728	1.332738118	0.41439332	0.45298357	1
gene53628	57.8558302	83.24139474	1.438772799	0.524838789	0.45302526	1
gene4774	2632.440274	3659.717599	1.390237657	0.475331528	0.45302928	1
gene47584	261.3843757	195.5204853	0.748019023	-0.41885314	0.45304217	1
gene70915	261.3843757	195.5204853	0.748019023	-0.41885314	0.45304217	1
gene35669	504.1722346	666.8990811	1.322760428	0.403551792	0.45306251	1
gene37554	504.1722346	666.8990811	1.322760428	0.403551792	0.45306251	1
gene36037	415.3222097	313.6071151	0.755093534	-0.40527273	0.45308579	1
gene62303	1318.286417	986.3137353	0.748178638	-0.41854532	0.45308857	1
gene14391	695.3031023	525.5822947	0.755903854	-0.40372535	0.45317352	1
gene72492	963.9194568	725.942396	0.753115202	-0.40905753	0.45321411	1
gene52513	492.8076966	372.6504299	0.756178186	-0.40320186	0.45324828	1
gene39004	528.9675904	699.8084697	1.322970409	0.403780794	0.45326147	1
gene33125	57.8558302	38.71692779	0.669196651	-0.57949787	0.45327257	1
gene69782	57.8558302	38.71692779	0.669196651	-0.57949787	0.45327257	1
gene55219	407.0570911	538.1652962	1.322088002	0.40281821	0.45331415	1
gene18904	142.5732959	103.5677818	0.726417813	-0.46112851	0.45334647	1
gene39108	509.3379338	385.2334315	0.756341529	-0.40289026	0.45337255	1
gene43504	1201.541617	901.1364942	0.749983589	-0.41506907	0.45339423	1
gene15559	1952.634269	1444.141406	0.739586224	-0.43520974	0.45341547	1
gene30249	1319.319557	987.2816585	0.748326403	-0.41826042	0.45343041	1
gene31340	348.1681211	262.3071857	0.753392312	-0.40852678	0.45348838	1

gene19254	1865.850524	1382.194322	0.740785129	-0.43287296	0.45352829	1
gene61018	774.8548688	1029.870279	1.329113774	0.410464607	0.45353306	1
gene48881	111.5791011	152.9318648	1.37061388	0.454822203	0.45354115	1
gene66447	111.5791011	152.9318648	1.37061388	0.454822203	0.45354115	1
gene520	9.298258426	17.4226175	1.873750621	0.905928957	0.4535563	1
gene5195	9.298258426	17.4226175	1.873750621	0.905928957	0.4535563	1
gene5245	9.298258426	17.4226175	1.873750621	0.905928957	0.4535563	1
gene7537	9.298258426	17.4226175	1.873750621	0.905928957	0.4535563	1
gene9133	9.298258426	17.4226175	1.873750621	0.905928957	0.4535563	1
gene9368	9.298258426	17.4226175	1.873750621	0.905928957	0.4535563	1
gene13609	9.298258426	17.4226175	1.873750621	0.905928957	0.4535563	1
gene19769	9.298258426	17.4226175	1.873750621	0.905928957	0.4535563	1
gene22176	9.298258426	17.4226175	1.873750621	0.905928957	0.4535563	1
gene22982	9.298258426	17.4226175	1.873750621	0.905928957	0.4535563	1
gene29182	9.298258426	17.4226175	1.873750621	0.905928957	0.4535563	1
gene30045	9.298258426	17.4226175	1.873750621	0.905928957	0.4535563	1
gene35385	9.298258426	17.4226175	1.873750621	0.905928957	0.4535563	1
gene39036	9.298258426	17.4226175	1.873750621	0.905928957	0.4535563	1
gene58128	9.298258426	17.4226175	1.873750621	0.905928957	0.4535563	1
gene60735	9.298258426	17.4226175	1.873750621	0.905928957	0.4535563	1
gene61692	9.298258426	17.4226175	1.873750621	0.905928957	0.4535563	1
gene37734	335.7704432	444.2767463	1.323156208	0.403983392	0.45360307	1
gene61163	285.1465917	213.911026	0.750179144	-0.41469294	0.45360632	1
gene22552	119.8442197	163.5790199	1.36493041	0.448827398	0.45370021	1
gene49904	119.8442197	163.5790199	1.36493041	0.448827398	0.45370021	1
gene68610	107.4465418	76.46593238	0.711664899	-0.49073001	0.45373963	1
gene59798	84.71746566	59.04331487	0.696943829	-0.52088571	0.4537664	1
gene5925	28.9279151	17.4226175	0.602276985	-0.73150096	0.4537791	1
gene71996	28.9279151	17.4226175	0.602276985	-0.73150096	0.4537791	1
gene41144	1193.276498	1602.88081	1.343260186	0.425738778	0.45378871	1
gene25703	128.1093383	174.226175	1.35998029	0.443585743	0.4538111	1
gene60850	125.0099188	90.0168571	0.720077718	-0.47377547	0.45382688	1
gene28879	148.7721348	108.4073978	0.728680797	-0.45664112	0.4538642	1
gene18893	302.7099687	227.4619507	0.751418765	-0.41231095	0.4538718	1
gene2773	1954.700549	1446.077253	0.739794775	-0.43480298	0.45389864	1
gene54235	1699.515012	1263.139769	0.743235429	-0.42810882	0.4539694	1
gene43432	256.2186766	191.6487925	0.747989159	-0.41891073	0.45397316	1
gene51345	2053.881972	2816.656496	1.371381868	0.455630353	0.45397365	1
gene29918	416.3553495	314.5750383	0.755544605	-0.40441116	0.45400492	1
gene26218	480.4100187	634.9576157	1.321699363	0.402394055	0.45402226	1
gene31038	76.45234706	107.4394746	1.405312966	0.490891457	0.45405835	1
gene68663	331.6378839	249.7241842	0.753002586	-0.40927328	0.45410475	1
gene5817	510.3710736	386.2013547	0.756706982	-0.40219334	0.4541446	1
gene34850	255.1855368	338.7731181	1.327556108	0.408772837	0.45416903	1
gene1846	1049.670062	789.8253268	0.752451037	-0.41033039	0.45418554	1
gene71718	231.4233208	307.7995759	1.330028343	0.41145699	0.45437586	1
gene40813	98.14828338	135.5092472	1.380658353	0.465356365	0.45438454	1
gene34162	279.9808926	210.0393332	0.750191669	-0.41466885	0.45440514	1

gene50536	543.431548	411.3673577	0.756981002	-0.401671	0.45447583	1
gene38329	131.2087578	94.85647307	0.722943153	-0.46804589	0.45452514	1
gene29753	268.6163545	356.1957356	1.326038901	0.4071231	0.45457086	1
gene44451	297.5442696	223.590258	0.751452072	-0.412247	0.45458651	1
gene10037	337.8367228	254.5638002	0.753511336	-0.40829888	0.4546149	1
gene29926	21.69593633	34.84523501	1.606071961	0.683536535	0.4546901	1
gene33199	21.69593633	34.84523501	1.606071961	0.683536535	0.4546901	1
gene37211	21.69593633	34.84523501	1.606071961	0.683536535	0.4546901	1
gene38422	21.69593633	34.84523501	1.606071961	0.683536535	0.4546901	1
gene46603	21.69593633	34.84523501	1.606071961	0.683536535	0.4546901	1
gene63106	21.69593633	34.84523501	1.606071961	0.683536535	0.4546901	1
gene53321	226.2576217	301.0241135	1.330448501	0.411912667	0.45469367	1
gene58765	326.4721847	245.8524914	0.753058003	-0.4091671	0.45471794	1
gene31485	1191.210218	1599.009118	1.342339994	0.42475013	0.45473852	1
gene42860	4274.099456	3050.893909	0.713809761	-0.48638847	0.4547481	1
gene3858	38.22617353	24.19807987	0.633023859	-0.65966822	0.45475871	1
gene10365	38.22617353	24.19807987	0.633023859	-0.65966822	0.45475871	1
gene38068	38.22617353	24.19807987	0.633023859	-0.65966822	0.45475871	1
gene57570	38.22617353	24.19807987	0.633023859	-0.65966822	0.45475871	1
gene65843	38.22617353	24.19807987	0.633023859	-0.65966822	0.45475871	1
gene73378	38.22617353	24.19807987	0.633023859	-0.65966822	0.45475871	1
gene54376	1804.895274	1339.605701	0.742206886	-0.43010671	0.45478445	1
gene39637	113.6453808	81.30554835	0.715432055	-0.48311333	0.45480871	1
gene48436	113.6453808	81.30554835	0.715432055	-0.48311333	0.45480871	1
gene35597	488.6751373	645.6047708	1.321132838	0.401775534	0.45482203	1
gene15794	71.28664793	100.6640122	1.412101918	0.497844218	0.45487003	1
gene20736	71.28664793	100.6640122	1.412101918	0.497844218	0.45487003	1
gene23563	71.28664793	100.6640122	1.412101918	0.497844218	0.45487003	1
gene53360	197.3297066	146.1564024	0.740671057	-0.43309513	0.45489972	1
gene41685	1880.314482	1393.8094	0.741263982	-0.43194068	0.45490775	1
gene72421	258.2849563	342.6448109	1.32661544	0.407750222	0.45496297	1
gene43692	2821.504862	2058.772635	0.729671837	-0.45468032	0.45501759	1
gene18980	137.4075967	99.69608905	0.725550053	-0.46285295	0.45511243	1
gene36654	137.4075967	99.69608905	0.725550053	-0.46285295	0.45511243	1
gene44797	888.5002496	670.7707739	0.754947198	-0.40555235	0.45515574	1
gene28386	79.55176653	55.17162209	0.693531074	-0.52796757	0.45517716	1
gene25345	203.5285455	150.9960184	0.741891109	-0.43072064	0.45521504	1
gene27217	711.8333395	943.7251148	1.325766949	0.406827193	0.4552209	1
gene42066	172.5343508	231.3336435	1.3407976	0.423091472	0.45531995	1
gene34067	180.7994694	241.9807987	1.338393301	0.420502129	0.45550767	1
gene69626	229.3570412	304.8958063	1.329350103	0.410721109	0.45552627	1
gene4696	102.2808427	72.5942396	0.709754023	-0.49460897	0.45555301	1
gene6557	143.6064357	104.535705	0.727931896	-0.45812461	0.45561163	1
gene54533	119.8442197	86.14516432	0.718809506	-0.47631861	0.45567551	1
gene39991	189.064588	252.6279538	1.336199214	0.418135115	0.4557379	1
gene14381	179.7663296	132.6054777	0.737654699	-0.43898246	0.45574085	1
gene50062	1116.824151	840.1573329	0.752273607	-0.41067062	0.45574561	1
gene71427	50.62385143	73.56216279	1.453112727	0.539146626	0.45590697	1

gene72451	558.9286454	738.5253975	1.32132322	0.40198342	0.45593273	1
gene7841	134.3081773	181.9695606	1.354865834	0.438149995	0.45593999	1
gene16397	79.55176653	111.3111674	1.39922936	0.484632467	0.45602364	1
gene29961	316.1407865	238.1091059	0.753174269	-0.40894438	0.45602689	1
gene3056	38.22617353	57.10746848	1.493936306	0.57911864	0.45610464	1
gene5692	38.22617353	57.10746848	1.493936306	0.57911864	0.45610464	1
gene65692	38.22617353	57.10746848	1.493936306	0.57911864	0.45610464	1
gene65818	38.22617353	57.10746848	1.493936306	0.57911864	0.45610464	1
gene10239	345.0687016	260.3713394	0.754549277	-0.40631297	0.45619339	1
gene51397	55.78955055	80.33762515	1.440012052	0.526080886	0.4561995	1
gene57547	55.78955055	80.33762515	1.440012052	0.526080886	0.4561995	1
gene62181	55.78955055	80.33762515	1.440012052	0.526080886	0.4561995	1
gene31840	251.0529775	332.965579	1.32627616	0.407381208	0.45623272	1
gene25411	219.0256429	291.3448816	1.330186172	0.411628179	0.45626147	1
gene58384	475.2443195	627.2142301	1.319772177	0.400288909	0.45633099	1
gene4412	778.9874281	589.4652255	0.756706982	-0.40219334	0.45640487	1
gene61077	1708.813271	1271.851078	0.744289092	-0.426065	0.45641482	1
gene26048	661.2094881	875.002568	1.323336376	0.404179824	0.45642468	1
gene5562	24.7953558	14.51884792	0.585547069	-0.77214295	0.45644167	1
gene8794	24.7953558	14.51884792	0.585547069	-0.77214295	0.45644167	1
gene12947	24.7953558	14.51884792	0.585547069	-0.77214295	0.45644167	1
gene33982	24.7953558	14.51884792	0.585547069	-0.77214295	0.45644167	1
gene65656	24.7953558	14.51884792	0.585547069	-0.77214295	0.45644167	1
gene38104	74.38606741	51.29992932	0.689644326	-0.53607559	0.45646348	1
gene59216	881.2682708	1172.154989	1.330077375	0.411510175	0.45649364	1
gene37729	4383.612278	6301.179997	1.437440083	0.523501821	0.45652216	1
gene48147	275.8483333	207.1355637	0.750903807	-0.41329999	0.45654193	1
gene27778	293.4117103	220.6864884	0.752139334	-0.41092815	0.45656687	1
gene39663	450.4489637	594.3048415	1.319361103	0.399839477	0.45663902	1
gene41806	87.81688513	121.9583225	1.388779872	0.473817944	0.45666828	1
gene5035	351.2675405	265.2109553	0.75501128	-0.4054299	0.45667456	1
gene29497	108.4796816	77.43385557	0.713809761	-0.48638847	0.45669423	1
gene3711	1587.935911	1184.73799	0.74608678	-0.42258465	0.45670973	1
gene7135	19.62965668	31.94146542	1.627204487	0.702395563	0.45674598	1
gene28888	19.62965668	31.94146542	1.627204487	0.702395563	0.45674598	1
gene35232	19.62965668	31.94146542	1.627204487	0.702395563	0.45674598	1
gene38407	19.62965668	31.94146542	1.627204487	0.702395563	0.45674598	1
gene38653	19.62965668	31.94146542	1.627204487	0.702395563	0.45674598	1
gene45381	19.62965668	31.94146542	1.627204487	0.702395563	0.45674598	1
gene26240	432.8855867	571.0746848	1.319227764	0.399693667	0.456754	1
gene408	234.5227403	175.1940982	0.747023926	-0.42077364	0.45676273	1
gene13817	339.9030025	256.4996466	0.75462601	-0.40616627	0.45679407	1
gene18475	1786.298758	1327.990623	0.743431421	-0.42772843	0.45679991	1
gene16679	204.5616854	151.9639416	0.742875878	-0.42880691	0.45686661	1
gene4020	20.6627965	11.61507834	0.562125186	-0.83103664	0.45694897	1
gene10713	20.6627965	11.61507834	0.562125186	-0.83103664	0.45694897	1
gene23102	20.6627965	11.61507834	0.562125186	-0.83103664	0.45694897	1
gene28915	20.6627965	11.61507834	0.562125186	-0.83103664	0.45694897	1

gene35757	20.6627965	11.61507834	0.562125186	-0.83103664	0.45694897	1
gene48386	20.6627965	11.61507834	0.562125186	-0.83103664	0.45694897	1
gene51163	20.6627965	11.61507834	0.562125186	-0.83103664	0.45694897	1
gene59741	20.6627965	11.61507834	0.562125186	-0.83103664	0.45694897	1
gene66131	20.6627965	11.61507834	0.562125186	-0.83103664	0.45694897	1
gene16768	132.2418976	95.82439627	0.724614498	-0.46471442	0.45696455	1
gene17387	132.2418976	95.82439627	0.724614498	-0.46471442	0.45696455	1
gene17765	96.08200373	132.6054777	1.380128146	0.464802229	0.4569933	1
gene39013	299.6105493	225.5261044	0.752730853	-0.40979399	0.45701294	1
gene20919	129.1424781	175.1940982	1.35659545	0.439990559	0.45701674	1
gene7596	74.38606741	104.535705	1.405312966	0.490891457	0.45702085	1
gene5586	368.8309176	278.7618801	0.75579857	-0.40392631	0.45708068	1
gene40781	235.5558801	312.6391919	1.327240024	0.408429297	0.45714904	1
gene20660	357.4663795	270.0505713	0.755457259	-0.40457796	0.45715082	1
gene16712	317.1739263	239.0770291	0.753772644	-0.40779866	0.4571791	1
gene15159	387.4274344	511.0634468	1.319120437	0.399576291	0.45719617	1
gene38393	880.235131	1170.219142	1.329439261	0.410817866	0.45720148	1
gene17856	186.9983083	249.7241842	1.335435526	0.417310325	0.45722813	1
gene35368	186.9983083	249.7241842	1.335435526	0.417310325	0.45722813	1
gene17117	267.5832147	354.2598892	1.323924184	0.404820507	0.4572739	1
gene49044	43.39187265	28.06977264	0.646890095	-0.62840747	0.45729842	1
gene50532	43.39187265	28.06977264	0.646890095	-0.62840747	0.45729842	1
gene57917	43.39187265	28.06977264	0.646890095	-0.62840747	0.45729842	1
gene60526	43.39187265	28.06977264	0.646890095	-0.62840747	0.45729842	1
gene67248	43.39187265	28.06977264	0.646890095	-0.62840747	0.45729842	1
gene50744	402.9245318	531.3898339	1.318832168	0.399260982	0.45736781	1
gene69628	288.2460112	216.8147956	0.752186629	-0.41083743	0.45736957	1
gene43960	97.11514356	68.72254682	0.707639862	-0.49891278	0.45740648	1
gene44924	836.8432583	1111.175827	1.327818342	0.409057786	0.45742216	1
gene62473	195.2634269	260.3713394	1.333436289	0.415148895	0.45743208	1
gene29875	138.4407366	100.6640122	0.727127107	-0.45972052	0.45744699	1
gene69366	770.7223095	583.6576864	0.757286612	-0.40108867	0.45748206	1
gene15412	85.75060548	60.01123807	0.699834569	-0.51491416	0.45751013	1
gene35831	2320.432047	3193.178619	1.376113825	0.460599807	0.45751208	1
gene3039	69.22036828	47.42823654	0.685177466	-0.54545039	0.45755566	1
gene8058	69.22036828	47.42823654	0.685177466	-0.54545039	0.45755566	1
gene39933	69.22036828	47.42823654	0.685177466	-0.54545039	0.45755566	1
gene18328	1232.535811	926.3024973	0.751542056	-0.41207426	0.45761299	1
gene64115	203.5285455	271.0184945	1.331599426	0.413160154	0.45768339	1
gene50947	2910.354887	2124.591412	0.730011114	-0.45400967	0.45774044	1
gene311	165.302372	221.6544116	1.340902788	0.42320465	0.45778501	1
gene14250	157.0372534	211.0072564	1.343676432	0.426185768	0.4577888	1
gene18761	276.8814731	208.1034868	0.751597731	-0.41196738	0.45782859	1
gene18365	82.65118601	115.1828602	1.393602025	0.478818625	0.45783265	1
gene13898	2926.885124	4079.796265	1.393903789	0.479130986	0.45788217	1
gene41248	624.0164543	473.3144422	0.758496733	-0.39878513	0.45791665	1
gene17961	140.5070162	189.7129461	1.350202654	0.43317596	0.45792261	1
gene46605	140.5070162	189.7129461	1.350202654	0.43317596	0.45792261	1

gene19301	11.36453808	20.32638709	1.788580139	0.838814761	0.45792615	1
gene22692	11.36453808	20.32638709	1.788580139	0.838814761	0.45792615	1
gene39874	11.36453808	20.32638709	1.788580139	0.838814761	0.45792615	1
gene49582	11.36453808	20.32638709	1.788580139	0.838814761	0.45792615	1
gene58250	11.36453808	20.32638709	1.788580139	0.838814761	0.45792615	1
gene67776	11.36453808	20.32638709	1.788580139	0.838814761	0.45792615	1
gene51912	69.22036828	97.76024266	1.412304573	0.498051249	0.45793634	1
gene55611	1968.131367	1459.628178	0.74163148	-0.43122561	0.4580284	1
gene43378	259.3180961	194.5525621	0.750246763	-0.41456291	0.458062	1
gene41207	1556.941716	2104.265025	1.351537442	0.43460148	0.45809262	1
gene24736	43.39187265	63.88293085	1.472232631	0.558005653	0.45814075	1
gene28156	43.39187265	63.88293085	1.472232631	0.558005653	0.45814075	1
gene28197	43.39187265	63.88293085	1.472232631	0.558005653	0.45814075	1
gene44948	43.39187265	63.88293085	1.472232631	0.558005653	0.45814075	1
gene61832	854.4066353	646.572694	0.756750553	-0.40211027	0.45818406	1
gene16810	358.4995193	271.0184945	0.755980078	-0.40357988	0.45818664	1
gene14704	1766.669101	2399.4816	1.35819526	0.441690903	0.45819533	1
gene54377	235.5558801	176.1620214	0.747856608	-0.41916642	0.45822689	1
gene59579	313.041367	413.3032041	1.320283029	0.400847233	0.45825959	1
gene43713	120.8773595	87.11308752	0.720673316	-0.47258267	0.45832528	1
gene26739	64.05466916	43.55654376	0.679990145	-0.55641426	0.45835495	1
gene46935	64.05466916	43.55654376	0.679990145	-0.55641426	0.45835495	1
gene238	17.56337703	29.03769584	1.653309372	0.725356711	0.45841836	1
gene30100	17.56337703	29.03769584	1.653309372	0.725356711	0.45841836	1
gene37546	17.56337703	29.03769584	1.653309372	0.725356711	0.45841836	1
gene56663	17.56337703	29.03769584	1.653309372	0.725356711	0.45841836	1
gene59918	17.56337703	29.03769584	1.653309372	0.725356711	0.45841836	1
gene38840	1025.907846	774.3385557	0.754783735	-0.40586476	0.45848297	1
gene12766	410.1565106	310.7033455	0.757523866	-0.40063675	0.4585011	1
gene15171	1158.149744	872.0987984	0.753010397	-0.40925831	0.45852081	1
gene25861	48.55757178	31.94146542	0.657806069	-0.60426578	0.4585368	1
gene46320	48.55757178	31.94146542	0.657806069	-0.60426578	0.4585368	1
gene22157	103.3139825	73.56216279	0.712025236	-0.48999972	0.45864156	1
gene29397	103.3139825	73.56216279	0.712025236	-0.48999972	0.45864156	1
gene69459	103.3139825	73.56216279	0.712025236	-0.48999972	0.45864156	1
gene19421	168.4017915	225.5261044	1.339214401	0.421386947	0.45864975	1
gene11527	64.05466916	90.9847803	1.420423858	0.506321498	0.45871353	1
gene7870	163.2360924	120.0224761	0.735269231	-0.44365548	0.45875033	1
gene30756	273.7820536	362.0032748	1.322231571	0.402974867	0.45875884	1
gene57898	58.88897003	39.68485098	0.673892767	-0.56940905	0.45882573	1
gene65697	58.88897003	39.68485098	0.673892767	-0.56940905	0.45882573	1
gene59992	4646.029793	6698.99643	1.441875478	0.527946577	0.45892454	1
gene1206	53.7232709	35.8131582	0.666622817	-0.5850574	0.45894851	1
gene4446	53.7232709	35.8131582	0.666622817	-0.5850574	0.45894851	1
gene50259	53.7232709	35.8131582	0.666622817	-0.5850574	0.45894851	1
gene65272	53.7232709	35.8131582	0.666622817	-0.5850574	0.45894851	1
gene68796	53.7232709	35.8131582	0.666622817	-0.5850574	0.45894851	1
gene52109	217.9925031	162.6110967	0.745948115	-0.42285281	0.45896223	1

gene27801	28.9279151	44.52446695	1.539152296	0.622135991	0.45899968	1
gene30022	28.9279151	44.52446695	1.539152296	0.622135991	0.45899968	1
gene33003	28.9279151	44.52446695	1.539152296	0.622135991	0.45899968	1
gene41919	28.9279151	44.52446695	1.539152296	0.622135991	0.45899968	1
gene48839	28.9279151	44.52446695	1.539152296	0.622135991	0.45899968	1
gene52580	28.9279151	44.52446695	1.539152296	0.622135991	0.45899968	1
gene61367	28.9279151	44.52446695	1.539152296	0.622135991	0.45899968	1
gene71118	268.6163545	355.2278124	1.322435535	0.403197396	0.45900718	1
gene34064	2499.165237	1838.086147	0.735480039	-0.44324191	0.45901099	1
gene11117	295.47799	222.6223348	0.753431194	-0.40845233	0.45901243	1
gene6390	80.58490636	56.13954529	0.696650872	-0.52149227	0.45915903	1
gene19222	80.58490636	56.13954529	0.696650872	-0.52149227	0.45915903	1
gene25484	58.88897003	84.20931793	1.42996758	0.515982438	0.45927148	1
gene3503	91.94944443	64.85085404	0.705288155	-0.50371528	0.45928766	1
gene46140	91.94944443	64.85085404	0.705288155	-0.50371528	0.45928766	1
gene69383	647.7786703	491.7049829	0.75906325	-0.39770799	0.45935798	1
gene8895	181.8326092	134.5413241	0.739918569	-0.43456159	0.45939324	1
gene67151	181.8326092	134.5413241	0.739918569	-0.43456159	0.45939324	1
gene536	411.1896504	311.6712687	0.757974498	-0.39977879	0.45942023	1
gene21511	319.2402059	241.0128755	0.754957775	-0.40553214	0.45946609	1
gene694	15.49709738	26.13392626	1.686375559	0.753925863	0.45946929	1
gene3149	15.49709738	26.13392626	1.686375559	0.753925863	0.45946929	1
gene11823	15.49709738	26.13392626	1.686375559	0.753925863	0.45946929	1
gene21250	15.49709738	26.13392626	1.686375559	0.753925863	0.45946929	1
gene38683	15.49709738	26.13392626	1.686375559	0.753925863	0.45946929	1
gene42764	15.49709738	26.13392626	1.686375559	0.753925863	0.45946929	1
gene54707	15.49709738	26.13392626	1.686375559	0.753925863	0.45946929	1
gene65193	15.49709738	26.13392626	1.686375559	0.753925863	0.45946929	1
gene59751	284.1134519	213.911026	0.752907068	-0.40945629	0.45947272	1
gene2020	53.7232709	77.43385557	1.441346632	0.527417333	0.45949272	1
gene15970	53.7232709	77.43385557	1.441346632	0.527417333	0.45949272	1
gene29170	53.7232709	77.43385557	1.441346632	0.527417333	0.45949272	1
gene18780	13.43081773	23.23015667	1.729615958	0.790451739	0.45951823	1
gene39045	13.43081773	23.23015667	1.729615958	0.790451739	0.45951823	1
gene44450	13.43081773	23.23015667	1.729615958	0.790451739	0.45951823	1
gene70041	13.43081773	23.23015667	1.729615958	0.790451739	0.45951823	1
gene70144	13.43081773	23.23015667	1.729615958	0.790451739	0.45951823	1
gene55901	188.0314482	139.38094	0.741263982	-0.43194068	0.45958245	1
gene5823	109.5128215	78.40177877	0.715914153	-0.4821415	0.45960348	1
gene31398	109.5128215	78.40177877	0.715914153	-0.4821415	0.45960348	1
gene71924	109.5128215	78.40177877	0.715914153	-0.4821415	0.45960348	1
gene35378	587.8565605	775.3064789	1.318870165	0.399302547	0.45966604	1
gene107	236.5890199	177.1299446	0.748682017	-0.41757499	0.45968148	1
gene29966	94.01572408	129.7017081	1.379574633	0.464223507	0.45969339	1
gene36373	266.5500749	200.3601013	0.751679028	-0.41181134	0.45972409	1
gene11047	974.2508551	1296.049158	1.330303331	0.411755241	0.45990821	1
gene4564	34.09361423	21.29431028	0.62458354	-0.67903354	0.45991869	1
gene12422	34.09361423	21.29431028	0.62458354	-0.67903354	0.45991869	1

gene52528	34.09361423	21.29431028	0.62458354	-0.67903354	0.45991869	1
gene57259	34.09361423	21.29431028	0.62458354	-0.67903354	0.45991869	1
gene67363	34.09361423	21.29431028	0.62458354	-0.67903354	0.45991869	1
gene71753	34.09361423	21.29431028	0.62458354	-0.67903354	0.45991869	1
gene52064	1014.543308	766.5951702	0.755606157	-0.40429364	0.45992135	1
gene51265	949.4554992	1262.171846	1.329363879	0.41073606	0.46002034	1
gene20829	5803.146397	8540.954269	1.471779908	0.557561945	0.46005201	1
gene21103	145.6727153	106.4715514	0.730895632	-0.45226268	0.46006623	1
gene57087	145.6727153	106.4715514	0.730895632	-0.45226268	0.46006623	1
gene57280	595.0885392	452.0201319	0.75958467	-0.39671731	0.46008596	1
gene66979	726.297297	960.1798091	1.322020353	0.402744388	0.46023011	1
gene11811	212.826804	158.7394039	0.745861898	-0.42301957	0.46031862	1
gene25494	151.8715543	111.3111674	0.732929665	-0.44825334	0.46032548	1
gene893	191.1308676	254.5638002	1.331882198	0.413466485	0.46038067	1
gene9638	166.3355118	222.6223348	1.338393301	0.420502129	0.46038168	1
gene28834	1050.703202	793.6970196	0.755396022	-0.40469491	0.46048921	1
gene49613	687.0379837	521.7106019	0.759362094	-0.39714011	0.46049351	1
gene35687	1827.624351	2481.755071	1.357913113	0.441391171	0.46052191	1
gene40707	1917.507515	1425.750866	0.743543821	-0.42751032	0.46060882	1
gene4406	439.0844257	577.8501472	1.31603426	0.396197047	0.46072281	1
gene64390	261.3843757	196.4884085	0.751722087	-0.4117287	0.46074786	1
gene26223	480.4100187	364.9070444	0.759574177	-0.39673724	0.46075674	1
gene35707	80.58490636	112.2790906	1.393301744	0.478507733	0.46078616	1
gene42820	80.58490636	112.2790906	1.393301744	0.478507733	0.46078616	1
gene44384	395.692553	300.0561903	0.758306387	-0.39914722	0.46086351	1
gene14598	231.4233208	173.2582518	0.748663753	-0.41761019	0.46091287	1
gene69615	105.3802622	144.220556	1.368572758	0.452672135	0.46091694	1
gene71709	105.3802622	144.220556	1.368572758	0.452672135	0.46091694	1
gene34375	590.9559799	449.1163623	0.75998277	-0.39596139	0.46092893	1
gene13806	121.9104994	88.08101071	0.722505536	-0.46891945	0.46093898	1
gene10907	176.6669101	130.6696313	0.739638403	-0.43510796	0.46105316	1
gene25925	97.11514356	133.5734009	1.37541269	0.459864562	0.46105849	1
gene22487	291.3454307	219.7185652	0.754151403	-0.40707391	0.46107374	1
gene23693	2211.952365	3028.631676	1.369211979	0.45334582	0.4611114	1
gene71385	1281.093383	964.0515019	0.752522427	-0.41019352	0.46113616	1
gene59570	4398.076235	3148.654152	0.715916229	-0.48213731	0.46116477	1
gene69012	277.9146129	366.8428908	1.319984174	0.400520632	0.46116648	1
gene60529	86.78374531	60.97916126	0.702656483	-0.50910854	0.46117892	1
gene43717	531.0338701	403.6239722	0.760071993	-0.39579202	0.46119762	1
gene42523	169.4349313	226.4940275	1.336761114	0.418741672	0.4612065	1
gene61252	435.9850062	331.0297326	0.759268617	-0.39731772	0.46124404	1
gene23203	447.3495443	339.7410413	0.759453196	-0.39696704	0.46128539	1
gene30941	189.064588	140.3488632	0.742332896	-0.42986179	0.46133302	1
gene62318	161.1698127	215.8468724	1.339251245	0.421426638	0.4613387	1
gene11090	243.8209987	182.9374838	0.750294211	-0.41447167	0.46134957	1
gene5166	128.1093383	92.92062669	0.725322821	-0.46330485	0.46139623	1
gene14528	128.1093383	92.92062669	0.725322821	-0.46330485	0.46139623	1
gene42832	41.325593	60.97916126	1.475578614	0.561280785	0.4614831	1

gene52697	41.325593	60.97916126	1.475578614	0.561280785	0.4614831	1
gene64639	2171.659912	1608.688349	0.740764399	-0.43291333	0.46157116	1
gene43775	5244.217752	7630.138543	1.45496219	0.540981662	0.46159718	1
gene8683	408.0902309	309.7354223	0.758987593	-0.39785179	0.46172815	1
gene13264	207.6611048	154.8677111	0.745771392	-0.42319464	0.46174656	1
gene28886	144.6395755	194.5525621	1.345085268	0.427697631	0.4617797	1
gene55077	908.1299062	688.1933914	0.757813818	-0.40008465	0.46179698	1
gene24040	1736.708046	2351.08544	1.353759744	0.436971722	0.46188697	1
gene38769	1384.407366	1858.412534	1.342388505	0.424802267	0.46193139	1
gene64020	6331.080848	9386.919142	1.482672448	0.568199913	0.46193996	1
gene70921	518.6361922	682.3858522	1.315731263	0.39586485	0.46195899	1
gene42310	585.7902808	771.4347861	1.316912915	0.397159946	0.46201474	1
gene7603	220.0587827	164.5469431	0.747740858	-0.41938973	0.46204667	1
gene33309	75.41920723	105.5036282	1.398896012	0.484288723	0.46205936	1
gene4567	136.3744569	183.905407	1.348532644	0.431390446	0.46208078	1
gene12874	668.4414668	508.1596772	0.760215669	-0.39551933	0.46208484	1
gene3532	437.018146	331.9976558	0.759688491	-0.39652013	0.46211188	1
gene20156	893.6659487	1184.73799	1.325705642	0.406760477	0.46212607	1
gene22464	70.25350811	48.39615973	0.688878905	-0.53767769	0.46213913	1
gene23447	407.0570911	535.2615266	1.314954434	0.395012807	0.46215963	1
gene55977	526.9013108	400.7202026	0.760522311	-0.39493752	0.46219857	1
gene2535	26.86163545	41.62069737	1.549447629	0.631753993	0.4622083	1
gene19475	26.86163545	41.62069737	1.549447629	0.631753993	0.4622083	1
gene62434	26.86163545	41.62069737	1.549447629	0.631753993	0.4622083	1
gene66427	26.86163545	41.62069737	1.549447629	0.631753993	0.4622083	1
gene72885	26.86163545	41.62069737	1.549447629	0.631753993	0.4622083	1
gene19208	465.9460611	612.6953822	1.314949161	0.395007023	0.46221585	1
gene54962	903.9973469	685.2896218	0.758065966	-0.3996047	0.46223307	1
gene56956	1844.154588	1374.450936	0.745301368	-0.42410419	0.46225483	1
gene8155	146.7058552	107.4394746	0.732346194	-0.4494023	0.46225654	1
gene66896	146.7058552	107.4394746	0.732346194	-0.4494023	0.46225654	1
gene48238	292.3785705	220.6864884	0.75479707	-0.40583927	0.46229557	1
gene7416	2236.747721	3061.541065	1.368746701	0.452855488	0.46234023	1
gene47508	128.1093383	173.2582518	1.352424844	0.435548424	0.46242533	1
gene38112	152.9046941	112.2790906	0.734307676	-0.44554341	0.46243368	1
gene32470	587.8565605	447.1805159	0.760696649	-0.39460684	0.46245992	1
gene17064	110.5459613	79.36970196	0.71797921	-0.47798603	0.46246845	1
gene41016	712.8664793	940.8213452	1.319772177	0.400288909	0.46247728	1
gene34767	754.1920723	573.0105312	0.759767375	-0.39637033	0.46249937	1
gene47614	238.6552996	179.065791	0.750311396	-0.41443862	0.46256235	1
gene55511	454.581523	345.5485805	0.760146559	-0.39565049	0.46257451	1
gene45388	420.4879088	319.4146542	0.75962863	-0.39663381	0.46258054	1
gene38122	46.49129213	67.75462362	1.457361594	0.543358877	0.46261581	1
gene21849	192.1640075	255.5317234	1.329758506	0.411164265	0.46268417	1
gene65633	183.8988889	244.8845682	1.331626144	0.4131891	0.4627119	1
gene69505	516.5699125	392.976817	0.760742752	-0.39451941	0.46276366	1
gene64727	119.8442197	162.6110967	1.356853898	0.440265384	0.46279969	1
gene71189	119.8442197	162.6110967	1.356853898	0.440265384	0.46279969	1

gene43055	208.6942447	276.8260337	1.326467024	0.407588811	0.46281486	1
gene52689	51.65699125	74.53008599	1.442787979	0.528859308	0.46291063	1
gene64947	51.65699125	74.53008599	1.442787979	0.528859308	0.46291063	1
gene57975	251.0529775	188.745023	0.751813521	-0.41155323	0.46295914	1
gene29950	81.61804618	57.10746848	0.699691688	-0.51520874	0.46305527	1
gene47411	81.61804618	57.10746848	0.699691688	-0.51520874	0.46305527	1
gene5916	833.7438388	633.0217693	0.759252111	-0.39734908	0.46307631	1
gene8896	39.25931335	25.16600306	0.641019949	-0.64155884	0.46312964	1
gene22854	39.25931335	25.16600306	0.641019949	-0.64155884	0.46312964	1
gene23301	39.25931335	25.16600306	0.641019949	-0.64155884	0.46312964	1
gene53488	39.25931335	25.16600306	0.641019949	-0.64155884	0.46312964	1
gene42754	1766.669101	1319.279314	0.746760847	-0.42128181	0.46315533	1
gene10154	111.5791011	151.9639416	1.361939109	0.445662203	0.46318276	1
gene13685	111.5791011	151.9639416	1.361939109	0.445662203	0.46318276	1
gene13402	2593.180961	1909.712463	0.73643625	-0.44136745	0.46320348	1
gene22124	202.4954057	150.9960184	0.745676268	-0.42337867	0.46324975	1
gene59888	70.25350811	98.72816585	1.405312966	0.490891457	0.46329002	1
gene54814	6968.52812	4833.808434	0.693662758	-0.52769367	0.46329309	1
gene9709	2048.716273	1522.543185	0.743169372	-0.42823705	0.46331614	1
gene15859	771.7554493	586.561456	0.760035393	-0.39586149	0.46342219	1
gene20175	952.5549187	722.0707032	0.758035772	-0.39966216	0.46343331	1
gene41787	994.9136516	1321.215161	1.327969677	0.409222205	0.46345188	1
gene62569	478.343739	363.9391212	0.760831786	-0.39435057	0.46350543	1
gene21858	578.558302	440.4050536	0.76121119	-0.39363133	0.46354893	1
gene27498	352.3006804	267.1468017	0.75829204	-0.39917452	0.46357548	1
gene42147	2315.266348	1713.224055	0.739968452	-0.43446433	0.46357848	1
gene49149	178.7331897	238.1091059	1.332204199	0.413815234	0.46357964	1
gene62510	740.7612546	563.3312993	0.760476194	-0.39502501	0.46377791	1
gene48964	99.18142321	70.65839321	0.712415601	-0.48920899	0.46382671	1
gene46857	95.04886391	130.6696313	1.374762684	0.459182598	0.46382986	1
gene65670	78.51862671	109.375321	1.392985659	0.478180406	0.4638508	1
gene13776	859.5723345	1137.309754	1.323111166	0.40393428	0.46388455	1
gene24104	421.5210486	553.6520673	1.313462445	0.393374951	0.46394672	1
gene27451	86.78374531	120.0224761	1.383006411	0.467807844	0.46397083	1
gene30008	86.78374531	120.0224761	1.383006411	0.467807844	0.46397083	1
gene25649	774.8548688	1023.094817	1.320369605	0.400941833	0.46397545	1
gene31508	10909.95655	17291.94787	1.584969453	0.664455036	0.4640067	1
gene34913	1222.204413	922.4308045	0.75472711	-0.405973	0.46405865	1
gene64540	993.8805117	1319.279314	1.327402337	0.40860572	0.46407498	1
gene36904	404.9908114	307.7995759	0.760016196	-0.39589793	0.46407596	1
gene12416	59.92210985	40.65277417	0.678426949	-0.55973462	0.46422152	1
gene24802	59.92210985	40.65277417	0.678426949	-0.55973462	0.46422152	1
gene52628	59.92210985	40.65277417	0.678426949	-0.55973462	0.46422152	1
gene60847	59.92210985	40.65277417	0.678426949	-0.55973462	0.46422152	1
gene67159	364.6983583	276.8260337	0.759054784	-0.39772408	0.46430448	1
gene63104	298.5774094	392.976817	1.316163931	0.396339191	0.46430453	1
gene55172	181.8326092	241.9807987	1.330788794	0.412281623	0.4643404	1
gene8771	65.08780898	91.95270349	1.412748484	0.498504641	0.46443099	1

gene10955	223.1582022	295.2165744	1.322902638	0.403706887	0.46445731	1
gene61472	223.1582022	295.2165744	1.322902638	0.403706887	0.46445731	1
gene11116	44.42501248	29.03769584	0.653633938	-0.6134452	0.46462191	1
gene21217	44.42501248	29.03769584	0.653633938	-0.6134452	0.46462191	1
gene61762	44.42501248	29.03769584	0.653633938	-0.6134452	0.46462191	1
gene62598	335.7704432	254.5638002	0.758148328	-0.39944796	0.4646399	1
gene10877	2023.920917	1506.088491	0.744143943	-0.42634638	0.46467018	1
gene11087	1004.21191	1332.830239	1.327240024	0.408429297	0.46467377	1
gene38032	106.413402	145.1884792	1.36438152	0.44824712	0.4646773	1
gene73085	106.413402	145.1884792	1.36438152	0.44824712	0.4646773	1
gene23338	1165.381723	880.8101071	0.755812529	-0.40389966	0.46469233	1
gene51800	1311.054438	988.2495817	0.753782263	-0.40778025	0.46469676	1
gene11188	264.4837952	199.3921781	0.753891852	-0.40757052	0.464718	1
gene46233	178.7331897	132.6054777	0.741918599	-0.43066719	0.4647199	1
gene65345	294.4448501	222.6223348	0.756074812	-0.4033991	0.46471991	1
gene23595	126.0430587	170.3544823	1.351557825	0.434623238	0.46472082	1
gene11104	87.81688513	61.94708446	0.705411999	-0.50346198	0.46477504	1
gene23107	87.81688513	61.94708446	0.705411999	-0.50346198	0.46477504	1
gene23579	87.81688513	61.94708446	0.705411999	-0.50346198	0.46477504	1
gene26379	87.81688513	61.94708446	0.705411999	-0.50346198	0.46477504	1
gene58723	87.81688513	61.94708446	0.705411999	-0.50346198	0.46477504	1
gene49190	197.3297066	147.1243256	0.745576164	-0.42357236	0.46483218	1
gene8475	54.75641073	36.7810814	0.671721921	-0.57406398	0.46484988	1
gene18422	54.75641073	36.7810814	0.671721921	-0.57406398	0.46484988	1
gene10046	699.4356616	921.4628813	1.31743766	0.397734696	0.46485589	1
gene46638	288.2460112	379.4258923	1.316326601	0.396517489	0.46486338	1
gene15892	203.5285455	151.9639416	0.746646821	-0.42150211	0.46488102	1
gene58964	1700.548152	1272.819001	0.748475719	-0.41797258	0.46488481	1
gene17149	270.6826342	204.2317941	0.754506453	-0.40639486	0.46493295	1
gene9248	201.4622659	267.1468017	1.326038901	0.4071231	0.46493314	1
gene9795	29.96105493	18.3905407	0.613814859	-0.70412453	0.46497085	1
gene11642	29.96105493	18.3905407	0.613814859	-0.70412453	0.46497085	1
gene20853	29.96105493	18.3905407	0.613814859	-0.70412453	0.46497085	1
gene46435	29.96105493	18.3905407	0.613814859	-0.70412453	0.46497085	1
gene41920	613.6850561	467.506903	0.761802652	-0.39251079	0.46497889	1
gene10632	1200.508477	906.9440334	0.75546658	-0.40456016	0.46498929	1
gene14783	39.25931335	58.07539168	1.479276806	0.564892039	0.4649915	1
gene14971	39.25931335	58.07539168	1.479276806	0.564892039	0.4649915	1
gene18358	39.25931335	58.07539168	1.479276806	0.564892039	0.4649915	1
gene48433	39.25931335	58.07539168	1.479276806	0.564892039	0.4649915	1
gene65696	39.25931335	58.07539168	1.479276806	0.564892039	0.4649915	1
gene59961	300.6436891	227.4619507	0.756583155	-0.40242944	0.46501158	1
gene63131	217.9925031	288.441112	1.32316987	0.403998288	0.46504315	1
gene27426	832.710699	633.0217693	0.760194111	-0.39556025	0.46508229	1
gene40293	98.14828338	134.5413241	1.370796507	0.455014421	0.46508301	1
gene42690	422.5541885	554.6199905	1.312541694	0.392363252	0.4650988	1
gene54159	480.4100187	365.8749676	0.761588962	-0.39291553	0.4650994	1
gene32904	1566.239975	2107.168795	1.345367778	0.428000611	0.46511689	1

gene70200	531.0338701	404.5918954	0.761894708	-0.39233646	0.4651335	1
gene18866	2386.552996	3270.612475	1.370433626	0.454632456	0.46514902	1
gene2023	1328.617815	1777.106985	1.337560708	0.419604373	0.46528304	1
gene67626	234.5227403	176.1620214	0.75115113	-0.41282489	0.46528645	1
gene56362	1962.965668	2664.692555	1.357483016	0.440934148	0.46537095	1
gene58449	412.2227902	313.6071151	0.760770929	-0.39446598	0.46537475	1
gene17036	240.7215792	181.0016374	0.751912803	-0.41136273	0.46540607	1
gene1371	24.7953558	38.71692779	1.561458851	0.642894551	0.46546511	1
gene19851	24.7953558	38.71692779	1.561458851	0.642894551	0.46546511	1
gene29850	24.7953558	38.71692779	1.561458851	0.642894551	0.46546511	1
gene48746	24.7953558	38.71692779	1.561458851	0.642894551	0.46546511	1
gene62099	24.7953558	38.71692779	1.561458851	0.642894551	0.46546511	1
gene65631	376.0628963	493.6408293	1.312654968	0.392487753	0.46547276	1
gene10068	475.2443195	362.0032748	0.761720361	-0.39266663	0.46548353	1
gene71768	921.560724	699.8084697	0.75937315	-0.39711911	0.46549283	1
gene46909	452.5152434	344.5806573	0.761478563	-0.39312467	0.46549935	1
gene38676	2017.722078	2742.12641	1.359020868	0.442567609	0.46550739	1
gene29418	246.9204182	185.8412534	0.752636233	-0.40997535	0.46553968	1
gene70245	962.886317	730.782012	0.75894942	-0.39792435	0.46567066	1
gene331	336.803583	255.5317234	0.758696571	-0.39840508	0.46571576	1
gene65592	109.5128215	149.060172	1.361120734	0.444795043	0.46575003	1
gene38825	360.565799	473.3144422	1.312699218	0.392536385	0.46578561	1
gene22386	319.2402059	241.9807987	0.757989734	-0.39974979	0.46589396	1
gene68458	295.47799	223.590258	0.756706982	-0.40219334	0.46592219	1
gene35965	360.565799	273.9222641	0.759701183	-0.39649603	0.46597435	1
gene40906	360.565799	273.9222641	0.759701183	-0.39649603	0.46597435	1
gene32813	649.84495	854.6761809	1.315200158	0.395282377	0.46597808	1
gene66573	259.3180961	341.6768877	1.317597549	0.397909776	0.4660047	1
gene44468	435.9850062	331.9976558	0.7614887	-0.39310547	0.46601947	1
gene16189	379.1623158	497.512522	1.312135994	0.391917253	0.46606589	1
gene8860	3486.84691	2537.894616	0.727848019	-0.45829086	0.46609493	1
gene37732	54.75641073	78.40177877	1.431828305	0.517858505	0.46613961	1
gene12153	526.9013108	401.6881258	0.762359321	-0.39145695	0.46616742	1
gene16696	526.9013108	401.6881258	0.762359321	-0.39145695	0.46616742	1
gene8792	120.8773595	163.5790199	1.353264338	0.436443673	0.46617808	1
gene11084	345.0687016	452.9880551	1.312747441	0.392589384	0.4662146	1
gene62227	271.715774	205.1997173	0.75519987	-0.40506958	0.46621672	1
gene65749	454.581523	596.2406879	1.311625435	0.391355784	0.466295	1
gene1758	13.43081773	6.775462362	0.504471321	-0.98715584	0.46635074	1
gene4261	13.43081773	6.775462362	0.504471321	-0.98715584	0.46635074	1
gene5869	13.43081773	6.775462362	0.504471321	-0.98715584	0.46635074	1
gene7517	13.43081773	6.775462362	0.504471321	-0.98715584	0.46635074	1
gene11539	13.43081773	6.775462362	0.504471321	-0.98715584	0.46635074	1
gene19867	13.43081773	6.775462362	0.504471321	-0.98715584	0.46635074	1
gene42292	13.43081773	6.775462362	0.504471321	-0.98715584	0.46635074	1
gene42561	13.43081773	6.775462362	0.504471321	-0.98715584	0.46635074	1
gene48694	13.43081773	6.775462362	0.504471321	-0.98715584	0.46635074	1
gene51813	13.43081773	6.775462362	0.504471321	-0.98715584	0.46635074	1

gene56003	13.43081773	6.775462362	0.504471321	-0.98715584	0.46635074	1
gene61124	13.43081773	6.775462362	0.504471321	-0.98715584	0.46635074	1
gene61316	13.43081773	6.775462362	0.504471321	-0.98715584	0.46635074	1
gene69841	13.43081773	6.775462362	0.504471321	-0.98715584	0.46635074	1
gene11579	32.02733458	48.39615973	1.511089211	0.595588836	0.4663964	1
gene31945	32.02733458	48.39615973	1.511089211	0.595588836	0.4663964	1
gene3739	49.5907116	71.6263164	1.444349437	0.530419822	0.4664592	1
gene4109	49.5907116	71.6263164	1.444349437	0.530419822	0.4664592	1
gene66417	307.8756679	233.2694899	0.757674328	-0.40035023	0.46646412	1
gene22521	331.6378839	251.6600306	0.758839816	-0.39813272	0.4664879	1
gene10957	713.8996191	939.853422	1.316506406	0.396714542	0.46648848	1
gene51145	192.1640075	143.2526328	0.745470677	-0.42377649	0.4664981	1
gene38192	5226.654375	7569.159382	1.44818441	0.534245325	0.46650331	1
gene42949	210.7605243	157.7714807	0.748581743	-0.41776823	0.46651735	1
gene72045	154.9709738	114.214937	0.737008578	-0.44024668	0.46658393	1
gene7745	71.28664793	49.36408293	0.692473056	-0.53017016	0.46660719	1
gene26870	71.28664793	49.36408293	0.692473056	-0.53017016	0.46660719	1
gene38812	71.28664793	49.36408293	0.692473056	-0.53017016	0.46660719	1
gene55908	71.28664793	49.36408293	0.692473056	-0.53017016	0.46660719	1
gene54200	1952.634269	1456.724408	0.74603034	-0.42269379	0.46667724	1
gene31338	112.6122409	152.9318648	1.358039441	0.44152538	0.46676654	1
gene513	631.2484331	829.5101778	1.314078791	0.394051781	0.46679251	1
gene19954	2814.272884	2070.387713	0.735674115	-0.44286126	0.46685025	1
gene69533	82.65118601	58.07539168	0.702656483	-0.50910854	0.46686869	1
gene68780	290.3122908	219.7185652	0.756835215	-0.40194888	0.46687332	1
gene71507	290.3122908	219.7185652	0.756835215	-0.40194888	0.46687332	1
gene35410	6349.677365	4450.510849	0.700903462	-0.51271234	0.46687846	1
gene30241	1327.584675	1001.800506	0.754603849	-0.40620864	0.4669294	1
gene48060	406.0239513	532.3577571	1.311148654	0.390831263	0.46694732	1
gene46636	100.214563	71.6263164	0.714729618	-0.48453052	0.4669546	1
gene19790	361.5989388	274.8901873	0.760207395	-0.39553504	0.46698434	1
gene36210	84.71746566	117.1187066	1.382462349	0.467240189	0.46699846	1
gene14569	976.3171347	741.4291671	0.759414273	-0.39704098	0.46703076	1
gene54833	649.84495	495.5766757	0.762607566	-0.39098725	0.46703977	1
gene36156	154.9709738	207.1355637	1.336608777	0.418577253	0.46704325	1
gene61616	483.5094381	633.9896925	1.311225061	0.390915333	0.46705106	1
gene46875	595.0885392	453.9559783	0.76283771	-0.39055193	0.46711836	1
gene44069	816.1804618	621.406691	0.76135943	-0.3933504	0.46722309	1
gene7362	768.6560299	585.5935328	0.76184081	-0.39243852	0.46725957	1
gene8276	1147.818346	869.1950288	0.757258352	-0.40114251	0.46726182	1
gene29417	1592.06847	2140.078183	1.344212402	0.42676112	0.467274	1
gene71206	998.013071	1322.183084	1.324815398	0.405791346	0.46727638	1
gene13790	302.7099687	229.3977971	0.757813818	-0.40008465	0.46736635	1
gene58290	1856.552266	1388.001861	0.74762337	-0.41961643	0.46740039	1
gene46587	1227.370112	928.2383437	0.756282343	-0.40300316	0.46751829	1
gene11664	584.757141	446.2125927	0.763073354	-0.39010635	0.46759299	1
gene70655	106.413402	76.46593238	0.718574267	-0.47679082	0.46761124	1
gene17076	780.0205679	594.3048415	0.761909193	-0.39230903	0.46763711	1

gene37868	900.8979275	685.2896218	0.760673991	-0.39464982	0.46776525	1
gene23303	205.5948252	271.9864177	1.322924434	0.403730656	0.46783616	1
gene50475	579.5914419	442.3408999	0.763194326	-0.38987765	0.46784303	1
gene21358	501.0728152	382.3296619	0.76302216	-0.39020314	0.46786729	1
gene493	96.08200373	131.6375545	1.370054218	0.454232987	0.46792379	1
gene45105	3781.29176	2744.062257	0.725694401	-0.46256596	0.46795413	1
gene25591	1267.662565	1689.993898	1.333157533	0.414847267	0.46801221	1
gene23369	1335.849794	1008.575969	0.755007018	-0.40543804	0.46802964	1
gene30802	9682.586441	6545.096642	0.675965733	-0.56497798	0.46808187	1
gene215	205.5948252	153.8997879	0.748558665	-0.41781271	0.468106	1
gene28467	230.390181	173.2582518	0.752020989	-0.41115517	0.46811187	1
gene55439	230.390181	173.2582518	0.752020989	-0.41115517	0.46811187	1
gene18571	138.4407366	185.8412534	1.342388505	0.424802267	0.46813396	1
gene50067	236.5890199	178.0978678	0.752773175	-0.40971288	0.46815415	1
gene3539	66.12094881	45.49239015	0.688017806	-0.53948219	0.46816078	1
gene50460	66.12094881	45.49239015	0.688017806	-0.53948219	0.46816078	1
gene18145	349.2012609	457.8276711	1.311071071	0.390745894	0.46818799	1
gene55158	193.1971473	144.220556	0.746494232	-0.42179698	0.46819152	1
gene11866	907.0967664	690.1292378	0.760811044	-0.39438991	0.4682176	1
gene59164	1110.625312	842.0931793	0.758215368	-0.3993204	0.46822826	1
gene32268	186.9983083	139.38094	0.745359363	-0.42399193	0.46825209	1
gene50802	507.2716541	387.1692779	0.763238542	-0.38979407	0.46825804	1
gene26849	1686.084195	1265.075615	0.750303941	-0.41445296	0.46829416	1
gene6340	1819.359232	1361.867935	0.748542625	-0.41784362	0.46829988	1
gene24073	88.85002496	62.91500765	0.708103433	-0.49796799	0.4683007	1
gene9811	118.8110799	86.14516432	0.725060023	-0.46382766	0.46837745	1
gene50604	107.4465418	146.1564024	1.360270884	0.443893978	0.46840306	1
gene6638	541.3652683	413.3032041	0.763446102	-0.38940179	0.46843362	1
gene1287	6.19883895	12.58300153	2.029896507	1.021406174	0.46844503	1
gene2014	6.19883895	12.58300153	2.029896507	1.021406174	0.46844503	1
gene2180	6.19883895	12.58300153	2.029896507	1.021406174	0.46844503	1
gene6087	6.19883895	12.58300153	2.029896507	1.021406174	0.46844503	1
gene10441	6.19883895	12.58300153	2.029896507	1.021406174	0.46844503	1
gene10514	6.19883895	12.58300153	2.029896507	1.021406174	0.46844503	1
gene10852	6.19883895	12.58300153	2.029896507	1.021406174	0.46844503	1
gene12710	6.19883895	12.58300153	2.029896507	1.021406174	0.46844503	1
gene15552	6.19883895	12.58300153	2.029896507	1.021406174	0.46844503	1
gene15680	6.19883895	12.58300153	2.029896507	1.021406174	0.46844503	1
gene19154	6.19883895	12.58300153	2.029896507	1.021406174	0.46844503	1
gene19526	6.19883895	12.58300153	2.029896507	1.021406174	0.46844503	1
gene20092	6.19883895	12.58300153	2.029896507	1.021406174	0.46844503	1
gene34052	6.19883895	12.58300153	2.029896507	1.021406174	0.46844503	1
gene35650	6.19883895	12.58300153	2.029896507	1.021406174	0.46844503	1
gene43825	6.19883895	12.58300153	2.029896507	1.021406174	0.46844503	1
gene46579	6.19883895	12.58300153	2.029896507	1.021406174	0.46844503	1
gene55366	6.19883895	12.58300153	2.029896507	1.021406174	0.46844503	1
gene59493	6.19883895	12.58300153	2.029896507	1.021406174	0.46844503	1
gene61237	6.19883895	12.58300153	2.029896507	1.021406174	0.46844503	1

gene64077	6.19883895	12.58300153	2.029896507	1.021406174	0.46844503	1
gene68103	6.19883895	12.58300153	2.029896507	1.021406174	0.46844503	1
gene70112	6.19883895	12.58300153	2.029896507	1.021406174	0.46844503	1
gene70852	6.19883895	12.58300153	2.029896507	1.021406174	0.46844503	1
gene71383	6.19883895	12.58300153	2.029896507	1.021406174	0.46844503	1
gene42435	325.4390449	426.8541288	1.311625435	0.391355784	0.46845373	1
gene45184	1205.674176	912.7515725	0.757046631	-0.40154593	0.46850588	1
gene24552	71.28664793	99.69608905	1.398524015	0.483905027	0.46856899	1
gene41438	71.28664793	99.69608905	1.398524015	0.483905027	0.46856899	1
gene53814	71.28664793	99.69608905	1.398524015	0.483905027	0.46856899	1
gene44696	267.5832147	202.2959477	0.756011351	-0.4035202	0.46861938	1
gene12593	118.8110799	160.6752503	1.352359144	0.435478337	0.46865153	1
gene35331	118.8110799	160.6752503	1.352359144	0.435478337	0.46865153	1
gene73588	37.1930337	55.17162209	1.483385909	0.568893969	0.4686767	1
gene44413	143.6064357	105.5036282	0.734672006	-0.44482779	0.46872006	1
gene11834	468.0123408	357.1636588	0.763150088	-0.38996128	0.46872518	1
gene8314	22.72907615	35.8131582	1.575653932	0.655950704	0.46873284	1
gene43796	22.72907615	35.8131582	1.575653932	0.655950704	0.46873284	1
gene43984	22.72907615	35.8131582	1.575653932	0.655950704	0.46873284	1
gene31747	333.7041635	437.501284	1.311045326	0.390717564	0.46881149	1
gene22088	279.9808926	211.9751796	0.757105878	-0.40143303	0.46892197	1
gene59710	1137.486947	862.4195664	0.758179747	-0.39938818	0.4689294	1
gene69671	464.9129213	608.8236894	1.30954349	0.389063972	0.4689851	1
gene1976	99.18142321	135.5092472	1.366276495	0.450249473	0.46906735	1
gene39316	830.6444194	633.0217693	0.762085141	-0.39197591	0.469117	1
gene55632	1152.984045	874.0346448	0.758063087	-0.39961018	0.46913088	1
gene62573	1068.266579	1416.071634	1.325578897	0.40662254	0.46914582	1
gene64650	1052.769482	799.5045588	0.75942984	-0.39701141	0.46917882	1
gene5926	35.12675405	22.26223348	0.633768593	-0.65797193	0.46923027	1
gene48321	35.12675405	22.26223348	0.633768593	-0.65797193	0.46923027	1
gene50263	35.12675405	22.26223348	0.633768593	-0.65797193	0.46923027	1
gene62570	35.12675405	22.26223348	0.633768593	-0.65797193	0.46923027	1
gene3418	659.1432084	503.3200612	0.763597432	-0.38911584	0.46929009	1
gene9490	95.04886391	67.75462362	0.71283991	-0.48834998	0.46931983	1
gene4699	203.5285455	269.0826481	1.322088002	0.40281821	0.46932814	1
gene52561	719.0653183	548.8124514	0.763230318	-0.38980961	0.46935545	1
gene19508	1143.685786	1518.671492	1.327874763	0.409119087	0.46939862	1
gene10698	253.1192571	332.965579	1.315449416	0.395555772	0.46941542	1
gene10470	283.0803121	371.6825067	1.312993136	0.392859374	0.46947587	1
gene4782	25.82849563	15.48677111	0.599600199	-0.73792723	0.46954143	1
gene8394	25.82849563	15.48677111	0.599600199	-0.73792723	0.46954143	1
gene24523	25.82849563	15.48677111	0.599600199	-0.73792723	0.46954143	1
gene45669	25.82849563	15.48677111	0.599600199	-0.73792723	0.46954143	1
gene68728	25.82849563	15.48677111	0.599600199	-0.73792723	0.46954143	1
gene69569	25.82849563	15.48677111	0.599600199	-0.73792723	0.46954143	1
gene18679	219.0256429	164.5469431	0.751267938	-0.41260056	0.46959554	1
gene54337	742.8275342	976.6345034	1.314752696	0.394791455	0.46964551	1
gene38563	250.0198377	188.745023	0.754920188	-0.40560397	0.46965165	1

gene6839	90.91630461	124.8620921	1.373374035	0.457724593	0.46967324	1
gene48501	90.91630461	124.8620921	1.373374035	0.457724593	0.46967324	1
gene25781	200.4291261	150.0280952	0.748534398	-0.41785948	0.46977771	1
gene7446	52.69013108	75.49800918	1.432868122	0.518905834	0.46978029	1
gene5145	42.35873283	61.94708446	1.462439509	0.548376952	0.46981176	1
gene44051	42.35873283	61.94708446	1.462439509	0.548376952	0.46981176	1
gene56360	547.5641073	717.2310872	1.309857746	0.38941014	0.46986	1
gene25263	358.4995193	469.4427494	1.309465492	0.388978041	0.46993281	1
gene14333	848.2077964	646.572694	0.762281008	-0.39160516	0.46994718	1
gene62795	647.7786703	849.8365649	1.311924279	0.391684453	0.46996101	1
gene18079	11076.29206	17476.8212	1.577858466	0.657967801	0.46997529	1
gene55341	463.8797815	354.2598892	0.763689006	-0.38894284	0.46998487	1
gene46280	2949.614201	2170.083802	0.735717845	-0.44277551	0.47001633	1
gene52007	2234.681442	1662.892048	0.74412935	-0.42637467	0.47005327	1
gene3904	655.0106491	500.4162916	0.763981917	-0.3883896	0.47007657	1
gene68966	621.9501747	475.2502886	0.764129199	-0.38811151	0.47008002	1
gene28070	286.1797315	375.5541995	1.312301879	0.392099633	0.47009365	1
gene70990	452.5152434	345.5485805	0.763617548	-0.38907784	0.47011709	1
gene29603	29.96105493	45.49239015	1.518384124	0.602536813	0.47012782	1
gene2197	47.52443195	68.72254682	1.446046675	0.53211412	0.47014467	1
gene7691	47.52443195	68.72254682	1.446046675	0.53211412	0.47014467	1
gene32765	47.52443195	68.72254682	1.446046675	0.53211412	0.47014467	1
gene54093	47.52443195	68.72254682	1.446046675	0.53211412	0.47014467	1
gene38082	492.8076966	376.5221227	0.764034583	-0.38829015	0.47017693	1
gene54664	347.1349812	264.2430321	0.76121119	-0.39363133	0.47021684	1
gene39394	175.6337703	130.6696313	0.743989217	-0.42664638	0.47022958	1
gene33475	1183.97824	1572.875191	1.3284663	0.409761631	0.4702517	1
gene22414	113.6453808	153.8997879	1.354210676	0.437452198	0.47031901	1
gene61716	353.3338202	269.0826481	0.761553615	-0.39298249	0.47046852	1
gene33410	190.0977278	251.6600306	1.323845548	0.404734814	0.47047042	1
gene54950	583.7240012	446.2125927	0.764423926	-0.38755516	0.47051937	1
gene64756	83.68432583	59.04331487	0.705548074	-0.50318371	0.47060195	1
gene73599	403.9576716	528.4860643	1.308270894	0.3876613	0.47060791	1
gene38849	595.0885392	454.9239015	0.764464229	-0.3874791	0.47064582	1
gene38552	1884.447041	1411.232018	0.748883883	-0.41718605	0.47076539	1
gene13474	113.6453808	82.27347154	0.723949104	-0.46603982	0.47080786	1
gene51167	94.01572408	128.7337849	1.3692793	0.453416752	0.47086576	1
gene15995	634.3478526	484.9295205	0.764453633	-0.3874991	0.47088875	1
gene71228	144.6395755	106.4715514	0.736116316	-0.44199435	0.47089798	1
gene31206	550.6635268	421.0465897	0.764616811	-0.38719118	0.47093836	1
gene15797	6966.461841	4861.878207	0.697897773	-0.51891237	0.47096398	1
gene41220	72.31978776	50.33200612	0.695964517	-0.52291434	0.47096424	1
gene69786	72.31978776	50.33200612	0.695964517	-0.52291434	0.47096424	1
gene10088	119.8442197	87.11308752	0.726886017	-0.46019894	0.47097721	1
gene11842	119.8442197	87.11308752	0.726886017	-0.46019894	0.47097721	1
gene32200	232.4564606	175.1940982	0.753664139	-0.40800635	0.47100379	1
gene39631	1821.425512	1365.739628	0.749819094	-0.41538553	0.47100944	1
gene70283	691.170543	906.9440334	1.3121856	0.391971794	0.47102842	1

gene38264	312.0082272	237.1411827	0.760047851	-0.39583784	0.4710421	1
gene29200	226.2576217	170.3544823	0.752922624	-0.40942648	0.47104336	1
gene63134	132.2418976	96.79231946	0.731933836	-0.45021485	0.47104556	1
gene9815	389.4937141	297.1524208	0.762919683	-0.39039691	0.47108904	1
gene67098	492.8076966	644.6368476	1.308090056	0.387461868	0.47111604	1
gene24755	40.29245318	26.13392626	0.648605984	-0.62458576	0.47113372	1
gene25700	40.29245318	26.13392626	0.648605984	-0.62458576	0.47113372	1
gene26580	40.29245318	26.13392626	0.648605984	-0.62458576	0.47113372	1
gene50903	40.29245318	26.13392626	0.648605984	-0.62458576	0.47113372	1
gene26418	105.3802622	143.2526328	1.359387706	0.44295698	0.47114577	1
gene62812	162.2029525	215.8468724	1.330720983	0.412208108	0.47116402	1
gene21702	348.1681211	265.2109553	0.761732448	-0.39264374	0.47125065	1
gene4532	2734.721117	3758.445765	1.374343344	0.458742469	0.47127316	1
gene41893	173.5674906	230.3657203	1.327240024	0.408429297	0.47128637	1
gene34931	262.4175156	344.5806573	1.313100829	0.392977701	0.47131299	1
gene2689	50.62385143	33.87731181	0.669196651	-0.57949787	0.47136844	1
gene15827	50.62385143	33.87731181	0.669196651	-0.57949787	0.47136844	1
gene31441	50.62385143	33.87731181	0.669196651	-0.57949787	0.47136844	1
gene57894	50.62385143	33.87731181	0.669196651	-0.57949787	0.47136844	1
gene62698	340.9361423	446.2125927	1.308786419	0.388229683	0.47138428	1
gene49858	60.95524968	86.14516432	1.413252587	0.499019338	0.47145537	1
gene45701	184.9320287	244.8845682	1.324186892	0.405106754	0.47149081	1
gene12411	1896.844719	1420.91125	0.749092024	-0.41678513	0.47152885	1
gene46874	528.9675904	404.5918954	0.764870859	-0.38671191	0.47159438	1
gene17085	465.9460611	356.1957356	0.764457016	-0.38749271	0.47159933	1
gene34269	367.7977777	481.0578277	1.307941094	0.387297567	0.47162429	1
gene35469	45.4581523	30.00561903	0.660071242	-0.59930635	0.47166401	1
gene54964	45.4581523	30.00561903	0.660071242	-0.59930635	0.47166401	1
gene58089	45.4581523	30.00561903	0.660071242	-0.59930635	0.47166401	1
gene65962	89.88316478	63.88293085	0.710732994	-0.49262042	0.47175799	1
gene11781	724.2310174	950.5005771	1.312427325	0.392237537	0.47185123	1
gene52500	182.865749	136.4771704	0.7463244	-0.42212524	0.47186078	1
gene10924	265.516935	348.4523501	1.312354521	0.392157504	0.47192787	1
gene64169	77.48548688	107.4394746	1.38657546	0.471526133	0.47194176	1
gene7218	20.6627965	32.90938862	1.592688028	0.671463703	0.47195281	1
gene11266	20.6627965	32.90938862	1.592688028	0.671463703	0.47195281	1
gene17422	20.6627965	32.90938862	1.592688028	0.671463703	0.47195281	1
gene18449	20.6627965	32.90938862	1.592688028	0.671463703	0.47195281	1
gene36211	20.6627965	32.90938862	1.592688028	0.671463703	0.47195281	1
gene51924	20.6627965	32.90938862	1.592688028	0.671463703	0.47195281	1
gene38603	777.9542883	594.3048415	0.763932856	-0.38848225	0.47197616	1
gene52993	176.6669101	131.6375545	0.745117206	-0.42446072	0.47204442	1
gene66221	343.0024219	261.3392626	0.761916668	-0.39229488	0.47206322	1
gene59876	108.4796816	147.1243256	1.356238545	0.439610952	0.47209458	1
gene42376	188.0314482	248.756261	1.322950301	0.403758866	0.47213838	1
gene29683	2876.261273	2121.687643	0.737654699	-0.43898246	0.47223849	1
gene49287	349.2012609	266.1788785	0.762250623	-0.39166267	0.4722796	1
gene45600	396.7256928	302.9599599	0.763650969	-0.3890147	0.47229101	1

gene53546	290.3122908	380.3938155	1.310291805	0.389888139	0.47231468	1
gene53714	312.0082272	408.4635881	1.309143646	0.388623406	0.47231592	1
gene28980	252.0861173	190.6808693	0.756411624	-0.40275656	0.47235063	1
gene45521	252.0861173	190.6808693	0.756411624	-0.40275656	0.47235063	1
gene42715	239.6884394	181.0016374	0.755153806	-0.40515758	0.47238558	1
gene41580	199.3959862	263.2751089	1.320363132	0.400934761	0.4724215	1
gene14775	252.0861173	331.0297326	1.313161296	0.393044134	0.47242156	1
gene39543	157.0372534	209.07141	1.331349126	0.412888945	0.47248558	1
gene64761	157.0372534	209.07141	1.331349126	0.412888945	0.47248558	1
gene41228	333.7041635	436.5333608	1.308144784	0.387522225	0.47249577	1
gene10759	35.12675405	52.26785251	1.487978435	0.573353618	0.47254997	1
gene32838	35.12675405	52.26785251	1.487978435	0.573353618	0.47254997	1
gene43303	35.12675405	52.26785251	1.487978435	0.573353618	0.47254997	1
gene67072	35.12675405	52.26785251	1.487978435	0.573353618	0.47254997	1
gene60282	725.2641572	554.6199905	0.764714463	-0.38700693	0.47267993	1
gene39857	361.5989388	275.8581105	0.762884182	-0.39046405	0.47272294	1
gene72484	1764.602821	2372.37975	1.344427041	0.426991466	0.47284953	1
gene7203	21.69593633	12.58300153	0.57997043	-0.78594875	0.47286409	1
gene9978	21.69593633	12.58300153	0.57997043	-0.78594875	0.47286409	1
gene35662	21.69593633	12.58300153	0.57997043	-0.78594875	0.47286409	1
gene48485	21.69593633	12.58300153	0.57997043	-0.78594875	0.47286409	1
gene53254	21.69593633	12.58300153	0.57997043	-0.78594875	0.47286409	1
gene55829	21.69593633	12.58300153	0.57997043	-0.78594875	0.47286409	1
gene62797	21.69593633	12.58300153	0.57997043	-0.78594875	0.47286409	1
gene63711	21.69593633	12.58300153	0.57997043	-0.78594875	0.47286409	1
gene65120	21.69593633	12.58300153	0.57997043	-0.78594875	0.47286409	1
gene71252	21.69593633	12.58300153	0.57997043	-0.78594875	0.47286409	1
gene71296	21.69593633	12.58300153	0.57997043	-0.78594875	0.47286409	1
gene24258	67.15408863	46.46031334	0.691846383	-0.53147636	0.47286443	1
gene55178	78.51862671	55.17162209	0.702656483	-0.50910854	0.47291779	1
gene57973	78.51862671	55.17162209	0.702656483	-0.50910854	0.47291779	1
gene23542	367.7977777	280.6977264	0.763184944	-0.38989538	0.47294953	1
gene61922	604.3867977	462.667287	0.765515211	-0.38549705	0.47297442	1
gene26532	202.4954057	151.9639416	0.750456244	-0.41416014	0.47301015	1
gene59502	202.4954057	151.9639416	0.750456244	-0.41416014	0.47301015	1
gene10380	100.214563	136.4771704	1.361849678	0.445567467	0.47301199	1
gene63573	102.2808427	73.56216279	0.71921741	-0.47550015	0.47305306	1
gene41397	1424.699819	1077.298516	0.756158246	-0.40323991	0.47310375	1
gene21125	400.8582521	523.6464483	1.306313255	0.385500898	0.47313077	1
gene48575	373.9966167	285.5373424	0.763475737	-0.38934579	0.47317919	1
gene59122	1124.05613	854.6761809	0.760350091	-0.39526426	0.47318649	1
gene33053	196.2965668	147.1243256	0.749500249	-0.41599914	0.47318941	1
gene7940	17.56337703	9.679231946	0.551103124	-0.85960579	0.47322629	1
gene15709	17.56337703	9.679231946	0.551103124	-0.85960579	0.47322629	1
gene18931	17.56337703	9.679231946	0.551103124	-0.85960579	0.47322629	1
gene27165	17.56337703	9.679231946	0.551103124	-0.85960579	0.47322629	1
gene36821	17.56337703	9.679231946	0.551103124	-0.85960579	0.47322629	1
gene40404	17.56337703	9.679231946	0.551103124	-0.85960579	0.47322629	1

gene57573	17.56337703	9.679231946	0.551103124	-0.85960579	0.47322629	1
gene38335	148.7721348	198.4242549	1.333746102	0.415484055	0.47323957	1
gene49684	765.5566104	1004.704276	1.312384038	0.392189953	0.47336232	1
gene2473	448.3826841	585.5935328	1.306012818	0.385169056	0.47336542	1
gene65225	133.2750374	97.76024266	0.73352253	-0.44708682	0.47338282	1
gene38694	80.58490636	111.3111674	1.381290522	0.466016789	0.4734005	1
gene57799	80.58490636	111.3111674	1.381290522	0.466016789	0.4734005	1
gene48307	213.8599438	281.6656496	1.317056596	0.397317342	0.47342208	1
gene3074	120.8773595	88.08101071	0.728680797	-0.45664112	0.47354087	1
gene20326	120.8773595	88.08101071	0.728680797	-0.45664112	0.47354087	1
gene42426	2892.79151	2135.238567	0.738123906	-0.43806508	0.47355407	1
gene23075	50.62385143	72.5942396	1.433992823	0.520037803	0.473569	1
gene34006	183.8988889	137.4450936	0.747394911	-0.42005735	0.47360712	1
gene64404	183.8988889	137.4450936	0.747394911	-0.42005735	0.47360712	1
gene15698	754.1920723	576.882224	0.764900939	-0.38665518	0.47360848	1
gene57403	326.4721847	248.756261	0.761952389	-0.39222724	0.47361611	1
gene166	64.05466916	90.0168571	1.405312966	0.490891457	0.47365508	1
gene6072	64.05466916	90.0168571	1.405312966	0.490891457	0.47365508	1
gene11419	64.05466916	90.0168571	1.405312966	0.490891457	0.47365508	1
gene46697	259.3180961	196.4884085	0.757711905	-0.40027868	0.47367606	1
gene17459	362.6320786	276.8260337	0.763379883	-0.38952693	0.47371629	1
gene55407	332.6710237	253.595877	0.762302271	-0.39156492	0.47378507	1
gene26672	1270.761985	964.0515019	0.758640496	-0.39851171	0.47382244	1
gene34979	114.6785206	154.8677111	1.350450898	0.433441185	0.47384048	1
gene69852	114.6785206	154.8677111	1.350450898	0.433441185	0.47384048	1
gene43017	284.1134519	215.8468724	0.759720707	-0.39645895	0.47384102	1
gene53310	284.1134519	215.8468724	0.759720707	-0.39645895	0.47384102	1
gene63579	689.1042633	527.5181411	0.76551281	-0.38550157	0.47384193	1
gene35825	605.4199375	791.7611732	1.307788403	0.387129135	0.47388608	1
gene41561	228.3239013	172.2903286	0.754587354	-0.40624017	0.4739597	1
gene15387	45.4581523	65.81877724	1.447898207	0.533960179	0.47397346	1
gene31518	45.4581523	65.81877724	1.447898207	0.533960179	0.47397346	1
gene37992	45.4581523	65.81877724	1.447898207	0.533960179	0.47397346	1
gene11149	103.3139825	140.3488632	1.358469201	0.441981857	0.47398238	1
gene49798	103.3139825	140.3488632	1.358469201	0.441981857	0.47398238	1
gene10005	27.89477528	42.58862056	1.526759766	0.610473073	0.47401127	1
gene10692	27.89477528	42.58862056	1.526759766	0.610473073	0.47401127	1
gene19006	27.89477528	42.58862056	1.526759766	0.610473073	0.47401127	1
gene41198	27.89477528	42.58862056	1.526759766	0.610473073	0.47401127	1
gene50176	27.89477528	42.58862056	1.526759766	0.610473073	0.47401127	1
gene62370	27.89477528	42.58862056	1.526759766	0.610473073	0.47401127	1
gene64333	27.89477528	42.58862056	1.526759766	0.610473073	0.47401127	1
gene73900	27.89477528	42.58862056	1.526759766	0.610473073	0.47401127	1
gene21316	759.3577714	995.9929673	1.311625435	0.391355784	0.47407407	1
gene71188	171.501211	127.7658617	0.744985187	-0.42471636	0.47409382	1
gene63454	521.7356117	681.417929	1.306059839	0.385220998	0.47413724	1
gene47769	1375.109107	1041.485357	0.757383797	-0.40090354	0.47423539	1
gene27543	215.9262234	162.6110967	0.753086374	-0.40911275	0.474236	1

gene4022	1238.73465	1643.533584	1.326784218	0.407933757	0.47425391	1
gene3704	84.71746566	60.01123807	0.708369137	-0.49742674	0.47425763	1
gene11559	84.71746566	60.01123807	0.708369137	-0.49742674	0.47425763	1
gene12167	84.71746566	60.01123807	0.708369137	-0.49742674	0.47425763	1
gene39593	84.71746566	60.01123807	0.708369137	-0.49742674	0.47425763	1
gene9	4.1325593	0.967923195	0.234218828	-2.09407104	0.47429138	1
gene162	4.1325593	0.967923195	0.234218828	-2.09407104	0.47429138	1
gene274	4.1325593	0.967923195	0.234218828	-2.09407104	0.47429138	1
gene380	4.1325593	0.967923195	0.234218828	-2.09407104	0.47429138	1
gene797	4.1325593	0.967923195	0.234218828	-2.09407104	0.47429138	1
gene2097	4.1325593	0.967923195	0.234218828	-2.09407104	0.47429138	1
gene2325	4.1325593	0.967923195	0.234218828	-2.09407104	0.47429138	1
gene3547	4.1325593	0.967923195	0.234218828	-2.09407104	0.47429138	1
gene3871	4.1325593	0.967923195	0.234218828	-2.09407104	0.47429138	1
gene4703	4.1325593	0.967923195	0.234218828	-2.09407104	0.47429138	1
gene5286	4.1325593	0.967923195	0.234218828	-2.09407104	0.47429138	1
gene5583	4.1325593	0.967923195	0.234218828	-2.09407104	0.47429138	1
gene5940	4.1325593	0.967923195	0.234218828	-2.09407104	0.47429138	1
gene7063	4.1325593	0.967923195	0.234218828	-2.09407104	0.47429138	1
gene7935	4.1325593	0.967923195	0.234218828	-2.09407104	0.47429138	1
gene8538	4.1325593	0.967923195	0.234218828	-2.09407104	0.47429138	1
gene10016	4.1325593	0.967923195	0.234218828	-2.09407104	0.47429138	1
gene10181	4.1325593	0.967923195	0.234218828	-2.09407104	0.47429138	1
gene11012	4.1325593	0.967923195	0.234218828	-2.09407104	0.47429138	1
gene14790	4.1325593	0.967923195	0.234218828	-2.09407104	0.47429138	1
gene15822	4.1325593	0.967923195	0.234218828	-2.09407104	0.47429138	1
gene16141	4.1325593	0.967923195	0.234218828	-2.09407104	0.47429138	1
gene16419	4.1325593	0.967923195	0.234218828	-2.09407104	0.47429138	1
gene16559	4.1325593	0.967923195	0.234218828	-2.09407104	0.47429138	1
gene16581	4.1325593	0.967923195	0.234218828	-2.09407104	0.47429138	1
gene18336	4.1325593	0.967923195	0.234218828	-2.09407104	0.47429138	1
gene20585	4.1325593	0.967923195	0.234218828	-2.09407104	0.47429138	1
gene20941	4.1325593	0.967923195	0.234218828	-2.09407104	0.47429138	1
gene21968	4.1325593	0.967923195	0.234218828	-2.09407104	0.47429138	1
gene24705	4.1325593	0.967923195	0.234218828	-2.09407104	0.47429138	1
gene25012	4.1325593	0.967923195	0.234218828	-2.09407104	0.47429138	1
gene27135	4.1325593	0.967923195	0.234218828	-2.09407104	0.47429138	1
gene28209	4.1325593	0.967923195	0.234218828	-2.09407104	0.47429138	1
gene30616	4.1325593	0.967923195	0.234218828	-2.09407104	0.47429138	1
gene31580	4.1325593	0.967923195	0.234218828	-2.09407104	0.47429138	1
gene32291	4.1325593	0.967923195	0.234218828	-2.09407104	0.47429138	1
gene33238	4.1325593	0.967923195	0.234218828	-2.09407104	0.47429138	1
gene33474	4.1325593	0.967923195	0.234218828	-2.09407104	0.47429138	1
gene34248	4.1325593	0.967923195	0.234218828	-2.09407104	0.47429138	1
gene34763	4.1325593	0.967923195	0.234218828	-2.09407104	0.47429138	1
gene36569	4.1325593	0.967923195	0.234218828	-2.09407104	0.47429138	1
gene37261	4.1325593	0.967923195	0.234218828	-2.09407104	0.47429138	1
gene39719	4.1325593	0.967923195	0.234218828	-2.09407104	0.47429138	1

gene41272	4.1325593	0.967923195	0.234218828	-2.09407104	0.47429138	1
gene42404	4.1325593	0.967923195	0.234218828	-2.09407104	0.47429138	1
gene42806	4.1325593	0.967923195	0.234218828	-2.09407104	0.47429138	1
gene42916	4.1325593	0.967923195	0.234218828	-2.09407104	0.47429138	1
gene43106	4.1325593	0.967923195	0.234218828	-2.09407104	0.47429138	1
gene44983	4.1325593	0.967923195	0.234218828	-2.09407104	0.47429138	1
gene45743	4.1325593	0.967923195	0.234218828	-2.09407104	0.47429138	1
gene45957	4.1325593	0.967923195	0.234218828	-2.09407104	0.47429138	1
gene49253	4.1325593	0.967923195	0.234218828	-2.09407104	0.47429138	1
gene49479	4.1325593	0.967923195	0.234218828	-2.09407104	0.47429138	1
gene50140	4.1325593	0.967923195	0.234218828	-2.09407104	0.47429138	1
gene51759	4.1325593	0.967923195	0.234218828	-2.09407104	0.47429138	1
gene54458	4.1325593	0.967923195	0.234218828	-2.09407104	0.47429138	1
gene54857	4.1325593	0.967923195	0.234218828	-2.09407104	0.47429138	1
gene56406	4.1325593	0.967923195	0.234218828	-2.09407104	0.47429138	1
gene56848	4.1325593	0.967923195	0.234218828	-2.09407104	0.47429138	1
gene57104	4.1325593	0.967923195	0.234218828	-2.09407104	0.47429138	1
gene57483	4.1325593	0.967923195	0.234218828	-2.09407104	0.47429138	1
gene58084	4.1325593	0.967923195	0.234218828	-2.09407104	0.47429138	1
gene59892	4.1325593	0.967923195	0.234218828	-2.09407104	0.47429138	1
gene60450	4.1325593	0.967923195	0.234218828	-2.09407104	0.47429138	1
gene61273	4.1325593	0.967923195	0.234218828	-2.09407104	0.47429138	1
gene61352	4.1325593	0.967923195	0.234218828	-2.09407104	0.47429138	1
gene61416	4.1325593	0.967923195	0.234218828	-2.09407104	0.47429138	1
gene63342	4.1325593	0.967923195	0.234218828	-2.09407104	0.47429138	1
gene63348	4.1325593	0.967923195	0.234218828	-2.09407104	0.47429138	1
gene63653	4.1325593	0.967923195	0.234218828	-2.09407104	0.47429138	1
gene63864	4.1325593	0.967923195	0.234218828	-2.09407104	0.47429138	1
gene65733	4.1325593	0.967923195	0.234218828	-2.09407104	0.47429138	1
gene67309	4.1325593	0.967923195	0.234218828	-2.09407104	0.47429138	1
gene71133	4.1325593	0.967923195	0.234218828	-2.09407104	0.47429138	1
gene71553	4.1325593	0.967923195	0.234218828	-2.09407104	0.47429138	1
gene72505	4.1325593	0.967923195	0.234218828	-2.09407104	0.47429138	1
gene74204	4.1325593	0.967923195	0.234218828	-2.09407104	0.47429138	1
gene74207	4.1325593	0.967923195	0.234218828	-2.09407104	0.47429138	1
gene74220	4.1325593	0.967923195	0.234218828	-2.09407104	0.47429138	1
gene47186	277.9146129	363.9391212	1.30953575	0.389055446	0.47430598	1
gene5463	165.302372	122.9262457	0.743644778	-0.42731445	0.47435728	1
gene14542	166.3355118	220.6864884	1.326755098	0.407902093	0.47442037	1
gene528	3.099419475	7.743385557	2.498334162	1.320966456	0.47442568	1
gene3797	3.099419475	7.743385557	2.498334162	1.320966456	0.47442568	1
gene4878	3.099419475	7.743385557	2.498334162	1.320966456	0.47442568	1
gene9950	3.099419475	7.743385557	2.498334162	1.320966456	0.47442568	1
gene11600	3.099419475	7.743385557	2.498334162	1.320966456	0.47442568	1
gene13598	3.099419475	7.743385557	2.498334162	1.320966456	0.47442568	1
gene13966	3.099419475	7.743385557	2.498334162	1.320966456	0.47442568	1
gene14097	3.099419475	7.743385557	2.498334162	1.320966456	0.47442568	1
gene15203	3.099419475	7.743385557	2.498334162	1.320966456	0.47442568	1

gene15910	3.099419475	7.743385557	2.498334162	1.320966456	0.47442568	1
gene16537	3.099419475	7.743385557	2.498334162	1.320966456	0.47442568	1
gene18528	3.099419475	7.743385557	2.498334162	1.320966456	0.47442568	1
gene18575	3.099419475	7.743385557	2.498334162	1.320966456	0.47442568	1
gene25173	3.099419475	7.743385557	2.498334162	1.320966456	0.47442568	1
gene32342	3.099419475	7.743385557	2.498334162	1.320966456	0.47442568	1
gene32367	3.099419475	7.743385557	2.498334162	1.320966456	0.47442568	1
gene33152	3.099419475	7.743385557	2.498334162	1.320966456	0.47442568	1
gene33362	3.099419475	7.743385557	2.498334162	1.320966456	0.47442568	1
gene35430	3.099419475	7.743385557	2.498334162	1.320966456	0.47442568	1
gene35508	3.099419475	7.743385557	2.498334162	1.320966456	0.47442568	1
gene37180	3.099419475	7.743385557	2.498334162	1.320966456	0.47442568	1
gene37769	3.099419475	7.743385557	2.498334162	1.320966456	0.47442568	1
gene38544	3.099419475	7.743385557	2.498334162	1.320966456	0.47442568	1
gene43177	3.099419475	7.743385557	2.498334162	1.320966456	0.47442568	1
gene44536	3.099419475	7.743385557	2.498334162	1.320966456	0.47442568	1
gene44753	3.099419475	7.743385557	2.498334162	1.320966456	0.47442568	1
gene44920	3.099419475	7.743385557	2.498334162	1.320966456	0.47442568	1
gene45006	3.099419475	7.743385557	2.498334162	1.320966456	0.47442568	1
gene45876	3.099419475	7.743385557	2.498334162	1.320966456	0.47442568	1
gene48982	3.099419475	7.743385557	2.498334162	1.320966456	0.47442568	1
gene49939	3.099419475	7.743385557	2.498334162	1.320966456	0.47442568	1
gene49984	3.099419475	7.743385557	2.498334162	1.320966456	0.47442568	1
gene50323	3.099419475	7.743385557	2.498334162	1.320966456	0.47442568	1
gene53228	3.099419475	7.743385557	2.498334162	1.320966456	0.47442568	1
gene53936	3.099419475	7.743385557	2.498334162	1.320966456	0.47442568	1
gene54953	3.099419475	7.743385557	2.498334162	1.320966456	0.47442568	1
gene55254	3.099419475	7.743385557	2.498334162	1.320966456	0.47442568	1
gene56977	3.099419475	7.743385557	2.498334162	1.320966456	0.47442568	1
gene64938	3.099419475	7.743385557	2.498334162	1.320966456	0.47442568	1
gene72281	3.099419475	7.743385557	2.498334162	1.320966456	0.47442568	1
gene73733	3.099419475	7.743385557	2.498334162	1.320966456	0.47442568	1
gene73909	3.099419475	7.743385557	2.498334162	1.320966456	0.47442568	1
gene38656	3274.020106	2404.321215	0.734363607	-0.44543353	0.47444122	1
gene23273	189.064588	249.7241842	1.320840602	0.401456374	0.47446344	1
gene21901	8048.159237	5563.622523	0.691291308	-0.53263431	0.4745208	1
gene27475	1376.142247	1042.453281	0.757518551	-0.40064688	0.47455199	1
gene10996	61.9883895	42.58862056	0.687041895	-0.54153002	0.47456727	1
gene21625	61.9883895	42.58862056	0.687041895	-0.54153002	0.47456727	1
gene28927	61.9883895	42.58862056	0.687041895	-0.54153002	0.47456727	1
gene73076	203.5285455	152.9318648	0.751402533	-0.41234211	0.47460746	1
gene13034	159.1035331	118.0866297	0.742199921	-0.43012025	0.47462958	1
gene13708	117.7779401	158.7394039	1.347785535	0.430590947	0.47464732	1
gene32066	2398.950674	1785.818294	0.744416429	-0.4258182	0.47470466	1
gene70233	9815.861478	6664.151195	0.678916589	-0.55869376	0.47476655	1
gene13850	264.4837952	346.5165037	1.310161567	0.389744734	0.47481579	1
gene23602	197.3297066	148.0922488	0.75048127	-0.41411203	0.47482849	1
gene53459	272.7489138	357.1636588	1.309496173	0.389011843	0.47483376	1

gene2066	1210.839875	1604.816657	1.3253748	0.406400394	0.47484812	1
gene61500	417.3884893	319.4146542	0.765269437	-0.38596031	0.47486005	1
gene59078	729.3967165	558.4916833	0.765689879	-0.38516791	0.47487036	1
gene4495	607.4862171	465.5710566	0.766389497	-0.3838503	0.47489388	1
gene35699	152.9046941	113.2470138	0.740637915	-0.43315969	0.47490605	1
gene749	516.5699125	395.8805866	0.766364004	-0.3838983	0.47493941	1
gene17929	339.9030025	259.4034162	0.763168946	-0.38992563	0.47500614	1
gene40309	339.9030025	259.4034162	0.763168946	-0.38992563	0.47500614	1
gene71205	340.9361423	445.2446695	1.305947403	0.385096793	0.47500764	1
gene3554	18.59651685	30.00561903	1.61350748	0.690200266	0.47503166	1
gene15433	18.59651685	30.00561903	1.61350748	0.690200266	0.47503166	1
gene57937	18.59651685	30.00561903	1.61350748	0.690200266	0.47503166	1
gene7558	1149.884625	1521.575262	1.323241679	0.404076582	0.47503787	1
gene31868	285.1465917	216.8147956	0.760362571	-0.39524058	0.47505409	1
gene36668	231.4233208	303.9278831	1.313298427	0.393194783	0.47509751	1
gene27782	90.91630461	64.85085404	0.713302793	-0.48741347	0.47514895	1
gene35563	543.431548	709.4877017	1.305569587	0.384679355	0.47517801	1
gene52866	58.88897003	83.24139474	1.413531171	0.499303697	0.47518617	1
gene44840	1628.228364	1228.294534	0.754374854	-0.40664651	0.47519941	1
gene42011	14205.67259	9280.44759	0.653291671	-0.61420085	0.47522414	1
gene28239	75.41920723	104.535705	1.386062104	0.4709919	0.47535681	1
gene30291	75.41920723	104.535705	1.386062104	0.4709919	0.47535681	1
gene50952	75.41920723	104.535705	1.386062104	0.4709919	0.47535681	1
gene59305	75.41920723	104.535705	1.386062104	0.4709919	0.47535681	1
gene69739	229.3570412	173.2582518	0.755408471	-0.40467113	0.47540287	1
gene74117	229.3570412	173.2582518	0.755408471	-0.40467113	0.47540287	1
gene1668	120.8773595	162.6110967	1.345256856	0.42788166	0.47541571	1
gene2087	30.99419475	19.35846389	0.62458354	-0.67903354	0.47550477	1
gene15544	30.99419475	19.35846389	0.62458354	-0.67903354	0.47550477	1
gene36438	30.99419475	19.35846389	0.62458354	-0.67903354	0.47550477	1
gene37599	30.99419475	19.35846389	0.62458354	-0.67903354	0.47550477	1
gene10490	1895.811579	1423.815019	0.751031925	-0.41305386	0.47552967	1
gene21334	714.932759	935.9817292	1.309188476	0.388672808	0.47554053	1
gene38397	223.1582022	168.4186359	0.754705111	-0.40601505	0.47556162	1
gene36009	528.9675904	405.5598186	0.766700694	-0.38326461	0.47557035	1
gene41341	976.3171347	745.3008599	0.763379883	-0.38952693	0.47557082	1
gene3426	67.15408863	93.88854988	1.398106233	0.483473986	0.4756082	1
gene61806	67.15408863	93.88854988	1.398106233	0.483473986	0.4756082	1
gene34443	806.8822034	617.5349982	0.765334761	-0.38583717	0.47562603	1
gene27822	172.5343508	228.4298739	1.323967505	0.404867714	0.4756442	1
gene46493	134.3081773	98.72816585	0.735086782	-0.44401351	0.47569119	1
gene5669	234.5227403	307.7995759	1.312450876	0.392263425	0.47569532	1
gene17920	97.11514356	69.69047001	0.717606621	-0.47873489	0.47570985	1
gene10558	3441.388757	2523.375768	0.733243451	-0.44763581	0.47577073	1
gene16951	161.1698127	213.911026	1.327240024	0.408429297	0.47579667	1
gene707	477.3105992	365.8749676	0.766534345	-0.38357766	0.47586617	1
gene33812	172.5343508	128.7337849	0.746134229	-0.4224929	0.47593092	1
gene46953	172.5343508	128.7337849	0.746134229	-0.4224929	0.47593092	1

gene62317	86.78374531	119.0545529	1.371853134	0.456126039	0.4759524	1
gene51228	98.14828338	133.5734009	1.360934662	0.444597805	0.47598614	1
gene66595	103.3139825	74.53008599	0.721393989	-0.47114069	0.47602631	1
gene7388	121.9104994	89.04893391	0.730445158	-0.45315214	0.47606921	1
gene1585	56.82269038	38.71692779	0.681363862	-0.55350266	0.47611658	1
gene64620	56.82269038	38.71692779	0.681363862	-0.55350266	0.47611658	1
gene69350	56.82269038	38.71692779	0.681363862	-0.55350266	0.47611658	1
gene52927	448.3826841	584.6256096	1.303854119	0.382782464	0.47612065	1
gene10617	109.5128215	79.36970196	0.724752599	-0.46443949	0.47616188	1
gene12441	115.7116604	84.20931793	0.727751357	-0.45848247	0.47616418	1
gene62965	115.7116604	84.20931793	0.727751357	-0.45848247	0.47616418	1
gene9463	166.3355118	123.8941689	0.744844968	-0.42498792	0.47625353	1
gene12824	2288.404713	1708.384439	0.746539469	-0.42170956	0.47633088	1
gene72670	353.3338202	270.0505713	0.764293017	-0.38780225	0.47634748	1
gene42763	643.646111	493.6408293	0.766944476	-0.38280596	0.4763952	1
gene34614	1890.64588	2542.734232	1.344902427	0.427501509	0.4764227	1
gene11909	968.0520161	739.4933207	0.763898332	-0.38854745	0.47646915	1
gene67361	1323.452116	1004.704276	0.759154233	-0.39753508	0.47647261	1
gene12826	273.7820536	358.131582	1.308090056	0.387461868	0.47652802	1
gene7475	160.1366729	119.0545529	0.743455892	-0.42768094	0.47658985	1
gene12456	160.1366729	119.0545529	0.743455892	-0.42768094	0.47658985	1
gene6862	33.0604744	49.36408293	1.493145026	0.578354299	0.47662309	1
gene59321	33.0604744	49.36408293	1.493145026	0.578354299	0.47662309	1
gene59146	221.0919226	290.3769584	1.313376604	0.393280661	0.47670457	1
gene26865	192.1640075	144.220556	0.750507641	-0.41406133	0.476742	1
gene49619	1633.394063	2183.634727	1.336869514	0.418858657	0.47677548	1
gene62515	78.51862671	108.4073978	1.380658353	0.465356365	0.47678777	1
gene38471	178.7331897	236.1732595	1.321373271	0.402038067	0.47682407	1
gene47272	1174.679981	894.3610318	0.761365688	-0.39333854	0.4768296	1
gene16786	385.3611548	502.352138	1.303587899	0.382487865	0.47683223	1
gene38545	621.9501747	477.186135	0.767241741	-0.38224688	0.47684853	1
gene6907	661.2094881	507.191754	0.767066661	-0.38257614	0.47685526	1
gene68213	435.9850062	568.1709153	1.303189117	0.382046461	0.47694999	1
gene29215	502.105955	385.2334315	0.767235337	-0.38225893	0.47699506	1
gene29679	3360.803851	4656.678489	1.385584728	0.470494934	0.47711696	1
gene43524	632.2815729	825.638485	1.305808235	0.384943045	0.47713745	1
gene6026	147.738995	109.375321	0.740328043	-0.43376342	0.47728626	1
gene68185	224.191342	294.2486512	1.312488915	0.392305238	0.47729133	1
gene27180	825.4787202	1082.138132	1.310921899	0.390581737	0.47734935	1
gene30219	70.25350811	97.76024266	1.391535388	0.476677598	0.47734952	1
gene6690	193.1971473	254.5638002	1.317637469	0.397953486	0.47735113	1
gene31751	61.9883895	87.11308752	1.405312966	0.490891457	0.47739752	1
gene16123	181.8326092	240.0449523	1.320142483	0.400693648	0.47739952	1
gene56372	68.18722846	47.42823654	0.695558943	-0.52375532	0.47744248	1
gene18459	51.65699125	34.84523501	0.674550224	-0.56800223	0.47746105	1
gene41743	48.55757178	69.69047001	1.435213242	0.521265106	0.4775142	1
gene62794	170.4680711	225.5261044	1.322981499	0.403792887	0.47756783	1
gene5102	8.265118601	15.48677111	1.873750621	0.905928957	0.4775733	1

gene8621	8.265118601	15.48677111	1.873750621	0.905928957	0.4775733	1
gene11724	8.265118601	15.48677111	1.873750621	0.905928957	0.4775733	1
gene13634	8.265118601	15.48677111	1.873750621	0.905928957	0.4775733	1
gene18770	8.265118601	15.48677111	1.873750621	0.905928957	0.4775733	1
gene26517	8.265118601	15.48677111	1.873750621	0.905928957	0.4775733	1
gene29895	8.265118601	15.48677111	1.873750621	0.905928957	0.4775733	1
gene31854	8.265118601	15.48677111	1.873750621	0.905928957	0.4775733	1
gene38188	8.265118601	15.48677111	1.873750621	0.905928957	0.4775733	1
gene39644	8.265118601	15.48677111	1.873750621	0.905928957	0.4775733	1
gene40508	8.265118601	15.48677111	1.873750621	0.905928957	0.4775733	1
gene48889	8.265118601	15.48677111	1.873750621	0.905928957	0.4775733	1
gene57708	8.265118601	15.48677111	1.873750621	0.905928957	0.4775733	1
gene58045	8.265118601	15.48677111	1.873750621	0.905928957	0.4775733	1
gene58978	8.265118601	15.48677111	1.873750621	0.905928957	0.4775733	1
gene59233	8.265118601	15.48677111	1.873750621	0.905928957	0.4775733	1
gene59664	8.265118601	15.48677111	1.873750621	0.905928957	0.4775733	1
gene62593	8.265118601	15.48677111	1.873750621	0.905928957	0.4775733	1
gene65626	8.265118601	15.48677111	1.873750621	0.905928957	0.4775733	1
gene65664	8.265118601	15.48677111	1.873750621	0.905928957	0.4775733	1
gene68027	8.265118601	15.48677111	1.873750621	0.905928957	0.4775733	1
gene11956	144.6395755	192.6167157	1.331701335	0.41327056	0.47767053	1
gene2508	38.22617353	56.13954529	1.468615352	0.554456586	0.47772934	1
gene6711	38.22617353	56.13954529	1.468615352	0.554456586	0.47772934	1
gene56907	38.22617353	56.13954529	1.468615352	0.554456586	0.47772934	1
gene60169	503.1390948	386.2013547	0.767583673	-0.38160407	0.47774	1
gene12374	313.041367	408.4635881	1.30482304	0.383854162	0.47778075	1
gene27141	104.3471223	141.3167864	1.354295004	0.437542033	0.47778873	1
gene2771	16.5302372	27.10184945	1.639531794	0.713283879	0.47781869	1
gene4791	16.5302372	27.10184945	1.639531794	0.713283879	0.47781869	1
gene6965	16.5302372	27.10184945	1.639531794	0.713283879	0.47781869	1
gene39988	16.5302372	27.10184945	1.639531794	0.713283879	0.47781869	1
gene41826	16.5302372	27.10184945	1.639531794	0.713283879	0.47781869	1
gene49111	16.5302372	27.10184945	1.639531794	0.713283879	0.47781869	1
gene49594	16.5302372	27.10184945	1.639531794	0.713283879	0.47781869	1
gene58318	16.5302372	27.10184945	1.639531794	0.713283879	0.47781869	1
gene71328	16.5302372	27.10184945	1.639531794	0.713283879	0.47781869	1
gene30884	560.994925	430.7258216	0.767789159	-0.38121791	0.47781901	1
gene28776	85.75060548	60.97916126	0.711122224	-0.49183055	0.47783819	1
gene59105	2962.011879	2190.410189	0.739500812	-0.43537636	0.47786961	1
gene12250	43.39187265	62.91500765	1.449926076	0.535979347	0.47795309	1
gene43217	509.3379338	391.0409706	0.767743662	-0.3813034	0.47801529	1
gene5632	25.82849563	39.68485098	1.53647551	0.619624771	0.47804171	1
gene62323	25.82849563	39.68485098	1.53647551	0.619624771	0.47804171	1
gene68607	25.82849563	39.68485098	1.53647551	0.619624771	0.47804171	1
gene14423	612.6519163	470.4106726	0.767826983	-0.38114683	0.47805759	1
gene39376	860.6054743	1128.598445	1.311400495	0.391108344	0.47807528	1
gene4371	36.15989388	23.23015667	0.642428784	-0.63839156	0.47808015	1
gene66773	36.15989388	23.23015667	0.642428784	-0.63839156	0.47808015	1

gene2936	81.61804618	112.2790906	1.375665013	0.460129204	0.47808669	1
gene26668	439.0844257	336.8372717	0.767135549	-0.38244658	0.47813004	1
gene50038	167.3686517	124.8620921	0.74603034	-0.42269379	0.4781314	1
gene4085	238.6552996	312.6391919	1.31000314	0.38957027	0.47819413	1
gene72990	391.5599937	300.0561903	0.766309621	-0.38400068	0.47829327	1
gene27176	2133.433739	2880.539427	1.35018931	0.433161701	0.47835006	1
gene15926	607.4862171	466.5389798	0.767982823	-0.38085405	0.47836506	1
gene9873	193.1971473	145.1884792	0.75150426	-0.41214681	0.47839777	1
gene9398	845.1083769	647.5406172	0.766221984	-0.38416568	0.47841231	1
gene1990	46.49129213	30.97354223	0.666222443	-0.58592414	0.47844087	1
gene38760	46.49129213	30.97354223	0.666222443	-0.58592414	0.47844087	1
gene49386	46.49129213	30.97354223	0.666222443	-0.58592414	0.47844087	1
gene18520	641.5798314	492.6729061	0.76790585	-0.38099866	0.47846962	1
gene39515	91.94944443	65.81877724	0.715814844	-0.48234163	0.47847552	1
gene20261	225.2244819	170.3544823	0.756376398	-0.40282375	0.4784819	1
gene53313	122.9436392	90.0168571	0.732179865	-0.44973	0.47856297	1
gene51760	1067.233439	814.9913299	0.763648608	-0.38901916	0.47865587	1
gene47703	590.9559799	453.9559783	0.768172239	-0.38049827	0.47869819	1
gene9118	306.842528	234.2374131	0.763379883	-0.38952693	0.478725	1
gene65383	14888.57802	9705.365873	0.651866542	-0.61735147	0.47873055	1
gene43080	241.7547191	316.5108846	1.309223191	0.388711063	0.47875237	1
gene1805	186.9983083	140.3488632	0.75053547	-0.41400784	0.47875616	1
gene19899	186.9983083	140.3488632	0.75053547	-0.41400784	0.47875616	1
gene7774	41.325593	27.10184945	0.655812718	-0.60864422	0.47879492	1
gene10370	41.325593	27.10184945	0.655812718	-0.60864422	0.47879492	1
gene34013	41.325593	27.10184945	0.655812718	-0.60864422	0.47879492	1
gene36216	41.325593	27.10184945	0.655812718	-0.60864422	0.47879492	1
gene53380	41.325593	27.10184945	0.655812718	-0.60864422	0.47879492	1
gene64239	41.325593	27.10184945	0.655812718	-0.60864422	0.47879492	1
gene61346	136.3744569	181.9695606	1.334337564	0.416123689	0.4787967	1
gene23002	552.7298064	720.1348568	1.302869591	0.381692686	0.47880589	1
gene32526	98.14828338	70.65839321	0.719914712	-0.47410209	0.47882031	1
gene27211	319.2402059	416.2069737	1.303742342	0.382658778	0.47882833	1
gene35480	263.4506554	200.3601013	0.760522311	-0.39493752	0.47884394	1
gene25510	150.8384145	200.3601013	1.328309516	0.409591355	0.47885417	1
gene29138	73.35292758	101.6319354	1.385519826	0.470427355	0.47890816	1
gene25905	110.5459613	80.33762515	0.726735054	-0.4604986	0.47892417	1
gene26101	110.5459613	80.33762515	0.726735054	-0.4604986	0.47892417	1
gene12751	154.9709738	115.1828602	0.743254413	-0.42807197	0.47894418	1
gene6652	104.3471223	75.49800918	0.723527468	-0.46688031	0.47895063	1
gene45756	212.826804	160.6752503	0.754957775	-0.40553214	0.47901429	1
gene39360	1376.142247	1045.35705	0.75962863	-0.39663381	0.47903642	1
gene40676	56.82269038	80.33762515	1.413830014	0.499608674	0.47907478	1
gene54637	56.82269038	80.33762515	1.413830014	0.499608674	0.47907478	1
gene61241	505.2053745	388.137201	0.768276073	-0.38030327	0.47922242	1
gene5457	808.948483	620.4387678	0.766969443	-0.38275899	0.47922256	1
gene15538	1643.725462	1242.813382	0.756095474	-0.40335968	0.47925036	1
gene38538	84.71746566	116.1507834	1.37103704	0.455267548	0.47927001	1

gene20483	522.7687515	401.6881258	0.768385877	-0.38009709	0.47928619	1
gene24751	244.8541385	320.3825774	1.308462987	0.387873115	0.47930267	1
gene57413	206.627965	155.8356343	0.754184625	-0.40701036	0.47932555	1
gene72814	3118.015992	2302.68928	0.738511055	-0.43730858	0.47932594	1
gene12738	985.6153931	754.0121686	0.765016632	-0.38643698	0.479357	1
gene33451	625.0495942	814.9913299	1.303882664	0.382814048	0.47935827	1
gene907	74.38606741	52.26785251	0.702656483	-0.50910854	0.47936182	1
gene48968	74.38606741	52.26785251	0.702656483	-0.50910854	0.47936182	1
gene47187	110.5459613	149.060172	1.34839998	0.431248511	0.47937639	1
gene54782	765.5566104	587.5293791	0.767453865	-0.38184807	0.4793783	1
gene10651	125.0099188	167.4507127	1.339499411	0.421693947	0.47941926	1
gene72264	2124.13548	1593.201578	0.750047063	-0.41494697	0.47942538	1
gene57388	1089.962515	832.4139474	0.763708784	-0.38890548	0.47942776	1
gene44827	1095.128215	836.2856402	0.763641763	-0.38903209	0.4794301	1
gene71962	238.6552996	181.0016374	0.758422871	-0.39892562	0.47945067	1
gene11180	1202.574756	916.6232653	0.762217285	-0.39172577	0.47946716	1
gene15207	368.8309176	282.6335728	0.766295772	-0.38402675	0.47958286	1
gene22844	517.6030524	397.816433	0.768574357	-0.37974325	0.47974157	1
gene44496	142.5732959	105.5036282	0.739995716	-0.43441118	0.47979758	1
gene36025	289.279151	220.6864884	0.762884182	-0.39046405	0.47984123	1
gene2085	10.33139825	4.839615973	0.468437655	-1.09407104	0.47984325	1
gene2820	10.33139825	4.839615973	0.468437655	-1.09407104	0.47984325	1
gene7029	10.33139825	4.839615973	0.468437655	-1.09407104	0.47984325	1
gene10028	10.33139825	4.839615973	0.468437655	-1.09407104	0.47984325	1
gene11372	10.33139825	4.839615973	0.468437655	-1.09407104	0.47984325	1
gene13789	10.33139825	4.839615973	0.468437655	-1.09407104	0.47984325	1
gene13963	10.33139825	4.839615973	0.468437655	-1.09407104	0.47984325	1
gene14865	10.33139825	4.839615973	0.468437655	-1.09407104	0.47984325	1
gene17425	10.33139825	4.839615973	0.468437655	-1.09407104	0.47984325	1
gene17867	10.33139825	4.839615973	0.468437655	-1.09407104	0.47984325	1
gene22175	10.33139825	4.839615973	0.468437655	-1.09407104	0.47984325	1
gene24004	10.33139825	4.839615973	0.468437655	-1.09407104	0.47984325	1
gene29128	10.33139825	4.839615973	0.468437655	-1.09407104	0.47984325	1
gene32896	10.33139825	4.839615973	0.468437655	-1.09407104	0.47984325	1
gene36968	10.33139825	4.839615973	0.468437655	-1.09407104	0.47984325	1
gene40457	10.33139825	4.839615973	0.468437655	-1.09407104	0.47984325	1
gene41001	10.33139825	4.839615973	0.468437655	-1.09407104	0.47984325	1
gene41141	10.33139825	4.839615973	0.468437655	-1.09407104	0.47984325	1
gene55907	10.33139825	4.839615973	0.468437655	-1.09407104	0.47984325	1
gene61681	10.33139825	4.839615973	0.468437655	-1.09407104	0.47984325	1
gene63643	10.33139825	4.839615973	0.468437655	-1.09407104	0.47984325	1
gene64567	10.33139825	4.839615973	0.468437655	-1.09407104	0.47984325	1
gene66206	10.33139825	4.839615973	0.468437655	-1.09407104	0.47984325	1
gene70415	10.33139825	4.839615973	0.468437655	-1.09407104	0.47984325	1
gene54586	247.953558	324.2542702	1.307721788	0.387055646	0.47984529	1
gene35760	320.2733458	244.8845682	0.764611141	-0.38720187	0.47991683	1
gene30876	638.4804119	832.4139474	1.303742342	0.382658778	0.47992619	1
gene54634	226.2576217	171.3224055	0.757200594	-0.40125255	0.47992674	1

gene30053	506.2385143	389.1051242	0.768620153	-0.37965729	0.47995994	1
gene73816	430.8193071	560.4275297	1.300841259	0.379444921	0.47997272	1
gene19830	142.5732959	189.7129461	1.330634499	0.412114344	0.47998279	1
gene2736	9366.445654	6415.394934	0.68493377	-0.5459636	0.47998713	1
gene37635	14.46395755	24.19807987	1.672991626	0.742430224	0.48007083	1
gene44215	14.46395755	24.19807987	1.672991626	0.742430224	0.48007083	1
gene51851	14.46395755	24.19807987	1.672991626	0.742430224	0.48007083	1
gene71915	14.46395755	24.19807987	1.672991626	0.742430224	0.48007083	1
gene5233	1756.337703	1326.054777	0.75501128	-0.4054299	0.48007857	1
gene5175	338.8698626	259.4034162	0.765495681	-0.38553386	0.48008934	1
gene52405	136.3744569	100.6640122	0.738144184	-0.43802544	0.48022348	1
gene21133	399.8251123	306.8316527	0.76741466	-0.38192177	0.48027331	1
gene10894	185.9651685	244.8845682	1.316830298	0.397069435	0.48031785	1
gene2860	251.0529775	328.125963	1.30699889	0.386257916	0.48038048	1
gene71548	252.0861173	191.6487925	0.760251277	-0.39545176	0.48041366	1
gene14302	278.9477528	363.9391212	1.304685618	0.383702211	0.48041853	1
gene3575	961.8531772	736.5895511	0.765802483	-0.38495576	0.48042275	1
gene6300	483.5094381	371.6825067	0.768718204	-0.37947326	0.48050299	1
gene36518	160.1366729	211.9751796	1.323714149	0.404591611	0.48051935	1
gene57533	1468.091691	1950.365237	1.328503695	0.409802241	0.48052568	1
gene5780	213.8599438	161.6431735	0.755836603	-0.40385371	0.4805263	1
gene66804	51.65699125	73.56216279	1.424050472	0.51000028	0.48053694	1
gene375	982.5159737	1290.241618	1.313201671	0.393088491	0.48053879	1
gene70613	80.58490636	57.10746848	0.708662094	-0.49683021	0.4805986	1
gene13697	594.0553994	456.8597479	0.769052429	-0.37884614	0.48063116	1
gene72306	130.175618	95.82439627	0.736116316	-0.44199435	0.48063627	1
gene48820	131.2087578	175.1940982	1.335231742	0.417090157	0.48066412	1
gene72269	1231.502671	938.8854988	0.762390144	-0.39139863	0.48068199	1
gene45383	200.4291261	263.2751089	1.313557137	0.393478956	0.48069021	1
gene61014	489.7082771	376.5221227	0.768870244	-0.37918795	0.48073153	1
gene8823	102.2808427	138.4130168	1.353264338	0.436443673	0.48078403	1
gene3948	30.99419475	46.46031334	1.499000497	0.584000862	0.48090798	1
gene12565	30.99419475	46.46031334	1.499000497	0.584000862	0.48090798	1
gene48063	30.99419475	46.46031334	1.499000497	0.584000862	0.48090798	1
gene51849	30.99419475	46.46031334	1.499000497	0.584000862	0.48090798	1
gene58979	767.62289	1002.76843	1.306329505	0.385518844	0.48093021	1
gene17037	308.9088077	236.1732595	0.764540387	-0.38733538	0.48097247	1
gene33509	10.33139825	18.3905407	1.78006309	0.831928375	0.48100709	1
gene33608	10.33139825	18.3905407	1.78006309	0.831928375	0.48100709	1
gene37194	10.33139825	18.3905407	1.78006309	0.831928375	0.48100709	1
gene44698	10.33139825	18.3905407	1.78006309	0.831928375	0.48100709	1
gene48675	10.33139825	18.3905407	1.78006309	0.831928375	0.48100709	1
gene48858	10.33139825	18.3905407	1.78006309	0.831928375	0.48100709	1
gene58426	10.33139825	18.3905407	1.78006309	0.831928375	0.48100709	1
gene68863	10.33139825	18.3905407	1.78006309	0.831928375	0.48100709	1
gene69016	10.33139825	18.3905407	1.78006309	0.831928375	0.48100709	1
gene5960	123.976779	90.9847803	0.73388566	-0.44637279	0.48102289	1
gene23438	123.976779	90.9847803	0.73388566	-0.44637279	0.48102289	1

gene26444	123.976779	90.9847803	0.73388566	-0.44637279	0.48102289	1
gene62605	123.976779	90.9847803	0.73388566	-0.44637279	0.48102289	1
gene55047	284.1134519	216.8147956	0.763127526	-0.39000393	0.48107316	1
gene1407	233.4896005	177.1299446	0.758620274	-0.39855017	0.48107799	1
gene26521	883.3345504	677.5462362	0.767032418	-0.38264054	0.48108509	1
gene22375	177.7000499	234.2374131	1.318161774	0.398527439	0.4811349	1
gene2314	346.1018414	265.2109553	0.766280105	-0.38405625	0.48118179	1
gene4856	4844.39264	3498.074425	0.722087305	-0.46975482	0.48119933	1
gene54884	201.4622659	151.9639416	0.754304737	-0.40678061	0.48125472	1
gene12182	484.542578	372.6504299	0.769076748	-0.37880052	0.48126641	1
gene49626	1792.497597	2397.545753	1.337544752	0.419587162	0.4812815	1
gene10015	59.92210985	84.20931793	1.405312966	0.490891457	0.48129869	1
gene29537	59.92210985	84.20931793	1.405312966	0.490891457	0.48129869	1
gene69235	1105.459613	844.9969489	0.764385183	-0.38762828	0.48132506	1
gene6960	12.3976779	21.29431028	1.717604736	0.780398075	0.48137701	1
gene38734	12.3976779	21.29431028	1.717604736	0.780398075	0.48137701	1
gene41837	12.3976779	21.29431028	1.717604736	0.780398075	0.48137701	1
gene55373	12.3976779	21.29431028	1.717604736	0.780398075	0.48137701	1
gene58490	12.3976779	21.29431028	1.717604736	0.780398075	0.48137701	1
gene68555	12.3976779	21.29431028	1.717604736	0.780398075	0.48137701	1
gene1046	57.8558302	39.68485098	0.685926567	-0.54387396	0.48149808	1
gene42158	57.8558302	39.68485098	0.685926567	-0.54387396	0.48149808	1
gene66357	3589.127752	2636.622782	0.734613801	-0.44494209	0.48153417	1
gene38559	495.907116	644.6368476	1.299914494	0.378416728	0.481585	1
gene50187	377.0960362	289.4090352	0.76746772	-0.38182202	0.48160264	1
gene1490	46.49129213	66.78670043	1.436542143	0.522600317	0.48162495	1
gene51033	180.7994694	238.1091059	1.316979008	0.39723235	0.48164908	1
gene543	514.5036329	395.8805866	0.769441771	-0.37811594	0.48166285	1
gene65026	622.9833145	811.1196371	1.301992555	0.380721198	0.48169601	1
gene39188	1132.321248	865.323336	0.764203036	-0.38797211	0.48170033	1
gene620	26.86163545	16.45469431	0.612572319	-0.70704792	0.48173464	1
gene5033	26.86163545	16.45469431	0.612572319	-0.70704792	0.48173464	1
gene10622	26.86163545	16.45469431	0.612572319	-0.70704792	0.48173464	1
gene22571	26.86163545	16.45469431	0.612572319	-0.70704792	0.48173464	1
gene22943	26.86163545	16.45469431	0.612572319	-0.70704792	0.48173464	1
gene26654	26.86163545	16.45469431	0.612572319	-0.70704792	0.48173464	1
gene29289	26.86163545	16.45469431	0.612572319	-0.70704792	0.48173464	1
gene37449	26.86163545	16.45469431	0.612572319	-0.70704792	0.48173464	1
gene39921	26.86163545	16.45469431	0.612572319	-0.70704792	0.48173464	1
gene66401	26.86163545	16.45469431	0.612572319	-0.70704792	0.48173464	1
gene73949	26.86163545	16.45469431	0.612572319	-0.70704792	0.48173464	1
gene42800	425.6536079	327.1580398	0.76860159	-0.37969213	0.48176807	1
gene33797	105.3802622	76.46593238	0.725619113	-0.46271564	0.48182726	1
gene39066	3097.353196	2292.042125	0.740000245	-0.43440235	0.48185243	1
gene7789	99.18142321	71.6263164	0.722174719	-0.46958018	0.48187644	1
gene13643	69.22036828	48.39615973	0.69916068	-0.51630404	0.48190001	1
gene25109	36.15989388	53.23577571	1.472232631	0.558005653	0.48201968	1
gene35963	36.15989388	53.23577571	1.472232631	0.558005653	0.48201968	1

gene38547	36.15989388	53.23577571	1.472232631	0.558005653	0.48201968	1
gene64398	36.15989388	53.23577571	1.472232631	0.558005653	0.48201968	1
gene69460	36.15989388	53.23577571	1.472232631	0.558005653	0.48201968	1
gene25783	485.5757178	373.6183531	0.769433766	-0.37813095	0.48202718	1
gene69712	122.9436392	164.5469431	1.338393301	0.420502129	0.48206806	1
gene57128	169.4349313	223.590258	1.319623151	0.400125993	0.48208026	1
gene56327	189.064588	142.2847096	0.752571971	-0.41009854	0.48211582	1
gene22697	41.325593	60.01123807	1.452156732	0.538197172	0.48213348	1
gene40157	41.325593	60.01123807	1.452156732	0.538197172	0.48213348	1
gene67484	41.325593	60.01123807	1.452156732	0.538197172	0.48213348	1
gene40918	1076.531698	823.7026386	0.765144808	-0.38619528	0.48214374	1
gene69443	1076.531698	823.7026386	0.765144808	-0.38619528	0.48214374	1
gene65973	240.7215792	182.9374838	0.759954651	-0.39601476	0.48219254	1
gene12695	23.76221598	36.7810814	1.547880948	0.630294514	0.48220667	1
gene19952	23.76221598	36.7810814	1.547880948	0.630294514	0.48220667	1
gene44239	23.76221598	36.7810814	1.547880948	0.630294514	0.48220667	1
gene60123	23.76221598	36.7810814	1.547880948	0.630294514	0.48220667	1
gene41701	432.8855867	562.3633761	1.299103951	0.377516876	0.48221489	1
gene48772	491.7745567	378.4579691	0.769576148	-0.37786401	0.48223859	1
gene49123	955.6543382	732.7178583	0.766718498	-0.38323111	0.48224386	1
gene19701	140.5070162	186.8091766	1.329536287	0.410923153	0.48236283	1
gene40308	140.5070162	186.8091766	1.329536287	0.410923153	0.48236283	1
gene63948	365.7314981	280.6977264	0.767496723	-0.3817675	0.48237908	1
gene50686	940.1572408	721.10278	0.767002315	-0.38269716	0.48245449	1
gene25495	371.930337	285.5373424	0.767717269	-0.381353	0.48245725	1
gene66254	2255.344238	3044.118447	1.349735617	0.432676843	0.48248584	1
gene14621	1072.399138	820.7988691	0.76538561	-0.38574132	0.48254717	1
gene63655	266.5500749	203.2638709	0.762572927	-0.39105278	0.48264017	1
gene61821	409.1233707	531.3898339	1.298849863	0.377234676	0.48265263	1
gene1467	2574.584444	1921.327541	0.746267051	-0.4222361	0.48267604	1
gene54504	319.2402059	415.2390505	1.300710383	0.379299766	0.48274607	1
gene29129	462.8466416	356.1957356	0.769576148	-0.37786401	0.48279106	1
gene65829	409.1233707	314.5750383	0.768900192	-0.37913176	0.483073	1
gene14984	97.11514356	131.6375545	1.355479173	0.438802946	0.48310212	1
gene63069	222.1250624	168.4186359	0.758215368	-0.3993204	0.48313402	1
gene18460	335.7704432	257.4675698	0.766796408	-0.38308452	0.48315485	1
gene16814	583.7240012	758.8517846	1.30001813	0.378531743	0.48316918	1
gene64177	63.02152933	88.08101071	1.39763366	0.482986259	0.48324273	1
gene54597	1021.775287	783.0498645	0.76636211	-0.38390186	0.48325078	1
gene57489	196.2965668	148.0922488	0.754431171	-0.40653881	0.48328386	1
gene69220	196.2965668	148.0922488	0.754431171	-0.40653881	0.48328386	1
gene33523	360.565799	276.8260337	0.767754553	-0.38128293	0.48328642	1
gene10504	366.7646379	281.6656496	0.767973846	-0.38087091	0.48334245	1
gene13698	481.4431585	370.7145835	0.770006961	-0.37705661	0.48334899	1
gene62230	52.69013108	35.8131582	0.679693853	-0.55704302	0.48335263	1
gene70352	52.69013108	35.8131582	0.679693853	-0.55704302	0.48335263	1
gene45902	297.5442696	387.1692779	1.301215709	0.379860145	0.48342512	1
gene18279	125.0099188	91.95270349	0.73556326	-0.44307867	0.48344968	1

gene24845	150.8384145	112.2790906	0.744366685	-0.42591461	0.48346494	1
gene23656	463.8797815	357.1636588	0.769948752	-0.37716567	0.48357936	1
gene40800	607.4862171	789.8253268	1.300153492	0.378681954	0.48361114	1
gene12206	114.6785206	153.8997879	1.34201058	0.424396046	0.48361192	1
gene11676	470.0786204	362.0032748	0.770090915	-0.37689932	0.48375311	1
gene58811	132.2418976	176.1620214	1.332119582	0.413723597	0.48377896	1
gene68101	639.5135517	492.6729061	0.770386968	-0.3763448	0.48386782	1
gene32407	267.5832147	204.2317941	0.76324591	-0.38978014	0.48389077	1
gene48194	446.3164044	343.6127341	0.769885961	-0.37728333	0.48389383	1
gene40431	118.8110799	87.11308752	0.733206765	-0.447708	0.4839117	1
gene21510	476.2774594	366.8428908	0.770229377	-0.37663995	0.48393018	1
gene30555	416.3553495	320.3825774	0.769493121	-0.37801967	0.48406052	1
gene29157	605.4199375	466.5389798	0.770603925	-0.37593856	0.48407802	1
gene70977	167.3686517	220.6864884	1.318565252	0.398968968	0.48413135	1
gene12848	183.8988889	138.4130168	0.752658255	-0.40993314	0.4843012	1
gene7796	428.7530274	330.0618094	0.769818026	-0.37741064	0.48430294	1
gene29070	135.3413171	180.0337142	1.330219907	0.411664766	0.48430532	1
gene1720	112.6122409	82.27347154	0.730590839	-0.45286443	0.48432305	1
gene67946	112.6122409	82.27347154	0.730590839	-0.45286443	0.48432305	1
gene21686	64.05466916	44.52446695	0.695101037	-0.5247054	0.48435628	1
gene30280	64.05466916	44.52446695	0.695101037	-0.5247054	0.48435628	1
gene56605	64.05466916	44.52446695	0.695101037	-0.5247054	0.48435628	1
gene52668	1093.061935	837.2535634	0.765970835	-0.38463863	0.48439291	1
gene24642	152.9046941	202.2959477	1.323019864	0.403834723	0.48439336	1
gene39703	138.4407366	102.5998586	0.74111032	-0.43223978	0.48464667	1
gene42816	4303.027371	6033.065272	1.402051335	0.487539174	0.48466148	1
gene59852	429.7861672	557.5237601	1.297211969	0.37541424	0.48467437	1
gene58709	287.2128714	373.6183531	1.300841259	0.379444921	0.48468754	1
gene25632	88.85002496	120.9903993	1.36173737	0.445448487	0.48470056	1
gene1439	49.5907116	70.65839321	1.424831202	0.510791015	0.48472907	1
gene47151	49.5907116	70.65839321	1.424831202	0.510791015	0.48472907	1
gene42111	225.2244819	294.2486512	1.306468323	0.385672145	0.48477607	1
gene3879	87.81688513	62.91500765	0.716434061	-0.48109417	0.4847833	1
gene24327	372.9634769	483.9615973	1.297611234	0.375858214	0.48480825	1
gene25884	138.4407366	183.905407	1.328405291	0.409695375	0.48481328	1
gene20848	247.953558	323.286347	1.303818141	0.382742654	0.48485392	1
gene13997	621.9501747	808.2158675	1.299486519	0.377941667	0.48486067	1
gene40626	94.01572408	67.75462362	0.720673316	-0.47258267	0.48494293	1
gene5435	47.52443195	31.94146542	0.672106201	-0.57323888	0.48496745	1
gene14694	343.0024219	445.2446695	1.29808025	0.376379576	0.48509112	1
gene73625	696.3362421	905.9761102	1.301061262	0.379688895	0.48512039	1
gene16488	362.6320786	278.7618801	0.768718204	-0.37947326	0.4852281	1
gene25448	583.7240012	757.8838614	1.298359944	0.376690397	0.48529532	1
gene36703	472.1449001	363.9391212	0.770820825	-0.37553255	0.48530303	1
gene9183	141.540156	187.7770998	1.326670148	0.407809716	0.48530466	1
gene56997	141.540156	187.7770998	1.326670148	0.407809716	0.48530466	1
gene35195	57.8558302	81.30554835	1.405312966	0.490891457	0.48536887	1
gene64622	57.8558302	81.30554835	1.405312966	0.490891457	0.48536887	1

gene42298	28.9279151	43.55654376	1.505692464	0.590427131	0.48541636	1
gene43723	28.9279151	43.55654376	1.505692464	0.590427131	0.48541636	1
gene11725	32.02733458	20.32638709	0.634657469	-0.65594993	0.48543831	1
gene14809	32.02733458	20.32638709	0.634657469	-0.65594993	0.48543831	1
gene41895	32.02733458	20.32638709	0.634657469	-0.65594993	0.48543831	1
gene51528	32.02733458	20.32638709	0.634657469	-0.65594993	0.48543831	1
gene53896	32.02733458	20.32638709	0.634657469	-0.65594993	0.48543831	1
gene10725	393.6262734	302.9599599	0.769663969	-0.37769938	0.48545581	1
gene32925	151.8715543	113.2470138	0.745676268	-0.42337867	0.48547976	1
gene53177	151.8715543	113.2470138	0.745676268	-0.42337867	0.48547976	1
gene22860	1521.814962	1157.636141	0.760694414	-0.39461108	0.48552835	1
gene54112	91.94944443	124.8620921	1.357942866	0.441422781	0.48556695	1
gene58525	412.2227902	317.4788078	0.770163163	-0.37676397	0.4856865	1
gene29781	460.780362	355.2278124	0.770926545	-0.37533469	0.48578467	1
gene44508	961.8531772	1258.300153	1.308203978	0.387587507	0.48583338	1
gene68812	126.0430587	92.92062669	0.737213359	-0.43984588	0.48584401	1
gene69335	1404.037022	1070.523053	0.762460702	-0.39126511	0.48588734	1
gene13283	44.42501248	63.88293085	1.437994663	0.524058321	0.48591095	1
gene20578	44.42501248	63.88293085	1.437994663	0.524058321	0.48591095	1
gene52163	44.42501248	63.88293085	1.437994663	0.524058321	0.48591095	1
gene70938	44.42501248	63.88293085	1.437994663	0.524058321	0.48591095	1
gene6346	861.6386141	663.0273883	0.769495909	-0.37801444	0.48592193	1
gene52060	878.1688513	675.6103899	0.769339961	-0.37830685	0.48597814	1
gene3902	1637.526623	2175.891342	1.328767002	0.410088152	0.4859864	1
gene58314	437.018146	336.8372717	0.770762667	-0.3756414	0.48608482	1
gene16840	165.302372	123.8941689	0.749500249	-0.41599914	0.48609993	1
gene3167	718.0321784	934.0458828	1.300841259	0.379444921	0.48611412	1
gene13452	42.35873283	28.06977264	0.662667903	-0.59364205	0.48613522	1
gene19974	42.35873283	28.06977264	0.662667903	-0.59364205	0.48613522	1
gene24577	42.35873283	28.06977264	0.662667903	-0.59364205	0.48613522	1
gene55612	42.35873283	28.06977264	0.662667903	-0.59364205	0.48613522	1
gene6499	145.6727153	108.4073978	0.744184644	-0.42626747	0.48613955	1
gene16601	145.6727153	108.4073978	0.744184644	-0.42626747	0.48613955	1
gene66075	363.6652184	279.7298033	0.769195923	-0.37857698	0.48619226	1
gene24963	574.4257427	745.3008599	1.297471204	0.37570252	0.48621395	1
gene59016	165.302372	217.7827188	1.317480906	0.397782053	0.4862374	1
gene17931	1732.575487	2306.560973	1.331290319	0.412825219	0.48627497	1
gene27034	80.58490636	110.3432442	1.3692793	0.453416752	0.48629461	1
gene62057	80.58490636	110.3432442	1.3692793	0.453416752	0.48629461	1
gene48308	223.1582022	291.3448816	1.305553095	0.384661131	0.48629753	1
gene59202	3212.031716	2380.123136	0.741002377	-0.43244993	0.48632933	1
gene27071	326.4721847	250.6921074	0.767881979	-0.3810435	0.4863372	1
gene64154	197.3297066	258.435493	1.309663392	0.38919606	0.4863565	1
gene11002	95.04886391	128.7337849	1.35439583	0.437649436	0.4863625	1
gene11067	217.9925031	165.5148663	0.759268617	-0.39731772	0.4864637	1
gene14742	965.9857365	742.3970903	0.768538356	-0.37981083	0.48646485	1
gene71843	198.3628464	150.0280952	0.756331631	-0.40290914	0.48647526	1
gene9896	21.69593633	33.87731181	1.561458851	0.642894551	0.48648156	1

gene10354	21.69593633	33.87731181	1.561458851	0.642894551	0.48648156	1
gene20450	21.69593633	33.87731181	1.561458851	0.642894551	0.48648156	1
gene30694	21.69593633	33.87731181	1.561458851	0.642894551	0.48648156	1
gene31964	21.69593633	33.87731181	1.561458851	0.642894551	0.48648156	1
gene32686	21.69593633	33.87731181	1.561458851	0.642894551	0.48648156	1
gene55136	21.69593633	33.87731181	1.561458851	0.642894551	0.48648156	1
gene60224	21.69593633	33.87731181	1.561458851	0.642894551	0.48648156	1
gene16533	37.1930337	24.19807987	0.650607855	-0.62013986	0.48650251	1
gene28276	37.1930337	24.19807987	0.650607855	-0.62013986	0.48650251	1
gene33964	37.1930337	24.19807987	0.650607855	-0.62013986	0.48650251	1
gene39280	37.1930337	24.19807987	0.650607855	-0.62013986	0.48650251	1
gene41536	37.1930337	24.19807987	0.650607855	-0.62013986	0.48650251	1
gene43085	37.1930337	24.19807987	0.650607855	-0.62013986	0.48650251	1
gene43902	37.1930337	24.19807987	0.650607855	-0.62013986	0.48650251	1
gene46197	34.09361423	50.33200612	1.476288368	0.561974555	0.48655488	1
gene66578	34.09361423	50.33200612	1.476288368	0.561974555	0.48655488	1
gene6715	1373.042828	1812.920144	1.320366785	0.400938752	0.4865723	1
gene43060	385.3611548	499.4483684	1.296052709	0.374124392	0.48659565	1
gene50899	521.7356117	402.656049	0.771762632	-0.3737709	0.48666995	1
gene689	678.7728651	523.6464483	0.771460492	-0.37433582	0.48667434	1
gene18294	150.8384145	199.3921781	1.321892562	0.402604925	0.48669501	1
gene47408	58.88897003	40.65277417	0.690329176	-0.53464363	0.48672168	1
gene69379	58.88897003	40.65277417	0.690329176	-0.53464363	0.48672168	1
gene69779	58.88897003	40.65277417	0.690329176	-0.53464363	0.48672168	1
gene38417	2011.523239	1518.671492	0.754985805	-0.40547858	0.48678009	1
gene60650	139.4738764	103.5677818	0.742560431	-0.42941965	0.48681861	1
gene5635	133.2750374	177.1299446	1.329055673	0.41040154	0.48686971	1
gene20877	1639.592902	1245.717151	0.759772227	-0.39636112	0.48688422	1
gene13823	113.6453808	83.24139474	0.732466152	-0.449166	0.48696161	1
gene53944	2853.532197	2126.527259	0.745226306	-0.42424949	0.48696804	1
gene56482	98.14828338	132.6054777	1.351072817	0.434105431	0.48709561	1
gene21634	252.0861173	328.125963	1.301642337	0.380333082	0.48714896	1
gene37849	364.6983583	280.6977264	0.769670935	-0.37768633	0.48715202	1
gene67586	1723.277228	1307.664236	0.758824068	-0.39816266	0.48718354	1
gene12595	83.68432583	114.214937	1.3648307	0.448722003	0.48728638	1
gene37206	350.2344007	453.9559783	1.296149029	0.374231606	0.48735394	1
gene33546	76.45234706	54.2036989	0.708986722	-0.49616949	0.48736298	1
gene41411	52.69013108	74.53008599	1.414498018	0.500290155	0.48736692	1
gene8749	2116.903502	1596.105348	0.753981155	-0.40739963	0.48737987	1
gene11232	22.72907615	13.55092472	0.59619338	-0.74614774	0.4874458	1
gene20375	22.72907615	13.55092472	0.59619338	-0.74614774	0.4874458	1
gene42257	22.72907615	13.55092472	0.59619338	-0.74614774	0.4874458	1
gene52650	22.72907615	13.55092472	0.59619338	-0.74614774	0.4874458	1
gene61653	22.72907615	13.55092472	0.59619338	-0.74614774	0.4874458	1
gene71390	22.72907615	13.55092472	0.59619338	-0.74614774	0.4874458	1
gene47555	876.1025717	674.6424667	0.770049636	-0.37697665	0.48747446	1
gene39076	469.0454806	362.0032748	0.77178715	-0.37372507	0.48747832	1
gene57386	244.8541385	186.8091766	0.762940654	-0.39035725	0.48757001	1

gene5650	3102.518895	2304.625126	0.742823881	-0.4289079	0.48761922	1
gene61606	19544.93921	33442.7143	1.711067706	0.774896848	0.48770857	1
gene11994	703.5682209	543.0049122	0.77178715	-0.37372507	0.48774871	1
gene62455	101.2477029	136.4771704	1.347953253	0.430770465	0.48777361	1
gene794	72.31978776	99.69608905	1.3785451	0.463146467	0.48778331	1
gene1842	139.4738764	184.8733302	1.325505069	0.406542188	0.48778813	1
gene53601	386.3942946	500.4162916	1.295092341	0.373054967	0.48783426	1
gene25036	219.0256429	166.4827895	0.760106384	-0.39572674	0.48792102	1
gene43261	20672.09476	13042.76505	0.630935819	-0.66443484	0.48792502	1
gene13683	82.65118601	59.04331487	0.714367424	-0.4852618	0.48794756	1
gene15496	82.65118601	59.04331487	0.714367424	-0.4852618	0.48794756	1
gene29911	82.65118601	59.04331487	0.714367424	-0.4852618	0.48794756	1
gene32895	238.6552996	181.9695606	0.762478608	-0.39123123	0.48795978	1
gene24123	59695.85223	30637.67288	0.513229508	-0.96232397	0.48797522	1
gene64793	493.8408364	381.3617387	0.772236135	-0.37288603	0.48801951	1
gene42932	975.2839949	750.1404758	0.769150811	-0.37866159	0.48804143	1
gene50267	651.9112296	845.9648721	1.297668814	0.375922231	0.4880543	1
gene19656	95.04886391	68.72254682	0.723023338	-0.46788588	0.48808729	1
gene42813	95.04886391	68.72254682	0.723023338	-0.46788588	0.48808729	1
gene60342	95.04886391	68.72254682	0.723023338	-0.46788588	0.48808729	1
gene23489	384.3280149	296.1844976	0.770655498	-0.37584201	0.48809914	1
gene8849	390.5268539	301.0241135	0.770815401	-0.3755427	0.48811584	1
gene30116	121.9104994	162.6110967	1.333856375	0.41560333	0.48812873	1
gene16068	1426.766098	1088.913594	0.763204	-0.38985936	0.48813402	1
gene34752	88.85002496	63.88293085	0.718997331	-0.47594168	0.48815228	1
gene47167	258.2849563	197.4563317	0.764490254	-0.38742999	0.48817469	1
gene54539	508.3047939	658.1877724	1.294868316	0.372805387	0.48828981	1
gene43389	252.0861173	192.6167157	0.76409093	-0.38818376	0.48851521	1
gene57082	744.8938139	574.9463776	0.771850117	-0.37360737	0.48858532	1
gene4972	1136.453808	872.0987984	0.76738605	-0.38197556	0.48868096	1
gene11043	227.2907615	296.1844976	1.303108387	0.381957086	0.48875381	1
gene65575	183.8988889	241.0128755	1.310572766	0.390197458	0.48878402	1
gene29597	271.715774	208.1034868	0.765886661	-0.38479718	0.48882079	1
gene7471	1194.309638	915.6553421	0.766681699	-0.38330035	0.48882197	1
gene32446	120.8773595	89.04893391	0.736688279	-0.44087381	0.48889517	1
gene42782	75.41920723	103.5677818	1.373228195	0.457571384	0.48896434	1
gene1916	140.5070162	104.535705	0.743989217	-0.42664638	0.48896477	1
gene47482	64.05466916	89.04893391	1.390202074	0.475294602	0.48899311	1
gene67354	206.627965	156.8035575	0.758869002	-0.39807723	0.48900651	1
gene60284	215.9262234	281.6656496	1.304453184	0.383445167	0.48901001	1
gene73885	326.4721847	422.9824361	1.29561554	0.373637678	0.48902242	1
gene14100	65.08780898	45.49239015	0.698938724	-0.51676212	0.48904654	1
gene26840	65.08780898	45.49239015	0.698938724	-0.51676212	0.48904654	1
gene50210	53.7232709	36.7810814	0.68463965	-0.54658325	0.48905312	1
gene53362	53.7232709	36.7810814	0.68463965	-0.54658325	0.48905312	1
gene5596	370.8971972	480.0899045	1.294401543	0.372285233	0.48905328	1
gene7477	148.7721348	196.4884085	1.320733945	0.401339872	0.48906212	1
gene18997	148.7721348	196.4884085	1.320733945	0.401339872	0.48906212	1

gene56376	148.7721348	196.4884085	1.320733945	0.401339872	0.48906212	1
gene58074	3646.983583	5034.168535	1.380365012	0.465049811	0.48906488	1
gene33058	47.52443195	67.75462362	1.425679821	0.511650018	0.48910716	1
gene36305	3286.417784	2437.230604	0.741607052	-0.43127313	0.48911491	1
gene57752	403.9576716	522.6785251	1.293894291	0.371719756	0.48916542	1
gene55457	186.9983083	244.8845682	1.309554992	0.389076644	0.48919124	1
gene27431	348.1681211	268.1147249	0.770072585	-0.37693366	0.4892075	1
gene72079	348.1681211	268.1147249	0.770072585	-0.37693366	0.4892075	1
gene35668	483.5094381	373.6183531	0.772721944	-0.37197873	0.48924601	1
gene11710	1395.771904	1066.65136	0.764201771	-0.38797449	0.48936207	1
gene28460	220.0587827	167.4507127	0.760936285	-0.39415244	0.48936777	1
gene9752	335.7704432	258.435493	0.769679101	-0.37767102	0.48937135	1
gene69757	153.9378339	115.1828602	0.748242698	-0.4184218	0.48944452	1
gene64698	2698.561223	2019.087784	0.748208996	-0.41848678	0.48949997	1
gene30965	437.018146	565.2671457	1.293463786	0.371239662	0.48955972	1
gene43613	114.6785206	84.20931793	0.734307676	-0.44554341	0.48956083	1
gene31254	4696.653645	6605.10788	1.406343405	0.491948919	0.48959211	1
gene35293	172.5343508	226.4940275	1.312747441	0.392589384	0.48960158	1
gene7602	55.78955055	78.40177877	1.405312966	0.490891457	0.48961939	1
gene146	7.231978776	2.903769584	0.40151799	-1.31646346	0.48965143	1
gene549	7.231978776	2.903769584	0.40151799	-1.31646346	0.48965143	1
gene1155	7.231978776	2.903769584	0.40151799	-1.31646346	0.48965143	1
gene1204	7.231978776	2.903769584	0.40151799	-1.31646346	0.48965143	1
gene3750	7.231978776	2.903769584	0.40151799	-1.31646346	0.48965143	1
gene4112	7.231978776	2.903769584	0.40151799	-1.31646346	0.48965143	1
gene5187	7.231978776	2.903769584	0.40151799	-1.31646346	0.48965143	1
gene7888	7.231978776	2.903769584	0.40151799	-1.31646346	0.48965143	1
gene8398	7.231978776	2.903769584	0.40151799	-1.31646346	0.48965143	1
gene8908	7.231978776	2.903769584	0.40151799	-1.31646346	0.48965143	1
gene11324	7.231978776	2.903769584	0.40151799	-1.31646346	0.48965143	1
gene15240	7.231978776	2.903769584	0.40151799	-1.31646346	0.48965143	1
gene15555	7.231978776	2.903769584	0.40151799	-1.31646346	0.48965143	1
gene15920	7.231978776	2.903769584	0.40151799	-1.31646346	0.48965143	1
gene17051	7.231978776	2.903769584	0.40151799	-1.31646346	0.48965143	1
gene18371	7.231978776	2.903769584	0.40151799	-1.31646346	0.48965143	1
gene19310	7.231978776	2.903769584	0.40151799	-1.31646346	0.48965143	1
gene19633	7.231978776	2.903769584	0.40151799	-1.31646346	0.48965143	1
gene20929	7.231978776	2.903769584	0.40151799	-1.31646346	0.48965143	1
gene23161	7.231978776	2.903769584	0.40151799	-1.31646346	0.48965143	1
gene23469	7.231978776	2.903769584	0.40151799	-1.31646346	0.48965143	1
gene24481	7.231978776	2.903769584	0.40151799	-1.31646346	0.48965143	1
gene24509	7.231978776	2.903769584	0.40151799	-1.31646346	0.48965143	1
gene26864	7.231978776	2.903769584	0.40151799	-1.31646346	0.48965143	1
gene28674	7.231978776	2.903769584	0.40151799	-1.31646346	0.48965143	1
gene33082	7.231978776	2.903769584	0.40151799	-1.31646346	0.48965143	1
gene34315	7.231978776	2.903769584	0.40151799	-1.31646346	0.48965143	1
gene38496	7.231978776	2.903769584	0.40151799	-1.31646346	0.48965143	1
gene38997	7.231978776	2.903769584	0.40151799	-1.31646346	0.48965143	1

gene39611	7.231978776	2.903769584	0.40151799	-1.31646346	0.48965143	1
gene40420	7.231978776	2.903769584	0.40151799	-1.31646346	0.48965143	1
gene42678	7.231978776	2.903769584	0.40151799	-1.31646346	0.48965143	1
gene43266	7.231978776	2.903769584	0.40151799	-1.31646346	0.48965143	1
gene43797	7.231978776	2.903769584	0.40151799	-1.31646346	0.48965143	1
gene44766	7.231978776	2.903769584	0.40151799	-1.31646346	0.48965143	1
gene47063	7.231978776	2.903769584	0.40151799	-1.31646346	0.48965143	1
gene48333	7.231978776	2.903769584	0.40151799	-1.31646346	0.48965143	1
gene53816	7.231978776	2.903769584	0.40151799	-1.31646346	0.48965143	1
gene60175	7.231978776	2.903769584	0.40151799	-1.31646346	0.48965143	1
gene64977	7.231978776	2.903769584	0.40151799	-1.31646346	0.48965143	1
gene70975	7.231978776	2.903769584	0.40151799	-1.31646346	0.48965143	1
gene73756	7.231978776	2.903769584	0.40151799	-1.31646346	0.48965143	1
gene40037	573.3926029	742.3970903	1.294744799	0.372667763	0.48971234	1
gene38491	154.9709738	204.2317941	1.31787127	0.398209455	0.48985825	1
gene26110	730.4298563	564.2992225	0.772557717	-0.37228537	0.48987764	1
gene55795	478.343739	369.7466604	0.772972719	-0.3715106	0.48988699	1
gene53837	134.3081773	178.0978678	1.326038901	0.4071231	0.48993657	1
gene15499	380.1954556	293.280728	0.771394617	-0.37445902	0.489937	1
gene3812	410.1565106	530.4219107	1.293218313	0.370965842	0.48999453	1
gene20624	78.51862671	107.4394746	1.368331046	0.45241731	0.49001662	1
gene64810	78.51862671	107.4394746	1.368331046	0.45241731	0.49001662	1
gene51623	520.7024718	402.656049	0.773293907	-0.37091125	0.49003277	1
gene22215	822.3793008	1069.55513	1.300561832	0.37913499	0.49011655	1
gene61492	355.4000998	273.9222641	0.770743352	-0.37567756	0.49012001	1
gene23066	4770.006572	3470.004653	0.727463285	-0.45905366	0.49015582	1
gene10719	26.86163545	40.65277417	1.513413963	0.597806661	0.49015891	1
gene44837	26.86163545	40.65277417	1.513413963	0.597806661	0.49015891	1
gene53471	26.86163545	40.65277417	1.513413963	0.597806661	0.49015891	1
gene33218	108.4796816	79.36970196	0.731655005	-0.45076456	0.49018278	1
gene44074	108.4796816	79.36970196	0.731655005	-0.45076456	0.49018278	1
gene65439	108.4796816	79.36970196	0.731655005	-0.45076456	0.49018278	1
gene24363	865.7711734	667.8670043	0.771412845	-0.37442493	0.49020123	1
gene45868	147.738995	110.3432442	0.746879618	-0.42105237	0.49024866	1
gene20966	194.2302871	147.1243256	0.757473655	-0.40073238	0.49027027	1
gene11480	343.0024219	264.2430321	0.770382409	-0.37635333	0.49028646	1
gene2512	454.581523	351.3561197	0.772922131	-0.37160502	0.49029772	1
gene66538	137.4075967	181.9695606	1.32430495	0.405235373	0.49034553	1
gene20832	650.8780898	503.3200612	0.773293907	-0.37091125	0.49035326	1
gene49947	1013.510168	780.1460949	0.769746688	-0.37754434	0.49036242	1
gene55628	178.7331897	234.2374131	1.310542342	0.390163966	0.49037631	1
gene37937	42.35873283	60.97916126	1.439588892	0.525656876	0.49040174	1
gene45504	42.35873283	60.97916126	1.439588892	0.525656876	0.49040174	1
gene67843	537.232709	694.9688538	1.293608602	0.371401177	0.49042098	1
gene23393	668.4414668	516.8709859	0.773247938	-0.37099701	0.4904579	1
gene45764	509.3379338	393.9447402	0.773444729	-0.3706299	0.49047153	1
gene3178	71.28664793	50.33200612	0.706050959	-0.50215578	0.49047257	1
gene62061	1939.203452	1469.307409	0.757686053	-0.4003279	0.49053886	1

gene11916	161.1698127	120.9903993	0.750701371	-0.41368898	0.49056299	1
gene26863	513.4704931	663.9953115	1.293151837	0.370891681	0.49060568	1
gene64150	286.1797315	219.7185652	0.767764244	-0.38126472	0.4906789	1
gene71743	102.2808427	74.53008599	0.728680797	-0.45664112	0.49073318	1
gene68452	1669.553957	1270.883155	0.76121119	-0.39363133	0.490749	1
gene68756	174.6006304	131.6375545	0.753935161	-0.40748764	0.49078409	1
gene43922	406.0239513	313.6071151	0.772385752	-0.37260654	0.49079293	1
gene10320	221.0919226	168.4186359	0.76175843	-0.39259453	0.49080407	1
gene4515	19.62965668	30.97354223	1.57789526	0.658001443	0.49082194	1
gene47342	19.62965668	30.97354223	1.57789526	0.658001443	0.49082194	1
gene52214	19.62965668	30.97354223	1.57789526	0.658001443	0.49082194	1
gene73496	19.62965668	30.97354223	1.57789526	0.658001443	0.49082194	1
gene46932	692.2036828	535.2615266	0.773271712	-0.37095266	0.49083218	1
gene19517	1009.377609	777.2423253	0.770021366	-0.37702962	0.49084715	1
gene32982	381.2285955	294.2486512	0.771843074	-0.37362054	0.49085348	1
gene27352	2031.152896	1537.062033	0.756743639	-0.40212345	0.49094173	1
gene14561	2370.022759	1783.882448	0.752685788	-0.40988036	0.49094262	1
gene21652	777.9542883	601.0803039	0.772642189	-0.37212764	0.49095418	1
gene73537	725.2641572	940.8213452	1.297211969	0.37541424	0.49096802	1
gene22749	141.540156	105.5036282	0.745397145	-0.4239188	0.49108563	1
gene60111	1279.027103	980.5061962	0.766603142	-0.38344818	0.49110734	1
gene20170	341.9692821	442.3408999	1.293510625	0.371291905	0.49111632	1
gene70466	234.5227403	179.065791	0.763532742	-0.38923807	0.49113229	1
gene44891	184.9320287	241.9807987	1.308485071	0.387897464	0.4911353	1
gene6162	96.08200373	69.69047001	0.725322821	-0.46330485	0.49117436	1
gene35006	96.08200373	69.69047001	0.725322821	-0.46330485	0.49117436	1
gene41505	96.08200373	69.69047001	0.725322821	-0.46330485	0.49117436	1
gene8182	37.1930337	54.2036989	1.457361594	0.543358877	0.49123603	1
gene20069	37.1930337	54.2036989	1.457361594	0.543358877	0.49123603	1
gene5287	48.55757178	32.90938862	0.677739586	-0.56119705	0.49125762	1
gene28329	48.55757178	32.90938862	0.677739586	-0.56119705	0.49125762	1
gene47283	48.55757178	32.90938862	0.677739586	-0.56119705	0.49125762	1
gene35238	1607.565568	1225.390764	0.762264874	-0.3916357	0.49126778	1
gene36040	4723.51528	3440.966957	0.728475881	-0.45704689	0.491268	1
gene47031	344.0355618	265.2109553	0.770882388	-0.37541733	0.49128552	1
gene14018	3714.137671	2742.12641	0.73829423	-0.43773221	0.49131679	1
gene5557	14.46395755	7.743385557	0.53535732	-0.90142597	0.49132049	1
gene6394	14.46395755	7.743385557	0.53535732	-0.90142597	0.49132049	1
gene14442	14.46395755	7.743385557	0.53535732	-0.90142597	0.49132049	1
gene16764	14.46395755	7.743385557	0.53535732	-0.90142597	0.49132049	1
gene17588	14.46395755	7.743385557	0.53535732	-0.90142597	0.49132049	1
gene24393	14.46395755	7.743385557	0.53535732	-0.90142597	0.49132049	1
gene32704	14.46395755	7.743385557	0.53535732	-0.90142597	0.49132049	1
gene35392	14.46395755	7.743385557	0.53535732	-0.90142597	0.49132049	1
gene38405	14.46395755	7.743385557	0.53535732	-0.90142597	0.49132049	1
gene41697	14.46395755	7.743385557	0.53535732	-0.90142597	0.49132049	1
gene46116	14.46395755	7.743385557	0.53535732	-0.90142597	0.49132049	1
gene50557	14.46395755	7.743385557	0.53535732	-0.90142597	0.49132049	1

gene52153	14.46395755	7.743385557	0.53535732	-0.90142597	0.49132049	1
gene53593	14.46395755	7.743385557	0.53535732	-0.90142597	0.49132049	1
gene67375	14.46395755	7.743385557	0.53535732	-0.90142597	0.49132049	1
gene58758	2308.034369	1739.357981	0.753610087	-0.40810982	0.49134624	1
gene23432	32.02733458	47.42823654	1.480867427	0.56644249	0.49135503	1
gene62051	32.02733458	47.42823654	1.480867427	0.56644249	0.49135503	1
gene11430	167.3686517	219.7185652	1.312782071	0.392627441	0.49136008	1
gene42817	167.3686517	219.7185652	1.312782071	0.392627441	0.49136008	1
gene5696	299.6105493	230.3657203	0.768883876	-0.37916237	0.49136104	1
gene69407	214.8930836	163.5790199	0.76121119	-0.39363133	0.4913904	1
gene13489	154.9709738	116.1507834	0.749500249	-0.41599914	0.49139518	1
gene43160	154.9709738	116.1507834	0.749500249	-0.41599914	0.49139518	1
gene48867	337.8367228	260.3713394	0.770701708	-0.37575551	0.49140505	1
gene61094	89.88316478	64.85085404	0.721501676	-0.47092535	0.49145502	1
gene50901	146.7058552	193.5846389	1.319542691	0.400038027	0.49149714	1
gene33616	83.68432583	60.01123807	0.717114435	-0.47972474	0.49150445	1
gene1620	18.59651685	10.64715514	0.572534912	-0.80456443	0.49155573	1
gene1635	18.59651685	10.64715514	0.572534912	-0.80456443	0.49155573	1
gene11647	18.59651685	10.64715514	0.572534912	-0.80456443	0.49155573	1
gene21994	18.59651685	10.64715514	0.572534912	-0.80456443	0.49155573	1
gene30019	18.59651685	10.64715514	0.572534912	-0.80456443	0.49155573	1
gene30908	18.59651685	10.64715514	0.572534912	-0.80456443	0.49155573	1
gene58453	18.59651685	10.64715514	0.572534912	-0.80456443	0.49155573	1
gene61907	18.59651685	10.64715514	0.572534912	-0.80456443	0.49155573	1
gene61944	18.59651685	10.64715514	0.572534912	-0.80456443	0.49155573	1
gene63066	18.59651685	10.64715514	0.572534912	-0.80456443	0.49155573	1
gene65295	18.59651685	10.64715514	0.572534912	-0.80456443	0.49155573	1
gene66760	168.4017915	126.7979385	0.752948869	-0.4093762	0.49158355	1
gene17090	670.5077465	518.8068323	0.773752183	-0.37005652	0.49159067	1
gene27489	699.4356616	541.0690658	0.773579466	-0.37037859	0.49161468	1
gene51691	170.4680711	223.590258	1.311625435	0.391355784	0.49172139	1
gene43648	5375.42651	3885.243703	0.722778685	-0.46837413	0.49172689	1
gene29590	1086.863096	836.2856402	0.769448924	-0.37810253	0.49174141	1
gene73597	382.2617353	295.2165744	0.772289107	-0.37278707	0.49176594	1
gene26056	50.62385143	71.6263164	1.414872918	0.500672478	0.49179376	1
gene32608	59.92210985	41.62069737	0.694579972	-0.52578728	0.49179436	1
gene62624	84.71746566	115.1828602	1.359611731	0.443194715	0.49180171	1
gene25999	195.2634269	148.0922488	0.758422871	-0.39892562	0.49186065	1
gene43602	195.2634269	148.0922488	0.758422871	-0.39892562	0.49186065	1
gene5223	135.3413171	100.6640122	0.743778873	-0.42705433	0.49195109	1
gene46367	135.3413171	100.6640122	0.743778873	-0.42705433	0.49195109	1
gene33929	1556.941716	2056.836789	1.321075007	0.401712381	0.49207518	1
gene49134	981.4828338	1279.594463	1.303735959	0.382651715	0.49207795	1
gene1062	339.9030025	439.4371304	1.292830976	0.37053367	0.492093	1
gene9860	544.4646878	703.6801625	1.292425713	0.370081359	0.49210365	1
gene63785	281.0140324	215.8468724	0.768099979	-0.38063398	0.49212502	1
gene58630	800.6833644	1039.549511	1.29832785	0.376654734	0.4921572	1
gene22645	1116.824151	1460.596101	1.307812066	0.387155239	0.49220261	1

gene16366	2274.973895	3050.893909	1.341067656	0.423382023	0.49224837	1
gene51595	194.2302871	253.595877	1.30564538	0.384763107	0.49225199	1
gene21414	660.1763482	511.0634468	0.774131712	-0.36934904	0.49229774	1
gene27995	277.9146129	360.0674284	1.295604519	0.373625405	0.49230863	1
gene12442	1523.881242	2011.344398	1.319882641	0.400409656	0.49234071	1
gene3437	162.2029525	121.9583225	0.751887192	-0.41141187	0.49243061	1
gene52078	689.1042633	892.4251855	1.295051029	0.373008946	0.49255861	1
gene41938	156.0041136	205.1997173	1.315348118	0.395444672	0.49256319	1
gene41765	1391.639344	1065.683437	0.765775588	-0.38500643	0.492607	1
gene64662	560.994925	724.9744728	1.292301303	0.369942477	0.49260988	1
gene59286	389.4937141	301.0241135	0.772860004	-0.37172099	0.49261957	1
gene9066	548.5972471	424.9182824	0.774554165	-0.36856196	0.49264525	1
gene9485	202.4954057	153.8997879	0.760016196	-0.39589793	0.49270551	1
gene21237	202.4954057	153.8997879	0.760016196	-0.39589793	0.49270551	1
gene9440	129.1424781	95.82439627	0.742005246	-0.43049871	0.49283893	1
gene24746	129.1424781	95.82439627	0.742005246	-0.43049871	0.49283893	1
gene55298	129.1424781	95.82439627	0.742005246	-0.43049871	0.49283893	1
gene50334	440.1175655	568.1709153	1.2909526	0.36843603	0.49286075	1
gene478	1211.873015	931.1421132	0.768349573	-0.38016526	0.4929238	1
gene4224	1280.060243	982.4420426	0.767496723	-0.3817675	0.49305843	1
gene57081	944.2898001	728.8461656	0.771845852	-0.37361534	0.49309624	1
gene63395	111.5791011	149.060172	1.335914795	0.417827995	0.4931129	1
gene2699	27.89477528	17.4226175	0.62458354	-0.67903354	0.49311423	1
gene13216	27.89477528	17.4226175	0.62458354	-0.67903354	0.49311423	1
gene14580	27.89477528	17.4226175	0.62458354	-0.67903354	0.49311423	1
gene16603	27.89477528	17.4226175	0.62458354	-0.67903354	0.49311423	1
gene18042	27.89477528	17.4226175	0.62458354	-0.67903354	0.49311423	1
gene20934	27.89477528	17.4226175	0.62458354	-0.67903354	0.49311423	1
gene39594	27.89477528	17.4226175	0.62458354	-0.67903354	0.49311423	1
gene71305	27.89477528	17.4226175	0.62458354	-0.67903354	0.49311423	1
gene697	43.39187265	29.03769584	0.669196651	-0.57949787	0.49317476	1
gene25267	43.39187265	29.03769584	0.669196651	-0.57949787	0.49317476	1
gene41431	43.39187265	29.03769584	0.669196651	-0.57949787	0.49317476	1
gene40873	543.431548	421.0465897	0.77479232	-0.36811844	0.4931856	1
gene10498	61.9883895	86.14516432	1.389698378	0.474771792	0.49321404	1
gene11161	702.5350811	543.9728354	0.774299889	-0.36903566	0.4932434	1
gene45438	2042.517434	1547.709188	0.757745889	-0.40021398	0.49331878	1
gene58416	459.7472222	593.3369183	1.290572057	0.368010694	0.49341736	1
gene54119	627.1158738	485.8974437	0.77481286	-0.3680802	0.4934611	1
gene27030	708.73392	548.8124514	0.774356124	-0.36893088	0.49346333	1
gene65724	927.7595629	1207.000224	1.300983867	0.379603072	0.49347588	1
gene72901	749.0263732	579.7859936	0.774052843	-0.36949604	0.49348891	1
gene6486	3731.701048	2758.581105	0.739228858	-0.43590702	0.49350948	1
gene229	209.7273845	159.7073271	0.761499637	-0.39308475	0.49351482	1
gene69470	209.7273845	159.7073271	0.761499637	-0.39308475	0.49351482	1
gene40752	471.1117602	364.9070444	0.774565772	-0.36854034	0.49351808	1
gene63796	912.2624655	704.6480857	0.77241815	-0.37254603	0.49353098	1
gene49927	1475.32367	1943.589775	1.317398896	0.397692247	0.49358078	1

gene9054	333.7041635	257.4675698	0.771544374	-0.37417896	0.49358482	1
gene68991	66.12094881	46.46031334	0.702656483	-0.50910854	0.4936057	1
gene23724	556.8623657	719.1669336	1.291462627	0.369005894	0.49361615	1
gene50240	256.2186766	196.4884085	0.766877774	-0.38293144	0.4936256	1
gene17775	1316.220137	1727.742902	1.312654968	0.392487753	0.49364143	1
gene23716	223.1582022	170.3544823	0.763379883	-0.38952693	0.49364576	1
gene65876	380.1954556	490.7370597	1.290749409	0.368208938	0.49366385	1
gene40803	45.4581523	64.85085404	1.426605587	0.512586528	0.49368371	1
gene57351	1297.62362	995.9929673	0.767551509	-0.38166453	0.49368557	1
gene63232	122.9436392	90.9847803	0.740052766	-0.43429995	0.49374072	1
gene5600	1451.561454	1111.175827	0.765503813	-0.38551853	0.49375636	1
gene23018	1140.586367	877.9063375	0.769697379	-0.37763676	0.49379555	1
gene62727	1240.80093	953.4043467	0.768378169	-0.38011156	0.49381996	1
gene11234	1006.27819	776.2744021	0.771431211	-0.37439058	0.49382928	1
gene22164	236.5890199	181.0016374	0.765046651	-0.38638037	0.49383929	1
gene42231	795.5176653	615.5991518	0.773834672	-0.36990272	0.49392689	1
gene43884	253.1192571	328.125963	1.296329512	0.374432481	0.4939432	1
gene56674	962.886317	743.3650135	0.772017423	-0.37329469	0.49395349	1
gene5734	1487.721348	1138.277677	0.765114837	-0.38625179	0.49395842	1
gene13613	1350.313751	1035.677818	0.766990499	-0.38271939	0.49400987	1
gene2688	53.7232709	75.49800918	1.405312966	0.490891457	0.49406272	1
gene59821	315.1076466	242.9487219	0.771002305	-0.37519292	0.49419059	1
gene41820	97.11514356	70.65839321	0.72757338	-0.45883534	0.49420572	1
gene53969	241.7547191	313.6071151	1.297211969	0.37541424	0.49424832	1
gene68426	241.7547191	313.6071151	1.297211969	0.37541424	0.49424832	1
gene72422	176.6669101	133.5734009	0.756074812	-0.4033991	0.49424846	1
gene2532	939.124101	1221.519072	1.300700376	0.379288667	0.49425483	1
gene35814	149.8052746	112.2790906	0.749500249	-0.41599914	0.49426651	1
gene6189	120.8773595	160.6752503	1.329241894	0.410603668	0.49433573	1
gene42104	886.4339699	685.2896218	0.773085921	-0.37129933	0.4943531	1
gene28864	531.0338701	685.2896218	1.290481946	0.367909958	0.49439442	1
gene68870	230.390181	176.1620214	0.764624693	-0.3871763	0.49442144	1
gene5689	490.7414169	380.3938155	0.775141047	-0.36746924	0.49443292	1
gene57344	97.11514356	130.6696313	1.345512414	0.428155702	0.49443939	1
gene65967	575.4588826	446.2125927	0.775403085	-0.36698162	0.49449542	1
gene48326	4471.429163	3275.452091	0.732529125	-0.44904197	0.49451766	1
gene1504	38.22617353	25.16600306	0.658344813	-0.60308469	0.49452825	1
gene10932	38.22617353	25.16600306	0.658344813	-0.60308469	0.49452825	1
gene35887	38.22617353	25.16600306	0.658344813	-0.60308469	0.49452825	1
gene50227	38.22617353	25.16600306	0.658344813	-0.60308469	0.49452825	1
gene66542	38.22617353	25.16600306	0.658344813	-0.60308469	0.49452825	1
gene9462	54.75641073	37.74900459	0.689398814	-0.53658928	0.49457185	1
gene66679	853.3734955	660.1236187	0.773545959	-0.37044109	0.49457374	1
gene26892	244.8541385	317.4788078	1.296603806	0.374737712	0.49462635	1
gene38691	150.8384145	198.4242549	1.315475608	0.395584497	0.49463933	1
gene58766	509.3379338	394.9126634	0.775345085	-0.36708954	0.49464234	1
gene52107	116.7448002	86.14516432	0.737892944	-0.43851657	0.49464487	1
gene46494	230.390181	299.0882671	1.298181484	0.376492085	0.49464867	1

gene15063	65.08780898	90.0168571	1.383006411	0.467807844	0.49465075	1
gene15055	90.91630461	65.81877724	0.723949104	-0.46603982	0.49469353	1
gene62272	90.91630461	65.81877724	0.723949104	-0.46603982	0.49469353	1
gene30355	79.55176653	108.4073978	1.362727725	0.446497338	0.494775	1
gene8205	507.2716541	654.3160796	1.289873137	0.367229179	0.49481141	1
gene2977	33.0604744	21.29431028	0.644101776	-0.63463942	0.49482278	1
gene11442	33.0604744	21.29431028	0.644101776	-0.63463942	0.49482278	1
gene15537	33.0604744	21.29431028	0.644101776	-0.63463942	0.49482278	1
gene43219	33.0604744	21.29431028	0.644101776	-0.63463942	0.49482278	1
gene44946	33.0604744	21.29431028	0.644101776	-0.63463942	0.49482278	1
gene69315	33.0604744	21.29431028	0.644101776	-0.63463942	0.49482278	1
gene69669	1857.585405	2465.300377	1.327153179	0.408334895	0.49489771	1
gene47270	100.214563	134.5413241	1.342532662	0.424957187	0.49495795	1
gene49681	100.214563	134.5413241	1.342532662	0.424957187	0.49495795	1
gene1417	460.780362	357.1636588	0.775127779	-0.36749394	0.49497691	1
gene30007	78.51862671	56.13954529	0.71498379	-0.48401756	0.49499591	1
gene24423	466.9792009	362.0032748	0.775202138	-0.36735555	0.49500062	1
gene65526	954.6211984	737.5574743	0.772617951	-0.3721729	0.49504777	1
gene11398	130.175618	96.79231946	0.743551834	-0.42749478	0.49511	1
gene71605	404.9908114	313.6071151	0.774356124	-0.36893088	0.49513949	1
gene13104	24.7953558	37.74900459	1.52242238	0.606368675	0.49514397	1
gene59342	24.7953558	37.74900459	1.52242238	0.606368675	0.49514397	1
gene68635	24.7953558	37.74900459	1.52242238	0.606368675	0.49514397	1
gene1484	488.6751373	630.1179997	1.289441495	0.366746316	0.49514439	1
gene4451	17.56337703	28.06977264	1.598199059	0.676447111	0.49514821	1
gene23551	17.56337703	28.06977264	1.598199059	0.676447111	0.49514821	1
gene23672	17.56337703	28.06977264	1.598199059	0.676447111	0.49514821	1
gene29824	17.56337703	28.06977264	1.598199059	0.676447111	0.49514821	1
gene45181	17.56337703	28.06977264	1.598199059	0.676447111	0.49514821	1
gene64011	17.56337703	28.06977264	1.598199059	0.676447111	0.49514821	1
gene64588	17.56337703	28.06977264	1.598199059	0.676447111	0.49514821	1
gene39436	40.29245318	58.07539168	1.441346632	0.527417333	0.49515853	1
gene59756	40.29245318	58.07539168	1.441346632	0.527417333	0.49515853	1
gene30611	491.7745567	381.3617387	0.775480824	-0.36683699	0.49516363	1
gene61582	180.7994694	236.1732595	1.306271862	0.385455182	0.49517248	1
gene46210	237.6221598	181.9695606	0.765793732	-0.38497224	0.49517917	1
gene64315	1034.172965	1347.349087	1.302827605	0.381646194	0.4951908	1
gene14515	157.0372534	118.0866297	0.75196571	-0.41126122	0.49523479	1
gene25857	143.6064357	107.4394746	0.748152227	-0.41859625	0.49525326	1
gene51792	341.9692821	264.2430321	0.772709848	-0.37200131	0.4954128	1
gene7698	5436.38176	7705.636553	1.417420059	0.503267371	0.49542079	1
gene7952	103.3139825	138.4130168	1.339731694	0.421944104	0.49543382	1
gene50294	103.3139825	138.4130168	1.339731694	0.421944104	0.49543382	1
gene10487	3288.484063	4487.29193	1.364547264	0.448422366	0.49543548	1
gene62649	1959.866248	2605.64924	1.329503604	0.410887688	0.49544294	1
gene65953	894.6990885	692.0650842	0.773517145	-0.37049483	0.49549771	1
gene53284	110.5459613	81.30554835	0.735490898	-0.44322061	0.49553561	1
gene18386	160.1366729	210.0393332	1.311625435	0.391355784	0.49554767	1

gene17315	1230.469532	946.6288844	0.769323303	-0.37833809	0.49556023	1
gene11964	335.7704432	259.4034162	0.772561795	-0.37227776	0.49560922	1
gene3054	1412.302141	1083.106055	0.766908173	-0.38287425	0.49562994	1
gene69976	133.2750374	176.1620214	1.321793074	0.402496341	0.49569395	1
gene40846	254.152397	329.0938862	1.294868316	0.372805387	0.49573695	1
gene40649	945.3229399	730.782012	0.77305012	-0.37136614	0.49575055	1
gene42642	359.5326591	463.6352102	1.289549637	0.366867307	0.49575811	1
gene68911	437.018146	338.7731181	0.775192337	-0.36737378	0.49576627	1
gene72367	186.9983083	243.916645	1.304378886	0.383362993	0.49579781	1
gene22318	456.6478027	588.4973023	1.288733459	0.365953911	0.49583078	1
gene11617	361.5989388	279.7298033	0.773591328	-0.37035647	0.49587184	1
gene24929	106.413402	142.2847096	1.33709389	0.419100774	0.49587233	1
gene43268	106.413402	142.2847096	1.33709389	0.419100774	0.49587233	1
gene10629	464.9129213	599.1444575	1.288724039	0.365943365	0.49588649	1
gene66306	68.18722846	93.88854988	1.376922805	0.461447679	0.49589601	1
gene56361	1413.335281	1084.073978	0.767032418	-0.38264054	0.49592576	1
gene393	177.7000499	134.5413241	0.757125978	-0.40139473	0.49595673	1
gene7047	177.7000499	134.5413241	0.757125978	-0.40139473	0.49595673	1
gene8726	56.82269038	79.36970196	1.396795918	0.482121248	0.49598677	1
gene33231	559.9617852	434.5975144	0.776119953	-0.36564845	0.49606709	1
gene19502	696.3362421	540.1011426	0.775632676	-0.36655451	0.49607921	1
gene39699	123.976779	91.95270349	0.741692954	-0.43110603	0.49611361	1
gene71190	362.6320786	467.506903	1.289204487	0.366481115	0.49612787	1
gene43448	35.12675405	51.29992932	1.460423278	0.54638657	0.49620337	1
gene48564	35.12675405	51.29992932	1.460423278	0.54638657	0.49620337	1
gene51256	35.12675405	51.29992932	1.460423278	0.54638657	0.49620337	1
gene58085	35.12675405	51.29992932	1.460423278	0.54638657	0.49620337	1
gene34177	2398.950674	1810.984297	0.75490685	-0.40562946	0.49620518	1
gene32695	150.8384145	113.2470138	0.750783639	-0.41353088	0.4962422	1
gene38672	626.082734	485.8974437	0.776091429	-0.36570147	0.49626534	1
gene2549	137.4075967	102.5998586	0.746682578	-0.42143302	0.49628295	1
gene2740	2909.321747	2179.763034	0.749234091	-0.41651155	0.49631095	1
gene55606	373.9966167	482.0257509	1.288850566	0.366085002	0.4963123	1
gene34742	104.3471223	76.46593238	0.732803461	-0.44850178	0.49639143	1
gene18562	343.0024219	265.2109553	0.773204323	-0.37107839	0.49640251	1
gene293	48.55757178	68.72254682	1.415279725	0.501087224	0.49642534	1
gene52819	48.55757178	68.72254682	1.415279725	0.501087224	0.49642534	1
gene26670	29.96105493	44.52446695	1.486078079	0.571509918	0.49644217	1
gene28252	29.96105493	44.52446695	1.486078079	0.571509918	0.49644217	1
gene29847	29.96105493	44.52446695	1.486078079	0.571509918	0.49644217	1
gene67951	225.2244819	172.2903286	0.764971584	-0.38652194	0.49644701	1
gene46063	1543.510899	2032.638709	1.316893007	0.397138136	0.49647082	1
gene12718	1229.436392	1607.720426	1.307688984	0.387019456	0.49648983	1
gene52469	238.6552996	182.9374838	0.766534345	-0.38357766	0.4965101	1
gene19917	375.0297565	290.3769584	0.774277116	-0.36907809	0.49651329	1
gene64716	213.8599438	277.7939569	1.298952725	0.377348926	0.49651942	1
gene45720	198.3628464	150.9960184	0.76121119	-0.39363133	0.49655384	1
gene47429	614.7181959	477.186135	0.776268115	-0.36537307	0.49656861	1

gene25964	1129.221829	1473.179102	1.304596727	0.383603914	0.49658363	1
gene26380	184.9320287	140.3488632	0.758921341	-0.39797773	0.49670876	1
gene51358	184.9320287	140.3488632	0.758921341	-0.39797773	0.49670876	1
gene13380	60.95524968	42.58862056	0.698686672	-0.51728247	0.49672265	1
gene32640	60.95524968	42.58862056	0.698686672	-0.51728247	0.49672265	1
gene43050	518.6361922	402.656049	0.776374759	-0.36517488	0.49681684	1
gene44916	1146.785206	1496.409259	1.304873181	0.383909599	0.49689504	1
gene44491	603.3536578	468.4748262	0.776451456	-0.36503236	0.49690219	1
gene48250	603.3536578	468.4748262	0.776451456	-0.36503236	0.49690219	1
gene64238	115.7116604	153.8997879	1.330028343	0.41145699	0.49700743	1
gene4051	380.1954556	489.7691365	1.288203552	0.365360575	0.49704093	1
gene73581	931.8921222	721.10278	0.773804996	-0.36995805	0.49705596	1
gene32465	117.7779401	87.11308752	0.739638403	-0.43510796	0.49713141	1
gene37251	117.7779401	87.11308752	0.739638403	-0.43510796	0.49713141	1
gene30717	266.5500749	344.5806573	1.292742677	0.370435132	0.49716742	1
gene55949	98.14828338	71.6263164	0.729776558	-0.45447329	0.49718294	1
gene11109	1273.861404	1666.763741	1.308434132	0.3878413	0.49718415	1
gene58489	1535.24578	1176.026681	0.766018508	-0.38454885	0.49722559	1
gene71505	281.0140324	362.971198	1.291647947	0.369212901	0.49725019	1
gene13259	144.6395755	108.4073978	0.749500249	-0.41599914	0.49730092	1
gene20319	144.6395755	108.4073978	0.749500249	-0.41599914	0.49730092	1
gene24265	144.6395755	108.4073978	0.749500249	-0.41599914	0.49730092	1
gene33582	49.5907116	33.87731181	0.683138247	-0.54975053	0.49732424	1
gene40571	118.8110799	157.7714807	1.327918919	0.40916706	0.4973377	1
gene47612	118.8110799	157.7714807	1.327918919	0.40916706	0.4973377	1
gene1624	5.165699125	10.64715514	2.061125684	1.04343248	0.49734482	1
gene5591	5.165699125	10.64715514	2.061125684	1.04343248	0.49734482	1
gene6580	5.165699125	10.64715514	2.061125684	1.04343248	0.49734482	1
gene8225	5.165699125	10.64715514	2.061125684	1.04343248	0.49734482	1
gene9891	5.165699125	10.64715514	2.061125684	1.04343248	0.49734482	1
gene11523	5.165699125	10.64715514	2.061125684	1.04343248	0.49734482	1
gene14074	5.165699125	10.64715514	2.061125684	1.04343248	0.49734482	1
gene14576	5.165699125	10.64715514	2.061125684	1.04343248	0.49734482	1
gene15551	5.165699125	10.64715514	2.061125684	1.04343248	0.49734482	1
gene16480	5.165699125	10.64715514	2.061125684	1.04343248	0.49734482	1
gene18837	5.165699125	10.64715514	2.061125684	1.04343248	0.49734482	1
gene20632	5.165699125	10.64715514	2.061125684	1.04343248	0.49734482	1
gene22734	5.165699125	10.64715514	2.061125684	1.04343248	0.49734482	1
gene23661	5.165699125	10.64715514	2.061125684	1.04343248	0.49734482	1
gene23968	5.165699125	10.64715514	2.061125684	1.04343248	0.49734482	1
gene24391	5.165699125	10.64715514	2.061125684	1.04343248	0.49734482	1
gene25269	5.165699125	10.64715514	2.061125684	1.04343248	0.49734482	1
gene25697	5.165699125	10.64715514	2.061125684	1.04343248	0.49734482	1
gene26753	5.165699125	10.64715514	2.061125684	1.04343248	0.49734482	1
gene27488	5.165699125	10.64715514	2.061125684	1.04343248	0.49734482	1
gene28005	5.165699125	10.64715514	2.061125684	1.04343248	0.49734482	1
gene28952	5.165699125	10.64715514	2.061125684	1.04343248	0.49734482	1
gene29587	5.165699125	10.64715514	2.061125684	1.04343248	0.49734482	1

gene32754	5.165699125	10.64715514	2.061125684	1.04343248	0.49734482	1
gene32801	5.165699125	10.64715514	2.061125684	1.04343248	0.49734482	1
gene32985	5.165699125	10.64715514	2.061125684	1.04343248	0.49734482	1
gene39296	5.165699125	10.64715514	2.061125684	1.04343248	0.49734482	1
gene39781	5.165699125	10.64715514	2.061125684	1.04343248	0.49734482	1
gene41458	5.165699125	10.64715514	2.061125684	1.04343248	0.49734482	1
gene43964	5.165699125	10.64715514	2.061125684	1.04343248	0.49734482	1
gene47103	5.165699125	10.64715514	2.061125684	1.04343248	0.49734482	1
gene52733	5.165699125	10.64715514	2.061125684	1.04343248	0.49734482	1
gene54728	5.165699125	10.64715514	2.061125684	1.04343248	0.49734482	1
gene54997	5.165699125	10.64715514	2.061125684	1.04343248	0.49734482	1
gene55881	5.165699125	10.64715514	2.061125684	1.04343248	0.49734482	1
gene56661	5.165699125	10.64715514	2.061125684	1.04343248	0.49734482	1
gene59685	5.165699125	10.64715514	2.061125684	1.04343248	0.49734482	1
gene60740	5.165699125	10.64715514	2.061125684	1.04343248	0.49734482	1
gene61262	5.165699125	10.64715514	2.061125684	1.04343248	0.49734482	1
gene69039	5.165699125	10.64715514	2.061125684	1.04343248	0.49734482	1
gene69249	5.165699125	10.64715514	2.061125684	1.04343248	0.49734482	1
gene72773	5.165699125	10.64715514	2.061125684	1.04343248	0.49734482	1
gene48134	1075.498558	830.478101	0.772179651	-0.37299156	0.4973516	1
gene37559	131.2087578	97.76024266	0.745074066	-0.42454425	0.49735179	1
gene60660	131.2087578	97.76024266	0.745074066	-0.42454425	0.49735179	1
gene43686	318.2070661	245.8524914	0.772617951	-0.3721729	0.49736454	1
gene72277	432.8855867	335.8693485	0.775884804	-0.36608562	0.4974164	1
gene22965	402.9245318	518.8068323	1.287602991	0.364687833	0.49748916	1
gene17067	292.3785705	225.5261044	0.771349637	-0.37454314	0.4975555	1
gene22980	3970.356348	2933.775203	0.738919872	-0.43651017	0.49756739	1
gene36600	369.8640574	286.5052656	0.774623162	-0.36843345	0.49758236	1
gene11822	59.92210985	83.24139474	1.389159943	0.474212716	0.49762409	1
gene39997	59.92210985	83.24139474	1.389159943	0.474212716	0.49762409	1
gene22009	240.7215792	311.6712687	1.294737554	0.37265969	0.49763361	1
gene39132	240.7215792	311.6712687	1.294737554	0.37265969	0.49763361	1
gene3759	1282.126523	1677.410896	1.308303718	0.387697496	0.49763495	1
gene3595	121.9104994	161.6431735	1.325916753	0.4069902	0.49764889	1
gene24297	121.9104994	161.6431735	1.325916753	0.4069902	0.49764889	1
gene56215	154.9709738	203.2638709	1.311625435	0.391355784	0.49766073	1
gene4730	239.6884394	183.905407	0.767268573	-0.38219643	0.49783216	1
gene1268	258.2849563	333.9335022	1.292887929	0.370597224	0.49786638	1
gene35016	165.302372	124.8620921	0.755355719	-0.40477188	0.49792156	1
gene38871	95.04886391	127.7658617	1.344212402	0.42676112	0.49795582	1
gene13550	67.15408863	47.42823654	0.70625985	-0.50172901	0.49803931	1
gene20395	67.15408863	47.42823654	0.70625985	-0.50172901	0.49803931	1
gene27242	67.15408863	47.42823654	0.70625985	-0.50172901	0.49803931	1
gene51491	458.7140823	356.1957356	0.776509267	-0.36492495	0.4980582	1
gene32019	495.907116	385.2334315	0.776825778	-0.36433702	0.49806131	1
gene42407	199.3959862	151.9639416	0.762121367	-0.39190733	0.4980928	1
gene10830	1548.676598	2037.478325	1.315625436	0.395748807	0.49812052	1
gene24009	151.8715543	114.214937	0.752049569	-0.41110034	0.49819623	1

gene47935	191.1308676	248.756261	1.301497053	0.380172045	0.49838879	1
gene34009	85.75060548	61.94708446	0.722409878	-0.46911047	0.49839573	1
gene45882	164.2692322	214.8789492	1.308090056	0.387461868	0.49842566	1
gene51012	125.0099188	92.92062669	0.743306032	-0.42797178	0.49845419	1
gene15335	43.39187265	61.94708446	1.427619521	0.513611534	0.49847662	1
gene22409	43.39187265	61.94708446	1.427619521	0.513611534	0.49847662	1
gene68363	43.39187265	61.94708446	1.427619521	0.513611534	0.49847662	1
gene60126	233.4896005	179.065791	0.766911206	-0.38286855	0.49849449	1
gene67057	726.297297	937.9175756	1.291368672	0.368900933	0.49850625	1
gene21339	546.5309675	703.6801625	1.287539416	0.364616598	0.49855766	1
gene46213	712.8664793	553.6520673	0.776656055	-0.36465226	0.49858635	1
gene26713	338.8698626	262.3071857	0.774064662	-0.36947401	0.49860828	1
gene34525	172.5343508	130.6696313	0.757354293	-0.40095974	0.49865096	1
gene66489	6177.143014	4444.70331	0.719540296	-0.47485261	0.49865753	1
gene43948	79.55176653	57.10746848	0.717865498	-0.47821453	0.49868238	1
gene54090	79.55176653	57.10746848	0.717865498	-0.47821453	0.49868238	1
gene38432	3045.696204	4127.224502	1.355100517	0.43839987	0.49868406	1
gene7744	51.65699125	72.5942396	1.405312966	0.490891457	0.49871266	1
gene45692	51.65699125	72.5942396	1.405312966	0.490891457	0.49871266	1
gene24708	206.627965	157.7714807	0.763553378	-0.38919908	0.49874059	1
gene14701	642.6129712	499.4483684	0.777214888	-0.36361456	0.49889068	1
gene13371	4149.089538	3061.541065	0.737882621	-0.43853676	0.49893967	1
gene51115	193.1971473	147.1243256	0.761524317	-0.39303799	0.49899532	1
gene20616	63.02152933	87.11308752	1.382275049	0.467044715	0.49902164	1
gene49133	546.5309675	424.9182824	0.777482536	-0.36311783	0.49909485	1
gene35121	1064.13402	822.7347154	0.773149528	-0.37118063	0.49914706	1
gene41502	105.3802622	77.43385557	0.734804165	-0.44456829	0.4991505	1
gene6998	200.4291261	260.3713394	1.299069374	0.377478477	0.49922944	1
gene23591	200.4291261	260.3713394	1.299069374	0.377478477	0.49922944	1
gene61351	672.5740261	867.2591824	1.289462793	0.366770146	0.49927755	1
gene73229	1097.194494	847.9007185	0.772789804	-0.37185203	0.49928154	1
gene29738	145.6727153	109.375321	0.75082915	-0.41344343	0.49932506	1
gene13032	15.49709738	25.16600306	1.623917205	0.699478079	0.49932507	1
gene23604	15.49709738	25.16600306	1.623917205	0.699478079	0.49932507	1
gene38029	15.49709738	25.16600306	1.623917205	0.699478079	0.49932507	1
gene52723	15.49709738	25.16600306	1.623917205	0.699478079	0.49932507	1
gene53756	15.49709738	25.16600306	1.623917205	0.699478079	0.49932507	1
gene64180	15.49709738	25.16600306	1.623917205	0.699478079	0.49932507	1
gene66778	15.49709738	25.16600306	1.623917205	0.699478079	0.49932507	1
gene72797	15.49709738	25.16600306	1.623917205	0.699478079	0.49932507	1
gene72474	943.2566603	730.782012	0.774743548	-0.36820926	0.49939814	1
gene65028	176.6669101	230.3657203	1.303955111	0.382894205	0.49941909	1
gene57084	497.9733957	387.1692779	0.777489884	-0.36310419	0.4994952	1
gene47755	485.5757178	377.4900459	0.777407173	-0.36325768	0.49949543	1
gene65616	281.0140324	216.8147956	0.771544374	-0.37417896	0.49950704	1
gene28511	132.2418976	98.72816585	0.746572513	-0.4216457	0.4995649	1
gene56699	179.7663296	234.2374131	1.30301049	0.381848698	0.4996658	1
gene34854	626.082734	486.8653669	0.777637428	-0.36283044	0.49967177	1

gene56410	626.082734	486.8653669	0.777637428	-0.36283044	0.49967177	1
gene60798	166.3355118	125.8300153	0.75648317	-0.40262011	0.49971554	1
gene7273	234.5227403	180.0337142	0.767659946	-0.38146072	0.49983363	1
gene53810	55.78955055	38.71692779	0.693981712	-0.52703045	0.49991754	1
gene32040	44.42501248	30.00561903	0.675421736	-0.56613949	0.49993206	1
gene68400	423.5873283	329.0938862	0.776920989	-0.36416021	0.50000915	1
gene60050	843.0420973	654.3160796	0.7761369	-0.36561695	0.5000165	1
gene12131	11408.96309	7799.525102	0.683631373	-0.54870949	0.50002787	1
gene56806	83.68432583	113.2470138	1.353264338	0.436443673	0.50010711	1
gene52254	307.8756679	238.1091059	0.773393713	-0.37072506	0.50012916	1
gene69537	307.8756679	238.1091059	0.773393713	-0.37072506	0.50012916	1
gene59508	38.22617353	55.17162209	1.443294398	0.529365605	0.50020823	1
gene63053	38.22617353	55.17162209	1.443294398	0.529365605	0.50020823	1
gene63125	38.22617353	55.17162209	1.443294398	0.529365605	0.50020823	1
gene66862	288.2460112	222.6223348	0.772334486	-0.3727023	0.5002102	1
gene25361	66.12094881	90.9847803	1.376035613	0.460517808	0.5002177	1
gene43342	66.12094881	90.9847803	1.376035613	0.460517808	0.5002177	1
gene30448	411.1896504	319.4146542	0.776806162	-0.36437345	0.5002367	1
gene40359	523.8018913	407.4956649	0.777957605	-0.36223656	0.5002612	1
gene39029	22.72907615	34.84523501	1.53306869	0.616422339	0.50037522	1
gene48975	22.72907615	34.84523501	1.53306869	0.616422339	0.50037522	1
gene50593	22.72907615	34.84523501	1.53306869	0.616422339	0.50037522	1
gene64779	22.72907615	34.84523501	1.53306869	0.616422339	0.50037522	1
gene70330	22.72907615	34.84523501	1.53306869	0.616422339	0.50037522	1
gene72675	173.5674906	131.6375545	0.758422871	-0.39892562	0.50037594	1
gene15525	139.4738764	104.535705	0.749500249	-0.41599914	0.50051018	1
gene65813	645.7123907	502.352138	0.777981258	-0.36219269	0.50061199	1
gene57798	54.75641073	76.46593238	1.39647452	0.48178925	0.50062801	1
gene67022	54.75641073	76.46593238	1.39647452	0.48178925	0.50062801	1
gene73917	119.8442197	158.7394039	1.324547853	0.405499966	0.50069362	1
gene73908	112.6122409	83.24139474	0.739186025	-0.43599061	0.50072414	1
gene17395	621.9501747	483.9615973	0.77813564	-0.36190644	0.50073784	1
gene46079	126.0430587	93.88854988	0.744892665	-0.42489554	0.50076315	1
gene604	383.2948751	492.6729061	1.285362623	0.362175426	0.50077186	1
gene60202	4252.40352	5880.133407	1.382778793	0.467570383	0.50078278	1
gene63539	214.8930836	164.5469431	0.765715398	-0.38511983	0.50080271	1
gene48148	886.4339699	1146.988986	1.293936181	0.371766463	0.50082687	1
gene59660	669.4746067	862.4195664	1.288203552	0.365360575	0.50082713	1
gene14921	23.76221598	14.51884792	0.611005637	-0.7107424	0.50085864	1
gene18793	23.76221598	14.51884792	0.611005637	-0.7107424	0.50085864	1
gene36750	23.76221598	14.51884792	0.611005637	-0.7107424	0.50085864	1
gene40726	23.76221598	14.51884792	0.611005637	-0.7107424	0.50085864	1
gene44211	23.76221598	14.51884792	0.611005637	-0.7107424	0.50085864	1
gene58509	23.76221598	14.51884792	0.611005637	-0.7107424	0.50085864	1
gene59552	23.76221598	14.51884792	0.611005637	-0.7107424	0.50085864	1
gene66567	23.76221598	14.51884792	0.611005637	-0.7107424	0.50085864	1
gene2868	195.2634269	253.595877	1.298737203	0.377109534	0.50090574	1
gene7323	195.2634269	253.595877	1.298737203	0.377109534	0.50090574	1

gene14459	512.4373532	398.7843562	0.778210944	-0.36176683	0.50091771	1
gene14464	92.98258426	67.75462362	0.728680797	-0.45664112	0.50098544	1
gene45487	92.98258426	67.75462362	0.728680797	-0.45664112	0.50098544	1
gene63497	92.98258426	67.75462362	0.728680797	-0.45664112	0.50098544	1
gene39718	1107.525892	856.6120273	0.773446502	-0.37062659	0.50099775	1
gene64943	1209.806735	1576.746884	1.303304766	0.382174485	0.50110544	1
gene23950	610.5856366	475.2502886	0.778351569	-0.36150615	0.50113413	1
gene63329	198.3628464	257.4675698	1.29796267	0.376248891	0.50115667	1
gene61905	1216.005574	938.8854988	0.772106246	-0.37312871	0.50116003	1
gene73879	168.4017915	219.7185652	1.304728193	0.38374929	0.501204	1
gene49873	406.0239513	315.5429615	0.777153566	-0.36372839	0.50121706	1
gene55249	406.0239513	315.5429615	0.777153566	-0.36372839	0.50121706	1
gene40722	69.22036828	94.85647307	1.370354932	0.45454961	0.50124369	1
gene56396	691.170543	890.4893391	1.288378604	0.365556607	0.50126292	1
gene55643	2404.116373	1820.663529	0.757310898	-0.40104241	0.50128119	1
gene6529	444.2501248	345.5485805	0.777824386	-0.36248363	0.50132712	1
gene25723	260.3512359	335.8693485	1.290062432	0.367440886	0.50137645	1
gene61701	473.1780399	607.8557662	1.284623788	0.361345917	0.50139101	1
gene7599	171.501211	223.590258	1.303724077	0.382638567	0.50141451	1
gene286	33.0604744	48.39615973	1.463867673	0.549785146	0.5014832	1
gene35428	33.0604744	48.39615973	1.463867673	0.549785146	0.5014832	1
gene40294	33.0604744	48.39615973	1.463867673	0.549785146	0.5014832	1
gene56679	33.0604744	48.39615973	1.463867673	0.549785146	0.5014832	1
gene56986	33.0604744	48.39615973	1.463867673	0.549785146	0.5014832	1
gene49553	61.9883895	43.55654376	0.702656483	-0.50910854	0.50151277	1
gene65226	847.1746566	1094.721133	1.29220241	0.369832071	0.50161709	1
gene1674	501.0728152	390.0730474	0.778475774	-0.36127595	0.50162763	1
gene29126	1233.568951	952.4364235	0.772098246	-0.37314366	0.50164505	1
gene16281	488.6751373	380.3938155	0.778418599	-0.36138191	0.50167372	1
gene1631	86.78374531	62.91500765	0.724963038	-0.46402065	0.50173487	1
gene4184	86.78374531	62.91500765	0.724963038	-0.46402065	0.50173487	1
gene42603	86.78374531	62.91500765	0.724963038	-0.46402065	0.50173487	1
gene59460	86.78374531	62.91500765	0.724963038	-0.46402065	0.50173487	1
gene63634	86.78374531	62.91500765	0.724963038	-0.46402065	0.50173487	1
gene47773	1292.457921	996.9608905	0.771368162	-0.3745085	0.50175727	1
gene65778	2383.453576	1806.144681	0.757784712	-0.40014006	0.50176911	1
gene63274	135.3413171	178.0978678	1.315916467	0.396067911	0.50177864	1
gene41656	27.89477528	41.62069737	1.49206068	0.577306209	0.50184026	1
gene49791	1629.261504	1249.588844	0.766966408	-0.3827647	0.50184359	1
gene65792	575.4588826	448.1484391	0.77876709	-0.36073618	0.50190829	1
gene36152	229.3570412	176.1620214	0.768068948	-0.38069227	0.50193168	1
gene34199	96.08200373	128.7337849	1.339832434	0.422052581	0.50199462	1
gene6000	296.5111298	229.3977971	0.773656615	-0.37023472	0.5020131	1
gene33564	296.5111298	229.3977971	0.773656615	-0.37023472	0.5020131	1
gene11396	153.9378339	116.1507834	0.754530452	-0.40634897	0.50204081	1
gene14192	180.7994694	235.2053363	1.300918289	0.379530348	0.50204857	1
gene64883	407.0570911	316.5108846	0.777558951	-0.36297604	0.50206137	1
gene51304	174.6006304	132.6054777	0.759478802	-0.3969184	0.50208467	1

gene20223	1553.842297	1193.449299	0.768063337	-0.38070281	0.50210373	1
gene33275	550.6635268	707.5518553	1.28490779	0.36166483	0.50212204	1
gene37804	39.25931335	26.13392626	0.665674563	-0.58711105	0.50218536	1
gene45911	39.25931335	26.13392626	0.665674563	-0.58711105	0.50218536	1
gene54629	39.25931335	26.13392626	0.665674563	-0.58711105	0.50218536	1
gene58769	39.25931335	26.13392626	0.665674563	-0.58711105	0.50218536	1
gene61575	39.25931335	26.13392626	0.665674563	-0.58711105	0.50218536	1
gene68723	39.25931335	26.13392626	0.665674563	-0.58711105	0.50218536	1
gene2254	1143.685786	884.6817999	0.773535713	-0.3704602	0.50220639	1
gene69226	215.9262234	165.5148663	0.766534345	-0.38357766	0.50222758	1
gene24275	57.8558302	80.33762515	1.38858305	0.473613466	0.50223659	1
gene39366	57.8558302	80.33762515	1.38858305	0.473613466	0.50223659	1
gene68676	57.8558302	80.33762515	1.38858305	0.473613466	0.50223659	1
gene10821	349.2012609	271.0184945	0.776109725	-0.36566746	0.50224065	1
gene12725	349.2012609	271.0184945	0.776109725	-0.36566746	0.50224065	1
gene6207	950.4886391	737.5574743	0.77597716	-0.36591391	0.50228336	1
gene6458	80.58490636	58.07539168	0.720673316	-0.47258267	0.50228621	1
gene28637	80.58490636	58.07539168	0.720673316	-0.47258267	0.50228621	1
gene18801	1160.216024	897.2648014	0.773360118	-0.37078773	0.50229166	1
gene36010	1347.214332	1038.581588	0.770910436	-0.37536484	0.50234558	1
gene54507	68.18722846	48.39615973	0.709754023	-0.49460897	0.50235265	1
gene53918	495.907116	386.2013547	0.778777602	-0.3607167	0.50236018	1
gene53271	778.9874281	605.9199198	0.777830165	-0.36247291	0.50236832	1
gene29322	899.8647876	698.8405465	0.77660617	-0.36474493	0.5023905	1
gene38955	426.6867478	331.9976558	0.778082885	-0.36200425	0.50245025	1
gene5673	814.1141822	633.0217693	0.777558951	-0.36297604	0.50250091	1
gene30481	74.38606741	53.23577571	0.71566864	-0.48263633	0.50254019	1
gene37221	74.38606741	53.23577571	0.71566864	-0.48263633	0.50254019	1
gene38174	74.38606741	53.23577571	0.71566864	-0.48263633	0.50254019	1
gene39516	74.38606741	53.23577571	0.71566864	-0.48263633	0.50254019	1
gene51602	74.38606741	53.23577571	0.71566864	-0.48263633	0.50254019	1
gene66819	737.6618351	950.5005771	1.28853159	0.365727907	0.50255833	1
gene61282	140.5070162	105.5036282	0.750878006	-0.41334956	0.50258579	1
gene41892	420.4879088	327.1580398	0.77804387	-0.36207659	0.50258783	1
gene5803	202.4954057	154.8677111	0.764796172	-0.38685279	0.50263561	1
gene52211	303.7431086	235.2053363	0.774356124	-0.36893088	0.50267283	1
gene26082	161.1698127	121.9583225	0.756706982	-0.40219334	0.50267647	1
gene42323	161.1698127	121.9583225	0.756706982	-0.40219334	0.50267647	1
gene51780	161.1698127	121.9583225	0.756706982	-0.40219334	0.50267647	1
gene54683	161.1698127	121.9583225	0.756706982	-0.40219334	0.50267647	1
gene28485	150.8384145	197.4563317	1.309058653	0.38852974	0.50268764	1
gene58297	216.9593633	280.6977264	1.293780191	0.371592529	0.50269674	1
gene43578	414.2890699	322.3184238	0.778003687	-0.3621511	0.50273827	1
gene69074	153.9378339	201.3280245	1.307852783	0.387200154	0.50286038	1
gene49090	284.1134519	219.7185652	0.773347984	-0.37081036	0.50294979	1
gene51379	284.1134519	219.7185652	0.773347984	-0.37081036	0.50294979	1
gene62469	220.0587827	284.5694192	1.293151837	0.370891681	0.50296019	1
gene2279	100.214563	73.56216279	0.734046635	-0.44605637	0.50298085	1

gene72016	100.214563	73.56216279	0.734046635	-0.44605637	0.50298085	1
gene61542	2222.283764	2955.069513	1.329744455	0.411149021	0.50298382	1
gene1521	444.2501248	570.1067616	1.283301298	0.359859931	0.50303424	1
gene1068	127.0761985	94.85647307	0.7464535	-0.4218757	0.50304114	1
gene50203	127.0761985	94.85647307	0.7464535	-0.4218757	0.50304114	1
gene15373	13.43081773	22.26223348	1.657548627	0.729051195	0.5031164	1
gene19572	13.43081773	22.26223348	1.657548627	0.729051195	0.5031164	1
gene29394	13.43081773	22.26223348	1.657548627	0.729051195	0.5031164	1
gene40144	13.43081773	22.26223348	1.657548627	0.729051195	0.5031164	1
gene45525	13.43081773	22.26223348	1.657548627	0.729051195	0.5031164	1
gene46412	13.43081773	22.26223348	1.657548627	0.729051195	0.5031164	1
gene51513	13.43081773	22.26223348	1.657548627	0.729051195	0.5031164	1
gene54668	13.43081773	22.26223348	1.657548627	0.729051195	0.5031164	1
gene59183	13.43081773	22.26223348	1.657548627	0.729051195	0.5031164	1
gene59814	13.43081773	22.26223348	1.657548627	0.729051195	0.5031164	1
gene11853	577.5251622	450.0842855	0.779332772	-0.35968861	0.50316118	1
gene44678	278.9477528	359.0995052	1.287336075	0.364388736	0.50316546	1
gene15381	50.62385143	34.84523501	0.688316555	-0.53885589	0.50317928	1
gene21643	50.62385143	34.84523501	0.688316555	-0.53885589	0.50317928	1
gene68985	50.62385143	34.84523501	0.688316555	-0.53885589	0.50317928	1
gene41602	350.2344007	271.9864177	0.77658396	-0.36478619	0.50319728	1
gene74034	583.7240012	454.9239015	0.779347604	-0.35966115	0.5032087	1
gene57078	1058.968321	820.7988691	0.77509294	-0.36755878	0.50323962	1
gene20434	113.6453808	84.20931793	0.7409832	-0.43248726	0.50325923	1
gene68087	643.646111	827.5743314	1.285759857	0.362621213	0.50327805	1
gene22079	772.7885892	995.9929673	1.288829806	0.366061764	0.50335139	1
gene5225	163.2360924	212.9431028	1.304509926	0.383507922	0.50337338	1
gene43146	277.9146129	214.8789492	0.773183342	-0.37111754	0.50347902	1
gene17431	78.51862671	106.4715514	1.356003739	0.439361157	0.50354011	1
gene3819	41.325593	59.04331487	1.428734849	0.514738199	0.50355021	1
gene15326	41.325593	59.04331487	1.428734849	0.514738199	0.50355021	1
gene21827	41.325593	59.04331487	1.428734849	0.514738199	0.50355021	1
gene30648	4055.073813	3005.401519	0.741145946	-0.43217043	0.50355052	1
gene47386	49.5907116	69.69047001	1.405312966	0.490891457	0.50358457	1
gene28305	60.95524968	84.20931793	1.381494102	0.466229403	0.50359062	1
gene68229	60.95524968	84.20931793	1.381494102	0.466229403	0.50359062	1
gene3051	6526.344275	4694.427494	0.719304299	-0.47532587	0.50359449	1
gene64869	1109.592172	859.5157968	0.774623162	-0.36843345	0.50361551	1
gene65388	196.2965668	150.0280952	0.764293017	-0.38780225	0.50364896	1
gene42373	627.1158738	488.8012133	0.779443216	-0.35948417	0.50366638	1
gene16291	34.09361423	22.26223348	0.652973701	-0.61490321	0.50370367	1
gene31923	34.09361423	22.26223348	0.652973701	-0.61490321	0.50370367	1
gene32209	34.09361423	22.26223348	0.652973701	-0.61490321	0.50370367	1
gene47461	34.09361423	22.26223348	0.652973701	-0.61490321	0.50370367	1
gene59982	34.09361423	22.26223348	0.652973701	-0.61490321	0.50370367	1
gene10986	169.4349313	220.6864884	1.302485188	0.381266966	0.50371613	1
gene31392	2558.054207	1935.846389	0.756765194	-0.40208236	0.5037511	1
gene10768	669.4746067	521.7106019	0.77928363	-0.35977958	0.50375849	1

gene7912	28.9279151	18.3905407	0.635736818	-0.65349845	0.50376086	1
gene68614	28.9279151	18.3905407	0.635736818	-0.65349845	0.50376086	1
gene43403	584.757141	455.8918247	0.779625921	-0.35914604	0.50382664	1
gene59709	746.9600935	581.72184	0.778785701	-0.3607017	0.50386327	1
gene26927	931.8921222	724.0065496	0.776920989	-0.36416021	0.50387767	1
gene18670	154.9709738	117.1187066	0.755746084	-0.4040265	0.50393206	1
gene57563	154.9709738	117.1187066	0.755746084	-0.4040265	0.50393206	1
gene64387	479.3768788	373.6183531	0.77938334	-0.359595	0.50395457	1
gene395	700.4688014	901.1364942	1.286476275	0.363424851	0.50403979	1
gene53606	253.1192571	326.1901166	1.28868155	0.365895799	0.50404496	1
gene37560	428.7530274	333.9335022	0.77884815	-0.36058602	0.50406147	1
gene63860	175.6337703	228.4298739	1.300603373	0.37918107	0.50406325	1
gene66569	1617.896966	1242.813382	0.768165964	-0.38051005	0.50408547	1
gene58370	1160.216024	898.2327246	0.77419438	-0.36923226	0.50410352	1
gene38954	615.7513357	480.0899045	0.779681467	-0.35904325	0.50410582	1
gene48136	81.61804618	110.3432442	1.351946651	0.435038223	0.50410607	1
gene48144	81.61804618	110.3432442	1.351946651	0.435038223	0.50410607	1
gene73261	81.61804618	110.3432442	1.351946651	0.435038223	0.50410607	1
gene44972	203.5285455	155.8356343	0.76566967	-0.38520598	0.50412569	1
gene56791	334.7373033	429.7578984	1.283866167	0.36049482	0.50424922	1
gene7736	273.7820536	352.3240428	1.286877785	0.363875047	0.50425014	1
gene27077	273.7820536	352.3240428	1.286877785	0.363875047	0.50425014	1
gene8376	182.865749	139.38094	0.762203643	-0.39175159	0.50426786	1
gene18378	182.865749	139.38094	0.762203643	-0.39175159	0.50426786	1
gene42057	182.865749	139.38094	0.762203643	-0.39175159	0.50426786	1
gene11060	235.5558801	303.9278831	1.290258103	0.367659691	0.50428518	1
gene21444	162.2029525	122.9262457	0.757854551	-0.40000711	0.50448925	1
gene24657	162.2029525	122.9262457	0.757854551	-0.40000711	0.50448925	1
gene66469	826.5118601	1065.683437	1.289374646	0.36667152	0.50451403	1
gene4928	682.9054244	532.3577571	0.779548292	-0.3592897	0.50451909	1
gene27078	107.4465418	79.36970196	0.738690149	-0.43695876	0.5045344	1
gene12056	337.8367228	433.6295912	1.283547826	0.360137053	0.50454609	1
gene16495	652.9443695	509.1276004	0.779741161	-0.3589328	0.50457479	1
gene33135	231.4233208	178.0978678	0.769576148	-0.37786401	0.5046168	1
gene32110	1189.143939	920.4949581	0.774082033	-0.36944163	0.50467799	1
gene45605	616.7844756	481.0578277	0.779944773	-0.35855612	0.50469513	1
gene17423	64.05466916	88.08101071	1.375091182	0.459527286	0.50473228	1
gene40110	378.129176	484.9295205	1.282444073	0.358895912	0.5047946	1
gene38889	338.8698626	263.2751089	0.776920989	-0.36416021	0.50482229	1
gene45717	701.5019412	546.876605	0.779579603	-0.35923175	0.50485619	1
gene61964	429.7861672	334.9014253	0.779228023	-0.35988253	0.50486229	1
gene61487	10317.96743	15523.5522	1.504516495	0.589299924	0.50488279	1
gene50139	297.5442696	382.3296619	1.284950513	0.361712798	0.50488467	1
gene46311	1079.631117	1399.616939	1.296384401	0.374493567	0.50489287	1
gene20337	2002.224981	2647.269937	1.322164074	0.402901219	0.50493456	1
gene19184	191.1308676	247.7883378	1.296432862	0.374547496	0.50496488	1
gene53456	3047.762484	2292.042125	0.752040927	-0.41111692	0.50499228	1
gene11238	87.81688513	63.88293085	0.727456124	-0.45906786	0.50500601	1

gene49734	87.81688513	63.88293085	0.727456124	-0.45906786	0.50500601		1
gene65970	87.81688513	63.88293085	0.727456124	-0.45906786	0.50500601		1
gene70380	87.81688513	63.88293085	0.727456124	-0.45906786	0.50500601		1
gene36672	2816.339163	3782.643845	1.343106645	0.425573862	0.50502437		1
gene48877	423.5873283	330.0618094	0.779206051	-0.35992321	0.50502895		1
gene48684	217.9925031	167.4507127	0.768148951	-0.380542	0.50504561		1
gene4016	952.5549187	1231.198304	1.292522121	0.370188972	0.50504901		1
gene57155	512.4373532	399.7522794	0.780099805	-0.35826938	0.50508577		1
gene6322	56.82269038	39.68485098	0.698397959	-0.51787875	0.50509836		1
gene72263	56.82269038	39.68485098	0.698397959	-0.51787875	0.50509836		1
gene40641	197.3297066	150.9960184	0.765196589	-0.38609765	0.50517454		1
gene63282	922.5938638	1191.513453	1.291482091	0.369027638	0.50527304		1
gene54527	128.1093383	95.82439627	0.747989159	-0.41891073	0.50528882		1
gene66930	941.1903806	731.7499351	0.777472815	-0.36313586	0.50532341		1
gene16615	259.3180961	200.3601013	0.772642189	-0.37212764	0.50534569		1
gene53654	259.3180961	200.3601013	0.772642189	-0.37212764	0.50534569		1
gene36284	395.692553	507.191754	1.281782409	0.358151376	0.50537785		1
gene34123	90.91630461	121.9583225	1.341435104	0.423777262	0.50538885		1
gene6615	791.385106	616.567075	0.779098659	-0.36012206	0.50541388		1
gene22706	176.6669101	134.5413241	0.761553615	-0.39298249	0.5054543		1
gene28710	372.9634769	478.1540582	1.282039899	0.358441161	0.50545447		1
gene59363	1841.055168	1410.264095	0.766008601	-0.3845675	0.50546174		1
gene33288	385.3611548	300.0561903	0.778636317	-0.36097846	0.50546347		1
gene37780	52.69013108	73.56216279	1.396127914	0.481431128	0.50549001		1
gene7702	7.231978776	13.55092472	1.873750621	0.905928957	0.50550201		1
gene26852	7.231978776	13.55092472	1.873750621	0.905928957	0.50550201		1
gene27137	7.231978776	13.55092472	1.873750621	0.905928957	0.50550201		1
gene29973	7.231978776	13.55092472	1.873750621	0.905928957	0.50550201		1
gene33203	7.231978776	13.55092472	1.873750621	0.905928957	0.50550201		1
gene37252	7.231978776	13.55092472	1.873750621	0.905928957	0.50550201		1
gene40897	7.231978776	13.55092472	1.873750621	0.905928957	0.50550201		1
gene41797	7.231978776	13.55092472	1.873750621	0.905928957	0.50550201		1
gene42544	7.231978776	13.55092472	1.873750621	0.905928957	0.50550201		1
gene48014	7.231978776	13.55092472	1.873750621	0.905928957	0.50550201		1
gene48174	7.231978776	13.55092472	1.873750621	0.905928957	0.50550201		1
gene49064	7.231978776	13.55092472	1.873750621	0.905928957	0.50550201		1
gene51116	7.231978776	13.55092472	1.873750621	0.905928957	0.50550201		1
gene54543	7.231978776	13.55092472	1.873750621	0.905928957	0.50550201		1
gene55849	7.231978776	13.55092472	1.873750621	0.905928957	0.50550201		1
gene56182	7.231978776	13.55092472	1.873750621	0.905928957	0.50550201		1
gene56479	7.231978776	13.55092472	1.873750621	0.905928957	0.50550201		1
gene63650	7.231978776	13.55092472	1.873750621	0.905928957	0.50550201		1
gene67801	7.231978776	13.55092472	1.873750621	0.905928957	0.50550201		1
gene72414	7.231978776	13.55092472	1.873750621	0.905928957	0.50550201		1
gene73850	7.231978776	13.55092472	1.873750621	0.905928957	0.50550201		1
gene40366	154.9709738	202.2959477	1.3053796	0.384469398	0.50556275		1
gene58697	981.4828338	762.7234774	0.777113416	-0.36380293	0.50557628		1
gene22668	36.15989388	52.26785251	1.445464765	0.531533442	0.50557969		1

gene23084	36.15989388	52.26785251	1.445464765	0.531533442	0.50557969	1
gene27442	36.15989388	52.26785251	1.445464765	0.531533442	0.50557969	1
gene62910	36.15989388	52.26785251	1.445464765	0.531533442	0.50557969	1
gene13289	3413.493982	2555.317234	0.748592863	-0.4177468	0.5056977	1
gene1016	156.0041136	118.0866297	0.756945615	-0.40173845	0.50580308	1
gene52960	156.0041136	118.0866297	0.756945615	-0.40173845	0.50580308	1
gene42665	101.2477029	74.53008599	0.736116316	-0.44199435	0.50580436	1
gene70156	101.2477029	74.53008599	0.736116316	-0.44199435	0.50580436	1
gene32290	81.61804618	59.04331487	0.72341005	-0.46711445	0.50581025	1
gene60621	81.61804618	59.04331487	0.72341005	-0.46711445	0.50581025	1
gene11587	20.6627965	31.94146542	1.545844263	0.628394981	0.50584779	1
gene12902	20.6627965	31.94146542	1.545844263	0.628394981	0.50584779	1
gene14773	20.6627965	31.94146542	1.545844263	0.628394981	0.50584779	1
gene33867	20.6627965	31.94146542	1.545844263	0.628394981	0.50584779	1
gene38041	20.6627965	31.94146542	1.545844263	0.628394981	0.50584779	1
gene59000	20.6627965	31.94146542	1.545844263	0.628394981	0.50584779	1
gene72940	20.6627965	31.94146542	1.545844263	0.628394981	0.50584779	1
gene62721	266.5500749	206.1676405	0.773466826	-0.37058868	0.50586569	1
gene29425	183.8988889	140.3488632	0.763184944	-0.38989538	0.50588554	1
gene1023	233.4896005	301.0241135	1.289239919	0.366520764	0.50594441	1
gene58784	232.4564606	179.065791	0.7703197	-0.37647077	0.50594542	1
gene117	2.06627965	5.807539168	2.810625932	1.490891457	0.50595729	1
gene326	2.06627965	5.807539168	2.810625932	1.490891457	0.50595729	1
gene1609	2.06627965	5.807539168	2.810625932	1.490891457	0.50595729	1
gene1722	2.06627965	5.807539168	2.810625932	1.490891457	0.50595729	1
gene1779	2.06627965	5.807539168	2.810625932	1.490891457	0.50595729	1
gene2732	2.06627965	5.807539168	2.810625932	1.490891457	0.50595729	1
gene4682	2.06627965	5.807539168	2.810625932	1.490891457	0.50595729	1
gene5704	2.06627965	5.807539168	2.810625932	1.490891457	0.50595729	1
gene9761	2.06627965	5.807539168	2.810625932	1.490891457	0.50595729	1
gene12031	2.06627965	5.807539168	2.810625932	1.490891457	0.50595729	1
gene15417	2.06627965	5.807539168	2.810625932	1.490891457	0.50595729	1
gene16561	2.06627965	5.807539168	2.810625932	1.490891457	0.50595729	1
gene17099	2.06627965	5.807539168	2.810625932	1.490891457	0.50595729	1
gene18357	2.06627965	5.807539168	2.810625932	1.490891457	0.50595729	1
gene18584	2.06627965	5.807539168	2.810625932	1.490891457	0.50595729	1
gene18601	2.06627965	5.807539168	2.810625932	1.490891457	0.50595729	1
gene19023	2.06627965	5.807539168	2.810625932	1.490891457	0.50595729	1
gene19449	2.06627965	5.807539168	2.810625932	1.490891457	0.50595729	1
gene19762	2.06627965	5.807539168	2.810625932	1.490891457	0.50595729	1
gene20412	2.06627965	5.807539168	2.810625932	1.490891457	0.50595729	1
gene21448	2.06627965	5.807539168	2.810625932	1.490891457	0.50595729	1
gene23391	2.06627965	5.807539168	2.810625932	1.490891457	0.50595729	1
gene23954	2.06627965	5.807539168	2.810625932	1.490891457	0.50595729	1
gene25660	2.06627965	5.807539168	2.810625932	1.490891457	0.50595729	1
gene26767	2.06627965	5.807539168	2.810625932	1.490891457	0.50595729	1
gene28583	2.06627965	5.807539168	2.810625932	1.490891457	0.50595729	1
gene28839	2.06627965	5.807539168	2.810625932	1.490891457	0.50595729	1

gene29231	2.06627965	5.807539168	2.810625932	1.490891457	0.50595729	1
gene29845	2.06627965	5.807539168	2.810625932	1.490891457	0.50595729	1
gene29906	2.06627965	5.807539168	2.810625932	1.490891457	0.50595729	1
gene29965	2.06627965	5.807539168	2.810625932	1.490891457	0.50595729	1
gene30328	2.06627965	5.807539168	2.810625932	1.490891457	0.50595729	1
gene31473	2.06627965	5.807539168	2.810625932	1.490891457	0.50595729	1
gene31733	2.06627965	5.807539168	2.810625932	1.490891457	0.50595729	1
gene31831	2.06627965	5.807539168	2.810625932	1.490891457	0.50595729	1
gene32170	2.06627965	5.807539168	2.810625932	1.490891457	0.50595729	1
gene32230	2.06627965	5.807539168	2.810625932	1.490891457	0.50595729	1
gene32934	2.06627965	5.807539168	2.810625932	1.490891457	0.50595729	1
gene33811	2.06627965	5.807539168	2.810625932	1.490891457	0.50595729	1
gene34279	2.06627965	5.807539168	2.810625932	1.490891457	0.50595729	1
gene34827	2.06627965	5.807539168	2.810625932	1.490891457	0.50595729	1
gene35090	2.06627965	5.807539168	2.810625932	1.490891457	0.50595729	1
gene35839	2.06627965	5.807539168	2.810625932	1.490891457	0.50595729	1
gene36131	2.06627965	5.807539168	2.810625932	1.490891457	0.50595729	1
gene36652	2.06627965	5.807539168	2.810625932	1.490891457	0.50595729	1
gene37621	2.06627965	5.807539168	2.810625932	1.490891457	0.50595729	1
gene37784	2.06627965	5.807539168	2.810625932	1.490891457	0.50595729	1
gene38474	2.06627965	5.807539168	2.810625932	1.490891457	0.50595729	1
gene38690	2.06627965	5.807539168	2.810625932	1.490891457	0.50595729	1
gene39702	2.06627965	5.807539168	2.810625932	1.490891457	0.50595729	1
gene40203	2.06627965	5.807539168	2.810625932	1.490891457	0.50595729	1
gene40552	2.06627965	5.807539168	2.810625932	1.490891457	0.50595729	1
gene41908	2.06627965	5.807539168	2.810625932	1.490891457	0.50595729	1
gene42564	2.06627965	5.807539168	2.810625932	1.490891457	0.50595729	1
gene42647	2.06627965	5.807539168	2.810625932	1.490891457	0.50595729	1
gene42717	2.06627965	5.807539168	2.810625932	1.490891457	0.50595729	1
gene43286	2.06627965	5.807539168	2.810625932	1.490891457	0.50595729	1
gene43514	2.06627965	5.807539168	2.810625932	1.490891457	0.50595729	1
gene43715	2.06627965	5.807539168	2.810625932	1.490891457	0.50595729	1
gene45134	2.06627965	5.807539168	2.810625932	1.490891457	0.50595729	1
gene45405	2.06627965	5.807539168	2.810625932	1.490891457	0.50595729	1
gene45623	2.06627965	5.807539168	2.810625932	1.490891457	0.50595729	1
gene47065	2.06627965	5.807539168	2.810625932	1.490891457	0.50595729	1
gene47824	2.06627965	5.807539168	2.810625932	1.490891457	0.50595729	1
gene48264	2.06627965	5.807539168	2.810625932	1.490891457	0.50595729	1
gene49199	2.06627965	5.807539168	2.810625932	1.490891457	0.50595729	1
gene49456	2.06627965	5.807539168	2.810625932	1.490891457	0.50595729	1
gene49504	2.06627965	5.807539168	2.810625932	1.490891457	0.50595729	1
gene49846	2.06627965	5.807539168	2.810625932	1.490891457	0.50595729	1
gene50152	2.06627965	5.807539168	2.810625932	1.490891457	0.50595729	1
gene50621	2.06627965	5.807539168	2.810625932	1.490891457	0.50595729	1
gene51107	2.06627965	5.807539168	2.810625932	1.490891457	0.50595729	1
gene52179	2.06627965	5.807539168	2.810625932	1.490891457	0.50595729	1
gene54151	2.06627965	5.807539168	2.810625932	1.490891457	0.50595729	1
gene54199	2.06627965	5.807539168	2.810625932	1.490891457	0.50595729	1

gene54433	2.06627965	5.807539168	2.810625932	1.490891457	0.50595729	1
gene54453	2.06627965	5.807539168	2.810625932	1.490891457	0.50595729	1
gene54632	2.06627965	5.807539168	2.810625932	1.490891457	0.50595729	1
gene55474	2.06627965	5.807539168	2.810625932	1.490891457	0.50595729	1
gene57698	2.06627965	5.807539168	2.810625932	1.490891457	0.50595729	1
gene58241	2.06627965	5.807539168	2.810625932	1.490891457	0.50595729	1
gene59761	2.06627965	5.807539168	2.810625932	1.490891457	0.50595729	1
gene62060	2.06627965	5.807539168	2.810625932	1.490891457	0.50595729	1
gene63804	2.06627965	5.807539168	2.810625932	1.490891457	0.50595729	1
gene63946	2.06627965	5.807539168	2.810625932	1.490891457	0.50595729	1
gene64161	2.06627965	5.807539168	2.810625932	1.490891457	0.50595729	1
gene66070	2.06627965	5.807539168	2.810625932	1.490891457	0.50595729	1
gene66570	2.06627965	5.807539168	2.810625932	1.490891457	0.50595729	1
gene68829	2.06627965	5.807539168	2.810625932	1.490891457	0.50595729	1
gene68859	2.06627965	5.807539168	2.810625932	1.490891457	0.50595729	1
gene68982	2.06627965	5.807539168	2.810625932	1.490891457	0.50595729	1
gene70063	2.06627965	5.807539168	2.810625932	1.490891457	0.50595729	1
gene71052	2.06627965	5.807539168	2.810625932	1.490891457	0.50595729	1
gene72279	2.06627965	5.807539168	2.810625932	1.490891457	0.50595729	1
gene72399	2.06627965	5.807539168	2.810625932	1.490891457	0.50595729	1
gene73071	2.06627965	5.807539168	2.810625932	1.490891457	0.50595729	1
gene73264	2.06627965	5.807539168	2.810625932	1.490891457	0.50595729	1
gene73957	2.06627965	5.807539168	2.810625932	1.490891457	0.50595729	1
gene74109	2.06627965	5.807539168	2.810625932	1.490891457	0.50595729	1
gene40484	211.7936641	162.6110967	0.767780742	-0.38123372	0.50602413	1
gene68264	211.7936641	162.6110967	0.767780742	-0.38123372	0.50602413	1
gene32841	135.3413171	101.6319354	0.750930593	-0.41324853	0.50603774	1
gene15217	353.3338202	274.8901873	0.777990024	-0.36217644	0.50603974	1
gene29732	488.6751373	381.3617387	0.780399307	-0.3577156	0.50604303	1
gene7386	424.6204681	543.9728354	1.281080109	0.357360693	0.50605714	1
gene33915	253.1192571	195.5204853	0.772444134	-0.3724975	0.50606853	1
gene45039	253.1192571	195.5204853	0.772444134	-0.3724975	0.50606853	1
gene27887	257.2518164	331.0297326	1.286792595	0.363779539	0.50608212	1
gene8429	11.36453808	19.35846389	1.703409656	0.768425433	0.50609397	1
gene18286	11.36453808	19.35846389	1.703409656	0.768425433	0.50609397	1
gene33839	11.36453808	19.35846389	1.703409656	0.768425433	0.50609397	1
gene37193	11.36453808	19.35846389	1.703409656	0.768425433	0.50609397	1
gene38337	11.36453808	19.35846389	1.703409656	0.768425433	0.50609397	1
gene47672	11.36453808	19.35846389	1.703409656	0.768425433	0.50609397	1
gene49860	11.36453808	19.35846389	1.703409656	0.768425433	0.50609397	1
gene50400	11.36453808	19.35846389	1.703409656	0.768425433	0.50609397	1
gene65349	11.36453808	19.35846389	1.703409656	0.768425433	0.50609397	1
gene65638	11.36453808	19.35846389	1.703409656	0.768425433	0.50609397	1
gene71738	11.36453808	19.35846389	1.703409656	0.768425433	0.50609397	1
gene9458	643.646111	502.352138	0.78047879	-0.35756867	0.50610774	1
gene30603	63.02152933	44.52446695	0.706496136	-0.50124642	0.50617036	1
gene25592	277.9146129	357.1636588	1.285156095	0.3619436	0.50617728	1
gene52476	170.4680711	221.6544116	1.300269371	0.37881053	0.50621232	1

gene35159	612.6519163	785.953634	1.28287142	0.359376579	0.50625642	1
gene69065	163.2360924	123.8941689	0.758987593	-0.39785179	0.50628358	1
gene57631	191.1308676	146.1564024	0.764692821	-0.38704776	0.50629439	1
gene33957	366.7646379	285.5373424	0.778530188	-0.36117511	0.5063126	1
gene5759	287.2128714	222.6223348	0.775112667	-0.36752207	0.5063354	1
gene37893	44.42501248	62.91500765	1.416206865	0.502032015	0.50636417	1
gene46182	44.42501248	62.91500765	1.416206865	0.502032015	0.50636417	1
gene11962	674.6403058	866.2912592	1.284078718	0.360733647	0.50639672	1
gene17897	45.4581523	30.97354223	0.681363862	-0.55350266	0.50642413	1
gene61993	45.4581523	30.97354223	0.681363862	-0.55350266	0.50642413	1
gene62502	103.3139825	137.4450936	1.330362941	0.411819886	0.50644804	1
gene64876	70.25350811	95.82439627	1.363980232	0.447822735	0.5065099	1
gene7939	69.22036828	49.36408293	0.713143893	-0.48773489	0.50655068	1
gene19189	69.22036828	49.36408293	0.713143893	-0.48773489	0.50655068	1
gene25658	69.22036828	49.36408293	0.713143893	-0.48773489	0.50655068	1
gene63817	69.22036828	49.36408293	0.713143893	-0.48773489	0.50655068	1
gene49332	260.3512359	201.3280245	0.773293907	-0.37091125	0.50655687	1
gene32080	121.9104994	90.9847803	0.7463244	-0.42212524	0.50672681	1
gene66643	121.9104994	90.9847803	0.7463244	-0.42212524	0.50672681	1
gene71391	246.9204182	190.6808693	0.772236135	-0.37288603	0.50684587	1
gene31495	226.2576217	174.226175	0.770034502	-0.37700501	0.50685174	1
gene25615	411.1896504	526.5502179	1.280553189	0.356767177	0.50685907	1
gene67635	3012.63573	4055.598186	1.346196005	0.428888481	0.50689185	1
gene19218	304.7762484	391.0409706	1.283042798	0.359569295	0.50696263	1
gene46199	1209.806735	1570.939345	1.29850438	0.376850881	0.50699573	1
gene45753	1229.436392	1597.073271	1.299028792	0.377433408	0.50704785	1
gene69496	439.0844257	342.6448109	0.780362024	-0.35778452	0.5070644	1
gene30625	55.78955055	77.43385557	1.387963423	0.472969549	0.50706637	1
gene64117	55.78955055	77.43385557	1.387963423	0.472969549	0.50706637	1
gene54721	115.7116604	152.9318648	1.321663385	0.402354783	0.50708652	1
gene19668	30.99419475	45.49239015	1.46777132	0.553627213	0.50710633	1
gene53790	30.99419475	45.49239015	1.46777132	0.553627213	0.50710633	1
gene2460	108.4796816	80.33762515	0.740577627	-0.43327713	0.50716149	1
gene43552	108.4796816	80.33762515	0.740577627	-0.43327713	0.50716149	1
gene49342	432.8855867	337.8051949	0.780356762	-0.35779425	0.50724573	1
gene67276	2252.244819	1716.127824	0.761963269	-0.39220664	0.50728574	1
gene34904	468.0123408	599.1444575	1.280189442	0.356357316	0.50730291	1
gene3834	121.9104994	160.6752503	1.317977132	0.398325339	0.50731709	1
gene72212	121.9104994	160.6752503	1.317977132	0.398325339	0.50731709	1
gene36725	595.0885392	762.7234774	1.281697474	0.358055775	0.50737794	1
gene9633	1235.635231	957.2760395	0.774723815	-0.36824601	0.50739116	1
gene3320	9.298258426	16.45469431	1.769653365	0.823466796	0.50743935	1
gene8677	9.298258426	16.45469431	1.769653365	0.823466796	0.50743935	1
gene24015	9.298258426	16.45469431	1.769653365	0.823466796	0.50743935	1
gene31225	9.298258426	16.45469431	1.769653365	0.823466796	0.50743935	1
gene37886	9.298258426	16.45469431	1.769653365	0.823466796	0.50743935	1
gene41638	9.298258426	16.45469431	1.769653365	0.823466796	0.50743935	1
gene46485	9.298258426	16.45469431	1.769653365	0.823466796	0.50743935	1

gene63341	9.298258426	16.45469431	1.769653365	0.823466796	0.50743935	1
gene66584	9.298258426	16.45469431	1.769653365	0.823466796	0.50743935	1
gene66683	9.298258426	16.45469431	1.769653365	0.823466796	0.50743935	1
gene17526	459.7472222	588.4973023	1.280045368	0.356194944	0.50745376	1
gene34654	459.7472222	588.4973023	1.280045368	0.356194944	0.50745376	1
gene71751	1298.65676	1689.025975	1.300594604	0.379171343	0.50756058	1
gene46511	25.82849563	38.71692779	1.499000497	0.584000862	0.50757508	1
gene74165	25.82849563	38.71692779	1.499000497	0.584000862	0.50757508	1
gene51875	615.7513357	481.0578277	0.781253405	-0.35613752	0.50758287	1
gene22541	131.2087578	172.2903286	1.313100829	0.392977701	0.50760165	1
gene22930	3579.829494	2677.275556	0.747877954	-0.41912524	0.50762223	1
gene39507	240.7215792	185.8412534	0.772017423	-0.37329469	0.50768046	1
gene62659	201.4622659	260.3713394	1.29240748	0.370061005	0.50770889	1
gene42264	772.7885892	603.0161503	0.780311923	-0.35787715	0.5077185	1
gene45472	76.45234706	103.5677818	1.354671057	0.437942577	0.50777806	1
gene46059	76.45234706	103.5677818	1.354671057	0.437942577	0.50777806	1
gene46803	76.45234706	103.5677818	1.354671057	0.437942577	0.50777806	1
gene64980	76.45234706	103.5677818	1.354671057	0.437942577	0.50777806	1
gene7377	1287.292222	1673.539204	1.300046077	0.378562757	0.50783516	1
gene4448	143.6064357	187.7770998	1.307581369	0.386900726	0.50792701	1
gene18531	143.6064357	187.7770998	1.307581369	0.386900726	0.50792701	1
gene33810	299.6105493	384.2655083	1.282549994	0.359015064	0.50797276	1
gene69412	295.47799	229.3977971	0.776361709	-0.36519913	0.50798656	1
gene57203	2265.675636	1726.774979	0.762145716	-0.39186124	0.50798808	1
gene19413	317.1739263	406.5277417	1.281718666	0.358079629	0.5080088	1
gene30677	423.5873283	542.036989	1.279634571	0.355731874	0.50801486	1
gene45439	423.5873283	542.036989	1.279634571	0.355731874	0.50801486	1
gene27110	207.6611048	268.1147249	1.291116722	0.368619432	0.5080509	1
gene2679	19.62965668	11.61507834	0.591710723	-0.75703606	0.50805509	1
gene8253	19.62965668	11.61507834	0.591710723	-0.75703606	0.50805509	1
gene9585	19.62965668	11.61507834	0.591710723	-0.75703606	0.50805509	1
gene10922	19.62965668	11.61507834	0.591710723	-0.75703606	0.50805509	1
gene12741	19.62965668	11.61507834	0.591710723	-0.75703606	0.50805509	1
gene23841	19.62965668	11.61507834	0.591710723	-0.75703606	0.50805509	1
gene35007	19.62965668	11.61507834	0.591710723	-0.75703606	0.50805509	1
gene42803	19.62965668	11.61507834	0.591710723	-0.75703606	0.50805509	1
gene47742	19.62965668	11.61507834	0.591710723	-0.75703606	0.50805509	1
gene53535	19.62965668	11.61507834	0.591710723	-0.75703606	0.50805509	1
gene55097	19.62965668	11.61507834	0.591710723	-0.75703606	0.50805509	1
gene55222	19.62965668	11.61507834	0.591710723	-0.75703606	0.50805509	1
gene69063	19.62965668	11.61507834	0.591710723	-0.75703606	0.50805509	1
gene18496	247.953558	191.6487925	0.772922131	-0.37160502	0.50810019	1
gene71551	1388.539925	1073.426823	0.773061547	-0.37134482	0.50816405	1
gene15164	832.710699	649.4764636	0.779954508	-0.35853812	0.50818349	1
gene17843	227.2907615	175.1940982	0.770792869	-0.37558487	0.50819732	1
gene64187	88.85002496	64.85085404	0.72989123	-0.45424661	0.50821129	1
gene11847	115.7116604	86.14516432	0.744481274	-0.42569253	0.50821568	1
gene73351	797.583945	622.3746142	0.780324903	-0.35785315	0.50824639	1

gene36853	382.2617353	298.1203439	0.779885394	-0.35866596	0.50837063	1
gene52362	382.2617353	298.1203439	0.779885394	-0.35866596	0.50837063	1
gene56958	58.88897003	81.30554835	1.380658353	0.465356365	0.5083719	1
gene71399	366.7646379	469.4427494	1.27995641	0.356094679	0.50842594	1
gene20646	206.627965	158.7394039	0.768237755	-0.38037523	0.50852547	1
gene41546	206.627965	158.7394039	0.768237755	-0.38037523	0.50852547	1
gene58353	1006.27819	783.0498645	0.778164401	-0.36185311	0.5085325	1
gene56297	2059.047671	2718.896254	1.320462994	0.401043871	0.5085398	1
gene7655	629.1821535	806.2800211	1.281473126	0.357803224	0.5085511	1
gene27902	102.2808427	75.49800918	0.738144184	-0.43802544	0.50857939	1
gene41427	329.5716042	256.4996466	0.778281998	-0.36163511	0.50858633	1
gene17647	2987.840374	2254.29312	0.754489142	-0.40642796	0.50866219	1
gene71370	143.6064357	108.4073978	0.754892337	-0.40565719	0.5086662	1
gene19611	868.8705929	677.5462362	0.779801091	-0.35882192	0.50867055	1
gene21232	82.65118601	111.3111674	1.346758259	0.429490913	0.50868091	1
gene28384	82.65118601	111.3111674	1.346758259	0.429490913	0.50868091	1
gene31256	47.52443195	66.78670043	1.405312966	0.490891457	0.50869554	1
gene58724	47.52443195	66.78670043	1.405312966	0.490891457	0.50869554	1
gene49740	580.6245817	453.9559783	0.781840784	-0.35505325	0.50871566	1
gene45583	691.170543	540.1011426	0.781429631	-0.35581213	0.5087945	1
gene11679	51.65699125	35.8131582	0.69328773	-0.52847387	0.50883385	1
gene23658	51.65699125	35.8131582	0.69328773	-0.52847387	0.50883385	1
gene39643	51.65699125	35.8131582	0.69328773	-0.52847387	0.50883385	1
gene68206	51.65699125	35.8131582	0.69328773	-0.52847387	0.50883385	1
gene69230	51.65699125	35.8131582	0.69328773	-0.52847387	0.50883385	1
gene33286	356.4332397	277.7939569	0.779371635	-0.35961667	0.50884166	1
gene53961	356.4332397	277.7939569	0.779371635	-0.35961667	0.50884166	1
gene28	213.8599438	164.5469431	0.769414506	-0.37816706	0.50886007	1
gene24091	1931.971473	1480.922488	0.766534345	-0.38357766	0.50891214	1
gene19949	39.25931335	56.13954529	1.42996758	0.515982438	0.50894514	1
gene38283	39.25931335	56.13954529	1.42996758	0.515982438	0.50894514	1
gene50218	39.25931335	56.13954529	1.42996758	0.515982438	0.50894514	1
gene73196	1678.852216	1292.177465	0.769679101	-0.37767102	0.5089548	1
gene52510	241.7547191	186.8091766	0.772721944	-0.37197873	0.5089578	1
gene58170	241.7547191	186.8091766	0.772721944	-0.37197873	0.5089578	1
gene25423	1597.23417	2088.778254	1.307747038	0.387083502	0.50897504	1
gene54666	180.7994694	234.2374131	1.295564715	0.373581082	0.50900137	1
gene61229	1560.041136	1203.128531	0.771215901	-0.3747933	0.50900896	1
gene68541	6373.439581	4611.186099	0.723500402	-0.46693428	0.50905552	1
gene32391	898.8316478	700.7763929	0.779652557	-0.35909675	0.5090586	1
gene59072	256.2186766	329.0938862	1.284425829	0.361123582	0.50937407	1
gene19269	569.2600436	445.2446695	0.782146357	-0.3544895	0.50937463	1
gene50795	1686.084195	1297.985004	0.769822176	-0.37740286	0.50945279	1
gene27885	40.29245318	27.10184945	0.672628428	-0.57211834	0.50949928	1
gene50215	40.29245318	27.10184945	0.672628428	-0.57211834	0.50949928	1
gene59638	40.29245318	27.10184945	0.672628428	-0.57211834	0.50949928	1
gene71473	40.29245318	27.10184945	0.672628428	-0.57211834	0.50949928	1
gene73087	40.29245318	27.10184945	0.672628428	-0.57211834	0.50949928	1

gene70022	899.8647876	1158.604064	1.287531282	0.364607485	0.50951167	1
gene27698	3318.445118	4483.420238	1.351060535	0.434092317	0.50954332	1
gene36520	461.8135018	361.0353516	0.781777385	-0.35517024	0.50959261	1
gene41304	4572.676866	3380.955719	0.739382165	-0.43560785	0.50961314	1
gene6748	290.3122908	225.5261044	0.77683967	-0.36431122	0.50966525	1
gene32542	235.5558801	302.9599599	1.286149001	0.363057789	0.5097597	1
gene47287	357.4663795	278.7618801	0.779826848	-0.35877427	0.50976677	1
gene66863	95.04886391	126.7979385	1.334028975	0.415790002	0.50976934	1
gene13067	165.302372	125.8300153	0.76121119	-0.39363133	0.50981806	1
gene32078	941.1903806	733.6857815	0.779529622	-0.35932425	0.50983927	1
gene14640	235.5558801	181.9695606	0.772511221	-0.37237221	0.50987666	1
gene16896	595.0885392	465.5710566	0.782355945	-0.35410296	0.50990664	1
gene25547	306.842528	392.976817	1.280711704	0.356945753	0.50998225	1
gene10564	96.08200373	70.65839321	0.735396749	-0.4434053	0.50998723	1
gene35283	947.3892196	738.5253975	0.779537472	-0.35930972	0.51001374	1
gene47281	265.516935	340.7089645	1.283191087	0.359736026	0.51001774	1
gene45445	341.9692821	437.501284	1.27935843	0.355420512	0.51004458	1
gene33999	101.2477029	134.5413241	1.328833349	0.410160185	0.51008164	1
gene42537	57.8558302	40.65277417	0.702656483	-0.50910854	0.51012199	1
gene42568	57.8558302	40.65277417	0.702656483	-0.50910854	0.51012199	1
gene65669	57.8558302	40.65277417	0.702656483	-0.50910854	0.51012199	1
gene51024	242.7878589	187.7770998	0.773420469	-0.37067515	0.51022666	1
gene59257	807.9153432	631.0859229	0.781128776	-0.35636769	0.51023724	1
gene37469	831.6775592	649.4764636	0.780923396	-0.35674706	0.51029763	1
gene34232	546.5309675	698.8405465	1.278684262	0.354660071	0.51035982	1
gene69588	110.5459613	146.1564024	1.322132448	0.40286671	0.51037731	1
gene30914	760.3909113	975.6665802	1.283111839	0.359646924	0.51039385	1
gene65347	634.3478526	812.0875603	1.280192811	0.356361112	0.51039625	1
gene43334	2668.600168	2024.895323	0.758785579	-0.39823584	0.51040922	1
gene72535	1648.891161	1270.883155	0.770750177	-0.37566478	0.51043108	1
gene18858	113.6453808	150.0280952	1.320142483	0.400693648	0.51044407	1
gene26395	113.6453808	150.0280952	1.320142483	0.400693648	0.51044407	1
gene52996	378.129176	295.2165744	0.780729426	-0.35710545	0.51045345	1
gene67798	677.7397253	868.2271056	1.281062734	0.357341127	0.51047892	1
gene32536	440.1175655	562.3633761	1.277757173	0.35361369	0.51051285	1
gene68246	639.5135517	500.4162916	0.782495211	-0.35384617	0.51053172	1
gene1943	119.8442197	157.7714807	1.316471342	0.396676116	0.51054596	1
gene20955	645.7123907	505.2559076	0.78247826	-0.35387743	0.51055572	1
gene25447	411.1896504	321.3505006	0.781514078	-0.35565623	0.51058387	1
gene59874	411.1896504	321.3505006	0.781514078	-0.35565623	0.51058387	1
gene938	122.9436392	161.6431735	1.314774596	0.394815486	0.51058529	1
gene23465	50.62385143	70.65839321	1.395753014	0.481043671	0.51058975	1
gene27175	50.62385143	70.65839321	1.395753014	0.481043671	0.51058975	1
gene57766	50.62385143	70.65839321	1.395753014	0.481043671	0.51058975	1
gene9032	881.2682708	1133.438061	1.286144184	0.363052386	0.51063469	1
gene13369	70.25350811	50.33200612	0.716434061	-0.48109417	0.51063811	1
gene36695	70.25350811	50.33200612	0.716434061	-0.48109417	0.51063811	1
gene38592	116.7448002	87.11308752	0.746183876	-0.42239691	0.51063881	1

gene23736	129.1424781	169.3865591	1.311625435	0.391355784	0.51064924	1
gene36222	1102.360193	857.5799504	0.777948946	-0.36225262	0.51066698	1
gene69145	358.4995193	279.7298033	0.780279437	-0.35793721	0.5106875	1
gene32369	135.3413171	177.1299446	1.308764747	0.388205793	0.51070257	1
gene51861	450.4489637	352.3240428	0.782161957	-0.35446073	0.51071817	1
gene8887	707.7007802	906.9440334	1.281536009	0.357874016	0.51076809	1
gene73209	394.6594132	504.2879844	1.277780201	0.353639691	0.51081367	1
gene67936	150.8384145	196.4884085	1.302641699	0.381440315	0.51084043	1
gene37146	465.9460611	595.2727647	1.277557242	0.353387934	0.51084981	1
gene15436	695.3031023	543.9728354	0.782353528	-0.35410742	0.5108998	1
gene7751	194.2302871	149.060172	0.767440414	-0.38187335	0.51090492	1
gene24607	194.2302871	149.060172	0.767440414	-0.38187335	0.51090492	1
gene28830	319.2402059	408.4635881	1.27948667	0.355565117	0.51092169	1
gene23029	1871.016223	1437.365944	0.768227408	-0.38039466	0.51092178	1
gene153	257.2518164	199.3921781	0.775085598	-0.36757245	0.51095157	1
gene26401	257.2518164	199.3921781	0.775085598	-0.36757245	0.51095157	1
gene6999	385.3611548	301.0241135	0.781148047	-0.35633209	0.51097834	1
gene39929	301.6768289	386.2013547	1.28018236	0.356349334	0.51103669	1
gene4157	338.8698626	264.2430321	0.779777317	-0.35886591	0.51105579	1
gene4314	463.8797815	362.971198	0.782468244	-0.35389589	0.51107382	1
gene10207	418.4216292	327.1580398	0.781886062	-0.3549697	0.51112117	1
gene28048	489.7082771	383.2975851	0.782705956	-0.35345767	0.51112755	1
gene45110	438.0512858	342.6448109	0.7822025	-0.35438595	0.51115592	1
gene13535	483.5094381	378.4579691	0.782731296	-0.35341097	0.51128073	1
gene37205	230.390181	296.1844976	1.285577781	0.362416899	0.51130509	1
gene53448	34.09361423	49.36408293	1.447898207	0.533960179	0.51130604	1
gene56235	34.09361423	49.36408293	1.447898207	0.533960179	0.51130604	1
gene63513	34.09361423	49.36408293	1.447898207	0.533960179	0.51130604	1
gene14289	103.3139825	76.46593238	0.740131495	-0.43414648	0.51130721	1
gene27403	426.6867478	544.9407586	1.277144794	0.352922097	0.511378	1
gene16887	1053.802622	820.7988691	0.778892415	-0.36050403	0.51140178	1
gene22285	1064.13402	1373.483013	1.290704918	0.368159209	0.51146057	1
gene55202	243.8209987	188.745023	0.774113075	-0.36938378	0.51148715	1
gene20013	18.59651685	29.03769584	1.561458851	0.642894551	0.51153998	1
gene32417	18.59651685	29.03769584	1.561458851	0.642894551	0.51153998	1
gene57774	18.59651685	29.03769584	1.561458851	0.642894551	0.51153998	1
gene59713	18.59651685	29.03769584	1.561458851	0.642894551	0.51153998	1
gene70545	18.59651685	29.03769584	1.561458851	0.642894551	0.51153998	1
gene43590	215.9262234	166.4827895	0.771017002	-0.37516542	0.51165331	1
gene29335	71.28664793	96.79231946	1.357790305	0.44126069	0.51169632	1
gene69024	71.28664793	96.79231946	1.357790305	0.44126069	0.51169632	1
gene4950	271.715774	211.0072564	0.776573451	-0.36480571	0.51171514	1
gene8948	11.36453808	5.807539168	0.511022897	-0.96854016	0.51173116	1
gene11983	11.36453808	5.807539168	0.511022897	-0.96854016	0.51173116	1
gene12828	11.36453808	5.807539168	0.511022897	-0.96854016	0.51173116	1
gene16372	11.36453808	5.807539168	0.511022897	-0.96854016	0.51173116	1
gene21308	11.36453808	5.807539168	0.511022897	-0.96854016	0.51173116	1
gene22636	11.36453808	5.807539168	0.511022897	-0.96854016	0.51173116	1

gene24637	11.36453808	5.807539168	0.511022897	-0.96854016	0.51173116	1
gene24815	11.36453808	5.807539168	0.511022897	-0.96854016	0.51173116	1
gene27251	11.36453808	5.807539168	0.511022897	-0.96854016	0.51173116	1
gene27484	11.36453808	5.807539168	0.511022897	-0.96854016	0.51173116	1
gene35942	11.36453808	5.807539168	0.511022897	-0.96854016	0.51173116	1
gene42024	11.36453808	5.807539168	0.511022897	-0.96854016	0.51173116	1
gene42543	11.36453808	5.807539168	0.511022897	-0.96854016	0.51173116	1
gene42897	11.36453808	5.807539168	0.511022897	-0.96854016	0.51173116	1
gene44839	11.36453808	5.807539168	0.511022897	-0.96854016	0.51173116	1
gene45940	11.36453808	5.807539168	0.511022897	-0.96854016	0.51173116	1
gene52113	11.36453808	5.807539168	0.511022897	-0.96854016	0.51173116	1
gene53637	11.36453808	5.807539168	0.511022897	-0.96854016	0.51173116	1
gene60658	11.36453808	5.807539168	0.511022897	-0.96854016	0.51173116	1
gene60968	11.36453808	5.807539168	0.511022897	-0.96854016	0.51173116	1
gene61556	11.36453808	5.807539168	0.511022897	-0.96854016	0.51173116	1
gene61609	11.36453808	5.807539168	0.511022897	-0.96854016	0.51173116	1
gene33304	42.35873283	60.01123807	1.416738275	0.502573262	0.51173626	1
gene62450	173.5674906	132.6054777	0.763999509	-0.38835638	0.51179254	1
gene1294	197.3297066	254.5638002	1.290042967	0.367419118	0.51180367	1
gene29061	671.5408863	859.5157968	1.279915809	0.356048915	0.5118196	1
gene2441	899.8647876	702.7122393	0.780908698	-0.35677421	0.51184938	1
gene10012	517.6030524	661.0915419	1.27721724	0.353003932	0.51185016	1
gene48309	131.2087578	98.72816585	0.752451037	-0.41033039	0.51185613	1
gene30686	314.0745068	401.6881258	1.278958072	0.354968969	0.51192777	1
gene6919	6209.170349	8781.967145	1.414354358	0.500143624	0.51197172	1
gene18154	1123.02299	1450.916869	1.291974325	0.3695774	0.51200965	1
gene5661	35.12675405	23.23015667	0.661323749	-0.59657138	0.51212148	1
gene11165	35.12675405	23.23015667	0.661323749	-0.59657138	0.51212148	1
gene56788	35.12675405	23.23015667	0.661323749	-0.59657138	0.51212148	1
gene66244	35.12675405	23.23015667	0.661323749	-0.59657138	0.51212148	1
gene18820	53.7232709	74.53008599	1.387296133	0.472275779	0.5121299	1
gene29849	53.7232709	74.53008599	1.387296133	0.472275779	0.5121299	1
gene43465	1519.748683	1175.058758	0.773192813	-0.37109987	0.5121333	1
gene46750	470.0786204	600.1123807	1.276621303	0.352330627	0.51214467	1
gene64374	258.2849563	200.3601013	0.775732757	-0.36636837	0.51215336	1
gene27149	230.390181	178.0978678	0.773027162	-0.37140899	0.51217734	1
gene4480	74.38606741	100.6640122	1.353264338	0.436443673	0.51219567	1
gene51597	74.38606741	100.6640122	1.353264338	0.436443673	0.51219567	1
gene25744	617.8176154	483.9615973	0.783340561	-0.35228843	0.51222302	1
gene24996	1805.928414	1389.937708	0.769652715	-0.37772048	0.51225353	1
gene19685	936.0246815	1204.096454	1.286393914	0.363332487	0.51228414	1
gene3768	599.2210985	469.4427494	0.783421596	-0.3521392	0.51228907	1
gene3278	110.5459613	82.27347154	0.744246742	-0.42614709	0.51229128	1
gene68038	536.1995692	420.0786665	0.783437158	-0.35211054	0.51230079	1
gene54865	1653.02372	2159.436647	1.306355511	0.385547565	0.51241196	1
gene9520	237.6221598	183.905407	0.773940474	-0.36970549	0.51245296	1
gene33038	265.516935	206.1676405	0.776476425	-0.36498597	0.51250305	1
gene51325	401.891392	512.9992932	1.276462506	0.352151161	0.51251073	1

gene15263	145.6727153	110.3432442	0.757473655	-0.40073238	0.51260221	1
gene48467	77.48548688	104.535705	1.349100447	0.431997768	0.51260289	1
gene40770	83.68432583	60.97916126	0.728680797	-0.45664112	0.51262967	1
gene15097	46.49129213	31.94146542	0.687041895	-0.54153002	0.51266666	1
gene34652	46.49129213	31.94146542	0.687041895	-0.54153002	0.51266666	1
gene47028	46.49129213	31.94146542	0.687041895	-0.54153002	0.51266666	1
gene51290	46.49129213	31.94146542	0.687041895	-0.54153002	0.51266666	1
gene48512	485.5757178	380.3938155	0.783387228	-0.35220249	0.51269972	1
gene45840	420.4879088	329.0938862	0.78264768	-0.35356509	0.51272477	1
gene46947	306.842528	239.0770291	0.779152194	-0.36002293	0.51272955	1
gene69369	306.842528	239.0770291	0.779152194	-0.36002293	0.51272955	1
gene16039	2561.153626	1949.397314	0.761140329	-0.39376563	0.51281752	1
gene16928	97.11514356	71.6263164	0.737540138	-0.43920653	0.51287818	1
gene858	600.2542384	470.4106726	0.783685716	-0.35165289	0.51288034	1
gene39517	1421.600399	1101.496595	0.774828564	-0.36805096	0.51291111	1
gene60831	80.58490636	108.4073978	1.345256856	0.42788166	0.51293249	1
gene34111	225.2244819	289.4090352	1.284980357	0.361746306	0.51293394	1
gene59134	340.9361423	266.1788785	0.780729426	-0.35710545	0.5129711	1
gene29217	367.7977777	287.4731888	0.78160665	-0.35548535	0.51299352	1
gene30039	825.4787202	1058.907975	1.282780463	0.359274287	0.51304409	1
gene37934	15.49709738	8.711308752	0.562125186	-0.83103664	0.51308888	1
gene50292	15.49709738	8.711308752	0.562125186	-0.83103664	0.51308888	1
gene57279	15.49709738	8.711308752	0.562125186	-0.83103664	0.51308888	1
gene64782	15.49709738	8.711308752	0.562125186	-0.83103664	0.51308888	1
gene74104	15.49709738	8.711308752	0.562125186	-0.83103664	0.51308888	1
gene66370	2647.937372	2013.280245	0.76032019	-0.39532099	0.51309336	1
gene62508	1087.896236	1403.488632	1.290094208	0.367476421	0.51310401	1
gene24572	569.2600436	446.2125927	0.783846676	-0.35135661	0.51314497	1
gene24740	24.7953558	15.48677111	0.62458354	-0.67903354	0.51324112	1
gene34665	24.7953558	15.48677111	0.62458354	-0.67903354	0.51324112	1
gene39760	24.7953558	15.48677111	0.62458354	-0.67903354	0.51324112	1
gene45103	24.7953558	15.48677111	0.62458354	-0.67903354	0.51324112	1
gene57684	24.7953558	15.48677111	0.62458354	-0.67903354	0.51324112	1
gene60042	24.7953558	15.48677111	0.62458354	-0.67903354	0.51324112	1
gene19390	224.191342	173.2582518	0.772814196	-0.3718065	0.51325462	1
gene10247	833.7438388	1069.55513	1.282834223	0.359334748	0.51326121	1
gene47756	56.82269038	78.40177877	1.379761821	0.464419246	0.51338126	1
gene9193	231.4233208	179.065791	0.773758627	-0.3700445	0.51348545	1
gene5800	903.9973469	706.5839321	0.781621688	-0.3554576	0.51352091	1
gene36199	164.2692322	212.9431028	1.296305461	0.374405715	0.51356042	1
gene4740	89.88316478	120.0224761	1.335316535	0.417181771	0.51356851	1
gene72351	125.0099188	93.88854988	0.751048803	-0.41302144	0.51357623	1
gene2672	266.5500749	207.1355637	0.777098126	-0.36383131	0.51367082	1
gene4742	23.76221598	35.8131582	1.507147239	0.591820366	0.51367366	1
gene22936	23.76221598	35.8131582	1.507147239	0.591820366	0.51367366	1
gene54715	23.76221598	35.8131582	1.507147239	0.591820366	0.51367366	1
gene63173	23.76221598	35.8131582	1.507147239	0.591820366	0.51367366	1
gene64612	240.7215792	308.7674991	1.282674782	0.359155426	0.51369135	1

gene8704	136.3744569	178.0978678	1.305947403	0.385096793	0.51369465	1
gene23534	29.96105493	19.35846389	0.646120904	-0.63012394	0.51374487	1
gene25475	29.96105493	19.35846389	0.646120904	-0.63012394	0.51374487	1
gene49425	29.96105493	19.35846389	0.646120904	-0.63012394	0.51374487	1
gene19327	77.48548688	56.13954529	0.724516907	-0.46490874	0.51375116	1
gene29902	77.48548688	56.13954529	0.724516907	-0.46490874	0.51375116	1
gene29928	77.48548688	56.13954529	0.724516907	-0.46490874	0.51375116	1
gene37534	77.48548688	56.13954529	0.724516907	-0.46490874	0.51375116	1
gene48465	77.48548688	56.13954529	0.724516907	-0.46490874	0.51375116	1
gene24329	96.08200373	127.7658617	1.329758506	0.411164265	0.5137835	1
gene21651	415.3222097	325.2221934	0.783059961	-0.35280531	0.51382944	1
gene54899	196.2965668	150.9960184	0.769223939	-0.37852443	0.51391433	1
gene37759	111.5791011	147.1243256	1.318565252	0.398968968	0.51391576	1
gene52121	586.8234206	749.1725526	1.276657554	0.352371593	0.51393893	1
gene40514	940.1572408	734.6537047	0.781415781	-0.3558377	0.51396228	1
gene19551	377.0960362	481.0578277	1.275690492	0.351278344	0.51398569	1
gene22290	132.2418976	99.69608905	0.753891852	-0.40757052	0.51398865	1
gene53844	468.0123408	366.8428908	0.783831662	-0.35138425	0.51400346	1
gene38800	395.692553	309.7354223	0.782767884	-0.35334353	0.51402765	1
gene48560	45.4581523	63.88293085	1.405312966	0.490891457	0.51406479	1
gene50707	45.4581523	63.88293085	1.405312966	0.490891457	0.51406479	1
gene51911	203.5285455	156.8035575	0.770425382	-0.37627286	0.51407006	1
gene55928	203.5285455	156.8035575	0.770425382	-0.37627286	0.51407006	1
gene29496	394.6594132	503.3200612	1.275327648	0.350867942	0.51415936	1
gene22181	315.1076466	245.8524914	0.780217472	-0.35805179	0.51418064	1
gene6746	380.1954556	484.9295205	1.275474268	0.351033794	0.51421552	1
gene67643	210.7605243	162.6110967	0.771544374	-0.37417896	0.51423273	1
gene34549	9395.373569	6616.722959	0.704253313	-0.50583365	0.51424179	1
gene29982	52.69013108	36.7810814	0.698063957	-0.51856887	0.51429832	1
gene56669	52.69013108	36.7810814	0.698063957	-0.51856887	0.51429832	1
gene60052	139.4738764	105.5036282	0.756440066	-0.40270232	0.51429953	1
gene43274	210.7605243	271.0184945	1.285907289	0.362786632	0.51433818	1
gene54308	210.7605243	271.0184945	1.285907289	0.362786632	0.51433818	1
gene46085	59.92210985	82.27347154	1.373006921	0.457338898	0.51439563	1
gene7494	217.9925031	168.4186359	0.772589119	-0.37222674	0.51440483	1
gene56333	486.6088576	620.4387678	1.275025635	0.350526254	0.51444622	1
gene21095	1576.571373	1219.583225	0.77356677	-0.37040227	0.51449227	1
gene31644	702.5350811	550.7482977	0.783944194	-0.35117714	0.51453324	1
gene13747	146.7058552	111.3111674	0.758737047	-0.39832811	0.51453623	1
gene24669	680.8391447	870.162952	1.27807421	0.353971607	0.51455004	1
gene19407	769.6891697	603.0161503	0.783454119	-0.35207931	0.51460741	1
gene12962	220.0587827	282.6335728	1.284354886	0.361043895	0.51465746	1
gene46304	37.1930337	53.23577571	1.43133728	0.517363669	0.51469448	1
gene56012	37.1930337	53.23577571	1.43133728	0.517363669	0.51469448	1
gene44513	1043.471223	1343.477394	1.287507853	0.364581232	0.5147674	1
gene4642	429.7861672	336.8372717	0.783732231	-0.35156727	0.51479698	1
gene51229	809.9816229	1037.613665	1.281033588	0.357308303	0.51480127	1
gene1906	432.8855867	551.7162209	1.274508179	0.349940632	0.51495231	1

gene51583	168.4017915	128.7337849	0.764444272	-0.38751676	0.51498854	1
gene10801	58.88897003	41.62069737	0.706765585	-0.5006963	0.51499565	1
gene19454	648.8118102	828.5422546	1.277014755	0.352775194	0.51504552	1
gene4402	65.08780898	46.46031334	0.713809761	-0.48638847	0.51508873	1
gene65948	65.08780898	46.46031334	0.713809761	-0.48638847	0.51508873	1
gene19110	63.02152933	86.14516432	1.366916437	0.45092505	0.51521453	1
gene49343	2123.102341	1629.014737	0.767280364	-0.38217426	0.51524592	1
gene65504	322.3396254	411.3673577	1.27619233	0.351845769	0.51525049	1
gene23540	1574.505093	1218.615302	0.773967202	-0.36965566	0.51529385	1
gene29585	118.8110799	89.04893391	0.749500249	-0.41599914	0.51537905	1
gene3958	302.7099687	236.1732595	0.780196504	-0.35809056	0.51546052	1
gene55916	453.5483832	577.8501472	1.274065058	0.349438948	0.51555611	1
gene54933	716.9990386	562.3633761	0.784329331	-0.35046854	0.51561325	1
gene54560	211.7936641	163.5790199	0.772350866	-0.37267171	0.51563204	1
gene29924	784.1531272	614.6312286	0.783815313	-0.35141434	0.51569088	1
gene23552	98.14828338	72.5942396	0.739638403	-0.43510796	0.51571688	1
gene46327	307.8756679	392.976817	1.276414014	0.352096353	0.51576979	1
gene27157	66.12094881	90.0168571	1.361396936	0.445087768	0.51587156	1
gene41333	869.9037327	1115.04752	1.281805651	0.358177535	0.51587413	1
gene3978	48.55757178	67.75462362	1.395346207	0.480623122	0.51594626	1
gene49579	48.55757178	67.75462362	1.395346207	0.480623122	0.51594626	1
gene54335	48.55757178	67.75462362	1.395346207	0.480623122	0.51594626	1
gene51370	1197.409057	1545.773342	1.290931727	0.368412703	0.51597735	1
gene28827	404.9908114	515.9030627	1.273863624	0.349210835	0.51602877	1
gene42830	680.8391447	534.2936034	0.784757468	-0.34968124	0.51602924	1
gene46360	177.7000499	229.3977971	1.290927027	0.368407451	0.51603199	1
gene34068	11669.31432	8069.575674	0.69152098	-0.53215507	0.51604489	1
gene37067	705.6345005	553.6520673	0.784615926	-0.34994147	0.51607095	1
gene13357	580.6245817	455.8918247	0.78517486	-0.34891411	0.51611311	1
gene65217	296.5111298	231.3336435	0.780185363	-0.35811116	0.51615644	1
gene15719	159.1035331	206.1676405	1.29580806	0.373852036	0.5161864	1
gene66700	508.3047939	647.5406172	1.273921916	0.349276852	0.51619614	1
gene28714	317.1739263	247.7883378	0.781238044	-0.35616589	0.51620045	1
gene66818	490.7414169	385.2334315	0.785002892	-0.34923013	0.51620337	1
gene27387	351.2675405	274.8901873	0.782566436	-0.35371486	0.51621133	1
gene42519	351.2675405	274.8901873	0.782566436	-0.35371486	0.51621133	1
gene249	140.5070162	106.4715514	0.757766795	-0.40017417	0.51630248	1
gene64191	140.5070162	106.4715514	0.757766795	-0.40017417	0.51630248	1
gene5973	1175.713121	916.6232653	0.779631739	-0.35913527	0.51637893	1
gene32361	69.22036828	93.88854988	1.356371719	0.439752608	0.51639411	1
gene54169	69.22036828	93.88854988	1.356371719	0.439752608	0.51639411	1
gene13892	211.7936641	271.9864177	1.284204694	0.360875177	0.51641187	1
gene12758	147.738995	112.2790906	0.75998277	-0.39596139	0.51644811	1
gene28033	1823.491791	2383.994828	1.307378975	0.386677401	0.51651152	1
gene41441	11987.52139	18170.82213	1.515811446	0.600090306	0.51652783	1
gene13238	659.1432084	841.1252561	1.276088785	0.35172871	0.51657914	1
gene69934	162.2029525	123.8941689	0.763821909	-0.38869179	0.51662281	1
gene57436	21315.74087	13784.19421	0.646667376	-0.62890427	0.51662397	1

gene3869	105.3802622	78.40177877	0.743989217	-0.42664638	0.5166262	1
gene58936	105.3802622	78.40177877	0.743989217	-0.42664638	0.5166262	1
gene58139	1268.695705	1639.661892	1.292399655	0.370052271	0.51663072	1
gene10013	137.4075967	179.065791	1.303172425	0.382027982	0.51666291	1
gene65140	3780.25862	2836.982883	0.750473226	-0.41412749	0.51669994	1
gene55512	1051.736342	821.7667922	0.781342965	-0.35597215	0.51671726	1
gene70949	176.6669101	135.5092472	0.767032418	-0.38264054	0.51672473	1
gene68450	584.757141	745.3008599	1.274547684	0.349985349	0.51674368	1
gene42880	183.8988889	141.3167864	0.768448289	-0.37997992	0.51676955	1
gene56836	653.9775093	834.3497938	1.275808085	0.351411326	0.51681075	1
gene9304	131.2087578	171.3224055	1.305723858	0.38484982	0.51684399	1
gene58921	988.7148126	773.3706325	0.782197882	-0.35439446	0.51692702	1
gene409	205.5948252	158.7394039	0.772098246	-0.37314366	0.51694064	1
gene28247	8561.62973	12449.42813	1.454095601	0.540122124	0.51695251	1
gene3287	601.2873782	472.346519	0.78555868	-0.34820905	0.51704623	1
gene57400	601.2873782	472.346519	0.78555868	-0.34820905	0.51704623	1
gene46989	233.4896005	299.0882671	1.280948987	0.357213022	0.51706248	1
gene5958	689.1042633	541.0690658	0.785177359	-0.34890952	0.51707518	1
gene43128	305.8093882	390.0730474	1.275543075	0.35111162	0.51709286	1
gene41460	75.41920723	101.6319354	1.347560378	0.430349915	0.51712172	1
gene10623	861.6386141	1103.432442	1.280620928	0.356843492	0.51713495	1
gene6610	78.51862671	57.10746848	0.727311096	-0.45935551	0.51731388	1
gene8168	78.51862671	57.10746848	0.727311096	-0.45935551	0.51731388	1
gene34907	78.51862671	57.10746848	0.727311096	-0.45935551	0.51731388	1
gene66415	115.7116604	151.9639416	1.313298427	0.393194783	0.51732753	1
gene71077	115.7116604	151.9639416	1.313298427	0.393194783	0.51732753	1
gene32690	399.8251123	313.6071151	0.784360725	-0.3504108	0.51735051	1
gene72979	78.51862671	105.5036282	1.343676432	0.426185768	0.5173608	1
gene12069	16.5302372	26.13392626	1.580977087	0.660816459	0.51740068	1
gene25797	16.5302372	26.13392626	1.580977087	0.660816459	0.51740068	1
gene34011	16.5302372	26.13392626	1.580977087	0.660816459	0.51740068	1
gene73921	16.5302372	26.13392626	1.580977087	0.660816459	0.51740068	1
gene7450	32.02733458	46.46031334	1.450645642	0.536695147	0.51742558	1
gene69999	51.65699125	71.6263164	1.38657546	0.471526133	0.51744562	1
gene40286	40.29245318	57.10746848	1.417324188	0.503169787	0.51745527	1
gene8845	109.5128215	144.220556	1.316928503	0.397177023	0.51750709	1
gene13472	1173.646841	915.6553421	0.780179616	-0.35812179	0.51751728	1
gene61929	570.2931834	448.1484391	0.785821139	-0.34772712	0.51753228	1
gene25958	315.1076466	401.6881258	1.274764767	0.35023105	0.51759946	1
gene57695	315.1076466	401.6881258	1.274764767	0.35023105	0.51759946	1
gene66209	714.932759	561.3954529	0.785242312	-0.34879018	0.51760683	1
gene56108	84.71746566	113.2470138	1.336761114	0.418741672	0.51765443	1
gene5462	103.3139825	136.4771704	1.320994188	0.401624119	0.51765626	1
gene61430	2446.475106	1870.995535	0.764771949	-0.38689849	0.51769009	1
gene280	2080.743608	1599.977041	0.76894483	-0.379048	0.51769969	1
gene20787	97.11514356	128.7337849	1.325578897	0.40662254	0.51775265	1
gene67037	926.7264231	725.942396	0.783340561	-0.35228843	0.51786945	1
gene50601	2567.352465	3401.282106	1.324820862	0.405797296	0.51793957	1

gene21576	621.9501747	488.8012133	0.785916997	-0.34755114	0.51797706	1
gene5844	447.3495443	351.3561197	0.785417408	-0.34846852	0.51800431	1
gene26125	447.3495443	351.3561197	0.785417408	-0.34846852	0.51800431	1
gene71405	447.3495443	351.3561197	0.785417408	-0.34846852	0.51800431	1
gene71550	263.4506554	205.1997173	0.778892415	-0.36050403	0.51805209	1
gene14503	2027.020337	1560.29219	0.769746688	-0.37754434	0.51806134	1
gene67669	697.3693819	547.8445282	0.785587298	-0.34815649	0.51810455	1
gene71868	2689.262965	2050.061326	0.762313449	-0.39154377	0.51810823	1
gene61938	368.8309176	469.4427494	1.272785786	0.34798963	0.51816876	1
gene5674	134.3081773	101.6319354	0.756706982	-0.40219334	0.51817219	1
gene61127	319.2402059	249.7241842	0.782245405	-0.35430682	0.5181988	1
gene4401	427.7198876	335.8693485	0.785255393	-0.34876615	0.5182357	1
gene46302	3503.377147	2641.462398	0.753976032	-0.40740943	0.51826835	1
gene10379	327.5053246	417.1748969	1.273795769	0.349133986	0.51828033	1
gene5022	441.1507053	346.5165037	0.785483282	-0.34834753	0.51832228	1
gene48340	188.0314482	241.9807987	1.286916636	0.363918601	0.5183366	1
gene50122	188.0314482	241.9807987	1.286916636	0.363918601	0.5183366	1
gene68955	177.7000499	136.4771704	0.768019877	-0.38078445	0.51833806	1
gene26126	148.7721348	113.2470138	0.76121119	-0.39363133	0.51833828	1
gene70503	148.7721348	113.2470138	0.76121119	-0.39363133	0.51833828	1
gene11354	163.2360924	124.8620921	0.764917184	-0.38662454	0.51836127	1
gene44539	72.31978776	52.26785251	0.722732383	-0.46846656	0.51849875	1
gene24370	99.18142321	73.56216279	0.741692954	-0.43110603	0.5185048	1
gene36251	99.18142321	73.56216279	0.741692954	-0.43110603	0.5185048	1
gene63272	392.5931335	499.4483684	1.272178054	0.347300604	0.51851455	1
gene7307	829.6112795	1060.843821	1.278723961	0.354704862	0.51851828	1
gene57780	2716.1246	2070.387713	0.762258003	-0.3916487	0.51858875	1
gene55070	698.4025218	548.8124514	0.785811096	-0.34774555	0.51861708	1
gene8105	235.5558801	182.9374838	0.776620323	-0.36471863	0.51862774	1
gene46455	47.52443195	32.90938862	0.692473056	-0.53017016	0.51867413	1
gene57466	47.52443195	32.90938862	0.692473056	-0.53017016	0.51867413	1
gene70624	47.52443195	32.90938862	0.692473056	-0.53017016	0.51867413	1
gene66588	952.5549187	746.2687831	0.783439116	-0.35210693	0.51873326	1
gene64785	520.7024718	409.4315113	0.786306064	-0.34683711	0.51876855	1
gene49229	604.3867977	475.2502886	0.786334663	-0.34678464	0.51878906	1
gene2019	1444.329475	1870.995535	1.295407708	0.373406233	0.51884307	1
gene65142	725.2641572	925.3345741	1.275858685	0.351468544	0.51887257	1
gene67767	1620.996386	1256.364307	0.775056822	-0.36762601	0.51888219	1
gene67868	157.0372534	203.2638709	1.294367206	0.372246961	0.51888908	1
gene2938	485.5757178	617.5349982	1.271758401	0.346824624	0.51890314	1
gene15230	869.9037327	682.3858522	0.784438354	-0.35026802	0.51893029	1
gene52591	234.5227403	300.0561903	1.279433244	0.355504875	0.51896251	1
gene16353	1914.408096	1477.050795	0.771544374	-0.37417896	0.51903788	1
gene62773	363.6652184	462.667287	1.272234087	0.347364146	0.51906808	1
gene33721	724.2310174	569.1388384	0.785852614	-0.34766933	0.51911334	1
gene64288	85.75060548	62.91500765	0.733697532	-0.44674266	0.51916097	1
gene68709	85.75060548	62.91500765	0.733697532	-0.44674266	0.51916097	1
gene11075	749.0263732	588.4973023	0.785683019	-0.34798071	0.51916534	1

gene5280	340.9361423	267.1468017	0.783568442	-0.3518688	0.51919317	1
gene67520	319.2402059	406.5277417	1.273422752	0.348711446	0.51921569	1
gene47574	106.413402	79.36970196	0.745861898	-0.42301957	0.51921972	1
gene21087	1467.058552	1140.213523	0.777210645	-0.36362243	0.51931716	1
gene26080	66.12094881	47.42823654	0.71729516	-0.4793612	0.51935804	1
gene40743	322.3396254	410.3994345	1.273189525	0.348447192	0.51937314	1
gene13599	449.4158239	353.291966	0.786113766	-0.34718998	0.51949397	1
gene11958	26.86163545	39.68485098	1.477380298	0.563041243	0.51952837	1
gene58344	26.86163545	39.68485098	1.477380298	0.563041243	0.51952837	1
gene63507	26.86163545	39.68485098	1.477380298	0.563041243	0.51952837	1
gene24222	454.581523	577.8501472	1.271169456	0.346156364	0.51953117	1
gene2043	53.7232709	37.74900459	0.702656483	-0.50910854	0.51958235	1
gene25883	53.7232709	37.74900459	0.702656483	-0.50910854	0.51958235	1
gene29232	53.7232709	37.74900459	0.702656483	-0.50910854	0.51958235	1
gene63174	138.4407366	180.0337142	1.300438864	0.378998577	0.51960759	1
gene28464	113.6453808	85.17724113	0.749500249	-0.41599914	0.51969002	1
gene10736	411.1896504	522.6785251	1.271137356	0.346119933	0.51971409	1
gene46981	43.39187265	60.97916126	1.405312966	0.490891457	0.51972362	1
gene54830	59.92210985	42.58862056	0.710732994	-0.49262042	0.51972609	1
gene22514	1220.138133	952.4364235	0.780597211	-0.35734979	0.51972743	1
gene18352	207.6611048	160.6752503	0.773737819	-0.3700833	0.51976589	1
gene11123	781.0537078	613.6633054	0.785686438	-0.34797444	0.51977799	1
gene5677	262.4175156	334.9014253	1.276215974	0.351872498	0.5198174	1
gene18074	193.1971473	149.060172	0.771544374	-0.37417896	0.51982123	1
gene25673	193.1971473	149.060172	0.771544374	-0.37417896	0.51982123	1
gene51567	193.1971473	149.060172	0.771544374	-0.37417896	0.51982123	1
gene44445	286.1797315	223.590258	0.781293129	-0.35606417	0.51985551	1
gene48503	132.2418976	172.2903286	1.302842229	0.381662388	0.51990279	1
gene60821	178.7331897	137.4450936	0.76899592	-0.37895215	0.51993639	1
gene14089	650.8780898	512.03137	0.78667784	-0.34615515	0.51993689	1
gene9956	4123.261042	3087.674991	0.748842957	-0.4172649	0.51993713	1
gene20255	548.5972471	431.6937448	0.786904687	-0.34573919	0.51995929	1
gene48268	1304.855599	1017.287278	0.779616747	-0.35916301	0.51997325	1
gene11347	171.501211	131.6375545	0.767560496	-0.38164763	0.52000794	1
gene37465	171.501211	131.6375545	0.767560496	-0.38164763	0.52000794	1
gene35217	1130.254969	883.7138767	0.78187126	-0.35499702	0.52001433	1
gene11394	129.1424781	168.4186359	1.304130433	0.383088168	0.52005881	1
gene46671	129.1424781	168.4186359	1.304130433	0.383088168	0.52005881	1
gene42577	36.15989388	24.19807987	0.669196651	-0.57949787	0.52011244	1
gene49611	36.15989388	24.19807987	0.669196651	-0.57949787	0.52011244	1
gene72919	36.15989388	24.19807987	0.669196651	-0.57949787	0.52011244	1
gene4005	21.69593633	32.90938862	1.516845741	0.601074375	0.5201632	1
gene33297	21.69593633	32.90938862	1.516845741	0.601074375	0.5201632	1
gene38944	21.69593633	32.90938862	1.516845741	0.601074375	0.5201632	1
gene44770	21.69593633	32.90938862	1.516845741	0.601074375	0.5201632	1
gene56213	21.69593633	32.90938862	1.516845741	0.601074375	0.5201632	1
gene38790	135.3413171	102.5998586	0.758082312	-0.39957359	0.5202243	1
gene44241	135.3413171	102.5998586	0.758082312	-0.39957359	0.5202243	1

gene35969	4228.641304	3163.173	0.748035308	-0.41882173	0.52028216	1
gene31710	2127.2349	2790.52257	1.311807441	0.391555964	0.52034483	1
gene59945	452.5152434	574.9463776	1.270556928	0.345461018	0.52037023	1
gene44237	122.9436392	160.6752503	1.306901694	0.386150625	0.52038412	1
gene38942	581.6577215	457.8276711	0.787108387	-0.34536578	0.52041707	1
gene16388	204.5616854	262.3071857	1.282288935	0.358721378	0.52049416	1
gene72641	595.0885392	756.9159382	1.271938356	0.347028753	0.52052601	1
gene49494	444.2501248	349.4202733	0.786539505	-0.34640887	0.52057695	1
gene36403	727.3304369	572.042608	0.786496177	-0.34648834	0.52059508	1
gene9508	189.064588	242.9487219	1.285003841	0.361772672	0.52060866	1
gene72066	189.064588	242.9487219	1.285003841	0.361772672	0.52060866	1
gene19892	4484.859981	3345.142561	0.74587447	-0.42299525	0.52066336	1
gene15160	229.3570412	293.280728	1.278708194	0.354687074	0.52073426	1
gene18495	179.7663296	231.3336435	1.286857467	0.363852269	0.52078419	1
gene64426	79.55176653	58.07539168	0.73003271	-0.45396699	0.52079467	1
gene35805	35.12675405	50.33200612	1.432868122	0.518905834	0.52083648	1
gene63844	35.12675405	50.33200612	1.432868122	0.518905834	0.52083648	1
gene67341	377.0960362	296.1844976	0.785435192	-0.34843585	0.52087214	1
gene6171	64.05466916	87.11308752	1.35998029	0.443585743	0.52087691	1
gene7485	113.6453808	149.060172	1.311625435	0.391355784	0.52089034	1
gene48045	424.6204681	333.9335022	0.786428181	-0.34661308	0.52093928	1
gene33628	1560.041136	1211.83984	0.776799927	-0.36438503	0.52097897	1
gene11988	241.7547191	308.7674991	1.277193265	0.352976851	0.52098431	1
gene43409	497.9733957	392.0088938	0.787208508	-0.34518228	0.52100568	1
gene62466	244.8541385	312.6391919	1.276838504	0.352576062	0.52105434	1
gene55884	215.9262234	167.4507127	0.775499659	-0.36680195	0.5211232	1
gene31632	516.5699125	656.251926	1.270402921	0.345286135	0.52112445	1
gene19981	640.5466916	504.2879844	0.787277479	-0.34505589	0.52116662	1
gene64988	2035.285455	2663.724632	1.308772008	0.388213797	0.52118718	1
gene66238	244.8541385	190.6808693	0.778752895	-0.36076247	0.521201	1
gene45655	100.214563	74.53008599	0.743705144	-0.42719734	0.52124336	1
gene23012	491.7745567	387.1692779	0.787290177	-0.34503262	0.52127012	1
gene54202	350.2344007	274.8901873	0.784874892	-0.34946539	0.52135902	1
gene48606	104.3471223	137.4450936	1.317191031	0.397464593	0.52138391	1
gene24704	1100.293914	861.4516432	0.782928663	-0.35304723	0.52151163	1
gene28725	101.2477029	133.5734009	1.319273397	0.399743569	0.52153609	1
gene15250	46.49129213	64.85085404	1.39490324	0.480165051	0.52158098	1
gene19960	46.49129213	64.85085404	1.39490324	0.480165051	0.52158098	1
gene73425	518.6361922	408.4635881	0.787572472	-0.34451541	0.52160246	1
gene3789	273.7820536	213.911026	0.781318655	-0.35601703	0.5216527	1
gene42896	357.4663795	280.6977264	0.785242312	-0.34879018	0.52166099	1
gene14267	154.9709738	200.3601013	1.292887929	0.370597224	0.52166723	1
gene35025	1342.048633	1731.614595	1.290277083	0.367680913	0.52177644	1
gene27670	412.2227902	324.2542702	0.786599572	-0.34629869	0.52177782	1
gene71481	2519.828033	1930.03885	0.765940701	-0.38469539	0.52178496	1
gene13964	95.04886391	125.8300153	1.323845548	0.404734814	0.52180455	1
gene20105	73.35292758	98.72816585	1.345933545	0.428607179	0.52183647	1
gene31267	398.7919725	313.6071151	0.786392748	-0.34667808	0.52188901	1

gene40909	158.0703932	120.9903993	0.765421005	-0.3856746	0.52192349	1
gene63097	158.0703932	120.9903993	0.765421005	-0.3856746	0.52192349	1
gene73868	371.930337	472.346519	1.269986532	0.344813198	0.52195684	1
gene32846	76.45234706	102.5998586	1.34201058	0.424396046	0.52197703	1
gene60598	344.0355618	270.0505713	0.784949585	-0.3493281	0.52198554	1
gene43642	1671.620237	2171.051726	1.298770904	0.377146969	0.52204568	1
gene45397	79.55176653	106.4715514	1.338393301	0.420502129	0.52205307	1
gene23904	82.65118601	110.3432442	1.335047318	0.416890876	0.52207594	1
gene52855	2062.147091	2698.569867	1.308621426	0.388047797	0.52213803	1
gene68524	844.0752371	1077.298516	1.276306268	0.351974566	0.52221415	1
gene8102	121.9104994	91.95270349	0.754264021	-0.40685848	0.52223625	1
gene35350	486.6088576	383.2975851	0.787691344	-0.34429767	0.5222385	1
gene73195	486.6088576	383.2975851	0.787691344	-0.34429767	0.5222385	1
gene6042	136.3744569	103.5677818	0.759436805	-0.39699818	0.52225066	1
gene939	519.669332	409.4315113	0.787869297	-0.34397178	0.52225509	1
gene41878	5577.921916	4111.737731	0.737145086	-0.43997949	0.52226499	1
gene15082	73.35292758	53.23577571	0.72574848	-0.46245845	0.52228018	1
gene20109	73.35292758	53.23577571	0.72574848	-0.46245845	0.52228018	1
gene22496	73.35292758	53.23577571	0.72574848	-0.46245845	0.52228018	1
gene38490	73.35292758	53.23577571	0.72574848	-0.46245845	0.52228018	1
gene71398	3832.948751	2885.379043	0.7527831	-0.40969385	0.52228756	1
gene6695	1741.873745	1350.252857	0.775172633	-0.36741046	0.52232057	1
gene39908	86.78374531	63.88293085	0.736116316	-0.44199435	0.52232443	1
gene30936	1189.143939	930.17419	0.782221697	-0.35435054	0.52241017	1
gene32016	224.191342	174.226175	0.777131594	-0.36376918	0.52241861	1
gene51104	453.5483832	357.1636588	0.787487448	-0.34467117	0.52243913	1
gene60983	2621.075736	2005.536859	0.76515792	-0.38617056	0.52244402	1
gene50150	253.1192571	197.4563317	0.780092095	-0.35828364	0.52246817	1
gene51032	253.1192571	197.4563317	0.780092095	-0.35828364	0.52246817	1
gene13723	1396.805044	1088.913594	0.7795745	-0.35924119	0.52246824	1
gene70647	216.9593633	168.4186359	0.776268115	-0.36537307	0.5224701	1
gene36482	202.4954057	259.4034162	1.281033588	0.357308303	0.52264371	1
gene11636	202.4954057	156.8035575	0.774356124	-0.36893088	0.52264846	1
gene44362	195.2634269	150.9960184	0.773293907	-0.37091125	0.52277375	1
gene61963	474.2111797	373.6183531	0.787873355	-0.34396435	0.52285314	1
gene56519	4078.836029	5527.809365	1.355241869	0.438550351	0.52286674	1
gene13950	188.0314482	145.1884792	0.772149981	-0.37304699	0.5229213	1
gene49496	188.0314482	145.1884792	0.772149981	-0.37304699	0.5229213	1
gene57844	188.0314482	145.1884792	0.772149981	-0.37304699	0.5229213	1
gene3720	20.6627965	12.58300153	0.608968952	-0.71555942	0.52299211	1
gene13037	20.6627965	12.58300153	0.608968952	-0.71555942	0.52299211	1
gene13695	20.6627965	12.58300153	0.608968952	-0.71555942	0.52299211	1
gene14857	20.6627965	12.58300153	0.608968952	-0.71555942	0.52299211	1
gene23935	20.6627965	12.58300153	0.608968952	-0.71555942	0.52299211	1
gene25287	20.6627965	12.58300153	0.608968952	-0.71555942	0.52299211	1
gene32749	20.6627965	12.58300153	0.608968952	-0.71555942	0.52299211	1
gene43850	20.6627965	12.58300153	0.608968952	-0.71555942	0.52299211	1
gene56738	20.6627965	12.58300153	0.608968952	-0.71555942	0.52299211	1

gene70463	20.6627965	12.58300153	0.608968952	-0.71555942	0.52299211	1
gene70530	20.6627965	12.58300153	0.608968952	-0.71555942	0.52299211	1
gene32743	987.6816728	775.3064789	0.784976071	-0.34927942	0.52302064	1
gene29866	501.0728152	394.9126634	0.788134282	-0.34348664	0.52302801	1
gene61151	2478.50244	1901.001154	0.766995878	-0.38270927	0.52303044	1
gene9954	848.2077964	1082.138132	1.275793663	0.351395018	0.52303621	1
gene7797	255.1855368	325.2221934	1.274453864	0.349879148	0.52304115	1
gene34893	30.99419475	20.32638709	0.655812718	-0.60864422	0.52312786	1
gene46218	30.99419475	20.32638709	0.655812718	-0.60864422	0.52312786	1
gene33301	180.7994694	232.3015667	1.284857569	0.36160844	0.52313822	1
gene13587	42.35873283	29.03769584	0.68551852	-0.54473245	0.5231881	1
gene69683	42.35873283	29.03769584	0.68551852	-0.54473245	0.5231881	1
gene74150	42.35873283	29.03769584	0.68551852	-0.54473245	0.5231881	1
gene33269	393.6262734	309.7354223	0.786876901	-0.34579014	0.52320056	1
gene28297	593.0222596	467.506903	0.788346298	-0.34309859	0.5232139	1
gene12487	650.8780898	512.9992932	0.788164944	-0.34343051	0.52324875	1
gene4741	1591.035331	1237.005843	0.777484836	-0.36311356	0.52328499	1
gene30404	527.9344506	416.2069737	0.788368657	-0.34305767	0.52330921	1
gene73698	94.01572408	69.69047001	0.741263982	-0.43194068	0.52331847	1
gene1663	349.2012609	443.3088231	1.269493764	0.344253308	0.5233186	1
gene13813	14.46395755	23.23015667	1.606071961	0.683536535	0.5233276	1
gene19582	14.46395755	23.23015667	1.606071961	0.683536535	0.5233276	1
gene20232	14.46395755	23.23015667	1.606071961	0.683536535	0.5233276	1
gene20666	14.46395755	23.23015667	1.606071961	0.683536535	0.5233276	1
gene30336	14.46395755	23.23015667	1.606071961	0.683536535	0.5233276	1
gene30460	14.46395755	23.23015667	1.606071961	0.683536535	0.5233276	1
gene32310	14.46395755	23.23015667	1.606071961	0.683536535	0.5233276	1
gene42527	14.46395755	23.23015667	1.606071961	0.683536535	0.5233276	1
gene51419	14.46395755	23.23015667	1.606071961	0.683536535	0.5233276	1
gene65806	14.46395755	23.23015667	1.606071961	0.683536535	0.5233276	1
gene66657	14.46395755	23.23015667	1.606071961	0.683536535	0.5233276	1
gene71172	14.46395755	23.23015667	1.606071961	0.683536535	0.5233276	1
gene71988	14.46395755	23.23015667	1.606071961	0.683536535	0.5233276	1
gene73855	14.46395755	23.23015667	1.606071961	0.683536535	0.5233276	1
gene384	127.0761985	165.5148663	1.302485188	0.381266966	0.52337519	1
gene18564	682.9054244	538.1652962	0.788052455	-0.34363643	0.52338582	1
gene71651	859.5723345	1096.65698	1.27581698	0.351421384	0.52339744	1
gene87	273.7820536	348.4523501	1.272736271	0.347933504	0.52345491	1
gene67503	795.5176653	626.2463069	0.787218605	-0.34516378	0.52346945	1
gene35735	1361.678289	1062.779668	0.780492482	-0.35754336	0.52347261	1
gene69833	801.7165043	631.0859229	0.787168431	-0.34525573	0.5234847	1
gene19963	67.15408863	48.39615973	0.720673316	-0.47258267	0.52350808	1
gene63316	38.22617353	54.2036989	1.417973443	0.503830513	0.52355793	1
gene52079	487.6419974	618.5029214	1.268354499	0.342958028	0.52360927	1
gene60203	502.105955	395.8805866	0.788440334	-0.34292651	0.52369744	1
gene35578	4126.360461	5592.660219	1.35534941	0.438664828	0.52379186	1
gene72889	346.1018414	271.9864177	0.785856604	-0.34766201	0.52382412	1
gene31550	120.8773595	157.7714807	1.30521945	0.384292391	0.52384115	1

gene59889	151.8715543	116.1507834	0.764796172	-0.38685279	0.52388222	1
gene71967	151.8715543	116.1507834	0.764796172	-0.38685279	0.52388222	1
gene54279	1043.471223	818.8630227	0.784749023	-0.34969677	0.52398253	1
gene13422	29.96105493	43.55654376	1.453772034	0.539801058	0.5239829	1
gene13425	29.96105493	43.55654376	1.453772034	0.539801058	0.5239829	1
gene70498	29.96105493	43.55654376	1.453772034	0.539801058	0.5239829	1
gene72378	29.96105493	43.55654376	1.453772034	0.539801058	0.5239829	1
gene15605	80.58490636	59.04331487	0.732684538	-0.44873592	0.52419643	1
gene31246	601.2873782	474.2823654	0.788778183	-0.34230845	0.52421583	1
gene49244	196.2965668	151.9639416	0.774154862	-0.3693059	0.52423138	1
gene47309	137.4075967	104.535705	0.760770929	-0.39446598	0.52425179	1
gene12740	108.4796816	81.30554835	0.749500249	-0.41599914	0.52428043	1
gene59028	394.6594132	500.4162916	1.267969989	0.342520599	0.52430501	1
gene58781	297.5442696	233.2694899	0.783982465	-0.35110671	0.52430508	1
gene44008	60.95524968	43.55654376	0.714565915	-0.484861	0.52431971	1
gene61860	60.95524968	43.55654376	0.714565915	-0.484861	0.52431971	1
gene34463	114.6785206	150.0280952	1.308249308	0.387637496	0.52432732	1
gene10977	778.9874281	613.6633054	0.787770487	-0.34415273	0.52436687	1
gene11478	818.2467415	1042.453281	1.274008472	0.349374871	0.52438991	1
gene39852	115.7116604	87.11308752	0.752846232	-0.40957287	0.52443613	1
gene52766	111.5791011	146.1564024	1.309890481	0.389446194	0.52457513	1
gene61488	111.5791011	146.1564024	1.309890481	0.389446194	0.52457513	1
gene68271	1789.398177	1388.001861	0.775680829	-0.36646495	0.52463206	1
gene33319	181.8326092	140.3488632	0.7718575	-0.37359357	0.52464357	1
gene67601	181.8326092	140.3488632	0.7718575	-0.37359357	0.52464357	1
gene41753	243.8209987	310.7033455	1.274309215	0.349715394	0.52465336	1
gene52372	54.75641073	38.71692779	0.707075706	-0.5000634	0.52469496	1
gene4866	25.82849563	16.45469431	0.637075211	-0.65046439	0.52471064	1
gene6632	25.82849563	16.45469431	0.637075211	-0.65046439	0.52471064	1
gene27960	25.82849563	16.45469431	0.637075211	-0.65046439	0.52471064	1
gene41706	25.82849563	16.45469431	0.637075211	-0.65046439	0.52471064	1
gene48897	25.82849563	16.45469431	0.637075211	-0.65046439	0.52471064	1
gene53842	25.82849563	16.45469431	0.637075211	-0.65046439	0.52471064	1
gene58995	25.82849563	16.45469431	0.637075211	-0.65046439	0.52471064	1
gene65501	25.82849563	16.45469431	0.637075211	-0.65046439	0.52471064	1
gene67848	25.82849563	16.45469431	0.637075211	-0.65046439	0.52471064	1
gene71122	25.82849563	16.45469431	0.637075211	-0.65046439	0.52471064	1
gene23474	203.5285455	260.3713394	1.279286592	0.3553395	0.52477565	1
gene15946	628.1490136	495.5766757	0.78894763	-0.34199856	0.52477723	1
gene6910	817.2136016	643.6689244	0.787638536	-0.3443944	0.52485265	1
gene39372	174.6006304	134.5413241	0.770566084	-0.37600941	0.5248831	1
gene16131	1110.625312	871.1308752	0.784360725	-0.3504108	0.52493619	1
gene7850	537.232709	423.9503593	0.789137281	-0.3416518	0.52497305	1
gene47522	276.8814731	216.8147956	0.783059961	-0.35280531	0.52497382	1
gene67332	226.2576217	176.1620214	0.778590441	-0.36106346	0.52501264	1
gene64648	55.78955055	76.46593238	1.37061388	0.454822203	0.52501307	1
gene45242	767.62289	976.6345034	1.272284238	0.347421015	0.52502164	1
gene4992	1668.520818	1297.017081	0.777345459	-0.36337221	0.52504416	1

gene67822	1373.042828	1072.4589	0.781081899	-0.35645427	0.52507027	1
gene26650	274.8151935	349.4202733	1.271473636	0.346501548	0.52510163	1
gene66475	291.3454307	228.4298739	0.784051678	-0.35097935	0.52520159	1
gene67103	291.3454307	228.4298739	0.784051678	-0.35097935	0.52520159	1
gene63982	211.7936641	164.5469431	0.776920989	-0.36416021	0.52528478	1
gene70467	211.7936641	164.5469431	0.776920989	-0.36416021	0.52528478	1
gene29976	774.8548688	610.7595358	0.788224428	-0.34332163	0.52529629	1
gene64530	501.0728152	634.9576157	1.267196297	0.341640024	0.52534904	1
gene18305	1374.075967	1073.426823	0.781199037	-0.35623792	0.52535342	1
gene46474	544.4646878	429.7578984	0.789321894	-0.34131433	0.52535726	1
gene67978	1845.187728	2399.4816	1.300399717	0.378955148	0.52539665	1
gene727	454.581523	575.9143008	1.266910932	0.341315101	0.5254194	1
gene33667	87.81688513	64.85085404	0.738478186	-0.43737279	0.5254227	1
gene56328	87.81688513	64.85085404	0.738478186	-0.43737279	0.5254227	1
gene9573	305.8093882	240.0449523	0.784949585	-0.3493281	0.5254879	1
gene13641	99.18142321	130.6696313	1.317480906	0.397782053	0.5255586	1
gene28250	178.7331897	229.3977971	1.283465021	0.360043978	0.52562184	1
gene19780	58.88897003	80.33762515	1.364221944	0.448078374	0.52565396	1
gene41924	58.88897003	80.33762515	1.364221944	0.448078374	0.52565396	1
gene54156	464.9129213	366.8428908	0.789057206	-0.3417982	0.52567135	1
gene40458	152.9046941	117.1187066	0.765958869	-0.38466117	0.52568925	1
gene56312	41.325593	58.07539168	1.405312966	0.490891457	0.52574683	1
gene57877	41.325593	58.07539168	1.405312966	0.490891457	0.52574683	1
gene58190	41.325593	58.07539168	1.405312966	0.490891457	0.52574683	1
gene51215	175.6337703	225.5261044	1.284070279	0.360724165	0.52577758	1
gene23206	923.6270036	726.9103192	0.787017179	-0.34553297	0.52591923	1
gene62147	1096.161354	860.48372	0.784997315	-0.34924038	0.52594232	1
gene3173	172.5343508	221.6544116	1.284697282	0.361428452	0.52594507	1
gene69179	172.5343508	221.6544116	1.284697282	0.361428452	0.52594507	1
gene48289	145.6727153	111.3111674	0.764118161	-0.38813234	0.5259653	1
gene40220	74.38606741	54.2036989	0.728680797	-0.45664112	0.52596749	1
gene53741	74.38606741	54.2036989	0.728680797	-0.45664112	0.52596749	1
gene62221	74.38606741	54.2036989	0.728680797	-0.45664112	0.52596749	1
gene24151	3238.893352	4317.905371	1.333142189	0.414830662	0.5259736	1
gene927	61.9883895	84.20931793	1.358469201	0.441981857	0.52611975	1
gene25671	61.9883895	84.20931793	1.358469201	0.441981857	0.52611975	1
gene39603	61.9883895	84.20931793	1.358469201	0.441981857	0.52611975	1
gene24965	95.04886391	70.65839321	0.743390192	-0.42780844	0.52616874	1
gene14476	411.1896504	324.2542702	0.788575952	-0.34267838	0.5262006	1
gene8076	89.88316478	119.0545529	1.324547853	0.405499966	0.52621594	1
gene22393	857.5060548	675.6103899	0.787878273	-0.34395534	0.52626856	1
gene68150	1079.631117	847.9007185	0.785361504	-0.34857121	0.5262973	1
gene71320	1439.163776	1123.758829	0.780841519	-0.35689833	0.5263898	1
gene21506	3170.706123	2414.000447	0.761344746	-0.39337822	0.52639233	1
gene18116	86.78374531	115.1828602	1.327240024	0.408429297	0.52639982	1
gene184	24.7953558	36.7810814	1.483385909	0.568893969	0.52641379	1
gene6469	24.7953558	36.7810814	1.483385909	0.568893969	0.52641379	1
gene16356	24.7953558	36.7810814	1.483385909	0.568893969	0.52641379	1

gene17780	24.7953558	36.7810814	1.483385909	0.568893969	0.52641379	1
gene32642	24.7953558	36.7810814	1.483385909	0.568893969	0.52641379	1
gene57600	24.7953558	36.7810814	1.483385909	0.568893969	0.52641379	1
gene62150	1733.608626	1347.349087	0.777193345	-0.36365455	0.52641856	1
gene9454	65.08780898	88.08101071	1.353264338	0.436443673	0.52644279	1
gene51794	65.08780898	88.08101071	1.353264338	0.436443673	0.52644279	1
gene9449	220.0587827	171.3224055	0.778530188	-0.36117511	0.52645058	1
gene44601	175.6337703	135.5092472	0.771544374	-0.37417896	0.52647723	1
gene27114	163.2360924	210.0393332	1.286721155	0.363699441	0.52651994	1
gene57576	163.2360924	210.0393332	1.286721155	0.363699441	0.52651994	1
gene53892	393.6262734	498.4804452	1.266380013	0.340710391	0.52653515	1
gene38995	2219.184344	1712.256131	0.77157003	-0.37413099	0.52654005	1
gene4155	83.68432583	111.3111674	1.330131614	0.411569005	0.52655714	1
gene6491	2229.515743	1719.999517	0.77146776	-0.37432223	0.52657017	1
gene32241	102.2808427	76.46593238	0.747607571	-0.41964692	0.52657774	1
gene38519	266.5500749	338.7731181	1.270954879	0.345912813	0.52662374	1
gene768	314.0745068	246.8204146	0.785865803	-0.34764512	0.52663299	1
gene12677	123.976779	93.88854988	0.757307543	-0.4010488	0.52664762	1
gene44438	68.18722846	91.95270349	1.348532644	0.431390446	0.5266492	1
gene54956	68.18722846	91.95270349	1.348532644	0.431390446	0.5266492	1
gene69452	116.7448002	88.08101071	0.754474808	-0.40645537	0.5267559	1
gene57689	71.28664793	95.82439627	1.344212402	0.42676112	0.52676026	1
gene62242	71.28664793	95.82439627	1.344212402	0.42676112	0.52676026	1
gene64699	526.9013108	416.2069737	0.789914478	-0.34023163	0.52676273	1
gene36122	74.38606741	99.69608905	1.340252181	0.422504482	0.52679341	1
gene62893	74.38606741	99.69608905	1.340252181	0.422504482	0.52679341	1
gene14153	3225.462534	2454.653222	0.761023635	-0.39398684	0.52686034	1
gene12519	19.62965668	30.00561903	1.528586033	0.612197754	0.52706824	1
gene45200	19.62965668	30.00561903	1.528586033	0.612197754	0.52706824	1
gene50403	19.62965668	30.00561903	1.528586033	0.612197754	0.52706824	1
gene55891	19.62965668	30.00561903	1.528586033	0.612197754	0.52706824	1
gene9874	121.9104994	158.7394039	1.302097889	0.380837912	0.52710074	1
gene66367	121.9104994	158.7394039	1.302097889	0.380837912	0.52710074	1
gene73225	1639.592902	1276.690694	0.778663223	-0.36092861	0.52710597	1
gene58026	4099.498826	3085.739145	0.752711313	-0.40983144	0.52711404	1
gene12969	534.1332896	422.0145129	0.790092138	-0.33990719	0.52711816	1
gene71525	297.5442696	377.4900459	1.268685317	0.343334269	0.52711905	1
gene44509	271.715774	212.9431028	0.783697979	-0.35163032	0.52712447	1
gene6319	1070.332859	841.1252561	0.785853904	-0.34766696	0.52713148	1
gene25788	9992.528388	7073.582706	0.707887176	-0.49840865	0.52721244	1
gene23100	286.1797315	224.5581812	0.78467535	-0.34983221	0.52721486	1
gene7414	195.2634269	249.7241842	1.278909154	0.354913788	0.52721562	1
gene51744	195.2634269	249.7241842	1.278909154	0.354913788	0.52721562	1
gene41763	293.4117103	230.3657203	0.785127901	-0.3490004	0.52729706	1
gene23847	385.3611548	487.8332901	1.265911948	0.34017706	0.52733279	1
gene2115	118.8110799	154.8677111	1.303478693	0.382367001	0.52741226	1
gene29239	33.0604744	47.42823654	1.43459032	0.520638801	0.52741576	1
gene35167	33.0604744	47.42823654	1.43459032	0.520638801	0.52741576	1

gene39151	33.0604744	47.42823654	1.43459032	0.520638801	0.52741576	1
gene40755	33.0604744	47.42823654	1.43459032	0.520638801	0.52741576	1
gene54981	44.42501248	61.94708446	1.394419067	0.479664202	0.52751895	1
gene32837	81.61804618	60.01123807	0.735269231	-0.44365548	0.52752193	1
gene67242	81.61804618	60.01123807	0.735269231	-0.44365548	0.52752193	1
gene71490	415.3222097	525.5822947	1.26548083	0.339685653	0.52752716	1
gene45389	316.1407865	400.7202026	1.267537185	0.342028071	0.52754113	1
gene38034	68.18722846	49.36408293	0.723949104	-0.46603982	0.52754395	1
gene56545	68.18722846	49.36408293	0.723949104	-0.46603982	0.52754395	1
gene61308	228.3239013	178.0978678	0.780022883	-0.35841165	0.5275695	1
gene37716	1165.381723	914.6874189	0.784882242	-0.34945188	0.52757868	1
gene62903	37.1930337	25.16600306	0.676632169	-0.56355633	0.5277091	1
gene66814	37.1930337	25.16600306	0.676632169	-0.56355633	0.5277091	1
gene25989	2095.207565	2733.415102	1.30460349	0.383611393	0.52773873	1
gene12948	548.5972471	433.6295912	0.790433407	-0.33928417	0.52782661	1
gene19732	829.6112795	1055.036282	1.271723647	0.346785199	0.52782964	1
gene9754	2983.707815	2279.459123	0.763968614	-0.38841473	0.52784624	1
gene65498	799.6502246	1016.319354	1.270954879	0.345912813	0.52786001	1
gene30866	454.581523	359.0995052	0.789956228	-0.34015538	0.52790566	1
gene16408	483.5094381	611.727459	1.265182044	0.339344985	0.52797437	1
gene14489	213.8599438	166.4827895	0.778466442	-0.36129325	0.52798055	1
gene5871	267.5832147	339.7410413	1.269664996	0.344447889	0.52830619	1
gene15648	1328.617815	1040.517434	0.783157822	-0.35262503	0.52834118	1
gene12839	233.4896005	297.1524208	1.272658055	0.34784484	0.52837672	1
gene18623	868.8705929	685.2896218	0.788713103	-0.34242748	0.52838068	1
gene50083	109.5128215	143.2526328	1.308090056	0.387461868	0.52838784	1
gene9747	5051.020605	3762.317458	0.744862821	-0.42495334	0.52841704	1
gene63363	582.6908613	460.7314406	0.790696184	-0.33880463	0.52842258	1
gene61918	88.85002496	65.81877724	0.740785129	-0.43287296	0.52845786	1
gene43827	132.2418976	100.6640122	0.76121119	-0.39363133	0.52851206	1
gene70125	1607.565568	1253.460537	0.779725917	-0.35896101	0.52854041	1
gene72403	1759.437122	1368.643397	0.777887075	-0.36236736	0.5285668	1
gene41573	769.6891697	607.8557662	0.789741873	-0.34054691	0.52857569	1
gene19943	635.3809924	502.352138	0.79063136	-0.33892292	0.52859886	1
gene26615	924.6601434	728.8461656	0.788231407	-0.34330886	0.52863397	1
gene2093	2995.072353	3968.485098	1.325004751	0.405997533	0.52867515	1
gene7166	106.413402	139.38094	1.30980626	0.389353431	0.52872265	1
gene21327	1015.576448	799.5045588	0.787242123	-0.34512068	0.52874509	1
gene43391	392.5931335	496.5445988	1.264781669	0.338888364	0.52878524	1
gene1	421.5210486	332.965579	0.789914478	-0.34023163	0.52878685	1
gene66305	1486.688208	1915.520002	1.2884477	0.365633977	0.52880372	1
gene24992	125.0099188	94.85647307	0.758791574	-0.39822444	0.52880738	1
gene922	330.604744	260.3713394	0.787560808	-0.34453678	0.5288204	1
gene43793	47.52443195	65.81877724	1.384946111	0.469829842	0.52891916	1
gene74026	96.08200373	71.6263164	0.745470677	-0.42377649	0.52896585	1
gene65762	3251.29103	2476.915455	0.761825205	-0.39246807	0.52900473	1
gene69755	676.7065854	857.5799504	1.267284771	0.341740749	0.52901519	1
gene232	2.06627965	0	0	#NAME?	0.52904185	1

gene336	2.06627965	0	0	#NAME?	0.52904185	1
gene339	2.06627965	0	0	#NAME?	0.52904185	1
gene343	2.06627965	0	0	#NAME?	0.52904185	1
gene690	2.06627965	0	0	#NAME?	0.52904185	1
gene723	2.06627965	0	0	#NAME?	0.52904185	1
gene1214	2.06627965	0	0	#NAME?	0.52904185	1
gene1347	2.06627965	0	0	#NAME?	0.52904185	1
gene1561	2.06627965	0	0	#NAME?	0.52904185	1
gene1807	2.06627965	0	0	#NAME?	0.52904185	1
gene2173	2.06627965	0	0	#NAME?	0.52904185	1
gene2175	2.06627965	0	0	#NAME?	0.52904185	1
gene2252	2.06627965	0	0	#NAME?	0.52904185	1
gene2338	2.06627965	0	0	#NAME?	0.52904185	1
gene2513	2.06627965	0	0	#NAME?	0.52904185	1
gene2652	2.06627965	0	0	#NAME?	0.52904185	1
gene2943	2.06627965	0	0	#NAME?	0.52904185	1
gene3403	2.06627965	0	0	#NAME?	0.52904185	1
gene3408	2.06627965	0	0	#NAME?	0.52904185	1
gene3718	2.06627965	0	0	#NAME?	0.52904185	1
gene4058	2.06627965	0	0	#NAME?	0.52904185	1
gene4060	2.06627965	0	0	#NAME?	0.52904185	1
gene4207	2.06627965	0	0	#NAME?	0.52904185	1
gene4421	2.06627965	0	0	#NAME?	0.52904185	1
gene4631	2.06627965	0	0	#NAME?	0.52904185	1
gene5079	2.06627965	0	0	#NAME?	0.52904185	1
gene5240	2.06627965	0	0	#NAME?	0.52904185	1
gene5521	2.06627965	0	0	#NAME?	0.52904185	1
gene5559	2.06627965	0	0	#NAME?	0.52904185	1
gene5687	2.06627965	0	0	#NAME?	0.52904185	1
gene5784	2.06627965	0	0	#NAME?	0.52904185	1
gene5985	2.06627965	0	0	#NAME?	0.52904185	1
gene6399	2.06627965	0	0	#NAME?	0.52904185	1
gene6794	2.06627965	0	0	#NAME?	0.52904185	1
gene6903	2.06627965	0	0	#NAME?	0.52904185	1
gene6917	2.06627965	0	0	#NAME?	0.52904185	1
gene7062	2.06627965	0	0	#NAME?	0.52904185	1
gene7693	2.06627965	0	0	#NAME?	0.52904185	1
gene7913	2.06627965	0	0	#NAME?	0.52904185	1
gene7989	2.06627965	0	0	#NAME?	0.52904185	1
gene8077	2.06627965	0	0	#NAME?	0.52904185	1
gene8344	2.06627965	0	0	#NAME?	0.52904185	1
gene8904	2.06627965	0	0	#NAME?	0.52904185	1
gene8945	2.06627965	0	0	#NAME?	0.52904185	1
gene9287	2.06627965	0	0	#NAME?	0.52904185	1
gene10039	2.06627965	0	0	#NAME?	0.52904185	1
gene10079	2.06627965	0	0	#NAME?	0.52904185	1
gene10517	2.06627965	0	0	#NAME?	0.52904185	1
gene10883	2.06627965	0	0	#NAME?	0.52904185	1

gene11251	2.06627965	0	0	#NAME?	0.52904185	1
gene11795	2.06627965	0	0	#NAME?	0.52904185	1
gene11911	2.06627965	0	0	#NAME?	0.52904185	1
gene11963	2.06627965	0	0	#NAME?	0.52904185	1
gene12398	2.06627965	0	0	#NAME?	0.52904185	1
gene12530	2.06627965	0	0	#NAME?	0.52904185	1
gene12585	2.06627965	0	0	#NAME?	0.52904185	1
gene12683	2.06627965	0	0	#NAME?	0.52904185	1
gene12749	2.06627965	0	0	#NAME?	0.52904185	1
gene13455	2.06627965	0	0	#NAME?	0.52904185	1
gene13484	2.06627965	0	0	#NAME?	0.52904185	1
gene13694	2.06627965	0	0	#NAME?	0.52904185	1
gene13774	2.06627965	0	0	#NAME?	0.52904185	1
gene13880	2.06627965	0	0	#NAME?	0.52904185	1
gene14064	2.06627965	0	0	#NAME?	0.52904185	1
gene14078	2.06627965	0	0	#NAME?	0.52904185	1
gene14652	2.06627965	0	0	#NAME?	0.52904185	1
gene14752	2.06627965	0	0	#NAME?	0.52904185	1
gene15290	2.06627965	0	0	#NAME?	0.52904185	1
gene15603	2.06627965	0	0	#NAME?	0.52904185	1
gene15814	2.06627965	0	0	#NAME?	0.52904185	1
gene15849	2.06627965	0	0	#NAME?	0.52904185	1
gene16165	2.06627965	0	0	#NAME?	0.52904185	1
gene16184	2.06627965	0	0	#NAME?	0.52904185	1
gene16218	2.06627965	0	0	#NAME?	0.52904185	1
gene16272	2.06627965	0	0	#NAME?	0.52904185	1
gene16355	2.06627965	0	0	#NAME?	0.52904185	1
gene16523	2.06627965	0	0	#NAME?	0.52904185	1
gene16598	2.06627965	0	0	#NAME?	0.52904185	1
gene16599	2.06627965	0	0	#NAME?	0.52904185	1
gene16728	2.06627965	0	0	#NAME?	0.52904185	1
gene16785	2.06627965	0	0	#NAME?	0.52904185	1
gene17215	2.06627965	0	0	#NAME?	0.52904185	1
gene17391	2.06627965	0	0	#NAME?	0.52904185	1
gene17593	2.06627965	0	0	#NAME?	0.52904185	1
gene17734	2.06627965	0	0	#NAME?	0.52904185	1
gene17771	2.06627965	0	0	#NAME?	0.52904185	1
gene17880	2.06627965	0	0	#NAME?	0.52904185	1
gene17903	2.06627965	0	0	#NAME?	0.52904185	1
gene18113	2.06627965	0	0	#NAME?	0.52904185	1
gene18579	2.06627965	0	0	#NAME?	0.52904185	1
gene18949	2.06627965	0	0	#NAME?	0.52904185	1
gene19002	2.06627965	0	0	#NAME?	0.52904185	1
gene19158	2.06627965	0	0	#NAME?	0.52904185	1
gene19331	2.06627965	0	0	#NAME?	0.52904185	1
gene19473	2.06627965	0	0	#NAME?	0.52904185	1
gene19514	2.06627965	0	0	#NAME?	0.52904185	1
gene19708	2.06627965	0	0	#NAME?	0.52904185	1

gene20182	2.06627965	0	0	#NAME?	0.52904185	1
gene20380	2.06627965	0	0	#NAME?	0.52904185	1
gene20567	2.06627965	0	0	#NAME?	0.52904185	1
gene20826	2.06627965	0	0	#NAME?	0.52904185	1
gene21063	2.06627965	0	0	#NAME?	0.52904185	1
gene21119	2.06627965	0	0	#NAME?	0.52904185	1
gene21349	2.06627965	0	0	#NAME?	0.52904185	1
gene21351	2.06627965	0	0	#NAME?	0.52904185	1
gene21777	2.06627965	0	0	#NAME?	0.52904185	1
gene22085	2.06627965	0	0	#NAME?	0.52904185	1
gene22231	2.06627965	0	0	#NAME?	0.52904185	1
gene22360	2.06627965	0	0	#NAME?	0.52904185	1
gene22676	2.06627965	0	0	#NAME?	0.52904185	1
gene22690	2.06627965	0	0	#NAME?	0.52904185	1
gene23026	2.06627965	0	0	#NAME?	0.52904185	1
gene23327	2.06627965	0	0	#NAME?	0.52904185	1
gene23505	2.06627965	0	0	#NAME?	0.52904185	1
gene23521	2.06627965	0	0	#NAME?	0.52904185	1
gene23525	2.06627965	0	0	#NAME?	0.52904185	1
gene23637	2.06627965	0	0	#NAME?	0.52904185	1
gene23786	2.06627965	0	0	#NAME?	0.52904185	1
gene23895	2.06627965	0	0	#NAME?	0.52904185	1
gene23962	2.06627965	0	0	#NAME?	0.52904185	1
gene23979	2.06627965	0	0	#NAME?	0.52904185	1
gene24188	2.06627965	0	0	#NAME?	0.52904185	1
gene24407	2.06627965	0	0	#NAME?	0.52904185	1
gene24603	2.06627965	0	0	#NAME?	0.52904185	1
gene24632	2.06627965	0	0	#NAME?	0.52904185	1
gene25050	2.06627965	0	0	#NAME?	0.52904185	1
gene25068	2.06627965	0	0	#NAME?	0.52904185	1
gene25142	2.06627965	0	0	#NAME?	0.52904185	1
gene25300	2.06627965	0	0	#NAME?	0.52904185	1
gene26406	2.06627965	0	0	#NAME?	0.52904185	1
gene26453	2.06627965	0	0	#NAME?	0.52904185	1
gene27018	2.06627965	0	0	#NAME?	0.52904185	1
gene27122	2.06627965	0	0	#NAME?	0.52904185	1
gene27474	2.06627965	0	0	#NAME?	0.52904185	1
gene27889	2.06627965	0	0	#NAME?	0.52904185	1
gene27911	2.06627965	0	0	#NAME?	0.52904185	1
gene28028	2.06627965	0	0	#NAME?	0.52904185	1
gene28370	2.06627965	0	0	#NAME?	0.52904185	1
gene28501	2.06627965	0	0	#NAME?	0.52904185	1
gene28621	2.06627965	0	0	#NAME?	0.52904185	1
gene29146	2.06627965	0	0	#NAME?	0.52904185	1
gene29383	2.06627965	0	0	#NAME?	0.52904185	1
gene29627	2.06627965	0	0	#NAME?	0.52904185	1
gene29707	2.06627965	0	0	#NAME?	0.52904185	1
gene29783	2.06627965	0	0	#NAME?	0.52904185	1

gene29981	2.06627965	0	0	#NAME?	0.52904185	1
gene30127	2.06627965	0	0	#NAME?	0.52904185	1
gene30213	2.06627965	0	0	#NAME?	0.52904185	1
gene30262	2.06627965	0	0	#NAME?	0.52904185	1
gene30311	2.06627965	0	0	#NAME?	0.52904185	1
gene30683	2.06627965	0	0	#NAME?	0.52904185	1
gene31745	2.06627965	0	0	#NAME?	0.52904185	1
gene31805	2.06627965	0	0	#NAME?	0.52904185	1
gene31914	2.06627965	0	0	#NAME?	0.52904185	1
gene31952	2.06627965	0	0	#NAME?	0.52904185	1
gene32206	2.06627965	0	0	#NAME?	0.52904185	1
gene32535	2.06627965	0	0	#NAME?	0.52904185	1
gene32548	2.06627965	0	0	#NAME?	0.52904185	1
gene32635	2.06627965	0	0	#NAME?	0.52904185	1
gene32729	2.06627965	0	0	#NAME?	0.52904185	1
gene33170	2.06627965	0	0	#NAME?	0.52904185	1
gene33363	2.06627965	0	0	#NAME?	0.52904185	1
gene33630	2.06627965	0	0	#NAME?	0.52904185	1
gene33846	2.06627965	0	0	#NAME?	0.52904185	1
gene33921	2.06627965	0	0	#NAME?	0.52904185	1
gene34119	2.06627965	0	0	#NAME?	0.52904185	1
gene34202	2.06627965	0	0	#NAME?	0.52904185	1
gene34342	2.06627965	0	0	#NAME?	0.52904185	1
gene35045	2.06627965	0	0	#NAME?	0.52904185	1
gene36221	2.06627965	0	0	#NAME?	0.52904185	1
gene36488	2.06627965	0	0	#NAME?	0.52904185	1
gene36659	2.06627965	0	0	#NAME?	0.52904185	1
gene36986	2.06627965	0	0	#NAME?	0.52904185	1
gene37148	2.06627965	0	0	#NAME?	0.52904185	1
gene37417	2.06627965	0	0	#NAME?	0.52904185	1
gene37473	2.06627965	0	0	#NAME?	0.52904185	1
gene37642	2.06627965	0	0	#NAME?	0.52904185	1
gene37661	2.06627965	0	0	#NAME?	0.52904185	1
gene37663	2.06627965	0	0	#NAME?	0.52904185	1
gene37973	2.06627965	0	0	#NAME?	0.52904185	1
gene38320	2.06627965	0	0	#NAME?	0.52904185	1
gene38535	2.06627965	0	0	#NAME?	0.52904185	1
gene38579	2.06627965	0	0	#NAME?	0.52904185	1
gene39166	2.06627965	0	0	#NAME?	0.52904185	1
gene39802	2.06627965	0	0	#NAME?	0.52904185	1
gene39905	2.06627965	0	0	#NAME?	0.52904185	1
gene39917	2.06627965	0	0	#NAME?	0.52904185	1
gene39956	2.06627965	0	0	#NAME?	0.52904185	1
gene40475	2.06627965	0	0	#NAME?	0.52904185	1
gene40900	2.06627965	0	0	#NAME?	0.52904185	1
gene40949	2.06627965	0	0	#NAME?	0.52904185	1
gene40951	2.06627965	0	0	#NAME?	0.52904185	1
gene41041	2.06627965	0	0	#NAME?	0.52904185	1

gene41083	2.06627965	0	0	#NAME?	0.52904185	1
gene41527	2.06627965	0	0	#NAME?	0.52904185	1
gene41669	2.06627965	0	0	#NAME?	0.52904185	1
gene41959	2.06627965	0	0	#NAME?	0.52904185	1
gene42120	2.06627965	0	0	#NAME?	0.52904185	1
gene42989	2.06627965	0	0	#NAME?	0.52904185	1
gene43269	2.06627965	0	0	#NAME?	0.52904185	1
gene43507	2.06627965	0	0	#NAME?	0.52904185	1
gene44264	2.06627965	0	0	#NAME?	0.52904185	1
gene44348	2.06627965	0	0	#NAME?	0.52904185	1
gene44394	2.06627965	0	0	#NAME?	0.52904185	1
gene44463	2.06627965	0	0	#NAME?	0.52904185	1
gene44500	2.06627965	0	0	#NAME?	0.52904185	1
gene44739	2.06627965	0	0	#NAME?	0.52904185	1
gene44890	2.06627965	0	0	#NAME?	0.52904185	1
gene44986	2.06627965	0	0	#NAME?	0.52904185	1
gene45213	2.06627965	0	0	#NAME?	0.52904185	1
gene45498	2.06627965	0	0	#NAME?	0.52904185	1
gene45628	2.06627965	0	0	#NAME?	0.52904185	1
gene45781	2.06627965	0	0	#NAME?	0.52904185	1
gene45800	2.06627965	0	0	#NAME?	0.52904185	1
gene45919	2.06627965	0	0	#NAME?	0.52904185	1
gene46223	2.06627965	0	0	#NAME?	0.52904185	1
gene46560	2.06627965	0	0	#NAME?	0.52904185	1
gene46648	2.06627965	0	0	#NAME?	0.52904185	1
gene47861	2.06627965	0	0	#NAME?	0.52904185	1
gene47912	2.06627965	0	0	#NAME?	0.52904185	1
gene48574	2.06627965	0	0	#NAME?	0.52904185	1
gene48806	2.06627965	0	0	#NAME?	0.52904185	1
gene49031	2.06627965	0	0	#NAME?	0.52904185	1
gene49567	2.06627965	0	0	#NAME?	0.52904185	1
gene49704	2.06627965	0	0	#NAME?	0.52904185	1
gene50125	2.06627965	0	0	#NAME?	0.52904185	1
gene50261	2.06627965	0	0	#NAME?	0.52904185	1
gene50347	2.06627965	0	0	#NAME?	0.52904185	1
gene50571	2.06627965	0	0	#NAME?	0.52904185	1
gene50709	2.06627965	0	0	#NAME?	0.52904185	1
gene50834	2.06627965	0	0	#NAME?	0.52904185	1
gene51106	2.06627965	0	0	#NAME?	0.52904185	1
gene51185	2.06627965	0	0	#NAME?	0.52904185	1
gene51195	2.06627965	0	0	#NAME?	0.52904185	1
gene51530	2.06627965	0	0	#NAME?	0.52904185	1
gene51756	2.06627965	0	0	#NAME?	0.52904185	1
gene52649	2.06627965	0	0	#NAME?	0.52904185	1
gene52653	2.06627965	0	0	#NAME?	0.52904185	1
gene52891	2.06627965	0	0	#NAME?	0.52904185	1
gene53095	2.06627965	0	0	#NAME?	0.52904185	1
gene53693	2.06627965	0	0	#NAME?	0.52904185	1

gene53833	2.06627965	0	0	#NAME?	0.52904185	1
gene54072	2.06627965	0	0	#NAME?	0.52904185	1
gene54220	2.06627965	0	0	#NAME?	0.52904185	1
gene54269	2.06627965	0	0	#NAME?	0.52904185	1
gene54400	2.06627965	0	0	#NAME?	0.52904185	1
gene54411	2.06627965	0	0	#NAME?	0.52904185	1
gene54649	2.06627965	0	0	#NAME?	0.52904185	1
gene55064	2.06627965	0	0	#NAME?	0.52904185	1
gene55480	2.06627965	0	0	#NAME?	0.52904185	1
gene55771	2.06627965	0	0	#NAME?	0.52904185	1
gene55802	2.06627965	0	0	#NAME?	0.52904185	1
gene55952	2.06627965	0	0	#NAME?	0.52904185	1
gene55955	2.06627965	0	0	#NAME?	0.52904185	1
gene57007	2.06627965	0	0	#NAME?	0.52904185	1
gene57097	2.06627965	0	0	#NAME?	0.52904185	1
gene57232	2.06627965	0	0	#NAME?	0.52904185	1
gene57298	2.06627965	0	0	#NAME?	0.52904185	1
gene57410	2.06627965	0	0	#NAME?	0.52904185	1
gene57941	2.06627965	0	0	#NAME?	0.52904185	1
gene58222	2.06627965	0	0	#NAME?	0.52904185	1
gene59366	2.06627965	0	0	#NAME?	0.52904185	1
gene59973	2.06627965	0	0	#NAME?	0.52904185	1
gene60571	2.06627965	0	0	#NAME?	0.52904185	1
gene60605	2.06627965	0	0	#NAME?	0.52904185	1
gene60657	2.06627965	0	0	#NAME?	0.52904185	1
gene60717	2.06627965	0	0	#NAME?	0.52904185	1
gene60969	2.06627965	0	0	#NAME?	0.52904185	1
gene61920	2.06627965	0	0	#NAME?	0.52904185	1
gene62054	2.06627965	0	0	#NAME?	0.52904185	1
gene62183	2.06627965	0	0	#NAME?	0.52904185	1
gene62540	2.06627965	0	0	#NAME?	0.52904185	1
gene62632	2.06627965	0	0	#NAME?	0.52904185	1
gene62686	2.06627965	0	0	#NAME?	0.52904185	1
gene62858	2.06627965	0	0	#NAME?	0.52904185	1
gene62975	2.06627965	0	0	#NAME?	0.52904185	1
gene63043	2.06627965	0	0	#NAME?	0.52904185	1
gene63437	2.06627965	0	0	#NAME?	0.52904185	1
gene63464	2.06627965	0	0	#NAME?	0.52904185	1
gene63500	2.06627965	0	0	#NAME?	0.52904185	1
gene63546	2.06627965	0	0	#NAME?	0.52904185	1
gene63556	2.06627965	0	0	#NAME?	0.52904185	1
gene63701	2.06627965	0	0	#NAME?	0.52904185	1
gene63726	2.06627965	0	0	#NAME?	0.52904185	1
gene63883	2.06627965	0	0	#NAME?	0.52904185	1
gene63898	2.06627965	0	0	#NAME?	0.52904185	1
gene64282	2.06627965	0	0	#NAME?	0.52904185	1
gene64425	2.06627965	0	0	#NAME?	0.52904185	1
gene64618	2.06627965	0	0	#NAME?	0.52904185	1

gene64849	2.06627965	0	0	#NAME?	0.52904185	1
gene64928	2.06627965	0	0	#NAME?	0.52904185	1
gene64992	2.06627965	0	0	#NAME?	0.52904185	1
gene65239	2.06627965	0	0	#NAME?	0.52904185	1
gene65362	2.06627965	0	0	#NAME?	0.52904185	1
gene65705	2.06627965	0	0	#NAME?	0.52904185	1
gene66016	2.06627965	0	0	#NAME?	0.52904185	1
gene66032	2.06627965	0	0	#NAME?	0.52904185	1
gene66400	2.06627965	0	0	#NAME?	0.52904185	1
gene66548	2.06627965	0	0	#NAME?	0.52904185	1
gene66711	2.06627965	0	0	#NAME?	0.52904185	1
gene66786	2.06627965	0	0	#NAME?	0.52904185	1
gene66810	2.06627965	0	0	#NAME?	0.52904185	1
gene67356	2.06627965	0	0	#NAME?	0.52904185	1
gene67443	2.06627965	0	0	#NAME?	0.52904185	1
gene67494	2.06627965	0	0	#NAME?	0.52904185	1
gene67611	2.06627965	0	0	#NAME?	0.52904185	1
gene68004	2.06627965	0	0	#NAME?	0.52904185	1
gene68013	2.06627965	0	0	#NAME?	0.52904185	1
gene68252	2.06627965	0	0	#NAME?	0.52904185	1
gene68494	2.06627965	0	0	#NAME?	0.52904185	1
gene68520	2.06627965	0	0	#NAME?	0.52904185	1
gene68633	2.06627965	0	0	#NAME?	0.52904185	1
gene68881	2.06627965	0	0	#NAME?	0.52904185	1
gene69580	2.06627965	0	0	#NAME?	0.52904185	1
gene70334	2.06627965	0	0	#NAME?	0.52904185	1
gene70622	2.06627965	0	0	#NAME?	0.52904185	1
gene70625	2.06627965	0	0	#NAME?	0.52904185	1
gene71048	2.06627965	0	0	#NAME?	0.52904185	1
gene71071	2.06627965	0	0	#NAME?	0.52904185	1
gene71123	2.06627965	0	0	#NAME?	0.52904185	1
gene71500	2.06627965	0	0	#NAME?	0.52904185	1
gene71730	2.06627965	0	0	#NAME?	0.52904185	1
gene71885	2.06627965	0	0	#NAME?	0.52904185	1
gene71956	2.06627965	0	0	#NAME?	0.52904185	1
gene72490	2.06627965	0	0	#NAME?	0.52904185	1
gene72684	2.06627965	0	0	#NAME?	0.52904185	1
gene72758	2.06627965	0	0	#NAME?	0.52904185	1
gene72804	2.06627965	0	0	#NAME?	0.52904185	1
gene73123	2.06627965	0	0	#NAME?	0.52904185	1
gene73300	2.06627965	0	0	#NAME?	0.52904185	1
gene73721	2.06627965	0	0	#NAME?	0.52904185	1
gene74088	2.06627965	0	0	#NAME?	0.52904185	1
gene74135	2.06627965	0	0	#NAME?	0.52904185	1
gene74246	2.06627965	0	0	#NAME?	0.52904185	1
gene2612	12.3976779	20.32638709	1.639531794	0.713283879	0.5291162	1
gene3813	12.3976779	20.32638709	1.639531794	0.713283879	0.5291162	1
gene14637	12.3976779	20.32638709	1.639531794	0.713283879	0.5291162	1

gene33427	12.3976779	20.32638709	1.639531794	0.713283879	0.5291162	1
gene37452	12.3976779	20.32638709	1.639531794	0.713283879	0.5291162	1
gene40863	12.3976779	20.32638709	1.639531794	0.713283879	0.5291162	1
gene44799	12.3976779	20.32638709	1.639531794	0.713283879	0.5291162	1
gene50042	12.3976779	20.32638709	1.639531794	0.713283879	0.5291162	1
gene52020	12.3976779	20.32638709	1.639531794	0.713283879	0.5291162	1
gene54276	12.3976779	20.32638709	1.639531794	0.713283879	0.5291162	1
gene55183	12.3976779	20.32638709	1.639531794	0.713283879	0.5291162	1
gene61924	12.3976779	20.32638709	1.639531794	0.713283879	0.5291162	1
gene63602	12.3976779	20.32638709	1.639531794	0.713283879	0.5291162	1
gene70992	12.3976779	20.32638709	1.639531794	0.713283879	0.5291162	1
gene72663	1306.921879	1677.410896	1.283482145	0.360063226	0.52913427	1
gene21839	735.5955555	933.0779596	1.268466011	0.343084862	0.52916437	1
gene31932	2531.192571	1947.461468	0.769384949	-0.37822249	0.52917035	1
gene9864	103.3139825	77.43385557	0.749500249	-0.41599914	0.52917614	1
gene27322	103.3139825	77.43385557	0.749500249	-0.41599914	0.52917614	1
gene61972	103.3139825	77.43385557	0.749500249	-0.41599914	0.52917614	1
gene64914	326.4721847	413.3032041	1.265967588	0.340240468	0.52918969	1
gene49765	184.9320287	143.2526328	0.774623162	-0.36843345	0.52922341	1
gene63636	154.9709738	119.0545529	0.768237755	-0.38037523	0.529244	1
gene67556	154.9709738	119.0545529	0.768237755	-0.38037523	0.529244	1
gene13563	404.9908114	512.03137	1.264303672	0.338343025	0.52927924	1
gene15466	214.8930836	167.4507127	0.779228023	-0.35988253	0.52931296	1
gene20794	975.2839949	768.5310165	0.788007412	-0.3437189	0.52939954	1
gene11471	196.2965668	250.6921074	1.277108976	0.352881636	0.52940572	1
gene23024	259.3180961	203.2638709	0.783839901	-0.35136908	0.52944446	1
gene24515	932.925262	735.6216279	0.788510782	-0.34279761	0.52945542	1
gene31520	252.0861173	197.4563317	0.783289194	-0.35238304	0.52955311	1
gene38314	75.41920723	55.17162209	0.731532777	-0.45100559	0.52956425	1
gene16430	524.8350311	415.2390505	0.791180134	-0.33792189	0.52960221	1
gene8395	43.39187265	30.00561903	0.691503206	-0.53219216	0.52960338	1
gene33588	43.39187265	30.00561903	0.691503206	-0.53219216	0.52960338	1
gene49443	43.39187265	30.00561903	0.691503206	-0.53219216	0.52960338	1
gene51511	43.39187265	30.00561903	0.691503206	-0.53219216	0.52960338	1
gene53494	43.39187265	30.00561903	0.691503206	-0.53219216	0.52960338	1
gene11590	55.78955055	39.68485098	0.711331254	-0.49140654	0.52964456	1
gene30656	55.78955055	39.68485098	0.711331254	-0.49140654	0.52964456	1
gene66297	55.78955055	39.68485098	0.711331254	-0.49140654	0.52964456	1
gene36759	1435.031217	1122.790906	0.782415666	-0.35399284	0.52968544	1
gene27062	1282.126523	1005.672199	0.784378282	-0.3503785	0.52970729	1
gene7406	915.361885	722.0707032	0.788836323	-0.34220211	0.52974936	1
gene44327	1372.009688	1762.588137	1.28467616	0.361404732	0.52978858	1
gene20378	734.5624156	580.7539168	0.790612077	-0.3389581	0.52988348	1
gene70578	772.7885892	980.5061962	1.268789692	0.343452956	0.52990453	1
gene9170	50.62385143	69.69047001	1.37663311	0.461144114	0.52996477	1
gene33639	50.62385143	69.69047001	1.37663311	0.461144114	0.52996477	1
gene54978	50.62385143	69.69047001	1.37663311	0.461144114	0.52996477	1
gene7235	274.8151935	348.4523501	1.267951548	0.342499617	0.52998893	1

gene36924	284.1134519	360.0674284	1.267336784	0.341799959	0.53002935	1
gene22128	49.5907116	34.84523501	0.702656483	-0.50910854	0.53003635	1
gene25909	49.5907116	34.84523501	0.702656483	-0.50910854	0.53003635	1
gene41946	49.5907116	34.84523501	0.702656483	-0.50910854	0.53003635	1
gene48993	49.5907116	34.84523501	0.702656483	-0.50910854	0.53003635	1
gene52032	49.5907116	34.84523501	0.702656483	-0.50910854	0.53003635	1
gene48156	848.2077964	669.8028507	0.78966835	-0.34068123	0.53003697	1
gene9244	170.4680711	131.6375545	0.772212377	-0.37293042	0.5300496	1
gene67574	94.01572408	123.8941689	1.317802635	0.398134316	0.5300609	1
gene49510	1007.311329	793.6970196	0.787936159	-0.34384935	0.53006353	1
gene26152	36.15989388	51.29992932	1.418696899	0.504566394	0.53008683	1
gene58590	140.5070162	107.4394746	0.764655584	-0.38711802	0.53010858	1
gene7815	1476.35681	1154.732371	0.782149927	-0.35448292	0.53024119	1
gene58581	423.5873283	334.9014253	0.79063136	-0.33892292	0.53030929	1
gene39809	1560.041136	1218.615302	0.781143057	-0.35634131	0.53032499	1
gene21864	122.9436392	159.7073271	1.299028792	0.377433408	0.53033072	1
gene3447	223.1582022	174.226175	0.780729426	-0.35710545	0.53034317	1
gene45799	1186.044519	932.1100364	0.785898018	-0.34758598	0.5303741	1
gene12110	315.1076466	398.7843562	1.2655496	0.339764051	0.53037692	1
gene74211	90.91630461	120.0224761	1.320142483	0.400693648	0.53038162	1
gene30628	282.0471722	221.6544116	0.785877092	-0.3476244	0.53038203	1
gene58951	296.5111298	233.2694899	0.786714111	-0.34608863	0.53039515	1
gene43821	911.2293257	719.1669336	0.789227161	-0.34148749	0.53051685	1
gene33993	133.2750374	101.6319354	0.762572927	-0.39105278	0.53053514	1
gene71720	215.9262234	168.4186359	0.779982316	-0.35848668	0.53063521	1
gene34485	87.81688513	116.1507834	1.322647497	0.403428616	0.53068945	1
gene48557	215.9262234	274.8901873	1.273074585	0.348316944	0.53072704	1
gene14063	53.7232709	73.56216279	1.3692793	0.453416752	0.53072755	1
gene29393	53.7232709	73.56216279	1.3692793	0.453416752	0.53072755	1
gene34227	82.65118601	60.97916126	0.737789307	-0.43871921	0.5307738	1
gene57694	82.65118601	60.97916126	0.737789307	-0.43871921	0.5307738	1
gene45597	339.9030025	429.7578984	1.264354523	0.338401049	0.53084606	1
gene14810	417.3884893	330.0618094	0.790778418	-0.3386546	0.53085744	1
gene35295	906.0636266	715.2952408	0.789453654	-0.34107352	0.53089575	1
gene50098	2254.311098	2943.454435	1.305700192	0.384823671	0.53090988	1
gene42965	1540.411479	1984.242549	1.288125008	0.365272609	0.53091456	1
gene23649	6735.03852	9440.154917	1.401648244	0.487124338	0.53093504	1
gene27359	1394.738764	1092.785287	0.783505352	-0.35198496	0.53093731	1
gene51065	445.2832646	352.3240428	0.791235761	-0.33782046	0.53099266	1
gene47938	3224.429394	4282.092213	1.3280155	0.409271985	0.531009	1
gene3820	27.89477528	40.65277417	1.457361594	0.543358877	0.53103033	1
gene29888	27.89477528	40.65277417	1.457361594	0.543358877	0.53103033	1
gene65545	27.89477528	40.65277417	1.457361594	0.543358877	0.53103033	1
gene71681	171.501211	219.7185652	1.281148768	0.357438013	0.53103438	1
gene6045	238.6552996	186.8091766	0.782757294	-0.35336305	0.53109183	1
gene13615	448.3826841	566.2350689	1.26283884	0.336670539	0.53109448	1
gene4796	81.61804618	108.4073978	1.328228289	0.409503131	0.53124565	1
gene18318	81.61804618	108.4073978	1.328228289	0.409503131	0.53124565	1

gene33045	81.61804618	108.4073978	1.328228289	0.409503131	0.53124565	1
gene67523	368.8309176	291.3448816	0.789914478	-0.34023163	0.53125554	1
gene55871	851.3072159	672.7066203	0.790204297	-0.3397024	0.53129785	1
gene35925	201.4622659	156.8035575	0.778327181	-0.36155135	0.5313396	1
gene65029	201.4622659	156.8035575	0.778327181	-0.36155135	0.5313396	1
gene61479	376.0628963	297.1524208	0.790166814	-0.33977084	0.53139577	1
gene4600	200.4291261	255.5317234	1.274923103	0.350410234	0.53140975	1
gene35370	89.88316478	66.78670043	0.74303904	-0.42849008	0.53143189	1
gene70212	89.88316478	66.78670043	0.74303904	-0.42849008	0.53143189	1
gene59386	459.7472222	363.9391212	0.791607004	-0.33714372	0.53146573	1
gene60635	78.51862671	104.535705	1.331349126	0.412888945	0.53148103	1
gene28457	148.7721348	114.214937	0.767717269	-0.381353	0.53149674	1
gene10966	516.5699125	652.3802332	1.262907919	0.336749453	0.53150389	1
gene8167	1553.842297	1214.743609	0.78176763	-0.35518825	0.53150591	1
gene3830	111.5791011	84.20931793	0.754705111	-0.40601505	0.53157136	1
gene62705	111.5791011	84.20931793	0.754705111	-0.40601505	0.53157136	1
gene12494	59.92210985	81.30554835	1.356853898	0.440265384	0.53161819	1
gene40577	501.0728152	396.8485098	0.791997685	-0.33643188	0.53164594	1
gene4466	75.41920723	100.6640122	1.33472647	0.416544116	0.53167702	1
gene27925	75.41920723	100.6640122	1.33472647	0.416544116	0.53167702	1
gene32792	97.11514356	72.5942396	0.747506897	-0.4198412	0.53171133	1
gene47361	97.11514356	72.5942396	0.747506897	-0.4198412	0.53171133	1
gene26194	712.8664793	564.2992225	0.791591748	-0.33717152	0.53171728	1
gene63191	104.3471223	78.40177877	0.751355447	-0.41243252	0.53173032	1
gene24688	194.2302871	150.9960184	0.777407173	-0.36325768	0.53175332	1
gene4101	1213.939294	954.3722699	0.78617792	-0.34707225	0.53175542	1
gene44373	901.9310673	1146.021062	1.270630433	0.345544479	0.53176658	1
gene12094	440.1175655	555.5879137	1.262362508	0.336126263	0.53176842	1
gene12698	72.31978776	96.79231946	1.338393301	0.420502129	0.53182334	1
gene45816	254.152397	199.3921781	0.784537862	-0.35008502	0.5318706	1
gene11039	69.22036828	92.92062669	1.342388505	0.424802267	0.53190755	1
gene56558	69.22036828	92.92062669	1.342388505	0.424802267	0.53190755	1
gene64274	69.22036828	92.92062669	1.342388505	0.424802267	0.53190755	1
gene68098	69.22036828	92.92062669	1.342388505	0.424802267	0.53190755	1
gene778	66.12094881	89.04893391	1.346758259	0.429490913	0.5319145	1
gene26298	66.12094881	89.04893391	1.346758259	0.429490913	0.5319145	1
gene2224	32.02733458	21.29431028	0.664879253	-0.58883574	0.53196408	1
gene46769	32.02733458	21.29431028	0.664879253	-0.58883574	0.53196408	1
gene49368	32.02733458	21.29431028	0.664879253	-0.58883574	0.53196408	1
gene19152	557.8955055	704.6480857	1.263046715	0.336908	0.53198204	1
gene23383	758.3246316	600.1123807	0.791366066	-0.33758289	0.53198543	1
gene50474	241.7547191	306.8316527	1.269185784	0.343903267	0.53200047	1
gene24814	141.540156	108.4073978	0.765912663	-0.3847482	0.53201354	1
gene51535	1263.530006	1617.399658	1.280064304	0.356216286	0.53202715	1
gene2780	582.6908613	461.6993638	0.792357311	-0.33577694	0.53213685	1
gene15180	668.4414668	529.4539875	0.792072326	-0.33629592	0.53217258	1
gene66691	39.25931335	55.17162209	1.405312966	0.490891457	0.5321798	1
gene12939	188.0314482	240.0449523	1.276621303	0.352330627	0.53218245	1

gene53386	188.0314482	240.0449523	1.276621303	0.352330627	0.53218245	1
gene49	16.5302372	9.679231946	0.585547069	-0.77214295	0.53218998	1
gene3804	16.5302372	9.679231946	0.585547069	-0.77214295	0.53218998	1
gene9826	16.5302372	9.679231946	0.585547069	-0.77214295	0.53218998	1
gene11830	16.5302372	9.679231946	0.585547069	-0.77214295	0.53218998	1
gene12752	16.5302372	9.679231946	0.585547069	-0.77214295	0.53218998	1
gene22971	16.5302372	9.679231946	0.585547069	-0.77214295	0.53218998	1
gene24941	16.5302372	9.679231946	0.585547069	-0.77214295	0.53218998	1
gene34999	16.5302372	9.679231946	0.585547069	-0.77214295	0.53218998	1
gene38516	16.5302372	9.679231946	0.585547069	-0.77214295	0.53218998	1
gene47389	16.5302372	9.679231946	0.585547069	-0.77214295	0.53218998	1
gene48928	16.5302372	9.679231946	0.585547069	-0.77214295	0.53218998	1
gene60303	16.5302372	9.679231946	0.585547069	-0.77214295	0.53218998	1
gene66616	16.5302372	9.679231946	0.585547069	-0.77214295	0.53218998	1
gene71766	16.5302372	9.679231946	0.585547069	-0.77214295	0.53218998	1
gene30857	1685.051055	2174.923418	1.29071663	0.368172299	0.53221979	1
gene49156	688.0711235	544.9407586	0.791983183	-0.3364583	0.53222801	1
gene73401	2424.779169	1871.963458	0.772013997	-0.37330109	0.53223087	1
gene59214	209.7273845	163.5790199	0.779960234	-0.35852752	0.53231531	1
gene43324	522.7687515	414.2711273	0.792455796	-0.33559763	0.53246802	1
gene66536	134.3081773	102.5998586	0.763913715	-0.3885184	0.53253225	1
gene5876	509.3379338	403.6239722	0.792448285	-0.33561131	0.53256449	1
gene35857	276.8814731	217.7827188	0.786555765	-0.34637904	0.53257872	1
gene61113	276.8814731	217.7827188	0.786555765	-0.34637904	0.53257872	1
gene29659	1169.514282	1493.505489	1.277030569	0.35279306	0.53272599	1
gene31683	356.4332397	281.6656496	0.790233958	-0.33964825	0.53275063	1
gene19305	104.3471223	136.4771704	1.307915038	0.387268826	0.53276098	1
gene47812	104.3471223	136.4771704	1.307915038	0.387268826	0.53276098	1
gene42173	874.036292	1109.239981	1.269100598	0.343806432	0.53285875	1
gene59375	3449.653876	2627.911473	0.761789898	-0.39253494	0.5328815	1
gene67233	225.2244819	176.1620214	0.782161957	-0.35446073	0.53289091	1
gene96	4.1325593	8.711308752	2.107969449	1.075853958	0.53290322	1
gene364	4.1325593	8.711308752	2.107969449	1.075853958	0.53290322	1
gene2778	4.1325593	8.711308752	2.107969449	1.075853958	0.53290322	1
gene2965	4.1325593	8.711308752	2.107969449	1.075853958	0.53290322	1
gene5255	4.1325593	8.711308752	2.107969449	1.075853958	0.53290322	1
gene7984	4.1325593	8.711308752	2.107969449	1.075853958	0.53290322	1
gene8117	4.1325593	8.711308752	2.107969449	1.075853958	0.53290322	1
gene11576	4.1325593	8.711308752	2.107969449	1.075853958	0.53290322	1
gene12013	4.1325593	8.711308752	2.107969449	1.075853958	0.53290322	1
gene12279	4.1325593	8.711308752	2.107969449	1.075853958	0.53290322	1
gene13203	4.1325593	8.711308752	2.107969449	1.075853958	0.53290322	1
gene13652	4.1325593	8.711308752	2.107969449	1.075853958	0.53290322	1
gene16836	4.1325593	8.711308752	2.107969449	1.075853958	0.53290322	1
gene18340	4.1325593	8.711308752	2.107969449	1.075853958	0.53290322	1
gene18835	4.1325593	8.711308752	2.107969449	1.075853958	0.53290322	1
gene23996	4.1325593	8.711308752	2.107969449	1.075853958	0.53290322	1
gene29362	4.1325593	8.711308752	2.107969449	1.075853958	0.53290322	1

gene30767	4.1325593	8.711308752	2.107969449	1.075853958	0.53290322	1
gene33974	4.1325593	8.711308752	2.107969449	1.075853958	0.53290322	1
gene37509	4.1325593	8.711308752	2.107969449	1.075853958	0.53290322	1
gene38347	4.1325593	8.711308752	2.107969449	1.075853958	0.53290322	1
gene39774	4.1325593	8.711308752	2.107969449	1.075853958	0.53290322	1
gene39950	4.1325593	8.711308752	2.107969449	1.075853958	0.53290322	1
gene40137	4.1325593	8.711308752	2.107969449	1.075853958	0.53290322	1
gene41861	4.1325593	8.711308752	2.107969449	1.075853958	0.53290322	1
gene42518	4.1325593	8.711308752	2.107969449	1.075853958	0.53290322	1
gene44326	4.1325593	8.711308752	2.107969449	1.075853958	0.53290322	1
gene44412	4.1325593	8.711308752	2.107969449	1.075853958	0.53290322	1
gene45872	4.1325593	8.711308752	2.107969449	1.075853958	0.53290322	1
gene49065	4.1325593	8.711308752	2.107969449	1.075853958	0.53290322	1
gene52683	4.1325593	8.711308752	2.107969449	1.075853958	0.53290322	1
gene55749	4.1325593	8.711308752	2.107969449	1.075853958	0.53290322	1
gene56075	4.1325593	8.711308752	2.107969449	1.075853958	0.53290322	1
gene56590	4.1325593	8.711308752	2.107969449	1.075853958	0.53290322	1
gene56891	4.1325593	8.711308752	2.107969449	1.075853958	0.53290322	1
gene57316	4.1325593	8.711308752	2.107969449	1.075853958	0.53290322	1
gene58827	4.1325593	8.711308752	2.107969449	1.075853958	0.53290322	1
gene59747	4.1325593	8.711308752	2.107969449	1.075853958	0.53290322	1
gene59820	4.1325593	8.711308752	2.107969449	1.075853958	0.53290322	1
gene65629	4.1325593	8.711308752	2.107969449	1.075853958	0.53290322	1
gene67063	4.1325593	8.711308752	2.107969449	1.075853958	0.53290322	1
gene68320	4.1325593	8.711308752	2.107969449	1.075853958	0.53290322	1
gene68898	4.1325593	8.711308752	2.107969449	1.075853958	0.53290322	1
gene70165	4.1325593	8.711308752	2.107969449	1.075853958	0.53290322	1
gene70247	4.1325593	8.711308752	2.107969449	1.075853958	0.53290322	1
gene70965	4.1325593	8.711308752	2.107969449	1.075853958	0.53290322	1
gene71757	4.1325593	8.711308752	2.107969449	1.075853958	0.53290322	1
gene72752	4.1325593	8.711308752	2.107969449	1.075853958	0.53290322	1
gene47114	760.3909113	602.0482271	0.791761472	-0.33686223	0.53290583	1
gene8281	1747.039444	1362.835858	0.78008305	-0.35830037	0.53294205	1
gene71444	444.2501248	560.4275297	1.2615135	0.335155645	0.53295628	1
gene14254	127.0761985	96.79231946	0.761687244	-0.39272936	0.53303834	1
gene51678	127.0761985	96.79231946	0.761687244	-0.39272936	0.53303834	1
gene72978	378.129176	299.0882671	0.7909685	-0.33830785	0.53305948	1
gene50236	76.45234706	56.13954529	0.734307676	-0.44554341	0.53307391	1
gene53630	76.45234706	56.13954529	0.734307676	-0.44554341	0.53307391	1
gene23186	381.2285955	481.0578277	1.261861868	0.335553992	0.53309778	1
gene16608	63.02152933	45.49239015	0.721854748	-0.47021953	0.53311797	1
gene19077	63.02152933	45.49239015	0.721854748	-0.47021953	0.53311797	1
gene19842	63.02152933	45.49239015	0.721854748	-0.47021953	0.53311797	1
gene24478	63.02152933	45.49239015	0.721854748	-0.47021953	0.53311797	1
gene53749	63.02152933	45.49239015	0.721854748	-0.47021953	0.53311797	1
gene41368	420.4879088	530.4219107	1.261443907	0.335076055	0.53314708	1
gene72338	533.1001497	672.7066203	1.26187663	0.335570869	0.5331856	1
gene46524	301.6768289	381.3617387	1.264139974	0.338156217	0.53318711	1

gene17811	702.5350811	888.5534927	1.264781669	0.338888364	0.53319495	1
gene30650	2822.538002	3716.825067	1.316837918	0.397077784	0.53325587	1
gene23459	317.1739263	400.7202026	1.263408399	0.337321069	0.53326591	1
gene40115	320.2733458	404.5918954	1.26327058	0.337163683	0.53329113	1
gene8876	390.5268539	492.6729061	1.261559612	0.335208379	0.53334618	1
gene14035	329.5716042	416.2069737	1.262872676	0.336709192	0.53338409	1
gene27529	306.842528	241.9807987	0.788615581	-0.34260588	0.53341129	1
gene27511	255.1855368	323.286347	1.266867829	0.341266018	0.53354011	1
gene20262	432.8855867	545.9086818	1.261092304	0.334673875	0.53357441	1
gene46546	357.4663795	282.6335728	0.790657777	-0.33887471	0.5336196	1
gene35673	188.0314482	146.1564024	0.777297648	-0.36346094	0.53368125	1
gene25479	618.8507552	490.7370597	0.792981273	-0.3346413	0.53371868	1
gene32660	1546.610318	1210.871916	0.782919849	-0.35306348	0.53379923	1
gene2267	22.72907615	33.87731181	1.490483449	0.575780355	0.53381738	1
gene22018	22.72907615	33.87731181	1.490483449	0.575780355	0.53381738	1
gene23836	22.72907615	33.87731181	1.490483449	0.575780355	0.53381738	1
gene44912	22.72907615	33.87731181	1.490483449	0.575780355	0.53381738	1
gene7986	165.302372	127.7658617	0.772922131	-0.37160502	0.53381917	1
gene24401	42.35873283	59.04331487	1.393887657	0.47911429	0.53382765	1
gene1763	684.971704	543.0049122	0.792740648	-0.33507914	0.53387965	1
gene35726	112.6122409	85.17724113	0.756376398	-0.40282375	0.53392557	1
gene54966	112.6122409	85.17724113	0.756376398	-0.40282375	0.53392557	1
gene67743	83.68432583	61.94708446	0.740247159	-0.43392105	0.53395456	1
gene21927	26660.17319	45874.71981	1.720720998	0.783013194	0.53407322	1
gene27237	518.6361922	411.3673577	0.793171329	-0.33429557	0.53409974	1
gene15059	226.2576217	177.1299446	0.78286841	-0.35315826	0.53415091	1
gene70516	192.1640075	244.8845682	1.274351901	0.34976372	0.53419098	1
gene43688	105.3802622	79.36970196	0.753174269	-0.40894438	0.53424144	1
gene70086	105.3802622	79.36970196	0.753174269	-0.40894438	0.53424144	1
gene35074	852.3403557	674.6424667	0.791517687	-0.33730651	0.53424392	1
gene71137	322.3396254	254.5638002	0.789737842	-0.34055427	0.53434831	1
gene4461	10.33139825	17.4226175	1.686375559	0.753925863	0.53435274	1
gene6682	10.33139825	17.4226175	1.686375559	0.753925863	0.53435274	1
gene16663	10.33139825	17.4226175	1.686375559	0.753925863	0.53435274	1
gene16773	10.33139825	17.4226175	1.686375559	0.753925863	0.53435274	1
gene19805	10.33139825	17.4226175	1.686375559	0.753925863	0.53435274	1
gene21398	10.33139825	17.4226175	1.686375559	0.753925863	0.53435274	1
gene25682	10.33139825	17.4226175	1.686375559	0.753925863	0.53435274	1
gene37472	10.33139825	17.4226175	1.686375559	0.753925863	0.53435274	1
gene45915	10.33139825	17.4226175	1.686375559	0.753925863	0.53435274	1
gene53829	10.33139825	17.4226175	1.686375559	0.753925863	0.53435274	1
gene56201	10.33139825	17.4226175	1.686375559	0.753925863	0.53435274	1
gene57820	10.33139825	17.4226175	1.686375559	0.753925863	0.53435274	1
gene66495	10.33139825	17.4226175	1.686375559	0.753925863	0.53435274	1
gene71664	10.33139825	17.4226175	1.686375559	0.753925863	0.53435274	1
gene72187	10.33139825	17.4226175	1.686375559	0.753925863	0.53435274	1
gene72246	10.33139825	17.4226175	1.686375559	0.753925863	0.53435274	1
gene42626	307.8756679	242.9487219	0.789113097	-0.34169601	0.53439499	1

gene1560	17.56337703	27.10184945	1.543088747	0.625821037	0.53440401	1
gene4816	17.56337703	27.10184945	1.543088747	0.625821037	0.53440401	1
gene17579	17.56337703	27.10184945	1.543088747	0.625821037	0.53440401	1
gene19015	17.56337703	27.10184945	1.543088747	0.625821037	0.53440401	1
gene34915	17.56337703	27.10184945	1.543088747	0.625821037	0.53440401	1
gene62835	17.56337703	27.10184945	1.543088747	0.625821037	0.53440401	1
gene73722	98.14828338	73.56216279	0.749500249	-0.41599914	0.53440668	1
gene4658	2196.455268	1703.544823	0.775588216	-0.36663721	0.53443319	1
gene36806	56.82269038	40.65277417	0.715432055	-0.48311333	0.53443905	1
gene66638	56.82269038	40.65277417	0.715432055	-0.48311333	0.53443905	1
gene70129	56.82269038	40.65277417	0.715432055	-0.48311333	0.53443905	1
gene36282	30.99419475	44.52446695	1.436542143	0.522600317	0.53448475	1
gene41325	30.99419475	44.52446695	1.436542143	0.522600317	0.53448475	1
gene47700	30.99419475	44.52446695	1.436542143	0.522600317	0.53448475	1
gene50980	30.99419475	44.52446695	1.436542143	0.522600317	0.53448475	1
gene52082	30.99419475	44.52446695	1.436542143	0.522600317	0.53448475	1
gene61290	30.99419475	44.52446695	1.436542143	0.522600317	0.53448475	1
gene16662	135.3413171	103.5677818	0.765234032	-0.38602706	0.53450391	1
gene17606	135.3413171	103.5677818	0.765234032	-0.38602706	0.53450391	1
gene65042	365.7314981	289.4090352	0.791315587	-0.33767492	0.53454846	1
gene12851	196.2965668	152.9318648	0.779085785	-0.3601459	0.53459748	1
gene16100	1241.83407	1586.426116	1.277486385	0.353307915	0.53460477	1
gene19733	802.7496441	635.9255389	0.792184143	-0.33609227	0.53467132	1
gene41099	302.7099687	382.3296619	1.26302303	0.336880945	0.53468868	1
gene58824	1299.6899	1022.126894	0.786439053	-0.34659313	0.53469448	1
gene21216	290.3122908	366.8428908	1.263614743	0.337556675	0.53474526	1
gene63238	553.7629462	439.4371304	0.793547371	-0.33361175	0.53478081	1
gene37793	271.715774	213.911026	0.787260242	-0.34508747	0.53486971	1
gene3061	8.265118601	3.871692779	0.468437655	-1.09407104	0.53488832	1
gene4246	8.265118601	3.871692779	0.468437655	-1.09407104	0.53488832	1
gene9713	8.265118601	3.871692779	0.468437655	-1.09407104	0.53488832	1
gene12051	8.265118601	3.871692779	0.468437655	-1.09407104	0.53488832	1
gene14589	8.265118601	3.871692779	0.468437655	-1.09407104	0.53488832	1
gene15042	8.265118601	3.871692779	0.468437655	-1.09407104	0.53488832	1
gene17687	8.265118601	3.871692779	0.468437655	-1.09407104	0.53488832	1
gene18057	8.265118601	3.871692779	0.468437655	-1.09407104	0.53488832	1
gene19779	8.265118601	3.871692779	0.468437655	-1.09407104	0.53488832	1
gene21028	8.265118601	3.871692779	0.468437655	-1.09407104	0.53488832	1
gene25824	8.265118601	3.871692779	0.468437655	-1.09407104	0.53488832	1
gene26090	8.265118601	3.871692779	0.468437655	-1.09407104	0.53488832	1
gene31365	8.265118601	3.871692779	0.468437655	-1.09407104	0.53488832	1
gene36278	8.265118601	3.871692779	0.468437655	-1.09407104	0.53488832	1
gene37680	8.265118601	3.871692779	0.468437655	-1.09407104	0.53488832	1
gene38284	8.265118601	3.871692779	0.468437655	-1.09407104	0.53488832	1
gene39294	8.265118601	3.871692779	0.468437655	-1.09407104	0.53488832	1
gene41747	8.265118601	3.871692779	0.468437655	-1.09407104	0.53488832	1
gene43023	8.265118601	3.871692779	0.468437655	-1.09407104	0.53488832	1
gene46515	8.265118601	3.871692779	0.468437655	-1.09407104	0.53488832	1

gene47396	8.265118601	3.871692779	0.468437655	-1.09407104	0.53488832	1
gene51326	8.265118601	3.871692779	0.468437655	-1.09407104	0.53488832	1
gene51657	8.265118601	3.871692779	0.468437655	-1.09407104	0.53488832	1
gene56395	8.265118601	3.871692779	0.468437655	-1.09407104	0.53488832	1
gene57630	8.265118601	3.871692779	0.468437655	-1.09407104	0.53488832	1
gene59452	8.265118601	3.871692779	0.468437655	-1.09407104	0.53488832	1
gene59637	8.265118601	3.871692779	0.468437655	-1.09407104	0.53488832	1
gene59817	8.265118601	3.871692779	0.468437655	-1.09407104	0.53488832	1
gene68422	8.265118601	3.871692779	0.468437655	-1.09407104	0.53488832	1
gene24595	613.6850561	774.3385557	1.26178493	0.335466026	0.53494003	1
gene833	38.22617353	26.13392626	0.683665767	-0.54863691	0.53494077	1
gene3524	38.22617353	26.13392626	0.683665767	-0.54863691	0.53494077	1
gene20269	38.22617353	26.13392626	0.683665767	-0.54863691	0.53494077	1
gene36945	38.22617353	26.13392626	0.683665767	-0.54863691	0.53494077	1
gene60315	38.22617353	26.13392626	0.683665767	-0.54863691	0.53494077	1
gene3378	182.865749	233.2694899	1.275632485	0.351212743	0.53496505	1
gene32277	182.865749	233.2694899	1.275632485	0.351212743	0.53496505	1
gene34053	574.4257427	455.8918247	0.79364797	-0.33342887	0.53500818	1
gene57639	680.8391447	540.1011426	0.793287441	-0.33408439	0.5350481	1
gene33481	5291.742184	3949.126634	0.746280997	-0.42220914	0.53507396	1
gene51805	150.8384145	116.1507834	0.770034502	-0.37700501	0.53508003	1
gene55314	128.1093383	97.76024266	0.763100051	-0.39005587	0.53511081	1
gene25225	45.4581523	62.91500765	1.384020345	0.468865151	0.53512696	1
gene53007	45.4581523	62.91500765	1.384020345	0.468865151	0.53512696	1
gene55905	45.4581523	62.91500765	1.384020345	0.468865151	0.53512696	1
gene69120	45.4581523	62.91500765	1.384020345	0.468865151	0.53512696	1
gene55343	361.5989388	455.8918247	1.26076649	0.334301094	0.53513804	1
gene33239	157.0372534	201.3280245	1.282039899	0.358441161	0.53514011	1
gene4372	134.3081773	173.2582518	1.290005236	0.367376921	0.53526562	1
gene29598	873.0031522	1106.336211	1.267276308	0.341731114	0.53527995	1
gene63659	345.0687016	272.9543409	0.791014484	-0.33822398	0.53528204	1
gene56178	257.2518164	202.2959477	0.786373253	-0.34671384	0.53529176	1
gene22720	70.25350811	51.29992932	0.730211639	-0.45361343	0.53529209	1
gene74115	85.75060548	113.2470138	1.320655558	0.401254245	0.5353439	1
gene55699	26.86163545	17.4226175	0.648605984	-0.62458576	0.53536732	1
gene61099	26.86163545	17.4226175	0.648605984	-0.62458576	0.53536732	1
gene62757	26.86163545	17.4226175	0.648605984	-0.62458576	0.53536732	1
gene66636	26.86163545	17.4226175	0.648605984	-0.62458576	0.53536732	1
gene71384	26.86163545	17.4226175	0.648605984	-0.62458576	0.53536732	1
gene73155	26.86163545	17.4226175	0.648605984	-0.62458576	0.53536732	1
gene8711	778.9874281	985.3458121	1.264905923	0.339030089	0.53538497	1
gene56404	2716.1246	3564.861126	1.312480703	0.392296212	0.53539405	1
gene8734	227.2907615	178.0978678	0.783568442	-0.3518688	0.5354018	1
gene64899	50.62385143	35.8131582	0.707436459	-0.49932752	0.53541495	1
gene32398	1250.099188	984.3778889	0.787439827	-0.34475841	0.53551593	1
gene70163	176.6669101	225.5261044	1.276561096	0.352262586	0.53555852	1
gene61157	243.8209987	308.7674991	1.266369594	0.340698521	0.53564285	1
gene21023	120.8773595	91.95270349	0.760710722	-0.39458015	0.53569873	1

gene19759	131.2087578	169.3865591	1.290969916	0.368455382	0.53572722	1
gene56288	240.7215792	304.8958063	1.266591085	0.34095083	0.53575027	1
gene21306	44.42501248	30.97354223	0.697209534	-0.5203358	0.53575666	1
gene24042	44.42501248	30.97354223	0.697209534	-0.5203358	0.53575666	1
gene53652	44.42501248	30.97354223	0.697209534	-0.5203358	0.53575666	1
gene10792	1765.635961	1379.290552	0.78118626	-0.35626152	0.53577691	1
gene12884	220.0587827	172.2903286	0.782928663	-0.35304723	0.53582514	1
gene63983	535.1664294	424.9182824	0.793992783	-0.3328022	0.53583564	1
gene14911	173.5674906	221.6544116	1.277050275	0.352815322	0.53587967	1
gene18327	1335.849794	1708.384439	1.278874651	0.354874865	0.53595479	1
gene13894	417.3884893	331.0297326	0.793097416	-0.33443001	0.53602206	1
gene69543	2554.954787	1973.595394	0.77245805	-0.37247151	0.53606933	1
gene1893	159.1035331	122.9262457	0.772617951	-0.3721729	0.53612562	1
gene34289	2575.617584	1989.082165	0.772273872	-0.37281553	0.53614362	1
gene32685	424.6204681	336.8372717	0.793266686	-0.33412213	0.5361455	1
gene24268	346.1018414	273.9222641	0.791449889	-0.33743009	0.53616876	1
gene61873	338.8698626	268.1147249	0.791202625	-0.33788088	0.53617281	1
gene30358	128.1093383	165.5148663	1.291981275	0.369585161	0.53620642	1
gene9438	450.4489637	567.2029921	1.259194798	0.332501486	0.5362243	1
gene22082	297.5442696	375.5541995	1.262179238	0.335916797	0.53623209	1
gene4921	367.7977777	291.3448816	0.792133339	-0.3361848	0.53623679	1
gene60710	367.7977777	291.3448816	0.792133339	-0.3361848	0.53623679	1
gene4553	113.6453808	86.14516432	0.758017297	-0.39969733	0.5362433	1
gene12544	113.6453808	86.14516432	0.758017297	-0.39969733	0.5362433	1
gene40207	113.6453808	86.14516432	0.758017297	-0.39969733	0.5362433	1
gene41142	113.6453808	86.14516432	0.758017297	-0.39969733	0.5362433	1
gene4004	317.1739263	250.6921074	0.790393177	-0.3393576	0.53627721	1
gene13967	285.1465917	360.0674284	1.262744984	0.336563311	0.53638022	1
gene18831	914.3287452	724.0065496	0.791844895	-0.33671023	0.53640553	1
gene42361	258.2849563	203.2638709	0.786975261	-0.34560981	0.53641775	1
gene53053	583.7240012	735.6216279	1.260221657	0.333677508	0.5364418	1
gene61596	423.5873283	533.3256802	1.259069015	0.332357366	0.53647846	1
gene41307	76.45234706	101.6319354	1.329350103	0.410721109	0.53648881	1
gene68257	76.45234706	101.6319354	1.329350103	0.410721109	0.53648881	1
gene18453	703.5682209	558.4916833	0.793798905	-0.33315452	0.5365115	1
gene42583	6963.362421	9742.146954	1.399057864	0.484455632	0.53656187	1
gene12667	742.8275342	589.4652255	0.793542509	-0.33362059	0.53657131	1
gene49273	167.3686517	213.911026	1.278082986	0.353981513	0.53657288	1
gene2548	21.69593633	13.55092472	0.62458354	-0.67903354	0.53658449	1
gene2927	21.69593633	13.55092472	0.62458354	-0.67903354	0.53658449	1
gene4517	21.69593633	13.55092472	0.62458354	-0.67903354	0.53658449	1
gene14805	21.69593633	13.55092472	0.62458354	-0.67903354	0.53658449	1
gene18128	21.69593633	13.55092472	0.62458354	-0.67903354	0.53658449	1
gene21035	21.69593633	13.55092472	0.62458354	-0.67903354	0.53658449	1
gene41182	21.69593633	13.55092472	0.62458354	-0.67903354	0.53658449	1
gene52487	21.69593633	13.55092472	0.62458354	-0.67903354	0.53658449	1
gene62878	21.69593633	13.55092472	0.62458354	-0.67903354	0.53658449	1
gene72966	21.69593633	13.55092472	0.62458354	-0.67903354	0.53658449	1

gene59368	707.7007802	893.3931087	1.262388192	0.336155616	0.53664152	1
gene12155	190.0977278	241.9807987	1.272928411	0.348151285	0.53667198	1
gene16794	190.0977278	241.9807987	1.272928411	0.348151285	0.53667198	1
gene65820	125.0099188	161.6431735	1.293042784	0.370770012	0.53670323	1
gene14021	106.413402	80.33762515	0.754957775	-0.40553214	0.53671064	1
gene2666	51.65699125	70.65839321	1.367837954	0.451897326	0.53674295	1
gene1498	1171.580562	1492.537566	1.273952142	0.349311082	0.53679658	1
gene43929	205.5948252	160.6752503	0.781514078	-0.35565623	0.53681945	1
gene38353	281.0140324	221.6544116	0.788766346	-0.3423301	0.53682861	1
gene46239	730.4298563	579.7859936	0.793759987	-0.33322526	0.53684729	1
gene1045	460.780362	365.8749676	0.794033335	-0.33272852	0.53686228	1
gene19500	638.4804119	507.191754	0.794373241	-0.33211107	0.53700053	1
gene22835	99.18142321	74.53008599	0.751452072	-0.412247	0.53705331	1
gene54343	99.18142321	74.53008599	0.751452072	-0.412247	0.53705331	1
gene42656	70.25350811	93.88854988	1.336425076	0.418378958	0.53708159	1
gene18751	34.09361423	48.39615973	1.419508047	0.505391027	0.53709188	1
gene65569	34.09361423	48.39615973	1.419508047	0.505391027	0.53709188	1
gene54005	1007.311329	1278.62654	1.269345934	0.344085299	0.53714594	1
gene38943	54.75641073	74.53008599	1.361120734	0.444795043	0.53716229	1
gene53533	54.75641073	74.53008599	1.361120734	0.444795043	0.53716229	1
gene54227	54.75641073	74.53008599	1.361120734	0.444795043	0.53716229	1
gene2818	91.94944443	68.72254682	0.747394911	-0.42005735	0.53720412	1
gene17402	251.0529775	317.4788078	1.264588897	0.338668458	0.53721722	1
gene32793	121.9104994	157.7714807	1.294158268	0.372014062	0.53721764	1
gene71421	121.9104994	157.7714807	1.294158268	0.372014062	0.53721764	1
gene23867	783.1199874	621.406691	0.793501253	-0.33369559	0.53722133	1
gene8473	182.865749	142.2847096	0.778082885	-0.36200425	0.53722608	1
gene70891	318.2070661	251.6600306	0.790868769	-0.33848977	0.53722631	1
gene67425	447.3495443	355.2278124	0.794072146	-0.33265801	0.53726879	1
gene68795	67.15408863	90.0168571	1.340452368	0.422719955	0.5372943	1
gene57429	645.7123907	814.0234067	1.260659418	0.334178567	0.53730811	1
gene5657	64.05466916	46.46031334	0.725322821	-0.46330485	0.53732517	1
gene7704	64.05466916	46.46031334	0.725322821	-0.46330485	0.53732517	1
gene16449	64.05466916	46.46031334	0.725322821	-0.46330485	0.53732517	1
gene30918	57.8558302	78.40177877	1.355123217	0.438424037	0.5373924	1
gene47591	57.8558302	78.40177877	1.355123217	0.438424037	0.5373924	1
gene54127	57.8558302	78.40177877	1.355123217	0.438424037	0.5373924	1
gene57527	531.0338701	422.0145129	0.794703571	-0.33151127	0.53744925	1
gene41577	60.95524968	82.27347154	1.349735617	0.432676843	0.53747177	1
gene25244	259.3180961	204.2317941	0.787572472	-0.34451541	0.53753665	1
gene14136	461.8135018	366.8428908	0.794352892	-0.33214803	0.53755458	1
gene26385	213.8599438	167.4507127	0.782992409	-0.35292977	0.53760384	1
gene20842	289.279151	228.4298739	0.789652048	-0.34071101	0.53769812	1
gene16790	469.0454806	372.6504299	0.794486772	-0.3319049	0.53770618	1
gene59496	640.5466916	807.2479443	1.260248402	0.333708125	0.53774637	1
gene70771	121.9104994	92.92062669	0.762203643	-0.39175159	0.53785417	1
gene16354	369.8640574	293.280728	0.792941953	-0.33471284	0.5379093	1
gene1449	175.6337703	136.4771704	0.777055405	-0.36391063	0.53791697	1

gene25179	924.6601434	732.7178583	0.792418559	-0.33566543	0.53792929	1
gene41671	384.3280149	304.8958063	0.793321836	-0.33402184	0.53796046	1
gene66090	191.1308676	149.060172	0.779885394	-0.35866596	0.53802215	1
gene49240	200.4291261	254.5638002	1.270093849	0.344935104	0.53804005	1
gene17156	646.7455305	814.9913299	1.260142191	0.333586533	0.5380549	1
gene34433	398.7919725	316.5108846	0.793674162	-0.33338126	0.53805663	1
gene48981	1875.148783	1464.467793	0.780987518	-0.3566286	0.53810237	1
gene54124	397.7588327	500.4162916	1.258089703	0.331234791	0.53813903	1
gene6592	8.265118601	14.51884792	1.756641208	0.812819552	0.53815904	1
gene9930	8.265118601	14.51884792	1.756641208	0.812819552	0.53815904	1
gene10274	8.265118601	14.51884792	1.756641208	0.812819552	0.53815904	1
gene17250	8.265118601	14.51884792	1.756641208	0.812819552	0.53815904	1
gene18753	8.265118601	14.51884792	1.756641208	0.812819552	0.53815904	1
gene28064	8.265118601	14.51884792	1.756641208	0.812819552	0.53815904	1
gene30394	8.265118601	14.51884792	1.756641208	0.812819552	0.53815904	1
gene30651	8.265118601	14.51884792	1.756641208	0.812819552	0.53815904	1
gene33341	8.265118601	14.51884792	1.756641208	0.812819552	0.53815904	1
gene34207	8.265118601	14.51884792	1.756641208	0.812819552	0.53815904	1
gene35051	8.265118601	14.51884792	1.756641208	0.812819552	0.53815904	1
gene38321	8.265118601	14.51884792	1.756641208	0.812819552	0.53815904	1
gene38628	8.265118601	14.51884792	1.756641208	0.812819552	0.53815904	1
gene43961	8.265118601	14.51884792	1.756641208	0.812819552	0.53815904	1
gene52547	8.265118601	14.51884792	1.756641208	0.812819552	0.53815904	1
gene53900	8.265118601	14.51884792	1.756641208	0.812819552	0.53815904	1
gene56693	8.265118601	14.51884792	1.756641208	0.812819552	0.53815904	1
gene60561	8.265118601	14.51884792	1.756641208	0.812819552	0.53815904	1
gene61805	8.265118601	14.51884792	1.756641208	0.812819552	0.53815904	1
gene62267	8.265118601	14.51884792	1.756641208	0.812819552	0.53815904	1
gene65315	8.265118601	14.51884792	1.756641208	0.812819552	0.53815904	1
gene67728	8.265118601	14.51884792	1.756641208	0.812819552	0.53815904	1
gene70789	8.265118601	14.51884792	1.756641208	0.812819552	0.53815904	1
gene74254	8.265118601	14.51884792	1.756641208	0.812819552	0.53815904	1
gene18439	206.627965	161.6431735	0.782290884	-0.35422294	0.53816192	1
gene35506	244.8541385	192.6167157	0.786659016	-0.34618967	0.5382233	1
gene18193	753.1589325	598.1765343	0.794223514	-0.33238302	0.53827662	1
gene6357	518.6361922	412.3352809	0.795037614	-0.33090498	0.53827979	1
gene40638	115.7116604	150.0280952	1.29656851	0.37469844	0.53829866	1
gene50590	115.7116604	150.0280952	1.29656851	0.37469844	0.53829866	1
gene12912	1370.976548	1079.234362	0.787201184	-0.3451957	0.53832427	1
gene64133	7496.462571	10543.58736	1.406475022	0.492083932	0.53836737	1
gene4496	12.3976779	6.775462362	0.546510598	-0.87167862	0.53843434	1
gene13303	12.3976779	6.775462362	0.546510598	-0.87167862	0.53843434	1
gene23970	12.3976779	6.775462362	0.546510598	-0.87167862	0.53843434	1
gene37467	12.3976779	6.775462362	0.546510598	-0.87167862	0.53843434	1
gene45152	12.3976779	6.775462362	0.546510598	-0.87167862	0.53843434	1
gene46725	12.3976779	6.775462362	0.546510598	-0.87167862	0.53843434	1
gene57199	12.3976779	6.775462362	0.546510598	-0.87167862	0.53843434	1
gene57274	12.3976779	6.775462362	0.546510598	-0.87167862	0.53843434	1

gene63531	12.3976779	6.775462362	0.546510598	-0.87167862	0.53843434	1
gene67316	12.3976779	6.775462362	0.546510598	-0.87167862	0.53843434	1
gene68707	12.3976779	6.775462362	0.546510598	-0.87167862	0.53843434	1
gene68853	12.3976779	6.775462362	0.546510598	-0.87167862	0.53843434	1
gene73619	12.3976779	6.775462362	0.546510598	-0.87167862	0.53843434	1
gene17209	900.8979275	714.3273176	0.79290594	-0.33477836	0.53844748	1
gene24161	1842.088308	2375.28352	1.289451493	0.366757503	0.53845266	1
gene13897	730.4298563	921.4628813	1.261535072	0.335180315	0.53849747	1
gene44953	9367.478794	13474.45879	1.438429602	0.524494616	0.53852027	1
gene70288	114.6785206	87.11308752	0.75962863	-0.39663381	0.53852541	1
gene60061	92.98258426	121.9583225	1.311625435	0.391355784	0.53856163	1
gene31442	721.1315979	573.0105312	0.794599117	-0.3317009	0.53857399	1
gene66250	152.9046941	118.0866297	0.772289107	-0.37278707	0.5385833	1
gene43624	194.2302871	246.8204146	1.270761725	0.345693542	0.53859497	1
gene14511	25.82849563	37.74900459	1.461525485	0.547474986	0.53862978	1
gene14595	25.82849563	37.74900459	1.461525485	0.547474986	0.53862978	1
gene21134	25.82849563	37.74900459	1.461525485	0.547474986	0.53862978	1
gene62532	25.82849563	37.74900459	1.461525485	0.547474986	0.53862978	1
gene65463	25.82849563	37.74900459	1.461525485	0.547474986	0.53862978	1
gene30208	260.3512359	205.1997173	0.788164944	-0.34343051	0.53864855	1
gene3662	168.4017915	130.6696313	0.775939675	-0.3659836	0.5386609	1
gene73413	132.2418976	170.3544823	1.288203552	0.365360575	0.53874907	1
gene7145	216.9593633	274.8901873	1.267012325	0.341430558	0.53891565	1
gene64481	1002.14563	793.6970196	0.791997685	-0.33643188	0.53892176	1
gene2937	6.19883895	11.61507834	1.873750621	0.905928957	0.53893662	1
gene4748	6.19883895	11.61507834	1.873750621	0.905928957	0.53893662	1
gene5131	6.19883895	11.61507834	1.873750621	0.905928957	0.53893662	1
gene6363	6.19883895	11.61507834	1.873750621	0.905928957	0.53893662	1
gene11107	6.19883895	11.61507834	1.873750621	0.905928957	0.53893662	1
gene12733	6.19883895	11.61507834	1.873750621	0.905928957	0.53893662	1
gene17176	6.19883895	11.61507834	1.873750621	0.905928957	0.53893662	1
gene17592	6.19883895	11.61507834	1.873750621	0.905928957	0.53893662	1
gene19443	6.19883895	11.61507834	1.873750621	0.905928957	0.53893662	1
gene21612	6.19883895	11.61507834	1.873750621	0.905928957	0.53893662	1
gene29523	6.19883895	11.61507834	1.873750621	0.905928957	0.53893662	1
gene30125	6.19883895	11.61507834	1.873750621	0.905928957	0.53893662	1
gene30459	6.19883895	11.61507834	1.873750621	0.905928957	0.53893662	1
gene33480	6.19883895	11.61507834	1.873750621	0.905928957	0.53893662	1
gene38330	6.19883895	11.61507834	1.873750621	0.905928957	0.53893662	1
gene40317	6.19883895	11.61507834	1.873750621	0.905928957	0.53893662	1
gene42983	6.19883895	11.61507834	1.873750621	0.905928957	0.53893662	1
gene44168	6.19883895	11.61507834	1.873750621	0.905928957	0.53893662	1
gene46936	6.19883895	11.61507834	1.873750621	0.905928957	0.53893662	1
gene48155	6.19883895	11.61507834	1.873750621	0.905928957	0.53893662	1
gene49876	6.19883895	11.61507834	1.873750621	0.905928957	0.53893662	1
gene50956	6.19883895	11.61507834	1.873750621	0.905928957	0.53893662	1
gene51374	6.19883895	11.61507834	1.873750621	0.905928957	0.53893662	1
gene53212	6.19883895	11.61507834	1.873750621	0.905928957	0.53893662	1

gene53819	6.19883895	11.61507834	1.873750621	0.905928957	0.53893662	1
gene57645	6.19883895	11.61507834	1.873750621	0.905928957	0.53893662	1
gene58945	6.19883895	11.61507834	1.873750621	0.905928957	0.53893662	1
gene59794	6.19883895	11.61507834	1.873750621	0.905928957	0.53893662	1
gene61272	6.19883895	11.61507834	1.873750621	0.905928957	0.53893662	1
gene61497	6.19883895	11.61507834	1.873750621	0.905928957	0.53893662	1
gene65121	6.19883895	11.61507834	1.873750621	0.905928957	0.53893662	1
gene65139	6.19883895	11.61507834	1.873750621	0.905928957	0.53893662	1
gene67528	6.19883895	11.61507834	1.873750621	0.905928957	0.53893662	1
gene68504	6.19883895	11.61507834	1.873750621	0.905928957	0.53893662	1
gene69880	6.19883895	11.61507834	1.873750621	0.905928957	0.53893662	1
gene71680	6.19883895	11.61507834	1.873750621	0.905928957	0.53893662	1
gene73407	6.19883895	11.61507834	1.873750621	0.905928957	0.53893662	1
gene74216	6.19883895	11.61507834	1.873750621	0.905928957	0.53893662	1
gene18076	504.1722346	633.9896925	1.257486329	0.330542716	0.53896946	1
gene61071	327.5053246	259.4034162	0.792058622	-0.33632088	0.53900572	1
gene63210	71.28664793	52.26785251	0.733206765	-0.447708	0.53901322	1
gene24754	37.1930337	52.26785251	1.405312966	0.490891457	0.5390688	1
gene14756	57.8558302	41.62069737	0.719386399	-0.47516121	0.5390858	1
gene30392	57.8558302	41.62069737	0.719386399	-0.47516121	0.5390858	1
gene47728	57.8558302	41.62069737	0.719386399	-0.47516121	0.5390858	1
gene49975	107.4465418	81.30554835	0.756706982	-0.40219334	0.53913901	1
gene42909	1506.317865	1929.070927	1.280653288	0.356879947	0.53917191	1
gene45012	130.175618	99.69608905	0.765858389	-0.38485044	0.53917275	1
gene3257	443.216985	352.3240428	0.794924506	-0.33111024	0.53928372	1
gene55537	622.9833145	495.5766757	0.795489484	-0.33008524	0.53937133	1
gene31815	3447.587596	2637.590705	0.765054007	-0.3863665	0.53941066	1
gene19435	161.1698127	124.8620921	0.774723815	-0.36824601	0.53945756	1
gene55762	5097.511897	3824.264542	0.750221798	-0.41461091	0.53948717	1
gene28603	634.3478526	798.5366356	1.258830833	0.33208442	0.53956638	1
gene2194	740.7612546	934.0458828	1.260927022	0.33448478	0.53965088	1
gene7506	408.0902309	324.2542702	0.794565137	-0.3317626	0.53965147	1
gene60125	86.78374531	114.214937	1.316086746	0.396254583	0.53965158	1
gene23624	100.214563	75.49800918	0.753363652	-0.40858167	0.53965259	1
gene63782	100.214563	75.49800918	0.753363652	-0.40858167	0.53965259	1
gene65971	291.3454307	230.3657203	0.790696184	-0.33880463	0.5397345	1
gene13169	261.3843757	206.1676405	0.788752732	-0.342355	0.53975351	1
gene55947	78.51862671	58.07539168	0.739638403	-0.43510796	0.53984472	1
gene55584	669.4746067	843.0611025	1.259287648	0.332607862	0.53985267	1
gene71464	181.8326092	231.3336435	1.272234087	0.347364146	0.5399019	1
gene24998	672.5740261	846.9327953	1.259241009	0.33255443	0.54000261	1
gene8176	92.98258426	69.69047001	0.749500249	-0.41599914	0.54000591	1
gene8981	92.98258426	69.69047001	0.749500249	-0.41599914	0.54000591	1
gene33224	92.98258426	69.69047001	0.749500249	-0.41599914	0.54000591	1
gene56628	92.98258426	69.69047001	0.749500249	-0.41599914	0.54000591	1
gene54406	106.413402	138.4130168	1.300710383	0.379299766	0.54004572	1
gene5366	1400.937603	1103.432442	0.787638536	-0.3443944	0.54009953	1
gene30074	184.9320287	144.220556	0.779857102	-0.3587183	0.54017169	1

gene18827	215.9262234	169.3865591	0.784464973	-0.35021906	0.54018729	1
gene52626	83.68432583	110.3432442	1.318565252	0.398968968	0.54019029	1
gene63641	684.971704	544.9407586	0.795566817	-0.32994499	0.54020957	1
gene30847	340.9361423	428.7899752	1.257684129	0.330769631	0.54023226	1
gene70798	340.9361423	428.7899752	1.257684129	0.330769631	0.54023226	1
gene17659	227.2907615	287.4731888	1.264781669	0.338888364	0.54026012	1
gene3143	570.2931834	453.9559783	0.796004567	-0.32915139	0.54028722	1
gene35224	33.0604744	22.26223348	0.67337913	-0.57050909	0.54030165	1
gene39496	33.0604744	22.26223348	0.67337913	-0.57050909	0.54030165	1
gene40724	33.0604744	22.26223348	0.67337913	-0.57050909	0.54030165	1
gene28867	231.4233208	181.9695606	0.786306064	-0.34683711	0.54031631	1
gene28670	262.4175156	331.0297326	1.261462033	0.335096785	0.54032103	1
gene34185	306.842528	242.9487219	0.791770044	-0.33684661	0.54034694	1
gene41194	394.6594132	313.6071151	0.794627227	-0.33164987	0.54036679	1
gene49484	2193.355849	1707.416515	0.778449387	-0.36132485	0.54037779	1
gene35374	365.7314981	290.3769584	0.793962128	-0.3328579	0.54042312	1
gene10735	122.9436392	158.7394039	1.29115589	0.368663198	0.54042576	1
gene8287	611.6187764	486.8653669	0.796027502	-0.32910982	0.54049826	1
gene29852	40.29245318	56.13954529	1.393301744	0.478507733	0.5405694	1
gene57064	51.65699125	36.7810814	0.712025236	-0.48999972	0.54060634	1
gene67226	51.65699125	36.7810814	0.712025236	-0.48999972	0.54060634	1
gene49452	175.6337703	223.590258	1.273048216	0.348287062	0.54066141	1
gene46937	1166.414863	922.4308045	0.790825661	-0.33856841	0.54066916	1
gene10270	771.7554493	613.6633054	0.795152539	-0.33069645	0.54069669	1
gene25581	584.757141	465.5710566	0.796178488	-0.3288362	0.540708	1
gene22886	80.58490636	106.4715514	1.321234413	0.401886451	0.54071854	1
gene46594	1130.254969	894.3610318	0.791291396	-0.33771902	0.5407225	1
gene11727	618.8507552	492.6729061	0.796109404	-0.32896139	0.54073168	1
gene2720	688.0711235	866.2912592	1.259014119	0.332294462	0.54075055	1
gene27959	115.7116604	88.08101071	0.76121119	-0.39363133	0.54077276	1
gene44344	115.7116604	88.08101071	0.76121119	-0.39363133	0.54077276	1
gene10643	459.7472222	365.8749676	0.79581768	-0.32949014	0.54087787	1
gene69942	3200.667178	4219.177205	1.318218037	0.398589016	0.54089047	1
gene56614	939.124101	745.3008599	0.793612749	-0.33349289	0.54093596	1
gene59258	285.1465917	225.5261044	0.790912853	-0.33840936	0.54098153	1
gene55184	162.2029525	125.8300153	0.775756627	-0.36632398	0.54109736	1
gene21614	440.1175655	552.6841441	1.255764794	0.328566272	0.54109827	1
gene45645	682.9054244	859.5157968	1.258616151	0.331838361	0.54115958	1
gene71679	1088.929376	862.4195664	0.791988522	-0.33644857	0.54116113	1
gene67855	381.2285955	302.9599599	0.794693692	-0.3315292	0.54117434	1
gene11300	146.7058552	113.2470138	0.771932474	-0.37345344	0.5412033	1
gene50066	837.8763981	665.9311579	0.794784481	-0.33136439	0.5412129	1
gene36456	77.48548688	102.5998586	1.324117106	0.405030721	0.54123028	1
gene46161	77.48548688	102.5998586	1.324117106	0.405030721	0.54123028	1
gene64151	77.48548688	102.5998586	1.324117106	0.405030721	0.54123028	1
gene65689	77.48548688	102.5998586	1.324117106	0.405030721	0.54123028	1
gene69291	77.48548688	102.5998586	1.324117106	0.405030721	0.54123028	1
gene70857	277.9146129	219.7185652	0.790597381	-0.33898492	0.5412576	1

gene7415	5118.174693	3843.623006	0.750975345	-0.41316255	0.54127245	1
gene35352	1571.405674	2012.312322	1.280581046	0.356798562	0.5412861	1
gene17908	895.7322283	711.4235481	0.794236855	-0.33235879	0.5412912	1
gene64855	1423.666679	1817.75976	1.276815554	0.352550132	0.5414248	1
gene63924	1083.763677	858.5478736	0.79219104	-0.33607971	0.54147237	1
gene69415	345.0687016	273.9222641	0.7938195	-0.33311709	0.54149379	1
gene29290	1289.358502	1018.255201	0.789737842	-0.34055427	0.54162772	1
gene15324	948.4223594	1199.256838	1.264475501	0.338539085	0.54163597	1
gene72031	116.7448002	150.9960184	1.293385385	0.371152213	0.54164616	1
gene18374	45.4581523	31.94146542	0.702656483	-0.50910854	0.54166412	1
gene49418	45.4581523	31.94146542	0.702656483	-0.50910854	0.54166412	1
gene23978	269.6494943	339.7410413	1.259935763	0.33335018	0.54173961	1
gene71155	1066.200299	844.9969489	0.792531149	-0.33546046	0.54176263	1
gene25991	20.6627965	30.97354223	1.499000497	0.584000862	0.54180007	1
gene36225	20.6627965	30.97354223	1.499000497	0.584000862	0.54180007	1
gene48116	20.6627965	30.97354223	1.499000497	0.584000862	0.54180007	1
gene73363	20.6627965	30.97354223	1.499000497	0.584000862	0.54180007	1
gene64538	149.8052746	191.6487925	1.27931939	0.355376487	0.54180848	1
gene32296	170.4680711	132.6054777	0.77789041	-0.36236117	0.54180862	1
gene22201	39.25931335	27.10184945	0.690329176	-0.53464363	0.54183389	1
gene24067	39.25931335	27.10184945	0.690329176	-0.53464363	0.54183389	1
gene52270	39.25931335	27.10184945	0.690329176	-0.53464363	0.54183389	1
gene57692	39.25931335	27.10184945	0.690329176	-0.53464363	0.54183389	1
gene71441	1592.06847	1251.524691	0.78609979	-0.34721563	0.5418671	1
gene48013	510.3710736	640.7651549	1.255488777	0.328249132	0.54187012	1
gene25613	411.1896504	327.1580398	0.795637827	-0.32981623	0.54192415	1
gene29142	97.11514356	126.7979385	1.30564538	0.384763107	0.54192664	1
gene55263	97.11514356	126.7979385	1.30564538	0.384763107	0.54192664	1
gene15501	564.0943445	708.5197785	1.256030636	0.328871654	0.54193867	1
gene61348	166.3355118	211.9751796	1.274383187	0.349799138	0.54194471	1
gene35868	865.7711734	688.1933914	0.794890628	-0.33117173	0.54206376	1
gene25414	316.1407865	250.6921074	0.792976162	-0.3346506	0.54207181	1
gene48305	123.976779	94.85647307	0.765114837	-0.38625179	0.54207377	1
gene60194	123.976779	94.85647307	0.765114837	-0.38625179	0.54207377	1
gene18727	225.2244819	177.1299446	0.78645955	-0.34655553	0.54209818	1
gene14318	28.9279151	41.62069737	1.438772799	0.524838789	0.5421057	1
gene37688	28.9279151	41.62069737	1.438772799	0.524838789	0.5421057	1
gene44171	28.9279151	41.62069737	1.438772799	0.524838789	0.5421057	1
gene48809	28.9279151	41.62069737	1.438772799	0.524838789	0.5421057	1
gene50081	28.9279151	41.62069737	1.438772799	0.524838789	0.5421057	1
gene3199	833.7438388	663.0273883	0.795241125	-0.33053573	0.54214439	1
gene41759	360.565799	286.5052656	0.794599117	-0.3317009	0.54216065	1
gene7891	15.49709738	24.19807987	1.561458851	0.642894551	0.54216986	1
gene16173	15.49709738	24.19807987	1.561458851	0.642894551	0.54216986	1
gene22745	15.49709738	24.19807987	1.561458851	0.642894551	0.54216986	1
gene47948	15.49709738	24.19807987	1.561458851	0.642894551	0.54216986	1
gene61771	15.49709738	24.19807987	1.561458851	0.642894551	0.54216986	1
gene62343	15.49709738	24.19807987	1.561458851	0.642894551	0.54216986	1

gene20349	71.28664793	94.85647307	1.330634499	0.412114344	0.54217323	1
gene61800	71.28664793	94.85647307	1.330634499	0.412114344	0.54217323	1
gene23124	4589.207103	6178.253751	1.346257341	0.428954212	0.54218381	1
gene37350	580.6245817	462.667287	0.796844125	-0.32763056	0.54219126	1
gene41520	101.2477029	76.46593238	0.75523622	-0.40500014	0.54220585	1
gene51080	194.2302871	151.9639416	0.782390552	-0.35403915	0.5422509	1
gene5334	353.3338202	280.6977264	0.794426433	-0.33201447	0.54225407	1
gene40230	202.4954057	256.4996466	1.26669366	0.341067662	0.54228278	1
gene27938	23105.13905	37966.78731	1.6432183	0.716524154	0.5422998	1
gene40127	2905.189188	3806.841925	1.310359387	0.389962549	0.54243076	1
gene73190	178.7331897	139.38094	0.779826848	-0.35877427	0.54245921	1
gene7072	3487.880049	2672.43594	0.766206378	-0.38419506	0.5425104	1
gene37059	46.49129213	63.88293085	1.374083789	0.45846998	0.54254742	1
gene31080	789.3188264	628.1821533	0.795853503	-0.3294252	0.54260271	1
gene65883	5336.167197	4001.394487	0.749863027	-0.415301	0.54263739	1
gene31	72.31978776	53.23577571	0.736116316	-0.44199435	0.54263786	1
gene49638	72.31978776	53.23577571	0.736116316	-0.44199435	0.54263786	1
gene60495	72.31978776	53.23577571	0.736116316	-0.44199435	0.54263786	1
gene62946	72.31978776	53.23577571	0.736116316	-0.44199435	0.54263786	1
gene73742	289.279151	363.9391212	1.258089703	0.331234791	0.54263899	1
gene30092	271.715774	214.8789492	0.790822506	-0.33857417	0.54264054	1
gene48599	1440.196916	1838.086147	1.276274186	0.351938301	0.54264064	1
gene34492	419.454769	333.9335022	0.796113257	-0.32895441	0.54268102	1
gene1880	373.9966167	469.4427494	1.255205872	0.327924007	0.54270978	1
gene34959	373.9966167	469.4427494	1.255205872	0.327924007	0.54270978	1
gene6587	217.9925031	171.3224055	0.785909621	-0.34756468	0.54273148	1
gene16941	217.9925031	171.3224055	0.785909621	-0.34756468	0.54273148	1
gene31528	294.4448501	233.2694899	0.792234912	-0.33599982	0.54274458	1
gene56938	94.01572408	70.65839321	0.751559315	-0.41204112	0.54275375	1
gene51237	1657.156279	2123.623489	1.281486553	0.357818341	0.54276593	1
gene42004	404.9908114	508.1596772	1.25474372	0.327392725	0.54280706	1
gene40315	1342.048633	1709.352362	1.273688837	0.349012869	0.54281494	1
gene64012	202.4954057	158.7394039	0.783916076	-0.35122888	0.54284864	1
gene69112	514.5036329	645.6047708	1.254810908	0.327469976	0.54288298	1
gene42625	65.08780898	87.11308752	1.338393301	0.420502129	0.54293817	1
gene60191	196.2965668	248.756261	1.267247131	0.341697898	0.54294671	1
gene51350	127.0761985	163.5790199	1.287251443	0.364293888	0.54296141	1
gene41211	609.5524968	485.8974437	0.797137976	-0.32709863	0.54297921	1
gene18097	116.7448002	89.04893391	0.76276574	-0.39068805	0.54298619	1
gene46807	116.7448002	89.04893391	0.76276574	-0.39068805	0.54298619	1
gene58330	116.7448002	89.04893391	0.76276574	-0.39068805	0.54298619	1
gene71493	361.5989388	287.4731888	0.795005621	-0.33096303	0.54299882	1
gene69820	4307.159931	3268.676628	0.758893721	-0.39803024	0.54300041	1
gene63372	2455.773364	1907.776617	0.776853697	-0.36428517	0.54301091	1
gene43784	895.7322283	1130.534291	1.262134213	0.335865332	0.54301927	1
gene57376	186.9983083	146.1564024	0.78159211	-0.35551219	0.54306444	1
gene72144	49.5907116	67.75462362	1.366276495	0.450249473	0.54308583	1
gene26255	86.78374531	64.85085404	0.747269593	-0.42029928	0.54309371	1

gene45355	354.36696	281.6656496	0.794841736	-0.33126047	0.54310638	1
gene1575	90.91630461	119.0545529	1.309496173	0.389011843	0.54323745	1
gene18759	90.91630461	119.0545529	1.309496173	0.389011843	0.54323745	1
gene42340	1259.397447	995.9929673	0.790848806	-0.33852619	0.54324577	1
gene28878	932.925262	741.4291671	0.794735867	-0.33145264	0.54328578	1
gene3694	6601.763482	4886.076287	0.740116834	-0.43417506	0.54332501	1
gene43491	171.501211	133.5734009	0.77884815	-0.36058602	0.54335921	1
gene24636	52.69013108	71.6263164	1.359387706	0.44295698	0.54337367	1
gene38936	52.69013108	71.6263164	1.359387706	0.44295698	0.54337367	1
gene46528	52.69013108	71.6263164	1.359387706	0.44295698	0.54337367	1
gene71851	52.69013108	71.6263164	1.359387706	0.44295698	0.54337367	1
gene26140	210.7605243	165.5148663	0.785321952	-0.34864387	0.5434259	1
gene40459	958.7537577	761.7555542	0.794526799	-0.33183221	0.54345042	1
gene37214	55.78955055	75.49800918	1.353264338	0.436443673	0.54346505	1
gene45955	140.5070162	180.0337142	1.281314763	0.357624927	0.54348189	1
gene60723	1021.775287	811.1196371	0.793833681	-0.33309132	0.54349673	1
gene69686	391.5599937	311.6712687	0.795973219	-0.3292082	0.54351284	1
gene49499	267.5832147	336.8372717	1.258813159	0.332064165	0.54353613	1
gene36804	677.7397253	851.7724113	1.256783953	0.329736666	0.54357494	1
gene17859	58.88897003	42.58862056	0.723201994	-0.46752944	0.54359174	1
gene642	107.4465418	139.38094	1.297211969	0.37541424	0.54363024	1
gene46790	678.7728651	541.0690658	0.797128309	-0.32711613	0.54363204	1
gene61824	362.6320786	288.441112	0.795409808	-0.33022974	0.54383297	1
gene69009	232.4564606	293.280728	1.261658752	0.335321749	0.54384531	1
gene67628	1787.331897	1402.520709	0.784700766	-0.34978549	0.54384909	1
gene925	109.5128215	83.24139474	0.760106384	-0.39572674	0.54387745	1
gene59871	492.8076966	392.976817	0.797424269	-0.32658058	0.54390389	1
gene3228	153.9378339	196.4884085	1.276414014	0.352096353	0.54394818	1
gene73051	250.0198377	197.4563317	0.789762659	-0.34050894	0.54395131	1
gene43310	7945.878395	5805.603321	0.730643364	-0.45276071	0.54398412	1
gene7705	140.5070162	108.4073978	0.771544374	-0.37417896	0.54399786	1
gene11227	140.5070162	108.4073978	0.771544374	-0.37417896	0.54399786	1
gene23561	4657.394331	6267.302685	1.345667178	0.428321634	0.54410717	1
gene66898	1030.040406	817.8950995	0.794041763	-0.33271321	0.54417073	1
gene65951	203.5285455	159.7073271	0.784692519	-0.34980065	0.5441844	1
gene43223	560.994925	703.6801625	1.254343188	0.326932123	0.54426971	1
gene151	1.033139825	3.871692779	3.747501243	1.905928957	0.5442996	1
gene453	1.033139825	3.871692779	3.747501243	1.905928957	0.5442996	1
gene501	1.033139825	3.871692779	3.747501243	1.905928957	0.5442996	1
gene986	1.033139825	3.871692779	3.747501243	1.905928957	0.5442996	1
gene1107	1.033139825	3.871692779	3.747501243	1.905928957	0.5442996	1
gene1242	1.033139825	3.871692779	3.747501243	1.905928957	0.5442996	1
gene1523	1.033139825	3.871692779	3.747501243	1.905928957	0.5442996	1
gene1799	1.033139825	3.871692779	3.747501243	1.905928957	0.5442996	1
gene2005	1.033139825	3.871692779	3.747501243	1.905928957	0.5442996	1
gene3186	1.033139825	3.871692779	3.747501243	1.905928957	0.5442996	1
gene3360	1.033139825	3.871692779	3.747501243	1.905928957	0.5442996	1
gene4310	1.033139825	3.871692779	3.747501243	1.905928957	0.5442996	1

gene5215	1.033139825	3.871692779	3.747501243	1.905928957	0.5442996	1
gene5831	1.033139825	3.871692779	3.747501243	1.905928957	0.5442996	1
gene6271	1.033139825	3.871692779	3.747501243	1.905928957	0.5442996	1
gene8142	1.033139825	3.871692779	3.747501243	1.905928957	0.5442996	1
gene8295	1.033139825	3.871692779	3.747501243	1.905928957	0.5442996	1
gene8472	1.033139825	3.871692779	3.747501243	1.905928957	0.5442996	1
gene8740	1.033139825	3.871692779	3.747501243	1.905928957	0.5442996	1
gene9350	1.033139825	3.871692779	3.747501243	1.905928957	0.5442996	1
gene9932	1.033139825	3.871692779	3.747501243	1.905928957	0.5442996	1
gene10642	1.033139825	3.871692779	3.747501243	1.905928957	0.5442996	1
gene10712	1.033139825	3.871692779	3.747501243	1.905928957	0.5442996	1
gene10781	1.033139825	3.871692779	3.747501243	1.905928957	0.5442996	1
gene11319	1.033139825	3.871692779	3.747501243	1.905928957	0.5442996	1
gene12666	1.033139825	3.871692779	3.747501243	1.905928957	0.5442996	1
gene12841	1.033139825	3.871692779	3.747501243	1.905928957	0.5442996	1
gene13549	1.033139825	3.871692779	3.747501243	1.905928957	0.5442996	1
gene13608	1.033139825	3.871692779	3.747501243	1.905928957	0.5442996	1
gene13786	1.033139825	3.871692779	3.747501243	1.905928957	0.5442996	1
gene13996	1.033139825	3.871692779	3.747501243	1.905928957	0.5442996	1
gene14051	1.033139825	3.871692779	3.747501243	1.905928957	0.5442996	1
gene14156	1.033139825	3.871692779	3.747501243	1.905928957	0.5442996	1
gene14681	1.033139825	3.871692779	3.747501243	1.905928957	0.5442996	1
gene14767	1.033139825	3.871692779	3.747501243	1.905928957	0.5442996	1
gene15034	1.033139825	3.871692779	3.747501243	1.905928957	0.5442996	1
gene15041	1.033139825	3.871692779	3.747501243	1.905928957	0.5442996	1
gene15089	1.033139825	3.871692779	3.747501243	1.905928957	0.5442996	1
gene15147	1.033139825	3.871692779	3.747501243	1.905928957	0.5442996	1
gene15316	1.033139825	3.871692779	3.747501243	1.905928957	0.5442996	1
gene16984	1.033139825	3.871692779	3.747501243	1.905928957	0.5442996	1
gene17618	1.033139825	3.871692779	3.747501243	1.905928957	0.5442996	1
gene17744	1.033139825	3.871692779	3.747501243	1.905928957	0.5442996	1
gene17852	1.033139825	3.871692779	3.747501243	1.905928957	0.5442996	1
gene17979	1.033139825	3.871692779	3.747501243	1.905928957	0.5442996	1
gene18102	1.033139825	3.871692779	3.747501243	1.905928957	0.5442996	1
gene18149	1.033139825	3.871692779	3.747501243	1.905928957	0.5442996	1
gene18578	1.033139825	3.871692779	3.747501243	1.905928957	0.5442996	1
gene18755	1.033139825	3.871692779	3.747501243	1.905928957	0.5442996	1
gene18914	1.033139825	3.871692779	3.747501243	1.905928957	0.5442996	1
gene20202	1.033139825	3.871692779	3.747501243	1.905928957	0.5442996	1
gene21025	1.033139825	3.871692779	3.747501243	1.905928957	0.5442996	1
gene21695	1.033139825	3.871692779	3.747501243	1.905928957	0.5442996	1
gene21753	1.033139825	3.871692779	3.747501243	1.905928957	0.5442996	1
gene22232	1.033139825	3.871692779	3.747501243	1.905928957	0.5442996	1
gene22505	1.033139825	3.871692779	3.747501243	1.905928957	0.5442996	1
gene22543	1.033139825	3.871692779	3.747501243	1.905928957	0.5442996	1
gene22920	1.033139825	3.871692779	3.747501243	1.905928957	0.5442996	1
gene23010	1.033139825	3.871692779	3.747501243	1.905928957	0.5442996	1
gene23698	1.033139825	3.871692779	3.747501243	1.905928957	0.5442996	1

gene24308	1.033139825	3.871692779	3.747501243	1.905928957	0.5442996	1
gene24635	1.033139825	3.871692779	3.747501243	1.905928957	0.5442996	1
gene25218	1.033139825	3.871692779	3.747501243	1.905928957	0.5442996	1
gene26542	1.033139825	3.871692779	3.747501243	1.905928957	0.5442996	1
gene26798	1.033139825	3.871692779	3.747501243	1.905928957	0.5442996	1
gene27706	1.033139825	3.871692779	3.747501243	1.905928957	0.5442996	1
gene28415	1.033139825	3.871692779	3.747501243	1.905928957	0.5442996	1
gene28598	1.033139825	3.871692779	3.747501243	1.905928957	0.5442996	1
gene28666	1.033139825	3.871692779	3.747501243	1.905928957	0.5442996	1
gene29822	1.033139825	3.871692779	3.747501243	1.905928957	0.5442996	1
gene30345	1.033139825	3.871692779	3.747501243	1.905928957	0.5442996	1
gene31105	1.033139825	3.871692779	3.747501243	1.905928957	0.5442996	1
gene31691	1.033139825	3.871692779	3.747501243	1.905928957	0.5442996	1
gene32808	1.033139825	3.871692779	3.747501243	1.905928957	0.5442996	1
gene33487	1.033139825	3.871692779	3.747501243	1.905928957	0.5442996	1
gene33755	1.033139825	3.871692779	3.747501243	1.905928957	0.5442996	1
gene34049	1.033139825	3.871692779	3.747501243	1.905928957	0.5442996	1
gene34997	1.033139825	3.871692779	3.747501243	1.905928957	0.5442996	1
gene35415	1.033139825	3.871692779	3.747501243	1.905928957	0.5442996	1
gene35697	1.033139825	3.871692779	3.747501243	1.905928957	0.5442996	1
gene35797	1.033139825	3.871692779	3.747501243	1.905928957	0.5442996	1
gene35815	1.033139825	3.871692779	3.747501243	1.905928957	0.5442996	1
gene36842	1.033139825	3.871692779	3.747501243	1.905928957	0.5442996	1
gene36905	1.033139825	3.871692779	3.747501243	1.905928957	0.5442996	1
gene37329	1.033139825	3.871692779	3.747501243	1.905928957	0.5442996	1
gene37974	1.033139825	3.871692779	3.747501243	1.905928957	0.5442996	1
gene38247	1.033139825	3.871692779	3.747501243	1.905928957	0.5442996	1
gene38437	1.033139825	3.871692779	3.747501243	1.905928957	0.5442996	1
gene38660	1.033139825	3.871692779	3.747501243	1.905928957	0.5442996	1
gene38680	1.033139825	3.871692779	3.747501243	1.905928957	0.5442996	1
gene38834	1.033139825	3.871692779	3.747501243	1.905928957	0.5442996	1
gene39210	1.033139825	3.871692779	3.747501243	1.905928957	0.5442996	1
gene39932	1.033139825	3.871692779	3.747501243	1.905928957	0.5442996	1
gene40154	1.033139825	3.871692779	3.747501243	1.905928957	0.5442996	1
gene40829	1.033139825	3.871692779	3.747501243	1.905928957	0.5442996	1
gene40850	1.033139825	3.871692779	3.747501243	1.905928957	0.5442996	1
gene41225	1.033139825	3.871692779	3.747501243	1.905928957	0.5442996	1
gene41299	1.033139825	3.871692779	3.747501243	1.905928957	0.5442996	1
gene41312	1.033139825	3.871692779	3.747501243	1.905928957	0.5442996	1
gene41761	1.033139825	3.871692779	3.747501243	1.905928957	0.5442996	1
gene42687	1.033139825	3.871692779	3.747501243	1.905928957	0.5442996	1
gene43529	1.033139825	3.871692779	3.747501243	1.905928957	0.5442996	1
gene44102	1.033139825	3.871692779	3.747501243	1.905928957	0.5442996	1
gene44341	1.033139825	3.871692779	3.747501243	1.905928957	0.5442996	1
gene44372	1.033139825	3.871692779	3.747501243	1.905928957	0.5442996	1
gene44416	1.033139825	3.871692779	3.747501243	1.905928957	0.5442996	1
gene45071	1.033139825	3.871692779	3.747501243	1.905928957	0.5442996	1
gene45161	1.033139825	3.871692779	3.747501243	1.905928957	0.5442996	1

gene45674	1.033139825	3.871692779	3.747501243	1.905928957	0.5442996	1
gene45714	1.033139825	3.871692779	3.747501243	1.905928957	0.5442996	1
gene45783	1.033139825	3.871692779	3.747501243	1.905928957	0.5442996	1
gene45889	1.033139825	3.871692779	3.747501243	1.905928957	0.5442996	1
gene46848	1.033139825	3.871692779	3.747501243	1.905928957	0.5442996	1
gene47540	1.033139825	3.871692779	3.747501243	1.905928957	0.5442996	1
gene47541	1.033139825	3.871692779	3.747501243	1.905928957	0.5442996	1
gene47595	1.033139825	3.871692779	3.747501243	1.905928957	0.5442996	1
gene47956	1.033139825	3.871692779	3.747501243	1.905928957	0.5442996	1
gene48466	1.033139825	3.871692779	3.747501243	1.905928957	0.5442996	1
gene49303	1.033139825	3.871692779	3.747501243	1.905928957	0.5442996	1
gene49577	1.033139825	3.871692779	3.747501243	1.905928957	0.5442996	1
gene49637	1.033139825	3.871692779	3.747501243	1.905928957	0.5442996	1
gene50091	1.033139825	3.871692779	3.747501243	1.905928957	0.5442996	1
gene50132	1.033139825	3.871692779	3.747501243	1.905928957	0.5442996	1
gene50498	1.033139825	3.871692779	3.747501243	1.905928957	0.5442996	1
gene50630	1.033139825	3.871692779	3.747501243	1.905928957	0.5442996	1
gene50639	1.033139825	3.871692779	3.747501243	1.905928957	0.5442996	1
gene50748	1.033139825	3.871692779	3.747501243	1.905928957	0.5442996	1
gene51415	1.033139825	3.871692779	3.747501243	1.905928957	0.5442996	1
gene51450	1.033139825	3.871692779	3.747501243	1.905928957	0.5442996	1
gene51613	1.033139825	3.871692779	3.747501243	1.905928957	0.5442996	1
gene51730	1.033139825	3.871692779	3.747501243	1.905928957	0.5442996	1
gene52063	1.033139825	3.871692779	3.747501243	1.905928957	0.5442996	1
gene52423	1.033139825	3.871692779	3.747501243	1.905928957	0.5442996	1
gene52726	1.033139825	3.871692779	3.747501243	1.905928957	0.5442996	1
gene52748	1.033139825	3.871692779	3.747501243	1.905928957	0.5442996	1
gene52820	1.033139825	3.871692779	3.747501243	1.905928957	0.5442996	1
gene52946	1.033139825	3.871692779	3.747501243	1.905928957	0.5442996	1
gene53421	1.033139825	3.871692779	3.747501243	1.905928957	0.5442996	1
gene54628	1.033139825	3.871692779	3.747501243	1.905928957	0.5442996	1
gene55582	1.033139825	3.871692779	3.747501243	1.905928957	0.5442996	1
gene55594	1.033139825	3.871692779	3.747501243	1.905928957	0.5442996	1
gene56307	1.033139825	3.871692779	3.747501243	1.905928957	0.5442996	1
gene56377	1.033139825	3.871692779	3.747501243	1.905928957	0.5442996	1
gene56847	1.033139825	3.871692779	3.747501243	1.905928957	0.5442996	1
gene56909	1.033139825	3.871692779	3.747501243	1.905928957	0.5442996	1
gene57287	1.033139825	3.871692779	3.747501243	1.905928957	0.5442996	1
gene57304	1.033139825	3.871692779	3.747501243	1.905928957	0.5442996	1
gene57588	1.033139825	3.871692779	3.747501243	1.905928957	0.5442996	1
gene57838	1.033139825	3.871692779	3.747501243	1.905928957	0.5442996	1
gene57969	1.033139825	3.871692779	3.747501243	1.905928957	0.5442996	1
gene57990	1.033139825	3.871692779	3.747501243	1.905928957	0.5442996	1
gene58266	1.033139825	3.871692779	3.747501243	1.905928957	0.5442996	1
gene59173	1.033139825	3.871692779	3.747501243	1.905928957	0.5442996	1
gene59476	1.033139825	3.871692779	3.747501243	1.905928957	0.5442996	1
gene59775	1.033139825	3.871692779	3.747501243	1.905928957	0.5442996	1
gene60136	1.033139825	3.871692779	3.747501243	1.905928957	0.5442996	1

gene60541	1.033139825	3.871692779	3.747501243	1.905928957	0.5442996	1
gene60661	1.033139825	3.871692779	3.747501243	1.905928957	0.5442996	1
gene60776	1.033139825	3.871692779	3.747501243	1.905928957	0.5442996	1
gene60840	1.033139825	3.871692779	3.747501243	1.905928957	0.5442996	1
gene60949	1.033139825	3.871692779	3.747501243	1.905928957	0.5442996	1
gene61228	1.033139825	3.871692779	3.747501243	1.905928957	0.5442996	1
gene61741	1.033139825	3.871692779	3.747501243	1.905928957	0.5442996	1
gene61779	1.033139825	3.871692779	3.747501243	1.905928957	0.5442996	1
gene62042	1.033139825	3.871692779	3.747501243	1.905928957	0.5442996	1
gene63491	1.033139825	3.871692779	3.747501243	1.905928957	0.5442996	1
gene63598	1.033139825	3.871692779	3.747501243	1.905928957	0.5442996	1
gene63713	1.033139825	3.871692779	3.747501243	1.905928957	0.5442996	1
gene64732	1.033139825	3.871692779	3.747501243	1.905928957	0.5442996	1
gene65442	1.033139825	3.871692779	3.747501243	1.905928957	0.5442996	1
gene65555	1.033139825	3.871692779	3.747501243	1.905928957	0.5442996	1
gene65643	1.033139825	3.871692779	3.747501243	1.905928957	0.5442996	1
gene66913	1.033139825	3.871692779	3.747501243	1.905928957	0.5442996	1
gene66971	1.033139825	3.871692779	3.747501243	1.905928957	0.5442996	1
gene67716	1.033139825	3.871692779	3.747501243	1.905928957	0.5442996	1
gene68430	1.033139825	3.871692779	3.747501243	1.905928957	0.5442996	1
gene68643	1.033139825	3.871692779	3.747501243	1.905928957	0.5442996	1
gene68758	1.033139825	3.871692779	3.747501243	1.905928957	0.5442996	1
gene69081	1.033139825	3.871692779	3.747501243	1.905928957	0.5442996	1
gene69208	1.033139825	3.871692779	3.747501243	1.905928957	0.5442996	1
gene69704	1.033139825	3.871692779	3.747501243	1.905928957	0.5442996	1
gene70207	1.033139825	3.871692779	3.747501243	1.905928957	0.5442996	1
gene70250	1.033139825	3.871692779	3.747501243	1.905928957	0.5442996	1
gene70612	1.033139825	3.871692779	3.747501243	1.905928957	0.5442996	1
gene71111	1.033139825	3.871692779	3.747501243	1.905928957	0.5442996	1
gene73299	1.033139825	3.871692779	3.747501243	1.905928957	0.5442996	1
gene73441	1.033139825	3.871692779	3.747501243	1.905928957	0.5442996	1
gene73593	1.033139825	3.871692779	3.747501243	1.905928957	0.5442996	1
gene73765	1.033139825	3.871692779	3.747501243	1.905928957	0.5442996	1
gene11155	646.7455305	812.0875603	1.255652373	0.32843711	0.54433718	1
gene34116	449.4158239	563.3312993	1.253474554	0.325932709	0.54435665	1
gene56542	385.3611548	306.8316527	0.796218428	-0.32876383	0.54436103	1
gene19297	252.0861173	317.4788078	1.259406155	0.332743624	0.54439518	1
gene30492	333.7041635	265.2109553	0.794748716	-0.33142932	0.54443538	1
gene48458	891.599669	709.4877017	0.795746933	-0.3296184	0.5445633	1
gene48130	248.9866978	313.6071151	1.259533613	0.332889623	0.54459206	1
gene43875	2392.751835	1862.284226	0.778302287	-0.3615975	0.54462849	1
gene42441	32.02733458	45.49239015	1.420423858	0.506321498	0.54463202	1
gene49797	32.02733458	45.49239015	1.420423858	0.506321498	0.54463202	1
gene51731	32.02733458	45.49239015	1.420423858	0.506321498	0.54463202	1
gene50074	102.2808427	77.43385557	0.757070958	-0.40149957	0.54471436	1
gene15224	134.3081773	172.2903286	1.282798502	0.359294575	0.5447154	1
gene27301	1029.007266	1300.888774	1.264217287	0.338244447	0.54475469	1
gene36589	444.2501248	354.2598892	0.797433404	-0.32656405	0.54487264	1

gene38451	172.5343508	134.5413241	0.77979442	-0.35883426	0.54489459	1
gene4649	242.7878589	305.8637295	1.25979829	0.333192758	0.54501322	1
gene32531	309.9419475	389.1051242	1.255412916	0.328161957	0.5450297	1
gene6217	101.2477029	131.6375545	1.300153492	0.378681954	0.54505167	1
gene6281	101.2477029	131.6375545	1.300153492	0.378681954	0.54505167	1
gene61460	726.297297	912.7515725	1.256718945	0.329662038	0.54505583	1
gene51431	251.0529775	198.4242549	0.79036806	-0.33940345	0.54507444	1
gene66923	251.0529775	198.4242549	0.79036806	-0.33940345	0.54507444	1
gene3648	147.738995	188.745023	1.277557242	0.353387934	0.5450861	1
gene53578	422.5541885	529.4539875	1.252984829	0.325368946	0.54515286	1
gene16237	117.7779401	90.0168571	0.764293017	-0.38780225	0.5451665	1
gene55672	117.7779401	90.0168571	0.764293017	-0.38780225	0.5451665	1
gene59081	268.6163545	337.8051949	1.257574936	0.33064437	0.54519302	1
gene70952	81.61804618	107.4394746	1.316369107	0.396564075	0.54524064	1
gene37407	1630.294644	2084.906561	1.27885261	0.354850001	0.54525185	1
gene44867	538.2658489	674.6424667	1.25336294	0.32580424	0.54527603	1
gene24014	27.89477528	18.3905407	0.659282626	-0.60103103	0.54529702	1
gene26437	27.89477528	18.3905407	0.659282626	-0.60103103	0.54529702	1
gene28230	27.89477528	18.3905407	0.659282626	-0.60103103	0.54529702	1
gene29371	27.89477528	18.3905407	0.659282626	-0.60103103	0.54529702	1
gene35904	27.89477528	18.3905407	0.659282626	-0.60103103	0.54529702	1
gene50193	27.89477528	18.3905407	0.659282626	-0.60103103	0.54529702	1
gene50766	27.89477528	18.3905407	0.659282626	-0.60103103	0.54529702	1
gene16597	732.496136	920.4949581	1.256655036	0.32958867	0.54532923	1
gene465	66.12094881	48.39615973	0.731933836	-0.45021485	0.54537632	1
gene2765	66.12094881	48.39615973	0.731933836	-0.45021485	0.54537632	1
gene60065	66.12094881	48.39615973	0.731933836	-0.45021485	0.54537632	1
gene4931	2764.682172	2142.01403	0.774777677	-0.36814571	0.54540171	1
gene20748	180.7994694	141.3167864	0.781621688	-0.3554576	0.54541678	1
gene42211	662.2426279	528.4860643	0.798024836	-0.32549445	0.54544474	1
gene15456	95.04886391	71.6263164	0.753573619	-0.40817963	0.54544926	1
gene50123	95.04886391	71.6263164	0.753573619	-0.40817963	0.54544926	1
gene57012	161.1698127	205.1997173	1.273189525	0.348447192	0.54545311	1
gene67138	9222.839218	13156.97998	1.426565038	0.512545522	0.54548704	1
gene58325	52.69013108	37.74900459	0.716434061	-0.48109417	0.54562041	1
gene5919	259.3180961	205.1997173	0.791305043	-0.33769414	0.54565639	1
gene38982	1521.814962	1941.653928	1.27588043	0.351493132	0.54569479	1
gene4753	401.891392	320.3825774	0.797186961	-0.32700998	0.54576182	1
gene16096	210.7605243	266.1788785	1.262944659	0.336791423	0.54577902	1
gene32560	98.14828338	127.7658617	1.30176359	0.380467468	0.54579003	1
gene54779	98.14828338	127.7658617	1.30176359	0.380467468	0.54579003	1
gene13084	191.1308676	241.9807987	1.266047717	0.340331781	0.5458945	1
gene64366	189.064588	148.0922488	0.783289194	-0.35238304	0.54590582	1
gene61037	923.6270036	1164.411603	1.26069463	0.334218863	0.54592208	1
gene57358	510.3710736	407.4956649	0.798430174	-0.32476185	0.54596596	1
gene6539	230.390181	290.3769584	1.260370373	0.333847747	0.54597436	1
gene58472	158.0703932	201.3280245	1.273660553	0.348980832	0.54600506	1
gene2743	87.81688513	65.81877724	0.749500249	-0.41599914	0.54601303	1

gene29247	1753.238283	1378.322629	0.786158186	-0.34710846	0.54612046	1
gene7642	73.35292758	54.2036989	0.738943907	-0.43646324	0.54616988	1
gene43711	73.35292758	54.2036989	0.738943907	-0.43646324	0.54616988	1
gene59284	110.5459613	84.20931793	0.76175843	-0.39259453	0.54618952	1
gene21841	252.0861173	199.3921781	0.7909685	-0.33830785	0.54619025	1
gene46618	3576.730074	2744.062257	0.767198586	-0.38232803	0.54620242	1
gene11418	267.5832147	211.9751796	0.792184143	-0.33609227	0.54624123	1
gene33984	453.5483832	362.0032748	0.79815801	-0.32525371	0.54626475	1
gene45916	236.5890199	186.8091766	0.789593602	-0.34081779	0.54626647	1
gene18033	2847.333358	2204.929037	0.774383874	-0.36887918	0.5463258	1
gene4593	197.3297066	154.8677111	0.784817014	-0.34957178	0.54637202	1
gene1431	417.3884893	332.965579	0.797735413	-0.32601777	0.54638362	1
gene70878	111.5791011	144.220556	1.292540938	0.370209975	0.54641414	1
gene22658	757.2914918	951.4685003	1.256409864	0.329307175	0.54642052	1
gene14258	35.12675405	49.36408293	1.405312966	0.490891457	0.54646813	1
gene44861	35.12675405	49.36408293	1.405312966	0.490891457	0.54646813	1
gene42262	221.0919226	174.226175	0.788025962	-0.34368493	0.54647621	1
gene3384	224.191342	282.6335728	1.260680142	0.334202283	0.5465225	1
gene2526	9576.173039	6909.035763	0.721481926	-0.47096484	0.54653581	1
gene17769	95.04886391	123.8941689	1.303478693	0.382367001	0.54654538	1
gene67922	75.41920723	99.69608905	1.321892562	0.402604925	0.54655325	1
gene42760	2197.488408	1717.095747	0.781390128	-0.35588507	0.54666844	1
gene44406	125.0099188	160.6752503	1.285300013	0.362105151	0.54675369	1
gene5695	692.2036828	552.6841441	0.798441496	-0.32474139	0.54676169	1
gene7719	3878.406903	2965.716668	0.764673935	-0.3870834	0.54676442	1
gene31448	479.3768788	600.1123807	1.251859251	0.324072367	0.54676442	1
gene22659	2464.038483	1918.423772	0.778568916	-0.36110335	0.54677198	1
gene61764	205.5948252	161.6431735	0.786221994	-0.34699137	0.54682343	1
gene26681	23.76221598	34.84523501	1.46641353	0.552292002	0.54685529	1
gene30907	23.76221598	34.84523501	1.46641353	0.552292002	0.54685529	1
gene38448	23.76221598	34.84523501	1.46641353	0.552292002	0.54685529	1
gene42831	23.76221598	34.84523501	1.46641353	0.552292002	0.54685529	1
gene58148	23.76221598	34.84523501	1.46641353	0.552292002	0.54685529	1
gene62549	23.76221598	34.84523501	1.46641353	0.552292002	0.54685529	1
gene57144	298.5774094	374.5862763	1.254570053	0.32719303	0.54697783	1
gene38677	134.3081773	103.5677818	0.771120448	-0.37497187	0.5469805	1
gene57685	266.5500749	334.9014253	1.25642968	0.329329929	0.54703177	1
gene49117	418.4216292	333.9335022	0.798078968	-0.32539659	0.54711596	1
gene38915	366.7646379	292.3128048	0.797003785	-0.32734152	0.54712997	1
gene61736	108.4796816	140.3488632	1.293780191	0.371592529	0.54717691	1
gene33115	103.3139825	78.40177877	0.758869002	-0.39807723	0.54717933	1
gene17178	2813.239744	2180.730958	0.775167123	-0.36742071	0.54722616	1
gene72267	263.4506554	331.0297326	1.256515123	0.329428035	0.54722963	1
gene57817	181.8326092	230.3657203	1.266910932	0.341315101	0.54722989	1
gene21640	3393.864325	2611.456779	0.769464106	-0.37807407	0.54726655	1
gene29779	46.49129213	32.90938862	0.707861346	-0.4984613	0.54734064	1
gene54877	46.49129213	32.90938862	0.707861346	-0.4984613	0.54734064	1
gene66251	46.49129213	32.90938862	0.707861346	-0.4984613	0.54734064	1

gene265	165.302372	210.0393332	1.27063714	0.345552094	0.54741804	1
gene7086	166.3355118	129.7017081	0.779759575	-0.35889873	0.54748876	1
gene9661	166.3355118	129.7017081	0.779759575	-0.35889873	0.54748876	1
gene26630	1453.627734	1148.924832	0.790384502	-0.33937344	0.54750382	1
gene45104	322.3396254	256.4996466	0.795743453	-0.32962471	0.54761337	1
gene4561	198.3628464	155.8356343	0.785608985	-0.34811667	0.54772255	1
gene61748	2040.451155	1599.009118	0.78365469	-0.35171001	0.54778305	1
gene39542	38.22617353	53.23577571	1.392652489	0.477835305	0.54779352	1
gene55564	38.22617353	53.23577571	1.392652489	0.477835305	0.54779352	1
gene4483	2616.943177	2034.574555	0.777462259	-0.36315545	0.54780443	1
gene50662	768.6560299	613.6633054	0.7983588	-0.32489082	0.54782393	1
gene5235	315.1076466	250.6921074	0.795576084	-0.32992819	0.54791606	1
gene54700	1245.966629	988.2495817	0.793158949	-0.33431808	0.5479574	1
gene16303	59.92210985	43.55654376	0.726886017	-0.46019894	0.54796334	1
gene23903	59.92210985	43.55654376	0.726886017	-0.46019894	0.54796334	1
gene47717	59.92210985	43.55654376	0.726886017	-0.46019894	0.54796334	1
gene48470	59.92210985	43.55654376	0.726886017	-0.46019894	0.54796334	1
gene22107	105.3802622	136.4771704	1.295092341	0.373054967	0.54796378	1
gene69018	808.948483	645.6047708	0.798078968	-0.32539659	0.54797849	1
gene12527	3701.739993	2838.91873	0.766914677	-0.38286202	0.54798034	1
gene14973	162.2029525	206.1676405	1.271047396	0.346017828	0.54798522	1
gene58143	292.3785705	232.3015667	0.794523232	-0.33183869	0.54802198	1
gene1089	96.08200373	72.5942396	0.755544605	-0.40441116	0.54809398	1
gene43000	88.85002496	116.1507834	1.307267875	0.386554798	0.54810066	1
gene55471	88.85002496	116.1507834	1.307267875	0.386554798	0.54810066	1
gene42351	612.6519163	489.7691365	0.799424801	-0.32296576	0.5481421	1
gene3778	11145.51243	7949.553198	0.713251476	-0.48751727	0.54815447	1
gene53542	208.6942447	263.2751089	1.261535072	0.335180315	0.54817302	1
gene1109	150.8384145	117.1187066	0.776451456	-0.36503236	0.54817729	1
gene56880	150.8384145	117.1187066	0.776451456	-0.36503236	0.54817729	1
gene13466	34.09361423	23.23015667	0.681363862	-0.55350266	0.54818288	1
gene18811	34.09361423	23.23015667	0.681363862	-0.55350266	0.54818288	1
gene24412	34.09361423	23.23015667	0.681363862	-0.55350266	0.54818288	1
gene28389	34.09361423	23.23015667	0.681363862	-0.55350266	0.54818288	1
gene66042	34.09361423	23.23015667	0.681363862	-0.55350266	0.54818288	1
gene66790	34.09361423	23.23015667	0.681363862	-0.55350266	0.54818288	1
gene70646	34.09361423	23.23015667	0.681363862	-0.55350266	0.54818288	1
gene71389	34.09361423	23.23015667	0.681363862	-0.55350266	0.54818288	1
gene29958	640.5466916	512.03137	0.799366193	-0.32307154	0.54823414	1
gene17831	783.1199874	625.2783837	0.798445186	-0.32473473	0.54829	1
gene48190	485.5757178	388.137201	0.799334042	-0.32312956	0.54829589	1
gene47983	449.4158239	359.0995052	0.799036185	-0.32366726	0.54833524	1
gene15363	2043.550574	1601.912887	0.783887077	-0.35128225	0.54834965	1
gene35143	66.12094881	88.08101071	1.332119582	0.413723597	0.54834983	1
gene51239	66.12094881	88.08101071	1.332119582	0.413723597	0.54834983	1
gene30411	1421.600399	1124.726752	0.791169412	-0.33794144	0.54835691	1
gene25110	254.152397	201.3280245	0.792154734	-0.33614583	0.54840025	1
gene12796	40.29245318	28.06977264	0.696650872	-0.52149227	0.54841239	1

gene21130	40.29245318	28.06977264	0.696650872	-0.52149227	0.54841239	1
gene26485	40.29245318	28.06977264	0.696650872	-0.52149227	0.54841239	1
gene61958	40.29245318	28.06977264	0.696650872	-0.52149227	0.54841239	1
gene15767	225.2244819	283.601496	1.259194798	0.332501486	0.54845021	1
gene4254	111.5791011	85.17724113	0.763379883	-0.38952693	0.54846476	1
gene9391	111.5791011	85.17724113	0.763379883	-0.38952693	0.54846476	1
gene13152	111.5791011	85.17724113	0.763379883	-0.38952693	0.54846476	1
gene14653	111.5791011	85.17724113	0.763379883	-0.38952693	0.54846476	1
gene61576	1326.551535	1683.218435	1.26886773	0.343541687	0.54857122	1
gene27827	300.6436891	239.0770291	0.795217188	-0.33057915	0.54860953	1
gene10254	662.2426279	529.4539875	0.79948642	-0.32285457	0.54873176	1
gene60489	328.5384644	411.3673577	1.25211323	0.324365032	0.54875844	1
gene12655	172.5343508	218.750642	1.267867187	0.342403627	0.54875853	1
gene34961	102.2808427	132.6054777	1.296484016	0.37460442	0.54877452	1
gene24047	316.1407865	251.6600306	0.796037846	-0.32909107	0.54883892	1
gene44034	63.02152933	84.20931793	1.336199214	0.418135115	0.54885876	1
gene63673	63.02152933	84.20931793	1.336199214	0.418135115	0.54885876	1
gene41192	493.8408364	617.5349982	1.250473741	0.322474762	0.54886024	1
gene72666	135.3413171	104.535705	0.772385752	-0.37260654	0.54886971	1
gene22821	88.85002496	66.78670043	0.751679028	-0.41181134	0.54887225	1
gene9155	223.1582022	176.1620214	0.789404197	-0.34116391	0.5489263	1
gene11553	1743.940025	1373.483013	0.787574684	-0.34451136	0.54893053	1
gene55806	464.9129213	371.6825067	0.799466932	-0.32288973	0.5489552	1
gene63601	457.6809425	365.8749676	0.799410536	-0.32299151	0.54898182	1
gene3630	22.72907615	14.51884792	0.638778621	-0.64661207	0.54901096	1
gene3886	22.72907615	14.51884792	0.638778621	-0.64661207	0.54901096	1
gene14396	22.72907615	14.51884792	0.638778621	-0.64661207	0.54901096	1
gene15094	22.72907615	14.51884792	0.638778621	-0.64661207	0.54901096	1
gene17256	22.72907615	14.51884792	0.638778621	-0.64661207	0.54901096	1
gene24923	22.72907615	14.51884792	0.638778621	-0.64661207	0.54901096	1
gene63888	22.72907615	14.51884792	0.638778621	-0.64661207	0.54901096	1
gene64102	22.72907615	14.51884792	0.638778621	-0.64661207	0.54901096	1
gene14663	246.9204182	195.5204853	0.791836037	-0.33672637	0.54903794	1
gene39878	607.4862171	485.8974437	0.79984933	-0.32219983	0.54906576	1
gene131	17.56337703	10.64715514	0.606213436	-0.72210227	0.54909792	1
gene20766	17.56337703	10.64715514	0.606213436	-0.72210227	0.54909792	1
gene27449	17.56337703	10.64715514	0.606213436	-0.72210227	0.54909792	1
gene29154	17.56337703	10.64715514	0.606213436	-0.72210227	0.54909792	1
gene36739	17.56337703	10.64715514	0.606213436	-0.72210227	0.54909792	1
gene37610	17.56337703	10.64715514	0.606213436	-0.72210227	0.54909792	1
gene50032	17.56337703	10.64715514	0.606213436	-0.72210227	0.54909792	1
gene54730	17.56337703	10.64715514	0.606213436	-0.72210227	0.54909792	1
gene10946	67.15408863	49.36408293	0.735086782	-0.44401351	0.54923092	1
gene56221	67.15408863	49.36408293	0.735086782	-0.44401351	0.54923092	1
gene71760	14283.15808	21402.71768	1.498458363	0.583478997	0.54927028	1
gene15609	59.92210985	80.33762515	1.340700876	0.422987393	0.54929614	1
gene46939	169.4349313	214.8789492	1.268209262	0.342792818	0.5493142	1
gene73480	524.8350311	656.251926	1.250396576	0.322385733	0.54931807	1

gene58588	752.1257927	601.0803039	0.799175231	-0.32341623	0.54935856	1
gene20521	313.041367	392.0088938	1.252259079	0.324533071	0.54937206	1
gene35591	81.61804618	60.97916126	0.747128412	-0.42057187	0.54942381	1
gene37773	81.61804618	60.97916126	0.747128412	-0.42057187	0.54942381	1
gene14675	235.5558801	296.1844976	1.257385285	0.330426785	0.549435	1
gene19585	235.5558801	296.1844976	1.257385285	0.330426785	0.549435	1
gene50809	235.5558801	296.1844976	1.257385285	0.330426785	0.549435	1
gene67831	540.3321285	675.6103899	1.250361313	0.322345047	0.54959423	1
gene48219	99.18142321	128.7337849	1.29796267	0.376248891	0.5496087	1
gene72436	99.18142321	128.7337849	1.29796267	0.376248891	0.5496087	1
gene639	56.82269038	76.46593238	1.345693628	0.428349991	0.54963979	1
gene47350	56.82269038	76.46593238	1.345693628	0.428349991	0.54963979	1
gene13300	944.2898001	753.0442454	0.797471545	-0.32649505	0.54965814	1
gene33073	277.9146129	348.4523501	1.253810825	0.32631969	0.54969898	1
gene2999	82.65118601	108.4073978	1.311625435	0.391355784	0.54969998	1
gene7081	82.65118601	108.4073978	1.311625435	0.391355784	0.54969998	1
gene46691	82.65118601	108.4073978	1.311625435	0.391355784	0.54969998	1
gene61600	82.65118601	108.4073978	1.311625435	0.391355784	0.54969998	1
gene67397	82.65118601	108.4073978	1.311625435	0.391355784	0.54969998	1
gene66255	2983.707815	2311.400589	0.774673907	-0.36833895	0.54973146	1
gene22703	183.8988889	144.220556	0.784238322	-0.35063595	0.54975137	1
gene64114	362.6320786	289.4090352	0.798078968	-0.32539659	0.54977748	1
gene37683	793.4513857	633.9896925	0.799027772	-0.32368245	0.54979711	1
gene12867	152.9046941	194.5525621	1.272377956	0.347527282	0.54983733	1
gene15820	112.6122409	145.1884792	1.289277951	0.366563322	0.54984419	1
gene25317	1053.802622	839.1894098	0.796344014	-0.3285363	0.54989327	1
gene38720	50.62385143	68.72254682	1.357513205	0.440966232	0.54992589	1
gene51974	50.62385143	68.72254682	1.357513205	0.440966232	0.54992589	1
gene10751	951.5217789	758.8517846	0.797513837	-0.32641855	0.54992767	1
gene35387	645.7123907	808.2158675	1.251665415	0.323848964	0.54994095	1
gene67874	332.6710237	265.2109553	0.797216879	-0.32695584	0.54999471	1
gene39155	602.320518	482.0257509	0.80028114	-0.32142118	0.55000929	1
gene19434	179.7663296	227.4619507	1.265320104	0.339502407	0.55003829	1
gene20393	377.0960362	471.3785958	1.250022675	0.321954265	0.55008802	1
gene41965	1191.210218	1506.088491	1.264334765	0.338378504	0.55011491	1
gene13408	209.7273845	264.2430321	1.259935763	0.33335018	0.55021531	1
gene24594	294.4448501	368.7787372	1.252454363	0.324758035	0.55031206	1
gene6593	13.43081773	21.29431028	1.585481295	0.664920857	0.55032641	1
gene7064	13.43081773	21.29431028	1.585481295	0.664920857	0.55032641	1
gene10178	13.43081773	21.29431028	1.585481295	0.664920857	0.55032641	1
gene21490	13.43081773	21.29431028	1.585481295	0.664920857	0.55032641	1
gene34368	13.43081773	21.29431028	1.585481295	0.664920857	0.55032641	1
gene49001	13.43081773	21.29431028	1.585481295	0.664920857	0.55032641	1
gene56577	13.43081773	21.29431028	1.585481295	0.664920857	0.55032641	1
gene63421	13.43081773	21.29431028	1.585481295	0.664920857	0.55032641	1
gene65607	13.43081773	21.29431028	1.585481295	0.664920857	0.55032641	1
gene66382	13.43081773	21.29431028	1.585481295	0.664920857	0.55032641	1
gene26547	867.8374531	693.0330074	0.79857467	-0.32450078	0.55034419	1

gene6676	26.86163545	38.71692779	1.441346632	0.527417333	0.55035325	1
gene10940	26.86163545	38.71692779	1.441346632	0.527417333	0.55035325	1
gene23457	26.86163545	38.71692779	1.441346632	0.527417333	0.55035325	1
gene48948	26.86163545	38.71692779	1.441346632	0.527417333	0.55035325	1
gene64501	26.86163545	38.71692779	1.441346632	0.527417333	0.55035325	1
gene10078	18.59651685	28.06977264	1.509410223	0.59398495	0.55042956	1
gene12953	18.59651685	28.06977264	1.509410223	0.59398495	0.55042956	1
gene22820	18.59651685	28.06977264	1.509410223	0.59398495	0.55042956	1
gene46154	18.59651685	28.06977264	1.509410223	0.59398495	0.55042956	1
gene52457	18.59651685	28.06977264	1.509410223	0.59398495	0.55042956	1
gene67759	18.59651685	28.06977264	1.509410223	0.59398495	0.55042956	1
gene918	53.7232709	38.71692779	0.720673316	-0.47258267	0.55046637	1
gene2538	53.7232709	38.71692779	0.720673316	-0.47258267	0.55046637	1
gene10621	1412.302141	1118.919213	0.792266174	-0.33594289	0.55050134	1
gene46214	828.5781397	662.0594651	0.799030814	-0.32367695	0.55051672	1
gene28035	302.7099687	241.0128755	0.796184138	-0.32882597	0.55051993	1
gene9129	616.7844756	771.4347861	1.250736386	0.322777749	0.55056139	1
gene21661	256.2186766	203.2638709	0.793321836	-0.33402184	0.55058188	1
gene26309	109.5128215	141.3167864	1.290413164	0.367833061	0.55068629	1
gene54559	109.5128215	141.3167864	1.290413164	0.367833061	0.55068629	1
gene49116	97.11514356	73.56216279	0.757473655	-0.40073238	0.55068941	1
gene16783	112.6122409	86.14516432	0.764971584	-0.38652194	0.5507041	1
gene39837	489.7082771	611.727459	1.249167081	0.320966456	0.55070542	1
gene63944	136.3744569	105.5036282	0.773631885	-0.37028084	0.55073485	1
gene68126	386.3942946	308.7674991	0.79909953	-0.32355289	0.55074474	1
gene56871	1771.8348	2263.972352	1.27775589	0.353612242	0.55081212	1
gene32076	775.8880086	620.4387678	0.799649899	-0.32255959	0.55085678	1
gene57417	240.7215792	190.6808693	0.792122044	-0.33620537	0.55087948	1
gene63330	176.6669101	138.4130168	0.783468827	-0.35205222	0.55088881	1
gene18587	514.5036329	642.7010012	1.249167081	0.320966456	0.55095222	1
gene4519	216.9593633	171.3224055	0.789652048	-0.34071101	0.55101843	1
gene47996	310.9750873	247.7883378	0.796810895	-0.32769072	0.55105198	1
gene71440	220.0587827	276.8260337	1.257964032	0.331090673	0.55106514	1
gene69320	184.9320287	145.1884792	0.785091043	-0.34906813	0.55116988	1
gene70784	184.9320287	145.1884792	0.785091043	-0.34906813	0.55116988	1
gene47928	742.8275342	594.3048415	0.800057637	-0.32182416	0.55118038	1
gene64971	439.0844257	351.3561197	0.800201736	-0.32156434	0.55122393	1
gene15388	1147.818346	1448.981022	1.262378344	0.336144361	0.55127498	1
gene22910	236.5890199	297.1524208	1.255985679	0.328820014	0.55127876	1
gene20166	3916.633077	3002.49775	0.766601745	-0.38345081	0.55129624	1
gene64835	76.45234706	100.6640122	1.316689626	0.396915309	0.55131557	1
gene16428	92.98258426	120.9903993	1.301215709	0.379860145	0.55134455	1
gene45596	92.98258426	120.9903993	1.301215709	0.379860145	0.55134455	1
gene5961	193.1971473	151.9639416	0.786574459	-0.34634475	0.55144014	1
gene11631	193.1971473	151.9639416	0.786574459	-0.34634475	0.55144014	1
gene3840	303.7431086	241.9807987	0.796662679	-0.3279591	0.55146698	1
gene55205	551.6966666	689.1613146	1.249167081	0.320966456	0.55149057	1
gene23374	119.8442197	153.8997879	1.284165297	0.360830917	0.55149541	1

gene33473	330.604744	413.3032041	1.250142993	0.322093121	0.55150466	1
gene14571	598.1879587	479.1219813	0.800955577	-0.32020587	0.55150811	1
gene13884	152.9046941	119.0545529	0.778619346	-0.3610099	0.5515466	1
gene49668	1329.650955	1684.186359	1.266637949	0.341004209	0.55158698	1
gene45420	233.4896005	293.280728	1.256076191	0.328923978	0.55159812	1
gene58050	1097.194494	874.0346448	0.796608668	-0.32805692	0.55160816	1
gene15386	878.1688513	1102.464519	1.255412916	0.328161957	0.55166134	1
gene9897	89.88316478	67.75462362	0.753807721	-0.40773152	0.55167329	1
gene45506	1084.796816	864.3554128	0.796790145	-0.32772829	0.55168712	1
gene71286	1265.596286	1005.672199	0.794623223	-0.33165714	0.55170854	1
gene56136	1630.294644	1288.305772	0.790228795	-0.33965768	0.55177861	1
gene18182	161.1698127	125.8300153	0.780729426	-0.35710545	0.55185016	1
gene61291	884.3676903	1110.207904	1.255369137	0.328111646	0.55192569	1
gene47242	1241.83407	987.2816585	0.795018982	-0.33093879	0.55194032	1
gene37242	3261.622428	4270.477135	1.309310697	0.388807487	0.55195625	1
gene41250	1260.430587	1001.800506	0.794808153	-0.33132142	0.55197637	1
gene64252	312.0082272	248.756261	0.797274685	-0.32685123	0.55197645	1
gene17195	105.3802622	80.33762515	0.762359321	-0.39145695	0.55198337	1
gene52459	105.3802622	80.33762515	0.762359321	-0.39145695	0.55198337	1
gene5034	531.0338701	663.0273883	1.248559509	0.320264585	0.55204247	1
gene55770	865.7711734	692.0650842	0.799362586	-0.32307805	0.55206235	1
gene39840	169.4349313	132.6054777	0.782633644	-0.35359097	0.5521136	1
gene42379	169.4349313	132.6054777	0.782633644	-0.35359097	0.5521136	1
gene4562	73.35292758	96.79231946	1.319542691	0.400038027	0.55211673	1
gene23706	129.1424781	99.69608905	0.771985256	-0.3733548	0.55212072	1
gene62430	5440.514319	4102.058499	0.753983587	-0.40739498	0.55215332	1
gene60951	648.8118102	811.1196371	1.250161641	0.322114641	0.55215501	1
gene54217	60.95524968	44.52446695	0.730445158	-0.45315214	0.55220671	1
gene22117	391.5599937	488.8012133	1.248343092	0.320014496	0.55222511	1
gene43108	1851.386567	1458.660254	0.787874494	-0.34396226	0.55223072	1
gene65736	89.88316478	117.1187066	1.30301049	0.381848698	0.55224405	1
gene30961	343.0024219	273.9222641	0.798601545	-0.32445223	0.55227028	1
gene64275	227.2907615	285.5373424	1.256264621	0.329140387	0.55227521	1
gene62919	2076.611048	1630.950583	0.785390497	-0.34851795	0.55231782	1
gene11974	628.1490136	784.9857109	1.249680719	0.321559547	0.55233257	1
gene53615	180.7994694	228.4298739	1.263443276	0.337360894	0.55234436	1
gene29588	177.7000499	139.38094	0.784360725	-0.3504108	0.55235165	1
gene21763	116.7448002	150.0280952	1.285094453	0.3618744	0.55235169	1
gene57785	919.4944443	734.6537047	0.798975686	-0.32377649	0.55242547	1
gene72835	1875.148783	1477.050795	0.78769792	-0.34428563	0.55243132	1
gene37657	103.3139825	133.5734009	1.292887929	0.370597224	0.55245581	1
gene56166	82.65118601	61.94708446	0.749500249	-0.41599914	0.55247374	1
gene34543	2464.038483	1925.199234	0.781318655	-0.35601703	0.55251345	1
gene3682	226.2576217	179.065791	0.791424349	-0.33747664	0.55253401	1
gene21581	964.9525966	1212.807763	1.256857349	0.329820916	0.55256066	1
gene41200	600.2542384	481.0578277	0.801423459	-0.31936336	0.5525731	1
gene17625	137.4075967	106.4715514	0.77485928	-0.36799377	0.55257639	1
gene6493	337.8367228	422.0145129	1.249167081	0.320966456	0.55259518	1

gene46064	441.1507053	353.291966	0.800841893	-0.32041065	0.55260517	1
gene41332	1213.939294	1533.19034	1.262987653	0.336840535	0.55265685	1
gene9388	7898.353963	5811.410861	0.735774933	-0.44266357	0.55267432	1
gene51198	536.1995692	429.7578984	0.801488705	-0.31924591	0.55267702	1
gene43220	207.6611048	261.3392626	1.258489223	0.331692863	0.55267788	1
gene48172	373.9966167	299.0882671	0.799708483	-0.3224539	0.55275139	1
gene38778	29.96105493	42.58862056	1.421465989	0.50737958	0.55277758	1
gene44319	29.96105493	42.58862056	1.421465989	0.50737958	0.55277758	1
gene51297	29.96105493	42.58862056	1.421465989	0.50737958	0.55277758	1
gene51572	29.96105493	42.58862056	1.421465989	0.50737958	0.55277758	1
gene52743	29.96105493	42.58862056	1.421465989	0.50737958	0.55277758	1
gene66959	29.96105493	42.58862056	1.421465989	0.50737958	0.55277758	1
gene54610	47.52443195	33.87731181	0.71283991	-0.48834998	0.55279991	1
gene65150	47.52443195	33.87731181	0.71283991	-0.48834998	0.55279991	1
gene26789	646.7455305	808.2158675	1.24966595	0.321542497	0.55280857	1
gene73661	1681.951635	1328.958546	0.790128871	-0.33984012	0.55287369	1
gene50004	70.25350811	92.92062669	1.322647497	0.403428616	0.55290407	1
gene8661	113.6453808	87.11308752	0.766534345	-0.38357766	0.55290843	1
gene49672	834.7769787	667.8670043	0.800054411	-0.32182997	0.55294215	1
gene3355	75.41920723	56.13954529	0.744366685	-0.42591461	0.55297042	1
gene20465	75.41920723	56.13954529	0.744366685	-0.42591461	0.55297042	1
gene39896	1565.206835	1238.941689	0.791551418	-0.33724503	0.55297146	1
gene5023	68.18722846	50.33200612	0.738144184	-0.43802544	0.55297812	1
gene9433	68.18722846	50.33200612	0.738144184	-0.43802544	0.55297812	1
gene46577	68.18722846	50.33200612	0.738144184	-0.43802544	0.55297812	1
gene49178	68.18722846	50.33200612	0.738144184	-0.43802544	0.55297812	1
gene48115	894.6990885	715.2952408	0.799481356	-0.3228637	0.55298028	1
gene59849	202.4954057	159.7073271	0.788696052	-0.34245867	0.5530129	1
gene12386	875.0694318	699.8084697	0.79971765	-0.32243737	0.55306455	1
gene37012	3579.829494	2758.581105	0.770590082	-0.37596448	0.55307815	1
gene65146	1012.477029	808.2158675	0.798256005	-0.32507659	0.55309113	1
gene58938	150.8384145	191.6487925	1.270556928	0.345461018	0.55318679	1
gene35599	98.14828338	74.53008599	0.759362094	-0.39714011	0.55323698	1
gene6718	210.7605243	166.4827895	0.789914478	-0.34023163	0.55323782	1
gene12871	210.7605243	166.4827895	0.789914478	-0.34023163	0.55323782	1
gene72848	210.7605243	166.4827895	0.789914478	-0.34023163	0.55323782	1
gene66237	113.6453808	146.1564024	1.28607429	0.362973982	0.55323932	1
gene46961	903.9973469	1134.405984	1.254877559	0.327546604	0.55325277	1
gene46904	254.152397	318.446731	1.252975517	0.325358225	0.55332238	1
gene11006	296.5111298	370.7145835	1.250255206	0.322222612	0.55333533	1
gene33826	4650.162353	3539.695123	0.761198181	-0.39365598	0.55337312	1
gene72111	321.3064856	256.4996466	0.798302114	-0.32499326	0.5533785	1
gene56472	14325.51681	21377.55168	1.492270887	0.577509447	0.55339882	1
gene23644	219.0256429	173.2582518	0.791040946	-0.33817572	0.55347226	1
gene29055	336.803583	269.0826481	0.79893048	-0.32385812	0.55347338	1
gene24912	174.6006304	220.6864884	1.263950123	0.337939535	0.55351802	1
gene19314	802.7496441	642.7010012	0.800624461	-0.3208024	0.55356461	1
gene4124	121.9104994	93.88854988	0.770143264	-0.37680125	0.55357168	1

gene19855	121.9104994	93.88854988	0.770143264	-0.37680125	0.55357168	1
gene55259	121.9104994	93.88854988	0.770143264	-0.37680125	0.55357168	1
gene25944	1424.699819	1130.534291	0.793524556	-0.33365323	0.55358148	1
gene50710	170.4680711	133.5734009	0.783568442	-0.3518688	0.55362444	1
gene64685	170.4680711	133.5734009	0.783568442	-0.3518688	0.55362444	1
gene26903	581.6577215	725.942396	1.248057696	0.31968463	0.55363422	1
gene7421	975.2839949	779.1781717	0.798924391	-0.32386912	0.55365495	1
gene8546	1110.625312	885.6497231	0.797433404	-0.32656405	0.5537884	1
gene9756	259.3180961	206.1676405	0.795037614	-0.33090498	0.55380241	1
gene34993	123.976779	158.7394039	1.280396258	0.356590366	0.55382438	1
gene27873	214.8930836	270.0505713	1.256674095	0.32961055	0.55382952	1
gene71947	147.738995	187.7770998	1.271005666	0.345970462	0.55393002	1
gene23664	1408.169582	1117.95129	0.793903877	-0.33296375	0.55396962	1
gene3767	309.9419475	387.1692779	1.249167081	0.320966456	0.55402448	1
gene52417	1180.87882	940.8213452	0.796712863	-0.32786823	0.55404787	1
gene11550	184.9320287	233.2694899	1.261379608	0.335002516	0.55405422	1
gene13745	383.2948751	306.8316527	0.80051071	-0.32100739	0.55405587	1
gene56149	673.607166	540.1011426	0.80180433	-0.31867789	0.55408696	1
gene62559	596.1216791	478.1540582	0.802108152	-0.31813132	0.55409749	1
gene23578	897.798508	718.1990104	0.799955674	-0.32200803	0.55411291	1
gene4021	406.0239513	325.2221934	0.800992632	-0.32013912	0.55411339	1
gene43144	1354.446311	1076.330592	0.794664642	-0.33158194	0.55419298	1
gene57460	284.1134519	355.2278124	1.250302687	0.322277401	0.55422001	1
gene32282	332.6710237	415.2390505	1.248197231	0.319845916	0.55422964	1
gene3641	322.3396254	257.4675698	0.798746258	-0.32419083	0.55427175	1
gene73414	1606.532428	1271.851078	0.7916747	-0.33702035	0.55430095	1
gene62416	106.413402	81.30554835	0.764053651	-0.38825415	0.55432466	1
gene22465	2635.539694	2056.836789	0.780423377	-0.3576711	0.55435024	1
gene38632	158.0703932	200.3601013	1.267537185	0.342028071	0.5543622	1
gene36992	138.4407366	107.4394746	0.776068354	-0.36574437	0.55439481	1
gene49971	1307.955019	1653.212816	1.263967639	0.337959527	0.55440562	1
gene34130	90.91630461	68.72254682	0.755888035	-0.40375554	0.55441798	1
gene43386	90.91630461	68.72254682	0.755888035	-0.40375554	0.55441798	1
gene33263	33.0604744	46.46031334	1.405312966	0.490891457	0.55444207	1
gene70280	33.0604744	46.46031334	1.405312966	0.490891457	0.55444207	1
gene60802	560.994925	450.0842855	0.802296537	-0.31779253	0.55444398	1
gene17313	632.2815729	507.191754	0.802161214	-0.31803588	0.55445713	1
gene27103	3995.151704	5280.98895	1.321849417	0.402557837	0.55453028	1
gene26487	275.8483333	219.7185652	0.796519459	-0.32821849	0.55453058	1
gene26734	28.9279151	19.35846389	0.669196651	-0.57949787	0.55457382	1
gene35526	28.9279151	19.35846389	0.669196651	-0.57949787	0.55457382	1
gene40869	28.9279151	19.35846389	0.669196651	-0.57949787	0.55457382	1
gene56192	28.9279151	19.35846389	0.669196651	-0.57949787	0.55457382	1
gene40739	515.5367727	642.7010012	1.24666374	0.318072383	0.55457484	1
gene5906	181.8326092	229.3977971	1.261587776	0.335240587	0.55463542	1
gene13552	146.7058552	114.214937	0.778530188	-0.36117511	0.55464909	1
gene23174	146.7058552	114.214937	0.778530188	-0.36117511	0.55464909	1
gene46898	146.7058552	114.214937	0.778530188	-0.36117511	0.55464909	1

gene25189	41.325593	29.03769584	0.702656483	-0.50910854	0.55469797	1
gene44579	41.325593	29.03769584	0.702656483	-0.50910854	0.55469797	1
gene44638	41.325593	29.03769584	0.702656483	-0.50910854	0.55469797	1
gene3127	144.6395755	183.905407	1.271473636	0.346501548	0.55470371	1
gene48586	481.4431585	386.2013547	0.802174354	-0.31801225	0.55475141	1
gene70944	819.2798813	656.251926	0.801010669	-0.32010664	0.5547656	1
gene72732	2869.029294	2232.99881	0.77831161	-0.36158022	0.55486117	1
gene56041	260.3512359	207.1355637	0.795600462	-0.32988398	0.55486234	1
gene59854	696.3362421	558.4916833	0.802043107	-0.31824832	0.55492472	1
gene57951	163.2360924	127.7658617	0.782705956	-0.35345767	0.55499276	1
gene10904	114.6785206	88.08101071	0.768068948	-0.38069227	0.5550786	1
gene27039	107.4465418	138.4130168	1.288203552	0.365360575	0.55511102	1
gene24952	920.5275841	736.5895511	0.800181943	-0.32160002	0.55514699	1
gene21512	54.75641073	39.68485098	0.724752599	-0.46443949	0.55515277	1
gene29044	54.75641073	39.68485098	0.724752599	-0.46443949	0.55515277	1
gene29236	54.75641073	39.68485098	0.724752599	-0.46443949	0.55515277	1
gene61185	54.75641073	39.68485098	0.724752599	-0.46443949	0.55515277	1
gene54545	268.6163545	213.911026	0.796344014	-0.3285363	0.55519167	1
gene21229	1082.730537	1361.867935	1.257808743	0.330912568	0.55523792	1
gene14626	178.7331897	225.5261044	1.261803164	0.335486874	0.55524164	1
gene3377	354.36696	283.601496	0.800304566	-0.32137896	0.55527075	1
gene45698	219.0256429	274.8901873	1.255059379	0.327755622	0.55537523	1
gene6875	2216.084925	1740.325904	0.785315529	-0.34865567	0.55537551	1
gene57258	697.3693819	559.4596065	0.802242859	-0.31788905	0.55538941	1
gene46041	3305.0143	4319.841218	1.30705674	0.386321771	0.55540252	1
gene31641	668.4414668	834.3497938	1.248201728	0.319851114	0.55545169	1
gene12192	83.68432583	62.91500765	0.751813521	-0.41155323	0.55545625	1
gene40510	83.68432583	62.91500765	0.751813521	-0.41155323	0.55545625	1
gene25490	1019.709007	814.9913299	0.79923912	-0.32330089	0.55546294	1
gene45748	165.302372	209.07141	1.264781669	0.338888364	0.55547344	1
gene26824	403.9576716	503.3200612	1.24597228	0.317271972	0.55547848	1
gene46070	676.7065854	543.0049122	0.802422976	-0.31756518	0.55552015	1
gene10031	570.2931834	457.8276711	0.802793518	-0.31689913	0.5555697	1
gene44831	204.5616854	161.6431735	0.790192813	-0.33972337	0.55559304	1
gene70879	204.5616854	161.6431735	0.790192813	-0.33972337	0.55559304	1
gene30915	35.12675405	24.19807987	0.688878905	-0.53767769	0.55564536	1
gene34860	35.12675405	24.19807987	0.688878905	-0.53767769	0.55564536	1
gene50989	35.12675405	24.19807987	0.688878905	-0.53767769	0.55564536	1
gene73139	35.12675405	24.19807987	0.688878905	-0.53767769	0.55564536	1
gene10198	9542.079424	13530.59834	1.417992634	0.503850038	0.55565922	1
gene73312	248.9866978	311.6712687	1.251758714	0.323956499	0.55568966	1
gene13607	57.8558302	77.43385557	1.338393301	0.420502129	0.55569032	1
gene32153	57.8558302	77.43385557	1.338393301	0.420502129	0.55569032	1
gene70945	57.8558302	77.43385557	1.338393301	0.420502129	0.55569032	1
gene36696	202.4954057	254.5638002	1.257133708	0.330138102	0.55569588	1
gene19957	606.4530773	486.8653669	0.802807975	-0.31687315	0.55573016	1
gene56587	719.0653183	576.882224	0.802266789	-0.31784602	0.55576485	1
gene6429	21.69593633	31.94146542	1.472232631	0.558005653	0.55579624	1

gene20710	21.69593633	31.94146542	1.472232631	0.558005653	0.55579624	1
gene38024	21.69593633	31.94146542	1.472232631	0.558005653	0.55579624	1
gene72834	21.69593633	31.94146542	1.472232631	0.558005653	0.55579624	1
gene26625	175.6337703	221.6544116	1.262026154	0.335741809	0.55587372	1
gene16576	131.2087578	101.6319354	0.77458195	-0.36851021	0.55595146	1
gene44559	131.2087578	101.6319354	0.77458195	-0.36851021	0.55595146	1
gene14741	77.48548688	101.6319354	1.311625435	0.391355784	0.55600671	1
gene50702	4093.299987	5412.626504	1.322313664	0.403064437	0.55607159	1
gene2063	393.6262734	315.5429615	0.801630843	-0.31899008	0.55607229	1
gene74184	382.2617353	476.2182118	1.245790954	0.317062001	0.55611709	1
gene40587	571.3263233	458.7955943	0.803035981	-0.31646346	0.55611828	1
gene47175	4439.401828	3393.53872	0.764413507	-0.38757482	0.55611913	1
gene4763	54.75641073	73.56216279	1.343443842	0.425936016	0.55621137	1
gene68148	54.75641073	73.56216279	1.343443842	0.425936016	0.55621137	1
gene57783	699.4356616	873.0667216	1.248244506	0.319900556	0.55622604	1
gene19051	76.45234706	57.10746848	0.746968153	-0.42088136	0.55624567	1
gene68484	76.45234706	57.10746848	0.746968153	-0.42088136	0.55624567	1
gene39171	3132.47995	4080.764189	1.302726356	0.38153407	0.55626746	1
gene1669	39.25931335	54.2036989	1.380658353	0.465356365	0.55627156	1
gene6027	39.25931335	54.2036989	1.380658353	0.465356365	0.55627156	1
gene24804	39.25931335	54.2036989	1.380658353	0.465356365	0.55627156	1
gene44080	39.25931335	54.2036989	1.380658353	0.465356365	0.55627156	1
gene38561	278.9477528	348.4523501	1.249167081	0.320966456	0.55630275	1
gene39927	61.9883895	45.49239015	0.73388566	-0.44637279	0.55632746	1
gene43032	90.91630461	118.0866297	1.298849863	0.377234676	0.55633464	1
gene56768	727.3304369	583.6576864	0.802465643	-0.31748847	0.55634326	1
gene3386	138.4407366	176.1620214	1.272472437	0.347634406	0.55634657	1
gene25107	147.738995	115.1828602	0.779637496	-0.35912462	0.55635151	1
gene38926	386.3942946	309.7354223	0.801604544	-0.31903741	0.55635241	1
gene64103	1716.045249	1357.996242	0.791352234	-0.33760811	0.55636752	1
gene42253	242.7878589	303.9278831	1.251824883	0.324032759	0.55638529	1
gene14692	480.4100187	598.1765343	1.24513751	0.316305079	0.55647294	1
gene15911	2969.243857	2310.432666	0.778121561	-0.36193254	0.55651084	1
gene38959	164.2692322	128.7337849	0.783675574	-0.35167156	0.55653953	1
gene58577	1297.62362	1637.726045	1.262096358	0.335822061	0.55655105	1
gene12802	172.5343508	135.5092472	0.785404452	-0.34849232	0.5566014	1
gene7239	51.65699125	69.69047001	1.349100447	0.431997768	0.55661107	1
gene47292	51.65699125	69.69047001	1.349100447	0.431997768	0.55661107	1
gene68258	404.9908114	504.2879844	1.245183768	0.316358675	0.55661422	1
gene20959	69.22036828	51.29992932	0.74111032	-0.43223978	0.55662253	1
gene11097	107.4465418	82.27347154	0.765715398	-0.38511983	0.5566269	1
gene58767	180.7994694	142.2847096	0.786975261	-0.34560981	0.55665786	1
gene28738	586.8234206	471.3785958	0.803271613	-0.3160402	0.55668658	1
gene59977	42.35873283	58.07539168	1.37103704	0.455267548	0.55668697	1
gene11518	254.152397	202.2959477	0.79596317	-0.32922642	0.55670603	1
gene67924	196.2965668	246.8204146	1.257385285	0.330426785	0.55675873	1
gene16843	3715.170811	2865.052656	0.771176563	-0.37486689	0.55676527	1
gene32124	292.3785705	364.9070444	1.248063576	0.319691427	0.556814	1

gene55274	48.55757178	65.81877724	1.355479173	0.438802946	0.55685063	1
gene34137	45.4581523	61.94708446	1.362727725	0.446497338	0.55687948	1
gene44345	45.4581523	61.94708446	1.362727725	0.446497338	0.55687948	1
gene4264	1389.573065	1105.368288	0.795473312	-0.33011457	0.55690763	1
gene45747	630.2152933	506.2238308	0.803255389	-0.31606934	0.55690876	1
gene61084	1209.806735	965.0194251	0.79766412	-0.32614671	0.55691923	1
gene39099	125.0099188	159.7073271	1.277557242	0.353387934	0.55694949	1
gene35927	595.0885392	741.4291671	1.245914042	0.317204537	0.55699101	1
gene60448	1217.038714	1533.19034	1.259771216	0.333161753	0.55705145	1
gene52494	842.0089574	1053.100436	1.250699801	0.322735549	0.55705236	1
gene4903	222.1250624	176.1620214	0.793075844	-0.33446925	0.55708397	1
gene19426	91.94944443	69.69047001	0.7579216	-0.39987947	0.5571081	1
gene44679	91.94944443	69.69047001	0.7579216	-0.39987947	0.5571081	1
gene65170	91.94944443	69.69047001	0.7579216	-0.39987947	0.5571081	1
gene69163	91.94944443	69.69047001	0.7579216	-0.39987947	0.5571081	1
gene15517	1031.073545	1294.113311	1.255112515	0.327816701	0.55717313	1
gene8755	1217.038714	970.8269642	0.797696041	-0.32608898	0.55718208	1
gene61336	580.6245817	466.5389798	0.803512277	-0.31560803	0.55721166	1
gene14531	115.7116604	89.04893391	0.769576148	-0.37786401	0.55721544	1
gene20656	206.627965	259.4034162	1.255412916	0.328161957	0.55725401	1
gene34823	859.5723345	689.1613146	0.801749064	-0.31877733	0.55727595	1
gene49295	500.0396753	622.3746142	1.244650464	0.315740647	0.55732985	1
gene53250	448.3826841	360.0674284	0.803035981	-0.31646346	0.55735362	1
gene67721	238.6552996	189.7129461	0.794924506	-0.33111024	0.5573835	1
gene18159	145.6727153	184.8733302	1.269100598	0.343806432	0.55745268	1
gene15705	562.0280648	699.8084697	1.245148621	0.316317952	0.55745789	1
gene47960	571.3263233	711.4235481	1.245214021	0.316393726	0.55753161	1
gene18598	377.0960362	469.4427494	1.244889112	0.31601724	0.55754908	1
gene3019	1477.38995	1174.090835	0.79470612	-0.33150664	0.55755148	1
gene61999	123.976779	95.82439627	0.772922131	-0.37160502	0.55759463	1
gene72989	123.976779	95.82439627	0.772922131	-0.37160502	0.55759463	1
gene9212	111.5791011	143.2526328	1.283866167	0.36049482	0.55759528	1
gene71562	1051.736342	1320.247237	1.255302479	0.32803504	0.55760688	1
gene23048	775.8880086	968.8911178	1.248751246	0.320486118	0.5577338	1
gene28262	941.1903806	754.0121686	0.801126089	-0.31989877	0.55774761	1
gene34003	1072.399138	857.5799504	0.79968355	-0.32249888	0.55779366	1
gene246	5.165699125	1.935846389	0.374750124	-1.41599914	0.55780734	1
gene402	5.165699125	1.935846389	0.374750124	-1.41599914	0.55780734	1
gene1123	5.165699125	1.935846389	0.374750124	-1.41599914	0.55780734	1
gene1814	5.165699125	1.935846389	0.374750124	-1.41599914	0.55780734	1
gene2609	5.165699125	1.935846389	0.374750124	-1.41599914	0.55780734	1
gene2631	5.165699125	1.935846389	0.374750124	-1.41599914	0.55780734	1
gene4071	5.165699125	1.935846389	0.374750124	-1.41599914	0.55780734	1
gene4393	5.165699125	1.935846389	0.374750124	-1.41599914	0.55780734	1
gene5829	5.165699125	1.935846389	0.374750124	-1.41599914	0.55780734	1
gene7069	5.165699125	1.935846389	0.374750124	-1.41599914	0.55780734	1
gene9281	5.165699125	1.935846389	0.374750124	-1.41599914	0.55780734	1
gene11135	5.165699125	1.935846389	0.374750124	-1.41599914	0.55780734	1

gene14218	5.165699125	1.935846389	0.374750124	-1.41599914	0.55780734	1
gene14453	5.165699125	1.935846389	0.374750124	-1.41599914	0.55780734	1
gene14997	5.165699125	1.935846389	0.374750124	-1.41599914	0.55780734	1
gene20190	5.165699125	1.935846389	0.374750124	-1.41599914	0.55780734	1
gene20763	5.165699125	1.935846389	0.374750124	-1.41599914	0.55780734	1
gene23718	5.165699125	1.935846389	0.374750124	-1.41599914	0.55780734	1
gene24095	5.165699125	1.935846389	0.374750124	-1.41599914	0.55780734	1
gene25419	5.165699125	1.935846389	0.374750124	-1.41599914	0.55780734	1
gene27467	5.165699125	1.935846389	0.374750124	-1.41599914	0.55780734	1
gene28020	5.165699125	1.935846389	0.374750124	-1.41599914	0.55780734	1
gene28533	5.165699125	1.935846389	0.374750124	-1.41599914	0.55780734	1
gene29112	5.165699125	1.935846389	0.374750124	-1.41599914	0.55780734	1
gene29946	5.165699125	1.935846389	0.374750124	-1.41599914	0.55780734	1
gene30428	5.165699125	1.935846389	0.374750124	-1.41599914	0.55780734	1
gene30495	5.165699125	1.935846389	0.374750124	-1.41599914	0.55780734	1
gene32537	5.165699125	1.935846389	0.374750124	-1.41599914	0.55780734	1
gene33775	5.165699125	1.935846389	0.374750124	-1.41599914	0.55780734	1
gene35177	5.165699125	1.935846389	0.374750124	-1.41599914	0.55780734	1
gene36169	5.165699125	1.935846389	0.374750124	-1.41599914	0.55780734	1
gene36661	5.165699125	1.935846389	0.374750124	-1.41599914	0.55780734	1
gene37437	5.165699125	1.935846389	0.374750124	-1.41599914	0.55780734	1
gene40564	5.165699125	1.935846389	0.374750124	-1.41599914	0.55780734	1
gene40696	5.165699125	1.935846389	0.374750124	-1.41599914	0.55780734	1
gene45761	5.165699125	1.935846389	0.374750124	-1.41599914	0.55780734	1
gene46424	5.165699125	1.935846389	0.374750124	-1.41599914	0.55780734	1
gene46900	5.165699125	1.935846389	0.374750124	-1.41599914	0.55780734	1
gene49857	5.165699125	1.935846389	0.374750124	-1.41599914	0.55780734	1
gene50525	5.165699125	1.935846389	0.374750124	-1.41599914	0.55780734	1
gene52969	5.165699125	1.935846389	0.374750124	-1.41599914	0.55780734	1
gene55197	5.165699125	1.935846389	0.374750124	-1.41599914	0.55780734	1
gene55703	5.165699125	1.935846389	0.374750124	-1.41599914	0.55780734	1
gene56840	5.165699125	1.935846389	0.374750124	-1.41599914	0.55780734	1
gene57810	5.165699125	1.935846389	0.374750124	-1.41599914	0.55780734	1
gene59474	5.165699125	1.935846389	0.374750124	-1.41599914	0.55780734	1
gene59804	5.165699125	1.935846389	0.374750124	-1.41599914	0.55780734	1
gene60078	5.165699125	1.935846389	0.374750124	-1.41599914	0.55780734	1
gene60976	5.165699125	1.935846389	0.374750124	-1.41599914	0.55780734	1
gene61402	5.165699125	1.935846389	0.374750124	-1.41599914	0.55780734	1
gene65219	5.165699125	1.935846389	0.374750124	-1.41599914	0.55780734	1
gene70873	5.165699125	1.935846389	0.374750124	-1.41599914	0.55780734	1
gene73165	5.165699125	1.935846389	0.374750124	-1.41599914	0.55780734	1
gene74160	5.165699125	1.935846389	0.374750124	-1.41599914	0.55780734	1
gene8828	132.2418976	102.5998586	0.775849867	-0.36615059	0.55782879	1
gene62038	190.0977278	239.0770291	1.25765327	0.330734232	0.55791565	1
gene4693	444.2501248	552.6841441	1.244083261	0.315083042	0.55793378	1
gene55300	71.28664793	93.88854988	1.317056596	0.397317342	0.55793772	1
gene68634	71.28664793	93.88854988	1.317056596	0.397317342	0.55793772	1
gene66546	441.1507053	548.8124514	1.244047544	0.315041622	0.5579934	1

gene44314	263.4506554	210.0393332	0.797262519	-0.32687325	0.55800238	1
gene39211	2007.39068	1583.522346	0.788846119	-0.3421842	0.55804127	1
gene5008	48.55757178	34.84523501	0.717606621	-0.47873489	0.55805453	1
gene23723	48.55757178	34.84523501	0.717606621	-0.47873489	0.55805453	1
gene31395	48.55757178	34.84523501	0.717606621	-0.47873489	0.55805453	1
gene35353	48.55757178	34.84523501	0.717606621	-0.47873489	0.55805453	1
gene59215	173.5674906	136.4771704	0.786306064	-0.34683711	0.558068	1
gene23330	165.302372	129.7017081	0.784633073	-0.34990995	0.55807031	1
gene18	653.9775093	525.5822947	0.80367029	-0.31532434	0.55807117	1
gene5291	190.0977278	150.0280952	0.789215615	-0.34150859	0.55807321	1
gene51372	2013.589519	1588.361962	0.788821131	-0.3422299	0.55813356	1
gene62682	1382.341086	1100.528672	0.796133952	-0.32891691	0.5581621	1
gene23782	276.8814731	345.5485805	1.248001813	0.31962003	0.55817586	1
gene60604	2229.515743	2858.277194	1.282017049	0.358415448	0.55818614	1
gene27898	100.214563	76.46593238	0.76302216	-0.39020314	0.55819399	1
gene38723	100.214563	76.46593238	0.76302216	-0.39020314	0.55819399	1
gene6885	604.3867977	485.8974437	0.803951121	-0.3148203	0.55829985	1
gene71694	396.7256928	318.446731	0.802687441	-0.31708977	0.55830488	1
gene11054	142.5732959	181.0016374	1.269533936	0.34429896	0.5583057	1
gene22739	916.3950248	734.6537047	0.801677972	-0.31890526	0.55839893	1
gene54344	186.9983083	235.2053363	1.257793925	0.330895573	0.55853135	1
gene50230	1042.438084	834.3497938	0.80038307	-0.32123744	0.55858573	1
gene9017	108.4796816	139.38094	1.284857569	0.36160844	0.55862707	1
gene63468	480.4100187	386.2013547	0.80389946	-0.31491301	0.558657	1
gene59788	2448.541385	3149.622075	1.286325849	0.363256149	0.55866319	1
gene68509	327.5053246	262.3071857	0.80092495	-0.32026103	0.55866674	1
gene68315	788.2856865	633.0217693	0.803035981	-0.31646346	0.5587039	1
gene2478	11.36453808	18.3905407	1.618239173	0.694424851	0.55874693	1
gene18134	11.36453808	18.3905407	1.618239173	0.694424851	0.55874693	1
gene46425	11.36453808	18.3905407	1.618239173	0.694424851	0.55874693	1
gene6886	224.191342	280.6977264	1.252045346	0.324286814	0.55880432	1
gene70984	863.7048938	693.0330074	0.802395601	-0.3176144	0.55881712	1
gene31531	288.2460112	230.3657203	0.799198294	-0.32337459	0.55882539	1
gene47737	288.2460112	230.3657203	0.799198294	-0.32337459	0.55882539	1
gene66759	288.2460112	230.3657203	0.799198294	-0.32337459	0.55882539	1
gene25492	1071.365999	1344.445317	1.254888917	0.327559662	0.55883032	1
gene15155	256.2186766	204.2317941	0.797099559	-0.32716817	0.55883333	1
gene16587	619.883895	498.4804452	0.804151308	-0.31446111	0.55885142	1
gene60297	312.0082272	249.7241842	0.800376921	-0.32124853	0.55885369	1
gene1784	108.4796816	83.24139474	0.767345493	-0.38205181	0.5588911	1
gene31865	108.4796816	83.24139474	0.767345493	-0.38205181	0.5588911	1
gene45516	108.4796816	83.24139474	0.767345493	-0.38205181	0.5588911	1
gene51363	68.18722846	90.0168571	1.320142483	0.400693648	0.55889593	1
gene55275	68.18722846	90.0168571	1.320142483	0.400693648	0.55889593	1
gene70584	68.18722846	90.0168571	1.320142483	0.400693648	0.55889593	1
gene12637	118.8110799	151.9639416	1.279038468	0.355059655	0.55889666	1
gene33513	118.8110799	151.9639416	1.279038468	0.355059655	0.55889666	1
gene28503	366.7646379	294.2486512	0.802281956	-0.31781874	0.55891818	1

gene65675	518.6361922	417.1748969	0.804369042	-0.31407054	0.55927937	1
gene54596	320.2733458	398.7843562	1.24513751	0.316305079	0.55930081	1
gene18367	24.7953558	35.8131582	1.444349437	0.530419822	0.55931808	1
gene43190	24.7953558	35.8131582	1.444349437	0.530419822	0.55931808	1
gene48821	24.7953558	35.8131582	1.444349437	0.530419822	0.55931808	1
gene57157	24.7953558	35.8131582	1.444349437	0.530419822	0.55931808	1
gene65563	24.7953558	35.8131582	1.444349437	0.530419822	0.55931808	1
gene5838	116.7448002	90.0168571	0.771056672	-0.37509119	0.55931976	1
gene56243	837.8763981	672.7066203	0.802870951	-0.31675998	0.55933039	1
gene24508	199.3959862	157.7714807	0.791247024	-0.33779993	0.55939048	1
gene14420	215.9262234	171.3224055	0.793430287	-0.33382463	0.55940342	1
gene7304	831.6775592	1038.581588	1.248779141	0.320518344	0.55942231	1
gene21698	264.4837952	330.0618094	1.247947191	0.319556886	0.55944839	1
gene58886	182.865749	144.220556	0.788669047	-0.34250807	0.55946211	1
gene40871	1071.365999	857.5799504	0.800454701	-0.32110833	0.55948267	1
gene10212	232.4564606	184.8733302	0.795303042	-0.33042341	0.55951682	1
gene45392	232.4564606	184.8733302	0.795303042	-0.33042341	0.55951682	1
gene10183	174.6006304	137.4450936	0.787197007	-0.34520336	0.5595203	1
gene28939	81.61804618	106.4715514	1.304509926	0.383507922	0.55952136	1
gene5191	613.6850561	763.6914006	1.244435387	0.315491326	0.55952485	1
gene6461	125.0099188	96.79231946	0.774277116	-0.36907809	0.55956354	1
gene23407	466.9792009	375.5541995	0.804220399	-0.31433716	0.55961597	1
gene59693	55.78955055	40.65277417	0.728680797	-0.45664112	0.55968762	1
gene62383	55.78955055	40.65277417	0.728680797	-0.45664112	0.55968762	1
gene6064	383.2948751	307.7995759	0.803035981	-0.31646346	0.55971392	1
gene7356	92.98258426	70.65839321	0.759909974	-0.39609958	0.55974532	1
gene4083	313.041367	250.6921074	0.800827411	-0.32043674	0.55975396	1
gene43098	313.041367	250.6921074	0.800827411	-0.32043674	0.55975396	1
gene19819	16.5302372	25.16600306	1.52242238	0.606368675	0.5597817	1
gene26558	16.5302372	25.16600306	1.52242238	0.606368675	0.5597817	1
gene35598	16.5302372	25.16600306	1.52242238	0.606368675	0.5597817	1
gene40370	16.5302372	25.16600306	1.52242238	0.606368675	0.5597817	1
gene43798	16.5302372	25.16600306	1.52242238	0.606368675	0.5597817	1
gene59673	16.5302372	25.16600306	1.52242238	0.606368675	0.5597817	1
gene8049	289.279151	231.3336435	0.799689997	-0.32248725	0.55978737	1
gene69113	414.2890699	514.9351395	1.242936821	0.313752966	0.55979077	1
gene7860	261.3843757	326.1901166	1.247932726	0.319540163	0.55979476	1
gene17533	804.8159237	646.572694	0.803379599	-0.31584627	0.55979881	1
gene30265	65.08780898	86.14516432	1.323522264	0.404382464	0.55983752	1
gene39131	65.08780898	86.14516432	1.323522264	0.404382464	0.55983752	1
gene53575	65.08780898	86.14516432	1.323522264	0.404382464	0.55983752	1
gene74105	65.08780898	86.14516432	1.323522264	0.404382464	0.55983752	1
gene18143	585.7902808	471.3785958	0.804688318	-0.31349801	0.55988388	1
gene53711	414.2890699	332.965579	0.803703508	-0.31526472	0.55988713	1
gene19065	512.4373532	412.3352809	0.804655005	-0.31355773	0.55997515	1
gene66994	577.5251622	718.1990104	1.243580466	0.31449986	0.56001279	1
gene67570	1548.676598	1231.198304	0.795000264	-0.33097276	0.56003868	1
gene12583	191.1308676	240.0449523	1.255919335	0.328743807	0.5601041	1

gene38627	191.1308676	240.0449523	1.255919335	0.328743807	0.5601041	1
gene26884	258.2849563	322.3184238	1.247917914	0.319523039	0.56015319	1
gene40548	70.25350811	52.26785251	0.743989217	-0.42664638	0.56016847	1
gene66663	146.7058552	185.8412534	1.266760984	0.341144338	0.56017954	1
gene64373	407.0570911	327.1580398	0.803715368	-0.31524343	0.56018644	1
gene56774	157.0372534	198.4242549	1.263548939	0.337481543	0.56026107	1
gene9626	1303.822459	1040.517434	0.798051473	-0.32544629	0.56027795	1
gene64485	842.0089574	1051.164589	1.248400721	0.320081095	0.5602814	1
gene4686	63.02152933	46.46031334	0.737213359	-0.43984588	0.56032539	1
gene42628	63.02152933	46.46031334	0.737213359	-0.43984588	0.56032539	1
gene55054	63.02152933	46.46031334	0.737213359	-0.43984588	0.56032539	1
gene19926	615.7513357	495.5766757	0.804832482	-0.31323956	0.56036171	1
gene3796	23.76221598	15.48677111	0.651739347	-0.617633	0.56041963	1
gene18530	23.76221598	15.48677111	0.651739347	-0.617633	0.56041963	1
gene19306	23.76221598	15.48677111	0.651739347	-0.617633	0.56041963	1
gene20629	23.76221598	15.48677111	0.651739347	-0.617633	0.56041963	1
gene36577	23.76221598	15.48677111	0.651739347	-0.617633	0.56041963	1
gene45495	23.76221598	15.48677111	0.651739347	-0.617633	0.56041963	1
gene66188	1483.588789	1180.866297	0.795952562	-0.32924564	0.56043277	1
gene68724	678.7728651	844.9969489	1.244889112	0.31601724	0.56046333	1
gene69791	395.692553	491.7049829	1.242644015	0.313413061	0.56046748	1
gene26423	438.0512858	352.3240428	0.804298616	-0.31419686	0.56046839	1
gene49163	399.8251123	321.3505006	0.803727657	-0.31522137	0.56050793	1
gene53642	177.7000499	223.590258	1.25824533	0.331413243	0.56053772	1
gene39201	684.971704	852.7403345	1.244927826	0.316062105	0.56057216	1
gene28417	1159.182884	927.2704205	0.799934534	-0.32204616	0.56060666	1
gene68703	3027.099687	2359.796749	0.779556999	-0.35927358	0.56061643	1
gene13221	78.51862671	102.5998586	1.306694512	0.385921898	0.56062818	1
gene73184	78.51862671	102.5998586	1.306694512	0.385921898	0.56062818	1
gene37989	453.5483832	364.9070444	0.804560347	-0.31372746	0.56066572	1
gene5966	42.35873283	30.00561903	0.708369137	-0.49742674	0.56071039	1
gene14142	42.35873283	30.00561903	0.708369137	-0.49742674	0.56071039	1
gene19479	42.35873283	30.00561903	0.708369137	-0.49742674	0.56071039	1
gene36989	42.35873283	30.00561903	0.708369137	-0.49742674	0.56071039	1
gene51651	42.35873283	30.00561903	0.708369137	-0.49742674	0.56071039	1
gene11745	290.3122908	232.3015667	0.800178201	-0.32160677	0.56074361	1
gene27098	268.6163545	334.9014253	1.246764837	0.318189371	0.56075035	1
gene56190	268.6163545	334.9014253	1.246764837	0.318189371	0.56075035	1
gene27948	238.6552996	298.1203439	1.249167081	0.320966456	0.56077454	1
gene48179	238.6552996	298.1203439	1.249167081	0.320966456	0.56077454	1
gene34382	4223.475605	3246.414395	0.76865944	-0.37958355	0.56078805	1
gene55545	1212.906155	1524.479032	1.256881273	0.329848377	0.56079958	1
gene3657	102.2808427	131.6375545	1.287020629	0.364035178	0.56080352	1
gene69703	842.0089574	676.5783131	0.803528641	-0.31557865	0.5608956	1
gene20374	258.2849563	206.1676405	0.798217765	-0.32514571	0.56093343	1
gene39528	258.2849563	206.1676405	0.798217765	-0.32514571	0.56093343	1
gene23790	112.6122409	144.220556	1.280682764	0.356913152	0.56099593	1
gene47318	112.6122409	144.220556	1.280682764	0.356913152	0.56099593	1

gene65469	122.9436392	156.8035575	1.275410087	0.350961196	0.56106338	1
gene33151	167.3686517	131.6375545	0.786512607	-0.3464582	0.56108493	1
gene43530	167.3686517	131.6375545	0.786512607	-0.3464582	0.56108493	1
gene70052	1854.485986	2357.860902	1.271436355	0.346459246	0.5610912	1
gene16636	13.43081773	7.743385557	0.576538653	-0.79451076	0.56114504	1
gene18625	13.43081773	7.743385557	0.576538653	-0.79451076	0.56114504	1
gene24896	13.43081773	7.743385557	0.576538653	-0.79451076	0.56114504	1
gene26417	13.43081773	7.743385557	0.576538653	-0.79451076	0.56114504	1
gene34444	13.43081773	7.743385557	0.576538653	-0.79451076	0.56114504	1
gene37888	13.43081773	7.743385557	0.576538653	-0.79451076	0.56114504	1
gene38219	13.43081773	7.743385557	0.576538653	-0.79451076	0.56114504	1
gene40386	13.43081773	7.743385557	0.576538653	-0.79451076	0.56114504	1
gene51447	13.43081773	7.743385557	0.576538653	-0.79451076	0.56114504	1
gene59259	13.43081773	7.743385557	0.576538653	-0.79451076	0.56114504	1
gene64913	13.43081773	7.743385557	0.576538653	-0.79451076	0.56114504	1
gene65996	13.43081773	7.743385557	0.576538653	-0.79451076	0.56114504	1
gene67599	13.43081773	7.743385557	0.576538653	-0.79451076	0.56114504	1
gene69497	13.43081773	7.743385557	0.576538653	-0.79451076	0.56114504	1
gene71791	13.43081773	7.743385557	0.576538653	-0.79451076	0.56114504	1
gene12143	85.75060548	64.85085404	0.756272841	-0.40302128	0.56122818	1
gene22698	85.75060548	64.85085404	0.756272841	-0.40302128	0.56122818	1
gene73954	85.75060548	64.85085404	0.756272841	-0.40302128	0.56122818	1
gene2995	150.8384145	118.0866297	0.78286841	-0.35315826	0.56134157	1
gene37667	142.5732959	111.3111674	0.780729426	-0.35710545	0.56144626	1
gene2013	134.3081773	104.535705	0.778327181	-0.36155135	0.56151	1
gene70049	428.7530274	532.3577571	1.241641978	0.312249239	0.56157127	1
gene56317	1321.385836	1055.036282	0.798431657	-0.32475917	0.56158236	1
gene41135	2078.677328	1641.597738	0.789731872	-0.34056518	0.56158542	1
gene37175	492.8076966	396.8485098	0.805280665	-0.3124364	0.56159821	1
gene70425	492.8076966	396.8485098	0.805280665	-0.3124364	0.56159821	1
gene587	27.89477528	39.68485098	1.422662509	0.508593459	0.56161346	1
gene14261	27.89477528	39.68485098	1.422662509	0.508593459	0.56161346	1
gene46903	27.89477528	39.68485098	1.422662509	0.508593459	0.56161346	1
gene67104	27.89477528	39.68485098	1.422662509	0.508593459	0.56161346	1
gene1345	58.88897003	78.40177877	1.331349126	0.412888945	0.56162032	1
gene9352	58.88897003	78.40177877	1.331349126	0.412888945	0.56162032	1
gene10646	58.88897003	78.40177877	1.331349126	0.412888945	0.56162032	1
gene12570	58.88897003	78.40177877	1.331349126	0.412888945	0.56162032	1
gene40107	291.3454307	233.2694899	0.800662943	-0.32073306	0.56169416	1
gene10892	219.0256429	273.9222641	1.250640155	0.322666745	0.56170254	1
gene10927	1117.857291	895.328955	0.800933145	-0.32024627	0.56174521	1
gene57464	75.41920723	98.72816585	1.309058653	0.38852974	0.56175245	1
gene31007	217.9925031	173.2582518	0.794789956	-0.33135445	0.56180341	1
gene21005	259.3180961	323.286347	1.2466787	0.318089695	0.56183999	1
gene12566	209.7273845	166.4827895	0.793805682	-0.33314221	0.56186257	1
gene22103	205.5948252	257.4675698	1.252305692	0.324586772	0.56190278	1
gene53209	201.4622659	159.7073271	0.792740648	-0.33507914	0.56195195	1
gene29607	150.8384145	190.6808693	1.264139974	0.338156217	0.56197509	1

gene8311	683.9385642	550.7482977	0.805259897	-0.31247361	0.5620157	1
gene28820	926.7264231	744.3329367	0.803185188	-0.31619543	0.56201651	1
gene57688	272.7489138	339.7410413	1.245618311	0.316862058	0.56202388	1
gene31924	119.8442197	152.9318648	1.276088785	0.35172871	0.56212105	1
gene48681	119.8442197	152.9318648	1.276088785	0.35172871	0.56212105	1
gene19655	1219.104994	1531.254494	1.256048086	0.328891697	0.56212323	1
gene27023	2859.731036	3694.562834	1.291926684	0.3695242	0.56214686	1
gene21978	523.8018913	422.0145129	0.80567581	-0.31172866	0.56219695	1
gene33945	1034.172965	829.5101778	0.802100041	-0.31814591	0.56220451	1
gene10967	184.9320287	146.1564024	0.790324983	-0.33948208	0.56221492	1
gene71750	448.3826841	361.0353516	0.805194679	-0.31259046	0.56221881	1
gene19318	94.01572408	71.6263164	0.761854648	-0.39241232	0.56233127	1
gene14418	407.0570911	505.2559076	1.241240894	0.311783134	0.5623637	1
gene38035	269.6494943	335.8693485	1.24557752	0.316814813	0.56237926	1
gene64192	55.78955055	74.53008599	1.335914795	0.417827995	0.56242722	1
gene15697	571.3263233	709.4877017	1.241825683	0.312462675	0.56244638	1
gene12549	546.5309675	678.5141594	1.241492614	0.312075679	0.56251244	1
gene9793	78.51862671	59.04331487	0.75196571	-0.41126122	0.5625617	1
gene62782	78.51862671	59.04331487	0.75196571	-0.41126122	0.5625617	1
gene28308	168.4017915	132.6054777	0.787435077	-0.34476711	0.56256927	1
gene50714	532.0670099	428.7899752	0.805894685	-0.31133678	0.56264526	1
gene33668	5824.842334	7854.696724	1.348482289	0.431336574	0.56265422	1
gene20598	85.75060548	111.3111674	1.29808025	0.376379576	0.56271362	1
gene6430	36.15989388	25.16600306	0.695964517	-0.52291434	0.56272263	1
gene15047	36.15989388	25.16600306	0.695964517	-0.52291434	0.56272263	1
gene19037	36.15989388	25.16600306	0.695964517	-0.52291434	0.56272263	1
gene26367	36.15989388	25.16600306	0.695964517	-0.52291434	0.56272263	1
gene34406	36.15989388	25.16600306	0.695964517	-0.52291434	0.56272263	1
gene40633	36.15989388	25.16600306	0.695964517	-0.52291434	0.56272263	1
gene53149	36.15989388	25.16600306	0.695964517	-0.52291434	0.56272263	1
gene53238	36.15989388	25.16600306	0.695964517	-0.52291434	0.56272263	1
gene60790	36.15989388	25.16600306	0.695964517	-0.52291434	0.56272263	1
gene62963	36.15989388	25.16600306	0.695964517	-0.52291434	0.56272263	1
gene14797	160.1366729	125.8300153	0.78576639	-0.34782764	0.56276717	1
gene26833	212.826804	266.1788785	1.25068306	0.322716238	0.56277878	1
gene15884	4390.844257	3373.212333	0.768237755	-0.38037523	0.56280931	1
gene50072	371.930337	299.0882671	0.804151308	-0.31446111	0.5628122	1
gene2632	158.0703932	199.3921781	1.261413817	0.335041641	0.56281805	1
gene53861	178.7331897	224.5581812	1.2563877	0.329281724	0.56284628	1
gene65781	313.041367	389.1051242	1.242983086	0.313806664	0.56286153	1
gene39170	1128.188689	904.0402638	0.801320092	-0.31954944	0.56287285	1
gene46198	147.738995	186.8091766	1.264454091	0.338514657	0.56288452	1
gene33682	72.31978776	94.85647307	1.311625435	0.391355784	0.56288989	1
gene24403	936.0246815	752.0763222	0.803479157	-0.31566749	0.56289335	1
gene11061	1003.17877	805.3120979	0.802760307	-0.31695881	0.56290648	1
gene73572	1257.331167	1005.672199	0.799846711	-0.32220456	0.56300098	1
gene1975	30.99419475	43.55654376	1.405312966	0.490891457	0.56306753	1
gene16134	30.99419475	43.55654376	1.405312966	0.490891457	0.56306753	1

gene59659	449.4158239	557.5237601	1.240552136	0.310982367	0.56309913	1
gene7949	127.0761985	161.6431735	1.272017698	0.347118744	0.56311285	1
gene19165	778.9874281	969.859041	1.24502528	0.316175037	0.56311505	1
gene11627	49.5907116	35.8131582	0.722174719	-0.46958018	0.56311617	1
gene29078	49.5907116	35.8131582	0.722174719	-0.46958018	0.56311617	1
gene50172	49.5907116	35.8131582	0.722174719	-0.46958018	0.56311617	1
gene61817	96.08200373	123.8941689	1.289462793	0.366770146	0.5631354	1
gene64188	52.69013108	70.65839321	1.341017602	0.423328174	0.56314657	1
gene9446	442.1838451	356.1957356	0.80553765	-0.31197607	0.56314975	1
gene71602	143.6064357	112.2790906	0.781852777	-0.35503112	0.56315571	1
gene56551	202.4954057	160.6752503	0.793476028	-0.33374146	0.56321673	1
gene56	29.96105493	20.32638709	0.678426949	-0.55973462	0.56326196	1
gene17708	29.96105493	20.32638709	0.678426949	-0.55973462	0.56326196	1
gene30547	29.96105493	20.32638709	0.678426949	-0.55973462	0.56326196	1
gene70679	29.96105493	20.32638709	0.678426949	-0.55973462	0.56326196	1
gene68461	110.5459613	85.17724113	0.770514274	-0.37610641	0.5633093	1
gene71792	209.7273845	262.3071857	1.250705464	0.322742081	0.56335121	1
gene35	194.2302871	153.8997879	0.792357311	-0.33577694	0.56337929	1
gene1121	127.0761985	98.72816585	0.776920989	-0.36416021	0.56341935	1
gene59272	1786.298758	1418.00748	0.793824367	-0.33310825	0.56342373	1
gene49749	349.2012609	280.6977264	0.803827929	-0.31504139	0.56349083	1
gene39618	293.4117103	235.2053363	0.801622185	-0.31900566	0.5635784	1
gene18151	175.6337703	220.6864884	1.256515123	0.329428035	0.56360358	1
gene14862	1559.007996	1968.755778	1.262825966	0.33665583	0.56362425	1
gene26149	1032.106685	828.5422546	0.802768034	-0.31694492	0.56364317	1
gene25228	49.5907116	66.78670043	1.346758259	0.429490913	0.56374625	1
gene56814	49.5907116	66.78670043	1.346758259	0.429490913	0.56374625	1
gene62987	659.1432084	531.3898339	0.806182673	-0.31082132	0.56380406	1
gene65082	144.6395755	182.9374838	1.264781669	0.338888364	0.56383063	1
gene27067	34.09361423	47.42823654	1.391117886	0.476244681	0.56393128	1
gene49654	34.09361423	47.42823654	1.391117886	0.476244681	0.56393128	1
gene58246	34.09361423	47.42823654	1.391117886	0.476244681	0.56393128	1
gene73214	34.09361423	47.42823654	1.391117886	0.476244681	0.56393128	1
gene28926	82.65118601	107.4394746	1.299914494	0.378416728	0.56393226	1
gene69727	82.65118601	107.4394746	1.299914494	0.378416728	0.56393226	1
gene54032	1953.667409	1547.709188	0.792207098	-0.33605047	0.56395229	1
gene70350	309.9419475	248.756261	0.80258985	-0.31726518	0.56397099	1
gene70883	309.9419475	248.756261	0.80258985	-0.31726518	0.56397099	1
gene32053	86.78374531	65.81877724	0.758422871	-0.39892562	0.5640219	1
gene55758	86.78374531	65.81877724	0.758422871	-0.39892562	0.5640219	1
gene36186	69.22036828	90.9847803	1.314422078	0.394428618	0.56403495	1
gene40234	69.22036828	90.9847803	1.314422078	0.394428618	0.56403495	1
gene72863	341.9692821	274.8901873	0.803844677	-0.31501133	0.56404058	1
gene8722	56.82269038	41.62069737	0.732466152	-0.449166	0.56407837	1
gene58849	56.82269038	41.62069737	0.732466152	-0.449166	0.56407837	1
gene40630	420.4879088	338.7731181	0.805666729	-0.31174492	0.5640898	1
gene6978	18.59651685	11.61507834	0.62458354	-0.67903354	0.56418001	1
gene36450	18.59651685	11.61507834	0.62458354	-0.67903354	0.56418001	1

gene37445	18.59651685	11.61507834	0.62458354	-0.67903354	0.56418001	1
gene66856	18.59651685	11.61507834	0.62458354	-0.67903354	0.56418001	1
gene74024	18.59651685	11.61507834	0.62458354	-0.67903354	0.56418001	1
gene74162	18.59651685	11.61507834	0.62458354	-0.67903354	0.56418001	1
gene67027	46.49129213	62.91500765	1.353264338	0.436443673	0.56418422	1
gene18465	64.05466916	47.42823654	0.740433713	-0.43355751	0.5642031	1
gene58877	64.05466916	47.42823654	0.740433713	-0.43355751	0.5642031	1
gene33221	4312.32563	3319.008634	0.769656311	-0.37771374	0.5642737	1
gene28866	286.1797315	229.3977971	0.801586457	-0.31906996	0.5643544	1
gene27968	92.98258426	120.0224761	1.290805984	0.368272171	0.56436114	1
gene28869	113.6453808	145.1884792	1.277557242	0.353387934	0.56436136	1
gene41405	113.6453808	145.1884792	1.277557242	0.353387934	0.56436136	1
gene14031	37.1930337	51.29992932	1.379288652	0.46392441	0.56437399	1
gene41378	37.1930337	51.29992932	1.379288652	0.46392441	0.56437399	1
gene57282	2008.42382	1590.297809	0.791813856	-0.33676678	0.56439251	1
gene25236	565.1274843	455.8918247	0.806706163	-0.30988482	0.56441027	1
gene25598	616.7844756	497.512522	0.806622964	-0.31003362	0.56441871	1
gene53084	43.39187265	59.04331487	1.360699856	0.444348871	0.56443155	1
gene53104	43.39187265	59.04331487	1.360699856	0.444348871	0.56443155	1
gene54106	43.39187265	59.04331487	1.360699856	0.444348871	0.56443155	1
gene66606	227.2907615	283.601496	1.247747573	0.319326098	0.56449988	1
gene62966	40.29245318	55.17162209	1.3692793	0.453416752	0.564513	1
gene23784	837.8763981	674.6424667	0.805181371	-0.3126143	0.56452613	1
gene72731	152.9046941	120.0224761	0.784949585	-0.3493281	0.56457415	1
gene5271	151.8715543	191.6487925	1.261913684	0.335613232	0.56461478	1
gene32458	845.1083769	680.4500058	0.805163012	-0.3126472	0.56463662	1
gene24613	213.8599438	267.1468017	1.249167081	0.320966456	0.56476455	1
gene67488	421.5210486	339.7410413	0.805988319	-0.31116917	0.56477994	1
gene57015	502.105955	622.3746142	1.239528446	0.30979138	0.56483416	1
gene39321	144.6395755	113.2470138	0.782960081	-0.35298934	0.56484461	1
gene52742	277.9146129	345.5485805	1.243362401	0.314246858	0.56485272	1
gene6743	1141.619507	915.6553421	0.802067008	-0.31820532	0.56488062	1
gene31820	2998.171772	3874.596548	1.292319734	0.369963053	0.56491705	1
gene18739	921.560724	741.4291671	0.804536422	-0.31377036	0.56492611	1
gene10402	733.5292758	591.4010719	0.806240584	-0.31071769	0.56495852	1
gene67237	2000.158701	1584.49027	0.792182275	-0.33609567	0.56498646	1
gene55818	319.2402059	256.4996466	0.803469118	-0.31568552	0.56505227	1
gene6901	131.2087578	166.4827895	1.268839003	0.343509025	0.56505623	1
gene52406	1016.609588	816.9271763	0.803580043	-0.31548636	0.56507029	1
gene16610	758.3246316	942.7571916	1.243210562	0.314070666	0.56509435	1
gene39566	1373.042828	1097.624903	0.799410536	-0.32299151	0.56510529	1
gene1284	66.12094881	87.11308752	1.317480906	0.397782053	0.56518047	1
gene32247	66.12094881	87.11308752	1.317480906	0.397782053	0.56518047	1
gene68062	66.12094881	87.11308752	1.317480906	0.397782053	0.56518047	1
gene32778	79.55176653	103.5677818	1.301891666	0.380609402	0.56518147	1
gene63074	79.55176653	103.5677818	1.301891666	0.380609402	0.56518147	1
gene70256	178.7331897	141.3167864	0.790657777	-0.33887471	0.56519095	1
gene72221	178.7331897	141.3167864	0.790657777	-0.33887471	0.56519095	1

gene1022	448.3826841	555.5879137	1.239093153	0.309284651	0.56524889	1
gene65667	647.7786703	522.6785251	0.806878258	-0.30957708	0.5652588	1
gene15259	103.3139825	79.36970196	0.768237755	-0.38037523	0.56530333	1
gene57072	287.2128714	230.3657203	0.802073108	-0.31819435	0.56530551	1
gene11501	234.5227403	292.3128048	1.246415612	0.317785209	0.56534403	1
gene13727	359.5326591	289.4090352	0.804958959	-0.31301287	0.56534946	1
gene64208	159.1035331	200.3601013	1.259306424	0.332629373	0.56535583	1
gene60727	618.8507552	499.4483684	0.807057864	-0.30925598	0.56541764	1
gene6414	170.4680711	134.5413241	0.789246474	-0.34145218	0.56549327	1
gene13826	170.4680711	134.5413241	0.789246474	-0.34145218	0.56549327	1
gene32356	170.4680711	134.5413241	0.789246474	-0.34145218	0.56549327	1
gene51640	110.5459613	141.3167864	1.278353228	0.354286529	0.56554685	1
gene13191	19.62965668	29.03769584	1.479276806	0.564892039	0.56556136	1
gene17419	19.62965668	29.03769584	1.479276806	0.564892039	0.56556136	1
gene33305	19.62965668	29.03769584	1.479276806	0.564892039	0.56556136	1
gene42358	19.62965668	29.03769584	1.479276806	0.564892039	0.56556136	1
gene45633	19.62965668	29.03769584	1.479276806	0.564892039	0.56556136	1
gene47959	19.62965668	29.03769584	1.479276806	0.564892039	0.56556136	1
gene51487	19.62965668	29.03769584	1.479276806	0.564892039	0.56556136	1
gene58520	19.62965668	29.03769584	1.479276806	0.564892039	0.56556136	1
gene59580	148.7721348	187.7770998	1.262179238	0.335916797	0.56556784	1
gene72297	367.7977777	296.1844976	0.8052917	-0.31241663	0.56560283	1
gene12092	79.55176653	60.01123807	0.754367133	-0.40666127	0.56560824	1
gene15521	79.55176653	60.01123807	0.754367133	-0.40666127	0.56560824	1
gene61382	89.88316478	116.1507834	1.292241808	0.369876056	0.56562728	1
gene36785	464.9129213	575.9143008	1.238757355	0.308893624	0.56575178	1
gene31158	138.4407366	175.1940982	1.26548083	0.339685653	0.56583857	1
gene5467	415.3222097	334.9014253	0.806365317	-0.31049451	0.56584277	1
gene34982	750.059513	604.9519966	0.806538663	-0.3101844	0.56590698	1
gene44490	312.0082272	387.1692779	1.240894451	0.311380407	0.56599555	1
gene24914	1389.573065	1111.175827	0.799652682	-0.32255457	0.56607325	1
gene39070	560.994925	452.9880551	0.807472643	-0.30851471	0.56614539	1
gene52759	128.1093383	162.6110967	1.269314937	0.344050069	0.56615181	1
gene31446	241.7547191	301.0241135	1.24516334	0.316335007	0.56616515	1
gene48668	1446.395755	1155.700294	0.799020801	-0.32369503	0.5661733	1
gene60719	6034.569718	8136.362374	1.34829205	0.431133028	0.56623363	1
gene45239	156.0041136	196.4884085	1.259507868	0.332860135	0.56627634	1
gene49157	63.02152933	83.24139474	1.320840602	0.401456374	0.56631723	1
gene28429	230.390181	183.905407	0.79823457	-0.32511533	0.56633043	1
gene26085	368.8309176	297.1524208	0.805660281	-0.31175646	0.56637106	1
gene21161	635.3809924	787.8894804	1.240026834	0.310371341	0.56640874	1
gene64091	76.45234706	99.69608905	1.304029149	0.382976118	0.56645917	1
gene4512	43.39187265	30.97354223	0.713809761	-0.48638847	0.56646761	1
gene7468	43.39187265	30.97354223	0.713809761	-0.48638847	0.56646761	1
gene12901	43.39187265	30.97354223	0.713809761	-0.48638847	0.56646761	1
gene32261	940.1572408	756.9159382	0.805095047	-0.31276898	0.56661294	1
gene10203	183.8988889	230.3657203	1.252675977	0.325013289	0.56662827	1
gene27910	2471.270462	1947.461468	0.788040604	-0.34365813	0.56673458	1

gene13676	87.81688513	66.78670043	0.760522311	-0.39493752	0.56675684	1
gene22768	440.1175655	355.2278124	0.807120279	-0.30914441	0.56677633	1
gene32500	321.3064856	258.435493	0.804327035	-0.31414588	0.56677997	1
gene53317	329.5716042	265.2109553	0.804714217	-0.31345157	0.56694229	1
gene5173	163.2360924	205.1997173	1.257073202	0.330068663	0.56695179	1
gene10348	163.2360924	205.1997173	1.257073202	0.330068663	0.56695179	1
gene13219	72.31978776	54.2036989	0.749500249	-0.41599914	0.56698109	1
gene57136	72.31978776	54.2036989	0.749500249	-0.41599914	0.56698109	1
gene68347	72.31978776	54.2036989	0.749500249	-0.41599914	0.56698109	1
gene57619	448.3826841	362.0032748	0.807353378	-0.30872782	0.56709195	1
gene10144	9.298258426	15.48677111	1.665556108	0.736003955	0.56710159	1
gene14901	9.298258426	15.48677111	1.665556108	0.736003955	0.56710159	1
gene22011	9.298258426	15.48677111	1.665556108	0.736003955	0.56710159	1
gene31797	9.298258426	15.48677111	1.665556108	0.736003955	0.56710159	1
gene35748	9.298258426	15.48677111	1.665556108	0.736003955	0.56710159	1
gene36155	9.298258426	15.48677111	1.665556108	0.736003955	0.56710159	1
gene49161	9.298258426	15.48677111	1.665556108	0.736003955	0.56710159	1
gene49676	9.298258426	15.48677111	1.665556108	0.736003955	0.56710159	1
gene58225	9.298258426	15.48677111	1.665556108	0.736003955	0.56710159	1
gene59625	9.298258426	15.48677111	1.665556108	0.736003955	0.56710159	1
gene61693	9.298258426	15.48677111	1.665556108	0.736003955	0.56710159	1
gene63607	9.298258426	15.48677111	1.665556108	0.736003955	0.56710159	1
gene65709	9.298258426	15.48677111	1.665556108	0.736003955	0.56710159	1
gene69181	9.298258426	15.48677111	1.665556108	0.736003955	0.56710159	1
gene23020	432.8855867	349.4202733	0.807188514	-0.30902245	0.56713271	1
gene12385	600.2542384	484.9295205	0.807873547	-0.3077986	0.56715211	1
gene68854	247.953558	198.4242549	0.800247661	-0.32148154	0.567182	1
gene46391	417.3884893	336.8372717	0.807011406	-0.30933903	0.56722681	1
gene9116	586.8234206	726.9103192	1.238720701	0.308850935	0.56727109	1
gene19766	586.8234206	726.9103192	1.238720701	0.308850935	0.56727109	1
gene19815	259.3180961	322.3184238	1.242946129	0.31376377	0.56729039	1
gene24085	239.6884394	191.6487925	0.799574619	-0.32269542	0.56729457	1
gene1110	120.8773595	93.88854988	0.776725685	-0.36452292	0.56742702	1
gene69902	59.92210985	79.36970196	1.324547853	0.405499966	0.56743333	1
gene43740	354.36696	285.5373424	0.805767396	-0.31156467	0.56748891	1
gene1489	112.6122409	87.11308752	0.77356677	-0.37040227	0.56758673	1
gene32716	112.6122409	87.11308752	0.77356677	-0.37040227	0.56758673	1
gene65698	503.1390948	406.5277417	0.807982814	-0.30760349	0.56759001	1
gene48320	142.5732959	180.0337142	1.262744984	0.336563311	0.56759042	1
gene31422	104.3471223	80.33762515	0.769907434	-0.37724309	0.56759089	1
gene1628	170.4680711	213.911026	1.254845113	0.327509302	0.56760319	1
gene23170	768.6560299	620.4387678	0.807173487	-0.30904931	0.56766677	1
gene44037	804.8159237	999.8646601	1.242351985	0.313073977	0.56771042	1
gene59465	154.9709738	121.9583225	0.786975261	-0.34560981	0.56773455	1
gene30955	356.4332397	441.3729768	1.238304759	0.308366419	0.56781897	1
gene10051	1620.996386	1293.145388	0.797747237	-0.32599639	0.56783949	1
gene46890	214.8930836	171.3224055	0.797244856	-0.32690521	0.56788669	1
gene22222	492.8076966	609.7916126	1.237382486	0.307291519	0.56791797	1

gene65286	434.9518664	538.1652962	1.23729851	0.307193606	0.56794322	1
gene17584	180.7994694	143.2526328	0.792328834	-0.33582879	0.56794606	1
gene70609	180.7994694	143.2526328	0.792328834	-0.33582879	0.56794606	1
gene26569	65.08780898	48.39615973	0.743551834	-0.42749478	0.56796613	1
gene54663	65.08780898	48.39615973	0.743551834	-0.42749478	0.56796613	1
gene68474	50.62385143	36.7810814	0.726556363	-0.46085337	0.5679956	1
gene1375	104.3471223	133.5734009	1.280087058	0.356241931	0.56805588	1
gene5005	104.3471223	133.5734009	1.280087058	0.356241931	0.56805588	1
gene12641	265.516935	212.9431028	0.801994429	-0.31833588	0.56807733	1
gene51995	265.516935	212.9431028	0.801994429	-0.31833588	0.56807733	1
gene57192	1257.331167	1008.575969	0.802156182	-0.31804493	0.56810369	1
gene68815	7233.011915	9881.527894	1.366170554	0.450137602	0.56811916	1
gene44503	426.6867478	344.5806573	0.807572907	-0.30833558	0.56818752	1
gene50397	248.9866978	199.3921781	0.800814581	-0.32045985	0.56823438	1
gene60227	992.8473719	799.5045588	0.805264315	-0.31246569	0.56825331	1
gene29087	1360.64515	1089.881517	0.801003493	-0.32011956	0.56828015	1
gene67792	94.01572408	120.9903993	1.286916636	0.363918601	0.56829889	1
gene59453	2267.741916	1793.56168	0.790902028	-0.3384291	0.5683306	1
gene1743	57.8558302	42.58862056	0.736116316	-0.44199435	0.56833201	1
gene34801	57.8558302	42.58862056	0.736116316	-0.44199435	0.56833201	1
gene62146	57.8558302	42.58862056	0.736116316	-0.44199435	0.56833201	1
gene62516	57.8558302	42.58862056	0.736116316	-0.44199435	0.56833201	1
gene36079	477.3105992	590.4331487	1.236999869	0.306845348	0.56839005	1
gene21835	691.170543	558.4916833	0.80803745	-0.30750594	0.56839214	1
gene12525	2658.26877	3409.025492	1.282423181	0.358872409	0.56844563	1
gene19936	56.82269038	75.49800918	1.328659532	0.409971462	0.56851336	1
gene47888	56.82269038	75.49800918	1.328659532	0.409971462	0.56851336	1
gene65474	56.82269038	75.49800918	1.328659532	0.409971462	0.56851336	1
gene71099	56.82269038	75.49800918	1.328659532	0.409971462	0.56851336	1
gene27069	890.5665292	718.1990104	0.806451834	-0.31033973	0.56852342	1
gene56512	2444.408826	1929.070927	0.789176878	-0.34157941	0.56854142	1
gene8827	1955.733689	1553.516727	0.794339606	-0.33217216	0.56856253	1
gene58858	138.4407366	108.4073978	0.783059961	-0.35280531	0.56859248	1
gene72304	138.4407366	108.4073978	0.783059961	-0.35280531	0.56859248	1
gene55789	640.5466916	517.8389091	0.808432728	-0.30680037	0.56870934	1
gene63565	640.5466916	517.8389091	0.808432728	-0.30680037	0.56870934	1
gene55406	224.191342	179.065791	0.798718583	-0.32424082	0.56878796	1
gene18706	403.9576716	326.1901166	0.807485882	-0.30849106	0.56879614	1
gene51806	750.059513	605.9199198	0.807829125	-0.30787793	0.56882231	1
gene68089	157.0372534	197.4563317	1.257385285	0.330426785	0.56883435	1
gene24892	380.1954556	306.8316527	0.807036613	-0.30929397	0.56885465	1
gene22537	190.0977278	150.9960184	0.794307329	-0.33223078	0.56888156	1
gene53008	190.0977278	150.9960184	0.794307329	-0.33223078	0.56888156	1
gene34422	528.9675904	654.3160796	1.236968184	0.306808393	0.56890008	1
gene64006	677.7397253	547.8445282	0.808340588	-0.30696481	0.56890504	1
gene69663	1069.299719	860.48372	0.804717054	-0.31344649	0.56892783	1
gene67745	1999.125562	1587.394039	0.794044191	-0.33270879	0.56893848	1
gene33459	111.5791011	142.2847096	1.275191395	0.350713799	0.56895167	1

gene60835	870.9368725	702.7122393	0.806846353	-0.30963413	0.5689785	1
gene63818	130.175618	101.6319354	0.780729426	-0.35710545	0.56900691	1
gene20642	497.9733957	402.656049	0.80858948	-0.30652066	0.56902033	1
gene29828	274.8151935	220.6864884	0.803035981	-0.31646346	0.56902985	1
gene45454	215.9262234	172.2903286	0.797912944	-0.32569674	0.56906341	1
gene31587	70.25350811	91.95270349	1.308869919	0.388321724	0.56908755	1
gene44758	70.25350811	91.95270349	1.308869919	0.388321724	0.56908755	1
gene1301	22.72907615	32.90938862	1.447898207	0.533960179	0.56911185	1
gene29402	22.72907615	32.90938862	1.447898207	0.533960179	0.56911185	1
gene32036	22.72907615	32.90938862	1.447898207	0.533960179	0.56911185	1
gene43985	22.72907615	32.90938862	1.447898207	0.533960179	0.56911185	1
gene50003	22.72907615	32.90938862	1.447898207	0.533960179	0.56911185	1
gene63930	22.72907615	32.90938862	1.447898207	0.533960179	0.56911185	1
gene16558	920.5275841	1145.053139	1.243909644	0.314881693	0.56912148	1
gene10689	129.1424781	163.5790199	1.26665542	0.341024108	0.56916276	1
gene61130	634.3478526	512.9992932	0.808703444	-0.30631734	0.56926735	1
gene11184	250.0198377	200.3601013	0.801376815	-0.31944732	0.56927976	1
gene18091	156.0041136	122.9262457	0.787967977	-0.3437911	0.56928849	1
gene5730	207.6611048	165.5148663	0.797043175	-0.32727022	0.56938652	1
gene40624	207.6611048	165.5148663	0.797043175	-0.32727022	0.56938652	1
gene66041	1064.13402	1327.0227	1.24704471	0.31851319	0.56939715	1
gene2846	372.9634769	301.0241135	0.807114187	-0.3091553	0.56940763	1
gene25271	332.6710237	268.1147249	0.805945531	-0.31124576	0.56944426	1
[truncated: 2,685,234 more chars]
