# Supplementary material for: Molecular Regulation of Host Defense Responses Mediated by Biological Anti-TMV Agent Ningnanmycin
Source: Viruses. 2019 Sep 3;11(9):815. doi: 10.3390/v11090815 (PMC6784071; doi:10.3390/v11090815)
Supplement: Supplementary file 1 [file viruses-11-00815-s001.zip › Supplementary file/Supplementary Table S3.pdf.pdf]

Table S3. Total Annotation of KEGGs of tobacco BY-2 transcriptome.

Gene_ID	KO_ID	KO_name	Definition	EC	Number Of Pathway	Pathway_ID;Pathway_Des
gene10004	K06689	UBE2D_E, UBC4, UBC5	ubiquitin-conjugating enzyme E2 D/E	EC:6.3.2.19	2	ko04120 Ubiquitin mediated proteolysis;ko04141 Protein processing in endoplasmic reticulum;
gene10007	K12900	FUSIP1	FUS-interacting serine-arginine-rich protein 1	--	1	ko03040 Spliceosome;
gene10009	K00789	metK	S-adenosylmethionine synthetase	EC:2.5.1.6	2	ko00270 Cysteine and methionine metabolism;ko01230 Biosynthesis of amino acids;
gene10011	K01100	E3.1.3.37	sedoheptulose-bisphosphatase	EC:3.1.3.37	2	ko00710 Carbon fixation in photosynthetic organisms;ko01200 Carbon metabolism;
gene10013	K07375	TUBB	tubulin beta	--	3	ko04145 Phagosome;ko04540 Gap junction;ko05130 Pathogenic Escherichia coli infection;
gene10015	K15053	CHMP7	charged multivesicular body protein 7	--	1	ko04144 Endocytosis;
gene10020	K05282	E1.14.11.12	gibberellin 20-oxidase	EC:1.14.11.12	1	ko00904 Diterpenoid biosynthesis;
gene10024	K15559	RTT103	regulator of Ty1 transposition protein 103	--		
gene10026	K02256	COX1	cytochrome c oxidase subunit 1	EC:1.9.3.1	6	ko00190 Oxidative phosphorylation;ko04260 Cardiac muscle contraction;ko04932 Non-alcoholic fatty liver disease (NAFLD);ko05010 Alzheimer's disease;ko05012 Parkinson's disease;ko05016 Huntington's disease;
gene10032	K02973	RP-S23e, RPS23	small subunit ribosomal protein S23e	--	1	ko03010 Ribosome;
gene10034	K11253	H3	histone H3	--	3	ko05034 Alcoholism;ko05202 Transcriptional misregulation in cancer;ko05322 Systemic lupus erythematosus;
gene10037	K17619	MDP1	magnesium-dependent phosphatase 1	EC:3.1.3.48		
gene1003	K16911	DDX21	ATP-dependent RNA helicase DDX21	EC:3.6.4.13		
gene10041	K06620	E2F3	transcription factor E2F3	--	13	ko04110 Cell cycle;ko05161 Hepatitis B;ko05166 HTLV-I infection;ko05200 Pathways in cancer;ko05206 MicroRNAs in cancer;ko05212 Pancreatic cancer;ko05214 Glioma;ko05215 Prostate cancer;ko05218 Melanoma;ko05219 Bladder cancer;ko05220 Chronic myeloid leukemia;ko05222 Small cell lung cancer;ko05223 Non-small cell lung cancer;
gene10048	K13176	THOC7	THO complex subunit 7	--	1	ko03013 RNA transport;
gene10051	K14290	XPO1, CRM1	exportin-1	--	5	ko03008 Ribosome biogenesis in eukaryotes;ko03013 RNA transport;ko05164 Influenza A;ko05166 HTLV-I infection;ko05169 Epstein-Barr virus infection;
gene10058	K14235	tRNA-Trp	tRNA Trp	--	1	ko00970 Aminoacyl-tRNA biosynthesis;
gene10061	K11253	H3	histone H3	--	3	ko05034 Alcoholism;ko05202 Transcriptional misregulation in cancer;ko05322 Systemic lupus erythematosus;
gene10062	K02868	RP-L11e, RPL11	large subunit ribosomal protein L11e	--	1	ko03010 Ribosome;
gene10070	K15216	RRN3, TIFIA	RNA polymerase I-specific transcription initiation factor RRN3	--		
gene10072	K14500	BSK	BR-signaling kinase	EC:2.7.11.1	1	ko04075 Plant hormone signal transduction;
gene10073	K03798	ftsH, hflB	cell division protease FtsH	EC:3.4.24.-		
gene10082	K00279	E1.5.99.12	cytokinin dehydrogenase	EC:1.5.99.12	1	ko00908 Zeatin biosynthesis;
gene10089	K15203	GTF3C6	general transcription factor 3C polypeptide 6	--		
gene10090	K08592	SENP1	sentrin-specific protease 1	EC:3.4.22.68		
gene10091	K10393	KIF2_24, MCAK	kinesin family member 2/24	--		
gene10094	K03844	ALG11	alpha-1,2-mannosyltransferase	EC:2.4.1.131	2	ko00510 N-Glycan biosynthesis;ko00513 Various types of N-glycan biosynthesis;
gene10095	K02912	RP-L32e, RPL32	large subunit ribosomal protein L32e	--	1	ko03010 Ribosome;
gene10103	K13024	HISPPD, VIP	inositol hexakisphosphate/diphosphoinositol-pentakisphosphate kinase	EC:2.7.4.21 2.7.4.24		
gene10106	K03671	trxA	thioredoxin 1	--		
gene10111	K03327	TC.MATE, SLC47A, norM, mdtK, dinF	multidrug resistance protein, MATE family	--		
gene10113	K12386	CTNS	cystinosin	--	1	ko04142 Lysosome;
gene10114	K07556	ATPeAF2, ATPAF2	ATP synthase mitochondrial F1 complex assembly factor 2	--		
gene10115	K02955	RP-S14e, RPS14	small subunit ribosomal protein S14e	--	1	ko03010 Ribosome;
gene10116	K00948	PRPS, prsA	ribose-phosphate pyrophosphokinase	EC:2.7.6.1	4	ko00030 Pentose phosphate pathway;ko00230 Purine metabolism;ko01200 Carbon metabolism;ko01230 Biosynthesis of amino acids;
gene10117	K03679	RRP4, EXOSC2	exosome complex component RRP4	--	1	ko03018 RNA degradation;

gene10121	K05956	RABGGTB	geranylgeranyl transferase type-2 subunit beta	EC:2.5.1.60		
gene10126	K07870	RHOT1, ARHT1	Ras homolog gene family, member T1	--		
gene10128	K14085	ALDH7A1	aldehyde dehydrogenase family 7 member A1	EC:1.2.1.31 1.2.1.8 1.2.1.3	15	ko00010 Glycolysis / Gluconeogenesis;ko00053 Ascorbate and aldarate metabolism;ko00071 Fatty acid degradation;ko00260 Glycine, serine and threonine metabolism;ko00280 Valine, leucine and isoleucine degradation;ko00300 Lysine biosynthesis;ko00310 Lysine degradation;ko00330 Arginine and proline metabolism;ko00340 Histidine metabolism;ko00380 Tryptophan metabolism;ko00410 beta-Alanine metabolism;ko00561 Glycerolipid metabolism;ko00620 Pyruvate metabolism;ko00640 Propanoate metabolism;ko01230 Biosynthesis of amino acids;
gene10130	K14303	NUP160	nuclear pore complex protein Nup160	--	1	ko03013 RNA transport;
gene10131	K11320	EP400	E1A-binding protein p400	EC:3.6.4.-		
gene10134	K09510	DNAJB4	DnaJ homolog subfamily B member 4	--		
gene10135	K06100	SYMPK	symplekin	--	2	ko03015 mRNA surveillance pathway;ko04530 Tight junction;
gene10137	K05305	FUK	fucokinase	EC:2.7.1.52	2	ko00051 Fructose and mannose metabolism;ko00520 Amino sugar and nucleotide sugar metabolism;
gene1013	K03012	RPB4, POLR2D	DNA-directed RNA polymerase II subunit RPB4	--	5	ko00230 Purine metabolism;ko00240 Pyrimidine metabolism;ko03020 RNA polymerase;ko05016 Huntington's disease;ko05169 Epstein-Barr virus infection;
gene10140	K02961	RP-S17, MRPS17, rpsQ	small subunit ribosomal protein S17	--	1	ko03010 Ribosome;
gene10141	K14321	NUPL2, CG1	nucleoporin-like protein 2	--	1	ko03013 RNA transport;
gene10154	K13250	SSR2	translocon-associated protein subunit beta	--	1	ko04141 Protein processing in endoplasmic reticulum;
gene10162	K08241	E2.1.1.141	jasmonate O-methyltransferase	EC:2.1.1.141	1	ko00592 alpha-Linolenic acid metabolism;
gene10165	K14684	SLC25A23S	solute carrier family 25 (mitochondrial phosphate transporter), member 23/24/25/41	--		
gene1016	K05607	AUH	methylglutaconyl-CoA hydratase	EC:4.2.1.18	1	ko00280 Valine, leucine and isoleucine degradation;
gene10172	K00430	E1.11.1.7	peroxidase	EC:1.11.1.7	2	ko00360 Phenylalanine metabolism;ko00940 Phenylpropanoid biosynthesis;
gene10177	K02293	PDS, crtP	15-cis-phytoene desaturase	EC:1.3.5.5	1	ko00906 Carotenoid biosynthesis;
gene1017	K05609	UCHL3, YUH1	ubiquitin carboxyl-terminal hydrolase L3	EC:3.4.19.12		
gene10181	K14638	SLC15A3_4, PHT	solute carrier family 15 (peptide/histidine transporter), member 3/4	--		
gene10182	K09285	OVM, ANT	AP2-like factor, ANT lineage	--		
gene10186	K12587	MTR3, EXOSC6	exosome complex component MTR3	--	1	ko03018 RNA degradation;
gene10187	K11643	CHD4, MI2B	chromodomain-helicase-DNA-binding protein 4	EC:3.6.4.12	1	ko05203 Viral carcinogenesis;
gene10189	K01115	PLD1_2	phospholipase D1/2	EC:3.1.4.4	7	ko00564 Glycerophospholipid metabolism;ko00565 Ether lipid metabolism;ko04014 Ras signaling pathway;ko04144 Endocytosis;ko04666 Fc gamma R-mediated phagocytosis;ko04724 Glutamatergic synapse;ko04912 GnRH signaling pathway;
gene10190	K12897	TRA2	transformer-2 protein	--	1	ko03040 Spliceosome;
gene10192	K17550	PPP1R7, SDS22	protein phosphatase 1 regulatory subunit 7	--		
gene10198	K00799	GST, gst	glutathione S-transferase	EC:2.5.1.18	4	ko00480 Glutathione metabolism;ko00980 Metabolism of xenobiotics by cytochrome P450;ko00982 Drug metabolism - cytochrome P450;ko05204 Chemical carcinogenesis;
gene1019	K09286	EREBP	EREBP-like factor	--		
gene101	K03066	PSMC5, RPT6	26S proteasome regulatory subunit T6	--	2	ko03050 Proteasome;ko05169 Epstein-Barr virus infection;
gene10202	K09286	EREBP	EREBP-like factor	--		
gene10203	K04733	IRAK4	interleukin-1 receptor-associated kinase 4	EC:2.7.11.1	11	ko04064 NF-kappa B signaling pathway;ko04210 Apoptosis;ko04620 Toll-like receptor signaling pathway;ko04722 Neurotrophin signaling pathway;ko05133 Pertussis;ko05140 Leishmaniasis;ko05142 Chagas disease (American trypanosomiasis);ko05145 Toxoplasmosis;ko05152 Tuberculosis;ko05162 Measles;ko05164 Influenza A;
gene10205	K14801	TSR4	pre-rRNA-processing protein TSR4	--		
gene10212	K03665	hflX	GTP-binding protein HflX	--		
gene10213	K00430	E1.11.1.7	peroxidase	EC:1.11.1.7	2	ko00360 Phenylalanine metabolism;ko00940 Phenylpropanoid biosynthesis;

gene10216	K02942	RP-LP1, RPLP1	large subunit ribosomal protein LP1	--	1	ko03010 Ribosome;
gene10217	K12890	SFRS1_9	splicing factor, arginine/serine-rich 1/9	--	2	ko03040 Spliceosome;ko05168 Herpes simplex infection;
gene10219	K03178	UBE1, UBA1	ubiquitin-activating enzyme E1	EC:6.3.2.19	2	ko04120 Ubiquitin mediated proteolysis;ko05012 Parkinson's disease;
gene10220	K08059	IFI30, GILT	interferon, gamma-inducible protein 30	--	1	ko04612 Antigen processing and presentation;
gene10223	K00382	DLD, lpd, pdhD	dihydrolipoamide dehydrogenase	EC:1.8.1.4	6	ko00010 Glycolysis / Gluconeogenesis;ko00020 Citrate cycle (TCA cycle);ko00260 Glycine, serine and threonine metabolism;ko00280 Valine, leucine and isoleucine degradation;ko00620 Pyruvate metabolism;ko01200 Carbon metabolism;
gene10227	K15559	RTT103	regulator of Ty1 transposition protein 103	--		
gene10229	K14297	NUP98, ADAR2	nuclear pore complex protein Nup98-Nup96	--	2	ko03013 RNA transport;ko05164 Influenza A;
gene10233	K13173	ARGLU1	arginine and glutamate-rich protein 1	--		
gene10234	K01982	LSUrRNA	large subunit ribosomal RNA	--	2	ko03008 Ribosome biogenesis in eukaryotes;ko03010 Ribosome;
gene10236	K06268	PPP3R, CNB	serine/threonine-protein phosphatase 2B regulatory subunit	--	18	ko04010 MAPK signaling pathway;ko04020 Calcium signaling pathway;ko04114 Oocyte meiosis;ko04210 Apoptosis;ko04310 Wnt signaling pathway;ko04360 Axon guidance;ko04370 VEGF signaling pathway;ko04380 Osteoclast differentiation;ko04650 Natural killer cell mediated cytotoxicity;ko04660 T cell receptor signaling pathway;ko04662 B cell receptor signaling pathway;ko04720 Long-term potentiation;ko04724 Glutamatergic synapse;ko05010 Alzheimer's disease;ko05014 Amyotrophic lateral sclerosis (ALS);ko05031 Amphetamine addiction;ko05152
gene10238	K00940	E2.7.4.6, ndk	nucleoside-diphosphate kinase	EC:2.7.4.6	2	ko00230 Purine metabolism;ko00240 Pyrimidine metabolism;
gene10241	K00975	glgC	glucose-1-phosphate adenylyltransferase	EC:2.7.7.27	2	ko00500 Starch and sucrose metabolism;ko00520 Amino sugar and nucleotide sugar metabolism;
gene10244	K08679	E5.1.3.6	UDP-glucuronate 4-epimerase	EC:5.1.3.6	2	ko00500 Starch and sucrose metabolism;ko00520 Amino sugar and nucleotide sugar metabolism;
gene10247	K05359	ADT, PDT	arogenate/prephenate dehydratase	EC:4.2.1.91 4.2.1.51	2	ko00400 Phenylalanine, tyrosine and tryptophan biosynthesis;ko01230 Biosynthesis of amino acids;
gene10252	K05909	E1.10.3.2				
gene10253	K02728	PSMA4	20S proteasome subunit alpha 3	EC:3.4.25.1	1	ko03050 Proteasome;
gene10254	K11863	ATXN3, MJD	Ataxin-3	EC:3.4.22.-	1	ko04141 Protein processing in endoplasmic reticulum;
gene10257	K14403	CPSF3, YSH1	cleavage and polyadenylation specificity factor subunit 3	EC:3.1.27.-	1	ko03015 mRNA surveillance pathway;
gene10258	K14403	CPSF3, YSH1	cleavage and polyadenylation specificity factor subunit 3	EC:3.1.27.-	1	ko03015 mRNA surveillance pathway;
gene10259	K02265	COX5B	cytochrome c oxidase subunit 5b	--	6	ko00190 Oxidative phosphorylation;ko04260 Cardiac muscle contraction;ko04932 Non-alcoholic fatty liver disease (NAFLD);ko05010 Alzheimer's disease;ko05012 Parkinson's disease;ko05016 Huntington's disease;
gene10263	K01392	THOP1	thimet oligopeptidase	EC:3.4.24.15	2	ko04614 Renin-angiotensin system;ko05143 African trypanosomiasis;
gene10264	K14137	PTAR1	protein prenyltransferase alpha subunit repeat containing protein 1	--		
gene10265	K14775	UTP30, RSL1D1	ribosome biogenesis protein UTP30	--		
gene10269	K10400	KIF15	kinesin family member 15	--		
gene10271	K10400	KIF15	kinesin family member 15	--		
gene10272	K13946	AUX1, LAX	auxin influx carrier (AUX1 LAX family)	--	1	ko04075 Plant hormone signal transduction;
gene10278	K10523	SPOP	speckle-type POZ protein	--		
gene1027	K05857	PLCD	phosphatidylinositol phospholipase C, delta	EC:3.1.4.11	3	ko00562 Inositol phosphate metabolism;ko04020 Calcium signaling pathway;ko04070 Phosphatidylinositol signaling system;
gene10283	K08202	SLC22A4_5, OCTN	MFS transporter, OCT family, solute carrier family 22 (organic cation transporter), member 4/5	--		
gene10286	K02938	RP-L8e, RPL8	large subunit ribosomal protein L8e	--	1	ko03010 Ribosome;
gene10287	K13947	PIN	auxin efflux carrier family	--		
gene10291	K00430	E1.11.1.7	peroxidase	EC:1.11.1.7	2	ko00360 Phenylalanine metabolism;ko00940 Phenylpropanoid biosynthesis;
gene10296	K10406	KIFC2_3	kinesin family member C2/C3	--		
gene10297	K01601	rbcL	ribulose-bisphosphate carboxylase large chain	EC:4.1.1.39	3	ko00630 Glyoxylate and dicarboxylate metabolism;ko00710 Carbon fixation in photosynthetic organisms;ko01200 Carbon metabolism;
gene1029	K02917	RP-L35Ae, RPL35A	large subunit ribosomal protein L35Ae	--	1	ko03010 Ribosome;

gene10302	K14293	KPNB1	importin subunit beta-1	--	1	ko03013 RNA transport;
gene10305	K03671	trxA	thioredoxin 1	--		
gene10306	K08901	psbQ	photosystem II oxygen-evolving enhancer protein 3	--	1	ko00195 Photosynthesis;
gene10311	K07766	E3.6.1.52	diphosphoinositol-polyphosphate diphosphatase	EC:3.6.1.52		
gene10315	K09285	OVM, ANT	AP2-like factor, ANT lineage	--		
gene10317	K16278	K16278, HOS1	E3 ubiquitin-protein ligase HOS1	EC:6.3.2.19		
gene10319	K11108	RCL1	RNA 3'-terminal phosphate cyclase-like protein	--	1	ko03008 Ribosome biogenesis in eukaryotes;
gene10323	K14230	tRNA-Met	tRNA Met	--	1	ko00970 Aminoacyl-tRNA biosynthesis;
gene10326	K02882	RP-L18Ae, RPL18A	large subunit ribosomal protein L18Ae	--	1	ko03010 Ribosome;
gene10329	K12847	USP39, SAD1	U4/U6.U5 tri-snRNP-associated protein 2	--	1	ko03040 Spliceosome;
gene10335	K13691	SGT1	pathogen-inducible salicylic acid glucosyltransferase	EC:2.4.1.-		
gene10336	K05391	CNGF	cyclic nucleotide gated channel, other eukaryote	--	1	ko04626 Plant-pathogen interaction;
gene10338	K05391	CNGF	cyclic nucleotide gated channel, other eukaryote	--	1	ko04626 Plant-pathogen interaction;
gene10339	K02114	ATPF1E, atpC	F-type H+-transporting ATPase subunit epsilon	EC:3.6.3.14	2	ko00190 Oxidative phosphorylation;ko00195 Photosynthesis;
gene10340	K01641	E2.3.3.10	hydroxymethylglutaryl-CoA synthase	EC:2.3.3.10	4	ko00072 Synthesis and degradation of ketone bodies;ko00280 Valine, leucine and isoleucine degradation;ko00650 Butanoate metabolism;ko00900 Terpenoid backbone biosynthesis;
gene10344	K02998	RP-SAe, RPSA	small subunit ribosomal protein SAe	--	1	ko03010 Ribosome;
gene10346	K10761	THG1	tRNA(His) guanylyltransferase	EC:2.7.7.79		
gene10351	K17583	NOM1	nucleolar MIF4G domain-containing protein 1	--		
gene10355	K01082	E3.1.3.7, cysQ, MET22, BPNT1	3'(2'), 5'-bisphosphate nucleotidase	EC:3.1.3.7	1	ko00920 Sulfur metabolism;
gene10356	K05931	CARM1, PRMT4	histone-arginine methyltransferase CARM1	EC:2.1.1.125		
gene10357	K12831	SF3B4, SAP49	splicing factor 3B subunit 4	--	1	ko03040 Spliceosome;
gene10362	K10999	CESA	cellulose synthase A	EC:2.4.1.12		
gene10367	K16871	POP2	4-aminobutyrate---pyruvate transaminase	EC:2.6.1.96	2	ko00250 Alanine, aspartate and glutamate metabolism;ko00650 Butanoate metabolism;
gene10370	K00975	glgC	glucose-1-phosphate adenylyltransferase	EC:2.7.7.27	2	ko00500 Starch and sucrose metabolism;ko00520 Amino sugar and nucleotide sugar metabolism;
gene10373	K11368	ENY2, DC6, SUS1	enhancer of yellow 2 transcription factor	--		
gene10374	K11650	SMARCD	SWI/SNF-related matrix-associated actin-dependent regulator of chromatin subfamily D	--		
gene1037	K10782	FATA	fatty acyl-ACP thioesterase A	EC:3.1.2.14	1	ko00061 Fatty acid biosynthesis;
gene10381	K11498	CENPE	centromeric protein E	--		
gene10385	K01188	E3.2.1.21	beta-glucosidase	EC:3.2.1.21	3	ko00460 Cyanoamino acid metabolism;ko00500 Starch and sucrose metabolism;ko00940 Phenylpropanoid biosynthesis;
gene10390	K16285	XERICO	RING/U-box domain-containing protein	--		
gene1039	K06628	CDC45	cell division control protein 45	--	3	ko04110 Cell cycle;ko04111 Cell cycle - yeast;ko04113 Meiosis - yeast;
gene10400	K12169	KPC1, RNF123	Kip1 ubiquitination-promoting complex protein 1	EC:6.3.2.19		
gene10402	K01304	E3.4.19.3, pcp	pyroglutamyl-peptidase	EC:3.4.19.3		
gene10404	K13152	ZMAT5	U11/U12 small nuclear ribonucleoprotein 20 kDa protein	--		
gene10407	K14496	PYL	abscisic acid receptor PYR/PYL family	--	1	ko04075 Plant hormone signal transduction;
gene1040	K13456	RIN4	RPM1-interacting protein 4	--	1	ko04626 Plant-pathogen interaction;
gene10412	K02155	ATPeVPL, ATP6L	V-type H+-transporting ATPase 16kDa proteolipid subunit	EC:3.6.3.14	9	ko00190 Oxidative phosphorylation;ko04142 Lysosome;ko04145 Phagosome;ko04721 Synaptic vesicle cycle;ko04966 Collecting duct acid secretion;ko05110 Vibrio cholerae infection;ko05120 Epithelial cell signaling in Helicobacter pylori infection;ko05152 Tuberculosis;ko05323 Rheumatoid arthritis;
gene10414	K14484	IAA	auxin-responsive protein IAA	--	1	ko04075 Plant hormone signal transduction;
gene10416	K00326	E1.6.2.2	cytochrome-b5 reductase	EC:1.6.2.2	1	ko00520 Amino sugar and nucleotide sugar metabolism;
gene10419	K15172	SUPT5H, SPT5	transcription elongation factor SPT5	--		
gene1041	K14009	BCAP31, BAP31	B-cell receptor-associated protein 31	--	1	ko04141 Protein processing in endoplasmic reticulum;
gene10423	K01696	trpB	tryptophan synthase beta chain	EC:4.2.1.20	3	ko00260 Glycine, serine and threonine metabolism;ko00400 Phenylalanine, tyrosine and tryptophan biosynthesis;ko01230 Biosynthesis of amino acids;

gene10430	K12382	PSAP, SGP1	saposin	--	1	ko04142 Lysosome;
gene10431	K15362	BRIP1, BACH1, FANCJ	fanconi anemia group J protein	EC:3.6.4.12	1	ko03460 Fanconi anemia pathway;
gene10432	K14803	PTC2_3	protein phosphatase 2C homolog 2/3	EC:3.1.3.16		
gene10433	K03018	RPC1, POLR3A	DNA-directed RNA polymerase III subunit RPC1	EC:2.7.7.6	5	ko00230 Purine metabolism;ko00240 Pyrimidine metabolism;ko03020 RNA polymerase;ko04623 Cytosolic DNA-sensing pathway;ko05169 Epstein-Barr virus infection;
gene10435	K03018	RPC1, POLR3A	DNA-directed RNA polymerase III subunit RPC1	EC:2.7.7.6	5	ko00230 Purine metabolism;ko00240 Pyrimidine metabolism;ko03020 RNA polymerase;ko04623 Cytosolic DNA-sensing pathway;ko05169 Epstein-Barr virus infection;
gene10438	K14509	ETR, ERS	ethylene receptor	EC:2.7.13.-	1	ko04075 Plant hormone signal transduction;
gene10443	K01934	E6.3.3.2	5-formyltetrahydrofolate cyclo-ligase	EC:6.3.3.2	1	ko00670 One carbon pool by folate;
gene10446	K15601	KDM3	lysine-specific demethylase 3	EC:1.14.11.-		
gene10449	K09753	CCR	cinnamoyl-CoA reductase	EC:1.2.1.44	1	ko00940 Phenylpropanoid biosynthesis;
gene1044	K05291	PIGS	phosphatidylinositol glycan, class S	--	1	ko00563 Glycosylphosphatidylinositol(GPI)-anchor biosynthesis;
gene10451	K12824	TCERG1, CA150	transcription elongation regulator 1	--	1	ko03040 Spliceosome;
gene10452	K14326	UPF1, RENT1	regulator of nonsense transcripts 1	EC:3.6.4.-	2	ko03013 RNA transport;ko03015 mRNA surveillance pathway;
gene10453	K09422	MYBP	myb proto-oncogene protein, plant	--		
gene10457	K11000	CALS	callose synthase	EC:2.4.1.-		
gene10472	K01188	E3.2.1.21	beta-glucosidase	EC:3.2.1.21	3	ko00460 Cyanoamino acid metabolism;ko00500 Starch and sucrose metabolism;ko00940 Phenylpropanoid biosynthesis;
gene10473	K09338	HD-ZIP	homeobox-leucine zipper protein	--		
gene10477	K09422	MYBP	myb proto-oncogene protein, plant	--		
gene10478	K00863	E2.7.1.29, DAK1, DAK2	dihydroxyacetone kinase	EC:2.7.1.29	4	ko00561 Glycerolipid metabolism;ko00680 Methane metabolism;ko01200 Carbon metabolism;ko04622 RIG-I-like receptor signaling pathway;
gene10480	K14496	PYL	abscisic acid receptor PYR/PYL family	--	1	ko04075 Plant hormone signal transduction;
gene10485	K09286	EREBP	EREBP-like factor	--		
gene10486	K00079	CBR1	carbonyl reductase 1	EC:1.1.1.184 1.1.1.189 1.1.1.197	3	ko00590 Arachidonic acid metabolism;ko00980 Metabolism of xenobiotics by cytochrome P450;ko05204 Chemical carcinogenesis;
gene1048	K00799	GST, gst	glutathione S-transferase	EC:2.5.1.18	4	ko00480 Glutathione metabolism;ko00980 Metabolism of xenobiotics by cytochrome P450;ko00982 Drug metabolism - cytochrome P450;ko05204 Chemical carcinogenesis;
gene10490	K09487	HSP90B, TRA1	heat shock protein 90kDa beta	--	8	ko04141 Protein processing in endoplasmic reticulum;ko04151 PI3K-Akt signaling pathway;ko04621 NOD-like receptor signaling pathway;ko04626 Plant-pathogen interaction;ko04915 Estrogen signaling pathway;ko04918 Thyroid hormone synthesis;ko05200 Pathways in cancer;ko05215 Prostate cancer;
gene10491	K09487	HSP90B, TRA1	heat shock protein 90kDa beta	--	8	ko04141 Protein processing in endoplasmic reticulum;ko04151 PI3K-Akt signaling pathway;ko04621 NOD-like receptor signaling pathway;ko04626 Plant-pathogen interaction;ko04915 Estrogen signaling pathway;ko04918 Thyroid hormone synthesis;ko05200 Pathways in cancer;ko05215 Prostate cancer;
gene10493	K10401	KIF18_19	kinesin family member 18/19	--		
gene10494	K03000	RPA12, ZNRD1	DNA-directed RNA polymerase I subunit RPA12	--	3	ko00230 Purine metabolism;ko00240 Pyrimidine metabolism;ko03020 RNA polymerase;
gene10498	K12391	AP1G1	AP-1 complex subunit gamma-1	--	1	ko04142 Lysosome;
gene1049	K11253	H3	histone H3	--	3	ko05034 Alcoholism;ko05202 Transcriptional misregulation in cancer;ko05322 Systemic lupus erythematosus;
gene10502	K17099	ANXA13	annexin A13	--		
gene10504	K04733	IRAK4	interleukin-1 receptor-associated kinase 4	EC:2.7.11.1	11	ko04064 NF-kappa B signaling pathway;ko04210 Apoptosis;ko04620 Toll-like receptor signaling pathway;ko04722 Neurotrophin signaling pathway;ko05133 Pertussis;ko05140 Leishmaniasis;ko05142 Chagas disease (American trypanosomiasis);ko05145 Toxoplasmosis;ko05152 Tuberculosis;ko05162 Measles;ko05164 Influenza A;
gene10505	K15176	CTR9	RNA polymerase-associated protein CTR9	--		
gene10512	K00654	E2.3.1.50	serine palmitoyltransferase	EC:2.3.1.50	1	ko00600 Sphingolipid metabolism;
gene10515	K14977	ylbA	ureidoglycine aminohydrolase	EC:3.5.3.-	1	ko00230 Purine metabolism;
gene10516	K06072	E1.14.99.29, DOHH	deoxyhypusine monooxygenase	EC:1.14.99.29		

gene1051	K05391	CNGF	cyclic nucleotide gated channel, other eukaryote	--	1	ko04626 Plant-pathogen interaction;
gene10524	K11254	H4	histone H4	--	3	ko05034 Alcoholism;ko05203 Viral carcinogenesis;ko05322 Systemic lupus erythematosus;
gene10528	K15289	SLC35F5	solute carrier family 35, member F5	--		
gene1052	K09422	MYBP	myb proto-oncogene protein, plant	--		
gene10530	K15272	SLC35A1_2_3	solute carrier family 35 (UDP-sugar transporter), member A1/2/3	--		
gene10532	K17095	ANXA7_11	annexin A7/11	--		
gene10534	K14235	tRNA-Trp	tRNA Trp	--	1	ko00970 Aminoacyl-tRNA biosynthesis;
gene10538	K13917	RNGTT	mRNA-capping enzyme	EC:2.7.7.50 3.1.3.33	1	ko03015 mRNA surveillance pathway;
gene1053	K01759	E4.4.1.5, GLO1, gloA	lactoylglutathione lyase	EC:4.4.1.5	2	ko00620 Pyruvate metabolism;ko04011 MAPK signaling pathway - yeast;
gene10558	K15920	XYL4	beta-D-xylosidase 4	EC:3.2.1.37	2	ko00500 Starch and sucrose metabolism;ko00520 Amino sugar and nucleotide sugar metabolism;
gene10559	K03255	TIF31, CLU1	protein TIF31	--		
gene10567	K00083	E1.1.1.195	cinnamyl-alcohol dehydrogenase	EC:1.1.1.195	1	ko00940 Phenylpropanoid biosynthesis;
gene10568	K06620	E2F3	transcription factor E2F3	--	13	ko04110 Cell cycle;ko05161 Hepatitis B;ko05166 HTLV-I infection;ko05200 Pathways in cancer;ko05206 MicroRNAs in cancer;ko05212 Pancreatic cancer;ko05214 Glioma;ko05215 Prostate cancer;ko05218 Melanoma;ko05219 Bladder cancer;ko05220 Chronic myeloid leukemia;ko05222 Small cell lung cancer;ko05223 Non-small cell lung cancer;
gene10572	K01365	CTSL	cathepsin L	EC:3.4.22.15	5	ko04142 Lysosome;ko04145 Phagosome;ko04612 Antigen processing and presentation;ko05205 Proteoglycans in cancer;ko05323 Rheumatoid arthritis;
gene10573	K10781	FATB	fatty acyl-ACP thioesterase B	EC:3.1.2.14	1	ko00061 Fatty acid biosynthesis;
gene10580	K15015	SLC32A, VGAT	solute carrier family 32 (vesicular inhibitory amino acid transporter)	--	5	ko04721 Synaptic vesicle cycle;ko04723 Retrograde endocannabinoid signaling;ko04727 GABAergic synapse;ko05032 Morphine addiction;ko05033 Nicotine addiction;
gene10581	K08679	E5.1.3.6	UDP-glucuronate 4-epimerase	EC:5.1.3.6	2	ko00500 Starch and sucrose metabolism;ko00520 Amino sugar and nucleotide sugar metabolism;
gene10583	K10573	UBE2A, UBC2, RAD6A	ubiquitin-conjugating enzyme E2 A	EC:6.3.2.19	1	ko04120 Ubiquitin mediated proteolysis;
gene10586	K03327	TC.MATE, SLC47A, norM, mdtK, dinF	multidrug resistance protein, MATE family	--		
gene10594	K11450	AOF2, LSD1	lysine-specific histone demethylase 1	EC:1.-.-.-		
gene10597	K08235	E2.4.1.207	xyloglucan:xyloglucosyl transferase	EC:2.4.1.207		
gene10598	K12842	SR140	U2-associated protein SR140	--	1	ko03040 Spliceosome;
gene10601	K13151	SNUPN, RNUT1	snurportin-1	--	1	ko03013 RNA transport;
gene10607	K13681	FUT	xyloglucan fucosyltransferase	EC:2.4.1.-		
gene10612	K12393	AP1M	AP-1 complex subunit mu	--	1	ko04142 Lysosome;
gene10617	K12456	APC13	anaphase-promoting complex subunit 13	--	4	ko04110 Cell cycle;ko04114 Oocyte meiosis;ko04120 Ubiquitin mediated proteolysis;ko04914 Progesterone-mediated oocyte maturation;
gene10627	K11253	H3	histone H3	--	3	ko05034 Alcoholism;ko05202 Transcriptional misregulation in cancer;ko05322 Systemic lupus erythematosus;
gene10629	K11253	H3	histone H3	--	3	ko05034 Alcoholism;ko05202 Transcriptional misregulation in cancer;ko05322 Systemic lupus erythematosus;
gene10632	K10355	ACTF	actin, other eukaryote	--		
gene10633	K10355	ACTF	actin, other eukaryote	--		
gene10636	K10355	ACTF	actin, other eukaryote	--		
gene10642	K08679	E5.1.3.6	UDP-glucuronate 4-epimerase	EC:5.1.3.6	2	ko00500 Starch and sucrose metabolism;ko00520 Amino sugar and nucleotide sugar metabolism;
gene10643	K05019	CLNS1A	chloride channel, nucleotide-sensitive, 1A	--	1	ko03013 RNA transport;
gene10644	K02953	RP-S13e, RPS13	small subunit ribosomal protein S13e	--	1	ko03010 Ribosome;
gene1064	K01982	LSUrRNA	large subunit ribosomal RNA	--	2	ko03008 Ribosome biogenesis in eukaryotes;ko03010 Ribosome;
gene10652	K13436	PTI1	pto-interacting protein 1	EC:2.7.11.1	1	ko04626 Plant-pathogen interaction;
gene10655	K12638	CYP90D1	cytochrome P450, family 90, subfamily D, polypeptide 1 (3-epi-6-deoxocathasterone 23-monooxygenase)	EC:1.14.13.11 2	1	ko00905 Brassinosteroid biosynthesis;
gene10657	K11145	K11145	ribonuclease III family protein	EC:3.1.26.-		

gene10659	K00968	PCYT1	choline-phosphate cytidylyltransferase	EC:2.7.7.15	2	ko00440 Phosphonate and phosphinate metabolism;ko00564 Glycerophospholipid metabolism;
gene1065	K07904	RAB11A	Ras-related protein Rab-11A	--	4	ko04144 Endocytosis;ko04961 Endocrine and other factor-regulated calcium reabsorption;ko04962 Vasopressin-regulated water reabsorption;ko04972 Pancreatic secretion;
gene10666	K02293	PDS, crtP	15-cis-phytoene desaturase	EC:1.3.5.5	1	ko00906 Carotenoid biosynthesis;
gene10670	K11251	H2A	histone H2A	--	2	ko05034 Alcoholism;ko05322 Systemic lupus erythematosus;
gene10671	K11418	HDAC11	histone deacetylase 11	EC:3.5.1.98	2	ko05034 Alcoholism;ko05203 Viral carcinogenesis;
gene10678	K08857	NEK	NIMA (never in mitosis gene a)-related kinase	EC:2.7.11.1		
gene1067	K03165	TOP3	DNA topoisomerase III	EC:5.99.1.2	2	ko03440 Homologous recombination;ko03460 Fanconi anemia pathway;
gene10681	K09264	K09264	MADS-box transcription factor, plant	--		
gene10683	K10591	NEDD4, RSP5	E3 ubiquitin-protein ligase NEDD4	EC:6.3.2.19	3	ko04120 Ubiquitin mediated proteolysis;ko04144 Endocytosis;ko05169 Epstein-Barr virus infection;
gene10685	K14445	SLC13A2_3_5	solute carrier family 13 (sodium-dependent dicarboxylate transporter), member 2/3/5	--		
gene1068	K13448	CML	calcium-binding protein CML	--	1	ko04626 Plant-pathogen interaction;
gene10690	K01051	E3.1.1.11	pectinesterase	EC:3.1.1.11	2	ko00040 Pentose and glucuronate interconversions;ko00500 Starch and sucrose metabolism;
gene10693	K01246	tag	DNA-3-methyladenine glycosylase I	EC:3.2.2.20	1	ko03410 Base excision repair;
gene10696	K08770	UBC	ubiquitin C	--	1	ko03320 PPAR signaling pathway;
gene10697	K08269	ULK1_2_3, ATG1	serine/threonine-protein kinase ULK/ATG1	EC:2.7.11.1	2	ko04140 Regulation of autophagy;ko04150 mTOR signaling pathway;
gene10698	K00930	argB	acetylglutamate kinase	EC:2.7.2.8	3	ko00330 Arginine and proline metabolism;ko01210 2-Oxocarboxylic acid metabolism;ko01230 Biosynthesis of amino acids;
gene10702	K00276	E1.4.3.21, AOC2, AOC3, tynA	primary-amine oxidase	EC:1.4.3.21	6	ko00260 Glycine, serine and threonine metabolism;ko00350 Tyrosine metabolism;ko00360 Phenylalanine metabolism;ko00410 beta-Alanine metabolism;ko00950 Isoquinoline alkaloid biosynthesis;ko00960 Tropane, piperidine and pyridine alkaloid biosynthesis;
gene10703	K14684	SLC25A23S	solute carrier family 25 (mitochondrial phosphate transporter), member 23/24/25/41	--		
gene1070	K09264	K09264	MADS-box transcription factor, plant	--		
gene10710	K03237	EIF2S1	translation initiation factor 2 subunit 1	--	7	ko03013 RNA transport;ko04141 Protein processing in endoplasmic reticulum;ko04932 Non-alcoholic fatty liver disease (NAFLD);ko05160 Hepatitis C;ko05162 Measles;ko05164 Influenza A;ko05168 Herpes simplex infection;
gene10715	K02973	RP-S23e, RPS23	small subunit ribosomal protein S23e	--	1	ko03010 Ribosome;
gene10717	K16287	ULP1C_D	ubiquitin-like-specific protease 1C/D	EC:3.4.22.68		
gene1071	K00434	E1.11.1.11	L-ascorbate peroxidase	EC:1.11.1.11	2	ko00053 Ascorbate and aldarate metabolism;ko00480 Glutathione metabolism;
gene10724	K15382	SLC50A, SWEET	solute carrier family 50 (sugar transporter)	--		
gene10725	K00031	IDH1, IDH2, icd	isocitrate dehydrogenase	EC:1.1.1.42	7	ko00020 Citrate cycle (TCA cycle);ko00480 Glutathione metabolism;ko00720 Carbon fixation pathways in prokaryotes;ko01200 Carbon metabolism;ko01210 2-Oxocarboxylic acid metabolism;ko01230 Biosynthesis of amino acids;ko04146 Peroxisome;
gene10726	K00962	pnp, PNPT1	polyribonucleotide nucleotidyltransferase	EC:2.7.7.8	3	ko00230 Purine metabolism;ko00240 Pyrimidine metabolism;ko03018 RNA degradation;
gene1072	K13379	RGP, UTM	reversibly glycosylated polypeptide / UDP-arabinopyranose mutase	EC:2.4.1.- 5.4.99.30		
gene10731	K07253	MIF	phenylpyruvate tautomerase	EC:5.3.2.1	2	ko00350 Tyrosine metabolism;ko00360 Phenylalanine metabolism;
gene10732	K14849	RRP1	ribosomal RNA-processing protein 1	--		
gene10736	K10570	ERCC8, CKN1, CSA	DNA excision repair protein ERCC-8	--	2	ko03420 Nucleotide excision repair;ko04120 Ubiquitin mediated proteolysis;
gene10741	K08819	CDK12_13	cyclin-dependent kinase 12/13	EC:2.7.11.22 2.7.11.23		
gene10742	K10143	RFWD2, COP1	E3 ubiquitin-protein ligase RFWD2	EC:6.3.2.19	3	ko04115 p53 signaling pathway;ko04120 Ubiquitin mediated proteolysis;ko04712 Circadian rhythm - plant;
gene10744	K08866	TTK, MPS1	serine/threonine-protein kinase TTK/MPS1	EC:2.7.12.1	2	ko04110 Cell cycle;ko04111 Cell cycle - yeast;

gene1074	K01047	PLA2G, SPLA2	secretory phospholipase A2	EC:3.1.1.4	9	ko00564 Glycerophospholipid metabolism;ko00565 Ether lipid metabolism;ko00590 Arachidonic acid metabolism;ko00591 Linoleic acid metabolism;ko00592 alpha-Linolenic acid metabolism;ko04014 Ras signaling pathway;ko04270 Vascular smooth muscle contraction;ko04972 Pancreatic secretion;ko04975 Fat digestion and absorption;
gene10751	K07760	CDK	cyclin-dependent kinase	EC:2.7.11.22		
gene10756	K09422	MYBP	myb proto-oncogene protein, plant	--		
gene10758	K03553	recA	recombination protein RecA	--	1	ko03440 Homologous recombination;
gene10759	K03541	psbR	photosystem II 10kDa protein	--	1	ko00195 Photosynthesis;
gene1075	K06699	PSME4	proteasome activator subunit 4	--	1	ko03050 Proteasome;
gene10764	K01592	E4.1.1.25	tyrosine decarboxylase	EC:4.1.1.25	2	ko00350 Tyrosine metabolism;ko00950 Isoquinoline alkaloid biosynthesis;
gene10768	K15639	CYP734A1, BAS1	cytochrome P450, family 734, subfamily A, polypeptide 1 (PHYB activation tagged suppressor 1)	EC:1.14.-.-	1	ko00905 Brassinosteroid biosynthesis;
gene10769	K11816	YUCCA	indole-3-pyruvate monooxygenase	EC:1.14.13.168	1	ko00380 Tryptophan metabolism;
gene10771	K02929	RP-L44e, RPL44	large subunit ribosomal protein L44e	--	1	ko03010 Ribosome;
gene1077	K15285	SLC35E3	solute carrier family 35, member E3	--		
gene10783	K14231	tRNA-Phe	tRNA Phe	--	1	ko00970 Aminoacyl-tRNA biosynthesis;
gene10785	K01184	E3.2.1.15	polygalacturonase	EC:3.2.1.15	2	ko00040 Pentose and glucuronate interconversions;ko00500 Starch and sucrose metabolism;
gene10787	K10706	SETX, ALS4	senataxin	EC:3.6.4.-		
gene10788	K10706	SETX, ALS4	senataxin	EC:3.6.4.-		
gene1078	K15285	SLC35E3	solute carrier family 35, member E3	--		
gene10796	K08247	E2.1.1.12	methionine S-methyltransferase	EC:2.1.1.12	1	ko00450 Selenocompound metabolism;
gene10797	K00430	E1.11.1.7	peroxidase	EC:1.11.1.7	2	ko00360 Phenylalanine metabolism;ko00940 Phenylpropanoid biosynthesis;
gene10799	K07198	PRKAA, AMPK	5'-AMP-activated protein kinase, catalytic alpha subunit	EC:2.7.11.11	8	ko04140 Regulation of autophagy;ko04150 mTOR signaling pathway;ko04151 PI3K-Akt signaling pathway;ko04710 Circadian rhythm;ko04910 Insulin signaling pathway;ko04920 Adipocytokine signaling pathway;ko04932 Non-alcoholic fatty liver disease (NAFLD);ko05410 Hypertrophic cardiomyopathy (HCM);
gene10800	K04733	IRAK4	interleukin-1 receptor-associated kinase 4	EC:2.7.11.1	11	ko04064 NF-kappa B signaling pathway;ko04210 Apoptosis;ko04620 Toll-like receptor signaling pathway;ko04722 Neurotrophin signaling pathway;ko05133 Pertussis;ko05140 Leishmaniasis;ko05142 Chagas disease (American trypanosomiasis);ko05145 Toxoplasmosis;ko05152 Tuberculosis;ko05162 Measles;ko05164 Influenza A;
gene10804	K14297	NUP98, ADAR2	nuclear pore complex protein Nup98-Nup96	--	2	ko03013 RNA transport;ko05164 Influenza A;
gene10807	K14638	SLC15A3_4, PHT	solute carrier family 15 (peptide/histidine transporter), member 3/4	--		
gene10809	K03163	TOP1	DNA topoisomerase I	EC:5.99.1.2		
gene10810	K10528	HPL	hydroperoxide lyase	EC:4.1.2.-	1	ko00592 alpha-Linolenic acid metabolism;
gene10811	K03165	TOP3	DNA topoisomerase III	EC:5.99.1.2	2	ko03440 Homologous recombination;ko03460 Fanconi anemia pathway;
gene10815	K01507	ppa	inorganic pyrophosphatase	EC:3.6.1.1	1	ko00190 Oxidative phosphorylation;
gene10817	K10843	ERCC3, XPB	DNA excision repair protein ERCC-3	EC:3.6.4.12	2	ko03022 Basal transcription factors;ko03420 Nucleotide excision repair;
gene10818	K06883	K06883				
gene10819	K10046	GME	GDP-D-mannose 3', 5'-epimerase	EC:5.1.3.18	2	ko00053 Ascorbate and aldarate metabolism;ko00520 Amino sugar and nucleotide sugar metabolism;
gene10822	K03320	amt, AMT, MEP	ammonium transporter, Amt family	--		
gene10823	K00818	E2.6.1.11, argD	acetylornithine aminotransferase	EC:2.6.1.11	3	ko00330 Arginine and proline metabolism;ko01210 2-Oxocarboxylic acid metabolism;ko01230 Biosynthesis of amino acids;
gene10824	K00627	DLAT, aceF, pdhC	pyruvate dehydrogenase E2 component (dihydrolipoamide acetyltransferase)	EC:2.3.1.12	4	ko00010 Glycolysis / Gluconeogenesis;ko00020 Citrate cycle (TCA cycle);ko00620 Pyruvate metabolism;ko01200 Carbon metabolism;
gene10826	K00565	RNMT	mRNA (guanine-N7-)-methyltransferase	EC:2.1.1.56	1	ko03015 mRNA surveillance pathway;
gene10830	K05359	ADT, PDT	arogenate/prephenate dehydratase	EC:4.2.1.91 4.2.1.51	2	ko00400 Phenylalanine, tyrosine and tryptophan biosynthesis;ko01230 Biosynthesis of amino acids;

gene10832	K10896	FANCM	fanconi anemia group M protein	--	1	ko03460 Fanconi anemia pathway;
gene10833	K10571	DET1	de-etiolated-1	--	1	ko04120 Ubiquitin mediated proteolysis;
gene10835	K00888	PI4K	phosphatidylinositol 4-kinase	EC:2.7.1.67	2	ko00562 Inositol phosphate metabolism;ko04070 Phosphatidylinositol signaling system;
gene10838	K03695	clpB	ATP-dependent Clp protease ATP-binding subunit ClpB	--		
gene10839	K06268	PPP3R, CNB	serine/threonine-protein phosphatase 2B regulatory subunit	--	18	ko04010 MAPK signaling pathway;ko04020 Calcium signaling pathway;ko04114 Oocyte meiosis;ko04210 Apoptosis;ko04310 Wnt signaling pathway;ko04360 Axon guidance;ko04370 VEGF signaling pathway;ko04380 Osteoclast differentiation;ko04650 Natural killer cell mediated cytotoxicity;ko04660 T cell receptor signaling pathway;ko04662 B cell receptor signaling pathway;ko04720 Long-term potentiation;ko04724 Glutamatergic synapse;ko05010 Alzheimer's disease;ko05014 Amyotrophic lateral sclerosis (ALS);ko05031 Amphetamine addiction;ko05152
gene10841	K05894	E1.3.1.42	12-oxophytodienoic acid reductase	EC:1.3.1.42	1	ko00592 alpha-Linolenic acid metabolism;
gene10843	K14945	QKI	protein quaking	--		
gene10844	K14328	UPF3, RENT3	regulator of nonsense transcripts 3	--	2	ko03013 RNA transport;ko03015 mRNA surveillance pathway;
gene10845	K03083	GSK3B	glycogen synthase kinase 3 beta	EC:2.7.11.26	29	ko04012 ErbB signaling pathway;ko04062 Chemokine signaling pathway;ko04110 Cell cycle;ko04151 PI3K-Akt signaling pathway;ko04310 Wnt signaling pathway;ko04340 Hedgehog signaling pathway;ko04360 Axon guidance;ko04390 Hippo signaling pathway;ko04510 Focal adhesion;ko04660 T cell receptor signaling pathway;ko04662 B cell receptor signaling pathway;ko04711 Circadian rhythm - fly;ko04722 Neurotrophin signaling pathway;ko04728 Dopaminergic synapse;ko04910 Insulin signaling pathway;ko04916 Melanogenesis;ko04917 Prolactin signaling pathway;ko04932 Non-alcoholic fatty liver disease (NAFLD);ko05010 Alzheimer's disease;ko05160 Hepatitis C;ko05162 Measles;ko05164 Influenza A;ko05166 HTLV-I infection;ko05169 Epstein-Barr virus infection;ko05200 Pathways in cancer;ko05210 Colorectal cancer;ko05213 Endometrial cancer;ko05215 Prostate
gene10846	K03283	HSPA1_8	heat shock 70kDa protein 1/8	--	11	ko03040 Spliceosome;ko04010 MAPK signaling pathway;ko04141 Protein processing in endoplasmic reticulum;ko04144 Endocytosis;ko04612 Antigen processing and presentation;ko04915 Estrogen signaling pathway;ko05134 Legionellosis;ko05145 Toxoplasmosis;ko05162 Measles;ko05164 Influenza A;ko05169 Epstein-Barr virus infection;
gene10854	K13947	PIN	auxin efflux carrier family	--		
gene10855	K01728	E4.2.2.2, pel	pectate lyase	EC:4.2.2.2	1	ko00040 Pentose and glucuronate interconversions;
gene10856	K09272	SSRP1	structure-specific recognition protein 1	--		
gene10857	K10704	UBE2V	ubiquitin-conjugating enzyme E2 variant	--		
gene10862	K01214	E3.2.1.68	isoamylase	EC:3.2.1.68		
gene10864	K17108	GBA2	non-lysosomal glucosylceramidase	EC:3.2.1.45	2	ko00511 Other glycan degradation;ko00600 Sphingolipid metabolism;
gene10865	K09919	K09919	hypothetical protein	--		
gene10866	K15404	K15404, CER1	aldehyde decarbonylase	EC:4.1.99.5	1	ko00073 Cutin, suberine and wax biosynthesis;
gene10867	K15404	K15404, CER1	aldehyde decarbonylase	EC:4.1.99.5	1	ko00073 Cutin, suberine and wax biosynthesis;
gene10873	K09716	K09716	hypothetical protein	--		
gene10879	K16810	TBCCD1	TBCC domain-containing protein 1	--		
gene10880	K12900	FUSIP1	FUS-interacting serine-arginine-rich protein 1	--	1	ko03040 Spliceosome;
gene10888	K09527	DNAJC7	DnaJ homolog subfamily C member 7	--		
gene10889	K10580	UBE2N, BLU, UBC13	ubiquitin-conjugating enzyme E2 N	EC:6.3.2.19	1	ko04120 Ubiquitin mediated proteolysis;
gene10895	K13248	PHOSPHO2	pyridoxal phosphate phosphatase PHOSPHO2	EC:3.1.3.74	1	ko00750 Vitamin B6 metabolism;
gene10897	K03364	CDH1	cell division cycle 20-like protein 1, cofactor of APC complex	--	4	ko04110 Cell cycle;ko04111 Cell cycle - yeast;ko04120 Ubiquitin mediated proteolysis;ko04914 Progesterone-mediated oocyte maturation;
gene10903	K02915	RP-L34e, RPL34	large subunit ribosomal protein L34e	--	1	ko03010 Ribosome;
gene10904	K09264	K09264	MADS-box transcription factor, plant	--		

gene10916	K11159	K11159	carotenoid cleavage dioxygenase	--		
gene10917	K03676	grxC, GLRX, GLRX2	glutaredoxin 3	--		
gene10918	K03676	grxC, GLRX, GLRX2	glutaredoxin 3	--		
gene10920	K15223	UAF30, SPP27	upstream activation factor subunit UAF30	--		
gene10923	K09522	DNAJC2	DnaJ homolog subfamily C member 2	--		
gene10926	K12188	SNF8, EAP30	ESCRT-II complex subunit VPS22	--	1	ko04144 Endocytosis;
gene10927	K12896	SFRS7	splicing factor, arginine/serine-rich 7	--	2	ko03040 Spliceosome;ko05168 Herpes simplex infection;
gene10928	K06940	K06940				
gene10931	K15440	TAD1, ADAT1	tRNA-specific adenosine deaminase 1	EC:3.5.4.34		
gene10932	K13667	RUMI, KTELC1	protein glucosyltransferase	EC:2.4.1.-	1	ko00514 Other types of O-glycan biosynthesis;
gene10933	K13667	RUMI, KTELC1	protein glucosyltransferase	EC:2.4.1.-	1	ko00514 Other types of O-glycan biosynthesis;
gene10935	K09872	PIP	aquaporin PIP	--		
gene10936	K00940	E2.7.4.6, ndk	nucleoside-diphosphate kinase	EC:2.7.4.6	2	ko00230 Purine metabolism;ko00240 Pyrimidine metabolism;
gene10937	K10406	KIFC2_3	kinesin family member C2/C3	--		
gene10938	K14219	tRNA-Arg	tRNA Arg	--	1	ko00970 Aminoacyl-tRNA biosynthesis;
gene10939	K14220	tRNA-Asn	tRNA Asn	--	1	ko00970 Aminoacyl-tRNA biosynthesis;
gene10945	K06276	PDPK1	3-phosphoinositide dependent protein kinase-1	EC:2.7.11.1	9	ko03320 PPAR signaling pathway;ko04150 mTOR signaling pathway;ko04151 PI3K-Akt signaling pathway;ko04510 Focal adhesion;ko04910 Insulin signaling pathway;ko04960 Aldosterone-regulated sodium reabsorption;ko05213 Endometrial cancer;ko05215 Prostate cancer;ko05223 Non-small cell lung cancer;
gene10947	K00279	E1.5.99.12	cytokinin dehydrogenase	EC:1.5.99.12	1	ko00908 Zeatin biosynthesis;
gene10954	K01517	ADPRM	manganese-dependent ADP-ribose/CDP-alcohol diphosphatase	EC:3.6.1.13 3.6.1.16 3.6.1.53	2	ko00230 Purine metabolism;ko00564 Glycerophospholipid metabolism;
gene10955	K03517	nadA	quinolinate synthase	EC:2.5.1.72	1	ko00760 Nicotinate and nicotinamide metabolism;
gene10956	K02723	psbY	photosystem II PsbY protein	--	1	ko00195 Photosynthesis;
gene10957	K08287	E2.7.12.1	dual-specificity kinase	EC:2.7.12.1		
gene10959	K11968	ARIH1	ariadne-1	--		
gene10961	K15340	DCLRE1A, SNM1A, PSO2	DNA cross-link repair 1A protein	--		
gene10964	K02898	RP-L26e, RPL26	large subunit ribosomal protein L26e	--	1	ko03010 Ribosome;
gene10965	K15040	VDAC2	voltage-dependent anion channel protein 2	--	4	ko04020 Calcium signaling pathway;ko05012 Parkinson's disease;ko05016 Huntington's disease;ko05166 HTLV-I infection;
gene10966	K13947	PIN	auxin efflux carrier family	--		
gene10967	K15285	SLC35E3	solute carrier family 35, member E3	--		
gene10969	K11322	EPC	enhancer of polycomb-like protein	--		
gene10970	K07748	E1.1.1.170, NSDHL, ERG26	sterol-4alpha-carboxylate 3-dehydrogenase (decarboxylating)	EC:1.1.1.170	1	ko00100 Steroid biosynthesis;
gene10971	K10523	SPOP	speckle-type POZ protein	--		
gene10973	K14641	APY1_2	apyrase	EC:3.6.1.5	2	ko00230 Purine metabolism;ko00240 Pyrimidine metabolism;
gene10975	K01802	E5.2.1.8	peptidylprolyl isomerase	EC:5.2.1.8		
gene10978	K16292	CEP, CYSEP	KDEL-tailed cysteine endopeptidase	EC:3.4.22.-		
gene10982	K01267	E3.4.11.21, DNPEP	aspartyl aminopeptidase	EC:3.4.11.21		
gene10985	K01597	MVD, mvaD	diphosphomevalonate decarboxylase	EC:4.1.1.33	1	ko00900 Terpenoid backbone biosynthesis;
gene1098	K02729	PSMA5	20S proteasome subunit alpha 5	EC:3.4.25.1	1	ko03050 Proteasome;
gene10993	K12193	VPS24, CHMP3	charged multivesicular body protein 3	--	1	ko04144 Endocytosis;
gene10994	K08235	E2.4.1.207	xyloglucan:xyloglucosyl transferase	EC:2.4.1.207		
gene10995	K14846	RPF1	ribosome production factor 1	--		
gene10997	K04733	IRAK4	interleukin-1 receptor-associated kinase 4	EC:2.7.11.1	11	ko04064 NF-kappa B signaling pathway;ko04210 Apoptosis;ko04620 Toll-like receptor signaling pathway;ko04722 Neurotrophin signaling pathway;ko05133 Pertussis;ko05140 Leishmaniasis;ko05142 Chagas disease (American trypanosomiasis);ko05145 Toxoplasmosis;ko05152 Tuberculosis;ko05162 Measles;ko05164 Influenza A;
gene10998	K05305	FUK	fucokinase	EC:2.7.1.52	2	ko00051 Fructose and mannose metabolism;ko00520 Amino sugar and nucleotide sugar metabolism;

gene10	K11584	PPP2R5	serine/threonine-protein phosphatase 2A regulatory subunit B'	--	5	ko03015 mRNA surveillance pathway;ko04113 Meiosis - yeast;ko04114 Oocyte meiosis;ko04151 PI3K-Akt signaling pathway;ko04728 Dopaminergic synapse;
gene11003	K02735	PSMB3	20S proteasome subunit beta 3	EC:3.4.25.1	1	ko03050 Proteasome;
gene11004	K08818	CDC2L	cell division cycle 2-like	EC:2.7.11.22		
gene11008	K14487	GH3	auxin responsive GH3 gene family	--	1	ko04075 Plant hormone signal transduction;
gene1100	K00902	E2.7.1.108	dolichol kinase	EC:2.7.1.108	1	ko00510 N-Glycan biosynthesis;
gene11012	K14638	SLC15A3_4, PHT	solute carrier family 15 (peptide/histidine transporter), member 3/4	--		
gene11014	K12832	SF3B5, SF3B10	splicing factor 3B subunit 5	--	1	ko03040 Spliceosome;
gene11016	K04120	E5.5.1.13	ent-copalyl diphosphate synthase	EC:5.5.1.13	1	ko00904 Diterpenoid biosynthesis;
gene11020	K08819	CDK12_13	cyclin-dependent kinase 12/13	EC:2.7.11.22 2.7.11.23		
gene11021	K08150	SLC2A13, ITR	MFS transporter, SP family, solute carrier family 2 (myo-inositol transporter), member 13	--		
gene11022	K02882	RP-L18Ae, RPL18A	large subunit ribosomal protein L18Ae	--	1	ko03010 Ribosome;
gene11025	K04733	IRAK4	interleukin-1 receptor-associated kinase 4	EC:2.7.11.1	11	ko04064 NF-kappa B signaling pathway;ko04210 Apoptosis;ko04620 Toll-like receptor signaling pathway;ko04722 Neurotrophin signaling pathway;ko05133 Pertussis;ko05140 Leishmaniasis;ko05142 Chagas disease (American trypanosomiasis);ko05145 Toxoplasmosis;ko05152 Tuberculosis;ko05162 Measles;ko05164 Influenza A;
gene11026	K04733	IRAK4	interleukin-1 receptor-associated kinase 4	EC:2.7.11.1	11	ko04064 NF-kappa B signaling pathway;ko04210 Apoptosis;ko04620 Toll-like receptor signaling pathway;ko04722 Neurotrophin signaling pathway;ko05133 Pertussis;ko05140 Leishmaniasis;ko05142 Chagas disease (American trypanosomiasis);ko05145 Toxoplasmosis;ko05152 Tuberculosis;ko05162 Measles;ko05164 Influenza A;
gene11027	K04733	IRAK4	interleukin-1 receptor-associated kinase 4	EC:2.7.11.1	11	ko04064 NF-kappa B signaling pathway;ko04210 Apoptosis;ko04620 Toll-like receptor signaling pathway;ko04722 Neurotrophin signaling pathway;ko05133 Pertussis;ko05140 Leishmaniasis;ko05142 Chagas disease (American trypanosomiasis);ko05145 Toxoplasmosis;ko05152 Tuberculosis;ko05162 Measles;ko05164 Influenza A;
gene11028	K04733	IRAK4	interleukin-1 receptor-associated kinase 4	EC:2.7.11.1	11	ko04064 NF-kappa B signaling pathway;ko04210 Apoptosis;ko04620 Toll-like receptor signaling pathway;ko04722 Neurotrophin signaling pathway;ko05133 Pertussis;ko05140 Leishmaniasis;ko05142 Chagas disease (American trypanosomiasis);ko05145 Toxoplasmosis;ko05152 Tuberculosis;ko05162 Measles;ko05164 Influenza A;
gene11029	K04733	IRAK4	interleukin-1 receptor-associated kinase 4	EC:2.7.11.1	11	ko04064 NF-kappa B signaling pathway;ko04210 Apoptosis;ko04620 Toll-like receptor signaling pathway;ko04722 Neurotrophin signaling pathway;ko05133 Pertussis;ko05140 Leishmaniasis;ko05142 Chagas disease (American trypanosomiasis);ko05145 Toxoplasmosis;ko05152 Tuberculosis;ko05162 Measles;ko05164 Influenza A;
gene11030	K04733	IRAK4	interleukin-1 receptor-associated kinase 4	EC:2.7.11.1	11	ko04064 NF-kappa B signaling pathway;ko04210 Apoptosis;ko04620 Toll-like receptor signaling pathway;ko04722 Neurotrophin signaling pathway;ko05133 Pertussis;ko05140 Leishmaniasis;ko05142 Chagas disease (American trypanosomiasis);ko05145 Toxoplasmosis;ko05152 Tuberculosis;ko05162 Measles;ko05164 Influenza A;
gene11033	K11418	HDAC11	histone deacetylase 11	EC:3.5.1.98	2	ko05034 Alcoholism;ko05203 Viral carcinogenesis;
gene11036	K16818	K16818, DAD1	phospholipase A1	EC:3.1.1.32	2	ko00564 Glycerophospholipid metabolism;ko00592 alpha-Linolenic acid metabolism;
gene11039	K02939	RP-L9, MRPL9, rplI	large subunit ribosomal protein L9	--	1	ko03010 Ribosome;
gene11042	K11252	H2B	histone H2B	--	3	ko05034 Alcoholism;ko05203 Viral carcinogenesis;ko05322 Systemic lupus erythematosus;
gene11043	K13071	PAO, ACD1	pheophorbide a oxygenase	EC:1.14.12.20	1	ko00860 Porphyrin and chlorophyll metabolism;
gene11047	K01164	POP1	ribonuclease P/MRP protein subunit POP1	EC:3.1.26.5	2	ko03008 Ribosome biogenesis in eukaryotes;ko03013 RNA transport;
gene11048	K08099	E3.1.1.14	chlorophyllase	EC:3.1.1.14	1	ko00860 Porphyrin and chlorophyll metabolism;
gene11054	K01179	E3.2.1.4	endoglucanase	EC:3.2.1.4	1	ko00500 Starch and sucrose metabolism;

gene11056	K08065	NFYB	nuclear transcription Y subunit beta	--	3	ko04612 Antigen processing and presentation;ko05152 Tuberculosis;ko05166 HTLV-I infection;
gene1105	K03327	TC.MATE, SLC47A, norM, mdtK, dinF	multidrug resistance protein, MATE family	--		
gene11061	K14841	NSA1, WDR74	ribosome biogenesis protein NSA1	--		
gene11066	K14638	SLC15A3_4, PHT	solute carrier family 15 (peptide/histidine transporter), member 3/4	--		
gene11067	K01476	E3.5.3.1, rocF, arg	arginase	EC:3.5.3.1	3	ko00330 Arginine and proline metabolism;ko01230 Biosynthesis of amino acids;ko05146 Amoebiasis;
gene11068	K10406	KIFC2_3	kinesin family member C2/C3	--		
gene11069	K08909	LHCA3	light-harvesting complex I chlorophyll a/b binding protein 3	--	1	ko00196 Photosynthesis - antenna proteins;
gene1106	K14961	RBBP5, SWD1, CPS50	COMPASS component SWD1	--		
gene11074	K09338	HD-ZIP	homeobox-leucine zipper protein	--		
gene11084	K14432	ABF	ABA responsive element binding factor	--	1	ko04075 Plant hormone signal transduction;
gene11087	K03002	RPA2, POLR1B	DNA-directed RNA polymerase I subunit RPA2	EC:2.7.7.6	3	ko00230 Purine metabolism;ko00240 Pyrimidine metabolism;ko03020 RNA polymerase;
gene11089	K02946	RP-S10, MRPS10, rpsJ	small subunit ribosomal protein S10	--	1	ko03010 Ribosome;
gene11097	K05868	CCNB	cyclin B	--	3	ko04110 Cell cycle;ko04115 p53 signaling pathway;ko04914 Progesterone-mediated oocyte maturation;
gene11098	K13448	CML	calcium-binding protein CML	--	1	ko04626 Plant-pathogen interaction;
gene11099	K13448	CML	calcium-binding protein CML	--	1	ko04626 Plant-pathogen interaction;
gene110	K11490	NCAPH2	condensin-2 complex subunit H2	--		
gene11106	K08145	SLC2A8, GLUT8	MFS transporter, SP family, solute carrier family 2 (facilitated glucose transporter), member 8	--		
gene11114	K09422	MYBP	myb proto-oncogene protein, plant	--		
gene11118	K10355	ACTF	actin, other eukaryote	--		
gene11120	K03347	CUL1, CDC53	cullin 1	--	9	ko04110 Cell cycle;ko04111 Cell cycle - yeast;ko04114 Oocyte meiosis;ko04120 Ubiquitin mediated proteolysis;ko04141 Protein processing in endoplasmic reticulum;ko04310 Wnt signaling pathway;ko04350 TGF-beta signaling pathway;ko04710 Circadian rhythm;ko05168 Herpes simplex infection;
gene11123	K00627	DLAT, aceF, pdhC	pyruvate dehydrogenase E2 component (dihydrolipoamide acetyltransferase)	EC:2.3.1.12	4	ko00010 Glycolysis / Gluconeogenesis;ko00020 Citrate cycle (TCA cycle);ko00620 Pyruvate metabolism;ko01200 Carbon metabolism;
gene11125	K14487	GH3	auxin responsive GH3 gene family	--	1	ko04075 Plant hormone signal transduction;
gene1112	K17080	PHB1	prohibitin 1	--		
gene11133	K06670	SCC1, MCD1, RAD21	cohesin complex subunit SCC1	--	2	ko04110 Cell cycle;ko04111 Cell cycle - yeast;
gene11136	K10268	FBXL2_20	F-box and leucine-rich repeat protein 2/20	--		
gene11137	K03327	TC.MATE, SLC47A, norM, mdtK, dinF	multidrug resistance protein, MATE family	--		
gene11138	K17408	DAP3, MRPS29	small subunit ribosomal protein S29, mitochondrial	--		
gene1113	K06630	YWHAE	14-3-3 protein epsilon	--	8	ko04110 Cell cycle;ko04114 Oocyte meiosis;ko04151 PI3K-Akt signaling pathway;ko04390 Hippo signaling pathway;ko04391 Hippo signaling pathway - fly;ko04722 Neurotrophin signaling pathway;ko05169 Epstein-Barr virus infection;ko05203 Viral carcinogenesis;
gene11143	K08150	SLC2A13, ITR	MFS transporter, SP family, solute carrier family 2 (myo-inositol transporter), member 13	--		
gene11144	K16570	TUBGCP3, GCP3	gamma-tubulin complex component 3	--		
gene11145	K08150	SLC2A13, ITR	MFS transporter, SP family, solute carrier family 2 (myo-inositol transporter), member 13	--		
gene11147	K14400	PCF11	pre-mRNA cleavage complex 2 protein Pcf11	--	1	ko03015 mRNA surveillance pathway;
gene11148	K09419	HSFF	heat shock transcription factor, other eukaryote	--		
gene11149	K14510	CTR1	serine/threonine-protein kinase CTR1	EC:2.7.11.1	1	ko04075 Plant hormone signal transduction;
gene1114	K01626	E2.5.1.54, aroF, aroG, aroH	3-deoxy-7-phosphoheptulonate synthase	EC:2.5.1.54	2	ko00400 Phenylalanine, tyrosine and tryptophan biosynthesis;ko01230 Biosynthesis of amino acids;

gene11154	K00847	E2.7.1.4, scrK	fructokinase	EC:2.7.1.4	3	ko00051 Fructose and mannose metabolism;ko00500 Starch and sucrose metabolism;ko00520 Amino sugar and nucleotide sugar metabolism;
gene11155	K13412	CPK	calcium-dependent protein kinase	EC:2.7.11.1	2	ko04626 Plant-pathogen interaction;ko05145 Toxoplasmosis;
gene11159	K00134	GAPDH, gapA	glyceraldehyde 3-phosphate dehydrogenase	EC:1.2.1.12	6	ko00010 Glycolysis / Gluconeogenesis;ko00710 Carbon fixation in photosynthetic organisms;ko01200 Carbon metabolism;ko01230 Biosynthesis of amino acids;ko04066 HIF-1 signaling pathway;ko05010 Alzheimer's disease;
gene11161	K13566	NIT2	omega-amidase	EC:3.5.1.3	1	ko00250 Alanine, aspartate and glutamate metabolism;
gene11166	K13525	VCP, CDC48	transitional endoplasmic reticulum ATPase	--	2	ko04141 Protein processing in endoplasmic reticulum;ko05134 Legionellosis;
gene11167	K03809	wrbA	Trp repressor binding protein	--		
gene11173	K09510	DNAJB4	DnaJ homolog subfamily B member 4	--		
gene11174	K13121	FRA10AC1	protein FRA10AC1	--		
gene11175	K01762	ACS	1-aminocyclopropane-1-carboxylate synthase	EC:4.4.1.14	1	ko00270 Cysteine and methionine metabolism;
gene11177	K10666	RNF5	E3 ubiquitin-protein ligase RNF5	EC:6.3.2.19	1	ko04141 Protein processing in endoplasmic reticulum;
gene11178	K03671	trxA	thioredoxin 1	--		
gene11179	K00979	kdsB	3-deoxy-manno-octulosonate cytidylyltransferase (CMP-KDO synthetase)	EC:2.7.7.38	1	ko00540 Lipopolysaccharide biosynthesis;
gene1117	K01213	E3.2.1.67	galacturan 1,4-alpha-galacturonidase	EC:3.2.1.67	2	ko00040 Pentose and glucuronate interconversions;ko00500 Starch and sucrose metabolism;
gene11180	K03514	POLS, TRF4	DNA polymerase sigma	EC:2.7.7.7	1	ko03018 RNA degradation;
gene11181	K10581	UBE2O	ubiquitin-conjugating enzyme E2 O	EC:6.3.2.19	1	ko04120 Ubiquitin mediated proteolysis;
gene11182	K06027	NSF	vesicle-fusing ATPase	EC:3.6.4.6	3	ko04721 Synaptic vesicle cycle;ko04727 GABAergic synapse;ko04962 Vasopressin-regulated water reabsorption;
gene11183	K02260	COX17	cytochrome c oxidase assembly protein subunit 17	--	1	ko00190 Oxidative phosphorylation;
gene11186	K02209	MCM5, CDC46	DNA replication licensing factor MCM5	EC:3.6.4.12	4	ko03030 DNA replication;ko04110 Cell cycle;ko04111 Cell cycle - yeast;ko04113 Meiosis - yeast;
gene11188	K16054	DEP1	methylthioribulose 1-phosphate dehydratase / enolase-phosphatase E1	EC:4.2.1.109 3.1.3.77	1	ko00270 Cysteine and methionine metabolism;
gene11190	K01581	E4.1.1.17, ODC1, speC, speF	ornithine decarboxylase	EC:4.1.1.17	2	ko00330 Arginine and proline metabolism;ko00480 Glutathione metabolism;
gene11192	K11000	CALS	callose synthase	EC:2.4.1.-		
gene11193	K02888	RP-L21, MRPL21, rplU	large subunit ribosomal protein L21	--	1	ko03010 Ribosome;
gene11194	K02882	RP-L18Ae, RPL18A	large subunit ribosomal protein L18Ae	--	1	ko03010 Ribosome;
gene11199	K11159	K11159	carotenoid cleavage dioxygenase	--		
gene11200	K11159	K11159	carotenoid cleavage dioxygenase	--		
gene11201	K08867	WNK, PRKWNK	WNK lysine deficient protein kinase	EC:2.7.11.1		
gene11204	K14492	ARR-A	two-component response regulator ARR-A family	--	1	ko04075 Plant hormone signal transduction;
gene11211	K14508	NPR1	regulatory protein NPR1	--	1	ko04075 Plant hormone signal transduction;
gene11213	K11820	UGT74B1	N-hydroxythioamide S-beta-glucosyltransferase	EC:2.4.1.195	3	ko00380 Tryptophan metabolism;ko00966 Glucosinolate biosynthesis;ko01210 2-Oxocarboxylic acid metabolism;
gene11214	K03512	POLL	DNA polymerase lambda	EC:2.7.7.7	2	ko03410 Base excision repair;ko03450 Non-homologous end-joining;
gene11220	K15472	CYP71D55	premnaspirodiene oxygenase	EC:1.14.13.12 1	1	ko00909 Sesquiterpenoid and triterpenoid biosynthesis;
gene1122	K00025	MDH1	malate dehydrogenase	EC:1.1.1.37	6	ko00020 Citrate cycle (TCA cycle);ko00620 Pyruvate metabolism;ko00630 Glyoxylate and dicarboxylate metabolism;ko00710 Carbon fixation in photosynthetic organisms;ko01200 Carbon metabolism;ko04964 Proximal tubule bicarbonate reclamation;
gene11234	K02880	RP-L17e, RPL17	large subunit ribosomal protein L17e	--	1	ko03010 Ribosome;
gene11236	K01537	E3.6.3.8	Ca2+-transporting ATPase	EC:3.6.3.8		
gene11238	K13963	SERPINB	serpin B	--	1	ko05146 Amoebiasis;
gene11239	K02954	RP-S14, MRPS14, rpsN	small subunit ribosomal protein S14	--	1	ko03010 Ribosome;
gene11244	K11826	AP2M1	AP-2 complex subunit mu-1	--	4	ko04144 Endocytosis;ko04721 Synaptic vesicle cycle;ko04961 Endocrine and other factor-regulated calcium reabsorption;ko05016 Huntington's disease;
gene11246	K01738	cysK	cysteine synthase A	EC:2.5.1.47	4	ko00270 Cysteine and methionine metabolism;ko00920 Sulfur metabolism;ko01200 Carbon metabolism;ko01230 Biosynthesis of amino acids;
gene11247	K01090	E3.1.3.16	protein phosphatase	EC:3.1.3.16		

gene11249	K04079	htpG, HSP90A	molecular chaperone HtpG	--	9	ko04141 Protein processing in endoplasmic reticulum;ko04151 PI3K-Akt signaling pathway;ko04612 Antigen processing and presentation;ko04621 NOD-like receptor signaling pathway;ko04626 Plant-pathogen interaction;ko04914 Progesterone-mediated oocyte maturation;ko04915 Estrogen signaling pathway;ko05200 Pathways in cancer;ko05215 Prostate cancer;
gene11250	K01414	prlC	oligopeptidase A	EC:3.4.24.70		
gene11257	K03253	EIF3B	translation initiation factor 3 subunit B	--	1	ko03013 RNA transport;
gene11265	K04730	IRAK1	interleukin-1 receptor-associated kinase 1	EC:2.7.11.1	11	ko04064 NF-kappa B signaling pathway;ko04210 Apoptosis;ko04620 Toll-like receptor signaling pathway;ko04722 Neurotrophin signaling pathway;ko05133 Pertussis;ko05140 Leishmaniasis;ko05142 Chagas disease (American trypanosomiasis);ko05145 Toxoplasmosis;ko05152 Tuberculosis;ko05162 Measles;ko05169 Epstein-Barr virus infection;
gene11267	K15191	LARP7	La-related protein 7	--		
gene11275	K05349	bglX	beta-glucosidase	EC:3.2.1.21	3	ko00460 Cyanoamino acid metabolism;ko00500 Starch and sucrose metabolism;ko00940 Phenylpropanoid biosynthesis;
gene11276	K02154	ATPeVI, ATP6N1A	V-type H+-transporting ATPase subunit I	EC:3.6.3.14	9	ko00190 Oxidative phosphorylation;ko04142 Lysosome;ko04145 Phagosome;ko04721 Synaptic vesicle cycle;ko04966 Collecting duct acid secretion;ko05110 Vibrio cholerae infection;ko05120 Epithelial cell signaling in Helicobacter pylori infection;ko05152 Tuberculosis;ko05323 Rheumatoid arthritis;
gene11278	K14638	SLC15A3_4, PHT	solute carrier family 15 (peptide/histidine transporter), member 3/4	--		
gene11288	K14236	tRNA-Tyr	tRNA Tyr	--	1	ko00970 Aminoacyl-tRNA biosynthesis;
gene11290	K14236	tRNA-Tyr	tRNA Tyr	--	1	ko00970 Aminoacyl-tRNA biosynthesis;
gene11291	K14236	tRNA-Tyr	tRNA Tyr	--	1	ko00970 Aminoacyl-tRNA biosynthesis;
gene11292	K14236	tRNA-Tyr	tRNA Tyr	--	1	ko00970 Aminoacyl-tRNA biosynthesis;
gene11293	K14236	tRNA-Tyr	tRNA Tyr	--	1	ko00970 Aminoacyl-tRNA biosynthesis;
gene11294	K14236	tRNA-Tyr	tRNA Tyr	--	1	ko00970 Aminoacyl-tRNA biosynthesis;
gene11297	K02926	RP-L4, MRPL4, rplD	large subunit ribosomal protein L4	--	1	ko03010 Ribosome;
gene11298	K02154	ATPeVI, ATP6N1A	V-type H+-transporting ATPase subunit I	EC:3.6.3.14	9	ko00190 Oxidative phosphorylation;ko04142 Lysosome;ko04145 Phagosome;ko04721 Synaptic vesicle cycle;ko04966 Collecting duct acid secretion;ko05110 Vibrio cholerae infection;ko05120 Epithelial cell signaling in Helicobacter pylori infection;ko05152 Tuberculosis;ko05323 Rheumatoid arthritis;
gene11299	K05236	COPA	coatomer protein complex, subunit alpha (xenin)	--	1	ko04080 Neuroactive ligand-receptor interaction;
gene112	K09419	HSFF	heat shock transcription factor, other eukaryote	--		
gene11300	K12586	RRP43, EXOSC8, OIP2	exosome complex component RRP43	--	1	ko03018 RNA degradation;
gene11301	K01507	ppa	inorganic pyrophosphatase	EC:3.6.1.1	1	ko00190 Oxidative phosphorylation;
gene11302	K04125	E1.14.11.13	gibberellin 2-oxidase	EC:1.14.11.13	1	ko00904 Diterpenoid biosynthesis;
gene11304	K11517	HAO	(S)-2-hydroxy-acid oxidase	EC:1.1.3.15	2	ko00630 Glyoxylate and dicarboxylate metabolism;ko04146 Peroxisome;
gene11319	K01115	PLD1_2	phospholipase D1/2	EC:3.1.4.4	7	ko00564 Glycerophospholipid metabolism;ko00565 Ether lipid metabolism;ko04014 Ras signaling pathway;ko04144 Endocytosis;ko04666 Fc gamma R-mediated phagocytosis;ko04724 Glutamatergic synapse;ko04912 GnRH signaling pathway;
gene1131	K14546	UTP5, WDR43	U3 small nucleolar RNA-associated protein 5	--	1	ko03008 Ribosome biogenesis in eukaryotes;
gene11322	K01674	cah	carbonic anhydrase	EC:4.2.1.1	1	ko00910 Nitrogen metabolism;
gene11323	K10144	RCHY1, PIRH2	RING finger and CHY zinc finger domain-containing protein 1	EC:6.3.2.19	3	ko04115 p53 signaling pathway;ko04120 Ubiquitin mediated proteolysis;ko05162 Measles;
gene11329	K17525	CHID1	chitinase domain-containing protein 1	--		
gene1132	K02867	RP-L11, MRPL11, rplK	large subunit ribosomal protein L11	--	1	ko03010 Ribosome;
gene11330	K09286	EREBP	EREBP-like factor	--		
gene11333	K10842	MNAT1	CDK-activating kinase assembly factor MAT1	--	2	ko03022 Basal transcription factors;ko03420 Nucleotide excision repair;
gene11339	K01555	FAH, fahA	fumarylacetoacetase	EC:3.7.1.2	2	ko00350 Tyrosine metabolism;ko00643 Styrene degradation;
gene1133	K02867	RP-L11, MRPL11, rplK	large subunit ribosomal protein L11	--	1	ko03010 Ribosome;
gene11349	K10580	UBE2N, BLU, UBC13	ubiquitin-conjugating enzyme E2 N	EC:6.3.2.19	1	ko04120 Ubiquitin mediated proteolysis;
gene1134	K01507	ppa	inorganic pyrophosphatase	EC:3.6.1.1	1	ko00190 Oxidative phosphorylation;

gene11351	K12603	CNOT6, CCR4	CCR4-NOT transcription complex subunit 6	--	1	ko03018 RNA degradation;
gene11352	K14411	MSI	RNA-binding protein Musashi	--	1	ko03015 mRNA surveillance pathway;
gene11353	K07904	RAB11A	Ras-related protein Rab-11A	--	4	ko04144 Endocytosis;ko04961 Endocrine and other factor-regulated calcium reabsorption;ko04962 Vasopressin-regulated water reabsorption;ko04972 Pancreatic secretion;
gene11361	K03327	TC.MATE, SLC47A, norM, mdtK, dinF	multidrug resistance protein, MATE family	--		
gene11363	K13379	RGP, UTM	reversibly glycosylated polypeptide / UDP-arabinopyranose mutase	EC:2.4.1.- 5.4.99.30		
gene11369	K00993	EPT1	ethanolaminephosphotransferase	EC:2.7.8.1	3	ko00440 Phosphonate and phosphinate metabolism;ko00564 Glycerophospholipid metabolism;ko00565 Ether lipid metabolism;
gene1136	K06670	SCC1, MCD1, RAD21	cohesin complex subunit SCC1	--	2	ko04110 Cell cycle;ko04111 Cell cycle - yeast;
gene11370	K01611	speD, AMD1	S-adenosylmethionine decarboxylase	EC:4.1.1.50	2	ko00270 Cysteine and methionine metabolism;ko00330 Arginine and proline metabolism;
gene11371	K01611	speD, AMD1	S-adenosylmethionine decarboxylase	EC:4.1.1.50	2	ko00270 Cysteine and methionine metabolism;ko00330 Arginine and proline metabolism;
gene11375	K12190	VPS36, EAP45	ESCRT-II complex subunit VPS36	--	1	ko04144 Endocytosis;
gene11378	K04730	IRAK1	interleukin-1 receptor-associated kinase 1	EC:2.7.11.1	11	ko04064 NF-kappa B signaling pathway;ko04210 Apoptosis;ko04620 Toll-like receptor signaling pathway;ko04722 Neurotrophin signaling pathway;ko05133 Pertussis;ko05140 Leishmaniasis;ko05142 Chagas disease (American trypanosomiasis);ko05145 Toxoplasmosis;ko05152 Tuberculosis;ko05162 Measles;ko05169 Epstein-Barr virus infection;
gene1137	K13153	SNRNP25	U11/U12 small nuclear ribonucleoprotein 25 kDa protein	--		
gene11381	K15601	KDM3	lysine-specific demethylase 3	EC:1.14.11.-		
gene11383	K15979	SND1	staphylococcal nuclease domain-containing protein 1	--	2	ko05169 Epstein-Barr virus infection;ko05203 Viral carcinogenesis;
gene11385	K12619	XRN2, RAT1	5'-3' exoribonuclease 2	EC:3.1.13.-	2	ko03008 Ribosome biogenesis in eukaryotes;ko03018 RNA degradation;
gene11386	K03541	psbR	photosystem II 10kDa protein	--	1	ko00195 Photosynthesis;
gene11390	K09527	DNAJC7	DnaJ homolog subfamily C member 7	--		
gene11395	K02936	RP-L7Ae, RPL7A	large subunit ribosomal protein L7Ae	--	1	ko03010 Ribosome;
gene11396	K01259	pip	proline iminopeptidase	EC:3.4.11.5	1	ko00330 Arginine and proline metabolism;
gene11397	K13118	DGCR14	protein DGCR14	--		
gene11398	K08867	WNK, PRKWNK	WNK lysine deficient protein kinase	EC:2.7.11.1		
gene11404	K08472	MLO	mlo protein	--		
gene11406	K00383	E1.8.1.7, GSR, gor	glutathione reductase (NADPH)	EC:1.8.1.7	2	ko00480 Glutathione metabolism;ko04918 Thyroid hormone synthesis;
gene11407	K03935	NDUFS2	NADH dehydrogenase (ubiquinone) Fe-S protein 2	EC:1.6.5.3 1.6.99.3	5	ko00190 Oxidative phosphorylation;ko04932 Non-alcoholic fatty liver disease (NAFLD);ko05010 Alzheimer's disease;ko05012 Parkinson's disease;ko05016 Huntington's disease;
gene11412	K02365	ESP1	separase	EC:3.4.22.49	4	ko04110 Cell cycle;ko04111 Cell cycle - yeast;ko04113 Meiosis - yeast;ko04114 Oocyte meiosis;
gene11416	K03686	dnaJ	molecular chaperone DnaJ	--		
gene11418	K09578	PIN1	peptidyl-prolyl cis-trans isomerase NIMA-interacting 1	EC:5.2.1.8	1	ko04622 RIG-I-like receptor signaling pathway;
gene11420	K16296	SCPL-I	serine carboxypeptidase-like clade I	EC:3.4.16.-		
gene11422	K14611	SLC23A1_2, SVCT1_2	solute carrier family 23 (nucleobase transporter), member 1/2	--		
gene11429	K12450	RHM	UDP-glucose 4,6-dehydratase	EC:4.2.1.76	1	ko00520 Amino sugar and nucleotide sugar metabolism;
gene11430	K12190	VPS36, EAP45	ESCRT-II complex subunit VPS36	--	1	ko04144 Endocytosis;
gene11431	K14404	CPSF4, YTH1	cleavage and polyadenylation specificity factor subunit 4	--	2	ko03015 mRNA surveillance pathway;ko05164 Influenza A;
gene11432	K17301	COPB1, SEC26	coatomer, subunit beta	--		
gene11435	K15397	KCS	3-ketoacyl-CoA synthase	EC:2.3.1.199	1	ko00062 Fatty acid elongation;
gene1143	K02516	PRMT5, HSL7	protein arginine N-methyltransferase 5	EC:2.1.1.125	2	ko03013 RNA transport;ko04111 Cell cycle - yeast;
gene11449	K11099	SNRPG, SMG	small nuclear ribonucleoprotein G	--	1	ko03040 Spliceosome;
gene11451	K07023	K07023	putative hydrolases of HD superfamily	--		
gene11453	K13459	RPS2	disease resistance protein RPS2	--	1	ko04626 Plant-pathogen interaction;

gene11455	K00847	E2.7.1.4, scrK	fructokinase	EC:2.7.1.4	3	ko00051 Fructose and mannose metabolism;ko00500 Starch and sucrose metabolism;ko00520 Amino sugar and nucleotide sugar metabolism;
gene11459	K13947	PIN	auxin efflux carrier family	--		
gene11461	K10999	CESA	cellulose synthase A	EC:2.4.1.12		
gene11465	K02894	RP-L23e, RPL23	large subunit ribosomal protein L23e	--	1	ko03010 Ribosome;
gene11466	K16302	CNNM	metal transporter CNNM	--		
gene11468	K13348	MPV17	protein Mpv17	--	1	ko04146 Peroxisome;
gene11470	K03039	PSMD13, RPN9	26S proteasome regulatory subunit N9	--	2	ko03050 Proteasome;ko05169 Epstein-Barr virus infection;
gene11471	K11324	DMAP1, SWC4, EAF2	DNA methyltransferase 1-associated protein 1	--		
gene11474	K01897	ACSL, fadD	long-chain acyl-CoA synthetase	EC:6.2.1.3	4	ko00071 Fatty acid degradation;ko03320 PPAR signaling pathway;ko04146 Peroxisome;ko04920 Adipocytokine signaling pathway;
gene11476	K12820	DHX15, PRP43	pre-mRNA-splicing factor ATP-dependent RNA helicase DHX15/PRP43	EC:3.6.4.13	1	ko03040 Spliceosome;
gene11489	K00962	pnp, PNPT1	polyribonucleotide nucleotidyltransferase	EC:2.7.7.8	3	ko00230 Purine metabolism;ko00240 Pyrimidine metabolism;ko03018 RNA degradation;
gene11490	K02996	RP-S9, MRPS9, rpsI	small subunit ribosomal protein S9	--	1	ko03010 Ribosome;
gene11492	K05542	DUS1	tRNA-dihydrouridine synthase 1	EC:1.3.1.88		
gene11494	K04371	MAPK1_3	mitogen-activated protein kinase 1/3	EC:2.7.11.24	71	ko04010 MAPK signaling pathway;ko04012 ErbB signaling pathway;ko04013 MAPK signaling pathway - fly;ko04014 Ras signaling pathway;ko04062 Chemokine signaling pathway;ko04066 HIF-1 signaling pathway;ko04114 Oocyte meiosis;ko04150 mTOR signaling pathway;ko04151 PI3K-Akt signaling pathway;ko04270 Vascular smooth muscle contraction;ko04320 Dorso-ventral axis formation;ko04350 TGF-beta signaling pathway;ko04360 Axon guidance;ko04370 VEGF signaling pathway;ko04380 Osteoclast differentiation;ko04510 Focal adhesion;ko04520 Adherens junction;ko04540 Gap junction;ko04620 Toll-like receptor signaling pathway;ko04621 NOD-like receptor signaling pathway;ko04650 Natural killer cell mediated cytotoxicity;ko04660 T cell receptor signaling pathway;ko04662 B cell receptor signaling pathway;ko04664 Fc epsilon RI signaling pathway;ko04666 Fc gamma R-mediated phagocytosis;ko04668 TNF signaling pathway;ko04713 Circadian entrainment;ko04720 Long-term potentiation;ko04722 Neurotrophin signaling pathway;ko04723 Retrograde endocannabinoid signaling;ko04724 Glutamatergic synapse;ko04725 Cholinergic synapse;ko04726 Serotonergic synapse;ko04730 Long-term depression;ko04810 Regulation of actin cytoskeleton;ko04910 Insulin signaling pathway;ko04912 GnRH signaling pathway;ko04914 Progesterone-mediated oocyte maturation;ko04915 Estrogen signaling pathway;ko04916 Melanogenesis;ko04917 Prolactin signaling pathway;ko04930 Type II diabetes mellitus;ko04960 Aldosterone-regulated sodium reabsorption;ko05010 Alzheimer's disease;ko05020 Prion diseases;ko05034 Alcoholism;ko05131 Shigellosis;ko05132 Salmonella infection;ko05133 Pertussis;ko05140 Leishmaniasis;ko05142 Chagas disease (American trypanosomiasis);ko05145 Toxoplasmosis;ko05152 Tuberculosis;ko05160 Hepatitis C;ko05161 Hepatitis B;ko05164 Influenza A;ko05200 Pathways in cancer;ko05203 Viral carcinogenesis;ko05205 Proteoglycans in cancer;ko05210 Colorectal cancer;ko05211 Renal cell carcinoma;ko05212
gene11495	K16670	MEIS2	homeobox protein Meis2	--		
gene11496	K13110	MFAP1	microfibrillar-associated protein 1	--		
gene11497	K05953	E2.5.1.43	nicotianamine synthase	EC:2.5.1.43		
gene11505	K00876	E2.7.1.48, udk	uridine kinase	EC:2.7.1.48	2	ko00240 Pyrimidine metabolism;ko00983 Drug metabolism - other enzymes;
gene1150	K00079	CBR1	carbonyl reductase 1	EC:1.1.1.184 1.1.1.189 1.1.1.197	3	ko00590 Arachidonic acid metabolism;ko00980 Metabolism of xenobiotics by cytochrome P450;ko05204 Chemical carcinogenesis;
gene11511	K12842	SR140	U2-associated protein SR140	--	1	ko03040 Spliceosome;
gene11517	K02882	RP-L18Ae, RPL18A	large subunit ribosomal protein L18Ae	--	1	ko03010 Ribosome;

gene11520	K04715	E2.7.1.138, CERK	ceramide kinase	EC:2.7.1.138	1	ko00600 Sphingolipid metabolism;
gene11521	K14153	thiDE	hydroxymethylpyrimidine kinase / phosphomethylpyrimidine kinase / thiamine-phosphate diphosphorylase	EC:2.7.1.49 2.7.4.7 2.5.1.3	1	ko00730 Thiamine metabolism;
gene11522	K14498	SNRK2	serine/threonine-protein kinase SRK2	EC:2.7.11.1	1	ko04075 Plant hormone signal transduction;
gene11525	K08176	PHO84	MFS transporter, PHS family, inorganic phosphate transporter	--		
gene11526	K05765	CFL	cofilin	--	4	ko04360 Axon guidance;ko04666 Fc gamma R-mediated phagocytosis;ko04810 Regulation of actin cytoskeleton;ko05133 Pertussis;
gene11527	K11344	EAF6	chromatin modification-related protein EAF6	--		
gene11546	K01889	FARSA, pheS	phenylalanyl-tRNA synthetase alpha chain	EC:6.1.1.20	1	ko00970 Aminoacyl-tRNA biosynthesis;
gene11547	K14677	ACY1	aminoacylase	EC:3.5.1.14	3	ko00330 Arginine and proline metabolism;ko01210 2-Oxocarboxylic acid metabolism;ko01230 Biosynthesis of amino acids;
gene11549	K00726	MGAT1	alpha-1,3-mannosyl-glycoprotein beta-1,2-N-acetylglucosaminyltransferase	EC:2.4.1.101	2	ko00510 N-Glycan biosynthesis;ko00513 Various types of N-glycan biosynthesis;
gene11550	K02737	PSMB5	20S proteasome subunit beta 5	EC:3.4.25.1	1	ko03050 Proteasome;
gene11553	K06630	YWHAE	14-3-3 protein epsilon	--	8	ko04110 Cell cycle;ko04114 Oocyte meiosis;ko04151 PI3K-Akt signaling pathway;ko04390 Hippo signaling pathway;ko04391 Hippo signaling pathway - fly;ko04722 Neurotrophin signaling pathway;ko05169 Epstein-Barr virus infection;ko05203 Viral carcinogenesis;
gene11554	K03926	cutA	periplasmic divalent cation tolerance protein	--		
gene11560	K00940	E2.7.4.6, ndk	nucleoside-diphosphate kinase	EC:2.7.4.6	2	ko00230 Purine metabolism;ko00240 Pyrimidine metabolism;
gene11567	K01982	LSUrRNA	large subunit ribosomal RNA	--	2	ko03008 Ribosome biogenesis in eukaryotes;ko03010 Ribosome;
gene11574	K13422	MYC2	transcription factor MYC2	--	2	ko04075 Plant hormone signal transduction;ko04626 Plant-pathogen interaction;
gene11583	K10838	XPC	xeroderma pigmentosum group C-complementing protein	--	1	ko03420 Nucleotide excision repair;
gene11585	K12881	THOC4, ALY	THO complex subunit 4	--	4	ko03013 RNA transport;ko03015 mRNA surveillance pathway;ko03040 Spliceosome;ko05168 Herpes simplex infection;
gene1158	K14232	tRNA-Pro	tRNA Pro	--	1	ko00970 Aminoacyl-tRNA biosynthesis;
gene11592	K14829	IPI3	pre-rRNA-processing protein IPI3	--		
gene11593	K14794	RRP12	ribosomal RNA-processing protein 12	--		
gene11596	K14963	WDR5, SWD3, CPS30	COMPASS component SWD3	--		
gene11598	K01772	hemH, FECH	ferrochelatase	EC:4.99.1.1	1	ko00860 Porphyrin and chlorophyll metabolism;
gene115	K09338	HD-ZIP	homeobox-leucine zipper protein	--		
gene11601	K13195	CIRBP	cold-inducible RNA-binding protein	--		
gene11607	K00485	E1.14.13.8	dimethylaniline monooxygenase (N-oxide forming)	EC:1.14.13.8	2	ko00680 Methane metabolism;ko00982 Drug metabolism - cytochrome P450;
gene11609	K17275	PLS1	plastin-1	--		
gene11614	K02331	POL5, MYBBP1A	DNA polymerase phi	EC:2.7.7.7		
gene11617	K07304	msrA	peptide-methionine (S)-S-oxide reductase	EC:1.8.4.11		
gene11618	K12877	MAGOH	protein mago nashi	--	3	ko03013 RNA transport;ko03015 mRNA surveillance pathway;ko03040 Spliceosome;
gene1161	K13963	SERPINB	serpin B	--	1	ko05146 Amoebiasis;
gene11628	K09873	TIP	aquaporin TIP	--		
gene11629	K01433	purU	formyltetrahydrofolate deformylase	EC:3.5.1.10	2	ko00630 Glyoxylate and dicarboxylate metabolism;ko00670 One carbon pool by folate;
gene1162	K13963	SERPINB	serpin B	--	1	ko05146 Amoebiasis;
gene11634	K14498	SNRK2	serine/threonine-protein kinase SRK2	EC:2.7.11.1	1	ko04075 Plant hormone signal transduction;
gene11636	K17361	ACOT9	acyl-coenzyme A thioesterase 9	EC:3.1.2.-		
gene11639	K01953	asnB, ASNS	asparagine synthase (glutamine-hydrolysing)	EC:6.3.5.4	1	ko00250 Alanine, aspartate and glutamate metabolism;
gene1163	K13963	SERPINB	serpin B	--	1	ko05146 Amoebiasis;
gene11641	K14567	UTP14	U3 small nucleolar RNA-associated protein 14	--	1	ko03008 Ribosome biogenesis in eukaryotes;
gene11642	K14567	UTP14	U3 small nucleolar RNA-associated protein 14	--	1	ko03008 Ribosome biogenesis in eukaryotes;
gene11649	K14293	KPNB1	importin subunit beta-1	--	1	ko03013 RNA transport;
gene11651	K01280	TPP2	tripeptidyl-peptidase II	EC:3.4.14.10		

gene11655	K02134	ATPeF1D, ATP5D	F-type H+-transporting ATPase subunit delta	EC:3.6.3.14	4	ko00190 Oxidative phosphorylation;ko05010 Alzheimer's disease;ko05012 Parkinson's disease;ko05016 Huntington's disease;
gene11659	K13161	HNRNPR	heterogeneous nuclear ribonucleoprotein R	--		
gene1165	K15718	LOX1_5	linoleate 9S-lipoxygenase	EC:1.13.11.58	1	ko00591 Linoleic acid metabolism;
gene11662	K15397	KCS	3-ketoacyl-CoA synthase	EC:2.3.1.199	1	ko00062 Fatty acid elongation;
gene11664	K07953	SAR1	GTP-binding protein SAR1	EC:3.6.5.-	2	ko04141 Protein processing in endoplasmic reticulum;ko05134 Legionellosis;
gene11667	K01166	E3.1.27.1	ribonuclease T2	EC:3.1.27.1		
gene11669	K12501	HST	homogentisate solanesyltransferase	--	1	ko00130 Ubiquinone and other terpenoid-quinone biosynthesis;
gene1166	K08738	CYC	cytochrome c	--	19	ko00920 Sulfur metabolism;ko02020 Two-component system;ko04115 p53 signaling pathway;ko04210 Apoptosis;ko04932 Non-alcoholic fatty liver disease (NAFLD);ko05010 Alzheimer's disease;ko05012 Parkinson's disease;ko05014 Amyotrophic lateral sclerosis (ALS);ko05016 Huntington's disease;ko05134 Legionellosis;ko05145 Toxoplasmosis;ko05152 Tuberculosis;ko05161 Hepatitis B;ko05164 Influenza A;ko05168 Herpes simplex infection;ko05200 Pathways in cancer;ko05210 Colorectal cancer;ko05222 Small cell lung cancer;ko05416 Viral myocarditis;
gene11673	K09540	SEC63	translocation protein SEC63	--	2	ko03060 Protein export;ko04141 Protein processing in endoplasmic reticulum;
gene11681	K03327	TC.MATE, SLC47A, norM, mdtK, dinF	multidrug resistance protein, MATE family	--		
gene11683	K11884	PNO1, DIM2	RNA-binding protein PNO1	--		
gene11686	K01074	PPT	palmitoyl-protein thioesterase	EC:3.1.2.22	2	ko00062 Fatty acid elongation;ko04142 Lysosome;
gene11692	K14487	GH3	auxin responsive GH3 gene family	--	1	ko04075 Plant hormone signal transduction;
gene116	K01214	E3.2.1.68	isoamylase	EC:3.2.1.68		
gene11700	K08900	BCS1	mitochondrial chaperone BCS1	--		
gene11709	K02639	petF	ferredoxin	--	1	ko00195 Photosynthesis;
gene11710	K10638	UHRF1, NP95	E3 ubiquitin-protein ligase UHRF1	EC:6.3.2.19		
gene11713	K02155	ATPeVPL, ATP6L	V-type H+-transporting ATPase 16kDa proteolipid subunit	EC:3.6.3.14	9	ko00190 Oxidative phosphorylation;ko04142 Lysosome;ko04145 Phagosome;ko04721 Synaptic vesicle cycle;ko04966 Collecting duct acid secretion;ko05110 Vibrio cholerae infection;ko05120 Epithelial cell signaling in Helicobacter pylori infection;ko05152 Tuberculosis;ko05323 Rheumatoid arthritis;
gene11717	K08341	GABARAP, ATG8, LC3	GABA(A) receptor-associated protein (autophagy-related protein 8)	--	2	ko04140 Regulation of autophagy;ko04727 GABAergic synapse;
gene11719	K02115	ATPF1G, atpG	F-type H+-transporting ATPase subunit gamma	EC:3.6.3.14	2	ko00190 Oxidative phosphorylation;ko00195 Photosynthesis;
gene11720	K16587	HAUS4	HAUS augmin-like complex subunit 4	--		
gene11721	K06947	GRC3, NOL9	polynucleotide 5'-hydroxyl-kinase GRC3/NOL9	EC:2.7.1.-		
gene11723	K01934	E6.3.3.2	5-formyltetrahydrofolate cyclo-ligase	EC:6.3.3.2	1	ko00670 One carbon pool by folate;
gene11729	K08287	E2.7.12.1	dual-specificity kinase	EC:2.7.12.1		
gene11733	K04773	sppA	protease IV	EC:3.4.21.-		
gene11743	K00943	E2.7.4.9, tmk	dTMP kinase	EC:2.7.4.9	1	ko00240 Pyrimidine metabolism;
gene11747	K11340	ACTL6A, INO80K	actin-like protein 6A	--		
gene11753	K03544	clpX, CLPX	ATP-dependent Clp protease ATP-binding subunit ClpX	--	1	ko04112 Cell cycle - Caulobacter;
gene11754	K01188	E3.2.1.21	beta-glucosidase	EC:3.2.1.21	3	ko00460 Cyanoamino acid metabolism;ko00500 Starch and sucrose metabolism;ko00940 Phenylpropanoid biosynthesis;
gene11755	K08916	LHCB5	light-harvesting complex II chlorophyll a/b binding protein 5	--	1	ko00196 Photosynthesis - antenna proteins;
gene11759	K02145	ATPeVA, ATP6A1	V-type H+-transporting ATPase subunit A	EC:3.6.3.14	7	ko00190 Oxidative phosphorylation;ko04145 Phagosome;ko04721 Synaptic vesicle cycle;ko04966 Collecting duct acid secretion;ko05110 Vibrio cholerae infection;ko05120 Epithelial cell signaling in Helicobacter pylori infection;ko05323 Rheumatoid arthritis;
gene11760	K08245	E3.4.23.40	phytepsin	EC:3.4.23.40		
gene11765	K13648	GAUT	alpha-1,4-galacturonosyltransferase	EC:2.4.1.43	2	ko00500 Starch and sucrose metabolism;ko00520 Amino sugar and nucleotide sugar metabolism;
gene11766	K14324	SAP18	histone deacetylase complex subunit SAP18	--	2	ko03013 RNA transport;ko03015 mRNA surveillance pathway;
gene11768	K13412	CPK	calcium-dependent protein kinase	EC:2.7.11.1	2	ko04626 Plant-pathogen interaction;ko05145 Toxoplasmosis;
gene11771	K01366	CTSH	cathepsin H	EC:3.4.22.16	1	ko04142 Lysosome;

gene11773	K00279	E1.5.99.12	cytokinin dehydrogenase	EC:1.5.99.12	1	ko00908 Zeatin biosynthesis;
gene11774	K10684	UBLE1A, SAE1	ubiquitin-like 1-activating enzyme E1 A	EC:6.3.2.19	1	ko04120 Ubiquitin mediated proteolysis;
gene11775	K12195	CHMP6, VPS20	charged multivesicular body protein 6	--	1	ko04144 Endocytosis;
gene11778	K17278	PGRMC1_2	membrane-associated progesterone receptor component	--		
gene11780	K03236	EIF1A	translation initiation factor 1A	--	1	ko03013 RNA transport;
gene11784	K02575	NRT, narK, nrtP, nasA	MFS transporter, NNP family, nitrate/nitrite transporter	--	1	ko00910 Nitrogen metabolism;
gene11786	K13946	AUX1, LAX	auxin influx carrier (AUX1 LAX family)	--	1	ko04075 Plant hormone signal transduction;
gene1178	K02894	RP-L23e, RPL23	large subunit ribosomal protein L23e	--	1	ko03010 Ribosome;
gene11794	K05956	RABGGTB	geranylgeranyl transferase type-2 subunit beta	EC:2.5.1.60		
gene11799	K04368	MAP2K1, MEK1	mitogen-activated protein kinase kinase 1	EC:2.7.12.2	53	ko04010 MAPK signaling pathway;ko04012 ErbB signaling pathway;ko04013 MAPK signaling pathway - fly;ko04014 Ras signaling pathway;ko04062 Chemokine signaling pathway;ko04066 HIF-1 signaling pathway;ko04114 Oocyte meiosis;ko04151 PI3K-Akt signaling pathway;ko04270 Vascular smooth muscle contraction;ko04320 Dorso-ventral axis formation;ko04370 VEGF signaling pathway;ko04380 Osteoclast differentiation;ko04510 Focal adhesion;ko04540 Gap junction;ko04620 Toll-like receptor signaling pathway;ko04626 Plant-pathogen interaction;ko04650 Natural killer cell mediated cytotoxicity;ko04660 T cell receptor signaling pathway;ko04662 B cell receptor signaling pathway;ko04664 Fc epsilon RI signaling pathway;ko04666 Fc gamma R-mediated phagocytosis;ko04668 TNF signaling pathway;ko04720 Long-term potentiation;ko04722 Neurotrophin signaling pathway;ko04725 Cholinergic synapse;ko04726 Serotonergic synapse;ko04730 Long-term depression;ko04810 Regulation of actin cytoskeleton;ko04910 Insulin signaling pathway;ko04912 GnRH signaling pathway;ko04914 Progesterone-mediated oocyte maturation;ko04915 Estrogen signaling pathway;ko04916 Melanogenesis;ko04917 Prolactin signaling pathway;ko05020 Prion diseases;ko05034 Alcoholism;ko05161 Hepatitis B;ko05164 Influenza A;ko05200 Pathways in cancer;ko05205 Proteoglycans in cancer;ko05206 MicroRNAs in cancer;ko05210 Colorectal cancer;ko05211 Renal cell carcinoma;ko05212 Pancreatic cancer;ko05213 Endometrial cancer;ko05214 Glioma;ko05215 Prostate cancer;ko05216 Thyroid cancer;ko05218 Melanoma;ko05219 Bladder cancer;ko05220
gene11804	K16911	DDX21	ATP-dependent RNA helicase DDX21	EC:3.6.4.13		
gene1180	K09286	EREBP	EREBP-like factor	--		
gene11822	K12837	U2AF2	splicing factor U2AF 65 kDa subunit	--	1	ko03040 Spliceosome;
gene11826	K02358	tuf, TUFM	elongation factor Tu	--	1	ko04626 Plant-pathogen interaction;
gene11827	K08867	WNK, PRKWNK	WNK lysine deficient protein kinase	EC:2.7.11.1		
gene11828	K02893	RP-L23Ae, RPL23A	large subunit ribosomal protein L23Ae	--	1	ko03010 Ribosome;
gene11833	K11322	EPC	enhancer of polycomb-like protein	--		
gene11834	K07748	E1.1.1.170, NSDHL, ERG26	sterol-4alpha-carboxylate 3-dehydrogenase (decarboxylating)	EC:1.1.1.170	1	ko00100 Steroid biosynthesis;
gene11835	K14488	SAUR	SAUR family protein	--	1	ko04075 Plant hormone signal transduction;
gene11836	K14488	SAUR	SAUR family protein	--	1	ko04075 Plant hormone signal transduction;
gene11837	K14488	SAUR	SAUR family protein	--	1	ko04075 Plant hormone signal transduction;
gene11838	K00434	E1.11.1.11	L-ascorbate peroxidase	EC:1.11.1.11	2	ko00053 Ascorbate and aldarate metabolism;ko00480 Glutathione metabolism;
gene11844	K13600	CAO	chlorophyllide a oxygenase	EC:1.14.13.12 2	1	ko00860 Porphyrin and chlorophyll metabolism;
gene11855	K11251	H2A	histone H2A	--	2	ko05034 Alcoholism;ko05322 Systemic lupus erythematosus;
gene11864	K08907	LHCA1	light-harvesting complex I chlorophyll a/b binding protein 1	--	1	ko00196 Photosynthesis - antenna proteins;
gene11871	K16546	FGFR10P	FGFR1 oncogene partner	--		

gene11883	K01115	PLD1_2	phospholipase D1/2	EC:3.1.4.4	7	ko00564 Glycerophospholipid metabolism;ko00565 Ether lipid metabolism;ko04014 Ras signaling pathway;ko04144 Endocytosis;ko04666 Fc gamma R-mediated phagocytosis;ko04724 Glutamatergic synapse;ko04912 GnRH signaling pathway;
gene11884	K02493	hemK, prmC	release factor glutamine methyltransferase	EC:2.1.1.-		
gene11885	K09534	DNAJC14	DnaJ homolog subfamily C member 14	--		
gene11889	K12666	OST1, RPN1	oligosaccharyltransferase complex subunit alpha (ribophorin I)	--	3	ko00510 N-Glycan biosynthesis;ko00513 Various types of N-glycan biosynthesis;ko04141 Protein processing in endoplasmic reticulum;
gene11894	K00679	E2.3.1.158	phospholipid:diacylglycerol acyltransferase	EC:2.3.1.158	1	ko00561 Glycerolipid metabolism;
gene11897	K08869	ADCK, ABC1	aarF domain-containing kinase	--		
gene11898	K08869	ADCK, ABC1	aarF domain-containing kinase	--		
gene11905	K14488	SAUR	SAUR family protein	--	1	ko04075 Plant hormone signal transduction;
gene11909	K13917	RNGTT	mRNA-capping enzyme	EC:2.7.7.50 3.1.3.33	1	ko03015 mRNA surveillance pathway;
gene11910	K08505	SFT1	protein transport protein SFT1	--	1	ko04130 SNARE interactions in vesicular transport;
gene11913	K14962	WDR82, SWD2, CPS35	COMPASS component SWD2	--	1	ko03015 mRNA surveillance pathway;
gene11917	K07375	TUBB	tubulin beta	--	3	ko04145 Phagosome;ko04540 Gap junction;ko05130 Pathogenic Escherichia coli infection;
gene11920	K11293	HIRA, HIR1	protein HIRA/HIR1	--		
gene11921	K13356	FAR	fatty acyl-CoA reductase	EC:1.2.1.-	2	ko00073 Cutin, suberine and wax biosynthesis;ko04146 Peroxisome;
gene1192	K05283	PIGW	phosphatidylinositol glycan, class W	EC:2.3.-.-	1	ko00563 Glycosylphosphatidylinositol(GPI)-anchor biosynthesis;
gene11935	K14845	RAI1, DOM3Z	RAT1-interacting protein	--		
gene11936	K12472	EPS15	epidermal growth factor receptor substrate 15	--	1	ko04144 Endocytosis;
gene11938	K00162	PDHB, pdhB	pyruvate dehydrogenase E1 component subunit beta	EC:1.2.4.1	6	ko00010 Glycolysis / Gluconeogenesis;ko00020 Citrate cycle (TCA cycle);ko00620 Pyruvate metabolism;ko00650 Butanoate metabolism;ko01200 Carbon metabolism;ko04066 HIF-1 signaling pathway;
gene11939	K15296	NAPA, SNAPA, SEC17	alpha-soluble NSF attachment protein	--	1	ko04721 Synaptic vesicle cycle;
gene11947	K14842	NSA2	ribosome biogenesis protein NSA2	--		
gene11948	K15363	FAN1, MTMR15	fanconi-associated nuclease 1	--	1	ko03460 Fanconi anemia pathway;
gene11950	K13155	SNRNP35	U11/U12 small nuclear ribonucleoprotein 35 kDa protein	--		
gene11951	K04730	IRAK1	interleukin-1 receptor-associated kinase 1	EC:2.7.11.1	11	ko04064 NF-kappa B signaling pathway;ko04210 Apoptosis;ko04620 Toll-like receptor signaling pathway;ko04722 Neurotrophin signaling pathway;ko05133 Pertussis;ko05140 Leishmaniasis;ko05142 Chagas disease (American trypanosomiasis);ko05145 Toxoplasmosis;ko05152 Tuberculosis;ko05162 Measles;ko05169 Epstein-Barr virus infection;
gene11953	K03327	TC.MATE, SLC47A, norM, mdtK, dinF	multidrug resistance protein, MATE family	--		
gene11957	K11253	H3	histone H3	--	3	ko05034 Alcoholism;ko05202 Transcriptional misregulation in cancer;ko05322 Systemic lupus erythematosus;
gene1195	K02929	RP-L44e, RPL44	large subunit ribosomal protein L44e	--	1	ko03010 Ribosome;
gene11960	K06630	YWHAE	14-3-3 protein epsilon	--	8	ko04110 Cell cycle;ko04114 Oocyte meiosis;ko04151 PI3K-Akt signaling pathway;ko04390 Hippo signaling pathway;ko04391 Hippo signaling pathway - fly;ko04722 Neurotrophin signaling pathway;ko05169 Epstein-Barr virus infection;ko05203 Viral carcinogenesis;
gene11964	K00166	E1.2.4.4A, bkdA1	2-oxoisovalerate dehydrogenase E1 component, alpha subunit	EC:1.2.4.4	1	ko00280 Valine, leucine and isoleucine degradation;
gene11965	K15422	SAL	3'(2'), 5'-bisphosphate nucleotidase / inositol polyphosphate 1-phosphatase	EC:3.1.3.7 3.1.3.57	3	ko00562 Inositol phosphate metabolism;ko00920 Sulfur metabolism;ko04070 Phosphatidylinositol signaling system;
gene11969	K02206	CDK2	cyclin-dependent kinase 2	EC:2.7.11.22	13	ko04110 Cell cycle;ko04114 Oocyte meiosis;ko04115 p53 signaling pathway;ko04151 PI3K-Akt signaling pathway;ko04914 Progesterone-mediated oocyte maturation;ko05161 Hepatitis B;ko05162 Measles;ko05168 Herpes simplex infection;ko05169 Epstein-Barr virus infection;ko05200 Pathways in cancer;ko05203 Viral carcinogenesis;ko05215 Prostate cancer;ko05222 Small cell lung cancer;
gene11971	K03695	clpB	ATP-dependent Clp protease ATP-binding subunit ClpB	--		
gene11974	K13466	EIX1_2	EIX receptor 1/2	--	1	ko04626 Plant-pathogen interaction;

gene11975	K13466	EIX1_2	EIX receptor 1/2	--	1	ko04626 Plant-pathogen interaction;
gene11976	K14488	SAUR	SAUR family protein	--	1	ko04075 Plant hormone signal transduction;
gene11990	K13341	PEX7, PTS2R	peroxin-7	--	1	ko04146 Peroxisome;
gene11995	K06133	LYS5, acpT	4'-phosphopantetheinyl transferase	EC:2.7.8.-	1	ko00770 Pantothenate and CoA biosynthesis;
gene11996	K13628	iscA, ISCA1	iron-sulfur cluster assembly protein	--		
gene11997	K06129	LYPLA3	lysophospholipase III	EC:3.1.1.5	2	ko00564 Glycerophospholipid metabolism;ko04142 Lysosome;
gene11	K03130	TAF5	transcription initiation factor TFIID subunit 5	--	2	ko03022 Basal transcription factors;ko05168 Herpes simplex infection;
gene12000	K15109	SLC25A20_29, CACT, CACL, CRC1	solute carrier family 25 (mitochondrial carnitine/acylcarnitine transporter), member 20/29	--		
gene12006	K02291	crtB	phytoene synthase	EC:2.5.1.32	1	ko00906 Carotenoid biosynthesis;
gene12010	K09422	MYBP	myb proto-oncogene protein, plant	--		
gene12011	K12386	CTNS	cystinosin	--	1	ko04142 Lysosome;
gene12015	K14516	ERF1	ethylene-responsive transcription factor 1	--	1	ko04075 Plant hormone signal transduction;
gene12016	K14568	EMG1, NEP1	rRNA small subunit pseudouridine methyltransferase Nep1	EC:2.1.1.260	1	ko03008 Ribosome biogenesis in eukaryotes;
gene12026	K05658	ABCB1	ATP-binding cassette, subfamily B (MDR/TAP), member 1	--	3	ko02010 ABC transporters;ko04976 Bile secretion;ko05206 MicroRNAs in cancer;
gene12027	K08331	ATG13	autophagy-related protein 13	--	1	ko04140 Regulation of autophagy;
gene1202	K01754	E4.3.1.19, ilvA, tdcB	threonine dehydratase	EC:4.3.1.19	4	ko00260 Glycine, serine and threonine metabolism;ko00290 Valine, leucine and isoleucine biosynthesis;ko01200 Carbon metabolism;ko01230 Biosynthesis of amino acids;
gene12034	K08819	CDK12_13	cyclin-dependent kinase 12/13	EC:2.7.11.22 2.7.11.23		
gene12042	K13171	SRRM1, SRM160	serine/arginine repetitive matrix protein 1	--	2	ko03013 RNA transport;ko03015 mRNA surveillance pathway;
gene12046	K13947	PIN	auxin efflux carrier family	--		
gene1204	K09284	AP2	AP2-like factor, euAP2 lineage	--		
gene12050	K13436	PTI1	pto-interacting protein 1	EC:2.7.11.1	1	ko04626 Plant-pathogen interaction;
gene12052	K15040	VDAC2	voltage-dependent anion channel protein 2	--	4	ko04020 Calcium signaling pathway;ko05012 Parkinson's disease;ko05016 Huntington's disease;ko05166 HTLV-I infection;
gene12056	K01598	PPCDC, coaC	phosphopantothenoylcysteine decarboxylase	EC:4.1.1.36	1	ko00770 Pantothenate and CoA biosynthesis;
gene1205	K02930	RP-L4e, RPL4	large subunit ribosomal protein L4e	--	1	ko03010 Ribosome;
gene12060	K07018	K07018				
gene12062	K09647	IMP1	mitochondrial inner membrane protease subunit 1	EC:3.4.21.-	1	ko03060 Protein export;
gene12063	K09286	EREBP	EREBP-like factor	--		
gene12069	K10727	CDT1	chromatin licensing and DNA replication factor 1	--		
gene12070	K13783	SLC37A1_2	MFS transporter, OPA family, solute carrier family 37 (glycerol-3-phosphate transporter), member 1/2	--		
gene12073	K12581	CNOT7_8, CAF1, POP2	CCR4-NOT transcription complex subunit 7/8	--	1	ko03018 RNA degradation;
gene12081	K01733	thrC	threonine synthase	EC:4.2.3.1	3	ko00260 Glycine, serine and threonine metabolism;ko00750 Vitamin B6 metabolism;ko01230 Biosynthesis of amino acids;
gene1208	K04733	IRAK4	interleukin-1 receptor-associated kinase 4	EC:2.7.11.1	11	ko04064 NF-kappa B signaling pathway;ko04210 Apoptosis;ko04620 Toll-like receptor signaling pathway;ko04722 Neurotrophin signaling pathway;ko05133 Pertussis;ko05140 Leishmaniasis;ko05142 Chagas disease (American trypanosomiasis);ko05145 Toxoplasmosis;ko05152 Tuberculosis;ko05162 Measles;ko05164 Influenza A;
gene12090	K04079	htpG, HSP90A	molecular chaperone HtpG	--	9	ko04141 Protein processing in endoplasmic reticulum;ko04151 PI3K-Akt signaling pathway;ko04612 Antigen processing and presentation;ko04621 NOD-like receptor signaling pathway;ko04626 Plant-pathogen interaction;ko04914 Progesterone-mediated oocyte maturation;ko04915 Estrogen signaling pathway;ko05200 Pathways in cancer;ko05215 Prostate cancer;
gene12095	K00901	E2.7.1.107, DGK, dgkA	diacylglycerol kinase (ATP dependent)	EC:2.7.1.107	3	ko00561 Glycerolipid metabolism;ko00564 Glycerophospholipid metabolism;ko04070 Phosphatidylinositol signaling system;
gene12101	K10581	UBE2O	ubiquitin-conjugating enzyme E2 O	EC:6.3.2.19	1	ko04120 Ubiquitin mediated proteolysis;
gene12103	K11000	CALS	callose synthase	EC:2.4.1.-		
gene12104	K15637	PGAM5	serine/threonine-protein phosphatase PGAM5	EC:3.1.3.16	1	ko04668 TNF signaling pathway;

gene12106	K03010	RPB2, POLR2B	DNA-directed RNA polymerase II subunit RPB2	EC:2.7.7.6	5	ko00230 Purine metabolism;ko00240 Pyrimidine metabolism;ko03020 RNA polymerase;ko05016 Huntington's disease;ko05169 Epstein-Barr virus infection;
gene12108	K03635	MOCS2, moaE	molybdopterin synthase catalytic subunit	EC:2.-.-.-	2	ko00790 Folate biosynthesis;ko04122 Sulfur relay system;
gene12113	K03262	EIF5	translation initiation factor 5	--	1	ko03013 RNA transport;
gene12126	K01979	SSUrRNA	small subunit ribosomal RNA	--	2	ko03008 Ribosome biogenesis in eukaryotes;ko03010 Ribosome;
gene12127	K01979	SSUrRNA	small subunit ribosomal RNA	--	2	ko03008 Ribosome biogenesis in eukaryotes;ko03010 Ribosome;
gene12131	K00873	PK, pyk	pyruvate kinase	EC:2.7.1.40	7	ko00010 Glycolysis / Gluconeogenesis;ko00230 Purine metabolism;ko00620 Pyruvate metabolism;ko01200 Carbon metabolism;ko01230 Biosynthesis of amino acids;ko04930 Type II diabetes mellitus;ko05203 Viral carcinogenesis;
gene12132	K09874	NIP	aquaporin NIP	--		
gene12148	K09338	HD-ZIP	homeobox-leucine zipper protein	--		
gene12150	K13420	FLS2	LRR receptor-like serine/threonine-protein kinase FLS2	EC:2.7.11.1	1	ko04626 Plant-pathogen interaction;
gene12157	K05543	DUS2	tRNA-dihydrouridine synthase 2	EC:1.3.1.91		
gene12162	K14709	SLC39A1_2_3, ZIP1_2_3	solute carrier family 39 (zinc transporter), member 1/2/3	--		
gene12164	K02693	psaE	photosystem I subunit IV	--	1	ko00195 Photosynthesis;
gene12165	K03676	grxC, GLRX, GLRX2	glutaredoxin 3	--		
gene12173	K08493	VTI1	vesicle transport through interaction with t-SNAREs 1	--	1	ko04130 SNARE interactions in vesicular transport;
gene12175	K02971	RP-S21e, RPS21	small subunit ribosomal protein S21e	--	1	ko03010 Ribosome;
gene1217	K09377	CSRP	cysteine and glycine-rich protein	--		
gene12180	K11786	STH1_SNF2	ATP-dependent helicase STH1/SNF2	EC:3.6.4.-		
gene12182	K15437	AIMP1, ARC1	aminoacyl tRNA synthase complex-interacting multifunctional protein 1	--		
gene12188	K12492	ARFGAP1	ADP-ribosylation factor GTPase-activating protein 1	--	1	ko04144 Endocytosis;
gene12193	K05754	ARPC5	actin related protein 2/3 complex, subunit 5	--	6	ko04666 Fc gamma R-mediated phagocytosis;ko04810 Regulation of actin cytoskeleton;ko05100 Bacterial invasion of epithelial cells;ko05130 Pathogenic Escherichia coli infection;ko05131 Shigellosis;ko05132 Salmonella infection;
gene12194	K09338	HD-ZIP	homeobox-leucine zipper protein	--		
gene12195	K15397	KCS	3-ketoacyl-CoA synthase	EC:2.3.1.199	1	ko00062 Fatty acid elongation;
gene12196	K00666	K00666	fatty-acyl-CoA synthase	EC:6.2.1.-		
gene12199	K07893	RAB6A	Ras-related protein Rab-6A	--		
gene12204	K01102	PDP	pyruvate dehydrogenase phosphatase	EC:3.1.3.43		
gene12208	K06100	SYMPK	symplekin	--	2	ko03015 mRNA surveillance pathway;ko04530 Tight junction;
gene12210	K08245	E3.4.23.40	phytepsin	EC:3.4.23.40		
gene12211	K03352	APC5	anaphase-promoting complex subunit 5	--	7	ko04110 Cell cycle;ko04111 Cell cycle - yeast;ko04113 Meiosis - yeast;ko04114 Oocyte meiosis;ko04120 Ubiquitin mediated proteolysis;ko04914 Progesterone-mediated oocyte maturation;ko05166 HTLV-I infection;
gene12215	K03934	NDUFS1	NADH dehydrogenase (ubiquinone) Fe-S protein 1	EC:1.6.5.3 1.6.99.3	5	ko00190 Oxidative phosphorylation;ko04932 Non-alcoholic fatty liver disease (NAFLD);ko05010 Alzheimer's disease;ko05012 Parkinson's disease;ko05016 Huntington's disease;
gene12216	K06171	NCSTN	nicastrin	--	2	ko04330 Notch signaling pathway;ko05010 Alzheimer's disease;
gene12217	K13104	ZNF830, CCDC16	zinc finger protein 830	--		
gene12224	K11518	TOM40	mitochondrial import receptor subunit TOM40	--	1	ko05014 Amyotrophic lateral sclerosis (ALS);
gene12226	K03364	CDH1	cell division cycle 20-like protein 1, cofactor of APC complex	--	4	ko04110 Cell cycle;ko04111 Cell cycle - yeast;ko04120 Ubiquitin mediated proteolysis;ko04914 Progesterone-mediated oocyte maturation;
gene12235	K12309	GLB1, ELNR1	beta-galactosidase	EC:3.2.1.23	6	ko00052 Galactose metabolism;ko00511 Other glycan degradation;ko00531 Glycosaminoglycan degradation;ko00600 Sphingolipid metabolism;ko00604 Glycosphingolipid biosynthesis - ganglio series;ko04142 Lysosome;
gene12236	K05665	ABCC1	ATP-binding cassette, subfamily C (CFTR/MRP), member 1	--	3	ko02010 ABC transporters;ko04977 Vitamin digestion and absorption;ko05206 MicroRNAs in cancer;
gene12240	K07766	E3.6.1.52	diphosphoinositol-polyphosphate diphosphatase	EC:3.6.1.52		
gene12250	K01051	E3.1.1.11	pectinesterase	EC:3.1.1.11	2	ko00040 Pentose and glucuronate interconversions;ko00500 Starch and sucrose metabolism;

gene12253	K07870	RHOT1, ARHT1	Ras homolog gene family, member T1	--		
gene12254	K12823	DDX5, DBP2	ATP-dependent RNA helicase DDX5/DBP2	EC:3.6.4.13	3	ko03040 Spliceosome;ko05202 Transcriptional misregulation in cancer;ko05205 Proteoglycans in cancer;
gene12271	K12862	PLRG1, PRL1, PRP46	pleiotropic regulator 1	--	1	ko03040 Spliceosome;
gene12273	K02882	RP-L18Ae, RPL18A	large subunit ribosomal protein L18Ae	--	1	ko03010 Ribosome;
gene12277	K09955	K09955	hypothetical protein	--		
gene12285	K05909	E1.10.3.2				
gene12287	K03008	RPB11, POLR2J	DNA-directed RNA polymerase II subunit RPB11	--	5	ko00230 Purine metabolism;ko00240 Pyrimidine metabolism;ko03020 RNA polymerase;ko05016 Huntington's disease;ko05169 Epstein-Barr virus infection;
gene12296	K14556	DIP2, UTP12, WDR3	U3 small nucleolar RNA-associated protein 12	--	1	ko03008 Ribosome biogenesis in eukaryotes;
gene12301	K15803	GERD	(-)-germacrene D synthase	EC:4.2.3.22 4.2.3.75	1	ko00909 Sesquiterpenoid and triterpenoid biosynthesis;
gene12302	K11000	CALS	callose synthase	EC:2.4.1.-		
gene12311	K11968	ARIH1	ariadne-1	--		
gene12314	K13459	RPS2	disease resistance protein RPS2	--	1	ko04626 Plant-pathogen interaction;
gene12319	K04125	E1.14.11.13	gibberellin 2-oxidase	EC:1.14.11.13	1	ko00904 Diterpenoid biosynthesis;
gene12320	K01895	ACSS, acs	acetyl-CoA synthetase	EC:6.2.1.1	6	ko00010 Glycolysis / Gluconeogenesis;ko00620 Pyruvate metabolism;ko00640 Propanoate metabolism;ko00680 Methane metabolism;ko00720 Carbon fixation pathways in prokaryotes;ko01200 Carbon metabolism;
gene12327	K12197	CHMP1, VPS46, DID2	charged multivesicular body protein 1	--	1	ko04144 Endocytosis;
gene12328	K02903	RP-L28e, RPL28	large subunit ribosomal protein L28e	--	1	ko03010 Ribosome;
gene12333	K16296	SCPL-I	serine carboxypeptidase-like clade I	EC:3.4.16.-		
gene12341	K02959	RP-S16, MRPS16, rpsP	small subunit ribosomal protein S16	--	1	ko03010 Ribosome;
gene12342	K11279	NAP1L1, NRP	nucleosome assembly protein 1-like 1	--		
gene12348	K02913	RP-L33, MRPL33, rpmG	large subunit ribosomal protein L33	--	1	ko03010 Ribosome;
gene12349	K10357	MYO5	myosin V	--		
gene12354	K10760	IPT	adenylate isopentenyltransferase (cytokinin synthase)	--	1	ko00908 Zeatin biosynthesis;
gene12356	K09422	MYBP	myb proto-oncogene protein, plant	--		
gene12357	K08232	E1.6.5.4	monodehydroascorbate reductase (NADH)	EC:1.6.5.4	1	ko00053 Ascorbate and aldarate metabolism;
gene12358	K01358	clpP, CLPP	ATP-dependent Clp protease, protease subunit	EC:3.4.21.92	1	ko04112 Cell cycle - Caulobacter;
gene12360	K02875	RP-L14e, RPL14	large subunit ribosomal protein L14e	--	1	ko03010 Ribosome;
gene12361	K08960	CSNK1E	casein kinase 1, epsilon	EC:2.7.11.1	6	ko04310 Wnt signaling pathway;ko04340 Hedgehog signaling pathway;ko04390 Hippo signaling pathway;ko04391 Hippo signaling pathway - fly;ko04710 Circadian rhythm;ko04711 Circadian rhythm - fly;
gene12363	K00927	PGK, pgk	phosphoglycerate kinase	EC:2.7.2.3	4	ko00010 Glycolysis / Gluconeogenesis;ko00710 Carbon fixation in photosynthetic organisms;ko01200 Carbon metabolism;ko01230 Biosynthesis of amino acids;
gene12368	K09264	K09264	MADS-box transcription factor, plant	--		
gene12371	K12813	DHX16	pre-mRNA-splicing factor ATP-dependent RNA helicase DHX16	EC:3.6.4.13	1	ko03040 Spliceosome;
gene12372	K10858	PMS2	DNA mismatch repair protein PMS2	--	2	ko03430 Mismatch repair;ko03460 Fanconi anemia pathway;
gene12373	K04797	pfdA, PFDN5	prefoldin alpha subunit	--		
gene12374	K17605	PPP2R4, PTPA	serine/threonine-protein phosphatase 2A activator	--		
gene12378	K00558	DNMT1, dcm	DNA (cytosine-5)-methyltransferase 1	EC:2.1.1.37	2	ko00270 Cysteine and methionine metabolism;ko05206 MicroRNAs in cancer;
gene12381	K01669	E4.1.99.3, phrB	deoxyribodipyrimidine photo-lyase	EC:4.1.99.3		
gene12384	K01836	E5.4.2.3	phosphoacetylglucosamine mutase	EC:5.4.2.3	1	ko00520 Amino sugar and nucleotide sugar metabolism;
gene12386	K02913	RP-L33, MRPL33, rpmG	large subunit ribosomal protein L33	--	1	ko03010 Ribosome;
gene12387	K01662	dxs	1-deoxy-D-xylulose-5-phosphate synthase	EC:2.2.1.7	2	ko00730 Thiamine metabolism;ko00900 Terpenoid backbone biosynthesis;
gene12390	K12741	HNRNPA1_3	heterogeneous nuclear ribonucleoprotein A1/A3	--	1	ko03040 Spliceosome;
gene12392	K07953	SAR1	GTP-binding protein SAR1	EC:3.6.5.-	2	ko04141 Protein processing in endoplasmic reticulum;ko05134 Legionellosis;
gene12397	K11253	H3	histone H3	--	3	ko05034 Alcoholism;ko05202 Transcriptional misregulation in cancer;ko05322 Systemic lupus erythematosus;
gene12398	K09338	HD-ZIP	homeobox-leucine zipper protein	--		

gene12399	K02927	RP-L40e, RPL40	large subunit ribosomal protein L40e	--	1	ko03010 Ribosome;
gene12400	K01761	E4.4.1.11	methionine-gamma-lyase	EC:4.4.1.11	2	ko00270 Cysteine and methionine metabolism;ko00450 Selenocompound metabolism;
gene12401	K07456	mutS2	DNA mismatch repair protein MutS2	--	1	ko03430 Mismatch repair;
gene12407	K06638	MAD1L	mitotic spindle assembly checkpoint protein MAD1	--	3	ko04110 Cell cycle;ko04914 Progesterone-mediated oocyte maturation;ko05203 Viral carcinogenesis;
gene12409	K14486	K14486, ARF	auxin response factor	--	1	ko04075 Plant hormone signal transduction;
gene1240	K12501	HST	homogentisate solanesyltransferase	--	1	ko00130 Ubiquinone and other terpenoid-quinone biosynthesis;
gene12411	K17302	COPB2, SEC27	coatomer, subunit beta'	--		
gene12414	K06617	E2.4.1.82	raffinose synthase	EC:2.4.1.82	1	ko00052 Galactose metabolism;
gene12418	K16911	DDX21	ATP-dependent RNA helicase DDX21	EC:3.6.4.13		
gene12419	K15305	VAC14, TAX1BP2	vacuole morphology and inheritance protein 14	--	2	ko05166 HTLV-I infection;ko05203 Viral carcinogenesis;
gene12423	K13422	MYC2	transcription factor MYC2	--	2	ko04075 Plant hormone signal transduction;ko04626 Plant-pathogen interaction;
gene12428	K15382	SLC50A, SWEET	solute carrier family 50 (sugar transporter)	--		
gene12429	K15382	SLC50A, SWEET	solute carrier family 50 (sugar transporter)	--		
gene12430	K00860	cysC	adenylylsulfate kinase	EC:2.7.1.25	2	ko00230 Purine metabolism;ko00920 Sulfur metabolism;
gene12433	K08790	STK38, NDR	serine/threonine kinase 38	EC:2.7.11.1		
gene12434	K04368	MAP2K1, MEK1	mitogen-activated protein kinase kinase 1	EC:2.7.12.2	53	ko04010 MAPK signaling pathway;ko04012 ErbB signaling pathway;ko04013 MAPK signaling pathway - fly;ko04014 Ras signaling pathway;ko04062 Chemokine signaling pathway;ko04066 HIF-1 signaling pathway;ko04114 Oocyte meiosis;ko04151 PI3K-Akt signaling pathway;ko04270 Vascular smooth muscle contraction;ko04320 Dorso-ventral axis formation;ko04370 VEGF signaling pathway;ko04380 Osteoclast differentiation;ko04510 Focal adhesion;ko04540 Gap junction;ko04620 Toll-like receptor signaling pathway;ko04626 Plant-pathogen interaction;ko04650 Natural killer cell mediated cytotoxicity;ko04660 T cell receptor signaling pathway;ko04662 B cell receptor signaling pathway;ko04664 Fc epsilon RI signaling pathway;ko04666 Fc gamma R-mediated phagocytosis;ko04668 TNF signaling pathway;ko04720 Long-term potentiation;ko04722 Neurotrophin signaling pathway;ko04725 Cholinergic synapse;ko04726 Serotonergic synapse;ko04730 Long-term depression;ko04810 Regulation of actin cytoskeleton;ko04910 Insulin signaling pathway;ko04912 GnRH signaling pathway;ko04914 Progesterone-mediated oocyte maturation;ko04915 Estrogen signaling pathway;ko04916 Melanogenesis;ko04917 Prolactin signaling pathway;ko05020 Prion diseases;ko05034 Alcoholism;ko05161 Hepatitis B;ko05164 Influenza A;ko05200 Pathways in cancer;ko05205 Proteoglycans in cancer;ko05206 MicroRNAs in cancer;ko05210 Colorectal cancer;ko05211 Renal cell carcinoma;ko05212 Pancreatic cancer;ko05213 Endometrial cancer;ko05214 Glioma;ko05215 Prostate cancer;ko05216 Thyroid cancer;ko05218 Melanoma;ko05219 Bladder cancer;ko05220
gene12435	K12602	WDR61, REC14, SKI8	WD repeat-containing protein 61	--	1	ko03018 RNA degradation;
gene12437	K00901	E2.7.1.107, DGK, dgkA	diacylglycerol kinase (ATP dependent)	EC:2.7.1.107	3	ko00561 Glycerolipid metabolism;ko00564 Glycerophospholipid metabolism;ko04070 Phosphatidylinositol signaling system;
gene12442	K11254	H4	histone H4	--	3	ko05034 Alcoholism;ko05203 Viral carcinogenesis;ko05322 Systemic lupus erythematosus;
gene12443	K11254	H4	histone H4	--	3	ko05034 Alcoholism;ko05203 Viral carcinogenesis;ko05322 Systemic lupus erythematosus;
gene12444	K02553	rraA, menG	regulator of ribonuclease activity A	--		
gene12445	K17095	ANXA7_11	annexin A7/11	--		
gene12453	K09490	HSPA5, BIP	heat shock 70kDa protein 5	--	4	ko03060 Protein export;ko04141 Protein processing in endoplasmic reticulum;ko04918 Thyroid hormone synthesis;ko05020 Prion diseases;
gene12454	K09422	MYBP	myb proto-oncogene protein, plant	--		
gene12456	K00099	dxr	1-deoxy-D-xylulose-5-phosphate reductoisomerase	EC:1.1.1.267	1	ko00900 Terpenoid backbone biosynthesis;
gene12457	K13343	PEX14	peroxin-14	--	1	ko04146 Peroxisome;

gene12463	K04730	IRAK1	interleukin-1 receptor-associated kinase 1	EC:2.7.11.1	11	ko04064 NF-kappa B signaling pathway;ko04210 Apoptosis;ko04620 Toll-like receptor signaling pathway;ko04722 Neurotrophin signaling pathway;ko05133 Pertussis;ko05140 Leishmaniasis;ko05142 Chagas disease (American trypanosomiasis);ko05145 Toxoplasmosis;ko05152 Tuberculosis;ko05162 Measles;ko05169 Epstein-Barr virus infection;
gene12472	K00891	E2.7.1.71, aroK, aroL	shikimate kinase	EC:2.7.1.71	2	ko00400 Phenylalanine, tyrosine and tryptophan biosynthesis;ko01230 Biosynthesis of amino acids;
gene12475	K04688	RPS6KB	p70 ribosomal S6 kinase	EC:2.7.11.1	9	ko04012 ErbB signaling pathway;ko04066 HIF-1 signaling pathway;ko04150 mTOR signaling pathway;ko04151 PI3K-Akt signaling pathway;ko04350 TGF-beta signaling pathway;ko04666 Fc gamma R-mediated phagocytosis;ko04910 Insulin signaling pathway;ko05205 Proteoglycans in cancer;ko05221 Acute myeloid leukemia;
gene12480	K09286	EREBP	EREBP-like factor	--		
gene12485	K14308	NUP54	nuclear pore complex protein Nup54	--	1	ko03013 RNA transport;
gene12486	K03108	SRP72	signal recognition particle subunit SRP72	--	1	ko03060 Protein export;
gene12488	K04733	IRAK4	interleukin-1 receptor-associated kinase 4	EC:2.7.11.1	11	ko04064 NF-kappa B signaling pathway;ko04210 Apoptosis;ko04620 Toll-like receptor signaling pathway;ko04722 Neurotrophin signaling pathway;ko05133 Pertussis;ko05140 Leishmaniasis;ko05142 Chagas disease (American trypanosomiasis);ko05145 Toxoplasmosis;ko05152 Tuberculosis;ko05162 Measles;ko05164 Influenza A;
gene12491	K01533	E3.6.3.4, ATP7, copA	Cu2+-exporting ATPase	EC:3.6.3.4		
gene12493	K00430	E1.11.1.7	peroxidase	EC:1.11.1.7	2	ko00360 Phenylalanine metabolism;ko00940 Phenylpropanoid biosynthesis;
gene12499	K01853	E5.4.99.8	cycloartenol synthase	EC:5.4.99.8	1	ko00100 Steroid biosynthesis;
gene124	K00510	HMOX, hmuO, ho	heme oxygenase	EC:1.14.99.3	2	ko00860 Porphyrin and chlorophyll metabolism;ko04978 Mineral absorption;
gene12502	K17609	NXN	nucleoredoxin	EC:1.8.1.8		
gene12507	K13436	PTI1	pto-interacting protein 1	EC:2.7.11.1	1	ko04626 Plant-pathogen interaction;
gene12511	K12471	EPN	epsin	--	1	ko04144 Endocytosis;
gene12513	K14220	tRNA-Asn	tRNA Asn	--	1	ko00970 Aminoacyl-tRNA biosynthesis;
gene12514	K14219	tRNA-Arg	tRNA Arg	--	1	ko00970 Aminoacyl-tRNA biosynthesis;
gene1252	K03627	MBF1	putative transcription factor	--		
gene12530	K15336	TRDMT1, DNMT2	tRNA (cytosine38-C5)-methyltransferase	EC:2.1.1.204		
gene12531	K11718	HUGT	UDP-glucose:glycoprotein glucosyltransferase	EC:2.4.1.-	1	ko04141 Protein processing in endoplasmic reticulum;
gene12536	K13420	FLS2	LRR receptor-like serine/threonine-protein kinase FLS2	EC:2.7.11.1	1	ko04626 Plant-pathogen interaction;
gene12537	K13420	FLS2	LRR receptor-like serine/threonine-protein kinase FLS2	EC:2.7.11.1	1	ko04626 Plant-pathogen interaction;
gene12541	K14315	NDC1, TMEM48	nucleoporin NDC1	--	1	ko03013 RNA transport;
gene12542	K08486	STX1B_2_3	syntaxin 1B/2/3	--	2	ko04130 SNARE interactions in vesicular transport;ko04721 Synaptic vesicle cycle;
gene12543	K01637	E4.1.3.1, aceA	isocitrate lyase	EC:4.1.3.1	2	ko00630 Glyoxylate and dicarboxylate metabolism;ko01200 Carbon metabolism;
gene12546	K02882	RP-L18Ae, RPL18A	large subunit ribosomal protein L18Ae	--	1	ko03010 Ribosome;
gene12549	K08238	XXT	xyloglucan 6-xylosyltransferase	EC:2.4.2.39		
gene12552	K08493	VTI1	vesicle transport through interaction with t-SNAREs 1	--	1	ko04130 SNARE interactions in vesicular transport;
gene12553	K12127	TOC1, APRR1	pseudo-response regulator 1	--	1	ko04712 Circadian rhythm - plant;
gene12559	K01783	rpe, RPE	ribulose-phosphate 3-epimerase	EC:5.1.3.1	5	ko00030 Pentose phosphate pathway;ko00040 Pentose and glucuronate interconversions;ko00710 Carbon fixation in photosynthetic organisms;ko01200 Carbon metabolism;ko01230 Biosynthesis of amino acids;
gene12560	K13681	FUT	xyloglucan fucosyltransferase	EC:2.4.1.-		
gene12562	K01883	CARS, cysS	cysteinyl-tRNA synthetase	EC:6.1.1.16	1	ko00970 Aminoacyl-tRNA biosynthesis;
gene12566	K14485	TIR1	transport inhibitor response 1	--	1	ko04075 Plant hormone signal transduction;
gene12567	K14638	SLC15A3_4, PHT	solute carrier family 15 (peptide/histidine transporter), member 3/4	--		
gene12571	K02154	ATPeVI, ATP6N1A	V-type H+-transporting ATPase subunit I	EC:3.6.3.14	9	ko00190 Oxidative phosphorylation;ko04142 Lysosome;ko04145 Phagosome;ko04721 Synaptic vesicle cycle;ko04966 Collecting duct acid secretion;ko05110 Vibrio cholerae infection;ko05120 Epithelial cell signaling in Helicobacter pylori infection;ko05152 Tuberculosis;ko05323 Rheumatoid arthritis;

gene12575	K00847	E2.7.1.4, scrK	fructokinase	EC:2.7.1.4	3	ko00051 Fructose and mannose metabolism;ko00500 Starch and sucrose metabolism;ko00520 Amino sugar and nucleotide sugar metabolism;
gene12579	K12900	FUSIP1	FUS-interacting serine-arginine-rich protein 1	--	1	ko03040 Spliceosome;
gene12582	K00586	DPH5	diphthine synthase	EC:2.1.1.98		
gene12584	K06966	K06966				
gene12586	K01051	E3.1.1.11	pectinesterase	EC:3.1.1.11	2	ko00040 Pentose and glucuronate interconversions;ko00500 Starch and sucrose metabolism;
gene1258	K03165	TOP3	DNA topoisomerase III	EC:5.99.1.2	2	ko03440 Homologous recombination;ko03460 Fanconi anemia pathway;
gene12595	K04773	sppA	protease IV	EC:3.4.21.-		
gene12597	K06268	PPP3R, CNB	serine/threonine-protein phosphatase 2B regulatory subunit	--	18	ko04010 MAPK signaling pathway;ko04020 Calcium signaling pathway;ko04114 Oocyte meiosis;ko04210 Apoptosis;ko04310 Wnt signaling pathway;ko04360 Axon guidance;ko04370 VEGF signaling pathway;ko04380 Osteoclast differentiation;ko04650 Natural killer cell mediated cytotoxicity;ko04660 T cell receptor signaling pathway;ko04662 B cell receptor signaling pathway;ko04720 Long-term potentiation;ko04724 Glutamatergic synapse;ko05010 Alzheimer's disease;ko05014 Amyotrophic lateral sclerosis (ALS);ko05031 Amphetamine addiction;ko05152
gene12609	K12667	SWP1, RPN2	oligosaccharyltransferase complex subunit delta (ribophorin II)	--	3	ko00510 N-Glycan biosynthesis;ko00513 Various types of N-glycan biosynthesis;ko04141 Protein processing in endoplasmic reticulum;
gene12610	K15450	TYW3	tRNA wybutosine-synthesizing protein 3	EC:2.1.1.-		
gene12616	K02985	RP-S3e, RPS3	small subunit ribosomal protein S3e	--	1	ko03010 Ribosome;
gene12622	K00888	PI4K	phosphatidylinositol 4-kinase	EC:2.7.1.67	2	ko00562 Inositol phosphate metabolism;ko04070 Phosphatidylinositol signaling system;
gene12622	K11262	ACAC	acetyl-CoA carboxylase / biotin carboxylase	EC:6.4.1.2 6.3.4.14	4	ko00061 Fatty acid biosynthesis;ko00620 Pyruvate metabolism;ko00640 Propanoate metabolism;ko04910 Insulin signaling pathway;
gene12629	K00626	E2.3.1.9, atoB	acetyl-CoA C-acetyltransferase	EC:2.3.1.9	14	ko00071 Fatty acid degradation;ko00072 Synthesis and degradation of ketone bodies;ko00280 Valine, leucine and isoleucine degradation;ko00310 Lysine degradation;ko00362 Benzoate degradation;ko00380 Tryptophan metabolism;ko00620 Pyruvate metabolism;ko00630 Glyoxylate and dicarboxylate metabolism;ko00640 Propanoate metabolism;ko00650 Butanoate metabolism;ko00720 Carbon fixation pathways in prokaryotes;ko00900 Terpenoid backbone biosynthesis;ko01200 Carbon
gene12630	K09264	K09264	MADS-box transcription factor, plant	--		
gene12635	K10666	RNF5	E3 ubiquitin-protein ligase RNF5	EC:6.3.2.19	1	ko04141 Protein processing in endoplasmic reticulum;
gene12638	K06669	SMC3, CSPG6	structural maintenance of chromosome 3 (chondroitin sulfate proteoglycan 6)	--	4	ko04110 Cell cycle;ko04111 Cell cycle - yeast;ko04113 Meiosis - yeast;ko04114 Oocyte meiosis;
gene1263	K13606	NOL, NYC1	chlorophyll(ide) b reductase	EC:1.1.1.294	1	ko00860 Porphyrin and chlorophyll metabolism;
gene12643	K12591	RRP6, EXOSC10	exosome complex exonuclease RRP6	EC:3.1.13.-	1	ko03018 RNA degradation;
gene12644	K09527	DNAJC7	DnaJ homolog subfamily C member 7	--		
gene12645	K07152	K07152				
gene12651	K05294	PGAP1	glycosylphosphatidylinositol deacylase	EC:3.-.-.-	1	ko00563 Glycosylphosphatidylinositol(GPI)-anchor biosynthesis;
gene12653	K11584	PPP2R5	serine/threonine-protein phosphatase 2A regulatory subunit B'	--	5	ko03015 mRNA surveillance pathway;ko04113 Meiosis - yeast;ko04114 Oocyte meiosis;ko04151 PI3K-Akt signaling pathway;ko04728 Dopaminergic synapse;
gene12661	K13447	RBOH	respiratory burst oxidase	EC:1.6.3.- 1.11.1.-	1	ko04626 Plant-pathogen interaction;
gene12662	K00626	E2.3.1.9, atoB	acetyl-CoA C-acetyltransferase	EC:2.3.1.9	14	ko00071 Fatty acid degradation;ko00072 Synthesis and degradation of ketone bodies;ko00280 Valine, leucine and isoleucine degradation;ko00310 Lysine degradation;ko00362 Benzoate degradation;ko00380 Tryptophan metabolism;ko00620 Pyruvate metabolism;ko00630 Glyoxylate and dicarboxylate metabolism;ko00640 Propanoate metabolism;ko00650 Butanoate metabolism;ko00720 Carbon fixation pathways in prokaryotes;ko00900 Terpenoid backbone biosynthesis;ko01200 Carbon

gene12664	K04733	IRAK4	interleukin-1 receptor-associated kinase 4	EC:2.7.11.1	11	ko04064 NF-kappa B signaling pathway;ko04210 Apoptosis;ko04620 Toll-like receptor signaling pathway;ko04722 Neurotrophin signaling pathway;ko05133 Pertussis;ko05140 Leishmaniasis;ko05142 Chagas disease (American trypanosomiasis);ko05145 Toxoplasmosis;ko05152 Tuberculosis;ko05162 Measles;ko05164 Influenza A;
gene12667	K12670	WBP1	oligosaccharyltransferase complex subunit beta	--	3	ko00510 N-Glycan biosynthesis;ko00513 Various types of N-glycan biosynthesis;ko04141 Protein processing in endoplasmic reticulum;
gene1266	K13459	RPS2	disease resistance protein RPS2	--	1	ko04626 Plant-pathogen interaction;
gene12674	K01051	E3.1.1.11	pectinesterase	EC:3.1.1.11	2	ko00040 Pentose and glucuronate interconversions;ko00500 Starch and sucrose metabolism;
gene12676	K09286	EREBP	EREBP-like factor	--		
gene1267	K13459	RPS2	disease resistance protein RPS2	--	1	ko04626 Plant-pathogen interaction;
gene12681	K07937	ARF1	ADP-ribosylation factor 1	--	2	ko05110 Vibrio cholerae infection;ko05134 Legionellosis;
gene12687	K01870	IARS, ileS	isoleucyl-tRNA synthetase	EC:6.1.1.5	1	ko00970 Aminoacyl-tRNA biosynthesis;
gene126	K01982	LSUrRNA	large subunit ribosomal RNA	--	2	ko03008 Ribosome biogenesis in eukaryotes;ko03010 Ribosome;
gene12701	K12850	PRPF38B	pre-mRNA-splicing factor 38B	--	1	ko03040 Spliceosome;
gene12703	K17637	EXOC2, SEC5	exocyst complex component 2	--	1	ko04014 Ras signaling pathway;
gene12704	K03841	FBP, fbp	fructose-1,6-bisphosphatase I	EC:3.1.3.11	7	ko00010 Glycolysis / Gluconeogenesis;ko00030 Pentose phosphate pathway;ko00051 Fructose and mannose metabolism;ko00680 Methane metabolism;ko00710 Carbon fixation in photosynthetic organisms;ko01200 Carbon metabolism;ko04910 Insulin signaling pathway;
gene12705	K16297	SCPL-II	serine carboxypeptidase-like clade II	EC:3.4.16.-		
gene12709	K12859	TXNL4A, DIB1	U5 snRNP protein, DIM1 family	--	1	ko03040 Spliceosome;
gene1270	K01051	E3.1.1.11	pectinesterase	EC:3.1.1.11	2	ko00040 Pentose and glucuronate interconversions;ko00500 Starch and sucrose metabolism;
gene12711	K01082	E3.1.3.7, cysQ, MET22, BPNT1	3'(2'), 5'-bisphosphate nucleotidase	EC:3.1.3.7	1	ko00920 Sulfur metabolism;
gene12713	K03062	PSMC1, RPT2	26S proteasome regulatory subunit T2	--	3	ko03050 Proteasome;ko05169 Epstein-Barr virus infection;ko05203 Viral carcinogenesis;
gene12717	K15777	DOPA	4,5-DOPA dioxygenase extradiol	EC:1.13.11.-	1	ko00965 Betalain biosynthesis;
gene1271	K06268	PPP3R, CNB	serine/threonine-protein phosphatase 2B regulatory subunit	--	18	ko04010 MAPK signaling pathway;ko04020 Calcium signaling pathway;ko04114 Oocyte meiosis;ko04210 Apoptosis;ko04310 Wnt signaling pathway;ko04360 Axon guidance;ko04370 VEGF signaling pathway;ko04380 Osteoclast differentiation;ko04650 Natural killer cell mediated cytotoxicity;ko04660 T cell receptor signaling pathway;ko04662 B cell receptor signaling pathway;ko04720 Long-term potentiation;ko04724 Glutamatergic synapse;ko05010 Alzheimer's disease;ko05014 Amyotrophic lateral sclerosis (ALS);ko05031 Amphetamine addiction;ko05152
gene12720	K00814	GPT, ALT	alanine transaminase	EC:2.6.1.2	4	ko00250 Alanine, aspartate and glutamate metabolism;ko00710 Carbon fixation in photosynthetic organisms;ko01210 2-Oxocarboxylic acid metabolism;ko01230 Biosynthesis of amino acids;
gene12725	K12603	CNOT6, CCR4	CCR4-NOT transcription complex subunit 6	--	1	ko03018 RNA degradation;
gene12734	K10398	KIF11, EG5	kinesin family member 11	--		
gene12738	K03943	NDUFV2	NADH dehydrogenase (ubiquinone) flavoprotein 2	EC:1.6.5.3 1.6.99.3	5	ko00190 Oxidative phosphorylation;ko04932 Non-alcoholic fatty liver disease (NAFLD);ko05010 Alzheimer's disease;ko05012 Parkinson's disease;ko05016 Huntington's disease;
gene12742	K08235	E2.4.1.207	xyloglucan:xyloglucosyl transferase	EC:2.4.1.207		
gene12743	K08235	E2.4.1.207	xyloglucan:xyloglucosyl transferase	EC:2.4.1.207		
gene12745	K08235	E2.4.1.207	xyloglucan:xyloglucosyl transferase	EC:2.4.1.207		
gene12749	K04733	IRAK4	interleukin-1 receptor-associated kinase 4	EC:2.7.11.1	11	ko04064 NF-kappa B signaling pathway;ko04210 Apoptosis;ko04620 Toll-like receptor signaling pathway;ko04722 Neurotrophin signaling pathway;ko05133 Pertussis;ko05140 Leishmaniasis;ko05142 Chagas disease (American trypanosomiasis);ko05145 Toxoplasmosis;ko05152 Tuberculosis;ko05162 Measles;ko05164 Influenza A;
gene12751	K00888	PI4K	phosphatidylinositol 4-kinase	EC:2.7.1.67	2	ko00562 Inositol phosphate metabolism;ko04070 Phosphatidylinositol signaling system;

gene12754	K13157	RNPC3	U11/U12 small nuclear ribonucleoprotein 65 kDa protein	--		
gene12755	K06130	LYPLA2	lysophospholipase II	EC:3.1.1.5	1	ko00564 Glycerophospholipid metabolism;
gene12756	K09338	HD-ZIP	homeobox-leucine zipper protein	--		
gene12758	K14662	NTAN1	protein N-terminal asparagine amidohydrolase	EC:3.5.1.-		
gene12766	K00895	E2.7.1.90, pfk	pyrophosphate--fructose-6-phosphate 1-phosphotransferase	EC:2.7.1.90	1	ko00051 Fructose and mannose metabolism;
gene12773	K02873	RP-L13e, RPL13	large subunit ribosomal protein L13e	--	1	ko03010 Ribosome;
gene12785	K15275	SLC35B1	solute carrier family 35 (UDP-galactose transporter), member B1	--		
gene12787	K01942	HLCS	biotin--protein ligase	EC:6.3.4.9 6.3.4.10 6.3.4.11 6.3.4.15	1	ko00780 Biotin metabolism;
gene12791	K08866	TTK, MPS1	serine/threonine-protein kinase TTK/MPS1	EC:2.7.12.1	2	ko04110 Cell cycle;ko04111 Cell cycle - yeast;
gene1279	K16284	SIS3	E3 ubiquitin-protein ligase SIS3	EC:6.3.2.19		
gene12802	K11979	UBR7	E3 ubiquitin-protein ligase UBR7	EC:6.3.2.19		
gene12804	K03884	ND6	NADH-ubiquinone oxidoreductase chain 6	EC:1.6.5.3	2	ko00190 Oxidative phosphorylation;ko05012 Parkinson's disease;
gene12805	K02986	RP-S4, rpsD	small subunit ribosomal protein S4	--	1	ko03010 Ribosome;
gene12806	K00591	COQ3	hexaprenyldihydroxybenzoate methyltransferase	EC:2.1.1.114	1	ko00130 Ubiquinone and other terpenoid-quinone biosynthesis;
gene12808	K09377	CSRP	cysteine and glycine-rich protein	--		
gene12811	K14497	PP2C	protein phosphatase 2C	EC:3.1.3.16	1	ko04075 Plant hormone signal transduction;
gene12813	K01056	PTH1, pth, spoVC	peptidyl-tRNA hydrolase, PTH1 family	EC:3.1.1.29		
gene12816	K09873	TIP	aquaporin TIP	--		
gene12819	K13606	NOL, NYC1	chlorophyll(ide) b reductase	EC:1.1.1.294	1	ko00860 Porphyrin and chlorophyll metabolism;
gene1281	K01874	MARS, metG	methionyl-tRNA synthetase	EC:6.1.1.10	2	ko00450 Selenocompound metabolism;ko00970 Aminoacyl-tRNA biosynthesis;
gene12823	K01580	E4.1.1.15, gadB, gadA, GAD	glutamate decarboxylase	EC:4.1.1.15	6	ko00250 Alanine, aspartate and glutamate metabolism;ko00410 beta-Alanine metabolism;ko00430 Taurine and hypotaurine metabolism;ko00650 Butanoate metabolism;ko04727 GABAergic synapse;ko04940 Type I diabetes mellitus;
gene12824	K01810	GPI, pgi	glucose-6-phosphate isomerase	EC:5.3.1.9	5	ko00010 Glycolysis / Gluconeogenesis;ko00030 Pentose phosphate pathway;ko00500 Starch and sucrose metabolism;ko00520 Amino sugar and nucleotide sugar metabolism;ko01200 Carbon metabolism;
gene12826	K07466	RFA1, RPA1, rpa	replication factor A1	--	5	ko03030 DNA replication;ko03420 Nucleotide excision repair;ko03430 Mismatch repair;ko03440 Homologous recombination;ko03460 Fanconi anemia pathway;
gene12828	K00815	TAT	tyrosine aminotransferase	EC:2.6.1.5	9	ko00130 Ubiquinone and other terpenoid-quinone biosynthesis;ko00270 Cysteine and methionine metabolism;ko00350 Tyrosine metabolism;ko00360 Phenylalanine metabolism;ko00400 Phenylalanine, tyrosine and tryptophan biosynthesis;ko00401 Novobiocin biosynthesis;ko00950 Isoquinoline alkaloid biosynthesis;ko00960 Tropane, piperidine and pyridine alkaloid biosynthesis;ko01230 Biosynthesis of amino acids;
gene12830	K00815	TAT	tyrosine aminotransferase	EC:2.6.1.5	9	ko00130 Ubiquinone and other terpenoid-quinone biosynthesis;ko00270 Cysteine and methionine metabolism;ko00350 Tyrosine metabolism;ko00360 Phenylalanine metabolism;ko00400 Phenylalanine, tyrosine and tryptophan biosynthesis;ko00401 Novobiocin biosynthesis;ko00950 Isoquinoline alkaloid biosynthesis;ko00960 Tropane, piperidine and pyridine alkaloid biosynthesis;ko01230 Biosynthesis of amino acids;
gene12831	K07910	RAB18	Ras-related protein Rab-18	--		
gene12834	K15472	CYP71D55	premnaspirodiene oxygenase	EC:1.14.13.121	1	ko00909 Sesquiterpenoid and triterpenoid biosynthesis;
gene12835	K13356	FAR	fatty acyl-CoA reductase	EC:1.2.1.-	2	ko00073 Cutin, suberine and wax biosynthesis;ko04146 Peroxisome;
gene12844	K14835	NOP2	ribosomal RNA methyltransferase Nop2	EC:2.1.1.-		
gene12845	K12817	PRPF18, PRP18	pre-mRNA-splicing factor 18	--	1	ko03040 Spliceosome;
gene12851	K11498	CENPE	centromeric protein E	--		

gene12856	K08150	SLC2A13, ITR	MFS transporter, SP family, solute carrier family 2 (myo-inositol transporter), member 13	--		
gene12857	K17413	MRPS35	small subunit ribosomal protein S35, mitochondrial	--		
gene12858	K17413	MRPS35	small subunit ribosomal protein S35, mitochondrial	--		
gene12861	K09873	TIP	aquaporin TIP	--		
gene12864	K16055	TPS	trehalose 6-phosphate synthase/phosphatase	EC:2.4.1.15 3.1.3.12	1	ko00500 Starch and sucrose metabolism;
gene12866	K01792	E5.1.3.15	glucose-6-phosphate 1-epimerase	EC:5.1.3.15	1	ko00010 Glycolysis / Gluconeogenesis;
gene12867	K11600	RRP41, EXOSC4, SKI6	exosome complex component RRP41	--	1	ko03018 RNA degradation;
gene12871	K10389	TUBG	tubulin gamma	--		
gene12873	K15472	CYP71D55	premnaspirodiene oxygenase	EC:1.14.13.12 1	1	ko00909 Sesquiterpenoid and triterpenoid biosynthesis;
gene12874	K06066	CIR	CBF1 interacting corepressor	--	1	ko04330 Notch signaling pathway;
gene12876	K09286	EREBP	EREBP-like factor	--		
gene12880	K15281	SLC35D	solute carrier family 35	--		
gene12886	K08235	E2.4.1.207	xyloglucan:xyloglucosyl transferase	EC:2.4.1.207		
gene12900	K04120	E5.5.1.13	ent-copalyl diphosphate synthase	EC:5.5.1.13	1	ko00904 Diterpenoid biosynthesis;
gene12902	K15601	KDM3	lysine-specific demethylase 3	EC:1.14.11.-		
gene12903	K15601	KDM3	lysine-specific demethylase 3	EC:1.14.11.-		
gene1290	K17616	CTDSPL2	CTD small phosphatase-like protein 2	EC:3.1.3.-		
gene12913	K12120	PHYA	phytochrome A	--	1	ko04712 Circadian rhythm - plant;
gene12917	K05277	E1.14.11.19	leucoanthocyanidin dioxygenase	EC:1.14.11.19	1	ko00941 Flavonoid biosynthesis;
gene12918	K15919	HPR2	hydroxypyruvate reductase 2	--	2	ko00260 Glycine, serine and threonine metabolism;ko00630 Glyoxylate and dicarboxylate metabolism;
gene1291	K02154	ATPeVI, ATP6N1A	V-type H+-transporting ATPase subunit I	EC:3.6.3.14	9	ko00190 Oxidative phosphorylation;ko04142 Lysosome;ko04145 Phagosome;ko04721 Synaptic vesicle cycle;ko04966 Collecting duct acid secretion;ko05110 Vibrio cholerae infection;ko05120 Epithelial cell signaling in Helicobacter pylori infection;ko05152 Tuberculosis;ko05323 Rheumatoid arthritis;
gene12920	K14168	CTU1, NCS6	cytoplasmic tRNA 2-thiolation protein 1	EC:2.7.7.-	1	ko04122 Sulfur relay system;
gene12921	K13101	GPKOW	G patch domain and KOW motifs-containing protein	--		
gene12925	K14431	TGA	transcription factor TGA	--	1	ko04075 Plant hormone signal transduction;
gene12926	K00895	E2.7.1.90, pfk	pyrophosphate--fructose-6-phosphate 1-phosphotransferase	EC:2.7.1.90	1	ko00051 Fructose and mannose metabolism;
gene12927	K14232	tRNA-Pro	tRNA Pro	--	1	ko00970 Aminoacyl-tRNA biosynthesis;
gene12929	K14232	tRNA-Pro	tRNA Pro	--	1	ko00970 Aminoacyl-tRNA biosynthesis;
gene12930	K14232	tRNA-Pro	tRNA Pro	--	1	ko00970 Aminoacyl-tRNA biosynthesis;
gene12931	K13448	CML	calcium-binding protein CML	--	1	ko04626 Plant-pathogen interaction;
gene12933	K11262	ACAC	acetyl-CoA carboxylase / biotin carboxylase	EC:6.4.1.2 6.3.4.14	4	ko00061 Fatty acid biosynthesis;ko00620 Pyruvate metabolism;ko00640 Propanoate metabolism;ko04910 Insulin signaling pathway;
gene12937	K00430	E1.11.1.7	peroxidase	EC:1.11.1.7	2	ko00360 Phenylalanine metabolism;ko00940 Phenylpropanoid biosynthesis;
gene12938	K10880	XRCC3	DNA-repair protein XRCC3	--	1	ko03440 Homologous recombination;
gene12939	K10575	UBE2G1, UBC7	ubiquitin-conjugating enzyme E2 G1	EC:6.3.2.19	3	ko04120 Ubiquitin mediated proteolysis;ko04141 Protein processing in endoplasmic reticulum;ko05012 Parkinson's disease;
gene1293	K14972	PAXIP1, PTIP	PAX-interacting protein 1	--		
gene12948	K01648	ACLY	ATP citrate (pro-S)-lyase	EC:2.3.3.8	2	ko00020 Citrate cycle (TCA cycle);ko00720 Carbon fixation pathways in prokaryotes;
gene1294	K00791	miaA, TRIT1	tRNA dimethylallyltransferase	EC:2.5.1.75	1	ko00908 Zeatin biosynthesis;
gene12950	K05349	bglX	beta-glucosidase	EC:3.2.1.21	3	ko00460 Cyanoamino acid metabolism;ko00500 Starch and sucrose metabolism;ko00940 Phenylpropanoid biosynthesis;
gene12953	K08869	ADCK, ABC1	aarF domain-containing kinase	--		
gene12954	K03037	PSMD6, RPN7	26S proteasome regulatory subunit N7	--	2	ko03050 Proteasome;ko05169 Epstein-Barr virus infection;
gene12955	K14509	ETR, ERS	ethylene receptor	EC:2.7.13.-	1	ko04075 Plant hormone signal transduction;
gene12957	K09553	STIP1	stress-induced-phosphoprotein 1	--	1	ko05020 Prion diseases;
gene12959	K14488	SAUR	SAUR family protein	--	1	ko04075 Plant hormone signal transduction;
gene12962	K09013	sufC	Fe-S cluster assembly ATP-binding protein	--		

gene12969	K02258	COX11	cytochrome c oxidase assembly protein subunit 11	--	1	ko00190 Oxidative phosphorylation;
gene12974	K13448	CML	calcium-binding protein CML	--	1	ko04626 Plant-pathogen interaction;
gene12978	K00799	GST, gst	glutathione S-transferase	EC:2.5.1.18	4	ko00480 Glutathione metabolism;ko00980 Metabolism of xenobiotics by cytochrome P450;ko00982 Drug metabolism - cytochrome P450;ko05204 Chemical carcinogenesis;
gene1297	K15803	GERD	(-)-germacrene D synthase	EC:4.2.3.22 4.2.3.75	1	ko00909 Sesquiterpenoid and triterpenoid biosynthesis;
gene12982	K06620	E2F3	transcription factor E2F3	--	13	ko04110 Cell cycle;ko05161 Hepatitis B;ko05166 HTLV-I infection;ko05200 Pathways in cancer;ko05206 MicroRNAs in cancer;ko05212 Pancreatic cancer;ko05214 Glioma;ko05215 Prostate cancer;ko05218 Melanoma;ko05219 Bladder cancer;ko05220 Chronic myeloid leukemia;ko05222 Small cell lung cancer;ko05223 Non-small cell lung cancer;
gene12985	K09060	GBF	plant G-box-binding factor	--		
gene12986	K08908	LHCA2	light-harvesting complex I chlorophyll a/b binding protein 2	--	1	ko00196 Photosynthesis - antenna proteins;
gene1298	K01051	E3.1.1.11	pectinesterase	EC:3.1.1.11	2	ko00040 Pentose and glucuronate interconversions;ko00500 Starch and sucrose metabolism;
gene12999	K01102	PDP	pyruvate dehydrogenase phosphatase	EC:3.1.3.43		
gene129	K06966	K06966				
gene13000	K15026	EIF2A	translation initiation factor 2A	--		
gene13006	K03787	surE	5'-nucleotidase	EC:3.1.3.5	3	ko00230 Purine metabolism;ko00240 Pyrimidine metabolism;ko00760 Nicotinate and nicotinamide metabolism;
gene13007	K09422	MYBP	myb proto-oncogene protein, plant	--		
gene1300	K12669	OST3, OST6	oligosaccharyltransferase complex subunit gamma	--	3	ko00510 N-Glycan biosynthesis;ko00513 Various types of N-glycan biosynthesis;ko04141 Protein processing in endoplasmic reticulum;
gene13010	K16900	TPC1, CCH1	two pore calcium channel protein, plant	--		
gene13013	K00963	UGP2, galU, galF	UTP--glucose-1-phosphate uridylyltransferase	EC:2.7.7.9	4	ko00040 Pentose and glucuronate interconversions;ko00052 Galactose metabolism;ko00500 Starch and sucrose metabolism;ko00520 Amino sugar and nucleotide sugar metabolism;
gene13017	K09264	K09264	MADS-box transcription factor, plant	--		
gene13018	K13648	GAUT	alpha-1,4-galacturonosyltransferase	EC:2.4.1.43	2	ko00500 Starch and sucrose metabolism;ko00520 Amino sugar and nucleotide sugar metabolism;
gene13020	K03232	EEF1B	elongation factor 1-beta	--		
gene13021	K03294	TC.APA	basic amino acid/polyamine antiporter, APA family	--		
gene13023	K14165	K14165	dual specificity phosphatase	EC:3.1.3.16 3.1.3.48		
gene13027	K05305	FUK	fucokinase	EC:2.7.1.52	2	ko00051 Fructose and mannose metabolism;ko00520 Amino sugar and nucleotide sugar metabolism;
gene1302	K08235	E2.4.1.207	xyloglucan:xyloglucosyl transferase	EC:2.4.1.207		
gene13030	K05857	PLCD	phosphatidylinositol phospholipase C, delta	EC:3.1.4.11	3	ko00562 Inositol phosphate metabolism;ko04020 Calcium signaling pathway;ko04070 Phosphatidylinositol signaling system;
gene13035	K00703	E2.4.1.21, glgA	starch synthase	EC:2.4.1.21	1	ko00500 Starch and sucrose metabolism;
gene13037	K02213	CDC6	cell division control protein 6	--	3	ko04110 Cell cycle;ko04111 Cell cycle - yeast;ko04113 Meiosis - yeast;
gene13038	K07437	CYP26A	cytochrome P450, family 26, subfamily A	--	1	ko00830 Retinol metabolism;
gene13040	K00166	E1.2.4.4A, bkdA1	2-oxoisovalerate dehydrogenase E1 component, alpha subunit	EC:1.2.4.4	1	ko00280 Valine, leucine and isoleucine degradation;
gene13041	K00166	E1.2.4.4A, bkdA1	2-oxoisovalerate dehydrogenase E1 component, alpha subunit	EC:1.2.4.4	1	ko00280 Valine, leucine and isoleucine degradation;
gene13043	K17535	TNNI3K	serine/threonine-protein kinase TNNI3K	EC:2.7.11.1		
gene13044	K02960	RP-S16e, RPS16	small subunit ribosomal protein S16e	--	1	ko03010 Ribosome;
gene13048	K01213	E3.2.1.67	galacturan 1,4-alpha-galacturonidase	EC:3.2.1.67	2	ko00040 Pentose and glucuronate interconversions;ko00500 Starch and sucrose metabolism;
gene1304	K04077	groEL, HSPD1	chaperonin GroEL	--	4	ko03018 RNA degradation;ko04940 Type I diabetes mellitus;ko05134 Legionellosis;ko05152 Tuberculosis;
gene13051	K14317	NUP214, CAN	nuclear pore complex protein Nup214	--	2	ko03013 RNA transport;ko05169 Epstein-Barr virus infection;
gene13065	K00771	XYLT	protein xylosyltransferase	EC:2.4.2.26	2	ko00532 Glycosaminoglycan biosynthesis - chondroitin sulfate / dermatan sulfate;ko00534 Glycosaminoglycan biosynthesis - heparan sulfate / heparin;

gene13073	K03841	FBP, fbp	fructose-1,6-bisphosphatase I	EC:3.1.3.11	7	ko00010 Glycolysis / Gluconeogenesis;ko00030 Pentose phosphate pathway;ko00051 Fructose and mannose metabolism;ko00680 Methane metabolism;ko00710 Carbon fixation in photosynthetic organisms;ko01200 Carbon metabolism;ko04910 Insulin signaling pathway;
gene13074	K12854	SNRNP200, BRR2	pre-mRNA-splicing helicase BRR2	EC:3.6.4.13	1	ko03040 Spliceosome;
gene13075	K16904	DCTPP1	dCTP diphosphatase	EC:3.6.1.12	1	ko00240 Pyrimidine metabolism;
gene13076	K00611	OTC, argF, argI	ornithine carbamoyltransferase	EC:2.1.3.3	2	ko00330 Arginine and proline metabolism;ko01230 Biosynthesis of amino acids;
gene13078	K02725	PSMA1	20S proteasome subunit alpha 6	EC:3.4.25.1	1	ko03050 Proteasome;
gene13079	K14220	tRNA-Asn	tRNA Asn	--	1	ko00970 Aminoacyl-tRNA biosynthesis;
gene1307	K14230	tRNA-Met	tRNA Met	--	1	ko00970 Aminoacyl-tRNA biosynthesis;
gene13083	K12657	ALDH18A1, P5CS	delta-1-pyrroline-5-carboxylate synthetase	EC:2.7.2.11 1.2.1.41	2	ko00330 Arginine and proline metabolism;ko01230 Biosynthesis of amino acids;
gene13084	K14292	TGS1	trimethylguanosine synthase	EC:2.1.1.-	1	ko03013 RNA transport;
gene13086	K09338	HD-ZIP	homeobox-leucine zipper protein	--		
gene13087	K03012	RPB4, POLR2D	DNA-directed RNA polymerase II subunit RPB4	--	5	ko00230 Purine metabolism;ko00240 Pyrimidine metabolism;ko03020 RNA polymerase;ko05016 Huntington's disease;ko05169 Epstein-Barr virus infection;
gene13088	K14509	ETR, ERS	ethylene receptor	EC:2.7.13.-	1	ko04075 Plant hormone signal transduction;
gene13092	K00565	RNMT	mRNA (guanine-N7-)-methyltransferase	EC:2.1.1.56	1	ko03015 mRNA surveillance pathway;
gene13097	K04564	SOD2	superoxide dismutase, Fe-Mn family	EC:1.15.1.1	2	ko04146 Peroxisome;ko05016 Huntington's disease;
gene1309	K14638	SLC15A3_4, PHT	solute carrier family 15 (peptide/histidine transporter), member 3/4	--		
gene130	K10843	ERCC3, XPB	DNA excision repair protein ERCC-3	EC:3.6.4.12	2	ko03022 Basal transcription factors;ko03420 Nucleotide excision repair;
gene13111	K08678	UXS1	UDP-glucuronate decarboxylase	EC:4.1.1.35	2	ko00500 Starch and sucrose metabolism;ko00520 Amino sugar and nucleotide sugar metabolism;
gene13112	K11254	H4	histone H4	--	3	ko05034 Alcoholism;ko05203 Viral carcinogenesis;ko05322 Systemic lupus erythematosus;
gene13115	K12881	THOC4, ALY	THO complex subunit 4	--	4	ko03013 RNA transport;ko03015 mRNA surveillance pathway;ko03040 Spliceosome;ko05168 Herpes simplex infection;
gene13116	K05305	FUK	fucokinase	EC:2.7.1.52	2	ko00051 Fructose and mannose metabolism;ko00520 Amino sugar and nucleotide sugar metabolism;
gene13117	K11252	H2B	histone H2B	--	3	ko05034 Alcoholism;ko05203 Viral carcinogenesis;ko05322 Systemic lupus erythematosus;
gene13119	K05765	CFL	cofilin	--	4	ko04360 Axon guidance;ko04666 Fc gamma R-mediated phagocytosis;ko04810 Regulation of actin cytoskeleton;ko05133 Pertussis;
gene13120	K11251	H2A	histone H2A	--	2	ko05034 Alcoholism;ko05322 Systemic lupus erythematosus;
gene13121	K13195	CIRBP	cold-inducible RNA-binding protein	--		
gene13123	K02949	RP-S11e, RPS11	small subunit ribosomal protein S11e	--	1	ko03010 Ribosome;
gene13125	K10950	ERO1L	ERO1-like protein alpha	EC:1.8.4.-	2	ko04141 Protein processing in endoplasmic reticulum;ko05110 Vibrio cholerae infection;
gene13126	K01881	PARS, proS	prolyl-tRNA synthetase	EC:6.1.1.15	1	ko00970 Aminoacyl-tRNA biosynthesis;
gene13134	K14570	REX1, REXO1, RNH70	RNA exonuclease 1	EC:3.1.-.-	1	ko03008 Ribosome biogenesis in eukaryotes;
gene13136	K01897	ACSL, fadD	long-chain acyl-CoA synthetase	EC:6.2.1.3	4	ko00071 Fatty acid degradation;ko03320 PPAR signaling pathway;ko04146 Peroxisome;ko04920 Adipocytokine signaling pathway;
gene13138	K01945	purD	phosphoribosylamine--glycine ligase	EC:6.3.4.13	1	ko00230 Purine metabolism;
gene13139	K01723	AOS	hydroperoxide dehydratase	EC:4.2.1.92	1	ko00592 alpha-Linolenic acid metabolism;
gene13145	K01184	E3.2.1.15	polygalacturonase	EC:3.2.1.15	2	ko00040 Pentose and glucuronate interconversions;ko00500 Starch and sucrose metabolism;
gene13146	K02940	RP-L9e, RPL9	large subunit ribosomal protein L9e	--	1	ko03010 Ribosome;
gene13147	K08235	E2.4.1.207	xyloglucan:xyloglucosyl transferase	EC:2.4.1.207		
gene13151	K02897	RP-L25, rplY	large subunit ribosomal protein L25	--	1	ko03010 Ribosome;
gene13152	K00860	cysC	adenylylsulfate kinase	EC:2.7.1.25	2	ko00230 Purine metabolism;ko00920 Sulfur metabolism;
gene13155	K03501	gidB, rsmG	16S rRNA (guanine527-N7)-methyltransferase	EC:2.1.1.170		
gene13157	K11126	TERT, EST2	telomerase reverse transcriptase	EC:2.7.7.49	1	ko05166 HTLV-I infection;
gene1315	K00734	B3GALT6	galactosylxylosylprotein 3-beta-galactosyltransferase	EC:2.4.1.134	2	ko00532 Glycosaminoglycan biosynthesis - chondroitin sulfate / dermatan sulfate;ko00534 Glycosaminoglycan biosynthesis - heparan sulfate / heparin;

gene13160	K03126	TAF12	transcription initiation factor TFIID subunit 12	--	1	ko03022 Basal transcription factors;
gene13161	K12891	SFRS2	splicing factor, arginine/serine-rich 2	--	2	ko03040 Spliceosome;ko05168 Herpes simplex infection;
gene13163	K10683	BARD1	BRCA1-associated RING domain protein 1	EC:6.3.2.19		
gene13164	K14638	SLC15A3_4, PHT	solute carrier family 15 (peptide/histidine transporter), member 3/4	--		
gene13166	K14832	MAK21, NOC1, CEBPZ	ribosome biogenesis protein MAK21	--		
gene13173	K01513	ENPP1_3	ectonucleotide pyrophosphatase/phosphodiesterase family member 1/3	EC:3.1.4.1 3.6.1.9	5	ko00230 Purine metabolism;ko00500 Starch and sucrose metabolism;ko00740 Riboflavin metabolism;ko00760 Nicotinate and nicotinamide metabolism;ko00770 Pantothenate and CoA biosynthesis;
gene13174	K01513	ENPP1_3	ectonucleotide pyrophosphatase/phosphodiesterase family member 1/3	EC:3.1.4.1 3.6.1.9	5	ko00230 Purine metabolism;ko00500 Starch and sucrose metabolism;ko00740 Riboflavin metabolism;ko00760 Nicotinate and nicotinamide metabolism;ko00770 Pantothenate and CoA biosynthesis;
gene13178	K00844	HK	hexokinase	EC:2.7.1.1	12	ko00010 Glycolysis / Gluconeogenesis;ko00051 Fructose and mannose metabolism;ko00052 Galactose metabolism;ko00500 Starch and sucrose metabolism;ko00520 Amino sugar and nucleotide sugar metabolism;ko00521 Streptomycin biosynthesis;ko00524 Butirosin and neomycin biosynthesis;ko01200 Carbon metabolism;ko04066 HIF-1 signaling pathway;ko04910 Insulin signaling pathway;ko04930 Type II diabetes mellitus;ko04973 Carbohydrate digestion and absorption;
gene13179	K05391	CNGF	cyclic nucleotide gated channel, other eukaryote	--	1	ko04626 Plant-pathogen interaction;
gene13181	K05391	CNGF	cyclic nucleotide gated channel, other eukaryote	--	1	ko04626 Plant-pathogen interaction;
gene13182	K09422	MYBP	myb proto-oncogene protein, plant	--		
gene13183	K14498	SNRK2	serine/threonine-protein kinase SRK2	EC:2.7.11.1	1	ko04075 Plant hormone signal transduction;
gene13189	K03106	SRP54, ffh	signal recognition particle subunit SRP54	--	2	ko03060 Protein export;ko03070 Bacterial secretion system;
gene1318	K12188	SNF8, EAP30	ESCRT-II complex subunit VPS22	--	1	ko04144 Endocytosis;
gene13200	K03676	grxC, GLRX, GLRX2	glutaredoxin 3	--		
gene13203	K15109	SLC25A20_29, CACT, CACL, CRC1	solute carrier family 25 (mitochondrial carnitine/acylcarnitine transporter), member 20/29	--		
gene13204	K06215	pdxS, pdx1	pyridoxine biosynthesis protein	EC:4.-.-.-	1	ko00750 Vitamin B6 metabolism;
gene13208	K05391	CNGF	cyclic nucleotide gated channel, other eukaryote	--	1	ko04626 Plant-pathogen interaction;
gene13215	K09569	FKBP2	FK506-binding protein 2	EC:5.2.1.8		
gene13218	K09571	FKBP4_5	FK506-binding protein 4/5	EC:5.2.1.8	1	ko04915 Estrogen signaling pathway;
gene13224	K02133	ATPeF1B, ATP5B	F-type H+-transporting ATPase subunit beta	EC:3.6.3.14	4	ko00190 Oxidative phosphorylation;ko05010 Alzheimer's disease;ko05012 Parkinson's disease;ko05016 Huntington's disease;
gene13227	K15153	MED31, SOH1	mediator of RNA polymerase II transcription subunit 31	--		
gene13229	K11593	ELF2C	eukaryotic translation initiation factor 2C	--		
gene13231	K09286	EREBP	EREBP-like factor	--		
gene13232	K06674	SMC2	structural maintenance of chromosome 2	--	1	ko04111 Cell cycle - yeast;
gene13233	K13427	NOA1	nitric-oxide synthase, plant	EC:1.14.13.39	2	ko00330 Arginine and proline metabolism;ko04626 Plant-pathogen interaction;
gene13237	K02879	RP-L17, MRPL17, rplQ	large subunit ribosomal protein L17	--	1	ko03010 Ribosome;
gene13253	K09422	MYBP	myb proto-oncogene protein, plant	--		
gene13258	K03319	TC.DASS	divalent anion:Na+ symporter, DASS family	--		
gene13259	K10738	MCM9	DNA helicase MCM9	EC:3.6.4.12		
gene13263	K02548	menA	1,4-dihydroxy-2-naphthoate octaprenyltransferase	EC:2.5.1.74	1	ko00130 Ubiquinone and other terpenoid-quinone biosynthesis;
gene13267	K03364	CDH1	cell division cycle 20-like protein 1, cofactor of APC complex	--	4	ko04110 Cell cycle;ko04111 Cell cycle - yeast;ko04120 Ubiquitin mediated proteolysis;ko04914 Progesterone-mediated oocyte maturation;
gene13272	K10643	CNOT4, NOT4, MOT2	CCR4-NOT transcription complex subunit 4	EC:6.3.2.19	1	ko03018 RNA degradation;
gene13273	K09284	AP2	AP2-like factor, euAP2 lineage	--		
gene13278	K15326	TSEN54	tRNA-splicing endonuclease subunit Sen54	--		
gene13282	K12885	RBMX, HNRNPG	heterogeneous nuclear ribonucleoprotein G	--	1	ko03040 Spliceosome;
gene13289	K00434	E1.11.1.11	L-ascorbate peroxidase	EC:1.11.1.11	2	ko00053 Ascorbate and aldarate metabolism;ko00480 Glutathione metabolism;
gene1328	K01551	arsA, ASNA1	arsenite-transporting ATPase	EC:3.6.3.16		

gene13290	K09422	MYBP	myb proto-oncogene protein, plant	--		
gene13293	K02934	RP-L6e, RPL6	large subunit ribosomal protein L6e	--	1	ko03010 Ribosome;
gene13294	K00601	E2.1.2.2	phosphoribosylglycinamide formyltransferase	EC:2.1.2.2	2	ko00230 Purine metabolism;ko00670 One carbon pool by folate;
gene13306	K12867	SYF1, XAB2	pre-mRNA-splicing factor SYF1	--	1	ko03040 Spliceosome;
gene13307	K12356	UGT72E	coniferyl-alcohol glucosyltransferase	EC:2.4.1.111	1	ko00940 Phenylpropanoid biosynthesis;
gene13311	K13116	DDX41, ABS	ATP-dependent RNA helicase DDX41	EC:3.6.4.13		
gene13313	K15083	RAD16	DNA repair protein RAD16	--		
gene13317	K03252	EIF3C	translation initiation factor 3 subunit C	--	1	ko03013 RNA transport;
gene13320	K14487	GH3	auxin responsive GH3 gene family	--	1	ko04075 Plant hormone signal transduction;
gene1332	K01100	E3.1.3.37	sedoheptulose-bisphosphatase	EC:3.1.3.37	2	ko00710 Carbon fixation in photosynthetic organisms;ko01200 Carbon metabolism;
gene13341	K10903	HUS1	HUS1 checkpoint protein	--		
gene13342	K17604	ZSWIM3	zinc finger SWIM domain-containing protein 3	--		
gene13343	K13946	AUX1, LAX	auxin influx carrier (AUX1 LAX family)	--	1	ko04075 Plant hormone signal transduction;
gene13344	K00658	DLST, sucB	2-oxoglutarate dehydrogenase E2 component (dihydrolipoamide succinyltransferase)	EC:2.3.1.61	3	ko00020 Citrate cycle (TCA cycle);ko00310 Lysine degradation;ko01200 Carbon metabolism;
gene13346	K09680	coaW	type II pantothenate kinase	EC:2.7.1.33	1	ko00770 Pantothenate and CoA biosynthesis;
gene1334	K13463	COI-1	coronatine-insensitive protein 1	--	2	ko04075 Plant hormone signal transduction;ko04626 Plant-pathogen interaction;
gene13350	K03327	TC.MATE, SLC47A, norM, mdtK, dinF	multidrug resistance protein, MATE family	--		
gene13351	K13025	EIF4A3, FAL1	ATP-dependent RNA helicase	EC:3.6.4.13	3	ko03013 RNA transport;ko03015 mRNA surveillance pathway;ko03040 Spliceosome;
gene13352	K03131	TAF6	transcription initiation factor TFIID subunit 6	--	2	ko03022 Basal transcription factors;ko05168 Herpes simplex infection;
gene13353	K08503	SYP5	syntaxin of plants SYP5	--	1	ko04130 SNARE interactions in vesicular transport;
gene13354	K15423	PPP4C	serine/threonine-protein phosphatase 4 catalytic subunit	EC:3.1.3.16		
gene13358	K01835	pgm	phosphoglucomutase	EC:5.4.2.2	7	ko00010 Glycolysis / Gluconeogenesis;ko00030 Pentose phosphate pathway;ko00052 Galactose metabolism;ko00230 Purine metabolism;ko00500 Starch and sucrose metabolism;ko00520 Amino sugar and nucleotide sugar metabolism;ko00521 Streptomycin biosynthesis;
gene13359	K12191	CHMP2A	charged multivesicular body protein 2A	--	1	ko04144 Endocytosis;
gene13363	K03949	NDUFA5	NADH dehydrogenase (ubiquinone) 1 alpha subcomplex subunit 5	--	5	ko00190 Oxidative phosphorylation;ko04932 Non-alcoholic fatty liver disease (NAFLD);ko05010 Alzheimer's disease;ko05012 Parkinson's disease;ko05016 Huntington's disease;
gene13365	K16810	TBCCD1	TBCC domain-containing protein 1	--		
gene13367	K02835	prfA, MTRF1, MRF1	peptide chain release factor 1	--		
gene13372	K07904	RAB11A	Ras-related protein Rab-11A	--	4	ko04144 Endocytosis;ko04961 Endocrine and other factor-regulated calcium reabsorption;ko04962 Vasopressin-regulated water reabsorption;ko04972 Pancreatic secretion;
gene13373	K14491	ARR-B	two-component response regulator ARR-B family	--	1	ko04075 Plant hormone signal transduction;
gene13376	K01939	E6.3.4.4, purA	adenylosuccinate synthase	EC:6.3.4.4	2	ko00230 Purine metabolism;ko00250 Alanine, aspartate and glutamate metabolism;
gene13381	K14398	CPSF6_7	cleavage and polyadenylation specificity factor subunit 6/7	--	1	ko03015 mRNA surveillance pathway;
gene13382	K17046	DEK	protein DEK	--		
gene13383	K05389	KCNKF	potassium channel subfamily K, other eukaryote	--		
gene13389	K08819	CDK12_13	cyclin-dependent kinase 12/13	EC:2.7.11.22 2.7.11.23		
gene13400	K00430	E1.11.1.7	peroxidase	EC:1.11.1.7	2	ko00360 Phenylalanine metabolism;ko00940 Phenylpropanoid biosynthesis;
gene1340	K09658	DPM2	dolichyl-phosphate mannosyltransferase polypeptide 2, regulatory subunit	--	2	ko00510 N-Glycan biosynthesis;ko00563 Glycosylphosphatidylinositol(GPI)-anchor biosynthesis;
gene13412	K13162	PCBP2_3_4	poly(rC)-binding protein 2/3/4	--		
gene13414	K03846	ALG9	alpha-1,2-mannosyltransferase	EC:2.4.1.259 2.4.1.261	2	ko00510 N-Glycan biosynthesis;ko00513 Various types of N-glycan biosynthesis;
gene13418	K05765	CFL	cofilin	--	4	ko04360 Axon guidance;ko04666 Fc gamma R-mediated phagocytosis;ko04810 Regulation of actin cytoskeleton;ko05133 Pertussis;
gene13419	K09422	MYBP	myb proto-oncogene protein, plant	--		
gene13423	K14664	ILR1	IAA-amino acid hydrolase	EC:3.5.1.-		

gene13431	K11498	CENPE	centromeric protein E	--		
gene13435	K05016	CLCN7	chloride channel 7	--		
gene13437	K09561	STUB1, CHIP	STIP1 homology and U-box containing protein 1	EC:6.3.2.19	2	ko04120 Ubiquitin mediated proteolysis;ko04141 Protein processing in endoplasmic reticulum;
gene13444	K11268	ESCO, ECO1	N-acetyltransferase	EC:2.3.1.-		
gene13446	K11438	PRMT7	protein arginine N-methyltransferase 7	EC:2.1.1.-		
gene13447	K10268	FBXL2_20	F-box and leucine-rich repeat protein 2/20	--		
gene13448	K02882	RP-L18Ae, RPL18A	large subunit ribosomal protein L18Ae	--	1	ko03010 Ribosome;
gene13449	K07119	K07119				
gene13451	K07119	K07119				
gene13453	K17616	CTDSPL2	CTD small phosphatase-like protein 2	EC:3.1.3.-		
gene13458	K09775	K09775	hypothetical protein	--		
gene13459	K10730	RECQL4	ATP-dependent DNA helicase Q4	EC:3.6.4.12		
gene13462	K09338	HD-ZIP	homeobox-leucine zipper protein	--		
gene13463	K00688	E2.4.1.1, glgP, PYG	starch phosphorylase	EC:2.4.1.1	2	ko00500 Starch and sucrose metabolism;ko04910 Insulin signaling pathway;
gene13464	K08193	SLC17A	MFS transporter, ACS family, solute carrier family 17 (sodium-dependent inorganic phosphate cotransporter), other	--		
gene13466	K07870	RHOT1, ARHT1	Ras homolog gene family, member T1	--		
gene13469	K02961	RP-S17, MRPS17, rpsQ	small subunit ribosomal protein S17	--	1	ko03010 Ribosome;
gene13470	K09338	HD-ZIP	homeobox-leucine zipper protein	--		
gene13472	K06174	ABCE1	ATP-binding cassette, sub-family E, member 1	--		
gene13475	K01724	E4.2.1.96, PCBD, phhB	4a-hydroxytetrahydrobiopterin dehydratase	EC:4.2.1.96		
gene13479	K05759	PFN	profilin	--	3	ko04810 Regulation of actin cytoskeleton;ko05131 Shigellosis;ko05132 Salmonella infection;
gene13481	K07904	RAB11A	Ras-related protein Rab-11A	--	4	ko04144 Endocytosis;ko04961 Endocrine and other factor-regulated calcium reabsorption;ko04962 Vasopressin-regulated water reabsorption;ko04972 Pancreatic secretion;
gene13489	K16296	SCPL-I	serine carboxypeptidase-like clade I	EC:3.4.16.-		
gene1348	K13181	DDX27, DRS1	ATP-dependent RNA helicase DDX27	EC:3.6.4.13		
gene13491	K09651	RHBDD1	rhomboid domain-containing protein 1	EC:3.4.21.-		
gene13492	K09873	TIP	aquaporin TIP	--		
gene13496	K07195	EXOC7, EXO70	exocyst complex component 7	--	1	ko04910 Insulin signaling pathway;
gene13501	K13525	VCP, CDC48	transitional endoplasmic reticulum ATPase	--	2	ko04141 Protein processing in endoplasmic reticulum;ko05134 Legionellosis;
gene13507	K15397	KCS	3-ketoacyl-CoA synthase	EC:2.3.1.199	1	ko00062 Fatty acid elongation;
gene13512	K14484	IAA	auxin-responsive protein IAA	--	1	ko04075 Plant hormone signal transduction;
gene13520	K00133	asd	aspartate-semialdehyde dehydrogenase	EC:1.2.1.11	5	ko00260 Glycine, serine and threonine metabolism;ko00270 Cysteine and methionine metabolism;ko00300 Lysine biosynthesis;ko01210 2-Oxocarboxylic acid metabolism;ko01230 Biosynthesis of amino acids;
gene13522	K00928	lysC	aspartate kinase	EC:2.7.2.4	5	ko00260 Glycine, serine and threonine metabolism;ko00270 Cysteine and methionine metabolism;ko00300 Lysine biosynthesis;ko01210 2-Oxocarboxylic acid metabolism;ko01230 Biosynthesis of amino acids;
gene13523	K08099	E3.1.1.14	chlorophyllase	EC:3.1.1.14	1	ko00860 Porphyrin and chlorophyll metabolism;
gene13530	K03327	TC.MATE, SLC47A, norM, mdtK, dinF	multidrug resistance protein, MATE family	--		
gene13531	K11978	UBR3	E3 ubiquitin-protein ligase UBR3	EC:6.3.2.19		
gene13532	K09422	MYBP	myb proto-oncogene protein, plant	--		

gene13533	K02183	CALM	calmodulin	--	25	ko04014 Ras signaling pathway;ko04020 Calcium signaling pathway;ko04070 Phosphatidylinositol signaling system;ko04114 Oocyte meiosis;ko04270 Vascular smooth muscle contraction;ko04626 Plant-pathogen interaction;ko04713 Circadian entrainment;ko04720 Long-term potentiation;ko04722 Neurotrophin signaling pathway;ko04728 Dopaminergic synapse;ko04740 Olfactory transduction;ko04744 Phototransduction;ko04745 Phototransduction - fly;ko04910 Insulin signaling pathway;ko04912 GnRH signaling pathway;ko04915 Estrogen signaling pathway;ko04916 Melanogenesis;ko04970 Salivary secretion;ko04971 Gastric acid secretion;ko05010 Alzheimer's disease;ko05031 Amphetamine addiction;ko05034 Alcoholism;ko05133 Pertussis;ko05152 Tuberculosis;ko05214 Glioma;
gene13535	K04564	SOD2	superoxide dismutase, Fe-Mn family	EC:1.15.1.1	2	ko04146 Peroxisome;ko05016 Huntington's disease;
gene13536	K08818	CDC2L	cell division cycle 2-like	EC:2.7.11.22		
gene13538	K00454	LOX2S	lipoxygenase	EC:1.13.11.12	2	ko00591 Linoleic acid metabolism;ko00592 alpha-Linolenic acid metabolism;
gene13539	K03094	SKP1, CBF3D	S-phase kinase-associated protein 1	--	9	ko04110 Cell cycle;ko04111 Cell cycle - yeast;ko04114 Oocyte meiosis;ko04120 Ubiquitin mediated proteolysis;ko04141 Protein processing in endoplasmic reticulum;ko04310 Wnt signaling pathway;ko04350 TGF-beta signaling pathway;ko04710 Circadian rhythm;ko05168 Herpes simplex infection;
gene13547	K00767	nadC, QPRT	nicotinate-nucleotide pyrophosphorylase (carboxylating)	EC:2.4.2.19	1	ko00760 Nicotinate and nicotinamide metabolism;
gene13549	K17099	ANXA13	annexin A13	--		
gene13551	K04733	IRAK4	interleukin-1 receptor-associated kinase 4	EC:2.7.11.1	11	ko04064 NF-kappa B signaling pathway;ko04210 Apoptosis;ko04620 Toll-like receptor signaling pathway;ko04722 Neurotrophin signaling pathway;ko05133 Pertussis;ko05140 Leishmaniasis;ko05142 Chagas disease (American trypanosomiasis);ko05145 Toxoplasmosis;ko05152 Tuberculosis;ko05162 Measles;ko05164 Influenza A;
gene13552	K15275	SLC35B1	solute carrier family 35 (UDP-galactose transporter), member B1	--		
gene13555	K03626	EGD2, NACA	nascent polypeptide-associated complex subunit alpha	--		
gene13556	K11292	SUPT6H, SPT6	transcription elongation factor SPT6	--		
gene13562	K07766	E3.6.1.52	diphosphoinositol-polyphosphate diphosphatase	EC:3.6.1.52		
gene13564	K13993	HSP20	HSP20 family protein	--	1	ko04141 Protein processing in endoplasmic reticulum;
gene13567	K01897	ACSL, fadD	long-chain acyl-CoA synthetase	EC:6.2.1.3	4	ko00071 Fatty acid degradation;ko03320 PPAR signaling pathway;ko04146 Peroxisome;ko04920 Adipocytokine signaling pathway;
gene1357	K03531	ftsZ	cell division protein FtsZ	--	1	ko04112 Cell cycle - Caulobacter;
gene13583	K10575	UBE2G1, UBC7	ubiquitin-conjugating enzyme E2 G1	EC:6.3.2.19	3	ko04120 Ubiquitin mediated proteolysis;ko04141 Protein processing in endoplasmic reticulum;ko05012 Parkinson's disease;
gene1358	K01982	LSUrRNA	large subunit ribosomal RNA	--	2	ko03008 Ribosome biogenesis in eukaryotes;ko03010 Ribosome;
gene13590	K08678	UXS1	UDP-glucuronate decarboxylase	EC:4.1.1.35	2	ko00500 Starch and sucrose metabolism;ko00520 Amino sugar and nucleotide sugar metabolism;
gene13591	K11254	H4	histone H4	--	3	ko05034 Alcoholism;ko05203 Viral carcinogenesis;ko05322 Systemic lupus erythematosus;
gene13597	K05286	PIGB	phosphatidylinositol glycan, class B	EC:2.4.1.-	1	ko00563 Glycosylphosphatidylinositol(GPI)-anchor biosynthesis;
gene13598	K02707	psbE	photosystem II cytochrome b559 subunit alpha	--	1	ko00195 Photosynthesis;
gene135	K00799	GST, gst	glutathione S-transferase	EC:2.5.1.18	4	ko00480 Glutathione metabolism;ko00980 Metabolism of xenobiotics by cytochrome P450;ko00982 Drug metabolism - cytochrome P450;ko05204 Chemical carcinogenesis;
gene13601	K00430	E1.11.1.7	peroxidase	EC:1.11.1.7	2	ko00360 Phenylalanine metabolism;ko00940 Phenylpropanoid biosynthesis;
gene13603	K03809	wrbA	Trp repressor binding protein	--		
gene13604	K00434	E1.11.1.11	L-ascorbate peroxidase	EC:1.11.1.11	2	ko00053 Ascorbate and aldarate metabolism;ko00480 Glutathione metabolism;
gene13607	K03544	clpX, CLPX	ATP-dependent Clp protease ATP-binding subunit ClpX	--	1	ko04112 Cell cycle - Caulobacter;
gene13609	K15402	CYP86B1	cytochrome P450, family 86, subfamily B, polypeptide 1 (fatty acid omega-hydroxylase)	EC:1.14.-.-	1	ko00073 Cutin, suberine and wax biosynthesis;

gene13610	K14439	SMARCAD1	SWI/SNF-related matrix-associated actin-dependent regulator of chromatin subfamily A containing DEAD/H box 1	EC:3.6.4.12		
gene13611	K15718	LOX1_5	linoleate 9S-lipoxygenase	EC:1.13.11.58	1	ko00591 Linoleic acid metabolism;
gene13620	K11971	RNF14, ARA54	E3 ubiquitin-protein ligase RNF14	EC:6.3.2.19		
gene1362	K08776	NPEPPS	puromycin-sensitive aminopeptidase	EC:3.4.11.-		
gene1363	K14497	PP2C	protein phosphatase 2C	EC:3.1.3.16	1	ko04075 Plant hormone signal transduction;
gene13641	K09591	DET2	steroid 5-alpha-reductase	EC:1.3.1.22	1	ko00905 Brassinosteroid biosynthesis;
gene13647	K11446	JARID1	histone demethylase JARID1	EC:1.14.11.-		
gene13648	K08857	NEK	NIMA (never in mitosis gene a)-related kinase	EC:2.7.11.1		
gene13650	K01728	E4.2.2.2, pel	pectate lyase	EC:4.2.2.2	1	ko00040 Pentose and glucuronate interconversions;
gene13651	K01728	E4.2.2.2, pel	pectate lyase	EC:4.2.2.2	1	ko00040 Pentose and glucuronate interconversions;
gene13654	K06997	K06997				
gene13658	K01869	LARS, leuS	leucyl-tRNA synthetase	EC:6.1.1.4	1	ko00970 Aminoacyl-tRNA biosynthesis;
gene13660	K15601	KDM3	lysine-specific demethylase 3	EC:1.14.11.-		
gene13664	K16292	CEP, CYSEP	KDEL-tailed cysteine endopeptidase	EC:3.4.22.-		
gene13666	K04420	MAP3K2, MEKK2	mitogen-activated protein kinase kinase kinase 2	EC:2.7.11.25	3	ko04010 MAPK signaling pathway;ko04540 Gap junction;ko04912 GnRH signaling pathway;
gene13673	K17506	PPM1L, PP2CE	protein phosphatase 1L	EC:3.1.3.16		
gene13674	K12118	CRY1	cryptochrome 1	--	1	ko04712 Circadian rhythm - plant;
gene13685	K12883	NCBP2, CBP20	nuclear cap-binding protein subunit 2	--	3	ko03013 RNA transport;ko03015 mRNA surveillance pathway;ko03040 Spliceosome;
gene13687	K12162	UFM1	ubiquitin-fold modifier 1	--		
gene13689	K14709	SLC39A1_2_3, ZIP1_2_3	solute carrier family 39 (zinc transporter), member 1/2/3	--		
gene13691	K15747	LUT5, CYP97A3	cytochrome P450, family 97, subfamily A (beta-ring hydroxylase)	EC:1.14.-.-	1	ko00906 Carotenoid biosynthesis;
gene13692	K05841	E2.4.1.173	sterol 3beta-glucosyltransferase	EC:2.4.1.173		
gene13699	K02641	petH	ferredoxin--NADP+ reductase	EC:1.18.1.2	1	ko00195 Photosynthesis;
gene13702	K02716	psbO	photosystem II oxygen-evolving enhancer protein 1	--	1	ko00195 Photosynthesis;
gene1370	K02877	RP-L15e, RPL15	large subunit ribosomal protein L15e	--	1	ko03010 Ribosome;
gene13712	K12196	VPS4	vacuolar protein-sorting-associated protein 4	--	1	ko04144 Endocytosis;
gene13713	K03878	ND1	NADH-ubiquinone oxidoreductase chain 1	EC:1.6.5.3	2	ko00190 Oxidative phosphorylation;ko05012 Parkinson's disease;
gene13717	K00962	pnp, PNPT1	polyribonucleotide nucleotidyltransferase	EC:2.7.7.8	3	ko00230 Purine metabolism;ko00240 Pyrimidine metabolism;ko03018 RNA degradation;
gene13719	K03320	amt, AMT, MEP	ammonium transporter, Amt family	--		
gene13723	K13249	SSR1	translocon-associated protein subunit alpha	--	1	ko04141 Protein processing in endoplasmic reticulum;
gene13725	K12373	HEXA_B	hexosaminidase	EC:3.2.1.52	6	ko00511 Other glycan degradation;ko00520 Amino sugar and nucleotide sugar metabolism;ko00531 Glycosaminoglycan degradation;ko00603 Glycosphingolipid biosynthesis - globo series;ko00604 Glycosphingolipid biosynthesis - ganglio series;ko04142 Lysosome;
gene13727	K00763	pncB, NAPRT1	nicotinate phosphoribosyltransferase	EC:6.3.4.21	1	ko00760 Nicotinate and nicotinamide metabolism;
gene13731	K07007	K07007				
gene13735	K15920	XYL4	beta-D-xylosidase 4	EC:3.2.1.37	2	ko00500 Starch and sucrose metabolism;ko00520 Amino sugar and nucleotide sugar metabolism;
gene13736	K00029	E1.1.1.40, maeB	malate dehydrogenase (oxaloacetate-decarboxylating)(NADP+)	EC:1.1.1.40	3	ko00620 Pyruvate metabolism;ko00710 Carbon fixation in photosynthetic organisms;ko01200 Carbon metabolism;
gene1373	K15397	KCS	3-ketoacyl-CoA synthase	EC:2.3.1.199	1	ko00062 Fatty acid elongation;
gene13745	K10406	KIFC2_3	kinesin family member C2/C3	--		
gene13746	K00799	GST, gst	glutathione S-transferase	EC:2.5.1.18	4	ko00480 Glutathione metabolism;ko00980 Metabolism of xenobiotics by cytochrome P450;ko00982 Drug metabolism - cytochrome P450;ko05204 Chemical carcinogenesis;
gene13749	K14218	tRNA-Ala	tRNA Ala	--	1	ko00970 Aminoacyl-tRNA biosynthesis;
gene13754	K02144	ATPeV54kD	V-type H+-transporting ATPase 54 kD subunit	EC:3.6.3.14	8	ko00190 Oxidative phosphorylation;ko04142 Lysosome;ko04145 Phagosome;ko04721 Synaptic vesicle cycle;ko05110 Vibrio cholerae infection;ko05120 Epithelial cell signaling in Helicobacter pylori infection;ko05152 Tuberculosis;ko05323 Rheumatoid arthritis;
gene13757	K00430	E1.11.1.7	peroxidase	EC:1.11.1.7	2	ko00360 Phenylalanine metabolism;ko00940 Phenylpropanoid biosynthesis;

gene13762	K08232	E1.6.5.4	monodehydroascorbate reductase (NADH)	EC:1.6.5.4	1	ko00053 Ascorbate and aldarate metabolism;
gene13765	K01251	E3.3.1.1, ahcY	adenosylhomocysteinase	EC:3.3.1.1	1	ko00270 Cysteine and methionine metabolism;
gene13769	K02358	tuf, TUFM	elongation factor Tu	--	1	ko04626 Plant-pathogen interaction;
gene13771	K00218	E1.3.1.33, por	protochlorophyllide reductase	EC:1.3.1.33	1	ko00860 Porphyrin and chlorophyll metabolism;
gene13777	K05236	COPA	coatomer protein complex, subunit alpha (xenin)	--	1	ko04080 Neuroactive ligand-receptor interaction;
gene13781	K06910	K06910				
gene13791	K07342	SEC61G, SSS1, secE	protein transport protein SEC61 subunit gamma and related proteins	--	4	ko03060 Protein export;ko04141 Protein processing in endoplasmic reticulum;ko04145 Phagosome;ko05110 Vibrio cholerae infection;
gene13795	K03006	RPB1, POLR2A	DNA-directed RNA polymerase II subunit RPB1	EC:2.7.7.6	6	ko00230 Purine metabolism;ko00240 Pyrimidine metabolism;ko03020 RNA polymerase;ko05016 Huntington's disease;ko05168 Herpes simplex infection;ko05169 Epstein-Barr virus infection;
gene1379	K02437	gcvH, GCSH	glycine cleavage system H protein	--	1	ko00630 Glyoxylate and dicarboxylate metabolism;
gene13800	K09422	MYBP	myb proto-oncogene protein, plant	--		
gene13801	K11253	H3	histone H3	--	3	ko05034 Alcoholism;ko05202 Transcriptional misregulation in cancer;ko05322 Systemic lupus erythematosus;
gene13802	K17525	CHID1	chitinase domain-containing protein 1	--		
gene13804	K03236	EIF1A	translation initiation factor 1A	--	1	ko03013 RNA transport;
gene13812	K06269	PPP1C	serine/threonine-protein phosphatase PP1 catalytic subunit	EC:3.1.3.16	14	ko03015 mRNA surveillance pathway;ko04113 Meiosis - yeast;ko04114 Oocyte meiosis;ko04270 Vascular smooth muscle contraction;ko04390 Hippo signaling pathway;ko04510 Focal adhesion;ko04720 Long-term potentiation;ko04728 Dopaminergic synapse;ko04810 Regulation of actin cytoskeleton;ko04910 Insulin signaling pathway;ko05031 Amphetamine addiction;ko05034 Alcoholism;ko05168 Herpes simplex infection;ko05205 Proteoglycans in
gene13817	K14496	PYL	abscisic acid receptor PYR/PYL family	--	1	ko04075 Plant hormone signal transduction;
gene13821	K07897	RAB7A	Ras-related protein Rab-7A	--	5	ko04144 Endocytosis;ko04145 Phagosome;ko05132 Salmonella infection;ko05146 Amoebiasis;ko05152 Tuberculosis;
gene13822	K14521	NAT10, KRE33	N-acetyltransferase 10	EC:2.3.1.-	1	ko03008 Ribosome biogenesis in eukaryotes;
gene13832	K15032	MTERFD	mTERF domain-containing protein, mitochondrial	--		
gene13834	K01620	ltaE	threonine aldolase	EC:4.1.2.5	2	ko00260 Glycine, serine and threonine metabolism;ko01230 Biosynthesis of amino acids;
gene13835	K12825	SF3A1, SAP114	splicing factor 3A subunit 1	--	1	ko03040 Spliceosome;
gene13838	K11274	WDHD1, CTF4	chromosome transmission fidelity protein 4	--		
gene13846	K10879	XRCC2	DNA-repair protein XRCC2	--	1	ko03440 Homologous recombination;
gene13850	K14611	SLC23A1_2, SVCT1_2	solute carrier family 23 (nucleobase transporter), member 1/2	--		
gene13854	K14490	AHP	histidine-containing phosphotransfer peotein	--	1	ko04075 Plant hormone signal transduction;
gene13862	K14320	AAAS	aladin	--	1	ko03013 RNA transport;
gene13863	K01611	speD, AMD1	S-adenosylmethionine decarboxylase	EC:4.1.1.50	2	ko00270 Cysteine and methionine metabolism;ko00330 Arginine and proline metabolism;
gene13872	K14006	SEC23	protein transport protein SEC23	--	1	ko04141 Protein processing in endoplasmic reticulum;
gene13873	K09667	OGT	polypeptide N-acetylglucosaminyltransferase	EC:2.4.1.-	1	ko00514 Other types of O-glycan biosynthesis;
gene13874	K01115	PLD1_2	phospholipase D1/2	EC:3.1.4.4	7	ko00564 Glycerophospholipid metabolism;ko00565 Ether lipid metabolism;ko04014 Ras signaling pathway;ko04144 Endocytosis;ko04666 Fc gamma R-mediated phagocytosis;ko04724 Glutamatergic synapse;ko04912 GnRH signaling pathway;
gene13875	K13989	DERL2_3	Derlin-2/3	--	1	ko04141 Protein processing in endoplasmic reticulum;
gene13876	K13106	BUD13, CWC26	pre-mRNA-splicing factor CWC26	--		
gene13878	K03686	dnaJ	molecular chaperone DnaJ	--		
gene1387	K09422	MYBP	myb proto-oncogene protein, plant	--		
gene13885	K17506	PPM1L, PP2CE	protein phosphatase 1L	EC:3.1.3.16		
gene13889	K16297	SCPL-II	serine carboxypeptidase-like clade II	EC:3.4.16.-		
gene1388	K10357	MYO5	myosin V	--		
gene13892	K03511	POLK	DNA polymerase kappa	EC:2.7.7.7	1	ko03460 Fanconi anemia pathway;

gene13897	K04424	ZAK, MLTK	sterile alpha motif and leucine zipper containing kinase AZK	EC:2.7.11.25	2	ko04010 MAPK signaling pathway;ko04530 Tight junction;
gene13905	K11253	H3	histone H3	--	3	ko05034 Alcoholism;ko05202 Transcriptional misregulation in cancer;ko05322 Systemic lupus erythematosus;
gene13907	K15015	SLC32A, VGAT	solute carrier family 32 (vesicular inhibitory amino acid transporter)	--	5	ko04721 Synaptic vesicle cycle;ko04723 Retrograde endocannabinoid signaling;ko04727 GABAergic synapse;ko05032 Morphine addiction;ko05033 Nicotine addiction;
gene13910	K03216	trmL, cspR	tRNA (cytidine/uridine-2'-O-)-methyltransferase	EC:2.1.1.207		
gene13911	K15095	E1.1.1.208	(+)-neomenthol dehydrogenase	EC:1.1.1.208	1	ko00902 Monoterpenoid biosynthesis;
gene13912	K11253	H3	histone H3	--	3	ko05034 Alcoholism;ko05202 Transcriptional misregulation in cancer;ko05322 Systemic lupus erythematosus;
gene13913	K11098	SNRPF, SMF	small nuclear ribonucleoprotein F	--	1	ko03040 Spliceosome;
gene13914	K11855	USP36_42	ubiquitin carboxyl-terminal hydrolase 36/42	EC:3.1.2.15		
gene13916	K00827	AGXT2	alanine-glyoxylate transaminase / (R)-3-amino-2-methylpropionate-pyruvate transaminase	EC:2.6.1.44 2.6.1.40	2	ko00250 Alanine, aspartate and glutamate metabolism;ko00260 Glycine, serine and threonine metabolism;
gene13918	K14802	DRS2, ATP8A	phospholipid-transporting ATPase	EC:3.6.3.1		
gene13919	K12937	UGAT	cyanidin-3-O-glucoside 2''-O-glucuronosyltransferase	EC:2.4.1.254	1	ko00942 Anthocyanin biosynthesis;
gene13923	K01179	E3.2.1.4	endoglucanase	EC:3.2.1.4	1	ko00500 Starch and sucrose metabolism;
gene13924	K10775	E4.3.1.24	phenylalanine ammonia-lyase	EC:4.3.1.24	2	ko00360 Phenylalanine metabolism;ko00940 Phenylpropanoid biosynthesis;
gene13927	K03243	EIF5B	translation initiation factor 5B	--	1	ko03013 RNA transport;
gene13928	K03243	EIF5B	translation initiation factor 5B	--	1	ko03013 RNA transport;
gene13931	K05658	ABCB1	ATP-binding cassette, subfamily B (MDR/TAP), member 1	--	3	ko02010 ABC transporters;ko04976 Bile secretion;ko05206 MicroRNAs in cancer;
gene13938	K10999	CESA	cellulose synthase A	EC:2.4.1.12		
gene1393	K04506	SIAH1	E3 ubiquitin-protein ligase SIAH1	EC:6.3.2.19	3	ko04115 p53 signaling pathway;ko04120 Ubiquitin mediated proteolysis;ko04310 Wnt signaling pathway;
gene13942	K14411	MSI	RNA-binding protein Musashi	--	1	ko03015 mRNA surveillance pathway;
gene13943	K14638	SLC15A3_4, PHT	solute carrier family 15 (peptide/histidine transporter), member 3/4	--		
gene13944	K10683	BARD1	BRCA1-associated RING domain protein 1	EC:6.3.2.19		
gene13946	K00679	E2.3.1.158	phospholipid:diacylglycerol acyltransferase	EC:2.3.1.158	1	ko00561 Glycerolipid metabolism;
gene13948	K12462	ARHGDI, RHOGDI	Rho GDP-dissociation inhibitor	--	2	ko04722 Neurotrophin signaling pathway;ko04962 Vasopressin-regulated water reabsorption;
gene13951	K01900	LSC2	succinyl-CoA synthetase beta subunit	EC:6.2.1.4 6.2.1.5	3	ko00020 Citrate cycle (TCA cycle);ko00640 Propanoate metabolism;ko01200 Carbon metabolism;
gene13954	K05941	E2.3.2.15	glutathione gamma-glutamylcysteinyltransferase	EC:2.3.2.15		
gene13956	K00102	E1.1.2.4, dld	D-lactate dehydrogenase (cytochrome)	EC:1.1.2.4	1	ko00620 Pyruvate metabolism;
gene13958	K12666	OST1, RPN1	oligosaccharyltransferase complex subunit alpha (ribophorin I)	--	3	ko00510 N-Glycan biosynthesis;ko00513 Various types of N-glycan biosynthesis;ko04141 Protein processing in endoplasmic reticulum;
gene13959	K10798	PARP	poly [ADP-ribose] polymerase	EC:2.4.2.30	1	ko03410 Base excision repair;
gene13961	K11247	SH3GL	endophilin-A	--	1	ko04144 Endocytosis;
gene13964	K02943	RP-LP2, RPLP2	large subunit ribosomal protein LP2	--	1	ko03010 Ribosome;
gene13965	K02943	RP-LP2, RPLP2	large subunit ribosomal protein LP2	--	1	ko03010 Ribosome;
gene13967	K02915	RP-L34e, RPL34	large subunit ribosomal protein L34e	--	1	ko03010 Ribosome;
gene13971	K08900	BCS1	mitochondrial chaperone BCS1	--		
gene1397	K01181	E3.2.1.8, xynA	endo-1,4-beta-xylanase	EC:3.2.1.8		
gene13980	K12874	AQR	intron-binding protein aquarius	--	1	ko03040 Spliceosome;
gene13982	K11593	ELF2C	eukaryotic translation initiation factor 2C	--		
gene13985	K05294	PGAP1	glycosylphosphatidylinositol deacylase	EC:3.-.-.-	1	ko00563 Glycosylphosphatidylinositol(GPI)-anchor biosynthesis;
gene13987	K05350	bglB	beta-glucosidase	EC:3.2.1.21	3	ko00460 Cyanoamino acid metabolism;ko00500 Starch and sucrose metabolism;ko00940 Phenylpropanoid biosynthesis;
gene13989	K14232	tRNA-Pro	tRNA Pro	--	1	ko00970 Aminoacyl-tRNA biosynthesis;
gene13997	K09422	MYBP	myb proto-oncogene protein, plant	--		
gene1399	K14232	tRNA-Pro	tRNA Pro	--	1	ko00970 Aminoacyl-tRNA biosynthesis;
gene13	K01951	E6.3.5.2, guaA	GMP synthase (glutamine-hydrolysing)	EC:6.3.5.2	2	ko00230 Purine metabolism;ko00983 Drug metabolism - other enzymes;
gene14006	K12605	CNOT2, NOT2	CCR4-NOT transcription complex subunit 2	--	1	ko03018 RNA degradation;

gene14008	K04706	PIAS1	E3 SUMO-protein ligase PIAS1	EC:6.3.2.-	3	ko04120 Ubiquitin mediated proteolysis;ko04630 Jak-STAT signaling pathway;ko05160 Hepatitis C;
gene1400	K14232	tRNA-Pro	tRNA Pro	--	1	ko00970 Aminoacyl-tRNA biosynthesis;
gene14010	K00454	LOX2S	lipoxygenase	EC:1.13.11.12	2	ko00591 Linoleic acid metabolism;ko00592 alpha-Linolenic acid metabolism;
gene14014	K14235	tRNA-Trp	tRNA Trp	--	1	ko00970 Aminoacyl-tRNA biosynthesis;
gene14017	K00432	E1.11.1.9	glutathione peroxidase	EC:1.11.1.9	3	ko00480 Glutathione metabolism;ko00590 Arachidonic acid metabolism;ko04918 Thyroid hormone synthesis;
gene14018	K06630	YWHAE	14-3-3 protein epsilon	--	8	ko04110 Cell cycle;ko04114 Oocyte meiosis;ko04151 PI3K-Akt signaling pathway;ko04390 Hippo signaling pathway;ko04391 Hippo signaling pathway - fly;ko04722 Neurotrophin signaling pathway;ko05169 Epstein-Barr virus infection;ko05203 Viral carcinogenesis;
gene14020	K00053	ilvC	ketol-acid reductoisomerase	EC:1.1.1.86	4	ko00290 Valine, leucine and isoleucine biosynthesis;ko00770 Pantothenate and CoA biosynthesis;ko01210 2-Oxocarboxylic acid metabolism;ko01230 Biosynthesis of amino acids;
gene14021	K06268	PPP3R, CNB	serine/threonine-protein phosphatase 2B regulatory subunit	--	18	ko04010 MAPK signaling pathway;ko04020 Calcium signaling pathway;ko04114 Oocyte meiosis;ko04210 Apoptosis;ko04310 Wnt signaling pathway;ko04360 Axon guidance;ko04370 VEGF signaling pathway;ko04380 Osteoclast differentiation;ko04650 Natural killer cell mediated cytotoxicity;ko04660 T cell receptor signaling pathway;ko04662 B cell receptor signaling pathway;ko04720 Long-term potentiation;ko04724 Glutamatergic synapse;ko05010 Alzheimer's disease;ko05014 Amyotrophic lateral sclerosis (ALS);ko05031 Amphetamine addiction;ko05152
gene14022	K01265	E3.4.11.18, map	methionyl aminopeptidase	EC:3.4.11.18		
gene14023	K13466	EIX1_2	EIX receptor 1/2	--	1	ko04626 Plant-pathogen interaction;
gene14024	K06184	ABCF1	ATP-binding cassette, subfamily F, member 1	--		
gene14029	K14508	NPR1	regulatory protein NPR1	--	1	ko04075 Plant hormone signal transduction;
gene14036	K13448	CML	calcium-binding protein CML	--	1	ko04626 Plant-pathogen interaction;
gene14040	K02995	RP-S8e, RPS8	small subunit ribosomal protein S8e	--	1	ko03010 Ribosome;
gene14041	K09843	E1.14.13.93	(+)-abscisic acid 8'-hydroxylase	EC:1.14.13.93	1	ko00906 Carotenoid biosynthesis;
gene14042	K04710	CERS	ceramide synthetase	EC:2.3.1.24	1	ko00600 Sphingolipid metabolism;
gene14045	K00079	CBR1	carbonyl reductase 1	EC:1.1.1.184 1.1.1.189 1.1.1.197	3	ko00590 Arachidonic acid metabolism;ko00980 Metabolism of xenobiotics by cytochrome P450;ko05204 Chemical carcinogenesis;
gene14047	K03404	chlD, bchD	magnesium chelatase subunit D	EC:6.6.1.1	1	ko00860 Porphyrin and chlorophyll metabolism;
gene14052	K04536	GNB1	guanine nucleotide-binding protein G(I)/G(S)/G(T) subunit beta-1	--	14	ko04014 Ras signaling pathway;ko04062 Chemokine signaling pathway;ko04151 PI3K-Akt signaling pathway;ko04713 Circadian entrainment;ko04723 Retrograde endocannabinoid signaling;ko04724 Glutamatergic synapse;ko04725 Cholinergic synapse;ko04726 Serotonergic synapse;ko04727 GABAergic synapse;ko04728 Dopaminergic synapse;ko04742 Taste transduction;ko04744 Phototransduction;ko05032 Morphine addiction;ko05034 Alcoholism;
gene14053	K17267	COPG	coatomer protein complex, subunit gamma	--		
gene14054	K09338	HD-ZIP	homeobox-leucine zipper protein	--		
gene14055	K00791	miaA, TRIT1	tRNA dimethylallyltransferase	EC:2.5.1.75	1	ko00908 Zeatin biosynthesis;
gene14059	K08241	E2.1.1.141	jasmonate O-methyltransferase	EC:2.1.1.141	1	ko00592 alpha-Linolenic acid metabolism;
gene14061	K03354	APC7	anaphase-promoting complex subunit 7	--	7	ko04110 Cell cycle;ko04111 Cell cycle - yeast;ko04113 Meiosis - yeast;ko04114 Oocyte meiosis;ko04120 Ubiquitin mediated proteolysis;ko04914 Progesterone-mediated oocyte maturation;ko05166 HTLV-I infection;
gene14066	K02295	CRY	cryptochrome	--	1	ko04710 Circadian rhythm;
gene14067	K00413	CYC1, CYT1, petC	ubiquinol-cytochrome c reductase cytochrome c1 subunit	--	7	ko00190 Oxidative phosphorylation;ko02020 Two-component system;ko04260 Cardiac muscle contraction;ko04932 Non-alcoholic fatty liver disease (NAFLD);ko05010 Alzheimer's disease;ko05012 Parkinson's disease;ko05016 Huntington's disease;
gene14076	K02918	RP-L35e, RPL35	large subunit ribosomal protein L35e	--	1	ko03010 Ribosome;
gene14077	K01728	E4.2.2.2, pel	pectate lyase	EC:4.2.2.2	1	ko00040 Pentose and glucuronate interconversions;
gene1407	K11778	DHDDS, RER2, SRT1	ditrans,polycis-polyprenyl diphosphate synthase	EC:2.5.1.87	1	ko00900 Terpenoid backbone biosynthesis;
gene14080	K14945	QKI	protein quaking	--		

gene14095	K03428	E2.1.1.11, chlM, bchM	magnesium-protoporphyrin O-methyltransferase	EC:2.1.1.11	1	ko00860 Porphyrin and chlorophyll metabolism;
gene1409	K11087	SNRPD1, SMD1	small nuclear ribonucleoprotein D1	--	2	ko03040 Spliceosome;ko05322 Systemic lupus erythematosus;
gene140	K01082	E3.1.3.7, cysQ, MET22, BPNT1	3'(2'), 5'-bisphosphate nucleotidase	EC:3.1.3.7	1	ko00920 Sulfur metabolism;
gene14101	K06316	RFT1	oligosaccharidyl-lipid flippase family	--	1	ko00510 N-Glycan biosynthesis;
gene14107	K12842	SR140	U2-associated protein SR140	--	1	ko03040 Spliceosome;
gene1410	K13917	RNGTT	mRNA-capping enzyme	EC:2.7.7.50 3.1.3.33	1	ko03015 mRNA surveillance pathway;
gene14111	K00873	PK, pyk	pyruvate kinase	EC:2.7.1.40	7	ko00010 Glycolysis / Gluconeogenesis;ko00230 Purine metabolism;ko00620 Pyruvate metabolism;ko01200 Carbon metabolism;ko01230 Biosynthesis of amino acids;ko04930 Type II diabetes mellitus;ko05203 Viral carcinogenesis;
gene14112	K03155	TIMELESS	timeless	--		
gene14114	K14771	NOC4, UTP19	U3 small nucleolar RNA-associated protein 19	--		
gene14115	K16732	PRC1	protein regulator of cytokinesis 1	--		
gene14119	K12373	HEXA_B	hexosaminidase	EC:3.2.1.52	6	ko00511 Other glycan degradation;ko00520 Amino sugar and nucleotide sugar metabolism;ko00531 Glycosaminoglycan degradation;ko00603 Glycosphingolipid biosynthesis - globo series;ko00604 Glycosphingolipid biosynthesis - ganglio series;ko04142 Lysosome;
gene1412	K00899	mtnK	5-methylthioribose kinase	EC:2.7.1.100	1	ko00270 Cysteine and methionine metabolism;
gene14135	K15687	MKRN	E3 ubiquitin-protein ligase makorin	EC:6.3.2.19		
gene14137	K03500	rsmB, sun	16S rRNA (cytosine967-C5)-methyltransferase	EC:2.1.1.176		
gene1413	K13150	COIL, CLN80	coilin	--		
gene14147	K00888	PI4K	phosphatidylinositol 4-kinase	EC:2.7.1.67	2	ko00562 Inositol phosphate metabolism;ko04070 Phosphatidylinositol signaling system;
gene14148	K00888	PI4K	phosphatidylinositol 4-kinase	EC:2.7.1.67	2	ko00562 Inositol phosphate metabolism;ko04070 Phosphatidylinositol signaling system;
gene14153	K03263	EIF5A	translation initiation factor 5A	--		
gene14158	K01246	tag	DNA-3-methyladenine glycosylase I	EC:3.2.2.20	1	ko03410 Base excision repair;
gene14159	K10727	CDT1	chromatin licensing and DNA replication factor 1	--		
gene1415	K02495	hemN, hemZ	oxygen-independent coproporphyrinogen III oxidase	EC:1.3.99.22	1	ko00860 Porphyrin and chlorophyll metabolism;
gene14163	K13106	BUD13, CWC26	pre-mRNA-splicing factor CWC26	--		
gene14164	K13989	DERL2_3	Derlin-2/3	--	1	ko04141 Protein processing in endoplasmic reticulum;
gene14167	K08835	OXSR1, STK39	serine/threonine-protein kinase OSR1/STK39	EC:2.7.11.1		
gene14173	K13950	pabAB	para-aminobenzoate synthetase	EC:2.6.1.85	1	ko00790 Folate biosynthesis;
gene14174	K14802	DRS2, ATP8A	phospholipid-transporting ATPase	EC:3.6.3.1		
gene14177	K13127	RNF113A, CWC24	RING finger protein 113A	--		
gene14179	K11806	WDSOF1	WD repeat and SOF domain-containing protein 1	--		
gene1417	K10389	TUBG	tubulin gamma	--		
gene14181	K03028	PSMD2, RPN1	26S proteasome regulatory subunit N1	--	2	ko03050 Proteasome;ko05169 Epstein-Barr virus infection;
gene14182	K10523	SPOP	speckle-type POZ protein	--		
gene14186	K14488	SAUR	SAUR family protein	--	1	ko04075 Plant hormone signal transduction;
gene14189	K02870	RP-L12e, RPL12	large subunit ribosomal protein L12e	--	1	ko03010 Ribosome;
gene14196	K00487	CYP73A	trans-cinnamate 4-monooxygenase	EC:1.14.13.11	5	ko00360 Phenylalanine metabolism;ko00940 Phenylpropanoid biosynthesis;ko00941 Flavonoid biosynthesis;ko00945 Stilbenoid, diarylheptanoid and gingerol biosynthesis;ko01220 Degradation of aromatic compounds;
gene14197	K12598	MTR4, SKIV2L2	ATP-dependent RNA helicase DOB1	EC:3.6.4.13	1	ko03018 RNA degradation;
gene14204	K09264	K09264	MADS-box transcription factor, plant	--		
gene1420	K05542	DUS1	tRNA-dihydrouridine synthase 1	EC:1.3.1.88		
gene14213	K03327	TC.MATE, SLC47A, norM, mdtK, dinF	multidrug resistance protein, MATE family	--		
gene14216	K14488	SAUR	SAUR family protein	--	1	ko04075 Plant hormone signal transduction;
gene14218	K08900	BCS1	mitochondrial chaperone BCS1	--		

gene14219	K02149	ATPeVD, ATP6M	V-type H+-transporting ATPase subunit D	EC:3.6.3.14	7	ko00190 Oxidative phosphorylation;ko04145 Phagosome;ko04721 Synaptic vesicle cycle;ko04966 Collecting duct acid secretion;ko05110 Vibrio cholerae infection;ko05120 Epithelial cell signaling in Helicobacter pylori infection;ko05323 Rheumatoid arthritis;
gene14220	K04125	E1.14.11.13	gibberellin 2-oxidase	EC:1.14.11.13	1	ko00904 Diterpenoid biosynthesis;
gene14224	K14611	SLC23A1_2, SVCT1_2	solute carrier family 23 (nucleobase transporter), member 1/2	--		
gene14228	K02695	psaH	photosystem I subunit VI	--	1	ko00195 Photosynthesis;
gene14239	K00517	E1.14.-.-			4	ko00624 Polycyclic aromatic hydrocarbon degradation;ko00627 Aminobenzoate degradation;ko00903 Limonene and pinene degradation;ko00945 Stilbenoid, diarylheptanoid and gingerol biosynthesis;
gene14242	K10406	KIFC2_3	kinesin family member C2/C3	--		
gene14246	K02717	psbP	photosystem II oxygen-evolving enhancer protein 2	--	1	ko00195 Photosynthesis;
gene14248	K10781	FATB	fatty acyl-ACP thioesterase B	EC:3.1.2.14	1	ko00061 Fatty acid biosynthesis;
gene14249	K08730	PTDSS2	phosphatidylserine synthase 2	EC:2.7.8.29	1	ko00564 Glycerophospholipid metabolism;
gene14250	K12737	SDCCAG10	peptidyl-prolyl cis-trans isomerase SDCCAG10	EC:5.2.1.8		
gene14251	K11643	CHD4, MI2B	chromodomain-helicase-DNA-binding protein 4	EC:3.6.4.12	1	ko05203 Viral carcinogenesis;
gene14255	K13337	PEX19	peroxin-19	--	1	ko04146 Peroxisome;
gene14256	K01115	PLD1_2	phospholipase D1/2	EC:3.1.4.4	7	ko00564 Glycerophospholipid metabolism;ko00565 Ether lipid metabolism;ko04014 Ras signaling pathway;ko04144 Endocytosis;ko04666 Fc gamma R-mediated phagocytosis;ko04724 Glutamatergic synapse;ko04912 GnRH signaling pathway;
gene14258	K13335	PEX16	peroxin-16	--	1	ko04146 Peroxisome;
gene14259	K01590	hdc, HDC	histidine decarboxylase	EC:4.1.1.22	1	ko00340 Histidine metabolism;
gene1425	K05387	GRIP	glutamate receptor, ionotropic, plant	--		
gene14260	K01590	hdc, HDC	histidine decarboxylase	EC:4.1.1.22	1	ko00340 Histidine metabolism;
gene14264	K17065	DNM1L	dynamin 1-like protein	EC:3.6.5.5	1	ko04668 TNF signaling pathway;
gene14266	K01369	LGMN	legumain	EC:3.4.22.34	2	ko04142 Lysosome;ko04612 Antigen processing and presentation;
gene1426	K08852	ERN1	serine/threonine-protein kinase/endoribonuclease IRE1	EC:2.7.11.1	3	ko04141 Protein processing in endoplasmic reticulum;ko04932 Non-alcoholic fatty liver disease (NAFLD);ko05010 Alzheimer's disease;
gene14276	K15188	CCNT	cyclin T	--	1	ko05202 Transcriptional misregulation in cancer;
gene1427	K05298	GAPA	glyceraldehyde-3-phosphate dehydrogenase (NADP+) (phosphorylating)	EC:1.2.1.13	2	ko00710 Carbon fixation in photosynthetic organisms;ko01200 Carbon metabolism;
gene14280	K12191	CHMP2A	charged multivesicular body protein 2A	--	1	ko04144 Endocytosis;
gene14292	K02936	RP-L7Ae, RPL7A	large subunit ribosomal protein L7Ae	--	1	ko03010 Ribosome;
gene14296	K13262	7-IOMT	isoflavone-7-O-methyltransferase	EC:2.1.1.150	1	ko00943 Isoflavonoid biosynthesis;
gene14297	K06689	UBE2D_E, UBC4, UBC5	ubiquitin-conjugating enzyme E2 D/E	EC:6.3.2.19	2	ko04120 Ubiquitin mediated proteolysis;ko04141 Protein processing in endoplasmic reticulum;
gene142	K16297	SCPL-II	serine carboxypeptidase-like clade II	EC:3.4.16.-		
gene14302	K13422	MYC2	transcription factor MYC2	--	2	ko04075 Plant hormone signal transduction;ko04626 Plant-pathogen interaction;
gene14304	K14841	NSA1, WDR74	ribosome biogenesis protein NSA1	--		
gene14305	K08738	CYC	cytochrome c	--	19	ko00920 Sulfur metabolism;ko02020 Two-component system;ko04115 p53 signaling pathway;ko04210 Apoptosis;ko04932 Non-alcoholic fatty liver disease (NAFLD);ko05010 Alzheimer's disease;ko05012 Parkinson's disease;ko05014 Amyotrophic lateral sclerosis (ALS);ko05016 Huntington's disease;ko05134 Legionellosis;ko05145 Toxoplasmosis;ko05152 Tuberculosis;ko05161 Hepatitis B;ko05164 Influenza A;ko05168 Herpes simplex infection;ko05200 Pathways in cancer;ko05210 Colorectal cancer;ko05222 Small cell lung cancer;ko05416 Viral myocarditis;
gene14306	K13617	PPME1	protein phosphatase methylesterase 1	EC:3.1.1.89		
gene1430	K01802	E5.2.1.8	peptidylprolyl isomerase	EC:5.2.1.8		
gene14310	K13950	pabAB	para-aminobenzoate synthetase	EC:2.6.1.85	1	ko00790 Folate biosynthesis;
gene14314	K09422	MYBP	myb proto-oncogene protein, plant	--		
gene14315	K08332	VAC8	vacuolar protein 8	--	1	ko04140 Regulation of autophagy;

gene14317	K00600	glyA, SHMT	glycine hydroxymethyltransferase	EC:2.1.2.1	7	ko00260 Glycine, serine and threonine metabolism;ko00460 Cyanoamino acid metabolism;ko00630 Glyoxylate and dicarboxylate metabolism;ko00670 One carbon pool by folate;ko00680 Methane metabolism;ko01200 Carbon metabolism;ko01230 Biosynthesis of amino acids;
gene1431	K12666	OST1, RPN1	oligosaccharyltransferase complex subunit alpha (ribophorin I)	--	3	ko00510 N-Glycan biosynthesis;ko00513 Various types of N-glycan biosynthesis;ko04141 Protein processing in endoplasmic reticulum;
gene14322	K14638	SLC15A3_4, PHT	solute carrier family 15 (peptide/histidine transporter), member 3/4	--		
gene14327	K07555	ATPeAF1, ATPAF1	ATP synthase mitochondrial F1 complex assembly factor 1	--		
gene14328	K13420	FLS2	LRR receptor-like serine/threonine-protein kinase FLS2	EC:2.7.11.1	1	ko04626 Plant-pathogen interaction;
gene14329	K13420	FLS2	LRR receptor-like serine/threonine-protein kinase FLS2	EC:2.7.11.1	1	ko04626 Plant-pathogen interaction;
gene14330	K02907	RP-L30, MRPL30, rpmD	large subunit ribosomal protein L30	--	1	ko03010 Ribosome;
gene14334	K06972	K06972				
gene14336	K00857	E2.7.1.21, tdk	thymidine kinase	EC:2.7.1.21	2	ko00240 Pyrimidine metabolism;ko00983 Drug metabolism - other enzymes;
gene14338	K16253	NRPD7, NRPE7	DNA-directed RNA polymerase IV and V subunit 7	--		
gene14339	K09699	E2.3.1.168, bkdB	2-oxoisovalerate dehydrogenase E2 component (dihydrolipoyl transacylase)	EC:2.3.1.168	1	ko00280 Valine, leucine and isoleucine degradation;
gene14340	K16253	NRPD7, NRPE7	DNA-directed RNA polymerase IV and V subunit 7	--		
gene14344	K14831	MAK16	protein MAK16	--		
gene14348	K03216	trmL, cspR	tRNA (cytidine/uridine-2'-O-)-methyltransferase	EC:2.1.1.207		
gene14349	K13096	SF4	splicing factor 4	--		
gene14353	K05391	CNGF	cyclic nucleotide gated channel, other eukaryote	--	1	ko04626 Plant-pathogen interaction;
gene14355	K00876	E2.7.1.48, udk	uridine kinase	EC:2.7.1.48	2	ko00240 Pyrimidine metabolism;ko00983 Drug metabolism - other enzymes;
gene14356	K02882	RP-L18Ae, RPL18A	large subunit ribosomal protein L18Ae	--	1	ko03010 Ribosome;
gene14359	K01188	E3.2.1.21	beta-glucosidase	EC:3.2.1.21	3	ko00460 Cyanoamino acid metabolism;ko00500 Starch and sucrose metabolism;ko00940 Phenylpropanoid biosynthesis;
gene14367	K09284	AP2	AP2-like factor, euAP2 lineage	--		
gene14369	K07359	CAMKK	calcium/calmodulin-dependent protein kinase kinase	EC:2.7.11.17	2	ko04920 Adipocytokine signaling pathway;ko05034 Alcoholism;
gene1436	K10534	NR	nitrate reductase (NAD(P)H)	EC:1.7.1.1 1.7.1.2 1.7.1.3	1	ko00910 Nitrogen metabolism;
gene14370	K14320	AAAS	aladin	--	1	ko03013 RNA transport;
gene14371	K00162	PDHB, pdhB	pyruvate dehydrogenase E1 component subunit beta	EC:1.2.4.1	6	ko00010 Glycolysis / Gluconeogenesis;ko00020 Citrate cycle (TCA cycle);ko00620 Pyruvate metabolism;ko00650 Butanoate metabolism;ko01200 Carbon metabolism;ko04066 HIF-1 signaling pathway;
gene14372	K17592	SACS	sacsin	--		
gene14377	K14774	UTP25, DEF	U3 small nucleolar RNA-associated protein 25	--		
gene14378	K02942	RP-LP1, RPLP1	large subunit ribosomal protein LP1	--	1	ko03010 Ribosome;
gene14384	K09422	MYBP	myb proto-oncogene protein, plant	--		
gene1438	K00417	QCR7, UQCRB	ubiquinol-cytochrome c reductase subunit 7	--	6	ko00190 Oxidative phosphorylation;ko04260 Cardiac muscle contraction;ko04932 Non-alcoholic fatty liver disease (NAFLD);ko05010 Alzheimer's disease;ko05012 Parkinson's disease;ko05016 Huntington's disease;
gene143	K02965	RP-S19, rpsS	small subunit ribosomal protein S19	--	1	ko03010 Ribosome;
gene14400	K08064	NFYA	nuclear transcription factor Y, alpha	--	2	ko04612 Antigen processing and presentation;ko05152 Tuberculosis;
gene14404	K09264	K09264	MADS-box transcription factor, plant	--		
gene14408	K12813	DHX16	pre-mRNA-splicing factor ATP-dependent RNA helicase DHX16	EC:3.6.4.13	1	ko03040 Spliceosome;
gene14411	K10683	BARD1	BRCA1-associated RING domain protein 1	EC:6.3.2.19		
gene14413	K01762	ACS	1-aminocyclopropane-1-carboxylate synthase	EC:4.4.1.14	1	ko00270 Cysteine and methionine metabolism;
gene14414	K00511	SQLE, ERG1	squalene monooxygenase	EC:1.14.13.132	1	ko00909 Sesquiterpenoid and triterpenoid biosynthesis;
gene14418	K14521	NAT10, KRE33	N-acetyltransferase 10	EC:2.3.1.-	1	ko03008 Ribosome biogenesis in eukaryotes;

gene1441	K01662	dxs	1-deoxy-D-xylulose-5-phosphate synthase	EC:2.2.1.7	2	ko00730 Thiamine metabolism;ko00900 Terpenoid backbone biosynthesis;
gene14420	K14835	NOP2	ribosomal RNA methyltransferase Nop2	EC:2.1.1.-		
gene14422	K15191	LARP7	La-related protein 7	--		
gene14424	K00382	DLD, lpd, pdhD	dihydrolipoamide dehydrogenase	EC:1.8.1.4	6	ko00010 Glycolysis / Gluconeogenesis;ko00020 Citrate cycle (TCA cycle);ko00260 Glycine, serine and threonine metabolism;ko00280 Valine, leucine and isoleucine degradation;ko00620 Pyruvate metabolism;ko01200 Carbon metabolism;
gene14425	K14803	PTC2_3	protein phosphatase 2C homolog 2/3	EC:3.1.3.16		
gene14426	K14413	GALT1	beta-1,3-galactosyltransferase	EC:2.4.1.-	1	ko00513 Various types of N-glycan biosynthesis;
gene14427	K09338	HD-ZIP	homeobox-leucine zipper protein	--		
gene1442	K14638	SLC15A3_4, PHT	solute carrier family 15 (peptide/histidine transporter), member 3/4	--		
gene14437	K00770	E2.4.2.24	1,4-beta-D-xylan synthase	EC:2.4.2.24	2	ko00500 Starch and sucrose metabolism;ko00520 Amino sugar and nucleotide sugar metabolism;
gene14441	K08869	ADCK, ABC1	aarF domain-containing kinase	--		
gene14446	K02206	CDK2	cyclin-dependent kinase 2	EC:2.7.11.22	13	ko04110 Cell cycle;ko04114 Oocyte meiosis;ko04115 p53 signaling pathway;ko04151 PI3K-Akt signaling pathway;ko04914 Progesterone-mediated oocyte maturation;ko05161 Hepatitis B;ko05162 Measles;ko05168 Herpes simplex infection;ko05169 Epstein-Barr virus infection;ko05200 Pathways in cancer;ko05203 Viral carcinogenesis;ko05215 Prostate cancer;ko05222 Small cell lung cancer;
gene14447	K14490	AHP	histidine-containing phosphotransfer peotein	--	1	ko04075 Plant hormone signal transduction;
gene14454	K11290	SET, TAF1, I2PP2A	template-activating factor I	--		
gene14456	K08237	E2.4.1.218	hydroquinone glucosyltransferase	EC:2.4.1.218		
gene14459	K03424	tatD	TatD DNase family protein	EC:3.1.21.-		
gene14462	K10666	RNF5	E3 ubiquitin-protein ligase RNF5	EC:6.3.2.19	1	ko04141 Protein processing in endoplasmic reticulum;
gene14465	K15015	SLC32A, VGAT	solute carrier family 32 (vesicular inhibitory amino acid transporter)	--	5	ko04721 Synaptic vesicle cycle;ko04723 Retrograde endocannabinoid signaling;ko04727 GABAergic synapse;ko05032 Morphine addiction;ko05033 Nicotine addiction;
gene14468	K05868	CCNB	cyclin B	--	3	ko04110 Cell cycle;ko04115 p53 signaling pathway;ko04914 Progesterone-mediated oocyte maturation;
gene1446	K08790	STK38, NDR	serine/threonine kinase 38	EC:2.7.11.1		
gene14471	K11498	CENPE	centromeric protein E	--		
gene14476	K12194	CHMP4, SNF7, VPS32	charged multivesicular body protein 4	--	1	ko04144 Endocytosis;
gene14477	K05759	PFN	profilin	--	3	ko04810 Regulation of actin cytoskeleton;ko05131 Shigellosis;ko05132 Salmonella infection;
gene14484	K15175	CDC73	parafibromin	--		
gene14489	K09458	fabF	3-oxoacyl-[acyl-carrier-protein] synthase II	EC:2.3.1.179	2	ko00061 Fatty acid biosynthesis;ko00780 Biotin metabolism;
gene1448	K00026	MDH2	malate dehydrogenase	EC:1.1.1.37	5	ko00020 Citrate cycle (TCA cycle);ko00620 Pyruvate metabolism;ko00630 Glyoxylate and dicarboxylate metabolism;ko00710 Carbon fixation in photosynthetic organisms;ko01200 Carbon metabolism;
gene14490	K12865	PQBP1, NPW38	polyglutamine-binding protein 1	--	1	ko03040 Spliceosome;
gene14494	K01213	E3.2.1.67	galacturan 1,4-alpha-galacturonidase	EC:3.2.1.67	2	ko00040 Pentose and glucuronate interconversions;ko00500 Starch and sucrose metabolism;
gene14499	K13348	MPV17	protein Mpv17	--	1	ko04146 Peroxisome;
gene1449	K00791	miaA, TRIT1	tRNA dimethylallyltransferase	EC:2.5.1.75	1	ko00908 Zeatin biosynthesis;
gene14505	K02907	RP-L30, MRPL30, rpmD	large subunit ribosomal protein L30	--	1	ko03010 Ribosome;
gene14515	K00549	metE	5-methyltetrahydropteroyltriglutamate--homocysteine methyltransferase	EC:2.1.1.14	3	ko00270 Cysteine and methionine metabolism;ko00450 Selenocompound metabolism;ko01230 Biosynthesis of amino acids;
gene1451	K00811	ASP5	aspartate aminotransferase, chloroplastic	EC:2.6.1.1	10	ko00250 Alanine, aspartate and glutamate metabolism;ko00270 Cysteine and methionine metabolism;ko00330 Arginine and proline metabolism;ko00350 Tyrosine metabolism;ko00360 Phenylalanine metabolism;ko00400 Phenylalanine, tyrosine and tryptophan biosynthesis;ko00950 Isoquinoline alkaloid biosynthesis;ko00960 Tropane, piperidine and pyridine alkaloid biosynthesis;ko01210 2-Oxocarboxylic acid metabolism;ko01230 Biosynthesis of amino acids;
gene14520	K11855	USP36_42	ubiquitin carboxyl-terminal hydrolase 36/42	EC:3.1.2.15		

gene14528	K16871	POP2	4-aminobutyrate---pyruvate transaminase	EC:2.6.1.96	2	ko00250 Alanine, aspartate and glutamate metabolism;ko00650 Butanoate metabolism;
gene14529	K02935	RP-L7, MRPL12, rplL	large subunit ribosomal protein L7/L12	--	1	ko03010 Ribosome;
gene1452	K03083	GSK3B	glycogen synthase kinase 3 beta	EC:2.7.11.26	29	ko04012 ErbB signaling pathway;ko04062 Chemokine signaling pathway;ko04110 Cell cycle;ko04151 PI3K-Akt signaling pathway;ko04310 Wnt signaling pathway;ko04340 Hedgehog signaling pathway;ko04360 Axon guidance;ko04390 Hippo signaling pathway;ko04510 Focal adhesion;ko04660 T cell receptor signaling pathway;ko04662 B cell receptor signaling pathway;ko04711 Circadian rhythm - fly;ko04722 Neurotrophin signaling pathway;ko04728 Dopaminergic synapse;ko04910 Insulin signaling pathway;ko04916 Melanogenesis;ko04917 Prolactin signaling pathway;ko04932 Non-alcoholic fatty liver disease (NAFLD);ko05010 Alzheimer's disease;ko05160 Hepatitis C;ko05162 Measles;ko05164 Influenza A;ko05166 HTLV-I infection;ko05169 Epstein-Barr virus infection;ko05200 Pathways in cancer;ko05210 Colorectal cancer;ko05213 Endometrial cancer;ko05215 Prostate
gene14530	K01114	plcC	phospholipase C	EC:3.1.4.3	3	ko00562 Inositol phosphate metabolism;ko00564 Glycerophospholipid metabolism;ko00565 Ether lipid metabolism;
gene14532	K01623	ALDO	fructose-bisphosphate aldolase, class I	EC:4.1.2.13	7	ko00010 Glycolysis / Gluconeogenesis;ko00030 Pentose phosphate pathway;ko00051 Fructose and mannose metabolism;ko00680 Methane metabolism;ko00710 Carbon fixation in photosynthetic organisms;ko01200 Carbon metabolism;ko01230 Biosynthesis of amino acids;
gene14533	K06269	PPP1C	serine/threonine-protein phosphatase PP1 catalytic subunit	EC:3.1.3.16	14	ko03015 mRNA surveillance pathway;ko04113 Meiosis - yeast;ko04114 Oocyte meiosis;ko04270 Vascular smooth muscle contraction;ko04390 Hippo signaling pathway;ko04510 Focal adhesion;ko04720 Long-term potentiation;ko04728 Dopaminergic synapse;ko04810 Regulation of actin cytoskeleton;ko04910 Insulin signaling pathway;ko05031 Amphetamine addiction;ko05034 Alcoholism;ko05168 Herpes simplex infection;ko05205 Proteoglycans in
gene14536	K06269	PPP1C	serine/threonine-protein phosphatase PP1 catalytic subunit	EC:3.1.3.16	14	ko03015 mRNA surveillance pathway;ko04113 Meiosis - yeast;ko04114 Oocyte meiosis;ko04270 Vascular smooth muscle contraction;ko04390 Hippo signaling pathway;ko04510 Focal adhesion;ko04720 Long-term potentiation;ko04728 Dopaminergic synapse;ko04810 Regulation of actin cytoskeleton;ko04910 Insulin signaling pathway;ko05031 Amphetamine addiction;ko05034 Alcoholism;ko05168 Herpes simplex infection;ko05205 Proteoglycans in
gene14538	K14638	SLC15A3_4, PHT	solute carrier family 15 (peptide/histidine transporter), member 3/4	--		
gene14540	K09419	HSFF	heat shock transcription factor, other eukaryote	--		
gene14542	K10666	RNF5	E3 ubiquitin-protein ligase RNF5	EC:6.3.2.19	1	ko04141 Protein processing in endoplasmic reticulum;
gene14549	K03844	ALG11	alpha-1,2-mannosyltransferase	EC:2.4.1.131	2	ko00510 N-Glycan biosynthesis;ko00513 Various types of N-glycan biosynthesis;
gene14553	K15436	TRPO3, MTR10	transportin-3	--		
gene14558	K08819	CDK12_13	cyclin-dependent kinase 12/13	EC:2.7.11.22 2.7.11.23		
gene1455	K10901	BLM, RECQL3, SGS1	bloom syndrome protein	EC:3.6.4.12	2	ko03440 Homologous recombination;ko03460 Fanconi anemia pathway;
gene14561	K07874	RAB1A	Ras-related protein Rab-1A	--	1	ko05134 Legionellosis;
gene14564	K00799	GST, gst	glutathione S-transferase	EC:2.5.1.18	4	ko00480 Glutathione metabolism;ko00980 Metabolism of xenobiotics by cytochrome P450;ko00982 Drug metabolism - cytochrome P450;ko05204 Chemical carcinogenesis;
gene14567	K03549	kup	KUP system potassium uptake protein	--		
gene14568	K03018	RPC1, POLR3A	DNA-directed RNA polymerase III subunit RPC1	EC:2.7.7.6	5	ko00230 Purine metabolism;ko00240 Pyrimidine metabolism;ko03020 RNA polymerase;ko04623 Cytosolic DNA-sensing pathway;ko05169 Epstein-Barr virus infection;
gene14569	K14485	TIR1	transport inhibitor response 1	--	1	ko04075 Plant hormone signal transduction;
gene1456	K15707	RNF170	RING finger protein 170	--		
gene14576	K10355	ACTF	actin, other eukaryote	--		
gene14577	K10355	ACTF	actin, other eukaryote	--		

gene14587	K00921	PIKFYVE, FAB1	1-phosphatidylinositol-3-phosphate 5-kinase	EC:2.7.1.150	4	ko00562 Inositol phosphate metabolism;ko04070 Phosphatidylinositol signaling system;ko04145 Phagosome;ko04810 Regulation of actin cytoskeleton;
gene14594	K14516	ERF1	ethylene-responsive transcription factor 1	--	1	ko04075 Plant hormone signal transduction;
gene14595	K06620	E2F3	transcription factor E2F3	--	13	ko04110 Cell cycle;ko05161 Hepatitis B;ko05166 HTLV-I infection;ko05200 Pathways in cancer;ko05206 MicroRNAs in cancer;ko05212 Pancreatic cancer;ko05214 Glioma;ko05215 Prostate cancer;ko05218 Melanoma;ko05219 Bladder cancer;ko05220 Chronic myeloid leukemia;ko05222 Small cell lung cancer;ko05223 Non-small cell lung cancer;
gene14597	K14231	tRNA-Phe	tRNA Phe	--	1	ko00970 Aminoacyl-tRNA biosynthesis;
gene1459	K09561	STUB1, CHIP	STIP1 homology and U-box containing protein 1	EC:6.3.2.19	2	ko04120 Ubiquitin mediated proteolysis;ko04141 Protein processing in endoplasmic reticulum;
gene14603	K04730	IRAK1	interleukin-1 receptor-associated kinase 1	EC:2.7.11.1	11	ko04064 NF-kappa B signaling pathway;ko04210 Apoptosis;ko04620 Toll-like receptor signaling pathway;ko04722 Neurotrophin signaling pathway;ko05133 Pertussis;ko05140 Leishmaniasis;ko05142 Chagas disease (American trypanosomiasis);ko05145 Toxoplasmosis;ko05152 Tuberculosis;ko05162 Measles;ko05169 Epstein-Barr virus infection;
gene14604	K07200	PRKAG	5'-AMP-activated protein kinase, regulatory gamma subunit	--	5	ko04710 Circadian rhythm;ko04910 Insulin signaling pathway;ko04920 Adipocytokine signaling pathway;ko04932 Non-alcoholic fatty liver disease (NAFLD);ko05410 Hypertrophic cardiomyopathy (HCM);
gene14608	K01814	hisA	phosphoribosylformimino-5-aminoimidazole carboxamide ribotide isomerase	EC:5.3.1.16	2	ko00340 Histidine metabolism;ko01230 Biosynthesis of amino acids;
gene14612	K12373	HEXA_B	hexosaminidase	EC:3.2.1.52	6	ko00511 Other glycan degradation;ko00520 Amino sugar and nucleotide sugar metabolism;ko00531 Glycosaminoglycan degradation;ko00603 Glycosphingolipid biosynthesis - globo series;ko00604 Glycosphingolipid biosynthesis - ganglio series;ko04142 Lysosome;
gene14618	K17619	MDP1	magnesium-dependent phosphatase 1	EC:3.1.3.48		
gene14620	K03978	engB	GTP-binding protein	--		
gene14626	K16240	SPA1	protein suppressor of PHYA-105 1	--	1	ko04712 Circadian rhythm - plant;
gene14627	K00963	UGP2, galU, galF	UTP--glucose-1-phosphate uridylyltransferase	EC:2.7.7.9	4	ko00040 Pentose and glucuronate interconversions;ko00052 Galactose metabolism;ko00500 Starch and sucrose metabolism;ko00520 Amino sugar and nucleotide sugar metabolism;
gene1462	K07765	MBTPS2	S2P endopeptidase	EC:3.4.24.85	1	ko04141 Protein processing in endoplasmic reticulum;
gene14639	K00430	E1.11.1.7	peroxidase	EC:1.11.1.7	2	ko00360 Phenylalanine metabolism;ko00940 Phenylpropanoid biosynthesis;
gene1463	K07765	MBTPS2	S2P endopeptidase	EC:3.4.24.85	1	ko04141 Protein processing in endoplasmic reticulum;
gene14640	K10904	TIPIN	TIMELESS-interacting protein	--		
gene14649	K02213	CDC6	cell division control protein 6	--	3	ko04110 Cell cycle;ko04111 Cell cycle - yeast;ko04113 Meiosis - yeast;
gene1464	K09667	OGT	polypeptide N-acetylglucosaminyltransferase	EC:2.4.1.-	1	ko00514 Other types of O-glycan biosynthesis;
gene14650	K07437	CYP26A	cytochrome P450, family 26, subfamily A	--	1	ko00830 Retinol metabolism;
gene14652	K01102	PDP	pyruvate dehydrogenase phosphatase	EC:3.1.3.43		
gene14658	K13436	PTI1	pto-interacting protein 1	EC:2.7.11.1	1	ko04626 Plant-pathogen interaction;
gene14665	K00799	GST, gst	glutathione S-transferase	EC:2.5.1.18	4	ko00480 Glutathione metabolism;ko00980 Metabolism of xenobiotics by cytochrome P450;ko00982 Drug metabolism - cytochrome P450;ko05204 Chemical carcinogenesis;
gene14668	K05275	E1.1.1.65	pyridoxine 4-dehydrogenase	EC:1.1.1.65	1	ko00750 Vitamin B6 metabolism;
gene14672	K08242	E2.1.1.143	24-methylenesterol C-methyltransferase	EC:2.1.1.143	1	ko00100 Steroid biosynthesis;
gene14673	K02147	ATPeVB, ATP6B1	V-type H+-transporting ATPase subunit B	EC:3.6.3.14	7	ko00190 Oxidative phosphorylation;ko04145 Phagosome;ko04721 Synaptic vesicle cycle;ko04966 Collecting duct acid secretion;ko05110 Vibrio cholerae infection;ko05120 Epithelial cell signaling in Helicobacter pylori infection;ko05323 Rheumatoid arthritis;
gene1467	K01673	cynT, can	carbonic anhydrase	EC:4.2.1.1	1	ko00910 Nitrogen metabolism;
gene14681	K10143	RFWD2, COP1	E3 ubiquitin-protein ligase RFWD2	EC:6.3.2.19	3	ko04115 p53 signaling pathway;ko04120 Ubiquitin mediated proteolysis;ko04712 Circadian rhythm - plant;
gene14682	K12124	GI	GIGANTEA	--	1	ko04712 Circadian rhythm - plant;
gene14683	K16911	DDX21	ATP-dependent RNA helicase DDX21	EC:3.6.4.13		

gene14693	K07198	PRKAA, AMPK	5'-AMP-activated protein kinase, catalytic alpha subunit	EC:2.7.11.11	8	ko04140 Regulation of autophagy;ko04150 mTOR signaling pathway;ko04151 PI3K-Akt signaling pathway;ko04710 Circadian rhythm;ko04910 Insulin signaling pathway;ko04920 Adipocytokine signaling pathway;ko04932 Non-alcoholic fatty liver disease (NAFLD);ko05410 Hypertrophic cardiomyopathy (HCM);
gene14698	K03327	TC.MATE, SLC47A, norM, mdtK, dinF	multidrug resistance protein, MATE family	--		
gene146	K00430	E1.11.1.7	peroxidase	EC:1.11.1.7	2	ko00360 Phenylalanine metabolism;ko00940 Phenylpropanoid biosynthesis;
gene14703	K05765	CFL	cofilin	--	4	ko04360 Axon guidance;ko04666 Fc gamma R-mediated phagocytosis;ko04810 Regulation of actin cytoskeleton;ko05133 Pertussis;
gene14704	K11251	H2A	histone H2A	--	2	ko05034 Alcoholism;ko05322 Systemic lupus erythematosus;
gene14706	K00454	LOX2S	lipoxygenase	EC:1.13.11.12	2	ko00591 Linoleic acid metabolism;ko00592 alpha-Linolenic acid metabolism;
gene14707	K01725	cynS	cyanate lyase	EC:4.2.1.104	1	ko00910 Nitrogen metabolism;
gene14710	K00384	E1.8.1.9, trxB	thioredoxin reductase (NADPH)	EC:1.8.1.9	2	ko00240 Pyrimidine metabolism;ko00450 Selenocompound metabolism;
gene14715	K14489	AHK2_3_4	arabidopsis histidine kinase 2/3/4 (cytokinin receptor)	EC:2.7.13.3	1	ko04075 Plant hormone signal transduction;
gene14718	K09338	HD-ZIP	homeobox-leucine zipper protein	--		
gene14724	K00863	E2.7.1.29, DAK1, DAK2	dihydroxyacetone kinase	EC:2.7.1.29	4	ko00561 Glycerolipid metabolism;ko00680 Methane metabolism;ko01200 Carbon metabolism;ko04622 RIG-I-like receptor signaling pathway;
gene14726	K10364	CAPZA	capping protein (actin filament) muscle Z-line, alpha	--		
gene14731	K14233	tRNA-Ser	tRNA Ser	--	1	ko00970 Aminoacyl-tRNA biosynthesis;
gene14732	K00586	DPH5	diphthine synthase	EC:2.1.1.98		
gene14734	K06966	K06966				
gene14741	K08869	ADCK, ABC1	aarF domain-containing kinase	--		
gene14742	K06126	COQ6	ubiquinone biosynthesis monooxygenase Coq6	EC:1.14.13.-	1	ko00130 Ubiquinone and other terpenoid-quinone biosynthesis;
gene14747	K15255	PIF1	ATP-dependent DNA helicase PIF1	EC:3.6.4.12		
gene1474	K01183	E3.2.1.14	chitinase	EC:3.2.1.14	1	ko00520 Amino sugar and nucleotide sugar metabolism;
gene14750	K05284	PIGM	phosphatidylinositol glycan, class M	EC:2.4.1.-	1	ko00563 Glycosylphosphatidylinositol(GPI)-anchor biosynthesis;
gene14756	K12456	APC13	anaphase-promoting complex subunit 13	--	4	ko04110 Cell cycle;ko04114 Oocyte meiosis;ko04120 Ubiquitin mediated proteolysis;ko04914 Progesterone-mediated oocyte maturation;
gene14764	K05349	bglX	beta-glucosidase	EC:3.2.1.21	3	ko00460 Cyanoamino acid metabolism;ko00500 Starch and sucrose metabolism;ko00940 Phenylpropanoid biosynthesis;
gene14765	K09495	CCT3, TRIC5	T-complex protein 1 subunit gamma	--		
gene14766	K16578	CLASP1_2	CLIP-associating protein 1/2	--		
gene14768	K03251	EIF3D	translation initiation factor 3 subunit D	--	1	ko03013 RNA transport;
gene1476	K15404	K15404, CER1	aldehyde decarbonylase	EC:4.1.99.5	1	ko00073 Cutin, suberine and wax biosynthesis;
gene14775	K14763	NAF1	H/ACA ribonucleoprotein complex non-core subunit NAF1	--		
gene14781	K15803	GERD	(-)-germacrene D synthase	EC:4.2.3.22 4.2.3.75	1	ko00909 Sesquiterpenoid and triterpenoid biosynthesis;
gene14788	K11267	PDS5	sister chromatid cohesion protein PDS5	--		
gene14789	K04498	EP300, CREBBP, KAT3	E1A/CREB-binding protein	EC:2.3.1.48	21	ko04066 HIF-1 signaling pathway;ko04110 Cell cycle;ko04310 Wnt signaling pathway;ko04330 Notch signaling pathway;ko04350 TGF-beta signaling pathway;ko04520 Adherens junction;ko04630 Jak-STAT signaling pathway;ko04720 Long-term potentiation;ko04916 Melanogenesis;ko05016 Huntington's disease;ko05152 Tuberculosis;ko05161 Hepatitis B;ko05164 Influenza A;ko05166 HTLV-I infection;ko05168 Herpes simplex infection;ko05169 Epstein-Barr virus infection;ko05200 Pathways in cancer;ko05203 Viral carcinogenesis;ko05206 MicroRNAs in cancer;ko05211 Renal cell carcinoma;ko05215 Prostate cancer;
gene14793	K00026	MDH2	malate dehydrogenase	EC:1.1.1.37	5	ko00020 Citrate cycle (TCA cycle);ko00620 Pyruvate metabolism;ko00630 Glyoxylate and dicarboxylate metabolism;ko00710 Carbon fixation in photosynthetic organisms;ko01200 Carbon metabolism;
gene14799	K01875	SARS, serS	seryl-tRNA synthetase	EC:6.1.1.11	1	ko00970 Aminoacyl-tRNA biosynthesis;
gene14800	K00434	E1.11.1.11	L-ascorbate peroxidase	EC:1.11.1.11	2	ko00053 Ascorbate and aldarate metabolism;ko00480 Glutathione metabolism;
gene14803	K12818	DHX8, PRP22	ATP-dependent RNA helicase DHX8/PRP22	EC:3.6.4.13	1	ko03040 Spliceosome;

gene14804	K14802	DRS2, ATP8A	phospholipid-transporting ATPase	EC:3.6.3.1		
gene14805	K13495	CISZOG	cis-zeatin O-glucosyltransferase	EC:2.4.1.215	1	ko00908 Zeatin biosynthesis;
gene14809	K10357	MYO5	myosin V	--		
gene1480	K12272	SRPRB, SRP102	signal recognition particle receptor subunit beta	--	1	ko03060 Protein export;
gene14810	K10355	ACTF	actin, other eukaryote	--		
gene1481	K13513	LCLAT1, AGPAT8	lysocardiolipin and lysophospholipid acyltransferase	EC:2.3.1.-2.3.1.51	2	ko00561 Glycerolipid metabolism;ko00564 Glycerophospholipid metabolism;
gene14820	K01673	cynT, can	carbonic anhydrase	EC:4.2.1.1	1	ko00910 Nitrogen metabolism;
gene14822	K17609	NXN	nucleoredoxin	EC:1.8.1.8		
gene14823	K17609	NXN	nucleoredoxin	EC:1.8.1.8		
gene14834	K04125	E1.14.11.13	gibberellin 2-oxidase	EC:1.14.11.13	1	ko00904 Diterpenoid biosynthesis;
gene14837	K01092	E3.1.3.25, IMPA, suhB	myo-inositol-1(or 4)-monophosphatase	EC:3.1.3.25	3	ko00521 Streptomycin biosynthesis;ko00562 Inositol phosphate metabolism;ko04070 Phosphatidylinositol signaling system;
gene14840	K00873	PK, pyk	pyruvate kinase	EC:2.7.1.40	7	ko00010 Glycolysis / Gluconeogenesis;ko00230 Purine metabolism;ko00620 Pyruvate metabolism;ko01200 Carbon metabolism;ko01230 Biosynthesis of amino acids;ko04930 Type II diabetes mellitus;ko05203 Viral carcinogenesis;
gene14843	K14803	PTC2_3	protein phosphatase 2C homolog 2/3	EC:3.1.3.16		
gene14847	K09487	HSP90B, TRA1	heat shock protein 90kDa beta	--	8	ko04141 Protein processing in endoplasmic reticulum;ko04151 PI3K-Akt signaling pathway;ko04621 NOD-like receptor signaling pathway;ko04626 Plant-pathogen interaction;ko04915 Estrogen signaling pathway;ko04918 Thyroid hormone synthesis;ko05200 Pathways in cancer;ko05215 Prostate cancer;
gene1484	K07198	PRKAA, AMPK	5'-AMP-activated protein kinase, catalytic alpha subunit	EC:2.7.11.11	8	ko04140 Regulation of autophagy;ko04150 mTOR signaling pathway;ko04151 PI3K-Akt signaling pathway;ko04710 Circadian rhythm;ko04910 Insulin signaling pathway;ko04920 Adipocytokine signaling pathway;ko04932 Non-alcoholic fatty liver disease (NAFLD);ko05410 Hypertrophic cardiomyopathy (HCM);
gene14851	K01920	E6.3.2.3, gshB	glutathione synthase	EC:6.3.2.3	1	ko00480 Glutathione metabolism;
gene14852	K00814	GPT, ALT	alanine transaminase	EC:2.6.1.2	4	ko00250 Alanine, aspartate and glutamate metabolism;ko00710 Carbon fixation in photosynthetic organisms;ko01210 2-Oxocarboxylic acid metabolism;ko01230 Biosynthesis of amino acids;
gene14861	K14709	SLC39A1_2_3, ZIP1_2_3	solute carrier family 39 (zinc transporter), member 1/2/3	--		
gene14865	K02873	RP-L13e, RPL13	large subunit ribosomal protein L13e	--	1	ko03010 Ribosome;
gene14866	K02948	RP-S11, MRPS11, rpsK	small subunit ribosomal protein S11	--	1	ko03010 Ribosome;
gene14869	K01807	rpiA	ribose 5-phosphate isomerase A	EC:5.3.1.6	4	ko00030 Pentose phosphate pathway;ko00710 Carbon fixation in photosynthetic organisms;ko01200 Carbon metabolism;ko01230 Biosynthesis of amino acids;
gene14870	K00422	E1.10.3.1	polyphenol oxidase	EC:1.10.3.1	2	ko00350 Tyrosine metabolism;ko00950 Isoquinoline alkaloid biosynthesis;
gene14873	K03135	TAF11	transcription initiation factor TFIID subunit 11	--	1	ko03022 Basal transcription factors;
gene14881	K15382	SLC50A, SWEET	solute carrier family 50 (sugar transporter)	--		
gene14882	K01658	trpG	anthranilate synthase component II	EC:4.1.3.27	2	ko00400 Phenylalanine, tyrosine and tryptophan biosynthesis;ko01230 Biosynthesis of amino acids;
gene14883	K03122	TFIIA1, GTF2A1, TOA1	transcription initiation factor TFIIA large subunit	--	2	ko03022 Basal transcription factors;ko05203 Viral carcinogenesis;
gene14884	K01874	MARS, metG	methionyl-tRNA synthetase	EC:6.1.1.10	2	ko00450 Selenocompound metabolism;ko00970 Aminoacyl-tRNA biosynthesis;
gene14885	K08678	UXS1	UDP-glucuronate decarboxylase	EC:4.1.1.35	2	ko00500 Starch and sucrose metabolism;ko00520 Amino sugar and nucleotide sugar metabolism;
gene14893	K00430	E1.11.1.7	peroxidase	EC:1.11.1.7	2	ko00360 Phenylalanine metabolism;ko00940 Phenylpropanoid biosynthesis;
gene14896	K02149	ATPeVD, ATP6M	V-type H+-transporting ATPase subunit D	EC:3.6.3.14	7	ko00190 Oxidative phosphorylation;ko04145 Phagosome;ko04721 Synaptic vesicle cycle;ko04966 Collecting duct acid secretion;ko05110 Vibrio cholerae infection;ko05120 Epithelial cell signaling in Helicobacter pylori infection;ko05323 Rheumatoid arthritis;
gene14897	K13076	SLD	delta8-fatty-acid desaturase	EC:1.14.19.4		
gene14900	K13425	WRKY22	WRKY transcription factor 22	--	1	ko04626 Plant-pathogen interaction;
gene14901	K10878	SPO11	meiotic recombination protein SPO11	--	1	ko04113 Meiosis - yeast;
gene14902	K03452	MHX	magnesium/proton exchanger	--		

gene14906	K01874	MARS, metG	methionyl-tRNA synthetase	EC:6.1.1.10	2	ko00450 Selenocompound metabolism;ko00970 Aminoacyl-tRNA biosynthesis;
gene1490	K11308	MYST1, MOF, KAT8	histone acetyltransferase MYST1	EC:2.3.1.48		
gene14914	K03122	TFIIA1, GTF2A1, TOA1	transcription initiation factor TFIIA large subunit	--	2	ko03022 Basal transcription factors;ko05203 Viral carcinogenesis;
gene14922	K01408	IDE, ide	insulysin	EC:3.4.24.56	1	ko05010 Alzheimer's disease;
gene14928	K10742	DNA2	DNA replication ATP-dependent helicase Dna2	EC:3.6.4.12	1	ko03030 DNA replication;
gene14929	K01489	E3.5.4.5, cdd	cytidine deaminase	EC:3.5.4.5	2	ko00240 Pyrimidine metabolism;ko00983 Drug metabolism - other enzymes;
gene1492	K05387	GRIP	glutamate receptor, ionotropic, plant	--		
gene14933	K04733	IRAK4	interleukin-1 receptor-associated kinase 4	EC:2.7.11.1	11	ko04064 NF-kappa B signaling pathway;ko04210 Apoptosis;ko04620 Toll-like receptor signaling pathway;ko04722 Neurotrophin signaling pathway;ko05133 Pertussis;ko05140 Leishmaniasis;ko05142 Chagas disease (American trypanosomiasis);ko05145 Toxoplasmosis;ko05152 Tuberculosis;ko05162 Measles;ko05164 Influenza A;
gene14935	K10400	KIF15	kinesin family member 15	--		
gene14939	K00876	E2.7.1.48, udk	uridine kinase	EC:2.7.1.48	2	ko00240 Pyrimidine metabolism;ko00983 Drug metabolism - other enzymes;
gene14940	K02882	RP-L18Ae, RPL18A	large subunit ribosomal protein L18Ae	--	1	ko03010 Ribosome;
gene14947	K00696	E2.4.1.14	sucrose-phosphate synthase	EC:2.4.1.14	1	ko00500 Starch and sucrose metabolism;
gene14949	K07904	RAB11A	Ras-related protein Rab-11A	--	4	ko04144 Endocytosis;ko04961 Endocrine and other factor-regulated calcium reabsorption;ko04962 Vasopressin-regulated water reabsorption;ko04972 Pancreatic secretion;
gene1494	K17553	PPP1R11	protein phosphatase 1 regulatory subunit 11	--		
gene14954	K01855	PUS3, DEG1	tRNA pseudouridine38/39 synthase	EC:5.4.99.45		
gene14956	K05674	ABCC10	ATP-binding cassette, subfamily C (CFTR/MRP), member 10	--	1	ko02010 ABC transporters;
gene14960	K02879	RP-L17, MRPL17, rplQ	large subunit ribosomal protein L17	--	1	ko03010 Ribosome;
gene14965	K04124	E1.14.11.15	gibberellin 3-beta-dioxygenase	EC:1.14.11.15	1	ko00904 Diterpenoid biosynthesis;
gene1496	K12845	SNU13, NHP2L	U4/U6 small nuclear ribonucleoprotein SNU13	--	2	ko03008 Ribosome biogenesis in eukaryotes;ko03040 Spliceosome;
gene14973	K00784	rnz	ribonuclease Z	EC:3.1.26.11	1	ko03013 RNA transport;
gene14974	K01188	E3.2.1.21	beta-glucosidase	EC:3.2.1.21	3	ko00460 Cyanoamino acid metabolism;ko00500 Starch and sucrose metabolism;ko00940 Phenylpropanoid biosynthesis;
gene14975	K09873	TIP	aquaporin TIP	--		
gene14985	K08235	E2.4.1.207	xyloglucan:xyloglucosyl transferase	EC:2.4.1.207		
gene14986	K02695	psaH	photosystem I subunit VI	--	1	ko00195 Photosynthesis;
gene14989	K02922	RP-L37e, RPL37	large subunit ribosomal protein L37e	--	1	ko03010 Ribosome;
gene1498	K06700	PSMF1	proteasome inhibitor subunit 1 (PI31)	--	1	ko03050 Proteasome;
gene14990	K06892	K06892				
gene14994	K08829	MAK	male germ cell-associated kinase	EC:2.7.11.22		
gene14998	K03363	CDC20	cell division cycle 20, cofactor of APC complex	--	7	ko04110 Cell cycle;ko04111 Cell cycle - yeast;ko04113 Meiosis - yeast;ko04114 Oocyte meiosis;ko04120 Ubiquitin mediated proteolysis;ko05166 HTLV-I infection;ko05203 Viral carcinogenesis;
gene149	K14638	SLC15A3_4, PHT	solute carrier family 15 (peptide/histidine transporter), member 3/4	--		
gene14	K14497	PP2C	protein phosphatase 2C	EC:3.1.3.16	1	ko04075 Plant hormone signal transduction;
gene15000	K12854	SNRNP200, BRR2	pre-mRNA-splicing helicase BRR2	EC:3.6.4.13	1	ko03040 Spliceosome;
gene15001	K13946	AUX1, LAX	auxin influx carrier (AUX1 LAX family)	--	1	ko04075 Plant hormone signal transduction;
gene15002	K01592	E4.1.1.25	tyrosine decarboxylase	EC:4.1.1.25	2	ko00350 Tyrosine metabolism;ko00950 Isoquinoline alkaloid biosynthesis;
gene15004	K02836	prfB	peptide chain release factor 2	--		
gene15005	K14191	DIM1	18S rRNA (adenine1779-N6/adenine1780-N6)-dimethyltransferase	EC:2.1.1.183		
gene15010	K17262	TBCB, CKAP1, ALF1	tubulin-folding cofactor B	--		
gene15013	K11228	STE11	mitogen-activated protein kinase kinase kinase	EC:2.7.11.1	1	ko04011 MAPK signaling pathway - yeast;
gene15014	K14574	SDO1, SBDS	ribosome maturation protein SDO1	--	1	ko03008 Ribosome biogenesis in eukaryotes;
gene15019	K00102	E1.1.2.4, dld	D-lactate dehydrogenase (cytochrome)	EC:1.1.2.4	1	ko00620 Pyruvate metabolism;
gene15020	K06287	maf	septum formation protein	--		

gene15021	K02933	RP-L6, MRPL6, rplF	large subunit ribosomal protein L6	--	1	ko03010 Ribosome;
gene15028	K00863	E2.7.1.29, DAK1, DAK2	dihydroxyacetone kinase	EC:2.7.1.29	4	ko00561 Glycerolipid metabolism;ko00680 Methane metabolism;ko01200 Carbon metabolism;ko04622 RIG-I-like receptor signaling pathway;
gene15034	K01823	idi, IDI	isopentenyl-diphosphate delta-isomerase	EC:5.3.3.2	1	ko00900 Terpenoid backbone biosynthesis;
gene15036	K01823	idi, IDI	isopentenyl-diphosphate delta-isomerase	EC:5.3.3.2	1	ko00900 Terpenoid backbone biosynthesis;
gene15039	K14500	BSK	BR-signaling kinase	EC:2.7.11.1	1	ko04075 Plant hormone signal transduction;
gene1503	K13348	MPV17	protein Mpv17	--	1	ko04146 Peroxisome;
gene15040	K03798	ftsH, hflB	cell division protease FtsH	EC:3.4.24.-		
gene15048	K01653	E2.2.1.6S, ilvH, ilvN	acetolactate synthase I/III small subunit	EC:2.2.1.6	6	ko00290 Valine, leucine and isoleucine biosynthesis;ko00650 Butanoate metabolism;ko00660 C5-Branched dibasic acid metabolism;ko00770 Pantothenate and CoA biosynthesis;ko01210 2-Oxocarboxylic acid metabolism;ko01230 Biosynthesis of amino acids;
gene15049	K14236	tRNA-Tyr	tRNA Tyr	--	1	ko00970 Aminoacyl-tRNA biosynthesis;
gene15056	K00434	E1.11.1.11	L-ascorbate peroxidase	EC:1.11.1.11	2	ko00053 Ascorbate and aldarate metabolism;ko00480 Glutathione metabolism;
gene15057	K03029	PSMD4, RPN10	26S proteasome regulatory subunit N10	--	2	ko03050 Proteasome;ko05169 Epstein-Barr virus infection;
gene15058	K03260	EIF4G	translation initiation factor 4G	--	2	ko03013 RNA transport;ko05416 Viral myocarditis;
gene1505	K03798	ftsH, hflB	cell division protease FtsH	EC:3.4.24.-		
gene15060	K02891	RP-L22e, RPL22	large subunit ribosomal protein L22e	--	1	ko03010 Ribosome;
gene15067	K00074	paaH, hbd, fadB, mmgB	3-hydroxybutyryl-CoA dehydrogenase	EC:1.1.1.157	3	ko00360 Phenylalanine metabolism;ko00362 Benzoate degradation;ko00650 Butanoate metabolism;
gene15069	K09539	DNAJC19	DnaJ homolog subfamily C member 19	--		
gene15072	K00671	E2.3.1.97, NMT	glycylpeptide N-tetradecanoyltransferase	EC:2.3.1.97		
gene15073	K03257	EIF4A	translation initiation factor 4A	--	1	ko03013 RNA transport;
gene15075	K02218	CSNK1, CK1	casein kinase 1	EC:2.7.11.1	1	ko04340 Hedgehog signaling pathway;
gene1507	K16055	TPS	trehalose 6-phosphate synthase/phosphatase	EC:2.4.1.15 3.1.3.12	1	ko00500 Starch and sucrose metabolism;
gene15082	K15280	SLC35C2	solute carrier family 35, member C2	--		
gene1508	K07904	RAB11A	Ras-related protein Rab-11A	--	4	ko04144 Endocytosis;ko04961 Endocrine and other factor-regulated calcium reabsorption;ko04962 Vasopressin-regulated water reabsorption;ko04972 Pancreatic secretion;
gene15090	K12349	ASAH2	neutral ceramidase	EC:3.5.1.23	1	ko00600 Sphingolipid metabolism;
gene15097	K03190	ureD, ureH	urease accessory protein	--		
gene15099	K02935	RP-L7, MRPL12, rplL	large subunit ribosomal protein L7/L12	--	1	ko03010 Ribosome;
gene1509	K13429	CERK1	chitin elicitor receptor kinase 1	--	1	ko04626 Plant-pathogen interaction;
gene15100	K01728	E4.2.2.2, pel	pectate lyase	EC:4.2.2.2	1	ko00040 Pentose and glucuronate interconversions;
gene15104	K11548	NUF2, CDCA1	kinetochore protein Nuf2	--		
gene15105	K04733	IRAK4	interleukin-1 receptor-associated kinase 4	EC:2.7.11.1	11	ko04064 NF-kappa B signaling pathway;ko04210 Apoptosis;ko04620 Toll-like receptor signaling pathway;ko04722 Neurotrophin signaling pathway;ko05133 Pertussis;ko05140 Leishmaniasis;ko05142 Chagas disease (American trypanosomiasis);ko05145 Toxoplasmosis;ko05152 Tuberculosis;ko05162 Measles;ko05164 Influenza A;
gene15109	K02868	RP-L11e, RPL11	large subunit ribosomal protein L11e	--	1	ko03010 Ribosome;
gene15110	K02868	RP-L11e, RPL11	large subunit ribosomal protein L11e	--	1	ko03010 Ribosome;
gene15112	K00912	lpxK	tetraacyldisaccharide 4'-kinase	EC:2.7.1.130	1	ko00540 Lipopolysaccharide biosynthesis;
gene15115	K05349	bglX	beta-glucosidase	EC:3.2.1.21	3	ko00460 Cyanoamino acid metabolism;ko00500 Starch and sucrose metabolism;ko00940 Phenylpropanoid biosynthesis;
gene1511	K03255	TIF31, CLU1	protein TIF31	--		
gene15120	K04688	RPS6KB	p70 ribosomal S6 kinase	EC:2.7.11.1	9	ko04012 ErbB signaling pathway;ko04066 HIF-1 signaling pathway;ko04150 mTOR signaling pathway;ko04151 PI3K-Akt signaling pathway;ko04350 TGF-beta signaling pathway;ko04666 Fc gamma R-mediated phagocytosis;ko04910 Insulin signaling pathway;ko05205 Proteoglycans in cancer;ko05221 Acute myeloid leukemia;
gene15135	K14332	psaO	photosystem I subunit PsaO	--	1	ko00195 Photosynthesis;
gene15137	K12611	DCP1B	mRNA-decapping enzyme 1B	EC:3.-.-.-	1	ko03018 RNA degradation;

gene15139	K00587	ICMT, STE14	protein-S-isoprenylcysteine O-methyltransferase	EC:2.1.1.100	1	ko00900 Terpenoid backbone biosynthesis;
gene15152	K04371	MAPK1_3	mitogen-activated protein kinase 1/3	EC:2.7.11.24	71	ko04010 MAPK signaling pathway;ko04012 ErbB signaling pathway;ko04013 MAPK signaling pathway - fly;ko04014 Ras signaling pathway;ko04062 Chemokine signaling pathway;ko04066 HIF-1 signaling pathway;ko04114 Oocyte meiosis;ko04150 mTOR signaling pathway;ko04151 PI3K-Akt signaling pathway;ko04270 Vascular smooth muscle contraction;ko04320 Dorso-ventral axis formation;ko04350 TGF-beta signaling pathway;ko04360 Axon guidance;ko04370 VEGF signaling pathway;ko04380 Osteoclast differentiation;ko04510 Focal adhesion;ko04520 Adherens junction;ko04540 Gap junction;ko04620 Toll-like receptor signaling pathway;ko04621 NOD-like receptor signaling pathway;ko04650 Natural killer cell mediated cytotoxicity;ko04660 T cell receptor signaling pathway;ko04662 B cell receptor signaling pathway;ko04664 Fc epsilon RI signaling pathway;ko04666 Fc gamma R-mediated phagocytosis;ko04668 TNF signaling pathway;ko04713 Circadian entrainment;ko04720 Long-term potentiation;ko04722 Neurotrophin signaling pathway;ko04723 Retrograde endocannabinoid signaling;ko04724 Glutamatergic synapse;ko04725 Cholinergic synapse;ko04726 Serotonergic synapse;ko04730 Long-term depression;ko04810 Regulation of actin cytoskeleton;ko04910 Insulin signaling pathway;ko04912 GnRH signaling pathway;ko04914 Progesterone-mediated oocyte maturation;ko04915 Estrogen signaling pathway;ko04916 Melanogenesis;ko04917 Prolactin signaling pathway;ko04930 Type II diabetes mellitus;ko04960 Aldosterone-regulated sodium reabsorption;ko05010 Alzheimer's disease;ko05020 Prion diseases;ko05034 Alcoholism;ko05131 Shigellosis;ko05132 Salmonella infection;ko05133 Pertussis;ko05140 Leishmaniasis;ko05142 Chagas disease (American trypanosomiasis);ko05145 Toxoplasmosis;ko05152 Tuberculosis;ko05160 Hepatitis C;ko05161 Hepatitis B;ko05164 Influenza A;ko05200 Pathways in cancer;ko05203 Viral carcinogenesis;ko05205 Proteoglycans in cancer;ko05210 Colorectal cancer;ko05211 Renal cell carcinoma;ko05212
gene15153	K11804	WDR42A	WD repeat-containing protein 42A	--		
gene15154	K11279	NAP1L1, NRP	nucleosome assembly protein 1-like 1	--		
gene15155	K02959	RP-S16, MRPS16, rpsP	small subunit ribosomal protein S16	--	1	ko03010 Ribosome;
gene15156	K09419	HSFF	heat shock transcription factor, other eukaryote	--		
gene15157	K14508	NPR1	regulatory protein NPR1	--	1	ko04075 Plant hormone signal transduction;
gene15159	K02218	CSNK1, CK1	casein kinase 1	EC:2.7.11.1	1	ko04340 Hedgehog signaling pathway;
gene15161	K11155	DGAT1	diacylglycerol O-acyltransferase 1	EC:2.3.1.20 2.3.1.75 2.3.1.76	3	ko00561 Glycerolipid metabolism;ko00830 Retinol metabolism;ko04975 Fat digestion and absorption;
gene15164	K12613	DCP2	mRNA-decapping enzyme subunit 2	EC:3.6.1.62	1	ko03018 RNA degradation;
gene15166	K03124	TFIIB, GTF2B, SUA7, tfb	transcription initiation factor TFIIB	--	3	ko03022 Basal transcription factors;ko05169 Epstein-Barr virus infection;ko05203 Viral carcinogenesis;
gene15169	K00053	ilvC	ketol-acid reductoisomerase	EC:1.1.1.86	4	ko00290 Valine, leucine and isoleucine biosynthesis;ko00770 Pantothenate and CoA biosynthesis;ko01210 2-Oxocarboxylic acid metabolism;ko01230 Biosynthesis of amino acids;
gene1516	K00079	CBR1	carbonyl reductase 1	EC:1.1.1.184 1.1.1.189 1.1.1.197	3	ko00590 Arachidonic acid metabolism;ko00980 Metabolism of xenobiotics by cytochrome P450;ko05204 Chemical carcinogenesis;
gene15171	K06630	YWHAE	14-3-3 protein epsilon	--	8	ko04110 Cell cycle;ko04114 Oocyte meiosis;ko04151 PI3K-Akt signaling pathway;ko04390 Hippo signaling pathway;ko04391 Hippo signaling pathway - fly;ko04722 Neurotrophin signaling pathway;ko05169 Epstein-Barr virus infection;ko05203 Viral carcinogenesis;
gene15177	K03007	RPB10, POLR2L	DNA-directed RNA polymerases I, II, and III subunit RPABC5	--	6	ko00230 Purine metabolism;ko00240 Pyrimidine metabolism;ko03020 RNA polymerase;ko04623 Cytosolic DNA-sensing pathway;ko05016 Huntington's disease;ko05169 Epstein-Barr virus infection;
gene15182	K07052	K07052				
gene15183	K03327	TC.MATE, SLC47A, norM, mdtK, dinF	multidrug resistance protein, MATE family	--		

gene15187	K13993	HSP20	HSP20 family protein	--	1	ko04141 Protein processing in endoplasmic reticulum;
gene1518	K14684	SLC25A23S	solute carrier family 25 (mitochondrial phosphate transporter), member 23/24/25/41	--		
gene15191	K05236	COPA	coatomer protein complex, subunit alpha (xenin)	--	1	ko04080 Neuroactive ligand-receptor interaction;
gene15195	K17428	MRPL47, NCM1	large subunit ribosomal protein L47, mitochondrial	--		
gene15196	K14611	SLC23A1_2, SVCT1_2	solute carrier family 23 (nucleobase transporter), member 1/2	--		
gene15198	K09377	CSRP	cysteine and glycine-rich protein	--		
gene1519	K02978	RP-S27e, RPS27	small subunit ribosomal protein S27e	--	1	ko03010 Ribosome;
gene15209	K02977	RP-S27Ae, RPS27A	small subunit ribosomal protein S27Ae	--	1	ko03010 Ribosome;
gene1520	K02978	RP-S27e, RPS27	small subunit ribosomal protein S27e	--	1	ko03010 Ribosome;
gene15210	K17601	WDR81	WD repeat-containing protein 81	--		
gene15211	K02893	RP-L23Ae, RPL23A	large subunit ribosomal protein L23Ae	--	1	ko03010 Ribosome;
gene15216	K09338	HD-ZIP	homeobox-leucine zipper protein	--		
gene15217	K00559	E2.1.1.41, SMT1, ERG6	sterol 24-C-methyltransferase	EC:2.1.1.41	1	ko00100 Steroid biosynthesis;
gene15218	K08332	VAC8	vacuolar protein 8	--	1	ko04140 Regulation of autophagy;
gene15219	K01188	E3.2.1.21	beta-glucosidase	EC:3.2.1.21	3	ko00460 Cyanoamino acid metabolism;ko00500 Starch and sucrose metabolism;ko00940 Phenylpropanoid biosynthesis;
gene1521	K13448	CML	calcium-binding protein CML	--	1	ko04626 Plant-pathogen interaction;
gene15223	K00981	E2.7.7.41, CDS1, CDS2, cdsA	phosphatidate cytidylyltransferase	EC:2.7.7.41	2	ko00564 Glycerophospholipid metabolism;ko04070 Phosphatidylinositol signaling system;
gene15226	K03687	GRPE	molecular chaperone GrpE	--		
gene1522	K07904	RAB11A	Ras-related protein Rab-11A	--	4	ko04144 Endocytosis;ko04961 Endocrine and other factor-regulated calcium reabsorption;ko04962 Vasopressin-regulated water reabsorption;ko04972 Pancreatic secretion;
gene15230	K04799	FEN1, RAD2	flap endonuclease-1	EC:3.-.-.-	3	ko03030 DNA replication;ko03410 Base excision repair;ko03450 Non-homologous end-joining;
gene15231	K13566	NIT2	omega-amidase	EC:3.5.1.3	1	ko00250 Alanine, aspartate and glutamate metabolism;
gene15235	K10268	FBXL2_20	F-box and leucine-rich repeat protein 2/20	--		
gene15241	K11671	NFRKB, INO80G	nuclear factor related to kappa-B-binding protein	--		
gene15242	K10398	KIF11, EG5	kinesin family member 11	--		
gene15243	K14487	GH3	auxin responsive GH3 gene family	--	1	ko04075 Plant hormone signal transduction;
gene15249	K00799	GST, gst	glutathione S-transferase	EC:2.5.1.18	4	ko00480 Glutathione metabolism;ko00980 Metabolism of xenobiotics by cytochrome P450;ko00982 Drug metabolism - cytochrome P450;ko05204 Chemical carcinogenesis;
gene15254	K08287	E2.7.12.1	dual-specificity kinase	EC:2.7.12.1		
gene15255	K01517	ADPRM	manganese-dependent ADP-ribose/CDP-alcohol diphosphatase	EC:3.6.1.13 3.6.1.16 3.6.1.53	2	ko00230 Purine metabolism;ko00564 Glycerophospholipid metabolism;
gene15256	K01369	LGMN	legumain	EC:3.4.22.34	2	ko04142 Lysosome;ko04612 Antigen processing and presentation;
gene15259	K02113	ATPF1D, atpH	F-type H+-transporting ATPase subunit delta	EC:3.6.3.14	2	ko00190 Oxidative phosphorylation;ko00195 Photosynthesis;
gene15262	K01922	PPCS, coaB	phosphopantothenate-cysteine ligase	EC:6.3.2.5	1	ko00770 Pantothenate and CoA biosynthesis;
gene15265	K14431	TGA	transcription factor TGA	--	1	ko04075 Plant hormone signal transduction;
gene15267	K14709	SLC39A1_2_3, ZIP1_2_3	solute carrier family 39 (zinc transporter), member 1/2/3	--		
gene15273	K10581	UBE2O	ubiquitin-conjugating enzyme E2 O	EC:6.3.2.19	1	ko04120 Ubiquitin mediated proteolysis;
gene15277	K08853	AAK	AP2-associated kinase	EC:2.7.11.1		
gene15281	K16302	CNNM	metal transporter CNNM	--		
gene15285	K03514	POLS, TRF4	DNA polymerase sigma	EC:2.7.7.7	1	ko03018 RNA degradation;
gene15289	K01723	AOS	hydroperoxide dehydratase	EC:4.2.1.92	1	ko00592 alpha-Linolenic acid metabolism;
gene1528	K10590	TRIP12	E3 ubiquitin-protein ligase TRIP12	EC:6.3.2.19	1	ko04120 Ubiquitin mediated proteolysis;
gene15292	K03801	lipB	lipoyl(octanoyl) transferase	EC:2.3.1.181	1	ko00785 Lipoic acid metabolism;
gene15297	K08235	E2.4.1.207	xyloglucan:xyloglucosyl transferase	EC:2.4.1.207		
gene15301	K10949	KDELR	ER lumen protein retaining receptor	--	1	ko05110 Vibrio cholerae infection;
gene15305	K08472	MLO	mlo protein	--		
gene1530	K10737	MCM8	DNA helicase MCM8	EC:3.6.4.12		

gene15313	K12580	CNOT3, NOT3	CCR4-NOT transcription complex subunit 3	--	1	ko03018 RNA degradation;
gene15319	K16285	XERICO	RING/U-box domain-containing protein	--		
gene1531	K15718	LOX1_5	linoleate 9S-lipoxygenase	EC:1.13.11.58	1	ko00591 Linoleic acid metabolism;
gene15327	K02873	RP-L13e, RPL13	large subunit ribosomal protein L13e	--	1	ko03010 Ribosome;
gene15329	K10527	MFP2	enoyl-CoA hydratase/3-hydroxyacyl-CoA dehydrogenase	EC:4.2.1.17 1.1.1.35 1.1.1.211	2	ko00071 Fatty acid degradation;ko00592 alpha-Linolenic acid metabolism;
gene15330	K16223	FT	protein FLOWERING LOCUS T	--	1	ko04712 Circadian rhythm - plant;
gene15333	K05868	CCNB	cyclin B	--	3	ko04110 Cell cycle;ko04115 p53 signaling pathway;ko04914 Progesterone-mediated oocyte maturation;
gene15336	K10591	NEDD4, RSP5	E3 ubiquitin-protein ligase NEDD4	EC:6.3.2.19	3	ko04120 Ubiquitin mediated proteolysis;ko04144 Endocytosis;ko05169 Epstein-Barr virus infection;
gene15341	K16296	SCPL-I	serine carboxypeptidase-like clade I	EC:3.4.16.-		
gene15343	K00036	G6PD, zwf	glucose-6-phosphate 1-dehydrogenase	EC:1.1.1.49	3	ko00030 Pentose phosphate pathway;ko00480 Glutathione metabolism;ko01200 Carbon metabolism;
gene15344	K15032	MTERFD	mTERF domain-containing protein, mitochondrial	--		
gene15346	K14798	LTV1	protein LTV1	--		
gene15347	K14572	MDN1, REA1	midasin	--	1	ko03008 Ribosome biogenesis in eukaryotes;
gene15349	K01807	rpiA	ribose 5-phosphate isomerase A	EC:5.3.1.6	4	ko00030 Pentose phosphate pathway;ko00710 Carbon fixation in photosynthetic organisms;ko01200 Carbon metabolism;ko01230 Biosynthesis of amino acids;
gene1534	K01183	E3.2.1.14	chitinase	EC:3.2.1.14	1	ko00520 Amino sugar and nucleotide sugar metabolism;
gene15350	K03135	TAF11	transcription initiation factor TFIID subunit 11	--	1	ko03022 Basal transcription factors;
gene15351	K14572	MDN1, REA1	midasin	--	1	ko03008 Ribosome biogenesis in eukaryotes;
gene1536	K15289	SLC35F5	solute carrier family 35, member F5	--		
gene15370	K14559	MPP10	U3 small nucleolar RNA-associated protein MPP10	--	1	ko03008 Ribosome biogenesis in eukaryotes;
gene15371	K08504	BET1	blocked early in transport 1	--	1	ko04130 SNARE interactions in vesicular transport;
gene15377	K14831	MAK16	protein MAK16	--		
gene15378	K04078	groES, HSPE1	chaperonin GroES	--		
gene15379	K14486	K14486, ARF	auxin response factor	--	1	ko04075 Plant hormone signal transduction;
gene15381	K16297	SCPL-II	serine carboxypeptidase-like clade II	EC:3.4.16.-		
gene15382	K14500	BSK	BR-signaling kinase	EC:2.7.11.1	1	ko04075 Plant hormone signal transduction;
gene15385	K06955	K06955				
gene15388	K13342	PEX5, PXR1	peroxin-5	--	1	ko04146 Peroxisome;
gene15392	K14638	SLC15A3_4, PHT	solute carrier family 15 (peptide/histidine transporter), member 3/4	--		
gene15393	K00850	pfkA, PFK	6-phosphofructokinase 1	EC:2.7.1.11	7	ko00010 Glycolysis / Gluconeogenesis;ko00030 Pentose phosphate pathway;ko00051 Fructose and mannose metabolism;ko00052 Galactose metabolism;ko00680 Methane metabolism;ko01200 Carbon metabolism;ko01230 Biosynthesis of amino acids;
gene15396	K03012	RPB4, POLR2D	DNA-directed RNA polymerase II subunit RPB4	--	5	ko00230 Purine metabolism;ko00240 Pyrimidine metabolism;ko03020 RNA polymerase;ko05016 Huntington's disease;ko05169 Epstein-Barr virus infection;
gene15397	K13216	PPP1R8, NIPP1	nuclear inhibitor of protein phosphatase 1	EC:3.1.4.-		
gene15406	K10875	RAD54L, RAD54	DNA repair and recombination protein RAD54 and RAD54-like protein	EC:3.6.4.-	1	ko03440 Homologous recombination;
gene15412	K15414	C1QBP	complement component 1 Q subcomponent-binding protein, mitochondrial	--	1	ko05168 Herpes simplex infection;
gene15413	K14638	SLC15A3_4, PHT	solute carrier family 15 (peptide/histidine transporter), member 3/4	--		
gene15418	K14488	SAUR	SAUR family protein	--	1	ko04075 Plant hormone signal transduction;
gene1541	K16675	ZDHHC14	probable palmitoyltransferase ZDHHC14	EC:2.3.1.-	1	ko04391 Hippo signaling pathway - fly;
gene15427	K12622	LSM3	U6 snRNA-associated Sm-like protein LSm3	--	2	ko03018 RNA degradation;ko03040 Spliceosome;
gene15428	K03007	RPB10, POLR2L	DNA-directed RNA polymerases I, II, and III subunit RPABC5	--	6	ko00230 Purine metabolism;ko00240 Pyrimidine metabolism;ko03020 RNA polymerase;ko04623 Cytosolic DNA-sensing pathway;ko05016 Huntington's disease;ko05169 Epstein-Barr virus infection;
gene15436	K12879	THOC2	THO complex subunit 2	--	2	ko03013 RNA transport;ko03040 Spliceosome;

gene15442	K01166	E3.1.27.1	ribonuclease T2	EC:3.1.27.1		
gene15444	K13354	SLC25A17, PMP34	solute carrier family 25 (peroxisomal adenine nucleotide transporter), member 17	--	1	ko04146 Peroxisome;
gene15447	K15472	CYP71D55	premnaspirodiene oxygenase	EC:1.14.13.121	1	ko00909 Sesquiterpenoid and triterpenoid biosynthesis;
gene15450	K00549	metE	5-methyltetrahydropteroyltriglutamate--homocysteine methyltransferase	EC:2.1.1.14	3	ko00270 Cysteine and methionine metabolism;ko00450 Selenocompound metabolism;ko01230 Biosynthesis of amino acids;
gene15452	K03327	TC.MATE, SLC47A, norM, mdtK, dinF	multidrug resistance protein, MATE family	--		
gene15456	K01183	E3.2.1.14	chitinase	EC:3.2.1.14	1	ko00520 Amino sugar and nucleotide sugar metabolism;
gene15465	K08235	E2.4.1.207	xyloglucan:xyloglucosyl transferase	EC:2.4.1.207		
gene15468	K02358	tuf, TUFM	elongation factor Tu	--	1	ko04626 Plant-pathogen interaction;
gene15469	K03239	EIF2B1	translation initiation factor eIF-2B subunit alpha	--	1	ko03013 RNA transport;
gene15470	K00789	metK	S-adenosylmethionine synthetase	EC:2.5.1.6	2	ko00270 Cysteine and methionine metabolism;ko01230 Biosynthesis of amino acids;
gene15471	K14682	argAB	amino-acid N-acetyltransferase	EC:2.3.1.1	3	ko00330 Arginine and proline metabolism;ko01210 2-Oxocarboxylic acid metabolism;ko01230 Biosynthesis of amino acids;
gene15472	K01051	E3.1.1.11	pectinesterase	EC:3.1.1.11	2	ko00040 Pentose and glucuronate interconversions;ko00500 Starch and sucrose metabolism;
gene15482	K00801	FDFT1	farnesyl-diphosphate farnesyltransferase	EC:2.5.1.21	1	ko00909 Sesquiterpenoid and triterpenoid biosynthesis;
gene15486	K12854	SNRNP200, BRR2	pre-mRNA-splicing helicase BRR2	EC:3.6.4.13	1	ko03040 Spliceosome;
gene15490	K07561	dph2	diphthamide synthase subunit DPH2	--		
gene15491	K00938	E2.7.4.2, mvaK2	phosphomevalonate kinase	EC:2.7.4.2	1	ko00900 Terpenoid backbone biosynthesis;
gene15492	K17498	SPN1, IWS1	transcription factor SPN1	--		
gene15494	K08867	WNK, PRKWNK	WNK lysine deficient protein kinase	EC:2.7.11.1		
gene15499	K07904	RAB11A	Ras-related protein Rab-11A	--	4	ko04144 Endocytosis;ko04961 Endocrine and other factor-regulated calcium reabsorption;ko04962 Vasopressin-regulated water reabsorption;ko04972 Pancreatic secretion;
gene15500	K14689	SLC30A2, ZNT2	solute carrier family 30 (zinc transporter), member 2	--		
gene15503	K15441	TAD2, ADAT2	tRNA-specific adenosine deaminase 2	EC:3.5.4.-		
gene15505	K03676	grxC, GLRX, GLRX2	glutaredoxin 3	--		
gene15507	K03263	EIF5A	translation initiation factor 5A	--		
gene15512	K13065	E2.3.1.133, HCT	shikimate O-hydroxycinnamoyltransferase	EC:2.3.1.133	3	ko00940 Phenylpropanoid biosynthesis;ko00941 Flavonoid biosynthesis;ko00945 Stilbenoid, diarylheptanoid and gingerol biosynthesis;
gene15516	K14484	IAA	auxin-responsive protein IAA	--	1	ko04075 Plant hormone signal transduction;
gene1551	K02725	PSMA1	20S proteasome subunit alpha 6	EC:3.4.25.1	1	ko03050 Proteasome;
gene15520	K00901	E2.7.1.107, DGK, dgkA	diacylglycerol kinase (ATP dependent)	EC:2.7.1.107	3	ko00561 Glycerolipid metabolism;ko00564 Glycerophospholipid metabolism;ko04070 Phosphatidylinositol signaling system;
gene15525	K12818	DHX8, PRP22	ATP-dependent RNA helicase DHX8/PRP22	EC:3.6.4.13	1	ko03040 Spliceosome;
gene15526	K15746	crtZ	beta-carotene 3-hydroxylase	EC:1.14.13.129	1	ko00906 Carotenoid biosynthesis;
gene15527	K04730	IRAK1	interleukin-1 receptor-associated kinase 1	EC:2.7.11.1	11	ko04064 NF-kappa B signaling pathway;ko04210 Apoptosis;ko04620 Toll-like receptor signaling pathway;ko04722 Neurotrophin signaling pathway;ko05133 Pertussis;ko05140 Leishmaniasis;ko05142 Chagas disease (American trypanosomiasis);ko05145 Toxoplasmosis;ko05152 Tuberculosis;ko05162 Measles;ko05169 Epstein-Barr virus infection;
gene15529	K12855	PRPF6, PRP6	pre-mRNA-processing factor 6	--	1	ko03040 Spliceosome;
gene15535	K09250	CNBP	cellular nucleic acid-binding protein	--		
gene15536	K02880	RP-L17e, RPL17	large subunit ribosomal protein L17e	--	1	ko03010 Ribosome;
gene15538	K17255	GDI1_2	Rab GDP dissociation inhibitor	--		
gene15539	K12471	EPN	epsin	--	1	ko04144 Endocytosis;
gene1553	K00611	OTC, argF, argI	ornithine carbamoyltransferase	EC:2.1.3.3	2	ko00330 Arginine and proline metabolism;ko01230 Biosynthesis of amino acids;
gene15540	K08054	CANX	calnexin	--	5	ko04141 Protein processing in endoplasmic reticulum;ko04145 Phagosome;ko04612 Antigen processing and presentation;ko04918 Thyroid hormone synthesis;ko05166 HTLV-I infection;

gene15560	K04733	IRAK4	interleukin-1 receptor-associated kinase 4	EC:2.7.11.1	11	ko04064 NF-kappa B signaling pathway;ko04210 Apoptosis;ko04620 Toll-like receptor signaling pathway;ko04722 Neurotrophin signaling pathway;ko05133 Pertussis;ko05140 Leishmaniasis;ko05142 Chagas disease (American trypanosomiasis);ko05145 Toxoplasmosis;ko05152 Tuberculosis;ko05162 Measles;ko05164 Influenza A;
gene15562	K13448	CML	calcium-binding protein CML	--	1	ko04626 Plant-pathogen interaction;
gene15567	K15172	SUPT5H, SPT5	transcription elongation factor SPT5	--		
gene15572	K12194	CHMP4, SNF7, VPS32	charged multivesicular body protein 4	--	1	ko04144 Endocytosis;
gene15577	K10994	RAD9A	cell cycle checkpoint control protein RAD9A	EC:3.1.11.2		
gene15580	K11251	H2A	histone H2A	--	2	ko05034 Alcoholism;ko05322 Systemic lupus erythematosus;
gene15585	K09285	OVM, ANT	AP2-like factor, ANT lineage	--		
gene1558	K13162	PCBP2_3_4	poly(rC)-binding protein 2/3/4	--		
gene15596	K02918	RP-L35e, RPL35	large subunit ribosomal protein L35e	--	1	ko03010 Ribosome;
gene15597	K00844	HK	hexokinase	EC:2.7.1.1	12	ko00010 Glycolysis / Gluconeogenesis;ko00051 Fructose and mannose metabolism;ko00052 Galactose metabolism;ko00500 Starch and sucrose metabolism;ko00520 Amino sugar and nucleotide sugar metabolism;ko00521 Streptomycin biosynthesis;ko00524 Butirosin and neomycin biosynthesis;ko01200 Carbon metabolism;ko04066 HIF-1 signaling pathway;ko04910 Insulin signaling pathway;ko04930 Type II diabetes mellitus;ko04973 Carbohydrate digestion and absorption;
gene15599	K11000	CALS	callose synthase	EC:2.4.1.-		
gene15601	K15103	UCP2_3, SLC25A8_9	solute carrier family 25 (mitochondrial uncoupling protein), member 8/9	--		
gene1560	K14763	NAF1	H/ACA ribonucleoprotein complex non-core subunit NAF1	--		
gene15611	K00011	E1.1.1.21, AKR1	aldehyde reductase	EC:1.1.1.21	5	ko00040 Pentose and glucuronate interconversions;ko00051 Fructose and mannose metabolism;ko00052 Galactose metabolism;ko00561 Glycerolipid metabolism;ko00620 Pyruvate metabolism;
gene15615	K07893	RAB6A	Ras-related protein Rab-6A	--		
gene15617	K13356	FAR	fatty acyl-CoA reductase	EC:1.2.1.-	2	ko00073 Cutin, suberine and wax biosynthesis;ko04146 Peroxisome;
gene15619	K09201	YY	transcription factor YY	--		
gene15620	K15139	MED22	mediator of RNA polymerase II transcription subunit 22	--		
gene15621	K02951	RP-S12e, RPS12	small subunit ribosomal protein S12e	--	1	ko03010 Ribosome;
gene15622	K05665	ABCC1	ATP-binding cassette, subfamily C (CFTR/MRP), member 1	--	3	ko02010 ABC transporters;ko04977 Vitamin digestion and absorption;ko05206 MicroRNAs in cancer;
gene15623	K09422	MYBP	myb proto-oncogene protein, plant	--		
gene15625	K02915	RP-L34e, RPL34	large subunit ribosomal protein L34e	--	1	ko03010 Ribosome;
gene15626	K00799	GST, gst	glutathione S-transferase	EC:2.5.1.18	4	ko00480 Glutathione metabolism;ko00980 Metabolism of xenobiotics by cytochrome P450;ko00982 Drug metabolism - cytochrome P450;ko05204 Chemical carcinogenesis;
gene15630	K02868	RP-L11e, RPL11	large subunit ribosomal protein L11e	--	1	ko03010 Ribosome;
gene15632	K03100	lepB	signal peptidase I	EC:3.4.21.89	1	ko03060 Protein export;
gene15634	K14638	SLC15A3_4, PHT	solute carrier family 15 (peptide/histidine transporter), member 3/4	--		
gene15635	K14638	SLC15A3_4, PHT	solute carrier family 15 (peptide/histidine transporter), member 3/4	--		
gene15639	K13963	SERPINB	serpin B	--	1	ko05146 Amoebiasis;
gene15640	K13963	SERPINB	serpin B	--	1	ko05146 Amoebiasis;
gene15641	K13963	SERPINB	serpin B	--	1	ko05146 Amoebiasis;
gene15643	K13337	PEX19	peroxin-19	--	1	ko04146 Peroxisome;
gene15644	K02907	RP-L30, MRPL30, rpmD	large subunit ribosomal protein L30	--	1	ko03010 Ribosome;
gene15645	K13420	FLS2	LRR receptor-like serine/threonine-protein kinase FLS2	EC:2.7.11.1	1	ko04626 Plant-pathogen interaction;
gene15647	K03294	TC.APA	basic amino acid/polyamine antiporter, APA family	--		
gene15653	K12826	SF3A2, SAP62	splicing factor 3A subunit 2	--	1	ko03040 Spliceosome;

gene15654	K00889	E2.7.1.68, PIP5K	1-phosphatidylinositol-4-phosphate 5-kinase	EC:2.7.1.68	5	ko00562 Inositol phosphate metabolism;ko04070 Phosphatidylinositol signaling system;ko04144 Endocytosis;ko04666 Fc gamma R-mediated phagocytosis;ko04810 Regulation of actin cytoskeleton;
gene15657	K01647	CS, gltA	citrate synthase	EC:2.3.3.1	5	ko00020 Citrate cycle (TCA cycle);ko00630 Glyoxylate and dicarboxylate metabolism;ko01200 Carbon metabolism;ko01210 2-Oxocarboxylic acid metabolism;ko01230 Biosynthesis of amino acids;
gene15659	K10882	EME1, MMS4	crossover junction endonuclease EME1	EC:3.1.22.-	2	ko03440 Homologous recombination;ko03460 Fanconi anemia pathway;
gene15660	K08819	CDK12_13	cyclin-dependent kinase 12/13	EC:2.7.11.22 2.7.11.23		
gene15661	K08819	CDK12_13	cyclin-dependent kinase 12/13	EC:2.7.11.22 2.7.11.23		
gene15666	K13162	PCBP2_3_4	poly(rC)-binding protein 2/3/4	--		
gene15667	K05349	bglX	beta-glucosidase	EC:3.2.1.21	3	ko00460 Cyanoamino acid metabolism;ko00500 Starch and sucrose metabolism;ko00940 Phenylpropanoid biosynthesis;
gene15672	K10685	UBLE1B, SAE2, UBA2	ubiquitin-like 1-activating enzyme E1 B	EC:6.3.2.19	1	ko04120 Ubiquitin mediated proteolysis;
gene15673	K10875	RAD54L, RAD54	DNA repair and recombination protein RAD54 and RAD54-like protein	EC:3.6.4.-	1	ko03440 Homologous recombination;
gene15675	K14573	NOP4, RBM28	nucleolar protein 4	--	1	ko03008 Ribosome biogenesis in eukaryotes;
gene15682	K14235	tRNA-Trp	tRNA Trp	--	1	ko00970 Aminoacyl-tRNA biosynthesis;
gene15686	K08492	STX18	syntaxin 18	--	2	ko04130 SNARE interactions in vesicular transport;ko04145 Phagosome;
gene15687	K14310	NUP205	nuclear pore complex protein Nup205	--	1	ko03013 RNA transport;
gene15696	K02320	POLA1	DNA polymerase alpha subunit A	EC:2.7.7.7	3	ko00230 Purine metabolism;ko00240 Pyrimidine metabolism;ko03030 DNA replication;
gene15697	K00763	pncB, NAPRT1	nicotinate phosphoribosyltransferase	EC:6.3.4.21	1	ko00760 Nicotinate and nicotinamide metabolism;
gene15698	K00670	E2.3.1.88	peptide alpha-N-acetyltransferase	EC:2.3.1.88		
gene15700	K09567	PPIH, CYPH	peptidyl-prolyl isomerase H (cyclophilin H)	EC:5.2.1.8	1	ko03040 Spliceosome;
gene15705	K03252	EIF3C	translation initiation factor 3 subunit C	--	1	ko03013 RNA transport;
gene15708	K09377	CSRP	cysteine and glycine-rich protein	--		
gene1570	K10683	BARD1	BRCA1-associated RING domain protein 1	EC:6.3.2.19		
gene15711	K14611	SLC23A1_2, SVCT1_2	solute carrier family 23 (nucleobase transporter), member 1/2	--		
gene15718	K14488	SAUR	SAUR family protein	--	1	ko04075 Plant hormone signal transduction;
gene15726	K10756	RFC3_5	replication factor C subunit 3/5	--	3	ko03030 DNA replication;ko03420 Nucleotide excision repair;ko03430 Mismatch repair;
gene15729	K15803	GERD	(-)-germacrene D synthase	EC:4.2.3.22 4.2.3.75	1	ko00909 Sesquiterpenoid and triterpenoid biosynthesis;
gene15730	K09422	MYBP	myb proto-oncogene protein, plant	--		
gene15732	K01102	PDP	pyruvate dehydrogenase phosphatase	EC:3.1.3.43		
gene15739	K13789	GGPS	geranylgeranyl diphosphate synthase, type II	EC:2.5.1.1 2.5.1.10 2.5.1.29	1	ko00900 Terpenoid backbone biosynthesis;
gene15745	K07253	MIF	phenylpyruvate tautomerase	EC:5.3.2.1	2	ko00350 Tyrosine metabolism;ko00360 Phenylalanine metabolism;
gene15746	K08287	E2.7.12.1	dual-specificity kinase	EC:2.7.12.1		
gene15748	K08790	STK38, NDR	serine/threonine kinase 38	EC:2.7.11.1		
gene15752	K00750	GYG1, GYG2	glycogenin glucosyltransferase	EC:2.4.1.186		
gene15753	K14219	tRNA-Arg	tRNA Arg	--	1	ko00970 Aminoacyl-tRNA biosynthesis;
gene15755	K01106	E3.1.3.56	inositol-1,4,5-trisphosphate 5-phosphatase	EC:3.1.3.56	2	ko00562 Inositol phosphate metabolism;ko04070 Phosphatidylinositol signaling system;
gene15757	K03884	ND6	NADH-ubiquinone oxidoreductase chain 6	EC:1.6.5.3	2	ko00190 Oxidative phosphorylation;ko05012 Parkinson's disease;
gene15759	K14220	tRNA-Asn	tRNA Asn	--	1	ko00970 Aminoacyl-tRNA biosynthesis;
gene15761	K15095	E1.1.1.208	(+)-neomenthol dehydrogenase	EC:1.1.1.208	1	ko00902 Monoterpenoid biosynthesis;
gene15763	K14537	NUG2, GNL2	nuclear GTP-binding protein	--	1	ko03008 Ribosome biogenesis in eukaryotes;
gene15764	K08472	MLO	mlo protein	--		
gene15776	K09422	MYBP	myb proto-oncogene protein, plant	--		
gene15777	K10523	SPOP	speckle-type POZ protein	--		
gene15779	K15376	GPHN	gephyrin	EC:2.10.1.1 2.7.7.75	1	ko04727 GABAergic synapse;
gene15780	K05868	CCNB	cyclin B	--	3	ko04110 Cell cycle;ko04115 p53 signaling pathway;ko04914 Progesterone-mediated oocyte maturation;

gene15784	K13420	FLS2	LRR receptor-like serine/threonine-protein kinase FLS2	EC:2.7.11.1	1	ko04626 Plant-pathogen interaction;
gene15785	K13420	FLS2	LRR receptor-like serine/threonine-protein kinase FLS2	EC:2.7.11.1	1	ko04626 Plant-pathogen interaction;
gene15789	K00919	ispE	4-diphosphocytidyl-2-C-methyl-D-erythritol kinase	EC:2.7.1.148	1	ko00900 Terpenoid backbone biosynthesis;
gene15792	K00284	E1.4.7.1	glutamate synthase (ferredoxin)	EC:1.4.7.1	2	ko00630 Glyoxylate and dicarboxylate metabolism;ko00910 Nitrogen metabolism;
gene15797	K12670	WBP1	oligosaccharyltransferase complex subunit beta	--	3	ko00510 N-Glycan biosynthesis;ko00513 Various types of N-glycan biosynthesis;ko04141 Protein processing in endoplasmic reticulum;
gene15798	K03043	rpoB	DNA-directed RNA polymerase subunit beta	EC:2.7.7.6	3	ko00230 Purine metabolism;ko00240 Pyrimidine metabolism;ko03020 RNA polymerase;
gene15800	K01533	E3.6.3.4, ATP7, copA	Cu2+-exporting ATPase	EC:3.6.3.4		
gene15801	K01853	E5.4.99.8	cycloartenol synthase	EC:5.4.99.8	1	ko00100 Steroid biosynthesis;
gene15802	K09548	PFDN1	prefoldin subunit 1	--		
gene15803	K12885	RBMX, HNRNPG	heterogeneous nuclear ribonucleoprotein G	--	1	ko03040 Spliceosome;
gene15805	K11838	USP7, UBP15	ubiquitin carboxyl-terminal hydrolase 7	EC:3.1.2.15	3	ko05168 Herpes simplex infection;ko05169 Epstein-Barr virus infection;ko05203 Viral carcinogenesis;
gene15806	K11838	USP7, UBP15	ubiquitin carboxyl-terminal hydrolase 7	EC:3.1.2.15	3	ko05168 Herpes simplex infection;ko05169 Epstein-Barr virus infection;ko05203 Viral carcinogenesis;
gene15813	K04733	IRAK4	interleukin-1 receptor-associated kinase 4	EC:2.7.11.1	11	ko04064 NF-kappa B signaling pathway;ko04210 Apoptosis;ko04620 Toll-like receptor signaling pathway;ko04722 Neurotrophin signaling pathway;ko05133 Pertussis;ko05140 Leishmaniasis;ko05142 Chagas disease (American trypanosomiasis);ko05145 Toxoplasmosis;ko05152 Tuberculosis;ko05162 Measles;ko05164 Influenza A;
gene15815	K06184	ABCF1	ATP-binding cassette, subfamily F, member 1	--		
gene15816	K06693	PSMD9	26S proteasome non-ATPase regulatory subunit 9	--		
gene15822	K00430	E1.11.1.7	peroxidase	EC:1.11.1.7	2	ko00360 Phenylalanine metabolism;ko00940 Phenylpropanoid biosynthesis;
gene15827	K14432	ABF	ABA responsive element binding factor	--	1	ko04075 Plant hormone signal transduction;
gene15830	K16075	MRS2, MFM1	magnesium transporter	--		
gene15833	K06883	K06883				
gene15836	K01369	LGMN	legumain	EC:3.4.22.34	2	ko04142 Lysosome;ko04612 Antigen processing and presentation;
gene15837	K01369	LGMN	legumain	EC:3.4.22.34	2	ko04142 Lysosome;ko04612 Antigen processing and presentation;
gene15840	K09264	K09264	MADS-box transcription factor, plant	--		
gene15847	K01739	metB	cystathionine gamma-synthase	EC:2.5.1.48	4	ko00270 Cysteine and methionine metabolism;ko00450 Selenocompound metabolism;ko00920 Sulfur metabolism;ko01230 Biosynthesis of amino acids;
gene15848	K14220	tRNA-Asn	tRNA Asn	--	1	ko00970 Aminoacyl-tRNA biosynthesis;
gene15849	K06269	PPP1C	serine/threonine-protein phosphatase PP1 catalytic subunit	EC:3.1.3.16	14	ko03015 mRNA surveillance pathway;ko04113 Meiosis - yeast;ko04114 Oocyte meiosis;ko04270 Vascular smooth muscle contraction;ko04390 Hippo signaling pathway;ko04510 Focal adhesion;ko04720 Long-term potentiation;ko04728 Dopaminergic synapse;ko04810 Regulation of actin cytoskeleton;ko04910 Insulin signaling pathway;ko05031 Amphetamine addiction;ko05034 Alcoholism;ko05168 Herpes simplex infection;ko05205 Proteoglycans in
gene1584	K17361	ACOT9	acyl-coenzyme A thioesterase 9	EC:3.1.2.-		
gene15854	K09286	EREBP	EREBP-like factor	--		
gene15855	K06110	EXOC3, SEC6L1	exocyst complex component 3	--	1	ko04530 Tight junction;
gene15856	K05909	E1.10.3.2				
gene15858	K11373	ELP1, IKI3, IKBKAP	elongator complex protein 1	--		
gene15861	K06901	pbuG	putative MFS transporter, AGZA family, xanthine/uracil permease	--		
gene15864	K16284	SIS3	E3 ubiquitin-protein ligase SIS3	EC:6.3.2.19		
gene15869	K08852	ERN1	serine/threonine-protein kinase/endoribonuclease IRE1	EC:2.7.11.1	3	ko04141 Protein processing in endoplasmic reticulum;ko04932 Non-alcoholic fatty liver disease (NAFLD);ko05010 Alzheimer's disease;
gene15872	K10752	RBBP4, HAT2, CAF1, MIS16	histone-binding protein RBBP4	--		
gene15873	K10752	RBBP4, HAT2, CAF1, MIS16	histone-binding protein RBBP4	--		
gene15874	K11643	CHD4, MI2B	chromodomain-helicase-DNA-binding protein 4	EC:3.6.4.12	1	ko05203 Viral carcinogenesis;

gene15875	K06883	K06883				
gene15882	K10591	NEDD4, RSP5	E3 ubiquitin-protein ligase NEDD4	EC:6.3.2.19	3	ko04120 Ubiquitin mediated proteolysis;ko04144 Endocytosis;ko05169 Epstein-Barr virus infection;
gene15883	K03327	TC.MATE, SLC47A, norM, mdtK, dinF	multidrug resistance protein, MATE family	--		
gene15885	K07766	E3.6.1.52	diphosphoinositol-polyphosphate diphosphatase	EC:3.6.1.52		
gene15889	K15718	LOX1_5	linoleate 9S-lipoxygenase	EC:1.13.11.58	1	ko00591 Linoleic acid metabolism;
gene1588	K14779	DDX52, ROK1	ATP-dependent RNA helicase DDX52/ROK1	EC:3.6.4.13		
gene15893	K00915	IPK2	1D-myo-Inositol-tetrakisphosphate 5-kinase / inositol-polyphosphate multikinase	EC:2.7.1.140 2.7.1.151	2	ko00562 Inositol phosphate metabolism;ko04070 Phosphatidylinositol signaling system;
gene15894	K15979	SND1	staphylococcal nuclease domain-containing protein 1	--	2	ko05169 Epstein-Barr virus infection;ko05203 Viral carcinogenesis;
gene15895	K00430	E1.11.1.7	peroxidase	EC:1.11.1.7	2	ko00360 Phenylalanine metabolism;ko00940 Phenylpropanoid biosynthesis;
gene15896	K01673	cynT, can	carbonic anhydrase	EC:4.2.1.1	1	ko00910 Nitrogen metabolism;
gene158	K09422	MYBP	myb proto-oncogene protein, plant	--		
gene15905	K10573	UBE2A, UBC2, RAD6A	ubiquitin-conjugating enzyme E2 A	EC:6.3.2.19	1	ko04120 Ubiquitin mediated proteolysis;
gene15906	K14496	PYL	abscisic acid receptor PYR/PYL family	--	1	ko04075 Plant hormone signal transduction;
gene15911	K02942	RP-LP1, RPLP1	large subunit ribosomal protein LP1	--	1	ko03010 Ribosome;
gene15912	K10364	CAPZA	capping protein (actin filament) muscle Z-line, alpha	--		
gene15915	K10364	CAPZA	capping protein (actin filament) muscle Z-line, alpha	--		
gene15919	K10733	GINS2, PSF2	GINS complex subunit 2	--		
gene15926	K14457	MOGAT2, MGAT2	2-acylglycerol O-acyltransferase 2	EC:2.3.1.22	1	ko04975 Fat digestion and absorption;
gene1592	K11835	USP4_11_15, UBP12	ubiquitin carboxyl-terminal hydrolase 4/11/15	EC:3.1.2.15		
gene15941	K11420	EHMT	euchromatic histone-lysine N-methyltransferase	EC:2.1.1.43	1	ko00310 Lysine degradation;
gene15946	K06685	MOB1, Mats	MOB kinase activator 1	--	3	ko04111 Cell cycle - yeast;ko04390 Hippo signaling pathway;ko04391 Hippo signaling pathway - fly;
gene15953	K14488	SAUR	SAUR family protein	--	1	ko04075 Plant hormone signal transduction;
gene15954	K14488	SAUR	SAUR family protein	--	1	ko04075 Plant hormone signal transduction;
gene15955	K14488	SAUR	SAUR family protein	--	1	ko04075 Plant hormone signal transduction;
gene15964	K02897	RP-L25, rplY	large subunit ribosomal protein L25	--	1	ko03010 Ribosome;
gene15976	K01627	kdsA	2-dehydro-3-deoxyphosphooctonate aldolase (KDO 8-P synthase)	EC:2.5.1.55	1	ko00540 Lipopolysaccharide biosynthesis;
gene15983	K01897	ACSL, fadD	long-chain acyl-CoA synthetase	EC:6.2.1.3	4	ko00071 Fatty acid degradation;ko03320 PPAR signaling pathway;ko04146 Peroxisome;ko04920 Adipocytokine signaling pathway;
gene15984	K05665	ABCC1	ATP-binding cassette, subfamily C (CFTR/MRP), member 1	--	3	ko02010 ABC transporters;ko04977 Vitamin digestion and absorption;ko05206 MicroRNAs in cancer;
gene15985	K05275	E1.1.1.65	pyridoxine 4-dehydrogenase	EC:1.1.1.65	1	ko00750 Vitamin B6 metabolism;
gene15987	K02434	gatB	aspartyl-tRNA(Asn)/glutamyl-tRNA(Gln) amidotransferase subunit B	EC:6.3.5.6 6.3.5.7	1	ko00970 Aminoacyl-tRNA biosynthesis;
gene15989	K15544	SSU72	RNA polymerase II subunit A C-terminal domain phosphatase SSU72	EC:3.1.3.16	1	ko03015 mRNA surveillance pathway;
gene15994	K07904	RAB11A	Ras-related protein Rab-11A	--	4	ko04144 Endocytosis;ko04961 Endocrine and other factor-regulated calcium reabsorption;ko04962 Vasopressin-regulated water reabsorption;ko04972 Pancreatic secretion;
gene15995	K15334	NCL1, TRM4	multisite-specific tRNA:(cytosine-C5)-methyltransferase	EC:2.1.1.202		
gene15996	K00430	E1.11.1.7	peroxidase	EC:1.11.1.7	2	ko00360 Phenylalanine metabolism;ko00940 Phenylpropanoid biosynthesis;
gene159	K03319	TC.DASS	divalent anion:Na+ symporter, DASS family	--		
gene15	K14396	PABPN1, PABP2	polyadenylate-binding protein 2	--	2	ko03015 mRNA surveillance pathway;ko05164 Influenza A;
gene1600	K01880	GARS, glyS1	glycyl-tRNA synthetase	EC:6.1.1.14	1	ko00970 Aminoacyl-tRNA biosynthesis;
gene16012	K08099	E3.1.1.14	chlorophyllase	EC:3.1.1.14	1	ko00860 Porphyrin and chlorophyll metabolism;
gene16013	K11789	VPRBP, DCAF1	HIV-1 Vpr-binding protein	--		
gene16014	K14963	WDR5, SWD3, CPS30	COMPASS component SWD3	--		
gene16017	K15203	GTF3C6	general transcription factor 3C polypeptide 6	--		

gene16021	K01184	E3.2.1.15	polygalacturonase	EC:3.2.1.15	2	ko00040 Pentose and glucuronate interconversions;ko00500 Starch and sucrose metabolism;
gene16022	K13447	RBOH	respiratory burst oxidase	EC:1.6.3.- 1.11.1.-	1	ko04626 Plant-pathogen interaction;
gene16024	K11816	YUCCA	indole-3-pyruvate monooxygenase	EC:1.14.13.168	1	ko00380 Tryptophan metabolism;
gene16030	K13051	iaaA, ASRGL1	beta-aspartyl-peptidase (threonine type)	EC:3.4.19.5		
gene16031	K00863	E2.7.1.29, DAK1, DAK2	dihydroxyacetone kinase	EC:2.7.1.29	4	ko00561 Glycerolipid metabolism;ko00680 Methane metabolism;ko01200 Carbon metabolism;ko04622 RIG-I-like receptor signaling pathway;
gene16034	K05863	SLC25A4S, ANT	solute carrier family 25 (mitochondrial adenine nucleotide translocator), member 4/5/6/31	--	4	ko04020 Calcium signaling pathway;ko05012 Parkinson's disease;ko05016 Huntington's disease;ko05166 HTLV-I infection;
gene16039	K03676	grxC, GLRX, GLRX2	glutaredoxin 3	--		
gene16040	K00031	IDH1, IDH2, icd	isocitrate dehydrogenase	EC:1.1.1.42	7	ko00020 Citrate cycle (TCA cycle);ko00480 Glutathione metabolism;ko00720 Carbon fixation pathways in prokaryotes;ko01200 Carbon metabolism;ko01210 2-Oxocarboxylic acid metabolism;ko01230 Biosynthesis of amino acids;ko04146 Peroxisome;
gene16041	K06888	K06888				
gene16042	K02835	prfA, MTRF1, MRF1	peptide chain release factor 1	--		
gene1604	K09286	EREBP	EREBP-like factor	--		
gene16053	K12580	CNOT3, NOT3	CCR4-NOT transcription complex subunit 3	--	1	ko03018 RNA degradation;
gene16058	K09060	GBF	plant G-box-binding factor	--		
gene16059	K09338	HD-ZIP	homeobox-leucine zipper protein	--		
gene16063	K03787	surE	5'-nucleotidase	EC:3.1.3.5	3	ko00230 Purine metabolism;ko00240 Pyrimidine metabolism;ko00760 Nicotinate and nicotinamide metabolism;
gene16069	K17613	CABIN1	calcineurin-binding protein cabin-1	--		
gene16070	K00654	E2.3.1.50	serine palmitoyltransferase	EC:2.3.1.50	1	ko00600 Sphingolipid metabolism;
gene16076	K00264	GLT1	glutamate synthase (NADPH/NADH)	EC:1.4.1.13 1.4.1.14	3	ko00250 Alanine, aspartate and glutamate metabolism;ko00910 Nitrogen metabolism;ko01230 Biosynthesis of amino acids;
gene16080	K14638	SLC15A3_4, PHT	solute carrier family 15 (peptide/histidine transporter), member 3/4	--		
gene16081	K00873	PK, pyk	pyruvate kinase	EC:2.7.1.40	7	ko00010 Glycolysis / Gluconeogenesis;ko00230 Purine metabolism;ko00620 Pyruvate metabolism;ko01200 Carbon metabolism;ko01230 Biosynthesis of amino acids;ko04930 Type II diabetes mellitus;ko05203 Viral carcinogenesis;
gene16083	K09338	HD-ZIP	homeobox-leucine zipper protein	--		
gene16087	K02604	ORC2	origin recognition complex subunit 2	--	3	ko04110 Cell cycle;ko04111 Cell cycle - yeast;ko04113 Meiosis - yeast;
gene1608	K03428	E2.1.1.11, chlM, bchM	magnesium-protoporphyrin O-methyltransferase	EC:2.1.1.11	1	ko00860 Porphyrin and chlorophyll metabolism;
gene16092	K14432	ABF	ABA responsive element binding factor	--	1	ko04075 Plant hormone signal transduction;
gene16099	K00858	ppnK, NADK	NAD+ kinase	EC:2.7.1.23	1	ko00760 Nicotinate and nicotinamide metabolism;
gene160	K09458	fabF	3-oxoacyl-[acyl-carrier-protein] synthase II	EC:2.3.1.179	2	ko00061 Fatty acid biosynthesis;ko00780 Biotin metabolism;
gene16100	K05933	E1.14.17.4	aminocyclopropanecarboxylate oxidase	EC:1.14.17.4	1	ko00270 Cysteine and methionine metabolism;
gene16113	K08235	E2.4.1.207	xyloglucan:xyloglucosyl transferase	EC:2.4.1.207		
gene16117	K01184	E3.2.1.15	polygalacturonase	EC:3.2.1.15	2	ko00040 Pentose and glucuronate interconversions;ko00500 Starch and sucrose metabolism;
gene16131	K00382	DLD, lpd, pdhD	dihydrolipoamide dehydrogenase	EC:1.8.1.4	6	ko00010 Glycolysis / Gluconeogenesis;ko00020 Citrate cycle (TCA cycle);ko00260 Glycine, serine and threonine metabolism;ko00280 Valine, leucine and isoleucine degradation;ko00620 Pyruvate metabolism;ko01200 Carbon metabolism;
gene16136	K13431	SRPR	signal recognition particle receptor subunit alpha	--	1	ko03060 Protein export;
gene1613	K09338	HD-ZIP	homeobox-leucine zipper protein	--		
gene16141	K12176	COPS2, CSN2, TRIP15	COP9 signalosome complex subunit 2	--		
gene16144	K15920	XYL4	beta-D-xylosidase 4	EC:3.2.1.37	2	ko00500 Starch and sucrose metabolism;ko00520 Amino sugar and nucleotide sugar metabolism;
gene16147	K01649	leuA	2-isopropylmalate synthase	EC:2.3.3.13	4	ko00290 Valine, leucine and isoleucine biosynthesis;ko00620 Pyruvate metabolism;ko01210 2-Oxocarboxylic acid metabolism;ko01230 Biosynthesis of amino acids;
gene16151	K09840	NCED	9-cis-epoxycarotenoid dioxygenase	EC:1.13.11.51	1	ko00906 Carotenoid biosynthesis;

gene16154	K03294	TC.APA	basic amino acid/polyamine antiporter, APA family	--		
gene16156	K14165	K14165	dual specificity phosphatase	EC:3.1.3.16 3.1.3.48		
gene16158	K11498	CENPE	centromeric protein E	--		
gene16159	K10624	RBBP6	E3 ubiquitin-protein ligase RBBP6	EC:6.3.2.19		
gene1615	K15631	ABA3	molybdenum cofactor sulfurtransferase	EC:2.8.1.9		
gene16160	K10624	RBBP6	E3 ubiquitin-protein ligase RBBP6	EC:6.3.2.19		
gene16166	K07889	RAB5C	Ras-related protein Rab-5C	--	6	ko04014 Ras signaling pathway;ko04144 Endocytosis;ko04145 Phagosome;ko04962 Vasopressin-regulated water reabsorption;ko05146 Amoebiasis;ko05152 Tuberculosis;
gene1616	K03039	PSMD13, RPN9	26S proteasome regulatory subunit N9	--	2	ko03050 Proteasome;ko05169 Epstein-Barr virus infection;
gene16171	K10573	UBE2A, UBC2, RAD6A	ubiquitin-conjugating enzyme E2 A	EC:6.3.2.19	1	ko04120 Ubiquitin mediated proteolysis;
gene16175	K09591	DET2	steroid 5-alpha-reductase	EC:1.3.1.22	1	ko00905 Brassinosteroid biosynthesis;
gene1617	K09486	HYOU1	hypoxia up-regulated 1	--	1	ko04141 Protein processing in endoplasmic reticulum;
gene16181	K08495	GOSR1, GOS1	golgi SNAP receptor complex member 1	--	1	ko04130 SNARE interactions in vesicular transport;
gene16184	K14516	ERF1	ethylene-responsive transcription factor 1	--	1	ko04075 Plant hormone signal transduction;
gene16185	K16296	SCPL-I	serine carboxypeptidase-like clade I	EC:3.4.16.-		
gene16186	K00051	E1.1.1.82	malate dehydrogenase (NADP+)	EC:1.1.1.82	2	ko00620 Pyruvate metabolism;ko00710 Carbon fixation in photosynthetic organisms;
gene16187	K17569	GPATCH2	G patch domain-containing protein 2	--		
gene16198	K17411	MRPS33	small subunit ribosomal protein S33, mitochondrial	--		
gene16201	K03348	APC1	anaphase-promoting complex subunit 1	--	7	ko04110 Cell cycle;ko04111 Cell cycle - yeast;ko04113 Meiosis - yeast;ko04114 Oocyte meiosis;ko04120 Ubiquitin mediated proteolysis;ko04914 Progesterone-mediated oocyte maturation;ko05166 HTLV-I infection;
gene16211	K15340	DCLRE1A, SNM1A, PSO2	DNA cross-link repair 1A protein	--		
gene16213	K15208	SNAPC1	snRNA-activating protein complex subunit 1	--		
gene16216	K15102	SLC25A3, PHC, PIC	solute carrier family 25 (mitochondrial phosphate transporter), member 3	--		
gene16218	K03549	kup	KUP system potassium uptake protein	--		
gene16222	K03327	TC.MATE, SLC47A, norM, mdtK, dinF	multidrug resistance protein, MATE family	--		
gene16226	K14490	AHP	histidine-containing phosphotransfer peotein	--	1	ko04075 Plant hormone signal transduction;
gene16229	K14638	SLC15A3_4, PHT	solute carrier family 15 (peptide/histidine transporter), member 3/4	--		
gene1622	K12856	PRPF8, PRP8	pre-mRNA-processing factor 8	--	1	ko03040 Spliceosome;
gene16230	K11826	AP2M1	AP-2 complex subunit mu-1	--	4	ko04144 Endocytosis;ko04721 Synaptic vesicle cycle;ko04961 Endocrine and other factor-regulated calcium reabsorption;ko05016 Huntington's disease;
gene16232	K11000	CALS	callose synthase	EC:2.4.1.-		
gene16233	K10256	FAD2	omega-6 fatty acid desaturase (delta-12 desaturase)	EC:1.14.19.-	1	ko01040 Biosynthesis of unsaturated fatty acids;
gene16237	K08869	ADCK, ABC1	aarF domain-containing kinase	--		
gene16242	K12890	SFRS1_9	splicing factor, arginine/serine-rich 1/9	--	2	ko03040 Spliceosome;ko05168 Herpes simplex infection;
gene16251	K13754	SLC24A6, NCKX6	solute carrier family 24 (sodium/potassium/calcium exchanger), member 6	--		
gene16252	K13456	RIN4	RPM1-interacting protein 4	--	1	ko04626 Plant-pathogen interaction;
gene16253	K12483	EHD1	EH domain-containing protein 1	--	1	ko04144 Endocytosis;
gene16255	K11147	DHRS4	dehydrogenase/reductase SDR family member 4	EC:1.1.-.-	2	ko00830 Retinol metabolism;ko04146 Peroxisome;
gene16264	K17086	TM9SF2_4	transmembrane 9 superfamily member 2/4	--		
gene16274	K03283	HSPA1_8	heat shock 70kDa protein 1/8	--	11	ko03040 Spliceosome;ko04010 MAPK signaling pathway;ko04141 Protein processing in endoplasmic reticulum;ko04144 Endocytosis;ko04612 Antigen processing and presentation;ko04915 Estrogen signaling pathway;ko05134 Legionellosis;ko05145 Toxoplasmosis;ko05162 Measles;ko05164 Influenza A;ko05169 Epstein-Barr virus infection;
gene1627	K14484	IAA	auxin-responsive protein IAA	--	1	ko04075 Plant hormone signal transduction;
gene16281	K00921	PIKFYVE, FAB1	1-phosphatidylinositol-3-phosphate 5-kinase	EC:2.7.1.150	4	ko00562 Inositol phosphate metabolism;ko04070 Phosphatidylinositol signaling system;ko04145 Phagosome;ko04810 Regulation of actin cytoskeleton;

gene16290	K03263	EIF5A	translation initiation factor 5A	--		
gene16293	K12178	COPS4, CSN4	COP9 signalosome complex subunit 4	--		
gene16295	K01551	arsA, ASNA1	arsenite-transporting ATPase	EC:3.6.3.16		
gene16296	K01728	E4.2.2.2, pel	pectate lyase	EC:4.2.2.2	1	ko00040 Pentose and glucuronate interconversions;
gene16301	K00013	hisD	histidinol dehydrogenase	EC:1.1.1.23	2	ko00340 Histidine metabolism;ko01230 Biosynthesis of amino acids;
gene16302	K13412	CPK	calcium-dependent protein kinase	EC:2.7.11.1	2	ko04626 Plant-pathogen interaction;ko05145 Toxoplasmosis;
gene16303	K08737	MSH6	DNA mismatch repair protein MSH6	--	3	ko03430 Mismatch repair;ko05200 Pathways in cancer;ko05210 Colorectal cancer;
gene16306	K08850	AURKX	aurora kinase, other	EC:2.7.11.1		
gene16307	K00850	pfkA, PFK	6-phosphofructokinase 1	EC:2.7.1.11	7	ko00010 Glycolysis / Gluconeogenesis;ko00030 Pentose phosphate pathway;ko00051 Fructose and mannose metabolism;ko00052 Galactose metabolism;ko00680 Methane metabolism;ko01200 Carbon metabolism;ko01230 Biosynthesis of amino acids;
gene1630	K01057	PGLS, pgl, devB	6-phosphogluconolactonase	EC:3.1.1.31	2	ko00030 Pentose phosphate pathway;ko01200 Carbon metabolism;
gene16314	K08150	SLC2A13, ITR	MFS transporter, SP family, solute carrier family 2 (myo-inositol transporter), member 13	--		
gene16318	K13269	E2.4.1.234	kaempferol 3-O-beta-D-galactosyltransferase	EC:2.4.1.234	1	ko00944 Flavone and flavonol biosynthesis;
gene16319	K03327	TC.MATE, SLC47A, norM, mdtK, dinF	multidrug resistance protein, MATE family	--		
gene1631	K09564	PPIE	peptidyl-prolyl isomerase E (cyclophilin E)	EC:5.2.1.8	1	ko03040 Spliceosome;
gene16320	K03327	TC.MATE, SLC47A, norM, mdtK, dinF	multidrug resistance protein, MATE family	--		
gene16321	K09419	HSFF	heat shock transcription factor, other eukaryote	--		
gene16331	K09874	NIP	aquaporin NIP	--		
gene16332	K08332	VAC8	vacuolar protein 8	--	1	ko04140 Regulation of autophagy;
gene16335	K01115	PLD1_2	phospholipase D1/2	EC:3.1.4.4	7	ko00564 Glycerophospholipid metabolism;ko00565 Ether lipid metabolism;ko04014 Ras signaling pathway;ko04144 Endocytosis;ko04666 Fc gamma R-mediated phagocytosis;ko04724 Glutamatergic synapse;ko04912 GnRH signaling pathway;
gene1633	K13379	RGP, UTM	reversibly glycosylated polypeptide / UDP-arabinopyranose mutase	EC:2.4.1.- 5.4.99.30		
gene16345	K04730	IRAK1	interleukin-1 receptor-associated kinase 1	EC:2.7.11.1	11	ko04064 NF-kappa B signaling pathway;ko04210 Apoptosis;ko04620 Toll-like receptor signaling pathway;ko04722 Neurotrophin signaling pathway;ko05133 Pertussis;ko05140 Leishmaniasis;ko05142 Chagas disease (American trypanosomiasis);ko05145 Toxoplasmosis;ko05152 Tuberculosis;ko05162 Measles;ko05169 Epstein-Barr virus infection;
gene16346	K14488	SAUR	SAUR family protein	--	1	ko04075 Plant hormone signal transduction;
gene16352	K14288	XPOT	exportin-T	--	1	ko03013 RNA transport;
gene16353	K09553	STIP1	stress-induced-phosphoprotein 1	--	1	ko05020 Prion diseases;
gene16354	K02355	fusA, GFM, EFG	elongation factor G	--		
gene16364	K16609	TTLL12	tubulin--tyrosine ligase-like protein 12	--		
gene16366	K01366	CTSH	cathepsin H	EC:3.4.22.16	1	ko04142 Lysosome;
gene16369	K15601	KDM3	lysine-specific demethylase 3	EC:1.14.11.-		
gene16377	K14404	CPSF4, YTH1	cleavage and polyadenylation specificity factor subunit 4	--	2	ko03015 mRNA surveillance pathway;ko05164 Influenza A;
gene16382	K03327	TC.MATE, SLC47A, norM, mdtK, dinF	multidrug resistance protein, MATE family	--		
gene16390	K17278	PGRMC1_2	membrane-associated progesterone receptor component	--		
gene16392	K16833	PPP1R2, IPP2	protein phosphatase inhibitor 2	--		
gene16397	K09419	HSFF	heat shock transcription factor, other eukaryote	--		
gene16411	K01115	PLD1_2	phospholipase D1/2	EC:3.1.4.4	7	ko00564 Glycerophospholipid metabolism;ko00565 Ether lipid metabolism;ko04014 Ras signaling pathway;ko04144 Endocytosis;ko04666 Fc gamma R-mediated phagocytosis;ko04724 Glutamatergic synapse;ko04912 GnRH signaling pathway;
gene16415	K01955	carB, CPA2	carbamoyl-phosphate synthase large subunit	EC:6.3.5.5	2	ko00240 Pyrimidine metabolism;ko00250 Alanine, aspartate and glutamate metabolism;

gene16416	K07374	TUBA	tubulin alpha	--	3	ko04145 Phagosome;ko04540 Gap junction;ko05130 Pathogenic Escherichia coli infection;
gene16417	K14638	SLC15A3_4, PHT	solute carrier family 15 (peptide/histidine transporter), member 3/4	--		
gene16427	K07874	RAB1A	Ras-related protein Rab-1A	--	1	ko05134 Legionellosis;
gene16444	K06268	PPP3R, CNB	serine/threonine-protein phosphatase 2B regulatory subunit	--	18	ko04010 MAPK signaling pathway;ko04020 Calcium signaling pathway;ko04114 Oocyte meiosis;ko04210 Apoptosis;ko04310 Wnt signaling pathway;ko04360 Axon guidance;ko04370 VEGF signaling pathway;ko04380 Osteoclast differentiation;ko04650 Natural killer cell mediated cytotoxicity;ko04660 T cell receptor signaling pathway;ko04662 B cell receptor signaling pathway;ko04720 Long-term potentiation;ko04724 Glutamatergic synapse;ko05010 Alzheimer's disease;ko05014 Amyotrophic lateral sclerosis (ALS);ko05031 Amphetamine addiction;ko05152
gene16448	K00434	E1.11.1.11	L-ascorbate peroxidase	EC:1.11.1.11	2	ko00053 Ascorbate and aldarate metabolism;ko00480 Glutathione metabolism;
gene16453	K10393	KIF2_24, MCAK	kinesin family member 2/24	--		
gene16458	K08873	SMG1	PI-3-kinase-related kinase SMG-1	--	1	ko03015 mRNA surveillance pathway;
gene16460	K08101	E1.3.7.4	phytochromobilin:ferredoxin oxidoreductase	EC:1.3.7.4	1	ko00860 Porphyrin and chlorophyll metabolism;
gene16463	K17255	GDI1_2	Rab GDP dissociation inhibitor	--		
gene16464	K08596	SENP7	sentrin-specific protease 7	EC:3.4.22.68		
gene16466	K03549	kup	KUP system potassium uptake protein	--		
gene16469	K04730	IRAK1	interleukin-1 receptor-associated kinase 1	EC:2.7.11.1	11	ko04064 NF-kappa B signaling pathway;ko04210 Apoptosis;ko04620 Toll-like receptor signaling pathway;ko04722 Neurotrophin signaling pathway;ko05133 Pertussis;ko05140 Leishmaniasis;ko05142 Chagas disease (American trypanosomiasis);ko05145 Toxoplasmosis;ko05152 Tuberculosis;ko05162 Measles;ko05169 Epstein-Barr virus infection;
gene16478	K05605	HIBCH	3-hydroxyisobutyryl-CoA hydrolase	EC:3.1.2.4	3	ko00280 Valine, leucine and isoleucine degradation;ko00410 beta-Alanine metabolism;ko00640 Propanoate metabolism;
gene16479	K08900	BCS1	mitochondrial chaperone BCS1	--		
gene1647	K14172	LHCB7	light-harvesting complex II chlorophyll a/b binding protein 7	--	1	ko00196 Photosynthesis - antenna proteins;
gene16483	K12188	SNF8, EAP30	ESCRT-II complex subunit VPS22	--	1	ko04144 Endocytosis;
gene16487	K11801	WDR23	WD repeat-containing protein 23	--		
gene16488	K08145	SLC2A8, GLUT8	MFS transporter, SP family, solute carrier family 2 (facilitated glucose transporter), member 8	--		
gene16498	K03671	trxA	thioredoxin 1	--		
gene16499	K04688	RPS6KB	p70 ribosomal S6 kinase	EC:2.7.11.1	9	ko04012 ErbB signaling pathway;ko04066 HIF-1 signaling pathway;ko04150 mTOR signaling pathway;ko04151 PI3K-Akt signaling pathway;ko04350 TGF-beta signaling pathway;ko04666 Fc gamma R-mediated phagocytosis;ko04910 Insulin signaling pathway;ko05205 Proteoglycans in cancer;ko05221 Acute myeloid leukemia;
gene16504	K13448	CML	calcium-binding protein CML	--	1	ko04626 Plant-pathogen interaction;
gene16513	K05305	FUK	fucokinase	EC:2.7.1.52	2	ko00051 Fructose and mannose metabolism;ko00520 Amino sugar and nucleotide sugar metabolism;
gene16514	K05305	FUK	fucokinase	EC:2.7.1.52	2	ko00051 Fructose and mannose metabolism;ko00520 Amino sugar and nucleotide sugar metabolism;
gene16519	K09422	MYBP	myb proto-oncogene protein, plant	--		
gene16528	K03549	kup	KUP system potassium uptake protein	--		
gene16529	K11778	DHDDS, RER2, SRT1	ditrans,polycis-polyprenyl diphosphate synthase	EC:2.5.1.87	1	ko00900 Terpenoid backbone biosynthesis;
gene16535	K02731	PSMA7	20S proteasome subunit alpha 4	EC:3.4.25.1	1	ko03050 Proteasome;
gene1653	K02976	RP-S26e, RPS26	small subunit ribosomal protein S26e	--	1	ko03010 Ribosome;
gene16542	K13420	FLS2	LRR receptor-like serine/threonine-protein kinase FLS2	EC:2.7.11.1	1	ko04626 Plant-pathogen interaction;
gene16547	K00820	E2.6.1.16, glmS	glucosamine--fructose-6-phosphate aminotransferase (isomerizing)	EC:2.6.1.16	2	ko00250 Alanine, aspartate and glutamate metabolism;ko00520 Amino sugar and nucleotide sugar metabolism;
gene1654	K14233	tRNA-Ser	tRNA Ser	--	1	ko00970 Aminoacyl-tRNA biosynthesis;

gene16550	K13648	GAUT	alpha-1,4-galacturonosyltransferase	EC:2.4.1.43	2	ko00500 Starch and sucrose metabolism;ko00520 Amino sugar and nucleotide sugar metabolism;
gene16553	K15168	MED25	mediator of RNA polymerase II transcription subunit 25	--		
gene16555	K00430	E1.11.1.7	peroxidase	EC:1.11.1.7	2	ko00360 Phenylalanine metabolism;ko00940 Phenylpropanoid biosynthesis;
gene16556	K02882	RP-L18Ae, RPL18A	large subunit ribosomal protein L18Ae	--	1	ko03010 Ribosome;
gene16559	K05909	E1.10.3.2				
gene16564	K01728	E4.2.2.2, pel	pectate lyase	EC:4.2.2.2	1	ko00040 Pentose and glucuronate interconversions;
gene16572	K10532	HGSNAT	heparan-alpha-glucosaminide N-acetyltransferase	EC:2.3.1.78	2	ko00531 Glycosaminoglycan degradation;ko04142 Lysosome;
gene16574	K01937	E6.3.4.2, pyrG	CTP synthase	EC:6.3.4.2	1	ko00240 Pyrimidine metabolism;
gene1657	K12890	SFRS1_9	splicing factor, arginine/serine-rich 1/9	--	2	ko03040 Spliceosome;ko05168 Herpes simplex infection;
gene16586	K09549	PFDN2	prefoldin subunit 2	--		
gene16588	K14709	SLC39A1_2_3, ZIP1_2_3	solute carrier family 39 (zinc transporter), member 1/2/3	--		
gene16590	K01051	E3.1.1.11	pectinesterase	EC:3.1.1.11	2	ko00040 Pentose and glucuronate interconversions;ko00500 Starch and sucrose metabolism;
gene16591	K01051	E3.1.1.11	pectinesterase	EC:3.1.1.11	2	ko00040 Pentose and glucuronate interconversions;ko00500 Starch and sucrose metabolism;
gene16596	K09422	MYBP	myb proto-oncogene protein, plant	--		
gene16597	K09680	coaW	type II pantothenate kinase	EC:2.7.1.33	1	ko00770 Pantothenate and CoA biosynthesis;
gene16598	K14790	NOP9	nucleolar protein 9	--		
gene16599	K14790	NOP9	nucleolar protein 9	--		
gene16600	K05765	CFL	cofilin	--	4	ko04360 Axon guidance;ko04666 Fc gamma R-mediated phagocytosis;ko04810 Regulation of actin cytoskeleton;ko05133 Pertussis;
gene16601	K07375	TUBB	tubulin beta	--	3	ko04145 Phagosome;ko04540 Gap junction;ko05130 Pathogenic Escherichia coli infection;
gene16604	K15305	VAC14, TAX1BP2	vacuole morphology and inheritance protein 14	--	2	ko05166 HTLV-I infection;ko05203 Viral carcinogenesis;
gene16605	K13448	CML	calcium-binding protein CML	--	1	ko04626 Plant-pathogen interaction;
gene16608	K01537	E3.6.3.8	Ca2+-transporting ATPase	EC:3.6.3.8		
gene16609	K14696	SLC30A9, ZNT9	solute carrier family 30 (zinc transporter), member 9	--		
gene1660	K00083	E1.1.1.195	cinnamyl-alcohol dehydrogenase	EC:1.1.1.195	1	ko00940 Phenylpropanoid biosynthesis;
gene16610	K01230	MAN1	mannosyl-oligosaccharide alpha-1,2-mannosidase	EC:3.2.1.113	3	ko00510 N-Glycan biosynthesis;ko00513 Various types of N-glycan biosynthesis;ko04141 Protein processing in endoplasmic reticulum;
gene16616	K01623	ALDO	fructose-bisphosphate aldolase, class I	EC:4.1.2.13	7	ko00010 Glycolysis / Gluconeogenesis;ko00030 Pentose phosphate pathway;ko00051 Fructose and mannose metabolism;ko00680 Methane metabolism;ko00710 Carbon fixation in photosynthetic organisms;ko01200 Carbon metabolism;ko01230 Biosynthesis of amino acids;
gene16617	K11498	CENPE	centromeric protein E	--		
gene16618	K09286	EREBP	EREBP-like factor	--		
gene16619	K02952	RP-S13, rpsM	small subunit ribosomal protein S13	--	1	ko03010 Ribosome;
gene16622	K04733	IRAK4	interleukin-1 receptor-associated kinase 4	EC:2.7.11.1	11	ko04064 NF-kappa B signaling pathway;ko04210 Apoptosis;ko04620 Toll-like receptor signaling pathway;ko04722 Neurotrophin signaling pathway;ko05133 Pertussis;ko05140 Leishmaniasis;ko05142 Chagas disease (American trypanosomiasis);ko05145 Toxoplasmosis;ko05152 Tuberculosis;ko05162 Measles;ko05164 Influenza A;
gene16623	K00006	GPD1	glycerol-3-phosphate dehydrogenase (NAD+)	EC:1.1.1.8	1	ko00564 Glycerophospholipid metabolism;
gene16624	K14792	RRP5, PDCD11	rRNA biogenesis protein RRP5	--		
gene16626	K01714	dapA	4-hydroxy-tetrahydrodipicolinate synthase	EC:4.3.3.7	2	ko00300 Lysine biosynthesis;ko01230 Biosynthesis of amino acids;
gene16629	K04733	IRAK4	interleukin-1 receptor-associated kinase 4	EC:2.7.11.1	11	ko04064 NF-kappa B signaling pathway;ko04210 Apoptosis;ko04620 Toll-like receptor signaling pathway;ko04722 Neurotrophin signaling pathway;ko05133 Pertussis;ko05140 Leishmaniasis;ko05142 Chagas disease (American trypanosomiasis);ko05145 Toxoplasmosis;ko05152 Tuberculosis;ko05162 Measles;ko05164 Influenza A;
gene16631	K02993	RP-S7e, RPS7	small subunit ribosomal protein S7e	--	1	ko03010 Ribosome;
gene16632	K11838	USP7, UBP15	ubiquitin carboxyl-terminal hydrolase 7	EC:3.1.2.15	3	ko05168 Herpes simplex infection;ko05169 Epstein-Barr virus infection;ko05203 Viral carcinogenesis;

gene16635	K12823	DDX5, DBP2	ATP-dependent RNA helicase DDX5/DBP2	EC:3.6.4.13	3	ko03040 Spliceosome;ko05202 Transcriptional misregulation in cancer;ko05205 Proteoglycans in cancer;
gene16636	K12842	SR140	U2-associated protein SR140	--	1	ko03040 Spliceosome;
gene16640	K07052	K07052				
gene16641	K14503	BZR1_2	brassinosteroid resistant 1/2	--	1	ko04075 Plant hormone signal transduction;
gene16642	K00487	CYP73A	trans-cinnamate 4-monooxygenase	EC:1.14.13.11	5	ko00360 Phenylalanine metabolism;ko00940 Phenylpropanoid biosynthesis;ko00941 Flavonoid biosynthesis;ko00945 Stilbenoid, diarylheptanoid and gingerol biosynthesis;ko01220 Degradation of aromatic compounds;
gene16649	K13680	CSLA	beta-mannan synthase	EC:2.4.1.32		
gene16655	K00547	mmuM	homocysteine S-methyltransferase	EC:2.1.1.10	1	ko00270 Cysteine and methionine metabolism;
gene16657	K11438	PRMT7	protein arginine N-methyltransferase 7	EC:2.1.1.-		
gene1665	K01507	ppa	inorganic pyrophosphatase	EC:3.6.1.1	1	ko00190 Oxidative phosphorylation;
gene16661	K00817	hisC	histidinol-phosphate aminotransferase	EC:2.6.1.9	7	ko00340 Histidine metabolism;ko00350 Tyrosine metabolism;ko00360 Phenylalanine metabolism;ko00400 Phenylalanine, tyrosine and tryptophan biosynthesis;ko00401 Novobiocin biosynthesis;ko00960 Tropane, piperidine and pyridine alkaloid biosynthesis;ko01230 Biosynthesis of amino acids;
gene16666	K13457	RPM1, RPS3	disease resistance protein RPM1	--	1	ko04626 Plant-pathogen interaction;
gene16671	K12591	RRP6, EXOSC10	exosome complex exonuclease RRP6	EC:3.1.13.-	1	ko03018 RNA degradation;
gene16673	K14220	tRNA-Asn	tRNA Asn	--	1	ko00970 Aminoacyl-tRNA biosynthesis;
gene16675	K03531	ftsZ	cell division protein FtsZ	--	1	ko04112 Cell cycle - Caulobacter;
gene16677	K00382	DLD, lpd, pdhD	dihydrolipoamide dehydrogenase	EC:1.8.1.4	6	ko00010 Glycolysis / Gluconeogenesis;ko00020 Citrate cycle (TCA cycle);ko00260 Glycine, serine and threonine metabolism;ko00280 Valine, leucine and isoleucine degradation;ko00620 Pyruvate metabolism;ko01200 Carbon metabolism;
gene16683	K07025	K07025	putative hydrolase of the HAD superfamily	--		
gene16689	K14508	NPR1	regulatory protein NPR1	--	1	ko04075 Plant hormone signal transduction;
gene16692	K01205	NAGLU	alpha-N-acetylglucosaminidase	EC:3.2.1.50	2	ko00531 Glycosaminoglycan degradation;ko04142 Lysosome;
gene16696	K00894	ETNK, EKI	ethanolamine kinase	EC:2.7.1.82	1	ko00564 Glycerophospholipid metabolism;
gene16698	K07889	RAB5C	Ras-related protein Rab-5C	--	6	ko04014 Ras signaling pathway;ko04144 Endocytosis;ko04145 Phagosome;ko04962 Vasopressin-regulated water reabsorption;ko05146 Amoebiasis;ko05152 Tuberculosis;
gene16704	K09840	NCED	9-cis-epoxycarotenoid dioxygenase	EC:1.13.11.51	1	ko00906 Carotenoid biosynthesis;
gene16708	K14561	IMP4	U3 small nucleolar ribonucleoprotein protein IMP4	--	1	ko03008 Ribosome biogenesis in eukaryotes;
gene16711	K12356	UGT72E	coniferyl-alcohol glucosyltransferase	EC:2.4.1.111	1	ko00940 Phenylpropanoid biosynthesis;
gene16712	K08518	STXBP5	syntaxin-binding protein 5	--		
gene16714	K13917	RNGTT	mRNA-capping enzyme	EC:2.7.7.50 3.1.3.33	1	ko03015 mRNA surveillance pathway;
gene16717	K01892	HARS, hisS	histidyl-tRNA synthetase	EC:6.1.1.21	1	ko00970 Aminoacyl-tRNA biosynthesis;
gene16722	K00430	E1.11.1.7	peroxidase	EC:1.11.1.7	2	ko00360 Phenylalanine metabolism;ko00940 Phenylpropanoid biosynthesis;
gene16723	K10579	UBE2M, UBC12	ubiquitin-conjugating enzyme E2 M	EC:6.3.2.19	1	ko04120 Ubiquitin mediated proteolysis;
gene16726	K11188	PRDX6	peroxiredoxin 6, 1-Cys peroxiredoxin	EC:1.11.1.7 1.11.1.15	2	ko00360 Phenylalanine metabolism;ko00940 Phenylpropanoid biosynthesis;
gene16728	K15404	K15404, CER1	aldehyde decarbonylase	EC:4.1.99.5	1	ko00073 Cutin, suberine and wax biosynthesis;
gene16731	K08511	ATVAMP72	vesicle-associated membrane protein 72	--		
gene16738	K12829	SF3B2, SAP145, CUS1	splicing factor 3B subunit 2	--	1	ko03040 Spliceosome;
gene1673	K01179	E3.2.1.4	endoglucanase	EC:3.2.1.4	1	ko00500 Starch and sucrose metabolism;
gene16745	K03327	TC.MATE, SLC47A, norM, mdtK, dinF	multidrug resistance protein, MATE family	--		
gene16747	K01051	E3.1.1.11	pectinesterase	EC:3.1.1.11	2	ko00040 Pentose and glucuronate interconversions;ko00500 Starch and sucrose metabolism;
gene16748	K00858	ppnK, NADK	NAD+ kinase	EC:2.7.1.23	1	ko00760 Nicotinate and nicotinamide metabolism;

gene1674	K00128	E1.2.1.3	aldehyde dehydrogenase (NAD+)	EC:1.2.1.3	15	ko00010 Glycolysis / Gluconeogenesis;ko00040 Pentose and glucuronate interconversions;ko00053 Ascorbate and aldarate metabolism;ko00071 Fatty acid degradation;ko00280 Valine, leucine and isoleucine degradation;ko00310 Lysine degradation;ko00330 Arginine and proline metabolism;ko00340 Histidine metabolism;ko00380 Tryptophan metabolism;ko00410 beta-Alanine metabolism;ko00561 Glycerolipid metabolism;ko00620 Pyruvate metabolism;ko00625 Chloroalkane and chloroalkene degradation;ko00640 Propanoate metabolism;ko00903 Limonene and pinene
gene16752	K01728	E4.2.2.2, pel	pectate lyase	EC:4.2.2.2	1	ko00040 Pentose and glucuronate interconversions;
gene16753	K01653	E2.2.1.6S, ilvH, ilvN	acetolactate synthase I/III small subunit	EC:2.2.1.6	6	ko00290 Valine, leucine and isoleucine biosynthesis;ko00650 Butanoate metabolism;ko00660 C5-Branched dibasic acid metabolism;ko00770 Pantothenate and CoA biosynthesis;ko01210 2-Oxocarboxylic acid metabolism;ko01230 Biosynthesis of amino acids;
gene16757	K02921	RP-L37Ae, RPL37A	large subunit ribosomal protein L37Ae	--	1	ko03010 Ribosome;
gene16767	K14222	tRNA-Cys	tRNA Cys	--	1	ko00970 Aminoacyl-tRNA biosynthesis;
gene16768	K14270	ACS10_12	aminotransferase	--		
gene16770	K03142	TFIIH2, GTF2H2, SSL1	transcription initiation factor TFIIH subunit 2	--	3	ko03022 Basal transcription factors;ko03420 Nucleotide excision repair;ko05203 Viral carcinogenesis;
gene16771	K11790	DTL, CDT2, DCAF2	denticleless	--		
gene16773	K07765	MBTPS2	S2P endopeptidase	EC:3.4.24.85	1	ko04141 Protein processing in endoplasmic reticulum;
gene16777	K14297	NUP98, ADAR2	nuclear pore complex protein Nup98-Nup96	--	2	ko03013 RNA transport;ko05164 Influenza A;
gene1677	K01102	PDP	pyruvate dehydrogenase phosphatase	EC:3.1.3.43		
gene16784	K03094	SKP1, CBF3D	S-phase kinase-associated protein 1	--	9	ko04110 Cell cycle;ko04111 Cell cycle - yeast;ko04114 Oocyte meiosis;ko04120 Ubiquitin mediated proteolysis;ko04141 Protein processing in endoplasmic reticulum;ko04310 Wnt signaling pathway;ko04350 TGF-beta signaling pathway;ko04710 Circadian rhythm;ko05168 Herpes simplex infection;
gene16788	K12883	NCBP2, CBP20	nuclear cap-binding protein subunit 2	--	3	ko03013 RNA transport;ko03015 mRNA surveillance pathway;ko03040 Spliceosome;
gene16790	K02639	petF	ferredoxin	--	1	ko00195 Photosynthesis;
gene16796	K07466	RFA1, RPA1, rpa	replication factor A1	--	5	ko03030 DNA replication;ko03420 Nucleotide excision repair;ko03430 Mismatch repair;ko03440 Homologous recombination;ko03460 Fanconi anemia pathway;
gene16797	K07466	RFA1, RPA1, rpa	replication factor A1	--	5	ko03030 DNA replication;ko03420 Nucleotide excision repair;ko03430 Mismatch repair;ko03440 Homologous recombination;ko03460 Fanconi anemia pathway;
gene16798	K07195	EXOC7, EXO70	exocyst complex component 7	--	1	ko04910 Insulin signaling pathway;
gene16799	K04505	PSEN1, PS1	presenilin 1	EC:3.4.23.-	4	ko04310 Wnt signaling pathway;ko04330 Notch signaling pathway;ko04722 Neurotrophin signaling pathway;ko05010 Alzheimer's disease;
gene167	K00645	fabD	[acyl-carrier-protein] S-malonyltransferase	EC:2.3.1.39	1	ko00061 Fatty acid biosynthesis;
gene16801	K02136	ATPeF1G, ATP5C1	F-type H+-transporting ATPase subunit gamma	EC:3.6.3.14	4	ko00190 Oxidative phosphorylation;ko05010 Alzheimer's disease;ko05012 Parkinson's disease;ko05016 Huntington's disease;
gene16802	K09506	DNAJA5	DnaJ homolog subfamily A member 5	--		
gene16803	K11407	HDAC6_10	histone deacetylase 6/10	EC:3.5.1.98	2	ko05034 Alcoholism;ko05203 Viral carcinogenesis;
gene16806	K01528	DNM	dynamin GTPase	EC:3.6.5.5	4	ko04144 Endocytosis;ko04721 Synaptic vesicle cycle;ko04961 Endocrine and other factor-regulated calcium reabsorption;ko05100 Bacterial invasion of epithelial cells;
gene16810	K13094	RBM5_10	RNA-binding protein 5/10	--		
gene16815	K03671	trxA	thioredoxin 1	--		
gene16818	K00815	TAT	tyrosine aminotransferase	EC:2.6.1.5	9	ko00130 Ubiquinone and other terpenoid-quinone biosynthesis;ko00270 Cysteine and methionine metabolism;ko00350 Tyrosine metabolism;ko00360 Phenylalanine metabolism;ko00400 Phenylalanine, tyrosine and tryptophan biosynthesis;ko00401 Novobiocin biosynthesis;ko00950 Isoquinoline alkaloid biosynthesis;ko00960 Tropane, piperidine and pyridine alkaloid biosynthesis;ko01230 Biosynthesis of amino acids;
gene16821	K12160	SUMO, SMT3	small ubiquitin-related modifier	--	1	ko03013 RNA transport;

gene16826	K13144	INTS7	integrator complex subunit 7	--		
gene1682	K11290	SET, TAF1, I2PP2A	template-activating factor I	--		
gene16830	K12600	SKI3, TTC37	superkiller protein 3	--	1	ko03018 RNA degradation;
gene16837	K13447	RBOH	respiratory burst oxidase	EC:1.6.3.- 1.11.1.-	1	ko04626 Plant-pathogen interaction;
gene16843	K10534	NR	nitrate reductase (NAD(P)H)	EC:1.7.1.1 1.7.1.2 1.7.1.3	1	ko00910 Nitrogen metabolism;
gene16846	K13628	iscA, ISCA1	iron-sulfur cluster assembly protein	--		
gene16847	K06133	LYS5, acpT	4'-phosphopantetheinyl transferase	EC:2.7.8.-	1	ko00770 Pantothenate and CoA biosynthesis;
gene1684	K01507	ppa	inorganic pyrophosphatase	EC:3.6.1.1	1	ko00190 Oxidative phosphorylation;
gene16853	K15255	PIF1	ATP-dependent DNA helicase PIF1	EC:3.6.4.12		
gene16856	K14856	SDA1, SDAD1	protein SDA1	--		
gene16859	K01623	ALDO	fructose-bisphosphate aldolase, class I	EC:4.1.2.13	7	ko00010 Glycolysis / Gluconeogenesis;ko00030 Pentose phosphate pathway;ko00051 Fructose and mannose metabolism;ko00680 Methane metabolism;ko00710 Carbon fixation in photosynthetic organisms;ko01200 Carbon metabolism;ko01230 Biosynthesis of amino acids;
gene1685	K10756	RFC3_5	replication factor C subunit 3/5	--	3	ko03030 DNA replication;ko03420 Nucleotide excision repair;ko03430 Mismatch repair;
gene16860	K07195	EXOC7, EXO70	exocyst complex component 7	--	1	ko04910 Insulin signaling pathway;
gene16863	K02726	PSMA2	20S proteasome subunit alpha 2	EC:3.4.25.1	1	ko03050 Proteasome;
gene16867	K15113	SLC25A28_37, MFRN	solute carrier family 25 (mitochondrial iron transporter), member 28/37	--		
gene16869	K14557	UTP6	U3 small nucleolar RNA-associated protein 6	--	1	ko03008 Ribosome biogenesis in eukaryotes;
gene16873	K13125	NOSIP	nitric oxide synthase-interacting protein	--		
gene16884	K05857	PLCD	phosphatidylinositol phospholipase C, delta	EC:3.1.4.11	3	ko00562 Inositol phosphate metabolism;ko04020 Calcium signaling pathway;ko04070 Phosphatidylinositol signaling system;
gene16885	K08956	AFG3	AFG3 family protein	EC:3.4.24.-		
gene16886	K03070	secA	preprotein translocase subunit SecA	--	2	ko03060 Protein export;ko03070 Bacterial secretion system;
gene16888	K02153	ATPeVH, ATP6H	V-type H+-transporting ATPase subunit H	EC:3.6.3.14	7	ko00190 Oxidative phosphorylation;ko04145 Phagosome;ko04721 Synaptic vesicle cycle;ko04966 Collecting duct acid secretion;ko05110 Vibrio cholerae infection;ko05120 Epithelial cell signaling in Helicobacter pylori infection;ko05323 Rheumatoid arthritis;
gene16891	K16570	TUBGCP3, GCP3	gamma-tubulin complex component 3	--		
gene16892	K08150	SLC2A13, ITR	MFS transporter, SP family, solute carrier family 2 (myo-inositol transporter), member 13	--		
gene16899	K00059	fabG	3-oxoacyl-[acyl-carrier protein] reductase	EC:1.1.1.100	3	ko00061 Fatty acid biosynthesis;ko00780 Biotin metabolism;ko01040 Biosynthesis of unsaturated fatty acids;
gene168	K15077	ELA1	elongin-A	--		
gene16901	K02321	POLA2	DNA polymerase alpha subunit B	--	3	ko00230 Purine metabolism;ko00240 Pyrimidine metabolism;ko03030 DNA replication;
gene16902	K02145	ATPeVA, ATP6A1	V-type H+-transporting ATPase subunit A	EC:3.6.3.14	7	ko00190 Oxidative phosphorylation;ko04145 Phagosome;ko04721 Synaptic vesicle cycle;ko04966 Collecting duct acid secretion;ko05110 Vibrio cholerae infection;ko05120 Epithelial cell signaling in Helicobacter pylori infection;ko05323 Rheumatoid arthritis;
gene16906	K01695	trpA	tryptophan synthase alpha chain	EC:4.2.1.20	3	ko00260 Glycine, serine and threonine metabolism;ko00400 Phenylalanine, tyrosine and tryptophan biosynthesis;ko01230 Biosynthesis of amino acids;
gene16911	K09377	CSRP	cysteine and glycine-rich protein	--		
gene16913	K09422	MYBP	myb proto-oncogene protein, plant	--		
gene16915	K14709	SLC39A1_2_3, ZIP1_2_3	solute carrier family 39 (zinc transporter), member 1/2/3	--		
gene16917	K05305	FUK	fucokinase	EC:2.7.1.52	2	ko00051 Fructose and mannose metabolism;ko00520 Amino sugar and nucleotide sugar metabolism;
gene16918	K14488	SAUR	SAUR family protein	--	1	ko04075 Plant hormone signal transduction;
gene16919	K01512	acyP	acylphosphatase	EC:3.6.1.7	2	ko00620 Pyruvate metabolism;ko00627 Aminobenzoate degradation;
gene16922	K14649	TAF8	transcription initiation factor TFIID subunit 8	--	1	ko03022 Basal transcription factors;
gene16925	K01897	ACSL, fadD	long-chain acyl-CoA synthetase	EC:6.2.1.3	4	ko00071 Fatty acid degradation;ko03320 PPAR signaling pathway;ko04146 Peroxisome;ko04920 Adipocytokine signaling pathway;

gene16926	K10875	RAD54L, RAD54	DNA repair and recombination protein RAD54 and RAD54-like protein	EC:3.6.4.-	1	ko03440 Homologous recombination;
gene16928	K14494	DELLA	DELLA protein	--	1	ko04075 Plant hormone signal transduction;
gene16929	K03086	SIG1, rpoD	RNA polymerase primary sigma factor	--		
gene16935	K08272	CAB39, MO25	calcium binding protein 39	--	1	ko04150 mTOR signaling pathway;
gene1693	K03098	APOD	apolipoprotein D and lipocalin family protein	--		
gene16942	K10398	KIF11, EG5	kinesin family member 11	--		
gene16943	K16329	psuG	pseudouridine-5'-phosphate glycosidase	EC:3.2.-.-	1	ko00240 Pyrimidine metabolism;
gene16945	K02966	RP-S19e, RPS19	small subunit ribosomal protein S19e	--	1	ko03010 Ribosome;
gene16948	K00430	E1.11.1.7	peroxidase	EC:1.11.1.7	2	ko00360 Phenylalanine metabolism;ko00940 Phenylpropanoid biosynthesis;
gene16950	K09285	OVM, ANT	AP2-like factor, ANT lineage	--		
gene16952	K14412	FUT13, FucTC	alpha-1,4-fucosyltransferase	EC:2.4.1.-	1	ko00513 Various types of N-glycan biosynthesis;
gene16958	K16585	HAUS2	HAUS augmin-like complex subunit 2	--		
gene1695	K14536	RIA1	ribosome assembly protein 1	EC:3.6.5.-	1	ko03008 Ribosome biogenesis in eukaryotes;
gene16962	K05605	HIBCH	3-hydroxyisobutyryl-CoA hydrolase	EC:3.1.2.4	3	ko00280 Valine, leucine and isoleucine degradation;ko00410 beta-Alanine metabolism;ko00640 Propanoate metabolism;
gene16966	K13519	LPT1, ALE1	lysophospholipid acyltransferase	EC:2.3.1.51 2.3.1.23	3	ko00561 Glycerolipid metabolism;ko00564 Glycerophospholipid metabolism;ko00565 Ether lipid metabolism;
gene16967	K03327	TC.MATE, SLC47A, norM, mdtK, dinF	multidrug resistance protein, MATE family	--		
gene16969	K01551	arsA, ASNA1	arsenite-transporting ATPase	EC:3.6.3.16		
gene16972	K08360	CYB561	cytochrome b-561	--		
gene16976	K13024	HISPPD, VIP	inositol hexakisphosphate/diphosphoinositol-pentakisphosphate kinase	EC:2.7.4.21 2.7.4.24		
gene16978	K08835	OXSR1, STK39	serine/threonine-protein kinase OSR1/STK39	EC:2.7.11.1		
gene1697	K06067	HDAC1_2	histone deacetylase 1/2	EC:3.5.1.98	9	ko04110 Cell cycle;ko04330 Notch signaling pathway;ko05016 Huntington's disease;ko05034 Alcoholism;ko05169 Epstein-Barr virus infection;ko05200 Pathways in cancer;ko05202 Transcriptional misregulation in cancer;ko05203 Viral carcinogenesis;ko05220 Chronic myeloid leukemia;
gene16984	K14709	SLC39A1_2_3, ZIP1_2_3	solute carrier family 39 (zinc transporter), member 1/2/3	--		
gene16990	K03938	NDUFS5	NADH dehydrogenase (ubiquinone) Fe-S protein 5	--	5	ko00190 Oxidative phosphorylation;ko04932 Non-alcoholic fatty liver disease (NAFLD);ko05010 Alzheimer's disease;ko05012 Parkinson's disease;ko05016 Huntington's disease;
gene16992	K05909	E1.10.3.2				
gene16993	K00685	ATE1, ate1	arginine-tRNA-protein transferase	EC:2.3.2.8		
gene16998	K00025	MDH1	malate dehydrogenase	EC:1.1.1.37	6	ko00020 Citrate cycle (TCA cycle);ko00620 Pyruvate metabolism;ko00630 Glyoxylate and dicarboxylate metabolism;ko00710 Carbon fixation in photosynthetic organisms;ko01200 Carbon metabolism;ko04964 Proximal tubule bicarbonate reclamation;
gene169	K00591	COQ3	hexaprenyldihydroxybenzoate methyltransferase	EC:2.1.1.114	1	ko00130 Ubiquinone and other terpenoid-quinone biosynthesis;
gene16	K01626	E2.5.1.54, aroF, aroG, aroH	3-deoxy-7-phosphoheptulonate synthase	EC:2.5.1.54	2	ko00400 Phenylalanine, tyrosine and tryptophan biosynthesis;ko01230 Biosynthesis of amino acids;
gene17006	K08150	SLC2A13, ITR	MFS transporter, SP family, solute carrier family 2 (myo-inositol transporter), member 13	--		
gene17010	K00799	GST, gst	glutathione S-transferase	EC:2.5.1.18	4	ko00480 Glutathione metabolism;ko00980 Metabolism of xenobiotics by cytochrome P450;ko00982 Drug metabolism - cytochrome P450;ko05204 Chemical carcinogenesis;
gene17012	K02693	psaE	photosystem I subunit IV	--	1	ko00195 Photosynthesis;
gene1701	K01104	E3.1.3.48	protein-tyrosine phosphatase	EC:3.1.3.48		
gene17023	K05909	E1.10.3.2				
gene17024	K04730	IRAK1	interleukin-1 receptor-associated kinase 1	EC:2.7.11.1	11	ko04064 NF-kappa B signaling pathway;ko04210 Apoptosis;ko04620 Toll-like receptor signaling pathway;ko04722 Neurotrophin signaling pathway;ko05133 Pertussis;ko05140 Leishmaniasis;ko05142 Chagas disease (American trypanosomiasis);ko05145 Toxoplasmosis;ko05152 Tuberculosis;ko05162 Measles;ko05169 Epstein-Barr virus infection;

gene17029	K07766	E3.6.1.52	diphosphoinositol-polyphosphate diphosphatase	EC:3.6.1.52		
gene1702	K01104	E3.1.3.48	protein-tyrosine phosphatase	EC:3.1.3.48		
gene17030	K01586	lysA	diaminopimelate decarboxylase	EC:4.1.1.20	2	ko00300 Lysine biosynthesis;ko01230 Biosynthesis of amino acids;
gene17033	K03363	CDC20	cell division cycle 20, cofactor of APC complex	--	7	ko04110 Cell cycle;ko04111 Cell cycle - yeast;ko04113 Meiosis - yeast;ko04114 Oocyte meiosis;ko04120 Ubiquitin mediated proteolysis;ko05166 HTLV-I infection;ko05203 Viral carcinogenesis;
gene17037	K07977	ARF	Arf/Sar family, other	--		
gene1703	K02291	crtB	phytoene synthase	EC:2.5.1.32	1	ko00906 Carotenoid biosynthesis;
gene17042	K00799	GST, gst	glutathione S-transferase	EC:2.5.1.18	4	ko00480 Glutathione metabolism;ko00980 Metabolism of xenobiotics by cytochrome P450;ko00982 Drug metabolism - cytochrome P450;ko05204 Chemical carcinogenesis;
gene17043	K00799	GST, gst	glutathione S-transferase	EC:2.5.1.18	4	ko00480 Glutathione metabolism;ko00980 Metabolism of xenobiotics by cytochrome P450;ko00982 Drug metabolism - cytochrome P450;ko05204 Chemical carcinogenesis;
gene17045	K07897	RAB7A	Ras-related protein Rab-7A	--	5	ko04144 Endocytosis;ko04145 Phagosome;ko05132 Salmonella infection;ko05146 Amoebiasis;ko05152 Tuberculosis;
gene17048	K15397	KCS	3-ketoacyl-CoA synthase	EC:2.3.1.199	1	ko00062 Fatty acid elongation;
gene1704	K04382	PPP2C	serine/threonine-protein phosphatase 2A catalytic subunit	EC:3.1.3.16	13	ko03015 mRNA surveillance pathway;ko04111 Cell cycle - yeast;ko04113 Meiosis - yeast;ko04114 Oocyte meiosis;ko04151 PI3K-Akt signaling pathway;ko04350 TGF-beta signaling pathway;ko04390 Hippo signaling pathway;ko04391 Hippo signaling pathway - fly;ko04530 Tight junction;ko04728 Dopaminergic synapse;ko04730 Long-term depression;ko05142 Chagas disease (American trypanosomiasis);ko05160 Hepatitis C;
gene17052	K00949	E2.7.6.2, THI80	thiamine pyrophosphokinase	EC:2.7.6.2	1	ko00730 Thiamine metabolism;
gene1705	K11594	DDX3X, bel	ATP-dependent RNA helicase	EC:3.6.4.13	3	ko04622 RIG-I-like receptor signaling pathway;ko05161 Hepatitis B;ko05203 Viral carcinogenesis;
gene17065	K02937	RP-L7e, RPL7	large subunit ribosomal protein L7e	--	1	ko03010 Ribosome;
gene17068	K09422	MYBP	myb proto-oncogene protein, plant	--		
gene1706	K03283	HSPA1_8	heat shock 70kDa protein 1/8	--	11	ko03040 Spliceosome;ko04010 MAPK signaling pathway;ko04141 Protein processing in endoplasmic reticulum;ko04144 Endocytosis;ko04612 Antigen processing and presentation;ko04915 Estrogen signaling pathway;ko05134 Legionellosis;ko05145 Toxoplasmosis;ko05162 Measles;ko05164 Influenza A;ko05169 Epstein-Barr virus infection;
gene17070	K12158	NEDD8	ubiquitin-like protein Nedd8	--		
gene17075	K00938	E2.7.4.2, mvaK2	phosphomevalonate kinase	EC:2.7.4.2	1	ko00900 Terpenoid backbone biosynthesis;
gene17076	K12189	VPS25, EAP20	ESCRT-II complex subunit VPS25	--	1	ko04144 Endocytosis;
gene17078	K14794	RRP12	ribosomal RNA-processing protein 12	--		
gene17079	K13628	iscA, ISCA1	iron-sulfur cluster assembly protein	--		
gene1707	K03217	yidC, spoIIIJ, OXA1	preprotein translocase subunit YidC	--	2	ko03060 Protein export;ko03070 Bacterial secretion system;
gene17083	K07300	chaA, CAX	Ca2+:H+ antiporter	--		
gene17085	K11672	ACTR5, ARP5, INO80M	actin-related protein 5	--		
gene17087	K09422	MYBP	myb proto-oncogene protein, plant	--		
gene17090	K09560	ST13	suppressor of tumorigenicity protein 13	--		
gene17092	K05391	CNGF	cyclic nucleotide gated channel, other eukaryote	--	1	ko04626 Plant-pathogen interaction;
gene17093	K11294	NCL, NSR1	nucleolin	--	1	ko05130 Pathogenic Escherichia coli infection;
gene17096	K07767	E3.6.4.3	microtubule-severing ATPase	EC:3.6.4.3		
gene170	K08770	UBC	ubiquitin C	--	1	ko03320 PPAR signaling pathway;
gene17104	K03868	RBX1, ROC1	RING-box protein 1	--	12	ko03420 Nucleotide excision repair;ko04066 HIF-1 signaling pathway;ko04110 Cell cycle;ko04111 Cell cycle - yeast;ko04114 Oocyte meiosis;ko04120 Ubiquitin mediated proteolysis;ko04141 Protein processing in endoplasmic reticulum;ko04310 Wnt signaling pathway;ko04350 TGF-beta signaling pathway;ko04710 Circadian rhythm;ko05200 Pathways in cancer;ko05211 Renal cell carcinoma;
gene17105	K14488	SAUR	SAUR family protein	--	1	ko04075 Plant hormone signal transduction;
gene17107	K02730	PSMA6	20S proteasome subunit alpha 1	EC:3.4.25.1	1	ko03050 Proteasome;
gene17109	K17541	SCYL2	SCY1-like protein 2	--		

gene17111	K14754	MX1	interferon-induced GTP-binding protein Mx1	--	2	ko05162 Measles;ko05164 Influenza A;
gene17118	K15746	crtZ	beta-carotene 3-hydroxylase	EC:1.14.13.129	1	ko00906 Carotenoid biosynthesis;
gene1711	K09500	CCT8	T-complex protein 1 subunit theta	--		
gene17121	K12900	FUSIP1	FUS-interacting serine-arginine-rich protein 1	--	1	ko03040 Spliceosome;
gene17122	K01188	E3.2.1.21	beta-glucosidase	EC:3.2.1.21	3	ko00460 Cyanoamino acid metabolism;ko00500 Starch and sucrose metabolism;ko00940 Phenylpropanoid biosynthesis;
gene17125	K02977	RP-S27Ae, RPS27A	small subunit ribosomal protein S27Ae	--	1	ko03010 Ribosome;
gene17126	K15446	TRM13, CCDC76	tRNA:m4X modification enzyme	EC:2.1.1.225		
gene17128	K02882	RP-L18Ae, RPL18A	large subunit ribosomal protein L18Ae	--	1	ko03010 Ribosome;
gene1712	K15078	SLX1	structure-specific endonuclease subunit SLX1	EC:3.6.1.-	1	ko03460 Fanconi anemia pathway;
gene17132	K01696	trpB	tryptophan synthase beta chain	EC:4.2.1.20	3	ko00260 Glycine, serine and threonine metabolism;ko00400 Phenylalanine, tyrosine and tryptophan biosynthesis;ko01230 Biosynthesis of amino acids;
gene17137	K04733	IRAK4	interleukin-1 receptor-associated kinase 4	EC:2.7.11.1	11	ko04064 NF-kappa B signaling pathway;ko04210 Apoptosis;ko04620 Toll-like receptor signaling pathway;ko04722 Neurotrophin signaling pathway;ko05133 Pertussis;ko05140 Leishmaniasis;ko05142 Chagas disease (American trypanosomiasis);ko05145 Toxoplasmosis;ko05152 Tuberculosis;ko05162 Measles;ko05164 Influenza A;
gene17141	K01426	E3.5.1.4, amiE	amidase	EC:3.5.1.4	5	ko00330 Arginine and proline metabolism;ko00360 Phenylalanine metabolism;ko00380 Tryptophan metabolism;ko00627 Aminobenzoate degradation;ko00643 Styrene degradation;
gene17142	K01426	E3.5.1.4, amiE	amidase	EC:3.5.1.4	5	ko00330 Arginine and proline metabolism;ko00360 Phenylalanine metabolism;ko00380 Tryptophan metabolism;ko00627 Aminobenzoate degradation;ko00643 Styrene degradation;
gene1714	K17479	GRXCR1	glutaredoxin domain-containing cysteine-rich protein 1	--		
gene17150	K11714	RGXT	rhamnogalacturonan II specific xylosyltransferase	EC:2.4.2.-		
gene17151	K11805	WDR68, HAN11	WD repeat-containing protein 68	--		
gene17152	K13963	SERPINB	serpin B	--	1	ko05146 Amoebiasis;
gene17154	K12385	NPC1	Niemann-Pick C1 protein	--	1	ko04142 Lysosome;
gene17155	K04733	IRAK4	interleukin-1 receptor-associated kinase 4	EC:2.7.11.1	11	ko04064 NF-kappa B signaling pathway;ko04210 Apoptosis;ko04620 Toll-like receptor signaling pathway;ko04722 Neurotrophin signaling pathway;ko05133 Pertussis;ko05140 Leishmaniasis;ko05142 Chagas disease (American trypanosomiasis);ko05145 Toxoplasmosis;ko05152 Tuberculosis;ko05162 Measles;ko05164 Influenza A;
gene17156	K04733	IRAK4	interleukin-1 receptor-associated kinase 4	EC:2.7.11.1	11	ko04064 NF-kappa B signaling pathway;ko04210 Apoptosis;ko04620 Toll-like receptor signaling pathway;ko04722 Neurotrophin signaling pathway;ko05133 Pertussis;ko05140 Leishmaniasis;ko05142 Chagas disease (American trypanosomiasis);ko05145 Toxoplasmosis;ko05152 Tuberculosis;ko05162 Measles;ko05164 Influenza A;
gene17158	K11723	BRD7_9	bromodomain-containing protein 7/9	--		
gene17160	K01087	otsB	trehalose 6-phosphate phosphatase	EC:3.1.3.12	1	ko00500 Starch and sucrose metabolism;
gene17161	K12472	EPS15	epidermal growth factor receptor substrate 15	--	1	ko04144 Endocytosis;
gene17164	K10688	UBE2W, UBC16	ubiquitin-conjugating enzyme E2 W	EC:6.3.2.19	1	ko04120 Ubiquitin mediated proteolysis;
gene17165	K00430	E1.11.1.7	peroxidase	EC:1.11.1.7	2	ko00360 Phenylalanine metabolism;ko00940 Phenylpropanoid biosynthesis;
gene17170	K12819	SLU7	pre-mRNA-processing factor SLU7	--	1	ko03040 Spliceosome;
gene17172	K15535	PWD	phosphoglucan, water dikinase	EC:2.7.9.5		
gene17173	K12385	NPC1	Niemann-Pick C1 protein	--	1	ko04142 Lysosome;
gene17176	K08900	BCS1	mitochondrial chaperone BCS1	--		
gene17177	K14664	ILR1	IAA-amino acid hydrolase	EC:3.5.1.-		
gene17178	K09571	FKBP4_5	FK506-binding protein 4/5	EC:5.2.1.8	1	ko04915 Estrogen signaling pathway;
gene17180	K02995	RP-S8e, RPS8	small subunit ribosomal protein S8e	--	1	ko03010 Ribosome;
gene17184	K11592	DICER1, DCR1	endoribonuclease Dicer	EC:3.1.26.-	1	ko05206 MicroRNAs in cancer;
gene17185	K03768	PPIB, ppiB	peptidyl-prolyl cis-trans isomerase B (cyclophilin B)	EC:5.2.1.8		

gene17188	K08678	UXS1	UDP-glucuronate decarboxylase	EC:4.1.1.35	2	ko00500 Starch and sucrose metabolism;ko00520 Amino sugar and nucleotide sugar metabolism;
gene1718	K09286	EREBP	EREBP-like factor	--		
gene17192	K03671	trxA	thioredoxin 1	--		
gene17193	K00901	E2.7.1.107, DGK, dgkA	diacylglycerol kinase (ATP dependent)	EC:2.7.1.107	3	ko00561 Glycerolipid metabolism;ko00564 Glycerophospholipid metabolism;ko04070 Phosphatidylinositol signaling system;
gene17194	K02966	RP-S19e, RPS19	small subunit ribosomal protein S19e	--	1	ko03010 Ribosome;
gene17195	K02160	accB, bccP	acetyl-CoA carboxylase biotin carboxyl carrier protein	--	6	ko00061 Fatty acid biosynthesis;ko00253 Tetracycline biosynthesis;ko00620 Pyruvate metabolism;ko00640 Propanoate metabolism;ko00720 Carbon fixation pathways in prokaryotes;ko01200 Carbon metabolism;
gene17200	K11835	USP4_11_15, UBP12	ubiquitin carboxyl-terminal hydrolase 4/11/15	EC:3.1.2.15		
gene17201	K01592	E4.1.1.25	tyrosine decarboxylase	EC:4.1.1.25	2	ko00350 Tyrosine metabolism;ko00950 Isoquinoline alkaloid biosynthesis;
gene17202	K17506	PPM1L, PP2CE	protein phosphatase 1L	EC:3.1.3.16		
gene17203	K04733	IRAK4	interleukin-1 receptor-associated kinase 4	EC:2.7.11.1	11	ko04064 NF-kappa B signaling pathway;ko04210 Apoptosis;ko04620 Toll-like receptor signaling pathway;ko04722 Neurotrophin signaling pathway;ko05133 Pertussis;ko05140 Leishmaniasis;ko05142 Chagas disease (American trypanosomiasis);ko05145 Toxoplasmosis;ko05152 Tuberculosis;ko05162 Measles;ko05164 Influenza A;
gene17207	K06638	MAD1L	mitotic spindle assembly checkpoint protein MAD1	--	3	ko04110 Cell cycle;ko04914 Progesterone-mediated oocyte maturation;ko05203 Viral carcinogenesis;
gene17209	K07198	PRKAA, AMPK	5'-AMP-activated protein kinase, catalytic alpha subunit	EC:2.7.11.11	8	ko04140 Regulation of autophagy;ko04150 mTOR signaling pathway;ko04151 PI3K-Akt signaling pathway;ko04710 Circadian rhythm;ko04910 Insulin signaling pathway;ko04920 Adipocytokine signaling pathway;ko04932 Non-alcoholic fatty liver disease (NAFLD);ko05410 Hypertrophic cardiomyopathy (HCM);
gene1720	K02871	RP-L13, MRPL13, rplM	large subunit ribosomal protein L13	--	1	ko03010 Ribosome;
gene17212	K08850	AURKX	aurora kinase, other	EC:2.7.11.1		
gene17213	K01184	E3.2.1.15	polygalacturonase	EC:3.2.1.15	2	ko00040 Pentose and glucuronate interconversions;ko00500 Starch and sucrose metabolism;
gene17218	K15032	MTERFD	mTERF domain-containing protein, mitochondrial	--		
gene17224	K09422	MYBP	myb proto-oncogene protein, plant	--		
gene17228	K02872	RP-L13Ae, RPL13A	large subunit ribosomal protein L13Ae	--	1	ko03010 Ribosome;
gene17229	K01051	E3.1.1.11	pectinesterase	EC:3.1.1.11	2	ko00040 Pentose and glucuronate interconversions;ko00500 Starch and sucrose metabolism;
gene17230	K11844	USP16_45	ubiquitin carboxyl-terminal hydrolase 16/45	EC:3.1.2.15		
gene17232	K01835	pgm	phosphoglucomutase	EC:5.4.2.2	7	ko00010 Glycolysis / Gluconeogenesis;ko00030 Pentose phosphate pathway;ko00052 Galactose metabolism;ko00230 Purine metabolism;ko00500 Starch and sucrose metabolism;ko00520 Amino sugar and nucleotide sugar metabolism;ko00521 Streptomycin biosynthesis;
gene17233	K14236	tRNA-Tyr	tRNA Tyr	--	1	ko00970 Aminoacyl-tRNA biosynthesis;
gene17235	K04711	YDC1	dihydroceramidase	EC:3.5.1.-	1	ko00600 Sphingolipid metabolism;
gene17236	K01052	LIPA	lysosomal acid lipase/cholesteryl ester hydrolase	EC:3.1.1.13	2	ko00100 Steroid biosynthesis;ko04142 Lysosome;
gene1723	K17086	TM9SF2_4	transmembrane 9 superfamily member 2/4	--		
gene17242	K16296	SCPL-I	serine carboxypeptidase-like clade I	EC:3.4.16.-		
gene17245	K12462	ARHGDI, RHOGDI	Rho GDP-dissociation inhibitor	--	2	ko04722 Neurotrophin signaling pathway;ko04962 Vasopressin-regulated water reabsorption;
gene17247	K15223	UAF30, SPP27	upstream activation factor subunit UAF30	--		
gene17248	K02689	psaA	photosystem I P700 chlorophyll a apoprotein A1	--	1	ko00195 Photosynthesis;
gene1724	K12309	GLB1, ELNR1	beta-galactosidase	EC:3.2.1.23	6	ko00052 Galactose metabolism;ko00511 Other glycan degradation;ko00531 Glycosaminoglycan degradation;ko00600 Sphingolipid metabolism;ko00604 Glycosphingolipid biosynthesis - ganglio series;ko04142 Lysosome;
gene17251	K14792	RRP5, PDCD11	rRNA biogenesis protein RRP5	--		
gene17255	K10896	FANCM	fanconi anemia group M protein	--	1	ko03460 Fanconi anemia pathway;
gene17259	K02492	hemA	glutamyl-tRNA reductase	EC:1.2.1.70	1	ko00860 Porphyrin and chlorophyll metabolism;

gene17265	K14403	CPSF3, YSH1	cleavage and polyadenylation specificity factor subunit 3	EC:3.1.27.-	1	ko03015 mRNA surveillance pathway;
gene17269	K13420	FLS2	LRR receptor-like serine/threonine-protein kinase FLS2	EC:2.7.11.1	1	ko04626 Plant-pathogen interaction;
gene17278	K07877	RAB2A	Ras-related protein Rab-2A	--		
gene17281	K13508	GPAT	glycerol-3-phosphate acyltransferase	EC:2.3.1.15	2	ko00561 Glycerolipid metabolism;ko00564 Glycerophospholipid metabolism;
gene17286	K16290	XCP	xylem cysteine proteinase	EC:3.4.22.-		
gene17287	K08176	PHO84	MFS transporter, PHS family, inorganic phosphate transporter	--		
gene17296	K11649	SMARCC	SWI/SNF related-matrix-associated actin-dependent regulator of chromatin subfamily C	--		
gene17297	K03549	kup	KUP system potassium uptake protein	--		
gene17299	K13508	GPAT	glycerol-3-phosphate acyltransferase	EC:2.3.1.15	2	ko00561 Glycerolipid metabolism;ko00564 Glycerophospholipid metabolism;
gene172	K03676	grxC, GLRX, GLRX2	glutaredoxin 3	--		
gene17300	K09503	DNAJA2	DnaJ homolog subfamily A member 2	--	1	ko04141 Protein processing in endoplasmic reticulum;
gene17305	K09754	CYP98A3, C3'H	coumaroylquinate(coumaroylshikimate) 3'-monooxygenase	EC:1.14.13.36	3	ko00940 Phenylpropanoid biosynthesis;ko00941 Flavonoid biosynthesis;ko00945 Stilbenoid, diarylheptanoid and gingerol biosynthesis;
gene17306	K00469	MIOX	inositol oxygenase	EC:1.13.99.1	2	ko00053 Ascorbate and aldarate metabolism;ko00562 Inositol phosphate metabolism;
gene17323	K00771	XYLT	protein xylosyltransferase	EC:2.4.2.26	2	ko00532 Glycosaminoglycan biosynthesis - chondroitin sulfate / dermatan sulfate;ko00534 Glycosaminoglycan biosynthesis - heparan sulfate / heparin;
gene17325	K10881	SHFM1, DSS1	26 proteasome complex subunit DSS1	--	3	ko03050 Proteasome;ko03440 Homologous recombination;ko05169 Epstein-Barr virus infection;
gene17326	K01183	E3.2.1.14	chitinase	EC:3.2.1.14	1	ko00520 Amino sugar and nucleotide sugar metabolism;
gene17330	K11308	MYST1, MOF, KAT8	histone acetyltransferase MYST1	EC:2.3.1.48		
gene17331	K14403	CPSF3, YSH1	cleavage and polyadenylation specificity factor subunit 3	EC:3.1.27.-	1	ko03015 mRNA surveillance pathway;
gene17332	K08488	STX7	syntaxin 7	--	2	ko04130 SNARE interactions in vesicular transport;ko04145 Phagosome;
gene17341	K08472	MLO	mlo protein	--		
gene17346	K00873	PK, pyk	pyruvate kinase	EC:2.7.1.40	7	ko00010 Glycolysis / Gluconeogenesis;ko00230 Purine metabolism;ko00620 Pyruvate metabolism;ko01200 Carbon metabolism;ko01230 Biosynthesis of amino acids;ko04930 Type II diabetes mellitus;ko05203 Viral carcinogenesis;
gene17349	K12619	XRN2, RAT1	5'-3' exoribonuclease 2	EC:3.1.13.-	2	ko03008 Ribosome biogenesis in eukaryotes;ko03018 RNA degradation;
gene1734	K09060	GBF	plant G-box-binding factor	--		
gene17351	K14376	PAP	poly(A) polymerase	EC:2.7.7.19	1	ko03015 mRNA surveillance pathway;
gene17353	K15304	RANBP3	Ran-binding protein 3	--	1	ko05166 HTLV-I infection;
gene1735	K02183	CALM	calmodulin	--	25	ko04014 Ras signaling pathway;ko04020 Calcium signaling pathway;ko04070 Phosphatidylinositol signaling system;ko04114 Oocyte meiosis;ko04270 Vascular smooth muscle contraction;ko04626 Plant-pathogen interaction;ko04713 Circadian entrainment;ko04720 Long-term potentiation;ko04722 Neurotrophin signaling pathway;ko04728 Dopaminergic synapse;ko04740 Olfactory transduction;ko04744 Phototransduction;ko04745 Phototransduction - fly;ko04910 Insulin signaling pathway;ko04912 GnRH signaling pathway;ko04915 Estrogen signaling pathway;ko04916 Melanogenesis;ko04970 Salivary secretion;ko04971 Gastric acid secretion;ko05010 Alzheimer's disease;ko05031 Amphetamine addiction;ko05034 Alcoholism;ko05133 Pertussis;ko05152 Tuberculosis;ko05214 Glioma;
gene1736	K02863	RP-L1, MRPL1, rplA	large subunit ribosomal protein L1	--	1	ko03010 Ribosome;
gene17370	K07204	RAPTOR	regulatory associated protein of mTOR	--	4	ko04150 mTOR signaling pathway;ko04151 PI3K-Akt signaling pathway;ko04910 Insulin signaling pathway;ko05206 MicroRNAs in cancer;
gene17374	K11366	USP22_27_51, UBP8	ubiquitin carboxyl-terminal hydrolase 22/27/51	EC:3.1.2.15		
gene17377	K00430	E1.11.1.7	peroxidase	EC:1.11.1.7	2	ko00360 Phenylalanine metabolism;ko00940 Phenylpropanoid biosynthesis;
gene17378	K07213	ATOX1, ATX1, copZ	copper chaperone	--	1	ko04978 Mineral absorption;
gene17383	K01180	E3.2.1.6	endo-1,3(4)-beta-glucanase	EC:3.2.1.6		

gene17385	K03364	CDH1	cell division cycle 20-like protein 1, cofactor of APC complex	--	4	ko04110 Cell cycle;ko04111 Cell cycle - yeast;ko04120 Ubiquitin mediated proteolysis;ko04914 Progesterone-mediated oocyte maturation;
gene17389	K06067	HDAC1_2	histone deacetylase 1/2	EC:3.5.1.98	9	ko04110 Cell cycle;ko04330 Notch signaling pathway;ko05016 Huntington's disease;ko05034 Alcoholism;ko05169 Epstein-Barr virus infection;ko05200 Pathways in cancer;ko05202 Transcriptional misregulation in cancer;ko05203 Viral carcinogenesis;ko05220 Chronic myeloid leukemia;
gene17390	K02262	COX3	cytochrome c oxidase subunit 3	--	6	ko00190 Oxidative phosphorylation;ko04260 Cardiac muscle contraction;ko04932 Non-alcoholic fatty liver disease (NAFLD);ko05010 Alzheimer's disease;ko05012 Parkinson's disease;ko05016 Huntington's disease;
gene17392	K13116	DDX41, ABS	ATP-dependent RNA helicase DDX41	EC:3.6.4.13		
gene17395	K14963	WDR5, SWD3, CPS30	COMPASS component SWD3	--		
gene17399	K02995	RP-S8e, RPS8	small subunit ribosomal protein S8e	--	1	ko03010 Ribosome;
gene1739	K07441	ALG14	beta-1,4-N-acetylglucosaminyltransferase	EC:2.4.1.141	2	ko00510 N-Glycan biosynthesis;ko00513 Various types of N-glycan biosynthesis;
gene17402	K04499	RUVBL1, RVB1, INO80H	RuvB-like protein 1 (pontin 52)	--	1	ko04310 Wnt signaling pathway;
gene17406	K10895	FANCI	fanconi anemia group I protein	--	1	ko03460 Fanconi anemia pathway;
gene1740	K15040	VDAC2	voltage-dependent anion channel protein 2	--	4	ko04020 Calcium signaling pathway;ko05012 Parkinson's disease;ko05016 Huntington's disease;ko05166 HTLV-I infection;
gene17415	K01061	E3.1.1.45	carboxymethylenebutenolidase	EC:3.1.1.45	3	ko00361 Chlorocyclohexane and chlorobenzene degradation;ko00364 Fluorobenzoate degradation;ko00623 Toluene degradation;
gene17419	K03671	trxA	thioredoxin 1	--		
gene17422	K15731	CTDSP	carboxy-terminal domain RNA polymerase II polypeptide A small phosphatase	EC:3.1.3.16		
gene17426	K01183	E3.2.1.14	chitinase	EC:3.2.1.14	1	ko00520 Amino sugar and nucleotide sugar metabolism;
gene17437	K00666	K00666	fatty-acyl-CoA synthase	EC:6.2.1.-		
gene17438	K09422	MYBP	myb proto-oncogene protein, plant	--		
gene17440	K05387	GRIP	glutamate receptor, ionotropic, plant	--		
gene17444	K14432	ABF	ABA responsive element binding factor	--	1	ko04075 Plant hormone signal transduction;
gene17445	K13174	THOC5	THO complex subunit 5	--	1	ko03013 RNA transport;
gene17446	K17095	ANXA7_11	annexin A7/11	--		
gene17447	K11159	K11159	carotenoid cleavage dioxygenase	--		
gene17450	K00128	E1.2.1.3	aldehyde dehydrogenase (NAD+)	EC:1.2.1.3	15	ko00010 Glycolysis / Gluconeogenesis;ko00040 Pentose and glucuronate interconversions;ko00053 Ascorbate and aldarate metabolism;ko00071 Fatty acid degradation;ko00280 Valine, leucine and isoleucine degradation;ko00310 Lysine degradation;ko00330 Arginine and proline metabolism;ko00340 Histidine metabolism;ko00380 Tryptophan metabolism;ko00410 beta-Alanine metabolism;ko00561 Glycerolipid metabolism;ko00620 Pyruvate metabolism;ko00625 Chloroalkane and chloroalkene degradation;ko00640 Propanoate metabolism;ko00903 Limonene and pinene
gene17456	K12200	PDCD6IP, ALIX, RIM20	programmed cell death 6-interacting protein	--	1	ko04144 Endocytosis;
gene17457	K00799	GST, gst	glutathione S-transferase	EC:2.5.1.18	4	ko00480 Glutathione metabolism;ko00980 Metabolism of xenobiotics by cytochrome P450;ko00982 Drug metabolism - cytochrome P450;ko05204 Chemical carcinogenesis;
gene17458	K14509	ETR, ERS	ethylene receptor	EC:2.7.13.-	1	ko04075 Plant hormone signal transduction;
gene17461	K11252	H2B	histone H2B	--	3	ko05034 Alcoholism;ko05203 Viral carcinogenesis;ko05322 Systemic lupus erythematosus;
gene17463	K04121	E4.2.3.19	ent-kaurene synthase	EC:4.2.3.19	1	ko00904 Diterpenoid biosynthesis;
gene17466	K02266	COX6A	cytochrome c oxidase subunit 6a	--	6	ko00190 Oxidative phosphorylation;ko04260 Cardiac muscle contraction;ko04932 Non-alcoholic fatty liver disease (NAFLD);ko05010 Alzheimer's disease;ko05012 Parkinson's disease;ko05016 Huntington's disease;
gene17467	K10606	FANCL, PHF9	E3 ubiquitin-protein ligase FANCL	EC:6.3.2.19	2	ko03460 Fanconi anemia pathway;ko04120 Ubiquitin mediated proteolysis;
gene17468	K00826	E2.6.1.42, ilvE	branched-chain amino acid aminotransferase	EC:2.6.1.42	5	ko00280 Valine, leucine and isoleucine degradation;ko00290 Valine, leucine and isoleucine biosynthesis;ko00770 Pantothenate and CoA biosynthesis;ko01210 2-Oxocarboxylic acid metabolism;ko01230 Biosynthesis of amino acids;

gene17469	K00921	PIKFYVE, FAB1	1-phosphatidylinositol-3-phosphate 5-kinase	EC:2.7.1.150	4	ko00562 Inositol phosphate metabolism;ko04070 Phosphatidylinositol signaling system;ko04145 Phagosome;ko04810 Regulation of actin cytoskeleton;
gene17473	K11699	RDR1, RDP1	RNA-dependent RNA polymerase 1	EC:2.7.7.48		
gene17474	K03165	TOP3	DNA topoisomerase III	EC:5.99.1.2	2	ko03440 Homologous recombination;ko03460 Fanconi anemia pathway;
gene17475	K02685	PRI2	DNA primase large subunit	EC:2.7.7.-	3	ko00230 Purine metabolism;ko00240 Pyrimidine metabolism;ko03030 DNA replication;
gene17477	K03531	ftsZ	cell division protein FtsZ	--	1	ko04112 Cell cycle - Caulobacter;
gene17478	K03131	TAF6	transcription initiation factor TFIID subunit 6	--	2	ko03022 Basal transcription factors;ko05168 Herpes simplex infection;
gene17482	K14213	PEPD	Xaa-Pro dipeptidase	EC:3.4.13.9		
gene17483	K11434	PRMT1	protein arginine N-methyltransferase 1	EC:2.1.1.-		
gene17484	K04520	APP	amyloid beta A4 protein	--	2	ko04726 Serotonergic synapse;ko05010 Alzheimer's disease;
gene17486	K01679	E4.2.1.2B, fumC	fumarate hydratase, class II	EC:4.2.1.2	5	ko00020 Citrate cycle (TCA cycle);ko00720 Carbon fixation pathways in prokaryotes;ko01200 Carbon metabolism;ko05200 Pathways in cancer;ko05211 Renal cell carcinoma;
gene17493	K14236	tRNA-Tyr	tRNA Tyr	--	1	ko00970 Aminoacyl-tRNA biosynthesis;
gene17494	K14563	NOP1, FBL	rRNA 2'-O-methyltransferase fibrillarin	EC:2.1.1.-	1	ko03008 Ribosome biogenesis in eukaryotes;
gene17496	K06013	STE24	STE24 endopeptidase	EC:3.4.24.84	1	ko00900 Terpenoid backbone biosynthesis;
gene17497	K04371	MAPK1_3	mitogen-activated protein kinase 1/3	EC:2.7.11.24	71	ko04010 MAPK signaling pathway;ko04012 ErbB signaling pathway;ko04013 MAPK signaling pathway - fly;ko04014 Ras signaling pathway;ko04062 Chemokine signaling pathway;ko04066 HIF-1 signaling pathway;ko04114 Oocyte meiosis;ko04150 mTOR signaling pathway;ko04151 PI3K-Akt signaling pathway;ko04270 Vascular smooth muscle contraction;ko04320 Dorso-ventral axis formation;ko04350 TGF-beta signaling pathway;ko04360 Axon guidance;ko04370 VEGF signaling pathway;ko04380 Osteoclast differentiation;ko04510 Focal adhesion;ko04520 Adherens junction;ko04540 Gap junction;ko04620 Toll-like receptor signaling pathway;ko04621 NOD-like receptor signaling pathway;ko04650 Natural killer cell mediated cytotoxicity;ko04660 T cell receptor signaling pathway;ko04662 B cell receptor signaling pathway;ko04664 Fc epsilon RI signaling pathway;ko04666 Fc gamma R-mediated phagocytosis;ko04668 TNF signaling pathway;ko04713 Circadian entrainment;ko04720 Long-term potentiation;ko04722 Neurotrophin signaling pathway;ko04723 Retrograde endocannabinoid signaling;ko04724 Glutamatergic synapse;ko04725 Cholinergic synapse;ko04726 Serotonergic synapse;ko04730 Long-term depression;ko04810 Regulation of actin cytoskeleton;ko04910 Insulin signaling pathway;ko04912 GnRH signaling pathway;ko04914 Progesterone-mediated oocyte maturation;ko04915 Estrogen signaling pathway;ko04916 Melanogenesis;ko04917 Prolactin signaling pathway;ko04930 Type II diabetes mellitus;ko04960 Aldosterone-regulated sodium reabsorption;ko05010 Alzheimer's disease;ko05020 Prion diseases;ko05034 Alcoholism;ko05131 Shigellosis;ko05132 Salmonella infection;ko05133 Pertussis;ko05140 Leishmaniasis;ko05142 Chagas disease (American trypanosomiasis);ko05145 Toxoplasmosis;ko05152 Tuberculosis;ko05160 Hepatitis C;ko05161 Hepatitis B;ko05164 Influenza A;ko05200 Pathways in cancer;ko05203 Viral carcinogenesis;ko05205 Proteoglycans in cancer;ko05210 Colorectal cancer;ko05211 Renal cell carcinoma;ko05212
gene174	K16833	PPP1R2, IPP2	protein phosphatase inhibitor 2	--		
gene17500	K00434	E1.11.1.11	L-ascorbate peroxidase	EC:1.11.1.11	2	ko00053 Ascorbate and aldarate metabolism;ko00480 Glutathione metabolism;
gene17502	K05605	HIBCH	3-hydroxyisobutyryl-CoA hydrolase	EC:3.1.2.4	3	ko00280 Valine, leucine and isoleucine degradation;ko00410 beta-Alanine metabolism;ko00640 Propanoate metabolism;
gene17503	K05605	HIBCH	3-hydroxyisobutyryl-CoA hydrolase	EC:3.1.2.4	3	ko00280 Valine, leucine and isoleucine degradation;ko00410 beta-Alanine metabolism;ko00640 Propanoate metabolism;
gene17504	K14498	SNRK2	serine/threonine-protein kinase SRK2	EC:2.7.11.1	1	ko04075 Plant hormone signal transduction;
gene17507	K13420	FLS2	LRR receptor-like serine/threonine-protein kinase FLS2	EC:2.7.11.1	1	ko04626 Plant-pathogen interaction;

gene17508	K13420	FLS2	LRR receptor-like serine/threonine-protein kinase FLS2	EC:2.7.11.1	1	ko04626 Plant-pathogen interaction;
gene17509	K09872	PIP	aquaporin PIP	--		
gene17510	K12193	VPS24, CHMP3	charged multivesicular body protein 3	--	1	ko04144 Endocytosis;
gene17511	K16298	SCPL-IV	serine carboxypeptidase-like clade IV	EC:3.4.16.-		
gene17512	K16298	SCPL-IV	serine carboxypeptidase-like clade IV	EC:3.4.16.-		
gene17516	K01369	LGMN	legumain	EC:3.4.22.34	2	ko04142 Lysosome;ko04612 Antigen processing and presentation;
gene17518	K00600	glyA, SHMT	glycine hydroxymethyltransferase	EC:2.1.2.1	7	ko00260 Glycine, serine and threonine metabolism;ko00460 Cyanoamino acid metabolism;ko00630 Glyoxylate and dicarboxylate metabolism;ko00670 One carbon pool by folate;ko00680 Methane metabolism;ko01200 Carbon metabolism;ko01230 Biosynthesis of amino acids;
gene17527	K14295	NUP50, NPAP60	nuclear pore complex protein Nup50	--	1	ko03013 RNA transport;
gene17533	K14297	NUP98, ADAR2	nuclear pore complex protein Nup98-Nup96	--	2	ko03013 RNA transport;ko05164 Influenza A;
gene17535	K15255	PIF1	ATP-dependent DNA helicase PIF1	EC:3.6.4.12		
gene17541	K10527	MFP2	enoyl-CoA hydratase/3-hydroxyacyl-CoA dehydrogenase	EC:4.2.1.17 1.1.1.35 1.1.1.211	2	ko00071 Fatty acid degradation;ko00592 alpha-Linolenic acid metabolism;
gene17542	K10527	MFP2	enoyl-CoA hydratase/3-hydroxyacyl-CoA dehydrogenase	EC:4.2.1.17 1.1.1.35 1.1.1.211	2	ko00071 Fatty acid degradation;ko00592 alpha-Linolenic acid metabolism;
gene17543	K01623	ALDO	fructose-bisphosphate aldolase, class I	EC:4.1.2.13	7	ko00010 Glycolysis / Gluconeogenesis;ko00030 Pentose phosphate pathway;ko00051 Fructose and mannose metabolism;ko00680 Methane metabolism;ko00710 Carbon fixation in photosynthetic organisms;ko01200 Carbon metabolism;ko01230 Biosynthesis of amino acids;
gene17551	K01115	PLD1_2	phospholipase D1/2	EC:3.1.4.4	7	ko00564 Glycerophospholipid metabolism;ko00565 Ether lipid metabolism;ko04014 Ras signaling pathway;ko04144 Endocytosis;ko04666 Fc gamma R-mediated phagocytosis;ko04724 Glutamatergic synapse;ko04912 GnRH signaling pathway;
gene17552	K01520	dut, DUT	dUTP pyrophosphatase	EC:3.6.1.23	1	ko00240 Pyrimidine metabolism;
gene17555	K14611	SLC23A1_2, SVCT1_2	solute carrier family 23 (nucleobase transporter), member 1/2	--		
gene17558	K10990	RMI1, BRAP75	RecQ-mediated genome instability protein 1	--	1	ko03460 Fanconi anemia pathway;
gene17559	K09422	MYBP	myb proto-oncogene protein, plant	--		
gene17560	K07119	K07119				
gene17568	K11407	HDAC6_10	histone deacetylase 6/10	EC:3.5.1.98	2	ko05034 Alcoholism;ko05203 Viral carcinogenesis;
gene17573	K03128	TAF2	transcription initiation factor TFIID subunit 2	--	1	ko03022 Basal transcription factors;
gene17574	K03136	TFIIE1, GTF2E1, TFA1, tfe	transcription initiation factor TFIIE subunit alpha	--	3	ko03022 Basal transcription factors;ko05169 Epstein-Barr virus infection;ko05203 Viral carcinogenesis;
gene17593	K03183	ubiE	ubiquinone/menaquinone biosynthesis methyltransferase	EC:2.1.1.163 2.1.1.201	1	ko00130 Ubiquinone and other terpenoid-quinone biosynthesis;
gene17595	K02553	rraA, menG	regulator of ribonuclease activity A	--		
gene17597	K14328	UPF3, RENT3	regulator of nonsense transcripts 3	--	2	ko03013 RNA transport;ko03015 mRNA surveillance pathway;
gene17598	K13495	CISZOG	cis-zeatin O-glucosyltransferase	EC:2.4.1.215	1	ko00908 Zeatin biosynthesis;
gene17599	K03686	dnaJ	molecular chaperone DnaJ	--		
gene17600	K09872	PIP	aquaporin PIP	--		
gene17601	K10268	FBXL2_20	F-box and leucine-rich repeat protein 2/20	--		
gene17602	K02877	RP-L15e, RPL15	large subunit ribosomal protein L15e	--	1	ko03010 Ribosome;
gene17606	K08504	BET1	blocked early in transport 1	--	1	ko04130 SNARE interactions in vesicular transport;
gene1760	K01246	tag	DNA-3-methyladenine glycosylase I	EC:3.2.2.20	1	ko03410 Base excision repair;
gene17612	K06630	YWHAE	14-3-3 protein epsilon	--	8	ko04110 Cell cycle;ko04114 Oocyte meiosis;ko04151 PI3K-Akt signaling pathway;ko04390 Hippo signaling pathway;ko04391 Hippo signaling pathway - fly;ko04722 Neurotrophin signaling pathway;ko05169 Epstein-Barr virus infection;ko05203 Viral carcinogenesis;
gene17619	K03100	lepB	signal peptidase I	EC:3.4.21.89	1	ko03060 Protein export;
gene17622	K09286	EREBP	EREBP-like factor	--		
gene17623	K17553	PPP1R11	protein phosphatase 1 regulatory subunit 11	--		

gene17625	K00550	OPI3	methylene-fatty-acyl-phospholipid synthase	EC:2.1.1.16	1	ko00564 Glycerophospholipid metabolism;
gene17627	K16075	MRS2, MFM1	magnesium transporter	--		
gene17632	K07195	EXOC7, EXO70	exocyst complex component 7	--	1	ko04910 Insulin signaling pathway;
gene17634	K12620	LSM1	U6 snRNA-associated Sm-like protein LSm1	--	1	ko03018 RNA degradation;
gene17636	K11979	UBR7	E3 ubiquitin-protein ligase UBR7	EC:6.3.2.19		
gene1763	K10643	CNOT4, NOT4, MOT2	CCR4-NOT transcription complex subunit 4	EC:6.3.2.19	1	ko03018 RNA degradation;
gene17641	K09419	HSFF	heat shock transcription factor, other eukaryote	--		
gene17644	K13496	UGT73C	UDP-glucosyl transferase 73C	EC:2.4.1.-		
gene17647	K01802	E5.2.1.8	peptidylprolyl isomerase	EC:5.2.1.8		
gene17648	K11253	H3	histone H3	--	3	ko05034 Alcoholism;ko05202 Transcriptional misregulation in cancer;ko05322 Systemic lupus erythematosus;
gene17651	K04125	E1.14.11.13	gibberellin 2-oxidase	EC:1.14.11.13	1	ko00904 Diterpenoid biosynthesis;
gene17659	K14861	URB1	nucleolar pre-ribosomal-associated protein 1	--		
gene17660	K14220	tRNA-Asn	tRNA Asn	--	1	ko00970 Aminoacyl-tRNA biosynthesis;
gene17661	K14219	tRNA-Arg	tRNA Arg	--	1	ko00970 Aminoacyl-tRNA biosynthesis;
gene17666	K03231	EEF1A	elongation factor 1-alpha	--	2	ko03013 RNA transport;ko05134 Legionellosis;
gene17671	K07198	PRKAA, AMPK	5'-AMP-activated protein kinase, catalytic alpha subunit	EC:2.7.11.11	8	ko04140 Regulation of autophagy;ko04150 mTOR signaling pathway;ko04151 PI3K-Akt signaling pathway;ko04710 Circadian rhythm;ko04910 Insulin signaling pathway;ko04920 Adipocytokine signaling pathway;ko04932 Non-alcoholic fatty liver disease (NAFLD);ko05410 Hypertrophic cardiomyopathy (HCM);
gene17684	K17408	DAP3, MRPS29	small subunit ribosomal protein S29, mitochondrial	--		
gene17693	K02433	gatA	aspartyl-tRNA(Asn)/glutamyl-tRNA(Gln) amidotransferase subunit A	EC:6.3.5.6 6.3.5.7	1	ko00970 Aminoacyl-tRNA biosynthesis;
gene17693	K15077	ELA1	elongin-A	--		
gene17695	K17279	REEP5_6	receptor expression-enhancing protein 5/6	--		
gene17700	K00434	E1.11.1.11	L-ascorbate peroxidase	EC:1.11.1.11	2	ko00053 Ascorbate and aldarate metabolism;ko00480 Glutathione metabolism;
gene17701	K09313	CUTL	homeobox protein cut-like	--		
gene17707	K12179	COPS6, CSN6	COP9 signalosome complex subunit 6	--		
gene17708	K07456	mutS2	DNA mismatch repair protein MutS2	--	1	ko03430 Mismatch repair;
gene17717	K08910	LHCA4	light-harvesting complex I chlorophyll a/b binding protein 4	--	1	ko00196 Photosynthesis - antenna proteins;
gene17724	K07466	RFA1, RPA1, rpa	replication factor A1	--	5	ko03030 DNA replication;ko03420 Nucleotide excision repair;ko03430 Mismatch repair;ko03440 Homologous recombination;ko03460 Fanconi anemia pathway;
gene17727	K03665	hflX	GTP-binding protein HflX	--		
gene17731	K14379	ACP5	tartrate-resistant acid phosphatase type 5	EC:3.1.3.2	3	ko00740 Riboflavin metabolism;ko04380 Osteoclast differentiation;ko05323 Rheumatoid arthritis;
gene17733	K17095	ANXA7_11	annexin A7/11	--		
gene17736	K12734	PPIL3	peptidyl-prolyl cis-trans isomerase-like 3	EC:5.2.1.8		
gene17738	K13269	E2.4.1.234	kaempferol 3-O-beta-D-galactosyltransferase	EC:2.4.1.234	1	ko00944 Flavone and flavonol biosynthesis;
gene17739	K03327	TC.MATE, SLC47A, norM, mdtK, dinF	multidrug resistance protein, MATE family	--		
gene1773	K05663	ABC.ATM	mitochondrial ABC transporter ATM	--	1	ko02010 ABC transporters;
gene17741	K00763	pncB, NAPRT1	nicotinate phosphoribosyltransferase	EC:6.3.4.21	1	ko00760 Nicotinate and nicotinamide metabolism;
gene1774	K03026	RPC53, POLR3D	DNA-directed RNA polymerase III subunit RPC4	--	5	ko00230 Purine metabolism;ko00240 Pyrimidine metabolism;ko03020 RNA polymerase;ko04623 Cytosolic DNA-sensing pathway;ko05169 Epstein-Barr virus infection;
gene17752	K03320	amt, AMT, MEP	ammonium transporter, Amt family	--		
gene17754	K09489	HSPA4	heat shock 70kDa protein 4	--	1	ko04612 Antigen processing and presentation;
gene17756	K04124	E1.14.11.15	gibberellin 3-beta-dioxygenase	EC:1.14.11.15	1	ko00904 Diterpenoid biosynthesis;
gene17757	K00434	E1.11.1.11	L-ascorbate peroxidase	EC:1.11.1.11	2	ko00053 Ascorbate and aldarate metabolism;ko00480 Glutathione metabolism;
gene17759	K10752	RBBP4, HAT2, CAF1, MIS16	histone-binding protein RBBP4	--		
gene17766	K05868	CCNB	cyclin B	--	3	ko04110 Cell cycle;ko04115 p53 signaling pathway;ko04914 Progesterone-mediated oocyte maturation;

gene17769	K08876	SCYL1	SCY1-like protein 1	--		
gene17775	K12605	CNOT2, NOT2	CCR4-NOT transcription complex subunit 2	--	1	ko03018 RNA degradation;
gene17785	K06941	rlmN	23S rRNA (adenine2503-C2)-methyltransferase	EC:2.1.1.192		
gene17786	K00721	DPM1	dolichol-phosphate mannosyltransferase	EC:2.4.1.83	1	ko00510 N-Glycan biosynthesis;
gene1778	K14413	GALT1	beta-1,3-galactosyltransferase	EC:2.4.1.-	1	ko00513 Various types of N-glycan biosynthesis;
gene17791	K01051	E3.1.1.11	pectinesterase	EC:3.1.1.11	2	ko00040 Pentose and glucuronate interconversions;ko00500 Starch and sucrose metabolism;
gene17793	K16743	ASPM, ASP	abnormal spindle-like microcephaly-associated protein	--		
gene1779	K00422	E1.10.3.1	polyphenol oxidase	EC:1.10.3.1	2	ko00350 Tyrosine metabolism;ko00950 Isoquinoline alkaloid biosynthesis;
gene17802	K15283	SLC35E1	solute carrier family 35, member E1	--		
gene17809	K02535	lpxC	UDP-3-O-[3-hydroxymyristoyl] N-acetylglucosamine deacetylase	EC:3.5.1.108	1	ko00540 Lipopolysaccharide biosynthesis;
gene1780	K11593	ELF2C	eukaryotic translation initiation factor 2C	--		
gene17811	K04523	UBQLN, DSK2	ubiquilin	--	1	ko04141 Protein processing in endoplasmic reticulum;
gene17820	K14516	ERF1	ethylene-responsive transcription factor 1	--	1	ko04075 Plant hormone signal transduction;
gene17821	K16240	SPA1	protein suppressor of PHYA-105 1	--	1	ko04712 Circadian rhythm - plant;
gene17826	K03978	engB	GTP-binding protein	--		
gene17831	K14684	SLC25A23S	solute carrier family 25 (mitochondrial phosphate transporter), member 23/24/25/41	--		
gene17832	K14684	SLC25A23S	solute carrier family 25 (mitochondrial phosphate transporter), member 23/24/25/41	--		
gene17833	K02732	PSMB1	20S proteasome subunit beta 6	EC:3.4.25.1	1	ko03050 Proteasome;
gene17834	K14488	SAUR	SAUR family protein	--	1	ko04075 Plant hormone signal transduction;
gene17839	K01520	dut, DUT	dUTP pyrophosphatase	EC:3.6.1.23	1	ko00240 Pyrimidine metabolism;
gene17841	K01738	cysK	cysteine synthase A	EC:2.5.1.47	4	ko00270 Cysteine and methionine metabolism;ko00920 Sulfur metabolism;ko01200 Carbon metabolism;ko01230 Biosynthesis of amino acids;
gene17847	K03231	EEF1A	elongation factor 1-alpha	--	2	ko03013 RNA transport;ko05134 Legionellosis;
gene17849	K00799	GST, gst	glutathione S-transferase	EC:2.5.1.18	4	ko00480 Glutathione metabolism;ko00980 Metabolism of xenobiotics by cytochrome P450;ko00982 Drug metabolism - cytochrome P450;ko05204 Chemical carcinogenesis;
gene17850	K01738	cysK	cysteine synthase A	EC:2.5.1.47	4	ko00270 Cysteine and methionine metabolism;ko00920 Sulfur metabolism;ko01200 Carbon metabolism;ko01230 Biosynthesis of amino acids;
gene17853	K10781	FATB	fatty acyl-ACP thioesterase B	EC:3.1.2.14	1	ko00061 Fatty acid biosynthesis;
gene17859	K16055	TPS	trehalose 6-phosphate synthase/phosphatase	EC:2.4.1.15 3.1.3.12	1	ko00500 Starch and sucrose metabolism;
gene1785	K03943	NDUFV2	NADH dehydrogenase (ubiquinone) flavoprotein 2	EC:1.6.5.3 1.6.99.3	5	ko00190 Oxidative phosphorylation;ko04932 Non-alcoholic fatty liver disease (NAFLD);ko05010 Alzheimer's disease;ko05012 Parkinson's disease;ko05016 Huntington's disease;
gene17867	K15442	TAD3, ADAT3	tRNA-specific adenosine deaminase 3	--		
gene17870	K09286	EREBP	EREBP-like factor	--		
gene17876	K13648	GAUT	alpha-1,4-galacturonosyltransferase	EC:2.4.1.43	2	ko00500 Starch and sucrose metabolism;ko00520 Amino sugar and nucleotide sugar metabolism;
gene1787	K14221	tRNA-Asp	tRNA Asp	--	1	ko00970 Aminoacyl-tRNA biosynthesis;
gene17882	K13429	CERK1	chitin elicitor receptor kinase 1	--	1	ko04626 Plant-pathogen interaction;
gene17883	K11649	SMARCC	SWI/SNF related-matrix-associated actin-dependent regulator of chromatin subfamily C	--		
gene17884	K13146	INTS9	integrator complex subunit 9	--		
gene17886	K10688	UBE2W, UBC16	ubiquitin-conjugating enzyme E2 W	EC:6.3.2.19	1	ko04120 Ubiquitin mediated proteolysis;
gene17887	K15115	SLC25A32, MFT	solute carrier family 25 (mitochondrial folate transporter), member 32	--		
gene17888	K08679	E5.1.3.6	UDP-glucuronate 4-epimerase	EC:5.1.3.6	2	ko00500 Starch and sucrose metabolism;ko00520 Amino sugar and nucleotide sugar metabolism;
gene1788	K14484	IAA	auxin-responsive protein IAA	--	1	ko04075 Plant hormone signal transduction;

gene17891	K15634	gpmB	probable phosphoglycerate mutase	EC:5.4.2.12	5	ko00010 Glycolysis / Gluconeogenesis;ko00260 Glycine, serine and threonine metabolism;ko00680 Methane metabolism;ko01200 Carbon metabolism;ko01230 Biosynthesis of amino acids;
gene17895	K10406	KIFC2_3	kinesin family member C2/C3	--		
gene17899	K06883	K06883				
gene17900	K09377	CSRP	cysteine and glycine-rich protein	--		
gene17902	K04794	PTH2	peptidyl-tRNA hydrolase, PTH2 family	EC:3.1.1.29		
gene17903	K14236	tRNA-Tyr	tRNA Tyr	--	1	ko00970 Aminoacyl-tRNA biosynthesis;
gene17909	K07513	ACAA1	acetyl-CoA acyltransferase 1	EC:2.3.1.16	7	ko00071 Fatty acid degradation;ko00280 Valine, leucine and isoleucine degradation;ko00362 Benzoate degradation;ko00592 alpha-Linolenic acid metabolism;ko01040 Biosynthesis of unsaturated fatty acids;ko03320 PPAR signaling pathway;ko04146 Peroxisome;
gene17911	K12624	LSM5	U6 snRNA-associated Sm-like protein LSm5	--	2	ko03018 RNA degradation;ko03040 Spliceosome;
gene17914	K04508	TBL1	transducin (beta)-like 1	--	1	ko04310 Wnt signaling pathway;
gene17915	K06627	CCNA	cyclin A	--	5	ko04110 Cell cycle;ko04914 Progesterone-mediated oocyte maturation;ko05161 Hepatitis B;ko05169 Epstein-Barr virus infection;ko05203 Viral carcinogenesis;
gene17917	K05658	ABCB1	ATP-binding cassette, subfamily B (MDR/TAP), member 1	--	3	ko02010 ABC transporters;ko04976 Bile secretion;ko05206 MicroRNAs in cancer;
gene1791	K16546	FGFR10P	FGFR1 oncogene partner	--		
gene17920	K16732	PRC1	protein regulator of cytokinesis 1	--		
gene17921	K17604	ZSWIM3	zinc finger SWIM domain-containing protein 3	--		
gene17923	K10563	mutM, fpg	formamidopyrimidine-DNA glycosylase	EC:3.2.2.23 4.2.99.18	1	ko03410 Base excision repair;
gene17929	K10268	FBXL2_20	F-box and leucine-rich repeat protein 2/20	--		
gene1792	K08235	E2.4.1.207	xyloglucan:xyloglucosyl transferase	EC:2.4.1.207		
gene17930	K02892	RP-L23, MRPL23, rplW	large subunit ribosomal protein L23	--	1	ko03010 Ribosome;
gene17932	K11592	DICER1, DCR1	endoribonuclease Dicer	EC:3.1.26.-	1	ko05206 MicroRNAs in cancer;
gene17933	K03352	APC5	anaphase-promoting complex subunit 5	--	7	ko04110 Cell cycle;ko04111 Cell cycle - yeast;ko04113 Meiosis - yeast;ko04114 Oocyte meiosis;ko04120 Ubiquitin mediated proteolysis;ko04914 Progesterone-mediated oocyte maturation;ko05166 HTLV-I infection;
gene17937	K11290	SET, TAF1, I2PP2A	template-activating factor I	--		
gene17948	K06442	tlyA	23S rRNA (cytidine1920-2'-O)/16S rRNA (cytidine1409-2'-O)-methyltransferase	EC:2.1.1.226 2.1.1.227		
gene17953	K05289	GAA1	glycosylphosphatidylinositol transamidase	--	1	ko00563 Glycosylphosphatidylinositol(GPI)-anchor biosynthesis;
gene17956	K13648	GAUT	alpha-1,4-galacturonosyltransferase	EC:2.4.1.43	2	ko00500 Starch and sucrose metabolism;ko00520 Amino sugar and nucleotide sugar metabolism;
gene1795	K09286	EREBP	EREBP-like factor	--		
gene17960	K16223	FT	protein FLOWERING LOCUS T	--	1	ko04712 Circadian rhythm - plant;
gene17963	K03950	NDUFA6	NADH dehydrogenase (ubiquinone) 1 alpha subcomplex subunit 6	--	5	ko00190 Oxidative phosphorylation;ko04932 Non-alcoholic fatty liver disease (NAFLD);ko05010 Alzheimer's disease;ko05012 Parkinson's disease;ko05016 Huntington's disease;
gene17965	K00826	E2.6.1.42, ilvE	branched-chain amino acid aminotransferase	EC:2.6.1.42	5	ko00280 Valine, leucine and isoleucine degradation;ko00290 Valine, leucine and isoleucine biosynthesis;ko00770 Pantothenate and CoA biosynthesis;ko01210 2-Oxocarboxylic acid metabolism;ko01230 Biosynthesis of amino acids;
gene17966	K12373	HEXA_B	hexosaminidase	EC:3.2.1.52	6	ko00511 Other glycan degradation;ko00520 Amino sugar and nucleotide sugar metabolism;ko00531 Glycosaminoglycan degradation;ko00603 Glycosphingolipid biosynthesis - globo series;ko00604 Glycosphingolipid biosynthesis - ganglio series;ko04142 Lysosome;
gene17971	K09422	MYBP	myb proto-oncogene protein, plant	--		
gene17977	K02903	RP-L28e, RPL28	large subunit ribosomal protein L28e	--	1	ko03010 Ribosome;
gene1797	K09286	EREBP	EREBP-like factor	--		
gene17986	K15196	BRF1, GTF3B	transcription factor IIIB 90 kDa subunit	--		
gene17987	K13600	CAO	chlorophyllide a oxygenase	EC:1.14.13.12 2	1	ko00860 Porphyrin and chlorophyll metabolism;
gene1798	K15382	SLC50A, SWEET	solute carrier family 50 (sugar transporter)	--		
gene18004	K11446	JARID1	histone demethylase JARID1	EC:1.14.11.-		

gene18006	K15472	CYP71D55	premnaspirodiene oxygenase	EC:1.14.13.121	1	ko00909 Sesquiterpenoid and triterpenoid biosynthesis;
gene18010	K09422	MYBP	myb proto-oncogene protein, plant	--		
gene18011	K16223	FT	protein FLOWERING LOCUS T	--	1	ko04712 Circadian rhythm - plant;
gene18012	K15400	HHT1	omega-hydroxypalmitate O-feruloyl transferase	EC:2.3.1.188	1	ko00073 Cutin, suberine and wax biosynthesis;
gene18016	K03189	ureG	urease accessory protein	--		
gene18018	K03189	ureG	urease accessory protein	--		
gene18019	K02882	RP-L18Ae, RPL18A	large subunit ribosomal protein L18Ae	--	1	ko03010 Ribosome;
gene1801	K04730	IRAK1	interleukin-1 receptor-associated kinase 1	EC:2.7.11.1	11	ko04064 NF-kappa B signaling pathway;ko04210 Apoptosis;ko04620 Toll-like receptor signaling pathway;ko04722 Neurotrophin signaling pathway;ko05133 Pertussis;ko05140 Leishmaniasis;ko05142 Chagas disease (American trypanosomiasis);ko05145 Toxoplasmosis;ko05152 Tuberculosis;ko05162 Measles;ko05169 Epstein-Barr virus infection;
gene18027	K00434	E1.11.1.11	L-ascorbate peroxidase	EC:1.11.1.11	2	ko00053 Ascorbate and aldarate metabolism;ko00480 Glutathione metabolism;
gene18029	K06700	PSMF1	proteasome inhibitor subunit 1 (PI31)	--	1	ko03050 Proteasome;
gene1802	K13420	FLS2	LRR receptor-like serine/threonine-protein kinase FLS2	EC:2.7.11.1	1	ko04626 Plant-pathogen interaction;
gene18032	K02906	RP-L3, MRPL3, rplC	large subunit ribosomal protein L3	--	1	ko03010 Ribosome;
gene18039	K12885	RBMX, HNRNPG	heterogeneous nuclear ribonucleoprotein G	--	1	ko03040 Spliceosome;
gene18040	K01728	E4.2.2.2, pel	pectate lyase	EC:4.2.2.2	1	ko00040 Pentose and glucuronate interconversions;
gene18048	K16292	CEP, CYSEP	KDEL-tailed cysteine endopeptidase	EC:3.4.22.-		
gene18057	K02349	POLQ	DNA polymerase theta	EC:2.7.7.7		
gene18059	K14223	tRNA-Gln	tRNA Gln	--	1	ko00970 Aminoacyl-tRNA biosynthesis;
gene18060	K14230	tRNA-Met	tRNA Met	--	1	ko00970 Aminoacyl-tRNA biosynthesis;
gene18061	K14292	TGS1	trimethylguanosine synthase	EC:2.1.1.-	1	ko03013 RNA transport;
gene18065	K14233	tRNA-Ser	tRNA Ser	--	1	ko00970 Aminoacyl-tRNA biosynthesis;
gene18068	K01873	VARS, valS	valyl-tRNA synthetase	EC:6.1.1.9	1	ko00970 Aminoacyl-tRNA biosynthesis;
gene1806	K09422	MYBP	myb proto-oncogene protein, plant	--		
gene18074	K02961	RP-S17, MRPS17, rpsQ	small subunit ribosomal protein S17	--	1	ko03010 Ribosome;
gene18075	K14321	NUPL2, CG1	nucleoporin-like protein 2	--	1	ko03013 RNA transport;
gene18076	K00161	PDHA, pdhA	pyruvate dehydrogenase E1 component subunit alpha	EC:1.2.4.1	6	ko00010 Glycolysis / Gluconeogenesis;ko00020 Citrate cycle (TCA cycle);ko00620 Pyruvate metabolism;ko00650 Butanoate metabolism;ko01200 Carbon metabolism;ko04066 HIF-1 signaling pathway;
gene18080	K14222	tRNA-Cys	tRNA Cys	--	1	ko00970 Aminoacyl-tRNA biosynthesis;
gene18081	K00430	E1.11.1.7	peroxidase	EC:1.11.1.7	2	ko00360 Phenylalanine metabolism;ko00940 Phenylpropanoid biosynthesis;
gene18084	K12400	AP4E1	AP-4 complex subunit epsilon-1	--	1	ko04142 Lysosome;
gene18095	K14944	NOVA	RNA-binding protein Nova	--		
gene18097	K08869	ADCK, ABC1	aarF domain-containing kinase	--		
gene1809	K14235	tRNA-Trp	tRNA Trp	--	1	ko00970 Aminoacyl-tRNA biosynthesis;
gene18102	K03018	RPC1, POLR3A	DNA-directed RNA polymerase III subunit RPC1	EC:2.7.7.6	5	ko00230 Purine metabolism;ko00240 Pyrimidine metabolism;ko03020 RNA polymerase;ko04623 Cytosolic DNA-sensing pathway;ko05169 Epstein-Barr virus infection;
gene18103	K03018	RPC1, POLR3A	DNA-directed RNA polymerase III subunit RPC1	EC:2.7.7.6	5	ko00230 Purine metabolism;ko00240 Pyrimidine metabolism;ko03020 RNA polymerase;ko04623 Cytosolic DNA-sensing pathway;ko05169 Epstein-Barr virus infection;
gene18104	K09872	PIP	aquaporin PIP	--		
gene18105	K04567	KARS, lysS	lysyl-tRNA synthetase, class II	EC:6.1.1.6	1	ko00970 Aminoacyl-tRNA biosynthesis;
gene18106	K14190	VTC2_5	GDP-L-galactose phosphorylase	EC:2.7.7.69	1	ko00053 Ascorbate and aldarate metabolism;
gene18107	K03265	ETF1, ERF1	peptide chain release factor subunit 1	--	1	ko03015 mRNA surveillance pathway;
gene18109	K02116	atpI	ATP synthase protein I	--		
gene18117	K02838	frr, MRRF, RRF	ribosome recycling factor	--		
gene18120	K11420	EHMT	euchromatic histone-lysine N-methyltransferase	EC:2.1.1.43	1	ko00310 Lysine degradation;
gene18122	K15111	SLC25A26	solute carrier family 25 (mitochondrial S-adenosylmethionine transporter), member 26	--		
gene18125	K16292	CEP, CYSEP	KDEL-tailed cysteine endopeptidase	EC:3.4.22.-		

gene1812	K14856	SDA1, SDAD1	protein SDA1	--		
gene18132	K17095	ANXA7_11	annexin A7/11	--		
gene18133	K03676	grxC, GLRX, GLRX2	glutaredoxin 3	--		
gene18135	K11000	CALS	callose synthase	EC:2.4.1.-		
gene18136	K14484	IAA	auxin-responsive protein IAA	--	1	ko04075 Plant hormone signal transduction;
gene18137	K02983	RP-S30e, RPS30	small subunit ribosomal protein S30e	--	1	ko03010 Ribosome;
gene18138	K14664	ILR1	IAA-amino acid hydrolase	EC:3.5.1.-		
gene18139	K11086	SNRPB, SMB	small nuclear ribonucleoprotein B and B'	--	2	ko03040 Spliceosome;ko05322 Systemic lupus erythematosus;
gene18140	K02920	RP-L36e, RPL36	large subunit ribosomal protein L36e	--	1	ko03010 Ribosome;
gene18141	K16055	TPS	trehalose 6-phosphate synthase/phosphatase	EC:2.4.1.15 3.1.3.12	1	ko00500 Starch and sucrose metabolism;
gene18142	K14837	NOP12	nucleolar protein 12	--		
gene18146	K14488	SAUR	SAUR family protein	--	1	ko04075 Plant hormone signal transduction;
gene18148	K00721	DPM1	dolichol-phosphate mannosyltransferase	EC:2.4.1.83	1	ko00510 N-Glycan biosynthesis;
gene1814	K01052	LIPA	lysosomal acid lipase/cholesteryl ester hydrolase	EC:3.1.1.13	2	ko00100 Steroid biosynthesis;ko04142 Lysosome;
gene18151	K00901	E2.7.1.107, DGK, dgkA	diacylglycerol kinase (ATP dependent)	EC:2.7.1.107	3	ko00561 Glycerolipid metabolism;ko00564 Glycerophospholipid metabolism;ko04070 Phosphatidylinositol signaling system;
gene18153	K12501	HST	homogentisate solanesyltransferase	--	1	ko00130 Ubiquinone and other terpenoid-quinone biosynthesis;
gene18154	K02979	RP-S28e, RPS28	small subunit ribosomal protein S28e	--	1	ko03010 Ribosome;
gene18156	K10706	SETX, ALS4	senataxin	EC:3.6.4.-		
gene18163	K14304	NUP85	nuclear pore complex protein Nup85	--	1	ko03013 RNA transport;
gene18165	K01590	hdc, HDC	histidine decarboxylase	EC:4.1.1.22	1	ko00340 Histidine metabolism;
gene18170	K14492	ARR-A	two-component response regulator ARR-A family	--	1	ko04075 Plant hormone signal transduction;
gene18173	K00793	ribE, RIB5	riboflavin synthase	EC:2.5.1.9	1	ko00740 Riboflavin metabolism;
gene18176	K00006	GPD1	glycerol-3-phosphate dehydrogenase (NAD+)	EC:1.1.1.8	1	ko00564 Glycerophospholipid metabolism;
gene18179	K08876	SCYL1	SCY1-like protein 1	--		
gene18180	K12852	EFTUD2	116 kDa U5 small nuclear ribonucleoprotein component	--	1	ko03040 Spliceosome;
gene18183	K01535	E3.6.3.6	H+-transporting ATPase	EC:3.6.3.6	1	ko00190 Oxidative phosphorylation;
gene18184	K14638	SLC15A3_4, PHT	solute carrier family 15 (peptide/histidine transporter), member 3/4	--		
gene18188	K06269	PPP1C	serine/threonine-protein phosphatase PP1 catalytic subunit	EC:3.1.3.16	14	ko03015 mRNA surveillance pathway;ko04113 Meiosis - yeast;ko04114 Oocyte meiosis;ko04270 Vascular smooth muscle contraction;ko04390 Hippo signaling pathway;ko04510 Focal adhesion;ko04720 Long-term potentiation;ko04728 Dopaminergic synapse;ko04810 Regulation of actin cytoskeleton;ko04910 Insulin signaling pathway;ko05031 Amphetamine addiction;ko05034 Alcoholism;ko05168 Herpes simplex infection;ko05205 Proteoglycans in
gene18193	K03350	APC3, CDC27	anaphase-promoting complex subunit 3	--	7	ko04110 Cell cycle;ko04111 Cell cycle - yeast;ko04113 Meiosis - yeast;ko04114 Oocyte meiosis;ko04120 Ubiquitin mediated proteolysis;ko04914 Progesterone-mediated oocyte maturation;ko05166 HTLV-I infection;
gene1819	K05768	GSN	gelsolin	--	3	ko04666 Fc gamma R-mediated phagocytosis;ko04810 Regulation of actin cytoskeleton;ko05203 Viral carcinogenesis;
gene18205	K11789	VPRBP, DCAF1	HIV-1 Vpr-binding protein	--		
gene18207	K03143	TFIIH3, GTF2H3, TFB4	transcription initiation factor TFIIH subunit 3	--	3	ko03022 Basal transcription factors;ko03420 Nucleotide excision repair;ko05203 Viral carcinogenesis;
gene18208	K01918	panC	pantoate--beta-alanine ligase	EC:6.3.2.1	2	ko00410 beta-Alanine metabolism;ko00770 Pantothenate and CoA biosynthesis;
gene18209	K08511	ATVAMP72	vesicle-associated membrane protein 72	--		
gene18212	K09597	SPPL2B	signal peptide peptidase-like 2B	EC:3.4.23.-		
gene18217	K09377	CSRP	cysteine and glycine-rich protein	--		
gene1821	K14801	TSR4	pre-rRNA-processing protein TSR4	--		
gene18220	K01303	APEH	acylaminoacyl-peptidase	EC:3.4.19.1		
gene18223	K10666	RNF5	E3 ubiquitin-protein ligase RNF5	EC:6.3.2.19	1	ko04141 Protein processing in endoplasmic reticulum;
gene18230	K14782	AATF, BFR2	protein AATF/BFR2	--		
gene18241	K01723	AOS	hydroperoxide dehydratase	EC:4.2.1.92	1	ko00592 alpha-Linolenic acid metabolism;
gene18242	K14230	tRNA-Met	tRNA Met	--	1	ko00970 Aminoacyl-tRNA biosynthesis;

gene18247	K06685	MOB1, Mats	MOB kinase activator 1	--	3	ko04111 Cell cycle - yeast;ko04390 Hippo signaling pathway;ko04391 Hippo signaling pathway - fly;
gene18248	K05928	E2.1.1.95	tocopherol O-methyltransferase	EC:2.1.1.95	1	ko00130 Ubiquinone and other terpenoid-quinone biosynthesis;
gene1824	K05909	E1.10.3.2				
gene18250	K09264	K09264	MADS-box transcription factor, plant	--		
gene18254	K08744	CRLS	cardiolipin synthase	EC:2.7.8.-	1	ko00564 Glycerophospholipid metabolism;
gene1825	K06119	SQD2	sulfoquinovosyltransferase	EC:2.4.1.-	1	ko00561 Glycerolipid metabolism;
gene18264	K03549	kup	KUP system potassium uptake protein	--		
gene18277	K00430	E1.11.1.7	peroxidase	EC:1.11.1.7	2	ko00360 Phenylalanine metabolism;ko00940 Phenylpropanoid biosynthesis;
gene18280	K13510	LPCAT1_2	lysophosphatidylcholine acyltransferase / lyso-PAF acetyltransferase	EC:2.3.1.23 2.3.1.67	2	ko00564 Glycerophospholipid metabolism;ko00565 Ether lipid metabolism;
gene18296	K08737	MSH6	DNA mismatch repair protein MSH6	--	3	ko03430 Mismatch repair;ko05200 Pathways in cancer;ko05210 Colorectal cancer;
gene18297	K11797	PHIP, WDR11	PH-interacting protein	--		
gene18300	K04460	PPP5C	serine/threonine-protein phosphatase 5	EC:3.1.3.16	1	ko04010 MAPK signaling pathway;
gene18301	K15255	PIF1	ATP-dependent DNA helicase PIF1	EC:3.6.4.12		
gene18305	K03879	ND2	NADH-ubiquinone oxidoreductase chain 2	EC:1.6.5.3	2	ko00190 Oxidative phosphorylation;ko05012 Parkinson's disease;
gene18306	K15397	KCS	3-ketoacyl-CoA synthase	EC:2.3.1.199	1	ko00062 Fatty acid elongation;
gene18307	K10609	CUL4	cullin 4	--	2	ko03420 Nucleotide excision repair;ko04120 Ubiquitin mediated proteolysis;
gene18311	K02977	RP-S27Ae, RPS27A	small subunit ribosomal protein S27Ae	--	1	ko03010 Ribosome;
gene18313	K02882	RP-L18Ae, RPL18A	large subunit ribosomal protein L18Ae	--	1	ko03010 Ribosome;
gene18315	K16075	MRS2, MFM1	magnesium transporter	--		
gene18317	K02721	psbW	photosystem II PsbW protein	--	1	ko00195 Photosynthesis;
gene18320	K13617	PPME1	protein phosphatase methylesterase 1	EC:3.1.1.89		
gene18322	K04708	E1.1.1.102	3-dehydrosphinganine reductase	EC:1.1.1.102	1	ko00600 Sphingolipid metabolism;
gene18324	K00799	GST, gst	glutathione S-transferase	EC:2.5.1.18	4	ko00480 Glutathione metabolism;ko00980 Metabolism of xenobiotics by cytochrome P450;ko00982 Drug metabolism - cytochrome P450;ko05204 Chemical carcinogenesis;
gene18328	K11353	NDUFA13	NADH dehydrogenase (ubiquinone) 1 alpha subcomplex subunit 13	--	5	ko00190 Oxidative phosphorylation;ko04932 Non-alcoholic fatty liver disease (NAFLD);ko05010 Alzheimer's disease;ko05012 Parkinson's disease;ko05016 Huntington's disease;
gene18329	K04077	groEL, HSPD1	chaperonin GroEL	--	4	ko03018 RNA degradation;ko04940 Type I diabetes mellitus;ko05134 Legionellosis;ko05152 Tuberculosis;
gene1832	K14230	tRNA-Met	tRNA Met	--	1	ko00970 Aminoacyl-tRNA biosynthesis;
gene1832	K16297	SCPL-II	serine carboxypeptidase-like clade II	EC:3.4.16.-		
gene18337	K02639	petF	ferredoxin	--	1	ko00195 Photosynthesis;
gene18338	K16075	MRS2, MFM1	magnesium transporter	--		
gene1833	K14230	tRNA-Met	tRNA Met	--	1	ko00970 Aminoacyl-tRNA biosynthesis;
gene18341	K01081	E3.1.3.5	5'-nucleotidase	EC:3.1.3.5	3	ko00230 Purine metabolism;ko00240 Pyrimidine metabolism;ko00760 Nicotinate and nicotinamide metabolism;
gene18342	K17479	GRXCR1	glutaredoxin domain-containing cysteine-rich protein 1	--		
gene18346	K09503	DNAJA2	DnaJ homolog subfamily A member 2	--	1	ko04141 Protein processing in endoplasmic reticulum;
gene18348	K00924	E2.7.1.-				
gene18350	K15040	VDAC2	voltage-dependent anion channel protein 2	--	4	ko04020 Calcium signaling pathway;ko05012 Parkinson's disease;ko05016 Huntington's disease;ko05166 HTLV-I infection;
gene18352	K11752	ribD	diaminohydroxyphosphoribosylaminopyrimidine deaminase / 5-amino-6-(5-phosphoribosylamino)uracil reductase	EC:3.5.4.26 1.1.1.193	1	ko00740 Riboflavin metabolism;
gene18359	K00688	E2.4.1.1, glgP, PYG	starch phosphorylase	EC:2.4.1.1	2	ko00500 Starch and sucrose metabolism;ko04910 Insulin signaling pathway;
gene18360	K02988	RP-S5, MRPS5, rpsE	small subunit ribosomal protein S5	--	1	ko03010 Ribosome;
gene18365	K00059	fabG	3-oxoacyl-[acyl-carrier protein] reductase	EC:1.1.1.100	3	ko00061 Fatty acid biosynthesis;ko00780 Biotin metabolism;ko01040 Biosynthesis of unsaturated fatty acids;
gene18366	K11137	TELO2, TEL2	telomere length regulation protein	--	1	ko03460 Fanconi anemia pathway;
gene18367	K11137	TELO2, TEL2	telomere length regulation protein	--	1	ko03460 Fanconi anemia pathway;
gene18373	K12592	C1D, LRP1	exosome complex protein LRP1	--	1	ko03018 RNA degradation;

gene18375	K01527	EGD1, BTF3	nascent polypeptide-associated complex subunit beta	--		
gene18376	K14777	DDX47, RRP3	ATP-dependent RNA helicase DDX47/RRP3	EC:3.6.4.13		
gene18377	K17499	PPM1G, PP2CG	protein phosphatase 1G	EC:3.1.3.16		
gene18379	K12446	E2.7.1.46	L-arabinokinase	EC:2.7.1.46	1	ko00520 Amino sugar and nucleotide sugar metabolism;
gene1837	K14488	SAUR	SAUR family protein	--	1	ko04075 Plant hormone signal transduction;
gene18381	K06063	SNW1, SKIIP, SKIP	SNW domain-containing protein 1	--	4	ko03040 Spliceosome;ko04330 Notch signaling pathway;ko05169 Epstein-Barr virus infection;ko05203 Viral carcinogenesis;
gene18383	K08235	E2.4.1.207	xyloglucan:xyloglucosyl transferase	EC:2.4.1.207		
gene18387	K10882	EME1, MMS4	crossover junction endonuclease EME1	EC:3.1.22.-	2	ko03440 Homologous recombination;ko03460 Fanconi anemia pathway;
gene1838	K10364	CAPZA	capping protein (actin filament) muscle Z-line, alpha	--		
gene18392	K02116	atpI	ATP synthase protein I	--		
gene18395	K01595	ppc	phosphoenolpyruvate carboxylase	EC:4.1.1.31	5	ko00620 Pyruvate metabolism;ko00680 Methane metabolism;ko00710 Carbon fixation in photosynthetic organisms;ko00720 Carbon fixation pathways in prokaryotes;ko01200 Carbon metabolism;
gene1839	K16055	TPS	trehalose 6-phosphate synthase/phosphatase	EC:2.4.1.15 3.1.3.12	1	ko00500 Starch and sucrose metabolism;
gene183	K09422	MYBP	myb proto-oncogene protein, plant	--		
gene18400	K11254	H4	histone H4	--	3	ko05034 Alcoholism;ko05203 Viral carcinogenesis;ko05322 Systemic lupus erythematosus;
gene18402	K05658	ABCB1	ATP-binding cassette, subfamily B (MDR/TAP), member 1	--	3	ko02010 ABC transporters;ko04976 Bile secretion;ko05206 MicroRNAs in cancer;
gene18403	K14821	BUD20	bud site selection protein 20	--		
gene18404	K10753	ASF1	histone chaperone ASF1	--		
gene18405	K12261	HACL1	2-hydroxyacyl-CoA lyase 1	EC:4.1.-.-	1	ko04146 Peroxisome;
gene18406	K01679	E4.2.1.2B, fumC	fumarate hydratase, class II	EC:4.2.1.2	5	ko00020 Citrate cycle (TCA cycle);ko00720 Carbon fixation pathways in prokaryotes;ko01200 Carbon metabolism;ko05200 Pathways in cancer;ko05211 Renal cell carcinoma;
gene18410	K03868	RBX1, ROC1	RING-box protein 1	--	12	ko03420 Nucleotide excision repair;ko04066 HIF-1 signaling pathway;ko04110 Cell cycle;ko04111 Cell cycle - yeast;ko04114 Oocyte meiosis;ko04120 Ubiquitin mediated proteolysis;ko04141 Protein processing in endoplasmic reticulum;ko04310 Wnt signaling pathway;ko04350 TGF-beta signaling pathway;ko04710 Circadian rhythm;ko05200 Pathways in cancer;ko05211 Renal cell carcinoma;
gene18411	K00721	DPM1	dolichol-phosphate mannosyltransferase	EC:2.4.1.83	1	ko00510 N-Glycan biosynthesis;
gene18413	K14232	tRNA-Pro	tRNA Pro	--	1	ko00970 Aminoacyl-tRNA biosynthesis;
gene18414	K14232	tRNA-Pro	tRNA Pro	--	1	ko00970 Aminoacyl-tRNA biosynthesis;
gene18415	K14232	tRNA-Pro	tRNA Pro	--	1	ko00970 Aminoacyl-tRNA biosynthesis;
gene18416	K14232	tRNA-Pro	tRNA Pro	--	1	ko00970 Aminoacyl-tRNA biosynthesis;
gene1841	K00799	GST, gst	glutathione S-transferase	EC:2.5.1.18	4	ko00480 Glutathione metabolism;ko00980 Metabolism of xenobiotics by cytochrome P450;ko00982 Drug metabolism - cytochrome P450;ko05204 Chemical carcinogenesis;
gene18424	K13412	CPK	calcium-dependent protein kinase	EC:2.7.11.1	2	ko04626 Plant-pathogen interaction;ko05145 Toxoplasmosis;
gene18425	K00434	E1.11.1.11	L-ascorbate peroxidase	EC:1.11.1.11	2	ko00053 Ascorbate and aldarate metabolism;ko00480 Glutathione metabolism;
gene18429	K00517	E1.14.-.-			4	ko00624 Polycyclic aromatic hydrocarbon degradation;ko00627 Aminobenzoate degradation;ko00903 Limonene and pinene degradation;ko00945 Stilbenoid, diarylheptanoid and gingerol biosynthesis;
gene1842	K00799	GST, gst	glutathione S-transferase	EC:2.5.1.18	4	ko00480 Glutathione metabolism;ko00980 Metabolism of xenobiotics by cytochrome P450;ko00982 Drug metabolism - cytochrome P450;ko05204 Chemical carcinogenesis;
gene18430	K09286	EREBP	EREBP-like factor	--		
gene18431	K14207	SLC38A2, SNAT2	solute carrier family 38 (sodium-coupled neutral amino acid transporter), member 2	--	3	ko04724 Glutamatergic synapse;ko04727 GABAergic synapse;ko04974 Protein digestion and absorption;
gene18437	K13448	CML	calcium-binding protein CML	--	1	ko04626 Plant-pathogen interaction;
gene18439	K15103	UCP2_3, SLC25A8_9	solute carrier family 25 (mitochondrial uncoupling protein), member 8/9	--		
gene18445	K06689	UBE2D_E, UBC4, UBC5	ubiquitin-conjugating enzyme E2 D/E	EC:6.3.2.19	2	ko04120 Ubiquitin mediated proteolysis;ko04141 Protein processing in endoplasmic reticulum;

gene18452	K01904	E6.2.1.12	4-coumarate--CoA ligase	EC:6.2.1.12	3	ko00130 Ubiquinone and other terpenoid-quinone biosynthesis;ko00360 Phenylalanine metabolism;ko00940 Phenylpropanoid biosynthesis;
gene18454	K14488	SAUR	SAUR family protein	--	1	ko04075 Plant hormone signal transduction;
gene18456	K05750	NCKAP1, NAP125	NCK-associated protein 1	--	1	ko04810 Regulation of actin cytoskeleton;
gene18460	K13545	ACD2	red chlorophyll catabolite reductase	EC:1.3.1.80	1	ko00860 Porphyrin and chlorophyll metabolism;
gene18465	K10355	ACTF	actin, other eukaryote	--		
gene1846	K15196	BRF1, GTF3B	transcription factor IIIB 90 kDa subunit	--		
gene18474	K00863	E2.7.1.29, DAK1, DAK2	dihydroxyacetone kinase	EC:2.7.1.29	4	ko00561 Glycerolipid metabolism;ko00680 Methane metabolism;ko01200 Carbon metabolism;ko04622 RIG-I-like receptor signaling pathway;
gene18478	K00218	E1.3.1.33, por	protochlorophyllide reductase	EC:1.3.1.33	1	ko00860 Porphyrin and chlorophyll metabolism;
gene1847	K13496	UGT73C	UDP-glucosyl transferase 73C	EC:2.4.1.-		
gene18480	K11838	USP7, UBP15	ubiquitin carboxyl-terminal hydrolase 7	EC:3.1.2.15	3	ko05168 Herpes simplex infection;ko05169 Epstein-Barr virus infection;ko05203 Viral carcinogenesis;
gene18482	K05909	E1.10.3.2				
gene18488	K10747	LIG1	DNA ligase 1	EC:6.5.1.1	4	ko03030 DNA replication;ko03410 Base excision repair;ko03420 Nucleotide excision repair;ko03430 Mismatch repair;
gene18499	K08739	MLH3	DNA mismatch repair protein MLH3	--	1	ko03430 Mismatch repair;
gene1849	K11662	ACTR6, ARP6	actin-related protein 6	--		
gene18502	K04733	IRAK4	interleukin-1 receptor-associated kinase 4	EC:2.7.11.1	11	ko04064 NF-kappa B signaling pathway;ko04210 Apoptosis;ko04620 Toll-like receptor signaling pathway;ko04722 Neurotrophin signaling pathway;ko05133 Pertussis;ko05140 Leishmaniasis;ko05142 Chagas disease (American trypanosomiasis);ko05145 Toxoplasmosis;ko05152 Tuberculosis;ko05162 Measles;ko05164 Influenza A;
gene18504	K14235	tRNA-Trp	tRNA Trp	--	1	ko00970 Aminoacyl-tRNA biosynthesis;
gene18509	K01859	E5.5.1.6	chalcone isomerase	EC:5.5.1.6	1	ko00941 Flavonoid biosynthesis;
gene1850	K03320	amt, AMT, MEP	ammonium transporter, Amt family	--		
gene18510	K00799	GST, gst	glutathione S-transferase	EC:2.5.1.18	4	ko00480 Glutathione metabolism;ko00980 Metabolism of xenobiotics by cytochrome P450;ko00982 Drug metabolism - cytochrome P450;ko05204 Chemical carcinogenesis;
gene18512	K07119	K07119				
gene18514	K00940	E2.7.4.6, ndk	nucleoside-diphosphate kinase	EC:2.7.4.6	2	ko00230 Purine metabolism;ko00240 Pyrimidine metabolism;
gene18516	K01183	E3.2.1.14	chitinase	EC:3.2.1.14	1	ko00520 Amino sugar and nucleotide sugar metabolism;
gene18517	K01183	E3.2.1.14	chitinase	EC:3.2.1.14	1	ko00520 Amino sugar and nucleotide sugar metabolism;
gene18519	K12123	PHYE	phytochrome E	--		
gene1851	K03850	ALG10	alpha-1,2-glucosyltransferase	EC:2.4.1.256	1	ko00510 N-Glycan biosynthesis;
gene18522	K02912	RP-L32e, RPL32	large subunit ribosomal protein L32e	--	1	ko03010 Ribosome;
gene18528	K10352	MYH	myosin heavy chain	--	2	ko04530 Tight junction;ko05416 Viral myocarditis;
gene1852	K01102	PDP	pyruvate dehydrogenase phosphatase	EC:3.1.3.43		
gene18533	K01681	ACO, acnA	aconitate hydratase	EC:4.2.1.3	6	ko00020 Citrate cycle (TCA cycle);ko00630 Glyoxylate and dicarboxylate metabolism;ko00720 Carbon fixation pathways in prokaryotes;ko01200 Carbon metabolism;ko01210 2-Oxocarboxylic acid metabolism;ko01230 Biosynthesis of amino acids;
gene18544	K01663	HIS7	glutamine amidotransferase / cyclase	EC:2.4.2.- 4.1.3.-	2	ko00340 Histidine metabolism;ko01230 Biosynthesis of amino acids;
gene18547	K08819	CDK12_13	cyclin-dependent kinase 12/13	EC:2.7.11.22 2.7.11.23		
gene18549	K01187	E3.2.1.20, malZ	alpha-glucosidase	EC:3.2.1.20	2	ko00052 Galactose metabolism;ko00500 Starch and sucrose metabolism;
gene18553	K01803	TPI, tpiA	triosephosphate isomerase (TIM)	EC:5.3.1.1	6	ko00010 Glycolysis / Gluconeogenesis;ko00051 Fructose and mannose metabolism;ko00562 Inositol phosphate metabolism;ko00710 Carbon fixation in photosynthetic organisms;ko01200 Carbon metabolism;ko01230 Biosynthesis of amino acids;
gene18555	K01915	glnA	glutamine synthetase	EC:6.3.1.2	8	ko00250 Alanine, aspartate and glutamate metabolism;ko00330 Arginine and proline metabolism;ko00630 Glyoxylate and dicarboxylate metabolism;ko00910 Nitrogen metabolism;ko01230 Biosynthesis of amino acids;ko02020 Two-component system;ko04724 Glutamatergic synapse;ko04727 GABAergic synapse;
gene18556	K07766	E3.6.1.52	diphosphoinositol-polyphosphate diphosphatase	EC:3.6.1.52		

gene18558	K02641	petH	ferredoxin--NADP+ reductase	EC:1.18.1.2	1	ko00195 Photosynthesis;
gene18560	K00645	fabD	[acyl-carrier-protein] S-malonyltransferase	EC:2.3.1.39	1	ko00061 Fatty acid biosynthesis;
gene18564	K11583	PPP2R3	serine/threonine-protein phosphatase 2A regulatory subunit B''	--	3	ko03015 mRNA surveillance pathway;ko04151 PI3K-Akt signaling pathway;ko04728 Dopaminergic synapse;
gene18566	K14709	SLC39A1_2_3, ZIP1_2_3	solute carrier family 39 (zinc transporter), member 1/2/3	--		
gene18567	K04121	E4.2.3.19	ent-kaurene synthase	EC:4.2.3.19	1	ko00904 Diterpenoid biosynthesis;
gene18574	K11366	USP22_27_51, UBP8	ubiquitin carboxyl-terminal hydrolase 22/27/51	EC:3.1.2.15		
gene18581	K12194	CHMP4, SNF7, VPS32	charged multivesicular body protein 4	--	1	ko04144 Endocytosis;
gene18585	K16252	NRPD2, NRPE2	DNA-directed RNA polymerase IV and V subunit 2	EC:2.7.7.6		
gene18587	K11000	CALS	callose synthase	EC:2.4.1.-		
gene1858	K01213	E3.2.1.67	galacturan 1,4-alpha-galacturonidase	EC:3.2.1.67	2	ko00040 Pentose and glucuronate interconversions;ko00500 Starch and sucrose metabolism;
gene18590	K02153	ATPeVH, ATP6H	V-type H+-transporting ATPase subunit H	EC:3.6.3.14	7	ko00190 Oxidative phosphorylation;ko04145 Phagosome;ko04721 Synaptic vesicle cycle;ko04966 Collecting duct acid secretion;ko05110 Vibrio cholerae infection;ko05120 Epithelial cell signaling in Helicobacter pylori infection;ko05323 Rheumatoid arthritis;
gene18595	K01530	E3.6.3.1	phospholipid-translocating ATPase	EC:3.6.3.1		
gene18597	K02914	RP-L34, MRPL34, rpmH	large subunit ribosomal protein L34	--	1	ko03010 Ribosome;
gene18598	K03787	surE	5'-nucleotidase	EC:3.1.3.5	3	ko00230 Purine metabolism;ko00240 Pyrimidine metabolism;ko00760 Nicotinate and nicotinamide metabolism;
gene18599	K12741	HNRNPA1_3	heterogeneous nuclear ribonucleoprotein A1/A3	--	1	ko03040 Spliceosome;
gene1859	K01213	E3.2.1.67	galacturan 1,4-alpha-galacturonidase	EC:3.2.1.67	2	ko00040 Pentose and glucuronate interconversions;ko00500 Starch and sucrose metabolism;
gene18602	K01176	E3.2.1.1, amyA, malS	alpha-amylase	EC:3.2.1.1	2	ko00500 Starch and sucrose metabolism;ko04973 Carbohydrate digestion and absorption;
gene18604	K10990	RMI1, BRAP75	RecQ-mediated genome instability protein 1	--	1	ko03460 Fanconi anemia pathway;
gene18605	K09422	MYBP	myb proto-oncogene protein, plant	--		
gene18607	K09775	K09775	hypothetical protein	--		
gene18608	K00685	ATE1, ate1	arginine-tRNA-protein transferase	EC:2.3.2.8		
gene18615	K17505	PPM1K, PP2CM	protein phosphatase 1K	EC:3.1.3.16		
gene18616	K16055	TPS	trehalose 6-phosphate synthase/phosphatase	EC:2.4.1.15 3.1.3.12	1	ko00500 Starch and sucrose metabolism;
gene18622	K01206	FUCA	alpha-L-fucosidase	EC:3.2.1.51	1	ko00511 Other glycan degradation;
gene18623	K09494	CCT2	T-complex protein 1 subunit beta	--		
gene18625	K16298	SCPL-IV	serine carboxypeptidase-like clade IV	EC:3.4.16.-		
gene18626	K16298	SCPL-IV	serine carboxypeptidase-like clade IV	EC:3.4.16.-		
gene18631	K09420	MYB	myb proto-oncogene protein	--	2	ko04151 PI3K-Akt signaling pathway;ko05166 HTLV-I infection;
gene18632	K14454	GOT1	aspartate aminotransferase, cytoplasmic	EC:2.6.1.1	11	ko00250 Alanine, aspartate and glutamate metabolism;ko00270 Cysteine and methionine metabolism;ko00330 Arginine and proline metabolism;ko00350 Tyrosine metabolism;ko00360 Phenylalanine metabolism;ko00400 Phenylalanine, tyrosine and tryptophan biosynthesis;ko00710 Carbon fixation in photosynthetic organisms;ko00950 Isoquinoline alkaloid biosynthesis;ko00960 Tropane, piperidine and pyridine alkaloid biosynthesis;ko01210 2-Oxocarboxylic acid metabolism;ko01230 Biosynthesis of amino acids;
gene18633	K15166	MED23	mediator of RNA polymerase II transcription subunit 23	--		
gene1863	K09872	PIP	aquaporin PIP	--		
gene18641	K11851	USP30	ubiquitin carboxyl-terminal hydrolase 30	EC:3.1.2.15		
gene18645	K14838	NOP15	nucleolar protein 15	--		
gene18646	K00924	E2.7.1.-				
gene18649	K01285	PRCP	lysosomal Pro-X carboxypeptidase	EC:3.4.16.2	1	ko04974 Protein digestion and absorption;
gene1864	K09597	SPPL2B	signal peptide peptidase-like 2B	EC:3.4.23.-		
gene18654	K03935	NDUFS2	NADH dehydrogenase (ubiquinone) Fe-S protein 2	EC:1.6.5.3 1.6.99.3	5	ko00190 Oxidative phosphorylation;ko04932 Non-alcoholic fatty liver disease (NAFLD);ko05010 Alzheimer's disease;ko05012 Parkinson's disease;ko05016 Huntington's disease;
gene18659	K13495	CISZOG	cis-zeatin O-glucosyltransferase	EC:2.4.1.215	1	ko00908 Zeatin biosynthesis;

gene18660	K03124	TFIIB, GTF2B, SUA7, tfb	transcription initiation factor TFIIB	--	3	ko03022 Basal transcription factors;ko05169 Epstein-Barr virus infection;ko05203 Viral carcinogenesis;
gene18663	K14509	ETR, ERS	ethylene receptor	EC:2.7.13.-	1	ko04075 Plant hormone signal transduction;
gene18667	K08150	SLC2A13, ITR	MFS transporter, SP family, solute carrier family 2 (myo-inositol transporter), member 13	--		
gene18669	K11423	SETD2, SET2	histone-lysine N-methyltransferase SETD2	EC:2.1.1.43	1	ko00310 Lysine degradation;
gene18672	K04506	SIAH1	E3 ubiquitin-protein ligase SIAH1	EC:6.3.2.19	3	ko04115 p53 signaling pathway;ko04120 Ubiquitin mediated proteolysis;ko04310 Wnt signaling pathway;
gene18674	K02942	RP-LP1, RPLP1	large subunit ribosomal protein LP1	--	1	ko03010 Ribosome;
gene18682	K09313	CUTL	homeobox protein cut-like	--		
gene18683	K00434	E1.11.1.11	L-ascorbate peroxidase	EC:1.11.1.11	2	ko00053 Ascorbate and aldarate metabolism;ko00480 Glutathione metabolism;
gene18685	K08735	MSH2	DNA mismatch repair protein MSH2	--	3	ko03430 Mismatch repair;ko05200 Pathways in cancer;ko05210 Colorectal cancer;
gene18687	K15559	RTT103	regulator of Ty1 transposition protein 103	--		
gene1868	K12639	CYP724B1, D11	cytochrome P450, family 724, subfamily B, polypeptide 1	EC:1.14.13.-	1	ko00905 Brassinosteroid biosynthesis;
gene18692	K11491	NCAPD3	condensin-2 complex subunit D3	--		
gene18693	K09518	DNAJB12	DnaJ homolog subfamily B member 12	--	1	ko04141 Protein processing in endoplasmic reticulum;
gene18694	K13120	FAM32A	protein FAM32A	--		
gene18695	K08678	UXS1	UDP-glucuronate decarboxylase	EC:4.1.1.35	2	ko00500 Starch and sucrose metabolism;ko00520 Amino sugar and nucleotide sugar metabolism;
gene18696	K03242	EIF2S3	translation initiation factor 2 subunit 3	--	1	ko03013 RNA transport;
gene18699	K10527	MFP2	enoyl-CoA hydratase/3-hydroxyacyl-CoA dehydrogenase	EC:4.2.1.17 1.1.1.35 1.1.1.211	2	ko00071 Fatty acid degradation;ko00592 alpha-Linolenic acid metabolism;
gene18706	K10666	RNF5	E3 ubiquitin-protein ligase RNF5	EC:6.3.2.19	1	ko04141 Protein processing in endoplasmic reticulum;
gene18708	K10635	RNF111	E3 ubiquitin-protein ligase Arkadia	EC:6.3.2.19		
gene18710	K02563	murG	UDP-N-acetylglucosamine--N-acetylmuramyl-(pentapeptide) pyrophosphoryl-undecaprenol N-acetylglucosamine transferase	EC:2.4.1.227	2	ko00550 Peptidoglycan biosynthesis;ko04112 Cell cycle - Caulobacter;
gene18711	K01408	IDE, ide	insulysin	EC:3.4.24.56	1	ko05010 Alzheimer's disease;
gene18715	K03671	trxA	thioredoxin 1	--		
gene1871	K14298	RAE1	mRNA export factor	--	2	ko03013 RNA transport;ko05164 Influenza A;
gene18725	K08341	GABARAP, ATG8, LC3	GABA(A) receptor-associated protein (autophagy-related protein 8)	--	2	ko04140 Regulation of autophagy;ko04727 GABAergic synapse;
gene18727	K02906	RP-L3, MRPL3, rplC	large subunit ribosomal protein L3	--	1	ko03010 Ribosome;
gene18728	K01881	PARS, proS	prolyl-tRNA synthetase	EC:6.1.1.15	1	ko00970 Aminoacyl-tRNA biosynthesis;
gene1872	K13416	BAK1	brassinosteroid insensitive 1-associated receptor kinase 1	EC:2.7.10.1 2.7.11.1	2	ko04075 Plant hormone signal transduction;ko04626 Plant-pathogen interaction;
gene18732	K00434	E1.11.1.11	L-ascorbate peroxidase	EC:1.11.1.11	2	ko00053 Ascorbate and aldarate metabolism;ko00480 Glutathione metabolism;
gene18735	K04506	SIAH1	E3 ubiquitin-protein ligase SIAH1	EC:6.3.2.19	3	ko04115 p53 signaling pathway;ko04120 Ubiquitin mediated proteolysis;ko04310 Wnt signaling pathway;
gene18736	K04506	SIAH1	E3 ubiquitin-protein ligase SIAH1	EC:6.3.2.19	3	ko04115 p53 signaling pathway;ko04120 Ubiquitin mediated proteolysis;ko04310 Wnt signaling pathway;
gene18739	K01810	GPI, pgi	glucose-6-phosphate isomerase	EC:5.3.1.9	5	ko00010 Glycolysis / Gluconeogenesis;ko00030 Pentose phosphate pathway;ko00500 Starch and sucrose metabolism;ko00520 Amino sugar and nucleotide sugar metabolism;ko01200 Carbon metabolism;
gene18740	K08900	BCS1	mitochondrial chaperone BCS1	--		
gene18741	K14488	SAUR	SAUR family protein	--	1	ko04075 Plant hormone signal transduction;
gene18742	K17506	PPM1L, PP2CE	protein phosphatase 1L	EC:3.1.3.16		
gene18751	K00938	E2.7.4.2, mvaK2	phosphomevalonate kinase	EC:2.7.4.2	1	ko00900 Terpenoid backbone biosynthesis;
gene18753	K11816	YUCCA	indole-3-pyruvate monooxygenase	EC:1.14.13.168	1	ko00380 Tryptophan metabolism;
gene1875	K00827	AGXT2	alanine-glyoxylate transaminase / (R)-3-amino-2-methylpropionate-pyruvate transaminase	EC:2.6.1.44 2.6.1.40	2	ko00250 Alanine, aspartate and glutamate metabolism;ko00260 Glycine, serine and threonine metabolism;
gene18761	K00767	nadC, QPRT	nicotinate-nucleotide pyrophosphorylase (carboxylating)	EC:2.4.2.19	1	ko00760 Nicotinate and nicotinamide metabolism;
gene18762	K06689	UBE2D_E, UBC4, UBC5	ubiquitin-conjugating enzyme E2 D/E	EC:6.3.2.19	2	ko04120 Ubiquitin mediated proteolysis;ko04141 Protein processing in endoplasmic reticulum;

gene18769	K13174	THOC5	THO complex subunit 5	--	1	ko03013 RNA transport;
gene1876	K05282	E1.14.11.12	gibberellin 20-oxidase	EC:1.14.11.12	1	ko00904 Diterpenoid biosynthesis;
gene18770	K17095	ANXA7_11	annexin A7/11	--		
gene18777	K02988	RP-S5, MRPS5, rpsE	small subunit ribosomal protein S5	--	1	ko03010 Ribosome;
gene1877	K14564	NOP56	nucleolar protein 56	--	1	ko03008 Ribosome biogenesis in eukaryotes;
gene18781	K09060	GBF	plant G-box-binding factor	--		
gene18783	K03283	HSPA1_8	heat shock 70kDa protein 1/8	--	11	ko03040 Spliceosome;ko04010 MAPK signaling pathway;ko04141 Protein processing in endoplasmic reticulum;ko04144 Endocytosis;ko04612 Antigen processing and presentation;ko04915 Estrogen signaling pathway;ko05134 Legionellosis;ko05145 Toxoplasmosis;ko05162 Measles;ko05164 Influenza A;ko05169 Epstein-Barr virus infection;
gene18784	K16818	K16818, DAD1	phospholipase A1	EC:3.1.1.32	2	ko00564 Glycerophospholipid metabolism;ko00592 alpha-Linolenic acid metabolism;
gene18785	K10268	FBXL2_20	F-box and leucine-rich repeat protein 2/20	--		
gene18787	K15892	FOLK	farnesol kinase	EC:2.7.1.-	1	ko00900 Terpenoid backbone biosynthesis;
gene1878	K05391	CNGF	cyclic nucleotide gated channel, other eukaryote	--	1	ko04626 Plant-pathogen interaction;
gene18796	K15728	LPIN	phosphatidate phosphatase LPIN	EC:3.1.3.4	2	ko00561 Glycerolipid metabolism;ko00564 Glycerophospholipid metabolism;
gene18799	K14236	tRNA-Tyr	tRNA Tyr	--	1	ko00970 Aminoacyl-tRNA biosynthesis;
gene187	K12741	HNRNPA1_3	heterogeneous nuclear ribonucleoprotein A1/A3	--	1	ko03040 Spliceosome;
gene18802	K03164	TOP2	DNA topoisomerase II	EC:5.99.1.3		
gene18807	K00559	E2.1.1.41, SMT1, ERG6	sterol 24-C-methyltransferase	EC:2.1.1.41	1	ko00100 Steroid biosynthesis;
gene1880	K05391	CNGF	cyclic nucleotide gated channel, other eukaryote	--	1	ko04626 Plant-pathogen interaction;
gene18813	K03231	EEF1A	elongation factor 1-alpha	--	2	ko03013 RNA transport;ko05134 Legionellosis;
gene18814	K03231	EEF1A	elongation factor 1-alpha	--	2	ko03013 RNA transport;ko05134 Legionellosis;
gene18816	K02736	PSMB4	20S proteasome subunit beta 7	EC:3.4.25.1	1	ko03050 Proteasome;
gene18827	K00763	pncB, NAPRT1	nicotinate phosphoribosyltransferase	EC:6.3.4.21	1	ko00760 Nicotinate and nicotinamide metabolism;
gene18835	K07767	E3.6.4.3	microtubule-severing ATPase	EC:3.6.4.3		
gene18841	K01578	E4.1.1.9, MLYCD	malonyl-CoA decarboxylase	EC:4.1.1.9	3	ko00410 beta-Alanine metabolism;ko00640 Propanoate metabolism;ko04146 Peroxisome;
gene18847	K13606	NOL, NYC1	chlorophyll(ide) b reductase	EC:1.1.1.294	1	ko00860 Porphyrin and chlorophyll metabolism;
gene18848	K02575	NRT, narK, nrtP, nasA	MFS transporter, NNP family, nitrate/nitrite transporter	--	1	ko00910 Nitrogen metabolism;
gene18855	K11816	YUCCA	indole-3-pyruvate monooxygenase	EC:1.14.13.168	1	ko00380 Tryptophan metabolism;
gene18856	K07297	ADIPOR	adiponectin receptor	--	2	ko04920 Adipocytokine signaling pathway;ko04932 Non-alcoholic fatty liver disease (NAFLD);
gene18857	K16903	TAA1	L-tryptophan---pyruvate aminotransferase	EC:2.6.1.99	1	ko00380 Tryptophan metabolism;
gene1885	K17279	REEP5_6	receptor expression-enhancing protein 5/6	--		
gene18866	K11252	H2B	histone H2B	--	3	ko05034 Alcoholism;ko05203 Viral carcinogenesis;ko05322 Systemic lupus erythematosus;
gene18867	K14156	CHK	choline/ethanolamine kinase	EC:2.7.1.32 2.7.1.82	1	ko00564 Glycerophospholipid metabolism;
gene18871	K14484	IAA	auxin-responsive protein IAA	--	1	ko04075 Plant hormone signal transduction;
gene18880	K09422	MYBP	myb proto-oncogene protein, plant	--		
gene18893	K00253	IVD, ivd	isovaleryl-CoA dehydrogenase	EC:1.3.8.4	1	ko00280 Valine, leucine and isoleucine degradation;
gene18897	K01520	dut, DUT	dUTP pyrophosphatase	EC:3.6.1.23	1	ko00240 Pyrimidine metabolism;
gene18902	K05663	ABC.ATM	mitochondrial ABC transporter ATM	--	1	ko02010 ABC transporters;
gene18903	K00924	E2.7.1.-				
gene18907	K01537	E3.6.3.8	Ca2+-transporting ATPase	EC:3.6.3.8		
gene18910	K10396	KIF5	kinesin family member 5	--	1	ko04728 Dopaminergic synapse;
gene18911	K14231	tRNA-Phe	tRNA Phe	--	1	ko00970 Aminoacyl-tRNA biosynthesis;
gene18913	K01262	pepP	Xaa-Pro aminopeptidase	EC:3.4.11.9		
gene18922	K13681	FUT	xyloglucan fucosyltransferase	EC:2.4.1.-		
gene18923	K01900	LSC2	succinyl-CoA synthetase beta subunit	EC:6.2.1.4 6.2.1.5	3	ko00020 Citrate cycle (TCA cycle);ko00640 Propanoate metabolism;ko01200 Carbon metabolism;
gene18927	K10590	TRIP12	E3 ubiquitin-protein ligase TRIP12	EC:6.3.2.19	1	ko04120 Ubiquitin mediated proteolysis;

gene18930	K01962	accA	acetyl-CoA carboxylase carboxyl transferase subunit alpha	EC:6.4.1.2	6	ko00061 Fatty acid biosynthesis;ko00253 Tetracycline biosynthesis;ko00620 Pyruvate metabolism;ko00640 Propanoate metabolism;ko00720 Carbon fixation pathways in prokaryotes;ko01200 Carbon metabolism;
gene18931	K09422	MYBP	myb proto-oncogene protein, plant	--		
gene1893	K11808	ADE2	phosphoribosylaminoimidazole carboxylase	EC:4.1.1.21	1	ko00230 Purine metabolism;
gene18944	K03942	NDUFV1	NADH dehydrogenase (ubiquinone) flavoprotein 1	EC:1.6.5.3 1.6.99.3	5	ko00190 Oxidative phosphorylation;ko04932 Non-alcoholic fatty liver disease (NAFLD);ko05010 Alzheimer's disease;ko05012 Parkinson's disease;ko05016 Huntington's disease;
gene18946	K00167	E1.2.4.4B, bkdA2	2-oxoisovalerate dehydrogenase E1 component, beta subunit	EC:1.2.4.4	1	ko00280 Valine, leucine and isoleucine degradation;
gene18947	K09775	K09775	hypothetical protein	--		
gene1894	K05391	CNGF	cyclic nucleotide gated channel, other eukaryote	--	1	ko04626 Plant-pathogen interaction;
gene18950	K09480	E2.4.1.241	digalactosyldiacylglycerol synthase	EC:2.4.1.241	1	ko00561 Glycerolipid metabolism;
gene18951	K08486	STX1B_2_3	syntaxin 1B/2/3	--	2	ko04130 SNARE interactions in vesicular transport;ko04721 Synaptic vesicle cycle;
gene18952	K08486	STX1B_2_3	syntaxin 1B/2/3	--	2	ko04130 SNARE interactions in vesicular transport;ko04721 Synaptic vesicle cycle;
gene18953	K08064	NFYA	nuclear transcription factor Y, alpha	--	2	ko04612 Antigen processing and presentation;ko05152 Tuberculosis;
gene18954	K01177	E3.2.1.2	beta-amylase	EC:3.2.1.2	1	ko00500 Starch and sucrose metabolism;
gene18956	K11507	CENPO	centromere protein O	--		
gene18957	K01568	E4.1.1.1, pdc	pyruvate decarboxylase	EC:4.1.1.1	1	ko00010 Glycolysis / Gluconeogenesis;
gene18958	K10863	APTX	aprataxin	EC:3.-.-.-		
gene18966	K02991	RP-S6e, RPS6	small subunit ribosomal protein S6e	--	6	ko03010 Ribosome;ko04066 HIF-1 signaling pathway;ko04150 mTOR signaling pathway;ko04151 PI3K-Akt signaling pathway;ko04910 Insulin signaling pathway;ko05205 Proteoglycans in cancer;
gene18969	K03850	ALG10	alpha-1,2-glucosyltransferase	EC:2.4.1.256	1	ko00510 N-Glycan biosynthesis;
gene1896	K01897	ACSL, fadD	long-chain acyl-CoA synthetase	EC:6.2.1.3	4	ko00071 Fatty acid degradation;ko03320 PPAR signaling pathway;ko04146 Peroxisome;ko04920 Adipocytokine signaling pathway;
gene18972	K04730	IRAK1	interleukin-1 receptor-associated kinase 1	EC:2.7.11.1	11	ko04064 NF-kappa B signaling pathway;ko04210 Apoptosis;ko04620 Toll-like receptor signaling pathway;ko04722 Neurotrophin signaling pathway;ko05133 Pertussis;ko05140 Leishmaniasis;ko05142 Chagas disease (American trypanosomiasis);ko05145 Toxoplasmosis;ko05152 Tuberculosis;ko05162 Measles;ko05169 Epstein-Barr virus infection;
gene18980	K04382	PPP2C	serine/threonine-protein phosphatase 2A catalytic subunit	EC:3.1.3.16	13	ko03015 mRNA surveillance pathway;ko04111 Cell cycle - yeast;ko04113 Meiosis - yeast;ko04114 Oocyte meiosis;ko04151 PI3K-Akt signaling pathway;ko04350 TGF-beta signaling pathway;ko04390 Hippo signaling pathway;ko04391 Hippo signaling pathway - fly;ko04530 Tight junction;ko04728 Dopaminergic synapse;ko04730 Long-term depression;ko05142 Chagas disease (American trypanosomiasis);ko05160 Hepatitis C;
gene18982	K02937	RP-L7e, RPL7	large subunit ribosomal protein L7e	--	1	ko03010 Ribosome;
gene18984	K01537	E3.6.3.8	Ca2+-transporting ATPase	EC:3.6.3.8		
gene18985	K00499	E1.14.15.7	choline monooxygenase	EC:1.14.15.7	1	ko00260 Glycine, serine and threonine metabolism;
gene18988	K10802	HMGB1	high mobility group protein B1	--	1	ko03410 Base excision repair;
gene18991	K12194	CHMP4, SNF7, VPS32	charged multivesicular body protein 4	--	1	ko04144 Endocytosis;
gene18994	K14229	tRNA-Lys	tRNA Lys	--	1	ko00970 Aminoacyl-tRNA biosynthesis;
gene19001	K09872	PIP	aquaporin PIP	--		
gene19002	K09872	PIP	aquaporin PIP	--		
gene19005	K00766	trpD	anthranilate phosphoribosyltransferase	EC:2.4.2.18	2	ko00400 Phenylalanine, tyrosine and tryptophan biosynthesis;ko01230 Biosynthesis of amino acids;
gene19006	K04354	PPP2R2	serine/threonine-protein phosphatase 2A regulatory subunit B	--	9	ko03015 mRNA surveillance pathway;ko04111 Cell cycle - yeast;ko04151 PI3K-Akt signaling pathway;ko04390 Hippo signaling pathway;ko04391 Hippo signaling pathway - fly;ko04530 Tight junction;ko04728 Dopaminergic synapse;ko05142 Chagas disease (American trypanosomiasis);ko05160 Hepatitis C;
gene19009	K14503	BZR1_2	brassinosteroid resistant 1/2	--	1	ko04075 Plant hormone signal transduction;
gene19011	K01184	E3.2.1.15	polygalacturonase	EC:3.2.1.15	2	ko00040 Pentose and glucuronate interconversions;ko00500 Starch and sucrose metabolism;

gene19013	K01184	E3.2.1.15	polygalacturonase	EC:3.2.1.15	2	ko00040 Pentose and glucuronate interconversions;ko00500 Starch and sucrose metabolism;
gene19014	K07119	K07119				
gene19017	K13065	E2.3.1.133, HCT	shikimate O-hydroxycinnamoyltransferase	EC:2.3.1.133	3	ko00940 Phenylpropanoid biosynthesis;ko00941 Flavonoid biosynthesis;ko00945 Stilbenoid, diarylheptanoid and gingerol biosynthesis;
gene19024	K10666	RNF5	E3 ubiquitin-protein ligase RNF5	EC:6.3.2.19	1	ko04141 Protein processing in endoplasmic reticulum;
gene19026	K13459	RPS2	disease resistance protein RPS2	--	1	ko04626 Plant-pathogen interaction;
gene19027	K13459	RPS2	disease resistance protein RPS2	--	1	ko04626 Plant-pathogen interaction;
gene19041	K13448	CML	calcium-binding protein CML	--	1	ko04626 Plant-pathogen interaction;
gene19043	K00939	E2.7.4.3, adk	adenylate kinase	EC:2.7.4.3	1	ko00230 Purine metabolism;
gene19048	K07304	msrA	peptide-methionine (S)-S-oxide reductase	EC:1.8.4.11		
gene19049	K00600	glyA, SHMT	glycine hydroxymethyltransferase	EC:2.1.2.1	7	ko00260 Glycine, serine and threonine metabolism;ko00460 Cyanoamino acid metabolism;ko00630 Glyoxylate and dicarboxylate metabolism;ko00670 One carbon pool by folate;ko00680 Methane metabolism;ko01200 Carbon metabolism;ko01230 Biosynthesis of amino acids;
gene19052	K12309	GLB1, ELNR1	beta-galactosidase	EC:3.2.1.23	6	ko00052 Galactose metabolism;ko00511 Other glycan degradation;ko00531 Glycosaminoglycan degradation;ko00600 Sphingolipid metabolism;ko00604 Glycosphingolipid biosynthesis - ganglio series;ko04142 Lysosome;
gene19053	K00434	E1.11.1.11	L-ascorbate peroxidase	EC:1.11.1.11	2	ko00053 Ascorbate and aldarate metabolism;ko00480 Glutathione metabolism;
gene19059	K09338	HD-ZIP	homeobox-leucine zipper protein	--		
gene1905	K00869	E2.7.1.36, MVK, mvaK1	mevalonate kinase	EC:2.7.1.36	2	ko00900 Terpenoid backbone biosynthesis;ko04146 Peroxisome;
gene19060	K02927	RP-L40e, RPL40	large subunit ribosomal protein L40e	--	1	ko03010 Ribosome;
gene19062	K00472	E1.14.11.2	prolyl 4-hydroxylase	EC:1.14.11.2	1	ko00330 Arginine and proline metabolism;
gene19065	K01853	E5.4.99.8	cycloartenol synthase	EC:5.4.99.8	1	ko00100 Steroid biosynthesis;
gene1906	K11000	CALS	callose synthase	EC:2.4.1.-		
gene19072	K12160	SUMO, SMT3	small ubiquitin-related modifier	--	1	ko03013 RNA transport;
gene19073	K09872	PIP	aquaporin PIP	--		
gene19075	K02737	PSMB5	20S proteasome subunit beta 5	EC:3.4.25.1	1	ko03050 Proteasome;
gene19076	K14855	RSA4, NLE1	ribosome assembly protein 4	--		
gene19077	K15032	MTERFD	mTERF domain-containing protein, mitochondrial	--		
gene19082	K14486	K14486, ARF	auxin response factor	--	1	ko04075 Plant hormone signal transduction;
gene19083	K00253	IVD, ivd	isovaleryl-CoA dehydrogenase	EC:1.3.8.4	1	ko00280 Valine, leucine and isoleucine degradation;
gene19089	K10666	RNF5	E3 ubiquitin-protein ligase RNF5	EC:6.3.2.19	1	ko04141 Protein processing in endoplasmic reticulum;
gene1908	K15397	KCS	3-ketoacyl-CoA synthase	EC:2.3.1.199	1	ko00062 Fatty acid elongation;
gene19090	K09422	MYBP	myb proto-oncogene protein, plant	--		
gene19093	K04646	CLTC	clathrin heavy chain	--	6	ko04142 Lysosome;ko04144 Endocytosis;ko04721 Synaptic vesicle cycle;ko04961 Endocrine and other factor-regulated calcium reabsorption;ko05016 Huntington's disease;ko05100 Bacterial invasion of epithelial cells;
gene19097	K03259	EIF4E	translation initiation factor 4E	--	5	ko03013 RNA transport;ko04066 HIF-1 signaling pathway;ko04150 mTOR signaling pathway;ko04151 PI3K-Akt signaling pathway;ko04910 Insulin signaling pathway;
gene19098	K04565	SOD1	superoxide dismutase, Cu-Zn family	EC:1.15.1.1	4	ko04146 Peroxisome;ko05014 Amyotrophic lateral sclerosis (ALS);ko05016 Huntington's disease;ko05020 Prion diseases;
gene19102	K00700	glgB	1,4-alpha-glucan branching enzyme	EC:2.4.1.18	1	ko00500 Starch and sucrose metabolism;
gene19103	K00721	DPM1	dolichol-phosphate mannosyltransferase	EC:2.4.1.83	1	ko00510 N-Glycan biosynthesis;
gene19108	K14799	TSR1	pre-rRNA-processing protein TSR1	--		
gene19109	K17497	PMM	phosphomannomutase	EC:5.4.2.8	2	ko00051 Fructose and mannose metabolism;ko00520 Amino sugar and nucleotide sugar metabolism;

gene1910	K04371	MAPK1_3	mitogen-activated protein kinase 1/3	EC:2.7.11.24	71	ko04010 MAPK signaling pathway;ko04012 ErbB signaling pathway;ko04013 MAPK signaling pathway - fly;ko04014 Ras signaling pathway;ko04062 Chemokine signaling pathway;ko04066 HIF-1 signaling pathway;ko04114 Oocyte meiosis;ko04150 mTOR signaling pathway;ko04151 PI3K-Akt signaling pathway;ko04270 Vascular smooth muscle contraction;ko04320 Dorso-ventral axis formation;ko04350 TGF-beta signaling pathway;ko04360 Axon guidance;ko04370 VEGF signaling pathway;ko04380 Osteoclast differentiation;ko04510 Focal adhesion;ko04520 Adherens junction;ko04540 Gap junction;ko04620 Toll-like receptor signaling pathway;ko04621 NOD-like receptor signaling pathway;ko04650 Natural killer cell mediated cytotoxicity;ko04660 T cell receptor signaling pathway;ko04662 B cell receptor signaling pathway;ko04664 Fc epsilon RI signaling pathway;ko04666 Fc gamma R-mediated phagocytosis;ko04668 TNF signaling pathway;ko04713 Circadian entrainment;ko04720 Long-term potentiation;ko04722 Neurotrophin signaling pathway;ko04723 Retrograde endocannabinoid signaling;ko04724 Glutamatergic synapse;ko04725 Cholinergic synapse;ko04726 Serotonergic synapse;ko04730 Long-term depression;ko04810 Regulation of actin cytoskeleton;ko04910 Insulin signaling pathway;ko04912 GnRH signaling pathway;ko04914 Progesterone-mediated oocyte maturation;ko04915 Estrogen signaling pathway;ko04916 Melanogenesis;ko04917 Prolactin signaling pathway;ko04930 Type II diabetes mellitus;ko04960 Aldosterone-regulated sodium reabsorption;ko05010 Alzheimer's disease;ko05020 Prion diseases;ko05034 Alcoholism;ko05131 Shigellosis;ko05132 Salmonella infection;ko05133 Pertussis;ko05140 Leishmaniasis;ko05142 Chagas disease (American trypanosomiasis);ko05145 Toxoplasmosis;ko05152 Tuberculosis;ko05160 Hepatitis C;ko05161 Hepatitis B;ko05164 Influenza A;ko05200 Pathways in cancer;ko05203 Viral carcinogenesis;ko05205 Proteoglycans in cancer;ko05210 Colorectal cancer;ko05211 Renal cell carcinoma;ko05212
gene19113	K14324	SAP18	histone deacetylase complex subunit SAP18	--	2	ko03013 RNA transport;ko03015 mRNA surveillance pathway;
gene19114	K07198	PRKAA, AMPK	5'-AMP-activated protein kinase, catalytic alpha subunit	EC:2.7.11.11	8	ko04140 Regulation of autophagy;ko04150 mTOR signaling pathway;ko04151 PI3K-Akt signaling pathway;ko04710 Circadian rhythm;ko04910 Insulin signaling pathway;ko04920 Adipocytokine signaling pathway;ko04932 Non-alcoholic fatty liver disease (NAFLD);ko05410 Hypertrophic cardiomyopathy (HCM);
gene19118	K00591	COQ3	hexaprenyldihydroxybenzoate methyltransferase	EC:2.1.1.114	1	ko00130 Ubiquinone and other terpenoid-quinone biosynthesis;
gene19124	K00815	TAT	tyrosine aminotransferase	EC:2.6.1.5	9	ko00130 Ubiquinone and other terpenoid-quinone biosynthesis;ko00270 Cysteine and methionine metabolism;ko00350 Tyrosine metabolism;ko00360 Phenylalanine metabolism;ko00400 Phenylalanine, tyrosine and tryptophan biosynthesis;ko00401 Novobiocin biosynthesis;ko00950 Isoquinoline alkaloid biosynthesis;ko00960 Tropane, piperidine and pyridine alkaloid biosynthesis;ko01230 Biosynthesis of amino acids;
gene19127	K15151	MED10, NUT2	mediator of RNA polymerase II transcription subunit 10	--		
gene1912	K15382	SLC50A, SWEET	solute carrier family 50 (sugar transporter)	--		
gene19132	K13648	GAUT	alpha-1,4-galacturonosyltransferase	EC:2.4.1.43	2	ko00500 Starch and sucrose metabolism;ko00520 Amino sugar and nucleotide sugar metabolism;
gene19133	K15191	LARP7	La-related protein 7	--		
gene19134	K00434	E1.11.1.11	L-ascorbate peroxidase	EC:1.11.1.11	2	ko00053 Ascorbate and aldarate metabolism;ko00480 Glutathione metabolism;
gene19135	K00430	E1.11.1.7	peroxidase	EC:1.11.1.7	2	ko00360 Phenylalanine metabolism;ko00940 Phenylpropanoid biosynthesis;
gene19137	K08341	GABARAP, ATG8, LC3	GABA(A) receptor-associated protein (autophagy-related protein 8)	--	2	ko04140 Regulation of autophagy;ko04727 GABAergic synapse;
gene19139	K15341	DCLRE1B, SNM1B	DNA cross-link repair 1B protein	EC:3.1.-.-		
gene1913	K11649	SMARCC	SWI/SNF related-matrix-associated actin-dependent regulator of chromatin subfamily C	--		
gene19141	K02877	RP-L15e, RPL15	large subunit ribosomal protein L15e	--	1	ko03010 Ribosome;

gene19142	K01193	E3.2.1.26, sacA	beta-fructofuranosidase	EC:3.2.1.26	2	ko00052 Galactose metabolism;ko00500 Starch and sucrose metabolism;
gene19150	K11824	AP2A	AP-2 complex subunit alpha	--	4	ko04144 Endocytosis;ko04721 Synaptic vesicle cycle;ko04961 Endocrine and other factor-regulated calcium reabsorption;ko05016 Huntington's disease;
gene19152	K10046	GME	GDP-D-mannose 3', 5'-epimerase	EC:5.1.3.18	2	ko00053 Ascorbate and aldarate metabolism;ko00520 Amino sugar and nucleotide sugar metabolism;
gene19154	K01897	ACSL, fadD	long-chain acyl-CoA synthetase	EC:6.2.1.3	4	ko00071 Fatty acid degradation;ko03320 PPAR signaling pathway;ko04146 Peroxisome;ko04920 Adipocytokine signaling pathway;
gene19158	K17285	SELENBP1	selenium-binding protein 1	--		
gene19160	K17285	SELENBP1	selenium-binding protein 1	--		
gene19162	K15601	KDM3	lysine-specific demethylase 3	EC:1.14.11.-		
gene19164	K11855	USP36_42	ubiquitin carboxyl-terminal hydrolase 36/42	EC:3.1.2.15		
gene19165	K02898	RP-L26e, RPL26	large subunit ribosomal protein L26e	--	1	ko03010 Ribosome;
gene19166	K15040	VDAC2	voltage-dependent anion channel protein 2	--	4	ko04020 Calcium signaling pathway;ko05012 Parkinson's disease;ko05016 Huntington's disease;ko05166 HTLV-I infection;
gene1916	K01738	cysK	cysteine synthase A	EC:2.5.1.47	4	ko00270 Cysteine and methionine metabolism;ko00920 Sulfur metabolism;ko01200 Carbon metabolism;ko01230 Biosynthesis of amino acids;
gene19172	K00888	PI4K	phosphatidylinositol 4-kinase	EC:2.7.1.67	2	ko00562 Inositol phosphate metabolism;ko04070 Phosphatidylinositol signaling system;
gene19173	K13420	FLS2	LRR receptor-like serine/threonine-protein kinase FLS2	EC:2.7.11.1	1	ko04626 Plant-pathogen interaction;
gene19177	K16055	TPS	trehalose 6-phosphate synthase/phosphatase	EC:2.4.1.15 3.1.3.12	1	ko00500 Starch and sucrose metabolism;
gene19179	K12581	CNOT7_8, CAF1, POP2	CCR4-NOT transcription complex subunit 7/8	--	1	ko03018 RNA degradation;
gene19182	K02936	RP-L7Ae, RPL7A	large subunit ribosomal protein L7Ae	--	1	ko03010 Ribosome;
gene19183	K15027	EIF2D	translation initiation factor 2D	--		
gene19184	K00696	E2.4.1.14	sucrose-phosphate synthase	EC:2.4.1.14	1	ko00500 Starch and sucrose metabolism;
gene19190	K13680	CSLA	beta-mannan synthase	EC:2.4.1.32		
gene1919	K16055	TPS	trehalose 6-phosphate synthase/phosphatase	EC:2.4.1.15 3.1.3.12	1	ko00500 Starch and sucrose metabolism;
gene19204	K14232	tRNA-Pro	tRNA Pro	--	1	ko00970 Aminoacyl-tRNA biosynthesis;
gene19210	K06943	NOG1	nucleolar GTP-binding protein	--	1	ko03008 Ribosome biogenesis in eukaryotes;
gene19211	K03781	katE, CAT, catB, srpA	catalase	EC:1.11.1.6	4	ko00380 Tryptophan metabolism;ko00630 Glyoxylate and dicarboxylate metabolism;ko04146 Peroxisome;ko05014 Amyotrophic lateral sclerosis (ALS);
gene19216	K14611	SLC23A1_2, SVCT1_2	solute carrier family 23 (nucleobase transporter), member 1/2	--		
gene19218	K12856	PRPF8, PRP8	pre-mRNA-processing factor 8	--	1	ko03040 Spliceosome;
gene1921	K13356	FAR	fatty acyl-CoA reductase	EC:1.2.1.-	2	ko00073 Cutin, suberine and wax biosynthesis;ko04146 Peroxisome;
gene19230	K13024	HISPPD, VIP	inositol hexakisphosphate/diphosphoinositol-pentakisphosphate kinase	EC:2.7.4.21 2.7.4.24		
gene19231	K03671	trxA	thioredoxin 1	--		
gene19233	K12824	TCERG1, CA150	transcription elongation regulator 1	--	1	ko03040 Spliceosome;
gene19239	K03231	EEF1A	elongation factor 1-alpha	--	2	ko03013 RNA transport;ko05134 Legionellosis;
gene1923	K15283	SLC35E1	solute carrier family 35, member E1	--		
gene19240	K13984	TXNDC5, ERP46	thioredoxin domain-containing protein 5	--	1	ko04141 Protein processing in endoplasmic reticulum;
gene19248	K00430	E1.11.1.7	peroxidase	EC:1.11.1.7	2	ko00360 Phenylalanine metabolism;ko00940 Phenylpropanoid biosynthesis;
gene19249	K00430	E1.11.1.7	peroxidase	EC:1.11.1.7	2	ko00360 Phenylalanine metabolism;ko00940 Phenylpropanoid biosynthesis;
gene19254	K07374	TUBA	tubulin alpha	--	3	ko04145 Phagosome;ko04540 Gap junction;ko05130 Pathogenic Escherichia coli infection;
gene19255	K02912	RP-L32e, RPL32	large subunit ribosomal protein L32e	--	1	ko03010 Ribosome;
gene19256	K08960	CSNK1E	casein kinase 1, epsilon	EC:2.7.11.1	6	ko04310 Wnt signaling pathway;ko04340 Hedgehog signaling pathway;ko04390 Hippo signaling pathway;ko04391 Hippo signaling pathway - fly;ko04710 Circadian rhythm;ko04711 Circadian rhythm - fly;
gene19258	K15040	VDAC2	voltage-dependent anion channel protein 2	--	4	ko04020 Calcium signaling pathway;ko05012 Parkinson's disease;ko05016 Huntington's disease;ko05166 HTLV-I infection;
gene19267	K03549	kup	KUP system potassium uptake protein	--		
gene19268	K00660	CHS	chalcone synthase	EC:2.3.1.74	2	ko00941 Flavonoid biosynthesis;ko04712 Circadian rhythm - plant;

gene19269	K11578	ZW10	centromere/kinetochore protein ZW10	--		
gene1926	K14772	UTP20	U3 small nucleolar RNA-associated protein 20	--		
gene19272	K13412	CPK	calcium-dependent protein kinase	EC:2.7.11.1	2	ko04626 Plant-pathogen interaction;ko05145 Toxoplasmosis;
gene19274	K12821	PRPF40, PRP40	pre-mRNA-processing factor 40	--	1	ko03040 Spliceosome;
gene19277	K12153	CYP79A2	cytochrome P450, family 79, subfamily A, polypeptide 2 (phenylalanine N-monooxygenase)	EC:1.14.13.124	3	ko00460 Cyanoamino acid metabolism;ko00966 Glucosinolate biosynthesis;ko01210 2-Oxocarboxylic acid metabolism;
gene19280	K08857	NEK	NIMA (never in mitosis gene a)-related kinase	EC:2.7.11.1		
gene19281	K11374	ELP2	elongator complex protein 2	--		
gene19283	K04733	IRAK4	interleukin-1 receptor-associated kinase 4	EC:2.7.11.1	11	ko04064 NF-kappa B signaling pathway;ko04210 Apoptosis;ko04620 Toll-like receptor signaling pathway;ko04722 Neurotrophin signaling pathway;ko05133 Pertussis;ko05140 Leishmaniasis;ko05142 Chagas disease (American trypanosomiasis);ko05145 Toxoplasmosis;ko05152 Tuberculosis;ko05162 Measles;ko05164 Influenza A;
gene19289	K02989	RP-S5e, RPS5	small subunit ribosomal protein S5e	--	1	ko03010 Ribosome;
gene19290	K15382	SLC50A, SWEET	solute carrier family 50 (sugar transporter)	--		
gene19291	K09422	MYBP	myb proto-oncogene protein, plant	--		
gene19292	K10144	RCHY1, PIRH2	RING finger and CHY zinc finger domain-containing protein 1	EC:6.3.2.19	3	ko04115 p53 signaling pathway;ko04120 Ubiquitin mediated proteolysis;ko05162 Measles;
gene19293	K01674	cah	carbonic anhydrase	EC:4.2.1.1	1	ko00910 Nitrogen metabolism;
gene19299	K13963	SERPINB	serpin B	--	1	ko05146 Amoebiasis;
gene19301	K09338	HD-ZIP	homeobox-leucine zipper protein	--		
gene19302	K15168	MED25	mediator of RNA polymerase II transcription subunit 25	--		
gene19307	K02957	RP-S15Ae, RPS15A	small subunit ribosomal protein S15Ae	--	1	ko03010 Ribosome;
gene19308	K12197	CHMP1, VPS46, DID2	charged multivesicular body protein 1	--	1	ko04144 Endocytosis;
gene19310	K00750	GYG1, GYG2	glycogenin glucosyltransferase	EC:2.4.1.186		
gene19311	K00750	GYG1, GYG2	glycogenin glucosyltransferase	EC:2.4.1.186		
gene19312	K08900	BCS1	mitochondrial chaperone BCS1	--		
gene19313	K15923	AXY8, FUC95A, afcA	alpha-L-fucosidase 2	EC:3.2.1.51	1	ko00511 Other glycan degradation;
gene19316	K04733	IRAK4	interleukin-1 receptor-associated kinase 4	EC:2.7.11.1	11	ko04064 NF-kappa B signaling pathway;ko04210 Apoptosis;ko04620 Toll-like receptor signaling pathway;ko04722 Neurotrophin signaling pathway;ko05133 Pertussis;ko05140 Leishmaniasis;ko05142 Chagas disease (American trypanosomiasis);ko05145 Toxoplasmosis;ko05152 Tuberculosis;ko05162 Measles;ko05164 Influenza A;
gene1931	K03327	TC.MATE, SLC47A, norM, mdtK, dinF	multidrug resistance protein, MATE family	--		
gene19321	K08818	CDC2L	cell division cycle 2-like	EC:2.7.11.22		
gene19326	K00899	mtnK	5-methylthioribose kinase	EC:2.7.1.100	1	ko00270 Cysteine and methionine metabolism;
gene19328	K00430	E1.11.1.7	peroxidase	EC:1.11.1.7	2	ko00360 Phenylalanine metabolism;ko00940 Phenylpropanoid biosynthesis;
gene19334	K02933	RP-L6, MRPL6, rplF	large subunit ribosomal protein L6	--	1	ko03010 Ribosome;
gene19335	K07767	E3.6.4.3	microtubule-severing ATPase	EC:3.6.4.3		
gene19337	K02267	COX6B	cytochrome c oxidase subunit 6b	--	6	ko00190 Oxidative phosphorylation;ko04260 Cardiac muscle contraction;ko04932 Non-alcoholic fatty liver disease (NAFLD);ko05010 Alzheimer's disease;ko05012 Parkinson's disease;ko05016 Huntington's disease;
gene19346	K13864	SLC7A2, ATRC2	solute carrier family 7 (cationic amino acid transporter), member 2	--		
gene19347	K13863	SLC7A1, ATRC1	solute carrier family 7 (cationic amino acid transporter), member 1	--	1	ko05206 MicroRNAs in cancer;
gene1934	K01845	hemL	glutamate-1-semialdehyde 2,1-aminomutase	EC:5.4.3.8	1	ko00860 Porphyrin and chlorophyll metabolism;
gene19350	K10532	HGSNAT	heparan-alpha-glucosaminide N-acetyltransferase	EC:2.3.1.78	2	ko00531 Glycosaminoglycan degradation;ko04142 Lysosome;
gene19353	K02983	RP-S30e, RPS30	small subunit ribosomal protein S30e	--	1	ko03010 Ribosome;
gene19356	K11667	INO80C, IES6	INO80 complex subunit C	--		
gene19357	K10706	SETX, ALS4	senataxin	EC:3.6.4.-		
gene19358	K14233	tRNA-Ser	tRNA Ser	--	1	ko00970 Aminoacyl-tRNA biosynthesis;
gene1935	K07542	PIGV	phosphatidylinositol glycan, class V	EC:2.4.1.-	1	ko00563 Glycosylphosphatidylinositol(GPI)-anchor biosynthesis;

gene19361	K15456	KTI12	protein KTI12	--		
gene19362	K02575	NRT, narK, nrtP, nasA	MFS transporter, NNP family, nitrate/nitrite transporter	--	1	ko00910 Nitrogen metabolism;
gene19363	K00487	CYP73A	trans-cinnamate 4-monooxygenase	EC:1.14.13.11	5	ko00360 Phenylalanine metabolism;ko00940 Phenylpropanoid biosynthesis;ko00941 Flavonoid biosynthesis;ko00945 Stilbenoid, diarylheptanoid and gingerol biosynthesis;ko01220 Degradation of aromatic compounds;
gene19365	K13993	HSP20	HSP20 family protein	--	1	ko04141 Protein processing in endoplasmic reticulum;
gene19368	K02990	RP-S6, MRPS6, rpsF	small subunit ribosomal protein S6	--	1	ko03010 Ribosome;
gene19370	K13093	HTATSF1	HIV Tat-specific factor 1	--		
gene19372	K15397	KCS	3-ketoacyl-CoA synthase	EC:2.3.1.199	1	ko00062 Fatty acid elongation;
gene19373	K12183	TSG101, STP22, VPS23	ESCRT-I complex subunit TSG101	--	1	ko04144 Endocytosis;
gene19378	K08905	psaG	photosystem I subunit V	--	1	ko00195 Photosynthesis;
gene19385	K14638	SLC15A3_4, PHT	solute carrier family 15 (peptide/histidine transporter), member 3/4	--		
gene19390	K14012	SHP1, UBX1, NSFL1C	UBX domain-containing protein 1	--	1	ko04141 Protein processing in endoplasmic reticulum;
gene19397	K00975	glgC	glucose-1-phosphate adenylyltransferase	EC:2.7.7.27	2	ko00500 Starch and sucrose metabolism;ko00520 Amino sugar and nucleotide sugar metabolism;
gene19402	K04125	E1.14.11.13	gibberellin 2-oxidase	EC:1.14.11.13	1	ko00904 Diterpenoid biosynthesis;
gene19404	K03626	EGD2, NACA	nascent polypeptide-associated complex subunit alpha	--		
gene19405	K05280	E1.14.13.21	flavonoid 3'-monooxygenase	EC:1.14.13.21	2	ko00941 Flavonoid biosynthesis;ko00944 Flavone and flavonol biosynthesis;
gene19411	K14498	SNRK2	serine/threonine-protein kinase SRK2	EC:2.7.11.1	1	ko04075 Plant hormone signal transduction;
gene19415	K03695	clpB	ATP-dependent Clp protease ATP-binding subunit ClpB	--		
gene19424	K14488	SAUR	SAUR family protein	--	1	ko04075 Plant hormone signal transduction;
gene19434	K10751	CHAF1B	chromatin assembly factor 1 subunit B	--		
gene19443	K01802	E5.2.1.8	peptidylprolyl isomerase	EC:5.2.1.8		
gene19444	K10703	PHS1, PAS2	very-long-chain (3R)-3-hydroxyacyl-[acyl-carrier protein] dehydratase	EC:4.2.1.134	2	ko00062 Fatty acid elongation;ko01040 Biosynthesis of unsaturated fatty acids;
gene19448	K17479	GRXCR1	glutaredoxin domain-containing cysteine-rich protein 1	--		
gene1944	K11498	CENPE	centromeric protein E	--		
gene19453	K15075	MET18, MMS19	DNA repair/transcription protein MET18/MMS19	--		
gene19454	K15174	PAF1	RNA polymerase II-associated factor 1	--		
gene19460	K12193	VPS24, CHMP3	charged multivesicular body protein 3	--	1	ko04144 Endocytosis;
gene19466	K00750	GYG1, GYG2	glycogenin glucosyltransferase	EC:2.4.1.186		
gene19467	K03253	EIF3B	translation initiation factor 3 subunit B	--	1	ko03013 RNA transport;
gene19470	K01176	E3.2.1.1, amyA, malS	alpha-amylase	EC:3.2.1.1	2	ko00500 Starch and sucrose metabolism;ko04973 Carbohydrate digestion and absorption;
gene19471	K08869	ADCK, ABC1	aarF domain-containing kinase	--		
gene19474	K03238	EIF2S2	translation initiation factor 2 subunit 2	--	1	ko03013 RNA transport;
gene19476	K13506	GPAT3_4, AGPAT9, AGPAT6	glycerol-3-phosphate O-acyltransferase 3/4	EC:2.3.1.15	2	ko00561 Glycerolipid metabolism;ko00564 Glycerophospholipid metabolism;
gene19481	K01904	E6.2.1.12	4-coumarate--CoA ligase	EC:6.2.1.12	3	ko00130 Ubiquinone and other terpenoid-quinone biosynthesis;ko00360 Phenylalanine metabolism;ko00940 Phenylpropanoid biosynthesis;
gene19483	K08679	E5.1.3.6	UDP-glucuronate 4-epimerase	EC:5.1.3.6	2	ko00500 Starch and sucrose metabolism;ko00520 Amino sugar and nucleotide sugar metabolism;
gene19485	K06693	PSMD9	26S proteasome non-ATPase regulatory subunit 9	--		
gene19486	K07937	ARF1	ADP-ribosylation factor 1	--	2	ko05110 Vibrio cholerae infection;ko05134 Legionellosis;
gene19495	K11984	SART1, HAF, SNU66	U4/U6.U5 tri-snRNP-associated protein 1	--	1	ko03040 Spliceosome;
gene19498	K14220	tRNA-Asn	tRNA Asn	--	1	ko00970 Aminoacyl-tRNA biosynthesis;
gene19499	K16284	SIS3	E3 ubiquitin-protein ligase SIS3	EC:6.3.2.19		
gene1949	K10364	CAPZA	capping protein (actin filament) muscle Z-line, alpha	--		
gene19500	K12734	PPIL3	peptidyl-prolyl cis-trans isomerase-like 3	EC:5.2.1.8		
gene19501	K03327	TC.MATE, SLC47A, norM, mdtK, dinF	multidrug resistance protein, MATE family	--		

gene19504	K01895	ACSS, acs	acetyl-CoA synthetase	EC:6.2.1.1	6	ko00010 Glycolysis / Gluconeogenesis;ko00620 Pyruvate metabolism;ko00640 Propanoate metabolism;ko00680 Methane metabolism;ko00720 Carbon fixation pathways in prokaryotes;ko01200 Carbon metabolism;
gene19505	K14777	DDX47, RRP3	ATP-dependent RNA helicase DDX47/RRP3	EC:3.6.4.13		
gene19508	K03347	CUL1, CDC53	cullin 1	--	9	ko04110 Cell cycle;ko04111 Cell cycle - yeast;ko04114 Oocyte meiosis;ko04120 Ubiquitin mediated proteolysis;ko04141 Protein processing in endoplasmic reticulum;ko04310 Wnt signaling pathway;ko04350 TGF-beta signaling pathway;ko04710 Circadian rhythm;ko05168 Herpes simplex infection;
gene19509	K14221	tRNA-Asp	tRNA Asp	--	1	ko00970 Aminoacyl-tRNA biosynthesis;
gene1950	K00432	E1.11.1.9	glutathione peroxidase	EC:1.11.1.9	3	ko00480 Glutathione metabolism;ko00590 Arachidonic acid metabolism;ko04918 Thyroid hormone synthesis;
gene19512	K01961	accC	acetyl-CoA carboxylase, biotin carboxylase subunit	EC:6.4.1.2 6.3.4.14	6	ko00061 Fatty acid biosynthesis;ko00253 Tetracycline biosynthesis;ko00620 Pyruvate metabolism;ko00640 Propanoate metabolism;ko00720 Carbon fixation pathways in prokaryotes;ko01200 Carbon metabolism;
gene19515	K11593	ELF2C	eukaryotic translation initiation factor 2C	--		
gene19517	K00602	purH	phosphoribosylaminoimidazolecarboxamide formyltransferase / IMP cyclohydrolase	EC:2.1.2.3 3.5.4.10	2	ko00230 Purine metabolism;ko00670 One carbon pool by folate;
gene19524	K00454	LOX2S	lipoxygenase	EC:1.13.11.12	2	ko00591 Linoleic acid metabolism;ko00592 alpha-Linolenic acid metabolism;
gene19526	K00588	E2.1.1.104	caffeoyl-CoA O-methyltransferase	EC:2.1.1.104	4	ko00360 Phenylalanine metabolism;ko00940 Phenylpropanoid biosynthesis;ko00941 Flavonoid biosynthesis;ko00945 Stilbenoid, diarylheptanoid and gingerol biosynthesis;
gene19527	K03327	TC.MATE, SLC47A, norM, mdtK, dinF	multidrug resistance protein, MATE family	--		
gene19529	K00915	IPK2	1D-myo-Inositol-tetrakisphosphate 5-kinase / inositol-polyphosphate multikinase	EC:2.7.1.140 2.7.1.151	2	ko00562 Inositol phosphate metabolism;ko04070 Phosphatidylinositol signaling system;
gene19533	K01876	DARS, aspS	aspartyl-tRNA synthetase	EC:6.1.1.12	1	ko00970 Aminoacyl-tRNA biosynthesis;
gene19535	K14790	NOP9	nucleolar protein 9	--		
gene19537	K08150	SLC2A13, ITR	MFS transporter, SP family, solute carrier family 2 (myo-inositol transporter), member 13	--		
gene1953	K09264	K09264	MADS-box transcription factor, plant	--		
gene19541	K05579	ndhH	NAD(P)H-quinone oxidoreductase subunit H	EC:1.6.5.3	1	ko00190 Oxidative phosphorylation;
gene19547	K05941	E2.3.2.15	glutathione gamma-glutamylcysteinyltransferase	EC:2.3.2.15		
gene19549	K12309	GLB1, ELNR1	beta-galactosidase	EC:3.2.1.23	6	ko00052 Galactose metabolism;ko00511 Other glycan degradation;ko00531 Glycosaminoglycan degradation;ko00600 Sphingolipid metabolism;ko00604 Glycosphingolipid biosynthesis - ganglio series;ko04142 Lysosome;
gene19557	K12200	PDCD6IP, ALIX, RIM20	programmed cell death 6-interacting protein	--	1	ko04144 Endocytosis;
gene19564	K14787	MRD1, RBM19	multiple RNA-binding domain-containing protein 1	--		
gene1956	K00423	E1.10.3.3	L-ascorbate oxidase	EC:1.10.3.3	1	ko00053 Ascorbate and aldarate metabolism;
gene19576	K14859	SSF1_2	ribosome biogenesis protein SSF1/2	--		
gene19577	K14514	EIN3	ethylene-insensitive protein 3	--	1	ko04075 Plant hormone signal transduction;
gene19581	K05906	PCYOX1, FCLY	prenylcysteine oxidase / farnesylcysteine lyase	EC:1.8.3.5 1.8.3.6	1	ko00900 Terpenoid backbone biosynthesis;
gene19588	K04733	IRAK4	interleukin-1 receptor-associated kinase 4	EC:2.7.11.1	11	ko04064 NF-kappa B signaling pathway;ko04210 Apoptosis;ko04620 Toll-like receptor signaling pathway;ko04722 Neurotrophin signaling pathway;ko05133 Pertussis;ko05140 Leishmaniasis;ko05142 Chagas disease (American trypanosomiasis);ko05145 Toxoplasmosis;ko05152 Tuberculosis;ko05162 Measles;ko05164 Influenza A;
gene19593	K01099	E3.1.3.36	phosphatidylinositol-bisphosphatase	EC:3.1.3.36	2	ko00562 Inositol phosphate metabolism;ko04070 Phosphatidylinositol signaling system;
gene19599	K13335	PEX16	peroxin-16	--	1	ko04146 Peroxisome;
gene19601	K12161	URM1	ubiquitin related modifier 1	--	1	ko04122 Sulfur relay system;
gene19602	K08150	SLC2A13, ITR	MFS transporter, SP family, solute carrier family 2 (myo-inositol transporter), member 13	--		
gene19603	K01551	arsA, ASNA1	arsenite-transporting ATPase	EC:3.6.3.16		
gene19605	K01183	E3.2.1.14	chitinase	EC:3.2.1.14	1	ko00520 Amino sugar and nucleotide sugar metabolism;

gene19608	K12184	VPS28	ESCRT-I complex subunit VPS28	--	1	ko04144 Endocytosis;
gene1960	K07375	TUBB	tubulin beta	--	3	ko04145 Phagosome;ko04540 Gap junction;ko05130 Pathogenic Escherichia coli infection;
gene19615	K09422	MYBP	myb proto-oncogene protein, plant	--		
gene19616	K08867	WNK, PRKWNK	WNK lysine deficient protein kinase	EC:2.7.11.1		
gene19617	K11864	BRCC3	BRCA1/BRCA2-containing complex subunit 3	--		
gene19628	K03963	NDUFB7	NADH dehydrogenase (ubiquinone) 1 beta subcomplex subunit 7	--	5	ko00190 Oxidative phosphorylation;ko04932 Non-alcoholic fatty liver disease (NAFLD);ko05010 Alzheimer's disease;ko05012 Parkinson's disease;ko05016 Huntington's disease;
gene19646	K09391	E2F7_8	transcription factor E2F7/8	--		
gene19647	K00275	pdxH, PNPO	pyridoxamine 5'-phosphate oxidase	EC:1.4.3.5	1	ko00750 Vitamin B6 metabolism;
gene19648	K03609	minD	septum site-determining protein MinD	--		
gene1964	K09571	FKBP4_5	FK506-binding protein 4/5	EC:5.2.1.8	1	ko04915 Estrogen signaling pathway;
gene19651	K07910	RAB18	Ras-related protein Rab-18	--		
gene19656	K02331	POL5, MYBBP1A	DNA polymerase phi	EC:2.7.7.7		
gene19657	K11423	SETD2, SET2	histone-lysine N-methyltransferase SETD2	EC:2.1.1.43	1	ko00310 Lysine degradation;
gene19661	K14521	NAT10, KRE33	N-acetyltransferase 10	EC:2.3.1.-	1	ko03008 Ribosome biogenesis in eukaryotes;
gene19662	K13117	DHX35	ATP-dependent RNA helicase DDX35	EC:3.6.4.13		
gene19663	K07904	RAB11A	Ras-related protein Rab-11A	--	4	ko04144 Endocytosis;ko04961 Endocrine and other factor-regulated calcium reabsorption;ko04962 Vasopressin-regulated water reabsorption;ko04972 Pancreatic secretion;
gene19668	K07393	ECM4	putative glutathione S-transferase	--		
gene19669	K17275	PLS1	plastin-1	--		
gene19671	K03531	ftsZ	cell division protein FtsZ	--	1	ko04112 Cell cycle - Caulobacter;
gene19672	K03131	TAF6	transcription initiation factor TFIID subunit 6	--	2	ko03022 Basal transcription factors;ko05168 Herpes simplex infection;
gene19675	K00517	E1.14.-.-			4	ko00624 Polycyclic aromatic hydrocarbon degradation;ko00627 Aminobenzoate degradation;ko00903 Limonene and pinene degradation;ko00945 Stilbenoid, diarylheptanoid and gingerol biosynthesis;
gene19678	K11778	DHDDS, RER2, SRT1	ditrans,polycis-polyprenyl diphosphate synthase	EC:2.5.1.87	1	ko00900 Terpenoid backbone biosynthesis;
gene19679	K11778	DHDDS, RER2, SRT1	ditrans,polycis-polyprenyl diphosphate synthase	EC:2.5.1.87	1	ko00900 Terpenoid backbone biosynthesis;
gene19687	K12158	NEDD8	ubiquitin-like protein Nedd8	--		
gene19691	K01126	E3.1.4.46, glpQ, ugpQ	glycerophosphoryl diester phosphodiesterase	EC:3.1.4.46	1	ko00564 Glycerophospholipid metabolism;
gene19699	K09561	STUB1, CHIP	STIP1 homology and U-box containing protein 1	EC:6.3.2.19	2	ko04120 Ubiquitin mediated proteolysis;ko04141 Protein processing in endoplasmic reticulum;
gene1969	K17086	TM9SF2_4	transmembrane 9 superfamily member 2/4	--		
gene19700	K14509	ETR, ERS	ethylene receptor	EC:2.7.13.-	1	ko04075 Plant hormone signal transduction;
gene19703	K06170	PEN2	presenilin enhancer 2	--	2	ko04330 Notch signaling pathway;ko05010 Alzheimer's disease;
gene19707	K14664	ILR1	IAA-amino acid hydrolase	EC:3.5.1.-		
gene19709	K13379	RGP, UTM	reversibly glycosylated polypeptide / UDP-arabinopyranose mutase	EC:2.4.1.- 5.4.99.30		
gene19717	K00759	E2.4.2.7, apt	adenine phosphoribosyltransferase	EC:2.4.2.7	1	ko00230 Purine metabolism;
gene1971	K03538	POP4, RPP29	ribonuclease P protein subunit POP4	EC:3.1.26.5	2	ko03008 Ribosome biogenesis in eukaryotes;ko03013 RNA transport;
gene19724	K14805	DDX24, MAK5	ATP-dependent RNA helicase DDX24/MAK5	EC:3.6.4.13		
gene1972	K09497	CCT5	T-complex protein 1 subunit epsilon	--		
gene19730	K15119	SLC25A39_40	solute carrier family 25, member 39/40	--		
gene19731	K17613	CABIN1	calcineurin-binding protein cabin-1	--		
gene19735	K11366	USP22_27_51, UBP8	ubiquitin carboxyl-terminal hydrolase 22/27/51	EC:3.1.2.15		
gene19740	K13065	E2.3.1.133, HCT	shikimate O-hydroxycinnamoyltransferase	EC:2.3.1.133	3	ko00940 Phenylpropanoid biosynthesis;ko00941 Flavonoid biosynthesis;ko00945 Stilbenoid, diarylheptanoid and gingerol biosynthesis;
gene19743	K10590	TRIP12	E3 ubiquitin-protein ligase TRIP12	EC:6.3.2.19	1	ko04120 Ubiquitin mediated proteolysis;
gene19744	K07556	ATPeAF2, ATPAF2	ATP synthase mitochondrial F1 complex assembly factor 2	--		

gene19745	K00235	SDHB, SDH2	succinate dehydrogenase (ubiquinone) iron-sulfur subunit	EC:1.3.5.1	7	ko00020 Citrate cycle (TCA cycle);ko00190 Oxidative phosphorylation;ko01200 Carbon metabolism;ko04932 Non-alcoholic fatty liver disease (NAFLD);ko05010 Alzheimer's disease;ko05012 Parkinson's disease;ko05016 Huntington's disease;
gene19748	K16546	FGFR10P	FGFR1 oncogene partner	--		
gene19749	K02735	PSMB3	20S proteasome subunit beta 3	EC:3.4.25.1	1	ko03050 Proteasome;
gene1974	K13508	GPAT	glycerol-3-phosphate acyltransferase	EC:2.3.1.15	2	ko00561 Glycerolipid metabolism;ko00564 Glycerophospholipid metabolism;
gene19752	K08762	DBI, ACBP	diazepam-binding inhibitor (GABA receptor modulator, acyl-CoA-binding protein)	--	1	ko03320 PPAR signaling pathway;
gene19756	K14498	SNRK2	serine/threonine-protein kinase SRK2	EC:2.7.11.1	1	ko04075 Plant hormone signal transduction;
gene19759	K02152	ATPeVG, ATP6G1	V-type H+-transporting ATPase subunit G	EC:3.6.3.14	7	ko00190 Oxidative phosphorylation;ko04145 Phagosome;ko04721 Synaptic vesicle cycle;ko04966 Collecting duct acid secretion;ko05110 Vibrio cholerae infection;ko05120 Epithelial cell signaling in Helicobacter pylori infection;ko05323 Rheumatoid arthritis;
gene1975	K15191	LARP7	La-related protein 7	--		
gene19764	K13105	PRCC	proline-rich protein PRCC	--	1	ko05202 Transcriptional misregulation in cancer;
gene1976	K14408	CSTF3, RNA14	cleavage stimulation factor subunit 3	--	1	ko03015 mRNA surveillance pathway;
gene19772	K10949	KDELR	ER lumen protein retaining receptor	--	1	ko05110 Vibrio cholerae infection;
gene19774	K00028	E1.1.1.39	malate dehydrogenase (decarboxylating)	EC:1.1.1.39	3	ko00620 Pyruvate metabolism;ko00710 Carbon fixation in photosynthetic organisms;ko01200 Carbon metabolism;
gene19784	K14318	NUP88	nuclear pore complex protein Nup88	--	1	ko03013 RNA transport;
gene19786	K07053	K07053				
gene19791	K01673	cynT, can	carbonic anhydrase	EC:4.2.1.1	1	ko00910 Nitrogen metabolism;
gene19796	K03798	ftsH, hflB	cell division protease FtsH	EC:3.4.24.-		
gene19799	K14638	SLC15A3_4, PHT	solute carrier family 15 (peptide/histidine transporter), member 3/4	--		
gene19804	K00326	E1.6.2.2	cytochrome-b5 reductase	EC:1.6.2.2	1	ko00520 Amino sugar and nucleotide sugar metabolism;
gene19806	K01464	E3.5.2.2, DPYS	dihydropyrimidinase	EC:3.5.2.2	4	ko00240 Pyrimidine metabolism;ko00410 beta-Alanine metabolism;ko00770 Pantothenate and CoA biosynthesis;ko00983 Drug metabolism - other enzymes;
gene19808	K01711	gmd, GMDS	GDPmannose 4,6-dehydratase	EC:4.2.1.47	2	ko00051 Fructose and mannose metabolism;ko00520 Amino sugar and nucleotide sugar metabolism;
gene19809	K14431	TGA	transcription factor TGA	--	1	ko04075 Plant hormone signal transduction;
gene19810	K13412	CPK	calcium-dependent protein kinase	EC:2.7.11.1	2	ko04626 Plant-pathogen interaction;ko05145 Toxoplasmosis;
gene19811	K14500	BSK	BR-signaling kinase	EC:2.7.11.1	1	ko04075 Plant hormone signal transduction;
gene19814	K03327	TC.MATE, SLC47A, norM, mdtK, dinF	multidrug resistance protein, MATE family	--		
gene19815	K03327	TC.MATE, SLC47A, norM, mdtK, dinF	multidrug resistance protein, MATE family	--		
gene19817	K02953	RP-S13e, RPS13	small subunit ribosomal protein S13e	--	1	ko03010 Ribosome;
gene19818	K13495	CISZOG	cis-zeatin O-glucosyltransferase	EC:2.4.1.215	1	ko00908 Zeatin biosynthesis;
gene19823	K14835	NOP2	ribosomal RNA methyltransferase Nop2	EC:2.1.1.-		
gene19829	K01177	E3.2.1.2	beta-amylase	EC:3.2.1.2	1	ko00500 Starch and sucrose metabolism;
gene1982	K12606	RCD1, CNOT9, CAF40	CCR4-NOT transcription complex subunit 9	--	1	ko03018 RNA degradation;
gene19832	K13447	RBOH	respiratory burst oxidase	EC:1.6.3.- 1.11.1.-	1	ko04626 Plant-pathogen interaction;
gene19833	K09580	PDIA1, P4HB	protein disulfide-isomerase A1	EC:5.3.4.1	1	ko04141 Protein processing in endoplasmic reticulum;
gene19835	K01640	E4.1.3.4, HMGCL, hmgL	hydroxymethylglutaryl-CoA lyase	EC:4.1.3.4	5	ko00072 Synthesis and degradation of ketone bodies;ko00280 Valine, leucine and isoleucine degradation;ko00281 Geraniol degradation;ko00650 Butanoate metabolism;ko04146 Peroxisome;
gene19840	K16573	TUBGCP6, GCP6	gamma-tubulin complex component 6	--		
gene19843	K05389	KCNKF	potassium channel subfamily K, other eukaryote	--		
gene19844	K05658	ABCB1	ATP-binding cassette, subfamily B (MDR/TAP), member 1	--	3	ko02010 ABC transporters;ko04976 Bile secretion;ko05206 MicroRNAs in cancer;
gene19849	K12837	U2AF2	splicing factor U2AF 65 kDa subunit	--	1	ko03040 Spliceosome;
gene1984	K15472	CYP71D55	premnaspirodiene oxygenase	EC:1.14.13.12 1	1	ko00909 Sesquiterpenoid and triterpenoid biosynthesis;
gene19852	K07901	RAB8A, MEL	Ras-related protein Rab-8A	--	1	ko04972 Pancreatic secretion;

gene19853	K03946	NDUFA2	NADH dehydrogenase (ubiquinone) 1 alpha subcomplex subunit 2	--	5	ko00190 Oxidative phosphorylation;ko04932 Non-alcoholic fatty liver disease (NAFLD);ko05010 Alzheimer's disease;ko05012 Parkinson's disease;ko05016 Huntington's disease;
gene19855	K09595	HM13	minor histocompatibility antigen H13	EC:3.4.23.-		
gene19857	K02918	RP-L35e, RPL35	large subunit ribosomal protein L35e	--	1	ko03010 Ribosome;
gene19858	K07195	EXOC7, EXO70	exocyst complex component 7	--	1	ko04910 Insulin signaling pathway;
gene19861	K15397	KCS	3-ketoacyl-CoA synthase	EC:2.3.1.199	1	ko00062 Fatty acid elongation;
gene19865	K03094	SKP1, CBF3D	S-phase kinase-associated protein 1	--	9	ko04110 Cell cycle;ko04111 Cell cycle - yeast;ko04114 Oocyte meiosis;ko04120 Ubiquitin mediated proteolysis;ko04141 Protein processing in endoplasmic reticulum;ko04310 Wnt signaling pathway;ko04350 TGF-beta signaling pathway;ko04710 Circadian rhythm;ko05168 Herpes simplex infection;
gene19866	K03424	tatD	TatD DNase family protein	EC:3.1.21.-		
gene19867	K15919	HPR2	hydroxypyruvate reductase 2	--	2	ko00260 Glycine, serine and threonine metabolism;ko00630 Glyoxylate and dicarboxylate metabolism;
gene19868	K15919	HPR2	hydroxypyruvate reductase 2	--	2	ko00260 Glycine, serine and threonine metabolism;ko00630 Glyoxylate and dicarboxylate metabolism;
gene19871	K06063	SNW1, SKIIP, SKIP	SNW domain-containing protein 1	--	4	ko03040 Spliceosome;ko04330 Notch signaling pathway;ko05169 Epstein-Barr virus infection;ko05203 Viral carcinogenesis;
gene19876	K09338	HD-ZIP	homeobox-leucine zipper protein	--		
gene19880	K09874	NIP	aquaporin NIP	--		
gene19887	K17362	ACOT13	acyl-coenzyme A thioesterase 13	EC:3.1.2.-		
gene19890	K07195	EXOC7, EXO70	exocyst complex component 7	--	1	ko04910 Insulin signaling pathway;
gene19891	K13162	PCBP2_3_4	poly(rC)-binding protein 2/3/4	--		
gene19892	K02145	ATPeVA, ATP6A1	V-type H+-transporting ATPase subunit A	EC:3.6.3.14	7	ko00190 Oxidative phosphorylation;ko04145 Phagosome;ko04721 Synaptic vesicle cycle;ko04966 Collecting duct acid secretion;ko05110 Vibrio cholerae infection;ko05120 Epithelial cell signaling in Helicobacter pylori infection;ko05323 Rheumatoid arthritis;
gene19899	K13140	INTS3	integrator complex subunit 3	--		
gene19905	K00006	GPD1	glycerol-3-phosphate dehydrogenase (NAD+)	EC:1.1.1.8	1	ko00564 Glycerophospholipid metabolism;
gene19911	K10532	HGSNAT	heparan-alpha-glucosaminide N-acetyltransferase	EC:2.3.1.78	2	ko00531 Glycosaminoglycan degradation;ko04142 Lysosome;
gene19914	K16075	MRS2, MFM1	magnesium transporter	--		
gene19922	K02151	ATPeVF, ATP6S14	V-type H+-transporting ATPase subunit F	EC:3.6.3.14	7	ko00190 Oxidative phosphorylation;ko04145 Phagosome;ko04721 Synaptic vesicle cycle;ko04966 Collecting duct acid secretion;ko05110 Vibrio cholerae infection;ko05120 Epithelial cell signaling in Helicobacter pylori infection;ko05323 Rheumatoid arthritis;
gene19925	K07739	ELP3, KAT9	elongator complex protein 3	EC:2.3.1.48		
gene19932	K01534	zntA	Cd2+/Zn2+-exporting ATPase	EC:3.6.3.3 3.6.3.5		
gene19933	K02880	RP-L17e, RPL17	large subunit ribosomal protein L17e	--	1	ko03010 Ribosome;
gene19936	K08864	TLK	tousled-like kinase	EC:2.7.11.1		
gene19938	K01519	ITPA	inosine triphosphate pyrophosphatase	EC:3.6.1.19	3	ko00230 Purine metabolism;ko00240 Pyrimidine metabolism;ko00983 Drug metabolism - other enzymes;
gene19941	K13065	E2.3.1.133, HCT	shikimate O-hydroxycinnamoyltransferase	EC:2.3.1.133	3	ko00940 Phenylpropanoid biosynthesis;ko00941 Flavonoid biosynthesis;ko00945 Stilbenoid, diarylheptanoid and gingerol biosynthesis;
gene19943	K13431	SRPR	signal recognition particle receptor subunit alpha	--	1	ko03060 Protein export;
gene19947	K10777	LIG4, DNL4	DNA ligase 4	EC:6.5.1.1	1	ko03450 Non-homologous end-joining;
gene19951	K14007	SEC24	protein transport protein SEC24	--	1	ko04141 Protein processing in endoplasmic reticulum;
gene19953	K14233	tRNA-Ser	tRNA Ser	--	1	ko00970 Aminoacyl-tRNA biosynthesis;
gene19954	K12667	SWP1, RPN2	oligosaccharyltransferase complex subunit delta (ribophorin II)	--	3	ko00510 N-Glycan biosynthesis;ko00513 Various types of N-glycan biosynthesis;ko04141 Protein processing in endoplasmic reticulum;
gene19960	K15634	gpmB	probable phosphoglycerate mutase	EC:5.4.2.12	5	ko00010 Glycolysis / Gluconeogenesis;ko00260 Glycine, serine and threonine metabolism;ko00680 Methane metabolism;ko01200 Carbon metabolism;ko01230 Biosynthesis of amino acids;
gene19969	K10143	RFWD2, COP1	E3 ubiquitin-protein ligase RFWD2	EC:6.3.2.19	3	ko04115 p53 signaling pathway;ko04120 Ubiquitin mediated proteolysis;ko04712 Circadian rhythm - plant;
gene19970	K02892	RP-L23, MRPL23, rplW	large subunit ribosomal protein L23	--	1	ko03010 Ribosome;

gene19976	K15095	E1.1.1.208	(+)-neomenthol dehydrogenase	EC:1.1.1.208	1	ko00902 Monoterpenoid biosynthesis;
gene1997	K13091	RBM39, RNPC2	RNA-binding protein 39	--		
gene19980	K02927	RP-L40e, RPL40	large subunit ribosomal protein L40e	--	1	ko03010 Ribosome;
gene19981	K01057	PGLS, pgl, devB	6-phosphogluconolactonase	EC:3.1.1.31	2	ko00030 Pentose phosphate pathway;ko01200 Carbon metabolism;
gene19985	K03231	EEF1A	elongation factor 1-alpha	--	2	ko03013 RNA transport;ko05134 Legionellosis;
gene19987	K09422	MYBP	myb proto-oncogene protein, plant	--		
gene1998	K11584	PPP2R5	serine/threonine-protein phosphatase 2A regulatory subunit B'	--	5	ko03015 mRNA surveillance pathway;ko04113 Meiosis - yeast;ko04114 Oocyte meiosis;ko04151 PI3K-Akt signaling pathway;ko04728 Dopaminergic synapse;
gene19990	K00700	glgB	1,4-alpha-glucan branching enzyme	EC:2.4.1.18	1	ko00500 Starch and sucrose metabolism;
gene19995	K03126	TAF12	transcription initiation factor TFIID subunit 12	--	1	ko03022 Basal transcription factors;
gene1	K12489	ACAP	Arf-GAP with coiled-coil, ANK repeat and PH domain-containing protein	--	1	ko04144 Endocytosis;
gene20002	K00430	E1.11.1.7	peroxidase	EC:1.11.1.7	2	ko00360 Phenylalanine metabolism;ko00940 Phenylpropanoid biosynthesis;
gene20006	K15501	PPP6R3, SAPS3	serine/threonine-protein phosphatase 6 regulatory subunit 3	--		
gene20020	K07933	RABL3	Rab-like protein 3	--		
gene20024	K12818	DHX8, PRP22	ATP-dependent RNA helicase DHX8/PRP22	EC:3.6.4.13	1	ko03040 Spliceosome;
gene20029	K01955	carB, CPA2	carbamoyl-phosphate synthase large subunit	EC:6.3.5.5	2	ko00240 Pyrimidine metabolism;ko00250 Alanine, aspartate and glutamate metabolism;
gene2002	K06130	LYPLA2	lysophospholipase II	EC:3.1.1.5	1	ko00564 Glycerophospholipid metabolism;
gene20031	K10084	EDEM1	ER degradation enhancer, mannosidase alpha-like 1	--	1	ko04141 Protein processing in endoplasmic reticulum;
gene20036	K14509	ETR, ERS	ethylene receptor	EC:2.7.13.-	1	ko04075 Plant hormone signal transduction;
gene20038	K07198	PRKAA, AMPK	5'-AMP-activated protein kinase, catalytic alpha subunit	EC:2.7.11.11	8	ko04140 Regulation of autophagy;ko04150 mTOR signaling pathway;ko04151 PI3K-Akt signaling pathway;ko04710 Circadian rhythm;ko04910 Insulin signaling pathway;ko04920 Adipocytokine signaling pathway;ko04932 Non-alcoholic fatty liver disease (NAFLD);ko05410 Hypertrophic cardiomyopathy (HCM);
gene2003	K15639	CYP734A1, BAS1	cytochrome P450, family 734, subfamily A, polypeptide 1 (PHYB activation tagged suppressor 1)	EC:1.14.-.-	1	ko00905 Brassinosteroid biosynthesis;
gene20041	K06173	truA, PUS1	tRNA pseudouridine38-40 synthase	EC:5.4.99.12		
gene20043	K00566	mnmA, trmU, TRMU	tRNA-specific 2-thiouridylase	EC:2.8.1.-	1	ko04122 Sulfur relay system;
gene20044	K03977	engA	GTP-binding protein	--		
gene2004	K03026	RPC53, POLR3D	DNA-directed RNA polymerase III subunit RPC4	--	5	ko00230 Purine metabolism;ko00240 Pyrimidine metabolism;ko03020 RNA polymerase;ko04623 Cytosolic DNA-sensing pathway;ko05169 Epstein-Barr virus infection;
gene20050	K14157	AASS	alpha-aminoadipic semialdehyde synthase	EC:1.5.1.8 1.5.1.9	1	ko00310 Lysine degradation;
gene20052	K09338	HD-ZIP	homeobox-leucine zipper protein	--		
gene20054	K15382	SLC50A, SWEET	solute carrier family 50 (sugar transporter)	--		
gene20058	K00122	FDH	formate dehydrogenase	EC:1.2.1.2	3	ko00630 Glyoxylate and dicarboxylate metabolism;ko00680 Methane metabolism;ko01200 Carbon metabolism;
gene20077	K00924	E2.7.1.-				
gene20078	K01897	ACSL, fadD	long-chain acyl-CoA synthetase	EC:6.2.1.3	4	ko00071 Fatty acid degradation;ko03320 PPAR signaling pathway;ko04146 Peroxisome;ko04920 Adipocytokine signaling pathway;
gene20083	K06911	K06911				
gene20087	K10143	RFWD2, COP1	E3 ubiquitin-protein ligase RFWD2	EC:6.3.2.19	3	ko04115 p53 signaling pathway;ko04120 Ubiquitin mediated proteolysis;ko04712 Circadian rhythm - plant;
gene20093	K03515	REV1	DNA repair protein REV1	EC:2.7.7.-	1	ko03460 Fanconi anemia pathway;
gene20098	K15382	SLC50A, SWEET	solute carrier family 50 (sugar transporter)	--		
gene2009	K06127	COQ5	ubiquinone biosynthesis methyltransferase	EC:2.1.1.201	1	ko00130 Ubiquinone and other terpenoid-quinone biosynthesis;
gene20103	K06001	K06001, trpB	tryptophan synthase beta chain	EC:4.2.1.20	3	ko00260 Glycine, serine and threonine metabolism;ko00400 Phenylalanine, tyrosine and tryptophan biosynthesis;ko01230 Biosynthesis of amino acids;
gene2010	K03439	trmB, METTL1	tRNA (guanine-N7-)-methyltransferase	EC:2.1.1.33		
gene20110	K03183	ubiE	ubiquinone/menaquinone biosynthesis methyltransferase	EC:2.1.1.163 2.1.1.201	1	ko00130 Ubiquinone and other terpenoid-quinone biosynthesis;

gene20111	K00830	AGXT	alanine-glyoxylate transaminase / serine-glyoxylate transaminase / serine-pyruvate transaminase	EC:2.6.1.44 2.6.1.45 2.6.1.51	6	ko00250 Alanine, aspartate and glutamate metabolism;ko00260 Glycine, serine and threonine metabolism;ko00630 Glyoxylate and dicarboxylate metabolism;ko00680 Methane metabolism;ko01200 Carbon metabolism;ko04146 Peroxisome;
gene20114	K07199	PRKAB	5'-AMP-activated protein kinase, regulatory beta subunit	--	5	ko04710 Circadian rhythm;ko04910 Insulin signaling pathway;ko04920 Adipocytokine signaling pathway;ko04932 Non-alcoholic fatty liver disease (NAFLD);ko05410 Hypertrophic cardiomyopathy (HCM);
gene20115	K02183	CALM	calmodulin	--	25	ko04014 Ras signaling pathway;ko04020 Calcium signaling pathway;ko04070 Phosphatidylinositol signaling system;ko04114 Oocyte meiosis;ko04270 Vascular smooth muscle contraction;ko04626 Plant-pathogen interaction;ko04713 Circadian entrainment;ko04720 Long-term potentiation;ko04722 Neurotrophin signaling pathway;ko04728 Dopaminergic synapse;ko04740 Olfactory transduction;ko04744 Phototransduction;ko04745 Phototransduction - fly;ko04910 Insulin signaling pathway;ko04912 GnRH signaling pathway;ko04915 Estrogen signaling pathway;ko04916 Melanogenesis;ko04970 Salivary secretion;ko04971 Gastric acid secretion;ko05010 Alzheimer's disease;ko05031 Amphetamine addiction;ko05034 Alcoholism;ko05133 Pertussis;ko05152 Tuberculosis;ko05214 Glioma;
gene20116	K03108	SRP72	signal recognition particle subunit SRP72	--	1	ko03060 Protein export;
gene20117	K08332	VAC8	vacuolar protein 8	--	1	ko04140 Regulation of autophagy;
gene20118	K03233	EEF1G	elongation factor 1-gamma	--	1	ko05134 Legionellosis;
gene2011	K02946	RP-S10, MRPS10, rpsJ	small subunit ribosomal protein S10	--	1	ko03010 Ribosome;
gene20122	K06965	PELO, DOM34, pelA	protein pelota	--	1	ko03015 mRNA surveillance pathway;
gene20123	K11808	ADE2	phosphoribosylaminoimidazole carboxylase	EC:4.1.1.21	1	ko00230 Purine metabolism;
gene20124	K13412	CPK	calcium-dependent protein kinase	EC:2.7.11.1	2	ko04626 Plant-pathogen interaction;ko05145 Toxoplasmosis;
gene20127	K01900	LSC2	succinyl-CoA synthetase beta subunit	EC:6.2.1.4 6.2.1.5	3	ko00020 Citrate cycle (TCA cycle);ko00640 Propanoate metabolism;ko01200 Carbon metabolism;
gene20130	K08472	MLO	mlo protein	--		
gene20132	K03070	secA	preprotein translocase subunit SecA	--	2	ko03060 Protein export;ko03070 Bacterial secretion system;
gene20135	K00231	PPOX, hemY	oxygen-dependent protoporphyrinogen oxidase	EC:1.3.3.4	1	ko00860 Porphyrin and chlorophyll metabolism;
gene20136	K01823	idi, IDI	isopentenyl-diphosphate delta-isomerase	EC:5.3.3.2	1	ko00900 Terpenoid backbone biosynthesis;
gene20143	K02883	RP-L18e, RPL18	large subunit ribosomal protein L18e	--	1	ko03010 Ribosome;
gene20144	K00873	PK, pyk	pyruvate kinase	EC:2.7.1.40	7	ko00010 Glycolysis / Gluconeogenesis;ko00230 Purine metabolism;ko00620 Pyruvate metabolism;ko01200 Carbon metabolism;ko01230 Biosynthesis of amino acids;ko04930 Type II diabetes mellitus;ko05203 Viral carcinogenesis;
gene20147	K00565	RNMT	mRNA (guanine-N7-)-methyltransferase	EC:2.1.1.56	1	ko03015 mRNA surveillance pathway;
gene2014	K15398	CYP86A4S	cytochrome P450, family 86, subfamily A, polypeptide 2/4/7/8 (fatty acid omega-hydroxylase)	EC:1.14.-.-	1	ko00073 Cutin, suberine and wax biosynthesis;
gene20151	K10639	CCNB1IP1, HEI10	E3 ubiquitin-protein ligase CCNP1IP1	EC:6.3.2.19		
gene20153	K01915	glnA	glutamine synthetase	EC:6.3.1.2	8	ko00250 Alanine, aspartate and glutamate metabolism;ko00330 Arginine and proline metabolism;ko00630 Glyoxylate and dicarboxylate metabolism;ko00910 Nitrogen metabolism;ko01230 Biosynthesis of amino acids;ko02020 Two-component system;ko04724 Glutamatergic synapse;ko04727 GABAergic synapse;
gene20154	K14950	ATP13A1	cation-transporting ATPase 13A1	EC:3.6.3.-		
gene2015	K08193	SLC17A	MFS transporter, ACS family, solute carrier family 17 (sodium-dependent inorganic phosphate cotransporter), other	--		
gene20167	K08486	STX1B_2_3	syntaxin 1B/2/3	--	2	ko04130 SNARE interactions in vesicular transport;ko04721 Synaptic vesicle cycle;
gene2016	K13508	GPAT	glycerol-3-phosphate acyltransferase	EC:2.3.1.15	2	ko00561 Glycerolipid metabolism;ko00564 Glycerophospholipid metabolism;
gene20172	K14497	PP2C	protein phosphatase 2C	EC:3.1.3.16	1	ko04075 Plant hormone signal transduction;
gene20173	K00599	E2.1.1.-			2	ko00350 Tyrosine metabolism;ko00624 Polycyclic aromatic hydrocarbon degradation;

gene20175	K00627	DLAT, aceF, pdhC	pyruvate dehydrogenase E2 component (dihydrolipoamide acetyltransferase)	EC:2.3.1.12	4	ko00010 Glycolysis / Gluconeogenesis;ko00020 Citrate cycle (TCA cycle);ko00620 Pyruvate metabolism;ko01200 Carbon metabolism;
gene20177	K09422	MYBP	myb proto-oncogene protein, plant	--		
gene20178	K00671	E2.3.1.97, NMT	glycylpeptide N-tetradecanoyltransferase	EC:2.3.1.97		
gene2017	K09872	PIP	aquaporin PIP	--		
gene20181	K14791	PWP1	periodic tryptophan protein 1	--		
gene2018	K09597	SPPL2B	signal peptide peptidase-like 2B	EC:3.4.23.-		
gene20192	K00549	metE	5-methyltetrahydropteroyltriglutamate--homocysteine methyltransferase	EC:2.1.1.14	3	ko00270 Cysteine and methionine metabolism;ko00450 Selenocompound metabolism;ko01230 Biosynthesis of amino acids;
gene20194	K13113	UBL5, HUB1	ubiquitin-like protein 5	--		
gene20195	K14232	tRNA-Pro	tRNA Pro	--	1	ko00970 Aminoacyl-tRNA biosynthesis;
gene20196	K14232	tRNA-Pro	tRNA Pro	--	1	ko00970 Aminoacyl-tRNA biosynthesis;
gene20197	K14235	tRNA-Trp	tRNA Trp	--	1	ko00970 Aminoacyl-tRNA biosynthesis;
gene20207	K01784	galE, GALE	UDP-glucose 4-epimerase	EC:5.1.3.2	2	ko00052 Galactose metabolism;ko00520 Amino sugar and nucleotide sugar metabolism;
gene20213	K06943	NOG1	nucleolar GTP-binding protein	--	1	ko03008 Ribosome biogenesis in eukaryotes;
gene20219	K00721	DPM1	dolichol-phosphate mannosyltransferase	EC:2.4.1.83	1	ko00510 N-Glycan biosynthesis;
gene20220	K10761	THG1	tRNA(His) guanylyltransferase	EC:2.7.7.79		
gene20223	K01414	prlC	oligopeptidase A	EC:3.4.24.70		
gene20228	K10689	PEX4	peroxin-4	EC:6.3.2.19		
gene20232	K08790	STK38, NDR	serine/threonine kinase 38	EC:2.7.11.1		
gene20234	K09286	EREBP	EREBP-like factor	--		
gene20235	K03231	EEF1A	elongation factor 1-alpha	--	2	ko03013 RNA transport;ko05134 Legionellosis;
gene20240	K13420	FLS2	LRR receptor-like serine/threonine-protein kinase FLS2	EC:2.7.11.1	1	ko04626 Plant-pathogen interaction;
gene20242	K14498	SNRK2	serine/threonine-protein kinase SRK2	EC:2.7.11.1	1	ko04075 Plant hormone signal transduction;
gene2024	K02998	RP-SAe, RPSA	small subunit ribosomal protein SAe	--	1	ko03010 Ribosome;
gene20250	K01704	leuD	3-isopropylmalate/(R)-2-methylmalate dehydratase small subunit	EC:4.2.1.33 4.2.1.35	4	ko00290 Valine, leucine and isoleucine biosynthesis;ko00660 C5-Branched dibasic acid metabolism;ko01210 2-Oxocarboxylic acid metabolism;ko01230 Biosynthesis of amino acids;
gene20251	K01704	leuD	3-isopropylmalate/(R)-2-methylmalate dehydratase small subunit	EC:4.2.1.33 4.2.1.35	4	ko00290 Valine, leucine and isoleucine biosynthesis;ko00660 C5-Branched dibasic acid metabolism;ko01210 2-Oxocarboxylic acid metabolism;ko01230 Biosynthesis of amino acids;
gene20252	K01704	leuD	3-isopropylmalate/(R)-2-methylmalate dehydratase small subunit	EC:4.2.1.33 4.2.1.35	4	ko00290 Valine, leucine and isoleucine biosynthesis;ko00660 C5-Branched dibasic acid metabolism;ko01210 2-Oxocarboxylic acid metabolism;ko01230 Biosynthesis of amino acids;
gene20257	K07964	HPSE	heparanase 1	EC:3.2.1.166	2	ko00531 Glycosaminoglycan degradation;ko05205 Proteoglycans in cancer;
gene20260	K00766	trpD	anthranilate phosphoribosyltransferase	EC:2.4.2.18	2	ko00400 Phenylalanine, tyrosine and tryptophan biosynthesis;ko01230 Biosynthesis of amino acids;
gene20261	K04354	PPP2R2	serine/threonine-protein phosphatase 2A regulatory subunit B	--	9	ko03015 mRNA surveillance pathway;ko04111 Cell cycle - yeast;ko04151 PI3K-Akt signaling pathway;ko04390 Hippo signaling pathway;ko04391 Hippo signaling pathway - fly;ko04530 Tight junction;ko04728 Dopaminergic synapse;ko05142 Chagas disease (American trypanosomiasis);ko05160 Hepatitis C;
gene20262	K10293	FBXO7	F-box protein 7	--		
gene2026	K15639	CYP734A1, BAS1	cytochrome P450, family 734, subfamily A, polypeptide 1 (PHYB activation tagged suppressor 1)	EC:1.14.-.-	1	ko00905 Brassinosteroid biosynthesis;
gene20270	K02938	RP-L8e, RPL8	large subunit ribosomal protein L8e	--	1	ko03010 Ribosome;
gene20271	K03453	TC.BASS	bile acid:Na+ symporter, BASS family	--		
gene20273	K14487	GH3	auxin responsive GH3 gene family	--	1	ko04075 Plant hormone signal transduction;
gene20278	K05954	FNTB	protein farnesyltransferase subunit beta	EC:2.5.1.58	1	ko00900 Terpenoid backbone biosynthesis;
gene2027	K11851	USP30	ubiquitin carboxyl-terminal hydrolase 30	EC:3.1.2.15		
gene20291	K14490	AHP	histidine-containing phosphotransfer peotein	--	1	ko04075 Plant hormone signal transduction;

gene20293	K08054	CANX	calnexin	--	5	ko04141 Protein processing in endoplasmic reticulum;ko04145 Phagosome;ko04612 Antigen processing and presentation;ko04918 Thyroid hormone synthesis;ko05166 HTLV-I infection;
gene20294	K13447	RBOH	respiratory burst oxidase	EC:1.6.3.- 1.11.1.-	1	ko04626 Plant-pathogen interaction;
gene20296	K00232	E1.3.3.6, ACOX1, ACOX3	acyl-CoA oxidase	EC:1.3.3.6	5	ko00071 Fatty acid degradation;ko00592 alpha-Linolenic acid metabolism;ko01040 Biosynthesis of unsaturated fatty acids;ko03320 PPAR signaling pathway;ko04146 Peroxisome;
gene2029	K00847	E2.7.1.4, scrK	fructokinase	EC:2.7.1.4	3	ko00051 Fructose and mannose metabolism;ko00500 Starch and sucrose metabolism;ko00520 Amino sugar and nucleotide sugar metabolism;
gene20301	K05309	PTGES2	microsomal prostaglandin-E synthase 2	EC:5.3.99.3	1	ko00590 Arachidonic acid metabolism;
gene20304	K04506	SIAH1	E3 ubiquitin-protein ligase SIAH1	EC:6.3.2.19	3	ko04115 p53 signaling pathway;ko04120 Ubiquitin mediated proteolysis;ko04310 Wnt signaling pathway;
gene20306	K01205	NAGLU	alpha-N-acetylglucosaminidase	EC:3.2.1.50	2	ko00531 Glycosaminoglycan degradation;ko04142 Lysosome;
gene20309	K03319	TC.DASS	divalent anion:Na+ symporter, DASS family	--		
gene2030	K13448	CML	calcium-binding protein CML	--	1	ko04626 Plant-pathogen interaction;
gene20312	K02988	RP-S5, MRPS5, rpsE	small subunit ribosomal protein S5	--	1	ko03010 Ribosome;
gene20317	K13448	CML	calcium-binding protein CML	--	1	ko04626 Plant-pathogen interaction;
gene20318	K13811	PAPSS	3'-phosphoadenosine 5'-phosphosulfate synthase	EC:2.7.7.4 2.7.1.25	3	ko00230 Purine metabolism;ko00450 Selenocompound metabolism;ko00920 Sulfur metabolism;
gene20319	K11420	EHMT	euchromatic histone-lysine N-methyltransferase	EC:2.1.1.43	1	ko00310 Lysine degradation;
gene20325	K13511	TAZ	monolysocardiolipin acyltransferase	EC:2.3.1.-	1	ko00564 Glycerophospholipid metabolism;
gene20326	K10364	CAPZA	capping protein (actin filament) muscle Z-line, alpha	--		
gene20327	K10364	CAPZA	capping protein (actin filament) muscle Z-line, alpha	--		
gene20328	K12623	LSM4	U6 snRNA-associated Sm-like protein LSm4	--	2	ko03018 RNA degradation;ko03040 Spliceosome;
gene20329	K10532	HGSNAT	heparan-alpha-glucosaminide N-acetyltransferase	EC:2.3.1.78	2	ko00531 Glycosaminoglycan degradation;ko04142 Lysosome;
gene20330	K03065	PSMC3, RPT5	26S proteasome regulatory subunit T5	--	2	ko03050 Proteasome;ko05169 Epstein-Barr virus infection;
gene20332	K08653	MBTPS1	membrane-bound transcription factor site-1 protease	EC:3.4.21.112	1	ko04141 Protein processing in endoplasmic reticulum;
gene20335	K06627	CCNA	cyclin A	--	5	ko04110 Cell cycle;ko04914 Progesterone-mediated oocyte maturation;ko05161 Hepatitis B;ko05169 Epstein-Barr virus infection;ko05203 Viral carcinogenesis;
gene20337	K13113	UBL5, HUB1	ubiquitin-like protein 5	--		
gene20346	K14638	SLC15A3_4, PHT	solute carrier family 15 (peptide/histidine transporter), member 3/4	--		
gene20347	K12158	NEDD8	ubiquitin-like protein Nedd8	--		
gene20348	K09422	MYBP	myb proto-oncogene protein, plant	--		
gene20350	K12882	NCBP1, CBP80	nuclear cap-binding protein subunit 1	--	3	ko03013 RNA transport;ko03015 mRNA surveillance pathway;ko03040 Spliceosome;
gene20351	K14778	DDX49, DBP8	ATP-dependent RNA helicase DDX49/DBP8	EC:3.6.4.13		
gene20352	K00134	GAPDH, gapA	glyceraldehyde 3-phosphate dehydrogenase	EC:1.2.1.12	6	ko00010 Glycolysis / Gluconeogenesis;ko00710 Carbon fixation in photosynthetic organisms;ko01200 Carbon metabolism;ko01230 Biosynthesis of amino acids;ko04066 HIF-1 signaling pathway;ko05010 Alzheimer's disease;
gene20356	K02150	ATPeVE, ATP6E	V-type H+-transporting ATPase subunit E	EC:3.6.3.14	7	ko00190 Oxidative phosphorylation;ko04145 Phagosome;ko04721 Synaptic vesicle cycle;ko04966 Collecting duct acid secretion;ko05110 Vibrio cholerae infection;ko05120 Epithelial cell signaling in Helicobacter pylori infection;ko05323 Rheumatoid arthritis;
gene20357	K15601	KDM3	lysine-specific demethylase 3	EC:1.14.11.-		
gene20358	K15109	SLC25A20_29, CACT, CACL, CRC1	solute carrier family 25 (mitochondrial carnitine/acylcarnitine transporter), member 20/29	--		
gene20365	K10869	RAD51L1, RAD51B	RAD51-like protein 1	--	1	ko03440 Homologous recombination;
gene20368	K03676	grxC, GLRX, GLRX2	glutaredoxin 3	--		
gene20369	K03676	grxC, GLRX, GLRX2	glutaredoxin 3	--		
gene20370	K07192	FLOT	flotillin	--	1	ko04910 Insulin signaling pathway;
gene20372	K02356	efp	elongation factor P	--		
gene20374	K15451	PPM2, LCMT2, TYW4	tRNA wybutosine-synthesizing protein 4	EC:2.1.1.-		

gene20378	K12861	BCAS2	pre-mRNA-splicing factor SPF27	--	1	ko03040 Spliceosome;
gene20381	K09840	NCED	9-cis-epoxycarotenoid dioxygenase	EC:1.13.11.51	1	ko00906 Carotenoid biosynthesis;
gene20384	K13201	TIA1, TIAL1	nucleolysin TIA-1/TIAR	--		
gene20390	K14325	RNPS1	RNA-binding protein with serine-rich domain 1	--	2	ko03013 RNA transport;ko03015 mRNA surveillance pathway;
gene20392	K10357	MYO5	myosin V	--		
gene20393	K17506	PPM1L, PP2CE	protein phosphatase 1L	EC:3.1.3.16		
gene20395	K11594	DDX3X, bel	ATP-dependent RNA helicase	EC:3.6.4.13	3	ko04622 RIG-I-like receptor signaling pathway;ko05161 Hepatitis B;ko05203 Viral carcinogenesis;
gene20397	K11426	SMYD	SET and MYND domain-containing protein	--		
gene20398	K09286	EREBP	EREBP-like factor	--		
gene203	K12816	CDC40, PRP17	pre-mRNA-processing factor 17	--	1	ko03040 Spliceosome;
gene20402	K13448	CML	calcium-binding protein CML	--	1	ko04626 Plant-pathogen interaction;
gene20403	K14820	BRX1, BRIX1	ribosome biogenesis protein BRX1	--		
gene20407	K16297	SCPL-II	serine carboxypeptidase-like clade II	EC:3.4.16.-		
gene2040	K12153	CYP79A2	cytochrome P450, family 79, subfamily A, polypeptide 2 (phenylalanine N-monooxygenase)	EC:1.14.13.124	3	ko00460 Cyanoamino acid metabolism;ko00966 Glucosinolate biosynthesis;ko01210 2-Oxocarboxylic acid metabolism;
gene20410	K11968	ARIH1	ariadne-1	--		
gene20416	K14233	tRNA-Ser	tRNA Ser	--	1	ko00970 Aminoacyl-tRNA biosynthesis;
gene20417	K14233	tRNA-Ser	tRNA Ser	--	1	ko00970 Aminoacyl-tRNA biosynthesis;
gene20419	K13648	GAUT	alpha-1,4-galacturonosyltransferase	EC:2.4.1.43	2	ko00500 Starch and sucrose metabolism;ko00520 Amino sugar and nucleotide sugar metabolism;
gene20422	K01893	NARS, asnS	asparaginyl-tRNA synthetase	EC:6.1.1.22	1	ko00970 Aminoacyl-tRNA biosynthesis;
gene20425	K07904	RAB11A	Ras-related protein Rab-11A	--	4	ko04144 Endocytosis;ko04961 Endocrine and other factor-regulated calcium reabsorption;ko04962 Vasopressin-regulated water reabsorption;ko04972 Pancreatic secretion;
gene20430	K12821	PRPF40, PRP40	pre-mRNA-processing factor 40	--	1	ko03040 Spliceosome;
gene20431	K03257	EIF4A	translation initiation factor 4A	--	1	ko03013 RNA transport;
gene20432	K02218	CSNK1, CK1	casein kinase 1	EC:2.7.11.1	1	ko04340 Hedgehog signaling pathway;
gene20435	K12896	SFRS7	splicing factor, arginine/serine-rich 7	--	2	ko03040 Spliceosome;ko05168 Herpes simplex infection;
gene20441	K11979	UBR7	E3 ubiquitin-protein ligase UBR7	EC:6.3.2.19		
gene20443	K00761	upp, UPRT	uracil phosphoribosyltransferase	EC:2.4.2.9	1	ko00240 Pyrimidine metabolism;
gene20446	K14488	SAUR	SAUR family protein	--	1	ko04075 Plant hormone signal transduction;
gene20450	K08835	OXSR1, STK39	serine/threonine-protein kinase OSR1/STK39	EC:2.7.11.1		
gene20458	K03257	EIF4A	translation initiation factor 4A	--	1	ko03013 RNA transport;
gene20460	K00827	AGXT2	alanine-glyoxylate transaminase / (R)-3-amino-2-methylpropionate-pyruvate transaminase	EC:2.6.1.44 2.6.1.40	2	ko00250 Alanine, aspartate and glutamate metabolism;ko00260 Glycine, serine and threonine metabolism;
gene20461	K14431	TGA	transcription factor TGA	--	1	ko04075 Plant hormone signal transduction;
gene20465	K15691	RFWD3	E3 ubiquitin-protein ligase RFWD3	EC:6.3.2.19		
gene20467	K13495	CISZOG	cis-zeatin O-glucosyltransferase	EC:2.4.1.215	1	ko00908 Zeatin biosynthesis;
gene20468	K03124	TFIIB, GTF2B, SUA7, tfb	transcription initiation factor TFIIB	--	3	ko03022 Basal transcription factors;ko05169 Epstein-Barr virus infection;ko05203 Viral carcinogenesis;
gene20469	K12607	CNOT10	CCR4-NOT transcription complex subunit 10	--	1	ko03018 RNA degradation;
gene20471	K07904	RAB11A	Ras-related protein Rab-11A	--	4	ko04144 Endocytosis;ko04961 Endocrine and other factor-regulated calcium reabsorption;ko04962 Vasopressin-regulated water reabsorption;ko04972 Pancreatic secretion;
gene20478	K15255	PIF1	ATP-dependent DNA helicase PIF1	EC:3.6.4.12		
gene20483	K12483	EHD1	EH domain-containing protein 1	--	1	ko04144 Endocytosis;
gene20484	K03687	GRPE	molecular chaperone GrpE	--		
gene20486	K00799	GST, gst	glutathione S-transferase	EC:2.5.1.18	4	ko00480 Glutathione metabolism;ko00980 Metabolism of xenobiotics by cytochrome P450;ko00982 Drug metabolism - cytochrome P450;ko05204 Chemical carcinogenesis;
gene20487	K12120	PHYA	phytochrome A	--	1	ko04712 Circadian rhythm - plant;
gene20492	K08486	STX1B_2_3	syntaxin 1B/2/3	--	2	ko04130 SNARE interactions in vesicular transport;ko04721 Synaptic vesicle cycle;
gene20495	K16280	RGLG	E3 ubiquitin-protein ligase RGLG	EC:6.3.2.19		

gene20498	K16280	RGLG	E3 ubiquitin-protein ligase RGLG	EC:6.3.2.19		
gene20502	K16280	RGLG	E3 ubiquitin-protein ligase RGLG	EC:6.3.2.19		
gene20503	K07195	EXOC7, EXO70	exocyst complex component 7	--	1	ko04910 Insulin signaling pathway;
gene20517	K15255	PIF1	ATP-dependent DNA helicase PIF1	EC:3.6.4.12		
gene20518	K14544	UTP22, NOL6	U3 small nucleolar RNA-associated protein 22	--	1	ko03008 Ribosome biogenesis in eukaryotes;
gene20525	K02934	RP-L6e, RPL6	large subunit ribosomal protein L6e	--	1	ko03010 Ribosome;
gene20528	K13648	GAUT	alpha-1,4-galacturonosyltransferase	EC:2.4.1.43	2	ko00500 Starch and sucrose metabolism;ko00520 Amino sugar and nucleotide sugar metabolism;
gene20534	K11423	SETD2, SET2	histone-lysine N-methyltransferase SETD2	EC:2.1.1.43	1	ko00310 Lysine degradation;
gene20537	K17262	TBCB, CKAP1, ALF1	tubulin-folding cofactor B	--		
gene20542	K15285	SLC35E3	solute carrier family 35, member E3	--		
gene20544	K05391	CNGF	cyclic nucleotide gated channel, other eukaryote	--	1	ko04626 Plant-pathogen interaction;
gene20545	K12587	MTR3, EXOSC6	exosome complex component MTR3	--	1	ko03018 RNA degradation;
gene20548	K16912	LAS1	ribosomal biogenesis protein LAS1	--		
gene2054	K13412	CPK	calcium-dependent protein kinase	EC:2.7.11.1	2	ko04626 Plant-pathogen interaction;ko05145 Toxoplasmosis;
gene20553	K13412	CPK	calcium-dependent protein kinase	EC:2.7.11.1	2	ko04626 Plant-pathogen interaction;ko05145 Toxoplasmosis;
gene20557	K01874	MARS, metG	methionyl-tRNA synthetase	EC:6.1.1.10	2	ko00450 Selenocompound metabolism;ko00970 Aminoacyl-tRNA biosynthesis;
gene20558	K03787	surE	5'-nucleotidase	EC:3.1.3.5	3	ko00230 Purine metabolism;ko00240 Pyrimidine metabolism;ko00760 Nicotinate and nicotinamide metabolism;
gene20559	K15192	BTAF1, MOT1	TATA-binding protein-associated factor	EC:3.6.4.-		
gene2055	K09875	SIP	aquaporin SIP	--		
gene20561	K00001	E1.1.1.1, adh	alcohol dehydrogenase	EC:1.1.1.1	9	ko00010 Glycolysis / Gluconeogenesis;ko00071 Fatty acid degradation;ko00350 Tyrosine metabolism;ko00625 Chloroalkane and chloroalkene degradation;ko00626 Naphthalene degradation;ko00830 Retinol metabolism;ko00980 Metabolism of xenobiotics by cytochrome P450;ko00982 Drug metabolism - cytochrome P450;ko01220 Degradation of aromatic compounds;
gene20563	K11835	USP4_11_15, UBP12	ubiquitin carboxyl-terminal hydrolase 4/11/15	EC:3.1.2.15		
gene20566	K00434	E1.11.1.11	L-ascorbate peroxidase	EC:1.11.1.11	2	ko00053 Ascorbate and aldarate metabolism;ko00480 Glutathione metabolism;
gene20569	K00472	E1.14.11.2	prolyl 4-hydroxylase	EC:1.14.11.2	1	ko00330 Arginine and proline metabolism;
gene20573	K01102	PDP	pyruvate dehydrogenase phosphatase	EC:3.1.3.43		
gene20578	K03453	TC.BASS	bile acid:Na+ symporter, BASS family	--		
gene20587	K03063	PSMC4, RPT3	26S proteasome regulatory subunit T3	--	2	ko03050 Proteasome;ko05169 Epstein-Barr virus infection;
gene20588	K02866	RP-L10e, RPL10	large subunit ribosomal protein L10e	--	1	ko03010 Ribosome;
gene20590	K14945	QKI	protein quaking	--		
gene20592	K11752	ribD	diaminohydroxyphosphoribosylaminopyrimidine deaminase / 5-amino-6-(5-phosphoribosylamino)uracil reductase	EC:3.5.4.26 1.1.1.193	1	ko00740 Riboflavin metabolism;
gene20594	K14231	tRNA-Phe	tRNA Phe	--	1	ko00970 Aminoacyl-tRNA biosynthesis;
gene20598	K15111	SLC25A26	solute carrier family 25 (mitochondrial S-adenosylmethionine transporter), member 26	--		
gene20599	K13917	RNGTT	mRNA-capping enzyme	EC:2.7.7.50 3.1.3.33	1	ko03015 mRNA surveillance pathway;
gene20602	K10591	NEDD4, RSP5	E3 ubiquitin-protein ligase NEDD4	EC:6.3.2.19	3	ko04120 Ubiquitin mediated proteolysis;ko04144 Endocytosis;ko05169 Epstein-Barr virus infection;
gene20605	K13091	RBM39, RNPC2	RNA-binding protein 39	--		
gene20608	K14788	NOL10, ENP2	ribosome biogenesis protein ENP2	--		
gene20609	K01719	hemD, UROS	uroporphyrinogen-III synthase	EC:4.2.1.75	1	ko00860 Porphyrin and chlorophyll metabolism;
gene20610	K16297	SCPL-II	serine carboxypeptidase-like clade II	EC:3.4.16.-		
gene20616	K14491	ARR-B	two-component response regulator ARR-B family	--	1	ko04075 Plant hormone signal transduction;
gene20620	K16297	SCPL-II	serine carboxypeptidase-like clade II	EC:3.4.16.-		
gene20621	K16297	SCPL-II	serine carboxypeptidase-like clade II	EC:3.4.16.-		
gene20622	K16297	SCPL-II	serine carboxypeptidase-like clade II	EC:3.4.16.-		

gene20623	K05391	CNGF	cyclic nucleotide gated channel, other eukaryote	--	1	ko04626 Plant-pathogen interaction;
gene20625	K01051	E3.1.1.11	pectinesterase	EC:3.1.1.11	2	ko00040 Pentose and glucuronate interconversions;ko00500 Starch and sucrose metabolism;
gene20629	K01534	zntA	Cd2+/Zn2+-exporting ATPase	EC:3.6.3.3 3.6.3.5		
gene2062	K08653	MBTPS1	membrane-bound transcription factor site-1 protease	EC:3.4.21.112	1	ko04141 Protein processing in endoplasmic reticulum;
gene20631	K12126	PIF3	phytochrome-interacting factor 3	--	2	ko04075 Plant hormone signal transduction;ko04712 Circadian rhythm - plant;
gene20638	K09338	HD-ZIP	homeobox-leucine zipper protein	--		
gene2063	K08065	NFYB	nuclear transcription Y subunit beta	--	3	ko04612 Antigen processing and presentation;ko05152 Tuberculosis;ko05166 HTLV-I infection;
gene20640	K07976	RAB	Rab family, other	--		
gene20641	K03327	TC.MATE, SLC47A, norM, mdtK, dinF	multidrug resistance protein, MATE family	--		
gene20642	K13162	PCBP2_3_4	poly(rC)-binding protein 2/3/4	--		
gene20643	K00412	CYTB, petB	ubiquinol-cytochrome c reductase cytochrome b subunit	--	7	ko00190 Oxidative phosphorylation;ko02020 Two-component system;ko04260 Cardiac muscle contraction;ko04932 Non-alcoholic fatty liver disease (NAFLD);ko05010 Alzheimer's disease;ko05012 Parkinson's disease;ko05016 Huntington's disease;
gene20645	K03841	FBP, fbp	fructose-1,6-bisphosphatase I	EC:3.1.3.11	7	ko00010 Glycolysis / Gluconeogenesis;ko00030 Pentose phosphate pathway;ko00051 Fructose and mannose metabolism;ko00680 Methane metabolism;ko00710 Carbon fixation in photosynthetic organisms;ko01200 Carbon metabolism;ko04910 Insulin signaling pathway;
gene2064	K14232	tRNA-Pro	tRNA Pro	--	1	ko00970 Aminoacyl-tRNA biosynthesis;
gene20651	K09841	ABA2	xanthoxin dehydrogenase	EC:1.1.1.288	1	ko00906 Carotenoid biosynthesis;
gene20655	K11279	NAP1L1, NRP	nucleosome assembly protein 1-like 1	--		
gene20656	K05863	SLC25A4S, ANT	solute carrier family 25 (mitochondrial adenine nucleotide translocator), member 4/5/6/31	--	4	ko04020 Calcium signaling pathway;ko05012 Parkinson's disease;ko05016 Huntington's disease;ko05166 HTLV-I infection;
gene20660	K08819	CDK12_13	cyclin-dependent kinase 12/13	EC:2.7.11.22 2.7.11.23		
gene20661	K08081	E1.1.1.206	tropine dehydrogenase	EC:1.1.1.206	1	ko00960 Tropane, piperidine and pyridine alkaloid biosynthesis;
gene20663	K14786	KRI1	protein KRI1	--		
gene20665	K08829	MAK	male germ cell-associated kinase	EC:2.7.11.22		
gene20668	K10597	UBE4B, UFD2	ubiquitin conjugation factor E4 B	EC:6.3.2.19	2	ko04120 Ubiquitin mediated proteolysis;ko04141 Protein processing in endoplasmic reticulum;
gene2066	K08472	MLO	mlo protein	--		
gene20675	K05658	ABCB1	ATP-binding cassette, subfamily B (MDR/TAP), member 1	--	3	ko02010 ABC transporters;ko04976 Bile secretion;ko05206 MicroRNAs in cancer;
gene20680	K11699	RDR1, RDP1	RNA-dependent RNA polymerase 1	EC:2.7.7.48		
gene20681	K02912	RP-L32e, RPL32	large subunit ribosomal protein L32e	--	1	ko03010 Ribosome;
gene20682	K07374	TUBA	tubulin alpha	--	3	ko04145 Phagosome;ko04540 Gap junction;ko05130 Pathogenic Escherichia coli infection;
gene20686	K11807	WDTC1	WD and tetratricopeptide repeats protein 1	--		
gene20690	K06889	K06889				
gene206	K14220	tRNA-Asn	tRNA Asn	--	1	ko00970 Aminoacyl-tRNA biosynthesis;
gene20703	K00658	DLST, sucB	2-oxoglutarate dehydrogenase E2 component (dihydrolipoamide succinyltransferase)	EC:2.3.1.61	3	ko00020 Citrate cycle (TCA cycle);ko00310 Lysine degradation;ko01200 Carbon metabolism;
gene20707	K10747	LIG1	DNA ligase 1	EC:6.5.1.1	4	ko03030 DNA replication;ko03410 Base excision repair;ko03420 Nucleotide excision repair;ko03430 Mismatch repair;
gene20710	K12126	PIF3	phytochrome-interacting factor 3	--	2	ko04075 Plant hormone signal transduction;ko04712 Circadian rhythm - plant;
gene20711	K00827	AGXT2	alanine-glyoxylate transaminase / (R)-3-amino-2-methylpropionate-pyruvate transaminase	EC:2.6.1.44 2.6.1.40	2	ko00250 Alanine, aspartate and glutamate metabolism;ko00260 Glycine, serine and threonine metabolism;
gene20716	K02144	ATPeV54kD	V-type H+-transporting ATPase 54 kD subunit	EC:3.6.3.14	8	ko00190 Oxidative phosphorylation;ko04142 Lysosome;ko04145 Phagosome;ko04721 Synaptic vesicle cycle;ko05110 Vibrio cholerae infection;ko05120 Epithelial cell signaling in Helicobacter pylori infection;ko05152 Tuberculosis;ko05323 Rheumatoid arthritis;
gene20717	K10908	POLRMT, RPO41	DNA-directed RNA polymerase, mitochondrial	EC:2.7.7.6		

gene20719	K01963	accD	acetyl-CoA carboxylase carboxyl transferase subunit beta	EC:6.4.1.2	6	ko00061 Fatty acid biosynthesis;ko00253 Tetracycline biosynthesis;ko00620 Pyruvate metabolism;ko00640 Propanoate metabolism;ko00720 Carbon fixation pathways in prokaryotes;ko01200 Carbon metabolism;
gene20727	K12189	VPS25, EAP20	ESCRT-II complex subunit VPS25	--	1	ko04144 Endocytosis;
gene2072	K03549	kup	KUP system potassium uptake protein	--		
gene20732	K03627	MBF1	putative transcription factor	--		
gene20734	K17260	ACTR2, ARP2	actin-related protein 2	--		
gene20745	K04646	CLTC	clathrin heavy chain	--	6	ko04142 Lysosome;ko04144 Endocytosis;ko04721 Synaptic vesicle cycle;ko04961 Endocrine and other factor-regulated calcium reabsorption;ko05016 Huntington's disease;ko05100 Bacterial invasion of epithelial cells;
gene2074	K10735	GINS4, SLD5	GINS complex subunit 4	--		
gene20751	K15803	GERD	(-)-germacrene D synthase	EC:4.2.3.22 4.2.3.75	1	ko00909 Sesquiterpenoid and triterpenoid biosynthesis;
gene20762	K13412	CPK	calcium-dependent protein kinase	EC:2.7.11.1	2	ko04626 Plant-pathogen interaction;ko05145 Toxoplasmosis;
gene20763	K11000	CALS	callose synthase	EC:2.4.1.-		
gene20764	K15015	SLC32A, VGAT	solute carrier family 32 (vesicular inhibitory amino acid transporter)	--	5	ko04721 Synaptic vesicle cycle;ko04723 Retrograde endocannabinoid signaling;ko04727 GABAergic synapse;ko05032 Morphine addiction;ko05033 Nicotine addiction;
gene20765	K15015	SLC32A, VGAT	solute carrier family 32 (vesicular inhibitory amino acid transporter)	--	5	ko04721 Synaptic vesicle cycle;ko04723 Retrograde endocannabinoid signaling;ko04727 GABAergic synapse;ko05032 Morphine addiction;ko05033 Nicotine addiction;
gene20768	K08238	XXT	xyloglucan 6-xylosyltransferase	EC:2.4.2.39		
gene2077	K17065	DNM1L	dynamin 1-like protein	EC:3.6.5.5	1	ko04668 TNF signaling pathway;
gene20782	K02925	RP-L3e, RPL3	large subunit ribosomal protein L3e	--	1	ko03010 Ribosome;
gene20784	K04569	CCS	copper chaperone for superoxide dismutase	--	1	ko05014 Amyotrophic lateral sclerosis (ALS);
gene2078	K02357	tsf, TSFM	elongation factor Ts	--		
gene20795	K01411	NRD1	nardilysin	EC:3.4.24.61		
gene20796	K00915	IPK2	1D-myo-Inositol-tetrakisphosphate 5-kinase / inositol-polyphosphate multikinase	EC:2.7.1.140 2.7.1.151	2	ko00562 Inositol phosphate metabolism;ko04070 Phosphatidylinositol signaling system;
gene20797	K14423	SMO1	4,4-dimethyl-9beta,19-cyclopropylsterol-4alpha-methyl oxidase	EC:1.14.13.72	1	ko00100 Steroid biosynthesis;
gene20798	K10666	RNF5	E3 ubiquitin-protein ligase RNF5	EC:6.3.2.19	1	ko04141 Protein processing in endoplasmic reticulum;
gene2079	K14209	SLC36A, PAT	solute carrier family 36 (proton-coupled amino acid transporter)	--		
gene207	K14230	tRNA-Met	tRNA Met	--	1	ko00970 Aminoacyl-tRNA biosynthesis;
gene20800	K03671	trxA	thioredoxin 1	--		
gene20802	K00979	kdsB	3-deoxy-manno-octulosonate cytidylyltransferase (CMP-KDO synthetase)	EC:2.7.7.38	1	ko00540 Lipopolysaccharide biosynthesis;
gene20807	K00134	GAPDH, gapA	glyceraldehyde 3-phosphate dehydrogenase	EC:1.2.1.12	6	ko00010 Glycolysis / Gluconeogenesis;ko00710 Carbon fixation in photosynthetic organisms;ko01200 Carbon metabolism;ko01230 Biosynthesis of amino acids;ko04066 HIF-1 signaling pathway;ko05010 Alzheimer's disease;
gene2080	K02867	RP-L11, MRPL11, rplK	large subunit ribosomal protein L11	--	1	ko03010 Ribosome;
gene20810	K02957	RP-S15Ae, RPS15A	small subunit ribosomal protein S15Ae	--	1	ko03010 Ribosome;
gene20811	K01507	ppa	inorganic pyrophosphatase	EC:3.6.1.1	1	ko00190 Oxidative phosphorylation;
gene2081	K14803	PTC2_3	protein phosphatase 2C homolog 2/3	EC:3.1.3.16		
gene20824	K00454	LOX2S	lipoxygenase	EC:1.13.11.12	2	ko00591 Linoleic acid metabolism;ko00592 alpha-Linolenic acid metabolism;
gene20829	K10527	MFP2	enoyl-CoA hydratase/3-hydroxyacyl-CoA dehydrogenase	EC:4.2.1.17 1.1.1.35 1.1.1.211	2	ko00071 Fatty acid degradation;ko00592 alpha-Linolenic acid metabolism;
gene20831	K01728	E4.2.2.2, pel	pectate lyase	EC:4.2.2.2	1	ko00040 Pentose and glucuronate interconversions;
gene20832	K01800	maiA, GSTZ1	maleylacetoacetate isomerase	EC:5.2.1.2	2	ko00350 Tyrosine metabolism;ko00643 Styrene degradation;
gene20835	K12619	XRN2, RAT1	5'-3' exoribonuclease 2	EC:3.1.13.-	2	ko03008 Ribosome biogenesis in eukaryotes;ko03018 RNA degradation;
gene20836	K04728	ATM, TEL1	ataxia telangiectasia mutated family protein	EC:2.7.11.1	7	ko04064 NF-kappa B signaling pathway;ko04110 Cell cycle;ko04115 p53 signaling pathway;ko04210 Apoptosis;ko05166 HTLV-I infection;ko05202 Transcriptional misregulation in cancer;ko05206 MicroRNAs in cancer;

gene20837	K04728	ATM, TEL1	ataxia telangiectasia mutated family protein	EC:2.7.11.1	7	ko04064 NF-kappa B signaling pathway;ko04110 Cell cycle;ko04115 p53 signaling pathway;ko04210 Apoptosis;ko05166 HTLV-I infection;ko05202 Transcriptional misregulation in cancer;ko05206 MicroRNAs in cancer;
gene2083	K06085	SSX2IP, ADIP	synovial sarcoma, X breakpoint 2 interacting protein	--	1	ko04520 Adherens junction;
gene20847	K17301	COPB1, SEC26	coatomer, subunit beta	--		
gene20849	K00033	E1.1.1.44, PGD, gnd	6-phosphogluconate dehydrogenase	EC:1.1.1.44	3	ko00030 Pentose phosphate pathway;ko00480 Glutathione metabolism;ko01200 Carbon metabolism;
gene2084	K06085	SSX2IP, ADIP	synovial sarcoma, X breakpoint 2 interacting protein	--	1	ko04520 Adherens junction;
gene20853	K12669	OST3, OST6	oligosaccharyltransferase complex subunit gamma	--	3	ko00510 N-Glycan biosynthesis;ko00513 Various types of N-glycan biosynthesis;ko04141 Protein processing in endoplasmic reticulum;
gene20855	K07512	MECR, NRBF1	mitochondrial trans-2-enoyl-CoA reductase	EC:1.3.1.38	1	ko00062 Fatty acid elongation;
gene20856	K09286	EREBP	EREBP-like factor	--		
gene20861	K08517	SEC22	vesicle transport protein SEC22	--	3	ko04130 SNARE interactions in vesicular transport;ko04145 Phagosome;ko05134 Legionellosis;
gene20865	K05610	UCHL5, UCH37	ubiquitin carboxyl-terminal hydrolase L5	EC:3.4.19.12		
gene20872	K15086	TPS14	(3S)-linalool synthase	EC:4.2.3.25	1	ko00902 Monoterpenoid biosynthesis;
gene20874	K01759	E4.4.1.5, GLO1, gloA	lactoylglutathione lyase	EC:4.4.1.5	2	ko00620 Pyruvate metabolism;ko04011 MAPK signaling pathway - yeast;
gene20877	K00797	speE, SRM	spermidine synthase	EC:2.5.1.16	4	ko00270 Cysteine and methionine metabolism;ko00330 Arginine and proline metabolism;ko00410 beta-Alanine metabolism;ko00480 Glutathione metabolism;
gene20878	K06689	UBE2D_E, UBC4, UBC5	ubiquitin-conjugating enzyme E2 D/E	EC:6.3.2.19	2	ko04120 Ubiquitin mediated proteolysis;ko04141 Protein processing in endoplasmic reticulum;
gene20885	K01537	E3.6.3.8	Ca2+-transporting ATPase	EC:3.6.3.8		
gene20888	K07904	RAB11A	Ras-related protein Rab-11A	--	4	ko04144 Endocytosis;ko04961 Endocrine and other factor-regulated calcium reabsorption;ko04962 Vasopressin-regulated water reabsorption;ko04972 Pancreatic secretion;
gene20894	K13420	FLS2	LRR receptor-like serine/threonine-protein kinase FLS2	EC:2.7.11.1	1	ko04626 Plant-pathogen interaction;
gene20895	K04730	IRAK1	interleukin-1 receptor-associated kinase 1	EC:2.7.11.1	11	ko04064 NF-kappa B signaling pathway;ko04210 Apoptosis;ko04620 Toll-like receptor signaling pathway;ko04722 Neurotrophin signaling pathway;ko05133 Pertussis;ko05140 Leishmaniasis;ko05142 Chagas disease (American trypanosomiasis);ko05145 Toxoplasmosis;ko05152 Tuberculosis;ko05162 Measles;ko05169 Epstein-Barr virus infection;
gene20897	K07393	ECM4	putative glutathione S-transferase	--		
gene20899	K08232	E1.6.5.4	monodehydroascorbate reductase (NADH)	EC:1.6.5.4	1	ko00053 Ascorbate and aldarate metabolism;
gene208	K14230	tRNA-Met	tRNA Met	--	1	ko00970 Aminoacyl-tRNA biosynthesis;
gene20903	K14315	NDC1, TMEM48	nucleoporin NDC1	--	1	ko03013 RNA transport;
gene20904	K01637	E4.1.3.1, aceA	isocitrate lyase	EC:4.1.3.1	2	ko00630 Glyoxylate and dicarboxylate metabolism;ko01200 Carbon metabolism;
gene20906	K14492	ARR-A	two-component response regulator ARR-A family	--	1	ko04075 Plant hormone signal transduction;
gene20907	K03020	RPC19, POLR1D	DNA-directed RNA polymerases I and III subunit RPAC2	--	5	ko00230 Purine metabolism;ko00240 Pyrimidine metabolism;ko03020 RNA polymerase;ko04623 Cytosolic DNA-sensing pathway;ko05169 Epstein-Barr virus infection;
gene20908	K02899	RP-L27, MRPL27, rpmA	large subunit ribosomal protein L27	--	1	ko03010 Ribosome;
gene20909	K13348	MPV17	protein Mpv17	--	1	ko04146 Peroxisome;
gene20912	K10523	SPOP	speckle-type POZ protein	--		
gene2091	K00434	E1.11.1.11	L-ascorbate peroxidase	EC:1.11.1.11	2	ko00053 Ascorbate and aldarate metabolism;ko00480 Glutathione metabolism;
gene20921	K01950	E6.3.5.1, NADSYN1, QNS1, nadE	NAD+ synthase (glutamine-hydrolysing)	EC:6.3.5.1	1	ko00760 Nicotinate and nicotinamide metabolism;
gene20923	K00344	E1.6.5.5, qor	NADPH2:quinone reductase	EC:1.6.5.5		
gene20924	K03781	katE, CAT, catB, srpA	catalase	EC:1.11.1.6	4	ko00380 Tryptophan metabolism;ko00630 Glyoxylate and dicarboxylate metabolism;ko04146 Peroxisome;ko05014 Amyotrophic lateral sclerosis (ALS);
gene20926	K00967	PCYT2	ethanolamine-phosphate cytidylyltransferase	EC:2.7.7.14	2	ko00440 Phosphonate and phosphinate metabolism;ko00564 Glycerophospholipid metabolism;
gene2092	K00029	E1.1.1.40, maeB	malate dehydrogenase (oxaloacetate-decarboxylating)(NADP+)	EC:1.1.1.40	3	ko00620 Pyruvate metabolism;ko00710 Carbon fixation in photosynthetic organisms;ko01200 Carbon metabolism;

gene20936	K00549	metE	5-methyltetrahydropteroyltriglutamate--homocysteine methyltransferase	EC:2.1.1.14	3	ko00270 Cysteine and methionine metabolism;ko00450 Selenocompound metabolism;ko01230 Biosynthesis of amino acids;
gene20940	K01979	SSUrRNA	small subunit ribosomal RNA	--	2	ko03008 Ribosome biogenesis in eukaryotes;ko03010 Ribosome;
gene20945	K14856	SDA1, SDAD1	protein SDA1	--		
gene20948	K01115	PLD1_2	phospholipase D1/2	EC:3.1.4.4	7	ko00564 Glycerophospholipid metabolism;ko00565 Ether lipid metabolism;ko04014 Ras signaling pathway;ko04144 Endocytosis;ko04666 Fc gamma R-mediated phagocytosis;ko04724 Glutamatergic synapse;ko04912 GnRH signaling pathway;
gene20955	K00924	E2.7.1.-				
gene20956	K14573	NOP4, RBM28	nucleolar protein 4	--	1	ko03008 Ribosome biogenesis in eukaryotes;
gene20957	K01069	E3.1.2.6, gloB	hydroxyacylglutathione hydrolase	EC:3.1.2.6	1	ko00620 Pyruvate metabolism;
gene20959	K00648	fabH	3-oxoacyl-[acyl-carrier-protein] synthase III	EC:2.3.1.180	1	ko00061 Fatty acid biosynthesis;
gene2095	K00261	GLUD1_2, gdhA	glutamate dehydrogenase (NAD(P)+)	EC:1.4.1.3	5	ko00250 Alanine, aspartate and glutamate metabolism;ko00330 Arginine and proline metabolism;ko00471 D-Glutamine and D-glutamate metabolism;ko00910 Nitrogen metabolism;ko04964 Proximal tubule bicarbonate reclamation;
gene20960	K15731	CTDSP	carboxy-terminal domain RNA polymerase II polypeptide A small phosphatase	EC:3.1.3.16		
gene20961	K13103	TFIP11	tuftelin-interacting protein 11	--		
gene20962	K08066	NFYC	nuclear transcription factor Y, gamma	--	2	ko04612 Antigen processing and presentation;ko05152 Tuberculosis;
gene20963	K07342	SEC61G, SSS1, secE	protein transport protein SEC61 subunit gamma and related proteins	--	4	ko03060 Protein export;ko04141 Protein processing in endoplasmic reticulum;ko04145 Phagosome;ko05110 Vibrio cholerae infection;
gene20965	K14944	NOVA	RNA-binding protein Nova	--		
gene20968	K08867	WNK, PRKWNK	WNK lysine deficient protein kinase	EC:2.7.11.1		
gene2096	K04371	MAPK1_3	mitogen-activated protein kinase 1/3	EC:2.7.11.24	71	ko04010 MAPK signaling pathway;ko04012 ErbB signaling pathway;ko04013 MAPK signaling pathway - fly;ko04014 Ras signaling pathway;ko04062 Chemokine signaling pathway;ko04066 HIF-1 signaling pathway;ko04114 Oocyte meiosis;ko04150 mTOR signaling pathway;ko04151 PI3K-Akt signaling pathway;ko04270 Vascular smooth muscle contraction;ko04320 Dorso-ventral axis formation;ko04350 TGF-beta signaling pathway;ko04360 Axon guidance;ko04370 VEGF signaling pathway;ko04380 Osteoclast differentiation;ko04510 Focal adhesion;ko04520 Adherens junction;ko04540 Gap junction;ko04620 Toll-like receptor signaling pathway;ko04621 NOD-like receptor signaling pathway;ko04650 Natural killer cell mediated cytotoxicity;ko04660 T cell receptor signaling pathway;ko04662 B cell receptor signaling pathway;ko04664 Fc epsilon RI signaling pathway;ko04666 Fc gamma R-mediated phagocytosis;ko04668 TNF signaling pathway;ko04713 Circadian entrainment;ko04720 Long-term potentiation;ko04722 Neurotrophin signaling pathway;ko04723 Retrograde endocannabinoid signaling;ko04724 Glutamatergic synapse;ko04725 Cholinergic synapse;ko04726 Serotonergic synapse;ko04730 Long-term depression;ko04810 Regulation of actin cytoskeleton;ko04910 Insulin signaling pathway;ko04912 GnRH signaling pathway;ko04914 Progesterone-mediated oocyte maturation;ko04915 Estrogen signaling pathway;ko04916 Melanogenesis;ko04917 Prolactin signaling pathway;ko04930 Type II diabetes mellitus;ko04960 Aldosterone-regulated sodium reabsorption;ko05010 Alzheimer's disease;ko05020 Prion diseases;ko05034 Alcoholism;ko05131 Shigellosis;ko05132 Salmonella infection;ko05133 Pertussis;ko05140 Leishmaniasis;ko05142 Chagas disease (American trypanosomiasis);ko05145 Toxoplasmosis;ko05152 Tuberculosis;ko05160 Hepatitis C;ko05161 Hepatitis B;ko05164 Influenza A;ko05200 Pathways in cancer;ko05203 Viral carcinogenesis;ko05205 Proteoglycans in cancer;ko05210 Colorectal cancer;ko05211 Renal cell carcinoma;ko05212
gene20970	K10752	RBBP4, HAT2, CAF1, MIS16	histone-binding protein RBBP4	--		
gene20972	K01177	E3.2.1.2	beta-amylase	EC:3.2.1.2	1	ko00500 Starch and sucrose metabolism;

gene20975	K02516	PRMT5, HSL7	protein arginine N-methyltransferase 5	EC:2.1.1.125	2	ko03013 RNA transport;ko04111 Cell cycle - yeast;
gene20979	K04392	RAC1	Ras-related C3 botulinum toxin substrate 1	--	33	ko04010 MAPK signaling pathway;ko04014 Ras signaling pathway;ko04062 Chemokine signaling pathway;ko04145 Phagosome;ko04151 PI3K-Akt signaling pathway;ko04310 Wnt signaling pathway;ko04360 Axon guidance;ko04370 VEGF signaling pathway;ko04380 Osteoclast differentiation;ko04510 Focal adhesion;ko04520 Adherens junction;ko04620 Toll-like receptor signaling pathway;ko04650 Natural killer cell mediated cytotoxicity;ko04662 B cell receptor signaling pathway;ko04664 Fc epsilon RI signaling pathway;ko04666 Fc gamma R-mediated phagocytosis;ko04670 Leukocyte transendothelial migration;ko04722 Neurotrophin signaling pathway;ko04810 Regulation of actin cytoskeleton;ko04932 Non-alcoholic fatty liver disease (NAFLD);ko04972 Pancreatic secretion;ko05014 Amyotrophic lateral sclerosis (ALS);ko05100 Bacterial invasion of epithelial cells;ko05120 Epithelial cell signaling in Helicobacter pylori infection;ko05131 Shigellosis;ko05132 Salmonella infection;ko05200 Pathways in cancer;ko05203 Viral carcinogenesis;ko05205 Proteoglycans in cancer;ko05210 Colorectal cancer;ko05211 Renal cell carcinoma;ko05212 Pancreatic
gene20981	K03247	EIF3H	translation initiation factor 3 subunit H	--	2	ko03013 RNA transport;ko05162 Measles;
gene20982	K01213	E3.2.1.67	galacturan 1,4-alpha-galacturonidase	EC:3.2.1.67	2	ko00040 Pentose and glucuronate interconversions;ko00500 Starch and sucrose metabolism;
gene20985	K02917	RP-L35Ae, RPL35A	large subunit ribosomal protein L35Ae	--	1	ko03010 Ribosome;
gene20986	K11643	CHD4, MI2B	chromodomain-helicase-DNA-binding protein 4	EC:3.6.4.12	1	ko05203 Viral carcinogenesis;
gene20991	K09286	EREBP	EREBP-like factor	--		
gene20993	K12180	COPS7, CSN7	COP9 signalosome complex subunit 7	--		
gene20994	K02365	ESP1	separase	EC:3.4.22.49	4	ko04110 Cell cycle;ko04111 Cell cycle - yeast;ko04113 Meiosis - yeast;ko04114 Oocyte meiosis;
gene20996	K10752	RBBP4, HAT2, CAF1, MIS16	histone-binding protein RBBP4	--		
gene20998	K07964	HPSE	heparanase 1	EC:3.2.1.166	2	ko00531 Glycosaminoglycan degradation;ko05205 Proteoglycans in cancer;
gene2099	K12450	RHM	UDP-glucose 4,6-dehydratase	EC:4.2.1.76	1	ko00520 Amino sugar and nucleotide sugar metabolism;
gene21000	K02267	COX6B	cytochrome c oxidase subunit 6b	--	6	ko00190 Oxidative phosphorylation;ko04260 Cardiac muscle contraction;ko04932 Non-alcoholic fatty liver disease (NAFLD);ko05010 Alzheimer's disease;ko05012 Parkinson's disease;ko05016 Huntington's disease;
gene21001	K06630	YWHAE	14-3-3 protein epsilon	--	8	ko04110 Cell cycle;ko04114 Oocyte meiosis;ko04151 PI3K-Akt signaling pathway;ko04390 Hippo signaling pathway;ko04391 Hippo signaling pathway - fly;ko04722 Neurotrophin signaling pathway;ko05169 Epstein-Barr virus infection;ko05203 Viral carcinogenesis;
gene21002	K14788	NOL10, ENP2	ribosome biogenesis protein ENP2	--		
gene21003	K12183	TSG101, STP22, VPS23	ESCRT-I complex subunit TSG101	--	1	ko04144 Endocytosis;
gene21005	K17086	TM9SF2_4	transmembrane 9 superfamily member 2/4	--		
gene21009	K01982	LSUrRNA	large subunit ribosomal RNA	--	2	ko03008 Ribosome biogenesis in eukaryotes;ko03010 Ribosome;
gene21010	K00784	rnz	ribonuclease Z	EC:3.1.26.11	1	ko03013 RNA transport;
gene21012	K14232	tRNA-Pro	tRNA Pro	--	1	ko00970 Aminoacyl-tRNA biosynthesis;
gene21014	K14232	tRNA-Pro	tRNA Pro	--	1	ko00970 Aminoacyl-tRNA biosynthesis;
gene21016	K14232	tRNA-Pro	tRNA Pro	--	1	ko00970 Aminoacyl-tRNA biosynthesis;
gene21017	K14232	tRNA-Pro	tRNA Pro	--	1	ko00970 Aminoacyl-tRNA biosynthesis;
gene21018	K09422	MYBP	myb proto-oncogene protein, plant	--		
gene21027	K12837	U2AF2	splicing factor U2AF 65 kDa subunit	--	1	ko03040 Spliceosome;
gene2103	K01307	GGH	gamma-glutamyl hydrolase	EC:3.4.19.9	1	ko00790 Folate biosynthesis;
gene21047	K02900	RP-L27Ae, RPL27A	large subunit ribosomal protein L27Ae	--	1	ko03010 Ribosome;
gene21048	K16903	TAA1	L-tryptophan---pyruvate aminotransferase	EC:2.6.1.99	1	ko00380 Tryptophan metabolism;
gene21049	K12393	AP1M	AP-1 complex subunit mu	--	1	ko04142 Lysosome;
gene2104	K03504	POLD3	DNA polymerase delta subunit 3	--	8	ko00230 Purine metabolism;ko00240 Pyrimidine metabolism;ko03030 DNA replication;ko03410 Base excision repair;ko03420 Nucleotide excision repair;ko03430 Mismatch repair;ko03440 Homologous recombination;ko05166 HTLV-I infection;

gene21051	K06067	HDAC1_2	histone deacetylase 1/2	EC:3.5.1.98	9	ko04110 Cell cycle;ko04330 Notch signaling pathway;ko05016 Huntington's disease;ko05034 Alcoholism;ko05169 Epstein-Barr virus infection;ko05200 Pathways in cancer;ko05202 Transcriptional misregulation in cancer;ko05203 Viral carcinogenesis;ko05220 Chronic myeloid leukemia;
gene21055	K10999	CESA	cellulose synthase A	EC:2.4.1.12		
gene2105	K10956	SEC61A	protein transport protein SEC61 subunit alpha	--	4	ko03060 Protein export;ko04141 Protein processing in endoplasmic reticulum;ko04145 Phagosome;ko05110 Vibrio cholerae infection;
gene21064	K11883	NOB1	RNA-binding protein NOB1	--	1	ko03008 Ribosome biogenesis in eukaryotes;
gene21065	K12235	SRR	serine racemase	EC:5.1.1.18	1	ko00260 Glycine, serine and threonine metabolism;
gene21069	K14773	UTP23	U3 small nucleolar RNA-associated protein 23	--		
gene2106	K03327	TC.MATE, SLC47A, norM, mdtK, dinF	multidrug resistance protein, MATE family	--		
gene21070	K09872	PIP	aquaporin PIP	--		
gene21077	K03686	dnaJ	molecular chaperone DnaJ	--		
gene2107	K03843	ALG2	alpha-1,3/alpha-1,6-mannosyltransferase	EC:2.4.1.132 2.4.1.257	2	ko00510 N-Glycan biosynthesis;ko00513 Various types of N-glycan biosynthesis;
gene21089	K04392	RAC1	Ras-related C3 botulinum toxin substrate 1	--	33	ko04010 MAPK signaling pathway;ko04014 Ras signaling pathway;ko04062 Chemokine signaling pathway;ko04145 Phagosome;ko04151 PI3K-Akt signaling pathway;ko04310 Wnt signaling pathway;ko04360 Axon guidance;ko04370 VEGF signaling pathway;ko04380 Osteoclast differentiation;ko04510 Focal adhesion;ko04520 Adherens junction;ko04620 Toll-like receptor signaling pathway;ko04650 Natural killer cell mediated cytotoxicity;ko04662 B cell receptor signaling pathway;ko04664 Fc epsilon RI signaling pathway;ko04666 Fc gamma R-mediated phagocytosis;ko04670 Leukocyte transendothelial migration;ko04722 Neurotrophin signaling pathway;ko04810 Regulation of actin cytoskeleton;ko04932 Non-alcoholic fatty liver disease (NAFLD);ko04972 Pancreatic secretion;ko05014 Amyotrophic lateral sclerosis (ALS);ko05100 Bacterial invasion of epithelial cells;ko05120 Epithelial cell signaling in Helicobacter pylori infection;ko05131 Shigellosis;ko05132 Salmonella infection;ko05200 Pathways in cancer;ko05203 Viral carcinogenesis;ko05205 Proteoglycans in cancer;ko05210 Colorectal cancer;ko05211 Renal cell carcinoma;ko05212 Pancreatic
gene21091	K03549	kup	KUP system potassium uptake protein	--		
gene21093	K12194	CHMP4, SNF7, VPS32	charged multivesicular body protein 4	--	1	ko04144 Endocytosis;
gene21095	K08967	mtnD, mtnZ, ADI1	1,2-dihydroxy-3-keto-5-methylthiopentene dioxygenase	EC:1.13.11.53 1.13.11.54	1	ko00270 Cysteine and methionine metabolism;
gene21097	K13448	CML	calcium-binding protein CML	--	1	ko04626 Plant-pathogen interaction;
gene21098	K13448	CML	calcium-binding protein CML	--	1	ko04626 Plant-pathogen interaction;
gene2109	K14677	ACY1	aminoacylase	EC:3.5.1.14	3	ko00330 Arginine and proline metabolism;ko01210 2-Oxocarboxylic acid metabolism;ko01230 Biosynthesis of amino acids;
gene210	K14721	RPC37, POLR3E	DNA-directed RNA polymerase III subunit RPC5	--	5	ko00230 Purine metabolism;ko00240 Pyrimidine metabolism;ko03020 RNA polymerase;ko04623 Cytosolic DNA-sensing pathway;ko05169 Epstein-Barr virus infection;
gene21101	K13719	OTU1, YOD1	ubiquitin thioesterase OTU1	EC:3.1.2.-	1	ko04141 Protein processing in endoplasmic reticulum;
gene21105	K11446	JARID1	histone demethylase JARID1	EC:1.14.11.-		
gene2110	K02931	RP-L5, MRPL5, rplE	large subunit ribosomal protein L5	--	1	ko03010 Ribosome;
gene21113	K08864	TLK	tousled-like kinase	EC:2.7.11.1		
gene21117	K02134	ATPeF1D, ATP5D	F-type H+-transporting ATPase subunit delta	EC:3.6.3.14	4	ko00190 Oxidative phosphorylation;ko05010 Alzheimer's disease;ko05012 Parkinson's disease;ko05016 Huntington's disease;
gene21118	K05868	CCNB	cyclin B	--	3	ko04110 Cell cycle;ko04115 p53 signaling pathway;ko04914 Progesterone-mediated oocyte maturation;
gene21119	K03327	TC.MATE, SLC47A, norM, mdtK, dinF	multidrug resistance protein, MATE family	--		
gene2111	K13192	RBM26	RNA-binding protein 26	--		
gene21122	K06941	rlmN	23S rRNA (adenine2503-C2)-methyltransferase	EC:2.1.1.192		
gene21123	K09550	PFDN4	prefoldin subunit 4	--		
gene21124	K03116	tatA	sec-independent protein translocase protein TatA	--	2	ko03060 Protein export;ko03070 Bacterial secretion system;

gene21125	K15156	MED14, RGR1	mediator of RNA polymerase II transcription subunit 14	--		
gene21127	K01728	E4.2.2.2, pel	pectate lyase	EC:4.2.2.2	1	ko00040 Pentose and glucuronate interconversions;
gene21132	K08869	ADCK, ABC1	aarF domain-containing kinase	--		
gene21136	K10999	CESA	cellulose synthase A	EC:2.4.1.12		
gene21137	K10999	CESA	cellulose synthase A	EC:2.4.1.12		
gene21142	K03327	TC.MATE, SLC47A, norM, mdtK, dinF	multidrug resistance protein, MATE family	--		
gene21146	K09422	MYBP	myb proto-oncogene protein, plant	--		
gene21147	K01409	gcp, QRI7, OSGEPL	O-sialoglycoprotein endopeptidase	EC:3.4.24.57		
gene21149	K02938	RP-L8e, RPL8	large subunit ribosomal protein L8e	--	1	ko03010 Ribosome;
gene21155	K01466	allB	allantoinase	EC:3.5.2.5	1	ko00230 Purine metabolism;
gene21157	K12592	C1D, LRP1	exosome complex protein LRP1	--	1	ko03018 RNA degradation;
gene21162	K14488	SAUR	SAUR family protein	--	1	ko04075 Plant hormone signal transduction;
gene21174	K15033	ICT1	peptidyl-tRNA hydrolase ICT1	EC:3.1.1.29		
gene2117	K03025	RPC34, POLR3F	DNA-directed RNA polymerase III subunit RPC6	--	5	ko00230 Purine metabolism;ko00240 Pyrimidine metabolism;ko03020 RNA polymerase;ko04623 Cytosolic DNA-sensing pathway;ko05169 Epstein-Barr virus infection;
gene21181	K17302	COPB2, SEC27	coatomer, subunit beta'	--		
gene21187	K02893	RP-L23Ae, RPL23A	large subunit ribosomal protein L23Ae	--	1	ko03010 Ribosome;
gene21189	K05605	HIBCH	3-hydroxyisobutyryl-CoA hydrolase	EC:3.1.2.4	3	ko00280 Valine, leucine and isoleucine degradation;ko00410 beta-Alanine metabolism;ko00640 Propanoate metabolism;
gene21195	K02872	RP-L13Ae, RPL13A	large subunit ribosomal protein L13Ae	--	1	ko03010 Ribosome;
gene21196	K01051	E3.1.1.11	pectinesterase	EC:3.1.1.11	2	ko00040 Pentose and glucuronate interconversions;ko00500 Starch and sucrose metabolism;
gene21198	K02518	infA	translation initiation factor IF-1	--		
gene21204	K02926	RP-L4, MRPL4, rplD	large subunit ribosomal protein L4	--	1	ko03010 Ribosome;
gene21206	K03686	dnaJ	molecular chaperone DnaJ	--		
gene21209	K03264	EIF6	translation initiation factor 6	--	1	ko03008 Ribosome biogenesis in eukaryotes;
gene21221	K01982	LSUrRNA	large subunit ribosomal RNA	--	2	ko03008 Ribosome biogenesis in eukaryotes;ko03010 Ribosome;
gene21224	K11446	JARID1	histone demethylase JARID1	EC:1.14.11.-		
gene21229	K16911	DDX21	ATP-dependent RNA helicase DDX21	EC:3.6.4.13		
gene21231	K13154	ZCRB1	U11/U12 small nuclear ribonucleoprotein 31 kDa protein	--		
gene21233	K08770	UBC	ubiquitin C	--	1	ko03320 PPAR signaling pathway;
gene21234	K05283	PIGW	phosphatidylinositol glycan, class W	EC:2.3.-.-	1	ko00563 Glycosylphosphatidylinositol(GPI)-anchor biosynthesis;
gene21237	K16276	K16276, BTS	zinc finger protein-like protein	--		
gene21238	K09872	PIP	aquaporin PIP	--		
gene2123	K09571	FKBP4_5	FK506-binding protein 4/5	EC:5.2.1.8	1	ko04915 Estrogen signaling pathway;
gene21243	K14802	DRS2, ATP8A	phospholipid-transporting ATPase	EC:3.6.3.1		
gene21245	K13993	HSP20	HSP20 family protein	--	1	ko04141 Protein processing in endoplasmic reticulum;
gene21247	K10754	RFC1	replication factor C subunit 1	--	3	ko03030 DNA replication;ko03420 Nucleotide excision repair;ko03430 Mismatch repair;
gene21248	K08237	E2.4.1.218	hydroquinone glucosyltransferase	EC:2.4.1.218		
gene2124	K14235	tRNA-Trp	tRNA Trp	--	1	ko00970 Aminoacyl-tRNA biosynthesis;
gene21255	K01507	ppa	inorganic pyrophosphatase	EC:3.6.1.1	1	ko00190 Oxidative phosphorylation;
gene21260	K16277	DRIP	E3 ubiquitin-protein ligase DRIP	EC:6.3.2.19		
gene21262	K00981	E2.7.7.41, CDS1, CDS2, cdsA	phosphatidate cytidylyltransferase	EC:2.7.7.41	2	ko00564 Glycerophospholipid metabolism;ko04070 Phosphatidylinositol signaling system;
gene21267	K10389	TUBG	tubulin gamma	--		
gene21268	K06130	LYPLA2	lysophospholipase II	EC:3.1.1.5	1	ko00564 Glycerophospholipid metabolism;
gene21269	K08907	LHCA1	light-harvesting complex I chlorophyll a/b binding protein 1	--	1	ko00196 Photosynthesis - antenna proteins;
gene21273	K01411	NRD1	nardilysin	EC:3.4.24.61		

gene21274	K04730	IRAK1	interleukin-1 receptor-associated kinase 1	EC:2.7.11.1	11	ko04064 NF-kappa B signaling pathway;ko04210 Apoptosis;ko04620 Toll-like receptor signaling pathway;ko04722 Neurotrophin signaling pathway;ko05133 Pertussis;ko05140 Leishmaniasis;ko05142 Chagas disease (American trypanosomiasis);ko05145 Toxoplasmosis;ko05152 Tuberculosis;ko05162 Measles;ko05169 Epstein-Barr virus infection;
gene21277	K01982	LSUrRNA	large subunit ribosomal RNA	--	2	ko03008 Ribosome biogenesis in eukaryotes;ko03010 Ribosome;
gene21279	K10808	RRM2	ribonucleoside-diphosphate reductase subunit M2	EC:1.17.4.1	4	ko00230 Purine metabolism;ko00240 Pyrimidine metabolism;ko00480 Glutathione metabolism;ko04115 p53 signaling pathway;
gene21280	K14516	ERF1	ethylene-responsive transcription factor 1	--	1	ko04075 Plant hormone signal transduction;
gene21282	K11095	SNRPC	U1 small nuclear ribonucleoprotein C	--	1	ko03040 Spliceosome;
gene21284	K02909	RP-L31, rpmE	large subunit ribosomal protein L31	--	1	ko03010 Ribosome;
gene21289	K02882	RP-L18Ae, RPL18A	large subunit ribosomal protein L18Ae	--	1	ko03010 Ribosome;
gene21290	K11279	NAP1L1, NRP	nucleosome assembly protein 1-like 1	--		
gene21295	K03452	MHX	magnesium/proton exchanger	--		
gene21298	K12890	SFRS1_9	splicing factor, arginine/serine-rich 1/9	--	2	ko03040 Spliceosome;ko05168 Herpes simplex infection;
gene2129	K03115	CSNK2B	casein kinase II subunit beta	--	9	ko03008 Ribosome biogenesis in eukaryotes;ko04064 NF-kappa B signaling pathway;ko04310 Wnt signaling pathway;ko04520 Adherens junction;ko04530 Tight junction;ko04712 Circadian rhythm - plant;ko05162 Measles;ko05168 Herpes simplex infection;ko05169 Epstein-Barr virus infection;
gene21300	K03242	EIF2S3	translation initiation factor 2 subunit 3	--	1	ko03013 RNA transport;
gene21301	K08678	UXS1	UDP-glucuronate decarboxylase	EC:4.1.1.35	2	ko00500 Starch and sucrose metabolism;ko00520 Amino sugar and nucleotide sugar metabolism;
gene2130	K11801	WDR23	WD repeat-containing protein 23	--		
gene21310	K00799	GST, gst	glutathione S-transferase	EC:2.5.1.18	4	ko00480 Glutathione metabolism;ko00980 Metabolism of xenobiotics by cytochrome P450;ko00982 Drug metabolism - cytochrome P450;ko05204 Chemical carcinogenesis;
gene21312	K02969	RP-S20e, RPS20	small subunit ribosomal protein S20e	--	1	ko03010 Ribosome;
gene21313	K12878	THOC1	THO complex subunit 1	--	2	ko03013 RNA transport;ko03040 Spliceosome;
gene21315	K07765	MBTPS2	S2P endopeptidase	EC:3.4.24.85	1	ko04141 Protein processing in endoplasmic reticulum;
gene21316	K10393	KIF2_24, MCAK	kinesin family member 2/24	--		
gene2131	K14235	tRNA-Trp	tRNA Trp	--	1	ko00970 Aminoacyl-tRNA biosynthesis;
gene21327	K05309	PTGES2	microsomal prostaglandin-E synthase 2	EC:5.3.99.3	1	ko00590 Arachidonic acid metabolism;
gene21336	K10753	ASF1	histone chaperone ASF1	--		
gene21338	K15707	RNF170	RING finger protein 170	--		
gene21346	K05391	CNGF	cyclic nucleotide gated channel, other eukaryote	--	1	ko04626 Plant-pathogen interaction;
gene21347	K02262	COX3	cytochrome c oxidase subunit 3	--	6	ko00190 Oxidative phosphorylation;ko04260 Cardiac muscle contraction;ko04932 Non-alcoholic fatty liver disease (NAFLD);ko05010 Alzheimer's disease;ko05012 Parkinson's disease;ko05016 Huntington's disease;
gene21348	K08334	BECN1, VPS30, ATG6	beclin 1	--	1	ko04140 Regulation of autophagy;
gene21350	K01528	DNM	dynamin GTPase	EC:3.6.5.5	4	ko04144 Endocytosis;ko04721 Synaptic vesicle cycle;ko04961 Endocrine and other factor-regulated calcium reabsorption;ko05100 Bacterial invasion of epithelial cells;
gene21353	K06268	PPP3R, CNB	serine/threonine-protein phosphatase 2B regulatory subunit	--	18	ko04010 MAPK signaling pathway;ko04020 Calcium signaling pathway;ko04114 Oocyte meiosis;ko04210 Apoptosis;ko04310 Wnt signaling pathway;ko04360 Axon guidance;ko04370 VEGF signaling pathway;ko04380 Osteoclast differentiation;ko04650 Natural killer cell mediated cytotoxicity;ko04660 T cell receptor signaling pathway;ko04662 B cell receptor signaling pathway;ko04720 Long-term potentiation;ko04724 Glutamatergic synapse;ko05010 Alzheimer's disease;ko05014 Amyotrophic lateral sclerosis (ALS);ko05031 Amphetamine addiction;ko05152
gene21355	K00889	E2.7.1.68, PIP5K	1-phosphatidylinositol-4-phosphate 5-kinase	EC:2.7.1.68	5	ko00562 Inositol phosphate metabolism;ko04070 Phosphatidylinositol signaling system;ko04144 Endocytosis;ko04666 Fc gamma R-mediated phagocytosis;ko04810 Regulation of actin cytoskeleton;
gene21355	K07910	RAB18	Ras-related protein Rab-18	--		

gene21356	K00889	E2.7.1.68, PIP5K	1-phosphatidylinositol-4-phosphate 5-kinase	EC:2.7.1.68	5	ko00562 Inositol phosphate metabolism;ko04070 Phosphatidylinositol signaling system;ko04144 Endocytosis;ko04666 Fc gamma R-mediated phagocytosis;ko04810 Regulation of actin cytoskeleton;
gene21357	K02893	RP-L23Ae, RPL23A	large subunit ribosomal protein L23Ae	--	1	ko03010 Ribosome;
gene21358	K10398	KIF11, EG5	kinesin family member 11	--		
gene21359	K07901	RAB8A, MEL	Ras-related protein Rab-8A	--	1	ko04972 Pancreatic secretion;
gene21361	K09264	K09264	MADS-box transcription factor, plant	--		
gene21362	K00847	E2.7.1.4, scrK	fructokinase	EC:2.7.1.4	3	ko00051 Fructose and mannose metabolism;ko00500 Starch and sucrose metabolism;ko00520 Amino sugar and nucleotide sugar metabolism;
gene21367	K07904	RAB11A	Ras-related protein Rab-11A	--	4	ko04144 Endocytosis;ko04961 Endocrine and other factor-regulated calcium reabsorption;ko04962 Vasopressin-regulated water reabsorption;ko04972 Pancreatic secretion;
gene21368	K12603	CNOT6, CCR4	CCR4-NOT transcription complex subunit 6	--	1	ko03018 RNA degradation;
gene21370	K04646	CLTC	clathrin heavy chain	--	6	ko04142 Lysosome;ko04144 Endocytosis;ko04721 Synaptic vesicle cycle;ko04961 Endocrine and other factor-regulated calcium reabsorption;ko05016 Huntington's disease;ko05100 Bacterial invasion of epithelial cells;
gene21371	K15501	PPP6R3, SAPS3	serine/threonine-protein phosphatase 6 regulatory subunit 3	--		
gene21375	K03231	EEF1A	elongation factor 1-alpha	--	2	ko03013 RNA transport;ko05134 Legionellosis;
gene21382	K00511	SQLE, ERG1	squalene monooxygenase	EC:1.14.13.132	1	ko00909 Sesquiterpenoid and triterpenoid biosynthesis;
gene21383	K13508	GPAT	glycerol-3-phosphate acyltransferase	EC:2.3.1.15	2	ko00561 Glycerolipid metabolism;ko00564 Glycerophospholipid metabolism;
gene21390	K14945	QKI	protein quaking	--		
gene21391	K01265	E3.4.11.18, map	methionyl aminopeptidase	EC:3.4.11.18		
gene21393	K04730	IRAK1	interleukin-1 receptor-associated kinase 1	EC:2.7.11.1	11	ko04064 NF-kappa B signaling pathway;ko04210 Apoptosis;ko04620 Toll-like receptor signaling pathway;ko04722 Neurotrophin signaling pathway;ko05133 Pertussis;ko05140 Leishmaniasis;ko05142 Chagas disease (American trypanosomiasis);ko05145 Toxoplasmosis;ko05152 Tuberculosis;ko05162 Measles;ko05169 Epstein-Barr virus infection;
gene21395	K08176	PHO84	MFS transporter, PHS family, inorganic phosphate transporter	--		
gene21396	K06269	PPP1C	serine/threonine-protein phosphatase PP1 catalytic subunit	EC:3.1.3.16	14	ko03015 mRNA surveillance pathway;ko04113 Meiosis - yeast;ko04114 Oocyte meiosis;ko04270 Vascular smooth muscle contraction;ko04390 Hippo signaling pathway;ko04510 Focal adhesion;ko04720 Long-term potentiation;ko04728 Dopaminergic synapse;ko04810 Regulation of actin cytoskeleton;ko04910 Insulin signaling pathway;ko05031 Amphetamine addiction;ko05034 Alcoholism;ko05168 Herpes simplex infection;ko05205 Proteoglycans in
gene21398	K11426	SMYD	SET and MYND domain-containing protein	--		
gene21399	K11426	SMYD	SET and MYND domain-containing protein	--		
gene2139	K05666	ABCC2	ATP-binding cassette, subfamily C (CFTR/MRP), member 2	--	2	ko02010 ABC transporters;ko04976 Bile secretion;
gene213	K13065	E2.3.1.133, HCT	shikimate O-hydroxycinnamoyltransferase	EC:2.3.1.133	3	ko00940 Phenylpropanoid biosynthesis;ko00941 Flavonoid biosynthesis;ko00945 Stilbenoid, diarylheptanoid and gingerol biosynthesis;
gene21400	K11426	SMYD	SET and MYND domain-containing protein	--		
gene21411	K13172	SRRM2, SRM300	serine/arginine repetitive matrix protein 2	--		
gene2141	K00419	QCR9, UCRC	ubiquinol-cytochrome c reductase subunit 9	--	6	ko00190 Oxidative phosphorylation;ko04260 Cardiac muscle contraction;ko04932 Non-alcoholic fatty liver disease (NAFLD);ko05010 Alzheimer's disease;ko05012 Parkinson's disease;ko05016 Huntington's disease;
gene21428	K00921	PIKFYVE, FAB1	1-phosphatidylinositol-3-phosphate 5-kinase	EC:2.7.1.150	4	ko00562 Inositol phosphate metabolism;ko04070 Phosphatidylinositol signaling system;ko04145 Phagosome;ko04810 Regulation of actin cytoskeleton;
gene2142	K03531	ftsZ	cell division protein FtsZ	--	1	ko04112 Cell cycle - Caulobacter;
gene21442	K07943	ARL2	ADP-ribosylation factor-like protein 2	--		
gene21443	K13416	BAK1	brassinosteroid insensitive 1-associated receptor kinase 1	EC:2.7.10.1 2.7.11.1	2	ko04075 Plant hormone signal transduction;ko04626 Plant-pathogen interaction;

gene21444	K01354	ptrB	oligopeptidase B	EC:3.4.21.83	2	ko05142 Chagas disease (American trypanosomiasis);ko05143 African trypanosomiasis;
gene21447	K01823	idi, IDI	isopentenyl-diphosphate delta-isomerase	EC:5.3.3.2	1	ko00900 Terpenoid backbone biosynthesis;
gene2144	K15397	KCS	3-ketoacyl-CoA synthase	EC:2.3.1.199	1	ko00062 Fatty acid elongation;
gene21450	K14326	UPF1, RENT1	regulator of nonsense transcripts 1	EC:3.6.4.-	2	ko03013 RNA transport;ko03015 mRNA surveillance pathway;
gene21458	K15305	VAC14, TAX1BP2	vacuole morphology and inheritance protein 14	--	2	ko05166 HTLV-I infection;ko05203 Viral carcinogenesis;
gene21464	K15777	DOPA	4,5-DOPA dioxygenase extradiol	EC:1.13.11.-	1	ko00965 Betalain biosynthesis;
gene21470	K11649	SMARCC	SWI/SNF related-matrix-associated actin-dependent regulator of chromatin subfamily C	--		
gene21471	K07897	RAB7A	Ras-related protein Rab-7A	--	5	ko04144 Endocytosis;ko04145 Phagosome;ko05132 Salmonella infection;ko05146 Amoebiasis;ko05152 Tuberculosis;
gene21475	K10704	UBE2V	ubiquitin-conjugating enzyme E2 variant	--		
gene21476	K00434	E1.11.1.11	L-ascorbate peroxidase	EC:1.11.1.11	2	ko00053 Ascorbate and aldarate metabolism;ko00480 Glutathione metabolism;
gene21477	K12581	CNOT7_8, CAF1, POP2	CCR4-NOT transcription complex subunit 7/8	--	1	ko03018 RNA degradation;
gene21480	K03327	TC.MATE, SLC47A, norM, mdtK, dinF	multidrug resistance protein, MATE family	--		
gene21488	K17361	ACOT9	acyl-coenzyme A thioesterase 9	EC:3.1.2.-		
gene21493	K00261	GLUD1_2, gdhA	glutamate dehydrogenase (NAD(P)+)	EC:1.4.1.3	5	ko00250 Alanine, aspartate and glutamate metabolism;ko00330 Arginine and proline metabolism;ko00471 D-Glutamine and D-glutamate metabolism;ko00910 Nitrogen metabolism;ko04964 Proximal tubule bicarbonate reclamation;
gene21497	K14516	ERF1	ethylene-responsive transcription factor 1	--	1	ko04075 Plant hormone signal transduction;
gene2149	K04733	IRAK4	interleukin-1 receptor-associated kinase 4	EC:2.7.11.1	11	ko04064 NF-kappa B signaling pathway;ko04210 Apoptosis;ko04620 Toll-like receptor signaling pathway;ko04722 Neurotrophin signaling pathway;ko05133 Pertussis;ko05140 Leishmaniasis;ko05142 Chagas disease (American trypanosomiasis);ko05145 Toxoplasmosis;ko05152 Tuberculosis;ko05162 Measles;ko05164 Influenza A;
gene21504	K11437	PRMT6	protein arginine N-methyltransferase 6	EC:2.1.1.-		
gene21510	K02553	rraA, menG	regulator of ribonuclease activity A	--		
gene21511	K12179	COPS6, CSN6	COP9 signalosome complex subunit 6	--		
gene21512	K07437	CYP26A	cytochrome P450, family 26, subfamily A	--	1	ko00830 Retinol metabolism;
gene21516	K01979	SSUrRNA	small subunit ribosomal RNA	--	2	ko03008 Ribosome biogenesis in eukaryotes;ko03010 Ribosome;
gene21519	K14964	ASH2	Set1/Ash2 histone methyltransferase complex subunit ASH2	--		
gene21520	K05909	E1.10.3.2				
gene21523	K11293	HIRA, HIR1	protein HIRA/HIR1	--		
gene21527	K05765	CFL	cofilin	--	4	ko04360 Axon guidance;ko04666 Fc gamma R-mediated phagocytosis;ko04810 Regulation of actin cytoskeleton;ko05133 Pertussis;
gene21528	K13667	RUMI, KTELC1	protein glucosyltransferase	EC:2.4.1.-	1	ko00514 Other types of O-glycan biosynthesis;
gene2152	K11699	RDR1, RDP1	RNA-dependent RNA polymerase 1	EC:2.7.7.48		
gene21538	K00036	G6PD, zwf	glucose-6-phosphate 1-dehydrogenase	EC:1.1.1.49	3	ko00030 Pentose phosphate pathway;ko00480 Glutathione metabolism;ko01200 Carbon metabolism;
gene21545	K00430	E1.11.1.7	peroxidase	EC:1.11.1.7	2	ko00360 Phenylalanine metabolism;ko00940 Phenylpropanoid biosynthesis;
gene21546	K00430	E1.11.1.7	peroxidase	EC:1.11.1.7	2	ko00360 Phenylalanine metabolism;ko00940 Phenylpropanoid biosynthesis;
gene21551	K07375	TUBB	tubulin beta	--	3	ko04145 Phagosome;ko04540 Gap junction;ko05130 Pathogenic Escherichia coli infection;
gene21562	K07297	ADIPOR	adiponectin receptor	--	2	ko04920 Adipocytokine signaling pathway;ko04932 Non-alcoholic fatty liver disease (NAFLD);
gene21564	K01057	PGLS, pgl, devB	6-phosphogluconolactonase	EC:3.1.1.31	2	ko00030 Pentose phosphate pathway;ko01200 Carbon metabolism;
gene21567	K03020	RPC19, POLR1D	DNA-directed RNA polymerases I and III subunit RPAC2	--	5	ko00230 Purine metabolism;ko00240 Pyrimidine metabolism;ko03020 RNA polymerase;ko04623 Cytosolic DNA-sensing pathway;ko05169 Epstein-Barr virus infection;

gene21569	K04733	IRAK4	interleukin-1 receptor-associated kinase 4	EC:2.7.11.1	11	ko04064 NF-kappa B signaling pathway;ko04210 Apoptosis;ko04620 Toll-like receptor signaling pathway;ko04722 Neurotrophin signaling pathway;ko05133 Pertussis;ko05140 Leishmaniasis;ko05142 Chagas disease (American trypanosomiasis);ko05145 Toxoplasmosis;ko05152 Tuberculosis;ko05162 Measles;ko05164 Influenza A;
gene21573	K04733	IRAK4	interleukin-1 receptor-associated kinase 4	EC:2.7.11.1	11	ko04064 NF-kappa B signaling pathway;ko04210 Apoptosis;ko04620 Toll-like receptor signaling pathway;ko04722 Neurotrophin signaling pathway;ko05133 Pertussis;ko05140 Leishmaniasis;ko05142 Chagas disease (American trypanosomiasis);ko05145 Toxoplasmosis;ko05152 Tuberculosis;ko05162 Measles;ko05164 Influenza A;
gene21574	K08245	E3.4.23.40	phytepsin	EC:3.4.23.40		
gene21575	K01087	otsB	trehalose 6-phosphate phosphatase	EC:3.1.3.12	1	ko00500 Starch and sucrose metabolism;
gene2157	K01193	E3.2.1.26, sacA	beta-fructofuranosidase	EC:3.2.1.26	2	ko00052 Galactose metabolism;ko00500 Starch and sucrose metabolism;
gene21581	K17506	PPM1L, PP2CE	protein phosphatase 1L	EC:3.1.3.16		
gene21589	K00485	E1.14.13.8	dimethylaniline monooxygenase (N-oxide forming)	EC:1.14.13.8	2	ko00680 Methane metabolism;ko00982 Drug metabolism - cytochrome P450;
gene21594	K14500	BSK	BR-signaling kinase	EC:2.7.11.1	1	ko04075 Plant hormone signal transduction;
gene2159	K02955	RP-S14e, RPS14	small subunit ribosomal protein S14e	--	1	ko03010 Ribosome;
gene215	K08794	CAMK1	calcium/calmodulin-dependent protein kinase I	EC:2.7.11.17		
gene21600	K01530	E3.6.3.1	phospholipid-translocating ATPase	EC:3.6.3.1		
gene21602	K00028	E1.1.1.39	malate dehydrogenase (decarboxylating)	EC:1.1.1.39	3	ko00620 Pyruvate metabolism;ko00710 Carbon fixation in photosynthetic organisms;ko01200 Carbon metabolism;
gene21603	K13679	WAXY	granule-bound starch synthase	EC:2.4.1.242		
gene21606	K00857	E2.7.1.21, tdk	thymidine kinase	EC:2.7.1.21	2	ko00240 Pyrimidine metabolism;ko00983 Drug metabolism - other enzymes;
gene21614	K12816	CDC40, PRP17	pre-mRNA-processing factor 17	--	1	ko03040 Spliceosome;
gene21615	K07952	ARFRP1	ADP-ribosylation factor related protein 1	--		
gene2161	K14754	MX1	interferon-induced GTP-binding protein Mx1	--	2	ko05162 Measles;ko05164 Influenza A;
gene21620	K01881	PARS, proS	prolyl-tRNA synthetase	EC:6.1.1.15	1	ko00970 Aminoacyl-tRNA biosynthesis;
gene21621	K13508	GPAT	glycerol-3-phosphate acyltransferase	EC:2.3.1.15	2	ko00561 Glycerolipid metabolism;ko00564 Glycerophospholipid metabolism;
gene21622	K12824	TCERG1, CA150	transcription elongation regulator 1	--	1	ko03040 Spliceosome;
gene21627	K00454	LOX2S	lipoxygenase	EC:1.13.11.12	2	ko00591 Linoleic acid metabolism;ko00592 alpha-Linolenic acid metabolism;
gene21628	K00454	LOX2S	lipoxygenase	EC:1.13.11.12	2	ko00591 Linoleic acid metabolism;ko00592 alpha-Linolenic acid metabolism;
gene21629	K00454	LOX2S	lipoxygenase	EC:1.13.11.12	2	ko00591 Linoleic acid metabolism;ko00592 alpha-Linolenic acid metabolism;
gene21630	K01979	SSUrRNA	small subunit ribosomal RNA	--	2	ko03008 Ribosome biogenesis in eukaryotes;ko03010 Ribosome;
gene21631	K08835	OXSR1, STK39	serine/threonine-protein kinase OSR1/STK39	EC:2.7.11.1		
gene21634	K09680	coaW	type II pantothenate kinase	EC:2.7.1.33	1	ko00770 Pantothenate and CoA biosynthesis;
gene21641	K12741	HNRNPA1_3	heterogeneous nuclear ribonucleoprotein A1/A3	--	1	ko03040 Spliceosome;
gene21654	K14555	UTP13, TBL3	U3 small nucleolar RNA-associated protein 13	--	1	ko03008 Ribosome biogenesis in eukaryotes;
gene21655	K09422	MYBP	myb proto-oncogene protein, plant	--		
gene21656	K01051	E3.1.1.11	pectinesterase	EC:3.1.1.11	2	ko00040 Pentose and glucuronate interconversions;ko00500 Starch and sucrose metabolism;
gene21657	K02958	RP-S15e, RPS15	small subunit ribosomal protein S15e	--	1	ko03010 Ribosome;
gene21658	K15032	MTERFD	mTERF domain-containing protein, mitochondrial	--		
gene21659	K15032	MTERFD	mTERF domain-containing protein, mitochondrial	--		
gene21665	K15889	PCME	prenylcysteine alpha-carboxyl methylesterase	EC:3.1.1.-	1	ko00900 Terpenoid backbone biosynthesis;
gene21669	K14944	NOVA	RNA-binding protein Nova	--		
gene21671	K01213	E3.2.1.67	galacturan 1,4-alpha-galacturonidase	EC:3.2.1.67	2	ko00040 Pentose and glucuronate interconversions;ko00500 Starch and sucrose metabolism;
gene21672	K08678	UXS1	UDP-glucuronate decarboxylase	EC:4.1.1.35	2	ko00500 Starch and sucrose metabolism;ko00520 Amino sugar and nucleotide sugar metabolism;
gene21674	K12862	PLRG1, PRL1, PRP46	pleiotropic regulator 1	--	1	ko03040 Spliceosome;
gene21675	K12862	PLRG1, PRL1, PRP46	pleiotropic regulator 1	--	1	ko03040 Spliceosome;
gene21680	K08492	STX18	syntaxin 18	--	2	ko04130 SNARE interactions in vesicular transport;ko04145 Phagosome;
gene21685	K00705	malQ	4-alpha-glucanotransferase	EC:2.4.1.25	1	ko00500 Starch and sucrose metabolism;

gene21686	K12845	SNU13, NHP2L	U4/U6 small nuclear ribonucleoprotein SNU13	--	2	ko03008 Ribosome biogenesis in eukaryotes;ko03040 Spliceosome;
gene21698	K01599	hemE, UROD	uroporphyrinogen decarboxylase	EC:4.1.1.37	1	ko00860 Porphyrin and chlorophyll metabolism;
gene21699	K07198	PRKAA, AMPK	5'-AMP-activated protein kinase, catalytic alpha subunit	EC:2.7.11.11	8	ko04140 Regulation of autophagy;ko04150 mTOR signaling pathway;ko04151 PI3K-Akt signaling pathway;ko04710 Circadian rhythm;ko04910 Insulin signaling pathway;ko04920 Adipocytokine signaling pathway;ko04932 Non-alcoholic fatty liver disease (NAFLD);ko05410 Hypertrophic cardiomyopathy (HCM);
gene2169	K02883	RP-L18e, RPL18	large subunit ribosomal protein L18e	--	1	ko03010 Ribosome;
gene21701	K00767	nadC, QPRT	nicotinate-nucleotide pyrophosphorylase (carboxylating)	EC:2.4.2.19	1	ko00760 Nicotinate and nicotinamide metabolism;
gene21702	K00767	nadC, QPRT	nicotinate-nucleotide pyrophosphorylase (carboxylating)	EC:2.4.2.19	1	ko00760 Nicotinate and nicotinamide metabolism;
gene2170	K07575	K07575	PUA domain protein	--		
gene21710	K01802	E5.2.1.8	peptidylprolyl isomerase	EC:5.2.1.8		
gene21715	K06875	PDCD5, TFAR19	programmed cell death protein 5	--		
gene21716	K02894	RP-L23e, RPL23	large subunit ribosomal protein L23e	--	1	ko03010 Ribosome;
gene21721	K12599	SKI2, SKIV2L	antiviral helicase SKI2	EC:3.6.4.-	1	ko03018 RNA degradation;
gene21726	K02108	ATPF0A, atpB	F-type H+-transporting ATPase subunit a	EC:3.6.3.14	2	ko00190 Oxidative phosphorylation;ko00195 Photosynthesis;
gene21729	K01895	ACSS, acs	acetyl-CoA synthetase	EC:6.2.1.1	6	ko00010 Glycolysis / Gluconeogenesis;ko00620 Pyruvate metabolism;ko00640 Propanoate metabolism;ko00680 Methane metabolism;ko00720 Carbon fixation pathways in prokaryotes;ko01200 Carbon metabolism;
gene21730	K09566	PPIG	peptidyl-prolyl isomerase G (cyclophilin G)	EC:5.2.1.8		
gene21733	K03217	yidC, spoIIIJ, OXA1	preprotein translocase subunit YidC	--	2	ko03060 Protein export;ko03070 Bacterial secretion system;
gene21738	K12844	PRPF31	U4/U6 small nuclear ribonucleoprotein PRP31	--	1	ko03040 Spliceosome;
gene21741	K00232	E1.3.3.6, ACOX1, ACOX3	acyl-CoA oxidase	EC:1.3.3.6	5	ko00071 Fatty acid degradation;ko00592 alpha-Linolenic acid metabolism;ko01040 Biosynthesis of unsaturated fatty acids;ko03320 PPAR signaling pathway;ko04146 Peroxisome;
gene21742	K00232	E1.3.3.6, ACOX1, ACOX3	acyl-CoA oxidase	EC:1.3.3.6	5	ko00071 Fatty acid degradation;ko00592 alpha-Linolenic acid metabolism;ko01040 Biosynthesis of unsaturated fatty acids;ko03320 PPAR signaling pathway;ko04146 Peroxisome;
gene21747	K09422	MYBP	myb proto-oncogene protein, plant	--		
gene21749	K04730	IRAK1	interleukin-1 receptor-associated kinase 1	EC:2.7.11.1	11	ko04064 NF-kappa B signaling pathway;ko04210 Apoptosis;ko04620 Toll-like receptor signaling pathway;ko04722 Neurotrophin signaling pathway;ko05133 Pertussis;ko05140 Leishmaniasis;ko05142 Chagas disease (American trypanosomiasis);ko05145 Toxoplasmosis;ko05152 Tuberculosis;ko05162 Measles;ko05169 Epstein-Barr virus infection;
gene21751	K09287	RAV	RAV-like factor	--		
gene21753	K16297	SCPL-II	serine carboxypeptidase-like clade II	EC:3.4.16.-		
gene21757	K15133	MED17	mediator of RNA polymerase II transcription subunit 17	--		
gene21763	K06966	K06966				
gene21766	K03129	TAF4	transcription initiation factor TFIID subunit 4	--	3	ko03022 Basal transcription factors;ko05016 Huntington's disease;ko05168 Herpes simplex infection;
gene21767	K11804	WDR42A	WD repeat-containing protein 42A	--		
gene21770	K10704	UBE2V	ubiquitin-conjugating enzyme E2 variant	--		
gene21771	K16675	ZDHHC14	probable palmitoyltransferase ZDHHC14	EC:2.3.1.-	1	ko04391 Hippo signaling pathway - fly;
gene21772	K03035	PSMD12, RPN5	26S proteasome regulatory subunit N5	--	2	ko03050 Proteasome;ko05169 Epstein-Barr virus infection;
gene21773	K09286	EREBP	EREBP-like factor	--		
gene21774	K10807	RRM1	ribonucleoside-diphosphate reductase subunit M1	EC:1.17.4.1	3	ko00230 Purine metabolism;ko00240 Pyrimidine metabolism;ko00480 Glutathione metabolism;

gene21775	K04392	RAC1	Ras-related C3 botulinum toxin substrate 1	--	33	ko04010 MAPK signaling pathway;ko04014 Ras signaling pathway;ko04062 Chemokine signaling pathway;ko04145 Phagosome;ko04151 PI3K-Akt signaling pathway;ko04310 Wnt signaling pathway;ko04360 Axon guidance;ko04370 VEGF signaling pathway;ko04380 Osteoclast differentiation;ko04510 Focal adhesion;ko04520 Adherens junction;ko04620 Toll-like receptor signaling pathway;ko04650 Natural killer cell mediated cytotoxicity;ko04662 B cell receptor signaling pathway;ko04664 Fc epsilon RI signaling pathway;ko04666 Fc gamma R-mediated phagocytosis;ko04670 Leukocyte transendothelial migration;ko04722 Neurotrophin signaling pathway;ko04810 Regulation of actin cytoskeleton;ko04932 Non-alcoholic fatty liver disease (NAFLD);ko04972 Pancreatic secretion;ko05014 Amyotrophic lateral sclerosis (ALS);ko05100 Bacterial invasion of epithelial cells;ko05120 Epithelial cell signaling in Helicobacter pylori infection;ko05131 Shigellosis;ko05132 Salmonella infection;ko05200 Pathways in cancer;ko05203 Viral carcinogenesis;ko05205 Proteoglycans in cancer;ko05210 Colorectal cancer;ko05211 Renal cell carcinoma;ko05212 Pancreatic
gene21778	K03259	EIF4E	translation initiation factor 4E	--	5	ko03013 RNA transport;ko04066 HIF-1 signaling pathway;ko04150 mTOR signaling pathway;ko04151 PI3K-Akt signaling pathway;ko04910 Insulin signaling pathway;
gene21783	K01762	ACS	1-aminocyclopropane-1-carboxylate synthase	EC:4.4.1.14	1	ko00270 Cysteine and methionine metabolism;
gene21784	K01770	ispF	2-C-methyl-D-erythritol 2,4-cyclodiphosphate synthase	EC:4.6.1.12	1	ko00900 Terpenoid backbone biosynthesis;
gene21785	K11135	PINX1	Pin2-interacting protein X1	--		
gene21788	K03327	TC.MATE, SLC47A, norM, mdtK, dinF	multidrug resistance protein, MATE family	--		
gene21790	K14488	SAUR	SAUR family protein	--	1	ko04075 Plant hormone signal transduction;
gene21791	K00140	mmsA, iolA, ALDH6A1	malonate-semialdehyde dehydrogenase (acetylating) / methylmalonate-semialdehyde dehydrogenase	EC:1.2.1.18 1.2.1.27	4	ko00280 Valine, leucine and isoleucine degradation;ko00410 beta-Alanine metabolism;ko00562 Inositol phosphate metabolism;ko00640 Propanoate metabolism;
gene21792	K00140	mmsA, iolA, ALDH6A1	malonate-semialdehyde dehydrogenase (acetylating) / methylmalonate-semialdehyde dehydrogenase	EC:1.2.1.18 1.2.1.27	4	ko00280 Valine, leucine and isoleucine degradation;ko00410 beta-Alanine metabolism;ko00562 Inositol phosphate metabolism;ko00640 Propanoate metabolism;
gene21794	K04706	PIAS1	E3 SUMO-protein ligase PIAS1	EC:6.3.2.-	3	ko04120 Ubiquitin mediated proteolysis;ko04630 Jak-STAT signaling pathway;ko05160 Hepatitis C;
gene21803	K02898	RP-L26e, RPL26	large subunit ribosomal protein L26e	--	1	ko03010 Ribosome;
gene21804	K12883	NCBP2, CBP20	nuclear cap-binding protein subunit 2	--	3	ko03013 RNA transport;ko03015 mRNA surveillance pathway;ko03040 Spliceosome;
gene21806	K04567	KARS, lysS	lysyl-tRNA synthetase, class II	EC:6.1.1.6	1	ko00970 Aminoacyl-tRNA biosynthesis;
gene2180	K05909	E1.10.3.2				
gene2181	K03083	GSK3B	glycogen synthase kinase 3 beta	EC:2.7.11.26	29	ko04012 ErbB signaling pathway;ko04062 Chemokine signaling pathway;ko04110 Cell cycle;ko04151 PI3K-Akt signaling pathway;ko04310 Wnt signaling pathway;ko04340 Hedgehog signaling pathway;ko04360 Axon guidance;ko04390 Hippo signaling pathway;ko04510 Focal adhesion;ko04660 T cell receptor signaling pathway;ko04662 B cell receptor signaling pathway;ko04711 Circadian rhythm - fly;ko04722 Neurotrophin signaling pathway;ko04728 Dopaminergic synapse;ko04910 Insulin signaling pathway;ko04916 Melanogenesis;ko04917 Prolactin signaling pathway;ko04932 Non-alcoholic fatty liver disease (NAFLD);ko05010 Alzheimer's disease;ko05160 Hepatitis C;ko05162 Measles;ko05164 Influenza A;ko05166 HTLV-I infection;ko05169 Epstein-Barr virus infection;ko05200 Pathways in cancer;ko05210 Colorectal cancer;ko05213 Endometrial cancer;ko05215 Prostate
gene21822	K03248	EIF3G	translation initiation factor 3 subunit G	--	1	ko03013 RNA transport;
gene21823	K11247	SH3GL	endophilin-A	--	1	ko04144 Endocytosis;
gene21825	K05909	E1.10.3.2				
gene2182	K03655	recG	ATP-dependent DNA helicase RecG	EC:3.6.4.12	1	ko03440 Homologous recombination;
gene21831	K11094	SNRPB2	U2 small nuclear ribonucleoprotein B''	--	1	ko03040 Spliceosome;
gene21832	K17408	DAP3, MRPS29	small subunit ribosomal protein S29, mitochondrial	--		
gene21836	K00574	E2.1.1.79, cfa	cyclopropane-fatty-acyl-phospholipid synthase	EC:2.1.1.79		

gene21839	K12854	SNRNP200, BRR2	pre-mRNA-splicing helicase BRR2	EC:3.6.4.13	1	ko03040 Spliceosome;
gene2183	K09645	CPVL	vitellogenic carboxypeptidase-like protein	EC:3.4.16.-		
gene21841	K09008	K09008	hypothetical protein	--		
gene21842	K09667	OGT	polypeptide N-acetylglucosaminyltransferase	EC:2.4.1.-	1	ko00514 Other types of O-glycan biosynthesis;
gene21843	K02257	COX10	protoheme IX farnesyltransferase	EC:2.5.1.-	2	ko00190 Oxidative phosphorylation;ko00860 Porphyrin and chlorophyll metabolism;
gene21846	K14182	E4.2.3.21	vetispiradiene synthase	EC:4.2.3.21	1	ko00909 Sesquiterpenoid and triterpenoid biosynthesis;
gene21847	K14182	E4.2.3.21	vetispiradiene synthase	EC:4.2.3.21	1	ko00909 Sesquiterpenoid and triterpenoid biosynthesis;
gene21848	K14182	E4.2.3.21	vetispiradiene synthase	EC:4.2.3.21	1	ko00909 Sesquiterpenoid and triterpenoid biosynthesis;
gene2184	K09645	CPVL	vitellogenic carboxypeptidase-like protein	EC:3.4.16.-		
gene21858	K12896	SFRS7	splicing factor, arginine/serine-rich 7	--	2	ko03040 Spliceosome;ko05168 Herpes simplex infection;
gene2185	K15032	MTERFD	mTERF domain-containing protein, mitochondrial	--		
gene21861	K02518	infA	translation initiation factor IF-1	--		
gene21863	K05285	PIGN	phosphatidylinositol glycan, class N	EC:2.7.-.-	1	ko00563 Glycosylphosphatidylinositol(GPI)-anchor biosynthesis;
gene21864	K03868	RBX1, ROC1	RING-box protein 1	--	12	ko03420 Nucleotide excision repair;ko04066 HIF-1 signaling pathway;ko04110 Cell cycle;ko04111 Cell cycle - yeast;ko04114 Oocyte meiosis;ko04120 Ubiquitin mediated proteolysis;ko04141 Protein processing in endoplasmic reticulum;ko04310 Wnt signaling pathway;ko04350 TGF-beta signaling pathway;ko04710 Circadian rhythm;ko05200 Pathways in cancer;ko05211 Renal cell carcinoma;
gene21865	K02435	gatC	aspartyl-tRNA(Asn)/glutamyl-tRNA(Gln) amidotransferase subunit C	EC:6.3.5.6 6.3.5.7	1	ko00970 Aminoacyl-tRNA biosynthesis;
gene21871	K03231	EEF1A	elongation factor 1-alpha	--	2	ko03013 RNA transport;ko05134 Legionellosis;
gene21875	K00164	OGDH, sucA	2-oxoglutarate dehydrogenase E1 component	EC:1.2.4.2	4	ko00020 Citrate cycle (TCA cycle);ko00310 Lysine degradation;ko00380 Tryptophan metabolism;ko01200 Carbon metabolism;
gene21877	K07901	RAB8A, MEL	Ras-related protein Rab-8A	--	1	ko04972 Pancreatic secretion;
gene2187	K15336	TRDMT1, DNMT2	tRNA (cytosine38-C5)-methyltransferase	EC:2.1.1.204		
gene21881	K03126	TAF12	transcription initiation factor TFIID subunit 12	--	1	ko03022 Basal transcription factors;
gene21882	K01240	URH1	uridine nucleosidase	EC:3.2.2.3	2	ko00240 Pyrimidine metabolism;ko00760 Nicotinate and nicotinamide metabolism;
gene21886	K15559	RTT103	regulator of Ty1 transposition protein 103	--		
gene21888	K14538	NUG1, GNL3	nuclear GTP-binding protein	--	1	ko03008 Ribosome biogenesis in eukaryotes;
gene21893	K17361	ACOT9	acyl-coenzyme A thioesterase 9	EC:3.1.2.-		
gene21895	K13691	SGT1	pathogen-inducible salicylic acid glucosyltransferase	EC:2.4.1.-		
gene21898	K17479	GRXCR1	glutaredoxin domain-containing cysteine-rich protein 1	--		
gene21901	K09503	DNAJA2	DnaJ homolog subfamily A member 2	--	1	ko04141 Protein processing in endoplasmic reticulum;
gene21906	K14220	tRNA-Asn	tRNA Asn	--	1	ko00970 Aminoacyl-tRNA biosynthesis;
gene21914	K00817	hisC	histidinol-phosphate aminotransferase	EC:2.6.1.9	7	ko00340 Histidine metabolism;ko00350 Tyrosine metabolism;ko00360 Phenylalanine metabolism;ko00400 Phenylalanine, tyrosine and tryptophan biosynthesis;ko00401 Novobiocin biosynthesis;ko00960 Tropane, piperidine and pyridine alkaloid biosynthesis;ko01230 Biosynthesis of amino acids;
gene21921	K14498	SNRK2	serine/threonine-protein kinase SRK2	EC:2.7.11.1	1	ko04075 Plant hormone signal transduction;
gene21926	K03113	EIF1, SUI1	translation initiation factor 1	--	1	ko03013 RNA transport;
gene21927	K08770	UBC	ubiquitin C	--	1	ko03320 PPAR signaling pathway;
gene21936	K14721	RPC37, POLR3E	DNA-directed RNA polymerase III subunit RPC5	--	5	ko00230 Purine metabolism;ko00240 Pyrimidine metabolism;ko03020 RNA polymerase;ko04623 Cytosolic DNA-sensing pathway;ko05169 Epstein-Barr virus infection;
gene21937	K12837	U2AF2	splicing factor U2AF 65 kDa subunit	--	1	ko03040 Spliceosome;
gene21939	K08737	MSH6	DNA mismatch repair protein MSH6	--	3	ko03430 Mismatch repair;ko05200 Pathways in cancer;ko05210 Colorectal cancer;
gene21941	K14512	MPK6	mitogen-activated protein kinase 6	EC:2.7.11.24	1	ko04075 Plant hormone signal transduction;
gene21949	K09422	MYBP	myb proto-oncogene protein, plant	--		
gene21950	K10867	NBN, NBS1	nijmegen breakage syndrome protein 1	--	1	ko03440 Homologous recombination;

gene21951	K04730	IRAK1	interleukin-1 receptor-associated kinase 1	EC:2.7.11.1	11	ko04064 NF-kappa B signaling pathway;ko04210 Apoptosis;ko04620 Toll-like receptor signaling pathway;ko04722 Neurotrophin signaling pathway;ko05133 Pertussis;ko05140 Leishmaniasis;ko05142 Chagas disease (American trypanosomiasis);ko05145 Toxoplasmosis;ko05152 Tuberculosis;ko05162 Measles;ko05169 Epstein-Barr virus infection;
gene21952	K04730	IRAK1	interleukin-1 receptor-associated kinase 1	EC:2.7.11.1	11	ko04064 NF-kappa B signaling pathway;ko04210 Apoptosis;ko04620 Toll-like receptor signaling pathway;ko04722 Neurotrophin signaling pathway;ko05133 Pertussis;ko05140 Leishmaniasis;ko05142 Chagas disease (American trypanosomiasis);ko05145 Toxoplasmosis;ko05152 Tuberculosis;ko05162 Measles;ko05169 Epstein-Barr virus infection;
gene21953	K04733	IRAK4	interleukin-1 receptor-associated kinase 4	EC:2.7.11.1	11	ko04064 NF-kappa B signaling pathway;ko04210 Apoptosis;ko04620 Toll-like receptor signaling pathway;ko04722 Neurotrophin signaling pathway;ko05133 Pertussis;ko05140 Leishmaniasis;ko05142 Chagas disease (American trypanosomiasis);ko05145 Toxoplasmosis;ko05152 Tuberculosis;ko05162 Measles;ko05164 Influenza A;
gene21954	K03809	wrbA	Trp repressor binding protein	--		
gene21968	K02183	CALM	calmodulin	--	25	ko04014 Ras signaling pathway;ko04020 Calcium signaling pathway;ko04070 Phosphatidylinositol signaling system;ko04114 Oocyte meiosis;ko04270 Vascular smooth muscle contraction;ko04626 Plant-pathogen interaction;ko04713 Circadian entrainment;ko04720 Long-term potentiation;ko04722 Neurotrophin signaling pathway;ko04728 Dopaminergic synapse;ko04740 Olfactory transduction;ko04744 Phototransduction;ko04745 Phototransduction - fly;ko04910 Insulin signaling pathway;ko04912 GnRH signaling pathway;ko04915 Estrogen signaling pathway;ko04916 Melanogenesis;ko04970 Salivary secretion;ko04971 Gastric acid secretion;ko05010 Alzheimer's disease;ko05031 Amphetamine addiction;ko05034 Alcoholism;ko05133 Pertussis;ko05152 Tuberculosis;ko05214 Glioma;
gene21974	K12818	DHX8, PRP22	ATP-dependent RNA helicase DHX8/PRP22	EC:3.6.4.13	1	ko03040 Spliceosome;
gene21977	K10406	KIFC2_3	kinesin family member C2/C3	--		
gene21978	K04382	PPP2C	serine/threonine-protein phosphatase 2A catalytic subunit	EC:3.1.3.16	13	ko03015 mRNA surveillance pathway;ko04111 Cell cycle - yeast;ko04113 Meiosis - yeast;ko04114 Oocyte meiosis;ko04151 PI3K-Akt signaling pathway;ko04350 TGF-beta signaling pathway;ko04390 Hippo signaling pathway;ko04391 Hippo signaling pathway - fly;ko04530 Tight junction;ko04728 Dopaminergic synapse;ko04730 Long-term depression;ko05142 Chagas disease (American trypanosomiasis);ko05160 Hepatitis C;
gene21990	K12845	SNU13, NHP2L	U4/U6 small nuclear ribonucleoprotein SNU13	--	2	ko03008 Ribosome biogenesis in eukaryotes;ko03040 Spliceosome;
gene22005	K02935	RP-L7, MRPL12, rplL	large subunit ribosomal protein L7/L12	--	1	ko03010 Ribosome;
gene22008	K08695	E1.3.1.77	anthocyanidin reductase	EC:1.3.1.77	1	ko00941 Flavonoid biosynthesis;
gene22009	K08695	E1.3.1.77	anthocyanidin reductase	EC:1.3.1.77	1	ko00941 Flavonoid biosynthesis;
gene2200	K01601	rbcL	ribulose-bisphosphate carboxylase large chain	EC:4.1.1.39	3	ko00630 Glyoxylate and dicarboxylate metabolism;ko00710 Carbon fixation in photosynthetic organisms;ko01200 Carbon metabolism;
gene2201	K01885	EARS, gltX	glutamyl-tRNA synthetase	EC:6.1.1.17	2	ko00860 Porphyrin and chlorophyll metabolism;ko00970 Aminoacyl-tRNA biosynthesis;
gene22021	K01623	ALDO	fructose-bisphosphate aldolase, class I	EC:4.1.2.13	7	ko00010 Glycolysis / Gluconeogenesis;ko00030 Pentose phosphate pathway;ko00051 Fructose and mannose metabolism;ko00680 Methane metabolism;ko00710 Carbon fixation in photosynthetic organisms;ko01200 Carbon metabolism;ko01230 Biosynthesis of amino acids;
gene22022	K01240	URH1	uridine nucleosidase	EC:3.2.2.3	2	ko00240 Pyrimidine metabolism;ko00760 Nicotinate and nicotinamide metabolism;
gene22027	K00232	E1.3.3.6, ACOX1, ACOX3	acyl-CoA oxidase	EC:1.3.3.6	5	ko00071 Fatty acid degradation;ko00592 alpha-Linolenic acid metabolism;ko01040 Biosynthesis of unsaturated fatty acids;ko03320 PPAR signaling pathway;ko04146 Peroxisome;
gene22035	K02721	psbW	photosystem II PsbW protein	--	1	ko00195 Photosynthesis;
gene22037	K03241	EIF2B3	translation initiation factor eIF-2B subunit gamma	--	1	ko03013 RNA transport;
gene2203	K14780	DHX37, DHR1	ATP-dependent RNA helicase DHX37/DHR1	EC:3.6.4.13		

gene22040	K00940	E2.7.4.6, ndk	nucleoside-diphosphate kinase	EC:2.7.4.6	2	ko00230 Purine metabolism;ko00240 Pyrimidine metabolism;
gene22044	K00517	E1.14.-.-			4	ko00624 Polycyclic aromatic hydrocarbon degradation;ko00627 Aminobenzoate degradation;ko00903 Limonene and pinene degradation;ko00945 Stilbenoid, diarylheptanoid and gingerol biosynthesis;
gene22046	K14566	UTP24, FCF1	U3 small nucleolar RNA-associated protein 24	--	1	ko03008 Ribosome biogenesis in eukaryotes;
gene22047	K01183	E3.2.1.14	chitinase	EC:3.2.1.14	1	ko00520 Amino sugar and nucleotide sugar metabolism;
gene2204	K00485	E1.14.13.8	dimethylaniline monooxygenase (N-oxide forming)	EC:1.14.13.8	2	ko00680 Methane metabolism;ko00982 Drug metabolism - cytochrome P450;
gene22055	K15128	MED6	mediator of RNA polymerase II transcription subunit 6	--		
gene22060	K05016	CLCN7	chloride channel 7	--		
gene22062	K03352	APC5	anaphase-promoting complex subunit 5	--	7	ko04110 Cell cycle;ko04111 Cell cycle - yeast;ko04113 Meiosis - yeast;ko04114 Oocyte meiosis;ko04120 Ubiquitin mediated proteolysis;ko04914 Progesterone-mediated oocyte maturation;ko05166 HTLV-I infection;
gene22063	K11592	DICER1, DCR1	endoribonuclease Dicer	EC:3.1.26.-	1	ko05206 MicroRNAs in cancer;
gene22064	K09264	K09264	MADS-box transcription factor, plant	--		
gene22068	K14169	CTU2, NCS2	cytoplasmic tRNA 2-thiolation protein 2	--	1	ko04122 Sulfur relay system;
gene22069	K05280	E1.14.13.21	flavonoid 3'-monooxygenase	EC:1.14.13.21	2	ko00941 Flavonoid biosynthesis;ko00944 Flavone and flavonol biosynthesis;
gene22071	K09540	SEC63	translocation protein SEC63	--	2	ko03060 Protein export;ko04141 Protein processing in endoplasmic reticulum;
gene22077	K17065	DNM1L	dynamin 1-like protein	EC:3.6.5.5	1	ko04668 TNF signaling pathway;
gene22080	K01674	cah	carbonic anhydrase	EC:4.2.1.1	1	ko00910 Nitrogen metabolism;
gene22087	K15442	TAD3, ADAT3	tRNA-specific adenosine deaminase 3	--		
gene22090	K13648	GAUT	alpha-1,4-galacturonosyltransferase	EC:2.4.1.43	2	ko00500 Starch and sucrose metabolism;ko00520 Amino sugar and nucleotide sugar metabolism;
gene22095	K05665	ABCC1	ATP-binding cassette, subfamily C (CFTR/MRP), member 1	--	3	ko02010 ABC transporters;ko04977 Vitamin digestion and absorption;ko05206 MicroRNAs in cancer;
gene22099	K00721	DPM1	dolichol-phosphate mannosyltransferase	EC:2.4.1.83	1	ko00510 N-Glycan biosynthesis;
gene22100	K08150	SLC2A13, ITR	MFS transporter, SP family, solute carrier family 2 (myo-inositol transporter), member 13	--		
gene22101	K05359	ADT, PDT	arogenate/prephenate dehydratase	EC:4.2.1.91 4.2.1.51	2	ko00400 Phenylalanine, tyrosine and tryptophan biosynthesis;ko01230 Biosynthesis of amino acids;
gene22104	K16903	TAA1	L-tryptophan---pyruvate aminotransferase	EC:2.6.1.99	1	ko00380 Tryptophan metabolism;
gene22108	K13412	CPK	calcium-dependent protein kinase	EC:2.7.11.1	2	ko04626 Plant-pathogen interaction;ko05145 Toxoplasmosis;
gene22109	K16587	HAUS4	HAUS augmin-like complex subunit 4	--		
gene2210	K15731	CTDSP	carboxy-terminal domain RNA polymerase II polypeptide A small phosphatase	EC:3.1.3.16		
gene22110	K00815	TAT	tyrosine aminotransferase	EC:2.6.1.5	9	ko00130 Ubiquinone and other terpenoid-quinone biosynthesis;ko00270 Cysteine and methionine metabolism;ko00350 Tyrosine metabolism;ko00360 Phenylalanine metabolism;ko00400 Phenylalanine, tyrosine and tryptophan biosynthesis;ko00401 Novobiocin biosynthesis;ko00950 Isoquinoline alkaloid biosynthesis;ko00960 Tropane, piperidine and pyridine alkaloid biosynthesis;ko01230 Biosynthesis of amino acids;
gene22115	K17525	CHID1	chitinase domain-containing protein 1	--		
gene22122	K10357	MYO5	myosin V	--		
gene22125	K02883	RP-L18e, RPL18	large subunit ribosomal protein L18e	--	1	ko03010 Ribosome;
gene22126	K04564	SOD2	superoxide dismutase, Fe-Mn family	EC:1.15.1.1	2	ko04146 Peroxisome;ko05016 Huntington's disease;
gene22127	K00555	TRMT1, trm1	tRNA (guanine26-N2/guanine27-N2)-dimethyltransferase	EC:2.1.1.215 2.1.1.216		
gene2212	K17302	COPB2, SEC27	coatomer, subunit beta'	--		
gene22132	K07964	HPSE	heparanase 1	EC:3.2.1.166	2	ko00531 Glycosaminoglycan degradation;ko05205 Proteoglycans in cancer;
gene22143	K14500	BSK	BR-signaling kinase	EC:2.7.11.1	1	ko04075 Plant hormone signal transduction;
gene22144	K14500	BSK	BR-signaling kinase	EC:2.7.11.1	1	ko04075 Plant hormone signal transduction;
gene22145	K02883	RP-L18e, RPL18	large subunit ribosomal protein L18e	--	1	ko03010 Ribosome;
gene22147	K15446	TRM13, CCDC76	tRNA:m4X modification enzyme	EC:2.1.1.225		
gene22149	K06892	K06892				

gene22150	K14611	SLC23A1_2, SVCT1_2	solute carrier family 23 (nucleobase transporter), member 1/2	--		
gene22157	K13528	MED20	mediator of RNA polymerase II transcription subunit 20	--		
gene22162	K06268	PPP3R, CNB	serine/threonine-protein phosphatase 2B regulatory subunit	--	18	ko04010 MAPK signaling pathway;ko04020 Calcium signaling pathway;ko04114 Oocyte meiosis;ko04210 Apoptosis;ko04310 Wnt signaling pathway;ko04360 Axon guidance;ko04370 VEGF signaling pathway;ko04380 Osteoclast differentiation;ko04650 Natural killer cell mediated cytotoxicity;ko04660 T cell receptor signaling pathway;ko04662 B cell receptor signaling pathway;ko04720 Long-term potentiation;ko04724 Glutamatergic synapse;ko05010 Alzheimer's disease;ko05014 Amyotrophic lateral sclerosis (ALS);ko05031 Amphetamine addiction;ko05152
gene22166	K08081	E1.1.1.206	tropine dehydrogenase	EC:1.1.1.206	1	ko00960 Tropane, piperidine and pyridine alkaloid biosynthesis;
gene22169	K09503	DNAJA2	DnaJ homolog subfamily A member 2	--	1	ko04141 Protein processing in endoplasmic reticulum;
gene22171	K09286	EREBP	EREBP-like factor	--		
gene22173	K01785	E5.1.3.3, galM	aldose 1-epimerase	EC:5.1.3.3	1	ko00010 Glycolysis / Gluconeogenesis;
gene22176	K00430	E1.11.1.7	peroxidase	EC:1.11.1.7	2	ko00360 Phenylalanine metabolism;ko00940 Phenylpropanoid biosynthesis;
gene22180	K01637	E4.1.3.1, aceA	isocitrate lyase	EC:4.1.3.1	2	ko00630 Glyoxylate and dicarboxylate metabolism;ko01200 Carbon metabolism;
gene22181	K08486	STX1B_2_3	syntaxin 1B/2/3	--	2	ko04130 SNARE interactions in vesicular transport;ko04721 Synaptic vesicle cycle;
gene22185	K14301	NUP107	nuclear pore complex protein Nup107	--	1	ko03013 RNA transport;
gene22192	K12741	HNRNPA1_3	heterogeneous nuclear ribonucleoprotein A1/A3	--	1	ko03040 Spliceosome;
gene22193	K11279	NAP1L1, NRP	nucleosome assembly protein 1-like 1	--		
gene22195	K01193	E3.2.1.26, sacA	beta-fructofuranosidase	EC:3.2.1.26	2	ko00052 Galactose metabolism;ko00500 Starch and sucrose metabolism;
gene22199	K03130	TAF5	transcription initiation factor TFIID subunit 5	--	2	ko03022 Basal transcription factors;ko05168 Herpes simplex infection;
gene22205	K10256	FAD2	omega-6 fatty acid desaturase (delta-12 desaturase)	EC:1.14.19.-	1	ko01040 Biosynthesis of unsaturated fatty acids;
gene22206	K02897	RP-L25, rplY	large subunit ribosomal protein L25	--	1	ko03010 Ribosome;
gene22211	K06891	clpS	ATP-dependent Clp protease adaptor protein ClpS	--		
gene22212	K10727	CDT1	chromatin licensing and DNA replication factor 1	--		
gene22218	K09419	HSFF	heat shock transcription factor, other eukaryote	--		
gene2221	K01885	EARS, gltX	glutamyl-tRNA synthetase	EC:6.1.1.17	2	ko00860 Porphyrin and chlorophyll metabolism;ko00970 Aminoacyl-tRNA biosynthesis;
gene22220	K14488	SAUR	SAUR family protein	--	1	ko04075 Plant hormone signal transduction;
gene22224	K12617	PATL1, PAT1	DNA topoisomerase 2-associated protein PAT1	--	1	ko03018 RNA degradation;
gene22227	K04567	KARS, lysS	lysyl-tRNA synthetase, class II	EC:6.1.1.6	1	ko00970 Aminoacyl-tRNA biosynthesis;
gene2222	K04688	RPS6KB	p70 ribosomal S6 kinase	EC:2.7.11.1	9	ko04012 ErbB signaling pathway;ko04066 HIF-1 signaling pathway;ko04150 mTOR signaling pathway;ko04151 PI3K-Akt signaling pathway;ko04350 TGF-beta signaling pathway;ko04666 Fc gamma R-mediated phagocytosis;ko04910 Insulin signaling pathway;ko05205 Proteoglycans in cancer;ko05221 Acute myeloid leukemia;
gene22232	K00423	E1.10.3.3	L-ascorbate oxidase	EC:1.10.3.3	1	ko00053 Ascorbate and aldarate metabolism;
gene22233	K13126	PABPC	polyadenylate-binding protein	--	3	ko03013 RNA transport;ko03015 mRNA surveillance pathway;ko03018 RNA degradation;
gene22239	K11978	UBR3	E3 ubiquitin-protein ligase UBR3	EC:6.3.2.19		
gene22241	K00726	MGAT1	alpha-1,3-mannosyl-glycoprotein beta-1,2-N-acetylglucosaminyltransferase	EC:2.4.1.101	2	ko00510 N-Glycan biosynthesis;ko00513 Various types of N-glycan biosynthesis;
gene22245	K06269	PPP1C	serine/threonine-protein phosphatase PP1 catalytic subunit	EC:3.1.3.16	14	ko03015 mRNA surveillance pathway;ko04113 Meiosis - yeast;ko04114 Oocyte meiosis;ko04270 Vascular smooth muscle contraction;ko04390 Hippo signaling pathway;ko04510 Focal adhesion;ko04720 Long-term potentiation;ko04728 Dopaminergic synapse;ko04810 Regulation of actin cytoskeleton;ko04910 Insulin signaling pathway;ko05031 Amphetamine addiction;ko05034 Alcoholism;ko05168 Herpes simplex infection;ko05205 Proteoglycans in
gene22246	K14689	SLC30A2, ZNT2	solute carrier family 30 (zinc transporter), member 2	--		
gene22247	K07904	RAB11A	Ras-related protein Rab-11A	--	4	ko04144 Endocytosis;ko04961 Endocrine and other factor-regulated calcium reabsorption;ko04962 Vasopressin-regulated water reabsorption;ko04972 Pancreatic secretion;

gene22248	K03872	TCEB1	transcription elongation factor B, polypeptide 1	--	4	ko04066 HIF-1 signaling pathway;ko04120 Ubiquitin mediated proteolysis;ko05200 Pathways in cancer;ko05211 Renal cell carcinoma;
gene22249	K14233	tRNA-Ser	tRNA Ser	--	1	ko00970 Aminoacyl-tRNA biosynthesis;
gene22252	K12669	OST3, OST6	oligosaccharyltransferase complex subunit gamma	--	3	ko00510 N-Glycan biosynthesis;ko00513 Various types of N-glycan biosynthesis;ko04141 Protein processing in endoplasmic reticulum;
gene22253	K08288	PRKCSH	protein kinase C substrate 80K-H	--	1	ko04141 Protein processing in endoplasmic reticulum;
gene22255	K12180	COPS7, CSN7	COP9 signalosome complex subunit 7	--		
gene2225	K06670	SCC1, MCD1, RAD21	cohesin complex subunit SCC1	--	2	ko04110 Cell cycle;ko04111 Cell cycle - yeast;
gene22263	K14232	tRNA-Pro	tRNA Pro	--	1	ko00970 Aminoacyl-tRNA biosynthesis;
gene22264	K00434	E1.11.1.11	L-ascorbate peroxidase	EC:1.11.1.11	2	ko00053 Ascorbate and aldarate metabolism;ko00480 Glutathione metabolism;
gene22265	K12349	ASAH2	neutral ceramidase	EC:3.5.1.23	1	ko00600 Sphingolipid metabolism;
gene22266	K00121	frmA, ADH5, adhC	S-(hydroxymethyl)glutathione dehydrogenase / alcohol dehydrogenase	EC:1.1.1.284 1.1.1.1	12	ko00010 Glycolysis / Gluconeogenesis;ko00071 Fatty acid degradation;ko00350 Tyrosine metabolism;ko00625 Chloroalkane and chloroalkene degradation;ko00626 Naphthalene degradation;ko00680 Methane metabolism;ko00830 Retinol metabolism;ko00980 Metabolism of xenobiotics by cytochrome P450;ko00982 Drug metabolism - cytochrome P450;ko01200 Carbon metabolism;ko01220 Degradation of aromatic compounds;ko05204 Chemical carcinogenesis;
gene2226	K12177	COPS3, CSN3	COP9 signalosome complex subunit 3	--		
gene22271	K17086	TM9SF2_4	transmembrane 9 superfamily member 2/4	--		
gene22274	K01762	ACS	1-aminocyclopropane-1-carboxylate synthase	EC:4.4.1.14	1	ko00270 Cysteine and methionine metabolism;
gene22285	K03178	UBE1, UBA1	ubiquitin-activating enzyme E1	EC:6.3.2.19	2	ko04120 Ubiquitin mediated proteolysis;ko05012 Parkinson's disease;
gene22287	K14234	tRNA-Thr	tRNA Thr	--	1	ko00970 Aminoacyl-tRNA biosynthesis;
gene22288	K09422	MYBP	myb proto-oncogene protein, plant	--		
gene22289	K08518	STXBP5	syntaxin-binding protein 5	--		
gene2228	K00413	CYC1, CYT1, petC	ubiquinol-cytochrome c reductase cytochrome c1 subunit	--	7	ko00190 Oxidative phosphorylation;ko02020 Two-component system;ko04260 Cardiac muscle contraction;ko04932 Non-alcoholic fatty liver disease (NAFLD);ko05010 Alzheimer's disease;ko05012 Parkinson's disease;ko05016 Huntington's disease;
gene22291	K01866	YARS, tyrS	tyrosyl-tRNA synthetase	EC:6.1.1.1	1	ko00970 Aminoacyl-tRNA biosynthesis;
gene22292	K04125	E1.14.11.13	gibberellin 2-oxidase	EC:1.14.11.13	1	ko00904 Diterpenoid biosynthesis;
gene22296	K10398	KIF11, EG5	kinesin family member 11	--		
gene22297	K10398	KIF11, EG5	kinesin family member 11	--		
gene22305	K03939	NDUFS6	NADH dehydrogenase (ubiquinone) Fe-S protein 6	--	5	ko00190 Oxidative phosphorylation;ko04932 Non-alcoholic fatty liver disease (NAFLD);ko05010 Alzheimer's disease;ko05012 Parkinson's disease;ko05016 Huntington's disease;
gene22309	K06630	YWHAE	14-3-3 protein epsilon	--	8	ko04110 Cell cycle;ko04114 Oocyte meiosis;ko04151 PI3K-Akt signaling pathway;ko04390 Hippo signaling pathway;ko04391 Hippo signaling pathway - fly;ko04722 Neurotrophin signaling pathway;ko05169 Epstein-Barr virus infection;ko05203 Viral carcinogenesis;
gene2230	K01723	AOS	hydroperoxide dehydratase	EC:4.2.1.92	1	ko00592 alpha-Linolenic acid metabolism;
gene22316	K07556	ATPeAF2, ATPAF2	ATP synthase mitochondrial F1 complex assembly factor 2	--		
gene22317	K12386	CTNS	cystinosin	--	1	ko04142 Lysosome;
gene22318	K03127	TAF13	transcription initiation factor TFIID subunit 13	--	2	ko03022 Basal transcription factors;ko05168 Herpes simplex infection;
gene22319	K02948	RP-S11, MRPS11, rpsK	small subunit ribosomal protein S11	--	1	ko03010 Ribosome;
gene22322	K06699	PSME4	proteasome activator subunit 4	--	1	ko03050 Proteasome;
gene22323	K15148	MED7	mediator of RNA polymerase II transcription subunit 7	--		
gene22324	K01051	E3.1.1.11	pectinesterase	EC:3.1.1.11	2	ko00040 Pentose and glucuronate interconversions;ko00500 Starch and sucrose metabolism;
gene22325	K01051	E3.1.1.11	pectinesterase	EC:3.1.1.11	2	ko00040 Pentose and glucuronate interconversions;ko00500 Starch and sucrose metabolism;
gene2232	K12585	DIS3, RRP44	exosome complex exonuclease DIS3/RRP44	EC:3.1.13.-	1	ko03018 RNA degradation;
gene22331	K01982	LSUrRNA	large subunit ribosomal RNA	--	2	ko03008 Ribosome biogenesis in eukaryotes;ko03010 Ribosome;
gene22336	K00869	E2.7.1.36, MVK, mvaK1	mevalonate kinase	EC:2.7.1.36	2	ko00900 Terpenoid backbone biosynthesis;ko04146 Peroxisome;

gene2233	K07088	K07088				
gene22340	K11593	ELF2C	eukaryotic translation initiation factor 2C	--		
gene22341	K12854	SNRNP200, BRR2	pre-mRNA-splicing helicase BRR2	EC:3.6.4.13	1	ko03040 Spliceosome;
gene22346	K10588	UBE3B	ubiquitin-protein ligase E3 B	EC:6.3.2.19	1	ko04120 Ubiquitin mediated proteolysis;
gene22349	K11420	EHMT	euchromatic histone-lysine N-methyltransferase	EC:2.1.1.43	1	ko00310 Lysine degradation;
gene22350	K11420	EHMT	euchromatic histone-lysine N-methyltransferase	EC:2.1.1.43	1	ko00310 Lysine degradation;
gene22353	K12188	SNF8, EAP30	ESCRT-II complex subunit VPS22	--	1	ko04144 Endocytosis;
gene22355	K02335	DPO1, polA	DNA polymerase I	EC:2.7.7.7	6	ko00230 Purine metabolism;ko00240 Pyrimidine metabolism;ko03030 DNA replication;ko03410 Base excision repair;ko03420 Nucleotide excision repair;ko03440 Homologous recombination;
gene22357	K15382	SLC50A, SWEET	solute carrier family 50 (sugar transporter)	--		
gene22358	K03093	SIGMA70	RNA polymerase sigma factor	--		
gene22366	K17491	SMEK, PPP4R3	protein phosphatase 4 regulatory subunit 3	--		
gene22367	K03122	TFIIA1, GTF2A1, TOA1	transcription initiation factor TFIIA large subunit	--	2	ko03022 Basal transcription factors;ko05203 Viral carcinogenesis;
gene22376	K14272	GGAT	glutamate--glyoxylate aminotransferase	EC:2.6.1.4 2.6.1.2 2.6.1.44	6	ko00250 Alanine, aspartate and glutamate metabolism;ko00260 Glycine, serine and threonine metabolism;ko00630 Glyoxylate and dicarboxylate metabolism;ko00710 Carbon fixation in photosynthetic organisms;ko01210 2-Oxocarboxylic acid metabolism;ko01230 Biosynthesis of amino acids;
gene22378	K00430	E1.11.1.7	peroxidase	EC:1.11.1.7	2	ko00360 Phenylalanine metabolism;ko00940 Phenylpropanoid biosynthesis;
gene22382	K03509	POLH	DNA polymerase eta	EC:2.7.7.7	1	ko03460 Fanconi anemia pathway;
gene22383	K15687	MKRN	E3 ubiquitin-protein ligase makorin	EC:6.3.2.19		
gene22384	K01892	HARS, hisS	histidyl-tRNA synthetase	EC:6.1.1.21	1	ko00970 Aminoacyl-tRNA biosynthesis;
gene22386	K13510	LPCAT1_2	lysophosphatidylcholine acyltransferase / lyso-PAF acetyltransferase	EC:2.3.1.23 2.3.1.67	2	ko00564 Glycerophospholipid metabolism;ko00565 Ether lipid metabolism;
gene2238	K14855	RSA4, NLE1	ribosome assembly protein 4	--		
gene22390	K05907	APR	adenylyl-sulfate reductase (glutathione)	EC:1.8.4.9	1	ko00920 Sulfur metabolism;
gene22393	K12394	AP1S1_2	AP-1 complex subunit sigma 1/2	--	1	ko04142 Lysosome;
gene22394	K00789	metK	S-adenosylmethionine synthetase	EC:2.5.1.6	2	ko00270 Cysteine and methionine metabolism;ko01230 Biosynthesis of amino acids;
gene22396	K02947	RP-S10e, RPS10	small subunit ribosomal protein S10e	--	1	ko03010 Ribosome;
gene22397	K09490	HSPA5, BIP	heat shock 70kDa protein 5	--	4	ko03060 Protein export;ko04141 Protein processing in endoplasmic reticulum;ko04918 Thyroid hormone synthesis;ko05020 Prion diseases;
gene22399	K09422	MYBP	myb proto-oncogene protein, plant	--		
gene223	K01246	tag	DNA-3-methyladenine glycosylase I	EC:3.2.2.20	1	ko03410 Base excision repair;
gene22402	K09422	MYBP	myb proto-oncogene protein, plant	--		
gene22404	K02900	RP-L27Ae, RPL27A	large subunit ribosomal protein L27Ae	--	1	ko03010 Ribosome;
gene2240	K11550	SPBC25, SPC25	kinetochore protein Spc25, animal type	--		
gene22411	K00648	fabH	3-oxoacyl-[acyl-carrier-protein] synthase III	EC:2.3.1.180	1	ko00061 Fatty acid biosynthesis;
gene22413	K02372	fabZ	3-hydroxyacyl-[acyl-carrier-protein] dehydratase	EC:4.2.1.59	2	ko00061 Fatty acid biosynthesis;ko00780 Biotin metabolism;
gene2241	K13418	SERK1	somatic embryogenesis receptor kinase 1	EC:2.7.10.1 2.7.11.1		
gene22420	K13420	FLS2	LRR receptor-like serine/threonine-protein kinase FLS2	EC:2.7.11.1	1	ko04626 Plant-pathogen interaction;
gene22421	K07936	RAN	GTP-binding nuclear protein Ran	--	4	ko03008 Ribosome biogenesis in eukaryotes;ko03013 RNA transport;ko05166 HTLV-I infection;ko05169 Epstein-Barr virus infection;
gene22425	K07198	PRKAA, AMPK	5'-AMP-activated protein kinase, catalytic alpha subunit	EC:2.7.11.11	8	ko04140 Regulation of autophagy;ko04150 mTOR signaling pathway;ko04151 PI3K-Akt signaling pathway;ko04710 Circadian rhythm;ko04910 Insulin signaling pathway;ko04920 Adipocytokine signaling pathway;ko04932 Non-alcoholic fatty liver disease (NAFLD);ko05410 Hypertrophic cardiomyopathy (HCM);
gene22426	K12761	SNF1	carbon catabolite-derepressing protein kinase	EC:2.7.11.1	1	ko04113 Meiosis - yeast;
gene22431	K05759	PFN	profilin	--	3	ko04810 Regulation of actin cytoskeleton;ko05131 Shigellosis;ko05132 Salmonella infection;
gene22434	K07300	chaA, CAX	Ca2+:H+ antiporter	--		
gene22435	K01183	E3.2.1.14	chitinase	EC:3.2.1.14	1	ko00520 Amino sugar and nucleotide sugar metabolism;

gene22446	K14638	SLC15A3_4, PHT	solute carrier family 15 (peptide/histidine transporter), member 3/4	--		
gene22447	K01231	MAN2	alpha-mannosidase II	EC:3.2.1.114	2	ko00510 N-Glycan biosynthesis;ko00513 Various types of N-glycan biosynthesis;
gene22448	K14004	SEC13	protein transport protein SEC13	--	2	ko03013 RNA transport;ko04141 Protein processing in endoplasmic reticulum;
gene22449	K06268	PPP3R, CNB	serine/threonine-protein phosphatase 2B regulatory subunit	--	18	ko04010 MAPK signaling pathway;ko04020 Calcium signaling pathway;ko04114 Oocyte meiosis;ko04210 Apoptosis;ko04310 Wnt signaling pathway;ko04360 Axon guidance;ko04370 VEGF signaling pathway;ko04380 Osteoclast differentiation;ko04650 Natural killer cell mediated cytotoxicity;ko04660 T cell receptor signaling pathway;ko04662 B cell receptor signaling pathway;ko04720 Long-term potentiation;ko04724 Glutamatergic synapse;ko05010 Alzheimer's disease;ko05014 Amyotrophic lateral sclerosis (ALS);ko05031 Amphetamine addiction;ko05152
gene22450	K07901	RAB8A, MEL	Ras-related protein Rab-8A	--	1	ko04972 Pancreatic secretion;
gene22451	K12191	CHMP2A	charged multivesicular body protein 2A	--	1	ko04144 Endocytosis;
gene22452	K00928	lysC	aspartate kinase	EC:2.7.2.4	5	ko00260 Glycine, serine and threonine metabolism;ko00270 Cysteine and methionine metabolism;ko00300 Lysine biosynthesis;ko01210 2-Oxocarboxylic acid metabolism;ko01230 Biosynthesis of amino acids;
gene22454	K13210	FUBP	far upstream element-binding protein	--		
gene22457	K14230	tRNA-Met	tRNA Met	--	1	ko00970 Aminoacyl-tRNA biosynthesis;
gene22458	K14230	tRNA-Met	tRNA Met	--	1	ko00970 Aminoacyl-tRNA biosynthesis;
gene2245	K02739	PSMB7	20S proteasome subunit beta 2	EC:3.4.25.1	1	ko03050 Proteasome;
gene22460	K00434	E1.11.1.11	L-ascorbate peroxidase	EC:1.11.1.11	2	ko00053 Ascorbate and aldarate metabolism;ko00480 Glutathione metabolism;
gene22465	K10802	HMGB1	high mobility group protein B1	--	1	ko03410 Base excision repair;
gene22472	K14963	WDR5, SWD3, CPS30	COMPASS component SWD3	--		
gene22473	K10401	KIF18_19	kinesin family member 18/19	--		
gene22475	K15193	SPTY2D1, SPT2	protein SPT2	--		
gene22479	K10884	XRCC6, KU70, G22P1	ATP-dependent DNA helicase 2 subunit 1	--	1	ko03450 Non-homologous end-joining;
gene22487	K08867	WNK, PRKWNK	WNK lysine deficient protein kinase	EC:2.7.11.1		
gene22489	K13356	FAR	fatty acyl-CoA reductase	EC:1.2.1.-	2	ko00073 Cutin, suberine and wax biosynthesis;ko04146 Peroxisome;
gene22490	K03046	rpoC	DNA-directed RNA polymerase subunit beta'	EC:2.7.7.6	3	ko00230 Purine metabolism;ko00240 Pyrimidine metabolism;ko03020 RNA polymerase;
gene22492	K09338	HD-ZIP	homeobox-leucine zipper protein	--		
gene22497	K01595	ppc	phosphoenolpyruvate carboxylase	EC:4.1.1.31	5	ko00620 Pyruvate metabolism;ko00680 Methane metabolism;ko00710 Carbon fixation in photosynthetic organisms;ko00720 Carbon fixation pathways in prokaryotes;ko01200 Carbon metabolism;
gene22499	K06664	PEX2, PXMP3	peroxin-2	--	1	ko04146 Peroxisome;
gene224	K13127	RNF113A, CWC24	RING finger protein 113A	--		
gene22500	K14233	tRNA-Ser	tRNA Ser	--	1	ko00970 Aminoacyl-tRNA biosynthesis;
gene22504	K00695	E2.4.1.13	sucrose synthase	EC:2.4.1.13	1	ko00500 Starch and sucrose metabolism;
gene22505	K15015	SLC32A, VGAT	solute carrier family 32 (vesicular inhibitory amino acid transporter)	--	5	ko04721 Synaptic vesicle cycle;ko04723 Retrograde endocannabinoid signaling;ko04727 GABAergic synapse;ko05032 Morphine addiction;ko05033 Nicotine addiction;
gene22515	K00232	E1.3.3.6, ACOX1, ACOX3	acyl-CoA oxidase	EC:1.3.3.6	5	ko00071 Fatty acid degradation;ko00592 alpha-Linolenic acid metabolism;ko01040 Biosynthesis of unsaturated fatty acids;ko03320 PPAR signaling pathway;ko04146 Peroxisome;
gene22516	K11835	USP4_11_15, UBP12	ubiquitin carboxyl-terminal hydrolase 4/11/15	EC:3.1.2.15		
gene22517	K08472	MLO	mlo protein	--		
gene22519	K05387	GRIP	glutamate receptor, ionotropic, plant	--		
gene2251	K10357	MYO5	myosin V	--		
gene22520	K14567	UTP14	U3 small nucleolar RNA-associated protein 14	--	1	ko03008 Ribosome biogenesis in eukaryotes;
gene22521	K12741	HNRNPA1_3	heterogeneous nuclear ribonucleoprotein A1/A3	--	1	ko03040 Spliceosome;
gene22525	K01897	ACSL, fadD	long-chain acyl-CoA synthetase	EC:6.2.1.3	4	ko00071 Fatty acid degradation;ko03320 PPAR signaling pathway;ko04146 Peroxisome;ko04920 Adipocytokine signaling pathway;
gene2252	K08914	LHCB3	light-harvesting complex II chlorophyll a/b binding protein 3	--	1	ko00196 Photosynthesis - antenna proteins;

gene22531	K03240	EIF2B5	translation initiation factor eIF-2B subunit epsilon	--	1	ko03013 RNA transport;
gene22533	K14289	XPO5	exportin-5	--	1	ko03013 RNA transport;
gene22535	K07119	K07119				
gene2253	K10661	MARCH6, DOA10	E3 ubiquitin-protein ligase MARCH6	EC:6.3.2.19	1	ko04141 Protein processing in endoplasmic reticulum;
gene22541	K00753	E2.4.1.214	glycoprotein 3-alpha-L-fucosyltransferase	EC:2.4.1.214	1	ko00513 Various types of N-glycan biosynthesis;
gene22544	K00967	PCYT2	ethanolamine-phosphate cytidylyltransferase	EC:2.7.7.14	2	ko00440 Phosphonate and phosphinate metabolism;ko00564 Glycerophospholipid metabolism;
gene22546	K14209	SLC36A, PAT	solute carrier family 36 (proton-coupled amino acid transporter)	--		
gene2254	K13126	PABPC	polyadenylate-binding protein	--	3	ko03013 RNA transport;ko03015 mRNA surveillance pathway;ko03018 RNA degradation;
gene22550	K04392	RAC1	Ras-related C3 botulinum toxin substrate 1	--	33	ko04010 MAPK signaling pathway;ko04014 Ras signaling pathway;ko04062 Chemokine signaling pathway;ko04145 Phagosome;ko04151 PI3K-Akt signaling pathway;ko04310 Wnt signaling pathway;ko04360 Axon guidance;ko04370 VEGF signaling pathway;ko04380 Osteoclast differentiation;ko04510 Focal adhesion;ko04520 Adherens junction;ko04620 Toll-like receptor signaling pathway;ko04650 Natural killer cell mediated cytotoxicity;ko04662 B cell receptor signaling pathway;ko04664 Fc epsilon RI signaling pathway;ko04666 Fc gamma R-mediated phagocytosis;ko04670 Leukocyte transendothelial migration;ko04722 Neurotrophin signaling pathway;ko04810 Regulation of actin cytoskeleton;ko04932 Non-alcoholic fatty liver disease (NAFLD);ko04972 Pancreatic secretion;ko05014 Amyotrophic lateral sclerosis (ALS);ko05100 Bacterial invasion of epithelial cells;ko05120 Epithelial cell signaling in Helicobacter pylori infection;ko05131 Shigellosis;ko05132 Salmonella infection;ko05200 Pathways in cancer;ko05203 Viral carcinogenesis;ko05205 Proteoglycans in cancer;ko05210 Colorectal cancer;ko05211 Renal cell carcinoma;ko05212 Pancreatic [illegible]
gene22557	K14484	IAA	auxin-responsive protein IAA	--	1	ko04075 Plant hormone signal transduction;
gene2255	K15255	PIF1	ATP-dependent DNA helicase PIF1	EC:3.6.4.12		
gene22561	K15084	SLC25A16, GDA, LEU5	solute carrier family 25 (mitochondrial carrier protein), member 16	--		
gene22562	K11252	H2B	histone H2B	--	3	ko05034 Alcoholism;ko05203 Viral carcinogenesis;ko05322 Systemic lupus erythematosus;
gene22563	K05305	FUK	fucokinase	EC:2.7.1.52	2	ko00051 Fructose and mannose metabolism;ko00520 Amino sugar and nucleotide sugar metabolism;
gene22567	K14164	glyQS	glycyl-tRNA synthetase	EC:6.1.1.14	1	ko00970 Aminoacyl-tRNA biosynthesis;
gene22570	K01535	E3.6.3.6	H+-transporting ATPase	EC:3.6.3.6	1	ko00190 Oxidative phosphorylation;
gene22572	K04733	IRAK4	interleukin-1 receptor-associated kinase 4	EC:2.7.11.1	11	ko04064 NF-kappa B signaling pathway;ko04210 Apoptosis;ko04620 Toll-like receptor signaling pathway;ko04722 Neurotrophin signaling pathway;ko05133 Pertussis;ko05140 Leishmaniasis;ko05142 Chagas disease (American trypanosomiasis);ko05145 Toxoplasmosis;ko05152 Tuberculosis;ko05162 Measles;ko05164 Influenza A;
gene22574	K14777	DDX47, RRP3	ATP-dependent RNA helicase DDX47/RRP3	EC:3.6.4.13		
gene22580	K15322	TSEN2	tRNA-splicing endonuclease subunit Sen2	EC:3.1.27.9		
gene22581	K15322	TSEN2	tRNA-splicing endonuclease subunit Sen2	EC:3.1.27.9		
gene22582	K15397	KCS	3-ketoacyl-CoA synthase	EC:2.3.1.199	1	ko00062 Fatty acid elongation;
gene22584	K09843	E1.14.13.93	(+)-abscisic acid 8'-hydroxylase	EC:1.14.13.93	1	ko00906 Carotenoid biosynthesis;
gene22585	K15718	LOX1_5	linoleate 9S-lipoxygenase	EC:1.13.11.58	1	ko00591 Linoleic acid metabolism;
gene22586	K15718	LOX1_5	linoleate 9S-lipoxygenase	EC:1.13.11.58	1	ko00591 Linoleic acid metabolism;
gene22587	K00088	guaB	IMP dehydrogenase	EC:1.1.1.205	2	ko00230 Purine metabolism;ko00983 Drug metabolism - other enzymes;
gene22595	K01369	LGMN	legumain	EC:3.4.22.34	2	ko04142 Lysosome;ko04612 Antigen processing and presentation;
gene22597	K04730	IRAK1	interleukin-1 receptor-associated kinase 1	EC:2.7.11.1	11	ko04064 NF-kappa B signaling pathway;ko04210 Apoptosis;ko04620 Toll-like receptor signaling pathway;ko04722 Neurotrophin signaling pathway;ko05133 Pertussis;ko05140 Leishmaniasis;ko05142 Chagas disease (American trypanosomiasis);ko05145 Toxoplasmosis;ko05152 Tuberculosis;ko05162 Measles;ko05169 Epstein-Barr virus infection;

gene22602	K04733	IRAK4	interleukin-1 receptor-associated kinase 4	EC:2.7.11.1	11	ko04064 NF-kappa B signaling pathway;ko04210 Apoptosis;ko04620 Toll-like receptor signaling pathway;ko04722 Neurotrophin signaling pathway;ko05133 Pertussis;ko05140 Leishmaniasis;ko05142 Chagas disease (American trypanosomiasis);ko05145 Toxoplasmosis;ko05152 Tuberculosis;ko05162 Measles;ko05164 Influenza A;
gene22604	K04733	IRAK4	interleukin-1 receptor-associated kinase 4	EC:2.7.11.1	11	ko04064 NF-kappa B signaling pathway;ko04210 Apoptosis;ko04620 Toll-like receptor signaling pathway;ko04722 Neurotrophin signaling pathway;ko05133 Pertussis;ko05140 Leishmaniasis;ko05142 Chagas disease (American trypanosomiasis);ko05145 Toxoplasmosis;ko05152 Tuberculosis;ko05162 Measles;ko05164 Influenza A;
gene22605	K13412	CPK	calcium-dependent protein kinase	EC:2.7.11.1	2	ko04626 Plant-pathogen interaction;ko05145 Toxoplasmosis;
gene22607	K09338	HD-ZIP	homeobox-leucine zipper protein	--		
gene22611	K01213	E3.2.1.67	galacturan 1,4-alpha-galacturonidase	EC:3.2.1.67	2	ko00040 Pentose and glucuronate interconversions;ko00500 Starch and sucrose metabolism;
gene22616	K01886	QARS, glnS	glutaminyl-tRNA synthetase	EC:6.1.1.18	1	ko00970 Aminoacyl-tRNA biosynthesis;
gene22617	K01749	hemC, HMBS	hydroxymethylbilane synthase	EC:2.5.1.61	1	ko00860 Porphyrin and chlorophyll metabolism;
gene2262	K09873	TIP	aquaporin TIP	--		
gene22630	K06228	FU	fused	EC:2.7.11.1	3	ko04340 Hedgehog signaling pathway;ko05200 Pathways in cancer;ko05217 Basal cell carcinoma;
gene22640	K02180	BUB3	cell cycle arrest protein BUB3	--	3	ko04110 Cell cycle;ko04111 Cell cycle - yeast;ko05166 HTLV-I infection;
gene22647	K01703	leuC	3-isopropylmalate/(R)-2-methylmalate dehydratase large subunit	EC:4.2.1.33 4.2.1.35	4	ko00290 Valine, leucine and isoleucine biosynthesis;ko00660 C5-Branched dibasic acid metabolism;ko01210 2-Oxocarboxylic acid metabolism;ko01230 Biosynthesis of amino acids;
gene22648	K04371	MAPK1_3	mitogen-activated protein kinase 1/3	EC:2.7.11.24	71	ko04010 MAPK signaling pathway;ko04012 ErbB signaling pathway;ko04013 MAPK signaling pathway - fly;ko04014 Ras signaling pathway;ko04062 Chemokine signaling pathway;ko04066 HIF-1 signaling pathway;ko04114 Oocyte meiosis;ko04150 mTOR signaling pathway;ko04151 PI3K-Akt signaling pathway;ko04270 Vascular smooth muscle contraction;ko04320 Dorso-ventral axis formation;ko04350 TGF-beta signaling pathway;ko04360 Axon guidance;ko04370 VEGF signaling pathway;ko04380 Osteoclast differentiation;ko04510 Focal adhesion;ko04520 Adherens junction;ko04540 Gap junction;ko04620 Toll-like receptor signaling pathway;ko04621 NOD-like receptor signaling pathway;ko04650 Natural killer cell mediated cytotoxicity;ko04660 T cell receptor signaling pathway;ko04662 B cell receptor signaling pathway;ko04664 Fc epsilon RI signaling pathway;ko04666 Fc gamma R-mediated phagocytosis;ko04668 TNF signaling pathway;ko04713 Circadian entrainment;ko04720 Long-term potentiation;ko04722 Neurotrophin signaling pathway;ko04723 Retrograde endocannabinoid signaling;ko04724 Glutamatergic synapse;ko04725 Cholinergic synapse;ko04726 Serotonergic synapse;ko04730 Long-term depression;ko04810 Regulation of actin cytoskeleton;ko04910 Insulin signaling pathway;ko04912 GnRH signaling pathway;ko04914 Progesterone-mediated oocyte maturation;ko04915 Estrogen signaling pathway;ko04916 Melanogenesis;ko04917 Prolactin signaling pathway;ko04930 Type II diabetes mellitus;ko04960 Aldosterone-regulated sodium reabsorption;ko05010 Alzheimer's disease;ko05020 Prion diseases;ko05034 Alcoholism;ko05131 Shigellosis;ko05132 Salmonella infection;ko05133 Pertussis;ko05140 Leishmaniasis;ko05142 Chagas disease (American trypanosomiasis);ko05145 Toxoplasmosis;ko05152 Tuberculosis;ko05160 Hepatitis C;ko05161 Hepatitis B;ko05164 Influenza A;ko05200 Pathways in cancer;ko05203 Viral carcinogenesis;ko05205 Proteoglycans in cancer;ko05210 Colorectal cancer;ko05211 Renal cell carcinoma;ko05212
gene22649	K05350	bglB	beta-glucosidase	EC:3.2.1.21	3	ko00460 Cyanoamino acid metabolism;ko00500 Starch and sucrose metabolism;ko00940 Phenylpropanoid biosynthesis;
gene22650	K13963	SERPINB	serpin B	--	1	ko05146 Amoebiasis;

gene22651	K13963	SERPINB	serpin B	--	1	ko05146 Amoebiasis;
gene22653	K15166	MED23	mediator of RNA polymerase II transcription subunit 23	--		
gene22657	K15559	RTT103	regulator of Ty1 transposition protein 103	--		
gene22659	K07937	ARF1	ADP-ribosylation factor 1	--	2	ko05110 Vibrio cholerae infection;ko05134 Legionellosis;
gene22668	K08679	E5.1.3.6	UDP-glucuronate 4-epimerase	EC:5.1.3.6	2	ko00500 Starch and sucrose metabolism;ko00520 Amino sugar and nucleotide sugar metabolism;
gene22669	K00800	aroA	3-phosphoshikimate 1-carboxyvinyltransferase	EC:2.5.1.19	2	ko00400 Phenylalanine, tyrosine and tryptophan biosynthesis;ko01230 Biosynthesis of amino acids;
gene22674	K00558	DNMT1, dcm	DNA (cytosine-5)-methyltransferase 1	EC:2.1.1.37	2	ko00270 Cysteine and methionine metabolism;ko05206 MicroRNAs in cancer;
gene22675	K14823	EBP2, EBNA1BP2	rRNA-processing protein EBP2	--		
gene22679	K08867	WNK, PRKWNK	WNK lysine deficient protein kinase	EC:2.7.11.1		
gene22681	K02717	psbP	photosystem II oxygen-evolving enhancer protein 2	--	1	ko00195 Photosynthesis;
gene22689	K03188	ureF	urease accessory protein	--		
gene22692	K11159	K11159	carotenoid cleavage dioxygenase	--		
gene22694	K05350	bglB	beta-glucosidase	EC:3.2.1.21	3	ko00460 Cyanoamino acid metabolism;ko00500 Starch and sucrose metabolism;ko00940 Phenylpropanoid biosynthesis;
gene22701	K06067	HDAC1_2	histone deacetylase 1/2	EC:3.5.1.98	9	ko04110 Cell cycle;ko04330 Notch signaling pathway;ko05016 Huntington's disease;ko05034 Alcoholism;ko05169 Epstein-Barr virus infection;ko05200 Pathways in cancer;ko05202 Transcriptional misregulation in cancer;ko05203 Viral carcinogenesis;ko05220 Chronic myeloid leukemia;
gene22703	K07893	RAB6A	Ras-related protein Rab-6A	--		
gene22706	K14399	CLP1, HERB	polyribonucleotide 5'-hydroxyl-kinase	EC:2.7.1.78	1	ko03015 mRNA surveillance pathway;
gene22707	K08857	NEK	NIMA (never in mitosis gene a)-related kinase	EC:2.7.11.1		
gene2270	K00430	E1.11.1.7	peroxidase	EC:1.11.1.7	2	ko00360 Phenylalanine metabolism;ko00940 Phenylpropanoid biosynthesis;
gene22711	K01895	ACSS, acs	acetyl-CoA synthetase	EC:6.2.1.1	6	ko00010 Glycolysis / Gluconeogenesis;ko00620 Pyruvate metabolism;ko00640 Propanoate metabolism;ko00680 Methane metabolism;ko00720 Carbon fixation pathways in prokaryotes;ko01200 Carbon metabolism;
gene22713	K08679	E5.1.3.6	UDP-glucuronate 4-epimerase	EC:5.1.3.6	2	ko00500 Starch and sucrose metabolism;ko00520 Amino sugar and nucleotide sugar metabolism;
gene22714	K00600	glyA, SHMT	glycine hydroxymethyltransferase	EC:2.1.2.1	7	ko00260 Glycine, serine and threonine metabolism;ko00460 Cyanoamino acid metabolism;ko00630 Glyoxylate and dicarboxylate metabolism;ko00670 One carbon pool by folate;ko00680 Methane metabolism;ko01200 Carbon metabolism;ko01230 Biosynthesis of amino acids;
gene2271	K00430	E1.11.1.7	peroxidase	EC:1.11.1.7	2	ko00360 Phenylalanine metabolism;ko00940 Phenylpropanoid biosynthesis;
gene22720	K06636	SMC1	structural maintenance of chromosome 1	--	4	ko04110 Cell cycle;ko04111 Cell cycle - yeast;ko04113 Meiosis - yeast;ko04114 Oocyte meiosis;
gene22722	K15026	EIF2A	translation initiation factor 2A	--		
gene22725	K01188	E3.2.1.21	beta-glucosidase	EC:3.2.1.21	3	ko00460 Cyanoamino acid metabolism;ko00500 Starch and sucrose metabolism;ko00940 Phenylpropanoid biosynthesis;
gene22727	K07195	EXOC7, EXO70	exocyst complex component 7	--	1	ko04910 Insulin signaling pathway;
gene22733	K04564	SOD2	superoxide dismutase, Fe-Mn family	EC:1.15.1.1	2	ko04146 Peroxisome;ko05016 Huntington's disease;
gene22734	K00555	TRMT1, trm1	tRNA (guanine26-N2/guanine27-N2)-dimethyltransferase	EC:2.1.1.215 2.1.1.216		
gene22739	K05539	dusA	tRNA-dihydrouridine synthase A	EC:1.-.-.-		
gene22743	K01899	LSC1	succinyl-CoA synthetase alpha subunit	EC:6.2.1.4 6.2.1.5	3	ko00020 Citrate cycle (TCA cycle);ko00640 Propanoate metabolism;ko01200 Carbon metabolism;
gene2274	K10747	LIG1	DNA ligase 1	EC:6.5.1.1	4	ko03030 DNA replication;ko03410 Base excision repair;ko03420 Nucleotide excision repair;ko03430 Mismatch repair;
gene22751	K10878	SPO11	meiotic recombination protein SPO11	--	1	ko04113 Meiosis - yeast;
gene22752	K05389	KCNKF	potassium channel subfamily K, other eukaryote	--		
gene22756	K14948	PTBP2, NPTB	olypyrimidine tract-binding protein 2	--		
gene22757	K08955	YME1	ATP-dependent metalloprotease	EC:3.4.24.-		
gene22767	K02875	RP-L14e, RPL14	large subunit ribosomal protein L14e	--	1	ko03010 Ribosome;

gene22768	K11982	RNF115_126	E3 ubiquitin-protein ligase RNF115/126	EC:6.3.2.19		
gene22769	K12501	HST	homogentisate solanesyltransferase	--	1	ko00130 Ubiquinone and other terpenoid-quinone biosynthesis;
gene2276	K00696	E2.4.1.14	sucrose-phosphate synthase	EC:2.4.1.14	1	ko00500 Starch and sucrose metabolism;
gene22776	K04713	SUR2	sphinganine C4-monooxygenase	EC:1.14.13.169	1	ko00600 Sphingolipid metabolism;
gene22778	K10999	CESA	cellulose synthase A	EC:2.4.1.12		
gene22788	K02910	RP-L31e, RPL31	large subunit ribosomal protein L31e	--	1	ko03010 Ribosome;
gene22789	K14232	tRNA-Pro	tRNA Pro	--	1	ko00970 Aminoacyl-tRNA biosynthesis;
gene22795	K08770	UBC	ubiquitin C	--	1	ko03320 PPAR signaling pathway;
gene22796	K04077	groEL, HSPD1	chaperonin GroEL	--	4	ko03018 RNA degradation;ko04940 Type I diabetes mellitus;ko05134 Legionellosis;ko05152 Tuberculosis;
gene2279	K10960	chlP, bchP	geranylgeranyl reductase	EC:1.3.1.83	2	ko00860 Porphyrin and chlorophyll metabolism;ko00900 Terpenoid backbone biosynthesis;
gene22802	K14791	PWP1	periodic tryptophan protein 1	--		
gene22807	K07964	HPSE	heparanase 1	EC:3.2.1.166	2	ko00531 Glycosaminoglycan degradation;ko05205 Proteoglycans in cancer;
gene22811	K04733	IRAK4	interleukin-1 receptor-associated kinase 4	EC:2.7.11.1	11	ko04064 NF-kappa B signaling pathway;ko04210 Apoptosis;ko04620 Toll-like receptor signaling pathway;ko04722 Neurotrophin signaling pathway;ko05133 Pertussis;ko05140 Leishmaniasis;ko05142 Chagas disease (American trypanosomiasis);ko05145 Toxoplasmosis;ko05152 Tuberculosis;ko05162 Measles;ko05164 Influenza A;
gene22817	K10396	KIF5	kinesin family member 5	--	1	ko04728 Dopaminergic synapse;
gene2281	K00913	ITPK1	inositol-1,3,4-trisphosphate 5/6-kinase / inositol-tetrakisphosphate 1-kinase	EC:2.7.1.159 2.7.1.134	2	ko00562 Inositol phosphate metabolism;ko04070 Phosphatidylinositol signaling system;
gene22823	K02470	gyrB	DNA gyrase subunit B	EC:5.99.1.3		
gene22829	K03086	SIG1, rpoD	RNA polymerase primary sigma factor	--		
gene22830	K00721	DPM1	dolichol-phosphate mannosyltransferase	EC:2.4.1.83	1	ko00510 N-Glycan biosynthesis;
gene22833	K00855	PRK, prkB	phosphoribulokinase	EC:2.7.1.19	2	ko00710 Carbon fixation in photosynthetic organisms;ko01200 Carbon metabolism;
gene22834	K00817	hisC	histidinol-phosphate aminotransferase	EC:2.6.1.9	7	ko00340 Histidine metabolism;ko00350 Tyrosine metabolism;ko00360 Phenylalanine metabolism;ko00400 Phenylalanine, tyrosine and tryptophan biosynthesis;ko00401 Novobiocin biosynthesis;ko00960 Tropane, piperidine and pyridine alkaloid biosynthesis;ko01230 Biosynthesis of amino acids;
gene2283	K05658	ABCB1	ATP-binding cassette, subfamily B (MDR/TAP), member 1	--	3	ko02010 ABC transporters;ko04976 Bile secretion;ko05206 MicroRNAs in cancer;
gene22842	K02437	gcvH, GCSH	glycine cleavage system H protein	--	1	ko00630 Glyoxylate and dicarboxylate metabolism;
gene22843	K14291	PHAX	phosphorylated adapter RNA export protein	--	1	ko03013 RNA transport;
gene22844	K00873	PK, pyk	pyruvate kinase	EC:2.7.1.40	7	ko00010 Glycolysis / Gluconeogenesis;ko00230 Purine metabolism;ko00620 Pyruvate metabolism;ko01200 Carbon metabolism;ko01230 Biosynthesis of amino acids;ko04930 Type II diabetes mellitus;ko05203 Viral carcinogenesis;
gene22846	K02207	UBE2R, UBC3, CDC34	ubiquitin-conjugating enzyme E2 R	EC:6.3.2.19	2	ko04120 Ubiquitin mediated proteolysis;ko05168 Herpes simplex infection;
gene22847	K10666	RNF5	E3 ubiquitin-protein ligase RNF5	EC:6.3.2.19	1	ko04141 Protein processing in endoplasmic reticulum;
gene22857	K07407	E3.2.1.22B, galA, rafA	alpha-galactosidase	EC:3.2.1.22	4	ko00052 Galactose metabolism;ko00561 Glycerolipid metabolism;ko00600 Sphingolipid metabolism;ko00603 Glycosphingolipid biosynthesis - globo series;
gene22859	K02865	RP-L10Ae, RPL10A	large subunit ribosomal protein L10Ae	--	1	ko03010 Ribosome;
gene2285	K07904	RAB11A	Ras-related protein Rab-11A	--	4	ko04144 Endocytosis;ko04961 Endocrine and other factor-regulated calcium reabsorption;ko04962 Vasopressin-regulated water reabsorption;ko04972 Pancreatic secretion;
gene22860	K00419	QCR9, UCRC	ubiquinol-cytochrome c reductase subunit 9	--	6	ko00190 Oxidative phosphorylation;ko04260 Cardiac muscle contraction;ko04932 Non-alcoholic fatty liver disease (NAFLD);ko05010 Alzheimer's disease;ko05012 Parkinson's disease;ko05016 Huntington's disease;
gene22863	K02116	atpI	ATP synthase protein I	--		
gene22864	K01595	ppc	phosphoenolpyruvate carboxylase	EC:4.1.1.31	5	ko00620 Pyruvate metabolism;ko00680 Methane metabolism;ko00710 Carbon fixation in photosynthetic organisms;ko00720 Carbon fixation pathways in prokaryotes;ko01200 Carbon metabolism;
gene22867	K15032	MTERFD	mTERF domain-containing protein, mitochondrial	--		
gene22869	K00430	E1.11.1.7	peroxidase	EC:1.11.1.7	2	ko00360 Phenylalanine metabolism;ko00940 Phenylpropanoid biosynthesis;

gene22871	K14793	RRP9	ribosomal RNA-processing protein 9	--		
gene22872	K15501	PPP6R3, SAPS3	serine/threonine-protein phosphatase 6 regulatory subunit 3	--		
gene22873	K07198	PRKAA, AMPK	5'-AMP-activated protein kinase, catalytic alpha subunit	EC:2.7.11.11	8	ko04140 Regulation of autophagy;ko04150 mTOR signaling pathway;ko04151 PI3K-Akt signaling pathway;ko04710 Circadian rhythm;ko04910 Insulin signaling pathway;ko04920 Adipocytokine signaling pathway;ko04932 Non-alcoholic fatty liver disease (NAFLD);ko05410 Hypertrophic cardiomyopathy (HCM);
gene22875	K00898	PDK2_3_4	pyruvate dehydrogenase kinase 2/3/4	EC:2.7.11.2		
gene22876	K08494	NSPN	novel plant SNARE	--		
gene2287	K14686	SLC31A1, CTR1	solute carrier family 31 (copper transporter), member 1	--	1	ko04978 Mineral absorption;
gene22880	K01052	LIPA	lysosomal acid lipase/cholesteryl ester hydrolase	EC:3.1.1.13	2	ko00100 Steroid biosynthesis;ko04142 Lysosome;
gene22888	K00889	E2.7.1.68, PIP5K	1-phosphatidylinositol-4-phosphate 5-kinase	EC:2.7.1.68	5	ko00562 Inositol phosphate metabolism;ko04070 Phosphatidylinositol signaling system;ko04144 Endocytosis;ko04666 Fc gamma R-mediated phagocytosis;ko04810 Regulation of actin cytoskeleton;
gene22891	K03495	gidA, mnmG, MTO1	tRNA uridine 5-carboxymethylaminomethyl modification enzyme	--		
gene22892	K14571	RIX7, NVL	ribosome biogenesis ATPase	--	1	ko03008 Ribosome biogenesis in eukaryotes;
gene22893	K08991	MUS81	crossover junction endonuclease MUS81	EC:3.1.22.-	2	ko03440 Homologous recombination;ko03460 Fanconi anemia pathway;
gene22899	K10896	FANCM	fanconi anemia group M protein	--	1	ko03460 Fanconi anemia pathway;
gene22900	K03549	kup	KUP system potassium uptake protein	--		
gene22901	K07393	ECM4	putative glutathione S-transferase	--		
gene22904	K03163	TOP1	DNA topoisomerase I	EC:5.99.1.2		
gene22905	K02153	ATPeVH, ATP6H	V-type H+-transporting ATPase subunit H	EC:3.6.3.14	7	ko00190 Oxidative phosphorylation;ko04145 Phagosome;ko04721 Synaptic vesicle cycle;ko04966 Collecting duct acid secretion;ko05110 Vibrio cholerae infection;ko05120 Epithelial cell signaling in Helicobacter pylori infection;ko05323 Rheumatoid arthritis;
gene22906	K10528	HPL	hydroperoxide lyase	EC:4.1.2.-	1	ko00592 alpha-Linolenic acid metabolism;
gene22907	K14508	NPR1	regulatory protein NPR1	--	1	ko04075 Plant hormone signal transduction;
gene22908	K02999	RPA1, POLR1A	DNA-directed RNA polymerase I subunit RPA1	EC:2.7.7.6	3	ko00230 Purine metabolism;ko00240 Pyrimidine metabolism;ko03020 RNA polymerase;
gene2290	K07374	TUBA	tubulin alpha	--	3	ko04145 Phagosome;ko04540 Gap junction;ko05130 Pathogenic Escherichia coli infection;
gene22913	K02982	RP-S3, rpsC	small subunit ribosomal protein S3	--	1	ko03010 Ribosome;
gene22920	K02983	RP-S30e, RPS30	small subunit ribosomal protein S30e	--	1	ko03010 Ribosome;
gene22923	K07767	E3.6.4.3	microtubule-severing ATPase	EC:3.6.4.3		
gene22924	K10695	RNF1_2	E3 ubiquitin-protein ligase RNF1/2	EC:6.3.2.19		
gene22929	K01363	CTSB	cathepsin B	EC:3.4.22.1	2	ko04142 Lysosome;ko04612 Antigen processing and presentation;
gene22930	K01363	CTSB	cathepsin B	EC:3.4.22.1	2	ko04142 Lysosome;ko04612 Antigen processing and presentation;
gene22932	K09872	PIP	aquaporin PIP	--		
gene22937	K00430	E1.11.1.7	peroxidase	EC:1.11.1.7	2	ko00360 Phenylalanine metabolism;ko00940 Phenylpropanoid biosynthesis;
gene22938	K17099	ANXA13	annexin A13	--		
gene22939	K01051	E3.1.1.11	pectinesterase	EC:3.1.1.11	2	ko00040 Pentose and glucuronate interconversions;ko00500 Starch and sucrose metabolism;
gene22940	K02883	RP-L18e, RPL18	large subunit ribosomal protein L18e	--	1	ko03010 Ribosome;
gene22947	K13162	PCBP2_3_4	poly(rC)-binding protein 2/3/4	--		
gene22954	K02372	fabZ	3-hydroxyacyl-[acyl-carrier-protein] dehydratase	EC:4.2.1.59	2	ko00061 Fatty acid biosynthesis;ko00780 Biotin metabolism;
gene22955	K08917	LHCB6	light-harvesting complex II chlorophyll a/b binding protein 6	--	1	ko00196 Photosynthesis - antenna proteins;
gene22956	K08917	LHCB6	light-harvesting complex II chlorophyll a/b binding protein 6	--	1	ko00196 Photosynthesis - antenna proteins;
gene22957	K08917	LHCB6	light-harvesting complex II chlorophyll a/b binding protein 6	--	1	ko00196 Photosynthesis - antenna proteins;
gene22961	K14292	TGS1	trimethylguanosine synthase	EC:2.1.1.-	1	ko03013 RNA transport;

gene22964	K13416	BAK1	brassinosteroid insensitive 1-associated receptor kinase 1	EC:2.7.10.1 2.7.11.1	2	ko04075 Plant hormone signal transduction;ko04626 Plant-pathogen interaction;
gene22965	K15920	XYL4	beta-D-xylosidase 4	EC:3.2.1.37	2	ko00500 Starch and sucrose metabolism;ko00520 Amino sugar and nucleotide sugar metabolism;
gene22968	K00975	glgC	glucose-1-phosphate adenylyltransferase	EC:2.7.7.27	2	ko00500 Starch and sucrose metabolism;ko00520 Amino sugar and nucleotide sugar metabolism;
gene22969	K00794	ribH, RIB4	6,7-dimethyl-8-ribityllumazine synthase	EC:2.5.1.78	1	ko00740 Riboflavin metabolism;
gene2296	K12471	EPN	epsin	--	1	ko04144 Endocytosis;
gene22981	K04733	IRAK4	interleukin-1 receptor-associated kinase 4	EC:2.7.11.1	11	ko04064 NF-kappa B signaling pathway;ko04210 Apoptosis;ko04620 Toll-like receptor signaling pathway;ko04722 Neurotrophin signaling pathway;ko05133 Pertussis;ko05140 Leishmaniasis;ko05142 Chagas disease (American trypanosomiasis);ko05145 Toxoplasmosis;ko05152 Tuberculosis;ko05162 Measles;ko05164 Influenza A;
gene22982	K13412	CPK	calcium-dependent protein kinase	EC:2.7.11.1	2	ko04626 Plant-pathogen interaction;ko05145 Toxoplasmosis;
gene22987	K11838	USP7, UBP15	ubiquitin carboxyl-terminal hydrolase 7	EC:3.1.2.15	3	ko05168 Herpes simplex infection;ko05169 Epstein-Barr virus infection;ko05203 Viral carcinogenesis;
gene2298	K12125	ELF3	protein EARLY FLOWERING 3	--	1	ko04712 Circadian rhythm - plant;
gene22990	K08910	LHCA4	light-harvesting complex I chlorophyll a/b binding protein 4	--	1	ko00196 Photosynthesis - antenna proteins;
gene22993	K13412	CPK	calcium-dependent protein kinase	EC:2.7.11.1	2	ko04626 Plant-pathogen interaction;ko05145 Toxoplasmosis;
gene22994	K02888	RP-L21, MRPL21, rplU	large subunit ribosomal protein L21	--	1	ko03010 Ribosome;
gene22	K02966	RP-S19e, RPS19	small subunit ribosomal protein S19e	--	1	ko03010 Ribosome;
gene23000	K03231	EEF1A	elongation factor 1-alpha	--	2	ko03013 RNA transport;ko05134 Legionellosis;
gene23001	K02736	PSMB4	20S proteasome subunit beta 7	EC:3.4.25.1	1	ko03050 Proteasome;
gene23002	K10706	SETX, ALS4	senataxin	EC:3.6.4.-		
gene23003	K08472	MLO	mlo protein	--		
gene23005	K01953	asnB, ASNS	asparagine synthase (glutamine-hydrolysing)	EC:6.3.5.4	1	ko00250 Alanine, aspartate and glutamate metabolism;
gene23007	K01369	LGMN	legumain	EC:3.4.22.34	2	ko04142 Lysosome;ko04612 Antigen processing and presentation;
gene23008	K01867	WARS, trpS	tryptophanyl-tRNA synthetase	EC:6.1.1.2	1	ko00970 Aminoacyl-tRNA biosynthesis;
gene2301	K08176	PHO84	MFS transporter, PHS family, inorganic phosphate transporter	--		
gene23020	K02884	RP-L19, MRPL19, rplS	large subunit ribosomal protein L19	--	1	ko03010 Ribosome;
gene23021	K14416	HBS1	elongation factor 1 alpha-like protein	--	2	ko03015 mRNA surveillance pathway;ko05134 Legionellosis;
gene23024	K02259	COX15	cytochrome c oxidase assembly protein subunit 15	--	3	ko00190 Oxidative phosphorylation;ko00860 Porphyrin and chlorophyll metabolism;ko02020 Two-component system;
gene23028	K02906	RP-L3, MRPL3, rplC	large subunit ribosomal protein L3	--	1	ko03010 Ribosome;
gene23029	K06675	SMC4	structural maintenance of chromosome 4	--	1	ko04111 Cell cycle - yeast;
gene23031	K00766	trpD	anthranilate phosphoribosyltransferase	EC:2.4.2.18	2	ko00400 Phenylalanine, tyrosine and tryptophan biosynthesis;ko01230 Biosynthesis of amino acids;
gene23032	K04354	PPP2R2	serine/threonine-protein phosphatase 2A regulatory subunit B	--	9	ko03015 mRNA surveillance pathway;ko04111 Cell cycle - yeast;ko04151 PI3K-Akt signaling pathway;ko04390 Hippo signaling pathway;ko04391 Hippo signaling pathway - fly;ko04530 Tight junction;ko04728 Dopaminergic synapse;ko05142 Chagas disease (American trypanosomiasis);ko05160 Hepatitis C;
gene23042	K08176	PHO84	MFS transporter, PHS family, inorganic phosphate transporter	--		
gene23043	K12119	CRY2	cryptochrome 2	--	1	ko04712 Circadian rhythm - plant;
gene23044	K09561	STUB1, CHIP	STIP1 homology and U-box containing protein 1	EC:6.3.2.19	2	ko04120 Ubiquitin mediated proteolysis;ko04141 Protein processing in endoplasmic reticulum;
gene2304	K05350	bglB	beta-glucosidase	EC:3.2.1.21	3	ko00460 Cyanoamino acid metabolism;ko00500 Starch and sucrose metabolism;ko00940 Phenylpropanoid biosynthesis;
gene23052	K01982	LSUrRNA	large subunit ribosomal RNA	--	2	ko03008 Ribosome biogenesis in eukaryotes;ko03010 Ribosome;
gene23054	K14315	NDC1, TMEM48	nucleoporin NDC1	--	1	ko03013 RNA transport;
gene23059	K04485	sms, radA	DNA repair protein RadA/Sms	--		
gene2305	K12619	XRN2, RAT1	5'-3' exoribonuclease 2	EC:3.1.13.-	2	ko03008 Ribosome biogenesis in eukaryotes;ko03018 RNA degradation;
gene23061	K11593	ELF2C	eukaryotic translation initiation factor 2C	--		

gene23064	K01803	TPI, tpiA	triosephosphate isomerase (TIM)	EC:5.3.1.1	6	ko00010 Glycolysis / Gluconeogenesis;ko00051 Fructose and mannose metabolism;ko00562 Inositol phosphate metabolism;ko00710 Carbon fixation in photosynthetic organisms;ko01200 Carbon metabolism;ko01230 Biosynthesis of amino acids;
gene23065	K00915	IPK2	1D-myo-Inositol-tetrakisphosphate 5-kinase / inositol-polyphosphate multikinase	EC:2.7.1.140 2.7.1.151	2	ko00562 Inositol phosphate metabolism;ko04070 Phosphatidylinositol signaling system;
gene23066	K10355	ACTF	actin, other eukaryote	--		
gene23070	K12184	VPS28	ESCRT-I complex subunit VPS28	--	1	ko04144 Endocytosis;
gene23071	K01945	purD	phosphoribosylamine--glycine ligase	EC:6.3.4.13	1	ko00230 Purine metabolism;
gene23079	K02830	HRAD1, RAD17	cell cycle checkpoint protein	EC:3.1.11.2	2	ko04111 Cell cycle - yeast;ko04113 Meiosis - yeast;
gene2307	K17338	REEP1_2_3_4	receptor expression-enhancing protein 1/2/3/4	--		
gene23083	K15082	RAD7	DNA repair protein RAD7	--		
gene23086	K14484	IAA	auxin-responsive protein IAA	--	1	ko04075 Plant hormone signal transduction;
gene23087	K02958	RP-S15e, RPS15	small subunit ribosomal protein S15e	--	1	ko03010 Ribosome;
gene23092	K15032	MTERFD	mTERF domain-containing protein, mitochondrial	--		
gene23093	K11253	H3	histone H3	--	3	ko05034 Alcoholism;ko05202 Transcriptional misregulation in cancer;ko05322 Systemic lupus erythematosus;
gene23094	K13800	CMPK1, UMPK	UMP-CMP kinase	EC:2.7.4.- 2.7.4.14	1	ko00240 Pyrimidine metabolism;
gene23099	K11279	NAP1L1, NRP	nucleosome assembly protein 1-like 1	--		
gene230	K13168	SFRS16	splicing factor, arginine/serine-rich 16	--		
gene23102	K12524	thrA	bifunctional aspartokinase / homoserine dehydrogenase 1	EC:2.7.2.4 1.1.1.3	4	ko00260 Glycine, serine and threonine metabolism;ko00270 Cysteine and methionine metabolism;ko00300 Lysine biosynthesis;ko01230 Biosynthesis of amino acids;
gene23103	K08245	E3.4.23.40	phytepsin	EC:3.4.23.40		
gene23104	K14009	BCAP31, BAP31	B-cell receptor-associated protein 31	--	1	ko04141 Protein processing in endoplasmic reticulum;
gene23109	K11423	SETD2, SET2	histone-lysine N-methyltransferase SETD2	EC:2.1.1.43	1	ko00310 Lysine degradation;
gene23110	K08342	ATG4	autophagy-related protein 4	EC:3.4.22.-	1	ko04140 Regulation of autophagy;
gene23114	K03537	POP5	ribonuclease P/MRP protein subunit POP5	EC:3.1.26.5	2	ko03008 Ribosome biogenesis in eukaryotes;ko03013 RNA transport;
gene23117	K01938	fhs	formate--tetrahydrofolate ligase	EC:6.3.4.3	3	ko00670 One carbon pool by folate;ko00720 Carbon fixation pathways in prokaryotes;ko01200 Carbon metabolism;
gene2311	K12585	DIS3, RRP44	exosome complex exonuclease DIS3/RRP44	EC:3.1.13.-	1	ko03018 RNA degradation;
gene23123	K13950	pabAB	para-aminobenzoate synthetase	EC:2.6.1.85	1	ko00790 Folate biosynthesis;
gene23124	K14486	K14486, ARF	auxin response factor	--	1	ko04075 Plant hormone signal transduction;
gene23125	K15382	SLC50A, SWEET	solute carrier family 50 (sugar transporter)	--		
gene23126	K15382	SLC50A, SWEET	solute carrier family 50 (sugar transporter)	--		
gene2312	K10268	FBXL2_20	F-box and leucine-rich repeat protein 2/20	--		
gene23132	K12855	PRPF6, PRP6	pre-mRNA-processing factor 6	--	1	ko03040 Spliceosome;
gene23140	K05909	E1.10.3.2				
gene2314	K00162	PDHB, pdhB	pyruvate dehydrogenase E1 component subunit beta	EC:1.2.4.1	6	ko00010 Glycolysis / Gluconeogenesis;ko00020 Citrate cycle (TCA cycle);ko00620 Pyruvate metabolism;ko00650 Butanoate metabolism;ko01200 Carbon metabolism;ko04066 HIF-1 signaling pathway;
gene23155	K03686	dnaJ	molecular chaperone DnaJ	--		
gene2315	K03549	kup	KUP system potassium uptake protein	--		
gene23168	K17505	PPM1K, PP2CM	protein phosphatase 1K	EC:3.1.3.16		
gene23169	K10364	CAPZA	capping protein (actin filament) muscle Z-line, alpha	--		
gene23170	K11251	H2A	histone H2A	--	2	ko05034 Alcoholism;ko05322 Systemic lupus erythematosus;
gene23172	K14484	IAA	auxin-responsive protein IAA	--	1	ko04075 Plant hormone signal transduction;
gene23175	K02721	psbW	photosystem II PsbW protein	--	1	ko00195 Photosynthesis;
gene23177	K16277	DRIP	E3 ubiquitin-protein ligase DRIP	EC:6.3.2.19		
gene23179	K11275	H1_5	histone H1/5	--		
gene23180	K13162	PCBP2_3_4	poly(rC)-binding protein 2/3/4	--		
gene23181	K03593	mrp	ATP-binding protein involved in chromosome partitioning	--		

gene23183	K15382	SLC50A, SWEET	solute carrier family 50 (sugar transporter)	--		
gene23185	K09651	RHBDD1	rhomboid domain-containing protein 1	EC:3.4.21.-		
gene2318	K02178	BUB1	checkpoint serine/threonine-protein kinase	EC:2.7.11.1	5	ko04110 Cell cycle;ko04111 Cell cycle - yeast;ko04113 Meiosis - yeast;ko04114 Oocyte meiosis;ko04914 Progesterone-mediated oocyte maturation;
gene23191	K15170	MED27	mediator of RNA polymerase II transcription subunit 27	--		
gene23192	K08064	NFYA	nuclear transcription factor Y, alpha	--	2	ko04612 Antigen processing and presentation;ko05152 Tuberculosis;
gene23193	K14803	PTC2_3	protein phosphatase 2C homolog 2/3	EC:3.1.3.16		
gene23194	K02999	RPA1, POLR1A	DNA-directed RNA polymerase I subunit RPA1	EC:2.7.7.6	3	ko00230 Purine metabolism;ko00240 Pyrimidine metabolism;ko03020 RNA polymerase;
gene23195	K05909	E1.10.3.2				
gene23198	K09540	SEC63	translocation protein SEC63	--	2	ko03060 Protein export;ko04141 Protein processing in endoplasmic reticulum;
gene2319	K12836	U2AF1	splicing factor U2AF 35 kDa subunit	--	2	ko03040 Spliceosome;ko05131 Shigellosis;
gene23201	K00679	E2.3.1.158	phospholipid:diacylglycerol acyltransferase	EC:2.3.1.158	1	ko00561 Glycerolipid metabolism;
gene23202	K14662	NTAN1	protein N-terminal asparagine amidohydrolase	EC:3.5.1.-		
gene23205	K08272	CAB39, MO25	calcium binding protein 39	--	1	ko04150 mTOR signaling pathway;
gene23211	K09422	MYBP	myb proto-oncogene protein, plant	--		
gene23212	K17046	DEK	protein DEK	--		
gene23221	K00873	PK, pyk	pyruvate kinase	EC:2.7.1.40	7	ko00010 Glycolysis / Gluconeogenesis;ko00230 Purine metabolism;ko00620 Pyruvate metabolism;ko01200 Carbon metabolism;ko01230 Biosynthesis of amino acids;ko04930 Type II diabetes mellitus;ko05203 Viral carcinogenesis;
gene23222	K09903	pyrH	uridylate kinase	EC:2.7.4.22	1	ko00240 Pyrimidine metabolism;
gene23225	K13103	TFIP11	tuftelin-interacting protein 11	--		
gene23226	K03130	TAF5	transcription initiation factor TFIID subunit 5	--	2	ko03022 Basal transcription factors;ko05168 Herpes simplex infection;
gene23227	K00262	E1.4.1.4, gdhA	glutamate dehydrogenase (NADP+)	EC:1.4.1.4	3	ko00250 Alanine, aspartate and glutamate metabolism;ko00330 Arginine and proline metabolism;ko00910 Nitrogen metabolism;
gene23228	K00262	E1.4.1.4, gdhA	glutamate dehydrogenase (NADP+)	EC:1.4.1.4	3	ko00250 Alanine, aspartate and glutamate metabolism;ko00330 Arginine and proline metabolism;ko00910 Nitrogen metabolism;
gene23230	K11841	USP10, UBP3	ubiquitin carboxyl-terminal hydrolase 10	EC:3.1.2.15		
gene23231	K08913	LHCB2	light-harvesting complex II chlorophyll a/b binding protein 2	--	1	ko00196 Photosynthesis - antenna proteins;
gene23236	K02881	RP-L18, MRPL18, rplR	large subunit ribosomal protein L18	--	1	ko03010 Ribosome;
gene23238	K05605	HIBCH	3-hydroxyisobutyryl-CoA hydrolase	EC:3.1.2.4	3	ko00280 Valine, leucine and isoleucine degradation;ko00410 beta-Alanine metabolism;ko00640 Propanoate metabolism;
gene23239	K03921	DESA1	acyl-[acyl-carrier-protein] desaturase	EC:1.14.19.2	2	ko00061 Fatty acid biosynthesis;ko01040 Biosynthesis of unsaturated fatty acids;
gene23241	K13093	HTATSF1	HIV Tat-specific factor 1	--		
gene23242	K14007	SEC24	protein transport protein SEC24	--	1	ko04141 Protein processing in endoplasmic reticulum;
gene23246	K12741	HNRNPA1_3	heterogeneous nuclear ribonucleoprotein A1/A3	--	1	ko03040 Spliceosome;
gene23247	K11593	ELF2C	eukaryotic translation initiation factor 2C	--		
gene23248	K10756	RFC3_5	replication factor C subunit 3/5	--	3	ko03030 DNA replication;ko03420 Nucleotide excision repair;ko03430 Mismatch repair;
gene2324	K02638	petE	plastocyanin	--	1	ko00195 Photosynthesis;
gene23253	K12598	MTR4, SKIV2L2	ATP-dependent RNA helicase DOB1	EC:3.6.4.13	1	ko03018 RNA degradation;
gene23258	K03327	TC.MATE, SLC47A, norM, mdtK, dinF	multidrug resistance protein, MATE family	--		
gene2325	K13412	CPK	calcium-dependent protein kinase	EC:2.7.11.1	2	ko04626 Plant-pathogen interaction;ko05145 Toxoplasmosis;
gene23262	K05658	ABCB1	ATP-binding cassette, subfamily B (MDR/TAP), member 1	--	3	ko02010 ABC transporters;ko04976 Bile secretion;ko05206 MicroRNAs in cancer;
gene23265	K04733	IRAK4	interleukin-1 receptor-associated kinase 4	EC:2.7.11.1	11	ko04064 NF-kappa B signaling pathway;ko04210 Apoptosis;ko04620 Toll-like receptor signaling pathway;ko04722 Neurotrophin signaling pathway;ko05133 Pertussis;ko05140 Leishmaniasis;ko05142 Chagas disease (American trypanosomiasis);ko05145 Toxoplasmosis;ko05152 Tuberculosis;ko05162 Measles;ko05164 Influenza A;

gene23269	K04733	IRAK4	interleukin-1 receptor-associated kinase 4	EC:2.7.11.1	11	ko04064 NF-kappa B signaling pathway;ko04210 Apoptosis;ko04620 Toll-like receptor signaling pathway;ko04722 Neurotrophin signaling pathway;ko05133 Pertussis;ko05140 Leishmaniasis;ko05142 Chagas disease (American trypanosomiasis);ko05145 Toxoplasmosis;ko05152 Tuberculosis;ko05162 Measles;ko05164 Influenza A;
gene23271	K11723	BRD7_9	bromodomain-containing protein 7/9	--		
gene23278	K13095	SF1	splicing factor 1	--		
gene23279	K15305	VAC14, TAX1BP2	vacuole morphology and inheritance protein 14	--	2	ko05166 HTLV-I infection;ko05203 Viral carcinogenesis;
gene23282	K16812	TPX2	targeting protein for Xklp2	--		
gene23286	K17081	PHB2	prohibitin 2	--		
gene2328	K08901	psbQ	photosystem II oxygen-evolving enhancer protein 3	--	1	ko00195 Photosynthesis;
gene23291	K01251	E3.3.1.1, ahcY	adenosylhomocysteinase	EC:3.3.1.1	1	ko00270 Cysteine and methionine metabolism;
gene23297	K09422	MYBP	myb proto-oncogene protein, plant	--		
gene23299	K03301	TC.AAA	ATP:ADP antiporter, AAA family	--		
gene23303	K13496	UGT73C	UDP-glucosyl transferase 73C	EC:2.4.1.-		
gene23305	K12876	RBM8A, Y14	RNA-binding protein 8A	--	3	ko03013 RNA transport;ko03015 mRNA surveillance pathway;ko03040 Spliceosome;
gene23309	K13947	PIN	auxin efflux carrier family	--		
gene23310	K10406	KIFC2_3	kinesin family member C2/C3	--		
gene23314	K02882	RP-L18Ae, RPL18A	large subunit ribosomal protein L18Ae	--	1	ko03010 Ribosome;
gene23315	K10357	MYO5	myosin V	--		
gene23316	K00565	RNMT	mRNA (guanine-N7-)-methyltransferase	EC:2.1.1.56	1	ko03015 mRNA surveillance pathway;
gene23319	K07119	K07119				
gene2331	K13162	PCBP2_3_4	poly(rC)-binding protein 2/3/4	--		
gene23320	K07119	K07119				
gene23324	K00514	ZDS, crtQ	zeta-carotene desaturase	EC:1.3.5.6	1	ko00906 Carotenoid biosynthesis;
gene23326	K06444	lcyE, crtL2	lycopene epsilon-cyclase	EC:5.5.1.18	1	ko00906 Carotenoid biosynthesis;
gene23327	K03257	EIF4A	translation initiation factor 4A	--	1	ko03013 RNA transport;
gene23328	K11155	DGAT1	diacylglycerol O-acyltransferase 1	EC:2.3.1.20 2.3.1.75 2.3.1.76	3	ko00561 Glycerolipid metabolism;ko00830 Retinol metabolism;ko04975 Fat digestion and absorption;
gene23331	K12194	CHMP4, SNF7, VPS32	charged multivesicular body protein 4	--	1	ko04144 Endocytosis;
gene23336	K02140	ATPeFG, ATP5L	F-type H+-transporting ATPase subunit g	EC:3.6.3.14	1	ko00190 Oxidative phosphorylation;
gene23338	K02155	ATPeVPL, ATP6L	V-type H+-transporting ATPase 16kDa proteolipid subunit	EC:3.6.3.14	9	ko00190 Oxidative phosphorylation;ko04142 Lysosome;ko04145 Phagosome;ko04721 Synaptic vesicle cycle;ko04966 Collecting duct acid secretion;ko05110 Vibrio cholerae infection;ko05120 Epithelial cell signaling in Helicobacter pylori infection;ko05152 Tuberculosis;ko05323 Rheumatoid arthritis;
gene23341	K13430	PBS1	serine/threonine-protein kinase PBS1	EC:2.7.11.1	1	ko04626 Plant-pathogen interaction;
gene23344	K01918	panC	pantoate--beta-alanine ligase	EC:6.3.2.1	2	ko00410 beta-Alanine metabolism;ko00770 Pantothenate and CoA biosynthesis;
gene23345	K15731	CTDSP	carboxy-terminal domain RNA polymerase II polypeptide A small phosphatase	EC:3.1.3.16		
gene2334	K14488	SAUR	SAUR family protein	--	1	ko04075 Plant hormone signal transduction;
gene23357	K06067	HDAC1_2	histone deacetylase 1/2	EC:3.5.1.98	9	ko04110 Cell cycle;ko04330 Notch signaling pathway;ko05016 Huntington's disease;ko05034 Alcoholism;ko05169 Epstein-Barr virus infection;ko05200 Pathways in cancer;ko05202 Transcriptional misregulation in cancer;ko05203 Viral carcinogenesis;ko05220 Chronic myeloid leukemia;
gene23361	K03676	grxC, GLRX, GLRX2	glutaredoxin 3	--		
gene23366	K09842	AAO3	abscisic-aldehyde oxidase	EC:1.2.3.14	1	ko00906 Carotenoid biosynthesis;

gene23369	K03083	GSK3B	glycogen synthase kinase 3 beta	EC:2.7.11.26	29	ko04012 ErbB signaling pathway;ko04062 Chemokine signaling pathway;ko04110 Cell cycle;ko04151 PI3K-Akt signaling pathway;ko04310 Wnt signaling pathway;ko04340 Hedgehog signaling pathway;ko04360 Axon guidance;ko04390 Hippo signaling pathway;ko04510 Focal adhesion;ko04660 T cell receptor signaling pathway;ko04662 B cell receptor signaling pathway;ko04711 Circadian rhythm - fly;ko04722 Neurotrophin signaling pathway;ko04728 Dopaminergic synapse;ko04910 Insulin signaling pathway;ko04916 Melanogenesis;ko04917 Prolactin signaling pathway;ko04932 Non-alcoholic fatty liver disease (NAFLD);ko05010 Alzheimer's disease;ko05160 Hepatitis C;ko05162 Measles;ko05164 Influenza A;ko05166 HTLV-I infection;ko05169 Epstein-Barr virus infection;ko05200 Pathways in cancer;ko05210 Colorectal cancer;ko05213 Endometrial cancer;ko05215 Prostate
gene23370	K01867	WARS, trpS	tryptophanyl-tRNA synthetase	EC:6.1.1.2	1	ko00970 Aminoacyl-tRNA biosynthesis;
gene23373	K01679	E4.2.1.2B, fumC	fumarate hydratase, class II	EC:4.2.1.2	5	ko00020 Citrate cycle (TCA cycle);ko00720 Carbon fixation pathways in prokaryotes;ko01200 Carbon metabolism;ko05200 Pathways in cancer;ko05211 Renal cell carcinoma;
gene23374	K09773	K09773	hypothetical protein	--		
gene23378	K00430	E1.11.1.7	peroxidase	EC:1.11.1.7	2	ko00360 Phenylalanine metabolism;ko00940 Phenylpropanoid biosynthesis;
gene2337	K09419	HSFF	heat shock transcription factor, other eukaryote	--		
gene23380	K13448	CML	calcium-binding protein CML	--	1	ko04626 Plant-pathogen interaction;
gene23381	K01530	E3.6.3.1	phospholipid-translocating ATPase	EC:3.6.3.1		
gene23383	K02918	RP-L35e, RPL35	large subunit ribosomal protein L35e	--	1	ko03010 Ribosome;
gene23388	K02519	infB, MTIF2	translation initiation factor IF-2	--		
gene23393	K07151	STT3	dolichyl-diphosphooligosaccharide--protein glycosyltransferase	EC:2.4.99.18	3	ko00510 N-Glycan biosynthesis;ko00513 Various types of N-glycan biosynthesis;ko04141 Protein processing in endoplasmic reticulum;
gene23394	K11982	RNF115_126	E3 ubiquitin-protein ligase RNF115/126	EC:6.3.2.19		
gene23399	K14398	CPSF6_7	cleavage and polyadenylation specificity factor subunit 6/7	--	1	ko03015 mRNA surveillance pathway;
gene23405	K15176	CTR9	RNA polymerase-associated protein CTR9	--		
gene2340	K04733	IRAK4	interleukin-1 receptor-associated kinase 4	EC:2.7.11.1	11	ko04064 NF-kappa B signaling pathway;ko04210 Apoptosis;ko04620 Toll-like receptor signaling pathway;ko04722 Neurotrophin signaling pathway;ko05133 Pertussis;ko05140 Leishmaniasis;ko05142 Chagas disease (American trypanosomiasis);ko05145 Toxoplasmosis;ko05152 Tuberculosis;ko05162 Measles;ko05164 Influenza A;
gene23413	K00899	mtnK	5-methylthioribose kinase	EC:2.7.1.100	1	ko00270 Cysteine and methionine metabolism;
gene23417	K05280	E1.14.13.21	flavonoid 3'-monooxygenase	EC:1.14.13.21	2	ko00941 Flavonoid biosynthesis;ko00944 Flavone and flavonol biosynthesis;
gene23419	K13457	RPM1, RPS3	disease resistance protein RPM1	--	1	ko04626 Plant-pathogen interaction;
gene23421	K17434	MRPL53	large subunit ribosomal protein L53, mitochondrial	--		
gene23424	K15404	K15404, CER1	aldehyde decarbonylase	EC:4.1.99.5	1	ko00073 Cutin, suberine and wax biosynthesis;
gene2342	K04733	IRAK4	interleukin-1 receptor-associated kinase 4	EC:2.7.11.1	11	ko04064 NF-kappa B signaling pathway;ko04210 Apoptosis;ko04620 Toll-like receptor signaling pathway;ko04722 Neurotrophin signaling pathway;ko05133 Pertussis;ko05140 Leishmaniasis;ko05142 Chagas disease (American trypanosomiasis);ko05145 Toxoplasmosis;ko05152 Tuberculosis;ko05162 Measles;ko05164 Influenza A;
gene23431	K00549	metE	5-methyltetrahydropteroyltriglutamate--homocysteine methyltransferase	EC:2.1.1.14	3	ko00270 Cysteine and methionine metabolism;ko00450 Selenocompound metabolism;ko01230 Biosynthesis of amino acids;
gene23432	K00549	metE	5-methyltetrahydropteroyltriglutamate--homocysteine methyltransferase	EC:2.1.1.14	3	ko00270 Cysteine and methionine metabolism;ko00450 Selenocompound metabolism;ko01230 Biosynthesis of amino acids;
gene23437	K10704	UBE2V	ubiquitin-conjugating enzyme E2 variant	--		
gene23438	K08829	MAK	male germ cell-associated kinase	EC:2.7.11.22		
gene23446	K04718	SPHK	sphingosine kinase	EC:2.7.1.91	5	ko00600 Sphingolipid metabolism;ko04020 Calcium signaling pathway;ko04370 VEGF signaling pathway;ko04666 Fc gamma R-mediated phagocytosis;ko05152 Tuberculosis;

gene23448	K03351	APC4	anaphase-promoting complex subunit 4	--	7	ko04110 Cell cycle;ko04111 Cell cycle - yeast;ko04113 Meiosis - yeast;ko04114 Oocyte meiosis;ko04120 Ubiquitin mediated proteolysis;ko04914 Progesterone-mediated oocyte maturation;ko05166 HTLV-I infection;
gene23449	K03245	EIF3J	translation initiation factor 3 subunit J	--	1	ko03013 RNA transport;
gene23451	K01188	E3.2.1.21	beta-glucosidase	EC:3.2.1.21	3	ko00460 Cyanoamino acid metabolism;ko00500 Starch and sucrose metabolism;ko00940 Phenylpropanoid biosynthesis;
gene23458	K12449	AXS	UDP-apiose/xylose synthase	--	1	ko00520 Amino sugar and nucleotide sugar metabolism;
gene23460	K08272	CAB39, MO25	calcium binding protein 39	--	1	ko04150 mTOR signaling pathway;
gene2346	K15032	MTERFD	mTERF domain-containing protein, mitochondrial	--		
gene23472	K14776	DDX10, DBP4	ATP-dependent RNA helicase DDX10/DBP4	EC:3.6.4.13		
gene23477	K14209	SLC36A, PAT	solute carrier family 36 (proton-coupled amino acid transporter)	--		
gene2347	K00915	IPK2	1D-myo-Inositol-tetrakisphosphate 5-kinase / inositol-polyphosphate multikinase	EC:2.7.1.140 2.7.1.151	2	ko00562 Inositol phosphate metabolism;ko04070 Phosphatidylinositol signaling system;
gene23481	K14652	ribBA	3,4-dihydroxy 2-butanone 4-phosphate synthase / GTP cyclohydrolase II	EC:4.1.99.12 3.5.4.25	1	ko00740 Riboflavin metabolism;
gene23488	K14512	MPK6	mitogen-activated protein kinase 6	EC:2.7.11.24	1	ko04075 Plant hormone signal transduction;
gene23489	K06889	K06889				
gene23492	K15361	WDR48, UAF1	WD repeat-containing protein 48	--	1	ko03460 Fanconi anemia pathway;
gene23493	K09131	K09131	hypothetical protein	--		
gene234	K08237	E2.4.1.218	hydroquinone glucosyltransferase	EC:2.4.1.218		
gene23502	K11096	SNRPD2, SMD2	small nuclear ribonucleoprotein D2	--	1	ko03040 Spliceosome;
gene23506	K16297	SCPL-II	serine carboxypeptidase-like clade II	EC:3.4.16.-		
gene23513	K11864	BRCC3	BRCA1/BRCA2-containing complex subunit 3	--		
gene23515	K05289	GAA1	glycosylphosphatidylinositol transamidase	--	1	ko00563 Glycosylphosphatidylinositol(GPI)-anchor biosynthesis;
gene23520	K07277	yaeT	outer membrane protein	--		
gene23524	K04567	KARS, lysS	lysyl-tRNA synthetase, class II	EC:6.1.1.6	1	ko00970 Aminoacyl-tRNA biosynthesis;
gene23527	K16296	SCPL-I	serine carboxypeptidase-like clade I	EC:3.4.16.-		
gene23530	K10143	RFWD2, COP1	E3 ubiquitin-protein ligase RFWD2	EC:6.3.2.19	3	ko04115 p53 signaling pathway;ko04120 Ubiquitin mediated proteolysis;ko04712 Circadian rhythm - plant;
gene23535	K10355	ACTF	actin, other eukaryote	--		
gene23537	K09285	OVM, ANT	AP2-like factor, ANT lineage	--		
gene23538	K12891	SFRS2	splicing factor, arginine/serine-rich 2	--	2	ko03040 Spliceosome;ko05168 Herpes simplex infection;
gene23540	K03137	TFIIE2, GTF2E2, TFA2	transcription initiation factor TFIIE subunit beta	--	3	ko03022 Basal transcription factors;ko05169 Epstein-Barr virus infection;ko05203 Viral carcinogenesis;
gene23541	K13566	NIT2	omega-amidase	EC:3.5.1.3	1	ko00250 Alanine, aspartate and glutamate metabolism;
gene23549	K02882	RP-L18Ae, RPL18A	large subunit ribosomal protein L18Ae	--	1	ko03010 Ribosome;
gene23552	K16578	CLASP1_2	CLIP-associating protein 1/2	--		
gene23554	K03043	rpoB	DNA-directed RNA polymerase subunit beta	EC:2.7.7.6	3	ko00230 Purine metabolism;ko00240 Pyrimidine metabolism;ko03020 RNA polymerase;
gene23559	K14802	DRS2, ATP8A	phospholipid-transporting ATPase	EC:3.6.3.1		
gene23560	K10357	MYO5	myosin V	--		
gene23566	K15186	EAF	ELL-associated factor	--		
gene23567	K01358	clpP, CLPP	ATP-dependent Clp protease, protease subunit	EC:3.4.21.92	1	ko04112 Cell cycle - Caulobacter;
gene23568	K00604	MTFMT, fmt	methionyl-tRNA formyltransferase	EC:2.1.2.9	2	ko00670 One carbon pool by folate;ko00970 Aminoacyl-tRNA biosynthesis;
gene2356	K07198	PRKAA, AMPK	5'-AMP-activated protein kinase, catalytic alpha subunit	EC:2.7.11.11	8	ko04140 Regulation of autophagy;ko04150 mTOR signaling pathway;ko04151 PI3K-Akt signaling pathway;ko04710 Circadian rhythm;ko04910 Insulin signaling pathway;ko04920 Adipocytokine signaling pathway;ko04932 Non-alcoholic fatty liver disease (NAFLD);ko05410 Hypertrophic cardiomyopathy (HCM);
gene23571	K02973	RP-S23e, RPS23	small subunit ribosomal protein S23e	--	1	ko03010 Ribosome;
gene23573	K08272	CAB39, MO25	calcium binding protein 39	--	1	ko04150 mTOR signaling pathway;
gene23574	K10047	VTC4	inositol-phosphate phosphatase / L-galactose 1-phosphate phosphatase	EC:3.1.3.25	3	ko00053 Ascorbate and aldarate metabolism;ko00562 Inositol phosphate metabolism;ko04070 Phosphatidylinositol signaling system;

gene23576	K00412	CYTB, petB	ubiquinol-cytochrome c reductase cytochrome b subunit	--	7	ko00190 Oxidative phosphorylation;ko02020 Two-component system;ko04260 Cardiac muscle contraction;ko04932 Non-alcoholic fatty liver disease (NAFLD);ko05010 Alzheimer's disease;ko05012 Parkinson's disease;ko05016 Huntington's disease;
gene23578	K00966	GMPP	mannose-1-phosphate guanylyltransferase	EC:2.7.7.13	2	ko00051 Fructose and mannose metabolism;ko00520 Amino sugar and nucleotide sugar metabolism;
gene23582	K08238	XXT	xyloglucan 6-xylosyltransferase	EC:2.4.2.39		
gene23587	K01537	E3.6.3.8	Ca2+-transporting ATPase	EC:3.6.3.8		
gene23593	K06636	SMC1	structural maintenance of chromosome 1	--	4	ko04110 Cell cycle;ko04111 Cell cycle - yeast;ko04113 Meiosis - yeast;ko04114 Oocyte meiosis;
gene23594	K14505	CYCD3	cyclin D3, plant	--	1	ko04075 Plant hormone signal transduction;
gene23595	K02737	PSMB5	20S proteasome subunit beta 5	EC:3.4.25.1	1	ko03050 Proteasome;
gene23596	K10775	E4.3.1.24	phenylalanine ammonia-lyase	EC:4.3.1.24	2	ko00360 Phenylalanine metabolism;ko00940 Phenylpropanoid biosynthesis;
gene23597	K08679	E5.1.3.6	UDP-glucuronate 4-epimerase	EC:5.1.3.6	2	ko00500 Starch and sucrose metabolism;ko00520 Amino sugar and nucleotide sugar metabolism;
gene23601	K01904	E6.2.1.12	4-coumarate--CoA ligase	EC:6.2.1.12	3	ko00130 Ubiquinone and other terpenoid-quinone biosynthesis;ko00360 Phenylalanine metabolism;ko00940 Phenylpropanoid biosynthesis;
gene23602	K01802	E5.2.1.8	peptidylprolyl isomerase	EC:5.2.1.8		
gene23604	K05656	ABCB9, TAPL	ATP-binding cassette, subfamily B (MDR/TAP), member 9	--	2	ko02010 ABC transporters;ko04142 Lysosome;
gene23608	K12823	DDX5, DBP2	ATP-dependent RNA helicase DDX5/DBP2	EC:3.6.4.13	3	ko03040 Spliceosome;ko05202 Transcriptional misregulation in cancer;ko05205 Proteoglycans in cancer;
gene23610	K08237	E2.4.1.218	hydroquinone glucosyltransferase	EC:2.4.1.218		
gene23612	K03106	SRP54, ffh	signal recognition particle subunit SRP54	--	2	ko03060 Protein export;ko03070 Bacterial secretion system;
gene23613	K11804	WDR42A	WD repeat-containing protein 42A	--		
gene23614	K06640	ATR	serine/threonine-protein kinase ATR	EC:2.7.11.1	4	ko03460 Fanconi anemia pathway;ko04110 Cell cycle;ko04115 p53 signaling pathway;ko05166 HTLV-I infection;
gene23618	K01188	E3.2.1.21	beta-glucosidase	EC:3.2.1.21	3	ko00460 Cyanoamino acid metabolism;ko00500 Starch and sucrose metabolism;ko00940 Phenylpropanoid biosynthesis;
gene23619	K02893	RP-L23Ae, RPL23A	large subunit ribosomal protein L23Ae	--	1	ko03010 Ribosome;
gene23624	K00555	TRMT1, trm1	tRNA (guanine26-N2/guanine27-N2)-dimethyltransferase	EC:2.1.1.215 2.1.1.216		
gene23631	K16732	PRC1	protein regulator of cytokinesis 1	--		
gene23632	K13141	INTS4	integrator complex subunit 4	--		
gene23634	K12261	HACL1	2-hydroxyacyl-CoA lyase 1	EC:4.1.-.-	1	ko04146 Peroxisome;
gene23635	K03327	TC.MATE, SLC47A, norM, mdtK, dinF	multidrug resistance protein, MATE family	--		
gene23638	K00485	E1.14.13.8	dimethylaniline monooxygenase (N-oxide forming)	EC:1.14.13.8	2	ko00680 Methane metabolism;ko00982 Drug metabolism - cytochrome P450;
gene23648	K00799	GST, gst	glutathione S-transferase	EC:2.5.1.18	4	ko00480 Glutathione metabolism;ko00980 Metabolism of xenobiotics by cytochrome P450;ko00982 Drug metabolism - cytochrome P450;ko05204 Chemical carcinogenesis;
gene23649	K00799	GST, gst	glutathione S-transferase	EC:2.5.1.18	4	ko00480 Glutathione metabolism;ko00980 Metabolism of xenobiotics by cytochrome P450;ko00982 Drug metabolism - cytochrome P450;ko05204 Chemical carcinogenesis;
gene23652	K08272	CAB39, MO25	calcium binding protein 39	--	1	ko04150 mTOR signaling pathway;
gene23655	K14831	MAK16	protein MAK16	--		
gene23656	K04078	groES, HSPE1	chaperonin GroES	--		
gene23657	K14486	K14486, ARF	auxin response factor	--	1	ko04075 Plant hormone signal transduction;
gene23659	K14806	DDX31, DBP7	ATP-dependent RNA helicase DDX31/DBP7	EC:3.6.4.13		
gene23660	K00939	E2.7.4.3, adk	adenylate kinase	EC:2.7.4.3	1	ko00230 Purine metabolism;
gene23670	K01802	E5.2.1.8	peptidylprolyl isomerase	EC:5.2.1.8		
gene23671	K14571	RIX7, NVL	ribosome biogenesis ATPase	--	1	ko03008 Ribosome biogenesis in eukaryotes;
gene23672	K03495	gidA, mnmG, MTO1	tRNA uridine 5-carboxymethylaminomethyl modification enzyme	--		
gene23673	K00326	E1.6.2.2	cytochrome-b5 reductase	EC:1.6.2.2	1	ko00520 Amino sugar and nucleotide sugar metabolism;
gene23680	K07466	RFA1, RPA1, rpa	replication factor A1	--	5	ko03030 DNA replication;ko03420 Nucleotide excision repair;ko03430 Mismatch repair;ko03440 Homologous recombination;ko03460 Fanconi anemia pathway;

gene23687	K10355	ACTF	actin, other eukaryote	--		
gene23689	K17637	EXOC2, SEC5	exocyst complex component 2	--	1	ko04014 Ras signaling pathway;
gene2368	K14493	GID1	gibberellin receptor GID1	EC:3.-.-.-	1	ko04075 Plant hormone signal transduction;
gene23690	K03841	FBP, fbp	fructose-1,6-bisphosphatase I	EC:3.1.3.11	7	ko00010 Glycolysis / Gluconeogenesis;ko00030 Pentose phosphate pathway;ko00051 Fructose and mannose metabolism;ko00680 Methane metabolism;ko00710 Carbon fixation in photosynthetic organisms;ko01200 Carbon metabolism;ko04910 Insulin signaling pathway;
gene23692	K03801	lipB	lipoyl(octanoyl) transferase	EC:2.3.1.181	1	ko00785 Lipoic acid metabolism;
gene23693	K11253	H3	histone H3	--	3	ko05034 Alcoholism;ko05202 Transcriptional misregulation in cancer;ko05322 Systemic lupus erythematosus;
gene23696	K10396	KIF5	kinesin family member 5	--	1	ko04728 Dopaminergic synapse;
gene23706	K16732	PRC1	protein regulator of cytokinesis 1	--		
gene23710	K10885	XRCC5, KU80, G22P2	ATP-dependent DNA helicase 2 subunit 2	--	1	ko03450 Non-homologous end-joining;
gene23711	K14826	FPR3_4	FK506-binding nuclear protein	EC:5.2.1.8		
gene23712	K01800	maiA, GSTZ1	maleylacetoacetate isomerase	EC:5.2.1.2	2	ko00350 Tyrosine metabolism;ko00643 Styrene degradation;
gene23719	K00799	GST, gst	glutathione S-transferase	EC:2.5.1.18	4	ko00480 Glutathione metabolism;ko00980 Metabolism of xenobiotics by cytochrome P450;ko00982 Drug metabolism - cytochrome P450;ko05204 Chemical carcinogenesis;
gene23727	K03083	GSK3B	glycogen synthase kinase 3 beta	EC:2.7.11.26	29	ko04012 ErbB signaling pathway;ko04062 Chemokine signaling pathway;ko04110 Cell cycle;ko04151 PI3K-Akt signaling pathway;ko04310 Wnt signaling pathway;ko04340 Hedgehog signaling pathway;ko04360 Axon guidance;ko04390 Hippo signaling pathway;ko04510 Focal adhesion;ko04660 T cell receptor signaling pathway;ko04662 B cell receptor signaling pathway;ko04711 Circadian rhythm - fly;ko04722 Neurotrophin signaling pathway;ko04728 Dopaminergic synapse;ko04910 Insulin signaling pathway;ko04916 Melanogenesis;ko04917 Prolactin signaling pathway;ko04932 Non-alcoholic fatty liver disease (NAFLD);ko05010 Alzheimer's disease;ko05160 Hepatitis C;ko05162 Measles;ko05164 Influenza A;ko05166 HTLV-I infection;ko05169 Epstein-Barr virus infection;ko05200 Pathways in cancer;ko05210 Colorectal cancer;ko05213 Endometrial cancer;ko05215 Prostate
gene23729	K08493	VTI1	vesicle transport through interaction with t-SNAREs 1	--	1	ko04130 SNARE interactions in vesicular transport;
gene2372	K14490	AHP	histidine-containing phosphotransfer peotein	--	1	ko04075 Plant hormone signal transduction;
gene23735	K02959	RP-S16, MRPS16, rpsP	small subunit ribosomal protein S16	--	1	ko03010 Ribosome;
gene23737	K01069	E3.1.2.6, gloB	hydroxyacylglutathione hydrolase	EC:3.1.2.6	1	ko00620 Pyruvate metabolism;
gene23742	K10772	APEX2	AP endonuclease 2	EC:4.2.99.18	1	ko03410 Base excision repair;
gene23745	K14488	SAUR	SAUR family protein	--	1	ko04075 Plant hormone signal transduction;
gene23751	K05643	ABCA3	ATP-binding cassette, subfamily A (ABC1), member 3	--	1	ko02010 ABC transporters;
gene23752	K09419	HSFF	heat shock transcription factor, other eukaryote	--		
gene23758	K00856	E2.7.1.20, ADK	adenosine kinase	EC:2.7.1.20	1	ko00230 Purine metabolism;
gene23760	K01051	E3.1.1.11	pectinesterase	EC:3.1.1.11	2	ko00040 Pentose and glucuronate interconversions;ko00500 Starch and sucrose metabolism;
gene23777	K09580	PDIA1, P4HB	protein disulfide-isomerase A1	EC:5.3.4.1	1	ko04141 Protein processing in endoplasmic reticulum;
gene23781	K14500	BSK	BR-signaling kinase	EC:2.7.11.1	1	ko04075 Plant hormone signal transduction;
gene23782	K03798	ftsH, hflB	cell division protease FtsH	EC:3.4.24.-		
gene23783	K11583	PPP2R3	serine/threonine-protein phosphatase 2A regulatory subunit B''	--	3	ko03015 mRNA surveillance pathway;ko04151 PI3K-Akt signaling pathway;ko04728 Dopaminergic synapse;
gene23784	K06927	K06927				
gene23785	K03133	TAF9B, TAF9	transcription initiation factor TFIID subunit 9B	--	2	ko03022 Basal transcription factors;ko05168 Herpes simplex infection;
gene2378	K02872	RP-L13Ae, RPL13A	large subunit ribosomal protein L13Ae	--	1	ko03010 Ribosome;
gene23791	K01762	ACS	1-aminocyclopropane-1-carboxylate synthase	EC:4.4.1.14	1	ko00270 Cysteine and methionine metabolism;
gene23797	K15192	BTAF1, MOT1	TATA-binding protein-associated factor	EC:3.6.4.-		
gene23798	K01873	VARS, valS	valyl-tRNA synthetase	EC:6.1.1.9	1	ko00970 Aminoacyl-tRNA biosynthesis;

gene2379	K01051	E3.1.1.11	pectinesterase	EC:3.1.1.11	2	ko00040 Pentose and glucuronate interconversions;ko00500 Starch and sucrose metabolism;
gene23807	K08866	TTK, MPS1	serine/threonine-protein kinase TTK/MPS1	EC:2.7.12.1	2	ko04110 Cell cycle;ko04111 Cell cycle - yeast;
gene2380	K07300	chaA, CAX	Ca2+:H+ antiporter	--		
gene23812	K12761	SNF1	carbon catabolite-derepressing protein kinase	EC:2.7.11.1	1	ko04113 Meiosis - yeast;
gene23816	K03319	TC.DASS	divalent anion:Na+ symporter, DASS family	--		
gene23817	K01867	WARS, trpS	tryptophanyl-tRNA synthetase	EC:6.1.1.2	1	ko00970 Aminoacyl-tRNA biosynthesis;
gene23819	K11841	USP10, UBP3	ubiquitin carboxyl-terminal hydrolase 10	EC:3.1.2.15		
gene23822	K13681	FUT	xyloglucan fucosyltransferase	EC:2.4.1.-		
gene23823	K01783	rpe, RPE	ribulose-phosphate 3-epimerase	EC:5.1.3.1	5	ko00030 Pentose phosphate pathway;ko00040 Pentose and glucuronate interconversions;ko00710 Carbon fixation in photosynthetic organisms;ko01200 Carbon metabolism;ko01230 Biosynthesis of amino acids;
gene23827	K12397	AP3B	AP-3 complex subunit beta	--	1	ko04142 Lysosome;
gene2382	K16302	CNNM	metal transporter CNNM	--		
gene23831	K11273	DDX11, CHL1, CTF1	chromosome transmission fidelity protein 1	EC:3.6.4.13		
gene23833	K15109	SLC25A20_29, CACT, CACL, CRC1	solute carrier family 25 (mitochondrial carnitine/acylcarnitine transporter), member 20/29	--		
gene23837	K04523	UBQLN, DSK2	ubiquilin	--	1	ko04141 Protein processing in endoplasmic reticulum;
gene23839	K11251	H2A	histone H2A	--	2	ko05034 Alcoholism;ko05322 Systemic lupus erythematosus;
gene2384	K00511	SQLE, ERG1	squalene monooxygenase	EC:1.14.13.132	1	ko00909 Sesquiterpenoid and triterpenoid biosynthesis;
gene23850	K07407	E3.2.1.22B, galA, rafA	alpha-galactosidase	EC:3.2.1.22	4	ko00052 Galactose metabolism;ko00561 Glycerolipid metabolism;ko00600 Sphingolipid metabolism;ko00603 Glycosphingolipid biosynthesis - globo series;
gene23851	K07407	E3.2.1.22B, galA, rafA	alpha-galactosidase	EC:3.2.1.22	4	ko00052 Galactose metabolism;ko00561 Glycerolipid metabolism;ko00600 Sphingolipid metabolism;ko00603 Glycosphingolipid biosynthesis - globo series;
gene23855	K12824	TCERG1, CA150	transcription elongation regulator 1	--	1	ko03040 Spliceosome;
gene2385	K15191	LARP7	La-related protein 7	--		
gene23860	K01868	TARS, thrS	threonyl-tRNA synthetase	EC:6.1.1.3	1	ko00970 Aminoacyl-tRNA biosynthesis;
gene23863	K02885	RP-L19e, RPL19	large subunit ribosomal protein L19e	--	1	ko03010 Ribosome;
gene23865	K02321	POLA2	DNA polymerase alpha subunit B	--	3	ko00230 Purine metabolism;ko00240 Pyrimidine metabolism;ko03030 DNA replication;
gene23868	K15397	KCS	3-ketoacyl-CoA synthase	EC:2.3.1.199	1	ko00062 Fatty acid elongation;
gene23882	K00036	G6PD, zwf	glucose-6-phosphate 1-dehydrogenase	EC:1.1.1.49	3	ko00030 Pentose phosphate pathway;ko00480 Glutathione metabolism;ko01200 Carbon metabolism;
gene23883	K15627	ASPSCR1, ASPL	tether containing UBX domain for GLUT4	--	1	ko05202 Transcriptional misregulation in cancer;
gene23890	K08902	psb27	photosystem II Psb27 protein	--	1	ko00195 Photosynthesis;
gene23891	K00162	PDHB, pdhB	pyruvate dehydrogenase E1 component subunit beta	EC:1.2.4.1	6	ko00010 Glycolysis / Gluconeogenesis;ko00020 Citrate cycle (TCA cycle);ko00620 Pyruvate metabolism;ko00650 Butanoate metabolism;ko01200 Carbon metabolism;ko04066 HIF-1 signaling pathway;
gene23893	K10684	UBLE1A, SAE1	ubiquitin-like 1-activating enzyme E1 A	EC:6.3.2.19	1	ko04120 Ubiquitin mediated proteolysis;
gene23900	K03022	RPC25, POLR3H	DNA-directed RNA polymerase III subunit RPC8	--	5	ko00230 Purine metabolism;ko00240 Pyrimidine metabolism;ko03020 RNA polymerase;ko04623 Cytosolic DNA-sensing pathway;ko05169 Epstein-Barr virus infection;
gene23902	K11824	AP2A	AP-2 complex subunit alpha	--	4	ko04144 Endocytosis;ko04721 Synaptic vesicle cycle;ko04961 Endocrine and other factor-regulated calcium reabsorption;ko05016 Huntington's disease;
gene23905	K01188	E3.2.1.21	beta-glucosidase	EC:3.2.1.21	3	ko00460 Cyanoamino acid metabolism;ko00500 Starch and sucrose metabolism;ko00940 Phenylpropanoid biosynthesis;
gene23907	K05310	GPI7	ethanolaminephosphotransferase	EC:2.7.-.-	1	ko00563 Glycosylphosphatidylinositol(GPI)-anchor biosynthesis;
gene23908	K01535	E3.6.3.6	H+-transporting ATPase	EC:3.6.3.6	1	ko00190 Oxidative phosphorylation;
gene23911	K07198	PRKAA, AMPK	5'-AMP-activated protein kinase, catalytic alpha subunit	EC:2.7.11.11	8	ko04140 Regulation of autophagy;ko04150 mTOR signaling pathway;ko04151 PI3K-Akt signaling pathway;ko04710 Circadian rhythm;ko04910 Insulin signaling pathway;ko04920 Adipocytokine signaling pathway;ko04932 Non-alcoholic fatty liver disease (NAFLD);ko05410 Hypertrophic cardiomyopathy (HCM);
gene23918	K13680	CSLA	beta-mannan synthase	EC:2.4.1.32		

gene2391	K10879	XRCC2	DNA-repair protein XRCC2	--	1	ko03440 Homologous recombination;
gene23925	K02899	RP-L27, MRPL27, rpmA	large subunit ribosomal protein L27	--	1	ko03010 Ribosome;
gene23926	K13348	MPV17	protein Mpv17	--	1	ko04146 Peroxisome;
gene23927	K02922	RP-L37e, RPL37	large subunit ribosomal protein L37e	--	1	ko03010 Ribosome;
gene23930	K11593	ELF2C	eukaryotic translation initiation factor 2C	--		
gene23934	K14229	tRNA-Lys	tRNA Lys	--	1	ko00970 Aminoacyl-tRNA biosynthesis;
gene23937	K00547	mmuM	homocysteine S-methyltransferase	EC:2.1.1.10	1	ko00270 Cysteine and methionine metabolism;
gene23938	K06268	PPP3R, CNB	serine/threonine-protein phosphatase 2B regulatory subunit	--	18	ko04010 MAPK signaling pathway;ko04020 Calcium signaling pathway;ko04114 Oocyte meiosis;ko04210 Apoptosis;ko04310 Wnt signaling pathway;ko04360 Axon guidance;ko04370 VEGF signaling pathway;ko04380 Osteoclast differentiation;ko04650 Natural killer cell mediated cytotoxicity;ko04660 T cell receptor signaling pathway;ko04662 B cell receptor signaling pathway;ko04720 Long-term potentiation;ko04724 Glutamatergic synapse;ko05010 Alzheimer's disease;ko05014 Amyotrophic lateral sclerosis (ALS);ko05031 Amphetamine addiction;ko05152
gene23943	K14509	ETR, ERS	ethylene receptor	EC:2.7.13.-	1	ko04075 Plant hormone signal transduction;
gene23944	K01679	E4.2.1.2B, fumC	fumarate hydratase, class II	EC:4.2.1.2	5	ko00020 Citrate cycle (TCA cycle);ko00720 Carbon fixation pathways in prokaryotes;ko01200 Carbon metabolism;ko05200 Pathways in cancer;ko05211 Renal cell carcinoma;
gene23947	K01590	hdc, HDC	histidine decarboxylase	EC:4.1.1.22	1	ko00340 Histidine metabolism;
gene23950	K08838	STK24_25_MST4	serine/threonine-protein kinase 24/25/MST4	EC:2.7.11.1		
gene23953	K15032	MTERFD	mTERF domain-containing protein, mitochondrial	--		
gene23966	K14486	K14486, ARF	auxin response factor	--	1	ko04075 Plant hormone signal transduction;
gene23973	K04733	IRAK4	interleukin-1 receptor-associated kinase 4	EC:2.7.11.1	11	ko04064 NF-kappa B signaling pathway;ko04210 Apoptosis;ko04620 Toll-like receptor signaling pathway;ko04722 Neurotrophin signaling pathway;ko05133 Pertussis;ko05140 Leishmaniasis;ko05142 Chagas disease (American trypanosomiasis);ko05145 Toxoplasmosis;ko05152 Tuberculosis;ko05162 Measles;ko05164 Influenza A;
gene23974	K12890	SFRS1_9	splicing factor, arginine/serine-rich 1/9	--	2	ko03040 Spliceosome;ko05168 Herpes simplex infection;
gene23975	K12450	RHM	UDP-glucose 4,6-dehydratase	EC:4.2.1.76	1	ko00520 Amino sugar and nucleotide sugar metabolism;
gene23978	K11855	USP36_42	ubiquitin carboxyl-terminal hydrolase 36/42	EC:3.1.2.15		
gene23979	K00430	E1.11.1.7	peroxidase	EC:1.11.1.7	2	ko00360 Phenylalanine metabolism;ko00940 Phenylpropanoid biosynthesis;
gene23987	K17279	REEP5_6	receptor expression-enhancing protein 5/6	--		
gene23991	K17545	ULK4	serine/threonine-protein kinase ULK4	EC:2.7.11.1		
gene2399	K01183	E3.2.1.14	chitinase	EC:3.2.1.14	1	ko00520 Amino sugar and nucleotide sugar metabolism;
gene23	K02892	RP-L23, MRPL23, rplW	large subunit ribosomal protein L23	--	1	ko03010 Ribosome;
gene24001	K16904	DCTPP1	dCTP diphosphatase	EC:3.6.1.12	1	ko00240 Pyrimidine metabolism;
gene24007	K04392	RAC1	Ras-related C3 botulinum toxin substrate 1	--	33	ko04010 MAPK signaling pathway;ko04014 Ras signaling pathway;ko04062 Chemokine signaling pathway;ko04145 Phagosome;ko04151 PI3K-Akt signaling pathway;ko04310 Wnt signaling pathway;ko04360 Axon guidance;ko04370 VEGF signaling pathway;ko04380 Osteoclast differentiation;ko04510 Focal adhesion;ko04520 Adherens junction;ko04620 Toll-like receptor signaling pathway;ko04650 Natural killer cell mediated cytotoxicity;ko04662 B cell receptor signaling pathway;ko04664 Fc epsilon RI signaling pathway;ko04666 Fc gamma R-mediated phagocytosis;ko04670 Leukocyte transendothelial migration;ko04722 Neurotrophin signaling pathway;ko04810 Regulation of actin cytoskeleton;ko04932 Non-alcoholic fatty liver disease (NAFLD);ko04972 Pancreatic secretion;ko05014 Amyotrophic lateral sclerosis (ALS);ko05100 Bacterial invasion of epithelial cells;ko05120 Epithelial cell signaling in Helicobacter pylori infection;ko05131 Shigellosis;ko05132 Salmonella infection;ko05200 Pathways in cancer;ko05203 Viral carcinogenesis;ko05205 Proteoglycans in cancer;ko05210 Colorectal cancer;ko05211 Renal cell carcinoma;ko05212 Pancreatic

gene24010	K10527	MFP2	enoyl-CoA hydratase/3-hydroxyacyl-CoA dehydrogenase	EC:4.2.1.17 1.1.1.35 1.1.1.211	2	ko00071 Fatty acid degradation;ko00592 alpha-Linolenic acid metabolism;
gene24011	K15633	gpmI	2,3-bisphosphoglycerate-independent phosphoglycerate mutase	EC:5.4.2.12	5	ko00010 Glycolysis / Gluconeogenesis;ko00260 Glycine, serine and threonine metabolism;ko00680 Methane metabolism;ko01200 Carbon metabolism;ko01230 Biosynthesis of amino acids;
gene24012	K12735	PPIL4	peptidyl-prolyl cis-trans isomerase-like 4	EC:5.2.1.8		
gene24013	K15356	VRG4, GONST1	GDP-mannose transporter	--		
gene24015	K02575	NRT, narK, nrtP, nasA	MFS transporter, NNP family, nitrate/nitrite transporter	--	1	ko00910 Nitrogen metabolism;
gene24017	K02575	NRT, narK, nrtP, nasA	MFS transporter, NNP family, nitrate/nitrite transporter	--	1	ko00910 Nitrogen metabolism;
gene2401	K03066	PSMC5, RPT6	26S proteasome regulatory subunit T6	--	2	ko03050 Proteasome;ko05169 Epstein-Barr virus infection;
gene24022	K17506	PPM1L, PP2CE	protein phosphatase 1L	EC:3.1.3.16		
gene24032	K09580	PDIA1, P4HB	protein disulfide-isomerase A1	EC:5.3.4.1	1	ko04141 Protein processing in endoplasmic reticulum;
gene24033	K15692	RNF13, RZF	E3 ubiquitin-protein ligase RNF13	EC:6.3.2.19		
gene24035	K13960	UBE2T, HSPC150	ubiquitin-conjugating enzyme E2 T	EC:6.3.2.19	1	ko03460 Fanconi anemia pathway;
gene24045	K13412	CPK	calcium-dependent protein kinase	EC:2.7.11.1	2	ko04626 Plant-pathogen interaction;ko05145 Toxoplasmosis;
gene24046	K03327	TC.MATE, SLC47A, norM, mdtK, dinF	multidrug resistance protein, MATE family	--		
gene24047	K09580	PDIA1, P4HB	protein disulfide-isomerase A1	EC:5.3.4.1	1	ko04141 Protein processing in endoplasmic reticulum;
gene24050	K08869	ADCK, ABC1	aarF domain-containing kinase	--		
gene24052	K14236	tRNA-Tyr	tRNA Tyr	--	1	ko00970 Aminoacyl-tRNA biosynthesis;
gene24068	K13495	CISZOG	cis-zeatin O-glucosyltransferase	EC:2.4.1.215	1	ko00908 Zeatin biosynthesis;
gene24070	K02873	RP-L13e, RPL13	large subunit ribosomal protein L13e	--	1	ko03010 Ribosome;
gene24076	K13162	PCBP2_3_4	poly(rC)-binding protein 2/3/4	--		
gene2407	K03236	EIF1A	translation initiation factor 1A	--	1	ko03013 RNA transport;
gene24080	K14508	NPR1	regulatory protein NPR1	--	1	ko04075 Plant hormone signal transduction;
gene24081	K09419	HSFF	heat shock transcription factor, other eukaryote	--		
gene24087	K06268	PPP3R, CNB	serine/threonine-protein phosphatase 2B regulatory subunit	--	18	ko04010 MAPK signaling pathway;ko04020 Calcium signaling pathway;ko04114 Oocyte meiosis;ko04210 Apoptosis;ko04310 Wnt signaling pathway;ko04360 Axon guidance;ko04370 VEGF signaling pathway;ko04380 Osteoclast differentiation;ko04650 Natural killer cell mediated cytotoxicity;ko04660 T cell receptor signaling pathway;ko04662 B cell receptor signaling pathway;ko04720 Long-term potentiation;ko04724 Glutamatergic synapse;ko05010 Alzheimer's disease;ko05014 Amyotrophic lateral sclerosis (ALS);ko05031 Amphetamine addiction;ko05152
gene24089	K00939	E2.7.4.3, adk	adenylate kinase	EC:2.7.4.3	1	ko00230 Purine metabolism;
gene2408	K01792	E5.1.3.15	glucose-6-phosphate 1-epimerase	EC:5.1.3.15	1	ko00010 Glycolysis / Gluconeogenesis;
gene24091	K01267	E3.4.11.21, DNPEP	aspartyl aminopeptidase	EC:3.4.11.21		
gene24093	K00430	E1.11.1.7	peroxidase	EC:1.11.1.7	2	ko00360 Phenylalanine metabolism;ko00940 Phenylpropanoid biosynthesis;
gene24094	K00430	E1.11.1.7	peroxidase	EC:1.11.1.7	2	ko00360 Phenylalanine metabolism;ko00940 Phenylpropanoid biosynthesis;
gene24095	K00430	E1.11.1.7	peroxidase	EC:1.11.1.7	2	ko00360 Phenylalanine metabolism;ko00940 Phenylpropanoid biosynthesis;
gene24096	K10880	XRCC3	DNA-repair protein XRCC3	--	1	ko03440 Homologous recombination;
gene24097	K00430	E1.11.1.7	peroxidase	EC:1.11.1.7	2	ko00360 Phenylalanine metabolism;ko00940 Phenylpropanoid biosynthesis;
gene24098	K00430	E1.11.1.7	peroxidase	EC:1.11.1.7	2	ko00360 Phenylalanine metabolism;ko00940 Phenylpropanoid biosynthesis;
gene24099	K00430	E1.11.1.7	peroxidase	EC:1.11.1.7	2	ko00360 Phenylalanine metabolism;ko00940 Phenylpropanoid biosynthesis;
gene2409	K01979	SSUrRNA	small subunit ribosomal RNA	--	2	ko03008 Ribosome biogenesis in eukaryotes;ko03010 Ribosome;
gene24100	K00430	E1.11.1.7	peroxidase	EC:1.11.1.7	2	ko00360 Phenylalanine metabolism;ko00940 Phenylpropanoid biosynthesis;
gene24106	K03135	TAF11	transcription initiation factor TFIID subunit 11	--	1	ko03022 Basal transcription factors;
gene24109	K10604	MGRN1	E3 ubiquitin-protein ligase MGRN1	EC:6.3.2.19	1	ko04120 Ubiquitin mediated proteolysis;
gene2410	K09286	EREBP	EREBP-like factor	--		
gene24112	K00888	PI4K	phosphatidylinositol 4-kinase	EC:2.7.1.67	2	ko00562 Inositol phosphate metabolism;ko04070 Phosphatidylinositol signaling system;

gene24114	K15378	SLC45A1_2_4	solute carrier family 45, member 1/2/4	--		
gene24117	K08596	SENP7	sentrin-specific protease 7	EC:3.4.22.68		
gene24118	K03246	EIF3I	translation initiation factor 3 subunit I	--	1	ko03013 RNA transport;
gene24122	K11420	EHMT	euchromatic histone-lysine N-methyltransferase	EC:2.1.1.43	1	ko00310 Lysine degradation;
gene24125	K01358	clpP, CLPP	ATP-dependent Clp protease, protease subunit	EC:3.4.21.92	1	ko04112 Cell cycle - Caulobacter;
gene24129	K10704	UBE2V	ubiquitin-conjugating enzyme E2 variant	--		
gene24130	K02898	RP-L26e, RPL26	large subunit ribosomal protein L26e	--	1	ko03010 Ribosome;
gene24131	K15040	VDAC2	voltage-dependent anion channel protein 2	--	4	ko04020 Calcium signaling pathway;ko05012 Parkinson's disease;ko05016 Huntington's disease;ko05166 HTLV-I infection;
gene24136	K08900	BCS1	mitochondrial chaperone BCS1	--		
gene24143	K01166	E3.1.27.1	ribonuclease T2	EC:3.1.27.1		
gene24145	K11517	HAO	(S)-2-hydroxy-acid oxidase	EC:1.1.3.15	2	ko00630 Glyoxylate and dicarboxylate metabolism;ko04146 Peroxisome;
gene24148	K00799	GST, gst	glutathione S-transferase	EC:2.5.1.18	4	ko00480 Glutathione metabolism;ko00980 Metabolism of xenobiotics by cytochrome P450;ko00982 Drug metabolism - cytochrome P450;ko05204 Chemical carcinogenesis;
gene24149	K00799	GST, gst	glutathione S-transferase	EC:2.5.1.18	4	ko00480 Glutathione metabolism;ko00980 Metabolism of xenobiotics by cytochrome P450;ko00982 Drug metabolism - cytochrome P450;ko05204 Chemical carcinogenesis;
gene24150	K00799	GST, gst	glutathione S-transferase	EC:2.5.1.18	4	ko00480 Glutathione metabolism;ko00980 Metabolism of xenobiotics by cytochrome P450;ko00982 Drug metabolism - cytochrome P450;ko05204 Chemical carcinogenesis;
gene24155	K08360	CYB561	cytochrome b-561	--		
gene24156	K08360	CYB561	cytochrome b-561	--		
gene24159	K00889	E2.7.1.68, PIP5K	1-phosphatidylinositol-4-phosphate 5-kinase	EC:2.7.1.68	5	ko00562 Inositol phosphate metabolism;ko04070 Phosphatidylinositol signaling system;ko04144 Endocytosis;ko04666 Fc gamma R-mediated phagocytosis;ko04810 Regulation of actin cytoskeleton;
gene24161	K10256	FAD2	omega-6 fatty acid desaturase (delta-12 desaturase)	EC:1.14.19.-	1	ko01040 Biosynthesis of unsaturated fatty acids;
gene24165	K02321	POLA2	DNA polymerase alpha subunit B	--	3	ko00230 Purine metabolism;ko00240 Pyrimidine metabolism;ko03030 DNA replication;
gene24168	K15283	SLC35E1	solute carrier family 35, member E1	--		
gene2416	K00915	IPK2	1D-myo-Inositol-tetrakisphosphate 5-kinase / inositol-polyphosphate multikinase	EC:2.7.1.140 2.7.1.151	2	ko00562 Inositol phosphate metabolism;ko04070 Phosphatidylinositol signaling system;
gene24174	K13800	CMPK1, UMPK	UMP-CMP kinase	EC:2.7.4.- 2.7.4.14	1	ko00240 Pyrimidine metabolism;
gene24178	K02350	POLZ1, rev3	DNA polymerase zeta	EC:2.7.7.7	1	ko03460 Fanconi anemia pathway;
gene24179	K02350	POLZ1, rev3	DNA polymerase zeta	EC:2.7.7.7	1	ko03460 Fanconi anemia pathway;
gene24180	K14537	NUG2, GNL2	nuclear GTP-binding protein	--	1	ko03008 Ribosome biogenesis in eukaryotes;
gene24181	K12471	EPN	epsin	--	1	ko04144 Endocytosis;
gene24182	K10666	RNF5	E3 ubiquitin-protein ligase RNF5	EC:6.3.2.19	1	ko04141 Protein processing in endoplasmic reticulum;
gene2418	K01835	pgm	phosphoglucomutase	EC:5.4.2.2	7	ko00010 Glycolysis / Gluconeogenesis;ko00030 Pentose phosphate pathway;ko00052 Galactose metabolism;ko00230 Purine metabolism;ko00500 Starch and sucrose metabolism;ko00520 Amino sugar and nucleotide sugar metabolism;ko00521 Streptomycin biosynthesis;
gene24193	K09338	HD-ZIP	homeobox-leucine zipper protein	--		
gene24194	K02917	RP-L35Ae, RPL35A	large subunit ribosomal protein L35Ae	--	1	ko03010 Ribosome;
gene24195	K02917	RP-L35Ae, RPL35A	large subunit ribosomal protein L35Ae	--	1	ko03010 Ribosome;
gene24196	K02906	RP-L3, MRPL3, rplC	large subunit ribosomal protein L3	--	1	ko03010 Ribosome;
gene2419	K03011	RPB3, POLR2C	DNA-directed RNA polymerase II subunit RPB3	--	5	ko00230 Purine metabolism;ko00240 Pyrimidine metabolism;ko03020 RNA polymerase;ko05016 Huntington's disease;ko05169 Epstein-Barr virus infection;
gene241	K00855	PRK, prkB	phosphoribulokinase	EC:2.7.1.19	2	ko00710 Carbon fixation in photosynthetic organisms;ko01200 Carbon metabolism;
gene24203	K03327	TC.MATE, SLC47A, norM, mdtK, dinF	multidrug resistance protein, MATE family	--		
gene24206	K11423	SETD2, SET2	histone-lysine N-methyltransferase SETD2	EC:2.1.1.43	1	ko00310 Lysine degradation;
gene24207	K02377	E1.1.1.271, fcl	GDP-L-fucose synthase	EC:1.1.1.271	2	ko00051 Fructose and mannose metabolism;ko00520 Amino sugar and nucleotide sugar metabolism;
gene24209	K17506	PPM1L, PP2CE	protein phosphatase 1L	EC:3.1.3.16		

gene24212	K08838	STK24_25_MST4	serine/threonine-protein kinase 24/25/MST4	EC:2.7.11.1		
gene24219	K11252	H2B	histone H2B	--	3	ko05034 Alcoholism;ko05203 Viral carcinogenesis;ko05322 Systemic lupus erythematosus;
gene2421	K04710	CERS	ceramide synthetase	EC:2.3.1.24	1	ko00600 Sphingolipid metabolism;
gene24221	K03946	NDUFA2	NADH dehydrogenase (ubiquinone) 1 alpha subcomplex subunit 2	--	5	ko00190 Oxidative phosphorylation;ko04932 Non-alcoholic fatty liver disease (NAFLD);ko05010 Alzheimer's disease;ko05012 Parkinson's disease;ko05016 Huntington's disease;
gene24225	K02918	RP-L35e, RPL35	large subunit ribosomal protein L35e	--	1	ko03010 Ribosome;
gene24231	K17279	REEP5_6	receptor expression-enhancing protein 5/6	--		
gene24235	K17046	DEK	protein DEK	--		
gene24237	K14508	NPR1	regulatory protein NPR1	--	1	ko04075 Plant hormone signal transduction;
gene24238	K11793	CRBN	cereblon	--		
gene2423	K08066	NFYC	nuclear transcription factor Y, gamma	--	2	ko04612 Antigen processing and presentation;ko05152 Tuberculosis;
gene24243	K07297	ADIPOR	adiponectin receptor	--	2	ko04920 Adipocytokine signaling pathway;ko04932 Non-alcoholic fatty liver disease (NAFLD);
gene24245	K01205	NAGLU	alpha-N-acetylglucosaminidase	EC:3.2.1.50	2	ko00531 Glycosaminoglycan degradation;ko04142 Lysosome;
gene24250	K01051	E3.1.1.11	pectinesterase	EC:3.1.1.11	2	ko00040 Pentose and glucuronate interconversions;ko00500 Starch and sucrose metabolism;
gene24253	K02924	RP-L39e, RPL39	large subunit ribosomal protein L39e	--	1	ko03010 Ribosome;
gene24255	K13420	FLS2	LRR receptor-like serine/threonine-protein kinase FLS2	EC:2.7.11.1	1	ko04626 Plant-pathogen interaction;
gene24260	K08908	LHCA2	light-harvesting complex I chlorophyll a/b binding protein 2	--	1	ko00196 Photosynthesis - antenna proteins;
gene24264	K10839	RAD23, HR23	UV excision repair protein RAD23	--	2	ko03420 Nucleotide excision repair;ko04141 Protein processing in endoplasmic reticulum;
gene24265	K03860	PIGQ, GPI1	phosphatidylinositol glycan, class Q	--	1	ko00563 Glycosylphosphatidylinositol(GPI)-anchor biosynthesis;
gene24268	K01633	folB	dihydroneopterin aldolase	EC:4.1.2.25	1	ko00790 Folate biosynthesis;
gene24272	K15306	RANBP1	Ran-binding protein 1	--	2	ko05166 HTLV-I infection;ko05203 Viral carcinogenesis;
gene24274	K12854	SNRNP200, BRR2	pre-mRNA-splicing helicase BRR2	EC:3.6.4.13	1	ko03040 Spliceosome;
gene24275	K12854	SNRNP200, BRR2	pre-mRNA-splicing helicase BRR2	EC:3.6.4.13	1	ko03040 Spliceosome;
gene24277	K02985	RP-S3e, RPS3	small subunit ribosomal protein S3e	--	1	ko03010 Ribosome;
gene2427	K14379	ACP5	tartrate-resistant acid phosphatase type 5	EC:3.1.3.2	3	ko00740 Riboflavin metabolism;ko04380 Osteoclast differentiation;ko05323 Rheumatoid arthritis;
gene24280	K12191	CHMP2A	charged multivesicular body protein 2A	--	1	ko04144 Endocytosis;
gene24292	K00864	E2.7.1.30, glpK	glycerol kinase	EC:2.7.1.30	3	ko00561 Glycerolipid metabolism;ko03320 PPAR signaling pathway;ko04626 Plant-pathogen interaction;
gene24296	K01052	LIPA	lysosomal acid lipase/cholesteryl ester hydrolase	EC:3.1.1.13	2	ko00100 Steroid biosynthesis;ko04142 Lysosome;
gene24305	K14769	UTP11	U3 small nucleolar RNA-associated protein 11	--		
gene24309	K14230	tRNA-Met	tRNA Met	--	1	ko00970 Aminoacyl-tRNA biosynthesis;
gene24314	K13035	NIT4	beta-cyano-L-alanine hydratase/nitrilase	EC:3.5.5.1 3.5.5.4	1	ko00460 Cyanoamino acid metabolism;
gene24315	K13035	NIT4	beta-cyano-L-alanine hydratase/nitrilase	EC:3.5.5.1 3.5.5.4	1	ko00460 Cyanoamino acid metabolism;
gene24317	K01051	E3.1.1.11	pectinesterase	EC:3.1.1.11	2	ko00040 Pentose and glucuronate interconversions;ko00500 Starch and sucrose metabolism;
gene2431	K02133	ATPeF1B, ATP5B	F-type H+-transporting ATPase subunit beta	EC:3.6.3.14	4	ko00190 Oxidative phosphorylation;ko05010 Alzheimer's disease;ko05012 Parkinson's disease;ko05016 Huntington's disease;
gene24324	K14484	IAA	auxin-responsive protein IAA	--	1	ko04075 Plant hormone signal transduction;
gene24325	K14233	tRNA-Ser	tRNA Ser	--	1	ko00970 Aminoacyl-tRNA biosynthesis;
gene24326	K11420	EHMT	euchromatic histone-lysine N-methyltransferase	EC:2.1.1.43	1	ko00310 Lysine degradation;
gene24327	K13436	PTI1	pto-interacting protein 1	EC:2.7.11.1	1	ko04626 Plant-pathogen interaction;
gene24328	K04733	IRAK4	interleukin-1 receptor-associated kinase 4	EC:2.7.11.1	11	ko04064 NF-kappa B signaling pathway;ko04210 Apoptosis;ko04620 Toll-like receptor signaling pathway;ko04722 Neurotrophin signaling pathway;ko05133 Pertussis;ko05140 Leishmaniasis;ko05142 Chagas disease (American trypanosomiasis);ko05145 Toxoplasmosis;ko05152 Tuberculosis;ko05162 Measles;ko05164 Influenza A;
gene24332	K12392	AP1B1	AP-1 complex subunit beta-1	--	1	ko04142 Lysosome;

gene24337	K02144	ATPeV54kD	V-type H+-transporting ATPase 54 kD subunit	EC:3.6.3.14	8	ko00190 Oxidative phosphorylation;ko04142 Lysosome;ko04145 Phagosome;ko04721 Synaptic vesicle cycle;ko05110 Vibrio cholerae infection;ko05120 Epithelial cell signaling in Helicobacter pylori infection;ko05152 Tuberculosis;ko05323 Rheumatoid arthritis;
gene24339	K01711	gmd, GMDS	GDPmannose 4,6-dehydratase	EC:4.2.1.47	2	ko00051 Fructose and mannose metabolism;ko00520 Amino sugar and nucleotide sugar metabolism;
gene24342	K11275	H1_5	histone H1/5	--		
gene24347	K10406	KIFC2_3	kinesin family member C2/C3	--		
gene24356	K13424	WRKY33	WRKY transcription factor 33	--	1	ko04626 Plant-pathogen interaction;
gene2435	K01537	E3.6.3.8	Ca2+-transporting ATPase	EC:3.6.3.8		
gene24366	K07964	HPSE	heparanase 1	EC:3.2.1.166	2	ko00531 Glycosaminoglycan degradation;ko05205 Proteoglycans in cancer;
gene24372	K09580	PDIA1, P4HB	protein disulfide-isomerase A1	EC:5.3.4.1	1	ko04141 Protein processing in endoplasmic reticulum;
gene24378	K03094	SKP1, CBF3D	S-phase kinase-associated protein 1	--	9	ko04110 Cell cycle;ko04111 Cell cycle - yeast;ko04114 Oocyte meiosis;ko04120 Ubiquitin mediated proteolysis;ko04141 Protein processing in endoplasmic reticulum;ko04310 Wnt signaling pathway;ko04350 TGF-beta signaling pathway;ko04710 Circadian rhythm;ko05168 Herpes simplex infection;
gene24382	K11498	CENPE	centromeric protein E	--		
gene2438	K14486	K14486, ARF	auxin response factor	--	1	ko04075 Plant hormone signal transduction;
gene24392	K01658	trpG	anthranilate synthase component II	EC:4.1.3.27	2	ko00400 Phenylalanine, tyrosine and tryptophan biosynthesis;ko01230 Biosynthesis of amino acids;
gene24395	K09716	K09716	hypothetical protein	--		
gene2439	K12446	E2.7.1.46	L-arabinokinase	EC:2.7.1.46	1	ko00520 Amino sugar and nucleotide sugar metabolism;
gene24412	K04371	MAPK1_3	mitogen-activated protein kinase 1/3	EC:2.7.11.24	71	ko04010 MAPK signaling pathway;ko04012 ErbB signaling pathway;ko04013 MAPK signaling pathway - fly;ko04014 Ras signaling pathway;ko04062 Chemokine signaling pathway;ko04066 HIF-1 signaling pathway;ko04114 Oocyte meiosis;ko04150 mTOR signaling pathway;ko04151 PI3K-Akt signaling pathway;ko04270 Vascular smooth muscle contraction;ko04320 Dorso-ventral axis formation;ko04350 TGF-beta signaling pathway;ko04360 Axon guidance;ko04370 VEGF signaling pathway;ko04380 Osteoclast differentiation;ko04510 Focal adhesion;ko04520 Adherens junction;ko04540 Gap junction;ko04620 Toll-like receptor signaling pathway;ko04621 NOD-like receptor signaling pathway;ko04650 Natural killer cell mediated cytotoxicity;ko04660 T cell receptor signaling pathway;ko04662 B cell receptor signaling pathway;ko04664 Fc epsilon RI signaling pathway;ko04666 Fc gamma R-mediated phagocytosis;ko04668 TNF signaling pathway;ko04713 Circadian entrainment;ko04720 Long-term potentiation;ko04722 Neurotrophin signaling pathway;ko04723 Retrograde endocannabinoid signaling;ko04724 Glutamatergic synapse;ko04725 Cholinergic synapse;ko04726 Serotonergic synapse;ko04730 Long-term depression;ko04810 Regulation of actin cytoskeleton;ko04910 Insulin signaling pathway;ko04912 GnRH signaling pathway;ko04914 Progesterone-mediated oocyte maturation;ko04915 Estrogen signaling pathway;ko04916 Melanogenesis;ko04917 Prolactin signaling pathway;ko04930 Type II diabetes mellitus;ko04960 Aldosterone-regulated sodium reabsorption;ko05010 Alzheimer's disease;ko05020 Prion diseases;ko05034 Alcoholism;ko05131 Shigellosis;ko05132 Salmonella infection;ko05133 Pertussis;ko05140 Leishmaniasis;ko05142 Chagas disease (American trypanosomiasis);ko05145 Toxoplasmosis;ko05152 Tuberculosis;ko05160 Hepatitis C;ko05161 Hepatitis B;ko05164 Influenza A;ko05200 Pathways in cancer;ko05203 Viral carcinogenesis;ko05205 Proteoglycans in cancer;ko05210 Colorectal cancer;ko05211 Renal cell carcinoma;ko05212
gene24414	K10756	RFC3_5	replication factor C subunit 3/5	--	3	ko03030 DNA replication;ko03420 Nucleotide excision repair;ko03430 Mismatch repair;
gene24415	K13082	DFR	bifunctional dihydroflavonol 4-reductase/flavanone 4-reductase	EC:1.1.1.219 1.1.1.234	1	ko00941 Flavonoid biosynthesis;

gene24416	K00588	E2.1.1.104	caffeoyl-CoA O-methyltransferase	EC:2.1.1.104	4	ko00360 Phenylalanine metabolism;ko00940 Phenylpropanoid biosynthesis;ko00941 Flavonoid biosynthesis;ko00945 Stilbenoid, diarylheptanoid and gingerol biosynthesis;
gene24424	K04498	EP300, CREBBP, KAT3	E1A/CREB-binding protein	EC:2.3.1.48	21	ko04066 HIF-1 signaling pathway;ko04110 Cell cycle;ko04310 Wnt signaling pathway;ko04330 Notch signaling pathway;ko04350 TGF-beta signaling pathway;ko04520 Adherens junction;ko04630 Jak-STAT signaling pathway;ko04720 Long-term potentiation;ko04916 Melanogenesis;ko05016 Huntington's disease;ko05152 Tuberculosis;ko05161 Hepatitis B;ko05164 Influenza A;ko05166 HTLV-I infection;ko05168 Herpes simplex infection;ko05169 Epstein-Barr virus infection;ko05200 Pathways in cancer;ko05203 Viral carcinogenesis;ko05206 MicroRNAs in cancer;ko05211 Renal cell carcinoma:ko05215 Prostate cancer:
gene24426	K08150	SLC2A13, ITR	MFS transporter, SP family, solute carrier family 2 (myo-inositol transporter), member 13	--		
gene24428	K02980	RP-S29e, RPS29	small subunit ribosomal protein S29e	--	1	ko03010 Ribosome;
gene24430	K15356	VRG4, GONST1	GDP-mannose transporter	--		
gene24433	K03574	MUTT, NUDT1, MTH1	7,8-dihydro-8-oxoguanine triphosphatase	EC:3.6.1.-		
gene24440	K12197	CHMP1, VPS46, DID2	charged multivesicular body protein 1	--	1	ko04144 Endocytosis;
gene24441	K02903	RP-L28e, RPL28	large subunit ribosomal protein L28e	--	1	ko03010 Ribosome;
gene24443	K12125	ELF3	protein EARLY FLOWERING 3	--	1	ko04712 Circadian rhythm - plant;
gene24445	K00599	E2.1.1.-			2	ko00350 Tyrosine metabolism;ko00624 Polycyclic aromatic hydrocarbon degradation;
gene24452	K08832	SRPK3, STK23	serine/threonine-protein kinase SRPK3	EC:2.7.11.1		
gene24453	K02896	RP-L24e, RPL24	large subunit ribosomal protein L24e	--	1	ko03010 Ribosome;
gene24455	K02891	RP-L22e, RPL22	large subunit ribosomal protein L22e	--	1	ko03010 Ribosome;
gene24461	K13153	SNRNP25	U11/U12 small nuclear ribonucleoprotein 25 kDa protein	--		
gene24469	K13832	aroDE, DHQ-SDH	3-dehydroquinate dehydratase / shikimate dehydrogenase	EC:4.2.1.10 1.1.1.25	2	ko00400 Phenylalanine, tyrosine and tryptophan biosynthesis;ko01230 Biosynthesis of amino acids;
gene2446	K09874	NIP	aquaporin NIP	--		
gene24471	K14558	PWP2, UTP1	periodic tryptophan protein 2	--	1	ko03008 Ribosome biogenesis in eukaryotes;
gene24474	K14516	ERF1	ethylene-responsive transcription factor 1	--	1	ko04075 Plant hormone signal transduction;
gene24475	K02727	PSMA3	20S proteasome subunit alpha 7	EC:3.4.25.1	1	ko03050 Proteasome;
gene24479	K14489	AHK2_3_4	arabidopsis histidine kinase 2/3/4 (cytokinin receptor)	EC:2.7.13.3	1	ko04075 Plant hormone signal transduction;
gene2447	K11982	RNF115_126	E3 ubiquitin-protein ligase RNF115/126	EC:6.3.2.19		
gene24483	K15201	GTP3C3, TFC4	general transcription factor 3C polypeptide 3 (transcription factor C subunit 4)	--		
gene24487	K14236	tRNA-Tyr	tRNA Tyr	--	1	ko00970 Aminoacyl-tRNA biosynthesis;
gene24488	K09422	MYBP	myb proto-oncogene protein, plant	--		
gene24496	K03252	EIF3C	translation initiation factor 3 subunit C	--	1	ko03013 RNA transport;
gene24498	K14944	NOVA	RNA-binding protein Nova	--		
gene244	K03549	kup	KUP system potassium uptake protein	--		
gene24501	K01674	cah	carbonic anhydrase	EC:4.2.1.1	1	ko00910 Nitrogen metabolism;
gene24504	K03097	CSNK2A	casein kinase II subunit alpha	EC:2.7.11.1	9	ko03008 Ribosome biogenesis in eukaryotes;ko04064 NF-kappa B signaling pathway;ko04310 Wnt signaling pathway;ko04520 Adherens junction;ko04530 Tight junction;ko04712 Circadian rhythm - plant;ko05162 Measles;ko05168 Herpes simplex infection;ko05169 Epstein-Barr virus infection;
gene24507	K12876	RBM8A, Y14	RNA-binding protein 8A	--	3	ko03013 RNA transport;ko03015 mRNA surveillance pathway;ko03040 Spliceosome;
gene24508	K01522	FHIT	bis(5'-adenosyl)-triphosphatase	EC:3.6.1.29	3	ko00230 Purine metabolism;ko05222 Small cell lung cancer;ko05223 Non-small cell lung cancer;
gene24509	K03654	recQ	ATP-dependent DNA helicase RecQ	EC:3.6.4.12	1	ko03018 RNA degradation;
gene2450	K08234	yaeR	glyoxylase I family protein	--		
gene24511	K00660	CHS	chalcone synthase	EC:2.3.1.74	2	ko00941 Flavonoid biosynthesis;ko04712 Circadian rhythm - plant;
gene24512	K12593	MPHOSPH6, MPP6	M-phase phosphoprotein 6, animal type	--	1	ko03018 RNA degradation;
gene24514	K00430	E1.11.1.7	peroxidase	EC:1.11.1.7	2	ko00360 Phenylalanine metabolism;ko00940 Phenylpropanoid biosynthesis;

gene24515	K13448	CML	calcium-binding protein CML	--	1	ko04626 Plant-pathogen interaction;
gene24520	K13098	TLS, FUS	RNA-binding protein FUS	--	1	ko05202 Transcriptional misregulation in cancer;
gene24521	K02987	RP-S4e, RPS4	small subunit ribosomal protein S4e	--	1	ko03010 Ribosome;
gene24527	K03016	RPB8, POLR2H	DNA-directed RNA polymerases I, II, and III subunit RPABC3	--	6	ko00230 Purine metabolism;ko00240 Pyrimidine metabolism;ko03020 RNA polymerase;ko04623 Cytosolic DNA-sensing pathway;ko05016 Huntington's disease;ko05169 Epstein-Barr virus infection;
gene24536	K12189	VPS25, EAP20	ESCRT-II complex subunit VPS25	--	1	ko04144 Endocytosis;
gene24538	K09422	MYBP	myb proto-oncogene protein, plant	--		
gene24547	K01802	E5.2.1.8	peptidylprolyl isomerase	EC:5.2.1.8		
gene24555	K14516	ERF1	ethylene-responsive transcription factor 1	--	1	ko04075 Plant hormone signal transduction;
gene24556	K11000	CALS	callose synthase	EC:2.4.1.-		
gene24559	K10752	RBBP4, HAT2, CAF1, MIS16	histone-binding protein RBBP4	--		
gene24564	K13946	AUX1, LAX	auxin influx carrier (AUX1 LAX family)	--	1	ko04075 Plant hormone signal transduction;
gene24567	K15032	MTERFD	mTERF domain-containing protein, mitochondrial	--		
gene24570	K11835	USP4_11_15, UBP12	ubiquitin carboxyl-terminal hydrolase 4/11/15	EC:3.1.2.15		
gene24572	K10684	UBLE1A, SAE1	ubiquitin-like 1-activating enzyme E1 A	EC:6.3.2.19	1	ko04120 Ubiquitin mediated proteolysis;
gene24577	K00784	rnz	ribonuclease Z	EC:3.1.26.11	1	ko03013 RNA transport;
gene24578	K10858	PMS2	DNA mismatch repair protein PMS2	--	2	ko03430 Mismatch repair;ko03460 Fanconi anemia pathway;
gene2457	K13648	GAUT	alpha-1,4-galacturonosyltransferase	EC:2.4.1.43	2	ko00500 Starch and sucrose metabolism;ko00520 Amino sugar and nucleotide sugar metabolism;
gene24584	K10999	CESA	cellulose synthase A	EC:2.4.1.12		
gene24586	K00276	E1.4.3.21, AOC2, AOC3, tynA	primary-amine oxidase	EC:1.4.3.21	6	ko00260 Glycine, serine and threonine metabolism;ko00350 Tyrosine metabolism;ko00360 Phenylalanine metabolism;ko00410 beta-Alanine metabolism;ko00950 Isoquinoline alkaloid biosynthesis;ko00960 Tropane, piperidine and pyridine alkaloid biosynthesis;
gene2458	K07952	ARFRP1	ADP-ribosylation factor related protein 1	--		
gene24590	K00430	E1.11.1.7	peroxidase	EC:1.11.1.7	2	ko00360 Phenylalanine metabolism;ko00940 Phenylpropanoid biosynthesis;
gene24594	K14292	TGS1	trimethylguanosine synthase	EC:2.1.1.-	1	ko03013 RNA transport;
gene24595	K14964	ASH2	Set1/Ash2 histone methyltransferase complex subunit ASH2	--		
gene24599	K03678	RRP45, EXOSC9	exosome complex component RRP45	--	1	ko03018 RNA degradation;
gene24600	K02922	RP-L37e, RPL37	large subunit ribosomal protein L37e	--	1	ko03010 Ribosome;
gene24602	K08235	E2.4.1.207	xyloglucan:xyloglucosyl transferase	EC:2.4.1.207		
gene24606	K03260	EIF4G	translation initiation factor 4G	--	2	ko03013 RNA transport;ko05416 Viral myocarditis;
gene24612	K12275	SEC62	translocation protein SEC62	--	2	ko03060 Protein export;ko04141 Protein processing in endoplasmic reticulum;
gene24613	K12275	SEC62	translocation protein SEC62	--	2	ko03060 Protein export;ko04141 Protein processing in endoplasmic reticulum;
gene24614	K08064	NFYA	nuclear transcription factor Y, alpha	--	2	ko04612 Antigen processing and presentation;ko05152 Tuberculosis;
gene24615	K08867	WNK, PRKWNK	WNK lysine deficient protein kinase	EC:2.7.11.1		
gene24619	K13448	CML	calcium-binding protein CML	--	1	ko04626 Plant-pathogen interaction;
gene24621	K03350	APC3, CDC27	anaphase-promoting complex subunit 3	--	7	ko04110 Cell cycle;ko04111 Cell cycle - yeast;ko04113 Meiosis - yeast;ko04114 Oocyte meiosis;ko04120 Ubiquitin mediated proteolysis;ko04914 Progesterone-mediated oocyte maturation;ko05166 HTLV-I infection;
gene24627	K00423	E1.10.3.3	L-ascorbate oxidase	EC:1.10.3.3	1	ko00053 Ascorbate and aldarate metabolism;
gene2462	K03103	MINPP1	multiple inositol-polyphosphate phosphatase	EC:3.1.3.62	1	ko00562 Inositol phosphate metabolism;
gene24634	K00029	E1.1.1.40, maeB	malate dehydrogenase (oxaloacetate-decarboxylating)(NADP+)	EC:1.1.1.40	3	ko00620 Pyruvate metabolism;ko00710 Carbon fixation in photosynthetic organisms;ko01200 Carbon metabolism;
gene24636	K11341	YEATS4, GAS41, YAF9	YEATS domain-containing protein 4	--		
gene24638	K11088	SNRPD3, SMD3	small nuclear ribonucleoprotein D3	--	2	ko03040 Spliceosome;ko05322 Systemic lupus erythematosus;
gene24639	K04733	IRAK4	interleukin-1 receptor-associated kinase 4	EC:2.7.11.1	11	ko04064 NF-kappa B signaling pathway;ko04210 Apoptosis;ko04620 Toll-like receptor signaling pathway;ko04722 Neurotrophin signaling pathway;ko05133 Pertussis;ko05140 Leishmaniasis;ko05142 Chagas disease (American trypanosomiasis);ko05145 Toxoplasmosis;ko05152 Tuberculosis;ko05162 Measles;ko05164 Influenza A;
gene2463	K00522	FTH1	ferritin heavy chain	EC:1.16.3.1	2	ko04978 Mineral absorption;

gene24640	K04733	IRAK4	interleukin-1 receptor-associated kinase 4	EC:2.7.11.1	11	ko04064 NF-kappa B signaling pathway;ko04210 Apoptosis;ko04620 Toll-like receptor signaling pathway;ko04722 Neurotrophin signaling pathway;ko05133 Pertussis;ko05140 Leishmaniasis;ko05142 Chagas disease (American trypanosomiasis);ko05145 Toxoplasmosis;ko05152 Tuberculosis;ko05162 Measles;ko05164 Influenza A;
gene24642	K05841	E2.4.1.173	sterol 3beta-glucosyltransferase	EC:2.4.1.173		
gene24643	K09872	PIP	aquaporin PIP	--		
gene2464	K01866	YARS, tyrS	tyrosyl-tRNA synthetase	EC:6.1.1.1	1	ko00970 Aminoacyl-tRNA biosynthesis;
gene24651	K13947	PIN	auxin efflux carrier family	--		
gene24653	K02324	POLE1	DNA polymerase epsilon subunit 1	EC:2.7.7.7	6	ko00230 Purine metabolism;ko00240 Pyrimidine metabolism;ko03030 DNA replication;ko03410 Base excision repair;ko03420 Nucleotide excision repair;ko05166 HTLV-I infection;
gene24655	K00670	E2.3.1.88	peptide alpha-N-acetyltransferase	EC:2.3.1.88		
gene24656	K06966	K06966				
gene24657	K01817	trpF	phosphoribosylanthranilate isomerase	EC:5.3.1.24	2	ko00400 Phenylalanine, tyrosine and tryptophan biosynthesis;ko01230 Biosynthesis of amino acids;
gene24658	K10257	FAD8, desB	omega-3 fatty acid desaturase (delta-15 desaturase)	EC:1.14.19.-	1	ko01040 Biosynthesis of unsaturated fatty acids;
gene24660	K15919	HPR2	hydroxypyruvate reductase 2	--	2	ko00260 Glycine, serine and threonine metabolism;ko00630 Glyoxylate and dicarboxylate metabolism;
gene24661	K15919	HPR2	hydroxypyruvate reductase 2	--	2	ko00260 Glycine, serine and threonine metabolism;ko00630 Glyoxylate and dicarboxylate metabolism;
gene24662	K02880	RP-L17e, RPL17	large subunit ribosomal protein L17e	--	1	ko03010 Ribosome;
gene24667	K07119	K07119				
gene24670	K13495	CISZOG	cis-zeatin O-glucosyltransferase	EC:2.4.1.215	1	ko00908 Zeatin biosynthesis;
gene24671	K14309	NUP93	nuclear pore complex protein Nup93	--	1	ko03013 RNA transport;
gene24672	K02880	RP-L17e, RPL17	large subunit ribosomal protein L17e	--	1	ko03010 Ribosome;
gene24675	K01689	ENO, eno	enolase	EC:4.2.1.11	6	ko00010 Glycolysis / Gluconeogenesis;ko00680 Methane metabolism;ko01200 Carbon metabolism;ko01230 Biosynthesis of amino acids;ko03018 RNA degradation;ko04066 HIF-1 signaling pathway;
gene24676	K10712	ADO	cysteamine dioxygenase	EC:1.13.11.19	1	ko00430 Taurine and hypotaurine metabolism;
gene24680	K10268	FBXL2_20	F-box and leucine-rich repeat protein 2/20	--		
gene24681	K04120	E5.5.1.13	ent-copalyl diphosphate synthase	EC:5.5.1.13	1	ko00904 Diterpenoid biosynthesis;
gene2468	K01867	WARS, trpS	tryptophanyl-tRNA synthetase	EC:6.1.1.2	1	ko00970 Aminoacyl-tRNA biosynthesis;
gene24695	K01213	E3.2.1.67	galacturan 1,4-alpha-galacturonidase	EC:3.2.1.67	2	ko00040 Pentose and glucuronate interconversions;ko00500 Starch and sucrose metabolism;
gene24696	K00858	ppnK, NADK	NAD+ kinase	EC:2.7.1.23	1	ko00760 Nicotinate and nicotinamide metabolism;
gene24697	K09872	PIP	aquaporin PIP	--		
gene24709	K14315	NDC1, TMEM48	nucleoporin NDC1	--	1	ko03013 RNA transport;
gene24713	K00600	glyA, SHMT	glycine hydroxymethyltransferase	EC:2.1.2.1	7	ko00260 Glycine, serine and threonine metabolism;ko00460 Cyanoamino acid metabolism;ko00630 Glyoxylate and dicarboxylate metabolism;ko00670 One carbon pool by folate;ko00680 Methane metabolism;ko01200 Carbon metabolism;ko01230 Biosynthesis of amino acids;
gene24716	K16912	LAS1	ribosomal biogenesis protein LAS1	--		
gene24719	K03301	TC.AAA	ATP:ADP antiporter, AAA family	--		
gene2471	K08244	E2.7.9.4	alpha-glucan, water dikinase	EC:2.7.9.4		
gene24720	K01772	hemH, FECH	ferrochelatase	EC:4.99.1.1	1	ko00860 Porphyrin and chlorophyll metabolism;
gene24738	K11420	EHMT	euchromatic histone-lysine N-methyltransferase	EC:2.1.1.43	1	ko00310 Lysine degradation;
gene24741	K12813	DHX16	pre-mRNA-splicing factor ATP-dependent RNA helicase DHX16	EC:3.6.4.13	1	ko03040 Spliceosome;
gene24747	K02148	ATPeVC, ATP6C	V-type H+-transporting ATPase subunit C	EC:3.6.3.14	7	ko00190 Oxidative phosphorylation;ko04145 Phagosome;ko04721 Synaptic vesicle cycle;ko04966 Collecting duct acid secretion;ko05110 Vibrio cholerae infection;ko05120 Epithelial cell signaling in Helicobacter pylori infection;ko05323 Rheumatoid arthritis;
gene24748	K00297	metF, MTHFR	methylenetetrahydrofolate reductase (NADPH)	EC:1.5.1.20	3	ko00670 One carbon pool by folate;ko00720 Carbon fixation pathways in prokaryotes;ko01200 Carbon metabolism;

gene24750	K00423	E1.10.3.3	L-ascorbate oxidase	EC:1.10.3.3	1	ko00053 Ascorbate and aldarate metabolism;
gene24758	K11799	WDR21A	WD repeat-containing protein 21A	--		
gene24760	K10739	RFA2, RPA2	replication factor A2	--	5	ko03030 DNA replication;ko03420 Nucleotide excision repair;ko03430 Mismatch repair;ko03440 Homologous recombination;ko03460 Fanconi anemia pathway;
gene24761	K01869	LARS, leuS	leucyl-tRNA synthetase	EC:6.1.1.4	1	ko00970 Aminoacyl-tRNA biosynthesis;
gene24762	K11778	DHDDS, RER2, SRT1	ditrans,polycis-polyprenyl diphosphate synthase	EC:2.5.1.87	1	ko00900 Terpenoid backbone biosynthesis;
gene24766	K10666	RNF5	E3 ubiquitin-protein ligase RNF5	EC:6.3.2.19	1	ko04141 Protein processing in endoplasmic reticulum;
gene24767	K03671	trxA	thioredoxin 1	--		
gene24768	K00979	kdsB	3-deoxy-manno-octulosonate cytidylyltransferase (CMP-KDO synthetase)	EC:2.7.7.38	1	ko00540 Lipopolysaccharide biosynthesis;
gene24771	K14327	UPF2, RENT2	regulator of nonsense transcripts 2	--	2	ko03013 RNA transport;ko03015 mRNA surveillance pathway;
gene24783	K07023	K07023	putative hydrolases of HD superfamily	--		
gene24789	K03549	kup	KUP system potassium uptake protein	--		
gene24790	K03549	kup	KUP system potassium uptake protein	--		
gene24793	K09873	TIP	aquaporin TIP	--		
gene24795	K07192	FLOT	flotillin	--	1	ko04910 Insulin signaling pathway;
gene24801	K14617	LMBRD1	LMBR1 domain-containing protein 1	--	1	ko04977 Vitamin digestion and absorption;
gene24803	K00565	RNMT	mRNA (guanine-N7-)-methyltransferase	EC:2.1.1.56	1	ko03015 mRNA surveillance pathway;
gene24806	K13412	CPK	calcium-dependent protein kinase	EC:2.7.11.1	2	ko04626 Plant-pathogen interaction;ko05145 Toxoplasmosis;
gene24807	K09835	crtISO, crtH	prolycopene isomerase	EC:5.2.1.13	1	ko00906 Carotenoid biosynthesis;
gene24811	K13648	GAUT	alpha-1,4-galacturonosyltransferase	EC:2.4.1.43	2	ko00500 Starch and sucrose metabolism;ko00520 Amino sugar and nucleotide sugar metabolism;
gene24816	K13366	MPAO	polyamine oxidase (propane-1,3-diamine-forming)	EC:1.5.3.14		
gene24824	K11824	AP2A	AP-2 complex subunit alpha	--	4	ko04144 Endocytosis;ko04721 Synaptic vesicle cycle;ko04961 Endocrine and other factor-regulated calcium reabsorption;ko05016 Huntington's disease;
gene24825	K00734	B3GALT6	galactosylxylosylprotein 3-beta-galactosyltransferase	EC:2.4.1.134	2	ko00532 Glycosaminoglycan biosynthesis - chondroitin sulfate / dermatan sulfate;ko00534 Glycosaminoglycan biosynthesis - heparan sulfate / heparin;
gene24827	K10396	KIF5	kinesin family member 5	--	1	ko04728 Dopaminergic synapse;
gene24829	K01640	E4.1.3.4, HMGCL, hmgL	hydroxymethylglutaryl-CoA lyase	EC:4.1.3.4	5	ko00072 Synthesis and degradation of ketone bodies;ko00280 Valine, leucine and isoleucine degradation;ko00281 Geraniol degradation;ko00650 Butanoate metabolism;ko04146 Peroxisome;
gene24836	K10696	BRE1	E3 ubiquitin-protein ligase BRE1	EC:6.3.2.19		
gene24837	K05757	ARPC1A_B	actin related protein 2/3 complex, subunit 1A/1B	--	6	ko04666 Fc gamma R-mediated phagocytosis;ko04810 Regulation of actin cytoskeleton;ko05100 Bacterial invasion of epithelial cells;ko05130 Pathogenic Escherichia coli infection;ko05131 Shigellosis;ko05132 Salmonella infection;
gene24839	K13420	FLS2	LRR receptor-like serine/threonine-protein kinase FLS2	EC:2.7.11.1	1	ko04626 Plant-pathogen interaction;
gene2483	K00616	E2.2.1.2, talA, talB	transaldolase	EC:2.2.1.2	3	ko00030 Pentose phosphate pathway;ko01200 Carbon metabolism;ko01230 Biosynthesis of amino acids;
gene24844	K09419	HSFF	heat shock transcription factor, other eukaryote	--		
gene24845	K00913	ITPK1	inositol-1,3,4-trisphosphate 5/6-kinase / inositol-tetrakisphosphate 1-kinase	EC:2.7.1.159 2.7.1.134	2	ko00562 Inositol phosphate metabolism;ko04070 Phosphatidylinositol signaling system;
gene24848	K10601	SYVN1, HRD1	E3 ubiquitin-protein ligase synoviolin	EC:6.3.2.19	2	ko04120 Ubiquitin mediated proteolysis;ko04141 Protein processing in endoplasmic reticulum;
gene2484	K01087	otsB	trehalose 6-phosphate phosphatase	EC:3.1.3.12	1	ko00500 Starch and sucrose metabolism;
gene24853	K06671	STAG1_2, SCC3, IRR1	cohesin complex subunit SA-1/2	--	3	ko04110 Cell cycle;ko04111 Cell cycle - yeast;ko04113 Meiosis - yeast;
gene24855	K11252	H2B	histone H2B	--	3	ko05034 Alcoholism;ko05203 Viral carcinogenesis;ko05322 Systemic lupus erythematosus;
gene24861	K00430	E1.11.1.7	peroxidase	EC:1.11.1.7	2	ko00360 Phenylalanine metabolism;ko00940 Phenylpropanoid biosynthesis;
gene24862	K03021	RPC2, POLR3B	DNA-directed RNA polymerase III subunit RPC2	EC:2.7.7.6	5	ko00230 Purine metabolism;ko00240 Pyrimidine metabolism;ko03020 RNA polymerase;ko04623 Cytosolic DNA-sensing pathway;ko05169 Epstein-Barr virus infection;
gene24873	K09510	DNAJB4	DnaJ homolog subfamily B member 4	--		
gene24875	K13153	SNRNP25	U11/U12 small nuclear ribonucleoprotein 25 kDa protein	--		

gene24879	K05356	SPS, sds	all-trans-nonaprenyl-diphosphate synthase	EC:2.5.1.84 2.5.1.85	1	ko00900 Terpenoid backbone biosynthesis;
gene2487	K01723	AOS	hydroperoxide dehydratase	EC:4.2.1.92	1	ko00592 alpha-Linolenic acid metabolism;
gene24883	K01051	E3.1.1.11	pectinesterase	EC:3.1.1.11	2	ko00040 Pentose and glucuronate interconversions;ko00500 Starch and sucrose metabolism;
gene24887	K15151	MED10, NUT2	mediator of RNA polymerase II transcription subunit 10	--		
gene2488	K14488	SAUR	SAUR family protein	--	1	ko04075 Plant hormone signal transduction;
gene24902	K16675	ZDHHC14	probable palmitoyltransferase ZDHHC14	EC:2.3.1.-	1	ko04391 Hippo signaling pathway - fly;
gene24903	K03035	PSMD12, RPN5	26S proteasome regulatory subunit N5	--	2	ko03050 Proteasome;ko05169 Epstein-Barr virus infection;
gene24904	K09286	EREBP	EREBP-like factor	--		
gene24906	K00517	E1.14.-.-			4	ko00624 Polycyclic aromatic hydrocarbon degradation;ko00627 Aminobenzoate degradation;ko00903 Limonene and pinene degradation;ko00945 Stilbenoid, diarylheptanoid and gingerol biosynthesis;
gene24907	K08245	E3.4.23.40	phytepsin	EC:3.4.23.40		
gene24909	K08900	BCS1	mitochondrial chaperone BCS1	--		
gene24911	K10841	ERCC6, CSB, RAD26	DNA excision repair protein ERCC-6	--	1	ko03420 Nucleotide excision repair;
gene24914	K10088	OS9	protein OS-9	--	1	ko04141 Protein processing in endoplasmic reticulum;
gene24915	K13525	VCP, CDC48	transitional endoplasmic reticulum ATPase	--	2	ko04141 Protein processing in endoplasmic reticulum;ko05134 Legionellosis;
gene2491	K14001	SIL1, SLS1	nucleotide exchange factor SIL1	--	1	ko04141 Protein processing in endoplasmic reticulum;
gene24923	K16900	TPC1, CCH1	two pore calcium channel protein, plant	--		
gene24929	K14505	CYCD3	cyclin D3, plant	--	1	ko04075 Plant hormone signal transduction;
gene24931	K03549	kup	KUP system potassium uptake protein	--		
gene24935	K16223	FT	protein FLOWERING LOCUS T	--	1	ko04712 Circadian rhythm - plant;
gene24936	K12878	THOC1	THO complex subunit 1	--	2	ko03013 RNA transport;ko03040 Spliceosome;
gene24937	K02969	RP-S20e, RPS20	small subunit ribosomal protein S20e	--	1	ko03010 Ribosome;
gene24939	K02958	RP-S15e, RPS15	small subunit ribosomal protein S15e	--	1	ko03010 Ribosome;
gene24941	K03019	RPC11, POLR3K	DNA-directed RNA polymerase III subunit RPC10	--	5	ko00230 Purine metabolism;ko00240 Pyrimidine metabolism;ko03020 RNA polymerase;ko04623 Cytosolic DNA-sensing pathway;ko05169 Epstein-Barr virus infection;
gene24943	K11490	NCAPH2	condensin-2 complex subunit H2	--		
gene24945	K07199	PRKAB	5'-AMP-activated protein kinase, regulatory beta subunit	--	5	ko04710 Circadian rhythm;ko04910 Insulin signaling pathway;ko04920 Adipocytokine signaling pathway;ko04932 Non-alcoholic fatty liver disease (NAFLD);ko05410 Hypertrophic cardiomyopathy (HCM);
gene24948	K13412	CPK	calcium-dependent protein kinase	EC:2.7.11.1	2	ko04626 Plant-pathogen interaction;ko05145 Toxoplasmosis;
gene2494	K04125	E1.14.11.13	gibberellin 2-oxidase	EC:1.14.11.13	1	ko00904 Diterpenoid biosynthesis;
gene24950	K08245	E3.4.23.40	phytepsin	EC:3.4.23.40		
gene24953	K14007	SEC24	protein transport protein SEC24	--	1	ko04141 Protein processing in endoplasmic reticulum;
gene24954	K04460	PPP5C	serine/threonine-protein phosphatase 5	EC:3.1.3.16	1	ko04010 MAPK signaling pathway;
gene24956	K00384	E1.8.1.9, trxB	thioredoxin reductase (NADPH)	EC:1.8.1.9	2	ko00240 Pyrimidine metabolism;ko00450 Selenocompound metabolism;
gene24957	K00021	E1.1.1.34	hydroxymethylglutaryl-CoA reductase (NADPH)	EC:1.1.1.34	2	ko00900 Terpenoid backbone biosynthesis;ko04976 Bile secretion;
gene2495	K13412	CPK	calcium-dependent protein kinase	EC:2.7.11.1	2	ko04626 Plant-pathogen interaction;ko05145 Toxoplasmosis;
gene24961	K07437	CYP26A	cytochrome P450, family 26, subfamily A	--	1	ko00830 Retinol metabolism;
gene24966	K14638	SLC15A3_4, PHT	solute carrier family 15 (peptide/histidine transporter), member 3/4	--		
gene24967	K14638	SLC15A3_4, PHT	solute carrier family 15 (peptide/histidine transporter), member 3/4	--		
gene24969	K16871	POP2	4-aminobutyrate---pyruvate transaminase	EC:2.6.1.96	2	ko00250 Alanine, aspartate and glutamate metabolism;ko00650 Butanoate metabolism;
gene24970	K01611	speD, AMD1	S-adenosylmethionine decarboxylase	EC:4.1.1.50	2	ko00270 Cysteine and methionine metabolism;ko00330 Arginine and proline metabolism;
gene24973	K17426	MRPL45	large subunit ribosomal protein L45, mitochondrial	--		
gene24974	K01920	E6.3.2.3, gshB	glutathione synthase	EC:6.3.2.3	1	ko00480 Glutathione metabolism;
gene24977	K00457	HPD, hppD	4-hydroxyphenylpyruvate dioxygenase	EC:1.13.11.27	3	ko00130 Ubiquinone and other terpenoid-quinone biosynthesis;ko00350 Tyrosine metabolism;ko00360 Phenylalanine metabolism;
gene24981	K12896	SFRS7	splicing factor, arginine/serine-rich 7	--	2	ko03040 Spliceosome;ko05168 Herpes simplex infection;
gene24982	K00306	PIPOX	sarcosine oxidase / L-pipecolate oxidase	EC:1.5.3.1 1.5.3.7	3	ko00260 Glycine, serine and threonine metabolism;ko00310 Lysine degradation;ko04146 Peroxisome;

gene2498	K01568	E4.1.1.1, pdc	pyruvate decarboxylase	EC:4.1.1.1	1	ko00010 Glycolysis / Gluconeogenesis;
gene24991	K01728	E4.2.2.2, pel	pectate lyase	EC:4.2.2.2	1	ko00040 Pentose and glucuronate interconversions;
gene24994	K02997	RP-S9e, RPS9	small subunit ribosomal protein S9e	--	1	ko03010 Ribosome;
gene24995	K05350	bglB	beta-glucosidase	EC:3.2.1.21	3	ko00460 Cyanoamino acid metabolism;ko00500 Starch and sucrose metabolism;ko00940 Phenylpropanoid biosynthesis;
gene24996	K02894	RP-L23e, RPL23	large subunit ribosomal protein L23e	--	1	ko03010 Ribosome;
gene24998	K12196	VPS4	vacuolar protein-sorting-associated protein 4	--	1	ko04144 Endocytosis;
gene24999	K00915	IPK2	1D-myo-Inositol-tetrakisphosphate 5-kinase / inositol-polyphosphate multikinase	EC:2.7.1.140 2.7.1.151	2	ko00562 Inositol phosphate metabolism;ko04070 Phosphatidylinositol signaling system;
gene2499	K02928	RP-L41e, RPL41	large subunit ribosomal protein L41e	--	1	ko03010 Ribosome;
gene25004	K06269	PPP1C	serine/threonine-protein phosphatase PP1 catalytic subunit	EC:3.1.3.16	14	ko03015 mRNA surveillance pathway;ko04113 Meiosis - yeast;ko04114 Oocyte meiosis;ko04270 Vascular smooth muscle contraction;ko04390 Hippo signaling pathway;ko04510 Focal adhesion;ko04720 Long-term potentiation;ko04728 Dopaminergic synapse;ko04810 Regulation of actin cytoskeleton;ko04910 Insulin signaling pathway;ko05031 Amphetamine addiction;ko05034 Alcoholism;ko05168 Herpes simplex infection;ko05205 Proteoglycans in
gene25007	K17505	PPM1K, PP2CM	protein phosphatase 1K	EC:3.1.3.16		
gene25012	K09571	FKBP4_5	FK506-binding protein 4/5	EC:5.2.1.8	1	ko04915 Estrogen signaling pathway;
gene25013	K14640	SLC20A, PIT	solute carrier family 20 (sodium-dependent phosphate transporter)	--		
gene25016	K08902	psb27	photosystem II Psb27 protein	--	1	ko00195 Photosynthesis;
gene25020	K04368	MAP2K1, MEK1	mitogen-activated protein kinase kinase 1	EC:2.7.12.2	53	ko04010 MAPK signaling pathway;ko04012 ErbB signaling pathway;ko04013 MAPK signaling pathway - fly;ko04014 Ras signaling pathway;ko04062 Chemokine signaling pathway;ko04066 HIF-1 signaling pathway;ko04114 Oocyte meiosis;ko04151 PI3K-Akt signaling pathway;ko04270 Vascular smooth muscle contraction;ko04320 Dorso-ventral axis formation;ko04370 VEGF signaling pathway;ko04380 Osteoclast differentiation;ko04510 Focal adhesion;ko04540 Gap junction;ko04620 Toll-like receptor signaling pathway;ko04626 Plant-pathogen interaction;ko04650 Natural killer cell mediated cytotoxicity;ko04660 T cell receptor signaling pathway;ko04662 B cell receptor signaling pathway;ko04664 Fc epsilon RI signaling pathway;ko04666 Fc gamma R-mediated phagocytosis;ko04668 TNF signaling pathway;ko04720 Long-term potentiation;ko04722 Neurotrophin signaling pathway;ko04725 Cholinergic synapse;ko04726 Serotonergic synapse;ko04730 Long-term depression;ko04810 Regulation of actin cytoskeleton;ko04910 Insulin signaling pathway;ko04912 GnRH signaling pathway;ko04914 Progesterone-mediated oocyte maturation;ko04915 Estrogen signaling pathway;ko04916 Melanogenesis;ko04917 Prolactin signaling pathway;ko05020 Prion diseases;ko05034 Alcoholism;ko05161 Hepatitis B;ko05164 Influenza A;ko05200 Pathways in cancer;ko05205 Proteoglycans in cancer;ko05206 MicroRNAs in cancer;ko05210 Colorectal cancer;ko05211 Renal cell carcinoma;ko05212 Pancreatic cancer;ko05213 Endometrial cancer;ko05214 Glioma;ko05215 Prostate cancer;ko05216 Thyroid cancer;ko05218 Melanoma;ko05219 Bladder cancer;ko05220
gene25022	K04733	IRAK4	interleukin-1 receptor-associated kinase 4	EC:2.7.11.1	11	ko04064 NF-kappa B signaling pathway;ko04210 Apoptosis;ko04620 Toll-like receptor signaling pathway;ko04722 Neurotrophin signaling pathway;ko05133 Pertussis;ko05140 Leishmaniasis;ko05142 Chagas disease (American trypanosomiasis);ko05145 Toxoplasmosis;ko05152 Tuberculosis;ko05162 Measles;ko05164 Influenza A;
gene25024	K14488	SAUR	SAUR family protein	--	1	ko04075 Plant hormone signal transduction;
gene25026	K12860	CDC5L, CDC5, CEF1	pre-mRNA-splicing factor CDC5/CEF1	--	1	ko03040 Spliceosome;
gene25027	K01823	idi, IDI	isopentenyl-diphosphate delta-isomerase	EC:5.3.3.2	1	ko00900 Terpenoid backbone biosynthesis;
gene25029	K02689	psaA	photosystem I P700 chlorophyll a apoprotein A1	--	1	ko00195 Photosynthesis;

gene25031	K16900	TPC1, CCH1	two pore calcium channel protein, plant	--		
gene25032	K16900	TPC1, CCH1	two pore calcium channel protein, plant	--		
gene25035	K10581	UBE2O	ubiquitin-conjugating enzyme E2 O	EC:6.3.2.19	1	ko04120 Ubiquitin mediated proteolysis;
gene25040	K05909	E1.10.3.2				
gene25042	K04515	CAMK2	calcium/calmodulin-dependent protein kinase (CaM kinase) II	EC:2.7.11.17	20	ko04012 ErbB signaling pathway;ko04020 Calcium signaling pathway;ko04066 HIF-1 signaling pathway;ko04114 Oocyte meiosis;ko04310 Wnt signaling pathway;ko04713 Circadian entrainment;ko04720 Long-term potentiation;ko04722 Neurotrophin signaling pathway;ko04725 Cholinergic synapse;ko04728 Dopaminergic synapse;ko04740 Olfactory transduction;ko04745 Phototransduction - fly;ko04911 Insulin secretion;ko04912 GnRH signaling pathway;ko04916 Melanogenesis;ko04971 Gastric acid secretion;ko05031 Amphetamine addiction;ko05152 Tuberculosis;ko05205 Proteoglycans in cancer;ko05214 Glioma;
gene25043	K02134	ATPeF1D, ATP5D	F-type H+-transporting ATPase subunit delta	EC:3.6.3.14	4	ko00190 Oxidative phosphorylation;ko05010 Alzheimer's disease;ko05012 Parkinson's disease;ko05016 Huntington's disease;
gene25044	K04564	SOD2	superoxide dismutase, Fe-Mn family	EC:1.15.1.1	2	ko04146 Peroxisome;ko05016 Huntington's disease;
gene2504	K07904	RAB11A	Ras-related protein Rab-11A	--	4	ko04144 Endocytosis;ko04961 Endocrine and other factor-regulated calcium reabsorption;ko04962 Vasopressin-regulated water reabsorption;ko04972 Pancreatic secretion;
gene25052	K01897	ACSL, fadD	long-chain acyl-CoA synthetase	EC:6.2.1.3	4	ko00071 Fatty acid degradation;ko03320 PPAR signaling pathway;ko04146 Peroxisome;ko04920 Adipocytokine signaling pathway;
gene25055	K07375	TUBB	tubulin beta	--	3	ko04145 Phagosome;ko04540 Gap junction;ko05130 Pathogenic Escherichia coli infection;
gene25059	K05350	bglB	beta-glucosidase	EC:3.2.1.21	3	ko00460 Cyanoamino acid metabolism;ko00500 Starch and sucrose metabolism;ko00940 Phenylpropanoid biosynthesis;
gene2505	K06911	K06911				
gene25060	K08819	CDK12_13	cyclin-dependent kinase 12/13	EC:2.7.11.22 2.7.11.23		
gene25066	K01369	LGMN	legumain	EC:3.4.22.34	2	ko04142 Lysosome;ko04612 Antigen processing and presentation;
gene25070	K00036	G6PD, zwf	glucose-6-phosphate 1-dehydrogenase	EC:1.1.1.49	3	ko00030 Pentose phosphate pathway;ko00480 Glutathione metabolism;ko01200 Carbon metabolism;
gene25076	K10875	RAD54L, RAD54	DNA repair and recombination protein RAD54 and RAD54-like protein	EC:3.6.4.-	1	ko03440 Homologous recombination;
gene25078	K10532	HGSNAT	heparan-alpha-glucosaminide N-acetyltransferase	EC:2.3.1.78	2	ko00531 Glycosaminoglycan degradation;ko04142 Lysosome;
gene25079	K10712	ADO	cysteamine dioxygenase	EC:1.13.11.19	1	ko00430 Taurine and hypotaurine metabolism;
gene2507	K15437	AIMP1, ARC1	aminoacyl tRNA synthase complex-interacting multifunctional protein 1	--		
gene25081	K06013	STE24	STE24 endopeptidase	EC:3.4.24.84	1	ko00900 Terpenoid backbone biosynthesis;
gene25084	K12195	CHMP6, VPS20	charged multivesicular body protein 6	--	1	ko04144 Endocytosis;
gene25088	K11290	SET, TAF1, I2PP2A	template-activating factor I	--		
gene25091	K05756	ARPC3	actin related protein 2/3 complex, subunit 3	--	6	ko04666 Fc gamma R-mediated phagocytosis;ko04810 Regulation of actin cytoskeleton;ko05100 Bacterial invasion of epithelial cells;ko05130 Pathogenic Escherichia coli infection;ko05131 Shigellosis;ko05132 Salmonella infection;
gene25092	K03715	E2.4.1.46	1,2-diacylglycerol 3-beta-galactosyltransferase	EC:2.4.1.46	1	ko00561 Glycerolipid metabolism;
gene25094	K02552	menF	menaquinone-specific isochorismate synthase	EC:5.4.4.2	2	ko00130 Ubiquinone and other terpenoid-quinone biosynthesis;ko01053 Biosynthesis of siderophore group nonribosomal peptides;
gene25096	K04733	IRAK4	interleukin-1 receptor-associated kinase 4	EC:2.7.11.1	11	ko04064 NF-kappa B signaling pathway;ko04210 Apoptosis;ko04620 Toll-like receptor signaling pathway;ko04722 Neurotrophin signaling pathway;ko05133 Pertussis;ko05140 Leishmaniasis;ko05142 Chagas disease (American trypanosomiasis);ko05145 Toxoplasmosis;ko05152 Tuberculosis;ko05162 Measles;ko05164 Influenza A;
gene250	K01177	E3.2.1.2	beta-amylase	EC:3.2.1.2	1	ko00500 Starch and sucrose metabolism;
gene25100	K02178	BUB1	checkpoint serine/threonine-protein kinase	EC:2.7.11.1	5	ko04110 Cell cycle;ko04111 Cell cycle - yeast;ko04113 Meiosis - yeast;ko04114 Oocyte meiosis;ko04914 Progesterone-mediated oocyte maturation;

gene25101	K05863	SLC25A4S, ANT	solute carrier family 25 (mitochondrial adenine nucleotide translocator), member 4/5/6/31	--	4	ko04020 Calcium signaling pathway;ko05012 Parkinson's disease;ko05016 Huntington's disease;ko05166 HTLV-I infection;
gene25104	K08472	MLO	mlo protein	--		
gene25108	K08790	STK38, NDR	serine/threonine kinase 38	EC:2.7.11.1		
gene2510	K06966	K06966				
gene25110	K11130	NOP10, NOLA3	H/ACA ribonucleoprotein complex subunit 3	--	1	ko03008 Ribosome biogenesis in eukaryotes;
gene25111	K08880	STK19	serine/threonine kinase 19	EC:2.7.11.1		
gene25112	K10875	RAD54L, RAD54	DNA repair and recombination protein RAD54 and RAD54-like protein	EC:3.6.4.-	1	ko03440 Homologous recombination;
gene25119	K10781	FATB	fatty acyl-ACP thioesterase B	EC:3.1.2.14	1	ko00061 Fatty acid biosynthesis;
gene2511	K10752	RBBP4, HAT2, CAF1, MIS16	histone-binding protein RBBP4	--		
gene25120	K11584	PPP2R5	serine/threonine-protein phosphatase 2A regulatory subunit B'	--	5	ko03015 mRNA surveillance pathway;ko04113 Meiosis - yeast;ko04114 Oocyte meiosis;ko04151 PI3K-Akt signaling pathway;ko04728 Dopaminergic synapse;
gene25124	K06130	LYPLA2	lysophospholipase II	EC:3.1.1.5	1	ko00564 Glycerophospholipid metabolism;
gene25125	K12275	SEC62	translocation protein SEC62	--	2	ko03060 Protein export;ko04141 Protein processing in endoplasmic reticulum;
gene2512	K17086	TM9SF2_4	transmembrane 9 superfamily member 2/4	--		
gene25132	K08770	UBC	ubiquitin C	--	1	ko03320 PPAR signaling pathway;
gene25133	K13110	MFAP1	microfibrillar-associated protein 1	--		
gene25135	K17506	PPM1L, PP2CE	protein phosphatase 1L	EC:3.1.3.16		
gene25137	K08867	WNK, PRKWNK	WNK lysine deficient protein kinase	EC:2.7.11.1		
gene25139	K11290	SET, TAF1, I2PP2A	template-activating factor I	--		
gene25140	K08908	LHCA2	light-harvesting complex I chlorophyll a/b binding protein 2	--	1	ko00196 Photosynthesis - antenna proteins;
gene25146	K00430	E1.11.1.7	peroxidase	EC:1.11.1.7	2	ko00360 Phenylalanine metabolism;ko00940 Phenylpropanoid biosynthesis;
gene25148	K14516	ERF1	ethylene-responsive transcription factor 1	--	1	ko04075 Plant hormone signal transduction;
gene25154	K02575	NRT, narK, nrtP, nasA	MFS transporter, NNP family, nitrate/nitrite transporter	--	1	ko00910 Nitrogen metabolism;
gene25157	K01183	E3.2.1.14	chitinase	EC:3.2.1.14	1	ko00520 Amino sugar and nucleotide sugar metabolism;
gene25159	K09422	MYBP	myb proto-oncogene protein, plant	--		
gene2515	K15255	PIF1	ATP-dependent DNA helicase PIF1	EC:3.6.4.12		
gene25162	K15202	GTF3C5, TFC1	general transcription factor 3C polypeptide 5 (transcription factor C subunit 1)	--		
gene25166	K00326	E1.6.2.2	cytochrome-b5 reductase	EC:1.6.2.2	1	ko00520 Amino sugar and nucleotide sugar metabolism;
gene25167	K06269	PPP1C	serine/threonine-protein phosphatase PP1 catalytic subunit	EC:3.1.3.16	14	ko03015 mRNA surveillance pathway;ko04113 Meiosis - yeast;ko04114 Oocyte meiosis;ko04270 Vascular smooth muscle contraction;ko04390 Hippo signaling pathway;ko04510 Focal adhesion;ko04720 Long-term potentiation;ko04728 Dopaminergic synapse;ko04810 Regulation of actin cytoskeleton;ko04910 Insulin signaling pathway;ko05031 Amphetamine addiction;ko05034 Alcoholism;ko05168 Herpes simplex infection;ko05205 Proteoglycans in
gene25171	K17086	TM9SF2_4	transmembrane 9 superfamily member 2/4	--		
gene25180	K07583	PUS10	tRNA pseudouridine synthase 10	EC:5.4.99.-		
gene25181	K01982	LSUrRNA	large subunit ribosomal RNA	--	2	ko03008 Ribosome biogenesis in eukaryotes;ko03010 Ribosome;
gene25182	K14516	ERF1	ethylene-responsive transcription factor 1	--	1	ko04075 Plant hormone signal transduction;
gene25184	K08960	CSNK1E	casein kinase 1, epsilon	EC:2.7.11.1	6	ko04310 Wnt signaling pathway;ko04340 Hedgehog signaling pathway;ko04390 Hippo signaling pathway;ko04391 Hippo signaling pathway - fly;ko04710 Circadian rhythm;ko04711 Circadian rhythm - fly;
gene25186	K08790	STK38, NDR	serine/threonine kinase 38	EC:2.7.11.1		
gene25188	K14709	SLC39A1_2_3, ZIP1_2_3	solute carrier family 39 (zinc transporter), member 1/2/3	--		
gene2518	K11131	DKC1, NOLA4, CBF5	H/ACA ribonucleoprotein complex subunit 4	EC:5.4.99.-	1	ko03008 Ribosome biogenesis in eukaryotes;
gene25190	K01982	LSUrRNA	large subunit ribosomal RNA	--	2	ko03008 Ribosome biogenesis in eukaryotes;ko03010 Ribosome;
gene25192	K05857	PLCD	phosphatidylinositol phospholipase C, delta	EC:3.1.4.11	3	ko00562 Inositol phosphate metabolism;ko04020 Calcium signaling pathway;ko04070 Phosphatidylinositol signaling system;

gene25195	K04733	IRAK4	interleukin-1 receptor-associated kinase 4	EC:2.7.11.1	11	ko04064 NF-kappa B signaling pathway;ko04210 Apoptosis;ko04620 Toll-like receptor signaling pathway;ko04722 Neurotrophin signaling pathway;ko05133 Pertussis;ko05140 Leishmaniasis;ko05142 Chagas disease (American trypanosomiasis);ko05145 Toxoplasmosis;ko05152 Tuberculosis;ko05162 Measles;ko05164 Influenza A;
gene25199	K14487	GH3	auxin responsive GH3 gene family	--	1	ko04075 Plant hormone signal transduction;
gene25201	K14517	ERF2	ethylene-responsive transcription factor 2	--	1	ko04075 Plant hormone signal transduction;
gene25202	K09579	PIN4	peptidyl-prolyl cis-trans isomerase NIMA-interacting 4	EC:5.2.1.8		
gene25203	K13681	FUT	xyloglucan fucosyltransferase	EC:2.4.1.-		
gene25206	K16296	SCPL-I	serine carboxypeptidase-like clade I	EC:3.4.16.-		
gene25211	K09540	SEC63	translocation protein SEC63	--	2	ko03060 Protein export;ko04141 Protein processing in endoplasmic reticulum;
gene25217	K15731	CTDSP	carboxy-terminal domain RNA polymerase II polypeptide A small phosphatase	EC:3.1.3.16		
gene25218	K13508	GPAT	glycerol-3-phosphate acyltransferase	EC:2.3.1.15	2	ko00561 Glycerolipid metabolism;ko00564 Glycerophospholipid metabolism;
gene2521	K14403	CPSF3, YSH1	cleavage and polyadenylation specificity factor subunit 3	EC:3.1.27.-	1	ko03015 mRNA surveillance pathway;
gene25220	K00276	E1.4.3.21, AOC2, AOC3, tynA	primary-amine oxidase	EC:1.4.3.21	6	ko00260 Glycine, serine and threonine metabolism;ko00350 Tyrosine metabolism;ko00360 Phenylalanine metabolism;ko00410 beta-Alanine metabolism;ko00950 Isoquinoline alkaloid biosynthesis;ko00960 Tropane, piperidine and pyridine alkaloid biosynthesis;
gene25227	K10088	OS9	protein OS-9	--	1	ko04141 Protein processing in endoplasmic reticulum;
gene25228	K13525	VCP, CDC48	transitional endoplasmic reticulum ATPase	--	2	ko04141 Protein processing in endoplasmic reticulum;ko05134 Legionellosis;
gene2522	K05863	SLC25A4S, ANT	solute carrier family 25 (mitochondrial adenine nucleotide translocator), member 4/5/6/31	--	4	ko04020 Calcium signaling pathway;ko05012 Parkinson's disease;ko05016 Huntington's disease;ko05166 HTLV-I infection;
gene25230	K03015	RPB7, POLR2G	DNA-directed RNA polymerase II subunit RPB7	--	5	ko00230 Purine metabolism;ko00240 Pyrimidine metabolism;ko03020 RNA polymerase;ko05016 Huntington's disease;ko05169 Epstein-Barr virus infection;
gene25231	K06269	PPP1C	serine/threonine-protein phosphatase PP1 catalytic subunit	EC:3.1.3.16	14	ko03015 mRNA surveillance pathway;ko04113 Meiosis - yeast;ko04114 Oocyte meiosis;ko04270 Vascular smooth muscle contraction;ko04390 Hippo signaling pathway;ko04510 Focal adhesion;ko04720 Long-term potentiation;ko04728 Dopaminergic synapse;ko04810 Regulation of actin cytoskeleton;ko04910 Insulin signaling pathway;ko05031 Amphetamine addiction;ko05034 Alcoholism;ko05168 Herpes simplex infection;ko05205 Proteoglycans in
gene25236	K00600	glyA, SHMT	glycine hydroxymethyltransferase	EC:2.1.2.1	7	ko00260 Glycine, serine and threonine metabolism;ko00460 Cyanoamino acid metabolism;ko00630 Glyoxylate and dicarboxylate metabolism;ko00670 One carbon pool by folate;ko00680 Methane metabolism;ko01200 Carbon metabolism;ko01230 Biosynthesis of amino acids;
gene25244	K06630	YWHAE	14-3-3 protein epsilon	--	8	ko04110 Cell cycle;ko04114 Oocyte meiosis;ko04151 PI3K-Akt signaling pathway;ko04390 Hippo signaling pathway;ko04391 Hippo signaling pathway - fly;ko04722 Neurotrophin signaling pathway;ko05169 Epstein-Barr virus infection;ko05203 Viral carcinogenesis;
gene25246	K00053	ilvC	ketol-acid reductoisomerase	EC:1.1.1.86	4	ko00290 Valine, leucine and isoleucine biosynthesis;ko00770 Pantothenate and CoA biosynthesis;ko01210 2-Oxocarboxylic acid metabolism;ko01230 Biosynthesis of amino acids;
gene25248	K08678	UXS1	UDP-glucuronate decarboxylase	EC:4.1.1.35	2	ko00500 Starch and sucrose metabolism;ko00520 Amino sugar and nucleotide sugar metabolism;
gene25251	K13496	UGT73C	UDP-glucosyl transferase 73C	EC:2.4.1.-		
gene25254	K10742	DNA2	DNA replication ATP-dependent helicase Dna2	EC:3.6.4.12	1	ko03030 DNA replication;
gene25256	K05349	bglX	beta-glucosidase	EC:3.2.1.21	3	ko00460 Cyanoamino acid metabolism;ko00500 Starch and sucrose metabolism;ko00940 Phenylpropanoid biosynthesis;
gene25260	K04078	groES, HSPE1	chaperonin GroES	--		
gene25263	K00737	MGAT3	beta-1,4-mannosyl-glycoprotein beta-1,4-N-acetylglucosaminyltransferase	EC:2.4.1.144	1	ko00510 N-Glycan biosynthesis;
gene25264	K14945	QKI	protein quaking	--		

gene25265	K12880	THOC3	THO complex subunit 3	--	2	ko03013 RNA transport;ko03040 Spliceosome;
gene2526	K00434	E1.11.1.11	L-ascorbate peroxidase	EC:1.11.1.11	2	ko00053 Ascorbate and aldarate metabolism;ko00480 Glutathione metabolism;
gene25279	K14400	PCF11	pre-mRNA cleavage complex 2 protein Pcf11	--	1	ko03015 mRNA surveillance pathway;
gene25282	K10666	RNF5	E3 ubiquitin-protein ligase RNF5	EC:6.3.2.19	1	ko04141 Protein processing in endoplasmic reticulum;
gene25283	K00430	E1.11.1.7	peroxidase	EC:1.11.1.7	2	ko00360 Phenylalanine metabolism;ko00940 Phenylpropanoid biosynthesis;
gene25284	K04710	CERS	ceramide synthetase	EC:2.3.1.24	1	ko00600 Sphingolipid metabolism;
gene2528	K09284	AP2	AP2-like factor, euAP2 lineage	--		
gene25290	K10950	ERO1L	ERO1-like protein alpha	EC:1.8.4.-	2	ko04141 Protein processing in endoplasmic reticulum;ko05110 Vibrio cholerae infection;
gene25295	K02889	RP-L21e, RPL21	large subunit ribosomal protein L21e	--	1	ko03010 Ribosome;
gene25296	K13436	PTI1	pto-interacting protein 1	EC:2.7.11.1	1	ko04626 Plant-pathogen interaction;
gene25298	K04506	SIAH1	E3 ubiquitin-protein ligase SIAH1	EC:6.3.2.19	3	ko04115 p53 signaling pathway;ko04120 Ubiquitin mediated proteolysis;ko04310 Wnt signaling pathway;
gene25299	K03652	MPG	DNA-3-methyladenine glycosylase	EC:3.2.2.21	1	ko03410 Base excision repair;
gene25307	K12837	U2AF2	splicing factor U2AF 65 kDa subunit	--	1	ko03040 Spliceosome;
gene25308	K02902	RP-L28, MRPL28, rpmB	large subunit ribosomal protein L28	--	1	ko03010 Ribosome;
gene25309	K03754	EIF2B2	translation initiation factor eIF-2B subunit beta	--	1	ko03013 RNA transport;
gene25314	K15119	SLC25A39_40	solute carrier family 25, member 39/40	--		
gene25317	K10688	UBE2W, UBC16	ubiquitin-conjugating enzyme E2 W	EC:6.3.2.19	1	ko04120 Ubiquitin mediated proteolysis;
gene25318	K15115	SLC25A32, MFT	solute carrier family 25 (mitochondrial folate transporter), member 32	--		
gene25322	K11253	H3	histone H3	--	3	ko05034 Alcoholism;ko05202 Transcriptional misregulation in cancer;ko05322 Systemic lupus erythematosus;
gene25324	K10143	RFWD2, COP1	E3 ubiquitin-protein ligase RFWD2	EC:6.3.2.19	3	ko04115 p53 signaling pathway;ko04120 Ubiquitin mediated proteolysis;ko04712 Circadian rhythm - plant;
gene25326	K02891	RP-L22e, RPL22	large subunit ribosomal protein L22e	--	1	ko03010 Ribosome;
gene25329	K01689	ENO, eno	enolase	EC:4.2.1.11	6	ko00010 Glycolysis / Gluconeogenesis;ko00680 Methane metabolism;ko01200 Carbon metabolism;ko01230 Biosynthesis of amino acids;ko03018 RNA degradation;ko04066 HIF-1 signaling pathway;
gene25336	K10140	DDB2	DNA damage-binding protein 2	--	4	ko03420 Nucleotide excision repair;ko04115 p53 signaling pathway;ko04120 Ubiquitin mediated proteolysis;ko05161 Hepatitis B;
gene25342	K09264	K09264	MADS-box transcription factor, plant	--		
gene25344	K07575	K07575	PUA domain protein	--		
gene25345	K02883	RP-L18e, RPL18	large subunit ribosomal protein L18e	--	1	ko03010 Ribosome;
gene25353	K09422	MYBP	myb proto-oncogene protein, plant	--		
gene25354	K11290	SET, TAF1, I2PP2A	template-activating factor I	--		
gene25355	K00021	E1.1.1.34	hydroxymethylglutaryl-CoA reductase (NADPH)	EC:1.1.1.34	2	ko00900 Terpenoid backbone biosynthesis;ko04976 Bile secretion;
gene25358	K11093	SNRP70	U1 small nuclear ribonucleoprotein 70kDa	--	1	ko03040 Spliceosome;
gene25359	K04730	IRAK1	interleukin-1 receptor-associated kinase 1	EC:2.7.11.1	11	ko04064 NF-kappa B signaling pathway;ko04210 Apoptosis;ko04620 Toll-like receptor signaling pathway;ko04722 Neurotrophin signaling pathway;ko05133 Pertussis;ko05140 Leishmaniasis;ko05142 Chagas disease (American trypanosomiasis);ko05145 Toxoplasmosis;ko05152 Tuberculosis;ko05162 Measles;ko05169 Epstein-Barr virus infection;
gene25360	K14782	AATF, BFR2	protein AATF/BFR2	--		
gene25361	K14709	SLC39A1_2_3, ZIP1_2_3	solute carrier family 39 (zinc transporter), member 1/2/3	--		
gene25364	K02918	RP-L35e, RPL35	large subunit ribosomal protein L35e	--	1	ko03010 Ribosome;
gene25365	K07195	EXOC7, EXO70	exocyst complex component 7	--	1	ko04910 Insulin signaling pathway;
gene25373	K01495	GCH1, folE	GTP cyclohydrolase I	EC:3.5.4.16	1	ko00790 Folate biosynthesis;
gene25377	K08829	MAK	male germ cell-associated kinase	EC:2.7.11.22		
gene25381	K01679	E4.2.1.2B, fumC	fumarate hydratase, class II	EC:4.2.1.2	5	ko00020 Citrate cycle (TCA cycle);ko00720 Carbon fixation pathways in prokaryotes;ko01200 Carbon metabolism;ko05200 Pathways in cancer;ko05211 Renal cell carcinoma;
gene25382	K14229	tRNA-Lys	tRNA Lys	--	1	ko00970 Aminoacyl-tRNA biosynthesis;
gene2538	K10728	TOPBP1	topoisomerase (DNA) II binding protein 1	--		
gene25390	K14497	PP2C	protein phosphatase 2C	EC:3.1.3.16	1	ko04075 Plant hormone signal transduction;

gene25391	K14497	PP2C	protein phosphatase 2C	EC:3.1.3.16	1	ko04075 Plant hormone signal transduction;
gene25392	K15472	CYP71D55	premnaspirodiene oxygenase	EC:1.14.13.12 1	1	ko00909 Sesquiterpenoid and triterpenoid biosynthesis;
gene25393	K00888	PI4K	phosphatidylinositol 4-kinase	EC:2.7.1.67	2	ko00562 Inositol phosphate metabolism;ko04070 Phosphatidylinositol signaling system;
gene25394	K09286	EREBP	EREBP-like factor	--		
gene25396	K14236	tRNA-Tyr	tRNA Tyr	--	1	ko00970 Aminoacyl-tRNA biosynthesis;
gene25397	K11647	SMARCA2_4	SWI/SNF-related matrix-associated actin-dependent regulator of chromatin subfamily A member 2/4	EC:3.6.4.-		
gene25398	K01940	E6.3.4.5, argG	argininosuccinate synthase	EC:6.3.4.5	3	ko00250 Alanine, aspartate and glutamate metabolism;ko00330 Arginine and proline metabolism;ko01230 Biosynthesis of amino acids;
gene25403	K10355	ACTF	actin, other eukaryote	--		
gene25409	K14190	VTC2_5	GDP-L-galactose phosphorylase	EC:2.7.7.69	1	ko00053 Ascorbate and aldarate metabolism;
gene2540	K11253	H3	histone H3	--	3	ko05034 Alcoholism;ko05202 Transcriptional misregulation in cancer;ko05322 Systemic lupus erythematosus;
gene25410	K12872	RBM22, SLT11	pre-mRNA-splicing factor RBM22/SLT11	--	1	ko03040 Spliceosome;
gene25416	K02896	RP-L24e, RPL24	large subunit ribosomal protein L24e	--	1	ko03010 Ribosome;
gene25418	K16584	HAUS1	HAUS augmin-like complex subunit 1	--		
gene2541	K01115	PLD1_2	phospholipase D1/2	EC:3.1.4.4	7	ko00564 Glycerophospholipid metabolism;ko00565 Ether lipid metabolism;ko04014 Ras signaling pathway;ko04144 Endocytosis;ko04666 Fc gamma R-mediated phagocytosis;ko04724 Glutamatergic synapse;ko04912 GnRH signaling pathway;
gene25420	K13126	PABPC	polyadenylate-binding protein	--	3	ko03013 RNA transport;ko03015 mRNA surveillance pathway;ko03018 RNA degradation;
gene25421	K03039	PSMD13, RPN9	26S proteasome regulatory subunit N9	--	2	ko03050 Proteasome;ko05169 Epstein-Barr virus infection;
gene25425	K03006	RPB1, POLR2A	DNA-directed RNA polymerase II subunit RPB1	EC:2.7.7.6	6	ko00230 Purine metabolism;ko00240 Pyrimidine metabolism;ko03020 RNA polymerase;ko05016 Huntington's disease;ko05168 Herpes simplex infection;ko05169 Epstein-Barr virus infection;
gene25426	K06269	PPP1C	serine/threonine-protein phosphatase PP1 catalytic subunit	EC:3.1.3.16	14	ko03015 mRNA surveillance pathway;ko04113 Meiosis - yeast;ko04114 Oocyte meiosis;ko04270 Vascular smooth muscle contraction;ko04390 Hippo signaling pathway;ko04510 Focal adhesion;ko04720 Long-term potentiation;ko04728 Dopaminergic synapse;ko04810 Regulation of actin cytoskeleton;ko04910 Insulin signaling pathway;ko05031 Amphetamine addiction;ko05034 Alcoholism;ko05168 Herpes simplex infection;ko05205 Proteoglycans in
gene25427	K08794	CAMK1	calcium/calmodulin-dependent protein kinase I	EC:2.7.11.17		
gene2542	K12451	UER1	3,5-epimerase/4-reductase	EC:5.1.3.- 1.1.1.-	2	ko00520 Amino sugar and nucleotide sugar metabolism;ko00523 Polyketide sugar unit biosynthesis;
gene25430	K13162	PCBP2_3_4	poly(rC)-binding protein 2/3/4	--		
gene25431	K10862	TDP1	tyrosyl-DNA phosphodiesterase 1	EC:3.1.4.-		
gene25432	K03113	EIF1, SUI1	translation initiation factor 1	--	1	ko03013 RNA transport;
gene25437	K10355	ACTF	actin, other eukaryote	--		
gene25441	K01858	E5.5.1.4, INO1	myo-inositol-1-phosphate synthase	EC:5.5.1.4	2	ko00521 Streptomycin biosynthesis;ko00562 Inositol phosphate metabolism;
gene25444	K02947	RP-S10e, RPS10	small subunit ribosomal protein S10e	--	1	ko03010 Ribosome;
gene25445	K15443	TRM82, WDR4	tRNA (guanine-N(7)-)-methyltransferase subunit TRM82	--		
gene25446	K03347	CUL1, CDC53	cullin 1	--	9	ko04110 Cell cycle;ko04111 Cell cycle - yeast;ko04114 Oocyte meiosis;ko04120 Ubiquitin mediated proteolysis;ko04141 Protein processing in endoplasmic reticulum;ko04310 Wnt signaling pathway;ko04350 TGF-beta signaling pathway;ko04710 Circadian rhythm;ko05168 Herpes simplex infection;
gene25448	K13339	PEX6, PXAAA1	peroxin-6	--	1	ko04146 Peroxisome;
gene25450	K03671	trxA	thioredoxin 1	--		
gene25451	K03671	trxA	thioredoxin 1	--		
gene25453	K14009	BCAP31, BAP31	B-cell receptor-associated protein 31	--	1	ko04141 Protein processing in endoplasmic reticulum;

gene25456	K04733	IRAK4	interleukin-1 receptor-associated kinase 4	EC:2.7.11.1	11	ko04064 NF-kappa B signaling pathway;ko04210 Apoptosis;ko04620 Toll-like receptor signaling pathway;ko04722 Neurotrophin signaling pathway;ko05133 Pertussis;ko05140 Leishmaniasis;ko05142 Chagas disease (American trypanosomiasis);ko05145 Toxoplasmosis;ko05152 Tuberculosis;ko05162 Measles;ko05164 Influenza A;
gene25457	K15289	SLC35F5	solute carrier family 35, member F5	--		
gene25459	K07999	MMP20	matrix metalloproteinase-20 (enamelysin)	EC:3.4.24.-		
gene25464	K05387	GRIP	glutamate receptor, ionotropic, plant	--		
gene25468	K15083	RAD16	DNA repair protein RAD16	--		
gene25470	K10355	ACTF	actin, other eukaryote	--		
gene25474	K09842	AAO3	abscisic-aldehyde oxidase	EC:1.2.3.14	1	ko00906 Carotenoid biosynthesis;
gene25476	K17479	GRXCR1	glutaredoxin domain-containing cysteine-rich protein 1	--		
gene25479	K11290	SET, TAF1, I2PP2A	template-activating factor I	--		
gene25481	K08150	SLC2A13, ITR	MFS transporter, SP family, solute carrier family 2 (myo-inositol transporter), member 13	--		
gene25482	K08150	SLC2A13, ITR	MFS transporter, SP family, solute carrier family 2 (myo-inositol transporter), member 13	--		
gene25485	K13412	CPK	calcium-dependent protein kinase	EC:2.7.11.1	2	ko04626 Plant-pathogen interaction;ko05145 Toxoplasmosis;
gene25488	K09250	CNBP	cellular nucleic acid-binding protein	--		
gene25489	K01623	ALDO	fructose-bisphosphate aldolase, class I	EC:4.1.2.13	7	ko00010 Glycolysis / Gluconeogenesis;ko00030 Pentose phosphate pathway;ko00051 Fructose and mannose metabolism;ko00680 Methane metabolism;ko00710 Carbon fixation in photosynthetic organisms;ko01200 Carbon metabolism;ko01230 Biosynthesis of amino acids;
gene2548	K14500	BSK	BR-signaling kinase	EC:2.7.11.1	1	ko04075 Plant hormone signal transduction;
gene25490	K03965	NDUFB9	NADH dehydrogenase (ubiquinone) 1 beta subcomplex subunit 9	--	5	ko00190 Oxidative phosphorylation;ko04932 Non-alcoholic fatty liver disease (NAFLD);ko05010 Alzheimer's disease;ko05012 Parkinson's disease;ko05016 Huntington's disease;
gene25491	K01583	E4.1.1.19	arginine decarboxylase	EC:4.1.1.19	1	ko00330 Arginine and proline metabolism;
gene25493	K00801	FDFT1	farnesyl-diphosphate farnesyltransferase	EC:2.5.1.21	1	ko00909 Sesquiterpenoid and triterpenoid biosynthesis;
gene25497	K03549	kup	KUP system potassium uptake protein	--		
gene254	K00058	serA, PHGDH	D-3-phosphoglycerate dehydrogenase	EC:1.1.1.95	4	ko00260 Glycine, serine and threonine metabolism;ko00680 Methane metabolism;ko01200 Carbon metabolism;ko01230 Biosynthesis of amino acids;
gene25504	K02948	RP-S11, MRPS11, rpsK	small subunit ribosomal protein S11	--	1	ko03010 Ribosome;
gene2550	K15404	K15404, CER1	aldehyde decarbonylase	EC:4.1.99.5	1	ko00073 Cutin, suberine and wax biosynthesis;
gene25513	K10591	NEDD4, RSP5	E3 ubiquitin-protein ligase NEDD4	EC:6.3.2.19	3	ko04120 Ubiquitin mediated proteolysis;ko04144 Endocytosis;ko05169 Epstein-Barr virus infection;
gene25514	K16675	ZDHHC14	probable palmitoyltransferase ZDHHC14	EC:2.3.1.-	1	ko04391 Hippo signaling pathway - fly;
gene25515	K11420	EHMT	euchromatic histone-lysine N-methyltransferase	EC:2.1.1.43	1	ko00310 Lysine degradation;
gene25516	K07760	CDK	cyclin-dependent kinase	EC:2.7.11.22		
gene25520	K12603	CNOT6, CCR4	CCR4-NOT transcription complex subunit 6	--	1	ko03018 RNA degradation;
gene25523	K01166	E3.1.27.1	ribonuclease T2	EC:3.1.27.1		
gene25524	K01102	PDP	pyruvate dehydrogenase phosphatase	EC:3.1.3.43		
gene25526	K03781	katE, CAT, catB, srpA	catalase	EC:1.11.1.6	4	ko00380 Tryptophan metabolism;ko00630 Glyoxylate and dicarboxylate metabolism;ko04146 Peroxisome;ko05014 Amyotrophic lateral sclerosis (ALS);
gene25530	K14802	DRS2, ATP8A	phospholipid-transporting ATPase	EC:3.6.3.1		
gene25531	K12196	VPS4	vacuolar protein-sorting-associated protein 4	--	1	ko04144 Endocytosis;
gene2553	K13335	PEX16	peroxin-16	--	1	ko04146 Peroxisome;
gene25547	K13157	RNPC3	U11/U12 small nuclear ribonucleoprotein 65 kDa protein	--		
gene25548	K00430	E1.11.1.7	peroxidase	EC:1.11.1.7	2	ko00360 Phenylalanine metabolism;ko00940 Phenylpropanoid biosynthesis;

gene2554	K04421	MAP3K3, MEKK3	mitogen-activated protein kinase kinase kinase 3	EC:2.7.11.25	4	ko04010 MAPK signaling pathway;ko04722 Neurotrophin signaling pathway;ko04912 GnRH signaling pathway;ko05166 HTLV-I infection;
gene25552	K13099	CD2BP2, PPP1R59	CD2 antigen cytoplasmic tail-binding protein 2	--		
gene25560	K08496	GOSR2, BOS1	golgi SNAP receptor complex member 2	--	1	ko04130 SNARE interactions in vesicular transport;
gene25562	K02908	RP-L30e, RPL30	large subunit ribosomal protein L30e	--	1	ko03010 Ribosome;
gene25565	K15196	BRF1, GTF3B	transcription factor IIIB 90 kDa subunit	--		
gene25570	K14492	ARR-A	two-component response regulator ARR-A family	--	1	ko04075 Plant hormone signal transduction;
gene25571	K01530	E3.6.3.1	phospholipid-translocating ATPase	EC:3.6.3.1		
gene25572	K01369	LGMN	legumain	EC:3.4.22.34	2	ko04142 Lysosome;ko04612 Antigen processing and presentation;
gene25574	K02986	RP-S4, rpsD	small subunit ribosomal protein S4	--	1	ko03010 Ribosome;
gene25575	K13963	SERPINB	serpin B	--	1	ko05146 Amoebiasis;
gene25576	K14638	SLC15A3_4, PHT	solute carrier family 15 (peptide/histidine transporter), member 3/4	--		
gene25577	K00558	DNMT1, dcm	DNA (cytosine-5)-methyltransferase 1	EC:2.1.1.37	2	ko00270 Cysteine and methionine metabolism;ko05206 MicroRNAs in cancer;
gene25578	K09285	OVM, ANT	AP2-like factor, ANT lineage	--		
gene25583	K14007	SEC24	protein transport protein SEC24	--	1	ko04141 Protein processing in endoplasmic reticulum;
gene25584	K09422	MYBP	myb proto-oncogene protein, plant	--		
gene25586	K17301	COPB1, SEC26	coatomer, subunit beta	--		
gene25587	K07195	EXOC7, EXO70	exocyst complex component 7	--	1	ko04910 Insulin signaling pathway;
gene25591	K10255	FAD6, desA	omega-6 fatty acid desaturase (delta-12 desaturase)	EC:1.14.19.-	2	ko01040 Biosynthesis of unsaturated fatty acids;ko02020 Two-component system;
gene25598	K13418	SERK1	somatic embryogenesis receptor kinase 1	EC:2.7.10.1 2.7.11.1		
gene2559	K16871	POP2	4-aminobutyrate---pyruvate transaminase	EC:2.6.1.96	2	ko00250 Alanine, aspartate and glutamate metabolism;ko00650 Butanoate metabolism;
gene25603	K03969	pspA	phage shock protein A	--		
gene25604	K15188	CCNT	cyclin T	--	1	ko05202 Transcriptional misregulation in cancer;
gene2560	K04123	KAO	ent-kaurenoic acid hydroxylase	EC:1.14.13.79	1	ko00904 Diterpenoid biosynthesis;
gene25613	K13151	SNUPN, RNUT1	snurportin-1	--	1	ko03013 RNA transport;
gene25614	K08730	PTDSS2	phosphatidylserine synthase 2	EC:2.7.8.29	1	ko00564 Glycerophospholipid metabolism;
gene25615	K14442	DHX36, RHAU	ATP-dependent RNA helicase DHX36	EC:3.6.4.13	1	ko03018 RNA degradation;
gene25616	K12619	XRN2, RAT1	5'-3' exoribonuclease 2	EC:3.1.13.-	2	ko03008 Ribosome biogenesis in eukaryotes;ko03018 RNA degradation;
gene25619	K05863	SLC25A4S, ANT	solute carrier family 25 (mitochondrial adenine nucleotide translocator), member 4/5/6/31	--	4	ko04020 Calcium signaling pathway;ko05012 Parkinson's disease;ko05016 Huntington's disease;ko05166 HTLV-I infection;
gene2561	K16871	POP2	4-aminobutyrate---pyruvate transaminase	EC:2.6.1.96	2	ko00250 Alanine, aspartate and glutamate metabolism;ko00650 Butanoate metabolism;
gene25621	K11126	TERT, EST2	telomerase reverse transcriptase	EC:2.7.7.49	1	ko05166 HTLV-I infection;
gene25622	K02137	ATPeF1O, ATP5O	F-type H+-transporting ATPase oligomycin sensitivity conferral protein	EC:3.6.3.14	4	ko00190 Oxidative phosphorylation;ko05010 Alzheimer's disease;ko05012 Parkinson's disease;ko05016 Huntington's disease;
gene25624	K09422	MYBP	myb proto-oncogene protein, plant	--		
gene25626	K09338	HD-ZIP	homeobox-leucine zipper protein	--		
gene25628	K13679	WAXY	granule-bound starch synthase	EC:2.4.1.242		
gene25630	K11835	USP4_11_15, UBP12	ubiquitin carboxyl-terminal hydrolase 4/11/15	EC:3.1.2.15		
gene25633	K03609	minD	septum site-determining protein MinD	--		
gene25634	K01595	ppc	phosphoenolpyruvate carboxylase	EC:4.1.1.31	5	ko00620 Pyruvate metabolism;ko00680 Methane metabolism;ko00710 Carbon fixation in photosynthetic organisms;ko00720 Carbon fixation pathways in prokaryotes;ko01200 Carbon metabolism;
gene25635	K16572	TUBGCP5, GCP5	gamma-tubulin complex component 5	--		
gene25637	K15472	CYP71D55	premnaspirodiene oxygenase	EC:1.14.13.12 1	1	ko00909 Sesquiterpenoid and triterpenoid biosynthesis;
gene25638	K13412	CPK	calcium-dependent protein kinase	EC:2.7.11.1	2	ko04626 Plant-pathogen interaction;ko05145 Toxoplasmosis;
gene2563	K11968	ARIH1	ariadne-1	--		
gene2564	K15272	SLC35A1_2_3	solute carrier family 35 (UDP-sugar transporter), member A1/2/3	--		
gene25656	K00430	E1.11.1.7	peroxidase	EC:1.11.1.7	2	ko00360 Phenylalanine metabolism;ko00940 Phenylpropanoid biosynthesis;
gene25658	K13422	MYC2	transcription factor MYC2	--	2	ko04075 Plant hormone signal transduction;ko04626 Plant-pathogen interaction;
gene25661	K12951	ctpD	cation-transporting P-type ATPase D	EC:3.6.3.-		

gene25664	K01681	ACO, acnA	aconitate hydratase	EC:4.2.1.3	6	ko00020 Citrate cycle (TCA cycle);ko00630 Glyoxylate and dicarboxylate metabolism;ko00720 Carbon fixation pathways in prokaryotes;ko01200 Carbon metabolism;ko01210 2-Oxocarboxylic acid metabolism;ko01230 Biosynthesis of amino acids;
gene25668	K03327	TC.MATE, SLC47A, norM, mdtK, dinF	multidrug resistance protein, MATE family	--		
gene25670	K05387	GRIP	glutamate receptor, ionotropic, plant	--		
gene25671	K05387	GRIP	glutamate receptor, ionotropic, plant	--		
gene25673	K07976	RAB	Rab family, other	--		
gene25679	K01728	E4.2.2.2, pel	pectate lyase	EC:4.2.2.2	1	ko00040 Pentose and glucuronate interconversions;
gene25681	K14232	tRNA-Pro	tRNA Pro	--	1	ko00970 Aminoacyl-tRNA biosynthesis;
gene25689	K10712	ADO	cysteamine dioxygenase	EC:1.13.11.19	1	ko00430 Taurine and hypotaurine metabolism;
gene2568	K00759	E2.4.2.7, apt	adenine phosphoribosyltransferase	EC:2.4.2.7	1	ko00230 Purine metabolism;
gene25690	K08770	UBC	ubiquitin C	--	1	ko03320 PPAR signaling pathway;
gene25698	K15235	JOSD	josephin	EC:3.4.19.12		
gene256	K09191	GTF3A	general transcription factor IIIA	--		
gene25700	K09510	DNAJB4	DnaJ homolog subfamily B member 4	--		
gene25701	K03671	trxA	thioredoxin 1	--		
gene25702	K03671	trxA	thioredoxin 1	--		
gene25709	K12181	COPS8, CSN8	COP9 signalosome complex subunit 8	--		
gene25712	K03834	tyrP	tyrosine-specific transport protein	--		
gene25713	K13176	THOC7	THO complex subunit 7	--	1	ko03013 RNA transport;
gene25714	K03232	EEF1B	elongation factor 1-beta	--		
gene25718	K00430	E1.11.1.7	peroxidase	EC:1.11.1.7	2	ko00360 Phenylalanine metabolism;ko00940 Phenylpropanoid biosynthesis;
gene25719	K00472	E1.14.11.2	prolyl 4-hydroxylase	EC:1.14.11.2	1	ko00330 Arginine and proline metabolism;
gene25722	K14638	SLC15A3_4, PHT	solute carrier family 15 (peptide/histidine transporter), member 3/4	--		
gene25730	K15032	MTERFD	mTERF domain-containing protein, mitochondrial	--		
gene25731	K09286	EREBP	EREBP-like factor	--		
gene25732	K11267	PDS5	sister chromatid cohesion protein PDS5	--		
gene25735	K12449	AXS	UDP-apiose/xylose synthase	--	1	ko00520 Amino sugar and nucleotide sugar metabolism;
gene25736	K05765	CFL	cofilin	--	4	ko04360 Axon guidance;ko04666 Fc gamma R-mediated phagocytosis;ko04810 Regulation of actin cytoskeleton;ko05133 Pertussis;
gene25737	K15382	SLC50A, SWEET	solute carrier family 50 (sugar transporter)	--		
gene25744	K09377	CSRP	cysteine and glycine-rich protein	--		
gene25746	K04728	ATM, TEL1	ataxia telangiectasia mutated family protein	EC:2.7.11.1	7	ko04064 NF-kappa B signaling pathway;ko04110 Cell cycle;ko04115 p53 signaling pathway;ko04210 Apoptosis;ko05166 HTLV-I infection;ko05202 Transcriptional misregulation in cancer;ko05206 MicroRNAs in cancer;
gene25753	K14509	ETR, ERS	ethylene receptor	EC:2.7.13.-	1	ko04075 Plant hormone signal transduction;
gene25754	K05391	CNGF	cyclic nucleotide gated channel, other eukaryote	--	1	ko04626 Plant-pathogen interaction;
gene25757	K12356	UGT72E	coniferyl-alcohol glucosyltransferase	EC:2.4.1.111	1	ko00940 Phenylpropanoid biosynthesis;
gene25758	K01262	pepP	Xaa-Pro aminopeptidase	EC:3.4.11.9		
gene25760	K09338	HD-ZIP	homeobox-leucine zipper protein	--		
gene25762	K09060	GBF	plant G-box-binding factor	--		
gene25765	K13126	PABPC	polyadenylate-binding protein	--	3	ko03013 RNA transport;ko03015 mRNA surveillance pathway;ko03018 RNA degradation;
gene25768	K10640	RNF25, AO7	E3 ubiquitin-protein ligase RNF25	EC:6.3.2.19		
gene25769	K16818	K16818, DAD1	phospholipase A1	EC:3.1.1.32	2	ko00564 Glycerophospholipid metabolism;ko00592 alpha-Linolenic acid metabolism;
gene25774	K01051	E3.1.1.11	pectinesterase	EC:3.1.1.11	2	ko00040 Pentose and glucuronate interconversions;ko00500 Starch and sucrose metabolism;
gene25779	K14398	CPSF6_7	cleavage and polyadenylation specificity factor subunit 6/7	--	1	ko03015 mRNA surveillance pathway;
gene25782	K17275	PLS1	plastin-1	--		
gene25783	K07393	ECM4	putative glutathione S-transferase	--		

gene25787	K12812	UAP56, BAT1, SUB2	ATP-dependent RNA helicase UAP56/SUB2	EC:3.6.4.13	4	ko03013 RNA transport;ko03015 mRNA surveillance pathway;ko03040 Spliceosome;ko05164 Influenza A;
gene25788	K00434	E1.11.1.11	L-ascorbate peroxidase	EC:1.11.1.11	2	ko00053 Ascorbate and aldarate metabolism;ko00480 Glutathione metabolism;
gene25791	K10206	E2.6.1.83	LL-diaminopimelate aminotransferase	EC:2.6.1.83	2	ko00300 Lysine biosynthesis;ko01230 Biosynthesis of amino acids;
gene25796	K15710	SHPRH	E3 ubiquitin-protein ligase SHPRH	EC:3.6.4.- 6.3.2.19		
gene25797	K01915	glnA	glutamine synthetase	EC:6.3.1.2	8	ko00250 Alanine, aspartate and glutamate metabolism;ko00330 Arginine and proline metabolism;ko00630 Glyoxylate and dicarboxylate metabolism;ko00910 Nitrogen metabolism;ko01230 Biosynthesis of amino acids;ko02020 Two-component system;ko04724 Glutamatergic synapse;ko04727 GABAergic synapse;
gene25802	K01187	E3.2.1.20, malZ	alpha-glucosidase	EC:3.2.1.20	2	ko00052 Galactose metabolism;ko00500 Starch and sucrose metabolism;
gene25804	K01597	MVD, mvaD	diphosphomevalonate decarboxylase	EC:4.1.1.33	1	ko00900 Terpenoid backbone biosynthesis;
gene25807	K16280	RGLG	E3 ubiquitin-protein ligase RGLG	EC:6.3.2.19		
gene25809	K16280	RGLG	E3 ubiquitin-protein ligase RGLG	EC:6.3.2.19		
gene25810	K16280	RGLG	E3 ubiquitin-protein ligase RGLG	EC:6.3.2.19		
gene25811	K07195	EXOC7, EXO70	exocyst complex component 7	--	1	ko04910 Insulin signaling pathway;
gene25817	K02701	psaN	photosystem I subunit PsaN	--	1	ko00195 Photosynthesis;
gene25820	K02927	RP-L40e, RPL40	large subunit ribosomal protein L40e	--	1	ko03010 Ribosome;
gene25821	K01761	E4.4.1.11	methionine-gamma-lyase	EC:4.4.1.11	2	ko00270 Cysteine and methionine metabolism;ko00450 Selenocompound metabolism;
gene25823	K00416	QCR6, UQCRH	ubiquinol-cytochrome c reductase subunit 6	--	6	ko00190 Oxidative phosphorylation;ko04260 Cardiac muscle contraction;ko04932 Non-alcoholic fatty liver disease (NAFLD);ko05010 Alzheimer's disease;ko05012 Parkinson's disease;ko05016 Huntington's disease;
gene25827	K10364	CAPZA	capping protein (actin filament) muscle Z-line, alpha	--		
gene25828	K02218	CSNK1, CK1	casein kinase 1	EC:2.7.11.1	1	ko04340 Hedgehog signaling pathway;
gene25829	K01530	E3.6.3.1	phospholipid-translocating ATPase	EC:3.6.3.1		
gene2582	K09286	EREBP	EREBP-like factor	--		
gene25830	K13963	SERPINB	serpin B	--	1	ko05146 Amoebiasis;
gene25836	K01897	ACSL, fadD	long-chain acyl-CoA synthetase	EC:6.2.1.3	4	ko00071 Fatty acid degradation;ko03320 PPAR signaling pathway;ko04146 Peroxisome;ko04920 Adipocytokine signaling pathway;
gene25839	K00547	mmuM	homocysteine S-methyltransferase	EC:2.1.1.10	1	ko00270 Cysteine and methionine metabolism;
gene2583	K02885	RP-L19e, RPL19	large subunit ribosomal protein L19e	--	1	ko03010 Ribosome;
gene25846	K07901	RAB8A, MEL	Ras-related protein Rab-8A	--	1	ko04972 Pancreatic secretion;
gene25847	K10398	KIF11, EG5	kinesin family member 11	--		
gene25850	K14496	PYL	abscisic acid receptor PYR/PYL family	--	1	ko04075 Plant hormone signal transduction;
gene25855	K13065	E2.3.1.133, HCT	shikimate O-hydroxycinnamoyltransferase	EC:2.3.1.133	3	ko00940 Phenylpropanoid biosynthesis;ko00941 Flavonoid biosynthesis;ko00945 Stilbenoid, diarylheptanoid and gingerol biosynthesis;
gene25856	K13065	E2.3.1.133, HCT	shikimate O-hydroxycinnamoyltransferase	EC:2.3.1.133	3	ko00940 Phenylpropanoid biosynthesis;ko00941 Flavonoid biosynthesis;ko00945 Stilbenoid, diarylheptanoid and gingerol biosynthesis;
gene2586	K00434	E1.11.1.11	L-ascorbate peroxidase	EC:1.11.1.11	2	ko00053 Ascorbate and aldarate metabolism;ko00480 Glutathione metabolism;
gene25875	K15095	E1.1.1.208	(+)-neomenthol dehydrogenase	EC:1.1.1.208	1	ko00902 Monoterpenoid biosynthesis;
gene25876	K15095	E1.1.1.208	(+)-neomenthol dehydrogenase	EC:1.1.1.208	1	ko00902 Monoterpenoid biosynthesis;
gene25881	K07119	K07119				
gene25883	K06640	ATR	serine/threonine-protein kinase ATR	EC:2.7.11.1	4	ko03460 Fanconi anemia pathway;ko04110 Cell cycle;ko04115 p53 signaling pathway;ko05166 HTLV-I infection;
gene25884	K06671	STAG1_2, SCC3, IRR1	cohesin complex subunit SA-1/2	--	3	ko04110 Cell cycle;ko04111 Cell cycle - yeast;ko04113 Meiosis - yeast;
gene25889	K15283	SLC35E1	solute carrier family 35, member E1	--		
gene25893	K04733	IRAK4	interleukin-1 receptor-associated kinase 4	EC:2.7.11.1	11	ko04064 NF-kappa B signaling pathway;ko04210 Apoptosis;ko04620 Toll-like receptor signaling pathway;ko04722 Neurotrophin signaling pathway;ko05133 Pertussis;ko05140 Leishmaniasis;ko05142 Chagas disease (American trypanosomiasis);ko05145 Toxoplasmosis;ko05152 Tuberculosis;ko05162 Measles;ko05164 Influenza A;
gene258	K09422	MYBP	myb proto-oncogene protein, plant	--		

gene25900	K14638	SLC15A3_4, PHT	solute carrier family 15 (peptide/histidine transporter), member 3/4	--		
gene25918	K03456	PPP2R1	serine/threonine-protein phosphatase 2A regulatory subunit A	--	13	ko03015 mRNA surveillance pathway;ko04111 Cell cycle - yeast;ko04113 Meiosis - yeast;ko04114 Oocyte meiosis;ko04151 PI3K-Akt signaling pathway;ko04350 TGF-beta signaling pathway;ko04390 Hippo signaling pathway;ko04391 Hippo signaling pathway - fly;ko04530 Tight junction;ko04728 Dopaminergic synapse;ko04730 Long-term depression;ko05142 Chagas disease (American trypanosomiasis);ko05160 Hepatitis C;
gene2591	K01115	PLD1_2	phospholipase D1/2	EC:3.1.4.4	7	ko00564 Glycerophospholipid metabolism;ko00565 Ether lipid metabolism;ko04014 Ras signaling pathway;ko04144 Endocytosis;ko04666 Fc gamma R-mediated phagocytosis;ko04724 Glutamatergic synapse;ko04912 GnRH signaling pathway;
gene25923	K09391	E2F7_8	transcription factor E2F7/8	--		
gene25925	K00275	pdxH, PNPO	pyridoxamine 5'-phosphate oxidase	EC:1.4.3.5	1	ko00750 Vitamin B6 metabolism;
gene25927	K14492	ARR-A	two-component response regulator ARR-A family	--	1	ko04075 Plant hormone signal transduction;
gene25931	K03531	ftsZ	cell division protein FtsZ	--	1	ko04112 Cell cycle - Caulobacter;
gene25934	K14232	tRNA-Pro	tRNA Pro	--	1	ko00970 Aminoacyl-tRNA biosynthesis;
gene25937	K08653	MBTPS1	membrane-bound transcription factor site-1 protease	EC:3.4.21.112	1	ko04141 Protein processing in endoplasmic reticulum;
gene25940	K02930	RP-L4e, RPL4	large subunit ribosomal protein L4e	--	1	ko03010 Ribosome;
gene25941	K12129	PRR7	pseudo-response regulator 7	--	1	ko04712 Circadian rhythm - plant;
gene25942	K14407	CSTF2, RNA15	cleavage stimulation factor subunit 2	--	1	ko03015 mRNA surveillance pathway;
gene25949	K15032	MTERFD	mTERF domain-containing protein, mitochondrial	--		
gene2594	K02933	RP-L6, MRPL6, rplF	large subunit ribosomal protein L6	--	1	ko03010 Ribosome;
gene25951	K14538	NUG1, GNL3	nuclear GTP-binding protein	--	1	ko03008 Ribosome biogenesis in eukaryotes;
gene25955	K07910	RAB18	Ras-related protein Rab-18	--		
gene25958	K12830	SF3B3, SAP130, RSE1	splicing factor 3B subunit 3	--	1	ko03040 Spliceosome;
gene25962	K03868	RBX1, ROC1	RING-box protein 1	--	12	ko03420 Nucleotide excision repair;ko04066 HIF-1 signaling pathway;ko04110 Cell cycle;ko04111 Cell cycle - yeast;ko04114 Oocyte meiosis;ko04120 Ubiquitin mediated proteolysis;ko04141 Protein processing in endoplasmic reticulum;ko04310 Wnt signaling pathway;ko04350 TGF-beta signaling pathway;ko04710 Circadian rhythm;ko05200 Pathways in cancer;ko05211 Renal cell carcinoma;
gene25963	K14488	SAUR	SAUR family protein	--	1	ko04075 Plant hormone signal transduction;
gene25967	K13754	SLC24A6, NCKX6	solute carrier family 24 (sodium/potassium/calcium exchanger), member 6	--		
gene25968	K10901	BLM, RECQL3, SGS1	bloom syndrome protein	EC:3.6.4.12	2	ko03440 Homologous recombination;ko03460 Fanconi anemia pathway;
gene25969	K15172	SUPT5H, SPT5	transcription elongation factor SPT5	--		
gene2596	K01184	E3.2.1.15	polygalacturonase	EC:3.2.1.15	2	ko00040 Pentose and glucuronate interconversions;ko00500 Starch and sucrose metabolism;
gene25976	K05933	E1.14.17.4	aminocyclopropanecarboxylate oxidase	EC:1.14.17.4	1	ko00270 Cysteine and methionine metabolism;
gene25977	K05933	E1.14.17.4	aminocyclopropanecarboxylate oxidase	EC:1.14.17.4	1	ko00270 Cysteine and methionine metabolism;
gene25978	K16833	PPP1R2, IPP2	protein phosphatase inhibitor 2	--		
gene25989	K00901	E2.7.1.107, DGK, dgkA	diacylglycerol kinase (ATP dependent)	EC:2.7.1.107	3	ko00561 Glycerolipid metabolism;ko00564 Glycerophospholipid metabolism;ko04070 Phosphatidylinositol signaling system;
gene25991	K03538	POP4, RPP29	ribonuclease P protein subunit POP4	EC:3.1.26.5	2	ko03008 Ribosome biogenesis in eukaryotes;ko03013 RNA transport;
gene25993	K11498	CENPE	centromeric protein E	--		
gene25998	K12741	HNRNPA1_3	heterogeneous nuclear ribonucleoprotein A1/A3	--	1	ko03040 Spliceosome;
gene26005	K15378	SLC45A1_2_4	solute carrier family 45, member 1/2/4	--		
gene26008	K14488	SAUR	SAUR family protein	--	1	ko04075 Plant hormone signal transduction;
gene26009	K14488	SAUR	SAUR family protein	--	1	ko04075 Plant hormone signal transduction;
gene26011	K02710	psbI	photosystem II PsbI protein	--	1	ko00195 Photosynthesis;
gene26015	K07897	RAB7A	Ras-related protein Rab-7A	--	5	ko04144 Endocytosis;ko04145 Phagosome;ko05132 Salmonella infection;ko05146 Amoebiasis;ko05152 Tuberculosis;
gene26019	K00648	fabH	3-oxoacyl-[acyl-carrier-protein] synthase III	EC:2.3.1.180	1	ko00061 Fatty acid biosynthesis;

gene26023	K00434	E1.11.1.11	L-ascorbate peroxidase	EC:1.11.1.11	2	ko00053 Ascorbate and aldarate metabolism;ko00480 Glutathione metabolism;
gene26024	K15731	CTDSP	carboxy-terminal domain RNA polymerase II polypeptide A small phosphatase	EC:3.1.3.16		
gene26026	K16871	POP2	4-aminobutyrate---pyruvate transaminase	EC:2.6.1.96	2	ko00250 Alanine, aspartate and glutamate metabolism;ko00650 Butanoate metabolism;
gene26029	K02901	RP-L27e, RPL27	large subunit ribosomal protein L27e	--	1	ko03010 Ribosome;
gene26032	K01520	dut, DUT	dUTP pyrophosphatase	EC:3.6.1.23	1	ko00240 Pyrimidine metabolism;
gene26034	K01738	cysK	cysteine synthase A	EC:2.5.1.47	4	ko00270 Cysteine and methionine metabolism;ko00920 Sulfur metabolism;ko01200 Carbon metabolism;ko01230 Biosynthesis of amino acids;
gene26036	K08675	PRSS15, PIM1	Lon-like ATP-dependent protease	EC:3.4.21.-		
gene26038	K16732	PRC1	protein regulator of cytokinesis 1	--		
gene26040	K08360	CYB561	cytochrome b-561	--		
gene26041	K08360	CYB561	cytochrome b-561	--		
gene26042	K08360	CYB561	cytochrome b-561	--		
gene26045	K00799	GST, gst	glutathione S-transferase	EC:2.5.1.18	4	ko00480 Glutathione metabolism;ko00980 Metabolism of xenobiotics by cytochrome P450;ko00982 Drug metabolism - cytochrome P450;ko05204 Chemical carcinogenesis;
gene26046	K00430	E1.11.1.7	peroxidase	EC:1.11.1.7	2	ko00360 Phenylalanine metabolism;ko00940 Phenylpropanoid biosynthesis;
gene26048	K03687	GRPE	molecular chaperone GrpE	--		
gene2604	K07999	MMP20	matrix metalloproteinase-20 (enamelysin)	EC:3.4.24.-		
gene26051	K09422	MYBP	myb proto-oncogene protein, plant	--		
gene26059	K15382	SLC50A, SWEET	solute carrier family 50 (sugar transporter)	--		
gene26061	K15382	SLC50A, SWEET	solute carrier family 50 (sugar transporter)	--		
gene26066	K07766	E3.6.1.52	diphosphoinositol-polyphosphate diphosphatase	EC:3.6.1.52		
gene26068	K03696	clpC	ATP-dependent Clp protease ATP-binding subunit ClpC	--		
gene2606	K03879	ND2	NADH-ubiquinone oxidoreductase chain 2	EC:1.6.5.3	2	ko00190 Oxidative phosphorylation;ko05012 Parkinson's disease;
gene26071	K06689	UBE2D_E, UBC4, UBC5	ubiquitin-conjugating enzyme E2 D/E	EC:6.3.2.19	2	ko04120 Ubiquitin mediated proteolysis;ko04141 Protein processing in endoplasmic reticulum;
gene26072	K02150	ATPeVE, ATP6E	V-type H+-transporting ATPase subunit E	EC:3.6.3.14	7	ko00190 Oxidative phosphorylation;ko04145 Phagosome;ko04721 Synaptic vesicle cycle;ko04966 Collecting duct acid secretion;ko05110 Vibrio cholerae infection;ko05120 Epithelial cell signaling in Helicobacter pylori infection;ko05323 Rheumatoid arthritis;
gene26089	K11423	SETD2, SET2	histone-lysine N-methyltransferase SETD2	EC:2.1.1.43	1	ko00310 Lysine degradation;
gene2608	K14857	SPB1, FTSJ3	AdoMet-dependent rRNA methyltransferase SPB1	EC:2.1.1.-		
gene26090	K10760	IPT	adenylate isopentenyltransferase (cytokinin synthase)	--	1	ko00908 Zeatin biosynthesis;
gene26106	K00413	CYC1, CYT1, petC	ubiquinol-cytochrome c reductase cytochrome c1 subunit	--	7	ko00190 Oxidative phosphorylation;ko02020 Two-component system;ko04260 Cardiac muscle contraction;ko04932 Non-alcoholic fatty liver disease (NAFLD);ko05010 Alzheimer's disease;ko05012 Parkinson's disease;ko05016 Huntington's disease;
gene26109	K07198	PRKAA, AMPK	5'-AMP-activated protein kinase, catalytic alpha subunit	EC:2.7.11.11	8	ko04140 Regulation of autophagy;ko04150 mTOR signaling pathway;ko04151 PI3K-Akt signaling pathway;ko04710 Circadian rhythm;ko04910 Insulin signaling pathway;ko04920 Adipocytokine signaling pathway;ko04932 Non-alcoholic fatty liver disease (NAFLD);ko05410 Hypertrophic cardiomyopathy (HCM);
gene26110	K00052	leuB	3-isopropylmalate dehydrogenase	EC:1.1.1.85	3	ko00290 Valine, leucine and isoleucine biosynthesis;ko01210 2-Oxocarboxylic acid metabolism;ko01230 Biosynthesis of amino acids;
gene26118	K02324	POLE1	DNA polymerase epsilon subunit 1	EC:2.7.7.7	6	ko00230 Purine metabolism;ko00240 Pyrimidine metabolism;ko03030 DNA replication;ko03410 Base excision repair;ko03420 Nucleotide excision repair;ko05166 HTLV-I infection;
gene26122	K16275	BAH, NLA	E3 ubiquitin-protein ligase BAH	EC:6.3.2.19		
gene26123	K14571	RIX7, NVL	ribosome biogenesis ATPase	--	1	ko03008 Ribosome biogenesis in eukaryotes;
gene26125	K11091	SNRPA	U1 small nuclear ribonucleoprotein A	--	1	ko03040 Spliceosome;
gene26126	K16571	TUBGCP4, GCP4	gamma-tubulin complex component 4	--		
gene26128	K03452	MHX	magnesium/proton exchanger	--		

gene2612	K03841	FBP, fbp	fructose-1,6-bisphosphatase I	EC:3.1.3.11	7	ko00010 Glycolysis / Gluconeogenesis;ko00030 Pentose phosphate pathway;ko00051 Fructose and mannose metabolism;ko00680 Methane metabolism;ko00710 Carbon fixation in photosynthetic organisms;ko01200 Carbon metabolism;ko04910 Insulin signaling pathway;
gene26133	K14802	DRS2, ATP8A	phospholipid-transporting ATPase	EC:3.6.3.1		
gene2613	K14509	ETR, ERS	ethylene receptor	EC:2.7.13.-	1	ko04075 Plant hormone signal transduction;
gene26141	K12188	SNF8, EAP30	ESCRT-II complex subunit VPS22	--	1	ko04144 Endocytosis;
gene26142	K04733	IRAK4	interleukin-1 receptor-associated kinase 4	EC:2.7.11.1	11	ko04064 NF-kappa B signaling pathway;ko04210 Apoptosis;ko04620 Toll-like receptor signaling pathway;ko04722 Neurotrophin signaling pathway;ko05133 Pertussis;ko05140 Leishmaniasis;ko05142 Chagas disease (American trypanosomiasis);ko05145 Toxoplasmosis;ko05152 Tuberculosis;ko05162 Measles;ko05164 Influenza A;
gene26146	K12188	SNF8, EAP30	ESCRT-II complex subunit VPS22	--	1	ko04144 Endocytosis;
gene26148	K11344	EAF6	chromatin modification-related protein EAF6	--		
gene26149	K05765	CFL	cofilin	--	4	ko04360 Axon guidance;ko04666 Fc gamma R-mediated phagocytosis;ko04810 Regulation of actin cytoskeleton;ko05133 Pertussis;
gene26153	K05909	E1.10.3.2				
gene26156	K08511	ATVAMP72	vesicle-associated membrane protein 72	--		
gene26157	K05016	CLCN7	chloride channel 7	--		
gene26159	K14496	PYL	abscisic acid receptor PYR/PYL family	--	1	ko04075 Plant hormone signal transduction;
gene2615	K11838	USP7, UBP15	ubiquitin carboxyl-terminal hydrolase 7	EC:3.1.2.15	3	ko05168 Herpes simplex infection;ko05169 Epstein-Barr virus infection;ko05203 Viral carcinogenesis;
gene26165	K11000	CALS	callose synthase	EC:2.4.1.-		
gene2616	K11838	USP7, UBP15	ubiquitin carboxyl-terminal hydrolase 7	EC:3.1.2.15	3	ko05168 Herpes simplex infection;ko05169 Epstein-Barr virus infection;ko05203 Viral carcinogenesis;
gene26179	K00161	PDHA, pdhA	pyruvate dehydrogenase E1 component subunit alpha	EC:1.2.4.1	6	ko00010 Glycolysis / Gluconeogenesis;ko00020 Citrate cycle (TCA cycle);ko00620 Pyruvate metabolism;ko00650 Butanoate metabolism;ko01200 Carbon metabolism;ko04066 HIF-1 signaling pathway;
gene26184	K00799	GST, gst	glutathione S-transferase	EC:2.5.1.18	4	ko00480 Glutathione metabolism;ko00980 Metabolism of xenobiotics by cytochrome P450;ko00982 Drug metabolism - cytochrome P450;ko05204 Chemical carcinogenesis;
gene26190	K09250	CNBP	cellular nucleic acid-binding protein	--		
gene26191	K02641	petH	ferredoxin--NADP+ reductase	EC:1.18.1.2	1	ko00195 Photosynthesis;
gene26193	K04420	MAP3K2, MEKK2	mitogen-activated protein kinase kinase kinase 2	EC:2.7.11.25	3	ko04010 MAPK signaling pathway;ko04540 Gap junction;ko04912 GnRH signaling pathway;
gene26200	K11266	MAU2	MAternally affected uncoordination	--		
gene26201	K14843	PES1, NOP7	pescadillo	--		
gene26202	K14611	SLC23A1_2, SVCT1_2	solute carrier family 23 (nucleobase transporter), member 1/2	--		
gene26204	K13420	FLS2	LRR receptor-like serine/threonine-protein kinase FLS2	EC:2.7.11.1	1	ko04626 Plant-pathogen interaction;
gene26219	K04733	IRAK4	interleukin-1 receptor-associated kinase 4	EC:2.7.11.1	11	ko04064 NF-kappa B signaling pathway;ko04210 Apoptosis;ko04620 Toll-like receptor signaling pathway;ko04722 Neurotrophin signaling pathway;ko05133 Pertussis;ko05140 Leishmaniasis;ko05142 Chagas disease (American trypanosomiasis);ko05145 Toxoplasmosis;ko05152 Tuberculosis;ko05162 Measles;ko05164 Influenza A;
gene2621	K02936	RP-L7Ae, RPL7A	large subunit ribosomal protein L7Ae	--	1	ko03010 Ribosome;
gene26221	K09422	MYBP	myb proto-oncogene protein, plant	--		
gene26223	K10636	AMFR, GP78	autocrine motility factor receptor	EC:6.3.2.19	1	ko04141 Protein processing in endoplasmic reticulum;
gene26224	K04733	IRAK4	interleukin-1 receptor-associated kinase 4	EC:2.7.11.1	11	ko04064 NF-kappa B signaling pathway;ko04210 Apoptosis;ko04620 Toll-like receptor signaling pathway;ko04722 Neurotrophin signaling pathway;ko05133 Pertussis;ko05140 Leishmaniasis;ko05142 Chagas disease (American trypanosomiasis);ko05145 Toxoplasmosis;ko05152 Tuberculosis;ko05162 Measles;ko05164 Influenza A;
gene26227	K11446	JARID1	histone demethylase JARID1	EC:1.14.11.-		
gene26231	K13066	E2.1.1.68, COMT	caffeic acid 3-O-methyltransferase	EC:2.1.1.68	1	ko00940 Phenylpropanoid biosynthesis;

gene26236	K03254	EIF3A	translation initiation factor 3 subunit A	--	1	ko03013 RNA transport;
gene26240	K01303	APEH	acylaminoacyl-peptidase	EC:3.4.19.1		
gene26241	K01303	APEH	acylaminoacyl-peptidase	EC:3.4.19.1		
gene26242	K00863	E2.7.1.29, DAK1, DAK2	dihydroxyacetone kinase	EC:2.7.1.29	4	ko00561 Glycerolipid metabolism;ko00680 Methane metabolism;ko01200 Carbon metabolism;ko04622 RIG-I-like receptor signaling pathway;
gene26246	K16750	BLOC1S2	biogenesis of lysosome-related organelles complex 1 subunit 2	--		
gene26247	K14207	SLC38A2, SNAT2	solute carrier family 38 (sodium-coupled neutral amino acid transporter), member 2	--	3	ko04724 Glutamatergic synapse;ko04727 GABAergic synapse;ko04974 Protein digestion and absorption;
gene26250	K02976	RP-S26e, RPS26	small subunit ribosomal protein S26e	--	1	ko03010 Ribosome;
gene26257	K01373	CTSF	cathepsin F	EC:3.4.22.41	1	ko04142 Lysosome;
gene2625	K01213	E3.2.1.67	galacturan 1,4-alpha-galacturonidase	EC:3.2.1.67	2	ko00040 Pentose and glucuronate interconversions;ko00500 Starch and sucrose metabolism;
gene26260	K01979	SSUrRNA	small subunit ribosomal RNA	--	2	ko03008 Ribosome biogenesis in eukaryotes;ko03010 Ribosome;
gene26261	K10777	LIG4, DNL4	DNA ligase 4	EC:6.5.1.1	1	ko03450 Non-homologous end-joining;
gene26262	K00422	E1.10.3.1	polyphenol oxidase	EC:1.10.3.1	2	ko00350 Tyrosine metabolism;ko00950 Isoquinoline alkaloid biosynthesis;
gene26269	K02995	RP-S8e, RPS8	small subunit ribosomal protein S8e	--	1	ko03010 Ribosome;
gene26270	K06911	K06911				
gene26271	K03714	E2.4.2.38	glycoprotein 2-beta-D-xylosyltransferase	EC:2.4.2.38	1	ko00513 Various types of N-glycan biosynthesis;
gene26274	K15109	SLC25A20_29, CACT, CACL, CRC1	solute carrier family 25 (mitochondrial carnitine/acylcarnitine transporter), member 20/29	--		
gene26282	K14018	PLAA, DOA1, UFD3	phospholipase A-2-activating protein	--	1	ko04141 Protein processing in endoplasmic reticulum;
gene26283	K10666	RNF5	E3 ubiquitin-protein ligase RNF5	EC:6.3.2.19	1	ko04141 Protein processing in endoplasmic reticulum;
gene26284	K07399	resB, ccs1	cytochrome c biogenesis protein	--		
gene26285	K03327	TC.MATE, SLC47A, norM, mdtK, dinF	multidrug resistance protein, MATE family	--		
gene26286	K03327	TC.MATE, SLC47A, norM, mdtK, dinF	multidrug resistance protein, MATE family	--		
gene26287	K03527	E1.17.1.2, lytB, ispH	4-hydroxy-3-methylbut-2-enyl diphosphate reductase	EC:1.17.1.2	1	ko00900 Terpenoid backbone biosynthesis;
gene2628	K06685	MOB1, Mats	MOB kinase activator 1	--	3	ko04111 Cell cycle - yeast;ko04390 Hippo signaling pathway;ko04391 Hippo signaling pathway - fly;
gene26292	K14724	SLC9A8, NHE8	solute carrier family 9 (sodium/hydrogen exchanger), member 8	--		
gene26296	K00640	cysE	serine O-acetyltransferase	EC:2.3.1.30	4	ko00270 Cysteine and methionine metabolism;ko00920 Sulfur metabolism;ko01200 Carbon metabolism;ko01230 Biosynthesis of amino acids;
gene26298	K09419	HSFF	heat shock transcription factor, other eukaryote	--		
gene26299	K00850	pfkA, PFK	6-phosphofructokinase 1	EC:2.7.1.11	7	ko00010 Glycolysis / Gluconeogenesis;ko00030 Pentose phosphate pathway;ko00051 Fructose and mannose metabolism;ko00052 Galactose metabolism;ko00680 Methane metabolism;ko01200 Carbon metabolism;ko01230 Biosynthesis of amino acids;
gene262	K15289	SLC35F5	solute carrier family 35, member F5	--		
gene26303	K17613	CABIN1	calcineurin-binding protein cabin-1	--		
gene26305	K03456	PPP2R1	serine/threonine-protein phosphatase 2A regulatory subunit A	--	13	ko03015 mRNA surveillance pathway;ko04111 Cell cycle - yeast;ko04113 Meiosis - yeast;ko04114 Oocyte meiosis;ko04151 PI3K-Akt signaling pathway;ko04350 TGF-beta signaling pathway;ko04390 Hippo signaling pathway;ko04391 Hippo signaling pathway - fly;ko04530 Tight junction;ko04728 Dopaminergic synapse;ko04730 Long-term depression;ko05142 Chagas disease (American [illegible]
gene26307	K08489	STX16	syntaxin 16	--	1	ko04130 SNARE interactions in vesicular transport;
gene26311	K06692	PSMD5	26S proteasome non-ATPase regulatory subunit 5	--		
gene26312	K05391	CNGF	cyclic nucleotide gated channel, other eukaryote	--	1	ko04626 Plant-pathogen interaction;
gene26317	K03107	SRP68	signal recognition particle subunit SRP68	--	1	ko03060 Protein export;
gene26321	K03878	ND1	NADH-ubiquinone oxidoreductase chain 1	EC:1.6.5.3	2	ko00190 Oxidative phosphorylation;ko05012 Parkinson's disease;

gene26324	K12486	SMAP	stromal membrane-associated protein	--	1	ko04144 Endocytosis;
gene26327	K15718	LOX1_5	linoleate 9S-lipoxygenase	EC:1.13.11.58	1	ko00591 Linoleic acid metabolism;
gene26329	K16252	NRPD2, NRPE2	DNA-directed RNA polymerase IV and V subunit 2	EC:2.7.7.6		
gene2632	K11838	USP7, UBP15	ubiquitin carboxyl-terminal hydrolase 7	EC:3.1.2.15	3	ko05168 Herpes simplex infection;ko05169 Epstein-Barr virus infection;ko05203 Viral carcinogenesis;
gene26330	K08874	TRRAP	transformation/transcription domain-associated protein	--	1	ko05166 HTLV-I infection;
gene26331	K03062	PSMC1, RPT2	26S proteasome regulatory subunit T2	--	3	ko03050 Proteasome;ko05169 Epstein-Barr virus infection;ko05203 Viral carcinogenesis;
gene26334	K08852	ERN1	serine/threonine-protein kinase/endoribonuclease IRE1	EC:2.7.11.1	3	ko04141 Protein processing in endoplasmic reticulum;ko04932 Non-alcoholic fatty liver disease (NAFLD);ko05010 Alzheimer's disease;
gene26335	K05387	GRIP	glutamate receptor, ionotropic, plant	--		
gene26336	K06883	K06883				
gene26338	K01590	hdc, HDC	histidine decarboxylase	EC:4.1.1.22	1	ko00340 Histidine metabolism;
gene26339	K13418	SERK1	somatic embryogenesis receptor kinase 1	EC:2.7.10.1 2.7.11.1		
gene26345	K14496	PYL	abscisic acid receptor PYR/PYL family	--	1	ko04075 Plant hormone signal transduction;
gene26349	K13347	PXMP2, PMP22	peroxisomal membrane protein 2	--	1	ko04146 Peroxisome;
gene2634	K10406	KIFC2_3	kinesin family member C2/C3	--		
gene26352	K14843	PES1, NOP7	pescadillo	--		
gene26357	K00161	PDHA, pdhA	pyruvate dehydrogenase E1 component subunit alpha	EC:1.2.4.1	6	ko00010 Glycolysis / Gluconeogenesis;ko00020 Citrate cycle (TCA cycle);ko00620 Pyruvate metabolism;ko00650 Butanoate metabolism;ko01200 Carbon metabolism;ko04066 HIF-1 signaling pathway;
gene26365	K02961	RP-S17, MRPS17, rpsQ	small subunit ribosomal protein S17	--	1	ko03010 Ribosome;
gene26367	K00703	E2.4.1.21, glgA	starch synthase	EC:2.4.1.21	1	ko00500 Starch and sucrose metabolism;
gene26373	K10773	NTH	endonuclease III	EC:4.2.99.18	1	ko03410 Base excision repair;
gene26380	K13162	PCBP2_3_4	poly(rC)-binding protein 2/3/4	--		
gene26387	K00873	PK, pyk	pyruvate kinase	EC:2.7.1.40	7	ko00010 Glycolysis / Gluconeogenesis;ko00230 Purine metabolism;ko00620 Pyruvate metabolism;ko01200 Carbon metabolism;ko01230 Biosynthesis of amino acids;ko04930 Type II diabetes mellitus;ko05203 Viral carcinogenesis;
gene26389	K09510	DNAJB4	DnaJ homolog subfamily B member 4	--		
gene26390	K03671	trxA	thioredoxin 1	--		
gene26397	K10999	CESA	cellulose synthase A	EC:2.4.1.12		
gene26399	K16276	K16276, BTS	zinc finger protein-like protein	--		
gene26401	K11866	STAMBP, AMSH	STAM-binding protein	EC:3.1.2.15	1	ko04144 Endocytosis;
gene26402	K04733	IRAK4	interleukin-1 receptor-associated kinase 4	EC:2.7.11.1	11	ko04064 NF-kappa B signaling pathway;ko04210 Apoptosis;ko04620 Toll-like receptor signaling pathway;ko04722 Neurotrophin signaling pathway;ko05133 Pertussis;ko05140 Leishmaniasis;ko05142 Chagas disease (American trypanosomiasis);ko05145 Toxoplasmosis;ko05152 Tuberculosis;ko05162 Measles;ko05164 Influenza A;
gene26404	K02993	RP-S7e, RPS7	small subunit ribosomal protein S7e	--	1	ko03010 Ribosome;
gene26405	K09422	MYBP	myb proto-oncogene protein, plant	--		
gene2640	K04730	IRAK1	interleukin-1 receptor-associated kinase 1	EC:2.7.11.1	11	ko04064 NF-kappa B signaling pathway;ko04210 Apoptosis;ko04620 Toll-like receptor signaling pathway;ko04722 Neurotrophin signaling pathway;ko05133 Pertussis;ko05140 Leishmaniasis;ko05142 Chagas disease (American trypanosomiasis);ko05145 Toxoplasmosis;ko05152 Tuberculosis;ko05162 Measles;ko05169 Epstein-Barr virus infection;
gene26410	K12486	SMAP	stromal membrane-associated protein	--	1	ko04144 Endocytosis;
gene26412	K00454	LOX2S	lipoxygenase	EC:1.13.11.12	2	ko00591 Linoleic acid metabolism;ko00592 alpha-Linolenic acid metabolism;
gene26413	K10268	FBXL2_20	F-box and leucine-rich repeat protein 2/20	--		
gene26414	K01051	E3.1.1.11	pectinesterase	EC:3.1.1.11	2	ko00040 Pentose and glucuronate interconversions;ko00500 Starch and sucrose metabolism;
gene26420	K16292	CEP, CYSEP	KDEL-tailed cysteine endopeptidase	EC:3.4.22.-		
gene26421	K08150	SLC2A13, ITR	MFS transporter, SP family, solute carrier family 2 (myo-inositol transporter), member 13	--		

gene2642	K15283	SLC35E1	solute carrier family 35, member E1	--		
gene26430	K01240	URH1	uridine nucleosidase	EC:3.2.2.3	2	ko00240 Pyrimidine metabolism;ko00760 Nicotinate and nicotinamide metabolism;
gene26431	K03126	TAF12	transcription initiation factor TFIID subunit 12	--	1	ko03022 Basal transcription factors;
gene26434	K09843	E1.14.13.93	(+)-abscisic acid 8'-hydroxylase	EC:1.14.13.93	1	ko00906 Carotenoid biosynthesis;
gene26437	K14270	ACS10_12	aminotransferase	--		
gene26440	K13993	HSP20	HSP20 family protein	--	1	ko04141 Protein processing in endoplasmic reticulum;
gene26441	K02882	RP-L18Ae, RPL18A	large subunit ribosomal protein L18Ae	--	1	ko03010 Ribosome;
gene26442	K08819	CDK12_13	cyclin-dependent kinase 12/13	EC:2.7.11.22 2.7.11.23		
gene26446	K09422	MYBP	myb proto-oncogene protein, plant	--		
gene26448	K09422	MYBP	myb proto-oncogene protein, plant	--		
gene26450	K00799	GST, gst	glutathione S-transferase	EC:2.5.1.18	4	ko00480 Glutathione metabolism;ko00980 Metabolism of xenobiotics by cytochrome P450;ko00982 Drug metabolism - cytochrome P450;ko05204 Chemical carcinogenesis;
gene26455	K14851	RRP17, NOL12	ribosomal RNA-processing protein 17	--		
gene26462	K15382	SLC50A, SWEET	solute carrier family 50 (sugar transporter)	--		
gene26467	K01177	E3.2.1.2	beta-amylase	EC:3.2.1.2	1	ko00500 Starch and sucrose metabolism;
gene26469	K07437	CYP26A	cytochrome P450, family 26, subfamily A	--	1	ko00830 Retinol metabolism;
gene26475	K03526	E1.17.7.1, gcpE, ispG	(E)-4-hydroxy-3-methylbut-2-enyl-diphosphate synthase	EC:1.17.7.1	1	ko00900 Terpenoid backbone biosynthesis;
gene26482	K14315	NDC1, TMEM48	nucleoporin NDC1	--	1	ko03013 RNA transport;
gene26483	K04733	IRAK4	interleukin-1 receptor-associated kinase 4	EC:2.7.11.1	11	ko04064 NF-kappa B signaling pathway;ko04210 Apoptosis;ko04620 Toll-like receptor signaling pathway;ko04722 Neurotrophin signaling pathway;ko05133 Pertussis;ko05140 Leishmaniasis;ko05142 Chagas disease (American trypanosomiasis);ko05145 Toxoplasmosis;ko05152 Tuberculosis;ko05162 Measles;ko05164 Influenza A;
gene26493	K09841	ABA2	xanthoxin dehydrogenase	EC:1.1.1.288	1	ko00906 Carotenoid biosynthesis;
gene26497	K11755	hisIE	phosphoribosyl-ATP pyrophosphohydrolase / phosphoribosyl-AMP cyclohydrolase	EC:3.6.1.31 3.5.4.19	2	ko00340 Histidine metabolism;ko01230 Biosynthesis of amino acids;
gene26499	K05658	ABCB1	ATP-binding cassette, subfamily B (MDR/TAP), member 1	--	3	ko02010 ABC transporters;ko04976 Bile secretion;ko05206 MicroRNAs in cancer;
gene26505	K02537	MAD2	mitotic spindle assembly checkpoint protein MAD2	--	6	ko04110 Cell cycle;ko04111 Cell cycle - yeast;ko04113 Meiosis - yeast;ko04114 Oocyte meiosis;ko04914 Progesterone-mediated oocyte maturation;ko05166 HTLV-I infection;
gene26529	K15166	MED23	mediator of RNA polymerase II transcription subunit 23	--		
gene26530	K08681	pdxT, pdx2	glutamine amidotransferase	EC:2.6.-.-	1	ko00750 Vitamin B6 metabolism;
gene26531	K07359	CAMKK	calcium/calmodulin-dependent protein kinase kinase	EC:2.7.11.17	2	ko04920 Adipocytokine signaling pathway;ko05034 Alcoholism;
gene26534	K02957	RP-S15Ae, RPS15A	small subunit ribosomal protein S15Ae	--	1	ko03010 Ribosome;
gene26535	K13832	aroDE, DHQ-SDH	3-dehydroquinate dehydratase / shikimate dehydrogenase	EC:4.2.1.10 1.1.1.25	2	ko00400 Phenylalanine, tyrosine and tryptophan biosynthesis;ko01230 Biosynthesis of amino acids;
gene26536	K06670	SCC1, MCD1, RAD21	cohesin complex subunit SCC1	--	2	ko04110 Cell cycle;ko04111 Cell cycle - yeast;
gene26537	K13153	SNRNP25	U11/U12 small nuclear ribonucleoprotein 25 kDa protein	--		
gene26539	K13457	RPM1, RPS3	disease resistance protein RPM1	--	1	ko04626 Plant-pathogen interaction;
gene26540	K13457	RPM1, RPS3	disease resistance protein RPM1	--	1	ko04626 Plant-pathogen interaction;
gene26541	K03549	kup	KUP system potassium uptake protein	--		
gene26544	K17087	TM9SF3	transmembrane 9 superfamily member 3	--		
gene26545	K01934	E6.3.3.2	5-formyltetrahydrofolate cyclo-ligase	EC:6.3.3.2	1	ko00670 One carbon pool by folate;
gene26547	K12817	PRPF18, PRP18	pre-mRNA-splicing factor 18	--	1	ko03040 Spliceosome;
gene26549	K09338	HD-ZIP	homeobox-leucine zipper protein	--		
gene26552	K14802	DRS2, ATP8A	phospholipid-transporting ATPase	EC:3.6.3.1		
gene26556	K14220	tRNA-Asn	tRNA Asn	--	1	ko00970 Aminoacyl-tRNA biosynthesis;
gene26559	K10140	DDB2	DNA damage-binding protein 2	--	4	ko03420 Nucleotide excision repair;ko04115 p53 signaling pathway;ko04120 Ubiquitin mediated proteolysis;ko05161 Hepatitis B;
gene2656	K05666	ABCC2	ATP-binding cassette, subfamily C (CFTR/MRP), member 2	--	2	ko02010 ABC transporters;ko04976 Bile secretion;

gene26570	K01760	metC	cystathionine beta-lyase	EC:4.4.1.8	3	ko00270 Cysteine and methionine metabolism;ko00450 Selenocompound metabolism;ko01230 Biosynthesis of amino acids;
gene26572	K14492	ARR-A	two-component response regulator ARR-A family	--	1	ko04075 Plant hormone signal transduction;
gene26574	K00164	OGDH, sucA	2-oxoglutarate dehydrogenase E1 component	EC:1.2.4.2	4	ko00020 Citrate cycle (TCA cycle);ko00310 Lysine degradation;ko00380 Tryptophan metabolism;ko01200 Carbon metabolism;
gene26575	K07901	RAB8A, MEL	Ras-related protein Rab-8A	--	1	ko04972 Pancreatic secretion;
gene26576	K05765	CFL	cofilin	--	4	ko04360 Axon guidance;ko04666 Fc gamma R-mediated phagocytosis;ko04810 Regulation of actin cytoskeleton;ko05133 Pertussis;
gene26579	K03941	NDUFS8	NADH dehydrogenase (ubiquinone) Fe-S protein 8	EC:1.6.5.3 1.6.99.3	5	ko00190 Oxidative phosphorylation;ko04932 Non-alcoholic fatty liver disease (NAFLD);ko05010 Alzheimer's disease;ko05012 Parkinson's disease;ko05016 Huntington's disease;
gene26584	K17261	CAP1_2, SRV2	adenylyl cyclase-associated protein	--		
gene26586	K04536	GNB1	guanine nucleotide-binding protein G(I)/G(S)/G(T) subunit beta-1	--	14	ko04014 Ras signaling pathway;ko04062 Chemokine signaling pathway;ko04151 PI3K-Akt signaling pathway;ko04713 Circadian entrainment;ko04723 Retrograde endocannabinoid signaling;ko04724 Glutamatergic synapse;ko04725 Cholinergic synapse;ko04726 Serotonergic synapse;ko04727 GABAergic synapse;ko04728 Dopaminergic synapse;ko04742 Taste transduction;ko04744 Phototransduction;ko05032 Morphine addiction;ko05034 Alcoholism;
gene26587	K15032	MTERFD	mTERF domain-containing protein, mitochondrial	--		
gene26588	K17267	COPG	coatomer protein complex, subunit gamma	--		
gene26590	K09419	HSFF	heat shock transcription factor, other eukaryote	--		
gene26599	K15166	MED23	mediator of RNA polymerase II transcription subunit 23	--		
gene2659	K01177	E3.2.1.2	beta-amylase	EC:3.2.1.2	1	ko00500 Starch and sucrose metabolism;
gene26600	K15166	MED23	mediator of RNA polymerase II transcription subunit 23	--		
gene26605	K11593	ELF2C	eukaryotic translation initiation factor 2C	--		
gene26607	K10355	ACTF	actin, other eukaryote	--		
gene26609	K01803	TPI, tpiA	triosephosphate isomerase (TIM)	EC:5.3.1.1	6	ko00010 Glycolysis / Gluconeogenesis;ko00051 Fructose and mannose metabolism;ko00562 Inositol phosphate metabolism;ko00710 Carbon fixation in photosynthetic organisms;ko01200 Carbon metabolism;ko01230 Biosynthesis of amino acids;
gene26611	K14409	SMG7, EST1C	protein SMG7	--	1	ko03015 mRNA surveillance pathway;
gene26617	K00968	PCYT1	choline-phosphate cytidylyltransferase	EC:2.7.7.15	2	ko00440 Phosphonate and phosphinate metabolism;ko00564 Glycerophospholipid metabolism;
gene26620	K01358	clpP, CLPP	ATP-dependent Clp protease, protease subunit	EC:3.4.21.92	1	ko04112 Cell cycle - Caulobacter;
gene26622	K11594	DDX3X, bel	ATP-dependent RNA helicase	EC:3.6.4.13	3	ko04622 RIG-I-like receptor signaling pathway;ko05161 Hepatitis B;ko05203 Viral carcinogenesis;
gene26623	K10290	FBXO3	F-box protein 3	--		
gene26628	K01183	E3.2.1.14	chitinase	EC:3.2.1.14	1	ko00520 Amino sugar and nucleotide sugar metabolism;
gene26629	K01183	E3.2.1.14	chitinase	EC:3.2.1.14	1	ko00520 Amino sugar and nucleotide sugar metabolism;
gene26630	K03104	SRP14	signal recognition particle subunit SRP14	--	1	ko03060 Protein export;
gene26634	K01649	leuA	2-isopropylmalate synthase	EC:2.3.3.13	4	ko00290 Valine, leucine and isoleucine biosynthesis;ko00620 Pyruvate metabolism;ko01210 2-Oxocarboxylic acid metabolism;ko01230 Biosynthesis of amino acids;
gene26637	K12581	CNOT7_8, CAF1, POP2	CCR4-NOT transcription complex subunit 7/8	--	1	ko03018 RNA degradation;
gene26638	K14780	DHX37, DHR1	ATP-dependent RNA helicase DHX37/DHR1	EC:3.6.4.13		
gene2663	K13076	SLD	delta8-fatty-acid desaturase	EC:1.14.19.4		
gene26640	K14219	tRNA-Arg	tRNA Arg	--	1	ko00970 Aminoacyl-tRNA biosynthesis;
gene26642	K13448	CML	calcium-binding protein CML	--	1	ko04626 Plant-pathogen interaction;
gene26643	K14503	BZR1_2	brassinosteroid resistant 1/2	--	1	ko04075 Plant hormone signal transduction;
gene26644	K01307	GGH	gamma-glutamyl hydrolase	EC:3.4.19.9	1	ko00790 Folate biosynthesis;
gene26646	K05607	AUH	methylglutaconyl-CoA hydratase	EC:4.2.1.18	1	ko00280 Valine, leucine and isoleucine degradation;
gene26647	K05609	UCHL3, YUH1	ubiquitin carboxyl-terminal hydrolase L3	EC:3.4.19.12		
gene26649	K03245	EIF3J	translation initiation factor 3 subunit J	--	1	ko03013 RNA transport;
gene26651	K15119	SLC25A39_40	solute carrier family 25, member 39/40	--		

gene26654	K00088	guaB	IMP dehydrogenase	EC:1.1.1.205	2	ko00230 Purine metabolism;ko00983 Drug metabolism - other enzymes;
gene26658	K01214	E3.2.1.68	isoamylase	EC:3.2.1.68		
gene26659	K02908	RP-L30e, RPL30	large subunit ribosomal protein L30e	--	1	ko03010 Ribosome;
gene26664	K14497	PP2C	protein phosphatase 2C	EC:3.1.3.16	1	ko04075 Plant hormone signal transduction;
gene26668	K03660	OGG1	N-glycosylase/DNA lyase	EC:3.2.2.- 4.2.99.18	1	ko03410 Base excision repair;
gene26669	K12347	SLC11A, NRAMP	natural resistance-associated macrophage protein	--	2	ko04142 Lysosome;ko04978 Mineral absorption;
gene26672	K10755	RFC2_4	replication factor C subunit 2/4	--	3	ko03030 DNA replication;ko03420 Nucleotide excision repair;ko03430 Mismatch repair;
gene26674	K02863	RP-L1, MRPL1, rplA	large subunit ribosomal protein L1	--	1	ko03010 Ribosome;
gene26676	K14488	SAUR	SAUR family protein	--	1	ko04075 Plant hormone signal transduction;
gene26678	K11855	USP36_42	ubiquitin carboxyl-terminal hydrolase 36/42	EC:3.1.2.15		
gene2667	K14004	SEC13	protein transport protein SEC13	--	2	ko03013 RNA transport;ko04141 Protein processing in endoplasmic reticulum;
gene26680	K01176	E3.2.1.1, amyA, malS	alpha-amylase	EC:3.2.1.1	2	ko00500 Starch and sucrose metabolism;ko04973 Carbohydrate digestion and absorption;
gene26681	K14488	SAUR	SAUR family protein	--	1	ko04075 Plant hormone signal transduction;
gene26682	K04523	UBQLN, DSK2	ubiquilin	--	1	ko04141 Protein processing in endoplasmic reticulum;
gene26685	K14409	SMG7, EST1C	protein SMG7	--	1	ko03015 mRNA surveillance pathway;
gene26687	K03652	MPG	DNA-3-methyladenine glycosylase	EC:3.2.2.21	1	ko03410 Base excision repair;
gene26690	K03676	grxC, GLRX, GLRX2	glutaredoxin 3	--		
gene26696	K14509	ETR, ERS	ethylene receptor	EC:2.7.13.-	1	ko04075 Plant hormone signal transduction;
gene26697	K00599	E2.1.1.-			2	ko00350 Tyrosine metabolism;ko00624 Polycyclic aromatic hydrocarbon degradation;
gene26698	K13496	UGT73C	UDP-glucosyl transferase 73C	EC:2.4.1.-		
gene26699	K09286	EREBP	EREBP-like factor	--		
gene26704	K14792	RRP5, PDCD11	rRNA biogenesis protein RRP5	--		
gene26706	K13680	CSLA	beta-mannan synthase	EC:2.4.1.32		
gene2670	K00384	E1.8.1.9, trxB	thioredoxin reductase (NADPH)	EC:1.8.1.9	2	ko00240 Pyrimidine metabolism;ko00450 Selenocompound metabolism;
gene26710	K04499	RUVBL1, RVB1, INO80H	RuvB-like protein 1 (pontin 52)	--	1	ko04310 Wnt signaling pathway;
gene26711	K15104	SLC25A11, OGC	solute carrier family 25 (mitochondrial oxoglutarate transporter), member 11	--		
gene26714	K14432	ABF	ABA responsive element binding factor	--	1	ko04075 Plant hormone signal transduction;
gene26715	K12856	PRPF8, PRP8	pre-mRNA-processing factor 8	--	1	ko03040 Spliceosome;
gene26720	K15191	LARP7	La-related protein 7	--		
gene26728	K15113	SLC25A28_37, MFRN	solute carrier family 25 (mitochondrial iron transporter), member 28/37	--		
gene2672	K03639	MOCS1, moaA	molybdenum cofactor biosynthesis protein	--	2	ko00790 Folate biosynthesis;ko04122 Sulfur relay system;
gene26734	K14182	E4.2.3.21	vetispiradiene synthase	EC:4.2.3.21	1	ko00909 Sesquiterpenoid and triterpenoid biosynthesis;
gene26735	K10730	RECQL4	ATP-dependent DNA helicase Q4	EC:3.6.4.12		
gene26737	K03695	clpB	ATP-dependent Clp protease ATP-binding subunit ClpB	--		
gene26741	K11279	NAP1L1, NRP	nucleosome assembly protein 1-like 1	--		
gene26742	K03553	recA	recombination protein RecA	--	1	ko03440 Homologous recombination;
gene26743	K00645	fabD	[acyl-carrier-protein] S-malonyltransferase	EC:2.3.1.39	1	ko00061 Fatty acid biosynthesis;
gene26745	K05605	HIBCH	3-hydroxyisobutyryl-CoA hydrolase	EC:3.1.2.4	3	ko00280 Valine, leucine and isoleucine degradation;ko00410 beta-Alanine metabolism;ko00640 Propanoate metabolism;
gene26751	K07213	ATOX1, ATX1, copZ	copper chaperone	--	1	ko04978 Mineral absorption;
gene26752	K00430	E1.11.1.7	peroxidase	EC:1.11.1.7	2	ko00360 Phenylalanine metabolism;ko00940 Phenylpropanoid biosynthesis;
gene26753	K08736	MSH3	DNA mismatch repair protein MSH3	--	3	ko03430 Mismatch repair;ko05200 Pathways in cancer;ko05210 Colorectal cancer;
gene26754	K03252	EIF3C	translation initiation factor 3 subunit C	--	1	ko03013 RNA transport;
gene26755	K08819	CDK12_13	cyclin-dependent kinase 12/13	EC:2.7.11.22 2.7.11.23		
gene26760	K14232	tRNA-Pro	tRNA Pro	--	1	ko00970 Aminoacyl-tRNA biosynthesis;
gene26763	K14232	tRNA-Pro	tRNA Pro	--	1	ko00970 Aminoacyl-tRNA biosynthesis;
gene26764	K14232	tRNA-Pro	tRNA Pro	--	1	ko00970 Aminoacyl-tRNA biosynthesis;
gene26769	K14235	tRNA-Trp	tRNA Trp	--	1	ko00970 Aminoacyl-tRNA biosynthesis;
gene26770	K14232	tRNA-Pro	tRNA Pro	--	1	ko00970 Aminoacyl-tRNA biosynthesis;
gene26771	K06947	GRC3, NOL9	polynucleotide 5'-hydroxyl-kinase GRC3/NOL9	EC:2.7.1.-		

gene26773	K01934	E6.3.3.2	5-formyltetrahydrofolate cyclo-ligase	EC:6.3.3.2	1	ko00670 One carbon pool by folate;
gene26776	K04733	IRAK4	interleukin-1 receptor-associated kinase 4	EC:2.7.11.1	11	ko04064 NF-kappa B signaling pathway;ko04210 Apoptosis;ko04620 Toll-like receptor signaling pathway;ko04722 Neurotrophin signaling pathway;ko05133 Pertussis;ko05140 Leishmaniasis;ko05142 Chagas disease (American trypanosomiasis);ko05145 Toxoplasmosis;ko05152 Tuberculosis;ko05162 Measles;ko05164 Influenza A;
gene26783	K11252	H2B	histone H2B	--	3	ko05034 Alcoholism;ko05203 Viral carcinogenesis;ko05322 Systemic lupus erythematosus;
gene26786	K08176	PHO84	MFS transporter, PHS family, inorganic phosphate transporter	--		
gene26787	K03549	kup	KUP system potassium uptake protein	--		
gene26790	K01551	arsA, ASNA1	arsenite-transporting ATPase	EC:3.6.3.16		
gene26794	K04733	IRAK4	interleukin-1 receptor-associated kinase 4	EC:2.7.11.1	11	ko04064 NF-kappa B signaling pathway;ko04210 Apoptosis;ko04620 Toll-like receptor signaling pathway;ko04722 Neurotrophin signaling pathway;ko05133 Pertussis;ko05140 Leishmaniasis;ko05142 Chagas disease (American trypanosomiasis);ko05145 Toxoplasmosis;ko05152 Tuberculosis;ko05162 Measles;ko05164 Influenza A;
gene26797	K14638	SLC15A3_4, PHT	solute carrier family 15 (peptide/histidine transporter), member 3/4	--		
gene26803	K00915	IPK2	1D-myo-Inositol-tetrakisphosphate 5-kinase / inositol-polyphosphate multikinase	EC:2.7.1.140 2.7.1.151	2	ko00562 Inositol phosphate metabolism;ko04070 Phosphatidylinositol signaling system;
gene26804	K17080	PHB1	prohibitin 1	--		
gene26805	K17435	MRPL54	large subunit ribosomal protein L54, mitochondrial	--		
gene26807	K13457	RPM1, RPS3	disease resistance protein RPM1	--	1	ko04626 Plant-pathogen interaction;
gene26811	K00164	OGDH, sucA	2-oxoglutarate dehydrogenase E1 component	EC:1.2.4.2	4	ko00020 Citrate cycle (TCA cycle);ko00310 Lysine degradation;ko00380 Tryptophan metabolism;ko01200 Carbon metabolism;
gene26813	K02955	RP-S14e, RPS14	small subunit ribosomal protein S14e	--	1	ko03010 Ribosome;
gene26814	K08238	XXT	xyloglucan 6-xylosyltransferase	EC:2.4.2.39		
gene26815	K04078	groES, HSPE1	chaperonin GroES	--		
gene2681	K12486	SMAP	stromal membrane-associated protein	--	1	ko04144 Endocytosis;
gene26821	K08960	CSNK1E	casein kinase 1, epsilon	EC:2.7.11.1	6	ko04310 Wnt signaling pathway;ko04340 Hedgehog signaling pathway;ko04390 Hippo signaling pathway;ko04391 Hippo signaling pathway - fly;ko04710 Circadian rhythm;ko04711 Circadian rhythm - fly;
gene26822	K02875	RP-L14e, RPL14	large subunit ribosomal protein L14e	--	1	ko03010 Ribosome;
gene26824	K09422	MYBP	myb proto-oncogene protein, plant	--		
gene26828	K14638	SLC15A3_4, PHT	solute carrier family 15 (peptide/histidine transporter), member 3/4	--		
gene26831	K12865	PQBP1, NPW38	polyglutamine-binding protein 1	--	1	ko03040 Spliceosome;
gene26835	K14487	GH3	auxin responsive GH3 gene family	--	1	ko04075 Plant hormone signal transduction;
gene26836	K14487	GH3	auxin responsive GH3 gene family	--	1	ko04075 Plant hormone signal transduction;
gene26837	K14802	DRS2, ATP8A	phospholipid-transporting ATPase	EC:3.6.3.1		
gene26842	K14769	UTP11	U3 small nucleolar RNA-associated protein 11	--		
gene26849	K09613	COPS5, CSN5	COP9 signalosome complex subunit 5	EC:3.4.-.-		
gene26855	K14411	MSI	RNA-binding protein Musashi	--	1	ko03015 mRNA surveillance pathway;
gene26857	K00574	E2.1.1.79, cfa	cyclopropane-fatty-acyl-phospholipid synthase	EC:2.1.1.79		
gene26858	K02152	ATPeVG, ATP6G1	V-type H+-transporting ATPase subunit G	EC:3.6.3.14	7	ko00190 Oxidative phosphorylation;ko04145 Phagosome;ko04721 Synaptic vesicle cycle;ko04966 Collecting duct acid secretion;ko05110 Vibrio cholerae infection;ko05120 Epithelial cell signaling in Helicobacter pylori infection;ko05323 Rheumatoid arthritis;
gene26860	K00799	GST, gst	glutathione S-transferase	EC:2.5.1.18	4	ko00480 Glutathione metabolism;ko00980 Metabolism of xenobiotics by cytochrome P450;ko00982 Drug metabolism - cytochrome P450;ko05204 Chemical carcinogenesis;
gene26870	K15601	KDM3	lysine-specific demethylase 3	EC:1.14.11.-		
gene26873	K10704	UBE2V	ubiquitin-conjugating enzyme E2 variant	--		
gene26875	K15472	CYP71D55	premnaspirodiene oxygenase	EC:1.14.13.12 1	1	ko00909 Sesquiterpenoid and triterpenoid biosynthesis;

gene26881	K15891	FLDH	farnesol dehydrogenase	EC:1.1.1.216	2	ko00900 Terpenoid backbone biosynthesis;ko00909 Sesquiterpenoid and triterpenoid biosynthesis;
gene26887	K11600	RRP41, EXOSC4, SKI6	exosome complex component RRP41	--	1	ko03018 RNA degradation;
gene26888	K01792	E5.1.3.15	glucose-6-phosphate 1-epimerase	EC:5.1.3.15	1	ko00010 Glycolysis / Gluconeogenesis;
gene2688	K13448	CML	calcium-binding protein CML	--	1	ko04626 Plant-pathogen interaction;
gene26891	K16912	LAS1	ribosomal biogenesis protein LAS1	--		
gene26895	K00142	AASDH	acyl-CoA synthetase	EC:6.2.1.-		
gene2689	K13448	CML	calcium-binding protein CML	--	1	ko04626 Plant-pathogen interaction;
gene26901	K13412	CPK	calcium-dependent protein kinase	EC:2.7.11.1	2	ko04626 Plant-pathogen interaction;ko05145 Toxoplasmosis;
gene26902	K13412	CPK	calcium-dependent protein kinase	EC:2.7.11.1	2	ko04626 Plant-pathogen interaction;ko05145 Toxoplasmosis;
gene26903	K14771	NOC4, UTP19	U3 small nucleolar RNA-associated protein 19	--		
gene26908	K08678	UXS1	UDP-glucuronate decarboxylase	EC:4.1.1.35	2	ko00500 Starch and sucrose metabolism;ko00520 Amino sugar and nucleotide sugar metabolism;
gene26917	K01183	E3.2.1.14	chitinase	EC:3.2.1.14	1	ko00520 Amino sugar and nucleotide sugar metabolism;
gene26919	K00555	TRMT1, trm1	tRNA (guanine26-N2/guanine27-N2)-dimethyltransferase	EC:2.1.1.215 2.1.1.216		
gene26920	K13379	RGP, UTM	reversibly glycosylated polypeptide / UDP-arabinopyranose mutase	EC:2.4.1.- 5.4.99.30		
gene26921	K12640	CYP85A2, BR6OX2	cytochrome P450, family 85, subfamily A, polypeptide 2 (brassinosteroid-6-oxidase 2)	EC:1.14.-.-	1	ko00905 Brassinosteroid biosynthesis;
gene26927	K09566	PPIG	peptidyl-prolyl isomerase G (cyclophilin G)	EC:5.2.1.8		
gene26929	K12502	VTE3, APG1	MPBQ/MSBQ methyltransferase	--	1	ko00130 Ubiquinone and other terpenoid-quinone biosynthesis;
gene26930	K03320	amt, AMT, MEP	ammonium transporter, Amt family	--		
gene26932	K11251	H2A	histone H2A	--	2	ko05034 Alcoholism;ko05322 Systemic lupus erythematosus;
gene26934	K17541	SCYL2	SCY1-like protein 2	--		
gene26940	K00801	FDFT1	farnesyl-diphosphate farnesyltransferase	EC:2.5.1.21	1	ko00909 Sesquiterpenoid and triterpenoid biosynthesis;
gene26946	K16675	ZDHHC14	probable palmitoyltransferase ZDHHC14	EC:2.3.1.-	1	ko04391 Hippo signaling pathway - fly;
gene26950	K01051	E3.1.1.11	pectinesterase	EC:3.1.1.11	2	ko00040 Pentose and glucuronate interconversions;ko00500 Starch and sucrose metabolism;
gene26951	K16055	TPS	trehalose 6-phosphate synthase/phosphatase	EC:2.4.1.15 3.1.3.12	1	ko00500 Starch and sucrose metabolism;
gene26954	K14754	MX1	interferon-induced GTP-binding protein Mx1	--	2	ko05162 Measles;ko05164 Influenza A;
gene26958	K11982	RNF115_126	E3 ubiquitin-protein ligase RNF115/126	EC:6.3.2.19		
gene26960	K02906	RP-L3, MRPL3, rplC	large subunit ribosomal protein L3	--	1	ko03010 Ribosome;
gene26968	K11320	EP400	E1A-binding protein p400	EC:3.6.4.-		
gene2696	K03327	TC.MATE, SLC47A, norM, mdtK, dinF	multidrug resistance protein, MATE family	--		
gene26973	K14506	JAR1	jasmonic acid-amino synthetase	--	1	ko04075 Plant hormone signal transduction;
gene26977	K08065	NFYB	nuclear transcription Y subunit beta	--	3	ko04612 Antigen processing and presentation;ko05152 Tuberculosis;ko05166 HTLV-I infection;
gene26980	K02931	RP-L5, MRPL5, rplE	large subunit ribosomal protein L5	--	1	ko03010 Ribosome;
gene26981	K13192	RBM26	RNA-binding protein 26	--		
gene26982	K16292	CEP, CYSEP	KDEL-tailed cysteine endopeptidase	EC:3.4.22.-		
gene26993	K14026	SEL1, SEL1L	SEL1 protein	--	1	ko04141 Protein processing in endoplasmic reticulum;
gene26996	K02934	RP-L6e, RPL6	large subunit ribosomal protein L6e	--	1	ko03010 Ribosome;
gene26997	K01092	E3.1.3.25, IMPA, suhB	myo-inositol-1(or 4)-monophosphatase	EC:3.1.3.25	3	ko00521 Streptomycin biosynthesis;ko00562 Inositol phosphate metabolism;ko04070 Phosphatidylinositol signaling system;
gene26	K06111	EXOC4, SEC8L1	exocyst complex component 4	--	1	ko04530 Tight junction;
gene27000	K01074	PPT	palmitoyl-protein thioesterase	EC:3.1.2.22	2	ko00062 Fatty acid elongation;ko04142 Lysosome;
gene27002	K08235	E2.4.1.207	xyloglucan:xyloglucosyl transferase	EC:2.4.1.207		
gene27010	K10357	MYO5	myosin V	--		
gene27011	K10357	MYO5	myosin V	--		
gene27012	K10357	MYO5	myosin V	--		
gene27017	K11340	ACTL6A, INO80K	actin-like protein 6A	--		
gene27022	K13448	CML	calcium-binding protein CML	--	1	ko04626 Plant-pathogen interaction;
gene27023	K13448	CML	calcium-binding protein CML	--	1	ko04626 Plant-pathogen interaction;

gene27026	K11000	CALS	callose synthase	EC:2.4.1.-		
gene27028	K06966	K06966				
gene27030	K11667	INO80C, IES6	INO80 complex subunit C	--		
gene27034	K03142	TFIIH2, GTF2H2, SSL1	transcription initiation factor TFIIH subunit 2	--	3	ko03022 Basal transcription factors;ko03420 Nucleotide excision repair;ko05203 Viral carcinogenesis;
gene27037	K13436	PTI1	pto-interacting protein 1	EC:2.7.11.1	1	ko04626 Plant-pathogen interaction;
gene27038	K12603	CNOT6, CCR4	CCR4-NOT transcription complex subunit 6	--	1	ko03018 RNA degradation;
gene27039	K11978	UBR3	E3 ubiquitin-protein ligase UBR3	EC:6.3.2.19		
gene27041	K01904	E6.2.1.12	4-coumarate--CoA ligase	EC:6.2.1.12	3	ko00130 Ubiquinone and other terpenoid-quinone biosynthesis;ko00360 Phenylalanine metabolism;ko00940 Phenylpropanoid biosynthesis;
gene27045	K10839	RAD23, HR23	UV excision repair protein RAD23	--	2	ko03420 Nucleotide excision repair;ko04141 Protein processing in endoplasmic reticulum;
gene27046	K04733	IRAK4	interleukin-1 receptor-associated kinase 4	EC:2.7.11.1	11	ko04064 NF-kappa B signaling pathway;ko04210 Apoptosis;ko04620 Toll-like receptor signaling pathway;ko04722 Neurotrophin signaling pathway;ko05133 Pertussis;ko05140 Leishmaniasis;ko05142 Chagas disease (American trypanosomiasis);ko05145 Toxoplasmosis;ko05152 Tuberculosis;ko05162 Measles;ko05164 Influenza A;
gene27047	K11713	PGTB1	geranylgeranyl transferase type-1 subunit beta	EC:2.5.1.59		
gene27051	K10703	PHS1, PAS2	very-long-chain (3R)-3-hydroxyacyl-[acyl-carrier protein] dehydratase	EC:4.2.1.134	2	ko00062 Fatty acid elongation;ko01040 Biosynthesis of unsaturated fatty acids;
gene27056	K07943	ARL2	ADP-ribosylation factor-like protein 2	--		
gene27059	K00511	SQLE, ERG1	squalene monooxygenase	EC:1.14.13.132	1	ko00909 Sesquiterpenoid and triterpenoid biosynthesis;
gene27060	K01762	ACS	1-aminocyclopropane-1-carboxylate synthase	EC:4.4.1.14	1	ko00270 Cysteine and methionine metabolism;
gene27062	K10704	UBE2V	ubiquitin-conjugating enzyme E2 variant	--		
gene27063	K02960	RP-S16e, RPS16	small subunit ribosomal protein S16e	--	1	ko03010 Ribosome;
gene27064	K07393	ECM4	putative glutathione S-transferase	--		
gene27068	K10742	DNA2	DNA replication ATP-dependent helicase Dna2	EC:3.6.4.12	1	ko03030 DNA replication;
gene27069	K11098	SNRPF, SMF	small nuclear ribonucleoprotein F	--	1	ko03040 Spliceosome;
gene27073	K16196	EIF2AK4	eukaryotic translation initiation factor 2-alpha kinase 4	EC:2.7.11.1	6	ko04141 Protein processing in endoplasmic reticulum;ko05160 Hepatitis C;ko05162 Measles;ko05164 Influenza A;ko05168 Herpes simplex infection;ko05169 Epstein-Barr virus infection;
gene27080	K11407	HDAC6_10	histone deacetylase 6/10	EC:3.5.1.98	2	ko05034 Alcoholism;ko05203 Viral carcinogenesis;
gene27085	K10406	KIFC2_3	kinesin family member C2/C3	--		
gene27088	K04730	IRAK1	interleukin-1 receptor-associated kinase 1	EC:2.7.11.1	11	ko04064 NF-kappa B signaling pathway;ko04210 Apoptosis;ko04620 Toll-like receptor signaling pathway;ko04722 Neurotrophin signaling pathway;ko05133 Pertussis;ko05140 Leishmaniasis;ko05142 Chagas disease (American trypanosomiasis);ko05145 Toxoplasmosis;ko05152 Tuberculosis;ko05162 Measles;ko05169 Epstein-Barr virus infection;
gene27089	K04730	IRAK1	interleukin-1 receptor-associated kinase 1	EC:2.7.11.1	11	ko04064 NF-kappa B signaling pathway;ko04210 Apoptosis;ko04620 Toll-like receptor signaling pathway;ko04722 Neurotrophin signaling pathway;ko05133 Pertussis;ko05140 Leishmaniasis;ko05142 Chagas disease (American trypanosomiasis);ko05145 Toxoplasmosis;ko05152 Tuberculosis;ko05162 Measles;ko05169 Epstein-Barr virus infection;
gene27096	K01074	PPT	palmitoyl-protein thioesterase	EC:3.1.2.22	2	ko00062 Fatty acid elongation;ko04142 Lysosome;
gene27097	K01074	PPT	palmitoyl-protein thioesterase	EC:3.1.2.22	2	ko00062 Fatty acid elongation;ko04142 Lysosome;
gene27098	K11647	SMARCA2_4	SWI/SNF-related matrix-associated actin-dependent regulator of chromatin subfamily A member 2/4	EC:3.6.4.-		
gene27101	K13354	SLC25A17, PMP34	solute carrier family 25 (peroxisomal adenine nucleotide transporter), member 17	--	1	ko04146 Peroxisome;
gene27108	K15271	HFM1, MER3	ATP-dependent DNA helicase HFM1/MER3	EC:3.6.4.12		
gene27109	K15271	HFM1, MER3	ATP-dependent DNA helicase HFM1/MER3	EC:3.6.4.12		
gene2710	K00517	E1.14.-.-			4	ko00624 Polycyclic aromatic hydrocarbon degradation;ko00627 Aminobenzoate degradation;ko00903 Limonene and pinene degradation;ko00945 Stilbenoid, diarylheptanoid and gingerol biosynthesis;

gene27110	K13095	SF1	splicing factor 1	--		
gene27113	K10754	RFC1	replication factor C subunit 1	--	3	ko03030 DNA replication;ko03420 Nucleotide excision repair;ko03430 Mismatch repair;
gene27116	K14324	SAP18	histone deacetylase complex subunit SAP18	--	2	ko03013 RNA transport;ko03015 mRNA surveillance pathway;
gene27122	K00430	E1.11.1.7	peroxidase	EC:1.11.1.7	2	ko00360 Phenylalanine metabolism;ko00940 Phenylpropanoid biosynthesis;
gene27123	K00430	E1.11.1.7	peroxidase	EC:1.11.1.7	2	ko00360 Phenylalanine metabolism;ko00940 Phenylpropanoid biosynthesis;
gene27126	K00430	E1.11.1.7	peroxidase	EC:1.11.1.7	2	ko00360 Phenylalanine metabolism;ko00940 Phenylpropanoid biosynthesis;
gene27127	K13024	HISPPD, VIP	inositol hexakisphosphate/diphosphoinositol-pentakisphosphate kinase	EC:2.7.4.21 2.7.4.24		
gene2712	K01251	E3.3.1.1, ahcY	adenosylhomocysteinase	EC:3.3.1.1	1	ko00270 Cysteine and methionine metabolism;
gene27131	K09419	HSFF	heat shock transcription factor, other eukaryote	--		
gene27132	K08081	E1.1.1.206	tropine dehydrogenase	EC:1.1.1.206	1	ko00960 Tropane, piperidine and pyridine alkaloid biosynthesis;
gene27134	K15634	gpmB	probable phosphoglycerate mutase	EC:5.4.2.12	5	ko00010 Glycolysis / Gluconeogenesis;ko00260 Glycine, serine and threonine metabolism;ko00680 Methane metabolism;ko01200 Carbon metabolism;ko01230 Biosynthesis of amino acids;
gene27138	K07766	E3.6.1.52	diphosphoinositol-polyphosphate diphosphatase	EC:3.6.1.52		
gene2713	K00600	glyA, SHMT	glycine hydroxymethyltransferase	EC:2.1.2.1	7	ko00260 Glycine, serine and threonine metabolism;ko00460 Cyanoamino acid metabolism;ko00630 Glyoxylate and dicarboxylate metabolism;ko00670 One carbon pool by folate;ko00680 Methane metabolism;ko01200 Carbon metabolism;ko01230 Biosynthesis of amino acids;
gene27146	K12593	MPHOSPH6, MPP6	M-phase phosphoprotein 6, animal type	--	1	ko03018 RNA degradation;
gene27147	K11423	SETD2, SET2	histone-lysine N-methyltransferase SETD2	EC:2.1.1.43	1	ko00310 Lysine degradation;
gene27149	K00600	glyA, SHMT	glycine hydroxymethyltransferase	EC:2.1.2.1	7	ko00260 Glycine, serine and threonine metabolism;ko00460 Cyanoamino acid metabolism;ko00630 Glyoxylate and dicarboxylate metabolism;ko00670 One carbon pool by folate;ko00680 Methane metabolism;ko01200 Carbon metabolism;ko01230 Biosynthesis of amino acids;
gene2714	K10866	RAD50	DNA repair protein RAD50	EC:3.6.-.-	2	ko03440 Homologous recombination;ko03450 Non-homologous end-joining;
gene27154	K15190	MEPCE, BCDIN3	7SK snRNA methylphosphate capping enzyme	EC:2.1.1.-		
gene27168	K00703	E2.4.1.21, glgA	starch synthase	EC:2.4.1.21	1	ko00500 Starch and sucrose metabolism;
gene27170	K12153	CYP79A2	cytochrome P450, family 79, subfamily A, polypeptide 2 (phenylalanine N-monooxygenase)	EC:1.14.13.124	3	ko00460 Cyanoamino acid metabolism;ko00966 Glucosinolate biosynthesis;ko01210 2-Oxocarboxylic acid metabolism;
gene27173	K04730	IRAK1	interleukin-1 receptor-associated kinase 1	EC:2.7.11.1	11	ko04064 NF-kappa B signaling pathway;ko04210 Apoptosis;ko04620 Toll-like receptor signaling pathway;ko04722 Neurotrophin signaling pathway;ko05133 Pertussis;ko05140 Leishmaniasis;ko05142 Chagas disease (American trypanosomiasis);ko05145 Toxoplasmosis;ko05152 Tuberculosis;ko05162 Measles;ko05169 Epstein-Barr virus infection;
gene27177	K06268	PPP3R, CNB	serine/threonine-protein phosphatase 2B regulatory subunit	--	18	ko04010 MAPK signaling pathway;ko04020 Calcium signaling pathway;ko04114 Oocyte meiosis;ko04210 Apoptosis;ko04310 Wnt signaling pathway;ko04360 Axon guidance;ko04370 VEGF signaling pathway;ko04380 Osteoclast differentiation;ko04650 Natural killer cell mediated cytotoxicity;ko04660 T cell receptor signaling pathway;ko04662 B cell receptor signaling pathway;ko04720 Long-term potentiation;ko04724 Glutamatergic synapse;ko05010 Alzheimer's disease;ko05014 Amyotrophic lateral sclerosis (ALS);ko05031 Amphetamine addiction;ko05152
gene27178	K01278	DPP4	dipeptidyl-peptidase 4	EC:3.4.14.5	1	ko04974 Protein digestion and absorption;
gene27179	K00915	IPK2	1D-myo-Inositol-tetrakisphosphate 5-kinase / inositol-polyphosphate multikinase	EC:2.7.1.140 2.7.1.151	2	ko00562 Inositol phosphate metabolism;ko04070 Phosphatidylinositol signaling system;
gene27180	K14766	NOP14, UTP2	nucleolar protein 14	--		
gene27183	K09264	K09264	MADS-box transcription factor, plant	--		
gene27185	K00854	xylB, XYLB	xylulokinase	EC:2.7.1.17	1	ko00040 Pentose and glucuronate interconversions;
gene27191	K03979	obg	GTP-binding protein	--		

gene27198	K12177	COPS3, CSN3	COP9 signalosome complex subunit 3	--		
gene27199	K00231	PPOX, hemY	oxygen-dependent protoporphyrinogen oxidase	EC:1.3.3.4	1	ko00860 Porphyrin and chlorophyll metabolism;
gene27206	K01988	A4GALT	lactosylceramide 4-alpha-galactosyltransferase	EC:2.4.1.228	1	ko00603 Glycosphingolipid biosynthesis - globo series;
gene27210	K10747	LIG1	DNA ligase 1	EC:6.5.1.1	4	ko03030 DNA replication;ko03410 Base excision repair;ko03420 Nucleotide excision repair;ko03430 Mismatch repair;
gene27218	K00297	metF, MTHFR	methylenetetrahydrofolate reductase (NADPH)	EC:1.5.1.20	3	ko00670 One carbon pool by folate;ko00720 Carbon fixation pathways in prokaryotes;ko01200 Carbon metabolism;
gene27224	K02893	RP-L23Ae, RPL23A	large subunit ribosomal protein L23Ae	--	1	ko03010 Ribosome;
gene27227	K10576	UBE2H, UBC8	ubiquitin-conjugating enzyme E2 H	EC:6.3.2.19	1	ko04120 Ubiquitin mediated proteolysis;
gene27228	K02202	CDK7	cyclin-dependent kinase 7	EC:2.7.11.22 2.7.11.23	3	ko03022 Basal transcription factors;ko03420 Nucleotide excision repair;ko04110 Cell cycle;
gene2722	K10900	WRN, RECQL2	werner syndrome ATP-dependent helicase	EC:3.6.4.12		
gene27232	K14638	SLC15A3_4, PHT	solute carrier family 15 (peptide/histidine transporter), member 3/4	--		
gene27237	K01227	E3.2.1.96	mannosyl-glycoprotein endo-beta-N-acetylglucosaminidase	EC:3.2.1.96	1	ko00511 Other glycan degradation;
gene2723	K11251	H2A	histone H2A	--	2	ko05034 Alcoholism;ko05322 Systemic lupus erythematosus;
gene27242	K09833	HPT, TPT	homogenitisate phytyltransferase	--	1	ko00130 Ubiquinone and other terpenoid-quinone biosynthesis;
gene2724	K00430	E1.11.1.7	peroxidase	EC:1.11.1.7	2	ko00360 Phenylalanine metabolism;ko00940 Phenylpropanoid biosynthesis;
gene27252	K14521	NAT10, KRE33	N-acetyltransferase 10	EC:2.3.1.-	1	ko03008 Ribosome biogenesis in eukaryotes;
gene2725	K02935	RP-L7, MRPL12, rplL	large subunit ribosomal protein L7/L12	--	1	ko03010 Ribosome;
gene27263	K10364	CAPZA	capping protein (actin filament) muscle Z-line, alpha	--		
gene27265	K14229	tRNA-Lys	tRNA Lys	--	1	ko00970 Aminoacyl-tRNA biosynthesis;
gene27270	K12890	SFRS1_9	splicing factor, arginine/serine-rich 1/9	--	2	ko03040 Spliceosome;ko05168 Herpes simplex infection;
gene27272	K14785	ESF2, ABT1	ESF2/ABP1 family protein	--		
gene27278	K03064	PSMC6, RPT4	26S proteasome regulatory subunit T4	--	2	ko03050 Proteasome;ko05169 Epstein-Barr virus infection;
gene27284	K02929	RP-L44e, RPL44	large subunit ribosomal protein L44e	--	1	ko03010 Ribosome;
gene27295	K01205	NAGLU	alpha-N-acetylglucosaminidase	EC:3.2.1.50	2	ko00531 Glycosaminoglycan degradation;ko04142 Lysosome;
gene27298	K01507	ppa	inorganic pyrophosphatase	EC:3.6.1.1	1	ko00190 Oxidative phosphorylation;
gene272	K15285	SLC35E3	solute carrier family 35, member E3	--		
gene27301	K14538	NUG1, GNL3	nuclear GTP-binding protein	--	1	ko03008 Ribosome biogenesis in eukaryotes;
gene27304	K01061	E3.1.1.45	carboxymethylenebutenolidase	EC:3.1.1.45	3	ko00361 Chlorocyclohexane and chlorobenzene degradation;ko00364 Fluorobenzoate degradation;ko00623 Toluene degradation;
gene27307	K10395	KIF4S	kinesin family member 4/7/21/27	--		
gene2730	K01613	psd, PISD	phosphatidylserine decarboxylase	EC:4.1.1.65	1	ko00564 Glycerophospholipid metabolism;
gene27314	K03809	wrbA	Trp repressor binding protein	--		
gene27317	K02921	RP-L37Ae, RPL37A	large subunit ribosomal protein L37Ae	--	1	ko03010 Ribosome;
gene2731	K00430	E1.11.1.7	peroxidase	EC:1.11.1.7	2	ko00360 Phenylalanine metabolism;ko00940 Phenylpropanoid biosynthesis;
gene27322	K07466	RFA1, RPA1, rpa	replication factor A1	--	5	ko03030 DNA replication;ko03420 Nucleotide excision repair;ko03430 Mismatch repair;ko03440 Homologous recombination;ko03460 Fanconi anemia pathway;
gene27324	K01673	cynT, can	carbonic anhydrase	EC:4.2.1.1	1	ko00910 Nitrogen metabolism;
gene27325	K01673	cynT, can	carbonic anhydrase	EC:4.2.1.1	1	ko00910 Nitrogen metabolism;
gene27327	K17609	NXN	nucleoredoxin	EC:1.8.1.8		
gene27328	K17609	NXN	nucleoredoxin	EC:1.8.1.8		
gene27331	K13100	CWC22	pre-mRNA-splicing factor CWC22	--		
gene27335	K11262	ACAC	acetyl-CoA carboxylase / biotin carboxylase	EC:6.4.1.2 6.3.4.14	4	ko00061 Fatty acid biosynthesis;ko00620 Pyruvate metabolism;ko00640 Propanoate metabolism;ko04910 Insulin signaling pathway;
gene27338	K10739	RFA2, RPA2	replication factor A2	--	5	ko03030 DNA replication;ko03420 Nucleotide excision repair;ko03430 Mismatch repair;ko03440 Homologous recombination;ko03460 Fanconi anemia pathway;
gene27339	K07297	ADIPOR	adiponectin receptor	--	2	ko04920 Adipocytokine signaling pathway;ko04932 Non-alcoholic fatty liver disease (NAFLD);
gene27340	K11593	ELF2C	eukaryotic translation initiation factor 2C	--		
gene27341	K14800	TSR2	pre-rRNA-processing protein TSR2	--		

gene27349	K04506	SIAH1	E3 ubiquitin-protein ligase SIAH1	EC:6.3.2.19	3	ko04115 p53 signaling pathway;ko04120 Ubiquitin mediated proteolysis;ko04310 Wnt signaling pathway;
gene27350	K08653	MBTPS1	membrane-bound transcription factor site-1 protease	EC:3.4.21.112	1	ko04141 Protein processing in endoplasmic reticulum;
gene27352	K13462	MIN7	guanine nucleotide-exchange factor	--	1	ko04626 Plant-pathogen interaction;
gene27356	K02218	CSNK1, CK1	casein kinase 1	EC:2.7.11.1	1	ko04340 Hedgehog signaling pathway;
gene27358	K08472	MLO	mlo protein	--		
gene27359	K02147	ATPeVB, ATP6B1	V-type H+-transporting ATPase subunit B	EC:3.6.3.14	7	ko00190 Oxidative phosphorylation;ko04145 Phagosome;ko04721 Synaptic vesicle cycle;ko04966 Collecting duct acid secretion;ko05110 Vibrio cholerae infection;ko05120 Epithelial cell signaling in Helicobacter pylori infection;ko05323 Rheumatoid arthritis;
gene2735	K01610	E4.1.1.49, pckA	phosphoenolpyruvate carboxykinase (ATP)	EC:4.1.1.49	4	ko00010 Glycolysis / Gluconeogenesis;ko00020 Citrate cycle (TCA cycle);ko00620 Pyruvate metabolism;ko00710 Carbon fixation in photosynthetic organisms;
gene27362	K14220	tRNA-Asn	tRNA Asn	--	1	ko00970 Aminoacyl-tRNA biosynthesis;
gene2736	K10775	E4.3.1.24	phenylalanine ammonia-lyase	EC:4.3.1.24	2	ko00360 Phenylalanine metabolism;ko00940 Phenylpropanoid biosynthesis;
gene27373	K00889	E2.7.1.68, PIP5K	1-phosphatidylinositol-4-phosphate 5-kinase	EC:2.7.1.68	5	ko00562 Inositol phosphate metabolism;ko04070 Phosphatidylinositol signaling system;ko04144 Endocytosis;ko04666 Fc gamma R-mediated phagocytosis;ko04810 Regulation of actin cytoskeleton;
gene27375	K12625	LSM6	U6 snRNA-associated Sm-like protein LSm6	--	2	ko03018 RNA degradation;ko03040 Spliceosome;
gene27378	K13464	JAZ	jasmonate ZIM domain-containing protein	--	2	ko04075 Plant hormone signal transduction;ko04626 Plant-pathogen interaction;
gene27380	K05868	CCNB	cyclin B	--	3	ko04110 Cell cycle;ko04115 p53 signaling pathway;ko04914 Progesterone-mediated oocyte maturation;
gene27381	K09291	TPR	nucleoprotein TPR	--	4	ko03013 RNA transport;ko04011 MAPK signaling pathway - yeast;ko05200 Pathways in cancer;ko05216 Thyroid cancer;
gene27383	K03255	TIF31, CLU1	protein TIF31	--		
gene27384	K13116	DDX41, ABS	ATP-dependent RNA helicase DDX41	EC:3.6.4.13		
gene27386	K11367	CHD1	chromodomain-helicase-DNA-binding protein 1	EC:3.6.4.12		
gene27394	K10624	RBBP6	E3 ubiquitin-protein ligase RBBP6	EC:6.3.2.19		
gene27396	K02962	RP-S17e, RPS17	small subunit ribosomal protein S17e	--	1	ko03010 Ribosome;
gene27399	K12492	ARFGAP1	ADP-ribosylation factor GTPase-activating protein 1	--	1	ko04144 Endocytosis;
gene27401	K03254	EIF3A	translation initiation factor 3 subunit A	--	1	ko03013 RNA transport;
gene27405	K02112	ATPF1B, atpD	F-type H+-transporting ATPase subunit beta	EC:3.6.3.14	2	ko00190 Oxidative phosphorylation;ko00195 Photosynthesis;
gene27409	K08678	UXS1	UDP-glucuronate decarboxylase	EC:4.1.1.35	2	ko00500 Starch and sucrose metabolism;ko00520 Amino sugar and nucleotide sugar metabolism;
gene2740	K14513	EIN2	ethylene-insensitive protein 2	--	1	ko04075 Plant hormone signal transduction;
gene27414	K12199	VTA1, LIP5	vacuolar protein sorting-associated protein VTA1	--	1	ko04144 Endocytosis;
gene27415	K16732	PRC1	protein regulator of cytokinesis 1	--		
gene27416	K00981	E2.7.7.41, CDS1, CDS2, cdsA	phosphatidate cytidylyltransferase	EC:2.7.7.41	2	ko00564 Glycerophospholipid metabolism;ko04070 Phosphatidylinositol signaling system;
gene27419	K03687	GRPE	molecular chaperone GrpE	--		
gene27422	K12483	EHD1	EH domain-containing protein 1	--	1	ko04144 Endocytosis;
gene27424	K13606	NOL, NYC1	chlorophyll(ide) b reductase	EC:1.1.1.294	1	ko00860 Porphyrin and chlorophyll metabolism;
gene27428	K13356	FAR	fatty acyl-CoA reductase	EC:1.2.1.-	2	ko00073 Cutin, suberine and wax biosynthesis;ko04146 Peroxisome;
gene27431	K00549	metE	5-methyltetrahydropteroyltriglutamate--homocysteine methyltransferase	EC:2.1.1.14	3	ko00270 Cysteine and methionine metabolism;ko00450 Selenocompound metabolism;ko01230 Biosynthesis of amino acids;
gene2743	K10572	IPPK	inositol-pentakisphosphate 2-kinase	EC:2.7.1.158	2	ko00562 Inositol phosphate metabolism;ko04070 Phosphatidylinositol signaling system;
gene27442	K00059	fabG	3-oxoacyl-[acyl-carrier protein] reductase	EC:1.1.1.100	3	ko00061 Fatty acid biosynthesis;ko00780 Biotin metabolism;ko01040 Biosynthesis of unsaturated fatty acids;
gene27444	K01099	E3.1.3.36	phosphatidylinositol-bisphosphatase	EC:3.1.3.36	2	ko00562 Inositol phosphate metabolism;ko04070 Phosphatidylinositol signaling system;
gene27445	K03231	EEF1A	elongation factor 1-alpha	--	2	ko03013 RNA transport;ko05134 Legionellosis;
gene27446	K17479	GRXCR1	glutaredoxin domain-containing cysteine-rich protein 1	--		
gene27450	K03671	trxA	thioredoxin 1	--		

gene27451	K04688	RPS6KB	p70 ribosomal S6 kinase	EC:2.7.11.1	9	ko04012 ErbB signaling pathway;ko04066 HIF-1 signaling pathway;ko04150 mTOR signaling pathway;ko04151 PI3K-Akt signaling pathway;ko04350 TGF-beta signaling pathway;ko04666 Fc gamma R-mediated phagocytosis;ko04910 Insulin signaling pathway;ko05205 Proteoglycans in cancer;ko05221 Acute myeloid leukemia;
gene27455	K08869	ADCK, ABC1	aarF domain-containing kinase	--		
gene27457	K06911	K06911				
gene2745	K00696	E2.4.1.14	sucrose-phosphate synthase	EC:2.4.1.14	1	ko00500 Starch and sucrose metabolism;
gene27462	K02942	RP-LP1, RPLP1	large subunit ribosomal protein LP1	--	1	ko03010 Ribosome;
gene27465	K08770	UBC	ubiquitin C	--	1	ko03320 PPAR signaling pathway;
gene27468	K03126	TAF12	transcription initiation factor TFIID subunit 12	--	1	ko03022 Basal transcription factors;
gene27469	K13379	RGP, UTM	reversibly glycosylated polypeptide / UDP-arabinopyranose mutase	EC:2.4.1.- 5.4.99.30		
gene2746	K13459	RPS2	disease resistance protein RPS2	--	1	ko04626 Plant-pathogen interaction;
gene27470	K13379	RGP, UTM	reversibly glycosylated polypeptide / UDP-arabinopyranose mutase	EC:2.4.1.- 5.4.99.30		
gene27477	K14220	tRNA-Asn	tRNA Asn	--	1	ko00970 Aminoacyl-tRNA biosynthesis;
gene27479	K12823	DDX5, DBP2	ATP-dependent RNA helicase DDX5/DBP2	EC:3.6.4.13	3	ko03040 Spliceosome;ko05202 Transcriptional misregulation in cancer;ko05205 Proteoglycans in cancer;
gene27485	K12893	SFRS4_5_6	splicing factor, arginine/serine-rich 4/5/6	--	2	ko03040 Spliceosome;ko05168 Herpes simplex infection;
gene27488	K01184	E3.2.1.15	polygalacturonase	EC:3.2.1.15	2	ko00040 Pentose and glucuronate interconversions;ko00500 Starch and sucrose metabolism;
gene27490	K14219	tRNA-Arg	tRNA Arg	--	1	ko00970 Aminoacyl-tRNA biosynthesis;
gene27494	K13496	UGT73C	UDP-glucosyl transferase 73C	EC:2.4.1.-		
gene27495	K03935	NDUFS2	NADH dehydrogenase (ubiquinone) Fe-S protein 2	EC:1.6.5.3 1.6.99.3	5	ko00190 Oxidative phosphorylation;ko04932 Non-alcoholic fatty liver disease (NAFLD);ko05010 Alzheimer's disease;ko05012 Parkinson's disease;ko05016 Huntington's disease;
gene27498	K03809	wrbA	Trp repressor binding protein	--		
gene27499	K09422	MYBP	myb proto-oncogene protein, plant	--		
gene27510	K15103	UCP2_3, SLC25A8_9	solute carrier family 25 (mitochondrial uncoupling protein), member 8/9	--		
gene27511	K13207	CUGBP, BRUNOL, CELF	CUG-BP- and ETR3-like factor	--		
gene27514	K08101	E1.3.7.4	phytochromobilin:ferredoxin oxidoreductase	EC:1.3.7.4	1	ko00860 Porphyrin and chlorophyll metabolism;
gene27520	K07953	SAR1	GTP-binding protein SAR1	EC:3.6.5.-	2	ko04141 Protein processing in endoplasmic reticulum;ko05134 Legionellosis;
gene27523	K12848	SNU23	U4/U6.U5 tri-snRNP component SNU23	--	1	ko03040 Spliceosome;
gene27524	K02894	RP-L23e, RPL23	large subunit ribosomal protein L23e	--	1	ko03010 Ribosome;
gene27527	K06063	SNW1, SKIIP, SKIP	SNW domain-containing protein 1	--	4	ko03040 Spliceosome;ko04330 Notch signaling pathway;ko05169 Epstein-Barr virus infection;ko05203 Viral carcinogenesis;
gene27534	K17398	DNMT3A	DNA (cytosine-5)-methyltransferase 3A	EC:2.1.1.37	2	ko00270 Cysteine and methionine metabolism;ko05206 MicroRNAs in cancer;
gene27541	K14500	BSK	BR-signaling kinase	EC:2.7.11.1	1	ko04075 Plant hormone signal transduction;
gene27547	K00873	PK, pyk	pyruvate kinase	EC:2.7.1.40	7	ko00010 Glycolysis / Gluconeogenesis;ko00230 Purine metabolism;ko00620 Pyruvate metabolism;ko01200 Carbon metabolism;ko01230 Biosynthesis of amino acids;ko04930 Type II diabetes mellitus;ko05203 Viral carcinogenesis;
gene27558	K03094	SKP1, CBF3D	S-phase kinase-associated protein 1	--	9	ko04110 Cell cycle;ko04111 Cell cycle - yeast;ko04114 Oocyte meiosis;ko04120 Ubiquitin mediated proteolysis;ko04141 Protein processing in endoplasmic reticulum;ko04310 Wnt signaling pathway;ko04350 TGF-beta signaling pathway;ko04710 Circadian rhythm;ko05168 Herpes simplex infection;
gene27560	K03257	EIF4A	translation initiation factor 4A	--	1	ko03013 RNA transport;
gene27563	K01115	PLD1_2	phospholipase D1/2	EC:3.1.4.4	7	ko00564 Glycerophospholipid metabolism;ko00565 Ether lipid metabolism;ko04014 Ras signaling pathway;ko04144 Endocytosis;ko04666 Fc gamma R-mediated phagocytosis;ko04724 Glutamatergic synapse;ko04912 GnRH signaling pathway;
gene27569	K03264	EIF6	translation initiation factor 6	--	1	ko03008 Ribosome biogenesis in eukaryotes;
gene27577	K01012	bioB	biotin synthase	EC:2.8.1.6	1	ko00780 Biotin metabolism;
gene27582	K09528	DNAJC8	DnaJ homolog subfamily C member 8	--		

gene27586	K08057	CALR	calreticulin	--	5	ko04141 Protein processing in endoplasmic reticulum;ko04145 Phagosome;ko04612 Antigen processing and presentation;ko05142 Chagas disease (American trypanosomiasis);ko05166 HTLV-I infection;
gene27588	K02291	crtB	phytoene synthase	EC:2.5.1.32	1	ko00906 Carotenoid biosynthesis;
gene27589	K00799	GST, gst	glutathione S-transferase	EC:2.5.1.18	4	ko00480 Glutathione metabolism;ko00980 Metabolism of xenobiotics by cytochrome P450;ko00982 Drug metabolism - cytochrome P450;ko05204 Chemical carcinogenesis;
gene27591	K07889	RAB5C	Ras-related protein Rab-5C	--	6	ko04014 Ras signaling pathway;ko04144 Endocytosis;ko04145 Phagosome;ko04962 Vasopressin-regulated water reabsorption;ko05146 Amoebiasis;ko05152 Tuberculosis;
gene27593	K12937	UGAT	cyanidin-3-O-glucoside 2''-O-glucuronosyltransferase	EC:2.4.1.254	1	ko00942 Anthocyanin biosynthesis;
gene27594	K01728	E4.2.2.2, pel	pectate lyase	EC:4.2.2.2	1	ko00040 Pentose and glucuronate interconversions;
gene27595	K09272	SSRP1	structure-specific recognition protein 1	--		
gene27597	K09286	EREBP	EREBP-like factor	--		
gene27598	K12818	DHX8, PRP22	ATP-dependent RNA helicase DHX8/PRP22	EC:3.6.4.13	1	ko03040 Spliceosome;
gene27599	K01188	E3.2.1.21	beta-glucosidase	EC:3.2.1.21	3	ko00460 Cyanoamino acid metabolism;ko00500 Starch and sucrose metabolism;ko00940 Phenylpropanoid biosynthesis;
gene275	K13420	FLS2	LRR receptor-like serine/threonine-protein kinase FLS2	EC:2.7.11.1	1	ko04626 Plant-pathogen interaction;
gene27608	K15718	LOX1_5	linoleate 9S-lipoxygenase	EC:1.13.11.58	1	ko00591 Linoleic acid metabolism;
gene27611	K12669	OST3, OST6	oligosaccharyltransferase complex subunit gamma	--	3	ko00510 N-Glycan biosynthesis;ko00513 Various types of N-glycan biosynthesis;ko04141 Protein processing in endoplasmic reticulum;
gene27613	K04640	GNA	guanine nucleotide-binding protein subunit alpha, other	--		
gene27614	K10802	HMGB1	high mobility group protein B1	--	1	ko03410 Base excision repair;
gene27617	K00855	PRK, prkB	phosphoribulokinase	EC:2.7.1.19	2	ko00710 Carbon fixation in photosynthetic organisms;ko01200 Carbon metabolism;
gene2761	K07052	K07052				
gene2762	K03327	TC.MATE, SLC47A, norM, mdtK, dinF	multidrug resistance protein, MATE family	--		
gene27630	K12176	COPS2, CSN2, TRIP15	COP9 signalosome complex subunit 2	--		
gene27631	K10583	UBE2S, E2EPF	ubiquitin-conjugating enzyme E2 S	EC:6.3.2.19	1	ko04120 Ubiquitin mediated proteolysis;
gene27632	K02183	CALM	calmodulin	--	25	ko04014 Ras signaling pathway;ko04020 Calcium signaling pathway;ko04070 Phosphatidylinositol signaling system;ko04114 Oocyte meiosis;ko04270 Vascular smooth muscle contraction;ko04626 Plant-pathogen interaction;ko04713 Circadian entrainment;ko04720 Long-term potentiation;ko04722 Neurotrophin signaling pathway;ko04728 Dopaminergic synapse;ko04740 Olfactory transduction;ko04744 Phototransduction;ko04745 Phototransduction - fly;ko04910 Insulin signaling pathway;ko04912 GnRH signaling pathway;ko04915 Estrogen signaling pathway;ko04916 Melanogenesis;ko04970 Salivary secretion;ko04971 Gastric acid secretion;ko05010 Alzheimer's disease;ko05031 Amphetamine addiction;ko05034 Alcoholism;ko05133 Pertussis;ko05152 Tuberculosis;ko05214 Glioma;
gene27634	K05658	ABCB1	ATP-binding cassette, subfamily B (MDR/TAP), member 1	--	3	ko02010 ABC transporters;ko04976 Bile secretion;ko05206 MicroRNAs in cancer;
gene27635	K12271	SRP43, CAO	signal recognition particle 43 kDa protein	--		
gene27636	K16732	PRC1	protein regulator of cytokinesis 1	--		
gene27639	K10884	XRCC6, KU70, G22P1	ATP-dependent DNA helicase 2 subunit 1	--	1	ko03450 Non-homologous end-joining;
gene27643	K12581	CNOT7_8, CAF1, POP2	CCR4-NOT transcription complex subunit 7/8	--	1	ko03018 RNA degradation;
gene27644	K17046	DEK	protein DEK	--		
gene27646	K17279	REEP5_6	receptor expression-enhancing protein 5/6	--		
gene2764	K11000	CALS	callose synthase	EC:2.4.1.-		
gene27652	K05658	ABCB1	ATP-binding cassette, subfamily B (MDR/TAP), member 1	--	3	ko02010 ABC transporters;ko04976 Bile secretion;ko05206 MicroRNAs in cancer;
gene27658	K07300	chaA, CAX	Ca2+:H+ antiporter	--		
gene27659	K14549	UTP15	U3 small nucleolar RNA-associated protein 15	--	1	ko03008 Ribosome biogenesis in eukaryotes;
gene27661	K15382	SLC50A, SWEET	solute carrier family 50 (sugar transporter)	--		

gene27662	K01638	E2.3.3.9, aceB, glcB	malate synthase	EC:2.3.3.9	3	ko00620 Pyruvate metabolism;ko00630 Glyoxylate and dicarboxylate metabolism;ko01200 Carbon metabolism;
gene27667	K06997	K06997				
gene27671	K01164	POP1	ribonuclease P/MRP protein subunit POP1	EC:3.1.26.5	2	ko03008 Ribosome biogenesis in eukaryotes;ko03013 RNA transport;
gene27672	K08099	E3.1.1.14	chlorophyllase	EC:3.1.1.14	1	ko00860 Porphyrin and chlorophyll metabolism;
gene27676	K09264	K09264	MADS-box transcription factor, plant	--		
gene27679	K08234	yaeR	glyoxylase I family protein	--		
gene27681	K14497	PP2C	protein phosphatase 2C	EC:3.1.3.16	1	ko04075 Plant hormone signal transduction;
gene27684	K04368	MAP2K1, MEK1	mitogen-activated protein kinase kinase 1	EC:2.7.12.2	53	ko04010 MAPK signaling pathway;ko04012 ErbB signaling pathway;ko04013 MAPK signaling pathway - fly;ko04014 Ras signaling pathway;ko04062 Chemokine signaling pathway;ko04066 HIF-1 signaling pathway;ko04114 Oocyte meiosis;ko04151 PI3K-Akt signaling pathway;ko04270 Vascular smooth muscle contraction;ko04320 Dorso-ventral axis formation;ko04370 VEGF signaling pathway;ko04380 Osteoclast differentiation;ko04510 Focal adhesion;ko04540 Gap junction;ko04620 Toll-like receptor signaling pathway;ko04626 Plant-pathogen interaction;ko04650 Natural killer cell mediated cytotoxicity;ko04660 T cell receptor signaling pathway;ko04662 B cell receptor signaling pathway;ko04664 Fc epsilon RI signaling pathway;ko04666 Fc gamma R-mediated phagocytosis;ko04668 TNF signaling pathway;ko04720 Long-term potentiation;ko04722 Neurotrophin signaling pathway;ko04725 Cholinergic synapse;ko04726 Serotonergic synapse;ko04730 Long-term depression;ko04810 Regulation of actin cytoskeleton;ko04910 Insulin signaling pathway;ko04912 GnRH signaling pathway;ko04914 Progesterone-mediated oocyte maturation;ko04915 Estrogen signaling pathway;ko04916 Melanogenesis;ko04917 Prolactin signaling pathway;ko05020 Prion diseases;ko05034 Alcoholism;ko05161 Hepatitis B;ko05164 Influenza A;ko05200 Pathways in cancer;ko05205 Proteoglycans in cancer;ko05206 MicroRNAs in cancer;ko05210 Colorectal cancer;ko05211 Renal cell carcinoma;ko05212 Pancreatic cancer;ko05213 Endometrial cancer;ko05214 Glioma;ko05215 Prostate cancer;ko05216 Thyroid cancer;ko05218 Melanoma;ko05219 Bladder cancer;ko05220
gene27685	K04499	RUVBL1, RVB1, INO80H	RuvB-like protein 1 (pontin 52)	--	1	ko04310 Wnt signaling pathway;
gene27687	K04499	RUVBL1, RVB1, INO80H	RuvB-like protein 1 (pontin 52)	--	1	ko04310 Wnt signaling pathway;
gene27688	K04499	RUVBL1, RVB1, INO80H	RuvB-like protein 1 (pontin 52)	--	1	ko04310 Wnt signaling pathway;
gene27691	K00422	E1.10.3.1	polyphenol oxidase	EC:1.10.3.1	2	ko00350 Tyrosine metabolism;ko00950 Isoquinoline alkaloid biosynthesis;
gene27693	K04688	RPS6KB	p70 ribosomal S6 kinase	EC:2.7.11.1	9	ko04012 ErbB signaling pathway;ko04066 HIF-1 signaling pathway;ko04150 mTOR signaling pathway;ko04151 PI3K-Akt signaling pathway;ko04350 TGF-beta signaling pathway;ko04666 Fc gamma R-mediated phagocytosis;ko04910 Insulin signaling pathway;ko05205 Proteoglycans in cancer;ko05221 Acute myeloid leukemia;
gene2769	K14498	SNRK2	serine/threonine-protein kinase SRK2	EC:2.7.11.1	1	ko04075 Plant hormone signal transduction;
gene276	K03626	EGD2, NACA	nascent polypeptide-associated complex subunit alpha	--		
gene27701	K15083	RAD16	DNA repair protein RAD16	--		
gene27711	K12169	KPC1, RNF123	Kip1 ubiquitination-promoting complex protein 1	EC:6.3.2.19		
gene27714	K11584	PPP2R5	serine/threonine-protein phosphatase 2A regulatory subunit B'	--	5	ko03015 mRNA surveillance pathway;ko04113 Meiosis - yeast;ko04114 Oocyte meiosis;ko04151 PI3K-Akt signaling pathway;ko04728 Dopaminergic synapse;
gene27715	K06627	CCNA	cyclin A	--	5	ko04110 Cell cycle;ko04914 Progesterone-mediated oocyte maturation;ko05161 Hepatitis B;ko05169 Epstein-Barr virus infection;ko05203 Viral carcinogenesis;
gene27722	K01835	pgm	phosphoglucomutase	EC:5.4.2.2	7	ko00010 Glycolysis / Gluconeogenesis;ko00030 Pentose phosphate pathway;ko00052 Galactose metabolism;ko00230 Purine metabolism;ko00500 Starch and sucrose metabolism;ko00520 Amino sugar and nucleotide sugar metabolism;ko00521 Streptomycin biosynthesis;

gene27724	K04730	IRAK1	interleukin-1 receptor-associated kinase 1	EC:2.7.11.1	11	ko04064 NF-kappa B signaling pathway;ko04210 Apoptosis;ko04620 Toll-like receptor signaling pathway;ko04722 Neurotrophin signaling pathway;ko05133 Pertussis;ko05140 Leishmaniasis;ko05142 Chagas disease (American trypanosomiasis);ko05145 Toxoplasmosis;ko05152 Tuberculosis;ko05162 Measles;ko05169 Epstein-Barr virus infection;
gene27726	K07942	ARL1	ADP-ribosylation factor-like protein 1	--		
gene27727	K07942	ARL1	ADP-ribosylation factor-like protein 1	--		
gene27728	K01805	xylA	xylose isomerase	EC:5.3.1.5	2	ko00040 Pentose and glucuronate interconversions;ko00051 Fructose and mannose metabolism;
gene27731	K13789	GGPS	geranylgeranyl diphosphate synthase, type II	EC:2.5.1.1 2.5.1.10 2.5.1.29	1	ko00900 Terpenoid backbone biosynthesis;
gene27735	K10949	KDELR	ER lumen protein retaining receptor	--	1	ko05110 Vibrio cholerae infection;
gene27739	K09338	HD-ZIP	homeobox-leucine zipper protein	--		
gene27741	K15032	MTERFD	mTERF domain-containing protein, mitochondrial	--		
gene27745	K00895	E2.7.1.90, pfk	pyrophosphate--fructose-6-phosphate 1-phosphotransferase	EC:2.7.1.90	1	ko00051 Fructose and mannose metabolism;
gene27747	K13691	SGT1	pathogen-inducible salicylic acid glucosyltransferase	EC:2.4.1.-		
gene27748	K13691	SGT1	pathogen-inducible salicylic acid glucosyltransferase	EC:2.4.1.-		
gene2774	K03549	kup	KUP system potassium uptake protein	--		
gene27750	K07375	TUBB	tubulin beta	--	3	ko04145 Phagosome;ko04540 Gap junction;ko05130 Pathogenic Escherichia coli infection;
gene27754	K16810	TBCCD1	TBCC domain-containing protein 1	--		
gene27755	K11420	EHMT	euchromatic histone-lysine N-methyltransferase	EC:2.1.1.43	1	ko00310 Lysine degradation;
gene27756	K01867	WARS, trpS	tryptophanyl-tRNA synthetase	EC:6.1.1.2	1	ko00970 Aminoacyl-tRNA biosynthesis;
gene27768	K03260	EIF4G	translation initiation factor 4G	--	2	ko03013 RNA transport;ko05416 Viral myocarditis;
gene27769	K12185	VPS37	ESCRT-I complex subunit VPS37	--	1	ko04144 Endocytosis;
gene27770	K09837	LUT1, CYP97C1	carotene epsilon-monooxygenase	EC:1.14.99.45	1	ko00906 Carotenoid biosynthesis;
gene27774	K12669	OST3, OST6	oligosaccharyltransferase complex subunit gamma	--	3	ko00510 N-Glycan biosynthesis;ko00513 Various types of N-glycan biosynthesis;ko04141 Protein processing in endoplasmic reticulum;
gene27776	K11799	WDR21A	WD repeat-containing protein 21A	--		
gene27778	K10400	KIF15	kinesin family member 15	--		
gene27779	K01476	E3.5.3.1, rocF, arg	arginase	EC:3.5.3.1	3	ko00330 Arginine and proline metabolism;ko01230 Biosynthesis of amino acids;ko05146 Amoebiasis;
gene27780	K15032	MTERFD	mTERF domain-containing protein, mitochondrial	--		
gene27784	K03881	ND4	NADH-ubiquinone oxidoreductase chain 4	EC:1.6.5.3	2	ko00190 Oxidative phosphorylation;ko05012 Parkinson's disease;
gene27787	K03549	kup	KUP system potassium uptake protein	--		
gene27788	K00794	ribH, RIB4	6,7-dimethyl-8-ribityllumazine synthase	EC:2.5.1.78	1	ko00740 Riboflavin metabolism;
gene27790	K00434	E1.11.1.11	L-ascorbate peroxidase	EC:1.11.1.11	2	ko00053 Ascorbate and aldarate metabolism;ko00480 Glutathione metabolism;
gene27792	K04730	IRAK1	interleukin-1 receptor-associated kinase 1	EC:2.7.11.1	11	ko04064 NF-kappa B signaling pathway;ko04210 Apoptosis;ko04620 Toll-like receptor signaling pathway;ko04722 Neurotrophin signaling pathway;ko05133 Pertussis;ko05140 Leishmaniasis;ko05142 Chagas disease (American trypanosomiasis);ko05145 Toxoplasmosis;ko05152 Tuberculosis;ko05162 Measles;ko05169 Epstein-Barr virus infection;
gene27799	K02138	ATPeFD, ATP5H	F-type H+-transporting ATPase subunit d	EC:3.6.3.14	4	ko00190 Oxidative phosphorylation;ko05010 Alzheimer's disease;ko05012 Parkinson's disease;ko05016 Huntington's disease;
gene27807	K10801	MBD4	methyl-CpG-binding domain protein 4	EC:3.2.2.-	1	ko03410 Base excision repair;
gene2780	K10577	UBE2I, UBC9	ubiquitin-conjugating enzyme E2 I	EC:6.3.2.19	4	ko03013 RNA transport;ko04064 NF-kappa B signaling pathway;ko04120 Ubiquitin mediated proteolysis;ko05206 MicroRNAs in cancer;
gene27810	K01214	E3.2.1.68	isoamylase	EC:3.2.1.68		
gene27811	K00799	GST, gst	glutathione S-transferase	EC:2.5.1.18	4	ko00480 Glutathione metabolism;ko00980 Metabolism of xenobiotics by cytochrome P450;ko00982 Drug metabolism - cytochrome P450;ko05204 Chemical carcinogenesis;
gene27824	K02997	RP-S9e, RPS9	small subunit ribosomal protein S9e	--	1	ko03010 Ribosome;
gene2783	K13126	PABPC	polyadenylate-binding protein	--	3	ko03013 RNA transport;ko03015 mRNA surveillance pathway;ko03018 RNA degradation;
gene27842	K08472	MLO	mlo protein	--		

gene27847	K13065	E2.3.1.133, HCT	shikimate O-hydroxycinnamoyltransferase	EC:2.3.1.133	3	ko00940 Phenylpropanoid biosynthesis;ko00941 Flavonoid biosynthesis;ko00945 Stilbenoid, diarylheptanoid and gingerol biosynthesis;
gene2784	K02926	RP-L4, MRPL4, rplD	large subunit ribosomal protein L4	--	1	ko03010 Ribosome;
gene27859	K12124	GI	GIGANTEA	--	1	ko04712 Circadian rhythm - plant;
gene2785	K01087	otsB	trehalose 6-phosphate phosphatase	EC:3.1.3.12	1	ko00500 Starch and sucrose metabolism;
gene27863	K07199	PRKAB	5'-AMP-activated protein kinase, regulatory beta subunit	--	5	ko04710 Circadian rhythm;ko04910 Insulin signaling pathway;ko04920 Adipocytokine signaling pathway;ko04932 Non-alcoholic fatty liver disease (NAFLD);ko05410 Hypertrophic cardiomyopathy (HCM);
gene27864	K14948	PTBP2, NPTB	olypyrimidine tract-binding protein 2	--		
gene2786	K02132	ATPeF1A, ATP5A1	F-type H+-transporting ATPase subunit alpha	EC:3.6.3.14	4	ko00190 Oxidative phosphorylation;ko05010 Alzheimer's disease;ko05012 Parkinson's disease;ko05016 Huntington's disease;
gene27875	K14516	ERF1	ethylene-responsive transcription factor 1	--	1	ko04075 Plant hormone signal transduction;
gene27884	K15707	RNF170	RING finger protein 170	--		
gene27894	K16818	K16818, DAD1	phospholipase A1	EC:3.1.1.32	2	ko00564 Glycerophospholipid metabolism;ko00592 alpha-Linolenic acid metabolism;
gene27895	K07195	EXOC7, EXO70	exocyst complex component 7	--	1	ko04910 Insulin signaling pathway;
gene27898	K15326	TSEN54	tRNA-splicing endonuclease subunit Sen54	--		
gene278	K14664	ILR1	IAA-amino acid hydrolase	EC:3.5.1.-		
gene27902	K01056	PTH1, pth, spoVC	peptidyl-tRNA hydrolase, PTH1 family	EC:3.1.1.29		
gene27905	K00227	SC5DL, ERG3	lathosterol oxidase	EC:1.14.21.6	1	ko00100 Steroid biosynthesis;
gene2790	K03019	RPC11, POLR3K	DNA-directed RNA polymerase III subunit RPC10	--	5	ko00230 Purine metabolism;ko00240 Pyrimidine metabolism;ko03020 RNA polymerase;ko04623 Cytosolic DNA-sensing pathway;ko05169 Epstein-Barr virus infection;
gene27910	K02136	ATPeF1G, ATP5C1	F-type H+-transporting ATPase subunit gamma	EC:3.6.3.14	4	ko00190 Oxidative phosphorylation;ko05010 Alzheimer's disease;ko05012 Parkinson's disease;ko05016 Huntington's disease;
gene27912	K15277	SLC35B3, PAPST2	solute carrier family 35 (adenosine 3'-phospho 5'-phosphosulfate transporter), member B3	--		
gene27921	K07192	FLOT	flotillin	--	1	ko04910 Insulin signaling pathway;
gene27923	K10364	CAPZA	capping protein (actin filament) muscle Z-line, alpha	--		
gene27925	K14488	SAUR	SAUR family protein	--	1	ko04075 Plant hormone signal transduction;
gene27928	K08850	AURKX	aurora kinase, other	EC:2.7.11.1		
gene27930	K13993	HSP20	HSP20 family protein	--	1	ko04141 Protein processing in endoplasmic reticulum;
gene27937	K02915	RP-L34e, RPL34	large subunit ribosomal protein L34e	--	1	ko03010 Ribosome;
gene27938	K03671	trxA	thioredoxin 1	--		
gene27940	K03952	NDUFA8	NADH dehydrogenase (ubiquinone) 1 alpha subcomplex subunit 8	--	5	ko00190 Oxidative phosphorylation;ko04932 Non-alcoholic fatty liver disease (NAFLD);ko05010 Alzheimer's disease;ko05012 Parkinson's disease;ko05016 Huntington's disease;
gene27941	K02892	RP-L23, MRPL23, rplW	large subunit ribosomal protein L23	--	1	ko03010 Ribosome;
gene27943	K09874	NIP	aquaporin NIP	--		
gene27945	K02937	RP-L7e, RPL7	large subunit ribosomal protein L7e	--	1	ko03010 Ribosome;
gene27948	K12856	PRPF8, PRP8	pre-mRNA-processing factor 8	--	1	ko03040 Spliceosome;
gene27951	K11423	SETD2, SET2	histone-lysine N-methyltransferase SETD2	EC:2.1.1.43	1	ko00310 Lysine degradation;
gene27953	K14566	UTP24, FCF1	U3 small nucleolar RNA-associated protein 24	--	1	ko03008 Ribosome biogenesis in eukaryotes;
gene27954	K01183	E3.2.1.14	chitinase	EC:3.2.1.14	1	ko00520 Amino sugar and nucleotide sugar metabolism;
gene27957	K15338	GEN1, GEN	flap endonuclease GEN	EC:3.1.-.-		
gene27958	K08736	MSH3	DNA mismatch repair protein MSH3	--	3	ko03430 Mismatch repair;ko05200 Pathways in cancer;ko05210 Colorectal cancer;
gene27961	K14397	NUDT21, CPSF5, CFIM25	cleavage and polyadenylation specificity factor subunit 5	--	1	ko03015 mRNA surveillance pathway;
gene27963	K03327	TC.MATE, SLC47A, norM, mdtK, dinF	multidrug resistance protein, MATE family	--		
gene27964	K14232	tRNA-Pro	tRNA Pro	--	1	ko00970 Aminoacyl-tRNA biosynthesis;
gene27966	K14231	tRNA-Phe	tRNA Phe	--	1	ko00970 Aminoacyl-tRNA biosynthesis;
gene27968	K02895	RP-L24, MRPL24, rplX	large subunit ribosomal protein L24	--	1	ko03010 Ribosome;
gene27969	K13606	NOL, NYC1	chlorophyll(ide) b reductase	EC:1.1.1.294	1	ko00860 Porphyrin and chlorophyll metabolism;
gene27974	K01193	E3.2.1.26, sacA	beta-fructofuranosidase	EC:3.2.1.26	2	ko00052 Galactose metabolism;ko00500 Starch and sucrose metabolism;

gene27978	K02960	RP-S16e, RPS16	small subunit ribosomal protein S16e	--	1	ko03010 Ribosome;
gene2797	K08900	BCS1	mitochondrial chaperone BCS1	--		
gene27980	K03809	wrbA	Trp repressor binding protein	--		
gene27981	K16292	CEP, CYSEP	KDEL-tailed cysteine endopeptidase	EC:3.4.22.-		
gene27982	K02873	RP-L13e, RPL13	large subunit ribosomal protein L13e	--	1	ko03010 Ribosome;
gene27988	K14492	ARR-A	two-component response regulator ARR-A family	--	1	ko04075 Plant hormone signal transduction;
gene27989	K13523	AGPAT3_4	lysophosphatidic acid acyltransferase / lysophosphatidylinositol acyltransferase	EC:2.3.1.51	2	ko00561 Glycerolipid metabolism;ko00564 Glycerophospholipid metabolism;
gene27991	K00106	XDH	xanthine dehydrogenase/oxidase	EC:1.17.1.4 1.17.3.2	4	ko00230 Purine metabolism;ko00232 Caffeine metabolism;ko00983 Drug metabolism - other enzymes;ko04146 Peroxisome;
gene27992	K12471	EPN	epsin	--	1	ko04144 Endocytosis;
gene27994	K10355	ACTF	actin, other eukaryote	--		
gene27996	K09287	RAV	RAV-like factor	--		
gene27997	K01265	E3.4.11.18, map	methionyl aminopeptidase	EC:3.4.11.18		
gene28008	K14315	NDC1, TMEM48	nucleoporin NDC1	--	1	ko03013 RNA transport;
gene28009	K01637	E4.1.3.1, aceA	isocitrate lyase	EC:4.1.3.1	2	ko00630 Glyoxylate and dicarboxylate metabolism;ko01200 Carbon metabolism;
gene28011	K07447	ruvX	putative holliday junction resolvase	EC:3.1.-.-		
gene28016	K12761	SNF1	carbon catabolite-derepressing protein kinase	EC:2.7.11.1	1	ko04113 Meiosis - yeast;
gene28017	K09273	UBTF	upstream-binding transcription factor	--		
gene28018	K14857	SPB1, FTSJ3	AdoMet-dependent rRNA methyltransferase SPB1	EC:2.1.1.-		
gene28024	K05350	bglB	beta-glucosidase	EC:3.2.1.21	3	ko00460 Cyanoamino acid metabolism;ko00500 Starch and sucrose metabolism;ko00940 Phenylpropanoid biosynthesis;
gene28025	K15813	LUP4	beta-amyrin synthase	EC:5.4.99.39	1	ko00909 Sesquiterpenoid and triterpenoid biosynthesis;
gene28026	K13280	SEC11, sipW	signal peptidase, endoplasmic reticulum-type	EC:3.4.-.-	1	ko03060 Protein export;
gene2802	K15283	SLC35E1	solute carrier family 35, member E1	--		
gene28032	K09422	MYBP	myb proto-oncogene protein, plant	--		
gene28034	K14488	SAUR	SAUR family protein	--	1	ko04075 Plant hormone signal transduction;
gene28037	K11366	USP22_27_51, UBP8	ubiquitin carboxyl-terminal hydrolase 22/27/51	EC:3.1.2.15		
gene28040	K09264	K09264	MADS-box transcription factor, plant	--		
gene28041	K13648	GAUT	alpha-1,4-galacturonosyltransferase	EC:2.4.1.43	2	ko00500 Starch and sucrose metabolism;ko00520 Amino sugar and nucleotide sugar metabolism;
gene28044	K15277	SLC35B3, PAPST2	solute carrier family 35 (adenosine 3'-phospho 5'-phosphosulfate transporter), member B3	--		
gene28046	K00948	PRPS, prsA	ribose-phosphate pyrophosphokinase	EC:2.7.6.1	4	ko00030 Pentose phosphate pathway;ko00230 Purine metabolism;ko01200 Carbon metabolism;ko01230 Biosynthesis of amino acids;
gene28048	K02739	PSMB7	20S proteasome subunit beta 2	EC:3.4.25.1	1	ko03050 Proteasome;
gene28054	K16302	CNNM	metal transporter CNNM	--		
gene28057	K08597	SENP8, NEDP1, DEN1	sentrin-specific protease 8	EC:3.4.22.68		
gene28059	K01595	ppc	phosphoenolpyruvate carboxylase	EC:4.1.1.31	5	ko00620 Pyruvate metabolism;ko00680 Methane metabolism;ko00710 Carbon fixation in photosynthetic organisms;ko00720 Carbon fixation pathways in prokaryotes;ko01200 Carbon metabolism;
gene28061	K09527	DNAJC7	DnaJ homolog subfamily C member 7	--		
gene28063	K14484	IAA	auxin-responsive protein IAA	--	1	ko04075 Plant hormone signal transduction;
gene28065	K11000	CALS	callose synthase	EC:2.4.1.-		
gene28068	K15283	SLC35E1	solute carrier family 35, member E1	--		
gene28069	K01696	trpB	tryptophan synthase beta chain	EC:4.2.1.20	3	ko00260 Glycine, serine and threonine metabolism;ko00400 Phenylalanine, tyrosine and tryptophan biosynthesis;ko01230 Biosynthesis of amino acids;
gene2806	K09286	EREBP	EREBP-like factor	--		
gene28070	K07976	RAB	Rab family, other	--		
gene28079	K01051	E3.1.1.11	pectinesterase	EC:3.1.1.11	2	ko00040 Pentose and glucuronate interconversions;ko00500 Starch and sucrose metabolism;
gene28080	K03125	TAF1	transcription initiation factor TFIID subunit 1	--	1	ko03022 Basal transcription factors;

gene28089	K03094	SKP1, CBF3D	S-phase kinase-associated protein 1	--	9	ko04110 Cell cycle;ko04111 Cell cycle - yeast;ko04114 Oocyte meiosis;ko04120 Ubiquitin mediated proteolysis;ko04141 Protein processing in endoplasmic reticulum;ko04310 Wnt signaling pathway;ko04350 TGF-beta signaling pathway;ko04710 Circadian rhythm;ko05168 Herpes simplex infection;
gene28092	K00721	DPM1	dolichol-phosphate mannosyltransferase	EC:2.4.1.83	1	ko00510 N-Glycan biosynthesis;
gene28095	K08869	ADCK, ABC1	aarF domain-containing kinase	--		
gene28096	K09422	MYBP	myb proto-oncogene protein, plant	--		
gene28100	K10357	MYO5	myosin V	--		
gene28102	K14301	NUP107	nuclear pore complex protein Nup107	--	1	ko03013 RNA transport;
gene2810	K08794	CAMK1	calcium/calmodulin-dependent protein kinase I	EC:2.7.11.17		
gene28111	K12830	SF3B3, SAP130, RSE1	splicing factor 3B subunit 3	--	1	ko03040 Spliceosome;
gene28112	K04733	IRAK4	interleukin-1 receptor-associated kinase 4	EC:2.7.11.1	11	ko04064 NF-kappa B signaling pathway;ko04210 Apoptosis;ko04620 Toll-like receptor signaling pathway;ko04722 Neurotrophin signaling pathway;ko05133 Pertussis;ko05140 Leishmaniasis;ko05142 Chagas disease (American trypanosomiasis);ko05145 Toxoplasmosis;ko05152 Tuberculosis;ko05162 Measles;ko05164 Influenza A;
gene28115	K03031	PSMD8, RPN12	26S proteasome regulatory subunit N12	--	2	ko03050 Proteasome;ko05169 Epstein-Barr virus infection;
gene28118	K00558	DNMT1, dcm	DNA (cytosine-5)-methyltransferase 1	EC:2.1.1.37	2	ko00270 Cysteine and methionine metabolism;ko05206 MicroRNAs in cancer;
gene28120	K13420	FLS2	LRR receptor-like serine/threonine-protein kinase FLS2	EC:2.7.11.1	1	ko04626 Plant-pathogen interaction;
gene28123	K08235	E2.4.1.207	xyloglucan:xyloglucosyl transferase	EC:2.4.1.207		
gene28124	K00784	rnz	ribonuclease Z	EC:3.1.26.11	1	ko03013 RNA transport;
gene28127	K17402	MRPS23	small subunit ribosomal protein S23, mitochondrial	--		
gene28129	K14007	SEC24	protein transport protein SEC24	--	1	ko04141 Protein processing in endoplasmic reticulum;
gene28130	K13093	HTATSF1	HIV Tat-specific factor 1	--		
gene28132	K03921	DESA1	acyl-[acyl-carrier-protein] desaturase	EC:1.14.19.2	2	ko00061 Fatty acid biosynthesis;ko01040 Biosynthesis of unsaturated fatty acids;
gene28133	K10750	CHAF1A	chromatin assembly factor 1 subunit A	--		
gene28146	K03327	TC.MATE, SLC47A, norM, mdtK, dinF	multidrug resistance protein, MATE family	--		
gene28148	K06892	K06892				
gene2814	K13429	CERK1	chitin elicitor receptor kinase 1	--	1	ko04626 Plant-pathogen interaction;
gene28150	K12448	UXE	UDP-arabinose 4-epimerase	EC:5.1.3.5	1	ko00520 Amino sugar and nucleotide sugar metabolism;
gene28151	K12617	PATL1, PAT1	DNA topoisomerase 2-associated protein PAT1	--	1	ko03018 RNA degradation;
gene28152	K07198	PRKAA, AMPK	5'-AMP-activated protein kinase, catalytic alpha subunit	EC:2.7.11.11	8	ko04140 Regulation of autophagy;ko04150 mTOR signaling pathway;ko04151 PI3K-Akt signaling pathway;ko04710 Circadian rhythm;ko04910 Insulin signaling pathway;ko04920 Adipocytokine signaling pathway;ko04932 Non-alcoholic fatty liver disease (NAFLD);ko05410 Hypertrophic [illegible]
gene28154	K07198	PRKAA, AMPK	5'-AMP-activated protein kinase, catalytic alpha subunit	EC:2.7.11.11	8	ko04140 Regulation of autophagy;ko04150 mTOR signaling pathway;ko04151 PI3K-Akt signaling pathway;ko04710 Circadian rhythm;ko04910 Insulin signaling pathway;ko04920 Adipocytokine signaling pathway;ko04932 Non-alcoholic fatty liver disease (NAFLD);ko05410 Hypertrophic [illegible]
gene28155	K15979	SND1	staphylococcal nuclease domain-containing protein 1	--	2	ko05169 Epstein-Barr virus infection;ko05203 Viral carcinogenesis;
gene28156	K13436	PTI1	pto-interacting protein 1	EC:2.7.11.1	1	ko04626 Plant-pathogen interaction;
gene28157	K14664	ILR1	IAA-amino acid hydrolase	EC:3.5.1.-		
gene28158	K12831	SF3B4, SAP49	splicing factor 3B subunit 4	--	1	ko03040 Spliceosome;
gene28159	K01739	metB	cystathionine gamma-synthase	EC:2.5.1.48	4	ko00270 Cysteine and methionine metabolism;ko00450 Selenocompound metabolism;ko00920 Sulfur metabolism;ko01230 Biosynthesis of amino acids;
gene2815	K13429	CERK1	chitin elicitor receptor kinase 1	--	1	ko04626 Plant-pathogen interaction;
gene28160	K00888	PI4K	phosphatidylinositol 4-kinase	EC:2.7.1.67	2	ko00562 Inositol phosphate metabolism;ko04070 Phosphatidylinositol signaling system;
gene28161	K00888	PI4K	phosphatidylinositol 4-kinase	EC:2.7.1.67	2	ko00562 Inositol phosphate metabolism;ko04070 Phosphatidylinositol signaling system;

gene28162	K00888	PI4K	phosphatidylinositol 4-kinase	EC:2.7.1.67	2	ko00562 Inositol phosphate metabolism;ko04070 Phosphatidylinositol signaling system;
gene28167	K09480	E2.4.1.241	digalactosyldiacylglycerol synthase	EC:2.4.1.241	1	ko00561 Glycerolipid metabolism;
gene28168	K15095	E1.1.1.208	(+)-neomenthol dehydrogenase	EC:1.1.1.208	1	ko00902 Monoterpenoid biosynthesis;
gene28170	K12614	DDX6, RCK, DHH1	ATP-dependent RNA helicase DDX6/DHH1	EC:3.6.4.13	1	ko03018 RNA degradation;
gene28184	K07119	K07119				
gene28186	K07119	K07119				
gene28188	K05282	E1.14.11.12	gibberellin 20-oxidase	EC:1.14.11.12	1	ko00904 Diterpenoid biosynthesis;
gene28189	K15919	HPR2	hydroxypyruvate reductase 2	--	2	ko00260 Glycine, serine and threonine metabolism;ko00630 Glyoxylate and dicarboxylate metabolism;
gene28190	K15919	HPR2	hydroxypyruvate reductase 2	--	2	ko00260 Glycine, serine and threonine metabolism;ko00630 Glyoxylate and dicarboxylate metabolism;
gene28191	K15919	HPR2	hydroxypyruvate reductase 2	--	2	ko00260 Glycine, serine and threonine metabolism;ko00630 Glyoxylate and dicarboxylate metabolism;
gene28195	K05387	GRIP	glutamate receptor, ionotropic, plant	--		
gene28197	K05387	GRIP	glutamate receptor, ionotropic, plant	--		
gene28200	K02135	ATPeF1E, ATP5E	F-type H+-transporting ATPase subunit epsilon	EC:3.6.3.14	4	ko00190 Oxidative phosphorylation;ko05010 Alzheimer's disease;ko05012 Parkinson's disease;ko05016 Huntington's disease;
gene28207	K00430	E1.11.1.7	peroxidase	EC:1.11.1.7	2	ko00360 Phenylalanine metabolism;ko00940 Phenylpropanoid biosynthesis;
gene28210	K08829	MAK	male germ cell-associated kinase	EC:2.7.11.22		
gene28219	K08065	NFYB	nuclear transcription Y subunit beta	--	3	ko04612 Antigen processing and presentation;ko05152 Tuberculosis;ko05166 HTLV-I infection;
gene28220	K10643	CNOT4, NOT4, MOT2	CCR4-NOT transcription complex subunit 4	EC:6.3.2.19	1	ko03018 RNA degradation;
gene28221	K00799	GST, gst	glutathione S-transferase	EC:2.5.1.18	4	ko00480 Glutathione metabolism;ko00980 Metabolism of xenobiotics by cytochrome P450;ko00982 Drug metabolism - cytochrome P450;ko05204 Chemical carcinogenesis;
gene28222	K02864	RP-L10, MRPL10, rplJ	large subunit ribosomal protein L10	--	1	ko03010 Ribosome;
gene28223	K16546	FGFR10P	FGFR1 oncogene partner	--		
gene28227	K13495	CISZOG	cis-zeatin O-glucosyltransferase	EC:2.4.1.215	1	ko00908 Zeatin biosynthesis;
gene28228	K03135	TAF11	transcription initiation factor TFIID subunit 11	--	1	ko03022 Basal transcription factors;
gene28231	K03031	PSMD8, RPN12	26S proteasome regulatory subunit N12	--	2	ko03050 Proteasome;ko05169 Epstein-Barr virus infection;
gene28232	K14376	PAP	poly(A) polymerase	EC:2.7.7.19	1	ko03015 mRNA surveillance pathway;
gene28233	K16282	RHA2	E3 ubiquitin-protein ligase RHA2	EC:6.3.2.19		
gene28237	K06632	WEE1	wee1-like protein kinase	EC:2.7.11.1	1	ko04110 Cell cycle;
gene28238	K01193	E3.2.1.26, sacA	beta-fructofuranosidase	EC:3.2.1.26	2	ko00052 Galactose metabolism;ko00500 Starch and sucrose metabolism;
gene28239	K10872	DMC1	meiotic recombination protein DMC1	--	1	ko04113 Meiosis - yeast;
gene28242	K08472	MLO	mlo protein	--		
gene28244	K15015	SLC32A, VGAT	solute carrier family 32 (vesicular inhibitory amino acid transporter)	--	5	ko04721 Synaptic vesicle cycle;ko04723 Retrograde endocannabinoid signaling;ko04727 GABAergic synapse;ko05032 Morphine addiction;ko05033 Nicotine addiction;
gene28245	K07752	CPD	carboxypeptidase D	EC:3.4.17.22		
gene28247	K14156	CHK	choline/ethanolamine kinase	EC:2.7.1.32 2.7.1.82	1	ko00564 Glycerophospholipid metabolism;
gene28248	K03953	NDUFA9	NADH dehydrogenase (ubiquinone) 1 alpha subcomplex subunit 9	--	5	ko00190 Oxidative phosphorylation;ko04932 Non-alcoholic fatty liver disease (NAFLD);ko05010 Alzheimer's disease;ko05012 Parkinson's disease;ko05016 Huntington's disease;
gene2824	K10268	FBXL2_20	F-box and leucine-rich repeat protein 2/20	--		
gene28251	K01979	SSUrRNA	small subunit ribosomal RNA	--	2	ko03008 Ribosome biogenesis in eukaryotes;ko03010 Ribosome;
gene28255	K03327	TC.MATE, SLC47A, norM, mdtK, dinF	multidrug resistance protein, MATE family	--		
gene28256	K15802	SSTLE1	germacrene C synthase	EC:4.2.3.60	1	ko00909 Sesquiterpenoid and triterpenoid biosynthesis;
gene28257	K10858	PMS2	DNA mismatch repair protein PMS2	--	2	ko03430 Mismatch repair;ko03460 Fanconi anemia pathway;
gene28260	K01102	PDP	pyruvate dehydrogenase phosphatase	EC:3.1.3.43		
gene28264	K10355	ACTF	actin, other eukaryote	--		

gene28265	K04730	IRAK1	interleukin-1 receptor-associated kinase 1	EC:2.7.11.1	11	ko04064 NF-kappa B signaling pathway;ko04210 Apoptosis;ko04620 Toll-like receptor signaling pathway;ko04722 Neurotrophin signaling pathway;ko05133 Pertussis;ko05140 Leishmaniasis;ko05142 Chagas disease (American trypanosomiasis);ko05145 Toxoplasmosis;ko05152 Tuberculosis;ko05162 Measles;ko05169 Epstein-Barr virus infection;
gene28266	K10773	NTH	endonuclease III	EC:4.2.99.18	1	ko03410 Base excision repair;
gene28267	K10773	NTH	endonuclease III	EC:4.2.99.18	1	ko03410 Base excision repair;
gene28270	K07252	E3.6.1.43	dolichyldiphosphatase	EC:3.6.1.43	1	ko00510 N-Glycan biosynthesis;
gene28272	K14779	DDX52, ROK1	ATP-dependent RNA helicase DDX52/ROK1	EC:3.6.4.13		
gene28278	K16297	SCPL-II	serine carboxypeptidase-like clade II	EC:3.4.16.-		
gene28281	K08150	SLC2A13, ITR	MFS transporter, SP family, solute carrier family 2 (myo-inositol transporter), member 13	--		
gene28282	K14411	MSI	RNA-binding protein Musashi	--	1	ko03015 mRNA surveillance pathway;
gene28283	K00857	E2.7.1.21, tdk	thymidine kinase	EC:2.7.1.21	2	ko00240 Pyrimidine metabolism;ko00983 Drug metabolism - other enzymes;
gene28290	K13117	DHX35	ATP-dependent RNA helicase DDX35	EC:3.6.4.13		
gene28292	K15172	SUPT5H, SPT5	transcription elongation factor SPT5	--		
gene28293	K15172	SUPT5H, SPT5	transcription elongation factor SPT5	--		
gene28294	K00939	E2.7.4.3, adk	adenylate kinase	EC:2.7.4.3	1	ko00230 Purine metabolism;
gene28297	K10403	KIF22	kinesin family member 22	--		
gene28301	K04554	UBE2J2, NCUBE2, UBC6	ubiquitin-conjugating enzyme E2 J2	EC:6.3.2.19	3	ko04120 Ubiquitin mediated proteolysis;ko04141 Protein processing in endoplasmic reticulum;ko05012 Parkinson's disease;
gene28304	K14165	K14165	dual specificity phosphatase	EC:3.1.3.16 3.1.3.48		
gene28306	K14552	NAN1, UTP17, WDR75	NET1-associated nuclear protein 1 (U3 small nucleolar RNA-associated protein 17)	--	1	ko03008 Ribosome biogenesis in eukaryotes;
gene28309	K14232	tRNA-Pro	tRNA Pro	--	1	ko00970 Aminoacyl-tRNA biosynthesis;
gene2830	K02083	allC	allantoate deiminase	EC:3.5.3.9	1	ko00230 Purine metabolism;
gene28310	K03283	HSPA1_8	heat shock 70kDa protein 1/8	--	11	ko03040 Spliceosome;ko04010 MAPK signaling pathway;ko04141 Protein processing in endoplasmic reticulum;ko04144 Endocytosis;ko04612 Antigen processing and presentation;ko04915 Estrogen signaling pathway;ko05134 Legionellosis;ko05145 Toxoplasmosis;ko05162 Measles;ko05164 Influenza A;ko05169 Epstein-Barr virus infection;
gene28313	K07910	RAB18	Ras-related protein Rab-18	--		
gene28314	K17506	PPM1L, PP2CE	protein phosphatase 1L	EC:3.1.3.16		
gene28315	K13648	GAUT	alpha-1,4-galacturonosyltransferase	EC:2.4.1.43	2	ko00500 Starch and sucrose metabolism;ko00520 Amino sugar and nucleotide sugar metabolism;
gene28318	K11666	INO80B, ZNHIT4, PAPA1	INO80 complex subunit B	--		
gene2831	K08770	UBC	ubiquitin C	--	1	ko03320 PPAR signaling pathway;
gene28320	K08282	E2.7.11.1	non-specific serine/threonine protein kinase	EC:2.7.11.1		
gene28322	K08176	PHO84	MFS transporter, PHS family, inorganic phosphate transporter	--		

gene28323	K04371	MAPK1_3	mitogen-activated protein kinase 1/3	EC:2.7.11.24	71	ko04010 MAPK signaling pathway;ko04012 ErbB signaling pathway;ko04013 MAPK signaling pathway - fly;ko04014 Ras signaling pathway;ko04062 Chemokine signaling pathway;ko04066 HIF-1 signaling pathway;ko04114 Oocyte meiosis;ko04150 mTOR signaling pathway;ko04151 PI3K-Akt signaling pathway;ko04270 Vascular smooth muscle contraction;ko04320 Dorso-ventral axis formation;ko04350 TGF-beta signaling pathway;ko04360 Axon guidance;ko04370 VEGF signaling pathway;ko04380 Osteoclast differentiation;ko04510 Focal adhesion;ko04520 Adherens junction;ko04540 Gap junction;ko04620 Toll-like receptor signaling pathway;ko04621 NOD-like receptor signaling pathway;ko04650 Natural killer cell mediated cytotoxicity;ko04660 T cell receptor signaling pathway;ko04662 B cell receptor signaling pathway;ko04664 Fc epsilon RI signaling pathway;ko04666 Fc gamma R-mediated phagocytosis;ko04668 TNF signaling pathway;ko04713 Circadian entrainment;ko04720 Long-term potentiation;ko04722 Neurotrophin signaling pathway;ko04723 Retrograde endocannabinoid signaling;ko04724 Glutamatergic synapse;ko04725 Cholinergic synapse;ko04726 Serotonergic synapse;ko04730 Long-term depression;ko04810 Regulation of actin cytoskeleton;ko04910 Insulin signaling pathway;ko04912 GnRH signaling pathway;ko04914 Progesterone-mediated oocyte maturation;ko04915 Estrogen signaling pathway;ko04916 Melanogenesis;ko04917 Prolactin signaling pathway;ko04930 Type II diabetes mellitus;ko04960 Aldosterone-regulated sodium reabsorption;ko05010 Alzheimer's disease;ko05020 Prion diseases;ko05034 Alcoholism;ko05131 Shigellosis;ko05132 Salmonella infection;ko05133 Pertussis;ko05140 Leishmaniasis;ko05142 Chagas disease (American trypanosomiasis);ko05145 Toxoplasmosis;ko05152 Tuberculosis;ko05160 Hepatitis C;ko05161 Hepatitis B;ko05164 Influenza A;ko05200 Pathways in cancer;ko05203 Viral carcinogenesis;ko05205 Proteoglycans in cancer;ko05210 Colorectal cancer;ko05211 Renal cell carcinoma;ko05212
gene28329	K06269	PPP1C	serine/threonine-protein phosphatase PP1 catalytic subunit	EC:3.1.3.16	14	ko03015 mRNA surveillance pathway;ko04113 Meiosis - yeast;ko04114 Oocyte meiosis;ko04270 Vascular smooth muscle contraction;ko04390 Hippo signaling pathway;ko04510 Focal adhesion;ko04720 Long-term potentiation;ko04728 Dopaminergic synapse;ko04810 Regulation of actin cytoskeleton;ko04910 Insulin signaling pathway;ko05031 Amphetamine addiction;ko05034 Alcoholism;ko05168 Herpes simplex infection;ko05205 Proteoglycans in
gene28330	K03015	RPB7, POLR2G	DNA-directed RNA polymerase II subunit RPB7	--	5	ko00230 Purine metabolism;ko00240 Pyrimidine metabolism;ko03020 RNA polymerase;ko05016 Huntington's disease;ko05169 Epstein-Barr virus infection;
gene28331	K01188	E3.2.1.21	beta-glucosidase	EC:3.2.1.21	3	ko00460 Cyanoamino acid metabolism;ko00500 Starch and sucrose metabolism;ko00940 Phenylpropanoid biosynthesis;
gene28340	K14487	GH3	auxin responsive GH3 gene family	--	1	ko04075 Plant hormone signal transduction;
gene28343	K08145	SLC2A8, GLUT8	MFS transporter, SP family, solute carrier family 2 (facilitated glucose transporter), member 8	--		
gene28345	K09422	MYBP	myb proto-oncogene protein, plant	--		
gene28347	K14236	tRNA-Tyr	tRNA Tyr	--	1	ko00970 Aminoacyl-tRNA biosynthesis;
gene28348	K02895	RP-L24, MRPL24, rplX	large subunit ribosomal protein L24	--	1	ko03010 Ribosome;
gene28355	K00327	E1.6.2.4	NADPH-ferrihemoprotein reductase	EC:1.6.2.4		
gene28361	K07253	MIF	phenylpyruvate tautomerase	EC:5.3.2.1	2	ko00350 Tyrosine metabolism;ko00360 Phenylalanine metabolism;
gene28369	K14638	SLC15A3_4, PHT	solute carrier family 15 (peptide/histidine transporter), member 3/4	--		
gene28374	K01853	E5.4.99.8	cycloartenol synthase	EC:5.4.99.8	1	ko00100 Steroid biosynthesis;
gene28385	K01183	E3.2.1.14	chitinase	EC:3.2.1.14	1	ko00520 Amino sugar and nucleotide sugar metabolism;
gene28386	K14496	PYL	abscisic acid receptor PYR/PYL family	--	1	ko04075 Plant hormone signal transduction;
gene28394	K00430	E1.11.1.7	peroxidase	EC:1.11.1.7	2	ko00360 Phenylalanine metabolism;ko00940 Phenylpropanoid biosynthesis;

gene28396	K16365	SGTA	small glutamine-rich tetratricopeptide repeat-containing protein alpha	--		
gene28398	K10144	RCHY1, PIRH2	RING finger and CHY zinc finger domain-containing protein 1	EC:6.3.2.19	3	ko04115 p53 signaling pathway;ko04120 Ubiquitin mediated proteolysis;ko05162 Measles;
gene2839	K12153	CYP79A2	cytochrome P450, family 79, subfamily A, polypeptide 2 (phenylalanine N-monooxygenase)	EC:1.14.13.124	3	ko00460 Cyanoamino acid metabolism;ko00966 Glucosinolate biosynthesis;ko01210 2-Oxocarboxylic acid metabolism;
gene28405	K03940	NDUFS7	NADH dehydrogenase (ubiquinone) Fe-S protein 7	EC:1.6.5.3 1.6.99.3	5	ko00190 Oxidative phosphorylation;ko04932 Non-alcoholic fatty liver disease (NAFLD);ko05010 Alzheimer's disease;ko05012 Parkinson's disease;ko05016 Huntington's disease;
gene28406	K04508	TBL1	transducin (beta)-like 1	--	1	ko04310 Wnt signaling pathway;
gene28409	K17302	COPB2, SEC27	coatomer, subunit beta'	--		
gene2840	K12153	CYP79A2	cytochrome P450, family 79, subfamily A, polypeptide 2 (phenylalanine N-monooxygenase)	EC:1.14.13.124	3	ko00460 Cyanoamino acid metabolism;ko00966 Glucosinolate biosynthesis;ko01210 2-Oxocarboxylic acid metabolism;
gene28412	K04730	IRAK1	interleukin-1 receptor-associated kinase 1	EC:2.7.11.1	11	ko04064 NF-kappa B signaling pathway;ko04210 Apoptosis;ko04620 Toll-like receptor signaling pathway;ko04722 Neurotrophin signaling pathway;ko05133 Pertussis;ko05140 Leishmaniasis;ko05142 Chagas disease (American trypanosomiasis);ko05145 Toxoplasmosis;ko05152 Tuberculosis;ko05162 Measles;ko05169 Epstein-Barr virus infection;
gene28413	K15601	KDM3	lysine-specific demethylase 3	EC:1.14.11.-		
gene28418	K03768	PPIB, ppiB	peptidyl-prolyl cis-trans isomerase B (cyclophilin B)	EC:5.2.1.8		
gene28423	K17087	TM9SF3	transmembrane 9 superfamily member 3	--		
gene28425	K09338	HD-ZIP	homeobox-leucine zipper protein	--		
gene28428	K04733	IRAK4	interleukin-1 receptor-associated kinase 4	EC:2.7.11.1	11	ko04064 NF-kappa B signaling pathway;ko04210 Apoptosis;ko04620 Toll-like receptor signaling pathway;ko04722 Neurotrophin signaling pathway;ko05133 Pertussis;ko05140 Leishmaniasis;ko05142 Chagas disease (American trypanosomiasis);ko05145 Toxoplasmosis;ko05152 Tuberculosis;ko05162 Measles;ko05164 Influenza A;
gene28431	K12846	SNRNP27	U4/U6.U5 tri-snRNP-associated protein 3	--	1	ko03040 Spliceosome;
gene28435	K05909	E1.10.3.2				
gene28436	K05909	E1.10.3.2				
gene28439	K12855	PRPF6, PRP6	pre-mRNA-processing factor 6	--	1	ko03040 Spliceosome;
gene28444	K07897	RAB7A	Ras-related protein Rab-7A	--	5	ko04144 Endocytosis;ko04145 Phagosome;ko05132 Salmonella infection;ko05146 Amoebiasis;ko05152 Tuberculosis;
gene28445	K13495	CISZOG	cis-zeatin O-glucosyltransferase	EC:2.4.1.215	1	ko00908 Zeatin biosynthesis;
gene28447	K13495	CISZOG	cis-zeatin O-glucosyltransferase	EC:2.4.1.215	1	ko00908 Zeatin biosynthesis;
gene2844	K00645	fabD	[acyl-carrier-protein] S-malonyltransferase	EC:2.3.1.39	1	ko00061 Fatty acid biosynthesis;
gene28452	K05283	PIGW	phosphatidylinositol glycan, class W	EC:2.3.-.-	1	ko00563 Glycosylphosphatidylinositol(GPI)-anchor biosynthesis;
gene28453	K17081	PHB2	prohibitin 2	--		
gene28459	K13993	HSP20	HSP20 family protein	--	1	ko04141 Protein processing in endoplasmic reticulum;
gene28461	K02886	RP-L2, MRPL2, rplB	large subunit ribosomal protein L2	--	1	ko03010 Ribosome;
gene28463	K01051	E3.1.1.11	pectinesterase	EC:3.1.1.11	2	ko00040 Pentose and glucuronate interconversions;ko00500 Starch and sucrose metabolism;
gene28464	K08486	STX1B_2_3	syntaxin 1B/2/3	--	2	ko04130 SNARE interactions in vesicular transport;ko04721 Synaptic vesicle cycle;
gene28468	K00975	glgC	glucose-1-phosphate adenylyltransferase	EC:2.7.7.27	2	ko00500 Starch and sucrose metabolism;ko00520 Amino sugar and nucleotide sugar metabolism;
gene28469	K10579	UBE2M, UBC12	ubiquitin-conjugating enzyme E2 M	EC:6.3.2.19	1	ko04120 Ubiquitin mediated proteolysis;
gene2846	K15077	ELA1	elongin-A	--		
gene28472	K14508	NPR1	regulatory protein NPR1	--	1	ko04075 Plant hormone signal transduction;
gene28476	K01939	E6.3.4.4, purA	adenylosuccinate synthase	EC:6.3.4.4	2	ko00230 Purine metabolism;ko00250 Alanine, aspartate and glutamate metabolism;
gene28479	K01166	E3.1.27.1	ribonuclease T2	EC:3.1.27.1		
gene28481	K14220	tRNA-Asn	tRNA Asn	--	1	ko00970 Aminoacyl-tRNA biosynthesis;
gene28483	K11294	NCL, NSR1	nucleolin	--	1	ko05130 Pathogenic Escherichia coli infection;
gene28486	K09873	TIP	aquaporin TIP	--		
gene2848	K00940	E2.7.4.6, ndk	nucleoside-diphosphate kinase	EC:2.7.4.6	2	ko00230 Purine metabolism;ko00240 Pyrimidine metabolism;

gene28495	K01627	kdsA	2-dehydro-3-deoxyphosphooctonate aldolase (KDO 8-P synthase)	EC:2.5.1.55	1	ko00540 Lipopolysaccharide biosynthesis;
gene28496	K00430	E1.11.1.7	peroxidase	EC:1.11.1.7	2	ko00360 Phenylalanine metabolism;ko00940 Phenylpropanoid biosynthesis;
gene28497	K03350	APC3, CDC27	anaphase-promoting complex subunit 3	--	7	ko04110 Cell cycle;ko04111 Cell cycle - yeast;ko04113 Meiosis - yeast;ko04114 Oocyte meiosis;ko04120 Ubiquitin mediated proteolysis;ko04914 Progesterone-mediated oocyte maturation;ko05166 HTLV-I infection;
gene28499	K13137	STRAP, UNRIP	serine-threonine kinase receptor-associated protein	--	1	ko03013 RNA transport;
gene28500	K13137	STRAP, UNRIP	serine-threonine kinase receptor-associated protein	--	1	ko03013 RNA transport;
gene28504	K04733	IRAK4	interleukin-1 receptor-associated kinase 4	EC:2.7.11.1	11	ko04064 NF-kappa B signaling pathway;ko04210 Apoptosis;ko04620 Toll-like receptor signaling pathway;ko04722 Neurotrophin signaling pathway;ko05133 Pertussis;ko05140 Leishmaniasis;ko05142 Chagas disease (American trypanosomiasis);ko05145 Toxoplasmosis;ko05152 Tuberculosis;ko05162 Measles;ko05164 Influenza A;
gene28506	K01728	E4.2.2.2, pel	pectate lyase	EC:4.2.2.2	1	ko00040 Pentose and glucuronate interconversions;
gene28511	K11647	SMARCA2_4	SWI/SNF-related matrix-associated actin-dependent regulator of chromatin subfamily A member 2/4	EC:3.6.4.-		
gene28512	K13429	CERK1	chitin elicitor receptor kinase 1	--	1	ko04626 Plant-pathogen interaction;
gene28513	K13412	CPK	calcium-dependent protein kinase	EC:2.7.11.1	2	ko04626 Plant-pathogen interaction;ko05145 Toxoplasmosis;
gene28514	K01213	E3.2.1.67	galacturan 1,4-alpha-galacturonidase	EC:3.2.1.67	2	ko00040 Pentose and glucuronate interconversions;ko00500 Starch and sucrose metabolism;
gene28515	K15255	PIF1	ATP-dependent DNA helicase PIF1	EC:3.6.4.12		
gene28522	K00430	E1.11.1.7	peroxidase	EC:1.11.1.7	2	ko00360 Phenylalanine metabolism;ko00940 Phenylpropanoid biosynthesis;
gene28523	K00655	plsC	1-acyl-sn-glycerol-3-phosphate acyltransferase	EC:2.3.1.51	2	ko00561 Glycerolipid metabolism;ko00564 Glycerophospholipid metabolism;
gene28524	K05391	CNGF	cyclic nucleotide gated channel, other eukaryote	--	1	ko04626 Plant-pathogen interaction;
gene28525	K09422	MYBP	myb proto-oncogene protein, plant	--		
gene28526	K09422	MYBP	myb proto-oncogene protein, plant	--		
gene28528	K03231	EEF1A	elongation factor 1-alpha	--	2	ko03013 RNA transport;ko05134 Legionellosis;
gene28535	K03188	ureF	urease accessory protein	--		
gene28537	K03294	TC.APA	basic amino acid/polyamine antiporter, APA family	--		
gene28538	K07195	EXOC7, EXO70	exocyst complex component 7	--	1	ko04910 Insulin signaling pathway;
gene28542	K04733	IRAK4	interleukin-1 receptor-associated kinase 4	EC:2.7.11.1	11	ko04064 NF-kappa B signaling pathway;ko04210 Apoptosis;ko04620 Toll-like receptor signaling pathway;ko04722 Neurotrophin signaling pathway;ko05133 Pertussis;ko05140 Leishmaniasis;ko05142 Chagas disease (American trypanosomiasis);ko05145 Toxoplasmosis;ko05152 Tuberculosis;ko05162 Measles;ko05164 Influenza A;
gene28543	K09872	PIP	aquaporin PIP	--		
gene28548	K15631	ABA3	molybdenum cofactor sulfurtransferase	EC:2.8.1.9		
gene28550	K03031	PSMD8, RPN12	26S proteasome regulatory subunit N12	--	2	ko03050 Proteasome;ko05169 Epstein-Barr virus infection;
gene28552	K09833	HPT, TPT	homogenitisate phytyltransferase	--	1	ko00130 Ubiquinone and other terpenoid-quinone biosynthesis;
gene28553	K01982	LSUrRNA	large subunit ribosomal RNA	--	2	ko03008 Ribosome biogenesis in eukaryotes;ko03010 Ribosome;

gene28555	K04392	RAC1	Ras-related C3 botulinum toxin substrate 1	--	33	ko04010 MAPK signaling pathway;ko04014 Ras signaling pathway;ko04062 Chemokine signaling pathway;ko04145 Phagosome;ko04151 PI3K-Akt signaling pathway;ko04310 Wnt signaling pathway;ko04360 Axon guidance;ko04370 VEGF signaling pathway;ko04380 Osteoclast differentiation;ko04510 Focal adhesion;ko04520 Adherens junction;ko04620 Toll-like receptor signaling pathway;ko04650 Natural killer cell mediated cytotoxicity;ko04662 B cell receptor signaling pathway;ko04664 Fc epsilon RI signaling pathway;ko04666 Fc gamma R-mediated phagocytosis;ko04670 Leukocyte transendothelial migration;ko04722 Neurotrophin signaling pathway;ko04810 Regulation of actin cytoskeleton;ko04932 Non-alcoholic fatty liver disease (NAFLD);ko04972 Pancreatic secretion;ko05014 Amyotrophic lateral sclerosis (ALS);ko05100 Bacterial invasion of epithelial cells;ko05120 Epithelial cell signaling in Helicobacter pylori infection;ko05131 Shigellosis;ko05132 Salmonella infection;ko05200 Pathways in cancer;ko05203 Viral carcinogenesis;ko05205 Proteoglycans in cancer;ko05210 Colorectal cancer;ko05211 Renal cell carcinoma;ko05212 Pancreatic
gene28556	K01061	E3.1.1.45	carboxymethylenebutenolidase	EC:3.1.1.45	3	ko00361 Chlorocyclohexane and chlorobenzene degradation;ko00364 Fluorobenzoate degradation;ko00623 Toluene degradation;
gene28561	K01762	ACS	1-aminocyclopropane-1-carboxylate synthase	EC:4.4.1.14	1	ko00270 Cysteine and methionine metabolism;
gene28562	K16055	TPS	trehalose 6-phosphate synthase/phosphatase	EC:2.4.1.15 3.1.3.12	1	ko00500 Starch and sucrose metabolism;
gene28565	K03798	ftsH, hflB	cell division protease FtsH	EC:3.4.24.-		
gene28567	K13348	MPV17	protein Mpv17	--	1	ko04146 Peroxisome;
gene2856	K15813	LUP4	beta-amyrin synthase	EC:5.4.99.39	1	ko00909 Sesquiterpenoid and triterpenoid biosynthesis;
gene28575	K00434	E1.11.1.11	L-ascorbate peroxidase	EC:1.11.1.11	2	ko00053 Ascorbate and aldarate metabolism;ko00480 Glutathione metabolism;
gene28579	K08235	E2.4.1.207	xyloglucan:xyloglucosyl transferase	EC:2.4.1.207		
gene28580	K12486	SMAP	stromal membrane-associated protein	--	1	ko04144 Endocytosis;
gene28581	K15747	LUT5, CYP97A3	cytochrome P450, family 97, subfamily A (beta-ring hydroxylase)	EC:1.14.-.-	1	ko00906 Carotenoid biosynthesis;
gene28584	K15191	LARP7	La-related protein 7	--		
gene28587	K15202	GTF3C5, TFC1	general transcription factor 3C polypeptide 5 (transcription factor C subunit 1)	--		
gene28588	K10355	ACTF	actin, other eukaryote	--		
gene28599	K03679	RRP4, EXOSC2	exosome complex component RRP4	--	1	ko03018 RNA degradation;
gene285	K14496	PYL	abscisic acid receptor PYR/PYL family	--	1	ko04075 Plant hormone signal transduction;
gene28604	K02358	tuf, TUFM	elongation factor Tu	--	1	ko04626 Plant-pathogen interaction;
gene28606	K10523	SPOP	speckle-type POZ protein	--		
gene28607	K12621	LSM2	U6 snRNA-associated Sm-like protein LSm2	--	2	ko03018 RNA degradation;ko03040 Spliceosome;
gene28614	K10666	RNF5	E3 ubiquitin-protein ligase RNF5	EC:6.3.2.19	1	ko04141 Protein processing in endoplasmic reticulum;
gene2861	K10268	FBXL2_20	F-box and leucine-rich repeat protein 2/20	--		
gene28621	K15401	CYP86A1	cytochrome P450, family 86, subfamily A, polypeptide 1 (fatty acid omega-hydroxylase)	EC:1.14.-.-	1	ko00073 Cutin, suberine and wax biosynthesis;
gene28624	K08193	SLC17A	MFS transporter, ACS family, solute carrier family 17 (sodium-dependent inorganic phosphate cotransporter), other	--		
gene28630	K04487	iscS, NFS1	cysteine desulfurase	EC:2.8.1.7	2	ko00730 Thiamine metabolism;ko04122 Sulfur relay system;
gene28634	K02266	COX6A	cytochrome c oxidase subunit 6a	--	6	ko00190 Oxidative phosphorylation;ko04260 Cardiac muscle contraction;ko04932 Non-alcoholic fatty liver disease (NAFLD);ko05010 Alzheimer's disease;ko05012 Parkinson's disease;ko05016 Huntington's disease;
gene28636	K07374	TUBA	tubulin alpha	--	3	ko04145 Phagosome;ko04540 Gap junction;ko05130 Pathogenic Escherichia coli infection;
gene28643	K05953	E2.5.1.43	nicotianamine synthase	EC:2.5.1.43		
gene28645	K04712	DEGS	sphingolipid delta-4 desaturase	EC:1.14.-.-	1	ko00600 Sphingolipid metabolism;
gene28646	K05577	ndhF	NAD(P)H-quinone oxidoreductase subunit 5	EC:1.6.5.3	1	ko00190 Oxidative phosphorylation;
gene28649	K00232	E1.3.3.6, ACOX1, ACOX3	acyl-CoA oxidase	EC:1.3.3.6	5	ko00071 Fatty acid degradation;ko00592 alpha-Linolenic acid metabolism;ko01040 Biosynthesis of unsaturated fatty acids;ko03320 PPAR signaling pathway;ko04146 Peroxisome;

gene28656	K10871	RAD51L3, RAD51D	RAD51-like protein 3	--	1	ko03440 Homologous recombination;
gene28658	K15263	LYER	cell growth-regulating nucleolar protein	--		
gene28661	K11665	INO80, INOC1	DNA helicase INO80	EC:3.6.4.12		
gene28665	K02324	POLE1	DNA polymerase epsilon subunit 1	EC:2.7.7.7	6	ko00230 Purine metabolism;ko00240 Pyrimidine metabolism;ko03030 DNA replication;ko03410 Base excision repair;ko03420 Nucleotide excision repair;ko05166 HTLV-I infection;
gene28672	K14498	SNRK2	serine/threonine-protein kinase SRK2	EC:2.7.11.1	1	ko04075 Plant hormone signal transduction;
gene28681	K03635	MOCS2, moaE	molybdopterin synthase catalytic subunit	EC:2.-.-.-	2	ko00790 Folate biosynthesis;ko04122 Sulfur relay system;
gene28684	K03754	EIF2B2	translation initiation factor eIF-2B subunit beta	--	1	ko03013 RNA transport;
gene28694	K03106	SRP54, ffh	signal recognition particle subunit SRP54	--	2	ko03060 Protein export;ko03070 Bacterial secretion system;
gene28698	K14411	MSI	RNA-binding protein Musashi	--	1	ko03015 mRNA surveillance pathway;
gene286	K05909	E1.10.3.2				
gene28710	K11346	ING4	inhibitor of growth protein 4	--		
gene28712	K11128	GAR1, NOLA1	H/ACA ribonucleoprotein complex subunit 1	--	1	ko03008 Ribosome biogenesis in eukaryotes;
gene28713	K00261	GLUD1_2, gdhA	glutamate dehydrogenase (NAD(P)+)	EC:1.4.1.3	5	ko00250 Alanine, aspartate and glutamate metabolism;ko00330 Arginine and proline metabolism;ko00471 D-Glutamine and D-glutamate metabolism;ko00910 Nitrogen metabolism;ko04964 Proximal tubule bicarbonate reclamation;
gene28722	K01610	E4.1.1.49, pckA	phosphoenolpyruvate carboxykinase (ATP)	EC:4.1.1.49	4	ko00010 Glycolysis / Gluconeogenesis;ko00020 Citrate cycle (TCA cycle);ko00620 Pyruvate metabolism;ko00710 Carbon fixation in photosynthetic organisms;
gene28723	K04120	E5.5.1.13	ent-copalyl diphosphate synthase	EC:5.5.1.13	1	ko00904 Diterpenoid biosynthesis;
gene28725	K12309	GLB1, ELNR1	beta-galactosidase	EC:3.2.1.23	6	ko00052 Galactose metabolism;ko00511 Other glycan degradation;ko00531 Glycosaminoglycan degradation;ko00600 Sphingolipid metabolism;ko00604 Glycosphingolipid biosynthesis - ganglio series;ko04142 Lysosome;
gene28727	K17086	TM9SF2_4	transmembrane 9 superfamily member 2/4	--		
gene28728	K10752	RBBP4, HAT2, CAF1, MIS16	histone-binding protein RBBP4	--		
gene28730	K01427	URE	urease	EC:3.5.1.5	2	ko00230 Purine metabolism;ko00330 Arginine and proline metabolism;
gene28737	K15601	KDM3	lysine-specific demethylase 3	EC:1.14.11.-		
gene28738	K14638	SLC15A3_4, PHT	solute carrier family 15 (peptide/histidine transporter), member 3/4	--		
gene28739	K02739	PSMB7	20S proteasome subunit beta 2	EC:3.4.25.1	1	ko03050 Proteasome;
gene2873	K05863	SLC25A4S, ANT	solute carrier family 25 (mitochondrial adenine nucleotide translocator), member 4/5/6/31	--	4	ko04020 Calcium signaling pathway;ko05012 Parkinson's disease;ko05016 Huntington's disease;ko05166 HTLV-I infection;
gene28740	K11654	SMARCA5, SNF2H, ISWI	SWI/SNF-related matrix-associated actin-dependent regulator of chromatin subfamily A member 5	EC:3.6.4.-		
gene28741	K00915	IPK2	1D-myo-Inositol-tetrakisphosphate 5-kinase / inositol-polyphosphate multikinase	EC:2.7.1.140 2.7.1.151	2	ko00562 Inositol phosphate metabolism;ko04070 Phosphatidylinositol signaling system;
gene28742	K00729	ALG5	dolichyl-phosphate beta-glucosyltransferase	EC:2.4.1.117	1	ko00510 N-Glycan biosynthesis;
gene28747	K04728	ATM, TEL1	ataxia telangiectasia mutated family protein	EC:2.7.11.1	7	ko04064 NF-kappa B signaling pathway;ko04110 Cell cycle;ko04115 p53 signaling pathway;ko04210 Apoptosis;ko05166 HTLV-I infection;ko05202 Transcriptional misregulation in cancer;ko05206 MicroRNAs in cancer;
gene28764	K07195	EXOC7, EXO70	exocyst complex component 7	--	1	ko04910 Insulin signaling pathway;
gene28770	K16281	RHA1	RING-H2 zinc finger protein RHA1	--		
gene28771	K08658	RCE1, FACE2	prenyl protein peptidase	EC:3.4.22.-	1	ko00900 Terpenoid backbone biosynthesis;
gene28773	K15287	SLC35F1_2	solute carrier family 35, member F1/2	--		
gene28778	K04459	DUSP, MKP	dual specificity phosphatase	EC:3.1.3.16 3.1.3.48	1	ko04010 MAPK signaling pathway;
gene28779	K14495	GID2, SLY1	F-box protein GID2	--	1	ko04075 Plant hormone signal transduction;
gene2877	K02155	ATPeVPL, ATP6L	V-type H+-transporting ATPase 16kDa proteolipid subunit	EC:3.6.3.14	9	ko00190 Oxidative phosphorylation;ko04142 Lysosome;ko04145 Phagosome;ko04721 Synaptic vesicle cycle;ko04966 Collecting duct acid secretion;ko05110 Vibrio cholerae infection;ko05120 Epithelial cell signaling in Helicobacter pylori infection;ko05152 Tuberculosis;ko05323 Rheumatoid arthritis;
gene28783	K04124	E1.14.11.15	gibberellin 3-beta-dioxygenase	EC:1.14.11.15	1	ko00904 Diterpenoid biosynthesis;

gene2878	K11426	SMYD	SET and MYND domain-containing protein	--		
gene28796	K01103	PFKFB	6-phosphofructo-2-kinase / fructose-2,6-bisphosphatase	EC:2.7.1.105 3.1.3.46	2	ko00051 Fructose and mannose metabolism;ko04066 HIF-1 signaling pathway;
gene28798	K05389	KCNKF	potassium channel subfamily K, other eukaryote	--		
gene28799	K09832	CYP710A	cytochrome P450, family 710, subfamily A	--	1	ko00100 Steroid biosynthesis;
gene28800	K02955	RP-S14e, RPS14	small subunit ribosomal protein S14e	--	1	ko03010 Ribosome;
gene28801	K06700	PSMF1	proteasome inhibitor subunit 1 (PI31)	--	1	ko03050 Proteasome;
gene28805	K14686	SLC31A1, CTR1	solute carrier family 31 (copper transporter), member 1	--	1	ko04978 Mineral absorption;
gene2880	K02877	RP-L15e, RPL15	large subunit ribosomal protein L15e	--	1	ko03010 Ribosome;
gene28812	K01507	ppa	inorganic pyrophosphatase	EC:3.6.1.1	1	ko00190 Oxidative phosphorylation;
gene28813	K13116	DDX41, ABS	ATP-dependent RNA helicase DDX41	EC:3.6.4.13		
gene28824	K11267	PDS5	sister chromatid cohesion protein PDS5	--		
gene28826	K04565	SOD1	superoxide dismutase, Cu-Zn family	EC:1.15.1.1	4	ko04146 Peroxisome;ko05014 Amyotrophic lateral sclerosis (ALS);ko05016 Huntington's disease;ko05020 Prion diseases;
gene28827	K03259	EIF4E	translation initiation factor 4E	--	5	ko03013 RNA transport;ko04066 HIF-1 signaling pathway;ko04150 mTOR signaling pathway;ko04151 PI3K-Akt signaling pathway;ko04910 Insulin signaling pathway;
gene28828	K13448	CML	calcium-binding protein CML	--	1	ko04626 Plant-pathogen interaction;
gene28829	K00797	speE, SRM	spermidine synthase	EC:2.5.1.16	4	ko00270 Cysteine and methionine metabolism;ko00330 Arginine and proline metabolism;ko00410 beta-Alanine metabolism;ko00480 Glutathione metabolism;
gene2882	K11253	H3	histone H3	--	3	ko05034 Alcoholism;ko05202 Transcriptional misregulation in cancer;ko05322 Systemic lupus erythematosus;
gene28831	K12822	RBM25, S164	RNA-binding protein 25	--	1	ko03040 Spliceosome;
gene28832	K15404	K15404, CER1	aldehyde decarbonylase	EC:4.1.99.5	1	ko00073 Cutin, suberine and wax biosynthesis;
gene28833	K11000	CALS	callose synthase	EC:2.4.1.-		
gene28834	K03152	thiJ	4-methyl-5(b-hydroxyethyl)-thiazole monophosphate biosynthesis	--		
gene28845	K02957	RP-S15Ae, RPS15A	small subunit ribosomal protein S15Ae	--	1	ko03010 Ribosome;
gene28846	K00948	PRPS, prsA	ribose-phosphate pyrophosphokinase	EC:2.7.6.1	4	ko00030 Pentose phosphate pathway;ko00230 Purine metabolism;ko01200 Carbon metabolism;ko01230 Biosynthesis of amino acids;
gene2884	K04730	IRAK1	interleukin-1 receptor-associated kinase 1	EC:2.7.11.1	11	ko04064 NF-kappa B signaling pathway;ko04210 Apoptosis;ko04620 Toll-like receptor signaling pathway;ko04722 Neurotrophin signaling pathway;ko05133 Pertussis;ko05140 Leishmaniasis;ko05142 Chagas disease (American trypanosomiasis);ko05145 Toxoplasmosis;ko05152 Tuberculosis;ko05162 Measles;ko05169 Epstein-Barr virus infection;
gene28850	K02868	RP-L11e, RPL11	large subunit ribosomal protein L11e	--	1	ko03010 Ribosome;
gene28852	K15687	MKRN	E3 ubiquitin-protein ligase makorin	EC:6.3.2.19		
gene28854	K12867	SYF1, XAB2	pre-mRNA-splicing factor SYF1	--	1	ko03040 Spliceosome;
gene2885	K13420	FLS2	LRR receptor-like serine/threonine-protein kinase FLS2	EC:2.7.11.1	1	ko04626 Plant-pathogen interaction;
gene28860	K04506	SIAH1	E3 ubiquitin-protein ligase SIAH1	EC:6.3.2.19	3	ko04115 p53 signaling pathway;ko04120 Ubiquitin mediated proteolysis;ko04310 Wnt signaling pathway;
gene28861	K03544	clpX, CLPX	ATP-dependent Clp protease ATP-binding subunit ClpX	--	1	ko04112 Cell cycle - Caulobacter;
gene28862	K15601	KDM3	lysine-specific demethylase 3	EC:1.14.11.-		
gene28864	K01689	ENO, eno	enolase	EC:4.2.1.11	6	ko00010 Glycolysis / Gluconeogenesis;ko00680 Methane metabolism;ko01200 Carbon metabolism;ko01230 Biosynthesis of amino acids;ko03018 RNA degradation;ko04066 HIF-1 signaling pathway;
gene28871	K14326	UPF1, RENT1	regulator of nonsense transcripts 1	EC:3.6.4.-	2	ko03013 RNA transport;ko03015 mRNA surveillance pathway;
gene28879	K02692	psaD	photosystem I subunit II	--	1	ko00195 Photosynthesis;
gene28883	K02981	RP-S2e, RPS2	small subunit ribosomal protein S2e	--	1	ko03010 Ribosome;
gene28885	K02981	RP-S2e, RPS2	small subunit ribosomal protein S2e	--	1	ko03010 Ribosome;
gene28892	K12873	BUD31, G10	bud site selection protein 31	--	1	ko03040 Spliceosome;
gene28893	K10865	MRE11	double-strand break repair protein MRE11	--	2	ko03440 Homologous recombination;ko03450 Non-homologous end-joining;
gene28895	K00083	E1.1.1.195	cinnamyl-alcohol dehydrogenase	EC:1.1.1.195	1	ko00940 Phenylpropanoid biosynthesis;
gene28897	K08241	E2.1.1.141	jasmonate O-methyltransferase	EC:2.1.1.141	1	ko00592 alpha-Linolenic acid metabolism;

gene28902	K11654	SMARCA5, SNF2H, ISWI	SWI/SNF-related matrix-associated actin-dependent regulator of chromatin subfamily A member 5	EC:3.6.4.-		
gene28903	K13430	PBS1	serine/threonine-protein kinase PBS1	EC:2.7.11.1	1	ko04626 Plant-pathogen interaction;
gene28905	K10706	SETX, ALS4	senataxin	EC:3.6.4.-		
gene28906	K11446	JARID1	histone demethylase JARID1	EC:1.14.11.-		
gene28910	K06892	K06892				
gene28911	K01870	IARS, ileS	isoleucyl-tRNA synthetase	EC:6.1.1.5	1	ko00970 Aminoacyl-tRNA biosynthesis;
gene28915	K08245	E3.4.23.40	phytepsin	EC:3.4.23.40		
gene28919	K07304	msrA	peptide-methionine (S)-S-oxide reductase	EC:1.8.4.11		
gene28921	K00227	SC5DL, ERG3	lathosterol oxidase	EC:1.14.21.6	1	ko00100 Steroid biosynthesis;
gene28922	K16675	ZDHHC14	probable palmitoyltransferase ZDHHC14	EC:2.3.1.-	1	ko04391 Hippo signaling pathway - fly;
gene28924	K15121	SLC25A44	solute carrier family 25, member 44	--		
gene28929	K16298	SCPL-IV	serine carboxypeptidase-like clade IV	EC:3.4.16.-		
gene28933	K06443	lcyB, crtL1, crtY	lycopene beta-cyclase	EC:5.5.1.19	1	ko00906 Carotenoid biosynthesis;
gene28938	K10949	KDELR	ER lumen protein retaining receptor	--	1	ko05110 Vibrio cholerae infection;
gene28943	K14404	CPSF4, YTH1	cleavage and polyadenylation specificity factor subunit 4	--	2	ko03015 mRNA surveillance pathway;ko05164 Influenza A;
gene2894	K03253	EIF3B	translation initiation factor 3 subunit B	--	1	ko03013 RNA transport;
gene28951	K09286	EREBP	EREBP-like factor	--		
gene28953	K01069	E3.1.2.6, gloB	hydroxyacylglutathione hydrolase	EC:3.1.2.6	1	ko00620 Pyruvate metabolism;
gene2895	K10742	DNA2	DNA replication ATP-dependent helicase Dna2	EC:3.6.4.12	1	ko03030 DNA replication;
gene28964	K09872	PIP	aquaporin PIP	--		
gene28965	K14503	BZR1_2	brassinosteroid resistant 1/2	--	1	ko04075 Plant hormone signal transduction;
gene2896	K00434	E1.11.1.11	L-ascorbate peroxidase	EC:1.11.1.11	2	ko00053 Ascorbate and aldarate metabolism;ko00480 Glutathione metabolism;
gene28978	K07252	E3.6.1.43	dolichyldiphosphatase	EC:3.6.1.43	1	ko00510 N-Glycan biosynthesis;
gene2898	K02492	hemA	glutamyl-tRNA reductase	EC:1.2.1.70	1	ko00860 Porphyrin and chlorophyll metabolism;
gene28993	K00655	plsC	1-acyl-sn-glycerol-3-phosphate acyltransferase	EC:2.3.1.51	2	ko00561 Glycerolipid metabolism;ko00564 Glycerophospholipid metabolism;
gene28998	K00434	E1.11.1.11	L-ascorbate peroxidase	EC:1.11.1.11	2	ko00053 Ascorbate and aldarate metabolism;ko00480 Glutathione metabolism;
gene29006	K12373	HEXA_B	hexosaminidase	EC:3.2.1.52	6	ko00511 Other glycan degradation;ko00520 Amino sugar and nucleotide sugar metabolism;ko00531 Glycosaminoglycan degradation;ko00603 Glycosphingolipid biosynthesis - globo series;ko00604 Glycosphingolipid biosynthesis - ganglio series;ko04142 Lysosome;
gene29017	K10610	DDB1	DNA damage-binding protein 1	--	4	ko03420 Nucleotide excision repair;ko04120 Ubiquitin mediated proteolysis;ko05161 Hepatitis B;ko05203 Viral carcinogenesis;
gene2901	K14484	IAA	auxin-responsive protein IAA	--	1	ko04075 Plant hormone signal transduction;
gene29020	K06911	K06911				
gene29021	K00921	PIKFYVE, FAB1	1-phosphatidylinositol-3-phosphate 5-kinase	EC:2.7.1.150	4	ko00562 Inositol phosphate metabolism;ko04070 Phosphatidylinositol signaling system;ko04145 Phagosome;ko04810 Regulation of actin cytoskeleton;
gene29024	K17479	GRXCR1	glutaredoxin domain-containing cysteine-rich protein 1	--		
gene29029	K12823	DDX5, DBP2	ATP-dependent RNA helicase DDX5/DBP2	EC:3.6.4.13	3	ko03040 Spliceosome;ko05202 Transcriptional misregulation in cancer;ko05205 Proteoglycans in cancer;
gene29030	K15283	SLC35E1	solute carrier family 35, member E1	--		
gene29031	K07442	TRM61, GCD14	tRNA (adenine57-N1/adenine58-N1)-methyltransferase	EC:2.1.1.219 2.1.1.220		
gene29032	K14809	DDX55, SPB4	ATP-dependent RNA helicase DDX55/SPB4	EC:3.6.4.13		
gene29033	K01227	E3.2.1.96	mannosyl-glycoprotein endo-beta-N-acetylglucosaminidase	EC:3.2.1.96	1	ko00511 Other glycan degradation;
gene29035	K14488	SAUR	SAUR family protein	--	1	ko04075 Plant hormone signal transduction;
gene29036	K14488	SAUR	SAUR family protein	--	1	ko04075 Plant hormone signal transduction;
gene29038	K14488	SAUR	SAUR family protein	--	1	ko04075 Plant hormone signal transduction;
gene29039	K15429	TRM5, TRMT5	tRNA (guanine37-N1)-methyltransferase	EC:2.1.1.228		
gene2903	K15718	LOX1_5	linoleate 9S-lipoxygenase	EC:1.13.11.58	1	ko00591 Linoleic acid metabolism;
gene29044	K15196	BRF1, GTF3B	transcription factor IIIB 90 kDa subunit	--		
gene29045	K13496	UGT73C	UDP-glucosyl transferase 73C	EC:2.4.1.-		

gene29050	K10691	UBR4, ZUBR1	E3 ubiquitin-protein ligase UBR4	EC:6.3.2.19	1	ko05203 Viral carcinogenesis;
gene29052	K14497	PP2C	protein phosphatase 2C	EC:3.1.3.16	1	ko04075 Plant hormone signal transduction;
gene29054	K03155	TIMELESS	timeless	--		
gene29056	K15190	MEPCE, BCDIN3	7SK snRNA methylphosphate capping enzyme	EC:2.1.1.-		
gene29058	K03243	EIF5B	translation initiation factor 5B	--	1	ko03013 RNA transport;
gene2905	K11000	CALS	callose synthase	EC:2.4.1.-		
gene29061	K01595	ppc	phosphoenolpyruvate carboxylase	EC:4.1.1.31	5	ko00620 Pyruvate metabolism;ko00680 Methane metabolism;ko00710 Carbon fixation in photosynthetic organisms;ko00720 Carbon fixation pathways in prokaryotes;ko01200 Carbon metabolism;
gene29062	K03846	ALG9	alpha-1,2-mannosyltransferase	EC:2.4.1.259 2.4.1.261	2	ko00510 N-Glycan biosynthesis;ko00513 Various types of N-glycan biosynthesis;
gene29063	K05605	HIBCH	3-hydroxyisobutyryl-CoA hydrolase	EC:3.1.2.4	3	ko00280 Valine, leucine and isoleucine degradation;ko00410 beta-Alanine metabolism;ko00640 Propanoate metabolism;
gene29066	K01187	E3.2.1.20, malZ	alpha-glucosidase	EC:3.2.1.20	2	ko00052 Galactose metabolism;ko00500 Starch and sucrose metabolism;
gene29070	K07904	RAB11A	Ras-related protein Rab-11A	--	4	ko04144 Endocytosis;ko04961 Endocrine and other factor-regulated calcium reabsorption;ko04962 Vasopressin-regulated water reabsorption;ko04972 Pancreatic secretion;
gene29071	K07874	RAB1A	Ras-related protein Rab-1A	--	1	ko05134 Legionellosis;
gene29072	K00430	E1.11.1.7	peroxidase	EC:1.11.1.7	2	ko00360 Phenylalanine metabolism;ko00940 Phenylpropanoid biosynthesis;
gene29073	K00434	E1.11.1.11	L-ascorbate peroxidase	EC:1.11.1.11	2	ko00053 Ascorbate and aldarate metabolism;ko00480 Glutathione metabolism;
gene29076	K14232	tRNA-Pro	tRNA Pro	--	1	ko00970 Aminoacyl-tRNA biosynthesis;
gene2907	K12871	CCDC12	coiled-coil domain-containing protein 12	--	1	ko03040 Spliceosome;
gene29085	K03327	TC.MATE, SLC47A, norM, mdtK, dinF	multidrug resistance protein, MATE family	--		
gene29087	K14835	NOP2	ribosomal RNA methyltransferase Nop2	EC:2.1.1.-		
gene29091	K01728	E4.2.2.2, pel	pectate lyase	EC:4.2.2.2	1	ko00040 Pentose and glucuronate interconversions;
gene29092	K09497	CCT5	T-complex protein 1 subunit epsilon	--		
gene29098	K05391	CNGF	cyclic nucleotide gated channel, other eukaryote	--	1	ko04626 Plant-pathogen interaction;
gene29099	K08235	E2.4.1.207	xyloglucan:xyloglucosyl transferase	EC:2.4.1.207		
gene29100	K00142	AASDH	acyl-CoA synthetase	EC:6.2.1.-		
gene29103	K07874	RAB1A	Ras-related protein Rab-1A	--	1	ko05134 Legionellosis;
gene29104	K03002	RPA2, POLR1B	DNA-directed RNA polymerase I subunit RPA2	EC:2.7.7.6	3	ko00230 Purine metabolism;ko00240 Pyrimidine metabolism;ko03020 RNA polymerase;
gene29107	K02915	RP-L34e, RPL34	large subunit ribosomal protein L34e	--	1	ko03010 Ribosome;
gene29111	K12623	LSM4	U6 snRNA-associated Sm-like protein LSm4	--	2	ko03018 RNA degradation;ko03040 Spliceosome;
gene29120	K00013	hisD	histidinol dehydrogenase	EC:1.1.1.23	2	ko00340 Histidine metabolism;ko01230 Biosynthesis of amino acids;
gene29123	K02901	RP-L27e, RPL27	large subunit ribosomal protein L27e	--	1	ko03010 Ribosome;
gene29125	K14315	NDC1, TMEM48	nucleoporin NDC1	--	1	ko03013 RNA transport;
gene29126	K10206	E2.6.1.83	LL-diaminopimelate aminotransferase	EC:2.6.1.83	2	ko00300 Lysine biosynthesis;ko01230 Biosynthesis of amino acids;
gene29127	K14315	NDC1, TMEM48	nucleoporin NDC1	--	1	ko03013 RNA transport;
gene29129	K05929	E2.1.1.103, NMT	phosphoethanolamine N-methyltransferase	EC:2.1.1.103	1	ko00564 Glycerophospholipid metabolism;
gene29130	K07375	TUBB	tubulin beta	--	3	ko04145 Phagosome;ko04540 Gap junction;ko05130 Pathogenic Escherichia coli infection;
gene29132	K09480	E2.4.1.241	digalactosyldiacylglycerol synthase	EC:2.4.1.241	1	ko00561 Glycerolipid metabolism;
gene29134	K02603	ORC1	origin recognition complex subunit 1	--	3	ko04110 Cell cycle;ko04111 Cell cycle - yeast;ko04113 Meiosis - yeast;
gene29137	K08498	STX6	syntaxin 6	--	1	ko04130 SNARE interactions in vesicular transport;
gene29139	K12741	HNRNPA1_3	heterogeneous nuclear ribonucleoprotein A1/A3	--	1	ko03040 Spliceosome;
gene2913	K12188	SNF8, EAP30	ESCRT-II complex subunit VPS22	--	1	ko04144 Endocytosis;
gene29140	K01188	E3.2.1.21	beta-glucosidase	EC:3.2.1.21	3	ko00460 Cyanoamino acid metabolism;ko00500 Starch and sucrose metabolism;ko00940 Phenylpropanoid biosynthesis;
gene29142	K10257	FAD8, desB	omega-3 fatty acid desaturase (delta-15 desaturase)	EC:1.14.19.-	1	ko01040 Biosynthesis of unsaturated fatty acids;
gene29144	K01597	MVD, mvaD	diphosphomevalonate decarboxylase	EC:4.1.1.33	1	ko00900 Terpenoid backbone biosynthesis;
gene29145	K01187	E3.2.1.20, malZ	alpha-glucosidase	EC:3.2.1.20	2	ko00052 Galactose metabolism;ko00500 Starch and sucrose metabolism;
gene29146	K12501	HST	homogentisate solanesyltransferase	--	1	ko00130 Ubiquinone and other terpenoid-quinone biosynthesis;

gene29148	K00995	E2.7.8.5, pgsA, PGS1	CDP-diacylglycerol--glycerol-3-phosphate 3-phosphatidyltransferase	EC:2.7.8.5	1	ko00564 Glycerophospholipid metabolism;
gene29150	K16546	FGFR10P	FGFR1 oncogene partner	--		
gene29151	K02864	RP-L10, MRPL10, rplJ	large subunit ribosomal protein L10	--	1	ko03010 Ribosome;
gene29152	K00799	GST, gst	glutathione S-transferase	EC:2.5.1.18	4	ko00480 Glutathione metabolism;ko00980 Metabolism of xenobiotics by cytochrome P450;ko00982 Drug metabolism - cytochrome P450;ko05204 Chemical carcinogenesis;
gene29156	K17095	ANXA7_11	annexin A7/11	--		
gene29164	K11594	DDX3X, bel	ATP-dependent RNA helicase	EC:3.6.4.13	3	ko04622 RIG-I-like receptor signaling pathway;ko05161 Hepatitis B;ko05203 Viral carcinogenesis;
gene29168	K13237	DECR2	peroxisomal 2,4-dienoyl-CoA reductase	EC:1.3.1.34	1	ko04146 Peroxisome;
gene2916	K03522	fixB, etfA	electron transfer flavoprotein alpha subunit	--		
gene29171	K02259	COX15	cytochrome c oxidase assembly protein subunit 15	--	3	ko00190 Oxidative phosphorylation;ko00860 Porphyrin and chlorophyll metabolism;ko02020 Two-component system;
gene29172	K00162	PDHB, pdhB	pyruvate dehydrogenase E1 component subunit beta	EC:1.2.4.1	6	ko00010 Glycolysis / Gluconeogenesis;ko00020 Citrate cycle (TCA cycle);ko00620 Pyruvate metabolism;ko00650 Butanoate metabolism;ko01200 Carbon metabolism;ko04066 HIF-1 signaling pathway;
gene29173	K00162	PDHB, pdhB	pyruvate dehydrogenase E1 component subunit beta	EC:1.2.4.1	6	ko00010 Glycolysis / Gluconeogenesis;ko00020 Citrate cycle (TCA cycle);ko00620 Pyruvate metabolism;ko00650 Butanoate metabolism;ko01200 Carbon metabolism;ko04066 HIF-1 signaling pathway;
gene29179	K14291	PHAX	phosphorylated adapter RNA export protein	--	1	ko03013 RNA transport;
gene29180	K01369	LGMN	legumain	EC:3.4.22.34	2	ko04142 Lysosome;ko04612 Antigen processing and presentation;
gene29182	K02926	RP-L4, MRPL4, rplD	large subunit ribosomal protein L4	--	1	ko03010 Ribosome;
gene29183	K01369	LGMN	legumain	EC:3.4.22.34	2	ko04142 Lysosome;ko04612 Antigen processing and presentation;
gene29185	K03676	grxC, GLRX, GLRX2	glutaredoxin 3	--		
gene29186	K06640	ATR	serine/threonine-protein kinase ATR	EC:2.7.11.1	4	ko03460 Fanconi anemia pathway;ko04110 Cell cycle;ko04115 p53 signaling pathway;ko05166 HTLV-I infection;
gene29187	K07976	RAB	Rab family, other	--		
gene29189	K15283	SLC35E1	solute carrier family 35, member E1	--		
gene2918	K01595	ppc	phosphoenolpyruvate carboxylase	EC:4.1.1.31	5	ko00620 Pyruvate metabolism;ko00680 Methane metabolism;ko00710 Carbon fixation in photosynthetic organisms;ko00720 Carbon fixation pathways in prokaryotes;ko01200 Carbon metabolism;
gene29190	K14442	DHX36, RHAU	ATP-dependent RNA helicase DHX36	EC:3.6.4.13	1	ko03018 RNA degradation;
gene29192	K01719	hemD, UROS	uroporphyrinogen-III synthase	EC:4.2.1.75	1	ko00860 Porphyrin and chlorophyll metabolism;
gene29195	K00025	MDH1	malate dehydrogenase	EC:1.1.1.37	6	ko00020 Citrate cycle (TCA cycle);ko00620 Pyruvate metabolism;ko00630 Glyoxylate and dicarboxylate metabolism;ko00710 Carbon fixation in photosynthetic organisms;ko01200 Carbon metabolism;ko04964 Proximal tubule bicarbonate reclamation;
gene29196	K11778	DHDDS, RER2, SRT1	ditrans,polycis-polyprenyl diphosphate synthase	EC:2.5.1.87	1	ko00900 Terpenoid backbone biosynthesis;
gene29207	K00297	metF, MTHFR	methylenetetrahydrofolate reductase (NADPH)	EC:1.5.1.20	3	ko00670 One carbon pool by folate;ko00720 Carbon fixation pathways in prokaryotes;ko01200 Carbon metabolism;
gene29210	K11982	RNF115_126	E3 ubiquitin-protein ligase RNF115/126	EC:6.3.2.19		
gene29215	K03695	clpB	ATP-dependent Clp protease ATP-binding subunit ClpB	--		
gene29216	K15177	LEO1	RNA polymerase-associated protein LEO1	--		
gene29217	K08507	USE1	unconventional SNARE in the endoplasmic reticulum protein 1	--	1	ko04130 SNARE interactions in vesicular transport;
gene2921	K04733	IRAK4	interleukin-1 receptor-associated kinase 4	EC:2.7.11.1	11	ko04064 NF-kappa B signaling pathway;ko04210 Apoptosis;ko04620 Toll-like receptor signaling pathway;ko04722 Neurotrophin signaling pathway;ko05133 Pertussis;ko05140 Leishmaniasis;ko05142 Chagas disease (American trypanosomiasis);ko05145 Toxoplasmosis;ko05152 Tuberculosis;ko05162 Measles;ko05164 Influenza A;
gene29225	K10754	RFC1	replication factor C subunit 1	--	3	ko03030 DNA replication;ko03420 Nucleotide excision repair;ko03430 Mismatch repair;
gene2922	K13436	PTI1	pto-interacting protein 1	EC:2.7.11.1	1	ko04626 Plant-pathogen interaction;
gene29231	K11000	CALS	callose synthase	EC:2.4.1.-		
gene29241	K01633	folB	dihydroneopterin aldolase	EC:4.1.2.25	1	ko00790 Folate biosynthesis;
gene29243	K02870	RP-L12e, RPL12	large subunit ribosomal protein L12e	--	1	ko03010 Ribosome;
gene29245	K01183	E3.2.1.14	chitinase	EC:3.2.1.14	1	ko00520 Amino sugar and nucleotide sugar metabolism;

gene29247	K10590	TRIP12	E3 ubiquitin-protein ligase TRIP12	EC:6.3.2.19	1	ko04120 Ubiquitin mediated proteolysis;
gene29248	K00434	E1.11.1.11	L-ascorbate peroxidase	EC:1.11.1.11	2	ko00053 Ascorbate and aldarate metabolism;ko00480 Glutathione metabolism;
gene29249	K09422	MYBP	myb proto-oncogene protein, plant	--		
gene2924	K00789	metK	S-adenosylmethionine synthetase	EC:2.5.1.6	2	ko00270 Cysteine and methionine metabolism;ko01230 Biosynthesis of amino acids;
gene29251	K13691	SGT1	pathogen-inducible salicylic acid glucosyltransferase	EC:2.4.1.-		
gene29253	K13691	SGT1	pathogen-inducible salicylic acid glucosyltransferase	EC:2.4.1.-		
gene29260	K10251	HSD17B12, KAR, IFA38	17beta-estradiol 17-dehydrogenase / very-long-chain 3-oxoacyl-CoA reductase	EC:1.1.1.62 1.1.1.330	3	ko00062 Fatty acid elongation;ko00140 Steroid hormone biosynthesis;ko01040 Biosynthesis of unsaturated fatty acids;
gene29265	K03217	yidC, spoIIIJ, OXA1	preprotein translocase subunit YidC	--	2	ko03060 Protein export;ko03070 Bacterial secretion system;
gene29271	K08518	STXBP5	syntaxin-binding protein 5	--		
gene29273	K15104	SLC25A11, OGC	solute carrier family 25 (mitochondrial oxoglutarate transporter), member 11	--		
gene29274	K14488	SAUR	SAUR family protein	--	1	ko04075 Plant hormone signal transduction;
gene29280	K02146	ATPeVAC39, ATP6D	V-type H+-transporting ATPase subunit AC39	EC:3.6.3.14	10	ko00190 Oxidative phosphorylation;ko04142 Lysosome;ko04145 Phagosome;ko04721 Synaptic vesicle cycle;ko04966 Collecting duct acid secretion;ko05110 Vibrio cholerae infection;ko05120 Epithelial cell signaling in Helicobacter pylori infection;ko05152 Tuberculosis;ko05203 Viral carcinogenesis;ko05323 Rheumatoid arthritis;
gene29282	K00549	metE	5-methyltetrahydropteroyltriglutamate--homocysteine methyltransferase	EC:2.1.1.14	3	ko00270 Cysteine and methionine metabolism;ko00450 Selenocompound metabolism;ko01230 Biosynthesis of amino acids;
gene29285	K14564	NOP56	nucleolar protein 56	--	1	ko03008 Ribosome biogenesis in eukaryotes;
gene29290	K02889	RP-L21e, RPL21	large subunit ribosomal protein L21e	--	1	ko03010 Ribosome;
gene29293	K05666	ABCC2	ATP-binding cassette, subfamily C (CFTR/MRP), member 2	--	2	ko02010 ABC transporters;ko04976 Bile secretion;
gene29294	K15718	LOX1_5	linoleate 9S-lipoxygenase	EC:1.13.11.58	1	ko00591 Linoleic acid metabolism;
gene29295	K11000	CALS	callose synthase	EC:2.4.1.-		
gene2929	K02945	RP-S1, rpsA	small subunit ribosomal protein S1	--	1	ko03010 Ribosome;
gene29300	K01533	E3.6.3.4, ATP7, copA	Cu2+-exporting ATPase	EC:3.6.3.4		
gene29302	K12891	SFRS2	splicing factor, arginine/serine-rich 2	--	2	ko03040 Spliceosome;ko05168 Herpes simplex infection;
gene29304	K09839	VDE, NPQ1	violaxanthin de-epoxidase	EC:1.10.99.3	1	ko00906 Carotenoid biosynthesis;
gene2930	K15285	SLC35E3	solute carrier family 35, member E3	--		
gene29317	K00454	LOX2S	lipoxygenase	EC:1.13.11.12	2	ko00591 Linoleic acid metabolism;ko00592 alpha-Linolenic acid metabolism;
gene29322	K12831	SF3B4, SAP49	splicing factor 3B subunit 4	--	1	ko03040 Spliceosome;
gene29324	K13066	E2.1.1.68, COMT	caffeic acid 3-O-methyltransferase	EC:2.1.1.68	1	ko00940 Phenylpropanoid biosynthesis;
gene29326	K02433	gatA	aspartyl-tRNA(Asn)/glutamyl-tRNA(Gln) amidotransferase subunit A	EC:6.3.5.6 6.3.5.7	1	ko00970 Aminoacyl-tRNA biosynthesis;
gene29327	K04733	IRAK4	interleukin-1 receptor-associated kinase 4	EC:2.7.11.1	11	ko04064 NF-kappa B signaling pathway;ko04210 Apoptosis;ko04620 Toll-like receptor signaling pathway;ko04722 Neurotrophin signaling pathway;ko05133 Pertussis;ko05140 Leishmaniasis;ko05142 Chagas disease (American trypanosomiasis);ko05145 Toxoplasmosis;ko05152 Tuberculosis;ko05162 Measles;ko05164 Influenza A;
gene29332	K07195	EXOC7, EXO70	exocyst complex component 7	--	1	ko04910 Insulin signaling pathway;
gene29333	K02918	RP-L35e, RPL35	large subunit ribosomal protein L35e	--	1	ko03010 Ribosome;
gene29339	K00600	glyA, SHMT	glycine hydroxymethyltransferase	EC:2.1.2.1	7	ko00260 Glycine, serine and threonine metabolism;ko00460 Cyanoamino acid metabolism;ko00630 Glyoxylate and dicarboxylate metabolism;ko00670 One carbon pool by folate;ko00680 Methane metabolism;ko01200 Carbon metabolism;ko01230 Biosynthesis of amino acids;
gene29343	K07765	MBTPS2	S2P endopeptidase	EC:3.4.24.85	1	ko04141 Protein processing in endoplasmic reticulum;
gene29347	K13993	HSP20	HSP20 family protein	--	1	ko04141 Protein processing in endoplasmic reticulum;
gene29352	K11426	SMYD	SET and MYND domain-containing protein	--		
gene29354	K05310	GPI7	ethanolaminephosphotransferase	EC:2.7.-.-	1	ko00563 Glycosylphosphatidylinositol(GPI)-anchor biosynthesis;

gene29356	K15918	GLYK	D-glycerate 3-kinase	EC:2.7.1.31	3	ko00260 Glycine, serine and threonine metabolism;ko00561 Glycerolipid metabolism;ko00630 Glyoxylate and dicarboxylate metabolism;
gene29357	K06627	CCNA	cyclin A	--	5	ko04110 Cell cycle;ko04914 Progesterone-mediated oocyte maturation;ko05161 Hepatitis B;ko05169 Epstein-Barr virus infection;ko05203 Viral carcinogenesis;
gene29358	K07767	E3.6.4.3	microtubule-severing ATPase	EC:3.6.4.3		
gene29359	K10406	KIFC2_3	kinesin family member C2/C3	--		
gene29361	K00276	E1.4.3.21, AOC2, AOC3, tynA	primary-amine oxidase	EC:1.4.3.21	6	ko00260 Glycine, serine and threonine metabolism;ko00350 Tyrosine metabolism;ko00360 Phenylalanine metabolism;ko00410 beta-Alanine metabolism;ko00950 Isoquinoline alkaloid biosynthesis;ko00960 Tropane, piperidine and pyridine alkaloid biosynthesis;
gene29362	K09422	MYBP	myb proto-oncogene protein, plant	--		
gene29370	K09503	DNAJA2	DnaJ homolog subfamily A member 2	--	1	ko04141 Protein processing in endoplasmic reticulum;
gene29375	K08150	SLC2A13, ITR	MFS transporter, SP family, solute carrier family 2 (myo-inositol transporter), member 13	--		
gene29383	K01051	E3.1.1.11	pectinesterase	EC:3.1.1.11	2	ko00040 Pentose and glucuronate interconversions;ko00500 Starch and sucrose metabolism;
gene29391	K11673	ACTR8, ARP8, INO80N	actin-related protein 8	--		
gene29394	K11446	JARID1	histone demethylase JARID1	EC:1.14.11.-		
gene29399	K10580	UBE2N, BLU, UBC13	ubiquitin-conjugating enzyme E2 N	EC:6.3.2.19	1	ko04120 Ubiquitin mediated proteolysis;
gene29400	K15430	TRM11, TRMT11	tRNA (guanine10-N2)-methyltransferase	EC:2.1.1.214		
gene29402	K15634	gpmB	probable phosphoglycerate mutase	EC:5.4.2.12	5	ko00010 Glycolysis / Gluconeogenesis;ko00260 Glycine, serine and threonine metabolism;ko00680 Methane metabolism;ko01200 Carbon metabolism;ko01230 Biosynthesis of amino acids;
gene29404	K15397	KCS	3-ketoacyl-CoA synthase	EC:2.3.1.199	1	ko00062 Fatty acid elongation;
gene29408	K15032	MTERFD	mTERF domain-containing protein, mitochondrial	--		
gene29413	K14638	SLC15A3_4, PHT	solute carrier family 15 (peptide/histidine transporter), member 3/4	--		
gene29417	K02183	CALM	calmodulin	--	25	ko04014 Ras signaling pathway;ko04020 Calcium signaling pathway;ko04070 Phosphatidylinositol signaling system;ko04114 Oocyte meiosis;ko04270 Vascular smooth muscle contraction;ko04626 Plant-pathogen interaction;ko04713 Circadian entrainment;ko04720 Long-term potentiation;ko04722 Neurotrophin signaling pathway;ko04728 Dopaminergic synapse;ko04740 Olfactory transduction;ko04744 Phototransduction;ko04745 Phototransduction - fly;ko04910 Insulin signaling pathway;ko04912 GnRH signaling pathway;ko04915 Estrogen signaling pathway;ko04916 Melanogenesis;ko04970 Salivary secretion;ko04971 Gastric acid secretion;ko05010 Alzheimer's disease;ko05031 Amphetamine addiction;ko05034 Alcoholism;ko05133 Pertussis;ko05152 Tuberculosis;ko05214 Glioma;
gene29423	K13412	CPK	calcium-dependent protein kinase	EC:2.7.11.1	2	ko04626 Plant-pathogen interaction;ko05145 Toxoplasmosis;
gene29424	K01102	PDP	pyruvate dehydrogenase phosphatase	EC:3.1.3.43		
gene29426	K00799	GST, gst	glutathione S-transferase	EC:2.5.1.18	4	ko00480 Glutathione metabolism;ko00980 Metabolism of xenobiotics by cytochrome P450;ko00982 Drug metabolism - cytochrome P450;ko05204 Chemical carcinogenesis;
gene29428	K05666	ABCC2	ATP-binding cassette, subfamily C (CFTR/MRP), member 2	--	2	ko02010 ABC transporters;ko04976 Bile secretion;
gene29429	K02519	infB, MTIF2	translation initiation factor IF-2	--		
gene29430	K02150	ATPeVE, ATP6E	V-type H+-transporting ATPase subunit E	EC:3.6.3.14	7	ko00190 Oxidative phosphorylation;ko04145 Phagosome;ko04721 Synaptic vesicle cycle;ko04966 Collecting duct acid secretion;ko05110 Vibrio cholerae infection;ko05120 Epithelial cell signaling in Helicobacter pylori infection;ko05323 Rheumatoid arthritis;
gene29431	K05016	CLCN7	chloride channel 7	--		
gene29433	K02983	RP-S30e, RPS30	small subunit ribosomal protein S30e	--	1	ko03010 Ribosome;
gene29441	K14821	BUD20	bud site selection protein 20	--		
gene29449	K14297	NUP98, ADAR2	nuclear pore complex protein Nup98-Nup96	--	2	ko03013 RNA transport;ko05164 Influenza A;

gene2944	K03327	TC.MATE, SLC47A, norM, mdtK, dinF	multidrug resistance protein, MATE family	--		
gene29451	K01535	E3.6.3.6	H+-transporting ATPase	EC:3.6.3.6	1	ko00190 Oxidative phosphorylation;
gene29453	K04733	IRAK4	interleukin-1 receptor-associated kinase 4	EC:2.7.11.1	11	ko04064 NF-kappa B signaling pathway;ko04210 Apoptosis;ko04620 Toll-like receptor signaling pathway;ko04722 Neurotrophin signaling pathway;ko05133 Pertussis;ko05140 Leishmaniasis;ko05142 Chagas disease (American trypanosomiasis);ko05145 Toxoplasmosis;ko05152 Tuberculosis;ko05162 Measles;ko05164 Influenza A;
gene29456	K17292	TBCA	tubulin-specific chaperone A	--		
gene29461	K17301	COPB1, SEC26	coatomer, subunit beta	--		
gene29465	K09458	fabF	3-oxoacyl-[acyl-carrier-protein] synthase II	EC:2.3.1.179	2	ko00061 Fatty acid biosynthesis;ko00780 Biotin metabolism;
gene29467	K04125	E1.14.11.13	gibberellin 2-oxidase	EC:1.14.11.13	1	ko00904 Diterpenoid biosynthesis;
gene29472	K08741	MSH5	DNA mismatch repair protein MSH5	--		
gene29474	K08486	STX1B_2_3	syntaxin 1B/2/3	--	2	ko04130 SNARE interactions in vesicular transport;ko04721 Synaptic vesicle cycle;
gene29477	K02893	RP-L23Ae, RPL23A	large subunit ribosomal protein L23Ae	--	1	ko03010 Ribosome;
gene29482	K11836	USP5_13, UBP14	ubiquitin carboxyl-terminal hydrolase 5/13	EC:3.1.2.15		
gene29486	K15407	QTRTD1	queuine tRNA-ribosyltransferase subunit QTRTD1	EC:2.4.2.29		
gene29488	K01895	ACSS, acs	acetyl-CoA synthetase	EC:6.2.1.1	6	ko00010 Glycolysis / Gluconeogenesis;ko00620 Pyruvate metabolism;ko00640 Propanoate metabolism;ko00680 Methane metabolism;ko00720 Carbon fixation pathways in prokaryotes;ko01200 Carbon metabolism;
gene29492	K02929	RP-L44e, RPL44	large subunit ribosomal protein L44e	--	1	ko03010 Ribosome;
gene29496	K04733	IRAK4	interleukin-1 receptor-associated kinase 4	EC:2.7.11.1	11	ko04064 NF-kappa B signaling pathway;ko04210 Apoptosis;ko04620 Toll-like receptor signaling pathway;ko04722 Neurotrophin signaling pathway;ko05133 Pertussis;ko05140 Leishmaniasis;ko05142 Chagas disease (American trypanosomiasis);ko05145 Toxoplasmosis;ko05152 Tuberculosis;ko05162 Measles;ko05164 Influenza A;
gene29497	K07198	PRKAA, AMPK	5'-AMP-activated protein kinase, catalytic alpha subunit	EC:2.7.11.11	8	ko04140 Regulation of autophagy;ko04150 mTOR signaling pathway;ko04151 PI3K-Akt signaling pathway;ko04710 Circadian rhythm;ko04910 Insulin signaling pathway;ko04920 Adipocytokine signaling pathway;ko04932 Non-alcoholic fatty liver disease (NAFLD);ko05410 Hypertrophic cardiomyopathy (HCM);
gene29499	K12861	BCAS2	pre-mRNA-splicing factor SPF27	--	1	ko03040 Spliceosome;
gene294	K13466	EIX1_2	EIX receptor 1/2	--	1	ko04626 Plant-pathogen interaction;
gene29500	K13336	PEX3	peroxin-3	--	1	ko04146 Peroxisome;
gene29501	K14753	RACK1	guanine nucleotide-binding protein subunit beta-2-like 1 protein	--	1	ko05162 Measles;
gene29503	K13464	JAZ	jasmonate ZIM domain-containing protein	--	2	ko04075 Plant hormone signal transduction;ko04626 Plant-pathogen interaction;
gene29506	K04733	IRAK4	interleukin-1 receptor-associated kinase 4	EC:2.7.11.1	11	ko04064 NF-kappa B signaling pathway;ko04210 Apoptosis;ko04620 Toll-like receptor signaling pathway;ko04722 Neurotrophin signaling pathway;ko05133 Pertussis;ko05140 Leishmaniasis;ko05142 Chagas disease (American trypanosomiasis);ko05145 Toxoplasmosis;ko05152 Tuberculosis;ko05162 Measles;ko05164 Influenza A;
gene29514	K17541	SCYL2	SCY1-like protein 2	--		
gene29519	K00799	GST, gst	glutathione S-transferase	EC:2.5.1.18	4	ko00480 Glutathione metabolism;ko00980 Metabolism of xenobiotics by cytochrome P450;ko00982 Drug metabolism - cytochrome P450;ko05204 Chemical carcinogenesis;
gene29521	K00811	ASP5	aspartate aminotransferase, chloroplastic	EC:2.6.1.1	10	ko00250 Alanine, aspartate and glutamate metabolism;ko00270 Cysteine and methionine metabolism;ko00330 Arginine and proline metabolism;ko00350 Tyrosine metabolism;ko00360 Phenylalanine metabolism;ko00400 Phenylalanine, tyrosine and tryptophan biosynthesis;ko00950 Isoquinoline alkaloid biosynthesis;ko00960 Tropane, piperidine and pyridine alkaloid biosynthesis;ko01210 2-Oxocarboxylic acid metabolism;ko01230 Biosynthesis of amino acids; [illegible]
gene29522	K09833	HPT, TPT	homogenitisate phytyltransferase	--	1	ko00130 Ubiquinone and other terpenoid-quinone biosynthesis;
gene29526	K09775	K09775	hypothetical protein	--		

gene29529	K02433	gatA	aspartyl-tRNA(Asn)/glutamyl-tRNA(Gln) amidotransferase subunit A	EC:6.3.5.6 6.3.5.7	1	ko00970 Aminoacyl-tRNA biosynthesis;
gene29530	K05290	PIGK	phosphatidylinositol glycan, class K	EC:2.6.-.-	1	ko00563 Glycosylphosphatidylinositol(GPI)-anchor biosynthesis;
gene29533	K00432	E1.11.1.9	glutathione peroxidase	EC:1.11.1.9	3	ko00480 Glutathione metabolism;ko00590 Arachidonic acid metabolism;ko04918 Thyroid hormone synthesis;
gene29534	K17637	EXOC2, SEC5	exocyst complex component 2	--	1	ko04014 Ras signaling pathway;
gene29541	K12462	ARHGDI, RHOGDI	Rho GDP-dissociation inhibitor	--	2	ko04722 Neurotrophin signaling pathway;ko04962 Vasopressin-regulated water reabsorption;
gene29545	K09580	PDIA1, P4HB	protein disulfide-isomerase A1	EC:5.3.4.1	1	ko04141 Protein processing in endoplasmic reticulum;
gene29554	K17618	UBLCP1	ubiquitin-like domain-containing CTD phosphatase 1	EC:3.1.3.16		
gene29559	K16075	MRS2, MFM1	magnesium transporter	--		
gene29561	K01115	PLD1_2	phospholipase D1/2	EC:3.1.4.4	7	ko00564 Glycerophospholipid metabolism;ko00565 Ether lipid metabolism;ko04014 Ras signaling pathway;ko04144 Endocytosis;ko04666 Fc gamma R-mediated phagocytosis;ko04724 Glutamatergic synapse;ko04912 GnRH signaling pathway;
gene29566	K15285	SLC35E3	solute carrier family 35, member E3	--		
gene29573	K00423	E1.10.3.3	L-ascorbate oxidase	EC:1.10.3.3	1	ko00053 Ascorbate and aldarate metabolism;
gene29576	K14799	TSR1	pre-rRNA-processing protein TSR1	--		
gene29579	K14564	NOP56	nucleolar protein 56	--	1	ko03008 Ribosome biogenesis in eukaryotes;
gene2957	K03294	TC.APA	basic amino acid/polyamine antiporter, APA family	--		
gene29581	K13464	JAZ	jasmonate ZIM domain-containing protein	--	2	ko04075 Plant hormone signal transduction;ko04626 Plant-pathogen interaction;
gene29582	K13993	HSP20	HSP20 family protein	--	1	ko04141 Protein processing in endoplasmic reticulum;
gene29584	K01183	E3.2.1.14	chitinase	EC:3.2.1.14	1	ko00520 Amino sugar and nucleotide sugar metabolism;
gene29588	K09838	ZEP, ABA1	zeaxanthin epoxidase	EC:1.14.13.90	1	ko00906 Carotenoid biosynthesis;
gene29590	K13168	SFRS16	splicing factor, arginine/serine-rich 16	--		
gene29596	K15979	SND1	staphylococcal nuclease domain-containing protein 1	--	2	ko05169 Epstein-Barr virus infection;ko05203 Viral carcinogenesis;
gene29599	K05747	WAS	Wiskott-Aldrich syndrome protein	--	8	ko04062 Chemokine signaling pathway;ko04520 Adherens junction;ko04666 Fc gamma R-mediated phagocytosis;ko04810 Regulation of actin cytoskeleton;ko05100 Bacterial invasion of epithelial cells;ko05130 Pathogenic Escherichia coli infection;ko05131 Shigellosis;ko05132 Salmonella infection;
gene29613	K11322	EPC	enhancer of polycomb-like protein	--		
gene29619	K12824	TCERG1, CA150	transcription elongation regulator 1	--	1	ko03040 Spliceosome;
gene29622	K08235	E2.4.1.207	xyloglucan:xyloglucosyl transferase	EC:2.4.1.207		
gene29623	K09422	MYBP	myb proto-oncogene protein, plant	--		
gene29626	K00670	E2.3.1.88	peptide alpha-N-acetyltransferase	EC:2.3.1.88		
gene29631	K14236	tRNA-Tyr	tRNA Tyr	--	1	ko00970 Aminoacyl-tRNA biosynthesis;
gene29636	K16675	ZDHHC14	probable palmitoyltransferase ZDHHC14	EC:2.3.1.-	1	ko04391 Hippo signaling pathway - fly;
gene2963	K14488	SAUR	SAUR family protein	--	1	ko04075 Plant hormone signal transduction;
gene29647	K13680	CSLA	beta-mannan synthase	EC:2.4.1.32		
gene2964	K16292	CEP, CYSEP	KDEL-tailed cysteine endopeptidase	EC:3.4.22.-		
gene29650	K00517	E1.14.-.-			4	ko00624 Polycyclic aromatic hydrocarbon degradation;ko00627 Aminobenzoate degradation;ko00903 Limonene and pinene degradation;ko00945 Stilbenoid, diarylheptanoid and gingerol biosynthesis;
gene29655	K00759	E2.4.2.7, apt	adenine phosphoribosyltransferase	EC:2.4.2.7	1	ko00230 Purine metabolism;
gene29667	K02732	PSMB1	20S proteasome subunit beta 6	EC:3.4.25.1	1	ko03050 Proteasome;
gene29668	K15378	SLC45A1_2_4	solute carrier family 45, member 1/2/4	--		
gene29674	K11254	H4	histone H4	--	3	ko05034 Alcoholism;ko05203 Viral carcinogenesis;ko05322 Systemic lupus erythematosus;
gene29677	K05305	FUK	fucokinase	EC:2.7.1.52	2	ko00051 Fructose and mannose metabolism;ko00520 Amino sugar and nucleotide sugar metabolism;
gene29679	K11252	H2B	histone H2B	--	3	ko05034 Alcoholism;ko05203 Viral carcinogenesis;ko05322 Systemic lupus erythematosus;
gene2967	K03139	TFIIF2, GTF2F2, TFG2	transcription initiation factor TFIIF subunit beta	EC:3.6.4.12	1	ko03022 Basal transcription factors;

gene29680	K00863	E2.7.1.29, DAK1, DAK2	dihydroxyacetone kinase	EC:2.7.1.29	4	ko00561 Glycerolipid metabolism;ko00680 Methane metabolism;ko01200 Carbon metabolism;ko04622 RIG-I-like receptor signaling pathway;
gene29685	K13114	PNN	pinin	--	2	ko03013 RNA transport;ko03015 mRNA surveillance pathway;
gene29689	K00913	ITPK1	inositol-1,3,4-trisphosphate 5/6-kinase / inositol-tetrakisphosphate 1-kinase	EC:2.7.1.159 2.7.1.134	2	ko00562 Inositol phosphate metabolism;ko04070 Phosphatidylinositol signaling system;
gene29693	K00799	GST, gst	glutathione S-transferase	EC:2.5.1.18	4	ko00480 Glutathione metabolism;ko00980 Metabolism of xenobiotics by cytochrome P450;ko00982 Drug metabolism - cytochrome P450;ko05204 Chemical carcinogenesis;
gene29694	K01923	purC	phosphoribosylaminoimidazole-succinocarboxamide synthase	EC:6.3.2.6	1	ko00230 Purine metabolism;
gene29699	K12823	DDX5, DBP2	ATP-dependent RNA helicase DDX5/DBP2	EC:3.6.4.13	3	ko03040 Spliceosome;ko05202 Transcriptional misregulation in cancer;ko05205 Proteoglycans in cancer;
gene29703	K11423	SETD2, SET2	histone-lysine N-methyltransferase SETD2	EC:2.1.1.43	1	ko00310 Lysine degradation;
gene29709	K02868	RP-L11e, RPL11	large subunit ribosomal protein L11e	--	1	ko03010 Ribosome;
gene29714	K02932	RP-L5e, RPL5	large subunit ribosomal protein L5e	--	1	ko03010 Ribosome;
gene29716	K02537	MAD2	mitotic spindle assembly checkpoint protein MAD2	--	6	ko04110 Cell cycle;ko04111 Cell cycle - yeast;ko04113 Meiosis - yeast;ko04114 Oocyte meiosis;ko04914 Progesterone-mediated oocyte maturation;ko05166 HTLV-I infection;
gene29717	K14235	tRNA-Trp	tRNA Trp	--	1	ko00970 Aminoacyl-tRNA biosynthesis;
gene29721	K11816	YUCCA	indole-3-pyruvate monooxygenase	EC:1.14.13.168	1	ko00380 Tryptophan metabolism;
gene29722	K05305	FUK	fucokinase	EC:2.7.1.52	2	ko00051 Fructose and mannose metabolism;ko00520 Amino sugar and nucleotide sugar metabolism;
gene29724	K03257	EIF4A	translation initiation factor 4A	--	1	ko03013 RNA transport;
gene29727	K09422	MYBP	myb proto-oncogene protein, plant	--		
gene29733	K15865	CDKAL1	CDK5 regulatory subunit-associated protein 1-like 1	--		
gene29734	K07512	MECR, NRBF1	mitochondrial trans-2-enoyl-CoA reductase	EC:1.3.1.38	1	ko00062 Fatty acid elongation;
gene29735	K04371	MAPK1_3	mitogen-activated protein kinase 1/3	EC:2.7.11.24	71	ko04010 MAPK signaling pathway;ko04012 ErbB signaling pathway;ko04013 MAPK signaling pathway - fly;ko04014 Ras signaling pathway;ko04062 Chemokine signaling pathway;ko04066 HIF-1 signaling pathway;ko04114 Oocyte meiosis;ko04150 mTOR signaling pathway;ko04151 PI3K-Akt signaling pathway;ko04270 Vascular smooth muscle contraction;ko04320 Dorso-ventral axis formation;ko04350 TGF-beta signaling pathway;ko04360 Axon guidance;ko04370 VEGF signaling pathway;ko04380 Osteoclast differentiation;ko04510 Focal adhesion;ko04520 Adherens junction;ko04540 Gap junction;ko04620 Toll-like receptor signaling pathway;ko04621 NOD-like receptor signaling pathway;ko04650 Natural killer cell mediated cytotoxicity;ko04660 T cell receptor signaling pathway;ko04662 B cell receptor signaling pathway;ko04664 Fc epsilon RI signaling pathway;ko04666 Fc gamma R-mediated phagocytosis;ko04668 TNF signaling pathway;ko04713 Circadian entrainment;ko04720 Long-term potentiation;ko04722 Neurotrophin signaling pathway;ko04723 Retrograde endocannabinoid signaling;ko04724 Glutamatergic synapse;ko04725 Cholinergic synapse;ko04726 Serotonergic synapse;ko04730 Long-term depression;ko04810 Regulation of actin cytoskeleton;ko04910 Insulin signaling pathway;ko04912 GnRH signaling pathway;ko04914 Progesterone-mediated oocyte maturation;ko04915 Estrogen signaling pathway;ko04916 Melanogenesis;ko04917 Prolactin signaling pathway;ko04930 Type II diabetes mellitus;ko04960 Aldosterone-regulated sodium reabsorption;ko05010 Alzheimer's disease;ko05020 Prion diseases;ko05034 Alcoholism;ko05131 Shigellosis;ko05132 Salmonella infection;ko05133 Pertussis;ko05140 Leishmaniasis;ko05142 Chagas disease (American trypanosomiasis);ko05145 Toxoplasmosis;ko05152 Tuberculosis;ko05160 Hepatitis C;ko05161 Hepatitis B;ko05164 Influenza A;ko05200 Pathways in cancer;ko05203 Viral carcinogenesis;ko05205 Proteoglycans in cancer;ko05210 Colorectal cancer;ko05211 Renal cell carcinoma;ko05212
gene29747	K00688	E2.4.1.1, glgP, PYG	starch phosphorylase	EC:2.4.1.1	2	ko00500 Starch and sucrose metabolism;ko04910 Insulin signaling pathway;

gene29748	K08193	SLC17A	MFS transporter, ACS family, solute carrier family 17 (sodium-dependent inorganic phosphate cotransporter), other	--		
gene29750	K10572	IPPK	inositol-pentakisphosphate 2-kinase	EC:2.7.1.158	2	ko00562 Inositol phosphate metabolism;ko04070 Phosphatidylinositol signaling system;
gene29754	K01803	TPI, tpiA	triosephosphate isomerase (TIM)	EC:5.3.1.1	6	ko00010 Glycolysis / Gluconeogenesis;ko00051 Fructose and mannose metabolism;ko00562 Inositol phosphate metabolism;ko00710 Carbon fixation in photosynthetic organisms;ko01200 Carbon metabolism;ko01230 Biosynthesis of amino acids;
gene29755	K01303	APEH	acylaminoacyl-peptidase	EC:3.4.19.1		
gene29756	K16818	K16818, DAD1	phospholipase A1	EC:3.1.1.32	2	ko00564 Glycerophospholipid metabolism;ko00592 alpha-Linolenic acid metabolism;
gene29761	K14556	DIP2, UTP12, WDR3	U3 small nucleolar RNA-associated protein 12	--	1	ko03008 Ribosome biogenesis in eukaryotes;
gene29762	K07466	RFA1, RPA1, rpa	replication factor A1	--	5	ko03030 DNA replication;ko03420 Nucleotide excision repair;ko03430 Mismatch repair;ko03440 Homologous recombination;ko03460 Fanconi anemia pathway;
gene29763	K02891	RP-L22e, RPL22	large subunit ribosomal protein L22e	--	1	ko03010 Ribosome;
gene29764	K00012	UGDH, ugd	UDPglucose 6-dehydrogenase	EC:1.1.1.22	4	ko00040 Pentose and glucuronate interconversions;ko00053 Ascorbate and aldarate metabolism;ko00500 Starch and sucrose metabolism;ko00520 Amino sugar and nucleotide sugar metabolism;
gene29766	K10579	UBE2M, UBC12	ubiquitin-conjugating enzyme E2 M	EC:6.3.2.19	1	ko04120 Ubiquitin mediated proteolysis;
gene29774	K08150	SLC2A13, ITR	MFS transporter, SP family, solute carrier family 2 (myo-inositol transporter), member 13	--		
gene29775	K14494	DELLA	DELLA protein	--	1	ko04075 Plant hormone signal transduction;
gene29776	K08272	CAB39, MO25	calcium binding protein 39	--	1	ko04150 mTOR signaling pathway;
gene29778	K12847	USP39, SAD1	U4/U6.U5 tri-snRNP-associated protein 2	--	1	ko03040 Spliceosome;
gene29780	K13496	UGT73C	UDP-glucosyl transferase 73C	EC:2.4.1.-		
gene29781	K01551	arsA, ASNA1	arsenite-transporting ATPase	EC:3.6.3.16		
gene29782	K08150	SLC2A13, ITR	MFS transporter, SP family, solute carrier family 2 (myo-inositol transporter), member 13	--		
gene29783	K01115	PLD1_2	phospholipase D1/2	EC:3.1.4.4	7	ko00564 Glycerophospholipid metabolism;ko00565 Ether lipid metabolism;ko04014 Ras signaling pathway;ko04144 Endocytosis;ko04666 Fc gamma R-mediated phagocytosis;ko04724 Glutamatergic synapse;ko04912 GnRH signaling pathway;
gene29787	K03046	rpoC	DNA-directed RNA polymerase subunit beta'	EC:2.7.7.6	3	ko00230 Purine metabolism;ko00240 Pyrimidine metabolism;ko03020 RNA polymerase;
gene29791	K01933	purM	phosphoribosylformylglycinamidine cyclo-ligase	EC:6.3.3.1	1	ko00230 Purine metabolism;
gene29795	K14484	IAA	auxin-responsive protein IAA	--	1	ko04075 Plant hormone signal transduction;
gene29799	K10357	MYO5	myosin V	--		
gene29800	K17583	NOM1	nucleolar MIF4G domain-containing protein 1	--		
gene29801	K03453	TC.BASS	bile acid:Na+ symporter, BASS family	--		
gene29805	K13430	PBS1	serine/threonine-protein kinase PBS1	EC:2.7.11.1	1	ko04626 Plant-pathogen interaction;
gene29812	K00472	E1.14.11.2	prolyl 4-hydroxylase	EC:1.14.11.2	1	ko00330 Arginine and proline metabolism;
gene29815	K00451	HGD, hmgA	homogentisate 1,2-dioxygenase	EC:1.13.11.5	2	ko00350 Tyrosine metabolism;ko00643 Styrene degradation;
gene29818	K02937	RP-L7e, RPL7	large subunit ribosomal protein L7e	--	1	ko03010 Ribosome;
gene2981	K01834	PGAM, gpmA	2,3-bisphosphoglycerate-dependent phosphoglycerate mutase	EC:5.4.2.11	5	ko00010 Glycolysis / Gluconeogenesis;ko00260 Glycine, serine and threonine metabolism;ko00680 Methane metabolism;ko01200 Carbon metabolism;ko01230 Biosynthesis of amino acids;
gene29820	K09874	NIP	aquaporin NIP	--		
gene29821	K10839	RAD23, HR23	UV excision repair protein RAD23	--	2	ko03420 Nucleotide excision repair;ko04141 Protein processing in endoplasmic reticulum;
gene29826	K03327	TC.MATE, SLC47A, norM, mdtK, dinF	multidrug resistance protein, MATE family	--		
gene29830	K05573	ndhB	NAD(P)H-quinone oxidoreductase subunit 2	EC:1.6.5.3	1	ko00190 Oxidative phosphorylation;
gene29837	K13508	GPAT	glycerol-3-phosphate acyltransferase	EC:2.3.1.15	2	ko00561 Glycerolipid metabolism;ko00564 Glycerophospholipid metabolism;
gene2983	K14514	EIN3	ethylene-insensitive protein 3	--	1	ko04075 Plant hormone signal transduction;

gene29840	K02998	RP-SAe, RPSA	small subunit ribosomal protein SAe	--	1	ko03010 Ribosome;
gene29841	K00128	E1.2.1.3	aldehyde dehydrogenase (NAD+)	EC:1.2.1.3	15	ko00010 Glycolysis / Gluconeogenesis;ko00040 Pentose and glucuronate interconversions;ko00053 Ascorbate and aldarate metabolism;ko00071 Fatty acid degradation;ko00280 Valine, leucine and isoleucine degradation;ko00310 Lysine degradation;ko00330 Arginine and proline metabolism;ko00340 Histidine metabolism;ko00380 Tryptophan metabolism;ko00410 beta-Alanine metabolism;ko00561 Glycerolipid metabolism;ko00620 Pyruvate metabolism;ko00625 Chloroalkane and chloroalkene degradation;ko00640 Propanoate metabolism;ko00903 Limonene and pinene
gene29842	K02603	ORC1	origin recognition complex subunit 1	--	3	ko04110 Cell cycle;ko04111 Cell cycle - yeast;ko04113 Meiosis - yeast;
gene29844	K10599	PRPF19, PRP19	pre-mRNA-processing factor 19	EC:6.3.2.19	2	ko03040 Spliceosome;ko04120 Ubiquitin mediated proteolysis;
gene29849	K04733	IRAK4	interleukin-1 receptor-associated kinase 4	EC:2.7.11.1	11	ko04064 NF-kappa B signaling pathway;ko04210 Apoptosis;ko04620 Toll-like receptor signaling pathway;ko04722 Neurotrophin signaling pathway;ko05133 Pertussis;ko05140 Leishmaniasis;ko05142 Chagas disease (American trypanosomiasis);ko05145 Toxoplasmosis;ko05152 Tuberculosis;ko05162 Measles;ko05164 Influenza A;
gene29853	K02885	RP-L19e, RPL19	large subunit ribosomal protein L19e	--	1	ko03010 Ribosome;
gene29855	K10742	DNA2	DNA replication ATP-dependent helicase Dna2	EC:3.6.4.12	1	ko03030 DNA replication;
gene29858	K07305	msrB	peptide-methionine (R)-S-oxide reductase	EC:1.8.4.12		
gene2985	K12617	PATL1, PAT1	DNA topoisomerase 2-associated protein PAT1	--	1	ko03018 RNA degradation;
gene29860	K10577	UBE2I, UBC9	ubiquitin-conjugating enzyme E2 I	EC:6.3.2.19	4	ko03013 RNA transport;ko04064 NF-kappa B signaling pathway;ko04120 Ubiquitin mediated proteolysis;ko05206 MicroRNAs in cancer;
gene29863	K13162	PCBP2_3_4	poly(rC)-binding protein 2/3/4	--		
gene29867	K02717	psbP	photosystem II oxygen-evolving enhancer protein 2	--	1	ko00195 Photosynthesis;
gene29868	K05658	ABCB1	ATP-binding cassette, subfamily B (MDR/TAP), member 1	--	3	ko02010 ABC transporters;ko04976 Bile secretion;ko05206 MicroRNAs in cancer;
gene29871	K14332	psaO	photosystem I subunit PsaO	--	1	ko00195 Photosynthesis;
gene29872	K12581	CNOT7_8, CAF1, POP2	CCR4-NOT transcription complex subunit 7/8	--	1	ko03018 RNA degradation;
gene29879	K07305	msrB	peptide-methionine (R)-S-oxide reductase	EC:1.8.4.12		
gene29881	K04043	dnaK	molecular chaperone DnaK	--	2	ko03018 RNA degradation;ko05152 Tuberculosis;
gene29884	K11584	PPP2R5	serine/threonine-protein phosphatase 2A regulatory subunit B'	--	5	ko03015 mRNA surveillance pathway;ko04113 Meiosis - yeast;ko04114 Oocyte meiosis;ko04151 PI3K-Akt signaling pathway;ko04728 Dopaminergic synapse;
gene29885	K14326	UPF1, RENT1	regulator of nonsense transcripts 1	EC:3.6.4.-	2	ko03013 RNA transport;ko03015 mRNA surveillance pathway;
gene29892	K09872	PIP	aquaporin PIP	--		
gene29893	K02870	RP-L12e, RPL12	large subunit ribosomal protein L12e	--	1	ko03010 Ribosome;
gene29894	K08960	CSNK1E	casein kinase 1, epsilon	EC:2.7.11.1	6	ko04310 Wnt signaling pathway;ko04340 Hedgehog signaling pathway;ko04390 Hippo signaling pathway;ko04391 Hippo signaling pathway - fly;ko04710 Circadian rhythm;ko04711 Circadian rhythm - fly;
gene29895	K01051	E3.1.1.11	pectinesterase	EC:3.1.1.11	2	ko00040 Pentose and glucuronate interconversions;ko00500 Starch and sucrose metabolism;
gene29896	K00249	ACADM, acd	acyl-CoA dehydrogenase	EC:1.3.8.7	5	ko00071 Fatty acid degradation;ko00280 Valine, leucine and isoleucine degradation;ko00410 beta-Alanine metabolism;ko00640 Propanoate metabolism;ko03320 PPAR signaling pathway;
gene2989	K01427	URE	urease	EC:3.5.1.5	2	ko00230 Purine metabolism;ko00330 Arginine and proline metabolism;
gene29900	K13217	PRPF39, PRP39	pre-mRNA-processing factor 39	--		
gene29901	K17616	CTDSPL2	CTD small phosphatase-like protein 2	EC:3.1.3.-		
gene29902	K04733	IRAK4	interleukin-1 receptor-associated kinase 4	EC:2.7.11.1	11	ko04064 NF-kappa B signaling pathway;ko04210 Apoptosis;ko04620 Toll-like receptor signaling pathway;ko04722 Neurotrophin signaling pathway;ko05133 Pertussis;ko05140 Leishmaniasis;ko05142 Chagas disease (American trypanosomiasis);ko05145 Toxoplasmosis;ko05152 Tuberculosis;ko05162 Measles;ko05164 Influenza A;
gene29905	K11648	SMARCB1, SNF5, INI1	SWI/SNF-related matrix-associated actin-dependent regulator of chromatin subfamily B member 1	--		

gene29908	K09458	fabF	3-oxoacyl-[acyl-carrier-protein] synthase II	EC:2.3.1.179	2	ko00061 Fatty acid biosynthesis;ko00780 Biotin metabolism;
gene29909	K14794	RRP12	ribosomal RNA-processing protein 12	--		
gene29911	K01533	E3.6.3.4, ATP7, copA	Cu2+-exporting ATPase	EC:3.6.3.4		
gene29913	K00873	PK, pyk	pyruvate kinase	EC:2.7.1.40	7	ko00010 Glycolysis / Gluconeogenesis;ko00230 Purine metabolism;ko00620 Pyruvate metabolism;ko01200 Carbon metabolism;ko01230 Biosynthesis of amino acids;ko04930 Type II diabetes mellitus;ko05203 Viral carcinogenesis;
gene29916	K03405	chlI, bchI	magnesium chelatase subunit I	EC:6.6.1.1	1	ko00860 Porphyrin and chlorophyll metabolism;
gene29918	K01070	frmB, ESD, fghA	S-formylglutathione hydrolase	EC:3.1.2.12	2	ko00680 Methane metabolism;ko01200 Carbon metabolism;
gene29929	K10666	RNF5	E3 ubiquitin-protein ligase RNF5	EC:6.3.2.19	1	ko04141 Protein processing in endoplasmic reticulum;
gene29937	K01530	E3.6.3.1	phospholipid-translocating ATPase	EC:3.6.3.1		
gene2993	K13728	MAD2L2	mitotic spindle assembly checkpoint protein MAD2B	--	5	ko04110 Cell cycle;ko04114 Oocyte meiosis;ko04914 Progesterone-mediated oocyte maturation;ko05100 Bacterial invasion of epithelial cells;ko05131 Shigellosis;
gene29941	K02520	infC, MTIF3	translation initiation factor IF-3	--		
gene29942	K03798	ftsH, hflB	cell division protease FtsH	EC:3.4.24.-		
gene29944	K11498	CENPE	centromeric protein E	--		
gene29945	K01087	otsB	trehalose 6-phosphate phosphatase	EC:3.1.3.12	1	ko00500 Starch and sucrose metabolism;
gene29952	K14641	APY1_2	apyrase	EC:3.6.1.5	2	ko00230 Purine metabolism;ko00240 Pyrimidine metabolism;
gene29955	K01728	E4.2.2.2, pel	pectate lyase	EC:4.2.2.2	1	ko00040 Pentose and glucuronate interconversions;
gene29956	K09264	K09264	MADS-box transcription factor, plant	--		
gene29957	K08912	LHCB1	light-harvesting complex II chlorophyll a/b binding protein 1	--	1	ko00196 Photosynthesis - antenna proteins;
gene29959	K17087	TM9SF3	transmembrane 9 superfamily member 3	--		
gene2995	K10884	XRCC6, KU70, G22P1	ATP-dependent DNA helicase 2 subunit 1	--	1	ko03450 Non-homologous end-joining;
gene29961	K11982	RNF115_126	E3 ubiquitin-protein ligase RNF115/126	EC:6.3.2.19		
gene29965	K03283	HSPA1_8	heat shock 70kDa protein 1/8	--	11	ko03040 Spliceosome;ko04010 MAPK signaling pathway;ko04141 Protein processing in endoplasmic reticulum;ko04144 Endocytosis;ko04612 Antigen processing and presentation;ko04915 Estrogen signaling pathway;ko05134 Legionellosis;ko05145 Toxoplasmosis;ko05162 Measles;ko05164 Influenza A;ko05169 Epstein-Barr virus infection;
gene29967	K08869	ADCK, ABC1	aarF domain-containing kinase	--		
gene29977	K01951	E6.3.5.2, guaA	GMP synthase (glutamine-hydrolysing)	EC:6.3.5.2	2	ko00230 Purine metabolism;ko00983 Drug metabolism - other enzymes;
gene29984	K14799	TSR1	pre-rRNA-processing protein TSR1	--		
gene29994	K08494	NSPN	novel plant SNARE	--		
gene29995	K00889	E2.7.1.68, PIP5K	1-phosphatidylinositol-4-phosphate 5-kinase	EC:2.7.1.68	5	ko00562 Inositol phosphate metabolism;ko04070 Phosphatidylinositol signaling system;ko04144 Endocytosis;ko04666 Fc gamma R-mediated phagocytosis;ko04810 Regulation of actin cytoskeleton;
gene29996	K02218	CSNK1, CK1	casein kinase 1	EC:2.7.11.1	1	ko04340 Hedgehog signaling pathway;
gene29999	K03032	PSMD1, RPN2	26S proteasome regulatory subunit N2	--	2	ko03050 Proteasome;ko05169 Epstein-Barr virus infection;
gene2999	K15227	TYRAAT	arogenate dehydrogenase (NADP+), plant	EC:1.3.1.78	2	ko00400 Phenylalanine, tyrosine and tryptophan biosynthesis;ko01230 Biosynthesis of amino acids;
gene2	K09422	MYBP	myb proto-oncogene protein, plant	--		
gene30000	K14484	IAA	auxin-responsive protein IAA	--	1	ko04075 Plant hormone signal transduction;
gene30002	K17604	ZSWIM3	zinc finger SWIM domain-containing protein 3	--		
gene30004	K08503	SYP5	syntaxin of plants SYP5	--	1	ko04130 SNARE interactions in vesicular transport;
gene30005	K03531	ftsZ	cell division protein FtsZ	--	1	ko04112 Cell cycle - Caulobacter;
gene30008	K01527	EGD1, BTF3	nascent polypeptide-associated complex subunit beta	--		
gene30009	K13648	GAUT	alpha-1,4-galacturonosyltransferase	EC:2.4.1.43	2	ko00500 Starch and sucrose metabolism;ko00520 Amino sugar and nucleotide sugar metabolism;
gene30012	K07870	RHOT1, ARHT1	Ras homolog gene family, member T1	--		
gene30016	K08065	NFYB	nuclear transcription Y subunit beta	--	3	ko04612 Antigen processing and presentation;ko05152 Tuberculosis;ko05166 HTLV-I infection;

gene30019	K11253	H3	histone H3	--	3	ko05034 Alcoholism;ko05202 Transcriptional misregulation in cancer;ko05322 Systemic lupus erythematosus;
gene30026	K14638	SLC15A3_4, PHT	solute carrier family 15 (peptide/histidine transporter), member 3/4	--		
gene30030	K10400	KIF15	kinesin family member 15	--		
gene30041	K13681	FUT	xyloglucan fucosyltransferase	EC:2.4.1.-		
gene30042	K09579	PIN4	peptidyl-prolyl cis-trans isomerase NIMA-interacting 4	EC:5.2.1.8		
gene30045	K08064	NFYA	nuclear transcription factor Y, alpha	--	2	ko04612 Antigen processing and presentation;ko05152 Tuberculosis;
gene30047	K12191	CHMP2A	charged multivesicular body protein 2A	--	1	ko04144 Endocytosis;
gene30051	K00366	nirA	ferredoxin-nitrite reductase	EC:1.7.7.1	1	ko00910 Nitrogen metabolism;
gene30052	K09598	SPPL3	signal peptide peptidase-like 3	EC:3.4.23.-		
gene30057	K11253	H3	histone H3	--	3	ko05034 Alcoholism;ko05202 Transcriptional misregulation in cancer;ko05322 Systemic lupus erythematosus;
gene30058	K16675	ZDHHC14	probable palmitoyltransferase ZDHHC14	EC:2.3.1.-	1	ko04391 Hippo signaling pathway - fly;
gene30059	K03426	E3.6.1.22, NUDT12, nudC	NAD+ diphosphatase	EC:3.6.1.22	2	ko00760 Nicotinate and nicotinamide metabolism;ko04146 Peroxisome;
gene30061	K03327	TC.MATE, SLC47A, norM, mdtK, dinF	multidrug resistance protein, MATE family	--		
gene30062	K13425	WRKY22	WRKY transcription factor 22	--	1	ko04626 Plant-pathogen interaction;
gene30068	K16570	TUBGCP3, GCP3	gamma-tubulin complex component 3	--		
gene30069	K05016	CLCN7	chloride channel 7	--		
gene3006	K02882	RP-L18Ae, RPL18A	large subunit ribosomal protein L18Ae	--	1	ko03010 Ribosome;
gene30070	K08282	E2.7.11.1	non-specific serine/threonine protein kinase	EC:2.7.11.1		
gene30072	K15188	CCNT	cyclin T	--	1	ko05202 Transcriptional misregulation in cancer;
gene30075	K05280	E1.14.13.21	flavonoid 3'-monooxygenase	EC:1.14.13.21	2	ko00941 Flavonoid biosynthesis;ko00944 Flavone and flavonol biosynthesis;
gene30077	K10639	CCNB1IP1, HEI10	E3 ubiquitin-protein ligase CCNP1IP1	EC:6.3.2.19		
gene30080	K09284	AP2	AP2-like factor, euAP2 lineage	--		
gene30087	K08242	E2.1.1.143	24-methylenesterol C-methyltransferase	EC:2.1.1.143	1	ko00100 Steroid biosynthesis;
gene30091	K07904	RAB11A	Ras-related protein Rab-11A	--	4	ko04144 Endocytosis;ko04961 Endocrine and other factor-regulated calcium reabsorption;ko04962 Vasopressin-regulated water reabsorption;ko04972 Pancreatic secretion;
gene30097	K12616	EDC4	enhancer of mRNA-decapping protein 4	--	1	ko03018 RNA degradation;
gene30099	K01551	arsA, ASNA1	arsenite-transporting ATPase	EC:3.6.3.16		
gene3009	K01551	arsA, ASNA1	arsenite-transporting ATPase	EC:3.6.3.16		
gene30100	K13717	OTUD3	OTU domain-containing protein 3	--		
gene30106	K10357	MYO5	myosin V	--		
gene30110	K10839	RAD23, HR23	UV excision repair protein RAD23	--	2	ko03420 Nucleotide excision repair;ko04141 Protein processing in endoplasmic reticulum;
gene30115	K14416	HBS1	elongation factor 1 alpha-like protein	--	2	ko03015 mRNA surveillance pathway;ko05134 Legionellosis;
gene30119	K09553	STIP1	stress-induced-phosphoprotein 1	--	1	ko05020 Prion diseases;
gene30120	K06966	K06966				
gene3012	K12446	E2.7.1.46	L-arabinokinase	EC:2.7.1.46	1	ko00520 Amino sugar and nucleotide sugar metabolism;
gene30131	K03347	CUL1, CDC53	cullin 1	--	9	ko04110 Cell cycle;ko04111 Cell cycle - yeast;ko04114 Oocyte meiosis;ko04120 Ubiquitin mediated proteolysis;ko04141 Protein processing in endoplasmic reticulum;ko04310 Wnt signaling pathway;ko04350 TGF-beta signaling pathway;ko04710 Circadian rhythm;ko05168 Herpes simplex infection;
gene30135	K00627	DLAT, aceF, pdhC	pyruvate dehydrogenase E2 component (dihydrolipoamide acetyltransferase)	EC:2.3.1.12	4	ko00010 Glycolysis / Gluconeogenesis;ko00020 Citrate cycle (TCA cycle);ko00620 Pyruvate metabolism;ko01200 Carbon metabolism;
gene30141	K07904	RAB11A	Ras-related protein Rab-11A	--	4	ko04144 Endocytosis;ko04961 Endocrine and other factor-regulated calcium reabsorption;ko04962 Vasopressin-regulated water reabsorption;ko04972 Pancreatic secretion;
gene30147	K05674	ABCC10	ATP-binding cassette, subfamily C (CFTR/MRP), member 10	--	1	ko02010 ABC transporters;
gene30153	K15445	TRM10, RG9MTD2	tRNA (guanine9-N1)-methyltransferase	EC:2.1.1.221		
gene30160	K13754	SLC24A6, NCKX6	solute carrier family 24 (sodium/potassium/calcium exchanger), member 6	--		

gene30163	K10604	MGRN1	E3 ubiquitin-protein ligase MGRN1	EC:6.3.2.19	1	ko04120 Ubiquitin mediated proteolysis;
gene30167	K09872	PIP	aquaporin PIP	--		
gene3016	K00789	metK	S-adenosylmethionine synthetase	EC:2.5.1.6	2	ko00270 Cysteine and methionine metabolism;ko01230 Biosynthesis of amino acids;
gene30172	K00016	LDH, ldh	L-lactate dehydrogenase	EC:1.1.1.27	4	ko00010 Glycolysis / Gluconeogenesis;ko00270 Cysteine and methionine metabolism;ko00620 Pyruvate metabolism;ko00640 Propanoate metabolism;
gene30176	K13993	HSP20	HSP20 family protein	--	1	ko04141 Protein processing in endoplasmic reticulum;
gene30180	K12403	AP4S1	AP-4 complex subunit sigma-1	--	1	ko04142 Lysosome;
gene30181	K00850	pfkA, PFK	6-phosphofructokinase 1	EC:2.7.1.11	7	ko00010 Glycolysis / Gluconeogenesis;ko00030 Pentose phosphate pathway;ko00051 Fructose and mannose metabolism;ko00052 Galactose metabolism;ko00680 Methane metabolism;ko01200 Carbon metabolism;ko01230 Biosynthesis of amino acids;
gene30182	K03652	MPG	DNA-3-methyladenine glycosylase	EC:3.2.2.21	1	ko03410 Base excision repair;
gene30188	K00797	speE, SRM	spermidine synthase	EC:2.5.1.16	4	ko00270 Cysteine and methionine metabolism;ko00330 Arginine and proline metabolism;ko00410 beta-Alanine metabolism;ko00480 Glutathione metabolism;
gene3018	K09422	MYBP	myb proto-oncogene protein, plant	--		
gene30194	K01613	psd, PISD	phosphatidylserine decarboxylase	EC:4.1.1.65	1	ko00564 Glycerophospholipid metabolism;
gene30197	K08472	MLO	mlo protein	--		
gene3019	K01759	E4.4.1.5, GLO1, gloA	lactoylglutathione lyase	EC:4.4.1.5	2	ko00620 Pyruvate metabolism;ko04011 MAPK signaling pathway - yeast;
gene30200	K01051	E3.1.1.11	pectinesterase	EC:3.1.1.11	2	ko00040 Pentose and glucuronate interconversions;ko00500 Starch and sucrose metabolism;
gene30207	K01551	arsA, ASNA1	arsenite-transporting ATPase	EC:3.6.3.16		
gene30210	K00059	fabG	3-oxoacyl-[acyl-carrier protein] reductase	EC:1.1.1.100	3	ko00061 Fatty acid biosynthesis;ko00780 Biotin metabolism;ko01040 Biosynthesis of unsaturated fatty acids;
gene30212	K00134	GAPDH, gapA	glyceraldehyde 3-phosphate dehydrogenase	EC:1.2.1.12	6	ko00010 Glycolysis / Gluconeogenesis;ko00710 Carbon fixation in photosynthetic organisms;ko01200 Carbon metabolism;ko01230 Biosynthesis of amino acids;ko04066 HIF-1 signaling pathway;ko05010 Alzheimer's disease;
gene30219	K11644	SIN3A	paired amphipathic helix protein Sin3a	--	2	ko05016 Huntington's disease;ko05202 Transcriptional misregulation in cancer;
gene30231	K13832	aroDE, DHQ-SDH	3-dehydroquinate dehydratase / shikimate dehydrogenase	EC:4.2.1.10 1.1.1.25	2	ko00400 Phenylalanine, tyrosine and tryptophan biosynthesis;ko01230 Biosynthesis of amino acids;
gene30236	K06316	RFT1	oligosaccharidyl-lipid flippase family	--	1	ko00510 N-Glycan biosynthesis;
gene30241	K12893	SFRS4_5_6	splicing factor, arginine/serine-rich 4/5/6	--	2	ko03040 Spliceosome;ko05168 Herpes simplex infection;
gene30242	K14232	tRNA-Pro	tRNA Pro	--	1	ko00970 Aminoacyl-tRNA biosynthesis;
gene30243	K17506	PPM1L, PP2CE	protein phosphatase 1L	EC:3.1.3.16		
gene30244	K14515	EBF1_2	EIN3-binding F-box protein	--	1	ko04075 Plant hormone signal transduction;
gene30246	K02935	RP-L7, MRPL12, rplL	large subunit ribosomal protein L7/L12	--	1	ko03010 Ribosome;
gene30249	K03265	ETF1, ERF1	peptide chain release factor subunit 1	--	1	ko03015 mRNA surveillance pathway;
gene3024	K11138	K11138, RIF1	telomere-associated protein RIF1	--		
gene30256	K14232	tRNA-Pro	tRNA Pro	--	1	ko00970 Aminoacyl-tRNA biosynthesis;
gene30258	K13811	PAPSS	3'-phosphoadenosine 5'-phosphosulfate synthase	EC:2.7.7.4 2.7.1.25	3	ko00230 Purine metabolism;ko00450 Selenocompound metabolism;ko00920 Sulfur metabolism;
gene3025	K12188	SNF8, EAP30	ESCRT-II complex subunit VPS22	--	1	ko04144 Endocytosis;
gene30262	K01979	SSUrRNA	small subunit ribosomal RNA	--	2	ko03008 Ribosome biogenesis in eukaryotes;ko03010 Ribosome;
gene30263	K02132	ATPeF1A, ATP5A1	F-type H+-transporting ATPase subunit alpha	EC:3.6.3.14	4	ko00190 Oxidative phosphorylation;ko05010 Alzheimer's disease;ko05012 Parkinson's disease;ko05016 Huntington's disease;
gene30265	K01408	IDE, ide	insulysin	EC:3.4.24.56	1	ko05010 Alzheimer's disease;
gene30266	K01408	IDE, ide	insulysin	EC:3.4.24.56	1	ko05010 Alzheimer's disease;
gene3026	K12896	SFRS7	splicing factor, arginine/serine-rich 7	--	2	ko03040 Spliceosome;ko05168 Herpes simplex infection;
gene30278	K09286	EREBP	EREBP-like factor	--		
gene30279	K07198	PRKAA, AMPK	5'-AMP-activated protein kinase, catalytic alpha subunit	EC:2.7.11.11	8	ko04140 Regulation of autophagy;ko04150 mTOR signaling pathway;ko04151 PI3K-Akt signaling pathway;ko04710 Circadian rhythm;ko04910 Insulin signaling pathway;ko04920 Adipocytokine signaling pathway;ko04932 Non-alcoholic fatty liver disease (NAFLD);ko05410 Hypertrophic cardiomyopathy (HCM);

gene3027	K06940	K06940				
gene30280	K07466	RFA1, RPA1, rpa	replication factor A1	--	5	ko03030 DNA replication;ko03420 Nucleotide excision repair;ko03430 Mismatch repair;ko03440 Homologous recombination;ko03460 Fanconi anemia pathway;
gene30281	K14664	ILR1	IAA-amino acid hydrolase	EC:3.5.1.-		
gene30285	K13459	RPS2	disease resistance protein RPS2	--	1	ko04626 Plant-pathogen interaction;
gene30288	K07195	EXOC7, EXO70	exocyst complex component 7	--	1	ko04910 Insulin signaling pathway;
gene30290	K10143	RFWD2, COP1	E3 ubiquitin-protein ligase RFWD2	EC:6.3.2.19	3	ko04115 p53 signaling pathway;ko04120 Ubiquitin mediated proteolysis;ko04712 Circadian rhythm - plant;
gene30291	K14559	MPP10	U3 small nucleolar RNA-associated protein MPP10	--	1	ko03008 Ribosome biogenesis in eukaryotes;
gene30293	K05917	CYP51	cytochrome P450, family 51 (sterol 14-demethylase)	EC:1.14.13.70	1	ko00100 Steroid biosynthesis;
gene30294	K15631	ABA3	molybdenum cofactor sulfurtransferase	EC:2.8.1.9		
gene30296	K12260	SRX1	sulfiredoxin	EC:1.8.98.2		
gene30305	K09264	K09264	MADS-box transcription factor, plant	--		
gene30307	K00627	DLAT, aceF, pdhC	pyruvate dehydrogenase E2 component (dihydrolipoamide acetyltransferase)	EC:2.3.1.12	4	ko00010 Glycolysis / Gluconeogenesis;ko00020 Citrate cycle (TCA cycle);ko00620 Pyruvate metabolism;ko01200 Carbon metabolism;
gene30312	K14439	SMARCAD1	SWI/SNF-related matrix-associated actin-dependent regulator of chromatin subfamily A containing DEAD/H box 1	EC:3.6.4.12		
gene30313	K09338	HD-ZIP	homeobox-leucine zipper protein	--		
gene30316	K15920	XYL4	beta-D-xylosidase 4	EC:3.2.1.37	2	ko00500 Starch and sucrose metabolism;ko00520 Amino sugar and nucleotide sugar metabolism;
gene30318	K14803	PTC2_3	protein phosphatase 2C homolog 2/3	EC:3.1.3.16		
gene30319	K13173	ARGLU1	arginine and glutamate-rich protein 1	--		
gene30322	K02879	RP-L17, MRPL17, rplQ	large subunit ribosomal protein L17	--	1	ko03010 Ribosome;
gene30326	K09286	EREBP	EREBP-like factor	--		
gene3032	K00759	E2.4.2.7, apt	adenine phosphoribosyltransferase	EC:2.4.2.7	1	ko00230 Purine metabolism;
gene30330	K01184	E3.2.1.15	polygalacturonase	EC:3.2.1.15	2	ko00040 Pentose and glucuronate interconversions;ko00500 Starch and sucrose metabolism;
gene30336	K15201	GTP3C3, TFC4	general transcription factor 3C polypeptide 3 (transcription factor C subunit 4)	--		
gene30337	K11423	SETD2, SET2	histone-lysine N-methyltransferase SETD2	EC:2.1.1.43	1	ko00310 Lysine degradation;
gene30340	K14767	UTP3, SAS10	U3 small nucleolar RNA-associated protein 3	--		
gene30344	K01876	DARS, aspS	aspartyl-tRNA synthetase	EC:6.1.1.12	1	ko00970 Aminoacyl-tRNA biosynthesis;
gene30348	K03231	EEF1A	elongation factor 1-alpha	--	2	ko03013 RNA transport;ko05134 Legionellosis;
gene30350	K03259	EIF4E	translation initiation factor 4E	--	5	ko03013 RNA transport;ko04066 HIF-1 signaling pathway;ko04150 mTOR signaling pathway;ko04151 PI3K-Akt signaling pathway;ko04910 Insulin signaling pathway;
gene30351	K01728	E4.2.2.2, pel	pectate lyase	EC:4.2.2.2	1	ko00040 Pentose and glucuronate interconversions;
gene30352	K01426	E3.5.1.4, amiE	amidase	EC:3.5.1.4	5	ko00330 Arginine and proline metabolism;ko00360 Phenylalanine metabolism;ko00380 Tryptophan metabolism;ko00627 Aminobenzoate degradation;ko00643 Styrene degradation;
gene30357	K13248	PHOSPHO2	pyridoxal phosphate phosphatase PHOSPHO2	EC:3.1.3.74	1	ko00750 Vitamin B6 metabolism;
gene30362	K14638	SLC15A3_4, PHT	solute carrier family 15 (peptide/histidine transporter), member 3/4	--		
gene30364	K14500	BSK	BR-signaling kinase	EC:2.7.11.1	1	ko04075 Plant hormone signal transduction;
gene30365	K08739	MLH3	DNA mismatch repair protein MLH3	--	1	ko03430 Mismatch repair;
gene30369	K00700	glgB	1,4-alpha-glucan branching enzyme	EC:2.4.1.18	1	ko00500 Starch and sucrose metabolism;
gene30372	K00327	E1.6.2.4	NADPH-ferrihemoprotein reductase	EC:1.6.2.4		

gene30377	K04371	MAPK1_3	mitogen-activated protein kinase 1/3	EC:2.7.11.24	71	ko04010 MAPK signaling pathway;ko04012 ErbB signaling pathway;ko04013 MAPK signaling pathway - fly;ko04014 Ras signaling pathway;ko04062 Chemokine signaling pathway;ko04066 HIF-1 signaling pathway;ko04114 Oocyte meiosis;ko04150 mTOR signaling pathway;ko04151 PI3K-Akt signaling pathway;ko04270 Vascular smooth muscle contraction;ko04320 Dorso-ventral axis formation;ko04350 TGF-beta signaling pathway;ko04360 Axon guidance;ko04370 VEGF signaling pathway;ko04380 Osteoclast differentiation;ko04510 Focal adhesion;ko04520 Adherens junction;ko04540 Gap junction;ko04620 Toll-like receptor signaling pathway;ko04621 NOD-like receptor signaling pathway;ko04650 Natural killer cell mediated cytotoxicity;ko04660 T cell receptor signaling pathway;ko04662 B cell receptor signaling pathway;ko04664 Fc epsilon RI signaling pathway;ko04666 Fc gamma R-mediated phagocytosis;ko04668 TNF signaling pathway;ko04713 Circadian entrainment;ko04720 Long-term potentiation;ko04722 Neurotrophin signaling pathway;ko04723 Retrograde endocannabinoid signaling;ko04724 Glutamatergic synapse;ko04725 Cholinergic synapse;ko04726 Serotonergic synapse;ko04730 Long-term depression;ko04810 Regulation of actin cytoskeleton;ko04910 Insulin signaling pathway;ko04912 GnRH signaling pathway;ko04914 Progesterone-mediated oocyte maturation;ko04915 Estrogen signaling pathway;ko04916 Melanogenesis;ko04917 Prolactin signaling pathway;ko04930 Type II diabetes mellitus;ko04960 Aldosterone-regulated sodium reabsorption;ko05010 Alzheimer's disease;ko05020 Prion diseases;ko05034 Alcoholism;ko05131 Shigellosis;ko05132 Salmonella infection;ko05133 Pertussis;ko05140 Leishmaniasis;ko05142 Chagas disease (American trypanosomiasis);ko05145 Toxoplasmosis;ko05152 Tuberculosis;ko05160 Hepatitis C;ko05161 Hepatitis B;ko05164 Influenza A;ko05200 Pathways in cancer;ko05203 Viral carcinogenesis;ko05205 Proteoglycans in cancer;ko05210 Colorectal cancer;ko05211 Renal cell carcinoma;ko05212
gene30379	K01051	E3.1.1.11	pectinesterase	EC:3.1.1.11	2	ko00040 Pentose and glucuronate interconversions;ko00500 Starch and sucrose metabolism;
gene30381	K02966	RP-S19e, RPS19	small subunit ribosomal protein S19e	--	1	ko03010 Ribosome;
gene30384	K12837	U2AF2	splicing factor U2AF 65 kDa subunit	--	1	ko03040 Spliceosome;
gene30386	K14753	RACK1	guanine nucleotide-binding protein subunit beta-2-like 1 protein	--	1	ko05162 Measles;
gene30394	K08873	SMG1	PI-3-kinase-related kinase SMG-1	--	1	ko03015 mRNA surveillance pathway;
gene30396	K13094	RBM5_10	RNA-binding protein 5/10	--		
gene30405	K13162	PCBP2_3_4	poly(rC)-binding protein 2/3/4	--		
gene30407	K01762	ACS	1-aminocyclopropane-1-carboxylate synthase	EC:4.4.1.14	1	ko00270 Cysteine and methionine metabolism;
gene30408	K00083	E1.1.1.195	cinnamyl-alcohol dehydrogenase	EC:1.1.1.195	1	ko00940 Phenylpropanoid biosynthesis;
gene3040	K08494	NSPN	novel plant SNARE	--		
gene30411	K09553	STIP1	stress-induced-phosphoprotein 1	--	1	ko05020 Prion diseases;
gene30412	K10949	KDELR	ER lumen protein retaining receptor	--	1	ko05110 Vibrio cholerae infection;
gene3041	K11339	MORF4L1, MRG15, EAF3	mortality factor 4-like protein 1	--		
gene30420	K00434	E1.11.1.11	L-ascorbate peroxidase	EC:1.11.1.11	2	ko00053 Ascorbate and aldarate metabolism;ko00480 Glutathione metabolism;
gene30421	K00434	E1.11.1.11	L-ascorbate peroxidase	EC:1.11.1.11	2	ko00053 Ascorbate and aldarate metabolism;ko00480 Glutathione metabolism;

gene30427	K04371	MAPK1_3	mitogen-activated protein kinase 1/3	EC:2.7.11.24	71	ko04010 MAPK signaling pathway;ko04012 ErbB signaling pathway;ko04013 MAPK signaling pathway - fly;ko04014 Ras signaling pathway;ko04062 Chemokine signaling pathway;ko04066 HIF-1 signaling pathway;ko04114 Oocyte meiosis;ko04150 mTOR signaling pathway;ko04151 PI3K-Akt signaling pathway;ko04270 Vascular smooth muscle contraction;ko04320 Dorso-ventral axis formation;ko04350 TGF-beta signaling pathway;ko04360 Axon guidance;ko04370 VEGF signaling pathway;ko04380 Osteoclast differentiation;ko04510 Focal adhesion;ko04520 Adherens junction;ko04540 Gap junction;ko04620 Toll-like receptor signaling pathway;ko04621 NOD-like receptor signaling pathway;ko04650 Natural killer cell mediated cytotoxicity;ko04660 T cell receptor signaling pathway;ko04662 B cell receptor signaling pathway;ko04664 Fc epsilon RI signaling pathway;ko04666 Fc gamma R-mediated phagocytosis;ko04668 TNF signaling pathway;ko04713 Circadian entrainment;ko04720 Long-term potentiation;ko04722 Neurotrophin signaling pathway;ko04723 Retrograde endocannabinoid signaling;ko04724 Glutamatergic synapse;ko04725 Cholinergic synapse;ko04726 Serotonergic synapse;ko04730 Long-term depression;ko04810 Regulation of actin cytoskeleton;ko04910 Insulin signaling pathway;ko04912 GnRH signaling pathway;ko04914 Progesterone-mediated oocyte maturation;ko04915 Estrogen signaling pathway;ko04916 Melanogenesis;ko04917 Prolactin signaling pathway;ko04930 Type II diabetes mellitus;ko04960 Aldosterone-regulated sodium reabsorption;ko05010 Alzheimer's disease;ko05020 Prion diseases;ko05034 Alcoholism;ko05131 Shigellosis;ko05132 Salmonella infection;ko05133 Pertussis;ko05140 Leishmaniasis;ko05142 Chagas disease (American trypanosomiasis);ko05145 Toxoplasmosis;ko05152 Tuberculosis;ko05160 Hepatitis C;ko05161 Hepatitis B;ko05164 Influenza A;ko05200 Pathways in cancer;ko05203 Viral carcinogenesis;ko05205 Proteoglycans in cancer;ko05210 Colorectal cancer;ko05211 Renal cell carcinoma;ko05212
gene3042	K08505	SFT1	protein transport protein SFT1	--	1	ko04130 SNARE interactions in vesicular transport;
gene30430	K02983	RP-S30e, RPS30	small subunit ribosomal protein S30e	--	1	ko03010 Ribosome;
gene30432	K01408	IDE, ide	insulysin	EC:3.4.24.56	1	ko05010 Alzheimer's disease;
gene30436	K14007	SEC24	protein transport protein SEC24	--	1	ko04141 Protein processing in endoplasmic reticulum;
gene30443	K10798	PARP	poly [ADP-ribose] polymerase	EC:2.4.2.30	1	ko03410 Base excision repair;
gene30448	K01880	GARS, glyS1	glycyl-tRNA synthetase	EC:6.1.1.14	1	ko00970 Aminoacyl-tRNA biosynthesis;
gene3044	K05868	CCNB	cyclin B	--	3	ko04110 Cell cycle;ko04115 p53 signaling pathway;ko04914 Progesterone-mediated oocyte maturation;
gene30450	K02873	RP-L13e, RPL13	large subunit ribosomal protein L13e	--	1	ko03010 Ribosome;
gene30454	K01652	E2.2.1.6L, ilvB, ilvG, ilvI	acetolactate synthase I/II/III large subunit	EC:2.2.1.6	6	ko00290 Valine, leucine and isoleucine biosynthesis;ko00650 Butanoate metabolism;ko00660 C5-Branched dibasic acid metabolism;ko00770 Pantothenate and CoA biosynthesis;ko01210 2-Oxocarboxylic acid metabolism;ko01230 Biosynthesis of amino acids;
gene3045	K09422	MYBP	myb proto-oncogene protein, plant	--		
gene30460	K00276	E1.4.3.21, AOC2, AOC3, tynA	primary-amine oxidase	EC:1.4.3.21	6	ko00260 Glycine, serine and threonine metabolism;ko00350 Tyrosine metabolism;ko00360 Phenylalanine metabolism;ko00410 beta-Alanine metabolism;ko00950 Isoquinoline alkaloid biosynthesis;ko00960 Tropane, piperidine and pyridine alkaloid biosynthesis;
gene30461	K00276	E1.4.3.21, AOC2, AOC3, tynA	primary-amine oxidase	EC:1.4.3.21	6	ko00260 Glycine, serine and threonine metabolism;ko00350 Tyrosine metabolism;ko00360 Phenylalanine metabolism;ko00410 beta-Alanine metabolism;ko00950 Isoquinoline alkaloid biosynthesis;ko00960 Tropane, piperidine and pyridine alkaloid biosynthesis;
gene3046	K14232	tRNA-Pro	tRNA Pro	--	1	ko00970 Aminoacyl-tRNA biosynthesis;
gene30470	K14498	SNRK2	serine/threonine-protein kinase SRK2	EC:2.7.11.1	1	ko04075 Plant hormone signal transduction;
gene30471	K14235	tRNA-Trp	tRNA Trp	--	1	ko00970 Aminoacyl-tRNA biosynthesis;

gene30475	K01099	E3.1.3.36	phosphatidylinositol-bisphosphatase	EC:3.1.3.36	2	ko00562 Inositol phosphate metabolism;ko04070 Phosphatidylinositol signaling system;
gene30482	K00799	GST, gst	glutathione S-transferase	EC:2.5.1.18	4	ko00480 Glutathione metabolism;ko00980 Metabolism of xenobiotics by cytochrome P450;ko00982 Drug metabolism - cytochrome P450;ko05204 Chemical carcinogenesis;
gene30483	K08150	SLC2A13, ITR	MFS transporter, SP family, solute carrier family 2 (myo-inositol transporter), member 13	--		
gene30484	K09955	K09955	hypothetical protein	--		
gene30488	K03327	TC.MATE, SLC47A, norM, mdtK, dinF	multidrug resistance protein, MATE family	--		
gene30489	K11498	CENPE	centromeric protein E	--		
gene30498	K00318	E1.5.99.8	proline dehydrogenase	EC:1.5.99.8	1	ko00330 Arginine and proline metabolism;
gene304	K02887	RP-L20, MRPL20, rplT	large subunit ribosomal protein L20	--	1	ko03010 Ribosome;
gene30501	K03032	PSMD1, RPN2	26S proteasome regulatory subunit N2	--	2	ko03050 Proteasome;ko05169 Epstein-Barr virus infection;
gene30503	K04733	IRAK4	interleukin-1 receptor-associated kinase 4	EC:2.7.11.1	11	ko04064 NF-kappa B signaling pathway;ko04210 Apoptosis;ko04620 Toll-like receptor signaling pathway;ko04722 Neurotrophin signaling pathway;ko05133 Pertussis;ko05140 Leishmaniasis;ko05142 Chagas disease (American trypanosomiasis);ko05145 Toxoplasmosis;ko05152 Tuberculosis;ko05162 Measles;ko05164 Influenza A;
gene30504	K17491	SMEK, PPP4R3	protein phosphatase 4 regulatory subunit 3	--		
gene30513	K00748	lpxB	lipid-A-disaccharide synthase	EC:2.4.1.182	1	ko00540 Lipopolysaccharide biosynthesis;
gene30514	K14488	SAUR	SAUR family protein	--	1	ko04075 Plant hormone signal transduction;
gene3051	K04079	htpG, HSP90A	molecular chaperone HtpG	--	9	ko04141 Protein processing in endoplasmic reticulum;ko04151 PI3K-Akt signaling pathway;ko04612 Antigen processing and presentation;ko04621 NOD-like receptor signaling pathway;ko04626 Plant-pathogen interaction;ko04914 Progesterone-mediated oocyte maturation;ko04915 Estrogen signaling pathway;ko05200 Pathways in cancer;ko05215 Prostate cancer;
gene30523	K01803	TPI, tpiA	triosephosphate isomerase (TIM)	EC:5.3.1.1	6	ko00010 Glycolysis / Gluconeogenesis;ko00051 Fructose and mannose metabolism;ko00562 Inositol phosphate metabolism;ko00710 Carbon fixation in photosynthetic organisms;ko01200 Carbon metabolism;ko01230 Biosynthesis of amino acids;
gene30525	K09580	PDIA1, P4HB	protein disulfide-isomerase A1	EC:5.3.4.1	1	ko04141 Protein processing in endoplasmic reticulum;
gene3052	K06268	PPP3R, CNB	serine/threonine-protein phosphatase 2B regulatory subunit	--	18	ko04010 MAPK signaling pathway;ko04020 Calcium signaling pathway;ko04114 Oocyte meiosis;ko04210 Apoptosis;ko04310 Wnt signaling pathway;ko04360 Axon guidance;ko04370 VEGF signaling pathway;ko04380 Osteoclast differentiation;ko04650 Natural killer cell mediated cytotoxicity;ko04660 T cell receptor signaling pathway;ko04662 B cell receptor signaling pathway;ko04720 Long-term potentiation;ko04724 Glutamatergic synapse;ko05010 Alzheimer's disease;ko05014 Amyotrophic lateral sclerosis (ALS);ko05031 Amphetamine addiction;ko05152
gene30540	K00861	RFK, FMN1	riboflavin kinase	EC:2.7.1.26	1	ko00740 Riboflavin metabolism;
gene30541	K10357	MYO5	myosin V	--		
gene30545	K01411	NRD1	nardilysin	EC:3.4.24.61		
gene30547	K05894	E1.3.1.42	12-oxophytodienoic acid reductase	EC:1.3.1.42	1	ko00592 alpha-Linolenic acid metabolism;
gene30548	K14427	SLC12A4_5_6, KCC	solute carrier family 12 (potassium/chloride transporter), member 4/5/6	--		
gene30549	K16297	SCPL-II	serine carboxypeptidase-like clade II	EC:3.4.16.-		
gene3054	K00787	FDPS	farnesyl diphosphate synthase	EC:2.5.1.1 2.5.1.10	3	ko00900 Terpenoid backbone biosynthesis;ko05164 Influenza A;ko05166 HTLV-I infection;
gene30553	K05933	E1.14.17.4	aminocyclopropanecarboxylate oxidase	EC:1.14.17.4	1	ko00270 Cysteine and methionine metabolism;
gene30556	K14709	SLC39A1_2_3, ZIP1_2_3	solute carrier family 39 (zinc transporter), member 1/2/3	--		
gene30557	K08235	E2.4.1.207	xyloglucan:xyloglucosyl transferase	EC:2.4.1.207		
gene30558	K03086	SIG1, rpoD	RNA polymerase primary sigma factor	--		
gene3055	K12863	CWC15	protein CWC15	--	1	ko03040 Spliceosome;

gene30560	K04730	IRAK1	interleukin-1 receptor-associated kinase 1	EC:2.7.11.1	11	ko04064 NF-kappa B signaling pathway;ko04210 Apoptosis;ko04620 Toll-like receptor signaling pathway;ko04722 Neurotrophin signaling pathway;ko05133 Pertussis;ko05140 Leishmaniasis;ko05142 Chagas disease (American trypanosomiasis);ko05145 Toxoplasmosis;ko05152 Tuberculosis;ko05162 Measles;ko05169 Epstein-Barr virus infection;
gene30562	K01674	cah	carbonic anhydrase	EC:4.2.1.1	1	ko00910 Nitrogen metabolism;
gene30564	K11643	CHD4, MI2B	chromodomain-helicase-DNA-binding protein 4	EC:3.6.4.12	1	ko05203 Viral carcinogenesis;
gene30568	K00029	E1.1.1.40, maeB	malate dehydrogenase (oxaloacetate-decarboxylating)(NADP+)	EC:1.1.1.40	3	ko00620 Pyruvate metabolism;ko00710 Carbon fixation in photosynthetic organisms;ko01200 Carbon metabolism;
gene30569	K15083	RAD16	DNA repair protein RAD16	--		
gene30573	K09560	ST13	suppressor of tumorigenicity protein 13	--		
gene30574	K13464	JAZ	jasmonate ZIM domain-containing protein	--	2	ko04075 Plant hormone signal transduction;ko04626 Plant-pathogen interaction;
gene30577	K03106	SRP54, ffh	signal recognition particle subunit SRP54	--	2	ko03060 Protein export;ko03070 Bacterial secretion system;
gene30579	K17604	ZSWIM3	zinc finger SWIM domain-containing protein 3	--		
gene30582	K12585	DIS3, RRP44	exosome complex exonuclease DIS3/RRP44	EC:3.1.13.-	1	ko03018 RNA degradation;
gene30586	K14325	RNPS1	RNA-binding protein with serine-rich domain 1	--	2	ko03013 RNA transport;ko03015 mRNA surveillance pathway;
gene30589	K12188	SNF8, EAP30	ESCRT-II complex subunit VPS22	--	1	ko04144 Endocytosis;
gene30590	K01792	E5.1.3.15	glucose-6-phosphate 1-epimerase	EC:5.1.3.15	1	ko00010 Glycolysis / Gluconeogenesis;
gene30591	K09540	SEC63	translocation protein SEC63	--	2	ko03060 Protein export;ko04141 Protein processing in endoplasmic reticulum;
gene30595	K15718	LOX1_5	linoleate 9S-lipoxygenase	EC:1.13.11.58	1	ko00591 Linoleic acid metabolism;
gene30596	K15718	LOX1_5	linoleate 9S-lipoxygenase	EC:1.13.11.58	1	ko00591 Linoleic acid metabolism;
gene30597	K05666	ABCC2	ATP-binding cassette, subfamily C (CFTR/MRP), member 2	--	2	ko02010 ABC transporters;ko04976 Bile secretion;
gene3059	K07119	K07119				
gene30603	K10268	FBXL2_20	F-box and leucine-rich repeat protein 2/20	--		
gene30609	K14775	UTP30, RSL1D1	ribosome biogenesis protein UTP30	--		
gene30613	K06196	ccdA	cytochrome c-type biogenesis protein	--		
gene30614	K10357	MYO5	myosin V	--		
gene30615	K08914	LHCB3	light-harvesting complex II chlorophyll a/b binding protein 3	--	1	ko00196 Photosynthesis - antenna proteins;
gene30621	K08515	VAMP7	vesicle-associated membrane protein 7	--	1	ko04130 SNARE interactions in vesicular transport;
gene30622	K03695	clpB	ATP-dependent Clp protease ATP-binding subunit ClpB	--		
gene3062	K15326	TSEN54	tRNA-splicing endonuclease subunit Sen54	--		
gene30646	K07904	RAB11A	Ras-related protein Rab-11A	--	4	ko04144 Endocytosis;ko04961 Endocrine and other factor-regulated calcium reabsorption;ko04962 Vasopressin-regulated water reabsorption;ko04972 Pancreatic secretion;
gene30647	K14498	SNRK2	serine/threonine-protein kinase SRK2	EC:2.7.11.1	1	ko04075 Plant hormone signal transduction;
gene30648	K06630	YWHAE	14-3-3 protein epsilon	--	8	ko04110 Cell cycle;ko04114 Oocyte meiosis;ko04151 PI3K-Akt signaling pathway;ko04390 Hippo signaling pathway;ko04391 Hippo signaling pathway - fly;ko04722 Neurotrophin signaling pathway;ko05169 Epstein-Barr virus infection;ko05203 Viral carcinogenesis;
gene30664	K13347	PXMP2, PMP22	peroxisomal membrane protein 2	--	1	ko04146 Peroxisome;
gene30665	K10398	KIF11, EG5	kinesin family member 11	--		
gene30666	K10398	KIF11, EG5	kinesin family member 11	--		
gene30667	K03809	wrbA	Trp repressor binding protein	--		
gene30671	K12839	SMNDC1, SPF30	survival of motor neuron-related-splicing factor 30	--	1	ko03040 Spliceosome;
gene30677	K15177	LEO1	RNA polymerase-associated protein LEO1	--		
gene30678	K03676	grxC, GLRX, GLRX2	glutaredoxin 3	--		
gene30682	K03676	grxC, GLRX, GLRX2	glutaredoxin 3	--		
gene306	K14347	SLC10A7, P7	solute carrier family 10 (sodium/bile acid cotransporter), member 7	--		
gene30700	K03233	EEF1G	elongation factor 1-gamma	--	1	ko05134 Legionellosis;
gene30701	K10691	UBR4, ZUBR1	E3 ubiquitin-protein ligase UBR4	EC:6.3.2.19	1	ko05203 Viral carcinogenesis;

gene30702	K14560	IMP3	U3 small nucleolar ribonucleoprotein protein IMP3	--	1	ko03008 Ribosome biogenesis in eukaryotes;
gene30705	K15925	XYL1	alpha-D-xyloside xylohydrolase	EC:3.2.1.177		
gene30709	K04733	IRAK4	interleukin-1 receptor-associated kinase 4	EC:2.7.11.1	11	ko04064 NF-kappa B signaling pathway;ko04210 Apoptosis;ko04620 Toll-like receptor signaling pathway;ko04722 Neurotrophin signaling pathway;ko05133 Pertussis;ko05140 Leishmaniasis;ko05142 Chagas disease (American trypanosomiasis);ko05145 Toxoplasmosis;ko05152 Tuberculosis;ko05162 Measles;ko05164 Influenza A;
gene30712	K14236	tRNA-Tyr	tRNA Tyr	--	1	ko00970 Aminoacyl-tRNA biosynthesis;
gene30717	K10685	UBLE1B, SAE2, UBA2	ubiquitin-like 1-activating enzyme E1 B	EC:6.3.2.19	1	ko04120 Ubiquitin mediated proteolysis;
gene30719	K03064	PSMC6, RPT4	26S proteasome regulatory subunit T4	--	2	ko03050 Proteasome;ko05169 Epstein-Barr virus infection;
gene30732	K15472	CYP71D55	premnaspirodiene oxygenase	EC:1.14.13.121	1	ko00909 Sesquiterpenoid and triterpenoid biosynthesis;
gene30739	K00789	metK	S-adenosylmethionine synthetase	EC:2.5.1.6	2	ko00270 Cysteine and methionine metabolism;ko01230 Biosynthesis of amino acids;
gene30741	K00021	E1.1.1.34	hydroxymethylglutaryl-CoA reductase (NADPH)	EC:1.1.1.34	2	ko00900 Terpenoid backbone biosynthesis;ko04976 Bile secretion;
gene30747	K11851	USP30	ubiquitin carboxyl-terminal hydrolase 30	EC:3.1.2.15		
gene30750	K17261	CAP1_2, SRV2	adenylyl cyclase-associated protein	--		
gene30751	K03941	NDUFS8	NADH dehydrogenase (ubiquinone) Fe-S protein 8	EC:1.6.5.3 1.6.99.3	5	ko00190 Oxidative phosphorylation;ko04932 Non-alcoholic fatty liver disease (NAFLD);ko05010 Alzheimer's disease;ko05012 Parkinson's disease;ko05016 Huntington's disease;
gene30756	K14307	NUPL1	nucleoporin p58/p45	--	1	ko03013 RNA transport;
gene30769	K03141	TFIIH1, GTF2H1, TFB1	transcription initiation factor TFIIH subunit 1	--	3	ko03022 Basal transcription factors;ko03420 Nucleotide excision repair;ko05203 Viral carcinogenesis;
gene30770	K10523	SPOP	speckle-type POZ protein	--		
gene30771	K10523	SPOP	speckle-type POZ protein	--		
gene30776	K07088	K07088				
gene30778	K04079	htpG, HSP90A	molecular chaperone HtpG	--	9	ko04141 Protein processing in endoplasmic reticulum;ko04151 PI3K-Akt signaling pathway;ko04612 Antigen processing and presentation;ko04621 NOD-like receptor signaling pathway;ko04626 Plant-pathogen interaction;ko04914 Progesterone-mediated oocyte maturation;ko04915 Estrogen signaling pathway;ko05200 Pathways in cancer;ko05215 Prostate cancer;
gene3077	K01870	IARS, ileS	isoleucyl-tRNA synthetase	EC:6.1.1.5	1	ko00970 Aminoacyl-tRNA biosynthesis;
gene30782	K12489	ACAP	Arf-GAP with coiled-coil, ANK repeat and PH domain-containing protein	--	1	ko04144 Endocytosis;
gene30783	K12489	ACAP	Arf-GAP with coiled-coil, ANK repeat and PH domain-containing protein	--	1	ko04144 Endocytosis;
gene30784	K00574	E2.1.1.79, cfa	cyclopropane-fatty-acyl-phospholipid synthase	EC:2.1.1.79		
gene3078	K00485	E1.14.13.8	dimethylaniline monooxygenase (N-oxide forming)	EC:1.14.13.8	2	ko00680 Methane metabolism;ko00982 Drug metabolism - cytochrome P450;
gene30794	K12827	SF3A3, SAP61, PRP9	splicing factor 3A subunit 3	--	1	ko03040 Spliceosome;
gene30798	K15687	MKRN	E3 ubiquitin-protein ligase makorin	EC:6.3.2.19		
gene30799	K15687	MKRN	E3 ubiquitin-protein ligase makorin	EC:6.3.2.19		
gene30802	K00434	E1.11.1.11	L-ascorbate peroxidase	EC:1.11.1.11	2	ko00053 Ascorbate and aldarate metabolism;ko00480 Glutathione metabolism;
gene30804	K10643	CNOT4, NOT4, MOT2	CCR4-NOT transcription complex subunit 4	EC:6.3.2.19	1	ko03018 RNA degradation;
gene30806	K03539	RPP1, RPP30	ribonuclease P/MRP protein subunit RPP1	EC:3.1.26.5	2	ko03008 Ribosome biogenesis in eukaryotes;ko03013 RNA transport;
gene3080	K04733	IRAK4	interleukin-1 receptor-associated kinase 4	EC:2.7.11.1	11	ko04064 NF-kappa B signaling pathway;ko04210 Apoptosis;ko04620 Toll-like receptor signaling pathway;ko04722 Neurotrophin signaling pathway;ko05133 Pertussis;ko05140 Leishmaniasis;ko05142 Chagas disease (American trypanosomiasis);ko05145 Toxoplasmosis;ko05152 Tuberculosis;ko05162 Measles;ko05164 Influenza A;
gene30812	K17424	MRPL43	large subunit ribosomal protein L43, mitochondrial	--		

gene30815	K04733	IRAK4	interleukin-1 receptor-associated kinase 4	EC:2.7.11.1	11	ko04064 NF-kappa B signaling pathway;ko04210 Apoptosis;ko04620 Toll-like receptor signaling pathway;ko04722 Neurotrophin signaling pathway;ko05133 Pertussis;ko05140 Leishmaniasis;ko05142 Chagas disease (American trypanosomiasis);ko05145 Toxoplasmosis;ko05152 Tuberculosis;ko05162 Measles;ko05164 Influenza A;
gene3081	K01835	pgm	phosphoglucomutase	EC:5.4.2.2	7	ko00010 Glycolysis / Gluconeogenesis;ko00030 Pentose phosphate pathway;ko00052 Galactose metabolism;ko00230 Purine metabolism;ko00500 Starch and sucrose metabolism;ko00520 Amino sugar and nucleotide sugar metabolism;ko00521 Streptomycin biosynthesis;
gene30821	K11699	RDR1, RDP1	RNA-dependent RNA polymerase 1	EC:2.7.7.48		
gene30823	K14487	GH3	auxin responsive GH3 gene family	--	1	ko04075 Plant hormone signal transduction;
gene30828	K01881	PARS, proS	prolyl-tRNA synthetase	EC:6.1.1.15	1	ko00970 Aminoacyl-tRNA biosynthesis;
gene30831	K01881	PARS, proS	prolyl-tRNA synthetase	EC:6.1.1.15	1	ko00970 Aminoacyl-tRNA biosynthesis;
gene30833	K09286	EREBP	EREBP-like factor	--		
gene30835	K15707	RNF170	RING finger protein 170	--		
gene30836	K10901	BLM, RECQL3, SGS1	bloom syndrome protein	EC:3.6.4.12	2	ko03440 Homologous recombination;ko03460 Fanconi anemia pathway;
gene30838	K02969	RP-S20e, RPS20	small subunit ribosomal protein S20e	--	1	ko03010 Ribosome;
gene30839	K12486	SMAP	stromal membrane-associated protein	--	1	ko04144 Endocytosis;
gene30843	K00434	E1.11.1.11	L-ascorbate peroxidase	EC:1.11.1.11	2	ko00053 Ascorbate and aldarate metabolism;ko00480 Glutathione metabolism;
gene30844	K14232	tRNA-Pro	tRNA Pro	--	1	ko00970 Aminoacyl-tRNA biosynthesis;
gene30849	K08869	ADCK, ABC1	aarF domain-containing kinase	--		
gene30851	K15378	SLC45A1_2_4	solute carrier family 45, member 1/2/4	--		
gene30852	K15451	PPM2, LCMT2, TYW4	tRNA wybutosine-synthesizing protein 4	EC:2.1.1.-		
gene30855	K07936	RAN	GTP-binding nuclear protein Ran	--	4	ko03008 Ribosome biogenesis in eukaryotes;ko03013 RNA transport;ko05166 HTLV-I infection;ko05169 Epstein-Barr virus infection;
gene30857	K13379	RGP, UTM	reversibly glycosylated polypeptide / UDP-arabinopyranose mutase	EC:2.4.1.- 5.4.99.30		
gene30860	K02894	RP-L23e, RPL23	large subunit ribosomal protein L23e	--	1	ko03010 Ribosome;
gene30862	K05868	CCNB	cyclin B	--	3	ko04110 Cell cycle;ko04115 p53 signaling pathway;ko04914 Progesterone-mediated oocyte maturation;
gene30866	K01358	clpP, CLPP	ATP-dependent Clp protease, protease subunit	EC:3.4.21.92	1	ko04112 Cell cycle - Caulobacter;
gene30867	K15559	RTT103	regulator of Ty1 transposition protein 103	--		
gene30872	K07199	PRKAB	5'-AMP-activated protein kinase, regulatory beta subunit	--	5	ko04710 Circadian rhythm;ko04910 Insulin signaling pathway;ko04920 Adipocytokine signaling pathway;ko04932 Non-alcoholic fatty liver disease (NAFLD);ko05410 Hypertrophic cardiomyopathy (HCM);
gene30876	K09014	sufB	Fe-S cluster assembly protein SufB	--		
gene30878	K09489	HSPA4	heat shock 70kDa protein 4	--	1	ko04612 Antigen processing and presentation;
gene30879	K03327	TC.MATE, SLC47A, norM, mdtK, dinF	multidrug resistance protein, MATE family	--		
gene30880	K03327	TC.MATE, SLC47A, norM, mdtK, dinF	multidrug resistance protein, MATE family	--		
gene30884	K12626	LSM7	U6 snRNA-associated Sm-like protein LSm7	--	2	ko03018 RNA degradation;ko03040 Spliceosome;
gene30887	K03120	TBP, tbp	transcription initiation factor TFIID TATA-box-binding protein	--	6	ko03022 Basal transcription factors;ko05016 Huntington's disease;ko05166 HTLV-I infection;ko05168 Herpes simplex infection;ko05169 Epstein-Barr virus infection;ko05203 Viral carcinogenesis;
gene30888	K00167	E1.2.4.4B, bkdA2	2-oxoisovalerate dehydrogenase E1 component, beta subunit	EC:1.2.4.4	1	ko00280 Valine, leucine and isoleucine degradation;
gene30890	K12897	TRA2	transformer-2 protein	--	1	ko03040 Spliceosome;
gene30891	K03320	amt, AMT, MEP	ammonium transporter, Amt family	--		
gene30894	K01102	PDP	pyruvate dehydrogenase phosphatase	EC:3.1.3.43		
gene30901	K01051	E3.1.1.11	pectinesterase	EC:3.1.1.11	2	ko00040 Pentose and glucuronate interconversions;ko00500 Starch and sucrose metabolism;
gene30902	K09534	DNAJC14	DnaJ homolog subfamily C member 14	--		
gene30903	K15405	MAH1, CYP96A15	cytochrome P450, family 96, subfamily A, polypeptide 15 (midchain alkane hydroxylase)	--	1	ko00073 Cutin, suberine and wax biosynthesis;

gene30909	K08066	NFYC	nuclear transcription factor Y, gamma	--	2	ko04612 Antigen processing and presentation;ko05152 Tuberculosis;
gene30910	K07342	SEC61G, SSS1, secE	protein transport protein SEC61 subunit gamma and related proteins	--	4	ko03060 Protein export;ko04141 Protein processing in endoplasmic reticulum;ko04145 Phagosome;ko05110 Vibrio cholerae infection;
gene30912	K03420	psmR	proteasome regulatory subunit	--	1	ko03050 Proteasome;
gene30913	K02147	ATPeVB, ATP6B1	V-type H+-transporting ATPase subunit B	EC:3.6.3.14	7	ko00190 Oxidative phosphorylation;ko04145 Phagosome;ko04721 Synaptic vesicle cycle;ko04966 Collecting duct acid secretion;ko05110 Vibrio cholerae infection;ko05120 Epithelial cell signaling in Helicobacter pylori infection;ko05323 Rheumatoid arthritis;
gene30914	K08242	E2.1.1.143	24-methylenesterol C-methyltransferase	EC:2.1.1.143	1	ko00100 Steroid biosynthesis;
gene30915	K02636	petC	cytochrome b6-f complex iron-sulfur subunit	EC:1.10.9.1	1	ko00195 Photosynthesis;
gene30921	K04733	IRAK4	interleukin-1 receptor-associated kinase 4	EC:2.7.11.1	11	ko04064 NF-kappa B signaling pathway;ko04210 Apoptosis;ko04620 Toll-like receptor signaling pathway;ko04722 Neurotrophin signaling pathway;ko05133 Pertussis;ko05140 Leishmaniasis;ko05142 Chagas disease (American trypanosomiasis);ko05145 Toxoplasmosis;ko05152 Tuberculosis;ko05162 Measles;ko05164 Influenza A;
gene30928	K02932	RP-L5e, RPL5	large subunit ribosomal protein L5e	--	1	ko03010 Ribosome;
gene3092	K14442	DHX36, RHAU	ATP-dependent RNA helicase DHX36	EC:3.6.4.13	1	ko03018 RNA degradation;
gene30930	K10798	PARP	poly [ADP-ribose] polymerase	EC:2.4.2.30	1	ko03410 Base excision repair;
gene30937	K09422	MYBP	myb proto-oncogene protein, plant	--		
gene3093	K09496	CCT4	T-complex protein 1 subunit delta	--		
gene30941	K03139	TFIIF2, GTF2F2, TFG2	transcription initiation factor TFIIF subunit beta	EC:3.6.4.12	1	ko03022 Basal transcription factors;
gene30945	K11228	STE11	mitogen-activated protein kinase kinase kinase	EC:2.7.11.1	1	ko04011 MAPK signaling pathway - yeast;
gene30946	K14574	SDO1, SBDS	ribosome maturation protein SDO1	--	1	ko03008 Ribosome biogenesis in eukaryotes;
gene3094	K17500	ILKAP	integrin-linked kinase-associated serine/threonine phosphatase 2C	EC:3.1.3.16		
gene30951	K03232	EEF1B	elongation factor 1-beta	--		
gene30955	K14832	MAK21, NOC1, CEBPZ	ribosome biogenesis protein MAK21	--		
gene30961	K09875	SIP	aquaporin SIP	--		
gene30963	K17479	GRXCR1	glutaredoxin domain-containing cysteine-rich protein 1	--		
gene30965	K11968	ARIH1	ariadne-1	--		
gene30968	K03527	E1.17.1.2, lytB, ispH	4-hydroxy-3-methylbut-2-enyl diphosphate reductase	EC:1.17.1.2	1	ko00900 Terpenoid backbone biosynthesis;
gene30969	K03327	TC.MATE, SLC47A, norM, mdtK, dinF	multidrug resistance protein, MATE family	--		
gene30970	K03327	TC.MATE, SLC47A, norM, mdtK, dinF	multidrug resistance protein, MATE family	--		
gene30971	K07399	resB, ccs1	cytochrome c biogenesis protein	--		
gene30972	K10666	RNF5	E3 ubiquitin-protein ligase RNF5	EC:6.3.2.19	1	ko04141 Protein processing in endoplasmic reticulum;
gene30973	K14018	PLAA, DOA1, UFD3	phospholipase A-2-activating protein	--	1	ko04141 Protein processing in endoplasmic reticulum;
gene30975	K01866	YARS, tyrS	tyrosyl-tRNA synthetase	EC:6.1.1.1	1	ko00970 Aminoacyl-tRNA biosynthesis;
gene30976	K13513	LCLAT1, AGPAT8	lysocardiolipin and lysophospholipid acyltransferase	EC:2.3.1.-2.3.1.51	2	ko00561 Glycerolipid metabolism;ko00564 Glycerophospholipid metabolism;
gene30982	K00430	E1.11.1.7	peroxidase	EC:1.11.1.7	2	ko00360 Phenylalanine metabolism;ko00940 Phenylpropanoid biosynthesis;
gene3098	K08065	NFYB	nuclear transcription Y subunit beta	--	3	ko04612 Antigen processing and presentation;ko05152 Tuberculosis;ko05166 HTLV-I infection;
gene30993	K10635	RNF111	E3 ubiquitin-protein ligase Arkadia	EC:6.3.2.19		
gene30995	K11426	SMYD	SET and MYND domain-containing protein	--		
gene30996	K11426	SMYD	SET and MYND domain-containing protein	--		
gene30999	K14432	ABF	ABA responsive element binding factor	--	1	ko04075 Plant hormone signal transduction;
gene30	K01598	PPCDC, coaC	phosphopantothenoylcysteine decarboxylase	EC:4.1.1.36	1	ko00770 Pantothenate and CoA biosynthesis;
gene31000	K02865	RP-L10Ae, RPL10A	large subunit ribosomal protein L10Ae	--	1	ko03010 Ribosome;
gene31001	K01193	E3.2.1.26, sacA	beta-fructofuranosidase	EC:3.2.1.26	2	ko00052 Galactose metabolism;ko00500 Starch and sucrose metabolism;
gene31002	K02877	RP-L15e, RPL15	large subunit ribosomal protein L15e	--	1	ko03010 Ribosome;
gene31006	K00430	E1.11.1.7	peroxidase	EC:1.11.1.7	2	ko00360 Phenylalanine metabolism;ko00940 Phenylpropanoid biosynthesis;

gene31011	K09060	GBF	plant G-box-binding factor	--		
gene31012	K03103	MINPP1	multiple inositol-polyphosphate phosphatase	EC:3.1.3.62	1	ko00562 Inositol phosphate metabolism;
gene31015	K04371	MAPK1_3	mitogen-activated protein kinase 1/3	EC:2.7.11.24	71	ko04010 MAPK signaling pathway;ko04012 ErbB signaling pathway;ko04013 MAPK signaling pathway - fly;ko04014 Ras signaling pathway;ko04062 Chemokine signaling pathway;ko04066 HIF-1 signaling pathway;ko04114 Oocyte meiosis;ko04150 mTOR signaling pathway;ko04151 PI3K-Akt signaling pathway;ko04270 Vascular smooth muscle contraction;ko04320 Dorso-ventral axis formation;ko04350 TGF-beta signaling pathway;ko04360 Axon guidance;ko04370 VEGF signaling pathway;ko04380 Osteoclast differentiation;ko04510 Focal adhesion;ko04520 Adherens junction;ko04540 Gap junction;ko04620 Toll-like receptor signaling pathway;ko04621 NOD-like receptor signaling pathway;ko04650 Natural killer cell mediated cytotoxicity;ko04660 T cell receptor signaling pathway;ko04662 B cell receptor signaling pathway;ko04664 Fc epsilon RI signaling pathway;ko04666 Fc gamma R-mediated phagocytosis;ko04668 TNF signaling pathway;ko04713 Circadian entrainment;ko04720 Long-term potentiation;ko04722 Neurotrophin signaling pathway;ko04723 Retrograde endocannabinoid signaling;ko04724 Glutamatergic synapse;ko04725 Cholinergic synapse;ko04726 Serotonergic synapse;ko04730 Long-term depression;ko04810 Regulation of actin cytoskeleton;ko04910 Insulin signaling pathway;ko04912 GnRH signaling pathway;ko04914 Progesterone-mediated oocyte maturation;ko04915 Estrogen signaling pathway;ko04916 Melanogenesis;ko04917 Prolactin signaling pathway;ko04930 Type II diabetes mellitus;ko04960 Aldosterone-regulated sodium reabsorption;ko05010 Alzheimer's disease;ko05020 Prion diseases;ko05034 Alcoholism;ko05131 Shigellosis;ko05132 Salmonella infection;ko05133 Pertussis;ko05140 Leishmaniasis;ko05142 Chagas disease (American trypanosomiasis);ko05145 Toxoplasmosis;ko05152 Tuberculosis;ko05160 Hepatitis C;ko05161 Hepatitis B;ko05164 Influenza A;ko05200 Pathways in cancer;ko05203 Viral carcinogenesis;ko05205 Proteoglycans in cancer;ko05210 Colorectal cancer;ko05211 Renal cell carcinoma;ko05212
gene31020	K06669	SMC3, CSPG6	structural maintenance of chromosome 3 (chondroitin sulfate proteoglycan 6)	--	4	ko04110 Cell cycle;ko04111 Cell cycle - yeast;ko04113 Meiosis - yeast;ko04114 Oocyte meiosis;
gene31021	K05349	bglX	beta-glucosidase	EC:3.2.1.21	3	ko00460 Cyanoamino acid metabolism;ko00500 Starch and sucrose metabolism;ko00940 Phenylpropanoid biosynthesis;
gene31022	K05349	bglX	beta-glucosidase	EC:3.2.1.21	3	ko00460 Cyanoamino acid metabolism;ko00500 Starch and sucrose metabolism;ko00940 Phenylpropanoid biosynthesis;
gene31027	K15404	K15404, CER1	aldehyde decarbonylase	EC:4.1.99.5	1	ko00073 Cutin, suberine and wax biosynthesis;
gene3102	K10577	UBE2I, UBC9	ubiquitin-conjugating enzyme E2 I	EC:6.3.2.19	4	ko03013 RNA transport;ko04064 NF-kappa B signaling pathway;ko04120 Ubiquitin mediated proteolysis;ko05206 MicroRNAs in cancer;
gene31031	K00261	GLUD1_2, gdhA	glutamate dehydrogenase (NAD(P)+)	EC:1.4.1.3	5	ko00250 Alanine, aspartate and glutamate metabolism;ko00330 Arginine and proline metabolism;ko00471 D-Glutamine and D-glutamate metabolism;ko00910 Nitrogen metabolism;ko04964 Proximal tubule bicarbonate reclamation;
gene31035	K02685	PRI2	DNA primase large subunit	EC:2.7.7.-	3	ko00230 Purine metabolism;ko00240 Pyrimidine metabolism;ko03030 DNA replication;
gene31041	K16904	DCTPP1	dCTP diphosphatase	EC:3.6.1.12	1	ko00240 Pyrimidine metabolism;
gene31045	K14508	NPR1	regulatory protein NPR1	--	1	ko04075 Plant hormone signal transduction;
gene31048	K10706	SETX, ALS4	senataxin	EC:3.6.4.-		
gene31049	K10706	SETX, ALS4	senataxin	EC:3.6.4.-		
gene31052	K14794	RRP12	ribosomal RNA-processing protein 12	--		
gene31061	K14510	CTR1	serine/threonine-protein kinase CTR1	EC:2.7.11.1	1	ko04075 Plant hormone signal transduction;
gene31063	K11228	STE11	mitogen-activated protein kinase kinase kinase	EC:2.7.11.1	1	ko04011 MAPK signaling pathway - yeast;
gene31067	K11131	DKC1, NOLA4, CBF5	H/ACA ribonucleoprotein complex subunit 4	EC:5.4.99.-	1	ko03008 Ribosome biogenesis in eukaryotes;
gene3106	K12259	SMOX	spermine oxidase	EC:1.5.3.16		

gene31075	K03129	TAF4	transcription initiation factor TFIID subunit 4	--	3	ko03022 Basal transcription factors;ko05016 Huntington's disease;ko05168 Herpes simplex infection;
gene31077	K13457	RPM1, RPS3	disease resistance protein RPM1	--	1	ko04626 Plant-pathogen interaction;
gene31079	K17434	MRPL53	large subunit ribosomal protein L53, mitochondrial	--		
gene31080	K00626	E2.3.1.9, atoB	acetyl-CoA C-acetyltransferase	EC:2.3.1.9	14	ko00071 Fatty acid degradation;ko00072 Synthesis and degradation of ketone bodies;ko00280 Valine, leucine and isoleucine degradation;ko00310 Lysine degradation;ko00362 Benzoate degradation;ko00380 Tryptophan metabolism;ko00620 Pyruvate metabolism;ko00630 Glyoxylate and dicarboxylate metabolism;ko00640 Propanoate metabolism;ko00650 Butanoate metabolism;ko00720 Carbon fixation pathways in prokaryotes;ko00900 Terpenoid backbone biosynthesis;ko01200 Carbon
gene3108	K00797	speE, SRM	spermidine synthase	EC:2.5.1.16	4	ko00270 Cysteine and methionine metabolism;ko00330 Arginine and proline metabolism;ko00410 beta-Alanine metabolism;ko00480 Glutathione metabolism;
gene31095	K08819	CDK12_13	cyclin-dependent kinase 12/13	EC:2.7.11.22 2.7.11.23		
gene3109	K14288	XPOT	exportin-T	--	1	ko03013 RNA transport;
gene31100	K00326	E1.6.2.2	cytochrome-b5 reductase	EC:1.6.2.2	1	ko00520 Amino sugar and nucleotide sugar metabolism;
gene31105	K11665	INO80, INOC1	DNA helicase INO80	EC:3.6.4.12		
gene31107	K14230	tRNA-Met	tRNA Met	--	1	ko00970 Aminoacyl-tRNA biosynthesis;
gene3110	K03627	MBF1	putative transcription factor	--		
gene31111	K08064	NFYA	nuclear transcription factor Y, alpha	--	2	ko04612 Antigen processing and presentation;ko05152 Tuberculosis;
gene31113	K14553	UTP18	U3 small nucleolar RNA-associated protein 18	--	1	ko03008 Ribosome biogenesis in eukaryotes;
gene31116	K14816	REI1	pre-60S factor REI1	--		
gene31118	K14222	tRNA-Cys	tRNA Cys	--	1	ko00970 Aminoacyl-tRNA biosynthesis;
gene31119	K08511	ATVAMP72	vesicle-associated membrane protein 72	--		
gene31120	K12834	PHF5A	PHD finger-like domain-containing protein 5A	--	1	ko03040 Spliceosome;
gene31122	K03671	trxA	thioredoxin 1	--		
gene31123	K02160	accB, bccP	acetyl-CoA carboxylase biotin carboxyl carrier protein	--	6	ko00061 Fatty acid biosynthesis;ko00253 Tetracycline biosynthesis;ko00620 Pyruvate metabolism;ko00640 Propanoate metabolism;ko00720 Carbon fixation pathways in prokaryotes;ko01200 Carbon metabolism;
gene31127	K02957	RP-S15Ae, RPS15A	small subunit ribosomal protein S15Ae	--	1	ko03010 Ribosome;
gene31128	K01285	PRCP	lysosomal Pro-X carboxypeptidase	EC:3.4.16.2	1	ko04974 Protein digestion and absorption;
gene31129	K00366	nirA	ferredoxin-nitrite reductase	EC:1.7.7.1	1	ko00910 Nitrogen metabolism;
gene31133	K14487	GH3	auxin responsive GH3 gene family	--	1	ko04075 Plant hormone signal transduction;
gene31134	K14820	BRX1, BRIX1	ribosome biogenesis protein BRX1	--		
gene31138	K08678	UXS1	UDP-glucuronate decarboxylase	EC:4.1.1.35	2	ko00500 Starch and sucrose metabolism;ko00520 Amino sugar and nucleotide sugar metabolism;
gene3113	K01599	hemE, UROD	uroporphyrinogen decarboxylase	EC:4.1.1.37	1	ko00860 Porphyrin and chlorophyll metabolism;
gene3114	K14236	tRNA-Tyr	tRNA Tyr	--	1	ko00970 Aminoacyl-tRNA biosynthesis;
gene31152	K07441	ALG14	beta-1,4-N-acetylglucosaminyltransferase	EC:2.4.1.141	2	ko00510 N-Glycan biosynthesis;ko00513 Various types of N-glycan biosynthesis;
gene31154	K15429	TRM5, TRMT5	tRNA (guanine37-N1)-methyltransferase	EC:2.1.1.228		
gene31156	K07937	ARF1	ADP-ribosylation factor 1	--	2	ko05110 Vibrio cholerae infection;ko05134 Legionellosis;
gene31157	K17609	NXN	nucleoredoxin	EC:1.8.1.8		
gene31159	K15014	SLC29A1_2_3, ENT1_2_3	solute carrier family 29 (equilibrative nucleoside transporter), member 1/2/3	--		
gene3115	K00434	E1.11.1.11	L-ascorbate peroxidase	EC:1.11.1.11	2	ko00053 Ascorbate and aldarate metabolism;ko00480 Glutathione metabolism;
gene31167	K02978	RP-S27e, RPS27	small subunit ribosomal protein S27e	--	1	ko03010 Ribosome;
gene3116	K14236	tRNA-Tyr	tRNA Tyr	--	1	ko00970 Aminoacyl-tRNA biosynthesis;
gene31171	K14006	SEC23	protein transport protein SEC23	--	1	ko04141 Protein processing in endoplasmic reticulum;
gene31172	K16075	MRS2, MFM1	magnesium transporter	--		
gene31173	K00981	E2.7.7.41, CDS1, CDS2, cdsA	phosphatidate cytidylyltransferase	EC:2.7.7.41	2	ko00564 Glycerophospholipid metabolism;ko04070 Phosphatidylinositol signaling system;
gene31174	K07192	FLOT	flotillin	--	1	ko04910 Insulin signaling pathway;
gene31178	K15115	SLC25A32, MFT	solute carrier family 25 (mitochondrial folate transporter), member 32	--		

gene31179	K14724	SLC9A8, NHE8	solute carrier family 9 (sodium/hydrogen exchanger), member 8	--		
gene31182	K01262	pepP	Xaa-Pro aminopeptidase	EC:3.4.11.9		
gene31185	K03237	EIF2S1	translation initiation factor 2 subunit 1	--	7	ko03013 RNA transport;ko04141 Protein processing in endoplasmic reticulum;ko04932 Non-alcoholic fatty liver disease (NAFLD);ko05160 Hepatitis C;ko05162 Measles;ko05164 Influenza A;ko05168 Herpes simplex infection;
gene31186	K14545	RRP7	ribosomal RNA-processing protein 7	--	1	ko03008 Ribosome biogenesis in eukaryotes;
gene311	K00799	GST, gst	glutathione S-transferase	EC:2.5.1.18	4	ko00480 Glutathione metabolism;ko00980 Metabolism of xenobiotics by cytochrome P450;ko00982 Drug metabolism - cytochrome P450;ko05204 Chemical carcinogenesis;
gene31200	K01051	E3.1.1.11	pectinesterase	EC:3.1.1.11	2	ko00040 Pentose and glucuronate interconversions;ko00500 Starch and sucrose metabolism;
gene31204	K00001	E1.1.1.1, adh	alcohol dehydrogenase	EC:1.1.1.1	9	ko00010 Glycolysis / Gluconeogenesis;ko00071 Fatty acid degradation;ko00350 Tyrosine metabolism;ko00625 Chloroalkane and chloroalkene degradation;ko00626 Naphthalene degradation;ko00830 Retinol metabolism;ko00980 Metabolism of xenobiotics by cytochrome P450;ko00982 Drug metabolism - cytochrome P450;ko01220 Degradation of aromatic compounds;
gene31205	K12871	CCDC12	coiled-coil domain-containing protein 12	--	1	ko03040 Spliceosome;
gene31208	K14638	SLC15A3_4, PHT	solute carrier family 15 (peptide/histidine transporter), member 3/4	--		
gene31211	K14516	ERF1	ethylene-responsive transcription factor 1	--	1	ko04075 Plant hormone signal transduction;
gene31213	K00327	E1.6.2.4	NADPH-ferrihemoprotein reductase	EC:1.6.2.4		
gene31214	K14514	EIN3	ethylene-insensitive protein 3	--	1	ko04075 Plant hormone signal transduction;
gene31215	K13448	CML	calcium-binding protein CML	--	1	ko04626 Plant-pathogen interaction;
gene31221	K10357	MYO5	myosin V	--		
gene31225	K14500	BSK	BR-signaling kinase	EC:2.7.11.1	1	ko04075 Plant hormone signal transduction;
gene31229	K14488	SAUR	SAUR family protein	--	1	ko04075 Plant hormone signal transduction;
gene31230	K11341	YEATS4, GAS41, YAF9	YEATS domain-containing protein 4	--		
gene31231	K13648	GAUT	alpha-1,4-galacturonosyltransferase	EC:2.4.1.43	2	ko00500 Starch and sucrose metabolism;ko00520 Amino sugar and nucleotide sugar metabolism;
gene31232	K04716	SGPP1	sphingosine-1-phosphate phosphatase 1	EC:3.1.3.-	1	ko00600 Sphingolipid metabolism;
gene31233	K13356	FAR	fatty acyl-CoA reductase	EC:1.2.1.-	2	ko00073 Cutin, suberine and wax biosynthesis;ko04146 Peroxisome;
gene31234	K00721	DPM1	dolichol-phosphate mannosyltransferase	EC:2.4.1.83	1	ko00510 N-Glycan biosynthesis;
gene31235	K11426	SMYD	SET and MYND domain-containing protein	--		
gene31239	K04733	IRAK4	interleukin-1 receptor-associated kinase 4	EC:2.7.11.1	11	ko04064 NF-kappa B signaling pathway;ko04210 Apoptosis;ko04620 Toll-like receptor signaling pathway;ko04722 Neurotrophin signaling pathway;ko05133 Pertussis;ko05140 Leishmaniasis;ko05142 Chagas disease (American trypanosomiasis);ko05145 Toxoplasmosis;ko05152 Tuberculosis;ko05162 Measles;ko05164 Influenza A;
gene31240	K03687	GRPE	molecular chaperone GrpE	--		
gene31241	K11648	SMARCB1, SNF5, INI1	SWI/SNF-related matrix-associated actin-dependent regulator of chromatin subfamily B member 1	--		
gene31242	K03249	EIF3F	translation initiation factor 3 subunit F	--	1	ko03013 RNA transport;
gene31245	K00859	coaE	dephospho-CoA kinase	EC:2.7.1.24	1	ko00770 Pantothenate and CoA biosynthesis;
gene31247	K11430	EZH2	histone-lysine N-methyltransferase EZH2	EC:2.1.1.43	1	ko05206 MicroRNAs in cancer;
gene31248	K01952	purL, PFAS	phosphoribosylformylglycinamidine synthase	EC:6.3.5.3	1	ko00230 Purine metabolism;
gene31251	K01602	rbcS	ribulose-bisphosphate carboxylase small chain	EC:4.1.1.39	3	ko00630 Glyoxylate and dicarboxylate metabolism;ko00710 Carbon fixation in photosynthetic organisms;ko01200 Carbon metabolism;
gene31253	K09510	DNAJB4	DnaJ homolog subfamily B member 4	--		
gene31254	K01535	E3.6.3.6	H+-transporting ATPase	EC:3.6.3.6	1	ko00190 Oxidative phosphorylation;
gene31255	K14638	SLC15A3_4, PHT	solute carrier family 15 (peptide/histidine transporter), member 3/4	--		

gene31259	K04733	IRAK4	interleukin-1 receptor-associated kinase 4	EC:2.7.11.1	11	ko04064 NF-kappa B signaling pathway;ko04210 Apoptosis;ko04620 Toll-like receptor signaling pathway;ko04722 Neurotrophin signaling pathway;ko05133 Pertussis;ko05140 Leishmaniasis;ko05142 Chagas disease (American trypanosomiasis);ko05145 Toxoplasmosis;ko05152 Tuberculosis;ko05162 Measles;ko05164 Influenza A;
gene3125	K12486	SMAP	stromal membrane-associated protein	--	1	ko04144 Endocytosis;
gene31262	K13347	PXMP2, PMP22	peroxisomal membrane protein 2	--	1	ko04146 Peroxisome;
gene31266	K10908	POLRMT, RPO41	DNA-directed RNA polymerase, mitochondrial	EC:2.7.7.6		
gene31268	K09419	HSFF	heat shock transcription factor, other eukaryote	--		
gene3126	K16675	ZDHHC14	probable palmitoyltransferase ZDHHC14	EC:2.3.1.-	1	ko04391 Hippo signaling pathway - fly;
gene31272	K14492	ARR-A	two-component response regulator ARR-A family	--	1	ko04075 Plant hormone signal transduction;
gene31277	K03966	NDUFB10	NADH dehydrogenase (ubiquinone) 1 beta subcomplex subunit 10	--	5	ko00190 Oxidative phosphorylation;ko04932 Non-alcoholic fatty liver disease (NAFLD);ko05010 Alzheimer's disease;ko05012 Parkinson's disease;ko05016 Huntington's disease;
gene3127	K09841	ABA2	xanthoxin dehydrogenase	EC:1.1.1.288	1	ko00906 Carotenoid biosynthesis;
gene31285	K05765	CFL	cofilin	--	4	ko04360 Axon guidance;ko04666 Fc gamma R-mediated phagocytosis;ko04810 Regulation of actin cytoskeleton;ko05133 Pertussis;
gene31286	K13667	RUMI, KTELC1	protein glucosyltransferase	EC:2.4.1.-	1	ko00514 Other types of O-glycan biosynthesis;
gene31287	K13667	RUMI, KTELC1	protein glucosyltransferase	EC:2.4.1.-	1	ko00514 Other types of O-glycan biosynthesis;
gene31288	K02984	RP-S3Ae, RPS3A	small subunit ribosomal protein S3Ae	--	1	ko03010 Ribosome;
gene3128	K09841	ABA2	xanthoxin dehydrogenase	EC:1.1.1.288	1	ko00906 Carotenoid biosynthesis;
gene31290	K02694	psaF	photosystem I subunit III	--	1	ko00195 Photosynthesis;
gene31292	K13356	FAR	fatty acyl-CoA reductase	EC:1.2.1.-	2	ko00073 Cutin, suberine and wax biosynthesis;ko04146 Peroxisome;
gene31295	K00430	E1.11.1.7	peroxidase	EC:1.11.1.7	2	ko00360 Phenylalanine metabolism;ko00940 Phenylpropanoid biosynthesis;
gene312	K00799	GST, gst	glutathione S-transferase	EC:2.5.1.18	4	ko00480 Glutathione metabolism;ko00980 Metabolism of xenobiotics by cytochrome P450;ko00982 Drug metabolism - cytochrome P450;ko05204 Chemical carcinogenesis;
gene31301	K10364	CAPZA	capping protein (actin filament) muscle Z-line, alpha	--		
gene31306	K09422	MYBP	myb proto-oncogene protein, plant	--		
gene31314	K01082	E3.1.3.7, cysQ, MET22, BPNT1	3'(2'), 5'-bisphosphate nucleotidase	EC:3.1.3.7	1	ko00920 Sulfur metabolism;
gene3131	K12124	GI	GIGANTEA	--	1	ko04712 Circadian rhythm - plant;
gene3132	K09264	K09264	MADS-box transcription factor, plant	--		
gene31331	K00430	E1.11.1.7	peroxidase	EC:1.11.1.7	2	ko00360 Phenylalanine metabolism;ko00940 Phenylpropanoid biosynthesis;
gene31335	K03124	TFIIB, GTF2B, SUA7, tfb	transcription initiation factor TFIIB	--	3	ko03022 Basal transcription factors;ko05169 Epstein-Barr virus infection;ko05203 Viral carcinogenesis;
gene31338	K04730	IRAK1	interleukin-1 receptor-associated kinase 1	EC:2.7.11.1	11	ko04064 NF-kappa B signaling pathway;ko04210 Apoptosis;ko04620 Toll-like receptor signaling pathway;ko04722 Neurotrophin signaling pathway;ko05133 Pertussis;ko05140 Leishmaniasis;ko05142 Chagas disease (American trypanosomiasis);ko05145 Toxoplasmosis;ko05152 Tuberculosis;ko05162 Measles;ko05169 Epstein-Barr virus infection;
gene31344	K01082	E3.1.3.7, cysQ, MET22, BPNT1	3'(2'), 5'-bisphosphate nucleotidase	EC:3.1.3.7	1	ko00920 Sulfur metabolism;
gene31346	K01082	E3.1.3.7, cysQ, MET22, BPNT1	3'(2'), 5'-bisphosphate nucleotidase	EC:3.1.3.7	1	ko00920 Sulfur metabolism;
gene31349	K10406	KIFC2_3	kinesin family member C2/C3	--		
gene3134	K16732	PRC1	protein regulator of cytokinesis 1	--		
gene31352	K17285	SELENBP1	selenium-binding protein 1	--		

gene31356	K04392	RAC1	Ras-related C3 botulinum toxin substrate 1	--	33	ko04010 MAPK signaling pathway;ko04014 Ras signaling pathway;ko04062 Chemokine signaling pathway;ko04145 Phagosome;ko04151 PI3K-Akt signaling pathway;ko04310 Wnt signaling pathway;ko04360 Axon guidance;ko04370 VEGF signaling pathway;ko04380 Osteoclast differentiation;ko04510 Focal adhesion;ko04520 Adherens junction;ko04620 Toll-like receptor signaling pathway;ko04650 Natural killer cell mediated cytotoxicity;ko04662 B cell receptor signaling pathway;ko04664 Fc epsilon RI signaling pathway;ko04666 Fc gamma R-mediated phagocytosis;ko04670 Leukocyte transendothelial migration;ko04722 Neurotrophin signaling pathway;ko04810 Regulation of actin cytoskeleton;ko04932 Non-alcoholic fatty liver disease (NAFLD);ko04972 Pancreatic secretion;ko05014 Amyotrophic lateral sclerosis (ALS);ko05100 Bacterial invasion of epithelial cells;ko05120 Epithelial cell signaling in Helicobacter pylori infection;ko05131 Shigellosis;ko05132 Salmonella infection;ko05200 Pathways in cancer;ko05203 Viral carcinogenesis;ko05205 Proteoglycans in cancer;ko05210 Colorectal cancer;ko05211 Renal cell carcinoma;ko05212 Pancreatic [illegible]
gene31359	K10752	RBBP4, HAT2, CAF1, MIS16	histone-binding protein RBBP4	--		
gene3135	K13458	RAR1	disease resistance protein	--	1	ko04626 Plant-pathogen interaction;
gene31360	K10728	TOPBP1	topoisomerase (DNA) II binding protein 1	--		
gene31362	K14516	ERF1	ethylene-responsive transcription factor 1	--	1	ko04075 Plant hormone signal transduction;
gene31363	K15893	HPR1	hydroxypyruvate reductase 1	--	2	ko00260 Glycine, serine and threonine metabolism;ko00630 Glyoxylate and dicarboxylate metabolism;
gene31365	K09338	HD-ZIP	homeobox-leucine zipper protein	--		
gene31367	K13206	CCDC55	coiled-coil domain-containing protein 55	--		
gene31368	K13421	UMPS	uridine monophosphate synthetase	EC:2.4.2.10 4.1.1.23	2	ko00240 Pyrimidine metabolism;ko00983 Drug metabolism - other enzymes;
gene31376	K14402	CPSF2, CFT2	cleavage and polyadenylation specificity factor subunit 2	--	1	ko03015 mRNA surveillance pathway;
gene31376	K17601	WDR81	WD repeat-containing protein 81	--		
gene31377	K12741	HNRNPA1_3	heterogeneous nuclear ribonucleoprotein A1/A3	--	1	ko03040 Spliceosome;
gene31383	K13162	PCBP2_3_4	poly(rC)-binding protein 2/3/4	--		
gene31389	K15505	RAD5	DNA repair protein RAD5	EC:3.6.4.-		
gene31392	K09498	CCT6	T-complex protein 1 subunit zeta	--		
gene31393	K13448	CML	calcium-binding protein CML	--	1	ko04626 Plant-pathogen interaction;
gene31394	K01148	PARN	poly(A)-specific ribonuclease	EC:3.1.13.4	1	ko03018 RNA degradation;
gene31396	K02115	ATPF1G, atpG	F-type H+-transporting ATPase subunit gamma	EC:3.6.3.14	2	ko00190 Oxidative phosphorylation;ko00195 Photosynthesis;
gene31399	K12818	DHX8, PRP22	ATP-dependent RNA helicase DHX8/PRP22	EC:3.6.4.13	1	ko03040 Spliceosome;
gene31402	K05356	SPS, sds	all-trans-nonaprenyl-diphosphate synthase	EC:2.5.1.84 2.5.1.85	1	ko00900 Terpenoid backbone biosynthesis;
gene31403	K09647	IMP1	mitochondrial inner membrane protease subunit 1	EC:3.4.21.-	1	ko03060 Protein export;
gene31404	K14236	tRNA-Tyr	tRNA Tyr	--	1	ko00970 Aminoacyl-tRNA biosynthesis;
gene31411	K14379	ACP5	tartrate-resistant acid phosphatase type 5	EC:3.1.3.2	3	ko00740 Riboflavin metabolism;ko04380 Osteoclast differentiation;ko05323 Rheumatoid arthritis;
gene31418	K04505	PSEN1, PS1	presenilin 1	EC:3.4.23.-	4	ko04310 Wnt signaling pathway;ko04330 Notch signaling pathway;ko04722 Neurotrophin signaling pathway;ko05010 Alzheimer's disease;
gene3141	K12825	SF3A1, SAP114	splicing factor 3A subunit 1	--	1	ko03040 Spliceosome;
gene31420	K01792	E5.1.3.15	glucose-6-phosphate 1-epimerase	EC:5.1.3.15	1	ko00010 Glycolysis / Gluconeogenesis;
gene31422	K15255	PIF1	ATP-dependent DNA helicase PIF1	EC:3.6.4.12		
gene31426	K08176	PHO84	MFS transporter, PHS family, inorganic phosphate transporter	--		
gene31430	K09142	K09142	hypothetical protein	--		
gene31437	K01802	E5.2.1.8	peptidylprolyl isomerase	EC:5.2.1.8		
gene31439	K08065	NFYB	nuclear transcription Y subunit beta	--	3	ko04612 Antigen processing and presentation;ko05152 Tuberculosis;ko05166 HTLV-I infection;
gene3143	K12124	GI	GIGANTEA	--	1	ko04712 Circadian rhythm - plant;
gene31443	K01187	E3.2.1.20, malZ	alpha-glucosidase	EC:3.2.1.20	2	ko00052 Galactose metabolism;ko00500 Starch and sucrose metabolism;

gene31444	K01188	E3.2.1.21	beta-glucosidase	EC:3.2.1.21	3	ko00460 Cyanoamino acid metabolism;ko00500 Starch and sucrose metabolism;ko00940 Phenylpropanoid biosynthesis;
gene31445	K00784	rnz	ribonuclease Z	EC:3.1.26.11	1	ko03013 RNA transport;
gene31447	K04733	IRAK4	interleukin-1 receptor-associated kinase 4	EC:2.7.11.1	11	ko04064 NF-kappa B signaling pathway;ko04210 Apoptosis;ko04620 Toll-like receptor signaling pathway;ko04722 Neurotrophin signaling pathway;ko05133 Pertussis;ko05140 Leishmaniasis;ko05142 Chagas disease (American trypanosomiasis);ko05145 Toxoplasmosis;ko05152 Tuberculosis;ko05162 Measles;ko05164 Influenza A;
gene31448	K00472	E1.14.11.2	prolyl 4-hydroxylase	EC:1.14.11.2	1	ko00330 Arginine and proline metabolism;
gene3144	K01246	tag	DNA-3-methyladenine glycosylase I	EC:3.2.2.20	1	ko03410 Base excision repair;
gene31458	K01915	glnA	glutamine synthetase	EC:6.3.1.2	8	ko00250 Alanine, aspartate and glutamate metabolism;ko00330 Arginine and proline metabolism;ko00630 Glyoxylate and dicarboxylate metabolism;ko00910 Nitrogen metabolism;ko01230 Biosynthesis of amino acids;ko02020 Two-component system;ko04724 Glutamatergic synapse;ko04727 GABAergic synapse;
gene31460	K00430	E1.11.1.7	peroxidase	EC:1.11.1.7	2	ko00360 Phenylalanine metabolism;ko00940 Phenylpropanoid biosynthesis;
gene31461	K14488	SAUR	SAUR family protein	--	1	ko04075 Plant hormone signal transduction;
gene31463	K13065	E2.3.1.133, HCT	shikimate O-hydroxycinnamoyltransferase	EC:2.3.1.133	3	ko00940 Phenylpropanoid biosynthesis;ko00941 Flavonoid biosynthesis;ko00945 Stilbenoid, diarylheptanoid and gingerol biosynthesis;
gene31469	K16281	RHA1	RING-H2 zinc finger protein RHA1	--		
gene31483	K17600	VPS54	vacuolar protein sorting-associated protein 54	--		
gene31489	K00679	E2.3.1.158	phospholipid:diacylglycerol acyltransferase	EC:2.3.1.158	1	ko00561 Glycerolipid metabolism;
gene31495	K15032	MTERFD	mTERF domain-containing protein, mitochondrial	--		
gene31498	K03963	NDUFB7	NADH dehydrogenase (ubiquinone) 1 beta subcomplex subunit 7	--	5	ko00190 Oxidative phosphorylation;ko04932 Non-alcoholic fatty liver disease (NAFLD);ko05010 Alzheimer's disease;ko05012 Parkinson's disease;ko05016 Huntington's disease;
gene31499	K00430	E1.11.1.7	peroxidase	EC:1.11.1.7	2	ko00360 Phenylalanine metabolism;ko00940 Phenylpropanoid biosynthesis;
gene314	K14232	tRNA-Pro	tRNA Pro	--	1	ko00970 Aminoacyl-tRNA biosynthesis;
gene31505	K01904	E6.2.1.12	4-coumarate--CoA ligase	EC:6.2.1.12	3	ko00130 Ubiquinone and other terpenoid-quinone biosynthesis;ko00360 Phenylalanine metabolism;ko00940 Phenylpropanoid biosynthesis;
gene31508	K13424	WRKY33	WRKY transcription factor 33	--	1	ko04626 Plant-pathogen interaction;
gene31512	K13464	JAZ	jasmonate ZIM domain-containing protein	--	2	ko04075 Plant hormone signal transduction;ko04626 Plant-pathogen interaction;
gene31515	K02866	RP-L10e, RPL10	large subunit ribosomal protein L10e	--	1	ko03010 Ribosome;
gene31517	K14945	QKI	protein quaking	--		
gene31520	K13254	SPAST	spastin	EC:3.6.4.3		
gene31528	K17302	COPB2, SEC27	coatomer, subunit beta'	--		
gene3152	K03129	TAF4	transcription initiation factor TFIID subunit 4	--	3	ko03022 Basal transcription factors;ko05016 Huntington's disease;ko05168 Herpes simplex infection;
gene31534	K11346	ING4	inhibitor of growth protein 4	--		
gene31535	K14220	tRNA-Asn	tRNA Asn	--	1	ko00970 Aminoacyl-tRNA biosynthesis;
gene31542	K05863	SLC25A4S, ANT	solute carrier family 25 (mitochondrial adenine nucleotide translocator), member 4/5/6/31	--	4	ko04020 Calcium signaling pathway;ko05012 Parkinson's disease;ko05016 Huntington's disease;ko05166 HTLV-I infection;
gene31548	K15277	SLC35B3, PAPST2	solute carrier family 35 (adenosine 3'-phospho 5'-phosphosulfate transporter), member B3	--		
gene31549	K14295	NUP50, NPAP60	nuclear pore complex protein Nup50	--	1	ko03013 RNA transport;
gene31551	K03952	NDUFA8	NADH dehydrogenase (ubiquinone) 1 alpha subcomplex subunit 8	--	5	ko00190 Oxidative phosphorylation;ko04932 Non-alcoholic fatty liver disease (NAFLD);ko05010 Alzheimer's disease;ko05012 Parkinson's disease;ko05016 Huntington's disease;
gene31554	K16250	NRPD1	DNA-directed RNA polymerase IV subunit 1	EC:2.7.7.6		
gene31556	K00696	E2.4.1.14	sucrose-phosphate synthase	EC:2.4.1.14	1	ko00500 Starch and sucrose metabolism;
gene31557	K14498	SNRK2	serine/threonine-protein kinase SRK2	EC:2.7.11.1	1	ko04075 Plant hormone signal transduction;

gene31558	K07904	RAB11A	Ras-related protein Rab-11A	--	4	ko04144 Endocytosis;ko04961 Endocrine and other factor-regulated calcium reabsorption;ko04962 Vasopressin-regulated water reabsorption;ko04972 Pancreatic secretion;
gene31563	K01184	E3.2.1.15	polygalacturonase	EC:3.2.1.15	2	ko00040 Pentose and glucuronate interconversions;ko00500 Starch and sucrose metabolism;
gene31567	K11000	CALS	callose synthase	EC:2.4.1.-		
gene31570	K08176	PHO84	MFS transporter, PHS family, inorganic phosphate transporter	--		
gene31576	K01051	E3.1.1.11	pectinesterase	EC:3.1.1.11	2	ko00040 Pentose and glucuronate interconversions;ko00500 Starch and sucrose metabolism;
gene31577	K01051	E3.1.1.11	pectinesterase	EC:3.1.1.11	2	ko00040 Pentose and glucuronate interconversions;ko00500 Starch and sucrose metabolism;
gene31579	K09338	HD-ZIP	homeobox-leucine zipper protein	--		
gene31581	K03500	rsmB, sun	16S rRNA (cytosine967-C5)-methyltransferase	EC:2.1.1.176		
gene31582	K09286	EREBP	EREBP-like factor	--		
gene31584	K09286	EREBP	EREBP-like factor	--		
gene31585	K04498	EP300, CREBBP, KAT3	E1A/CREB-binding protein	EC:2.3.1.48	21	ko04066 HIF-1 signaling pathway;ko04110 Cell cycle;ko04310 Wnt signaling pathway;ko04330 Notch signaling pathway;ko04350 TGF-beta signaling pathway;ko04520 Adherens junction;ko04630 Jak-STAT signaling pathway;ko04720 Long-term potentiation;ko04916 Melanogenesis;ko05016 Huntington's disease;ko05152 Tuberculosis;ko05161 Hepatitis B;ko05164 Influenza A;ko05166 HTLV-I infection;ko05168 Herpes simplex infection;ko05169 Epstein-Barr virus infection;ko05200 Pathways in cancer;ko05203 Viral carcinogenesis;ko05206 MicroRNAs in cancer;ko05211 Renal cell carcinoma;ko05215 Prostate cancer;
gene3158	K12153	CYP79A2	cytochrome P450, family 79, subfamily A, polypeptide 2 (phenylalanine N-monooxygenase)	EC:1.14.13.124	3	ko00460 Cyanoamino acid metabolism;ko00966 Glucosinolate biosynthesis;ko01210 2-Oxocarboxylic acid metabolism;
gene31591	K01551	arsA, ASNA1	arsenite-transporting ATPase	EC:3.6.3.16		
gene31592	K01613	psd, PISD	phosphatidylserine decarboxylase	EC:4.1.1.65	1	ko00564 Glycerophospholipid metabolism;
gene31593	K01704	leuD	3-isopropylmalate/(R)-2-methylmalate dehydratase small subunit	EC:4.2.1.33 4.2.1.35	4	ko00290 Valine, leucine and isoleucine biosynthesis;ko00660 C5-Branched dibasic acid metabolism;ko01210 2-Oxocarboxylic acid metabolism;ko01230 Biosynthesis of amino acids;
gene31598	K10527	MFP2	enoyl-CoA hydratase/3-hydroxyacyl-CoA dehydrogenase	EC:4.2.1.17 1.1.1.35 1.1.1.211	2	ko00071 Fatty acid degradation;ko00592 alpha-Linolenic acid metabolism;
gene31599	K10527	MFP2	enoyl-CoA hydratase/3-hydroxyacyl-CoA dehydrogenase	EC:4.2.1.17 1.1.1.35 1.1.1.211	2	ko00071 Fatty acid degradation;ko00592 alpha-Linolenic acid metabolism;
gene3159	K12153	CYP79A2	cytochrome P450, family 79, subfamily A, polypeptide 2 (phenylalanine N-monooxygenase)	EC:1.14.13.124	3	ko00460 Cyanoamino acid metabolism;ko00966 Glucosinolate biosynthesis;ko01210 2-Oxocarboxylic acid metabolism;
gene31600	K11648	SMARCB1, SNF5, INI1	SWI/SNF-related matrix-associated actin-dependent regulator of chromatin subfamily B member 1	--		
gene31601	K03687	GRPE	molecular chaperone GrpE	--		
gene31603	K10696	BRE1	E3 ubiquitin-protein ligase BRE1	EC:6.3.2.19		
gene31607	K01528	DNM	dynamin GTPase	EC:3.6.5.5	4	ko04144 Endocytosis;ko04721 Synaptic vesicle cycle;ko04961 Endocrine and other factor-regulated calcium reabsorption;ko05100 Bacterial invasion of epithelial cells;
gene31611	K01723	AOS	hydroperoxide dehydratase	EC:4.2.1.92	1	ko00592 alpha-Linolenic acid metabolism;
gene31612	K10760	IPT	adenylate isopentenyltransferase (cytokinin synthase)	--	1	ko00908 Zeatin biosynthesis;
gene31616	K11593	ELF2C	eukaryotic translation initiation factor 2C	--		
gene31619	K00921	PIKFYVE, FAB1	1-phosphatidylinositol-3-phosphate 5-kinase	EC:2.7.1.150	4	ko00562 Inositol phosphate metabolism;ko04070 Phosphatidylinositol signaling system;ko04145 Phagosome;ko04810 Regulation of actin cytoskeleton;
gene31621	K12125	ELF3	protein EARLY FLOWERING 3	--	1	ko04712 Circadian rhythm - plant;
gene31626	K00600	glyA, SHMT	glycine hydroxymethyltransferase	EC:2.1.2.1	7	ko00260 Glycine, serine and threonine metabolism;ko00460 Cyanoamino acid metabolism;ko00630 Glyoxylate and dicarboxylate metabolism;ko00670 One carbon pool by folate;ko00680 Methane metabolism;ko01200 Carbon metabolism;ko01230 Biosynthesis of amino acids;
gene31632	K11866	STAMBP, AMSH	STAM-binding protein	EC:3.1.2.15	1	ko04144 Endocytosis;

gene31635	K15029	EIF3L	translation initiation factor 3 subunit L	--		
gene31636	K03327	TC.MATE, SLC47A, norM, mdtK, dinF	multidrug resistance protein, MATE family	--		
gene3163	K01982	LSUrRNA	large subunit ribosomal RNA	--	2	ko03008 Ribosome biogenesis in eukaryotes;ko03010 Ribosome;
gene31641	K03238	EIF2S2	translation initiation factor 2 subunit 2	--	1	ko03013 RNA transport;
gene31644	K03661	ATPeVPF, ATP6F	V-type H+-transporting ATPase 21kDa proteolipid subunit	EC:3.6.3.14	8	ko00190 Oxidative phosphorylation;ko04142 Lysosome;ko04145 Phagosome;ko04721 Synaptic vesicle cycle;ko05110 Vibrio cholerae infection;ko05120 Epithelial cell signaling in Helicobacter pylori infection;ko05152 Tuberculosis;ko05323 Rheumatoid arthritis;
gene31651	K02180	BUB3	cell cycle arrest protein BUB3	--	3	ko04110 Cell cycle;ko04111 Cell cycle - yeast;ko05166 HTLV-I infection;
gene31652	K02180	BUB3	cell cycle arrest protein BUB3	--	3	ko04110 Cell cycle;ko04111 Cell cycle - yeast;ko05166 HTLV-I infection;
gene31655	K09285	OVM, ANT	AP2-like factor, ANT lineage	--		
gene31656	K12448	UXE	UDP-arabinose 4-epimerase	EC:5.1.3.5	1	ko00520 Amino sugar and nucleotide sugar metabolism;
gene31658	K02875	RP-L14e, RPL14	large subunit ribosomal protein L14e	--	1	ko03010 Ribosome;
gene31659	K04733	IRAK4	interleukin-1 receptor-associated kinase 4	EC:2.7.11.1	11	ko04064 NF-kappa B signaling pathway;ko04210 Apoptosis;ko04620 Toll-like receptor signaling pathway;ko04722 Neurotrophin signaling pathway;ko05133 Pertussis;ko05140 Leishmaniasis;ko05142 Chagas disease (American trypanosomiasis);ko05145 Toxoplasmosis;ko05152 Tuberculosis;ko05162 Measles;ko05164 Influenza A;
gene31662	K17560	URI1	unconventional prefoldin RPB5 interactor 1	--		
gene31675	K11583	PPP2R3	serine/threonine-protein phosphatase 2A regulatory subunit B''	--	3	ko03015 mRNA surveillance pathway;ko04151 PI3K-Akt signaling pathway;ko04728 Dopaminergic synapse;
gene31679	K04567	KARS, lysS	lysyl-tRNA synthetase, class II	EC:6.1.1.6	1	ko00970 Aminoacyl-tRNA biosynthesis;
gene31680	K04567	KARS, lysS	lysyl-tRNA synthetase, class II	EC:6.1.1.6	1	ko00970 Aminoacyl-tRNA biosynthesis;
gene31684	K14498	SNRK2	serine/threonine-protein kinase SRK2	EC:2.7.11.1	1	ko04075 Plant hormone signal transduction;
gene31689	K08494	NSPN	novel plant SNARE	--		
gene31691	K14497	PP2C	protein phosphatase 2C	EC:3.1.3.16	1	ko04075 Plant hormone signal transduction;
gene31696	K13448	CML	calcium-binding protein CML	--	1	ko04626 Plant-pathogen interaction;
gene31698	K01462	PDF, def	peptide deformylase	EC:3.5.1.88		
gene31699	K03283	HSPA1_8	heat shock 70kDa protein 1/8	--	11	ko03040 Spliceosome;ko04010 MAPK signaling pathway;ko04141 Protein processing in endoplasmic reticulum;ko04144 Endocytosis;ko04612 Antigen processing and presentation;ko04915 Estrogen signaling pathway;ko05134 Legionellosis;ko05145 Toxoplasmosis;ko05162 Measles;ko05164 Influenza A;ko05169 Epstein-Barr virus infection;
gene3169	K00366	nirA	ferredoxin-nitrite reductase	EC:1.7.7.1	1	ko00910 Nitrogen metabolism;
gene31710	K11275	H1_5	histone H1/5	--		
gene31719	K02144	ATPeV54kD	V-type H+-transporting ATPase 54 kD subunit	EC:3.6.3.14	8	ko00190 Oxidative phosphorylation;ko04142 Lysosome;ko04145 Phagosome;ko04721 Synaptic vesicle cycle;ko05110 Vibrio cholerae infection;ko05120 Epithelial cell signaling in Helicobacter pylori infection;ko05152 Tuberculosis;ko05323 Rheumatoid arthritis;
gene3171	K00145	argC	N-acetyl-gamma-glutamyl-phosphate reductase	EC:1.2.1.38	3	ko00330 Arginine and proline metabolism;ko01210 2-Oxocarboxylic acid metabolism;ko01230 Biosynthesis of amino acids;
gene31720	K11644	SIN3A	paired amphipathic helix protein Sin3a	--	2	ko05016 Huntington's disease;ko05202 Transcriptional misregulation in cancer;
gene31721	K11644	SIN3A	paired amphipathic helix protein Sin3a	--	2	ko05016 Huntington's disease;ko05202 Transcriptional misregulation in cancer;
gene31725	K13566	NIT2	omega-amidase	EC:3.5.1.3	1	ko00250 Alanine, aspartate and glutamate metabolism;
gene3172	K10400	KIF15	kinesin family member 15	--		
gene31734	K01102	PDP	pyruvate dehydrogenase phosphatase	EC:3.1.3.43		
gene31739	K08857	NEK	NIMA (never in mitosis gene a)-related kinase	EC:2.7.11.1		
gene3173	K03005	RPA49, POLR1E	DNA-directed RNA polymerase I subunit RPA49	--	3	ko00230 Purine metabolism;ko00240 Pyrimidine metabolism;ko03020 RNA polymerase;
gene31741	K13496	UGT73C	UDP-glucosyl transferase 73C	EC:2.4.1.-		
gene31743	K12199	VTA1, LIP5	vacuolar protein sorting-associated protein VTA1	--	1	ko04144 Endocytosis;
gene31744	K16732	PRC1	protein regulator of cytokinesis 1	--		
gene31756	K05544	DUS3	tRNA-dihydrouridine synthase 3	EC:1.3.1.89		
gene31757	K00764	purF, PPAT	amidophosphoribosyltransferase	EC:2.4.2.14	2	ko00230 Purine metabolism;ko00250 Alanine, aspartate and glutamate metabolism;

gene31758	K09578	PIN1	peptidyl-prolyl cis-trans isomerase NIMA-interacting 1	EC:5.2.1.8	1	ko04622 RIG-I-like receptor signaling pathway;
gene31759	K02834	rbfA	ribosome-binding factor A	--		
gene31760	K14488	SAUR	SAUR family protein	--	1	ko04075 Plant hormone signal transduction;
gene31763	K14327	UPF2, RENT2	regulator of nonsense transcripts 2	--	2	ko03013 RNA transport;ko03015 mRNA surveillance pathway;
gene31767	K00688	E2.4.1.1, glgP, PYG	starch phosphorylase	EC:2.4.1.1	2	ko00500 Starch and sucrose metabolism;ko04910 Insulin signaling pathway;
gene31768	K09510	DNAJB4	DnaJ homolog subfamily B member 4	--		
gene3176	K14409	SMG7, EST1C	protein SMG7	--	1	ko03015 mRNA surveillance pathway;
gene31772	K17609	NXN	nucleoredoxin	EC:1.8.1.8		
gene31773	K17609	NXN	nucleoredoxin	EC:1.8.1.8		
gene31774	K17609	NXN	nucleoredoxin	EC:1.8.1.8		
gene31775	K17609	NXN	nucleoredoxin	EC:1.8.1.8		
gene31776	K02941	RP-LP0, RPLP0	large subunit ribosomal protein LP0	--	1	ko03010 Ribosome;
gene31780	K05894	E1.3.1.42	12-oxophytodienoic acid reductase	EC:1.3.1.42	1	ko00592 alpha-Linolenic acid metabolism;
gene31781	K05894	E1.3.1.42	12-oxophytodienoic acid reductase	EC:1.3.1.42	1	ko00592 alpha-Linolenic acid metabolism;
gene31782	K05350	bglB	beta-glucosidase	EC:3.2.1.21	3	ko00460 Cyanoamino acid metabolism;ko00500 Starch and sucrose metabolism;ko00940 Phenylpropanoid biosynthesis;
gene31783	K06287	maf	septum formation protein	--		
gene31788	K13414	MEKK1P	mitogen-activated protein kinase kinase kinase 1, plant	EC:2.7.11.25	1	ko04626 Plant-pathogen interaction;
gene31790	K08678	UXS1	UDP-glucuronate decarboxylase	EC:4.1.1.35	2	ko00500 Starch and sucrose metabolism;ko00520 Amino sugar and nucleotide sugar metabolism;
gene31791	K12489	ACAP	Arf-GAP with coiled-coil, ANK repeat and PH domain-containing protein	--	1	ko04144 Endocytosis;
gene31792	K04125	E1.14.11.13	gibberellin 2-oxidase	EC:1.14.11.13	1	ko00904 Diterpenoid biosynthesis;
gene31795	K02133	ATPeF1B, ATP5B	F-type H+-transporting ATPase subunit beta	EC:3.6.3.14	4	ko00190 Oxidative phosphorylation;ko05010 Alzheimer's disease;ko05012 Parkinson's disease;ko05016 Huntington's disease;
gene31797	K00695	E2.4.1.13	sucrose synthase	EC:2.4.1.13	1	ko00500 Starch and sucrose metabolism;
gene31798	K12858	DDX23, PRP28	ATP-dependent RNA helicase DDX23/PRP28	EC:3.6.4.13	1	ko03040 Spliceosome;
gene31802	K15398	CYP86A4S	cytochrome P450, family 86, subfamily A, polypeptide 2/4/7/8 (fatty acid omega-hydroxylase)	EC:1.14.-.-	1	ko00073 Cutin, suberine and wax biosynthesis;
gene31804	K10717	CYP735A	cytokinin trans-hydroxylase	--	1	ko00908 Zeatin biosynthesis;
gene31805	K15397	KCS	3-ketoacyl-CoA synthase	EC:2.3.1.199	1	ko00062 Fatty acid elongation;
gene31809	K11271	DSCC1, DCC1	sister chromatid cohesion protein DCC1	--		
gene3180	K01939	E6.3.4.4, purA	adenylosuccinate synthase	EC:6.3.4.4	2	ko00230 Purine metabolism;ko00250 Alanine, aspartate and glutamate metabolism;
gene31810	K10742	DNA2	DNA replication ATP-dependent helicase Dna2	EC:3.6.4.12	1	ko03030 DNA replication;
gene31816	K10746	EXO1	exonuclease 1	EC:3.1.-.-	1	ko03430 Mismatch repair;
gene31818	K15382	SLC50A, SWEET	solute carrier family 50 (sugar transporter)	--		
gene31821	K14219	tRNA-Arg	tRNA Arg	--	1	ko00970 Aminoacyl-tRNA biosynthesis;
gene31823	K09537	DNAJC17	DnaJ homolog subfamily C member 17	--		
gene31824	K15196	BRF1, GTF3B	transcription factor IIIB 90 kDa subunit	--		
gene31825	K02921	RP-L37Ae, RPL37A	large subunit ribosomal protein L37Ae	--	1	ko03010 Ribosome;
gene31835	K03256	TRM6, GCD10	tRNA (adenine-N(1)-)-methyltransferase non-catalytic subunit	--		
gene31836	K06620	E2F3	transcription factor E2F3	--	13	ko04110 Cell cycle;ko05161 Hepatitis B;ko05166 HTLV-I infection;ko05200 Pathways in cancer;ko05206 MicroRNAs in cancer;ko05212 Pancreatic cancer;ko05214 Glioma;ko05215 Prostate cancer;ko05218 Melanoma;ko05219 Bladder cancer;ko05220 Chronic myeloid leukemia;ko05222 Small cell lung cancer;ko05223 Non-small cell lung cancer;
gene31837	K17338	REEP1_2_3_4	receptor expression-enhancing protein 1/2/3/4	--		
gene31840	K08832	SRPK3, STK23	serine/threonine-protein kinase SRPK3	EC:2.7.11.1		
gene3184	K17065	DNM1L	dynamin 1-like protein	EC:3.6.5.5	1	ko04668 TNF signaling pathway;
gene31851	K14293	KPNB1	importin subunit beta-1	--	1	ko03013 RNA transport;
gene31855	K10418	DYNLL	dynein light chain LC8-type	--	1	ko04962 Vasopressin-regulated water reabsorption;

gene3185	K09422	MYBP	myb proto-oncogene protein, plant	--		
gene31861	K11599	POMP, UMP1	proteasome maturation protein	--	1	ko03050 Proteasome;
gene31862	K03680	EIF2B4	translation initiation factor eIF-2B subunit delta	--	1	ko03013 RNA transport;
gene31864	K04498	EP300, CREBBP, KAT3	E1A/CREB-binding protein	EC:2.3.1.48	21	ko04066 HIF-1 signaling pathway;ko04110 Cell cycle;ko04310 Wnt signaling pathway;ko04330 Notch signaling pathway;ko04350 TGF-beta signaling pathway;ko04520 Adherens junction;ko04630 Jak-STAT signaling pathway;ko04720 Long-term potentiation;ko04916 Melanogenesis;ko05016 Huntington's disease;ko05152 Tuberculosis;ko05161 Hepatitis B;ko05164 Influenza A;ko05166 HTLV-I infection;ko05168 Herpes simplex infection;ko05169 Epstein-Barr virus infection;ko05200 Pathways in cancer;ko05203 Viral carcinogenesis;ko05206 MicroRNAs in cancer;ko05211 Renal cell carcinoma;ko05215 Prostate cancer;
gene31868	K10523	SPOP	speckle-type POZ protein	--		
gene31872	K02150	ATPeVE, ATP6E	V-type H+-transporting ATPase subunit E	EC:3.6.3.14	7	ko00190 Oxidative phosphorylation;ko04145 Phagosome;ko04721 Synaptic vesicle cycle;ko04966 Collecting duct acid secretion;ko05110 Vibrio cholerae infection;ko05120 Epithelial cell signaling in Helicobacter pylori infection;ko05323 Rheumatoid arthritis;
gene31878	K00844	HK	hexokinase	EC:2.7.1.1	12	ko00010 Glycolysis / Gluconeogenesis;ko00051 Fructose and mannose metabolism;ko00052 Galactose metabolism;ko00500 Starch and sucrose metabolism;ko00520 Amino sugar and nucleotide sugar metabolism;ko00521 Streptomycin biosynthesis;ko00524 Butirosin and neomycin biosynthesis;ko01200 Carbon metabolism;ko04066 HIF-1 signaling pathway;ko04910 Insulin signaling pathway;ko04930 Type II diabetes mellitus;ko04973 Carbohydrate digestion and absorption;
gene31879	K14376	PAP	poly(A) polymerase	EC:2.7.7.19	1	ko03015 mRNA surveillance pathway;
gene31881	K00915	IPK2	1D-myo-Inositol-tetrakisphosphate 5-kinase / inositol-polyphosphate multikinase	EC:2.7.1.140 2.7.1.151	2	ko00562 Inositol phosphate metabolism;ko04070 Phosphatidylinositol signaling system;
gene31883	K01586	lysA	diaminopimelate decarboxylase	EC:4.1.1.20	2	ko00300 Lysine biosynthesis;ko01230 Biosynthesis of amino acids;
gene31885	K01723	AOS	hydroperoxide dehydratase	EC:4.2.1.92	1	ko00592 alpha-Linolenic acid metabolism;
gene31886	K01858	E5.5.1.4, INO1	myo-inositol-1-phosphate synthase	EC:5.5.1.4	2	ko00521 Streptomycin biosynthesis;ko00562 Inositol phosphate metabolism;
gene31888	K07950	ARL5B	ADP-ribosylation factor-like protein 5B	--		
gene31896	K13496	UGT73C	UDP-glucosyl transferase 73C	EC:2.4.1.-		
gene31897	K13496	UGT73C	UDP-glucosyl transferase 73C	EC:2.4.1.-		
gene31898	K02873	RP-L13e, RPL13	large subunit ribosomal protein L13e	--	1	ko03010 Ribosome;
gene318	K13963	SERPINB	serpin B	--	1	ko05146 Amoebiasis;
gene31903	K14498	SNRK2	serine/threonine-protein kinase SRK2	EC:2.7.11.1	1	ko04075 Plant hormone signal transduction;
gene31904	K15334	NCL1, TRM4	multisite-specific tRNA:(cytosine-C5)-methyltransferase	EC:2.1.1.202		
gene3190	K02256	COX1	cytochrome c oxidase subunit 1	EC:1.9.3.1	6	ko00190 Oxidative phosphorylation;ko04260 Cardiac muscle contraction;ko04932 Non-alcoholic fatty liver disease (NAFLD);ko05010 Alzheimer's disease;ko05012 Parkinson's disease;ko05016 Huntington's disease;
gene3191	K03006	RPB1, POLR2A	DNA-directed RNA polymerase II subunit RPB1	EC:2.7.7.6	6	ko00230 Purine metabolism;ko00240 Pyrimidine metabolism;ko03020 RNA polymerase;ko05016 Huntington's disease;ko05168 Herpes simplex infection;ko05169 Epstein-Barr virus infection;
gene31920	K04122	E1.14.13.78	ent-kaurene oxidase	EC:1.14.13.78	1	ko00904 Diterpenoid biosynthesis;
gene31923	K00703	E2.4.1.21, glgA	starch synthase	EC:2.4.1.21	1	ko00500 Starch and sucrose metabolism;
gene31924	K00558	DNMT1, dcm	DNA (cytosine-5)-methyltransferase 1	EC:2.1.1.37	2	ko00270 Cysteine and methionine metabolism;ko05206 MicroRNAs in cancer;
gene3192	K17302	COPB2, SEC27	coatomer, subunit beta'	--		
gene31932	K01568	E4.1.1.1, pdc	pyruvate decarboxylase	EC:4.1.1.1	1	ko00010 Glycolysis / Gluconeogenesis;
gene31935	K09422	MYBP	myb proto-oncogene protein, plant	--		
gene31936	K14412	FUT13, FucTC	alpha-1,4-fucosyltransferase	EC:2.4.1.-	1	ko00513 Various types of N-glycan biosynthesis;
gene31944	K01602	rbcS	ribulose-bisphosphate carboxylase small chain	EC:4.1.1.39	3	ko00630 Glyoxylate and dicarboxylate metabolism;ko00710 Carbon fixation in photosynthetic organisms;ko01200 Carbon metabolism;
gene3194	K09489	HSPA4	heat shock 70kDa protein 4	--	1	ko04612 Antigen processing and presentation;

gene31950	K00915	IPK2	1D-myo-Inositol-tetrakisphosphate 5-kinase / inositol-polyphosphate multikinase	EC:2.7.1.140 2.7.1.151	2	ko00562 Inositol phosphate metabolism;ko04070 Phosphatidylinositol signaling system;
gene31955	K09264	K09264	MADS-box transcription factor, plant	--		
gene31958	K10728	TOPBP1	topoisomerase (DNA) II binding protein 1	--		
gene31965	K02716	psbO	photosystem II oxygen-evolving enhancer protein 1	--	1	ko00195 Photosynthesis;
gene31971	K12616	EDC4	enhancer of mRNA-decapping protein 4	--	1	ko03018 RNA degradation;
gene31986	K13457	RPM1, RPS3	disease resistance protein RPM1	--	1	ko04626 Plant-pathogen interaction;
gene31987	K05350	bglB	beta-glucosidase	EC:3.2.1.21	3	ko00460 Cyanoamino acid metabolism;ko00500 Starch and sucrose metabolism;ko00940 Phenylpropanoid biosynthesis;
gene31988	K01358	clpP, CLPP	ATP-dependent Clp protease, protease subunit	EC:3.4.21.92	1	ko04112 Cell cycle - Caulobacter;
gene31990	K00924	E2.7.1.-				
gene31991	K15188	CCNT	cyclin T	--	1	ko05202 Transcriptional misregulation in cancer;
gene31993	K13462	MIN7	guanine nucleotide-exchange factor	--	1	ko04626 Plant-pathogen interaction;
gene31994	K05305	FUK	fucokinase	EC:2.7.1.52	2	ko00051 Fructose and mannose metabolism;ko00520 Amino sugar and nucleotide sugar metabolism;
gene31995	K05305	FUK	fucokinase	EC:2.7.1.52	2	ko00051 Fructose and mannose metabolism;ko00520 Amino sugar and nucleotide sugar metabolism;
gene31997	K15178	RTF1	RNA polymerase-associated protein RTF1	--		
gene3199	K14396	PABPN1, PABP2	polyadenylate-binding protein 2	--	2	ko03015 mRNA surveillance pathway;ko05164 Influenza A;
gene32007	K14221	tRNA-Asp	tRNA Asp	--	1	ko00970 Aminoacyl-tRNA biosynthesis;
gene32011	K16055	TPS	trehalose 6-phosphate synthase/phosphatase	EC:2.4.1.15 3.1.3.12	1	ko00500 Starch and sucrose metabolism;
gene32012	K14236	tRNA-Tyr	tRNA Tyr	--	1	ko00970 Aminoacyl-tRNA biosynthesis;
gene32014	K09338	HD-ZIP	homeobox-leucine zipper protein	--		
gene32021	K12844	PRPF31	U4/U6 small nuclear ribonucleoprotein PRP31	--	1	ko03040 Spliceosome;
gene32023	K11462	EED	polycomb protein EED	--		
gene32026	K05658	ABCB1	ATP-binding cassette, subfamily B (MDR/TAP), member 1	--	3	ko02010 ABC transporters;ko04976 Bile secretion;ko05206 MicroRNAs in cancer;
gene32027	K08770	UBC	ubiquitin C	--	1	ko03320 PPAR signaling pathway;
gene32029	K03113	EIF1, SUI1	translation initiation factor 1	--	1	ko03013 RNA transport;
gene32032	K07766	E3.6.1.52	diphosphoinositol-polyphosphate diphosphatase	EC:3.6.1.52		
gene32035	K09419	HSFF	heat shock transcription factor, other eukaryote	--		
gene32036	K13448	CML	calcium-binding protein CML	--	1	ko04626 Plant-pathogen interaction;
gene32037	K09286	EREBP	EREBP-like factor	--		
gene32039	K13025	EIF4A3, FAL1	ATP-dependent RNA helicase	EC:3.6.4.13	3	ko03013 RNA transport;ko03015 mRNA surveillance pathway;ko03040 Spliceosome;
gene32040	K03327	TC.MATE, SLC47A, norM, mdtK, dinF	multidrug resistance protein, MATE family	--		
gene32043	K06632	WEE1	wee1-like protein kinase	EC:2.7.11.1	1	ko04110 Cell cycle;
gene32044	K03549	kup	KUP system potassium uptake protein	--		
gene32045	K13412	CPK	calcium-dependent protein kinase	EC:2.7.11.1	2	ko04626 Plant-pathogen interaction;ko05145 Toxoplasmosis;
gene32046	K09841	ABA2	xanthoxin dehydrogenase	EC:1.1.1.288	1	ko00906 Carotenoid biosynthesis;
gene32047	K17491	SMEK, PPP4R3	protein phosphatase 4 regulatory subunit 3	--		
gene32048	K10578	UBE2J1, NCUBE1, UBC6	ubiquitin-conjugating enzyme E2 J1	EC:6.3.2.19	3	ko04120 Ubiquitin mediated proteolysis;ko04141 Protein processing in endoplasmic reticulum;ko05012 Parkinson's disease;
gene32050	K14405	FIP1L1, FIP1	pre-mRNA 3'-end-processing factor FIP1	--	1	ko03015 mRNA surveillance pathway;
gene32052	K01648	ACLY	ATP citrate (pro-S)-lyase	EC:2.3.3.8	2	ko00020 Citrate cycle (TCA cycle);ko00720 Carbon fixation pathways in prokaryotes;
gene32053	K01648	ACLY	ATP citrate (pro-S)-lyase	EC:2.3.3.8	2	ko00020 Citrate cycle (TCA cycle);ko00720 Carbon fixation pathways in prokaryotes;
gene32065	K10728	TOPBP1	topoisomerase (DNA) II binding protein 1	--		
gene32068	K09286	EREBP	EREBP-like factor	--		
gene3206	K09286	EREBP	EREBP-like factor	--		
gene32071	K00814	GPT, ALT	alanine transaminase	EC:2.6.1.2	4	ko00250 Alanine, aspartate and glutamate metabolism;ko00710 Carbon fixation in photosynthetic organisms;ko01210 2-Oxocarboxylic acid metabolism;ko01230 Biosynthesis of amino acids;
gene32072	K07937	ARF1	ADP-ribosylation factor 1	--	2	ko05110 Vibrio cholerae infection;ko05134 Legionellosis;

gene32073	K01661	menB	naphthoate synthase	EC:4.1.3.36	1	ko00130 Ubiquinone and other terpenoid-quinone biosynthesis;
gene32076	K00869	E2.7.1.36, MVK, mvaK1	mevalonate kinase	EC:2.7.1.36	2	ko00900 Terpenoid backbone biosynthesis;ko04146 Peroxisome;
gene3207	K01517	ADPRM	manganese-dependent ADP-ribose/CDP-alcohol diphosphatase	EC:3.6.1.13 3.6.1.16 3.6.1.53	2	ko00230 Purine metabolism;ko00564 Glycerophospholipid metabolism;
gene32081	K13379	RGP, UTM	reversibly glycosylated polypeptide / UDP-arabinopyranose mutase	EC:2.4.1.- 5.4.99.30		
gene32082	K00555	TRMT1, trm1	tRNA (guanine26-N2/guanine27-N2)-dimethyltransferase	EC:2.1.1.215 2.1.1.216		
gene32084	K09286	EREBP	EREBP-like factor	--		
gene32085	K14309	NUP93	nuclear pore complex protein Nup93	--	1	ko03013 RNA transport;
gene32087	K01872	AARS, alaS	alanyl-tRNA synthetase	EC:6.1.1.7	1	ko00970 Aminoacyl-tRNA biosynthesis;
gene32089	K09419	HSFF	heat shock transcription factor, other eukaryote	--		
gene3208	K08287	E2.7.12.1	dual-specificity kinase	EC:2.7.12.1		
gene32091	K03260	EIF4G	translation initiation factor 4G	--	2	ko03013 RNA transport;ko05416 Viral myocarditis;
gene32094	K03260	EIF4G	translation initiation factor 4G	--	2	ko03013 RNA transport;ko05416 Viral myocarditis;
gene32102	K04794	PTH2	peptidyl-tRNA hydrolase, PTH2 family	EC:3.1.1.29		
gene32103	K01883	CARS, cysS	cysteinyl-tRNA synthetase	EC:6.1.1.16	1	ko00970 Aminoacyl-tRNA biosynthesis;
gene32109	K05658	ABCB1	ATP-binding cassette, subfamily B (MDR/TAP), member 1	--	3	ko02010 ABC transporters;ko04976 Bile secretion;ko05206 MicroRNAs in cancer;
gene32113	K12479	VPS45	vacuolar protein sorting-associated protein 45	--	1	ko04144 Endocytosis;
gene32116	K08737	MSH6	DNA mismatch repair protein MSH6	--	3	ko03430 Mismatch repair;ko05200 Pathways in cancer;ko05210 Colorectal cancer;
gene32117	K06228	FU	fused	EC:2.7.11.1	3	ko04340 Hedgehog signaling pathway;ko05200 Pathways in cancer;ko05217 Basal cell carcinoma;
gene32119	K07977	ARF	Arf/Sar family, other	--		
gene32120	K14290	XPO1, CRM1	exportin-1	--	5	ko03008 Ribosome biogenesis in eukaryotes;ko03013 RNA transport;ko05164 Influenza A;ko05166 HTLV-I infection;ko05169 Epstein-Barr virus infection;
gene32121	K10601	SYVN1, HRD1	E3 ubiquitin-protein ligase synoviolin	EC:6.3.2.19	2	ko04120 Ubiquitin mediated proteolysis;ko04141 Protein processing in endoplasmic reticulum;
gene32123	K14402	CPSF2, CFT2	cleavage and polyadenylation specificity factor subunit 2	--	1	ko03015 mRNA surveillance pathway;
gene32125	K02920	RP-L36e, RPL36	large subunit ribosomal protein L36e	--	1	ko03010 Ribosome;
gene32130	K06892	K06892				
gene32132	K00430	E1.11.1.7	peroxidase	EC:1.11.1.7	2	ko00360 Phenylalanine metabolism;ko00940 Phenylpropanoid biosynthesis;
gene32134	K09422	MYBP	myb proto-oncogene protein, plant	--		
gene32138	K13519	LPT1, ALE1	lysophospholipid acyltransferase	EC:2.3.1.51 2.3.1.23	3	ko00561 Glycerolipid metabolism;ko00564 Glycerophospholipid metabolism;ko00565 Ether lipid metabolism;
gene3213	K00789	metK	S-adenosylmethionine synthetase	EC:2.5.1.6	2	ko00270 Cysteine and methionine metabolism;ko01230 Biosynthesis of amino acids;
gene32141	K09680	coaW	type II pantothenate kinase	EC:2.7.1.33	1	ko00770 Pantothenate and CoA biosynthesis;
gene32147	K13947	PIN	auxin efflux carrier family	--		
gene32148	K12598	MTR4, SKIV2L2	ATP-dependent RNA helicase DOB1	EC:3.6.4.13	1	ko03018 RNA degradation;
gene32156	K14499	BKI1	BRI1 kinase inhibitor 1	--	1	ko04075 Plant hormone signal transduction;
gene32162	K02684	PRI1	DNA primase small subunit	EC:2.7.7.-	3	ko00230 Purine metabolism;ko00240 Pyrimidine metabolism;ko03030 DNA replication;
gene32164	K14315	NDC1, TMEM48	nucleoporin NDC1	--	1	ko03013 RNA transport;
gene32166	K07767	E3.6.4.3	microtubule-severing ATPase	EC:3.6.4.3		
gene32168	K13448	CML	calcium-binding protein CML	--	1	ko04626 Plant-pathogen interaction;
gene32174	K01937	E6.3.4.2, pyrG	CTP synthase	EC:6.3.4.2	1	ko00240 Pyrimidine metabolism;
gene32176	K13412	CPK	calcium-dependent protein kinase	EC:2.7.11.1	2	ko04626 Plant-pathogen interaction;ko05145 Toxoplasmosis;
gene32177	K17362	ACOT13	acyl-coenzyme A thioesterase 13	EC:3.1.2.-		
gene3217	K16904	DCTPP1	dCTP diphosphatase	EC:3.6.1.12	1	ko00240 Pyrimidine metabolism;
gene32181	K01530	E3.6.3.1	phospholipid-translocating ATPase	EC:3.6.3.1		
gene32184	K00430	E1.11.1.7	peroxidase	EC:1.11.1.7	2	ko00360 Phenylalanine metabolism;ko00940 Phenylpropanoid biosynthesis;
gene32186	K13917	RNGTT	mRNA-capping enzyme	EC:2.7.7.50 3.1.3.33	1	ko03015 mRNA surveillance pathway;
gene32189	K06944	K06944				
gene32196	K00430	E1.11.1.7	peroxidase	EC:1.11.1.7	2	ko00360 Phenylalanine metabolism;ko00940 Phenylpropanoid biosynthesis;

gene32197	K15305	VAC14, TAX1BP2	vacuole morphology and inheritance protein 14	--	2	ko05166 HTLV-I infection;ko05203 Viral carcinogenesis;
gene3219	K07204	RAPTOR	regulatory associated protein of mTOR	--	4	ko04150 mTOR signaling pathway;ko04151 PI3K-Akt signaling pathway;ko04910 Insulin signaling pathway;ko05206 MicroRNAs in cancer;
gene32203	K00128	E1.2.1.3	aldehyde dehydrogenase (NAD+)	EC:1.2.1.3	15	ko00010 Glycolysis / Gluconeogenesis;ko00040 Pentose and glucuronate interconversions;ko00053 Ascorbate and aldarate metabolism;ko00071 Fatty acid degradation;ko00280 Valine, leucine and isoleucine degradation;ko00310 Lysine degradation;ko00330 Arginine and proline metabolism;ko00340 Histidine metabolism;ko00380 Tryptophan metabolism;ko00410 beta-Alanine metabolism;ko00561 Glycerolipid metabolism;ko00620 Pyruvate metabolism;ko00625 Chloroalkane and chloroalkene degradation;ko00640 Propanoate metabolism;ko00903 Limonene and pinene
gene32204	K05391	CNGF	cyclic nucleotide gated channel, other eukaryote	--	1	ko04626 Plant-pathogen interaction;
gene32205	K03549	kup	KUP system potassium uptake protein	--		
gene32213	K08900	BCS1	mitochondrial chaperone BCS1	--		
gene32226	K12449	AXS	UDP-apiose/xylose synthase	--	1	ko00520 Amino sugar and nucleotide sugar metabolism;
gene32228	K00859	coaE	dephospho-CoA kinase	EC:2.7.1.24	1	ko00770 Pantothenate and CoA biosynthesis;
gene32229	K00228	hemF, CPOX	coproporphyrinogen III oxidase	EC:1.3.3.3	1	ko00860 Porphyrin and chlorophyll metabolism;
gene32230	K00423	E1.10.3.3	L-ascorbate oxidase	EC:1.10.3.3	1	ko00053 Ascorbate and aldarate metabolism;
gene32232	K10768	ALKBH6	alkylated DNA repair protein alkB homolog 6	EC:1.14.11.-		
gene32233	K08770	UBC	ubiquitin C	--	1	ko03320 PPAR signaling pathway;
gene32237	K03242	EIF2S3	translation initiation factor 2 subunit 3	--	1	ko03013 RNA transport;
gene32238	K08678	UXS1	UDP-glucuronate decarboxylase	EC:4.1.1.35	2	ko00500 Starch and sucrose metabolism;ko00520 Amino sugar and nucleotide sugar metabolism;
gene32239	K08245	E3.4.23.40	phytepsin	EC:3.4.23.40		
gene32241	K01051	E3.1.1.11	pectinesterase	EC:3.1.1.11	2	ko00040 Pentose and glucuronate interconversions;ko00500 Starch and sucrose metabolism;
gene32242	K11290	SET, TAF1, I2PP2A	template-activating factor I	--		
gene32250	K13343	PEX14	peroxin-14	--	1	ko04146 Peroxisome;
gene32251	K03681	RRP40, EXOSC3	exosome complex component RRP40	--	1	ko03018 RNA degradation;
gene32253	K05275	E1.1.1.65	pyridoxine 4-dehydrogenase	EC:1.1.1.65	1	ko00750 Vitamin B6 metabolism;
gene32254	K03143	TFIIH3, GTF2H3, TFB4	transcription initiation factor TFIIH subunit 3	--	3	ko03022 Basal transcription factors;ko03420 Nucleotide excision repair;ko05203 Viral carcinogenesis;
gene32258	K13111	SMU1	WD40 repeat-containing protein SMU1	--		
gene32260	K05894	E1.3.1.42	12-oxophytodienoic acid reductase	EC:1.3.1.42	1	ko00592 alpha-Linolenic acid metabolism;
gene32264	K03233	EEF1G	elongation factor 1-gamma	--	1	ko05134 Legionellosis;
gene32265	K10706	SETX, ALS4	senataxin	EC:3.6.4.-		
gene32270	K01640	E4.1.3.4, HMGCL, hmgL	hydroxymethylglutaryl-CoA lyase	EC:4.1.3.4	5	ko00072 Synthesis and degradation of ketone bodies;ko00280 Valine, leucine and isoleucine degradation;ko00281 Geraniol degradation;ko00650 Butanoate metabolism;ko04146 Peroxisome;
gene32272	K15356	VRG4, GONST1	GDP-mannose transporter	--		
gene32275	K00753	E2.4.1.214	glycoprotein 3-alpha-L-fucosyltransferase	EC:2.4.1.214	1	ko00513 Various types of N-glycan biosynthesis;
gene32278	K14432	ABF	ABA responsive element binding factor	--	1	ko04075 Plant hormone signal transduction;
gene32279	K14219	tRNA-Arg	tRNA Arg	--	1	ko00970 Aminoacyl-tRNA biosynthesis;
gene32280	K10635	RNF111	E3 ubiquitin-protein ligase Arkadia	EC:6.3.2.19		
gene32282	K16276	K16276, BTS	zinc finger protein-like protein	--		
gene32283	K16276	K16276, BTS	zinc finger protein-like protein	--		
gene3228	K14794	RRP12	ribosomal RNA-processing protein 12	--		
gene32290	K10357	MYO5	myosin V	--		
gene32296	K00261	GLUD1_2, gdhA	glutamate dehydrogenase (NAD(P)+)	EC:1.4.1.3	5	ko00250 Alanine, aspartate and glutamate metabolism;ko00330 Arginine and proline metabolism;ko00471 D-Glutamine and D-glutamate metabolism;ko00910 Nitrogen metabolism;ko04964 Proximal tubule bicarbonate reclamation;
gene32298	K13495	CISZOG	cis-zeatin O-glucosyltransferase	EC:2.4.1.215	1	ko00908 Zeatin biosynthesis;

gene32304	K04120	E5.5.1.13	ent-copalyl diphosphate synthase	EC:5.5.1.13	1	ko00904 Diterpenoid biosynthesis;
gene32308	K03013	RPB5, POLR2E	DNA-directed RNA polymerases I, II, and III subunit RPABC1	--	6	ko00230 Purine metabolism;ko00240 Pyrimidine metabolism;ko03020 RNA polymerase;ko04623 Cytosolic DNA-sensing pathway;ko05016 Huntington's disease;ko05169 Epstein-Barr virus infection;
gene32309	K11129	NHP2, NOLA2	H/ACA ribonucleoprotein complex subunit 2	--	1	ko03008 Ribosome biogenesis in eukaryotes;
gene32310	K08869	ADCK, ABC1	aarF domain-containing kinase	--		
gene32312	K14494	DELLA	DELLA protein	--	1	ko04075 Plant hormone signal transduction;
gene32313	K10406	KIFC2_3	kinesin family member C2/C3	--		
gene32315	K01883	CARS, cysS	cysteinyl-tRNA synthetase	EC:6.1.1.16	1	ko00970 Aminoacyl-tRNA biosynthesis;
gene32331	K08064	NFYA	nuclear transcription factor Y, alpha	--	2	ko04612 Antigen processing and presentation;ko05152 Tuberculosis;
gene32334	K10352	MYH	myosin heavy chain	--	2	ko04530 Tight junction;ko05416 Viral myocarditis;
gene32335	K06636	SMC1	structural maintenance of chromosome 1	--	4	ko04110 Cell cycle;ko04111 Cell cycle - yeast;ko04113 Meiosis - yeast;ko04114 Oocyte meiosis;
gene32338	K06130	LYPLA2	lysophospholipase II	EC:3.1.1.5	1	ko00564 Glycerophospholipid metabolism;
gene32342	K00059	fabG	3-oxoacyl-[acyl-carrier protein] reductase	EC:1.1.1.100	3	ko00061 Fatty acid biosynthesis;ko00780 Biotin metabolism;ko01040 Biosynthesis of unsaturated fatty acids;
gene32360	K00366	nirA	ferredoxin-nitrite reductase	EC:1.7.7.1	1	ko00910 Nitrogen metabolism;
gene3236	K00215	dapB	4-hydroxy-tetrahydrodipicolinate reductase	EC:1.17.1.8	2	ko00300 Lysine biosynthesis;ko01230 Biosynthesis of amino acids;
gene32370	K11826	AP2M1	AP-2 complex subunit mu-1	--	4	ko04144 Endocytosis;ko04721 Synaptic vesicle cycle;ko04961 Endocrine and other factor-regulated calcium reabsorption;ko05016 Huntington's disease;
gene32380	K13422	MYC2	transcription factor MYC2	--	2	ko04075 Plant hormone signal transduction;ko04626 Plant-pathogen interaction;
gene32383	K05868	CCNB	cyclin B	--	3	ko04110 Cell cycle;ko04115 p53 signaling pathway;ko04914 Progesterone-mediated oocyte maturation;
gene32385	K00430	E1.11.1.7	peroxidase	EC:1.11.1.7	2	ko00360 Phenylalanine metabolism;ko00940 Phenylpropanoid biosynthesis;
gene32386	K00430	E1.11.1.7	peroxidase	EC:1.11.1.7	2	ko00360 Phenylalanine metabolism;ko00940 Phenylpropanoid biosynthesis;
gene32388	K00430	E1.11.1.7	peroxidase	EC:1.11.1.7	2	ko00360 Phenylalanine metabolism;ko00940 Phenylpropanoid biosynthesis;
gene32389	K02997	RP-S9e, RPS9	small subunit ribosomal protein S9e	--	1	ko03010 Ribosome;
gene32390	K01723	AOS	hydroperoxide dehydratase	EC:4.2.1.92	1	ko00592 alpha-Linolenic acid metabolism;
gene32391	K07901	RAB8A, MEL	Ras-related protein Rab-8A	--	1	ko04972 Pancreatic secretion;
gene32395	K09873	TIP	aquaporin TIP	--		
gene32398	K09528	DNAJC8	DnaJ homolog subfamily C member 8	--		
gene32400	K00422	E1.10.3.1	polyphenol oxidase	EC:1.10.3.1	2	ko00350 Tyrosine metabolism;ko00950 Isoquinoline alkaloid biosynthesis;
gene32403	K15403	ACE, HTH	fatty acid omega-hydroxy dehydrogenase	EC:1.1.-.-	1	ko00073 Cutin, suberine and wax biosynthesis;
gene32407	K12309	GLB1, ELNR1	beta-galactosidase	EC:3.2.1.23	6	ko00052 Galactose metabolism;ko00511 Other glycan degradation;ko00531 Glycosaminoglycan degradation;ko00600 Sphingolipid metabolism;ko00604 Glycosphingolipid biosynthesis - ganglio series;ko04142 Lysosome;
gene32412	K15728	LPIN	phosphatidate phosphatase LPIN	EC:3.1.3.4	2	ko00561 Glycerolipid metabolism;ko00564 Glycerophospholipid metabolism;
gene32416	K01802	E5.2.1.8	peptidylprolyl isomerase	EC:5.2.1.8		
gene32421	K15111	SLC25A26	solute carrier family 25 (mitochondrial S-adenosylmethionine transporter), member 26	--		
gene32426	K05391	CNGF	cyclic nucleotide gated channel, other eukaryote	--	1	ko04626 Plant-pathogen interaction;
gene32427	K09338	HD-ZIP	homeobox-leucine zipper protein	--		
gene32430	K17262	TBCB, CKAP1, ALF1	tubulin-folding cofactor B	--		
gene32431	K02940	RP-L9e, RPL9	large subunit ribosomal protein L9e	--	1	ko03010 Ribosome;
gene32433	K15746	crtZ	beta-carotene 3-hydroxylase	EC:1.14.13.129	1	ko00906 Carotenoid biosynthesis;
gene32439	K14488	SAUR	SAUR family protein	--	1	ko04075 Plant hormone signal transduction;
gene32441	K05573	ndhB	NAD(P)H-quinone oxidoreductase subunit 2	EC:1.6.5.3	1	ko00190 Oxidative phosphorylation;
gene32443	K11578	ZW10	centromere/kinetochore protein ZW10	--		
gene32447	K00599	E2.1.1.-			2	ko00350 Tyrosine metabolism;ko00624 Polycyclic aromatic hydrocarbon degradation;
gene32448	K14509	ETR, ERS	ethylene receptor	EC:2.7.13.-	1	ko04075 Plant hormone signal transduction;

gene3245	K07198	PRKAA, AMPK	5'-AMP-activated protein kinase, catalytic alpha subunit	EC:2.7.11.11	8	ko04140 Regulation of autophagy;ko04150 mTOR signaling pathway;ko04151 PI3K-Akt signaling pathway;ko04710 Circadian rhythm;ko04910 Insulin signaling pathway;ko04920 Adipocytokine signaling pathway;ko04932 Non-alcoholic fatty liver disease (NAFLD);ko05410 Hypertrophic cardiomyopathy (HCM);
gene32463	K00011	E1.1.1.21, AKR1	aldehyde reductase	EC:1.1.1.21	5	ko00040 Pentose and glucuronate interconversions;ko00051 Fructose and mannose metabolism;ko00052 Galactose metabolism;ko00561 Glycerolipid metabolism;ko00620 Pyruvate metabolism;
gene32465	K00966	GMPP	mannose-1-phosphate guanylyltransferase	EC:2.7.7.13	2	ko00051 Fructose and mannose metabolism;ko00520 Amino sugar and nucleotide sugar metabolism;
gene3246	K07198	PRKAA, AMPK	5'-AMP-activated protein kinase, catalytic alpha subunit	EC:2.7.11.11	8	ko04140 Regulation of autophagy;ko04150 mTOR signaling pathway;ko04151 PI3K-Akt signaling pathway;ko04710 Circadian rhythm;ko04910 Insulin signaling pathway;ko04920 Adipocytokine signaling pathway;ko04932 Non-alcoholic fatty liver disease (NAFLD);ko05410 Hypertrophic cardiomyopathy (HCM);
gene32470	K01866	YARS, tyrS	tyrosyl-tRNA synthetase	EC:6.1.1.1	1	ko00970 Aminoacyl-tRNA biosynthesis;
gene32477	K02929	RP-L44e, RPL44	large subunit ribosomal protein L44e	--	1	ko03010 Ribosome;
gene32480	K12236	NFX1	transcriptional repressor NF-X1	EC:6.3.2.-		
gene32488	K07374	TUBA	tubulin alpha	--	3	ko04145 Phagosome;ko04540 Gap junction;ko05130 Pathogenic Escherichia coli infection;
gene32489	K13832	aroDE, DHQ-SDH	3-dehydroquinate dehydratase / shikimate dehydrogenase	EC:4.2.1.10 1.1.1.25	2	ko00400 Phenylalanine, tyrosine and tryptophan biosynthesis;ko01230 Biosynthesis of amino acids;
gene3248	K01762	ACS	1-aminocyclopropane-1-carboxylate synthase	EC:4.4.1.14	1	ko00270 Cysteine and methionine metabolism;
gene32490	K14962	WDR82, SWD2, CPS35	COMPASS component SWD2	--	1	ko03015 mRNA surveillance pathway;
gene32491	K02875	RP-L14e, RPL14	large subunit ribosomal protein L14e	--	1	ko03010 Ribosome;
gene32493	K07374	TUBA	tubulin alpha	--	3	ko04145 Phagosome;ko04540 Gap junction;ko05130 Pathogenic Escherichia coli infection;
gene32497	K00966	GMPP	mannose-1-phosphate guanylyltransferase	EC:2.7.7.13	2	ko00051 Fructose and mannose metabolism;ko00520 Amino sugar and nucleotide sugar metabolism;
gene32500	K00861	RFK, FMN1	riboflavin kinase	EC:2.7.1.26	1	ko00740 Riboflavin metabolism;
gene32501	K14219	tRNA-Arg	tRNA Arg	--	1	ko00970 Aminoacyl-tRNA biosynthesis;
gene32505	K00434	E1.11.1.11	L-ascorbate peroxidase	EC:1.11.1.11	2	ko00053 Ascorbate and aldarate metabolism;ko00480 Glutathione metabolism;
gene32509	K08202	SLC22A4_5, OCTN	MFS transporter, OCT family, solute carrier family 22 (organic cation transporter), member 4/5	--		
gene3250	K14488	SAUR	SAUR family protein	--	1	ko04075 Plant hormone signal transduction;
gene32511	K10406	KIFC2_3	kinesin family member C2/C3	--		
gene32516	K11816	YUCCA	indole-3-pyruvate monooxygenase	EC:1.14.13.168	1	ko00380 Tryptophan metabolism;
gene32520	K00432	E1.11.1.9	glutathione peroxidase	EC:1.11.1.9	3	ko00480 Glutathione metabolism;ko00590 Arachidonic acid metabolism;ko04918 Thyroid hormone synthesis;
gene32525	K09338	HD-ZIP	homeobox-leucine zipper protein	--		
gene3252	K14488	SAUR	SAUR family protein	--	1	ko04075 Plant hormone signal transduction;
gene32540	K01530	E3.6.3.1	phospholipid-translocating ATPase	EC:3.6.3.1		
gene32542	K14709	SLC39A1_2_3, ZIP1_2_3	solute carrier family 39 (zinc transporter), member 1/2/3	--		
gene32549	K14638	SLC15A3_4, PHT	solute carrier family 15 (peptide/histidine transporter), member 3/4	--		
gene32550	K13691	SGT1	pathogen-inducible salicylic acid glucosyltransferase	EC:2.4.1.-		
gene32551	K02639	petF	ferredoxin	--	1	ko00195 Photosynthesis;
gene32556	K03327	TC.MATE, SLC47A, norM, mdtK, dinF	multidrug resistance protein, MATE family	--		
gene32557	K14230	tRNA-Met	tRNA Met	--	1	ko00970 Aminoacyl-tRNA biosynthesis;
gene32559	K10755	RFC2_4	replication factor C subunit 2/4	--	3	ko03030 DNA replication;ko03420 Nucleotide excision repair;ko03430 Mismatch repair;
gene32571	K14315	NDC1, TMEM48	nucleoporin NDC1	--	1	ko03013 RNA transport;
gene32572	K01728	E4.2.2.2, pel	pectate lyase	EC:4.2.2.2	1	ko00040 Pentose and glucuronate interconversions;
gene32577	K14293	KPNB1	importin subunit beta-1	--	1	ko03013 RNA transport;
gene32578	K02873	RP-L13e, RPL13	large subunit ribosomal protein L13e	--	1	ko03010 Ribosome;
gene32579	K02948	RP-S11, MRPS11, rpsK	small subunit ribosomal protein S11	--	1	ko03010 Ribosome;
gene3257	K14496	PYL	abscisic acid receptor PYR/PYL family	--	1	ko04075 Plant hormone signal transduction;

gene32583	K07052	K07052				
gene32586	K16287	ULP1C_D	ubiquitin-like-specific protease 1C/D	EC:3.4.22.68		
gene32587	K16287	ULP1C_D	ubiquitin-like-specific protease 1C/D	EC:3.4.22.68		
gene32589	K12822	RBM25, S164	RNA-binding protein 25	--	1	ko03040 Spliceosome;
gene3258	K12158	NEDD8	ubiquitin-like protein Nedd8	--		
gene32591	K02331	POL5, MYBBP1A	DNA polymerase phi	EC:2.7.7.7		
gene32593	K16903	TAA1	L-tryptophan---pyruvate aminotransferase	EC:2.6.1.99	1	ko00380 Tryptophan metabolism;
gene32597	K17604	ZSWIM3	zinc finger SWIM domain-containing protein 3	--		
gene32598	K08515	VAMP7	vesicle-associated membrane protein 7	--	1	ko04130 SNARE interactions in vesicular transport;
gene32600	K05298	GAPA	glyceraldehyde-3-phosphate dehydrogenase (NADP+) (phosphorylating)	EC:1.2.1.13	2	ko00710 Carbon fixation in photosynthetic organisms;ko01200 Carbon metabolism;
gene32603	K14801	TSR4	pre-rRNA-processing protein TSR4	--		
gene32604	K06158	ABCF3	ATP-binding cassette, subfamily F, member 3	--		
gene32606	K04564	SOD2	superoxide dismutase, Fe-Mn family	EC:1.15.1.1	2	ko04146 Peroxisome;ko05016 Huntington's disease;
gene32609	K02937	RP-L7e, RPL7	large subunit ribosomal protein L7e	--	1	ko03010 Ribosome;
gene32610	K12581	CNOT7_8, CAF1, POP2	CCR4-NOT transcription complex subunit 7/8	--	1	ko03018 RNA degradation;
gene32615	K01728	E4.2.2.2, pel	pectate lyase	EC:4.2.2.2	1	ko00040 Pentose and glucuronate interconversions;
gene32617	K14709	SLC39A1_2_3, ZIP1_2_3	solute carrier family 39 (zinc transporter), member 1/2/3	--		
gene32623	K04508	TBL1	transducin (beta)-like 1	--	1	ko04310 Wnt signaling pathway;
gene32624	K08150	SLC2A13, ITR	MFS transporter, SP family, solute carrier family 2 (myo-inositol transporter), member 13	--		
gene32626	K14411	MSI	RNA-binding protein Musashi	--	1	ko03015 mRNA surveillance pathway;
gene32627	K00943	E2.7.4.9, tmk	dTMP kinase	EC:2.7.4.9	1	ko00240 Pyrimidine metabolism;
gene3262	K01051	E3.1.1.11	pectinesterase	EC:3.1.1.11	2	ko00040 Pentose and glucuronate interconversions;ko00500 Starch and sucrose metabolism;
gene32636	K03456	PPP2R1	serine/threonine-protein phosphatase 2A regulatory subunit A	--	13	ko03015 mRNA surveillance pathway;ko04111 Cell cycle - yeast;ko04113 Meiosis - yeast;ko04114 Oocyte meiosis;ko04151 PI3K-Akt signaling pathway;ko04350 TGF-beta signaling pathway;ko04390 Hippo signaling pathway;ko04391 Hippo signaling pathway - fly;ko04530 Tight junction;ko04728 Dopaminergic synapse;ko04730 Long-term depression;ko05142 Chagas disease (American trypanosomiasis);ko05160 Hepatitis C;
gene32638	K13412	CPK	calcium-dependent protein kinase	EC:2.7.11.1	2	ko04626 Plant-pathogen interaction;ko05145 Toxoplasmosis;
gene3263	K00275	pdxH, PNPO	pyridoxamine 5'-phosphate oxidase	EC:1.4.3.5	1	ko00750 Vitamin B6 metabolism;
gene32648	K00753	E2.4.1.214	glycoprotein 3-alpha-L-fucosyltransferase	EC:2.4.1.214	1	ko00513 Various types of N-glycan biosynthesis;
gene32651	K14432	ABF	ABA responsive element binding factor	--	1	ko04075 Plant hormone signal transduction;
gene32653	K14219	tRNA-Arg	tRNA Arg	--	1	ko00970 Aminoacyl-tRNA biosynthesis;
gene32654	K10635	RNF111	E3 ubiquitin-protein ligase Arkadia	EC:6.3.2.19		
gene32655	K12121	PHYB	phytochrome B	--	1	ko04712 Circadian rhythm - plant;
gene32659	K03033	PSMD3, RPN3	26S proteasome regulatory subunit N3	--	2	ko03050 Proteasome;ko05169 Epstein-Barr virus infection;
gene3265	K10590	TRIP12	E3 ubiquitin-protein ligase TRIP12	EC:6.3.2.19	1	ko04120 Ubiquitin mediated proteolysis;
gene32660	K03033	PSMD3, RPN3	26S proteasome regulatory subunit N3	--	2	ko03050 Proteasome;ko05169 Epstein-Barr virus infection;
gene32661	K02520	infC, MTIF3	translation initiation factor IF-3	--		
gene32664	K00549	metE	5-methyltetrahydropteroyltriglutamate--homocysteine methyltransferase	EC:2.1.1.14	3	ko00270 Cysteine and methionine metabolism;ko00450 Selenocompound metabolism;ko01230 Biosynthesis of amino acids;
gene32665	K01082	E3.1.3.7, cysQ, MET22, BPNT1	3'(2'), 5'-bisphosphate nucleotidase	EC:3.1.3.7	1	ko00920 Sulfur metabolism;
gene32669	K00088	guaB	IMP dehydrogenase	EC:1.1.1.205	2	ko00230 Purine metabolism;ko00983 Drug metabolism - other enzymes;
gene32681	K00430	E1.11.1.7	peroxidase	EC:1.11.1.7	2	ko00360 Phenylalanine metabolism;ko00940 Phenylpropanoid biosynthesis;
gene32686	K00279	E1.5.99.12	cytokinin dehydrogenase	EC:1.5.99.12	1	ko00908 Zeatin biosynthesis;
gene32687	K03250	EIF3E, INT6	translation initiation factor 3 subunit E	--	2	ko03013 RNA transport;ko05160 Hepatitis C;
gene32689	K01103	PFKFB	6-phosphofructo-2-kinase / fructose-2,6-bisphosphatase	EC:2.7.1.105 3.1.3.46	2	ko00051 Fructose and mannose metabolism;ko04066 HIF-1 signaling pathway;
gene3268	K11985	TRAIP, TRIP	TRAF-interacting protein	--		

gene32690	K01733	thrC	threonine synthase	EC:4.2.3.1	3	ko00260 Glycine, serine and threonine metabolism;ko00750 Vitamin B6 metabolism;ko01230 Biosynthesis of amino acids;
gene32695	K00966	GMPP	mannose-1-phosphate guanylyltransferase	EC:2.7.7.13	2	ko00051 Fructose and mannose metabolism;ko00520 Amino sugar and nucleotide sugar metabolism;
gene32697	K14004	SEC13	protein transport protein SEC13	--	2	ko03013 RNA transport;ko04141 Protein processing in endoplasmic reticulum;
gene32699	K09422	MYBP	myb proto-oncogene protein, plant	--		
gene3269	K00430	E1.11.1.7	peroxidase	EC:1.11.1.7	2	ko00360 Phenylalanine metabolism;ko00940 Phenylpropanoid biosynthesis;
gene32705	K12611	DCP1B	mRNA-decapping enzyme 1B	EC:3.-.-.-	1	ko03018 RNA degradation;
gene32706	K14332	psaO	photosystem I subunit PsaO	--	1	ko00195 Photosynthesis;
gene32712	K02923	RP-L38e, RPL38	large subunit ribosomal protein L38e	--	1	ko03010 Ribosome;
gene32713	K16615	PARP7	actin-related protein 7, plant	--		
gene32718	K10610	DDB1	DNA damage-binding protein 1	--	4	ko03420 Nucleotide excision repair;ko04120 Ubiquitin mediated proteolysis;ko05161 Hepatitis B;ko05203 Viral carcinogenesis;
gene32725	K17255	GDI1_2	Rab GDP dissociation inhibitor	--		
gene32726	K02049	ABC.SN.A	NitT/TauT family transport system ATP-binding protein	--		
gene32730	K00891	E2.7.1.71, aroK, aroL	shikimate kinase	EC:2.7.1.71	2	ko00400 Phenylalanine, tyrosine and tryptophan biosynthesis;ko01230 Biosynthesis of amino acids;
gene32731	K17080	PHB1	prohibitin 1	--		
gene32739	K15730	PTGES3	cytosolic prostaglandin-E synthase	EC:5.3.99.3	1	ko00590 Arachidonic acid metabolism;
gene32741	K04713	SUR2	sphinganine C4-monooxygenase	EC:1.14.13.169	1	ko00600 Sphingolipid metabolism;
gene32746	K13832	aroDE, DHQ-SDH	3-dehydroquinate dehydratase / shikimate dehydrogenase	EC:4.2.1.10 1.1.1.25	2	ko00400 Phenylalanine, tyrosine and tryptophan biosynthesis;ko01230 Biosynthesis of amino acids;
gene32748	K14232	tRNA-Pro	tRNA Pro	--	1	ko00970 Aminoacyl-tRNA biosynthesis;
gene3274	K10643	CNOT4, NOT4, MOT2	CCR4-NOT transcription complex subunit 4	EC:6.3.2.19	1	ko03018 RNA degradation;
gene32761	K12863	CWC15	protein CWC15	--	1	ko03040 Spliceosome;
gene32766	K15507	MRM1, PET56	21S rRNA (GM2251-2'-O)-methyltransferase	EC:2.1.1.-		
gene32767	K01535	E3.6.3.6	H+-transporting ATPase	EC:3.6.3.6	1	ko00190 Oxidative phosphorylation;
gene3276	K01537	E3.6.3.8	Ca2+-transporting ATPase	EC:3.6.3.8		
gene32773	K09458	fabF	3-oxoacyl-[acyl-carrier-protein] synthase II	EC:2.3.1.179	2	ko00061 Fatty acid biosynthesis;ko00780 Biotin metabolism;
gene32781	K13344	PEX13	peroxin-13	--	1	ko04146 Peroxisome;
gene32782	K10268	FBXL2_20	F-box and leucine-rich repeat protein 2/20	--		
gene32787	K15170	MED27	mediator of RNA polymerase II transcription subunit 27	--		
gene32790	K05016	CLCN7	chloride channel 7	--		
gene32791	K01052	LIPA	lysosomal acid lipase/cholesteryl ester hydrolase	EC:3.1.1.13	2	ko00100 Steroid biosynthesis;ko04142 Lysosome;
gene32796	K15920	XYL4	beta-D-xylosidase 4	EC:3.2.1.37	2	ko00500 Starch and sucrose metabolism;ko00520 Amino sugar and nucleotide sugar metabolism;
gene32797	K13416	BAK1	brassinosteroid insensitive 1-associated receptor kinase 1	EC:2.7.10.1 2.7.11.1	2	ko04075 Plant hormone signal transduction;ko04626 Plant-pathogen interaction;
gene32798	K14298	RAE1	mRNA export factor	--	2	ko03013 RNA transport;ko05164 Influenza A;
gene3279	K01363	CTSB	cathepsin B	EC:3.4.22.1	2	ko04142 Lysosome;ko04612 Antigen processing and presentation;
gene32802	K04565	SOD1	superoxide dismutase, Cu-Zn family	EC:1.15.1.1	4	ko04146 Peroxisome;ko05014 Amyotrophic lateral sclerosis (ALS);ko05016 Huntington's disease;ko05020 Prion diseases;
gene32804	K08150	SLC2A13, ITR	MFS transporter, SP family, solute carrier family 2 (myo-inositol transporter), member 13	--		
gene32805	K00943	E2.7.4.9, tmk	dTMP kinase	EC:2.7.4.9	1	ko00240 Pyrimidine metabolism;
gene32807	K08867	WNK, PRKWNK	WNK lysine deficient protein kinase	EC:2.7.11.1		
gene32809	K13407	CYP94A1	cytochrome P450, family 94, subfamily A, polypeptide 1 (fatty acid omega-hydroxylase)	EC:1.14.-.-	1	ko00073 Cutin, suberine and wax biosynthesis;
gene3280	K06874	K06874	zinc finger protein	--		
gene32811	K13464	JAZ	jasmonate ZIM domain-containing protein	--	2	ko04075 Plant hormone signal transduction;ko04626 Plant-pathogen interaction;
gene32820	K01213	E3.2.1.67	galacturan 1,4-alpha-galacturonidase	EC:3.2.1.67	2	ko00040 Pentose and glucuronate interconversions;ko00500 Starch and sucrose metabolism;
gene32821	K12812	UAP56, BAT1, SUB2	ATP-dependent RNA helicase UAP56/SUB2	EC:3.6.4.13	4	ko03013 RNA transport;ko03015 mRNA surveillance pathway;ko03040 Spliceosome;ko05164 Influenza A;

gene32822	K01883	CARS, cysS	cysteinyl-tRNA synthetase	EC:6.1.1.16	1	ko00970 Aminoacyl-tRNA biosynthesis;
gene32824	K02937	RP-L7e, RPL7	large subunit ribosomal protein L7e	--	1	ko03010 Ribosome;
gene32825	K08081	E1.1.1.206	tropine dehydrogenase	EC:1.1.1.206	1	ko00960 Tropane, piperidine and pyridine alkaloid biosynthesis;
gene32829	K01623	ALDO	fructose-bisphosphate aldolase, class I	EC:4.1.2.13	7	ko00010 Glycolysis / Gluconeogenesis;ko00030 Pentose phosphate pathway;ko00051 Fructose and mannose metabolism;ko00680 Methane metabolism;ko00710 Carbon fixation in photosynthetic organisms;ko01200 Carbon metabolism;ko01230 Biosynthesis of amino acids;
gene32833	K08819	CDK12_13	cyclin-dependent kinase 12/13	EC:2.7.11.22 2.7.11.23		
gene32835	K02138	ATPeFD, ATP5H	F-type H+-transporting ATPase subunit d	EC:3.6.3.14	4	ko00190 Oxidative phosphorylation;ko05010 Alzheimer's disease;ko05012 Parkinson's disease;ko05016 Huntington's disease;
gene3283	K09840	NCED	9-cis-epoxycarotenoid dioxygenase	EC:1.13.11.51	1	ko00906 Carotenoid biosynthesis;
gene32840	K10579	UBE2M, UBC12	ubiquitin-conjugating enzyme E2 M	EC:6.3.2.19	1	ko04120 Ubiquitin mediated proteolysis;
gene32841	K13789	GGPS	geranylgeranyl diphosphate synthase, type II	EC:2.5.1.1 2.5.1.10 2.5.1.29	1	ko00900 Terpenoid backbone biosynthesis;
gene32842	K13789	GGPS	geranylgeranyl diphosphate synthase, type II	EC:2.5.1.1 2.5.1.10 2.5.1.29	1	ko00900 Terpenoid backbone biosynthesis;
gene32843	K09422	MYBP	myb proto-oncogene protein, plant	--		
gene32846	K10525	E5.3.99.6, aoc	allene oxide cyclase	EC:5.3.99.6	1	ko00592 alpha-Linolenic acid metabolism;
gene3284	K17275	PLS1	plastin-1	--		
gene32852	K02925	RP-L3e, RPL3	large subunit ribosomal protein L3e	--	1	ko03010 Ribosome;
gene32856	K17479	GRXCR1	glutaredoxin domain-containing cysteine-rich protein 1	--		
gene32860	K12169	KPC1, RNF123	Kip1 ubiquitination-promoting complex protein 1	EC:6.3.2.19		
gene32861	K00434	E1.11.1.11	L-ascorbate peroxidase	EC:1.11.1.11	2	ko00053 Ascorbate and aldarate metabolism;ko00480 Glutathione metabolism;
gene32867	K02134	ATPeF1D, ATP5D	F-type H+-transporting ATPase subunit delta	EC:3.6.3.14	4	ko00190 Oxidative phosphorylation;ko05010 Alzheimer's disease;ko05012 Parkinson's disease;ko05016 Huntington's disease;
gene32869	K09338	HD-ZIP	homeobox-leucine zipper protein	--		
gene32871	K15421	SERGEF	secretion-regulating guanine nucleotide exchange factor	--		
gene32876	K08511	ATVAMP72	vesicle-associated membrane protein 72	--		
gene32877	K14289	XPO5	exportin-5	--	1	ko03013 RNA transport;
gene32879	K03240	EIF2B5	translation initiation factor eIF-2B subunit epsilon	--	1	ko03013 RNA transport;
gene32880	K14568	EMG1, NEP1	rRNA small subunit pseudouridine methyltransferase Nep1	EC:2.1.1.260	1	ko03008 Ribosome biogenesis in eukaryotes;
gene32881	K10406	KIFC2_3	kinesin family member C2/C3	--		
gene32889	K07437	CYP26A	cytochrome P450, family 26, subfamily A	--	1	ko00830 Retinol metabolism;
gene32895	K14401	CPSF1, CFT1	cleavage and polyadenylation specificity factor subunit 1	--	1	ko03015 mRNA surveillance pathway;
gene32899	K16732	PRC1	protein regulator of cytokinesis 1	--		
gene32903	K02942	RP-LP1, RPLP1	large subunit ribosomal protein LP1	--	1	ko03010 Ribosome;
gene32906	K14791	PWP1	periodic tryptophan protein 1	--		
gene32907	K14484	IAA	auxin-responsive protein IAA	--	1	ko04075 Plant hormone signal transduction;
gene32910	K03781	katE, CAT, catB, srpA	catalase	EC:1.11.1.6	4	ko00380 Tryptophan metabolism;ko00630 Glyoxylate and dicarboxylate metabolism;ko04146 Peroxisome;ko05014 Amyotrophic lateral sclerosis (ALS);
gene32913	K11723	BRD7_9	bromodomain-containing protein 7/9	--		
gene32914	K15362	BRIP1, BACH1, FANCJ	fanconi anemia group J protein	EC:3.6.4.12	1	ko03460 Fanconi anemia pathway;
gene32915	K14803	PTC2_3	protein phosphatase 2C homolog 2/3	EC:3.1.3.16		
gene32917	K01876	DARS, aspS	aspartyl-tRNA synthetase	EC:6.1.1.12	1	ko00970 Aminoacyl-tRNA biosynthesis;
gene32918	K02836	prfB	peptide chain release factor 2	--		
gene32919	K11165	DHRS7	dehydrogenase/reductase SDR family member 7	EC:1.1.-.-		
gene32923	K09595	HM13	minor histocompatibility antigen H13	EC:3.4.23.-		
gene32924	K00423	E1.10.3.3	L-ascorbate oxidase	EC:1.10.3.3	1	ko00053 Ascorbate and aldarate metabolism;
gene32925	K12176	COPS2, CSN2, TRIP15	COP9 signalosome complex subunit 2	--		
gene32927	K13420	FLS2	LRR receptor-like serine/threonine-protein kinase FLS2	EC:2.7.11.1	1	ko04626 Plant-pathogen interaction;
gene3292	K02923	RP-L38e, RPL38	large subunit ribosomal protein L38e	--	1	ko03010 Ribosome;

gene32934	K04730	IRAK1	interleukin-1 receptor-associated kinase 1	EC:2.7.11.1	11	ko04064 NF-kappa B signaling pathway;ko04210 Apoptosis;ko04620 Toll-like receptor signaling pathway;ko04722 Neurotrophin signaling pathway;ko05133 Pertussis;ko05140 Leishmaniasis;ko05142 Chagas disease (American trypanosomiasis);ko05145 Toxoplasmosis;ko05152 Tuberculosis;ko05162 Measles;ko05169 Epstein-Barr virus infection;
gene32936	K05305	FUK	fucokinase	EC:2.7.1.52	2	ko00051 Fructose and mannose metabolism;ko00520 Amino sugar and nucleotide sugar metabolism;
gene32937	K11816	YUCCA	indole-3-pyruvate monooxygenase	EC:1.14.13.168	1	ko00380 Tryptophan metabolism;
gene32949	K13648	GAUT	alpha-1,4-galacturonosyltransferase	EC:2.4.1.43	2	ko00500 Starch and sucrose metabolism;ko00520 Amino sugar and nucleotide sugar metabolism;
gene32953	K05752	C3ORF10, HSPC300	chromosome 3 open reading frame 10	--	1	ko04810 Regulation of actin cytoskeleton;
gene32956	K14508	NPR1	regulatory protein NPR1	--	1	ko04075 Plant hormone signal transduction;
gene32959	K01148	PARN	poly(A)-specific ribonuclease	EC:3.1.13.4	1	ko03018 RNA degradation;
gene32965	K13691	SGT1	pathogen-inducible salicylic acid glucosyltransferase	EC:2.4.1.-		
gene32967	K05391	CNGF	cyclic nucleotide gated channel, other eukaryote	--	1	ko04626 Plant-pathogen interaction;
gene32968	K01893	NARS, asnS	asparaginyl-tRNA synthetase	EC:6.1.1.22	1	ko00970 Aminoacyl-tRNA biosynthesis;
gene3296	K01519	ITPA	inosine triphosphate pyrophosphatase	EC:3.6.1.19	3	ko00230 Purine metabolism;ko00240 Pyrimidine metabolism;ko00983 Drug metabolism - other enzymes;
gene32972	K01611	speD, AMD1	S-adenosylmethionine decarboxylase	EC:4.1.1.50	2	ko00270 Cysteine and methionine metabolism;ko00330 Arginine and proline metabolism;
gene32978	K01623	ALDO	fructose-bisphosphate aldolase, class I	EC:4.1.2.13	7	ko00010 Glycolysis / Gluconeogenesis;ko00030 Pentose phosphate pathway;ko00051 Fructose and mannose metabolism;ko00680 Methane metabolism;ko00710 Carbon fixation in photosynthetic organisms;ko01200 Carbon metabolism;ko01230 Biosynthesis of amino acids;
gene32979	K12183	TSG101, STP22, VPS23	ESCRT-I complex subunit TSG101	--	1	ko04144 Endocytosis;
gene32983	K14297	NUP98, ADAR2	nuclear pore complex protein Nup98-Nup96	--	2	ko03013 RNA transport;ko05164 Influenza A;
gene32985	K01184	E3.2.1.15	polygalacturonase	EC:3.2.1.15	2	ko00040 Pentose and glucuronate interconversions;ko00500 Starch and sucrose metabolism;
gene3298	K06890	K06890				
gene32990	K03327	TC.MATE, SLC47A, norM, mdtK, dinF	multidrug resistance protein, MATE family	--		
gene329	K00430	E1.11.1.7	peroxidase	EC:1.11.1.7	2	ko00360 Phenylalanine metabolism;ko00940 Phenylpropanoid biosynthesis;
gene33004	K09285	OVM, ANT	AP2-like factor, ANT lineage	--		
gene33005	K09458	fabF	3-oxoacyl-[acyl-carrier-protein] synthase II	EC:2.3.1.179	2	ko00061 Fatty acid biosynthesis;ko00780 Biotin metabolism;
gene33007	K10520	ABTB1, BPOZ	ankyrin repeat and BTB/POZ domain-containing protein 1	--		
gene33008	K17275	PLS1	plastin-1	--		
gene3300	K08099	E3.1.1.14	chlorophyllase	EC:3.1.1.14	1	ko00860 Porphyrin and chlorophyll metabolism;
gene33013	K13448	CML	calcium-binding protein CML	--	1	ko04626 Plant-pathogen interaction;
gene33019	K00083	E1.1.1.195	cinnamyl-alcohol dehydrogenase	EC:1.1.1.195	1	ko00940 Phenylpropanoid biosynthesis;
gene33035	K12309	GLB1, ELNR1	beta-galactosidase	EC:3.2.1.23	6	ko00052 Galactose metabolism;ko00511 Other glycan degradation;ko00531 Glycosaminoglycan degradation;ko00600 Sphingolipid metabolism;ko00604 Glycosphingolipid biosynthesis - ganglio series;ko04142 Lysosome;
gene33038	K09264	K09264	MADS-box transcription factor, plant	--		
gene33040	K01673	cynT, can	carbonic anhydrase	EC:4.2.1.1	1	ko00910 Nitrogen metabolism;
gene33043	K09422	MYBP	myb proto-oncogene protein, plant	--		
gene33049	K09754	CYP98A3, C3'H	coumaroylquinate(coumaroylshikimate) 3'-monooxygenase	EC:1.14.13.36	3	ko00940 Phenylpropanoid biosynthesis;ko00941 Flavonoid biosynthesis;ko00945 Stilbenoid, diarylheptanoid and gingerol biosynthesis;
gene33050	K09571	FKBP4_5	FK506-binding protein 4/5	EC:5.2.1.8	1	ko04915 Estrogen signaling pathway;
gene33051	K09548	PFDN1	prefoldin subunit 1	--		
gene33054	K15028	EIF3K	translation initiation factor 3 subunit K	--		
gene33056	K10747	LIG1	DNA ligase 1	EC:6.5.1.1	4	ko03030 DNA replication;ko03410 Base excision repair;ko03420 Nucleotide excision repair;ko03430 Mismatch repair;
gene33058	K03064	PSMC6, RPT4	26S proteasome regulatory subunit T4	--	2	ko03050 Proteasome;ko05169 Epstein-Barr virus infection;
gene33059	K00430	E1.11.1.7	peroxidase	EC:1.11.1.7	2	ko00360 Phenylalanine metabolism;ko00940 Phenylpropanoid biosynthesis;

gene33066	K07767	E3.6.4.3	microtubule-severing ATPase	EC:3.6.4.3		
gene33069	K04125	E1.14.11.13	gibberellin 2-oxidase	EC:1.14.11.13	1	ko00904 Diterpenoid biosynthesis;
gene33070	K09286	EREBP	EREBP-like factor	--		
gene33071	K06689	UBE2D_E, UBC4, UBC5	ubiquitin-conjugating enzyme E2 D/E	EC:6.3.2.19	2	ko04120 Ubiquitin mediated proteolysis;ko04141 Protein processing in endoplasmic reticulum;
gene33073	K13719	OTU1, YOD1	ubiquitin thioesterase OTU1	EC:3.1.2.-	1	ko04141 Protein processing in endoplasmic reticulum;
gene33080	K01051	E3.1.1.11	pectinesterase	EC:3.1.1.11	2	ko00040 Pentose and glucuronate interconversions;ko00500 Starch and sucrose metabolism;
gene33086	K12259	SMOX	spermine oxidase	EC:1.5.3.16		
gene33093	K09338	HD-ZIP	homeobox-leucine zipper protein	--		
gene33094	K02927	RP-L40e, RPL40	large subunit ribosomal protein L40e	--	1	ko03010 Ribosome;
gene33095	K01761	E4.4.1.11	methionine-gamma-lyase	EC:4.4.1.11	2	ko00270 Cysteine and methionine metabolism;ko00450 Selenocompound metabolism;
gene33099	K04733	IRAK4	interleukin-1 receptor-associated kinase 4	EC:2.7.11.1	11	ko04064 NF-kappa B signaling pathway;ko04210 Apoptosis;ko04620 Toll-like receptor signaling pathway;ko04722 Neurotrophin signaling pathway;ko05133 Pertussis;ko05140 Leishmaniasis;ko05142 Chagas disease (American trypanosomiasis);ko05145 Toxoplasmosis;ko05152 Tuberculosis;ko05162 Measles;ko05164 Influenza A;
gene33111	K06630	YWHAE	14-3-3 protein epsilon	--	8	ko04110 Cell cycle;ko04114 Oocyte meiosis;ko04151 PI3K-Akt signaling pathway;ko04390 Hippo signaling pathway;ko04391 Hippo signaling pathway - fly;ko04722 Neurotrophin signaling pathway;ko05169 Epstein-Barr virus infection;ko05203 Viral carcinogenesis;
gene33113	K07955	ARL8	ADP-ribosylation factor-like protein 8	--		
gene33114	K13464	JAZ	jasmonate ZIM domain-containing protein	--	2	ko04075 Plant hormone signal transduction;ko04626 Plant-pathogen interaction;
gene33117	K01904	E6.2.1.12	4-coumarate--CoA ligase	EC:6.2.1.12	3	ko00130 Ubiquinone and other terpenoid-quinone biosynthesis;ko00360 Phenylalanine metabolism;ko00940 Phenylpropanoid biosynthesis;
gene3311	K14153	thiDE	hydroxymethylpyrimidine kinase / phosphomethylpyrimidine kinase / thiamine-phosphate diphosphorylase	EC:2.7.1.49 2.7.4.7 2.5.1.3	1	ko00730 Thiamine metabolism;
gene33120	K07195	EXOC7, EXO70	exocyst complex component 7	--	1	ko04910 Insulin signaling pathway;
gene33121	K01759	E4.4.1.5, GLO1, gloA	lactoylglutathione lyase	EC:4.4.1.5	2	ko00620 Pyruvate metabolism;ko04011 MAPK signaling pathway - yeast;
gene33122	K05359	ADT, PDT	arogenate/prephenate dehydratase	EC:4.2.1.91 4.2.1.51	2	ko00400 Phenylalanine, tyrosine and tryptophan biosynthesis;ko01230 Biosynthesis of amino acids;
gene33123	K00721	DPM1	dolichol-phosphate mannosyltransferase	EC:2.4.1.83	1	ko00510 N-Glycan biosynthesis;
gene33125	K08064	NFYA	nuclear transcription factor Y, alpha	--	2	ko04612 Antigen processing and presentation;ko05152 Tuberculosis;
gene33126	K12275	SEC62	translocation protein SEC62	--	2	ko03060 Protein export;ko04141 Protein processing in endoplasmic reticulum;
gene3312	K12840	RBM17, SPF45	splicing factor 45	--	1	ko03040 Spliceosome;
gene33137	K04077	groEL, HSPD1	chaperonin GroEL	--	4	ko03018 RNA degradation;ko04940 Type I diabetes mellitus;ko05134 Legionellosis;ko05152 Tuberculosis;
gene33147	K15015	SLC32A, VGAT	solute carrier family 32 (vesicular inhibitory amino acid transporter)	--	5	ko04721 Synaptic vesicle cycle;ko04723 Retrograde endocannabinoid signaling;ko04727 GABAergic synapse;ko05032 Morphine addiction;ko05033 Nicotine addiction;
gene33149	K09753	CCR	cinnamoyl-CoA reductase	EC:1.2.1.44	1	ko00940 Phenylpropanoid biosynthesis;
gene33151	K15192	BTAF1, MOT1	TATA-binding protein-associated factor	EC:3.6.4.-		
gene33155	K08901	psbQ	photosystem II oxygen-evolving enhancer protein 3	--	1	ko00195 Photosynthesis;
gene33156	K06630	YWHAE	14-3-3 protein epsilon	--	8	ko04110 Cell cycle;ko04114 Oocyte meiosis;ko04151 PI3K-Akt signaling pathway;ko04390 Hippo signaling pathway;ko04391 Hippo signaling pathway - fly;ko04722 Neurotrophin signaling pathway;ko05169 Epstein-Barr virus infection;ko05203 Viral carcinogenesis;
gene33157	K10575	UBE2G1, UBC7	ubiquitin-conjugating enzyme E2 G1	EC:6.3.2.19	3	ko04120 Ubiquitin mediated proteolysis;ko04141 Protein processing in endoplasmic reticulum;ko05012 Parkinson's disease;
gene33162	K09286	EREBP	EREBP-like factor	--		
gene33163	K09286	EREBP	EREBP-like factor	--		
gene33165	K14684	SLC25A23S	solute carrier family 25 (mitochondrial phosphate transporter), member 23/24/25/41	--		

gene33166	K00264	GLT1	glutamate synthase (NADPH/NADH)	EC:1.4.1.13 1.4.1.14	3	ko00250 Alanine, aspartate and glutamate metabolism;ko00910 Nitrogen metabolism;ko01230 Biosynthesis of amino acids;
gene33169	K04733	IRAK4	interleukin-1 receptor-associated kinase 4	EC:2.7.11.1	11	ko04064 NF-kappa B signaling pathway;ko04210 Apoptosis;ko04620 Toll-like receptor signaling pathway;ko04722 Neurotrophin signaling pathway;ko05133 Pertussis;ko05140 Leishmaniasis;ko05142 Chagas disease (American trypanosomiasis);ko05145 Toxoplasmosis;ko05152 Tuberculosis;ko05162 Measles;ko05164 Influenza A;
gene33175	K03695	clpB	ATP-dependent Clp protease ATP-binding subunit ClpB	--		
gene33177	K10398	KIF11, EG5	kinesin family member 11	--		
gene33182	K14220	tRNA-Asn	tRNA Asn	--	1	ko00970 Aminoacyl-tRNA biosynthesis;
gene33183	K14219	tRNA-Arg	tRNA Arg	--	1	ko00970 Aminoacyl-tRNA biosynthesis;
gene33186	K01583	E4.1.1.19	arginine decarboxylase	EC:4.1.1.19	1	ko00330 Arginine and proline metabolism;
gene33187	K00011	E1.1.1.21, AKR1	aldehyde reductase	EC:1.1.1.21	5	ko00040 Pentose and glucuronate interconversions;ko00051 Fructose and mannose metabolism;ko00052 Galactose metabolism;ko00561 Glycerolipid metabolism;ko00620 Pyruvate metabolism;
gene33188	K12345	SRD5A3	3-oxo-5-alpha-steroid 4-dehydrogenase 3	EC:1.3.1.22 1.3.1.94	1	ko00140 Steroid hormone biosynthesis;
gene33191	K03327	TC.MATE, SLC47A, norM, mdtK, dinF	multidrug resistance protein, MATE family	--		
gene33192	K12869	CRN, CRNKL1, CLF1, SYF3	crooked neck	--	1	ko03040 Spliceosome;
gene33194	K14684	SLC25A23S	solute carrier family 25 (mitochondrial phosphate transporter), member 23/24/25/41	--		
gene33195	K00383	E1.8.1.7, GSR, gor	glutathione reductase (NADPH)	EC:1.8.1.7	2	ko00480 Glutathione metabolism;ko04918 Thyroid hormone synthesis;
gene33196	K07442	TRM61, GCD14	tRNA (adenine57-N1/adenine58-N1)-methyltransferase	EC:2.1.1.219 2.1.1.220		
gene33198	K12819	SLU7	pre-mRNA-processing factor SLU7	--	1	ko03040 Spliceosome;
gene33204	K00276	E1.4.3.21, AOC2, AOC3, tynA	primary-amine oxidase	EC:1.4.3.21	6	ko00260 Glycine, serine and threonine metabolism;ko00350 Tyrosine metabolism;ko00360 Phenylalanine metabolism;ko00410 beta-Alanine metabolism;ko00950 Isoquinoline alkaloid biosynthesis;ko00960 Tropane, piperidine and pyridine alkaloid biosynthesis;
gene33209	K11650	SMARCD	SWI/SNF-related matrix-associated actin-dependent regulator of chromatin subfamily D	--		
gene33210	K02880	RP-L17e, RPL17	large subunit ribosomal protein L17e	--	1	ko03010 Ribosome;
gene33212	K01749	hemC, HMBS	hydroxymethylbilane synthase	EC:2.5.1.61	1	ko00860 Porphyrin and chlorophyll metabolism;
gene33216	K06928	K06928	nucleoside-triphosphatase THEP1	EC:3.6.1.15	2	ko00230 Purine metabolism;ko00730 Thiamine metabolism;
gene3321	K09422	MYBP	myb proto-oncogene protein, plant	--		
gene33221	K02136	ATPeF1G, ATP5C1	F-type H+-transporting ATPase subunit gamma	EC:3.6.3.14	4	ko00190 Oxidative phosphorylation;ko05010 Alzheimer's disease;ko05012 Parkinson's disease;ko05016 Huntington's disease;
gene33229	K08819	CDK12_13	cyclin-dependent kinase 12/13	EC:2.7.11.22 2.7.11.23		
gene3322	K01533	E3.6.3.4, ATP7, copA	Cu2+-exporting ATPase	EC:3.6.3.4		
gene33230	K10143	RFWD2, COP1	E3 ubiquitin-protein ligase RFWD2	EC:6.3.2.19	3	ko04115 p53 signaling pathway;ko04120 Ubiquitin mediated proteolysis;ko04712 Circadian rhythm - plant;
gene33234	K15032	MTERFD	mTERF domain-containing protein, mitochondrial	--		
gene33236	K02975	RP-S25e, RPS25	small subunit ribosomal protein S25e	--	1	ko03010 Ribosome;
gene3323	K11339	MORF4L1, MRG15, EAF3	mortality factor 4-like protein 1	--		
gene33240	K02350	POLZ1, rev3	DNA polymerase zeta	EC:2.7.7.7	1	ko03460 Fanconi anemia pathway;
gene33241	K00276	E1.4.3.21, AOC2, AOC3, tynA	primary-amine oxidase	EC:1.4.3.21	6	ko00260 Glycine, serine and threonine metabolism;ko00350 Tyrosine metabolism;ko00360 Phenylalanine metabolism;ko00410 beta-Alanine metabolism;ko00950 Isoquinoline alkaloid biosynthesis;ko00960 Tropane, piperidine and pyridine alkaloid biosynthesis;
gene33244	K06875	PDCD5, TFAR19	programmed cell death protein 5	--		
gene33246	K00679	E2.3.1.158	phospholipid:diacylglycerol acyltransferase	EC:2.3.1.158	1	ko00561 Glycerolipid metabolism;
gene33251	K00940	E2.7.4.6, ndk	nucleoside-diphosphate kinase	EC:2.7.4.6	2	ko00230 Purine metabolism;ko00240 Pyrimidine metabolism;
gene33256	K11364	SGF29	SAGA-associated factor 29	--		

gene33261	K14640	SLC20A, PIT	solute carrier family 20 (sodium-dependent phosphate transporter)	--		
gene33264	K10532	HGSNAT	heparan-alpha-glucosaminide N-acetyltransferase	EC:2.3.1.78	2	ko00531 Glycosaminoglycan degradation;ko04142 Lysosome;
gene33265	K06689	UBE2D_E, UBC4, UBC5	ubiquitin-conjugating enzyme E2 D/E	EC:6.3.2.19	2	ko04120 Ubiquitin mediated proteolysis;ko04141 Protein processing in endoplasmic reticulum;
gene33269	K16616	PARP8	actin-related protein 8, plant	--		
gene33278	K09422	MYBP	myb proto-oncogene protein, plant	--		
gene33279	K09422	MYBP	myb proto-oncogene protein, plant	--		
gene33281	K01528	DNM	dynamin GTPase	EC:3.6.5.5	4	ko04144 Endocytosis;ko04721 Synaptic vesicle cycle;ko04961 Endocrine and other factor-regulated calcium reabsorption;ko05100 Bacterial invasion of epithelial cells;
gene33287	K16223	FT	protein FLOWERING LOCUS T	--	1	ko04712 Circadian rhythm - plant;
gene33288	K10750	CHAF1A	chromatin assembly factor 1 subunit A	--		
gene33301	K14611	SLC23A1_2, SVCT1_2	solute carrier family 23 (nucleobase transporter), member 1/2	--		
gene33303	K13420	FLS2	LRR receptor-like serine/threonine-protein kinase FLS2	EC:2.7.11.1	1	ko04626 Plant-pathogen interaction;
gene33304	K04730	IRAK1	interleukin-1 receptor-associated kinase 1	EC:2.7.11.1	11	ko04064 NF-kappa B signaling pathway;ko04210 Apoptosis;ko04620 Toll-like receptor signaling pathway;ko04722 Neurotrophin signaling pathway;ko05133 Pertussis;ko05140 Leishmaniasis;ko05142 Chagas disease (American trypanosomiasis);ko05145 Toxoplasmosis;ko05152 Tuberculosis;ko05162 Measles;ko05169 Epstein-Barr virus infection;
gene33306	K04730	IRAK1	interleukin-1 receptor-associated kinase 1	EC:2.7.11.1	11	ko04064 NF-kappa B signaling pathway;ko04210 Apoptosis;ko04620 Toll-like receptor signaling pathway;ko04722 Neurotrophin signaling pathway;ko05133 Pertussis;ko05140 Leishmaniasis;ko05142 Chagas disease (American trypanosomiasis);ko05145 Toxoplasmosis;ko05152 Tuberculosis;ko05162 Measles;ko05169 Epstein-Barr virus infection;
gene33307	K04730	IRAK1	interleukin-1 receptor-associated kinase 1	EC:2.7.11.1	11	ko04064 NF-kappa B signaling pathway;ko04210 Apoptosis;ko04620 Toll-like receptor signaling pathway;ko04722 Neurotrophin signaling pathway;ko05133 Pertussis;ko05140 Leishmaniasis;ko05142 Chagas disease (American trypanosomiasis);ko05145 Toxoplasmosis;ko05152 Tuberculosis;ko05162 Measles;ko05169 Epstein-Barr virus infection;
gene33308	K13430	PBS1	serine/threonine-protein kinase PBS1	EC:2.7.11.1	1	ko04626 Plant-pathogen interaction;
gene33309	K11654	SMARCA5, SNF2H, ISWI	SWI/SNF-related matrix-associated actin-dependent regulator of chromatin subfamily A member 5	EC:3.6.4.-		
gene33316	K01102	PDP	pyruvate dehydrogenase phosphatase	EC:3.1.3.43		
gene33323	K09338	HD-ZIP	homeobox-leucine zipper protein	--		
gene33326	K08145	SLC2A8, GLUT8	MFS transporter, SP family, solute carrier family 2 (facilitated glucose transporter), member 8	--		
gene33327	K13436	PTI1	pto-interacting protein 1	EC:2.7.11.1	1	ko04626 Plant-pathogen interaction;
gene33329	K12393	AP1M	AP-1 complex subunit mu	--	1	ko04142 Lysosome;
gene33335	K01057	PGLS, pgl, devB	6-phosphogluconolactonase	EC:3.1.1.31	2	ko00030 Pentose phosphate pathway;ko01200 Carbon metabolism;
gene33336	K01057	PGLS, pgl, devB	6-phosphogluconolactonase	EC:3.1.1.31	2	ko00030 Pentose phosphate pathway;ko01200 Carbon metabolism;
gene33337	K02927	RP-L40e, RPL40	large subunit ribosomal protein L40e	--	1	ko03010 Ribosome;
gene33338	K05275	E1.1.1.65	pyridoxine 4-dehydrogenase	EC:1.1.1.65	1	ko00750 Vitamin B6 metabolism;
gene33339	K05275	E1.1.1.65	pyridoxine 4-dehydrogenase	EC:1.1.1.65	1	ko00750 Vitamin B6 metabolism;
gene33346	K14235	tRNA-Trp	tRNA Trp	--	1	ko00970 Aminoacyl-tRNA biosynthesis;
gene33355	K04730	IRAK1	interleukin-1 receptor-associated kinase 1	EC:2.7.11.1	11	ko04064 NF-kappa B signaling pathway;ko04210 Apoptosis;ko04620 Toll-like receptor signaling pathway;ko04722 Neurotrophin signaling pathway;ko05133 Pertussis;ko05140 Leishmaniasis;ko05142 Chagas disease (American trypanosomiasis);ko05145 Toxoplasmosis;ko05152 Tuberculosis;ko05162 Measles;ko05169 Epstein-Barr virus infection;

gene33356	K14454	GOT1	aspartate aminotransferase, cytoplasmic	EC:2.6.1.1	11	ko00250 Alanine, aspartate and glutamate metabolism;ko00270 Cysteine and methionine metabolism;ko00330 Arginine and proline metabolism;ko00350 Tyrosine metabolism;ko00360 Phenylalanine metabolism;ko00400 Phenylalanine, tyrosine and tryptophan biosynthesis;ko00710 Carbon fixation in photosynthetic organisms;ko00950 Isoquinoline alkaloid biosynthesis;ko00960 Tropane, piperidine and pyridine alkaloid biosynthesis;ko01210 2-Oxocarboxylic acid metabolism;ko01230 Biosynthesis of amino acids;
gene33373	K11087	SNRPD1, SMD1	small nuclear ribonucleoprotein D1	--	2	ko03040 Spliceosome;ko05322 Systemic lupus erythematosus;
gene33374	K09285	OVM, ANT	AP2-like factor, ANT lineage	--		
gene33377	K14611	SLC23A1_2, SVCT1_2	solute carrier family 23 (nucleobase transporter), member 1/2	--		
gene33378	K14843	PES1, NOP7	pescadillo	--		
gene33382	K14515	EBF1_2	EIN3-binding F-box protein	--	1	ko04075 Plant hormone signal transduction;
gene33389	K09285	OVM, ANT	AP2-like factor, ANT lineage	--		
gene33390	K02640	petG	cytochrome b6-f complex subunit 5	--	1	ko00195 Photosynthesis;
gene33392	K04733	IRAK4	interleukin-1 receptor-associated kinase 4	EC:2.7.11.1	11	ko04064 NF-kappa B signaling pathway;ko04210 Apoptosis;ko04620 Toll-like receptor signaling pathway;ko04722 Neurotrophin signaling pathway;ko05133 Pertussis;ko05140 Leishmaniasis;ko05142 Chagas disease (American trypanosomiasis);ko05145 Toxoplasmosis;ko05152 Tuberculosis;ko05162 Measles;ko05164 Influenza A;
gene33395	K00430	E1.11.1.7	peroxidase	EC:1.11.1.7	2	ko00360 Phenylalanine metabolism;ko00940 Phenylpropanoid biosynthesis;
gene33396	K11816	YUCCA	indole-3-pyruvate monooxygenase	EC:1.14.13.168	1	ko00380 Tryptophan metabolism;
gene33397	K10581	UBE2O	ubiquitin-conjugating enzyme E2 O	EC:6.3.2.19	1	ko04120 Ubiquitin mediated proteolysis;
gene33414	K13447	RBOH	respiratory burst oxidase	EC:1.6.3.- 1.11.1.-	1	ko04626 Plant-pathogen interaction;
gene33415	K08235	E2.4.1.207	xyloglucan:xyloglucosyl transferase	EC:2.4.1.207		
gene33419	K05765	CFL	cofilin	--	4	ko04360 Axon guidance;ko04666 Fc gamma R-mediated phagocytosis;ko04810 Regulation of actin cytoskeleton;ko05133 Pertussis;
gene33420	K17479	GRXCR1	glutaredoxin domain-containing cysteine-rich protein 1	--		
gene33424	K06316	RFT1	oligosaccharidyl-lipid flippase family	--	1	ko00510 N-Glycan biosynthesis;
gene33429	K04382	PPP2C	serine/threonine-protein phosphatase 2A catalytic subunit	EC:3.1.3.16	13	ko03015 mRNA surveillance pathway;ko04111 Cell cycle - yeast;ko04113 Meiosis - yeast;ko04114 Oocyte meiosis;ko04151 PI3K-Akt signaling pathway;ko04350 TGF-beta signaling pathway;ko04390 Hippo signaling pathway;ko04391 Hippo signaling pathway - fly;ko04530 Tight junction;ko04728 Dopaminergic synapse;ko04730 Long-term depression;ko05142 Chagas disease (American trypanosomiasis);ko05160 Hepatitis C;
gene3342	K08150	SLC2A13, ITR	MFS transporter, SP family, solute carrier family 2 (myo-inositol transporter), member 13	--		
gene33431	K14724	SLC9A8, NHE8	solute carrier family 9 (sodium/hydrogen exchanger), member 8	--		
gene33433	K02896	RP-L24e, RPL24	large subunit ribosomal protein L24e	--	1	ko03010 Ribosome;
gene33434	K14792	RRP5, PDCD11	rRNA biogenesis protein RRP5	--		
gene33438	K12881	THOC4, ALY	THO complex subunit 4	--	4	ko03013 RNA transport;ko03015 mRNA surveillance pathway;ko03040 Spliceosome;ko05168 Herpes simplex infection;
gene33441	K13513	LCLAT1, AGPAT8	lysocardiolipin and lysophospholipid acyltransferase	EC:2.3.1.- 2.3.1.51	2	ko00561 Glycerolipid metabolism;ko00564 Glycerophospholipid metabolism;
gene33445	K14484	IAA	auxin-responsive protein IAA	--	1	ko04075 Plant hormone signal transduction;
gene33446	K14233	tRNA-Ser	tRNA Ser	--	1	ko00970 Aminoacyl-tRNA biosynthesis;
gene33447	K00549	metE	5-methyltetrahydropteroyltriglutamate--homocysteine methyltransferase	EC:2.1.1.14	3	ko00270 Cysteine and methionine metabolism;ko00450 Selenocompound metabolism;ko01230 Biosynthesis of amino acids;
gene33448	K00549	metE	5-methyltetrahydropteroyltriglutamate--homocysteine methyltransferase	EC:2.1.1.14	3	ko00270 Cysteine and methionine metabolism;ko00450 Selenocompound metabolism;ko01230 Biosynthesis of amino acids;
gene33451	K14852	RRS1	regulator of ribosome biosynthesis	--		
gene33454	K13280	SEC11, sipW	signal peptidase, endoplasmic reticulum-type	EC:3.4.-.-	1	ko03060 Protein export;

gene3345	K08903	psb28	photosystem II 13kDa protein	--	1	ko00195 Photosynthesis;
gene33465	K11979	UBR7	E3 ubiquitin-protein ligase UBR7	EC:6.3.2.19		
gene33472	K14556	DIP2, UTP12, WDR3	U3 small nucleolar RNA-associated protein 12	--	1	ko03008 Ribosome biogenesis in eukaryotes;
gene33476	K14232	tRNA-Pro	tRNA Pro	--	1	ko00970 Aminoacyl-tRNA biosynthesis;
gene3347	K11824	AP2A	AP-2 complex subunit alpha	--	4	ko04144 Endocytosis;ko04721 Synaptic vesicle cycle;ko04961 Endocrine and other factor-regulated calcium reabsorption;ko05016 Huntington's disease;
gene33480	K12271	SRP43, CAO	signal recognition particle 43 kDa protein	--		
gene33481	K05658	ABCB1	ATP-binding cassette, subfamily B (MDR/TAP), member 1	--	3	ko02010 ABC transporters;ko04976 Bile secretion;ko05206 MicroRNAs in cancer;
gene33486	K08913	LHCB2	light-harvesting complex II chlorophyll a/b binding protein 2	--	1	ko00196 Photosynthesis - antenna proteins;
gene33488	K02894	RP-L23e, RPL23	large subunit ribosomal protein L23e	--	1	ko03010 Ribosome;
gene33490	K01900	LSC2	succinyl-CoA synthetase beta subunit	EC:6.2.1.4 6.2.1.5	3	ko00020 Citrate cycle (TCA cycle);ko00640 Propanoate metabolism;ko01200 Carbon metabolism;
gene33495	K07748	E1.1.1.170, NSDHL, ERG26	sterol-4alpha-carboxylate 3-dehydrogenase (decarboxylating)	EC:1.1.1.170	1	ko00100 Steroid biosynthesis;
gene33496	K02728	PSMA4	20S proteasome subunit alpha 3	EC:3.4.25.1	1	ko03050 Proteasome;
gene33498	K09480	E2.4.1.241	digalactosyldiacylglycerol synthase	EC:2.4.1.241	1	ko00561 Glycerolipid metabolism;
gene33506	K08998	K08998	hypothetical protein	--		
gene3350	K03358	APC11	anaphase-promoting complex subunit 11	--	7	ko04110 Cell cycle;ko04111 Cell cycle - yeast;ko04113 Meiosis - yeast;ko04114 Oocyte meiosis;ko04120 Ubiquitin mediated proteolysis;ko04914 Progesterone-mediated oocyte maturation;ko05166 HTLV-I infection;
gene33513	K12462	ARHGDI, RHOGDI	Rho GDP-dissociation inhibitor	--	2	ko04722 Neurotrophin signaling pathway;ko04962 Vasopressin-regulated water reabsorption;
gene33514	K03549	kup	KUP system potassium uptake protein	--		
gene33525	K04733	IRAK4	interleukin-1 receptor-associated kinase 4	EC:2.7.11.1	11	ko04064 NF-kappa B signaling pathway;ko04210 Apoptosis;ko04620 Toll-like receptor signaling pathway;ko04722 Neurotrophin signaling pathway;ko05133 Pertussis;ko05140 Leishmaniasis;ko05142 Chagas disease (American trypanosomiasis);ko05145 Toxoplasmosis;ko05152 Tuberculosis;ko05162 Measles;ko05164 Influenza A;
gene33533	K01213	E3.2.1.67	galacturan 1,4-alpha-galacturonidase	EC:3.2.1.67	2	ko00040 Pentose and glucuronate interconversions;ko00500 Starch and sucrose metabolism;
gene33539	K00942	E2.7.4.8, gmk	guanylate kinase	EC:2.7.4.8	1	ko00230 Purine metabolism;
gene33543	K00549	metE	5-methyltetrahydropteroyltriglutamate--homocysteine methyltransferase	EC:2.1.1.14	3	ko00270 Cysteine and methionine metabolism;ko00450 Selenocompound metabolism;ko01230 Biosynthesis of amino acids;
gene33544	K13065	E2.3.1.133, HCT	shikimate O-hydroxycinnamoyltransferase	EC:2.3.1.133	3	ko00940 Phenylpropanoid biosynthesis;ko00941 Flavonoid biosynthesis;ko00945 Stilbenoid, diarylheptanoid and gingerol biosynthesis;
gene33545	K10268	FBXL2_20	F-box and leucine-rich repeat protein 2/20	--		
gene33546	K10268	FBXL2_20	F-box and leucine-rich repeat protein 2/20	--		
gene33551	K02876	RP-L15, MRPL15, rplO	large subunit ribosomal protein L15	--	1	ko03010 Ribosome;
gene33556	K01051	E3.1.1.11	pectinesterase	EC:3.1.1.11	2	ko00040 Pentose and glucuronate interconversions;ko00500 Starch and sucrose metabolism;
gene33558	K08235	E2.4.1.207	xyloglucan:xyloglucosyl transferase	EC:2.4.1.207		
gene33559	K13449	PR1	pathogenesis-related protein 1	--	2	ko04075 Plant hormone signal transduction;ko04626 Plant-pathogen interaction;
gene33560	K13449	PR1	pathogenesis-related protein 1	--	2	ko04075 Plant hormone signal transduction;ko04626 Plant-pathogen interaction;
gene33561	K13449	PR1	pathogenesis-related protein 1	--	2	ko04075 Plant hormone signal transduction;ko04626 Plant-pathogen interaction;
gene33565	K01979	SSUrRNA	small subunit ribosomal RNA	--	2	ko03008 Ribosome biogenesis in eukaryotes;ko03010 Ribosome;
gene33566	K01979	SSUrRNA	small subunit ribosomal RNA	--	2	ko03008 Ribosome biogenesis in eukaryotes;ko03010 Ribosome;
gene33573	K10525	E5.3.99.6, aoc	allene oxide cyclase	EC:5.3.99.6	1	ko00592 alpha-Linolenic acid metabolism;
gene33574	K09422	MYBP	myb proto-oncogene protein, plant	--		
gene33575	K05543	DUS2	tRNA-dihydrouridine synthase 2	EC:1.3.1.91		
gene33578	K09422	MYBP	myb proto-oncogene protein, plant	--		

gene33588	K07904	RAB11A	Ras-related protein Rab-11A	--	4	ko04144 Endocytosis;ko04961 Endocrine and other factor-regulated calcium reabsorption;ko04962 Vasopressin-regulated water reabsorption;ko04972 Pancreatic secretion;
gene33592	K02256	COX1	cytochrome c oxidase subunit 1	EC:1.9.3.1	6	ko00190 Oxidative phosphorylation;ko04260 Cardiac muscle contraction;ko04932 Non-alcoholic fatty liver disease (NAFLD);ko05010 Alzheimer's disease;ko05012 Parkinson's disease;ko05016 Huntington's disease;
gene33594	K13076	SLD	delta8-fatty-acid desaturase	EC:1.14.19.4		
gene33595	K06118	E3.13.1.1, sqd1, sqdb	UDP-sulfoquinovose synthase	EC:3.13.1.1	2	ko00520 Amino sugar and nucleotide sugar metabolism;ko00561 Glycerolipid metabolism;
gene33596	K06889	K06889				
gene33597	K08955	YME1	ATP-dependent metalloprotease	EC:3.4.24.-		
gene3359	K02890	RP-L22, MRPL22, rplV	large subunit ribosomal protein L22	--	1	ko03010 Ribosome;
gene335	K04733	IRAK4	interleukin-1 receptor-associated kinase 4	EC:2.7.11.1	11	ko04064 NF-kappa B signaling pathway;ko04210 Apoptosis;ko04620 Toll-like receptor signaling pathway;ko04722 Neurotrophin signaling pathway;ko05133 Pertussis;ko05140 Leishmaniasis;ko05142 Chagas disease (American trypanosomiasis);ko05145 Toxoplasmosis;ko05152 Tuberculosis;ko05162 Measles;ko05164 Influenza A;
gene33600	K02836	prfB	peptide chain release factor 2	--		
gene33601	K14191	DIM1	18S rRNA (adenine1779-N6/adenine1780-N6)-dimethyltransferase	EC:2.1.1.183		
gene33605	K00434	E1.11.1.11	L-ascorbate peroxidase	EC:1.11.1.11	2	ko00053 Ascorbate and aldarate metabolism;ko00480 Glutathione metabolism;
gene33609	K14850	RRP8	ribosomal RNA-processing protein 8	EC:2.1.1.-		
gene33612	K00600	glyA, SHMT	glycine hydroxymethyltransferase	EC:2.1.2.1	7	ko00260 Glycine, serine and threonine metabolism;ko00460 Cyanoamino acid metabolism;ko00630 Glyoxylate and dicarboxylate metabolism;ko00670 One carbon pool by folate;ko00680 Methane metabolism;ko01200 Carbon metabolism;ko01230 Biosynthesis of amino acids;
gene33613	K08511	ATVAMP72	vesicle-associated membrane protein 72	--		
gene33624	K05275	E1.1.1.65	pyridoxine 4-dehydrogenase	EC:1.1.1.65	1	ko00750 Vitamin B6 metabolism;
gene3362	K00627	DLAT, aceF, pdhC	pyruvate dehydrogenase E2 component (dihydrolipoamide acetyltransferase)	EC:2.3.1.12	4	ko00010 Glycolysis / Gluconeogenesis;ko00020 Citrate cycle (TCA cycle);ko00620 Pyruvate metabolism;ko01200 Carbon metabolism;
gene33631	K11827	AP2S1	AP-2 complex subunit sigma-1	--	4	ko04144 Endocytosis;ko04721 Synaptic vesicle cycle;ko04961 Endocrine and other factor-regulated calcium reabsorption;ko05016 Huntington's disease;
gene33634	K14638	SLC15A3_4, PHT	solute carrier family 15 (peptide/histidine transporter), member 3/4	--		
gene33635	K14638	SLC15A3_4, PHT	solute carrier family 15 (peptide/histidine transporter), member 3/4	--		
gene33637	K15075	MET18, MMS19	DNA repair/transcription protein MET18/MMS19	--		
gene33638	K15174	PAF1	RNA polymerase II-associated factor 1	--		
gene33639	K08867	WNK, PRKWNK	WNK lysine deficient protein kinase	EC:2.7.11.1		
gene3363	K13495	CISZOG	cis-zeatin O-glucosyltransferase	EC:2.4.1.215	1	ko00908 Zeatin biosynthesis;
gene33644	K00943	E2.7.4.9, tmk	dTMP kinase	EC:2.7.4.9	1	ko00240 Pyrimidine metabolism;
gene33645	K08150	SLC2A13, ITR	MFS transporter, SP family, solute carrier family 2 (myo-inositol transporter), member 13	--		
gene33647	K11498	CENPE	centromeric protein E	--		
gene33649	K01979	SSUrRNA	small subunit ribosomal RNA	--	2	ko03008 Ribosome biogenesis in eukaryotes;ko03010 Ribosome;
gene33650	K08867	WNK, PRKWNK	WNK lysine deficient protein kinase	EC:2.7.11.1		
gene33652	K02974	RP-S24e, RPS24	small subunit ribosomal protein S24e	--	1	ko03010 Ribosome;
gene33658	K09842	AAO3	abscisic-aldehyde oxidase	EC:1.2.3.14	1	ko00906 Carotenoid biosynthesis;
gene33663	K03231	EEF1A	elongation factor 1-alpha	--	2	ko03013 RNA transport;ko05134 Legionellosis;
gene33667	K17422	MRPL41	large subunit ribosomal protein L41, mitochondrial	--		
gene33672	K03283	HSPA1_8	heat shock 70kDa protein 1/8	--	11	ko03040 Spliceosome;ko04010 MAPK signaling pathway;ko04141 Protein processing in endoplasmic reticulum;ko04144 Endocytosis;ko04612 Antigen processing and presentation;ko04915 Estrogen signaling pathway;ko05134 Legionellosis;ko05145 Toxoplasmosis;ko05162 Measles;ko05164 Influenza A;ko05169 Epstein-Barr virus infection;

gene33675	K16297	SCPL-II	serine carboxypeptidase-like clade II	EC:3.4.16.-		
gene33676	K15402	CYP86B1	cytochrome P450, family 86, subfamily B, polypeptide 1 (fatty acid omega-hydroxylase)	EC:1.14.-.-	1	ko00073 Cutin, suberine and wax biosynthesis;
gene33679	K11407	HDAC6_10	histone deacetylase 6/10	EC:3.5.1.98	2	ko05034 Alcoholism;ko05203 Viral carcinogenesis;
gene3367	K02890	RP-L22, MRPL22, rplV	large subunit ribosomal protein L22	--	1	ko03010 Ribosome;
gene33680	K11407	HDAC6_10	histone deacetylase 6/10	EC:3.5.1.98	2	ko05034 Alcoholism;ko05203 Viral carcinogenesis;
gene3369	K08232	E1.6.5.4	monodehydroascorbate reductase (NADH)	EC:1.6.5.4	1	ko00053 Ascorbate and aldarate metabolism;
gene33708	K14015	NPLOC4, NPL4	nuclear protein localization protein 4 homolog	--	1	ko04141 Protein processing in endoplasmic reticulum;
gene33709	K11251	H2A	histone H2A	--	2	ko05034 Alcoholism;ko05322 Systemic lupus erythematosus;
gene3370	K14232	tRNA-Pro	tRNA Pro	--	1	ko00970 Aminoacyl-tRNA biosynthesis;
gene33714	K02993	RP-S7e, RPS7	small subunit ribosomal protein S7e	--	1	ko03010 Ribosome;
gene33715	K01188	E3.2.1.21	beta-glucosidase	EC:3.2.1.21	3	ko00460 Cyanoamino acid metabolism;ko00500 Starch and sucrose metabolism;ko00940 Phenylpropanoid biosynthesis;
gene33717	K11593	ELF2C	eukaryotic translation initiation factor 2C	--		
gene3371	K02935	RP-L7, MRPL12, rplL	large subunit ribosomal protein L7/L12	--	1	ko03010 Ribosome;
gene33720	K02995	RP-S8e, RPS8	small subunit ribosomal protein S8e	--	1	ko03010 Ribosome;
gene33723	K15014	SLC29A1_2_3, ENT1_2_3	solute carrier family 29 (equilibrative nucleoside transporter), member 1/2/3	--		
gene33725	K16055	TPS	trehalose 6-phosphate synthase/phosphatase	EC:2.4.1.15 3.1.3.12	1	ko00500 Starch and sucrose metabolism;
gene33730	K01255	CARP, pepA	leucyl aminopeptidase	EC:3.4.11.1	1	ko00480 Glutathione metabolism;
gene33731	K05391	CNGF	cyclic nucleotide gated channel, other eukaryote	--	1	ko04626 Plant-pathogen interaction;
gene33734	K13431	SRPR	signal recognition particle receptor subunit alpha	--	1	ko03060 Protein export;
gene33737	K01057	PGLS, pgl, devB	6-phosphogluconolactonase	EC:3.1.1.31	2	ko00030 Pentose phosphate pathway;ko01200 Carbon metabolism;
gene33738	K01057	PGLS, pgl, devB	6-phosphogluconolactonase	EC:3.1.1.31	2	ko00030 Pentose phosphate pathway;ko01200 Carbon metabolism;
gene3373	K06662	HRAD17, RAD24	cell cycle checkpoint protein	--	2	ko04111 Cell cycle - yeast;ko04113 Meiosis - yeast;
gene33749	K17099	ANXA13	annexin A13	--		
gene3374	K01735	aroB	3-dehydroquinate synthase	EC:4.2.3.4	2	ko00400 Phenylalanine, tyrosine and tryptophan biosynthesis;ko01230 Biosynthesis of amino acids;
gene3375	K04392	RAC1	Ras-related C3 botulinum toxin substrate 1	--	33	ko04010 MAPK signaling pathway;ko04014 Ras signaling pathway;ko04062 Chemokine signaling pathway;ko04145 Phagosome;ko04151 PI3K-Akt signaling pathway;ko04310 Wnt signaling pathway;ko04360 Axon guidance;ko04370 VEGF signaling pathway;ko04380 Osteoclast differentiation;ko04510 Focal adhesion;ko04520 Adherens junction;ko04620 Toll-like receptor signaling pathway;ko04650 Natural killer cell mediated cytotoxicity;ko04662 B cell receptor signaling pathway;ko04664 Fc epsilon RI signaling pathway;ko04666 Fc gamma R-mediated phagocytosis;ko04670 Leukocyte transendothelial migration;ko04722 Neurotrophin signaling pathway;ko04810 Regulation of actin cytoskeleton;ko04932 Non-alcoholic fatty liver disease (NAFLD);ko04972 Pancreatic secretion;ko05014 Amyotrophic lateral sclerosis (ALS);ko05100 Bacterial invasion of epithelial cells;ko05120 Epithelial cell signaling in Helicobacter pylori infection;ko05131 Shigellosis;ko05132 Salmonella infection;ko05200 Pathways in cancer;ko05203 Viral carcinogenesis;ko05205 Proteoglycans in cancer;ko05210 Colorectal cancer;ko05211 Renal cell carcinoma;ko05212 Pancreatic
gene33762	K08342	ATG4	autophagy-related protein 4	EC:3.4.22.-	1	ko04140 Regulation of autophagy;
gene33764	K11423	SETD2, SET2	histone-lysine N-methyltransferase SETD2	EC:2.1.1.43	1	ko00310 Lysine degradation;
gene3376	K06689	UBE2D_E, UBC4, UBC5	ubiquitin-conjugating enzyme E2 D/E	EC:6.3.2.19	2	ko04120 Ubiquitin mediated proteolysis;ko04141 Protein processing in endoplasmic reticulum;
gene33772	K00991	ispD	2-C-methyl-D-erythritol 4-phosphate cytidylyltransferase	EC:2.7.7.60	1	ko00900 Terpenoid backbone biosynthesis;
gene33778	K15084	SLC25A16, GDA, LEU5	solute carrier family 25 (mitochondrial carrier protein), member 16	--		
gene33780	K14497	PP2C	protein phosphatase 2C	EC:3.1.3.16	1	ko04075 Plant hormone signal transduction;
gene33781	K14497	PP2C	protein phosphatase 2C	EC:3.1.3.16	1	ko04075 Plant hormone signal transduction;

gene33786	K00799	GST, gst	glutathione S-transferase	EC:2.5.1.18	4	ko00480 Glutathione metabolism;ko00980 Metabolism of xenobiotics by cytochrome P450;ko00982 Drug metabolism - cytochrome P450;ko05204 Chemical carcinogenesis;
gene33795	K02917	RP-L35Ae, RPL35A	large subunit ribosomal protein L35Ae	--	1	ko03010 Ribosome;
gene3379	K10781	FATB	fatty acyl-ACP thioesterase B	EC:3.1.2.14	1	ko00061 Fatty acid biosynthesis;
gene33802	K14488	SAUR	SAUR family protein	--	1	ko04075 Plant hormone signal transduction;
gene33803	K10706	SETX, ALS4	senataxin	EC:3.6.4.-		
gene33805	K14172	LHCB7	light-harvesting complex II chlorophyll a/b binding protein 7	--	1	ko00196 Photosynthesis - antenna proteins;
gene3380	K08730	PTDSS2	phosphatidylserine synthase 2	EC:2.7.8.29	1	ko00564 Glycerophospholipid metabolism;
gene33817	K03549	kup	KUP system potassium uptake protein	--		
gene3381	K01623	ALDO	fructose-bisphosphate aldolase, class I	EC:4.1.2.13	7	ko00010 Glycolysis / Gluconeogenesis;ko00030 Pentose phosphate pathway;ko00051 Fructose and mannose metabolism;ko00680 Methane metabolism;ko00710 Carbon fixation in photosynthetic organisms;ko01200 Carbon metabolism;ko01230 Biosynthesis of amino acids;
gene33823	K00948	PRPS, prsA	ribose-phosphate pyrophosphokinase	EC:2.7.6.1	4	ko00030 Pentose phosphate pathway;ko00230 Purine metabolism;ko01200 Carbon metabolism;ko01230 Biosynthesis of amino acids;
gene33826	K07213	ATOX1, ATX1, copZ	copper chaperone	--	1	ko04978 Mineral absorption;
gene33827	K00430	E1.11.1.7	peroxidase	EC:1.11.1.7	2	ko00360 Phenylalanine metabolism;ko00940 Phenylpropanoid biosynthesis;
gene33835	K00454	LOX2S	lipoxygenase	EC:1.13.11.12	2	ko00591 Linoleic acid metabolism;ko00592 alpha-Linolenic acid metabolism;
gene33837	K08497	SEC20	protein transport protein SEC20	--	1	ko04130 SNARE interactions in vesicular transport;
gene33839	K02434	gatB	aspartyl-tRNA(Asn)/glutamyl-tRNA(Gln) amidotransferase subunit B	EC:6.3.5.6 6.3.5.7	1	ko00970 Aminoacyl-tRNA biosynthesis;
gene3383	K10734	GINS3	GINS complex subunit 3	--		
gene33841	K09754	CYP98A3, C3'H	coumaroylquinate(coumaroylshikimate) 3'-monooxygenase	EC:1.14.13.36	3	ko00940 Phenylpropanoid biosynthesis;ko00941 Flavonoid biosynthesis;ko00945 Stilbenoid, diarylheptanoid and gingerol biosynthesis;
gene33842	K16297	SCPL-II	serine carboxypeptidase-like clade II	EC:3.4.16.-		
gene33843	K11266	MAU2	MAternally affected uncoordination	--		
gene33847	K14234	tRNA-Thr	tRNA Thr	--	1	ko00970 Aminoacyl-tRNA biosynthesis;
gene33854	K00940	E2.7.4.6, ndk	nucleoside-diphosphate kinase	EC:2.7.4.6	2	ko00230 Purine metabolism;ko00240 Pyrimidine metabolism;
gene33857	K13179	DDX18, HAS1	ATP-dependent RNA helicase DDX18/HAS1	EC:3.6.4.13		
gene33861	K14548	UTP4, CIRH1A	U3 small nucleolar RNA-associated protein 4	--	1	ko03008 Ribosome biogenesis in eukaryotes;
gene33862	K16296	SCPL-I	serine carboxypeptidase-like clade I	EC:3.4.16.-		
gene33864	K09753	CCR	cinnamoyl-CoA reductase	EC:1.2.1.44	1	ko00940 Phenylpropanoid biosynthesis;
gene33870	K01599	hemE, UROD	uroporphyrinogen decarboxylase	EC:4.1.1.37	1	ko00860 Porphyrin and chlorophyll metabolism;
gene33871	K09377	CSRP	cysteine and glycine-rich protein	--		
gene33876	K03357	APC10, DOC1	anaphase-promoting complex subunit 10	--	7	ko04110 Cell cycle;ko04111 Cell cycle - yeast;ko04113 Meiosis - yeast;ko04114 Oocyte meiosis;ko04120 Ubiquitin mediated proteolysis;ko04914 Progesterone-mediated oocyte maturation;ko05166 HTLV-I infection;
gene33880	K00863	E2.7.1.29, DAK1, DAK2	dihydroxyacetone kinase	EC:2.7.1.29	4	ko00561 Glycerolipid metabolism;ko00680 Methane metabolism;ko01200 Carbon metabolism;ko04622 RIG-I-like receptor signaling pathway;
gene33881	K00863	E2.7.1.29, DAK1, DAK2	dihydroxyacetone kinase	EC:2.7.1.29	4	ko00561 Glycerolipid metabolism;ko00680 Methane metabolism;ko01200 Carbon metabolism;ko04622 RIG-I-like receptor signaling pathway;
gene33884	K01051	E3.1.1.11	pectinesterase	EC:3.1.1.11	2	ko00040 Pentose and glucuronate interconversions;ko00500 Starch and sucrose metabolism;
gene3388	K11984	SART1, HAF, SNU66	U4/U6.U5 tri-snRNP-associated protein 1	--	1	ko03040 Spliceosome;
gene33890	K05929	E2.1.1.103, NMT	phosphoethanolamine N-methyltransferase	EC:2.1.1.103	1	ko00564 Glycerophospholipid metabolism;
gene33891	K13448	CML	calcium-binding protein CML	--	1	ko04626 Plant-pathogen interaction;
gene33893	K14510	CTR1	serine/threonine-protein kinase CTR1	EC:2.7.11.1	1	ko04075 Plant hormone signal transduction;

gene33900	K04733	IRAK4	interleukin-1 receptor-associated kinase 4	EC:2.7.11.1	11	ko04064 NF-kappa B signaling pathway;ko04210 Apoptosis;ko04620 Toll-like receptor signaling pathway;ko04722 Neurotrophin signaling pathway;ko05133 Pertussis;ko05140 Leishmaniasis;ko05142 Chagas disease (American trypanosomiasis);ko05145 Toxoplasmosis;ko05152 Tuberculosis;ko05162 Measles;ko05164 Influenza A;
gene33904	K01507	ppa	inorganic pyrophosphatase	EC:3.6.1.1	1	ko00190 Oxidative phosphorylation;
gene33905	K07901	RAB8A, MEL	Ras-related protein Rab-8A	--	1	ko04972 Pancreatic secretion;
gene33906	K00261	GLUD1_2, gdhA	glutamate dehydrogenase (NAD(P)+)	EC:1.4.1.3	5	ko00250 Alanine, aspartate and glutamate metabolism;ko00330 Arginine and proline metabolism;ko00471 D-Glutamine and D-glutamate metabolism;ko00910 Nitrogen metabolism;ko04964 Proximal tubule bicarbonate reclamation;
gene3390	K15015	SLC32A, VGAT	solute carrier family 32 (vesicular inhibitory amino acid transporter)	--	5	ko04721 Synaptic vesicle cycle;ko04723 Retrograde endocannabinoid signaling;ko04727 GABAergic synapse;ko05032 Morphine addiction;ko05033 Nicotine addiction;
gene33915	K04771	degP, htrA	serine protease Do	EC:3.4.21.107	1	ko02020 Two-component system;
gene33916	K01673	cynT, can	carbonic anhydrase	EC:4.2.1.1	1	ko00910 Nitrogen metabolism;
gene33920	K14677	ACY1	aminoacylase	EC:3.5.1.14	3	ko00330 Arginine and proline metabolism;ko01210 2-Oxocarboxylic acid metabolism;ko01230 Biosynthesis of amino acids;
gene33923	K14230	tRNA-Met	tRNA Met	--	1	ko00970 Aminoacyl-tRNA biosynthesis;
gene33925	K11982	RNF115_126	E3 ubiquitin-protein ligase RNF115/126	EC:6.3.2.19		
gene33928	K02925	RP-L3e, RPL3	large subunit ribosomal protein L3e	--	1	ko03010 Ribosome;
gene33929	K08064	NFYA	nuclear transcription factor Y, alpha	--	2	ko04612 Antigen processing and presentation;ko05152 Tuberculosis;
gene33931	K17604	ZSWIM3	zinc finger SWIM domain-containing protein 3	--		
gene33933	K09419	HSFF	heat shock transcription factor, other eukaryote	--		
gene33935	K04079	htpG, HSP90A	molecular chaperone HtpG	--	9	ko04141 Protein processing in endoplasmic reticulum;ko04151 PI3K-Akt signaling pathway;ko04612 Antigen processing and presentation;ko04621 NOD-like receptor signaling pathway;ko04626 Plant-pathogen interaction;ko04914 Progesterone-mediated oocyte maturation;ko04915 Estrogen signaling pathway;ko05200 Pathways in cancer;ko05215 Prostate cancer;
gene33941	K13448	CML	calcium-binding protein CML	--	1	ko04626 Plant-pathogen interaction;
gene33944	K00630	ATS1	glycerol-3-phosphate O-acyltransferase	EC:2.3.1.15	2	ko00561 Glycerolipid metabolism;ko00564 Glycerophospholipid metabolism;
gene33945	K12668	OST2, DAD1	oligosaccharyltransferase complex subunit epsilon	--	3	ko00510 N-Glycan biosynthesis;ko00513 Various types of N-glycan biosynthesis;ko04141 Protein processing in endoplasmic reticulum;
gene33950	K13192	RBM26	RNA-binding protein 26	--		
gene33951	K06617	E2.4.1.82	raffinose synthase	EC:2.4.1.82	1	ko00052 Galactose metabolism;
gene33956	K01051	E3.1.1.11	pectinesterase	EC:3.1.1.11	2	ko00040 Pentose and glucuronate interconversions;ko00500 Starch and sucrose metabolism;
gene33957	K02872	RP-L13Ae, RPL13A	large subunit ribosomal protein L13Ae	--	1	ko03010 Ribosome;
gene3395	K03881	ND4	NADH-ubiquinone oxidoreductase chain 4	EC:1.6.5.3	2	ko00190 Oxidative phosphorylation;ko05012 Parkinson's disease;
gene33968	K14219	tRNA-Arg	tRNA Arg	--	1	ko00970 Aminoacyl-tRNA biosynthesis;
gene3396	K15501	PPP6R3, SAPS3	serine/threonine-protein phosphatase 6 regulatory subunit 3	--		
gene33970	K10256	FAD2	omega-6 fatty acid desaturase (delta-12 desaturase)	EC:1.14.19.-	1	ko01040 Biosynthesis of unsaturated fatty acids;
gene33972	K08486	STX1B_2_3	syntaxin 1B/2/3	--	2	ko04130 SNARE interactions in vesicular transport;ko04721 Synaptic vesicle cycle;
gene33975	K00416	QCR6, UQCRH	ubiquinol-cytochrome c reductase subunit 6	--	6	ko00190 Oxidative phosphorylation;ko04260 Cardiac muscle contraction;ko04932 Non-alcoholic fatty liver disease (NAFLD);ko05010 Alzheimer's disease;ko05012 Parkinson's disease;ko05016 Huntington's disease;
gene33979	K13114	PNN	pinin	--	2	ko03013 RNA transport;ko03015 mRNA surveillance pathway;
gene33980	K11547	NDC80, HEC1, TID3	kinetochore protein NDC80	--		
gene33983	K02925	RP-L3e, RPL3	large subunit ribosomal protein L3e	--	1	ko03010 Ribosome;
gene33984	K12181	COPS8, CSN8	COP9 signalosome complex subunit 8	--		
gene33987	K12947	SPCS2, SPC2	signal peptidase complex subunit 2	EC:3.4.-.-	1	ko03060 Protein export;
gene33989	K11168	DHRS12	dehydrogenase/reductase SDR family member 12	EC:1.1.-.-		
gene33994	K13117	DHX35	ATP-dependent RNA helicase DDX35	EC:3.6.4.13		
gene33999	K07976	RAB	Rab family, other	--		

gene3399	K14488	SAUR	SAUR family protein	--	1	ko04075 Plant hormone signal transduction;
gene34003	K02135	ATPeF1E, ATP5E	F-type H+-transporting ATPase subunit epsilon	EC:3.6.3.14	4	ko00190 Oxidative phosphorylation;ko05010 Alzheimer's disease;ko05012 Parkinson's disease;ko05016 Huntington's disease;
gene34004	K07300	chaA, CAX	Ca2+:H+ antiporter	--		
gene34007	K01535	E3.6.3.6	H+-transporting ATPase	EC:3.6.3.6	1	ko00190 Oxidative phosphorylation;
gene34010	K01762	ACS	1-aminocyclopropane-1-carboxylate synthase	EC:4.4.1.14	1	ko00270 Cysteine and methionine metabolism;
gene34011	K17422	MRPL41	large subunit ribosomal protein L41, mitochondrial	--		
gene34012	K13525	VCP, CDC48	transitional endoplasmic reticulum ATPase	--	2	ko04141 Protein processing in endoplasmic reticulum;ko05134 Legionellosis;
gene34013	K13356	FAR	fatty acyl-CoA reductase	EC:1.2.1.-	2	ko00073 Cutin, suberine and wax biosynthesis;ko04146 Peroxisome;
gene34015	K00968	PCYT1	choline-phosphate cytidylyltransferase	EC:2.7.7.15	2	ko00440 Phosphonate and phosphinate metabolism;ko00564 Glycerophospholipid metabolism;
gene34018	K06630	YWHAE	14-3-3 protein epsilon	--	8	ko04110 Cell cycle;ko04114 Oocyte meiosis;ko04151 PI3K-Akt signaling pathway;ko04390 Hippo signaling pathway;ko04391 Hippo signaling pathway - fly;ko04722 Neurotrophin signaling pathway;ko05169 Epstein-Barr virus infection;ko05203 Viral carcinogenesis;
gene34020	K15283	SLC35E1	solute carrier family 35, member E1	--		
gene34026	K14638	SLC15A3_4, PHT	solute carrier family 15 (peptide/histidine transporter), member 3/4	--		
gene34030	K02933	RP-L6, MRPL6, rplF	large subunit ribosomal protein L6	--	1	ko03010 Ribosome;
gene34032	K00784	rnz	ribonuclease Z	EC:3.1.26.11	1	ko03013 RNA transport;
gene34033	K14563	NOP1, FBL	rRNA 2'-O-methyltransferase fibrillarin	EC:2.1.1.-	1	ko03008 Ribosome biogenesis in eukaryotes;
gene34034	K03327	TC.MATE, SLC47A, norM, mdtK, dinF	multidrug resistance protein, MATE family	--		
gene34035	K03327	TC.MATE, SLC47A, norM, mdtK, dinF	multidrug resistance protein, MATE family	--		
gene34039	K02698	psaK	photosystem I subunit X	--	1	ko00195 Photosynthesis;
gene34048	K02699	psaL	photosystem I subunit XI	--	1	ko00195 Photosynthesis;
gene34049	K02699	psaL	photosystem I subunit XI	--	1	ko00195 Photosynthesis;
gene34050	K01824	EBP	cholestenol delta-isomerase	EC:5.3.3.5	1	ko00100 Steroid biosynthesis;
gene34051	K01148	PARN	poly(A)-specific ribonuclease	EC:3.1.13.4	1	ko03018 RNA degradation;
gene34052	K13448	CML	calcium-binding protein CML	--	1	ko04626 Plant-pathogen interaction;
gene34054	K01590	hdc, HDC	histidine decarboxylase	EC:4.1.1.22	1	ko00340 Histidine metabolism;
gene3405	K14611	SLC23A1_2, SVCT1_2	solute carrier family 23 (nucleobase transporter), member 1/2	--		
gene34060	K14306	NUP62	nuclear pore complex protein Nup62	--	1	ko03013 RNA transport;
gene34062	K02355	fusA, GFM, EFG	elongation factor G	--		
gene34064	K06630	YWHAE	14-3-3 protein epsilon	--	8	ko04110 Cell cycle;ko04114 Oocyte meiosis;ko04151 PI3K-Akt signaling pathway;ko04390 Hippo signaling pathway;ko04391 Hippo signaling pathway - fly;ko04722 Neurotrophin signaling pathway;ko05169 Epstein-Barr virus infection;ko05203 Viral carcinogenesis;
gene34066	K04957	HCN4	hyperpolarization activated cyclic nucleotide-gated potassium channel 4	--		
gene34067	K10357	MYO5	myosin V	--		
gene34068	K00031	IDH1, IDH2, icd	isocitrate dehydrogenase	EC:1.1.1.42	7	ko00020 Citrate cycle (TCA cycle);ko00480 Glutathione metabolism;ko00720 Carbon fixation pathways in prokaryotes;ko01200 Carbon metabolism;ko01210 2-Oxocarboxylic acid metabolism;ko01230 Biosynthesis of amino acids;ko04146 Peroxisome;
gene3406	K05907	APR	adenylyl-sulfate reductase (glutathione)	EC:1.8.4.9	1	ko00920 Sulfur metabolism;
gene34072	K08838	STK24_25_MST4	serine/threonine-protein kinase 24/25/MST4	EC:2.7.11.1		
gene34074	K01057	PGLS, pgl, devB	6-phosphogluconolactonase	EC:3.1.1.31	2	ko00030 Pentose phosphate pathway;ko01200 Carbon metabolism;
gene34076	K13941	folKP	2-amino-4-hydroxy-6-hydroxymethyldihydropteridine diphosphokinase / dihydropteroate synthase	EC:2.7.6.3 2.5.1.15	1	ko00790 Folate biosynthesis;
gene34078	K15718	LOX1_5	linoleate 9S-lipoxygenase	EC:1.13.11.58	1	ko00591 Linoleic acid metabolism;
gene34083	K02990	RP-S6, MRPS6, rpsF	small subunit ribosomal protein S6	--	1	ko03010 Ribosome;
gene34084	K00166	E1.2.4.4A, bkdA1	2-oxoisovalerate dehydrogenase E1 component, alpha subunit	EC:1.2.4.4	1	ko00280 Valine, leucine and isoleucine degradation;

gene34086	K00430	E1.11.1.7	peroxidase	EC:1.11.1.7	2	ko00360 Phenylalanine metabolism;ko00940 Phenylpropanoid biosynthesis;
gene3408	K01051	E3.1.1.11	pectinesterase	EC:3.1.1.11	2	ko00040 Pentose and glucuronate interconversions;ko00500 Starch and sucrose metabolism;
gene34091	K01051	E3.1.1.11	pectinesterase	EC:3.1.1.11	2	ko00040 Pentose and glucuronate interconversions;ko00500 Starch and sucrose metabolism;
gene34093	K02945	RP-S1, rpsA	small subunit ribosomal protein S1	--	1	ko03010 Ribosome;
gene34097	K00760	hprT, hpt, HPRT1	hypoxanthine phosphoribosyltransferase	EC:2.4.2.8	2	ko00230 Purine metabolism;ko00983 Drug metabolism - other enzymes;
gene34101	K08511	ATVAMP72	vesicle-associated membrane protein 72	--		
gene34106	K03242	EIF2S3	translation initiation factor 2 subunit 3	--	1	ko03013 RNA transport;
gene34107	K09422	MYBP	myb proto-oncogene protein, plant	--		
gene34109	K05765	CFL	cofilin	--	4	ko04360 Axon guidance;ko04666 Fc gamma R-mediated phagocytosis;ko04810 Regulation of actin cytoskeleton;ko05133 Pertussis;
gene3410	K00472	E1.14.11.2	prolyl 4-hydroxylase	EC:1.14.11.2	1	ko00330 Arginine and proline metabolism;
gene34114	K14498	SNRK2	serine/threonine-protein kinase SRK2	EC:2.7.11.1	1	ko04075 Plant hormone signal transduction;
gene34115	K16585	HAUS2	HAUS augmin-like complex subunit 2	--		
gene34116	K06268	PPP3R, CNB	serine/threonine-protein phosphatase 2B regulatory subunit	--	18	ko04010 MAPK signaling pathway;ko04020 Calcium signaling pathway;ko04114 Oocyte meiosis;ko04210 Apoptosis;ko04310 Wnt signaling pathway;ko04360 Axon guidance;ko04370 VEGF signaling pathway;ko04380 Osteoclast differentiation;ko04650 Natural killer cell mediated cytotoxicity;ko04660 T cell receptor signaling pathway;ko04662 B cell receptor signaling pathway;ko04720 Long-term potentiation;ko04724 Glutamatergic synapse;ko05010 Alzheimer's disease;ko05014 Amyotrophic lateral sclerosis (ALS);ko05031 Amphetamine addiction;ko05152
gene34117	K00430	E1.11.1.7	peroxidase	EC:1.11.1.7	2	ko00360 Phenylalanine metabolism;ko00940 Phenylpropanoid biosynthesis;
gene34118	K02921	RP-L37Ae, RPL37A	large subunit ribosomal protein L37Ae	--	1	ko03010 Ribosome;
gene34119	K01177	E3.2.1.2	beta-amylase	EC:3.2.1.2	1	ko00500 Starch and sucrose metabolism;
gene34128	K01673	cynT, can	carbonic anhydrase	EC:4.2.1.1	1	ko00910 Nitrogen metabolism;
gene34129	K09422	MYBP	myb proto-oncogene protein, plant	--		
gene34131	K12501	HST	homogentisate solanesyltransferase	--	1	ko00130 Ubiquinone and other terpenoid-quinone biosynthesis;
gene34134	K02865	RP-L10Ae, RPL10A	large subunit ribosomal protein L10Ae	--	1	ko03010 Ribosome;
gene34135	K02639	petF	ferredoxin	--	1	ko00195 Photosynthesis;
gene34136	K17583	NOM1	nucleolar MIF4G domain-containing protein 1	--		
gene34137	K17583	NOM1	nucleolar MIF4G domain-containing protein 1	--		
gene34139	K02146	ATPeVAC39, ATP6D	V-type H+-transporting ATPase subunit AC39	EC:3.6.3.14	10	ko00190 Oxidative phosphorylation;ko04142 Lysosome;ko04145 Phagosome;ko04721 Synaptic vesicle cycle;ko04966 Collecting duct acid secretion;ko05110 Vibrio cholerae infection;ko05120 Epithelial cell signaling in Helicobacter pylori infection;ko05152 Tuberculosis;ko05203 Viral carcinogenesis;ko05323 Rheumatoid arthritis;
gene34140	K07512	MECR, NRBF1	mitochondrial trans-2-enoyl-CoA reductase	EC:1.3.1.38	1	ko00062 Fatty acid elongation;
gene34141	K07512	MECR, NRBF1	mitochondrial trans-2-enoyl-CoA reductase	EC:1.3.1.38	1	ko00062 Fatty acid elongation;
gene34148	K14431	TGA	transcription factor TGA	--	1	ko04075 Plant hormone signal transduction;
gene34154	K05758	ARPC2	actin related protein 2/3 complex, subunit 2	--	6	ko04666 Fc gamma R-mediated phagocytosis;ko04810 Regulation of actin cytoskeleton;ko05100 Bacterial invasion of epithelial cells;ko05130 Pathogenic Escherichia coli infection;ko05131 Shigellosis;ko05132 Salmonella infection;
gene34156	K03426	E3.6.1.22, NUDT12, nudC	NAD+ diphosphatase	EC:3.6.1.22	2	ko00760 Nicotinate and nicotinamide metabolism;ko04146 Peroxisome;
gene34157	K01674	cah	carbonic anhydrase	EC:4.2.1.1	1	ko00910 Nitrogen metabolism;
gene34158	K01674	cah	carbonic anhydrase	EC:4.2.1.1	1	ko00910 Nitrogen metabolism;
gene34166	K11099	SNRPG, SMG	small nuclear ribonucleoprotein G	--	1	ko03040 Spliceosome;
gene34170	K02962	RP-S17e, RPS17	small subunit ribosomal protein S17e	--	1	ko03010 Ribosome;
gene34174	K00434	E1.11.1.11	L-ascorbate peroxidase	EC:1.11.1.11	2	ko00053 Ascorbate and aldarate metabolism;ko00480 Glutathione metabolism;

gene34177	K09487	HSP90B, TRA1	heat shock protein 90kDa beta	--	8	ko04141 Protein processing in endoplasmic reticulum;ko04151 PI3K-Akt signaling pathway;ko04621 NOD-like receptor signaling pathway;ko04626 Plant-pathogen interaction;ko04915 Estrogen signaling pathway;ko04918 Thyroid hormone synthesis;ko05200 Pathways in cancer;ko05215 Prostate cancer;
gene34178	K09487	HSP90B, TRA1	heat shock protein 90kDa beta	--	8	ko04141 Protein processing in endoplasmic reticulum;ko04151 PI3K-Akt signaling pathway;ko04621 NOD-like receptor signaling pathway;ko04626 Plant-pathogen interaction;ko04915 Estrogen signaling pathway;ko04918 Thyroid hormone synthesis;ko05200 Pathways in cancer;ko05215 Prostate cancer;
gene3417	K08489	STX16	syntaxin 16	--	1	ko04130 SNARE interactions in vesicular transport;
gene34181	K10401	KIF18_19	kinesin family member 18/19	--		
gene34182	K03000	RPA12, ZNRD1	DNA-directed RNA polymerase I subunit RPA12	--	3	ko00230 Purine metabolism;ko00240 Pyrimidine metabolism;ko03020 RNA polymerase;
gene34186	K04078	groES, HSPE1	chaperonin GroES	--		
gene34190	K00799	GST, gst	glutathione S-transferase	EC:2.5.1.18	4	ko00480 Glutathione metabolism;ko00980 Metabolism of xenobiotics by cytochrome P450;ko00982 Drug metabolism - cytochrome P450;ko05204 Chemical carcinogenesis;
gene34191	K00799	GST, gst	glutathione S-transferase	EC:2.5.1.18	4	ko00480 Glutathione metabolism;ko00980 Metabolism of xenobiotics by cytochrome P450;ko00982 Drug metabolism - cytochrome P450;ko05204 Chemical carcinogenesis;
gene34193	K00799	GST, gst	glutathione S-transferase	EC:2.5.1.18	4	ko00480 Glutathione metabolism;ko00980 Metabolism of xenobiotics by cytochrome P450;ko00982 Drug metabolism - cytochrome P450;ko05204 Chemical carcinogenesis;
gene34200	K01262	pepP	Xaa-Pro aminopeptidase	EC:3.4.11.9		
gene34205	K08472	MLO	mlo protein	--		
gene34209	K02940	RP-L9e, RPL9	large subunit ribosomal protein L9e	--	1	ko03010 Ribosome;
gene34211	K01184	E3.2.1.15	polygalacturonase	EC:3.2.1.15	2	ko00040 Pentose and glucuronate interconversions;ko00500 Starch and sucrose metabolism;
gene34213	K09527	DNAJC7	DnaJ homolog subfamily C member 7	--		
gene34227	K08906	petJ	cytochrome c6	--	1	ko00195 Photosynthesis;
gene3422	K10635	RNF111	E3 ubiquitin-protein ligase Arkadia	EC:6.3.2.19		
gene34232	K11723	BRD7_9	bromodomain-containing protein 7/9	--		
gene34236	K10999	CESA	cellulose synthase A	EC:2.4.1.12		
gene34237	K02520	infC, MTIF3	translation initiation factor IF-3	--		
gene34238	K02989	RP-S5e, RPS5	small subunit ribosomal protein S5e	--	1	ko03010 Ribosome;
gene34239	K12188	SNF8, EAP30	ESCRT-II complex subunit VPS22	--	1	ko04144 Endocytosis;
gene34243	K13993	HSP20	HSP20 family protein	--	1	ko04141 Protein processing in endoplasmic reticulum;
gene34244	K13993	HSP20	HSP20 family protein	--	1	ko04141 Protein processing in endoplasmic reticulum;
gene34249	K10268	FBXL2_20	F-box and leucine-rich repeat protein 2/20	--		
gene34258	K02635	petB	cytochrome b6	--	1	ko00195 Photosynthesis;
gene3425	K08081	E1.1.1.206	tropine dehydrogenase	EC:1.1.1.206	1	ko00960 Tropane, piperidine and pyridine alkaloid biosynthesis;
gene34264	K00469	MIOX	inositol oxygenase	EC:1.13.99.1	2	ko00053 Ascorbate and aldarate metabolism;ko00562 Inositol phosphate metabolism;
gene34270	K00279	E1.5.99.12	cytokinin dehydrogenase	EC:1.5.99.12	1	ko00908 Zeatin biosynthesis;
gene34271	K01956	carA, CPA1	carbamoyl-phosphate synthase small subunit	EC:6.3.5.5	2	ko00240 Pyrimidine metabolism;ko00250 Alanine, aspartate and glutamate metabolism;
gene34272	K01945	purD	phosphoribosylamine--glycine ligase	EC:6.3.4.13	1	ko00230 Purine metabolism;
gene34273	K12184	VPS28	ESCRT-I complex subunit VPS28	--	1	ko04144 Endocytosis;
gene34279	K00079	CBR1	carbonyl reductase 1	EC:1.1.1.184 1.1.1.189 1.1.1.197	3	ko00590 Arachidonic acid metabolism;ko00980 Metabolism of xenobiotics by cytochrome P450;ko05204 Chemical carcinogenesis;
gene34280	K14684	SLC25A23S	solute carrier family 25 (mitochondrial phosphate transporter), member 23/24/25/41	--		
gene34281	K14233	tRNA-Ser	tRNA Ser	--	1	ko00970 Aminoacyl-tRNA biosynthesis;
gene34283	K07200	PRKAG	5'-AMP-activated protein kinase, regulatory gamma subunit	--	5	ko04710 Circadian rhythm;ko04910 Insulin signaling pathway;ko04920 Adipocytokine signaling pathway;ko04932 Non-alcoholic fatty liver disease (NAFLD);ko05410 Hypertrophic cardiomyopathy (HCM);
gene34289	K15979	SND1	staphylococcal nuclease domain-containing protein 1	--	2	ko05169 Epstein-Barr virus infection;ko05203 Viral carcinogenesis;
gene3428	K02915	RP-L34e, RPL34	large subunit ribosomal protein L34e	--	1	ko03010 Ribosome;

gene34294	K07513	ACAA1	acetyl-CoA acyltransferase 1	EC:2.3.1.16	7	ko00071 Fatty acid degradation;ko00280 Valine, leucine and isoleucine degradation;ko00362 Benzoate degradation;ko00592 alpha-Linolenic acid metabolism;ko01040 Biosynthesis of unsaturated fatty acids;ko03320 PPAR signaling pathway;ko04146 Peroxisome;
gene34296	K00051	E1.1.1.82	malate dehydrogenase (NADP+)	EC:1.1.1.82	2	ko00620 Pyruvate metabolism;ko00710 Carbon fixation in photosynthetic organisms;
gene34298	K14182	E4.2.3.21	vetispiradiene synthase	EC:4.2.3.21	1	ko00909 Sesquiterpenoid and triterpenoid biosynthesis;
gene34299	K15472	CYP71D55	premnaspirodiene oxygenase	EC:1.14.13.12 1	1	ko00909 Sesquiterpenoid and triterpenoid biosynthesis;
gene3429	K03327	TC.MATE, SLC47A, norM, mdtK, dinF	multidrug resistance protein, MATE family	--		
gene34312	K15223	UAF30, SPP27	upstream activation factor subunit UAF30	--		
gene34313	K14498	SNRK2	serine/threonine-protein kinase SRK2	EC:2.7.11.1	1	ko04075 Plant hormone signal transduction;
gene34315	K08900	BCS1	mitochondrial chaperone BCS1	--		
gene34316	K02732	PSMB1	20S proteasome subunit beta 6	EC:3.4.25.1	1	ko03050 Proteasome;
gene34317	K15425	PPP4R2	serine/threonine-protein phosphatase 4 regulatory subunit 2	--		
gene34318	K14209	SLC36A, PAT	solute carrier family 36 (proton-coupled amino acid transporter)	--		
gene34319	K08507	USE1	unconventional SNARE in the endoplasmic reticulum protein 1	--	1	ko04130 SNARE interactions in vesicular transport;
gene3432	K03869	CUL3	cullin 3	--	1	ko04120 Ubiquitin mediated proteolysis;
gene34330	K09499	CCT7	T-complex protein 1 subunit eta	--		
gene34334	K15191	LARP7	La-related protein 7	--		
gene34336	K00430	E1.11.1.7	peroxidase	EC:1.11.1.7	2	ko00360 Phenylalanine metabolism;ko00940 Phenylpropanoid biosynthesis;
gene34341	K01488	E3.5.4.4, ADA, add	adenosine deaminase	EC:3.5.4.4	2	ko00230 Purine metabolism;ko05340 Primary immunodeficiency;
gene34342	K00826	E2.6.1.42, ilvE	branched-chain amino acid aminotransferase	EC:2.6.1.42	5	ko00280 Valine, leucine and isoleucine degradation;ko00290 Valine, leucine and isoleucine biosynthesis;ko00770 Pantothenate and CoA biosynthesis;ko01210 2-Oxocarboxylic acid metabolism;ko01230 Biosynthesis of amino acids;
gene34343	K01979	SSUrRNA	small subunit ribosomal RNA	--	2	ko03008 Ribosome biogenesis in eukaryotes;ko03010 Ribosome;
gene34346	K14638	SLC15A3_4, PHT	solute carrier family 15 (peptide/histidine transporter), member 3/4	--		
gene34347	K06966	K06966				
gene34350	K14488	SAUR	SAUR family protein	--	1	ko04075 Plant hormone signal transduction;
gene34351	K08770	UBC	ubiquitin C	--	1	ko03320 PPAR signaling pathway;
gene34354	K14682	argAB	amino-acid N-acetyltransferase	EC:2.3.1.1	3	ko00330 Arginine and proline metabolism;ko01210 2-Oxocarboxylic acid metabolism;ko01230 Biosynthesis of amino acids;
gene34359	K15032	MTERFD	mTERF domain-containing protein, mitochondrial	--		
gene3435	K00750	GYG1, GYG2	glycogenin glucosyltransferase	EC:2.4.1.186		
gene34365	K11091	SNRPA	U1 small nuclear ribonucleoprotein A	--	1	ko03040 Spliceosome;
gene34366	K16571	TUBGCP4, GCP4	gamma-tubulin complex component 4	--		
gene34368	K03452	MHX	magnesium/proton exchanger	--		
gene3436	K00750	GYG1, GYG2	glycogenin glucosyltransferase	EC:2.4.1.186		
gene34370	K03955	NDUFAB1	NADH dehydrogenase (ubiquinone) 1 alpha/beta subcomplex 1	--	5	ko00190 Oxidative phosphorylation;ko04932 Non-alcoholic fatty liver disease (NAFLD);ko05010 Alzheimer's disease;ko05012 Parkinson's disease;ko05016 Huntington's disease;
gene34371	K08057	CALR	calreticulin	--	5	ko04141 Protein processing in endoplasmic reticulum;ko04145 Phagosome;ko04612 Antigen processing and presentation;ko05142 Chagas disease (American trypanosomiasis);ko05166 HTLV-I infection;
gene34372	K16297	SCPL-II	serine carboxypeptidase-like clade II	EC:3.4.16.-		
gene3437	K11228	STE11	mitogen-activated protein kinase kinase kinase	EC:2.7.11.1	1	ko04011 MAPK signaling pathway - yeast;
gene34382	K10775	E4.3.1.24	phenylalanine ammonia-lyase	EC:4.3.1.24	2	ko00360 Phenylalanine metabolism;ko00940 Phenylpropanoid biosynthesis;
gene34384	K09578	PIN1	peptidyl-prolyl cis-trans isomerase NIMA-interacting 1	EC:5.2.1.8	1	ko04622 RIG-I-like receptor signaling pathway;
gene34387	K01867	WARS, trpS	tryptophanyl-tRNA synthetase	EC:6.1.1.2	1	ko00970 Aminoacyl-tRNA biosynthesis;
gene34389	K13172	SRRM2, SRM300	serine/arginine repetitive matrix protein 2	--		

gene3438	K10406	KIFC2_3	kinesin family member C2/C3	--		
gene34392	K12621	LSM2	U6 snRNA-associated Sm-like protein LSm2	--	2	ko03018 RNA degradation;ko03040 Spliceosome;
gene34393	K15688	MUL1	E3 ubiquitin-protein ligase MUL1	EC:6.3.2.19		
gene34395	K01988	A4GALT	lactosylceramide 4-alpha-galactosyltransferase	EC:2.4.1.228	1	ko00603 Glycosphingolipid biosynthesis - globo series;
gene34396	K12394	AP1S1_2	AP-1 complex subunit sigma 1/2	--	1	ko04142 Lysosome;
gene34412	K00208	fabI	enoyl-[acyl-carrier protein] reductase I	EC:1.3.1.9 1.3.1.10	2	ko00061 Fatty acid biosynthesis;ko00780 Biotin metabolism;
gene34413	K04733	IRAK4	interleukin-1 receptor-associated kinase 4	EC:2.7.11.1	11	ko04064 NF-kappa B signaling pathway;ko04210 Apoptosis;ko04620 Toll-like receptor signaling pathway;ko04722 Neurotrophin signaling pathway;ko05133 Pertussis;ko05140 Leishmaniasis;ko05142 Chagas disease (American trypanosomiasis);ko05145 Toxoplasmosis;ko05152 Tuberculosis;ko05162 Measles;ko05164 Influenza A;
gene34414	K12309	GLB1, ELNR1	beta-galactosidase	EC:3.2.1.23	6	ko00052 Galactose metabolism;ko00511 Other glycan degradation;ko00531 Glycosaminoglycan degradation;ko00600 Sphingolipid metabolism;ko00604 Glycosphingolipid biosynthesis - ganglio series;ko04142 Lysosome;
gene34416	K10398	KIF11, EG5	kinesin family member 11	--		
gene34417	K12890	SFRS1_9	splicing factor, arginine/serine-rich 1/9	--	2	ko03040 Spliceosome;ko05168 Herpes simplex infection;
gene34418	K02942	RP-LP1, RPLP1	large subunit ribosomal protein LP1	--	1	ko03010 Ribosome;
gene34423	K14207	SLC38A2, SNAT2	solute carrier family 38 (sodium-coupled neutral amino acid transporter), member 2	--	3	ko04724 Glutamatergic synapse;ko04727 GABAergic synapse;ko04974 Protein digestion and absorption;
gene34434	K01188	E3.2.1.21	beta-glucosidase	EC:3.2.1.21	3	ko00460 Cyanoamino acid metabolism;ko00500 Starch and sucrose metabolism;ko00940 Phenylpropanoid biosynthesis;
gene34437	K14488	SAUR	SAUR family protein	--	1	ko04075 Plant hormone signal transduction;
gene34438	K14235	tRNA-Trp	tRNA Trp	--	1	ko00970 Aminoacyl-tRNA biosynthesis;
gene34443	K08959	CSNK1D	casein kinase 1, delta	EC:2.7.11.1	4	ko04340 Hedgehog signaling pathway;ko04390 Hippo signaling pathway;ko04540 Gap junction;ko04710 Circadian rhythm;
gene34446	K01759	E4.4.1.5, GLO1, gloA	lactoylglutathione lyase	EC:4.4.1.5	2	ko00620 Pyruvate metabolism;ko04011 MAPK signaling pathway - yeast;
gene34448	K09873	TIP	aquaporin TIP	--		
gene3444	K04459	DUSP, MKP	dual specificity phosphatase	EC:3.1.3.16 3.1.3.48	1	ko04010 MAPK signaling pathway;
gene34452	K10364	CAPZA	capping protein (actin filament) muscle Z-line, alpha	--		
gene34453	K10364	CAPZA	capping protein (actin filament) muscle Z-line, alpha	--		
gene34454	K12623	LSM4	U6 snRNA-associated Sm-like protein LSm4	--	2	ko03018 RNA degradation;ko03040 Spliceosome;
gene34459	K12832	SF3B5, SF3B10	splicing factor 3B subunit 5	--	1	ko03040 Spliceosome;
gene34461	K03097	CSNK2A	casein kinase II subunit alpha	EC:2.7.11.1	9	ko03008 Ribosome biogenesis in eukaryotes;ko04064 NF-kappa B signaling pathway;ko04310 Wnt signaling pathway;ko04520 Adherens junction;ko04530 Tight junction;ko04712 Circadian rhythm - plant;ko05162 Measles;ko05168 Herpes simplex infection;ko05169 Epstein-Barr virus infection;
gene34465	K01188	E3.2.1.21	beta-glucosidase	EC:3.2.1.21	3	ko00460 Cyanoamino acid metabolism;ko00500 Starch and sucrose metabolism;ko00940 Phenylpropanoid biosynthesis;
gene34466	K00799	GST, gst	glutathione S-transferase	EC:2.5.1.18	4	ko00480 Glutathione metabolism;ko00980 Metabolism of xenobiotics by cytochrome P450;ko00982 Drug metabolism - cytochrome P450;ko05204 Chemical carcinogenesis;
gene34469	K00799	GST, gst	glutathione S-transferase	EC:2.5.1.18	4	ko00480 Glutathione metabolism;ko00980 Metabolism of xenobiotics by cytochrome P450;ko00982 Drug metabolism - cytochrome P450;ko05204 Chemical carcinogenesis;
gene3446	K12828	SF3B1, SAP155	splicing factor 3B subunit 1	--	1	ko03040 Spliceosome;
gene34475	K14568	EMG1, NEP1	rRNA small subunit pseudouridine methyltransferase Nep1	EC:2.1.1.260	1	ko03008 Ribosome biogenesis in eukaryotes;
gene34476	K10406	KIFC2_3	kinesin family member C2/C3	--		
gene34487	K10570	ERCC8, CKN1, CSA	DNA excision repair protein ERCC-8	--	2	ko03420 Nucleotide excision repair;ko04120 Ubiquitin mediated proteolysis;
gene34492	K14848	RRB1, GRWD1	ribosome assembly protein RRB1	--		
gene34498	K07407	E3.2.1.22B, galA, rafA	alpha-galactosidase	EC:3.2.1.22	4	ko00052 Galactose metabolism;ko00561 Glycerolipid metabolism;ko00600 Sphingolipid metabolism;ko00603 Glycosphingolipid biosynthesis - globo series;
gene34499	K14833	NOC2	nucleolar complex protein 2	--		

gene34509	K13384	E2.1.1.140	(S)-coclaurine N-methyltransferase	EC:2.1.1.140	1	ko00950 Isoquinoline alkaloid biosynthesis;
gene34511	K03955	NDUFAB1	NADH dehydrogenase (ubiquinone) 1 alpha/beta subcomplex 1	--	5	ko00190 Oxidative phosphorylation;ko04932 Non-alcoholic fatty liver disease (NAFLD);ko05010 Alzheimer's disease;ko05012 Parkinson's disease;ko05016 Huntington's disease;
gene34515	K07198	PRKAA, AMPK	5'-AMP-activated protein kinase, catalytic alpha subunit	EC:2.7.11.11	8	ko04140 Regulation of autophagy;ko04150 mTOR signaling pathway;ko04151 PI3K-Akt signaling pathway;ko04710 Circadian rhythm;ko04910 Insulin signaling pathway;ko04920 Adipocytokine signaling pathway;ko04932 Non-alcoholic fatty liver disease (NAFLD);ko05410 Hypertrophic cardiomyopathy (HCM);
gene3451	K01392	THOP1	thimet oligopeptidase	EC:3.4.24.15	2	ko04614 Renin-angiotensin system;ko05143 African trypanosomiasis;
gene34522	K00857	E2.7.1.21, tdk	thymidine kinase	EC:2.7.1.21	2	ko00240 Pyrimidine metabolism;ko00983 Drug metabolism - other enzymes;
gene34529	K06630	YWHAE	14-3-3 protein epsilon	--	8	ko04110 Cell cycle;ko04114 Oocyte meiosis;ko04151 PI3K-Akt signaling pathway;ko04390 Hippo signaling pathway;ko04391 Hippo signaling pathway - fly;ko04722 Neurotrophin signaling pathway;ko05169 Epstein-Barr virus infection;ko05203 Viral carcinogenesis;
gene34531	K00737	MGAT3	beta-1,4-mannosyl-glycoprotein beta-1,4-N-acetylglucosaminyltransferase	EC:2.4.1.144	1	ko00510 N-Glycan biosynthesis;
gene34535	K00434	E1.11.1.11	L-ascorbate peroxidase	EC:1.11.1.11	2	ko00053 Ascorbate and aldarate metabolism;ko00480 Glutathione metabolism;
gene34538	K10365	CAPZB	capping protein (actin filament) muscle Z-line, beta	--		
gene3453	K15095	E1.1.1.208	(+)-neomenthol dehydrogenase	EC:1.1.1.208	1	ko00902 Monoterpenoid biosynthesis;
gene34543	K15633	gpmI	2,3-bisphosphoglycerate-independent phosphoglycerate mutase	EC:5.4.2.12	5	ko00010 Glycolysis / Gluconeogenesis;ko00260 Glycine, serine and threonine metabolism;ko00680 Methane metabolism;ko01200 Carbon metabolism;ko01230 Biosynthesis of amino acids;
gene34545	K02717	psbP	photosystem II oxygen-evolving enhancer protein 2	--	1	ko00195 Photosynthesis;
gene34547	K02958	RP-S15e, RPS15	small subunit ribosomal protein S15e	--	1	ko03010 Ribosome;
gene3454	K15095	E1.1.1.208	(+)-neomenthol dehydrogenase	EC:1.1.1.208	1	ko00902 Monoterpenoid biosynthesis;
gene34550	K01051	E3.1.1.11	pectinesterase	EC:3.1.1.11	2	ko00040 Pentose and glucuronate interconversions;ko00500 Starch and sucrose metabolism;
gene34561	K05391	CNGF	cyclic nucleotide gated channel, other eukaryote	--	1	ko04626 Plant-pathogen interaction;
gene34573	K11838	USP7, UBP15	ubiquitin carboxyl-terminal hydrolase 7	EC:3.1.2.15	3	ko05168 Herpes simplex infection;ko05169 Epstein-Barr virus infection;ko05203 Viral carcinogenesis;
gene34578	K02699	psaL	photosystem I subunit XI	--	1	ko00195 Photosynthesis;
gene3457	K15255	PIF1	ATP-dependent DNA helicase PIF1	EC:3.6.4.12		
gene34584	K00789	metK	S-adenosylmethionine synthetase	EC:2.5.1.6	2	ko00270 Cysteine and methionine metabolism;ko01230 Biosynthesis of amino acids;
gene34591	K03327	TC.MATE, SLC47A, norM, mdtK, dinF	multidrug resistance protein, MATE family	--		
gene34592	K03327	TC.MATE, SLC47A, norM, mdtK, dinF	multidrug resistance protein, MATE family	--		
gene34596	K01114	plcC	phospholipase C	EC:3.1.4.3	3	ko00562 Inositol phosphate metabolism;ko00564 Glycerophospholipid metabolism;ko00565 Ether lipid metabolism;
gene34597	K08193	SLC17A	MFS transporter, ACS family, solute carrier family 17 (sodium-dependent inorganic phosphate cotransporter), other	--		
gene34599	K14221	tRNA-Asp	tRNA Asp	--	1	ko00970 Aminoacyl-tRNA biosynthesis;
gene3459	K12885	RBMX, HNRNPG	heterogeneous nuclear ribonucleoprotein G	--	1	ko03040 Spliceosome;
gene3460	K01728	E4.2.2.2, pel	pectate lyase	EC:4.2.2.2	1	ko00040 Pentose and glucuronate interconversions;
gene34610	K14831	MAK16	protein MAK16	--		
gene34619	K02126	ATPeF0A, MTATP6	F-type H+-transporting ATPase subunit a	EC:3.6.3.14	4	ko00190 Oxidative phosphorylation;ko05010 Alzheimer's disease;ko05012 Parkinson's disease;ko05016 Huntington's disease;
gene34624	K11253	H3	histone H3	--	3	ko05034 Alcoholism;ko05202 Transcriptional misregulation in cancer;ko05322 Systemic lupus erythematosus;
gene34629	K14638	SLC15A3_4, PHT	solute carrier family 15 (peptide/histidine transporter), member 3/4	--		
gene34634	K07562	NMD3	nonsense-mediated mRNA decay protein 3	--	2	ko03008 Ribosome biogenesis in eukaryotes;ko03013 RNA transport;
gene34639	K08829	MAK	male germ cell-associated kinase	EC:2.7.11.22		

gene34643	K04392	RAC1	Ras-related C3 botulinum toxin substrate 1	--	33	ko04010 MAPK signaling pathway;ko04014 Ras signaling pathway;ko04062 Chemokine signaling pathway;ko04145 Phagosome;ko04151 PI3K-Akt signaling pathway;ko04310 Wnt signaling pathway;ko04360 Axon guidance;ko04370 VEGF signaling pathway;ko04380 Osteoclast differentiation;ko04510 Focal adhesion;ko04520 Adherens junction;ko04620 Toll-like receptor signaling pathway;ko04650 Natural killer cell mediated cytotoxicity;ko04662 B cell receptor signaling pathway;ko04664 Fc epsilon RI signaling pathway;ko04666 Fc gamma R-mediated phagocytosis;ko04670 Leukocyte transendothelial migration;ko04722 Neurotrophin signaling pathway;ko04810 Regulation of actin cytoskeleton;ko04932 Non-alcoholic fatty liver disease (NAFLD);ko04972 Pancreatic secretion;ko05014 Amyotrophic lateral sclerosis (ALS);ko05100 Bacterial invasion of epithelial cells;ko05120 Epithelial cell signaling in Helicobacter pylori infection;ko05131 Shigellosis;ko05132 Salmonella infection;ko05200 Pathways in cancer;ko05203 Viral carcinogenesis;ko05205 Proteoglycans in cancer;ko05210 Colorectal cancer;ko05211 Renal cell carcinoma;ko05212 Pancreatic
gene34646	K14232	tRNA-Pro	tRNA Pro	--	1	ko00970 Aminoacyl-tRNA biosynthesis;
gene34650	K00967	PCYT2	ethanolamine-phosphate cytidylyltransferase	EC:2.7.7.14	2	ko00440 Phosphonate and phosphinate metabolism;ko00564 Glycerophospholipid metabolism;
gene34651	K13412	CPK	calcium-dependent protein kinase	EC:2.7.11.1	2	ko04626 Plant-pathogen interaction;ko05145 Toxoplasmosis;
gene34659	K09422	MYBP	myb proto-oncogene protein, plant	--		
gene34660	K09422	MYBP	myb proto-oncogene protein, plant	--		
gene34661	K14312	NUP155	nuclear pore complex protein Nup155	--	1	ko03013 RNA transport;
gene34662	K00695	E2.4.1.13	sucrose synthase	EC:2.4.1.13	1	ko00500 Starch and sucrose metabolism;
gene34663	K01531	E3.6.3.2, mgtA, mgtB	Mg2+-importing ATPase	EC:3.6.3.2		
gene34669	K05546	GANAB	alpha 1,3-glucosidase	EC:3.2.1.84	2	ko00510 N-Glycan biosynthesis;ko04141 Protein processing in endoplasmic reticulum;
gene3466	K00472	E1.14.11.2	prolyl 4-hydroxylase	EC:1.14.11.2	1	ko00330 Arginine and proline metabolism;
gene34670	K03549	kup	KUP system potassium uptake protein	--		
gene34673	K14301	NUP107	nuclear pore complex protein Nup107	--	1	ko03013 RNA transport;
gene34674	K07466	RFA1, RPA1, rpa	replication factor A1	--	5	ko03030 DNA replication;ko03420 Nucleotide excision repair;ko03430 Mismatch repair;ko03440 Homologous recombination;ko03460 Fanconi anemia pathway;
gene34675	K02895	RP-L24, MRPL24, rplX	large subunit ribosomal protein L24	--	1	ko03010 Ribosome;
gene3467	K14182	E4.2.3.21	vetispiradiene synthase	EC:4.2.3.21	1	ko00909 Sesquiterpenoid and triterpenoid biosynthesis;
gene34680	K13141	INTS4	integrator complex subunit 4	--		
gene34683	K14638	SLC15A3_4, PHT	solute carrier family 15 (peptide/histidine transporter), member 3/4	--		
gene34684	K01254	E3.3.2.6, LTA4H	leukotriene-A4 hydrolase	EC:3.3.2.6	1	ko00590 Arachidonic acid metabolism;
gene34685	K14225	tRNA-Gly	tRNA Gly	--	1	ko00970 Aminoacyl-tRNA biosynthesis;
gene34686	K14230	tRNA-Met	tRNA Met	--	1	ko00970 Aminoacyl-tRNA biosynthesis;
gene34687	K14230	tRNA-Met	tRNA Met	--	1	ko00970 Aminoacyl-tRNA biosynthesis;
gene34688	K14230	tRNA-Met	tRNA Met	--	1	ko00970 Aminoacyl-tRNA biosynthesis;
gene34701	K15083	RAD16	DNA repair protein RAD16	--		
gene34703	K01051	E3.1.1.11	pectinesterase	EC:3.1.1.11	2	ko00040 Pentose and glucuronate interconversions;ko00500 Starch and sucrose metabolism;
gene34706	K12858	DDX23, PRP28	ATP-dependent RNA helicase DDX23/PRP28	EC:3.6.4.13	1	ko03040 Spliceosome;
gene34707	K00695	E2.4.1.13	sucrose synthase	EC:2.4.1.13	1	ko00500 Starch and sucrose metabolism;
gene34710	K05928	E2.1.1.95	tocopherol O-methyltransferase	EC:2.1.1.95	1	ko00130 Ubiquinone and other terpenoid-quinone biosynthesis;
gene34718	K01176	E3.2.1.1, amyA, malS	alpha-amylase	EC:3.2.1.1	2	ko00500 Starch and sucrose metabolism;ko04973 Carbohydrate digestion and absorption;
gene34720	K04077	groEL, HSPD1	chaperonin GroEL	--	4	ko03018 RNA degradation;ko04940 Type I diabetes mellitus;ko05134 Legionellosis;ko05152 Tuberculosis;
gene34721	K15281	SLC35D	solute carrier family 35	--		

gene34723	K02155	ATPeVPL, ATP6L	V-type H+-transporting ATPase 16kDa proteolipid subunit	EC:3.6.3.14	9	ko00190 Oxidative phosphorylation;ko04142 Lysosome;ko04145 Phagosome;ko04721 Synaptic vesicle cycle;ko04966 Collecting duct acid secretion;ko05110 Vibrio cholerae infection;ko05120 Epithelial cell signaling in Helicobacter pylori infection;ko05152 Tuberculosis;ko05323 Rheumatoid arthritis;
gene34726	K14229	tRNA-Lys	tRNA Lys	--	1	ko00970 Aminoacyl-tRNA biosynthesis;
gene34730	K00573	E2.1.1.77, pcm	protein-L-isoaspartate(D-aspartate) O-methyltransferase	EC:2.1.1.77		
gene34731	K11294	NCL, NSR1	nucleolin	--	1	ko05130 Pathogenic Escherichia coli infection;
gene34732	K01609	trpC	indole-3-glycerol phosphate synthase	EC:4.1.1.48	2	ko00400 Phenylalanine, tyrosine and tryptophan biosynthesis;ko01230 Biosynthesis of amino acids;
gene34734	K05350	bglB	beta-glucosidase	EC:3.2.1.21	3	ko00460 Cyanoamino acid metabolism;ko00500 Starch and sucrose metabolism;ko00940 Phenylpropanoid biosynthesis;
gene34737	K01736	aroC	chorismate synthase	EC:4.2.3.5	2	ko00400 Phenylalanine, tyrosine and tryptophan biosynthesis;ko01230 Biosynthesis of amino acids;
gene34740	K14638	SLC15A3_4, PHT	solute carrier family 15 (peptide/histidine transporter), member 3/4	--		
gene34742	K04733	IRAK4	interleukin-1 receptor-associated kinase 4	EC:2.7.11.1	11	ko04064 NF-kappa B signaling pathway;ko04210 Apoptosis;ko04620 Toll-like receptor signaling pathway;ko04722 Neurotrophin signaling pathway;ko05133 Pertussis;ko05140 Leishmaniasis;ko05142 Chagas disease (American trypanosomiasis);ko05145 Toxoplasmosis;ko05152 Tuberculosis;ko05162 Measles;ko05164 Influenza A;
gene34743	K15287	SLC35F1_2	solute carrier family 35, member F1/2	--		
gene34744	K04733	IRAK4	interleukin-1 receptor-associated kinase 4	EC:2.7.11.1	11	ko04064 NF-kappa B signaling pathway;ko04210 Apoptosis;ko04620 Toll-like receptor signaling pathway;ko04722 Neurotrophin signaling pathway;ko05133 Pertussis;ko05140 Leishmaniasis;ko05142 Chagas disease (American trypanosomiasis);ko05145 Toxoplasmosis;ko05152 Tuberculosis;ko05162 Measles;ko05164 Influenza A;
gene34753	K05387	GRIP	glutamate receptor, ionotropic, plant	--		
gene34755	K09422	MYBP	myb proto-oncogene protein, plant	--		
gene34760	K02986	RP-S4, rpsD	small subunit ribosomal protein S4	--	1	ko03010 Ribosome;
gene34761	K00434	E1.11.1.11	L-ascorbate peroxidase	EC:1.11.1.11	2	ko00053 Ascorbate and aldarate metabolism;ko00480 Glutathione metabolism;
gene34762	K03245	EIF3J	translation initiation factor 3 subunit J	--	1	ko03013 RNA transport;
gene34765	K01803	TPI, tpiA	triosephosphate isomerase (TIM)	EC:5.3.1.1	6	ko00010 Glycolysis / Gluconeogenesis;ko00051 Fructose and mannose metabolism;ko00562 Inositol phosphate metabolism;ko00710 Carbon fixation in photosynthetic organisms;ko01200 Carbon metabolism;ko01230 Biosynthesis of amino acids;
gene34767	K13119	FAM50, XAP5	protein FAM50	--		
gene34768	K13249	SSR1	translocon-associated protein subunit alpha	--	1	ko04141 Protein processing in endoplasmic reticulum;
gene34779	K10999	CESA	cellulose synthase A	EC:2.4.1.12		
gene34783	K13691	SGT1	pathogen-inducible salicylic acid glucosyltransferase	EC:2.4.1.-		
gene34786	K14497	PP2C	protein phosphatase 2C	EC:3.1.3.16	1	ko04075 Plant hormone signal transduction;
gene34787	K14506	JAR1	jasmonic acid-amino synthetase	--	1	ko04075 Plant hormone signal transduction;
gene34789	K00721	DPM1	dolichol-phosphate mannosyltransferase	EC:2.4.1.83	1	ko00510 N-Glycan biosynthesis;
gene3478	K01087	otsB	trehalose 6-phosphate phosphatase	EC:3.1.3.12	1	ko00500 Starch and sucrose metabolism;
gene34795	K01408	IDE, ide	insulysin	EC:3.4.24.56	1	ko05010 Alzheimer's disease;
gene34797	K01904	E6.2.1.12	4-coumarate--CoA ligase	EC:6.2.1.12	3	ko00130 Ubiquinone and other terpenoid-quinone biosynthesis;ko00360 Phenylalanine metabolism;ko00940 Phenylpropanoid biosynthesis;
gene34804	K07056	rsmI	16S rRNA (cytidine1402-2'-O)-methyltransferase	EC:2.1.1.198		
gene34805	K10688	UBE2W, UBC16	ubiquitin-conjugating enzyme E2 W	EC:6.3.2.19	1	ko04120 Ubiquitin mediated proteolysis;
gene34807	K09833	HPT, TPT	homogenitisate phytyltransferase	--	1	ko00130 Ubiquinone and other terpenoid-quinone biosynthesis;

gene34817	K06268	PPP3R, CNB	serine/threonine-protein phosphatase 2B regulatory subunit	--	18	ko04010 MAPK signaling pathway;ko04020 Calcium signaling pathway;ko04114 Oocyte meiosis;ko04210 Apoptosis;ko04310 Wnt signaling pathway;ko04360 Axon guidance;ko04370 VEGF signaling pathway;ko04380 Osteoclast differentiation;ko04650 Natural killer cell mediated cytotoxicity;ko04660 T cell receptor signaling pathway;ko04662 B cell receptor signaling pathway;ko04720 Long-term potentiation;ko04724 Glutamatergic synapse;ko05010 Alzheimer's disease;ko05014 Amyotrophic lateral sclerosis (ALS);ko05031 Amphetamine addiction;ko05152
gene34820	K00166	E1.2.4.4A, bkdA1	2-oxoisovalerate dehydrogenase E1 component, alpha subunit	EC:1.2.4.4	1	ko00280 Valine, leucine and isoleucine degradation;
gene34821	K02990	RP-S6, MRPS6, rpsF	small subunit ribosomal protein S6	--	1	ko03010 Ribosome;
gene34823	K14945	QKI	protein quaking	--		
gene34824	K06689	UBE2D_E, UBC4, UBC5	ubiquitin-conjugating enzyme E2 D/E	EC:6.3.2.19	2	ko04120 Ubiquitin mediated proteolysis;ko04141 Protein processing in endoplasmic reticulum;
gene34828	K16616	PARP8	actin-related protein 8, plant	--		
gene34835	K09338	HD-ZIP	homeobox-leucine zipper protein	--		
gene34836	K11430	EZH2	histone-lysine N-methyltransferase EZH2	EC:2.1.1.43	1	ko05206 MicroRNAs in cancer;
gene34848	K07297	ADIPOR	adiponectin receptor	--	2	ko04920 Adipocytokine signaling pathway;ko04932 Non-alcoholic fatty liver disease (NAFLD);
gene34849	K09487	HSP90B, TRA1	heat shock protein 90kDa beta	--	8	ko04141 Protein processing in endoplasmic reticulum;ko04151 PI3K-Akt signaling pathway;ko04621 NOD-like receptor signaling pathway;ko04626 Plant-pathogen interaction;ko04915 Estrogen signaling pathway;ko04918 Thyroid hormone synthesis;ko05200 Pathways in cancer;ko05215 Prostate cancer;
gene3484	K00827	AGXT2	alanine-glyoxylate transaminase / (R)-3-amino-2-methylpropionate-pyruvate transaminase	EC:2.6.1.44 2.6.1.40	2	ko00250 Alanine, aspartate and glutamate metabolism;ko00260 Glycine, serine and threonine metabolism;
gene34851	K14489	AHK2_3_4	arabidopsis histidine kinase 2/3/4 (cytokinin receptor)	EC:2.7.13.3	1	ko04075 Plant hormone signal transduction;
gene34854	K08496	GOSR2, BOS1	golgi SNAP receptor complex member 2	--	1	ko04130 SNARE interactions in vesicular transport;
gene34861	K11314	TADA2A, ADA2	transcriptional adapter 2-alpha	--		
gene34863	K14558	PWP2, UTP1	periodic tryptophan protein 2	--	1	ko03008 Ribosome biogenesis in eukaryotes;
gene34864	K06215	pdxS, pdx1	pyridoxine biosynthesis protein	EC:4.-.-.-	1	ko00750 Vitamin B6 metabolism;
gene34865	K15109	SLC25A20_29, CACT, CACL, CRC1	solute carrier family 25 (mitochondrial carnitine/acylcarnitine transporter), member 20/29	--		
gene34866	K15109	SLC25A20_29, CACT, CACL, CRC1	solute carrier family 25 (mitochondrial carnitine/acylcarnitine transporter), member 20/29	--		
gene34867	K04733	IRAK4	interleukin-1 receptor-associated kinase 4	EC:2.7.11.1	11	ko04064 NF-kappa B signaling pathway;ko04210 Apoptosis;ko04620 Toll-like receptor signaling pathway;ko04722 Neurotrophin signaling pathway;ko05133 Pertussis;ko05140 Leishmaniasis;ko05142 Chagas disease (American trypanosomiasis);ko05145 Toxoplasmosis;ko05152 Tuberculosis;ko05162 Measles;ko05164 Influenza A;
gene34871	K05758	ARPC2	actin related protein 2/3 complex, subunit 2	--	6	ko04666 Fc gamma R-mediated phagocytosis;ko04810 Regulation of actin cytoskeleton;ko05100 Bacterial invasion of epithelial cells;ko05130 Pathogenic Escherichia coli infection;ko05131 Shigellosis;ko05132 Salmonella infection;
gene34872	K05758	ARPC2	actin related protein 2/3 complex, subunit 2	--	6	ko04666 Fc gamma R-mediated phagocytosis;ko04810 Regulation of actin cytoskeleton;ko05100 Bacterial invasion of epithelial cells;ko05130 Pathogenic Escherichia coli infection;ko05131 Shigellosis;ko05132 Salmonella infection;
gene34877	K14485	TIR1	transport inhibitor response 1	--	1	ko04075 Plant hormone signal transduction;
gene34882	K03676	grxC, GLRX, GLRX2	glutaredoxin 3	--		
gene34883	K13148	CPSF3L, INTS11	integrator complex subunit 11	EC:3.1.27.-		
gene34884	K13148	CPSF3L, INTS11	integrator complex subunit 11	EC:3.1.27.-		
gene34889	K12883	NCBP2, CBP20	nuclear cap-binding protein subunit 2	--	3	ko03013 RNA transport;ko03015 mRNA surveillance pathway;ko03040 Spliceosome;
gene3488	K07195	EXOC7, EXO70	exocyst complex component 7	--	1	ko04910 Insulin signaling pathway;
gene34896	K00213	DHCR7	7-dehydrocholesterol reductase	EC:1.3.1.21	1	ko00100 Steroid biosynthesis;

gene348	K00430	E1.11.1.7	peroxidase	EC:1.11.1.7	2	ko00360 Phenylalanine metabolism;ko00940 Phenylpropanoid biosynthesis;
gene34903	K12399	AP3S	AP-3 complex subunit sigma	--	1	ko04142 Lysosome;
gene34904	K14544	UTP22, NOL6	U3 small nucleolar RNA-associated protein 22	--	1	ko03008 Ribosome biogenesis in eukaryotes;
gene34913	K10599	PRPF19, PRP19	pre-mRNA-processing factor 19	EC:6.3.2.19	2	ko03040 Spliceosome;ko04120 Ubiquitin mediated proteolysis;
gene34921	K12598	MTR4, SKIV2L2	ATP-dependent RNA helicase DOB1	EC:3.6.4.13	1	ko03018 RNA degradation;
gene34922	K00472	E1.14.11.2	prolyl 4-hydroxylase	EC:1.14.11.2	1	ko00330 Arginine and proline metabolism;
gene34934	K13424	WRKY33	WRKY transcription factor 33	--	1	ko04626 Plant-pathogen interaction;
gene34939	K03963	NDUFB7	NADH dehydrogenase (ubiquinone) 1 beta subcomplex subunit 7	--	5	ko00190 Oxidative phosphorylation;ko04932 Non-alcoholic fatty liver disease (NAFLD);ko05010 Alzheimer's disease;ko05012 Parkinson's disease;ko05016 Huntington's disease;
gene34941	K08472	MLO	mlo protein	--		
gene34943	K03327	TC.MATE, SLC47A, norM, mdtK, dinF	multidrug resistance protein, MATE family	--		
gene34944	K03327	TC.MATE, SLC47A, norM, mdtK, dinF	multidrug resistance protein, MATE family	--		
gene34946	K14775	UTP30, RSL1D1	ribosome biogenesis protein UTP30	--		
gene34947	K14775	UTP30, RSL1D1	ribosome biogenesis protein UTP30	--		
gene34948	K07999	MMP20	matrix metalloproteinase-20 (enamelysin)	EC:3.4.24.-		
gene34949	K08235	E2.4.1.207	xyloglucan:xyloglucosyl transferase	EC:2.4.1.207		
gene3494	K09060	GBF	plant G-box-binding factor	--		
gene34951	K17087	TM9SF3	transmembrane 9 superfamily member 3	--		
gene34953	K03083	GSK3B	glycogen synthase kinase 3 beta	EC:2.7.11.26	29	ko04012 ErbB signaling pathway;ko04062 Chemokine signaling pathway;ko04110 Cell cycle;ko04151 PI3K-Akt signaling pathway;ko04310 Wnt signaling pathway;ko04340 Hedgehog signaling pathway;ko04360 Axon guidance;ko04390 Hippo signaling pathway;ko04510 Focal adhesion;ko04660 T cell receptor signaling pathway;ko04662 B cell receptor signaling pathway;ko04711 Circadian rhythm - fly;ko04722 Neurotrophin signaling pathway;ko04728 Dopaminergic synapse;ko04910 Insulin signaling pathway;ko04916 Melanogenesis;ko04917 Prolactin signaling pathway;ko04932 Non-alcoholic fatty liver disease (NAFLD);ko05010 Alzheimer's disease;ko05160 Hepatitis C;ko05162 Measles;ko05164 Influenza A;ko05166 HTLV-I infection;ko05169 Epstein-Barr virus infection;ko05200 Pathways in cancer;ko05210 Colorectal cancer;ko05213 Endometrial cancer;ko05215 Prostate
gene34955	K12823	DDX5, DBP2	ATP-dependent RNA helicase DDX5/DBP2	EC:3.6.4.13	3	ko03040 Spliceosome;ko05202 Transcriptional misregulation in cancer;ko05205 Proteoglycans in cancer;
gene34957	K00766	trpD	anthranilate phosphoribosyltransferase	EC:2.4.2.18	2	ko00400 Phenylalanine, tyrosine and tryptophan biosynthesis;ko01230 Biosynthesis of amino acids;
gene34958	K02886	RP-L2, MRPL2, rplB	large subunit ribosomal protein L2	--	1	ko03010 Ribosome;
gene34961	K15505	RAD5	DNA repair protein RAD5	EC:3.6.4.-		
gene34969	K11251	H2A	histone H2A	--	2	ko05034 Alcoholism;ko05322 Systemic lupus erythematosus;
gene34974	K10523	SPOP	speckle-type POZ protein	--		
gene34977	K01845	hemL	glutamate-1-semialdehyde 2,1-aminomutase	EC:5.4.3.8	1	ko00860 Porphyrin and chlorophyll metabolism;
gene34982	K09775	K09775	hypothetical protein	--		
gene34985	K12606	RCD1, CNOT9, CAF40	CCR4-NOT transcription complex subunit 9	--	1	ko03018 RNA degradation;
gene34986	K06997	K06997				
gene34987	K02882	RP-L18Ae, RPL18A	large subunit ribosomal protein L18Ae	--	1	ko03010 Ribosome;
gene34988	K08341	GABARAP, ATG8, LC3	GABA(A) receptor-associated protein (autophagy-related protein 8)	--	2	ko04140 Regulation of autophagy;ko04727 GABAergic synapse;
gene3498	K09286	EREBP	EREBP-like factor	--		
gene34992	K01590	hdc, HDC	histidine decarboxylase	EC:4.1.1.22	1	ko00340 Histidine metabolism;
gene34996	K02938	RP-L8e, RPL8	large subunit ribosomal protein L8e	--	1	ko03010 Ribosome;
gene34997	K11593	ELF2C	eukaryotic translation initiation factor 2C	--		
gene35002	K09422	MYBP	myb proto-oncogene protein, plant	--		

gene35003	K11584	PPP2R5	serine/threonine-protein phosphatase 2A regulatory subunit B'	--	5	ko03015 mRNA surveillance pathway;ko04113 Meiosis - yeast;ko04114 Oocyte meiosis;ko04151 PI3K-Akt signaling pathway;ko04728 Dopaminergic synapse;
gene35006	K10960	chlP, bchP	geranylgeranyl reductase	EC:1.3.1.83	2	ko00860 Porphyrin and chlorophyll metabolism;ko00900 Terpenoid backbone biosynthesis;
gene35008	K09286	EREBP	EREBP-like factor	--		
gene35010	K09489	HSPA4	heat shock 70kDa protein 4	--	1	ko04612 Antigen processing and presentation;
gene35013	K00276	E1.4.3.21, AOC2, AOC3, tynA	primary-amine oxidase	EC:1.4.3.21	6	ko00260 Glycine, serine and threonine metabolism;ko00350 Tyrosine metabolism;ko00360 Phenylalanine metabolism;ko00410 beta-Alanine metabolism;ko00950 Isoquinoline alkaloid biosynthesis;ko00960 Tropane, piperidine and pyridine alkaloid biosynthesis;
gene35014	K00276	E1.4.3.21, AOC2, AOC3, tynA	primary-amine oxidase	EC:1.4.3.21	6	ko00260 Glycine, serine and threonine metabolism;ko00350 Tyrosine metabolism;ko00360 Phenylalanine metabolism;ko00410 beta-Alanine metabolism;ko00950 Isoquinoline alkaloid biosynthesis;ko00960 Tropane, piperidine and pyridine alkaloid biosynthesis;
gene35027	K08956	AFG3	AFG3 family protein	EC:3.4.24.-		
gene35028	K03097	CSNK2A	casein kinase II subunit alpha	EC:2.7.11.1	9	ko03008 Ribosome biogenesis in eukaryotes;ko04064 NF-kappa B signaling pathway;ko04310 Wnt signaling pathway;ko04520 Adherens junction;ko04530 Tight junction;ko04712 Circadian rhythm - plant;ko05162 Measles;ko05168 Herpes simplex infection;ko05169 Epstein-Barr virus infection;
gene35030	K14849	RRP1	ribosomal RNA-processing protein 1	--		
gene35032	K15102	SLC25A3, PHC, PIC	solute carrier family 25 (mitochondrial phosphate transporter), member 3	--		
gene35033	K00966	GMPP	mannose-1-phosphate guanylyltransferase	EC:2.7.7.13	2	ko00051 Fructose and mannose metabolism;ko00520 Amino sugar and nucleotide sugar metabolism;
gene35038	K13130	SIP1, GEMIN2	survival of motor neuron protein-interacting protein 1	--	1	ko03013 RNA transport;
gene35039	K14514	EIN3	ethylene-insensitive protein 3	--	1	ko04075 Plant hormone signal transduction;
gene35047	K02908	RP-L30e, RPL30	large subunit ribosomal protein L30e	--	1	ko03010 Ribosome;
gene35054	K13107	RBMX2, IST3	RNA-binding motif protein, X-linked 2	--		
gene35060	K15015	SLC32A, VGAT	solute carrier family 32 (vesicular inhibitory amino acid transporter)	--	5	ko04721 Synaptic vesicle cycle;ko04723 Retrograde endocannabinoid signaling;ko04727 GABAergic synapse;ko05032 Morphine addiction;ko05033 Nicotine addiction;
gene35063	K11984	SART1, HAF, SNU66	U4/U6.U5 tri-snRNP-associated protein 1	--	1	ko03040 Spliceosome;
gene35064	K02927	RP-L40e, RPL40	large subunit ribosomal protein L40e	--	1	ko03010 Ribosome;
gene35067	K13963	SERPINB	serpin B	--	1	ko05146 Amoebiasis;
gene35068	K09422	MYBP	myb proto-oncogene protein, plant	--		
gene35070	K03145	TFIIS	transcription elongation factor S-II	--		
gene35072	K08150	SLC2A13, ITR	MFS transporter, SP family, solute carrier family 2 (myo-inositol transporter), member 13	--		
gene35073	K02980	RP-S29e, RPS29	small subunit ribosomal protein S29e	--	1	ko03010 Ribosome;
gene35075	K10523	SPOP	speckle-type POZ protein	--		
gene35076	K09422	MYBP	myb proto-oncogene protein, plant	--		
gene35078	K14432	ABF	ABA responsive element binding factor	--	1	ko04075 Plant hormone signal transduction;
gene3507	K09419	HSFF	heat shock transcription factor, other eukaryote	--		
gene35083	K04125	E1.14.11.13	gibberellin 2-oxidase	EC:1.14.11.13	1	ko00904 Diterpenoid biosynthesis;
gene35088	K13993	HSP20	HSP20 family protein	--	1	ko04141 Protein processing in endoplasmic reticulum;
gene35097	K01807	rpiA	ribose 5-phosphate isomerase A	EC:5.3.1.6	4	ko00030 Pentose phosphate pathway;ko00710 Carbon fixation in photosynthetic organisms;ko01200 Carbon metabolism;ko01230 Biosynthesis of amino acids;
gene35110	K15040	VDAC2	voltage-dependent anion channel protein 2	--	4	ko04020 Calcium signaling pathway;ko05012 Parkinson's disease;ko05016 Huntington's disease;ko05166 HTLV-I infection;
gene35112	K13436	PTI1	pto-interacting protein 1	EC:2.7.11.1	1	ko04626 Plant-pathogen interaction;
gene35113	K11094	SNRPB2	U2 small nuclear ribonucleoprotein B''	--	1	ko03040 Spliceosome;
gene35119	K10527	MFP2	enoyl-CoA hydratase/3-hydroxyacyl-CoA dehydrogenase	EC:4.2.1.17 1.1.1.35 1.1.1.211	2	ko00071 Fatty acid degradation;ko00592 alpha-Linolenic acid metabolism;
gene35120	K08657	TASP1	taspase, threonine aspartase, 1	EC:3.4.25.-		

gene35122	K11353	NDUFA13	NADH dehydrogenase (ubiquinone) 1 alpha subcomplex subunit 13	--	5	ko00190 Oxidative phosphorylation;ko04932 Non-alcoholic fatty liver disease (NAFLD);ko05010 Alzheimer's disease;ko05012 Parkinson's disease;ko05016 Huntington's disease;
gene35124	K04077	groEL, HSPD1	chaperonin GroEL	--	4	ko03018 RNA degradation;ko04940 Type I diabetes mellitus;ko05134 Legionellosis;ko05152 Tuberculosis;
gene35126	K01181	E3.2.1.8, xynA	endo-1,4-beta-xylanase	EC:3.2.1.8		
gene35130	K03259	EIF4E	translation initiation factor 4E	--	5	ko03013 RNA transport;ko04066 HIF-1 signaling pathway;ko04150 mTOR signaling pathway;ko04151 PI3K-Akt signaling pathway;ko04910 Insulin signaling pathway;
gene35131	K00472	E1.14.11.2	prolyl 4-hydroxylase	EC:1.14.11.2	1	ko00330 Arginine and proline metabolism;
gene35133	K01051	E3.1.1.11	pectinesterase	EC:3.1.1.11	2	ko00040 Pentose and glucuronate interconversions;ko00500 Starch and sucrose metabolism;
gene35135	K01051	E3.1.1.11	pectinesterase	EC:3.1.1.11	2	ko00040 Pentose and glucuronate interconversions;ko00500 Starch and sucrose metabolism;
gene35138	K01193	E3.2.1.26, sacA	beta-fructofuranosidase	EC:3.2.1.26	2	ko00052 Galactose metabolism;ko00500 Starch and sucrose metabolism;
gene35139	K09287	RAV	RAV-like factor	--		
gene35141	K14638	SLC15A3_4, PHT	solute carrier family 15 (peptide/histidine transporter), member 3/4	--		
gene35142	K01476	E3.5.3.1, rocF, arg	arginase	EC:3.5.3.1	3	ko00330 Arginine and proline metabolism;ko01230 Biosynthesis of amino acids;ko05146 Amoebiasis;
gene35150	K01265	E3.4.11.18, map	methionyl aminopeptidase	EC:3.4.11.18		
gene35151	K01051	E3.1.1.11	pectinesterase	EC:3.1.1.11	2	ko00040 Pentose and glucuronate interconversions;ko00500 Starch and sucrose metabolism;
gene35152	K01051	E3.1.1.11	pectinesterase	EC:3.1.1.11	2	ko00040 Pentose and glucuronate interconversions;ko00500 Starch and sucrose metabolism;
gene35154	K09422	MYBP	myb proto-oncogene protein, plant	--		
gene35155	K04371	MAPK1_3	mitogen-activated protein kinase 1/3	EC:2.7.11.24	71	ko04010 MAPK signaling pathway;ko04012 ErbB signaling pathway;ko04013 MAPK signaling pathway - fly;ko04014 Ras signaling pathway;ko04062 Chemokine signaling pathway;ko04066 HIF-1 signaling pathway;ko04114 Oocyte meiosis;ko04150 mTOR signaling pathway;ko04151 PI3K-Akt signaling pathway;ko04270 Vascular smooth muscle contraction;ko04320 Dorso-ventral axis formation;ko04350 TGF-beta signaling pathway;ko04360 Axon guidance;ko04370 VEGF signaling pathway;ko04380 Osteoclast differentiation;ko04510 Focal adhesion;ko04520 Adherens junction;ko04540 Gap junction;ko04620 Toll-like receptor signaling pathway;ko04621 NOD-like receptor signaling pathway;ko04650 Natural killer cell mediated cytotoxicity;ko04660 T cell receptor signaling pathway;ko04662 B cell receptor signaling pathway;ko04664 Fc epsilon RI signaling pathway;ko04666 Fc gamma R-mediated phagocytosis;ko04668 TNF signaling pathway;ko04713 Circadian entrainment;ko04720 Long-term potentiation;ko04722 Neurotrophin signaling pathway;ko04723 Retrograde endocannabinoid signaling;ko04724 Glutamatergic synapse;ko04725 Cholinergic synapse;ko04726 Serotonergic synapse;ko04730 Long-term depression;ko04810 Regulation of actin cytoskeleton;ko04910 Insulin signaling pathway;ko04912 GnRH signaling pathway;ko04914 Progesterone-mediated oocyte maturation;ko04915 Estrogen signaling pathway;ko04916 Melanogenesis;ko04917 Prolactin signaling pathway;ko04930 Type II diabetes mellitus;ko04960 Aldosterone-regulated sodium reabsorption;ko05010 Alzheimer's disease;ko05020 Prion diseases;ko05034 Alcoholism;ko05131 Shigellosis;ko05132 Salmonella infection;ko05133 Pertussis;ko05140 Leishmaniasis;ko05142 Chagas disease (American trypanosomiasis);ko05145 Toxoplasmosis;ko05152 Tuberculosis;ko05160 Hepatitis C;ko05161 Hepatitis B;ko05164 Influenza A;ko05200 Pathways in cancer;ko05203 Viral carcinogenesis;ko05205 Proteoglycans in cancer;ko05210 Colorectal cancer;ko05211 Renal cell carcinoma;ko05212
gene35163	K03234	EEF2	elongation factor 2	--		

gene35164	K04354	PPP2R2	serine/threonine-protein phosphatase 2A regulatory subunit B	--	9	ko03015 mRNA surveillance pathway;ko04111 Cell cycle - yeast;ko04151 PI3K-Akt signaling pathway;ko04390 Hippo signaling pathway;ko04391 Hippo signaling pathway - fly;ko04530 Tight junction;ko04728 Dopaminergic synapse;ko05142 Chagas disease (American trypanosomiasis);ko05160 Hepatitis C;
gene35165	K13248	PHOSPHO2	pyridoxal phosphate phosphatase PHOSPHO2	EC:3.1.3.74	1	ko00750 Vitamin B6 metabolism;
gene35168	K02155	ATPeVPL, ATP6L	V-type H+-transporting ATPase 16kDa proteolipid subunit	EC:3.6.3.14	9	ko00190 Oxidative phosphorylation;ko04142 Lysosome;ko04145 Phagosome;ko04721 Synaptic vesicle cycle;ko04966 Collecting duct acid secretion;ko05110 Vibrio cholerae infection;ko05120 Epithelial cell signaling in Helicobacter pylori infection;ko05152 Tuberculosis;ko05323 Rheumatoid arthritis;
gene35176	K03063	PSMC4, RPT3	26S proteasome regulatory subunit T3	--	2	ko03050 Proteasome;ko05169 Epstein-Barr virus infection;
gene35181	K01507	ppa	inorganic pyrophosphatase	EC:3.6.1.1	1	ko00190 Oxidative phosphorylation;
gene35183	K12821	PRPF40, PRP40	pre-mRNA-processing factor 40	--	1	ko03040 Spliceosome;
gene35187	K17065	DNM1L	dynamin 1-like protein	EC:3.6.5.5	1	ko04668 TNF signaling pathway;
gene35188	K17065	DNM1L	dynamin 1-like protein	EC:3.6.5.5	1	ko04668 TNF signaling pathway;
gene3518	K11982	RNF115_126	E3 ubiquitin-protein ligase RNF115/126	EC:6.3.2.19		
gene35197	K01867	WARS, trpS	tryptophanyl-tRNA synthetase	EC:6.1.1.2	1	ko00970 Aminoacyl-tRNA biosynthesis;
gene35198	K11863	ATXN3, MJD	Ataxin-3	EC:3.4.22.-	1	ko04141 Protein processing in endoplasmic reticulum;
gene35201	K12153	CYP79A2	cytochrome P450, family 79, subfamily A, polypeptide 2 (phenylalanine N-monooxygenase)	EC:1.14.13.124	3	ko00460 Cyanoamino acid metabolism;ko00966 Glucosinolate biosynthesis;ko01210 2-Oxocarboxylic acid metabolism;
gene35202	K00011	E1.1.1.21, AKR1	aldehyde reductase	EC:1.1.1.21	5	ko00040 Pentose and glucuronate interconversions;ko00051 Fructose and mannose metabolism;ko00052 Galactose metabolism;ko00561 Glycerolipid metabolism;ko00620 Pyruvate metabolism;
gene3520	K05909	E1.10.3.2				
gene35215	K05284	PIGM	phosphatidylinositol glycan, class M	EC:2.4.1.-	1	ko00563 Glycosylphosphatidylinositol(GPI)-anchor biosynthesis;
gene35217	K11290	SET, TAF1, I2PP2A	template-activating factor I	--		
gene3521	K15015	SLC32A, VGAT	solute carrier family 32 (vesicular inhibitory amino acid transporter)	--	5	ko04721 Synaptic vesicle cycle;ko04723 Retrograde endocannabinoid signaling;ko04727 GABAergic synapse;ko05032 Morphine addiction;ko05033 Nicotine addiction;
gene35220	K03798	ftsH, hflB	cell division protease FtsH	EC:3.4.24.-		
gene35221	K13496	UGT73C	UDP-glucosyl transferase 73C	EC:2.4.1.-		
gene35222	K13496	UGT73C	UDP-glucosyl transferase 73C	EC:2.4.1.-		
gene35226	K08678	UXS1	UDP-glucuronate decarboxylase	EC:4.1.1.35	2	ko00500 Starch and sucrose metabolism;ko00520 Amino sugar and nucleotide sugar metabolism;
gene3522	K03544	clpX, CLPX	ATP-dependent Clp protease ATP-binding subunit ClpX	--	1	ko04112 Cell cycle - Caulobacter;
gene35232	K04733	IRAK4	interleukin-1 receptor-associated kinase 4	EC:2.7.11.1	11	ko04064 NF-kappa B signaling pathway;ko04210 Apoptosis;ko04620 Toll-like receptor signaling pathway;ko04722 Neurotrophin signaling pathway;ko05133 Pertussis;ko05140 Leishmaniasis;ko05142 Chagas disease (American trypanosomiasis);ko05145 Toxoplasmosis;ko05152 Tuberculosis;ko05162 Measles;ko05164 Influenza A;
gene35235	K01887	RARS, argS	arginyl-tRNA synthetase	EC:6.1.1.19	1	ko00970 Aminoacyl-tRNA biosynthesis;
gene35238	K12893	SFRS4_5_6	splicing factor, arginine/serine-rich 4/5/6	--	2	ko03040 Spliceosome;ko05168 Herpes simplex infection;
gene35244	K05353	PMT	putrescine N-methyltransferase	EC:2.1.1.53	1	ko00960 Tropane, piperidine and pyridine alkaloid biosynthesis;
gene35249	K03242	EIF2S3	translation initiation factor 2 subunit 3	--	1	ko03013 RNA transport;
gene35253	K00652	bioF	8-amino-7-oxononanoate synthase	EC:2.3.1.47	1	ko00780 Biotin metabolism;
gene35256	K11971	RNF14, ARA54	E3 ubiquitin-protein ligase RNF14	EC:6.3.2.19		
gene3525	K02327	POLD1	DNA polymerase delta subunit 1	EC:2.7.7.7	8	ko00230 Purine metabolism;ko00240 Pyrimidine metabolism;ko03030 DNA replication;ko03410 Base excision repair;ko03420 Nucleotide excision repair;ko03430 Mismatch repair;ko03440 Homologous recombination;ko05166 HTLV-I infection;
gene35268	K03626	EGD2, NACA	nascent polypeptide-associated complex subunit alpha	--		
gene35269	K00430	E1.11.1.7	peroxidase	EC:1.11.1.7	2	ko00360 Phenylalanine metabolism;ko00940 Phenylpropanoid biosynthesis;
gene35274	K02873	RP-L13e, RPL13	large subunit ribosomal protein L13e	--	1	ko03010 Ribosome;
gene35285	K01890	FARSB, pheT	phenylalanyl-tRNA synthetase beta chain	EC:6.1.1.20	1	ko00970 Aminoacyl-tRNA biosynthesis;

gene35286	K14235	tRNA-Trp	tRNA Trp	--	1	ko00970 Aminoacyl-tRNA biosynthesis;
gene35287	K14498	SNRK2	serine/threonine-protein kinase SRK2	EC:2.7.11.1	1	ko04075 Plant hormone signal transduction;
gene35295	K11593	ELF2C	eukaryotic translation initiation factor 2C	--		
gene35299	K00797	speE, SRM	spermidine synthase	EC:2.5.1.16	4	ko00270 Cysteine and methionine metabolism;ko00330 Arginine and proline metabolism;ko00410 beta-Alanine metabolism;ko00480 Glutathione metabolism;
gene35300	K14497	PP2C	protein phosphatase 2C	EC:3.1.3.16	1	ko04075 Plant hormone signal transduction;
gene35302	K04730	IRAK1	interleukin-1 receptor-associated kinase 1	EC:2.7.11.1	11	ko04064 NF-kappa B signaling pathway;ko04210 Apoptosis;ko04620 Toll-like receptor signaling pathway;ko04722 Neurotrophin signaling pathway;ko05133 Pertussis;ko05140 Leishmaniasis;ko05142 Chagas disease (American trypanosomiasis);ko05145 Toxoplasmosis;ko05152 Tuberculosis;ko05162 Measles;ko05169 Epstein-Barr virus infection;
gene35303	K04730	IRAK1	interleukin-1 receptor-associated kinase 1	EC:2.7.11.1	11	ko04064 NF-kappa B signaling pathway;ko04210 Apoptosis;ko04620 Toll-like receptor signaling pathway;ko04722 Neurotrophin signaling pathway;ko05133 Pertussis;ko05140 Leishmaniasis;ko05142 Chagas disease (American trypanosomiasis);ko05145 Toxoplasmosis;ko05152 Tuberculosis;ko05162 Measles;ko05169 Epstein-Barr virus infection;
gene35304	K04730	IRAK1	interleukin-1 receptor-associated kinase 1	EC:2.7.11.1	11	ko04064 NF-kappa B signaling pathway;ko04210 Apoptosis;ko04620 Toll-like receptor signaling pathway;ko04722 Neurotrophin signaling pathway;ko05133 Pertussis;ko05140 Leishmaniasis;ko05142 Chagas disease (American trypanosomiasis);ko05145 Toxoplasmosis;ko05152 Tuberculosis;ko05162 Measles;ko05169 Epstein-Barr virus infection;
gene35306	K04733	IRAK4	interleukin-1 receptor-associated kinase 4	EC:2.7.11.1	11	ko04064 NF-kappa B signaling pathway;ko04210 Apoptosis;ko04620 Toll-like receptor signaling pathway;ko04722 Neurotrophin signaling pathway;ko05133 Pertussis;ko05140 Leishmaniasis;ko05142 Chagas disease (American trypanosomiasis);ko05145 Toxoplasmosis;ko05152 Tuberculosis;ko05162 Measles;ko05164 Influenza A;
gene35307	K04730	IRAK1	interleukin-1 receptor-associated kinase 1	EC:2.7.11.1	11	ko04064 NF-kappa B signaling pathway;ko04210 Apoptosis;ko04620 Toll-like receptor signaling pathway;ko04722 Neurotrophin signaling pathway;ko05133 Pertussis;ko05140 Leishmaniasis;ko05142 Chagas disease (American trypanosomiasis);ko05145 Toxoplasmosis;ko05152 Tuberculosis;ko05162 Measles;ko05169 Epstein-Barr virus infection;
gene35310	K04733	IRAK4	interleukin-1 receptor-associated kinase 4	EC:2.7.11.1	11	ko04064 NF-kappa B signaling pathway;ko04210 Apoptosis;ko04620 Toll-like receptor signaling pathway;ko04722 Neurotrophin signaling pathway;ko05133 Pertussis;ko05140 Leishmaniasis;ko05142 Chagas disease (American trypanosomiasis);ko05145 Toxoplasmosis;ko05152 Tuberculosis;ko05162 Measles;ko05164 Influenza A;
gene35315	K07199	PRKAB	5'-AMP-activated protein kinase, regulatory beta subunit	--	5	ko04710 Circadian rhythm;ko04910 Insulin signaling pathway;ko04920 Adipocytokine signaling pathway;ko04932 Non-alcoholic fatty liver disease (NAFLD);ko05410 Hypertrophic cardiomyopathy (HCM);
gene35316	K15131	MED11	mediator of RNA polymerase II transcription subunit 11	--		
gene35317	K00736	MGAT2	alpha-1,6-mannosyl-glycoprotein beta-1,2-N-acetylglucosaminyltransferase	EC:2.4.1.143	2	ko00510 N-Glycan biosynthesis;ko00513 Various types of N-glycan biosynthesis;
gene35318	K14800	TSR2	pre-rRNA-processing protein TSR2	--		
gene35320	K07937	ARF1	ADP-ribosylation factor 1	--	2	ko05110 Vibrio cholerae infection;ko05134 Legionellosis;
gene35325	K06269	PPP1C	serine/threonine-protein phosphatase PP1 catalytic subunit	EC:3.1.3.16	14	ko03015 mRNA surveillance pathway;ko04113 Meiosis - yeast;ko04114 Oocyte meiosis;ko04270 Vascular smooth muscle contraction;ko04390 Hippo signaling pathway;ko04510 Focal adhesion;ko04720 Long-term potentiation;ko04728 Dopaminergic synapse;ko04810 Regulation of actin cytoskeleton;ko04910 Insulin signaling pathway;ko05031 Amphetamine addiction;ko05034 Alcoholism;ko05168 Herpes simplex infection;ko05205 Proteoglycans in
gene35326	K14440	SMARCAL1, HARP	SWI/SNF-related matrix-associated actin-dependent regulator of chromatin subfamily A-like protein 1	EC:3.6.4.12		
gene35328	K05658	ABCB1	ATP-binding cassette, subfamily B (MDR/TAP), member 1	--	3	ko02010 ABC transporters;ko04976 Bile secretion;ko05206 MicroRNAs in cancer;

gene35329	K01188	E3.2.1.21	beta-glucosidase	EC:3.2.1.21	3	ko00460 Cyanoamino acid metabolism;ko00500 Starch and sucrose metabolism;ko00940 Phenylpropanoid biosynthesis;
gene3532	K15033	ICT1	peptidyl-tRNA hydrolase ICT1	EC:3.1.1.29		
gene35330	K02988	RP-S5, MRPS5, rpsE	small subunit ribosomal protein S5	--	1	ko03010 Ribosome;
gene35331	K14007	SEC24	protein transport protein SEC24	--	1	ko04141 Protein processing in endoplasmic reticulum;
gene35334	K10636	AMFR, GP78	autocrine motility factor receptor	EC:6.3.2.19	1	ko04141 Protein processing in endoplasmic reticulum;
gene35336	K03029	PSMD4, RPN10	26S proteasome regulatory subunit N10	--	2	ko03050 Proteasome;ko05169 Epstein-Barr virus infection;
gene35340	K04710	CERS	ceramide synthetase	EC:2.3.1.24	1	ko00600 Sphingolipid metabolism;
gene35341	K08856	STK16	serine/threonine kinase 16	EC:2.7.11.1		
gene35343	K01653	E2.2.1.6S, ilvH, ilvN	acetolactate synthase I/III small subunit	EC:2.2.1.6	6	ko00290 Valine, leucine and isoleucine biosynthesis;ko00650 Butanoate metabolism;ko00660 C5-Branched dibasic acid metabolism;ko00770 Pantothenate and CoA biosynthesis;ko01210 2-Oxocarboxylic acid metabolism;ko01230 Biosynthesis of amino acids;
gene35347	K10807	RRM1	ribonucleoside-diphosphate reductase subunit M1	EC:1.17.4.1	3	ko00230 Purine metabolism;ko00240 Pyrimidine metabolism;ko00480 Glutathione metabolism;
gene35349	K13342	PEX5, PXR1	peroxin-5	--	1	ko04146 Peroxisome;
gene3534	K00413	CYC1, CYT1, petC	ubiquinol-cytochrome c reductase cytochrome c1 subunit	--	7	ko00190 Oxidative phosphorylation;ko02020 Two-component system;ko04260 Cardiac muscle contraction;ko04932 Non-alcoholic fatty liver disease (NAFLD);ko05010 Alzheimer's disease;ko05012 Parkinson's disease;ko05016 Huntington's disease;
gene35350	K12179	COPS6, CSN6	COP9 signalosome complex subunit 6	--		
gene35351	K02553	rraA, menG	regulator of ribonuclease activity A	--		
gene35362	K05391	CNGF	cyclic nucleotide gated channel, other eukaryote	--	1	ko04626 Plant-pathogen interaction;
gene35363	K09338	HD-ZIP	homeobox-leucine zipper protein	--		
gene35364	K00654	E2.3.1.50	serine palmitoyltransferase	EC:2.3.1.50	1	ko00600 Sphingolipid metabolism;
gene35368	K00161	PDHA, pdhA	pyruvate dehydrogenase E1 component subunit alpha	EC:1.2.4.1	6	ko00010 Glycolysis / Gluconeogenesis;ko00020 Citrate cycle (TCA cycle);ko00620 Pyruvate metabolism;ko00650 Butanoate metabolism;ko01200 Carbon metabolism;ko04066 HIF-1 signaling pathway;
gene3536	K12177	COPS3, CSN3	COP9 signalosome complex subunit 3	--		
gene35370	K05853	ATP2A	Ca2+ transporting ATPase, sarcoplasmic/endoplasmic reticulum	EC:3.6.3.8	3	ko04020 Calcium signaling pathway;ko04972 Pancreatic secretion;ko05010 Alzheimer's disease;
gene35373	K01358	clpP, CLPP	ATP-dependent Clp protease, protease subunit	EC:3.4.21.92	1	ko04112 Cell cycle - Caulobacter;
gene35376	K02698	psaK	photosystem I subunit X	--	1	ko00195 Photosynthesis;
gene3537	K06670	SCC1, MCD1, RAD21	cohesin complex subunit SCC1	--	2	ko04110 Cell cycle;ko04111 Cell cycle - yeast;
gene35381	K10908	POLRMT, RPO41	DNA-directed RNA polymerase, mitochondrial	EC:2.7.7.6		
gene35385	K09872	PIP	aquaporin PIP	--		
gene35386	K03327	TC.MATE, SLC47A, norM, mdtK, dinF	multidrug resistance protein, MATE family	--		
gene35387	K14486	K14486, ARF	auxin response factor	--	1	ko04075 Plant hormone signal transduction;
gene35389	K07955	ARL8	ADP-ribosylation factor-like protein 8	--		
gene35394	K09873	TIP	aquaporin TIP	--		
gene35396	K01433	purU	formyltetrahydrofolate deformylase	EC:3.5.1.10	2	ko00630 Glyoxylate and dicarboxylate metabolism;ko00670 One carbon pool by folate;
gene35398	K02923	RP-L38e, RPL38	large subunit ribosomal protein L38e	--	1	ko03010 Ribosome;
gene35400	K01885	EARS, gltX	glutamyl-tRNA synthetase	EC:6.1.1.17	2	ko00860 Porphyrin and chlorophyll metabolism;ko00970 Aminoacyl-tRNA biosynthesis;
gene35404	K03008	RPB11, POLR2J	DNA-directed RNA polymerase II subunit RPB11	--	5	ko00230 Purine metabolism;ko00240 Pyrimidine metabolism;ko03020 RNA polymerase;ko05016 Huntington's disease;ko05169 Epstein-Barr virus infection;
gene3540	K10144	RCHY1, PIRH2	RING finger and CHY zinc finger domain-containing protein 1	EC:6.3.2.19	3	ko04115 p53 signaling pathway;ko04120 Ubiquitin mediated proteolysis;ko05162 Measles;
gene35410	K06689	UBE2D_E, UBC4, UBC5	ubiquitin-conjugating enzyme E2 D/E	EC:6.3.2.19	2	ko04120 Ubiquitin mediated proteolysis;ko04141 Protein processing in endoplasmic reticulum;
gene35411	K03063	PSMC4, RPT3	26S proteasome regulatory subunit T3	--	2	ko03050 Proteasome;ko05169 Epstein-Barr virus infection;
gene35414	K17279	REEP5_6	receptor expression-enhancing protein 5/6	--		
gene35417	K10781	FATB	fatty acyl-ACP thioesterase B	EC:3.1.2.14	1	ko00061 Fatty acid biosynthesis;
gene35419	K05387	GRIP	glutamate receptor, ionotropic, plant	--		

gene35421	K05387	GRIP	glutamate receptor, ionotropic, plant	--		
gene35425	K09286	EREBP	EREBP-like factor	--		
gene35426	K05658	ABCB1	ATP-binding cassette, subfamily B (MDR/TAP), member 1	--	3	ko02010 ABC transporters;ko04976 Bile secretion;ko05206 MicroRNAs in cancer;
gene3542	K14485	TIR1	transport inhibitor response 1	--	1	ko04075 Plant hormone signal transduction;
gene35431	K14794	RRP12	ribosomal RNA-processing protein 12	--		
gene35436	K09060	GBF	plant G-box-binding factor	--		
gene35438	K09338	HD-ZIP	homeobox-leucine zipper protein	--		
gene35439	K17278	PGRMC1_2	membrane-associated progesterone receptor component	--		
gene35443	K00889	E2.7.1.68, PIP5K	1-phosphatidylinositol-4-phosphate 5-kinase	EC:2.7.1.68	5	ko00562 Inositol phosphate metabolism;ko04070 Phosphatidylinositol signaling system;ko04144 Endocytosis;ko04666 Fc gamma R-mediated phagocytosis;ko04810 Regulation of actin cytoskeleton;
gene35444	K08336	ATG12	autophagy-related protein 12	--	2	ko04140 Regulation of autophagy;ko04622 RIG-I-like receptor signaling pathway;
gene35448	K09523	DNAJC3	DnaJ homolog subfamily C member 3	--	2	ko04141 Protein processing in endoplasmic reticulum;ko05164 Influenza A;
gene35449	K09645	CPVL	vitellogenic carboxypeptidase-like protein	EC:3.4.16.-		
gene35453	K14219	tRNA-Arg	tRNA Arg	--	1	ko00970 Aminoacyl-tRNA biosynthesis;
gene35459	K00799	GST, gst	glutathione S-transferase	EC:2.5.1.18	4	ko00480 Glutathione metabolism;ko00980 Metabolism of xenobiotics by cytochrome P450;ko00982 Drug metabolism - cytochrome P450;ko05204 Chemical carcinogenesis;
gene35462	K03857	PIGA, GPI3	phosphatidylinositol glycan, class A	EC:2.4.1.198	1	ko00563 Glycosylphosphatidylinositol(GPI)-anchor biosynthesis;
gene35465	K13508	GPAT	glycerol-3-phosphate acyltransferase	EC:2.3.1.15	2	ko00561 Glycerolipid metabolism;ko00564 Glycerophospholipid metabolism;
gene35473	K00799	GST, gst	glutathione S-transferase	EC:2.5.1.18	4	ko00480 Glutathione metabolism;ko00980 Metabolism of xenobiotics by cytochrome P450;ko00982 Drug metabolism - cytochrome P450;ko05204 Chemical carcinogenesis;
gene35474	K01102	PDP	pyruvate dehydrogenase phosphatase	EC:3.1.3.43		
gene35479	K01181	E3.2.1.8, xynA	endo-1,4-beta-xylanase	EC:3.2.1.8		
gene3547	K03014	RPB6, POLR2F	DNA-directed RNA polymerases I, II, and III subunit RPABC2	--	6	ko00230 Purine metabolism;ko00240 Pyrimidine metabolism;ko03020 RNA polymerase;ko04623 Cytosolic DNA-sensing pathway;ko05016 Huntington's disease;ko05169 Epstein-Barr virus infection;
gene35481	K00771	XYLT	protein xylosyltransferase	EC:2.4.2.26	2	ko00532 Glycosaminoglycan biosynthesis - chondroitin sulfate / dermatan sulfate;ko00534 Glycosaminoglycan biosynthesis - heparan sulfate / heparin;
gene35483	K13789	GGPS	geranylgeranyl diphosphate synthase, type II	EC:2.5.1.1 2.5.1.10 2.5.1.29	1	ko00900 Terpenoid backbone biosynthesis;
gene35489	K04392	RAC1	Ras-related C3 botulinum toxin substrate 1	--	33	ko04010 MAPK signaling pathway;ko04014 Ras signaling pathway;ko04062 Chemokine signaling pathway;ko04145 Phagosome;ko04151 PI3K-Akt signaling pathway;ko04310 Wnt signaling pathway;ko04360 Axon guidance;ko04370 VEGF signaling pathway;ko04380 Osteoclast differentiation;ko04510 Focal adhesion;ko04520 Adherens junction;ko04620 Toll-like receptor signaling pathway;ko04650 Natural killer cell mediated cytotoxicity;ko04662 B cell receptor signaling pathway;ko04664 Fc epsilon RI signaling pathway;ko04666 Fc gamma R-mediated phagocytosis;ko04670 Leukocyte transendothelial migration;ko04722 Neurotrophin signaling pathway;ko04810 Regulation of actin cytoskeleton;ko04932 Non-alcoholic fatty liver disease (NAFLD);ko04972 Pancreatic secretion;ko05014 Amyotrophic lateral sclerosis (ALS);ko05100 Bacterial invasion of epithelial cells;ko05120 Epithelial cell signaling in Helicobacter pylori infection;ko05131 Shigellosis;ko05132 Salmonella infection;ko05200 Pathways in cancer;ko05203 Viral carcinogenesis;ko05205 Proteoglycans in cancer;ko05210 Colorectal cancer;ko05211 Renal cell carcinoma;ko05212 Pancreatic
gene3548	K13025	EIF4A3, FAL1	ATP-dependent RNA helicase	EC:3.6.4.13	3	ko03013 RNA transport;ko03015 mRNA surveillance pathway;ko03040 Spliceosome;
gene35492	K14232	tRNA-Pro	tRNA Pro	--	1	ko00970 Aminoacyl-tRNA biosynthesis;
gene35494	K03115	CSNK2B	casein kinase II subunit beta	--	9	ko03008 Ribosome biogenesis in eukaryotes;ko04064 NF-kappa B signaling pathway;ko04310 Wnt signaling pathway;ko04520 Adherens junction;ko04530 Tight junction;ko04712 Circadian rhythm - plant;ko05162 Measles;ko05168 Herpes simplex infection;ko05169 Epstein-Barr virus infection;

gene35496	K09537	DNAJC17	DnaJ homolog subfamily C member 17	--		
gene35497	K15196	BRF1, GTF3B	transcription factor IIIB 90 kDa subunit	--		
gene35498	K02921	RP-L37Ae, RPL37A	large subunit ribosomal protein L37Ae	--	1	ko03010 Ribosome;
gene3549	K16313	NRK	Nik-related protein kinase	EC:2.7.11.1		
gene354	K14491	ARR-B	two-component response regulator ARR-B family	--	1	ko04075 Plant hormone signal transduction;
gene35500	K00844	HK	hexokinase	EC:2.7.1.1	12	ko00010 Glycolysis / Gluconeogenesis;ko00051 Fructose and mannose metabolism;ko00052 Galactose metabolism;ko00500 Starch and sucrose metabolism;ko00520 Amino sugar and nucleotide sugar metabolism;ko00521 Streptomycin biosynthesis;ko00524 Butirosin and neomycin biosynthesis;ko01200 Carbon metabolism;ko04066 HIF-1 signaling pathway;ko04910 Insulin signaling pathway;ko04930 Type II diabetes mellitus;ko04973 Carbohydrate digestion and absorption;
gene35503	K01885	EARS, gltX	glutamyl-tRNA synthetase	EC:6.1.1.17	2	ko00860 Porphyrin and chlorophyll metabolism;ko00970 Aminoacyl-tRNA biosynthesis;
gene35505	K16900	TPC1, CCH1	two pore calcium channel protein, plant	--		
gene35508	K11360	SPT8	transcriptional activator SPT8	--		
gene35510	K00784	rnz	ribonuclease Z	EC:3.1.26.11	1	ko03013 RNA transport;
gene35514	K01102	PDP	pyruvate dehydrogenase phosphatase	EC:3.1.3.43		
gene35519	K03327	TC.MATE, SLC47A, norM, mdtK, dinF	multidrug resistance protein, MATE family	--		
gene35524	K05282	E1.14.11.12	gibberellin 20-oxidase	EC:1.14.11.12	1	ko00904 Diterpenoid biosynthesis;
gene35528	K16275	BAH, NLA	E3 ubiquitin-protein ligase BAH	EC:6.3.2.19		
gene35532	K16584	HAUS1	HAUS augmin-like complex subunit 1	--		
gene35533	K15148	MED7	mediator of RNA polymerase II transcription subunit 7	--		
gene35535	K05752	C3ORF10, HSPC300	chromosome 3 open reading frame 10	--	1	ko04810 Regulation of actin cytoskeleton;
gene35537	K00059	fabG	3-oxoacyl-[acyl-carrier protein] reductase	EC:1.1.1.100	3	ko00061 Fatty acid biosynthesis;ko00780 Biotin metabolism;ko01040 Biosynthesis of unsaturated fatty acids;
gene35538	K09422	MYBP	myb proto-oncogene protein, plant	--		
gene35543	K00921	PIKFYVE, FAB1	1-phosphatidylinositol-3-phosphate 5-kinase	EC:2.7.1.150	4	ko00562 Inositol phosphate metabolism;ko04070 Phosphatidylinositol signaling system;ko04145 Phagosome;ko04810 Regulation of actin cytoskeleton;
gene35558	K03265	ETF1, ERF1	peptide chain release factor subunit 1	--	1	ko03015 mRNA surveillance pathway;
gene35559	K01373	CTSF	cathepsin F	EC:3.4.22.41	1	ko04142 Lysosome;
gene35564	K03347	CUL1, CDC53	cullin 1	--	9	ko04110 Cell cycle;ko04111 Cell cycle - yeast;ko04114 Oocyte meiosis;ko04120 Ubiquitin mediated proteolysis;ko04141 Protein processing in endoplasmic reticulum;ko04310 Wnt signaling pathway;ko04350 TGF-beta signaling pathway;ko04710 Circadian rhythm;ko05168 Herpes simplex infection;
gene35566	K00588	E2.1.1.104	caffeoyl-CoA O-methyltransferase	EC:2.1.1.104	4	ko00360 Phenylalanine metabolism;ko00940 Phenylpropanoid biosynthesis;ko00941 Flavonoid biosynthesis;ko00945 Stilbenoid, diarylheptanoid and gingerol biosynthesis;
gene35568	K05747	WAS	Wiskott-Aldrich syndrome protein	--	8	ko04062 Chemokine signaling pathway;ko04520 Adherens junction;ko04666 Fc gamma R-mediated phagocytosis;ko04810 Regulation of actin cytoskeleton;ko05100 Bacterial invasion of epithelial cells;ko05130 Pathogenic Escherichia coli infection;ko05131 Shigellosis;ko05132 Salmonella infection;
gene35569	K17279	REEP5_6	receptor expression-enhancing protein 5/6	--		
gene35570	K09287	RAV	RAV-like factor	--		
gene35574	K05666	ABCC2	ATP-binding cassette, subfamily C (CFTR/MRP), member 2	--	2	ko02010 ABC transporters;ko04976 Bile secretion;
gene35578	K00430	E1.11.1.7	peroxidase	EC:1.11.1.7	2	ko00360 Phenylalanine metabolism;ko00940 Phenylpropanoid biosynthesis;
gene35579	K00430	E1.11.1.7	peroxidase	EC:1.11.1.7	2	ko00360 Phenylalanine metabolism;ko00940 Phenylpropanoid biosynthesis;
gene3557	K03644	lipA	lipoic acid synthetase	EC:2.8.1.8	1	ko00785 Lipoic acid metabolism;
gene35586	K14315	NDC1, TMEM48	nucleoporin NDC1	--	1	ko03013 RNA transport;
gene35587	K11752	ribD	diaminohydroxyphosphoribosylaminopyrimidine deaminase / 5-amino-6-(5-phosphoribosylamino)uracil reductase	EC:3.5.4.26 1.1.1.193	1	ko00740 Riboflavin metabolism;

gene35588	K01114	plcC	phospholipase C	EC:3.1.4.3	3	ko00562 Inositol phosphate metabolism;ko00564 Glycerophospholipid metabolism;ko00565 Ether lipid metabolism;
gene35591	K11253	H3	histone H3	--	3	ko05034 Alcoholism;ko05202 Transcriptional misregulation in cancer;ko05322 Systemic lupus erythematosus;
gene35595	K09286	EREBP	EREBP-like factor	--		
gene355	K15292	STXBP1, MUNC18-1	syntaxin-binding protein 1	--	1	ko04721 Synaptic vesicle cycle;
gene35607	K13356	FAR	fatty acyl-CoA reductase	EC:1.2.1.-	2	ko00073 Cutin, suberine and wax biosynthesis;ko04146 Peroxisome;
gene3560	K15472	CYP71D55	premnaspirodiene oxygenase	EC:1.14.13.12 1	1	ko00909 Sesquiterpenoid and triterpenoid biosynthesis;
gene35611	K04079	htpG, HSP90A	molecular chaperone HtpG	--	9	ko04141 Protein processing in endoplasmic reticulum;ko04151 PI3K-Akt signaling pathway;ko04612 Antigen processing and presentation;ko04621 NOD-like receptor signaling pathway;ko04626 Plant-pathogen interaction;ko04914 Progesterone-mediated oocyte maturation;ko04915 Estrogen signaling pathway;ko05200 Pathways in cancer;ko05215 Prostate cancer;
gene35612	K06268	PPP3R, CNB	serine/threonine-protein phosphatase 2B regulatory subunit	--	18	ko04010 MAPK signaling pathway;ko04020 Calcium signaling pathway;ko04114 Oocyte meiosis;ko04210 Apoptosis;ko04310 Wnt signaling pathway;ko04360 Axon guidance;ko04370 VEGF signaling pathway;ko04380 Osteoclast differentiation;ko04650 Natural killer cell mediated cytotoxicity;ko04660 T cell receptor signaling pathway;ko04662 B cell receptor signaling pathway;ko04720 Long-term potentiation;ko04724 Glutamatergic synapse;ko05010 Alzheimer's disease;ko05014 Amyotrophic lateral sclerosis (ALS);ko05031 Amphetamine addiction;ko05152
gene35617	K07195	EXOC7, EXO70	exocyst complex component 7	--	1	ko04910 Insulin signaling pathway;
gene3561	K14182	E4.2.3.21	vetispiradiene synthase	EC:4.2.3.21	1	ko00909 Sesquiterpenoid and triterpenoid biosynthesis;
gene35622	K02694	psaF	photosystem I subunit III	--	1	ko00195 Photosynthesis;
gene35624	K01530	E3.6.3.1	phospholipid-translocating ATPase	EC:3.6.3.1		
gene3562	K11498	CENPE	centromeric protein E	--		
gene35630	K08507	USE1	unconventional SNARE in the endoplasmic reticulum protein 1	--	1	ko04130 SNARE interactions in vesicular transport;
gene35631	K07889	RAB5C	Ras-related protein Rab-5C	--	6	ko04014 Ras signaling pathway;ko04144 Endocytosis;ko04145 Phagosome;ko04962 Vasopressin-regulated water reabsorption;ko05146 Amoebiasis;ko05152 Tuberculosis;
gene35633	K04733	IRAK4	interleukin-1 receptor-associated kinase 4	EC:2.7.11.1	11	ko04064 NF-kappa B signaling pathway;ko04210 Apoptosis;ko04620 Toll-like receptor signaling pathway;ko04722 Neurotrophin signaling pathway;ko05133 Pertussis;ko05140 Leishmaniasis;ko05142 Chagas disease (American trypanosomiasis);ko05145 Toxoplasmosis;ko05152 Tuberculosis;ko05162 Measles;ko05164 Influenza A;
gene35635	K16055	TPS	trehalose 6-phosphate synthase/phosphatase	EC:2.4.1.15 3.1.3.12	1	ko00500 Starch and sucrose metabolism;
gene35637	K03259	EIF4E	translation initiation factor 4E	--	5	ko03013 RNA transport;ko04066 HIF-1 signaling pathway;ko04150 mTOR signaling pathway;ko04151 PI3K-Akt signaling pathway;ko04910 Insulin signaling pathway;
gene35641	K05928	E2.1.1.95	tocopherol O-methyltransferase	EC:2.1.1.95	1	ko00130 Ubiquinone and other terpenoid-quinone biosynthesis;
gene35642	K02882	RP-L18Ae, RPL18A	large subunit ribosomal protein L18Ae	--	1	ko03010 Ribosome;
gene35646	K10579	UBE2M, UBC12	ubiquitin-conjugating enzyme E2 M	EC:6.3.2.19	1	ko04120 Ubiquitin mediated proteolysis;
gene35651	K14315	NDC1, TMEM48	nucleoporin NDC1	--	1	ko03013 RNA transport;
gene35652	K15360	STRA13, CENPX, MHF2	centromere protein X	--	1	ko03460 Fanconi anemia pathway;
gene35659	K08857	NEK	NIMA (never in mitosis gene a)-related kinase	EC:2.7.11.1		
gene35665	K00451	HGD, hmgA	homogentisate 1,2-dioxygenase	EC:1.13.11.5	2	ko00350 Tyrosine metabolism;ko00643 Styrene degradation;
gene35668	K07904	RAB11A	Ras-related protein Rab-11A	--	4	ko04144 Endocytosis;ko04961 Endocrine and other factor-regulated calcium reabsorption;ko04962 Vasopressin-regulated water reabsorption;ko04972 Pancreatic secretion;
gene35671	K07937	ARF1	ADP-ribosylation factor 1	--	2	ko05110 Vibrio cholerae infection;ko05134 Legionellosis;
gene35672	K08150	SLC2A13, ITR	MFS transporter, SP family, solute carrier family 2 (myo-inositol transporter), member 13	--		
gene35676	K01183	E3.2.1.14	chitinase	EC:3.2.1.14	1	ko00520 Amino sugar and nucleotide sugar metabolism;
gene3567	K02919	RP-L36, MRPL36, rpmJ	large subunit ribosomal protein L36	--	1	ko03010 Ribosome;

gene35680	K01953	asnB, ASNS	asparagine synthase (glutamine-hydrolysing)	EC:6.3.5.4	1	ko00250 Alanine, aspartate and glutamate metabolism;
gene35681	K01953	asnB, ASNS	asparagine synthase (glutamine-hydrolysing)	EC:6.3.5.4	1	ko00250 Alanine, aspartate and glutamate metabolism;
gene35683	K01230	MAN1	mannosyl-oligosaccharide alpha-1,2-mannosidase	EC:3.2.1.113	3	ko00510 N-Glycan biosynthesis;ko00513 Various types of N-glycan biosynthesis;ko04141 Protein processing in endoplasmic reticulum;
gene35684	K14696	SLC30A9, ZNT9	solute carrier family 30 (zinc transporter), member 9	--		
gene35685	K00430	E1.11.1.7	peroxidase	EC:1.11.1.7	2	ko00360 Phenylalanine metabolism;ko00940 Phenylpropanoid biosynthesis;
gene35686	K15455	KTI11, DPH3	diphthamide biosynthesis protein 3	--		
gene35687	K11252	H2B	histone H2B	--	3	ko05034 Alcoholism;ko05203 Viral carcinogenesis;ko05322 Systemic lupus erythematosus;
gene3568	K14515	EBF1_2	EIN3-binding F-box protein	--	1	ko04075 Plant hormone signal transduction;
gene356	K01669	E4.1.99.3, phrB	deoxyribodipyrimidine photo-lyase	EC:4.1.99.3		
gene35700	K08472	MLO	mlo protein	--		
gene35703	K06067	HDAC1_2	histone deacetylase 1/2	EC:3.5.1.98	9	ko04110 Cell cycle;ko04330 Notch signaling pathway;ko05016 Huntington's disease;ko05034 Alcoholism;ko05169 Epstein-Barr virus infection;ko05200 Pathways in cancer;ko05202 Transcriptional misregulation in cancer;ko05203 Viral carcinogenesis;ko05220 Chronic myeloid leukemia;
gene35704	K12200	PDCD6IP, ALIX, RIM20	programmed cell death 6-interacting protein	--	1	ko04144 Endocytosis;
gene35705	K00939	E2.7.4.3, adk	adenylate kinase	EC:2.7.4.3	1	ko00230 Purine metabolism;
gene3570	K07198	PRKAA, AMPK	5'-AMP-activated protein kinase, catalytic alpha subunit	EC:2.7.11.11	8	ko04140 Regulation of autophagy;ko04150 mTOR signaling pathway;ko04151 PI3K-Akt signaling pathway;ko04710 Circadian rhythm;ko04910 Insulin signaling pathway;ko04920 Adipocytokine signaling pathway;ko04932 Non-alcoholic fatty liver disease (NAFLD);ko05410 Hypertrophic cardiomyopathy (HCM);
gene35710	K03234	EEF2	elongation factor 2	--		
gene35712	K06173	truA, PUS1	tRNA pseudouridine38-40 synthase	EC:5.4.99.12		
gene35723	K04683	TFDP1	transcription factor Dp-1	--	2	ko04110 Cell cycle;ko04350 TGF-beta signaling pathway;
gene35729	K09874	NIP	aquaporin NIP	--		
gene35732	K00013	hisD	histidinol dehydrogenase	EC:1.1.1.23	2	ko00340 Histidine metabolism;ko01230 Biosynthesis of amino acids;
gene35735	K10609	CUL4	cullin 4	--	2	ko03420 Nucleotide excision repair;ko04120 Ubiquitin mediated proteolysis;
gene35737	K00232	E1.3.3.6, ACOX1, ACOX3	acyl-CoA oxidase	EC:1.3.3.6	5	ko00071 Fatty acid degradation;ko00592 alpha-Linolenic acid metabolism;ko01040 Biosynthesis of unsaturated fatty acids;ko03320 PPAR signaling pathway;ko04146 Peroxisome;
gene35739	K06678	YCG1, CAPG	condensin complex subunit 3	--	1	ko04111 Cell cycle - yeast;
gene35741	K07204	RAPTOR	regulatory associated protein of mTOR	--	4	ko04150 mTOR signaling pathway;ko04151 PI3K-Akt signaling pathway;ko04910 Insulin signaling pathway;ko05206 MicroRNAs in cancer;
gene35743	K02200	ccmH	cytochrome c-type biogenesis protein CcmH	--		
gene35745	K11097	SNRPE, SME	small nuclear ribonucleoprotein E	--	1	ko03040 Spliceosome;
gene35751	K14780	DHX37, DHR1	ATP-dependent RNA helicase DHX37/DHR1	EC:3.6.4.13		
gene35754	K04730	IRAK1	interleukin-1 receptor-associated kinase 1	EC:2.7.11.1	11	ko04064 NF-kappa B signaling pathway;ko04210 Apoptosis;ko04620 Toll-like receptor signaling pathway;ko04722 Neurotrophin signaling pathway;ko05133 Pertussis;ko05140 Leishmaniasis;ko05142 Chagas disease (American trypanosomiasis);ko05145 Toxoplasmosis;ko05152 Tuberculosis;ko05162 Measles;ko05169 Epstein-Barr virus infection;
gene35760	K05655	ABCB8	ATP-binding cassette, subfamily B (MDR/TAP), member 8	--	1	ko02010 ABC transporters;
gene35762	K13412	CPK	calcium-dependent protein kinase	EC:2.7.11.1	2	ko04626 Plant-pathogen interaction;ko05145 Toxoplasmosis;
gene35763	K08960	CSNK1E	casein kinase 1, epsilon	EC:2.7.11.1	6	ko04310 Wnt signaling pathway;ko04340 Hedgehog signaling pathway;ko04390 Hippo signaling pathway;ko04391 Hippo signaling pathway - fly;ko04710 Circadian rhythm;ko04711 Circadian rhythm - fly;
gene35770	K03327	TC.MATE, SLC47A, norM, mdtK, dinF	multidrug resistance protein, MATE family	--		
gene35771	K09495	CCT3, TRIC5	T-complex protein 1 subunit gamma	--		
gene35774	K13051	iaaA, ASRGL1	beta-aspartyl-peptidase (threonine type)	EC:3.4.19.5		
gene35785	K01213	E3.2.1.67	galacturan 1,4-alpha-galacturonidase	EC:3.2.1.67	2	ko00040 Pentose and glucuronate interconversions;ko00500 Starch and sucrose metabolism;

gene35786	K12812	UAP56, BAT1, SUB2	ATP-dependent RNA helicase UAP56/SUB2	EC:3.6.4.13	4	ko03013 RNA transport;ko03015 mRNA surveillance pathway;ko03040 Spliceosome;ko05164 Influenza A;
gene35797	K17479	GRXCR1	glutaredoxin domain-containing cysteine-rich protein 1	--		
gene3579	K08064	NFYA	nuclear transcription factor Y, alpha	--	2	ko04612 Antigen processing and presentation;ko05152 Tuberculosis;
gene35806	K06890	K06890				
gene35808	K02943	RP-LP2, RPLP2	large subunit ribosomal protein LP2	--	1	ko03010 Ribosome;
gene35809	K12736	PPWD1	peptidylprolyl isomerase domain and WD repeat-containing protein 1	EC:5.2.1.8		
gene35813	K13600	CAO	chlorophyllide a oxygenase	EC:1.14.13.122	1	ko00860 Porphyrin and chlorophyll metabolism;
gene35814	K10523	SPOP	speckle-type POZ protein	--		
gene35815	K16252	NRPD2, NRPE2	DNA-directed RNA polymerase IV and V subunit 2	EC:2.7.7.6		
gene35820	K00475	E1.14.11.9	naringenin 3-dioxygenase	EC:1.14.11.9	1	ko00941 Flavonoid biosynthesis;
gene35821	K01358	clpP, CLPP	ATP-dependent Clp protease, protease subunit	EC:3.4.21.92	1	ko04112 Cell cycle - Caulobacter;
gene35823	K00430	E1.11.1.7	peroxidase	EC:1.11.1.7	2	ko00360 Phenylalanine metabolism;ko00940 Phenylpropanoid biosynthesis;
gene35827	K07407	E3.2.1.22B, galA, rafA	alpha-galactosidase	EC:3.2.1.22	4	ko00052 Galactose metabolism;ko00561 Glycerolipid metabolism;ko00600 Sphingolipid metabolism;ko00603 Glycosphingolipid biosynthesis - globo series;
gene35828	K07513	ACAA1	acetyl-CoA acyltransferase 1	EC:2.3.1.16	7	ko00071 Fatty acid degradation;ko00280 Valine, leucine and isoleucine degradation;ko00362 Benzoate degradation;ko00592 alpha-Linolenic acid metabolism;ko01040 Biosynthesis of unsaturated fatty acids;ko03320 PPAR signaling pathway;ko04146 Peroxisome;
gene3582	K08695	E1.3.1.77	anthocyanidin reductase	EC:1.3.1.77	1	ko00941 Flavonoid biosynthesis;
gene35832	K12823	DDX5, DBP2	ATP-dependent RNA helicase DDX5/DBP2	EC:3.6.4.13	3	ko03040 Spliceosome;ko05202 Transcriptional misregulation in cancer;ko05205 Proteoglycans in cancer;
gene35833	K05677	ABCD3, PMP70	ATP-binding cassette, subfamily D (ALD), member 3	--	2	ko02010 ABC transporters;ko04146 Peroxisome;
gene3583	K08695	E1.3.1.77	anthocyanidin reductase	EC:1.3.1.77	1	ko00941 Flavonoid biosynthesis;
gene35842	K00558	DNMT1, dcm	DNA (cytosine-5)-methyltransferase 1	EC:2.1.1.37	2	ko00270 Cysteine and methionine metabolism;ko05206 MicroRNAs in cancer;
gene35843	K14823	EBP2, EBNA1BP2	rRNA-processing protein EBP2	--		
gene35844	K14696	SLC30A9, ZNT9	solute carrier family 30 (zinc transporter), member 9	--		
gene35845	K00966	GMPP	mannose-1-phosphate guanylyltransferase	EC:2.7.7.13	2	ko00051 Fructose and mannose metabolism;ko00520 Amino sugar and nucleotide sugar metabolism;
gene35849	K02183	CALM	calmodulin	--	25	ko04014 Ras signaling pathway;ko04020 Calcium signaling pathway;ko04070 Phosphatidylinositol signaling system;ko04114 Oocyte meiosis;ko04270 Vascular smooth muscle contraction;ko04626 Plant-pathogen interaction;ko04713 Circadian entrainment;ko04720 Long-term potentiation;ko04722 Neurotrophin signaling pathway;ko04728 Dopaminergic synapse;ko04740 Olfactory transduction;ko04744 Phototransduction;ko04745 Phototransduction - fly;ko04910 Insulin signaling pathway;ko04912 GnRH signaling pathway;ko04915 Estrogen signaling pathway;ko04916 Melanogenesis;ko04970 Salivary secretion;ko04971 Gastric acid secretion;ko05010 Alzheimer's disease;ko05031 Amphetamine addiction;ko05034 Alcoholism;ko05133 Pertussis;ko05152 Tuberculosis;ko05214 Glioma;
gene35854	K00789	metK	S-adenosylmethionine synthetase	EC:2.5.1.6	2	ko00270 Cysteine and methionine metabolism;ko01230 Biosynthesis of amino acids;
gene3585	K16196	EIF2AK4	eukaryotic translation initiation factor 2-alpha kinase 4	EC:2.7.11.1	6	ko04141 Protein processing in endoplasmic reticulum;ko05160 Hepatitis C;ko05162 Measles;ko05164 Influenza A;ko05168 Herpes simplex infection;ko05169 Epstein-Barr virus infection;
gene35860	K14006	SEC23	protein transport protein SEC23	--	1	ko04141 Protein processing in endoplasmic reticulum;
gene35864	K05857	PLCD	phosphatidylinositol phospholipase C, delta	EC:3.1.4.11	3	ko00562 Inositol phosphate metabolism;ko04020 Calcium signaling pathway;ko04070 Phosphatidylinositol signaling system;
gene35866	K01601	rbcL	ribulose-bisphosphate carboxylase large chain	EC:4.1.1.39	3	ko00630 Glyoxylate and dicarboxylate metabolism;ko00710 Carbon fixation in photosynthetic organisms;ko01200 Carbon metabolism;
gene35868	K01792	E5.1.3.15	glucose-6-phosphate 1-epimerase	EC:5.1.3.15	1	ko00010 Glycolysis / Gluconeogenesis;

gene35869	K00430	E1.11.1.7	peroxidase	EC:1.11.1.7	2	ko00360 Phenylalanine metabolism;ko00940 Phenylpropanoid biosynthesis;
gene35878	K02912	RP-L32e, RPL32	large subunit ribosomal protein L32e	--	1	ko03010 Ribosome;
gene3587	K14514	EIN3	ethylene-insensitive protein 3	--	1	ko04075 Plant hormone signal transduction;
gene35881	K07025	K07025	putative hydrolase of the HAD superfamily	--		
gene35883	K02873	RP-L13e, RPL13	large subunit ribosomal protein L13e	--	1	ko03010 Ribosome;
gene35886	K03241	EIF2B3	translation initiation factor eIF-2B subunit gamma	--	1	ko03013 RNA transport;
gene35887	K06892	K06892				
gene35889	K15200	GTF3C2	general transcription factor 3C polypeptide 2	--		
gene35891	K01762	ACS	1-aminocyclopropane-1-carboxylate synthase	EC:4.4.1.14	1	ko00270 Cysteine and methionine metabolism;
gene35894	K14236	tRNA-Tyr	tRNA Tyr	--	1	ko00970 Aminoacyl-tRNA biosynthesis;
gene35898	K06630	YWHAE	14-3-3 protein epsilon	--	8	ko04110 Cell cycle;ko04114 Oocyte meiosis;ko04151 PI3K-Akt signaling pathway;ko04390 Hippo signaling pathway;ko04391 Hippo signaling pathway - fly;ko04722 Neurotrophin signaling pathway;ko05169 Epstein-Barr virus infection;ko05203 Viral carcinogenesis;
gene3589	K14514	EIN3	ethylene-insensitive protein 3	--	1	ko04075 Plant hormone signal transduction;
gene35900	K12873	BUD31, G10	bud site selection protein 31	--	1	ko03040 Spliceosome;
gene35901	K15172	SUPT5H, SPT5	transcription elongation factor SPT5	--		
gene35902	K00430	E1.11.1.7	peroxidase	EC:1.11.1.7	2	ko00360 Phenylalanine metabolism;ko00940 Phenylpropanoid biosynthesis;
gene35908	K15382	SLC50A, SWEET	solute carrier family 50 (sugar transporter)	--		
gene35909	K15382	SLC50A, SWEET	solute carrier family 50 (sugar transporter)	--		
gene35915	K09422	MYBP	myb proto-oncogene protein, plant	--		
gene35917	K09422	MYBP	myb proto-oncogene protein, plant	--		
gene35918	K09422	MYBP	myb proto-oncogene protein, plant	--		
gene35919	K14486	K14486, ARF	auxin response factor	--	1	ko04075 Plant hormone signal transduction;
gene35920	K12446	E2.7.1.46	L-arabinokinase	EC:2.7.1.46	1	ko00520 Amino sugar and nucleotide sugar metabolism;
gene35934	K13413	MKK4_5P	mitogen-activated protein kinase kinase 4/5, plant	EC:2.7.12.2	1	ko04626 Plant-pathogen interaction;
gene35936	K08248	E4.1.2.10	mandelonitrile lyase	EC:4.1.2.10	1	ko00460 Cyanoamino acid metabolism;
gene35937	K00058	serA, PHGDH	D-3-phosphoglycerate dehydrogenase	EC:1.1.1.95	4	ko00260 Glycine, serine and threonine metabolism;ko00680 Methane metabolism;ko01200 Carbon metabolism;ko01230 Biosynthesis of amino acids;
gene35942	K14638	SLC15A3_4, PHT	solute carrier family 15 (peptide/histidine transporter), member 3/4	--		
gene35943	K14638	SLC15A3_4, PHT	solute carrier family 15 (peptide/histidine transporter), member 3/4	--		
gene3594	K02908	RP-L30e, RPL30	large subunit ribosomal protein L30e	--	1	ko03010 Ribosome;
gene35953	K12462	ARHGDI, RHOGDI	Rho GDP-dissociation inhibitor	--	2	ko04722 Neurotrophin signaling pathway;ko04962 Vasopressin-regulated water reabsorption;
gene35956	K01507	ppa	inorganic pyrophosphatase	EC:3.6.1.1	1	ko00190 Oxidative phosphorylation;
gene35957	K01507	ppa	inorganic pyrophosphatase	EC:3.6.1.1	1	ko00190 Oxidative phosphorylation;
gene35958	K12829	SF3B2, SAP145, CUS1	splicing factor 3B subunit 2	--	1	ko03040 Spliceosome;
gene35959	K01537	E3.6.3.8	Ca2+-transporting ATPase	EC:3.6.3.8		
gene35963	K14432	ABF	ABA responsive element binding factor	--	1	ko04075 Plant hormone signal transduction;
gene35964	K01982	LSUrRNA	large subunit ribosomal RNA	--	2	ko03008 Ribosome biogenesis in eukaryotes;ko03010 Ribosome;
gene35969	K02943	RP-LP2, RPLP2	large subunit ribosomal protein LP2	--	1	ko03010 Ribosome;
gene3596	K00021	E1.1.1.34	hydroxymethylglutaryl-CoA reductase (NADPH)	EC:1.1.1.34	2	ko00900 Terpenoid backbone biosynthesis;ko04976 Bile secretion;
gene35970	K13335	PEX16	peroxin-16	--	1	ko04146 Peroxisome;
gene35971	K11320	EP400	E1A-binding protein p400	EC:3.6.4.-		
gene3597	K08901	psbQ	photosystem II oxygen-evolving enhancer protein 3	--	1	ko00195 Photosynthesis;
gene35985	K02879	RP-L17, MRPL17, rplQ	large subunit ribosomal protein L17	--	1	ko03010 Ribosome;
gene35994	K01728	E4.2.2.2, pel	pectate lyase	EC:4.2.2.2	1	ko00040 Pentose and glucuronate interconversions;
gene35	K11498	CENPE	centromeric protein E	--		
gene3600	K08912	LHCB1	light-harvesting complex II chlorophyll a/b binding protein 1	--	1	ko00196 Photosynthesis - antenna proteins;
gene36011	K05909	E1.10.3.2				

gene36012	K14638	SLC15A3_4, PHT	solute carrier family 15 (peptide/histidine transporter), member 3/4	--		
gene36013	K14638	SLC15A3_4, PHT	solute carrier family 15 (peptide/histidine transporter), member 3/4	--		
gene36018	K10418	DYNLL	dynein light chain LC8-type	--	1	ko04962 Vasopressin-regulated water reabsorption;
gene3601	K11251	H2A	histone H2A	--	2	ko05034 Alcoholism;ko05322 Systemic lupus erythematosus;
gene36020	K03242	EIF2S3	translation initiation factor 2 subunit 3	--	1	ko03013 RNA transport;
gene36023	K14810	DDX56, DBP9	ATP-dependent RNA helicase DDX56/DBP9	EC:3.6.4.13		
gene36025	K00770	E2.4.2.24	1,4-beta-D-xylan synthase	EC:2.4.2.24	2	ko00500 Starch and sucrose metabolism;ko00520 Amino sugar and nucleotide sugar metabolism;
gene36029	K15397	KCS	3-ketoacyl-CoA synthase	EC:2.3.1.199	1	ko00062 Fatty acid elongation;
gene3602	K02895	RP-L24, MRPL24, rplX	large subunit ribosomal protein L24	--	1	ko03010 Ribosome;
gene36032	K00232	E1.3.3.6, ACOX1, ACOX3	acyl-CoA oxidase	EC:1.3.3.6	5	ko00071 Fatty acid degradation;ko00592 alpha-Linolenic acid metabolism;ko01040 Biosynthesis of unsaturated fatty acids;ko03320 PPAR signaling pathway;ko04146 Peroxisome;
gene36034	K09490	HSPA5, BIP	heat shock 70kDa protein 5	--	4	ko03060 Protein export;ko04141 Protein processing in endoplasmic reticulum;ko04918 Thyroid hormone synthesis;ko05020 Prion diseases;
gene36039	K09518	DNAJB12	DnaJ homolog subfamily B member 12	--	1	ko04141 Protein processing in endoplasmic reticulum;
gene36040	K02908	RP-L30e, RPL30	large subunit ribosomal protein L30e	--	1	ko03010 Ribosome;
gene36045	K12626	LSM7	U6 snRNA-associated Sm-like protein LSm7	--	2	ko03018 RNA degradation;ko03040 Spliceosome;
gene36047	K02960	RP-S16e, RPS16	small subunit ribosomal protein S16e	--	1	ko03010 Ribosome;
gene36048	K08235	E2.4.1.207	xyloglucan:xyloglucosyl transferase	EC:2.4.1.207		
gene3604	K02938	RP-L8e, RPL8	large subunit ribosomal protein L8e	--	1	ko03010 Ribosome;
gene36054	K14944	NOVA	RNA-binding protein Nova	--		
gene36059	K12188	SNF8, EAP30	ESCRT-II complex subunit VPS22	--	1	ko04144 Endocytosis;
gene36060	K02349	POLQ	DNA polymerase theta	EC:2.7.7.7		
gene36061	K03016	RPB8, POLR2H	DNA-directed RNA polymerases I, II, and III subunit RPABC3	--	6	ko00230 Purine metabolism;ko00240 Pyrimidine metabolism;ko03020 RNA polymerase;ko04623 Cytosolic DNA-sensing pathway;ko05016 Huntington's disease;ko05169 Epstein-Barr virus infection;
gene36067	K00430	E1.11.1.7	peroxidase	EC:1.11.1.7	2	ko00360 Phenylalanine metabolism;ko00940 Phenylpropanoid biosynthesis;
gene36069	K10999	CESA	cellulose synthase A	EC:2.4.1.12		
gene3606	K00851	E2.7.1.12, gntK, idnK	gluconokinase	EC:2.7.1.12	2	ko00030 Pentose phosphate pathway;ko01200 Carbon metabolism;
gene36072	K10355	ACTF	actin, other eukaryote	--		
gene36076	K03245	EIF3J	translation initiation factor 3 subunit J	--	1	ko03013 RNA transport;
gene36077	K03351	APC4	anaphase-promoting complex subunit 4	--	7	ko04110 Cell cycle;ko04111 Cell cycle - yeast;ko04113 Meiosis - yeast;ko04114 Oocyte meiosis;ko04120 Ubiquitin mediated proteolysis;ko04914 Progesterone-mediated oocyte maturation;ko05166 HTLV-I infection;
gene36079	K04718	SPHK	sphingosine kinase	EC:2.7.1.91	5	ko00600 Sphingolipid metabolism;ko04020 Calcium signaling pathway;ko04370 VEGF signaling pathway;ko04666 Fc gamma R-mediated phagocytosis;ko05152 Tuberculosis;
gene3607	K14487	GH3	auxin responsive GH3 gene family	--	1	ko04075 Plant hormone signal transduction;
gene36082	K03781	katE, CAT, catB, srpA	catalase	EC:1.11.1.6	4	ko00380 Tryptophan metabolism;ko00630 Glyoxylate and dicarboxylate metabolism;ko04146 Peroxisome;ko05014 Amyotrophic lateral sclerosis (ALS);
gene36084	K12493	ARFGAP2_3	ADP-ribosylation factor GTPase-activating protein 2/3	--	1	ko04144 Endocytosis;
gene36087	K05868	CCNB	cyclin B	--	3	ko04110 Cell cycle;ko04115 p53 signaling pathway;ko04914 Progesterone-mediated oocyte maturation;
gene3608	K14487	GH3	auxin responsive GH3 gene family	--	1	ko04075 Plant hormone signal transduction;
gene36093	K09286	EREBP	EREBP-like factor	--		
gene36096	K07766	E3.6.1.52	diphosphoinositol-polyphosphate diphosphatase	EC:3.6.1.52		
gene36097	K14221	tRNA-Asp	tRNA Asp	--	1	ko00970 Aminoacyl-tRNA biosynthesis;
gene36098	K14222	tRNA-Cys	tRNA Cys	--	1	ko00970 Aminoacyl-tRNA biosynthesis;
gene36113	K07478	ycaJ	putative ATPase	--		
gene36114	K07478	ycaJ	putative ATPase	--		
gene36115	K03696	clpC	ATP-dependent Clp protease ATP-binding subunit ClpC	--		
gene36116	K14165	K14165	dual specificity phosphatase	EC:3.1.3.16 3.1.3.48		

gene36117	K16297	SCPL-II	serine carboxypeptidase-like clade II	EC:3.4.16.-		
gene36124	K11279	NAP1L1, NRP	nucleosome assembly protein 1-like 1	--		
gene36125	K05863	SLC25A4S, ANT	solute carrier family 25 (mitochondrial adenine nucleotide translocator), member 4/5/6/31	--	4	ko04020 Calcium signaling pathway;ko05012 Parkinson's disease;ko05016 Huntington's disease;ko05166 HTLV-I infection;
gene36129	K17362	ACOT13	acyl-coenzyme A thioesterase 13	EC:3.1.2.-		
gene36132	K14847	RPF2	ribosome production factor 2	--		
gene36138	K08819	CDK12_13	cyclin-dependent kinase 12/13	EC:2.7.11.22 2.7.11.23		
gene36141	K07874	RAB1A	Ras-related protein Rab-1A	--	1	ko05134 Legionellosis;
gene36142	K07904	RAB11A	Ras-related protein Rab-11A	--	4	ko04144 Endocytosis;ko04961 Endocrine and other factor-regulated calcium reabsorption;ko04962 Vasopressin-regulated water reabsorption;ko04972 Pancreatic secretion;
gene36143	K01358	clpP, CLPP	ATP-dependent Clp protease, protease subunit	EC:3.4.21.92	1	ko04112 Cell cycle - Caulobacter;
gene3614	K10753	ASF1	histone chaperone ASF1	--		
gene36150	K10085	EDEM2	ER degradation enhancer, mannosidase alpha-like 2	--	1	ko04141 Protein processing in endoplasmic reticulum;
gene36154	K13348	MPV17	protein Mpv17	--	1	ko04146 Peroxisome;
gene36162	K00012	UGDH, ugd	UDPglucose 6-dehydrogenase	EC:1.1.1.22	4	ko00040 Pentose and glucuronate interconversions;ko00053 Ascorbate and aldarate metabolism;ko00500 Starch and sucrose metabolism;ko00520 Amino sugar and nucleotide sugar metabolism;
gene36170	K02881	RP-L18, MRPL18, rplR	large subunit ribosomal protein L18	--	1	ko03010 Ribosome;
gene36178	K12882	NCBP1, CBP80	nuclear cap-binding protein subunit 1	--	3	ko03013 RNA transport;ko03015 mRNA surveillance pathway;ko03040 Spliceosome;
gene36184	K01762	ACS	1-aminocyclopropane-1-carboxylate synthase	EC:4.4.1.14	1	ko00270 Cysteine and methionine metabolism;
gene36185	K01762	ACS	1-aminocyclopropane-1-carboxylate synthase	EC:4.4.1.14	1	ko00270 Cysteine and methionine metabolism;
gene36186	K11985	TRAIP, TRIP	TRAF-interacting protein	--		
gene36187	K13425	WRKY22	WRKY transcription factor 22	--	1	ko04626 Plant-pathogen interaction;
gene36188	K03327	TC.MATE, SLC47A, norM, mdtK, dinF	multidrug resistance protein, MATE family	--		
gene36189	K02732	PSMB1	20S proteasome subunit beta 6	EC:3.4.25.1	1	ko03050 Proteasome;
gene36191	K08900	BCS1	mitochondrial chaperone BCS1	--		
gene36197	K12605	CNOT2, NOT2	CCR4-NOT transcription complex subunit 2	--	1	ko03018 RNA degradation;
gene36199	K04733	IRAK4	interleukin-1 receptor-associated kinase 4	EC:2.7.11.1	11	ko04064 NF-kappa B signaling pathway;ko04210 Apoptosis;ko04620 Toll-like receptor signaling pathway;ko04722 Neurotrophin signaling pathway;ko05133 Pertussis;ko05140 Leishmaniasis;ko05142 Chagas disease (American trypanosomiasis);ko05145 Toxoplasmosis;ko05152 Tuberculosis;ko05162 Measles;ko05164 Influenza A;
gene361	K15718	LOX1_5	linoleate 9S-lipoxygenase	EC:1.13.11.58	1	ko00591 Linoleic acid metabolism;
gene36201	K01979	SSUrRNA	small subunit ribosomal RNA	--	2	ko03008 Ribosome biogenesis in eukaryotes;ko03010 Ribosome;
gene36202	K07253	MIF	phenylpyruvate tautomerase	EC:5.3.2.1	2	ko00350 Tyrosine metabolism;ko00360 Phenylalanine metabolism;
gene36207	K11252	H2B	histone H2B	--	3	ko05034 Alcoholism;ko05203 Viral carcinogenesis;ko05322 Systemic lupus erythematosus;
gene36208	K16282	RHA2	E3 ubiquitin-protein ligase RHA2	EC:6.3.2.19		
gene3621	K12608	CAF16	CCR4-NOT complex subunit CAF16	--	1	ko03018 RNA degradation;
gene36222	K13415	BRI1	protein brassinosteroid insensitive 1	EC:2.7.10.1 2.7.11.1	1	ko04075 Plant hormone signal transduction;
gene36223	K15306	RANBP1	Ran-binding protein 1	--	2	ko05166 HTLV-I infection;ko05203 Viral carcinogenesis;
gene36226	K11663	ZNHIT1, VPS71	zinc finger HIT domain-containing protein 1	--		
gene3622	K02882	RP-L18Ae, RPL18A	large subunit ribosomal protein L18Ae	--	1	ko03010 Ribosome;
gene36231	K02838	frr, MRRF, RRF	ribosome recycling factor	--		
gene36233	K06276	PDPK1	3-phosphoinositide dependent protein kinase-1	EC:2.7.11.1	9	ko03320 PPAR signaling pathway;ko04150 mTOR signaling pathway;ko04151 PI3K-Akt signaling pathway;ko04510 Focal adhesion;ko04910 Insulin signaling pathway;ko04960 Aldosterone-regulated sodium reabsorption;ko05213 Endometrial cancer;ko05215 Prostate cancer;ko05223 Non-small cell lung cancer;
gene36237	K09422	MYBP	myb proto-oncogene protein, plant	--		
gene36248	K05768	GSN	gelsolin	--	3	ko04666 Fc gamma R-mediated phagocytosis;ko04810 Regulation of actin cytoskeleton;ko05203 Viral carcinogenesis;

gene36250	K03155	TIMELESS	timeless	--		
gene36254	K08288	PRKCSH	protein kinase C substrate 80K-H	--	1	ko04141 Protein processing in endoplasmic reticulum;
gene36258	K13126	PABPC	polyadenylate-binding protein	--	3	ko03013 RNA transport;ko03015 mRNA surveillance pathway;ko03018 RNA degradation;
gene36263	K01510	ENTPD1_3_8	apyrase	EC:3.6.1.5	3	ko00230 Purine metabolism;ko00240 Pyrimidine metabolism;ko05169 Epstein-Barr virus infection;
gene36266	K13126	PABPC	polyadenylate-binding protein	--	3	ko03013 RNA transport;ko03015 mRNA surveillance pathway;ko03018 RNA degradation;
gene36267	K12854	SNRNP200, BRR2	pre-mRNA-splicing helicase BRR2	EC:3.6.4.13	1	ko03040 Spliceosome;
gene36272	K02325	POLE2	DNA polymerase epsilon subunit 2	EC:2.7.7.7	6	ko00230 Purine metabolism;ko00240 Pyrimidine metabolism;ko03030 DNA replication;ko03410 Base excision repair;ko03420 Nucleotide excision repair;ko05166 HTLV-I infection;
gene36273	K09422	MYBP	myb proto-oncogene protein, plant	--		
gene36274	K11593	ELF2C	eukaryotic translation initiation factor 2C	--		
gene36275	K00695	E2.4.1.13	sucrose synthase	EC:2.4.1.13	1	ko00500 Starch and sucrose metabolism;
gene36276	K01581	E4.1.1.17, ODC1, speC, speF	ornithine decarboxylase	EC:4.1.1.17	2	ko00330 Arginine and proline metabolism;ko00480 Glutathione metabolism;
gene36278	K12854	SNRNP200, BRR2	pre-mRNA-splicing helicase BRR2	EC:3.6.4.13	1	ko03040 Spliceosome;
gene36279	K12854	SNRNP200, BRR2	pre-mRNA-splicing helicase BRR2	EC:3.6.4.13	1	ko03040 Spliceosome;
gene36281	K12200	PDCD6IP, ALIX, RIM20	programmed cell death 6-interacting protein	--	1	ko04144 Endocytosis;
gene36282	K01530	E3.6.3.1	phospholipid-translocating ATPase	EC:3.6.3.1		
gene36283	K12826	SF3A2, SAP62	splicing factor 3A subunit 2	--	1	ko03040 Spliceosome;
gene36284	K00889	E2.7.1.68, PIP5K	1-phosphatidylinositol-4-phosphate 5-kinase	EC:2.7.1.68	5	ko00562 Inositol phosphate metabolism;ko04070 Phosphatidylinositol signaling system;ko04144 Endocytosis;ko04666 Fc gamma R-mediated phagocytosis;ko04810 Regulation of actin cytoskeleton;
gene36285	K02893	RP-L23Ae, RPL23A	large subunit ribosomal protein L23Ae	--	1	ko03010 Ribosome;
gene36287	K05609	UCHL3, YUH1	ubiquitin carboxyl-terminal hydrolase L3	EC:3.4.19.12		
gene36289	K00688	E2.4.1.1, glgP, PYG	starch phosphorylase	EC:2.4.1.1	2	ko00500 Starch and sucrose metabolism;ko04910 Insulin signaling pathway;
gene36290	K01537	E3.6.3.8	Ca2+-transporting ATPase	EC:3.6.3.8		
gene36292	K05956	RABGGTB	geranylgeranyl transferase type-2 subunit beta	EC:2.5.1.60		
gene36297	K11000	CALS	callose synthase	EC:2.4.1.-		
gene36298	K01652	E2.2.1.6L, ilvB, ilvG, ilvI	acetolactate synthase I/II/III large subunit	EC:2.2.1.6	6	ko00290 Valine, leucine and isoleucine biosynthesis;ko00650 Butanoate metabolism;ko00660 C5-Branched dibasic acid metabolism;ko00770 Pantothenate and CoA biosynthesis;ko01210 2-Oxocarboxylic acid metabolism;ko01230 Biosynthesis of amino acids;
gene362	K15813	LUP4	beta-amyrin synthase	EC:5.4.99.39	1	ko00909 Sesquiterpenoid and triterpenoid biosynthesis;
gene36300	K04730	IRAK1	interleukin-1 receptor-associated kinase 1	EC:2.7.11.1	11	ko04064 NF-kappa B signaling pathway;ko04210 Apoptosis;ko04620 Toll-like receptor signaling pathway;ko04722 Neurotrophin signaling pathway;ko05133 Pertussis;ko05140 Leishmaniasis;ko05142 Chagas disease (American trypanosomiasis);ko05145 Toxoplasmosis;ko05152 Tuberculosis;ko05162 Measles;ko05169 Epstein-Barr virus infection;
gene36301	K04733	IRAK4	interleukin-1 receptor-associated kinase 4	EC:2.7.11.1	11	ko04064 NF-kappa B signaling pathway;ko04210 Apoptosis;ko04620 Toll-like receptor signaling pathway;ko04722 Neurotrophin signaling pathway;ko05133 Pertussis;ko05140 Leishmaniasis;ko05142 Chagas disease (American trypanosomiasis);ko05145 Toxoplasmosis;ko05152 Tuberculosis;ko05162 Measles;ko05164 Influenza A;
gene36303	K09490	HSPA5, BIP	heat shock 70kDa protein 5	--	4	ko03060 Protein export;ko04141 Protein processing in endoplasmic reticulum;ko04918 Thyroid hormone synthesis;ko05020 Prion diseases:
gene36304	K09832	CYP710A	cytochrome P450, family 710, subfamily A	--	1	ko00100 Steroid biosynthesis;
gene36305	K02955	RP-S14e, RPS14	small subunit ribosomal protein S14e	--	1	ko03010 Ribosome;
gene36306	K17302	COPB2, SEC27	coatomer, subunit beta'	--		
gene36308	K15304	RANBP3	Ran-binding protein 3	--	1	ko05166 HTLV-I infection;
gene36317	K14538	NUG1, GNL3	nuclear GTP-binding protein	--	1	ko03008 Ribosome biogenesis in eukaryotes;
gene36318	K13343	PEX14	peroxin-14	--	1	ko04146 Peroxisome;

gene36321	K01051	E3.1.1.11	pectinesterase	EC:3.1.1.11	2	ko00040 Pentose and glucuronate interconversions;ko00500 Starch and sucrose metabolism;
gene36325	K05605	HIBCH	3-hydroxyisobutyryl-CoA hydrolase	EC:3.1.2.4	3	ko00280 Valine, leucine and isoleucine degradation;ko00410 beta-Alanine metabolism;ko00640 Propanoate metabolism;
gene36328	K01982	LSUrRNA	large subunit ribosomal RNA	--	2	ko03008 Ribosome biogenesis in eukaryotes;ko03010 Ribosome;
gene36329	K14235	tRNA-Trp	tRNA Trp	--	1	ko00970 Aminoacyl-tRNA biosynthesis;
gene36331	K17095	ANXA7_11	annexin A7/11	--		
gene36333	K15272	SLC35A1_2_3	solute carrier family 35 (UDP-sugar transporter), member A1/2/3	--		
gene36340	K15263	LYER	cell growth-regulating nucleolar protein	--		
gene36343	K12602	WDR61, REC14, SKI8	WD repeat-containing protein 61	--	1	ko03018 RNA degradation;
gene36344	K10357	MYO5	myosin V	--		
gene36346	K12191	CHMP2A	charged multivesicular body protein 2A	--	1	ko04144 Endocytosis;
gene36349	K13566	NIT2	omega-amidase	EC:3.5.1.3	1	ko00250 Alanine, aspartate and glutamate metabolism;
gene36350	K02882	RP-L18Ae, RPL18A	large subunit ribosomal protein L18Ae	--	1	ko03010 Ribosome;
gene36357	K02923	RP-L38e, RPL38	large subunit ribosomal protein L38e	--	1	ko03010 Ribosome;
gene36362	K01623	ALDO	fructose-bisphosphate aldolase, class I	EC:4.1.2.13	7	ko00010 Glycolysis / Gluconeogenesis;ko00030 Pentose phosphate pathway;ko00051 Fructose and mannose metabolism;ko00680 Methane metabolism;ko00710 Carbon fixation in photosynthetic organisms;ko01200 Carbon metabolism;ko01230 Biosynthesis of amino acids;
gene36365	K09873	TIP	aquaporin TIP	--		
gene36368	K11000	CALS	callose synthase	EC:2.4.1.-		
gene36369	K02888	RP-L21, MRPL21, rplU	large subunit ribosomal protein L21	--	1	ko03010 Ribosome;
gene3636	K01649	leuA	2-isopropylmalate synthase	EC:2.3.3.13	4	ko00290 Valine, leucine and isoleucine biosynthesis;ko00620 Pyruvate metabolism;ko01210 2-Oxocarboxylic acid metabolism;ko01230 Biosynthesis of amino acids;
gene36375	K01102	PDP	pyruvate dehydrogenase phosphatase	EC:3.1.3.43		
gene36376	K02915	RP-L34e, RPL34	large subunit ribosomal protein L34e	--	1	ko03010 Ribosome;
gene36377	K00799	GST, gst	glutathione S-transferase	EC:2.5.1.18	4	ko00480 Glutathione metabolism;ko00980 Metabolism of xenobiotics by cytochrome P450;ko00982 Drug metabolism - cytochrome P450;ko05204 Chemical carcinogenesis;
gene36378	K00799	GST, gst	glutathione S-transferase	EC:2.5.1.18	4	ko00480 Glutathione metabolism;ko00980 Metabolism of xenobiotics by cytochrome P450;ko00982 Drug metabolism - cytochrome P450;ko05204 Chemical carcinogenesis;
gene36379	K01431	E3.5.1.6	beta-ureidopropionase	EC:3.5.1.6	4	ko00240 Pyrimidine metabolism;ko00410 beta-Alanine metabolism;ko00770 Pantothenate and CoA biosynthesis;ko00983 Drug metabolism - other enzymes;
gene36382	K00423	E1.10.3.3	L-ascorbate oxidase	EC:1.10.3.3	1	ko00053 Ascorbate and aldarate metabolism;
gene36384	K01802	E5.2.1.8	peptidylprolyl isomerase	EC:5.2.1.8		
gene36386	K05665	ABCC1	ATP-binding cassette, subfamily C (CFTR/MRP), member 1	--	3	ko02010 ABC transporters;ko04977 Vitamin digestion and absorption;ko05206 MicroRNAs in cancer;
gene36387	K02951	RP-S12e, RPS12	small subunit ribosomal protein S12e	--	1	ko03010 Ribosome;
gene36388	K15139	MED22	mediator of RNA polymerase II transcription subunit 22	--		
gene36389	K09201	YY	transcription factor YY	--		
gene36390	K02882	RP-L18Ae, RPL18A	large subunit ribosomal protein L18Ae	--	1	ko03010 Ribosome;
gene36391	K08341	GABARAP, ATG8, LC3	GABA(A) receptor-associated protein (autophagy-related protein 8)	--	2	ko04140 Regulation of autophagy;ko04727 GABAergic synapse;
gene36392	K15446	TRM13, CCDC76	tRNA:m4X modification enzyme	EC:2.1.1.225		
gene36396	K00688	E2.4.1.1, glgP, PYG	starch phosphorylase	EC:2.4.1.1	2	ko00500 Starch and sucrose metabolism;ko04910 Insulin signaling pathway;
gene36399	K02147	ATPeVB, ATP6B1	V-type H+-transporting ATPase subunit B	EC:3.6.3.14	7	ko00190 Oxidative phosphorylation;ko04145 Phagosome;ko04721 Synaptic vesicle cycle;ko04966 Collecting duct acid secretion;ko05110 Vibrio cholerae infection;ko05120 Epithelial cell signaling in Helicobacter pylori infection;ko05323 Rheumatoid arthritis;
gene3639	K03942	NDUFV1	NADH dehydrogenase (ubiquinone) flavoprotein 1	EC:1.6.5.3 1.6.99.3	5	ko00190 Oxidative phosphorylation;ko04932 Non-alcoholic fatty liver disease (NAFLD);ko05010 Alzheimer's disease;ko05012 Parkinson's disease;ko05016 Huntington's disease;
gene36401	K01102	PDP	pyruvate dehydrogenase phosphatase	EC:3.1.3.43		

gene36402	K02995	RP-S8e, RPS8	small subunit ribosomal protein S8e	--	1	ko03010 Ribosome;
gene36405	K07456	mutS2	DNA mismatch repair protein MutS2	--	1	ko03430 Mismatch repair;
gene3640	K03942	NDUFV1	NADH dehydrogenase (ubiquinone) flavoprotein 1	EC:1.6.5.3 1.6.99.3	5	ko00190 Oxidative phosphorylation;ko04932 Non-alcoholic fatty liver disease (NAFLD);ko05010 Alzheimer's disease;ko05012 Parkinson's disease;ko05016 Huntington's disease;
gene36411	K01733	thrC	threonine synthase	EC:4.2.3.1	3	ko00260 Glycine, serine and threonine metabolism;ko00750 Vitamin B6 metabolism;ko01230 Biosynthesis of amino acids;
gene36418	K03354	APC7	anaphase-promoting complex subunit 7	--	7	ko04110 Cell cycle;ko04111 Cell cycle - yeast;ko04113 Meiosis - yeast;ko04114 Oocyte meiosis;ko04120 Ubiquitin mediated proteolysis;ko04914 Progesterone-mediated oocyte maturation;ko05166 HTLV-I infection;
gene3641	K01427	URE	urease	EC:3.5.1.5	2	ko00230 Purine metabolism;ko00330 Arginine and proline metabolism;
gene36423	K15891	FLDH	farnesol dehydrogenase	EC:1.1.1.216	2	ko00900 Terpenoid backbone biosynthesis;ko00909 Sesquiterpenoid and triterpenoid biosynthesis;
gene36426	K15925	XYL1	alpha-D-xyloside xylohydrolase	EC:3.2.1.177		
gene36432	K00454	LOX2S	lipoxygenase	EC:1.13.11.12	2	ko00591 Linoleic acid metabolism;ko00592 alpha-Linolenic acid metabolism;
gene36436	K05931	CARM1, PRMT4	histone-arginine methyltransferase CARM1	EC:2.1.1.125		
gene36442	K00666	K00666	fatty-acyl-CoA synthase	EC:6.2.1.-		
gene36444	K00600	glyA, SHMT	glycine hydroxymethyltransferase	EC:2.1.2.1	7	ko00260 Glycine, serine and threonine metabolism;ko00460 Cyanoamino acid metabolism;ko00630 Glyoxylate and dicarboxylate metabolism;ko00670 One carbon pool by folate;ko00680 Methane metabolism;ko01200 Carbon metabolism;ko01230 Biosynthesis of amino acids;
gene36445	K11420	EHMT	euchromatic histone-lysine N-methyltransferase	EC:2.1.1.43	1	ko00310 Lysine degradation;
gene36451	K10364	CAPZA	capping protein (actin filament) muscle Z-line, alpha	--		
gene36452	K04730	IRAK1	interleukin-1 receptor-associated kinase 1	EC:2.7.11.1	11	ko04064 NF-kappa B signaling pathway;ko04210 Apoptosis;ko04620 Toll-like receptor signaling pathway;ko04722 Neurotrophin signaling pathway;ko05133 Pertussis;ko05140 Leishmaniasis;ko05142 Chagas disease (American trypanosomiasis);ko05145 Toxoplasmosis;ko05152 Tuberculosis;ko05162 Measles;ko05169 Epstein-Barr virus infection;
gene36453	K01102	PDP	pyruvate dehydrogenase phosphatase	EC:3.1.3.43		
gene36469	K01739	metB	cystathionine gamma-synthase	EC:2.5.1.48	4	ko00270 Cysteine and methionine metabolism;ko00450 Selenocompound metabolism;ko00920 Sulfur metabolism;ko01230 Biosynthesis of amino acids;
gene3646	K04733	IRAK4	interleukin-1 receptor-associated kinase 4	EC:2.7.11.1	11	ko04064 NF-kappa B signaling pathway;ko04210 Apoptosis;ko04620 Toll-like receptor signaling pathway;ko04722 Neurotrophin signaling pathway;ko05133 Pertussis;ko05140 Leishmaniasis;ko05142 Chagas disease (American trypanosomiasis);ko05145 Toxoplasmosis;ko05152 Tuberculosis;ko05162 Measles;ko05164 Influenza A;
gene36473	K02951	RP-S12e, RPS12	small subunit ribosomal protein S12e	--	1	ko03010 Ribosome;
gene36480	K00600	glyA, SHMT	glycine hydroxymethyltransferase	EC:2.1.2.1	7	ko00260 Glycine, serine and threonine metabolism;ko00460 Cyanoamino acid metabolism;ko00630 Glyoxylate and dicarboxylate metabolism;ko00670 One carbon pool by folate;ko00680 Methane metabolism;ko01200 Carbon metabolism;ko01230 Biosynthesis of amino acids;
gene36481	K03010	RPB2, POLR2B	DNA-directed RNA polymerase II subunit RPB2	EC:2.7.7.6	5	ko00230 Purine metabolism;ko00240 Pyrimidine metabolism;ko03020 RNA polymerase;ko05016 Huntington's disease;ko05169 Epstein-Barr virus infection;
gene36483	K03635	MOCS2, moaE	molybdopterin synthase catalytic subunit	EC:2.-.-.-	2	ko00790 Folate biosynthesis;ko04122 Sulfur relay system;
gene36485	K03127	TAF13	transcription initiation factor TFIID subunit 13	--	2	ko03022 Basal transcription factors;ko05168 Herpes simplex infection;
gene36489	K01184	E3.2.1.15	polygalacturonase	EC:3.2.1.15	2	ko00040 Pentose and glucuronate interconversions;ko00500 Starch and sucrose metabolism;
gene3648	K05864	PPID, CYPD	peptidyl-prolyl isomerase D	EC:5.2.1.8		
gene36491	K01673	cynT, can	carbonic anhydrase	EC:4.2.1.1	1	ko00910 Nitrogen metabolism;
gene36502	K09493	CCT1, TCP1	T-complex protein 1 subunit alpha	--		
gene36504	K05353	PMT	putrescine N-methyltransferase	EC:2.1.1.53	1	ko00960 Tropane, piperidine and pyridine alkaloid biosynthesis;
gene36505	K05353	PMT	putrescine N-methyltransferase	EC:2.1.1.53	1	ko00960 Tropane, piperidine and pyridine alkaloid biosynthesis;

gene36512	K04733	IRAK4	interleukin-1 receptor-associated kinase 4	EC:2.7.11.1	11	ko04064 NF-kappa B signaling pathway;ko04210 Apoptosis;ko04620 Toll-like receptor signaling pathway;ko04722 Neurotrophin signaling pathway;ko05133 Pertussis;ko05140 Leishmaniasis;ko05142 Chagas disease (American trypanosomiasis);ko05145 Toxoplasmosis;ko05152 Tuberculosis;ko05162 Measles;ko05164 Influenza A;
gene36513	K11251	H2A	histone H2A	--	2	ko05034 Alcoholism;ko05322 Systemic lupus erythematosus;
gene36514	K15397	KCS	3-ketoacyl-CoA synthase	EC:2.3.1.199	1	ko00062 Fatty acid elongation;
gene36519	K14786	KRI1	protein KRI1	--		
gene36526	K01952	purL, PFAS	phosphoribosylformylglycinamidine synthase	EC:6.3.5.3	1	ko00230 Purine metabolism;
gene36527	K01938	fhs	formate--tetrahydrofolate ligase	EC:6.3.4.3	3	ko00670 One carbon pool by folate;ko00720 Carbon fixation pathways in prokaryotes;ko01200 Carbon metabolism;
gene36528	K11430	EZH2	histone-lysine N-methyltransferase EZH2	EC:2.1.1.43	1	ko05206 MicroRNAs in cancer;
gene36529	K13717	OTUD3	OTU domain-containing protein 3	--		
gene36530	K02208	CDK8_11	cyclin-dependent kinase 8/11	EC:2.7.11.22 2.7.11.23		
gene36534	K00940	E2.7.4.6, ndk	nucleoside-diphosphate kinase	EC:2.7.4.6	2	ko00230 Purine metabolism;ko00240 Pyrimidine metabolism;
gene3653	K13412	CPK	calcium-dependent protein kinase	EC:2.7.11.1	2	ko04626 Plant-pathogen interaction;ko05145 Toxoplasmosis;
gene36541	K01177	E3.2.1.2	beta-amylase	EC:3.2.1.2	1	ko00500 Starch and sucrose metabolism;
gene36546	K17275	PLS1	plastin-1	--		
gene36547	K10520	ABTB1, BPOZ	ankyrin repeat and BTB/POZ domain-containing protein 1	--		
gene36559	K10949	KDELR	ER lumen protein retaining receptor	--	1	ko05110 Vibrio cholerae infection;
gene36562	K01244	MTN	5'-methylthioadenosine nucleosidase	EC:3.2.2.16	1	ko00270 Cysteine and methionine metabolism;
gene36567	K16075	MRS2, MFM1	magnesium transporter	--		
gene36568	K16075	MRS2, MFM1	magnesium transporter	--		
gene36574	K01809	E5.3.1.8, manA	mannose-6-phosphate isomerase	EC:5.3.1.8	2	ko00051 Fructose and mannose metabolism;ko00520 Amino sugar and nucleotide sugar metabolism;
gene36575	K01602	rbcS	ribulose-bisphosphate carboxylase small chain	EC:4.1.1.39	3	ko00630 Glyoxylate and dicarboxylate metabolism;ko00710 Carbon fixation in photosynthetic organisms;ko01200 Carbon metabolism;
gene3657	K11518	TOM40	mitochondrial import receptor subunit TOM40	--	1	ko05014 Amyotrophic lateral sclerosis (ALS);
gene36582	K14431	TGA	transcription factor TGA	--	1	ko04075 Plant hormone signal transduction;
gene36584	K00419	QCR9, UCRC	ubiquinol-cytochrome c reductase subunit 9	--	6	ko00190 Oxidative phosphorylation;ko04260 Cardiac muscle contraction;ko04932 Non-alcoholic fatty liver disease (NAFLD);ko05010 Alzheimer's disease;ko05012 Parkinson's disease;ko05016 Huntington's disease;
gene36585	K02865	RP-L10Ae, RPL10A	large subunit ribosomal protein L10Ae	--	1	ko03010 Ribosome;
gene36586	K02639	petF	ferredoxin	--	1	ko00195 Photosynthesis;
gene36588	K08493	VTI1	vesicle transport through interaction with t-SNAREs 1	--	1	ko04130 SNARE interactions in vesicular transport;
gene36590	K12741	HNRNPA1_3	heterogeneous nuclear ribonucleoprotein A1/A3	--	1	ko03040 Spliceosome;
gene36591	K14431	TGA	transcription factor TGA	--	1	ko04075 Plant hormone signal transduction;
gene36594	K00511	SQLE, ERG1	squalene monooxygenase	EC:1.14.13.132	1	ko00909 Sesquiterpenoid and triterpenoid biosynthesis;
gene36599	K13436	PTI1	pto-interacting protein 1	EC:2.7.11.1	1	ko04626 Plant-pathogen interaction;
gene36600	K11975	RNF144	E3 ubiquitin-protein ligase RNF144	EC:6.3.2.19		
gene36608	K09872	PIP	aquaporin PIP	--		
gene36609	K14488	SAUR	SAUR family protein	--	1	ko04075 Plant hormone signal transduction;
gene36612	K08235	E2.4.1.207	xyloglucan:xyloglucosyl transferase	EC:2.4.1.207		
gene36615	K13448	CML	calcium-binding protein CML	--	1	ko04626 Plant-pathogen interaction;
gene36618	K11446	JARID1	histone demethylase JARID1	EC:1.14.11.-		
gene36619	K14236	tRNA-Tyr	tRNA Tyr	--	1	ko00970 Aminoacyl-tRNA biosynthesis;
gene3661	K09580	PDIA1, P4HB	protein disulfide-isomerase A1	EC:5.3.4.1	1	ko04141 Protein processing in endoplasmic reticulum;
gene36622	K12829	SF3B2, SAP145, CUS1	splicing factor 3B subunit 2	--	1	ko03040 Spliceosome;
gene3662	K17108	GBA2	non-lysosomal glucosylceramidase	EC:3.2.1.45	2	ko00511 Other glycan degradation;ko00600 Sphingolipid metabolism;
gene36636	K14638	SLC15A3_4, PHT	solute carrier family 15 (peptide/histidine transporter), member 3/4	--		
gene36637	K17087	TM9SF3	transmembrane 9 superfamily member 3	--		
gene36642	K02902	RP-L28, MRPL28, rpmB	large subunit ribosomal protein L28	--	1	ko03010 Ribosome;

gene36643	K05658	ABCB1	ATP-binding cassette, subfamily B (MDR/TAP), member 1	--	3	ko02010 ABC transporters;ko04976 Bile secretion;ko05206 MicroRNAs in cancer;
gene36646	K07877	RAB2A	Ras-related protein Rab-2A	--		
gene36648	K14785	ESF2, ABT1	ESF2/ABP1 family protein	--		
gene36649	K00787	FDPS	farnesyl diphosphate synthase	EC:2.5.1.1 2.5.1.10	3	ko00900 Terpenoid backbone biosynthesis;ko05164 Influenza A;ko05166 HTLV-I infection;
gene36651	K03283	HSPA1_8	heat shock 70kDa protein 1/8	--	11	ko03040 Spliceosome;ko04010 MAPK signaling pathway;ko04141 Protein processing in endoplasmic reticulum;ko04144 Endocytosis;ko04612 Antigen processing and presentation;ko04915 Estrogen signaling pathway;ko05134 Legionellosis;ko05145 Toxoplasmosis;ko05162 Measles;ko05164 Influenza A;ko05169 Epstein-Barr virus infection;
gene36655	K11593	ELF2C	eukaryotic translation initiation factor 2C	--		
gene36657	K16292	CEP, CYSEP	KDEL-tailed cysteine endopeptidase	EC:3.4.22.-		
gene36664	K02639	petF	ferredoxin	--	1	ko00195 Photosynthesis;
gene36670	K14207	SLC38A2, SNAT2	solute carrier family 38 (sodium-coupled neutral amino acid transporter), member 2	--	3	ko04724 Glutamatergic synapse;ko04727 GABAergic synapse;ko04974 Protein digestion and absorption;
gene36672	K11252	H2B	histone H2B	--	3	ko05034 Alcoholism;ko05203 Viral carcinogenesis;ko05322 Systemic lupus erythematosus;
gene36678	K00454	LOX2S	lipoxygenase	EC:1.13.11.12	2	ko00591 Linoleic acid metabolism;ko00592 alpha-Linolenic acid metabolism;
gene36680	K14233	tRNA-Ser	tRNA Ser	--	1	ko00970 Aminoacyl-tRNA biosynthesis;
gene36688	K04082	hscB, HSCB, HSC20	molecular chaperone HscB	--		
gene36691	K11666	INO80B, ZNHIT4, PAPA1	INO80 complex subunit B	--		
gene36695	K01358	clpP, CLPP	ATP-dependent Clp protease, protease subunit	EC:3.4.21.92	1	ko04112 Cell cycle - Caulobacter;
gene366	K06671	STAG1_2, SCC3, IRR1	cohesin complex subunit SA-1/2	--	3	ko04110 Cell cycle;ko04111 Cell cycle - yeast;ko04113 Meiosis - yeast;
gene36703	K11251	H2A	histone H2A	--	2	ko05034 Alcoholism;ko05322 Systemic lupus erythematosus;
gene36707	K10743	RNASEH2A	ribonuclease H2 subunit A	EC:3.1.26.4	1	ko03030 DNA replication;
gene36710	K01982	LSUrRNA	large subunit ribosomal RNA	--	2	ko03008 Ribosome biogenesis in eukaryotes;ko03010 Ribosome;
gene3671	K03260	EIF4G	translation initiation factor 4G	--	2	ko03013 RNA transport;ko05416 Viral myocarditis;
gene36725	K00966	GMPP	mannose-1-phosphate guanylyltransferase	EC:2.7.7.13	2	ko00051 Fructose and mannose metabolism;ko00520 Amino sugar and nucleotide sugar metabolism;
gene36727	K15414	C1QBP	complement component 1 Q subcomponent-binding protein, mitochondrial	--	1	ko05168 Herpes simplex infection;
gene36736	K09422	MYBP	myb proto-oncogene protein, plant	--		
gene36739	K14488	SAUR	SAUR family protein	--	1	ko04075 Plant hormone signal transduction;
gene36740	K15398	CYP86A4S	cytochrome P450, family 86, subfamily A, polypeptide 2/4/7/8 (fatty acid omega-hydroxylase)	EC:1.14.-.-	1	ko00073 Cutin, suberine and wax biosynthesis;
gene36743	K14998	SURF1	surfeit locus 1 family protein	--		
gene36747	K14641	APY1_2	apyrase	EC:3.6.1.5	2	ko00230 Purine metabolism;ko00240 Pyrimidine metabolism;
gene36750	K00826	E2.6.1.42, ilvE	branched-chain amino acid aminotransferase	EC:2.6.1.42	5	ko00280 Valine, leucine and isoleucine degradation;ko00290 Valine, leucine and isoleucine biosynthesis;ko00770 Pantothenate and CoA biosynthesis;ko01210 2-Oxocarboxylic acid metabolism;ko01230 Biosynthesis of amino acids;
gene36756	K12637	CYP90C1, ROT3	cytochrome P450, family 90, subfamily C, polypeptide 1 (3-epi-6-deoxocathasterone 23-monooxygenase)	EC:1.14.13.11 2	1	ko00905 Brassinosteroid biosynthesis;
gene36758	K14191	DIM1	18S rRNA (adenine1779-N6/adenine1780-N6)-dimethyltransferase	EC:2.1.1.183		
gene3675	K10389	TUBG	tubulin gamma	--		
gene36760	K03671	trxA	thioredoxin 1	--		
gene36764	K01214	E3.2.1.68	isoamylase	EC:3.2.1.68		
gene36765	K13420	FLS2	LRR receptor-like serine/threonine-protein kinase FLS2	EC:2.7.11.1	1	ko04626 Plant-pathogen interaction;
gene36766	K13420	FLS2	LRR receptor-like serine/threonine-protein kinase FLS2	EC:2.7.11.1	1	ko04626 Plant-pathogen interaction;
gene36767	K16292	CEP, CYSEP	KDEL-tailed cysteine endopeptidase	EC:3.4.22.-		
gene36770	K07018	K07018				

gene36780	K15422	SAL	3'(2'), 5'-bisphosphate nucleotidase / inositol polyphosphate 1-phosphatase	EC:3.1.3.7 3.1.3.57	3	ko00562 Inositol phosphate metabolism;ko00920 Sulfur metabolism;ko04070 Phosphatidylinositol signaling system;
gene36781	K00166	E1.2.4.4A, bkdA1	2-oxoisovalerate dehydrogenase E1 component, alpha subunit	EC:1.2.4.4	1	ko00280 Valine, leucine and isoleucine degradation;
gene36785	K02260	COX17	cytochrome c oxidase assembly protein subunit 17	--	1	ko00190 Oxidative phosphorylation;
gene36791	K14496	PYL	abscisic acid receptor PYR/PYL family	--	1	ko04075 Plant hormone signal transduction;
gene36801	K11434	PRMT1	protein arginine N-methyltransferase 1	EC:2.1.1.-		
gene36802	K12837	U2AF2	splicing factor U2AF 65 kDa subunit	--	1	ko03040 Spliceosome;
gene36806	K14486	K14486, ARF	auxin response factor	--	1	ko04075 Plant hormone signal transduction;
gene36811	K09754	CYP98A3, C3'H	coumaroylquinate(coumaroylshikimate) 3'-monooxygenase	EC:1.14.13.36	3	ko00940 Phenylpropanoid biosynthesis;ko00941 Flavonoid biosynthesis;ko00945 Stilbenoid, diarylheptanoid and gingerol biosynthesis;
gene36812	K05236	COPA	coatomer protein complex, subunit alpha (xenin)	--	1	ko04080 Neuroactive ligand-receptor interaction;
gene3681	K07342	SEC61G, SSS1, secE	protein transport protein SEC61 subunit gamma and related proteins	--	4	ko03060 Protein export;ko04141 Protein processing in endoplasmic reticulum;ko04145 Phagosome;ko05110 Vibrio cholerae infection;
gene36823	K05280	E1.14.13.21	flavonoid 3'-monooxygenase	EC:1.14.13.21	2	ko00941 Flavonoid biosynthesis;ko00944 Flavone and flavonol biosynthesis;
gene3682	K06941	rlmN	23S rRNA (adenine2503-C2)-methyltransferase	EC:2.1.1.192		
gene36830	K07342	SEC61G, SSS1, secE	protein transport protein SEC61 subunit gamma and related proteins	--	4	ko03060 Protein export;ko04141 Protein processing in endoplasmic reticulum;ko04145 Phagosome;ko05110 Vibrio cholerae infection;
gene36834	K11792	DDA1	DET1- and DDB1-associated protein 1	--		
gene36835	K01762	ACS	1-aminocyclopropane-1-carboxylate synthase	EC:4.4.1.14	1	ko00270 Cysteine and methionine metabolism;
gene36838	K04079	htpG, HSP90A	molecular chaperone HtpG	--	9	ko04141 Protein processing in endoplasmic reticulum;ko04151 PI3K-Akt signaling pathway;ko04612 Antigen processing and presentation;ko04621 NOD-like receptor signaling pathway;ko04626 Plant-pathogen interaction;ko04914 Progesterone-mediated oocyte maturation;ko04915 Estrogen signaling pathway;ko05200 Pathways in cancer;ko05215 Prostate cancer;
gene3683	K00721	DPM1	dolichol-phosphate mannosyltransferase	EC:2.4.1.83	1	ko00510 N-Glycan biosynthesis;
gene36844	K01187	E3.2.1.20, malZ	alpha-glucosidase	EC:3.2.1.20	2	ko00052 Galactose metabolism;ko00500 Starch and sucrose metabolism;
gene36850	K12844	PRPF31	U4/U6 small nuclear ribonucleoprotein PRP31	--	1	ko03040 Spliceosome;
gene36854	K01893	NARS, asnS	asparaginyl-tRNA synthetase	EC:6.1.1.22	1	ko00970 Aminoacyl-tRNA biosynthesis;
gene36855	K14948	PTBP2, NPTB	olypyrimidine tract-binding protein 2	--		
gene3685	K02895	RP-L24, MRPL24, rplX	large subunit ribosomal protein L24	--	1	ko03010 Ribosome;
gene36862	K14431	TGA	transcription factor TGA	--	1	ko04075 Plant hormone signal transduction;
gene36868	K00164	OGDH, sucA	2-oxoglutarate dehydrogenase E1 component	EC:1.2.4.2	4	ko00020 Citrate cycle (TCA cycle);ko00310 Lysine degradation;ko00380 Tryptophan metabolism;ko01200 Carbon metabolism;
gene36874	K06276	PDPK1	3-phosphoinositide dependent protein kinase-1	EC:2.7.11.1	9	ko03320 PPAR signaling pathway;ko04150 mTOR signaling pathway;ko04151 PI3K-Akt signaling pathway;ko04510 Focal adhesion;ko04910 Insulin signaling pathway;ko04960 Aldosterone-regulated sodium reabsorption;ko05213 Endometrial cancer;ko05215 Prostate cancer;ko05223 Non-small cell lung cancer;
gene36876	K02838	frr, MRRF, RRF	ribosome recycling factor	--		
gene36877	K14220	tRNA-Asn	tRNA Asn	--	1	ko00970 Aminoacyl-tRNA biosynthesis;
gene36893	K16297	SCPL-II	serine carboxypeptidase-like clade II	EC:3.4.16.-		
gene36894	K16297	SCPL-II	serine carboxypeptidase-like clade II	EC:3.4.16.-		
gene36895	K00670	E2.3.1.88	peptide alpha-N-acetyltransferase	EC:2.3.1.88		
gene36898	K01652	E2.2.1.6L, ilvB, ilvG, ilvI	acetolactate synthase I/II/III large subunit	EC:2.2.1.6	6	ko00290 Valine, leucine and isoleucine biosynthesis;ko00650 Butanoate metabolism;ko00660 C5-Branched dibasic acid metabolism;ko00770 Pantothenate and CoA biosynthesis;ko01210 2-Oxocarboxylic acid metabolism;ko01230 Biosynthesis of amino acids;
gene36900	K13128	ZCCHC8	zinc finger CCHC domain-containing protein 8	--		
gene36904	K06130	LYPLA2	lysophospholipase II	EC:3.1.1.5	1	ko00564 Glycerophospholipid metabolism;
gene36905	K09338	HD-ZIP	homeobox-leucine zipper protein	--		
gene36906	K05275	E1.1.1.65	pyridoxine 4-dehydrogenase	EC:1.1.1.65	1	ko00750 Vitamin B6 metabolism;
gene36915	K11778	DHDDS, RER2, SRT1	ditrans,polycis-polyprenyl diphosphate synthase	EC:2.5.1.87	1	ko00900 Terpenoid backbone biosynthesis;
gene36916	K00559	E2.1.1.41, SMT1, ERG6	sterol 24-C-methyltransferase	EC:2.1.1.41	1	ko00100 Steroid biosynthesis;

gene36917	K05658	ABCB1	ATP-binding cassette, subfamily B (MDR/TAP), member 1	--	3	ko02010 ABC transporters;ko04976 Bile secretion;ko05206 MicroRNAs in cancer;
gene36919	K07408	CYP1A1	cytochrome P450, family 1, subfamily A, polypeptide 1	EC:1.14.14.1	6	ko00140 Steroid hormone biosynthesis;ko00380 Tryptophan metabolism;ko00830 Retinol metabolism;ko00980 Metabolism of xenobiotics by cytochrome P450;ko04913 Ovarian steroidogenesis;ko05204 Chemical carcinogenesis;
gene3691	K11303	HAT1, KAT1	histone acetyltransferase 1	EC:2.3.1.48	1	ko05034 Alcoholism;
gene36926	K04371	MAPK1_3	mitogen-activated protein kinase 1/3	EC:2.7.11.24	71	ko04010 MAPK signaling pathway;ko04012 ErbB signaling pathway;ko04013 MAPK signaling pathway - fly;ko04014 Ras signaling pathway;ko04062 Chemokine signaling pathway;ko04066 HIF-1 signaling pathway;ko04114 Oocyte meiosis;ko04150 mTOR signaling pathway;ko04151 PI3K-Akt signaling pathway;ko04270 Vascular smooth muscle contraction;ko04320 Dorso-ventral axis formation;ko04350 TGF-beta signaling pathway;ko04360 Axon guidance;ko04370 VEGF signaling pathway;ko04380 Osteoclast differentiation;ko04510 Focal adhesion;ko04520 Adherens junction;ko04540 Gap junction;ko04620 Toll-like receptor signaling pathway;ko04621 NOD-like receptor signaling pathway;ko04650 Natural killer cell mediated cytotoxicity;ko04660 T cell receptor signaling pathway;ko04662 B cell receptor signaling pathway;ko04664 Fc epsilon RI signaling pathway;ko04666 Fc gamma R-mediated phagocytosis;ko04668 TNF signaling pathway;ko04713 Circadian entrainment;ko04720 Long-term potentiation;ko04722 Neurotrophin signaling pathway;ko04723 Retrograde endocannabinoid signaling;ko04724 Glutamatergic synapse;ko04725 Cholinergic synapse;ko04726 Serotonergic synapse;ko04730 Long-term depression;ko04810 Regulation of actin cytoskeleton;ko04910 Insulin signaling pathway;ko04912 GnRH signaling pathway;ko04914 Progesterone-mediated oocyte maturation;ko04915 Estrogen signaling pathway;ko04916 Melanogenesis;ko04917 Prolactin signaling pathway;ko04930 Type II diabetes mellitus;ko04960 Aldosterone-regulated sodium reabsorption;ko05010 Alzheimer's disease;ko05020 Prion diseases;ko05034 Alcoholism;ko05131 Shigellosis;ko05132 Salmonella infection;ko05133 Pertussis;ko05140 Leishmaniasis;ko05142 Chagas disease (American trypanosomiasis);ko05145 Toxoplasmosis;ko05152 Tuberculosis;ko05160 Hepatitis C;ko05161 Hepatitis B;ko05164 Influenza A;ko05200 Pathways in cancer;ko05203 Viral carcinogenesis;ko05205 Proteoglycans in cancer;ko05210 Colorectal cancer;ko05211 Renal cell carcinoma;ko05212
gene36929	K01246	tag	DNA-3-methyladenine glycosylase I	EC:3.2.2.20	1	ko03410 Base excision repair;
gene3692	K02910	RP-L31e, RPL31	large subunit ribosomal protein L31e	--	1	ko03010 Ribosome;
gene36934	K00021	E1.1.1.34	hydroxymethylglutaryl-CoA reductase (NADPH)	EC:1.1.1.34	2	ko00900 Terpenoid backbone biosynthesis;ko04976 Bile secretion;
gene36935	K00384	E1.8.1.9, trxB	thioredoxin reductase (NADPH)	EC:1.8.1.9	2	ko00240 Pyrimidine metabolism;ko00450 Selenocompound metabolism;
gene36936	K03165	TOP3	DNA topoisomerase III	EC:5.99.1.2	2	ko03440 Homologous recombination;ko03460 Fanconi anemia pathway;
gene36937	K09286	EREBP	EREBP-like factor	--		
gene36938	K00434	E1.11.1.11	L-ascorbate peroxidase	EC:1.11.1.11	2	ko00053 Ascorbate and aldarate metabolism;ko00480 Glutathione metabolism;
gene36939	K15731	CTDSP	carboxy-terminal domain RNA polymerase II polypeptide A small phosphatase	EC:3.1.3.16		
gene36940	K10661	MARCH6, DOA10	E3 ubiquitin-protein ligase MARCH6	EC:6.3.2.19	1	ko04141 Protein processing in endoplasmic reticulum;
gene36945	K10684	UBLE1A, SAE1	ubiquitin-like 1-activating enzyme E1 A	EC:6.3.2.19	1	ko04120 Ubiquitin mediated proteolysis;
gene36947	K02998	RP-SAe, RPSA	small subunit ribosomal protein SAe	--	1	ko03010 Ribosome;
gene36952	K15728	LPIN	phosphatidate phosphatase LPIN	EC:3.1.3.4	2	ko00561 Glycerolipid metabolism;ko00564 Glycerophospholipid metabolism;
gene36953	K15728	LPIN	phosphatidate phosphatase LPIN	EC:3.1.3.4	2	ko00561 Glycerolipid metabolism;ko00564 Glycerophospholipid metabolism;
gene36955	K14499	BKI1	BRI1 kinase inhibitor 1	--	1	ko04075 Plant hormone signal transduction;
gene36962	K08873	SMG1	PI-3-kinase-related kinase SMG-1	--	1	ko03015 mRNA surveillance pathway;
gene36969	K15397	KCS	3-ketoacyl-CoA synthase	EC:2.3.1.199	1	ko00062 Fatty acid elongation;

gene36971	K00737	MGAT3	beta-1,4-mannosyl-glycoprotein beta-1,4-N-acetylglucosaminyltransferase	EC:2.4.1.144	1	ko00510 N-Glycan biosynthesis;
gene36972	K12844	PRPF31	U4/U6 small nuclear ribonucleoprotein PRP31	--	1	ko03040 Spliceosome;
gene36977	K14972	PAXIP1, PTIP	PAX-interacting protein 1	--		
gene36979	K08065	NFYB	nuclear transcription Y subunit beta	--	3	ko04612 Antigen processing and presentation;ko05152 Tuberculosis;ko05166 HTLV-I infection;
gene36983	K14512	MPK6	mitogen-activated protein kinase 6	EC:2.7.11.24	1	ko04075 Plant hormone signal transduction;
gene36987	K14496	PYL	abscisic acid receptor PYR/PYL family	--	1	ko04075 Plant hormone signal transduction;
gene36990	K14803	PTC2_3	protein phosphatase 2C homolog 2/3	EC:3.1.3.16		
gene36991	K16297	SCPL-II	serine carboxypeptidase-like clade II	EC:3.4.16.-		
gene37003	K08472	MLO	mlo protein	--		
gene37005	K14753	RACK1	guanine nucleotide-binding protein subunit beta-2-like 1 protein	--	1	ko05162 Measles;
gene37007	K08343	ATG3	autophagy-related protein 3	--	1	ko04140 Regulation of autophagy;
gene37011	K15414	C1QBP	complement component 1 Q subcomponent-binding protein, mitochondrial	--	1	ko05168 Herpes simplex infection;
gene37012	K10573	UBE2A, UBC2, RAD6A	ubiquitin-conjugating enzyme E2 A	EC:6.3.2.19	1	ko04120 Ubiquitin mediated proteolysis;
gene37018	K15382	SLC50A, SWEET	solute carrier family 50 (sugar transporter)	--		
gene37021	K14833	NOC2	nucleolar complex protein 2	--		
gene37033	K17081	PHB2	prohibitin 2	--		
gene37036	K00939	E2.7.4.3, adk	adenylate kinase	EC:2.7.4.3	1	ko00230 Purine metabolism;
gene3703	K08150	SLC2A13, ITR	MFS transporter, SP family, solute carrier family 2 (myo-inositol transporter), member 13	--		
gene37044	K08054	CANX	calnexin	--	5	ko04141 Protein processing in endoplasmic reticulum;ko04145 Phagosome;ko04612 Antigen processing and presentation;ko04918 Thyroid hormone synthesis;ko05166 HTLV-I infection;
gene37045	K13447	RBOH	respiratory burst oxidase	EC:1.6.3.-1.11.1.-	1	ko04626 Plant-pathogen interaction;
gene3704	K13993	HSP20	HSP20 family protein	--	1	ko04141 Protein processing in endoplasmic reticulum;
gene37054	K15193	SPTY2D1, SPT2	protein SPT2	--		
gene37065	K00514	ZDS, crtQ	zeta-carotene desaturase	EC:1.3.5.6	1	ko00906 Carotenoid biosynthesis;
gene37076	K05909	E1.10.3.2				
gene37090	K03872	TCEB1	transcription elongation factor B, polypeptide 1	--	4	ko04066 HIF-1 signaling pathway;ko04120 Ubiquitin mediated proteolysis;ko05200 Pathways in cancer;ko05211 Renal cell carcinoma;
gene37097	K00430	E1.11.1.7	peroxidase	EC:1.11.1.7	2	ko00360 Phenylalanine metabolism;ko00940 Phenylpropanoid biosynthesis;
gene37102	K14539	LSG1	large subunit GTPase 1	EC:3.6.1.-	1	ko03008 Ribosome biogenesis in eukaryotes;
gene37103	K14611	SLC23A1_2, SVCT1_2	solute carrier family 23 (nucleobase transporter), member 1/2	--		
gene37105	K13161	HNRNPR	heterogeneous nuclear ribonucleoprotein R	--		
gene37106	K00627	DLAT, aceF, pdhC	pyruvate dehydrogenase E2 component (dihydrolipoamide acetyltransferase)	EC:2.3.1.12	4	ko00010 Glycolysis / Gluconeogenesis;ko00020 Citrate cycle (TCA cycle);ko00620 Pyruvate metabolism;ko01200 Carbon metabolism;
gene37111	K00599	E2.1.1.-			2	ko00350 Tyrosine metabolism;ko00624 Polycyclic aromatic hydrocarbon degradation;
gene37112	K14497	PP2C	protein phosphatase 2C	EC:3.1.3.16	1	ko04075 Plant hormone signal transduction;
gene37115	K16297	SCPL-II	serine carboxypeptidase-like clade II	EC:3.4.16.-		
gene37118	K02259	COX15	cytochrome c oxidase assembly protein subunit 15	--	3	ko00190 Oxidative phosphorylation;ko00860 Porphyrin and chlorophyll metabolism;ko02020 Two-component system;
gene37120	K13420	FLS2	LRR receptor-like serine/threonine-protein kinase FLS2	EC:2.7.11.1	1	ko04626 Plant-pathogen interaction;
gene37128	K12619	XRN2, RAT1	5'-3' exoribonuclease 2	EC:3.1.13.-	2	ko03008 Ribosome biogenesis in eukaryotes;ko03018 RNA degradation;
gene37129	K00083	E1.1.1.195	cinnamyl-alcohol dehydrogenase	EC:1.1.1.195	1	ko00940 Phenylpropanoid biosynthesis;
gene37137	K01868	TARS, thrS	threonyl-tRNA synthetase	EC:6.1.1.3	1	ko00970 Aminoacyl-tRNA biosynthesis;
gene37138	K00083	E1.1.1.195	cinnamyl-alcohol dehydrogenase	EC:1.1.1.195	1	ko00940 Phenylpropanoid biosynthesis;
gene37145	K15498	PPP6C	serine/threonine-protein phosphatase 6 catalytic subunit	EC:3.1.3.16		
gene37147	K03801	lipB	lipoyl(octanoyl) transferase	EC:2.3.1.181	1	ko00785 Lipoic acid metabolism;
gene37151	K01809	E5.3.1.8, manA	mannose-6-phosphate isomerase	EC:5.3.1.8	2	ko00051 Fructose and mannose metabolism;ko00520 Amino sugar and nucleotide sugar metabolism;

gene37153	K15803	GERD	(-)-germacrene D synthase	EC:4.2.3.22 4.2.3.75	1	ko00909 Sesquiterpenoid and triterpenoid biosynthesis;
gene37155	K02133	ATPeF1B, ATP5B	F-type H+-transporting ATPase subunit beta	EC:3.6.3.14	4	ko00190 Oxidative phosphorylation;ko05010 Alzheimer's disease;ko05012 Parkinson's disease;ko05016 Huntington's disease;
gene37158	K06272	ILK	integrin-linked kinase	EC:2.7.11.1	4	ko03320 PPAR signaling pathway;ko04510 Focal adhesion;ko05100 Bacterial invasion of epithelial cells;ko05213 Endometrial cancer;
gene3715	K05389	KCNKF	potassium channel subfamily K, other eukaryote	--		
gene37160	K10881	SHFM1, DSS1	26 proteasome complex subunit DSS1	--	3	ko03050 Proteasome;ko03440 Homologous recombination;ko05169 Epstein-Barr virus infection;
gene37161	K11338	RUVBL2, RVB2, INO80J	RuvB-like protein 2	EC:3.6.4.12		
gene37163	K14797	ENP1, BYSL	essential nuclear protein 1	--		
gene37165	K16251	NRPE1	DNA-directed RNA polymerase V subunit 1	EC:2.7.7.6		
gene3716	K14007	SEC24	protein transport protein SEC24	--	1	ko04141 Protein processing in endoplasmic reticulum;
gene37171	K00430	E1.11.1.7	peroxidase	EC:1.11.1.7	2	ko00360 Phenylalanine metabolism;ko00940 Phenylpropanoid biosynthesis;
gene37172	K00799	GST, gst	glutathione S-transferase	EC:2.5.1.18	4	ko00480 Glutathione metabolism;ko00980 Metabolism of xenobiotics by cytochrome P450;ko00982 Drug metabolism - cytochrome P450;ko05204 Chemical carcinogenesis;
gene37173	K07766	E3.6.1.52	diphosphoinositol-polyphosphate diphosphatase	EC:3.6.1.52		
gene37189	K00021	E1.1.1.34	hydroxymethylglutaryl-CoA reductase (NADPH)	EC:1.1.1.34	2	ko00900 Terpenoid backbone biosynthesis;ko04976 Bile secretion;
gene3718	K08737	MSH6	DNA mismatch repair protein MSH6	--	3	ko03430 Mismatch repair;ko05200 Pathways in cancer;ko05210 Colorectal cancer;
gene37191	K00888	PI4K	phosphatidylinositol 4-kinase	EC:2.7.1.67	2	ko00562 Inositol phosphate metabolism;ko04070 Phosphatidylinositol signaling system;
gene37193	K05610	UCHL5, UCH37	ubiquitin carboxyl-terminal hydrolase L5	EC:3.4.19.12		
gene37196	K10867	NBN, NBS1	nijmegen breakage syndrome protein 1	--	1	ko03440 Homologous recombination;
gene37197	K01663	HIS7	glutamine amidotransferase / cyclase	EC:2.4.2.- 4.1.3.-	2	ko00340 Histidine metabolism;ko01230 Biosynthesis of amino acids;
gene37198	K00658	DLST, sucB	2-oxoglutarate dehydrogenase E2 component (dihydrolipoamide succinyltransferase)	EC:2.3.1.61	3	ko00020 Citrate cycle (TCA cycle);ko00310 Lysine degradation;ko01200 Carbon metabolism;
gene37199	K08739	MLH3	DNA mismatch repair protein MLH3	--	1	ko03430 Mismatch repair;
gene37200	K12449	AXS	UDP-apiose/xylose synthase	--	1	ko00520 Amino sugar and nucleotide sugar metabolism;
gene37204	K01052	LIPA	lysosomal acid lipase/cholesteryl ester hydrolase	EC:3.1.1.13	2	ko00100 Steroid biosynthesis;ko04142 Lysosome;
gene37207	K01246	tag	DNA-3-methyladenine glycosylase I	EC:3.2.2.20	1	ko03410 Base excision repair;
gene3721	K03255	TIF31, CLU1	protein TIF31	--		
gene37226	K15095	E1.1.1.208	(+)-neomenthol dehydrogenase	EC:1.1.1.208	1	ko00902 Monoterpenoid biosynthesis;
gene37228	K12883	NCBP2, CBP20	nuclear cap-binding protein subunit 2	--	3	ko03013 RNA transport;ko03015 mRNA surveillance pathway;ko03040 Spliceosome;
gene37229	K03681	RRP40, EXOSC3	exosome complex component RRP40	--	1	ko03018 RNA degradation;
gene37232	K10950	ERO1L	ERO1-like protein alpha	EC:1.8.4.-	2	ko04141 Protein processing in endoplasmic reticulum;ko05110 Vibrio cholerae infection;
gene37236	K17413	MRPS35	small subunit ribosomal protein S35, mitochondrial	--		
gene37241	K03348	APC1	anaphase-promoting complex subunit 1	--	7	ko04110 Cell cycle;ko04111 Cell cycle - yeast;ko04113 Meiosis - yeast;ko04114 Oocyte meiosis;ko04120 Ubiquitin mediated proteolysis;ko04914 Progesterone-mediated oocyte maturation;ko05166 HTLV-I infection;
gene37242	K02941	RP-LP0, RPLP0	large subunit ribosomal protein LP0	--	1	ko03010 Ribosome;
gene37243	K08490	STX5	syntaxin 5	--	1	ko04130 SNARE interactions in vesicular transport;
gene37245	K10532	HGSNAT	heparan-alpha-glucosaminide N-acetyltransferase	EC:2.3.1.78	2	ko00531 Glycosaminoglycan degradation;ko04142 Lysosome;
gene37246	K09422	MYBP	myb proto-oncogene protein, plant	--		
gene37247	K16296	SCPL-I	serine carboxypeptidase-like clade I	EC:3.4.16.-		
gene37255	K14560	IMP3	U3 small nucleolar ribonucleoprotein protein IMP3	--	1	ko03008 Ribosome biogenesis in eukaryotes;
gene37260	K14826	FPR3_4	FK506-binding nuclear protein	EC:5.2.1.8		
gene37264	K00921	PIKFYVE, FAB1	1-phosphatidylinositol-3-phosphate 5-kinase	EC:2.7.1.150	4	ko00562 Inositol phosphate metabolism;ko04070 Phosphatidylinositol signaling system;ko04145 Phagosome;ko04810 Regulation of actin cytoskeleton;
gene37265	K01177	E3.2.1.2	beta-amylase	EC:3.2.1.2	1	ko00500 Starch and sucrose metabolism;
gene37267	K13091	RBM39, RNPC2	RNA-binding protein 39	--		
gene3726	K12849	PRPF38A	pre-mRNA-splicing factor 38A	--	1	ko03040 Spliceosome;
gene37271	K03257	EIF4A	translation initiation factor 4A	--	1	ko03013 RNA transport;

gene37272	K03094	SKP1, CBF3D	S-phase kinase-associated protein 1	--	9	ko04110 Cell cycle;ko04111 Cell cycle - yeast;ko04114 Oocyte meiosis;ko04120 Ubiquitin mediated proteolysis;ko04141 Protein processing in endoplasmic reticulum;ko04310 Wnt signaling pathway;ko04350 TGF-beta signaling pathway;ko04710 Circadian rhythm;ko05168 Herpes simplex infection;
gene37277	K14398	CPSF6_7	cleavage and polyadenylation specificity factor subunit 6/7	--	1	ko03015 mRNA surveillance pathway;
gene37278	K17046	DEK	protein DEK	--		
gene37279	K05389	KCNKF	potassium channel subfamily K, other eukaryote	--		
gene3727	K14234	tRNA-Thr	tRNA Thr	--	1	ko00970 Aminoacyl-tRNA biosynthesis;
gene37286	K03884	ND6	NADH-ubiquinone oxidoreductase chain 6	EC:1.6.5.3	2	ko00190 Oxidative phosphorylation;ko05012 Parkinson's disease;
gene37289	K00430	E1.11.1.7	peroxidase	EC:1.11.1.7	2	ko00360 Phenylalanine metabolism;ko00940 Phenylpropanoid biosynthesis;
gene37292	K08869	ADCK, ABC1	aarF domain-containing kinase	--		
gene37293	K00799	GST, gst	glutathione S-transferase	EC:2.5.1.18	4	ko00480 Glutathione metabolism;ko00980 Metabolism of xenobiotics by cytochrome P450;ko00982 Drug metabolism - cytochrome P450;ko05204 Chemical carcinogenesis;
gene37296	K11294	NCL, NSR1	nucleolin	--	1	ko05130 Pathogenic Escherichia coli infection;
gene37297	K11885	DDI1	DNA damage-inducible protein 1	--		
gene3729	K17506	PPM1L, PP2CE	protein phosphatase 1L	EC:3.1.3.16		
gene37303	K08505	SFT1	protein transport protein SFT1	--	1	ko04130 SNARE interactions in vesicular transport;
gene37304	K16292	CEP, CYSEP	KDEL-tailed cysteine endopeptidase	EC:3.4.22.-		
gene37308	K04733	IRAK4	interleukin-1 receptor-associated kinase 4	EC:2.7.11.1	11	ko04064 NF-kappa B signaling pathway;ko04210 Apoptosis;ko04620 Toll-like receptor signaling pathway;ko04722 Neurotrophin signaling pathway;ko05133 Pertussis;ko05140 Leishmaniasis;ko05142 Chagas disease (American trypanosomiasis);ko05145 Toxoplasmosis;ko05152 Tuberculosis;ko05162 Measles;ko05164 Influenza A;
gene37312	K10752	RBBP4, HAT2, CAF1, MIS16	histone-binding protein RBBP4	--		
gene37315	K01568	E4.1.1.1, pdc	pyruvate decarboxylase	EC:4.1.1.1	1	ko00010 Glycolysis / Gluconeogenesis;
gene37316	K00574	E2.1.1.79, cfa	cyclopropane-fatty-acyl-phospholipid synthase	EC:2.1.1.79		
gene37321	K02978	RP-S27e, RPS27	small subunit ribosomal protein S27e	--	1	ko03010 Ribosome;
gene37322	K01778	dapF	diaminopimelate epimerase	EC:5.1.1.7	2	ko00300 Lysine biosynthesis;ko01230 Biosynthesis of amino acids;
gene37324	K14484	IAA	auxin-responsive protein IAA	--	1	ko04075 Plant hormone signal transduction;
gene37326	K01649	leuA	2-isopropylmalate synthase	EC:2.3.3.13	4	ko00290 Valine, leucine and isoleucine biosynthesis;ko00620 Pyruvate metabolism;ko01210 2-Oxocarboxylic acid metabolism;ko01230 Biosynthesis of amino acids;
gene37327	K01649	leuA	2-isopropylmalate synthase	EC:2.3.3.13	4	ko00290 Valine, leucine and isoleucine biosynthesis;ko00620 Pyruvate metabolism;ko01210 2-Oxocarboxylic acid metabolism;ko01230 Biosynthesis of amino acids;
gene37329	K08960	CSNK1E	casein kinase 1, epsilon	EC:2.7.11.1	6	ko04310 Wnt signaling pathway;ko04340 Hedgehog signaling pathway;ko04390 Hippo signaling pathway;ko04391 Hippo signaling pathway - fly;ko04710 Circadian rhythm;ko04711 Circadian rhythm - fly;
gene37330	K04733	IRAK4	interleukin-1 receptor-associated kinase 4	EC:2.7.11.1	11	ko04064 NF-kappa B signaling pathway;ko04210 Apoptosis;ko04620 Toll-like receptor signaling pathway;ko04722 Neurotrophin signaling pathway;ko05133 Pertussis;ko05140 Leishmaniasis;ko05142 Chagas disease (American trypanosomiasis);ko05145 Toxoplasmosis;ko05152 Tuberculosis;ko05162 Measles;ko05164 Influenza A;
gene37333	K08235	E2.4.1.207	xyloglucan:xyloglucosyl transferase	EC:2.4.1.207		
gene37335	K00981	E2.7.7.41, CDS1, CDS2, cdsA	phosphatidate cytidylyltransferase	EC:2.7.7.41	2	ko00564 Glycerophospholipid metabolism;ko04070 Phosphatidylinositol signaling system;
gene37337	K03086	SIG1, rpoD	RNA polymerase primary sigma factor	--		
gene37338	K16298	SCPL-IV	serine carboxypeptidase-like clade IV	EC:3.4.16.-		
gene37339	K04122	E1.14.13.78	ent-kaurene oxidase	EC:1.14.13.78	1	ko00904 Diterpenoid biosynthesis;
gene37344	K09285	OVM, ANT	AP2-like factor, ANT lineage	--		
gene37348	K12619	XRN2, RAT1	5'-3' exoribonuclease 2	EC:3.1.13.-	2	ko03008 Ribosome biogenesis in eukaryotes;ko03018 RNA degradation;
gene37360	K06617	E2.4.1.82	raffinose synthase	EC:2.4.1.82	1	ko00052 Galactose metabolism;
gene37364	K14638	SLC15A3_4, PHT	solute carrier family 15 (peptide/histidine transporter), member 3/4	--		

gene37365	K09338	HD-ZIP	homeobox-leucine zipper protein	--		
gene37370	K02641	petH	ferredoxin--NADP+ reductase	EC:1.18.1.2	1	ko00195 Photosynthesis;
gene37371	K09250	CNBP	cellular nucleic acid-binding protein	--		
gene37373	K00051	E1.1.1.82	malate dehydrogenase (NADP+)	EC:1.1.1.82	2	ko00620 Pyruvate metabolism;ko00710 Carbon fixation in photosynthetic organisms;
gene37380	K00800	aroA	3-phosphoshikimate 1-carboxyvinyltransferase	EC:2.5.1.19	2	ko00400 Phenylalanine, tyrosine and tryptophan biosynthesis;ko01230 Biosynthesis of amino acids;
gene37383	K00588	E2.1.1.104	caffeoyl-CoA O-methyltransferase	EC:2.1.1.104	4	ko00360 Phenylalanine metabolism;ko00940 Phenylpropanoid biosynthesis;ko00941 Flavonoid biosynthesis;ko00945 Stilbenoid, diarylheptanoid and gingerol biosynthesis;
gene37385	K03134	TAF10	transcription initiation factor TFIID subunit 10	--	2	ko03022 Basal transcription factors;ko05168 Herpes simplex infection;
gene37388	K08912	LHCB1	light-harvesting complex II chlorophyll a/b binding protein 1	--	1	ko00196 Photosynthesis - antenna proteins;
gene3738	K01184	E3.2.1.15	polygalacturonase	EC:3.2.1.15	2	ko00040 Pentose and glucuronate interconversions;ko00500 Starch and sucrose metabolism;
gene37393	K10643	CNOT4, NOT4, MOT2	CCR4-NOT transcription complex subunit 4	EC:6.3.2.19	1	ko03018 RNA degradation;
gene37394	K02266	COX6A	cytochrome c oxidase subunit 6a	--	6	ko00190 Oxidative phosphorylation;ko04260 Cardiac muscle contraction;ko04932 Non-alcoholic fatty liver disease (NAFLD);ko05010 Alzheimer's disease;ko05012 Parkinson's disease;ko05016 Huntington's disease;
gene37396	K12192	CHMP2B	charged multivesicular body protein 2B	--	1	ko04144 Endocytosis;
gene37403	K03327	TC.MATE, SLC47A, norM, mdtK, dinF	multidrug resistance protein, MATE family	--		
gene37404	K14638	SLC15A3_4, PHT	solute carrier family 15 (peptide/histidine transporter), member 3/4	--		
gene37407	K12448	UXE	UDP-arabinose 4-epimerase	EC:5.1.3.5	1	ko00520 Amino sugar and nucleotide sugar metabolism;
gene37409	K10706	SETX, ALS4	senataxin	EC:3.6.4.-		
gene37422	K08912	LHCB1	light-harvesting complex II chlorophyll a/b binding protein 1	--	1	ko00196 Photosynthesis - antenna proteins;
gene37423	K07441	ALG14	beta-1,4-N-acetylglucosaminyltransferase	EC:2.4.1.141	2	ko00510 N-Glycan biosynthesis;ko00513 Various types of N-glycan biosynthesis;
gene37425	K00382	DLD, lpd, pdhD	dihydrolipoamide dehydrogenase	EC:1.8.1.4	6	ko00010 Glycolysis / Gluconeogenesis;ko00020 Citrate cycle (TCA cycle);ko00260 Glycine, serine and threonine metabolism;ko00280 Valine, leucine and isoleucine degradation;ko00620 Pyruvate metabolism;ko01200 Carbon metabolism;
gene37426	K02135	ATPeF1E, ATP5E	F-type H+-transporting ATPase subunit epsilon	EC:3.6.3.14	4	ko00190 Oxidative phosphorylation;ko05010 Alzheimer's disease;ko05012 Parkinson's disease;ko05016 Huntington's disease;
gene37429	K15382	SLC50A, SWEET	solute carrier family 50 (sugar transporter)	--		
gene37433	K04710	CERS	ceramide synthetase	EC:2.3.1.24	1	ko00600 Sphingolipid metabolism;
gene37436	K01613	psd, PISD	phosphatidylserine decarboxylase	EC:4.1.1.65	1	ko00564 Glycerophospholipid metabolism;
gene37437	K03531	ftsZ	cell division protein FtsZ	--	1	ko04112 Cell cycle - Caulobacter;
gene37438	K02875	RP-L14e, RPL14	large subunit ribosomal protein L14e	--	1	ko03010 Ribosome;
gene3743	K01115	PLD1_2	phospholipase D1/2	EC:3.1.4.4	7	ko00564 Glycerophospholipid metabolism;ko00565 Ether lipid metabolism;ko04014 Ras signaling pathway;ko04144 Endocytosis;ko04666 Fc gamma R-mediated phagocytosis;ko04724 Glutamatergic synapse;ko04912 GnRH signaling pathway;
gene37441	K17497	PMM	phosphomannomutase	EC:5.4.2.8	2	ko00051 Fructose and mannose metabolism;ko00520 Amino sugar and nucleotide sugar metabolism;
gene37444	K02575	NRT, narK, nrtP, nasA	MFS transporter, NNP family, nitrate/nitrite transporter	--	1	ko00910 Nitrogen metabolism;
gene37452	K11000	CALS	callose synthase	EC:2.4.1.-		
gene37454	K02355	fusA, GFM, EFG	elongation factor G	--		
gene37456	K01611	speD, AMD1	S-adenosylmethionine decarboxylase	EC:4.1.1.50	2	ko00270 Cysteine and methionine metabolism;ko00330 Arginine and proline metabolism;
gene37467	K10355	ACTF	actin, other eukaryote	--		
gene37469	K03254	EIF3A	translation initiation factor 3 subunit A	--	1	ko03013 RNA transport;
gene37471	K12616	EDC4	enhancer of mRNA-decapping protein 4	--	1	ko03018 RNA degradation;
gene37477	K15095	E1.1.1.208	(+)-neomenthol dehydrogenase	EC:1.1.1.208	1	ko00902 Monoterpenoid biosynthesis;
gene37478	K11294	NCL, NSR1	nucleolin	--	1	ko05130 Pathogenic Escherichia coli infection;
gene37479	K07304	msrA	peptide-methionine (S)-S-oxide reductase	EC:1.8.4.11		
gene37480	K12877	MAGOH	protein mago nashi	--	3	ko03013 RNA transport;ko03015 mRNA surveillance pathway;ko03040 Spliceosome;

gene37484	K00311	E1.5.5.1, etf	electron-transferring-flavoprotein dehydrogenase	EC:1.5.5.1		
gene37485	K03437	spoU	RNA methyltransferase, TrmH family	--		
gene3748	K00915	IPK2	1D-myo-Inositol-tetrakisphosphate 5-kinase / inositol-polyphosphate multikinase	EC:2.7.1.140 2.7.1.151	2	ko00562 Inositol phosphate metabolism;ko04070 Phosphatidylinositol signaling system;
gene37491	K02437	gcvH, GCSH	glycine cleavage system H protein	--	1	ko00630 Glyoxylate and dicarboxylate metabolism;
gene37492	K05909	E1.10.3.2				
gene37496	K08150	SLC2A13, ITR	MFS transporter, SP family, solute carrier family 2 (myo-inositol transporter), member 13	--		
gene3749	K13448	CML	calcium-binding protein CML	--	1	ko04626 Plant-pathogen interaction;
gene374	K13963	SERPINB	serpin B	--	1	ko05146 Amoebiasis;
gene37506	K13947	PIN	auxin efflux carrier family	--		
gene37508	K01369	LGMN	legumain	EC:3.4.22.34	2	ko04142 Lysosome;ko04612 Antigen processing and presentation;
gene37509	K04730	IRAK1	interleukin-1 receptor-associated kinase 1	EC:2.7.11.1	11	ko04064 NF-kappa B signaling pathway;ko04210 Apoptosis;ko04620 Toll-like receptor signaling pathway;ko04722 Neurotrophin signaling pathway;ko05133 Pertussis;ko05140 Leishmaniasis;ko05142 Chagas disease (American trypanosomiasis);ko05145 Toxoplasmosis;ko05152 Tuberculosis;ko05162 Measles;ko05169 Epstein-Barr virus infection;
gene37510	K10885	XRCC5, KU80, G22P2	ATP-dependent DNA helicase 2 subunit 2	--	1	ko03450 Non-homologous end-joining;
gene37512	K01662	dxs	1-deoxy-D-xylulose-5-phosphate synthase	EC:2.2.1.7	2	ko00730 Thiamine metabolism;ko00900 Terpenoid backbone biosynthesis;
gene37516	K03686	dnaJ	molecular chaperone DnaJ	--		
gene37518	K03106	SRP54, ffh	signal recognition particle subunit SRP54	--	2	ko03060 Protein export;ko03070 Bacterial secretion system;
gene37520	K15172	SUPT5H, SPT5	transcription elongation factor SPT5	--		
gene37521	K15382	SLC50A, SWEET	solute carrier family 50 (sugar transporter)	--		
gene37524	K13415	BRI1	protein brassinosteroid insensitive 1	EC:2.7.10.1 2.7.11.1	1	ko04075 Plant hormone signal transduction;
gene37530	K10885	XRCC5, KU80, G22P2	ATP-dependent DNA helicase 2 subunit 2	--	1	ko03450 Non-homologous end-joining;
gene37538	K16732	PRC1	protein regulator of cytokinesis 1	--		
gene3753	K15014	SLC29A1_2_3, ENT1_2_3	solute carrier family 29 (equilibrative nucleoside transporter), member 1/2/3	--		
gene37542	K14502	BIN2	protein brassinosteroid insensitive 2	EC:2.7.11.1	1	ko04075 Plant hormone signal transduction;
gene37543	K10683	BARD1	BRCA1-associated RING domain protein 1	EC:6.3.2.19		
gene37544	K00083	E1.1.1.195	cinnamyl-alcohol dehydrogenase	EC:1.1.1.195	1	ko00940 Phenylpropanoid biosynthesis;
gene37548	K03348	APC1	anaphase-promoting complex subunit 1	--	7	ko04110 Cell cycle;ko04111 Cell cycle - yeast;ko04113 Meiosis - yeast;ko04114 Oocyte meiosis;ko04120 Ubiquitin mediated proteolysis;ko04914 Progesterone-mediated oocyte maturation;ko05166 HTLV-I infection;
gene37552	K01528	DNM	dynamin GTPase	EC:3.6.5.5	4	ko04144 Endocytosis;ko04721 Synaptic vesicle cycle;ko04961 Endocrine and other factor-regulated calcium reabsorption;ko05100 Bacterial invasion of epithelial cells;
gene37553	K00029	E1.1.1.40, maeB	malate dehydrogenase (oxaloacetate-decarboxylating)(NADP+)	EC:1.1.1.40	3	ko00620 Pyruvate metabolism;ko00710 Carbon fixation in photosynthetic organisms;ko01200 Carbon metabolism;
gene37558	K13947	PIN	auxin efflux carrier family	--		
gene37560	K14724	SLC9A8, NHE8	solute carrier family 9 (sodium/hydrogen exchanger), member 8	--		
gene37563	K15505	RAD5	DNA repair protein RAD5	EC:3.6.4.-		
gene37576	K11137	TELO2, TEL2	telomere length regulation protein	--	1	ko03460 Fanconi anemia pathway;
gene37584	K10736	MCM10	minichromosome maintenance protein 10	--		
gene37587	K08912	LHCB1	light-harvesting complex II chlorophyll a/b binding protein 1	--	1	ko00196 Photosynthesis - antenna proteins;
gene37588	K11885	DDI1	DNA damage-inducible protein 1	--		
gene37594	K11593	ELF2C	eukaryotic translation initiation factor 2C	--		
gene37597	K09489	HSPA4	heat shock 70kDa protein 4	--	1	ko04612 Antigen processing and presentation;
gene375	K13963	SERPINB	serpin B	--	1	ko05146 Amoebiasis;
gene37600	K01728	E4.2.2.2, pel	pectate lyase	EC:4.2.2.2	1	ko00040 Pentose and glucuronate interconversions;
gene37601	K11644	SIN3A	paired amphipathic helix protein Sin3a	--	2	ko05016 Huntington's disease;ko05202 Transcriptional misregulation in cancer;
gene37602	K11644	SIN3A	paired amphipathic helix protein Sin3a	--	2	ko05016 Huntington's disease;ko05202 Transcriptional misregulation in cancer;

gene37614	K08912	LHCB1	light-harvesting complex II chlorophyll a/b binding protein 1	--	1	ko00196 Photosynthesis - antenna proteins;
gene37615	K12858	DDX23, PRP28	ATP-dependent RNA helicase DDX23/PRP28	EC:3.6.4.13	1	ko03040 Spliceosome;
gene37616	K09264	K09264	MADS-box transcription factor, plant	--		
gene37621	K01592	E4.1.1.25	tyrosine decarboxylase	EC:4.1.1.25	2	ko00350 Tyrosine metabolism;ko00950 Isoquinoline alkaloid biosynthesis;
gene37622	K12852	EFTUD2	116 kDa U5 small nuclear ribonucleoprotein component	--	1	ko03040 Spliceosome;
gene37624	K01880	GARS, glyS1	glycyl-tRNA synthetase	EC:6.1.1.14	1	ko00970 Aminoacyl-tRNA biosynthesis;
gene37625	K10638	UHRF1, NP95	E3 ubiquitin-protein ligase UHRF1	EC:6.3.2.19		
gene37627	K09422	MYBP	myb proto-oncogene protein, plant	--		
gene37629	K11593	ELF2C	eukaryotic translation initiation factor 2C	--		
gene3762	K10949	KDELR	ER lumen protein retaining receptor	--	1	ko05110 Vibrio cholerae infection;
gene37630	K14488	SAUR	SAUR family protein	--	1	ko04075 Plant hormone signal transduction;
gene37631	K09422	MYBP	myb proto-oncogene protein, plant	--		
gene37636	K08835	OXSR1, STK39	serine/threonine-protein kinase OSR1/STK39	EC:2.7.11.1		
gene37637	K03934	NDUFS1	NADH dehydrogenase (ubiquinone) Fe-S protein 1	EC:1.6.5.3 1.6.99.3	5	ko00190 Oxidative phosphorylation;ko04932 Non-alcoholic fatty liver disease (NAFLD);ko05010 Alzheimer's disease;ko05012 Parkinson's disease;ko05016 Huntington's disease;
gene3763	K02291	crtB	phytoene synthase	EC:2.5.1.32	1	ko00906 Carotenoid biosynthesis;
gene37645	K15216	RRN3, TIFIA	RNA polymerase I-specific transcription initiation factor RRN3	--		
gene37646	K02836	prfB	peptide chain release factor 2	--		
gene37648	K00108	E1.1.99.1, betA, CHDH	choline dehydrogenase	EC:1.1.99.1	1	ko00260 Glycine, serine and threonine metabolism;
gene3764	K04733	IRAK4	interleukin-1 receptor-associated kinase 4	EC:2.7.11.1	11	ko04064 NF-kappa B signaling pathway;ko04210 Apoptosis;ko04620 Toll-like receptor signaling pathway;ko04722 Neurotrophin signaling pathway;ko05133 Pertussis;ko05140 Leishmaniasis;ko05142 Chagas disease (American trypanosomiasis);ko05145 Toxoplasmosis;ko05152 Tuberculosis;ko05162 Measles;ko05164 Influenza A;
gene37650	K09264	K09264	MADS-box transcription factor, plant	--		
gene37653	K16732	PRC1	protein regulator of cytokinesis 1	--		
gene37656	K14827	IPI1, TEX10	pre-rRNA-processing protein IPI1	--		
gene37661	K03327	TC.MATE, SLC47A, norM, mdtK, dinF	multidrug resistance protein, MATE family	--		
gene37663	K12501	HST	homogentisate solanesyltransferase	--	1	ko00130 Ubiquinone and other terpenoid-quinone biosynthesis;
gene37668	K04730	IRAK1	interleukin-1 receptor-associated kinase 1	EC:2.7.11.1	11	ko04064 NF-kappa B signaling pathway;ko04210 Apoptosis;ko04620 Toll-like receptor signaling pathway;ko04722 Neurotrophin signaling pathway;ko05133 Pertussis;ko05140 Leishmaniasis;ko05142 Chagas disease (American trypanosomiasis);ko05145 Toxoplasmosis;ko05152 Tuberculosis;ko05162 Measles;ko05169 Epstein-Barr virus infection;
gene37669	K12616	EDC4	enhancer of mRNA-decapping protein 4	--	1	ko03018 RNA degradation;
gene37670	K11816	YUCCA	indole-3-pyruvate monooxygenase	EC:1.14.13.168	1	ko00380 Tryptophan metabolism;
gene37672	K01568	E4.1.1.1, pdc	pyruvate decarboxylase	EC:4.1.1.1	1	ko00010 Glycolysis / Gluconeogenesis;
gene37674	K14842	NSA2	ribosome biogenesis protein NSA2	--		
gene37678	K09422	MYBP	myb proto-oncogene protein, plant	--		
gene37679	K13065	E2.3.1.133, HCT	shikimate O-hydroxycinnamoyltransferase	EC:2.3.1.133	3	ko00940 Phenylpropanoid biosynthesis;ko00941 Flavonoid biosynthesis;ko00945 Stilbenoid, diarylheptanoid and gingerol biosynthesis;
gene3767	K07359	CAMKK	calcium/calmodulin-dependent protein kinase kinase	EC:2.7.11.17	2	ko04920 Adipocytokine signaling pathway;ko05034 Alcoholism;
gene37683	K03115	CSNK2B	casein kinase II subunit beta	--	9	ko03008 Ribosome biogenesis in eukaryotes;ko04064 NF-kappa B signaling pathway;ko04310 Wnt signaling pathway;ko04520 Adherens junction;ko04530 Tight junction;ko04712 Circadian rhythm - plant;ko05162 Measles;ko05168 Herpes simplex infection;ko05169 Epstein-Barr virus infection;
gene37684	K02921	RP-L37Ae, RPL37A	large subunit ribosomal protein L37Ae	--	1	ko03010 Ribosome;
gene37691	K01408	IDE, ide	insulysin	EC:3.4.24.56	1	ko05010 Alzheimer's disease;

gene37693	K03126	TAF12	transcription initiation factor TFIID subunit 12	--	1	ko03022 Basal transcription factors;
gene37698	K11778	DHDDS, RER2, SRT1	ditrans,polycis-polyprenyl diphosphate synthase	EC:2.5.1.87	1	ko00900 Terpenoid backbone biosynthesis;
gene376	K14611	SLC23A1_2, SVCT1_2	solute carrier family 23 (nucleobase transporter), member 1/2	--		
gene37704	K03138	TFIIF1, GTF2F1, TFG1	transcription initiation factor TFIIF subunit alpha	--	1	ko03022 Basal transcription factors;
gene37708	K13091	RBM39, RNPC2	RNA-binding protein 39	--		
gene37709	K15272	SLC35A1_2_3	solute carrier family 35 (UDP-sugar transporter), member A1/2/3	--		
gene37713	K00924	E2.7.1.-				
gene37714	K17279	REEP5_6	receptor expression-enhancing protein 5/6	--		
gene37716	K02979	RP-S28e, RPS28	small subunit ribosomal protein S28e	--	1	ko03010 Ribosome;
gene37718	K08880	STK19	serine/threonine kinase 19	EC:2.7.11.1		
gene37721	K04730	IRAK1	interleukin-1 receptor-associated kinase 1	EC:2.7.11.1	11	ko04064 NF-kappa B signaling pathway;ko04210 Apoptosis;ko04620 Toll-like receptor signaling pathway;ko04722 Neurotrophin signaling pathway;ko05133 Pertussis;ko05140 Leishmaniasis;ko05142 Chagas disease (American trypanosomiasis);ko05145 Toxoplasmosis;ko05152 Tuberculosis;ko05162 Measles;ko05169 Epstein-Barr virus infection;
gene37727	K08960	CSNK1E	casein kinase 1, epsilon	EC:2.7.11.1	6	ko04310 Wnt signaling pathway;ko04340 Hedgehog signaling pathway;ko04390 Hippo signaling pathway;ko04391 Hippo signaling pathway - fly;ko04710 Circadian rhythm;ko04711 Circadian rhythm - fly;
gene37729	K00083	E1.1.1.195	cinnamyl-alcohol dehydrogenase	EC:1.1.1.195	1	ko00940 Phenylpropanoid biosynthesis;
gene37733	K00817	hisC	histidinol-phosphate aminotransferase	EC:2.6.1.9	7	ko00340 Histidine metabolism;ko00350 Tyrosine metabolism;ko00360 Phenylalanine metabolism;ko00400 Phenylalanine, tyrosine and tryptophan biosynthesis;ko00401 Novobiocin biosynthesis;ko00960 Tropane, piperidine and pyridine alkaloid biosynthesis;ko01230 Biosynthesis of amino acids;
gene37735	K08912	LHCB1	light-harvesting complex II chlorophyll a/b binding protein 1	--	1	ko00196 Photosynthesis - antenna proteins;
gene37741	K02435	gatC	aspartyl-tRNA(Asn)/glutamyl-tRNA(Gln) amidotransferase subunit C	EC:6.3.5.6 6.3.5.7	1	ko00970 Aminoacyl-tRNA biosynthesis;
gene37749	K08790	STK38, NDR	serine/threonine kinase 38	EC:2.7.11.1		
gene3774	K03456	PPP2R1	serine/threonine-protein phosphatase 2A regulatory subunit A	--	13	ko03015 mRNA surveillance pathway;ko04111 Cell cycle - yeast;ko04113 Meiosis - yeast;ko04114 Oocyte meiosis;ko04151 PI3K-Akt signaling pathway;ko04350 TGF-beta signaling pathway;ko04390 Hippo signaling pathway;ko04391 Hippo signaling pathway - fly;ko04530 Tight junction;ko04728 Dopaminergic synapse;ko04730 Long-term depression;ko05142 Chagas disease (American trypanosomiasis);ko05160 Hepatitis C;
gene37750	K15902	PCC1, LAGE3	polarized growth chromatin-associated controller 1	--		
gene37751	K09338	HD-ZIP	homeobox-leucine zipper protein	--		
gene37753	K11262	ACAC	acetyl-CoA carboxylase / biotin carboxylase	EC:6.4.1.2 6.3.4.14	4	ko00061 Fatty acid biosynthesis;ko00620 Pyruvate metabolism;ko00640 Propanoate metabolism;ko04910 Insulin signaling pathway;
gene37758	K13416	BAK1	brassinosteroid insensitive 1-associated receptor kinase 1	EC:2.7.10.1 2.7.11.1	2	ko04075 Plant hormone signal transduction;ko04626 Plant-pathogen interaction;
gene37759	K15208	SNAPC1	snRNA-activating protein complex subunit 1	--		
gene37764	K06689	UBE2D_E, UBC4, UBC5	ubiquitin-conjugating enzyme E2 D/E	EC:6.3.2.19	2	ko04120 Ubiquitin mediated proteolysis;ko04141 Protein processing in endoplasmic reticulum;
gene37765	K15202	GTF3C5, TFC1	general transcription factor 3C polypeptide 5 (transcription factor C subunit 1)	--		
gene37769	K07870	RHOT1, ARHT1	Ras homolog gene family, member T1	--		
gene37771	K00430	E1.11.1.7	peroxidase	EC:1.11.1.7	2	ko00360 Phenylalanine metabolism;ko00940 Phenylpropanoid biosynthesis;
gene37775	K11346	ING4	inhibitor of growth protein 4	--		
gene37779	K14842	NSA2	ribosome biogenesis protein NSA2	--		
gene37781	K11826	AP2M1	AP-2 complex subunit mu-1	--	4	ko04144 Endocytosis;ko04721 Synaptic vesicle cycle;ko04961 Endocrine and other factor-regulated calcium reabsorption;ko05016 Huntington's disease;
gene37782	K10610	DDB1	DNA damage-binding protein 1	--	4	ko03420 Nucleotide excision repair;ko04120 Ubiquitin mediated proteolysis;ko05161 Hepatitis B;ko05203 Viral carcinogenesis;

gene37789	K02115	ATPF1G, atpG	F-type H+-transporting ATPase subunit gamma	EC:3.6.3.14	2	ko00190 Oxidative phosphorylation;ko00195 Photosynthesis;
gene3778	K00025	MDH1	malate dehydrogenase	EC:1.1.1.37	6	ko00020 Citrate cycle (TCA cycle);ko00620 Pyruvate metabolism;ko00630 Glyoxylate and dicarboxylate metabolism;ko00710 Carbon fixation in photosynthetic organisms;ko01200 Carbon metabolism;ko04964 Proximal tubule bicarbonate reclamation;
gene37790	K14944	NOVA	RNA-binding protein Nova	--		
gene37791	K13692	IAGLU	UDP-glucose:(indol-3-yl)acetate beta-D-glucosyltransferase	EC:2.4.1.121		
gene37792	K10532	HGSNAT	heparan-alpha-glucosaminide N-acetyltransferase	EC:2.3.1.78	2	ko00531 Glycosaminoglycan degradation;ko04142 Lysosome;
gene37793	K14826	FPR3_4	FK506-binding nuclear protein	EC:5.2.1.8		
gene37794	K01056	PTH1, pth, spoVC	peptidyl-tRNA hydrolase, PTH1 family	EC:3.1.1.29		
gene37796	K02180	BUB3	cell cycle arrest protein BUB3	--	3	ko04110 Cell cycle;ko04111 Cell cycle - yeast;ko05166 HTLV-I infection;
gene37797	K04125	E1.14.11.13	gibberellin 2-oxidase	EC:1.14.11.13	1	ko00904 Diterpenoid biosynthesis;
gene37799	K12900	FUSIP1	FUS-interacting serine-arginine-rich protein 1	--	1	ko03040 Spliceosome;
gene37803	K13116	DDX41, ABS	ATP-dependent RNA helicase DDX41	EC:3.6.4.13		
gene37805	K02706	psbD	photosystem II P680 reaction center D2 protein	--	1	ko00195 Photosynthesis;
gene37806	K08739	MLH3	DNA mismatch repair protein MLH3	--	1	ko03430 Mismatch repair;
gene37810	K17422	MRPL41	large subunit ribosomal protein L41, mitochondrial	--		
gene37815	K11262	ACAC	acetyl-CoA carboxylase / biotin carboxylase	EC:6.4.1.2 6.3.4.14	4	ko00061 Fatty acid biosynthesis;ko00620 Pyruvate metabolism;ko00640 Propanoate metabolism;ko04910 Insulin signaling pathway;
gene3781	K16251	NRPE1	DNA-directed RNA polymerase V subunit 1	EC:2.7.7.6		
gene37820	K01177	E3.2.1.2	beta-amylase	EC:3.2.1.2	1	ko00500 Starch and sucrose metabolism;
gene37823	K01930	FPGS	folylpolyglutamate synthase	EC:6.3.2.17	1	ko00790 Folate biosynthesis;
gene37824	K10357	MYO5	myosin V	--		
gene37828	K10739	RFA2, RPA2	replication factor A2	--	5	ko03030 DNA replication;ko03420 Nucleotide excision repair;ko03430 Mismatch repair;ko03440 Homologous recombination;ko03460 Fanconi anemia pathway;
gene3782	K14506	JAR1	jasmonic acid-amino synthetase	--	1	ko04075 Plant hormone signal transduction;
gene37830	K01533	E3.6.3.4, ATP7, copA	Cu2+-exporting ATPase	EC:3.6.3.4		
gene37831	K15208	SNAPC1	snRNA-activating protein complex subunit 1	--		
gene37833	K06617	E2.4.1.82	raffinose synthase	EC:2.4.1.82	1	ko00052 Galactose metabolism;
gene37834	K11138	K11138, RIF1	telomere-associated protein RIF1	--		
gene37835	K12607	CNOT10	CCR4-NOT transcription complex subunit 10	--	1	ko03018 RNA degradation;
gene37837	K05894	E1.3.1.42	12-oxophytodienoic acid reductase	EC:1.3.1.42	1	ko00592 alpha-Linolenic acid metabolism;
gene37843	K00876	E2.7.1.48, udk	uridine kinase	EC:2.7.1.48	2	ko00240 Pyrimidine metabolism;ko00983 Drug metabolism - other enzymes;
gene37849	K11699	RDR1, RDP1	RNA-dependent RNA polymerase 1	EC:2.7.7.48		
gene3784	K13993	HSP20	HSP20 family protein	--	1	ko04141 Protein processing in endoplasmic reticulum;
gene37853	K01890	FARSB, pheT	phenylalanyl-tRNA synthetase beta chain	EC:6.1.1.20	1	ko00970 Aminoacyl-tRNA biosynthesis;
gene37854	K01756	purB, ADSL	adenylosuccinate lyase	EC:4.3.2.2	2	ko00230 Purine metabolism;ko00250 Alanine, aspartate and glutamate metabolism;
gene37855	K00083	E1.1.1.195	cinnamyl-alcohol dehydrogenase	EC:1.1.1.195	1	ko00940 Phenylpropanoid biosynthesis;
gene37862	K03363	CDC20	cell division cycle 20, cofactor of APC complex	--	7	ko04110 Cell cycle;ko04111 Cell cycle - yeast;ko04113 Meiosis - yeast;ko04114 Oocyte meiosis;ko04120 Ubiquitin mediated proteolysis;ko05166 HTLV-I infection;ko05203 Viral carcinogenesis;
gene37879	K11600	RRP41, EXOSC4, SKI6	exosome complex component RRP41	--	1	ko03018 RNA degradation;
gene37881	K10256	FAD2	omega-6 fatty acid desaturase (delta-12 desaturase)	EC:1.14.19.-	1	ko01040 Biosynthesis of unsaturated fatty acids;
gene37882	K05658	ABCB1	ATP-binding cassette, subfamily B (MDR/TAP), member 1	--	3	ko02010 ABC transporters;ko04976 Bile secretion;ko05206 MicroRNAs in cancer;
gene37888	K14948	PTBP2, NPTB	olypyrimidine tract-binding protein 2	--		
gene37893	K01703	leuC	3-isopropylmalate/(R)-2-methylmalate dehydratase large subunit	EC:4.2.1.33 4.2.1.35	4	ko00290 Valine, leucine and isoleucine biosynthesis;ko00660 C5-Branched dibasic acid metabolism;ko01210 2-Oxocarboxylic acid metabolism;ko01230 Biosynthesis of amino acids;
gene37895	K01762	ACS	1-aminocyclopropane-1-carboxylate synthase	EC:4.4.1.14	1	ko00270 Cysteine and methionine metabolism;
gene37897	K01899	LSC1	succinyl-CoA synthetase alpha subunit	EC:6.2.1.4 6.2.1.5	3	ko00020 Citrate cycle (TCA cycle);ko00640 Propanoate metabolism;ko01200 Carbon metabolism;

gene37899	K14488	SAUR	SAUR family protein	--	1	ko04075 Plant hormone signal transduction;
gene37904	K15710	SHPRH	E3 ubiquitin-protein ligase SHPRH	EC:3.6.4.- 6.3.2.19		
gene37906	K06877	K06877	DEAD/DEAH box helicase domain-containing protein	--		
gene37908	K02295	CRY	cryptochrome	--	1	ko04710 Circadian rhythm;
gene37910	K13495	CISZOG	cis-zeatin O-glucosyltransferase	EC:2.4.1.215	1	ko00908 Zeatin biosynthesis;
gene37911	K09422	MYBP	myb proto-oncogene protein, plant	--		
gene37913	K03026	RPC53, POLR3D	DNA-directed RNA polymerase III subunit RPC4	--	5	ko00230 Purine metabolism;ko00240 Pyrimidine metabolism;ko03020 RNA polymerase;ko04623 Cytosolic DNA-sensing pathway;ko05169 Epstein-Barr virus infection;
gene37917	K14498	SNRK2	serine/threonine-protein kinase SRK2	EC:2.7.11.1	1	ko04075 Plant hormone signal transduction;
gene37919	K05933	E1.14.17.4	aminocyclopropanecarboxylate oxidase	EC:1.14.17.4	1	ko00270 Cysteine and methionine metabolism;
gene37920	K12657	ALDH18A1, P5CS	delta-1-pyrroline-5-carboxylate synthetase	EC:2.7.2.11 1.2.1.41	2	ko00330 Arginine and proline metabolism;ko01230 Biosynthesis of amino acids;
gene37921	K05016	CLCN7	chloride channel 7	--		
gene37924	K11252	H2B	histone H2B	--	3	ko05034 Alcoholism;ko05203 Viral carcinogenesis;ko05322 Systemic lupus erythematosus;
gene37927	K11663	ZNHIT1, VPS71	zinc finger HIT domain-containing protein 1	--		
gene37931	K11885	DDI1	DNA damage-inducible protein 1	--		
gene37933	K17086	TM9SF2_4	transmembrane 9 superfamily member 2/4	--		
gene37936	K05909	E1.10.3.2				
gene37937	K14802	DRS2, ATP8A	phospholipid-transporting ATPase	EC:3.6.3.1		
gene37938	K00130	betB, gbsA	betaine-aldehyde dehydrogenase	EC:1.2.1.8	1	ko00260 Glycine, serine and threonine metabolism;
gene37939	K00966	GMPP	mannose-1-phosphate guanylyltransferase	EC:2.7.7.13	2	ko00051 Fructose and mannose metabolism;ko00520 Amino sugar and nucleotide sugar metabolism;
gene37940	K10686	UBE1C, UBA3	ubiquitin-activating enzyme E1 C	EC:6.3.2.19	1	ko04120 Ubiquitin mediated proteolysis;
gene37942	K14488	SAUR	SAUR family protein	--	1	ko04075 Plant hormone signal transduction;
gene37943	K08912	LHCB1	light-harvesting complex II chlorophyll a/b binding protein 1	--	1	ko00196 Photosynthesis - antenna proteins;
gene37945	K00855	PRK, prkB	phosphoribulokinase	EC:2.7.1.19	2	ko00710 Carbon fixation in photosynthetic organisms;ko01200 Carbon metabolism;
gene37946	K01466	allB	allantoinase	EC:3.5.2.5	1	ko00230 Purine metabolism;
gene37949	K02935	RP-L7, MRPL12, rplL	large subunit ribosomal protein L7/L12	--	1	ko03010 Ribosome;
gene37951	K10256	FAD2	omega-6 fatty acid desaturase (delta-12 desaturase)	EC:1.14.19.-	1	ko01040 Biosynthesis of unsaturated fatty acids;
gene37952	K00083	E1.1.1.195	cinnamyl-alcohol dehydrogenase	EC:1.1.1.195	1	ko00940 Phenylpropanoid biosynthesis;
gene37953	K10683	BARD1	BRCA1-associated RING domain protein 1	EC:6.3.2.19		
gene37960	K13412	CPK	calcium-dependent protein kinase	EC:2.7.11.1	2	ko04626 Plant-pathogen interaction;ko05145 Toxoplasmosis;
gene37961	K13484	TTHL	5-hydroxyisourate hydrolase / 2-oxo-4-hydroxy-4-carboxy-5-ureidoimidazoline decarboxylase	EC:3.5.2.17	1	ko00230 Purine metabolism;
gene37970	K13436	PTI1	pto-interacting protein 1	EC:2.7.11.1	1	ko04626 Plant-pathogen interaction;
gene37972	K01703	leuC	3-isopropylmalate/(R)-2-methylmalate dehydratase large subunit	EC:4.2.1.33 4.2.1.35	4	ko00290 Valine, leucine and isoleucine biosynthesis;ko00660 C5-Branched dibasic acid metabolism;ko01210 2-Oxocarboxylic acid metabolism;ko01230 Biosynthesis of amino acids;
gene37978	K08869	ADCK, ABC1	aarF domain-containing kinase	--		
gene37982	K12600	SKI3, TTC37	superkiller protein 3	--	1	ko03018 RNA degradation;
gene37983	K06617	E2.4.1.82	raffinose synthase	EC:2.4.1.82	1	ko00052 Galactose metabolism;
gene37990	K15171	SUPT4H1, SPT4	transcription elongation factor SPT4	--		
gene37991	K15171	SUPT4H1, SPT4	transcription elongation factor SPT4	--		
gene37992	K03141	TFIIH1, GTF2H1, TFB1	transcription initiation factor TFIIH subunit 1	--	3	ko03022 Basal transcription factors;ko03420 Nucleotide excision repair;ko05203 Viral carcinogenesis;
gene37994	K00826	E2.6.1.42, ilvE	branched-chain amino acid aminotransferase	EC:2.6.1.42	5	ko00280 Valine, leucine and isoleucine degradation;ko00290 Valine, leucine and isoleucine biosynthesis;ko00770 Pantothenate and CoA biosynthesis;ko01210 2-Oxocarboxylic acid metabolism;ko01230 Biosynthesis of amino acids;
gene37999	K00469	MIOX	inositol oxygenase	EC:1.13.99.1	2	ko00053 Ascorbate and aldarate metabolism;ko00562 Inositol phosphate metabolism;
gene38000	K10895	FANCI	fanconi anemia group I protein	--	1	ko03460 Fanconi anemia pathway;
gene38004	K06184	ABCF1	ATP-binding cassette, subfamily F, member 1	--		

gene38006	K03259	EIF4E	translation initiation factor 4E	--	5	ko03013 RNA transport;ko04066 HIF-1 signaling pathway;ko04150 mTOR signaling pathway;ko04151 PI3K-Akt signaling pathway;ko04910 Insulin signaling pathway;
gene38008	K15404	K15404, CER1	aldehyde decarbonylase	EC:4.1.99.5	1	ko00073 Cutin, suberine and wax biosynthesis;
gene38010	K03453	TC.BASS	bile acid:Na+ symporter, BASS family	--		
gene38012	K05906	PCYOX1, FCLY	prenylcysteine oxidase / farnesylcysteine lyase	EC:1.8.3.5 1.8.3.6	1	ko00900 Terpenoid backbone biosynthesis;
gene38013	K13161	HNRNPR	heterogeneous nuclear ribonucleoprotein R	--		
gene38017	K00083	E1.1.1.195	cinnamyl-alcohol dehydrogenase	EC:1.1.1.195	1	ko00940 Phenylpropanoid biosynthesis;
gene38019	K01528	DNM	dynamin GTPase	EC:3.6.5.5	4	ko04144 Endocytosis;ko04721 Synaptic vesicle cycle;ko04961 Endocrine and other factor-regulated calcium reabsorption;ko05100 Bacterial invasion of epithelial cells;
gene38022	K00648	fabH	3-oxoacyl-[acyl-carrier-protein] synthase III	EC:2.3.1.180	1	ko00061 Fatty acid biosynthesis;
gene38026	K09285	OVM, ANT	AP2-like factor, ANT lineage	--		
gene38028	K03141	TFIIH1, GTF2H1, TFB1	transcription initiation factor TFIIH subunit 1	--	3	ko03022 Basal transcription factors;ko03420 Nucleotide excision repair;ko05203 Viral carcinogenesis;
gene38032	K02324	POLE1	DNA polymerase epsilon subunit 1	EC:2.7.7.7	6	ko00230 Purine metabolism;ko00240 Pyrimidine metabolism;ko03030 DNA replication;ko03410 Base excision repair;ko03420 Nucleotide excision repair;ko05166 HTLV-I infection;
gene38035	K09773	K09773	hypothetical protein	--		
gene38036	K09518	DNAJB12	DnaJ homolog subfamily B member 12	--	1	ko04141 Protein processing in endoplasmic reticulum;
gene38037	K03544	clpX, CLPX	ATP-dependent Clp protease ATP-binding subunit ClpX	--	1	ko04112 Cell cycle - Caulobacter;
gene3803	K09580	PDIA1, P4HB	protein disulfide-isomerase A1	EC:5.3.4.1	1	ko04141 Protein processing in endoplasmic reticulum;
gene38044	K13356	FAR	fatty acyl-CoA reductase	EC:1.2.1.-	2	ko00073 Cutin, suberine and wax biosynthesis;ko04146 Peroxisome;
gene38046	K10398	KIF11, EG5	kinesin family member 11	--		
gene38048	K01184	E3.2.1.15	polygalacturonase	EC:3.2.1.15	2	ko00040 Pentose and glucuronate interconversions;ko00500 Starch and sucrose metabolism;
gene38050	K01184	E3.2.1.15	polygalacturonase	EC:3.2.1.15	2	ko00040 Pentose and glucuronate interconversions;ko00500 Starch and sucrose metabolism;
gene38051	K01285	PRCP	lysosomal Pro-X carboxypeptidase	EC:3.4.16.2	1	ko04974 Protein digestion and absorption;
gene38054	K00800	aroA	3-phosphoshikimate 1-carboxyvinyltransferase	EC:2.5.1.19	2	ko00400 Phenylalanine, tyrosine and tryptophan biosynthesis;ko01230 Biosynthesis of amino acids;
gene38061	K07950	ARL5B	ADP-ribosylation factor-like protein 5B	--		
gene38064	K12502	VTE3, APG1	MPBQ/MSBQ methyltransferase	--	1	ko00130 Ubiquinone and other terpenoid-quinone biosynthesis;
gene38065	K13448	CML	calcium-binding protein CML	--	1	ko04626 Plant-pathogen interaction;
gene38066	K13811	PAPSS	3'-phosphoadenosine 5'-phosphosulfate synthase	EC:2.7.7.4 2.7.1.25	3	ko00230 Purine metabolism;ko00450 Selenocompound metabolism;ko00920 Sulfur metabolism;
gene38069	K14485	TIR1	transport inhibitor response 1	--	1	ko04075 Plant hormone signal transduction;
gene38072	K13946	AUX1, LAX	auxin influx carrier (AUX1 LAX family)	--	1	ko04075 Plant hormone signal transduction;
gene38074	K15382	SLC50A, SWEET	solute carrier family 50 (sugar transporter)	--		
gene38075	K09422	MYBP	myb proto-oncogene protein, plant	--		
gene38080	K12272	SRPRB, SRP102	signal recognition particle receptor subunit beta	--	1	ko03060 Protein export;
gene38085	K09419	HSFF	heat shock transcription factor, other eukaryote	--		
gene38086	K01662	dxs	1-deoxy-D-xylulose-5-phosphate synthase	EC:2.2.1.7	2	ko00730 Thiamine metabolism;ko00900 Terpenoid backbone biosynthesis;
gene38087	K08247	E2.1.1.12	methionine S-methyltransferase	EC:2.1.1.12	1	ko00450 Selenocompound metabolism;
gene38090	K00574	E2.1.1.79, cfa	cyclopropane-fatty-acyl-phospholipid synthase	EC:2.1.1.79		
gene38091	K01689	ENO, eno	enolase	EC:4.2.1.11	6	ko00010 Glycolysis / Gluconeogenesis;ko00680 Methane metabolism;ko01200 Carbon metabolism;ko01230 Biosynthesis of amino acids;ko03018 RNA degradation;ko04066 HIF-1 signaling pathway;
gene38092	K14484	IAA	auxin-responsive protein IAA	--	1	ko04075 Plant hormone signal transduction;
gene38093	K08472	MLO	mlo protein	--		
gene38096	K14617	LMBRD1	LMBR1 domain-containing protein 1	--	1	ko04977 Vitamin digestion and absorption;
gene38098	K12581	CNOT7_8, CAF1, POP2	CCR4-NOT transcription complex subunit 7/8	--	1	ko03018 RNA degradation;
gene3809	K00008	E1.1.1.14, gutB	L-iditol 2-dehydrogenase	EC:1.1.1.14	1	ko00051 Fructose and mannose metabolism;
gene38101	K07752	CPD	carboxypeptidase D	EC:3.4.17.22		
gene38102	K14826	FPR3_4	FK506-binding nuclear protein	EC:5.2.1.8		

gene38113	K00873	PK, pyk	pyruvate kinase	EC:2.7.1.40	7	ko00010 Glycolysis / Gluconeogenesis;ko00230 Purine metabolism;ko00620 Pyruvate metabolism;ko01200 Carbon metabolism;ko01230 Biosynthesis of amino acids;ko04930 Type II diabetes mellitus;ko05203 Viral carcinogenesis;
gene38117	K12600	SKI3, TTC37	superkiller protein 3	--	1	ko03018 RNA degradation;
gene3811	K08064	NFYA	nuclear transcription factor Y, alpha	--	2	ko04612 Antigen processing and presentation;ko05152 Tuberculosis;
gene38121	K00510	HMOX, hmuO, ho	heme oxygenase	EC:1.14.99.3	2	ko00860 Porphyrin and chlorophyll metabolism;ko04978 Mineral absorption;
gene38122	K00161	PDHA, pdhA	pyruvate dehydrogenase E1 component subunit alpha	EC:1.2.4.1	6	ko00010 Glycolysis / Gluconeogenesis;ko00020 Citrate cycle (TCA cycle);ko00620 Pyruvate metabolism;ko00650 Butanoate metabolism;ko01200 Carbon metabolism;ko04066 HIF-1 signaling pathway;
gene38127	K09560	ST13	suppressor of tumorigenicity protein 13	--		
gene38130	K14411	MSI	RNA-binding protein Musashi	--	1	ko03015 mRNA surveillance pathway;
gene38135	K09584	PDIA6, TXNDC7	protein disulfide-isomerase A6	EC:5.3.4.1	1	ko04141 Protein processing in endoplasmic reticulum;
gene38139	K03103	MINPP1	multiple inositol-polyphosphate phosphatase	EC:3.1.3.62	1	ko00562 Inositol phosphate metabolism;
gene38140	K02266	COX6A	cytochrome c oxidase subunit 6a	--	6	ko00190 Oxidative phosphorylation;ko04260 Cardiac muscle contraction;ko04932 Non-alcoholic fatty liver disease (NAFLD);ko05010 Alzheimer's disease;ko05012 Parkinson's disease;ko05016 Huntington's disease;
gene38141	K13448	CML	calcium-binding protein CML	--	1	ko04626 Plant-pathogen interaction;
gene38143	K01850	E5.4.99.5	chorismate mutase	EC:5.4.99.5	2	ko00400 Phenylalanine, tyrosine and tryptophan biosynthesis;ko01230 Biosynthesis of amino acids;
gene38145	K00888	PI4K	phosphatidylinositol 4-kinase	EC:2.7.1.67	2	ko00562 Inositol phosphate metabolism;ko04070 Phosphatidylinositol signaling system;
gene38147	K10256	FAD2	omega-6 fatty acid desaturase (delta-12 desaturase)	EC:1.14.19.-	1	ko01040 Biosynthesis of unsaturated fatty acids;
gene38148	K14401	CPSF1, CFT1	cleavage and polyadenylation specificity factor subunit 1	--	1	ko03015 mRNA surveillance pathway;
gene38153	K08912	LHCB1	light-harvesting complex II chlorophyll a/b binding protein 1	--	1	ko00196 Photosynthesis - antenna proteins;
gene38154	K08912	LHCB1	light-harvesting complex II chlorophyll a/b binding protein 1	--	1	ko00196 Photosynthesis - antenna proteins;
gene38157	K01099	E3.1.3.36	phosphatidylinositol-bisphosphatase	EC:3.1.3.36	2	ko00562 Inositol phosphate metabolism;ko04070 Phosphatidylinositol signaling system;
gene38159	K00826	E2.6.1.42, ilvE	branched-chain amino acid aminotransferase	EC:2.6.1.42	5	ko00280 Valine, leucine and isoleucine degradation;ko00290 Valine, leucine and isoleucine biosynthesis;ko00770 Pantothenate and CoA biosynthesis;ko01210 2-Oxocarboxylic acid metabolism;ko01230 Biosynthesis of amino acids;
gene38162	K13457	RPM1, RPS3	disease resistance protein RPM1	--	1	ko04626 Plant-pathogen interaction;
gene38163	K03234	EEF2	elongation factor 2	--		
gene38164	K15397	KCS	3-ketoacyl-CoA synthase	EC:2.3.1.199	1	ko00062 Fatty acid elongation;
gene38166	K17497	PMM	phosphomannomutase	EC:5.4.2.8	2	ko00051 Fructose and mannose metabolism;ko00520 Amino sugar and nucleotide sugar metabolism;
gene38167	K15901	CGI121, TPRKB	protein CGI121	--		
gene38173	K08912	LHCB1	light-harvesting complex II chlorophyll a/b binding protein 1	--	1	ko00196 Photosynthesis - antenna proteins;
gene38178	K05280	E1.14.13.21	flavonoid 3'-monooxygenase	EC:1.14.13.21	2	ko00941 Flavonoid biosynthesis;ko00944 Flavone and flavonol biosynthesis;
gene38179	K08150	SLC2A13, ITR	MFS transporter, SP family, solute carrier family 2 (myo-inositol transporter), member 13	--		
gene38190	K16240	SPA1	protein suppressor of PHYA-105 1	--	1	ko04712 Circadian rhythm - plant;
gene38192	K11254	H4	histone H4	--	3	ko05034 Alcoholism;ko05203 Viral carcinogenesis;ko05322 Systemic lupus erythematosus;
gene38197	K01895	ACSS, acs	acetyl-CoA synthetase	EC:6.2.1.1	6	ko00010 Glycolysis / Gluconeogenesis;ko00620 Pyruvate metabolism;ko00640 Propanoate metabolism;ko00680 Methane metabolism;ko00720 Carbon fixation pathways in prokaryotes;ko01200 Carbon metabolism;
gene38198	K02997	RP-S9e, RPS9	small subunit ribosomal protein S9e	--	1	ko03010 Ribosome;
gene38199	K01113	phoD	alkaline phosphatase D	EC:3.1.3.1	3	ko00627 Aminobenzoate degradation;ko00790 Folate biosynthesis;ko02020 Two-component system;
gene38200	K11816	YUCCA	indole-3-pyruvate monooxygenase	EC:1.14.13.168	1	ko00380 Tryptophan metabolism;
gene38201	K01265	E3.4.11.18, map	methionyl aminopeptidase	EC:3.4.11.18		
gene38202	K13800	CMPK1, UMPK	UMP-CMP kinase	EC:2.7.4.- 2.7.4.14	1	ko00240 Pyrimidine metabolism;

gene38203	K02881	RP-L18, MRPL18, rplR	large subunit ribosomal protein L18	--	1	ko03010 Ribosome;
gene38206	K02992	RP-S7, MRPS7, rpsG	small subunit ribosomal protein S7	--	1	ko03010 Ribosome;
gene38208	K12391	AP1G1	AP-1 complex subunit gamma-1	--	1	ko04142 Lysosome;
gene38209	K08869	ADCK, ABC1	aarF domain-containing kinase	--		
gene3821	K12815	DHX38, PRP16	pre-mRNA-splicing factor ATP-dependent RNA helicase PRP16	EC:3.6.4.13	1	ko03040 Spliceosome;
gene38223	K14484	IAA	auxin-responsive protein IAA	--	1	ko04075 Plant hormone signal transduction;
gene38225	K10706	SETX, ALS4	senataxin	EC:3.6.4.-		
gene38229	K00264	GLT1	glutamate synthase (NADPH/NADH)	EC:1.4.1.13 1.4.1.14	3	ko00250 Alanine, aspartate and glutamate metabolism;ko00910 Nitrogen metabolism;ko01230 Biosynthesis of amino acids;
gene3822	K13111	SMU1	WD40 repeat-containing protein SMU1	--		
gene38230	K14230	tRNA-Met	tRNA Met	--	1	ko00970 Aminoacyl-tRNA biosynthesis;
gene38231	K13783	SLC37A1_2	MFS transporter, OPA family, solute carrier family 37 (glycerol-3-phosphate transporter), member 1/2	--		
gene38233	K13457	RPM1, RPS3	disease resistance protein RPM1	--	1	ko04626 Plant-pathogen interaction;
gene38234	K00083	E1.1.1.195	cinnamyl-alcohol dehydrogenase	EC:1.1.1.195	1	ko00940 Phenylpropanoid biosynthesis;
gene38235	K00901	E2.7.1.107, DGK, dgkA	diacylglycerol kinase (ATP dependent)	EC:2.7.1.107	3	ko00561 Glycerolipid metabolism;ko00564 Glycerophospholipid metabolism;ko04070 Phosphatidylinositol signaling system;
gene38237	K12946	SPCS1	signal peptidase complex subunit 1	EC:3.4.-.-	1	ko03060 Protein export;
gene38240	K07374	TUBA	tubulin alpha	--	3	ko04145 Phagosome;ko04540 Gap junction;ko05130 Pathogenic Escherichia coli infection;
gene38241	K16290	XCP	xylem cysteine proteinase	EC:3.4.22.-		
gene38245	K08679	E5.1.3.6	UDP-glucuronate 4-epimerase	EC:5.1.3.6	2	ko00500 Starch and sucrose metabolism;ko00520 Amino sugar and nucleotide sugar metabolism;
gene38249	K12600	SKI3, TTC37	superkiller protein 3	--	1	ko03018 RNA degradation;
gene38254	K00423	E1.10.3.3	L-ascorbate oxidase	EC:1.10.3.3	1	ko00053 Ascorbate and aldarate metabolism;
gene38255	K07195	EXOC7, EXO70	exocyst complex component 7	--	1	ko04910 Insulin signaling pathway;
gene38264	K15601	KDM3	lysine-specific demethylase 3	EC:1.14.11.-		
gene38265	K14488	SAUR	SAUR family protein	--	1	ko04075 Plant hormone signal transduction;
gene38273	K11446	JARID1	histone demethylase JARID1	EC:1.14.11.-		
gene38276	K02893	RP-L23Ae, RPL23A	large subunit ribosomal protein L23Ae	--	1	ko03010 Ribosome;
gene38277	K02145	ATPeVA, ATP6A1	V-type H+-transporting ATPase subunit A	EC:3.6.3.14	7	ko00190 Oxidative phosphorylation;ko04145 Phagosome;ko04721 Synaptic vesicle cycle;ko04966 Collecting duct acid secretion;ko05110 Vibrio cholerae infection;ko05120 Epithelial cell signaling in Helicobacter pylori infection;ko05323 Rheumatoid arthritis;
gene38282	K02966	RP-S19e, RPS19	small subunit ribosomal protein S19e	--	1	ko03010 Ribosome;
gene38292	K09285	OVM, ANT	AP2-like factor, ANT lineage	--		
gene38294	K09419	HSFF	heat shock transcription factor, other eukaryote	--		
gene38296	K13162	PCBP2_3_4	poly(rC)-binding protein 2/3/4	--		
gene38301	K09419	HSFF	heat shock transcription factor, other eukaryote	--		
gene38303	K04733	IRAK4	interleukin-1 receptor-associated kinase 4	EC:2.7.11.1	11	ko04064 NF-kappa B signaling pathway;ko04210 Apoptosis;ko04620 Toll-like receptor signaling pathway;ko04722 Neurotrophin signaling pathway;ko05133 Pertussis;ko05140 Leishmaniasis;ko05142 Chagas disease (American trypanosomiasis);ko05145 Toxoplasmosis;ko05152 Tuberculosis;ko05162 Measles;ko05164 Influenza A;
gene38304	K04733	IRAK4	interleukin-1 receptor-associated kinase 4	EC:2.7.11.1	11	ko04064 NF-kappa B signaling pathway;ko04210 Apoptosis;ko04620 Toll-like receptor signaling pathway;ko04722 Neurotrophin signaling pathway;ko05133 Pertussis;ko05140 Leishmaniasis;ko05142 Chagas disease (American trypanosomiasis);ko05145 Toxoplasmosis;ko05152 Tuberculosis;ko05162 Measles;ko05164 Influenza A;

gene38305	K04733	IRAK4	interleukin-1 receptor-associated kinase 4	EC:2.7.11.1	11	ko04064 NF-kappa B signaling pathway;ko04210 Apoptosis;ko04620 Toll-like receptor signaling pathway;ko04722 Neurotrophin signaling pathway;ko05133 Pertussis;ko05140 Leishmaniasis;ko05142 Chagas disease (American trypanosomiasis);ko05145 Toxoplasmosis;ko05152 Tuberculosis;ko05162 Measles;ko05164 Influenza A;
gene38308	K12600	SKI3, TTC37	superkiller protein 3	--	1	ko03018 RNA degradation;
gene38314	K07297	ADIPOR	adiponectin receptor	--	2	ko04920 Adipocytokine signaling pathway;ko04932 Non-alcoholic fatty liver disease (NAFLD);
gene38319	K04733	IRAK4	interleukin-1 receptor-associated kinase 4	EC:2.7.11.1	11	ko04064 NF-kappa B signaling pathway;ko04210 Apoptosis;ko04620 Toll-like receptor signaling pathway;ko04722 Neurotrophin signaling pathway;ko05133 Pertussis;ko05140 Leishmaniasis;ko05142 Chagas disease (American trypanosomiasis);ko05145 Toxoplasmosis;ko05152 Tuberculosis;ko05162 Measles;ko05164 Influenza A;
gene38326	K15188	CCNT	cyclin T	--	1	ko05202 Transcriptional misregulation in cancer;
gene38327	K08901	psbQ	photosystem II oxygen-evolving enhancer protein 3	--	1	ko00195 Photosynthesis;
gene38330	K02021	ABC.MR	putative ABC transport system ATP-binding protein	--		
gene38332	K10669	TRPT1, TPT1	2'-phosphotransferase	EC:2.7.1.160		
gene38339	K00083	E1.1.1.195	cinnamyl-alcohol dehydrogenase	EC:1.1.1.195	1	ko00940 Phenylpropanoid biosynthesis;
gene38341	K11423	SETD2, SET2	histone-lysine N-methyltransferase SETD2	EC:2.1.1.43	1	ko00310 Lysine degradation;
gene38342	K07904	RAB11A	Ras-related protein Rab-11A	--	4	ko04144 Endocytosis;ko04961 Endocrine and other factor-regulated calcium reabsorption;ko04962 Vasopressin-regulated water reabsorption;ko04972 Pancreatic secretion;
gene38344	K14432	ABF	ABA responsive element binding factor	--	1	ko04075 Plant hormone signal transduction;
gene3834	K11878	PSMG4, PAC4	proteasome assembly chaperone 4	--		
gene38351	K08176	PHO84	MFS transporter, PHS family, inorganic phosphate transporter	--		
gene38358	K01285	PRCP	lysosomal Pro-X carboxypeptidase	EC:3.4.16.2	1	ko04974 Protein digestion and absorption;
gene38367	K12733	PPIL1	peptidyl-prolyl cis-trans isomerase-like 1	EC:5.2.1.8	1	ko03040 Spliceosome;
gene38369	K11594	DDX3X, bel	ATP-dependent RNA helicase	EC:3.6.4.13	3	ko04622 RIG-I-like receptor signaling pathway;ko05161 Hepatitis B;ko05203 Viral carcinogenesis;
gene38371	K10740	RPA3	replication factor A3	--	5	ko03030 DNA replication;ko03420 Nucleotide excision repair;ko03430 Mismatch repair;ko03440 Homologous recombination;ko03460 Fanconi anemia pathway;
gene38374	K08735	MSH2	DNA mismatch repair protein MSH2	--	3	ko03430 Mismatch repair;ko05200 Pathways in cancer;ko05210 Colorectal cancer;
gene38376	K01759	E4.4.1.5, GLO1, gloA	lactoylglutathione lyase	EC:4.4.1.5	2	ko00620 Pyruvate metabolism;ko04011 MAPK signaling pathway - yeast;
gene38378	K08592	SENP1	sentrin-specific protease 1	EC:3.4.22.68		
gene38382	K14638	SLC15A3_4, PHT	solute carrier family 15 (peptide/histidine transporter), member 3/4	--		
gene38386	K03013	RPB5, POLR2E	DNA-directed RNA polymerases I, II, and III subunit RPABC1	--	6	ko00230 Purine metabolism;ko00240 Pyrimidine metabolism;ko03020 RNA polymerase;ko04623 Cytosolic DNA-sensing pathway;ko05016 Huntington's disease;ko05169 Epstein-Barr virus infection;
gene38387	K07199	PRKAB	5'-AMP-activated protein kinase, regulatory beta subunit	--	5	ko04710 Circadian rhythm;ko04910 Insulin signaling pathway;ko04920 Adipocytokine signaling pathway;ko04932 Non-alcoholic fatty liver disease (NAFLD);ko05410 Hypertrophic cardiomyopathy (HCM);
gene38388	K15131	MED11	mediator of RNA polymerase II transcription subunit 11	--		
gene38389	K00736	MGAT2	alpha-1,6-mannosyl-glycoprotein beta-1,2-N-acetylglucosaminyltransferase	EC:2.4.1.143	2	ko00510 N-Glycan biosynthesis;ko00513 Various types of N-glycan biosynthesis;
gene38390	K12120	PHYA	phytochrome A	--	1	ko04712 Circadian rhythm - plant;
gene38396	K01193	E3.2.1.26, sacA	beta-fructofuranosidase	EC:3.2.1.26	2	ko00052 Galactose metabolism;ko00500 Starch and sucrose metabolism;
gene38399	K17434	MRPL53	large subunit ribosomal protein L53, mitochondrial	--		
gene383	K10143	RFWD2, COP1	E3 ubiquitin-protein ligase RFWD2	EC:6.3.2.19	3	ko04115 p53 signaling pathway;ko04120 Ubiquitin mediated proteolysis;ko04712 Circadian rhythm - plant;
gene38403	K14502	BIN2	protein brassinosteroid insensitive 2	EC:2.7.11.1	1	ko04075 Plant hormone signal transduction;
gene38404	K03834	tyrP	tyrosine-specific transport protein	--		
gene38409	K14803	PTC2_3	protein phosphatase 2C homolog 2/3	EC:3.1.3.16		
gene38411	K12733	PPIL1	peptidyl-prolyl cis-trans isomerase-like 1	EC:5.2.1.8	1	ko03040 Spliceosome;

gene38414	K00652	bioF	8-amino-7-oxononanoate synthase	EC:2.3.1.47	1	ko00780 Biotin metabolism;
gene38415	K00025	MDH1	malate dehydrogenase	EC:1.1.1.37	6	ko00020 Citrate cycle (TCA cycle);ko00620 Pyruvate metabolism;ko00630 Glyoxylate and dicarboxylate metabolism;ko00710 Carbon fixation in photosynthetic organisms;ko01200 Carbon metabolism;ko04964 Proximal tubule bicarbonate reclamation;
gene38421	K10885	XRCC5, KU80, G22P2	ATP-dependent DNA helicase 2 subunit 2	--	1	ko03450 Non-homologous end-joining;
gene38422	K14826	FPR3_4	FK506-binding nuclear protein	EC:5.2.1.8		
gene38423	K04730	IRAK1	interleukin-1 receptor-associated kinase 1	EC:2.7.11.1	11	ko04064 NF-kappa B signaling pathway;ko04210 Apoptosis;ko04620 Toll-like receptor signaling pathway;ko04722 Neurotrophin signaling pathway;ko05133 Pertussis;ko05140 Leishmaniasis;ko05142 Chagas disease (American trypanosomiasis);ko05145 Toxoplasmosis;ko05152 Tuberculosis;ko05162 Measles;ko05169 Epstein-Barr virus infection;
gene38431	K15029	EIF3L	translation initiation factor 3 subunit L	--		
gene38432	K14454	GOT1	aspartate aminotransferase, cytoplasmic	EC:2.6.1.1	11	ko00250 Alanine, aspartate and glutamate metabolism;ko00270 Cysteine and methionine metabolism;ko00330 Arginine and proline metabolism;ko00350 Tyrosine metabolism;ko00360 Phenylalanine metabolism;ko00400 Phenylalanine, tyrosine and tryptophan biosynthesis;ko00710 Carbon fixation in photosynthetic organisms;ko00950 Isoquinoline alkaloid biosynthesis;ko00960 Tropane, piperidine and pyridine alkaloid biosynthesis;ko01210 2-Oxocarboxylic acid metabolism;ko01230 Biosynthesis of amino acids;
gene38433	K09420	MYB	myb proto-oncogene protein	--	2	ko04151 PI3K-Akt signaling pathway;ko05166 HTLV-I infection;
gene38435	K11090	LA, SSB	lupus La protein	--	1	ko05322 Systemic lupus erythematosus;
gene38436	K00912	lpxK	tetraacyldisaccharide 4'-kinase	EC:2.7.1.130	1	ko00540 Lipopolysaccharide biosynthesis;
gene3844	K02882	RP-L18Ae, RPL18A	large subunit ribosomal protein L18Ae	--	1	ko03010 Ribosome;
gene38454	K10398	KIF11, EG5	kinesin family member 11	--		
gene38456	K14803	PTC2_3	protein phosphatase 2C homolog 2/3	EC:3.1.3.16		
gene3845	K08286	E2.7.11.-	protein-serine/threonine kinase	EC:2.7.11.-		
gene38461	K09874	NIP	aquaporin NIP	--		
gene38463	K00901	E2.7.1.107, DGK, dgkA	diacylglycerol kinase (ATP dependent)	EC:2.7.1.107	3	ko00561 Glycerolipid metabolism;ko00564 Glycerophospholipid metabolism;ko04070 Phosphatidylinositol signaling system;
gene38464	K14234	tRNA-Thr	tRNA Thr	--	1	ko00970 Aminoacyl-tRNA biosynthesis;
gene38466	K08912	LHCB1	light-harvesting complex II chlorophyll a/b binding protein 1	--	1	ko00196 Photosynthesis - antenna proteins;
gene38467	K08912	LHCB1	light-harvesting complex II chlorophyll a/b binding protein 1	--	1	ko00196 Photosynthesis - antenna proteins;
gene38469	K07053	K07053				
gene38470	K01703	leuC	3-isopropylmalate/(R)-2-methylmalate dehydratase large subunit	EC:4.2.1.33 4.2.1.35	4	ko00290 Valine, leucine and isoleucine biosynthesis;ko00660 C5-Branched dibasic acid metabolism;ko01210 2-Oxocarboxylic acid metabolism;ko01230 Biosynthesis of amino acids;
gene38479	K14803	PTC2_3	protein phosphatase 2C homolog 2/3	EC:3.1.3.16		
gene38480	K12852	EFTUD2	116 kDa U5 small nuclear ribonucleoprotein component	--	1	ko03040 Spliceosome;
gene38484	K12192	CHMP2B	charged multivesicular body protein 2B	--	1	ko04144 Endocytosis;
gene38485	K02503	HINT1, hinT, hit	histidine triad (HIT) family protein	--		
gene38489	K09419	HSFF	heat shock transcription factor, other eukaryote	--		
gene3848	K00685	ATE1, ate1	arginine-tRNA-protein transferase	EC:2.3.2.8		
gene38490	K10268	FBXL2_20	F-box and leucine-rich repeat protein 2/20	--		
gene38497	K07441	ALG14	beta-1,4-N-acetylglucosaminyltransferase	EC:2.4.1.141	2	ko00510 N-Glycan biosynthesis;ko00513 Various types of N-glycan biosynthesis;
gene38499	K04730	IRAK1	interleukin-1 receptor-associated kinase 1	EC:2.7.11.1	11	ko04064 NF-kappa B signaling pathway;ko04210 Apoptosis;ko04620 Toll-like receptor signaling pathway;ko04722 Neurotrophin signaling pathway;ko05133 Pertussis;ko05140 Leishmaniasis;ko05142 Chagas disease (American trypanosomiasis);ko05145 Toxoplasmosis;ko05152 Tuberculosis;ko05162 Measles;ko05169 Epstein-Barr virus infection;
gene38505	K00888	PI4K	phosphatidylinositol 4-kinase	EC:2.7.1.67	2	ko00562 Inositol phosphate metabolism;ko04070 Phosphatidylinositol signaling system;
gene38507	K08869	ADCK, ABC1	aarF domain-containing kinase	--		

gene38508	K12309	GLB1, ELNR1	beta-galactosidase	EC:3.2.1.23	6	ko00052 Galactose metabolism;ko00511 Other glycan degradation;ko00531 Glycosaminoglycan degradation;ko00600 Sphingolipid metabolism;ko00604 Glycosphingolipid biosynthesis - ganglio series;ko04142 Lysosome;
gene38513	K01193	E3.2.1.26, sacA	beta-fructofuranosidase	EC:3.2.1.26	2	ko00052 Galactose metabolism;ko00500 Starch and sucrose metabolism;
gene38516	K05658	ABCB1	ATP-binding cassette, subfamily B (MDR/TAP), member 1	--	3	ko02010 ABC transporters;ko04976 Bile secretion;ko05206 MicroRNAs in cancer;
gene38518	K14294	WIBG, PYM	partner of Y14 and mago	--	2	ko03013 RNA transport;ko03015 mRNA surveillance pathway;
gene38519	K14815	MRT4	mRNA turnover protein 4	--		
gene38521	K07198	PRKAA, AMPK	5'-AMP-activated protein kinase, catalytic alpha subunit	EC:2.7.11.11	8	ko04140 Regulation of autophagy;ko04150 mTOR signaling pathway;ko04151 PI3K-Akt signaling pathway;ko04710 Circadian rhythm;ko04910 Insulin signaling pathway;ko04920 Adipocytokine signaling pathway;ko04932 Non-alcoholic fatty liver disease (NAFLD);ko05410 Hypertrophic cardiomyopathy (HCM);
gene38523	K11450	AOF2, LSD1	lysine-specific histone demethylase 1	EC:1.-.-.-		
gene38526	K03106	SRP54, ffh	signal recognition particle subunit SRP54	--	2	ko03060 Protein export;ko03070 Bacterial secretion system;
gene38528	K16055	TPS	trehalose 6-phosphate synthase/phosphatase	EC:2.4.1.15 3.1.3.12	1	ko00500 Starch and sucrose metabolism;
gene38534	K01246	tag	DNA-3-methyladenine glycosylase I	EC:3.2.2.20	1	ko03410 Base excision repair;
gene38539	K02180	BUB3	cell cycle arrest protein BUB3	--	3	ko04110 Cell cycle;ko04111 Cell cycle - yeast;ko05166 HTLV-I infection;
gene38545	K14431	TGA	transcription factor TGA	--	1	ko04075 Plant hormone signal transduction;
gene38551	K13162	PCBP2_3_4	poly(rC)-binding protein 2/3/4	--		
gene38554	K10357	MYO5	myosin V	--		
gene38558	K15084	SLC25A16, GDA, LEU5	solute carrier family 25 (mitochondrial carrier protein), member 16	--		
gene38559	K00640	cysE	serine O-acetyltransferase	EC:2.3.1.30	4	ko00270 Cysteine and methionine metabolism;ko00920 Sulfur metabolism;ko01200 Carbon metabolism;ko01230 Biosynthesis of amino acids;
gene38561	K05016	CLCN7	chloride channel 7	--		
gene38562	K11416	SIRT6, SIR2L6	mono-ADP-ribosyltransferase sirtuin 6	EC:2.4.2.31		
gene38572	K17479	GRXCR1	glutaredoxin domain-containing cysteine-rich protein 1	--		
gene38592	K11975	RNF144	E3 ubiquitin-protein ligase RNF144	EC:6.3.2.19		
gene38595	K12126	PIF3	phytochrome-interacting factor 3	--	2	ko04075 Plant hormone signal transduction;ko04712 Circadian rhythm - plant;
gene38599	K14502	BIN2	protein brassinosteroid insensitive 2	EC:2.7.11.1	1	ko04075 Plant hormone signal transduction;
gene3859	K16278	K16278, HOS1	E3 ubiquitin-protein ligase HOS1	EC:6.3.2.19		
gene385	K03661	ATPeVPF, ATP6F	V-type H+-transporting ATPase 21kDa proteolipid subunit	EC:3.6.3.14	8	ko00190 Oxidative phosphorylation;ko04142 Lysosome;ko04145 Phagosome;ko04721 Synaptic vesicle cycle;ko05110 Vibrio cholerae infection;ko05120 Epithelial cell signaling in Helicobacter pylori infection;ko05152 Tuberculosis;ko05323 Rheumatoid arthritis;
gene38605	K01193	E3.2.1.26, sacA	beta-fructofuranosidase	EC:3.2.1.26	2	ko00052 Galactose metabolism;ko00500 Starch and sucrose metabolism;
gene38607	K15199	GTF3C1	general transcription factor 3C polypeptide 1	--		
gene38608	K02021	ABC.MR	putative ABC transport system ATP-binding protein	--		
gene38609	K08880	STK19	serine/threonine kinase 19	EC:2.7.11.1		
gene38611	K10406	KIFC2_3	kinesin family member C2/C3	--		
gene38620	K01800	maiA, GSTZ1	maleylacetoacetate isomerase	EC:5.2.1.2	2	ko00350 Tyrosine metabolism;ko00643 Styrene degradation;
gene38624	K15199	GTF3C1	general transcription factor 3C polypeptide 1	--		
gene38627	K04506	SIAH1	E3 ubiquitin-protein ligase SIAH1	EC:6.3.2.19	3	ko04115 p53 signaling pathway;ko04120 Ubiquitin mediated proteolysis;ko04310 Wnt signaling pathway;
gene38632	K14972	PAXIP1, PTIP	PAX-interacting protein 1	--		
gene38633	K11375	ELP4	elongator complex protein 4	--		
gene38634	K00083	E1.1.1.195	cinnamyl-alcohol dehydrogenase	EC:1.1.1.195	1	ko00940 Phenylpropanoid biosynthesis;
gene38636	K12611	DCP1B	mRNA-decapping enzyme 1B	EC:3.-.-.-	1	ko03018 RNA degradation;
gene38640	K15472	CYP71D55	premnaspirodiene oxygenase	EC:1.14.13.121	1	ko00909 Sesquiterpenoid and triterpenoid biosynthesis;
gene38641	K14638	SLC15A3_4, PHT	solute carrier family 15 (peptide/histidine transporter), member 3/4	--		
gene38645	K00888	PI4K	phosphatidylinositol 4-kinase	EC:2.7.1.67	2	ko00562 Inositol phosphate metabolism;ko04070 Phosphatidylinositol signaling system;

gene38646	K02894	RP-L23e, RPL23	large subunit ribosomal protein L23e	--	1	ko03010 Ribosome;
gene38648	K14823	EBP2, EBNA1BP2	rRNA-processing protein EBP2	--		
gene38649	K00558	DNMT1, dcm	DNA (cytosine-5)-methyltransferase 1	EC:2.1.1.37	2	ko00270 Cysteine and methionine metabolism;ko05206 MicroRNAs in cancer;
gene38650	K01179	E3.2.1.4	endoglucanase	EC:3.2.1.4	1	ko00500 Starch and sucrose metabolism;
gene38651	K10398	KIF11, EG5	kinesin family member 11	--		
gene38653	K12386	CTNS	cystinosin	--	1	ko04142 Lysosome;
gene38656	K03265	ETF1, ERF1	peptide chain release factor subunit 1	--	1	ko03015 mRNA surveillance pathway;
gene38661	K15472	CYP71D55	premnaspirodiene oxygenase	EC:1.14.13.12 1	1	ko00909 Sesquiterpenoid and triterpenoid biosynthesis;
gene38662	K15472	CYP71D55	premnaspirodiene oxygenase	EC:1.14.13.12 1	1	ko00909 Sesquiterpenoid and triterpenoid biosynthesis;
gene38663	K09264	K09264	MADS-box transcription factor, plant	--		
gene38672	K13946	AUX1, LAX	auxin influx carrier (AUX1 LAX family)	--	1	ko04075 Plant hormone signal transduction;
gene38676	K14484	IAA	auxin-responsive protein IAA	--	1	ko04075 Plant hormone signal transduction;
gene3868	K01739	metB	cystathionine gamma-synthase	EC:2.5.1.48	4	ko00270 Cysteine and methionine metabolism;ko00450 Selenocompound metabolism;ko00920 Sulfur metabolism;ko01230 Biosynthesis of amino acids;
gene38700	K14397	NUDT21, CPSF5, CFIM25	cleavage and polyadenylation specificity factor subunit 5	--	1	ko03015 mRNA surveillance pathway;
gene38701	K11314	TADA2A, ADA2	transcriptional adapter 2-alpha	--		
gene38707	K14638	SLC15A3_4, PHT	solute carrier family 15 (peptide/histidine transporter), member 3/4	--		
gene38711	K01082	E3.1.3.7, cysQ, MET22, BPNT1	3'(2'), 5'-bisphosphate nucleotidase	EC:3.1.3.7	1	ko00920 Sulfur metabolism;
gene38714	K00059	fabG	3-oxoacyl-[acyl-carrier protein] reductase	EC:1.1.1.100	3	ko00061 Fatty acid biosynthesis;ko00780 Biotin metabolism;ko01040 Biosynthesis of unsaturated fatty acids;
gene38716	K10268	FBXL2_20	F-box and leucine-rich repeat protein 2/20	--		
gene38724	K15449	TYW1	tRNA wybutosine-synthesizing protein 1	--		
gene38725	K16302	CNNM	metal transporter CNNM	--		
gene38726	K16302	CNNM	metal transporter CNNM	--		
gene38730	K13425	WRKY22	WRKY transcription factor 22	--	1	ko04626 Plant-pathogen interaction;
gene38733	K04733	IRAK4	interleukin-1 receptor-associated kinase 4	EC:2.7.11.1	11	ko04064 NF-kappa B signaling pathway;ko04210 Apoptosis;ko04620 Toll-like receptor signaling pathway;ko04722 Neurotrophin signaling pathway;ko05133 Pertussis;ko05140 Leishmaniasis;ko05142 Chagas disease (American trypanosomiasis);ko05145 Toxoplasmosis;ko05152 Tuberculosis;ko05162 Measles;ko05164 Influenza A;
gene38738	K02732	PSMB1	20S proteasome subunit beta 6	EC:3.4.25.1	1	ko03050 Proteasome;
gene38741	K11000	CALS	callose synthase	EC:2.4.1.-		
gene38753	K02899	RP-L27, MRPL27, rpmA	large subunit ribosomal protein L27	--	1	ko03010 Ribosome;
gene38756	K00721	DPM1	dolichol-phosphate mannosyltransferase	EC:2.4.1.83	1	ko00510 N-Glycan biosynthesis;
gene3875	K14709	SLC39A1_2_3, ZIP1_2_3	solute carrier family 39 (zinc transporter), member 1/2/3	--		
gene38767	K14638	SLC15A3_4, PHT	solute carrier family 15 (peptide/histidine transporter), member 3/4	--		
gene38769	K14297	NUP98, ADAR2	nuclear pore complex protein Nup98-Nup96	--	2	ko03013 RNA transport;ko05164 Influenza A;
gene38770	K14297	NUP98, ADAR2	nuclear pore complex protein Nup98-Nup96	--	2	ko03013 RNA transport;ko05164 Influenza A;
gene38775	K03676	grxC, GLRX, GLRX2	glutaredoxin 3	--		
gene38788	K07195	EXOC7, EXO70	exocyst complex component 7	--	1	ko04910 Insulin signaling pathway;
gene38789	K02575	NRT, narK, nrtP, nasA	MFS transporter, NNP family, nitrate/nitrite transporter	--	1	ko00910 Nitrogen metabolism;
gene3878	K09338	HD-ZIP	homeobox-leucine zipper protein	--		
gene38790	K16569	TUBGCP2, GCP2	gamma-tubulin complex component 2	--		
gene38791	K03921	DESA1	acyl-[acyl-carrier-protein] desaturase	EC:1.14.19.2	2	ko00061 Fatty acid biosynthesis;ko01040 Biosynthesis of unsaturated fatty acids;
gene38794	K00430	E1.11.1.7	peroxidase	EC:1.11.1.7	2	ko00360 Phenylalanine metabolism;ko00940 Phenylpropanoid biosynthesis;
gene38799	K12175	GPS1, COPS1, CSN1	COP9 signalosome complex subunit 1	--		
gene38816	K02896	RP-L24e, RPL24	large subunit ribosomal protein L24e	--	1	ko03010 Ribosome;
gene38817	K12830	SF3B3, SAP130, RSE1	splicing factor 3B subunit 3	--	1	ko03040 Spliceosome;
gene38821	K02922	RP-L37e, RPL37	large subunit ribosomal protein L37e	--	1	ko03010 Ribosome;

gene38822	K03978	engB	GTP-binding protein	--		
gene38824	K02899	RP-L27, MRPL27, rpmA	large subunit ribosomal protein L27	--	1	ko03010 Ribosome;
gene38828	K15178	RTF1	RNA polymerase-associated protein RTF1	--		
gene38835	K16573	TUBGCP6, GCP6	gamma-tubulin complex component 6	--		
gene38840	K10579	UBE2M, UBC12	ubiquitin-conjugating enzyme E2 M	EC:6.3.2.19	1	ko04120 Ubiquitin mediated proteolysis;
gene38841	K13789	GGPS	geranylgeranyl diphosphate synthase, type II	EC:2.5.1.1 2.5.1.10 2.5.1.29	1	ko00900 Terpenoid backbone biosynthesis;
gene38842	K00434	E1.11.1.11	L-ascorbate peroxidase	EC:1.11.1.11	2	ko00053 Ascorbate and aldarate metabolism;ko00480 Glutathione metabolism;
gene38849	K01358	clpP, CLPP	ATP-dependent Clp protease, protease subunit	EC:3.4.21.92	1	ko04112 Cell cycle - Caulobacter;
gene38850	K10845	TTDA, GTF2H5, TFB5	TFIIH basal transcription factor complex TTD-A subunit	--	2	ko03022 Basal transcription factors;ko03420 Nucleotide excision repair;
gene38854	K00432	E1.11.1.9	glutathione peroxidase	EC:1.11.1.9	3	ko00480 Glutathione metabolism;ko00590 Arachidonic acid metabolism;ko04918 Thyroid hormone synthesis;
gene38858	K10406	KIFC2_3	kinesin family member C2/C3	--		
gene38859	K10406	KIFC2_3	kinesin family member C2/C3	--		
gene38861	K02883	RP-L18e, RPL18	large subunit ribosomal protein L18e	--	1	ko03010 Ribosome;
gene38864	K13148	CPSF3L, INTS11	integrator complex subunit 11	EC:3.1.27.-		
gene38865	K15305	VAC14, TAX1BP2	vacuole morphology and inheritance protein 14	--	2	ko05166 HTLV-I infection;ko05203 Viral carcinogenesis;
gene38866	K13448	CML	calcium-binding protein CML	--	1	ko04626 Plant-pathogen interaction;
gene38869	K09286	EREBP	EREBP-like factor	--		
gene38871	K01087	otsB	trehalose 6-phosphate phosphatase	EC:3.1.3.12	1	ko00500 Starch and sucrose metabolism;
gene38873	K10747	LIG1	DNA ligase 1	EC:6.5.1.1	4	ko03030 DNA replication;ko03410 Base excision repair;ko03420 Nucleotide excision repair;ko03430 Mismatch repair;
gene38877	K08835	OXSR1, STK39	serine/threonine-protein kinase OSR1/STK39	EC:2.7.11.1		
gene38884	K17292	TBCA	tubulin-specific chaperone A	--		
gene38885	K13947	PIN	auxin efflux carrier family	--		
gene38888	K09422	MYBP	myb proto-oncogene protein, plant	--		
gene38889	K12386	CTNS	cystinosin	--	1	ko04142 Lysosome;
gene3888	K06890	K06890				
gene38896	K12667	SWP1, RPN2	oligosaccharyltransferase complex subunit delta (ribophorin II)	--	3	ko00510 N-Glycan biosynthesis;ko00513 Various types of N-glycan biosynthesis;ko04141 Protein processing in endoplasmic reticulum;
gene38897	K00898	PDK2_3_4	pyruvate dehydrogenase kinase 2/3/4	EC:2.7.11.2		
gene38901	K13116	DDX41, ABS	ATP-dependent RNA helicase DDX41	EC:3.6.4.13		
gene38902	K13116	DDX41, ABS	ATP-dependent RNA helicase DDX41	EC:3.6.4.13		
gene38906	K06268	PPP3R, CNB	serine/threonine-protein phosphatase 2B regulatory subunit	--	18	ko04010 MAPK signaling pathway;ko04020 Calcium signaling pathway;ko04114 Oocyte meiosis;ko04210 Apoptosis;ko04310 Wnt signaling pathway;ko04360 Axon guidance;ko04370 VEGF signaling pathway;ko04380 Osteoclast differentiation;ko04650 Natural killer cell mediated cytotoxicity;ko04660 T cell receptor signaling pathway;ko04662 B cell receptor signaling pathway;ko04720 Long-term potentiation;ko04724 Glutamatergic synapse;ko05010 Alzheimer's disease;ko05014 Amyotrophic lateral sclerosis (ALS);ko05031 Amphetamine addiction;ko05152
gene38911	K03283	HSPA1_8	heat shock 70kDa protein 1/8	--	11	ko03040 Spliceosome;ko04010 MAPK signaling pathway;ko04141 Protein processing in endoplasmic reticulum;ko04144 Endocytosis;ko04612 Antigen processing and presentation;ko04915 Estrogen signaling pathway;ko05134 Legionellosis;ko05145 Toxoplasmosis;ko05162 Measles;ko05164 Influenza A;ko05169 Epstein-Barr virus infection;
gene38913	K14766	NOP14, UTP2	nucleolar protein 14	--		
gene38915	K07955	ARL8	ADP-ribosylation factor-like protein 8	--		
gene38916	K14682	argAB	amino-acid N-acetyltransferase	EC:2.3.1.1	3	ko00330 Arginine and proline metabolism;ko01210 2-Oxocarboxylic acid metabolism;ko01230 Biosynthesis of amino acids;
gene3891	K08915	LHCB4	light-harvesting complex II chlorophyll a/b binding protein 4	--	1	ko00196 Photosynthesis - antenna proteins;
gene38923	K01592	E4.1.1.25	tyrosine decarboxylase	EC:4.1.1.25	2	ko00350 Tyrosine metabolism;ko00950 Isoquinoline alkaloid biosynthesis;

gene38927	K12402	AP4M1	AP-4 complex subunit mu-1	--	1	ko04142 Lysosome;
gene3892	K14795	RRP36	ribosomal RNA-processing protein 36	--		
gene38932	K14664	ILR1	IAA-amino acid hydrolase	EC:3.5.1.-		
gene38940	K09422	MYBP	myb proto-oncogene protein, plant	--		
gene38942	K12837	U2AF2	splicing factor U2AF 65 kDa subunit	--	1	ko03040 Spliceosome;
gene38950	K01880	GARS, glyS1	glycyl-tRNA synthetase	EC:6.1.1.14	1	ko00970 Aminoacyl-tRNA biosynthesis;
gene38952	K16292	CEP, CYSEP	KDEL-tailed cysteine endopeptidase	EC:3.4.22.-		
gene38953	K16292	CEP, CYSEP	KDEL-tailed cysteine endopeptidase	EC:3.4.22.-		
gene38958	K02912	RP-L32e, RPL32	large subunit ribosomal protein L32e	--	1	ko03010 Ribosome;
gene3895	K09613	COPS5, CSN5	COP9 signalosome complex subunit 5	EC:3.4.-.-		
gene38968	K10802	HMGB1	high mobility group protein B1	--	1	ko03410 Base excision repair;
gene38974	K07305	msrB	peptide-methionine (R)-S-oxide reductase	EC:1.8.4.12		
gene38978	K01507	ppa	inorganic pyrophosphatase	EC:3.6.1.1	1	ko00190 Oxidative phosphorylation;
gene38979	K11498	CENPE	centromeric protein E	--		
gene38981	K07071	K07071				
gene38982	K14016	UFD1	ubiquitin fusion degradation protein 1	--	1	ko04141 Protein processing in endoplasmic reticulum;
gene38983	K11320	EP400	E1A-binding protein p400	EC:3.6.4.-		
gene38984	K15472	CYP71D55	premnaspirodiene oxygenase	EC:1.14.13.121	1	ko00909 Sesquiterpenoid and triterpenoid biosynthesis;
gene38987	K14233	tRNA-Ser	tRNA Ser	--	1	ko00970 Aminoacyl-tRNA biosynthesis;
gene38990	K01188	E3.2.1.21	beta-glucosidase	EC:3.2.1.21	3	ko00460 Cyanoamino acid metabolism;ko00500 Starch and sucrose metabolism;ko00940 Phenylpropanoid biosynthesis;
gene38991	K15200	GTF3C2	general transcription factor 3C polypeptide 2	--		
gene38992	K10575	UBE2G1, UBC7	ubiquitin-conjugating enzyme E2 G1	EC:6.3.2.19	3	ko04120 Ubiquitin mediated proteolysis;ko04141 Protein processing in endoplasmic reticulum;ko05012 Parkinson's disease;
gene38995	K06630	YWHAE	14-3-3 protein epsilon	--	8	ko04110 Cell cycle;ko04114 Oocyte meiosis;ko04151 PI3K-Akt signaling pathway;ko04390 Hippo signaling pathway;ko04391 Hippo signaling pathway - fly;ko04722 Neurotrophin signaling pathway;ko05169 Epstein-Barr virus infection;ko05203 Viral carcinogenesis;
gene39000	K01602	rbcS	ribulose-bisphosphate carboxylase small chain	EC:4.1.1.39	3	ko00630 Glyoxylate and dicarboxylate metabolism;ko00710 Carbon fixation in photosynthetic organisms;ko01200 Carbon metabolism;
gene39001	K01602	rbcS	ribulose-bisphosphate carboxylase small chain	EC:4.1.1.39	3	ko00630 Glyoxylate and dicarboxylate metabolism;ko00710 Carbon fixation in photosynthetic organisms;ko01200 Carbon metabolism;
gene39006	K05933	E1.14.17.4	aminocyclopropanecarboxylate oxidase	EC:1.14.17.4	1	ko00270 Cysteine and methionine metabolism;
gene39009	K11092	SNRPA1	U2 small nuclear ribonucleoprotein A'	--	1	ko03040 Spliceosome;
gene39013	K11092	SNRPA1	U2 small nuclear ribonucleoprotein A'	--	1	ko03040 Spliceosome;
gene39014	K03696	clpC	ATP-dependent Clp protease ATP-binding subunit ClpC	--		
gene39016	K13447	RBOH	respiratory burst oxidase	EC:1.6.3.- 1.11.1.-	1	ko04626 Plant-pathogen interaction;
gene3901	K14396	PABPN1, PABP2	polyadenylate-binding protein 2	--	2	ko03015 mRNA surveillance pathway;ko05164 Influenza A;
gene39020	K00915	IPK2	1D-myo-Inositol-tetrakisphosphate 5-kinase / inositol-polyphosphate multikinase	EC:2.7.1.140 2.7.1.151	2	ko00562 Inositol phosphate metabolism;ko04070 Phosphatidylinositol signaling system;
gene39022	K13162	PCBP2_3_4	poly(rC)-binding protein 2/3/4	--		
gene39028	K15191	LARP7	La-related protein 7	--		
gene39029	K01000	mraY	phospho-N-acetylmuramoyl-pentapeptide-transferase	EC:2.7.8.13	1	ko00550 Peptidoglycan biosynthesis;
gene39033	K16276	K16276, BTS	zinc finger protein-like protein	--		
gene39034	K11294	NCL, NSR1	nucleolin	--	1	ko05130 Pathogenic Escherichia coli infection;
gene39035	K00573	E2.1.1.77, pcm	protein-L-isoaspartate(D-aspartate) O-methyltransferase	EC:2.1.1.77		
gene39040	K11322	EPC	enhancer of polycomb-like protein	--		
gene39043	K13963	SERPINB	serpin B	--	1	ko05146 Amoebiasis;
gene39046	K00558	DNMT1, dcm	DNA (cytosine-5)-methyltransferase 1	EC:2.1.1.37	2	ko00270 Cysteine and methionine metabolism;ko05206 MicroRNAs in cancer;
gene39047	K16276	K16276, BTS	zinc finger protein-like protein	--		

gene39048	K17479	GRXCR1	glutaredoxin domain-containing cysteine-rich protein 1	--		
gene39052	K03940	NDUFS7	NADH dehydrogenase (ubiquinone) Fe-S protein 7	EC:1.6.5.3 1.6.99.3	5	ko00190 Oxidative phosphorylation;ko04932 Non-alcoholic fatty liver disease (NAFLD);ko05010 Alzheimer's disease;ko05012 Parkinson's disease;ko05016 Huntington's disease;
gene39053	K13210	FUBP	far upstream element-binding protein	--		
gene39055	K02140	ATPeFG, ATP5L	F-type H+-transporting ATPase subunit g	EC:3.6.3.14	1	ko00190 Oxidative phosphorylation;
gene39056	K01772	hemH, FECH	ferrochelatase	EC:4.99.1.1	1	ko00860 Porphyrin and chlorophyll metabolism;
gene39057	K03132	TAF7	transcription initiation factor TFIID subunit 7	--	1	ko03022 Basal transcription factors;
gene39062	K14709	SLC39A1_2_3, ZIP1_2_3	solute carrier family 39 (zinc transporter), member 1/2/3	--		
gene39066	K17255	GDI1_2	Rab GDP dissociation inhibitor	--		
gene39067	K08472	MLO	mlo protein	--		
gene39069	K15437	AIMP1, ARC1	aminoacyl tRNA synthase complex-interacting multifunctional protein 1	--		
gene3906	K02604	ORC2	origin recognition complex subunit 2	--	3	ko04110 Cell cycle;ko04111 Cell cycle - yeast;ko04113 Meiosis - yeast;
gene39071	K10624	RBBP6	E3 ubiquitin-protein ligase RBBP6	EC:6.3.2.19		
gene39072	K14641	APY1_2	apyrase	EC:3.6.1.5	2	ko00230 Purine metabolism;ko00240 Pyrimidine metabolism;
gene39080	K14230	tRNA-Met	tRNA Met	--	1	ko00970 Aminoacyl-tRNA biosynthesis;
gene39083	K01979	SSUrRNA	small subunit ribosomal RNA	--	2	ko03008 Ribosome biogenesis in eukaryotes;ko03010 Ribosome;
gene39084	K01979	SSUrRNA	small subunit ribosomal RNA	--	2	ko03008 Ribosome biogenesis in eukaryotes;ko03010 Ribosome;
gene39088	K09566	PPIG	peptidyl-prolyl isomerase G (cyclophilin G)	EC:5.2.1.8		
gene3909	K14190	VTC2_5	GDP-L-galactose phosphorylase	EC:2.7.7.69	1	ko00053 Ascorbate and aldarate metabolism;
gene39100	K09286	EREBP	EREBP-like factor	--		
gene39104	K14829	IPI3	pre-rRNA-processing protein IPI3	--		
gene39107	K03352	APC5	anaphase-promoting complex subunit 5	--	7	ko04110 Cell cycle;ko04111 Cell cycle - yeast;ko04113 Meiosis - yeast;ko04114 Oocyte meiosis;ko04120 Ubiquitin mediated proteolysis;ko04914 Progesterone-mediated oocyte maturation;ko05166 HTLV-I infection;
gene3910	K13448	CML	calcium-binding protein CML	--	1	ko04626 Plant-pathogen interaction;
gene39112	K14005	SEC31	protein transport protein SEC31	--	1	ko04141 Protein processing in endoplasmic reticulum;
gene39113	K15078	SLX1	structure-specific endonuclease subunit SLX1	EC:3.6.1.-	1	ko03460 Fanconi anemia pathway;
gene39114	K07408	CYP1A1	cytochrome P450, family 1, subfamily A, polypeptide 1	EC:1.14.14.1	6	ko00140 Steroid hormone biosynthesis;ko00380 Tryptophan metabolism;ko00830 Retinol metabolism;ko00980 Metabolism of xenobiotics by cytochrome P450;ko04913 Ovarian steroidogenesis;ko05204 Chemical carcinogenesis;
gene39117	K15192	BTAF1, MOT1	TATA-binding protein-associated factor	EC:3.6.4.-		
gene3911	K02979	RP-S28e, RPS28	small subunit ribosomal protein S28e	--	1	ko03010 Ribosome;
gene39120	K00434	E1.11.1.11	L-ascorbate peroxidase	EC:1.11.1.11	2	ko00053 Ascorbate and aldarate metabolism;ko00480 Glutathione metabolism;
gene39124	K00861	RFK, FMN1	riboflavin kinase	EC:2.7.1.26	1	ko00740 Riboflavin metabolism;
gene39127	K12116	FKF1	flavin-binding kelch repeat F-box protein 1	--	1	ko04712 Circadian rhythm - plant;
gene39128	K11699	RDR1, RDP1	RNA-dependent RNA polymerase 1	EC:2.7.7.48		
gene39132	K15108	SLC25A19, DNC, TPC1	solute carrier family 25 (mitochondrial thiamine pyrophosphate transporter), member 19	--		
gene39134	K17398	DNMT3A	DNA (cytosine-5)-methyltransferase 3A	EC:2.1.1.37	2	ko00270 Cysteine and methionine metabolism;ko05206 MicroRNAs in cancer;
gene39141	K02873	RP-L13e, RPL13	large subunit ribosomal protein L13e	--	1	ko03010 Ribosome;
gene39145	K01681	ACO, acnA	aconitate hydratase	EC:4.2.1.3	6	ko00020 Citrate cycle (TCA cycle);ko00630 Glyoxylate and dicarboxylate metabolism;ko00720 Carbon fixation pathways in prokaryotes;ko01200 Carbon metabolism;ko01210 2-Oxocarboxylic acid metabolism;ko01230 Biosynthesis of amino acids;
gene39152	K14864	FTSJ1, TRM7	tRNA (cytidine32/guanosine34-2'-O)-methyltransferase	EC:2.1.1.205		
gene39155	K02908	RP-L30e, RPL30	large subunit ribosomal protein L30e	--	1	ko03010 Ribosome;
gene39159	K05391	CNGF	cyclic nucleotide gated channel, other eukaryote	--	1	ko04626 Plant-pathogen interaction;
gene39160	K10956	SEC61A	protein transport protein SEC61 subunit alpha	--	4	ko03060 Protein export;ko04141 Protein processing in endoplasmic reticulum;ko04145 Phagosome;ko05110 Vibrio cholerae infection;

gene39162	K05349	bglX	beta-glucosidase	EC:3.2.1.21	3	ko00460 Cyanoamino acid metabolism;ko00500 Starch and sucrose metabolism;ko00940 Phenylpropanoid biosynthesis;
gene39170	K02732	PSMB1	20S proteasome subunit beta 6	EC:3.4.25.1	1	ko03050 Proteasome;
gene39175	K13993	HSP20	HSP20 family protein	--	1	ko04141 Protein processing in endoplasmic reticulum;
gene39176	K17408	DAP3, MRPS29	small subunit ribosomal protein S29, mitochondrial	--		
gene39189	K02951	RP-S12e, RPS12	small subunit ribosomal protein S12e	--	1	ko03010 Ribosome;
gene39192	K02939	RP-L9, MRPL9, rplI	large subunit ribosomal protein L9	--	1	ko03010 Ribosome;
gene39195	K00626	E2.3.1.9, atoB	acetyl-CoA C-acetyltransferase	EC:2.3.1.9	14	ko00071 Fatty acid degradation;ko00072 Synthesis and degradation of ketone bodies;ko00280 Valine, leucine and isoleucine degradation;ko00310 Lysine degradation;ko00362 Benzoate degradation;ko00380 Tryptophan metabolism;ko00620 Pyruvate metabolism;ko00630 Glyoxylate and dicarboxylate metabolism;ko00640 Propanoate metabolism;ko00650 Butanoate metabolism;ko00720 Carbon fixation pathways in prokaryotes;ko00900 Terpenoid backbone biosynthesis;ko01200 Carbon
gene39198	K04371	MAPK1_3	mitogen-activated protein kinase 1/3	EC:2.7.11.24	71	ko04010 MAPK signaling pathway;ko04012 ErbB signaling pathway;ko04013 MAPK signaling pathway - fly;ko04014 Ras signaling pathway;ko04062 Chemokine signaling pathway;ko04066 HIF-1 signaling pathway;ko04114 Oocyte meiosis;ko04150 mTOR signaling pathway;ko04151 PI3K-Akt signaling pathway;ko04270 Vascular smooth muscle contraction;ko04320 Dorso-ventral axis formation;ko04350 TGF-beta signaling pathway;ko04360 Axon guidance;ko04370 VEGF signaling pathway;ko04380 Osteoclast differentiation;ko04510 Focal adhesion;ko04520 Adherens junction;ko04540 Gap junction;ko04620 Toll-like receptor signaling pathway;ko04621 NOD-like receptor signaling pathway;ko04650 Natural killer cell mediated cytotoxicity;ko04660 T cell receptor signaling pathway;ko04662 B cell receptor signaling pathway;ko04664 Fc epsilon RI signaling pathway;ko04666 Fc gamma R-mediated phagocytosis;ko04668 TNF signaling pathway;ko04713 Circadian entrainment;ko04720 Long-term potentiation;ko04722 Neurotrophin signaling pathway;ko04723 Retrograde endocannabinoid signaling;ko04724 Glutamatergic synapse;ko04725 Cholinergic synapse;ko04726 Serotonergic synapse;ko04730 Long-term depression;ko04810 Regulation of actin cytoskeleton;ko04910 Insulin signaling pathway;ko04912 GnRH signaling pathway;ko04914 Progesterone-mediated oocyte maturation;ko04915 Estrogen signaling pathway;ko04916 Melanogenesis;ko04917 Prolactin signaling pathway;ko04930 Type II diabetes mellitus;ko04960 Aldosterone-regulated sodium reabsorption;ko05010 Alzheimer's disease;ko05020 Prion diseases;ko05034 Alcoholism;ko05131 Shigellosis;ko05132 Salmonella infection;ko05133 Pertussis;ko05140 Leishmaniasis;ko05142 Chagas disease (American trypanosomiasis);ko05145 Toxoplasmosis;ko05152 Tuberculosis;ko05160 Hepatitis C;ko05161 Hepatitis B;ko05164 Influenza A;ko05200 Pathways in cancer;ko05203 Viral carcinogenesis;ko05205 Proteoglycans in cancer;ko05210 Colorectal cancer;ko05211 Renal cell carcinoma;ko05212
gene391	K07877	RAB2A	Ras-related protein Rab-2A	--		
gene39200	K00457	HPD, hppD	4-hydroxyphenylpyruvate dioxygenase	EC:1.13.11.27	3	ko00130 Ubiquinone and other terpenoid-quinone biosynthesis;ko00350 Tyrosine metabolism;ko00360 Phenylalanine metabolism;
gene39204	K14492	ARR-A	two-component response regulator ARR-A family	--	1	ko04075 Plant hormone signal transduction;
gene39208	K15404	K15404, CER1	aldehyde decarbonylase	EC:4.1.99.5	1	ko00073 Cutin, suberine and wax biosynthesis;
gene39212	K09549	PFDN2	prefoldin subunit 2	--		
gene39213	K11878	PSMG4, PAC4	proteasome assembly chaperone 4	--		
gene39214	K03921	DESA1	acyl-[acyl-carrier-protein] desaturase	EC:1.14.19.2	2	ko00061 Fatty acid biosynthesis;ko01040 Biosynthesis of unsaturated fatty acids;
gene39217	K04565	SOD1	superoxide dismutase, Cu-Zn family	EC:1.15.1.1	4	ko04146 Peroxisome;ko05014 Amyotrophic lateral sclerosis (ALS);ko05016 Huntington's disease;ko05020 Prion diseases;

gene39223	K00558	DNMT1, dcm	DNA (cytosine-5)-methyltransferase 1	EC:2.1.1.37	2	ko00270 Cysteine and methionine metabolism;ko05206 MicroRNAs in cancer;
gene39224	K09495	CCT3, TRIC5	T-complex protein 1 subunit gamma	--		
gene39230	K16292	CEP, CYSEP	KDEL-tailed cysteine endopeptidase	EC:3.4.22.-		
gene39236	K07897	RAB7A	Ras-related protein Rab-7A	--	5	ko04144 Endocytosis;ko04145 Phagosome;ko05132 Salmonella infection;ko05146 Amoebiasis;ko05152 Tuberculosis;
gene39239	K02924	RP-L39e, RPL39	large subunit ribosomal protein L39e	--	1	ko03010 Ribosome;
gene39240	K01535	E3.6.3.6	H+-transporting ATPase	EC:3.6.3.6	1	ko00190 Oxidative phosphorylation;
gene39243	K02210	MCM7, CDC47	DNA replication licensing factor MCM7	EC:3.6.4.12	4	ko03030 DNA replication;ko04110 Cell cycle;ko04111 Cell cycle - yeast;ko04113 Meiosis - yeast;
gene39244	K11797	PHIP, WDR11	PH-interacting protein	--		
gene39247	K17065	DNM1L	dynamin 1-like protein	EC:3.6.5.5	1	ko04668 TNF signaling pathway;
gene39252	K06966	K06966				
gene39262	K04733	IRAK4	interleukin-1 receptor-associated kinase 4	EC:2.7.11.1	11	ko04064 NF-kappa B signaling pathway;ko04210 Apoptosis;ko04620 Toll-like receptor signaling pathway;ko04722 Neurotrophin signaling pathway;ko05133 Pertussis;ko05140 Leishmaniasis;ko05142 Chagas disease (American trypanosomiasis);ko05145 Toxoplasmosis;ko05152 Tuberculosis;ko05162 Measles;ko05164 Influenza A;
gene39264	K00434	E1.11.1.11	L-ascorbate peroxidase	EC:1.11.1.11	2	ko00053 Ascorbate and aldarate metabolism;ko00480 Glutathione metabolism;
gene39265	K13379	RGP, UTM	reversibly glycosylated polypeptide / UDP-arabinopyranose mutase	EC:2.4.1.- 5.4.99.30		
gene39269	K12812	UAP56, BAT1, SUB2	ATP-dependent RNA helicase UAP56/SUB2	EC:3.6.4.13	4	ko03013 RNA transport;ko03015 mRNA surveillance pathway;ko03040 Spliceosome;ko05164 Influenza A;
gene3926	K15172	SUPT5H, SPT5	transcription elongation factor SPT5	--		
gene39272	K11975	RNF144	E3 ubiquitin-protein ligase RNF144	EC:6.3.2.19		
gene39274	K13989	DERL2_3	Derlin-2/3	--	1	ko04141 Protein processing in endoplasmic reticulum;
gene39279	K03857	PIGA, GPI3	phosphatidylinositol glycan, class A	EC:2.4.1.198	1	ko00563 Glycosylphosphatidylinositol(GPI)-anchor biosynthesis;
gene39285	K14677	ACY1	aminoacylase	EC:3.5.1.14	3	ko00330 Arginine and proline metabolism;ko01210 2-Oxocarboxylic acid metabolism;ko01230 Biosynthesis of amino acids;
gene39287	K11517	HAO	(S)-2-hydroxy-acid oxidase	EC:1.1.3.15	2	ko00630 Glyoxylate and dicarboxylate metabolism;ko04146 Peroxisome;
gene3928	K12386	CTNS	cystinosin	--	1	ko04142 Lysosome;
gene39290	K07192	FLOT	flotillin	--	1	ko04910 Insulin signaling pathway;
gene39291	K14516	ERF1	ethylene-responsive transcription factor 1	--	1	ko04075 Plant hormone signal transduction;
gene39296	K11294	NCL, NSR1	nucleolin	--	1	ko05130 Pathogenic Escherichia coli infection;
gene39298	K00434	E1.11.1.11	L-ascorbate peroxidase	EC:1.11.1.11	2	ko00053 Ascorbate and aldarate metabolism;ko00480 Glutathione metabolism;
gene3929	K07556	ATPeAF2, ATPAF2	ATP synthase mitochondrial F1 complex assembly factor 2	--		
gene392	K00975	glgC	glucose-1-phosphate adenylyltransferase	EC:2.7.7.27	2	ko00500 Starch and sucrose metabolism;ko00520 Amino sugar and nucleotide sugar metabolism;
gene39300	K08065	NFYB	nuclear transcription Y subunit beta	--	3	ko04612 Antigen processing and presentation;ko05152 Tuberculosis;ko05166 HTLV-I infection;
gene39301	K08653	MBTPS1	membrane-bound transcription factor site-1 protease	EC:3.4.21.112	1	ko04141 Protein processing in endoplasmic reticulum;
gene39305	K08472	MLO	mlo protein	--		
gene3930	K00235	SDHB, SDH2	succinate dehydrogenase (ubiquinone) iron-sulfur subunit	EC:1.3.5.1	7	ko00020 Citrate cycle (TCA cycle);ko00190 Oxidative phosphorylation;ko01200 Carbon metabolism;ko04932 Non-alcoholic fatty liver disease (NAFLD);ko05010 Alzheimer's disease;ko05012 Parkinson's disease;ko05016 Huntington's disease;
gene39310	K10878	SPO11	meiotic recombination protein SPO11	--	1	ko04113 Meiosis - yeast;
gene39311	K11135	PINX1	Pin2-interacting protein X1	--		
gene39312	K01770	ispF	2-C-methyl-D-erythritol 2,4-cyclodiphosphate synthase	EC:4.6.1.12	1	ko00900 Terpenoid backbone biosynthesis;
gene39313	K04733	IRAK4	interleukin-1 receptor-associated kinase 4	EC:2.7.11.1	11	ko04064 NF-kappa B signaling pathway;ko04210 Apoptosis;ko04620 Toll-like receptor signaling pathway;ko04722 Neurotrophin signaling pathway;ko05133 Pertussis;ko05140 Leishmaniasis;ko05142 Chagas disease (American trypanosomiasis);ko05145 Toxoplasmosis;ko05152 Tuberculosis;ko05162 Measles;ko05164 Influenza A;
gene39316	K15901	CGI121, TPRKB	protein CGI121	--		

gene39323	K02717	psbP	photosystem II oxygen-evolving enhancer protein 2	--	1	ko00195 Photosynthesis;
gene39324	K14406	CSTF1	cleavage stimulation factor subunit 1	--	1	ko03015 mRNA surveillance pathway;
gene39329	K00434	E1.11.1.11	L-ascorbate peroxidase	EC:1.11.1.11	2	ko00053 Ascorbate and aldarate metabolism;ko00480 Glutathione metabolism;
gene39330	K00847	E2.7.1.4, scrK	fructokinase	EC:2.7.1.4	3	ko00051 Fructose and mannose metabolism;ko00500 Starch and sucrose metabolism;ko00520 Amino sugar and nucleotide sugar metabolism;
gene39331	K09264	K09264	MADS-box transcription factor, plant	--		
gene39333	K01115	PLD1_2	phospholipase D1/2	EC:3.1.4.4	7	ko00564 Glycerophospholipid metabolism;ko00565 Ether lipid metabolism;ko04014 Ras signaling pathway;ko04144 Endocytosis;ko04666 Fc gamma R-mediated phagocytosis;ko04724 Glutamatergic synapse;ko04912 GnRH signaling pathway;
gene39334	K08150	SLC2A13, ITR	MFS transporter, SP family, solute carrier family 2 (myo-inositol transporter), member 13	--		
gene39335	K01551	arsA, ASNA1	arsenite-transporting ATPase	EC:3.6.3.16		
gene39349	K15032	MTERFD	mTERF domain-containing protein, mitochondrial	--		
gene3934	K10251	HSD17B12, KAR, IFA38	17beta-estradiol 17-dehydrogenase / very-long-chain 3-oxoacyl-CoA reductase	EC:1.1.1.62 1.1.1.330	3	ko00062 Fatty acid elongation;ko00140 Steroid hormone biosynthesis;ko01040 Biosynthesis of unsaturated fatty acids;
gene39353	K09338	HD-ZIP	homeobox-leucine zipper protein	--		
gene39360	K08493	VTI1	vesicle transport through interaction with t-SNAREs 1	--	1	ko04130 SNARE interactions in vesicular transport;
gene39364	K00472	E1.14.11.2	prolyl 4-hydroxylase	EC:1.14.11.2	1	ko00330 Arginine and proline metabolism;
gene39365	K13508	GPAT	glycerol-3-phosphate acyltransferase	EC:2.3.1.15	2	ko00561 Glycerolipid metabolism;ko00564 Glycerophospholipid metabolism;
gene39368	K00826	E2.6.1.42, ilvE	branched-chain amino acid aminotransferase	EC:2.6.1.42	5	ko00280 Valine, leucine and isoleucine degradation;ko00290 Valine, leucine and isoleucine biosynthesis;ko00770 Pantothenate and CoA biosynthesis;ko01210 2-Oxocarboxylic acid metabolism;ko01230 Biosynthesis of amino acids;
gene39370	K04043	dnaK	molecular chaperone DnaK	--	2	ko03018 RNA degradation;ko05152 Tuberculosis;
gene39371	K04043	dnaK	molecular chaperone DnaK	--	2	ko03018 RNA degradation;ko05152 Tuberculosis;
gene39372	K07305	msrB	peptide-methionine (R)-S-oxide reductase	EC:1.8.4.12		
gene39373	K13811	PAPSS	3'-phosphoadenosine 5'-phosphosulfate synthase	EC:2.7.7.4 2.7.1.25	3	ko00230 Purine metabolism;ko00450 Selenocompound metabolism;ko00920 Sulfur metabolism;
gene39375	K08245	E3.4.23.40	phytepsin	EC:3.4.23.40		
gene39376	K11251	H2A	histone H2A	--	2	ko05034 Alcoholism;ko05322 Systemic lupus erythematosus;
gene39377	K01537	E3.6.3.8	Ca2+-transporting ATPase	EC:3.6.3.8		
gene39379	K14492	ARR-A	two-component response regulator ARR-A family	--	1	ko04075 Plant hormone signal transduction;
gene3937	K00670	E2.3.1.88	peptide alpha-N-acetyltransferase	EC:2.3.1.88		
gene39381	K07955	ARL8	ADP-ribosylation factor-like protein 8	--		
gene39383	K03644	lipA	lipoic acid synthetase	EC:2.8.1.8	1	ko00785 Lipoic acid metabolism;
gene39385	K01507	ppa	inorganic pyrophosphatase	EC:3.6.1.1	1	ko00190 Oxidative phosphorylation;
gene39389	K08869	ADCK, ABC1	aarF domain-containing kinase	--		
gene39392	K05765	CFL	cofilin	--	4	ko04360 Axon guidance;ko04666 Fc gamma R-mediated phagocytosis;ko04810 Regulation of actin cytoskeleton;ko05133 Pertussis;
gene39394	K03023	RPC82, POLR3C	DNA-directed RNA polymerase III subunit RPC3	--	5	ko00230 Purine metabolism;ko00240 Pyrimidine metabolism;ko03020 RNA polymerase;ko04623 Cytosolic DNA-sensing pathway;ko05169 Epstein-Barr virus infection;
gene39395	K02920	RP-L36e, RPL36	large subunit ribosomal protein L36e	--	1	ko03010 Ribosome;
gene393	K07300	chaA, CAX	Ca2+:H+ antiporter	--		
gene39408	K02912	RP-L32e, RPL32	large subunit ribosomal protein L32e	--	1	ko03010 Ribosome;
gene39410	K16075	MRS2, MFM1	magnesium transporter	--		
gene39414	K03005	RPA49, POLR1E	DNA-directed RNA polymerase I subunit RPA49	--	3	ko00230 Purine metabolism;ko00240 Pyrimidine metabolism;ko03020 RNA polymerase;
gene39415	K13513	LCLAT1, AGPAT8	lysocardiolipin and lysophospholipid acyltransferase	EC:2.3.1.- 2.3.1.51	2	ko00561 Glycerolipid metabolism;ko00564 Glycerophospholipid metabolism;
gene39416	K01866	YARS, tyrS	tyrosyl-tRNA synthetase	EC:6.1.1.1	1	ko00970 Aminoacyl-tRNA biosynthesis;
gene39418	K14232	tRNA-Pro	tRNA Pro	--	1	ko00970 Aminoacyl-tRNA biosynthesis;
gene39419	K01188	E3.2.1.21	beta-glucosidase	EC:3.2.1.21	3	ko00460 Cyanoamino acid metabolism;ko00500 Starch and sucrose metabolism;ko00940 Phenylpropanoid biosynthesis;

gene3941	K13448	CML	calcium-binding protein CML	--	1	ko04626 Plant-pathogen interaction;
gene39420	K07195	EXOC7, EXO70	exocyst complex component 7	--	1	ko04910 Insulin signaling pathway;
gene39421	K02716	psbO	photosystem II oxygen-evolving enhancer protein 1	--	1	ko00195 Photosynthesis;
gene39422	K00434	E1.11.1.11	L-ascorbate peroxidase	EC:1.11.1.11	2	ko00053 Ascorbate and aldarate metabolism;ko00480 Glutathione metabolism;
gene39433	K17301	COPB1, SEC26	coatomer, subunit beta	--		
gene39435	K01887	RARS, argS	arginyl-tRNA synthetase	EC:6.1.1.19	1	ko00970 Aminoacyl-tRNA biosynthesis;
gene39436	K10949	KDELR	ER lumen protein retaining receptor	--	1	ko05110 Vibrio cholerae infection;
gene39446	K11094	SNRPB2	U2 small nuclear ribonucleoprotein B''	--	1	ko03040 Spliceosome;
gene39453	K04798	pfdB, PFDN6	prefoldin beta subunit	--		
gene39456	K11251	H2A	histone H2A	--	2	ko05034 Alcoholism;ko05322 Systemic lupus erythematosus;
gene39462	K07119	K07119				
gene39465	K09842	AAO3	abscisic-aldehyde oxidase	EC:1.2.3.14	1	ko00906 Carotenoid biosynthesis;
gene39467	K01885	EARS, gltX	glutamyl-tRNA synthetase	EC:6.1.1.17	2	ko00860 Porphyrin and chlorophyll metabolism;ko00970 Aminoacyl-tRNA biosynthesis;
gene39468	K06129	LYPLA3	lysophospholipase III	EC:3.1.1.5	2	ko00564 Glycerophospholipid metabolism;ko04142 Lysosome;
gene39469	K01583	E4.1.1.19	arginine decarboxylase	EC:4.1.1.19	1	ko00330 Arginine and proline metabolism;
gene39482	K08910	LHCA4	light-harvesting complex I chlorophyll a/b binding protein 4	--	1	ko00196 Photosynthesis - antenna proteins;
gene39484	K11975	RNF144	E3 ubiquitin-protein ligase RNF144	EC:6.3.2.19		
gene39485	K11975	RNF144	E3 ubiquitin-protein ligase RNF144	EC:6.3.2.19		
gene39488	K14506	JAR1	jasmonic acid-amino synthetase	--	1	ko04075 Plant hormone signal transduction;
gene39492	K08911	LHCA5	light-harvesting complex I chlorophyll a/b binding protein 5	--	1	ko00196 Photosynthesis - antenna proteins;
gene39493	K14398	CPSF6_7	cleavage and polyadenylation specificity factor subunit 6/7	--	1	ko03015 mRNA surveillance pathway;
gene39495	K09422	MYBP	myb proto-oncogene protein, plant	--		
gene39499	K14500	BSK	BR-signaling kinase	EC:2.7.11.1	1	ko04075 Plant hormone signal transduction;
gene39501	K14315	NDC1, TMEM48	nucleoporin NDC1	--	1	ko03013 RNA transport;
gene39502	K16292	CEP, CYSEP	KDEL-tailed cysteine endopeptidase	EC:3.4.22.-		
gene39507	K13617	PPME1	protein phosphatase methylesterase 1	EC:3.1.1.89		
gene39508	K08738	CYC	cytochrome c	--	19	ko00920 Sulfur metabolism;ko02020 Two-component system;ko04115 p53 signaling pathway;ko04210 Apoptosis;ko04932 Non-alcoholic fatty liver disease (NAFLD);ko05010 Alzheimer's disease;ko05012 Parkinson's disease;ko05014 Amyotrophic lateral sclerosis (ALS);ko05016 Huntington's disease;ko05134 Legionellosis;ko05145 Toxoplasmosis;ko05152 Tuberculosis;ko05161 Hepatitis B;ko05164 Influenza A;ko05168 Herpes simplex infection;ko05200 Pathways in cancer;ko05210 Colorectal cancer;ko05222 Small cell lung cancer;ko05416 Viral myocarditis;
gene39509	K14841	NSA1, WDR74	ribosome biogenesis protein NSA1	--		
gene39517	K08963	mtnA	methylthioribose-1-phosphate isomerase	EC:5.3.1.23	1	ko00270 Cysteine and methionine metabolism;
gene39522	K14567	UTP14	U3 small nucleolar RNA-associated protein 14	--	1	ko03008 Ribosome biogenesis in eukaryotes;
gene39525	K14775	UTP30, RSL1D1	ribosome biogenesis protein UTP30	--		
gene39526	K03965	NDUFB9	NADH dehydrogenase (ubiquinone) 1 beta subcomplex subunit 9	--	5	ko00190 Oxidative phosphorylation;ko04932 Non-alcoholic fatty liver disease (NAFLD);ko05010 Alzheimer's disease;ko05012 Parkinson's disease;ko05016 Huntington's disease;
gene39528	K14016	UFD1	ubiquitin fusion degradation protein 1	--	1	ko04141 Protein processing in endoplasmic reticulum;
gene39529	K02155	ATPeVPL, ATP6L	V-type H+-transporting ATPase 16kDa proteolipid subunit	EC:3.6.3.14	9	ko00190 Oxidative phosphorylation;ko04142 Lysosome;ko04145 Phagosome;ko04721 Synaptic vesicle cycle;ko04966 Collecting duct acid secretion;ko05110 Vibrio cholerae infection;ko05120 Epithelial cell signaling in Helicobacter pylori infection;ko05152 Tuberculosis;ko05323 Rheumatoid arthritis;
gene3952	K13337	PEX19	peroxin-19	--	1	ko04146 Peroxisome;
gene39530	K15802	SSTLE1	germacrene C synthase	EC:4.2.3.60	1	ko00909 Sesquiterpenoid and triterpenoid biosynthesis;
gene39533	K03327	TC.MATE, SLC47A, norM, mdtK, dinF	multidrug resistance protein, MATE family	--		
gene39536	K01530	E3.6.3.1	phospholipid-translocating ATPase	EC:3.6.3.1		

gene39537	K11991	tadA	tRNA(adenine34) deaminase	EC:3.5.4.33		
gene39540	K03217	yidC, spoIIIJ, OXA1	preprotein translocase subunit YidC	--	2	ko03060 Protein export;ko03070 Bacterial secretion system;
gene39544	K13162	PCBP2_3_4	poly(rC)-binding protein 2/3/4	--		
gene3954	K01602	rbcS	ribulose-bisphosphate carboxylase small chain	EC:4.1.1.39	3	ko00630 Glyoxylate and dicarboxylate metabolism;ko00710 Carbon fixation in photosynthetic organisms;ko01200 Carbon metabolism;
gene39556	K08912	LHCB1	light-harvesting complex II chlorophyll a/b binding protein 1	--	1	ko00196 Photosynthesis - antenna proteins;
gene39559	K13993	HSP20	HSP20 family protein	--	1	ko04141 Protein processing in endoplasmic reticulum;
gene39561	K08247	E2.1.1.12	methionine S-methyltransferase	EC:2.1.1.12	1	ko00450 Selenocompound metabolism;
gene39567	K13173	ARGLU1	arginine and glutamate-rich protein 1	--		
gene3956	K12846	SNRNP27	U4/U6.U5 tri-snRNP-associated protein 3	--	1	ko03040 Spliceosome;
gene39570	K13917	RNGTT	mRNA-capping enzyme	EC:2.7.7.50 3.1.3.33	1	ko03015 mRNA surveillance pathway;
gene39578	K15285	SLC35E3	solute carrier family 35, member E3	--		
gene39580	K02976	RP-S26e, RPS26	small subunit ribosomal protein S26e	--	1	ko03010 Ribosome;
gene39581	K09420	MYB	myb proto-oncogene protein	--	2	ko04151 PI3K-Akt signaling pathway;ko05166 HTLV-I infection;
gene39582	K01784	galE, GALE	UDP-glucose 4-epimerase	EC:5.1.3.2	2	ko00052 Galactose metabolism;ko00520 Amino sugar and nucleotide sugar metabolism;
gene39585	K09419	HSFF	heat shock transcription factor, other eukaryote	--		
gene39587	K11155	DGAT1	diacylglycerol O-acyltransferase 1	EC:2.3.1.20 2.3.1.75 2.3.1.76	3	ko00561 Glycerolipid metabolism;ko00830 Retinol metabolism;ko04975 Fat digestion and absorption;
gene39592	K09422	MYBP	myb proto-oncogene protein, plant	--		
gene39593	K02935	RP-L7, MRPL12, rplL	large subunit ribosomal protein L7/L12	--	1	ko03010 Ribosome;
gene39596	K07955	ARL8	ADP-ribosylation factor-like protein 8	--		
gene39597	K12847	USP39, SAD1	U4/U6.U5 tri-snRNP-associated protein 2	--	1	ko03040 Spliceosome;
gene39598	K01535	E3.6.3.6	H+-transporting ATPase	EC:3.6.3.6	1	ko00190 Oxidative phosphorylation;
gene39599	K14638	SLC15A3_4, PHT	solute carrier family 15 (peptide/histidine transporter), member 3/4	--		
gene3959	K16297	SCPL-II	serine carboxypeptidase-like clade II	EC:3.4.16.-		
gene39602	K01166	E3.1.27.1	ribonuclease T2	EC:3.1.27.1		
gene39603	K01166	E3.1.27.1	ribonuclease T2	EC:3.1.27.1		
gene39609	K10640	RNF25, AO7	E3 ubiquitin-protein ligase RNF25	EC:6.3.2.19		
gene39614	K13098	TLS, FUS	RNA-binding protein FUS	--	1	ko05202 Transcriptional misregulation in cancer;
gene3961	K01466	allB	allantoinase	EC:3.5.2.5	1	ko00230 Purine metabolism;
gene39626	K13993	HSP20	HSP20 family protein	--	1	ko04141 Protein processing in endoplasmic reticulum;
gene39627	K15255	PIF1	ATP-dependent DNA helicase PIF1	EC:3.6.4.12		
gene39635	K00850	pfkA, PFK	6-phosphofructokinase 1	EC:2.7.1.11	7	ko00010 Glycolysis / Gluconeogenesis;ko00030 Pentose phosphate pathway;ko00051 Fructose and mannose metabolism;ko00052 Galactose metabolism;ko00680 Methane metabolism;ko01200 Carbon metabolism;ko01230 Biosynthesis of amino acids;
gene39636	K08850	AURKX	aurora kinase, other	EC:2.7.11.1		
gene39637	K02206	CDK2	cyclin-dependent kinase 2	EC:2.7.11.22	13	ko04110 Cell cycle;ko04114 Oocyte meiosis;ko04115 p53 signaling pathway;ko04151 PI3K-Akt signaling pathway;ko04914 Progesterone-mediated oocyte maturation;ko05161 Hepatitis B;ko05162 Measles;ko05168 Herpes simplex infection;ko05169 Epstein-Barr virus infection;ko05200 Pathways in cancer;ko05203 Viral carcinogenesis;ko05215 Prostate cancer;ko05222 Small cell lung cancer;
gene39638	K01867	WARS, trpS	tryptophanyl-tRNA synthetase	EC:6.1.1.2	1	ko00970 Aminoacyl-tRNA biosynthesis;
gene39639	K11793	CRBN	cereblon	--		
gene39641	K16860	PLD3_4	phospholipase D3/4	EC:3.1.4.4	2	ko00564 Glycerophospholipid metabolism;ko00565 Ether lipid metabolism;
gene39644	K11490	NCAPH2	condensin-2 complex subunit H2	--		
gene39645	K01051	E3.1.1.11	pectinesterase	EC:3.1.1.11	2	ko00040 Pentose and glucuronate interconversions;ko00500 Starch and sucrose metabolism;
gene39649	K14491	ARR-B	two-component response regulator ARR-B family	--	1	ko04075 Plant hormone signal transduction;
gene3964	K02695	psaH	photosystem I subunit VI	--	1	ko00195 Photosynthesis;

gene39651	K14638	SLC15A3_4, PHT	solute carrier family 15 (peptide/histidine transporter), member 3/4	--		
gene39652	K14638	SLC15A3_4, PHT	solute carrier family 15 (peptide/histidine transporter), member 3/4	--		
gene39654	K00434	E1.11.1.11	L-ascorbate peroxidase	EC:1.11.1.11	2	ko00053 Ascorbate and aldarate metabolism;ko00480 Glutathione metabolism;
gene39657	K15363	FAN1, MTMR15	fanconi-associated nuclease 1	--	1	ko03460 Fanconi anemia pathway;
gene39659	K01728	E4.2.2.2, pel	pectate lyase	EC:4.2.2.2	1	ko00040 Pentose and glucuronate interconversions;
gene3965	K07375	TUBB	tubulin beta	--	3	ko04145 Phagosome;ko04540 Gap junction;ko05130 Pathogenic Escherichia coli infection;
gene39660	K02552	menF	menaquinone-specific isochorismate synthase	EC:5.4.4.2	2	ko00130 Ubiquinone and other terpenoid-quinone biosynthesis;ko01053 Biosynthesis of siderophore group nonribosomal peptides;
gene39661	K17602	YLPM1	YLP motif-containing protein 1	--		
gene39665	K00948	PRPS, prsA	ribose-phosphate pyrophosphokinase	EC:2.7.6.1	4	ko00030 Pentose phosphate pathway;ko00230 Purine metabolism;ko01200 Carbon metabolism;ko01230 Biosynthesis of amino acids;
gene39672	K10599	PRPF19, PRP19	pre-mRNA-processing factor 19	EC:6.3.2.19	2	ko03040 Spliceosome;ko04120 Ubiquitin mediated proteolysis;
gene39675	K00053	ilvC	ketol-acid reductoisomerase	EC:1.1.1.86	4	ko00290 Valine, leucine and isoleucine biosynthesis;ko00770 Pantothenate and CoA biosynthesis;ko01210 2-Oxocarboxylic acid metabolism;ko01230 Biosynthesis of amino acids;
gene39679	K12885	RBMX, HNRNPG	heterogeneous nuclear ribonucleoprotein G	--	1	ko03040 Spliceosome;
gene39681	K07976	RAB	Rab family, other	--		
gene39684	K02962	RP-S17e, RPS17	small subunit ribosomal protein S17e	--	1	ko03010 Ribosome;
gene39687	K10406	KIFC2_3	kinesin family member C2/C3	--		
gene39691	K13789	GGPS	geranylgeranyl diphosphate synthase, type II	EC:2.5.1.1 2.5.1.10 2.5.1.29	1	ko00900 Terpenoid backbone biosynthesis;
gene3969	K06950	K06950	uncharacterized protein	--		
gene39707	K12486	SMAP	stromal membrane-associated protein	--	1	ko04144 Endocytosis;
gene3970	K15053	CHMP7	charged multivesicular body protein 7	--	1	ko04144 Endocytosis;
gene39710	K04733	IRAK4	interleukin-1 receptor-associated kinase 4	EC:2.7.11.1	11	ko04064 NF-kappa B signaling pathway;ko04210 Apoptosis;ko04620 Toll-like receptor signaling pathway;ko04722 Neurotrophin signaling pathway;ko05133 Pertussis;ko05140 Leishmaniasis;ko05142 Chagas disease (American trypanosomiasis);ko05145 Toxoplasmosis;ko05152 Tuberculosis;ko05162 Measles;ko05164 Influenza A;
gene3971	K15235	JOSD	josephin	EC:3.4.19.12		
gene39720	K00430	E1.11.1.7	peroxidase	EC:1.11.1.7	2	ko00360 Phenylalanine metabolism;ko00940 Phenylpropanoid biosynthesis;
gene39722	K13457	RPM1, RPS3	disease resistance protein RPM1	--	1	ko04626 Plant-pathogen interaction;
gene39723	K13457	RPM1, RPS3	disease resistance protein RPM1	--	1	ko04626 Plant-pathogen interaction;
gene39724	K13457	RPM1, RPS3	disease resistance protein RPM1	--	1	ko04626 Plant-pathogen interaction;
gene39725	K12153	CYP79A2	cytochrome P450, family 79, subfamily A, polypeptide 2 (phenylalanine N-monooxygenase)	EC:1.14.13.12 4	3	ko00460 Cyanoamino acid metabolism;ko00966 Glucosinolate biosynthesis;ko01210 2-Oxocarboxylic acid metabolism;
gene39727	K03013	RPB5, POLR2E	DNA-directed RNA polymerases I, II, and III subunit RPABC1	--	6	ko00230 Purine metabolism;ko00240 Pyrimidine metabolism;ko03020 RNA polymerase;ko04623 Cytosolic DNA-sensing pathway;ko05016 Huntington's disease;ko05169 Epstein-Barr virus infection;
gene39732	K16547	NEDD1	protein NEDD1	--		
gene39733	K15414	C1QBP	complement component 1 Q subcomponent-binding protein, mitochondrial	--	1	ko05168 Herpes simplex infection;
gene39740	K02974	RP-S24e, RPS24	small subunit ribosomal protein S24e	--	1	ko03010 Ribosome;
gene39741	K12115	ZTL	clock-associated PAS protein ZTL	--	1	ko04712 Circadian rhythm - plant;
gene39742	K12133	LHY	MYB-related transcription factor LHY	--	1	ko04712 Circadian rhythm - plant;
gene39747	K14641	APY1_2	apyrase	EC:3.6.1.5	2	ko00230 Purine metabolism;ko00240 Pyrimidine metabolism;
gene39748	K14232	tRNA-Pro	tRNA Pro	--	1	ko00970 Aminoacyl-tRNA biosynthesis;
gene3974	K09422	MYBP	myb proto-oncogene protein, plant	--		
gene39757	K11982	RNF115_126	E3 ubiquitin-protein ligase RNF115/126	EC:6.3.2.19		
gene39759	K13464	JAZ	jasmonate ZIM domain-containing protein	--	2	ko04075 Plant hormone signal transduction;ko04626 Plant-pathogen interaction;

gene39763	K04554	UBE2J2, NCUBE2, UBC6	ubiquitin-conjugating enzyme E2 J2	EC:6.3.2.19	3	ko04120 Ubiquitin mediated proteolysis;ko04141 Protein processing in endoplasmic reticulum;ko05012 Parkinson's disease;
gene39766	K12818	DHX8, PRP22	ATP-dependent RNA helicase DHX8/PRP22	EC:3.6.4.13	1	ko03040 Spliceosome;
gene39772	K15280	SLC35C2	solute carrier family 35, member C2	--		
gene39782	K07300	chaA, CAX	Ca2+:H+ antiporter	--		
gene39785	K10143	RFWD2, COP1	E3 ubiquitin-protein ligase RFWD2	EC:6.3.2.19	3	ko04115 p53 signaling pathway;ko04120 Ubiquitin mediated proteolysis;ko04712 Circadian rhythm - plant;
gene39786	K11147	DHRS4	dehydrogenase/reductase SDR family member 4	EC:1.1.-.-	2	ko00830 Retinol metabolism;ko04146 Peroxisome;
gene39789	K01115	PLD1_2	phospholipase D1/2	EC:3.1.4.4	7	ko00564 Glycerophospholipid metabolism;ko00565 Ether lipid metabolism;ko04014 Ras signaling pathway;ko04144 Endocytosis;ko04666 Fc gamma R-mediated phagocytosis;ko04724 Glutamatergic synapse;ko04912 GnRH signaling pathway;
gene39790	K01520	dut, DUT	dUTP pyrophosphatase	EC:3.6.1.23	1	ko00240 Pyrimidine metabolism;
gene39796	K09873	TIP	aquaporin TIP	--		
gene3979	K04567	KARS, lysS	lysyl-tRNA synthetase, class II	EC:6.1.1.6	1	ko00970 Aminoacyl-tRNA biosynthesis;
gene397	K07199	PRKAB	5'-AMP-activated protein kinase, regulatory beta subunit	--	5	ko04710 Circadian rhythm;ko04910 Insulin signaling pathway;ko04920 Adipocytokine signaling pathway;ko04932 Non-alcoholic fatty liver disease (NAFLD);ko05410 Hypertrophic cardiomyopathy (HCM);
gene39809	K00088	guaB	IMP dehydrogenase	EC:1.1.1.205	2	ko00230 Purine metabolism;ko00983 Drug metabolism - other enzymes;
gene3980	K12818	DHX8, PRP22	ATP-dependent RNA helicase DHX8/PRP22	EC:3.6.4.13	1	ko03040 Spliceosome;
gene39816	K17428	MRPL47, NCM1	large subunit ribosomal protein L47, mitochondrial	--		
gene39819	K01412	E3.4.24.64	mitochondrial processing peptidase	EC:3.4.24.64		
gene39829	K09422	MYBP	myb proto-oncogene protein, plant	--		
gene39833	K05391	CNGF	cyclic nucleotide gated channel, other eukaryote	--	1	ko04626 Plant-pathogen interaction;
gene39837	K00278	nadB	L-aspartate oxidase	EC:1.4.3.16	2	ko00250 Alanine, aspartate and glutamate metabolism;ko00760 Nicotinate and nicotinamide metabolism;
gene39839	K11982	RNF115_126	E3 ubiquitin-protein ligase RNF115/126	EC:6.3.2.19		
gene39840	K13176	THOC7	THO complex subunit 7	--	1	ko03013 RNA transport;
gene39845	K09840	NCED	9-cis-epoxycarotenoid dioxygenase	EC:1.13.11.51	1	ko00906 Carotenoid biosynthesis;
gene39847	K15287	SLC35F1_2	solute carrier family 35, member F1/2	--		
gene39848	K13495	CISZOG	cis-zeatin O-glucosyltransferase	EC:2.4.1.215	1	ko00908 Zeatin biosynthesis;
gene39850	K08486	STX1B_2_3	syntaxin 1B/2/3	--	2	ko04130 SNARE interactions in vesicular transport;ko04721 Synaptic vesicle cycle;
gene39852	K02914	RP-L34, MRPL34, rpmH	large subunit ribosomal protein L34	--	1	ko03010 Ribosome;
gene39854	K01728	E4.2.2.2, pel	pectate lyase	EC:4.2.2.2	1	ko00040 Pentose and glucuronate interconversions;
gene39855	K15382	SLC50A, SWEET	solute carrier family 50 (sugar transporter)	--		
gene39857	K13354	SLC25A17, PMP34	solute carrier family 25 (peroxisomal adenine nucleotide transporter), member 17	--	1	ko04146 Peroxisome;
gene39859	K01114	plcC	phospholipase C	EC:3.1.4.3	3	ko00562 Inositol phosphate metabolism;ko00564 Glycerophospholipid metabolism;ko00565 Ether lipid metabolism;
gene39860	K02112	ATPF1B, atpD	F-type H+-transporting ATPase subunit beta	EC:3.6.3.14	2	ko00190 Oxidative phosphorylation;ko00195 Photosynthesis;
gene39863	K14561	IMP4	U3 small nucleolar ribonucleoprotein protein IMP4	--	1	ko03008 Ribosome biogenesis in eukaryotes;
gene39871	K00799	GST, gst	glutathione S-transferase	EC:2.5.1.18	4	ko00480 Glutathione metabolism;ko00980 Metabolism of xenobiotics by cytochrome P450;ko00982 Drug metabolism - cytochrome P450;ko05204 Chemical carcinogenesis;
gene39872	K00799	GST, gst	glutathione S-transferase	EC:2.5.1.18	4	ko00480 Glutathione metabolism;ko00980 Metabolism of xenobiotics by cytochrome P450;ko00982 Drug metabolism - cytochrome P450;ko05204 Chemical carcinogenesis;
gene39873	K02915	RP-L34e, RPL34	large subunit ribosomal protein L34e	--	1	ko03010 Ribosome;
gene39876	K13115	CCDC130	coiled-coil domain-containing protein 130	--		
gene39878	K02437	gcvH, GCSH	glycine cleavage system H protein	--	1	ko00630 Glyoxylate and dicarboxylate metabolism;
gene39881	K08911	LHCA5	light-harvesting complex I chlorophyll a/b binding protein 5	--	1	ko00196 Photosynthesis - antenna proteins;
gene39889	K08770	UBC	ubiquitin C	--	1	ko03320 PPAR signaling pathway;
gene39892	K02889	RP-L21e, RPL21	large subunit ribosomal protein L21e	--	1	ko03010 Ribosome;

gene39896	K00895	E2.7.1.90, pfk	pyrophosphate--fructose-6-phosphate 1-phosphotransferase	EC:2.7.1.90	1	ko00051 Fructose and mannose metabolism;
gene39897	K14220	tRNA-Asn	tRNA Asn	--	1	ko00970 Aminoacyl-tRNA biosynthesis;
gene39898	K09422	MYBP	myb proto-oncogene protein, plant	--		
gene398	K02183	CALM	calmodulin	--	25	ko04014 Ras signaling pathway;ko04020 Calcium signaling pathway;ko04070 Phosphatidylinositol signaling system;ko04114 Oocyte meiosis;ko04270 Vascular smooth muscle contraction;ko04626 Plant-pathogen interaction;ko04713 Circadian entrainment;ko04720 Long-term potentiation;ko04722 Neurotrophin signaling pathway;ko04728 Dopaminergic synapse;ko04740 Olfactory transduction;ko04744 Phototransduction;ko04745 Phototransduction - fly;ko04910 Insulin signaling pathway;ko04912 GnRH signaling pathway;ko04915 Estrogen signaling pathway;ko04916 Melanogenesis;ko04970 Salivary secretion;ko04971 Gastric acid secretion;ko05010 Alzheimer's disease;ko05031 Amphetamine addiction;ko05034 Alcoholism;ko05133 Pertussis;ko05152 Tuberculosis;ko05214 Glioma;
gene39909	K01148	PARN	poly(A)-specific ribonuclease	EC:3.1.13.4	1	ko03018 RNA degradation;
gene39910	K13448	CML	calcium-binding protein CML	--	1	ko04626 Plant-pathogen interaction;
gene39912	K00454	LOX2S	lipoxygenase	EC:1.13.11.12	2	ko00591 Linoleic acid metabolism;ko00592 alpha-Linolenic acid metabolism;
gene39914	K01148	PARN	poly(A)-specific ribonuclease	EC:3.1.13.4	1	ko03018 RNA degradation;
gene39916	K03549	kup	KUP system potassium uptake protein	--		
gene39917	K03549	kup	KUP system potassium uptake protein	--		
gene39920	K11277	NPM2	nucleophosmin 2	--		
gene39922	K01802	E5.2.1.8	peptidylprolyl isomerase	EC:5.2.1.8		
gene39925	K01188	E3.2.1.21	beta-glucosidase	EC:3.2.1.21	3	ko00460 Cyanoamino acid metabolism;ko00500 Starch and sucrose metabolism;ko00940 Phenylpropanoid biosynthesis;
gene39929	K12830	SF3B3, SAP130, RSE1	splicing factor 3B subunit 3	--	1	ko03040 Spliceosome;
gene39931	K01366	CTSH	cathepsin H	EC:3.4.22.16	1	ko04142 Lysosome;
gene39939	K15014	SLC29A1_2_3, ENT1_2_3	solute carrier family 29 (equilibrative nucleoside transporter), member 1/2/3	--		
gene39941	K17609	NXN	nucleoredoxin	EC:1.8.1.8		
gene39948	K04120	E5.5.1.13	ent-copalyl diphosphate synthase	EC:5.5.1.13	1	ko00904 Diterpenoid biosynthesis;
gene39951	K05841	E2.4.1.173	sterol 3beta-glucosyltransferase	EC:2.4.1.173		
gene39952	K15747	LUT5, CYP97A3	cytochrome P450, family 97, subfamily A (beta-ring hydroxylase)	EC:1.14.-.-	1	ko00906 Carotenoid biosynthesis;
gene39955	K01886	QARS, glnS	glutaminyl-tRNA synthetase	EC:6.1.1.18	1	ko00970 Aminoacyl-tRNA biosynthesis;
gene39956	K09422	MYBP	myb proto-oncogene protein, plant	--		
gene39959	K14611	SLC23A1_2, SVCT1_2	solute carrier family 23 (nucleobase transporter), member 1/2	--		
gene3995	K02324	POLE1	DNA polymerase epsilon subunit 1	EC:2.7.7.7	6	ko00230 Purine metabolism;ko00240 Pyrimidine metabolism;ko03030 DNA replication;ko03410 Base excision repair;ko03420 Nucleotide excision repair;ko05166 HTLV-I infection;
gene39961	K13448	CML	calcium-binding protein CML	--	1	ko04626 Plant-pathogen interaction;
gene39965	K12874	AQR	intron-binding protein aquarius	--	1	ko03040 Spliceosome;
gene39966	K08332	VAC8	vacuolar protein 8	--	1	ko04140 Regulation of autophagy;
gene3996	K02980	RP-S29e, RPS29	small subunit ribosomal protein S29e	--	1	ko03010 Ribosome;
gene39971	K00679	E2.3.1.158	phospholipid:diacylglycerol acyltransferase	EC:2.3.1.158	1	ko00561 Glycerolipid metabolism;
gene39972	K12449	AXS	UDP-apiose/xylose synthase	--	1	ko00520 Amino sugar and nucleotide sugar metabolism;
gene39973	K11824	AP2A	AP-2 complex subunit alpha	--	4	ko04144 Endocytosis;ko04721 Synaptic vesicle cycle;ko04961 Endocrine and other factor-regulated calcium reabsorption;ko05016 Huntington's disease;
gene39975	K13680	CSLA	beta-mannan synthase	EC:2.4.1.32		
gene39977	K00721	DPM1	dolichol-phosphate mannosyltransferase	EC:2.4.1.83	1	ko00510 N-Glycan biosynthesis;
gene39980	K00677	lpxA	UDP-N-acetylglucosamine acyltransferase	EC:2.3.1.129	1	ko00540 Lipopolysaccharide biosynthesis;
gene39982	K00430	E1.11.1.7	peroxidase	EC:1.11.1.7	2	ko00360 Phenylalanine metabolism;ko00940 Phenylpropanoid biosynthesis;

gene39984	K00430	E1.11.1.7	peroxidase	EC:1.11.1.7	2	ko00360 Phenylalanine metabolism;ko00940 Phenylpropanoid biosynthesis;
gene3999	K12836	U2AF1	splicing factor U2AF 35 kDa subunit	--	2	ko03040 Spliceosome;ko05131 Shigellosis;
gene399	K03108	SRP72	signal recognition particle subunit SRP72	--	1	ko03060 Protein export;
gene40005	K16279	KEG	E3 ubiquitin-protein ligase KEG	EC:2.7.11.1 6.3.2.19		
gene40010	K13412	CPK	calcium-dependent protein kinase	EC:2.7.11.1	2	ko04626 Plant-pathogen interaction;ko05145 Toxoplasmosis;
gene40019	K12813	DHX16	pre-mRNA-splicing factor ATP-dependent RNA helicase DHX16	EC:3.6.4.13	1	ko03040 Spliceosome;
gene4001	K05236	COPA	coatomer protein complex, subunit alpha (xenin)	--	1	ko04080 Neuroactive ligand-receptor interaction;
gene40021	K14222	tRNA-Cys	tRNA Cys	--	1	ko00970 Aminoacyl-tRNA biosynthesis;
gene40028	K14310	NUP205	nuclear pore complex protein Nup205	--	1	ko03013 RNA transport;
gene40031	K12356	UGT72E	coniferyl-alcohol glucosyltransferase	EC:2.4.1.111	1	ko00940 Phenylpropanoid biosynthesis;
gene40032	K10581	UBE2O	ubiquitin-conjugating enzyme E2 O	EC:6.3.2.19	1	ko04120 Ubiquitin mediated proteolysis;
gene40033	K14494	DELLA	DELLA protein	--	1	ko04075 Plant hormone signal transduction;
gene40035	K11968	ARIH1	ariadne-1	--		
gene4003	K00695	E2.4.1.13	sucrose synthase	EC:2.4.1.13	1	ko00500 Starch and sucrose metabolism;
gene40040	K01100	E3.1.3.37	sedoheptulose-bisphosphatase	EC:3.1.3.37	2	ko00710 Carbon fixation in photosynthetic organisms;ko01200 Carbon metabolism;
gene40048	K11927	rhlE	ATP-dependent RNA helicase RhlE	EC:3.6.4.13	1	ko03018 RNA degradation;
gene4004	K17427	MRPL46	large subunit ribosomal protein L46, mitochondrial	--		
gene40054	K12599	SKI2, SKIV2L	antiviral helicase SKI2	EC:3.6.4.-	1	ko03018 RNA degradation;
gene40056	K09422	MYBP	myb proto-oncogene protein, plant	--		
gene4005	K00889	E2.7.1.68, PIP5K	1-phosphatidylinositol-4-phosphate 5-kinase	EC:2.7.1.68	5	ko00562 Inositol phosphate metabolism;ko04070 Phosphatidylinositol signaling system;ko04144 Endocytosis;ko04666 Fc gamma R-mediated phagocytosis;ko04810 Regulation of actin cytoskeleton;
gene40060	K01183	E3.2.1.14	chitinase	EC:3.2.1.14	1	ko00520 Amino sugar and nucleotide sugar metabolism;
gene40064	K14561	IMP4	U3 small nucleolar ribonucleoprotein protein IMP4	--	1	ko03008 Ribosome biogenesis in eukaryotes;
gene40068	K04730	IRAK1	interleukin-1 receptor-associated kinase 1	EC:2.7.11.1	11	ko04064 NF-kappa B signaling pathway;ko04210 Apoptosis;ko04620 Toll-like receptor signaling pathway;ko04722 Neurotrophin signaling pathway;ko05133 Pertussis;ko05140 Leishmaniasis;ko05142 Chagas disease (American trypanosomiasis);ko05145 Toxoplasmosis;ko05152 Tuberculosis;ko05162 Measles;ko05169 Epstein-Barr virus infection;
gene40069	K03875	SKP2, FBXL1	F-box and leucine-rich repeat protein 1 (S-phase kinase-associated protein 2)	--	7	ko04110 Cell cycle;ko04120 Ubiquitin mediated proteolysis;ko05168 Herpes simplex infection;ko05169 Epstein-Barr virus infection;ko05200 Pathways in cancer;ko05203 Viral carcinogenesis;ko05222 Small cell lung cancer;
gene4006	K17413	MRPS35	small subunit ribosomal protein S35, mitochondrial	--		
gene40078	K03145	TFIIS	transcription elongation factor S-II	--		
gene4007	K09647	IMP1	mitochondrial inner membrane protease subunit 1	EC:3.4.21.-	1	ko03060 Protein export;
gene40080	K08876	SCYL1	SCY1-like protein 1	--		
gene40084	K08851	TP53RK, PRPK, BUD32	TP53 regulating kinase	EC:2.7.11.1		
gene40086	K10747	LIG1	DNA ligase 1	EC:6.5.1.1	4	ko03030 DNA replication;ko03410 Base excision repair;ko03420 Nucleotide excision repair;ko03430 Mismatch repair;
gene40094	K06910	K06910				
gene40101	K08657	TASP1	taspase, threonine aspartase, 1	EC:3.4.25.-		
gene40105	K11592	DICER1, DCR1	endoribonuclease Dicer	EC:3.1.26.-	1	ko05206 MicroRNAs in cancer;
gene40106	K09285	OVM, ANT	AP2-like factor, ANT lineage	--		
gene40107	K04730	IRAK1	interleukin-1 receptor-associated kinase 1	EC:2.7.11.1	11	ko04064 NF-kappa B signaling pathway;ko04210 Apoptosis;ko04620 Toll-like receptor signaling pathway;ko04722 Neurotrophin signaling pathway;ko05133 Pertussis;ko05140 Leishmaniasis;ko05142 Chagas disease (American trypanosomiasis);ko05145 Toxoplasmosis;ko05152 Tuberculosis;ko05162 Measles;ko05169 Epstein-Barr virus infection;
gene40111	K13947	PIN	auxin efflux carrier family	--		
gene40112	K14487	GH3	auxin responsive GH3 gene family	--	1	ko04075 Plant hormone signal transduction;
gene40114	K14431	TGA	transcription factor TGA	--	1	ko04075 Plant hormone signal transduction;

gene40117	K07437	CYP26A	cytochrome P450, family 26, subfamily A	--	1	ko00830 Retinol metabolism;
gene40122	K03955	NDUFAB1	NADH dehydrogenase (ubiquinone) 1 alpha/beta subcomplex 1	--	5	ko00190 Oxidative phosphorylation;ko04932 Non-alcoholic fatty liver disease (NAFLD);ko05010 Alzheimer's disease;ko05012 Parkinson's disease;ko05016 Huntington's disease;
gene40126	K04078	groES, HSPE1	chaperonin GroES	--		
gene40127	K11253	H3	histone H3	--	3	ko05034 Alcoholism;ko05202 Transcriptional misregulation in cancer;ko05322 Systemic lupus erythematosus;
gene40128	K13457	RPM1, RPS3	disease resistance protein RPM1	--	1	ko04626 Plant-pathogen interaction;
gene40129	K00485	E1.14.13.8	dimethylaniline monooxygenase (N-oxide forming)	EC:1.14.13.8	2	ko00680 Methane metabolism;ko00982 Drug metabolism - cytochrome P450;
gene40130	K00729	ALG5	dolichyl-phosphate beta-glucosyltransferase	EC:2.4.1.117	1	ko00510 N-Glycan biosynthesis;
gene40131	K00915	IPK2	1D-myo-Inositol-tetrakisphosphate 5-kinase / inositol-polyphosphate multikinase	EC:2.7.1.140 2.7.1.151	2	ko00562 Inositol phosphate metabolism;ko04070 Phosphatidylinositol signaling system;
gene40133	K07178	RIOK1	RIO kinase 1	EC:2.7.11.1	1	ko03008 Ribosome biogenesis in eukaryotes;
gene40134	K11498	CENPE	centromeric protein E	--		
gene40135	K00695	E2.4.1.13	sucrose synthase	EC:2.4.1.13	1	ko00500 Starch and sucrose metabolism;
gene40136	K04482	RAD51	DNA repair protein RAD51	--	4	ko03440 Homologous recombination;ko03460 Fanconi anemia pathway;ko05200 Pathways in cancer;ko05212 Pancreatic cancer;
gene40139	K15902	PCC1, LAGE3	polarized growth chromatin-associated controller 1	--		
gene40145	K01602	rbcS	ribulose-bisphosphate carboxylase small chain	EC:4.1.1.39	3	ko00630 Glyoxylate and dicarboxylate metabolism;ko00710 Carbon fixation in photosynthetic organisms;ko01200 Carbon metabolism;
gene40150	K15404	K15404, CER1	aldehyde decarbonylase	EC:4.1.99.5	1	ko00073 Cutin, suberine and wax biosynthesis;
gene40152	K14638	SLC15A3_4, PHT	solute carrier family 15 (peptide/histidine transporter), member 3/4	--		
gene40153	K08819	CDK12_13	cyclin-dependent kinase 12/13	EC:2.7.11.22 2.7.11.23		
gene40156	K08232	E1.6.5.4	monodehydroascorbate reductase (NADH)	EC:1.6.5.4	1	ko00053 Ascorbate and aldarate metabolism;
gene40162	K09286	EREBP	EREBP-like factor	--		
gene40168	K11252	H2B	histone H2B	--	3	ko05034 Alcoholism;ko05203 Viral carcinogenesis;ko05322 Systemic lupus erythematosus;
gene40174	K13280	SEC11, sipW	signal peptidase, endoplasmic reticulum-type	EC:3.4.-.-	1	ko03060 Protein export;
gene40179	K01611	speD, AMD1	S-adenosylmethionine decarboxylase	EC:4.1.1.50	2	ko00270 Cysteine and methionine metabolism;ko00330 Arginine and proline metabolism;
gene40181	K09873	TIP	aquaporin TIP	--		
gene40182	K14236	tRNA-Tyr	tRNA Tyr	--	1	ko00970 Aminoacyl-tRNA biosynthesis;
gene40184	K12193	VPS24, CHMP3	charged multivesicular body protein 3	--	1	ko04144 Endocytosis;
gene40188	K01114	plcC	phospholipase C	EC:3.1.4.3	3	ko00562 Inositol phosphate metabolism;ko00564 Glycerophospholipid metabolism;ko00565 Ether lipid metabolism;
gene40193	K01915	glnA	glutamine synthetase	EC:6.3.1.2	8	ko00250 Alanine, aspartate and glutamate metabolism;ko00330 Arginine and proline metabolism;ko00630 Glyoxylate and dicarboxylate metabolism;ko00910 Nitrogen metabolism;ko01230 Biosynthesis of amino acids;ko02020 Two-component system;ko04724 Glutamatergic synapse;ko04727 GABAergic synapse;
gene40198	K02875	RP-L14e, RPL14	large subunit ribosomal protein L14e	--	1	ko03010 Ribosome;
gene401	K08332	VAC8	vacuolar protein 8	--	1	ko04140 Regulation of autophagy;
gene40205	K01626	E2.5.1.54, aroF, aroG, aroH	3-deoxy-7-phosphoheptulonate synthase	EC:2.5.1.54	2	ko00400 Phenylalanine, tyrosine and tryptophan biosynthesis;ko01230 Biosynthesis of amino acids;
gene40209	K02940	RP-L9e, RPL9	large subunit ribosomal protein L9e	--	1	ko03010 Ribosome;
gene40212	K14664	ILR1	IAA-amino acid hydrolase	EC:3.5.1.-		
gene40213	K00059	fabG	3-oxoacyl-[acyl-carrier protein] reductase	EC:1.1.1.100	3	ko00061 Fatty acid biosynthesis;ko00780 Biotin metabolism;ko01040 Biosynthesis of unsaturated fatty acids;
gene40217	K08237	E2.4.1.218	hydroquinone glucosyltransferase	EC:2.4.1.218		
gene40221	K01438	argE	acetylornithine deacetylase	EC:3.5.1.16	3	ko00330 Arginine and proline metabolism;ko01210 2-Oxocarboxylic acid metabolism;ko01230 Biosynthesis of amino acids;
gene40227	K09458	fabF	3-oxoacyl-[acyl-carrier-protein] synthase II	EC:2.3.1.179	2	ko00061 Fatty acid biosynthesis;ko00780 Biotin metabolism;
gene40228	K02882	RP-L18Ae, RPL18A	large subunit ribosomal protein L18Ae	--	1	ko03010 Ribosome;
gene40229	K01184	E3.2.1.15	polygalacturonase	EC:3.2.1.15	2	ko00040 Pentose and glucuronate interconversions;ko00500 Starch and sucrose metabolism;

gene4022	K01102	PDP	pyruvate dehydrogenase phosphatase	EC:3.1.3.43		
gene40232	K08081	E1.1.1.206	tropine dehydrogenase	EC:1.1.1.206	1	ko00960 Tropane, piperidine and pyridine alkaloid biosynthesis;
gene40234	K16276	K16276, BTS	zinc finger protein-like protein	--		
gene40238	K05572	ndhA	NAD(P)H-quinone oxidoreductase subunit 1	EC:1.6.5.3	1	ko00190 Oxidative phosphorylation;
gene40239	K01087	otsB	trehalose 6-phosphate phosphatase	EC:3.1.3.12	1	ko00500 Starch and sucrose metabolism;
gene40243	K08244	E2.7.9.4	alpha-glucan, water dikinase	EC:2.7.9.4		
gene40255	K16075	MRS2, MFM1	magnesium transporter	--		
gene40256	K08515	VAMP7	vesicle-associated membrane protein 7	--	1	ko04130 SNARE interactions in vesicular transport;
gene4025	K10573	UBE2A, UBC2, RAD6A	ubiquitin-conjugating enzyme E2 A	EC:6.3.2.19	1	ko04120 Ubiquitin mediated proteolysis;
gene40260	K00759	E2.4.2.7, apt	adenine phosphoribosyltransferase	EC:2.4.2.7	1	ko00230 Purine metabolism;
gene40263	K05282	E1.14.11.12	gibberellin 20-oxidase	EC:1.14.11.12	1	ko00904 Diterpenoid biosynthesis;
gene4026	K09338	HD-ZIP	homeobox-leucine zipper protein	--		
gene40270	K11649	SMARCC	SWI/SNF related-matrix-associated actin-dependent regulator of chromatin subfamily C	--		
gene40272	K16297	SCPL-II	serine carboxypeptidase-like clade II	EC:3.4.16.-		
gene40273	K07432	ALG13	beta-1,4-N-acetylglucosaminyltransferase	EC:2.4.1.141	2	ko00510 N-Glycan biosynthesis;ko00513 Various types of N-glycan biosynthesis;
gene40279	K11380	NTO1	NuA3 HAT complex component NTO1	--		
gene40281	K04371	MAPK1_3	mitogen-activated protein kinase 1/3	EC:2.7.11.24	71	ko04010 MAPK signaling pathway;ko04012 ErbB signaling pathway;ko04013 MAPK signaling pathway - fly;ko04014 Ras signaling pathway;ko04062 Chemokine signaling pathway;ko04066 HIF-1 signaling pathway;ko04114 Oocyte meiosis;ko04150 mTOR signaling pathway;ko04151 PI3K-Akt signaling pathway;ko04270 Vascular smooth muscle contraction;ko04320 Dorso-ventral axis formation;ko04350 TGF-beta signaling pathway;ko04360 Axon guidance;ko04370 VEGF signaling pathway;ko04380 Osteoclast differentiation;ko04510 Focal adhesion;ko04520 Adherens junction;ko04540 Gap junction;ko04620 Toll-like receptor signaling pathway;ko04621 NOD-like receptor signaling pathway;ko04650 Natural killer cell mediated cytotoxicity;ko04660 T cell receptor signaling pathway;ko04662 B cell receptor signaling pathway;ko04664 Fc epsilon RI signaling pathway;ko04666 Fc gamma R-mediated phagocytosis;ko04668 TNF signaling pathway;ko04713 Circadian entrainment;ko04720 Long-term potentiation;ko04722 Neurotrophin signaling pathway;ko04723 Retrograde endocannabinoid signaling;ko04724 Glutamatergic synapse;ko04725 Cholinergic synapse;ko04726 Serotonergic synapse;ko04730 Long-term depression;ko04810 Regulation of actin cytoskeleton;ko04910 Insulin signaling pathway;ko04912 GnRH signaling pathway;ko04914 Progesterone-mediated oocyte maturation;ko04915 Estrogen signaling pathway;ko04916 Melanogenesis;ko04917 Prolactin signaling pathway;ko04930 Type II diabetes mellitus;ko04960 Aldosterone-regulated sodium reabsorption;ko05010 Alzheimer's disease;ko05020 Prion diseases;ko05034 Alcoholism;ko05131 Shigellosis;ko05132 Salmonella infection;ko05133 Pertussis;ko05140 Leishmaniasis;ko05142 Chagas disease (American trypanosomiasis);ko05145 Toxoplasmosis;ko05152 Tuberculosis;ko05160 Hepatitis C;ko05161 Hepatitis B;ko05164 Influenza A;ko05200 Pathways in cancer;ko05203 Viral carcinogenesis;ko05205 Proteoglycans in cancer;ko05210 Colorectal cancer;ko05211 Renal cell carcinoma;ko05212
gene40282	K00852	rbsK, RBKS	ribokinase	EC:2.7.1.15	1	ko00030 Pentose phosphate pathway;
gene40283	K07305	msrB	peptide-methionine (R)-S-oxide reductase	EC:1.8.4.12		
gene40288	K02918	RP-L35e, RPL35	large subunit ribosomal protein L35e	--	1	ko03010 Ribosome;
gene40290	K00028	E1.1.1.39	malate dehydrogenase (decarboxylating)	EC:1.1.1.39	3	ko00620 Pyruvate metabolism;ko00710 Carbon fixation in photosynthetic organisms;ko01200 Carbon metabolism;
gene40293	K01102	PDP	pyruvate dehydrogenase phosphatase	EC:3.1.3.43		
gene40294	K09591	DET2	steroid 5-alpha-reductase	EC:1.3.1.22	1	ko00905 Brassinosteroid biosynthesis;

gene40297	K03349	APC2	anaphase-promoting complex subunit 2	--	7	ko04110 Cell cycle;ko04111 Cell cycle - yeast;ko04113 Meiosis - yeast;ko04114 Oocyte meiosis;ko04120 Ubiquitin mediated proteolysis;ko04914 Progesterone-mediated oocyte maturation;ko05166 HTLV-I infection;
gene40299	K13096	SF4	splicing factor 4	--		
gene4029	K10908	POLRMT, RPO41	DNA-directed RNA polymerase, mitochondrial	EC:2.7.7.6		
gene40310	K12818	DHX8, PRP22	ATP-dependent RNA helicase DHX8/PRP22	EC:3.6.4.13	1	ko03040 Spliceosome;
gene40315	K02973	RP-S23e, RPS23	small subunit ribosomal protein S23e	--	1	ko03010 Ribosome;
gene40325	K13175	THOC6	THO complex subunit 6	--	1	ko03013 RNA transport;
gene40328	K11644	SIN3A	paired amphipathic helix protein Sin3a	--	2	ko05016 Huntington's disease;ko05202 Transcriptional misregulation in cancer;
gene40337	K03648	UNG, UDG	uracil-DNA glycosylase	EC:3.2.2.27	2	ko03410 Base excision repair;ko05340 Primary immunodeficiency;
gene40345	K12236	NFX1	transcriptional repressor NF-X1	EC:6.3.2.-		
gene40353	K13162	PCBP2_3_4	poly(rC)-binding protein 2/3/4	--		
gene40354	K03027	RPC40, POLR1C	DNA-directed RNA polymerases I and III subunit RPAC1	--	5	ko00230 Purine metabolism;ko00240 Pyrimidine metabolism;ko03020 RNA polymerase;ko04623 Cytosolic DNA-sensing pathway;ko05169 Epstein-Barr virus infection;
gene40360	K10753	ASF1	histone chaperone ASF1	--		
gene40361	K09338	HD-ZIP	homeobox-leucine zipper protein	--		
gene40364	K13947	PIN	auxin efflux carrier family	--		
gene40377	K04043	dnaK	molecular chaperone DnaK	--	2	ko03018 RNA degradation;ko05152 Tuberculosis;
gene40380	K09264	K09264	MADS-box transcription factor, plant	--		
gene40381	K09264	K09264	MADS-box transcription factor, plant	--		
gene40382	K15378	SLC45A1_2_4	solute carrier family 45, member 1/2/4	--		
gene40387	K14209	SLC36A, PAT	solute carrier family 36 (proton-coupled amino acid transporter)	--		
gene40389	K12193	VPS24, CHMP3	charged multivesicular body protein 3	--	1	ko04144 Endocytosis;
gene40390	K00026	MDH2	malate dehydrogenase	EC:1.1.1.37	5	ko00020 Citrate cycle (TCA cycle);ko00620 Pyruvate metabolism;ko00630 Glyoxylate and dicarboxylate metabolism;ko00710 Carbon fixation in photosynthetic organisms;ko01200 Carbon metabolism;
gene40395	K11380	NTO1	NuA3 HAT complex component NTO1	--		

gene40397	K04371	MAPK1_3	mitogen-activated protein kinase 1/3	EC:2.7.11.24	71	ko04010 MAPK signaling pathway;ko04012 ErbB signaling pathway;ko04013 MAPK signaling pathway - fly;ko04014 Ras signaling pathway;ko04062 Chemokine signaling pathway;ko04066 HIF-1 signaling pathway;ko04114 Oocyte meiosis;ko04150 mTOR signaling pathway;ko04151 PI3K-Akt signaling pathway;ko04270 Vascular smooth muscle contraction;ko04320 Dorso-ventral axis formation;ko04350 TGF-beta signaling pathway;ko04360 Axon guidance;ko04370 VEGF signaling pathway;ko04380 Osteoclast differentiation;ko04510 Focal adhesion;ko04520 Adherens junction;ko04540 Gap junction;ko04620 Toll-like receptor signaling pathway;ko04621 NOD-like receptor signaling pathway;ko04650 Natural killer cell mediated cytotoxicity;ko04660 T cell receptor signaling pathway;ko04662 B cell receptor signaling pathway;ko04664 Fc epsilon RI signaling pathway;ko04666 Fc gamma R-mediated phagocytosis;ko04668 TNF signaling pathway;ko04713 Circadian entrainment;ko04720 Long-term potentiation;ko04722 Neurotrophin signaling pathway;ko04723 Retrograde endocannabinoid signaling;ko04724 Glutamatergic synapse;ko04725 Cholinergic synapse;ko04726 Serotonergic synapse;ko04730 Long-term depression;ko04810 Regulation of actin cytoskeleton;ko04910 Insulin signaling pathway;ko04912 GnRH signaling pathway;ko04914 Progesterone-mediated oocyte maturation;ko04915 Estrogen signaling pathway;ko04916 Melanogenesis;ko04917 Prolactin signaling pathway;ko04930 Type II diabetes mellitus;ko04960 Aldosterone-regulated sodium reabsorption;ko05010 Alzheimer's disease;ko05020 Prion diseases;ko05034 Alcoholism;ko05131 Shigellosis;ko05132 Salmonella infection;ko05133 Pertussis;ko05140 Leishmaniasis;ko05142 Chagas disease (American trypanosomiasis);ko05145 Toxoplasmosis;ko05152 Tuberculosis;ko05160 Hepatitis C;ko05161 Hepatitis B;ko05164 Influenza A;ko05200 Pathways in cancer;ko05203 Viral carcinogenesis;ko05205 Proteoglycans in cancer;ko05210 Colorectal cancer;ko05211 Renal cell carcinoma;ko05212
gene4039	K04733	IRAK4	interleukin-1 receptor-associated kinase 4	EC:2.7.11.1	11	ko04064 NF-kappa B signaling pathway;ko04210 Apoptosis;ko04620 Toll-like receptor signaling pathway;ko04722 Neurotrophin signaling pathway;ko05133 Pertussis;ko05140 Leishmaniasis;ko05142 Chagas disease (American trypanosomiasis);ko05145 Toxoplasmosis;ko05152 Tuberculosis;ko05162 Measles;ko05164 Influenza A;
gene40401	K11498	CENPE	centromeric protein E	--		
gene40406	K09874	NIP	aquaporin NIP	--		
gene40411	K15083	RAD16	DNA repair protein RAD16	--		
gene40414	K03327	TC.MATE, SLC47A, norM, mdtK, dinF	multidrug resistance protein, MATE family	--		
gene40419	K01057	PGLS, pgl, devB	6-phosphogluconolactonase	EC:3.1.1.31	2	ko00030 Pentose phosphate pathway;ko01200 Carbon metabolism;
gene4041	K04733	IRAK4	interleukin-1 receptor-associated kinase 4	EC:2.7.11.1	11	ko04064 NF-kappa B signaling pathway;ko04210 Apoptosis;ko04620 Toll-like receptor signaling pathway;ko04722 Neurotrophin signaling pathway;ko05133 Pertussis;ko05140 Leishmaniasis;ko05142 Chagas disease (American trypanosomiasis);ko05145 Toxoplasmosis;ko05152 Tuberculosis;ko05162 Measles;ko05164 Influenza A;
gene40420	K03135	TAF11	transcription initiation factor TFIID subunit 11	--	1	ko03022 Basal transcription factors;

gene40430	K04392	RAC1	Ras-related C3 botulinum toxin substrate 1	--	33	ko04010 MAPK signaling pathway;ko04014 Ras signaling pathway;ko04062 Chemokine signaling pathway;ko04145 Phagosome;ko04151 PI3K-Akt signaling pathway;ko04310 Wnt signaling pathway;ko04360 Axon guidance;ko04370 VEGF signaling pathway;ko04380 Osteoclast differentiation;ko04510 Focal adhesion;ko04520 Adherens junction;ko04620 Toll-like receptor signaling pathway;ko04650 Natural killer cell mediated cytotoxicity;ko04662 B cell receptor signaling pathway;ko04664 Fc epsilon RI signaling pathway;ko04666 Fc gamma R-mediated phagocytosis;ko04670 Leukocyte transendothelial migration;ko04722 Neurotrophin signaling pathway;ko04810 Regulation of actin cytoskeleton;ko04932 Non-alcoholic fatty liver disease (NAFLD);ko04972 Pancreatic secretion;ko05014 Amyotrophic lateral sclerosis (ALS);ko05100 Bacterial invasion of epithelial cells;ko05120 Epithelial cell signaling in Helicobacter pylori infection;ko05131 Shigellosis;ko05132 Salmonella infection;ko05200 Pathways in cancer;ko05203 Viral carcinogenesis;ko05205 Proteoglycans in cancer;ko05210 Colorectal cancer;ko05211 Renal cell carcinoma;ko05212 Pancreatic
gene40431	K01807	rpiA	ribose 5-phosphate isomerase A	EC:5.3.1.6	4	ko00030 Pentose phosphate pathway;ko00710 Carbon fixation in photosynthetic organisms;ko01200 Carbon metabolism;ko01230 Biosynthesis of amino acids;
gene40434	K08857	NEK	NIMA (never in mitosis gene a)-related kinase	EC:2.7.11.1		
gene40439	K10578	UBE2J1, NCUBE1, UBC6	ubiquitin-conjugating enzyme E2 J1	EC:6.3.2.19	3	ko04120 Ubiquitin mediated proteolysis;ko04141 Protein processing in endoplasmic reticulum;ko05012 Parkinson's disease;
gene40442	K14405	FIP1L1, FIP1	pre-mRNA 3'-end-processing factor FIP1	--	1	ko03015 mRNA surveillance pathway;
gene40450	K01610	E4.1.1.49, pckA	phosphoenolpyruvate carboxykinase (ATP)	EC:4.1.1.49	4	ko00010 Glycolysis / Gluconeogenesis;ko00020 Citrate cycle (TCA cycle);ko00620 Pyruvate metabolism;ko00710 Carbon fixation in photosynthetic organisms;
gene40453	K10727	CDT1	chromatin licensing and DNA replication factor 1	--		
gene40455	K01893	NARS, asnS	asparaginyl-tRNA synthetase	EC:6.1.1.22	1	ko00970 Aminoacyl-tRNA biosynthesis;
gene40459	K15498	PPP6C	serine/threonine-protein phosphatase 6 catalytic subunit	EC:3.1.3.16		
gene4045	K06890	K06890				
gene40466	K10899	RECQL	ATP-dependent DNA helicase Q1	EC:3.6.4.12		
gene40467	K01897	ACSL, fadD	long-chain acyl-CoA synthetase	EC:6.2.1.3	4	ko00071 Fatty acid degradation;ko03320 PPAR signaling pathway;ko04146 Peroxisome;ko04920 Adipocytokine signaling pathway;
gene40469	K00600	glyA, SHMT	glycine hydroxymethyltransferase	EC:2.1.2.1	7	ko00260 Glycine, serine and threonine metabolism;ko00460 Cyanoamino acid metabolism;ko00630 Glyoxylate and dicarboxylate metabolism;ko00670 One carbon pool by folate;ko00680 Methane metabolism;ko01200 Carbon metabolism;ko01230 Biosynthesis of amino acids;
gene4046	K11816	YUCCA	indole-3-pyruvate monooxygenase	EC:1.14.13.168	1	ko00380 Tryptophan metabolism;
gene4047	K01006	ppdK	pyruvate,orthophosphate dikinase	EC:2.7.9.1	3	ko00620 Pyruvate metabolism;ko00710 Carbon fixation in photosynthetic organisms;ko01200 Carbon metabolism;
gene40480	K08730	PTDSS2	phosphatidylserine synthase 2	EC:2.7.8.29	1	ko00564 Glycerophospholipid metabolism;
gene40481	K03327	TC.MATE, SLC47A, norM, mdtK, dinF	multidrug resistance protein, MATE family	--		
gene40482	K02133	ATPeF1B, ATP5B	F-type H+-transporting ATPase subunit beta	EC:3.6.3.14	4	ko00190 Oxidative phosphorylation;ko05010 Alzheimer's disease;ko05012 Parkinson's disease;ko05016 Huntington's disease;
gene40487	K09422	MYBP	myb proto-oncogene protein, plant	--		
gene40499	K07199	PRKAB	5'-AMP-activated protein kinase, regulatory beta subunit	--	5	ko04710 Circadian rhythm;ko04910 Insulin signaling pathway;ko04920 Adipocytokine signaling pathway;ko04932 Non-alcoholic fatty liver disease (NAFLD);ko05410 Hypertrophic cardiomyopathy (HCM);
gene40502	K10742	DNA2	DNA replication ATP-dependent helicase Dna2	EC:3.6.4.12	1	ko03030 DNA replication;
gene40503	K10742	DNA2	DNA replication ATP-dependent helicase Dna2	EC:3.6.4.12	1	ko03030 DNA replication;
gene40506	K06685	MOB1, Mats	MOB kinase activator 1	--	3	ko04111 Cell cycle - yeast;ko04390 Hippo signaling pathway;ko04391 Hippo signaling pathway - fly;
gene4050	K00434	E1.11.1.11	L-ascorbate peroxidase	EC:1.11.1.11	2	ko00053 Ascorbate and aldarate metabolism;ko00480 Glutathione metabolism;
gene40514	K14319	RANGAP1	Ran GTPase-activating protein 1	--	1	ko03013 RNA transport;

gene40515	K11647	SMARCA2_4	SWI/SNF-related matrix-associated actin-dependent regulator of chromatin subfamily A member 2/4	EC:3.6.4.-		
gene40516	K09142	K09142	hypothetical protein	--		
gene40517	K01507	ppa	inorganic pyrophosphatase	EC:3.6.1.1	1	ko00190 Oxidative phosphorylation;
gene40522	K04733	IRAK4	interleukin-1 receptor-associated kinase 4	EC:2.7.11.1	11	ko04064 NF-kappa B signaling pathway;ko04210 Apoptosis;ko04620 Toll-like receptor signaling pathway;ko04722 Neurotrophin signaling pathway;ko05133 Pertussis;ko05140 Leishmaniasis;ko05142 Chagas disease (American trypanosomiasis);ko05145 Toxoplasmosis;ko05152 Tuberculosis;ko05162 Measles;ko05164 Influenza A;
gene40529	K13963	SERPINB	serpin B	--	1	ko05146 Amoebiasis;
gene40532	K03671	trxA	thioredoxin 1	--		
gene40533	K01969	E6.4.1.4B	3-methylcrotonyl-CoA carboxylase beta subunit	EC:6.4.1.4	1	ko00280 Valine, leucine and isoleucine degradation;
gene40534	K03152	thiJ	4-methyl-5(b-hydroxyethyl)-thiazole monophosphate biosynthesis	--		
gene40535	K14432	ABF	ABA responsive element binding factor	--	1	ko04075 Plant hormone signal transduction;
gene40536	K00555	TRMT1, trm1	tRNA (guanine26-N2/guanine27-N2)-dimethyltransferase	EC:2.1.1.215 2.1.1.216		
gene40537	K13379	RGP, UTM	reversibly glycosylated polypeptide / UDP-arabinopyranose mutase	EC:2.4.1.- 5.4.99.30		
gene40538	K12640	CYP85A2, BR6OX2	cytochrome P450, family 85, subfamily A, polypeptide 2 (brassinosteroid-6-oxidase 2)	EC:1.14.-.-	1	ko00905 Brassinosteroid biosynthesis;
gene40543	K11816	YUCCA	indole-3-pyruvate monooxygenase	EC:1.14.13.16 8	1	ko00380 Tryptophan metabolism;
gene40549	K03134	TAF10	transcription initiation factor TFIID subunit 10	--	2	ko03022 Basal transcription factors;ko05168 Herpes simplex infection;
gene40553	K15436	TRPO3, MTR10	transportin-3	--		
gene40558	K13137	STRAP, UNRIP	serine-threonine kinase receptor-associated protein	--	1	ko03013 RNA transport;
gene40568	K03139	TFIIF2, GTF2F2, TFG2	transcription initiation factor TFIIF subunit beta	EC:3.6.4.12	1	ko03022 Basal transcription factors;
gene4056	K01177	E3.2.1.2	beta-amylase	EC:3.2.1.2	1	ko00500 Starch and sucrose metabolism;
gene40575	K14066	GPS	geranyl diphosphate synthase	EC:2.5.1.1	1	ko00900 Terpenoid backbone biosynthesis;
gene40581	K14403	CPSF3, YSH1	cleavage and polyadenylation specificity factor subunit 3	EC:3.1.27.-	1	ko03015 mRNA surveillance pathway;
gene40582	K12260	SRX1	sulfiredoxin	EC:1.8.98.2		
gene40583	K05750	NCKAP1, NAP125	NCK-associated protein 1	--	1	ko04810 Regulation of actin cytoskeleton;
gene40585	K02437	gcvH, GCSH	glycine cleavage system H protein	--	1	ko00630 Glyoxylate and dicarboxylate metabolism;
gene40587	K02907	RP-L30, MRPL30, rpmD	large subunit ribosomal protein L30	--	1	ko03010 Ribosome;
gene40590	K13420	FLS2	LRR receptor-like serine/threonine-protein kinase FLS2	EC:2.7.11.1	1	ko04626 Plant-pathogen interaction;
gene40591	K13420	FLS2	LRR receptor-like serine/threonine-protein kinase FLS2	EC:2.7.11.1	1	ko04626 Plant-pathogen interaction;
gene40597	K01392	THOP1	thimet oligopeptidase	EC:3.4.24.15	2	ko04614 Renin-angiotensin system;ko05143 African trypanosomiasis;
gene40600	K15382	SLC50A, SWEET	solute carrier family 50 (sugar transporter)	--		
gene40602	K15361	WDR48, UAF1	WD repeat-containing protein 48	--	1	ko03460 Fanconi anemia pathway;
gene40605	K14488	SAUR	SAUR family protein	--	1	ko04075 Plant hormone signal transduction;
gene40608	K14505	CYCD3	cyclin D3, plant	--	1	ko04075 Plant hormone signal transduction;
gene40614	K00940	E2.7.4.6, ndk	nucleoside-diphosphate kinase	EC:2.7.4.6	2	ko00230 Purine metabolism;ko00240 Pyrimidine metabolism;
gene40615	K00940	E2.7.4.6, ndk	nucleoside-diphosphate kinase	EC:2.7.4.6	2	ko00230 Purine metabolism;ko00240 Pyrimidine metabolism;
gene40619	K14236	tRNA-Tyr	tRNA Tyr	--	1	ko00970 Aminoacyl-tRNA biosynthesis;
gene4061	K11968	ARIH1	ariadne-1	--		
gene40622	K06268	PPP3R, CNB	serine/threonine-protein phosphatase 2B regulatory subunit	--	18	ko04010 MAPK signaling pathway;ko04020 Calcium signaling pathway;ko04114 Oocyte meiosis;ko04210 Apoptosis;ko04310 Wnt signaling pathway;ko04360 Axon guidance;ko04370 VEGF signaling pathway;ko04380 Osteoclast differentiation;ko04650 Natural killer cell mediated cytotoxicity;ko04660 T cell receptor signaling pathway;ko04662 B cell receptor signaling pathway;ko04720 Long-term potentiation;ko04724 Glutamatergic synapse;ko05010 Alzheimer's disease;ko05014 Amyotrophic lateral sclerosis (ALS);ko05031 Amphetamine addiction;ko05152

gene40627	K00430	E1.11.1.7	peroxidase	EC:1.11.1.7	2	ko00360 Phenylalanine metabolism;ko00940 Phenylpropanoid biosynthesis;
gene40637	K03868	RBX1, ROC1	RING-box protein 1	--	12	ko03420 Nucleotide excision repair;ko04066 HIF-1 signaling pathway;ko04110 Cell cycle;ko04111 Cell cycle - yeast;ko04114 Oocyte meiosis;ko04120 Ubiquitin mediated proteolysis;ko04141 Protein processing in endoplasmic reticulum;ko04310 Wnt signaling pathway;ko04350 TGF-beta signaling pathway;ko04710 Circadian rhythm;ko05200 Pathways in cancer;ko05211 Renal cell carcinoma;
gene40640	K02985	RP-S3e, RPS3	small subunit ribosomal protein S3e	--	1	ko03010 Ribosome;
gene40642	K13346	PEX10	peroxin-10	--	1	ko04146 Peroxisome;
gene40643	K10624	RBBP6	E3 ubiquitin-protein ligase RBBP6	EC:6.3.2.19		
gene40644	K08472	MLO	mlo protein	--		
gene40649	K14972	PAXIP1, PTIP	PAX-interacting protein 1	--		
gene4064	K00850	pfkA, PFK	6-phosphofructokinase 1	EC:2.7.1.11	7	ko00010 Glycolysis / Gluconeogenesis;ko00030 Pentose phosphate pathway;ko00051 Fructose and mannose metabolism;ko00052 Galactose metabolism;ko00680 Methane metabolism;ko01200 Carbon metabolism;ko01230 Biosynthesis of amino acids;
gene40652	K11426	SMYD	SET and MYND domain-containing protein	--		
gene4065	K14488	SAUR	SAUR family protein	--	1	ko04075 Plant hormone signal transduction;
gene40663	K09338	HD-ZIP	homeobox-leucine zipper protein	--		
gene40666	K08912	LHCB1	light-harvesting complex II chlorophyll a/b binding protein 1	--	1	ko00196 Photosynthesis - antenna proteins;
gene40667	K14510	CTR1	serine/threonine-protein kinase CTR1	EC:2.7.11.1	1	ko04075 Plant hormone signal transduction;
gene4066	K15283	SLC35E1	solute carrier family 35, member E1	--		
gene40679	K07874	RAB1A	Ras-related protein Rab-1A	--	1	ko05134 Legionellosis;
gene4067	K01070	frmB, ESD, fghA	S-formylglutathione hydrolase	EC:3.1.2.12	2	ko00680 Methane metabolism;ko01200 Carbon metabolism;
gene40687	K14491	ARR-B	two-component response regulator ARR-B family	--	1	ko04075 Plant hormone signal transduction;
gene4068	K09564	PPIE	peptidyl-prolyl isomerase E (cyclophilin E)	EC:5.2.1.8	1	ko03040 Spliceosome;
gene40691	K01254	E3.3.2.6, LTA4H	leukotriene-A4 hydrolase	EC:3.3.2.6	1	ko00590 Arachidonic acid metabolism;
gene40695	K12161	URM1	ubiquitin related modifier 1	--	1	ko04122 Sulfur relay system;
gene4069	K01057	PGLS, pgl, devB	6-phosphogluconolactonase	EC:3.1.1.31	2	ko00030 Pentose phosphate pathway;ko01200 Carbon metabolism;
gene406	K05863	SLC25A4S, ANT	solute carrier family 25 (mitochondrial adenine nucleotide translocator), member 4/5/6/31	--	4	ko04020 Calcium signaling pathway;ko05012 Parkinson's disease;ko05016 Huntington's disease;ko05166 HTLV-I infection;
gene40702	K00430	E1.11.1.7	peroxidase	EC:1.11.1.7	2	ko00360 Phenylalanine metabolism;ko00940 Phenylpropanoid biosynthesis;
gene40703	K00430	E1.11.1.7	peroxidase	EC:1.11.1.7	2	ko00360 Phenylalanine metabolism;ko00940 Phenylpropanoid biosynthesis;
gene40704	K07390	grxD, GLRX5	monothiol glutaredoxin	--		
gene40705	K06130	LYPLA2	lysophospholipase II	EC:3.1.1.5	1	ko00564 Glycerophospholipid metabolism;
gene40707	K02265	COX5B	cytochrome c oxidase subunit 5b	--	6	ko00190 Oxidative phosphorylation;ko04260 Cardiac muscle contraction;ko04932 Non-alcoholic fatty liver disease (NAFLD);ko05010 Alzheimer's disease;ko05012 Parkinson's disease;ko05016 Huntington's disease;
gene40708	K14498	SNRK2	serine/threonine-protein kinase SRK2	EC:2.7.11.1	1	ko04075 Plant hormone signal transduction;
gene40716	K13832	aroDE, DHQ-SDH	3-dehydroquinate dehydratase / shikimate dehydrogenase	EC:4.2.1.10 1.1.1.25	2	ko00400 Phenylalanine, tyrosine and tryptophan biosynthesis;ko01230 Biosynthesis of amino acids;
gene40718	K00134	GAPDH, gapA	glyceraldehyde 3-phosphate dehydrogenase	EC:1.2.1.12	6	ko00010 Glycolysis / Gluconeogenesis;ko00710 Carbon fixation in photosynthetic organisms;ko01200 Carbon metabolism;ko01230 Biosynthesis of amino acids;ko04066 HIF-1 signaling pathway;ko05010 Alzheimer's disease;
gene40719	K11968	ARIH1	ariadne-1	--		
gene40729	K08790	STK38, NDR	serine/threonine kinase 38	EC:2.7.11.1		
gene4072	K08867	WNK, PRKWNK	WNK lysine deficient protein kinase	EC:2.7.11.1		
gene40735	K14674	TGL4	TAG lipase / steryl ester hydrolase / phospholipase A2 / LPA acyltransferase	EC:3.1.1.3 3.1.1.13 3.1.1.4 2.3.1.51	7	ko00100 Steroid biosynthesis;ko00561 Glycerolipid metabolism;ko00564 Glycerophospholipid metabolism;ko00565 Ether lipid metabolism;ko00590 Arachidonic acid metabolism;ko00591 Linoleic acid metabolism;ko00592 alpha-Linolenic acid metabolism;
gene40737	K15920	XYL4	beta-D-xylosidase 4	EC:3.2.1.37	2	ko00500 Starch and sucrose metabolism;ko00520 Amino sugar and nucleotide sugar metabolism;

gene40738	K15920	XYL4	beta-D-xylosidase 4	EC:3.2.1.37	2	ko00500 Starch and sucrose metabolism;ko00520 Amino sugar and nucleotide sugar metabolism;
gene40739	K14165	K14165	dual specificity phosphatase	EC:3.1.3.16 3.1.3.48		
gene40740	K01074	PPT	palmitoyl-protein thioesterase	EC:3.1.2.22	2	ko00062 Fatty acid elongation;ko04142 Lysosome;
gene40741	K01074	PPT	palmitoyl-protein thioesterase	EC:3.1.2.22	2	ko00062 Fatty acid elongation;ko04142 Lysosome;
gene40745	K00472	E1.14.11.2	prolyl 4-hydroxylase	EC:1.14.11.2	1	ko00330 Arginine and proline metabolism;
gene40756	K15425	PPP4R2	serine/threonine-protein phosphatase 4 regulatory subunit 2	--		
gene40764	K01648	ACLY	ATP citrate (pro-S)-lyase	EC:2.3.3.8	2	ko00020 Citrate cycle (TCA cycle);ko00720 Carbon fixation pathways in prokaryotes;
gene40773	K09422	MYBP	myb proto-oncogene protein, plant	--		
gene40775	K01762	ACS	1-aminocyclopropane-1-carboxylate synthase	EC:4.4.1.14	1	ko00270 Cysteine and methionine metabolism;
gene40776	K09422	MYBP	myb proto-oncogene protein, plant	--		
gene4077	K14787	MRD1, RBM19	multiple RNA-binding domain-containing protein 1	--		
gene40780	K05749	CYFIP	cytoplasmic FMR1 interacting protein	--	2	ko03013 RNA transport;ko04810 Regulation of actin cytoskeleton;
gene40781	K06176	truD, PUS7	tRNA pseudouridine13 synthase	EC:5.4.99.27		
gene40788	K03364	CDH1	cell division cycle 20-like protein 1, cofactor of APC complex	--	4	ko04110 Cell cycle;ko04111 Cell cycle - yeast;ko04120 Ubiquitin mediated proteolysis;ko04914 Progesterone-mediated oocyte maturation;
gene40793	K14765	NGDN, LCP5	U3 small nucleolar ribonucleoprotein protein LCP5	--		
gene40799	K14319	RANGAP1	Ran GTPase-activating protein 1	--	1	ko03013 RNA transport;
gene40800	K00942	E2.7.4.8, gmk	guanylate kinase	EC:2.7.4.8	1	ko00230 Purine metabolism;
gene40805	K15332	TRMT2A	tRNA (uracil-5-)-methyltransferase	--		
gene40809	K13783	SLC37A1_2	MFS transporter, OPA family, solute carrier family 37 (glycerol-3-phosphate transporter), member 1/2	--		
gene40814	K08145	SLC2A8, GLUT8	MFS transporter, SP family, solute carrier family 2 (facilitated glucose transporter), member 8	--		
gene40818	K14496	PYL	abscisic acid receptor PYR/PYL family	--	1	ko04075 Plant hormone signal transduction;
gene40819	K09286	EREBP	EREBP-like factor	--		
gene40821	K04523	UBQLN, DSK2	ubiquilin	--	1	ko04141 Protein processing in endoplasmic reticulum;
gene40822	K05868	CCNB	cyclin B	--	3	ko04110 Cell cycle;ko04115 p53 signaling pathway;ko04914 Progesterone-mediated oocyte maturation;
gene40825	K12885	RBMX, HNRNPG	heterogeneous nuclear ribonucleoprotein G	--	1	ko03040 Spliceosome;
gene40828	K01365	CTSL	cathepsin L	EC:3.4.22.15	5	ko04142 Lysosome;ko04145 Phagosome;ko04612 Antigen processing and presentation;ko05205 Proteoglycans in cancer;ko05323 Rheumatoid arthritis;

gene40831	K04371	MAPK1_3	mitogen-activated protein kinase 1/3	EC:2.7.11.24	71	ko04010 MAPK signaling pathway;ko04012 ErbB signaling pathway;ko04013 MAPK signaling pathway - fly;ko04014 Ras signaling pathway;ko04062 Chemokine signaling pathway;ko04066 HIF-1 signaling pathway;ko04114 Oocyte meiosis;ko04150 mTOR signaling pathway;ko04151 PI3K-Akt signaling pathway;ko04270 Vascular smooth muscle contraction;ko04320 Dorso-ventral axis formation;ko04350 TGF-beta signaling pathway;ko04360 Axon guidance;ko04370 VEGF signaling pathway;ko04380 Osteoclast differentiation;ko04510 Focal adhesion;ko04520 Adherens junction;ko04540 Gap junction;ko04620 Toll-like receptor signaling pathway;ko04621 NOD-like receptor signaling pathway;ko04650 Natural killer cell mediated cytotoxicity;ko04660 T cell receptor signaling pathway;ko04662 B cell receptor signaling pathway;ko04664 Fc epsilon RI signaling pathway;ko04666 Fc gamma R-mediated phagocytosis;ko04668 TNF signaling pathway;ko04713 Circadian entrainment;ko04720 Long-term potentiation;ko04722 Neurotrophin signaling pathway;ko04723 Retrograde endocannabinoid signaling;ko04724 Glutamatergic synapse;ko04725 Cholinergic synapse;ko04726 Serotonergic synapse;ko04730 Long-term depression;ko04810 Regulation of actin cytoskeleton;ko04910 Insulin signaling pathway;ko04912 GnRH signaling pathway;ko04914 Progesterone-mediated oocyte maturation;ko04915 Estrogen signaling pathway;ko04916 Melanogenesis;ko04917 Prolactin signaling pathway;ko04930 Type II diabetes mellitus;ko04960 Aldosterone-regulated sodium reabsorption;ko05010 Alzheimer's disease;ko05020 Prion diseases;ko05034 Alcoholism;ko05131 Shigellosis;ko05132 Salmonella infection;ko05133 Pertussis;ko05140 Leishmaniasis;ko05142 Chagas disease (American trypanosomiasis);ko05145 Toxoplasmosis;ko05152 Tuberculosis;ko05160 Hepatitis C;ko05161 Hepatitis B;ko05164 Influenza A;ko05200 Pathways in cancer;ko05203 Viral carcinogenesis;ko05205 Proteoglycans in cancer;ko05210 Colorectal cancer;ko05211 Renal cell carcinoma;ko05212
gene40836	K13648	GAUT	alpha-1,4-galacturonosyltransferase	EC:2.4.1.43	2	ko00500 Starch and sucrose metabolism;ko00520 Amino sugar and nucleotide sugar metabolism;
gene40837	K04733	IRAK4	interleukin-1 receptor-associated kinase 4	EC:2.7.11.1	11	ko04064 NF-kappa B signaling pathway;ko04210 Apoptosis;ko04620 Toll-like receptor signaling pathway;ko04722 Neurotrophin signaling pathway;ko05133 Pertussis;ko05140 Leishmaniasis;ko05142 Chagas disease (American trypanosomiasis);ko05145 Toxoplasmosis;ko05152 Tuberculosis;ko05162 Measles;ko05164 Influenza A;
gene40839	K10364	CAPZA	capping protein (actin filament) muscle Z-line, alpha	--		
gene4083	K00128	E1.2.1.3	aldehyde dehydrogenase (NAD+)	EC:1.2.1.3	15	ko00010 Glycolysis / Gluconeogenesis;ko00040 Pentose and glucuronate interconversions;ko00053 Ascorbate and aldarate metabolism;ko00071 Fatty acid degradation;ko00280 Valine, leucine and isoleucine degradation;ko00310 Lysine degradation;ko00330 Arginine and proline metabolism;ko00340 Histidine metabolism;ko00380 Tryptophan metabolism;ko00410 beta-Alanine metabolism;ko00561 Glycerolipid metabolism;ko00620 Pyruvate metabolism;ko00625 Chloroalkane and chloroalkene degradation;ko00640 Propanoate metabolism;ko00903 Limonene and pinene
gene40842	K09014	sufB	Fe-S cluster assembly protein SufB	--		
gene40845	K05350	bglB	beta-glucosidase	EC:3.2.1.21	3	ko00460 Cyanoamino acid metabolism;ko00500 Starch and sucrose metabolism;ko00940 Phenylpropanoid biosynthesis;
gene40846	K15283	SLC35E1	solute carrier family 35, member E1	--		
gene40853	K01887	RARS, argS	arginyl-tRNA synthetase	EC:6.1.1.19	1	ko00970 Aminoacyl-tRNA biosynthesis;
gene40854	K01301	E3.4.17.21	glutamate carboxypeptidase II	EC:3.4.17.21		
gene40855	K01051	E3.1.1.11	pectinesterase	EC:3.1.1.11	2	ko00040 Pentose and glucuronate interconversions;ko00500 Starch and sucrose metabolism;

gene40856	K04710	CERS	ceramide synthetase	EC:2.3.1.24	1	ko00600 Sphingolipid metabolism;
gene40859	K09843	E1.14.13.93	(+)-abscisic acid 8'-hydroxylase	EC:1.14.13.93	1	ko00906 Carotenoid biosynthesis;
gene40861	K00771	XYLT	protein xylosyltransferase	EC:2.4.2.26	2	ko00532 Glycosaminoglycan biosynthesis - chondroitin sulfate / dermatan sulfate;ko00534 Glycosaminoglycan biosynthesis - heparan sulfate / heparin;
gene40866	K14236	tRNA-Tyr	tRNA Tyr	--	1	ko00970 Aminoacyl-tRNA biosynthesis;
gene40867	K01301	E3.4.17.21	glutamate carboxypeptidase II	EC:3.4.17.21		
gene40868	K01887	RARS, argS	arginyl-tRNA synthetase	EC:6.1.1.19	1	ko00970 Aminoacyl-tRNA biosynthesis;
gene40871	K05759	PFN	profilin	--	3	ko04810 Regulation of actin cytoskeleton;ko05131 Shigellosis;ko05132 Salmonella infection;
gene40873	K11842	USP12_46	ubiquitin carboxyl-terminal hydrolase 12/46	EC:3.1.2.15		
gene40874	K08819	CDK12_13	cyclin-dependent kinase 12/13	EC:2.7.11.22 2.7.11.23		
gene40880	K00472	E1.14.11.2	prolyl 4-hydroxylase	EC:1.14.11.2	1	ko00330 Arginine and proline metabolism;
gene4088	K05933	E1.14.17.4	aminocyclopropanecarboxylate oxidase	EC:1.14.17.4	1	ko00270 Cysteine and methionine metabolism;
gene40892	K15053	CHMP7	charged multivesicular body protein 7	--	1	ko04144 Endocytosis;
gene40895	K00901	E2.7.1.107, DGK, dgkA	diacylglycerol kinase (ATP dependent)	EC:2.7.1.107	3	ko00561 Glycerolipid metabolism;ko00564 Glycerophospholipid metabolism;ko04070 Phosphatidylinositol signaling system;
gene40898	K07056	rsmI	16S rRNA (cytidine1402-2'-O)-methyltransferase	EC:2.1.1.198		
gene40899	K10688	UBE2W, UBC16	ubiquitin-conjugating enzyme E2 W	EC:6.3.2.19	1	ko04120 Ubiquitin mediated proteolysis;
gene40900	K08835	OXSR1, STK39	serine/threonine-protein kinase OSR1/STK39	EC:2.7.11.1		
gene40903	K13354	SLC25A17, PMP34	solute carrier family 25 (peroxisomal adenine nucleotide transporter), member 17	--	1	ko04146 Peroxisome;
gene40909	K05868	CCNB	cyclin B	--	3	ko04110 Cell cycle;ko04115 p53 signaling pathway;ko04914 Progesterone-mediated oocyte maturation;
gene40910	K14220	tRNA-Asn	tRNA Asn	--	1	ko00970 Aminoacyl-tRNA biosynthesis;
gene40912	K00799	GST, gst	glutathione S-transferase	EC:2.5.1.18	4	ko00480 Glutathione metabolism;ko00980 Metabolism of xenobiotics by cytochrome P450;ko00982 Drug metabolism - cytochrome P450;ko05204 Chemical carcinogenesis;
gene40918	K13510	LPCAT1_2	lysophosphatidylcholine acyltransferase / lyso-PAF acetyltransferase	EC:2.3.1.23 2.3.1.67	2	ko00564 Glycerophospholipid metabolism;ko00565 Ether lipid metabolism;
gene40927	K14492	ARR-A	two-component response regulator ARR-A family	--	1	ko04075 Plant hormone signal transduction;
gene40936	K09874	NIP	aquaporin NIP	--		
gene40937	K12118	CRY1	cryptochrome 1	--	1	ko04712 Circadian rhythm - plant;
gene40938	K03883	ND5	NADH-ubiquinone oxidoreductase chain 5	EC:1.6.5.3	2	ko00190 Oxidative phosphorylation;ko05012 Parkinson's disease;
gene40939	K03879	ND2	NADH-ubiquinone oxidoreductase chain 2	EC:1.6.5.3	2	ko00190 Oxidative phosphorylation;ko05012 Parkinson's disease;
gene40942	K09422	MYBP	myb proto-oncogene protein, plant	--		
gene40944	K11968	ARIH1	ariadne-1	--		
gene40946	K11968	ARIH1	ariadne-1	--		
gene4094	K04733	IRAK4	interleukin-1 receptor-associated kinase 4	EC:2.7.11.1	11	ko04064 NF-kappa B signaling pathway;ko04210 Apoptosis;ko04620 Toll-like receptor signaling pathway;ko04722 Neurotrophin signaling pathway;ko05133 Pertussis;ko05140 Leishmaniasis;ko05142 Chagas disease (American trypanosomiasis);ko05145 Toxoplasmosis;ko05152 Tuberculosis;ko05162 Measles;ko05164 Influenza A;
gene40960	K11251	H2A	histone H2A	--	2	ko05034 Alcoholism;ko05322 Systemic lupus erythematosus;
gene40969	K02734	PSMB2	20S proteasome subunit beta 4	EC:3.4.25.1	1	ko03050 Proteasome;
gene40980	K10849	ERCC1	DNA excision repair protein ERCC-1	--	2	ko03420 Nucleotide excision repair;ko03460 Fanconi anemia pathway;
gene40987	K01426	E3.5.1.4, amiE	amidase	EC:3.5.1.4	5	ko00330 Arginine and proline metabolism;ko00360 Phenylalanine metabolism;ko00380 Tryptophan metabolism;ko00627 Aminobenzoate degradation;ko00643 Styrene degradation;
gene40988	K01426	E3.5.1.4, amiE	amidase	EC:3.5.1.4	5	ko00330 Arginine and proline metabolism;ko00360 Phenylalanine metabolism;ko00380 Tryptophan metabolism;ko00627 Aminobenzoate degradation;ko00643 Styrene degradation;
gene40993	K00921	PIKFYVE, FAB1	1-phosphatidylinositol-3-phosphate 5-kinase	EC:2.7.1.150	4	ko00562 Inositol phosphate metabolism;ko04070 Phosphatidylinositol signaling system;ko04145 Phagosome;ko04810 Regulation of actin cytoskeleton;

gene409	K02183	CALM	calmodulin	--	25	ko04014 Ras signaling pathway;ko04020 Calcium signaling pathway;ko04070 Phosphatidylinositol signaling system;ko04114 Oocyte meiosis;ko04270 Vascular smooth muscle contraction;ko04626 Plant-pathogen interaction;ko04713 Circadian entrainment;ko04720 Long-term potentiation;ko04722 Neurotrophin signaling pathway;ko04728 Dopaminergic synapse;ko04740 Olfactory transduction;ko04744 Phototransduction;ko04745 Phototransduction - fly;ko04910 Insulin signaling pathway;ko04912 GnRH signaling pathway;ko04915 Estrogen signaling pathway;ko04916 Melanogenesis;ko04970 Salivary secretion;ko04971 Gastric acid secretion;ko05010 Alzheimer's disease;ko05031 Amphetamine addiction;ko05034 Alcoholism;ko05133 Pertussis;ko05152 Tuberculosis;ko05214 Glioma;
gene40	K17086	TM9SF2_4	transmembrane 9 superfamily member 2/4	--		
gene41002	K04532	APPBP1	amyloid beta precursor protein binding protein 1	--	1	ko05010 Alzheimer's disease;
gene41003	K01681	ACO, acnA	aconitate hydratase	EC:4.2.1.3	6	ko00020 Citrate cycle (TCA cycle);ko00630 Glyoxylate and dicarboxylate metabolism;ko00720 Carbon fixation pathways in prokaryotes;ko01200 Carbon metabolism;ko01210 2-Oxocarboxylic acid metabolism;ko01230 Biosynthesis of amino acids;
gene41004	K10523	SPOP	speckle-type POZ protein	--		
gene41005	K12869	CRN, CRNKL1, CLF1, SYF3	crooked neck	--	1	ko03040 Spliceosome;
gene41019	K06268	PPP3R, CNB	serine/threonine-protein phosphatase 2B regulatory subunit	--	18	ko04010 MAPK signaling pathway;ko04020 Calcium signaling pathway;ko04114 Oocyte meiosis;ko04210 Apoptosis;ko04310 Wnt signaling pathway;ko04360 Axon guidance;ko04370 VEGF signaling pathway;ko04380 Osteoclast differentiation;ko04650 Natural killer cell mediated cytotoxicity;ko04660 T cell receptor signaling pathway;ko04662 B cell receptor signaling pathway;ko04720 Long-term potentiation;ko04724 Glutamatergic synapse;ko05010 Alzheimer's disease;ko05014 Amyotrophic lateral sclerosis (ALS);ko05031 Amphetamine addiction;ko05152
gene4101	K13989	DERL2_3	Derlin-2/3	--	1	ko04141 Protein processing in endoplasmic reticulum;
gene41020	K01507	ppa	inorganic pyrophosphatase	EC:3.6.1.1	1	ko00190 Oxidative phosphorylation;
gene41027	K14641	APY1_2	apyrase	EC:3.6.1.5	2	ko00230 Purine metabolism;ko00240 Pyrimidine metabolism;
gene41029	K05658	ABCB1	ATP-binding cassette, subfamily B (MDR/TAP), member 1	--	3	ko02010 ABC transporters;ko04976 Bile secretion;ko05206 MicroRNAs in cancer;
gene41032	K09264	K09264	MADS-box transcription factor, plant	--		
gene41033	K01689	ENO, eno	enolase	EC:4.2.1.11	6	ko00010 Glycolysis / Gluconeogenesis;ko00680 Methane metabolism;ko01200 Carbon metabolism;ko01230 Biosynthesis of amino acids;ko03018 RNA degradation;ko04066 HIF-1 signaling pathway;
gene41038	K07893	RAB6A	Ras-related protein Rab-6A	--		
gene41040	K08341	GABARAP, ATG8, LC3	GABA(A) receptor-associated protein (autophagy-related protein 8)	--	2	ko04140 Regulation of autophagy;ko04727 GABAergic synapse;
gene41041	K14638	SLC15A3_4, PHT	solute carrier family 15 (peptide/histidine transporter), member 3/4	--		
gene41045	K03327	TC.MATE, SLC47A, norM, mdtK, dinF	multidrug resistance protein, MATE family	--		
gene41047	K09422	MYBP	myb proto-oncogene protein, plant	--		
gene4104	K09828	DHCR24	delta24-sterol reductase	EC:1.3.1.72	1	ko00100 Steroid biosynthesis;
gene41050	K17267	COPG	coatomer protein complex, subunit gamma	--		
gene41051	K04536	GNB1	guanine nucleotide-binding protein G(I)/G(S)/G(T) subunit beta-1	--	14	ko04014 Ras signaling pathway;ko04062 Chemokine signaling pathway;ko04151 PI3K-Akt signaling pathway;ko04713 Circadian entrainment;ko04723 Retrograde endocannabinoid signaling;ko04724 Glutamatergic synapse;ko04725 Cholinergic synapse;ko04726 Serotonergic synapse;ko04727 GABAergic synapse;ko04728 Dopaminergic synapse;ko04742 Taste transduction;ko04744 Phototransduction;ko05032 Morphine addiction;ko05034 Alcoholism;
gene41061	K14326	UPF1, RENT1	regulator of nonsense transcripts 1	EC:3.6.4.-	2	ko03013 RNA transport;ko03015 mRNA surveillance pathway;

gene41065	K01466	allB	allantoinase	EC:3.5.2.5	1	ko00230 Purine metabolism;
gene4106	K02735	PSMB3	20S proteasome subunit beta 3	EC:3.4.25.1	1	ko03050 Proteasome;
gene41071	K03144	TFIIH4, GTF2H4, TFB2	transcription initiation factor TFIIH subunit 4	--	3	ko03022 Basal transcription factors;ko03420 Nucleotide excision repair;ko05203 Viral carcinogenesis;
gene41073	K04712	DEGS	sphingolipid delta-4 desaturase	EC:1.14.-.-	1	ko00600 Sphingolipid metabolism;
gene41077	K14638	SLC15A3_4, PHT	solute carrier family 15 (peptide/histidine transporter), member 3/4	--		
gene41085	K09338	HD-ZIP	homeobox-leucine zipper protein	--		
gene41086	K15255	PIF1	ATP-dependent DNA helicase PIF1	EC:3.6.4.12		
gene41089	K02974	RP-S24e, RPS24	small subunit ribosomal protein S24e	--	1	ko03010 Ribosome;
gene4108	K11414	SIRT4, SIR2L4	NAD-dependent deacetylase sirtuin 4	EC:3.5.1.-		
gene41100	K01166	E3.1.27.1	ribonuclease T2	EC:3.1.27.1		
gene41103	K00051	E1.1.1.82	malate dehydrogenase (NADP+)	EC:1.1.1.82	2	ko00620 Pyruvate metabolism;ko00710 Carbon fixation in photosynthetic organisms;
gene41106	K15455	KTI11, DPH3	diphthamide biosynthesis protein 3	--		
gene4110	K14972	PAXIP1, PTIP	PAX-interacting protein 1	--		
gene41113	K09490	HSPA5, BIP	heat shock 70kDa protein 5	--	4	ko03060 Protein export;ko04141 Protein processing in endoplasmic reticulum;ko04918 Thyroid hormone synthesis;ko05020 Prion diseases;
gene41114	K14572	MDN1, REA1	midasin	--	1	ko03008 Ribosome biogenesis in eukaryotes;
gene41120	K07203	FRAP	FKBP12-rapamycin complex-associated protein	--	13	ko04012 ErbB signaling pathway;ko04066 HIF-1 signaling pathway;ko04150 mTOR signaling pathway;ko04151 PI3K-Akt signaling pathway;ko04910 Insulin signaling pathway;ko04920 Adipocytokine signaling pathway;ko04930 Type II diabetes mellitus;ko05200 Pathways in cancer;ko05205 Proteoglycans in cancer;ko05206 MicroRNAs in cancer;ko05214 Glioma;ko05215 Prostate cancer;ko05221 Acute myeloid leukemia;
gene41121	K12657	ALDH18A1, P5CS	delta-1-pyrroline-5-carboxylate synthetase	EC:2.7.2.11 1.2.1.41	2	ko00330 Arginine and proline metabolism;ko01230 Biosynthesis of amino acids;
gene41127	K05284	PIGM	phosphatidylinositol glycan, class M	EC:2.4.1.-	1	ko00563 Glycosylphosphatidylinositol(GPI)-anchor biosynthesis;
gene41132	K08955	YME1	ATP-dependent metalloprotease	EC:3.4.24.-		
gene41135	K17046	DEK	protein DEK	--		
gene41137	K01738	cysK	cysteine synthase A	EC:2.5.1.47	4	ko00270 Cysteine and methionine metabolism;ko00920 Sulfur metabolism;ko01200 Carbon metabolism;ko01230 Biosynthesis of amino acids;
gene41139	K05389	KCNKF	potassium channel subfamily K, other eukaryote	--		
gene41140	K10878	SPO11	meiotic recombination protein SPO11	--	1	ko04113 Meiosis - yeast;
gene41142	K12483	EHD1	EH domain-containing protein 1	--	1	ko04144 Endocytosis;
gene41144	K12199	VTA1, LIP5	vacuolar protein sorting-associated protein VTA1	--	1	ko04144 Endocytosis;
gene41146	K00815	TAT	tyrosine aminotransferase	EC:2.6.1.5	9	ko00130 Ubiquinone and other terpenoid-quinone biosynthesis;ko00270 Cysteine and methionine metabolism;ko00350 Tyrosine metabolism;ko00360 Phenylalanine metabolism;ko00400 Phenylalanine, tyrosine and tryptophan biosynthesis;ko00401 Novobiocin biosynthesis;ko00950 Isoquinoline alkaloid biosynthesis;ko00960 Tropane, piperidine and pyridine alkaloid biosynthesis;ko01230 Biosynthesis of amino acids;
gene41156	K11294	NCL, NSR1	nucleolin	--	1	ko05130 Pathogenic Escherichia coli infection;
gene4115	K05019	CLNS1A	chloride channel, nucleotide-sensitive, 1A	--	1	ko03013 RNA transport;
gene41161	K11843	USP14, UBP6	ubiquitin carboxyl-terminal hydrolase 14	EC:3.1.2.15		
gene41162	K01728	E4.2.2.2, pel	pectate lyase	EC:4.2.2.2	1	ko00040 Pentose and glucuronate interconversions;
gene41163	K04354	PPP2R2	serine/threonine-protein phosphatase 2A regulatory subunit B	--	9	ko03015 mRNA surveillance pathway;ko04111 Cell cycle - yeast;ko04151 PI3K-Akt signaling pathway;ko04390 Hippo signaling pathway;ko04391 Hippo signaling pathway - fly;ko04530 Tight junction;ko04728 Dopaminergic synapse;ko05142 Chagas disease (American trypanosomiasis);ko05160 Hepatitis C;
gene4116	K08679	E5.1.3.6	UDP-glucuronate 4-epimerase	EC:5.1.3.6	2	ko00500 Starch and sucrose metabolism;ko00520 Amino sugar and nucleotide sugar metabolism;
gene41170	K02968	RP-S20, rpsT	small subunit ribosomal protein S20	--	1	ko03010 Ribosome;
gene41171	K01102	PDP	pyruvate dehydrogenase phosphatase	EC:3.1.3.43		

gene41174	K10684	UBLE1A, SAE1	ubiquitin-like 1-activating enzyme E1 A	EC:6.3.2.19	1	ko04120 Ubiquitin mediated proteolysis;
gene41175	K13379	RGP, UTM	reversibly glycosylated polypeptide / UDP-arabinopyranose mutase	EC:2.4.1.- 5.4.99.30		
gene41179	K02998	RP-SAe, RPSA	small subunit ribosomal protein SAe	--	1	ko03010 Ribosome;
gene41183	K01649	leuA	2-isopropylmalate synthase	EC:2.3.3.13	4	ko00290 Valine, leucine and isoleucine biosynthesis;ko00620 Pyruvate metabolism;ko01210 2-Oxocarboxylic acid metabolism;ko01230 Biosynthesis of amino acids;
gene41184	K01649	leuA	2-isopropylmalate synthase	EC:2.3.3.13	4	ko00290 Valine, leucine and isoleucine biosynthesis;ko00620 Pyruvate metabolism;ko01210 2-Oxocarboxylic acid metabolism;ko01230 Biosynthesis of amino acids;
gene41191	K04733	IRAK4	interleukin-1 receptor-associated kinase 4	EC:2.7.11.1	11	ko04064 NF-kappa B signaling pathway;ko04210 Apoptosis;ko04620 Toll-like receptor signaling pathway;ko04722 Neurotrophin signaling pathway;ko05133 Pertussis;ko05140 Leishmaniasis;ko05142 Chagas disease (American trypanosomiasis);ko05145 Toxoplasmosis;ko05152 Tuberculosis;ko05162 Measles;ko05164 Influenza A;
gene41193	K15436	TRPO3, MTR10	transportin-3	--		
gene41196	K15340	DCLRE1A, SNM1A, PSO2	DNA cross-link repair 1A protein	--		
gene41200	K11420	EHMT	euchromatic histone-lysine N-methyltransferase	EC:2.1.1.43	1	ko00310 Lysine degradation;
gene41202	K14493	GID1	gibberellin receptor GID1	EC:3.-.-.-	1	ko04075 Plant hormone signal transduction;
gene41209	K16241	HY5	transcription factor HY5	--	1	ko04712 Circadian rhythm - plant;
gene41210	K09422	MYBP	myb proto-oncogene protein, plant	--		
gene41213	K09286	EREBP	EREBP-like factor	--		
gene41216	K10949	KDELR	ER lumen protein retaining receptor	--	1	ko05110 Vibrio cholerae infection;
gene4121	K09284	AP2	AP2-like factor, euAP2 lineage	--		
gene41220	K03255	TIF31, CLU1	protein TIF31	--		
gene41221	K07297	ADIPOR	adiponectin receptor	--	2	ko04920 Adipocytokine signaling pathway;ko04932 Non-alcoholic fatty liver disease (NAFLD);
gene41226	K08678	UXS1	UDP-glucuronate decarboxylase	EC:4.1.1.35	2	ko00500 Starch and sucrose metabolism;ko00520 Amino sugar and nucleotide sugar metabolism;
gene41228	K00430	E1.11.1.7	peroxidase	EC:1.11.1.7	2	ko00360 Phenylalanine metabolism;ko00940 Phenylpropanoid biosynthesis;
gene41234	K11087	SNRPD1, SMD1	small nuclear ribonucleoprotein D1	--	2	ko03040 Spliceosome;ko05322 Systemic lupus erythematosus;
gene41241	K14724	SLC9A8, NHE8	solute carrier family 9 (sodium/hydrogen exchanger), member 8	--		
gene41251	K14488	SAUR	SAUR family protein	--	1	ko04075 Plant hormone signal transduction;
gene41262	K00430	E1.11.1.7	peroxidase	EC:1.11.1.7	2	ko00360 Phenylalanine metabolism;ko00940 Phenylpropanoid biosynthesis;
gene41266	K06911	K06911				
gene41267	K02995	RP-S8e, RPS8	small subunit ribosomal protein S8e	--	1	ko03010 Ribosome;
gene41268	K01411	NRD1	nardilysin	EC:3.4.24.61		
gene4126	K09264	K09264	MADS-box transcription factor, plant	--		
gene41271	K09419	HSFF	heat shock transcription factor, other eukaryote	--		
gene41273	K01464	E3.5.2.2, DPYS	dihydropyrimidinase	EC:3.5.2.2	4	ko00240 Pyrimidine metabolism;ko00410 beta-Alanine metabolism;ko00770 Pantothenate and CoA biosynthesis;ko00983 Drug metabolism - other enzymes;
gene41275	K01711	gmd, GMDS	GDPmannose 4,6-dehydratase	EC:4.2.1.47	2	ko00051 Fructose and mannose metabolism;ko00520 Amino sugar and nucleotide sugar metabolism;
gene41276	K14431	TGA	transcription factor TGA	--	1	ko04075 Plant hormone signal transduction;
gene41278	K15025	EIF1AD	probable RNA-binding protein EIF1AD	--		
gene41279	K15710	SHPRH	E3 ubiquitin-protein ligase SHPRH	EC:3.6.4.- 6.3.2.19		
gene41281	K12639	CYP724B1, D11	cytochrome P450, family 724, subfamily B, polypeptide 1	EC:1.14.13.-	1	ko00905 Brassinosteroid biosynthesis;
gene41287	K08057	CALR	calreticulin	--	5	ko04141 Protein processing in endoplasmic reticulum;ko04145 Phagosome;ko04612 Antigen processing and presentation;ko05142 Chagas disease (American trypanosomiasis);ko05166 HTLV-I infection;

gene41289	K07198	PRKAA, AMPK	5'-AMP-activated protein kinase, catalytic alpha subunit	EC:2.7.11.11	8	ko04140 Regulation of autophagy;ko04150 mTOR signaling pathway;ko04151 PI3K-Akt signaling pathway;ko04710 Circadian rhythm;ko04910 Insulin signaling pathway;ko04920 Adipocytokine signaling pathway;ko04932 Non-alcoholic fatty liver disease (NAFLD);ko05410 Hypertrophic cardiomyopathy (HCM);
gene41290	K04733	IRAK4	interleukin-1 receptor-associated kinase 4	EC:2.7.11.1	11	ko04064 NF-kappa B signaling pathway;ko04210 Apoptosis;ko04620 Toll-like receptor signaling pathway;ko04722 Neurotrophin signaling pathway;ko05133 Pertussis;ko05140 Leishmaniasis;ko05142 Chagas disease (American trypanosomiasis);ko05145 Toxoplasmosis;ko05152 Tuberculosis;ko05162 Measles;ko05164 Influenza A;
gene41294	K00472	E1.14.11.2	prolyl 4-hydroxylase	EC:1.14.11.2	1	ko00330 Arginine and proline metabolism;
gene412	K15637	PGAM5	serine/threonine-protein phosphatase PGAM5	EC:3.1.3.16	1	ko04668 TNF signaling pathway;
gene41301	K14805	DDX24, MAK5	ATP-dependent RNA helicase DDX24/MAK5	EC:3.6.4.13		
gene41303	K03320	amt, AMT, MEP	ammonium transporter, Amt family	--		
gene41304	K00434	E1.11.1.11	L-ascorbate peroxidase	EC:1.11.1.11	2	ko00053 Ascorbate and aldarate metabolism;ko00480 Glutathione metabolism;
gene41305	K13680	CSLA	beta-mannan synthase	EC:2.4.1.32		
gene41307	K01679	E4.2.1.2B, fumC	fumarate hydratase, class II	EC:4.2.1.2	5	ko00020 Citrate cycle (TCA cycle);ko00720 Carbon fixation pathways in prokaryotes;ko01200 Carbon metabolism;ko05200 Pathways in cancer;ko05211 Renal cell carcinoma;
gene41314	K01528	DNM	dynamin GTPase	EC:3.6.5.5	4	ko04144 Endocytosis;ko04721 Synaptic vesicle cycle;ko04961 Endocrine and other factor-regulated calcium reabsorption;ko05100 Bacterial invasion of epithelial cells;
gene41318	K01188	E3.2.1.21	beta-glucosidase	EC:3.2.1.21	3	ko00460 Cyanoamino acid metabolism;ko00500 Starch and sucrose metabolism;ko00940 Phenylpropanoid biosynthesis;
gene4131	K17108	GBA2	non-lysosomal glucosylceramidase	EC:3.2.1.45	2	ko00511 Other glycan degradation;ko00600 Sphingolipid metabolism;
gene41320	K12741	HNRNPA1_3	heterogeneous nuclear ribonucleoprotein A1/A3	--	1	ko03040 Spliceosome;
gene41324	K02987	RP-S4e, RPS4	small subunit ribosomal protein S4e	--	1	ko03010 Ribosome;
gene41329	K13508	GPAT	glycerol-3-phosphate acyltransferase	EC:2.3.1.15	2	ko00561 Glycerolipid metabolism;ko00564 Glycerophospholipid metabolism;
gene4132	K00383	E1.8.1.7, GSR, gor	glutathione reductase (NADPH)	EC:1.8.1.7	2	ko00480 Glutathione metabolism;ko04918 Thyroid hormone synthesis;
gene41331	K05605	HIBCH	3-hydroxyisobutyryl-CoA hydrolase	EC:3.1.2.4	3	ko00280 Valine, leucine and isoleucine degradation;ko00410 beta-Alanine metabolism;ko00640 Propanoate metabolism;
gene41332	K09843	E1.14.13.93	(+)-abscisic acid 8'-hydroxylase	EC:1.14.13.93	1	ko00906 Carotenoid biosynthesis;
gene41333	K12400	AP4E1	AP-4 complex subunit epsilon-1	--	1	ko04142 Lysosome;
gene41341	K03941	NDUFS8	NADH dehydrogenase (ubiquinone) Fe-S protein 8	EC:1.6.5.3 1.6.99.3	5	ko00190 Oxidative phosphorylation;ko04932 Non-alcoholic fatty liver disease (NAFLD);ko05010 Alzheimer's disease;ko05012 Parkinson's disease;ko05016 Huntington's disease;
gene41343	K12193	VPS24, CHMP3	charged multivesicular body protein 3	--	1	ko04144 Endocytosis;
gene41350	K05389	KCNKF	potassium channel subfamily K, other eukaryote	--		
gene41357	K06669	SMC3, CSPG6	structural maintenance of chromosome 3 (chondroitin sulfate proteoglycan 6)	--	4	ko04110 Cell cycle;ko04111 Cell cycle - yeast;ko04113 Meiosis - yeast;ko04114 Oocyte meiosis;
gene41365	K03353	APC6, CDC16	anaphase-promoting complex subunit 6	--	7	ko04110 Cell cycle;ko04111 Cell cycle - yeast;ko04113 Meiosis - yeast;ko04114 Oocyte meiosis;ko04120 Ubiquitin mediated proteolysis;ko04914 Progesterone-mediated oocyte maturation;ko05166 HTLV-I infection;
gene41367	K05663	ABC.ATM	mitochondrial ABC transporter ATM	--	1	ko02010 ABC transporters;
gene41368	K09422	MYBP	myb proto-oncogene protein, plant	--		
gene4136	K10632	BRAP	BRCA1-associated protein	EC:6.3.2.19	1	ko04014 Ras signaling pathway;
gene41370	K00771	XYLT	protein xylosyltransferase	EC:2.4.2.26	2	ko00532 Glycosaminoglycan biosynthesis - chondroitin sulfate / dermatan sulfate;ko00534 Glycosaminoglycan biosynthesis - heparan sulfate / heparin;
gene41372	K14229	tRNA-Lys	tRNA Lys	--	1	ko00970 Aminoacyl-tRNA biosynthesis;
gene41374	K02965	RP-S19, rpsS	small subunit ribosomal protein S19	--	1	ko03010 Ribosome;
gene41377	K01895	ACSS, acs	acetyl-CoA synthetase	EC:6.2.1.1	6	ko00010 Glycolysis / Gluconeogenesis;ko00620 Pyruvate metabolism;ko00640 Propanoate metabolism;ko00680 Methane metabolism;ko00720 Carbon fixation pathways in prokaryotes;ko01200 Carbon metabolism;
gene4137	K14498	SNRK2	serine/threonine-protein kinase SRK2	EC:2.7.11.1	1	ko04075 Plant hormone signal transduction;
gene41382	K00430	E1.11.1.7	peroxidase	EC:1.11.1.7	2	ko00360 Phenylalanine metabolism;ko00940 Phenylpropanoid biosynthesis;

gene4138	K00430	E1.11.1.7	peroxidase	EC:1.11.1.7	2	ko00360 Phenylalanine metabolism;ko00940 Phenylpropanoid biosynthesis;
gene41397	K00033	E1.1.1.44, PGD, gnd	6-phosphogluconate dehydrogenase	EC:1.1.1.44	3	ko00030 Pentose phosphate pathway;ko00480 Glutathione metabolism;ko01200 Carbon metabolism;
gene41398	K02150	ATPeVE, ATP6E	V-type H+-transporting ATPase subunit E	EC:3.6.3.14	7	ko00190 Oxidative phosphorylation;ko04145 Phagosome;ko04721 Synaptic vesicle cycle;ko04966 Collecting duct acid secretion;ko05110 Vibrio cholerae infection;ko05120 Epithelial cell signaling in Helicobacter pylori infection;ko05323 Rheumatoid arthritis;
gene4139	K00430	E1.11.1.7	peroxidase	EC:1.11.1.7	2	ko00360 Phenylalanine metabolism;ko00940 Phenylpropanoid biosynthesis;
gene41406	K02739	PSMB7	20S proteasome subunit beta 2	EC:3.4.25.1	1	ko03050 Proteasome;
gene41409	K11320	EP400	E1A-binding protein p400	EC:3.6.4.-		
gene41410	K11786	STH1_SNF2	ATP-dependent helicase STH1/SNF2	EC:3.6.4.-		
gene41411	K02935	RP-L7, MRPL12, rplL	large subunit ribosomal protein L7/L12	--	1	ko03010 Ribosome;
gene4141	K11548	NUF2, CDCA1	kinetochore protein Nuf2	--		
gene41424	K12842	SR140	U2-associated protein SR140	--	1	ko03040 Spliceosome;
gene41427	K12603	CNOT6, CCR4	CCR4-NOT transcription complex subunit 6	--	1	ko03018 RNA degradation;
gene4142	K15121	SLC25A44	solute carrier family 25, member 44	--		
gene41430	K14006	SEC23	protein transport protein SEC23	--	1	ko04141 Protein processing in endoplasmic reticulum;
gene41440	K14544	UTP22, NOL6	U3 small nucleolar RNA-associated protein 22	--	1	ko03008 Ribosome biogenesis in eukaryotes;
gene41441	K14569	BMS1	ribosome biogenesis protein BMS1	--	1	ko03008 Ribosome biogenesis in eukaryotes;
gene41443	K17108	GBA2	non-lysosomal glucosylceramidase	EC:3.2.1.45	2	ko00511 Other glycan degradation;ko00600 Sphingolipid metabolism;
gene41444	K00383	E1.8.1.7, GSR, gor	glutathione reductase (NADPH)	EC:1.8.1.7	2	ko00480 Glutathione metabolism;ko04918 Thyroid hormone synthesis;
gene41448	K00861	RFK, FMN1	riboflavin kinase	EC:2.7.1.26	1	ko00740 Riboflavin metabolism;
gene4144	K14684	SLC25A23S	solute carrier family 25 (mitochondrial phosphate transporter), member 23/24/25/41	--		
gene41452	K13484	TTHL	5-hydroxyisourate hydrolase / 2-oxo-4-hydroxy-4-carboxy-5-ureidoimidazoline decarboxylase	EC:3.5.2.17	1	ko00230 Purine metabolism;
gene41453	K05605	HIBCH	3-hydroxyisobutyryl-CoA hydrolase	EC:3.1.2.4	3	ko00280 Valine, leucine and isoleucine degradation;ko00410 beta-Alanine metabolism;ko00640 Propanoate metabolism;
gene41457	K04798	pfdB, PFDN6	prefoldin beta subunit	--		
gene41463	K13416	BAK1	brassinosteroid insensitive 1-associated receptor kinase 1	EC:2.7.10.1 2.7.11.1	2	ko04075 Plant hormone signal transduction;ko04626 Plant-pathogen interaction;
gene41470	K13083	CYP75A	cytochrome P450, family 75, subfamily A (flavonoid 3',5'-hydroxylase)	EC:1.14.13.88	2	ko00941 Flavonoid biosynthesis;ko00944 Flavone and flavonol biosynthesis;
gene41474	K00485	E1.14.13.8	dimethylaniline monooxygenase (N-oxide forming)	EC:1.14.13.8	2	ko00680 Methane metabolism;ko00982 Drug metabolism - cytochrome P450;
gene41475	K03564	BCP, PRXQ, DOT5	peroxiredoxin Q/BCP	EC:1.11.1.15		
gene41477	K13447	RBOH	respiratory burst oxidase	EC:1.6.3.- 1.11.1.-	1	ko04626 Plant-pathogen interaction;
gene41478	K13447	RBOH	respiratory burst oxidase	EC:1.6.3.- 1.11.1.-	1	ko04626 Plant-pathogen interaction;
gene41479	K11000	CALS	callose synthase	EC:2.4.1.-		
gene41480	K14234	tRNA-Thr	tRNA Thr	--	1	ko00970 Aminoacyl-tRNA biosynthesis;
gene41482	K11592	DICER1, DCR1	endoribonuclease Dicer	EC:3.1.26.-	1	ko05206 MicroRNAs in cancer;
gene41484	K09285	OVM, ANT	AP2-like factor, ANT lineage	--		
gene41489	K00831	serC, PSAT1	phosphoserine aminotransferase	EC:2.6.1.52	5	ko00260 Glycine, serine and threonine metabolism;ko00680 Methane metabolism;ko00750 Vitamin B6 metabolism;ko01200 Carbon metabolism;ko01230 Biosynthesis of amino acids;
gene4148	K03531	ftsZ	cell division protein FtsZ	--	1	ko04112 Cell cycle - Caulobacter;
gene41491	K00640	cysE	serine O-acetyltransferase	EC:2.3.1.30	4	ko00270 Cysteine and methionine metabolism;ko00920 Sulfur metabolism;ko01200 Carbon metabolism;ko01230 Biosynthesis of amino acids;
gene41492	K01358	clpP, CLPP	ATP-dependent Clp protease, protease subunit	EC:3.4.21.92	1	ko04112 Cell cycle - Caulobacter;
gene41496	K08770	UBC	ubiquitin C	--	1	ko03320 PPAR signaling pathway;
gene41501	K11592	DICER1, DCR1	endoribonuclease Dicer	EC:3.1.26.-	1	ko05206 MicroRNAs in cancer;
gene41504	K00430	E1.11.1.7	peroxidase	EC:1.11.1.7	2	ko00360 Phenylalanine metabolism;ko00940 Phenylpropanoid biosynthesis;
gene41509	K14487	GH3	auxin responsive GH3 gene family	--	1	ko04075 Plant hormone signal transduction;

gene41516	K13963	SERPINB	serpin B	--	1	ko05146 Amoebiasis;
gene41532	K15382	SLC50A, SWEET	solute carrier family 50 (sugar transporter)	--		
gene41534	K05391	CNGF	cyclic nucleotide gated channel, other eukaryote	--	1	ko04626 Plant-pathogen interaction;
gene41535	K14494	DELLA	DELLA protein	--	1	ko04075 Plant hormone signal transduction;
gene4153	K00327	E1.6.2.4	NADPH-ferrihemoprotein reductase	EC:1.6.2.4		
gene41542	K00975	glgC	glucose-1-phosphate adenylyltransferase	EC:2.7.7.27	2	ko00500 Starch and sucrose metabolism;ko00520 Amino sugar and nucleotide sugar metabolism;
gene41543	K06067	HDAC1_2	histone deacetylase 1/2	EC:3.5.1.98	9	ko04110 Cell cycle;ko04330 Notch signaling pathway;ko05016 Huntington's disease;ko05034 Alcoholism;ko05169 Epstein-Barr virus infection;ko05200 Pathways in cancer;ko05202 Transcriptional misregulation in cancer;ko05203 Viral carcinogenesis;ko05220 Chronic myeloid leukemia;
gene41549	K09286	EREBP	EREBP-like factor	--		
gene41554	K01728	E4.2.2.2, pel	pectate lyase	EC:4.2.2.2	1	ko00040 Pentose and glucuronate interconversions;
gene41565	K08900	BCS1	mitochondrial chaperone BCS1	--		
gene41566	K13998	DHFR-TS	dihydrofolate reductase / thymidylate synthase	EC:1.5.1.3 2.1.1.45	3	ko00240 Pyrimidine metabolism;ko00670 One carbon pool by folate;ko00790 Folate biosynthesis;
gene41567	K11713	PGTB1	geranylgeranyl transferase type-1 subunit beta	EC:2.5.1.59		
gene4156	K02964	RP-S18e, RPS18	small subunit ribosomal protein S18e	--	1	ko03010 Ribosome;
gene41578	K01057	PGLS, pgl, devB	6-phosphogluconolactonase	EC:3.1.1.31	2	ko00030 Pentose phosphate pathway;ko01200 Carbon metabolism;
gene41597	K12857	SNRNP40, PRP8BP	Prp8 binding protein	--	1	ko03040 Spliceosome;
gene41598	K02891	RP-L22e, RPL22	large subunit ribosomal protein L22e	--	1	ko03010 Ribosome;
gene41599	K02155	ATPeVPL, ATP6L	V-type H+-transporting ATPase 16kDa proteolipid subunit	EC:3.6.3.14	9	ko00190 Oxidative phosphorylation;ko04142 Lysosome;ko04145 Phagosome;ko04721 Synaptic vesicle cycle;ko04966 Collecting duct acid secretion;ko05110 Vibrio cholerae infection;ko05120 Epithelial cell signaling in Helicobacter pylori infection;ko05152 Tuberculosis;ko05323 Rheumatoid arthritis;
gene415	K05933	E1.14.17.4	aminocyclopropanecarboxylate oxidase	EC:1.14.17.4	1	ko00270 Cysteine and methionine metabolism;
gene41601	K02221	yggT	YggT family protein	--		
gene41603	K00234	SDHA, SDH1	succinate dehydrogenase (ubiquinone) flavoprotein subunit	EC:1.3.5.1	7	ko00020 Citrate cycle (TCA cycle);ko00190 Oxidative phosphorylation;ko01200 Carbon metabolism;ko04932 Non-alcoholic fatty liver disease (NAFLD);ko05010 Alzheimer's disease;ko05012 Parkinson's disease;ko05016 Huntington's disease;
gene41605	K03239	EIF2B1	translation initiation factor eIF-2B subunit alpha	--	1	ko03013 RNA transport;
gene41606	K00921	PIKFYVE, FAB1	1-phosphatidylinositol-3-phosphate 5-kinase	EC:2.7.1.150	4	ko00562 Inositol phosphate metabolism;ko04070 Phosphatidylinositol signaling system;ko04145 Phagosome;ko04810 Regulation of actin cytoskeleton;
gene41609	K01051	E3.1.1.11	pectinesterase	EC:3.1.1.11	2	ko00040 Pentose and glucuronate interconversions;ko00500 Starch and sucrose metabolism;
gene4160	K14235	tRNA-Trp	tRNA Trp	--	1	ko00970 Aminoacyl-tRNA biosynthesis;
gene41611	K17600	VPS54	vacuolar protein sorting-associated protein 54	--		
gene41612	K17600	VPS54	vacuolar protein sorting-associated protein 54	--		
gene41616	K00454	LOX2S	lipoxygenase	EC:1.13.11.12	2	ko00591 Linoleic acid metabolism;ko00592 alpha-Linolenic acid metabolism;
gene41620	K14488	SAUR	SAUR family protein	--	1	ko04075 Plant hormone signal transduction;
gene41622	K11088	SNRPD3, SMD3	small nuclear ribonucleoprotein D3	--	2	ko03040 Spliceosome;ko05322 Systemic lupus erythematosus;
gene41626	K10590	TRIP12	E3 ubiquitin-protein ligase TRIP12	EC:6.3.2.19	1	ko04120 Ubiquitin mediated proteolysis;
gene41631	K11254	H4	histone H4	--	3	ko05034 Alcoholism;ko05203 Viral carcinogenesis;ko05322 Systemic lupus erythematosus;
gene41633	K03946	NDUFA2	NADH dehydrogenase (ubiquinone) 1 alpha subcomplex subunit 2	--	5	ko00190 Oxidative phosphorylation;ko04932 Non-alcoholic fatty liver disease (NAFLD);ko05010 Alzheimer's disease;ko05012 Parkinson's disease;ko05016 Huntington's disease;
gene41636	K02209	MCM5, CDC46	DNA replication licensing factor MCM5	EC:3.6.4.12	4	ko03030 DNA replication;ko04110 Cell cycle;ko04111 Cell cycle - yeast;ko04113 Meiosis - yeast;
gene41639	K13495	CISZOG	cis-zeatin O-glucosyltransferase	EC:2.4.1.215	1	ko00908 Zeatin biosynthesis;
gene4163	K10257	FAD8, desB	omega-3 fatty acid desaturase (delta-15 desaturase)	EC:1.14.19.-	1	ko01040 Biosynthesis of unsaturated fatty acids;

gene41642	K04371	MAPK1_3	mitogen-activated protein kinase 1/3	EC:2.7.11.24	71	ko04010 MAPK signaling pathway;ko04012 ErbB signaling pathway;ko04013 MAPK signaling pathway - fly;ko04014 Ras signaling pathway;ko04062 Chemokine signaling pathway;ko04066 HIF-1 signaling pathway;ko04114 Oocyte meiosis;ko04150 mTOR signaling pathway;ko04151 PI3K-Akt signaling pathway;ko04270 Vascular smooth muscle contraction;ko04320 Dorso-ventral axis formation;ko04350 TGF-beta signaling pathway;ko04360 Axon guidance;ko04370 VEGF signaling pathway;ko04380 Osteoclast differentiation;ko04510 Focal adhesion;ko04520 Adherens junction;ko04540 Gap junction;ko04620 Toll-like receptor signaling pathway;ko04621 NOD-like receptor signaling pathway;ko04650 Natural killer cell mediated cytotoxicity;ko04660 T cell receptor signaling pathway;ko04662 B cell receptor signaling pathway;ko04664 Fc epsilon RI signaling pathway;ko04666 Fc gamma R-mediated phagocytosis;ko04668 TNF signaling pathway;ko04713 Circadian entrainment;ko04720 Long-term potentiation;ko04722 Neurotrophin signaling pathway;ko04723 Retrograde endocannabinoid signaling;ko04724 Glutamatergic synapse;ko04725 Cholinergic synapse;ko04726 Serotonergic synapse;ko04730 Long-term depression;ko04810 Regulation of actin cytoskeleton;ko04910 Insulin signaling pathway;ko04912 GnRH signaling pathway;ko04914 Progesterone-mediated oocyte maturation;ko04915 Estrogen signaling pathway;ko04916 Melanogenesis;ko04917 Prolactin signaling pathway;ko04930 Type II diabetes mellitus;ko04960 Aldosterone-regulated sodium reabsorption;ko05010 Alzheimer's disease;ko05020 Prion diseases;ko05034 Alcoholism;ko05131 Shigellosis;ko05132 Salmonella infection;ko05133 Pertussis;ko05140 Leishmaniasis;ko05142 Chagas disease (American trypanosomiasis);ko05145 Toxoplasmosis;ko05152 Tuberculosis;ko05160 Hepatitis C;ko05161 Hepatitis B;ko05164 Influenza A;ko05200 Pathways in cancer;ko05203 Viral carcinogenesis;ko05205 Proteoglycans in cancer;ko05210 Colorectal cancer;ko05211 Renal cell carcinoma;ko05212
gene41644	K05016	CLCN7	chloride channel 7	--		
gene41645	K01052	LIPA	lysosomal acid lipase/cholesteryl ester hydrolase	EC:3.1.1.13	2	ko00100 Steroid biosynthesis;ko04142 Lysosome;
gene41650	K09872	PIP	aquaporin PIP	--		
gene41657	K01945	purD	phosphoribosylamine--glycine ligase	EC:6.3.4.13	1	ko00230 Purine metabolism;
gene41658	K13528	MED20	mediator of RNA polymerase II transcription subunit 20	--		
gene41659	K00318	E1.5.99.8	proline dehydrogenase	EC:1.5.99.8	1	ko00330 Arginine and proline metabolism;
gene41663	K11437	PRMT6	protein arginine N-methyltransferase 6	EC:2.1.1.-		
gene41666	K12761	SNF1	carbon catabolite-derepressing protein kinase	EC:2.7.11.1	1	ko04113 Meiosis - yeast;
gene41667	K12382	PSAP, SGP1	saposin	--	1	ko04142 Lysosome;
gene41671	K00108	E1.1.99.1, betA, CHDH	choline dehydrogenase	EC:1.1.99.1	1	ko00260 Glycine, serine and threonine metabolism;
gene41673	K08150	SLC2A13, ITR	MFS transporter, SP family, solute carrier family 2 (myo-inositol transporter), member 13	--		
gene41675	K01641	E2.3.3.10	hydroxymethylglutaryl-CoA synthase	EC:2.3.3.10	4	ko00072 Synthesis and degradation of ketone bodies;ko00280 Valine, leucine and isoleucine degradation;ko00650 Butanoate metabolism;ko00900 Terpenoid backbone biosynthesis;
gene41677	K10770	ALKBH8	alkylated DNA repair protein alkB homolog 8	EC:1.14.11.- 2.1.1.229		
gene41678	K13628	iscA, ISCA1	iron-sulfur cluster assembly protein	--		
gene41684	K13157	RNPC3	U11/U12 small nuclear ribonucleoprotein 65 kDa protein	--		
gene41685	K06963	TAN1, THUMPD1	tRNA acetyltransferase TAN1	--		
gene41686	K06688	UBE2C, UBC11	ubiquitin-conjugating enzyme E2 C	EC:6.3.2.19	1	ko04120 Ubiquitin mediated proteolysis;
gene41687	K11841	USP10, UBP3	ubiquitin carboxyl-terminal hydrolase 10	EC:3.1.2.15		
gene41699	K03118	tatC	sec-independent protein translocase protein TatC	--	2	ko03060 Protein export;ko03070 Bacterial secretion system;
gene41700	K02606	ORC4	origin recognition complex subunit 4	--	3	ko04110 Cell cycle;ko04111 Cell cycle - yeast;ko04113 Meiosis - yeast;

gene41706	K13648	GAUT	alpha-1,4-galacturonosyltransferase	EC:2.4.1.43	2	ko00500 Starch and sucrose metabolism;ko00520 Amino sugar and nucleotide sugar metabolism;
gene41710	K03695	clpB	ATP-dependent Clp protease ATP-binding subunit ClpB	--		
gene41712	K13648	GAUT	alpha-1,4-galacturonosyltransferase	EC:2.4.1.43	2	ko00500 Starch and sucrose metabolism;ko00520 Amino sugar and nucleotide sugar metabolism;
gene41717	K00012	UGDH, ugd	UDPglucose 6-dehydrogenase	EC:1.1.1.22	4	ko00040 Pentose and glucuronate interconversions;ko00053 Ascorbate and aldarate metabolism;ko00500 Starch and sucrose metabolism;ko00520 Amino sugar and nucleotide sugar metabolism;
gene41723	K01595	ppc	phosphoenolpyruvate carboxylase	EC:4.1.1.31	5	ko00620 Pyruvate metabolism;ko00680 Methane metabolism;ko00710 Carbon fixation in photosynthetic organisms;ko00720 Carbon fixation pathways in prokaryotes;ko01200 Carbon metabolism;
gene41724	K08900	BCS1	mitochondrial chaperone BCS1	--		
gene41734	K01792	E5.1.3.15	glucose-6-phosphate 1-epimerase	EC:5.1.3.15	1	ko00010 Glycolysis / Gluconeogenesis;
gene41735	K12188	SNF8, EAP30	ESCRT-II complex subunit VPS22	--	1	ko04144 Endocytosis;
gene41737	K06689	UBE2D_E, UBC4, UBC5	ubiquitin-conjugating enzyme E2 D/E	EC:6.3.2.19	2	ko04120 Ubiquitin mediated proteolysis;ko04141 Protein processing in endoplasmic reticulum;
gene4173	K01051	E3.1.1.11	pectinesterase	EC:3.1.1.11	2	ko00040 Pentose and glucuronate interconversions;ko00500 Starch and sucrose metabolism;
gene41749	K13648	GAUT	alpha-1,4-galacturonosyltransferase	EC:2.4.1.43	2	ko00500 Starch and sucrose metabolism;ko00520 Amino sugar and nucleotide sugar metabolism;
gene41753	K04681	RBL1	retinoblastoma-like protein 1	--	3	ko04110 Cell cycle;ko04350 TGF-beta signaling pathway;ko05203 Viral carcinogenesis;
gene41756	K06442	tlyA	23S rRNA (cytidine1920-2'-O)/16S rRNA (cytidine1409-2'-O)-methyltransferase	EC:2.1.1.226 2.1.1.227		
gene41760	K00549	metE	5-methyltetrahydropteroyltriglutamate--homocysteine methyltransferase	EC:2.1.1.14	3	ko00270 Cysteine and methionine metabolism;ko00450 Selenocompound metabolism;ko01230 Biosynthesis of amino acids;
gene41764	K03320	amt, AMT, MEP	ammonium transporter, Amt family	--		
gene41765	K00121	frmA, ADH5, adhC	S-(hydroxymethyl)glutathione dehydrogenase / alcohol dehydrogenase	EC:1.1.1.284 1.1.1.1	12	ko00010 Glycolysis / Gluconeogenesis;ko00071 Fatty acid degradation;ko00350 Tyrosine metabolism;ko00625 Chloroalkane and chloroalkene degradation;ko00626 Naphthalene degradation;ko00680 Methane metabolism;ko00830 Retinol metabolism;ko00980 Metabolism of xenobiotics by cytochrome P450;ko00982 Drug metabolism - cytochrome P450;ko01200 Carbon metabolism;ko01220 Degradation of aromatic compounds;ko05204 Chemical carcinogenesis;
gene41773	K11643	CHD4, MI2B	chromodomain-helicase-DNA-binding protein 4	EC:3.6.4.12	1	ko05203 Viral carcinogenesis;
gene41779	K12272	SRPRB, SRP102	signal recognition particle receptor subunit beta	--	1	ko03060 Protein export;
gene4177	K11517	HAO	(S)-2-hydroxy-acid oxidase	EC:1.1.3.15	2	ko00630 Glyoxylate and dicarboxylate metabolism;ko04146 Peroxisome;
gene41783	K14497	PP2C	protein phosphatase 2C	EC:3.1.3.16	1	ko04075 Plant hormone signal transduction;
gene41784	K12885	RBMX, HNRNPG	heterogeneous nuclear ribonucleoprotein G	--	1	ko03040 Spliceosome;
gene41787	K13431	SRPR	signal recognition particle receptor subunit alpha	--	1	ko03060 Protein export;
gene41790	K13065	E2.3.1.133, HCT	shikimate O-hydroxycinnamoyltransferase	EC:2.3.1.133	3	ko00940 Phenylpropanoid biosynthesis;ko00941 Flavonoid biosynthesis;ko00945 Stilbenoid, diarylheptanoid and gingerol biosynthesis;
gene41792	K03260	EIF4G	translation initiation factor 4G	--	2	ko03013 RNA transport;ko05416 Viral myocarditis;
gene41793	K00430	E1.11.1.7	peroxidase	EC:1.11.1.7	2	ko00360 Phenylalanine metabolism;ko00940 Phenylpropanoid biosynthesis;
gene41794	K13495	CISZOG	cis-zeatin O-glucosyltransferase	EC:2.4.1.215	1	ko00908 Zeatin biosynthesis;
gene41795	K01412	E3.4.24.64	mitochondrial processing peptidase	EC:3.4.24.64		
gene41800	K10144	RCHY1, PIRH2	RING finger and CHY zinc finger domain-containing protein 1	EC:6.3.2.19	3	ko04115 p53 signaling pathway;ko04120 Ubiquitin mediated proteolysis;ko05162 Measles;
gene41803	K01102	PDP	pyruvate dehydrogenase phosphatase	EC:3.1.3.43		
gene41805	K05931	CARM1, PRMT4	histone-arginine methyltransferase CARM1	EC:2.1.1.125		
gene41806	K03526	E1.17.7.1, gcpE, ispG	(E)-4-hydroxy-3-methylbut-2-enyl-diphosphate synthase	EC:1.17.7.1	1	ko00900 Terpenoid backbone biosynthesis;
gene41808	K03842	ALG1	beta-1,4-mannosyltransferase	EC:2.4.1.142	2	ko00510 N-Glycan biosynthesis;ko00513 Various types of N-glycan biosynthesis;
gene4180	K15322	TSEN2	tRNA-splicing endonuclease subunit Sen2	EC:3.1.27.9		
gene41814	K01061	E3.1.1.45	carboxymethylenebutenolidase	EC:3.1.1.45	3	ko00361 Chlorocyclohexane and chlorobenzene degradation;ko00364 Fluorobenzoate degradation;ko00623 Toluene degradation;
gene41816	K15707	RNF170	RING finger protein 170	--		

gene41819	K13832	aroDE, DHQ-SDH	3-dehydroquinate dehydratase / shikimate dehydrogenase	EC:4.2.1.10 1.1.1.25	2	ko00400 Phenylalanine, tyrosine and tryptophan biosynthesis;ko01230 Biosynthesis of amino acids;
gene41820	K03686	dnaJ	molecular chaperone DnaJ	--		
gene41822	K04125	E1.14.11.13	gibberellin 2-oxidase	EC:1.14.11.13	1	ko00904 Diterpenoid biosynthesis;
gene41824	K01259	pip	proline iminopeptidase	EC:3.4.11.5	1	ko00330 Arginine and proline metabolism;
gene41825	K14503	BZR1_2	brassinosteroid resistant 1/2	--	1	ko04075 Plant hormone signal transduction;
gene41828	K01180	E3.2.1.6	endo-1,3(4)-beta-glucanase	EC:3.2.1.6		
gene41829	K01180	E3.2.1.6	endo-1,3(4)-beta-glucanase	EC:3.2.1.6		
gene41830	K13424	WRKY33	WRKY transcription factor 33	--	1	ko04626 Plant-pathogen interaction;
gene41831	K10581	UBE2O	ubiquitin-conjugating enzyme E2 O	EC:6.3.2.19	1	ko04120 Ubiquitin mediated proteolysis;
gene41839	K17087	TM9SF3	transmembrane 9 superfamily member 3	--		
gene41847	K08738	CYC	cytochrome c	--	19	ko00920 Sulfur metabolism;ko02020 Two-component system;ko04115 p53 signaling pathway;ko04210 Apoptosis;ko04932 Non-alcoholic fatty liver disease (NAFLD);ko05010 Alzheimer's disease;ko05012 Parkinson's disease;ko05014 Amyotrophic lateral sclerosis (ALS);ko05016 Huntington's disease;ko05134 Legionellosis;ko05145 Toxoplasmosis;ko05152 Tuberculosis;ko05161 Hepatitis B;ko05164 Influenza A;ko05168 Herpes simplex infection;ko05200 Pathways in cancer;ko05210 Colorectal cancer;ko05222 Small cell lung cancer;ko05416 Viral myocarditis;
gene41848	K15718	LOX1_5	linoleate 9S-lipoxygenase	EC:1.13.11.58	1	ko00591 Linoleic acid metabolism;
gene4184	K02835	prfA, MTRF1, MRF1	peptide chain release factor 1	--		
gene41850	K00873	PK, pyk	pyruvate kinase	EC:2.7.1.40	7	ko00010 Glycolysis / Gluconeogenesis;ko00230 Purine metabolism;ko00620 Pyruvate metabolism;ko01200 Carbon metabolism;ko01230 Biosynthesis of amino acids;ko04930 Type II diabetes mellitus;ko05203 Viral carcinogenesis;
gene41857	K05759	PFN	profilin	--	3	ko04810 Regulation of actin cytoskeleton;ko05131 Shigellosis;ko05132 Salmonella infection;
gene41860	K10268	FBXL2_20	F-box and leucine-rich repeat protein 2/20	--		
gene41862	K17263	CAND1	cullin-associated NEDD8-dissociated protein 1	--		
gene41864	K05391	CNGF	cyclic nucleotide gated channel, other eukaryote	--	1	ko04626 Plant-pathogen interaction;
gene41867	K11518	TOM40	mitochondrial import receptor subunit TOM40	--	1	ko05014 Amyotrophic lateral sclerosis (ALS);
gene41868	K13288	orn, REX2, REXO2	oligoribonuclease	EC:3.1.-.-	1	ko03008 Ribosome biogenesis in eukaryotes;
gene41870	K03257	EIF4A	translation initiation factor 4A	--	1	ko03013 RNA transport;
gene41871	K13175	THOC6	THO complex subunit 6	--	1	ko03013 RNA transport;
gene41873	K09519	DNAJB13	DnaJ homolog subfamily B member 13	--		
gene41874	K00128	E1.2.1.3	aldehyde dehydrogenase (NAD+)	EC:1.2.1.3	15	ko00010 Glycolysis / Gluconeogenesis;ko00040 Pentose and glucuronate interconversions;ko00053 Ascorbate and aldarate metabolism;ko00071 Fatty acid degradation;ko00280 Valine, leucine and isoleucine degradation;ko00310 Lysine degradation;ko00330 Arginine and proline metabolism;ko00340 Histidine metabolism;ko00380 Tryptophan metabolism;ko00410 beta-Alanine metabolism;ko00561 Glycerolipid metabolism;ko00620 Pyruvate metabolism;ko00625 Chloroalkane and chloroalkene degradation;ko00640 Propanoate metabolism;ko00903 Limonene and pinene
gene41877	K00016	LDH, ldh	L-lactate dehydrogenase	EC:1.1.1.27	4	ko00010 Glycolysis / Gluconeogenesis;ko00270 Cysteine and methionine metabolism;ko00620 Pyruvate metabolism;ko00640 Propanoate metabolism;
gene41878	K06630	YWHAE	14-3-3 protein epsilon	--	8	ko04110 Cell cycle;ko04114 Oocyte meiosis;ko04151 PI3K-Akt signaling pathway;ko04390 Hippo signaling pathway;ko04391 Hippo signaling pathway - fly;ko04722 Neurotrophin signaling pathway;ko05169 Epstein-Barr virus infection;ko05203 Viral carcinogenesis;
gene41882	K00826	E2.6.1.42, ilvE	branched-chain amino acid aminotransferase	EC:2.6.1.42	5	ko00280 Valine, leucine and isoleucine degradation;ko00290 Valine, leucine and isoleucine biosynthesis;ko00770 Pantothenate and CoA biosynthesis;ko01210 2-Oxocarboxylic acid metabolism;ko01230 Biosynthesis of amino acids;

gene41884	K12881	THOC4, ALY	THO complex subunit 4	--	4	ko03013 RNA transport;ko03015 mRNA surveillance pathway;ko03040 Spliceosome;ko05168 Herpes simplex infection;
gene41885	K05305	FUK	fucokinase	EC:2.7.1.52	2	ko00051 Fructose and mannose metabolism;ko00520 Amino sugar and nucleotide sugar metabolism;
gene41886	K11252	H2B	histone H2B	--	3	ko05034 Alcoholism;ko05203 Viral carcinogenesis;ko05322 Systemic lupus erythematosus;
gene4188	K04371	MAPK1_3	mitogen-activated protein kinase 1/3	EC:2.7.11.24	71	ko04010 MAPK signaling pathway;ko04012 ErbB signaling pathway;ko04013 MAPK signaling pathway - fly;ko04014 Ras signaling pathway;ko04062 Chemokine signaling pathway;ko04066 HIF-1 signaling pathway;ko04114 Oocyte meiosis;ko04150 mTOR signaling pathway;ko04151 PI3K-Akt signaling pathway;ko04270 Vascular smooth muscle contraction;ko04320 Dorso-ventral axis formation;ko04350 TGF-beta signaling pathway;ko04360 Axon guidance;ko04370 VEGF signaling pathway;ko04380 Osteoclast differentiation;ko04510 Focal adhesion;ko04520 Adherens junction;ko04540 Gap junction;ko04620 Toll-like receptor signaling pathway;ko04621 NOD-like receptor signaling pathway;ko04650 Natural killer cell mediated cytotoxicity;ko04660 T cell receptor signaling pathway;ko04662 B cell receptor signaling pathway;ko04664 Fc epsilon RI signaling pathway;ko04666 Fc gamma R-mediated phagocytosis;ko04668 TNF signaling pathway;ko04713 Circadian entrainment;ko04720 Long-term potentiation;ko04722 Neurotrophin signaling pathway;ko04723 Retrograde endocannabinoid signaling;ko04724 Glutamatergic synapse;ko04725 Cholinergic synapse;ko04726 Serotonergic synapse;ko04730 Long-term depression;ko04810 Regulation of actin cytoskeleton;ko04910 Insulin signaling pathway;ko04912 GnRH signaling pathway;ko04914 Progesterone-mediated oocyte maturation;ko04915 Estrogen signaling pathway;ko04916 Melanogenesis;ko04917 Prolactin signaling pathway;ko04930 Type II diabetes mellitus;ko04960 Aldosterone-regulated sodium reabsorption;ko05010 Alzheimer's disease;ko05020 Prion diseases;ko05034 Alcoholism;ko05131 Shigellosis;ko05132 Salmonella infection;ko05133 Pertussis;ko05140 Leishmaniasis;ko05142 Chagas disease (American trypanosomiasis);ko05145 Toxoplasmosis;ko05152 Tuberculosis;ko05160 Hepatitis C;ko05161 Hepatitis B;ko05164 Influenza A;ko05200 Pathways in cancer;ko05203 Viral carcinogenesis;ko05205 Proteoglycans in cancer;ko05210 Colorectal cancer;ko05211 Renal cell carcinoma;ko05212
gene41892	K09510	DNAJB4	DnaJ homolog subfamily B member 4	--		
gene4189	K06013	STE24	STE24 endopeptidase	EC:3.4.24.84	1	ko00900 Terpenoid backbone biosynthesis;
gene41903	K09422	MYBP	myb proto-oncogene protein, plant	--		
gene41904	K03283	HSPA1_8	heat shock 70kDa protein 1/8	--	11	ko03040 Spliceosome;ko04010 MAPK signaling pathway;ko04141 Protein processing in endoplasmic reticulum;ko04144 Endocytosis;ko04612 Antigen processing and presentation;ko04915 Estrogen signaling pathway;ko05134 Legionellosis;ko05145 Toxoplasmosis;ko05162 Measles;ko05164 Influenza A;ko05169 Epstein-Barr virus infection;
gene41905	K03252	EIF3C	translation initiation factor 3 subunit C	--	1	ko03013 RNA transport;
gene41908	K04730	IRAK1	interleukin-1 receptor-associated kinase 1	EC:2.7.11.1	11	ko04064 NF-kappa B signaling pathway;ko04210 Apoptosis;ko04620 Toll-like receptor signaling pathway;ko04722 Neurotrophin signaling pathway;ko05133 Pertussis;ko05140 Leishmaniasis;ko05142 Chagas disease (American trypanosomiasis);ko05145 Toxoplasmosis;ko05152 Tuberculosis;ko05162 Measles;ko05169 Epstein-Barr virus infection;
gene41909	K04733	IRAK4	interleukin-1 receptor-associated kinase 4	EC:2.7.11.1	11	ko04064 NF-kappa B signaling pathway;ko04210 Apoptosis;ko04620 Toll-like receptor signaling pathway;ko04722 Neurotrophin signaling pathway;ko05133 Pertussis;ko05140 Leishmaniasis;ko05142 Chagas disease (American trypanosomiasis);ko05145 Toxoplasmosis;ko05152 Tuberculosis;ko05162 Measles;ko05164 Influenza A;

gene41918	K00799	GST, gst	glutathione S-transferase	EC:2.5.1.18	4	ko00480 Glutathione metabolism;ko00980 Metabolism of xenobiotics by cytochrome P450;ko00982 Drug metabolism - cytochrome P450;ko05204 Chemical carcinogenesis;
gene4191	K14563	NOP1, FBL	rRNA 2'-O-methyltransferase fibrillarin	EC:2.1.1.-	1	ko03008 Ribosome biogenesis in eukaryotes;
gene41921	K01738	cysK	cysteine synthase A	EC:2.5.1.47	4	ko00270 Cysteine and methionine metabolism;ko00920 Sulfur metabolism;ko01200 Carbon metabolism;ko01230 Biosynthesis of amino acids;
gene41928	K00799	GST, gst	glutathione S-transferase	EC:2.5.1.18	4	ko00480 Glutathione metabolism;ko00980 Metabolism of xenobiotics by cytochrome P450;ko00982 Drug metabolism - cytochrome P450;ko05204 Chemical carcinogenesis;
gene41929	K00799	GST, gst	glutathione S-transferase	EC:2.5.1.18	4	ko00480 Glutathione metabolism;ko00980 Metabolism of xenobiotics by cytochrome P450;ko00982 Drug metabolism - cytochrome P450;ko05204 Chemical carcinogenesis;
gene4192	K14236	tRNA-Tyr	tRNA Tyr	--	1	ko00970 Aminoacyl-tRNA biosynthesis;
gene41931	K09422	MYBP	myb proto-oncogene protein, plant	--		
gene41932	K09422	MYBP	myb proto-oncogene protein, plant	--		
gene41933	K01951	E6.3.5.2, guaA	GMP synthase (glutamine-hydrolysing)	EC:6.3.5.2	2	ko00230 Purine metabolism;ko00983 Drug metabolism - other enzymes;
gene41937	K10532	HGSNAT	heparan-alpha-glucosaminide N-acetyltransferase	EC:2.3.1.78	2	ko00531 Glycosaminoglycan degradation;ko04142 Lysosome;
gene4193	K11340	ACTL6A, INO80K	actin-like protein 6A	--		
gene41940	K11279	NAP1L1, NRP	nucleosome assembly protein 1-like 1	--		
gene41941	K11423	SETD2, SET2	histone-lysine N-methyltransferase SETD2	EC:2.1.1.43	1	ko00310 Lysine degradation;
gene41944	K15292	STXBP1, MUNC18-1	syntaxin-binding protein 1	--	1	ko04721 Synaptic vesicle cycle;
gene41945	K05954	FNTB	protein farnesyltransferase subunit beta	EC:2.5.1.58	1	ko00900 Terpenoid backbone biosynthesis;
gene41948	K11643	CHD4, MI2B	chromodomain-helicase-DNA-binding protein 4	EC:3.6.4.12	1	ko05203 Viral carcinogenesis;
gene4194	K08852	ERN1	serine/threonine-protein kinase/endoribonuclease IRE1	EC:2.7.11.1	3	ko04141 Protein processing in endoplasmic reticulum;ko04932 Non-alcoholic fatty liver disease (NAFLD);ko05010 Alzheimer's disease;
gene41952	K11000	CALS	callose synthase	EC:2.4.1.-		
gene41954	K00430	E1.11.1.7	peroxidase	EC:1.11.1.7	2	ko00360 Phenylalanine metabolism;ko00940 Phenylpropanoid biosynthesis;
gene4195	K05387	GRIP	glutamate receptor, ionotropic, plant	--		
gene41964	K08343	ATG3	autophagy-related protein 3	--	1	ko04140 Regulation of autophagy;
gene41968	K14230	tRNA-Met	tRNA Met	--	1	ko00970 Aminoacyl-tRNA biosynthesis;
gene4196	K12126	PIF3	phytochrome-interacting factor 3	--	2	ko04075 Plant hormone signal transduction;ko04712 Circadian rhythm - plant;
gene4197	K12126	PIF3	phytochrome-interacting factor 3	--	2	ko04075 Plant hormone signal transduction;ko04712 Circadian rhythm - plant;
gene41985	K14379	ACP5	tartrate-resistant acid phosphatase type 5	EC:3.1.3.2	3	ko00740 Riboflavin metabolism;ko04380 Osteoclast differentiation;ko05323 Rheumatoid arthritis;
gene41989	K09286	EREBP	EREBP-like factor	--		
gene4198	K10765	ALKBH1	alkylated DNA repair protein alkB homolog 1	EC:1.14.11.- 4.2.99.18		
gene41993	K03939	NDUFS6	NADH dehydrogenase (ubiquinone) Fe-S protein 6	--	5	ko00190 Oxidative phosphorylation;ko04932 Non-alcoholic fatty liver disease (NAFLD);ko05010 Alzheimer's disease;ko05012 Parkinson's disease;ko05016 Huntington's disease;
gene41996	K08272	CAB39, MO25	calcium binding protein 39	--	1	ko04150 mTOR signaling pathway;
gene41998	K12847	USP39, SAD1	U4/U6.U5 tri-snRNP-associated protein 2	--	1	ko03040 Spliceosome;
gene42001	K12462	ARHGDI, RHOGDI	Rho GDP-dissociation inhibitor	--	2	ko04722 Neurotrophin signaling pathway;ko04962 Vasopressin-regulated water reabsorption;
gene42004	K10591	NEDD4, RSP5	E3 ubiquitin-protein ligase NEDD4	EC:6.3.2.19	3	ko04120 Ubiquitin mediated proteolysis;ko04144 Endocytosis;ko05169 Epstein-Barr virus infection;
gene42008	K06943	NOG1	nucleolar GTP-binding protein	--	1	ko03008 Ribosome biogenesis in eukaryotes;
gene42009	K01528	DNM	dynamin GTPase	EC:3.6.5.5	4	ko04144 Endocytosis;ko04721 Synaptic vesicle cycle;ko04961 Endocrine and other factor-regulated calcium reabsorption;ko05100 Bacterial invasion of epithelial cells;
gene42012	K10398	KIF11, EG5	kinesin family member 11	--		
gene42013	K12837	U2AF2	splicing factor U2AF 65 kDa subunit	--	1	ko03040 Spliceosome;
gene42024	K15078	SLX1	structure-specific endonuclease subunit SLX1	EC:3.6.1.-	1	ko03460 Fanconi anemia pathway;
gene42025	K14005	SEC31	protein transport protein SEC31	--	1	ko04141 Protein processing in endoplasmic reticulum;
gene42028	K02877	RP-L15e, RPL15	large subunit ribosomal protein L15e	--	1	ko03010 Ribosome;
gene42031	K03921	DESA1	acyl-[acyl-carrier-protein] desaturase	EC:1.14.19.2	2	ko00061 Fatty acid biosynthesis;ko01040 Biosynthesis of unsaturated fatty acids;
gene42038	K08332	VAC8	vacuolar protein 8	--	1	ko04140 Regulation of autophagy;

gene42039	K09874	NIP	aquaporin NIP	--		
gene4204	K00083	E1.1.1.195	cinnamyl-alcohol dehydrogenase	EC:1.1.1.195	1	ko00940 Phenylpropanoid biosynthesis;
gene42050	K14709	SLC39A1_2_3, ZIP1_2_3	solute carrier family 39 (zinc transporter), member 1/2/3	--		
gene42052	K00021	E1.1.1.34	hydroxymethylglutaryl-CoA reductase (NADPH)	EC:1.1.1.34	2	ko00900 Terpenoid backbone biosynthesis;ko04976 Bile secretion;
gene42055	K09264	K09264	MADS-box transcription factor, plant	--		
gene42056	K12349	ASAH2	neutral ceramidase	EC:3.5.1.23	1	ko00600 Sphingolipid metabolism;
gene42057	K00965	galT, GALT	UDPglucose--hexose-1-phosphate uridylyltransferase	EC:2.7.7.12	3	ko00052 Galactose metabolism;ko00520 Amino sugar and nucleotide sugar metabolism;ko04917 Prolactin signaling pathway;
gene42058	K03687	GRPE	molecular chaperone GrpE	--		
gene42062	K04354	PPP2R2	serine/threonine-protein phosphatase 2A regulatory subunit B	--	9	ko03015 mRNA surveillance pathway;ko04111 Cell cycle - yeast;ko04151 PI3K-Akt signaling pathway;ko04390 Hippo signaling pathway;ko04391 Hippo signaling pathway - fly;ko04530 Tight junction;ko04728 Dopaminergic synapse;ko05142 Chagas disease (American trypanosomiasis);ko05160 Hepatitis C;
gene42064	K13157	RNPC3	U11/U12 small nuclear ribonucleoprotein 65 kDa protein	--		
gene42066	K14792	RRP5, PDCD11	rRNA biogenesis protein RRP5	--		
gene42069	K14232	tRNA-Pro	tRNA Pro	--	1	ko00970 Aminoacyl-tRNA biosynthesis;
gene42070	K02935	RP-L7, MRPL12, rplL	large subunit ribosomal protein L7/L12	--	1	ko03010 Ribosome;
gene42074	K14803	PTC2_3	protein phosphatase 2C homolog 2/3	EC:3.1.3.16		
gene42076	K14516	ERF1	ethylene-responsive transcription factor 1	--	1	ko04075 Plant hormone signal transduction;
gene42082	K03124	TFIIB, GTF2B, SUA7, tfb	transcription initiation factor TFIIB	--	3	ko03022 Basal transcription factors;ko05169 Epstein-Barr virus infection;ko05203 Viral carcinogenesis;
gene42083	K03124	TFIIB, GTF2B, SUA7, tfb	transcription initiation factor TFIIB	--	3	ko03022 Basal transcription factors;ko05169 Epstein-Barr virus infection;ko05203 Viral carcinogenesis;
gene42085	K09419	HSFF	heat shock transcription factor, other eukaryote	--		
gene42096	K10405	KIFC1	kinesin family member C1	--		
gene42097	K10695	RNF1_2	E3 ubiquitin-protein ligase RNF1/2	EC:6.3.2.19		
gene42098	K16812	TPX2	targeting protein for Xklp2	--		
gene4209	K00208	fabI	enoyl-[acyl-carrier protein] reductase I	EC:1.3.1.9 1.3.1.10	2	ko00061 Fatty acid biosynthesis;ko00780 Biotin metabolism;
gene42103	K09391	E2F7_8	transcription factor E2F7/8	--		
gene42104	K14004	SEC13	protein transport protein SEC13	--	2	ko03013 RNA transport;ko04141 Protein processing in endoplasmic reticulum;
gene42105	K01231	MAN2	alpha-mannosidase II	EC:3.2.1.114	2	ko00510 N-Glycan biosynthesis;ko00513 Various types of N-glycan biosynthesis;
gene42106	K14638	SLC15A3_4, PHT	solute carrier family 15 (peptide/histidine transporter), member 3/4	--		
gene42115	K03138	TFIIF1, GTF2F1, TFG1	transcription initiation factor TFIIF subunit alpha	--	1	ko03022 Basal transcription factors;
gene42116	K01555	FAH, fahA	fumarylacetoacetase	EC:3.7.1.2	2	ko00350 Tyrosine metabolism;ko00643 Styrene degradation;
gene42118	K03955	NDUFAB1	NADH dehydrogenase (ubiquinone) 1 alpha/beta subcomplex 1	--	5	ko00190 Oxidative phosphorylation;ko04932 Non-alcoholic fatty liver disease (NAFLD);ko05010 Alzheimer's disease;ko05012 Parkinson's disease;ko05016 Huntington's disease;
gene4211	K10666	RNF5	E3 ubiquitin-protein ligase RNF5	EC:6.3.2.19	1	ko04141 Protein processing in endoplasmic reticulum;
gene42128	K01915	glnA	glutamine synthetase	EC:6.3.1.2	8	ko00250 Alanine, aspartate and glutamate metabolism;ko00330 Arginine and proline metabolism;ko00630 Glyoxylate and dicarboxylate metabolism;ko00910 Nitrogen metabolism;ko01230 Biosynthesis of amino acids;ko02020 Two-component system;ko04724 Glutamatergic synapse;ko04727 GABAergic synapse;
gene4212	K05605	HIBCH	3-hydroxyisobutyryl-CoA hydrolase	EC:3.1.2.4	3	ko00280 Valine, leucine and isoleucine degradation;ko00410 beta-Alanine metabolism;ko00640 Propanoate metabolism;
gene42130	K17506	PPM1L, PP2CE	protein phosphatase 1L	EC:3.1.3.16		
gene42133	K10597	UBE4B, UFD2	ubiquitin conjugation factor E4 B	EC:6.3.2.19	2	ko04120 Ubiquitin mediated proteolysis;ko04141 Protein processing in endoplasmic reticulum;
gene42134	K05391	CNGF	cyclic nucleotide gated channel, other eukaryote	--	1	ko04626 Plant-pathogen interaction;
gene42140	K15032	MTERFD	mTERF domain-containing protein, mitochondrial	--		
gene42141	K17087	TM9SF3	transmembrane 9 superfamily member 3	--		
gene42146	K01880	GARS, glyS1	glycyl-tRNA synthetase	EC:6.1.1.14	1	ko00970 Aminoacyl-tRNA biosynthesis;

gene42147	K00411	UQCRFS1, RIP1, petA	ubiquinol-cytochrome c reductase iron-sulfur subunit	EC:1.10.2.2	7	ko00190 Oxidative phosphorylation;ko02020 Two-component system;ko04260 Cardiac muscle contraction;ko04932 Non-alcoholic fatty liver disease (NAFLD);ko05010 Alzheimer's disease;ko05012 Parkinson's disease;ko05016 Huntington's disease;
gene4214	K14209	SLC36A, PAT	solute carrier family 36 (proton-coupled amino acid transporter)	--		
gene42152	K04733	IRAK4	interleukin-1 receptor-associated kinase 4	EC:2.7.11.1	11	ko04064 NF-kappa B signaling pathway;ko04210 Apoptosis;ko04620 Toll-like receptor signaling pathway;ko04722 Neurotrophin signaling pathway;ko05133 Pertussis;ko05140 Leishmaniasis;ko05142 Chagas disease (American trypanosomiasis);ko05145 Toxoplasmosis;ko05152 Tuberculosis;ko05162 Measles;ko05164 Influenza A;
gene42158	K14638	SLC15A3_4, PHT	solute carrier family 15 (peptide/histidine transporter), member 3/4	--		
gene42160	K09489	HSPA4	heat shock 70kDa protein 4	--	1	ko04612 Antigen processing and presentation;
gene42161	K03327	TC.MATE, SLC47A, norM, mdtK, dinF	multidrug resistance protein, MATE family	--		
gene42162	K03327	TC.MATE, SLC47A, norM, mdtK, dinF	multidrug resistance protein, MATE family	--		
gene42163	K03327	TC.MATE, SLC47A, norM, mdtK, dinF	multidrug resistance protein, MATE family	--		
gene42164	K01183	E3.2.1.14	chitinase	EC:3.2.1.14	1	ko00520 Amino sugar and nucleotide sugar metabolism;
gene42168	K16290	XCP	xylem cysteine proteinase	EC:3.4.22.-		
gene4216	K01728	E4.2.2.2, pel	pectate lyase	EC:4.2.2.2	1	ko00040 Pentose and glucuronate interconversions;
gene42171	K07052	K07052				
gene42176	K13963	SERPINB	serpin B	--	1	ko05146 Amoebiasis;
gene42178	K15472	CYP71D55	premnaspirodiene oxygenase	EC:1.14.13.121	1	ko00909 Sesquiterpenoid and triterpenoid biosynthesis;
gene42179	K12883	NCBP2, CBP20	nuclear cap-binding protein subunit 2	--	3	ko03013 RNA transport;ko03015 mRNA surveillance pathway;ko03040 Spliceosome;
gene42182	K02909	RP-L31, rpmE	large subunit ribosomal protein L31	--	1	ko03010 Ribosome;
gene42183	K01915	glnA	glutamine synthetase	EC:6.3.1.2	8	ko00250 Alanine, aspartate and glutamate metabolism;ko00330 Arginine and proline metabolism;ko00630 Glyoxylate and dicarboxylate metabolism;ko00910 Nitrogen metabolism;ko01230 Biosynthesis of amino acids;ko02020 Two-component system;ko04724 Glutamatergic synapse;ko04727 GABAergic synapse;
gene42187	K00006	GPD1	glycerol-3-phosphate dehydrogenase (NAD+)	EC:1.1.1.8	1	ko00564 Glycerophospholipid metabolism;
gene42188	K01930	FPGS	folylpolyglutamate synthase	EC:6.3.2.17	1	ko00790 Folate biosynthesis;
gene42189	K01142	E3.1.11.2, xthA	exodeoxyribonuclease III	EC:3.1.11.2	1	ko03410 Base excision repair;
gene42190	K01001	ALG7	UDP-N-acetylglucosamine--dolichyl-phosphate N-acetylglucosaminephosphotransferase	EC:2.7.8.15	1	ko00510 N-Glycan biosynthesis;
gene42192	K01188	E3.2.1.21	beta-glucosidase	EC:3.2.1.21	3	ko00460 Cyanoamino acid metabolism;ko00500 Starch and sucrose metabolism;ko00940 Phenylpropanoid biosynthesis;
gene42195	K14487	GH3	auxin responsive GH3 gene family	--	1	ko04075 Plant hormone signal transduction;
gene42198	K13347	PXMP2, PMP22	peroxisomal membrane protein 2	--	1	ko04146 Peroxisome;
gene421	K05933	E1.14.17.4	aminocyclopropanecarboxylate oxidase	EC:1.14.17.4	1	ko00270 Cysteine and methionine metabolism;
gene42206	K13960	UBE2T, HSPC150	ubiquitin-conjugating enzyme E2 T	EC:6.3.2.19	1	ko03460 Fanconi anemia pathway;
gene42207	K06041	E5.3.1.13	arabinose-5-phosphate isomerase	EC:5.3.1.13		
gene42209	K12309	GLB1, ELNR1	beta-galactosidase	EC:3.2.1.23	6	ko00052 Galactose metabolism;ko00511 Other glycan degradation;ko00531 Glycosaminoglycan degradation;ko00600 Sphingolipid metabolism;ko00604 Glycosphingolipid biosynthesis - ganglio series;ko04142 Lysosome;
gene42210	K15186	EAF	ELL-associated factor	--		
gene42211	K01358	clpP, CLPP	ATP-dependent Clp protease, protease subunit	EC:3.4.21.92	1	ko04112 Cell cycle - Caulobacter;
gene42212	K00604	MTFMT, fmt	methionyl-tRNA formyltransferase	EC:2.1.2.9	2	ko00670 One carbon pool by folate;ko00970 Aminoacyl-tRNA biosynthesis;
gene42213	K14220	tRNA-Asn	tRNA Asn	--	1	ko00970 Aminoacyl-tRNA biosynthesis;
gene42218	K12890	SFRS1_9	splicing factor, arginine/serine-rich 1/9	--	2	ko03040 Spliceosome;ko05168 Herpes simplex infection;
gene4221	K00430	E1.11.1.7	peroxidase	EC:1.11.1.7	2	ko00360 Phenylalanine metabolism;ko00940 Phenylpropanoid biosynthesis;
gene42224	K11583	PPP2R3	serine/threonine-protein phosphatase 2A regulatory subunit B"	--	3	ko03015 mRNA surveillance pathway;ko04151 PI3K-Akt signaling pathway;ko04728 Dopaminergic synapse;

gene42225	K17525	CHID1	chitinase domain-containing protein 1	--		
gene42227	K00915	IPK2	1D-myo-Inositol-tetrakisphosphate 5-kinase / inositol-polyphosphate multikinase	EC:2.7.1.140 2.7.1.151	2	ko00562 Inositol phosphate metabolism;ko04070 Phosphatidylinositol signaling system;
gene42229	K00517	E1.14.-.-			4	ko00624 Polycyclic aromatic hydrocarbon degradation;ko00627 Aminobenzoate degradation;ko00903 Limonene and pinene degradation;ko00945 Stilbenoid, diarylheptanoid and gingerol biosynthesis;
gene42231	K13811	PAPSS	3'-phosphoadenosine 5'-phosphosulfate synthase	EC:2.7.7.4 2.7.1.25	3	ko00230 Purine metabolism;ko00450 Selenocompound metabolism;ko00920 Sulfur metabolism;
gene42235	K09191	GTF3A	general transcription factor IIIA	--		
gene42237	K11420	EHMT	euchromatic histone-lysine N-methyltransferase	EC:2.1.1.43	1	ko00310 Lysine degradation;
gene42238	K16675	ZDHHC14	probable palmitoyltransferase ZDHHC14	EC:2.3.1.-	1	ko04391 Hippo signaling pathway - fly;
gene42239	K16675	ZDHHC14	probable palmitoyltransferase ZDHHC14	EC:2.3.1.-	1	ko04391 Hippo signaling pathway - fly;
gene42240	K04035	E1.14.13.81, acsF, chlE	magnesium-protoporphyrin IX monomethyl ester (oxidative) cyclase	EC:1.14.13.81	1	ko00860 Porphyrin and chlorophyll metabolism;
gene42241	K02727	PSMA3	20S proteasome subunit alpha 7	EC:3.4.25.1	1	ko03050 Proteasome;
gene4224	K10999	CESA	cellulose synthase A	EC:2.4.1.12		
gene42257	K13459	RPS2	disease resistance protein RPS2	--	1	ko04626 Plant-pathogen interaction;
gene42260	K02896	RP-L24e, RPL24	large subunit ribosomal protein L24e	--	1	ko03010 Ribosome;
gene42265	K01082	E3.1.3.7, cysQ, MET22, BPNT1	3'(2'), 5'-bisphosphate nucleotidase	EC:3.1.3.7	1	ko00920 Sulfur metabolism;
gene42269	K10601	SYVN1, HRD1	E3 ubiquitin-protein ligase synoviolin	EC:6.3.2.19	2	ko04120 Ubiquitin mediated proteolysis;ko04141 Protein processing in endoplasmic reticulum;
gene4226	K10704	UBE2V	ubiquitin-conjugating enzyme E2 variant	--		
gene42270	K07977	ARF	Arf/Sar family, other	--		
gene4227	K06890	K06890				
gene42281	K12818	DHX8, PRP22	ATP-dependent RNA helicase DHX8/PRP22	EC:3.6.4.13	1	ko03040 Spliceosome;
gene42287	K09422	MYBP	myb proto-oncogene protein, plant	--		
gene42295	K17506	PPM1L, PP2CE	protein phosphatase 1L	EC:3.1.3.16		
gene42305	K01054	MGLL	acylglycerol lipase	EC:3.1.1.23	2	ko00561 Glycerolipid metabolism;ko04723 Retrograde endocannabinoid signaling;
gene42306	K00276	E1.4.3.21, AOC2, AOC3, tynA	primary-amine oxidase	EC:1.4.3.21	6	ko00260 Glycine, serine and threonine metabolism;ko00350 Tyrosine metabolism;ko00360 Phenylalanine metabolism;ko00410 beta-Alanine metabolism;ko00950 Isoquinoline alkaloid biosynthesis;ko00960 Tropane, piperidine and pyridine alkaloid biosynthesis;
gene42307	K13137	STRAP, UNRIP	serine-threonine kinase receptor-associated protein	--	1	ko03013 RNA transport;
gene42312	K08272	CAB39, MO25	calcium binding protein 39	--	1	ko04150 mTOR signaling pathway;
gene42314	K14488	SAUR	SAUR family protein	--	1	ko04075 Plant hormone signal transduction;
gene42324	K11136	RTEL1	regulator of telomere elongation helicase 1	EC:3.6.4.12		
gene42327	K13947	PIN	auxin efflux carrier family	--		
gene42328	K08081	E1.1.1.206	tropine dehydrogenase	EC:1.1.1.206	1	ko00960 Tropane, piperidine and pyridine alkaloid biosynthesis;
gene42333	K02738	PSMB6	20S proteasome subunit beta 1	EC:3.4.25.1	1	ko03050 Proteasome;
gene42335	K12486	SMAP	stromal membrane-associated protein	--	1	ko04144 Endocytosis;
gene42339	K01051	E3.1.1.11	pectinesterase	EC:3.1.1.11	2	ko00040 Pentose and glucuronate interconversions;ko00500 Starch and sucrose metabolism;
gene42342	K03457	TC.NCS1	nucleobase:cation symporter-1, NCS1 family	--		
gene42346	K14498	SNRK2	serine/threonine-protein kinase SRK2	EC:2.7.11.1	1	ko04075 Plant hormone signal transduction;
gene42347	K14235	tRNA-Trp	tRNA Trp	--	1	ko00970 Aminoacyl-tRNA biosynthesis;
gene42349	K06694	PSMD10	26S proteasome non-ATPase regulatory subunit 10	--		
gene42357	K14488	SAUR	SAUR family protein	--	1	ko04075 Plant hormone signal transduction;
gene42360	K05277	E1.14.11.19	leucoanthocyanidin dioxygenase	EC:1.14.11.19	1	ko00941 Flavonoid biosynthesis;
gene42361	K15919	HPR2	hydroxypyruvate reductase 2	--	2	ko00260 Glycine, serine and threonine metabolism;ko00630 Glyoxylate and dicarboxylate metabolism;
gene42362	K00020	E1.1.1.31, mmsB	3-hydroxyisobutyrate dehydrogenase	EC:1.1.1.31	1	ko00280 Valine, leucine and isoleucine degradation;
gene42367	K09286	EREBP	EREBP-like factor	--		
gene42373	K06943	NOG1	nucleolar GTP-binding protein	--	1	ko03008 Ribosome biogenesis in eukaryotes;

gene42375	K01528	DNM	dynamin GTPase	EC:3.6.5.5	4	ko04144 Endocytosis;ko04721 Synaptic vesicle cycle;ko04961 Endocrine and other factor-regulated calcium reabsorption;ko05100 Bacterial invasion of epithelial cells;
gene42376	K09841	ABA2	xanthoxin dehydrogenase	EC:1.1.1.288	1	ko00906 Carotenoid biosynthesis;
gene42379	K03842	ALG1	beta-1,4-mannosyltransferase	EC:2.4.1.142	2	ko00510 N-Glycan biosynthesis;ko00513 Various types of N-glycan biosynthesis;
gene4237	K15272	SLC35A1_2_3	solute carrier family 35 (UDP-sugar transporter), member A1/2/3	--		
gene42380	K00889	E2.7.1.68, PIP5K	1-phosphatidylinositol-4-phosphate 5-kinase	EC:2.7.1.68	5	ko00562 Inositol phosphate metabolism;ko04070 Phosphatidylinositol signaling system;ko04144 Endocytosis;ko04666 Fc gamma R-mediated phagocytosis;ko04810 Regulation of actin cytoskeleton;
gene42387	K02150	ATPeVE, ATP6E	V-type H+-transporting ATPase subunit E	EC:3.6.3.14	7	ko00190 Oxidative phosphorylation;ko04145 Phagosome;ko04721 Synaptic vesicle cycle;ko04966 Collecting duct acid secretion;ko05110 Vibrio cholerae infection;ko05120 Epithelial cell signaling in Helicobacter pylori infection;ko05323 Rheumatoid arthritis;
gene42388	K12188	SNF8, EAP30	ESCRT-II complex subunit VPS22	--	1	ko04144 Endocytosis;
gene42389	K06689	UBE2D_E, UBC4, UBC5	ubiquitin-conjugating enzyme E2 D/E	EC:6.3.2.19	2	ko04120 Ubiquitin mediated proteolysis;ko04141 Protein processing in endoplasmic reticulum;
gene42398	K12396	AP3D1	AP-3 complex subunit delta-1	--	1	ko04142 Lysosome;
gene42399	K01892	HARS, hisS	histidyl-tRNA synthetase	EC:6.1.1.21	1	ko00970 Aminoacyl-tRNA biosynthesis;
gene42406	K05391	CNGF	cyclic nucleotide gated channel, other eukaryote	--	1	ko04626 Plant-pathogen interaction;
gene42407	K11827	AP2S1	AP-2 complex subunit sigma-1	--	4	ko04144 Endocytosis;ko04721 Synaptic vesicle cycle;ko04961 Endocrine and other factor-regulated calcium reabsorption;ko05016 Huntington's disease;
gene42409	K13691	SGT1	pathogen-inducible salicylic acid glucosyltransferase	EC:2.4.1.-		
gene42410	K13691	SGT1	pathogen-inducible salicylic acid glucosyltransferase	EC:2.4.1.-		
gene42411	K09422	MYBP	myb proto-oncogene protein, plant	--		
gene42415	K12188	SNF8, EAP30	ESCRT-II complex subunit VPS22	--	1	ko04144 Endocytosis;
gene42416	K02335	DPO1, polA	DNA polymerase I	EC:2.7.7.7	6	ko00230 Purine metabolism;ko00240 Pyrimidine metabolism;ko03030 DNA replication;ko03410 Base excision repair;ko03420 Nucleotide excision repair;ko03440 Homologous recombination;
gene42417	K04733	IRAK4	interleukin-1 receptor-associated kinase 4	EC:2.7.11.1	11	ko04064 NF-kappa B signaling pathway;ko04210 Apoptosis;ko04620 Toll-like receptor signaling pathway;ko04722 Neurotrophin signaling pathway;ko05133 Pertussis;ko05140 Leishmaniasis;ko05142 Chagas disease (American trypanosomiasis);ko05145 Toxoplasmosis;ko05152 Tuberculosis;ko05162 Measles;ko05164 Influenza A;
gene42419	K14009	BCAP31, BAP31	B-cell receptor-associated protein 31	--	1	ko04141 Protein processing in endoplasmic reticulum;
gene42421	K09377	CSRP	cysteine and glycine-rich protein	--		
gene42422	K03564	BCP, PRXQ, DOT5	peroxiredoxin Q/BCP	EC:1.11.1.15		
gene42426	K00826	E2.6.1.42, ilvE	branched-chain amino acid aminotransferase	EC:2.6.1.42	5	ko00280 Valine, leucine and isoleucine degradation;ko00290 Valine, leucine and isoleucine biosynthesis;ko00770 Pantothenate and CoA biosynthesis;ko01210 2-Oxocarboxylic acid metabolism;ko01230 Biosynthesis of amino acids;
gene42427	K12309	GLB1, ELNR1	beta-galactosidase	EC:3.2.1.23	6	ko00052 Galactose metabolism;ko00511 Other glycan degradation;ko00531 Glycosaminoglycan degradation;ko00600 Sphingolipid metabolism;ko00604 Glycosphingolipid biosynthesis - ganglio series;ko04142 Lysosome;
gene42428	K12259	SMOX	spermine oxidase	EC:1.5.3.16		
gene4242	K00703	E2.4.1.21, glgA	starch synthase	EC:2.4.1.21	1	ko00500 Starch and sucrose metabolism;
gene42431	K01956	carA, CPA1	carbamoyl-phosphate synthase small subunit	EC:6.3.5.5	2	ko00240 Pyrimidine metabolism;ko00250 Alanine, aspartate and glutamate metabolism;
gene42432	K00279	E1.5.99.12	cytokinin dehydrogenase	EC:1.5.99.12	1	ko00908 Zeatin biosynthesis;
gene42434	K02911	RP-L32, MRPL32, rpmF	large subunit ribosomal protein L32	--	1	ko03010 Ribosome;
gene42437	K00592	E2.1.1.127	[ribulose-bisphosphate carboxylase]-lysine N-methyltransferase	EC:2.1.1.127		
gene42445	K08504	BET1	blocked early in transport 1	--	1	ko04130 SNARE interactions in vesicular transport;
gene42448	K13065	E2.3.1.133, HCT	shikimate O-hydroxycinnamoyltransferase	EC:2.3.1.133	3	ko00940 Phenylpropanoid biosynthesis;ko00941 Flavonoid biosynthesis;ko00945 Stilbenoid, diarylheptanoid and gingerol biosynthesis;
gene42450	K11876	PSMG2, PAC2	proteasome assembly chaperone 2	--		

gene42466	K08235	E2.4.1.207	xyloglucan:xyloglucosyl transferase	EC:2.4.1.207		
gene4247	K00750	GYG1, GYG2	glycogenin glucosyltransferase	EC:2.4.1.186		
gene42482	K00915	IPK2	1D-myo-Inositol-tetrakisphosphate 5-kinase / inositol-polyphosphate multikinase	EC:2.7.1.140 2.7.1.151	2	ko00562 Inositol phosphate metabolism;ko04070 Phosphatidylinositol signaling system;
gene42483	K15382	SLC50A, SWEET	solute carrier family 50 (sugar transporter)	--		
gene42484	K01087	otsB	trehalose 6-phosphate phosphatase	EC:3.1.3.12	1	ko00500 Starch and sucrose metabolism;
gene42489	K14431	TGA	transcription factor TGA	--	1	ko04075 Plant hormone signal transduction;
gene42490	K08913	LHCB2	light-harvesting complex II chlorophyll a/b binding protein 2	--	1	ko00196 Photosynthesis - antenna proteins;
gene42491	K14300	NUP133	nuclear pore complex protein Nup133	--	1	ko03013 RNA transport;
gene42494	K08511	ATVAMP72	vesicle-associated membrane protein 72	--		
gene42495	K14638	SLC15A3_4, PHT	solute carrier family 15 (peptide/histidine transporter), member 3/4	--		
gene42496	K02049	ABC.SN.A	NitT/TauT family transport system ATP-binding protein	--		
gene42497	K11426	SMYD	SET and MYND domain-containing protein	--		
gene42498	K11426	SMYD	SET and MYND domain-containing protein	--		
gene42499	K11426	SMYD	SET and MYND domain-containing protein	--		
gene42504	K15498	PPP6C	serine/threonine-protein phosphatase 6 catalytic subunit	EC:3.1.3.16		
gene42505	K02866	RP-L10e, RPL10	large subunit ribosomal protein L10e	--	1	ko03010 Ribosome;
gene42506	K02109	ATPF0B, atpF	F-type H+-transporting ATPase subunit b	EC:3.6.3.14	2	ko00190 Oxidative phosphorylation;ko00195 Photosynthesis;
gene42510	K10357	MYO5	myosin V	--		
gene42511	K10143	RFWD2, COP1	E3 ubiquitin-protein ligase RFWD2	EC:6.3.2.19	3	ko04115 p53 signaling pathway;ko04120 Ubiquitin mediated proteolysis;ko04712 Circadian rhythm - plant;
gene42513	K06966	K06966				
gene42515	K04802	PCNA	proliferating cell nuclear antigen	--	7	ko03030 DNA replication;ko03410 Base excision repair;ko03420 Nucleotide excision repair;ko03430 Mismatch repair;ko04110 Cell cycle;ko05161 Hepatitis B;ko05166 HTLV-I infection;
gene42516	K01689	ENO, eno	enolase	EC:4.2.1.11	6	ko00010 Glycolysis / Gluconeogenesis;ko00680 Methane metabolism;ko01200 Carbon metabolism;ko01230 Biosynthesis of amino acids;ko03018 RNA degradation;ko04066 HIF-1 signaling pathway;
gene42518	K13448	CML	calcium-binding protein CML	--	1	ko04626 Plant-pathogen interaction;
gene42519	K00847	E2.7.1.4, scrK	fructokinase	EC:2.7.1.4	3	ko00051 Fructose and mannose metabolism;ko00500 Starch and sucrose metabolism;ko00520 Amino sugar and nucleotide sugar metabolism;
gene42525	K14944	NOVA	RNA-binding protein Nova	--		
gene42526	K11252	H2B	histone H2B	--	3	ko05034 Alcoholism;ko05203 Viral carcinogenesis;ko05322 Systemic lupus erythematosus;
gene42528	K09523	DNAJC3	DnaJ homolog subfamily C member 3	--	2	ko04141 Protein processing in endoplasmic reticulum;ko05164 Influenza A;
gene42529	K09645	CPVL	vitellogenic carboxypeptidase-like protein	EC:3.4.16.-		
gene42530	K09645	CPVL	vitellogenic carboxypeptidase-like protein	EC:3.4.16.-		
gene4253	K00454	LOX2S	lipoxygenase	EC:1.13.11.12	2	ko00591 Linoleic acid metabolism;ko00592 alpha-Linolenic acid metabolism;
gene42540	K08900	BCS1	mitochondrial chaperone BCS1	--		
gene42545	K09422	MYBP	myb proto-oncogene protein, plant	--		
gene42549	K09490	HSPA5, BIP	heat shock 70kDa protein 5	--	4	ko03060 Protein export;ko04141 Protein processing in endoplasmic reticulum;ko04918 Thyroid hormone synthesis;ko05020 Prion diseases;
gene42558	K03347	CUL1, CDC53	cullin 1	--	9	ko04110 Cell cycle;ko04111 Cell cycle - yeast;ko04114 Oocyte meiosis;ko04120 Ubiquitin mediated proteolysis;ko04141 Protein processing in endoplasmic reticulum;ko04310 Wnt signaling pathway;ko04350 TGF-beta signaling pathway;ko04710 Circadian rhythm;ko05168 Herpes simplex infection;
gene42564	K11642	CHD3, MI2A	chromodomain-helicase-DNA-binding protein 3	EC:3.6.4.12		
gene42567	K00981	E2.7.7.41, CDS1, CDS2, cdsA	phosphatidate cytidylyltransferase	EC:2.7.7.41	2	ko00564 Glycerophospholipid metabolism;ko04070 Phosphatidylinositol signaling system;
gene42568	K16075	MRS2, MFM1	magnesium transporter	--		

gene42569	K14638	SLC15A3_4, PHT	solute carrier family 15 (peptide/histidine transporter), member 3/4	--		
gene42574	K09422	MYBP	myb proto-oncogene protein, plant	--		
gene42579	K02739	PSMB7	20S proteasome subunit beta 2	EC:3.4.25.1	1	ko03050 Proteasome;
gene42588	K00999	CDIPT	CDP-diacylglycerol--inositol 3-phosphatidyltransferase	EC:2.7.8.11	3	ko00562 Inositol phosphate metabolism;ko00564 Glycerophospholipid metabolism;ko04070 Phosphatidylinositol signaling system;
gene42589	K11594	DDX3X, bel	ATP-dependent RNA helicase	EC:3.6.4.13	3	ko04622 RIG-I-like receptor signaling pathway;ko05161 Hepatitis B;ko05203 Viral carcinogenesis;
gene4258	K05350	bglB	beta-glucosidase	EC:3.2.1.21	3	ko00460 Cyanoamino acid metabolism;ko00500 Starch and sucrose metabolism;ko00940 Phenylpropanoid biosynthesis;
gene42596	K11252	H2B	histone H2B	--	3	ko05034 Alcoholism;ko05203 Viral carcinogenesis;ko05322 Systemic lupus erythematosus;
gene42598	K11252	H2B	histone H2B	--	3	ko05034 Alcoholism;ko05203 Viral carcinogenesis;ko05322 Systemic lupus erythematosus;
gene4259	K05350	bglB	beta-glucosidase	EC:3.2.1.21	3	ko00460 Cyanoamino acid metabolism;ko00500 Starch and sucrose metabolism;ko00940 Phenylpropanoid biosynthesis;
gene42601	K14496	PYL	abscisic acid receptor PYR/PYL family	--	1	ko04075 Plant hormone signal transduction;
gene42606	K14219	tRNA-Arg	tRNA Arg	--	1	ko00970 Aminoacyl-tRNA biosynthesis;
gene42613	K02933	RP-L6, MRPL6, rplF	large subunit ribosomal protein L6	--	1	ko03010 Ribosome;
gene42620	K13436	PTI1	pto-interacting protein 1	EC:2.7.11.1	1	ko04626 Plant-pathogen interaction;
gene42622	K01507	ppa	inorganic pyrophosphatase	EC:3.6.1.1	1	ko00190 Oxidative phosphorylation;
gene42624	K03715	E2.4.1.46	1,2-diacylglycerol 3-beta-galactosyltransferase	EC:2.4.1.46	1	ko00561 Glycerolipid metabolism;
gene42626	K10576	UBE2H, UBC8	ubiquitin-conjugating enzyme E2 H	EC:6.3.2.19	1	ko04120 Ubiquitin mediated proteolysis;
gene42627	K08247	E2.1.1.12	methionine S-methyltransferase	EC:2.1.1.12	1	ko00450 Selenocompound metabolism;
gene4262	K13495	CISZOG	cis-zeatin O-glucosyltransferase	EC:2.4.1.215	1	ko00908 Zeatin biosynthesis;
gene42630	K15283	SLC35E1	solute carrier family 35, member E1	--		
gene42634	K11835	USP4_11_15, UBP12	ubiquitin carboxyl-terminal hydrolase 4/11/15	EC:3.1.2.15		
gene42635	K05282	E1.14.11.12	gibberellin 20-oxidase	EC:1.14.11.12	1	ko00904 Diterpenoid biosynthesis;
gene42638	K04120	E5.5.1.13	ent-copalyl diphosphate synthase	EC:5.5.1.13	1	ko00904 Diterpenoid biosynthesis;
gene42639	K04121	E4.2.3.19	ent-kaurene synthase	EC:4.2.3.19	1	ko00904 Diterpenoid biosynthesis;
gene42642	K11584	PPP2R5	serine/threonine-protein phosphatase 2A regulatory subunit B'	--	5	ko03015 mRNA surveillance pathway;ko04113 Meiosis - yeast;ko04114 Oocyte meiosis;ko04151 PI3K-Akt signaling pathway;ko04728 Dopaminergic synapse;
gene42644	K01762	ACS	1-aminocyclopropane-1-carboxylate synthase	EC:4.4.1.14	1	ko00270 Cysteine and methionine metabolism;
gene42645	K11792	DDA1	DET1- and DDB1-associated protein 1	--		
gene42646	K04706	PIAS1	E3 SUMO-protein ligase PIAS1	EC:6.3.2.-	3	ko04120 Ubiquitin mediated proteolysis;ko04630 Jak-STAT signaling pathway;ko05160 Hepatitis C;
gene42647	K04706	PIAS1	E3 SUMO-protein ligase PIAS1	EC:6.3.2.-	3	ko04120 Ubiquitin mediated proteolysis;ko04630 Jak-STAT signaling pathway;ko05160 Hepatitis C;
gene42648	K04706	PIAS1	E3 SUMO-protein ligase PIAS1	EC:6.3.2.-	3	ko04120 Ubiquitin mediated proteolysis;ko04630 Jak-STAT signaling pathway;ko05160 Hepatitis C;
gene4264	K14411	MSI	RNA-binding protein Musashi	--	1	ko03015 mRNA surveillance pathway;
gene42651	K08150	SLC2A13, ITR	MFS transporter, SP family, solute carrier family 2 (myo-inositol transporter), member 13	--		
gene42653	K02991	RP-S6e, RPS6	small subunit ribosomal protein S6e	--	6	ko03010 Ribosome;ko04066 HIF-1 signaling pathway;ko04150 mTOR signaling pathway;ko04151 PI3K-Akt signaling pathway;ko04910 Insulin signaling pathway;ko05205 Proteoglycans in cancer;
gene42654	K17499	PPM1G, PP2CG	protein phosphatase 1G	EC:3.1.3.16		
gene42658	K14495	GID2, SLY1	F-box protein GID2	--	1	ko04075 Plant hormone signal transduction;
gene42663	K13356	FAR	fatty acyl-CoA reductase	EC:1.2.1.-	2	ko00073 Cutin, suberine and wax biosynthesis;ko04146 Peroxisome;
gene42665	K05288	PIGO	phosphatidylinositol glycan, class O	--	1	ko00563 Glycosylphosphatidylinositol(GPI)-anchor biosynthesis;
gene42667	K00799	GST, gst	glutathione S-transferase	EC:2.5.1.18	4	ko00480 Glutathione metabolism;ko00980 Metabolism of xenobiotics by cytochrome P450;ko00982 Drug metabolism - cytochrome P450;ko05204 Chemical carcinogenesis;
gene42670	K13126	PABPC	polyadenylate-binding protein	--	3	ko03013 RNA transport;ko03015 mRNA surveillance pathway;ko03018 RNA degradation;
gene42674	K06955	K06955				

gene42675	K17426	MRPL45	large subunit ribosomal protein L45, mitochondrial	--		
gene42680	K17604	ZSWIM3	zinc finger SWIM domain-containing protein 3	--		
gene42684	K10712	ADO	cysteamine dioxygenase	EC:1.13.11.19	1	ko00430 Taurine and hypotaurine metabolism;
gene42689	K02987	RP-S4e, RPS4	small subunit ribosomal protein S4e	--	1	ko03010 Ribosome;
gene42692	K09480	E2.4.1.241	digalactosyldiacylglycerol synthase	EC:2.4.1.241	1	ko00561 Glycerolipid metabolism;
gene42695	K13800	CMPK1, UMPK	UMP-CMP kinase	EC:2.7.4.- 2.7.4.14	1	ko00240 Pyrimidine metabolism;
gene42702	K01213	E3.2.1.67	galacturan 1,4-alpha-galacturonidase	EC:3.2.1.67	2	ko00040 Pentose and glucuronate interconversions;ko00500 Starch and sucrose metabolism;
gene42703	K14686	SLC31A1, CTR1	solute carrier family 31 (copper transporter), member 1	--	1	ko04978 Mineral absorption;
gene42704	K14826	FPR3_4	FK506-binding nuclear protein	EC:5.2.1.8		
gene42706	K10770	ALKBH8	alkylated DNA repair protein alkB homolog 8	EC:1.14.11.- 2.1.1.229		
gene4270	K08245	E3.4.23.40	phytepsin	EC:3.4.23.40		
gene42713	K01551	arsA, ASNA1	arsenite-transporting ATPase	EC:3.6.3.16		
gene42716	K08360	CYB561	cytochrome b-561	--		
gene4271	K08193	SLC17A	MFS transporter, ACS family, solute carrier family 17 (sodium-dependent inorganic phosphate cotransporter), other	--		
gene42725	K09191	GTF3A	general transcription factor IIIA	--		
gene42727	K09872	PIP	aquaporin PIP	--		
gene42728	K09597	SPPL2B	signal peptide peptidase-like 2B	EC:3.4.23.-		
gene4272	K13153	SNRNP25	U11/U12 small nuclear ribonucleoprotein 25 kDa protein	--		
gene42732	K03542	psbS	photosystem II 22kDa protein	--	1	ko00195 Photosynthesis;
gene42737	K09531	DNAJC11	DnaJ homolog subfamily C member 11	--		
gene42739	K16586	HAUS3	HAUS augmin-like complex subunit 3	--		
gene42744	K16276	K16276, BTS	zinc finger protein-like protein	--		
gene42747	K13511	TAZ	monolysocardiolipin acyltransferase	EC:2.3.1.-	1	ko00564 Glycerophospholipid metabolism;
gene42749	K12602	WDR61, REC14, SKI8	WD repeat-containing protein 61	--	1	ko03018 RNA degradation;
gene4274	K14233	tRNA-Ser	tRNA Ser	--	1	ko00970 Aminoacyl-tRNA biosynthesis;
gene42754	K01648	ACLY	ATP citrate (pro-S)-lyase	EC:2.3.3.8	2	ko00020 Citrate cycle (TCA cycle);ko00720 Carbon fixation pathways in prokaryotes;
gene42760	K03083	GSK3B	glycogen synthase kinase 3 beta	EC:2.7.11.26	29	ko04012 ErbB signaling pathway;ko04062 Chemokine signaling pathway;ko04110 Cell cycle;ko04151 PI3K-Akt signaling pathway;ko04310 Wnt signaling pathway;ko04340 Hedgehog signaling pathway;ko04360 Axon guidance;ko04390 Hippo signaling pathway;ko04510 Focal adhesion;ko04660 T cell receptor signaling pathway;ko04662 B cell receptor signaling pathway;ko04711 Circadian rhythm - fly;ko04722 Neurotrophin signaling pathway;ko04728 Dopaminergic synapse;ko04910 Insulin signaling pathway;ko04916 Melanogenesis;ko04917 Prolactin signaling pathway;ko04932 Non-alcoholic fatty liver disease (NAFLD);ko05010 Alzheimer's disease;ko05160 Hepatitis C;ko05162 Measles;ko05164 Influenza A;ko05166 HTLV-I infection;ko05169 Epstein-Barr virus infection;ko05200 Pathways in cancer;ko05210 Colorectal cancer;ko05213 Endometrial cancer;ko05215 Prostate
gene42765	K15627	ASPSCR1, ASPL	tether containing UBX domain for GLUT4	--	1	ko05202 Transcriptional misregulation in cancer;
gene42770	K14376	PAP	poly(A) polymerase	EC:2.7.7.19	1	ko03015 mRNA surveillance pathway;
gene42773	K16296	SCPL-I	serine carboxypeptidase-like clade I	EC:3.4.16.-		
gene42775	K09286	EREBP	EREBP-like factor	--		
gene42781	K14641	APY1_2	apyrase	EC:3.6.1.5	2	ko00230 Purine metabolism;ko00240 Pyrimidine metabolism;
gene42787	K02913	RP-L33, MRPL33, rpmG	large subunit ribosomal protein L33	--	1	ko03010 Ribosome;
gene42788	K10357	MYO5	myosin V	--		
gene42789	K03549	kup	KUP system potassium uptake protein	--		
gene4279	K11654	SMARCA5, SNF2H, ISWI	SWI/SNF-related matrix-associated actin-dependent regulator of chromatin subfamily A member 5	EC:3.6.4.-		

gene42803	K11271	DSCC1, DCC1	sister chromatid cohesion protein DCC1	--		
gene42806	K15404	K15404, CER1	aldehyde decarbonylase	EC:4.1.99.5	1	ko00073 Cutin, suberine and wax biosynthesis;
gene42810	K01623	ALDO	fructose-bisphosphate aldolase, class I	EC:4.1.2.13	7	ko00010 Glycolysis / Gluconeogenesis;ko00030 Pentose phosphate pathway;ko00051 Fructose and mannose metabolism;ko00680 Methane metabolism;ko00710 Carbon fixation in photosynthetic organisms;ko01200 Carbon metabolism;ko01230 Biosynthesis of amino acids;
gene42811	K00876	E2.7.1.48, udk	uridine kinase	EC:2.7.1.48	2	ko00240 Pyrimidine metabolism;ko00983 Drug metabolism - other enzymes;
gene42815	K00051	E1.1.1.82	malate dehydrogenase (NADP+)	EC:1.1.1.82	2	ko00620 Pyruvate metabolism;ko00710 Carbon fixation in photosynthetic organisms;
gene42820	K16280	RGLG	E3 ubiquitin-protein ligase RGLG	EC:6.3.2.19		
gene42824	K03676	grxC, GLRX, GLRX2	glutaredoxin 3	--		
gene42828	K05909	E1.10.3.2				
gene42831	K08857	NEK	NIMA (never in mitosis gene a)-related kinase	EC:2.7.11.1		
gene42832	K08857	NEK	NIMA (never in mitosis gene a)-related kinase	EC:2.7.11.1		
gene42840	K14510	CTR1	serine/threonine-protein kinase CTR1	EC:2.7.11.1	1	ko04075 Plant hormone signal transduction;
gene42844	K07904	RAB11A	Ras-related protein Rab-11A	--	4	ko04144 Endocytosis;ko04961 Endocrine and other factor-regulated calcium reabsorption;ko04962 Vasopressin-regulated water reabsorption;ko04972 Pancreatic secretion;
gene42848	K13448	CML	calcium-binding protein CML	--	1	ko04626 Plant-pathogen interaction;
gene4284	K01814	hisA	phosphoribosylformimino-5-aminoimidazole carboxamide ribotide isomerase	EC:5.3.1.16	2	ko00340 Histidine metabolism;ko01230 Biosynthesis of amino acids;
gene42860	K14454	GOT1	aspartate aminotransferase, cytoplasmic	EC:2.6.1.1	11	ko00250 Alanine, aspartate and glutamate metabolism;ko00270 Cysteine and methionine metabolism;ko00330 Arginine and proline metabolism;ko00350 Tyrosine metabolism;ko00360 Phenylalanine metabolism;ko00400 Phenylalanine, tyrosine and tryptophan biosynthesis;ko00710 Carbon fixation in photosynthetic organisms;ko00950 Isoquinoline alkaloid biosynthesis;ko00960 Tropane, piperidine and pyridine alkaloid biosynthesis;ko01210 2-Oxocarboxylic acid metabolism;ko01230 Biosynthesis of amino acids;
gene42866	K13128	ZCCHC8	zinc finger CCHC domain-containing protein 8	--		
gene42873	K08869	ADCK, ABC1	aarF domain-containing kinase	--		
gene42886	K13116	DDX41, ABS	ATP-dependent RNA helicase DDX41	EC:3.6.4.13		
gene4288	K10355	ACTF	actin, other eukaryote	--		
gene42893	K01179	E3.2.1.4	endoglucanase	EC:3.2.1.4	1	ko00500 Starch and sucrose metabolism;
gene4289	K10355	ACTF	actin, other eukaryote	--		
gene42906	K11092	SNRPA1	U2 small nuclear ribonucleoprotein A'	--	1	ko03040 Spliceosome;
gene42907	K03696	clpC	ATP-dependent Clp protease ATP-binding subunit ClpC	--		
gene42909	K00913	ITPK1	inositol-1,3,4-trisphosphate 5/6-kinase / inositol-tetrakisphosphate 1-kinase	EC:2.7.1.159 2.7.1.134	2	ko00562 Inositol phosphate metabolism;ko04070 Phosphatidylinositol signaling system;
gene42913	K11971	RNF14, ARA54	E3 ubiquitin-protein ligase RNF14	EC:6.3.2.19		
gene42925	K01652	E2.2.1.6L, ilvB, ilvG, ilvI	acetolactate synthase I/II/III large subunit	EC:2.2.1.6	6	ko00290 Valine, leucine and isoleucine biosynthesis;ko00650 Butanoate metabolism;ko00660 C5-Branched dibasic acid metabolism;ko00770 Pantothenate and CoA biosynthesis;ko01210 2-Oxocarboxylic acid metabolism;ko01230 Biosynthesis of amino acids;
gene42935	K09422	MYBP	myb proto-oncogene protein, plant	--		
gene42941	K06966	K06966				
gene42944	K01214	E3.2.1.68	isoamylase	EC:3.2.1.68		
gene42945	K13420	FLS2	LRR receptor-like serine/threonine-protein kinase FLS2	EC:2.7.11.1	1	ko04626 Plant-pathogen interaction;
gene42947	K13420	FLS2	LRR receptor-like serine/threonine-protein kinase FLS2	EC:2.7.11.1	1	ko04626 Plant-pathogen interaction;
gene4294	K02883	RP-L18e, RPL18	large subunit ribosomal protein L18e	--	1	ko03010 Ribosome;
gene42953	K02905	RP-L29e, RPL29	large subunit ribosomal protein L29e	--	1	ko03010 Ribosome;
gene42961	K01304	E3.4.19.3, pcp	pyroglutamyl-peptidase	EC:3.4.19.3		
gene42962	K12827	SF3A3, SAP61, PRP9	splicing factor 3A subunit 3	--	1	ko03040 Spliceosome;
gene42963	K00939	E2.7.4.3, adk	adenylate kinase	EC:2.7.4.3	1	ko00230 Purine metabolism;

gene42964	K01535	E3.6.3.6	H+-transporting ATPase	EC:3.6.3.6	1	ko00190 Oxidative phosphorylation;
gene42965	K02924	RP-L39e, RPL39	large subunit ribosomal protein L39e	--	1	ko03010 Ribosome;
gene42966	K12834	PHF5A	PHD finger-like domain-containing protein 5A	--	1	ko03040 Spliceosome;
gene42977	K14431	TGA	transcription factor TGA	--	1	ko04075 Plant hormone signal transduction;
gene42980	K13095	SF1	splicing factor 1	--		
gene42988	K12669	OST3, OST6	oligosaccharyltransferase complex subunit gamma	--	3	ko00510 N-Glycan biosynthesis;ko00513 Various types of N-glycan biosynthesis;ko04141 Protein processing in endoplasmic reticulum;
gene42992	K10706	SETX, ALS4	senataxin	EC:3.6.4.-		
gene42995	K13754	SLC24A6, NCKX6	solute carrier family 24 (sodium/potassium/calcium exchanger), member 6	--		
gene42996	K10901	BLM, RECQL3, SGS1	bloom syndrome protein	EC:3.6.4.12	2	ko03440 Homologous recombination;ko03460 Fanconi anemia pathway;
gene4299	K07179	RIOK2	RIO kinase 2	EC:2.7.11.1	1	ko03008 Ribosome biogenesis in eukaryotes;
gene43000	K04733	IRAK4	interleukin-1 receptor-associated kinase 4	EC:2.7.11.1	11	ko04064 NF-kappa B signaling pathway;ko04210 Apoptosis;ko04620 Toll-like receptor signaling pathway;ko04722 Neurotrophin signaling pathway;ko05133 Pertussis;ko05140 Leishmaniasis;ko05142 Chagas disease (American trypanosomiasis);ko05145 Toxoplasmosis;ko05152 Tuberculosis;ko05162 Measles;ko05164 Influenza A;
gene43002	K01681	ACO, acnA	aconitate hydratase	EC:4.2.1.3	6	ko00020 Citrate cycle (TCA cycle);ko00630 Glyoxylate and dicarboxylate metabolism;ko00720 Carbon fixation pathways in prokaryotes;ko01200 Carbon metabolism;ko01210 2-Oxocarboxylic acid metabolism;ko01230 Biosynthesis of amino acids;
gene43010	K00700	glgB	1,4-alpha-glucan branching enzyme	EC:2.4.1.18	1	ko00500 Starch and sucrose metabolism;
gene43012	K00029	E1.1.1.40, maeB	malate dehydrogenase (oxaloacetate-decarboxylating)(NADP+)	EC:1.1.1.40	3	ko00620 Pyruvate metabolism;ko00710 Carbon fixation in photosynthetic organisms;ko01200 Carbon metabolism;
gene43016	K15332	TRMT2A	tRNA (uracil-5-)-methyltransferase	--		
gene43018	K00430	E1.11.1.7	peroxidase	EC:1.11.1.7	2	ko00360 Phenylalanine metabolism;ko00940 Phenylpropanoid biosynthesis;
gene4301	K00430	E1.11.1.7	peroxidase	EC:1.11.1.7	2	ko00360 Phenylalanine metabolism;ko00940 Phenylpropanoid biosynthesis;
gene43031	K12893	SFRS4_5_6	splicing factor, arginine/serine-rich 4/5/6	--	2	ko03040 Spliceosome;ko05168 Herpes simplex infection;
gene43034	K08867	WNK, PRKWNK	WNK lysine deficient protein kinase	EC:2.7.11.1		
gene43035	K01051	E3.1.1.11	pectinesterase	EC:3.1.1.11	2	ko00040 Pentose and glucuronate interconversions;ko00500 Starch and sucrose metabolism;
gene4303	K11584	PPP2R5	serine/threonine-protein phosphatase 2A regulatory subunit B'	--	5	ko03015 mRNA surveillance pathway;ko04113 Meiosis - yeast;ko04114 Oocyte meiosis;ko04151 PI3K-Akt signaling pathway;ko04728 Dopaminergic synapse;
gene43041	K01183	E3.2.1.14	chitinase	EC:3.2.1.14	1	ko00520 Amino sugar and nucleotide sugar metabolism;
gene43043	K09286	EREBP	EREBP-like factor	--		
gene43045	K01188	E3.2.1.21	beta-glucosidase	EC:3.2.1.21	3	ko00460 Cyanoamino acid metabolism;ko00500 Starch and sucrose metabolism;ko00940 Phenylpropanoid biosynthesis;
gene43046	K01188	E3.2.1.21	beta-glucosidase	EC:3.2.1.21	3	ko00460 Cyanoamino acid metabolism;ko00500 Starch and sucrose metabolism;ko00940 Phenylpropanoid biosynthesis;
gene43047	K01188	E3.2.1.21	beta-glucosidase	EC:3.2.1.21	3	ko00460 Cyanoamino acid metabolism;ko00500 Starch and sucrose metabolism;ko00940 Phenylpropanoid biosynthesis;
gene43054	K02923	RP-L38e, RPL38	large subunit ribosomal protein L38e	--	1	ko03010 Ribosome;
gene43056	K15382	SLC50A, SWEET	solute carrier family 50 (sugar transporter)	--		
gene43059	K03106	SRP54, ffh	signal recognition particle subunit SRP54	--	2	ko03060 Protein export;ko03070 Bacterial secretion system;
gene43063	K15472	CYP71D55	premnaspirodiene oxygenase	EC:1.14.13.12 1	1	ko00909 Sesquiterpenoid and triterpenoid biosynthesis;
gene43066	K09284	AP2	AP2-like factor, euAP2 lineage	--		
gene43067	K00873	PK, pyk	pyruvate kinase	EC:2.7.1.40	7	ko00010 Glycolysis / Gluconeogenesis;ko00230 Purine metabolism;ko00620 Pyruvate metabolism;ko01200 Carbon metabolism;ko01230 Biosynthesis of amino acids;ko04930 Type II diabetes mellitus;ko05203 Viral carcinogenesis;
gene4306	K14012	SHP1, UBX1, NSFL1C	UBX domain-containing protein 1	--	1	ko04141 Protein processing in endoplasmic reticulum;
gene43074	K15378	SLC45A1_2_4	solute carrier family 45, member 1/2/4	--		
gene43076	K11699	RDR1, RDP1	RNA-dependent RNA polymerase 1	EC:2.7.7.48		

gene43078	K17525	CHID1	chitinase domain-containing protein 1	--		
gene43083	K00430	E1.11.1.7	peroxidase	EC:1.11.1.7	2	ko00360 Phenylalanine metabolism;ko00940 Phenylpropanoid biosynthesis;
gene43084	K03626	EGD2, NACA	nascent polypeptide-associated complex subunit alpha	--		
gene4308	K00895	E2.7.1.90, pfk	pyrophosphate--fructose-6-phosphate 1-phosphotransferase	EC:2.7.1.90	1	ko00051 Fructose and mannose metabolism;
gene43092	K03294	TC.APA	basic amino acid/polyamine antiporter, APA family	--		
gene4309	K14638	SLC15A3_4, PHT	solute carrier family 15 (peptide/histidine transporter), member 3/4	--		
gene430	K13179	DDX18, HAS1	ATP-dependent RNA helicase DDX18/HAS1	EC:3.6.4.13		
gene43103	K02437	gcvH, GCSH	glycine cleavage system H protein	--	1	ko00630 Glyoxylate and dicarboxylate metabolism;
gene43104	K07359	CAMKK	calcium/calmodulin-dependent protein kinase kinase	EC:2.7.11.17	2	ko04920 Adipocytokine signaling pathway;ko05034 Alcoholism;
gene43108	K01802	E5.2.1.8	peptidylprolyl isomerase	EC:5.2.1.8		
gene4310	K00666	K00666	fatty-acyl-CoA synthase	EC:6.2.1.-		
gene43112	K01102	PDP	pyruvate dehydrogenase phosphatase	EC:3.1.3.43		
gene43120	K01358	clpP, CLPP	ATP-dependent Clp protease, protease subunit	EC:3.4.21.92	1	ko04112 Cell cycle - Caulobacter;
gene43121	K08496	GOSR2, BOS1	golgi SNAP receptor complex member 2	--	1	ko04130 SNARE interactions in vesicular transport;
gene43124	K14489	AHK2_3_4	arabidopsis histidine kinase 2/3/4 (cytokinin receptor)	EC:2.7.13.3	1	ko04075 Plant hormone signal transduction;
gene43126	K05275	E1.1.1.65	pyridoxine 4-dehydrogenase	EC:1.1.1.65	1	ko00750 Vitamin B6 metabolism;
gene4312	K00666	K00666	fatty-acyl-CoA synthase	EC:6.2.1.-		
gene43130	K12398	AP3M	AP-3 complex subunit mu	--	1	ko04142 Lysosome;
gene43138	K02838	frr, MRRF, RRF	ribosome recycling factor	--		
gene43139	K11128	GAR1, NOLA1	H/ACA ribonucleoprotein complex subunit 1	--	1	ko03008 Ribosome biogenesis in eukaryotes;
gene4313	K08739	MLH3	DNA mismatch repair protein MLH3	--	1	ko03430 Mismatch repair;
gene43140	K00261	GLUD1_2, gdhA	glutamate dehydrogenase (NAD(P)+)	EC:1.4.1.3	5	ko00250 Alanine, aspartate and glutamate metabolism;ko00330 Arginine and proline metabolism;ko00471 D-Glutamine and D-glutamate metabolism;ko00910 Nitrogen metabolism;ko04964 Proximal tubule bicarbonate reclamation;
gene43141	K11851	USP30	ubiquitin carboxyl-terminal hydrolase 30	EC:3.1.2.15		
gene43142	K12121	PHYB	phytochrome B	--	1	ko04712 Circadian rhythm - plant;
gene43144	K01648	ACLY	ATP citrate (pro-S)-lyase	EC:2.3.3.8	2	ko00020 Citrate cycle (TCA cycle);ko00720 Carbon fixation pathways in prokaryotes;
gene43148	K02991	RP-S6e, RPS6	small subunit ribosomal protein S6e	--	6	ko03010 Ribosome;ko04066 HIF-1 signaling pathway;ko04150 mTOR signaling pathway;ko04151 PI3K-Akt signaling pathway;ko04910 Insulin signaling pathway;ko05205 Proteoglycans in cancer;
gene43149	K05917	CYP51	cytochrome P450, family 51 (sterol 14-demethylase)	EC:1.14.13.70	1	ko00100 Steroid biosynthesis;
gene43152	K13345	PEX12, PAF3	peroxin-12	--	1	ko04146 Peroxisome;
gene43155	K00889	E2.7.1.68, PIP5K	1-phosphatidylinositol-4-phosphate 5-kinase	EC:2.7.1.68	5	ko00562 Inositol phosphate metabolism;ko04070 Phosphatidylinositol signaling system;ko04144 Endocytosis;ko04666 Fc gamma R-mediated phagocytosis;ko04810 Regulation of actin cytoskeleton;
gene43157	K16251	NRPE1	DNA-directed RNA polymerase V subunit 1	EC:2.7.7.6		
gene43162	K13343	PEX14	peroxin-14	--	1	ko04146 Peroxisome;
gene43163	K00099	dxr	1-deoxy-D-xylulose-5-phosphate reductoisomerase	EC:1.1.1.267	1	ko00900 Terpenoid backbone biosynthesis;
gene43165	K02900	RP-L27Ae, RPL27A	large subunit ribosomal protein L27Ae	--	1	ko03010 Ribosome;
gene43168	K14563	NOP1, FBL	rRNA 2'-O-methyltransferase fibrillarin	EC:2.1.1.-	1	ko03008 Ribosome biogenesis in eukaryotes;
gene43170	K03327	TC.MATE, SLC47A, norM, mdtK, dinF	multidrug resistance protein, MATE family	--		
gene43171	K03327	TC.MATE, SLC47A, norM, mdtK, dinF	multidrug resistance protein, MATE family	--		
gene43172	K03327	TC.MATE, SLC47A, norM, mdtK, dinF	multidrug resistance protein, MATE family	--		
gene43174	K16297	SCPL-II	serine carboxypeptidase-like clade II	EC:3.4.16.-		
gene43179	K07466	RFA1, RPA1, rpa	replication factor A1	--	5	ko03030 DNA replication;ko03420 Nucleotide excision repair;ko03430 Mismatch repair;ko03440 Homologous recombination;ko03460 Fanconi anemia pathway;

gene4317	K00485	E1.14.13.8	dimethylaniline monooxygenase (N-oxide forming)	EC:1.14.13.8	2	ko00680 Methane metabolism;ko00982 Drug metabolism - cytochrome P450;
gene43184	K13495	CISZOG	cis-zeatin O-glucosyltransferase	EC:2.4.1.215	1	ko00908 Zeatin biosynthesis;
gene43185	K13495	CISZOG	cis-zeatin O-glucosyltransferase	EC:2.4.1.215	1	ko00908 Zeatin biosynthesis;
gene43187	K11095	SNRPC	U1 small nuclear ribonucleoprotein C	--	1	ko03040 Spliceosome;
gene4318	K00485	E1.14.13.8	dimethylaniline monooxygenase (N-oxide forming)	EC:1.14.13.8	2	ko00680 Methane metabolism;ko00982 Drug metabolism - cytochrome P450;
gene43191	K14232	tRNA-Pro	tRNA Pro	--	1	ko00970 Aminoacyl-tRNA biosynthesis;
gene43193	K13065	E2.3.1.133, HCT	shikimate O-hydroxycinnamoyltransferase	EC:2.3.1.133	3	ko00940 Phenylpropanoid biosynthesis;ko00941 Flavonoid biosynthesis;ko00945 Stilbenoid, diarylheptanoid and gingerol biosynthesis;
gene43198	K02935	RP-L7, MRPL12, rplL	large subunit ribosomal protein L7/L12	--	1	ko03010 Ribosome;
gene4319	K00030	IDH3	isocitrate dehydrogenase (NAD+)	EC:1.1.1.41	4	ko00020 Citrate cycle (TCA cycle);ko01200 Carbon metabolism;ko01210 2-Oxocarboxylic acid metabolism;ko01230 Biosynthesis of amino acids;
gene43204	K14780	DHX37, DHR1	ATP-dependent RNA helicase DHX37/DHR1	EC:3.6.4.13		
gene43209	K09286	EREBP	EREBP-like factor	--		
gene43212	K09527	DNAJC7	DnaJ homolog subfamily C member 7	--		
gene43213	K13448	CML	calcium-binding protein CML	--	1	ko04626 Plant-pathogen interaction;
gene43217	K03768	PPIB, ppiB	peptidyl-prolyl cis-trans isomerase B (cyclophilin B)	EC:5.2.1.8		
gene43218	K05305	FUK	fucokinase	EC:2.7.1.52	2	ko00051 Fructose and mannose metabolism;ko00520 Amino sugar and nucleotide sugar metabolism;
gene43224	K00652	bioF	8-amino-7-oxononanoate synthase	EC:2.3.1.47	1	ko00780 Biotin metabolism;
gene43225	K00344	E1.6.5.5, qor	NADPH2:quinone reductase	EC:1.6.5.5		
gene43233	K07874	RAB1A	Ras-related protein Rab-1A	--	1	ko05134 Legionellosis;
gene43236	K06911	K06911				
gene43237	K09597	SPPL2B	signal peptide peptidase-like 2B	EC:3.4.23.-		
gene43239	K13457	RPM1, RPS3	disease resistance protein RPM1	--	1	ko04626 Plant-pathogen interaction;
gene4323	K14767	UTP3, SAS10	U3 small nucleolar RNA-associated protein 3	--		
gene43243	K05387	GRIP	glutamate receptor, ionotropic, plant	--		
gene43247	K14709	SLC39A1_2_3, ZIP1_2_3	solute carrier family 39 (zinc transporter), member 1/2/3	--		
gene43250	K03253	EIF3B	translation initiation factor 3 subunit B	--	1	ko03013 RNA transport;
gene43251	K00327	E1.6.2.4	NADPH-ferrihemoprotein reductase	EC:1.6.2.4		
gene43261	K01568	E4.1.1.1, pdc	pyruvate decarboxylase	EC:4.1.1.1	1	ko00010 Glycolysis / Gluconeogenesis;
gene43265	K08678	UXS1	UDP-glucuronate decarboxylase	EC:4.1.1.35	2	ko00500 Starch and sucrose metabolism;ko00520 Amino sugar and nucleotide sugar metabolism;
gene43268	K00799	GST, gst	glutathione S-transferase	EC:2.5.1.18	4	ko00480 Glutathione metabolism;ko00980 Metabolism of xenobiotics by cytochrome P450;ko00982 Drug metabolism - cytochrome P450;ko05204 Chemical carcinogenesis;
gene43269	K00799	GST, gst	glutathione S-transferase	EC:2.5.1.18	4	ko00480 Glutathione metabolism;ko00980 Metabolism of xenobiotics by cytochrome P450;ko00982 Drug metabolism - cytochrome P450;ko05204 Chemical carcinogenesis;
gene43272	K13420	FLS2	LRR receptor-like serine/threonine-protein kinase FLS2	EC:2.7.11.1	1	ko04626 Plant-pathogen interaction;
gene43273	K14488	SAUR	SAUR family protein	--	1	ko04075 Plant hormone signal transduction;
gene43276	K10755	RFC2_4	replication factor C subunit 2/4	--	3	ko03030 DNA replication;ko03420 Nucleotide excision repair;ko03430 Mismatch repair;
gene43278	K07198	PRKAA, AMPK	5'-AMP-activated protein kinase, catalytic alpha subunit	EC:2.7.11.11	8	ko04140 Regulation of autophagy;ko04150 mTOR signaling pathway;ko04151 PI3K-Akt signaling pathway;ko04710 Circadian rhythm;ko04910 Insulin signaling pathway;ko04920 Adipocytokine signaling pathway;ko04932 Non-alcoholic fatty liver disease (NAFLD);ko05410 Hypertrophic cardiomyopathy (HCM);
gene43280	K00898	PDK2_3_4	pyruvate dehydrogenase kinase 2/3/4	EC:2.7.11.2		
gene43285	K00995	E2.7.8.5, pgsA, PGS1	CDP-diacylglycerol--glycerol-3-phosphate 3-phosphatidyltransferase	EC:2.7.8.5	1	ko00564 Glycerophospholipid metabolism;
gene43287	K01102	PDP	pyruvate dehydrogenase phosphatase	EC:3.1.3.43		
gene43288	K10523	SPOP	speckle-type POZ protein	--		
gene43289	K09422	MYBP	myb proto-oncogene protein, plant	--		
gene4328	K14819	DUSP12, YVH1	dual specificity phosphatase 12	EC:3.1.3.16 3.1.3.48		

gene43293	K14484	IAA	auxin-responsive protein IAA	--	1	ko04075 Plant hormone signal transduction;
gene43294	K14484	IAA	auxin-responsive protein IAA	--	1	ko04075 Plant hormone signal transduction;
gene43297	K02183	CALM	calmodulin	--	25	ko04014 Ras signaling pathway;ko04020 Calcium signaling pathway;ko04070 Phosphatidylinositol signaling system;ko04114 Oocyte meiosis;ko04270 Vascular smooth muscle contraction;ko04626 Plant-pathogen interaction;ko04713 Circadian entrainment;ko04720 Long-term potentiation;ko04722 Neurotrophin signaling pathway;ko04728 Dopaminergic synapse;ko04740 Olfactory transduction;ko04744 Phototransduction;ko04745 Phototransduction - fly;ko04910 Insulin signaling pathway;ko04912 GnRH signaling pathway;ko04915 Estrogen signaling pathway;ko04916 Melanogenesis;ko04970 Salivary secretion;ko04971 Gastric acid secretion;ko05010 Alzheimer's disease;ko05031 Amphetamine addiction;ko05034 Alcoholism;ko05133 Pertussis;ko05152 Tuberculosis;ko05214 Glioma;
gene4329	K01365	CTSL	cathepsin L	EC:3.4.22.15	5	ko04142 Lysosome;ko04145 Phagosome;ko04612 Antigen processing and presentation;ko05205 Proteoglycans in cancer;ko05323 Rheumatoid arthritis;
gene43300	K02953	RP-S13e, RPS13	small subunit ribosomal protein S13e	--	1	ko03010 Ribosome;
gene43301	K07466	RFA1, RPA1, rpa	replication factor A1	--	5	ko03030 DNA replication;ko03420 Nucleotide excision repair;ko03430 Mismatch repair;ko03440 Homologous recombination;ko03460 Fanconi anemia pathway;
gene43302	K16833	PPP1R2, IPP2	protein phosphatase inhibitor 2	--		
gene43303	K17267	COPG	coatomer protein complex, subunit gamma	--		
gene43304	K12196	VPS4	vacuolar protein-sorting-associated protein 4	--	1	ko04144 Endocytosis;
gene43308	K10355	ACTF	actin, other eukaryote	--		
gene43310	K00033	E1.1.1.44, PGD, gnd	6-phosphogluconate dehydrogenase	EC:1.1.1.44	3	ko00030 Pentose phosphate pathway;ko00480 Glutathione metabolism;ko01200 Carbon metabolism;
gene43318	K14510	CTR1	serine/threonine-protein kinase CTR1	EC:2.7.11.1	1	ko04075 Plant hormone signal transduction;
gene43325	K03062	PSMC1, RPT2	26S proteasome regulatory subunit T2	--	3	ko03050 Proteasome;ko05169 Epstein-Barr virus infection;ko05203 Viral carcinogenesis;
gene4332	K01183	E3.2.1.14	chitinase	EC:3.2.1.14	1	ko00520 Amino sugar and nucleotide sugar metabolism;
gene43333	K03014	RPB6, POLR2F	DNA-directed RNA polymerases I, II, and III subunit RPABC2	--	6	ko00230 Purine metabolism;ko00240 Pyrimidine metabolism;ko03020 RNA polymerase;ko04623 Cytosolic DNA-sensing pathway;ko05016 Huntington's disease;ko05169 Epstein-Barr virus infection;
gene43334	K13025	EIF4A3, FAL1	ATP-dependent RNA helicase	EC:3.6.4.13	3	ko03013 RNA transport;ko03015 mRNA surveillance pathway;ko03040 Spliceosome;
gene43335	K16313	NRK	Nik-related protein kinase	EC:2.7.11.1		
gene43336	K08341	GABARAP, ATG8, LC3	GABA(A) receptor-associated protein (autophagy-related protein 8)	--	2	ko04140 Regulation of autophagy;ko04727 GABAergic synapse;
gene43337	K00721	DPM1	dolichol-phosphate mannosyltransferase	EC:2.4.1.83	1	ko00510 N-Glycan biosynthesis;
gene4333	K01183	E3.2.1.14	chitinase	EC:3.2.1.14	1	ko00520 Amino sugar and nucleotide sugar metabolism;
gene43340	K15032	MTERFD	mTERF domain-containing protein, mitochondrial	--		
gene43341	K14808	DDX54, DBP10	ATP-dependent RNA helicase DDX54/DBP10	EC:3.6.4.13		
gene43343	K03234	EEF2	elongation factor 2	--		
gene43344	K03234	EEF2	elongation factor 2	--		
gene43349	K14233	tRNA-Ser	tRNA Ser	--	1	ko00970 Aminoacyl-tRNA biosynthesis;
gene4334	K12347	SLC11A, NRAMP	natural resistance-associated macrophage protein	--	2	ko04142 Lysosome;ko04978 Mineral absorption;
gene43351	K01620	ltaE	threonine aldolase	EC:4.1.2.5	2	ko00260 Glycine, serine and threonine metabolism;ko01230 Biosynthesis of amino acids;
gene43352	K12860	CDC5L, CDC5, CEF1	pre-mRNA-splicing factor CDC5/CEF1	--	1	ko03040 Spliceosome;
gene43361	K02917	RP-L35Ae, RPL35A	large subunit ribosomal protein L35Ae	--	1	ko03010 Ribosome;
gene43365	K01100	E3.1.3.37	sedoheptulose-bisphosphatase	EC:3.1.3.37	2	ko00710 Carbon fixation in photosynthetic organisms;ko01200 Carbon metabolism;
gene43367	K05909	E1.10.3.2				
gene43368	K14219	tRNA-Arg	tRNA Arg	--	1	ko00970 Aminoacyl-tRNA biosynthesis;
gene43370	K13463	COI-1	coronatine-insensitive protein 1	--	2	ko04075 Plant hormone signal transduction;ko04626 Plant-pathogen interaction;
gene43372	K16223	FT	protein FLOWERING LOCUS T	--	1	ko04712 Circadian rhythm - plant;

gene43375	K01647	CS, gltA	citrate synthase	EC:2.3.3.1	5	ko00020 Citrate cycle (TCA cycle);ko00630 Glyoxylate and dicarboxylate metabolism;ko01200 Carbon metabolism;ko01210 2-Oxocarboxylic acid metabolism;ko01230 Biosynthesis of amino acids;
gene43377	K11000	CALS	callose synthase	EC:2.4.1.-		
gene43379	K13162	PCBP2_3_4	poly(rC)-binding protein 2/3/4	--		
gene43382	K10134	EI24	etoposide-induced 2.4 mRNA	--	1	ko04115 p53 signaling pathway;
gene43385	K12309	GLB1, ELNR1	beta-galactosidase	EC:3.2.1.23	6	ko00052 Galactose metabolism;ko00511 Other glycan degradation;ko00531 Glycosaminoglycan degradation;ko00600 Sphingolipid metabolism;ko00604 Glycosphingolipid biosynthesis - ganglio series;ko04142 Lysosome;
gene43391	K06067	HDAC1_2	histone deacetylase 1/2	EC:3.5.1.98	9	ko04110 Cell cycle;ko04330 Notch signaling pathway;ko05016 Huntington's disease;ko05034 Alcoholism;ko05169 Epstein-Barr virus infection;ko05200 Pathways in cancer;ko05202 Transcriptional misregulation in cancer;ko05203 Viral carcinogenesis;ko05220 Chronic myeloid leukemia;
gene43394	K06067	HDAC1_2	histone deacetylase 1/2	EC:3.5.1.98	9	ko04110 Cell cycle;ko04330 Notch signaling pathway;ko05016 Huntington's disease;ko05034 Alcoholism;ko05169 Epstein-Barr virus infection;ko05200 Pathways in cancer;ko05202 Transcriptional misregulation in cancer;ko05203 Viral carcinogenesis;ko05220 Chronic myeloid leukemia;
gene43397	K13412	CPK	calcium-dependent protein kinase	EC:2.7.11.1	2	ko04626 Plant-pathogen interaction;ko05145 Toxoplasmosis;
gene43398	K13412	CPK	calcium-dependent protein kinase	EC:2.7.11.1	2	ko04626 Plant-pathogen interaction;ko05145 Toxoplasmosis;
gene43400	K00799	GST, gst	glutathione S-transferase	EC:2.5.1.18	4	ko00480 Glutathione metabolism;ko00980 Metabolism of xenobiotics by cytochrome P450;ko00982 Drug metabolism - cytochrome P450;ko05204 Chemical carcinogenesis;
gene43404	K00432	E1.11.1.9	glutathione peroxidase	EC:1.11.1.9	3	ko00480 Glutathione metabolism;ko00590 Arachidonic acid metabolism;ko04918 Thyroid hormone synthesis;
gene43414	K06883	K06883				
gene43421	K04730	IRAK1	interleukin-1 receptor-associated kinase 1	EC:2.7.11.1	11	ko04064 NF-kappa B signaling pathway;ko04210 Apoptosis;ko04620 Toll-like receptor signaling pathway;ko04722 Neurotrophin signaling pathway;ko05133 Pertussis;ko05140 Leishmaniasis;ko05142 Chagas disease (American trypanosomiasis);ko05145 Toxoplasmosis;ko05152 Tuberculosis;ko05162 Measles;ko05169 Epstein-Barr virus infection;
gene43427	K14638	SLC15A3_4, PHT	solute carrier family 15 (peptide/histidine transporter), member 3/4	--		
gene43428	K13496	UGT73C	UDP-glucosyl transferase 73C	EC:2.4.1.-		
gene43435	K06961	KRR1	ribosomal RNA assembly protein	--		
gene43440	K14318	NUP88	nuclear pore complex protein Nup88	--	1	ko03013 RNA transport;
gene4344	K11790	DTL, CDT2, DCAF2	denticleless	--		
gene43453	K12877	MAGOH	protein mago nashi	--	3	ko03013 RNA transport;ko03015 mRNA surveillance pathway;ko03040 Spliceosome;
gene43455	K02605	ORC3	origin recognition complex subunit 3	--	3	ko04110 Cell cycle;ko04111 Cell cycle - yeast;ko04113 Meiosis - yeast;
gene43461	K08066	NFYC	nuclear transcription factor Y, gamma	--	2	ko04612 Antigen processing and presentation;ko05152 Tuberculosis;
gene43465	K02984	RP-S3Ae, RPS3A	small subunit ribosomal protein S3Ae	--	1	ko03010 Ribosome;
gene43467	K00737	MGAT3	beta-1,4-mannosyl-glycoprotein beta-1,4-N-acetylglucosaminyltransferase	EC:2.4.1.144	1	ko00510 N-Glycan biosynthesis;
gene43468	K14484	IAA	auxin-responsive protein IAA	--	1	ko04075 Plant hormone signal transduction;
gene43472	K01930	FPGS	folylpolyglutamate synthase	EC:6.3.2.17	1	ko00790 Folate biosynthesis;
gene43481	K03327	TC.MATE, SLC47A, norM, mdtK, dinF	multidrug resistance protein, MATE family	--		
gene43482	K12893	SFRS4_5_6	splicing factor, arginine/serine-rich 4/5/6	--	2	ko03040 Spliceosome;ko05168 Herpes simplex infection;
gene43488	K04523	UBQLN, DSK2	ubiquilin	--	1	ko04141 Protein processing in endoplasmic reticulum;
gene43489	K02684	PRI1	DNA primase small subunit	EC:2.7.7.-	3	ko00230 Purine metabolism;ko00240 Pyrimidine metabolism;ko03030 DNA replication;
gene43490	K09286	EREBP	EREBP-like factor	--		
gene43492	K13210	FUBP	far upstream element-binding protein	--		
gene43495	K01307	GGH	gamma-glutamyl hydrolase	EC:3.4.19.9	1	ko00790 Folate biosynthesis;
gene434	K07305	msrB	peptide-methionine (R)-S-oxide reductase	EC:1.8.4.12		

gene43502	K03327	TC.MATE, SLC47A, norM, mdtK, dinF	multidrug resistance protein, MATE family	--		
gene43505	K04043	dnaK	molecular chaperone DnaK	--	2	ko03018 RNA degradation;ko05152 Tuberculosis;
gene43506	K09422	MYBP	myb proto-oncogene protein, plant	--		
gene43515	K01184	E3.2.1.15	polygalacturonase	EC:3.2.1.15	2	ko00040 Pentose and glucuronate interconversions;ko00500 Starch and sucrose metabolism;
gene43516	K01184	E3.2.1.15	polygalacturonase	EC:3.2.1.15	2	ko00040 Pentose and glucuronate interconversions;ko00500 Starch and sucrose metabolism;
gene43517	K01184	E3.2.1.15	polygalacturonase	EC:3.2.1.15	2	ko00040 Pentose and glucuronate interconversions;ko00500 Starch and sucrose metabolism;
gene43519	K15176	CTR9	RNA polymerase-associated protein CTR9	--		
gene43520	K02962	RP-S17e, RPS17	small subunit ribosomal protein S17e	--	1	ko03010 Ribosome;
gene43523	K01824	EBP	cholestenol delta-isomerase	EC:5.3.3.5	1	ko00100 Steroid biosynthesis;
gene43524	K13105	PRCC	proline-rich protein PRCC	--	1	ko05202 Transcriptional misregulation in cancer;
gene43532	K15193	SPTY2D1, SPT2	protein SPT2	--		
gene43533	K13993	HSP20	HSP20 family protein	--	1	ko04141 Protein processing in endoplasmic reticulum;
gene43534	K09419	HSFF	heat shock transcription factor, other eukaryote	--		
gene43535	K15280	SLC35C2	solute carrier family 35, member C2	--		
gene43537	K09584	PDIA6, TXNDC7	protein disulfide-isomerase A6	EC:5.3.4.1	1	ko04141 Protein processing in endoplasmic reticulum;
gene43544	K00658	DLST, sucB	2-oxoglutarate dehydrogenase E2 component (dihydrolipoamide succinyltransferase)	EC:2.3.1.61	3	ko00020 Citrate cycle (TCA cycle);ko00310 Lysine degradation;ko01200 Carbon metabolism;
gene4354	K05909	E1.10.3.2				
gene43559	K01945	purD	phosphoribosylamine--glycine ligase	EC:6.3.4.13	1	ko00230 Purine metabolism;
gene43561	K01897	ACSL, fadD	long-chain acyl-CoA synthetase	EC:6.2.1.3	4	ko00071 Fatty acid degradation;ko03320 PPAR signaling pathway;ko04146 Peroxisome;ko04920 Adipocytokine signaling pathway;
gene4356	K04382	PPP2C	serine/threonine-protein phosphatase 2A catalytic subunit	EC:3.1.3.16	13	ko03015 mRNA surveillance pathway;ko04111 Cell cycle - yeast;ko04113 Meiosis - yeast;ko04114 Oocyte meiosis;ko04151 PI3K-Akt signaling pathway;ko04350 TGF-beta signaling pathway;ko04390 Hippo signaling pathway;ko04391 Hippo signaling pathway - fly;ko04530 Tight junction;ko04728 Dopaminergic synapse;ko04730 Long-term depression;ko05142 Chagas disease (American trypanosomiasis);ko05160 Hepatitis C;
gene43572	K11968	ARIH1	ariadne-1	--		
gene43573	K09338	HD-ZIP	homeobox-leucine zipper protein	--		
gene43578	K09560	ST13	suppressor of tumorigenicity protein 13	--		
gene43579	K09422	MYBP	myb proto-oncogene protein, plant	--		
gene4357	K00721	DPM1	dolichol-phosphate mannosyltransferase	EC:2.4.1.83	1	ko00510 N-Glycan biosynthesis;
gene43581	K01881	PARS, proS	prolyl-tRNA synthetase	EC:6.1.1.15	1	ko00970 Aminoacyl-tRNA biosynthesis;
gene43583	K02469	gyrA	DNA gyrase subunit A	EC:5.99.1.3		
gene43584	K12356	UGT72E	coniferyl-alcohol glucosyltransferase	EC:2.4.1.111	1	ko00940 Phenylpropanoid biosynthesis;
gene43585	K00294	E1.2.1.88	1-pyrroline-5-carboxylate dehydrogenase	EC:1.2.1.88	2	ko00250 Alanine, aspartate and glutamate metabolism;ko00330 Arginine and proline metabolism;
gene4359	K05282	E1.14.11.12	gibberellin 20-oxidase	EC:1.14.11.12	1	ko00904 Diterpenoid biosynthesis;
gene43602	K03363	CDC20	cell division cycle 20, cofactor of APC complex	--	7	ko04110 Cell cycle;ko04111 Cell cycle - yeast;ko04113 Meiosis - yeast;ko04114 Oocyte meiosis;ko04120 Ubiquitin mediated proteolysis;ko05166 HTLV-I infection;ko05203 Viral carcinogenesis;

gene43608	K02183	CALM	calmodulin	--	25	ko04014 Ras signaling pathway;ko04020 Calcium signaling pathway;ko04070 Phosphatidylinositol signaling system;ko04114 Oocyte meiosis;ko04270 Vascular smooth muscle contraction;ko04626 Plant-pathogen interaction;ko04713 Circadian entrainment;ko04720 Long-term potentiation;ko04722 Neurotrophin signaling pathway;ko04728 Dopaminergic synapse;ko04740 Olfactory transduction;ko04744 Phototransduction;ko04745 Phototransduction - fly;ko04910 Insulin signaling pathway;ko04912 GnRH signaling pathway;ko04915 Estrogen signaling pathway;ko04916 Melanogenesis;ko04970 Salivary secretion;ko04971 Gastric acid secretion;ko05010 Alzheimer's disease;ko05031 Amphetamine addiction;ko05034 Alcoholism;ko05133 Pertussis;ko05152 Tuberculosis;ko05214 Glioma;
gene43609	K15601	KDM3	lysine-specific demethylase 3	EC:1.14.11.-		
gene43612	K00013	hisD	histidinol dehydrogenase	EC:1.1.1.23	2	ko00340 Histidine metabolism;ko01230 Biosynthesis of amino acids;
gene43615	K01982	LSUrRNA	large subunit ribosomal RNA	--	2	ko03008 Ribosome biogenesis in eukaryotes;ko03010 Ribosome;
gene43616	K14219	tRNA-Arg	tRNA Arg	--	1	ko00970 Aminoacyl-tRNA biosynthesis;
gene43620	K00413	CYC1, CYT1, petC	ubiquinol-cytochrome c reductase cytochrome c1 subunit	--	7	ko00190 Oxidative phosphorylation;ko02020 Two-component system;ko04260 Cardiac muscle contraction;ko04932 Non-alcoholic fatty liver disease (NAFLD);ko05010 Alzheimer's disease;ko05012 Parkinson's disease;ko05016 Huntington's disease;
gene43623	K13348	MPV17	protein Mpv17	--	1	ko04146 Peroxisome;
gene43626	K00847	E2.7.1.4, scrK	fructokinase	EC:2.7.1.4	3	ko00051 Fructose and mannose metabolism;ko00500 Starch and sucrose metabolism;ko00520 Amino sugar and nucleotide sugar metabolism;
gene43628	K09422	MYBP	myb proto-oncogene protein, plant	--		
gene43630	K14821	BUD20	bud site selection protein 20	--		
gene43633	K03945	NDUFA1	NADH dehydrogenase (ubiquinone) 1 alpha subcomplex subunit 1	--	5	ko00190 Oxidative phosphorylation;ko04932 Non-alcoholic fatty liver disease (NAFLD);ko05010 Alzheimer's disease;ko05012 Parkinson's disease;ko05016 Huntington's disease;
gene43636	K14945	QKI	protein quaking	--		
gene43642	K04688	RPS6KB	p70 ribosomal S6 kinase	EC:2.7.11.1	9	ko04012 ErbB signaling pathway;ko04066 HIF-1 signaling pathway;ko04150 mTOR signaling pathway;ko04151 PI3K-Akt signaling pathway;ko04350 TGF-beta signaling pathway;ko04666 Fc gamma R-mediated phagocytosis;ko04910 Insulin signaling pathway;ko05205 Proteoglycans in cancer;ko05221 Acute myeloid leukemia;
gene43644	K14802	DRS2, ATP8A	phospholipid-transporting ATPase	EC:3.6.3.1		
gene43645	K00510	HMOX, hmuO, ho	heme oxygenase	EC:1.14.99.3	2	ko00860 Porphyrin and chlorophyll metabolism;ko04978 Mineral absorption;
gene43648	K07936	RAN	GTP-binding nuclear protein Ran	--	4	ko03008 Ribosome biogenesis in eukaryotes;ko03013 RNA transport;ko05166 HTLV-I infection;ko05169 Epstein-Barr virus infection;
gene43649	K03283	HSPA1_8	heat shock 70kDa protein 1/8	--	11	ko03040 Spliceosome;ko04010 MAPK signaling pathway;ko04141 Protein processing in endoplasmic reticulum;ko04144 Endocytosis;ko04612 Antigen processing and presentation;ko04915 Estrogen signaling pathway;ko05134 Legionellosis;ko05145 Toxoplasmosis;ko05162 Measles;ko05164 Influenza A;ko05169 Epstein-Barr virus infection;
gene43653	K12830	SF3B3, SAP130, RSE1	splicing factor 3B subunit 3	--	1	ko03040 Spliceosome;
gene43661	K00921	PIKFYVE, FAB1	1-phosphatidylinositol-3-phosphate 5-kinase	EC:2.7.1.150	4	ko00562 Inositol phosphate metabolism;ko04070 Phosphatidylinositol signaling system;ko04145 Phagosome;ko04810 Regulation of actin cytoskeleton;
gene43662	K16302	CNNM	metal transporter CNNM	--		
gene43663	K11254	H4	histone H4	--	3	ko05034 Alcoholism;ko05203 Viral carcinogenesis;ko05322 Systemic lupus erythematosus;
gene43666	K10875	RAD54L, RAD54	DNA repair and recombination protein RAD54 and RAD54-like protein	EC:3.6.4.-	1	ko03440 Homologous recombination;
gene43667	K03260	EIF4G	translation initiation factor 4G	--	2	ko03013 RNA transport;ko05416 Viral myocarditis;
gene43670	K09338	HD-ZIP	homeobox-leucine zipper protein	--		
gene43671	K10357	MYO5	myosin V	--		
gene43678	K08873	SMG1	PI-3-kinase-related kinase SMG-1	--	1	ko03015 mRNA surveillance pathway;

gene43680	K17362	ACOT13	acyl-coenzyme A thioesterase 13	EC:3.1.2.-		
gene43681	K17362	ACOT13	acyl-coenzyme A thioesterase 13	EC:3.1.2.-		
gene43682	K17362	ACOT13	acyl-coenzyme A thioesterase 13	EC:3.1.2.-		
gene43684	K04802	PCNA	proliferating cell nuclear antigen	--	7	ko03030 DNA replication;ko03410 Base excision repair;ko03420 Nucleotide excision repair;ko03430 Mismatch repair;ko04110 Cell cycle;ko05161 Hepatitis B;ko05166 HTLV-I infection;
gene43685	K01689	ENO, eno	enolase	EC:4.2.1.11	6	ko00010 Glycolysis / Gluconeogenesis;ko00680 Methane metabolism;ko01200 Carbon metabolism;ko01230 Biosynthesis of amino acids;ko03018 RNA degradation;ko04066 HIF-1 signaling pathway;
gene43686	K11420	EHMT	euchromatic histone-lysine N-methyltransferase	EC:2.1.1.43	1	ko00310 Lysine degradation;
gene43689	K14822	CGR1	rRNA-processing protein CGR1	--		
gene4368	K09874	NIP	aquaporin NIP	--		
gene43692	K15102	SLC25A3, PHC, PIC	solute carrier family 25 (mitochondrial phosphate transporter), member 3	--		
gene43694	K04733	IRAK4	interleukin-1 receptor-associated kinase 4	EC:2.7.11.1	11	ko04064 NF-kappa B signaling pathway;ko04210 Apoptosis;ko04620 Toll-like receptor signaling pathway;ko04722 Neurotrophin signaling pathway;ko05133 Pertussis;ko05140 Leishmaniasis;ko05142 Chagas disease (American trypanosomiasis);ko05145 Toxoplasmosis;ko05152 Tuberculosis;ko05162 Measles;ko05164 Influenza A;
gene43696	K09587	CYP90B1, DWF4	cytochrome P450, family 90, subfamily B, polypeptide 1 (steroid 22-alpha-hydroxylase)	EC:1.14.13.-	1	ko00905 Brassinosteroid biosynthesis;
gene43699	K15296	NAPA, SNAPA, SEC17	alpha-soluble NSF attachment protein	--	1	ko04721 Synaptic vesicle cycle;
gene43702	K01979	SSUrRNA	small subunit ribosomal RNA	--	2	ko03008 Ribosome biogenesis in eukaryotes;ko03010 Ribosome;
gene43703	K14638	SLC15A3_4, PHT	solute carrier family 15 (peptide/histidine transporter), member 3/4	--		
gene43704	K00208	fabI	enoyl-[acyl-carrier protein] reductase I	EC:1.3.1.9 1.3.1.10	2	ko00061 Fatty acid biosynthesis;ko00780 Biotin metabolism;
gene43706	K14220	tRNA-Asn	tRNA Asn	--	1	ko00970 Aminoacyl-tRNA biosynthesis;
gene4370	K03879	ND2	NADH-ubiquinone oxidoreductase chain 2	EC:1.6.5.3	2	ko00190 Oxidative phosphorylation;ko05012 Parkinson's disease;
gene43712	K13210	FUBP	far upstream element-binding protein	--		
gene4371	K09660	MPDU1	mannose-P-dolichol utilization defect 1	--		
gene43721	K00766	trpD	anthranilate phosphoribosyltransferase	EC:2.4.2.18	2	ko00400 Phenylalanine, tyrosine and tryptophan biosynthesis;ko01230 Biosynthesis of amino acids;
gene43722	K04733	IRAK4	interleukin-1 receptor-associated kinase 4	EC:2.7.11.1	11	ko04064 NF-kappa B signaling pathway;ko04210 Apoptosis;ko04620 Toll-like receptor signaling pathway;ko04722 Neurotrophin signaling pathway;ko05133 Pertussis;ko05140 Leishmaniasis;ko05142 Chagas disease (American trypanosomiasis);ko05145 Toxoplasmosis;ko05152 Tuberculosis;ko05162 Measles;ko05164 Influenza A;
gene43724	K11982	RNF115_126	E3 ubiquitin-protein ligase RNF115/126	EC:6.3.2.19		
gene43725	K07151	STT3	dolichyl-diphosphooligosaccharide--protein glycosyltransferase	EC:2.4.99.18	3	ko00510 N-Glycan biosynthesis;ko00513 Various types of N-glycan biosynthesis;ko04141 Protein processing in endoplasmic reticulum;
gene43727	K09422	MYBP	myb proto-oncogene protein, plant	--		
gene43728	K10703	PHS1, PAS2	very-long-chain (3R)-3-hydroxyacyl-[acyl-carrier protein] dehydratase	EC:4.2.1.134	2	ko00062 Fatty acid elongation;ko01040 Biosynthesis of unsaturated fatty acids;
gene43730	K02146	ATPeVAC39, ATP6D	V-type H+-transporting ATPase subunit AC39	EC:3.6.3.14	10	ko00190 Oxidative phosphorylation;ko04142 Lysosome;ko04145 Phagosome;ko04721 Synaptic vesicle cycle;ko04966 Collecting duct acid secretion;ko05110 Vibrio cholerae infection;ko05120 Epithelial cell signaling in Helicobacter pylori infection;ko05152 Tuberculosis;ko05203 Viral carcinogenesis;ko05323 Rheumatoid arthritis;
gene43736	K14406	CSTF1	cleavage stimulation factor subunit 1	--	1	ko03015 mRNA surveillance pathway;
gene43737	K03648	UNG, UDG	uracil-DNA glycosylase	EC:3.2.2.27	2	ko03410 Base excision repair;ko05340 Primary immunodeficiency;
gene43739	K04733	IRAK4	interleukin-1 receptor-associated kinase 4	EC:2.7.11.1	11	ko04064 NF-kappa B signaling pathway;ko04210 Apoptosis;ko04620 Toll-like receptor signaling pathway;ko04722 Neurotrophin signaling pathway;ko05133 Pertussis;ko05140 Leishmaniasis;ko05142 Chagas disease (American trypanosomiasis);ko05145 Toxoplasmosis;ko05152 Tuberculosis;ko05162 Measles;ko05164 Influenza A;

gene43741	K14489	AHK2_3_4	arabidopsis histidine kinase 2/3/4 (cytokinin receptor)	EC:2.7.13.3	1	ko04075 Plant hormone signal transduction;
gene43745	K03039	PSMD13, RPN9	26S proteasome regulatory subunit N9	--	2	ko03050 Proteasome;ko05169 Epstein-Barr virus infection;
gene43746	K02703	psbA	photosystem II P680 reaction center D1 protein	--	1	ko00195 Photosynthesis;
gene43747	K15688	MUL1	E3 ubiquitin-protein ligase MUL1	EC:6.3.2.19		
gene43752	K14638	SLC15A3_4, PHT	solute carrier family 15 (peptide/histidine transporter), member 3/4	--		
gene43753	K16296	SCPL-I	serine carboxypeptidase-like clade I	EC:3.4.16.-		
gene43754	K00588	E2.1.1.104	caffeoyl-CoA O-methyltransferase	EC:2.1.1.104	4	ko00360 Phenylalanine metabolism;ko00940 Phenylpropanoid biosynthesis;ko00941 Flavonoid biosynthesis;ko00945 Stilbenoid, diarylheptanoid and gingerol biosynthesis;
gene43755	K09571	FKBP4_5	FK506-binding protein 4/5	EC:5.2.1.8	1	ko04915 Estrogen signaling pathway;
gene43758	K02930	RP-L4e, RPL4	large subunit ribosomal protein L4e	--	1	ko03010 Ribosome;
gene43770	K01859	E5.5.1.6	chalcone isomerase	EC:5.5.1.6	1	ko00941 Flavonoid biosynthesis;
gene43772	K13418	SERK1	somatic embryogenesis receptor kinase 1	EC:2.7.10.1 2.7.11.1		
gene43774	K00799	GST, gst	glutathione S-transferase	EC:2.5.1.18	4	ko00480 Glutathione metabolism;ko00980 Metabolism of xenobiotics by cytochrome P450;ko00982 Drug metabolism - cytochrome P450;ko05204 Chemical carcinogenesis;
gene43775	K00799	GST, gst	glutathione S-transferase	EC:2.5.1.18	4	ko00480 Glutathione metabolism;ko00980 Metabolism of xenobiotics by cytochrome P450;ko00982 Drug metabolism - cytochrome P450;ko05204 Chemical carcinogenesis;
gene43776	K00799	GST, gst	glutathione S-transferase	EC:2.5.1.18	4	ko00480 Glutathione metabolism;ko00980 Metabolism of xenobiotics by cytochrome P450;ko00982 Drug metabolism - cytochrome P450;ko05204 Chemical carcinogenesis;
gene43777	K00799	GST, gst	glutathione S-transferase	EC:2.5.1.18	4	ko00480 Glutathione metabolism;ko00980 Metabolism of xenobiotics by cytochrome P450;ko00982 Drug metabolism - cytochrome P450;ko05204 Chemical carcinogenesis;
gene43779	K00799	GST, gst	glutathione S-transferase	EC:2.5.1.18	4	ko00480 Glutathione metabolism;ko00980 Metabolism of xenobiotics by cytochrome P450;ko00982 Drug metabolism - cytochrome P450;ko05204 Chemical carcinogenesis;
gene4377	K02960	RP-S16e, RPS16	small subunit ribosomal protein S16e	--	1	ko03010 Ribosome;
gene43782	K02154	ATPeVI, ATP6N1A	V-type H+-transporting ATPase subunit I	EC:3.6.3.14	9	ko00190 Oxidative phosphorylation;ko04142 Lysosome;ko04145 Phagosome;ko04721 Synaptic vesicle cycle;ko04966 Collecting duct acid secretion;ko05110 Vibrio cholerae infection;ko05120 Epithelial cell signaling in Helicobacter pylori infection;ko05152 Tuberculosis;ko05323 Rheumatoid arthritis;
gene43784	K01647	CS, gltA	citrate synthase	EC:2.3.3.1	5	ko00020 Citrate cycle (TCA cycle);ko00630 Glyoxylate and dicarboxylate metabolism;ko01200 Carbon metabolism;ko01210 2-Oxocarboxylic acid metabolism;ko01230 Biosynthesis of amino acids;
gene43786	K00968	PCYT1	choline-phosphate cytidylyltransferase	EC:2.7.7.15	2	ko00440 Phosphonate and phosphinate metabolism;ko00564 Glycerophospholipid metabolism;
gene4378	K08235	E2.4.1.207	xyloglucan:xyloglucosyl transferase	EC:2.4.1.207		
gene43791	K01213	E3.2.1.67	galacturan 1,4-alpha-galacturonidase	EC:3.2.1.67	2	ko00040 Pentose and glucuronate interconversions;ko00500 Starch and sucrose metabolism;
gene43795	K01115	PLD1_2	phospholipase D1/2	EC:3.1.4.4	7	ko00564 Glycerophospholipid metabolism;ko00565 Ether lipid metabolism;ko04014 Ras signaling pathway;ko04144 Endocytosis;ko04666 Fc gamma R-mediated phagocytosis;ko04724 Glutamatergic synapse;ko04912 GnRH signaling pathway;
gene43797	K04733	IRAK4	interleukin-1 receptor-associated kinase 4	EC:2.7.11.1	11	ko04064 NF-kappa B signaling pathway;ko04210 Apoptosis;ko04620 Toll-like receptor signaling pathway;ko04722 Neurotrophin signaling pathway;ko05133 Pertussis;ko05140 Leishmaniasis;ko05142 Chagas disease (American trypanosomiasis);ko05145 Toxoplasmosis;ko05152 Tuberculosis;ko05162 Measles;ko05164 Influenza A;
gene43798	K04730	IRAK1	interleukin-1 receptor-associated kinase 1	EC:2.7.11.1	11	ko04064 NF-kappa B signaling pathway;ko04210 Apoptosis;ko04620 Toll-like receptor signaling pathway;ko04722 Neurotrophin signaling pathway;ko05133 Pertussis;ko05140 Leishmaniasis;ko05142 Chagas disease (American trypanosomiasis);ko05145 Toxoplasmosis;ko05152 Tuberculosis;ko05162 Measles;ko05169 Epstein-Barr virus infection;
gene43799	K10867	NBN, NBS1	nijmegen breakage syndrome protein 1	--	1	ko03440 Homologous recombination;
gene43802	K00799	GST, gst	glutathione S-transferase	EC:2.5.1.18	4	ko00480 Glutathione metabolism;ko00980 Metabolism of xenobiotics by cytochrome P450;ko00982 Drug metabolism - cytochrome P450;ko05204 Chemical carcinogenesis;

gene43808	K09286	EREBP	EREBP-like factor	--		
gene43811	K02915	RP-L34e, RPL34	large subunit ribosomal protein L34e	--	1	ko03010 Ribosome;
gene43818	K05864	PPID, CYPD	peptidyl-prolyl isomerase D	EC:5.2.1.8		
gene43821	K14319	RANGAP1	Ran GTPase-activating protein 1	--	1	ko03013 RNA transport;
gene43822	K16281	RHA1	RING-H2 zinc finger protein RHA1	--		
gene43823	K00053	ilvC	ketol-acid reductoisomerase	EC:1.1.1.86	4	ko00290 Valine, leucine and isoleucine biosynthesis;ko00770 Pantothenate and CoA biosynthesis;ko01210 2-Oxocarboxylic acid metabolism;ko01230 Biosynthesis of amino acids;
gene43825	K14638	SLC15A3_4, PHT	solute carrier family 15 (peptide/histidine transporter), member 3/4	--		
gene43826	K00895	E2.7.1.90, pfk	pyrophosphate--fructose-6-phosphate 1-phosphotransferase	EC:2.7.1.90	1	ko00051 Fructose and mannose metabolism;
gene43828	K14012	SHP1, UBX1, NSFL1C	UBX domain-containing protein 1	--	1	ko04141 Protein processing in endoplasmic reticulum;
gene4382	K01681	ACO, acnA	aconitate hydratase	EC:4.2.1.3	6	ko00020 Citrate cycle (TCA cycle);ko00630 Glyoxylate and dicarboxylate metabolism;ko00720 Carbon fixation pathways in prokaryotes;ko01200 Carbon metabolism;ko01210 2-Oxocarboxylic acid metabolism;ko01230 Biosynthesis of amino acids;
gene43830	K11584	PPP2R5	serine/threonine-protein phosphatase 2A regulatory subunit B'	--	5	ko03015 mRNA surveillance pathway;ko04113 Meiosis - yeast;ko04114 Oocyte meiosis;ko04151 PI3K-Akt signaling pathway;ko04728 Dopaminergic synapse;
gene43832	K00430	E1.11.1.7	peroxidase	EC:1.11.1.7	2	ko00360 Phenylalanine metabolism;ko00940 Phenylpropanoid biosynthesis;
gene4383	K06947	GRC3, NOL9	polynucleotide 5'-hydroxyl-kinase GRC3/NOL9	EC:2.7.1.-		
gene43844	K04730	IRAK1	interleukin-1 receptor-associated kinase 1	EC:2.7.11.1	11	ko04064 NF-kappa B signaling pathway;ko04210 Apoptosis;ko04620 Toll-like receptor signaling pathway;ko04722 Neurotrophin signaling pathway;ko05133 Pertussis;ko05140 Leishmaniasis;ko05142 Chagas disease (American trypanosomiasis);ko05145 Toxoplasmosis;ko05152 Tuberculosis;ko05162 Measles;ko05169 Epstein-Barr virus infection;
gene43845	K04733	IRAK4	interleukin-1 receptor-associated kinase 4	EC:2.7.11.1	11	ko04064 NF-kappa B signaling pathway;ko04210 Apoptosis;ko04620 Toll-like receptor signaling pathway;ko04722 Neurotrophin signaling pathway;ko05133 Pertussis;ko05140 Leishmaniasis;ko05142 Chagas disease (American trypanosomiasis);ko05145 Toxoplasmosis;ko05152 Tuberculosis;ko05162 Measles;ko05164 Influenza A;
gene43849	K02879	RP-L17, MRPL17, rplQ	large subunit ribosomal protein L17	--	1	ko03010 Ribosome;
gene43852	K03955	NDUFAB1	NADH dehydrogenase (ubiquinone) 1 alpha/beta subcomplex 1	--	5	ko00190 Oxidative phosphorylation;ko04932 Non-alcoholic fatty liver disease (NAFLD);ko05010 Alzheimer's disease;ko05012 Parkinson's disease;ko05016 Huntington's disease;
gene43854	K08057	CALR	calreticulin	--	5	ko04141 Protein processing in endoplasmic reticulum;ko04145 Phagosome;ko04612 Antigen processing and presentation;ko05142 Chagas disease (American trypanosomiasis);ko05166 HTLV-I infection;
gene43855	K00963	UGP2, galU, galF	UTP--glucose-1-phosphate uridylyltransferase	EC:2.7.7.9	4	ko00040 Pentose and glucuronate interconversions;ko00052 Galactose metabolism;ko00500 Starch and sucrose metabolism;ko00520 Amino sugar and nucleotide sugar metabolism;
gene4385	K08869	ADCK, ABC1	aarF domain-containing kinase	--		
gene43860	K03354	APC7	anaphase-promoting complex subunit 7	--	7	ko04110 Cell cycle;ko04111 Cell cycle - yeast;ko04113 Meiosis - yeast;ko04114 Oocyte meiosis;ko04120 Ubiquitin mediated proteolysis;ko04914 Progesterone-mediated oocyte maturation;ko05166 HTLV-I infection;
gene43864	K05894	E1.3.1.42	12-oxophytodienoic acid reductase	EC:1.3.1.42	1	ko00592 alpha-Linolenic acid metabolism;
gene43869	K07893	RAB6A	Ras-related protein Rab-6A	--		
gene43873	K15015	SLC32A, VGAT	solute carrier family 32 (vesicular inhibitory amino acid transporter)	--	5	ko04721 Synaptic vesicle cycle;ko04723 Retrograde endocannabinoid signaling;ko04727 GABAergic synapse;ko05032 Morphine addiction;ko05033 Nicotine addiction;
gene43875	K03113	EIF1, SUI1	translation initiation factor 1	--	1	ko03013 RNA transport;
gene43879	K02694	psaF	photosystem I subunit III	--	1	ko00195 Photosynthesis;
gene43882	K00915	IPK2	1D-myo-Inositol-tetrakisphosphate 5-kinase / inositol-polyphosphate multikinase	EC:2.7.1.140 2.7.1.151	2	ko00562 Inositol phosphate metabolism;ko04070 Phosphatidylinositol signaling system;
gene43884	K11793	CRBN	cereblon	--		

gene43887	K14638	SLC15A3_4, PHT	solute carrier family 15 (peptide/histidine transporter), member 3/4	--		
gene43891	K03283	HSPA1_8	heat shock 70kDa protein 1/8	--	11	ko03040 Spliceosome;ko04010 MAPK signaling pathway;ko04141 Protein processing in endoplasmic reticulum;ko04144 Endocytosis;ko04612 Antigen processing and presentation;ko04915 Estrogen signaling pathway;ko05134 Legionellosis;ko05145 Toxoplasmosis;ko05162 Measles;ko05164 Influenza A;ko05169 Epstein-Barr virus infection;
gene43894	K02997	RP-S9e, RPS9	small subunit ribosomal protein S9e	--	1	ko03010 Ribosome;
gene43897	K00600	glyA, SHMT	glycine hydroxymethyltransferase	EC:2.1.2.1	7	ko00260 Glycine, serine and threonine metabolism;ko00460 Cyanoamino acid metabolism;ko00630 Glyoxylate and dicarboxylate metabolism;ko00670 One carbon pool by folate;ko00680 Methane metabolism;ko01200 Carbon metabolism;ko01230 Biosynthesis of amino acids;
gene43899	K01369	LGMN	legumain	EC:3.4.22.34	2	ko04142 Lysosome;ko04612 Antigen processing and presentation;
gene43902	K03364	CDH1	cell division cycle 20-like protein 1, cofactor of APC complex	--	4	ko04110 Cell cycle;ko04111 Cell cycle - yeast;ko04120 Ubiquitin mediated proteolysis;ko04914 Progesterone-mediated oocyte maturation;
gene43904	K03327	TC.MATE, SLC47A, norM, mdtK, dinF	multidrug resistance protein, MATE family	--		
gene43911	K01213	E3.2.1.67	galacturan 1,4-alpha-galacturonidase	EC:3.2.1.67	2	ko00040 Pentose and glucuronate interconversions;ko00500 Starch and sucrose metabolism;
gene4391	K12347	SLC11A, NRAMP	natural resistance-associated macrophage protein	--	2	ko04142 Lysosome;ko04978 Mineral absorption;
gene43920	K00006	GPD1	glycerol-3-phosphate dehydrogenase (NAD+)	EC:1.1.1.8	1	ko00564 Glycerophospholipid metabolism;
gene43922	K08876	SCYL1	SCY1-like protein 1	--		
gene43923	K11097	SNRPE, SME	small nuclear ribonucleoprotein E	--	1	ko03040 Spliceosome;
gene43927	K08235	E2.4.1.207	xyloglucan:xyloglucosyl transferase	EC:2.4.1.207		
gene43929	K01102	PDP	pyruvate dehydrogenase phosphatase	EC:3.1.3.43		
gene43932	K14457	MOGAT2, MGAT2	2-acylglycerol O-acyltransferase 2	EC:2.3.1.22	1	ko04975 Fat digestion and absorption;
gene43935	K14488	SAUR	SAUR family protein	--	1	ko04075 Plant hormone signal transduction;
gene43941	K01772	hemH, FECH	ferrochelatase	EC:4.99.1.1	1	ko00860 Porphyrin and chlorophyll metabolism;
gene43944	K14497	PP2C	protein phosphatase 2C	EC:3.1.3.16	1	ko04075 Plant hormone signal transduction;
gene43946	K07407	E3.2.1.22B, galA, rafA	alpha-galactosidase	EC:3.2.1.22	4	ko00052 Galactose metabolism;ko00561 Glycerolipid metabolism;ko00600 Sphingolipid metabolism;ko00603 Glycosphingolipid biosynthesis - globo series;
gene4394	K03538	POP4, RPP29	ribonuclease P protein subunit POP4	EC:3.1.26.5	2	ko03008 Ribosome biogenesis in eukaryotes;ko03013 RNA transport;
gene43956	K01191	E3.2.1.24	alpha-mannosidase	EC:3.2.1.24	1	ko00511 Other glycan degradation;
gene43959	K00434	E1.11.1.11	L-ascorbate peroxidase	EC:1.11.1.11	2	ko00053 Ascorbate and aldarate metabolism;ko00480 Glutathione metabolism;
gene43963	K08232	E1.6.5.4	monodehydroascorbate reductase (NADH)	EC:1.6.5.4	1	ko00053 Ascorbate and aldarate metabolism;
gene43964	K13407	CYP94A1	cytochrome P450, family 94, subfamily A, polypeptide 1 (fatty acid omega-hydroxylase)	EC:1.14.-.-	1	ko00073 Cutin, suberine and wax biosynthesis;
gene43967	K01528	DNM	dynamin GTPase	EC:3.6.5.5	4	ko04144 Endocytosis;ko04721 Synaptic vesicle cycle;ko04961 Endocrine and other factor-regulated calcium reabsorption;ko05100 Bacterial invasion of epithelial cells;
gene43972	K13094	RBM5_10	RNA-binding protein 5/10	--		
gene43974	K13800	CMPK1, UMPK	UMP-CMP kinase	EC:2.7.4.- 2.7.4.14	1	ko00240 Pyrimidine metabolism;
gene43978	K15227	TYRAAT	arogenate dehydrogenase (NADP+), plant	EC:1.3.1.78	2	ko00400 Phenylalanine, tyrosine and tryptophan biosynthesis;ko01230 Biosynthesis of amino acids;
gene43979	K02692	psaD	photosystem I subunit II	--	1	ko00195 Photosynthesis;
gene43980	K14785	ESF2, ABT1	ESF2/ABP1 family protein	--		
gene43982	K01366	CTSH	cathepsin H	EC:3.4.22.16	1	ko04142 Lysosome;
gene43984	K01835	pgm	phosphoglucomutase	EC:5.4.2.2	7	ko00010 Glycolysis / Gluconeogenesis;ko00030 Pentose phosphate pathway;ko00052 Galactose metabolism;ko00230 Purine metabolism;ko00500 Starch and sucrose metabolism;ko00520 Amino sugar and nucleotide sugar metabolism;ko00521 Streptomycin biosynthesis;
gene43985	K00860	cysC	adenylylsulfate kinase	EC:2.7.1.25	2	ko00230 Purine metabolism;ko00920 Sulfur metabolism;
gene43993	K00454	LOX2S	lipoxygenase	EC:1.13.11.12	2	ko00591 Linoleic acid metabolism;ko00592 alpha-Linolenic acid metabolism;
gene43996	K03189	ureG	urease accessory protein	--		

gene44000	K13126	PABPC	polyadenylate-binding protein	--	3	ko03013 RNA transport;ko03015 mRNA surveillance pathway;ko03018 RNA degradation;
gene44001	K01754	E4.3.1.19, ilvA, tdcB	threonine dehydratase	EC:4.3.1.19	4	ko00260 Glycine, serine and threonine metabolism;ko00290 Valine, leucine and isoleucine biosynthesis;ko01200 Carbon metabolism;ko01230 Biosynthesis of amino acids;
gene44002	K00434	E1.11.1.11	L-ascorbate peroxidase	EC:1.11.1.11	2	ko00053 Ascorbate and aldarate metabolism;ko00480 Glutathione metabolism;
gene44003	K00430	E1.11.1.7	peroxidase	EC:1.11.1.7	2	ko00360 Phenylalanine metabolism;ko00940 Phenylpropanoid biosynthesis;
gene44008	K01115	PLD1_2	phospholipase D1/2	EC:3.1.4.4	7	ko00564 Glycerophospholipid metabolism;ko00565 Ether lipid metabolism;ko04014 Ras signaling pathway;ko04144 Endocytosis;ko04666 Fc gamma R-mediated phagocytosis;ko04724 Glutamatergic synapse;ko04912 GnRH signaling pathway;
gene4400	K12471	EPN	epsin	--	1	ko04144 Endocytosis;
gene44011	K00787	FDPS	farnesyl diphosphate synthase	EC:2.5.1.1 2.5.1.10	3	ko00900 Terpenoid backbone biosynthesis;ko05164 Influenza A;ko05166 HTLV-I infection;
gene44012	K11090	LA, SSB	lupus La protein	--	1	ko05322 Systemic lupus erythematosus;
gene44013	K09529	DNAJC9	DnaJ homolog subfamily C member 9	--		
gene44016	K00434	E1.11.1.11	L-ascorbate peroxidase	EC:1.11.1.11	2	ko00053 Ascorbate and aldarate metabolism;ko00480 Glutathione metabolism;
gene4401	K13993	HSP20	HSP20 family protein	--	1	ko04141 Protein processing in endoplasmic reticulum;
gene44026	K01114	plcC	phospholipase C	EC:3.1.4.3	3	ko00562 Inositol phosphate metabolism;ko00564 Glycerophospholipid metabolism;ko00565 Ether lipid metabolism;
gene44030	K01087	otsB	trehalose 6-phosphate phosphatase	EC:3.1.3.12	1	ko00500 Starch and sucrose metabolism;
gene44031	K00128	E1.2.1.3	aldehyde dehydrogenase (NAD+)	EC:1.2.1.3	15	ko00010 Glycolysis / Gluconeogenesis;ko00040 Pentose and glucuronate interconversions;ko00053 Ascorbate and aldarate metabolism;ko00071 Fatty acid degradation;ko00280 Valine, leucine and isoleucine degradation;ko00310 Lysine degradation;ko00330 Arginine and proline metabolism;ko00340 Histidine metabolism;ko00380 Tryptophan metabolism;ko00410 beta-Alanine metabolism;ko00561 Glycerolipid metabolism;ko00620 Pyruvate metabolism;ko00625 Chloroalkane and chloroalkene degradation;ko00640 Propanoate metabolism;ko00903 Limonene and pinene
gene44032	K12813	DHX16	pre-mRNA-splicing factor ATP-dependent RNA helicase DHX16	EC:3.6.4.13	1	ko03040 Spliceosome;
gene44033	K10364	CAPZA	capping protein (actin filament) muscle Z-line, alpha	--		
gene44038	K09872	PIP	aquaporin PIP	--		
gene44039	K13448	CML	calcium-binding protein CML	--	1	ko04626 Plant-pathogen interaction;
gene44044	K13126	PABPC	polyadenylate-binding protein	--	3	ko03013 RNA transport;ko03015 mRNA surveillance pathway;ko03018 RNA degradation;
gene44049	K08955	YME1	ATP-dependent metalloprotease	EC:3.4.24.-		
gene4404	K04733	IRAK4	interleukin-1 receptor-associated kinase 4	EC:2.7.11.1	11	ko04064 NF-kappa B signaling pathway;ko04210 Apoptosis;ko04620 Toll-like receptor signaling pathway;ko04722 Neurotrophin signaling pathway;ko05133 Pertussis;ko05140 Leishmaniasis;ko05142 Chagas disease (American trypanosomiasis);ko05145 Toxoplasmosis;ko05152 Tuberculosis;ko05162 Measles;ko05164 Influenza A;
gene44053	K07071	K07071				
gene44054	K14232	tRNA-Pro	tRNA Pro	--	1	ko00970 Aminoacyl-tRNA biosynthesis;
gene44056	K13832	aroDE, DHQ-SDH	3-dehydroquinate dehydratase / shikimate dehydrogenase	EC:4.2.1.10 1.1.1.25	2	ko00400 Phenylalanine, tyrosine and tryptophan biosynthesis;ko01230 Biosynthesis of amino acids;
gene4405	K04123	KAO	ent-kaurenoic acid hydroxylase	EC:1.14.13.79	1	ko00904 Diterpenoid biosynthesis;
gene44060	K02923	RP-L38e, RPL38	large subunit ribosomal protein L38e	--	1	ko03010 Ribosome;
gene44062	K15108	SLC25A19, DNC, TPC1	solute carrier family 25 (mitochondrial thiamine pyrophosphate transporter), member 19	--		
gene44063	K17398	DNMT3A	DNA (cytosine-5)-methyltransferase 3A	EC:2.1.1.37	2	ko00270 Cysteine and methionine metabolism;ko05206 MicroRNAs in cancer;
gene44065	K12200	PDCD6IP, ALIX, RIM20	programmed cell death 6-interacting protein	--	1	ko04144 Endocytosis;
gene4406	K12617	PATL1, PAT1	DNA topoisomerase 2-associated protein PAT1	--	1	ko03018 RNA degradation;
gene44072	K05387	GRIP	glutamate receptor, ionotropic, plant	--		

gene44080	K00783	rlmH	23S rRNA (pseudouridine1915-N3)-methyltransferase	EC:2.1.1.177		
gene44082	K15281	SLC35D	solute carrier family 35	--		
gene44086	K04382	PPP2C	serine/threonine-protein phosphatase 2A catalytic subunit	EC:3.1.3.16	13	ko03015 mRNA surveillance pathway;ko04111 Cell cycle - yeast;ko04113 Meiosis - yeast;ko04114 Oocyte meiosis;ko04151 PI3K-Akt signaling pathway;ko04350 TGF-beta signaling pathway;ko04390 Hippo signaling pathway;ko04391 Hippo signaling pathway - fly;ko04530 Tight junction;ko04728 Dopaminergic synapse;ko04730 Long-term depression;ko05142 Chagas disease (American trypanosomiasis);ko05160 Hepatitis C;
gene44087	K14652	ribBA	3,4-dihydroxy 2-butanone 4-phosphate synthase / GTP cyclohydrolase II	EC:4.1.99.12 3.5.4.25	1	ko00740 Riboflavin metabolism;
gene44088	K13065	E2.3.1.133, HCT	shikimate O-hydroxycinnamoyltransferase	EC:2.3.1.133	3	ko00940 Phenylpropanoid biosynthesis;ko00941 Flavonoid biosynthesis;ko00945 Stilbenoid, diarylheptanoid and gingerol biosynthesis;
gene44089	K15472	CYP71D55	premnaspirodiene oxygenase	EC:1.14.13.121	1	ko00909 Sesquiterpenoid and triterpenoid biosynthesis;
gene44098	K05857	PLCD	phosphatidylinositol phospholipase C, delta	EC:3.1.4.11	3	ko00562 Inositol phosphate metabolism;ko04020 Calcium signaling pathway;ko04070 Phosphatidylinositol signaling system;
gene440	K12819	SLU7	pre-mRNA-processing factor SLU7	--	1	ko03040 Spliceosome;
gene44100	K05857	PLCD	phosphatidylinositol phospholipase C, delta	EC:3.1.4.11	3	ko00562 Inositol phosphate metabolism;ko04020 Calcium signaling pathway;ko04070 Phosphatidylinositol signaling system;
gene44101	K15683	NFXL1, OZFP	NF-X1-type zinc finger protein NFXL1	--		
gene44103	K11320	EP400	E1A-binding protein p400	EC:3.6.4.-		
gene44107	K11752	ribD	diaminohydroxyphosphoribosylaminopyrimidine deaminase / 5-amino-6-(5-phosphoribosylamino)uracil reductase	EC:3.5.4.26 1.1.1.193	1	ko00740 Riboflavin metabolism;
gene44108	K08770	UBC	ubiquitin C	--	1	ko03320 PPAR signaling pathway;
gene4410	K01890	FARSB, pheT	phenylalanyl-tRNA synthetase beta chain	EC:6.1.1.20	1	ko00970 Aminoacyl-tRNA biosynthesis;
gene44111	K17087	TM9SF3	transmembrane 9 superfamily member 3	--		
gene44112	K14638	SLC15A3_4, PHT	solute carrier family 15 (peptide/histidine transporter), member 3/4	--		
gene44115	K05349	bglX	beta-glucosidase	EC:3.2.1.21	3	ko00460 Cyanoamino acid metabolism;ko00500 Starch and sucrose metabolism;ko00940 Phenylpropanoid biosynthesis;
gene44116	K05349	bglX	beta-glucosidase	EC:3.2.1.21	3	ko00460 Cyanoamino acid metabolism;ko00500 Starch and sucrose metabolism;ko00940 Phenylpropanoid biosynthesis;
gene44118	K06669	SMC3, CSPG6	structural maintenance of chromosome 3 (chondroitin sulfate proteoglycan 6)	--	4	ko04110 Cell cycle;ko04111 Cell cycle - yeast;ko04113 Meiosis - yeast;ko04114 Oocyte meiosis;
gene4411	K14488	SAUR	SAUR family protein	--	1	ko04075 Plant hormone signal transduction;
gene44120	K07375	TUBB	tubulin beta	--	3	ko04145 Phagosome;ko04540 Gap junction;ko05130 Pathogenic Escherichia coli infection;
gene44121	K05929	E2.1.1.103, NMT	phosphoethanolamine N-methyltransferase	EC:2.1.1.103	1	ko00564 Glycerophospholipid metabolism;
gene44126	K02331	POL5, MYBBP1A	DNA polymerase phi	EC:2.7.7.7		
gene4412	K06119	SQD2	sulfoquinovosyltransferase	EC:2.4.1.-	1	ko00561 Glycerolipid metabolism;
gene44131	K15397	KCS	3-ketoacyl-CoA synthase	EC:2.3.1.199	1	ko00062 Fatty acid elongation;
gene44133	K08248	E4.1.2.10	mandelonitrile lyase	EC:4.1.2.10	1	ko00460 Cyanoamino acid metabolism;
gene44135	K07300	chaA, CAX	Ca2+:H+ antiporter	--		
gene44138	K00895	E2.7.1.90, pfk	pyrophosphate--fructose-6-phosphate 1-phosphotransferase	EC:2.7.1.90	1	ko00051 Fructose and mannose metabolism;
gene44140	K10268	FBXL2_20	F-box and leucine-rich repeat protein 2/20	--		
gene44141	K01051	E3.1.1.11	pectinesterase	EC:3.1.1.11	2	ko00040 Pentose and glucuronate interconversions;ko00500 Starch and sucrose metabolism;
gene44142	K01051	E3.1.1.11	pectinesterase	EC:3.1.1.11	2	ko00040 Pentose and glucuronate interconversions;ko00500 Starch and sucrose metabolism;
gene44143	K01051	E3.1.1.11	pectinesterase	EC:3.1.1.11	2	ko00040 Pentose and glucuronate interconversions;ko00500 Starch and sucrose metabolism;
gene44154	K00422	E1.10.3.1	polyphenol oxidase	EC:1.10.3.1	2	ko00350 Tyrosine metabolism;ko00950 Isoquinoline alkaloid biosynthesis;
gene44157	K09645	CPVL	vitellogenic carboxypeptidase-like protein	EC:3.4.16.-		
gene44160	K00434	E1.11.1.11	L-ascorbate peroxidase	EC:1.11.1.11	2	ko00053 Ascorbate and aldarate metabolism;ko00480 Glutathione metabolism;

gene44161	K03190	ureD, ureH	urease accessory protein	--		
gene44168	K05298	GAPA	glyceraldehyde-3-phosphate dehydrogenase (NADP+) (phosphorylating)	EC:1.2.1.13	2	ko00710 Carbon fixation in photosynthetic organisms;ko01200 Carbon metabolism;
gene44169	K11778	DHDDS, RER2, SRT1	ditrans,polycis-polyprenyl diphosphate synthase	EC:2.5.1.87	1	ko00900 Terpenoid backbone biosynthesis;
gene44176	K11975	RNF144	E3 ubiquitin-protein ligase RNF144	EC:6.3.2.19		
gene44185	K05909	E1.10.3.2				
gene44186	K05909	E1.10.3.2				
gene44187	K11269	CTF18, CHL12	chromosome transmission fidelity protein 18	--		
gene44189	K01728	E4.2.2.2, pel	pectate lyase	EC:4.2.2.2	1	ko00040 Pentose and glucuronate interconversions;
gene44191	K14816	REI1	pre-60S factor REI1	--		
gene44193	K03134	TAF10	transcription initiation factor TFIID subunit 10	--	2	ko03022 Basal transcription factors;ko05168 Herpes simplex infection;
gene44197	K15121	SLC25A44	solute carrier family 25, member 44	--		
gene44199	K08064	NFYA	nuclear transcription factor Y, alpha	--	2	ko04612 Antigen processing and presentation;ko05152 Tuberculosis;
gene441	K11344	EAF6	chromatin modification-related protein EAF6	--		
gene44204	K13051	iaaA, ASRGL1	beta-aspartyl-peptidase (threonine type)	EC:3.4.19.5		
gene44205	K13947	PIN	auxin efflux carrier family	--		
gene44206	K09874	NIP	aquaporin NIP	--		
gene44217	K03942	NDUFV1	NADH dehydrogenase (ubiquinone) flavoprotein 1	EC:1.6.5.3 1.6.99.3	5	ko00190 Oxidative phosphorylation;ko04932 Non-alcoholic fatty liver disease (NAFLD);ko05010 Alzheimer's disease;ko05012 Parkinson's disease;ko05016 Huntington's disease;
gene44222	K01176	E3.2.1.1, amyA, malS	alpha-amylase	EC:3.2.1.1	2	ko00500 Starch and sucrose metabolism;ko04973 Carbohydrate digestion and absorption;
gene44223	K06173	truA, PUS1	tRNA pseudouridine38-40 synthase	EC:5.4.99.12		
gene44226	K07943	ARL2	ADP-ribosylation factor-like protein 2	--		
gene44229	K01179	E3.2.1.4	endoglucanase	EC:3.2.1.4	1	ko00500 Starch and sucrose metabolism;
gene44240	K07195	EXOC7, EXO70	exocyst complex component 7	--	1	ko04910 Insulin signaling pathway;
gene44241	K00734	B3GALT6	galactosylxylosylprotein 3-beta-galactosyltransferase	EC:2.4.1.134	2	ko00532 Glycosaminoglycan biosynthesis - chondroitin sulfate / dermatan sulfate;ko00534 Glycosaminoglycan biosynthesis - heparan sulfate / heparin;
gene44242	K14810	DDX56, DBP9	ATP-dependent RNA helicase DDX56/DBP9	EC:3.6.4.13		
gene44246	K12619	XRN2, RAT1	5'-3' exoribonuclease 2	EC:3.1.13.-	2	ko03008 Ribosome biogenesis in eukaryotes;ko03018 RNA degradation;
gene44249	K13098	TLS, FUS	RNA-binding protein FUS	--	1	ko05202 Transcriptional misregulation in cancer;
gene4424	K00797	speE, SRM	spermidine synthase	EC:2.5.1.16	4	ko00270 Cysteine and methionine metabolism;ko00330 Arginine and proline metabolism;ko00410 beta-Alanine metabolism;ko00480 Glutathione metabolism;
gene44254	K05909	E1.10.3.2				
gene44262	K00430	E1.11.1.7	peroxidase	EC:1.11.1.7	2	ko00360 Phenylalanine metabolism;ko00940 Phenylpropanoid biosynthesis;
gene44263	K08343	ATG3	autophagy-related protein 3	--	1	ko04140 Regulation of autophagy;
gene44268	K12179	COPS6, CSN6	COP9 signalosome complex subunit 6	--		
gene44285	K13412	CPK	calcium-dependent protein kinase	EC:2.7.11.1	2	ko04626 Plant-pathogen interaction;ko05145 Toxoplasmosis;
gene44286	K02960	RP-S16e, RPS16	small subunit ribosomal protein S16e	--	1	ko03010 Ribosome;
gene4428	K09122	K09122	hypothetical protein	--		
gene44293	K13249	SSR1	translocon-associated protein subunit alpha	--	1	ko04141 Protein processing in endoplasmic reticulum;
gene44297	K00434	E1.11.1.11	L-ascorbate peroxidase	EC:1.11.1.11	2	ko00053 Ascorbate and aldarate metabolism;ko00480 Glutathione metabolism;
gene44298	K14484	IAA	auxin-responsive protein IAA	--	1	ko04075 Plant hormone signal transduction;
gene442	K05765	CFL	cofilin	--	4	ko04360 Axon guidance;ko04666 Fc gamma R-mediated phagocytosis;ko04810 Regulation of actin cytoskeleton;ko05133 Pertussis;
gene44305	K10526	OPCL1	OPC-8:0 CoA ligase 1	EC:6.2.1.-	1	ko00592 alpha-Linolenic acid metabolism;
gene44306	K14182	E4.2.3.21	vetispiradiene synthase	EC:4.2.3.21	1	ko00909 Sesquiterpenoid and triterpenoid biosynthesis;
gene4430	K13379	RGP, UTM	reversibly glycosylated polypeptide / UDP-arabinopyranose mutase	EC:2.4.1.- 5.4.99.30		
gene44310	K01209	E3.2.1.55, abfA	alpha-N-arabinofuranosidase	EC:3.2.1.55	1	ko00520 Amino sugar and nucleotide sugar metabolism;
gene44318	K11498	CENPE	centromeric protein E	--		
gene4431	K12640	CYP85A2, BR6OX2	cytochrome P450, family 85, subfamily A, polypeptide 2 (brassinosteroid-6-oxidase 2)	EC:1.14.-.-	1	ko00905 Brassinosteroid biosynthesis;

gene44328	K03868	RBX1, ROC1	RING-box protein 1	--	12	ko03420 Nucleotide excision repair;ko04066 HIF-1 signaling pathway;ko04110 Cell cycle;ko04111 Cell cycle - yeast;ko04114 Oocyte meiosis;ko04120 Ubiquitin mediated proteolysis;ko04141 Protein processing in endoplasmic reticulum;ko04310 Wnt signaling pathway;ko04350 TGF-beta signaling pathway;ko04710 Circadian rhythm;ko05200 Pathways in cancer;ko05211 Renal cell carcinoma;
gene44330	K00430	E1.11.1.7	peroxidase	EC:1.11.1.7	2	ko00360 Phenylalanine metabolism;ko00940 Phenylpropanoid biosynthesis;
gene44333	K03239	EIF2B1	translation initiation factor eIF-2B subunit alpha	--	1	ko03013 RNA transport;
gene44339	K09338	HD-ZIP	homeobox-leucine zipper protein	--		
gene4433	K13356	FAR	fatty acyl-CoA reductase	EC:1.2.1.-	2	ko00073 Cutin, suberine and wax biosynthesis;ko04146 Peroxisome;
gene44341	K01507	ppa	inorganic pyrophosphatase	EC:3.6.1.1	1	ko00190 Oxidative phosphorylation;
gene44342	K10843	ERCC3, XPB	DNA excision repair protein ERCC-3	EC:3.6.4.12	2	ko03022 Basal transcription factors;ko03420 Nucleotide excision repair;
gene44343	K16302	CNNM	metal transporter CNNM	--		
gene44344	K01188	E3.2.1.21	beta-glucosidase	EC:3.2.1.21	3	ko00460 Cyanoamino acid metabolism;ko00500 Starch and sucrose metabolism;ko00940 Phenylpropanoid biosynthesis;
gene44348	K03626	EGD2, NACA	nascent polypeptide-associated complex subunit alpha	--		
gene44349	K03327	TC.MATE, SLC47A, norM, mdtK, dinF	multidrug resistance protein, MATE family	--		
gene4434	K14236	tRNA-Tyr	tRNA Tyr	--	1	ko00970 Aminoacyl-tRNA biosynthesis;
gene44355	K00696	E2.4.1.14	sucrose-phosphate synthase	EC:2.4.1.14	1	ko00500 Starch and sucrose metabolism;
gene44356	K04711	YDC1	dihydroceramidase	EC:3.5.1.-	1	ko00600 Sphingolipid metabolism;
gene44357	K02705	psbC	photosystem II CP43 chlorophyll apoprotein	--	1	ko00195 Photosynthesis;
gene44359	K15397	KCS	3-ketoacyl-CoA synthase	EC:2.3.1.199	1	ko00062 Fatty acid elongation;
gene4435	K00819	E2.6.1.13, rocD	ornithine--oxo-acid transaminase	EC:2.6.1.13	1	ko00330 Arginine and proline metabolism;
gene44367	K01115	PLD1_2	phospholipase D1/2	EC:3.1.4.4	7	ko00564 Glycerophospholipid metabolism;ko00565 Ether lipid metabolism;ko04014 Ras signaling pathway;ko04144 Endocytosis;ko04666 Fc gamma R-mediated phagocytosis;ko04724 Glutamatergic synapse;ko04912 GnRH signaling pathway;
gene44368	K08472	MLO	mlo protein	--		
gene44369	K02639	petF	ferredoxin	--	1	ko00195 Photosynthesis;
gene44370	K17087	TM9SF3	transmembrane 9 superfamily member 3	--		
gene44372	K13648	GAUT	alpha-1,4-galacturonosyltransferase	EC:2.4.1.43	2	ko00500 Starch and sucrose metabolism;ko00520 Amino sugar and nucleotide sugar metabolism;
gene44375	K08901	psbQ	photosystem II oxygen-evolving enhancer protein 3	--	1	ko00195 Photosynthesis;
gene44378	K08912	LHCB1	light-harvesting complex II chlorophyll a/b binding protein 1	--	1	ko00196 Photosynthesis - antenna proteins;
gene44379	K11251	H2A	histone H2A	--	2	ko05034 Alcoholism;ko05322 Systemic lupus erythematosus;
gene44385	K01183	E3.2.1.14	chitinase	EC:3.2.1.14	1	ko00520 Amino sugar and nucleotide sugar metabolism;
gene44386	K14500	BSK	BR-signaling kinase	EC:2.7.11.1	1	ko04075 Plant hormone signal transduction;
gene4438	K08867	WNK, PRKWNK	WNK lysine deficient protein kinase	EC:2.7.11.1		
gene44390	K00033	E1.1.1.44, PGD, gnd	6-phosphogluconate dehydrogenase	EC:1.1.1.44	3	ko00030 Pentose phosphate pathway;ko00480 Glutathione metabolism;ko01200 Carbon metabolism;
gene44392	K04710	CERS	ceramide synthetase	EC:2.3.1.24	1	ko00600 Sphingolipid metabolism;
gene44393	K15429	TRM5, TRMT5	tRNA (guanine37-N1)-methyltransferase	EC:2.1.1.228		
gene44396	K13341	PEX7, PTS2R	peroxin-7	--	1	ko04146 Peroxisome;
gene443	K08176	PHO84	MFS transporter, PHS family, inorganic phosphate transporter	--		
gene44404	K11982	RNF115_126	E3 ubiquitin-protein ligase RNF115/126	EC:6.3.2.19		
gene44407	K08681	pdxT, pdx2	glutamine amidotransferase	EC:2.6.-.-	1	ko00750 Vitamin B6 metabolism;
gene44413	K09264	K09264	MADS-box transcription factor, plant	--		
gene44417	K11450	AOF2, LSD1	lysine-specific histone demethylase 1	EC:1.-.-.-		
gene44425	K09419	HSFF	heat shock transcription factor, other eukaryote	--		
gene44428	K10364	CAPZA	capping protein (actin filament) muscle Z-line, alpha	--		

gene44430	K07748	E1.1.1.170, NSDHL, ERG26	sterol-4alpha-carboxylate 3-dehydrogenase (decarboxylating)	EC:1.1.1.170	1	ko00100 Steroid biosynthesis;
gene44432	K11790	DTL, CDT2, DCAF2	denticleless	--		
gene44434	K10706	SETX, ALS4	senataxin	EC:3.6.4.-		
gene44435	K08498	STX6	syntaxin 6	--	1	ko04130 SNARE interactions in vesicular transport;
gene44440	K02548	menA	1,4-dihydroxy-2-naphthoate octaprenyltransferase	EC:2.5.1.74	1	ko00130 Ubiquinone and other terpenoid-quinone biosynthesis;
gene44445	K11498	CENPE	centromeric protein E	--		
gene44451	K14485	TIR1	transport inhibitor response 1	--	1	ko04075 Plant hormone signal transduction;
gene44453	K00434	E1.11.1.11	L-ascorbate peroxidase	EC:1.11.1.11	2	ko00053 Ascorbate and aldarate metabolism;ko00480 Glutathione metabolism;
gene44460	K14213	PEPD	Xaa-Pro dipeptidase	EC:3.4.13.9		
gene44464	K15032	MTERFD	mTERF domain-containing protein, mitochondrial	--		
gene44466	K09422	MYBP	myb proto-oncogene protein, plant	--		
gene44467	K02257	COX10	protoheme IX farnesyltransferase	EC:2.5.1.-	2	ko00190 Oxidative phosphorylation;ko00860 Porphyrin and chlorophyll metabolism;
gene44468	K09008	K09008	hypothetical protein	--		
gene44470	K03363	CDC20	cell division cycle 20, cofactor of APC complex	--	7	ko04110 Cell cycle;ko04111 Cell cycle - yeast;ko04113 Meiosis - yeast;ko04114 Oocyte meiosis;ko04120 Ubiquitin mediated proteolysis;ko05166 HTLV-I infection;ko05203 Viral carcinogenesis;
gene44474	K13124	MORG1	mitogen-activated protein kinase organizer 1	--		
gene44477	K12830	SF3B3, SAP130, RSE1	splicing factor 3B subunit 3	--	1	ko03040 Spliceosome;
gene44479	K02918	RP-L35e, RPL35	large subunit ribosomal protein L35e	--	1	ko03010 Ribosome;
gene44480	K01728	E4.2.2.2, pel	pectate lyase	EC:4.2.2.2	1	ko00040 Pentose and glucuronate interconversions;
gene44483	K15505	RAD5	DNA repair protein RAD5	EC:3.6.4.-		
gene44490	K13195	CIRBP	cold-inducible RNA-binding protein	--		
gene44491	K05864	PPID, CYPD	peptidyl-prolyl isomerase D	EC:5.2.1.8		
gene44498	K10884	XRCC6, KU70, G22P1	ATP-dependent DNA helicase 2 subunit 1	--	1	ko03450 Non-homologous end-joining;
gene444	K01778	dapF	diaminopimelate epimerase	EC:5.1.1.7	2	ko00300 Lysine biosynthesis;ko01230 Biosynthesis of amino acids;
gene44501	K09422	MYBP	myb proto-oncogene protein, plant	--		
gene44513	K11968	ARIH1	ariadne-1	--		
gene44514	K13412	CPK	calcium-dependent protein kinase	EC:2.7.11.1	2	ko04626 Plant-pathogen interaction;ko05145 Toxoplasmosis;
gene4451	K09839	VDE, NPQ1	violaxanthin de-epoxidase	EC:1.10.99.3	1	ko00906 Carotenoid biosynthesis;
gene44520	K03263	EIF5A	translation initiation factor 5A	--		
gene44521	K00863	E2.7.1.29, DAK1, DAK2	dihydroxyacetone kinase	EC:2.7.1.29	4	ko00561 Glycerolipid metabolism;ko00680 Methane metabolism;ko01200 Carbon metabolism;ko04622 RIG-I-like receptor signaling pathway;
gene44522	K05765	CFL	cofilin	--	4	ko04360 Axon guidance;ko04666 Fc gamma R-mediated phagocytosis;ko04810 Regulation of actin cytoskeleton;ko05133 Pertussis;
gene44523	K16277	DRIP	E3 ubiquitin-protein ligase DRIP	EC:6.3.2.19		
gene44525	K03453	TC.BASS	bile acid:Na+ symporter, BASS family	--		
gene44530	K01874	MARS, metG	methionyl-tRNA synthetase	EC:6.1.1.10	2	ko00450 Selenocompound metabolism;ko00970 Aminoacyl-tRNA biosynthesis;
gene44532	K17479	GRXCR1	glutaredoxin domain-containing cysteine-rich protein 1	--		
gene44535	K15445	TRM10, RG9MTD2	tRNA (guanine9-N1)-methyltransferase	EC:2.1.1.221		
gene44540	K14209	SLC36A, PAT	solute carrier family 36 (proton-coupled amino acid transporter)	--		
gene44546	K01054	MGLL	acylglycerol lipase	EC:3.1.1.23	2	ko00561 Glycerolipid metabolism;ko04723 Retrograde endocannabinoid signaling;
gene44548	K07767	E3.6.4.3	microtubule-severing ATPase	EC:3.6.4.3		
gene44549	K01444	AGA, aspG	N4-(beta-N-acetylglucosaminyl)-L-asparaginase	EC:3.5.1.26	2	ko00511 Other glycan degradation;ko04142 Lysosome;
gene44557	K06901	pbuG	putative MFS transporter, AGZA family, xanthine/uracil permease	--		
gene44559	K03301	TC.AAA	ATP:ADP antiporter, AAA family	--		
gene4455	K08065	NFYB	nuclear transcription Y subunit beta	--	3	ko04612 Antigen processing and presentation;ko05152 Tuberculosis;ko05166 HTLV-I infection;

gene44560	K07198	PRKAA, AMPK	5'-AMP-activated protein kinase, catalytic alpha subunit	EC:2.7.11.11	8	ko04140 Regulation of autophagy;ko04150 mTOR signaling pathway;ko04151 PI3K-Akt signaling pathway;ko04710 Circadian rhythm;ko04910 Insulin signaling pathway;ko04920 Adipocytokine signaling pathway;ko04932 Non-alcoholic fatty liver disease (NAFLD);ko05410 Hypertrophic cardiomyopathy (HCM);
gene44562	K02888	RP-L21, MRPL21, rplU	large subunit ribosomal protein L21	--	1	ko03010 Ribosome;
gene44568	K02949	RP-S11e, RPS11	small subunit ribosomal protein S11e	--	1	ko03010 Ribosome;
gene44574	K04733	IRAK4	interleukin-1 receptor-associated kinase 4	EC:2.7.11.1	11	ko04064 NF-kappa B signaling pathway;ko04210 Apoptosis;ko04620 Toll-like receptor signaling pathway;ko04722 Neurotrophin signaling pathway;ko05133 Pertussis;ko05140 Leishmaniasis;ko05142 Chagas disease (American trypanosomiasis);ko05145 Toxoplasmosis;ko05152 Tuberculosis;ko05162 Measles;ko05164 Influenza A;
gene4457	K14500	BSK	BR-signaling kinase	EC:2.7.11.1	1	ko04075 Plant hormone signal transduction;
gene44586	K01870	IARS, ileS	isoleucyl-tRNA synthetase	EC:6.1.1.5	1	ko00970 Aminoacyl-tRNA biosynthesis;
gene44590	K13495	CISZOG	cis-zeatin O-glucosyltransferase	EC:2.4.1.215	1	ko00908 Zeatin biosynthesis;
gene44599	K03978	engB	GTP-binding protein	--		
gene44607	K01115	PLD1_2	phospholipase D1/2	EC:3.1.4.4	7	ko00564 Glycerophospholipid metabolism;ko00565 Ether lipid metabolism;ko04014 Ras signaling pathway;ko04144 Endocytosis;ko04666 Fc gamma R-mediated phagocytosis;ko04724 Glutamatergic synapse;ko04912 GnRH signaling pathway;
gene44608	K12897	TRA2	transformer-2 protein	--	1	ko03040 Spliceosome;
gene44612	K09490	HSPA5, BIP	heat shock 70kDa protein 5	--	4	ko03060 Protein export;ko04141 Protein processing in endoplasmic reticulum;ko04918 Thyroid hormone synthesis;ko05020 Prion diseases;
gene44613	K02947	RP-S10e, RPS10	small subunit ribosomal protein S10e	--	1	ko03010 Ribosome;
gene44619	K12130	PRR5	pseudo-response regulator 5	--	1	ko04712 Circadian rhythm - plant;
gene4461	K01456	E3.5.1.52, NGLY1, PNG1	peptide-N4-(N-acetyl-beta-glucosaminyl)asparagine amidase	EC:3.5.1.52	1	ko04141 Protein processing in endoplasmic reticulum;
gene44631	K01213	E3.2.1.67	galacturan 1,4-alpha-galacturonidase	EC:3.2.1.67	2	ko00040 Pentose and glucuronate interconversions;ko00500 Starch and sucrose metabolism;
gene44632	K06100	SYMPK	symplekin	--	2	ko03015 mRNA surveillance pathway;ko04530 Tight junction;
gene44635	K09264	K09264	MADS-box transcription factor, plant	--		
gene44639	K01115	PLD1_2	phospholipase D1/2	EC:3.1.4.4	7	ko00564 Glycerophospholipid metabolism;ko00565 Ether lipid metabolism;ko04014 Ras signaling pathway;ko04144 Endocytosis;ko04666 Fc gamma R-mediated phagocytosis;ko04724 Glutamatergic synapse;ko04912 GnRH signaling pathway;
gene44640	K12451	UER1	3,5-epimerase/4-reductase	EC:5.1.3.- 1.1.1.-	2	ko00520 Amino sugar and nucleotide sugar metabolism;ko00523 Polyketide sugar unit biosynthesis;
gene44643	K09481	SEC61B, SBH2	protein transport protein SEC61 subunit beta	--	4	ko03060 Protein export;ko04141 Protein processing in endoplasmic reticulum;ko04145 Phagosome;ko05110 Vibrio cholerae infection;
gene44644	K10706	SETX, ALS4	senataxin	EC:3.6.4.-		
gene44649	K02519	infB, MTIF2	translation initiation factor IF-2	--		
gene44650	K10950	ERO1L	ERO1-like protein alpha	EC:1.8.4.-	2	ko04141 Protein processing in endoplasmic reticulum;ko05110 Vibrio cholerae infection;
gene44651	K01897	ACSL, fadD	long-chain acyl-CoA synthetase	EC:6.2.1.3	4	ko00071 Fatty acid degradation;ko03320 PPAR signaling pathway;ko04146 Peroxisome;ko04920 Adipocytokine signaling pathway;
gene44652	K15191	LARP7	La-related protein 7	--		
gene44653	K03259	EIF4E	translation initiation factor 4E	--	5	ko03013 RNA transport;ko04066 HIF-1 signaling pathway;ko04150 mTOR signaling pathway;ko04151 PI3K-Akt signaling pathway;ko04910 Insulin signaling pathway;
gene44661	K12840	RBM17, SPF45	splicing factor 45	--	1	ko03040 Spliceosome;
gene44662	K15501	PPP6R3, SAPS3	serine/threonine-protein phosphatase 6 regulatory subunit 3	--		
gene44663	K14793	RRP9	ribosomal RNA-processing protein 9	--		
gene44664	K14156	CHK	choline/ethanolamine kinase	EC:2.7.1.32 2.7.1.82	1	ko00564 Glycerophospholipid metabolism;
gene44665	K11446	JARID1	histone demethylase JARID1	EC:1.14.11.-		
gene44676	K03843	ALG2	alpha-1,3/alpha-1,6-mannosyltransferase	EC:2.4.1.132 2.4.1.257	2	ko00510 N-Glycan biosynthesis;ko00513 Various types of N-glycan biosynthesis;
gene44677	K14515	EBF1_2	EIN3-binding F-box protein	--	1	ko04075 Plant hormone signal transduction;

gene44679	K07305	msrB	peptide-methionine (R)-S-oxide reductase	EC:1.8.4.12		
gene44683	K01006	ppdK	pyruvate,orthophosphate dikinase	EC:2.7.9.1	3	ko00620 Pyruvate metabolism;ko00710 Carbon fixation in photosynthetic organisms;ko01200 Carbon metabolism;
gene4468	K14487	GH3	auxin responsive GH3 gene family	--	1	ko04075 Plant hormone signal transduction;
gene44691	K00016	LDH, ldh	L-lactate dehydrogenase	EC:1.1.1.27	4	ko00010 Glycolysis / Gluconeogenesis;ko00270 Cysteine and methionine metabolism;ko00620 Pyruvate metabolism;ko00640 Propanoate metabolism;
gene44692	K13130	SIP1, GEMIN2	survival of motor neuron protein-interacting protein 1	--	1	ko03013 RNA transport;
gene44697	K01079	serB, PSPH	phosphoserine phosphatase	EC:3.1.3.3	4	ko00260 Glycine, serine and threonine metabolism;ko00680 Methane metabolism;ko01200 Carbon metabolism;ko01230 Biosynthesis of amino acids;
gene446	K13448	CML	calcium-binding protein CML	--	1	ko04626 Plant-pathogen interaction;
gene44700	K00703	E2.4.1.21, glgA	starch synthase	EC:2.4.1.21	1	ko00500 Starch and sucrose metabolism;
gene44707	K06173	truA, PUS1	tRNA pseudouridine38-40 synthase	EC:5.4.99.12		
gene4470	K15889	PCME	prenylcysteine alpha-carboxyl methylesterase	EC:3.1.1.-	1	ko00900 Terpenoid backbone biosynthesis;
gene44712	K09955	K09955	hypothetical protein	--		
gene44713	K00026	MDH2	malate dehydrogenase	EC:1.1.1.37	5	ko00020 Citrate cycle (TCA cycle);ko00620 Pyruvate metabolism;ko00630 Glyoxylate and dicarboxylate metabolism;ko00710 Carbon fixation in photosynthetic organisms;ko01200 Carbon metabolism;
gene44717	K14003	PREB, SEC12	prolactin regulatory element-binding protein	--	1	ko04141 Protein processing in endoplasmic reticulum;
gene44718	K03859	PIGC, GPI2	phosphatidylinositol glycan, class C	--	1	ko00563 Glycosylphosphatidylinositol(GPI)-anchor biosynthesis;
gene44723	K09539	DNAJC19	DnaJ homolog subfamily C member 19	--		
gene4472	K01438	argE	acetylornithine deacetylase	EC:3.5.1.16	3	ko00330 Arginine and proline metabolism;ko01210 2-Oxocarboxylic acid metabolism;ko01230 Biosynthesis of amino acids;
gene44731	K13496	UGT73C	UDP-glucosyl transferase 73C	EC:2.4.1.-		
gene44732	K04733	IRAK4	interleukin-1 receptor-associated kinase 4	EC:2.7.11.1	11	ko04064 NF-kappa B signaling pathway;ko04210 Apoptosis;ko04620 Toll-like receptor signaling pathway;ko04722 Neurotrophin signaling pathway;ko05133 Pertussis;ko05140 Leishmaniasis;ko05142 Chagas disease (American trypanosomiasis);ko05145 Toxoplasmosis;ko05152 Tuberculosis;ko05162 Measles;ko05164 Influenza A;
gene44737	K01115	PLD1_2	phospholipase D1/2	EC:3.1.4.4	7	ko00564 Glycerophospholipid metabolism;ko00565 Ether lipid metabolism;ko04014 Ras signaling pathway;ko04144 Endocytosis;ko04666 Fc gamma R-mediated phagocytosis;ko04724 Glutamatergic synapse;ko04912 GnRH signaling pathway;
gene44738	K14638	SLC15A3_4, PHT	solute carrier family 15 (peptide/histidine transporter), member 3/4	--		
gene44742	K14442	DHX36, RHAU	ATP-dependent RNA helicase DHX36	EC:3.6.4.13	1	ko03018 RNA degradation;
gene44744	K13993	HSP20	HSP20 family protein	--	1	ko04141 Protein processing in endoplasmic reticulum;
gene44747	K12160	SUMO, SMT3	small ubiquitin-related modifier	--	1	ko03013 RNA transport;
gene44752	K08193	SLC17A	MFS transporter, ACS family, solute carrier family 17 (sodium-dependent inorganic phosphate cotransporter), other	--		
gene44756	K02987	RP-S4e, RPS4	small subunit ribosomal protein S4e	--	1	ko03010 Ribosome;
gene44758	K02213	CDC6	cell division control protein 6	--	3	ko04110 Cell cycle;ko04111 Cell cycle - yeast;ko04113 Meiosis - yeast;
gene44759	K02941	RP-LP0, RPLP0	large subunit ribosomal protein LP0	--	1	ko03010 Ribosome;
gene4475	K01897	ACSL, fadD	long-chain acyl-CoA synthetase	EC:6.2.1.3	4	ko00071 Fatty acid degradation;ko03320 PPAR signaling pathway;ko04146 Peroxisome;ko04920 Adipocytokine signaling pathway;
gene44762	K12188	SNF8, EAP30	ESCRT-II complex subunit VPS22	--	1	ko04144 Endocytosis;
gene44763	K03869	CUL3	cullin 3	--	1	ko04120 Ubiquitin mediated proteolysis;
gene44770	K08850	AURKX	aurora kinase, other	EC:2.7.11.1		
gene44772	K14492	ARR-A	two-component response regulator ARR-A family	--	1	ko04075 Plant hormone signal transduction;
gene44775	K01188	E3.2.1.21	beta-glucosidase	EC:3.2.1.21	3	ko00460 Cyanoamino acid metabolism;ko00500 Starch and sucrose metabolism;ko00940 Phenylpropanoid biosynthesis;

gene44777	K01940	E6.3.4.5, argG	argininosuccinate synthase	EC:6.3.4.5	3	ko00250 Alanine, aspartate and glutamate metabolism;ko00330 Arginine and proline metabolism;ko01230 Biosynthesis of amino acids;
gene4477	K05391	CNGF	cyclic nucleotide gated channel, other eukaryote	--	1	ko04626 Plant-pathogen interaction;
gene44782	K12121	PHYB	phytochrome B	--	1	ko04712 Circadian rhythm - plant;
gene44787	K00574	E2.1.1.79, cfa	cyclopropane-fatty-acyl-phospholipid synthase	EC:2.1.1.79		
gene44789	K00574	E2.1.1.79, cfa	cyclopropane-fatty-acyl-phospholipid synthase	EC:2.1.1.79		
gene44797	K03676	grxC, GLRX, GLRX2	glutaredoxin 3	--		
gene4479	K15692	RNF13, RZF	E3 ubiquitin-protein ligase RNF13	EC:6.3.2.19		
gene44800	K01883	CARS, cysS	cysteinyl-tRNA synthetase	EC:6.1.1.16	1	ko00970 Aminoacyl-tRNA biosynthesis;
gene44803	K03639	MOCS1, moaA	molybdenum cofactor biosynthesis protein	--	2	ko00790 Folate biosynthesis;ko04122 Sulfur relay system;
gene44804	K09595	HM13	minor histocompatibility antigen H13	EC:3.4.23.-		
gene44805	K00423	E1.10.3.3	L-ascorbate oxidase	EC:1.10.3.3	1	ko00053 Ascorbate and aldarate metabolism;
gene44808	K11816	YUCCA	indole-3-pyruvate monooxygenase	EC:1.14.13.168	1	ko00380 Tryptophan metabolism;
gene4480	K04730	IRAK1	interleukin-1 receptor-associated kinase 1	EC:2.7.11.1	11	ko04064 NF-kappa B signaling pathway;ko04210 Apoptosis;ko04620 Toll-like receptor signaling pathway;ko04722 Neurotrophin signaling pathway;ko05133 Pertussis;ko05140 Leishmaniasis;ko05142 Chagas disease (American trypanosomiasis);ko05145 Toxoplasmosis;ko05152 Tuberculosis;ko05162 Measles;ko05169 Epstein-Barr virus infection;
gene44810	K00844	HK	hexokinase	EC:2.7.1.1	12	ko00010 Glycolysis / Gluconeogenesis;ko00051 Fructose and mannose metabolism;ko00052 Galactose metabolism;ko00500 Starch and sucrose metabolism;ko00520 Amino sugar and nucleotide sugar metabolism;ko00521 Streptomycin biosynthesis;ko00524 Butirosin and neomycin biosynthesis;ko01200 Carbon metabolism;ko04066 HIF-1 signaling pathway;ko04910 Insulin signaling pathway;ko04930 Type II diabetes mellitus;ko04973 Carbohydrate digestion and absorption;
gene44815	K03046	rpoC	DNA-directed RNA polymerase subunit beta'	EC:2.7.7.6	3	ko00230 Purine metabolism;ko00240 Pyrimidine metabolism;ko03020 RNA polymerase;
gene44826	K03262	EIF5	translation initiation factor 5	--	1	ko03013 RNA transport;
gene44828	K03070	secA	preprotein translocase subunit SecA	--	2	ko03060 Protein export;ko03070 Bacterial secretion system;
gene44831	K00231	PPOX, hemY	oxygen-dependent protoporphyrinogen oxidase	EC:1.3.3.4	1	ko00860 Porphyrin and chlorophyll metabolism;
gene44832	K12875	ACIN1, ACINUS	apoptotic chromatin condensation inducer in the nucleus	--	3	ko03013 RNA transport;ko03015 mRNA surveillance pathway;ko03040 Spliceosome;
gene44833	K10904	TIPIN	TIMELESS-interacting protein	--		
gene44834	K10577	UBE2I, UBC9	ubiquitin-conjugating enzyme E2 I	EC:6.3.2.19	4	ko03013 RNA transport;ko04064 NF-kappa B signaling pathway;ko04120 Ubiquitin mediated proteolysis;ko05206 MicroRNAs in cancer;
gene44840	K00392	sir	sulfite reductase (ferredoxin)	EC:1.8.7.1	1	ko00920 Sulfur metabolism;
gene44853	K13412	CPK	calcium-dependent protein kinase	EC:2.7.11.1	2	ko04626 Plant-pathogen interaction;ko05145 Toxoplasmosis;
gene44856	K01246	tag	DNA-3-methyladenine glycosylase I	EC:3.2.2.20	1	ko03410 Base excision repair;
gene44858	K11254	H4	histone H4	--	3	ko05034 Alcoholism;ko05203 Viral carcinogenesis;ko05322 Systemic lupus erythematosus;
gene4485	K14807	DDX51, DBP6	ATP-dependent RNA helicase DDX51/DBP6	EC:3.6.4.13		
gene44862	K00430	E1.11.1.7	peroxidase	EC:1.11.1.7	2	ko00360 Phenylalanine metabolism;ko00940 Phenylpropanoid biosynthesis;
gene44864	K02943	RP-LP2, RPLP2	large subunit ribosomal protein LP2	--	1	ko03010 Ribosome;
gene44865	K12736	PPWD1	peptidylprolyl isomerase domain and WD repeat-containing protein 1	EC:5.2.1.8		
gene44866	K14846	RPF1	ribosome production factor 1	--		
gene44868	K00876	E2.7.1.48, udk	uridine kinase	EC:2.7.1.48	2	ko00240 Pyrimidine metabolism;ko00983 Drug metabolism - other enzymes;
gene44869	K16190	GLCAK	glucuronokinase	EC:2.7.1.43	3	ko00040 Pentose and glucuronate interconversions;ko00053 Ascorbate and aldarate metabolism;ko00520 Amino sugar and nucleotide sugar metabolism;
gene44878	K15191	LARP7	La-related protein 7	--		
gene44879	K01000	mraY	phospho-N-acetylmuramoyl-pentapeptide-transferase	EC:2.7.8.13	1	ko00550 Peptidoglycan biosynthesis;
gene4487	K11778	DHDDS, RER2, SRT1	ditrans,polycis-polyprenyl diphosphate synthase	EC:2.5.1.87	1	ko00900 Terpenoid backbone biosynthesis;
gene44881	K08490	STX5	syntaxin 5	--	1	ko04130 SNARE interactions in vesicular transport;
gene4488	K00423	E1.10.3.3	L-ascorbate oxidase	EC:1.10.3.3	1	ko00053 Ascorbate and aldarate metabolism;

gene44893	K15919	HPR2	hydroxypyruvate reductase 2	--	2	ko00260 Glycine, serine and threonine metabolism;ko00630 Glyoxylate and dicarboxylate metabolism;
gene44894	K10839	RAD23, HR23	UV excision repair protein RAD23	--	2	ko03420 Nucleotide excision repair;ko04141 Protein processing in endoplasmic reticulum;
gene44895	K08835	OXSR1, STK39	serine/threonine-protein kinase OSR1/STK39	EC:2.7.11.1		
gene44896	K01897	ACSL, fadD	long-chain acyl-CoA synthetase	EC:6.2.1.3	4	ko00071 Fatty acid degradation;ko03320 PPAR signaling pathway;ko04146 Peroxisome;ko04920 Adipocytokine signaling pathway;
gene44897	K12820	DHX15, PRP43	pre-mRNA-splicing factor ATP-dependent RNA helicase DHX15/PRP43	EC:3.6.4.13	1	ko03040 Spliceosome;
gene4489	K16743	ASPM, ASP	abnormal spindle-like microcephaly-associated protein	--		
gene448	K02910	RP-L31e, RPL31	large subunit ribosomal protein L31e	--	1	ko03010 Ribosome;
gene44900	K08511	ATVAMP72	vesicle-associated membrane protein 72	--		
gene44905	K14611	SLC23A1_2, SVCT1_2	solute carrier family 23 (nucleobase transporter), member 1/2	--		
gene44910	K14492	ARR-A	two-component response regulator ARR-A family	--	1	ko04075 Plant hormone signal transduction;
gene44915	K11000	CALS	callose synthase	EC:2.4.1.-		
gene44916	K01652	E2.2.1.6L, ilvB, ilvG, ilvI	acetolactate synthase I/II/III large subunit	EC:2.2.1.6	6	ko00290 Valine, leucine and isoleucine biosynthesis;ko00650 Butanoate metabolism;ko00660 C5-Branched dibasic acid metabolism;ko00770 Pantothenate and CoA biosynthesis;ko01210 2-Oxocarboxylic acid metabolism;ko01230 Biosynthesis of amino acids;
gene44917	K02868	RP-L11e, RPL11	large subunit ribosomal protein L11e	--	1	ko03010 Ribosome;
gene44918	K14488	SAUR	SAUR family protein	--	1	ko04075 Plant hormone signal transduction;
gene44919	K14844	PUF6	pumilio homology domain family member 6	--		
gene44924	K11000	CALS	callose synthase	EC:2.4.1.-		
gene44925	K06268	PPP3R, CNB	serine/threonine-protein phosphatase 2B regulatory subunit	--	18	ko04010 MAPK signaling pathway;ko04020 Calcium signaling pathway;ko04114 Oocyte meiosis;ko04210 Apoptosis;ko04310 Wnt signaling pathway;ko04360 Axon guidance;ko04370 VEGF signaling pathway;ko04380 Osteoclast differentiation;ko04650 Natural killer cell mediated cytotoxicity;ko04660 T cell receptor signaling pathway;ko04662 B cell receptor signaling pathway;ko04720 Long-term potentiation;ko04724 Glutamatergic synapse;ko05010 Alzheimer's disease;ko05014 Amyotrophic lateral sclerosis (ALS);ko05031 Amphetamine addiction;ko05152
gene44927	K14498	SNRK2	serine/threonine-protein kinase SRK2	EC:2.7.11.1	1	ko04075 Plant hormone signal transduction;
gene44928	K10632	BRAP	BRCA1-associated protein	EC:6.3.2.19	1	ko04014 Ras signaling pathway;
gene44933	K08790	STK38, NDR	serine/threonine kinase 38	EC:2.7.11.1		
gene44941	K14488	SAUR	SAUR family protein	--	1	ko04075 Plant hormone signal transduction;
gene44945	K03953	NDUFA9	NADH dehydrogenase (ubiquinone) 1 alpha subcomplex subunit 9	--	5	ko00190 Oxidative phosphorylation;ko04932 Non-alcoholic fatty liver disease (NAFLD);ko05010 Alzheimer's disease;ko05012 Parkinson's disease;ko05016 Huntington's disease;
gene44949	K14569	BMS1	ribosome biogenesis protein BMS1	--	1	ko03008 Ribosome biogenesis in eukaryotes;
gene44950	K00281	GLDC, gcvP	glycine dehydrogenase	EC:1.4.4.2	2	ko00260 Glycine, serine and threonine metabolism;ko01200 Carbon metabolism;
gene44953	K03781	katE, CAT, catB, srpA	catalase	EC:1.11.1.6	4	ko00380 Tryptophan metabolism;ko00630 Glyoxylate and dicarboxylate metabolism;ko04146 Peroxisome;ko05014 Amyotrophic lateral sclerosis (ALS);
gene44956	K05868	CCNB	cyclin B	--	3	ko04110 Cell cycle;ko04115 p53 signaling pathway;ko04914 Progesterone-mediated oocyte maturation;
gene44959	K04730	IRAK1	interleukin-1 receptor-associated kinase 1	EC:2.7.11.1	11	ko04064 NF-kappa B signaling pathway;ko04210 Apoptosis;ko04620 Toll-like receptor signaling pathway;ko04722 Neurotrophin signaling pathway;ko05133 Pertussis;ko05140 Leishmaniasis;ko05142 Chagas disease (American trypanosomiasis);ko05145 Toxoplasmosis;ko05152 Tuberculosis;ko05162 Measles;ko05169 Epstein-Barr virus infection;
gene44963	K00600	glyA, SHMT	glycine hydroxymethyltransferase	EC:2.1.2.1	7	ko00260 Glycine, serine and threonine metabolism;ko00460 Cyanoamino acid metabolism;ko00630 Glyoxylate and dicarboxylate metabolism;ko00670 One carbon pool by folate;ko00680 Methane metabolism;ko01200 Carbon metabolism;ko01230 Biosynthesis of amino acids;

gene44964	K03676	grxC, GLRX, GLRX2	glutaredoxin 3	--		
gene44968	K00387	SUOX	sulfite oxidase	EC:1.8.3.1	1	ko00920 Sulfur metabolism;
gene4496	K03267	ERF3, GSPT	peptide chain release factor subunit 3	--	1	ko03015 mRNA surveillance pathway;
gene44973	K10808	RRM2	ribonucleoside-diphosphate reductase subunit M2	EC:1.17.4.1	4	ko00230 Purine metabolism;ko00240 Pyrimidine metabolism;ko00480 Glutathione metabolism;ko04115 p53 signaling pathway;
gene44976	K00873	PK, pyk	pyruvate kinase	EC:2.7.1.40	7	ko00010 Glycolysis / Gluconeogenesis;ko00230 Purine metabolism;ko00620 Pyruvate metabolism;ko01200 Carbon metabolism;ko01230 Biosynthesis of amino acids;ko04930 Type II diabetes mellitus;ko05203 Viral carcinogenesis;
gene44979	K08511	ATVAMP72	vesicle-associated membrane protein 72	--		
gene4497	K13094	RBM5_10	RNA-binding protein 5/10	--		
gene44983	K12194	CHMP4, SNF7, VPS32	charged multivesicular body protein 4	--	1	ko04144 Endocytosis;
gene44	K05298	GAPA	glyceraldehyde-3-phosphate dehydrogenase (NADP+) (phosphorylating)	EC:1.2.1.13	2	ko00710 Carbon fixation in photosynthetic organisms;ko01200 Carbon metabolism;
gene45000	K10740	RPA3	replication factor A3	--	5	ko03030 DNA replication;ko03420 Nucleotide excision repair;ko03430 Mismatch repair;ko03440 Homologous recombination;ko03460 Fanconi anemia pathway;
gene45006	K10206	E2.6.1.83	LL-diaminopimelate aminotransferase	EC:2.6.1.83	2	ko00300 Lysine biosynthesis;ko01230 Biosynthesis of amino acids;
gene45009	K03124	TFIIB, GTF2B, SUA7, tfb	transcription initiation factor TFIIB	--	3	ko03022 Basal transcription factors;ko05169 Epstein-Barr virus infection;ko05203 Viral carcinogenesis;
gene4500	K03265	ETF1, ERF1	peptide chain release factor subunit 1	--	1	ko03015 mRNA surveillance pathway;
gene45025	K01205	NAGLU	alpha-N-acetylglucosaminidase	EC:3.2.1.50	2	ko00531 Glycosaminoglycan degradation;ko04142 Lysosome;
gene45027	K05643	ABCA3	ATP-binding cassette, subfamily A (ABC1), member 3	--	1	ko02010 ABC transporters;
gene45028	K01206	FUCA	alpha-L-fucosidase	EC:3.2.1.51	1	ko00511 Other glycan degradation;
gene45038	K05765	CFL	cofilin	--	4	ko04360 Axon guidance;ko04666 Fc gamma R-mediated phagocytosis;ko04810 Regulation of actin cytoskeleton;ko05133 Pertussis;
gene45040	K09338	HD-ZIP	homeobox-leucine zipper protein	--		
gene45042	K04706	PIAS1	E3 SUMO-protein ligase PIAS1	EC:6.3.2.-	3	ko04120 Ubiquitin mediated proteolysis;ko04630 Jak-STAT signaling pathway;ko05160 Hepatitis C;
gene45046	K02997	RP-S9e, RPS9	small subunit ribosomal protein S9e	--	1	ko03010 Ribosome;
gene45047	K09422	MYBP	myb proto-oncogene protein, plant	--		
gene45050	K12188	SNF8, EAP30	ESCRT-II complex subunit VPS22	--	1	ko04144 Endocytosis;
gene45052	K07976	RAB	Rab family, other	--		
gene45053	K13108	SNIP1	smad nuclear-interacting protein 1	--		
gene45055	K05933	E1.14.17.4	aminocyclopropanecarboxylate oxidase	EC:1.14.17.4	1	ko00270 Cysteine and methionine metabolism;
gene45062	K14786	KRI1	protein KRI1	--		
gene45064	K01979	SSUrRNA	small subunit ribosomal RNA	--	2	ko03008 Ribosome biogenesis in eukaryotes;ko03010 Ribosome;
gene45065	K01982	LSUrRNA	large subunit ribosomal RNA	--	2	ko03008 Ribosome biogenesis in eukaryotes;ko03010 Ribosome;
gene45066	K01979	SSUrRNA	small subunit ribosomal RNA	--	2	ko03008 Ribosome biogenesis in eukaryotes;ko03010 Ribosome;
gene45067	K14231	tRNA-Phe	tRNA Phe	--	1	ko00970 Aminoacyl-tRNA biosynthesis;
gene45068	K14234	tRNA-Thr	tRNA Thr	--	1	ko00970 Aminoacyl-tRNA biosynthesis;
gene45069	K01462	PDF, def	peptide deformylase	EC:3.5.1.88		
gene45070	K00430	E1.11.1.7	peroxidase	EC:1.11.1.7	2	ko00360 Phenylalanine metabolism;ko00940 Phenylpropanoid biosynthesis;
gene45071	K14516	ERF1	ethylene-responsive transcription factor 1	--	1	ko04075 Plant hormone signal transduction;
gene45073	K02265	COX5B	cytochrome c oxidase subunit 5b	--	6	ko00190 Oxidative phosphorylation;ko04260 Cardiac muscle contraction;ko04932 Non-alcoholic fatty liver disease (NAFLD);ko05010 Alzheimer's disease;ko05012 Parkinson's disease;ko05016 Huntington's disease;
gene45077	K06130	LYPLA2	lysophospholipase II	EC:3.1.1.5	1	ko00564 Glycerophospholipid metabolism;
gene45078	K07390	grxD, GLRX5	monothiol glutaredoxin	--		
gene45088	K12623	LSM4	U6 snRNA-associated Sm-like protein LSm4	--	2	ko03018 RNA degradation;ko03040 Spliceosome;
gene45089	K11320	EP400	E1A-binding protein p400	EC:3.6.4.-		

gene45091	K02327	POLD1	DNA polymerase delta subunit 1	EC:2.7.7.7	8	ko00230 Purine metabolism;ko00240 Pyrimidine metabolism;ko03030 DNA replication;ko03410 Base excision repair;ko03420 Nucleotide excision repair;ko03430 Mismatch repair;ko03440 Homologous recombination;ko05166 HTLV-I infection;
gene45093	K13993	HSP20	HSP20 family protein	--	1	ko04141 Protein processing in endoplasmic reticulum;
gene45095	K09486	HYOU1	hypoxia up-regulated 1	--	1	ko04141 Protein processing in endoplasmic reticulum;
gene45096	K12448	UXE	UDP-arabinose 4-epimerase	EC:5.1.3.5	1	ko00520 Amino sugar and nucleotide sugar metabolism;
gene45097	K12617	PATL1, PAT1	DNA topoisomerase 2-associated protein PAT1	--	1	ko03018 RNA degradation;
gene4509	K15119	SLC25A39_40	solute carrier family 25, member 39/40	--		
gene45105	K07375	TUBB	tubulin beta	--	3	ko04145 Phagosome;ko04540 Gap junction;ko05130 Pathogenic Escherichia coli infection;
gene45107	K13420	FLS2	LRR receptor-like serine/threonine-protein kinase FLS2	EC:2.7.11.1	1	ko04626 Plant-pathogen interaction;
gene45108	K13420	FLS2	LRR receptor-like serine/threonine-protein kinase FLS2	EC:2.7.11.1	1	ko04626 Plant-pathogen interaction;
gene45110	K12898	HNRNPF_H	heterogeneous nuclear ribonucleoprotein F/H	--		
gene45111	K10802	HMGB1	high mobility group protein B1	--	1	ko03410 Base excision repair;
gene45112	K06928	K06928	nucleoside-triphosphatase THEP1	EC:3.6.1.15	2	ko00230 Purine metabolism;ko00730 Thiamine metabolism;
gene45115	K14209	SLC36A, PAT	solute carrier family 36 (proton-coupled amino acid transporter)	--		
gene45119	K10838	XPC	xeroderma pigmentosum group C-complementing protein	--	1	ko03420 Nucleotide excision repair;
gene45123	K01869	LARS, leuS	leucyl-tRNA synthetase	EC:6.1.1.4	1	ko00970 Aminoacyl-tRNA biosynthesis;
gene45126	K03144	TFIIH4, GTF2H4, TFB2	transcription initiation factor TFIIH subunit 4	--	3	ko03022 Basal transcription factors;ko03420 Nucleotide excision repair;ko05203 Viral carcinogenesis;
gene45128	K04712	DEGS	sphingolipid delta-4 desaturase	EC:1.14.-.-	1	ko00600 Sphingolipid metabolism;
gene45131	K11434	PRMT1	protein arginine N-methyltransferase 1	EC:2.1.1.-		
gene45132	K12837	U2AF2	splicing factor U2AF 65 kDa subunit	--	1	ko03040 Spliceosome;
gene45139	K00472	E1.14.11.2	prolyl 4-hydroxylase	EC:1.14.11.2	1	ko00330 Arginine and proline metabolism;
gene45148	K01102	PDP	pyruvate dehydrogenase phosphatase	EC:3.1.3.43		
gene4514	K13420	FLS2	LRR receptor-like serine/threonine-protein kinase FLS2	EC:2.7.11.1	1	ko04626 Plant-pathogen interaction;
gene45156	K02991	RP-S6e, RPS6	small subunit ribosomal protein S6e	--	6	ko03010 Ribosome;ko04066 HIF-1 signaling pathway;ko04150 mTOR signaling pathway;ko04151 PI3K-Akt signaling pathway;ko04910 Insulin signaling pathway;ko05205 Proteoglycans in cancer;
gene45157	K17499	PPM1G, PP2CG	protein phosphatase 1G	EC:3.1.3.16		
gene45160	K00030	IDH3	isocitrate dehydrogenase (NAD+)	EC:1.1.1.41	4	ko00020 Citrate cycle (TCA cycle);ko01200 Carbon metabolism;ko01210 2-Oxocarboxylic acid metabolism;ko01230 Biosynthesis of amino acids;
gene45163	K04382	PPP2C	serine/threonine-protein phosphatase 2A catalytic subunit	EC:3.1.3.16	13	ko03015 mRNA surveillance pathway;ko04111 Cell cycle - yeast;ko04113 Meiosis - yeast;ko04114 Oocyte meiosis;ko04151 PI3K-Akt signaling pathway;ko04350 TGF-beta signaling pathway;ko04390 Hippo signaling pathway;ko04391 Hippo signaling pathway - fly;ko04530 Tight junction;ko04728 Dopaminergic synapse;ko04730 Long-term depression;ko05142 Chagas disease (American trypanosomiasis);ko05160 Hepatitis C;
gene45188	K09842	AAO3	abscisic-aldehyde oxidase	EC:1.2.3.14	1	ko00906 Carotenoid biosynthesis;
gene45189	K13420	FLS2	LRR receptor-like serine/threonine-protein kinase FLS2	EC:2.7.11.1	1	ko04626 Plant-pathogen interaction;
gene4518	K07904	RAB11A	Ras-related protein Rab-11A	--	4	ko04144 Endocytosis;ko04961 Endocrine and other factor-regulated calcium reabsorption;ko04962 Vasopressin-regulated water reabsorption;ko04972 Pancreatic secretion;
gene45192	K01648	ACLY	ATP citrate (pro-S)-lyase	EC:2.3.3.8	2	ko00020 Citrate cycle (TCA cycle);ko00720 Carbon fixation pathways in prokaryotes;
gene45203	K16297	SCPL-II	serine carboxypeptidase-like clade II	EC:3.4.16.-		
gene45204	K07195	EXOC7, EXO70	exocyst complex component 7	--	1	ko04910 Insulin signaling pathway;
gene45207	K01802	E5.2.1.8	peptidylprolyl isomerase	EC:5.2.1.8		
gene45210	K01537	E3.6.3.8	Ca2+-transporting ATPase	EC:3.6.3.8		
gene4522	K10601	SYVN1, HRD1	E3 ubiquitin-protein ligase synoviolin	EC:6.3.2.19	2	ko04120 Ubiquitin mediated proteolysis;ko04141 Protein processing in endoplasmic reticulum;

gene45240	K08909	LHCA3	light-harvesting complex I chlorophyll a/b binding protein 3	--	1	ko00196 Photosynthesis - antenna proteins;
gene45241	K07877	RAB2A	Ras-related protein Rab-2A	--		
gene45242	K06689	UBE2D_E, UBC4, UBC5	ubiquitin-conjugating enzyme E2 D/E	EC:6.3.2.19	2	ko04120 Ubiquitin mediated proteolysis;ko04141 Protein processing in endoplasmic reticulum;
gene45247	K03363	CDC20	cell division cycle 20, cofactor of APC complex	--	7	ko04110 Cell cycle;ko04111 Cell cycle - yeast;ko04113 Meiosis - yeast;ko04114 Oocyte meiosis;ko04120 Ubiquitin mediated proteolysis;ko05166 HTLV-I infection;ko05203 Viral carcinogenesis;
gene45254	K03627	MBF1	putative transcription factor	--		
gene45263	K13420	FLS2	LRR receptor-like serine/threonine-protein kinase FLS2	EC:2.7.11.1	1	ko04626 Plant-pathogen interaction;
gene45269	K03283	HSPA1_8	heat shock 70kDa protein 1/8	--	11	ko03040 Spliceosome;ko04010 MAPK signaling pathway;ko04141 Protein processing in endoplasmic reticulum;ko04144 Endocytosis;ko04612 Antigen processing and presentation;ko04915 Estrogen signaling pathway;ko05134 Legionellosis;ko05145 Toxoplasmosis;ko05162 Measles;ko05164 Influenza A;ko05169 Epstein-Barr virus infection;
gene45270	K06268	PPP3R, CNB	serine/threonine-protein phosphatase 2B regulatory subunit	--	18	ko04010 MAPK signaling pathway;ko04020 Calcium signaling pathway;ko04114 Oocyte meiosis;ko04210 Apoptosis;ko04310 Wnt signaling pathway;ko04360 Axon guidance;ko04370 VEGF signaling pathway;ko04380 Osteoclast differentiation;ko04650 Natural killer cell mediated cytotoxicity;ko04660 T cell receptor signaling pathway;ko04662 B cell receptor signaling pathway;ko04720 Long-term potentiation;ko04724 Glutamatergic synapse;ko05010 Alzheimer's disease;ko05014 Amyotrophic lateral sclerosis (ALS);ko05031 Amphetamine addiction;ko05152
gene45271	K01835	pgm	phosphoglucomutase	EC:5.4.2.2	7	ko00010 Glycolysis / Gluconeogenesis;ko00030 Pentose phosphate pathway;ko00052 Galactose metabolism;ko00230 Purine metabolism;ko00500 Starch and sucrose metabolism;ko00520 Amino sugar and nucleotide sugar metabolism;ko00521 Streptomycin biosynthesis;
gene45272	K10357	MYO5	myosin V	--		
gene45276	K15356	VRG4, GONST1	GDP-mannose transporter	--		
gene45279	K00208	fabI	enoyl-[acyl-carrier protein] reductase I	EC:1.3.1.9 1.3.1.10	2	ko00061 Fatty acid biosynthesis;ko00780 Biotin metabolism;
gene45286	K00059	fabG	3-oxoacyl-[acyl-carrier protein] reductase	EC:1.1.1.100	3	ko00061 Fatty acid biosynthesis;ko00780 Biotin metabolism;ko01040 Biosynthesis of unsaturated fatty acids;
gene45287	K01426	E3.5.1.4, amiE	amidase	EC:3.5.1.4	5	ko00330 Arginine and proline metabolism;ko00360 Phenylalanine metabolism;ko00380 Tryptophan metabolism;ko00627 Aminobenzoate degradation;ko00643 Styrene degradation;
gene45289	K00606	panB	3-methyl-2-oxobutanoate hydroxymethyltransferase	EC:2.1.2.11	1	ko00770 Pantothenate and CoA biosynthesis;
gene45294	K03505	POLD4	DNA polymerase delta subunit 4	--	8	ko00230 Purine metabolism;ko00240 Pyrimidine metabolism;ko03030 DNA replication;ko03410 Base excision repair;ko03420 Nucleotide excision repair;ko03430 Mismatch repair;ko03440 Homologous recombination;ko05166 HTLV-I infection;
gene45295	K15688	MUL1	E3 ubiquitin-protein ligase MUL1	EC:6.3.2.19		
gene45299	K08336	ATG12	autophagy-related protein 12	--	2	ko04140 Regulation of autophagy;ko04622 RIG-I-like receptor signaling pathway;
gene452	K07119	K07119				
gene45300	K03260	EIF4G	translation initiation factor 4G	--	2	ko03013 RNA transport;ko05416 Viral myocarditis;
gene45308	K08518	STXBP5	syntaxin-binding protein 5	--		
gene45311	K00770	E2.4.2.24	1,4-beta-D-xylan synthase	EC:2.4.2.24	2	ko00500 Starch and sucrose metabolism;ko00520 Amino sugar and nucleotide sugar metabolism;
gene45314	K08489	STX16	syntaxin 16	--	1	ko04130 SNARE interactions in vesicular transport;
gene4531	K10357	MYO5	myosin V	--		
gene45321	K14232	tRNA-Pro	tRNA Pro	--	1	ko00970 Aminoacyl-tRNA biosynthesis;
gene45323	K13051	iaaA, ASRGL1	beta-aspartyl-peptidase (threonine type)	EC:3.4.19.5		
gene45326	K01537	E3.6.3.8	Ca2+-transporting ATPase	EC:3.6.3.8		
gene45329	K00765	hisG	ATP phosphoribosyltransferase	EC:2.4.2.17	2	ko00340 Histidine metabolism;ko01230 Biosynthesis of amino acids;
gene45330	K14824	ERB1, BOP1	ribosome biogenesis protein ERB1	--		

gene45334	K13989	DERL2_3	Derlin-2/3	--	1	ko04141 Protein processing in endoplasmic reticulum;
gene45335	K07195	EXOC7, EXO70	exocyst complex component 7	--	1	ko04910 Insulin signaling pathway;
gene45344	K15601	KDM3	lysine-specific demethylase 3	EC:1.14.11.-		
gene45354	K04733	IRAK4	interleukin-1 receptor-associated kinase 4	EC:2.7.11.1	11	ko04064 NF-kappa B signaling pathway;ko04210 Apoptosis;ko04620 Toll-like receptor signaling pathway;ko04722 Neurotrophin signaling pathway;ko05133 Pertussis;ko05140 Leishmaniasis;ko05142 Chagas disease (American trypanosomiasis);ko05145 Toxoplasmosis;ko05152 Tuberculosis;ko05162 Measles;ko05164 Influenza A;
gene45355	K11251	H2A	histone H2A	--	2	ko05034 Alcoholism;ko05322 Systemic lupus erythematosus;
gene45356	K15397	KCS	3-ketoacyl-CoA synthase	EC:2.3.1.199	1	ko00062 Fatty acid elongation;
gene45365	K14492	ARR-A	two-component response regulator ARR-A family	--	1	ko04075 Plant hormone signal transduction;
gene45367	K01760	metC	cystathionine beta-lyase	EC:4.4.1.8	3	ko00270 Cysteine and methionine metabolism;ko00450 Selenocompound metabolism;ko01230 Biosynthesis of amino acids;
gene45368	K06677	YCS4, CNAP1, CAPD2	condensin complex subunit 1	--	1	ko04111 Cell cycle - yeast;
gene4536	K10400	KIF15	kinesin family member 15	--		
gene45370	K13989	DERL2_3	Derlin-2/3	--	1	ko04141 Protein processing in endoplasmic reticulum;
gene45371	K16275	BAH, NLA	E3 ubiquitin-protein ligase BAH	EC:6.3.2.19		
gene45375	K08851	TP53RK, PRPK, BUD32	TP53 regulating kinase	EC:2.7.11.1		
gene45381	K06617	E2.4.1.82	raffinose synthase	EC:2.4.1.82	1	ko00052 Galactose metabolism;
gene45382	K13412	CPK	calcium-dependent protein kinase	EC:2.7.11.1	2	ko04626 Plant-pathogen interaction;ko05145 Toxoplasmosis;
gene45384	K03327	TC.MATE, SLC47A, norM, mdtK, dinF	multidrug resistance protein, MATE family	--		
gene45389	K07583	PUS10	tRNA pseudouridine synthase 10	EC:5.4.99.-		
gene45390	K01322	E3.4.21.26, PREP	prolyl oligopeptidase	EC:3.4.21.26		
gene45391	K08832	SRPK3, STK23	serine/threonine-protein kinase SRPK3	EC:2.7.11.1		
gene45392	K12472	EPS15	epidermal growth factor receptor substrate 15	--	1	ko04144 Endocytosis;
gene45393	K14209	SLC36A, PAT	solute carrier family 36 (proton-coupled amino acid transporter)	--		
gene45399	K14638	SLC15A3_4, PHT	solute carrier family 15 (peptide/histidine transporter), member 3/4	--		
gene4539	K02727	PSMA3	20S proteasome subunit alpha 7	EC:3.4.25.1	1	ko03050 Proteasome;
gene453	K07119	K07119				
gene45400	K01535	E3.6.3.6	H+-transporting ATPase	EC:3.6.3.6	1	ko00190 Oxidative phosphorylation;
gene45407	K08829	MAK	male germ cell-associated kinase	EC:2.7.11.22		
gene45415	K09843	E1.14.13.93	(+)-abscisic acid 8'-hydroxylase	EC:1.14.13.93	1	ko00906 Carotenoid biosynthesis;
gene45417	K05868	CCNB	cyclin B	--	3	ko04110 Cell cycle;ko04115 p53 signaling pathway;ko04914 Progesterone-mediated oocyte maturation;
gene45418	K13811	PAPSS	3'-phosphoadenosine 5'-phosphosulfate synthase	EC:2.7.7.4 2.7.1.25	3	ko00230 Purine metabolism;ko00450 Selenocompound metabolism;ko00920 Sulfur metabolism;
gene45427	K02896	RP-L24e, RPL24	large subunit ribosomal protein L24e	--	1	ko03010 Ribosome;
gene4542	K09285	OVM, ANT	AP2-like factor, ANT lineage	--		
gene45430	K02527	kdtA, waaA	3-deoxy-D-manno-octulosonic-acid transferase	EC:2.4.99.12 2.4.99.13 2.4.99.14 2.4.99.15	1	ko00540 Lipopolysaccharide biosynthesis;
gene45435	K08241	E2.1.1.141	jasmonate O-methyltransferase	EC:2.1.1.141	1	ko00592 alpha-Linolenic acid metabolism;
gene45436	K15032	MTERFD	mTERF domain-containing protein, mitochondrial	--		
gene45437	K09422	MYBP	myb proto-oncogene protein, plant	--		
gene45447	K08286	E2.7.11.-	protein-serine/threonine kinase	EC:2.7.11.-		
gene45448	K02882	RP-L18Ae, RPL18A	large subunit ribosomal protein L18Ae	--	1	ko03010 Ribosome;
gene45450	K01455	E3.5.1.49	formamidase	EC:3.5.1.49	3	ko00630 Glyoxylate and dicarboxylate metabolism;ko00910 Nitrogen metabolism;ko01200 Carbon metabolism;
gene45458	K09510	DNAJB4	DnaJ homolog subfamily B member 4	--		
gene45463	K15014	SLC29A1_2_3, ENT1_2_3	solute carrier family 29 (equilibrative nucleoside transporter), member 1/2/3	--		
gene45465	K17506	PPM1L, PP2CE	protein phosphatase 1L	EC:3.1.3.16		

gene45469	K07195	EXOC7, EXO70	exocyst complex component 7	--	1	ko04910 Insulin signaling pathway;
gene45470	K12620	LSM1	U6 snRNA-associated Sm-like protein LSm1	--	1	ko03018 RNA degradation;
gene45471	K16911	DDX21	ATP-dependent RNA helicase DDX21	EC:3.6.4.13		
gene45474	K02924	RP-L39e, RPL39	large subunit ribosomal protein L39e	--	1	ko03010 Ribosome;
gene45475	K15030	EIF3M	translation initiation factor 3 subunit M	--		
gene45478	K14232	tRNA-Pro	tRNA Pro	--	1	ko00970 Aminoacyl-tRNA biosynthesis;
gene45481	K14505	CYCD3	cyclin D3, plant	--	1	ko04075 Plant hormone signal transduction;
gene45483	K09660	MPDU1	mannose-P-dolichol utilization defect 1	--		
gene45487	K13648	GAUT	alpha-1,4-galacturonosyltransferase	EC:2.4.1.43	2	ko00500 Starch and sucrose metabolism;ko00520 Amino sugar and nucleotide sugar metabolism;
gene4548	K03521	fixA, etfB	electron transfer flavoprotein beta subunit	--		
gene45490	K01658	trpG	anthranilate synthase component II	EC:4.1.3.27	2	ko00400 Phenylalanine, tyrosine and tryptophan biosynthesis;ko01230 Biosynthesis of amino acids;
gene45501	K14231	tRNA-Phe	tRNA Phe	--	1	ko00970 Aminoacyl-tRNA biosynthesis;
gene45502	K04733	IRAK4	interleukin-1 receptor-associated kinase 4	EC:2.7.11.1	11	ko04064 NF-kappa B signaling pathway;ko04210 Apoptosis;ko04620 Toll-like receptor signaling pathway;ko04722 Neurotrophin signaling pathway;ko05133 Pertussis;ko05140 Leishmaniasis;ko05142 Chagas disease (American trypanosomiasis);ko05145 Toxoplasmosis;ko05152 Tuberculosis;ko05162 Measles;ko05164 Influenza A;
gene45503	K06617	E2.4.1.82	raffinose synthase	EC:2.4.1.82	1	ko00052 Galactose metabolism;
gene45506	K07953	SAR1	GTP-binding protein SAR1	EC:3.6.5.-	2	ko04141 Protein processing in endoplasmic reticulum;ko05134 Legionellosis;
gene45512	K04371	MAPK1_3	mitogen-activated protein kinase 1/3	EC:2.7.11.24	71	ko04010 MAPK signaling pathway;ko04012 ErbB signaling pathway;ko04013 MAPK signaling pathway - fly;ko04014 Ras signaling pathway;ko04062 Chemokine signaling pathway;ko04066 HIF-1 signaling pathway;ko04114 Oocyte meiosis;ko04150 mTOR signaling pathway;ko04151 PI3K-Akt signaling pathway;ko04270 Vascular smooth muscle contraction;ko04320 Dorso-ventral axis formation;ko04350 TGF-beta signaling pathway;ko04360 Axon guidance;ko04370 VEGF signaling pathway;ko04380 Osteoclast differentiation;ko04510 Focal adhesion;ko04520 Adherens junction;ko04540 Gap junction;ko04620 Toll-like receptor signaling pathway;ko04621 NOD-like receptor signaling pathway;ko04650 Natural killer cell mediated cytotoxicity;ko04660 T cell receptor signaling pathway;ko04662 B cell receptor signaling pathway;ko04664 Fc epsilon RI signaling pathway;ko04666 Fc gamma R-mediated phagocytosis;ko04668 TNF signaling pathway;ko04713 Circadian entrainment;ko04720 Long-term potentiation;ko04722 Neurotrophin signaling pathway;ko04723 Retrograde endocannabinoid signaling;ko04724 Glutamatergic synapse;ko04725 Cholinergic synapse;ko04726 Serotonergic synapse;ko04730 Long-term depression;ko04810 Regulation of actin cytoskeleton;ko04910 Insulin signaling pathway;ko04912 GnRH signaling pathway;ko04914 Progesterone-mediated oocyte maturation;ko04915 Estrogen signaling pathway;ko04916 Melanogenesis;ko04917 Prolactin signaling pathway;ko04930 Type II diabetes mellitus;ko04960 Aldosterone-regulated sodium reabsorption;ko05010 Alzheimer's disease;ko05020 Prion diseases;ko05034 Alcoholism;ko05131 Shigellosis;ko05132 Salmonella infection;ko05133 Pertussis;ko05140 Leishmaniasis;ko05142 Chagas disease (American trypanosomiasis);ko05145 Toxoplasmosis;ko05152 Tuberculosis;ko05160 Hepatitis C;ko05161 Hepatitis B;ko05164 Influenza A;ko05200 Pathways in cancer;ko05203 Viral carcinogenesis;ko05205 Proteoglycans in cancer;ko05210 Colorectal cancer;ko05211 Renal cell carcinoma;ko05212
gene45517	K08235	E2.4.1.207	xyloglucan:xyloglucosyl transferase	EC:2.4.1.207		
gene4551	K01051	E3.1.1.11	pectinesterase	EC:3.1.1.11	2	ko00040 Pentose and glucuronate interconversions;ko00500 Starch and sucrose metabolism;
gene45522	K14753	RACK1	guanine nucleotide-binding protein subunit beta-2-like 1 protein	--	1	ko05162 Measles;

gene45523	K05857	PLCD	phosphatidylinositol phospholipase C, delta	EC:3.1.4.11	3	ko00562 Inositol phosphate metabolism;ko04020 Calcium signaling pathway;ko04070 Phosphatidylinositol signaling system;
gene45526	K15305	VAC14, TAX1BP2	vacuole morphology and inheritance protein 14	--	2	ko05166 HTLV-I infection;ko05203 Viral carcinogenesis;
gene4552	K04482	RAD51	DNA repair protein RAD51	--	4	ko03440 Homologous recombination;ko03460 Fanconi anemia pathway;ko05200 Pathways in cancer;ko05212 Pancreatic cancer;
gene45530	K03575	mutY	A/G-specific adenine glycosylase	EC:3.2.2.-	1	ko03410 Base excision repair;
gene45534	K15216	RRN3, TIFIA	RNA polymerase I-specific transcription initiation factor RRN3	--		
gene45545	K04733	IRAK4	interleukin-1 receptor-associated kinase 4	EC:2.7.11.1	11	ko04064 NF-kappa B signaling pathway;ko04210 Apoptosis;ko04620 Toll-like receptor signaling pathway;ko04722 Neurotrophin signaling pathway;ko05133 Pertussis;ko05140 Leishmaniasis;ko05142 Chagas disease (American trypanosomiasis);ko05145 Toxoplasmosis;ko05152 Tuberculosis;ko05162 Measles;ko05164 Influenza A;
gene45553	K13696	ABHD1_3	abhydrolase domain-containing protein 1/3	--		
gene45554	K13696	ABHD1_3	abhydrolase domain-containing protein 1/3	--		
gene45557	K14962	WDR82, SWD2, CPS35	COMPASS component SWD2	--	1	ko03015 mRNA surveillance pathway;
gene45558	K01184	E3.2.1.15	polygalacturonase	EC:3.2.1.15	2	ko00040 Pentose and glucuronate interconversions;ko00500 Starch and sucrose metabolism;
gene45560	K04733	IRAK4	interleukin-1 receptor-associated kinase 4	EC:2.7.11.1	11	ko04064 NF-kappa B signaling pathway;ko04210 Apoptosis;ko04620 Toll-like receptor signaling pathway;ko04722 Neurotrophin signaling pathway;ko05133 Pertussis;ko05140 Leishmaniasis;ko05142 Chagas disease (American trypanosomiasis);ko05145 Toxoplasmosis;ko05152 Tuberculosis;ko05162 Measles;ko05164 Influenza A;
gene45565	K03256	TRM6, GCD10	tRNA (adenine-N(1)-)-methyltransferase non-catalytic subunit	--		
gene45566	K06620	E2F3	transcription factor E2F3	--	13	ko04110 Cell cycle;ko05161 Hepatitis B;ko05166 HTLV-I infection;ko05200 Pathways in cancer;ko05206 MicroRNAs in cancer;ko05212 Pancreatic cancer;ko05214 Glioma;ko05215 Prostate cancer;ko05218 Melanoma;ko05219 Bladder cancer;ko05220 Chronic myeloid leukemia;ko05222 Small cell lung cancer;ko05223 Non-small cell lung cancer;
gene45567	K17338	REEP1_2_3_4	receptor expression-enhancing protein 1/2/3/4	--		
gene45568	K13066	E2.1.1.68, COMT	caffeic acid 3-O-methyltransferase	EC:2.1.1.68	1	ko00940 Phenylpropanoid biosynthesis;
gene45574	K10293	FBXO7	F-box protein 7	--		
gene45578	K01623	ALDO	fructose-bisphosphate aldolase, class I	EC:4.1.2.13	7	ko00010 Glycolysis / Gluconeogenesis;ko00030 Pentose phosphate pathway;ko00051 Fructose and mannose metabolism;ko00680 Methane metabolism;ko00710 Carbon fixation in photosynthetic organisms;ko01200 Carbon metabolism;ko01230 Biosynthesis of amino acids;
gene45579	K08150	SLC2A13, ITR	MFS transporter, SP family, solute carrier family 2 (myo-inositol transporter), member 13	--		
gene45582	K03083	GSK3B	glycogen synthase kinase 3 beta	EC:2.7.11.26	29	ko04012 ErbB signaling pathway;ko04062 Chemokine signaling pathway;ko04110 Cell cycle;ko04151 PI3K-Akt signaling pathway;ko04310 Wnt signaling pathway;ko04340 Hedgehog signaling pathway;ko04360 Axon guidance;ko04390 Hippo signaling pathway;ko04510 Focal adhesion;ko04660 T cell receptor signaling pathway;ko04662 B cell receptor signaling pathway;ko04711 Circadian rhythm - fly;ko04722 Neurotrophin signaling pathway;ko04728 Dopaminergic synapse;ko04910 Insulin signaling pathway;ko04916 Melanogenesis;ko04917 Prolactin signaling pathway;ko04932 Non-alcoholic fatty liver disease (NAFLD);ko05010 Alzheimer's disease;ko05160 Hepatitis C;ko05162 Measles;ko05164 Influenza A;ko05166 HTLV-I infection;ko05169 Epstein-Barr virus infection;ko05200 Pathways in cancer;ko05210 Colorectal cancer;ko05213 Endometrial cancer;ko05215 Prostate
gene45583	K07937	ARF1	ADP-ribosylation factor 1	--	2	ko05110 Vibrio cholerae infection;ko05134 Legionellosis;
gene45587	K15472	CYP71D55	premnaspirodiene oxygenase	EC:1.14.13.121	1	ko00909 Sesquiterpenoid and triterpenoid biosynthesis;

gene4558	K16750	BLOC1S2	biogenesis of lysosome-related organelles complex 1 subunit 2	--		
gene45593	K11883	NOB1	RNA-binding protein NOB1	--	1	ko03008 Ribosome biogenesis in eukaryotes;
gene45594	K12235	SRR	serine racemase	EC:5.1.1.18	1	ko00260 Glycine, serine and threonine metabolism;
gene45595	K01285	PRCP	lysosomal Pro-X carboxypeptidase	EC:3.4.16.2	1	ko04974 Protein digestion and absorption;
gene45597	K08516	YKT6	synaptobrevin homolog YKT6	--	1	ko04130 SNARE interactions in vesicular transport;
gene455	K03549	kup	KUP system potassium uptake protein	--		
gene45603	K06890	K06890				
gene45605	K00284	E1.4.7.1	glutamate synthase (ferredoxin)	EC:1.4.7.1	2	ko00630 Glyoxylate and dicarboxylate metabolism;ko00910 Nitrogen metabolism;
gene45608	K15341	DCLRE1B, SNM1B	DNA cross-link repair 1B protein	EC:3.1.-.-		
gene45610	K13508	GPAT	glycerol-3-phosphate acyltransferase	EC:2.3.1.15	2	ko00561 Glycerolipid metabolism;ko00564 Glycerophospholipid metabolism;
gene45611	K00868	pdxK, pdxY	pyridoxine kinase	EC:2.7.1.35	1	ko00750 Vitamin B6 metabolism;
gene45612	K13205	AAR2, C20orf4	A1 cistron-splicing factor AAR2	--		
gene45616	K00770	E2.4.2.24	1,4-beta-D-xylan synthase	EC:2.4.2.24	2	ko00500 Starch and sucrose metabolism;ko00520 Amino sugar and nucleotide sugar metabolism;
gene4561	K12486	SMAP	stromal membrane-associated protein	--	1	ko04144 Endocytosis;
gene45623	K01074	PPT	palmitoyl-protein thioesterase	EC:3.1.2.22	2	ko00062 Fatty acid elongation;ko04142 Lysosome;
gene45624	K08867	WNK, PRKWNK	WNK lysine deficient protein kinase	EC:2.7.11.1		
gene45625	K01982	LSUrRNA	large subunit ribosomal RNA	--	2	ko03008 Ribosome biogenesis in eukaryotes;ko03010 Ribosome;
gene45626	K10639	CCNB1IP1, HEI10	E3 ubiquitin-protein ligase CCNP1IP1	EC:6.3.2.19		
gene45630	K11096	SNRPD2, SMD2	small nuclear ribonucleoprotein D2	--	1	ko03040 Spliceosome;
gene45637	K13420	FLS2	LRR receptor-like serine/threonine-protein kinase FLS2	EC:2.7.11.1	1	ko04626 Plant-pathogen interaction;
gene45645	K07198	PRKAA, AMPK	5'-AMP-activated protein kinase, catalytic alpha subunit	EC:2.7.11.11	8	ko04140 Regulation of autophagy;ko04150 mTOR signaling pathway;ko04151 PI3K-Akt signaling pathway;ko04710 Circadian rhythm;ko04910 Insulin signaling pathway;ko04920 Adipocytokine signaling pathway;ko04932 Non-alcoholic fatty liver disease (NAFLD);ko05410 Hypertrophic cardiomyopathy (HCM);
gene45654	K01689	ENO, eno	enolase	EC:4.2.1.11	6	ko00010 Glycolysis / Gluconeogenesis;ko00680 Methane metabolism;ko01200 Carbon metabolism;ko01230 Biosynthesis of amino acids;ko03018 RNA degradation;ko04066 HIF-1 signaling pathway;
gene45655	K10712	ADO	cysteamine dioxygenase	EC:1.13.11.19	1	ko00430 Taurine and hypotaurine metabolism;
gene45657	K11129	NHP2, NOLA2	H/ACA ribonucleoprotein complex subunit 2	--	1	ko03008 Ribosome biogenesis in eukaryotes;
gene45659	K00921	PIKFYVE, FAB1	1-phosphatidylinositol-3-phosphate 5-kinase	EC:2.7.1.150	4	ko00562 Inositol phosphate metabolism;ko04070 Phosphatidylinositol signaling system;ko04145 Phagosome;ko04810 Regulation of actin cytoskeleton;
gene4565	K01537	E3.6.3.8	Ca2+-transporting ATPase	EC:3.6.3.8		
gene45666	K02109	ATPF0B, atpF	F-type H+-transporting ATPase subunit b	EC:3.6.3.14	2	ko00190 Oxidative phosphorylation;ko00195 Photosynthesis;
gene45667	K02109	ATPF0B, atpF	F-type H+-transporting ATPase subunit b	EC:3.6.3.14	2	ko00190 Oxidative phosphorylation;ko00195 Photosynthesis;
gene45670	K08081	E1.1.1.206	tropine dehydrogenase	EC:1.1.1.206	1	ko00960 Tropane, piperidine and pyridine alkaloid biosynthesis;
gene45672	K11816	YUCCA	indole-3-pyruvate monooxygenase	EC:1.14.13.168	1	ko00380 Tryptophan metabolism;
gene45674	K10527	MFP2	enoyl-CoA hydratase/3-hydroxyacyl-CoA dehydrogenase	EC:4.2.1.17 1.1.1.35 1.1.1.211	2	ko00071 Fatty acid degradation;ko00592 alpha-Linolenic acid metabolism;
gene45682	K12261	HACL1	2-hydroxyacyl-CoA lyase 1	EC:4.1.-.-	1	ko04146 Peroxisome;
gene45688	K04730	IRAK1	interleukin-1 receptor-associated kinase 1	EC:2.7.11.1	11	ko04064 NF-kappa B signaling pathway;ko04210 Apoptosis;ko04620 Toll-like receptor signaling pathway;ko04722 Neurotrophin signaling pathway;ko05133 Pertussis;ko05140 Leishmaniasis;ko05142 Chagas disease (American trypanosomiasis);ko05145 Toxoplasmosis;ko05152 Tuberculosis;ko05162 Measles;ko05169 Epstein-Barr virus infection;
gene4568	K13436	PTI1	pto-interacting protein 1	EC:2.7.11.1	1	ko04626 Plant-pathogen interaction;
gene45693	K10802	HMGB1	high mobility group protein B1	--	1	ko03410 Base excision repair;
gene45694	K14066	GPS	geranyl diphosphate synthase	EC:2.5.1.1	1	ko00900 Terpenoid backbone biosynthesis;
gene45699	K00700	glgB	1,4-alpha-glucan branching enzyme	EC:2.4.1.18	1	ko00500 Starch and sucrose metabolism;
gene4569	K03327	TC.MATE, SLC47A, norM, mdtK, dinF	multidrug resistance protein, MATE family	--		

gene456	K03549	kup	KUP system potassium uptake protein	--		
gene45703	K12598	MTR4, SKIV2L2	ATP-dependent RNA helicase DOB1	EC:3.6.4.13	1	ko03018 RNA degradation;
gene45704	K00487	CYP73A	trans-cinnamate 4-monooxygenase	EC:1.14.13.11	5	ko00360 Phenylalanine metabolism;ko00940 Phenylpropanoid biosynthesis;ko00941 Flavonoid biosynthesis;ko00945 Stilbenoid, diarylheptanoid and gingerol biosynthesis;ko01220 Degradation of aromatic compounds;
gene45706	K17609	NXN	nucleoredoxin	EC:1.8.1.8		
gene45709	K03262	EIF5	translation initiation factor 5	--	1	ko03013 RNA transport;
gene45713	K08857	NEK	NIMA (never in mitosis gene a)-related kinase	EC:2.7.11.1		
gene45716	K01807	rpiA	ribose 5-phosphate isomerase A	EC:5.3.1.6	4	ko00030 Pentose phosphate pathway;ko00710 Carbon fixation in photosynthetic organisms;ko01200 Carbon metabolism;ko01230 Biosynthesis of amino acids;
gene45718	K01456	E3.5.1.52, NGLY1, PNG1	peptide-N4-(N-acetyl-beta-glucosaminyl)asparagine amidase	EC:3.5.1.52	1	ko04141 Protein processing in endoplasmic reticulum;
gene4571	K03495	gidA, mnmG, MTO1	tRNA uridine 5-carboxymethylaminomethyl modification enzyme	--		
gene45722	K01979	SSUrRNA	small subunit ribosomal RNA	--	2	ko03008 Ribosome biogenesis in eukaryotes;ko03010 Ribosome;
gene45723	K02355	fusA, GFM, EFG	elongation factor G	--		
gene45724	K05605	HIBCH	3-hydroxyisobutyryl-CoA hydrolase	EC:3.1.2.4	3	ko00280 Valine, leucine and isoleucine degradation;ko00410 beta-Alanine metabolism;ko00640 Propanoate metabolism;
gene45726	K15865	CDKAL1	CDK5 regulatory subunit-associated protein 1-like 1	--		
gene45729	K01051	E3.1.1.11	pectinesterase	EC:3.1.1.11	2	ko00040 Pentose and glucuronate interconversions;ko00500 Starch and sucrose metabolism;
gene45730	K00249	ACADM, acd	acyl-CoA dehydrogenase	EC:1.3.8.7	5	ko00071 Fatty acid degradation;ko00280 Valine, leucine and isoleucine degradation;ko00410 beta-Alanine metabolism;ko00640 Propanoate metabolism;ko03320 PPAR signaling pathway;
gene45734	K09420	MYB	myb proto-oncogene protein	--	2	ko04151 PI3K-Akt signaling pathway;ko05166 HTLV-I infection;
gene45735	K01784	galE, GALE	UDP-glucose 4-epimerase	EC:5.1.3.2	2	ko00052 Galactose metabolism;ko00520 Amino sugar and nucleotide sugar metabolism;
gene45747	K11996	MOCS3, UBA4, moeB	adenylyltransferase and sulfurtransferase	--	1	ko04122 Sulfur relay system;
gene45748	K00700	glgB	1,4-alpha-glucan branching enzyme	EC:2.4.1.18	1	ko00500 Starch and sucrose metabolism;
gene45749	K05305	FUK	fucokinase	EC:2.7.1.52	2	ko00051 Fructose and mannose metabolism;ko00520 Amino sugar and nucleotide sugar metabolism;
gene45751	K01469	E3.5.2.9	5-oxoprolinase (ATP-hydrolysing)	EC:3.5.2.9	1	ko00480 Glutathione metabolism;
gene45752	K05350	bglB	beta-glucosidase	EC:3.2.1.21	3	ko00460 Cyanoamino acid metabolism;ko00500 Starch and sucrose metabolism;ko00940 Phenylpropanoid biosynthesis;
gene45755	K13412	CPK	calcium-dependent protein kinase	EC:2.7.11.1	2	ko04626 Plant-pathogen interaction;ko05145 Toxoplasmosis;
gene45756	K02888	RP-L21, MRPL21, rplU	large subunit ribosomal protein L21	--	1	ko03010 Ribosome;
gene4575	K01982	LSUrRNA	large subunit ribosomal RNA	--	2	ko03008 Ribosome biogenesis in eukaryotes;ko03010 Ribosome;
gene45767	K09872	PIP	aquaporin PIP	--		
gene4576	K10760	IPT	adenylate isopentenyltransferase (cytokinin synthase)	--	1	ko00908 Zeatin biosynthesis;
gene45770	K14326	UPF1, RENT1	regulator of nonsense transcripts 1	EC:3.6.4.-	2	ko03013 RNA transport;ko03015 mRNA surveillance pathway;
gene45778	K15047	HNRNPUL1, E1BAP5	heterogeneous nuclear ribonucleoprotein U-like protein 1	--	1	ko05164 Influenza A;
gene45784	K12869	CRN, CRNKL1, CLF1, SYF3	crooked neck	--	1	ko03040 Spliceosome;
gene45786	K06689	UBE2D_E, UBC4, UBC5	ubiquitin-conjugating enzyme E2 D/E	EC:6.3.2.19	2	ko04120 Ubiquitin mediated proteolysis;ko04141 Protein processing in endoplasmic reticulum;
gene45794	K04733	IRAK4	interleukin-1 receptor-associated kinase 4	EC:2.7.11.1	11	ko04064 NF-kappa B signaling pathway;ko04210 Apoptosis;ko04620 Toll-like receptor signaling pathway;ko04722 Neurotrophin signaling pathway;ko05133 Pertussis;ko05140 Leishmaniasis;ko05142 Chagas disease (American trypanosomiasis);ko05145 Toxoplasmosis;ko05152 Tuberculosis;ko05162 Measles;ko05164 Influenza A;
gene45799	K07374	TUBA	tubulin alpha	--	3	ko04145 Phagosome;ko04540 Gap junction;ko05130 Pathogenic Escherichia coli infection;
gene45809	K15920	XYL4	beta-D-xylosidase 4	EC:3.2.1.37	2	ko00500 Starch and sucrose metabolism;ko00520 Amino sugar and nucleotide sugar metabolism;
gene4580	K14485	TIR1	transport inhibitor response 1	--	1	ko04075 Plant hormone signal transduction;

gene45811	K07203	FRAP	FKBP12-rapamycin complex-associated protein	--	13	ko04012 ErbB signaling pathway;ko04066 HIF-1 signaling pathway;ko04150 mTOR signaling pathway;ko04151 PI3K-Akt signaling pathway;ko04910 Insulin signaling pathway;ko04920 Adipocytokine signaling pathway;ko04930 Type II diabetes mellitus;ko05200 Pathways in cancer;ko05205 Proteoglycans in cancer;ko05206 MicroRNAs in cancer;ko05214 Glioma;ko05215 Prostate cancer;ko05221 Acute myeloid leukemia;
gene45812	K04040	chlG, bchG	chlorophyll synthase	EC:2.5.1.62	1	ko00860 Porphyrin and chlorophyll metabolism;
gene45814	K00850	pfkA, PFK	6-phosphofructokinase 1	EC:2.7.1.11	7	ko00010 Glycolysis / Gluconeogenesis;ko00030 Pentose phosphate pathway;ko00051 Fructose and mannose metabolism;ko00052 Galactose metabolism;ko00680 Methane metabolism;ko01200 Carbon metabolism;ko01230 Biosynthesis of amino acids;
gene45815	K08850	AURKX	aurora kinase, other	EC:2.7.11.1		
gene45819	K02183	CALM	calmodulin	--	25	ko04014 Ras signaling pathway;ko04020 Calcium signaling pathway;ko04070 Phosphatidylinositol signaling system;ko04114 Oocyte meiosis;ko04270 Vascular smooth muscle contraction;ko04626 Plant-pathogen interaction;ko04713 Circadian entrainment;ko04720 Long-term potentiation;ko04722 Neurotrophin signaling pathway;ko04728 Dopaminergic synapse;ko04740 Olfactory transduction;ko04744 Phototransduction;ko04745 Phototransduction - fly;ko04910 Insulin signaling pathway;ko04912 GnRH signaling pathway;ko04915 Estrogen signaling pathway;ko04916 Melanogenesis;ko04970 Salivary secretion;ko04971 Gastric acid secretion;ko05010 Alzheimer's disease;ko05031 Amphetamine addiction;ko05034 Alcoholism;ko05133 Pertussis;ko05152 Tuberculosis;ko05214 Glioma;
gene45824	K14230	tRNA-Met	tRNA Met	--	1	ko00970 Aminoacyl-tRNA biosynthesis;
gene4582	K09422	MYBP	myb proto-oncogene protein, plant	--		
gene45836	K01537	E3.6.3.8	Ca2+-transporting ATPase	EC:3.6.3.8		
gene45839	K03320	amt, AMT, MEP	ammonium transporter, Amt family	--		
gene4583	K09377	CSRP	cysteine and glycine-rich protein	--		
gene45841	K17506	PPM1L, PP2CE	protein phosphatase 1L	EC:3.1.3.16		
gene45845	K01115	PLD1_2	phospholipase D1/2	EC:3.1.4.4	7	ko00564 Glycerophospholipid metabolism;ko00565 Ether lipid metabolism;ko04014 Ras signaling pathway;ko04144 Endocytosis;ko04666 Fc gamma R-mediated phagocytosis;ko04724 Glutamatergic synapse;ko04912 GnRH signaling pathway;
gene45847	K01213	E3.2.1.67	galacturan 1,4-alpha-galacturonidase	EC:3.2.1.67	2	ko00040 Pentose and glucuronate interconversions;ko00500 Starch and sucrose metabolism;
gene45848	K06688	UBE2C, UBC11	ubiquitin-conjugating enzyme E2 C	EC:6.3.2.19	1	ko04120 Ubiquitin mediated proteolysis;
gene45849	K06963	TAN1, THUMPD1	tRNA acetyltransferase TAN1	--		
gene45851	K12160	SUMO, SMT3	small ubiquitin-related modifier	--	1	ko03013 RNA transport;
gene45853	K06689	UBE2D_E, UBC4, UBC5	ubiquitin-conjugating enzyme E2 D/E	EC:6.3.2.19	2	ko04120 Ubiquitin mediated proteolysis;ko04141 Protein processing in endoplasmic reticulum;
gene45854	K13434	PTI6	pathogenesis-related genes transcriptional activator PTI6	--	1	ko04626 Plant-pathogen interaction;
gene45855	K03238	EIF2S2	translation initiation factor 2 subunit 2	--	1	ko03013 RNA transport;
gene45856	K14219	tRNA-Arg	tRNA Arg	--	1	ko00970 Aminoacyl-tRNA biosynthesis;
gene45859	K01653	E2.2.1.6S, ilvH, ilvN	acetolactate synthase I/III small subunit	EC:2.2.1.6	6	ko00290 Valine, leucine and isoleucine biosynthesis;ko00650 Butanoate metabolism;ko00660 C5-Branched dibasic acid metabolism;ko00770 Pantothenate and CoA biosynthesis;ko01210 2-Oxocarboxylic acid metabolism;ko01230 Biosynthesis of amino acids;
gene45860	K07119	K07119				
gene45864	K14003	PREB, SEC12	prolactin regulatory element-binding protein	--	1	ko04141 Protein processing in endoplasmic reticulum;
gene45867	K04125	E1.14.11.13	gibberellin 2-oxidase	EC:1.14.11.13	1	ko00904 Diterpenoid biosynthesis;
gene45868	K11855	USP36_42	ubiquitin carboxyl-terminal hydrolase 36/42	EC:3.1.2.15		
gene45871	K14431	TGA	transcription factor TGA	--	1	ko04075 Plant hormone signal transduction;
gene45877	K12400	AP4E1	AP-4 complex subunit epsilon-1	--	1	ko04142 Lysosome;
gene45882	K14771	NOC4, UTP19	U3 small nucleolar RNA-associated protein 19	--		

gene45884	K09680	coaW	type II pantothenate kinase	EC:2.7.1.33	1	ko00770 Pantothenate and CoA biosynthesis;
gene45885	K09519	DNAJB13	DnaJ homolog subfamily B member 13	--		
gene45892	K00128	E1.2.1.3	aldehyde dehydrogenase (NAD+)	EC:1.2.1.3	15	ko00010 Glycolysis / Gluconeogenesis;ko00040 Pentose and glucuronate interconversions;ko00053 Ascorbate and aldarate metabolism;ko00071 Fatty acid degradation;ko00280 Valine, leucine and isoleucine degradation;ko00310 Lysine degradation;ko00330 Arginine and proline metabolism;ko00340 Histidine metabolism;ko00380 Tryptophan metabolism;ko00410 beta-Alanine metabolism;ko00561 Glycerolipid metabolism;ko00620 Pyruvate metabolism;ko00625 Chloroalkane and chloroalkene degradation;ko00640 Propanoate metabolism;ko00903 Limonene and pinene
gene45893	K02960	RP-S16e, RPS16	small subunit ribosomal protein S16e	--	1	ko03010 Ribosome;
gene45900	K09840	NCED	9-cis-epoxycarotenoid dioxygenase	EC:1.13.11.51	1	ko00906 Carotenoid biosynthesis;
gene45901	K01687	ilvD	dihydroxy-acid dehydratase	EC:4.2.1.9	4	ko00290 Valine, leucine and isoleucine biosynthesis;ko00770 Pantothenate and CoA biosynthesis;ko01210 2-Oxocarboxylic acid metabolism;ko01230 Biosynthesis of amino acids;
gene45902	K08869	ADCK, ABC1	aarF domain-containing kinase	--		
gene45907	K09338	HD-ZIP	homeobox-leucine zipper protein	--		
gene45908	K01696	trpB	tryptophan synthase beta chain	EC:4.2.1.20	3	ko00260 Glycine, serine and threonine metabolism;ko00400 Phenylalanine, tyrosine and tryptophan biosynthesis;ko01230 Biosynthesis of amino acids;
gene45913	K11975	RNF144	E3 ubiquitin-protein ligase RNF144	EC:6.3.2.19		
gene45915	K02935	RP-L7, MRPL12, rplL	large subunit ribosomal protein L7/L12	--	1	ko03010 Ribosome;
gene45916	K09131	K09131	hypothetical protein	--		
gene45917	K01438	argE	acetylornithine deacetylase	EC:3.5.1.16	3	ko00330 Arginine and proline metabolism;ko01210 2-Oxocarboxylic acid metabolism;ko01230 Biosynthesis of amino acids;
gene45921	K01835	pgm	phosphoglucomutase	EC:5.4.2.2	7	ko00010 Glycolysis / Gluconeogenesis;ko00030 Pentose phosphate pathway;ko00052 Galactose metabolism;ko00230 Purine metabolism;ko00500 Starch and sucrose metabolism;ko00520 Amino sugar and nucleotide sugar metabolism;ko00521 Streptomycin biosynthesis;
gene45922	K00847	E2.7.1.4, scrK	fructokinase	EC:2.7.1.4	3	ko00051 Fructose and mannose metabolism;ko00500 Starch and sucrose metabolism;ko00520 Amino sugar and nucleotide sugar metabolism;
gene45924	K02266	COX6A	cytochrome c oxidase subunit 6a	--	6	ko00190 Oxidative phosphorylation;ko04260 Cardiac muscle contraction;ko04932 Non-alcoholic fatty liver disease (NAFLD);ko05010 Alzheimer's disease;ko05012 Parkinson's disease;ko05016 Huntington's disease;
gene45926	K01230	MAN1	mannosyl-oligosaccharide alpha-1,2-mannosidase	EC:3.2.1.113	3	ko00510 N-Glycan biosynthesis;ko00513 Various types of N-glycan biosynthesis;ko04141 Protein processing in endoplasmic reticulum;
gene4592	K11266	MAU2	MAternally affected uncoordination	--		
gene45930	K00863	E2.7.1.29, DAK1, DAK2	dihydroxyacetone kinase	EC:2.7.1.29	4	ko00561 Glycerolipid metabolism;ko00680 Methane metabolism;ko01200 Carbon metabolism;ko04622 RIG-I-like receptor signaling pathway;
gene45931	K13993	HSP20	HSP20 family protein	--	1	ko04141 Protein processing in endoplasmic reticulum;
gene45933	K06699	PSME4	proteasome activator subunit 4	--	1	ko03050 Proteasome;
gene45936	K00940	E2.7.4.6, ndk	nucleoside-diphosphate kinase	EC:2.7.4.6	2	ko00230 Purine metabolism;ko00240 Pyrimidine metabolism;
gene45938	K01784	galE, GALE	UDP-glucose 4-epimerase	EC:5.1.3.2	2	ko00052 Galactose metabolism;ko00520 Amino sugar and nucleotide sugar metabolism;
gene45943	K10393	KIF2_24, MCAK	kinesin family member 2/24	--		
gene45944	K08592	SENP1	sentrin-specific protease 1	EC:3.4.22.68		
gene45945	K08592	SENP1	sentrin-specific protease 1	EC:3.4.22.68		
gene4594	K01937	E6.3.4.2, pyrG	CTP synthase	EC:6.3.4.2	1	ko00240 Pyrimidine metabolism;
gene45952	K00791	miaA, TRIT1	tRNA dimethylallyltransferase	EC:2.5.1.75	1	ko00908 Zeatin biosynthesis;
gene45955	K11664	VPS72, TCFL1, YL1	vacuolar protein sorting-associated protein 72	--		
gene45956	K09285	OVM, ANT	AP2-like factor, ANT lineage	--		
gene45958	K00261	GLUD1_2, gdhA	glutamate dehydrogenase (NAD(P)+)	EC:1.4.1.3	5	ko00250 Alanine, aspartate and glutamate metabolism;ko00330 Arginine and proline metabolism;ko00471 D-Glutamine and D-glutamate metabolism;ko00910 Nitrogen metabolism;ko04964 Proximal tubule bicarbonate reclamation;

gene45959	K15153	MED31, SOH1	mediator of RNA polymerase II transcription subunit 31	--		
gene45960	K12883	NCBP2, CBP20	nuclear cap-binding protein subunit 2	--	3	ko03013 RNA transport;ko03015 mRNA surveillance pathway;ko03040 Spliceosome;
gene45963	K02976	RP-S26e, RPS26	small subunit ribosomal protein S26e	--	1	ko03010 Ribosome;
gene45966	K05544	DUS3	tRNA-dihydrouridine synthase 3	EC:1.3.1.89		
gene45967	K00764	purF, PPAT	amidophosphoribosyltransferase	EC:2.4.2.14	2	ko00230 Purine metabolism;ko00250 Alanine, aspartate and glutamate metabolism;
gene45968	K09578	PIN1	peptidyl-prolyl cis-trans isomerase NIMA-interacting 1	EC:5.2.1.8	1	ko04622 RIG-I-like receptor signaling pathway;
gene45970	K02834	rbfA	ribosome-binding factor A	--		
gene45971	K03301	TC.AAA	ATP:ADP antiporter, AAA family	--		
gene45974	K14236	tRNA-Tyr	tRNA Tyr	--	1	ko00970 Aminoacyl-tRNA biosynthesis;
gene45977	K07937	ARF1	ADP-ribosylation factor 1	--	2	ko05110 Vibrio cholerae infection;ko05134 Legionellosis;
gene45978	K14505	CYCD3	cyclin D3, plant	--	1	ko04075 Plant hormone signal transduction;
gene45979	K10268	FBXL2_20	F-box and leucine-rich repeat protein 2/20	--		
gene4598	K13146	INTS9	integrator complex subunit 9	--		
gene45990	K10770	ALKBH8	alkylated DNA repair protein alkB homolog 8	EC:1.14.11.-2.1.1.229		
gene45994	K03671	trxA	thioredoxin 1	--		
gene45995	K01969	E6.4.1.4B	3-methylcrotonyl-CoA carboxylase beta subunit	EC:6.4.1.4	1	ko00280 Valine, leucine and isoleucine degradation;
gene45996	K03152	thiJ	4-methyl-5(b-hydroxyethyl)-thiazole monophosphate biosynthesis	--		
gene45997	K14432	ABF	ABA responsive element binding factor	--	1	ko04075 Plant hormone signal transduction;
gene45998	K02535	lpxC	UDP-3-O-[3-hydroxymyristoyl] N-acetylglucosamine deacetylase	EC:3.5.1.108	1	ko00540 Lipopolysaccharide biosynthesis;
gene459	K14961	RBBP5, SWD1, CPS50	COMPASS component SWD1	--		
gene4600	K12761	SNF1	carbon catabolite-derepressing protein kinase	EC:2.7.11.1	1	ko04113 Meiosis - yeast;
gene46016	K15382	SLC50A, SWEET	solute carrier family 50 (sugar transporter)	--		
gene46018	K12900	FUSIP1	FUS-interacting serine-arginine-rich protein 1	--	1	ko03040 Spliceosome;
gene46020	K01256	pepN	aminopeptidase N	EC:3.4.11.2	1	ko00480 Glutathione metabolism;
gene46027	K15104	SLC25A11, OGC	solute carrier family 25 (mitochondrial oxoglutarate transporter), member 11	--		
gene46031	K13993	HSP20	HSP20 family protein	--	1	ko04141 Protein processing in endoplasmic reticulum;
gene46032	K14497	PP2C	protein phosphatase 2C	EC:3.1.3.16	1	ko04075 Plant hormone signal transduction;
gene46035	K03065	PSMC3, RPT5	26S proteasome regulatory subunit T5	--	2	ko03050 Proteasome;ko05169 Epstein-Barr virus infection;
gene46036	K10532	HGSNAT	heparan-alpha-glucosaminide N-acetyltransferase	EC:2.3.1.78	2	ko00531 Glycosaminoglycan degradation;ko04142 Lysosome;
gene46037	K12623	LSM4	U6 snRNA-associated Sm-like protein LSm4	--	2	ko03018 RNA degradation;ko03040 Spliceosome;
gene46041	K02866	RP-L10e, RPL10	large subunit ribosomal protein L10e	--	1	ko03010 Ribosome;
gene46042	K02109	ATPF0B, atpF	F-type H+-transporting ATPase subunit b	EC:3.6.3.14	2	ko00190 Oxidative phosphorylation;ko00195 Photosynthesis;
gene46044	K03147	thiC	thiamine biosynthesis protein ThiC	--	1	ko00730 Thiamine metabolism;
gene46045	K04733	IRAK4	interleukin-1 receptor-associated kinase 4	EC:2.7.11.1	11	ko04064 NF-kappa B signaling pathway;ko04210 Apoptosis;ko04620 Toll-like receptor signaling pathway;ko04722 Neurotrophin signaling pathway;ko05133 Pertussis;ko05140 Leishmaniasis;ko05142 Chagas disease (American trypanosomiasis);ko05145 Toxoplasmosis;ko05152 Tuberculosis;ko05162 Measles;ko05164 Influenza A;
gene46047	K03327	TC.MATE, SLC47A, norM, mdtK, dinF	multidrug resistance protein, MATE family	--		
gene46050	K09264	K09264	MADS-box transcription factor, plant	--		
gene46051	K11341	YEATS4, GAS41, YAF9	YEATS domain-containing protein 4	--		
gene46053	K11088	SNRPD3, SMD3	small nuclear ribonucleoprotein D3	--	2	ko03040 Spliceosome;ko05322 Systemic lupus erythematosus;
gene46054	K07304	msrA	peptide-methionine (S)-S-oxide reductase	EC:1.8.4.11		
gene46056	K01490	E3.5.4.6, AMPD	AMP deaminase	EC:3.5.4.6	1	ko00230 Purine metabolism;
gene46058	K00962	pnp, PNPT1	polyribonucleotide nucleotidyltransferase	EC:2.7.7.8	3	ko00230 Purine metabolism;ko00240 Pyrimidine metabolism;ko03018 RNA degradation;
gene4605	K01176	E3.2.1.1, amyA, malS	alpha-amylase	EC:3.2.1.1	2	ko00500 Starch and sucrose metabolism;ko04973 Carbohydrate digestion and absorption;
gene46062	K12836	U2AF1	splicing factor U2AF 35 kDa subunit	--	2	ko03040 Spliceosome;ko05131 Shigellosis;

gene46064	K01968	E6.4.1.4A	3-methylcrotonyl-CoA carboxylase alpha subunit	EC:6.4.1.4	1	ko00280 Valine, leucine and isoleucine degradation;
gene46069	K15040	VDAC2	voltage-dependent anion channel protein 2	--	4	ko04020 Calcium signaling pathway;ko05012 Parkinson's disease;ko05016 Huntington's disease;ko05166 HTLV-I infection;
gene46071	K00915	IPK2	1D-myo-Inositol-tetrakisphosphate 5-kinase / inositol-polyphosphate multikinase	EC:2.7.1.140 2.7.1.151	2	ko00562 Inositol phosphate metabolism;ko04070 Phosphatidylinositol signaling system;
gene46074	K12589	RRP42, EXOSC7	exosome complex component RRP42	--	1	ko03018 RNA degradation;
gene46076	K04733	IRAK4	interleukin-1 receptor-associated kinase 4	EC:2.7.11.1	11	ko04064 NF-kappa B signaling pathway;ko04210 Apoptosis;ko04620 Toll-like receptor signaling pathway;ko04722 Neurotrophin signaling pathway;ko05133 Pertussis;ko05140 Leishmaniasis;ko05142 Chagas disease (American trypanosomiasis);ko05145 Toxoplasmosis;ko05152 Tuberculosis;ko05162 Measles;ko05164 Influenza A;
gene4607	K04371	MAPK1_3	mitogen-activated protein kinase 1/3	EC:2.7.11.24	71	ko04010 MAPK signaling pathway;ko04012 ErbB signaling pathway;ko04013 MAPK signaling pathway - fly;ko04014 Ras signaling pathway;ko04062 Chemokine signaling pathway;ko04066 HIF-1 signaling pathway;ko04114 Oocyte meiosis;ko04150 mTOR signaling pathway;ko04151 PI3K-Akt signaling pathway;ko04270 Vascular smooth muscle contraction;ko04320 Dorso-ventral axis formation;ko04350 TGF-beta signaling pathway;ko04360 Axon guidance;ko04370 VEGF signaling pathway;ko04380 Osteoclast differentiation;ko04510 Focal adhesion;ko04520 Adherens junction;ko04540 Gap junction;ko04620 Toll-like receptor signaling pathway;ko04621 NOD-like receptor signaling pathway;ko04650 Natural killer cell mediated cytotoxicity;ko04660 T cell receptor signaling pathway;ko04662 B cell receptor signaling pathway;ko04664 Fc epsilon RI signaling pathway;ko04666 Fc gamma R-mediated phagocytosis;ko04668 TNF signaling pathway;ko04713 Circadian entrainment;ko04720 Long-term potentiation;ko04722 Neurotrophin signaling pathway;ko04723 Retrograde endocannabinoid signaling;ko04724 Glutamatergic synapse;ko04725 Cholinergic synapse;ko04726 Serotonergic synapse;ko04730 Long-term depression;ko04810 Regulation of actin cytoskeleton;ko04910 Insulin signaling pathway;ko04912 GnRH signaling pathway;ko04914 Progesterone-mediated oocyte maturation;ko04915 Estrogen signaling pathway;ko04916 Melanogenesis;ko04917 Prolactin signaling pathway;ko04930 Type II diabetes mellitus;ko04960 Aldosterone-regulated sodium reabsorption;ko05010 Alzheimer's disease;ko05020 Prion diseases;ko05034 Alcoholism;ko05131 Shigellosis;ko05132 Salmonella infection;ko05133 Pertussis;ko05140 Leishmaniasis;ko05142 Chagas disease (American trypanosomiasis);ko05145 Toxoplasmosis;ko05152 Tuberculosis;ko05160 Hepatitis C;ko05161 Hepatitis B;ko05164 Influenza A;ko05200 Pathways in cancer;ko05203 Viral carcinogenesis;ko05205 Proteoglycans in cancer;ko05210 Colorectal cancer;ko05211 Renal cell carcinoma;ko05212
gene46082	K14494	DELLA	DELLA protein	--	1	ko04075 Plant hormone signal transduction;
gene46083	K03017	RPB9, POLR2I	DNA-directed RNA polymerase II subunit RPB9	--	5	ko00230 Purine metabolism;ko00240 Pyrimidine metabolism;ko03020 RNA polymerase;ko05016 Huntington's disease;ko05169 Epstein-Barr virus infection;
gene46084	K02905	RP-L29e, RPL29	large subunit ribosomal protein L29e	--	1	ko03010 Ribosome;
gene46085	K00382	DLD, lpd, pdhD	dihydrolipoamide dehydrogenase	EC:1.8.1.4	6	ko00010 Glycolysis / Gluconeogenesis;ko00020 Citrate cycle (TCA cycle);ko00260 Glycine, serine and threonine metabolism;ko00280 Valine, leucine and isoleucine degradation;ko00620 Pyruvate metabolism;ko01200 Carbon metabolism;
gene46089	K16283	SDIR1	E3 ubiquitin-protein ligase SDIR1	EC:6.3.2.19		
gene46090	K10807	RRM1	ribonucleoside-diphosphate reductase subunit M1	EC:1.17.4.1	3	ko00230 Purine metabolism;ko00240 Pyrimidine metabolism;ko00480 Glutathione metabolism;
gene46094	K08235	E2.4.1.207	xyloglucan:xyloglucosyl transferase	EC:2.4.1.207		
gene46095	K08341	GABARAP, ATG8, LC3	GABA(A) receptor-associated protein (autophagy-related protein 8)	--	2	ko04140 Regulation of autophagy;ko04727 GABAergic synapse;
gene46096	K07541	PIGX	phosphatidylinositol glycan, class X	--	1	ko00563 Glycosylphosphatidylinositol(GPI)-anchor biosynthesis;

gene4609	K09533	DNAJC13	DnaJ homolog subfamily C member 13	--		
gene46101	K09580	PDIA1, P4HB	protein disulfide-isomerase A1	EC:5.3.4.1	1	ko04141 Protein processing in endoplasmic reticulum;
gene46113	K15436	TRPO3, MTR10	transportin-3	--		
gene46114	K11490	NCAPH2	condensin-2 complex subunit H2	--		
gene46123	K05605	HIBCH	3-hydroxyisobutyryl-CoA hydrolase	EC:3.1.2.4	3	ko00280 Valine, leucine and isoleucine degradation;ko00410 beta-Alanine metabolism;ko00640 Propanoate metabolism;
gene4612	K00889	E2.7.1.68, PIP5K	1-phosphatidylinositol-4-phosphate 5-kinase	EC:2.7.1.68	5	ko00562 Inositol phosphate metabolism;ko04070 Phosphatidylinositol signaling system;ko04144 Endocytosis;ko04666 Fc gamma R-mediated phagocytosis;ko04810 Regulation of actin cytoskeleton;
gene46137	K01087	otsB	trehalose 6-phosphate phosphatase	EC:3.1.3.12	1	ko00500 Starch and sucrose metabolism;
gene46145	K06662	HRAD17, RAD24	cell cycle checkpoint protein	--	2	ko04111 Cell cycle - yeast;ko04113 Meiosis - yeast;
gene46148	K00434	E1.11.1.11	L-ascorbate peroxidase	EC:1.11.1.11	2	ko00053 Ascorbate and aldarate metabolism;ko00480 Glutathione metabolism;
gene46149	K00434	E1.11.1.11	L-ascorbate peroxidase	EC:1.11.1.11	2	ko00053 Ascorbate and aldarate metabolism;ko00480 Glutathione metabolism;
gene46153	K02912	RP-L32e, RPL32	large subunit ribosomal protein L32e	--	1	ko03010 Ribosome;
gene46155	K15227	TYRAAT	arogenate dehydrogenase (NADP+), plant	EC:1.3.1.78	2	ko00400 Phenylalanine, tyrosine and tryptophan biosynthesis;ko01230 Biosynthesis of amino acids;
gene46165	K01051	E3.1.1.11	pectinesterase	EC:3.1.1.11	2	ko00040 Pentose and glucuronate interconversions;ko00500 Starch and sucrose metabolism;
gene46166	K01102	PDP	pyruvate dehydrogenase phosphatase	EC:3.1.3.43		
gene46170	K09422	MYBP	myb proto-oncogene protein, plant	--		
gene46176	K07198	PRKAA, AMPK	5'-AMP-activated protein kinase, catalytic alpha subunit	EC:2.7.11.11	8	ko04140 Regulation of autophagy;ko04150 mTOR signaling pathway;ko04151 PI3K-Akt signaling pathway;ko04710 Circadian rhythm;ko04910 Insulin signaling pathway;ko04920 Adipocytokine signaling pathway;ko04932 Non-alcoholic fatty liver disease (NAFLD);ko05410 Hypertrophic cardiomyopathy (HCM);
gene46179	K09422	MYBP	myb proto-oncogene protein, plant	--		
gene4617	K08237	E2.4.1.218	hydroquinone glucosyltransferase	EC:2.4.1.218		
gene46182	K00517	E1.14.-.-			4	ko00624 Polycyclic aromatic hydrocarbon degradation;ko00627 Aminobenzoate degradation;ko00903 Limonene and pinene degradation;ko00945 Stilbenoid, diarylheptanoid and gingerol biosynthesis;
gene46185	K11968	ARIH1	ariadne-1	--		
gene46186	K11968	ARIH1	ariadne-1	--		
gene46188	K10579	UBE2M, UBC12	ubiquitin-conjugating enzyme E2 M	EC:6.3.2.19	1	ko04120 Ubiquitin mediated proteolysis;
gene46191	K12121	PHYB	phytochrome B	--	1	ko04712 Circadian rhythm - plant;
gene4619	K00850	pfkA, PFK	6-phosphofructokinase 1	EC:2.7.1.11	7	ko00010 Glycolysis / Gluconeogenesis;ko00030 Pentose phosphate pathway;ko00051 Fructose and mannose metabolism;ko00052 Galactose metabolism;ko00680 Methane metabolism;ko01200 Carbon metabolism;ko01230 Biosynthesis of amino acids;
gene46202	K13199	SERBP1	plasminogen activator inhibitor 1 RNA-binding protein	--		
gene46208	K01179	E3.2.1.4	endoglucanase	EC:3.2.1.4	1	ko00500 Starch and sucrose metabolism;
gene46214	K02145	ATPeVA, ATP6A1	V-type H+-transporting ATPase subunit A	EC:3.6.3.14	7	ko00190 Oxidative phosphorylation;ko04145 Phagosome;ko04721 Synaptic vesicle cycle;ko04966 Collecting duct acid secretion;ko05110 Vibrio cholerae infection;ko05120 Epithelial cell signaling in Helicobacter pylori infection;ko05323 Rheumatoid arthritis;
gene46218	K08916	LHCB5	light-harvesting complex II chlorophyll a/b binding protein 5	--	1	ko00196 Photosynthesis - antenna proteins;
gene46219	K03544	clpX, CLPX	ATP-dependent Clp protease ATP-binding subunit ClpX	--	1	ko04112 Cell cycle - Caulobacter;
gene4621	K10364	CAPZA	capping protein (actin filament) muscle Z-line, alpha	--		
gene46220	K01188	E3.2.1.21	beta-glucosidase	EC:3.2.1.21	3	ko00460 Cyanoamino acid metabolism;ko00500 Starch and sucrose metabolism;ko00940 Phenylpropanoid biosynthesis;
gene46221	K15201	GTP3C3, TFC4	general transcription factor 3C polypeptide 3 (transcription factor C subunit 4)	--		

gene46225	K14692	SLC30A5_7, ZNT5_7, MTP, MSC2	solute carrier family 30 (zinc transporter), member 5/7	--		
gene4622	K02942	RP-LP1, RPLP1	large subunit ribosomal protein LP1	--	1	ko03010 Ribosome;
gene46236	K13076	SLD	delta8-fatty-acid desaturase	EC:1.14.19.4		
gene46238	K01183	E3.2.1.14	chitinase	EC:3.2.1.14	1	ko00520 Amino sugar and nucleotide sugar metabolism;
gene46241	K14431	TGA	transcription factor TGA	--	1	ko04075 Plant hormone signal transduction;
gene46248	K07766	E3.6.1.52	diphosphoinositol-polyphosphate diphosphatase	EC:3.6.1.52		
gene46249	K13152	ZMAT5	U11/U12 small nuclear ribonucleoprotein 20 kDa protein	--		
gene46251	K01304	E3.4.19.3, pcp	pyroglutamyl-peptidase	EC:3.4.19.3		
gene46256	K13448	CML	calcium-binding protein CML	--	1	ko04626 Plant-pathogen interaction;
gene46263	K13946	AUX1, LAX	auxin influx carrier (AUX1 LAX family)	--	1	ko04075 Plant hormone signal transduction;
gene46265	K15285	SLC35E3	solute carrier family 35, member E3	--		
gene46272	K10364	CAPZA	capping protein (actin filament) muscle Z-line, alpha	--		
gene46280	K01590	hdc, HDC	histidine decarboxylase	EC:4.1.1.22	1	ko00340 Histidine metabolism;
gene46284	K00863	E2.7.1.29, DAK1, DAK2	dihydroxyacetone kinase	EC:2.7.1.29	4	ko00561 Glycerolipid metabolism;ko00680 Methane metabolism;ko01200 Carbon metabolism;ko04622 RIG-I-like receptor signaling pathway;
gene46285	K01303	APEH	acylaminoacyl-peptidase	EC:3.4.19.1		
gene46287	K15802	SSTLE1	germacrene C synthase	EC:4.2.3.60	1	ko00909 Sesquiterpenoid and triterpenoid biosynthesis;
gene4628	K14709	SLC39A1_2_3, ZIP1_2_3	solute carrier family 39 (zinc transporter), member 1/2/3	--		
gene46291	K15113	SLC25A28_37, MFRN	solute carrier family 25 (mitochondrial iron transporter), member 28/37	--		
gene46295	K12275	SEC62	translocation protein SEC62	--	2	ko03060 Protein export;ko04141 Protein processing in endoplasmic reticulum;
gene46296	K08064	NFYA	nuclear transcription factor Y, alpha	--	2	ko04612 Antigen processing and presentation;ko05152 Tuberculosis;
gene46298	K14497	PP2C	protein phosphatase 2C	EC:3.1.3.16	1	ko04075 Plant hormone signal transduction;
gene46308	K01466	allB	allantoinase	EC:3.5.2.5	1	ko00230 Purine metabolism;
gene46310	K01674	cah	carbonic anhydrase	EC:4.2.1.1	1	ko00910 Nitrogen metabolism;
gene46313	K14231	tRNA-Phe	tRNA Phe	--	1	ko00970 Aminoacyl-tRNA biosynthesis;
gene46314	K03671	trxA	thioredoxin 1	--		
gene46317	K14684	SLC25A23S	solute carrier family 25 (mitochondrial phosphate transporter), member 23/24/25/41	--		
gene46328	K10400	KIF15	kinesin family member 15	--		
gene46331	K08867	WNK, PRKWNK	WNK lysine deficient protein kinase	EC:2.7.11.1		
gene46338	K09273	UBTF	upstream-binding transcription factor	--		
gene46341	K00873	PK, pyk	pyruvate kinase	EC:2.7.1.40	7	ko00010 Glycolysis / Gluconeogenesis;ko00230 Purine metabolism;ko00620 Pyruvate metabolism;ko01200 Carbon metabolism;ko01230 Biosynthesis of amino acids;ko04930 Type II diabetes mellitus;ko05203 Viral carcinogenesis;
gene46345	K15032	MTERFD	mTERF domain-containing protein, mitochondrial	--		
gene46347	K15109	SLC25A20_29, CACT, CACL, CRC1	solute carrier family 25 (mitochondrial carnitine/acylcarnitine transporter), member 20/29	--		
gene46348	K01188	E3.2.1.21	beta-glucosidase	EC:3.2.1.21	3	ko00460 Cyanoamino acid metabolism;ko00500 Starch and sucrose metabolism;ko00940 Phenylpropanoid biosynthesis;
gene46352	K13946	AUX1, LAX	auxin influx carrier (AUX1 LAX family)	--	1	ko04075 Plant hormone signal transduction;
gene46362	K05756	ARPC3	actin related protein 2/3 complex, subunit 3	--	6	ko04666 Fc gamma R-mediated phagocytosis;ko04810 Regulation of actin cytoskeleton;ko05100 Bacterial invasion of epithelial cells;ko05130 Pathogenic Escherichia coli infection;ko05131 Shigellosis;ko05132 Salmonella infection;
gene46365	K05305	FUK	fucokinase	EC:2.7.1.52	2	ko00051 Fructose and mannose metabolism;ko00520 Amino sugar and nucleotide sugar metabolism;
gene46368	K02866	RP-L10e, RPL10	large subunit ribosomal protein L10e	--	1	ko03010 Ribosome;
gene4636	K02603	ORC1	origin recognition complex subunit 1	--	3	ko04110 Cell cycle;ko04111 Cell cycle - yeast;ko04113 Meiosis - yeast;
gene46370	K13172	SRRM2, SRM300	serine/arginine repetitive matrix protein 2	--		

gene46372	K16278	K16278, HOS1	E3 ubiquitin-protein ligase HOS1	EC:6.3.2.19		
gene4637	K09422	MYBP	myb proto-oncogene protein, plant	--		
gene46381	K03686	dnaJ	molecular chaperone DnaJ	--		
gene46386	K00434	E1.11.1.11	L-ascorbate peroxidase	EC:1.11.1.11	2	ko00053 Ascorbate and aldarate metabolism;ko00480 Glutathione metabolism;
gene46388	K10400	KIF15	kinesin family member 15	--		
gene46389	K00145	argC	N-acetyl-gamma-glutamyl-phosphate reductase	EC:1.2.1.38	3	ko00330 Arginine and proline metabolism;ko01210 2-Oxocarboxylic acid metabolism;ko01230 Biosynthesis of amino acids;
gene46390	K03254	EIF3A	translation initiation factor 3 subunit A	--	1	ko03013 RNA transport;
gene46392	K00130	betB, gbsA	betaine-aldehyde dehydrogenase	EC:1.2.1.8	1	ko00260 Glycine, serine and threonine metabolism;
gene46396	K01358	clpP, CLPP	ATP-dependent Clp protease, protease subunit	EC:3.4.21.92	1	ko04112 Cell cycle - Caulobacter;
gene46404	K14484	IAA	auxin-responsive protein IAA	--	1	ko04075 Plant hormone signal transduction;
gene46405	K05759	PFN	profilin	--	3	ko04810 Regulation of actin cytoskeleton;ko05131 Shigellosis;ko05132 Salmonella infection;
gene46406	K15128	MED6	mediator of RNA polymerase II transcription subunit 6	--		
gene46407	K09286	EREBP	EREBP-like factor	--		
gene46409	K12735	PPIL4	peptidyl-prolyl cis-trans isomerase-like 4	EC:5.2.1.8		
gene46410	K17279	REEP5_6	receptor expression-enhancing protein 5/6	--		
gene46411	K14232	tRNA-Pro	tRNA Pro	--	1	ko00970 Aminoacyl-tRNA biosynthesis;
gene46413	K10436	MAPRE	microtubule-associated protein, RP/EB family	--		
gene46414	K14546	UTP5, WDR43	U3 small nucleolar RNA-associated protein 5	--	1	ko03008 Ribosome biogenesis in eukaryotes;
gene4641	K01904	E6.2.1.12	4-coumarate--CoA ligase	EC:6.2.1.12	3	ko00130 Ubiquinone and other terpenoid-quinone biosynthesis;ko00360 Phenylalanine metabolism;ko00940 Phenylpropanoid biosynthesis;
gene46430	K14432	ABF	ABA responsive element binding factor	--	1	ko04075 Plant hormone signal transduction;
gene46432	K01982	LSUrRNA	large subunit ribosomal RNA	--	2	ko03008 Ribosome biogenesis in eukaryotes;ko03010 Ribosome;
gene46433	K13628	iscA, ISCA1	iron-sulfur cluster assembly protein	--		
gene46434	K03531	ftsZ	cell division protein FtsZ	--	1	ko04112 Cell cycle - Caulobacter;
gene46435	K09338	HD-ZIP	homeobox-leucine zipper protein	--		
gene46439	K02991	RP-S6e, RPS6	small subunit ribosomal protein S6e	--	6	ko03010 Ribosome;ko04066 HIF-1 signaling pathway;ko04150 mTOR signaling pathway;ko04151 PI3K-Akt signaling pathway;ko04910 Insulin signaling pathway;ko05205 Proteoglycans in cancer;
gene46441	K01669	E4.1.99.3, phrB	deoxyribodipyrimidine photo-lyase	EC:4.1.99.3		
gene46442	K08869	ADCK, ABC1	aarF domain-containing kinase	--		
gene46448	K04730	IRAK1	interleukin-1 receptor-associated kinase 1	EC:2.7.11.1	11	ko04064 NF-kappa B signaling pathway;ko04210 Apoptosis;ko04620 Toll-like receptor signaling pathway;ko04722 Neurotrophin signaling pathway;ko05133 Pertussis;ko05140 Leishmaniasis;ko05142 Chagas disease (American trypanosomiasis);ko05145 Toxoplasmosis;ko05152 Tuberculosis;ko05162 Measles;ko05169 Epstein-Barr virus infection;
gene4644	K06100	SYMPK	symplekin	--	2	ko03015 mRNA surveillance pathway;ko04530 Tight junction;
gene46451	K14777	DDX47, RRP3	ATP-dependent RNA helicase DDX47/RRP3	EC:3.6.4.13		
gene46452	K14497	PP2C	protein phosphatase 2C	EC:3.1.3.16	1	ko04075 Plant hormone signal transduction;
gene46455	K13917	RNGTT	mRNA-capping enzyme	EC:2.7.7.50 3.1.3.33	1	ko03015 mRNA surveillance pathway;
gene46456	K11087	SNRPD1, SMD1	small nuclear ribonucleoprotein D1	--	2	ko03040 Spliceosome;ko05322 Systemic lupus erythematosus;
gene46459	K04708	E1.1.1.102	3-dehydrosphinganine reductase	EC:1.1.1.102	1	ko00600 Sphingolipid metabolism;
gene46462	K09338	HD-ZIP	homeobox-leucine zipper protein	--		
gene46463	K02265	COX5B	cytochrome c oxidase subunit 5b	--	6	ko00190 Oxidative phosphorylation;ko04260 Cardiac muscle contraction;ko04932 Non-alcoholic fatty liver disease (NAFLD);ko05010 Alzheimer's disease;ko05012 Parkinson's disease;ko05016 Huntington's disease;
gene46470	K14221	tRNA-Asp	tRNA Asp	--	1	ko00970 Aminoacyl-tRNA biosynthesis;
gene46474	K12857	SNRNP40, PRP8BP	Prp8 binding protein	--	1	ko03040 Spliceosome;
gene46476	K17361	ACOT9	acyl-coenzyme A thioesterase 9	EC:3.1.2.-		
gene46477	K13066	E2.1.1.68, COMT	caffeic acid 3-O-methyltransferase	EC:2.1.1.68	1	ko00940 Phenylpropanoid biosynthesis;

gene46484	K04733	IRAK4	interleukin-1 receptor-associated kinase 4	EC:2.7.11.1	11	ko04064 NF-kappa B signaling pathway;ko04210 Apoptosis;ko04620 Toll-like receptor signaling pathway;ko04722 Neurotrophin signaling pathway;ko05133 Pertussis;ko05140 Leishmaniasis;ko05142 Chagas disease (American trypanosomiasis);ko05145 Toxoplasmosis;ko05152 Tuberculosis;ko05162 Measles;ko05164 Influenza A;
gene46485	K04733	IRAK4	interleukin-1 receptor-associated kinase 4	EC:2.7.11.1	11	ko04064 NF-kappa B signaling pathway;ko04210 Apoptosis;ko04620 Toll-like receptor signaling pathway;ko04722 Neurotrophin signaling pathway;ko05133 Pertussis;ko05140 Leishmaniasis;ko05142 Chagas disease (American trypanosomiasis);ko05145 Toxoplasmosis;ko05152 Tuberculosis;ko05162 Measles;ko05164 Influenza A;
gene46486	K04733	IRAK4	interleukin-1 receptor-associated kinase 4	EC:2.7.11.1	11	ko04064 NF-kappa B signaling pathway;ko04210 Apoptosis;ko04620 Toll-like receptor signaling pathway;ko04722 Neurotrophin signaling pathway;ko05133 Pertussis;ko05140 Leishmaniasis;ko05142 Chagas disease (American trypanosomiasis);ko05145 Toxoplasmosis;ko05152 Tuberculosis;ko05162 Measles;ko05164 Influenza A;
gene46493	K13156	SNRNP48	U11/U12 small nuclear ribonucleoprotein 48 kDa protein	--		
gene4649	K10635	RNF111	E3 ubiquitin-protein ligase Arkadia	EC:6.3.2.19		
gene46510	K10590	TRIP12	E3 ubiquitin-protein ligase TRIP12	EC:6.3.2.19	1	ko04120 Ubiquitin mediated proteolysis;
gene46511	K13436	PTI1	pto-interacting protein 1	EC:2.7.11.1	1	ko04626 Plant-pathogen interaction;
gene46516	K03013	RPB5, POLR2E	DNA-directed RNA polymerases I, II, and III subunit RPABC1	--	6	ko00230 Purine metabolism;ko00240 Pyrimidine metabolism;ko03020 RNA polymerase;ko04623 Cytosolic DNA-sensing pathway;ko05016 Huntington's disease;ko05169 Epstein-Barr virus infection;
gene46519	K00878	thiM	hydroxyethylthiazole kinase	EC:2.7.1.50	1	ko00730 Thiamine metabolism;
gene46520	K04077	groEL, HSPD1	chaperonin GroEL	--	4	ko03018 RNA degradation;ko04940 Type I diabetes mellitus;ko05134 Legionellosis;ko05152 Tuberculosis;
gene46523	K01177	E3.2.1.2	beta-amylase	EC:3.2.1.2	1	ko00500 Starch and sucrose metabolism;
gene46527	K02937	RP-L7e, RPL7	large subunit ribosomal protein L7e	--	1	ko03010 Ribosome;
gene46530	K07408	CYP1A1	cytochrome P450, family 1, subfamily A, polypeptide 1	EC:1.14.14.1	6	ko00140 Steroid hormone biosynthesis;ko00380 Tryptophan metabolism;ko00830 Retinol metabolism;ko00980 Metabolism of xenobiotics by cytochrome P450;ko04913 Ovarian steroidogenesis;ko05204 Chemical carcinogenesis;
gene46532	K00430	E1.11.1.7	peroxidase	EC:1.11.1.7	2	ko00360 Phenylalanine metabolism;ko00940 Phenylpropanoid biosynthesis;
gene46537	K05894	E1.3.1.42	12-oxophytodienoic acid reductase	EC:1.3.1.42	1	ko00592 alpha-Linolenic acid metabolism;
gene46541	K16302	CNNM	metal transporter CNNM	--		
gene46543	K06944	K06944				
gene46546	K00679	E2.3.1.158	phospholipid:diacylglycerol acyltransferase	EC:2.3.1.158	1	ko00561 Glycerolipid metabolism;
gene46548	K14236	tRNA-Tyr	tRNA Tyr	--	1	ko00970 Aminoacyl-tRNA biosynthesis;
gene46549	K11446	JARID1	histone demethylase JARID1	EC:1.14.11.-		
gene4654	K15084	SLC25A16, GDA, LEU5	solute carrier family 25 (mitochondrial carrier protein), member 16	--		
gene46550	K05349	bglX	beta-glucosidase	EC:3.2.1.21	3	ko00460 Cyanoamino acid metabolism;ko00500 Starch and sucrose metabolism;ko00940 Phenylpropanoid biosynthesis;
gene46551	K00863	E2.7.1.29, DAK1, DAK2	dihydroxyacetone kinase	EC:2.7.1.29	4	ko00561 Glycerolipid metabolism;ko00680 Methane metabolism;ko01200 Carbon metabolism;ko04622 RIG-I-like receptor signaling pathway;
gene46554	K05275	E1.1.1.65	pyridoxine 4-dehydrogenase	EC:1.1.1.65	1	ko00750 Vitamin B6 metabolism;
gene46567	K09527	DNAJC7	DnaJ homolog subfamily C member 7	--		
gene46568	K07152	K07152				
gene46570	K00235	SDHB, SDH2	succinate dehydrogenase (ubiquinone) iron-sulfur subunit	EC:1.3.5.1	7	ko00020 Citrate cycle (TCA cycle);ko00190 Oxidative phosphorylation;ko01200 Carbon metabolism;ko04932 Non-alcoholic fatty liver disease (NAFLD);ko05010 Alzheimer's disease;ko05012 Parkinson's disease;ko05016 Huntington's disease;
gene46575	K16052	ynaI, mscMJ, MSL	MscS family membrane protein	--		
gene46582	K12836	U2AF1	splicing factor U2AF 35 kDa subunit	--	2	ko03040 Spliceosome;ko05131 Shigellosis;

gene46586	K00030	IDH3	isocitrate dehydrogenase (NAD+)	EC:1.1.1.41	4	ko00020 Citrate cycle (TCA cycle);ko01200 Carbon metabolism;ko01210 2-Oxocarboxylic acid metabolism;ko01230 Biosynthesis of amino acids;
gene46592	K08867	WNK, PRKWNK	WNK lysine deficient protein kinase	EC:2.7.11.1		
gene46601	K12620	LSM1	U6 snRNA-associated Sm-like protein LSm1	--	1	ko03018 RNA degradation;
gene46605	K03858	PIGH, GPI15	phosphatidylinositol glycan, class H	--	1	ko00563 Glycosylphosphatidylinositol(GPI)-anchor biosynthesis;
gene46613	K03319	TC.DASS	divalent anion:Na+ symporter, DASS family	--		
gene46614	K10738	MCM9	DNA helicase MCM9	EC:3.6.4.12		
gene46620	K07277	yaeT	outer membrane protein	--		
gene46623	K03363	CDC20	cell division cycle 20, cofactor of APC complex	--	7	ko04110 Cell cycle;ko04111 Cell cycle - yeast;ko04113 Meiosis - yeast;ko04114 Oocyte meiosis;ko04120 Ubiquitin mediated proteolysis;ko05166 HTLV-I infection;ko05203 Viral carcinogenesis;
gene46633	K12881	THOC4, ALY	THO complex subunit 4	--	4	ko03013 RNA transport;ko03015 mRNA surveillance pathway;ko03040 Spliceosome;ko05168 Herpes simplex infection;
gene46634	K11593	ELF2C	eukaryotic translation initiation factor 2C	--		
gene46635	K13429	CERK1	chitin elicitor receptor kinase 1	--	1	ko04626 Plant-pathogen interaction;
gene46637	K14488	SAUR	SAUR family protein	--	1	ko04075 Plant hormone signal transduction;
gene46638	K07904	RAB11A	Ras-related protein Rab-11A	--	4	ko04144 Endocytosis;ko04961 Endocrine and other factor-regulated calcium reabsorption;ko04962 Vasopressin-regulated water reabsorption;ko04972 Pancreatic secretion;
gene46641	K00873	PK, pyk	pyruvate kinase	EC:2.7.1.40	7	ko00010 Glycolysis / Gluconeogenesis;ko00230 Purine metabolism;ko00620 Pyruvate metabolism;ko01200 Carbon metabolism;ko01230 Biosynthesis of amino acids;ko04930 Type II diabetes mellitus;ko05203 Viral carcinogenesis;
gene46643	K13237	DECR2	peroxisomal 2,4-dienoyl-CoA reductase	EC:1.3.1.34	1	ko04146 Peroxisome;
gene46645	K08237	E2.4.1.218	hydroquinone glucosyltransferase	EC:2.4.1.218		
gene46646	K08472	MLO	mlo protein	--		
gene46647	K17095	ANXA7_11	annexin A7/11	--		
gene46649	K15026	EIF2A	translation initiation factor 2A	--		
gene46656	K02876	RP-L15, MRPL15, rplO	large subunit ribosomal protein L15	--	1	ko03010 Ribosome;
gene46658	K00430	E1.11.1.7	peroxidase	EC:1.11.1.7	2	ko00360 Phenylalanine metabolism;ko00940 Phenylpropanoid biosynthesis;
gene46660	K07047	K07047				
gene46678	K09872	PIP	aquaporin PIP	--		
gene46679	K08770	UBC	ubiquitin C	--	1	ko03320 PPAR signaling pathway;
gene46681	K00799	GST, gst	glutathione S-transferase	EC:2.5.1.18	4	ko00480 Glutathione metabolism;ko00980 Metabolism of xenobiotics by cytochrome P450;ko00982 Drug metabolism - cytochrome P450;ko05204 Chemical carcinogenesis;
gene46682	K00799	GST, gst	glutathione S-transferase	EC:2.5.1.18	4	ko00480 Glutathione metabolism;ko00980 Metabolism of xenobiotics by cytochrome P450;ko00982 Drug metabolism - cytochrome P450;ko05204 Chemical carcinogenesis;
gene46692	K05349	bglX	beta-glucosidase	EC:3.2.1.21	3	ko00460 Cyanoamino acid metabolism;ko00500 Starch and sucrose metabolism;ko00940 Phenylpropanoid biosynthesis;
gene46693	K01759	E4.4.1.5, GLO1, gloA	lactoylglutathione lyase	EC:4.4.1.5	2	ko00620 Pyruvate metabolism;ko04011 MAPK signaling pathway - yeast;
gene46695	K16054	DEP1	methylthioribulose 1-phosphate dehydratase / enolase-phosphatase E1	EC:4.2.1.109 3.1.3.77	1	ko00270 Cysteine and methionine metabolism;
gene46699	K07071	K07071				
gene46700	K14016	UFD1	ubiquitin fusion degradation protein 1	--	1	ko04141 Protein processing in endoplasmic reticulum;
gene46709	K06966	K06966				
gene46711	K00430	E1.11.1.7	peroxidase	EC:1.11.1.7	2	ko00360 Phenylalanine metabolism;ko00940 Phenylpropanoid biosynthesis;
gene46712	K00799	GST, gst	glutathione S-transferase	EC:2.5.1.18	4	ko00480 Glutathione metabolism;ko00980 Metabolism of xenobiotics by cytochrome P450;ko00982 Drug metabolism - cytochrome P450;ko05204 Chemical carcinogenesis;
gene46719	K01689	ENO, eno	enolase	EC:4.2.1.11	6	ko00010 Glycolysis / Gluconeogenesis;ko00680 Methane metabolism;ko01200 Carbon metabolism;ko01230 Biosynthesis of amino acids;ko03018 RNA degradation;ko04066 HIF-1 signaling pathway;
gene46727	K14638	SLC15A3_4, PHT	solute carrier family 15 (peptide/histidine transporter), member 3/4	--		

gene46733	K16871	POP2	4-aminobutyrate---pyruvate transaminase	EC:2.6.1.96	2	ko00250 Alanine, aspartate and glutamate metabolism;ko00650 Butanoate metabolism;
gene46736	K05349	bglX	beta-glucosidase	EC:3.2.1.21	3	ko00460 Cyanoamino acid metabolism;ko00500 Starch and sucrose metabolism;ko00940 Phenylpropanoid biosynthesis;
gene46740	K14842	NSA2	ribosome biogenesis protein NSA2	--		
gene46746	K02704	psbB	photosystem II CP47 chlorophyll apoprotein	--	1	ko00195 Photosynthesis;
gene46747	K13338	PEX1	peroxin-1	--	1	ko04146 Peroxisome;
gene4674	K00430	E1.11.1.7	peroxidase	EC:1.11.1.7	2	ko00360 Phenylalanine metabolism;ko00940 Phenylpropanoid biosynthesis;
gene46750	K08331	ATG13	autophagy-related protein 13	--	1	ko04140 Regulation of autophagy;
gene46751	K01251	E3.3.1.1, ahcY	adenosylhomocysteinase	EC:3.3.1.1	1	ko00270 Cysteine and methionine metabolism;
gene46754	K02729	PSMA5	20S proteasome subunit alpha 5	EC:3.4.25.1	1	ko03050 Proteasome;
gene46758	K10803	XRCC1	DNA-repair protein XRCC1	--	1	ko03410 Base excision repair;
gene46759	K00670	E2.3.1.88	peptide alpha-N-acetyltransferase	EC:2.3.1.88		
gene46771	K13416	BAK1	brassinosteroid insensitive 1-associated receptor kinase 1	EC:2.7.10.1 2.7.11.1	2	ko04075 Plant hormone signal transduction;ko04626 Plant-pathogen interaction;
gene46775	K07195	EXOC7, EXO70	exocyst complex component 7	--	1	ko04910 Insulin signaling pathway;
gene46779	K14795	RRP36	ribosomal RNA-processing protein 36	--		
gene46783	K14413	GALT1	beta-1,3-galactosyltransferase	EC:2.4.1.-	1	ko00513 Various types of N-glycan biosynthesis;
gene46787	K15404	K15404, CER1	aldehyde decarbonylase	EC:4.1.99.5	1	ko00073 Cutin, suberine and wax biosynthesis;
gene46792	K06634	CCNH	cyclin H	--	3	ko03022 Basal transcription factors;ko03420 Nucleotide excision repair;ko04110 Cell cycle;
gene46793	K03327	TC.MATE, SLC47A, norM, mdtK, dinF	multidrug resistance protein, MATE family	--		
gene46795	K09699	E2.3.1.168, bkdB	2-oxoisovalerate dehydrogenase E2 component (dihydrolipoyl transacylase)	EC:2.3.1.168	1	ko00280 Valine, leucine and isoleucine degradation;
gene46796	K16253	NRPD7, NRPE7	DNA-directed RNA polymerase IV and V subunit 7	--		
gene46801	K01214	E3.2.1.68	isoamylase	EC:3.2.1.68		
gene46804	K13211	GCFC	GC-rich sequence DNA-binding factor	--		
gene46808	K12813	DHX16	pre-mRNA-splicing factor ATP-dependent RNA helicase DHX16	EC:3.6.4.13	1	ko03040 Spliceosome;
gene46810	K00012	UGDH, ugd	UDPglucose 6-dehydrogenase	EC:1.1.1.22	4	ko00040 Pentose and glucuronate interconversions;ko00053 Ascorbate and aldarate metabolism;ko00500 Starch and sucrose metabolism;ko00520 Amino sugar and nucleotide sugar metabolism;
gene46811	K00901	E2.7.1.107, DGK, dgkA	diacylglycerol kinase (ATP dependent)	EC:2.7.1.107	3	ko00561 Glycerolipid metabolism;ko00564 Glycerophospholipid metabolism;ko04070 Phosphatidylinositol signaling system;
gene46812	K02966	RP-S19e, RPS19	small subunit ribosomal protein S19e	--	1	ko03010 Ribosome;
gene46817	K12338	UGT75C1	anthocyanidin 3-O-glucoside 5-O-glucosyltransferase	EC:2.4.1.298	1	ko00942 Anthocyanin biosynthesis;
gene46820	K05359	ADT, PDT	arogenate/prephenate dehydratase	EC:4.2.1.91 4.2.1.51	2	ko00400 Phenylalanine, tyrosine and tryptophan biosynthesis;ko01230 Biosynthesis of amino acids;
gene46823	K02138	ATPeFD, ATP5H	F-type H+-transporting ATPase subunit d	EC:3.6.3.14	4	ko00190 Oxidative phosphorylation;ko05010 Alzheimer's disease;ko05012 Parkinson's disease;ko05016 Huntington's disease;
gene46830	K13140	INTS3	integrator complex subunit 3	--		
gene46831	K12854	SNRNP200, BRR2	pre-mRNA-splicing helicase BRR2	EC:3.6.4.13	1	ko03040 Spliceosome;
gene46837	K08488	STX7	syntaxin 7	--	2	ko04130 SNARE interactions in vesicular transport;ko04145 Phagosome;
gene46838	K07541	PIGX	phosphatidylinositol glycan, class X	--	1	ko00563 Glycosylphosphatidylinositol(GPI)-anchor biosynthesis;
gene46856	K17600	VPS54	vacuolar protein sorting-associated protein 54	--		
gene46858	K01535	E3.6.3.6	H+-transporting ATPase	EC:3.6.3.6	1	ko00190 Oxidative phosphorylation;
gene4685	K02728	PSMA4	20S proteasome subunit alpha 3	EC:3.4.25.1	1	ko03050 Proteasome;
gene46860	K02209	MCM5, CDC46	DNA replication licensing factor MCM5	EC:3.6.4.12	4	ko03030 DNA replication;ko04110 Cell cycle;ko04111 Cell cycle - yeast;ko04113 Meiosis - yeast;
gene46864	K11816	YUCCA	indole-3-pyruvate monooxygenase	EC:1.14.13.168	1	ko00380 Tryptophan metabolism;
gene46874	K12667	SWP1, RPN2	oligosaccharyltransferase complex subunit delta (ribophorin II)	--	3	ko00510 N-Glycan biosynthesis;ko00513 Various types of N-glycan biosynthesis;ko04141 Protein processing in endoplasmic reticulum;
gene46881	K16252	NRPD2, NRPE2	DNA-directed RNA polymerase IV and V subunit 2	EC:2.7.7.6		
gene46883	K09285	OVM, ANT	AP2-like factor, ANT lineage	--		
gene4688	K00281	GLDC, gcvP	glycine dehydrogenase	EC:1.4.4.2	2	ko00260 Glycine, serine and threonine metabolism;ko01200 Carbon metabolism;

gene46890	K04382	PPP2C	serine/threonine-protein phosphatase 2A catalytic subunit	EC:3.1.3.16	13	ko03015 mRNA surveillance pathway;ko04111 Cell cycle - yeast;ko04113 Meiosis - yeast;ko04114 Oocyte meiosis;ko04151 PI3K-Akt signaling pathway;ko04350 TGF-beta signaling pathway;ko04390 Hippo signaling pathway;ko04391 Hippo signaling pathway - fly;ko04530 Tight junction;ko04728 Dopaminergic synapse;ko04730 Long-term depression;ko05142 Chagas disease (American trypanosomiasis);ko05160 Hepatitis C;
gene46894	K09422	MYBP	myb proto-oncogene protein, plant	--		
gene46899	K00434	E1.11.1.11	L-ascorbate peroxidase	EC:1.11.1.11	2	ko00053 Ascorbate and aldarate metabolism;ko00480 Glutathione metabolism;
gene4689	K08241	E2.1.1.141	jasmonate O-methyltransferase	EC:2.1.1.141	1	ko00592 alpha-Linolenic acid metabolism;
gene468	K05658	ABCB1	ATP-binding cassette, subfamily B (MDR/TAP), member 1	--	3	ko02010 ABC transporters;ko04976 Bile secretion;ko05206 MicroRNAs in cancer;
gene46900	K00434	E1.11.1.11	L-ascorbate peroxidase	EC:1.11.1.11	2	ko00053 Ascorbate and aldarate metabolism;ko00480 Glutathione metabolism;
gene46906	K08235	E2.4.1.207	xyloglucan:xyloglucosyl transferase	EC:2.4.1.207		
gene46907	K08235	E2.4.1.207	xyloglucan:xyloglucosyl transferase	EC:2.4.1.207		
gene46908	K08235	E2.4.1.207	xyloglucan:xyloglucosyl transferase	EC:2.4.1.207		
gene46909	K00412	CYTB, petB	ubiquinol-cytochrome c reductase cytochrome b subunit	--	7	ko00190 Oxidative phosphorylation;ko02020 Two-component system;ko04260 Cardiac muscle contraction;ko04932 Non-alcoholic fatty liver disease (NAFLD);ko05010 Alzheimer's disease;ko05012 Parkinson's disease;ko05016 Huntington's disease;
gene46910	K14484	IAA	auxin-responsive protein IAA	--	1	ko04075 Plant hormone signal transduction;
gene46913	K11254	H4	histone H4	--	3	ko05034 Alcoholism;ko05203 Viral carcinogenesis;ko05322 Systemic lupus erythematosus;
gene4691	K13095	SF1	splicing factor 1	--		
gene4692	K13095	SF1	splicing factor 1	--		
gene46933	K03064	PSMC6, RPT4	26S proteasome regulatory subunit T4	--	2	ko03050 Proteasome;ko05169 Epstein-Barr virus infection;
gene46941	K09875	SIP	aquaporin SIP	--		
gene46945	K09510	DNAJB4	DnaJ homolog subfamily B member 4	--		
gene46946	K00688	E2.4.1.1, glgP, PYG	starch phosphorylase	EC:2.4.1.1	2	ko00500 Starch and sucrose metabolism;ko04910 Insulin signaling pathway;
gene4694	K10696	BRE1	E3 ubiquitin-protein ligase BRE1	EC:6.3.2.19		
gene46952	K10960	chlP, bchP	geranylgeranyl reductase	EC:1.3.1.83	2	ko00860 Porphyrin and chlorophyll metabolism;ko00900 Terpenoid backbone biosynthesis;
gene46953	K00026	MDH2	malate dehydrogenase	EC:1.1.1.37	5	ko00020 Citrate cycle (TCA cycle);ko00620 Pyruvate metabolism;ko00630 Glyoxylate and dicarboxylate metabolism;ko00710 Carbon fixation in photosynthetic organisms;ko01200 Carbon metabolism;
gene46957	K15082	RAD7	DNA repair protein RAD7	--		
gene46959	K02221	yggT	YggT family protein	--		
gene46960	K11808	ADE2	phosphoribosylaminoimidazole carboxylase	EC:4.1.1.21	1	ko00230 Purine metabolism;
gene46961	K13412	CPK	calcium-dependent protein kinase	EC:2.7.11.1	2	ko04626 Plant-pathogen interaction;ko05145 Toxoplasmosis;
gene46968	K00430	E1.11.1.7	peroxidase	EC:1.11.1.7	2	ko00360 Phenylalanine metabolism;ko00940 Phenylpropanoid biosynthesis;
gene46969	K00695	E2.4.1.13	sucrose synthase	EC:2.4.1.13	1	ko00500 Starch and sucrose metabolism;
gene4696	K05863	SLC25A4S, ANT	solute carrier family 25 (mitochondrial adenine nucleotide translocator), member 4/5/6/31	--	4	ko04020 Calcium signaling pathway;ko05012 Parkinson's disease;ko05016 Huntington's disease;ko05166 HTLV-I infection;
gene46974	K14488	SAUR	SAUR family protein	--	1	ko04075 Plant hormone signal transduction;
gene46979	K01551	arsA, ASNA1	arsenite-transporting ATPase	EC:3.6.3.16		
gene46983	K14759	PHYLLO	isochorismate synthase / 2-succinyl-5-enolpyruvyl-6-hydroxy-3-cyclohexene-1-carboxylate synthase / 2-succinyl-6-hydroxy-2,4-cyclohexadiene-1-carboxylate synthase / O-succinylbenzoate synthase	EC:5.4.4.2 2.2.1.9 4.2.99.20 4.2.1.113	1	ko00130 Ubiquinone and other terpenoid-quinone biosynthesis;
gene46985	K12355	REF1	coniferyl-aldehyde dehydrogenase	EC:1.2.1.68	1	ko00940 Phenylpropanoid biosynthesis;
gene46987	K02882	RP-L18Ae, RPL18A	large subunit ribosomal protein L18Ae	--	1	ko03010 Ribosome;
gene46988	K02882	RP-L18Ae, RPL18A	large subunit ribosomal protein L18Ae	--	1	ko03010 Ribosome;
gene46989	K06874	K06874	zinc finger protein	--		
gene46990	K01363	CTSB	cathepsin B	EC:3.4.22.1	2	ko04142 Lysosome;ko04612 Antigen processing and presentation;
gene46991	K01363	CTSB	cathepsin B	EC:3.4.22.1	2	ko04142 Lysosome;ko04612 Antigen processing and presentation;

gene46993	K14486	K14486, ARF	auxin response factor	--	1	ko04075 Plant hormone signal transduction;
gene46998	K07375	TUBB	tubulin beta	--	3	ko04145 Phagosome;ko04540 Gap junction;ko05130 Pathogenic Escherichia coli infection;
gene4699	K02928	RP-L41e, RPL41	large subunit ribosomal protein L41e	--	1	ko03010 Ribosome;
gene46	K11778	DHDDS, RER2, SRT1	ditrans,polycis-polyprenyl diphosphate synthase	EC:2.5.1.87	1	ko00900 Terpenoid backbone biosynthesis;
gene47002	K00721	DPM1	dolichol-phosphate mannosyltransferase	EC:2.4.1.83	1	ko00510 N-Glycan biosynthesis;
gene47003	K14232	tRNA-Pro	tRNA Pro	--	1	ko00970 Aminoacyl-tRNA biosynthesis;
gene47004	K14232	tRNA-Pro	tRNA Pro	--	1	ko00970 Aminoacyl-tRNA biosynthesis;
gene47005	K14232	tRNA-Pro	tRNA Pro	--	1	ko00970 Aminoacyl-tRNA biosynthesis;
gene47006	K14232	tRNA-Pro	tRNA Pro	--	1	ko00970 Aminoacyl-tRNA biosynthesis;
gene47007	K14232	tRNA-Pro	tRNA Pro	--	1	ko00970 Aminoacyl-tRNA biosynthesis;
gene47008	K14232	tRNA-Pro	tRNA Pro	--	1	ko00970 Aminoacyl-tRNA biosynthesis;
gene47009	K14232	tRNA-Pro	tRNA Pro	--	1	ko00970 Aminoacyl-tRNA biosynthesis;
gene4700	K10257	FAD8, desB	omega-3 fatty acid desaturase (delta-15 desaturase)	EC:1.14.19.-	1	ko01040 Biosynthesis of unsaturated fatty acids;
gene47010	K14232	tRNA-Pro	tRNA Pro	--	1	ko00970 Aminoacyl-tRNA biosynthesis;
gene47011	K14232	tRNA-Pro	tRNA Pro	--	1	ko00970 Aminoacyl-tRNA biosynthesis;
gene47012	K14232	tRNA-Pro	tRNA Pro	--	1	ko00970 Aminoacyl-tRNA biosynthesis;
gene47014	K01580	E4.1.1.15, gadB, gadA, GAD	glutamate decarboxylase	EC:4.1.1.15	6	ko00250 Alanine, aspartate and glutamate metabolism;ko00410 beta-Alanine metabolism;ko00430 Taurine and hypotaurine metabolism;ko00650 Butanoate metabolism;ko04727 GABAergic synapse;ko04940 Type I diabetes mellitus;
gene47016	K01087	otsB	trehalose 6-phosphate phosphatase	EC:3.1.3.12	1	ko00500 Starch and sucrose metabolism;
gene4701	K01817	trpF	phosphoribosylanthranilate isomerase	EC:5.3.1.24	2	ko00400 Phenylalanine, tyrosine and tryptophan biosynthesis;ko01230 Biosynthesis of amino acids;
gene47020	K14222	tRNA-Cys	tRNA Cys	--	1	ko00970 Aminoacyl-tRNA biosynthesis;
gene47021	K01937	E6.3.4.2, pyrG	CTP synthase	EC:6.3.4.2	1	ko00240 Pyrimidine metabolism;
gene47024	K14233	tRNA-Ser	tRNA Ser	--	1	ko00970 Aminoacyl-tRNA biosynthesis;
gene47025	K12864	CTNNBL1	beta-catenin-like protein 1	--	1	ko03040 Spliceosome;
gene47026	K10706	SETX, ALS4	senataxin	EC:3.6.4.-		
gene4702	K13420	FLS2	LRR receptor-like serine/threonine-protein kinase FLS2	EC:2.7.11.1	1	ko04626 Plant-pathogen interaction;
gene47038	K13412	CPK	calcium-dependent protein kinase	EC:2.7.11.1	2	ko04626 Plant-pathogen interaction;ko05145 Toxoplasmosis;
gene47039	K00652	bioF	8-amino-7-oxononanoate synthase	EC:2.3.1.47	1	ko00780 Biotin metabolism;
gene47040	K08343	ATG3	autophagy-related protein 3	--	1	ko04140 Regulation of autophagy;
gene47042	K14852	RRS1	regulator of ribosome biosynthesis	--		
gene47044	K05288	PIGO	phosphatidylinositol glycan, class O	--	1	ko00563 Glycosylphosphatidylinositol(GPI)-anchor biosynthesis;
gene47046	K08065	NFYB	nuclear transcription Y subunit beta	--	3	ko04612 Antigen processing and presentation;ko05152 Tuberculosis;ko05166 HTLV-I infection;
gene47047	K01190	lacZ	beta-galactosidase	EC:3.2.1.23	3	ko00052 Galactose metabolism;ko00511 Other glycan degradation;ko00600 Sphingolipid metabolism;
gene47057	K08869	ADCK, ABC1	aarF domain-containing kinase	--		
gene47058	K06950	K06950	uncharacterized protein	--		
gene47059	K03283	HSPA1_8	heat shock 70kDa protein 1/8	--	11	ko03040 Spliceosome;ko04010 MAPK signaling pathway;ko04141 Protein processing in endoplasmic reticulum;ko04144 Endocytosis;ko04612 Antigen processing and presentation;ko04915 Estrogen signaling pathway;ko05134 Legionellosis;ko05145 Toxoplasmosis;ko05162 Measles;ko05164 Influenza A;ko05169 Epstein-Barr virus infection;
gene47061	K12116	FKF1	flavin-binding kelch repeat F-box protein 1	--	1	ko04712 Circadian rhythm - plant;
gene47065	K01056	PTH1, pth, spoVC	peptidyl-tRNA hydrolase, PTH1 family	EC:3.1.1.29		
gene47067	K09284	AP2	AP2-like factor, euAP2 lineage	--		
gene47072	K01102	PDP	pyruvate dehydrogenase phosphatase	EC:3.1.3.43		
gene47076	K01307	GGH	gamma-glutamyl hydrolase	EC:3.4.19.9	1	ko00790 Folate biosynthesis;
gene47084	K02993	RP-S7e, RPS7	small subunit ribosomal protein S7e	--	1	ko03010 Ribosome;
gene47087	K09646	SCPEP1	serine carboxypeptidase 1	EC:3.4.16.-		
gene47093	K14948	PTBP2, NPTB	olypyrimidine tract-binding protein 2	--		

gene47099	K02921	RP-L37Ae, RPL37A	large subunit ribosomal protein L37Ae	--	1	ko03010 Ribosome;
gene47101	K03115	CSNK2B	casein kinase II subunit beta	--	9	ko03008 Ribosome biogenesis in eukaryotes;ko04064 NF-kappa B signaling pathway;ko04310 Wnt signaling pathway;ko04520 Adherens junction;ko04530 Tight junction;ko04712 Circadian rhythm - plant;ko05162 Measles;ko05168 Herpes simplex infection;ko05169 Epstein-Barr virus infection;
gene47104	K14512	MPK6	mitogen-activated protein kinase 6	EC:2.7.11.24	1	ko04075 Plant hormone signal transduction;
gene47110	K10143	RFWD2, COP1	E3 ubiquitin-protein ligase RFWD2	EC:6.3.2.19	3	ko04115 p53 signaling pathway;ko04120 Ubiquitin mediated proteolysis;ko04712 Circadian rhythm - plant;
gene47113	K05665	ABCC1	ATP-binding cassette, subfamily C (CFTR/MRP), member 1	--	3	ko02010 ABC transporters;ko04977 Vitamin digestion and absorption;ko05206 MicroRNAs in cancer;
gene47118	K13065	E2.3.1.133, HCT	shikimate O-hydroxycinnamoyltransferase	EC:2.3.1.133	3	ko00940 Phenylpropanoid biosynthesis;ko00941 Flavonoid biosynthesis;ko00945 Stilbenoid, diarylheptanoid and gingerol biosynthesis;
gene4711	K12153	CYP79A2	cytochrome P450, family 79, subfamily A, polypeptide 2 (phenylalanine N-monooxygenase)	EC:1.14.13.124	3	ko00460 Cyanoamino acid metabolism;ko00966 Glucosinolate biosynthesis;ko01210 2-Oxocarboxylic acid metabolism;
gene47120	K07195	EXOC7, EXO70	exocyst complex component 7	--	1	ko04910 Insulin signaling pathway;
gene47121	K02908	RP-L30e, RPL30	large subunit ribosomal protein L30e	--	1	ko03010 Ribosome;
gene47122	K14638	SLC15A3_4, PHT	solute carrier family 15 (peptide/histidine transporter), member 3/4	--		
gene4712	K12153	CYP79A2	cytochrome P450, family 79, subfamily A, polypeptide 2 (phenylalanine N-monooxygenase)	EC:1.14.13.124	3	ko00460 Cyanoamino acid metabolism;ko00966 Glucosinolate biosynthesis;ko01210 2-Oxocarboxylic acid metabolism;
gene47137	K08065	NFYB	nuclear transcription Y subunit beta	--	3	ko04612 Antigen processing and presentation;ko05152 Tuberculosis;ko05166 HTLV-I infection;
gene47144	K01728	E4.2.2.2, pel	pectate lyase	EC:4.2.2.2	1	ko00040 Pentose and glucuronate interconversions;
gene47145	K01519	ITPA	inosine triphosphate pyrophosphatase	EC:3.6.1.19	3	ko00230 Purine metabolism;ko00240 Pyrimidine metabolism;ko00983 Drug metabolism - other enzymes;
gene47148	K12863	CWC15	protein CWC15	--	1	ko03040 Spliceosome;
gene47155	K13356	FAR	fatty acyl-CoA reductase	EC:1.2.1.-	2	ko00073 Cutin, suberine and wax biosynthesis;ko04146 Peroxisome;
gene47157	K16223	FT	protein FLOWERING LOCUS T	--	1	ko04712 Circadian rhythm - plant;
gene47158	K13424	WRKY33	WRKY transcription factor 33	--	1	ko04626 Plant-pathogen interaction;
gene47161	K04711	YDC1	dihydroceramidase	EC:3.5.1.-	1	ko00600 Sphingolipid metabolism;
gene47164	K14236	tRNA-Tyr	tRNA Tyr	--	1	ko00970 Aminoacyl-tRNA biosynthesis;
gene47165	K01835	pgm	phosphoglucomutase	EC:5.4.2.2	7	ko00010 Glycolysis / Gluconeogenesis;ko00030 Pentose phosphate pathway;ko00052 Galactose metabolism;ko00230 Purine metabolism;ko00500 Starch and sucrose metabolism;ko00520 Amino sugar and nucleotide sugar metabolism;ko00521 Streptomycin biosynthesis;
gene47167	K11844	USP16_45	ubiquitin carboxyl-terminal hydrolase 16/45	EC:3.1.2.15		
gene47169	K01051	E3.1.1.11	pectinesterase	EC:3.1.1.11	2	ko00040 Pentose and glucuronate interconversions;ko00500 Starch and sucrose metabolism;
gene47170	K02872	RP-L13Ae, RPL13A	large subunit ribosomal protein L13Ae	--	1	ko03010 Ribosome;
gene47172	K03032	PSMD1, RPN2	26S proteasome regulatory subunit N2	--	2	ko03050 Proteasome;ko05169 Epstein-Barr virus infection;
gene47175	K02144	ATPeV54kD	V-type H+-transporting ATPase 54 kD subunit	EC:3.6.3.14	8	ko00190 Oxidative phosphorylation;ko04142 Lysosome;ko04145 Phagosome;ko04721 Synaptic vesicle cycle;ko05110 Vibrio cholerae infection;ko05120 Epithelial cell signaling in Helicobacter pylori infection;ko05152 Tuberculosis;ko05323 Rheumatoid arthritis;
gene47176	K10908	POLRMT, RPO41	DNA-directed RNA polymerase, mitochondrial	EC:2.7.7.6		
gene47178	K00873	PK, pyk	pyruvate kinase	EC:2.7.1.40	7	ko00010 Glycolysis / Gluconeogenesis;ko00230 Purine metabolism;ko00620 Pyruvate metabolism;ko01200 Carbon metabolism;ko01230 Biosynthesis of amino acids;ko04930 Type II diabetes mellitus;ko05203 Viral carcinogenesis;
gene47179	K14638	SLC15A3_4, PHT	solute carrier family 15 (peptide/histidine transporter), member 3/4	--		
gene4717	K12669	OST3, OST6	oligosaccharyltransferase complex subunit gamma	--	3	ko00510 N-Glycan biosynthesis;ko00513 Various types of N-glycan biosynthesis;ko04141 Protein processing in endoplasmic reticulum;
gene47183	K07766	E3.6.1.52	diphosphoinositol-polyphosphate diphosphatase	EC:3.6.1.52		
gene47187	K13429	CERK1	chitin elicitor receptor kinase 1	--	1	ko04626 Plant-pathogen interaction;

gene47188	K13429	CERK1	chitin elicitor receptor kinase 1	--	1	ko04626 Plant-pathogen interaction;
gene4718	K05275	E1.1.1.65	pyridoxine 4-dehydrogenase	EC:1.1.1.65	1	ko00750 Vitamin B6 metabolism;
gene47196	K12129	PRR7	pseudo-response regulator 7	--	1	ko04712 Circadian rhythm - plant;
gene47197	K00318	E1.5.99.8	proline dehydrogenase	EC:1.5.99.8	1	ko00330 Arginine and proline metabolism;
gene47198	K01611	speD, AMD1	S-adenosylmethionine decarboxylase	EC:4.1.1.50	2	ko00270 Cysteine and methionine metabolism;ko00330 Arginine and proline metabolism;
gene47199	K01611	speD, AMD1	S-adenosylmethionine decarboxylase	EC:4.1.1.50	2	ko00270 Cysteine and methionine metabolism;ko00330 Arginine and proline metabolism;
gene4719	K08235	E2.4.1.207	xyloglucan:xyloglucosyl transferase	EC:2.4.1.207		
gene47202	K03320	amt, AMT, MEP	ammonium transporter, Amt family	--		
gene47205	K15340	DCLRE1A, SNM1A, PSO2	DNA cross-link repair 1A protein	--		
gene47210	K07375	TUBB	tubulin beta	--	3	ko04145 Phagosome;ko04540 Gap junction;ko05130 Pathogenic Escherichia coli infection;
gene47211	K07375	TUBB	tubulin beta	--	3	ko04145 Phagosome;ko04540 Gap junction;ko05130 Pathogenic Escherichia coli infection;
gene47213	K05765	CFL	cofilin	--	4	ko04360 Axon guidance;ko04666 Fc gamma R-mediated phagocytosis;ko04810 Regulation of actin cytoskeleton;ko05133 Pertussis;
gene47214	K13667	RUMI, KTELC1	protein glucosyltransferase	EC:2.4.1.-	1	ko00514 Other types of O-glycan biosynthesis;
gene47222	K12937	UGAT	cyanidin-3-O-glucoside 2''-O-glucuronosyltransferase	EC:2.4.1.254	1	ko00942 Anthocyanin biosynthesis;
gene47223	K12937	UGAT	cyanidin-3-O-glucoside 2''-O-glucuronosyltransferase	EC:2.4.1.254	1	ko00942 Anthocyanin biosynthesis;
gene47227	K08900	BCS1	mitochondrial chaperone BCS1	--		
gene4723	K15115	SLC25A32, MFT	solute carrier family 25 (mitochondrial folate transporter), member 32	--		
gene47242	K01899	LSC1	succinyl-CoA synthetase alpha subunit	EC:6.2.1.4 6.2.1.5	3	ko00020 Citrate cycle (TCA cycle);ko00640 Propanoate metabolism;ko01200 Carbon metabolism;
gene47245	K02695	psaH	photosystem I subunit VI	--	1	ko00195 Photosynthesis;
gene4724	K10406	KIFC2_3	kinesin family member C2/C3	--		
gene47250	K14236	tRNA-Tyr	tRNA Tyr	--	1	ko00970 Aminoacyl-tRNA biosynthesis;
gene47251	K14236	tRNA-Tyr	tRNA Tyr	--	1	ko00970 Aminoacyl-tRNA biosynthesis;
gene47252	K14236	tRNA-Tyr	tRNA Tyr	--	1	ko00970 Aminoacyl-tRNA biosynthesis;
gene47253	K14236	tRNA-Tyr	tRNA Tyr	--	1	ko00970 Aminoacyl-tRNA biosynthesis;
gene47254	K14236	tRNA-Tyr	tRNA Tyr	--	1	ko00970 Aminoacyl-tRNA biosynthesis;
gene47255	K14236	tRNA-Tyr	tRNA Tyr	--	1	ko00970 Aminoacyl-tRNA biosynthesis;
gene47256	K14236	tRNA-Tyr	tRNA Tyr	--	1	ko00970 Aminoacyl-tRNA biosynthesis;
gene47257	K14236	tRNA-Tyr	tRNA Tyr	--	1	ko00970 Aminoacyl-tRNA biosynthesis;
gene47258	K14236	tRNA-Tyr	tRNA Tyr	--	1	ko00970 Aminoacyl-tRNA biosynthesis;
gene47260	K14290	XPO1, CRM1	exportin-1	--	5	ko03008 Ribosome biogenesis in eukaryotes;ko03013 RNA transport;ko05164 Influenza A;ko05166 HTLV-I infection;ko05169 Epstein-Barr virus infection;
gene47265	K13288	orn, REX2, REXO2	oligoribonuclease	EC:3.1.-.-	1	ko03008 Ribosome biogenesis in eukaryotes;
gene47267	K10084	EDEM1	ER degradation enhancer, mannosidase alpha-like 1	--	1	ko04141 Protein processing in endoplasmic reticulum;
gene47269	K10773	NTH	endonuclease III	EC:4.2.99.18	1	ko03410 Base excision repair;
gene47272	K03875	SKP2, FBXL1	F-box and leucine-rich repeat protein 1 (S-phase kinase-associated protein 2)	--	7	ko04110 Cell cycle;ko04120 Ubiquitin mediated proteolysis;ko05168 Herpes simplex infection;ko05169 Epstein-Barr virus infection;ko05200 Pathways in cancer;ko05203 Viral carcinogenesis;ko05222 Small cell lung cancer;
gene47275	K06627	CCNA	cyclin A	--	5	ko04110 Cell cycle;ko04914 Progesterone-mediated oocyte maturation;ko05161 Hepatitis B;ko05169 Epstein-Barr virus infection;ko05203 Viral carcinogenesis;
gene47277	K10755	RFC2_4	replication factor C subunit 2/4	--	3	ko03030 DNA replication;ko03420 Nucleotide excision repair;ko03430 Mismatch repair;
gene47286	K14304	NUP85	nuclear pore complex protein Nup85	--	1	ko03013 RNA transport;
gene47295	K03549	kup	KUP system potassium uptake protein	--		
gene4730	K01930	FPGS	folylpolyglutamate synthase	EC:6.3.2.17	1	ko00790 Folate biosynthesis;
gene47310	K10882	EME1, MMS4	crossover junction endonuclease EME1	EC:3.1.22.-	2	ko03440 Homologous recombination;ko03460 Fanconi anemia pathway;
gene47311	K10839	RAD23, HR23	UV excision repair protein RAD23	--	2	ko03420 Nucleotide excision repair;ko04141 Protein processing in endoplasmic reticulum;
gene47314	K02896	RP-L24e, RPL24	large subunit ribosomal protein L24e	--	1	ko03010 Ribosome;
gene47317	K14792	RRP5, PDCD11	rRNA biogenesis protein RRP5	--		

gene4731	K15601	KDM3	lysine-specific demethylase 3	EC:1.14.11.-		
gene47321	K00254	DHODH	dihydroorotate dehydrogenase	EC:1.3.5.2	1	ko00240 Pyrimidine metabolism;
gene47323	K05350	bglB	beta-glucosidase	EC:3.2.1.21	3	ko00460 Cyanoamino acid metabolism;ko00500 Starch and sucrose metabolism;ko00940 Phenylpropanoid biosynthesis;
gene47325	K03921	DESA1	acyl-[acyl-carrier-protein] desaturase	EC:1.14.19.2	2	ko00061 Fatty acid biosynthesis;ko01040 Biosynthesis of unsaturated fatty acids;
gene47326	K10364	CAPZA	capping protein (actin filament) muscle Z-line, alpha	--		
gene47329	K09286	EREBP	EREBP-like factor	--		
gene47331	K16055	TPS	trehalose 6-phosphate synthase/phosphatase	EC:2.4.1.15 3.1.3.12	1	ko00500 Starch and sucrose metabolism;
gene47336	K08235	E2.4.1.207	xyloglucan:xyloglucosyl transferase	EC:2.4.1.207		
gene47339	K08339	ATG5	autophagy-related protein 5	--	3	ko04140 Regulation of autophagy;ko04622 RIG-I-like receptor signaling pathway;ko05131 Shigellosis;
gene47345	K09841	ABA2	xanthoxin dehydrogenase	EC:1.1.1.288	1	ko00906 Carotenoid biosynthesis;
gene4734	K15332	TRMT2A	tRNA (uracil-5-)-methyltransferase	--		
gene47352	K10839	RAD23, HR23	UV excision repair protein RAD23	--	2	ko03420 Nucleotide excision repair;ko04141 Protein processing in endoplasmic reticulum;
gene47356	K03347	CUL1, CDC53	cullin 1	--	9	ko04110 Cell cycle;ko04111 Cell cycle - yeast;ko04114 Oocyte meiosis;ko04120 Ubiquitin mediated proteolysis;ko04141 Protein processing in endoplasmic reticulum;ko04310 Wnt signaling pathway;ko04350 TGF-beta signaling pathway;ko04710 Circadian rhythm;ko05168 Herpes simplex infection;
gene47357	K13783	SLC37A1_2	MFS transporter, OPA family, solute carrier family 37 (glycerol-3-phosphate transporter), member 1/2	--		
gene47359	K05546	GANAB	alpha 1,3-glucosidase	EC:3.2.1.84	2	ko00510 N-Glycan biosynthesis;ko04141 Protein processing in endoplasmic reticulum;
gene47360	K12619	XRN2, RAT1	5'-3' exoribonuclease 2	EC:3.1.13.-	2	ko03008 Ribosome biogenesis in eukaryotes;ko03018 RNA degradation;
gene47363	K00799	GST, gst	glutathione S-transferase	EC:2.5.1.18	4	ko00480 Glutathione metabolism;ko00980 Metabolism of xenobiotics by cytochrome P450;ko00982 Drug metabolism - cytochrome P450;ko05204 Chemical carcinogenesis;
gene47367	K03039	PSMD13, RPN9	26S proteasome regulatory subunit N9	--	2	ko03050 Proteasome;ko05169 Epstein-Barr virus infection;
gene47369	K08900	BCS1	mitochondrial chaperone BCS1	--		
gene4736	K08678	UXS1	UDP-glucuronate decarboxylase	EC:4.1.1.35	2	ko00500 Starch and sucrose metabolism;ko00520 Amino sugar and nucleotide sugar metabolism;
gene47370	K14182	E4.2.3.21	vetispiradiene synthase	EC:4.2.3.21	1	ko00909 Sesquiterpenoid and triterpenoid biosynthesis;
gene47371	K17278	PGRMC1_2	membrane-associated progesterone receptor component	--		
gene47373	K00434	E1.11.1.11	L-ascorbate peroxidase	EC:1.11.1.11	2	ko00053 Ascorbate and aldarate metabolism;ko00480 Glutathione metabolism;
gene47377	K10751	CHAF1B	chromatin assembly factor 1 subunit B	--		
gene47378	K08900	BCS1	mitochondrial chaperone BCS1	--		
gene4737	K13120	FAM32A	protein FAM32A	--		
gene47383	K02894	RP-L23e, RPL23	large subunit ribosomal protein L23e	--	1	ko03010 Ribosome;
gene47398	K00809	E2.5.1.46, dys1	deoxyhypusine synthase	EC:2.5.1.46		
gene47399	K00605	gcvT, AMT	aminomethyltransferase	EC:2.1.2.10	3	ko00260 Glycine, serine and threonine metabolism;ko00670 One carbon pool by folate;ko01200 Carbon metabolism;
gene4739	K09518	DNAJB12	DnaJ homolog subfamily B member 12	--	1	ko04141 Protein processing in endoplasmic reticulum;
gene47400	K13347	PXMP2, PMP22	peroxisomal membrane protein 2	--	1	ko04146 Peroxisome;
gene4740	K11491	NCAPD3	condensin-2 complex subunit D3	--		
gene47415	K11838	USP7, UBP15	ubiquitin carboxyl-terminal hydrolase 7	EC:3.1.2.15	3	ko05168 Herpes simplex infection;ko05169 Epstein-Barr virus infection;ko05203 Viral carcinogenesis;
gene47418	K01533	E3.6.3.4, ATP7, copA	Cu2+-exporting ATPase	EC:3.6.3.4		
gene4741	K13108	SNIP1	smad nuclear-interacting protein 1	--		
gene47423	K15083	RAD16	DNA repair protein RAD16	--		
gene47424	K00430	E1.11.1.7	peroxidase	EC:1.11.1.7	2	ko00360 Phenylalanine metabolism;ko00940 Phenylpropanoid biosynthesis;
gene47427	K10803	XRCC1	DNA-repair protein XRCC1	--	1	ko03410 Base excision repair;
gene47429	K01230	MAN1	mannosyl-oligosaccharide alpha-1,2-mannosidase	EC:3.2.1.113	3	ko00510 N-Glycan biosynthesis;ko00513 Various types of N-glycan biosynthesis;ko04141 Protein processing in endoplasmic reticulum;
gene4742	K07976	RAB	Rab family, other	--		
gene47433	K12663	ECH1	delta(3,5)-Delta(2,4)-dienoyl-CoA isomerase	EC:5.3.3.-	1	ko04146 Peroxisome;

gene47435	K09584	PDIA6, TXNDC7	protein disulfide-isomerase A6	EC:5.3.4.1	1	ko04141 Protein processing in endoplasmic reticulum;
gene47436	K12848	SNU23	U4/U6.U5 tri-snRNP component SNU23	--	1	ko03040 Spliceosome;
gene47438	K07953	SAR1	GTP-binding protein SAR1	EC:3.6.5.-	2	ko04141 Protein processing in endoplasmic reticulum;ko05134 Legionellosis;
gene47439	K00382	DLD, lpd, pdhD	dihydrolipoamide dehydrogenase	EC:1.8.1.4	6	ko00010 Glycolysis / Gluconeogenesis;ko00020 Citrate cycle (TCA cycle);ko00260 Glycine, serine and threonine metabolism;ko00280 Valine, leucine and isoleucine degradation;ko00620 Pyruvate metabolism;ko01200 Carbon metabolism;
gene47440	K12844	PRPF31	U4/U6 small nuclear ribonucleoprotein PRP31	--	1	ko03040 Spliceosome;
gene47442	K12862	PLRG1, PRL1, PRP46	pleiotropic regulator 1	--	1	ko03040 Spliceosome;
gene47443	K13459	RPS2	disease resistance protein RPS2	--	1	ko04626 Plant-pathogen interaction;
gene47444	K14003	PREB, SEC12	prolactin regulatory element-binding protein	--	1	ko04141 Protein processing in endoplasmic reticulum;
gene47448	K14324	SAP18	histone deacetylase complex subunit SAP18	--	2	ko03013 RNA transport;ko03015 mRNA surveillance pathway;
gene47451	K09422	MYBP	myb proto-oncogene protein, plant	--		
gene47455	K11835	USP4_11_15, UBP12	ubiquitin carboxyl-terminal hydrolase 4/11/15	EC:3.1.2.15		
gene4745	K00773	tgt, QTRT1	queuine tRNA-ribosyltransferase	EC:2.4.2.29		
gene47466	K16302	CNNM	metal transporter CNNM	--		
gene4746	K09338	HD-ZIP	homeobox-leucine zipper protein	--		
gene47470	K05765	CFL	cofilin	--	4	ko04360 Axon guidance;ko04666 Fc gamma R-mediated phagocytosis;ko04810 Regulation of actin cytoskeleton;ko05133 Pertussis;
gene47471	K00620	argJ	glutamate N-acetyltransferase / amino-acid N-acetyltransferase	EC:2.3.1.35 2.3.1.1	3	ko00330 Arginine and proline metabolism;ko01210 2-Oxocarboxylic acid metabolism;ko01230 Biosynthesis of amino acids;
gene47473	K11251	H2A	histone H2A	--	2	ko05034 Alcoholism;ko05322 Systemic lupus erythematosus;
gene47474	K10839	RAD23, HR23	UV excision repair protein RAD23	--	2	ko03420 Nucleotide excision repair;ko04141 Protein processing in endoplasmic reticulum;
gene47475	K15095	E1.1.1.208	(+)-neomenthol dehydrogenase	EC:1.1.1.208	1	ko00902 Monoterpenoid biosynthesis;
gene47476	K02874	RP-L14, MRPL14, rplN	large subunit ribosomal protein L14	--	1	ko03010 Ribosome;
gene47477	K15095	E1.1.1.208	(+)-neomenthol dehydrogenase	EC:1.1.1.208	1	ko00902 Monoterpenoid biosynthesis;
gene47479	K01755	argH, ASL	argininosuccinate lyase	EC:4.3.2.1	3	ko00250 Alanine, aspartate and glutamate metabolism;ko00330 Arginine and proline metabolism;ko01230 Biosynthesis of amino acids;
gene47480	K10395	KIF4S	kinesin family member 4/7/21/27	--		
gene47481	K14686	SLC31A1, CTR1	solute carrier family 31 (copper transporter), member 1	--	1	ko04978 Mineral absorption;
gene47482	K16052	ynaI, mscMJ, MSL	MscS family membrane protein	--		
gene47489	K00927	PGK, pgk	phosphoglycerate kinase	EC:2.7.2.3	4	ko00010 Glycolysis / Gluconeogenesis;ko00710 Carbon fixation in photosynthetic organisms;ko01200 Carbon metabolism;ko01230 Biosynthesis of amino acids;
gene47491	K03100	lepB	signal peptidase I	EC:3.4.21.89	1	ko03060 Protein export;
gene47492	K01626	E2.5.1.54, aroF, aroG, aroH	3-deoxy-7-phosphoheptulonate synthase	EC:2.5.1.54	2	ko00400 Phenylalanine, tyrosine and tryptophan biosynthesis;ko01230 Biosynthesis of amino acids;
gene47499	K01834	PGAM, gpmA	2,3-bisphosphoglycerate-dependent phosphoglycerate mutase	EC:5.4.2.11	5	ko00010 Glycolysis / Gluconeogenesis;ko00260 Glycine, serine and threonine metabolism;ko00680 Methane metabolism;ko01200 Carbon metabolism;ko01230 Biosynthesis of amino acids;
gene47500	K07119	K07119				
gene47505	K13993	HSP20	HSP20 family protein	--	1	ko04141 Protein processing in endoplasmic reticulum;
gene4750	K15104	SLC25A11, OGC	solute carrier family 25 (mitochondrial oxoglutarate transporter), member 11	--		
gene47514	K14641	APY1_2	apyrase	EC:3.6.1.5	2	ko00230 Purine metabolism;ko00240 Pyrimidine metabolism;
gene47515	K09377	CSRP	cysteine and glycine-rich protein	--		
gene47517	K03257	EIF4A	translation initiation factor 4A	--	1	ko03013 RNA transport;
gene47518	K03257	EIF4A	translation initiation factor 4A	--	1	ko03013 RNA transport;
gene47520	K00232	E1.3.3.6, ACOX1, ACOX3	acyl-CoA oxidase	EC:1.3.3.6	5	ko00071 Fatty acid degradation;ko00592 alpha-Linolenic acid metabolism;ko01040 Biosynthesis of unsaturated fatty acids;ko03320 PPAR signaling pathway;ko04146 Peroxisome;
gene47524	K03405	chlI, bchI	magnesium chelatase subunit I	EC:6.6.1.1	1	ko00860 Porphyrin and chlorophyll metabolism;
gene47531	K12606	RCD1, CNOT9, CAF40	CCR4-NOT transcription complex subunit 9	--	1	ko03018 RNA degradation;

gene47538	K03327	TC.MATE, SLC47A, norM, mdtK, dinF	multidrug resistance protein, MATE family	--		
gene47539	K14488	SAUR	SAUR family protein	--	1	ko04075 Plant hormone signal transduction;
gene4753	K13201	TIA1, TIAL1	nucleolysin TIA-1/TIAR	--		
gene47540	K14488	SAUR	SAUR family protein	--	1	ko04075 Plant hormone signal transduction;
gene47541	K14488	SAUR	SAUR family protein	--	1	ko04075 Plant hormone signal transduction;
gene47542	K14488	SAUR	SAUR family protein	--	1	ko04075 Plant hormone signal transduction;
gene47543	K14488	SAUR	SAUR family protein	--	1	ko04075 Plant hormone signal transduction;
gene47544	K14488	SAUR	SAUR family protein	--	1	ko04075 Plant hormone signal transduction;
gene47545	K14488	SAUR	SAUR family protein	--	1	ko04075 Plant hormone signal transduction;
gene47546	K14488	SAUR	SAUR family protein	--	1	ko04075 Plant hormone signal transduction;
gene47547	K14488	SAUR	SAUR family protein	--	1	ko04075 Plant hormone signal transduction;
gene47548	K14488	SAUR	SAUR family protein	--	1	ko04075 Plant hormone signal transduction;
gene4754	K13464	JAZ	jasmonate ZIM domain-containing protein	--	2	ko04075 Plant hormone signal transduction;ko04626 Plant-pathogen interaction;
gene47553	K12897	TRA2	transformer-2 protein	--	1	ko03040 Spliceosome;
gene47554	K03320	amt, AMT, MEP	ammonium transporter, Amt family	--		
gene47566	K04077	groEL, HSPD1	chaperonin GroEL	--	4	ko03018 RNA degradation;ko04940 Type I diabetes mellitus;ko05134 Legionellosis;ko05152 Tuberculosis;
gene47568	K05909	E1.10.3.2				
gene47569	K09286	EREBP	EREBP-like factor	--		
gene47570	K00128	E1.2.1.3	aldehyde dehydrogenase (NAD+)	EC:1.2.1.3	15	ko00010 Glycolysis / Gluconeogenesis;ko00040 Pentose and glucuronate interconversions;ko00053 Ascorbate and aldarate metabolism;ko00071 Fatty acid degradation;ko00280 Valine, leucine and isoleucine degradation;ko00310 Lysine degradation;ko00330 Arginine and proline metabolism;ko00340 Histidine metabolism;ko00380 Tryptophan metabolism;ko00410 beta-Alanine metabolism;ko00561 Glycerolipid metabolism;ko00620 Pyruvate metabolism;ko00625 Chloroalkane and chloroalkene degradation;ko00640 Propanoate metabolism;ko00903 Limonene and pinene
gene47588	K08150	SLC2A13, ITR	MFS transporter, SP family, solute carrier family 2 (myo-inositol transporter), member 13	--		
gene47593	K13495	CISZOG	cis-zeatin O-glucosyltransferase	EC:2.4.1.215	1	ko00908 Zeatin biosynthesis;
gene47594	K01070	frmB, ESD, fghA	S-formylglutathione hydrolase	EC:3.1.2.12	2	ko00680 Methane metabolism;ko01200 Carbon metabolism;
gene47595	K11665	INO80, INOC1	DNA helicase INO80	EC:3.6.4.12		
gene47597	K16280	RGLG	E3 ubiquitin-protein ligase RGLG	EC:6.3.2.19		
gene47600	K09518	DNAJB12	DnaJ homolog subfamily B member 12	--	1	ko04141 Protein processing in endoplasmic reticulum;
gene47601	K15188	CCNT	cyclin T	--	1	ko05202 Transcriptional misregulation in cancer;
gene47606	K01723	AOS	hydroperoxide dehydratase	EC:4.2.1.92	1	ko00592 alpha-Linolenic acid metabolism;
gene47607	K03539	RPP1, RPP30	ribonuclease P/MRP protein subunit RPP1	EC:3.1.26.5	2	ko03008 Ribosome biogenesis in eukaryotes;ko03013 RNA transport;
gene47616	K05909	E1.10.3.2				
gene47618	K05765	CFL	cofilin	--	4	ko04360 Axon guidance;ko04666 Fc gamma R-mediated phagocytosis;ko04810 Regulation of actin cytoskeleton;ko05133 Pertussis;
gene4761	K10999	CESA	cellulose synthase A	EC:2.4.1.12		
gene47621	K15639	CYP734A1, BAS1	cytochrome P450, family 734, subfamily A, polypeptide 1 (PHYB activation tagged suppressor 1)	EC:1.14.-.-	1	ko00905 Brassinosteroid biosynthesis;

gene47627	K02183	CALM	calmodulin	--	25	ko04014 Ras signaling pathway;ko04020 Calcium signaling pathway;ko04070 Phosphatidylinositol signaling system;ko04114 Oocyte meiosis;ko04270 Vascular smooth muscle contraction;ko04626 Plant-pathogen interaction;ko04713 Circadian entrainment;ko04720 Long-term potentiation;ko04722 Neurotrophin signaling pathway;ko04728 Dopaminergic synapse;ko04740 Olfactory transduction;ko04744 Phototransduction;ko04745 Phototransduction - fly;ko04910 Insulin signaling pathway;ko04912 GnRH signaling pathway;ko04915 Estrogen signaling pathway;ko04916 Melanogenesis;ko04970 Salivary secretion;ko04971 Gastric acid secretion;ko05010 Alzheimer's disease;ko05031 Amphetamine addiction;ko05034 Alcoholism;ko05133 Pertussis;ko05152 Tuberculosis;ko05214 Glioma;
gene4762	K08678	UXS1	UDP-glucuronate decarboxylase	EC:4.1.1.35	2	ko00500 Starch and sucrose metabolism;ko00520 Amino sugar and nucleotide sugar metabolism;
gene47633	K09842	AAO3	abscisic-aldehyde oxidase	EC:1.2.3.14	1	ko00906 Carotenoid biosynthesis;
gene47634	K01188	E3.2.1.21	beta-glucosidase	EC:3.2.1.21	3	ko00460 Cyanoamino acid metabolism;ko00500 Starch and sucrose metabolism;ko00940 Phenylpropanoid biosynthesis;
gene47635	K10745	RNASEH2C	ribonuclease H2 subunit C	--	1	ko03030 DNA replication;
gene47644	K11836	USP5_13, UBP14	ubiquitin carboxyl-terminal hydrolase 5/13	EC:3.1.2.15		
gene47645	K00901	E2.7.1.107, DGK, dgkA	diacylglycerol kinase (ATP dependent)	EC:2.7.1.107	3	ko00561 Glycerolipid metabolism;ko00564 Glycerophospholipid metabolism;ko04070 Phosphatidylinositol signaling system;
gene47648	K03453	TC.BASS	bile acid:Na+ symporter, BASS family	--		
gene47653	K07904	RAB11A	Ras-related protein Rab-11A	--	4	ko04144 Endocytosis;ko04961 Endocrine and other factor-regulated calcium reabsorption;ko04962 Vasopressin-regulated water reabsorption;ko04972 Pancreatic secretion;
gene47654	K06207	typA, bipA	GTP-binding protein	--		
gene47656	K00517	E1.14.-.-			4	ko00624 Polycyclic aromatic hydrocarbon degradation;ko00627 Aminobenzoate degradation;ko00903 Limonene and pinene degradation;ko00945 Stilbenoid, diarylheptanoid and gingerol biosynthesis;
gene47657	K14791	PWP1	periodic tryptophan protein 1	--		
gene47665	K00430	E1.11.1.7	peroxidase	EC:1.11.1.7	2	ko00360 Phenylalanine metabolism;ko00940 Phenylpropanoid biosynthesis;
gene4766	K00858	ppnK, NADK	NAD+ kinase	EC:2.7.1.23	1	ko00760 Nicotinate and nicotinamide metabolism;
gene47670	K10770	ALKBH8	alkylated DNA repair protein alkB homolog 8	EC:1.14.11.- 2.1.1.229		
gene47684	K01213	E3.2.1.67	galacturan 1,4-alpha-galacturonidase	EC:3.2.1.67	2	ko00040 Pentose and glucuronate interconversions;ko00500 Starch and sucrose metabolism;
gene47688	K00915	IPK2	1D-myo-Inositol-tetrakisphosphate 5-kinase / inositol-polyphosphate multikinase	EC:2.7.1.140 2.7.1.151	2	ko00562 Inositol phosphate metabolism;ko04070 Phosphatidylinositol signaling system;
gene47697	K09874	NIP	aquaporin NIP	--		
gene47700	K13495	CISZOG	cis-zeatin O-glucosyltransferase	EC:2.4.1.215	1	ko00908 Zeatin biosynthesis;
gene47702	K16732	PRC1	protein regulator of cytokinesis 1	--		
gene47708	K10755	RFC2_4	replication factor C subunit 2/4	--	3	ko03030 DNA replication;ko03420 Nucleotide excision repair;ko03430 Mismatch repair;
gene47710	K06966	K06966				
gene47711	K09489	HSPA4	heat shock 70kDa protein 4	--	1	ko04612 Antigen processing and presentation;
gene47715	K13356	FAR	fatty acyl-CoA reductase	EC:1.2.1.-	2	ko00073 Cutin, suberine and wax biosynthesis;ko04146 Peroxisome;
gene47716	K13356	FAR	fatty acyl-CoA reductase	EC:1.2.1.-	2	ko00073 Cutin, suberine and wax biosynthesis;ko04146 Peroxisome;
gene4771	K09843	E1.14.13.93	(+)-abscisic acid 8'-hydroxylase	EC:1.14.13.93	1	ko00906 Carotenoid biosynthesis;
gene47724	K05770	BZRP	benzodiazapine receptor	--	2	ko04080 Neuroactive ligand-receptor interaction;ko05166 HTLV-I infection;
gene47726	K00052	leuB	3-isopropylmalate dehydrogenase	EC:1.1.1.85	3	ko00290 Valine, leucine and isoleucine biosynthesis;ko01210 2-Oxocarboxylic acid metabolism;ko01230 Biosynthesis of amino acids;
gene47730	K14411	MSI	RNA-binding protein Musashi	--	1	ko03015 mRNA surveillance pathway;

gene47733	K06268	PPP3R, CNB	serine/threonine-protein phosphatase 2B regulatory subunit	--	18	ko04010 MAPK signaling pathway;ko04020 Calcium signaling pathway;ko04114 Oocyte meiosis;ko04210 Apoptosis;ko04310 Wnt signaling pathway;ko04360 Axon guidance;ko04370 VEGF signaling pathway;ko04380 Osteoclast differentiation;ko04650 Natural killer cell mediated cytotoxicity;ko04660 T cell receptor signaling pathway;ko04662 B cell receptor signaling pathway;ko04720 Long-term potentiation;ko04724 Glutamatergic synapse;ko05010 Alzheimer's disease;ko05014 Amyotrophic lateral sclerosis (ALS);ko05031 Amphetamine addiction;ko05152
gene47734	K00799	GST, gst	glutathione S-transferase	EC:2.5.1.18	4	ko00480 Glutathione metabolism;ko00980 Metabolism of xenobiotics by cytochrome P450;ko00982 Drug metabolism - cytochrome P450;ko05204 Chemical carcinogenesis;
gene47736	K04733	IRAK4	interleukin-1 receptor-associated kinase 4	EC:2.7.11.1	11	ko04064 NF-kappa B signaling pathway;ko04210 Apoptosis;ko04620 Toll-like receptor signaling pathway;ko04722 Neurotrophin signaling pathway;ko05133 Pertussis;ko05140 Leishmaniasis;ko05142 Chagas disease (American trypanosomiasis);ko05145 Toxoplasmosis;ko05152 Tuberculosis;ko05162 Measles;ko05164 Influenza A;
gene47737	K00472	E1.14.11.2	prolyl 4-hydroxylase	EC:1.14.11.2	1	ko00330 Arginine and proline metabolism;
gene47739	K11713	PGTB1	geranylgeranyl transferase type-1 subunit beta	EC:2.5.1.59		
gene47740	K01188	E3.2.1.21	beta-glucosidase	EC:3.2.1.21	3	ko00460 Cyanoamino acid metabolism;ko00500 Starch and sucrose metabolism;ko00940 Phenylpropanoid biosynthesis;
gene47741	K02993	RP-S7e, RPS7	small subunit ribosomal protein S7e	--	1	ko03010 Ribosome;
gene47743	K14416	HBS1	elongation factor 1 alpha-like protein	--	2	ko03015 mRNA surveillance pathway;ko05134 Legionellosis;
gene47745	K13459	RPS2	disease resistance protein RPS2	--	1	ko04626 Plant-pathogen interaction;
gene47746	K02889	RP-L21e, RPL21	large subunit ribosomal protein L21e	--	1	ko03010 Ribosome;
gene4774	K01176	E3.2.1.1, amyA, malS	alpha-amylase	EC:3.2.1.1	2	ko00500 Starch and sucrose metabolism;ko04973 Carbohydrate digestion and absorption;
gene47753	K03283	HSPA1_8	heat shock 70kDa protein 1/8	--	11	ko03040 Spliceosome;ko04010 MAPK signaling pathway;ko04141 Protein processing in endoplasmic reticulum;ko04144 Endocytosis;ko04612 Antigen processing and presentation;ko04915 Estrogen signaling pathway;ko05134 Legionellosis;ko05145 Toxoplasmosis;ko05162 Measles;ko05164 Influenza A;ko05169 Epstein-Barr virus infection;
gene47755	K11755	hisIE	phosphoribosyl-ATP pyrophosphohydrolase / phosphoribosyl-AMP cyclohydrolase	EC:3.6.1.31 3.5.4.19	2	ko00340 Histidine metabolism;ko01230 Biosynthesis of amino acids;
gene47757	K01648	ACLY	ATP citrate (pro-S)-lyase	EC:2.3.3.8	2	ko00020 Citrate cycle (TCA cycle);ko00720 Carbon fixation pathways in prokaryotes;
gene47759	K00863	E2.7.1.29, DAK1, DAK2	dihydroxyacetone kinase	EC:2.7.1.29	4	ko00561 Glycerolipid metabolism;ko00680 Methane metabolism;ko01200 Carbon metabolism;ko04622 RIG-I-like receptor signaling pathway;
gene4775	K01176	E3.2.1.1, amyA, malS	alpha-amylase	EC:3.2.1.1	2	ko00500 Starch and sucrose metabolism;ko04973 Carbohydrate digestion and absorption;
gene47761	K14977	ylbA	ureidoglycine aminohydrolase	EC:3.5.3.-	1	ko00230 Purine metabolism;
gene47762	K06072	E1.14.99.29, DOHH	deoxyhypusine monooxygenase	EC:1.14.99.29		
gene47763	K08145	SLC2A8, GLUT8	MFS transporter, SP family, solute carrier family 2 (facilitated glucose transporter), member 8	--		
gene47764	K15397	KCS	3-ketoacyl-CoA synthase	EC:2.3.1.199	1	ko00062 Fatty acid elongation;
gene47765	K06287	maf	septum formation protein	--		
gene47771	K01069	E3.1.2.6, gloB	hydroxyacylglutathione hydrolase	EC:3.1.2.6	1	ko00620 Pyruvate metabolism;
gene47773	K01919	gshA	glutamate--cysteine ligase	EC:6.3.2.2	1	ko00480 Glutathione metabolism;
gene47774	K11446	JARID1	histone demethylase JARID1	EC:1.14.11.-		
gene47780	K03347	CUL1, CDC53	cullin 1	--	9	ko04110 Cell cycle;ko04111 Cell cycle - yeast;ko04114 Oocyte meiosis;ko04120 Ubiquitin mediated proteolysis;ko04141 Protein processing in endoplasmic reticulum;ko04310 Wnt signaling pathway;ko04350 TGF-beta signaling pathway;ko04710 Circadian rhythm;ko05168 Herpes simplex infection;
gene47782	K06634	CCNH	cyclin H	--	3	ko03022 Basal transcription factors;ko03420 Nucleotide excision repair;ko04110 Cell cycle;
gene47783	K06634	CCNH	cyclin H	--	3	ko03022 Basal transcription factors;ko03420 Nucleotide excision repair;ko04110 Cell cycle;

gene47788	K09553	STIP1	stress-induced-phosphoprotein 1	--	1	ko05020 Prion diseases;
gene47791	K09560	ST13	suppressor of tumorigenicity protein 13	--		
gene47792	K09422	MYBP	myb proto-oncogene protein, plant	--		
gene47795	K11593	ELF2C	eukaryotic translation initiation factor 2C	--		
gene47798	K00253	IVD, ivd	isovaleryl-CoA dehydrogenase	EC:1.3.8.4	1	ko00280 Valine, leucine and isoleucine degradation;
gene4779	K11090	LA, SSB	lupus La protein	--	1	ko05322 Systemic lupus erythematosus;
gene47801	K03978	engB	GTP-binding protein	--		
gene47802	K02922	RP-L37e, RPL37	large subunit ribosomal protein L37e	--	1	ko03010 Ribosome;
gene47803	K04710	CERS	ceramide synthetase	EC:2.3.1.24	1	ko00600 Sphingolipid metabolism;
gene47810	K01623	ALDO	fructose-bisphosphate aldolase, class I	EC:4.1.2.13	7	ko00010 Glycolysis / Gluconeogenesis;ko00030 Pentose phosphate pathway;ko00051 Fructose and mannose metabolism;ko00680 Methane metabolism;ko00710 Carbon fixation in photosynthetic organisms;ko01200 Carbon metabolism;ko01230 Biosynthesis of amino acids;
gene47811	K00876	E2.7.1.48, udk	uridine kinase	EC:2.7.1.48	2	ko00240 Pyrimidine metabolism;ko00983 Drug metabolism - other enzymes;
gene47812	K05349	bglX	beta-glucosidase	EC:3.2.1.21	3	ko00460 Cyanoamino acid metabolism;ko00500 Starch and sucrose metabolism;ko00940 Phenylpropanoid biosynthesis;
gene47814	K03676	grxC, GLRX, GLRX2	glutaredoxin 3	--		
gene47818	K14309	NUP93	nuclear pore complex protein Nup93	--	1	ko03013 RNA transport;
gene47820	K00863	E2.7.1.29, DAK1, DAK2	dihydroxyacetone kinase	EC:2.7.1.29	4	ko00561 Glycerolipid metabolism;ko00680 Methane metabolism;ko01200 Carbon metabolism;ko04622 RIG-I-like receptor signaling pathway;
gene47821	K00863	E2.7.1.29, DAK1, DAK2	dihydroxyacetone kinase	EC:2.7.1.29	4	ko00561 Glycerolipid metabolism;ko00680 Methane metabolism;ko01200 Carbon metabolism;ko04622 RIG-I-like receptor signaling pathway;
gene4782	K11517	HAO	(S)-2-hydroxy-acid oxidase	EC:1.1.3.15	2	ko00630 Glyoxylate and dicarboxylate metabolism;ko04146 Peroxisome;
gene47837	K02153	ATPeVH, ATP6H	V-type H+-transporting ATPase subunit H	EC:3.6.3.14	7	ko00190 Oxidative phosphorylation;ko04145 Phagosome;ko04721 Synaptic vesicle cycle;ko04966 Collecting duct acid secretion;ko05110 Vibrio cholerae infection;ko05120 Epithelial cell signaling in Helicobacter pylori infection;ko05323 Rheumatoid arthritis;
gene47843	K00499	E1.14.15.7	choline monooxygenase	EC:1.14.15.7	1	ko00260 Glycine, serine and threonine metabolism;
gene47844	K01537	E3.6.3.8	Ca2+-transporting ATPase	EC:3.6.3.8		
gene47850	K00029	E1.1.1.40, maeB	malate dehydrogenase (oxaloacetate-decarboxylating)(NADP+)	EC:1.1.1.40	3	ko00620 Pyruvate metabolism;ko00710 Carbon fixation in photosynthetic organisms;ko01200 Carbon metabolism;
gene47851	K13436	PTI1	pto-interacting protein 1	EC:2.7.11.1	1	ko04626 Plant-pathogen interaction;
gene4785	K14432	ABF	ABA responsive element binding factor	--	1	ko04075 Plant hormone signal transduction;
gene4786	K02865	RP-L10Ae, RPL10A	large subunit ribosomal protein L10Ae	--	1	ko03010 Ribosome;
gene47873	K12471	EPN	epsin	--	1	ko04144 Endocytosis;
gene47875	K14232	tRNA-Pro	tRNA Pro	--	1	ko00970 Aminoacyl-tRNA biosynthesis;
gene47878	K16292	CEP, CYSEP	KDEL-tailed cysteine endopeptidase	EC:3.4.22.-		
gene47879	K16292	CEP, CYSEP	KDEL-tailed cysteine endopeptidase	EC:3.4.22.-		
gene47882	K06892	K06892				
gene47884	K02932	RP-L5e, RPL5	large subunit ribosomal protein L5e	--	1	ko03010 Ribosome;
gene47891	K09550	PFDN4	prefoldin subunit 4	--		
gene47892	K03116	tatA	sec-independent protein translocase protein TatA	--	2	ko03060 Protein export;ko03070 Bacterial secretion system;
gene47893	K15156	MED14, RGR1	mediator of RNA polymerase II transcription subunit 14	--		
gene47895	K15639	CYP734A1, BAS1	cytochrome P450, family 734, subfamily A, polypeptide 1 (PHYB activation tagged suppressor 1)	EC:1.14.-.-	1	ko00905 Brassinosteroid biosynthesis;
gene47896	K03358	APC11	anaphase-promoting complex subunit 11	--	7	ko04110 Cell cycle;ko04111 Cell cycle - yeast;ko04113 Meiosis - yeast;ko04114 Oocyte meiosis;ko04120 Ubiquitin mediated proteolysis;ko04914 Progesterone-mediated oocyte maturation;ko05166 HTLV-I infection;
gene47899	K15289	SLC35F5	solute carrier family 35, member F5	--		
gene4789	K12191	CHMP2A	charged multivesicular body protein 2A	--	1	ko04144 Endocytosis;
gene478	K06675	SMC4	structural maintenance of chromosome 4	--	1	ko04111 Cell cycle - yeast;

gene47902	K16297	SCPL-II	serine carboxypeptidase-like clade II	EC:3.4.16.-		
gene47905	K03768	PPIB, ppiB	peptidyl-prolyl cis-trans isomerase B (cyclophilin B)	EC:5.2.1.8		
gene47907	K14492	ARR-A	two-component response regulator ARR-A family	--	1	ko04075 Plant hormone signal transduction;
gene47909	K01760	metC	cystathionine beta-lyase	EC:4.4.1.8	3	ko00270 Cysteine and methionine metabolism;ko00450 Selenocompound metabolism;ko01230 Biosynthesis of amino acids;
gene47910	K06677	YCS4, CNAP1, CAPD2	condensin complex subunit 1	--	1	ko04111 Cell cycle - yeast;
gene47911	K06063	SNW1, SKIIP, SKIP	SNW domain-containing protein 1	--	4	ko03040 Spliceosome;ko04330 Notch signaling pathway;ko05169 Epstein-Barr virus infection;ko05203 Viral carcinogenesis;
gene47914	K13691	SGT1	pathogen-inducible salicylic acid glucosyltransferase	EC:2.4.1.-		
gene47915	K01623	ALDO	fructose-bisphosphate aldolase, class I	EC:4.1.2.13	7	ko00010 Glycolysis / Gluconeogenesis;ko00030 Pentose phosphate pathway;ko00051 Fructose and mannose metabolism;ko00680 Methane metabolism;ko00710 Carbon fixation in photosynthetic organisms;ko01200 Carbon metabolism;ko01230 Biosynthesis of amino acids;
gene4791	K01187	E3.2.1.20, malZ	alpha-glucosidase	EC:3.2.1.20	2	ko00052 Galactose metabolism;ko00500 Starch and sucrose metabolism;
gene47920	K15032	MTERFD	mTERF domain-containing protein, mitochondrial	--		
gene47921	K00915	IPK2	1D-myo-Inositol-tetrakisphosphate 5-kinase / inositol-polyphosphate multikinase	EC:2.7.1.140 2.7.1.151	2	ko00562 Inositol phosphate metabolism;ko04070 Phosphatidylinositol signaling system;
gene47926	K15262	BCP1, BCCIP	protein BCP1	--		
gene47927	K15262	BCP1, BCCIP	protein BCP1	--		
gene47929	K06630	YWHAE	14-3-3 protein epsilon	--	8	ko04110 Cell cycle;ko04114 Oocyte meiosis;ko04151 PI3K-Akt signaling pathway;ko04390 Hippo signaling pathway;ko04391 Hippo signaling pathway - fly;ko04722 Neurotrophin signaling pathway;ko05169 Epstein-Barr virus infection;ko05203 Viral carcinogenesis;
gene4792	K17600	VPS54	vacuolar protein sorting-associated protein 54	--		
gene47936	K14236	tRNA-Tyr	tRNA Tyr	--	1	ko00970 Aminoacyl-tRNA biosynthesis;
gene47938	K13412	CPK	calcium-dependent protein kinase	EC:2.7.11.1	2	ko04626 Plant-pathogen interaction;ko05145 Toxoplasmosis;
gene47940	K11155	DGAT1	diacylglycerol O-acyltransferase 1	EC:2.3.1.20 2.3.1.75 2.3.1.76	3	ko00561 Glycerolipid metabolism;ko00830 Retinol metabolism;ko04975 Fat digestion and absorption;
gene47942	K02684	PRI1	DNA primase small subunit	EC:2.7.7.-	3	ko00230 Purine metabolism;ko00240 Pyrimidine metabolism;ko03030 DNA replication;
gene47944	K03294	TC.APA	basic amino acid/polyamine antiporter, APA family	--		
gene47948	K09284	AP2	AP2-like factor, euAP2 lineage	--		
gene47952	K06874	K06874	zinc finger protein	--		
gene47953	K00863	E2.7.1.29, DAK1, DAK2	dihydroxyacetone kinase	EC:2.7.1.29	4	ko00561 Glycerolipid metabolism;ko00680 Methane metabolism;ko01200 Carbon metabolism;ko04622 RIG-I-like receptor signaling pathway;
gene47955	K08511	ATVAMP72	vesicle-associated membrane protein 72	--		
gene47958	K14638	SLC15A3_4, PHT	solute carrier family 15 (peptide/histidine transporter), member 3/4	--		
gene47959	K15032	MTERFD	mTERF domain-containing protein, mitochondrial	--		
gene47966	K08496	GOSR2, BOS1	golgi SNAP receptor complex member 2	--	1	ko04130 SNARE interactions in vesicular transport;
gene47967	K13420	FLS2	LRR receptor-like serine/threonine-protein kinase FLS2	EC:2.7.11.1	1	ko04626 Plant-pathogen interaction;
gene47969	K13420	FLS2	LRR receptor-like serine/threonine-protein kinase FLS2	EC:2.7.11.1	1	ko04626 Plant-pathogen interaction;
gene4797	K12184	VPS28	ESCRT-I complex subunit VPS28	--	1	ko04144 Endocytosis;
gene47983	K10405	KIFC1	kinesin family member C1	--		
gene47985	K13648	GAUT	alpha-1,4-galacturonosyltransferase	EC:2.4.1.43	2	ko00500 Starch and sucrose metabolism;ko00520 Amino sugar and nucleotide sugar metabolism;
gene47988	K03113	EIF1, SUI1	translation initiation factor 1	--	1	ko03013 RNA transport;
gene4798	K15382	SLC50A, SWEET	solute carrier family 50 (sugar transporter)	--		
gene47990	K16871	POP2	4-aminobutyrate---pyruvate transaminase	EC:2.6.1.96	2	ko00250 Alanine, aspartate and glutamate metabolism;ko00650 Butanoate metabolism;
gene47992	K16903	TAA1	L-tryptophan---pyruvate aminotransferase	EC:2.6.1.99	1	ko00380 Tryptophan metabolism;
gene47993	K10908	POLRMT, RPO41	DNA-directed RNA polymerase, mitochondrial	EC:2.7.7.6		

gene47994	K02144	ATPeV54kD	V-type H+-transporting ATPase 54 kD subunit	EC:3.6.3.14	8	ko00190 Oxidative phosphorylation;ko04142 Lysosome;ko04145 Phagosome;ko04721 Synaptic vesicle cycle;ko05110 Vibrio cholerae infection;ko05120 Epithelial cell signaling in Helicobacter pylori infection;ko05152 Tuberculosis;ko05323 Rheumatoid arthritis;
gene47995	K02144	ATPeV54kD	V-type H+-transporting ATPase 54 kD subunit	EC:3.6.3.14	8	ko00190 Oxidative phosphorylation;ko04142 Lysosome;ko04145 Phagosome;ko04721 Synaptic vesicle cycle;ko05110 Vibrio cholerae infection;ko05120 Epithelial cell signaling in Helicobacter pylori infection;ko05152 Tuberculosis;ko05323 Rheumatoid arthritis;
gene47996	K08486	STX1B_2_3	syntaxin 1B/2/3	--	2	ko04130 SNARE interactions in vesicular transport;ko04721 Synaptic vesicle cycle;
gene4799	K00915	IPK2	1D-myo-Inositol-tetrakisphosphate 5-kinase / inositol-polyphosphate multikinase	EC:2.7.1.140 2.7.1.151	2	ko00562 Inositol phosphate metabolism;ko04070 Phosphatidylinositol signaling system;
gene48010	K01183	E3.2.1.14	chitinase	EC:3.2.1.14	1	ko00520 Amino sugar and nucleotide sugar metabolism;
gene48011	K14500	BSK	BR-signaling kinase	EC:2.7.11.1	1	ko04075 Plant hormone signal transduction;
gene48013	K02962	RP-S17e, RPS17	small subunit ribosomal protein S17e	--	1	ko03010 Ribosome;
gene48017	K08202	SLC22A4_5, OCTN	MFS transporter, OCT family, solute carrier family 22 (organic cation transporter), member 4/5	--		
gene48019	K08202	SLC22A4_5, OCTN	MFS transporter, OCT family, solute carrier family 22 (organic cation transporter), member 4/5	--		
gene4801	K01537	E3.6.3.8	Ca2+-transporting ATPase	EC:3.6.3.8		
gene48025	K02978	RP-S27e, RPS27	small subunit ribosomal protein S27e	--	1	ko03010 Ribosome;
gene48026	K09286	EREBP	EREBP-like factor	--		
gene48030	K15889	PCME	prenylcysteine alpha-carboxyl methylesterase	EC:3.1.1.-	1	ko00900 Terpenoid backbone biosynthesis;
gene48032	K14864	FTSJ1, TRM7	tRNA (cytidine32/guanosine34-2'-O)-methyltransferase	EC:2.1.1.205		
gene48036	K00430	E1.11.1.7	peroxidase	EC:1.11.1.7	2	ko00360 Phenylalanine metabolism;ko00940 Phenylpropanoid biosynthesis;
gene4803	K09458	fabF	3-oxoacyl-[acyl-carrier-protein] synthase II	EC:2.3.1.179	2	ko00061 Fatty acid biosynthesis;ko00780 Biotin metabolism;
gene48040	K08832	SRPK3, STK23	serine/threonine-protein kinase SRPK3	EC:2.7.11.1		
gene48043	K03327	TC.MATE, SLC47A, norM, mdtK, dinF	multidrug resistance protein, MATE family	--		
gene48045	K03118	tatC	sec-independent protein translocase protein TatC	--	2	ko03060 Protein export;ko03070 Bacterial secretion system;
gene48046	K02606	ORC4	origin recognition complex subunit 4	--	3	ko04110 Cell cycle;ko04111 Cell cycle - yeast;ko04113 Meiosis - yeast;
gene48049	K03549	kup	KUP system potassium uptake protein	--		
gene4804	K02541	MCM3	DNA replication licensing factor MCM3	EC:3.6.4.12	4	ko03030 DNA replication;ko04110 Cell cycle;ko04111 Cell cycle - yeast;ko04113 Meiosis - yeast;
gene48054	K02069	ABC.X2.P	putative ABC transport system permease protein	--		
gene48069	K01051	E3.1.1.11	pectinesterase	EC:3.1.1.11	2	ko00040 Pentose and glucuronate interconversions;ko00500 Starch and sucrose metabolism;
gene48075	K08238	XXT	xyloglucan 6-xylosyltransferase	EC:2.4.2.39		
gene48077	K10364	CAPZA	capping protein (actin filament) muscle Z-line, alpha	--		
gene48086	K00430	E1.11.1.7	peroxidase	EC:1.11.1.7	2	ko00360 Phenylalanine metabolism;ko00940 Phenylpropanoid biosynthesis;
gene48089	K01874	MARS, metG	methionyl-tRNA synthetase	EC:6.1.1.10	2	ko00450 Selenocompound metabolism;ko00970 Aminoacyl-tRNA biosynthesis;
gene48091	K07198	PRKAA, AMPK	5'-AMP-activated protein kinase, catalytic alpha subunit	EC:2.7.11.11	8	ko04140 Regulation of autophagy;ko04150 mTOR signaling pathway;ko04151 PI3K-Akt signaling pathway;ko04710 Circadian rhythm;ko04910 Insulin signaling pathway;ko04920 Adipocytokine signaling pathway;ko04932 Non-alcoholic fatty liver disease (NAFLD);ko05410 Hypertrophic cardiomyopathy (HCM);
gene48100	K12830	SF3B3, SAP130, RSE1	splicing factor 3B subunit 3	--	1	ko03040 Spliceosome;
gene48101	K09422	MYBP	myb proto-oncogene protein, plant	--		
gene48102	K14232	tRNA-Pro	tRNA Pro	--	1	ko00970 Aminoacyl-tRNA biosynthesis;
gene48112	K03841	FBP, fbp	fructose-1,6-bisphosphatase I	EC:3.1.3.11	7	ko00010 Glycolysis / Gluconeogenesis;ko00030 Pentose phosphate pathway;ko00051 Fructose and mannose metabolism;ko00680 Methane metabolism;ko00710 Carbon fixation in photosynthetic organisms;ko01200 Carbon metabolism;ko04910 Insulin signaling pathway;

gene48113	K03841	FBP, fbp	fructose-1,6-bisphosphatase I	EC:3.1.3.11	7	ko00010 Glycolysis / Gluconeogenesis;ko00030 Pentose phosphate pathway;ko00051 Fructose and mannose metabolism;ko00680 Methane metabolism;ko00710 Carbon fixation in photosynthetic organisms;ko01200 Carbon metabolism;ko04910 Insulin signaling pathway;
gene48115	K07375	TUBB	tubulin beta	--	3	ko04145 Phagosome;ko04540 Gap junction;ko05130 Pathogenic Escherichia coli infection;
gene48118	K03002	RPA2, POLR1B	DNA-directed RNA polymerase I subunit RPA2	EC:2.7.7.6	3	ko00230 Purine metabolism;ko00240 Pyrimidine metabolism;ko03020 RNA polymerase;
gene4811	K12655	OTUD5, DUBA	OTU domain-containing protein 5	EC:3.1.2.15	1	ko04622 RIG-I-like receptor signaling pathway;
gene48124	K10661	MARCH6, DOA10	E3 ubiquitin-protein ligase MARCH6	EC:6.3.2.19	1	ko04141 Protein processing in endoplasmic reticulum;
gene48125	K13126	PABPC	polyadenylate-binding protein	--	3	ko03013 RNA transport;ko03015 mRNA surveillance pathway;ko03018 RNA degradation;
gene48129	K01885	EARS, gltX	glutamyl-tRNA synthetase	EC:6.1.1.17	2	ko00860 Porphyrin and chlorophyll metabolism;ko00970 Aminoacyl-tRNA biosynthesis;
gene4812	K03352	APC5	anaphase-promoting complex subunit 5	--	7	ko04110 Cell cycle;ko04111 Cell cycle - yeast;ko04113 Meiosis - yeast;ko04114 Oocyte meiosis;ko04120 Ubiquitin mediated proteolysis;ko04914 Progesterone-mediated oocyte maturation;ko05166 HTLV-I infection;
gene48134	K02969	RP-S20e, RPS20	small subunit ribosomal protein S20e	--	1	ko03010 Ribosome;
gene48138	K11275	H1_5	histone H1/5	--		
gene48139	K14225	tRNA-Gly	tRNA Gly	--	1	ko00970 Aminoacyl-tRNA biosynthesis;
gene48140	K14230	tRNA-Met	tRNA Met	--	1	ko00970 Aminoacyl-tRNA biosynthesis;
gene48149	K11593	ELF2C	eukaryotic translation initiation factor 2C	--		
gene4814	K12489	ACAP	Arf-GAP with coiled-coil, ANK repeat and PH domain-containing protein	--	1	ko04144 Endocytosis;
gene48151	K05605	HIBCH	3-hydroxyisobutyryl-CoA hydrolase	EC:3.1.2.4	3	ko00280 Valine, leucine and isoleucine degradation;ko00410 beta-Alanine metabolism;ko00640 Propanoate metabolism;
gene48155	K05933	E1.14.17.4	aminocyclopropanecarboxylate oxidase	EC:1.14.17.4	1	ko00270 Cysteine and methionine metabolism;
gene48159	K14455	GOT2	aspartate aminotransferase, mitochondrial	EC:2.6.1.1	12	ko00250 Alanine, aspartate and glutamate metabolism;ko00270 Cysteine and methionine metabolism;ko00330 Arginine and proline metabolism;ko00350 Tyrosine metabolism;ko00360 Phenylalanine metabolism;ko00400 Phenylalanine, tyrosine and tryptophan biosynthesis;ko00710 Carbon fixation in photosynthetic organisms;ko00950 Isoquinoline alkaloid biosynthesis;ko00960 Tropane, piperidine and pyridine alkaloid biosynthesis;ko01210 2-Oxocarboxylic acid metabolism;ko01230 Biosynthesis of amino
gene4815	K11511	APITD1, CENPS, MHF1	centromere protein S	--	1	ko03460 Fanconi anemia pathway;
gene48163	K12126	PIF3	phytochrome-interacting factor 3	--	2	ko04075 Plant hormone signal transduction;ko04712 Circadian rhythm - plant;
gene48166	K09422	MYBP	myb proto-oncogene protein, plant	--		
gene48169	K13420	FLS2	LRR receptor-like serine/threonine-protein kinase FLS2	EC:2.7.11.1	1	ko04626 Plant-pathogen interaction;
gene48170	K06699	PSME4	proteasome activator subunit 4	--	1	ko03050 Proteasome;
gene48172	K12880	THOC3	THO complex subunit 3	--	2	ko03013 RNA transport;ko03040 Spliceosome;
gene48174	K12890	SFRS1_9	splicing factor, arginine/serine-rich 1/9	--	2	ko03040 Spliceosome;ko05168 Herpes simplex infection;
gene48175	K14320	AAAS	aladin	--	1	ko03013 RNA transport;
gene48176	K11982	RNF115_126	E3 ubiquitin-protein ligase RNF115/126	EC:6.3.2.19		
gene48177	K10575	UBE2G1, UBC7	ubiquitin-conjugating enzyme E2 G1	EC:6.3.2.19	3	ko04120 Ubiquitin mediated proteolysis;ko04141 Protein processing in endoplasmic reticulum;ko05012 Parkinson's disease;
gene48182	K09422	MYBP	myb proto-oncogene protein, plant	--		
gene48183	K09422	MYBP	myb proto-oncogene protein, plant	--		
gene48184	K13628	iscA, ISCA1	iron-sulfur cluster assembly protein	--		
gene48189	K08909	LHCA3	light-harvesting complex I chlorophyll a/b binding protein 3	--	1	ko00196 Photosynthesis - antenna proteins;
gene48190	K10406	KIFC2_3	kinesin family member C2/C3	--		
gene48198	K13336	PEX3	peroxin-3	--	1	ko04146 Peroxisome;
gene48199	K14411	MSI	RNA-binding protein Musashi	--	1	ko03015 mRNA surveillance pathway;

gene48201	K08150	SLC2A13, ITR	MFS transporter, SP family, solute carrier family 2 (myo-inositol transporter), member 13	--		
gene48209	K15397	KCS	3-ketoacyl-CoA synthase	EC:2.3.1.199	1	ko00062 Fatty acid elongation;
gene48210	K03868	RBX1, ROC1	RING-box protein 1	--	12	ko03420 Nucleotide excision repair;ko04066 HIF-1 signaling pathway;ko04110 Cell cycle;ko04111 Cell cycle - yeast;ko04114 Oocyte meiosis;ko04120 Ubiquitin mediated proteolysis;ko04141 Protein processing in endoplasmic reticulum;ko04310 Wnt signaling pathway;ko04350 TGF-beta signaling pathway;ko04710 Circadian rhythm;ko05200 Pathways in cancer;ko05211 Renal cell carcinoma;
gene48211	K14488	SAUR	SAUR family protein	--	1	ko04075 Plant hormone signal transduction;
gene48216	K05757	ARPC1A_B	actin related protein 2/3 complex, subunit 1A/1B	--	6	ko04666 Fc gamma R-mediated phagocytosis;ko04810 Regulation of actin cytoskeleton;ko05100 Bacterial invasion of epithelial cells;ko05130 Pathogenic Escherichia coli infection;ko05131 Shigellosis;ko05132 Salmonella infection;
gene48219	K11262	ACAC	acetyl-CoA carboxylase / biotin carboxylase	EC:6.4.1.2 6.3.4.14	4	ko00061 Fatty acid biosynthesis;ko00620 Pyruvate metabolism;ko00640 Propanoate metabolism;ko04910 Insulin signaling pathway;
gene48223	K10999	CESA	cellulose synthase A	EC:2.4.1.12		
gene48224	K01834	PGAM, gpmA	2,3-bisphosphoglycerate-dependent phosphoglycerate mutase	EC:5.4.2.11	5	ko00010 Glycolysis / Gluconeogenesis;ko00260 Glycine, serine and threonine metabolism;ko00680 Methane metabolism;ko01200 Carbon metabolism;ko01230 Biosynthesis of amino acids;
gene48226	K10706	SETX, ALS4	senataxin	EC:3.6.4.-		
gene48228	K03553	recA	recombination protein RecA	--	1	ko03440 Homologous recombination;
gene48231	K05768	GSN	gelsolin	--	3	ko04666 Fc gamma R-mediated phagocytosis;ko04810 Regulation of actin cytoskeleton;ko05203 Viral carcinogenesis;
gene48238	K12861	BCAS2	pre-mRNA-splicing factor SPF27	--	1	ko03040 Spliceosome;
gene48239	K13336	PEX3	peroxin-3	--	1	ko04146 Peroxisome;
gene4823	K13407	CYP94A1	cytochrome P450, family 94, subfamily A, polypeptide 1 (fatty acid omega-hydroxylase)	EC:1.14.-.-	1	ko00073 Cutin, suberine and wax biosynthesis;
gene48240	K14753	RACK1	guanine nucleotide-binding protein subunit beta-2-like 1 protein	--	1	ko05162 Measles;
gene48241	K09422	MYBP	myb proto-oncogene protein, plant	--		
gene48248	K15414	C1QBP	complement component 1 Q subcomponent-binding protein, mitochondrial	--	1	ko05168 Herpes simplex infection;
gene48249	K00011	E1.1.1.21, AKR1	aldehyde reductase	EC:1.1.1.21	5	ko00040 Pentose and glucuronate interconversions;ko00051 Fructose and mannose metabolism;ko00052 Galactose metabolism;ko00561 Glycerolipid metabolism;ko00620 Pyruvate metabolism;
gene48250	K13458	RAR1	disease resistance protein	--	1	ko04626 Plant-pathogen interaction;
gene48252	K16732	PRC1	protein regulator of cytokinesis 1	--		
gene48253	K13448	CML	calcium-binding protein CML	--	1	ko04626 Plant-pathogen interaction;
gene48260	K15718	LOX1_5	linoleate 9S-lipoxygenase	EC:1.13.11.58	1	ko00591 Linoleic acid metabolism;
gene48261	K15718	LOX1_5	linoleate 9S-lipoxygenase	EC:1.13.11.58	1	ko00591 Linoleic acid metabolism;
gene48264	K01982	LSUrRNA	large subunit ribosomal RNA	--	2	ko03008 Ribosome biogenesis in eukaryotes;ko03010 Ribosome;
gene48265	K14220	tRNA-Asn	tRNA Asn	--	1	ko00970 Aminoacyl-tRNA biosynthesis;
gene48266	K12815	DHX38, PRP16	pre-mRNA-splicing factor ATP-dependent RNA helicase PRP16	EC:3.6.4.13	1	ko03040 Spliceosome;
gene48268	K00759	E2.4.2.7, apt	adenine phosphoribosyltransferase	EC:2.4.2.7	1	ko00230 Purine metabolism;
gene48270	K03294	TC.APA	basic amino acid/polyamine antiporter, APA family	--		
gene48271	K15456	KTI12	protein KTI12	--		
gene48272	K08597	SENP8, NEDP1, DEN1	sentrin-specific protease 8	EC:3.4.22.68		
gene48273	K12881	THOC4, ALY	THO complex subunit 4	--	4	ko03013 RNA transport;ko03015 mRNA surveillance pathway;ko03040 Spliceosome;ko05168 Herpes simplex infection;
gene48280	K14012	SHP1, UBX1, NSFL1C	UBX domain-containing protein 1	--	1	ko04141 Protein processing in endoplasmic reticulum;
gene48283	K02575	NRT, narK, nrtP, nasA	MFS transporter, NNP family, nitrate/nitrite transporter	--	1	ko00910 Nitrogen metabolism;
gene48284	K02575	NRT, narK, nrtP, nasA	MFS transporter, NNP family, nitrate/nitrite transporter	--	1	ko00910 Nitrogen metabolism;

gene48285	K02575	NRT, narK, nrtP, nasA	MFS transporter, NNP family, nitrate/nitrite transporter	--	1	ko00910 Nitrogen metabolism;
gene48288	K03639	MOCS1, moaA	molybdenum cofactor biosynthesis protein	--	2	ko00790 Folate biosynthesis;ko04122 Sulfur relay system;
gene48289	K04506	SIAH1	E3 ubiquitin-protein ligase SIAH1	EC:6.3.2.19	3	ko04115 p53 signaling pathway;ko04120 Ubiquitin mediated proteolysis;ko04310 Wnt signaling pathway;
gene4828	K09286	EREBP	EREBP-like factor	--		
gene48292	K01528	DNM	dynamin GTPase	EC:3.6.5.5	4	ko04144 Endocytosis;ko04721 Synaptic vesicle cycle;ko04961 Endocrine and other factor-regulated calcium reabsorption;ko05100 Bacterial invasion of epithelial cells;
gene48294	K08334	BECN1, VPS30, ATG6	beclin 1	--	1	ko04140 Regulation of autophagy;
gene48297	K03283	HSPA1_8	heat shock 70kDa protein 1/8	--	11	ko03040 Spliceosome;ko04010 MAPK signaling pathway;ko04141 Protein processing in endoplasmic reticulum;ko04144 Endocytosis;ko04612 Antigen processing and presentation;ko04915 Estrogen signaling pathway;ko05134 Legionellosis;ko05145 Toxoplasmosis;ko05162 Measles;ko05164 Influenza A;ko05169 Epstein-Barr virus infection;
gene48301	K03841	FBP, fbp	fructose-1,6-bisphosphatase I	EC:3.1.3.11	7	ko00010 Glycolysis / Gluconeogenesis;ko00030 Pentose phosphate pathway;ko00051 Fructose and mannose metabolism;ko00680 Methane metabolism;ko00710 Carbon fixation in photosynthetic organisms;ko01200 Carbon metabolism;ko04910 Insulin signaling pathway;
gene48306	K02138	ATPeFD, ATP5H	F-type H+-transporting ATPase subunit d	EC:3.6.3.14	4	ko00190 Oxidative phosphorylation;ko05010 Alzheimer's disease;ko05012 Parkinson's disease;ko05016 Huntington's disease;
gene48307	K10393	KIF2_24, MCAK	kinesin family member 2/24	--		
gene48308	K08592	SENP1	sentrin-specific protease 1	EC:3.4.22.68		
gene48309	K11450	AOF2, LSD1	lysine-specific histone demethylase 1	EC:1.-.-.-		
gene4830	K10364	CAPZA	capping protein (actin filament) muscle Z-line, alpha	--		
gene48318	K16280	RGLG	E3 ubiquitin-protein ligase RGLG	EC:6.3.2.19		
gene48320	K01366	CTSH	cathepsin H	EC:3.4.22.16	1	ko04142 Lysosome;
gene48322	K16609	TTLL12	tubulin--tyrosine ligase-like protein 12	--		
gene48328	K09571	FKBP4_5	FK506-binding protein 4/5	EC:5.2.1.8	1	ko04915 Estrogen signaling pathway;
gene48329	K09569	FKBP2	FK506-binding protein 2	EC:5.2.1.8		
gene4832	K07560	dtd	D-tyrosyl-tRNA(Tyr) deacylase	EC:3.1.-.-		
gene48337	K00432	E1.11.1.9	glutathione peroxidase	EC:1.11.1.9	3	ko00480 Glutathione metabolism;ko00590 Arachidonic acid metabolism;ko04918 Thyroid hormone synthesis;
gene4833	K00430	E1.11.1.7	peroxidase	EC:1.11.1.7	2	ko00360 Phenylalanine metabolism;ko00940 Phenylpropanoid biosynthesis;
gene48340	K00128	E1.2.1.3	aldehyde dehydrogenase (NAD+)	EC:1.2.1.3	15	ko00010 Glycolysis / Gluconeogenesis;ko00040 Pentose and glucuronate interconversions;ko00053 Ascorbate and aldarate metabolism;ko00071 Fatty acid degradation;ko00280 Valine, leucine and isoleucine degradation;ko00310 Lysine degradation;ko00330 Arginine and proline metabolism;ko00340 Histidine metabolism;ko00380 Tryptophan metabolism;ko00410 beta-Alanine metabolism;ko00561 Glycerolipid metabolism;ko00620 Pyruvate metabolism;ko00625 Chloroalkane and chloroalkene degradation;ko00640 Propanoate metabolism;ko00903 Limonene and pinene
gene48348	K14509	ETR, ERS	ethylene receptor	EC:2.7.13.-	1	ko04075 Plant hormone signal transduction;
gene48349	K13495	CISZOG	cis-zeatin O-glucosyltransferase	EC:2.4.1.215	1	ko00908 Zeatin biosynthesis;
gene4834	K04733	IRAK4	interleukin-1 receptor-associated kinase 4	EC:2.7.11.1	11	ko04064 NF-kappa B signaling pathway;ko04210 Apoptosis;ko04620 Toll-like receptor signaling pathway;ko04722 Neurotrophin signaling pathway;ko05133 Pertussis;ko05140 Leishmaniasis;ko05142 Chagas disease (American trypanosomiasis);ko05145 Toxoplasmosis;ko05152 Tuberculosis;ko05162 Measles;ko05164 Influenza A;
gene48351	K01280	TPP2	tripeptidyl-peptidase II	EC:3.4.14.10		
gene48352	K14220	tRNA-Asn	tRNA Asn	--	1	ko00970 Aminoacyl-tRNA biosynthesis;
gene48358	K08867	WNK, PRKWNK	WNK lysine deficient protein kinase	EC:2.7.11.1		

gene48364	K09487	HSP90B, TRA1	heat shock protein 90kDa beta	--	8	ko04141 Protein processing in endoplasmic reticulum;ko04151 PI3K-Akt signaling pathway;ko04621 NOD-like receptor signaling pathway;ko04626 Plant-pathogen interaction;ko04915 Estrogen signaling pathway;ko04918 Thyroid hormone synthesis;ko05200 Pathways in cancer;ko05215 Prostate cancer;
gene48365	K10908	POLRMT, RPO41	DNA-directed RNA polymerase, mitochondrial	EC:2.7.7.6		
gene48373	K01962	accA	acetyl-CoA carboxylase carboxyl transferase subunit alpha	EC:6.4.1.2	6	ko00061 Fatty acid biosynthesis;ko00253 Tetracycline biosynthesis;ko00620 Pyruvate metabolism;ko00640 Propanoate metabolism;ko00720 Carbon fixation pathways in prokaryotes;ko01200 Carbon metabolism;
gene48375	K09377	CSRP	cysteine and glycine-rich protein	--		
gene48376	K14231	tRNA-Phe	tRNA Phe	--	1	ko00970 Aminoacyl-tRNA biosynthesis;
gene48382	K09422	MYBP	myb proto-oncogene protein, plant	--		
gene48383	K01979	SSUrRNA	small subunit ribosomal RNA	--	2	ko03008 Ribosome biogenesis in eukaryotes;ko03010 Ribosome;
gene48387	K07955	ARL8	ADP-ribosylation factor-like protein 8	--		
gene48390	K00059	fabG	3-oxoacyl-[acyl-carrier protein] reductase	EC:1.1.1.100	3	ko00061 Fatty acid biosynthesis;ko00780 Biotin metabolism;ko01040 Biosynthesis of unsaturated fatty acids;
gene48395	K09564	PPIE	peptidyl-prolyl isomerase E (cyclophilin E)	EC:5.2.1.8	1	ko03040 Spliceosome;
gene48397	K08835	OXSR1, STK39	serine/threonine-protein kinase OSR1/STK39	EC:2.7.11.1		
gene48401	K07019	K07019				
gene48403	K01620	ltaE	threonine aldolase	EC:4.1.2.5	2	ko00260 Glycine, serine and threonine metabolism;ko01230 Biosynthesis of amino acids;
gene48404	K12860	CDC5L, CDC5, CEF1	pre-mRNA-splicing factor CDC5/CEF1	--	1	ko03040 Spliceosome;
gene48406	K00939	E2.7.4.3, adk	adenylate kinase	EC:2.7.4.3	1	ko00230 Purine metabolism;
gene48407	K17553	PPP1R11	protein phosphatase 1 regulatory subunit 11	--		
gene4840	K12124	GI	GIGANTEA	--	1	ko04712 Circadian rhythm - plant;
gene48411	K12890	SFRS1_9	splicing factor, arginine/serine-rich 1/9	--	2	ko03040 Spliceosome;ko05168 Herpes simplex infection;
gene48413	K10638	UHRF1, NP95	E3 ubiquitin-protein ligase UHRF1	EC:6.3.2.19		
gene48417	K05868	CCNB	cyclin B	--	3	ko04110 Cell cycle;ko04115 p53 signaling pathway;ko04914 Progesterone-mediated oocyte maturation;
gene48421	K05909	E1.10.3.2				
gene48422	K05909	E1.10.3.2				
gene48423	K05909	E1.10.3.2				
gene48424	K05909	E1.10.3.2				
gene48425	K09286	EREBP	EREBP-like factor	--		
gene48426	K01254	E3.3.2.6, LTA4H	leukotriene-A4 hydrolase	EC:3.3.2.6	1	ko00590 Arachidonic acid metabolism;
gene48427	K07198	PRKAA, AMPK	5'-AMP-activated protein kinase, catalytic alpha subunit	EC:2.7.11.11	8	ko04140 Regulation of autophagy;ko04150 mTOR signaling pathway;ko04151 PI3K-Akt signaling pathway;ko04710 Circadian rhythm;ko04910 Insulin signaling pathway;ko04920 Adipocytokine signaling pathway;ko04932 Non-alcoholic fatty liver disease (NAFLD);ko05410 Hypertrophic cardiomyopathy (HCM);
gene48429	K12176	COPS2, CSN2, TRIP15	COP9 signalosome complex subunit 2	--		
gene4842	K12188	SNF8, EAP30	ESCRT-II complex subunit VPS22	--	1	ko04144 Endocytosis;
gene48431	K01052	LIPA	lysosomal acid lipase/cholesteryl ester hydrolase	EC:3.1.1.13	2	ko00100 Steroid biosynthesis;ko04142 Lysosome;
gene48434	K12402	AP4M1	AP-4 complex subunit mu-1	--	1	ko04142 Lysosome;
gene48438	K02918	RP-L35e, RPL35	large subunit ribosomal protein L35e	--	1	ko03010 Ribosome;
gene48439	K03015	RPB7, POLR2G	DNA-directed RNA polymerase II subunit RPB7	--	5	ko00230 Purine metabolism;ko00240 Pyrimidine metabolism;ko03020 RNA polymerase;ko05016 Huntington's disease;ko05169 Epstein-Barr virus infection;
gene4843	K09875	SIP	aquaporin SIP	--		
gene48440	K14424	SMO2	4-alpha-methyl-delta7-sterol-4alpha-methyl oxidase	EC:1.14.13.72	1	ko00100 Steroid biosynthesis;
gene48442	K14487	GH3	auxin responsive GH3 gene family	--	1	ko04075 Plant hormone signal transduction;
gene48443	K14487	GH3	auxin responsive GH3 gene family	--	1	ko04075 Plant hormone signal transduction;
gene48444	K14487	GH3	auxin responsive GH3 gene family	--	1	ko04075 Plant hormone signal transduction;
gene48449	K16578	CLASP1_2	CLIP-associating protein 1/2	--		
gene48453	K05909	E1.10.3.2				

gene48459	K00033	E1.1.1.44, PGD, gnd	6-phosphogluconate dehydrogenase	EC:1.1.1.44	3	ko00030 Pentose phosphate pathway;ko00480 Glutathione metabolism;ko01200 Carbon metabolism;
gene48460	K00033	E1.1.1.44, PGD, gnd	6-phosphogluconate dehydrogenase	EC:1.1.1.44	3	ko00030 Pentose phosphate pathway;ko00480 Glutathione metabolism;ko01200 Carbon metabolism;
gene48465	K09122	K09122	hypothetical protein	--		
gene48473	K09338	HD-ZIP	homeobox-leucine zipper protein	--		
gene48476	K07735	algH	putative transcriptional regulator	--		
gene48478	K14397	NUDT21, CPSF5, CFIM25	cleavage and polyadenylation specificity factor subunit 5	--	1	ko03015 mRNA surveillance pathway;
gene48479	K02183	CALM	calmodulin	--	25	ko04014 Ras signaling pathway;ko04020 Calcium signaling pathway;ko04070 Phosphatidylinositol signaling system;ko04114 Oocyte meiosis;ko04270 Vascular smooth muscle contraction;ko04626 Plant-pathogen interaction;ko04713 Circadian entrainment;ko04720 Long-term potentiation;ko04722 Neurotrophin signaling pathway;ko04728 Dopaminergic synapse;ko04740 Olfactory transduction;ko04744 Phototransduction;ko04745 Phototransduction - fly;ko04910 Insulin signaling pathway;ko04912 GnRH signaling pathway;ko04915 Estrogen signaling pathway;ko04916 Melanogenesis;ko04970 Salivary secretion;ko04971 Gastric acid secretion;ko05010 Alzheimer's disease;ko05031 Amphetamine addiction;ko05034 Alcoholism;ko05133 Pertussis;ko05152 Tuberculosis;ko05214 Glioma;
gene4847	K10206	E2.6.1.83	LL-diaminopimelate aminotransferase	EC:2.6.1.83	2	ko00300 Lysine biosynthesis;ko01230 Biosynthesis of amino acids;
gene48481	K11275	H1_5	histone H1/5	--		
gene48482	K13162	PCBP2_3_4	poly(rC)-binding protein 2/3/4	--		
gene48483	K03593	mrp	ATP-binding protein involved in chromosome partitioning	--		
gene48484	K01937	E6.3.4.2, pyrG	CTP synthase	EC:6.3.4.2	1	ko00240 Pyrimidine metabolism;
gene4848	K12830	SF3B3, SAP130, RSE1	splicing factor 3B subunit 3	--	1	ko03040 Spliceosome;
gene48492	K08511	ATVAMP72	vesicle-associated membrane protein 72	--		
gene48493	K08511	ATVAMP72	vesicle-associated membrane protein 72	--		
gene48497	K10949	KDELR	ER lumen protein retaining receptor	--	1	ko05110 Vibrio cholerae infection;
gene48499	K13207	CUGBP, BRUNOL, CELF	CUG-BP- and ETR3-like factor	--		
gene48503	K08678	UXS1	UDP-glucuronate decarboxylase	EC:4.1.1.35	2	ko00500 Starch and sucrose metabolism;ko00520 Amino sugar and nucleotide sugar metabolism;
gene48509	K15710	SHPRH	E3 ubiquitin-protein ligase SHPRH	EC:3.6.4.- 6.3.2.19		
gene48511	K02990	RP-S6, MRPS6, rpsF	small subunit ribosomal protein S6	--	1	ko03010 Ribosome;
gene48514	K14332	psaO	photosystem I subunit PsaO	--	1	ko00195 Photosynthesis;
gene48515	K12611	DCP1B	mRNA-decapping enzyme 1B	EC:3.-.-.-	1	ko03018 RNA degradation;
gene48517	K12655	OTUD5, DUBA	OTU domain-containing protein 5	EC:3.1.2.15	1	ko04622 RIG-I-like receptor signaling pathway;
gene48519	K14594	NSY	neoxanthin synthase	EC:5.3.99.9	1	ko00906 Carotenoid biosynthesis;
gene4851	K03327	TC.MATE, SLC47A, norM, mdtK, dinF	multidrug resistance protein, MATE family	--		
gene48522	K17604	ZSWIM3	zinc finger SWIM domain-containing protein 3	--		
gene48527	K01940	E6.3.4.5, argG	argininosuccinate synthase	EC:6.3.4.5	3	ko00250 Alanine, aspartate and glutamate metabolism;ko00330 Arginine and proline metabolism;ko01230 Biosynthesis of amino acids;
gene48529	K01188	E3.2.1.21	beta-glucosidase	EC:3.2.1.21	3	ko00460 Cyanoamino acid metabolism;ko00500 Starch and sucrose metabolism;ko00940 Phenylpropanoid biosynthesis;
gene48532	K14232	tRNA-Pro	tRNA Pro	--	1	ko00970 Aminoacyl-tRNA biosynthesis;
gene48534	K00863	E2.7.1.29, DAK1, DAK2	dihydroxyacetone kinase	EC:2.7.1.29	4	ko00561 Glycerolipid metabolism;ko00680 Methane metabolism;ko01200 Carbon metabolism;ko04622 RIG-I-like receptor signaling pathway;
gene48535	K02949	RP-S11e, RPS11	small subunit ribosomal protein S11e	--	1	ko03010 Ribosome;
gene48538	K10839	RAD23, HR23	UV excision repair protein RAD23	--	2	ko03420 Nucleotide excision repair;ko04141 Protein processing in endoplasmic reticulum;
gene4853	K01188	E3.2.1.21	beta-glucosidase	EC:3.2.1.21	3	ko00460 Cyanoamino acid metabolism;ko00500 Starch and sucrose metabolism;ko00940 Phenylpropanoid biosynthesis;
gene48542	K13429	CERK1	chitin elicitor receptor kinase 1	--	1	ko04626 Plant-pathogen interaction;

gene48543	K07195	EXOC7, EXO70	exocyst complex component 7	--	1	ko04910 Insulin signaling pathway;
gene48544	K14816	REI1	pre-60S factor REI1	--		
gene48552	K10592	HUWE1, MULE, ARF-BP1	E3 ubiquitin-protein ligase HUWE1	EC:6.3.2.19	1	ko04120 Ubiquitin mediated proteolysis;
gene48554	K13065	E2.3.1.133, HCT	shikimate O-hydroxycinnamoyltransferase	EC:2.3.1.133	3	ko00940 Phenylpropanoid biosynthesis;ko00941 Flavonoid biosynthesis;ko00945 Stilbenoid, diarylheptanoid and gingerol biosynthesis;
gene48561	K10798	PARP	poly [ADP-ribose] polymerase	EC:2.4.2.30	1	ko03410 Base excision repair;
gene48563	K14555	UTP13, TBL3	U3 small nucleolar RNA-associated protein 13	--	1	ko03008 Ribosome biogenesis in eukaryotes;
gene48564	K01602	rbcS	ribulose-bisphosphate carboxylase small chain	EC:4.1.1.39	3	ko00630 Glyoxylate and dicarboxylate metabolism;ko00710 Carbon fixation in photosynthetic organisms;ko01200 Carbon metabolism;
gene4856	K03263	EIF5A	translation initiation factor 5A	--		
gene48580	K15326	TSEN54	tRNA-splicing endonuclease subunit Sen54	--		
gene48583	K09588	CYP90A1, CPD	cytochrome P450, family 90, subfamily A, polypeptide 1	EC:1.14.-.-	1	ko00905 Brassinosteroid biosynthesis;
gene48584	K07937	ARF1	ADP-ribosylation factor 1	--	2	ko05110 Vibrio cholerae infection;ko05134 Legionellosis;
gene48586	K00058	serA, PHGDH	D-3-phosphoglycerate dehydrogenase	EC:1.1.1.95	4	ko00260 Glycine, serine and threonine metabolism;ko00680 Methane metabolism;ko01200 Carbon metabolism;ko01230 Biosynthesis of amino acids;
gene48588	K03257	EIF4A	translation initiation factor 4A	--	1	ko03013 RNA transport;
gene48593	K07897	RAB7A	Ras-related protein Rab-7A	--	5	ko04144 Endocytosis;ko04145 Phagosome;ko05132 Salmonella infection;ko05146 Amoebiasis;ko05152 Tuberculosis;
gene48594	K15210	SNAPC3	snRNA-activating protein complex subunit 3	--		
gene48597	K01873	VARS, valS	valyl-tRNA synthetase	EC:6.1.1.9	1	ko00970 Aminoacyl-tRNA biosynthesis;
gene48603	K01115	PLD1_2	phospholipase D1/2	EC:3.1.4.4	7	ko00564 Glycerophospholipid metabolism;ko00565 Ether lipid metabolism;ko04014 Ras signaling pathway;ko04144 Endocytosis;ko04666 Fc gamma R-mediated phagocytosis;ko04724 Glutamatergic synapse;ko04912 GnRH signaling pathway;
gene48609	K03064	PSMC6, RPT4	26S proteasome regulatory subunit T4	--	2	ko03050 Proteasome;ko05169 Epstein-Barr virus infection;
gene48615	K13119	FAM50, XAP5	protein FAM50	--		
gene48618	K14611	SLC23A1_2, SVCT1_2	solute carrier family 23 (nucleobase transporter), member 1/2	--		
gene4861	K11498	CENPE	centromeric protein E	--		
gene48620	K05909	E1.10.3.2				
gene48623	K08832	SRPK3, STK23	serine/threonine-protein kinase SRPK3	EC:2.7.11.1		
gene48630	K00430	E1.11.1.7	peroxidase	EC:1.11.1.7	2	ko00360 Phenylalanine metabolism;ko00940 Phenylpropanoid biosynthesis;
gene48635	K17268	COPE	coatomer protein complex, subunit epsilon	--		
gene48639	K03521	fixA, etfB	electron transfer flavoprotein beta subunit	--		
gene48645	K09422	MYBP	myb proto-oncogene protein, plant	--		
gene48646	K14233	tRNA-Ser	tRNA Ser	--	1	ko00970 Aminoacyl-tRNA biosynthesis;
gene48653	K09286	EREBP	EREBP-like factor	--		
gene48663	K13162	PCBP2_3_4	poly(rC)-binding protein 2/3/4	--		
gene48667	K05275	E1.1.1.65	pyridoxine 4-dehydrogenase	EC:1.1.1.65	1	ko00750 Vitamin B6 metabolism;
gene48670	K02437	gcvH, GCSH	glycine cleavage system H protein	--	1	ko00630 Glyoxylate and dicarboxylate metabolism;
gene48678	K02206	CDK2	cyclin-dependent kinase 2	EC:2.7.11.22	13	ko04110 Cell cycle;ko04114 Oocyte meiosis;ko04115 p53 signaling pathway;ko04151 PI3K-Akt signaling pathway;ko04914 Progesterone-mediated oocyte maturation;ko05161 Hepatitis B;ko05162 Measles;ko05168 Herpes simplex infection;ko05169 Epstein-Barr virus infection;ko05200 Pathways in cancer;ko05203 Viral carcinogenesis;ko05215 Prostate cancer;ko05222 Small cell lung cancer;
gene48680	K14486	K14486, ARF	auxin response factor	--	1	ko04075 Plant hormone signal transduction;
gene48682	K15730	PTGES3	cytosolic prostaglandin-E synthase	EC:5.3.99.3	1	ko00590 Arachidonic acid metabolism;
gene48683	K06276	PDPK1	3-phosphoinositide dependent protein kinase-1	EC:2.7.11.1	9	ko03320 PPAR signaling pathway;ko04150 mTOR signaling pathway;ko04151 PI3K-Akt signaling pathway;ko04510 Focal adhesion;ko04910 Insulin signaling pathway;ko04960 Aldosterone-regulated sodium reabsorption;ko05213 Endometrial cancer;ko05215 Prostate cancer;ko05223 Non-small cell lung cancer;

gene48686	K09338	HD-ZIP	homeobox-leucine zipper protein	--		
gene48692	K02685	PRI2	DNA primase large subunit	EC:2.7.7.-	3	ko00230 Purine metabolism;ko00240 Pyrimidine metabolism;ko03030 DNA replication;
gene48699	K14638	SLC15A3_4, PHT	solute carrier family 15 (peptide/histidine transporter), member 3/4	--		
gene4869	K10706	SETX, ALS4	senataxin	EC:3.6.4.-		
gene48704	K03283	HSPA1_8	heat shock 70kDa protein 1/8	--	11	ko03040 Spliceosome;ko04010 MAPK signaling pathway;ko04141 Protein processing in endoplasmic reticulum;ko04144 Endocytosis;ko04612 Antigen processing and presentation;ko04915 Estrogen signaling pathway;ko05134 Legionellosis;ko05145 Toxoplasmosis;ko05162 Measles;ko05164 Influenza A;ko05169 Epstein-Barr virus infection;
gene48711	K17262	TBCB, CKAP1, ALF1	tubulin-folding cofactor B	--		
gene48713	K08235	E2.4.1.207	xyloglucan:xyloglucosyl transferase	EC:2.4.1.207		
gene48714	K06268	PPP3R, CNB	serine/threonine-protein phosphatase 2B regulatory subunit	--	18	ko04010 MAPK signaling pathway;ko04020 Calcium signaling pathway;ko04114 Oocyte meiosis;ko04210 Apoptosis;ko04310 Wnt signaling pathway;ko04360 Axon guidance;ko04370 VEGF signaling pathway;ko04380 Osteoclast differentiation;ko04650 Natural killer cell mediated cytotoxicity;ko04660 T cell receptor signaling pathway;ko04662 B cell receptor signaling pathway;ko04720 Long-term potentiation;ko04724 Glutamatergic synapse;ko05010 Alzheimer's disease;ko05014 Amyotrophic lateral sclerosis (ALS);ko05031 Amphetamine addiction;ko05152
gene48716	K10590	TRIP12	E3 ubiquitin-protein ligase TRIP12	EC:6.3.2.19	1	ko04120 Ubiquitin mediated proteolysis;
gene48719	K13648	GAUT	alpha-1,4-galacturonosyltransferase	EC:2.4.1.43	2	ko00500 Starch and sucrose metabolism;ko00520 Amino sugar and nucleotide sugar metabolism;
gene4871	K02736	PSMB4	20S proteasome subunit beta 7	EC:3.4.25.1	1	ko03050 Proteasome;
gene48722	K01110	PTEN	phosphatidylinositol-3,4,5-trisphosphate 3-phosphatase and dual-specificity protein phosphatase PTEN	EC:3.1.3.16 3.1.3.48 3.1.3.67	15	ko00562 Inositol phosphate metabolism;ko04070 Phosphatidylinositol signaling system;ko04115 p53 signaling pathway;ko04150 mTOR signaling pathway;ko04151 PI3K-Akt signaling pathway;ko04510 Focal adhesion;ko04530 Tight junction;ko05161 Hepatitis B;ko05200 Pathways in cancer;ko05206 MicroRNAs in cancer;ko05213 Endometrial cancer;ko05214 Glioma;ko05215 Prostate cancer;ko05218 Melanoma;ko05222 Small cell lung cancer;
gene48725	K08819	CDK12_13	cyclin-dependent kinase 12/13	EC:2.7.11.22 2.7.11.23		
gene48729	K13447	RBOH	respiratory burst oxidase	EC:1.6.3.- 1.11.1.-	1	ko04626 Plant-pathogen interaction;
gene48730	K09458	fabF	3-oxoacyl-[acyl-carrier-protein] synthase II	EC:2.3.1.179	2	ko00061 Fatty acid biosynthesis;ko00780 Biotin metabolism;
gene48731	K13993	HSP20	HSP20 family protein	--	1	ko04141 Protein processing in endoplasmic reticulum;
gene48733	K15397	KCS	3-ketoacyl-CoA synthase	EC:2.3.1.199	1	ko00062 Fatty acid elongation;
gene48735	K05917	CYP51	cytochrome P450, family 51 (sterol 14-demethylase)	EC:1.14.13.70	1	ko00100 Steroid biosynthesis;
gene48738	K13095	SF1	splicing factor 1	--		
gene48746	K10268	FBXL2_20	F-box and leucine-rich repeat protein 2/20	--		
gene4874	K08059	IFI30, GILT	interferon, gamma-inducible protein 30	--	1	ko04612 Antigen processing and presentation;
gene48752	K06901	pbuG	putative MFS transporter, AGZA family, xanthine/uracil permease	--		
gene48756	K03549	kup	KUP system potassium uptake protein	--		
gene48758	K03511	POLK	DNA polymerase kappa	EC:2.7.7.7	1	ko03460 Fanconi anemia pathway;
gene48763	K17623	HDHD1	pseudouridine-5'-monophosphatase	EC:3.1.3.-		
gene48769	K11420	EHMT	euchromatic histone-lysine N-methyltransferase	EC:2.1.1.43	1	ko00310 Lysine degradation;
gene48773	K13783	SLC37A1_2	MFS transporter, OPA family, solute carrier family 37 (glycerol-3-phosphate transporter), member 1/2	--		
gene48775	K01188	E3.2.1.21	beta-glucosidase	EC:3.2.1.21	3	ko00460 Cyanoamino acid metabolism;ko00500 Starch and sucrose metabolism;ko00940 Phenylpropanoid biosynthesis;
gene48779	K12883	NCBP2, CBP20	nuclear cap-binding protein subunit 2	--	3	ko03013 RNA transport;ko03015 mRNA surveillance pathway;ko03040 Spliceosome;
gene48780	K14231	tRNA-Phe	tRNA Phe	--	1	ko00970 Aminoacyl-tRNA biosynthesis;
gene48786	K15683	NFXL1, OZFP	NF-X1-type zinc finger protein NFXL1	--		

gene48787	K05857	PLCD	phosphatidylinositol phospholipase C, delta	EC:3.1.4.11	3	ko00562 Inositol phosphate metabolism;ko04020 Calcium signaling pathway;ko04070 Phosphatidylinositol signaling system;
gene48788	K00430	E1.11.1.7	peroxidase	EC:1.11.1.7	2	ko00360 Phenylalanine metabolism;ko00940 Phenylpropanoid biosynthesis;
gene48789	K01409	gcp, QRI7, OSGEPL	O-sialoglycoprotein endopeptidase	EC:3.4.24.57		
gene48791	K09422	MYBP	myb proto-oncogene protein, plant	--		
gene48798	K06970	rlmF	23S rRNA (adenine1618-N6)-methyltransferase	EC:2.1.1.181		
gene487	K07874	RAB1A	Ras-related protein Rab-1A	--	1	ko05134 Legionellosis;
gene48802	K15283	SLC35E1	solute carrier family 35, member E1	--		
gene48804	K15436	TRPO3, MTR10	transportin-3	--		
gene48806	K15436	TRPO3, MTR10	transportin-3	--		
gene48809	K15131	MED11	mediator of RNA polymerase II transcription subunit 11	--		
gene48812	K00850	pfkA, PFK	6-phosphofructokinase 1	EC:2.7.1.11	7	ko00010 Glycolysis / Gluconeogenesis;ko00030 Pentose phosphate pathway;ko00051 Fructose and mannose metabolism;ko00052 Galactose metabolism;ko00680 Methane metabolism;ko01200 Carbon metabolism;ko01230 Biosynthesis of amino acids;
gene48815	K10571	DET1	de-etiolated-1	--	1	ko04120 Ubiquitin mediated proteolysis;
gene4881	K13192	RBM26	RNA-binding protein 26	--		
gene48822	K01873	VARS, valS	valyl-tRNA synthetase	EC:6.1.1.9	1	ko00970 Aminoacyl-tRNA biosynthesis;
gene48825	K15032	MTERFD	mTERF domain-containing protein, mitochondrial	--		
gene48827	K03574	MUTT, NUDT1, MTH1	7,8-dihydro-8-oxoguanine triphosphatase	EC:3.6.1.-		
gene48831	K07437	CYP26A	cytochrome P450, family 26, subfamily A	--	1	ko00830 Retinol metabolism;
gene48833	K07437	CYP26A	cytochrome P450, family 26, subfamily A	--	1	ko00830 Retinol metabolism;
gene48835	K10746	EXO1	exonuclease 1	EC:3.1.-.-	1	ko03430 Mismatch repair;
gene48837	K05933	E1.14.17.4	aminocyclopropanecarboxylate oxidase	EC:1.14.17.4	1	ko00270 Cysteine and methionine metabolism;
gene4883	K15188	CCNT	cyclin T	--	1	ko05202 Transcriptional misregulation in cancer;
gene48840	K00819	E2.6.1.13, rocD	ornithine--oxo-acid transaminase	EC:2.6.1.13	1	ko00330 Arginine and proline metabolism;
gene48841	K01184	E3.2.1.15	polygalacturonase	EC:3.2.1.15	2	ko00040 Pentose and glucuronate interconversions;ko00500 Starch and sucrose metabolism;
gene48849	K01495	GCH1, folE	GTP cyclohydrolase I	EC:3.5.4.16	1	ko00790 Folate biosynthesis;
gene48859	K12585	DIS3, RRP44	exosome complex exonuclease DIS3/RRP44	EC:3.1.13.-	1	ko03018 RNA degradation;
gene4885	K01602	rbcS	ribulose-bisphosphate carboxylase small chain	EC:4.1.1.39	3	ko00630 Glyoxylate and dicarboxylate metabolism;ko00710 Carbon fixation in photosynthetic organisms;ko01200 Carbon metabolism;
gene48860	K13462	MIN7	guanine nucleotide-exchange factor	--	1	ko04626 Plant-pathogen interaction;
gene48862	K15161	CCNC, SSN8	cyclin C	--		
gene48865	K08908	LHCA2	light-harvesting complex I chlorophyll a/b binding protein 2	--	1	ko00196 Photosynthesis - antenna proteins;
gene48867	K09705	K09705	hypothetical protein	--		
gene4886	K01602	rbcS	ribulose-bisphosphate carboxylase small chain	EC:4.1.1.39	3	ko00630 Glyoxylate and dicarboxylate metabolism;ko00710 Carbon fixation in photosynthetic organisms;ko01200 Carbon metabolism;
gene48870	K01051	E3.1.1.11	pectinesterase	EC:3.1.1.11	2	ko00040 Pentose and glucuronate interconversions;ko00500 Starch and sucrose metabolism;
gene48873	K12669	OST3, OST6	oligosaccharyltransferase complex subunit gamma	--	3	ko00510 N-Glycan biosynthesis;ko00513 Various types of N-glycan biosynthesis;ko04141 Protein processing in endoplasmic reticulum;
gene48875	K08235	E2.4.1.207	xyloglucan:xyloglucosyl transferase	EC:2.4.1.207		
gene48878	K04125	E1.14.11.13	gibberellin 2-oxidase	EC:1.14.11.13	1	ko00904 Diterpenoid biosynthesis;
gene4887	K00968	PCYT1	choline-phosphate cytidylyltransferase	EC:2.7.7.15	2	ko00440 Phosphonate and phosphinate metabolism;ko00564 Glycerophospholipid metabolism;
gene48880	K01897	ACSL, fadD	long-chain acyl-CoA synthetase	EC:6.2.1.3	4	ko00071 Fatty acid degradation;ko03320 PPAR signaling pathway;ko04146 Peroxisome;ko04920 Adipocytokine signaling pathway;
gene48881	K12602	WDR61, REC14, SKI8	WD repeat-containing protein 61	--	1	ko03018 RNA degradation;

gene48884	K04498	EP300, CREBBP, KAT3	E1A/CREB-binding protein	EC:2.3.1.48	21	ko04066 HIF-1 signaling pathway;ko04110 Cell cycle;ko04310 Wnt signaling pathway;ko04330 Notch signaling pathway;ko04350 TGF-beta signaling pathway;ko04520 Adherens junction;ko04630 Jak-STAT signaling pathway;ko04720 Long-term potentiation;ko04916 Melanogenesis;ko05016 Huntington's disease;ko05152 Tuberculosis;ko05161 Hepatitis B;ko05164 Influenza A;ko05166 HTLV-I infection;ko05168 Herpes simplex infection;ko05169 Epstein-Barr virus infection;ko05200 Pathways in cancer;ko05203 Viral carcinogenesis;ko05206 MicroRNAs in cancer;ko05211 Renal cell carcinoma;ko05215 Prostate cancer;
gene48889	K09422	MYBP	myb proto-oncogene protein, plant	--		
gene48890	K01904	E6.2.1.12	4-coumarate--CoA ligase	EC:6.2.1.12	3	ko00130 Ubiquinone and other terpenoid-quinone biosynthesis;ko00360 Phenylalanine metabolism;ko00940 Phenylpropanoid biosynthesis;
gene48891	K00574	E2.1.1.79, cfa	cyclopropane-fatty-acyl-phospholipid synthase	EC:2.1.1.79		
gene48892	K00574	E2.1.1.79, cfa	cyclopropane-fatty-acyl-phospholipid synthase	EC:2.1.1.79		
gene48894	K10534	NR	nitrate reductase (NAD(P)H)	EC:1.7.1.1 1.7.1.2 1.7.1.3	1	ko00910 Nitrogen metabolism;
gene488	K07904	RAB11A	Ras-related protein Rab-11A	--	4	ko04144 Endocytosis;ko04961 Endocrine and other factor-regulated calcium reabsorption;ko04962 Vasopressin-regulated water reabsorption;ko04972 Pancreatic secretion;
gene48900	K14638	SLC15A3_4, PHT	solute carrier family 15 (peptide/histidine transporter), member 3/4	--		
gene48901	K14489	AHK2_3_4	arabidopsis histidine kinase 2/3/4 (cytokinin receptor)	EC:2.7.13.3	1	ko04075 Plant hormone signal transduction;
gene48902	K02132	ATPeF1A, ATP5A1	F-type H+-transporting ATPase subunit alpha	EC:3.6.3.14	4	ko00190 Oxidative phosphorylation;ko05010 Alzheimer's disease;ko05012 Parkinson's disease;ko05016 Huntington's disease;
gene48903	K14233	tRNA-Ser	tRNA Ser	--	1	ko00970 Aminoacyl-tRNA biosynthesis;
gene48904	K03878	ND1	NADH-ubiquinone oxidoreductase chain 1	EC:1.6.5.3	2	ko00190 Oxidative phosphorylation;ko05012 Parkinson's disease;
gene48908	K10579	UBE2M, UBC12	ubiquitin-conjugating enzyme E2 M	EC:6.3.2.19	1	ko04120 Ubiquitin mediated proteolysis;
gene48911	K06689	UBE2D_E, UBC4, UBC5	ubiquitin-conjugating enzyme E2 D/E	EC:6.3.2.19	2	ko04120 Ubiquitin mediated proteolysis;ko04141 Protein processing in endoplasmic reticulum;
gene48915	K14219	tRNA-Arg	tRNA Arg	--	1	ko00970 Aminoacyl-tRNA biosynthesis;
gene48924	K15281	SLC35D	solute carrier family 35	--		
gene48925	K06970	rlmF	23S rRNA (adenine1618-N6)-methyltransferase	EC:2.1.1.181		
gene48930	K00588	E2.1.1.104	caffeoyl-CoA O-methyltransferase	EC:2.1.1.104	4	ko00360 Phenylalanine metabolism;ko00940 Phenylpropanoid biosynthesis;ko00941 Flavonoid biosynthesis;ko00945 Stilbenoid, diarylheptanoid and gingerol biosynthesis;
gene48931	K00588	E2.1.1.104	caffeoyl-CoA O-methyltransferase	EC:2.1.1.104	4	ko00360 Phenylalanine metabolism;ko00940 Phenylpropanoid biosynthesis;ko00941 Flavonoid biosynthesis;ko00945 Stilbenoid, diarylheptanoid and gingerol biosynthesis;
gene48940	K13447	RBOH	respiratory burst oxidase	EC:1.6.3.- 1.11.1.-	1	ko04626 Plant-pathogen interaction;
gene48941	K08235	E2.4.1.207	xyloglucan:xyloglucosyl transferase	EC:2.4.1.207		
gene48943	K02984	RP-S3Ae, RPS3A	small subunit ribosomal protein S3Ae	--	1	ko03010 Ribosome;
gene48945	K12125	ELF3	protein EARLY FLOWERING 3	--	1	ko04712 Circadian rhythm - plant;
gene48957	K05359	ADT, PDT	arogenate/prephenate dehydratase	EC:4.2.1.91 4.2.1.51	2	ko00400 Phenylalanine, tyrosine and tryptophan biosynthesis;ko01230 Biosynthesis of amino acids;
gene48958	K02154	ATPeVI, ATP6N1A	V-type H+-transporting ATPase subunit I	EC:3.6.3.14	9	ko00190 Oxidative phosphorylation;ko04142 Lysosome;ko04145 Phagosome;ko04721 Synaptic vesicle cycle;ko04966 Collecting duct acid secretion;ko05110 Vibrio cholerae infection;ko05120 Epithelial cell signaling in Helicobacter pylori infection;ko05152 Tuberculosis;ko05323 Rheumatoid arthritis;
gene48960	K01409	gcp, QRI7, OSGEPL	O-sialoglycoprotein endopeptidase	EC:3.4.24.57		
gene48961	K10870	RAD51L2, RAD51C	RAD51-like protein 2	--	2	ko03440 Homologous recombination;ko03460 Fanconi anemia pathway;
gene48962	K00615	E2.2.1.1, tktA, tktB	transketolase	EC:2.2.1.1	5	ko00030 Pentose phosphate pathway;ko00710 Carbon fixation in photosynthetic organisms;ko01051 Biosynthesis of ansamycins;ko01200 Carbon metabolism;ko01230 Biosynthesis of amino acids;
gene48963	K00253	IVD, ivd	isovaleryl-CoA dehydrogenase	EC:1.3.8.4	1	ko00280 Valine, leucine and isoleucine degradation;
gene48971	K10738	MCM9	DNA helicase MCM9	EC:3.6.4.12		
gene48973	K13412	CPK	calcium-dependent protein kinase	EC:2.7.11.1	2	ko04626 Plant-pathogen interaction;ko05145 Toxoplasmosis;

gene48978	K03243	EIF5B	translation initiation factor 5B	--	1	ko03013 RNA transport;
gene48983	K00789	metK	S-adenosylmethionine synthetase	EC:2.5.1.6	2	ko00270 Cysteine and methionine metabolism;ko01230 Biosynthesis of amino acids;
gene48984	K03671	trxA	thioredoxin 1	--		
gene48988	K12741	HNRNPA1_3	heterogeneous nuclear ribonucleoprotein A1/A3	--	1	ko03040 Spliceosome;
gene48989	K03787	surE	5'-nucleotidase	EC:3.1.3.5	3	ko00230 Purine metabolism;ko00240 Pyrimidine metabolism;ko00760 Nicotinate and nicotinamide metabolism;
gene48996	K00981	E2.7.7.41, CDS1, CDS2, cdsA	phosphatidate cytidylyltransferase	EC:2.7.7.41	2	ko00564 Glycerophospholipid metabolism;ko04070 Phosphatidylinositol signaling system;
gene4899	K15404	K15404, CER1	aldehyde decarbonylase	EC:4.1.99.5	1	ko00073 Cutin, suberine and wax biosynthesis;
gene49002	K02958	RP-S15e, RPS15	small subunit ribosomal protein S15e	--	1	ko03010 Ribosome;
gene49004	K14572	MDN1, REA1	midasin	--	1	ko03008 Ribosome biogenesis in eukaryotes;
gene49005	K14709	SLC39A1_2_3, ZIP1_2_3	solute carrier family 39 (zinc transporter), member 1/2/3	--		
gene49012	K09872	PIP	aquaporin PIP	--		
gene49013	K09872	PIP	aquaporin PIP	--		
gene49018	K14315	NDC1, TMEM48	nucleoporin NDC1	--	1	ko03013 RNA transport;
gene49025	K04382	PPP2C	serine/threonine-protein phosphatase 2A catalytic subunit	EC:3.1.3.16	13	ko03015 mRNA surveillance pathway;ko04111 Cell cycle - yeast;ko04113 Meiosis - yeast;ko04114 Oocyte meiosis;ko04151 PI3K-Akt signaling pathway;ko04350 TGF-beta signaling pathway;ko04390 Hippo signaling pathway;ko04391 Hippo signaling pathway - fly;ko04530 Tight junction;ko04728 Dopaminergic synapse;ko04730 Long-term depression;ko05142 Chagas disease (American trypanosomiasis);ko05160 Hepatitis C;
gene49026	K00721	DPM1	dolichol-phosphate mannosyltransferase	EC:2.4.1.83	1	ko00510 N-Glycan biosynthesis;
gene49031	K01354	ptrB	oligopeptidase B	EC:3.4.21.83	2	ko05142 Chagas disease (American trypanosomiasis);ko05143 African trypanosomiasis;
gene49034	K08851	TP53RK, PRPK, BUD32	TP53 regulating kinase	EC:2.7.11.1		
gene49039	K12524	thrA	bifunctional aspartokinase / homoserine dehydrogenase 1	EC:2.7.2.4 1.1.1.3	4	ko00260 Glycine, serine and threonine metabolism;ko00270 Cysteine and methionine metabolism;ko00300 Lysine biosynthesis;ko01230 Biosynthesis of amino acids;
gene4903	K17619	MDP1	magnesium-dependent phosphatase 1	EC:3.1.3.48		
gene49040	K02201	E2.7.7.3B	pantetheine-phosphate adenylyltransferase	EC:2.7.7.3	1	ko00770 Pantothenate and CoA biosynthesis;
gene49043	K02868	RP-L11e, RPL11	large subunit ribosomal protein L11e	--	1	ko03010 Ribosome;
gene49048	K07904	RAB11A	Ras-related protein Rab-11A	--	4	ko04144 Endocytosis;ko04961 Endocrine and other factor-regulated calcium reabsorption;ko04962 Vasopressin-regulated water reabsorption;ko04972 Pancreatic secretion;
gene4904	K05754	ARPC5	actin related protein 2/3 complex, subunit 5	--	6	ko04666 Fc gamma R-mediated phagocytosis;ko04810 Regulation of actin cytoskeleton;ko05100 Bacterial invasion of epithelial cells;ko05130 Pathogenic Escherichia coli infection;ko05131 Shigellosis;ko05132 Salmonella infection;
gene49050	K16052	ynaI, mscMJ, MSL	MscS family membrane protein	--		
gene49051	K14492	ARR-A	two-component response regulator ARR-A family	--	1	ko04075 Plant hormone signal transduction;
gene49053	K05349	bglX	beta-glucosidase	EC:3.2.1.21	3	ko00460 Cyanoamino acid metabolism;ko00500 Starch and sucrose metabolism;ko00940 Phenylpropanoid biosynthesis;
gene49056	K14948	PTBP2, NPTB	olypyrimidine tract-binding protein 2	--		
gene4905	K09338	HD-ZIP	homeobox-leucine zipper protein	--		
gene49062	K10908	POLRMT, RPO41	DNA-directed RNA polymerase, mitochondrial	EC:2.7.7.6		
gene49063	K09487	HSP90B, TRA1	heat shock protein 90kDa beta	--	8	ko04141 Protein processing in endoplasmic reticulum;ko04151 PI3K-Akt signaling pathway;ko04621 NOD-like receptor signaling pathway;ko04626 Plant-pathogen interaction;ko04915 Estrogen signaling pathway;ko04918 Thyroid hormone synthesis;ko05200 Pathways in cancer;ko05215 Prostate cancer;
gene49066	K14006	SEC23	protein transport protein SEC23	--	1	ko04141 Protein processing in endoplasmic reticulum;
gene49070	K13407	CYP94A1	cytochrome P450, family 94, subfamily A, polypeptide 1 (fatty acid omega-hydroxylase)	EC:1.14.-.-	1	ko00073 Cutin, suberine and wax biosynthesis;
gene49072	K12493	ARFGAP2_3	ADP-ribosylation factor GTPase-activating protein 2/3	--	1	ko04144 Endocytosis;

gene49080	K13162	PCBP2_3_4	poly(rC)-binding protein 2/3/4	--		
gene49081	K05909	E1.10.3.2				
gene49082	K10881	SHFM1, DSS1	26 proteasome complex subunit DSS1	--	3	ko03050 Proteasome;ko03440 Homologous recombination;ko05169 Epstein-Barr virus infection;
gene49088	K15322	TSEN2	tRNA-splicing endonuclease subunit Sen2	EC:3.1.27.9		
gene49089	K01955	carB, CPA2	carbamoyl-phosphate synthase large subunit	EC:6.3.5.5	2	ko00240 Pyrimidine metabolism;ko00250 Alanine, aspartate and glutamate metabolism;
gene49090	K13124	MORG1	mitogen-activated protein kinase organizer 1	--		
gene49094	K03686	dnaJ	molecular chaperone DnaJ	--		
gene49095	K03881	ND4	NADH-ubiquinone oxidoreductase chain 4	EC:1.6.5.3	2	ko00190 Oxidative phosphorylation;ko05012 Parkinson's disease;
gene49100	K15472	CYP71D55	premnaspirodiene oxygenase	EC:1.14.13.12 1	1	ko00909 Sesquiterpenoid and triterpenoid biosynthesis;
gene49102	K12619	XRN2, RAT1	5'-3' exoribonuclease 2	EC:3.1.13.-	2	ko03008 Ribosome biogenesis in eukaryotes;ko03018 RNA degradation;
gene49107	K06892	K06892				
gene49115	K09873	TIP	aquaporin TIP	--		
gene49116	K13427	NOA1	nitric-oxide synthase, plant	EC:1.14.13.39	2	ko00330 Arginine and proline metabolism;ko04626 Plant-pathogen interaction;
gene49117	K06674	SMC2	structural maintenance of chromosome 2	--	1	ko04111 Cell cycle - yeast;
gene4911	K02938	RP-L8e, RPL8	large subunit ribosomal protein L8e	--	1	ko03010 Ribosome;
gene49125	K14488	SAUR	SAUR family protein	--	1	ko04075 Plant hormone signal transduction;
gene49127	K09422	MYBP	myb proto-oncogene protein, plant	--		
gene49128	K09286	EREBP	EREBP-like factor	--		
gene49132	K14803	PTC2_3	protein phosphatase 2C homolog 2/3	EC:3.1.3.16		
gene49135	K10781	FATB	fatty acyl-ACP thioesterase B	EC:3.1.2.14	1	ko00061 Fatty acid biosynthesis;
gene49137	K00913	ITPK1	inositol-1,3,4-trisphosphate 5/6-kinase / inositol-tetrakisphosphate 1-kinase	EC:2.7.1.159 2.7.1.134	2	ko00562 Inositol phosphate metabolism;ko04070 Phosphatidylinositol signaling system;
gene49138	K00696	E2.4.1.14	sucrose-phosphate synthase	EC:2.4.1.14	1	ko00500 Starch and sucrose metabolism;
gene49139	K04711	YDC1	dihydroceramidase	EC:3.5.1.-	1	ko00600 Sphingolipid metabolism;
gene49140	K00131	E1.2.1.9, gapN	glyceraldehyde-3-phosphate dehydrogenase (NADP)	EC:1.2.1.9	1	ko00010 Glycolysis / Gluconeogenesis;
gene4914	K01893	NARS, asnS	asparaginyl-tRNA synthetase	EC:6.1.1.22	1	ko00970 Aminoacyl-tRNA biosynthesis;
gene49156	K13430	PBS1	serine/threonine-protein kinase PBS1	EC:2.7.11.1	1	ko04626 Plant-pathogen interaction;
gene49158	K01358	clpP, CLPP	ATP-dependent Clp protease, protease subunit	EC:3.4.21.92	1	ko04112 Cell cycle - Caulobacter;
gene49160	K01528	DNM	dynamin GTPase	EC:3.6.5.5	4	ko04144 Endocytosis;ko04721 Synaptic vesicle cycle;ko04961 Endocrine and other factor-regulated calcium reabsorption;ko05100 Bacterial invasion of epithelial cells;
gene49163	K10258	TER, TSC13, CER10	very-long-chain enoyl-CoA reductase	EC:1.3.1.93	2	ko00062 Fatty acid elongation;ko01040 Biosynthesis of unsaturated fatty acids;
gene49164	K13102	KIN	DNA/RNA-binding protein KIN17	--		
gene49168	K00864	E2.7.1.30, glpK	glycerol kinase	EC:2.7.1.30	3	ko00561 Glycerolipid metabolism;ko03320 PPAR signaling pathway;ko04626 Plant-pathogen interaction;
gene49178	K03549	kup	KUP system potassium uptake protein	--		
gene49189	K09422	MYBP	myb proto-oncogene protein, plant	--		
gene4918	K12829	SF3B2, SAP145, CUS1	splicing factor 3B subunit 2	--	1	ko03040 Spliceosome;
gene49193	K00784	rnz	ribonuclease Z	EC:3.1.26.11	1	ko03013 RNA transport;
gene49196	K00006	GPD1	glycerol-3-phosphate dehydrogenase (NAD+)	EC:1.1.1.8	1	ko00564 Glycerophospholipid metabolism;
gene4919	K05747	WAS	Wiskott-Aldrich syndrome protein	--	8	ko04062 Chemokine signaling pathway;ko04520 Adherens junction;ko04666 Fc gamma R-mediated phagocytosis;ko04810 Regulation of actin cytoskeleton;ko05100 Bacterial invasion of epithelial cells;ko05130 Pathogenic Escherichia coli infection;ko05131 Shigellosis;ko05132 Salmonella infection;
gene49200	K03139	TFIIF2, GTF2F2, TFG2	transcription initiation factor TFIIF subunit beta	EC:3.6.4.12	1	ko03022 Basal transcription factors;
gene49208	K16055	TPS	trehalose 6-phosphate synthase/phosphatase	EC:2.4.1.15 3.1.3.12	1	ko00500 Starch and sucrose metabolism;
gene49213	K11434	PRMT1	protein arginine N-methyltransferase 1	EC:2.1.1.-		
gene49215	K01184	E3.2.1.15	polygalacturonase	EC:3.2.1.15	2	ko00040 Pentose and glucuronate interconversions;ko00500 Starch and sucrose metabolism;
gene49223	K01728	E4.2.2.2, pel	pectate lyase	EC:4.2.2.2	1	ko00040 Pentose and glucuronate interconversions;
gene49225	K02717	psbP	photosystem II oxygen-evolving enhancer protein 2	--	1	ko00195 Photosynthesis;

gene49229	K01962	accA	acetyl-CoA carboxylase carboxyl transferase subunit alpha	EC:6.4.1.2	6	ko00061 Fatty acid biosynthesis;ko00253 Tetracycline biosynthesis;ko00620 Pyruvate metabolism;ko00640 Propanoate metabolism;ko00720 Carbon fixation pathways in prokaryotes;ko01200 Carbon metabolism;
gene49236	K09338	HD-ZIP	homeobox-leucine zipper protein	--		
gene49241	K05863	SLC25A4S, ANT	solute carrier family 25 (mitochondrial adenine nucleotide translocator), member 4/5/6/31	--	4	ko04020 Calcium signaling pathway;ko05012 Parkinson's disease;ko05016 Huntington's disease;ko05166 HTLV-I infection;
gene49244	K03424	tatD	TatD DNase family protein	EC:3.1.21.-		
gene49246	K00434	E1.11.1.11	L-ascorbate peroxidase	EC:1.11.1.11	2	ko00053 Ascorbate and aldarate metabolism;ko00480 Glutathione metabolism;
gene49249	K04733	IRAK4	interleukin-1 receptor-associated kinase 4	EC:2.7.11.1	11	ko04064 NF-kappa B signaling pathway;ko04210 Apoptosis;ko04620 Toll-like receptor signaling pathway;ko04722 Neurotrophin signaling pathway;ko05133 Pertussis;ko05140 Leishmaniasis;ko05142 Chagas disease (American trypanosomiasis);ko05145 Toxoplasmosis;ko05152 Tuberculosis;ko05162 Measles;ko05164 Influenza A;
gene49250	K00472	E1.14.11.2	prolyl 4-hydroxylase	EC:1.14.11.2	1	ko00330 Arginine and proline metabolism;
gene49256	K03294	TC.APA	basic amino acid/polyamine antiporter, APA family	--		
gene49258	K14427	SLC12A4_5_6, KCC	solute carrier family 12 (potassium/chloride transporter), member 4/5/6	--		
gene49262	K02912	RP-L32e, RPL32	large subunit ribosomal protein L32e	--	1	ko03010 Ribosome;
gene49264	K01792	E5.1.3.15	glucose-6-phosphate 1-epimerase	EC:5.1.3.15	1	ko00010 Glycolysis / Gluconeogenesis;
gene49265	K08486	STX1B_2_3	syntaxin 1B/2/3	--	2	ko04130 SNARE interactions in vesicular transport;ko04721 Synaptic vesicle cycle;
gene49269	K12489	ACAP	Arf-GAP with coiled-coil, ANK repeat and PH domain-containing protein	--	1	ko04144 Endocytosis;
gene49277	K14638	SLC15A3_4, PHT	solute carrier family 15 (peptide/histidine transporter), member 3/4	--		
gene4927	K15338	GEN1, GEN	flap endonuclease GEN	EC:3.1.-.-		
gene49283	K09286	EREBP	EREBP-like factor	--		
gene49284	K13379	RGP, UTM	reversibly glycosylated polypeptide / UDP-arabinopyranose mutase	EC:2.4.1.- 5.4.99.30		
gene49287	K10739	RFA2, RPA2	replication factor A2	--	5	ko03030 DNA replication;ko03420 Nucleotide excision repair;ko03430 Mismatch repair;ko03440 Homologous recombination;ko03460 Fanconi anemia pathway;
gene49295	K01090	E3.1.3.16	protein phosphatase	EC:3.1.3.16		
gene492	K01530	E3.6.3.1	phospholipid-translocating ATPase	EC:3.6.3.1		
gene49302	K14228	tRNA-Leu	tRNA Leu	--	1	ko00970 Aminoacyl-tRNA biosynthesis;
gene49305	K15505	RAD5	DNA repair protein RAD5	EC:3.6.4.-		
gene49309	K09285	OVM, ANT	AP2-like factor, ANT lineage	--		
gene49313	K09828	DHCR24	delta24-sterol reductase	EC:1.3.1.72	1	ko00100 Steroid biosynthesis;
gene49316	K09286	EREBP	EREBP-like factor	--		
gene4931	K05863	SLC25A4S, ANT	solute carrier family 25 (mitochondrial adenine nucleotide translocator), member 4/5/6/31	--	4	ko04020 Calcium signaling pathway;ko05012 Parkinson's disease;ko05016 Huntington's disease;ko05166 HTLV-I infection;
gene49328	K05758	ARPC2	actin related protein 2/3 complex, subunit 2	--	6	ko04666 Fc gamma R-mediated phagocytosis;ko04810 Regulation of actin cytoskeleton;ko05100 Bacterial invasion of epithelial cells;ko05130 Pathogenic Escherichia coli infection;ko05131 Shigellosis;ko05132 Salmonella infection;
gene49332	K11498	CENPE	centromeric protein E	--		
gene49333	K05863	SLC25A4S, ANT	solute carrier family 25 (mitochondrial adenine nucleotide translocator), member 4/5/6/31	--	4	ko04020 Calcium signaling pathway;ko05012 Parkinson's disease;ko05016 Huntington's disease;ko05166 HTLV-I infection;
gene49336	K02729	PSMA5	20S proteasome subunit alpha 5	EC:3.4.25.1	1	ko03050 Proteasome;
gene49340	K00679	E2.3.1.158	phospholipid:diacylglycerol acyltransferase	EC:2.3.1.158	1	ko00561 Glycerolipid metabolism;
gene49343	K01733	thrC	threonine synthase	EC:4.2.3.1	3	ko00260 Glycine, serine and threonine metabolism;ko00750 Vitamin B6 metabolism;ko01230 Biosynthesis of amino acids;
gene49352	K02941	RP-LP0, RPLP0	large subunit ribosomal protein LP0	--	1	ko03010 Ribosome;
gene49353	K08490	STX5	syntaxin 5	--	1	ko04130 SNARE interactions in vesicular transport;
gene49354	K14807	DDX51, DBP6	ATP-dependent RNA helicase DDX51/DBP6	EC:3.6.4.13		
gene49358	K16911	DDX21	ATP-dependent RNA helicase DDX21	EC:3.6.4.13		

gene49360	K00600	glyA, SHMT	glycine hydroxymethyltransferase	EC:2.1.2.1	7	ko00260 Glycine, serine and threonine metabolism;ko00460 Cyanoamino acid metabolism;ko00630 Glyoxylate and dicarboxylate metabolism;ko00670 One carbon pool by folate;ko00680 Methane metabolism;ko01200 Carbon metabolism;ko01230 Biosynthesis of amino acids;
gene49363	K01537	E3.6.3.8	Ca2+-transporting ATPase	EC:3.6.3.8		
gene49365	K08332	VAC8	vacuolar protein 8	--	1	ko04140 Regulation of autophagy;
gene49366	K09422	MYBP	myb proto-oncogene protein, plant	--		
gene49369	K15276	SLC35B2, PAPST1	solute carrier family 35 (adenosine 3'-phospho 5'-phosphosulfate transporter), member B2	--		
gene49372	K10871	RAD51L3, RAD51D	RAD51-like protein 3	--	1	ko03440 Homologous recombination;
gene49377	K08596	SENP7	sentrin-specific protease 7	EC:3.4.22.68		
gene49380	K14233	tRNA-Ser	tRNA Ser	--	1	ko00970 Aminoacyl-tRNA biosynthesis;
gene49381	K10706	SETX, ALS4	senataxin	EC:3.6.4.-		
gene49386	K03347	CUL1, CDC53	cullin 1	--	9	ko04110 Cell cycle;ko04111 Cell cycle - yeast;ko04114 Oocyte meiosis;ko04120 Ubiquitin mediated proteolysis;ko04141 Protein processing in endoplasmic reticulum;ko04310 Wnt signaling pathway;ko04350 TGF-beta signaling pathway;ko04710 Circadian rhythm;ko05168 Herpes simplex infection;
gene4938	K02868	RP-L11e, RPL11	large subunit ribosomal protein L11e	--	1	ko03010 Ribosome;
gene49398	K03434	PIGL	N-acetylglucosaminylphosphatidylinositol deacetylase	EC:3.5.1.89	1	ko00563 Glycosylphosphatidylinositol(GPI)-anchor biosynthesis;
gene49404	K13436	PTI1	pto-interacting protein 1	EC:2.7.11.1	1	ko04626 Plant-pathogen interaction;
gene49407	K01762	ACS	1-aminocyclopropane-1-carboxylate synthase	EC:4.4.1.14	1	ko00270 Cysteine and methionine metabolism;
gene49409	K02140	ATPeFG, ATP5L	F-type H+-transporting ATPase subunit g	EC:3.6.3.14	1	ko00190 Oxidative phosphorylation;
gene4940	K09286	EREBP	EREBP-like factor	--		
gene49410	K02140	ATPeFG, ATP5L	F-type H+-transporting ATPase subunit g	EC:3.6.3.14	1	ko00190 Oxidative phosphorylation;
gene49411	K03066	PSMC5, RPT6	26S proteasome regulatory subunit T6	--	2	ko03050 Proteasome;ko05169 Epstein-Barr virus infection;
gene49414	K08145	SLC2A8, GLUT8	MFS transporter, SP family, solute carrier family 2 (facilitated glucose transporter), member 8	--		
gene4941	K00430	E1.11.1.7	peroxidase	EC:1.11.1.7	2	ko00360 Phenylalanine metabolism;ko00940 Phenylpropanoid biosynthesis;
gene49420	K14509	ETR, ERS	ethylene receptor	EC:2.7.13.-	1	ko04075 Plant hormone signal transduction;
gene49422	K00232	E1.3.3.6, ACOX1, ACOX3	acyl-CoA oxidase	EC:1.3.3.6	5	ko00071 Fatty acid degradation;ko00592 alpha-Linolenic acid metabolism;ko01040 Biosynthesis of unsaturated fatty acids;ko03320 PPAR signaling pathway;ko04146 Peroxisome;
gene49426	K14484	IAA	auxin-responsive protein IAA	--	1	ko04075 Plant hormone signal transduction;
gene4942	K04392	RAC1	Ras-related C3 botulinum toxin substrate 1	--	33	ko04010 MAPK signaling pathway;ko04014 Ras signaling pathway;ko04062 Chemokine signaling pathway;ko04145 Phagosome;ko04151 PI3K-Akt signaling pathway;ko04310 Wnt signaling pathway;ko04360 Axon guidance;ko04370 VEGF signaling pathway;ko04380 Osteoclast differentiation;ko04510 Focal adhesion;ko04520 Adherens junction;ko04620 Toll-like receptor signaling pathway;ko04650 Natural killer cell mediated cytotoxicity;ko04662 B cell receptor signaling pathway;ko04664 Fc epsilon RI signaling pathway;ko04666 Fc gamma R-mediated phagocytosis;ko04670 Leukocyte transendothelial migration;ko04722 Neurotrophin signaling pathway;ko04810 Regulation of actin cytoskeleton;ko04932 Non-alcoholic fatty liver disease (NAFLD);ko04972 Pancreatic secretion;ko05014 Amyotrophic lateral sclerosis (ALS);ko05100 Bacterial invasion of epithelial cells;ko05120 Epithelial cell signaling in Helicobacter pylori infection;ko05131 Shigellosis;ko05132 Salmonella infection;ko05200 Pathways in cancer;ko05203 Viral carcinogenesis;ko05205 Proteoglycans in cancer;ko05210 Colorectal cancer;ko05211 Renal cell carcinoma;ko05212 Pancreatic
gene49431	K09873	TIP	aquaporin TIP	--		
gene49432	K01188	E3.2.1.21	beta-glucosidase	EC:3.2.1.21	3	ko00460 Cyanoamino acid metabolism;ko00500 Starch and sucrose metabolism;ko00940 Phenylpropanoid biosynthesis;

gene49434	K00166	E1.2.4.4A, bkdA1	2-oxoisovalerate dehydrogenase E1 component, alpha subunit	EC:1.2.4.4	1	ko00280 Valine, leucine and isoleucine degradation;
gene49438	K08486	STX1B_2_3	syntaxin 1B/2/3	--	2	ko04130 SNARE interactions in vesicular transport;ko04721 Synaptic vesicle cycle;
gene49439	K14236	tRNA-Tyr	tRNA Tyr	--	1	ko00970 Aminoacyl-tRNA biosynthesis;
gene49447	K14684	SLC25A23S	solute carrier family 25 (mitochondrial phosphate transporter), member 23/24/25/41	--		
gene49448	K08956	AFG3	AFG3 family protein	EC:3.4.24.-		
gene49451	K04120	E5.5.1.13	ent-copalyl diphosphate synthase	EC:5.5.1.13	1	ko00904 Diterpenoid biosynthesis;
gene49452	K03680	EIF2B4	translation initiation factor eIF-2B subunit delta	--	1	ko03013 RNA transport;
gene49453	K11599	POMP, UMP1	proteasome maturation protein	--	1	ko03050 Proteasome;
gene49461	K13412	CPK	calcium-dependent protein kinase	EC:2.7.11.1	2	ko04626 Plant-pathogen interaction;ko05145 Toxoplasmosis;
gene49462	K10772	APEX2	AP endonuclease 2	EC:4.2.99.18	1	ko03410 Base excision repair;
gene49463	K10772	APEX2	AP endonuclease 2	EC:4.2.99.18	1	ko03410 Base excision repair;
gene49466	K15430	TRM11, TRMT11	tRNA (guanine10-N2)-methyltransferase	EC:2.1.1.214		
gene49467	K00430	E1.11.1.7	peroxidase	EC:1.11.1.7	2	ko00360 Phenylalanine metabolism;ko00940 Phenylpropanoid biosynthesis;
gene49468	K15296	NAPA, SNAPA, SEC17	alpha-soluble NSF attachment protein	--	1	ko04721 Synaptic vesicle cycle;
gene4946	K02184	FMN2	formin 2	--	1	ko04320 Dorso-ventral axis formation;
gene49478	K08506	SYP7	syntaxin of plants SYP7	--	1	ko04130 SNARE interactions in vesicular transport;
gene49479	K04646	CLTC	clathrin heavy chain	--	6	ko04142 Lysosome;ko04144 Endocytosis;ko04721 Synaptic vesicle cycle;ko04961 Endocrine and other factor-regulated calcium reabsorption;ko05016 Huntington's disease;ko05100 Bacterial invasion of epithelial cells;
gene49482	K02908	RP-L30e, RPL30	large subunit ribosomal protein L30e	--	1	ko03010 Ribosome;
gene49483	K01051	E3.1.1.11	pectinesterase	EC:3.1.1.11	2	ko00040 Pentose and glucuronate interconversions;ko00500 Starch and sucrose metabolism;
gene49486	K01714	dapA	4-hydroxy-tetrahydrodipicolinate synthase	EC:4.3.3.7	2	ko00300 Lysine biosynthesis;ko01230 Biosynthesis of amino acids;
gene49487	K13154	ZCRB1	U11/U12 small nuclear ribonucleoprotein 31 kDa protein	--		
gene49488	K16298	SCPL-IV	serine carboxypeptidase-like clade IV	EC:3.4.16.-		
gene4948	K04708	E1.1.1.102	3-dehydrosphinganine reductase	EC:1.1.1.102	1	ko00600 Sphingolipid metabolism;
gene49494	K08286	E2.7.11.-	protein-serine/threonine kinase	EC:2.7.11.-		
gene49499	K13648	GAUT	alpha-1,4-galacturonosyltransferase	EC:2.4.1.43	2	ko00500 Starch and sucrose metabolism;ko00520 Amino sugar and nucleotide sugar metabolism;
gene49504	K10760	IPT	adenylate isopentenyltransferase (cytokinin synthase)	--	1	ko00908 Zeatin biosynthesis;
gene49505	K12842	SR140	U2-associated protein SR140	--	1	ko03040 Spliceosome;
gene49506	K02923	RP-L38e, RPL38	large subunit ribosomal protein L38e	--	1	ko03010 Ribosome;
gene49507	K14638	SLC15A3_4, PHT	solute carrier family 15 (peptide/histidine transporter), member 3/4	--		
gene4950	K08851	TP53RK, PRPK, BUD32	TP53 regulating kinase	EC:2.7.11.1		
gene49510	K03661	ATPeVPF, ATP6F	V-type H+-transporting ATPase 21kDa proteolipid subunit	EC:3.6.3.14	8	ko00190 Oxidative phosphorylation;ko04142 Lysosome;ko04145 Phagosome;ko04721 Synaptic vesicle cycle;ko05110 Vibrio cholerae infection;ko05120 Epithelial cell signaling in Helicobacter pylori infection;ko05152 Tuberculosis;ko05323 Rheumatoid arthritis;
gene49514	K17479	GRXCR1	glutaredoxin domain-containing cysteine-rich protein 1	--		
gene49517	K14490	AHP	histidine-containing phosphotransfer peotein	--	1	ko04075 Plant hormone signal transduction;
gene4951	K15360	STRA13, CENPX, MHF2	centromere protein X	--	1	ko03460 Fanconi anemia pathway;
gene49524	K00434	E1.11.1.11	L-ascorbate peroxidase	EC:1.11.1.11	2	ko00053 Ascorbate and aldarate metabolism;ko00480 Glutathione metabolism;
gene49527	K08235	E2.4.1.207	xyloglucan:xyloglucosyl transferase	EC:2.4.1.207		
gene4952	K14315	NDC1, TMEM48	nucleoporin NDC1	--	1	ko03013 RNA transport;
gene49531	K14514	EIN3	ethylene-insensitive protein 3	--	1	ko04075 Plant hormone signal transduction;
gene49533	K12795	SUGT1, SGT1	suppressor of G2 allele of SKP1	--	2	ko04621 NOD-like receptor signaling pathway;ko04626 Plant-pathogen interaction;
gene49534	K14484	IAA	auxin-responsive protein IAA	--	1	ko04075 Plant hormone signal transduction;
gene49545	K17411	MRPS33	small subunit ribosomal protein S33, mitochondrial	--		

gene49549	K02470	gyrB	DNA gyrase subunit B	EC:5.99.1.3		
gene49550	K04733	IRAK4	interleukin-1 receptor-associated kinase 4	EC:2.7.11.1	11	ko04064 NF-kappa B signaling pathway;ko04210 Apoptosis;ko04620 Toll-like receptor signaling pathway;ko04722 Neurotrophin signaling pathway;ko05133 Pertussis;ko05140 Leishmaniasis;ko05142 Chagas disease (American trypanosomiasis);ko05145 Toxoplasmosis;ko05152 Tuberculosis;ko05162 Measles;ko05164 Influenza A;
gene49551	K03781	katE, CAT, catB, srpA	catalase	EC:1.11.1.6	4	ko00380 Tryptophan metabolism;ko00630 Glyoxylate and dicarboxylate metabolism;ko04146 Peroxisome;ko05014 Amyotrophic lateral sclerosis (ALS);
gene49554	K12133	LHY	MYB-related transcription factor LHY	--	1	ko04712 Circadian rhythm - plant;
gene49561	K02915	RP-L34e, RPL34	large subunit ribosomal protein L34e	--	1	ko03010 Ribosome;
gene49562	K00411	UQCRFS1, RIP1, petA	ubiquinol-cytochrome c reductase iron-sulfur subunit	EC:1.10.2.2	7	ko00190 Oxidative phosphorylation;ko02020 Two-component system;ko04260 Cardiac muscle contraction;ko04932 Non-alcoholic fatty liver disease (NAFLD);ko05010 Alzheimer's disease;ko05012 Parkinson's disease;ko05016 Huntington's disease;
gene49565	K00517	E1.14.-.-			4	ko00624 Polycyclic aromatic hydrocarbon degradation;ko00627 Aminobenzoate degradation;ko00903 Limonene and pinene degradation;ko00945 Stilbenoid, diarylheptanoid and gingerol biosynthesis;
gene49568	K10956	SEC61A	protein transport protein SEC61 subunit alpha	--	4	ko03060 Protein export;ko04141 Protein processing in endoplasmic reticulum;ko04145 Phagosome;ko05110 Vibrio cholerae infection;
gene49571	K04733	IRAK4	interleukin-1 receptor-associated kinase 4	EC:2.7.11.1	11	ko04064 NF-kappa B signaling pathway;ko04210 Apoptosis;ko04620 Toll-like receptor signaling pathway;ko04722 Neurotrophin signaling pathway;ko05133 Pertussis;ko05140 Leishmaniasis;ko05142 Chagas disease (American trypanosomiasis);ko05145 Toxoplasmosis;ko05152 Tuberculosis;ko05162 Measles;ko05164 Influenza A;
gene49573	K04733	IRAK4	interleukin-1 receptor-associated kinase 4	EC:2.7.11.1	11	ko04064 NF-kappa B signaling pathway;ko04210 Apoptosis;ko04620 Toll-like receptor signaling pathway;ko04722 Neurotrophin signaling pathway;ko05133 Pertussis;ko05140 Leishmaniasis;ko05142 Chagas disease (American trypanosomiasis);ko05145 Toxoplasmosis;ko05152 Tuberculosis;ko05162 Measles;ko05164 Influenza A;
gene49581	K16732	PRC1	protein regulator of cytokinesis 1	--		
gene49582	K16732	PRC1	protein regulator of cytokinesis 1	--		
gene49583	K00036	G6PD, zwf	glucose-6-phosphate 1-dehydrogenase	EC:1.1.1.49	3	ko00030 Pentose phosphate pathway;ko00480 Glutathione metabolism;ko01200 Carbon metabolism;
gene49593	K02870	RP-L12e, RPL12	large subunit ribosomal protein L12e	--	1	ko03010 Ribosome;
gene49595	K15095	E1.1.1.208	(+)-neomenthol dehydrogenase	EC:1.1.1.208	1	ko00902 Monoterpenoid biosynthesis;
gene49597	K15281	SLC35D	solute carrier family 35	--		
gene49601	K05609	UCHL3, YUH1	ubiquitin carboxyl-terminal hydrolase L3	EC:3.4.19.12		
gene49613	K02149	ATPeVD, ATP6M	V-type H+-transporting ATPase subunit D	EC:3.6.3.14	7	ko00190 Oxidative phosphorylation;ko04145 Phagosome;ko04721 Synaptic vesicle cycle;ko04966 Collecting duct acid secretion;ko05110 Vibrio cholerae infection;ko05120 Epithelial cell signaling in Helicobacter pylori infection;ko05323 Rheumatoid arthritis;
gene49616	K06630	YWHAE	14-3-3 protein epsilon	--	8	ko04110 Cell cycle;ko04114 Oocyte meiosis;ko04151 PI3K-Akt signaling pathway;ko04390 Hippo signaling pathway;ko04391 Hippo signaling pathway - fly;ko04722 Neurotrophin signaling pathway;ko05169 Epstein-Barr virus infection;ko05203 Viral carcinogenesis;
gene49627	K06677	YCS4, CNAP1, CAPD2	condensin complex subunit 1	--	1	ko04111 Cell cycle - yeast;
gene49630	K01760	metC	cystathionine beta-lyase	EC:4.4.1.8	3	ko00270 Cysteine and methionine metabolism;ko00450 Selenocompound metabolism;ko01230 Biosynthesis of amino acids;
gene49632	K14492	ARR-A	two-component response regulator ARR-A family	--	1	ko04075 Plant hormone signal transduction;
gene49646	K14488	SAUR	SAUR family protein	--	1	ko04075 Plant hormone signal transduction;
gene49647	K13066	E2.1.1.68, COMT	caffeic acid 3-O-methyltransferase	EC:2.1.1.68	1	ko00940 Phenylpropanoid biosynthesis;
gene49654	K08064	NFYA	nuclear transcription factor Y, alpha	--	2	ko04612 Antigen processing and presentation;ko05152 Tuberculosis;
gene49655	K08486	STX1B_2_3	syntaxin 1B/2/3	--	2	ko04130 SNARE interactions in vesicular transport;ko04721 Synaptic vesicle cycle;
gene49656	K09480	E2.4.1.241	digalactosyldiacylglycerol synthase	EC:2.4.1.241	1	ko00561 Glycerolipid metabolism;

gene49667	K03787	surE	5'-nucleotidase	EC:3.1.3.5	3	ko00230 Purine metabolism;ko00240 Pyrimidine metabolism;ko00760 Nicotinate and nicotinamide metabolism;
gene4966	K03355	APC8, CDC23	anaphase-promoting complex subunit 8	--	7	ko04110 Cell cycle;ko04111 Cell cycle - yeast;ko04113 Meiosis - yeast;ko04114 Oocyte meiosis;ko04120 Ubiquitin mediated proteolysis;ko04914 Progesterone-mediated oocyte maturation;ko05166 HTLV-I infection;
gene49671	K02155	ATPeVPL, ATP6L	V-type H+-transporting ATPase 16kDa proteolipid subunit	EC:3.6.3.14	9	ko00190 Oxidative phosphorylation;ko04142 Lysosome;ko04145 Phagosome;ko04721 Synaptic vesicle cycle;ko04966 Collecting duct acid secretion;ko05110 Vibrio cholerae infection;ko05120 Epithelial cell signaling in Helicobacter pylori infection;ko05152 Tuberculosis;ko05323 Rheumatoid arthritis;
gene49672	K14016	UFD1	ubiquitin fusion degradation protein 1	--	1	ko04141 Protein processing in endoplasmic reticulum;
gene49673	K03965	NDUFB9	NADH dehydrogenase (ubiquinone) 1 beta subcomplex subunit 9	--	5	ko00190 Oxidative phosphorylation;ko04932 Non-alcoholic fatty liver disease (NAFLD);ko05010 Alzheimer's disease;ko05012 Parkinson's disease;ko05016 Huntington's disease;
gene49674	K11498	CENPE	centromeric protein E	--		
gene49676	K09500	CCT8	T-complex protein 1 subunit theta	--		
gene49677	K06995	K06995				
gene49678	K14231	tRNA-Phe	tRNA Phe	--	1	ko00970 Aminoacyl-tRNA biosynthesis;
gene49679	K02890	RP-L22, MRPL22, rplV	large subunit ribosomal protein L22	--	1	ko03010 Ribosome;
gene49687	K00278	nadB	L-aspartate oxidase	EC:1.4.3.16	2	ko00250 Alanine, aspartate and glutamate metabolism;ko00760 Nicotinate and nicotinamide metabolism;
gene49688	K04733	IRAK4	interleukin-1 receptor-associated kinase 4	EC:2.7.11.1	11	ko04064 NF-kappa B signaling pathway;ko04210 Apoptosis;ko04620 Toll-like receptor signaling pathway;ko04722 Neurotrophin signaling pathway;ko05133 Pertussis;ko05140 Leishmaniasis;ko05142 Chagas disease (American trypanosomiasis);ko05145 Toxoplasmosis;ko05152 Tuberculosis;ko05162 Measles;ko05164 Influenza A;
gene4968	K02932	RP-L5e, RPL5	large subunit ribosomal protein L5e	--	1	ko03010 Ribosome;
gene49690	K00485	E1.14.13.8	dimethylaniline monooxygenase (N-oxide forming)	EC:1.14.13.8	2	ko00680 Methane metabolism;ko00982 Drug metabolism - cytochrome P450;
gene49698	K11267	PDS5	sister chromatid cohesion protein PDS5	--		
gene4969	K06883	K06883				
gene49702	K14561	IMP4	U3 small nucleolar ribonucleoprotein protein IMP4	--	1	ko03008 Ribosome biogenesis in eukaryotes;
gene49706	K17506	PPM1L, PP2CE	protein phosphatase 1L	EC:3.1.3.16		
gene4970	K02934	RP-L6e, RPL6	large subunit ribosomal protein L6e	--	1	ko03010 Ribosome;
gene49720	K13192	RBM26	RNA-binding protein 26	--		
gene49721	K15255	PIF1	ATP-dependent DNA helicase PIF1	EC:3.6.4.12		
gene49728	K03809	wrbA	Trp repressor binding protein	--		
gene49735	K08176	PHO84	MFS transporter, PHS family, inorganic phosphate transporter	--		
gene49736	K16290	XCP	xylem cysteine proteinase	EC:3.4.22.-		
gene49738	K16282	RHA2	E3 ubiquitin-protein ligase RHA2	EC:6.3.2.19		
gene4973	K14236	tRNA-Tyr	tRNA Tyr	--	1	ko00970 Aminoacyl-tRNA biosynthesis;
gene49742	K10878	SPO11	meiotic recombination protein SPO11	--	1	ko04113 Meiosis - yeast;
gene49749	K08507	USE1	unconventional SNARE in the endoplasmic reticulum protein 1	--	1	ko04130 SNARE interactions in vesicular transport;
gene4974	K05755	ARPC4	actin related protein 2/3 complex, subunit 4	--	6	ko04666 Fc gamma R-mediated phagocytosis;ko04810 Regulation of actin cytoskeleton;ko05100 Bacterial invasion of epithelial cells;ko05130 Pathogenic Escherichia coli infection;ko05131 Shigellosis;ko05132 Salmonella infection;
gene49752	K01103	PFKFB	6-phosphofructo-2-kinase / fructose-2,6-bisphosphatase	EC:2.7.1.105 3.1.3.46	2	ko00051 Fructose and mannose metabolism;ko04066 HIF-1 signaling pathway;
gene49756	K12115	ZTL	clock-associated PAS protein ZTL	--	1	ko04712 Circadian rhythm - plant;
gene4975	K13456	RIN4	RPM1-interacting protein 4	--	1	ko04626 Plant-pathogen interaction;
gene49767	K08857	NEK	NIMA (never in mitosis gene a)-related kinase	EC:2.7.11.1		
gene49771	K05853	ATP2A	Ca2+ transporting ATPase, sarcoplasmic/endoplasmic reticulum	EC:3.6.3.8	3	ko04020 Calcium signaling pathway;ko04972 Pancreatic secretion;ko05010 Alzheimer's disease;
gene49776	K02128	ATPeF0C, ATP5G	F-type H+-transporting ATPase subunit c	EC:3.6.3.14	4	ko00190 Oxidative phosphorylation;ko05010 Alzheimer's disease;ko05012 Parkinson's disease;ko05016 Huntington's disease;

gene49778	K05349	bglX	beta-glucosidase	EC:3.2.1.21	3	ko00460 Cyanoamino acid metabolism;ko00500 Starch and sucrose metabolism;ko00940 Phenylpropanoid biosynthesis;
gene4977	K08518	STXBP5	syntaxin-binding protein 5	--		
gene49780	K02927	RP-L40e, RPL40	large subunit ribosomal protein L40e	--	1	ko03010 Ribosome;
gene49781	K10527	MFP2	enoyl-CoA hydratase/3-hydroxyacyl-CoA dehydrogenase	EC:4.2.1.17 1.1.1.35 1.1.1.211	2	ko00071 Fatty acid degradation;ko00592 alpha-Linolenic acid metabolism;
gene49786	K13424	WRKY33	WRKY transcription factor 33	--	1	ko04626 Plant-pathogen interaction;
gene49789	K08900	BCS1	mitochondrial chaperone BCS1	--		
gene49791	K00294	E1.2.1.88	1-pyrroline-5-carboxylate dehydrogenase	EC:1.2.1.88	2	ko00250 Alanine, aspartate and glutamate metabolism;ko00330 Arginine and proline metabolism;
gene49793	K00278	nadB	L-aspartate oxidase	EC:1.4.3.16	2	ko00250 Alanine, aspartate and glutamate metabolism;ko00760 Nicotinate and nicotinamide metabolism;
gene49795	K00430	E1.11.1.7	peroxidase	EC:1.11.1.7	2	ko00360 Phenylalanine metabolism;ko00940 Phenylpropanoid biosynthesis;
gene49796	K03164	TOP2	DNA topoisomerase II	EC:5.99.1.3		
gene49798	K01213	E3.2.1.67	galacturan 1,4-alpha-galacturonidase	EC:3.2.1.67	2	ko00040 Pentose and glucuronate interconversions;ko00500 Starch and sucrose metabolism;
gene49799	K02983	RP-S30e, RPS30	small subunit ribosomal protein S30e	--	1	ko03010 Ribosome;
gene4979	K13993	HSP20	HSP20 family protein	--	1	ko04141 Protein processing in endoplasmic reticulum;
gene49804	K08073	PNKP	bifunctional polynucleotide phosphatase/kinase	EC:3.1.3.32 2.7.1.78		
gene49806	K03627	MBF1	putative transcription factor	--		
gene4980	K09493	CCT1, TCP1	T-complex protein 1 subunit alpha	--		
gene49812	K05305	FUK	fucokinase	EC:2.7.1.52	2	ko00051 Fructose and mannose metabolism;ko00520 Amino sugar and nucleotide sugar metabolism;
gene49814	K14488	SAUR	SAUR family protein	--	1	ko04075 Plant hormone signal transduction;
gene49817	K09597	SPPL2B	signal peptide peptidase-like 2B	EC:3.4.23.-		
gene4981	K02109	ATPF0B, atpF	F-type H+-transporting ATPase subunit b	EC:3.6.3.14	2	ko00190 Oxidative phosphorylation;ko00195 Photosynthesis;
gene49822	K06627	CCNA	cyclin A	--	5	ko04110 Cell cycle;ko04914 Progesterone-mediated oocyte maturation;ko05161 Hepatitis B;ko05169 Epstein-Barr virus infection;ko05203 Viral carcinogenesis;
gene49823	K09422	MYBP	myb proto-oncogene protein, plant	--		
gene4982	K02109	ATPF0B, atpF	F-type H+-transporting ATPase subunit b	EC:3.6.3.14	2	ko00190 Oxidative phosphorylation;ko00195 Photosynthesis;
gene49834	K14503	BZR1_2	brassinosteroid resistant 1/2	--	1	ko04075 Plant hormone signal transduction;
gene49838	K02149	ATPeVD, ATP6M	V-type H+-transporting ATPase subunit D	EC:3.6.3.14	7	ko00190 Oxidative phosphorylation;ko04145 Phagosome;ko04721 Synaptic vesicle cycle;ko04966 Collecting duct acid secretion;ko05110 Vibrio cholerae infection;ko05120 Epithelial cell signaling in Helicobacter pylori infection;ko05323 Rheumatoid arthritis;
gene4984	K17279	REEP5_6	receptor expression-enhancing protein 5/6	--		
gene49856	K14431	TGA	transcription factor TGA	--	1	ko04075 Plant hormone signal transduction;
gene4985	K00279	E1.5.99.12	cytokinin dehydrogenase	EC:1.5.99.12	1	ko00908 Zeatin biosynthesis;
gene49863	K11269	CTF18, CHL12	chromosome transmission fidelity protein 18	--		
gene49866	K12183	TSG101, STP22, VPS23	ESCRT-I complex subunit TSG101	--	1	ko04144 Endocytosis;
gene49872	K14674	TGL4	TAG lipase / steryl ester hydrolase / phospholipase A2 / LPA acyltransferase	EC:3.1.1.3 3.1.1.13 3.1.1.4 2.3.1.51	7	ko00100 Steroid biosynthesis;ko00561 Glycerolipid metabolism;ko00564 Glycerophospholipid metabolism;ko00565 Ether lipid metabolism;ko00590 Arachidonic acid metabolism;ko00591 Linoleic acid metabolism;ko00592 alpha-Linolenic acid metabolism;
gene49876	K15285	SLC35E3	solute carrier family 35, member E3	--		
gene49877	K15501	PPP6R3, SAPS3	serine/threonine-protein phosphatase 6 regulatory subunit 3	--		
gene49880	K00924	E2.7.1.-				
gene49882	K16196	EIF2AK4	eukaryotic translation initiation factor 2-alpha kinase 4	EC:2.7.11.1	6	ko04141 Protein processing in endoplasmic reticulum;ko05160 Hepatitis C;ko05162 Measles;ko05164 Influenza A;ko05168 Herpes simplex infection;ko05169 Epstein-Barr virus infection;
gene49885	K17619	MDP1	magnesium-dependent phosphatase 1	EC:3.1.3.48		
gene49889	K12124	GI	GIGANTEA	--	1	ko04712 Circadian rhythm - plant;
gene49890	K00422	E1.10.3.1	polyphenol oxidase	EC:1.10.3.1	2	ko00350 Tyrosine metabolism;ko00950 Isoquinoline alkaloid biosynthesis;

gene49895	K14638	SLC15A3_4, PHT	solute carrier family 15 (peptide/histidine transporter), member 3/4	--		
gene49896	K10257	FAD8, desB	omega-3 fatty acid desaturase (delta-15 desaturase)	EC:1.14.19.-	1	ko01040 Biosynthesis of unsaturated fatty acids;
gene49903	K04649	HIP2, UBC1	ubiquitin-conjugating enzyme (huntingtin interacting protein 2)	EC:6.3.2.19	1	ko04120 Ubiquitin mediated proteolysis;
gene49909	K15032	MTERFD	mTERF domain-containing protein, mitochondrial	--		
gene49912	K13412	CPK	calcium-dependent protein kinase	EC:2.7.11.1	2	ko04626 Plant-pathogen interaction;ko05145 Toxoplasmosis;
gene49917	K03094	SKP1, CBF3D	S-phase kinase-associated protein 1	--	9	ko04110 Cell cycle;ko04111 Cell cycle - yeast;ko04114 Oocyte meiosis;ko04120 Ubiquitin mediated proteolysis;ko04141 Protein processing in endoplasmic reticulum;ko04310 Wnt signaling pathway;ko04350 TGF-beta signaling pathway;ko04710 Circadian rhythm;ko05168 Herpes simplex infection;
gene49919	K08770	UBC	ubiquitin C	--	1	ko03320 PPAR signaling pathway;
gene4991	K01251	E3.3.1.1, ahcY	adenosylhomocysteinase	EC:3.3.1.1	1	ko00270 Cysteine and methionine metabolism;
gene49920	K07573	CSL4, EXOSC1	exosome complex component CSL4	--	1	ko03018 RNA degradation;
gene49921	K03655	recG	ATP-dependent DNA helicase RecG	EC:3.6.4.12	1	ko03440 Homologous recombination;
gene49927	K08596	SENP7	sentrin-specific protease 7	EC:3.4.22.68		
gene49928	K03246	EIF3I	translation initiation factor 3 subunit I	--	1	ko03013 RNA transport;
gene4992	K00134	GAPDH, gapA	glyceraldehyde 3-phosphate dehydrogenase	EC:1.2.1.12	6	ko00010 Glycolysis / Gluconeogenesis;ko00710 Carbon fixation in photosynthetic organisms;ko01200 Carbon metabolism;ko01230 Biosynthesis of amino acids;ko04066 HIF-1 signaling pathway;ko05010 Alzheimer's disease;
gene49930	K15201	GTP3C3, TFC4	general transcription factor 3C polypeptide 3 (transcription factor C subunit 4)	--		
gene49938	K17616	CTDSPL2	CTD small phosphatase-like protein 2	EC:3.1.3.-		
gene49949	K17479	GRXCR1	glutaredoxin domain-containing cysteine-rich protein 1	--		
gene49953	K14950	ATP13A1	cation-transporting ATPase 13A1	EC:3.6.3.-		
gene49956	K16302	CNNM	metal transporter CNNM	--		
gene49959	K00511	SQLE, ERG1	squalene monooxygenase	EC:1.14.13.132	1	ko00909 Sesquiterpenoid and triterpenoid biosynthesis;
gene4995	K08202	SLC22A4_5, OCTN	MFS transporter, OCT family, solute carrier family 22 (organic cation transporter), member 4/5	--		
gene49961	K16302	CNNM	metal transporter CNNM	--		
gene49962	K14007	SEC24	protein transport protein SEC24	--	1	ko04141 Protein processing in endoplasmic reticulum;
gene49963	K04460	PPP5C	serine/threonine-protein phosphatase 5	EC:3.1.3.16	1	ko04010 MAPK signaling pathway;
gene49964	K15397	KCS	3-ketoacyl-CoA synthase	EC:2.3.1.199	1	ko00062 Fatty acid elongation;
gene49965	K15397	KCS	3-ketoacyl-CoA synthase	EC:2.3.1.199	1	ko00062 Fatty acid elongation;
gene49974	K01530	E3.6.3.1	phospholipid-translocating ATPase	EC:3.6.3.1		
gene49975	K15472	CYP71D55	premnaspirodiene oxygenase	EC:1.14.13.121	1	ko00909 Sesquiterpenoid and triterpenoid biosynthesis;
gene4997	K01426	E3.5.1.4, amiE	amidase	EC:3.5.1.4	5	ko00330 Arginine and proline metabolism;ko00360 Phenylalanine metabolism;ko00380 Tryptophan metabolism;ko00627 Aminobenzoate degradation;ko00643 Styrene degradation;
gene49983	K13102	KIN	DNA/RNA-binding protein KIN17	--		
gene49984	K10144	RCHY1, PIRH2	RING finger and CHY zinc finger domain-containing protein 1	EC:6.3.2.19	3	ko04115 p53 signaling pathway;ko04120 Ubiquitin mediated proteolysis;ko05162 Measles;
gene49987	K03671	trxA	thioredoxin 1	--		
gene49988	K00789	metK	S-adenosylmethionine synthetase	EC:2.5.1.6	2	ko00270 Cysteine and methionine metabolism;ko01230 Biosynthesis of amino acids;
gene49992	K12741	HNRNPA1_3	heterogeneous nuclear ribonucleoprotein A1/A3	--	1	ko03040 Spliceosome;
gene49995	K00472	E1.14.11.2	prolyl 4-hydroxylase	EC:1.14.11.2	1	ko00330 Arginine and proline metabolism;
gene49998	K13717	OTUD3	OTU domain-containing protein 3	--		
gene499	K02493	hemK, prmC	release factor glutamine methyltransferase	EC:2.1.1.-		
gene49	K04499	RUVBL1, RVB1, INO80H	RuvB-like protein 1 (pontin 52)	--	1	ko04310 Wnt signaling pathway;
gene50000	K06617	E2.4.1.82	raffinose synthase	EC:2.4.1.82	1	ko00052 Galactose metabolism;
gene50002	K13116	DDX41, ABS	ATP-dependent RNA helicase DDX41	EC:3.6.4.13		

gene50003	K01507	ppa	inorganic pyrophosphatase	EC:3.6.1.1	1	ko00190 Oxidative phosphorylation;
gene50004	K03255	TIF31, CLU1	protein TIF31	--		
gene50005	K03255	TIF31, CLU1	protein TIF31	--		
gene50007	K01613	psd, PISD	phosphatidylserine decarboxylase	EC:4.1.1.65	1	ko00564 Glycerophospholipid metabolism;
gene50008	K02987	RP-S4e, RPS4	small subunit ribosomal protein S4e	--	1	ko03010 Ribosome;
gene5000	K04733	IRAK4	interleukin-1 receptor-associated kinase 4	EC:2.7.11.1	11	ko04064 NF-kappa B signaling pathway;ko04210 Apoptosis;ko04620 Toll-like receptor signaling pathway;ko04722 Neurotrophin signaling pathway;ko05133 Pertussis;ko05140 Leishmaniasis;ko05142 Chagas disease (American trypanosomiasis);ko05145 Toxoplasmosis;ko05152 Tuberculosis;ko05162 Measles;ko05164 Influenza A;
gene50010	K02928	RP-L41e, RPL41	large subunit ribosomal protein L41e	--	1	ko03010 Ribosome;
gene50020	K14674	TGL4	TAG lipase / steryl ester hydrolase / phospholipase A2 / LPA acyltransferase	EC:3.1.1.3 3.1.1.13 3.1.1.4 2.3.1.51	7	ko00100 Steroid biosynthesis;ko00561 Glycerolipid metabolism;ko00564 Glycerophospholipid metabolism;ko00565 Ether lipid metabolism;ko00590 Arachidonic acid metabolism;ko00591 Linoleic acid metabolism;ko00592 alpha-Linolenic acid metabolism;
gene50023	K17108	GBA2	non-lysosomal glucosylceramidase	EC:3.2.1.45	2	ko00511 Other glycan degradation;ko00600 Sphingolipid metabolism;
gene50037	K13456	RIN4	RPM1-interacting protein 4	--	1	ko04626 Plant-pathogen interaction;
gene50038	K09313	CUTL	homeobox protein cut-like	--		
gene50039	K00434	E1.11.1.11	L-ascorbate peroxidase	EC:1.11.1.11	2	ko00053 Ascorbate and aldarate metabolism;ko00480 Glutathione metabolism;
gene50042	K06891	clpS	ATP-dependent Clp protease adaptor protein ClpS	--		
gene50045	K11251	H2A	histone H2A	--	2	ko05034 Alcoholism;ko05322 Systemic lupus erythematosus;
gene50046	K01177	E3.2.1.2	beta-amylase	EC:3.2.1.2	1	ko00500 Starch and sucrose metabolism;
gene5004	K00695	E2.4.1.13	sucrose synthase	EC:2.4.1.13	1	ko00500 Starch and sucrose metabolism;
gene50052	K08960	CSNK1E	casein kinase 1, epsilon	EC:2.7.11.1	6	ko04310 Wnt signaling pathway;ko04340 Hedgehog signaling pathway;ko04390 Hippo signaling pathway;ko04391 Hippo signaling pathway - fly;ko04710 Circadian rhythm;ko04711 Circadian rhythm - fly;
gene50058	K02221	yggT	YggT family protein	--		
gene5005	K15340	DCLRE1A, SNM1A, PSO2	DNA cross-link repair 1A protein	--		
gene50060	K14775	UTP30, RSL1D1	ribosome biogenesis protein UTP30	--		
gene50062	K09498	CCT6	T-complex protein 1 subunit zeta	--		
gene50075	K03283	HSPA1_8	heat shock 70kDa protein 1/8	--	11	ko03040 Spliceosome;ko04010 MAPK signaling pathway;ko04141 Protein processing in endoplasmic reticulum;ko04144 Endocytosis;ko04612 Antigen processing and presentation;ko04915 Estrogen signaling pathway;ko05134 Legionellosis;ko05145 Toxoplasmosis;ko05162 Measles;ko05164 Influenza A;ko05169 Epstein-Barr virus infection;
gene50076	K03252	EIF3C	translation initiation factor 3 subunit C	--	1	ko03013 RNA transport;
gene50077	K12614	DDX6, RCK, DHH1	ATP-dependent RNA helicase DDX6/DHH1	EC:3.6.4.13	1	ko03018 RNA degradation;
gene50081	K09487	HSP90B, TRA1	heat shock protein 90kDa beta	--	8	ko04141 Protein processing in endoplasmic reticulum;ko04151 PI3K-Akt signaling pathway;ko04621 NOD-like receptor signaling pathway;ko04626 Plant-pathogen interaction;ko04915 Estrogen signaling pathway;ko04918 Thyroid hormone synthesis;ko05200 Pathways in cancer;ko05215 Prostate cancer;
gene50085	K17479	GRXCR1	glutaredoxin domain-containing cysteine-rich protein 1	--		
gene50089	K16297	SCPL-II	serine carboxypeptidase-like clade II	EC:3.4.16.-		
gene50090	K16297	SCPL-II	serine carboxypeptidase-like clade II	EC:3.4.16.-		
gene50091	K16297	SCPL-II	serine carboxypeptidase-like clade II	EC:3.4.16.-		
gene50094	K12199	VTA1, LIP5	vacuolar protein sorting-associated protein VTA1	--	1	ko04144 Endocytosis;
gene50096	K01188	E3.2.1.21	beta-glucosidase	EC:3.2.1.21	3	ko00460 Cyanoamino acid metabolism;ko00500 Starch and sucrose metabolism;ko00940 Phenylpropanoid biosynthesis;
gene50097	K03131	TAF6	transcription initiation factor TFIID subunit 6	--	2	ko03022 Basal transcription factors;ko05168 Herpes simplex infection;
gene500	K09534	DNAJC14	DnaJ homolog subfamily C member 14	--		
gene50100	K14721	RPC37, POLR3E	DNA-directed RNA polymerase III subunit RPC5	--	5	ko00230 Purine metabolism;ko00240 Pyrimidine metabolism;ko03020 RNA polymerase;ko04623 Cytosolic DNA-sensing pathway;ko05169 Epstein-Barr virus infection;

gene50101	K14721	RPC37, POLR3E	DNA-directed RNA polymerase III subunit RPC5	--	5	ko00230 Purine metabolism;ko00240 Pyrimidine metabolism;ko03020 RNA polymerase;ko04623 Cytosolic DNA-sensing pathway;ko05169 Epstein-Barr virus infection;
gene50105	K10775	E4.3.1.24	phenylalanine ammonia-lyase	EC:4.3.1.24	2	ko00360 Phenylalanine metabolism;ko00940 Phenylpropanoid biosynthesis;
gene50106	K00856	E2.7.1.20, ADK	adenosine kinase	EC:2.7.1.20	1	ko00230 Purine metabolism;
gene50107	K14488	SAUR	SAUR family protein	--	1	ko04075 Plant hormone signal transduction;
gene50109	K14950	ATP13A1	cation-transporting ATPase 13A1	EC:3.6.3.-		
gene5010	K14516	ERF1	ethylene-responsive transcription factor 1	--	1	ko04075 Plant hormone signal transduction;
gene50115	K02717	psbP	photosystem II oxygen-evolving enhancer protein 2	--	1	ko00195 Photosynthesis;
gene50122	K02214	CDC7	cell division control protein 7	EC:2.7.11.1	3	ko04110 Cell cycle;ko04111 Cell cycle - yeast;ko04113 Meiosis - yeast;
gene50134	K00430	E1.11.1.7	peroxidase	EC:1.11.1.7	2	ko00360 Phenylalanine metabolism;ko00940 Phenylpropanoid biosynthesis;
gene50136	K00915	IPK2	1D-myo-Inositol-tetrakisphosphate 5-kinase / inositol-polyphosphate multikinase	EC:2.7.1.140 2.7.1.151	2	ko00562 Inositol phosphate metabolism;ko04070 Phosphatidylinositol signaling system;
gene50138	K14496	PYL	abscisic acid receptor PYR/PYL family	--	1	ko04075 Plant hormone signal transduction;
gene50146	K00122	FDH	formate dehydrogenase	EC:1.2.1.2	3	ko00630 Glyoxylate and dicarboxylate metabolism;ko00680 Methane metabolism;ko01200 Carbon metabolism;
gene50149	K14484	IAA	auxin-responsive protein IAA	--	1	ko04075 Plant hormone signal transduction;
gene5014	K09480	E2.4.1.241	digalactosyldiacylglycerol synthase	EC:2.4.1.241	1	ko00561 Glycerolipid metabolism;
gene50152	K14638	SLC15A3_4, PHT	solute carrier family 15 (peptide/histidine transporter), member 3/4	--		
gene50158	K10355	ACTF	actin, other eukaryote	--		
gene50159	K11662	ACTR6, ARP6	actin-related protein 6	--		
gene50160	K10355	ACTF	actin, other eukaryote	--		
gene50165	K04035	E1.14.13.81, acsF, chlE	magnesium-protoporphyrin IX monomethyl ester (oxidative) cyclase	EC:1.14.13.81	1	ko00860 Porphyrin and chlorophyll metabolism;
gene50166	K16675	ZDHHC14	probable palmitoyltransferase ZDHHC14	EC:2.3.1.-	1	ko04391 Hippo signaling pathway - fly;
gene50167	K01873	VARS, valS	valyl-tRNA synthetase	EC:6.1.1.9	1	ko00970 Aminoacyl-tRNA biosynthesis;
gene50173	K01188	E3.2.1.21	beta-glucosidase	EC:3.2.1.21	3	ko00460 Cyanoamino acid metabolism;ko00500 Starch and sucrose metabolism;ko00940 Phenylpropanoid biosynthesis;
gene50174	K10580	UBE2N, BLU, UBC13	ubiquitin-conjugating enzyme E2 N	EC:6.3.2.19	1	ko04120 Ubiquitin mediated proteolysis;
gene50177	K02716	psbO	photosystem II oxygen-evolving enhancer protein 1	--	1	ko00195 Photosynthesis;
gene50178	K00434	E1.11.1.11	L-ascorbate peroxidase	EC:1.11.1.11	2	ko00053 Ascorbate and aldarate metabolism;ko00480 Glutathione metabolism;
gene50182	K01807	rpiA	ribose 5-phosphate isomerase A	EC:5.3.1.6	4	ko00030 Pentose phosphate pathway;ko00710 Carbon fixation in photosynthetic organisms;ko01200 Carbon metabolism;ko01230 Biosynthesis of amino acids;
gene50183	K15544	SSU72	RNA polymerase II subunit A C-terminal domain phosphatase SSU72	EC:3.1.3.16	1	ko03015 mRNA surveillance pathway;
gene50187	K14641	APY1_2	apyrase	EC:3.6.1.5	2	ko00230 Purine metabolism;ko00240 Pyrimidine metabolism;
gene50189	K00430	E1.11.1.7	peroxidase	EC:1.11.1.7	2	ko00360 Phenylalanine metabolism;ko00940 Phenylpropanoid biosynthesis;
gene50190	K17506	PPM1L, PP2CE	protein phosphatase 1L	EC:3.1.3.16		
gene50197	K01728	E4.2.2.2, pel	pectate lyase	EC:4.2.2.2	1	ko00040 Pentose and glucuronate interconversions;
gene50203	K03020	RPC19, POLR1D	DNA-directed RNA polymerases I and III subunit RPAC2	--	5	ko00230 Purine metabolism;ko00240 Pyrimidine metabolism;ko03020 RNA polymerase;ko04623 Cytosolic DNA-sensing pathway;ko05169 Epstein-Barr virus infection;
gene50207	K07375	TUBB	tubulin beta	--	3	ko04145 Phagosome;ko04540 Gap junction;ko05130 Pathogenic Escherichia coli infection;
gene50209	K13420	FLS2	LRR receptor-like serine/threonine-protein kinase FLS2	EC:2.7.11.1	1	ko04626 Plant-pathogen interaction;
gene5020	K05933	E1.14.17.4	aminocyclopropanecarboxylate oxidase	EC:1.14.17.4	1	ko00270 Cysteine and methionine metabolism;
gene50210	K13420	FLS2	LRR receptor-like serine/threonine-protein kinase FLS2	EC:2.7.11.1	1	ko04626 Plant-pathogen interaction;
gene50212	K08235	E2.4.1.207	xyloglucan:xyloglucosyl transferase	EC:2.4.1.207		
gene50222	K09527	DNAJC7	DnaJ homolog subfamily C member 7	--		
gene50225	K13356	FAR	fatty acyl-CoA reductase	EC:1.2.1.-	2	ko00073 Cutin, suberine and wax biosynthesis;ko04146 Peroxisome;
gene50226	K13356	FAR	fatty acyl-CoA reductase	EC:1.2.1.-	2	ko00073 Cutin, suberine and wax biosynthesis;ko04146 Peroxisome;
gene50229	K15404	K15404, CER1	aldehyde decarbonylase	EC:4.1.99.5	1	ko00073 Cutin, suberine and wax biosynthesis;

gene5022	K05931	CARM1, PRMT4	histone-arginine methyltransferase CARM1	EC:2.1.1.125		
gene50231	K09422	MYBP	myb proto-oncogene protein, plant	--		
gene50237	K02727	PSMA3	20S proteasome subunit alpha 7	EC:3.4.25.1	1	ko03050 Proteasome;
gene50244	K04794	PTH2	peptidyl-tRNA hydrolase, PTH2 family	EC:3.1.1.29		
gene50245	K01184	E3.2.1.15	polygalacturonase	EC:3.2.1.15	2	ko00040 Pentose and glucuronate interconversions;ko00500 Starch and sucrose metabolism;
gene50246	K01897	ACSL, fadD	long-chain acyl-CoA synthetase	EC:6.2.1.3	4	ko00071 Fatty acid degradation;ko03320 PPAR signaling pathway;ko04146 Peroxisome;ko04920 Adipocytokine signaling pathway;
gene50247	K15032	MTERFD	mTERF domain-containing protein, mitochondrial	--		
gene50255	K12251	aguB	N-carbamoylputrescine amidase	EC:3.5.1.53	1	ko00330 Arginine and proline metabolism;
gene50266	K14416	HBS1	elongation factor 1 alpha-like protein	--	2	ko03015 mRNA surveillance pathway;ko05134 Legionellosis;
gene50267	K16276	K16276, BTS	zinc finger protein-like protein	--		
gene5026	K03036	PSMD11, RPN6	26S proteasome regulatory subunit N6	--	2	ko03050 Proteasome;ko05169 Epstein-Barr virus infection;
gene50273	K04506	SIAH1	E3 ubiquitin-protein ligase SIAH1	EC:6.3.2.19	3	ko04115 p53 signaling pathway;ko04120 Ubiquitin mediated proteolysis;ko04310 Wnt signaling pathway;
gene50276	K11975	RNF144	E3 ubiquitin-protein ligase RNF144	EC:6.3.2.19		
gene50278	K02725	PSMA1	20S proteasome subunit alpha 6	EC:3.4.25.1	1	ko03050 Proteasome;
gene50279	K00611	OTC, argF, argI	ornithine carbamoyltransferase	EC:2.1.3.3	2	ko00330 Arginine and proline metabolism;ko01230 Biosynthesis of amino acids;
gene50289	K05841	E2.4.1.173	sterol 3beta-glucosyltransferase	EC:2.4.1.173		
gene5028	K09872	PIP	aquaporin PIP	--		
gene50292	K16911	DDX21	ATP-dependent RNA helicase DDX21	EC:3.6.4.13		
gene50296	K16911	DDX21	ATP-dependent RNA helicase DDX21	EC:3.6.4.13		
gene50299	K01206	FUCA	alpha-L-fucosidase	EC:3.2.1.51	1	ko00511 Other glycan degradation;
gene5029	K12868	SYF2	pre-mRNA-splicing factor SYF2	--	1	ko03040 Spliceosome;
gene50301	K07198	PRKAA, AMPK	5'-AMP-activated protein kinase, catalytic alpha subunit	EC:2.7.11.11	8	ko04140 Regulation of autophagy;ko04150 mTOR signaling pathway;ko04151 PI3K-Akt signaling pathway;ko04710 Circadian rhythm;ko04910 Insulin signaling pathway;ko04920 Adipocytokine signaling pathway;ko04932 Non-alcoholic fatty liver disease (NAFLD);ko05410 Hypertrophic cardiomyopathy (HCM);
gene50309	K03016	RPB8, POLR2H	DNA-directed RNA polymerases I, II, and III subunit RPABC3	--	6	ko00230 Purine metabolism;ko00240 Pyrimidine metabolism;ko03020 RNA polymerase;ko04623 Cytosolic DNA-sensing pathway;ko05016 Huntington's disease;ko05169 Epstein-Barr virus infection;
gene50315	K15382	SLC50A, SWEET	solute carrier family 50 (sugar transporter)	--		
gene50319	K11842	USP12_46	ubiquitin carboxyl-terminal hydrolase 12/46	EC:3.1.2.15		
gene50321	K15542	PFS2	polyadenylation factor subunit 2	--	1	ko03015 mRNA surveillance pathway;
gene50322	K07936	RAN	GTP-binding nuclear protein Ran	--	4	ko03008 Ribosome biogenesis in eukaryotes;ko03013 RNA transport;ko05166 HTLV-I infection;ko05169 Epstein-Barr virus infection;
gene50324	K03627	MBF1	putative transcription factor	--		
gene50327	K15277	SLC35B3, PAPST2	solute carrier family 35 (adenosine 3'-phospho 5'-phosphosulfate transporter), member B3	--		
gene50334	K03005	RPA49, POLR1E	DNA-directed RNA polymerase I subunit RPA49	--	3	ko00230 Purine metabolism;ko00240 Pyrimidine metabolism;ko03020 RNA polymerase;
gene50339	K08235	E2.4.1.207	xyloglucan:xyloglucosyl transferase	EC:2.4.1.207		
gene50344	K16315	GSG2	serine/threonine-protein kinase haspin	EC:2.7.11.1		
gene50353	K07195	EXOC7, EXO70	exocyst complex component 7	--	1	ko04910 Insulin signaling pathway;
gene50354	K12741	HNRNPA1_3	heterogeneous nuclear ribonucleoprotein A1/A3	--	1	ko03040 Spliceosome;
gene50355	K01188	E3.2.1.21	beta-glucosidase	EC:3.2.1.21	3	ko00460 Cyanoamino acid metabolism;ko00500 Starch and sucrose metabolism;ko00940 Phenylpropanoid biosynthesis;
gene50356	K14863	YTM1, WDR12	ribosome biogenesis protein YTM1	--		
gene50363	K01373	CTSF	cathepsin F	EC:3.4.22.41	1	ko04142 Lysosome;
gene50365	K16833	PPP1R2, IPP2	protein phosphatase inhibitor 2	--		
gene50370	K14500	BSK	BR-signaling kinase	EC:2.7.11.1	1	ko04075 Plant hormone signal transduction;

gene50379	K15639	CYP734A1, BAS1	cytochrome P450, family 734, subfamily A, polypeptide 1 (PHYB activation tagged suppressor 1)	EC:1.14.-.-	1	ko00905 Brassinosteroid biosynthesis;
gene50381	K10268	FBXL2_20	F-box and leucine-rich repeat protein 2/20	--		
gene50382	K14685	SLC40A1, FPN1	solute carrier family 40 (iron-regulated transporter), member 1	--	1	ko04978 Mineral absorption;
gene50388	K01609	trpC	indole-3-glycerol phosphate synthase	EC:4.1.1.48	2	ko00400 Phenylalanine, tyrosine and tryptophan biosynthesis;ko01230 Biosynthesis of amino acids;
gene50390	K00573	E2.1.1.77, pcm	protein-L-isoaspartate(D-aspartate) O-methyltransferase	EC:2.1.1.77		
gene50391	K12449	AXS	UDP-apiose/xylose synthase	--	1	ko00520 Amino sugar and nucleotide sugar metabolism;
gene50393	K00026	MDH2	malate dehydrogenase	EC:1.1.1.37	5	ko00020 Citrate cycle (TCA cycle);ko00620 Pyruvate metabolism;ko00630 Glyoxylate and dicarboxylate metabolism;ko00710 Carbon fixation in photosynthetic organisms;ko01200 Carbon metabolism;
gene50397	K15305	VAC14, TAX1BP2	vacuole morphology and inheritance protein 14	--	2	ko05166 HTLV-I infection;ko05203 Viral carcinogenesis;
gene5039	K15710	SHPRH	E3 ubiquitin-protein ligase SHPRH	EC:3.6.4.- 6.3.2.19		
gene50403	K13508	GPAT	glycerol-3-phosphate acyltransferase	EC:2.3.1.15	2	ko00561 Glycerolipid metabolism;ko00564 Glycerophospholipid metabolism;
gene50404	K08193	SLC17A	MFS transporter, ACS family, solute carrier family 17 (sodium-dependent inorganic phosphate cotransporter), other	--		
gene50405	K15398	CYP86A4S	cytochrome P450, family 86, subfamily A, polypeptide 2/4/7/8 (fatty acid omega-hydroxylase)	EC:1.14.-.-	1	ko00073 Cutin, suberine and wax biosynthesis;
gene50406	K02607	ORC5	origin recognition complex subunit 5	--	3	ko04110 Cell cycle;ko04111 Cell cycle - yeast;ko04113 Meiosis - yeast;
gene50409	K13034	ATCYSC1	L-3-cyanoalanine synthase/ cysteine synthase	EC:2.5.1.47 4.4.1.9	5	ko00270 Cysteine and methionine metabolism;ko00460 Cyanoamino acid metabolism;ko00920 Sulfur metabolism;ko01200 Carbon metabolism;ko01230 Biosynthesis of amino acids;
gene50411	K17086	TM9SF2_4	transmembrane 9 superfamily member 2/4	--		
gene50417	K15175	CDC73	parafibromin	--		
gene50423	K03679	RRP4, EXOSC2	exosome complex component RRP4	--	1	ko03018 RNA degradation;
gene50429	K08245	E3.4.23.40	phytepsin	EC:3.4.23.40		
gene50431	K05387	GRIP	glutamate receptor, ionotropic, plant	--		
gene50436	K09872	PIP	aquaporin PIP	--		
gene50439	K11498	CENPE	centromeric protein E	--		
gene50443	K00430	E1.11.1.7	peroxidase	EC:1.11.1.7	2	ko00360 Phenylalanine metabolism;ko00940 Phenylpropanoid biosynthesis;
gene50445	K10364	CAPZA	capping protein (actin filament) muscle Z-line, alpha	--		
gene50449	K09422	MYBP	myb proto-oncogene protein, plant	--		
gene50450	K03257	EIF4A	translation initiation factor 4A	--	1	ko03013 RNA transport;
gene50453	K08762	DBI, ACBP	diazepam-binding inhibitor (GABA receptor modulator, acyl-CoA-binding protein)	--	1	ko03320 PPAR signaling pathway;
gene50456	K00059	fabG	3-oxoacyl-[acyl-carrier protein] reductase	EC:1.1.1.100	3	ko00061 Fatty acid biosynthesis;ko00780 Biotin metabolism;ko01040 Biosynthesis of unsaturated fatty acids;
gene50459	K02949	RP-S11e, RPS11	small subunit ribosomal protein S11e	--	1	ko03010 Ribosome;
gene5045	K15535	PWD	phosphoglucan, water dikinase	EC:2.7.9.5		
gene50461	K00134	GAPDH, gapA	glyceraldehyde 3-phosphate dehydrogenase	EC:1.2.1.12	6	ko00010 Glycolysis / Gluconeogenesis;ko00710 Carbon fixation in photosynthetic organisms;ko01200 Carbon metabolism;ko01230 Biosynthesis of amino acids;ko04066 HIF-1 signaling pathway;ko05010 Alzheimer's disease;
gene50471	K01661	menB	naphthoate synthase	EC:4.1.3.36	1	ko00130 Ubiquinone and other terpenoid-quinone biosynthesis;
gene50472	K07937	ARF1	ADP-ribosylation factor 1	--	2	ko05110 Vibrio cholerae infection;ko05134 Legionellosis;
gene50474	K04507	CACYBP, SIP	calcyclin binding protein	--	1	ko04310 Wnt signaling pathway;
gene50476	K01785	E5.1.3.3, galM	aldose 1-epimerase	EC:5.1.3.3	1	ko00010 Glycolysis / Gluconeogenesis;
gene50477	K01785	E5.1.3.3, galM	aldose 1-epimerase	EC:5.1.3.3	1	ko00010 Glycolysis / Gluconeogenesis;
gene50478	K01785	E5.1.3.3, galM	aldose 1-epimerase	EC:5.1.3.3	1	ko00010 Glycolysis / Gluconeogenesis;
gene50479	K01785	E5.1.3.3, galM	aldose 1-epimerase	EC:5.1.3.3	1	ko00010 Glycolysis / Gluconeogenesis;
gene50480	K01785	E5.1.3.3, galM	aldose 1-epimerase	EC:5.1.3.3	1	ko00010 Glycolysis / Gluconeogenesis;

gene50482	K09338	HD-ZIP	homeobox-leucine zipper protein	--		
gene50485	K12356	UGT72E	coniferyl-alcohol glucosyltransferase	EC:2.4.1.111	1	ko00940 Phenylpropanoid biosynthesis;
gene50487	K10355	ACTF	actin, other eukaryote	--		
gene50490	K01102	PDP	pyruvate dehydrogenase phosphatase	EC:3.1.3.43		
gene50492	K09591	DET2	steroid 5-alpha-reductase	EC:1.3.1.22	1	ko00905 Brassinosteroid biosynthesis;
gene50494	K09422	MYBP	myb proto-oncogene protein, plant	--		
gene50495	K00863	E2.7.1.29, DAK1, DAK2	dihydroxyacetone kinase	EC:2.7.1.29	4	ko00561 Glycerolipid metabolism;ko00680 Methane metabolism;ko01200 Carbon metabolism;ko04622 RIG-I-like receptor signaling pathway;
gene504	K03178	UBE1, UBA1	ubiquitin-activating enzyme E1	EC:6.3.2.19	2	ko04120 Ubiquitin mediated proteolysis;ko05012 Parkinson's disease;
gene50501	K13171	SRRM1, SRM160	serine/arginine repetitive matrix protein 1	--	2	ko03013 RNA transport;ko03015 mRNA surveillance pathway;
gene50502	K08288	PRKCSH	protein kinase C substrate 80K-H	--	1	ko04141 Protein processing in endoplasmic reticulum;
gene50503	K00392	sir	sulfite reductase (ferredoxin)	EC:1.8.7.1	1	ko00920 Sulfur metabolism;
gene50508	K03115	CSNK2B	casein kinase II subunit beta	--	9	ko03008 Ribosome biogenesis in eukaryotes;ko04064 NF-kappa B signaling pathway;ko04310 Wnt signaling pathway;ko04520 Adherens junction;ko04530 Tight junction;ko04712 Circadian rhythm - plant;ko05162 Measles;ko05168 Herpes simplex infection;ko05169 Epstein-Barr virus infection;
gene50510	K02692	psaD	photosystem I subunit II	--	1	ko00195 Photosynthesis;
gene50511	K16302	CNNM	metal transporter CNNM	--		
gene50512	K01188	E3.2.1.21	beta-glucosidase	EC:3.2.1.21	3	ko00460 Cyanoamino acid metabolism;ko00500 Starch and sucrose metabolism;ko00940 Phenylpropanoid biosynthesis;
gene50517	K14488	SAUR	SAUR family protein	--	1	ko04075 Plant hormone signal transduction;
gene50519	K12619	XRN2, RAT1	5'-3' exoribonuclease 2	EC:3.1.13.-	2	ko03008 Ribosome biogenesis in eukaryotes;ko03018 RNA degradation;
gene50524	K03189	ureG	urease accessory protein	--		
gene50526	K02882	RP-L18Ae, RPL18A	large subunit ribosomal protein L18Ae	--	1	ko03010 Ribosome;
gene50528	K02923	RP-L38e, RPL38	large subunit ribosomal protein L38e	--	1	ko03010 Ribosome;
gene5052	K09264	K09264	MADS-box transcription factor, plant	--		
gene50532	K15378	SLC45A1_2_4	solute carrier family 45, member 1/2/4	--		
gene50534	K11290	SET, TAF1, I2PP2A	template-activating factor I	--		
gene50537	K16055	TPS	trehalose 6-phosphate synthase/phosphatase	EC:2.4.1.15 3.1.3.12	1	ko00500 Starch and sucrose metabolism;
gene50541	K03070	secA	preprotein translocase subunit SecA	--	2	ko03060 Protein export;ko03070 Bacterial secretion system;
gene50543	K14442	DHX36, RHAU	ATP-dependent RNA helicase DHX36	EC:3.6.4.13	1	ko03018 RNA degradation;
gene50545	K07897	RAB7A	Ras-related protein Rab-7A	--	5	ko04144 Endocytosis;ko04145 Phagosome;ko05132 Salmonella infection;ko05146 Amoebiasis;ko05152 Tuberculosis;
gene50547	K08288	PRKCSH	protein kinase C substrate 80K-H	--	1	ko04141 Protein processing in endoplasmic reticulum;
gene50553	K14488	SAUR	SAUR family protein	--	1	ko04075 Plant hormone signal transduction;
gene50555	K15382	SLC50A, SWEET	solute carrier family 50 (sugar transporter)	--		
gene5055	K00759	E2.4.2.7, apt	adenine phosphoribosyltransferase	EC:2.4.2.7	1	ko00230 Purine metabolism;
gene50561	K00430	E1.11.1.7	peroxidase	EC:1.11.1.7	2	ko00360 Phenylalanine metabolism;ko00940 Phenylpropanoid biosynthesis;
gene50563	K00006	GPD1	glycerol-3-phosphate dehydrogenase (NAD+)	EC:1.1.1.8	1	ko00564 Glycerophospholipid metabolism;
gene50568	K00434	E1.11.1.11	L-ascorbate peroxidase	EC:1.11.1.11	2	ko00053 Ascorbate and aldarate metabolism;ko00480 Glutathione metabolism;
gene50573	K03439	trmB, METTL1	tRNA (guanine-N7-)-methyltransferase	EC:2.1.1.33		
gene50574	K06127	COQ5	ubiquinone biosynthesis methyltransferase	EC:2.1.1.201	1	ko00130 Ubiquinone and other terpenoid-quinone biosynthesis;
gene50578	K10666	RNF5	E3 ubiquitin-protein ligase RNF5	EC:6.3.2.19	1	ko04141 Protein processing in endoplasmic reticulum;
gene5057	K15083	RAD16	DNA repair protein RAD16	--		
gene50580	K13691	SGT1	pathogen-inducible salicylic acid glucosyltransferase	EC:2.4.1.-		
gene50586	K06671	STAG1_2, SCC3, IRR1	cohesin complex subunit SA-1/2	--	3	ko04110 Cell cycle;ko04111 Cell cycle - yeast;ko04113 Meiosis - yeast;
gene50588	K09422	MYBP	myb proto-oncogene protein, plant	--		
gene5058	K00430	E1.11.1.7	peroxidase	EC:1.11.1.7	2	ko00360 Phenylalanine metabolism;ko00940 Phenylpropanoid biosynthesis;
gene50595	K09422	MYBP	myb proto-oncogene protein, plant	--		
gene50596	K11253	H3	histone H3	--	3	ko05034 Alcoholism;ko05202 Transcriptional misregulation in cancer;ko05322 Systemic lupus erythematosus;

gene505	K14498	SNRK2	serine/threonine-protein kinase SRK2	EC:2.7.11.1	1	ko04075 Plant hormone signal transduction;
gene50600	K00430	E1.11.1.7	peroxidase	EC:1.11.1.7	2	ko00360 Phenylalanine metabolism;ko00940 Phenylpropanoid biosynthesis;
gene50607	K02116	atpI	ATP synthase protein I	--		
gene50608	K00218	E1.3.1.33, por	protochlorophyllide reductase	EC:1.3.1.33	1	ko00860 Porphyrin and chlorophyll metabolism;
gene50611	K15403	ACE, HTH	fatty acid omega-hydroxy dehydrogenase	EC:1.1.-.-	1	ko00073 Cutin, suberine and wax biosynthesis;
gene50615	K12309	GLB1, ELNR1	beta-galactosidase	EC:3.2.1.23	6	ko00052 Galactose metabolism;ko00511 Other glycan degradation;ko00531 Glycosaminoglycan degradation;ko00600 Sphingolipid metabolism;ko00604 Glycosphingolipid biosynthesis - ganglio series;ko04142 Lysosome;
gene5061	K00423	E1.10.3.3	L-ascorbate oxidase	EC:1.10.3.3	1	ko00053 Ascorbate and aldarate metabolism;
gene50623	K04733	IRAK4	interleukin-1 receptor-associated kinase 4	EC:2.7.11.1	11	ko04064 NF-kappa B signaling pathway;ko04210 Apoptosis;ko04620 Toll-like receptor signaling pathway;ko04722 Neurotrophin signaling pathway;ko05133 Pertussis;ko05140 Leishmaniasis;ko05142 Chagas disease (American trypanosomiasis);ko05145 Toxoplasmosis;ko05152 Tuberculosis;ko05162 Measles;ko05164 Influenza A;
gene50624	K12891	SFRS2	splicing factor, arginine/serine-rich 2	--	2	ko03040 Spliceosome;ko05168 Herpes simplex infection;
gene50627	K09597	SPPL2B	signal peptide peptidase-like 2B	EC:3.4.23.-		
gene50629	K00814	GPT, ALT	alanine transaminase	EC:2.6.1.2	4	ko00250 Alanine, aspartate and glutamate metabolism;ko00710 Carbon fixation in photosynthetic organisms;ko01210 2-Oxocarboxylic acid metabolism;ko01230 Biosynthesis of amino acids;
gene5062	K05275	E1.1.1.65	pyridoxine 4-dehydrogenase	EC:1.1.1.65	1	ko00750 Vitamin B6 metabolism;
gene50631	K10706	SETX, ALS4	senataxin	EC:3.6.4.-		
gene50632	K14821	BUD20	bud site selection protein 20	--		
gene50644	K10849	ERCC1	DNA excision repair protein ERCC-1	--	2	ko03420 Nucleotide excision repair;ko03460 Fanconi anemia pathway;
gene50645	K10400	KIF15	kinesin family member 15	--		
gene50657	K01762	ACS	1-aminocyclopropane-1-carboxylate synthase	EC:4.4.1.14	1	ko00270 Cysteine and methionine metabolism;
gene50659	K03437	spoU	RNA methyltransferase, TrmH family	--		
gene50660	K07874	RAB1A	Ras-related protein Rab-1A	--	1	ko05134 Legionellosis;
gene50661	K13199	SERBP1	plasminogen activator inhibitor 1 RNA-binding protein	--		
gene50663	K14491	ARR-B	two-component response regulator ARR-B family	--	1	ko04075 Plant hormone signal transduction;
gene50664	K12486	SMAP	stromal membrane-associated protein	--	1	ko04144 Endocytosis;
gene50665	K14516	ERF1	ethylene-responsive transcription factor 1	--	1	ko04075 Plant hormone signal transduction;
gene50666	K14516	ERF1	ethylene-responsive transcription factor 1	--	1	ko04075 Plant hormone signal transduction;
gene50667	K14516	ERF1	ethylene-responsive transcription factor 1	--	1	ko04075 Plant hormone signal transduction;
gene50669	K06062	PCAF, KAT2, GCN5	histone acetyltransferase	EC:2.3.1.48	3	ko04330 Notch signaling pathway;ko05166 HTLV-I infection;ko05203 Viral carcinogenesis;
gene50670	K03680	EIF2B4	translation initiation factor eIF-2B subunit delta	--	1	ko03013 RNA transport;
gene50671	K03111	ssb	single-strand DNA-binding protein	--	3	ko03030 DNA replication;ko03430 Mismatch repair;ko03440 Homologous recombination;
gene50676	K07555	ATPeAF1, ATPAF1	ATP synthase mitochondrial F1 complex assembly factor 1	--		
gene50678	K06063	SNW1, SKIIP, SKIP	SNW domain-containing protein 1	--	4	ko03040 Spliceosome;ko04330 Notch signaling pathway;ko05169 Epstein-Barr virus infection;ko05203 Viral carcinogenesis;
gene50679	K07151	STT3	dolichyl-diphosphooligosaccharide--protein glycosyltransferase	EC:2.4.99.18	3	ko00510 N-Glycan biosynthesis;ko00513 Various types of N-glycan biosynthesis;ko04141 Protein processing in endoplasmic reticulum;
gene50682	K15255	PIF1	ATP-dependent DNA helicase PIF1	EC:3.6.4.12		
gene50683	K04460	PPP5C	serine/threonine-protein phosphatase 5	EC:3.1.3.16	1	ko04010 MAPK signaling pathway;
gene50685	K12345	SRD5A3	3-oxo-5-alpha-steroid 4-dehydrogenase 3	EC:1.3.1.22 1.3.1.94	1	ko00140 Steroid hormone biosynthesis;
gene50686	K00011	E1.1.1.21, AKR1	aldehyde reductase	EC:1.1.1.21	5	ko00040 Pentose and glucuronate interconversions;ko00051 Fructose and mannose metabolism;ko00052 Galactose metabolism;ko00561 Glycerolipid metabolism;ko00620 Pyruvate metabolism;
gene50687	K16732	PRC1	protein regulator of cytokinesis 1	--		

gene50692	K00001	E1.1.1.1, adh	alcohol dehydrogenase	EC:1.1.1.1	9	ko00010 Glycolysis / Gluconeogenesis;ko00071 Fatty acid degradation;ko00350 Tyrosine metabolism;ko00625 Chloroalkane and chloroalkene degradation;ko00626 Naphthalene degradation;ko00830 Retinol metabolism;ko00980 Metabolism of xenobiotics by cytochrome P450;ko00982 Drug metabolism - cytochrome P450;ko01220 Degradation of aromatic compounds;
gene50693	K01638	E2.3.3.9, aceB, glcB	malate synthase	EC:2.3.3.9	3	ko00620 Pyruvate metabolism;ko00630 Glyoxylate and dicarboxylate metabolism;ko01200 Carbon metabolism;
gene5069	K13412	CPK	calcium-dependent protein kinase	EC:2.7.11.1	2	ko04626 Plant-pathogen interaction;ko05145 Toxoplasmosis;
gene50701	K04371	MAPK1_3	mitogen-activated protein kinase 1/3	EC:2.7.11.24	71	ko04010 MAPK signaling pathway;ko04012 ErbB signaling pathway;ko04013 MAPK signaling pathway - fly;ko04014 Ras signaling pathway;ko04062 Chemokine signaling pathway;ko04066 HIF-1 signaling pathway;ko04114 Oocyte meiosis;ko04150 mTOR signaling pathway;ko04151 PI3K-Akt signaling pathway;ko04270 Vascular smooth muscle contraction;ko04320 Dorso-ventral axis formation;ko04350 TGF-beta signaling pathway;ko04360 Axon guidance;ko04370 VEGF signaling pathway;ko04380 Osteoclast differentiation;ko04510 Focal adhesion;ko04520 Adherens junction;ko04540 Gap junction;ko04620 Toll-like receptor signaling pathway;ko04621 NOD-like receptor signaling pathway;ko04650 Natural killer cell mediated cytotoxicity;ko04660 T cell receptor signaling pathway;ko04662 B cell receptor signaling pathway;ko04664 Fc epsilon RI signaling pathway;ko04666 Fc gamma R-mediated phagocytosis;ko04668 TNF signaling pathway;ko04713 Circadian entrainment;ko04720 Long-term potentiation;ko04722 Neurotrophin signaling pathway;ko04723 Retrograde endocannabinoid signaling;ko04724 Glutamatergic synapse;ko04725 Cholinergic synapse;ko04726 Serotonergic synapse;ko04730 Long-term depression;ko04810 Regulation of actin cytoskeleton;ko04910 Insulin signaling pathway;ko04912 GnRH signaling pathway;ko04914 Progesterone-mediated oocyte maturation;ko04915 Estrogen signaling pathway;ko04916 Melanogenesis;ko04917 Prolactin signaling pathway;ko04930 Type II diabetes mellitus;ko04960 Aldosterone-regulated sodium reabsorption;ko05010 Alzheimer's disease;ko05020 Prion diseases;ko05034 Alcoholism;ko05131 Shigellosis;ko05132 Salmonella infection;ko05133 Pertussis;ko05140 Leishmaniasis;ko05142 Chagas disease (American trypanosomiasis);ko05145 Toxoplasmosis;ko05152 Tuberculosis;ko05160 Hepatitis C;ko05161 Hepatitis B;ko05164 Influenza A;ko05200 Pathways in cancer;ko05203 Viral carcinogenesis;ko05205 Proteoglycans in cancer;ko05210 Colorectal cancer;ko05211 Renal cell carcinoma;ko05212
gene50702	K10523	SPOP	speckle-type POZ protein	--		
gene50704	K12188	SNF8, EAP30	ESCRT-II complex subunit VPS22	--	1	ko04144 Endocytosis;
gene50705	K01338	lon	ATP-dependent Lon protease	EC:3.4.21.53	1	ko04112 Cell cycle - Caulobacter;
gene50707	K12309	GLB1, ELNR1	beta-galactosidase	EC:3.2.1.23	6	ko00052 Galactose metabolism;ko00511 Other glycan degradation;ko00531 Glycosaminoglycan degradation;ko00600 Sphingolipid metabolism;ko00604 Glycosphingolipid biosynthesis - ganglio series;ko04142 Lysosome;
gene50708	K01889	FARSA, pheS	phenylalanyl-tRNA synthetase alpha chain	EC:6.1.1.20	1	ko00970 Aminoacyl-tRNA biosynthesis;
gene5070	K13412	CPK	calcium-dependent protein kinase	EC:2.7.11.1	2	ko04626 Plant-pathogen interaction;ko05145 Toxoplasmosis;
gene50713	K03883	ND5	NADH-ubiquinone oxidoreductase chain 5	EC:1.6.5.3	2	ko00190 Oxidative phosphorylation;ko05012 Parkinson's disease;
gene50717	K03456	PPP2R1	serine/threonine-protein phosphatase 2A regulatory subunit A	--	13	ko03015 mRNA surveillance pathway;ko04111 Cell cycle - yeast;ko04113 Meiosis - yeast;ko04114 Oocyte meiosis;ko04151 PI3K-Akt signaling pathway;ko04350 TGF-beta signaling pathway;ko04390 Hippo signaling pathway;ko04391 Hippo signaling pathway - fly;ko04530 Tight junction;ko04728 Dopaminergic synapse;ko04730 Long-term depression;ko05142 Chagas disease (American trypanosomiasis);ko05160 Hepatitis C;
gene5071	K14431	TGA	transcription factor TGA	--	1	ko04075 Plant hormone signal transduction;
gene50720	K07760	CDK	cyclin-dependent kinase	EC:2.7.11.22		

gene50725	K16298	SCPL-IV	serine carboxypeptidase-like clade IV	EC:3.4.16.-		
gene50726	K16298	SCPL-IV	serine carboxypeptidase-like clade IV	EC:3.4.16.-		
gene50731	K13667	RUMI, KTELC1	protein glucosyltransferase	EC:2.4.1.-	1	ko00514 Other types of O-glycan biosynthesis;
gene50732	K13667	RUMI, KTELC1	protein glucosyltransferase	EC:2.4.1.-	1	ko00514 Other types of O-glycan biosynthesis;
gene50733	K15440	TAD1, ADAT1	tRNA-specific adenosine deaminase 1	EC:3.5.4.34		
gene50735	K01188	E3.2.1.21	beta-glucosidase	EC:3.2.1.21	3	ko00460 Cyanoamino acid metabolism;ko00500 Starch and sucrose metabolism;ko00940 Phenylpropanoid biosynthesis;
gene50736	K02993	RP-S7e, RPS7	small subunit ribosomal protein S7e	--	1	ko03010 Ribosome;
gene50742	K05391	CNGF	cyclic nucleotide gated channel, other eukaryote	--	1	ko04626 Plant-pathogen interaction;
gene50744	K08869	ADCK, ABC1	aarF domain-containing kinase	--		
gene50752	K00967	PCYT2	ethanolamine-phosphate cytidylyltransferase	EC:2.7.7.14	2	ko00440 Phosphonate and phosphinate metabolism;ko00564 Glycerophospholipid metabolism;
gene50763	K00858	ppnK, NADK	NAD+ kinase	EC:2.7.1.23	1	ko00760 Nicotinate and nicotinamide metabolism;
gene50772	K02995	RP-S8e, RPS8	small subunit ribosomal protein S8e	--	1	ko03010 Ribosome;
gene5077	K12606	RCD1, CNOT9, CAF40	CCR4-NOT transcription complex subunit 9	--	1	ko03018 RNA degradation;
gene5078	K03327	TC.MATE, SLC47A, norM, mdtK, dinF	multidrug resistance protein, MATE family	--		
gene50791	K01011	TST, MPST, sseA	thiosulfate/3-mercaptopyruvate sulfurtransferase	EC:2.8.1.1 2.8.1.2	3	ko00270 Cysteine and methionine metabolism;ko00920 Sulfur metabolism;ko04122 Sulfur relay system;
gene50797	K02262	COX3	cytochrome c oxidase subunit 3	--	6	ko00190 Oxidative phosphorylation;ko04260 Cardiac muscle contraction;ko04932 Non-alcoholic fatty liver disease (NAFLD);ko05010 Alzheimer's disease;ko05012 Parkinson's disease;ko05016 Huntington's disease;
gene50798	K15711	SMARCA3, HLTF	SWI/SNF-related matrix-associated actin-dependent regulator of chromatin subfamily A3	EC:3.6.4.- 6.3.2.19		
gene50799	K00873	PK, pyk	pyruvate kinase	EC:2.7.1.40	7	ko00010 Glycolysis / Gluconeogenesis;ko00230 Purine metabolism;ko00620 Pyruvate metabolism;ko01200 Carbon metabolism;ko01230 Biosynthesis of amino acids;ko04930 Type II diabetes mellitus;ko05203 Viral carcinogenesis;
gene507	K00645	fabD	[acyl-carrier-protein] S-malonyltransferase	EC:2.3.1.39	1	ko00061 Fatty acid biosynthesis;
gene50804	K03979	obg	GTP-binding protein	--		
gene50805	K04730	IRAK1	interleukin-1 receptor-associated kinase 1	EC:2.7.11.1	11	ko04064 NF-kappa B signaling pathway;ko04210 Apoptosis;ko04620 Toll-like receptor signaling pathway;ko04722 Neurotrophin signaling pathway;ko05133 Pertussis;ko05140 Leishmaniasis;ko05142 Chagas disease (American trypanosomiasis);ko05145 Toxoplasmosis;ko05152 Tuberculosis;ko05162 Measles;ko05169 Epstein-Barr virus infection;
gene50806	K11583	PPP2R3	serine/threonine-protein phosphatase 2A regulatory subunit B''	--	3	ko03015 mRNA surveillance pathway;ko04151 PI3K-Akt signaling pathway;ko04728 Dopaminergic synapse;
gene50811	K13993	HSP20	HSP20 family protein	--	1	ko04141 Protein processing in endoplasmic reticulum;
gene50812	K13993	HSP20	HSP20 family protein	--	1	ko04141 Protein processing in endoplasmic reticulum;
gene50820	K03115	CSNK2B	casein kinase II subunit beta	--	9	ko03008 Ribosome biogenesis in eukaryotes;ko04064 NF-kappa B signaling pathway;ko04310 Wnt signaling pathway;ko04520 Adherens junction;ko04530 Tight junction;ko04712 Circadian rhythm - plant;ko05162 Measles;ko05168 Herpes simplex infection;ko05169 Epstein-Barr virus infection;
gene50823	K02921	RP-L37Ae, RPL37A	large subunit ribosomal protein L37Ae	--	1	ko03010 Ribosome;
gene50825	K01802	E5.2.1.8	peptidylprolyl isomerase	EC:5.2.1.8		
gene50828	K04506	SIAH1	E3 ubiquitin-protein ligase SIAH1	EC:6.3.2.19	3	ko04115 p53 signaling pathway;ko04120 Ubiquitin mediated proteolysis;ko04310 Wnt signaling pathway;
gene50829	K08064	NFYA	nuclear transcription factor Y, alpha	--	2	ko04612 Antigen processing and presentation;ko05152 Tuberculosis;
gene5082	K00225	E1.3.2.3	L-galactono-1,4-lactone dehydrogenase	EC:1.3.2.3	1	ko00053 Ascorbate and aldarate metabolism;
gene50830	K02975	RP-S25e, RPS25	small subunit ribosomal protein S25e	--	1	ko03010 Ribosome;
gene50836	K15111	SLC25A26	solute carrier family 25 (mitochondrial S-adenosylmethionine transporter), member 26	--		
gene50838	K11978	UBR3	E3 ubiquitin-protein ligase UBR3	EC:6.3.2.19		

gene50839	K00915	IPK2	1D-myo-Inositol-tetrakisphosphate 5-kinase / inositol-polyphosphate multikinase	EC:2.7.1.140 2.7.1.151	2	ko00562 Inositol phosphate metabolism;ko04070 Phosphatidylinositol signaling system;
gene50841	K01648	ACLY	ATP citrate (pro-S)-lyase	EC:2.3.3.8	2	ko00020 Citrate cycle (TCA cycle);ko00720 Carbon fixation pathways in prokaryotes;
gene50844	K12875	ACIN1, ACINUS	apoptotic chromatin condensation inducer in the nucleus	--	3	ko03013 RNA transport;ko03015 mRNA surveillance pathway;ko03040 Spliceosome;
gene50845	K15559	RTT103	regulator of Ty1 transposition protein 103	--		
gene50849	K09422	MYBP	myb proto-oncogene protein, plant	--		
gene50850	K10355	ACTF	actin, other eukaryote	--		
gene50851	K08337	ATG7	autophagy-related protein 7	--	1	ko04140 Regulation of autophagy;
gene50853	K06100	SYMPK	symplekin	--	2	ko03015 mRNA surveillance pathway;ko04530 Tight junction;
gene50855	K03327	TC.MATE, SLC47A, norM, mdtK, dinF	multidrug resistance protein, MATE family	--		
gene50856	K15378	SLC45A1_2_4	solute carrier family 45, member 1/2/4	--		
gene50859	K01265	E3.4.11.18, map	methionyl aminopeptidase	EC:3.4.11.18		
gene50864	K14803	PTC2_3	protein phosphatase 2C homolog 2/3	EC:3.1.3.16		
gene50869	K11816	YUCCA	indole-3-pyruvate monooxygenase	EC:1.14.13.168	1	ko00380 Tryptophan metabolism;
gene50872	K07766	E3.6.1.52	diphosphoinositol-polyphosphate diphosphatase	EC:3.6.1.52		
gene50873	K02690	psaB	photosystem I P700 chlorophyll a apoprotein A2	--	1	ko00195 Photosynthesis;
gene50876	K16278	K16278, HOS1	E3 ubiquitin-protein ligase HOS1	EC:6.3.2.19		
gene50878	K15397	KCS	3-ketoacyl-CoA synthase	EC:2.3.1.199	1	ko00062 Fatty acid elongation;
gene50880	K00873	PK, pyk	pyruvate kinase	EC:2.7.1.40	7	ko00010 Glycolysis / Gluconeogenesis;ko00230 Purine metabolism;ko00620 Pyruvate metabolism;ko01200 Carbon metabolism;ko01230 Biosynthesis of amino acids;ko04930 Type II diabetes mellitus;ko05203 Viral carcinogenesis;
gene50881	K00820	E2.6.1.16, glmS	glucosamine--fructose-6-phosphate aminotransferase (isomerizing)	EC:2.6.1.16	2	ko00250 Alanine, aspartate and glutamate metabolism;ko00520 Amino sugar and nucleotide sugar metabolism;
gene50885	K09422	MYBP	myb proto-oncogene protein, plant	--		
gene50888	K13963	SERPINB	serpin B	--	1	ko05146 Amoebiasis;
gene50891	K14839	NOP16	nucleolar protein 16	--		
gene50892	K05665	ABCC1	ATP-binding cassette, subfamily C (CFTR/MRP), member 1	--	3	ko02010 ABC transporters;ko04977 Vitamin digestion and absorption;ko05206 MicroRNAs in cancer;
gene50894	K15730	PTGES3	cytosolic prostaglandin-E synthase	EC:5.3.99.3	1	ko00590 Arachidonic acid metabolism;
gene50896	K10858	PMS2	DNA mismatch repair protein PMS2	--	2	ko03430 Mismatch repair;ko03460 Fanconi anemia pathway;
gene50898	K05305	FUK	fucokinase	EC:2.7.1.52	2	ko00051 Fructose and mannose metabolism;ko00520 Amino sugar and nucleotide sugar metabolism;
gene50899	K00949	E2.7.6.2, THI80	thiamine pyrophosphokinase	EC:2.7.6.2	1	ko00730 Thiamine metabolism;
gene50900	K03063	PSMC4, RPT3	26S proteasome regulatory subunit T3	--	2	ko03050 Proteasome;ko05169 Epstein-Barr virus infection;
gene50904	K10896	FANCM	fanconi anemia group M protein	--	1	ko03460 Fanconi anemia pathway;
gene50908	K02713	psbL	photosystem II PsbL protein	--	1	ko00195 Photosynthesis;
gene50910	K02917	RP-L35Ae, RPL35A	large subunit ribosomal protein L35Ae	--	1	ko03010 Ribosome;
gene50913	K10418	DYNLL	dynein light chain LC8-type	--	1	ko04962 Vasopressin-regulated water reabsorption;
gene50917	K16732	PRC1	protein regulator of cytokinesis 1	--		
gene5091	K01652	E2.2.1.6L, ilvB, ilvG, ilvI	acetolactate synthase I/II/III large subunit	EC:2.2.1.6	6	ko00290 Valine, leucine and isoleucine biosynthesis;ko00650 Butanoate metabolism;ko00660 C5-Branched dibasic acid metabolism;ko00770 Pantothenate and CoA biosynthesis;ko01210 2-Oxocarboxylic acid metabolism;ko01230 Biosynthesis of amino acids;
gene50920	K15414	C1QBP	complement component 1 Q subcomponent-binding protein, mitochondrial	--	1	ko05168 Herpes simplex infection;
gene50923	K02739	PSMB7	20S proteasome subunit beta 2	EC:3.4.25.1	1	ko03050 Proteasome;
gene50924	K09422	MYBP	myb proto-oncogene protein, plant	--		
gene50925	K02946	RP-S10, MRPS10, rpsJ	small subunit ribosomal protein S10	--	1	ko03010 Ribosome;
gene50926	K06685	MOB1, Mats	MOB kinase activator 1	--	3	ko04111 Cell cycle - yeast;ko04390 Hippo signaling pathway;ko04391 Hippo signaling pathway - fly;
gene50928	K08245	E3.4.23.40	phytepsin	EC:3.4.23.40		
gene50929	K14009	BCAP31, BAP31	B-cell receptor-associated protein 31	--	1	ko04141 Protein processing in endoplasmic reticulum;

gene50934	K09528	DNAJC8	DnaJ homolog subfamily C member 8	--		
gene50937	K10755	RFC2_4	replication factor C subunit 2/4	--	3	ko03030 DNA replication;ko03420 Nucleotide excision repair;ko03430 Mismatch repair;
gene50940	K13420	FLS2	LRR receptor-like serine/threonine-protein kinase FLS2	EC:2.7.11.1	1	ko04626 Plant-pathogen interaction;
gene50941	K13420	FLS2	LRR receptor-like serine/threonine-protein kinase FLS2	EC:2.7.11.1	1	ko04626 Plant-pathogen interaction;
gene50943	K12818	DHX8, PRP22	ATP-dependent RNA helicase DHX8/PRP22	EC:3.6.4.13	1	ko03040 Spliceosome;
gene50947	K00921	PIKFYVE, FAB1	1-phosphatidylinositol-3-phosphate 5-kinase	EC:2.7.1.150	4	ko00562 Inositol phosphate metabolism;ko04070 Phosphatidylinositol signaling system;ko04145 Phagosome;ko04810 Regulation of actin cytoskeleton;
gene50948	K14768	UTP7, WDR46	U3 small nucleolar RNA-associated protein 7	--		
gene5094	K14948	PTBP2, NPTB	olypyrimidine tract-binding protein 2	--		
gene50950	K14839	NOP16	nucleolar protein 16	--		
gene50951	K05665	ABCC1	ATP-binding cassette, subfamily C (CFTR/MRP), member 1	--	3	ko02010 ABC transporters;ko04977 Vitamin digestion and absorption;ko05206 MicroRNAs in cancer;
gene50954	K12493	ARFGAP2_3	ADP-ribosylation factor GTPase-activating protein 2/3	--	1	ko04144 Endocytosis;
gene50957	K14689	SLC30A2, ZNT2	solute carrier family 30 (zinc transporter), member 2	--		
gene50961	K02716	psbO	photosystem II oxygen-evolving enhancer protein 1	--	1	ko00195 Photosynthesis;
gene50962	K15121	SLC25A44	solute carrier family 25, member 44	--		
gene50965	K06685	MOB1, Mats	MOB kinase activator 1	--	3	ko04111 Cell cycle - yeast;ko04390 Hippo signaling pathway;ko04391 Hippo signaling pathway - fly;
gene50966	K01695	trpA	tryptophan synthase alpha chain	EC:4.2.1.20	3	ko00260 Glycine, serine and threonine metabolism;ko00400 Phenylalanine, tyrosine and tryptophan biosynthesis;ko01230 Biosynthesis of amino acids;
gene50969	K05605	HIBCH	3-hydroxyisobutyryl-CoA hydrolase	EC:3.1.2.4	3	ko00280 Valine, leucine and isoleucine degradation;ko00410 beta-Alanine metabolism;ko00640 Propanoate metabolism;
gene50973	K01188	E3.2.1.21	beta-glucosidase	EC:3.2.1.21	3	ko00460 Cyanoamino acid metabolism;ko00500 Starch and sucrose metabolism;ko00940 Phenylpropanoid biosynthesis;
gene50977	K02977	RP-S27Ae, RPS27A	small subunit ribosomal protein S27Ae	--	1	ko03010 Ribosome;
gene50980	K15168	MED25	mediator of RNA polymerase II transcription subunit 25	--		
gene50983	K12489	ACAP	Arf-GAP with coiled-coil, ANK repeat and PH domain-containing protein	--	1	ko04144 Endocytosis;
gene50988	K14766	NOP14, UTP2	nucleolar protein 14	--		
gene50992	K09422	MYBP	myb proto-oncogene protein, plant	--		
gene50999	K16585	HAUS2	HAUS augmin-like complex subunit 2	--		
gene5099	K14235	tRNA-Trp	tRNA Trp	--	1	ko00970 Aminoacyl-tRNA biosynthesis;
gene50	K13495	CISZOG	cis-zeatin O-glucosyltransferase	EC:2.4.1.215	1	ko00908 Zeatin biosynthesis;
gene51002	K08472	MLO	mlo protein	--		
gene51008	K01240	URH1	uridine nucleosidase	EC:3.2.2.3	2	ko00240 Pyrimidine metabolism;ko00760 Nicotinate and nicotinamide metabolism;
gene5100	K10848	ERCC4, XPF	DNA excision repair protein ERCC-4	EC:3.1.-.-	2	ko03420 Nucleotide excision repair;ko03460 Fanconi anemia pathway;
gene51010	K10405	KIFC1	kinesin family member C1	--		
gene51013	K02889	RP-L21e, RPL21	large subunit ribosomal protein L21e	--	1	ko03010 Ribosome;
gene51017	K13411	DUOX, THOX	dual oxidase	EC:1.6.3.1		
gene51018	K07541	PIGX	phosphatidylinositol glycan, class X	--	1	ko00563 Glycosylphosphatidylinositol(GPI)-anchor biosynthesis;
gene51024	K14486	K14486, ARF	auxin response factor	--	1	ko04075 Plant hormone signal transduction;
gene51028	K14293	KPNB1	importin subunit beta-1	--	1	ko03013 RNA transport;
gene5102	K03327	TC.MATE, SLC47A, norM, mdtK, dinF	multidrug resistance protein, MATE family	--		
gene51032	K02884	RP-L19, MRPL19, rplS	large subunit ribosomal protein L19	--	1	ko03010 Ribosome;
gene51034	K02214	CDC7	cell division control protein 7	EC:2.7.11.1	3	ko04110 Cell cycle;ko04111 Cell cycle - yeast;ko04113 Meiosis - yeast;
gene51038	K00111	glpA, glpD	glycerol-3-phosphate dehydrogenase	EC:1.1.5.3	1	ko00564 Glycerophospholipid metabolism;
gene51041	K14235	tRNA-Trp	tRNA Trp	--	1	ko00970 Aminoacyl-tRNA biosynthesis;
gene51059	K14007	SEC24	protein transport protein SEC24	--	1	ko04141 Protein processing in endoplasmic reticulum;
gene51065	K01011	TST, MPST, sseA	thiosulfate/3-mercaptopyruvate sulfurtransferase	EC:2.8.1.1 2.8.1.2	3	ko00270 Cysteine and methionine metabolism;ko00920 Sulfur metabolism;ko04122 Sulfur relay system;

gene51067	K00927	PGK, pgk	phosphoglycerate kinase	EC:2.7.2.3	4	ko00010 Glycolysis / Gluconeogenesis;ko00710 Carbon fixation in photosynthetic organisms;ko01200 Carbon metabolism;ko01230 Biosynthesis of amino acids;
gene51069	K17255	GDI1_2	Rab GDP dissociation inhibitor	--		
gene51070	K08596	SENP7	sentrin-specific protease 7	EC:3.4.22.68		
gene51076	K01942	HLCS	biotin--protein ligase	EC:6.3.4.9 6.3.4.10 6.3.4.11 6.3.4.15	1	ko00780 Biotin metabolism;
gene51079	K01735	aroB	3-dehydroquinate synthase	EC:4.2.3.4	2	ko00400 Phenylalanine, tyrosine and tryptophan biosynthesis;ko01230 Biosynthesis of amino acids;
gene51080	K04392	RAC1	Ras-related C3 botulinum toxin substrate 1	--	33	ko04010 MAPK signaling pathway;ko04014 Ras signaling pathway;ko04062 Chemokine signaling pathway;ko04145 Phagosome;ko04151 PI3K-Akt signaling pathway;ko04310 Wnt signaling pathway;ko04360 Axon guidance;ko04370 VEGF signaling pathway;ko04380 Osteoclast differentiation;ko04510 Focal adhesion;ko04520 Adherens junction;ko04620 Toll-like receptor signaling pathway;ko04650 Natural killer cell mediated cytotoxicity;ko04662 B cell receptor signaling pathway;ko04664 Fc epsilon RI signaling pathway;ko04666 Fc gamma R-mediated phagocytosis;ko04670 Leukocyte transendothelial migration;ko04722 Neurotrophin signaling pathway;ko04810 Regulation of actin cytoskeleton;ko04932 Non-alcoholic fatty liver disease (NAFLD);ko04972 Pancreatic secretion;ko05014 Amyotrophic lateral sclerosis (ALS);ko05100 Bacterial invasion of epithelial cells;ko05120 Epithelial cell signaling in Helicobacter pylori infection;ko05131 Shigellosis;ko05132 Salmonella infection;ko05200 Pathways in cancer;ko05203 Viral carcinogenesis;ko05205 Proteoglycans in cancer;ko05210 Colorectal cancer;ko05211 Renal cell carcinoma;ko05212 Pancreatic
gene51081	K06689	UBE2D_E, UBC4, UBC5	ubiquitin-conjugating enzyme E2 D/E	EC:6.3.2.19	2	ko04120 Ubiquitin mediated proteolysis;ko04141 Protein processing in endoplasmic reticulum;
gene51083	K13211	GCFC	GC-rich sequence DNA-binding factor	--		
gene51084	K01183	E3.2.1.14	chitinase	EC:3.2.1.14	1	ko00520 Amino sugar and nucleotide sugar metabolism;
gene51086	K06949	rsgA, engC	ribosome biogenesis GTPase	EC:3.6.1.-		
gene51091	K12524	thrA	bifunctional aspartokinase / homoserine dehydrogenase 1	EC:2.7.2.4 1.1.1.3	4	ko00260 Glycine, serine and threonine metabolism;ko00270 Cysteine and methionine metabolism;ko00300 Lysine biosynthesis;ko01230 Biosynthesis of amino acids;
gene51092	K01728	E4.2.2.2, pel	pectate lyase	EC:4.2.2.2	1	ko00040 Pentose and glucuronate interconversions;
gene51097	K08472	MLO	mlo protein	--		
gene51099	K00058	serA, PHGDH	D-3-phosphoglycerate dehydrogenase	EC:1.1.1.95	4	ko00260 Glycine, serine and threonine metabolism;ko00680 Methane metabolism;ko01200 Carbon metabolism;ko01230 Biosynthesis of amino acids;
gene510	K12356	UGT72E	coniferyl-alcohol glucosyltransferase	EC:2.4.1.111	1	ko00940 Phenylpropanoid biosynthesis;
gene51104	K10406	KIFC2_3	kinesin family member C2/C3	--		
gene51105	K11228	STE11	mitogen-activated protein kinase kinase kinase	EC:2.7.11.1	1	ko04011 MAPK signaling pathway - yeast;
gene51106	K00750	GYG1, GYG2	glycogenin glucosyltransferase	EC:2.4.1.186		
gene51107	K00750	GYG1, GYG2	glycogenin glucosyltransferase	EC:2.4.1.186		
gene51108	K01051	E3.1.1.11	pectinesterase	EC:3.1.1.11	2	ko00040 Pentose and glucuronate interconversions;ko00500 Starch and sucrose metabolism;
gene51111	K14638	SLC15A3_4, PHT	solute carrier family 15 (peptide/histidine transporter), member 3/4	--		
gene51112	K00826	E2.6.1.42, ilvE	branched-chain amino acid aminotransferase	EC:2.6.1.42	5	ko00280 Valine, leucine and isoleucine degradation;ko00290 Valine, leucine and isoleucine biosynthesis;ko00770 Pantothenate and CoA biosynthesis;ko01210 2-Oxocarboxylic acid metabolism;ko01230 Biosynthesis of amino acids;
gene51114	K03676	grxC, GLRX, GLRX2	glutaredoxin 3	--		
gene51123	K08505	SFT1	protein transport protein SFT1	--	1	ko04130 SNARE interactions in vesicular transport;
gene5112	K03027	RPC40, POLR1C	DNA-directed RNA polymerases I and III subunit RPAC1	--	5	ko00230 Purine metabolism;ko00240 Pyrimidine metabolism;ko03020 RNA polymerase;ko04623 Cytosolic DNA-sensing pathway;ko05169 Epstein-Barr virus infection;
gene51132	K08269	ULK1_2_3, ATG1	serine/threonine-protein kinase ULK/ATG1	EC:2.7.11.1	2	ko04140 Regulation of autophagy;ko04150 mTOR signaling pathway;

gene51135	K00761	upp, UPRT	uracil phosphoribosyltransferase	EC:2.4.2.9	1	ko00240 Pyrimidine metabolism;
[truncated: 1,236,669 more chars]
