# Supplementary material for: Molecular Regulation of Host Defense Responses Mediated by Biological Anti-TMV Agent Ningnanmycin
Source: Viruses. 2019 Sep 3;11(9):815. doi: 10.3390/v11090815 (PMC6784071; doi:10.3390/v11090815)
Supplement: Supplementary file 1 [file viruses-11-00815-s001.zip › Supplementary file/Supplementary Table S4.pdf.pdf]

Table S4. Annotation of DEGs of NNM-responsive tobacco BY-2 transcriptome.

Gene ID	Gene Symbol	Treatment_mean_fpkm	Control_mean_fpkm	logFC(T/C)	p-value	regulate	NR_Description
gene35747	LOC107770811	32.67	0.68	5.698486877	6.36E-14	Ups	uncharacterized LOC107770811
gene27870	LOC107762028	10.4	0.26	5.429490913	1.32E-13	Ups	acylsugar acyltransferase 3-like
gene30562	LOC107765027	12.53	0.37	5.178947451	2.55E-12	Ups	alpha carbonic anhydrase 7
gene26522	LOC107760521	28.59	1.46	4.401505139	2.58E-12	Ups	probable E3 ubiquitin-protein ligase RNF217
gene6013	LOC107797745	27.58	1.15	4.68728867	3.03E-12	Ups	kiwellin-like
gene60199	LOC107797978	18.93	1.02	4.324277669	8.24E-12	Ups	uncharacterized LOC107797978
gene26669	LOC107760691	8.61	0.35	4.717241892	2.13E-11	Ups	metal transporter Nramp5-like
gene46157	LOC107782365	17.14	1.1	4.064358319	5.91E-11	Ups	uncharacterized LOC107782365
gene73356	LOC107812574	6.98	0.33	4.494893388	8.61E-11	Ups	protein GAMETE EXPRESSED 1
gene65284	LOC107803612	28.29	1.92	3.98470607	9.39E-11	Ups	pirin-like protein At1g50590
gene41266	LOC107776902	37.28	2.61	3.944238314	1.11E-10	Ups	pirin-like protein At1g50590
gene7862	LOC107813754	89.36	5.88	4.031177328	2.12E-10	Ups	transcription factor HBP-1b(c38)-like
gene9211	LOC107815256	13.22	0.84	4.078819417	2.21E-10	Ups	UDP-glycosyltransferase 74B1-like
gene57923	LOC107795454	9.15	0.57	4.113821808	2.22E-10	Ups	UDP-glucosyl transferase 73B2-like
gene2435	LOC107832175	10.81	0.8	3.859278863	2.50E-10	Ups	calcium-transporting ATPase 12, plasma membrane-type-like
gene25977	LOC107759913	13.33	0.85	4.082806719	4.74E-10	Ups	1-aminocyclopropane-1-carboxylate oxidase-like
gene15622	LOC107822384	2.93	0.17	4.185478449	4.96E-10	Ups	putative ABC transporter C family member 15
gene22012	LOC107829465	13.21	0.89	4.000446555	8.89E-10	Ups	protein NTM1-like 9
gene55266	LOC107792517	37.53	3.08	3.713283879	1.14E-09	Ups	uncharacterized LOC107792517
gene6932	LOC107808010	17.54	1.31	3.849578102	1.23E-09	Ups	dehydration-responsive element-binding protein 1D-like
gene52615	LOC107789568	63.49	5.39	3.665189591	1.76E-09	Ups	probable WRKY transcription factor 70
gene29775	LOC107764158	6.66	0.51	3.822405601	1.85E-09	Ups	DELLA protein GAI-like
gene55108	LOC107792337	63.18	5.32	3.67502232	1.92E-09	Ups	probable WRKY transcription factor 40
gene66576	LOC107805050	88.36	7.7	3.626558323	2.66E-09	Ups	uncharacterized LOC107805050
gene62238	LOC107800237	12.23	1.01	3.710336595	3.03E-09	Ups	calcium-transporting ATPase 12, plasma membrane-type-like
gene34157	LOC107769052	73.65	6.59	3.588502254	3.16E-09	Ups	bifunctional monodehydroascorbate reductase and carbonic anhydrase nectarin-3-like
gene44150	LOC107780121	59.98	5.6	3.528251985	3.95E-09	Ups	uncharacterized N-acetyltransferase p20-like
gene55356	LOC107792617	24.5	2.22	3.567932493	6.06E-09	Ups	putative calcium-transporting ATPase 13, plasma membrane-type
gene38266	LOC107773587	7.74	0.33	4.668429643	7.43E-09	Ups	probable WRKY transcription factor 70
gene11213	LOC107817482	43.77	4.2	3.488256397	7.51E-09	Ups	UDP-glycosyltransferase 74B1-like
gene30821	LOC107765311	153.86	9.86	4.069447445	1.01E-08	Ups	RNA-dependent RNA polymerase 1-like
gene47369	LOC107783715	36.71	3.57	3.466334353	1.17E-08	Ups	AAA-ATPase At3g28610-like
gene37225	LOC107772450	23.52	1.68	3.915913045	1.17E-08	Ups	patatin-like protein 2
gene61808	LOC107799761	18.18	1.44	3.766071293	1.24E-08	Ups	uncharacterized LOC107799761
gene51723	LOC107788560	8.11	0.74	3.564548499	1.25E-08	Ups	uncharacterized LOC107788560
gene69152	LOC107807916	9.56	0.9	3.514738199	1.25E-08	Ups	probable WRKY transcription factor 51
gene53544	LOC107790605	3.95	0.1	5.387055646	1.77E-08	Ups	aspartic proteinase CDR1-like
gene21997	LOC107829449	48.44	5.22	3.320596866	2.13E-08	Ups	blue copper protein-like
gene62216	LOC107800213	104.65	8	3.814605066	2.36E-08	Ups	uncharacterized LOC107800213
gene43463	LOC107779355	63.41	6.51	3.390619527	2.41E-08	Ups	cyclin-L1-1-like
gene56099	LOC107793436	24.75	2.74	3.27838461	2.62E-08	Ups	uncharacterized LOC107793436
gene32294	LOC107766960	17.48	2.01	3.229257047	3.88E-08	Ups	uncharacterized LOC107766960
gene25640	LOC107759538	114.81	9.32	3.729447844	3.94E-08	Ups	lysine histidine transporter-like 8
gene40997	LOC107776602	0.37	8.99	-4.486388466	5.93E-08	Down	uncharacterized LOC107776602

gene69731	LOC107808552	1.35	0.12	3.622135991	8.60E-08	Ups	uncharacterized LOC107808552
gene62237	LOC107800236	17.84	2.13	3.169440365	9.47E-08	Ups	putative calcium-transporting ATPase 13, plasma membrane-type
gene8820	LOC107814822	28.8	3.69	3.071222509	1.36E-07	Ups	patatin-like protein 2
gene53443	LOC107790492	5.26	0.35	4.00108619	1.41E-07	Ups	putative receptor-like protein kinase At1g72540
gene55625	LOC107792912	9.72	0.95	3.466643911	1.50E-07	Ups	acetylajmalan esterase-like
gene15013	LOC107821703	25.94	3.36	3.056643354	1.59E-07	Ups	mitogen-activated protein kinase kinase kinase YODA-like
gene56617	LOC107794001	7.91	0.63	3.747231211	1.95E-07	Ups	uncharacterized LOC107794001
gene70199	LOC107809059	19.86	2.34	3.19390972	1.96E-07	Ups	bifunctional monodehydroascorbate reductase and carbonic anhydrase nectarin-3-like
gene36021	LOC107771114	4.97	0.41	3.700344823	2.03E-07	Ups	flavin-dependent oxidoreductase FOX1-like
gene23519	LOC107831128	9.2	1.07	3.212590295	2.55E-07	Ups	patatin-like protein 2
gene39271	LOC107774700	28.48	3.39	3.177733572	2.67E-07	Ups	probable calcium-binding protein CML45
gene65110	LOC107803418	99.3	12.5	3.095578612	2.77E-07	Ups	uncharacterized LOC107803418
gene58626	LOC107796233	3.56	0.45	3.094590521	2.90E-07	Ups	uncharacterized LOC107796233
gene14215	LOC107820820	38.76	5.08	3.03795735	3.27E-07	Ups	uncharacterized LOC107820820
gene6464	LOC107802864	1.95	0.06	5.021406174	4.02E-07	Ups	calmodulin-binding protein 60 A-like
gene65625	LOC107803997	9.46	0.87	3.549785146	4.09E-07	Ups	dehydration-responsive element-binding protein 1A-like
gene73767	LOC107813031	45.62	6.44	2.931464049	4.24E-07	Ups	uncharacterized LOC107813031
gene12960	LOC107819419	10.68	0.93	3.620174474	4.25E-07	Ups	LOB domain-containing protein 4-like
gene35571	LOC107770617	18.87	2.68	2.919639337	4.76E-07	Ups	BRO1 domain-containing protein BROX-like
gene6841	LOC107807021	4.21	0.28	4.01212836	5.03E-07	Ups	G-type lectin S-receptor-like serine/threonine-protein kinase RLK1
gene66632	LOC107805115	7.7	1.1	2.918509017	5.13E-07	Ups	AAA-ATPase At3g28510-like
gene52148	LOC107789039	38.45	5.51	2.908024375	5.67E-07	Ups	heavy metal-associated isoprenylated plant protein 20-like
gene20340	LOC107827601	15.95	2.28	2.913335548	5.76E-07	Ups	LRR receptor-like serine/threonine-protein kinase FLS2
gene29803	LOC107764191	57.43	6.78	3.187329245	5.95E-07	Ups	uncharacterized LOC107764191
gene1828	LOC107825406	31.47	4.62	2.875283639	6.30E-07	Ups	glucan endo-1,3-beta-glucosidase, acidic
gene21954	LOC107829401	61.44	8.91	2.891384409	6.41E-07	Ups	probable NAD(P)H dehydrogenase (quinone) FQR1-like 1
gene70231	LOC107809096	12.54	1.07	3.660816459	6.55E-07	Ups	uncharacterized LOC107809096
gene28148	LOC107762339	45.08	6.59	2.880569507	7.04E-07	Ups	1-aminocyclopropane-1-carboxylate oxidase homolog
gene15937	LOC107822733	5.83	0.63	3.309651143	7.90E-07	Ups	uncharacterized LOC107822733
gene20680	LOC107827981	256.35	15.3	4.172983901	8.07E-07	Ups	RNA-dependent RNA polymerase 1-like
gene73687	LOC107812940	185.19	21.33	3.223787387	8.65E-07	Ups	uncharacterized LOC107812940
gene45913	LOC107782090	258.89	23.85	3.546092707	8.98E-07	Ups	probable E3 ubiquitin-protein ligase RNF217
gene46761	LOC107783040	19.89	3.02	2.824151025	1.04E-06	Ups	methyl-CpG-binding domain-containing protein 11-like
gene53708	LOC107790787	20.43	2.94	2.902869157	1.08E-06	Ups	tyramine N-feruloyltransferase 4/11-like
gene66626	LOC107805106	46.47	6.94	2.850052458	1.12E-06	Ups	probable inactive poly [ADP-ribose] polymerase SRO2
gene55318	LOC107792573	2.73	0	Inf	1.19E-06	Ups	40S ribosomal protein S15-like
gene27213	LOC107761299	5.35	0.75	2.938677539	1.27E-06	Ups	LRR receptor-like serine/threonine-protein kinase FLS2
gene60170	LOC107797944	15.36	2.26	2.873037075	1.29E-06	Ups	myb-related protein 315-like
gene4961	LOC107786057	8.2	1.07	3.040771499	1.33E-06	Ups	glucan endo-1,3-beta-glucosidase 11-like
gene7209	LOC107811242	8.06	0.95	3.184464456	1.39E-06	Ups	25.3 kDa vesicle transport protein-like
gene34547	LOC107769479	63.17	9.96	2.770999377	1.40E-06	Ups	40S ribosomal protein S15-like
gene38225	LOC107773543	8.65	1.3	2.844528412	1.41E-06	Ups	uncharacterized LOC107773543
gene37100	LOC107772311	9.09	0.66	3.885751075	1.47E-06	Ups	patatin-like protein 3
gene19174	LOC107826318	3.77	0.42	3.279387352	1.59E-06	Ups	guanylate-binding protein 1-like
gene24185	LOC107831870	45.95	7.35	2.750758584	1.65E-06	Ups	16.9 kDa class I heat shock protein 1-like
gene58165	LOC107795723	8.01	1.02	3.075853958	1.73E-06	Ups	protein HYPER-SENSITIVITY-RELATED 4-like
gene144	LOC107820639	2.85	0.11	4.828761096	1.76E-06	Ups	S-type anion channel SLAH4-like

gene52022	LOC107788900	18.84	2.69	2.916181929	1.98E-06	Ups	probable RNA-dependent RNA polymerase 1
gene39484	LOC107774935	358.74	31.44	3.618060578	1.99E-06	Ups	uncharacterized protein DDB_G0292642-like
gene47571	LOC107783945	14.08	2.3	2.720897063	2.03E-06	Ups	U-box domain-containing protein 19-like
gene30901	LOC107765400	10.06	1.54	2.814395407	2.12E-06	Ups	probable pectinesterase/pectinesterase inhibitor 41
gene10267	LOC107816427	109.71	16.51	2.838086311	2.14E-06	Ups	uncharacterized LOC107816427
gene66830	LOC107805331	45.31	7.34	2.731589533	2.16E-06	Ups	pirin-like protein At1g50590
gene64258	LOC107802466	10.94	1.5	2.970884398	2.22E-06	Ups	Bifunctional monodehydroascorbate reductase and carbonic anhydrase nectarin-3-like
gene60778	LOC107798618	1.71	14.94	-3.025483958	2.25E-06	Down	basic form of pathogenesis-related protein 1-like
gene193	LOC107825163	0.39	4.42	-3.383577661	2.27E-06	Down	fatty-acid-binding protein 1-like
gene15043	LOC107821734	9.84	0.29	5.209709705	2.36E-06	Ups	uncharacterized LOC107821734
gene64611	LOC107802866	11.62	1.66	2.910116754	2.45E-06	Ups	transcription factor MYB1R1-like
gene4787	LOC107784032	4.87	34.11	-2.70092615	2.50E-06	Down	uncharacterized LOC107784032
gene54251	LOC107791385	5.26	0.49	3.536406117	2.93E-06	Ups	bidirectional sugar transporter SWEET12-like
gene949	LOC107815394	24.97	4.01	2.745771731	2.93E-06	Ups	uncharacterized N-acetyltransferase p20-like
gene65354	LOC107803692	37.33	6.29	2.6756515	3.10E-06	Ups	zinc finger protein 225-like
gene20752	LOC107828059	2.75	0	Inf	3.18E-06	Ups	chaperone protein dnaJ 20, chloroplastic-like
gene29428	LOC107763769	0.29	0	Inf	3.18E-06	Ups	ABC transporter C family member 10-like
gene59326	LOC107797012	4.16	0.28	3.981217084	3.32E-06	Ups	24-methylenesterol C-methyltransferase 2-like
gene59115	LOC107796774	5.72	0	Inf	3.76E-06	Ups	uncharacterized LOC107796774
gene56330	LOC107793695	60.93	9.28	2.821241297	3.85E-06	Ups	cytochrome P450 81E8-like
gene514	LOC107787610	92.01	14.2	2.802283688	3.90E-06	Ups	trihelix transcription factor GT-3b-like
gene35340	LOC107770358	25.86	4.49	2.631753993	3.96E-06	Ups	protein ASC1-like
gene51332	LOC107788122	145.95	23.87	2.718482785	4.23E-06	Ups	zeatin O-xylosyltransferase-like
gene31321	LOC107765872	7.22	1.17	2.734092341	4.45E-06	Ups	molybdenum cofactor sulfurase-like
gene46729	LOC107783006	72.38	9.5	3.035211974	4.83E-06	Ups	uncharacterized LOC107783006
gene51610	LOC107788434	6.51	0.96	2.868454251	4.87E-06	Ups	flavin-dependent oxidoreductase FOX1-like
gene70652	LOC107809561	157.96	16.36	3.37717268	5.05E-06	Ups	AAA-ATPase At3g28610-like
gene43970	LOC107779922	32.15	5.72	2.595447388	5.23E-06	Ups	expansin-like B1
gene57029	LOC107794460	3.15	0.18	4.198710706	5.37E-06	Ups	WAT1-related protein At4g30420-like
gene22937	LOC107830487	1.99	14.05	-2.714080491	5.39E-06	Down	lignin-forming anionic peroxidase [
gene36215	LOC107771327	2.71	0.34	3.110287455	5.47E-06	Ups	receptor-like protein 12
gene26270	LOC107760242	85.02	13.81	2.727707814	5.51E-06	Ups	pirin-like protein At1g505
gene47913	LOC107784327	7.51	1.32	2.616199684	5.63E-06	Ups	uncharacterized LOC107784327
gene28058	LOC107762237	69.04	12.37	2.586216416	5.67E-06	Ups	sigma factor binding protein 1, chloroplastic-like
gene36497	LOC107771638	92.74	16.44	2.601877792	5.76E-06	Ups	metal tolerance protein 9-like
gene33460	LOC107768268	32.11	5.79	2.577306209	5.92E-06	Ups	blue copper protein-like
gene54020	LOC107791128	111.84	18.67	2.688712644	6.33E-06	Ups	zinc finger protein ZAT12-like
gene32217	LOC107766873	7.48	0.77	3.383976253	6.75E-06	Ups	uncharacterized LOC107766873
gene31636	LOC107766225	1.87	0.06	5.055676076	6.82E-06	Ups	protein DETOXIFICATION 49-like
gene39485	LOC107774939	56.08	9.93	2.603087157	6.90E-06	Ups	probable E3 ubiquitin-protein ligase RNF217
gene20310	LOC107827567	7.5	1.34	2.594284607	7.41E-06	Ups	uncharacterized LOC107827567
gene45836	LOC107782005	25.97	4.2	2.735310185	7.72E-06	Ups	putative calcium-transporting ATPase 13, plasma membrane-type
gene51331	LOC107788121	95.27	16.68	2.619781851	7.87E-06	Ups	zeatin O-glucosyltransferase-like
gene19660	LOC107826851	57.78	10.74	2.533374789	8.14E-06	Ups	BON1-associated protein 2-like
gene15701	LOC107822470	77.39	14.39	2.532906513	8.18E-06	Ups	sigma factor binding protein 1, chloroplastic-like
gene200	LOC107825896	3.4	22.32	-2.607561789	8.44E-06	Down	probable boron transporter 2
gene8577	LOC107814550	207.33	32.91	2.761143517	8.55E-06	Ups	60S acidic ribosomal protein P1-like

gene55937	LOC107793256	18.73	3.51	2.519883661	9.04E-06	Ups	probable trehalose-phosphate phosphatase F
gene52857	LOC107789836	6.46	0.66	3.395313797	9.08E-06	Ups	ribonuclease 3-like protein 3
gene70847	LOC107809780	0.65	7.02	-3.3267318	9.33E-06	Down	lichenase-like
gene6546	LOC107803778	17.48	2.59	2.861534837	9.51E-06	Ups	zinc finger protein ZAT12-like
gene62255	LOC107800257	1.91	12.83	-2.63972062	9.55E-06	Down	uncharacterized LOC107800257
gene11780	LOC107818117	4.49	0.15	4.993391798	1.03E-05	Ups	uncharacterized LOC107800257
gene65671	LOC107804043	159.88	28.27	2.605661645	1.04E-05	Ups	uncharacterized LOC107800257
gene64709	LOC107802973	8.61	1.18	2.967040471	1.04E-05	Ups	NAC domain-containing protein 83-like
gene64105	LOC107802299	4.29	0.41	3.490891457	1.05E-05	Ups	patatin-like protein 2
gene58998	LOC107796647	28.95	5.37	2.536514074	1.16E-05	Ups	uncharacterized LOC107796647
gene18936	LOC107826051	37.37	7.18	2.485200345	1.19E-05	Ups	zinc finger protein ZAT11-like
gene59778	LOC107797514	2.19	0.12	4.280968388	1.22E-05	Ups	1-aminocyclopropane-1-carboxylate oxidase homolog 1-like
gene43236	LOC107779098	127.81	19.67	2.805920501	1.25E-05	Ups	pirin-like protein
gene23487	LOC107831090	13.03	2.28	2.622135991	1.26E-05	Ups	mitogen-activated protein kinase kinase kinase 2-like
gene66871	LOC107805380	4.56	0.35	3.799013753	1.28E-05	Ups	uncharacterized LOC107805380
gene30283	LOC107764716	28.85	5.47	2.504966643	1.44E-05	Ups	putative pectinesterase/pectinesterase inhibitor
gene26500	LOC107760497	2.98	0.39	3.03717349	1.49E-05	Ups	cytochrome P450 89A2-like
gene66186	LOC107804615	28.61	5.57	2.467807844	1.49E-05	Ups	uncharacterized LOC107804615
gene73157	LOC107812348	63.61	12.18	2.490891457	1.49E-05	Ups	probable WRKY transcription factor 70
gene39135	LOC107774549	4.71	0.1	5.713283879	1.52E-05	Ups	nudix hydrolase 10-like
gene58347	LOC107795924	6.89	1.13	2.708248289	1.55E-05	Ups	ankyrin repeat-containing protein At2g01680-like
gene64255	LOC107802464	6.57	1.09	2.698486877	1.55E-05	Ups	17.5 kDa class I heat shock protein-like
gene50415	LOC107787103	31.7	6.18	2.465563277	1.77E-05	Ups	uncharacterized N-acetyltransferase p20-like
gene4477	LOC107780788	8.62	1.68	2.462167168	1.79E-05	Ups	cyclic nucleotide-gated ion channel 1-like
gene7482	LOC107813329	0.88	0.04	4.490891457	1.83E-05	Ups	receptor-like protein 12
gene59917	LOC107797667	53.24	10.64	2.428462253	1.84E-05	Ups	defensin-like protein 19
gene52727	LOC107789688	51.39	9.86	2.487548014	1.85E-05	Ups	eukaryotic initiation factor 4A-9-like
gene25637	LOC107759533	0.25	2.88	-3.441994347	1.87E-05	Down	premnaspirodiene oxygenase-like
gene35738	LOC107770801	46.71	9.17	2.454113912	1.87E-05	Ups	transcription factor HBP-1b(c38)-like
gene55235	LOC107792480	76.84	14.95	2.467432485	2.00E-05	Ups	dehydration-responsive element-binding protein 1E-like
gene25330	LOC107759193	0.55	3.52	-2.57211834	2.12E-05	Down	RNA polymerase II-associated factor 1 homolog
gene12379	LOC107818786	3.42	0.53	2.790936434	2.13E-05	Ups	protein ENHANCED DISEASE RESISTANCE 2-like
gene2505	LOC107759024	244.28	32.76	3.00441882	2.13E-05	Ups	pirin-like protein
gene71476	LOC107810481	10.41	2.05	2.453640062	2.13E-05	Ups	uncharacterized LOC107810481
gene57045	LOC107794474	217.73	31.65	2.888226955	2.18E-05	Ups	probable WRKY transcription factor 70
gene42417	LOC107778182	8.13	1.47	2.576176645	2.20E-05	Ups	serine/threonine-protein kinase CDL1-like
gene9044	LOC107815073	19.44	3.96	2.403551792	2.21E-05	Ups	patatin-like protein 2
gene2356	LOC107831360	4.77	27.01	-2.395451761	2.24E-05	Down	CBL-interacting serine/threonine-protein kinase 25-like
gene39458	LOC107774907	1.68	0.3	2.566025787	2.27E-05	Ups	histone-lysine N-methyltransferase SUVR4-like
gene53657	LOC107790729	2.84	0.14	4.450249473	2.38E-05	Ups	uncharacterized LOC107790729
gene21637	LOC107829049	18.45	3.71	2.420118383	2.43E-05	Ups	probable inactive poly [ADP-ribose] polymerase SRO2
gene47036	LOC107783350	5.8	1.2	2.380609402	2.44E-05	Ups	trihelix transcription factor GT-3b-like
gene64812	LOC107803088	16.49	3.43	2.373054967	2.63E-05	Ups	uncharacterized LOC107803088
gene33632	LOC107768460	19.37	4.01	2.376634278	2.65E-05	Ups	probable alkaline/neutral invertase D
gene31807	LOC107766414	11.34	2.15	2.502668625	2.65E-05	Ups	NAC domain-containing protein 30-like
gene55647	LOC107792937	1.26	0.06	4.429490913	2.71E-05	Ups	amino acid permease 6-like
gene234	LOC107829970	61.64	11.54	2.522675167	2.83E-05	Ups	hydroquinone glucosyltransferase-like

gene3107	LOC107765573	3.44	0.08	5.606368675	2.86E-05	Ups	disease resistance-like protein CSA1
gene15024	LOC107821714	4.89	0.77	2.767222686	2.90E-05	Ups	geraniol 8-hydroxylase-like
gene33951	LOC107768816	13	2.74	2.350147481	3.02E-05	Ups	galactinol--sucrose galactosyltransferase-like
gene57953	LOC107795490	55.14	11.52	2.365360575	3.03E-05	Ups	galactinol--sucrose galactosyltransferase-like
gene52737	LOC107789703	4.7	0.75	2.760589593	3.05E-05	Ups	galactinol--sucrose galactosyltransferase-like
gene51729	LOC107788569	8.12	1.42	2.619839315	3.13E-05	Ups	protein ODORANT1-like
gene32109	LOC107766745	2.12	0.35	2.726107919	3.15E-05	Ups	ABC transporter B family member 9-like
gene70396	LOC107809275	6.37	0.58	3.564140439	3.21E-05	Ups	16.9 kDa class I heat shock protein 1-like
gene2728	LOC107761367	3.38	0.43	3.095753516	3.21E-05	Ups	NAC domain-containing protein 104-like
gene59530	LOC107797237	20.88	4.41	2.35057621	3.30E-05	Ups	U-box domain-containing protein 19-like
gene65437	LOC107803783	2.35	0.18	3.780398075	3.36E-05	Ups	AAA-ATPase At3g28580-like
gene34314	LOC107769220	69.69	14.73	2.348258148	3.37E-05	Ups	GDSL esterase/lipase At1g28640-like
gene57796	LOC107795312	47.61	9.42	2.442797169	3.47E-05	Ups	zeatin O-glucosyltransferase-like
gene21377	LOC107828757	11.48	2.39	2.370930833	3.51E-05	Ups	receptor protein kinase CLAVATA1-like
gene36437	LOC107771572	61.65	12.8	2.373387373	3.56E-05	Ups	uncharacterized LOC107771572
gene47663	LOC107784049	3.47	0.56	2.74838768	3.80E-05	Ups	uncharacterized LOC107784049
gene49251	LOC107785811	7.59	1.63	2.329565585	3.93E-05	Ups	receptor-like protein 12
gene61004	LOC107798865	14.37	3.08	2.326260756	3.94E-05	Ups	alkane hydroxylase MAH1-like
gene15755	LOC107822530	3.36	0.37	3.304478333	4.05E-05	Ups	type IV inositol polyphosphate 5-phosphatase 11-like
gene64304	LOC107802518	14.09	2.55	2.574307466	4.13E-05	Ups	probable WRKY transcription factor 50
gene71871	LOC107810915	75.55	15.6	2.382057701	4.17E-05	Ups	probable WRKY transcription factor 40
gene71714	LOC107810743	8.59	1.49	2.636870763	4.18E-05	Ups	OPA3-like protein
gene71515	LOC107810524	2.8	0.52	2.543358877	4.27E-05	Ups	RR receptor-like serine/threonine-protein kinase FLS2
gene14316	LOC107820928	80.09	16.81	2.358348678	4.28E-05	Ups	uncharacterized protein At1g66480-like
gene54986	LOC107792203	59.4	12.16	2.393814143	4.39E-05	Ups	extensin-like
gene33562	LOC107768384	38.65	256.64	-2.625271135	4.41E-05	Down	pathogenesis-related leaf protein 4-like
gene31050	LOC107765563	6.84	1.26	2.544529421	4.52E-05	Ups	putative mediator of RNA polymerase II transcription subunit 24
gene40162	LOC107775685	11.18	2.13	2.495692444	4.59E-05	Ups	dehydration-responsive element-binding protein 1B-like
gene7315	LOC107812389	3.52	0.22	4.075853958	4.63E-05	Ups	zinc finger protein ZAT3-like
gene29212	LOC107763524	5.43	0.35	4.075853958	4.63E-05	Ups	uncharacterized LOC107763524
gene29307	LOC107763631	2.88	0.18	4.075853958	4.63E-05	Ups	probable WRKY transcription factor 70
gene61837	LOC107799793	31.11	6.85	2.289751585	4.66E-05	Ups	probable WRKY transcription factor 40
gene70960	LOC107809904	5.27	0.5	3.505841799	4.74E-05	Ups	NAC domain-containing protein 78-like
gene58681	LOC107796294	25.38	5.58	2.292456291	4.84E-05	Ups	uncharacterized LOC107796294
gene21401	LOC107828788	6.99	0.87	3.120941848	4.86E-05	Ups	uncharacterized LOC107828788
gene18935	LOC107826050	98.46	20.57	2.364975908	4.87E-05	Ups	zinc finger protein ZAT11-like
gene8819	LOC107814823	5.19	0.9	2.628394981	4.89E-05	Ups	patatin-like protein 2
gene12974	LOC107819435	54.69	12.02	2.291987389	4.93E-05	Ups	probable calcium-binding protein CML44
gene47227	LOC107783559	11.46	2.53	2.284870657	5.06E-05	Ups	protein HYPER-SENSITIVITY-RELATED 4-like
gene71106	LOC107810068	0.38	5.03	-3.626566124	5.07E-05	Down	uncharacterized LOC107810068
gene46645	LOC107782911	43.73	9.28	2.343196403	5.12E-05	Ups	UDP-glucose flavonoid 3-O-glucosyltransferase 6-like
gene52197	LOC107789095	4.82	0.76	2.76007809	5.22E-05	Ups	myb-related protein 306-like
gene67451	LOC107806023	5.95	0.74	3.10980129	5.26E-05	Ups	uncharacterized LOC107806023
gene41660	LOC107777339	6.66	1.19	2.591293354	5.41E-05	Ups	uncharacterized LOC107777339
gene14303	LOC107820920	2.12	0.05	5.490891457	5.49E-05	Ups	ethylene-responsive transcription factor ERF022-like
gene2912	LOC107763415	3.24	0.57	2.6001901	5.52E-05	Ups	uncharacterized LOC107763415
gene20284	LOC107827538	47.37	9.13	2.481332629	5.61E-05	Ups	uncharacterized LOC107827538

gene50080	LOC107786733	2.84	0.29	3.394215438	5.65E-05	Ups	G-box-binding factor 1-like
gene22758	LOC107830290	11.52	2.33	2.414270176	5.88E-05	Ups	uncharacterized LOC107830290
gene44547	LOC107780567	7.67	39.78	-2.268424404	5.95E-05	Down	protein CDI-like
gene48355	LOC107784799	14.24	2.91	2.397256385	5.96E-05	Ups	cytochrome P450 714C2-like
gene19398	LOC107826566	14.48	3.28	2.248724799	6.16E-05	Ups	phosphatidylinositol/phosphatidylcholine transfer protein SFH12-like
gene38456	LOC107773797	32.64	7.38	2.249996029	6.17E-05	Ups	probable protein phosphatase 2C 27
gene30945	LOC107765447	11.33	2.55	2.256868138	6.20E-05	Ups	mitogen-activated protein kinase kinase kinase YODA-like
gene58659	LOC107796272	1.26	0.1	3.831928375	6.32E-05	Ups	uncharacterized LOC107796272
gene55161	LOC107792398	233.33	37.1	2.7589937	6.35E-05	Ups	probable E3 ubiquitin-protein ligase RNF217
gene58048	LOC107795596	0.63	5.3	-2.957569043	6.40E-05	Down	cationic peroxidase 1-like
gene71914	LOC107810961	29.05	6.62	2.239620501	6.49E-05	Ups	uncharacterized LOC107810961
gene31887	LOC107766503	0.65	4.5	-2.679033544	6.74E-05	Down	uncharacterized LOC107766503
gene67583	LOC107806172	5.31	0.77	2.886820134	7.04E-05	Ups	ethylene-responsive transcription factor ERF109-like
gene294	LOC107763443	1.35	0.15	3.28444058	7.18E-05	Ups	leucine-rich repeat receptor protein kinase EMS1-like
gene31132	LOC107765657	21.5	4.8	2.270203342	7.35E-05	Ups	ethylene-responsive transcription factor ERF109-like
gene59697	LOC107797421	126.06	26.82	2.338888364	7.61E-05	Ups	NAC domain-containing protein 78-like
gene1327	LOC107819628	0.22	1.89	-3.000961639	7.69E-05	Down	uncharacterized LOC107819628
gene66233	LOC107804671	1.1	0.05	4.660816459	7.71E-05	Ups	protein GAMETE EXPRESSED 1-like
gene62254	LOC107800256	2.73	13.63	-2.212157113	7.74E-05	Down	phenylalanine ammonia-lyase G4-like
gene72888	LOC107812045	2.68	0.3	3.270501389	7.87E-05	Ups	glucan endo-1,3-beta-glucosidase 4-like
gene41481	LOC107777144	5.6	0.43	3.793454227	8.01E-05	Ups	uncharacterized LOC107777144
gene32453	LOC107767137	23.25	4.52	2.468865151	8.02E-05	Ups	protein SRC2 homolog
gene7104	LOC107810130	73.24	17.01	2.212516505	8.09E-05	Ups	uncharacterized LOC107810130
gene1196	LOC107818110	3.52	0.51	2.895875292	8.20E-05	Ups	uncharacterized LOC107818110
gene72706	LOC107811842	16.86	3.7	2.294626064	8.32E-05	Ups	uncharacterized LOC107811842
gene73601	LOC107812848	129.85	22.64	2.625774757	8.67E-05	Ups	protein SAR DEFICIENT 1-like
gene19991	LOC107827217	37.22	8.68	2.205585159	8.96E-05	Ups	uncharacterized LOC107827217
gene63867	LOC107802043	3.89	0.17	4.633849411	8.97E-05	Ups	LOB domain-containing protein 1-like
gene25365	LOC107759230	11.79	2.74	2.209345875	9.11E-05	Ups	exocyst complex component EXO70A1-like
gene60291	LOC107798082	7.02	1.6	2.236845835	9.13E-05	Ups	exocyst complex component EXO70B1-like
gene69436	LOC107808229	217.91	36.21	2.695249956	9.43E-05	Ups	putative calcium-binding protein CML19
gene49184	LOC107785737	10.89	2.49	2.233918804	9.44E-05	Ups	DELLA protein GAI-like
gene48183	LOC107784613	12.28	2.51	2.396499087	9.70E-05	Ups	transcription factor WER-like
gene39912	LOC107775404	0.4	0	Inf	9.85E-05	Ups	linoleate 9S-lipoxygenase 5, chloroplastic-like
gene46125	LOC107782328	29.04	6.83	2.194498455	9.85E-05	Ups	putative protein phosphatase 2C 53
gene43760	LOC107779690	7.73	1.35	2.61780357	0.000100246	Ups	putative GEM-like protein 8
gene44032	LOC107779991	2.81	0.65	2.211113788	0.000100718	Ups	pre-mRNA-splicing factor ATP-dependent RNA helicase DEAH1-like
gene56526	LOC107793903	135.95	27.55	2.408991204	0.000104547	Ups	putative calcium-binding protein CML19
gene36363	LOC107771492	4.03	0.66	2.722217003	0.000108421	Ups	NAC domain-containing protein 78-like
gene42730	LOC107778531	0.48	0.05	3.290592807	0.000110217	Ups	receptor-like protein 12
gene26635	LOC107760651	0	1.49	#NAME?	0.000110227	Down	putative expansin-B2
gene17299	LOC107824226	2.93	0.41	2.953234671	0.000115121	Ups	glycerol-3-phosphate acyltransferase 5-like
gene37372	LOC107772607	11.49	2.69	2.200549706	0.000118304	Ups	thaumatin-like protein 1b
gene29250	LOC107763570	3.81	20.68	-2.334122131	0.000120201	Down	uncharacterized LOC107763570
gene39785	LOC107775268	10.66	2.46	2.224376483	0.000120596	Ups	E3 ubiquitin-protein ligase COP1-like
gene5336	LOC107790020	18.65	4.41	2.185628816	0.000120662	Ups	uncharacterized protein At1g66480-like
gene7723	LOC107813601	20.51	4.89	2.175389632	0.000121076	Ups	uncharacterized LOC107813601

gene37332	LOC107772566	18.12	4.41	2.143955794	0.000123747	Ups	uncharacterized LOC107772566
gene55329	LOC107792587	7.46	1.43	2.490891457	0.000124009	Ups	25.3 kDa vesicle transport protein-like
gene49302	LOC107785865	10.4	2.5	2.15959284	0.000124652	Ups	pleiotropic drug resistance protein 1-like
gene5565	LOC107792571	162.18	33.44	2.383739105	0.000125999	Ups	calmodulin-like
gene4346	LOC107779406	31.02	7.31	2.190641716	0.00012683	Ups	ABC transporter C family member 3-like
gene37596	LOC107772851	5.78	0.52	3.568893969	0.00012716	Ups	E3 ubiquitin ligase BIG BROTHER-related-like
gene64822	LOC107803099	10.01	2.28	2.23935269	0.000127317	Ups	glucan endo-1,3-beta-glucosidase 11-like
gene61234	LOC107799125	8.37	1.97	2.190941732	0.000128205	Ups	cysteine-rich repeat secretory protein 3-like
gene44434	LOC107780443	0.92	0.1	3.348872452	0.000129166	Ups	uncharacterized LOC107780443
gene31873	LOC107766487	2.67	0.22	3.713283879	0.000129551	Ups	acid phosphatase 1
gene12343	LOC107818746	112.14	26.74	2.174327655	0.000131298	Ups	uncharacterized LOC107818746
gene48354	LOC107784803	8.57	1.94	2.25273172	0.000134479	Ups	cytokinin hydroxylase-like
gene13050	LOC107819520	74.59	16.45	2.286658405	0.00013648	Ups	uncharacterized LOC107819520
gene61314	LOC107799213	40.29	9.93	2.126195077	0.000140053	Ups	protein ariadne-1-like
gene63370	LOC107801499	0.65	0.03	4.549785146	0.000141897	Ups	transcription factor LHW-like
gene46514	LOC107782765	431.06	65.38	2.826894737	0.000142146	Ups	probable WRKY transcription factor 70
gene2209	LOC107829628	32.31	7.19	2.274399637	0.000147767	Ups	glutamic acid-rich protein-like
gene24652	LOC107832392	12.08	2.46	2.402354783	0.000149054	Ups	uncharacterized LOC107832392
gene49421	LOC107785995	0.28	2.79	-3.198407703	0.000149474	Down	trans-resveratrol di-O-methyltransferase-like
gene64111	LOC107802306	8.27	1.79	2.310912791	0.000149764	Ups	uncharacterized LOC107802306
gene39498	LOC107774952	1.19	0.03	5.298246379	0.000151386	Ups	dirigent protein 24-like
gene63450	LOC107801586	9.96	2.48	2.113442202	0.000151544	Ups	flap endonuclease GEN-like 1
gene62983	LOC107801068	17.75	4.08	2.227857052	0.000152509	Ups	ethylene-responsive transcription factor ERF022-like
gene30629	LOC107765095	23.39	5.84	2.108709786	0.000157687	Ups	E3 ubiquitin-protein ligase ATL6-like
gene62478	LOC107800503	17.81	4.45	2.106152559	0.00015906	Ups	mitogen-activated protein kinase kinase 5-like
gene48382	LOC107784834	2.33	0.22	3.530419822	0.000159871	Ups	transcription factor MYB44-like
gene67338	LOC107805895	32.03	7.89	2.126617072	0.000159956	Ups	putative calcium-binding protein CML19
gene53681	LOC107790755	5.05	23.42	-2.107617575	0.000160432	Down	chitotriosidase-1-like
gene28512	LOC107762745	5.27	1.07	2.410549349	0.000161439	Ups	chitin elicitor receptor kinase 1-like
gene51746	LOC107788588	6.84	1.42	2.371309842	0.000163775	Ups	patatin-like protein 2
gene23618	LOC107831236	21.9	5.49	2.102326169	0.000167432	Ups	beta-glucosidase 12-like
gene43954	LOC107779902	14.59	3.53	2.153056972	0.000167441	Ups	F-box protein At1g61340-like
gene51777	LOC107788621	1.17	6.37	-2.338958102	0.00016814	Down	protein trichome birefringence-like 34
gene63451	LOC107801584	9.29	2.22	2.169662173	0.000169205	Ups	myb-like protein X
gene11085	LOC107817341	12.47	2.99	2.166889034	0.000169812	Ups	probable magnesium transporter NIPA8
gene60908	LOC107798759	7.69	1.94	2.094638252	0.000172248	Ups	uncharacterized LOC107798759
gene25606	LOC107759499	11.61	2.82	2.145219903	0.000172673	Ups	microtubule-associated protein futsch-like
gene41474	LOC107777132	92.9	18.86	2.406088616	0.000172955	Ups	probable flavin-containing monooxygenase 1
gene50271	LOC107786947	19.67	4.88	2.115382322	0.000173894	Ups	uncharacterized LOC107786947
gene16798	LOC107823682	2.99	0.62	2.38758074	0.000177275	Ups	exocyst complex component EXO70B1-like
gene73439	LOC107812671	9.88	2.42	2.133413847	0.000179348	Ups	ubiquitin carboxyl-terminal hydrolase 12-like
gene72558	LOC107811676	40.96	10.31	2.096896374	0.000181128	Ups	ethylene-responsive transcription factor ERF109-like
gene36869	LOC107772055	153.87	33.2	2.318299606	0.00018249	Ups	zinc finger protein ZAT11-like
gene51622	LOC107788448	17.13	4.31	2.097227609	0.000183325	Ups	protein ELC-like
gene14443	LOC107821072	2.6	0.16	4.102326169	0.000184409	Ups	dirigent protein 22-like
gene31743	LOC107766345	13.56	3.42	2.094590521	0.000185162	Ups	protein HOMOLOG OF MAMMALIAN LYST-INTERACTING PROTEIN 5-like
gene1834	LOC107825461	109.04	20.93	2.487444789	0.000185664	Ups	cytochrome P450 CYP72A219-like

gene33303	LOC107768096	4.66	1.15	2.127300724	0.000187135	Ups	probable LRR receptor-like serine/threonine-protein kinase At3g47570
gene21810	LOC107829246	169.36	29.48	2.628434616	0.000187487	Ups	cannabidiolic acid synthase-like
gene43654	LOC107779568	5.97	1.16	2.468865151	0.000189672	Ups	caffeoylshikimate esterase-like
gene51407	LOC107788209	2.68	0.4	2.836666294	0.000193153	Ups	probable pectinesterase/pectinesterase inhibitor 33
gene8832	LOC107814836	115.1	25.82	2.262250098	0.000194042	Ups	ethylene-responsive transcription factor ERF109-like
gene20704	LOC107828008	4.42	0.52	3.195435574	0.000198278	Ups	uncharacterized LOC107828008
gene64025	LOC107802210	25.43	6.46	2.082806719	0.000199054	Ups	uncharacterized LOC107802210
gene44624	LOC107780651	58.43	15.01	2.066875346	0.000206502	Ups	uncharacterized LOC107780651
gene57434	LOC107794908	67.18	17.01	2.087728058	0.0002089	Ups	calcium-binding protein PBP1-like
gene7255	LOC107811832	19.2	4.92	2.069484433	0.000209701	Ups	disease susceptibility protein LOV1-like
gene8308	LOC107814244	7.97	1.11	2.946956225	0.000210507	Ups	non-specific lipid-transfer protein-like
gene18070	LOC107825088	3.03	0.32	3.365360575	0.000210905	Ups	diacylglycerol kinase theta-like
gene51949	LOC107788814	0.49	3.66	-2.782127037	0.000211213	Down	bifunctional L-3-cyanoalanine synthase/cysteine synthase 2, mitochondrial
gene58956	LOC107796596	3.14	0.2	4.075853958	0.000212755	Ups	uncharacterized LOC107796596
gene69514	LOC107808322	3.37	0.22	4.075853958	0.000212755	Ups	cell wall / vacuolar inhibitor of fructosidase 1-like
gene3992	LOC107775435	5.86	1.47	2.097746564	0.00021278	Ups	probable LRR receptor-like serine/threonine-protein kinase At1g67720
gene60752	LOC107798591	17.9	4.5	2.096112797	0.000213384	Ups	nudix hydrolase 10-like
gene41176	LOC107776805	1.81	0.28	2.7842504	0.000216669	Ups	ABC transporter G family member 11-like
gene43536	LOC107779438	6.75	30.37	-2.063075891	0.000217762	Down	G-type lectin S-receptor-like serine/threonine-protein kinase At5g35370
gene38274	LOC107773596	0.72	0	Inf	0.000219621	Ups	uncharacterized LOC107773596
gene8547	LOC107814515	51.26	13.27	2.05626323	0.000221495	Ups	VQ motif-containing protein 22-like
gene57173	LOC107794617	14.68	3.63	2.123682483	0.00022288	Ups	glutaredoxin-C9-like
gene59409	LOC107797101	10.09	2.56	2.085749994	0.000223687	Ups	myosin heavy chain kinase C-like
gene68123	LOC107806768	105.68	25.66	2.14778346	0.000225646	Ups	zinc finger protein ZAT12-like
gene6840	LOC107807014	2.82	0.4	2.933409693	0.000229086	Ups	G-type lectin S-receptor-like serine/threonine-protein kinase RLK1
gene712	LOC107809144	30.62	7.83	2.073223702	0.000229202	Ups	zinc finger protein ZAT11-like
gene43227	LOC107779088	8.86	1.97	2.273300022	0.000236014	Ups	acid phosphatase 1-like
gene27852	LOC107762012	1.92	0.23	3.16226871	0.000241859	Ups	calcium uniporter protein 2, mitochondrial-like
gene29442	LOC107763786	4.72	0.96	2.40600256	0.000245764	Ups	cytokinin hydroxylase-like
gene30557	LOC107765015	17.46	4.39	2.097624581	0.000246061	Ups	probable xyloglucan endotransglucosylase/hydrolase protein 23
gene24255	LOC107831947	1.9	0.27	2.919734756	0.000249356	Ups	probable L-type lectin-domain containing receptor kinase S.5
gene50979	LOC107787731	23	5.55	2.157061273	0.000252022	Ups	uncharacterized LOC107787731
gene24939	LOC107832709	143.7	35	2.143640582	0.000252185	Ups	40S ribosomal protein S15-3-like
gene12644	LOC107819074	18.64	4.83	2.05344958	0.000253481	Ups	TPR repeat-containing thioredoxin TTL4-like
gene35917	LOC107771000	1.44	0.04	5.191331176	0.000255948	Ups	transcription factor WER-like
gene37891	LOC107773174	20.89	5.45	2.045332013	0.000256135	Ups	protein YLS9-like
gene54930	LOC107792142	3.26	0.67	2.387055646	0.000257938	Ups	protein BREVIS RADIX-like
gene34450	LOC107769368	4.78	1.06	2.282077443	0.000258901	Ups	ankyrin repeat-containing protein At3g12360-like
gene5297	LOC107789564	12.15	3.17	2.044878308	0.000258929	Ups	U-box domain-containing protein 18-like
gene56401	LOC107793766	7.28	1.87	2.068481969	0.000259953	Ups	proline-rich receptor-like protein kinase PERK2
gene9990	LOC107816124	16.2	4.26	2.033905776	0.000260445	Ups	G-type lectin S-receptor-like serine/threonine-protein kinase RLK1
gene22678	LOC107830199	0.91	0.1	3.327392725	0.000262933	Ups	receptor-like protein 12
gene45541	LOC107781673	18.17	4.77	2.034698772	0.000266655	Ups	NAC domain-containing protein 100-like
gene49185	LOC107785738	17.67	4.66	2.027733794	0.000267834	Ups	uncharacterized LOC107785738
gene73206	LOC107812409	18.06	4.77	2.026862667	0.00026799	Ups	L-ascorbate oxidase homolog
gene5578	LOC107792699	1.41	0	Inf	0.00026979	Ups	uncharacterized LOC107792699
gene1868	LOC107825897	3.41	0.64	2.527417333	0.000270405	Ups	cytochrome P450 724B1-like

gene13280	LOC107819779	21.15	5.55	2.034631295	0.000271204	Ups	uncharacterized LOC107819779
gene15881	LOC107822671	0.67	4.27	-2.569155926	0.000273041	Down	protein NtpR-like
gene67645	LOC107806239	11.43	3.01	2.032081556	0.000273203	Ups	calmodulin-binding protein 60 D-like
gene73530	LOC107812771	3.39	0.67	2.443585743	0.00027345	Ups	calmodulin-binding protein 60 D-like
gene30667	LOC107765136	76.56	19.68	2.066280631	0.000274311	Ups	probable NAD(P)H dehydrogenase (quinone) FQR1-like 1
gene18757	LOC107825844	2.43	11.28	-2.106454768	0.00027798	Down	probable acyl-activating enzyme 6
gene48791	LOC107785294	6.46	1.14	2.606368675	0.000278569	Ups	transcription factor MYB114-like
gene59636	LOC107797353	41.3	10.68	2.05681151	0.000279328	Ups	patatin-like protein 2
gene53278	LOC107790305	7.77	1.97	2.082806719	0.000283216	Ups	exocyst complex component EXO70B1-like
gene71635	LOC107810655	19.42	4.88	2.099662615	0.000283381	Ups	uncharacterized LOC107810655
gene25610	LOC107759503	32.67	8.67	2.019349588	0.000285816	Ups	protein PLANT CADMIUM RESISTANCE 8-like
gene20701	LOC107828005	4.84	0.65	3.00108619	0.000286222	Ups	CASP-like protein 2D1
gene54550	LOC107791720	42.73	213.92	-2.21769726	0.000289016	Down	early nodulin-93-like
gene2148	LOC107828940	8.26	2.18	2.028785704	0.000289532	Ups	G-type lectin S-receptor-like serine/threonine-protein kinase At4g27290
gene50374	LOC107787059	15.13	3.99	2.027065394	0.00029049	Ups	cytochrome P450 81E8-like
gene48551	LOC107785025	15.42	4.1	2.017889977	0.000290612	Ups	uncharacterized LOC107785025
gene34386	LOC107769300	3.65	0.53	2.891989766	0.000295614	Ups	uncharacterized N-acetyltransferase p20-like
gene36512	LOC107771655	9.84	2.62	2.013659196	0.0002988	Ups	G-type lectin S-receptor-like serine/threonine-protein kinase CES101
gene71229	LOC107810202	28.33	7.26	2.069461856	0.0003016	Ups	UPF0496 protein At4g34320-like
gene36406	LOC107771538	14.64	66.57	-2.078928916	0.000307015	Down	stem-specific protein TSJT1-like
gene12748	LOC107819186	3.17	0.29	3.561280785	0.000307329	Ups	uncharacterized LOC107819186
gene10997	LOC107817243	1.36	0.07	4.397782053	0.000311148	Ups	serine/threonine-protein kinase-like protein At5g23170
gene54236	LOC107791371	24.08	5.61	2.209178248	0.000315826	Ups	F-box protein PP2-B10-like
gene5263	LOC107789265	2.49	0.45	2.58604869	0.00031798	Ups	ankyrin repeat domain-containing protein 13C-like
gene40521	LOC107776080	45.91	11.59	2.091795502	0.000318931	Ups	protein SAR DEFICIENT 1-like
gene65387	LOC107803728	0.84	0.11	3.04343248	0.000321187	Ups	ARF guanine-nucleotide exchange factor GNL2-like
gene39085	LOC107774492	215.06	38.11	2.60234155	0.000326021	Ups	lysine histidine transporter-like 8
gene56714	LOC107794110	29.68	7.65	2.06181101	0.000326448	Ups	uncharacterized protein At1g04910-like
gene37159	LOC107772377	58.82	15.37	2.042495361	0.000326681	Ups	uncharacterized LOC107772377
gene23565	LOC107831182	40.19	10.85	1.995196295	0.000327132	Ups	uncharacterized LOC107831182
gene49690	LOC107786297	4.7	0.98	2.36160844	0.00033266	Ups	probable flavin-containing monooxygenase 1
gene36671	LOC107771832	0.06	1.32	-4.415999138	0.000337205	Down	uncharacterized LOC107771832
gene60163	LOC107797939	20.58	5.43	2.028451089	0.000337962	Ups	ethylene-responsive transcription factor 1B-like
gene22180	LOC107829652	1.97	8.8	-2.052578914	0.000346138	Down	isocitrate lyase-like
gene30840	LOC107765331	4.72	0.79	2.678518461	0.000347229	Ups	uncharacterized LOC107765331
gene64147	LOC107802346	2.36	0.37	2.780398075	0.000348491	Ups	uncharacterized LOC107802346
gene45497	LOC107781623	89.35	23.92	2.007419502	0.000350314	Ups	uncharacterized LOC107781623
gene61392	LOC107799301	2.93	0.51	2.617423863	0.0003573	Ups	cytochrome P450 87A3-like
gene31582	LOC107766165	5.15	0.85	2.701109165	0.000367031	Ups	ethylene-responsive transcription factor 14-like
gene4990	LOC107786338	14.74	4.01	1.985112795	0.000371639	Ups	disease resistance protein TAO1-like
gene59211	LOC107796884	11.44	3.11	1.98267378	0.000371942	Ups	protein NRT1/ PTR FAMILY 4.6-like
