# Supplementary material for: Molecular Regulation of Host Defense Responses Mediated by Biological Anti-TMV Agent Ningnanmycin
Source: Viruses. 2019 Sep 3;11(9):815. doi: 10.3390/v11090815 (PMC6784071; doi:10.3390/v11090815)
Supplement: Supplementary file 1 [file viruses-11-00815-s001.zip › Supplementary file/Supplementary Table S5.pdf.pdf]

Table S5. List of DEGs involved in the KEGG pathways.

PathWay	Significant Pathway definition	DEGs with pathway annotation	Genelist	seqs_kos_list	Pathwaylink
ko00380	Tryptophan metabolism	2(2.74%)	gene9211,gene11213,	K11820,	http://www.kegg.jp/kegg-bin/show_pathway?scale=1.0&query=&map=ko00380&use=reference&multi_query=K11820+red%0D%0A
ko04744	Phototransduction	1(1.37%)	gene5565,	K02183,	http://www.kegg.jp/kegg-bin/show_pathway?scale=1.0&query=&map=ko04744&use=reference&multi_query=K02183+red%0D%0A
ko00624	Polycyclic aromatic hydrocarbon degradation	1(1.37%)	gene56330,	K00517,	http://www.kegg.jp/kegg-bin/show_pathway?scale=1.0&query=&map=ko00624&use=reference&multi_query=K00517+red%0D%0A
ko04910	Insulin signaling pathway	6(8.22%)	gene2356,gene25365,gene60291,gene5565,gene16798,gene53278,	K07198,K07195,K02183,	http://www.kegg.jp/kegg-bin/show_pathway?scale=1.0&query=&map=ko04910&use=reference&multi_query=K07198+blue%0D%0AK07195+red%0D%0AK02183+red%0D%0A
ko00966	Glucosinolate biosynthesis	2(2.74%)	gene9211,gene11213,	K11820,	http://www.kegg.jp/kegg-bin/show_pathway?scale=1.0&query=&map=ko00966&use=reference&multi_query=K11820+red%0D%0A
ko04011	MAPK signaling pathway - yeast	2(2.74%)	gene15013,gene30945,	K11228,	http://www.kegg.jp/kegg-bin/show_pathway?scale=1.0&query=&map=ko04011&use=reference&multi_query=K11228+red%0D%0A
ko00905	Brassinosteroid biosynthesis	1(1.37%)	gene1868,	K12639,	http://www.kegg.jp/kegg-bin/show_pathway?scale=1.0&query=&map=ko00905&use=reference&multi_query=K12639+red%0D%0A
ko04971	Gastric acid secretion	1(1.37%)	gene5565,	K02183,	http://www.kegg.jp/kegg-bin/show_pathway?scale=1.0&query=&map=ko04971&use=reference&multi_query=K02183+red%0D%0A
ko00908	Zeatin biosynthesis	3(4.11%)	gene51332,gene51331,gene57796,	K13495,	http://www.kegg.jp/kegg-bin/show_pathway?scale=1.0&query=&map=ko00908&use=reference&multi_query=K13495+red%0D%0A
ko04740	Olfactory transduction	1(1.37%)	gene5565,	K02183,	http://www.kegg.jp/kegg-bin/show_pathway?scale=1.0&query=&map=ko04740&use=reference&multi_query=K02183+red%0D%0A
ko04745	Phototransduction - fly	1(1.37%)	gene5565,	K02183,	http://www.kegg.jp/kegg-bin/show_pathway?scale=1.0&query=&map=ko04745&use=reference&multi_query=K02183+red%0D%0A
ko04978	Mineral absorption	1(1.37%)	gene26669,	K12347,	http://www.kegg.jp/kegg-bin/show_pathway?scale=1.0&query=&map=ko04978&use=reference&multi_query=K12347+red%0D%0A
ko04977	Vitamin digestion and absorption	1(1.37%)	gene15622,	K05665,	http://www.kegg.jp/kegg-bin/show_pathway?scale=1.0&query=&map=ko04977&use=reference&multi_query=K05665+red%0D%0A
ko04626	Plant-pathogen interaction	12(16.44%)	gene60778,gene4477,gene71515,gene12974,gene294,gene69436,gene56526,gene5565,gene67338,gene28512,gene33303,gene24255,	K13449,K05391,K13420,K13448,K13466,K02183,K13429,	http://www.kegg.jp/kegg-bin/show_pathway?scale=1.0&query=&map=ko04626&use=reference&multi_query=K13449+blue%0D%0AK05391+red%0D%0AK13420+red%0D%0AK13448+red%0D%0AK13466+red%0D%0AK02183+red%0D%0AK13429+red%0D%0A
ko02010	ABC transporters	3(4.11%)	gene15622,gene29428,gene32109,	K05665,K05666,K05658,	http://www.kegg.jp/kegg-bin/show_pathway?scale=1.0&query=&map=ko02010&use=reference&multi_query=K05665+red%0D%0AK05666+red%0D%0AK05658+red%0D%0A
ko04970	Salivary secretion	1(1.37%)	gene5565,	K02183,	http://www.kegg.jp/kegg-bin/show_pathway?scale=1.0&query=&map=ko04970&use=reference&multi_query=K02183+red%0D%0A
ko00627	Aminobenzoate degradation	1(1.37%)	gene56330,	K00517,	http://www.kegg.jp/kegg-bin/show_pathway?scale=1.0&query=&map=ko00627&use=reference&multi_query=K00517+red%0D%0A
ko04976	Bile secretion	2(2.74%)	gene29428,gene32109,	K05666,K05658,	http://www.kegg.jp/kegg-bin/show_pathway?scale=1.0&query=&map=ko04976&use=reference&multi_query=K05666+red%0D%0AK05658+red%0D%0A
ko00910	Nitrogen metabolism	4(5.48%)	gene30562,gene34157,gene70199,gene52737,	K01674,K02575,	http://www.kegg.jp/kegg-bin/show_pathway?scale=1.0&query=&map=ko00910&use=reference&multi_query=K01674+red%0D%0AK02575+red%0D%0A
