# Supplementary figures and images for: Molecular Regulation of Host Defense Responses Mediated by Biological Anti-TMV Agent Ningnanmycin
Source: Viruses. 2019 Sep 3;11(9):815. doi: 10.3390/v11090815 (PMC6784071; doi:10.3390/v11090815)

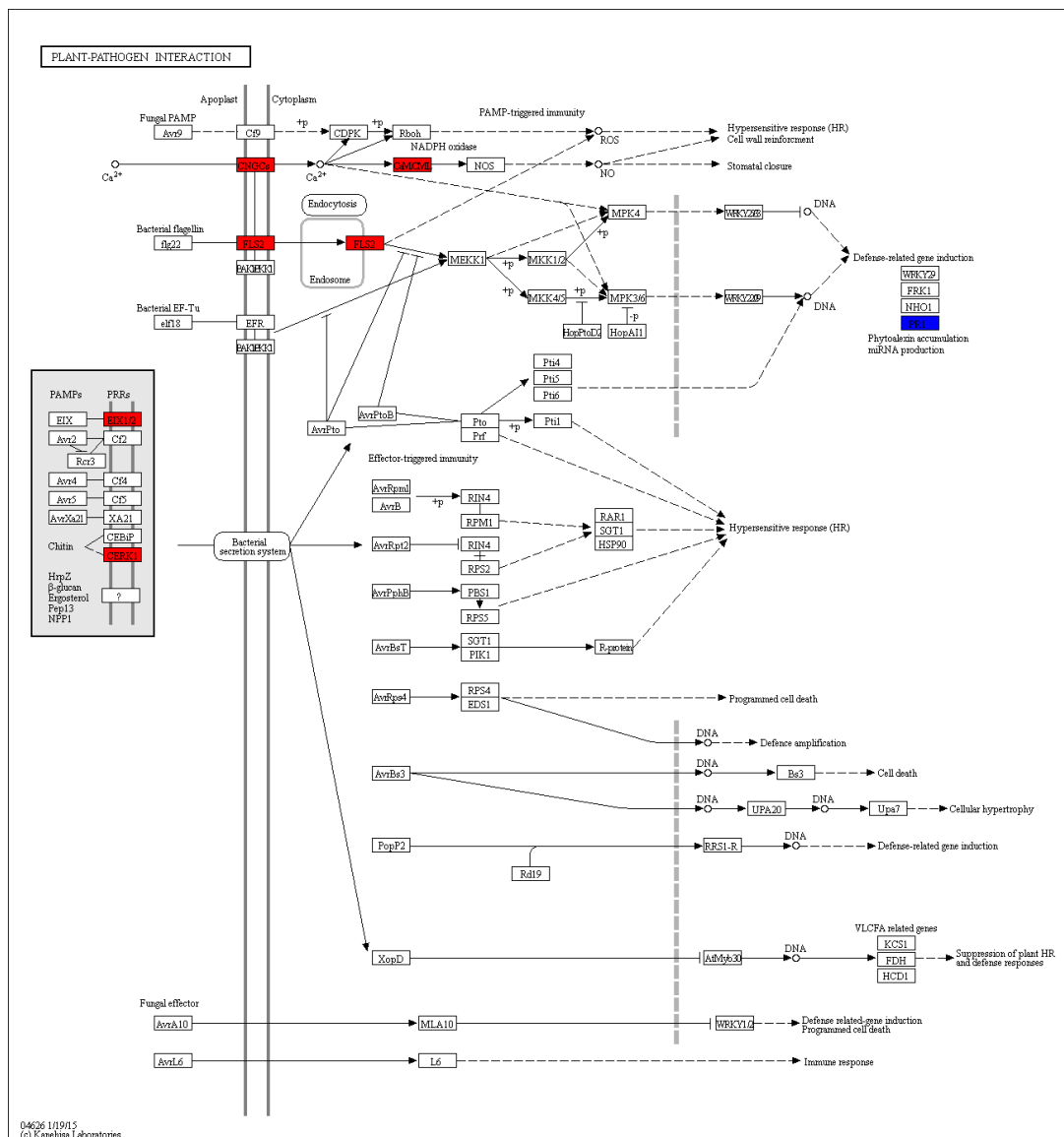

1

2 **Figure S1.** NNM induced KEGG pathway involved in plant-pathogen interaction.

3

Supplement: Supplementary file 1 [file viruses-11-00815-s001.zip › Supplementary file/Supplementary Figure S1.pdf]
