# Supplementary material for: Molecular Regulation of Host Defense Responses Mediated by Biological Anti-TMV Agent Ningnanmycin
Source: Viruses. 2019 Sep 3;11(9):815. doi: 10.3390/v11090815 (PMC6784071; doi:10.3390/v11090815)
Supplement: Supplementary file 1 [file Supplementary Figure S2.pdf › Supplementary file/Supplementary Figure S2.pdf]

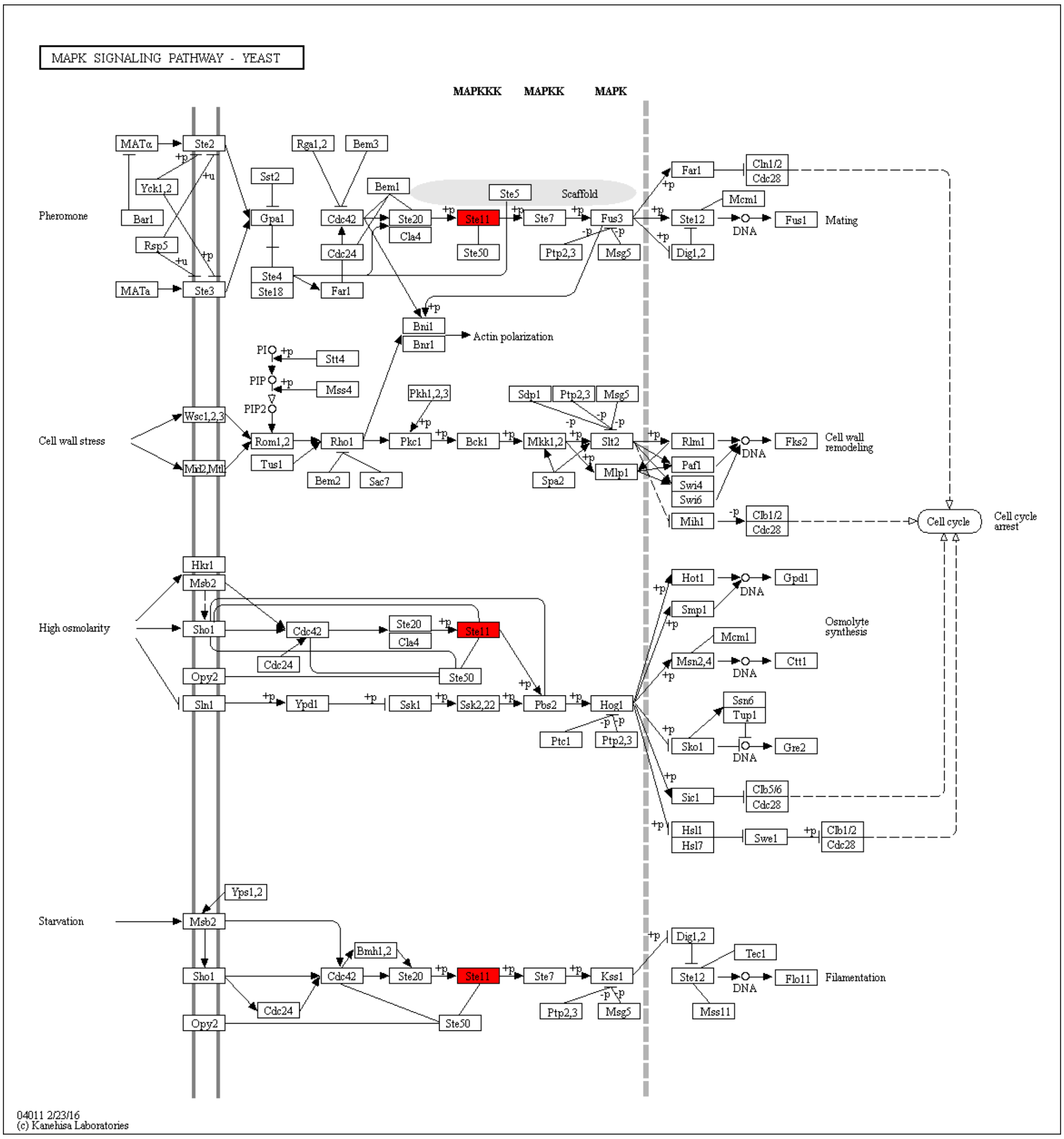

Figure S2. NNM induced KEGG pathway involved in MAPK signaling pathway.
